
JOURNAL INFORMATION
==============================
NLM Title Abbreviation: Innov Aging
Journal ID: Innov Aging
NLM Journal ID: 101703706
Journal ID: innovateage

Innovation in Aging

EISSN: 2399-5300
Publisher: Oxford University Press

ARTICLE INFORMATION
==============================
PMCID: PMC8679246
PMID: 34926835
DOI: 10.1093/geroni/igab046
Article ID: igab046
Article version: 1
Subjects: Full GSA 2021 Abstract Book PDF, AcademicSubjects/SOC02600

Program Abstracts from The GSA 2021 Annual Scientific Meeting, “Disruption to Transformation: Aging in the “New Normal””

Publication date: 2021
Electronic publication date: 2021 Dec 17
Volume: 5
Issue: Suppl 1
Issue title: Program Abstracts from The GSA 2021 Annual Scientific Meeting, “Disruption to Transformation: Aging in the “New Normal””
First page: NP
Last page: NP
Copyright: © The Author(s) 2021. Published by Oxford University Press on behalf of The Gerontological Society of America.
Copyright year: 2021
License: This is an Open Access article distributed under the terms of the Creative Commons Attribution License (https://creativecommons.org/licenses/by/4.0/), which permits unrestricted reuse, distribution, and reproduction in any medium, provided the original work is properly cited.
License URL: https://creativecommons.org/licenses/by/4.0/

Erratum in: Correction to: Program Abstracts from The GSA 2021 Annual Scientific Meeting, “Disruption to Transformation: Aging in the ‘New Normal’” 5 Suppl 1 4 4 2022 1 1 Innovation in Aging PMC8982022 35386422
Page count: 1112

==============================

ISSN 2399-5300 | ONLINE
Vol. 5 | No. S1

### INNOVATION IN AGING™

An Open Access Journal of
The Gerontological Society of America

**Program Abstracts from**
**The GSA 2021 Annual Scientific Meeting,**
**"Disruption to Transformation:**
**Aging in the "New Normal""**

OXFORD
UNIVERSITY PRESS

THE GERONTOLOGICAL SOCIETY OF AMERICA®

### INNOVATION IN AGING
### An Open Access Journal of The Gerontological Society of America

**Steven M. Albert, PhD, MS, FGSA**
**Editor-in-Chief**
University of Pittsburgh, USA
E-mail: smalbert@pitt.edu

**Karen J. Jung, MSW**
**Managing Editor**
The Gerontological Society of America
1220 L Street NW, Suite 901
Washington, DC 20005, USA
Email: ia@geron.org

**Christine E. Bishop, PhD, FGSA**
**Associate Editor, Economics of Aging**
Brandeis University, USA

**Howard B. Degenholtz, PhD, FGSA**
**Associate Editor, Long-Term Services and Supports**
University of Pittsburgh, USA

**Min-Ah Lee, PhD**
**Associate Editor Relationships & Well-being**
Chung-Ang University, South Korea

**Karen D. Lincoln, PhD, MSW, MA, FGSA**
**Associate Editor, Mental Health and Social Determinants of Health**
University of Southern California, USA

**Lena K. Makaroun, MD, MS**
**Associate Editor, Geriatrics**
**VA Pittsburgh Healthcare System, USA**
University of Pittsburgh, USA

**Richard Pak, PhD**
**Associate Editor, Technology**
Clemson University, USA

**Alison Phinney, PhD, RN**
**Associate Editor, Qualitative Research**
University of British Columbia, Canada

**J. Tina Savla, PhD, FGSA**
**Associate Editor, Methodology**
Virginia Tech, USA

**Jennifer Tehan Stanley, PhD, FGSA**
**Associate Editor, Psychological Sciences**
University of Akron, USA

**Roland J. Thorpe, Jr., PhD, FGSA**
**Associate Editor, Minority Health/Health Disparities**
Johns Hopkins Bloomberg School of Public Health, USA

**Elaine C. Wiersma, PhD**
**Deputy Associate Editor, Qualitative Research**
Lakehead University, Canada

### PROGRAM, PUBLICATIONS, AND PRODUCTS COMMITTEE

**Ishan Williams, PhD, FGSA, Chair**
University of Virginia

**Tamara Baker, PhD, FGSA**
University of North Carolina at Chapel Hill, USA

**Dana Bradley, FAGHE, FGSA, PhD**
University of Maryland Baltimore County, USA

**Cynthia Brown, MD, FGSA**
The University of Alabama, Birmingham, USA

**Marilyn Gugliucci, PhD, FGSA, FAGHE**
University of New England, USA

**Stephen Helfand, MD, FGSA**
Brown University, USA

**Judith Howe, PhD, FGSA, FAGHE**
Ichan School of Medicine at Mount Sinai, USA

**Judie Lieu**
The Gerontological Society of America, USA

**Robert T. Mankowski, PhD**
University of Florida, USA

**Megan McCutcheon, MS**
The Gerontological Society of America, USA

**Christina Mueller, FAAN, FGSA, PhD, RN**
University of Minnesota, USA

**Viviana Perez, PhD, FGSA**
Oregon State University / Linus Pauling Institute, USA

**Darina Petrovsky, PhD**
University of Pennsylvania, USA

**James Powers, MD, FGSA**
Vanderbilt University, USA

**Philip Rozario, PhD, FGSA**
Adelphi University, USA

**Debra Umberson, PhD, FGSA**
University of Texas, USA

**Danielle Waldron, MS**
University of Massachusetts, Boston, USA

**Deborah Waldrop, PhD, FGSA**
University of Buffalo, USA

**Fellow of The Gerontological Society of America (FGSA)**

### OUP PRODUCTION DEPARTMENT

**Winnie Titchener**, *Journal Manager - Production*

**INNOVATION IN AGING**
**An Open Access Journal of The Gerontological Society of America**

### EDITORIAL BOARD

**Kathleen Abrahamson, RN, PhD, FGSA**
Purdue University, USA

**Tamara A. Baker, PhD, FGSA**
University of North Carolina at Chapel Hill, USA

**Scott R. Beach, PhD, FGSA**
University of Pittsburgh, USA

**Julie Blaskewicz Boron, PhD, FGSA**
University of Nebraska, Omaha, USA

**Sara J. Czaja, PhD, FGSA**
Weill Cornell Medicine, USA

**Adam Davey, PhD, FGSA**
University of Delaware, Newark, USA

**Nancy R. Gee, PhD**
Virginia Commonwealth University, USA

**Megan Gilligan, PhD, FGSA**
Iowa State University, USA

**Ellen L. Idler, PhD, FGSA**
Emory University, USA

**Mary Ann Johnson, PhD**
University of Nebraska Lincoln, USA

**Yoshinori Kamo, PhD**
Louisiana State University, USA

**Giyeon Kim, PhD, FGSA**
Chung-Ang University, South Korea

**Amanda N. Leggett, PhD**
University of Michigan, USA

**Hui Liu, PhD, FGSA**
Michigan State University, USA

**Mariana López-Ortega, PhD, MPP**
National Institute of Geriatrics, National Institutes of Health, Mexico

**Meghan K. Mattos, PhD, RN**
University of Virginia, USA

**Anne Collins McLaughlin, PhD**
North Carolina State University, USA

**David A. Nace, MD, MPH**
University of Pittsburgh, USA

**Marcia Ory, PhD, FGSA**
Texas A&M, USA

**Karl Pillemer, PhD, FGSA**
Cornell University

**Karen A. Roberto, PhD, FGSA**
Virginia Tech, USA

**Laura P. Sands, PhD, FGSA**
Virginia Tech, USA

**J. Jill Suitor, PhD, FGSA**
Purdue University, USA

**Robert Joseph Taylor, MSW, PhD**
University of Michigan, USA

**Noah J. Webster, PhD**
University of Michigan, USA

**Richard Winett, PhD**
Virginia Tech, USA

**Jeremy B. Yorgason, PhD, FGSA**
Brigham Young University, USA

**Anna Zajacova, PhD, FGSA**
Western University, Canada

**Fellow of The Gerontological Society of America (FGSA)**

### Abstracts

Program Abstracts from The GSA 2021 Annual Scientific Meeting, "Disruption to Transformation: Aging in the "New Normal"

Abstracts are arranged numerically by session and in the order of presentation within each session.

Abstracts are published as received.

### Session 1000 (Symposium)

### A SIMULATION MODEL FOR GERIATRIC EDUCATION: THEORY, VIRTUAL TRANSFORMATION AND QUALITY IMPROVEMENT

Chair: Jennifer Drost Discussant: Susan Fosnight

Delivery of effective healthcare for the geriatric population is often complex due to the interplay between physical, social, and emotional variables. It is well established that it is the interplay between chronic medical conditions, social determinants of health, function and geriatric syndromes that drives outcomes. This complexity makes it especially important for the healthcare team to take an interprofessional team approach to avoid fragmented care which can lead to patient dissatisfaction, an ineffective plan of care, and low-quality outcomes. However, effective teamwork is not innate to healthcare; it must be learned and developed over time through purposeful education. The literature on team training supports active learning pedagogies such as simulation-based education that has emerged as an effective way to translate teamwork education into practice. Participation in active learning such as simulation, provides learners with authentic experiences that become cognitive frames that can transition into real practice. Education of adult learners should be a scaffolding of experiences that build on one another. This approach can lead the learner from the acquisition of basic knowledge, skills, and attitudes, to higher levels of competency and clinical judgement. Simulation simultaneously engages cognitive, perceptual-motor, and affective learning, and when combined with effective debriefing can lead to higher levels of learning. Effective models with scaffolding of experiences using simulations for geriatric team training are lacking in the literature. We describe here the theoretical framework for such training, adaptions of in-person and virtual training models due to COVID-19 restrictions through rapid cycle quality improvement.

### GERIATRIC INTERPROFESSIONAL EDUCATION THEORETICAL FRAMEWORK

Jennifer Drost, *Summa Health System, Akron, Ohio, United States*

The literature is lacking in theoretically grounded techniques to teach interprofessional skills specific to caring for older adults. This presentation details how Wagner's Chronic Care Model and the Constructivist/Active Learning theoretical frameworks were used in the design of an interprofessional education. The content of the education was modeled after Wagner's chronic illness care model that advocates changes in processes and organizational structures to promote interprofessional team practice. The educational intervention follows a Constructivist/Active learning framework delivered in a simulation format. Constructivist approaches encompass active learning and guided experiential learning procedures, methods well-suited to our scaffolded simulation educational experience.

### GERIATRIC INTERPROFESSIONAL EDUCATION: IN-PERSON SIMULATION

Diane Brown, *The University of Akron, Akron, Ohio, United States*

Our in-person geriatric interprofessional training model is layered with scaffolds of active learning, tabletop team meeting simulation, assessment of older adult community members at risk for falls, and reflective feedback. The first step addresses knowledge acquisition via online didactic content. The second step reinforces the knowledge gained in the online didactics through in-person posters and interactive skills practice, followed by a profession-specific huddle to communicate patient assessment findings. The third step is an interprofessional team meeting simulation based on a case study of a complex geriatric patient. The fourth step is performing a supervised assessment on an older adult. The assessment incorporates the assessment tools practiced during the poster/skills session and team skills learned in the didactics and simulation. This is followed by the design of an interprofessional team-developed patient-centered plan of care. The event ends with a reflective debrief focused on interprofessional collaborative competencies.

### ADAPTATION OF GERIATRIC INTERPROFESSIONAL EDUCATION FROM IN-PERSON TO VIRTUAL SIMULATION

Cynthia Hovland, *Cleveland State University, Cleveland, Ohio, United States*

We modified an in-person simulation-enhanced interprofessional education model as necessitated by COVID-19 restrictions to a fully virtual education experience. Online prework remained unchanged but adjustments were made related to previously in-person activities. Diverging from the in-person training we held live virtual poster sessions with learner-presenter interaction. In preparation for their role in the team meeting simulation, learners were moved into preassigned profession-specific breakout rooms for a live virtual huddle with facilitators. Next, learners were moved to preassigned interprofessional breakout rooms where they began the simulated team meeting. After initial discussion, a standardized patient joined the breakout room to present the patient/caregiver perspective. The event ends with a virtual reflective debrief focused on interprofessional collaborative competencies.

© The Author(s) 2021. Published by Oxford University Press on behalf of The Gerontological Society of America.
This is an Open Access article distributed under the terms of the Creative Commons Attribution License (https://creativecommons.org/licenses/by/4.0/), which permits unrestricted reuse, distribution, and reproduction in any medium, provided the original work is properly cited.

### GERIATRIC INTERPROFESSIONAL SIMULATION EDUCATION RESULTS AND RAPID CYCLE QUALITY IMPROVEMENT

Denise Kropp, *Northeast Ohio Medical University, Rootstown, Ohio, United States*

Following a geriatric interprofessional education event, we measured learner progression in interprofessional collaborative competencies using the Interprofessional Socialization and Valuing Scale (ISVS). We also measured student satisfaction with an investigator generated assessment tool. Through Rapid Cycle Quality Improvement (RCQI) processes, we implemented a number of variations of both the in-person and the virtual events. Variations included differences in case studies, pre work requirements, geriatric didactic topics, poster topics and presentation format, facilitator training, standardized patient or patient presence, huddle format, and demonstration of how to effectively perform teamwork. Results showed gains in interprofessional collaborative competencies between pre- and post-education using this geriatric simulation model. Learner satisfaction was high for all simulation variations. Results of education variations and comparisons of the delivery methods will be presented.

### Session 1005 (Paper)

### ADRD AND CAREGIVING

### A 10-YEAR BEHAVIORAL AND SOCIAL SCIENCES RESEARCH AGENDA FOR ADRD: REFLECTION ON A NEW NATIONAL ACADEMIES REPORT

Tia Powell,[1] David Reuben,[2] Vincent Mor,[3] Deborah Blacker,[4] and Molly Checksfield,[5] *1. Montefiore Medical Center, Bronx, New York, United States, 2. UCLA, Los Angeles, California, United States, 3. Providence, Rhode Island, United States, 4. Mass General Hospital, Charlestown, Massachusetts, United States, 5. National Academies of Sciences, Engineering and Medicine, Washington, District of Columbia, United States*

The National Academies of Sciences, Engineering, and Medicine (NASEM) was charged with developing a ten-year agenda for research in the behavioral and social sciences that would substantially contribute to reducing the impact of Alzheimer's disease and related dementias (AD/ADRD). The report, expected to be publicly released in June 2021, has been developed by a committee of individuals with expertise across a range of disciplines and fields, including dementia research. The committee was charged with assessing the role of the social and behavioral sciences in reducing the impact of dementia. The committee held several evidence-gathering sessions, reviewed published literature, commissioned several papers, and engaged individuals living with dementia and caregivers as a part of an Advisory Panel to the committee. This presentation will engage attendees in a discussion about the ten-year behavioral and social science research agenda related to dementia produced by this NASEM committee.

### CREATING A PERSON- AND FAMILY-CENTERED PROGRAM OF RESEARCH: LESSONS LEARNED FROM OVER 30 YEARS OF APPLIED RESEARCH

Carol Whitlatch,[1] and Silvia Orsulic-Jeras,[2] *1. Benjamin Rose Institute on Aging, Shaker Heights, Ohio, United States, 2. Benjamin Rose Institute on Aging, Cleveland, Ohio, United States*

Approximately 6 million Americans are living with Alzheimer's Disease or related dementia. Due to these alarming statistics, there is an increased need for families to seek out services and supports to not only cope with these devastating diagnoses, but to plan effectively for their future care needs. A plethora of research has shown that both the family care partner and person living with dementia are at-risk for negative outcomes such as depression, anxiety, social isolation, and worsening physical and mental health. Moreover, further and encouraging research supports the development and implementation of empowerment-based, person- and family-centered interventions. When utilized effectively these interventions improve quality of care and well-being in persons living with dementia and their care partners. The purpose of this paper is to provide guidance for researchers interested in making their work more person- and family-centered. Strategies discussed are based on over 30 years of applied research and include: 1) placing individuals at the center of their own care, 2) including persons with dementia as co-investigators, 3) convening diverse professionals and individuals in advisory councils from the start, and 4) conducting focus groups to obtain participant and stakeholder feedback. Demonstrations of select person-and-family-centered, evidence-programs will be included and supplemented with case examples to illustrate person-centered principles in practice.

### FAMILY CAREGIVER NEEDS AND PREFERENCES FOR VIRTUAL TRAINING TO MANAGE BEHAVIORAL SYMPTOMS OF DEMENTIA

Magaly Ramirez,[1] Miriana Duran,[1] Chester Pabiniak,[2] Kelly Hansen,[2] James Ralston,[2] Susan McCurry,[3] Linda Teri,[4] and Robert Penfold,[2] *1. University of Washington School of Public Health, Seattle, Washington, United States, 2. Kaiser Permanente Washington Health Research Institute, Seattle, Washington, United States, 3. University of Washington School of Nursing, Seattle, Washington, United States, 4. University of Washington, Seattle, Washington, United States*

STAR-Caregivers Virtual Training and Follow-up (STAR-VTF) is adapted from an evidence-based, in-person program that teaches family caregivers to manage behavioral and psychological symptoms of dementia (BPSD). The study objective was to understand the needs of family caregivers for improving BPSD management and the extent to which caregivers perceived that STAR-VTF could address those needs. We conducted 15 semi-structured interviews with family caregivers of people with dementia. We showed caregivers prototypes of STAR-VTF online self-directed materials. We obtained caregiver feedback, focusing on needs and preferences and perceived barriers to using STAR-VTF. We used a hybrid approach of inductive and deductive coding and aggregated codes to develop themes. The idea of a virtual training program for learning to manage BPSD appealed to caregivers. They said healthcare providers did not provide adequate education in the early disease stages about the personality and behavior symptoms that can affect people with dementia. Caregivers found it unexpected and frustrating when the person with dementia began experiencing BPSD, symptoms they felt unprepared to manage. Accordingly, caregivers expressed a strong desire for the healthcare organization to offer programs such as STAR-VTF much sooner. Many were interested in the virtual aspect of the training due to the convenience of receiving help from home and the perception that help from a virtual program would be timelier than traditional service modalities. Given caregivers' limited time, they suggested dividing the STAR-VTF content into chunks to review as time permitted. Caregivers reported a preference for having the same coach for the program duration.

### LONGITUDINAL EFFECTS OF CULTURAL AND PSYCHOSOCIAL FACTORS ON BIOMARKERS OF CARDIOVASCULAR RISK IN CAREGIVERS

Rosa Romero-Moreno,[1] Carlos Vara-García,[2] Samara Barrera-Caballero,[3] María del Sequeros Chaparro,[4] Javier olazarán,[5] Brent Mausbach,[6] Roland von Känel,[7] and Andrés Losada-Baltar,[2] *1. Universidad Rey Juan Carlos de Madrid, Madrid, Madrid, Spain, 2. Universidad Rey Juan Carlos, Madrid, Madrid, Spain, 3. Rey Juan Carlos University, Alcorcón, Madrid, Madrid, Spain, 4. Universidad Autónoma de Madrid, Madrid, Madrid, Spain, 5. HGU Gregorio Marañón, Madrid, Madrid, Spain, 6. University of California San Diego, La Jolla, California, United States, 7. University Hospital Zurich, Zurich, Zurich, Switzerland*

Dysfunctional thoughts about caregiving (DTAC) and familism (i.e. familistic obligation) were associated with worse caregiver emotional and cardiovascular health in cross-sectional studies. The aim of this study was to longitudinally examine the effects of familism and DTAC on cardiovascular health, considering caregiver kinship adjusting for well-established predictors of cardiovascular health. Study participants were 80 family dementia caregivers. Individual interviews and collection of blood samples were conducted in three yearly assessments. Linear mixed (random effects) regression analysis was performed to examine longitudinal associations of familism, DTAC, and circulating levels of cytokine interleukin (IL)-6, a cytokine and biomarker of cardiovascular disease risk (CVD). Caregiver age, gender, alcohol consumption, body mass index (BMI), hours caring, frequency and reaction of behavioral problems and caregivers' transitions were used as covariates. Results showed that increases in DTAC, in familism and higher caregiver age were independently and significantly associated with higher levels of IL-6 over time in the group of spousal caregivers. No significant effects were found for any of the other covariates in spousal caregivers. In contrast, increases in BMI and in frequency of behavioral problems were significantly associated with increases in IL-6 over time in adult child caregivers. No significant effects were found for any of the rest of predictors in adult child caregivers. Findings suggest that high level of obligation familism and DTAC may a profile of increased vulnerability for CVD in spousal caregivers. In contrast, problem behaviors of the care recipient may characterize adult child caregivers in terms of an increased CVD risk.

### THE EFFECTS OF REMOTE ACTIVITY MONITORING ON FAMILY CAREGIVERS OF PEOPLE LIVING WITH DEMENTIA OVER AN 18-MONTH PERIOD

Joseph Gaugler,[1] Rachel Zmora,[1] Lauren Mitchell,[2] Jessica Finlay,[3] Christina Rosebush,[4] Colleen Peterson,[5] Manka Nkimbeng,[1] and Zachary Baker,[6] *1. University of Minnesota, Minneapolis, Minnesota, United States, 2. Emmanuel College, Boston, Massachusetts, United States, 3. University of Michigan, Ann Arbor, Michigan, United States, 4. University of Minnesota, Twin Cities, Minneapolis, Minnesota, United States, 5. University of Minnesota, University of Minnesota, Minnesota, United States, 6. University of Minnesota, University of Minnesota/Minneapolis, Minnesota, United States*

Technology interventions for older persons and long-term care are generally utilized as real-time data capture tools to complement clinical or family care for older persons or as interventions themselves designed to improve important dementia care outcomes. Although research on novel technological interventions for people with Alzheimer's disease and related dementias (ADRD) and their family caregivers has grown considerably in the past two decades, much of this work continues to focus on design, feasibility, and acceptability (with a need for conceptual refinement in these areas) and less on controlled outcome studies. The objective of this experimental mixed methods demonstration was to determine the 18-month effectiveness of remote activity monitoring (RAM) technology in improving outcomes among family caregivers of community-dwelling persons with dementia. We used an embedded experimental mixed methods design, collecting qualitative data within the structure of a traditional randomized controlled trial ([QUAN+qual]→QUAN) over an 18-month period for 171 dementia caregivers. Change in caregiver self-efficacy, sense of competence, and caregiver distress served as the main quantitative outcomes of interest. Individual growth curve models indicated that the RAM technology did not have direct effects on caregiving outcomes, and although the qualitative findings indicated several potential moderators of RAM effectiveness on caregiving outcomes, the inclusion of these qualitatively-identified moderators did not result in statistically significant ($p < .05$) effects. Ensuring effective human care management alongside RAM technology may help to overcome the barriers reported by dementia caregivers in this demonstration study.

### Session 1010 (Symposium)

### ADVANCING DEMENTIA CARE INTERVENTIONS WITH TECHNOLOGY SOLUTIONS

Chair: Jinmyoung Cho Discussant: Elena Fazio

Over the past two decades, a number of interventions have been developed and tested to help meet the complex care needs of persons living with dementia (PLWD) and the family care support system. Despite the large foundation of empirical evidence, they are often not readily available as part of dementia care support services. Interventions leveraging technology-based solutions have the potential to bolster their desirability, efficacy, and feasibility. While progress has been made, there is still a need to design and test new innovative solutions in real-world settings. This symposium will highlight three such innovative technology solutions for dementia care and explore lessons learned in their development and testing. Smith et al. demonstrate the feasibility of using a novel in-situ sensor system to assess daily functions for PLWD in home or assisted care settings. Results of detecting and classifying diverse forms of functional assessment and environmental conditions will be discussed in the presentation. Czaja et al. describe a randomized controlled trial evaluating the feasibility and efficacy of an innovative dyadic intervention (DT) delivered through an interactive technology. Recruitment challenges and lessons learned from the feasibility of implementing a dyadic intervention will be presented. Stevens et al. introduce an online approach to delivering REACH II, GamePlan4Care (GP4C). Qualitative thematic analyses from GP4C user test sessions related to both the content and technical features will be discussed. Discussant Dr. Elena Fazio will address the role of technology solutions as a strategy within dementia care interventions and unique challenges and contributions of each project.

### TECHNOLOGY INNOVATIONS IN DEMENTIA CARE: IMPROVEMENTS IN FUNCTIONAL ASSESSMENTS THROUGH BACKGROUND SENSING

Matthew Smith,[1] Marcia Ory,[2] Gang Han,[3] Ashley Wilson,[4] Zane Foster,[5] and John Fitch,[5] *1. Texas A&M School of Public Health, College Station, Texas, United States, 2. Texas A&M University, College Station, Texas, United States, 3. Texas A&M University School of Public Health, College Station, Texas, United States, 4. Texas A&M Center for Population Health and Aging, Texas A&M University School of Public Health, College Station, Texas, United States, 5. Birkeland Current LLC, Waco, Texas, United States*

Technological innovations are becoming commonplace in research among persons living with dementia (PLWD) and their caregivers. However, few studies attempt to validate technology's ability to appropriately monitor functional assessment in dementia care research. Bringing together industry, academia, and health care, we demonstrate the feasibility of using a novel in-situ sensor system to continuously and accurately assess daily functions for PWLDs in home or assisted care settings. Phase1 revealed a high accuracy (~85%) of detecting and classifying ADLs between sensors and human loggers across 26 defined activities. Phase 2, which will target 140 PLWDs, has already demonstrated the value of such sensors in detecting safety concerns (e.g., no heat). Technology-driven research for PLWD and their caregivers have practical applications for assessing diverse forms of functional assessment and environmental conditions which can improve measurement precision over time and space and the ability to better tailor care plans for PLWDs and their caregivers.

### A DYADIC TECHNOLOGY-BASED INTERVENTION FOR INFORMAL CAREGIVERS AND PATIENTS WITH DEMENTIA

Sara Czaja,[1] David Loewenstein,[2] and Sarah Weingast,[3] *1. Weill Cornell Medicine/Center on Aging and Behavioral Research, New York, New York, United States, 2. University of Miami Miller School of Medicine, Miami, Florida, United States, 3. Weill Cornell Medicine, Weill Cornell Medicine, New York, United States*

Most intervention programs in the dementia domain have exclusively focused on the caregiver (CG) or the patient (CR), despite evidence of a reciprocal interaction between the dyad. This presentation will describe a randomized controlled trial that is evaluating the feasibility and efficacy of an innovative dyadic intervention (DT) that is delivered through an interactive technology that includes an evidenced-based CG component, an evidenced-based cognitive training component for the CR and a dyadic component. The program is designed to: be synergistic and emphasize issues important to CGs in the earlier stages of caregiving. The sample involves 200 informal CGs and CRs with early-stage dementia. Data will be presented regarding factors influencing the feasibility of implementing a dyadic intervention such as recruitment challenges (e.g., mutual consent and eligibility), and mutual engagement of both the CG and CR. Strategies implemented to maintain the trial during the COVID-19 pandemic will also be discussed.

### USER INTERFACE AND USER EXPERIENCE TESTING OF AN ONLINE TRANSLATION OF THE REACH II INTERVENTION: GAMEPLAN4CARE

Alan Stevens, Thomas Birchfield, Kira Swensen, Joseph Banda, and Jinmyoung Cho, *Baylor Scott & White Health Research Institute, Temple, Texas, United States*

GamePlan4Care (GP4C) is a web-based adaptation of the Resources for Enhancing Alzheimer's Caregiver Health II (REACH II) caregiver intervention, redesigned and reformatted for online delivery. The goal of GP4C is to create an online family caregiver support platform that facilitates self-directed exposure to evidence-based skills-training and support for dementia caregivers. This approach of utilizing technology enhanced with live support has the potential for scalability and sustainability. In preparation for an ongoing randomized clinical trial, the GP4C platform underwent industry standard user interface/user experience (UI/UX) testing with dementia caregivers as part of an iterative design process. Testing of caregiver's reaction to technical and content-related aspects of the system was conducted with 31 caregivers. The thematic analysis revealed three themes for technical aspects (logical flow, suggestions on features, innovative resource) and two themes for content aspects (satisfaction and engagement). We will discuss technical and content modifications resulting from UI/UX.

### Session 1015 (Symposium)

### AGING BIOMARKERS FOR CLINICAL TRIALS AND DRUG DISCOVERY

Chair: Margarita Meer Co-Chair: Raghav Sehgal
Discussant: Morgan Levine

Developing targeted therapies first requires a working definition of the condition of interest. Unfortunately for aging, this very initial step poses a challenge since chronological age is often not indicative of biological age nor modifiable. This symposium will demonstrate the enormous progress being made towards developing more reliable and valid measures for quantifying biological aging. First, Dr. Albert T. Higgins Chen will show how inaccuracy caused by noise at individual CpG sites can lead to high technical variability in the most widely applied biomarkers of aging—epigenetic clocks. He will further discuss how this can be overcome through novel statistical techniques. Second, Dr. Benoit Lehallier, will discuss plasma proteomic clocks and share insights into their potential roles in Alzheimer's disease and utilization in clinical trials. Third, quantifying the multifactorial aging process can be facilitated by projects incorporating multimodal biomarker data. Pei-Lun Kuo from the Baltimore Longitudinal Study of Aging will present an analysis of longitudinal trajectories of more than 30 phenotypes, which when combined into a single summarized score yield important insights. Fourth, our ability to uncover aging mechanisms and perform drug screens, requires valid and reliable measures that can be applied in vitro. Christopher Minteer who developed in cellulo epigenetic markers will demonstarte how epigenetic aging changes that can be induced in culture shed light on aging in vivo. Finally, a summarizing discussion will be held by Dr. Nir Barzilai, an expert in the field, who is leading the Targeting Aging with Metformin (TAME) clinical trial.

### A COMPUTATIONAL SOLUTION TO BOLSTER EPIGENETIC CLOCK RELIABILITY FOR CLINICAL TRIALS AND LONGITUDINAL TRACKING

Albert Higgins-Chen,[1] Kyra Thrush,[1] Tina Hu-Seliger,[2] Yunzhang Wang,[3] Sara Hagg,[3] and Morgan Levine,[1]
*1. Yale University, New Haven, Connecticut, United States, 2. Elysium Health, New York, New York, United States, 3. Karolinska Institutet, Stockholm, Stockholms Lan, Sweden*

Epigenetic clocks are widely used aging biomarkers, but they are calculated from methylation data for individual CpGs that can be surprisingly unreliable. We report that technical noise causes six major epigenetic clocks to deviate by 3 to 9 years between replicates. We present a novel computational solution: we perform principal component analysis followed by biological age prediction using principal components, extracting shared age-related changes across CpGs while ignoring noise from individual CpGs. Our novel principal-component versions of six clocks show agreement between most technical replicates within 1 year, and increased stability in short- and long-term longitudinal studies. This requires only one additional step compared to traditional clocks, does not require prior knowledge of CpG reliabilities, and can improve the reliability of any existing or future epigenetic biomarker. The extremely high reliability of principal component epigenetic clocks makes them particularly useful for personalized medicine and clinical trials evaluating novel aging interventions.

### DEEP LONGITUDINAL PROTEOMICS PROFILING REVEALS BIOLOGICAL PATHWAYS RESPONDING TO GRF6019 IN TWO AD CLINICAL TRIALS

Benoit Lehallier, Tibor Nanasi, Jonas Hannestad, and Steven Braithwaite, *R&D, Alkahest, California, United States*

Blood has been widely investigated to discover biomarkers and gain insights into the biology of aging and age-related diseases. Its protein composition provides insights into complex biological processes, as proteins are often direct regulators of cellular pathways. In clinical trials, selected proteins have been used as primary and secondary endpoints, but recent methodological developments allow the measurement of thousands of proteins with very high sensitivity and specificity. In two phase 2 clinical trials testing the safety, tolerability, and feasibility of infusions of the plasma fraction GRF6019 in Alzheimer's disease (AD), we measured more than 7000 proteins in plasma over the course of the clinical trials. Differential trajectories analysis revealed groups of proteins and pathways that were responding to GRF6019. Several pathways were relevant to the biology of aging and AD and our study suggests that deep proteomics profiling can inform on specific biological processes responding to treatment in clinical trials.

### LONGITUDINAL PROFILING IN PHENOTYPIC METRIC OF AGING: INSIGHTS FROM THE BALTIMORE LONGITUDINAL STUDY OF AGING

Pei-Lun Kuo,[1] Morgan Levine,[2] Jennifer Schrack,[3] Michelle Shardell,[4] and Luigi Ferrucci,[5] *1. National Institute on Aging, National Institute on Aging, Maryland, United States, 2. Yale University, New Haven, Connecticut, United States, 3. Johns Hopkins Bloomberg School of Public Health, Baltimore, Maryland, United States, 4. University of Maryland School of Medicine, Baltimore, Maryland, United States, 5. National Institute on Aging, Baltimore, Maryland, United States*

It remains challenging to quantify the pace of aging across lifespan due to lack of comprehensive longitudinal measurements across wide range of age. In Baltimore Longitudinal Study of Aging, we have measured the longitudinal trajectories of more than 30 phenotypes across four pre-identified domain - body composition, energy regulation, homeostatic mechanisms and neurodegeneration/neuroplasticity, among participants with age between 20+ and 90+. We implemented a two-stage approach to summarize the longitudinal trajectories of these phenotypes across four domains into a summarized score. We demonstrated that higher summarized score (denoting for slower longitudinal phenotypic decline) is associated with slower decline in both cognitive and physical functions, across different stages of adulthood. Our results imply that deep longitudinal profiling contains rich information and may potentially replace diseases as an early endpoint in trials targeting at aging. Further, understanding the underpinning of longitudinal phenotypic trajectories may provide clues to the biological mechanisms of aging.

### A DNAMCULTURE EPIGENETIC FINGERPRINT RECAPITULATES PHYSIOLOGICAL AGING

Christopher Minteer,[1] Marco Morselli,[2] Margarita Meer,[3] Jian Cao,[4] Sabine Lang,[5] Matteo Pellegrini,[6] Qin Yan,[5] and Morgan Levine,[5] *1. Yale University, Yale University, Connecticut, United States, 2. UCLA, Los Angeles, California, United States, 3. Yale School of Medicine, New Haven, Connecticut, United States, 4. Rutgers University, Rutgers University, New Jersey, United States, 5. Yale University, New Haven, Connecticut, United States, 6. UCLA, UCLA, California, United States*

Aging elicits dramatic changes to DNA methylation (DNAm), however the causes and consequences of such alterations to the epigenome remain unclear. The utility of biomarkers of aging based on DNAm patterns would be greatly enhanced if in vitro models existed that recapitulated physiological phenotypes such that modulation could garnish mechanistic insights. Using DNAm from serially passaged mouse embryonic fibroblasts, we developed a marker of culture aging and asked if culture phenotypes, like exhaustive replication, are epigenetically analogous to physiological aging. Our measure, termed DNAmCULTURE, accurately estimated passage number and was shown to strongly increase with age when examined in multiple tissues. Furthermore, we observed epigenetic alterations indicative of early cultured cells in animals undergoing caloric restriction and in lung and kidney fibroblasts re-programmed to iPSCs. This study identifies culture-derived alterations to the methylome as physiologically relevant and implicates culture aging as an important feature in known epigenetic aging phenomena.

### Session 1020 (Symposium)

### AN EXPLORATION ON TRUST

Chair: Elena Portacolone

"Trust is a form of love," explained a study participant. As a form of love, trust nourishes connections and accelerates progress. As a result, the purpose of this session is to reflect upon the notion of trust and examine how trust moves science and social justice forward. Trust must be seen as sustained or broken over multiple generations. Moreover, trust between older adults and medical and social support institutions has profound implications for this historical moment. In the COVID-19 pandemic, trust can be viewed as a facilitator of emergency responses in the State of Washington as noted in Dr. Berridge's paper. On the other hand, distrust and a related sense of abandonment contributes to Black Americans' limited uptake of COVID-19 vaccinations, as noted in Dr. Johnson's work. On a related note, Dr. Perry's work shows that lack of trust over time has led those aging with hemophilia to withdraw from care at different points in their own trajectories. Finally, on a positive note, Dr. Kotwal's work illustrates the role of a peer outreach intervention in facilitating trusting relationships among diverse, low-income older adults which led to sustained reductions, over a 2-year period, in loneliness, barriers to socializing, and depression. This symposium on trust highlights how researchers work, either consciously or unconsciously, within a continuum of trust in their participants' communities. At a broader level, systemic attention to building trust from academia, government, and national advocacy organizations holds the potential to foster meaningful scientific engagement and empowerment of historically marginalized communities.

### THE SIGNIFICANCE OF TRUST IN WASHINGTON STATE'S AGING NETWORK COVID RESPONSE

Clara Berridge,[1] Ian Johnson,[2] Callie Freitag,[1] Carolyn Parsey,[1] and Magaly Ramirez,[3] *1. University of Washington, Seattle, Washington, United States, 2. University of Washington, SEATTLE, Washington, United States, 3. University of Washington School of Public Health, Seattle, Washington, United States*

In late summer of 2020, we interviewed 45 senior leaders of social services and health care organizations serving older adults throughout Washington State about service demand, new challenges, and organizational adaptations. These organizations work with people made particularly vulnerable in the pandemic. A significant share reported that half or more of their clients live at or below the poverty line (54%), are people of color (29%), or have limited English language proficiency (20%). The state's aging network leveraged strong partnerships, expertise, and community knowledge to provide trusted essential services to older Washingtonians and their caregivers. The role of trust as an enabler of emergency response and connection in the context of gentrification, the digital divide, employment loss, and language service gaps will be discussed, as will lack of trust as a barrier to service access, particularly for Latinx immigrant and migrant older adults.

### A PEER INTERVENTION FACILITATES TRUST AND IMPROVES PSYCHOSOCIAL WELL-BEING IN DIVERSE, LOW-INCOME OLDER ADULTS

Ashwin Kotwal,[1] Shannon Fuller,[2] Janet Myers,[2] Daniel Hill,[3] Soe Han Tha,[2] Alexander Smith,[4] and Carla Perissinotto,[2] *1. University of California San Francisco, University of California San Francisco, California, United States, 2. University of California San Francisco, San Francisco, California, United States, 3. Curry Senior Center, San Francisco, California, United States, 4. UCSF, UCSF, California, United States*

We evaluate a peer outreach intervention to improve the psychosocial well-being of diverse, low-income older adults. Participants (N=74, Age 58-96 years) were recruited from an urban senior center and matched with peers who were >55 years old, received mental health training, and connected participants with health or social activities. We conducted surveys at baseline and 6-month follow-up for 2 years with validated measures of loneliness, social interaction, barriers to socializing, and depression, and thematically analyzed qualitative, semi-structured interviews conducted among a subset of participants (n=15) and peers (n=6). Participants were 58% male, 18% African-American, 19% Latinx, and 8% Asian. Over 2 years, participants experienced sustained reductions in loneliness ($p=0.015$), depression ($p<0.001$), and barriers to socializing ($p<0.001$). Qualitative interviews detailed the role of longitudinal relationships, program flexibility, and the matching process in facilitating trust, motivation, and improved mood. Results can inform larger efficacy studies and implementation of peer-driven community programs.

### CHANGING TIME HORIZONS AND TRUST: EXPERIENCES OF AGING WITH HEMOPHILIA

Tam Perry,[1] and Sara Schwartz,[2] *1. Wayne State University, Detroit, Michigan, United States, 2. University of Southern California, Los Angeles, California, United States*

Trust among those who have experienced a lifetime of medical encounters warrants attention to how trust is both cumulative and complex. This study of a historically isolated cohort incorporates interviews (n=25 older adults/professionals) and focus groups uses a lens of trust to highlight the experiences of those aging with hemophilia, individuals who never expected to age. Understood through the lens of trust, the data show evidence of the absence of safe spaces particularly during the early 80s - blood contamination concerns and homophobia-leading often to social withdrawal. Over time, however, some individuals and families created trusted venues to begin demanding research, treatment and policy change. Advocacy re-engaged the community to organize, educate and advance safety protocols for blood product manufacturing and distribution. This presentation will illuminate how experiences with medical providers, contaminated blood supplies, stigma and uncertain in other spheres of one's life make trust a co-constructed, fragile concept.

### FACTORS RELATED TO COVID-19 VACCINE UPTAKE IN BLACK AMERICAN COMMUNITIES

Julene Johnson,[1] Orlando Harris,[2] Carl V. Hill,[3] Peter Lichtenberg,[4] Sahru Keiser,[2] Tam Perry,[4] and Elena Portacolone,[5] *1. University California San Francisco, San Francisco, California, United States, 2. University of California, San Francisco, San Francisco, California, United States, 3. Alzheimer's Association, Chicago, Illinois, United States, 4. Wayne State University, Detroit, Michigan, United States, 5. University of California, Berkeley, California, United States*

Black/African Americans represent 13% of the population, yet account for about a quarter of COVID-19 deaths. Black Americans receive COVID-19 vaccines at lower rates than whites. To address this gap, we examined effects of the COVID-19 pandemic among Black Americans, emphasizing understanding trust and vaccines. Data were collected (July to September 2020) using 8 virtual focus groups in Detroit and San Francisco with 33 older Black Americans and 11 caregivers. Content analysis was used to identify themes. The first theme pointed to a sense of feeling abandoned by healthcare providers and the government, which exacerbated uncertainty and fear. The second theme emphasized distrust towards healthcare providers and government. The third theme pointed to a reluctance in receiving the vaccine because of distrust of pharmaceutical companies and government, as well as misinformation. These findings suggest that underlying systemic issues need to be addressed to accelerate vaccine uptake among older Black Americans.

### Session 1025 (Symposium)

### ANALYSIS OF BEST PRACTICE CAREGIVING: A NEW ONLINE DATABASE OF EVIDENCE-BASED DEMENTIA CAREGIVING PROGRAMS

Chair: Sara Powers Discussant: Sandy Markwood

Best Practice Caregiving (BPC) is a free online database providing comprehensive information on research and implementation characteristics for 44 evidence-based dementia caregiving programs. Programs eligible for BPC have research-tested positive outcomes for family/friend caregivers and demonstrated feasibility in community implementations. This symposium presents results from analyses of the BPC database that includes surveys of 44 program developers and 324 healthcare or community delivery-organizations, and content analysis of 231 published studies. Findings show the most common of 19 types of assistance provided by programs were: Supporting Caregiver/Individual-with-Dementia (IWD) Communication, Encouraging Positive Caregiver-IWD Activities, and Strengthening Coping (93.2%). Least common were: Getting a Dementia Diagnosis (29.5%) and Monitoring Service Benefits (20.5%). Methods of delivering the types of assistance were: information/referral (M=11.1), skills training (M=7.5), and direct provision of care (M=3.8). The most common types of organizations that delivered programs were healthcare organizations (23.8%) and Area Agencies on Aging (23.8%). The greatest delivery-challenges were program marketing (69.8%) and caregiver engagement (66.3%). Most organizations 'strongly agreed' that programs had positive impacts on caregivers (59.5%) but were less certain about IWD benefits (25.1% 'strongly agreed'). Published research studies found the most improved caregiver outcomes were: 1) Strain and/or burden (84.1%), 2) Depressive symptomology (79.5%), and 3) Caregiving efficacy (63.6%). Least common improved outcomes were 1) Access to support information/Community service use (9.1%); 2) Unmet needs (6.8%); and 3) Respite/break from care (2.3%). Overall, results highlight strengths of evidence-based dementia caregiving programs, along with gaps and challenges to be addressed by existing and new developing programs.

### DESCRIPTION AND DEMO OF THE BEST PRACTICE CAREGIVING DATABASE ON 44 RESEARCH PROVEN DEMENTIA CAREGIVING PROGRAMS

David Bass, Alyssa Ciancibello, Rachel Schaffer, and Sara Powers, *Benjamin Rose Institute on Aging, Cleveland, Ohio, United States*

A major advance in family caregiving has been the development, testing, and community delivery of research-proven, evidence-based support programs for family or friend caregivers of persons living with dementia. This presentation showcases and demos Best Practice Caregiving (BPC), a new online resource with comprehensive profiles for 44 of the top evidence-based dementia caregiving programs that are ready for scaling in communities. For these 44 programs, BPC is a database that presents key research findings with links to all its published articles, comprehensive program descriptions including all implementation features, and survey data on program delivery experiences from 324 healthcare and community organizations that offered the program as a regular part of their service portfolio 2019. BPC enables professionals to make side-by-side comparisons of the 44 programs, with the goal of increasing implementations of these evidence-based programs by healthcare and community service organizations.

### TYPES OF ASSISTANCES OF EVIDENCE-BASED DEMENTIA CAREGIVING PROGRAMS: DATA RESULTS AND FUTURE DIRECTIONS

Alyssa Ciancibello, David Bass, Rachel Schaffer, and Sara Powers, *Benjamin Rose Institute on Aging, Cleveland, Ohio, United States*

A key feature displayed in Best Practice Caregiving are the types of assistances. Data on 54 areas of care were collected for all 44 programs. These were analyzed through factor analysis and grouped into 19 types of assistance. Types were analyzed by the number of assistances provided, delivery method, and recipient of assistance. On average, programs delivered 11.8 types of assistances, with the most common types being Supporting CG/Individual-with-Dementia (IWD) Communication, Encouraging Positive CG-IWD Activities, and Assisting with Coping (93.2%), with the least common being Getting a Dementia Diagnosis (29.5%) and Monitoring Benefits of Services (20.5%). Assistance was delivered most often through information/referral delivery (M=11.07, SD=5.41) than direct (M=3.77, SD=4.54) or skills training (M= 7.50, SD=4.54). Results of the data show the breadth and characteristics of assistances programs provide to support caregivers of persons with dementia, along with gaps in types of assistances and future directions for programs.

### EXPERIENCES FROM HEALTHCARE AND COMMUNITY ORGANIZATIONS DELIVERING EVIDENCE-BASED DEMENTIA CAREGIVING PROGRAMS

Rachel Schaffer, Alyssa Ciancibello, David Bass, and Sara Powers, *Benjamin Rose Institute on Aging, Cleveland, Ohio, United States*

Best Practice Caregiving surveyed 324 healthcare and community organizations that replicated one or more of the 44 evidence-based programs about delivery organization characteristics, delivery staff, caregivers and persons with dementia served, funding sources, delivery challenges, perceived impact, and satisfaction. 211 (65.1%) organizations completed surveys about 30 different evidence-based programs. The most common types of organizations that delivered programs were healthcare organizations (23.8%) and Area Agencies on Aging (23.8%). Results showed on average organizations delivered programs for 49 months and served 68 families/year. The most common program delivery challenges were marketing (69.8%) and engaging participants (66.3%). Organizations generally agreed that programs had positive impacts on caregivers (59.5% strongly agree) but were less positive about benefits for persons with dementia (25.1% strongly agree). Discussion provides insights into successes and challenges organizations face when adopting evidence-based dementia caregiving programs in their communities.

### OUTCOME PRIORITIES AMONG EVIDENCE-BASED DEMENTIA CAREGIVING PROGRAMS: A CLOSER LOOK

Sara Powers,[1] Alyssa Ciancibello,[1] Rachel Schaffer,[1] David Bass,[1] and Morgan Minyo,[2] *1. Benjamin Rose Institute on Aging, Cleveland, Ohio, United States, 2. Cleveland State University, Cleveland, Ohio, United States*

Currently, the Best Practice Caregiving website provides information on 231 published studies from 44 dementia caregiving evidence-based programs that have demonstrated beneficial outcomes for dementia caregivers within health care and community-based settings. Across all programs, a total of 34 biopsychosocial outcomes were identified. Supported by the commonly used stress-related frameworks (e.g., Stress-Health Process, Cognitive Behavioral Theory) for which the programs were developed, the most frequently utilized program outcomes included: 1) Caregiver stress, strain, and/or burden (84.1%); 2) Caregiver depressive symptomology (79.5%); and 3) Caregiving efficacy, skills, and/or confidence (63.6%). The least common programmatic outcomes included: 1) Access to support information/Community service use (9.1%); 2) Unmet needs (6.8%); and 3) Respite/break from care (2.3%). The lesser utilized outcomes provide critical insight into current evidence-based programmatic priorities and ways in which professionals can seek to fill gaps in dementia caregiving interventions. Discussion will also focus on future directions of caregiver-related outcome assessments.

### Session 1030 (Symposium)

### ASPECTS OF GRIT AMONG DEMENTIA FAMILY CAREGIVERS

Chair: Jyoti Savla
Co-Chair: Karen Roberto
Discussant: Jennifer Margrett

Dementia family caregivers often show deep devotion and a strong sense of purpose and duty toward their relatives needing care. The concept of grit, which includes aspects of commitment, purpose, perseverance, and resilience, is a novel theoretical approach to expanding understanding of dementia family caregiver strengths amidst the challenges they face. Multiple psychosocial and contextual factors are likely to interact with and influence grit among these caregivers. This symposium addresses commitment to the family dementia caregiver role, willingness to embrace the stressful work of caregiving, and perseverance in finding ways to sustain caregiving roles under typical and adverse circumstances. Blieszner focuses on associations between grit and stressors and strains that challenge caregiver well-being and jeopardize continued caregiving. Wilks considers the impact of spiritual support on sustaining resilience among Caucasian and African American dementia caregivers. McCann explores caregivers' responses to changes in informal support and social interactions available to assist with home care tasks over the course of the COVID-19 pandemic. Albers examines the strengths and resources caregivers draw upon to manage the challenges of caring for and supporting a relative in long-term residential care during the pandemic. Collectively, these presentations provide new insights into the range of influences on aspects of grit and circumstances in which grit sustains caregiving. Discussant Margrett considers the value of the concept of grit for furthering understanding of caregivers' abilities to manage typical and unique challenges in their caregiver roles and offers suggestions for future research and interventions to enhance grit among dementia family caregivers.

### CARING BEYOND THE CHALLENGES: GRIT IN DEMENTIA FAMILY CAREGIVERS

Rosemary Blieszner, Jyoti Savla, Karen Roberto, Brandy McCann, and Emily Hoyt, *Virginia Tech, Blacksburg, Virginia, United States*

Scholars and practitioners recognize the importance of family caregivers for persons with dementia (PwD) persevering through difficulties and remaining committed to providing care (i.e., possessing grit). Based on Pearlin's stress process model, we examined how grit is associated with stressors and strains that interfere with caregiver well-being and jeopardize continued caregiving. The sample included 158 family caregivers of PwD from rural Appalachia. SEM analysis revealed that grit and family and friend affectual solidarity contributed significantly to mastery. Grit and family solidarity were associated indirectly with role overload through their effect on mastery. Results demonstrate the value of acknowledging the role of grit in enhancing caregivers' confidence about managing difficulties they face and reducing their sense of being overwhelmed by caregiving responsibilities. Thus, strengthening dementia caregivers' commitment to and perseverance in their role is crucial for sustaining their motivation to provide care, despite the challenges they face.

### SPIRITUAL SUPPORT AMONG AFRICAN AMERICAN AND CAUCASIAN ADRD CAREGIVERS: A RISK AND RESILIENCE STUDY

Scott Wilks,[1] Wanda Spurlock,[2] Sandra Brown,[2] Jennifer Geiger,[3] Sarah Choate,[4] Katherine Kirsch,[4]

Alisha Thompson,[4] and Cassie Slaton,[4] *1. Louisiana State University, Baton Rouge, Louisiana, United States, 2. Southern University College of Nursing and Allied health, Baton Rouge, Louisiana, United States, 3. East Tennessee State University Dept. of Social Work, Johnson City, Tennessee, United States, 4. Louisiana State University School of Social Work, Baton Rouge, Louisiana, United States*

Research shows African Americans at greater risk of developing Alzheimer's disease and related dementias (ADRD) compared to Caucasians, suggesting African American ADRD caregivers are rising in numbers at a greater rate than Caucasian counterparts. A recent study indicated spiritual wellbeing differences among these caregiver groups. Using a quasi-follow-up of members of a larger caregiver sample, the purpose of this study was to test spiritual support as a moderator via a risk-and-resilience framework. Secondary data analysis from a sample of 691 ADRD caregivers examined data on demographics and standardized measures of spiritual support, caregiver burden, and psychological resilience. One-third of the sample reported as African American. Resilience negatively regressed, though not significantly, on caregiving burden among both groups. Spiritual support positively, significantly impacted resilience among both groups, slightly stronger among African Americans. Spiritual support did not significantly moderate risk with either group. Implications for professional healthcare practice are discussed.

### RESILIENCE AMONG DEMENTIA FAMILY CAREGIVERS IN A CHANGING SOCIAL WORLD

Brandy McCann, Karen Roberto, Tina Savla, Rosemary Blieszner, and Emily Hoyt, *Virginia Tech, Blacksburg, Virginia, United States*

Dementia caregivers must manage the social worlds of their loved ones as well as their own. In a mixed methods study, we interviewed 50 family caregivers prior to the pandemic, twice during early phases of the pandemic, and again during the vaccine roll-out phase. Findings revealed how implementation of stay-at-home orders altered reliance on informal support as well as social ties and interactions. Using content analysis, we identified three ways in which caregivers' managed changes in their social world: rethinking family visits (fewer people, higher quality); reinventing public spaces (church services, exercise venues); and reconsidering self-care (setting boundaries, solace in nature). Caregivers showed varying degrees of resilience in the ways they managed adverse social situations and cared for themselves. Findings reinforce the need for inclusive programs and services to help caregivers learn to maintain supportive social connections that reinforce their care decisions and routines, particularly during times of duress.

### NAVIGATING COVID-19 AND LONG-TERM CARE: DEMENTIA CAREGIVERS' CHALLENGES AND STRENGTHS

Lauren Mitchell,[1] Elizabeth Albers,[2] Robyn Birkeland,[3] Henry Stabler,[2] Jinhee Cha,[3] and Joseph Gaugler,[2] *1. Emmanuel College, Boston, Massachusetts, United States, 2. University of Minnesota, Minneapolis, Minnesota, United States, 3. University of Minnesota, University of Minnesota, Minnesota, United States*

Persons with dementia living in long-term care settings have been especially affected by the COVID-19 pandemic, and their family caregivers have had to cope with numerous additional stressors during this time. We conducted 20 semi-structured interviews and gathered open-ended survey data from N=104 caregivers participating in an ongoing intervention trial at the start of the COVID-19 pandemic. Open-ended questions explored the difficulties caregivers have experienced in caring for and supporting a relative in long-term residential care. Caregivers provided their perspectives about services and supports that have facilitated coping with uncertainty, anxiety, and loss during the pandemic, and identified resources and strengths they have found helpful in caring for their relatives. Thematic analysis was used to identify themes reflecting the key challenges and supports that have emerged for caregivers, and to highlight caregivers' recommendations for promoting their and their relatives' well-being during this crisis.

### Session 1035 (Symposium)

### ASSOCIATIONS AMONG PERSONALITY TRAITS, STRESS EXPERIENCES, AND PHYSICAL AND COGNITIVE OUTCOMES IN OLDER ADULTS

Chair: Eileen Graham Discussant: David Almeida

Understanding between and within person variability in personality traits, and the processes of general and perceived stress are essential to understanding how to optimize cognitive health in older adults. It is well known that there is large variation in cognitive change: the pace and direction of change differs greatly across individuals. Personality traits and stress experiences are key factors that may account for some of these individual differences. The goal of our symposium is to present novel research in this area and discuss the implications for understanding personality, stress, and cognitive decline. First, Ferguson and colleagues will present a novel approach to assessing daily variability in personality. Their results demonstrate that daily personality assessments are able to capture within-person variability in personality, which could potentially help predict health trajectories in later adulthood. This is an important step in the study of change processes. Second, Luo and colleagues will present the factor structure of general and perceived stress, and show the predictive utility of these factors on physical and cognitive health outcomes. Third, Lawson and colleagues will discuss the extent to which personality is associated with cognitive function in a large sample of Mexican-origin adults. Fourth, Graham and colleagues will present results from a coordinated analysis that addressed associations among personality traits and cognitive decline both pre- and post- dementia diagnosis. Discussant David Almeida will contextualize these new findings and propose next steps.

### A COORDINATED ANALYSIS OF THE ASSOCIATIONS AMONG PERSONALITY TRAITS, COGNITIVE DECLINE, AND DEMENTIA.

Eileen Graham,[1] Kathryn Jackson,[2] Bryan James,[3] Emily Willroth,[1] and Daniel Mroczek,[1] *1. Northwestern University, Chicago, Illinois, United States, 2. Northwestern university, Chicago, Illinois, United States, 3. Rush University, Chicago, Illinois, United States*

There are considerable individual differences in the rates of cognitive decline across later adulthood. Personality traits are among the factors that may account for some of these differences. The current project investigated whether personality traits were associated with trajectories of cognitive decline, and whether the associations were different before and after dementia diagnosis. The data were analyzed using linear mixed effect regression models. Across study aims was a focus on replicability and generalizability. Each question was address in four independent longitudinal studies (EAS, MAP, ROS, SATSA), and then meta-analyzed using random effects meta-analysis, providing estimates of heterogeneity. As expected, we detected evidence for cognitive decline in all four samples. Results also indicated that neuroticism and openness were associated with total cognitive function. and openness was associated with decline post dementia diagnosis.

### ASSESSING PERSONALITY IN DAILY LIFE: VARIABILITY BETWEEN AND WITHIN PERSONS

Giselle Ferguson,[1] Giancarlo Pasquini,[2] Andreas Neubauer,[3] and Stacey Scott,[2] *1. Stony Brook University, Stony Brook University, New York, United States, 2. Stony Brook University, Stony Brook, New York, United States, 3. Leibniz Institute for Research and Information in Education, Frankfurt, Mecklenburg-Vorpommern, Germany*

Trait personality measures may not be able to detect subtle personality changes and fluctuations which may be indicative of cognitive impairment. Measuring personality in daily life may allow sufficient sensitivity to capture this within-person variability. Eighty-six older adults from the Einstein Aging Study completed items assessing daily extraversion and neuroticism for a median of 17 days. Using separate unconditional models, we calculated the proportions of variance in daily extraversion and neuroticism that were due to between-person and within-person variability. Variability in daily extraversion was relatively evenly related to between-person differences and within-person fluctuation (Intra-Class Correlation [ICC] = 0.576), but the majority of variability in daily neuroticism was at the between-person level (ICC = 0.730). Thus, although these daily assessments were sensitive enough to capture within-person variability in personality in daily life, different traits may exhibit more or less of this variability.

### THE ROLES OF GENERAL AND DOMAIN-SPECIFIC PERCEIVED STRESS IN HEALTHY AGING

Jing Luo,[1] Bo Zhang,[2] Emily Willroth,[1] Daniel Mroczek,[1] and Brent Roberts,[3], *1. Northwestern University, Chicago, Illinois, United States, 2. Texas A&M University, College Station, Texas, United States, 3. University of Illinois-Urbana-Champaign, Champaign, Illinois, United States*

Theoretical and empirical evidence suggests the existence of a general perceived stress factor overarching different life domains. The present study investigated the general perceived stress relative to domain-specific perceived stress as predictors of 26 diverse health outcomes, including mental and physical health, health behaviors, cognitive functioning, and physiological health indicators. A bifactor exploratory structural equational modelling approach was conducted in two samples from the Health and Retirement Study. Across the two samples, perceived stress was well-represented by a bifactor structure where there was a robust general perceived stress factor representing a general propensity towards stress perception. Meanwhile, after controlling for the general factor, specific factors representing perceived stress in different life domains were still clearly present. The general perceived stress factor was the most robust predictor of the majority of health outcomes. Age, sex, personality traits, and stressor exposure were found as possible diathesis underlying the general perceived stress factor.

### PERSONALITY AND SOCIOECONOMIC PREDICTORS OF MIDLIFE COGNITIVE FUNCTIONING IN MEXICAN-ORIGIN ADULTS

Katherine Lawson,[1] Angelina Sutin,[2] and Richard Robins,[3] *1. University of California, Davis, University of California, Davis, California, United States, 2. Florida State University College of Medicine, Tallahassee, Florida, United States, 3. University of California, Davis, Davis, California, United States*

The present study aims to identify personality and socioeconomic (e.g., education, per capita income, economic stress) factors that contribute to midlife cognitive functioning across middle adulthood. Specifically, we examined how the growth trajectories of personality and socioeconomic factors across 12 years predict subsequent cognitive functioning, using data from a large sample of Mexican-origin adults (N=1,110; median age at Time 1=37 years, age range at Time 1=26-65). Personality was assessed using the Big Five Inventory, which assesses the Big Five domains as well as specific facets of each domain; economic stress was assessed using measures of negative economic events (e.g., job loss) and economic hardship (e.g., difficulty paying bills). Cognitive functioning was assessed using the NIH Cognitive Toolbox with measures of memory, language, and executive function. Findings from this work will help identify intervention targets for promoting healthy cognitive aging in midlife and beyond in Mexican-origin adults.

### Session 1040 (Symposium)

### BENCHMARKING AGE-FRIENDLY UNIVERSITY PRACTICES: AFU INVENTORY AND CAMPUS CLIMATE SURVEY (ICCS) STUDY INSIGHTS

Chair: Joann Montepare Co-Chair: Nina Silverstein

The Age-Friendly University (AFU) initiative endorsed by GSA's Academy for Gerontology in Higher Education (AGHE) provides institutions of higher education with guiding principles for addressing the needs of aging populations. Benchmarks are now needed for assessing age-friendly academic, workplace, and physical campus environments, perceptions of campus constituents, and recommendations for advancing age inclusivity. This symposium will discuss what the AFU Inventory and Campus Climate Survey (ICCS) administered to a national sample of colleges and universities is revealing about the study of age-friendliness in higher education. The sample includes data from over 10,000 faculty, staff, students, and older learners surveyed in 2020-21. Whitbourne will introduce the conceptual model that served as the foundation for the ICCS, with special attention to the need to assess and compare "objective" age-friendly practices with "subjective" perceptions of these practices. Bowen will describe the utility of examining age-friendliness across institutional units with different functions: outreach-engagement, personnel, physical environment, research, services-resources, student affairs, and teaching-learning. Beaulieu will present data demonstrating the importance of assessing perceptions of specific constituent groups including faculty, staff, students, and lifelong learners. Montepare will discuss insights gained about the definition and manifestation of what it means to be ageist, age-friendly, and age-inclusive in higher education. Silverstein will describe strengths and challenges observed across campuses along with recommendations and promising new directions for advancing age inclusivity in higher education.

### TOWARD A MODEL OF AGE INCLUSIVITY IN HIGHER EDUCATION

Susan Whitbourne, *University of Massachusetts Amherst, Amherst, Massachusetts, United States*

The AFU principles clearly state the aspiration of promoting age inclusivity in higher education within the context of the UN Sustainability Development Goals. With these principles as a starting point, the Age-Friendly Campus Climate Inventory and Survey were developed to assess the extent to which AFU principles are put into practice (Inventory) and how campus constituencies perceive these practices. Based on social ecological models, a framework for measuring age inclusivity was developed in which practices ("objective environment") are compared to perceptions ("subjective environment"). Participating campuses (N=29) completed the inventory for each major executive unit, providing scores that were grouped by major campus functions, including research, teaching, community engagement, and support. By comparing these scores with perceptions of each function by samples of constituencies of faculty, staff, and students, it is possible to test the person-environment match as conceptualized by social ecological models providing important clarification for the AFU principles.

### TAKING A CAMPUS-WIDE VIEW OF AGE-FRIENDLY PRACTICES IN HIGHER EDUCATION

Lauren Bowen,[1] Nina Silverstein,[2] Susan Whitbourne,[3] and Joann Montepare,[4] *1. University of Massachusetts, Boston, University of Massachusetts Boston, Massachusetts, United States, 2. University of Massachusetts Boston, Boston, Massachusetts, United States, 3. University of Massachusetts Amherst, Amherst, Massachusetts, United States, 4. Lasell University, Newton, Massachusetts, United States*

The first AFU principle is to "encourage the participation of older adults in all the core activities of the university, including educational and research programs." As this suggests, a crucial goal of age inclusivity in higher education is to resist the siloing of older adults and age-inclusive efforts in age-specific programs and cohorts. In response, the Age-Friendly Inventory and Campus Climate Survey (ICCS) assessment was designed to assess age-inclusivity across seven areas of institutional activity: outreach & engagement, personnel, physical environment, research, services & resources, student affairs, and teaching & learning. By restructuring and expanding the "pillars" of institutional activity outlined by AFU principles, the ICCS presents two key advantages for benchmarking AFU practices: (1) it traces age-inclusivity across many facets of institutional operations; and (2) it prompts participants and report readers to recognize their role in current and potential age-inclusive efforts, regardless of their role or department on campus.

### EXPLORING PERCEPTIONS OF AGE FRIENDLINESS ACROSS DIFFERENT CAMPUS CONSTITUENT GROUPS

Celeste Beaulieu,[1] Nina Silverstein,[1] Lauren Bowen,[2] Susan Whitbourne,[3] and Joann Montepare,[4] *1. University of Massachusetts Boston, Boston, Massachusetts, United States, 2. University of Massachusetts, Boston, University of Massachusetts Boston, Massachusetts, United States, 3. University of Massachusetts Amherst, Amherst, Massachusetts, United States, 4. Lasell University, Newton, Massachusetts, United States*

College campuses are typically considered as environments for adults ages 18-24, even though campuses are comprised of faculty, staff, students, and lifelong learners of all ages. Each group may experience the campus environment differently due to their differing roles. Faculty, staff and students from 21 participating designated Age Friendly Universities across the country answered survey questions on age friendliness, AFU awareness, and on campus practice items. Crosstab analyses show that constituent groups are equally aware of their university as an AFU (6% of each group). Students perceived their university as more age friendly (M=3.47, SD=0.73) compared to faculty and staff, the latter having the lowest perceived friendliness (M=3.27, SD=0.63). Specific age friendly practices show that staff members had markedly different perceptions of the institution's age friendly practices. AFUs need to consider higher education environments as workplaces as well as learning centers to make policies age friendly for all groups.

### ON BEING AGEIST, AGE-FRIENDLY, AGE-INCLUSIVE: DIFFERENTIATING CORE CONSTRUCTS AND THEIR IMPLICATIONS

Joann Montepare, *Lasell University, Newton, Massachusetts, United States*

The pioneering Age-Friendly University (AFU) initiative has called for institutions of higher education to respond to the needs of older, more age-diverse populations through new approaches to programs, practices, and partnerships. In exploring in more detail what it means for a campus to be age-friendly, the national AFU Inventory and Campus Climate Survey (ICCS) study has raised questions about how core theoretical concepts are defined and manifested. Using observations from the ICCS study, this presentation will discuss tensions among constructs (e.g., does being age-friendly indicate the absence of ageist attitudes; are age-inclusive practices by design age-friendly?) and how differentiating these constructs better can help higher education focus its efforts in more intentional and productive ways.

### OBSERVATIONS AND RECOMMENDATIONS TOWARD INCREASING AGE-INCLUSIVITY

Nina Silverstein, *University of Massachusetts Boston, Boston, Massachusetts, United States*

Some areas of current campus practice better align with AFU principles than others. It may be that age-friendly practices already implemented by campus administrators are either not sufficiently publicized or that the campus constituents are not aware that these accommodations are in place. Specifically, staff members in these institutions appear to have markedly different perceptions of an institutions' overall age-friendliness than did students and faculty. Lower ratings of age-friendliness by staff suggest that the experiences of ageism could contribute to negative outcomes such as stress and burnout. In line with Sustainable Development Goals (SDGs) in the evaluation of campus sustainability efforts, aging should be addressed along with other elements of diversity, equity, and inclusion. There is also a need to support faculty development for designing courses and materials for age-diverse learners. Finally, campuses might organize age-inclusivity task forces and regularly reassess their age-friendly progress.

### Session 1045 (Paper)

### COGNITIVE FUNCTION

### EFFECT OF AN ELEARNING MEMORY PROGRAM ON REDUCING NEGATIVE IMPACT OF AGE-RELATED MEMORY CHANGES

Lynn Zhu,[1] Danielle D'Amico,[2] Iris Yusupov,[3] Jordan Lass,[1] Brian Levine,[1] Susan Vandermorris,[1] and Angela Troyer,[1] *1. Baycrest, Toronto, Ontario, Canada, 2. Ryerson University, Toronto, Ontario, Canada, 3. York University, Toronto, Ontario, Canada*

Age-related memory changes pose considerable concerns for aging adults, and can adversely affect their daily living and cause worry even when changes experienced are not clinically significant. The Memory and Aging Program® is a validated psychoeducation and memory strategy-training program that teaches the public about memory changes during aging and trains them to use evidence-based strategies to support brain health. The program has been offered in-person for over 20 years, and a self-guided eLearning version was recently developed to improve program accessibility. This study evaluated the self-reported impacts of memory changes in older adults who completed this eLearning against a control group. We randomized 202 older adults, without neurological or psychiatric diagnoses (71.6 years; 69 % female; 15.6 years of education), into the eLearning program or a control group that received no intervention. All participants reported their perceived impact of memory changes using the Memory Impact Questionnaire at pre-, post-, and 6-8 weeks follow-up. A significant reduction in negative impact of memory changes on daily living and a significant improvement in positive coping with memory changes relative to controls was observed at post-test (13.4 versus 2.5 points reduction and 7.4 versus 0.1 point improvement, respectively, both $p < 0.05$), but these did not persist at follow-up. The adoption of digital tools has hastened across all ages. Our study showed that self-guided digital tools, such as the eLearning Memory and Aging Program®, may be a promising avenue to help aging individuals reduce the impact of memory changes on daily living.

### HYPERTENSIVE RETINOPATHY IS ASSOCIATED WITH WORSE COGNITIVE FUNCTION IN WOMEN: THE SWAN STUDY

Sayoko Moroi,[1] Michelle Hood,[2] Carrie Karvonen Gutierrez,[2] Joshua Ehrlich,[3] Brenda Gillespie,[2] Sarah Dougherty Wood,[2] and David Musch,[2], *1. The Ohio State University, Columbus, Ohio, United States, 2. University of Michigan, Ann Arbor, Michigan, United States, 3. University of Michigan, University of Michigan, Michigan, United States*

Based on the 2018 American Academy of Neurology guideline, the prevalence of mild cognitive impairment (CI) increases from 6.7% at 60-64 years to 25.2% at 80-84 years. There is interest to identify potential biomarkers in the retina for CI and dementia. The aims of this analysis was to test whether hypertensive retinopathy (HR) was associated with cognitive function using data from the Study of Women's Health Across the Nation (SWAN), Michigan cohort. SWAN, launched in 1996/97, is a longitudinal study of women traversing midlife and into late adulthood. Starting in 2000, cognitive function tests were administered: East Boston Memory Test immediate (EBMTi) and 10-minute delay (EBMTd) for verbal episodic memory; digit span backwards (DSB) for working memory; and symbol digits modalities test (SDMT) for perception speed, motor speed, and visual scanning. Z-scores were calculated for EBMTi, EBMTd, DSB and SDMT and averaged at follow-up visit 15 (2015/16). Eye exams were performed on 255 women (66 + 2.6 years) at follow-up visit 16 (2016/17). HR was based on presence/absence of arteriovenous nicking. Logistic regression showed a statistically significant association of lower average cognitive Z-score with HR (p-value 0.03, beta=-0.21 [95% confidence interval: -0.40 to -0.02]) adjusting for measured hypertension or anti-hypertensive drugs, years of measured hypertension, race, education, and age. Preliminary results indicate that HR is associated with lower cognitive test scores in women in their 60s-70s. This association between a simple ophthalmic exam finding of systemic hypertension, i.e., arteriovenous nicking, and lower cognitive function is consistent with a cerebrovascular mechanism of CI.

### LONGITUDINAL MEASUREMENT PROPERTIES OF THE MONTREAL COGNITIVE ASSESSMENT

Hao Luo,[1] Björn Andersson,[2] Gloria HY Wong,[1] and Terry YS Lum,[1] *1. The University of Hong Kong, Hong Kong, Not Applicable, Hong Kong, 2. University of Oslo, Oslo, Oslo, Norway*

**Background:** The Montreal Cognitive Assessment (MoCA) has started to be widely used in longitudinal investigations to measure changes in cognition. However, the longitudinal measurement properties of MoCA have not been investigated. We aimed to examine the measurement invariance of individual MoCA items across four time points.

**Methods:** We used longitudinal data collected between 2014 and 2017 from a cohort study on health and well-being of older adults in Hong Kong. The Cantonese version of the MoCA was used. We applied multiple group confirmatory factor analysis of ordinal variables to examine measurement invariance by educational level and across time points. Invariant items were identified by sequential model comparisons.

**Results:** We included 1029 participants that answered MoCA items across all time points. We found that items Cube, Clock Hand and Clock Number had significantly different item parameters between participants with and without formal education at all time points. The selected model (RMSEA=0.031; SRMR=0.064) indicated that eight items (Trail, Cube, Clock Shape, Clock Number, Clock Hand, Abstraction, Short-term Memory, and Orientation) did not exhibit measurement invariance over time. However, the differences in item parameter estimate over time were marginal. Accounting for the lack of measurement invariance did not substantially affect classification properties based on cutoff values at the 2nd ( major neurocognitive disorder) and 7th (mild cognitive impairment) percentile.

**Conclusion:** Our findings support using MoCA to assess changes in cognition over time in the study population. Future research should examine the longitudinal measurement properties of the test in other populations with different characteristics.

### PLASMA LONG CHAIN FATTY ACIDS AND COGNITIVE FUNCTION IN OLDER ADULTS: A COMPARISON OF STATISTICAL ANALYSES

Caroline Duchaine,[1] Pierre-Hugues Carmichael,[2] Nancy Presse,[3] Alexandra Fiocco,[4] Pierrette Gaudreau,[5] Guylaine Ferland,[5] and Danielle Laurin,[1] *1. Laval University, Quebec, Quebec, Canada, 2. Centre intégré universitaire de santé et services sociaux de la Capitale-Nationale, Quebec, Quebec, Canada, 3. Université de Sherbrooke, Sherbrooke, Quebec, Canada, 4. Ryerson University, Toronto, Ontario, Canada, 5. Université de Montréal, Montreal, Quebec, Canada*

Omega-3 fatty acids (FAs) have been suggested as modifiable protective factors for cognitive decline because of their neuroprotective properties. However, the evidence is still inconsistent regarding types of omega-3 FAs, and the probable interrelation with other circulating long chain FAs (LCFAs). This study aimed to evaluate associations between 14 plasma LCFAs and four cognitive domains using a principal component analysis (PCA) and to compare results with those obtained using standard methods. A group of 386 healthy older adults aged 77 ± 4 years (53% women), selected from the NutCog Study, a sub-study from the Québec cohort on Nutrition and Successful Aging (NuAge), underwent a cognitive evaluation and fasting blood sampling. Verbal and non-verbal episodic memory, executive functioning, and processing speed were evaluated using validated tests. LCFAs circulating concentrations were measured by high-performance liquid chromatography using published procedures. Linear regressions adjusted for age, sex, education, and BMI were used to evaluate cross-sectional associations between LCFAs, using PCA or a more standard grouping (omega-3, omega-6, monounsaturated, and saturated LCFAs), and cognitive performance. Higher scoring on the omega-3 PCA factor and higher concentrations of total omega-3 FAs were both associated with better episodic non-verbal memory and processing speed. Higher eicosapentaenoic acid (EPA omega-3) was also associated with these two cognitive domains and with episodic verbal memory. The associations with total omega-3 FAs taken separately were of smaller magnitude than those with PCA. These results suggest that omega-3 FAs should be considered in combination with other LCFAs when evaluating the association with cognitive function.

### SOCIAL PARTICIPATION AND THE RISK FOR DEVELOPING MILD COGNITIVE IMPAIRMENT AND DEMENTIA AMONG OLDER ADULTS.

Hrafnhildur Eymundsdottir,[1] Sigurveig Sigurdardottir,[2] Alfons Ramel,[2] Palmi Jonsson,[3] Vilmundur Gudnason,[4] Lenore Launer,[5] and Milan Chang,[1] *1. The Icelandic Gerontological Research Institute, Reykjavik, Hofuoborgarsvaoio, Iceland, 2. University of Iceland, Reykjavik, Hofuoborgarsvaoio, Iceland, 3. University hospital Landspitali, Reykjavik, Hofuoborgarsvaoio, Iceland, 4. University of Iceland, Kopavogur, Hofuoborgarsvaoio, Iceland, 5. National Institute on Aging, Bethesda, Maryland, United States*

**Introduction:** We aim to investigate the longitudinal associations between social participation and the risk of developing mild cognitive impairment (MCI|) and dementia over 5 years of follow-up among cognitively normal older adults.

**Methods:** A total of 2802 participants had complete follow-up data from Age-Gene/Environment-Susceptibility-Reykjavik-Study. Social participation was assessed by a questionnaire asking the frequency of contact with children, relatives, friends and neighbors. MCI and dementia were diagnosed according to international guidelines and by a team composed of a geriatrician, neurologist, neuropsychologist, and neuroradiologist. Logistic regression analysis was used to assess the associations.

**Results:**At baseline 8% (n=225) reported no social participation. Among cognitively normal participants at baseline, 5.6% (n=243) developed mild cognitive impairment and 2.4% (n= 103) developed dementia during a mean follow-up time of 5.2 years. After full adjustment with covariates including age, gender, education, marital status, vitamin D levels, depression and APOE ε4, those with no social participation at baseline were significantly more likely to develop MCI at follow-up (OR=1.953, P=0.001). However, social participation at baseline was not associated with higher dementia diagnosis at follow-up (OR= 1.490, P=0.194).

**Conclusions:** Community-dwelling old adults who are socially inactive are more likely to develop MCI than those who are socially active. Social participation might independently indicate impending changes in cognitive function among older adults.

### Session 1050 (Paper)

### COVID-19 AND SOCIAL HEALTH

### CHICAGO OLDER ADULTS' LIKELIHOOD OF BEING HOME BEFORE AND DURING THE COVID-19 PANDEMIC

Ellen Compernolle,[1] Laura Finch,[2] Louise Hawkley,[1] and Kathleen Cagney,[3] *1. NORC at the University of Chicago,*

*Chicago, Illinois, United States, 2. NORC at The University of Chicago, Chicago, Illinois, United States, 3. University of Chicago, Chicago, Illinois, United States*

Staying at home has particularly been emphasized for older adults during the COVID-19 pandemic, given their elevated risk of infection and complications. However, little is known about the extent to which this population is indeed spending more time at home during the pandemic, compared to before it began. The present investigation addresses this question, also examining differences by gender and race/ethnicity. We analyzed ecological momentary assessments among 98 older adults (age 65-88 in 2020) who participated in two waves of the Chicago Health and Activity Space in Real Time study. Pre-pandemic data were collected from July-October 2019, and pandemic data were collected from June-September 2020. Participants responded to smartphone "pings" (five per day for 7 days in each wave; n=1,910 and n=2,437 before and during the pandemic, respectively) by reporting their momentary location (e.g., home). Findings suggest that respondents were indeed at home more often in mid-2020 than 1 year prior. Multilevel logistic regression models revealed that net of demographics, marital and employment status, and physical health, respondents were more likely to be momentarily at home during versus before the pandemic (B=0.70, SE=0.08, p<.001). This effect was larger among women than men (B=0.50, SE=0.16, p=.002), but did not differ by race/ethnicity. Additional analyses examine whether and how the observed increased reports of being at home may be associated with increased reports of momentary loneliness across the two waves. Findings characterize where Chicago older adults are spending their time amid the pandemic and how this may relate to their well-being.

### CREATIVE HOBBIES AS A PROTECTIVE FACTOR AGAINST STRESS DURING THE COVID-19 PANDEMIC IN OLDER ADULTS

Sarah Israel,[1] Darby Mackenstadt,[2] and Carolyn Adams-Price,[1] *1. Mississippi State University, Mississippi State University, Mississippi, United States, 2. Mississippi State University, Starkville, Mississippi, United States*

The COVID-19 pandemic dramatically impacted our way of life, leading to increased rates of anxiety and depression (Panchal et al., 2021). The implications may be worse for older adults who account for 80% of all COVID deaths (Freed et al., 2020). Meanwhile, prior to the pandemic, Adams-Price and colleagues (2018) found that creative hobby participation provided slightly different benefits for middle-aged and older adults. Specifically, evidence suggested that middle-aged adults may use their creative hobby more for stress relief than older adults. Using a sample of 239 women, aged 40 to 84 years old (M = 59.7), we examined whether the degree to which viewing one's creative hobby as a component of one's identity related to perceived stress, health anxiety, and depressive symptoms. In addition, we wanted to know whether these relationships were moderated by age. Single moderation models suggest that viewing one's creative hobby as a part of their identity was related to higher health anxiety and reporting more depressive symptoms. In addition, age was related to reporting lower perceived stress, health anxiety, and depressive symptoms. Lastly, age provided a significant moderation effect to the relationship between degree of identity associated with one's creative hobby and perceived stress such that middle-aged adults with a high degree of identification with their creative hobby reported the most perceived stress while older adults with a high degree of identification with their creative hobby reported the least perceived stress. Implications for older adult's well-being and adaptiveness to the COVID-19 pandemic will be discussed.

### UNDERSTANDING THE COMPLEXITIES OF COMMUNITY-DWELLING OLDER ADULTS' LIVED EXPERIENCES DURING COVID-19

Bo Xie,[1] Kristina Shiroma,[2] Atami Sagna De Main,[1] Nathan Davis,[1] Karen Fingerman,[1] and Valerie Danesh,[1] *1. The University of Texas at Austin, Austin, Texas, United States, 2. The University of Texas at Austin, The University of Texas at Austin, Texas, United States*

Since December 2019, COVID-19 has spurred rapid and extensive research, but this research has focused on some perspectives with others understudied. In particular, studies have not yet explored the complexities of community-dwelling older adults' lived experiences during the pandemic. This study aimed to address this gap. Community-dwelling older adults living in Central Texas (N = 200; age, 65–92 years, M = 73.6± 6.33) responded to open- and closed-ended questions over the telephone during June–August 2020. Data were analyzed using inductive thematic analysis. We identified three key themes. (1) Positive experiences, with 4 subthemes: perception that the pandemic has not changed one's lifestyle; adjusting well—particularly with the aid of technology; being positive in perspective; and a "loner advantage" (being a "loner" pre-pandemic was advantageous during the pandemic). (2) Mixed experiences, with 4 subthemes: doing okay but unhappy about changing lifestyle routines; doing okay but unhappy about loss of in-person interactions with family and friends; doing okay but frustrated by witnessing absence of social distancing or facemask use by others; and maintaining physical health with fluctuating symptoms of depression or anxiety. (3) Negative experiences, with 3 subthemes: bitter about others/society/government not caring for older adults; feeling isolated, bored, and powerless; and worsening as time goes by. A thematic map was subsequently developed. These findings reveal the complexities of community-dwelling older adults' lived experiences, illustrating effective coping and resilience during the pandemic and dissatisfaction owing to the pandemic's effects on their lives and to their observations of others' behaviors.

### USING ECOLOGICAL AND TWITTER-BASED ASSESSMENTS TO EXAMINE IMPACTS IN TEMPORAL AND COMMUNITY CONTEXT

Isabella Bouklas,[1] Giselle Ferguson,[2] Giancarlo Pasquini,[1] Huy Vu,[1] Mohammadzaman Zamani,[1] Ruixue Zhaoyang,[3] Stacey Scott,[1] and H. Andrew Schwartz,[1] *1. Stony Brook University, Stony Brook, New York, United States, 2. Stony Brook University, Stony Brook University, New York, United States, 3. The Pennsylvania State University, University Park, Pennsylvania, United States*

In March 2020, Bronx County (NY) saw one of the first U.S. COVID-19 outbreaks. This outbreak coincided with the ongoing Einstein Aging Study (EAS), which involved older adults living in Bronx County completing annual two-week intensive data collection “bursts.” Thus, it serves as a natural experiment to study pre-COVID to early pandemic-related changes in the daily well-being of participants who were at risk both due to their age and their location. We examined within-person change in self-reported negative thoughts, affect, stress, and loneliness from a subsample of 78 EAS participants. Participants’ data from a two-week “burst” of momentary surveys during 2019 were compared with their data from the corresponding timeframe during the early COVID-19 period (February-June 2020). Personality and mild cognitive impairment were examined as predictors of change. Average momentary loneliness significantly increased from 2019 to 2020. Participants with greater neuroticism increased more in thought unpleasantness and depressed feelings. To understand the community context, community distress markers were analyzed using Artificial Intelligence (AI)-based assessments of public Twitter posts from Bronx County during the same periods. These Twitter posts also showed a surge of COVID-related topics at the onset of the Bronx outbreak. Language analysis showed a 2019-2020 increase in Bronx community markers of anxiety, depressivity, and negatively-valenced affect extracted from Twitter. We observed 2019-2020 change in both individuals’ well-being (via intensive reports) and in their communities (via Twitter). Contextualizing these with the increased COVID-19 discussion online suggests that these may reflect common pandemic effects.

### Session 1055 (Paper)

### COVID-19 OUTCOMES FOR OLDER ADULTS

### AGE DIFFERENCES IN BECOMING COVID LONG-HAULERS AND IN POST-ACUTE SEQUELAE OF SARS-COV-2

Qiao Wu, and Eileen Crimmins, *University of Southern California, Los Angeles, California, United States*

People who have had COVID-19 can suffer from the continuation of Post-Acute Sequelae of SARS-CoV-2 (PASC), also known as “long COVID”, for months after infection. Understanding PASC is important for treatment, care, and projecting future health of the population. Since older adults are at higher risk of severe illness and consequences from COVID, we hypothesize that they are more likely to become COVID long-haulers and report more symptoms at the time of diagnosis and three months after. We use a nationally representative sample of adults from the Understanding America Study COVID-19 Survey, from March to December 2020, to estimate the prevalence of long COVID and identify the most common long-term symptoms and how they vary by age. We use multilevel models to examine the determinants of symptom count and change over time. Among the 608 people with a COVID diagnosis, 83 (13.7%) aged over 65; almost half (47.9%) reported symptoms three months after diagnosis; the proportion did not differ across age groups. The most common symptoms were fatigue (25.0%), runny/stuffy nose (18.9%), body aches (16.4%), sneezing (15.1%), and headache (13.6%). These symptoms were consistent across age groups, while people aged 65 and older reported significantly less cough ($\chi 2=3.96$; P=0.05) and headache ($\chi 2=4.24$; P=0.04) compared to their younger counterparts. Neither the mean at the time of the diagnosis nor the rate of change of the symptom count varied across age groups. Our analyses suggest that age is not a significant determinant of PASC symptom count or becoming a COVID long-hauler.

### BEYOND CHRONOLOGICAL AGE: FRAILTY AND MULTIMORBIDITY PREDICT IN-HOSPITAL MORTALITY IN PATIENTS WITH COVID-19

Alberto Zucchelli,[1] Alessandra Marengoni,[2] Davide Vetrano,[3] Luigi Ferrucci,[4] Laura Fratiglioni,[5] Roberto Bernabei,[6] Graziano Onder,[7] and Giuseppe Romanelli,[8] *1. Università degli Studi di Brescia, Karolinska Institutet, Stockholms Lan, Sweden, 2. University of Brescia, Brescia, Lombardia, Italy, 3. Karolinska Institutet, Solna, Stockholms Lan, Sweden, 4. National Institute on Aging, Baltimore, Maryland, United States, 5. Karolinska Institutet, 17175, Stockholms Lan, Sweden, 6. Catholic University of Rome, Rome, Lazio, Italy, 7. Italian National Institute of Health, Rome, Lazio, Italy, 8. Università degli Studi di Brescia, Università degli Studi di Brescia, Lombardia, Italy*

**Background:** We evaluated whether frailty and multimorbidity predict in-hospital mortality in patients with COVID-19 beyond chronological age.

**Methods:** 165 patients admitted from March 8th to April 17th, 2020, with COVID-19 in an acute geriatric ward in Italy were included. Pre-disease frailty was assessed with the Clinical Frailty Scale (CFS). Multimorbidity was defined as the co-occurrence of ≥2 of these in the same patient. The hazard (HR) of in-hospital mortality as a function of CFS score and number of chronic diseases in the whole population and in those aged 70+ years were calculated.

**Results:** Among the 165 patients, 112 were discharged, 11 were transferred to intensive care units and 42 died. Patients who died were older (81.0 vs. 65.2 years, p<0.001), more frequently multimorbid (97.6 vs. 52.8%; p<0.001) and more likely frail (37.5 vs. 4.1%; p<0.001). Less than 2.0% of patients without multimorbidity and frailty, 28% of those with multimorbidity only and 75% of those with both multimorbidity and frailty died. Each unitary increment in the CFS was associated with a higher risk of in-hospital death in the whole sample (HR=1.3; 95%CI=1.05-1.62) and in patients aged 70+ years (HR=1.29;95%CI=1.04-1.62), whereas the number of chronic diseases was not significantly associated with higher risk of death. The CFS addition to age and sex increased mortality prediction by 9.4% in those aged 70+ years.

**Conclusions** Frailty identifies patients with COVID-19 at risk of in-hospital death independently of age. Multimorbidity contributes to prognosis because of the very low probability of death in its absence.

### COVID-19 CASES, HOSPITALIZATIONS, AND DEATHS IN NURSING HOMES: FACTORS IMPACTING THE SECOND SURGE

Sean Fleming,[1] Danya Qato,[2] Alexandra Wallem,[2] Paulina Kepczynska,[2] Linda Wastila,[2] Tham Le,[2] and Jeanne Yang,[2] *1. University of Maryland Baltimore,*

*University of Maryland Baltimore, Maryland, United States, 2. University of Maryland School of Pharmacy, Baltimore, Maryland, United States*

As of March 2021, over 128,000 nursing home (NH) residents have died due to COVID-19 complications, accounting for one-third of all U.S. COVID-19 deaths. Early studies highlighted factors which heightened residents' risk—facility size and profit status, CMS Five-Star quality rating, race, and high Medicaid share. Despite improved nationwide social distancing and access to protective equipment, between October-December 2020 nursing home cases, hospitalizations, and deaths peaked to highest levels since the pandemic's advent. The purpose of this study is to quantify previously unexamined associations between resident, facility, and geographic characteristics and COVID-19 infections, hospitalizations, and fatalities in nursing homes during this second surge. In this cross-sectional study, we constructed a novel dataset with linked facility- and county-level data from the CMS Nursing Home COVID-19 Public File, Nursing Home Compare, Long-Term Care Focus, and The New York Times. Multivariable logistic regression evaluated the odds of COVID-19 infections, hospitalizations, and deaths in nursing homes. Among 13,156 nursing homes, 80.5% reported ≥1 COVID-19 cases; on average, nursing homes reported 4.5 hospitalizations and 3.0 deaths. Facilities with higher acuity patients, chain status, >150 beds, high percentage white residents, low Medicaid share, high surrounding county case rates, and occupancy rates >75% were significantly ($p < .001$) related to increased odds of all outcomes. N95 mask shortages continued to increase risk of cases and hospitalizations. Five-Star ratings, high influenza vaccination rates, and clinical staff shortages were not significant factors. Findings demonstrate that through 2020, nursing homes continued to face challenges protecting their residents from COVID-19-related morbidity and mortality.

### FACTORS ASSOCIATED WITH NURSING HOME RESIDENTS' COVID-19 INFECTIONS: A SYSTEMATIC REVIEW OF LITERATURE

CHENG YIN,[1] Xiaoli Li,[2] Rongfang Zhan,[1] and Liam ONeill,[3] *1. University of North Texas, Corinth, Texas, United States, 2. University of North Texas, denton, Texas, United States, 3. University of North Texas, University of North Texas, Texas, United States*

**Background:** Nursing homes were impacted disproportionately by the coronavirus because of their resident's vulnerabilities and settings. Even many previous studies illustrated factors related to nursing home residents' Covid-19 infections, there's no such study epitomizing those factors systematically, while some factors were controversial in different studies. The article aims to summarize major types of factors and provide crucially influential implications for nursing homes to prevent and manage their resident infections.

**Methods:** All articles published between 01 January 2020 - 15 January 2021 in English version were searched through three electronic databases (PubMed, Web of Science, and Scopus). Two authors screened and evaluated a total of 121 studies independently based on selection and extraction criteria.

**Results:** Seventeen identified studies were included in the research, which involved five major types of factors (nursing home's residence, nursing home, staff, resident, and others).

**Conclusion:** nursing home's county infection rate, size, and staff residence were the strongest significant factors in many studies. Per-capital income, symptom-based screening and testing, and asymptomatic individuals have impacted resident's infections variously since the beginning of the pandemic. Nursing home's star rating and a total count of fines became factors when considered its locations. Other factors, including nursing home's type, historical health deficiencies, staffing level, and staff working different facilities, etc., were also significant factors. The value of factors suggests healthcare systems reflect appropriate measures and allocate more resources to nursing homes in high prevalence counties on the basis of universal allocation.

### NO INCREASE IN SHORT-TERM MORTALITY FOLLOWING COVID-19 VACCINATION AMONG NURSING HOME RESIDENTS

Barbara Bardenheier,[1] Stefan Gravenstein,[2] Roee Gutman,[3] Neil Sarkar,[4] Richard Feifer,[5] Elizabeth White,[2] and Vincent Mor,[2] *1. Brown University School of Public Health, Smithfield, Rhode Island, United States, 2. Brown University, Providence, Rhode Island, United States, 3. Brown University, providence, Rhode Island, United States, 4. Rhode Island Quality Institute, Providence, Rhode Island, United States, 5. Genesis HealthCare, Kennett Square, Pennsylvania, United States*

Reports of fatal adverse events following mRNA-based vaccination for COVID-19 in Norwegian nursing home (NH) residents have raised concern regarding vaccine safety in very old and frail persons. A limitation of these reports, however, is the absence of contemporaneous control groups, particularly given the high baseline mortality in this population. Using electronic health records' data on resident deaths, hospital transfer, vaccination, and daily census from Genesis Healthcare, a large NH provider spanning 24 U.S. states, we compared 7-day mortality and hospitalization rates for vaccinated versus unvaccinated NH residents. Between December 18, 2020 and December 31, 2020, 7006 residents across 118 NHs were vaccinated with the first dose. Mortality and hospital transfer rates within 7 days of vaccination were compared to rates for: (1) unvaccinated residents in the same facility within 7 days of the vaccine clinic (n=4414), and (2) residents in 166 yet-to-be-vaccinated facilities between December 25, 2020 and January 1, 2021 (n=17,076). We excluded residents with a positive SARS-CoV-2 diagnostic test within 20 days prior to their 7-day observation window. Mortality rates per 100,000 residents were lower among vaccinated (587, 95%CI: 431, 798) versus unvaccinated residents within the same facilities (984, 95%CI: 705, 1382), and compared to residents in not-yet-vaccinated facilities (912, 95%CI: 770-1080), with overlapping 95% CIs. Hospital transfers were lower among vaccinated residents than in either comparison group, but with overlapping CIs. Our findings suggest that short term mortality rates appear unrelated to vaccination for COVID-19 in NH residents, and should dispel concerns raised by previous reports.

### Session 1060 (Paper)

### DEMENTIA AND COGNITIVE IMPAIRMENT: POLICY AND PROGRAMS

### BUILDING A MODEL OF ADVOCACY: IMPROVING THE DEMENTIA CAPABILITY OF MANAGED CARE HEALTH PLANS IN CALIFORNIA

Brooke Hollister,[1] Jarmin Yeh,[2] Leslie Ross,[2] Jennifer Schlesinger,[3] and Debra Cherry,[3] *1. University of California, San Francisco, California, United States, 2. University of California, San Francisco, San Francisco, California, United States, 3. Alzheimer's Los Angeles, Los Angeles, California, United States*

Given the growing prevalence of Alzheimer's Disease and related dementias, and the intensity of this population's care needs, it is imperative that health plans (HPs) increase their dementia-capability. The Dementia Cal MediConnect (Dementia CMC) project proposes an innovative model of health care advocacy that can create dementia-capable systems change. The Dementia CMC project was a partnership (2013 – 2018) between local Alzheimer's organizations and ten managed care HPs in California. It used the following model of health care advocacy: 1) Identify dementia-capable best practices to set as systems change indicators; 2) Identify and leverage public policies in support of systems change indicators; 3) Identify and engage champions; 4) Develop and advocate for a value and business case to improve dementia care; 5) Identify gaps in dementia-capable practices; 6) Provide technical assistance, tools, and staff training to address the gaps in dementia-capable practices; and 7) Track systems change. Systems change data was collected through participant observation with HPs and interviews with key informants. HPs reported making systems changes toward more dementia-capable practices such as: better pathways for identification and diagnosis; better identification, assessment, support, and engagement of caregivers; and improved systems of referral to Alzheimer's organizations. Some indicators of systems change were inconclusive as a result of variability in HP practices and the lack of common record systems between HPs and providers. The application of this advocacy model has led to systems changes that can be replicated to improve care for people living with dementia and their caregivers.

### COGNITIVE IMPAIRMENT TRENDS AMONG OLDER ADULTS IN A MEDICAID HOME AND COMMUNITY-BASED SERVICE PROGRAM

Richard Fortinsky,[1] Julie Robison,[2] David Steffens,[1] James Grady,[1] and Deborah Migneault,[3] *1. University of Connecticut School of Medicine, Farmington, Connecticut, United States, 2. University of Connecticut, University of Connecticut, Connecticut, United States, 3. UConn Health, Farmington, Connecticut, United States*

Cognitive impairment (CI) is an important risk factor for nursing home admission, but little is known about CI among older adults in Medicaid HCBS programs. Racial and ethnic group CI disparities are found among community-dwelling older adults, but these CI trends have not been explored in Medicaid HCBS populations. In this study, we determined how CI is associated with older adults' racial and ethnic group identification and educational attainment in Connecticut's Medicaid HCBS program. The study cohort includes program enrollees age >65 during January-March 2019 (N=3,520). CI measures include: Cognitive Performance Scale (CPS), ranging from 0-8 (cognitively intact to very severe impairment); and a dichotomous measure incorporating Alzheimer's disease or other dementia diagnosis (ADRD) and CPS score signifying moderate or severe CI. Study cohort characteristics: 75.7% female; age, mean(sd)=79.1(8.2); Non-Hispanic White=47.8%; Non-Hispanic Black=15.9%; Non-Hispanic Other=2.7%; Hispanic=33.6%; HS education=21.7%; mean(sd) CPS score=2.7(1.9); 36.1% with ADRD/high CPS2 score. In multivariate regression models adjusting for age and sex, CPS scores were not independently associated with race and ethnicity, and the likelihood of having ADRD/high CPS scores did not differ by race and ethnicity (all p-values >0.05). In these same models, persons with more than high school education had significantly lower CPS scores (b=-.12; p<.001), and significantly lower likelihood of having ADRD/high CPS scores (AOR=0.61; p<.001), than persons with less than high school education. We conclude that educational level is independently associated with CI, but race and ethnicity are not in this cohort. Policy and practice implications will be discussed.

### DO INDIVIDUALS SKIMP ON HEALTH CARE AFTER SPOUSE'S DEMENTIA DIAGNOSIS?

Yi Chen, *University of Southern California, Irvine, California, United States*

Dementia is a costly disease that places great burden on individuals and families. The substantial time and financial resources taken away by living with persons with dementia (PWDs) may make their spouses forgo essential health care, thus deteriorating long-term health and increasing downstream healthcare costs. However, such negative externality is understudied. This paper studied the impacts of spouse's incident dementia diagnosis on an individual's use of needed care, defined as annual flu shot and regular doctor visits for those with preexisting conditions. Using HRS linked to Medicare claims, I employed a fixed effects approach to compare the use of flu shot and doctor visit during 1 year before and after the index, for individuals whose spouse had dementia (N=691) and otherwise similar controls (N=5,073). After adjusting for time-varying health, caregiving roles, and other socio-demographic factors, spouse's dementia onset was associated with greater likelihood of getting flu shot and seeing doctors. Among those transitioning into caregiving, spouses of PWDs had a marginally higher risk of skimping on doctor visits, compared to controls (p=0.053). In this broadly representative sample, there lacks evidence for rationed health care ensuing spouse's dementia incidence, at least within a 1-year time frame. However, for new spousal caregivers, the impact of dementia is more profound and complex than deprivation of time. This group may face a trade-off between caring for spouses with dementia and caring for themselves, for whom policy support merits further study and consideration.

### PREDICTORS OF DISENROLLMENT AMONG MEDICARE FEE-FOR-SERVICE BENEFICIARIES WITH DEMENTIA

Maricruz Rivera-Hernandez,[1] Aaron Castillo,[2] and Amal Trivedi,[2] *1. Brown University School of Public Health, Providence, Rhode Island, United States, 2. Brown University, Providence, Rhode Island, United States*

Medicare enrollment among people with Alzheimer's Disease and Related Dementias (ADRD) has reached an all-time high with about 12% of beneficiaries having an ADRD diagnosis. The federal government has special interest in providing healthcare alternatives for Medicare beneficiaries. However, limited studies have focused on understanding disenrollment from fee-for-service, especially among those with high-needs. In this study we identified predictors of disenrollment among beneficiaries with ADRD. We used the 2017-2018 Medicare Master Beneficiary Summary File to determine enrollment, sociodemographic, clinical characteristics and healthcare utilization. We included all fee-for-service beneficiaries enrolled in 2017 who survived the first quarter of 2018. Our primary outcome was disenrollment from fee-for-service between 2017 and 2018. Regression models included age, sex, race/ethnicity, dually eligibility to Medicare and Medicaid, chronic and disabling conditions (categorized by quartiles), total health care costs including outpatient, inpatient, post-acute care and other costs (categorized by quartiles) and county fixed-effects. There were 1,797,047 beneficiaries enrolled in fee-for-service with an ADRD diagnosis. Stronger predictors of disenrollment included race/ethnicity and dual eligibility. Disenrollment rates were 7.9% (95% CI, 7.2 – 8.5) among African Americans, 6.6 (95% CI, 6.2 – 7.0) among Hispanics and 4.3 (95% CI, 4.2 – 4.3) among Whites. Duals were 1.9% (95% CI, 1.4 – 2.3) more likely to disenroll from fee-for-service to Medicare Advantage (MA). The inclusion of MA special need plans and additional benefits for those with ADRD and complex chronic conditions may be valuable for those beneficiaries with ADRD, and who may not have Medigap coverage when enrolling in fee-for-service.

### RURAL AND URBAN DIFFERENCE IN LONGITUDINAL TRENDS IN PREVALENCE OF DEMENTIA IN MEDICARE CLAIMS AND SURVEY DATA

Nasim B. Ferdows, *University of Oklahoma Health Sciences Center, Oklahoma City, Oklahoma, United States*

Shortage of physicians in rural areas can lead to lower diagnosis and underestimation of dementia prevalence in these communities. We used data from the nationally representative Health and Retirement Study and a 20-percent sample of Medicare claims to study rural-urban differences in dementia prevalence. The survey dementia diagnosis is free from medical assessment while the claims diagnosis needs a physician diagnosis. We estimated the trends in dementia prevalence from (2002-2016) based on cognitive tests (using survey data) and diagnosis codes (using claims data) utilizing ordinary least squares regression. Dementia prevalence based on diagnosis codes declined in both urban and rural areas over the course of the study, with a sharper decline in urban areas. Dementia prevalence using diagnosis codes showed significantly higher rates in urban areas during all years (0.024 vs 0.018 in 2002 and 0.017 vs 0.013 in 2014 in rural vs urban areas, respectively). Dementia in the cognitive test sample was higher in rural areas (0.11 vs 0.08 in 2000 and 0.08 vs 0.7 in 2014 in rural vs urban areas), a difference that was significant only in 2004. Our results indicate lower dementia prevalence rates in rural areas in claims based sample compared to survey sample which its dementia prevalence is free medical assessment. Claims data are valuable sources for tracking dementia in the US population, however they are based on medical diagnosis.In rural areas, where there is shortage of physicians and a lack of access to health care services, claims based studies may underestimate dementia rates.

### Session 1065 (Symposium)

### DIVERSITY OF ACTIVITIES, EMOTIONS, AND PLEASANT EVENTS AND THEIR ASSOCIATIONS WITH MENTAL AND COGNITIVE HEALTH

Chair: Soomi Lee Co-Chair: Emily Urban-Wojcik
Discussant: David Almeida

The COVID-19 pandemic dramatically changed the structure of our daily lives. One of the most significant changes is a limited opportunity to engage in face-to-face social interactions and enjoy diverse daily activities. This raises a public health concern, because diverse experiences are critical sources of health by increasing social integration, cognitive reserve, and psychological resources. Recently, two lines of research have consistently shown that activity diversity or emodiversity is associated with multiple health outcomes. However, still more integrated efforts are needed to better understand diversity of daily experiences in various aspects and their contributions to health. This symposium brings together different endeavors towards understanding how diversity of daily experiences – activity diversity, emodiversity, and variety in positive experiences – are associated with health and well-being across adulthood. The topic of this symposium is timely to discuss potential prevention approaches to protect population well-being as the pandemic evolves. Paper 1 examines activity diversity (breadth and evenness of daily activity participation) and how it is related to positive and negative emodiversity (rich and balanced emotional experiences) differently by age groups. Paper 2 investigates the longitudinal relationship between activity variety across cognitive, physical, and social domains and cognitive functioning. Paper 3 examines variety in pleasant events and its associations with mental health outcomes. Paper 4 examines whether and how negative emodiversity is associated with mental illness during COVID-19. The discussant, Dr. David Almeida will integrate key findings from these studies, discuss their theoretical and methodological contributions, and consider opportunities for future research.

### RICH AND BALANCED EXPERIENCES OF DAILY EMOTIONS ARE ASSOCIATED WITH ACTIVITY DIVERSITY ACROSS ADULTHOOD

Soomi Lee,[1] Emily Urban-Wojcik,[2] Susan Charles,[3] and David Almeida,[4] *1. University of South Florida, Tampa, Florida, United States, 2. University of Wisconsin—Madison, Madison, Wisconsin, United States, 3. University of California, Irvine, Irvine, California, United States,*

*4. Pennsylvania State University, University Park, Pennsylvania, United States*

We examined whether the diversity of daily activities ("activity diversity") is associated with the diversity of daily emotions ("emodiversity") and if the association differs by age. Two samples of adults from the Midlife in the United States Study provided activity and emotion data for eight days. Greater activity diversity was associated with greater positive and negative emodiversity in each sample. Age moderated the association between activity diversity and positive emodiversity in an older sample, such that association was stronger for younger adults than for older adults. Results from data combining the two samples revealed that the associations of activity diversity with positive or negative emodiversity were significant when age < 70, 71 years, respectively. Broad and even participation of daily activities may provide more opportunities to experience rich and balanced emotions in adulthood. The weaker associations in older age may suggest the need to promote active lifestyles in later life.

### MORE NEGATIVE EMODIVERSITY IS ASSOCIATED WITH WORSE MENTAL ILLNESS DURING (BUT NOT BEFORE) COVID-19

Emily Urban-Wojcik,[1] Alexandra Barnes,[2] Dan Fitch,[3] Andrew Kirvin-Quamme,[3] Elizabeth Nord,[3] Lauren Gresham,[3] Richard Davidson,[4] and Stacey Schaefer,[4] *1. University of Wisconsin--Madison, Madison, Wisconsin, United States, 2. Center for Healthy Minds, University of Wisconsin--Madison, Madison, Wisconsin, United States, 3. Center For Healthy Minds, University Of Wisconsin--Madison, Madison, Wisconsin, United States, 4. University of Wisconsin-Madison, Madison, Wisconsin, United States*

Relations between negative emodiversity (NED; the variety and relative abundance of negative emotions) with depression and anxiety were examined before and during the COVID-19 pandemic. Forty-five individuals (ages 25-65) participated in two ecological momentary assessments (EMA): pre-pandemic and during-pandemic (Fall, 2020). Participants reported how much they felt 6 negative emotions several times each day for 10 days (resulting up to 91 EMA "events"). Each event's NED was computed and then averaged using an adaptation of Shannon's entropy. Participants with higher levels of average NED had higher levels of concurrent depression and anxiety. When adjusting for average levels of negative emotion and other covariates, NED was a significant predictor of depression and anxiety only during the pandemic. These findings, which did not vary by age, suggest that having more diverse negative emotions on a moment-to-moment basis may hold greater significance for mental illness outcomes during times of extreme chronic stress.

### THE LONGITUDINAL RELATIONSHIP BETWEEN ENGAGEMENT IN VARIOUS ACTIVITIES AND COGNITIVE FUNCTIONING

Sangha Jeon,[1] Soomi Lee,[2] and Susan Charles,[1]
*1. University of California, Irvine, Irvine, California, United States, 2. University of South Florida, Tampa, Florida, United States*

Recent studies indicate that engaging in more diverse activities is related to higher cognitive functioning. Questions remain, however, regarding whether activity variety within different domains is important. We examined how overall activity variety across domains, as well as variety within cognitive, physical, and social domains are related to cognitive functioning. Data were drawn from Waves 2-3 of Midlife Development in the United States (MIDUS). In cross-sectional analyses (N = 3349), activity variety overall and within each domain were positively related to cognitive functioning regardless of activity frequency. In longitudinal analyses (n = 2054), participants with consistently higher activity variety overall and within the social domain over time (vs. those with consistently low or decreasing activity variety) exhibited better cognitive functioning at W3 after adjusting for cognitive functioning at W2. Findings suggest that engaging in a variety of activities that involve multiple cognitive processes might be beneficial for cognitive health.

### VARIETY IN POSITIVE EXPERIENCE AND MENTAL HEALTH: EVIDENCE FROM TWO NATIONAL SURVEYS OF U.S. ADULTS

Anthony Ong,[1] and Soomi Lee,[2] *1. Cornell University, Ithaca, New York, United States, 2. University of South Florida, Tampa, Florida, United States*

Scheduling of pleasant activities is a core treatment component for various psychiatric disorders, but little is known about whether variety in positive experiences is associated with improved mental health and well-being. Here we demonstrate the benefits of diverse positive experiences. Using data from the Midlife in the United States (MIDUS) II (N = 1,233, Mage = 57yrs) and Refresher (N = 855, Mage = 52yrs) cohorts, we show that greater variety of positive events is associated with lower depression and anxiety, and fewer visits to mental health professionals. These associations remained robust to differences in sociodemographics (age, gender, race, education), personality (openness, conscientiousness, extraversion, agreeableness, neuroticism), and the frequency of positive events, and the results replicated across the two cohorts. The findings highlight the importance of activity diversity in older adults and suggest that efforts to increase engagement in diverse positive experiences could have beneficial effects on mental health outcomes.

### Session 1070 (Paper)

### END-OF-LIFE CARE PRACTICE

### CHANGE OF HEALTH AND CHANGE OF PREFERENCES ON LIFE-SUSTAINING TREATMENT: EVIDENCE FROM A LONGITUDINAL STUDY

Yifan Lou,[1] Mercedes Bern-Klug,[2] and Jinyu Liu,[3]
*1. Columbia University, New York, New York, United States, 2. University of Iowa, Iowa City, Iowa, United States, 3. Columbia University, Columbia University, New York, United States*

**Background:** Decision-making for end-of-life (EoL) care is not a one-off choice. Older adults may change their preferences for life-sustaining treatments along their health continuum. Guided by prospect theory, we hypothesize that perceived change in health status is a driver behind preference changes.

**Method:** Health and Retirement Study Wave 2012 to 2018 data. Sample is limited to 5,646 older adults who reported whether they requested to limit treatment in living will during two waves of data. Two possible preference changes were tested: from limited to default care and from default to limited care. Change in health status was indicated by changes (1=same, 2=improve, 3=decline) in physical pain, general health, activities of daily living, instrumental activities of daily living (IADL), and number of diagnoses. Multilevel logistic regression models were used to understand how change of health status was related to changes in EoL preferences.

**Results:** 700 older adults changed their preferences some time in 8 years. Those who changed their preferences are more likely to be older and not married, and to have lower socioeconomic background. Older adults who experienced deteriorated pain levels were more likely to change their preferences from default to limited care (OR=3.77, p<.05) and less likely to change from limited to default care (OR=0.63, p<.05). Change in IADL is also a significant predictor of change of preferences.

**Implication:** The findings highlight the importance of periodic reassessment of EoL care preferences with older adults. We discuss policy and practice implications regarding health changes as underlying mechanisms of preference changes.

### CLINICAL OUTCOMES OF HOME-BASED PALLIATIVE CARE: AN ADVANCE ILLNESS MANAGEMENT PROGRAM

Cara Wallace,[1] and Yit Mui Khoo,[2] *1. Saint Louis University, St. Louis, Missouri, United States, 2. Saint Louis University, Saint Louis, Missouri, United States*

Prior studies have reported evidence that patients with chronic medical conditions benefit from home-based palliative care to manage symptoms. The purpose of this study is to evaluate the efficacy of an advanced illness management program provided by a visiting nurse agency. This program aims to reduce the burden of illness and to manage symptoms of patients who have difficulty leaving their home due to severity of their medical conditions. Data for this study were collected from patients who received home-based palliative care from the agency. Although the program has enrolled close to 500 patients from 2016 to 2019, the analytic sample for this study was restricted to patients enrolled between 2018 – 2019 who completed both baseline and follow-up assessments for three measures: Rapid Geriatric Assessment, Integrated Palliative Care Outcome Scale-Patient Version (IPOS), and Brief Illness Perception Questionnaire (BIPQ; N=96, capturing 33.8% of eligible patients). Paired sample t-tests were used to compare the symptoms and health outcomes between baseline and follow-up assessments. Average age of the participants were 79.9 years. Results from the RGA measure showed that patients' scores on frailty and sarcopenia were significantly lower at follow-up, indicating improvement. Comparison of scores between baseline and follow-up on the IPOS measure showed that patients experienced improvement in the following symptoms: pain, weakness, nausea, poor appetite, constipation, sore/dry mouth, drowsiness, and mobility. Findings suggest that palliative care services can effectively managed the symptoms and health outcomes of homebound chronically ill patients. Other implications include reduced emergency room visits and hospital admissions.

### CULTURE DIFFERENCES IN ADVANCE CARE PLANNING AND IMPLICATIONS FOR SOCIAL WORK PRACTICE

Jung-Hwa Ha,[1] Changsook Lee,[1] and Jennifer Yoo,[2], *1. Seoul National University, Seoul, Seoul-t'ukpyolsi, Republic of Korea, 2. University of Georgia, Atlanta, Georgia, United States*

Advance care planning (ACP) is the process of making plans and decisions regarding end-of-life care (EOLC) in advance while one has the physical and cognitive capacity to do so. However, even if health practitioners recognize the importance of ACP, they may be constrained by social and cultural factors in engaging their clients in ACP. This study examined cultural differences in ACP and various strategies that social workers use to initiate conversations on ACP in a range of settings. Using the case study method, we conducted in-depth interviews with 7 social workers who work in South Korea, 2 Korean-American social workers working in the Korean-American communities in the US, and 3 American social workers serving diverse populations in the US. Their practice sites include: university hospitals, day care centers, a community senior center, a nursing home, and a hospice agency. Social workers in both countries emphasized the need to build rapport with their clients early on and to empower them to take the lead in their ACP while they were still healthy. In Korean and Korean-American communities, social workers recognized their clients' reluctance to speak about EOLC and highlighted the importance of communicating with their family due to their clients' preference for family-centered decision-making. When doing this, a step-by-step approach in giving relevant information was recommended. We identified relationship-building, empowerment, and culturally sensitive approaches as common strategies in initiating discussions on ACP in both countries.

### DEVELOPMENT AND FORMATIVE EVALUATION OF A DEATH EDUCATION PROGRAM FOR COMMUNITY-DWELLING CHINESE OLDER ADULTS

Mandong Liu,[1] and Iris Chi,[2] *1. University of Southern California, Hangzhou, China (People's Republic), 2. University of Southern California, University of Southern California, California, United States*

Planning for end-of-life (EOL) care in advance can enhance one's quality of life at EOL. Culturally sensitive educational programs are needed in Chinese populations to enlighten the public and encourage advance planning due to a culture of death-denying and avoidance. This study describes the team's efforts to develop and formatively evaluate a death education program designed for community-dwelling Chinese older adults. The program was designed based on the Knowledge-Attitude-Behavior Model, as a 2-session 3-hour program spreading over two days with 1.5 hours for each day. The content paid attention to discussing the importance of making plans for EOL in Chinese culture and discussing how to have death-related conversations with the family and health care professionals. In 2020, semi-structured interviews were conducted by phone with 12 health care professionals and researchers, and four Chinese older adults in China to obtain their feedback on program content and delivery. The directed content analysis method was used to analyze the data. Although they confirmed multiple challenges in conducting death education in China, such as family avoidance even if an older adult initiates the death-related conversation, health care professionals not feeling comfortable with such discussions, etc., they also felt the urgency and importance of delivering death education among older adults and in society as a whole. Detailed suggestions were categorized into relationship building, program preparation (e.g., setting, materials), multiple ways of recruitment, target population, length, various formats of content delivery, content (e.g., pay attention to spiritual care), and general support from the public.

### Session 1075 (Paper)

### HEALTH AND LONG-TERM CARE

### A COMPARATIVE ASSESSMENT OF LONG-TERM CARE FINANCING AND SERVICE DELIVERY MODELS

Larry Polivka,[1] and LuMarie Polivka-West,[2] *1. Florida State University, Tallahassee, Florida, United States, 2. Florida State University, Florida State University, Florida, United States*

The face of public long term care (LTC) funded largely through the Medicaid program is changing rapidly in the U.S. Over the last decade, most states have moved to managed LTC programs in various forms, with a growing number transferring all their programs, home and community based (HCBS) and nursing home services, to a Medicaid (MLTC) model. The amount of rigorously conducted and reported evaluation results on these programs are still very limited. Enough information is available, however, from other sources for at least preliminary comparison of relative cost-effectiveness of MLTC vs. traditional, non-profit models of public LTC services delivery and financing, as discussed in this paper. This comparison will show that, at this point, the MLTC programs are not more cost-effective than the traditional model of LTC administration. In fact, these initial assessments seem to indicate that the traditional model may be superior to the corporate for-profit MLTC model.

### ARE NURSING HOME PREFERRED NETWORKS GOOD FOR PATIENTS' OUTCOMES? EVIDENCE FROM THE VETERANS HEALTH ADMINISTRATION

Portia Cornell,[1] Emily Corneau,[1] Kate Magid,[2] Patience Moyo,[3] James Rudolph,[3] Cari Levy,[2] and Vincent Mor,[4], *1. Providence VA Medical Center, Providence, Rhode Island, United States, 2. Rocky Mountain VA Medical Center, Aurora, Colorado, United States, 3. Brown University School of Public Health, Providence, Rhode Island, United States, 4. Brown University, Providence, Rhode Island, United States*

In the Veterans' Administration (VA), medical centers contract with community nursing homes to provide care to Veterans. As a purchaser, the VA could pursue a strategy of selecting a high-quality network; alternatively, it could focus resources on oversight by its nursing-home coordinators. The question of whether narrow networks are good for Veterans' outcomes, conditional on quality, therefore, needs empirical investigation. We examined the effect of network concentration on hospital admissions, conditional on Veterans' clinical acuity. We operationalized network concentration as the number of Veterans already in residence at the time of admission, and controlled for publicly reported quality measure (star rating). We identified 93,805 VA-paid admissions to nursing homes between 2013 to 2016. To address selectin bias, we estimated effects using a distance-based instrumental variable (IV) for each measure, with the log of distance to the nearest nursing home with a specified number of Veterans at the facility in the previous month (1-4, 5-9, and 10-13, and 14+ Veterans). Going to a facility with 10-13 or 14+ Veterans had a higher hospitalization probability (6.2 and 3.3 percentage points higher, respectively), than going to a facility with 1-4 Veterans. If quality rating improves outcomes, then broader networks are beneficial if consumers (Veterans) choose based on quality, given a broader choice set. Conditional on quality, concentrated networks do not seem to lead to fewer hospital admissions. Our results suggest that the VA could do more in its oversight role to work with these nursing homes to decrease hospital admissions.

### EXPLORING THE ROLE OF CONTEXTUAL FACTORS IN MEDICAID NURSING HOMES' PERFORMANCE: A QUALITATIVE PERSPECTIVE

Justin Lord,[1] Midge Ray,[2] Amy Landry,[2] Heather Lee,[3] Nataliya Ivankova,[2] Ganisher Davlyatov,[4] Ivan Herbey,[3] and Robert Weech-Maldonado,[2], *1. Louisiana State University at Shreveport, Bossier City, Louisiana, United States, 2. University of Alabama at Birmingham, Birmingham, Alabama, United States, 3. UAB, Birmingham, Alabama, United States, 4. University of Oklahoma Health Sciences Center, Oklahoma City, Oklahoma, United States*

This study explored the role of tested contextual factors (structural, market, and management) in high Medicaid (under resourced) nursing homes performance. Four nursing homes in geographically diverse states were purposefully selected for site visits based on high and low performance (quality/ profitability) indicators. Eight nursing home administrators and directors of nursing, and twenty-one nursing staff (RNs, LPNs, and CNAs) and providers of support services were interviewed. Data were analyzed using an inductive thematic approach with NVivo 12 Plus. Within and across case analysis was used to compare participants' perspectives across nursing homes and across administrators and staff. Several themes provide insight into varied influences of contextual factors on these nursing homes' performance: focus on quality care, team-based approach, community support and engagement, and staffing retention. Providing quality care to residents was strategic priority in all facilities, which was enhanced by an adopted team-based leadership approach, open-door policy and home-like atmosphere. Community reputation and availability of local training opportunities for CNAs affected nursing staffing which some facilities addressed using creative retention strategies. These research findings will facilitate interventions, such as leadership training and organizational development activities, aimed at improving the performance of low performing facilities in terms of lower costs and better quality.

### HOME HEALTH AGENCY OWNERSHIP AND QUALITY OF CARE OUTCOMES AMONG MEDICARE BENEFICIARIES

Rashmita Basu,[1] Huabin Luo,[1] and Bei Wu,[2] *1. East Carolina University, Greenville, North Carolina, United States, 2. New York University, New York, New York, United States*

Medicare restructured home healthcare reimbursement from a cost-basis to a 60- day risk-based prospective payment system (PPS) in 2000 to implement the value-based payment model for home healthcare services. Currently home healthcare market in the U.S. is dominated by the presence of for-profit (FP) agencies instead of being primarily served by not-for-profit (NFP) agencies. Using data from the 2016-2018 OASIS for beneficiaries participated in the Medicare Current Beneficiary Survey (MCBS) (N=6,115), the current study examines whether home health agency ownership status is associated with length of stay (LOS) and discharge outcome Medicare home health care patients. Our first outcome variable is discharge status (modeled via ordered probit) with three categories: discharge to the community, inpatient hospital and other long-term care facilities. The second outcome variable is LOS and two dummy variables LOS ≤ 30 days and LOS ≥ 99 days were modeled via binary probit. The key independent variable was the ownership status of the agency (FP vs. NFP). Patient level covariates includes demographics (age, gender, race/ethnicity, marital status), comorbidity index, agency characteristics (metropolitan statistical area, hospital-based). Patients in FP agencies were 5.1% ($p<0.01$) less likely to discharge to community, 15.3% ($p<0.001$) less likely to have LOS ≤ 30 days but 7.5% ($p<0.001$) more likely to have LOS ≥ 99 higher compared to patients from NFP agencies under the PPS. Our results have important implications for clinicians, patients and healthcare professionals to be cognizant about the influence of agency ownership on the delivery of healthcare services in home healthcare sector.

### QUALITY IMPROVEMENT EFFORTS IN VA COMMUNITY LIVING CENTERS INCREASED FOLLOWING PUBLIC REPORTING OF PERFORMANCE

Heather Davila,[1] Whitney Mills,[2] Valerie Clark,[3] Christine Hartmann,[4] David Mohr,[5] Dan Berlowitz,[6] Amy Baughman,[7] and Camilla Pimentel,[3] *1. VA Boston Healthcare System, VA Boston Healthcare System, Massachusetts, United States, 2. Providence VA Medical Center, Providence VAMC/Brown University, Rhode Island, United States, 3. VA Bedford Healthcare System, Bedford, Massachusetts, United States, 4. VA Bedford Healthcare System, VA Bedford Healthcare System, Massachusetts, United States, 5. VA Boston Healthcare System, Boston, Massachusetts, United States, 6. UMass-Lowell, UMass-Lowell, Massachusetts, United States, 7. Massachusetts General Hospital, Massachusetts General Hospital/Boston, Massachusetts, United States*

In 2018, the US Department of Veterans Affairs (VA) began publicly reporting performance ratings for its 134 Community Living Centers (CLCs; nursing homes) based on health inspections, staffing, and clinical quality measures. CLCs operate within a large, integrated healthcare system with unique financial and market incentives. Although public reporting has led to quality improvements in non-VA nursing homes, we do not know whether CLCs respond to public reporting differently than private sector nursing homes. To address this knowledge gap, we used a comparative case study approach involving 3 purposively selected CLCs with varied (low, medium, high) performance ratings. We conducted semi-structured interviews with personnel (n=12) responsible for quality measurement and improvement. Interviews focused on opinions of public reporting, actions taken to improve performance ratings, and motivations for change. Participants indicated public reporting improved transparency and provided an "outside perspective" on their performance. Strategies to improve performance ratings involved 1) data/information, 2) individual roles, and 3) teamwork/communication. All 3 CLCs made changes in these areas, yet respondents in the higher performing CLCs described implementing more strategies immediately after learning their ratings. Respondents in all 3 CLCs described being motivated to deliver good care and achieve public ratings that reflected the care they provided. This meant addressing internal weaknesses that contributed to lower scores for 2 CLCs. Our findings suggest public reporting may improve internal data collection, reporting, and quality improvement efforts in CLCs. They highlight the potential positive impact of public reporting in prompting quality improvement in nursing homes.

### Session 1080 (Paper)

### INFORMATION TECHNOLOGIES AND COVID-19

### COVID-19 AND BRAIN HEALTH: GLOBAL COUNCIL ON BRAIN HEALTH RECOMMENDATIONS

Sarah Lock, and Lindsay Chura, *AARP, Washington, District of Columbia, United States*

With growing evidence that the coronavirus directly harms the brain and indirectly harms mental well-being due to social isolation and new, increased stressors, the GCBH recognized the urgent need to inform adults age 50+ about ways to their protect brain health as the pandemic continues. In our latest report, the GCBH describes the known neurological symptoms occurring in the short and long term for adults, providing 10 recommendations to protect brain health and urging research in 11 different areas. Calling for an all-of-society approach to protect the brain health of everyone, the GCBH described the negative effects of COVID-19 on people living with Alzheimer's disease and other dementias and to the impact of health care inequalities. For example, people with dementia were twice as likely to catch the virus as those without dementia; African Americans with dementia had nearly three times the risk of COVID-19 as Caucasians with dementia. The GCBH also points out that caregivers for those living with dementia have experienced particular stress and provided resources and guidance. The Council spotlights the disproportionate toll of COVID-19 on the vulnerable, including racial and ethnic minorities and those living in low- to middle-income countries. After attending this session, participants will be able to identify the neurological impacts of COVID-19, understand the various ways to mitigate risks to brain health, and learn which areas of research will be critical in the future. These recommendations were developed and put forth by the Global Council on Brain Health Governance Committee and Issue Experts.

### EVERYDAY LIFE EXPERIENCES OF SWEDES AGE 70+ DURING THE COVID-19 PANDEMIC

Torbjorn Bildtgard,[1] and Peter Öberg,[2] *1. Stockholms University, Stockholm, Stockholms Lan, Sweden, 2. University of Gävle, Gävle, Gavleborgs Lan, Sweden*

As many other countries Sweden has been hit hard by the Corona pandemic, with high numbers of dead in the older population. Since march 16, 2020, the authorities have encouraged people 70+ to voluntarily quarantine and avoid contacts outside the household. How has this affected older people's everyday lives? This study reports on results from a web-survey on the everyday life experiences of Swedes 70+ carried out between May 28 and July 13, 2020 (n=1 926). The presentation focuses answers to an open-ended question: "Describe with your own words how your life has been affected by the Corona pandemic". A qualitative content analysis was used to investigate changes in the everyday lives of the respondents and their appreciations of these changes. Results show that older Swedes have mostly adhered to public recommendations of self-isolation and withdrawn from social and family contacts, as well as paid and volunteer work. The vast majority (76%) of the respondents describe what they see as negative life changes, such as loss of structure in their everyday life, loss of contact with children/grandchildren and friends, loss of meaningful activities, loss of abilities due to forced unemployment and experiences of ageism. Experiences of loneliness, depression and drop in quality of life are common. Some positive changes were reported. We argue that the experience of the 70+ population during Corona needs to be understood in relation to the promise of the third age, where everyday restrictions are experienced as a forced disengagement into a fourth age life style.

### INFORMATION SEEKING DURING THE PANDEMIC: THE ROLE OF AGE, AGENCY, AND FAKE NEWS CONCERNS

Ann Pearman, MacKenzie Hughes, and Clara Coblenz, *Georgia Institute of Technology, Atlanta, Georgia, United States*

COVID-19 brought rapid changes to the way in which people understand and process news, including both information and misinformation about the pandemic. This cross-sectional study was designed to examine persons' experiences during the earliest months of the pandemic. The sample included 871 adults ages 20-79 (M=38.27 years, SD=11.40). Online surveys were collected between March and May, 2020 using Amazon Mechanical Turk. Participants completed a series of questionnaires, including a measure of agency from the Midlife Development Inventory, a questionnaire that assessed level of skepticism about the COVID-19 pandemic (i.e. fake news beliefs), a depression scale, a question about their level of anxiety about developing COVID-19, and questions about the frequency in which they sought information about the pandemic from different sources (e.g., TV, social media). A multiple regression using information seeking frequency as the outcome variable revealed several significant relationships. Specifically, younger adults, people with higher agency, and people with higher fake news beliefs all reported higher levels of COVID-19-related information seeking. In addition, there was a significant 3-way interaction between age, agency, and fake news beliefs. Disentangling this interaction revealed that older adults with low agency were least likely to engage in information seeking. There were, however, no age differences in information seeking in participants with high agency and fake news beliefs, but large age differences in participants with low agency but high fake news beliefs. Findings suggest agency is an important predictor of information seeking behavior, particularly for older adults with high levels of skepticism about the pandemic.

### MOVING ONLINE: EVIDENCE-BASED PROGRAMMING DURING COVID

Susan Hughes,[1] Andrew DeMott,[2] Gerald Stapleton,[1] and Gail Huber,[3] *1. University of Illinois at Chicago, Chicago, Illinois, United States, 2. UIC, Chicago, Illinois, United States, 3. Northwestern University, Chicago, Illinois, United States*

The COVID pandemic disrupted the way evidence-based health promotion programs (EBPs) are delivered to older adults who were the most at-risk group in terms of mortality and faced unprecedented threats to their independence and physical and mental health. Many organizations stopped in-person EBPS causing older adults to lose access to key social networks and health resources. It is a top public health priority to find new ways to keep older adults connected to their EBPs. Fit & Strong! (F&S!) is a group exercise/health education EBP for older adults with arthritis offered by CBOs in 32 states. CBOs stopped offering F&S! in-person in March 2020. Since the lockdown, we have worked closely with our provider network to develop and pilot a version that is remote/online and live, titled "F&S! @Home". Instructors deliver F&S! @Home to older adults with minimal technological resources. We created a staging website for both providers/instructors and participants that is used to initiate the classes, enable providers to manage participants, collect data, and share support materials. The pilot began September 2020; since that time 15 classes have been offered to 147 participants. Administration on Community Living falls and arthritis outcomes data are being collected. Preliminary analyses of 45 participants and 8 instructors demonstrate a high rating of the program (mean score of 90.2 out of 100) with no adverse outcomes to date. This presentation will review the process of creating the online adaptation, lessons learned, and will review pre/post outcomes and participant and instructor evaluation feedback.

### STRATEGIES OLDER ADULTS USED DURING COVID-19 TO STAY CONNECTED

Brad Doebbeling,[1] Haley Harelson,[1] Michelle Houchins,[1] Hallie Wine,[1] Claire Pishko,[1] Cheryl Der Ananian,[1] Allie Peckham,[2] and M. Aaron Guest,[1] *1. Arizona State University, Phoenix, Arizona, United States, 2. Arizona State University, Pheonix, Arizona, United States*

An unintended consequence of the physical distancing guidelines to prevent the spread of COVID-19 may be increased feelings of social isolation and loneliness in older adults.

**Purpose:** The purpose of this study was to identify successful strategies used in avoiding social isolation and feelings of loneliness in older adults (50+) during the pandemic.

**Methods:** Older adults (n=22) selected from a longitudinal study, Aging In the Time of COVID-19, who did not report loneliness, participated in a semi-structured interview via zoom. Individuals were asked a series of questions about how their lives were impacted by the pandemic and what they did to avoid social isolation and loneliness. Each interview was recorded and transcribed verbatim. transcripts were analyzed and categorized to identify common strategies.

**Results:**Participants were primarily female and white (100%) with a mean age of 64.7 years. Preliminary findings (n=5) suggest older adults developed several effective strategies for combating social isolation and feelings of loneliness, including purposely reaching out and "checking in" on others; engaging in exercise, either alone or with others, and engaging in outdoor activities, such as socially distanced in-person encounters. Other effective strategies included virtual events (e.g. community or local events, museums or concerts, etc.), using technology to communicate with friends and family, and practicing gratitude consistently.

**Conclusions:**Although older adults have been encouraged to stay at home and physically distanced throughout the pandemic, they have found ways to remain socially connected with friends, family, and community, despite not being physically together.

### Session 1085 (Symposium)

### INJUSTICE SQUARED? AN INTERSECTIONAL LENS TO RESEARCH ON PRODUCTIVE ENGAGEMENT IN LATER LIFE

Chair: Christina Matz Co-Chair: Cal Halvorsen Discussant: Jacquelyn James

Social inequalities over the life course shape later life opportunities and outcomes in important ways. However, research on paid and unpaid work in later life has not always captured (and has sometimes mischaracterized) the variety and complexity of lived experiences in later life—in particular for low-income workers, workers of color, women, and others marginalized due to their social position. Further, statistics often obscure the most important information: how the most marginalized older workers are faring. Intersectionality, a term coined by legal scholar, Dr. Kimberlé Crenshaw, describes the overlapping and intersecting social identities that often influence how we move around in society. Some identities garner privilege and power and others oppression and marginalization; we must look at their intersection to better understand complexity and inform solutions. This symposium will apply an intersectional lens to research on paid and unpaid work in later life. The first paper is a scoping review that assesses the extent to which race and ethnicity are investigated in studies of the longitudinal association between workplace demands and cognitive health. The second paper explores how older Black and Hispanic adults' work engagement is impacted by COVID-19. The third paper considers gender differences in volunteer engagement among Asian-American older adults. The final paper examines the Senior Community Service Employment Program's role in participant financial, physical, and mental well-being. A discussant will reflect on these studies and the need for continued research that considers intersectionality in opportunities and experiences for paid and unpaid work in later life.

### WORKPLACE DEMANDS AND COGNITIVE HEALTH INEQUITIES ACROSS RACE AND ETHNICITY: A SCOPING REVIEW

Ernest Gonzales,[1] Rachel Krutchen,[1] Cliff Whetung,[2] and Jane Lee,[3], *1. New York University, New York, New York, United States, 2. NYU, New York, New York, United States, 3. University of Hawai'i, Myron B. Thompson School of Social Work, Honolulu, Hawaii, United States*

This PRISMA informed scoping review sought to understand the longitudinal association between workplace demands with cognitive health; and to review how race and ethnicity are investigated in this area of research and evidence of moderating effects. Peer-reviewed articles were drawn from five databases. Inclusion criteria were populations aged 18+, broad conceptualization of workplace demands (e.g., occupational complexity, mental work demands), and cognitive health outcomes (e.g., cognitive functioning, ADRD). The majority of studies drew from theories that did not interrogate heterogeneity and diverse aging experiences. Consequently, the majority of studies (85%) did not investigate inequities by race and ethnicity although variables and methods are available. Cognitive health inequities are evidenced but findings are mixed and more rigorous causal research is needed. We discuss integrating emerging critical theories (e.g. Critical Race Theory, critical gerontology, minority stress) to sharpen the focus on racial health inequities in an emerging area of prevention research.

### THE EFFECTS OF THE COVID-19 PANDEMIC ON THE WORK AND FINANCIAL OUTCOMES OF OLDER BLACK AND HISPANIC ADULTS

Kendra Jason,[1] Dawn Carr,[2] and Zhao Chen,[3] *1. UNC Charlotte, Charlotte, North Carolina, United States, 2. Florida State University, Tallahassee, Florida, United States, 3. The University of Arizona, Tucson, Arizona, United States*

This study investigates how older Black and Hispanic adults' work engagement is impacted by the effects of COVID-19. Using intersectionality and cumulative (dis)advantage as complementing theoretical frameworks, data from the Health and Retirement Study, and series of logistic regression models, we measure work engagement changes pre- and post- COVID-19. Preliminary findings suggest that net of other controls, there were no substantial or significant reductions in resilience as the result of COVID-19 itself for any racial/ethnic group. White older adults, and to some degree Hispanics older adults, experienced erosion in resilience related to financial hardships, but the resilience of Black older adults remained stable in the face of increased hardship. Future work in this area will improve our limited understanding of older Black and Hispanic adults' experiences of managing and coping with COVID-19- related work and financial risks– information that will be critical for planning intervention and support services.

### EXPLORING GENDER DIFFERENCES IN VOLUNTEER BEHAVIOR AMONG OLDER ASIAN AMERICANS

Patrick Ho Lam Lai, Christina Matz, and Cal Halvorsen, *Boston College, Chestnut Hill, Massachusetts, United States*

Prior research has documented the health and well-being effects of volunteerism in later life, and that positive outcomes increase in the first 100 volunteer hours/year and slightly increase between 100-200 hours. Given this, it seems that using an intersectional lens to explore disparities in volunteer behaviors and what might explain them is important from a health equity standpoint. Using data from 268,194 individuals aged 65-85 from the most recently available Volunteer Supplement of the Current Population Survey, this study found that White older adults were most likely to spend any time in volunteer activities, while Asian and Hispanic older adults were least, across all racial/ethnic groups. Further, the percentage of older Asian women volunteering in the 100-200 hour range (27%) was significantly higher than that of older Asian men (19%). Social and cultural factors that may explain these racial/gender differences and implications for recruiting older adults as volunteers are discussed.

### A COMMUNITY-BASED SYSTEM DYNAMICS STUDY OF THE SENIOR COMMUNITY SERVICE EMPLOYMENT PROGRAM

Cal Halvorsen, Kelsey Werner, and Elizabeth McColloch, *Boston College, Chestnut Hill, Massachusetts, United States*

The Senior Community Service Employment Program (SCSEP), the only federal workforce-training program that targets older adults, engages people aged 55 years and older with incomes at or below 125% of the federal poverty level with multiple barriers to employment. This study examined SCSEP's role in participant financial, physical, and mental well-being. To do so, we held five sessions (four virtual, one telephone) over a combined nine hours in August and September 2020 using a form of participatory research called community-based system dynamics with 15 Massachusetts SCSEP participants and case managers. Through structured activities, respondents identified how program, policy, and organizational factors influence and are influenced by participant well-being (e.g., SCSEP participation results in less social isolation, decreased isolation subsequently increases desire to participate) as well as program and policy recommendations to strengthen the program (e.g., reconsider benchmarks of success). These findings highlight the benefits and potential of this long-running program.

### Session 1090 (Symposium)

### METHODOLOGICAL APPROACHES TO TRANSFORM, DEVELOP, AND EVALUATE CANCER CARE INTERVENTIONS

Chair: Sean Halpin

Cancer diagnoses in older adults are often abrupt and unexpected, requiring patients to make quick therapy choices. Further, reflecting on traumatic therapy is often difficult. In our symposium, we bring together researchers from varied disciplines to report on patients' cancer therapy choices. Carrion will discuss her mixed-methods approach to older Latino adults' cancer therapy preferences. Next, Blackberry will present how patient-derived photographs (photovoice) can improve supportive care for older Australians in facilitating empowerment, patient-centered care, and shared decision making. Halpin, who will apply conversation analysis to examine how multiple myeloma patients interacted with supplemental material during in-person nurse-led education. Last, Seaman will use his work with head and neck cancer survivors to illustrate the conceptual and methodological challenges of investigating those who discontinue care. Understanding how patients from diverse populaces with various cancer diagnoses navigate their therapy may help inform future cancer-related health services' approaches.

### A MIXED-METHODS APPROACH TO THE EXAMINATION OF CANCER TREATMENT PREFERENCES AMONG OLDER LATINO ADULTS

Iraida Carrion,[1] Malinee Neelamegam,[2] and Tania Estape,[3] *1. University of South Florida, Tampa, Florida, United States, 2. Department of Epidemiology of Microbial Diseases Yale School of Public Health, New Haven, Connecticut, United States, 3. FEFOC, Fundacion Contra El Cáncer, Barcelona, Catalonia, Spain*

Given the growing population of Latino immigrants 60 years and older and the current lack of relevant data, there is an urgent need to understand this population's cancer treatment preferences to ensure effective interventions and psychosocial care. A study comprising 200 surveys with areas focused on cancer knowledge, attitudes, prevention, early diagnosis, and treatment was developed and administered in Spanish. The survey included a qualitative component consisting of open-ended questions. The mixed-method study gathered quantitative data regarding treatment preferences as well as the voices of older Latino men and women with a history of cancer, including their cancer treatment trajectory. Additionally, the survey data highlighted the lack of knowledge regarding available cancer treatments. The findings suggest that, while older Latino/as have knowledge about the causes of cancer, they lack knowledge regarding cancer diagnoses, which could potentially cause them to avoid treatment.

### WHAT HAPPENS NEXT? CONDUCTING RESEARCH WITH CANCER SURVIVORS WHO DISCONTINUE THEIR POST-TREATMENT CARE

Aaron Seaman, and Nitin Pagedar, *University of Iowa, Iowa City, Iowa, United States*

Due to improvements in screening, diagnosis, and treatment, more cancer patients are surviving and living longer. For them, survivorship care provides critical support: surveillance and screening for recurrence and new cancers; physical and psychological symptom management; social and financial management support; management of other chronic conditions; and preventive health and health promotion support. Yet, our pilot data indicates that a substantial number of survivors discontinue their survivorship care with the treating oncology team, a team that often provides critical multidisciplinary support and expertise. While it is important to understand the experiences, outcomes, and needs of these survivors, they can be challenging to engage in research. In this presentation, we will discuss survivors who discontinue, why they might do so, and methods for engaging them in research, drawing upon our work with head and neck cancer survivors.

### USING CONVERSATION ANALYSIS TO EXAMINE WHEN EDUCATIONAL VIDEOS ARE INTRODUCED IN PATIENT EDUCATION

Sean Halpin,[1] Michael Konomos,[2] and Kathryn Roulston,[3] *1. University of Georgia, Decatur, Georgia, United States,*

*2. Emory University, Atlanta, Georgia, United States, 3. University of Georgia, University of Georgia, Georgia, United States*

As the seas of advanced therapies have swelled in the last few decades, multiple myeloma patients have been empowered, encouraged, and sometimes required, to engage in their care. We applied a conversation analysis approach to 12 nurse-led education visits (1011 minutes of audio) containing reference to educational videos. We indexed extracts based on whether the nurse or patient first mention the video. Patients oriented toward the video to demonstrate knowledge (n=15; 88%) and clarify information (n=2; 12%). Nurses oriented toward the video either through positive (n=14; 39%), negative (n=13= 36%), or neutral (n=9; 25%) assessments. Videos created opportunities for patients to pursue their topics of interest during in-person visits. Also, nurses often oriented toward the videos pre-emptively to achieve their teaching goals either by acknowledging the patients' exposure to upcoming information or by positively or negatively assessing the videos in ways that enable them to re-orient talk to educational scripts.

### GERIATRIC ONCOLOGY IN THE INSTAGRAM ERA: PHOTOVOICE TO ENABLE PATIENT-CENTERED CARE AND SHARED DECISION MAKING

Irene Blackberry,[1] Christopher Steer,[2] Tshepo Rasekaba,[1] Kim Young,[1] Nicole Webb,[2] Darren Jayasuriya,[3] Kylie Owen,[1] and Mira Kapur,[3] *1. La Trobe University, Wodonga, Victoria, Australia, 2. Border Medical Oncology and Haematology, Albury, New South Wales, Australia, 3. UNSW, albury, New South Wales, Australia*

Evidence shows that multidimensional assessment of older adults with cancer yields more holistic care and results in better communication about age-related concerns; as well as enables personalised, patient-centered supportive care. Geriatric assessment (GA) captures clinical, physical and psychological factors, with limited opportunity to gather information about the patient's environment, personal contexts and priorities. We trialed the feasibility and acceptability of geriatric assessment (GA)-guided enhanced supportive care (ESC) among 20 adults aged over 70 years in a regional cancer center. We then studied the impact of the integration of four patient-derived photographs (with PhotoVoice analysis) to this ESC on patient satisfaction with communication with the oncologist regarding age-related concerns and on facilitating empowerment, patient-centered care and shared decision making. The use of PhotoVoice analysis of patient-derived photographs is a novel strategy that can facilitate gathering patient-centered information during the assessment process.

### Session 1095 (Paper)

### MOBILITY I

### FACTORS ASSOCIATED WITH OLDER ADULTS' IN-HOSPITAL MOBILITY: A COMPARISON BETWEEN ISRAEL AND DENMARK

Mette Merete Pedersen,[1] Janne Petersen,[2] Ove Andersen,[3] Efrat Shadmi,[4] Ksenya Shulyaev,[5] Maayan Agmon,[6] Efrat Gil,[7] and Anna Zisberg,[8] *1. Copenhagen University Hospital, Amager and Hvidovre, Hvidovre, Hovedstaden, Denmark, 2. Copenhagen University Hospital Frederiksberg, Frederiksberg, Hovedstaden, Denmark, 3. Copenhagen University Hospital Hvidovre, Hvidovre, Hovedstaden, Denmark, 4. University of Haifa, Mount Carmel, Israel, Haifa, Hefa, Israel, 5. Faculty of Social Welfare and Health Science, University of Haifa, Haifa, Hefa, Israel, 6. The Cheryl Spencer Department of Nursing, University of Haifa, Israel, Haifa, HaZafon, Israel, 7. Clalit health services, Haifa and West Galilee, Israel, Haifa, HaZafon, Israel, 8. University of Haifa, Haifa, HaZafon, Israel*

Low levels of in-hospital mobility and excessive bed rest are widely described across the globe as a major risk factor for hospital associated disabilities. Different predictors of in-hospital and post-discharge mobility limitations have been proposed across studies, including age, admission diagnosis, physical performance, cognitive impairment, performance of activities of daily living, and length of stay. However, it is unknown whether similar risk factors across countries are associated with in-hospital mobility given different mobility measurement methods, variations in measurement of predictors and differences in populations studied. In the current study, we investigated the relationship between in-hospital mobility and a set of similar risk factors in functionally independent older adults (65+) hospitalized in acute care settings in Israel (N=206) and Denmark (N=113). In Israel, mobility was measured via ActiGraph and in Denmark by ActivPal for up to seven hospital days. Parallel analysis of covariance (ANCOVA) in each sample showed that community-mobility before hospitalization, mobility performance at admission and length of stay were associated with in-hospital mobility in both countries, whereas age and self-reported health status were associated with mobility only in Denmark. This comparison indicates that despite slightly different measurement approaches, similar risks are attributed to older adults' low in-hospital mobility and emphasizes the contribution of commonly used pre-hospitalization mobility measures as strong and consistent risk factors. This knowledge can support a better understanding of the need of both standard risk assessments and country-based tailored approaches.

### MACHINE LEARNING PREDICTION MODELS FOR MOBILITY LIMITATION OVER TIME IN OLDER ADULTS: THE HEALTH ABC STUDY

Jaime Speiser,[1] Kathryn Callahan,[1] Edward Ip,[1] Michael Miller,[2] Janet Tooze,[1] Stephen Krtichevsky,[3] and Denise Houston,[1] *1. Wake Forest School of Medicine, Winston-Salem, North Carolina, United States, 2. Wake Forest School of Medicine, Winston Salem, North Carolina, United States, 3. Wake Forest School of Medicine, Wake Forest School of Medicine, North Carolina, United States*

Mobility limitation in older adults is common and associated with poor health outcomes and loss of independence. Identification of at-risk individuals remains challenging because of time-consuming clinical assessments and limitations of statistical models for dynamic outcomes over time. Therefore, we aimed to develop machine learning models for predicting mobility limitation in older adults using repeated measures and variable selection. We used nine years of follow-up data from the Health, Aging, and Body Composition study to model mobility limitation, defined as self-report of any difficulty walking ¼ mile or up a flight of stairs, assessed annually. We considered 46 predictors for modeling, including demographic, lifestyle, chronic condition and physical function variables. We developed three models with Binary Mixed Model Forest, using: 1) all 46 predictors, 2) an automated variable selection algorithm, and 3) the top five most important predictors. Area under the receiver operating curve ranged from 0.78 to 0.84 for the models for two validation datasets (with and without previous annual visit data for participants). Across the three models, the most important predictors of mobility limitation were ease of getting up from chair, gait speed, self-reported health status, body mass index and depression. Longitudinal, machine learning models predicting mobility limitation had good performance for identifying at-risk older adults based on current and previous annual visit data. Future studies should evaluate the utility and efficiency of the prediction models as a tool in a clinical setting for identifying at-risk older adults who may benefit from interventions aimed to prevent mobility limitation.

### PHYSICAL AND COGNITIVE CORRELATES OF GPS-DERIVED LIFE-SPACE CHARACTERISTICS IN OLDER ADULTS

Kyle Moored,[1] Breanna Crane,[2] Michelle Carlson,[3] and Andrea Rosso,[1] *1. University of Pittsburgh, Pittsburgh, Pennsylvania, United States, 2. Johns Hopkins Bloomberg School of Public Health, Baltimore, Maryland, United States, 3. Johns Hopkins University, Baltimore, Maryland, United States*

Life-space mobility, movement within one's living environment, is important for functional independence in later life. It is unclear which life-space characteristics (i.e., space, duration, shape) are most affected by physical and cognitive limitations. GPS-derived measures mitigate recall bias and offer novel ways to characterize life-space. We examined associations between physical and cognitive performance and GPS-derived life-space characteristics. Participants were 164 community-dwelling adults (Age: M=77.3±6.5) from baseline data of a clinical trial to improve walking in older adults. Participants carried a portable GPS for 7 days, which passively collected real-time location. Standard deviational ellipses (SDEs) and minimum convex polygons (MCPs) were derived for each day. Area and compactness of these measures quantified activity space and shape, respectively. For each measure, 7-day medians and median absolute deviations (MAD) were computed to capture both central tendency and variability of weekly activity. Activity duration was quantified as percentage of time outside home. Adjusting for age and sex, percent time outside home was associated with lower mobility performance (i.e., 6-minute walk (6MWT), figure 8 walk, $\rho$'s=.17-.18, p's<.05) and executive functioning (i.e., Trail Making Test, Part A: $\rho$=.16, p=.04, Part B: $\rho$=.19, p=.01). Median MCP and SDE areas, but not compactness, were associated with 6MWT performance ($\rho$'s=.18-.20, p's<.05). MCP area MAD was associated with greater global cognition (3MSE, $\rho$=.15, p=.05). Life-space characteristics were differentially associated with performance measures, suggesting physical and cognitive limitations may constrain life-space mobility via different mechanisms. Variation in these associations by neighborhood walkability and active versus passive travel will also be examined.

### Session 1100 (Symposium)

### MOBILITY, PHYSICAL ACTIVITY, AND SOCIAL ENGAGEMENT OF COMMUNITY-LIVING OLDER ADULTS

Chair: Wenjun Li Discussant: Lien Quach

Mobility, physical activity and social engagement are important to healthy aging and independent living among older adults. This symposium includes four related studies on these issues. Dr. Lien Quach and her team examined racial and ethnic disparities in social engagement among community-living older adults using data from the national Health and Retirement Study. The analysis found that Asians and Hispanics had significantly lower social engagement score compared with non-Hispanic Whites, advocating for further investigations of the causes of racial disparities in social engagement. Dr. Su-I Hou's study examined the impact of physical activity and social relationship on social engagement. The study found positive impacts of more physical activity, better social relationships and volunteers on social engagement. The results have important implications to promotion of social engagement among older adults participating in aging-in-community programs. Dr. Ladda Thiamwong's study demonstrated the benefits of using assistive health technology (AHT) to assess the relationships between fall risks, body compositions and objectively measured physical activity in older adults. Dr. Thiamwong' will discuss the research protocol and preliminary results. Dr. Li's Health Aging and Neighborhood Study examined variations of older adults' driving behaviors by sex, age, race, income, health status and housing density of the neighborhoods. The study found substantial differences in mobility and driving patterns by both personal characteristics and neighborhood living environment. The findings have important implications to community programs that support older adults aging in place.

### DRIVING HABITS OF OLDER ADULTS IN MASSACHUSETTS: VARIATIONS BY SEX, AGE, RACE, INCOME, HOUSING DENSITY, AND HEALTH

Wenjun Li, Elizabeth ProcterGray, Kevin Kane, Jie Cheng, and Anthony Clarke, *University of Massachusetts Lowell, Lowell, Massachusetts, United States*

Maintaining ability to drive is critical to independent living among older adults residing in suburban and rural communities. We administrated structured questionnaire about driving behaviors to 370 persons age 65 and older living in Central Massachusetts between 2018 and 2020. Of them, 307 were active drivers. Driving in the past year was strongly associated with being male, White race, higher income, non-urban resident, and good-to-excellent health. Advancing age was associated with lower frequency of driving, less miles driven, lower percentage of the day spent in transportation. Men and women drove with nearly equal frequency (~26 days/month), but men drove significantly more miles. Non-White drivers were significantly more likely to avoid driving out of town or in difficult conditions, even after controlling for age, sex, income, and density of residential area. In conclusion, driving behaviors differed significantly by age, sex, income, race, and housing density. Further investigation is warranted.

### RACE, ETHNICITY, AND SOCIAL ENGAGEMENT AMONG COMMUNITY-DWELLING OLDER ADULTS: THE HEALTH AND RETIREMENT STUDY

Lien Quach, *University of Massachusetts Boston, Newton, Massachusetts, United States*

Social engagement is crucial for older adults. This study examines the relationship between race, ethnicity, and social engagement among community-dwelling older adults using data came from the Health and Retirement Study (2014) (n=6221). Race and ethnic status were categorized as: non-Hispanic white (NHW), non-Hispanic black (NHB), non-Hispanic "Asians and other race" (NHA) and Hispanic (any race). Social engagement was based on self-report and included keeping in touch with friends, family and participating in social activities. Covariates included age, sex, education, number of comorbidities, physical function, and alcohol consumptions. The mean age was 74.6, 60% were female. Race and ethnicity distribution were 78.6% NHW, 11.9% NHB, 7.89% Hispanics, and 1.7% NHA. The social engagement (SE) score averaged 3.3. Hispanics, Asians and other races had a lower SE score compared with NHW (b=-0.29, p<.0001; b=-0.27, p=0.04). Understanding racial and ethnic disparities in SE can help target appropriate social intervention.

### PHYSICAL ACTIVITY AND SOCIAL RELATIONSHIPS ON SOCIAL ENGAGEMENT AMONG COMMUNITY-DWELLING OLDER ADULTS

Su-I Hou, *School of Global Health Management & Informatics, University of Central Florida, University of Central Florida, Florida, United States*

This study examined physical activity (FITNESS) and social relationships (FRIENDS) on social engagement among community older adults. Members from two Florida aging-in-village programs participated. Three five-Likert scales were used: A 5-item FITNESS (weight, endurance, strength, flexibility, health), 4-item FRIEND (family, friends, neighbors, communication), and a 3-item social engagement scales (social-leisure activities, stay involved, healthy independent) (Cronbach alphas: .82~.92). Among the 96 participants, 79% were females, 91% were whites, 56% were married, 86% had college education, and 46% living alone. Mean age was 70.7 (SD=10.10). Participants reported at least 30-min. physical activity about 4.2 days per week. Overall social engagement was high (mean=4.38), FITNESS was median (mean=3.46), and FRINED was high (mean=4.19). FITNESS was significant to more 30-min. physical activity. Yet, higher FITNESS, FRIENDS, age, and volunteers were all significant to social engagement. Results has implications on promoting social engagement among older adults participating in aging-in-community programs.

### USING ASSISTIVE HEALTH TECHNOLOGY TO ASSESS FALL RISK APPRAISAL, BODY COMPOSITION, AND PHYSICAL ACTIVITY

Ladda Thiamwong,[1] Joon-Hyuk Park,[2] Renoa Choudhury,[2] Oscar Garcia,[2] Maxine Furtado,[2] Nicole Stallworth,[2] and Jeffrey Stout,[2] *1. College of Nursing, University of Central Florida, Orlando, Florida, United States, 2. University of Central Florida, Orlando, Florida, United States*

One-third of older adults have a discrepancy between perceived and physiological fall risks or maladaptive fall risk appraisal (FRA). Older adults who report high fear of falling and overestimate their physiological fall risk are less likely to participate in physical activity (PA). Limited data suggest the interrelation between fall risk appraisal, body composition, and objective measured PA. This cross-sectional study examines the feasibility of recruitment and acceptability of Assistive Health Technology (AHT), including the BTrackS Balance System (BBS), Bioelectrical Impedance Analysis (InBody s10), and ActiGraph GT9X Link wireless activity monitor. This study demonstrates the benefits of using AHT to study the associations among FRA, body composition, and PA in older adults. We hypothesize that rational FRA is associated with higher levels of PA and skeletal muscle mass and lower levels of percent of body fat and body mass index. Topics presentation included research protocol and preliminary results.

### Session 1105 (Symposium)

### NEW ISSUES IN LIFE COURSE RESEARCH: WHICH EARLY-LIFE FACTORS MATTER FOR LATE-LIFE OUTCOMES?

Chair: Jacqui Smith Discussant: Katrina Walsemann

The increased availability of retrospective information about the lives of participants in population panel studies has expanded the range of precursors to include in life course research. However, this also challenges researchers to select among many potential precursors to a late-life outcome and to determine the relative role of factors from different periods in the life course. Each paper in this symposium uses life course information from the Health and Retirement Study (HRS) to examine different late-life outcomes. Speakers will discuss what guided the particular selection of factors and outcome to examine in their study. Sonnega, Helppie-McFall, and Lee focus on indicators of childhood financial and social adversity as potential predictors of early retirement due to poor health. Park, Larkina, and Smith ask if decisions taken in early adulthood about how to balance work-and family-life by individuals and their partners are related to the categories of important life accomplishments older adults report in their life review. Two papers examine precursors of late-life health outcomes. Williams-Farrelly and Smith identified different profiles of physical activity in early- and mid-adulthood. They discuss associations between these profiles and cognitive aging. Whereas social losses, relocation, and multimorbidity are well-documented precursors of Major Depression in old age, Bergmans and Smith asked if poor health in childhood played a distal role. The session concludes with an integrative discussion of issues by Walsemann.

### LIFE COURSE ADVERSITY AND EARLY RETIREMENT DUE TO POOR HEALTH

Amanda Sonnega,[1] Brooke Helppie-McFall,[1] and Haena Lee,[2] *1. University of Michigan, Ann Arbor, Michigan, United States, 2. University of Southern California, Los Angeles, California, United States*

The relationship between life adversity and physical and mental health is well documented. The present research investigates life course adversity and early retirement due to poor health. Data are from the Health and Retirement Study, including the Life History Mail Survey (LHMS), HRS core surveys, and HRS Psychosocial Leave-Behind surveys. We create measures of childhood financial and social adversity and young-mid adulthood financial and social adversity. Early retirement is defined as self-report of "full" retirement between age 50 and 62. We use a Cox proportional hazards model competing risk framework comparing early retirement when poor health is a major reason for retirement with early retirement for any other reason. Models include covariates for age, gender, marital status, cohort, log household income, and log household wealth. Childhood financial adversity and homelessness in young-mid adulthood increases the instantaneous hazard of early retirement due to poor health.

### PERCEIVED ACCOMPLISHMENT IN LATER LIFE: THE INFLUENCE OF PAST DECISIONS BETWEEN FAMILY AND WORK

Jeongsoo Park,[1] Marina Larkina,[2] and Jacqui Smith,[3]
*1. Ajou University, Suwon-si, Kyonggi-do, Republic of Korea, 2. University of Michigan, Ann Arbor, Michigan, United States, 3. University of Michigan, University of Michigan, Michigan, United States*

Whereas previous studies have investigated life regrets, little attention has been paid to the important accomplishments older adults include in their autobiographic life narratives. Phenomenon such as the memory positivity effect suggest that accomplishments should be observed. We used a Health and Retirement Study 2017 Life History Mail Survey (N = 2,165) to examine the characteristics of participants over age 65 who reported accomplishments (max = 3), what was reported, and whether early-life decisions about balancing family and work are associated with the reports. Women, whites, and people with at least high school education and normal cognitive status were more likely to report accomplishments (67%). We categorized reports as family-related (39%), personal (19%), combined family/personal (25%) or other (16%). Multinominal logistic regression models revealed that participants who themselves favored family over work in early life or whose spouse decided for family, were more likely to report family-related accomplishments.

### DO LIFE COURSE PHYSICAL ACTIVITY PROFILES REDUCE THE EFFECTS OF CHILDHOOD EXPOSURES ON COGNITION?

Monica Williams-Farrelly, and Jacqui Smith, *University of Michigan, University of Michigan, Michigan, United States*

Although physical activity throughout life is one of the most reliable predictors of healthy aging, can less consistent or favorable trajectories also improve cognition trajectories among older adults? Drawing from accumulation theories, we use longitudinal data from the Health and Retirement Study and Life History Mail Survey (N=9,309) to examine the early antecedents of cognitive decline and the extent to which different life course physical activity profiles can slow such a decline. Results from latent class analysis reveal seven distinct profiles: consistently low, consistently high, consistently average (reference), improvers, decliners, midlife motivators, and previously athletic "couch potatoes." Growth curve modeling analyses show that membership in the consistently high class and midlife motivators were associated with better cognition initially and over time, with no difference between the two classes. Additionally, though poor health and learning problems in childhood were associated with worse initial cognition, physical activity does not mediate the relationship.

### ASSOCIATIONS OF MENTAL HEALTH AND CHRONIC PHYSICAL ILLNESS DURING CHILDHOOD WITH MAJOR DEPRESSION IN LATER LIFE

Rachel Bergmans, and Jacqui Smith, *University of Michigan, University of Michigan, Michigan, United States*

While poor health in childhood has implications for mental health years later, less is known regarding its long-term impact. We determined whether childhood chronic physical illness burden was associated with major depression (MD) in later life (i.e., >50 years), and tested mediation by childhood mental health status using path analysis. Data came from the 2016 U.S. Health and Retirement Study (n=18,047). One standard deviation increase in childhood chronic physical illness burden was associated with 1.21 (95% CI = 1.12, 1.30) times higher odds of MD in later life. Childhood mental health status explained 57.8% (95% CI: 35.2, 80.4) of this association. Results indicated that the relationship of chronic physical illness burden in childhood with MD in later life was mediated by childhood mental health status. Whether greater screening for psychiatric-related symptoms in childhood or review of health histories in later life can reduce the burden of MD requires further study.

### Session 1110 (Symposium)

### OPTIMIZING THE GERIATRIC MENTAL HEALTH WORKFORCE THROUGH INNOVATIVE APPROACHES

Chair: Ana Jessica Alfaro Co-Chair: Rachel Rodriguez
Discussant: Michele Karel

The drastic demand for geriatrics-trained providers in medical and mental healthcare persists years after the Institute of Medicine first highlighted this need (2008; 2012). New innovative approaches must instead optimize the current workforce through leveraging existing geriatric experts' knowledge and skills related to working aging adults. This symposium will highlight four approaches spanning post-licensure education to using technology to deliver specialized services and training. First, Dr. Gregg will discuss the evaluation of an advanced topics workshop in Geropsychology which has significantly enhanced depth of Geropsychology competencies for psychologists working in primarily rural areas. Next, Dr. Asghar-Ali will describe the multi-modal interactive geriatric educational opportunities for interprofessional staff developed by the South East Texas Geriatric Workforce Enhancement Program (SETx GWEP). He will discuss how these training opportunities have been tailored to address the impact of COVID-19 and healthcare disparities among older adults. Third, Dr. Filips will present an evaluation of a consultation model in which a geriatric psychiatrist provides tele-consultation in a 5-state region to rural aging Veterans with complex medical and behavioral comorbidities. Finally, Dr. Beaudreau will describe adaptations to a national VA Problem Solving Training program for mental health clinicians of older Veterans with complex comorbidities. Dr. Karel, VA National Geriatric Mental Health Director, will serve as discussant and comment on the ways in which these novel approaches are meeting the ever-growing need for competent geriatric mental health providers.

### VA PROBLEM-SOLVING TRAINING DURING COVID-19 FOR CLINICIANS OF PATIENTS WITH COMPLEX COMORBIDITIES

Sherry Beaudreau,[1] Marcela Otero,[2] Jessica Walker,[3] Christine Gould,[4] and Julie Wetherell,[5], *1. VA Palo Alto / Stanford University School of Medicine, VA Palo Alto, California, United States, 2. VA Palo Alto, Palo Alto, California, United States, 3. VA Central Office, Salisbury, North Carolina, United States, 4. VA Palo Alto Health Care System, Palo Alto, California, United States, 5. VA San Diego / UCSD, La Jolla Village, California, United States*

To address the shortage of mental health providers in geriatrics, VA has implemented clinician training in a VA Problem Solving Training (PST) protocol adapted to the needs of mostly older patients with complex comorbidities. This presentation will summarize PST implementation adaptations during COVID-19, and compare Veteran treatment outcomes before (2019) and during COVID-19 (2020). Sixty-one clinicians attended a workshop and small-group consultation for two training cases. Consultants provided ongoing feedback to program leadership about pandemic-related implementation challenges. Program adaptations during COVID-19 addressed challenges related to delivering treatment by telephone, video, or in-person and recruitment barriers. Veterans in both cohorts ($N = 122$) had significant reductions in mental health symptoms from baseline to posttreatment in paired t-test comparisons ($ps < .01$). Flexibilities afforded to clinicians in the training during the pandemic did not diminish the effectiveness of the intervention, thus supporting continued implementation of the training program with added flexibility.

### EVALUATION OF AN ADVANCED TOPICS WORKSHOP IN GEROPSYCHOLOGY: THE VALUE OF DEPTH IN TRAINING

Jeffrey Gregg,[1] Rachel Rodriguez,[1] Priyanka Mehta,[2] and Christine Gould,[3] *1. Durham VA Health Care System, Durham, North Carolina, United States, 2. VA Palo Alto GRECC, Palo Alto, California, United States, 3. VA Palo Alto Health Care System, Palo Alto, California, United States*

The Geriatric Scholars Program- Psychology Track (GSP-P) was implemented to address the dire shortage of mental health providers with geriatrics expertise within the VA, a large integrated healthcare system. One hundred and five psychologists participated in the GSP-P introductory geropsychology competencies course. Though they exhibited significant improvements in confidence, knowledge, and skills across geropsychology domains, increased depth (in addition to breadth) of training is needed. In 2019, GSP-P implemented an advanced workshop for graduates of the introductory course. Twenty-one psychologists participated in the workshop, which included 3.5 days of expert-led seminars followed by completion of an individualized learning plan over six months. Results from our evaluation indicated significant improvements in four of five geropsychology domains on the Pikes Peak Geropsychology Knowledge and Skill Assessment Tool. Our findings demonstrate continued enhancement of geropsychology competencies through advanced coursework is feasible and improves depth of training, particularly when combined with individualized learning plans.

### VIRTUAL GERIATRIC MENTAL HEALTH CARE OFFERED WHERE NEEDED MOST: AN EVALUATION OF A TELEHEALTH CONSULTATION MODEL

Julie Filips,[1] Chalise Carlson,[2] Ana Alfaro,[2] Ranak Trevedi,[3] Anita Savell,[4] and Christine Gould,[2] *1. VAMC, Minneapolis, MN, Minnesota, United States, 2. VA Palo Alto Health Care System, Palo Alto, California, United States, 3. VA Palo Alto Center for Innovation to Implementation, Menlo Park, California, United States, 4. CRH Operations for VISN 23, Minneapolis, Minnesota, United States*

Many VA facilities serving large rural populations do not have geriatric mental health specialists available to assist with managing the aging Veteran population's complex medical and behavioral comorbidities. We applied mixed-methods to evaluate an innovative model utilizing a geriatric psychiatrist who provides cross-facility consultation in a 5-state region. During a 3-month period, the consultant completed 135 consults and 20 e-consults to settings ranging from outpatient to long-term care. Leadership stakeholder and provider interviews highlight the importance of the availability of the consultant, collaboration with local care teams, staff education, person-centered approach, and work ethic/passion. The core challenges that the consultant helps manage include complex comorbidities, medication questions, and dementia with behavioral disturbance. Initial provider survey responses ($n = 11$) show high satisfaction with services (100%) and strong agreement (80%) that providers could follow through with recommendations. Next steps include replication of this model in other VA facilities.

### THE SOUTH EAST TEXAS GERIATRIC WORKFORCE ENHANCEMENT PROGRAM: REACH, TEACH, INNOVATE

Ali Asghar-Ali, *Baylor College of Medicine, Michael E. DeBakey VA Medical Center/Houston, Texas, United States*

Through collaboration between academic and community partners, the South East Texas Geriatric Workforce Enhancement Program (SETx GWEP) aims to promulgate the 4Ms framework via a range of educational initiatives. The faculty and audience is interprofessional and diverse, representing the residents of South East Texas. Specific initiatives focus on Alzheimer's disease and related dementias, elder abuse, geriatric mental health, patient priorities, transitions of care, and geriatric dental care. Training modalities include online modules, Project ECHO sessions, webinars, discussion forums, and simulation. During the COVID19 pandemic the SETx GWEP adapted to meet the needs of its stakeholders, including increasing the number of online activities, hosting town hall meetings, and developing training to address the impact of COVID19 on the older adult population. The SETxGWEP trained over 1000 people in 2020. To address healthcare disparities among older adults, SETx GWEP developed training on the practice of cultural humility in older adult care.

### Session 1115 (Symposium)

### ORAL HEALTH AND DENTAL CARE AMONG OLDER ADULTS IN CHINA: WHAT ARE THE RISK AND PROTECTIVE FACTORS?

Chair: Weiyu Mao Co-Chair: Yaolin Pei Discussant: Huabin Luo

Oral health is a global public health concern. The four papers in this symposium capture various understudied risk and protective factors in oral health and dental care among older adults in China. The first paper examined the relationship between social isolation, loneliness, and tooth loss among Chinese older adults, using the Chinese Longitudinal Healthy Longevity Survey. The findings suggest that higher levels of social isolation, rather than loneliness, were associated with fewer remaining teeth and accelerated tooth loss over time. The second paper investigated urban-rural disparities in dental care utilization among Chinese adults aged 18 to 65 years old, using the 2019 New Era and Living Conditions in Megacities Survey. The findings demonstrate urban residents were more likely to visit dentists than rural residents. Besides socioeconomic status, health attitudes/behaviors, and oral health needs, health insurance coverage was considered an important enabling factor to promote dental care use in this population. The third paper examined the relationship between denture use and cognitive decline among Chinese older adults, using data from the Chinese Longitudinal Healthy Longevity Survey. The findings indicate that denture use is protective against cognitive decline over time in later life. The fourth paper examined the prevalence of self-reported orofacial pain symptoms and their correlates at the last year of life among Chinese older adults. Low socioeconomic status, smoking, chronic conditions, oral hygiene practice, and natural teeth condition were associated with such symptoms. This symposium offers valuable insights to improve oral health and dental care in older adults in China.

### SOCIAL ISOLATION AND ACCELERATED TOOTH LOSS AMONG CHINESE OLDER ADULTS

Xiang Qi,[1] Yaolin Pei,[2] Katherine Wang,[3] Shuyu Han,[4] and Bei Wu,[2] *1. Rory Meyers College of nursing, New York, New York, United States, 2. New York University, New York, New York, United States, 3. Duke University, Durham, North Carolina, United States, 4. Fudan University, Shanghai, Shanghai, China (People's Republic)*

Social isolation and loneliness in older adults are major global public health concerns. Tooth loss is also a common problem in this population. This study examined the effects of social isolation and loneliness on the number of remaining teeth and the rate of tooth loss among Chinese older adults. We included 4,268 older adults age 65+ from three waves of the Chinese Longitudinal Healthy Longevity Survey (2011/2012, 2014, 2018). Linear mixed-effect models showed higher levels of social isolation were associated with fewer remaining teeth ($\beta$ = -1.59, P < 0.05) and accelerated tooth loss ($\beta$=-0.10, P<0.05) controlled for sociodemographic, lifestyle, oral hygiene behavior, and health status. Loneliness was neither associated with the number of remaining teeth ($\beta$=0.64, P>0.05) nor with the rate of tooth loss ($\beta$=-0.09, P>0.05) before and after controlling for covariates. These findings expand our knowledge regarding the correlation between social connection and tooth loss in non-Western populations.

### DISPARITIES IN DENTAL SERVICES USE AMONG URBAN AND RURAL ADULTS IN CHINA'S MEGACITIES

Xiaomin Qu,[1] Xiang Qi,[2] and Bei Wu,[3], *1. East China University of Political Science and Law, East China University of Political Science and Law, Shanghai, China (People's Republic), 2. Rory Meyers College of nursing, New York, New York, United States, 3. New York University, New York, New York, United States*

Using data from the '2019 New Era and Living Conditions in Megacities Survey' that included 4,049 residents aged 18-65, we examined the urban-rural disparities in dental visits among adults living in China's 10 megacities. All of China's megacities are metropolitan regions that include urban, peri-urban and rural land, and all have rural populations within the city boundaries. The results show that 43.3% (n=595) rural and 23.8% urban (n=637) residents had never visited dentists. Urban residents were more likely to visit dentists than rural residents after controlling for covariates (OR=1.57, 95%CI=1.30 to 1.91). The rates of visits were similar across age groups. Higher socioeconomic status, having urban insurances, having positive attitudes towards healthy diets and visited physicians regularly, and having poorer oral health was associated with higher odds of visiting dentists (P<0.05). These findings can help develop policies to increase dental care access to underserved populations in Chinese megacities.

### DENTURE USE AND COGNITIVE DECLINE IN CHINESE OLDER ADULTS: A PROPENSITY SCORE ANALYSIS

Bei Wu,[1] and Xiang Qi,[2] *1. New York University, New York, New York, United States, 2. Rory Meyers College of nursing, New York, New York, United States*

Using data from the Chinese Longitudinal Healthy Longevity Survey (2011 to 2018), we examined the effect of denture use on cognitive decline (assessed by the Mini-Mental State Examination [MMSE]) among 1,316 cognitively normal older adults with severe tooth loss (≤9 remaining teeth) at baseline. We generated propensity scores for weighted and matched analyses using 18 covariates, classified as socio-demographics, health-related behaviors, health status, and oral health conditions. The results show that non-denture users had worse cognitive decline than denture users. In the kernel-based matched data, the difference in the declined score of cognitive function between denture and non-denture users was 2.25 (95%CI=1.37 to 3.13). In the weighted data, the difference in cognitive function score was 2.14 (95% CI=1.35 to 2.94). Using dentures is beneficial for cognitive health in older adults with severe tooth loss, suggesting that prosthodontic rehabilitation with dentures might have benefits beyond restoring oral functioning.

### OROFACIAL PAIN SYMPTOMS AMONG CHINESE OLDER ADULTS IN THE LAST YEAR OF LIFE

Yaolin Pei,[1] Xiang Qi,[2] Xi Chen,[3] and Bei Wu,[1]
*1. New York University, New York, New York, United States, 2. Rory Meyers College of nursing, New York, New York, United States, 3. University of Iowa, Iowa City, Iowa, United States*

The aims of this study were to examine the prevalence of orofacial pain symptoms and its associated factors in Chinese older adults in the last year of life. We retrospectively followed 1,646 participants (60 years or older) in the last year of life to death from the Chinese Longitudinal Healthy Longevity Survey (CLHLS). The 6-month prevalence of toothache and jaw joint pain or facial pain for older adults in the last year of life to death were 14.1% and 4.5%, respectively. Older adults who had lower socioeconomic status, were smokers, and had any chronic disease tended to have orofacial pain symptoms. This study generated interesting but counterintutive findings that Chinese older adults who brusehed their teeth at least daily and those who had at least one natural teeth were more likely to have orofacial pain. It is important to include dental care as a part of end-of-life medical treatment.

### Session 1120 (Symposium)

### PRESIDENTIAL SYMPOSIUM: PHYSIOLOGICAL VS. MOLECULAR CLOCKS, STUDIES FROM MICE TO HUMANS

Chair: Blanka Rogina

Aging is associated with a functional decline in metabolic, physiological, proliferative, and tissue homeostasis leading to deterioration at the organismal level, and an increased risk for disease and death. Genetic, pharmacological and nutritional interventions have been successfully used to preserve metabolic health, which leads to preserved healthspan and extended longevity. However, the rate at which animals in a population become impaired by age-related frailty and disease is highly variable and several aging clocks that measure different age-modulated processes in the organism are being use as potential markers of the rate of aging. These molecular clocks allow to a more accurate quantification of the biological age of animals. Nevertheless, there is still room for further discussion in terms of the strengths and weaknesses of these biomarkers, in order to probe their biological significance, cellular mechanisms, and epidemiological potential to further explore their long-term benefit of increasing healthspan. This symposium will discuss new approaches to delineate physiological versus molecular clocks based on studies in mice and humans. We will also discuss species-specific metabolic mechanisms based on longitudinal studies in mice, monkeys and humans.

### INTEGRATIVE ANALYSIS OF MOLECULAR AND PHYSIOLOGICAL DATA TO QUANTIFY BIOLOGICAL AGING

Daniel Belsky, *Columbia University Mailman School of Public Health, New York, New York, United States*

Development of reliable and valid measurements to quantify biological aging is a critical frontier geroscience. Originating in accumulations of molecular changes, biological aging undermines resilience within cellular networks and organ systems, driving disease, disability, and mortality. Measurements of biological aging have been proposed at several molecular and physiological levels of analysis. But agreement between measures implemented at different levels of analysis is low. The timing at which aging processes manifest at different levels of biological organization may vary, with the result that signs of aging manifest in one level of analysis are not yet observable in another. And different aging processes may be most apparent in different molecular levels of analysis. In midlife humans, aging-related changes are manifest at multiple molecular and physiological levels, making this population ideal for development of measurements that integrate information across levels of analysis to more precisely quantify the state and pace of biological aging.

### AGING ACROSS THE LIFESPAN: RETHINKING THE DAVID BARKER HYPOTHESIS

Luigi Ferrucci, *National Institute on Aging, Baltimore, Maryland, United States*

For many years, the scope of geriatric medicine has been the care of older persons affected by multiple disease with the aim of improving their functional status and optimize quality of life. As our knowledge of the mechanisms of aging grows rapidly, it is becoming clear that accomplishing this scope requires taking a life-course perspective. Research have failed to establish a clear-cut distinction between normal aging and pathology leading to the hypothesis that aging is at the root of chronic diseases, and difference in health can be ascribed to different aging rates. Research in model organisms, suggest that aging can be modified with consequent changes on healthspan and longevity. Interventions that modulate aging may ultimately prevent most-age-associated diseases and their consequences. From this perspective, geriatric medicine is the leading and most promising branch of biomedical science. Challenges remain: first, demonstrating that certain interventions slow down the aging rate requires the valid measure of aging, and while many tools were developed, “epigenetic clocks” being the most promising, the underline mechanism that drive their changes with aging and validity in clinical applications are unclear. We do not know whether variability in the rate of biological aging assessed in old age are already detectable in younger individuals and person-specific rates remain constant during life. In 1986, David Barker stated the hypothesis that the period of gestation, characterized by a strong epigenetic imprinting, affects health and wellbeing across life, perhaps by setting the aging rate. Perhaps pediatric and geriatric medicine are more connected than previously believed.

### EVOLUTION OF NATURAL LIFESPAN VARIATION AND MOLECULAR STRATEGIES OF EXTENDED LIFESPAN

Alaattin Kaya, *Virginia Commonwealth University, Richmond, Virginia, United States*

To understand the genetic basis and the selective forces acting on longevity, it is useful to employ ecologically diverse individuals of the same species, widely different in lifespan. This way, we may capture the experiment of Nature that modifies the genotype arriving at different lifespans. Here, we analyzed 76 ecologically diverse wild yeast isolates and discovered wide diversity of lifespan. We sequenced the genomes of these organisms and analyzed how their replicative lifespan is shaped by nutrients and transcriptional and metabolite patterns. By identifying genes, proteins and metabolites that correlate with longevity across these isolates, we found that long-lived strains elevate intermediary metabolites, differentially regulate genes involved in NAD metabolism and adjust control of epigenetic landscape through conserved, rare histone modifier. Our data further offer insights into the evolution and mechanisms by which caloric restriction regulates lifespan by modulating the availability of nutrients without decreasing fitness.

### FRAILTY IN PRECLINICAL MODELS: IMPACT OF HEALTHSPAN ON DISEASE EXPRESSION OVER THE LIFE COURSE

Susan Howlett, *Dalhousie University, Halifax, Nova Scotia, Canada*

People age at different rates. This heterogeneity in aging has led to the concept of “frailty”, a state of heightened vulnerability to adverse health outcomes at any age. Frailty challenges health care providers, as frail patients are more likely than non-frail patients to experience diseases, hospitalization, and death. We showed that frailty occurs not only in humans, but also in aging rodents. It can be measured with a “frailty index” (FI) based on age-related health deficit accumulation as originally established in humans. We found that maladaptive changes in heart structure and function in late life are correlated more so with frailty than age and are closely graded by FI score, especially in male mice. Adverse effects of frailty originate at cellular/subcellular levels and scale up to organ and system levels, predisposing towards cardiovascular disease. Poor overall health, quantified with an FI, may drive maladaptive cardiac remodeling, especially in older males.

### Session 1125 (Symposium)

### RECRUITING ELIGIBLE AND INTERESTED STUDY PARTICIPANTS WITH COGNITIVE IMPAIRMENT

Chair: Meghan Mattos Discussant: Jennifer Lingler

Recruiting and enrolling older adults with cognitive impairment is challenging under the best of circumstances. This symposium will begin with an introduction to best practices for recruitment of older adults living with cognitive impairment, followed by four presentations describing recruitment successes and challenges across multiple settings. The first presentation describes COVID-19 pandemic-related factors that have influenced recruitment and enrollment of older adults with cognitive impairment in an intervention study of a physical activity smartphone app. Strategies and procedural alterations to facilitate achievement of enrollment goals for technology-based interventions are discussed. The second presentation describes researchers’ recruiting experiences with older adults with mild cognitive impairment (oaMCI)-care partner dyads for a pilot, platform trial of biopsychosocial interventions. There were differences in study disinterest between oaMCI and study partners that may require specialized communication messaging and strategies for dyad engagement. The third presentation features recruitment adaptations for an Internet-delivered behavioral intervention study with oaMCI and insomnia. Anticipated concerns of oaMCI using technology or accessing the Internet were not significant barriers to recruitment, while fewer oaMCI endorsed sleep concerns than expected. The last presentation demonstrates the potential for telephone-based outreach to increase dementia knowledge and cognitive risk. Working with faith-based health educators to reach rural, ethnically-diverse older adults, researchers will describe how to promote inclusivity and successfully recruit oaMCI within the community. Presenters and participants are encouraged to dialogue on how recruitment and retention barriers may be avoided as well as to share success stories from their own research with oaMCI.

### LESSONS LEARNED FROM CLINICAL RECRUITMENT OF OLDER ADULTS WITH MCI FOR AN INTERNET-DELIVERED INTERVENTION STUDY

Meghan Mattos,[1] Mark Quigg,[2] Carol Manning,[3] Eric Davis,[3] Ann Sollinger,[4] Laura Barnes,[5] and Lee Ritterband,[3] *1. University of Virginia, School of Nursing, Charlottesville, Virginia, United States, 2. University of Virginia, Charlottesville, Virginia, United States, 3. University of Virginia, School of Medicine, Charlottesville, Virginia, United States, 4. Carilion Clinic, Roanoke, Virginia, United States, 5. University of Virginia, School of Engineering & Applied Science, Charlottesville, Virginia, United States*

Clinical research involving participants with mild cognitive impairment (MCI) presents challenges to recruitment that may be further compounded by concerns when delivering a behavioral intervention via the Internet. The purpose of this talk is to describe recruitment adaptations for an Internet-delivered behavioral intervention study with older adults living with MCI and insomnia. Over the course of study recruitment, unforeseen barriers to recruitment were discovered, including fewer older adults with MCI endorsing sleep concerns than expected. The most substantive changes made to improve clinical recruitment were related to eligibility criteria, yielding 50% of the overall sample. Anticipated concerns of older adults with MCI using technology or accessing the Internet were not significant barriers to recruitment. Study findings support Internet-delivered intervention use in this population, which in the context of the COVID-19 pandemic, presents a potentially efficient and effective method for recruiting and delivering behavioral interventions in this difficult-to-enroll population.

### THE DYAD DILEMMA: STRATEGIES TO RECRUIT STUDY PARTNERS FOR MILD COGNITIVE IMPAIRMENT CLINICAL TRIALS

Allison Gibson,[1] Caitlin Pope,[2] Elizabeth Rhodus,[2] Kelly Parsons,[2] Julia Johnson,[2] Dawn Oaks,[3] and Greg Jicha,[3] *1. University of Kentucky, University of Kentucky, Kentucky, United States, 2. University of Kentucky, Lexington, Kentucky, United States, 3. University Of Kentucky, Sanders-Brown Center On Aging, Lexington, Kentucky, United States*

Mild cognitive impairment (MCI) research faces challenges to successful enrollment, especially with clinical trial studies. This study explores researchers' experiences recruiting from a U.S. Alzheimer's Disease Center for a pilot, platform trial of biopsychosocial interventions for MCI dyads. Individuals with MCI that met the inclusion criteria for the study were invited to participate (n=39). Thematic analysis of recruitment case notes was utilized to track participants' and study partners' interest in participation. In most cases, participants with MCI were interested and willing to enroll and study partners were not. Recruiting persons with MCI and their study partners for clinical trials research may require specialized communication messaging such as education about how interventions address the needs of MCI, along with training on the relationship of MCI to cognitive decline. This presentation highlights effective strategies to engage study partners into recruitment for MCI research such as creating more flexible participation roles and offerings.

### RECRUITING OLDER ADULTS WITH COGNITIVE IMPAIRMENTS FOR A SMARTPHONE INTERVENTION DURING COVID-19

Stacey Schepens Niemiec,[1] Lon Schneider,[1] Jeanine Blanchard,[1] Rafael Wagas,[2] Liberty Teodoro,[2] and Karen Dagerman,[2] *1. University of Southern California, Los Angeles, California, United States, 2. University of Southern California, los angeles, California, United States*

The Coronavirus disease 2019 (COVID-19) pandemic has introduced numerous challenges to the global scientific community and has been particularly disruptive to the conduct of ongoing clinical trials. Gerontological studies that focus on older adults with cognitive impairments have endured additional challenges ranging from increased vulnerability of this group to COVID-19, thereby prohibiting study participation, to difficulties in participant engagement as a product of a worsening Digital Divide. The purpose of this talk is to describe the pandemic-related factors that have influenced recruitment and enrollment of older adults with mild cognitive impairment and mild dementia in an ongoing feasibility study of a physical activity smartphone app. We discuss the changes we made to recruitment procedures and the impact those changes have had on the success of enrolling individuals in the study. We conclude with a discussion of feasible strategies and procedural alterations moving forward that may facilitate achievement of enrollment goals.

### PROMOTING INCLUSIVITY IN DEMENTIA EDUCATION AND SCREENING IN AN ETHNICALLY DIVERSE, RURAL COMMUNITY DURING A PANDEMIC

Lisa Wiese,[1] Ishan Williams,[2] Nancy Schoenberg,[3] James Galvin,[4] and Jennifer Lingler,[5] *1. Florida Atlantic University, C.E. Lynn College of Nursing, Florida, United States, 2. University of Virginia, Charlottesville, Virginia, United States, 3. University of Kentucky, Lexington, Kentucky, United States, 4. University of Miami Miller School of Medicine, Miami, Florida, United States, 5. University of Pittsburgh, Pittsburgh, Virginia, United States*

Rural, ethnically diverse older adults experience disparities in dementia detection/management. The Covid-19 quarantine exacerbated these disparities, and threatened faith-based dementia education and screening activities. We investigated the effectiveness of a telephone-based outreach for increasing dementia knowledge and detecting cognitive risk among a rural, diverse, underserved community of 89% African American, Hispanic, and Haitian Creole residents, Faith-based health educators, trained using virtual Alzheimer's Association resources, contacted church congregants who responded to radio worship service announcements. Participants completed telephone measures of basic dementia knowledge and cognitive risk. Of the estimated 120 persons across five churches who received an invitation, 75% (n = 90) participated in dementia education and memory screening via telephone. Twelve (80%) of the 15 participants assessed as being at risk followed up with their provider. Rural residents are known for preferring face-to-face contact. Their willingness to complete health-promoting research activities by telephone highlighted the community's interest in dementia awareness.

### Session 1130 (Symposium)

### SLEEP AND HEALTH IN OLDER ADULTS: NEW RESEARCH FROM EARLY-CAREER INVESTIGATORS

Chair: Christopher Kaufmann Discussant: Amy Berkley

Sleep and circadian patterns change as people age and are linked to a number of health and psychosocial outcomes. As such, there is a need to continue generating new knowledge about sleep in older adults by encouraging early-career scientists to research this topic. In this symposium, sponsored by the Sleep, Circadian Rhythms and Aging Interest Group, we will showcase studies by early-career researchers at the masters through junior faculty level who conduct work in sleep and its impact on health outcomes in older adults. Our symposium will have five presentations. The first will examine how sleep and loneliness may mediate relationships between marital quality and depressive symptoms. The second study will assess links between personality characteristics and objectively measured chronotype. Our third presentation will determine the longitudinal association of sleep duration with body mass index. The fourth will evaluate how an intervention to reduce functional disability in low-income older adults impacts sleep quality. Finally, our fifth presentation will focus on understanding how sleep duration and changes in sleep patterns may impact speech-in-noise performance. Overall, our symposium will highlight multidisciplinary studies of sleep and health outcomes that are of importance to older populations and promote the work of the next generation of sleep, circadian rhythms, and aging scientists.

### SLEEP AND LONELINESS AS MECHANISMS THROUGH WHICH MARITAL QUALITY PREDICTS DEPRESSION AMONG OLDER ADULTS

Christina Marini,[1] Lynn Martire,[2] and Orfeu Buxton,[3] *1. Adelphi University, Garden City, New York, United States, 2. The Pennsylvania State University, University Park, Pennsylvania, United States, 3. The Pennsylvania State University, The Pennsylvania State University, Pennsylvania, United States*

Pathways through which spousal support and strain influence older adults' well-being are poorly understood.

We examined sleep quality and loneliness as mechanisms through which support and strain predict depressive symptoms across ten years utilizing National Social Life, Health, and Aging Project data. Our sample included partnered participants at waves 1 and 2 (N = 1,293; 39% female, M age = 66, SD = 6.93). Support (e.g., rely on spouse) and strain (e.g., spouse criticizes you) were measured at W1, loneliness (UCLA) and sleep quality (restless sleep) were measured at W2, and depression (CES-D) was measured at W3. We estimated latent-variable structural equation models, controlling for age, gender, and W1 depression. Indirect effects of support and strain on depressive symptoms through loneliness were significant. There was an additional trend-level indirect effect of spousal strain on depressive symptoms through restless sleep. Findings highlight multiple pathways through which marital quality predicts later-life well-being.

### LINKS BETWEEN PERSONALITY AND SLEEP MIDPOINT IN OLDER ADULTS IN THE NATIONAL SOCIAL LIFE, HEALTH, AND AGING PROJECT

Krishna Patel,[1] Darlynn Rojo-Wissar,[2] Katherine Duggan,[3] Garrett Hisler,[4] Brant Hasler,[4] and Adam Spira,[5], *1. Johns Hopkins Bloomberg School of Public Health, Johns Hopkins Bloomberg School of Public Health, Maryland, United States, 2. Department of Mental Health, Johns Hopkins Bloomberg School of Public Health, Baltimore, Maryland, United States, 3. Department of Psychology, North Dakota State University, Fargo, North Dakota, United States, 4. Department of Psychiatry, University of Pittsburgh, Pittsburgh, Pennsylvania, United States, 5. Johns Hopkins Bloomberg School of Public Health, Baltimore, Maryland, United States*

Chronotype has been linked to poor cognitive outcomes and mortality among older adults. Although previous studies indicate an association between personality and sleep, little is known about associations between personality and chronotype in older adults. We examined the association between personality and objective sleep midpoint (a measure of chronotype) in 463 older adults aged 73.5 ±7.7 from the National Social Life, Health, and Aging Project who completed the Midlife Developmental Inventory Personality scale and three nights of wrist actigraphy, from which we derived participants' average sleep midpoints. After adjusting for demographics, higher conscientiousness was associated with earlier sleep midpoint (B=-0.53, SE=0.02, p<0.01). Associations for other traits were not significant. Findings link conscientiousness to chronotype and raise the possibility that earlier sleep timing may partially account for associations of conscientiousness with health outcomes. Further studies are needed investigating the role of personality in links of sleep and circadian factors with health.

### HABITUAL SLEEP, SLEEP DURATION DIFFERENTIAL, AND WEIGHT CHANGE AMONG ADULTS

Yin Liu,[1] Mari Palta,[2] Jodi Barnet,[2] Max Roberts,[1] Erika Hagen,[2] Paul Peppard,[2] and Eric Reither,[1] *1. Utah State University, Logan, Utah, United States, 2. University of Wisconsin-Madison, Madison, Wisconsin, United States*

We assessed longitudinal associations between diary-measured sleep duration and clinically assessed body mass index (BMI) among 784 men and women enrolled in the Wisconsin Sleep Cohort Study (mean [SD] age = 51.1 [8.0] years at baseline). The outcome was BMI (kg/m2). Key predictors were habitual sleep duration (defined as average weekday nighttime sleep duration) and sleep duration differential (defined as the difference between average weekday and average weekend nighttime sleep duration) at each data collection wave. Men with shorter habitual sleep duration on weekdays had higher BMI than men with longer habitual sleep duration on weekdays. Participants with larger differentials between weekday and weekend sleep duration experienced more rapid BMI gain over time for both men and women. Inadequate sleep, characterized as shorter habitual sleep during weekdays and larger weekday-weekend sleep differential, is positively associated with BMI levels and trajectories among men and women in mid-to-late life.

### EFFECT OF A BIOBEHAVIORAL ENVIRONMENTAL APPROACH ON SLEEP IN LOW-INCOME OLDER ADULTS

Junxin Li,[1] Safiyyah Okoye,[1] Lena Sciarratta,[1] and Sarah Szanton,[2], *1. Johns Hopkins University, Baltimore, Maryland, United States, 2. Johns Hopkins School of Nursing, Baltimore, Maryland, United States*

Low socioeconomic status and disability are independent risk factors for disturbed sleep. The CAPABLE intervention used a multidisciplinary team approach of occupational therapist, nurse, and handyworker to reduce functional disability in low-income older adults. The 6-month intervention may benefit sleep as the intervention addressed multiple individual factors associated with sleep quality, including pain, depression, communication, mobility, strength, and balance. This study examined the effect of the CAPABLE intervention on actigraphy-measured sleep in a sub-sample of 73 older adults from the CAPABLE trial (26 intervention vs. 47 control). The sample was aged 75.8±7.45 years, 86.3% female, and 84.9% African American. No significant group differences in sleep parameters were found at 6-month, controlling for baseline values. The intervention resulted in a 5.56% increase in sleep efficiency (95% CI= [1.39, 9.71], Cohen's d=0.54), and 7.39 minutes decrease in sleep onset latency (95% CI= [0.10, 14.5], Cohen's d=0.41) within the intervention group at 6-months.

### ASSOCIATIONS OF HABITUAL SLEEP DURATION AND SLEEP STAGES WITH SPEECH-IN-NOISE PERFORMANCE

Kening Jiang,[1] Adam Spira,[2] Kelsie Full,[3] Emmanuel Garcia,[1] Frank Lin,[4] Nicholas Reed,[2] Pamela Lutsey,[5] and Jennifer Deal,[6] *1. Johns Hopkins Cochlear Center for Hearing and Public Health, Baltimore, Maryland, United States, 2. Johns Hopkins Bloomberg School of Public Health, Baltimore, Maryland, United States, 3. Division of Epidemiology and Community Health, University of Minnesota, Minneapolis, Minnesota, United States, 4. Johns Hopkins University, Johns Hopkins University, Maryland, United States, 5. School of Public Health, University of Minnesota, Minneapolis, Minnesota, United States, 6. Johns Hopkins University, Baltimore, Maryland, United States*

Speech-in-noise performance involves central auditory and cortical processing and is fundamental to communication. We investigated cross-temporal associations of habitual sleep duration and stages (1996-1998) with speech-in-noise performance (2016-2017) in a subset of the Atherosclerosis Risk in Communities Study participated in the Sleep Heart Health Study(N=755, 61±5 years, 53% female). Speech-in-noise performance was measured by Quick Speech-in-Noise Test; range:0-30; lower scores=worse performance. Time spent in each stage (stage 1;2;3/4;rapid eye movement (REM)) was measured by polysomnography. Habitual sleep duration was calculated by self-reported duration on weekdays and weekends. In models adjusting for demographic and disease covariates, every 10-minute increase in REM sleep was associated with better speech-in-noise performance (0.10 points,95% CI:0.00,0.21); every 1-hour increase in habitual sleep duration was associated with worse speech-in-noise performance (-1.28 points,95% CI:-2.49,-0.08) among participants sleep >8 hours. Long sleep duration might be a risk marker of speech-in-noise performance, but REM sleep might be a protective factor.

### Session 1135 (Symposium)

### SOCIAL CONNECTION IN TIMES OF PHYSICAL DISTANCING

Chair: Jeongeun Lee

COVID-19 has been especially devastating to older adults. To prevent the spread of the virus, physical distancing has become the norm. As a result, there are fewer opportunities available for face-to-face interaction and social activities, which may be particularly harmful to older adults, given their existing loneliness levels. Thus, this symposium brings together a collection of papers that exemplify the interplay of social connection, activities, and mental health outcomes among older adults facing loneliness. The first paper will discuss how activity diversity is linked to higher loneliness and depressive symptoms among heterosexual and LGBTQ older adults. The second paper will present findings on the changes in social connectedness due to physical distancing and their associated impact on the mental health outcomes among older adults. The third paper will present qualitative findings on the effect of physical distancing on older adults' social connectedness using a mixed-method study. The final paper discusses the challenges faced by older adults in their use of digital media for social connection in the present pandemic and highlights some of the population's untapped strengths, which can be leveraged to help them live prosperous online lives. All papers will address practical tips and recommendations for actions, which key stakeholders can take to support older adults during the pandemic. The discussant, Dr. Kahana, will integrate the four papers and highlight the potential and limits of the current effort to address these issues and consider future inquiry routes.

### DIVERSITY OF ACTIVITIES AND LONELINESS AMONG HETEROSEXUAL AND LGBT OLDER ADULTS

Jeongeun Lee, and Joseph Svec, *Iowa State University, Ames, Iowa, United States*

The extant literature highlights the physiological and psychological benefits of active lifestyles among older adults, though there is a considerable gap in scholarship for sexual minority groups. Utilizing the Social Integration Model, we hypothesize that social activities enhance individual psychological well-being, but those effects differ by one's social identities. Using a national AARP foundation survey of adults (45+), this study examines whether individuals' activities predict loneliness and depressive symptoms of heterosexual (n=2905) and LGBTQ adults (n=318). We utilize an index of diverse activities, which includes, social technology use, meeting with friends, and volunteer activities. Multiple linear regression is used to study cross-sectional associations of loneliness and depressive symptoms on the diverse activity index. Results show that a wider array of activities correspond with higher psychological well-being and lower loneliness, and this association is higher for LGBTQ older adults. We discuss implications for counseling and wellness programming for LGBT older adults.

### CAN WE REPLACE OUR HUMAN CONNECTION WITH TECHNOLOGY?

Nicholas Cone,[1] and Jeongeun Lee,[2] *1. Iowa State University, Iowa State University, Iowa, United States, 2. Iowa State University, Ames, Iowa, United States*

The COVID-19 pandemic has led to social distancing protocols, subsequently increasing social isolation for older adults. The purpose of this study was to explore the relationship between social connectedness and mental health outcomes. Leveraging NHATS, a nationally representative study (n = 2,558, Mage = 79.20, SDage = 6.25), we examined the association between the method of social connectedness and mental health outcomes. Descriptive analyses revealed older adults are using various methods (e.g., in-person, phone, and video calls) to remain connected with their social networks during COVID-19. Findings from all of the linear regression analyses indicated phone or video calls are associated with negative affect, whereas in-person visits are associated with lower levels of negative affect. These findings suggest substituting in-person visits with video calls or phones may not be sufficient to relieve their loneliness and negative affect. Future studies should investigate this effect on physical or emotional health outcomes.

### SOCIAL CONNECTION PLANNING FOR LONELY OR SOCIALLY ISOLATED OLDER ADULTS

Emily Bower,[1] Aurora Newman,[1] Paige Reohr,[1] and Kimberly Vandorden,[2] *1. Pacific University, Hillsboro, Oregon, United States, 2. University of Rochester Medical Center, Rochester, New York, United States*

Social connections are important for maintaining health and well-being with age. Behavioral interventions to promote connectedness hold promise, but there is limited evidence to guide effective modifications in the context of physical distancing or quarantine restrictions, such as those required during the COVID-19 pandemic. We present evidence for a brief (1-2 session) social connection intervention, "Connections Planning," to enhance social connectedness for older adults. We first describe a cognitive-behavioral model of loneliness, which served as the framework for developing the intervention. We then present two case examples to demonstrate the application of the intervention with older adults in a community mental health clinic during physical distancing restrictions. Finally, we present initial findings from a pilot study to examine the feasibility and acceptability of the intervention delivered remotely with up to 10 community-dwelling older adults who endorse clinically significant loneliness. Recommendations for adapting the intervention during physical distancing restrictions are provided.

### PHYSICAL DISTANCING AND SOCIAL CONNECTEDNESS AMONG OLDER ADULTS

Julie Lutz,[1] Emily Bower,[2] Ellen Beckwith,[1] Julie Choi,[1] and Kim Van Orden,[3], *1. University of Rochester Medical Center, Rochester, New York, United States, 2. Pacific University, Hillsboro, Oregon, United States, 3. University of Rochester School of Medicine & Dentistry, Rochester, New York, United States*

The COVID-19 pandemic has significantly impacted older adults; due to elevated risk, many older adults have followed physical distancing guidelines. These efforts, while critical to public health, have also impacted the social interactions and connectedness of older adults. In this mixed-methods study, we conducted qualitative interviews and administered questionnaires to 23 adults age 60 and older to examine how physical distancing has affected their social connectedness; what strategies and supports they have utilized to maintain or improve social connectedness despite physical distancing; and what types of supports, programs, and interventions they feel could promote and foster social connectedness among older adults during physical distancing. The results may have implications not only for the pandemic, but also for older adults who cannot leave their homes or experience barriers to typical social activities for any reason (e.g., being homebound, having functional impairments).

### Session 1140 (Paper)

### SOCIAL ISOLATION AND LONELINESS (SRPP PAPER)

### COVID-19 AND OLDER ADULTS: COPING WITH LONG-TERM PANDEMIC PRECAUTIONS

Kerstin Emerson, Deborah Kim, George Mois, and Jenay Beer, *University of Georgia, Athens, Georgia, United States*

Studies conducted at the beginning of Covid-19 precautions suggested that older adults were stressed, but hopeful. Less is known how coping has changed for older adults after experiencing months-long pandemic precautions. We explore differences in coping between the initial pandemic declaration in March 2020, and 9 months later, via an internet survey fielded in November 2020 (n= 781). We present summary data, using chi-square tests for subgroup analyses. A majority of respondents (aged M=66 yrs, range 60-89) were women (64%) and White (94%). When asked to compare their feelings to the beginning of the pandemic, 44.8% were more frustrated, 38.7% were more stressed, and 32.7% were more anxious. However, 38.3% were more appreciative. Women were significantly more likely than men to report increases in feeling frustrated, angry, scared, stressed, sad, and hopeless. Introverts were significantly more likely than extroverts to report an increase in loneliness and stress. Since the first few weeks of the pandemic, respondents reported more communication through video calls (45.2%), texting (40.2%), and phone calls (28.8%). Additionally, 61.5% spent more time on computers/tablets, 47.2% spent more time watching TV, and 24.5% spent more time praying. Extroverts were significantly more likely than introverts to report an increase in time with TV, phones, and computers/tablets. Women were significantly more likely than men to report increased texting and praying. These data provide further understanding of the impact of long-term pandemic precautions on older adults and suggest particular subgroups of older adults may benefit from public health and mental health interventions.

### INTEGRATING ASSESSMENT FOR SOCIAL DISCONNECTEDNESS INTO AGING SERVICES: CHARTING A PATH FORWARD

Alice Prendergast, and Kristi Fuller, *Georgia State University, Atlanta, Georgia, United States*

Social disconnectedness poses a serious threat to the health and well-being of older adults. Although research demonstrates that social disconnectedness was prevalent among older adults long before the COVID-19 pandemic, the crisis has brought significant attention to this issue, as well as resources to address it. The crisis also shed light on the current gap in evidence and guidance on how best to assess for and address social disconnectedness, especially in practice settings. Researchers from the Georgia Health Policy Center conducted a review of existing assessment tools and processes for social disconnectedness and formulated recommendations for the Georgia Division of Aging Services (DAS) in November 2020. These recommendations involve the use of evidence-based assessments paired with person-centered counseling to address social disconnectedness among at-risk individuals. In this session, researchers from Georgia State University will define social disconnectedness, explain how it differs from related constructs, and discuss the necessity of a holistic approach to assessment; summarize the review and recommendations made to DAS; and present preliminary data from DAS's initial implementation of the assessment process.

### THE GREAT MIGRATION AND THE URBAN-RURAL DIVIDE: LONELY LIFE EXPECTANCY IN CHINA

Xueqing Wang, and James Raymo, *Princeton University, Princeton University, New Jersey, United States*

After decades of below replacement fertility, China is now experiencing rapid population aging and the lives of the growing older population are being shaped by massive social and economic change. Of particular importance, is the large-scale migration of working-age adults from rural areas to large cities in search of job opportunities. The departure of migrants from their rural hometowns has resulted in a large population of left-behind older men and women. This distinctively Chinese demographic phenomenon has spurred scholarly interest in the emotional well-being of this older left-behind population, but careful demographic description of aging, migration, grandparenting, and loneliness has yet to be conducted. We bridge this gap by describing the average remaining life spent lonely by older men and women in China. We use Sullivan's method to calculate lonely life expectancy by urban/rural residence and by the migration status of adult children (as proxied by the presence or absence of coresiding children). We use data from the Harmonized version of the Chinese Health and Retirement Longitudinal Study and focus the analysis on adults aged 55-100. Preliminary results show that, at age 55, women on average spend 9% more of remaining life lonely than men and that rural men and women spend more of their remaining life lonely than their urban counterparts. We will extend these life table analyses by conducting multivariate analyses of the correlates of loneliness in urban and rural China to better understand the role of migration and grandparenting responsibilities.

### THE LENGTH OF MEMBERSHIP AND LONELINESS OF OLDER ADULTS IN VILLAGE PROGRAMS

Qiuchang (Katy) Cao,[1] Christine Happel,[2] Katie White,[3] and Holly Dabelko-Schoeny,[4] *1. The Ohio State University, Columbus, Ohio, United States, 2. Clintonville-Beechwold Community Resources Center, Clintonville-Beechwold Community Resources Center, Ohio, United States, 3. Ohio State University, Age-Friendly Columbus and Franklin County, Ohio, United States, 4. The Ohio State University, The Ohio State University, Ohio, United States*

Villages are consumer-driven programs supporting older adults to age in their own homes while staying socially connected through service referrals, coordination, and the organization of social activities. Although previous studies demonstrated an increase of perceived social support among Village members over time, few studies tested how Village membership influence older adults' loneliness. To address this gap, a total of 112 members from four Village programs in a Midwest Metropolitan area completed a cross-sectional pilot survey on their social well-being between January and March 2020. The age of participants ranged from 51 to 92 years old (M=72.30, SD=8.38), over 74% of participants were female and over 88% of participants identified as White/Caucasian. The relationship between the 20-item UCLA loneliness scale and length of Village membership was roughly linear according to the Loess Curve. The scores of the UCLA scale range from 20-80 and higher scores indicate higher loneliness. The Cronbach's alpha of the UCLA loneliness scale was 0.86 in the sample, indicating good internal consistency. The average loneliness score of the sample was 38.45, resembling the average of community-living older adults. Regression results suggested that a one-year increase in village membership was associated with approximately two points reduction in loneliness, holding all else constant. Being female, a racial/ethnic minority, retired, a driver, and having higher frequencies of socializing with friends and neighbors were associated with lower levels of loneliness among Village members. This pilot study provides initial support for the social impact of Villages and informs future larger sample longitudinal studies.

### Session 1145 (Symposium)

### SOCIAL ISOLATION AND WELL-BEING AMONG MIDDLE-AGE AND OLDER ADULTS: BEFORE AND DURING THE COVID-19 OUTBREAK

Chair: Lydia Li

This symposium brings together five studies that examined the relationship between social isolation and well-being. Two used pre-COVID data from the Health and Retirement Study (HRS). One aimed to identify patterns of social isolation trajectory in a 9-year period, where social isolation was conceptualized as a multidimensional construct. It identified four distinct patterns, and the pattern had a gradient relationship with health outcomes. Another examined the association between self-perceptions of aging (SPA) and social well-being among older adults. It found that positive SPA predicted increased social connectedness and reduced loneliness in four years. Two other studies were based on a longitudinal survey (COVID-19 Coping Survey) that began in April 2020. One reports that adults 55+ with comorbidity at pandemic onset had persistently elevated depressive symptoms in a 6-month period, regardless of their social isolation level. Another paper suggests that physical isolation at pandemic onset was associated with elevated symptoms of depression, anxiety, and loneliness throughout the following six months. The fifth paper was based on two-wave data—2019 survey and 2020 COVID supplement—from the National Aging and Health Trend Study (NAHTS). It found that older adults who were very socially isolated and completely homebound before the pandemic experienced less psychological distress during the outbreak than those who were very socially integrated and not homebound. The five studies highlight the multiple dimensions of social isolation, their antecedents and development over time, and their role in shaping mental health in a pandemic context.

### SOCIAL ISOLATION PATTERNS AND HEALTH OUTCOMES IN MIDLIFE AND LATER LIFE

Meng Sha Luo, *Zhejiang University, Hangzhou, Zhejiang, China (People's Republic)*

Conceptualizing social isolation as a multidimensional construct encompassing social networks, social contacts, perceived support and loneliness, this research aims to: (1) identify patterns of social isolation trajectory among middle-aged and older adults in the U.S.; (2) investigate how different patterns of social isolation trajectory are related to adults' physical, mental, cognitive, and overall health. Latent class growth modeling was used to examine social isolation trajectory patterns over nine years in a national sample of 6,457 adults aged 51+. Four patterns of social isolation trajectory were identified: severely isolated, moderately isolated, subjectively integrated, and objectively integrated. The objectively integrated group reported the best physical, mental, cognitive, and overall health, whereas the severely isolated group reported the worst. The moderately isolated and subjectively integrated groups fell in between, with the latter displaying relatively better health outcomes. Findings support a close relationship between poor health and long-term social isolation.

### EFFECTS OF SELF-PERCEPTIONS OF AGING ON SOCIAL DISCONNECTEDNESS AND LONELINESS IN OLDER ADULTS

Rita Hu, *Social Work, Ann Arbor, Michigan, United States*

Research shows that self-perceptions of aging (SPA) predict physical, mental, cognitive, and emotional well-being in older adults. Few studies have examined SPA's effects on social well-being. Using data from the 2014–2018 Health and Retirement Study, we examined SPA's effects on older adults' social connectedness and loneliness (age 65+, N = 3,808). SPA was measured by eight items. Social connectedness was operationalized by network size, social contact, and social participation. The UCLA Loneliness Scale assessed loneliness. Linear regression results show that more positive SPA is correlated with increased social connectedness (b = 0.05 SE = 0.01 p = 0.0003) and decreased loneliness (b = -0.09 SE = 0.02 p < 0.0001) in four years, controlling for sociodemographic and health characteristics. Further, loneliness is a mediator between SPA and social connectedness. Findings suggest that older adults with negative SPA are at risk of both objective and subjective social isolation.

### ASSOCIATIONS BETWEEN COMORBIDITY AND DEPRESSIVE SYMPTOMS DURING COVID-19: VARIATION BY SOCIAL ISOLATION?

Jianjia Cheng,[1] and Lindsay Kobayashi,[2] *1. School of Public Health, Ann Arbor, Michigan, United States, 2. University of Michigan, Ann Arbor, Michigan, United States*

Adults with comorbidities are at high COVID-19 risk and may experience elevated depressive symptoms during the pandemic. We aimed to investigate the associations between comorbidity at pandemic onset and subsequent depressive symptoms and whether social isolation modified this association. Data were from monthly online questionnaires in the COVID-19 Coping Study of US adults aged ≥55 from April/May-September/October 2020 (n=4,383). Depressive symptoms were measured by the 8-item CES-D, and social isolation as "high" vs. "low" based on contact with family, friends, social organizations, and living alone. In multivariable mixed-effects models, comorbidity (≥2 vs. <2 chronic conditions) was associated with greater depressive symptoms at baseline (β=0.50; 95% CI: 0.36-0.64), this association varied negligibly by social isolation. Differences in depressive symptoms by comorbidity status at pandemic onset were consistent over the six-month follow-up. This study indicates that middle-aged and older US adults with comorbidities experienced persistently elevated depressive symptoms during the COVID-19 pandemic.

### PHYSICAL ISOLATION AND MENTAL HEALTH AMONG OLDER U.S. ADULTS IN THE COVID-19 COPING STUDY

Carly Joseph,[1] Brendan O'Shea,[2] Jessica Finlay,[2] and Lindsay Kobayashi,[2] *1. University of Michigan, Central Michigan University, Ann Arbor, Michigan, United States, 2. University of Michigan, Ann Arbor, Michigan, United States*

The ongoing COVID-19 pandemic has set an urgent need to understand the impact of physical isolation on mental health. We aimed to investigate the relationships between physical isolation during the period when many US states had shelter-in-place orders (April-May 2020) and subsequent longitudinal trajectories of mental health in middle-aged and older adults (aged 55+, N=3,978) over a six-month follow-up (April to October 2020). We used population and attrition-weighted multivariable linear mixed-effects models. At baseline, 7 days/week of physical isolation (vs. 0 days/week) was associated with elevated depressive symptoms (β=0.82; 95% CI: 0.04-1.60), and all of 1-3, 4-6, and 7 days/week of physical isolation (vs. 0 days/week) were associated with elevated anxiety symptoms and loneliness. Physical isolation was not associated with changes in mental health symptoms over time. These findings highlight the need to prioritize opportunities for in-person connection for middle-aged and older adults when safe to do so.

### PRE-PANDEMIC SOCIAL ISOLATION: PROTECTION OR VULNERABILITY IN THE TIME OF COVID?

Lydia Li, *University of Michigan, Ann Arbor, Michigan, United States*

How do older adults at risk of social isolation before the pandemic fare during the COVID-19 outbreak? Using data from two waves (Round 9 [2019] and COVID-19 Supplement) of the National Health and Aging Trend Study (NHATS), we examined the relationship between pre-pandemic social isolation and psychological distress during the outbreak among community-living older adults (age 65+). Results show that the most socially integrated respondents had more PTSD (β=1.47, SE=.37, p<.001) and depression/anxiety (β=.34, SE=.11, p=.002) symptoms than the most isolated. Older adults who were not homebound had more PTSD (β=2.0, SE= .76, p=.01) and depression/anxiety (β=1.05, SE=.20, p<.001) than the completely homebound. With shelter-in-place and social distancing requirements, older adults who have been socially active and integrated may experience high-stress levels and may need extra support to adjust to the changes. Relatively, those who have been very isolated and homebound may experience fewer changes in their lives.

### Session 1150 (Paper)

### SOCIAL PARTICIPATION

### EXPERIENCE DESIGN STUDIO FOR SOCIAL CONNECTION OF OLDER ADULTS

G. Mauricio Mejía, Cheryl Der Ananian, and Brad Doebbeling, *Arizona State University, Phoenix, Arizona, United States*

Social isolation and loneliness are pressing health concerns in older adults, likely exacerbated by social distancing guidelines enacted during COVID-19. Creating effective interventions to address health issues is challenging. Design is an alternative approach to create innovative interventions and to test their preliminary potential. In the present case study, we describe the processes and outcomes of a four-week project in a graduate design studio. Students were asked to develop a prototype for an intervention using digital technologies to increase social connectedness among older adults. This was an interdisciplinary process guided by faculty with expertise in design (Mejía), healthcare redesign (Doebbeling), and gerontology (Der Ananian). In the first week, the faculty helped the students understand the design goals, the implications of social isolation and loneliness, and technology use in older adults. In the second week, students conducted user interviews. In the third week, students set the problem by defining a specific potential audience and context. They also prototyped two preliminary concepts using storyboards and received feedback from the faculty. In the last week, students presented refined prototypes with storyboards, user flows, and interface mockups. Student design ideas included an audio story-sharing app that facilitates conversations and new friendships, an assistance digital service for immigrant older adults that need support with language or cultural challenges, and an art and crafts subscription service with a sharing platform to connect older adults with similar interests. The students' design projects provided innovative technological approaches for improving social connections and could be used in future R&D.

### LATE WORKING LIFE PATTERNS IN SWEDEN

Gülin Öylü,[1] Susanne Kelfve,[2] and Andreas Motel-Klingebiel,[3], *1. Linköping University, Norrköping, Ostergotlands Lan, Sweden, 2. Linköping University, Norrkoping, Ostergotlands Lan, Sweden, 3. Linköping University, Linkoping University, Ostergotlands Lan, Sweden*

Late working life patterns differ across different social groups and birth cohorts. The mechanisms of these participation differences and role of working life policies can be understood better by using additional working life indicators and historical perspective. This paper investigates how late working life patterns of different age, gender, education groups and birth cohorts are structured in Sweden using participation, employment type, employment break and exit trajectories of different groups. Using Swedish National Registry Data, employment trajectories of the age groups of 60-68 of the birth cohorts 1930, 1935, 1940, 1945 and 1950 are followed. Results show that for all birth cohorts, participation is higher for younger age groups, men and higher educated; leaving the working life before 61 is more common among primary educated; changing employment type in late working life is observed more among higher educated men and usage of sickness compensation is higher among primary educated and women. However, the peak age that individuals exit, and experience employment breaks differs over the years. In addition, although higher educated individuals have lower shares in usage of unemployment and sickness compensation for all birth cohorts, the structure of benefits usage of the other education and gender groups change over the years. Overall, results give important insights how changes in working life policies affect working life patterns of different groups over the years.

### POLITICAL CONTEXT AND POLITICAL PARTICIPATION ACROSS THE LIFESPAN IN AFRICA

Eugene Dim, and Markus Schafer, *University of Toronto, Toronto, Ontario, Canada*

Gerontologists have long documented how age is associated with political participation. However, few studies have considered how macrocontextual factors shape participation across the life span. Moreover, very few studies have dealt with political engagement and aging in emerging democracies, including those in Africa. This study addresses those gaps, integrating the most recent three waves of Afrobarometer survey data (2011–2018) with country-level data from the freedom house (i.e. freedom index). Findings reveal that, at the individual level, an age gap widens for engagement in protests and shrinks for electoral and non-electoral political participation. When the political context is considered, however, we find that political freedom softens the drop-off of protest behavior at later ages. For electoral and non-electoral political participation, we find that freer countries lessen the expected growth in engagement across the life span. The study implies that political oppression shapes the links between age and political behaviour, but the processes seem different depending on whether they are engaging in risky (where the age gap widens) or non-risky (where the age gap shrinks) political forms of engagement.

### PROTECTIVE AND EXACERBATING COGNITION AND ATTRIBUTION FACTORS FROM THE COGNITIVE DISCREPANCY THEORY OF LONELINESS

Jillian Minahan, *Fordham University, New Providence, New Jersey, United States*

According to the cognitive discrepancy theory, although the discrepancy between actual and desired social resources may result in loneliness, Perlman and Peplau (1998) suggested that cognitive processing and attributional style also impact the interpretation of social information. Previous empirical research investigating predictors of loneliness have not assessed a wide range of cognition and attribution factors, so this study filled this gap by examining how protective (optimism, sense of mastery, and purpose in life) and exacerbating (depression, control constraints, negative self-perceptions of aging (SPA), and experiences of age-based discrimination) factors influence and moderate the experience of loneliness cross-sectionally and longitudinally using a sample of 3,345 Americans aged 50 years and older from the 2008 and 2012 waves of the Health and Retirement Study. Optimism ($\beta$s = -.15, -.13), mastery ($\beta$s = -.08, -.07), purpose in life ($\beta$s = -.19, -.18), depression ($\beta$s = .22,.14), control constraints ($\beta$s = .18, .17), negative SPA ($\beta$s = .13, .14), and experiences of ageism ($\beta$s = .07, .06) were significantly related to loneliness cross-sectionally and longitudinally, respectively. Optimism buffered the negative impact of poor functional social resources (e.g., low social support) on loneliness cross-sectionally while control constraints, negative SPA, and experiencing ageism exacerbated the relationship between low functional social resources and loneliness cross-sectionally. None of the protective or exacerbating factors modulated the relationship between functional social resources and loneliness longitudinally. These findings have important implications for the development of interventions that target loneliness. Targeting maladaptive cognitions may be particularly effective in reducing loneliness.

### THE ROLE OF COMMUNITY SUPPORT AND SOCIAL NETWORKS AMONG MARGINALIZED GROUPS IN LATER LIFE

Samuel Van Vleet, *Miami University, Oxford, Ohio, United States*

As the aging population in the United States continues to grow, so does the need for advancement and critical research to better understand later life experiences. The presence of cumulative disadvantages among racial minorities can often lead to later life health disparities. The goal of this study is to assess the role that social networks and community support play in later life health for marginalized communities. Data from the National Health and Aging Trends Study were analyzed using general linear regression techniques. This allowed for better understanding into the relationships between community support, social networks, race/ethnicity and self-reported health. The final sample included 3,857

participants aged 65 and older. After controlling for other variables, community support and social networks had statistically significant positive relationship with later life health. Race/ethnicity was the strongest predictor of worse later life health. The results of this study show the importance of later life social support for predicting health scores. White participants not only maintained higher health scores as compared to Black and Hispanic participants, but they also reported higher levels of social networks and community support. Findings from this study help build upon the literature regarding community support and social networks in later life.

### Session 1155 (Paper)

### TECHNOLOGY USE AND APPLICATIONS

### A LONGITUDINAL STUDY OF OLDER ADULTS' ACCEPTANCE OF ROBOT COMPANIONS AND THEIR EFFECTS ON WELL-BEING

Claudia Rebola,[1] and Bertram Malle,[2] *1. University of Cincinnati, Cincinnati, Ohio, United States, 2. Brown University, Brown University, Rhode Island, United States*

Robotic animal-like companions for older adults are promising technologies that have shown to have health benefits, especially for individuals with dementia, and good adoption rates in some previous studies. Our project, Affordable Robotic Intelligence for Elderly Support, aims to design new capabilities for companionship and smart care, but at high affordability. In a 6-month longitudinal study of baseline acceptance and well-being, we assessed the impact of an Ageless Innovation Joy for All™ robotic pet on user acceptance and emotional well-being (depression, loneliness, positive emotions). Nineteen participants from independent and assisted living facilities completed three standardized in-person surveys, each 3 months apart, including the CES-D, measures of Loneliness, Emotions, Attitude towards Technology (ATI), and various measures of evaluation of and engagement with robotic technology. The measures showed modest to very good reliability and meaningful construct validity. Participants in this sample showed little depression or loneliness, and these levels did not further decrease over the six months. People welcomed the pet and expressed positive evaluations of it, and these sentiments were stable over time. Attitudes toward technology varied but were unrelated to well-being measures and to robot evaluations. Our current conclusion, on the basis of a small sample, is that the selected robotic pet companion is appreciated and seen as beneficial, and for adults who are already low in depression and loneliness, the robot companion helps maintain the adult's emotional well-being but does not further increase it.

### ASSOCIATIONS BETWEEN ACTIGRAPHY-ASSESSED HEALTH BEHAVIORS AND COGNITIVE PERFORMANCE IN OLDER ADULTS

Alex Laffer, Hilary Hicks, Genna Losinski, and Amber Watts, *University of Kansas, Lawrence, Kansas, United States*

Lifestyle behaviors are important determinants of healthy brain aging. Research has not fully explored how sleep quality and physical activity may differentially influence specific domains of cognitive function. The present study aimed to estimate the relative influence of sleep quality and physical activity on cognitive performance in three domains in a sample of older adults. Older adults (ages 60-89, M = 74.74) without cognitive impairment (N= 160) wore an accelerometer for 7 days in a free-living environment. We used average vector magnitude counts per minute to measure total physical activity (TPA), and average wake after sleep onset (WASO) to measure sleep quality. We created cognitive composite scores (executive function, attention, and verbal memory) from neuropsychological data using confirmatory factor analysis. We regressed cognitive scores onto TPA and WASO with age and education entered as covariates. Higher amounts of physical activity and better sleep quality were associated with better executive function ($R2 = 20.3\%$, $F (4, 155) = 11.12$, $p < .001$). Neither physical activity nor sleep quality was associated with verbal memory or attention. Results suggest that more physical activity and improved ability to stay asleep may benefit executive function, but not other cognitive domains. Future studies should clarify the interaction and mechanisms of action between health behaviors and cognitive performance in older adults.

### INTERNET USE AND COGNITIVE FUNCTIONING IN LATER LIFE: FOCUS ON ASYMMETRIC EFFECTS AND CONTEXTUAL FACTORS

Yijung Kim,[1] and Sae Hwang Han,[2] *1. The University of Texas at Austin, Austin, Texas, United States, 2. University of Texas at Austin, Austin, Texas, United States*

Despite the emerging literature linking information communicative technology (ICT) use and cognitive functioning in later life, whether the association varies as a function of social environment and birth cohort remain an open question. Using nine waves of panel data from the U.S. Health and Retirement Study (2002-2018), we examined within-person asymmetric effects of transitioning into and out of Internet use on cognitive functioning, and whether the associations vary depending on living arrangement and across birth cohorts. Results from the multilevel models indicated that transitioning into Internet use was associated with improved cognitive functioning at a given wave and decelerated cognitive decline over time. Similarly, ceasing to use the Internet was associated with worse cognitive functioning and accelerated cognitive decline. Further, such linkages between Internet use and cognitive functioning were moderated by living arrangement and birth cohort. The detrimental effect of ceasing Internet use was worse for those older adults who live alone. Transitioning into and out of Internet use was unrelated to changes in cognitive functioning among recent HRS cohorts, namely, the War Babies (b:1942-47) and Early Baby Boomers (b:1948-53). These findings highlight the interplay between technology, social environment, and cognitive functioning in later life. The salubrious effects of adopting an ICT technology, such as the Internet, as well as deleterious effects of ceasing to use such technology, underscores the importance of promoting digital literacy and access to ICT technologies among the older adult population.

### MOTOR BEHAVIOR CHANGES ARE PREDICTIVE OF ACUTE EVENTS IN SKILLED NURSING

Mary (Libbey) Bowen,[1] Meredeth Rowe,[2] Pamela Cacchione,[3] and Ming Ji,[2], *1. University of*

*Delaware School of Nursing, Newark, Delaware, United States, 2. University of South Florida, Tampa, Florida, United States, 3. University of Pennsylvania, University of Pennsylvania School of Nursing, Pennsylvania, United States*

**Background:** Common acute medical conditions among older adults with dementia in skilled nursing include falls, delirium, and pneumonia. This study utilized a sensor technology to examine how motor behaviors may predict these acute events.

**Methods:** Radio frequency identification (RFID) technology continuously measured time and distance travelled, gait speed, and continuous walking with little/no breaks (paths) across 3 long-term facilities for up to 1 year (N=51). Change point analysis estimates the probability of whether a sudden change occurred and provides the location of the change point (in days prior to the event) in a time series model.

**Results:** Gait speed had very low probability to detect a change point across all events (22 falls, 10 delirium and 8 pneumonia). Sensitivity estimates ranged from 63% (number of paths) to 90% (distance travelled) for a fall; 37.5% (number of paths) to 100% (rest of the motor behaviors) for pneumonia. Except for gait speed, all other motor behaviors had high probability (100%) to detect a delirium change point. There was intra-individual variability in the location of the change points (mean of 10 days). Linear regression models for time and distance travelled using baseline predictors of age, ethnicity, gait and balance explained 89% and 90% of the variance in change point locations.

**Conclusions:** Prior to an acute event there is a significant change in motor behavior, suggesting these are an early signal that may be used to prevent a fall or provide for the earlier recognition and treatment of delirium and pneumonia.

### PRELIMINARY EFFICACY OF AN MHEALTH MINDFULNESS INTERVENTION WITH CAREGIVERS OF OLDER ADULTS WITH MCI OR DEMENTIA

Elissa Kozlov,[1] Danielle Llaneza,[1] XinQi Dong,[2] and Paul Duberstein,[3] *1. Rutgers University, New Brunswick, New Jersey, United States, 2. Rutgers University, Rutgers Institute for Health, New Jersey, United States, 3. Rutgers University School of Public Health, Piscataway, New Jersey, United States*

Decades of research have documented the negative effects of caregiving on unpaid caregivers. Caregivers are more likely to suffer from high levels of stress and anxiety, and caregivers of older adults with dementia are at especially high risk. Mindfulness Therapy (MT) is a promising, non-pharmacological technique with proven efficacy and effectiveness in managing stress and anxiety in diverse populations. Mindfulness Coach is an m-health delivered mindfulness therapy intervention developed by the Veterans Affairs National Center for PTSD. The objective of this paper is to report the preliminary efficacy of an 8-week pilot trial of mHealth-delivered mindfulness therapy to alleviate anxiety and caregiver stress in caregivers of persons with dementia. Sixty caregivers of patients with mild cognitive impairment or dementia were recruited to participate in this single group pre-post design study. After receiving an orientation to using the app, participants were instructed to use the app daily to learn about and practice mindfulness skills. At the end of the 8 weeks, there was a significant reduction between baseline anxiety on the Hospital Anxiety and Depression Scale Anxiety subscale (mean = 14.45, SD = 3.36) 15.42, SD = 3.12) and 8 weeks (mean = (t(55)=2.6, p=.012) and perceived stress measured by the perceived stress scale at baseline (mean = 23.59, SD = 3.99) and 8 weeks (mean = 21.12, SD = 3.09), (t(56)=5.94, p<.001). This study offers preliminary evidence that mHealth Mindfulness Therapy strategies may help caregivers manage the stress and anxiety associated with caregiving.

### Session 1160 (Symposium)

### THE LONG AND WINDING ROAD: EARLY YEARS OF THE ACCREDITATION FOR GERONTOLOGY EDUCATION COUNCIL

Chair: Robert Maiden Co-Chair: Donna Schafer

The long road of establishing an accreditation entity began in August 2010 when the AGHE Accreditation Task Force was convened. After numerous meetings complete with loud and vigorous debates, AGEC, the Accreditation for Gerontology Education Council emerged in 2016. Over the subsequent years, the Standards hit the hard road of reality leading to various revisions to the Handbook. The symposium's first presentation concerns the history of AGEC and its further development into an independent entity. The key purpose of AGEC is assuring gerontology programs educational quality and enhancement governed by the principle of self-evaluation and peer review that engenders trust. The next presentation discusses the marketing aspect of AGEC built on getting feedback from the public. One of the outcomes of conducting focus groups and surveying the public is the discovery that prospective students really see the value of accreditation. The penultimate presentation focuses on refinements to procedures alluded to in the first presentation in response to the feedback received in meeting with institutions and faculty about what accreditation offers to students, stake-holders, and ultimately the older adults served by the graduates in the work force. The key goal is to clarify the expectations and simplify the application process. On no other issue has more time been spent than on the assessment of students' competency. Our last presentation explains competency-based education consisting of well-articulated student learning outcome measures that are consonant with the program's mission that lead to "closing the loop" of continuous and durable improvements in the learning environment.

### REFINING THE LOOP OF CONTINUOUS IMPROVEMENT

Robert Maiden,[1] and Jan Abushakrah,[2] *1. Alfred University, Alfred, New York, United States, 2. Portland Community College, Portland, Oregon, United States*

Academic assessment of student competency is essential to measure learning within a gerontology program. In its self-evaluation, a program must assess its student learning outcomes. JoAnn Damron-Rodriguez et al. (2019, p. 423) proposed a systematic approach that has several levels. The key is to utilize a competency-based education model. Moreover, to satisfy workforce goals the gerontology program must adopt the AGHE competencies that reflect the knowledge, skills, and attitudes necessary to serve older adults at an acceptable level of care. The next step involves generating well-articulated quantitative or qualitative measures of student learning outcomes (SLOs) consistent with the program's mission statement that include twelve competency domains. SLO measures include test grades, assignments, projects, portfolios, field experiences, essay questions, multiple choice items, and so on. The program's enhancement loop requires the evaluation of SLOs, faculty discussion of them, and a continuous modification cycle "closing the loop" to reach the program's goals.

### GOOD TROUBLE ALONG THE WINDING ROAD: DISRUPTION AND ACCOMPLISHMENT

Harvey Sterns,[1] and Joseph Ruby,[2] *1. The University of Akron, Akron, Ohio, United States, 2. Joe Ruby Consulting, Akron, Ohio, United States*

The establishment of a accreditation body for gerontology degree programs was seen as disruptive. Many key leaders were against creating such a body and wanted this to be delayed or to never happen. In 2012, the AGHE Accreditation Task Force was established with a Competency Work Group and an Organization Work Group..There have been 5 programs evaluated with a number of schools/university currently in process. The task force filed documents for creating a legal entity and obtaining non-profit status for the new Accreditation for Gerontology Education Council. The Task Force developed the dimensions for program evaluation based on the Competencies and shared information with AGHE members. The Task Force obtained start-up funding for the organization and identified the first programs for accreditation and has been providing outreach and guidance to new programs. There is continual refinement of the process.

### IF YOU BUILD IT, WILL THEY COME?: GETTING THE WORD OUT ABOUT GERONTOLOGY ACCREDITATION

M. Aaron Guest,[1] and Phillip Randall,[2] *1. Arizona State University, Phoenix, Arizona, United States, 2. The Thorndike Group, Minneapolis, Minnesota, United States*

The development of AGEC introduced a new facet to gerontological education: accreditation. The presence of such a new organization requires continuing marketing and education. Throughout its first three years, AGEC has continually worked to provide information and to differentiate between accreditation and credentialing for the broader community. Through informal and formal feedback processes, including focus groups and interviews, AGEC has refined its message and delivery. While schools and departments of Gerontology remain the decision-makers behind seeking accreditation, students have become one of the largest drivers and constituencies AGEC engages as they seek clarification on and the availability of accredited programs of Gerontology. Prospective students, many coming from the health sciences, see the value of accreditation. Furthermore, emerging and international programs see accreditation as an opportunity to engage the field. There is an opportunity to further refine the messages around accreditation and differentiate among the organizations active in gerontological education.

### REFINING THE CONTENT OF THE AGEC ACCREDITATION HANDBOOK

Donna Schafer,[1] and Donna Weinreich,[2] *1. Executive Director, National Association for Professional Gerontologists, National Assn. for Professional Gerontologists, California, United States, 2. Western Michigan University, Plainwell, Michigan, United States*

The AGEC Accreditation Handbook and Self-Evaluation Guide has been revised several times since the first drafts were produced in 2016 and 2017. The Handbook contains information for accreditation applicants, AGEC reviewers, and members of the Board of Governors responsible for administering AGEC policies and procedures. It includes the accreditation standards, eligibility for accreditation, timelines, and self-study and site visit guidelines. It is the central document for the AGEC accreditation process. Handbook revisions, drawn from the experience of accrediting programs, have been for the purposes of clarifying expectations and simplifying the application process. This presentation highlights modifications included in the 2020 Handbook, which simplify the self-study outline and site visit guidelines, and the 2021 modifications, which clarify the "Mapping Course Matrix" (displaying AGHE competencies covered in program courses) and add an "Assessment Matrix" for measuring acquisition of the competencies and improving the program as needed.

### Session 1165 (Symposium)

### THE RELATIONSHIPS BETWEEN PHYSICAL ACTIVITY BEHAVIOR, DAILY EXPERIENCES, AND WELL-BEING IN EVERYDAY LIFE

Chair: Erica O'Brien Co-Chair: David Almeida Discussant: Christiane Hoppmann

A physically active lifestyle has several health benefits in both the short- and long-term. This includes higher levels of emotional and psychological well-being and, in many cases, maintained well-being despite stressful experiences that can have countervailing, negative impacts on health. This symposium presents innovative research that (a) links physical activity to positive and negative events or experiences in middle-aged and older adults, and that (b) uses different assessment and analytical techniques to unpack these links on multiple timescales – between moments, days, and years. Lee and colleagues investigate the influence of daily positive experiences on the likelihood and extent of physical activity. O'Brien and Almeida examine how daily stressor experiences predict engagement in different intensities of physical activity and highlight moderating factors such as perceived stressor control. Puterman and colleagues characterize the real-time associations between physical activity and negative responses to stressful experiences by using intensive, within-day assessment methods and accelerometry data. Lastly, Benson and colleagues report on whether the potential for daily physical activity to protect against negative stressor responses differs with age and changes across the adult lifespan. Together, these papers consider the nature and mechanisms of the relationship between physical activity and everyday experiences (e.g., stressors and positive events). They also underscore the importance of assessing relationships on different timescales to obtain a clearer understanding of intrapersonal and developmental processes. As discussant, Hoppmann will assess the strengths and limitations of these papers, elaborate on underlying and overarching themes, and identify future directions in this field.

### POSITIVE EVENTS AND PHYSICAL ACTIVITY IN DAILY LIFE FOR MIDDLE-AGED AND OLDER ADULTS

Sun Ah Lee,[1] Lizbeth Benson,[2] Erica O'Brien,[2] David Conroy,[3] and David Almeida,[2] *1. The Pennsylvania State University, University Park, Pennsylvania, United States, 2. Pennsylvania State University, University Park, Pennsylvania, United States, 3. Pennsylvania State University, Univeristy Park, Pennsylvania, United States*

Previous studies reveal that positive affective well-being is positively associated with physical activity. The present study extends this work by examining the relationship between positive events and physical activity in daily life. Participants (N=1,016, ages 43-90, 56% women) from the third wave of the National Study of Daily Experiences reported their experiences of positive events and physical activity in eight daily diary interviews. Results from multilevel model analyses showed that on days when participants experienced more positive events than usual, they were more likely to engage in physical activity and reported engaging in greater physical activity than usual. Further, participants who experienced more positive events on average across the study period also reported engaging in greater daily physical activity. These results were invariant across age. Our findings highlight the importance of naturally-occurring positive experiences in daily life across middle- and later adulthood.

### WALKING IT OFF: MORE STRESSORS AND PERCEIVED STRESSOR CONTROL PREDICT MORE PHYSICAL ACTIVITY IN DAILY LIFE

Erica O'Brien, and David Almeida, *Pennsylvania State University, University Park, Pennsylvania, United States*

Research shows that, while the experience of stress relates to lower levels of physical activity (PA), people who perceive a greater sense of control engage in higher levels of PA. This study explores whether a sense of control specifically over stressful situations moderates the negative association between stressor exposure and PA in daily life. We used 8-day diary data from up to 1,236 participants (Age: Range = 43-91, M = 62.47, SD = 10.20) in the National Study of Daily Experiences. Somewhat contrary to hypotheses, people reported spending more time on light PA (but not moderate-to-vigorous PA) on days when they also experienced more stressors than usual. Perceived stressor control appears to magnify this effect, with people reporting even more light PA on days when they feel greater control. Initial findings suggest that a physically active lifestyle may help middle-aged and older adults cope with daily stressors.

### ACCELEROMETRY-BASED PHYSICAL ACTIVITY AND AFFECTIVE RESPONSES TO DAILY STRESSORS: AN ANALYSIS OF THE AAPECS STUDY

Eli Puterman,[1] Benjamin Hives,[2] A. Janet Tomiyama,[3] Carissa Low,[4] Geralyn Ruissen,[2] Mark Beauchamp,[2] and Aidan Wright,[4] *1. University of British Columbia, University of British Columbia, British Columbia, Canada, 2. University of British Columbia, Vancouver, British Columbia, Canada, 3. University of California Los Angeles, Los Angeles, California, United States, 4. University of Pittsburgh, Pittsburgh, Pennsylvania, United States*

Evidence suggests that physical activity on a daily basis dampens the extent to which one experiences elevations in negative affect in response to daily stressors. Yet, these studies primarily relied solely on end-of-day recall of stressors and negative affect, and self-reported physical activity. More intensive assessments throughout the day and accelerometry-based physical activity measurements are required to answer whether any type of body movement (e.g. light, moderate, vigorous) reconfigures the end-of-day recall of the intensity of the affective experience of a stressor or, rather, mitigates the actual experience of a stressor in real-time. This presentation will summarize results addressing this question using data from the University of Pittsburgh's Assessment of Personality, Ecological Context, and Stress (AAPECS) study. AAPECS includes172 participants who wore accelerometers to assess movement-based activities and completed ecological momentary assessment 6 times daily for 14 days, with additional 'bursts' of affective assessments following reported stressors at any time.

### AGE-RELATED CHANGE IN THE EXTENT PHYSICAL ACTIVITY ATTENUATES NEGATIVE AFFECT REACTIVITY TO STRESSORS IN DAILY LIFE

Lizbeth Benson,[1] Nilam Ram,[2] David Conroy,[3] Zita Oravecz,[1] Timothy Brick,[1] and David Almeida,[1] *1. Pennsylvania State University, University Park, Pennsylvania, United States, 2. Stanford University, Stanford, California, United States, 3. Pennsylvania State University, Univeristy Park, Pennsylvania, United States*

Theories suggest that with increasing age, adults more effectively regulate their emotions and seek to limit high physiological arousal. Prior research indicates physical activity attenuates negative affect reactivity to stress, but also increases physiological arousal. The present study extends prior work by examining age-related differences and changes over time in the extent of attenuation. Participants (n=3,484; MedianAge=53.42 years, SD=13.3; 56% female), from the National Study of Daily Experiences completed 8 end-of-day assessments of their negative emotions, stress, and physical activity across 3 measurement bursts spaced approximately 10 years apart. Results from three-level multilevel models suggest that when full random effects are specified, physical activity does not attenuate negative affect reactivity to stress. Additionally, extent of attenuation did not differ with age or change over time. Discussion pertains to how these findings advance theoretical understanding of socioemotional development and to methodological nuances of random effects and non-normally distributed data.

### Session 1170 (Symposium)

### A NEW TYPE OF COP: THE ROLE OF CIRCULATING OSTEOPROGENITOR (COP) CELLS IN HEALTH AND DISEASE

Chair: Gustavo Duque

Circulating osteogenic progenitor (COP) cells are a population of cells in the peripheral blood with the capacity for bone formation and broader differentiation into mesoderm-like cells in vitro. While some of their biological characteristics are documented in vitro, their role in the aging process and the pathogenesis of musculoskeletal diseases remains yet to be thoroughly evaluated. This translational session will go from bench to bedside, reviewing the current evidence on COP cells. In this session, we will provide an overview of the role of COP cells in the aging process and a number of physiological and pathological conditions and identify areas for future research. In addition, we will suggest possible areas for clinical utilization in the management of musculoskeletal diseases, which include novel diagnostic and therapeutic uses.

### COP CELLS AND TISSUE LOSS SYNDROMES: FRAILTY, SARCOPENIA, AND OSTEOPOROSIS

Gustavo Duque, *The University of Melbourne, St Albans, Victoria, Australia*

COP cells have been identified as having a potential role in the pathogenesis of tissue loss syndromes such as osteoporosis and frailty. This is based on the hypothesis that their dysregulation may cause a decrease in bone and muscle formation, which also increase the risk of adverse outcomes such as frailty and disability. Whereas high numbers of COP cells have been associated with osteoporosis and fracture healing, a low percentage of COP (%COP) cells have been associated with frailty and disability. In addition, low expression of lamin A (a protein of the inner nuclear envelope) in COP cells has also been associated with frailty and disability in older persons. In this session, the evidence on quantification methods for COP cells in clinical settings and the potential clinical use of COP cells in tissue loss syndromes will be discussed. This discussion will include current evidence supporting the use of COP cells as a biomarker or as a novel therapeutic approach to these age-related conditions.

### COP CELLS IN STATES OF BONE ANABOLISM AND ABNORMAL CALCIFICATION/OSSIFICATION

Robert Pignolo, *Mayo Clinic College of Medicine, Rochester, Minnesota, United States*

Circulating osteogenic progenitor (COP) cells are a population of cells in the peripheral blood with the capacity for bone formation, as well as broader differentiation into mesoderm-like cells in vitro. There are several pathologies of accelerated bone formation and physiological responses to injury in which COP cells have been theorized to play a role. These include fracture, vascular calcification, and subtypes of heterotopic ossification (HO). Overall, the available studies suggest COP cells are likely to be mobilized in response to fracture, home to the site of injury, undergo a maturation process, and contribute to the osteogenesis and angiogenesis required for fracture healing. HO is the pathological process of bone formation in nonskeletal tissue and can be acquired or hereditary. COP cells may seed sites of injury and inflammation that precede the formation of endochondral bone identified in both genetic and nongenetic forms of HO. Vascular calcification is a common occurrence in older adults and is strongly associated with poorer cardiovascular health outcomes. It appears that COP cells, particularly those expressing hematopoietic and vascular markers such as CD45 and CD34, contribute to the calcification and ossification of atherosclerotic plaques and aortic valves, and that they correlate to the severity of the calcification. Whether COP cells are attracted to sites of injury and inflammation and so are highly associated with fracture, vascular calcification/ossification and HO, or whether they underlie these processes at a more mechanistic level, remains to be more clearly demonstrated.

### THE BIOLOGY OF COP CELLS: MESENCHYMAL OF HEMATOPOIETIC?

Meghan McGee-Lawrence, *Medical College of Georgia, Augusta University, Augusta, Georgia, United States*

Circulating osteogenic precursor (COP) cells constitute a recently discovered population of circulating progenitor cells with the capacity to form not only bone but other mesenchymal tissues. A small but growing body of literature explores these cells, but with a great deal of disagreement and contradiction within it, mainly whether these cells are from mesenchymal or hematopoietic origin. This session will discuss the origins and biological characterization of these cells, including the identification strategies used to isolate these cells from the peripheral blood. It also examines the available knowledge on the in vitro and in vivo behaviour of these cells in plastic adherence, differentiation capacity, proliferation, and cellular homing. We will also review the profound and exciting implications for future use of COP cells in clinical practice, particularly in comparison with other types of stem cells.

### THE KYNURENINE PATHWAY METABOLITES QA AND KYNA INDUCE SENESCENCE IN BONE MARROW STEM CELLS THROUGH THE AHR PATHWAY

Dmitry Kondrikov,[1] Ahmed Elmansi,[2] Xing-ming Shi,[3] Meghan McGee-Lawrence,[4] Sadanand Fulzele,[5] Mark Hamrick,[5] Carlos Isales,[5] and William Hill,[2]
*1. Medical University of South Carolina, Meddical University of South Carolina, South Carolina, United States, 2. Medical University of South Carolina, Charleston, South Carolina, United States, 3. Augusta University, Augusta, Georgia, United States, 4. Medical College of Georgia, Augusta University, Augusta, Georgia, United States, 5. Medical College of Georgia, Augusta, Georgia, United States*

Cell senescence is emerging as a critical factor in the pathophysiology of aging bone loss. We have shown that the essential amino acid tryptophan is metabolized by IDO-1 in the periphery to generate kynurenine (KYN), and that KYN can signal though the aryl hydrocarbon receptor (AhR) transcription factor pathway to inhibit osteogenesis in bone marrow MSCs via epigenetic regulation of osteogenic genes, while also upregulating osteoclastogenic transcription factors and genes driving osteoclast activity. Further, we recently showed that KYN acting via AhR inhibits MSC autophagy while inducing senescence. Here we demonstrate that KYN metabolites downstream from KYN act via the AhR signaling pathway to inhibit autophagy and induce SASP expression and drive senescence in murine and human bone marrow MSCs. We focused on two of these metabolites, quinolinic acid (QA) and kynurenic acid (KYNA) and investigated their effects on BMSC cellular function.

We demonstrated that both kynurenine pathway metabolites QA and KYNA increase biomarkers for senescence including beta-galactosidase, p21/Cdkn1 and other SASPs such as PAI-1 and TIMP-2, as well as nuclear DNA damage leading to senescent markers like H2A Ser139 phosphorylation, and the accumulation of senescence-associated hetero chromatin foci (SAHF) with H3K9-me3 labeling. Then upon treatment with the AhR inhibitor 3'4'-DMF the disruption of autophagy and induction of senescent biomarkers was blocked. Like KYN, the effects of QA and KYNA were mediated through the AhR receptor. Therefore, this presents novel therapeutic targets linked to KYN metabolite signaling via AhR to prevent senescence and bone loss.

### Session 1175 (Symposium)

### A TRANSITIONAL CARE MODEL FOR VETERANS WITH COMPLEX NEEDS DURING COVID: THE BEHAVIORAL RECOVERY OUTREACH (BRO) TEAM

Chair: Kathleen Matthews Discussant: Latrice Vinson

The Veterans Health Administration's Care for Patients with Complex Problems (CP)2 Program developed a national infrastructure to disseminate promising practice models to improve care for Veterans with complex medical, mental health, and/or neurocognitive conditions, who may also have behaviors disruptive to care. A strategic priority is improving safe and effective transitions to community care for Veterans with complex care needs, many of whom have historically been limited to VA settings as a result of behavioral concerns. The Behavioral Recovery Outreach (BRO) Team was the first model identified for national dissemination and evaluation at partner sites. Developed at VA Central Iowa, BRO is an interdisciplinary team model that identifies Veterans in long-term VA care settings with complex care needs to engage in individualized behavioral programing to manage/stabilize behaviors and safely transition them to more appropriate and less costly community settings. This symposium will describe the BRO team model, highlight the facilitators and barriers to nationally disseminating the BRO model with VA partner facilities, discuss adaptations in continuing community transitions following the COVID-19 pandemic, and describe program outcomes. The first speaker will discuss development of the BRO model and outcomes of a regional dissemination. The second speaker will present results from the program evaluation of the national dissemination. The final speaker will describe BRO Team expansion and lessons learned from the perspective of a VA partner facility. The (CP)2 Program Director will integrate findings and highlight implications for scaling and evaluating promising models for national dissemination for policy, practice, and future research.

### EVALUATING IMPLEMENTATION OF THE BEHAVIORAL RECOVERY OUTREACH (BRO) TEAM: ONE YEAR OF IMPLEMENTATION

Jenefer Jedele,[1] Cameron Griffin,[2] Kathleen Matthews,[3] and Latrice Vinson,[4] *1. Veterans Health Administration, Ann Arbor, Michigan, United States, 2. VA, Ann Arbor, Michigan, United States, 3. VISN 23, Des Moines, Iowa, United States, 4. Department of Veterans Affairs, Department of Veterans Affairs, District of Columbia, United States*

We present evaluation results after one year of implementation by nine BRO Teams. Monthly checklists documented consistent composition across teams: a psychologist, social worker and nurse. Social workers were recognized as having a critical role in implementation, serving as a referral source and liaison between the CLC, Veteran/family, and community facility. Early implementation focused on team and program development with barriers including unprotected time for Team members. In the first year, the nine teams enrolled 70 Veterans, discharging 86% to community facilities. Characteristics of the Veterans suggest Teams are reaching the complex Veteran targeted by the model. Barriers to successful discharge include community facility inexperience/training and confidence to manage complex residents. COVID emerged as the leading barrier to outreach to internal and external partners and providing transitional support to the Veteran after discharge. We discuss the impact of these preliminary findings on future implementation and dissemination of the model.

### THE BEHAVIORAL RECOVERY OUTREACH (BRO) TEAM: A TRANSITIONAL CARE MODEL FOR VETERANS WITH COMPLEX CARE NEEDS

Kathleen Matthews,[1] Grant Bauste,[2] and Emily Luitjens,[3] *1. VISN 23, Des Moines, Iowa, United States, 2. Minneapolis VA Medical Center, Minneapolis, Minnesota, United States, 3. St. Cloud VA Health Care System, St. Cloud, Minnesota, United States*

In 2012, VA Central Iowa developed a novel program known as the Behavioral Recovery Outreach (BRO) Team to address unmet needs of our aging Veteran population with complex medical, psychological, neurocognitive and behavioral concerns. BRO Teams provide evidence-informed treatments in inpatient VA settings, and transitional care/support post-discharge to ensure successful placement and stability in the community. We will discuss how implementation science informed the expansion of this model from a local pilot to a nationally disseminated program. We will explore the challenges of ensuring program fidelity while fostering innovation and adaptation. Given the challenges of national dissemination, we will highlight the predicted and unforeseen aspects of program evaluation and policy implications. Finally, we will discuss the impacts of the COVID-19 pandemic on delivery of care methods and community-based interactions, as well as how this program has improved the lives and quality of care for this high-risk Veteran population.

### ROLLING OUT BEHAVIORAL RECOVERY OUTREACH (BRO) TEAMS: PERSPECTIVES FROM AN INAUGURAL PARTNER SITE

Trisha Gaudig, *Sioux Falls VA, Sioux Falls, South Dakota, United States*

The Sioux Falls VA Community Living Center (CLC) is a partner site for the Behavioral Recovery Outreach (BRO) Team dissemination. This CLC is home to over 55 Veterans requiring a variety of specialty needs such as dementia care, short-term rehabilitation, respite, hospice, and/or psychosocial needs. Many of the Veterans followed by the BRO Team on the CLC experienced frequent rehospitalizations and difficult placement in the community due to behavioral concerns. Local leadership encouraged participation in the BRO Team dissemination due to the growing need in this VA system to open access to dementia and mental health care, successfully discharge Veterans to appropriate community settings, and reduce unnecessary rehospitalizations. This presentation will discuss BRO Team development, including several factors facilitating successful BRO Team implementation (e.g., leadership support, community outreach approaches, staff partner buy-in), and identify barriers impacting successful implementation with a case example to illustrate strategies to overcome such barriers.

### Session 1180 (Symposium)

### ADVANCING THE SCIENCE ON UNEXPECTED EPISODES OF CLARITY AND LUCIDITY IN PEOPLE WITH DEMENTIA

Chair: Joan Griffin Discussant: Basil Eldadah

People with late-stage Alzheimer's disease and related dementias (ADRD) have been reported, largely by way of anecdote, to exhibit unexpected episodes of spontaneous, meaningful, and relevant communication or behavior. These episodes of lucidity (EL) are characterized by spontaneous mental clarity in people living with dementia (PLWD) who are assumed to have lost coherent cognitive capacity. Given the transient nature and limited understanding of underlying mechanisms responsible for this phenomenon, these episodes are frequently overlooked and have received little scientific attention. Few studies have documented EL among PLWD with precision; scientific understanding is limited to anecdotes and case studies, which have not operationalized EL. Thus, there is a critical need for an evidence-based understanding and systematic operationalization of EL. Precise and robust operationalizations of EL will allow future research to assess if EL has different effects on ADRD prognosis or alters how family members manage and adapt to ADRD progression in their care recipient. The National Institute on Aging (NIA) has funded six studies to advance the scientific understanding of EL in dementia. These studies use a variety of methodological approaches to capture EL experiences, and together, they will provide evidence-based operational definitions of EL, novel approaches for measurement of this phenomenon, and estimates of its prevalence. This symposium will provide an overview of the funded studies and three different methodological approaches that are being used to better operationalize and understand EL.

### WHAT IS PARADOXICAL LUCIDITY?

Andrew Peterson,[1] and Jason Karlawish,[2] *1. George Mason University, George Mason University, Virginia, United States, 2. University of Pennsylvania, Philadelphia, Pennsylvania, United States*

In this presentation, I provide a conceptual background from which the other symposium speakers can describe detailed methods for investigating paradoxical lucidity (PL) in dementia. First, I outline the clinical and ethical significance of studying PL. Second, I describes how PL is understudied and so difficult to measure. A working definition of PL has been formulated from case reports, but aspects of this definition remain vague. I argue that this vagueness challenges the measurement of PL and the generalizability of study results. I conclude by proposing ways to address these problems.

### EARLY DESCRIPTIONS OF FAMILY CAREGIVERS' EXPERIENCES WITH UNEXPECTED LUCIDITY

Kyungmin Kim,[1] Joan Griffin,[2] Lauren Bangerter,[3] Virginia Biggar,[4] Dawn Finnie,[5] Theresa Frangiosa,[4] Joseph Gaugler,[6] and Maria Lapid,[7] *1. Seoul National University, Seoul, Seoul-t'ukpyolsi, Republic of Korea, 2. Mayo Clinic College of Medicine, Rochester, Minnesota, United States, 3. UnitedHealth Group, Minnetonka, Minnesota, United States, 4. UsAgainstAlzheimer's, Washington, District of Columbia, United States, 5. Mayo Clinic, Rocheste, Minnesota, United States, 6. University of Minnesota, Minneapolis, Minnesota, United States, 7. Mayo Clinic, Rochester, Minnesota, United States*

To develop an operational definition of and typologies for episodes of lucidity (EL), we conducted a cross-sectional study of former/current family caregivers from UsAgainstAlzheimer's A_LIST (N = 538). More than 60% of caregivers (n = 294, 62%) reported witnessing EL with their care recipient over the course of their dementia. Most episodes happened in late stages of dementia (71%). Only 10% happened within 7 days before death. The majority of episodes (71%) lasted <30 minutes. About half the episodes were characterized by uncharacteristic speech and communication. Caregivers perceived these experiences positively (M = 4.10; range = 1–5), but also expressed desire to know why/when EL occurs and how to best respond to it. Data will be used to refine definitions and typologies, and then a prospective, demographically diverse survey will be administered to family caregivers to assess predictors of EL, linking EL to caregiver well-being and bereavement response.

### CHARACTERIZING EPISODES OF LUCIDITY IN DEMENTIA: OBSERVATIONAL AND APPLIED COMPUTATIONAL LINGUISTICS APPROACHES

Andrea Gilmore Bykovskyi,[1] Kim Mueller,[1] Nicole Werner,[2] Erica Smith,[1] Laura Block,[1] and Clark Benson,[1] *1. University of Wisconsin-Madison, Madison, Wisconsin, United States, 2. Industrial and Systems Engineering, Madison, Wisconsin, United States*

Though episodes of lucidity (EL) in Alzheimer's disease and related dementia (ADRD), reportedly more common near end of life, have significant implications for care, they are poorly understood due to underdeveloped methodological approaches for capturing and measuring these events. This prospective observational study addresses these gaps through audiovisual observation among persons with ADRD surrounding end of life to inform data-driven definitions for EL and distinguish EL from routine fluctuations in ADRD. Audiovisual observation is well-suited to addressing gaps in operationalization of EL, providing an objective data source to assess verbal and nonverbal communication, the primary means through which EL are evidenced. Our study is designed to establish optimal procedures for capturing audiovisual data of targeted populations and timeframes to maximize opportunities for detecting EL. Operationalization of EL will be informed by computational linguistic and behavioral-event coding of linguistic and non-linguistic communication features of EL and associated temporal qualities.

### DEVELOPMENT OF A STAFF INFORMANT MEASURE OF LUCIDITY

Jeanne Teresi,[1] José Luchsinger,[2] Mildred Ramirez,[3] Stephanie Silver,[3] Davangere Devanand,[4] Julie Ellis,[5] Gabriel Boratgis,[3] and Paloma Gonzalez-Lopez,[3]
*1. Research Division, HHAR, Riverdale, New York, United States, 2. Columbia University, New York, New York, United States, 3. Research Division, Hebrew Home at Riverdale by RiverSpring Health, Riverdale, New York, United States, 4. Columbia University Irving Medical Center, New York, New York, United States, 5. La Trobe University, Nursing and Midwifery, Bundoora, Victoria, Australia*

Lucidity Measure Development: An existing questionnaire measuring lucidity length, degree, content, coinciding circumstances, and time from lucid episode to death was expanded to include time of day, expressive and receptive communication and speech the month prior to and during the lucid event. Pilot Study: 33 interviews with staff were conducted; 73% reported ever witnessing paradoxical lucidity. Among 29 events reported, 31% lasted several days, 20.7%, 1 day, and 24.1% less. In 78.6% the patient engaged in unexpected activity. 20% died within 3 days and 17% within 3 months after the event. Qualitative Analyses: To refine the measure, 10 family caregivers and 20 LTSS staff caregivers completed a web-based focus-group type exercise using QualtricsXM. A content-thematic analysis with an inductive approach was applied to make qualitative inferences by analyzing the meaning and semantic relationship of words, phrases, and concepts. Using the reduction method of selection, conceptual content categories will be developed.

### Session 1185 (Symposium)

### ANNUAL REVIEW OF GERONTOLOGY AND GERIATRICS SYMPOSIUM: BLACK OLDER ADULTS IN THE ERA OF BLACK LIVES MATTER

Chair: Jessica Kelley Co-Chair: Roland Thorpe, Jr.
Discussant: Linda Chatters

Our renewed urgency and engagement in a national dialogue on issues of systemic racism and racial justice provides a much-needed opportunity to expand the discourses, perspectives, and practices used in the study of aging. This symposium features contributions from the 2021 (Vol 41) Annual Review of Gerontology and Geriatrics focusing on the continued development and maturation of scholarship on the lives of older Black Americans. Building on the scholarship and research contributions of prior generations of eminent African American gerontologists, the volume asks: "What do we know about the lived experience of Black older adults and what is there still to be learned?" The contributing authors continue a tradition of research that examines the life histories and contemporary experiences of older Black adults within their relevant social and personal contexts. Symposium presenters from a range of social science fields (sociology, psychology, social work), explore aspects of physical health, stress, cognition, and social well-being in the context of intersecting social dimensions of marriage, family, gender, and neighborhood.

### CANCER AND CAREGIVING AMONG OLDER BLACK AMERICANS AND FAMILIES

Katrina Ellis, *University of Michigan, ANN ARBOR, Michigan, United States*

Cancer disproportionately affects Black Americans and consequently, their families. In addition, cancer is often just one of the significant health concerns facing Black families at any one time. Research on family support after an adult cancer diagnosis overwhelmingly focuses on a single (i.e., primary) caregiver and spousal family caregivers, limiting understanding of the complexity of caregiving within family systems facing multiple health challenges. This presentation presents a framework for a broader focus on the role of family systems in providing cancer care, highlighting both the unique strengths and challenges facing Black families who provide care. A family comorbidity lens is used to underscore the need to better understand the nature and impact of concurrent health challenges within families. Ultimately, this perspective reflects the lived experiences of older Black adults and their families after a cancer diagnosis and has implications for future research and interventions to address health issues interdependently.

### GENDER, STRESS, AND MENTAL HEALTH AMONG OLDER AFRICAN AMERICANS

Christy Erving, *Vanderbilt University, Nashville, Tennessee, United States*

Across studies on social stress exposure on the mental health of older African Americans, most investigate singular stress exposures (discrimination). Furthermore, this research rarely assesses gender differences in the psychological effects of specific stress exposures. I use the National Survey of American of Life to assess: (1) gendered patterns of stress exposure among older African Americans; (2) gendered nuances in the individual, collective, and cumulative effects of stress exposure on mental health. I find gender patterns of stress exposure differed by type of stressor. Women and men shared some stress predictors of mental health (everyday discrimination). Other stress predictors were specific to women (health-related mobility challenges) or to men (perceived neighborhood crime). Study findings challenge gerontologists to consider how race-gender groups are at distinct risks for stressors that elicit poor mental health and provide a call for tailored strategies for improving the psychological health of African American women and men.

### MARITAL AND ROMANTIC SATISFACTION AMONG OLDER AFRICAN AMERICANS

Antonius Skipper,[1] and Robert Taylor,[2], *1. Georgia State University, Atlanta, Georgia, United States, 2. University of Michigan, Ann Arbor, Michigan, United States*

There remains a lack of knowledge on marital satisfaction of African Americans generally, but particularly older African Americans. In addition, only a handful of studies investigate satisfaction among couples who are unmarried. With data from the National Survey of American Life, this study examined the correlates of romantic and marital satisfaction among older African Americans. Findings reveal that married older African Americans were slightly more satisfied with their relationship than individuals who were either remarried or unmarried but in a romantic relationship. Among older African American married adults, older age was associated with higher marital satisfaction, and men had higher levels of marital satisfaction than women. Also, married older African Americans with lower family incomes reported higher marital satisfaction. Given the limited research on older African Americans couples, either married or unmarried, this study offers valuable implications for individuals and professionals engaging these couples in practical settings.

### CONTEXTUAL CORRELATES OF COGNITIVE AGING IN BLACK OLDER ADULTS: EXAMINING THE NEIGHBORHOOD

Ketlyne Sol,[1] and Tanisha Hill-Jarrrett,[2] *1. University of Michigan, Ann Arbor, Michigan, United States, 2. University Of South Florida Morsani College Of Medicine, Tampa, Florida, United States*

Black older adults have a unique history that includes enslavement and legalized segregation. This history shapes the present-day experiences of older Blacks, in part, through the neighborhoods in which they live. The neighborhood is a reflection of both the physical and social contexts, and reflects the most natural and intimate context through which a person experiences life. Combined, the unique history and neighborhoods of Black older adults may contribute to their disproportionately experiencing impairments in cognitive function in older age. There is growing interest in how lived experiences across the life course affect cognitive trajectory and, ultimately, cognitive outcomes of older Black adults. In this presentation, we will review recent literature on psychosocial and physical contextual factors and their influence on cognitive aging in older blacks through the lens of the neighborhood.

### Session 1190 (Symposium)

### APPLYING PRINCIPLES OF IMPLEMENTATION SCIENCE TO AGING PROGRAMS AND POLICIES

Chair: Jaime Hughes Discussant: Nancy Morrow-Howell

Implementation science, defined by NIH as "the scientific study of the use of strategies to adopt and integrate evidence-based health interventions," continues to grow within research, education, and practice-based settings. Building on principles from organizational psychology, intervention science, health economics, and health services research, implementation science aims to explore how, and under what conditions, evidence-based interventions are successfully implemented and sustained in real-world settings. Applying implementation science to aging programs and settings may help to accelerate the translation of effective programs and policies into practice. This interdisciplinary symposium will provide an introduction to key principles and applications of implementation science. The first three presentations will focus on largescale spread of interventions while the last two presentations will focus on broader applications of implementation science. The first two presentations will focus on adapting interventions from delivery in one setting or population to another. The third presentation will discuss the role of implementation strategies in scaling an intervention from a controlled research setting into a large integrated healthcare system. The third presentation will focus on the intersection of implementation science and policy. The final presentation will discuss the role of implementation science in alleviating health disparities and advancing health equity. Each presentation will utilize examples from ongoing research studies to demonstrate principles. The session will close with an interactive discussion on the role of implementation science within aging, including challenges and considerations for aging programs, policies, and populations as well as opportunities for further training and education.

### ADAPTING MONTESSORI PROGRAMMING FOR AGING AND DEMENTIA WITH IMPLEMENTATION STRATEGIES

Natalie Douglas, *Central Michigan University, Mt Pleasant, Michigan, United States*

There is a need to translate research findings to support the wider adoption of person-centered care into typical long-term care environments across the world. Montessori for Aging and Dementia is one mechanism to support person-centeredness, dignity and autonomy of older adults living in long-term care environments. In this presentation, strategies used to support the implementation of Montessori for Aging and Dementia in a long-term care community of 20 people living with severe dementia will be highlighted. Implementation support was provided through capturing and sharing local knowledge, ongoing training and consultation, and tailoring communication supports. Through the use of these iterative strategies, the program was successfully adapted to include people living with severe dementia. While key findings of the project included improvements on a variety of observational and staff administered measures, the focus of this presentation will be on the relationships between the Montessori program's fidelity, local needs and implementation strategies.

### ADAPTING NUTRITION PROGRAMMING FOR INTERGENERATIONAL IMPLEMENTATION

Shannon Jarrott,[1] Rachel Scrivano,[1] Jill Juris Naar,[2] and Alicia Bunger,[1] *1. The Ohio State University, Columbus, Ohio, United States, 2. Appalachian State University, Boone, North Carolina, United States*

Practitioners frequently tailor programming to meet participant characteristics and logistic constraints, or to incorporate diverse participants, such as intergenerational programming. Adapted programming may be responsive but reduce impact on outcomes. With growing interest in and limited availability of intergenerational protocol, implementation science guides program tailoring to ensure that youth and older adults mutually benefit from adapted programming. We integrated guidelines for tailoring interventions (Framework for Reporting Adaptations and Modifications-Expanded: FRAME) and evidence-based intergenerational practice. We illustrate how program fidelity can be supported in intergenerational settings using examples from an adapted USDA-approved preschool nutrition curriculum delivered intergenerationally. Program acceptability, appropriateness, and feasibility were rated favorably by program stakeholders, and observational implementation data suggest fidelity can be maintained using evidence-based intergenerational strategies. Our findings support the potential for protocol developed for one age group to benefit youth and older adults when it is adapted using implementation and intergenerational guidelines.

### IMPLEMENTATION STRATEGIES FOR WIDESPREAD SCALING OF EFFECTIVE PROGRAMS INTO HEALTHCARE SYSTEMS

Jaime Hughes, *Duke University School of Medicine, Raleigh, North Carolina, United States*

Translation of effective evidence-based programs into practice is critical to promoting and preserving older adults' function and independence. This presentation will provide an introduction to implementation strategies, defined as the "methods or techniques used to enhance the adoption, implementation, and sustainability of a clinical program or practice." Some examples of implementation strategies include education and training, stakeholder engagement, patient and/or consumer involvement, adaptation, and technical assistance. Application of these implementation strategies will be illustrated using examples from local and national scale out of evidence-based health promotion programs for older adults within the VA Healthcare System. This presentation will close with guidance on how to select, track, and evaluate implementation strategies.

### ELIMINATING DISPARITIES AND ACHIEVING HEALTH EQUITY USING IMPLEMENTATION SCIENCE

Beth Prusaczyk,[1] and Ana Baumann,[2] *1. Washington University School of Medicine in St. Louis, Saint Louis, Missouri, United States, 2. Washington University in St. Louis, Saint Louis, Missouri, United States*

Eliminating health disparities and achieving equity are central to aging services, programs, and research, as we work to ensure older adults are treated equitably compared to their younger counterparts. Additionally, aging services, programs, and research are not immune from the structural racism and other inequities that plague all facets of our lives, and we must work to eliminate disparities within them as well. This presentation will discuss how Implementation science can be used to advance both of these fronts. Implementation science frameworks can be used to ensure multiple levels of context are considered, which is critical when working against something as pervasive and structural as racism. Implementation science can also guide the adaptation of evidence-based interventions for different populations, including for older adults or for different racial or ethnic groups. Furthermore, there are important ways health equity research can improve implementation science that advance the shared goal of eliminating disparities.

### USING IMPLEMENTATION SCIENCE TO INFORM AND EVALUATE HEALTH POLICY

Julie Bobitt,[1] and Beth Prusaczyk,[2] *1. Center for Dissemination and Implementation Science, University of Illinois at Chicago, Illinois, United States, 2. Washington University School of Medicine in St. Louis, Saint Louis, Missouri, United States*

Public health policies can be a tool for the promotion and protection of older adult's well-being but how can we ensure that policies will be effective and applied as intended? This presentation will discuss how implementation science can be used to both inform and evaluate health policies. Scientific evidence developed by applying dissemination and implementation frameworks can be used to inform policy makers as they develop legislation. When used to evaluate policy, D&I frameworks can be applied to examine policy diffusion, how a state, community, or individual organization chooses to carry out the policy, and the impact that policy has on the intended population. D&I frameworks are an effective way to measure the difference between policy intent and what actually happens when a policy is implemented. Examples of how D&I frameworks have been used to inform and evaluate policy will be shared.

### Session 1195 (Symposium)

### ASIAN OLDER ADULTS, TRAUMA, RESILIENCE, AND HEALTH: RECENT FINDINGS FROM THE RUTGERS ASIAN RCMAR

Chair: XinQi Dong Co-Chair: Melissa Simon Discussant: Bei Wu

U.S. Asians are the fastest growing group of older adults in the nation. However, there remains a dearth of disaggregated research for this population assessing health outcomes such as cognition, suicidality, mortality, and the influence of nutrition on chronic conditions. Drawing on the research of Rutgers Asian RCMAR Scientists, this symposium will examine these areas to provide a better understanding of the health of diverse groups of U.S. Asian older adults. Session 1 will assess the association between living in an ethnic enclave and better cognition among Chinese older immigrants and examine the influence of moderating factors. Session 2 will explore the prevalence of traumatic experience and discuss the association among trauma experience, lifetime mental disorder, and risk of endorsed suicide ideation among aging Asians. Session 3 will assess the relationship between family types and 6-year mortality among U.S. Chinese older adults in Chicago. Session 4 will examine the association between ultra-processed foods and cardiometabolic health (obesity, hypertension, high cholesterol, and diabetes) among U.S. adults 50 or older reporting a single ethnicity. In summation, this symposium describes key research areas such as cognition, suicidal ideation, mortality, and nutrition on the overall health of U.S. older adults. The symposium addresses both risk and protective factors that influence these health outcomes and aims to inform interventions to improve the health of U.S. Asian older adults in the areas of trauma, resilience, and health.

### IS LIVING IN AN ETHNIC ENCLAVE ASSOCIATED WITH BETTER COGNITIVE HEALTH OF OLDER IMMIGRANTS? RESULTS FROM PINE

Man Guo,[1] Yi Wang,[2] HANZHANG XU,[3] Mengting Li,[4] Bei Wu,[5] and XinQi Dong,[6], *1. The University of Iowa, Iowa City, Iowa, United States, 2. University of Iowa, Iowa City, Iowa, United States, 3. Duke University School of Medicine, Duke University, North Carolina, United States, 4. Rutgers, The State University of New Jersey, New Brunswick, New Jersey, United States, 5. New York University, New York,*

*New York, United States, 6. Rutgers University, Rutgers Institute for Health, New Jersey, United States*

This study addressed three questions: 1) Is living in Chinatown associated with better cognition among Chinese older immigrants? 2) Is the association moderated by education, acculturation level, and social engagement? 3) Does the association vary by preferred language (Mandarin, Cantonese, Taishanese), an important indicator of heterogeneity among Chinese immigrants? Data were derived from the Population Study of Chinese Elderly in Chicago (N = 3,055). Results showed that Chinese older immigrants who lived in Chinatown had significantly poorer cognition than those who didn't, and such a difference was largely due to educational differences between the two groups. Higher education or acculturation buffered the influence of Chinatown residence on cognitive health, but only among those who speak Mandarin. The findings indicate that living in an ethnic enclave may have a negative impact on cognitive function of Chinese older immigrants. The findings also reveal the sources of heterogeneity within the population.

### TRAUMA EXPOSURE, MENTAL DISORDER, AND RISK OF SUICIDE IDEATION AMONG AGING U.S. ASIANS

Ping Ma,[1] Ruike Li,[1] Yuki Shigemoto,[2] and Lei-Shih Chen,[1] *1. Texas A&M University, College Station, Texas, United States, 2. Prairie View A&M University, Prairie View, Texas, United States*

This project aims to explore the prevalence of traumatic experience and examine the association among trauma experience, lifetime mental disorder, and risk of endorsed suicide ideation among aging Asians. Data were drawn from the National Latino and Asian American Survey and participants aged 55 and above were included in the study. Descriptive analyses and weighted logistic regressions were employed to analyze the association among outcomes of interest. Approximately 74% of 376 aging Asians experienced trauma exposures. Specifically, 17% of respondents with trauma exposure reported a lifetime mental disorder, and 8% had suicide ideation. After adjusting for demographic, social support, and family factors, aging Asians with traumatic experience had a significantly higher risk of experiencing discrimination and mental disorders (p-values<.05). Assaultive or interpersonal violence exposure was positively associated with increased odds of suicide ideation. Culturally tailored mental health intervention and suicide screening are warranted for aging Asians with previous trauma exposure.

### TYPOLOGY OF FAMILY RELATIONSHIPS AND 6-YEAR MORTALITY RISK

Mengting Li,[1] and XinQi Dong,[2] *1. Rutgers, The State University of New Jersey, New Brunswick, New Jersey, United States, 2. Rutgers University, Rutgers Institute for Health, New Jersey, United States*

Durkheim's "family protection" thesis indicated that family could protect individuals from death. However, there are heterogeneities in family types. It remains unclear whether all family types play a protective role in older adults' later life. This study aims to test the relationship between family types and 6-year mortality. Data were derived from a prospective cohort study from 2011 to 2017 of 3,018 U.S. Chinese older adults in Chicago. Family typology was clustered by Latent Class Analysis, including tight-knit (high solidarity and low conflict), unobligated ambivalent (high solidarity and high conflict), commanding conflicted (low solidarity and high conflict), and detached (low solidarity and low conflict). Cox model was used. The result showed that older adults in detached type have higher mortality risk than those in tight-knit type after controlling age, gender, education, income, and medical conditions. Future study could explore the mechanisms through which family types affect mortality risk.

### ASSOCIATIONS BETWEEN ULTRA-PROCESSED FOOD INTAKE AND CARDIOMETABOLIC HEALTH AMONG DIVERSE U.S. ADULTS 50 OR OLDER

Tali Elfassy,[1] Filippa Juul,[2] Robert Mesa,[1] Latha Palaniappan,[3] Malathi Srinivasan,[3] and Stella Yi,[2]
*1. University of Miami, Miami, Florida, United States,*
*2. New York University, New York, New York, United States,*
*3. Stanford University, Stanford, California, United States*

Asian American (AA) diets are naturally adapted to the NOVA dietary recommendations, favoring minimally processed foods. Yet the relationship between dietary intake and metabolic health, among AAs is largely unknown. We examined the association between ultra-processed foods and cardiometabolic health (obesity, hypertension, high cholesterol, and diabetes), among US adults 50 or older reporting a single ethnicity, using the National Health and Nutrition Examination Survey (2001-2018). From multivariable adjusted logistic regression models, the highest compared to the lowest quartile of ultra-processed food intake was associated with obesity only, among AAs (OR: 2.15, 95% CI: 1.04, 4.45), followed by non-Hispanic blacks (OR: 1.73, 95% CI: 1.40, 2.14), non-Hispanic whites (OR: 1.41, 95% CI: 1.19, 1.68), and Hispanics (OR: 1.34, 95% CI: 1.08, 1.65). AAs are more likely than other ethnic/racial groups to be obese when consuming ultra-processed foods. Cultural adaptation of current North American-focused dietary recommendations should drive AA preventive dietary recommendations.

### Session 1200 (Paper)

### BODY WEIGHT AND OBESITY

### LONG-TERM VARIABILITY IN BODY WEIGHT IN RELATION TO THE RISK OF DEMENTIA: A PROSPECTIVE COHORT STUDY

Hui Chen,[1] Tianjing Zhou,[1] Yuan Ma,[2] and Changzheng Yuan,[1] *1. Zhejiang University, Hangzhou, Zhejiang, China (People's Republic), 2. Harvard T. H. Chan School of Public Health, Boston, Massachusetts, United States*

The prospective association of body weight variability with dementia remains unclear. We aimed to investigate whether long-term variability in body weight is associated with the risk of late-life dementia and to explore their potential temporal relationship using data from a nationwide prospective cohort study of the United States. A total of 5,556 participants free of dementia in 2008 (55.66% women; mean [SD] age, 71.1 [3.1] years) were followed up to 8 years for doctor-diagnosed dementia reported biennially. Body weight variability was assessed as the coefficient of variation utilizing the body weight information collected over 16 years before 2008. Cox proportion hazard model was applied to estimate hazard ratio (HR) of dementia associated with body weight variability. Higher body weight variability was associated with an increased incidence of dementia after controlling for sociodemographic factors, lifestyle, mean body weight, and body weight change. The multi-variable adjusted HR of dementia of the highest quartile of body weight variability was 2.01 (95% CI 1.01-1.87) compared with the lowest. Every 1% increment in variability was associated with a 6.2% higher risk of dementia (HR=1.06, 95%CI 1.04,1.09, p-trend<0.001). Such association was observed for both Alzheimer's disease and other types of dementia, with stronger association observed when body weight variability was assessed closer to dementia assessment.

### OCCUPATIONAL DIFFERENCES IN METABOLIC SYNDROME INCIDENCE AMONG OLDER WORKERS

Katharina Runge,[1] Sander K.R. van Zon,[2] Ute Bültmann,[2] and Kène Henkens,[1] *1. Netherlands Interdisciplinary Demographic Institute (NIDI), The Hague, Zuid-Holland, Netherlands, 2. University Medical Center Groningen (UMCG), Groningen, Groningen, Netherlands*

This study investigates whether the incidence of metabolic syndrome (MetS), and its components, differs by occupational group among older workers (45-65 years) and whether health behaviors (smoking, leisure-time physical activity, diet quality) can explain these differences. We analyzed data from older workers (N=23 051) from two comprehensive measurement waves of the Lifelines Cohort Study and Biobank. MetS components were determined by physical measurements, blood markers, medication use, and self-reports. Occupational group and health behaviors were assessed by questionnaires. The association between occupational groups and MetS incidence was examined using Cox regression analysis. Health behaviors were subsequently added to the model to examine whether they can explain differences in MetS incidence between occupational groups. Low skilled white-collar (HR: 1.25, 95% CI: 1.13, 1.39) and low skilled blue-collar (HR: 1.45, 95% CI: 1.25, 1.69) workers had a significantly higher MetS incidence risk during 3.65 years follow-up than high skilled white-collar workers. Health behaviors reduced the strength of the association between occupational group and MetS incidence most among low skilled blue-collar workers (i.e. 10.3% reduction) as unhealthy behaviors were more prevalent in this occupational group. Similar occupational differences were observed on MetS component level. To conclude, MetS incidence in older workers differs between occupational groups and health behaviors only explain a small part of these differences. Health promotion tailored to occupational groups may be beneficial specifically among older low skilled blue-collar workers. Research into other factors that contribute to occupational differences is needed, as well as studies spanning the entire working life course.

### REMOTE MONITORING ACTIVITY TRAJECTORY-ASSOCIATED WEIGHT LOSS AND FUNCTIONAL ABILITY IN OBESE OLDER ADULTS

Curtis Petersen,[1] Anna Kahkoska,[2] Karen Fortuna,[1] and John Batsis,[3] *1. Dartmouth College, Lebanon, New Hampshire, United States, 2. University of North Carolina at Chapel Hill, University of North Carolina at Chapel Hill/Chapel Hill, North Carolina, United States, 3. University of North Carolina at Chapel Hill, Chapel Hill, North Carolina, United States*

Functional decline in older adults can often be mitigated by physical activity. As older adults increasing adopt wearable technology, an understanding of how remotely monitored activity is associated with clinical outcomes is needed. Data was analyzed from two cohorts of older adults with obesity (□65 years, BMI (□30kg/m2)) who completed weekly dietary and exercise-based weight loss interventions (n=93). Follow-up time varied between cohorts (n=37: 12-weeks; n=56: 16 weeks). All participants were provided a Fitbit to monitor physical activity. Baseline and follow up weight, 6-minute walk distance, grip strength, and Late Life Function and Disability Instrument (LLFDI) were collected. We used k-means clustering for longitudinal data to identify physical activity trajectories from Fitbit steps at the daily level. Linear regression models tested for differences in each outcome between trajectories, adjusting for age and sex. Baseline characteristics did not vary across cohorts: mean age 72.7±4.5 years, 76.5% were female, and mean BMI was 36.4±5.1 kg/m2. Two physical activity trajectories were identified, termed high and low activity based on differences in mean daily steps (7,476±4,117 vs. 2,960±2,453, p <0.001). Participants in the high activity group experienced a 2.4% reduction in weight (p <0.001) and a 4.74% increase in LLFDI score (p=0.007) relative to the low activity cluster. Other outcomes were not significantly different between trajectories. These results demonstrate the potential for remote monitoring data to elucidate longitudinal trends in weight and functional ability. As such, older adults' use of wearable technology may facilitate improvements in weight and functional ability in the community.

### THE ASSOCIATION OF MEAL TIMING WITH BODY COMPOSITION AND CARDIOMETABOLIC HEALTH IN OBESE OLDER ADULTS

Samaneh Farsijani,[1] Nancy W. Glynn,[2] and Anne Newman,[1] *1. University of Pittsburgh, Pittsburgh, Pennsylvania, United States, 2. University of Pittsburgh Graduate School of Public Health, Pittsburgh, Pennsylvania, United States*

**Objectives:** To determine the association between eating window and time of last calorie intake with body composition and cardiometabolic health in obese older adults.

**Methods:** We performed a cross-sectional analysis on 36 community-dwelling, overweight-to-obese (BMI 28.0-39.9 kg/m2) older adults, recruited to participate in a weight loss and exercise trial. Time of food intake were extracted from three 24-hour food recalls. Eating window was calculated as the time elapsed between the first and last food intake. We recorded the time of last calorie intake either from food or drink. Blood glucose, triglycerides, high-density (HDL) & low-density (LDL) lipoprotein cholesterols were measured as markers of cardiometabolic health. Total fat and lean mass were assessed by DXA. Partial correlation was used to determine the relationships between eating window and last calorie intake with body composition and cardiometabolic markers, while controlling for sex, age, and total calorie intake.

**Results:** On average, participants' eating window was 12.0±1.1 hours. Time of last calorie intake in 86% of participants was between 6:00-8:00 PM. After controlling for potential confounders, longer eating windows were associated with higher triglyceride levels (P=0.032) and lower HDL (P=0.035), while no association was observed with the other cardiometabolic markers. We observed negative trends, though not statistically significant, between longer eating windows and greater weight, BMI, and fat mass. No association was observed between time of last calorie intake, body composition and cardiometabolic markers.

**Conclusions:** Our results suggest that timing of food intake may influence cardiometabolic risk and obesity in older adults.

### TRAJECTORIES OF BODY MASS INDEX AND MULTIMORBIDITY IN OLD AGE: 12-YEAR RESULTS FROM A POPULATION-BASED STUDY

Amaia Calderón-Larrañaga,[1] Xiaonan Hu,[1] Jie Guo,[1] Luigi Ferrucci,[2] Weili Xu,[1] and Davide Vetrano,[1]
*1. Karolinska Institutet, Solna, Stockholms Lan, Sweden, 2. National Institute on Aging, Baltimore, Maryland, United States*

We aimed to study the association of long-terms trajectories of body mass index (BMI) with contemporaneous changes in multimorbidity development in older adults. Twelve-year BMI trajectories (2001–2013) were identified in subjects aged 60+ years from the Swedish National Study on Aging and Care-Kungsholmen (SNAC-K) using growth mixture models (N=2,189). Information on chronic diseases and multimorbidity was ascertained based on clinical examinations, lab tests, medications, and inpatient and outpatient medical records. Linear mixed models were used to study the association between BMI trajectories and the speed of chronic diseases accumulation, in general and by groups of cardiovascular and neuropsychiatric diseases. Eighty percent of the study population was included in a stable BMI trajectory, 18% in a slow-decline trajectory with an accelerated BMI decline from age 78 onwards, and 2% in a fast-decline trajectory that reached underweight values before age 85. A significantly higher yearly rate of chronic disease accumulation was observed in the fast-decline versus stable trajectories ($\beta$=0.221, 95% CI 0.090-0.352) after adjusting for age, sex, education and time to death. Subjects in the slow-decline trajectory showed a significantly higher rate of cardiovascular diseases accumulation ($\beta$=0.016, 95% CI 0.000-0.031); those in the fast-decline trajectory showed a faster accumulation of both cardiovascular ($\beta$=0.020, 95% CI -0.025, 0.064) and neuropsychiatric diseases ($\beta$=0.102, 95% CI 0.064-0.139), even if the former association did not reach statistical significance. Carefully monitoring older adults with sustained weight loss seems relevant given their likelihood to develop a phenotype of rapidly accumulating chronic -especially neuropsychiatric- diseases.

### Session 1205 (Symposium)

### BUILDING CAREGIVER RESILIENCE: PITFALLS AND POTENTIAL FOR INTERVENTIONS

Chair: Jeongeun Lee Co-Chair: Natasha Peterson
Discussant: Steven Zarit

Informal caregivers provide substantial practical and emotional support for individuals with chronic and acute conditions. Consequently, many experience caregiver burden and are at high-risk for psychological morbidity and associated breakdown in the provision of care for care recipients. Many psychosocial interventions have been designed to help caregivers. However, more work is needed to identify which, or what kind of, interventions are optimal for identifying suitable strategies and care management. The main objectives of this symposium are to (1) address psychosocial and demographic factors contributing to caregiver resilience, (2) understand the role of cognitive and behavioral factors that have implications for caregivers' psychological well-being, and (3) specify different caregiving styles and adaptive outcomes. This symposium assembles a panel of experts and brings together empirical research on various challenges that need to be addressed and potential opportunities for creating effective psychosocial intervention targets for caregivers. The first session will discuss several psychosocial and demographic factors associated with resilience among caregivers. The second session will share how caregiving appraisals are closely related to positive and negative affect and whether the level and changes in caregivers' activity participation moderate this linkage. The third session will identify caregiving styles and strategies utilizing k-modes machine learning analysis and share how caregivers adapt to care situations. The final session will present caregivers' stress experiences related to dementia patients' behavior and psychosocial symptoms in dementia during the day. The session will conclude with Dr. Zarit, who will integrate the four papers and offer insight on implications across studies.

### CAREGIVING APPRAISALS AND EMOTIONAL VALENCE: BUFFERING EFFECT OF ACTIVITY PARTICIPATION

Jeongeun Lee,[1] and Nicholas Cone,[2] *1. Iowa State University, Ames, Iowa, United States, 2. Iowa State University, Iowa State University, Iowa, United States*

Caregiving activities often lead to positive and negative appraisal for caregivers. Caregivers may limit social participation due to caregiving activities. Changes in level of activity participation could have profound consequences for caregiver's valence. However, little is known about how activity participation could moderate the association between these caregiving appraisals and emotional valence. Data came from the National Study of Caregiving (Round 1 and 2), a nationally representative study of caregivers. Referencing Lawton's two-factor model (1990), we examined both the level and changes in activity restriction interacting with positive and negative caregiving appraisals to predict both valence across two waves. Consistent with two factor models, findings revealed level and changes in activity restriction moderated the relationship between caregiving appraisal and outcomes for both valences. These findings highlight the role of activity restriction as a target to reduce negative valence and improve positive valence for caregivers.

### TEMPORAL PATTERNS OF DAILY BEHAVIORAL AND PSYCHOLOGICAL SYMPTOMS OF DEMENTIA THROUGHOUT THE DAY

Caitlin Connelly,[1] Kyungmin Kim,[2] Yin Liu,[3] and Steven Zarit,[4] *1. University of Massachusetts Boston,*

*Asheville, North Carolina, United States, 2. Seoul National University, Seoul, Seoul-t'ukpyolsi, Republic of Korea, 3. Utah State University, Logan, Utah, United States, 4. Pennsylvania State University, University Park, Pennsylvania, United States*

Behavioral and psychological symptoms of dementia (BPSD) are taxing for both the person with dementia (PWD) and their family caregivers. Yet, little is known about how BPSD fluctuates throughout the day (i.e., morning, daytime, evening, and night; e.g., sundowning) and how caregivers perceive BPSD at different times of the day. Using 8-day daily diary data from 173 family caregivers whose relatives were using Adult Day Services (ADS), this study investigated temporal patterns of BPSD and caregivers' stress responses to BPSD throughout the day. Overall, the number of BPSD was highest in the evening, and caregivers' stress reactivity to BPSD increased throughout the phases of the day (i.e., most stressful at night). However, caregivers showed lower reactivity to BPSD in the mornings and at night on days when the PWD used ADS. Our findings about fluctuations of (caregiver reactions to) BPSD throughout the day suggest target windows for just-in-time adaptive intervention.

### FACTORS OF CAREGIVING RESILIENCE BY RACE-ETHNICITY IN A NATIONAL SAMPLE OF CAREGIVERS

Jinmyoung Cho,[1] and Natasha Peterson,[2] *1. Baylor Scott & White Health Research Institute, Temple, Texas, United States, 2. Iowa State University, Iowa State University, Iowa, United States*

Despite heavy burdens and responsibilities, some caregivers are more likely to cope better with their care responsibilities than others, and this could vary by cultural beliefs and norms on caregiving. This study examined contributing factors of resilience with three racial-ethnic groups (White, Blacks, Hispanic). A total of 2,652 caregivers were included from Round 7 of the National Study of Caregiving. Caregiving resilience was defined by higher levels of care demands and higher levels of psychological well-being. Five domains of contributing factors were included: sociodemographic characteristics, context of care, caregivers' psychological attributes, informal and formal support. Multiple logistic regressions showed that caregivers with higher psychological attribute levels were more likely to be resilient in all three groups. However, unique predictors have also been observed by race-ethnic groups (e.g., Blacks using formal support were more resilient). These findings suggest the need for culturally specific programs to facilitate resilience among caregivers.

### COGNITIVE-BEHAVIORAL STYLES OF DEMENTIA CARE MANAGEMENT: TARGETING AND TAILORING TO STYLE

Amanda Leggett,[1] Hyungjung Koo,[2] Cathleen Connell,[3] Laura Gitlin,[4] and Helen Kale,[5] *1. University of Michigan, Ypsilanti, Michigan, United States, 2. University of Michigan, Ann Arbor, Michigan, United States, 3. University of Michigan School of Public Health, Ann Arbor, Michigan, United States, 4. Drexel University, College of Nursing and Health Professions, Drexel University, Pennsylvania, United States, 5. UC Davis, Sacramento, California, United States*

Despite an extensive literature on the stress process of caregiving, little attention has focused on how caregivers provide care (caregiving styles). To explore caregiving styles among 100 primary caregivers for persons living with dementia, we utilize k-modes machine learning analysis. This technique clusters caregiver's use of behavioral (Dementia Management Strategies Scale; criticism, active management, encouragement) and cognitive (Caregiver Readiness Scale; understanding, adaptability) approaches into style profiles. Three styles were identified: Managers (n=25; high use of criticism, moderate use of active management and encouragement, poor understanding and adaptability), Adapters (n=48; low use of criticism, high use of adaptive management and encouragement, good understanding and adaptability), and Avoiders (n=27; low use of all behavioral strategies, moderate adaptability and understanding). Styles differ by demographic and care characteristics. Findings suggest that caregivers have variable approaches to care that are measurable, thus, targeting/tailoring interventions to caregiver style could be an effective approach.

### Session 1210 (Paper)

### CARDIOVASCULAR HEALTH AND AGING

### ARTERIAL STIFFNESS AND AGE MODERATE THE ASSOCIATION BETWEEN PHYSICAL ACTIVITY AND COGNITION IN OLDER ADULTS

Adrian Noriega de la Colina,[1] Atef Badji,[2] Maxime Lamarre-Cliche,[2] Louis Bherer,[2] Hélène Girouard,[2] and Navin Kaushal,[3] *1. University of Montreal, Montreal, Quebec, Canada, 2. Université de Montréal, Montreal, Quebec, Canada, 3. University of Indiana, Bloomington, Indiana, United States*

**Background:** Evidence supports that time spent on physical activity has beneficial effects on cognition in older adults. Nevertheless, this beneficial effect is likely to change in function of individual modifying factors like age and level of arterial stiffness. This study aims to reveal whether arterial stiffness and age modulate the positive impact of physical activity on cognition by developing a double moderation model.

**Methods:** 110 healthy older adults aged 60 to 75 years old were examined for arterial stiffness (carotid-femoral Pulse Wave Velocity [cf-PWV]), global cognition (composite score of Montreal Cognitive Assessment, and Mini-Mental State Examination), and self-reported physical activity (PACED diary). Using PROCESS macro for SPSS, we evaluated if cf-PWV (moderator 1), and age (moderator 2) moderate the relationship between physical activity (X) and global cognition (Y). The threshold for high stiffness was set at 8.5 m/s based on previous studies that reported this cut-off more appropriate for classifying cerebrovascular risk groups.

**Results:** The interaction of arterial stiffness x age moderated the effect of physical activity on global cognition ($\beta$=-.89, SE=.42, p=.037) (Model: $R2$=.15, p=.018). Physical activity had a positive effect on cognition in younger-older adults (aged 60 to 68.5 years) with cf-PWV>8.5 m/s ($\beta$=.57, SE=.222, p=.011, 95% CI.133 to 1.014) and on older-older adults (aged 68.6 to 75 years) with cf-PWV<8.5 m/s ($\beta$=.49, SE=.190, p=.010, 95% CI=.116 to .869).

**Conclusions:** Identifying the right age groups and arterial stiffness levels at which physical activity can have beneficial effects on cognition is a key step in providing tailored behavioral interventions.

### CAROTID INTIMA MEDIA THICKNESS AND COMORBID CARDIOMETABOLIC DYSFUNCTION IN WOMEN: THE SWAN STUDY

Aleda Leis,[1] Emma Barinas-Mitchell,[2] Ana Baylin,[1] Samar El Khoudary,[2] Elizabeth Jackson,[3] and Carrie Karvonen Gutierrez,[1] *1. University of Michigan, Ann Arbor, Michigan, United States, 2. University of Pittsburgh, Pittsburgh, Pennsylvania, United States, 3. University of Alabama, Birmingham, Alabama, United States*

Metabolic syndrome (MetS) and obesity are risk factors for atherosclerosis but their combined impact is unknown. The aim of this study was to quantify the added risk of obesity on carotid artery intima media thickness (cIMT), an early indicator for atherosclerosis, beyond MetS alone. The Study of Women's Health Across the Nation (SWAN) is a multi-center, multi-ethnic cohort of women traversing the midlife into early late adulthood. cIMT was assessed between 2005-2007 and MetS, obesity and covariates were measured at the same time. This cross-sectional analysis is restricted to 1,433 women with a body mass index ≥18.5 kg/m2 and free of cardiovascular disease (CVD) when cIMT was measured. Mean maximum cIMT was related to obesity, MetS and their interaction using multivariable linear regression models. The average age was 60 years (standard deviation 2.7) and the prevalence of obesity and MetS were 44% and 35%, respectively. Both conditions occurred in 24% of women. After adjustment for age, race, smoking, family history of heart disease, and antilipemic medications, obese women had a 0.051mm (95% confidence interval (CI): 0.033,0.070; p<0.001) larger maximum cIMT versus women not obese and women with MetS had a 0.066mm (95%CI: 0.042,0.090; p<0.001) larger maximum cIMT versus women without MetS. There was a statistically significant antagonistic interaction between obesity and MetS; women with both had a mean cIMT of 0.972mm (95%CI: 0.955,0.989) and MetS alone a cIMT of 0.961mm (95%CI:0.938,0.983). This suggests that there is only a small risk of obesity on augmenting cIMT beyond MetS alone.

### EPIDEMIOLOGY OF PERIPHERAL VASCULAR DISEASE IN THE LONG LIFE FAMILY STUDY (LLFS)

Allison Kuipers,[1] Ryan Cvejkus,[1] Emma Barinas-Mitchell,[1] Mary Feitosa,[2] Joanne Murabito,[3] and Joseph Zmuda,[1] *1. University of Pittsburgh, Pittsburgh, Pennsylvania, United States, 2. Washington University School of Medicine in St. Louis, St. Louis, Missouri, United States, 3. Boston University, Framingham, Massachusetts, United States*

Atherosclerotic occlusion of peripheral arteries is a major contributor to morbidity and mortality in older adults. Our aim was to describe the epidemiology of peripheral artery disease (PAD) and other peripheral vascular disease (OPD) in the LLFS. 3248 individuals from 509 families (1182 probands, mean age 89; 2066 offspring, mean age 60) had doppler ankle-brachial index (ABI) assessment. Measures were performed twice for each posterior tibial artery and minimum of the mean ABI was used. PAD was defined as any ABI<0.9. OPD was defined as any ABI >1.4 or ≥1 non-compressible artery. Stepwise linear or logistic regression determined significant independent clinical and demographic predictors (P<0.05) after adjustment for age, sex, study center, and familial relatedness. Overall, ABI had a median of 1.2 with 7.4% PAD (18.1% probands, 1.2% offspring; P<0.001). OPD prevalence was 10.6% and was more common than PAD in offspring (8.1%). Age-adjusted OPD was higher in men (13.3%) than women (8.3%, P<0.001), while age-adjusted PAD did not did not differ by sex (P=0.45). Predictors of PAD included greater age and systolic blood pressure, lower diastolic blood pressure, prevalent kidney disease, antihypertensive use, and current smoking. Predictors of OPD included greater age, male sex, and current smoking. In these exceptionally long-lived families, PAD was low compared to other epidemiologic studies. However, OPD including non-compressible arteries, a marker of arterial stiffness, was more prevalent than PAD. These findings in long-lived families highlight a need for more epidemiologic research in other peripheral vascular disease in adults from the general population.

### POSITIVE PSYCHOLOGY, ACTIVATION, AND SELF-CARE ADHERENCE IN A DIVERSE SAMPLE OF ADULTS WITH HEART FAILURE

Jocelyn McGee,[1] Rebecca Meraz,[2] Elizabeth Perry Caldwell,[3] and Kathryn Osteen,[4] *1. Baylor University, Waco, Texas, United States, 2. Baylor University, Dallas, Texas, United States, 3. Baylor University Louise Herrington School of Nursing, Dallas, Texas, United States, 4. Baylor University Louise Herrington School of Nursing, Baylor University Louise Herrington School of Nursing, Texas, United States*

Heart failure (HF) self-care is vital to health and wellbeing, yet more than half of all persons with HF do not adhere to the self-care recommendations of taking medications as prescribed, weighing daily, eating low salt foods, or exercising. It has been suggested that disparities in HF among racial/ethnic groups may be reflective of underlying determinants of health, such as poor engagement in self-care activities, rather than genetic or physiological differences. The purpose of this study was to examine direct and indirect effects of perceived social support, positive psychological (PP) characteristics, and patient activation on self-care behaviors in a diverse sample of older adults with HF. A nationwide survey was conducted in cooperation with the recruitment and sampling company Qualtrics. Stratified random sampling was used where 49% of the 174 respondents were persons of color (POC). The mean age was 60. Logistic regression statistical models were used with a lasso procedure. In this study, PP characteristics and activation level were most predictive of HF self-care adherence, particularly medication adherence. Respondents who were resilient, hopeful, and activated also reported higher medication and self-care adherence. Perceived social support and health literacy levels were not associated with self-care adherence. There were no differences in predictive variables by race/ethnicity, gender, or age. Interventions aimed at increasing resilience, hope, and engagement in care or activation may improve HF

self-care adherence among persons with HF. Further research is needed to understand the impact of PP characteristics and patient activation level on HF self-care adherence in POC.

### Session 1215 (Symposium)

### COHORT DIFFERENCES AND SIMILARITIES IN WOMEN'S ATTITUDES ABOUT SELF AND AGING

Chair: Aurora Sherman Discussant: Jamila Bookwala

This panel focuses on four complementing and international views of women's aging, with a special emphasis on cohort comparisons and using three different studies of women, with contrasting methodological frameworks. In so doing, we present evidence related to trends in social percepetions of aging, attitudes about aging and identity, and ideas about control and objectification. Dr. Newton presents data on older Canadian women showing the connection between physical aging and identity maintenance, using both qualitative and quantitative data and using the lifecourse perspective. Dr. Ryan, using data from the Health and Retirement Study to compare cohorts of women from the 2008 and 2018 HRS waves, reports cohort differences in negative self-perceptions of aging, and that both cohort and negative self-perfections are associated with life satisfaction, using the life course developmental framework. Ms. Tran compares younger and older cohorts of women on a measure of self-objectification, finding that the older cohort reported lower objectification, consistent with a selection, optimization, and compensation (SOC) model. Finally, Dr. Sherman, using the same data set as Ms. Tran, shows that control beliefs are associated with objectification, regardless of cohort, consistent with objectification theory predictions of consistency over time regarding the impact of objectification experiences. Dr. Jamila Bookwala will provide discussion of this group of papers.

### CONTROL BELIEFS, AGE, AND OBJECTIFICATION EXPERIENCES IN YOUNGER AND OLDER WOMEN

Aurora Sherman, *Oregon State University, Corvallis, Oregon, United States*

Control beliefs show age-related patterns; mastery decreases in adulthood, while constraints beliefs often increase. However, there is a great deal of individual variation. This paper addresses antecedents of control beliefs, with attention to experiences and beliefs related to sexual objectification, which have particular impact for women. In this study, younger women (N = 132, M = 20.93) and older women (N = 86, M = 67.83) were surveyed regarding their experiences with sexual objectification and constraints beliefs. Multiple regression analyses revealed higher self-objectification was associated with higher constraints (R2 = .09**) and lower mastery (R2 = .11**) but reports of body evaluation and sexual advances were not associated with control beliefs. Further, there were no interactions of either objectification scale with age. These results suggest that objectification may be an important part of the aging experience across the life course, not just in young adulthood.

### AGE, ATTITUDES TO AGING, AND IDENTITY IN OLDER CANADIAN WOMEN

Nicky Newton, *Wilfried Laurier University, Waterloo, Ontario, Canada*

The life course perspective emphasizes social structure, personal agency, and their interdependencies (Settersten et al., 2020), serving as the theoretical framework for this study. Given stereotypical societal views of gender and aging (e.g., Sontag, 1979), physical aging is often the focus when examining women's aging attitudes and concomitant changes in a sense of personal identity. Additionally, studies of midlife women have found relationships between age and identity (e.g., Stewart et al., 2001). Using quantitative and qualitative data, the present study examines associations between age, personal identity, and attitudes to physical, psychological and social aging in older Canadian women (N = 190, Mage = 70.38). Results show that while attitudes to physical aging contribute to identity maintenance, attitudes to social and psychological aging are also important for older women's identity maintenance. Interactions between age and attitudes to aging associated with personal identity are discussed with reference to the life course perspective.

### COHORT DIFFERENCES IN WELL-BEING AMONG MIDLIFE AND OLDER WOMEN: ROLE OF SELF-PERCEPTIONS OF AGING

Lindsay Ryan, *University of Michigan, Ann Arbor, Michigan, United States*

The current study examines how cohort differences across two age-matched groups of midlife and older women from the Health and Retirement Study are associated with well-being and self-perceptions of aging (SPA). Women aged 51–60 (n=2318) and 61–70 (n=1650) were selected from the 2008 and 2018 waves. No significant cohort differences were identified for life satisfaction (Diener, Emmons, Larsen & Griffin, 1985) or positive SPA (Lawton, 1975; Liang & Bollen, 1983). The 2008 cohort of midlife women reported significantly higher negative SPA compared to 2018 (p<.05). Linear regression analyses find that cohort and SPA are significantly associated with life satisfaction in both age groups, and that the association of negative SPA differs by cohort for the midlife women (p<.01). Implications are discussed within the life course developmental framework.

### COHORT DIFFERENCES IN SELF-OBJECTIFICATION

Sydney Tran, *Oregon State University, Corvallis, Oregon, United States*

Sexual objectification socializes women to engage in self-objectification—the tendency to view one's body as an object to be used by others and evaluating one's value in terms of attractiveness to others (Noll & Fredrickson, 1998)—and leads to negative psychological consequences. As women age, their bodies move further away from the thin ideal (Guo, Zeller, Chumlea, & Siervogel; 1999) potentially making them more susceptible to body i concerns and dissatisfaction. However, may also begin using selection, optimization, and compensation (SOC) strategies, countering the impacts of sexual objectification, and promoting successful aging. We compared self-objectification between women in early adulthood (N = 132, M = 20.93) and women in late middle age or late adulthood (N = 86, M = 67.83). Results showed that older women had significantly lower levels of self-objectification than younger women. Our findings support the idea the

SOC strategies protect against the consequences of sexual objectification.

### Session 1220 (Symposium)

### COVID-19 IN ASSISTED LIVING: EVIDENCE ON POLICIES, PROVIDER EXPERIENCES, AND RESIDENT MORTALITY

Chair: Kali Thomas Discussant: Lindsay Schwartz

The devastating effects of Coronavirus disease 2019 (COVID-19) among older adults residing in long-term care settings has been well documented. Assisted living settings in the U.S. have 811,000 residents; most are 80 years or older, and many have one or more chronic illnesses, making them highly susceptible to poor outcomes if exposed to COVID-19. This symposium highlights five studies that focus on various levels of COVID-19 response in assisted living: national organizations, states, assisted living operators and healthcare providers, and residents. The first study compares the sometimes conflicting guidance provided by national long-term care industry-related organizations and recommends assisted living-specific actions for the future. The second study describes the state regulatory response to COVID-19 in assisted living, identifying the themes and implications for the function of the care networks of assisted living residents. The third study presents findings from interviews with key stakeholders, including policymakers and industry leaders, that reflect on the challenges responding to changing recommendations and policies. The fourth study reports results from a survey with administrators and medical and mental health care providers who treat their residents that illustrates the care practices that were implemented in response to COVID-19 in assisted living. The fifth presentation documents the national excess assisted living resident mortality that was attributable to COVID-19. This symposium culminates with a leading assisted living industry expert reflecting on providers' experiences and posing areas to consider when preparing for and responding to future pandemic events in assisted living settings.

### COVID-19 RECOMMENDATIONS FOR ASSISTED LIVING: IMPLICATIONS FOR THE FUTURE

Andrew Vipperman,[1] Sheryl Zimmerman,[2] and Philip Sloane,[3] *1. University of Virginia, Charlottesville, Virginia, United States, 2. Cecil G. Sheps Center for Health Services Research, Chapel Hill, North Carolina, United States, 3. UNC Medical School, Sheps Center, Chapel Hill, North Carolina, United States*

Similar to nursing homes, COVID-19 has challenged assisted living (AL), given its congregate nature and vulnerable residents. However, COVID-19 recommendations have not consistently recognized differences between nursing homes and AL, and in so doing present implications for the future of AL. This project examined COVID-19 recommendations from six key organizations and compared them across nursing homes and AL. Differences include recommending more flexible visitation and group activities for AL, while similarities suggest that AL may best integrate health care into offered services (e.g., work with consulting clinicians who know residents and the AL community). Primary points to be discussed are that COVID-19 may accelerate the closer coordination of social work and medical care into AL, because recommendations suggest AL would benefit from the services and expertise of nurses, social workers, and physicians. There seems to be an unmet need to mitigate loneliness in AL, which warrants specific attention moving forward.

### STATE REGULATORY RESPONSE TO COVID-19 LIMITED ASSISTED LIVING RESIDENT CAREGIVER NETWORKS

Lindsey Smith,[1] Bethany Linscott Lowe,[2] Sarah Dys,[3] Kali Thomas,[4] Sheryl Zimmerman,[5] and Paula Carder,[1] *1. OHSU-PSU School of Public Health, Portland, Oregon, United States, 2. Oregon Health & Science University - Portland State University School of Public Health, Portland, Oregon, United States, 3. Portland State University, Portland, Oregon, United States, 4. Brown University, Brown University/Providence, Rhode Island, United States, 5. Cecil G. Sheps Center for Health Services Research, Chapel Hill, North Carolina, United States*

This paper describes a qualitative content analysis of assisted living emergency rules, revised regulations, and executive orders responding to the COVID-19 pandemic. Using key search terms, we identified 36 states that enacted policies between February and October 2020. The following themes occurred most frequently: testing, infection control, access restrictions, suspension of requirements, and reporting. The convoys of care model recognizes internal, external, formal, and informal caregivers as essential members of an AL resident's care network. We found that non-staff care providers, including external formal caregivers (e.g. home health and hospice) and informal caregivers (e.g. family), were most often addressed in policies limiting access. Informal caregivers were the least often specifically addressed in these policies. Given the importance of these network members in the AL context, these policies have implications for the wellbeing of the resident and care network.

### COVID-19 RESPONSE IN U.S. ASSISTED LIVING SETTINGS: KEY STAKEHOLDER PERSPECTIVES

Sarah Dys,[1] Jaclyn Winfree,[2] Paula Carder,[3] Sheryl Zimmerman,[4] and Kali Thomas,[5] *1. Portland State University, Portland, Oregon, United States, 2. Institute on Aging, Portland State University, Portland, Oregon, United States, 3. OHSU-PSU School of Public Health, Portland, Oregon, United States, 4. Cecil G. Sheps Center for Health Services Research, Chapel Hill, North Carolina, United States, 5. Brown University, Brown University/Providence, Rhode Island, United States*

Unique regulatory requirements and scope of services within assisted living (AL) pose distinctive challenges to COVID-19 response. To identify COVID-19 issues specific to AL, we recruited stakeholders with expertise in AL operations, policy, practice, and research (n=42) to participate in remote interviews between July and September 2020. Using thematic analysis, we derived the following overarching themes: 1) Policymakers lack an understanding of the AL context; 2) AL administrators were left to coordinate guidelines with little support; 3) AL organizations faced limited knowledge of and disparate access to resources; 4) State-level regulatory requirements conflicted with COVID-19

guidelines resulting in confusion; and 5) AL operators struggled to balance public health priorities with promoting their residents' wellbeing. To develop evidence-informed policy and avoid unintended consequences, AL operators, direct care workers, residents, and clinicians practicing in these settings should have opportunities to provide feedback through the policy development process, both state and national.

### SEVEN-STATE STUDY OF ASSISTED LIVING AND HEALTHCARE PROVIDERS' RESPONSES TO COVID-19

Sheryl Zimmerman,[1] Philip Sloane,[2] Johanna Hickey,[3] Christopher Wretman,[3] Paula Carder,[4] and Kali Thomas,[5]
*1. Cecil G. Sheps Center for Health Services Research, Chapel Hill, North Carolina, United States, 2. UNC Medical School, Sheps Center, Chapel Hill, North Carolina, United States, 3. University of North Carolina at Chapel Hill School, Chapel Hill, North Carolina, United States, 4. OHSU-PSU School of Public Health, Portland, Oregon, United States, 5. Brown University, Brown University/Providence, Rhode Island, United States*

COVID-19 has inordinately affected assisted living (AL), such that the proportion of fatalities to cases has been 21% in AL versus 2.5% for the general population. Understanding how AL administrators and medical and mental health providers have responded to COVID-19 can inform health care going forward. Using a seven-state stratified random sample of 250 communities, administrators were interviewed and providers completed questionnaires regarding COVID-19 practices. Preliminary data indicate that 79%, 44%, and 62% of administrators reported serving meals in rooms to segregate residents, using telemedicine, and providing extra pay for staff, respectively. Perceived use/effectiveness of practices differed based on dementia case-mix (e.g., face coverings, social distancing). Providers reported less access to patients (82%), more telehealth (63%), and less ability to provide care (43%). However, they uniformly reported high confidence in AL staff ability to prevent (94%) and respond to outbreaks (96%). Discussion will summarize points important for future care.

### EXCESS MORTALITY ATTRIBUTABLE TO COVID-19 AMONG ASSISTED LIVING RESIDENTS

Kali Thomas,[1] Wenhan Zhang,[2] David Dosa,[2] Paula Carder,[3] Philip Sloane,[4] and Sheryl Zimmerman,[5]
*1. Brown University, Brown University/Providence, Rhode Island, United States, 2. Brown University, Providence, Rhode Island, United States, 3. OHSU-PSU School of Public Health, Portland, Oregon, United States, 4. UNC Medical School, Sheps Center, Chapel Hill, North Carolina, United States, 5. Cecil G. Sheps Center for Health Services Research, Chapel Hill, North Carolina, United States*

This study examines the excess mortality attributable to COVID-19 among a national cohort of assisted living (AL) residents. To do this, we compare the weekly rate of all-cause mortality during 1/1/20-8/11/20 with the same weeks in 2019 and calculated adjusted incidence rate ratios (IRRs) and 95% confidence intervals (CIs). All-cause mortality rates, nationally, were 14% higher in 2020 compared with 2019 (mean, 2.309 vs. 2.020, respectively, per 1000 residents per week; adjusted IRR, 1.169; 95% CI 1.165-1.173). Among the 10 states with the highest community spread, the excess mortality attributable to COVID-19 was 24% higher, with 2.388 deaths per 1000 residents per week in 2020 during January-August vs 1.928 in 2019 (adjusted IRR, 1.241; 95% CI 1.233-1.250). These results suggest that AL residents suffered excess mortality due to COVID-19.

### Session 1225 (Symposium)

### COVID-19, POLICY, AND NURSING HOMES: A HEALTH AND AGING POLICY FELLOWS SYMPOSIUM

Chair: Nancy Kusmaul Co-Chair: Toni Miles Discussant: Lori Frank

COVID-19 significantly impacted older adults, particularly those in long term care. This symposium focuses on policy, and how policies drove many of the outcomes older adults in care experienced during 2020. We begin with a case study of one nursing home describing their operations, how those were impacted by policies at the local, state and federal levels, and operational factors that proved uncontrollable. From there we look more broadly at a national effort as implemented in one state which leveraged clinical and regulatory experts to partner with nursing homes and disseminate emerging and evidence based practice cohorts to address the realities of the COVID-19 pandemic over an extended period. Then we move from the federal to two state level projects. One looks at the experience of embedding advanced practice nurses (APRNs) into long term care facilities in one state over a five year period including during COVID-19. The final presentation describes hands on support provided by one state government to nursing homes and assisted livings during COVID-19, including the coordination of staff testing and the implementation of the use of the antigen testing machines issued through federal policy. We conclude with a discussion of the interplay of federal, state, and local policy on nursing home experiences in COVID-19 and recommendations for more effective policy interventions.

### COLLABORATION WITH THE STATE DEPARTMENT OF HEALTH

Kathleen Unroe, *Geriatrics, Indiana University, Indiana, United States*

COVID-19 disproportionately affected older adults, creating opportunities for experts in geriatrics and gerontology to support public policy. In Indiana, the Probari team, composed of a geriatrician and a team of nurses with geriatrics and palliative care expertise, supported the state government response to long-term care facilities during the pandemic. The team was involved in helping coordinate all staff testing (534 nursing homes) by the State Department of Health in June and in August, prior to the Federal mandated testing and the distribution of antigen machines. The Probari team also fielded surveys on behalf of the State regarding staff attitudes towards testing and willingness to be vaccinated, to inform state policy and resource efforts. In addition, Probari collaborated with the State Department of Health and the Indiana National Guard by training over 1600 service members to provide non-clinical support in nursing facilities, and monitoring and evaluating that 3 month deployment.

### UTILIZING PEER COHORT NETWORKS TO DISSEMINATE INFORMATION AND TO PREVENT AND MITIGATE COVID-19 IN NURSING HOMES

Rajean Moone, *University of Minnesota, Woodbury, Minnesota, United States*

The COVID-19 pandemic disproportionately impacted residents, families, and staff of nursing homes and senior care communities. Even with federally mandated emergency planning, the pandemic highlighted the lack of preparation to meet the daily challenges faced in senior care. In response, the federal CARES Act including funding for a nationwide network of nursing home cohorts led by academic health centers to disseminate clinical guidance in infection control and pandemic mitigation strategies. We present a case study of a successful diffusion model as implemented in Minnesota with seven cohorts comprised of 242 nursing homes and 544 employees. Experts in geriatric care, long term care regulatory management, and public health led ninety-minute sessions held over the span of sixteen weeks. The session format included foundational evidence-based practices in pandemic management (including infection control, social isolation, leadership, and other topics), individual case studies, peer to peer knowledge diffusion, and expert guidance.

### COVID-19 IN LONG-TERM CARE FACILITIES WITH ADVANCED PRACTICE REGISTERED NURSES

Tracie Harrison, *The University of Texas at Austin School of Nursing, Austin, Texas, United States*

The purpose of our report is to give our analytical description of COVID-19 response. The study placed two APRNs in each of five participating nursing facilities (NF), 5-7 days/week for up to two years with the purpose of quantifying if employment of a full-time, salaried APRN, as part of the NF care team, improved the quality of care for the NF residents. Secondarily, we studied policies that influenced ability to provide APRN care in the NF. Resident data collected and evaluated to determine if this model of care reduced the rates of adverse events among NF residents. Data included re-hospitalizations, inappropriate prescribing (antipsychotics, antibiotics, opioids and polypharmacy), observational data, and clinical outcomes negatively affecting the quality of life for NF residents (such as falls and pressure injuries). Factors influencing APRN employment in the NF setting were NF mission, environmental aesthetics, resources, NF administrator interactions, state law, and medical director support.

### CASE STUDY OF AN OUTBREAK: RESIDENT, STAFF, AND COMMUNITY INDICATORS

Toni Miles, *College of Public Health, University of Georgia, Georgia, United States*

Outbreak investigation is not infection control. We present a self-study of factors influencing outcomes inside a single nursing home during the early stage of the outbreak - February to May 2020. We examine 3 sources of influence: Practice / Operations; Local, State & Federal Policies; Uncontrollable operational factors. Outcomes of interest include: mortality and resident / staff health. Data consists of clinical records, review of communications, and interviews with staff present during the critical period. Infection control is different from outbreak investigation. There must be a balance between staff empowerment and adherence to guidelines. In an outbreak, staff need the confidence to make decisions based on incomplete knowledge. The presentation concludes with lessons learned – what worked and what actions need improvement. There are areas requiring further analyses of policy and ethics.

### Session 1230 (Paper)

### DISPARITIES AND ALZHEIMER'S DISEASE

### CAUSES OF THE RACIAL DISPARITIES IN THE RISK OF ALZHEIMER'S DISEASE

Stanislav Kolpakov Nikitin,[1] Arseniy Yashkin,[2] Julia Kravchenko,[1] and Igor Akushevich,[1] *1. Duke University, Durham, North Carolina, United States, 2. Duke University, Morrisville, North Carolina, United States*

The risk of Alzheimer's disease (AD) is not uniform across race-specific subpopulations: Blacks face approximately 50% higher risk of AD onset compared to Whites(Hazard Ratio=1.50; 95%CI:1.46-1.54). We used Blinder-Oaxaca decomposition, modified for censored data, to explain the disparities in the risk of AD between these races in Medicare beneficiaries aged 65+. This approach measures the contributions to the total difference in AD risks from the differences in the prevalence and the difference in magnitude of the effects of each potential explanatory variable. We used hypertension, diabetes mellitus, depression, cerebrovascular and renal diseases as the potential causes of the racial disparities in AD risk. We found that the greatest contribution was due to the impact of arterial hypertension, of which 24% of the effect was due to differences in prevalence and 76% due to the differences in effect magnitude. Unexpectedly, the contributions of other studied diseases explained only a small part of the racial disparity in AD risk. The remaining incidence rates, which could not be explained by the contributions of hypertension and other included diseases in the age-specific analysis, were lower for the Black population, although initially, the total age-specific incidence rates of AD were higher for the Blacks when compared to the Whites. Therefore, our results suggest that targeted interventions in the Black subpopulation are urgently needed to mitigate the adverse health effects of hypertension, independent of the possible causes, such as access to hypertension care, or race-related differences in adherence to antihypertensive treatment.

### DOCUMENTATION OF DEMENTIA AS A CAUSE OF DEATH AMONG MEXICAN-AMERICAN DECEDENTS WITH DEMENTIA

Brian Downer,[1] Lin-Na Chou,[1] Soham Al Snih,[1] Cheyanne Barba,[2] Yong-Fang Kuo,[1] Mukaila Raji,[1] Kyriakos Markides,[1] and Kenneth Ottenbacher,[1]
*1. University of Texas Medical Branch, Galveston, Texas, United States, 2. University of Alabama, Birmingham, Birmingham, Alabama, United States*

There is lack of data on the frequency and correlates of dementia being documented as a cause of death in Hispanic populations. We investigated characteristics associated with dementia as a cause of death among Mexican-American decedents diagnosed with dementia. Data came from the

Hispanic Established Populations for the Epidemiologic Study of the Elderly, Medicare claims files, and the National Death Index. Of the 744 decedents diagnosed with dementia before death, 26.9% had dementia documented as a cause of death. More health comorbidities (OR=0.38, 95% CI=0.25-0.57), older age at death (OR=1.05, 95% CI=1.01-1.08), and longer dementia duration (OR=1.09, 95% CI=1.03-1.16) were associated with dementia as a cause of death. In the last year of life, any ER admission with (OR=0.56, 95% CI=0.32-0.98) or without (OR=0.31, 95% CI=0.14-0.70) a hospitalization, more physician visits (OR=0.95, 95% CI=0.92-0.98) and seeing a medical specialist (OR=0.41, 95% CI=0.24-0.70) were associated with lower odds for dementia as a cause of death. In the last 30-days of life, any hospitalization with an ICU stay (OR=0.57, 95% CI=0.37-0.88) and ER admission with (OR=0.58, 95% CI=0.40-0.84) or without (OR=0.48, 95% CI=0.25-0.94) a hospitalization were associated with lower odds for dementia as a cause of death. Receiving hospice care in the last 30-days of life was associated with 2.09 (95% CI=1.38-3.16) higher odds for dementia as a cause of death. The possible under-documentation of dementia as a cause of death on death certificates may result in underestimation of healthcare resource need of dementia care for Mexican-Americans.

### GEOGRAPHIC DISPARITIES OF ALZHEIMER'S DISEASE MORTALITY IN FEMALES WITH BREAST CANCER

Igor Akushevich,[1] Arseniy Yashkin,[2] and Julia Kravchenko,[1] *1. Duke University, Durham, North Carolina, United States, 2. Duke University, Morrisville, North Carolina, United States*

Our estimates showed significant gaps in mortality rates between the West and East parts of the U.S. when these rates are based on death certificate data. These geographic disparities were persistent over time and could not be fully explained by differences in demographic and socioeconomic characteristics, comorbidities, and/or differences in AD coding between these regions. However, incidence and incidence-based mortality rates based on Medicare data do not reproduce these geographic disparities. Death certificate-based patterns hold for the subset of the population with breast cancer, e.g., for subpopulation for which breast cancer was listed as a secondary cause of death. Therefore, SEER-Medicare data, which contains both death-certificate records and Medicare administrative claims for the same individuals can be used to resolve this inconsistency in findings. Analysis of breast cancer patients from two SEER registries in NJ and WA states in SEER-Medicare data (2000-2013) showed that the fraction of deceased individuals with an underlying cause AD among those who had a Medicare diagnosis of AD is 2.5-3.5 times (depending on the Medicare ascertainment algorithm) higher in WA comparing to NJ ($p<0.0001$). The odds ratio of not-having AD as an underlying cause is 1.3 for WA vs. NJ and increases with age, for non-white races, and unmarried individuals. Our findings do not support the hypothesis of higher rates of AD in WA state but show that AD is likely underrepresented in death certificate in NJ and possibly other East coast states.

### NEIGHBORHOOD DEPRIVATION AND INCIDENT ALZHEIMER'S DISEASE: A REGIONAL COHORT STUDY OF ELECTRONIC MEDICAL RECORDS

Jarrod Dalton,[1] Elizabeth Pfoh,[2] Kristen Berg,[3] Douglas Gunzler,[3] Lyla Mourany,[2] Nikolas Krieger,[2] Eva Kahana,[4] and Adam Perzynski,[3] *1. Cleveland Clinic and Case Western Reserve University, Cleveland, Ohio, United States, 2. Cleveland Clinic, Cleveland, Ohio, United States, 3. Case Western Reserve University at MetroHealth, Cleveland, Ohio, United States, 4. Case Western University, Case Western reserve University, Ohio, United States*

The prevalence of Alzheimer's disease (AD) is anticipated to increase drastically. Neighborhood socioeconomic position (SEP) has been related to multiple processes of health. Understanding whether SEP is related to AD can inform who is at greatest risk of developing this disease. We analyzed electronic medical records of 394892 patients from the two largest health systems in Northeast Ohio to evaluate the relationship between Ohio Area Deprivation Index quintiles (defined at the census tract level) and hazard for a composite outcome of AD diagnosis or primary AD death. We included residents of Cuyahoga and neighboring counties, and used the first outpatient visit beyond age 60 occurring between 2005 and 2015 as study baseline. Outcome data were censored at the earlier of a) the beginning of any 3-year time period without visits or b) non-AD death. We estimated a Cox proportional hazards regression model, adjusting ADI quintile effects for the interaction between age at baseline, sex and race as well as birth year. We used quadratic terms for continuous predictors. After adjusting for these factors, ADI quintile was significantly related ($\chi2 = 83.0$ on 4 d.f.; $p < 0.0001$) to the composite time-to-event outcome. Compared to the lowest-deprivation quintile, ADI quintiles 4 (adjusted hazard ratio [95% confidence interval]: 1.18 [1.10, 1.26]) and 5 (1.37 [1.28, 1.47]) had significantly higher hazard for the composite outcome. In conclusion, neighborhood deprivation may be a risk factor for AD independent of demographic factors. Preventive efforts should target individuals living in neighborhoods with high levels of deprivation.

### RACIAL DIFFERENCES IN THE EFFECT OF ALZHEIMER'S DISEASE ON ADHERENCE TO MEDICATION THERAPY FOR CHRONIC DISEASES

Arseniy Yashkin,[1] Anatoliy Yashin,[2] Galina Gorbunova,[2] and Igor Akushevich,[2] *1. Duke University, Morrisville, North Carolina, United States, 2. Duke University, Durham, North Carolina, United States*

Multiple dementia (the presence of one or more types of dementia in a single individual) and multi-morbidity (the presence of multiple chronic diseases in an individual) present a major challenge to the U.S. healthcare system. The reduction in cognitive function associated with neurocognitive disorders such as Alzheimer's Disease (AD) and Related Dementias (ADRD) reduce the ability of the affected individual to take care of him/herself. This can manifest as reduced adherence to medication regimens designed to manage other chronic conditions, in reduced ability to engage in healthy behavior such as exercise, or in other ways. The result is an increase in the probability of otherwise avoidable adverse health outcomes and related healthcare costs. In this study, we showcase two high impact chronic conditions common in the elderly:

hypertension and type 2 diabetes mellitus (T2D). Using a 5% sample of the total Medicare population we identify groups of individuals with AD/ADRD and i) hypertension, ii) T2D or iii) both. Each group is then propensity-score-matched to similar individuals with hypertension, T2D or both but without a diagnosis of AD/ADRD. The primary explanatory variable of interest is the medication possession ratio (MPR) calculated at 1-year intervals for prescribed diabetes and/or hypertension medications. MPRs were compared between the two groups using t-tests and standardized differences each year after baseline and until death/censoring. A Cox proportional hazard model was then used to estimate differences in survival between these two groups and across race/ethnicity-related strata. Reduced adherence with time and notable race/ethnicity-related differences were identified.

### Session 1235 (Symposium)

### EMBODYING THE AGING EXPERIENCE: HOW VIRTUAL REALITY IS TRANSFORMING MEDICAL AND NURSING EDUCATION

Chair: Marilyn Gugliucci Co-Chair: Pamela Saunders Discussant: Erin Washington

Virtual reality (VR) has long been standard in healthcare education. Recent advances in VR hardware and software applications have coalesced to allow for higher fidelity, more highly realistic simulations that are also deployable at scale — not just in highly specialized, single location simulation labs. In tandem, there has been an examination in both the corporate and academic sectors around the efficacy of VR training and learning. While VR has been long proven to be effective in training students and workers in hard skills, its lack of realism has been a barrier to explore efficacy in simulations related to soft skills and emotional intelligence. This symposium will discuss the implementation of virtual reality "labs", where learners embody in a live 360 film environment the first-person point of view of an older adult — interacting with gaze, voice, and natural hand motions – into four university's medical and nursing curriculum. Lab outcomes include decreased ageism and stereotyping, and increased empathy, sensitivity, cultural competency, and disease knowledge. The first paper reports outcomes of increased understanding, comfort, compassion and empathy of students and informal caregivers after experiencing various labs. The second discusses comparative data on knowledge and attitudes of medical students experiencing the virtual labs individually vs. the group distance mode. The third reports the results of an initial study on how embodying an older adult with sensory impairment affects participant empathy using a standardized scale. The fourth discusses how one university transitioned to delivering immersive labs to nursing students remotely during COVID19.

### THE EFFECTS OF A VR TEACHING TOOL ON UNDERSTANDING, COMFORTABILITY, COMPASSION, AND EMPATHY OF STUDENTS AND CAREGIVERS

Carrie Elzie, *Eastern Virginia Medical School, Norfolk, Virginia, United States*

Empathetic care giving is associated with improved patient satisfaction, compliance and outcomes; clinical competence, career satisfaction, and burnout reduction; as well as diminished medical errors and litigation claims. Unfortunately, recent studies have shown erosion in empathy and compassion across the health professions. Virtual reality shows promise as a teaching tool to combat this decline as it has been dubbed the ultimate empathy machine, allowing users to vividly and viscerally experience any situation from any perspective. Embodied Labs allows users to virtually walk in the shoes of different patients, experiencing symptoms, family dynamics, support networks and various components of the health care systems. We have demonstrated that the high level of immersion and presence afforded by these virtual labs are effective pedagogical tools to increase understanding, comfortability, compassion and empathy within various populations including informal caregivers, high school students, health professional students and medical students.

### VIRTUALLY TRANSFORMATIVE EXPERIENCES IN GERIATRIC EDUCATION: LESSONS PRE AND POST PANDEMIC

Pamela Saunders, *Georgetown University, WASHINGTON, District of Columbia, United States*

Georgetown University medical students have the option of selecting a two-week rotation in Geriatrics during their third-year. Since Fall 2019, the curriculum has included three immersive virtual reality (VR) labs: hearing & vision loss, Alzheimer's disease, and end-of-life conversations created by Embodied Labs. The curricular goals include increasing empathy and sensitivity of learners to the perspective of older adults, decreasing ageism & stereotyping, and increasing clinical knowledge. In each lab, students are immersed in a live film, first-person point of view of an older adult. They interact with the immersive environment via gaze, voice, and natural hand motions. Pre-pandemic, students viewed the labs in-person using a commercial VR headset. Since the pandemic, March 2020, students accessed the VR labs through the virtual modality of Zoom. This abstract summarizes data on knowledge and attitudes examining differences in knowledge and attitudes pre and post-pandemic.

### IMPACT OF VIRTUAL REALITY ON HEALTHCARE PROVIDER EMPATHY FOR OLDER ADULTS WITH SENSORY IMPAIRMENT

Suzanne Dutton, *Sibley Memorial Hospital/Johns Hopkins, washington, District of Columbia, United States*

Virtual reality (VR) is an innovative technology that can simulate dual sensory impairment so that healthcare providers and others can experience this affliction common in older adults. This study investigated whether VR simulation could increase empathy among healthcare workers. Healthcare providers experienced a 7-minute scenario from the viewpoint of "Alfred", a 74-year-old with macular degeneration and high frequency hearing loss on a commercial VR headset (Oculus Rift). Using a one-group pre/post-test study design, we measured knowledge, changes in empathy, and assessed participants' self-reported behavior change. Results showed that participants increased their knowledge and that 9 of 14 empathy items had statistically significant increases. Additionally, 97% of participants agreed or strongly agreed that they would utilize the information learned in their work with patients. In conclusion, evidence suggests VR is an effective intervention to increase empathy and positively change behavior to support persons with sensory impairment.

### REMOTE DELIVERY OF VIRTUAL REALITY PATIENT SIMULATIONS FOR NURSING EDUCATION

Jaime Hannans, *California State University Channel Islands, Simi Valley, California, United States*

In the midst of rapid transfers to online teaching for experiential learning opportunities in nursing clinical labs this past spring due to the pandemic, nursing simulations with immersive virtual reality (VR) in VR headsets were deemed impossible. In partnership with Embodied Labs, nursing faculty pivoted to facilitating VR using remote learning approaches in groups. In this new VR approach nursing students engaged in active learning, critical discourse, and reflection guided by faculty delivered VR scenarios remotely with in-session debriefing during discussion pause points. Complex scenarios focused on patient or family perspectives (e.g. during end-of-life care or navigating community and healthcare needs as a LGBTQ individual). These were valuable online learning opportunities for undergraduate nursing education. Student feedback was positive, and faculty perceptions indicated using VR remote learning offers rich, engaging discussion through complex topics important to nursing clinical practice.

### Session 1240 (Paper)

### END-OF-LIFE CARE POLICY

### AN EVALUATION OF THE SERVICE INTENSITY ADD-ON PAYMENT POLICY REFORM IN THE MEDICARE HOSPICE BENEFIT

Thomas Christian,[1] Pedro Gozalo,[2] Joan Teno,[3] and Michael Plotzke,[4], *1. Abt Associates, Cambridge, Massachusetts, United States, 2. Brown University, Providence, Rhode Island, United States, 3. Oregon Health and Science University, Portland, Oregon, United States, 4. Abt Associates, Saint Louis, Missouri, United States*

In 2016, the Centers for Medicare & Medicaid Services (CMS) implemented the Service Intensity Add-On (SIA) payment, which incentivized skilled nurse and medical social worker (SN/MSW) visits in the last seven days of life. Little is known about the impact of this initiative. Using 100% Medicare hospice claims, we identified a 10% random sample of Medicare hospice beneficiaries utilizing routine home care service during calendar years 2012-2018. We compared the provision of SN/MSW visits on service dates before and after the SIA's implementation relative to beneficiaries' date of death. We also determined hospice providers' success in providing SN/MSW visits in the last days of life and categorized all providers into quintiles according to the average rate of these visits in the period prior to the SIA's implementation. Cumulative over the last seven days of life, we calculated an increase of 15.7 SN/MSW minutes (95% confidence internal [CI] 14.9-16.5 minutes) per beneficiary after the SIA was implemented. The per-minute increase was greatest on days nearer to death (4.0 minutes day of death, 95% CI 3.6-4.2). There was no detectable visit increase on days which were ineligible for the SIA. Additionally, those providers in the quintile providing the lowest rate of SN/MSW visits pre-SIA exhibited a 14-percentage point increase in rates of these visits, the third, fourth, and fifth quintiles exhibited little change over time. Further monitoring is needed to ensure beneficiaries receive adequate end-of-life care.

### CHALLENGES IN IMPLEMENTING AN EXPLICIT PROTOCOL FOR LIVE DISCHARGE FROM HOSPICE

Stephanie Wladkowski,[1] Susan Enguidanos,[2] Tracy Schroepfer,[3] and Keegan Pabst,[4] *1. Eastern Michigan University School of Social Work, Eastern Michigan University, Michigan, United States, 2. University of Southern California, Los Angeles, California, United States, 3. University of Wisconsin-Madison, Madison, Wisconsin, United States, 4. Eastern Michigan University, Ypsilanti, Michigan, United States*

A live discharge from hospice can occur when a patient stabilized under hospice care and no longer meets the life expectancy hospice eligibility criteria. In 2018, 220,000 hospice patients across the United States were discharged alive from hospice care, with 1 in 6 discharges due to stabilization, with a life expectancy exceeding hospice's six-month criteria. Hospice practitioners prepare patients and their caregivers upon enrollment for the possibility of a live discharge should their condition stabilize, however, there is no explicit discharge process available within hospice to guide practitioners in transitioning patients (and caregivers) out of hospice care. This transition process largely falls within the domain of hospice social workers, yet there is no research documenting the challenges and facilitators to conducting a live discharge from hospice. This study aimed to understand social workers' perspectives on the live discharge process. To better understand challenges and facilitators to the live discharge process, we conducted focus group interviews with hospice social workers at four hospice agencies across the U.S. We asked participants to discuss specific tasks associated with the live discharge process for a patient and their caregiver including identifying concrete services needed post-discharge; assessing the psychosocial and grief risk of patient and caregiver; and developing a post-discharge follow-up plan. Using constant comparison analysis we identified several themes including the need for clear professional roles during a live discharge, interprofessional education, and the need for dedicated time for live discharge follow-up. Policy implications and opportunities also will be discussed.

### CONSTRUCTION AND PERFORMANCE OF THE HOSPICE CARE INDEX CLAIMS-BASED QUALITY MEASURE

Michael Plotzke,[1] Thomas Christian,[2] Kim Groover,[3] Zinnia Harrison,[4] Ihsan Abdur-Rahman,[5] and Cindy Massuda,[5] *1. Abt Associates, Saint Louis, Missouri, United States, 2. Abt Associates, Cambridge, Massachusetts, United States, 3. Abt Associates, Atlanta, Georgia, United States, 4. Abt Associates, Rockville, Maryland, United States, 5. CMS, Baltimore, Maryland, United States*

As part of the Medicare Hospice Benefit (MHB), hospices submit claims containing information that allows policy makers to assess hospice quality, help policy makers improve the MHB, and increase patients' experiences of care. We examine ten different hospice quality indicators related to the provision of services and patterns of live discharge. We calculated indicators using 100% Medicare fee-for-service (FFS) claims from October 1, 2018 through September 30, 2019. A hospice's total score among all ten indicators is referred to as their Hospice Care Index (HCI), with a possible high score of 10. We examined all hospices with at least 20 discharges. After exclusion, we examined 4,155 hospices representing 1,562,003 beneficiaries. Most hospices earn a high HCI score: over 85% of hospices had scores of eight or more. At the same time, there were some lower scoring hospices: one in ten hospices scored seven on the index, and the remaining 4.9% scored six or lower. We find that on average hospices with higher HCI scores have better Consumer Assessment of Healthcare Providers and Systems (CAHPS®) Hospice ratings. Among hospices with a score of ten, 85.1% of caregivers reported they would definitely recommend the hospice vs. 82.9% of caregivers of patients receiving treatment from hospices with a score of seven or less. Using the HCI, the Centers for Medicare and Medicaid Services and hospice patient caregivers can assess hospices across a broad set of indicators. Policymakers and hospices should monitor these ten indicators to understand their performance relative to peers.

### DEATH WITH DIGNITY: A POLICY ANALYSIS

Zainab Suntai, *University of Alabama, Tuscaloosa, Alabama, United States*

Death with dignity is one of many titles attributed to the practice of providing a patient with terminal illness a means to die in light of extreme suffering as a result of a terminal illness. The purpose of the Oregon Death with Dignity Act is to provide individuals suffering from a terminal illness with the right to make a written request for life-ending medication to end their life in a "humane and dignified manner." Deborah Stone's policy goals of equity, efficiency, welfare, security, and liberty provides a framework to analyze whether a policy is doing what it says it wants to do. As such, the goal of this presentation is to apply the policy goals framework to determine if the Death with Dignity Act is accomplishing its stated goals. Applying a theory of the policy process, the Death with Dignity Act was analyzed using the most recently available data from the Oregon Health Authority. Results showed that since the Death with Dignity Act passed in 1997, 2,518 people have received prescriptions for life-ending medications, and of those, 1657 or 66% have used the medication to end their lives. Based on the data aggregated between 1997 and 2019, the Death with Dignity Act has mostly met its purpose of providing individuals with a terminal illness with the right to die on their own terms and with dignity. However, there are still several issues regarding equity, especially for low-income BIPOC populations. Implications for practice, policy and research are discussed.

### IMPACTS OF COVID-19 ON THE UTILIZATION OF THE MEDICARE HOSPICE BENEFIT

Michael Plotzke,[1] Betty Fout,[2] and Thomas Christian,[3]
*1. Abt Associates, Saint Louis, Missouri, United States,*
*2. Abt Associates, Rockville, Maryland, United States,*
*3. Abt Associates, Cambridge, Massachusetts, United States*

The Coronavirus Disease 2019 (COVID-19) Public Health Emergency (PHE) has had a substantial impact on the provision and utilization of healthcare services. Given the high mortality rate associated with COVID-19 amongst older adults, COVID-19 is likely to have a profound impact on all hospice users due to disruptions in providing services. Our work describes how Medicare beneficiaries have utilized the Medicare Hospice Benefit (MHB) during the PHE and how that compares to utilization of the MHB prior to the PHE. We conducted a retrospective analysis of 100% Part A and Part B Fee-for-Service (FFS) Medicare claims from January 1, 2019 – December 31, 2020. We identified approximately 42.3 million unique Medicare FFS beneficiaries from January 2019 through December 2020. Of these, 1.6 million (3.8%) had at least one hospice claim and 1.7 million (4.0%) had at least one Medicare Part A or Part B claim with a COVID-19 diagnosis during the same time period. The rate of COVID-19 amongst FFS Medicare patients who utilized hospice was 8.3%. Average per-beneficiary per-month hospice visits fell by 28.2% for aides and 15.4% for nurses from December 2019 (7.1 aide visits, 6.5 skilled nursing visits) through December 2020 (5.1 aide visits, 5.5 skilled nursing visits). CMS should continue to monitor the prevalence of COVID-19 amongst hospice users and measures of hospice utilization amongst all hospice users in order to better understand how the PHE impacts the provision of the MHB and ensure beneficiaries continue to have access to needed services.

### Session 1245 (Symposium)

### FAMILY CAREGIVING POLICIES: WHERE WE ARE NOW

Chair: Pamela Nadash Co-Chair: Rani Snyder Discussant: Eileen Tell

This session reviews prospects for advancing family caregiving policy under the Biden Administration, by reporting on the RAISE (Recognize, Assist, Include, Support, and Engage) Family Caregivers Act, enacted in January 2018. The Act directs the Secretary of HHS to develop a national family caregiving strategy, and supports research and consensus-building activities, in collaboration with The John A. Hartford Foundation. It aims to identify actions that communities, providers, government, and others may take to recognize and support family caregivers. To this end, the Administration for Community Living (ACL) has convened an Advisory Council, comprising 15 voting members from various stakeholder groups, to guide the effort; the project also commissioned primary data collection on caregiver priorities and recommendations, using a Request for Information (RFI) in the Federal Register garnering roughly 1600 responses, 12 focus groups with diverse family caregivers, and listening sessions with stakeholder groups. Wendy Fox-Grage, of the National Academy on State Health Policy, which supports RAISE Act activities, will describe the project's scope of work and activities to date. Pamela Nadash from the LeadingAge LTSS Center @UMass Boston, who leads the data analysis component, will present findings from the commissioned research, while Molly Evans, (MA Executive Office of Elder Affairs) will review the current state of state-level policies aimed at supporting family caregivers. The symposium will conclude with Grace Whiting,

CEO of the National Alliance for Caregiving, who will present an advocate's perspective on the status of family caregiving policy. Eileen Tell, of ET Consultants, will act as discussant.

### FINDINGS FROM RAISE ACT RESEARCH: FAMILY CAREGIVER PRIORITIES

Pamela Nadash,[1] Eileen Tell,[2] Carol Regan,[3] Taylor Jansen,[4] Andrew Alberth,[5] and Marc Cohen,[1]
*1. University of Massachusetts Boston, University of Massachusetts Boston, Massachusetts, United States, 2. ET Consulting, Belmont, Massachusetts, United States, 3. Community Catalyst, Boston, Massachusetts, United States, 4. UMass Boston, Boston, Massachusetts, United States, 5. University of Massachusetts Boston, Boston, Massachusetts, United States*

To understand the needs and policy priorities of family caregivers, the Advisory Council commissioned research: first, through a request for information (RFI) in the Federal Register, which garnered roughly 1600 responses. Qualitative analysis revealed that family caregivers have diverse needs spanning their financial security as well as their needs for caregiver-focused supports; recommendations were similarly diverse, including requests for caregiver pay, improved access to respite, and other major policy changes. These findings fed into 12 focus groups focusing on diverse populations of caregivers, yielding more depth around caregiver priorities. Six stakeholder listening sessions built on these results, aiming to develop concrete suggestions for a national caregiver strategy – a key outcome of the Advisory Council. Such strategies ranged from a major publicity campaign creating awareness of family caregivers, to suggestions on implementing caregiver assessments, to more ambitious goals such as improved financing for long term services and supports more broadly.

### ACTIVITIES UNDER THE RAISE FAMILY CAREGIVING ACT: DEVELOPING A NATIONAL STRATEGY TO SUPPORT FAMILY CAREGIVERS

Wendy Fox-Grage, *NASHP, Washington, DC, District of Columbia, United States*

This presentation describes the unique collaboration between The John A. Hartford Foundation, the Administration or Community Living (ACL), and the National Academy for State Health Policy (NASHP) in supporting the RAISE Act Family Caregiver Resource and Dissemination Center, and the goals and activities of the RAISE Act Family Caregiving Advisory Council. Most importantly, she will present the the development of recommendations for a national strategy to support family caregivers involving all levels of government as well as private-sector actors. These recommendations fall into five primary areas, which Fox-Grage will discuss in detail. She will also discuss the Center's development of family caregiving resources for state and federal policymakers and other stakeholders as well as next steps in turning the Council's recommendations into concrete action.

### STATE-LEVEL POLICY ACTIONS TO SUPPORT FAMILY CAREGIVERS

Molly Evans, *Commonwealth of Massachusetts, Boston, Massachusetts, United States*

The stresses created by the growing need for family caregivers have failed to prompt federal policy action; in its absence, states are stepping up. This review of state policies that support employment among family caregivers found six main categories of legislative action: paid leave; expanding federally mandated unpaid leave; paid sick time; unemployment insurance for job loss attributable to caregiving duties; establishing family caregivers as a protected classification in employment discrimination; and flexible or alternative work schedules. Despite the demand for policies that support and empower working caregivers, a minority of states have passed such legislation; to date, 9 states have implemented paid family leave; 14 have implemented mandatory sick leave legislation; and 14 have expanded FMLA. This study discusses state-level policy actions, reviews the status and importance of these policies, and finds that despite gaps in caregiver support legislation at the state level, there is significant and promising momentum.

### FAMILY CAREGIVERS AS ADVOCATES

C. Grace Whiting, *National Alliance for Caregiving, Washington, District of Columbia, United States*

This presentation discusses the growing influence of family caregiver advocacy and its prospects for impacting policy under the Biden administration, at both the federal and states levels. In particular, it will describe the National Alliance for Caregiving's 50-state unified strategy for establishing the caregiver support infrastructure that is needed to coordinate efforts and to support caregivers across the nation and the lifespan. Historically, family caregivers have had difficulty acting as effective advocates, given the multiple roles they often play and their widely divergent interests, based on the varying needs of their care recipient and their divergent life circumstances. However, the Biden administration has indicated receptivity to caregiver issues, and the public has become increasingly aware of the caregiver role (in part, due to the pandemic), resulting in improved prospects for policy action at both the state and federal levels. This presentation reviews recent developments and discusses strategies for moving forward.

### Session 1250 (Symposium)

### IMPACT OF COVID-19 ON OLDER EMPLOYEES OF A LARGE STATE UNIVERSITY: FINDINGS FROM A MIXED-METHODS STUDY

Chair: Zhao Chen Co-Chair: Amanda Sokan

Burgeoning research on the effects of COVID-19 and university experiences in the U.S. tends to focus on transmission of COVID-19 virus or student-related consequences of COVID-19. However, none to our knowledge examine the effects on older university employees. Universities employ a higher percentage of older adults with diverse job responsibilities and socioeconomic status, presenting a unique closed community for understanding the pandemic's consequences for older adults. Our aims are to: 1) understand older university employees' concerns related to COVID-19, 2) develop intervention strategies to mitigate the adverse impact of the COVID-19 pandemic on the health and wellbeing of older employees, and 3) test the interventions within the target population to help reduce stress and promote wellbeing. Using a community participatory approach, we sought input from employees aged 50 and older at the University of Arizona. Mixed methods were used to collect qualitative (six focus groups; N=

24) and quantitative (online survey; N=1030) data. We conducted and evaluated a set of interventions (i.e., virtual Tai Chi and Qigong, walking exercises, and meditation) using focus group feedback, process evaluation, and outcome assessment with validated questionnaires on sleep quality, mindfulness and psychological wellbeing. Findings show that a significant percentage of older employees worried about getting COVID-19 and had experienced undesirable changes in sleep quality, weight, and physical activity, and concerns about caregiving; however, we also observed psychological resilience in this population. The study highlights the importance of developing immediate and effective programs for promoting health and wellbeing for older employees during the pandemic.

### UNDESIRABLE WEIGHT CHANGE AND REDUCED SLEEP QUALITY IN UNIVERSITY OLDER EMPLOYEES DURING THE COVID-19 PANDEMIC

Hamza Butt,[1] Kathie Insel,[2] Kendra Jason,[3] Mark Wager,[1] Dagoberto Robles,[1] and Yunjia Yang,[1] *1. University of Arizona, Tucson, Arizona, United States, 2. Behavioral and Social Science, Tucson, Arizona, United States, 3. UNC Charlotte, Charlotte, North Carolina, United States*

This study investigated the occurrence of undesirable weight change (UDWC) and reduced sleep quality (RSQ), and major factors associated with these changes during COVID-19 pandemic amongst university older employees (age 50+). Participants (n = 846) were recruited throughout campus and completed an online survey. Summary statistics were used to describe characteristics of the study participants and frequency and level of UDWC and RSQ. Proportional odds models were used to assess major factors associated with UDWC and RSQ. The results showed 416 (43.2%) participants reported UDWC and 474 (49.2%) RSQ. Age was inversely, and obesity positively associated with UDWC and RSQ. With each 5-year increase in age, the OR (95% CI) was 0.87 (0.78, 0.97) for reporting UDWC and 0.90 (0.81, 1.00) for reporting RSQ. Obese individuals were significantly more likely to report a worse UDWC and RSQ (OR (95% CI) = 1.58 (1.18, 2.11) and 1.56 (1.16, 2.10) respectively).

### IN THEIR OWN WORDS: A STUDY ON COVID-19-RELATED CONCERNS AND PERCEPTIONS OF RISK AMONG OLDER UNIVERSITY EMPLOYEES

Amanda Sokan,[1] Nicole Yuan,[2] Mariana Felix,[2] Mark Wager,[2] Lisa O'Neill,[3] and Zhao Chen,[4]
*1. University of Arizona, Phoenix, Arizona, United States, 2. University of Arizona, Tucson, Arizona, United States, 3. University of Arizona, Center on Aging, Tucson, Arizona, United States, 4. The University of Arizona, Tucson, Arizona, United States*

The purpose of this qualitative study was to examine COVID-19-related concerns and risk perceptions among older employees aged 50+ related to reopening a large state university campus during the COVID-19 pandemic. Recruitment focused on older employees from diverse backgrounds and job classifications. Six focus group interviews, with a total of 24 participants, were conducted using Zoom video conferencing. Interviews were transcribed using Zoom and were double-checked for accuracy. Transcripts were coded and analyzed using ATLAS.ti 9 after establishing inter-rater reliability among two coders. During the campus reopening, older employees reported several concerns and perceptions of risk focused on COVID-19 exposure and transmission to others, individual health and health of other household members, mental health and stress, and job security. Findings were used to inform the development of intervention strategies and resources to promote the health and well-being among older employees during the pandemic.

### CAREGIVER CHALLENGES OF OLDER UNIVERSITY EMPLOYEES DURING THE COVID-19 PANDEMIC

Linda Phillips,[1] Yunjia Yang,[2] Lisa O'Neill,[3] Mindy Fain,[4] Mark Wager,[2] and Amanda Sokan,[5] *1. Un diversity of Arizona, Tucson, Arizona, United States, 2. University of Arizona, Tucson, Arizona, United States, 3. University of Arizona, Center on Aging, Tucson, Arizona, United States, 4. University of Arizona Center on Aging, Tucson, Arizona, United States, 5. University of Arizona, Phoenix, Arizona, United States*

The COVID-19 pandemic has created numerous challenges for older employees who are also caregivers. Some challenges are associated with disruptions in community-based support services leading to the intensification of caregiver responsibilities. Other challenges are related to caregivers' concerns about their health or the risk of bringing the virus to the care recipient. This study investigated the impacts of those challenges on older (age 55+) working caregivers, from a major university, with a sample that included 57 male and 80 female caregivers. The investigation explored the association of gender and perception of COVID risk, vulnerability, loneliness, resilience, and interpersonal change. Although literature suggests that female caregivers report more risk-perception, stress, and overburden than male caregivers, our findings showed no significant differences based on gender. These findings suggest the importance of understanding that both male and female older working caregivers have been affected by stress and overburden due to the recent pandemic.

### Session 1255 (Symposium)

### IMPROVING CARE FOR PERSONS WITH COGNITIVE IMPAIRMENT IN COMMUNITY-BASED LONG-TERM CARE AND ACUTE CARE SETTINGS

Chair: Jing Wang Co-Chair: Bei Wu

The COVID-19 pandemic has highlighted the importance of providing person-centered care for our vulnerable populations across the globe. This symposium focuses on improving care for persons with cognitive impairment and dementia in various care settings. The first study explored dyadic experiences of living with early-onset dementia pre and during COVID-19 in China through a person-centered care lens. The second concept analysis presented four interrelated dimensions of Asian American caregiver support, including individual, family, community, and professional health system. The third study investigated undergraduate nursing students' attitudes toward pursuing jobs of providing care for older adults with dementia in rapidly-aging urban areas in China and its associated factors. The fourth study examined the impact of social isolation on cognitive function and Quality of Life among acute ischemic stroke

(AIS) patients in China. The last study explored an association between perceived control and cognitive function among acute ischemic stroke (AIS) patients in China. The last two studies suggested that perceived control and social isolation may be potential targets in cognitive interventions for AIS patients. This symposium presents the understanding of dementia caregiver support, the empirical evidence of living with dementia, the attitudes towards dementia care among the next generation of nursing workforce, and the impact of social factors on cognitive functions longitudinally. The presenters emphasize the importance of improving care in long-term care and acute care settings. There is a need to design person and family-centered care to improve health and wellbeing of persons with cognitive impairment.

### LIVING WITH EARLY-ONSET DEMENTIA IN CHINA: THROUGH A PERSON-CENTERED CARE LENS

Jing Wang,[1] Ding Ding,[2] and Qianhua Zhao,[3] *1. Fudan University, Shanghai, Shanghai, China (People's Republic), 2. Fudan University Affiliated Huashan Hospital, Shanghai, Shanghai, China (People's Republic), 3. Fudan University Affiliated Huashan Hospital, shanghai, Shanghai, China (People's Republic)*

We conducted semi-structured interviews with 35 dyads of persons with early-onset dementia (EOD) and their primary informal care partners to explore their dyadic experiences of living EOD in Shanghai, China. Many of them are in their 50s and still need to make familial, financial, and social commitments. They experienced significant disruptions of their "normal" family life and family dynamics, social stigma, and felt marginalized when there was very limited age-appropriate support for them. During COVID-19 pandemic, many persons with EDO and their care partners had decreased social networking opportunities, physical exercises and experienced an increased level of social isolation. The pandemic further complicated their family dynamics, relationships, and communications. Care partners used their strengths to adaptively deal with multiple challenges, cope with the stress, social isolation, and normalize their family life by facilitating collaborative work with persons with EOD.

### CAREGIVER SUPPORT FOR ASIAN AMERICAN ALZHEIMER'S DISEASE AND RELATED DEMENTIAS CAREGIVERS: A CONCEPTUAL SYNTHESIS

Mengyao Hu, Allison Squires, and Bei Wu, *New York University, New York, New York, United States*

Informal caregiver support has been defined as different types of interventions. However, it has not been well explained in the social context and not well discussed as an integrative concept for dementia caregivers who are Asian Americans. Therefore, the aim of this study was to conduct a dimensional analysis--a type of evidence synthesis--to explore caregiver support in the context of Asian American dementia caregivers. A synthesis of 40 articles produced four interrelated dimensions of caregiver support: Individual (language, information, psychological issue, and culture); Family (family member support, availability of extended family, and decision making); Community (bilingual and bicultural help, and religion and spiritual source); and Professional healthcare system (expectations from healthcare professionals and caregivers for caregiver interventions, communication concordance, initiative in seeking help, and trust). The findings provide guidance for future studies on this population in promoting caregiver's health and developing caregiver interventions.

### PERCEIVED CONTROL AND COGNITIVE FUNCTION AMONG ACUTE ISCHEMIC STROKE PATIENTS IN CHINA

Zhijian Liu,[1] Xiangjing Kong,[2] Jing Wang,[3] Qin Wang,[4] Bei Wu,[5] and Juan Li,[1], *1. Department of clinical nursing School of Nursing Second Military Medical University, Shanghai, Shanghai, China (People's Republic), 2. School of Nursing Second Military Medical University, Shanghai, Shanghai, China (People's Republic), 3. Fudan University, shanghai, Shanghai, China (People's Republic), 4. Linyi People Hospital, Linyi, China, Linyi, Shandong, China (People's Republic), 5. New York University, New York, New York, United States*

This study explored an association between perceived control and cognitive function among 437 acute ischemic stroke (AIS) patients in China. We collected data from one stroke center in each of the three cities (Shanghai, Nanjing, and Linyi) from June to December, 2020. Cognitive function was assessed by the Montreal Cognitive Assessment (MoCA), and perceived control was assessed by Perceived Control in Health Care Questionnaire at acute stage. Hierarchical linear regression was used. The average of perceived control and MoCA were 81.36±0.877 and 19.66±0.304, respectively. A number of 374 (85.6%) patients were in cognitive impairment and 63(14.4%) were cognitively normal. Perceived control was positively associated with cognitive function ($\beta$=0.103, $p < 0.001$). After controlling for stroke severity, age, gender and education, the association was still significant ($\beta$=0.041, p=0.014). These findings suggest that perceived control may be a potential target in cognitive interventions for AIS patients.

### THE IMPACT OF SOCIAL ISOLATION ON COGNITION AND QUALITY OF LIFE AMONG STROKE PATIENTS IN CHINA

Xiangjing Kong,[1] Juan Li,[2] Zhijian Liu,[2] Jing Wang,[3] and Bei Wu,[4], *1. School of Nursing Second Military Medical University, Shanghai, Shanghai, China (People's Republic), 2. Department of clinical nursing School of Nursing Second Military Medical University, Shanghai, Shanghai, China (People's Republic), 3. Fudan University, shanghai, Shanghai, China (People's Republic), 4. New York University, New York, New York, United States*

This study examined the impactof social isolation on cognitive function and Quality of Life (QoL) among acute ischemic stroke (AIS) patients in China. We conducted in-person interviews among 206 AIS patients during the acute stage and at 3-month after onset in three cities between May 2020 and February 2021. The data was collected during and post-COVID-19 period in China. We conducted bivariate and multipleregression analyses.Results show that over time, average level of social isolation decreased, and cognitive function and QoL increased.After controllingfor covariates, social isolation was negatively associated with cognitive function (β=-0.438, p<0.01) and QoL (β=-2.521, p<0.01). These findings suggest that addressing the issue of social isolation could potentially impact patients' cognitive function and QoL.Future studies are needed to further examine the linkages between long-term social isolation and changes in cognitive function and QoL among AIS patients.

### UNDERGRADUATE NURSING STUDENTS' WILLINGNESS OF PROVIDING CARE FOR OLDER ADULTS WITH DEMENTIA AS THEIR FUTURE WORK

Wenlin Liu, and Jing Wang, *Fudan University, shanghai, Shanghai, China (People's Republic)*

This study examines how undergraduate nursing students' knowledge of dementia care are associated with their willingness of providing care for older adults with dementia across care settings as nurses in urban China, controlling for factors such as their socio-demographic characteristics, willingness of being a nurse, and years of studying nursing. We surveyed 320 undergraduate students from Shanghai, China and found that students with a better knowledge of dementia care, a longer year of nursing study, have no experience of being cared for by grandparents during childhood, and being the only child at home tended to be less willing to provide care for older adults with dementia in their future work. In order to prepare high-quality future dementia care workforce, nursing educators not only need to disseminate knowledge of dementia care, they should also tailor teaching to students' characteristics and motivate students to take the leadership in dementia care across settings.

### Session 1260 (Symposium)

### INFUSING REPRESENTATIVENESS AND CULTIVATING HARMONIZATION IN ALZHEIMER'S TRIALS: WORLD WIDE FINGERS

Chair: Rema Raman Discussant: Neelum Aggarwal

World Wide Fingers is a network involving over 30 countries organized to conduct randomized controlled clinical trials to slow the progression of cognitive decline and reduce dementia risks. Trials are designed to parallel the successful Finnish Geriatric Intervention Study to Prevent Cognitive Impairment and Disability (FINGER) trial of a multidomain lifestyle intervention featuring increased physical activity, improved diet, cognitive training, and metabolic risk factor monitoring. While FINGER found that its intervention significantly benefited cognitive function, it is not clear whether this approach might be successfully tailored to other cultures and environments to yield similar results. This is the goal of World Wide FINGERS. It infuses representativeness by enrolling cohorts that reflect the communities in which it is conducted. For findings across the many trials to be integrated, it is necessary for protocols to be harmonized as much as possible. The COVID-19 pandemic presents special challenges towards harmonization as its disruptions of trial protocols and conduct vary among countries and over time. This symposium is organized to provide the scientific background and framework for the World Wide FINGERS. Novel grassroots efforts towards enrolling representative cohorts in the US will be described. Plans for harmonization and federated data analyses spanning international boundaries and regulations will be outlined. Integrated approaches to challenges of COVID-19 pandemic across trials will be presented. The conclusion of this session will be a discussion of how World Wide FINGERS may serve as a model for collaborative approaches to identify effective, translatable approaches to reduce risks for Alzheimer's disease.

### THE SCIENCE BEHIND MULTIDOMAIN INTERVENTIONS TO SLOW COGNITIVE DECLINE

Laura Baker, *Wake Forest School of Medicine, Winston-Salem, North Carolina, United States*

The spotlight on interventions to protect brain health and prevent Alzheimer's disease (AD) has recently widened to include risk modification. In the last 20 years, evidence continues to build to support cognition-enhancing effects of individual lifestyle components, which include, among others, physical exercise, diet, cognitive training, and cardiovascular risk management. A recent evolution of lifestyle trials is to combine these components as part of intervention delivery. The potential benefit of this approach on cognition in older adults, first showcased in the FINGER trial, is now under investigation by multiple groups across the nation and the globe. The multidomain approach offers important opportunities to boost lifestyle intervention 'dose', to examine inter-component synergistic effects, and for intervention tailoring to meet specific needs and limitations. Harmonization and data-sharing will be essential to meaningfully address the question of whether multidomain lifestyle modification can indeed be 'medicine' to protect brain health and reduce AD risk.

### WORLD-WIDE FINGERS: AN INTERNATIONAL NETWORK OF LINKED MULTIDOMAIN TRIALS

Francesca Mangialasche,[1] Alina Solomon,[2] Tiia Ngandu,[3] and Miia Kivipelto,[2] *1. Division of Clinical Geriatrics, Stockholm, Stockholms Lan, Sweden, 2. Karolinska Institutet, Stockholm, Stockholms Lan, Sweden, 3. Finnish Institute for Health and Wefare, Public Health Promotoin Unit, Uusimaa, Finland*

Risk reduction and prevention of dementia in older adults is a growing research area. In the Finnish Geriatric Intervention Study to Prevent Cognitive Impairment and Disability (FINGER randomized controlled trial) a 2-year multidomain intervention -dietary counseling, exercise, cognitive training, vascular and metabolic risk monitoring- improved cognition in older adults from the general population who had increased dementia risk. The intervention was associated also with improvement of other clinical outcomes (e.g., multimorbidity, functional status). The FINGER model is being adapted and tested in different populations and settings through the World-Wide FINGERS, the first global network of multidomain prevention trials, including over thirty countries. The network goal is to identify effective and feasible solution for dementia risk reduction across the spectrum of cognitive decline - from at-risk asymptomatic states to early-symptomatic stages. Through the World-Wide FINGERS-SARS-CoV-2 initiative, the network aims to assess the effects of the COVID-19 pandemic in older adults.

### GRASSROOTS APPROACHES TO COMMUNITY-BASED REPRESENTATIVE RECRUITMENT IN MULTIDOMAIN TRIALS

Marcus Hill, *Division of Gerontology and Geriatric Medicine, Winston-Salem, North Carolina, United States*

U.S. POINTER is a randomized, controlled, multi-domain clinical trial to slow the progression of cognitive decline within the American population via tailored and culturally-appropriate healthy lifestyle interventions. For findings to be broadly relevant across the American population, incorporating an inclusive and robust recruitment effort has been essential to form a diverse and properly representative participant cohort. As such, the trial's inclusive enrollment goal is 30% from traditionally underrepresented communities that include those at elevated risk for Alzheimer's disease and related disorders. To accomplish this goal, U.S. POINTER developed and deployed a grassroots recruitment strategy in partnership with outreach specialists at each site that includes a mix of evidence-based and innovative community engagement approaches. While the COVID-19 pandemic continues to present unique challenges for recruitment, our team has been able to strategize ways to continue working within the community to support trial recruitment. An overview of these methods will be presented.

### HARMONIZING DATA COLLECTION AND ANALYSIS IN WORLDWIDE FINGERS TRIALS

Markku Peltonen, *Population Health Unit, Helsinki, Uusimaa, Finland*

One of the goals of the World-Wide FINGERS (WW-FINGERS): A Global Approach to Dementia Prevention network is to prospectively facilitate data sharing and joint analyses across clinical trials on prevention of cognitive impairment and dementia. The aim with prospectively harmonizing studies in different countries and settings regarding interventions, outcomes, measurements, data collection, and establishing best practices for responsible data sharing and access to data for remote joint analyses, is to increase the use of clinical trial data. By utilizing federated database system which connects multiple autonomous, decentralized databases and enables data-analyses to be conducted without individual level data being transferred, could be a feasible and acceptable technical solution for countries around the world, given wide variations in data protection and sharing regulations. Ultimately, prospectively harmonizing clinical studies and establishing a culture of harmonization and sharing, will promote international joint initiatives to identify globally implementable and effective preventive strategies.

### MAINTAINING INTEGRITY OF WORLDWIDE FINGER CLINICAL TRIALS DURING THE COVID-19 PANDEMIC

Susanne Roehr, *Institute of Social Medicine, Occupational Health and Public Health (ISAP), Leipzig, Sachsen, Germany*

The COVID-19 pandemic presents challenges to the conduct of randomized clinical trials of lifestyle interventions. World-Wide FINGERS international network convened a forum for researchers to discuss statistical design and analysis issues they faced during the pandemic. We report experiences of three trials that, at various stages of conduct, altered designs and analysis plans to navigate these issues. We provide recommendations for future trials to consider as they develop and launch behavioral intervention trials. The pandemic led researchers to change recruitment plans, interrupt timelines for assessments and intervention delivery, and move to remote intervention and assessments protocols. The necessity of these changes add emphasis to the importance, in study design and analysis, of intention to treat approaches, flexibility, within site stratification, interim power projections, and sensitivity analyses. Robust approaches to study design and analysis are critical to negotiate issues related to the intervention.

### Session 1265 (Symposium)

### INTEGRATING HUMANITIES AND ARTS INTO GERONTOLOGY AND GERIATRICS CURRICULA

Chair: Desmond O'Neill Co-Chair: Dana Bradley
Discussant: James Powers

Humanities, Arts and Cultural Gerontology (HACG) has been an integral element of GSA for over 4 decades, and is included as a notable feature of AGHE guidelines on curricula for gerontology and geriatrics. However, as with many interdisciplinary areas, the degree to which HACG has been successfully inserted into curricula, the extent to which this has involved engagement of faculty in arts and humanities, and the facilitators and barriers of such deeper joint working are unknown. The HACG Advisory Panel and AGHE would like to convene a round-table/symposium at the 2021 Phoenix GSA Meeting to consider the range of experiences of educators of programs in gerontology/geriatrics, from those who can relate success stories in integrating HACG into their curricula, to those who can give insights into challenges and opportunities in attempts to develop such elements in their curricula. Co-convened by Des O'Neill, Chair HACG AP and Dana Burr Bradley AGHE Program Chair, we invite lively discussion which we consider will aid in the development of a road map towards substantive and rewarding initiatives in incorporating scholarship and education in HACG in gerontology and geriatrics educational program

### A DECADE OF TEACHING THE COURSE AGING & THE ARTS: REFLECTING ON OPPORTUNITIES AND CHALLENGES

Jacqueline Eaton, *University of Utah, University of Utah, Utah, United States*

In 2010, the University of Utah Gerontology Interdisciplinary Program first offered GERON 5240/6240: Aging and the Arts. This course was developed to enrich program curricula by addressing a gap in content specific to the arts and humanities. The purpose of this presentation is to focus on identifying the opportunities and challenges experienced teaching this course over the past decade. Opportunities will highlight competency mapping, internal and external partnerships, the benefits of bridging disciplines, and innovation in teaching and problem-solving. Challenges experienced include addressing various needs

(online learning, undergraduate and graduate levels, multiple disciplines), tuition differentials, and varying levels of enrollment. A stand-alone course is one method of increasing humanities, arts, and cultural gerontology within curricula. It has the potential of enhancing student interest in gerontology while also demonstrating how the arts and humanities can improve work across disciplines.

### INTERDISCIPLINARY GERONTOLOGY IN HIGHER EDUCATION: A CASE STUDY FROM SOUP TO NUTS

Justine McGovern, *Lehman College, City University of New York, Bronx, New York, United States*

Through the lens of a multi-year joint project initiated by faculty in Social Work and Digital Arts at Lehman College, the City University of New York's senior college in the Bronx, NY, this paper provides a guide on how to initiate, implement and evaluate interdisciplinary collaborations in gerontology. The paper also suggests ways to ensure that these collaborations can support tenure and promotion processes, funding initiatives, and pedagogical enhancements. The paper focuses on how to make use of campus resources, including departmental Chairs, research offices, and campus-wide committees to identify appropriate collaborators and funding sources; how to nurture productive interdisciplinary relationships, such as clarifying disciplinary expectations and participants' professional needs; and how to maximize return on the effort for tenure and promotion, such as producing publishable content, identifying appropriate opportunities for interdisciplinary publishing and presenting, advocating for interdisciplinary collaborations, and developing interdisciplinary syllabi, an example of evidence-based high-impact pedagogy.

### COMING SOON TO A CLASS NEAR YOU: CINEMATIC INSIGHTS ON LATER LIFE

Candace Brown,[1] and Margaret Perkinson,[2] *1. University of North Carolina, Charlotte, Charlotte, North Carolina, United States, 2. University of Hawaii at Manoa, Honolulu, Hawaii, United States*

Cinema can enhance gerontological education by reinforcing a variety of learning styles, connecting course content to current culture, and providing an alternative, tangible view of what students are learning. The presenters discuss their use of film to teach gerontological concepts in the classroom. In an Introduction to Aging course, the films, "Young at Heart" and "Sunset Story" were used to break through ageist stereotypes, examine examples of resilience at the end of life, and convey the impact of residential context on the experience of aging, i.e., within a retirement home for "retired rebels." In a course on Health and Aging, movies, such as "Red," "Driving Miss Daisy," and "Somethings Gotta Give" are used to compare the social and psychological aspects of aging of the characters to learned concepts in the classroom. Students expressed how watching and writing about the films increased their understanding by bringing abstract gerontological concepts to life.

### LESSONS FOR HUMANITIES AND ARTS IN GERONTOLOGY AND GERIATRICS CURRICULA FROM THE MEDICAL AND HEALTH HUMANITIES

Desmond O'Neill, *Trinity College Dublin, Dublin, Dublin, Ireland*

The role of Humanities, Arts and Cultural Gerontology in gerontology and geriatrics curricula finds a metaphor in the rapidly evolving field of medical and health humanities, with which this author has been involved for three decades. Behind the call for increasing humanities and arts scholarship in the pedagogy of both fields lies the challenge of establishing an interdisciplinary nexus of scholarship that avoids the challenges of dilettantism and gestures such as providing lists of novels and movies. This presentation draws on the presenter's bibliometric research in the medical and health humanities which indicates authorship in the majority to be either solely from the humanities or from healthcare, with little indication of joint working in either authorship or acknowledgements (the scholar's courtesy), and explores the background issues of academic culture with a view to proposing solutions to elevate the inclusion of humanities and arts as a significant element of gerontology education.

### Session 1270 (Paper)

### LONG-TERM CARE I (BSS PAPER)

### FACTORS ASSOCIATED WITH THE QUALITY OF STAFF-RESIDENT INTERACTIONS IN ASSISTED LIVING

Anju Paudel,[1] Elizabeth Galik,[2] Barbara Resnick,[3] Kelly Doran,[3] Marie Boltz,[4] and Shijun Zhu,[2] *1. University of Maryland, University of Marland, Maryland, United States, 2. University of Maryland, Baltimore, Maryland, United States, 3. University of Maryland School of Nursing, Baltimore, Maryland, United States, 4. Pennsylvania State University, University Park, Pennsylvania, United States*

Care interactions are essential to understand and respond to resident needs in assisted living (AL). The factors that influence care interactions in AL have not been directly examined. In this study, we explored the factors associated with the quality of care interactions in AL. It was hypothesized that resident functional status, agitation, depression, and resistiveness to care as well as facility size and ownership would be significantly associated with the quality of care interactions in AL after controlling for resident demographics (age, gender, marital status), comorbidities, and cognition. To test the hypothesis, we utilized baseline data including 379 residents from the second and third cohorts recruited in a randomized trial titled 'Dissemination and Implementation of Function Focused Care for Assisted Living Using the Evidence Integration Triangle'. Regression analysis was performed using a stepwise method. The care interactions were mostly positive (mean=6.3; range = 0-7). Resident agitation and facility ownership were significantly associated with care interactions and accounted for 8.2% of the variance. Increased resident agitation was associated with negative or neutral interaction while for-profit ownership was associated with positive interactions. To promote positive care interactions, staff should be educated about strategies to minimize resident agitation (e.g., calm posture and respectful listening) and encouraged to engage with residents using resident-centered care and communication approach. Findings also suggest the need to work towards optimizing care interactions in nonprofit stings. Future research could further explore the impact of facility-level factors (e.g., staffing ratios, staff longevity, and job satisfaction) on care interactions.

### LAUNCHING THE LIVING ALONE WITH COGNITIVE IMPAIRMENT (LACI) PROJECT: BRIDGING RESEARCH AND POLICY

Carrie Graham,[1] Elena Portacolone,[2] Julene Johnson,[3] Jacqueline Torres,[3] M Maria Glymour,[3] Sahru Keiser,[4] and Thi Tran,[5] *1. UCSF, University California san francisco, California, United States, 2. University of California, Berkeley, California, United States, 3. University California San Francisco, San Francisco, California, United States, 4. University of California, San Francisco, San Francisco, California, United States, 5. UCSF/ San Francisco, UCSF Institute of Health and Aging/ San Francisco, California, United States*

The Living Alone with Cognitive Impairment (LACI) Project bridges research and policy to develop policy recommendations to address the needs of people living alone with cognitive impairment (PLACI) through new expansions of long-term services and supports. There are an estimated 4.3 million PLACI in the United States. Access to formal LTSS is critical to them because they lack cohabitants to assist with activities of daily living and navigating LTSS, especially during the COVID-19 pandemic. To bridge research with policy, seventeen Policy Advisory Group (PAG) members were recruited, including representatives from state and local government, and LTSS policy experts. Between November 2020-January 2021, a total of 17 individual meetings were conducted with PAG members and one webinar convening of the group. The PAG identified preliminary recommendations in three areas, including: 1) important areas of inquiry for qualitative and quantitative research, 2) best practices for addressing equity across diverse racial/ethnic minority groups, and 3) preliminary policy recommendations that leverage existing innovations. The LACI Project team is actively incorporating the PAG feedback by: a) modifying research questions for the quantitative and qualitative research, b) convening a diverse Community Advisory Group, and c) crafting preliminary policy recommendations based on PAG input. To conclude, engaging the expertise of the PAG to develop policy recommendations to increase LTSS for PLACI is a promising method of bridging research and policy. The engagement of policy experts ensures that fore-coming research is designed to address the most important policy gaps and all policy recommendations are actionable and timely.

### LOWER WAGES OF NURSES IN LONG-TERM CARE: DOES RACIAL AND ETHNIC DIVERSITY EXPLAIN THE DIFFERENCE?

Joanne Spetz, Laura Wagner, and Timothy Bates, *University of California, San Francisco, San Francisco, California, United States*

Registered nurses (RNs) are a key component of the long-term care (LTC) workforce and prior research demonstrates their importance to ensuring patient safety in LTC settings. RNs who work in LTC settings earn less than those who work in hospitals and also are more likely to be from racial and ethnic minority groups. This study seeks to measure wage differences between Registered Nurses (RNs) working in LTC and other settings (e.g., hospitals) and whether differences are associated with the characteristics of the RN workforce between and within settings. We used the 2018 National Sample Survey of Registered Nurses (NSSRN) public-use file to examine RN employment and earnings. Our study population included a sample of 15,373 employed RNs who provided patient care. Characteristics such as race/ ethnicity, type of RN degree completed, census region, and union status were included in bivariate analyses and multiple regression analyses to examine the effect of these characteristics on wages. Logistic regression was used to predict RN employment in LTC settings. We found that RNs in LTC experienced lower wages compared to those in non-LTC settings, yet this difference was not associated with racial/ ethnic or international educational differences. LTC nurses were also significantly less likely to be represented by a labor union, and there was not a statistically significant wage difference for LTC RNs who were unionized. Because RNs in LTC earn lower wages than RNs in other settings, policies to minimize pay inequities are needed to support the RN workforce caring for frail older adults.

### WHOLE TEAM, WHOLE PERSON HIGH-INVOLVEMENT QUALITY IMPROVEMENT TRAINING FOR VA COMMUNITY LIVING CENTER LEADERS

A. Lynn Snow,[1] Valerie Clark,[2] Shibei Zhao,[2] Ryann Engle,[3] Corilyn Ott,[4] Princess Nash,[5] Lisa Minor,[6] and Christine Hartmann,[7] *1. University of Alabama, Tuscaloosa, Alabama, United States, 2. VA Bedford Healthcare System, Bedford, Massachusetts, United States, 3. VA Boston Healthcare Center, CHOIR, VA Boston Healthcare Center, CHOIR, Massachusetts, United States, 4. Birmingham VA Medical Center, Birmingham VA Medical Center, Alabama, United States, 5. Tuscaloosa VA Medical Center, Tuscaloosa, Alabama, United States, 6. Geriatrics and Extended Care, Washington, District of Columbia, United States, 7. VA Bedford Healthcare System, VA Bedford Healthcare System, Massachusetts, United States*

Long-term care is a challenging environment for quality improvement due to the high resident acuity, wide variation in resident needs, and wide variation in types and backgrounds of the large staff across three daily shifts. We report results from a learning collaborative undertaken to improve care quality and staff quality improvement skills in the VA CLCs through development of high functioning relationally coordinated teams operating in accord with person-centered care principles. The collaborative included 27 CLCs. Over 9 months leadership teams completed action assignments supported by 5 workshops and regular group coaching calls. Evaluation included fidelity monitoring (attendance, mid- and final progress reports), satisfaction questionnaires, and review of the VA quality measures (CLC Compare). Pre-post participant evaluations revealed a significant increase in positive responses to the question "to what extent do you think applying these new skills/knowledge will improve quality in your CLC?" and positive responses trending toward significance in ratings of abilities to apply new skills. Open-ended survey comments were positive and indicated change in understanding and practice: "utilizing the daily huddle to facilitate real time communication afforded the team a proactive approach to providing care and reducing acute exacerbations. We are able to avert, evaluate as a real time team and make it happen in the now not as a look back."; "definitely unified front-line staff and CLC leadership." Some changes were achieved in CLC Compare quality scores (e.g., falls with major injury rate had a 9.6 reduction (average rate = 3.39 pre, 3.07 post)).

### Session 1275 (Paper)

### LONG-TERM CARE I (SRPP PAPER)

### NURSING HOME SATISFACTION SURVEYS: DIFFERENCES BY RACE, AGE, AND GENDER

Nicholas Castle,[1] John Harris,[2] and David Wolf,[3] *1. West Virginia University, Allison Park, Pennsylvania, United States, 2. University of Pittsburgh, Pittsburgh, Pennsylvania, United States, 3. Bellarmine, Louisville, Kentucky, United States*

Nursing home satisfaction information has gained substantial traction as a quality indicator representing the consumers perspective. However, very little research has examined differences in satisfaction related to race, age and gender. As a quality metric, satisfaction measures are variously used for quality improvement, benchmarking, public reporting, and for adjustment to payments. As such, valid comparisons among facilities are important. To our knowledge, no adjustment to satisfaction scores are currently used for nursing homes. However, in many other settings this is a common practice. In this research, nursing home resident, family, and discharge satisfaction scores were examined from >4,000 participants. The data were collected in 2020 and come from 420 facilities. Satisfaction information came from the CoreQ surveys, which include 23 individual questions four of which can be combined to produce an overall satisfaction score. These CoreQ nursing home surveys are endorsed by NQF. Generally lower overall satisfaction scores were found for family members compared to current residents or discharged residents. Minorities (Black, Asian, Hispanic) had lower overall satisfaction scores compared to whites; however, the differences were not significant at conventional levels. Participants of the lowest age (<65 years) were significantly (p=<.05) less satisfied than older participants (>75 years) and males were significantly (p=<.05) less satisfied than females. The findings indicate that some case-mix adjustment may be applicable for nursing home satisfaction scores.

### QUALITY-OF-LIFE SCORES FOR NURSING HOME RESIDENTS ARE STABLE OVER TIME: EVIDENCE FROM MINNESOTA

Weiwen Ng,[1] Tetyana Shippee,[2] John Bowblis,[3] Odichinma Akosionu,[1] Mark Woodhouse,[1] and Yinfei Duan,[1], *1. University of Minnesota, Minneapolis, Minnesota, United States, 2. University of Minnesota, University of Minnesota, Minnesota, United States, 3. Miami University, Oxford, Ohio, United States*

**Objective:** Quality of life (QoL) is a multidimensional construct that assesses the quality of lived experience in nursing homes (NHs). QoL is directly important to NH residents. However, QoL is only publicly reported in a few states, partly because of concerns regarding measure stability. To address these concerns, we tested the stability of Minnesota's NH QoL measure over one year.

**Study Design:** A pair of two-year cohorts of Minnesota NH residents who responded to the 2012-2013 (N = 4,448) or 2014-2015 (N = 4,644) QoL survey in consecutive years. Stability was measured using the intra-class correlation (ICC) from hierarchical linear models. Models were fit without any covariates, then individual and facility-level characteristics were added. Principal Findings: Overall QoL had ICCs of 0.602 and 0.614 in the earlier and later cohort respectively. Domain-level ICCs were lower, ranging from 0.374 (positive mood) to 0.571 (lack of negative mood) in the 2012-2013 cohort, with similar trends for the later cohort. Adjusting for important covariates reduces the ICCs slightly, but they remained at 0.565 or higher for the summary score.

**Conclusions:** Person-reported summary QoL has adequate stability over a period of one year. Our results provide impetus to assess and report NH QoL on a national level. Consumers can be confident that if an NH's QoL scores improve from year to year, that represents a real improvement, and not just the scores varying due to which residents were sampled. Some caution, however, is warranted when presenting facility-level domain scores, as these are less stable.

### Session 1280 (Paper)

### MOBILITY II

### MOBILITY AND MUSCLE STRENGTH TRAJECTORIES: THE EFFECT OF MEDITERRANEAN DIET, PHYSICAL ACTIVITY, AND SOCIAL SUPPORT

Marguerita Saadeh,[1] Federica Prinelli,[2] Davide Vetrano,[3] Weili Xu,[3] Anna-Karin Welmer,[4] Serhiy Dekhtyar,[1] Laura Fratiglioni,[4] and Amaia Calderón-Larrañaga,[3] *1. Aging Research Center, Karolinska Institutet, Stockholm, Stockholms Lan, Sweden, 2. Aging Research Center, Dept NVS, Karolinska Institutet, Neurobiology, Care Sciences and Society, Stockholms Lan, Sweden, 3. Karolinska Institutet, Solna, Stockholms Lan, Sweden, 4. Karolinska Institutet, 17175, Stockholms Lan, Sweden*

Decline in physical function varies substantially across older individuals due to several biological and extrinsic factors. We aimed to determine the effect of modifiable factors -such as dietary patterns, physical activity and social support- and their interaction with mobility and muscle strength decline after age 60. We analyzed data from 1686 individuals aged 60+ from the population-based Swedish National study on Aging and Care in Kungsholmen. The Mediterranean Diet Score was calculated based on a validated food frequency questionnaire. Physical activity was categorized based on current recommendations, and social support was measured according to participants' perceived material and psychological support. Participants' physical function was assessed over 12 years through changes in walking speed (m/s) and chair stand time (s). Linear mixed models adjusted for socio-demographic and clinical factors were used. Subjects with high adherence to Mediterranean diet were <78 years (82.3%), women (56.1%), married (61.1%), with university education (52.8%), high levels of social support (39.3%) and health-enhancing physical activity (51.5%). One-point (over nine) increase in the MDS was associated with a slower annual worsening in walking speed (β*time=0.001; p=0.024) and chair stand time (β*time=-0.014; p=0.008). The protective effect of Mediterranean diet was highest among subjects reporting high social support (β*time=-0.065, p=0.026 for chair stands) and high physical activity (β*time=0.010, p=0.001 for walking speed), beyond the effect of each exposure individually. A higher adherence to Mediterranean diet, especially in combination with recommended levels of physical activity and high social support, contribute to delay the decline in physical function observed with aging.

### REGIONAL MICROSTRUCTURAL INTEGRITY IN RELATION TO GAIT SPEED: THE ATHEROSCLEROSIS RISK IN COMMUNITIES STUDY

Kevin Sullivan,[1] Chad Blackshear,[2] Timothy Hughes,[3] Rebecca Gottesman,[4] Prashanthi Vemuri,[5] Thomas Mosley,[1] Michael Griswold,[1] and B. Gwen Windham,[2] *1. The University of Mississippi Medical Center, Jackson, Mississippi, United States, 2. University of Mississippi Medical Center, Jackson, Mississippi, United States, 3. Wake Forest School of Medicine, Winston-Salem, North Carolina, United States, 4. Johns Hopkins University, Baltimore, Maryland, United States, 5. The Mayo Clinic, Mayo Clinic, Minnesota, United States*

Brain imaging-based biomarkers of neuropathology are associated with mobility in older adults, but the relation of regional microstructural integrity to gait speed in the context of a broader neuropathological profile is less understood. We examined cross-sectional associations of microstructural integrity with 4-meter usual-pace gait speed (cm/s) in a subsample of ARIC study participants who completed 3T MRI brain scans with diffusion tensor imaging(2011-13; n=1785; mean age=76.2±5.3, 60% Female, 28% Black). We considered total brain and six regional averages of fractional anisotropy (FA; lower=worse microstructural integrity) and mean diffusivity (MD; higher=worse microstructural integrity): frontal, temporal, parietal, occipital, anterior and posterior corpus callosum. Associations were tested in multivariable linear regression models adjusted for demographics, cardiovascular risk factors, and with and without additional neuropathological indices: total brain volume, white matter hyperintensities, infarcts, and microhemorrhages. When modeled separately, all neuropathology indices were associated with slower gait speed. Every standard deviation(SD) higher total brain FA was associated with +2.56 cm/s gait speed (95%CI: 1.64,3.48) and every SD higher MD was associated with -4.27 cm/s gait speed (-5.34,-3.20). All regional estimates were comparable. When adjusted for all other neuropathology indices, only posterior corpus callosum FA (β=1.72; 0.67,2.77), total MD (β=-1.63; -3.02,-0.25), frontal lobe MD (β=-1.76; -3.03,-0.48), and temporal lobe MD (β=-1.40; -2.78,-0.02) remained significantly associated with gait speed. Microstructural integrity is an informative measure of brain pathology in relation to mobility, with regional measures tied to executive, memory, and somatosensory function being more informative when a broader neuropathological profile is considered.

### THE CORTICAL DYNAMICS OF DUAL-TASK STANDING IN OLDER ADULTS

Melike Kahya,[1] On-Yee Lo,[2] Junhong Zhou,[3] Alvaro Pascual-Leone,[4] Lewis Lipsitz,[5] Jeffrey Hausdorff,[6] Christoph Michel,[7] and Brad Manor,[8] *1. Marcus Institute for Aging Research, Harvard Medical School, Boston, Massachusetts, United States, 2. Hebrew SeniorLife/ Harvard Medical School, Boston, Massachusetts, United States, 3. Harvard Medical School/Hebrew SeniorLife, Roslindale, Massachusetts, United States, 4. Harvard Medical School, Boston, Massachusetts, United States, 5. Hebrew SeniorLife, Boston, Massachusetts, United States, 6. Tel Aviv University, Tel Aviv University, Tel Aviv, Israel, 7. University of Geneva, University of Geneva, Geneve, Switzerland, 8. Hinda and Arthur Marcus Institute for Aging Research, Harvard Medical School, Boston, Massachusetts, United States*

In older adults, the extent to which performing a cognitive task when standing diminishes postural control is predictive of future falls and cognitive decline. The cortical control of such "dual-tasking," however, remains poorly understood. Electroencephalogram (EEG) studies have demonstrated that the level of attention and cognitive inhibitory activity during cognitive task performance can be quantified by changes in brain activity in specific frequency bands; namely, an increase in theta/beta ratio and a decrease in alpha-band power, respectively. We hypothesized that in older adults, dual-tasking would increase theta/beta ratio and decrease alpha-band power, and, that greater alpha-band power during quiet standing would predict worse dual-task performance. To test this hypothesis, we recorded postural sway and EEG (32-channels) in 30 older adults without overt disease as they completed trials of standing, with and without verbalized serial subtractions, on four separate visits. Postural sway speed, as well as absolute theta/beta power ratio and alpha-band power, were calculated. The theta/beta power ratio and alpha-band power demonstrated high test-retest reliability during quiet and dual-task standing across visits (intra-class correlation coefficients >0.70). Compared with quiet standing, dual-tasking increased theta/beta power ratio (p<0.0001) and decreased alpha-band power (p=0.002). Participants who exhibited greater alpha-band power during quiet standing demonstrated a greater dual-task cost (i.e., percent increase, indicative of worse performance) to postural sway speed (r=0.3, p=0.01). These results suggest that in older adults, dual-tasking while standing increases EEG-derived metrics related to attention, and, that greater cognitive inhibitory activity during quiet standing is associated with worse dual-task standing performance.

### VALIDATION OF THE INTEGRATED CARE FOR OLDER PEOPLE SCREENING TOOL: FOCUS ON THE CHAIR RISE TEST TO ASSESS LOCOMOTION

Emmanuel Gonzalez-Bautista,[1] Philipe de Souto Barreto,[2] Aaron Salinas-Rodriguez,[3] Sandrine Sourdet,[1] Yves Rolland,[1] Leocadio Rodriguez-Mañas,[4] Sandrine Andrieu,[5] and Bruno Vellas,[2] *1. Toulouse University Hospital (CHU Toulouse), Toulouse, Midi-Pyrenees, France, 2. CHU Toulouse: Centre Hospitalier Universitaire de Toulouse, Gérontopôle de Toulouse, Institut du Vieillissement, Midi-Pyrenees, France, 3. National Institute of Public Health of Mexico, Cuernavaca, Morelos, Mexico, 4. Getafe University Hospital, Getafe, Madrid, Spain, 5. University of Toulouse III, INSERM, UPS, Toulouse, Midi-Pyrenees, France*

The Integrated Care for Older People (ICOPE) is a function- and person-centered healthcare pathway developed by the World Health Organization (WHO). ICOPE's first step (Step 1) consists of screening for impairments in the intrinsic capacity (IC) domains (namely sensorial, cognition, nutrition, psychological, and locomotion). For instance, the ICOPE Step1 tool suggests a cut-point of 14 seconds for five-repetition chair rise time as a marker of impaired locomotion. Given the lack of validation of this tool in the literature, we aimed to validate the ICOPE screening tool concerning incident health outcomes, focusing on the locomotion assessment. First, we analyzed the five-domain screening tool's ability to identify older adults (OA) at higher risk of incident outcomes (frailty, disability, dementia) using longitudinal data from the Multidomain Alzheimer Preventive Trial (MAPT). For the locomotion assessment (chair rise test), we derived and cross-validated age-specific cut points from two population-based cohorts using ROC (receiver operating characteristic) analysis. We further verified those cut points among OA real-life users of the health system and clinical trial participants. In conclusion, the ICOPE Step 1 screening tool was able to identify OA at higher risk of incident frailty, disability, and dementia. New chair-rise-time cut points for age groups 70-79 years old and 80 years and older were valid in populations from different settings. The ICOPE Step 1 tool provides a practical and integrative way of screening older adults for impairments in IC and detecting those at higher risk of functional decline.

### Session 1285 (Symposium)

### RECOGNIZING AND INTERVENING ON ELDER ABUSE, NEGLECT, AND EXPLOITATION IN THE AGE OF COVID-19

Chair: Pi-Ju Liu Discussant: Pamela Teaster

The rapid outbreak of coronavirus disease 2019 (COVID-19) caused by the severe acute respiratory syndrome coronavirus 2 (SARS-CoV-2) has led to a global pandemic. Public health measures to prevent the spread of COVID-19, such as social distancing and self-quarantine, have drastically altered people's lives and led to social isolation, financial instability, and disrupted access to healthcare and social services. Older adults have not only borne the brunt of the highest COVID-19 mortality rates, but recent studies also describe growing reports of elder mistreatment. It is necessary to attend to these age-related disparities during the remainder of the COVID-19 pandemic and future health crises. This symposium includes four presentations on researchers' findings in elder mistreatment during the COVID-19 pandemic. Dr. E-Shien Chang will compare prevalence of elder mistreatment before and since the pandemic, and highlight associated risk and protective factors during the pandemic. Dr. Lena Makaroun will examine changes in elder mistreatment risk factors among caregivers during the pandemic. Dr. Pamela Teaster will present Adult Protective Services' (APS) policy and practice changes in response to the pandemic to demonstrate the pandemic's impact on service providers. Lastly, Dr. Pi-Ju (Marian) Liu will appraise elder mistreatment victims' awareness of COVID-19 and their unmet needs during the pandemic. Following the four presentations, Dr. Pamela Teaster will moderate a discussion on how elder mistreatment is a growing concern, especially during the pandemic, and what service providers are doing to protect older adults.

### ELDER ABUSE DURING THE COVID-19 PANDEMIC: PREVALENCE, RISK, AND PROTECTIVE FACTORS

E-Shien Chang,[1] and Becca Levy,[2] *1. Weill Cornell Medicine, New York City, New York, United States, 2. Yale University, Woodbridge, Connecticut, United States*

It has been assumed that the pandemic has brought with it a surge in elder abuse due to heightened health and interpersonal stressors. However, empirical evidence is lacking. This study aimed to estimate the prevalence of, and risk and resilience factors of elder abuse during the pandemic. In a web-based survey of a socio-demographically diverse sample of 897 older persons, one in five older persons (n = 191; 21%) reported elder abuse, an increase of 84% from prevalence estimates before the pandemic. In the multivariate logistic regression models, sense of community was a persistent protective factor for elder abuse (OR= 0.89, 95% CI 0.85–0.93). At the relational level, physical distancing was associated with reduced risk of elder abuse (OR= 0.94, 95% CI 0.90–0.98). At the individual level, financial strain was associated with increased risk of abuse (OR= 1.08, 95% CI: 1.02–1.14). Implications for prevention strategies will be discussed.

### CHANGES IN ELDER MISTREATMENT RISK FACTORS REPORTED BY CAREGIVERS OF OLDER ADULTS DURING THE COVID-19 PANDEMIC

Lena Makaroun,[1] Scott Beach,[2] Tony Rosen,[3] and Ann-Marie Rosland,[1], *1. University of Pittsburgh, Pittsburgh, Pennsylvania, United States, 2. University of Pittsburgh, University of Pittsburgh, Pennsylvania, United States, 3. Weill Cornell Medical College / NewYork-Presbyterian Hospital, PELHAM, New York, United States*

This study aimed to assess how the COVID-19 pandemic has impacted caregiver (CG)-related risk factors for elder mistreatment (EM) in a community sample of CGs. A non-probability sample of 433 CGs caring for care recipients (CRs) age ≥60 years completed a survey on COVID-19 impacts in April-May 2020. Compared to before COVID-19, over 40% of caregivers reported doing worse financially,

16% were experiencing new financial hardship, 19.4% were a lot more worried about their financial situation, 15% reported drinking more alcohol, and 64% had somewhat or greatly increased feelings of social isolation and loneliness. CGs reported that COVID-19 had made caregiving more physically (18.7%), emotionally (48.5%) and financially (14.5%) difficult and interfered with their own healthcare (19%). Differences found between younger and older caregivers will be presented and implications of these increased CG-related EM risk factors for healthcare and social service providers discussed.

### A NATIONWIDE STUDY ON HOW COVID-19 CHANGED APS POLICIES AND PRACTICES

Pamela Teaster, *Virginia Tech, BLACKSBURG, West Virginia, United States*

The purpose of this inquiry by the Virginia Tech Center for Gerontology and WRMA, Inc., was to explore changes being implemented by APS programs across the country in response to the COVID-19 pandemic. With input from the Administration for Community Living, the research team used a three-step process (e.g., telephone interviews with state-level APS administrators, a national online survey, and in-depth interviews with local and APS) to capture information on changes caused by efforts to mitigate the spread of COVID-19. This presentation concerns changes in APS policy and practice that the pandemic caused, including modifications in-person visits and adjustments to timeline requirements. Discussion of alterations in policy and practices during the first five months of the pandemic can eludicate APS and other services and planning for older adults in future emergency situations.

### ELDER ABUSE VICTIMS DURING THE COVID-19 PANDEMIC: ADMINISTRATIVE DATA FROM SAN FRANCISCO ADULT PROTECTIVE SERVICES

Pi-Ju Liu,[1] Aining Wang,[1] Laura Schwab-Reese,[1] and Sara Stratton,[2] *1. Purdue University, West Lafayette, Indiana, United States, 2. San Francisco Adult Protective Services, Department of Aging and Adult Services, City and County of San Francisco, San Francisco, California, United States*

This study examined elder mistreatment victims' experiences at the beginning of the COVID-19 pandemic. San Francisco Adult Protective Services (APS) caseworkers conducted phone interviews to inquire about clients' awareness of COVID-19 and unmet needs. Nine-hundred-and-thirty-four (71%) of 1,313 APS' past clients or their collaterals were interviewed, with 741 (79%) responding positively to COVID-19-awareness questions, and 697 (75%) having no unmet needs. Binary logistic regression with Firth adjusted maximum likelihood estimation method revealed that older persons ($p < .05$), self-neglectors ($p < .05$), and victims of neglect ($p < .05$) were less aware of COVID-19. Unmet needs varied by mistreatment type. Victims of isolation were more likely to have medical needs ($p < .05$), while victims of emotional abuse were more likely to report loneliness ($p < .001$). Collaboration between service providers is key in assisting victims experiencing unmet needs to live safely in a public health crisis.

### Session 1290 (Paper)

RESEARCH METHODS

### AGING IN THE AFTERMATH OF ADVERSITY: LATER-LIFE IMPACT OF INSTITUTIONAL CHILD ABUSE AND DISCLOSURE

Shauna Rohner,[1] Andreas Maercker,[2] Alan Carr,[3] and Myriam Thoma,[4] *1. University of Zurich, Zürich, Zurich, Switzerland, 2. University of Zurich, University of Zürich, Zurich, Switzerland, 3. University College Dublin, Dublin, Dublin, Ireland, 4. University of Zurich, Zurich, Zurich, Switzerland*

Until the 1990's in Ireland, many children in institutional care experienced abuse and neglect, with lasting negative effects, including trauma symptoms and psychopathology. While trauma disclosure can be important for recovery, findings are inconsistent and often lack consideration of wider social and interpersonal contexts. As survivors of this historical adversity enter later-life stages, research is needed on the long-term impact and to clarify the role of disclosure. Therefore, this study aimed to examine the later-life impact of institutional child abuse on health and well-being, and the role of trauma disclosure and socio-interpersonal contexts in an older adult sample. Qualitative semi-structured interviews (60-120 minutes) were conducted with 17 Irish older adults, aged 50-77 years (mean age=60.7 years), who experienced childhood institutional abuse. Audio-recorded interviews were transcribed and analysed using Framework Analysis. Themes for 'childhood and related later-life adversity' included detrimental perceptions and interactions, re-exposure and reminders, failure of system and society, and cycle of abuse. Disclosure themes included successful, unsuccessful, and non-disclosure, as well as evidence of socio-interpersonal interactions (e.g., non-disclosure influenced by shame or fear, compounded by socio-cultural values, (lack of) social acknowledgment, or the power of the church in society). Results suggest that childhood institutional abuse can have long-term negative impacts into later life, including social, psychological, physical health, and socio-economic aspects. Disclosure results emphasize the need to consider the complex social, cultural, and interpersonal contexts within which an individual is embedded. This may enhance understanding and facilitate targeted health and social care services for this older adult population.

### CHILDHOOD EXPOSURE TO FAMILY VIOLENCE AND LATER-LIFE SIBLING RELATIONSHIPS

Jooyoung Kong, and Jaime Goldberg, *University of Wisconsin-Madison, Madison, Wisconsin, United States*

There has been a growth in research examining the long-term effects of childhood adversity on later life outcomes; however, only a few studies have examined the impact that childhood adversity has on sibling relationships in late adulthood. To address this gap in the literature, the current study examines the latent class structure of childhood exposure to family violence and investigates whether a latent class membership is associated with aspects of later-life sibling relationships, including geographical proximity, frequency of contact, perceived closeness, similarity in outlook, and exchange of support. Using data from 3,921 adult participants in the Wisconsin Longitudinal Study and the Bolck, Croon, and Hagenaars (BCH) approach of latent class analysis (LCA), we identified five latent classes (prevalence rate noted): "Never experienced violence (75%)," "experienced sibling violence (7%)," "experienced parental abuse & witnessed domestic violence (4%)," "experienced father's abuse & witnessed domestic violence (10%)," "experienced mother's abuse & witnessed domestic violence (5%)". Childhood exposure to family violence was a significant predictor of later-life sibling relationships. Specifically, adults who experienced sibling violence and their mother's and/or father's abuse in childhood showed significantly lower perceived closeness and similarity in outlook with their siblings in adulthood than those who did not experience violence. Our findings suggest that childhood exposure to family violence may have a long-term negative impact on the emotional aspect of sibling relationships. Future research may explore how the impact of childhood adversity on sibling relationships affects other aspects of adult lives, such as individual well-being or caregiving for aging parents.

### INVESTIGATING SEQUENTIAL AND SIMULTANEOUS CHANGES IN TRAJECTORIES OF COGNITIVE DECLINE AND DEPRESSIVE SYMPTOMS

Matthias Klee,[1] and Anja Leist,[2] *1. University of Luxembourg, Esch-sur-Alzette, Grevenmacher, Luxembourg, 2. University of Luxembourg, Esch-sur-Alzette, Diekirch, Luxembourg*

Background. The role of depression as risk factor or early symptom of cognitive decline and dementia is still debated. Exploiting longitudinal trajectories of memory recall in a large European sample, we sought to better understand the nature of simultaneous versus sequential changes in depressive symptoms alongside memory recall at older ages. Method. A total of 4,865 respondents to the SHARE survey, mean age at t1 61.5 years (SD = 7.53), completed the EURO-D depression scale and a delayed recall task across six waves spanning ~13 years. We applied k-means clustering to distinguish trajectories of depressive symptoms and delayed recall. Clusters indicating depressive and recall trajectories were included in logistic regressions to assess likelihood of parallel versus sequential change, controlling for age, gender, employment status and education. Results. Analyses revealed six distinct trajectories each for depressive symptoms and delayed recall. Visual inspections indicated that only declining recall trajectories showed increases in depressive symptoms, occurring simultaneously rather than sequentially. Using grouped declining recall trajectories as outcome, the low-increasing depressive symptoms trajectory was associated with cognitive decline (OR = 1.52 [1.11, 2.06]), whereas the stable-high depressive symptoms trajectory was associated with cognitive decline in respondents aged 60-69 years (OR = 1.78 [1.01, 3.16]). Discussion. Distinguishing trajectories in depression and recall incorporates longitudinal information able to further elucidate relationships between depression and cognition. While the findings suggest depression as a co-morbidity, attention needs to be given to a comparatively small high-stable depressive symptoms trajectory group with elevated risk of cognitive decline in their 60s.

### PRE-STATISTICAL HARMONIZATION OF BEHAVIORAL INSTRUMENTS ACROSS EIGHT SURVEYS AND TRIALS

Diefei Chen,[1] Eric Jutkowitz,[2] Skylar Iosepovici,[3] John Lin,[4] and Alden Gross,[1] *1. Johns Hopkins Bloomberg School of Public Health, Baltimore, Maryland, United States, 2. Brown University, Brown University, Rhode Island, United States, 3. Brown University School of Public Health, Providence, Rhode Island, United States, 4. Brown University, Sugar Land, Texas, United States*

Data harmonization methods facilitate further use of existing studies and research resources. Most statistical harmonization methods require pooling data across studies, which is complex and requires careful scrutiny of source data. Most methods (e.g., item response theory) require datasets to have common items for linking a common construct across studies: this necessitates the qualitative process of pre-statistical harmonization. Here, we document pre-statistical harmonization of items measuring behavioral and psychological symptoms (e.g., agitation, wandering, etc.) which represent problematic behaviors among people with dementia administered in a national survey (ADAMS), evaluations conducted at Alzheimer's Disease Research Centers (NACC), and in six randomized trials (COPE, TAP, ALZQOL, ACT, REACH, ADSPlus). We describe our approach to review question content and scoring procedures to establish comparability across items prior to data pooling. We identified 327 items from 15 instruments across these eight studies. We found considerable cross-study heterogeneity in administration and coding procedures for items that measure the same domain. For example, eight items were coded as count variables in some studies but as categorical variables in others. Moreover, of the 359 items, 191 are conditionally dependent on values of another item. These issues around item response heterogeneity and conditional dependency needed to be resolved prior to estimation of item response theory models for statistical co-calibration. We leveraged several rigorous data transformation procedures to address these issues, including re-coding and winsorization. This study provides guidelines for how future research may acknowledge and address similar issues in pooling behavioral and related instruments.

### TRENDS IN DEMENTIA PREVALENCE IN THE UNITED STATES, 1995-2019: AN AGE-PERIOD-COHORT-INTERACTION ANALYSIS

Mark Lee,[1] and Liying Luo,[2] *1. University of Minnesota, Minneapolis, Minnesota, United States, 2. Pennsylvania State University, University Park, Pennsylvania, United States*

Previous studies have indicated that age-specific dementia prevalence has declined in the United States and other high-income countries. However, these studies have been limited by estimating temporal change in dementia rates on a strictly period basis, with little attention to possible cohort effects. Distinguishing age, period, and cohort effects is both methodologically and theoretically important for identifying the etiological factors driving dementia decline in the population. In this study, we apply the novel Age-Period-Cohort-Interaction (APC-I) model, which defines cohort effects as the interaction between age and period main effects. The APC-I model improves on earlier APC models (e.g., the accounting method) by solving the linear dependence between predictors through theoretical clarification instead of statistical manipulation. We use the APC-I model to estimate period and cohort trends in dementia prevalence using data from the Health and Retirement Study (HRS). Our analysis points to significant period and cohort effects. Dementia prevalence declined significantly between the periods 1995-1999 and 2015-2019. At the same time, cohorts born in the 1940s had significantly lower odds of dementia than would be expected given age and period main effects. This cohort's unique protection from dementia has been relatively stable as they have aged. Our study identifies the periods during which and cohorts for whom dementia risk has declined in the United States. Further research is needed to specify the period factors (e.g., broad based improvements in nutrition) and cohort factors (e.g., increases in educational attainment) that are responsible for these trends.

### Session 1295 (Symposium)

### STRESS, COGNITION, AND MINORITY HEALTH

Chair: Toni Antonucci Co-Chair: Laura Zahodne
Discussant: Melissa Gerald

This symposium examines the many factors influencing cognition and health among ethnically and racially diverse groups. Kindratt et al. use representative, national data to examine cognitive limitations and diabetes among foreign born Non-Hispanic Whites, Blacks, Hispanics, Asians and Arab Americans. Results indicate that prevalence of cognitive limitations was highest among non-Hispanic Whites and Arab-Americans, lowest among Blacks and Asians. Diminich et al. investigate the association of stressors and metabolic risk factors with cognitive/emotional functioning in a population of Hispanic/Latina(o) immigrants. They find a link between components of metabolic syndrome that are associated with domain specific deficits in cognition. These impairments are linked to posttraumatic stress, immigration related trauma and emotional health and wellbeing. Arevalo et al. examine cross-sectional and prospective associations of sleep duration and insomnia symptoms with measures of cognitive functioning among older Latinos from Puerto Rican ancestry with a longitudinal sample of older adults from the Boston Puerto Rican Health Study. Findings indicate that hours of sleep and insomnia symptoms are significantly associated with a number of global and specific cognitive factors. Finally, Munoz and colleagues, using a regional racially and ethnically diverse sample of people living in a large northeastern city, identified four stress profiles. These profiles (which focus on different types of stress) were differentially associated with working memory performance. In sum, these four papers document the experiences of stress and their association with cognitive functioning in diverse minority groups each of whom are disproportionately at risk for ADRD/RD. Gerald, from NIA, will serve as discussant.

### DISPARITIES IN COGNITION AMONG U.S. AND FOREIGN-BORN MINORITY POPULATIONS WITH AND WITHOUT DIABETES

Tiffany Kindratt,[1] Florence Dallo,[2] Laura Zahodne,[3] and Kristine Ajrouch,[4] *1. University of Texas at Arlington, Arlington, Texas, United States, 2. Department of Public and Environmental Wellness, Rochester, Michigan, United States, 3. Clinical Science, Ann Arbor, Michigan, United States, 4. Eastern Michigan University, Ypsilnati, Michigan, United States*

Adults with cognitive limitations and diabetes may be less able to adhere to treatment recommendations. Our aims were to: 1) estimate and compare the prevalence of cognitive limitations and diabetes among foreign-born non-Hispanic whites, blacks, Hispanics, Asians, and Arab Americans to US-born non-Hispanic whites; and 2) examine associations after controlling for covariates. We linked 2002-2016 National Health Interview Survey and 2003-2017 Medical Expenditure Panel Survey data (ages >=45 years, n=122,898). The prevalence of cognitive limitations was highest among foreign-born non-Hispanic whites (9.71%) and Arab Americans (9.40%) and lowest among foreign-born blacks (5.19%). Foreign-born non-Hispanic whites had higher odds (OR=1.36; 95% CI=1.05-1.49) of cognitive limitations than their US-born counterparts. Foreign-born Hispanics with diabetes had greater odds of cognitive limitations (OR=1.91; 95% CI=1.63, 2.24) compared to US-born non-Hispanic whites. Additional findings will be discussed focused on stressors that may contribute to cognition disparities using the immigrant health paradox framework.

### SLEEP AND COGNITION: RESULTS FROM A LONGITUDINAL COHORT OF OLDER PUERTO RICAN ADULTS

Sandra Arévalo, *California State University, Long Beach, Long Beach, California, United States*

We examined cross-sectional and prospective associations of sleep duration and insomnia symptoms with measures of cognitive function among older adults aged 45-75 y from the Boston Puerto Rican Health Study, a longitudinal cohort of 1500 participants of Puerto Rican ancestry. We found, statistically significant cross-sectional associations of sleep duration (hours) and an executive function domain before (F=6.20; Prob>F=0.0001) and after (F=2.33; Prob>F=0.05) controlling for covariates (age, sex, education, smoking, drinking, mental and health conditions and medication use); between sleep duration and global cognition before (F=5.38; Prob>F=0.0003) and a trend after controlling for covariates (F=2.20; Prob>F=0.0669). In longitudinal associations, sleep duration (time2) was significantly associated with global condition at time3 (F=2.42; Prob>F=0.0475) after controlling for time2 global cognition. In conclusion, we found hours of sleep and insomnia symptoms significantly associated with various cognitive factors. A public health focus on sleep hygiene may improve cognitive health outcomes in older Puerto Rican adults.

### IMMIGRATION-RELATED TRAUMA ASSOCIATED WITH METABOLIC RISK AND COGNITION IN HISPANIC AND LATINO IMMIGRANT POPULATIONS

Erica Diminich,[1] Kristine Ajrouch,[2] Toni Antonucci,[3] Sean Clouston,[4] Irving Vega,[5] Laura Zahodne,[6] Noah Webster,[3] and RIchard Gonzalez,[3] *1. Stony Brook University, Stony Brook, New York, United States, 2. Eastern Michigan University, Ypsilnati, Michigan, United States, 3. University of Michigan, Ann Arbor, Michigan, United States, 4. Renaissance School of Medicine, Stony Brook University, Renaissance School of Medicine, Stony Brook University, New York, United States, 5. Michigan State University, Grand Rapids, Michigan, United States, 6. Clinical Science, Ann Arbor, Michigan, United States*

Recent immigrant and undocumented Hispanic/Latino adults in the United States (U.S.) are an underserved segment of the aging population. In this cross-sectional pilot study, we examined associations between self-reported stressors metabolic syndrome, emotional reactivity, and cognitive functioning in a heterogenous sample (N=80) of Hispanic/Latino adults (43.8% Central America; 43.8% South America; 7.5% Caribbean; mean years in the U.S.=18.1, SD=12.8). Participants (Meducation=10.2 years, SD=5.34; Mage=48.6 years, SD=12.3) underwent blood draw, anthropometrics and NIH-toolbox cognitive and behavioral measures. Linear regressions indicated that, elevated glucose was inversely associated with working memory (r=-.30), whereas higher HDL and controlled glucose were associated with better episodic memory (r=.27) and executive functioning (r=.32). Results further revealed associations between immigration-related trauma and elevated posttraumatic stress symptomatology. Implications for mental health and early detection of modifiable risk factors to promote healthy aging in vulnerable Hispanic/Latino immigrant populations are discussed.

### PSYCHOSOCIAL STRESS PROFILES AND COGNITIVE FUNCTION IN A RACIALLY AND ETHNICALLY DIVERSE SAMPLE OF ADULTS

Elizabeth Munoz,[1] Laura Zahodne,[2] RIchard Gonzalez,[3] Noah Webster,[3] Martin Sliwinski,[4] and Stacey Scott,[5] *1. The University of Texas at Austin, The University of Texas at Austin, Texas, United States, 2. Clinical Science, Ann Arbor, Michigan, United States, 3. University of Michigan, Ann Arbor, Michigan, United States, 4. The Pennsylvania State University, University Park, Pennsylvania, United States, 5. Stony Brook University, Stony Brook, New York, United States*

We developed comprehensive multi-domain profiles of psychosocial stress in urban-dwelling, racially and ethnically diverse adults (age range: 25-65; N=256; 63% Non-Hispanic Black; 25% Hispanic; 9% Non-Hispanic White) and evaluated associations with cognitive function. Participants completed psychosocial stress measures tapping into ten domains and tasks of processing speed, working memory, and episodic memory. Latent profile analyses controlling for age yielded four-profiles: high neighborhood stress, moderate versus high work stress and daily discrimination, and high health and relationship stress. Profiles significantly differed in income, age, and employment status. The profile with moderate work stress and daily discrimination and the profile with high neighborhood stress each had significantly lower working memory than the other profiles. The finding of lower working memory among individuals in the moderate work stress and daily discrimination profile was not due to sociodemographic variables. Results highlight the potentially cumulative influence of different contextual stressors on cognition.

### Session 1300 (Paper)

### TEACHING AND LEARNING DURING THE COVID-19 PANDEMIC

### GENERATING COMMUNITY ENGAGED LEARNING IN GERONTOLOGY COURSES DURING THE COVID-19 PANDEMIC

Katarina Felsted, and Samantha Whitehead, *University of Utah, Salt Lake City, Utah, United States*

This presentation describes the core traits of a community-engaged learning (CEL course), how one gerontology program incorporated a theoretical framework to continue to provide students opportunities during the COVID-19 pandemic, and how generalizable this is across gerontology programs. Caregiving and Aging Families, a gerontology course enrolling both undergraduate and graduate students, champions community-engaged learning in two critical ways: students attend caregiver support groups in the community, and students form a partnership with a caregiver mentor in the community. This partnership allows students an intimate look at the caregiver's role and burden while enlisting the student to prepare a service care plan and compendium of resources for the caregiver. Ensuring the safety of older adults during the COVID-19 pandemic placed restrictive parameters on these experiences. While students typically attend support groups and identify and partner with a caregiver mentor in person, this needed modification during the pandemic. This was created through the application of Baltes' Theory of Selection, Optimization, and Compensation (SOC model), aided by a CEL teaching assistant, funded through the campus Community Service Center. This allowed for identifying, coordinating, and communicating with community partners throughout the semester and provided ongoing communication, technical assistance, and problem-solving for both partners and students. Caregiver groups with a robust online, synchronous presence were identified and approached. The gerontology program communities of interest disseminated a call for community caregivers with basic technological familiarity. The caregiver mentor-student partnerships were founded and maintained, with additional benefits stemming from a shared pandemic experience.

### GENERATION TO GENERATION PROJECT: PAIRING STUDENTS WITH OLDER ADULT MENTORS DURING THE COVID-19 PANDEMIC

Pamela Elfenbein, *University of North Georgia, Gainesville, Georgia, United States*

To meet the needs of older adults isolated in the midst of the COVID-19 pandemic, we began pairing Human Services and Gerontology students with community dwelling adults 55 years of age and older, recruited through senior centers and other organizations serving older adults. Students paired with isolated older adults were tasked with building supportive relationships, assuring that basic needs were being met and that all available community resources were in place, and then documenting their mentor's life stories with an emphasis on the resilience, strength and wisdom of their older adult mentors (to focus and build on their strengths, the older adults we engage with are referred to as our "mentors"). Oral histories and supporting artifacts are archived in the university's library permanent repository. The oral histories portray unique perspectives into life, strength, and resilience during the COVID-19 pandemic and quarantine. The older adult mentors participating in the Generation to Generation project reported feeling strengthened and connected through their participation in the Generation to Generation project. To determine if social Isolation and loneliness can be ameliorated through participation in the Generation to Generation oral history project, researchers utilized the UCLA Loneliness Scale in pre- and post- participation interviews; findings will be shared.

### HIGH DEATH ANXIETY AND AMBIGUOUS LOSS: LESSONS LEARNED FROM TEACHING THROUGH THE COVID-19 PANDEMIC

Raven Weaver,[1] Cory Bolkan,[2] and Autumn Decker,[1] *1. Washington State University, Pullman, Washington, United States, 2. Washington State University, Vancouver, Washington, United States*

For gerontological educators, topics such as mortality, loss, and end-of-life issues often emerge or are central in their courses. However, the COVID-19 pandemic has raised our awareness of loss and death on a global scale and teaching during the pandemic has raised questions about how educators, communities, or systems of higher education can support students' learning while simultaneously experiencing losses during intense times of uncertainty. In this mixed-method study of 246 students enrolled in undergraduate thanatology courses, we explored their levels of death anxiety and their experiences with pandemic-related losses. We found that students' death anxiety increased significantly during the pandemic, in comparison to the years prior ($p < .001$). We also conducted a content analysis in a subset of students' written narratives ($n = 44$) regarding their pandemic experiences. We identified three themes. Participants desired: (a) more flexibility from instructors, no questions asked; (b) more compassion and understanding; and (c) specific, targeted support resources. The voices of students were filtered through the authors' interpretation as educators to provide several teaching recommendations that support student learning during challenging times. The recommendations align with a trauma-informed approach, given the high rates of death anxiety and ambiguous loss among students, and have immediate implications for educators teaching during the pandemic, and for years to come. Finally, we also advocate for more university and community-based thanatology, and gerontology education offerings in general, to help normalize conversations about death, loss, and bereavement.

### INTERGENERATIONAL COMMUNICATION AS INTERVENTION: SOCIAL ISOLATION IN OLDER ADULTS DURING COVID-19

Laura Kirk,[1] Penny Kessler,[2] Stephanie Gingerich,[3] Sharon McGill,[3] and Hanna Pryor,[4] *1. University of Minnesota, Minneapolis, Minnesota, United States, 2. University of Minnesota, University of Minnesota School of Nursing, Minneapolis, Minnesota, United States, 3. University of Minnesota School of Nursing, Minneapolis, Minnesota, United States, 4. Presbyterian Homes and Services, Roseville, Minnesota, United States*

Social isolation and loneliness are prevalent and impactful in the lives of older adult across care settings, and the emergence of a deadly global pandemic requiring social distancing and quarantining exacerbated these experiences significantly in 2020. A semester-long communication-focused clinical project was developed and piloted for sophomore bachelor of nursing science (BSN) students during fall 2020. Affording preclinical nursing students the opportunity to develop communication skills early in their program of study holds potential, and learning the story of older adults appears to be mutually beneficial; older adults serve as mentors and share their story, and preclinical nursing students have an opportunity to learn about their mentor's life, challenging some prevalent stereotypes about aging. A pre- post-clinical survey of student attitudes toward older adults suggests a dramatic positive shift in perspective, and unsolicited, anecdotal comments in student reflections support this finding: "…it helped me feel much more open to working with older adults in the future"; "This conversation taught me that my assumptions about the older generation are not always correct". Of older adult participants, 96% reported the experience enriched and enhanced their social connectedness, with 88% requesting to participate in the program again. Narrative comments from residents indicated that their involvement enabled them to feel engaged and purposeful: "I was a mentor"; "Conversations were so alive. Connections with curious young people fill my heart and soul." Intergenerational sharing of life stories has the potential for both healing and growth and may provide an antidote to ageism.

### SOCIAL RETRIBUTION OF STUDENTS OF MASTER IN GERONTOLOGY DURING THE COVID-19 PANDEMIC: ENHANCING COMPETENCIES

Elva Dolores Arias-Merino,[1] Ma. Guadalupe Díaz-González,[2] Neyda Ma. Mendoza-Ruvalcaba,[3] Martha Elena Vazquez-Arias,[4] and José Rosario Gonzalez-Ulloa,[5] *1. Universidad de Guadalajara, Zapopan, Jalisco, Jalisco, Mexico, 2. Desarrollo Integral de la Familia, DIF Zapopan, Zapopan, Jalisco, Mexico, 3. University of*

*Guadalajara CUTONALA, Guadalajara, Jalisco, Mexico, 4. Universidad de Guadalajara, El Palomar, Jalisco, Mexico, 5. Universidad de Guadalajara, Tlaquepaque, Jalisco, Mexico*

**Introduction:** In response to the COVID-19 pandemic older adults were called to lockdown and social isolation. Master in Gerontology (MAGE) in a social retribution action delivered a companion program called "I´m with you, You´re with me". MAGE competencies encompass those proposed by AGHE. The aim is to analyze competencies acquired by students in gerontology in the companion program during the COVID-19 Pandemic.

**Method:** Participated 16 students of MAGE and 16 older adults selected by their high vulnerability conditions reported by social workers from the Metropolitan Center of Older Adults from DIF-Zapopan city. The program was designed to provide emotional support, was delivered by telephone for 3 months (Ago-Nov 2020). Experiences were obtained through an online-questionnaire, data were analyzed qualitative and quantitatively.

**Results:** Students reported higher development in the competencies: 1) Interactional, that capture the process of knowing and doing across the fields of gerontology, related to stereotypes and discrimination, autonomy and self-determination, and the use of the technology to enhance the communication; 2) Fundamental, that represent the essential orientation to the field of gerontology, related to identify the impact of public policy and the application of intervention strategies and the use of technologies with older adults, families and caregivers; 3) Contextual, related to promote solid social networks for the wellbeing. The meaning of participating in this program was mainly centered in a gratifying professional experience, "small actions that make big gestures".

**Conclusion:** Gerontologist promote social solidarity through the transference and applying of the knowledge to enhance social development.

### Session 1305 (Symposium)

### THE WAKE FOREST IASDR: A RESOURCE FOR STUDYING THE EFFECTS OF CALORIC RESTRICTION ON HEALTH OUTCOMES IN OLDER ADULTS

Chair: Denise Houston Co-Chair: Jamie Justice Discussant: Anne Newman

Over the past 25+ years, a focus of the Wake Forest Claude D. Pepper Older Americans Independence Center (OAIC) has been to study the consequences of and treatments for geriatric obesity. The Wake Forest OAIC has provided support for 18 clinical trials of caloric restriction (CR), with and without various exercise regimens, in 2,545 adults (71% women, 21% African American) with a mean±SD age of 67.5±5.9 years and BMI ≥27 kg/m2. A priority of the Wake Forest OAIC is to collate and store common data (e.g., demographics, physical performance, cognitive function), biospecimens (blood, muscle, adipose), and images (DXA, CT) from these trials in the Integrated Aging Studies Databank and Repository (IASDR; https://www.peppercenter.org/public/dspIASDR.cfm). This IASDR serves as a resource for the scientific community to foster new scientific questions and analyses. This symposium will provide an overview of CR trials and participants included in the IASDR and how the IASDR supports secondary analyses of CR by highlighting several secondary analyses using data and/or samples from the IASDR. Justice and colleagues examined the effect of CR on a geroscience-guided biomarker index using blood samples from the biorepository. Weaver and colleagues examined the effect of different exercise regimens on CT-derived muscle and bone measures during CR. Miller and colleagues pooled data from 11 trials to determine if CR-induced appendicular lean mass loss is associated with changes in physical performance. Finally, Hsieh and colleagues pooled data from eight trials to examine whether the effect of CR on gait speed differed by baseline BMI and inflammation.

### THE FIRST EVALUATION OF A GEROSCIENCE BIOMARKER INDEX (TAME-BI) IN A TRIAL OF CALORIC RESTRICTION AND EXERCISE

Jamie Justice,[1] Mark Espeland,[2] Denise Houston,[2] Stephen Kritchevsky,[3] Barbara Nicklas,[2] Nicholas Pajewski,[2] and Dalane Kitzman,[2] *1. Wake Forest School of Medicine, Wake Forest School of Medicine, North Carolina, United States, 2. Wake Forest School of Medicine, Winston-Salem, North Carolina, United States, 3. Wake Forest School of Medicine, Winston Salem, North Carolina, United States*

We leveraged the WF OAIC biorepository to measure a consensus-derived panel of blood-based biomarkers of aging and constructed a geroscience-guided biomarker index (TAME-BI), testing it for the first time in a clinical trial. We measured IL-6, TNF-α-receptor-I, growth differentiating factor-15, cystatin C, and N-terminal pro-B-type natriuretic peptide in a 20-week randomized trial of caloric restriction (CR), aerobic exercise (EX), CR+EX, or attention-control in 88 patients (67±5years) with heart failure with preserved ejection fraction (HFpEF). We calculated TAME-BI (analyte levels ranked, binned by quintile, and summed) and found a time×treatment interaction for improved TAME-BI with intervention ($p \leq 0.05$) and detected associations between change in TAME-BI and change in six-minute walk distance (r= -0.24), usual walk speed (r= -0.23), and left ventricular relative wall thickness (r= 0.31). In sum, CR+EX intervention improved TAME-BI and changes in TAME-BI were associated with changes in key functional measures in older HFpEF patients.

### EXERCISE MODALITY AFFECTS OLDER ADULT CT-DERIVED MUSCLE AND BONE LOSS DURING CALORIC RESTRICTION

Ashley Weaver,[1] Diana Madrid,[1] Katelyn Greene,[1] Michael Walkup,[1] Walter Ambrosius,[1] Anthony Marsh,[2] W. Jack Rejeski,[3] and Kristen Beavers,[4] *1. Wake Forest School of Medicine, Winston Salem, North Carolina, United States, 2. Wake Forest University, Winston-Salem, North Carolina, United States, 3. Department of Health and Exercise Science, Wake Forest University, North Carolina, United States, 4. Wake Forest University, Winston Salem, North Carolina, United States*

Caloric restriction (CR) can exacerbate muscle and bone loss. We examined 18-month changes in computed tomography (CT)-derived trunk muscle, and volumetric bone mineral density (vBMD) and finite element-estimated bone strength of the spine and hip in 55 older adults randomized to CR alone or CR plus aerobic (CR+AT) or resistance (CR+RT) training. Trunk muscle area loss trended higher with CR+AT [-16.8 cm2 (95% CI: -26.4,-7.1) vs CR: -6.7 (-12.8,-0.5), CR+RT: -9.0 (-14.5,-3.4)]. Spine vBMD loss trended higher with CR+AT [−0.014 g/cm3 (−0.027,−0.001) vs. CR: −0.005 (−0.022,0.012), CR+RT: −0.004 (−0.019,0.011)], and similarly for vertebral bone strength. Hip vBMD losses trended lower with CR+RT [−0.015 g/cm3 (−0.024,−0.006) vs. CR: −0.027 (−0.036,−0.019), CR+AT: −0.029 (−0.037,−0.020)]. Hip vBMD and trunk muscle losses were positively correlated (r=0.53), and spine vBMD loss tended to increase with trunk muscle loss (r=0.21) and fat infiltration (r=0.17). Collectively, aerobic training was less effective at preserving muscle-bone health during CR.

### APPENDICULAR LEAN MASS LOSS DOES NOT IMPACT PHYSICAL PERFORMANCE CHANGE DURING CALORIC RESTRICTION IN OLDER ADULTS

Daniel Beavers,[1] Ryan Miller,[2] Kristen Beavers,[3] and Barbara Nicklas,[1] *1. Wake Forest School of Medicine, Winston-Salem, North Carolina, United States, 2. Wake Forest School of Medicine, Winston Salem, North Carolina, United States, 3. Wake Forest University, Winston Salem, North Carolina, United States*

Data from 11 six-month randomized controlled trials were pooled, with 902 participants randomized to caloric restriction (CR; n=762) or Non-CR (n=140) to determine if CR-induced appendicular lean mass (ALM) loss was associated with change in physical performance among older adults. After adjusting for age, sex, race, body mass index, exercise assignment and baseline value of the outcome, CR had significant ALM loss [-0.77 kg (95% CI: -0.89, -0.65)], while Non-CR had ALM gain [+0.28 kg (0.08, 0.49)]; p<0.001. Both groups experienced similar improvements in the Short Physical Performance Battery (SPPB) score [CR: +0.45 (0.35, 0.55) vs Non-CR: +0.50 (0.30, 0.69); p=0.63] and sit-to-stand time [CR: -1.42 s (-1.81, -1.03) vs Non-CR: -1.85 s (-2.49, -1.21); p=0.19]. Change in SPPB score and sit-to-stand time was not associated with change in ALM (both p>0.15). In spite of significant ALM loss, CR resulted in overall improvements in physical performance in older adults.

### EFFECT OF BASELINE BMI AND IL-6 ON GAIT SPEED RESPONSE TO CALORIC RESTRICTION IN OLDER ADULTS

Katherine Hsieh,[1] Rebecca Neiberg,[1] Kristen Beavers,[2] and Daniel Beavers,[3] *1. Wake Forest School of Medicine, Winston Salem, North Carolina, United States, 2. Wake Forest University, Winston Salem, North Carolina, United States, 3. Wake Forest School of Medicine, Winston-Salem, North Carolina, United States*

We examined whether the effect of caloric restriction (CR) on gait speed change in older adults (67.3±5.27 years) varied by BMI and interleukin 6 (IL-6). Data from eight six-month randomized controlled trials were pooled, with 1268 participants randomized to CR (n=710) and non-CR (n=558) conditions. Baseline BMI/IL-6 subgroups were constructed using BMI≥35 kg/m2 and IL-6>2.5 pg/dL, and participants were jointly classified as high/high (n=395), high/low (n=208), low/high (n=271), or low/low (n=344). Overall treatment effects showed significant improvements in gait speed in CR versus non-CR [mean difference: 0.02 m/s (95% CI: 0.01, 0.04)]; however, CR assignment significantly interacted with BMI/IL-6 subgroup (p=0.03). Greatest gait speed improvement was observed in the high/high CR subgroup [+0.06 m/s (0.03, 0.09)] and appeared to be driven by no gait speed change among the high/high non-CR subgroup. Gait speed response to CR was greatest in older adults with elevated baseline BMI and IL-6.

### Session 1310 (Symposium)

### TOWARD A MODEL FOR MEASURING SOCIAL AND STRUCTURAL DETERMINANTS OF ALZHEIMER'S DISEASE AND RELATED DEMENTIAS

Chair: Shana Stites Co-Chair: Joyce Balls-Berry Discussant: Lisa Barnes

Social and structural determinants of health (SSDoH) are conditions in the environments in which individuals are born, live, learn, work, play, worship, and age that affect health, functioning, and quality-of-life outcomes across the life course. Growing evidence suggests that SSDoH can help to explain heterogeneity in cognitive, functional, and interventional outcomes in Alzheimer's disease and related disorders research and clinical practice. The National Institute on Aging (NIA) has prioritized collecting SSDoH data in order to elucidate disease mechanisms and aid discovery of a disease modifying treatments. However, a major nexus of ADRD research, the national network of Alzheimer's Disease Research Centers (ADRCs), does not routinely collect SSDoH data. We describe a model for feasibly gathering and analyzing SSDoH data across Alzheimer's Disease Research Centers (ADRCs). We lay out theoretical underpinnings of key constructs and their measure, empirical evidence for their importance, and their potential for elucidating disease and prevention mechanisms. Toward a goal of translation, we describe a general approach to measuring SSDoH along with core set of measures. We also describe empirical support and rationales for assessing SSDoH in standing geographically and culturally diverse research cohorts, and guiding considerations in selecting modules to serve unique communities. We specifically address SSDoH in Black, Hispanic/Latin, and refugee populations with an eye toward conveying how geographic proximity, socioeconomic status, ethnoracial factors, and sex\gender\sexual orientation affect populations in ways directly relevant to Alzheimer's disease (AD) and Alzheimer's disease related dementias (ADRD).

### ASSESSING SSDOH IN ALZHEIMER'S RESEARCH: CORE MEASURES AND THOSE IN SEXUAL AND GENDER MINORITY POPULATIONS

Shana Stites,[1] and Sharnita Midgett,[2] *1. University of Pennsylvania, Philadelphia, Pennsylvania, United States, 2. University Pennsylvania, Philadelphia, Pennsylvania, United States*

Social and structural determinants of health (SSDoH) are conditions that can impact on Alzheimer's disease and Alzheimer's disease related dementias (AD/ADRD) outcomes. We will describe theoretical underpinnings of core SSDoH constructs and their measure, empirical evidence for their importance, and their potential for elucidating disease and prevention mechanisms. We focus on a core set of SSDoH measures that are important across a broad range of socially and culturally heterogeneous populations. We outline a rationale for universal implementation of a set of SSDoH measures and juxtapose the approach with alternatives, such as investigator-initiated grants, aimed at collecting SSDoH data. We also speak very briefly about the evidence supporting assessing SSDoH with respect to sex, gender, and sexual orientation and considerations in doing this.

### SSDOH IN LATINXS: FACTORS OF INFLUENCE

David Marquez, *Rush University, University of Illinois at Chicago, Illinois, United States*

Research with Latinxs/os/as regarding Alzheimer's disease and related dementias (ADRD) is lacking. This is staggering because among Latinxs in the United States, the number diagnosed with ADRD is expected to grow by more than 800% from 2012 to 2060 (Wu et al., 2016). Older Latinxs have a high risk and prevalence of ADRD - partially attributed to their longer life spans and the presence of adverse risk factors such as metabolic syndrome, type 2 diabetes mellitus, and other cardiovascular conditions (Chin et al., 2011). What is often missing in the discussion is the role of social and structural determinants of health (SSDOH) in this population. Overall Latinxs have low levels of formal education, work in physically demanding jobs, and experience immigration stress. How these and other SSDoH influence Latinxs will be discussed; as well as potential resilience factors like familial relationships, and religiosity or spirituality.

### AGING, SOCIAL DETERMINANTS OF HEALTH IN THE CASE OF PERSONS WITH DISABILITIES AND REFUGEES

Jean-Francois Trani, *Brown school, Washington University in St Louis, Missouri, United States*

Structural and social determinants of health differentially impact on social groups. Among those particularly disadvantaged during the life course are both persons with disabilities and refugees. Because of the way society treats these two populations, both persons with disabilities and refugees may face physical, social, economic and environmental barriers that impede them from benefiting from the same opportunities accessible to other social groups. As a result, these populations have less access to education, higher unemployment, are more likely to be deprived and excluded from social benefits. In other words, public stigma —prejudice and discrimination voiced and practiced by the general population— translates to a life course characterized by daily stressors that result in a higher likelihood of cognitive disorders and dementia. Measuring and analyzing SSDoH inclusive of disability and refugee experiences are essential to efforts aimed at recruitment and retention and knowledge generation in ADRD research.

### SSDOH AND AGING IN PERSONS OF AFRICAN ANCESTRY

Joyce Balls-Berry, *Knight Alzheimer's Disease Research Center, Washington University in St. Louis, Missouri, United States*

Persons of African Ancestry (Black) encompasses a broad spectrum of individuals across the African diaspora. The diversity of the Black community must be considered in the context of SSDoH especially as it relates to diseases of aging. Blacks report higher levels of discrimination as a barrier to Alzheimer's Disease or related dementia (ADRD) care, are less likely to receive timely diagnoses of ADRD, and many do not trust that a future cure for ADRD will be shared equally and equitability with their community compared to their white counterparts. Once diagnosed, older Blacks, are twice as likely as their white counterparts to have ADRD. A key to addressing the Black community's ADRD needs is speaking openly about the historical underpinnings related to social injustice and racism as a link to appropriate ADRD diagnoses. Ultimately, SSDoH impact treatment, healthcare policy, and the future of biomedical research for the Black community.

### Session 1315 (Symposium)

### UNDERSTANDING THE ABNORMAL TODAY FOR LIFE TOMORROW: A STUDY OF THE GENERATIONS DURING THE COVID-19 PANDEMIC

Chair: Lisa D'Ambrosio Discussant: Lisa D'Ambrosio

The spread of COVID-19 in the United States in early 2020 abruptly transformed American life, with widespread closures of public spaces and businesses, limitations on social activities, and the need for individuals to physically distance from each other. Some changes wrought by the virus may persist post-pandemic - such as Americans' adoption of new technologies or disease prevention behaviors. Since the onset of COVID-related safer-at-home orders, the MIT AgeLab has sought to understand how the pandemic affects people's attitudes and behaviors. This symposium will present findings drawn from three waves of national, online surveys conducted in 2020: March (N=1202), May-June (N=1,387), and November-December 2020 (N=1444). The surveys explored participants' COVID-19-related attitudes and behaviors across a range of domains. Each presentation in this symposium will highlight a different focus of cross-generational research conducted over time, with a particular focus on experiences of adults ages 55 and over. The first will focus on participants' overall health, wellbeing, and perceptions of the COVID-19 vaccine. The second will present experiences of family caregivers of older adults and children. The third will center on the impact of the pandemic on the generations' retirement and longevity planning experiences. The fourth and final presentation will focus on participants' attitudes and experiences using and adopting technology. This symposium will deepen attendees' understandings of multigenerational attitudes and experiences during the COVID-19 pandemic, with a particular focus on the experiences of adults ages 55 and over.

### SELF-REPORTED HEALTH, WELL-BEING, AND COVID-19 VACCINATION WILLINGNESS ACROSS THE GENERATIONS

Taylor Patskanick, *MIT, Somerville, Massachusetts, United States*

The COVID-19 pandemic has impacted the generations' health and wellbeing across a range of dimensions. In the first survey, the 50+ adopted the smallest number of health behaviors (e.g., handwashing, mask-wearing, etc.) in response to the pandemic compared to younger age groups. In the first and second surveys, the Baby Boomer generation reported less intense worry than other generations, especially regarding their socioemotional health and family members' health. For younger generations, worries tended to increase from March to June—especially those related to socio-emotional health and COVID-19 in general. In the third survey wave, older generations self-reported better psychological wellbeing, less personal burnout, and better cognitive health compared to younger generations. Willingness to get the COVID-19 vaccine did not vary by generation in this sample; however, implications of this (including additional factors that may be influential such as psychological wellbeing) will be discussed.

### PERCEPTIONS AND IMPACTS OF COVID-19 ON FAMILY CAREGIVERS

Alexa Balmuth, *MIT AgeLab, Cambridge, Massachusetts, United States*

For many family caregivers, COVID-19 has presented new obstacles to providing elder-care while balancing additional responsibilities such as work or childcare. Three survey waves explored impacts over the course of the pandemic. Family caregivers demonstrated resilience, taking a variety of measures to care for and protect family; caregivers were also more confident in their ability to protect loved ones age 60+ from contracting COVID-19 than non-caregivers. However, COVID-19's toll on caregivers was evident. Caregivers reported higher personal burnout than non-caregivers, and across all three survey waves, consistently reported greater degrees of worry in regards to COVID-19 in general, as well as its impacts on domains including the health and wellbeing of themselves and family members, and near and far term finances. This presentation will highlight how caregivers' perceptions and impacts of COVID-19 have evolved over the course of the pandemic, and implications of these findings for life tomorrow.

### THE IMPACTS OF COVID-19 ON TECHNOLOGY USE AND EXPERIENCE ACROSS GENERATIONS

Chaiwoo Lee,[1] Shabnam FakhrHosseini,[2] and John Rudnik,[3] *1. Massachusetts Institute of Technology, Cambridge, Massachusetts, United States, 2. MIT AgeLab, Cambridge, Massachusetts, United States, 3. MIT, Cambridge, Massachusetts, United States*

Among its many downstream effects, the COVID-19 pandemic has influenced how people think about and interact with technology. With limitations and restrictions around in-person interactions and use of public spaces, people are increasingly relying on technology to support everyday activities including work, communication and care. Results from the survey series showed an increased adoption of and interest in home, health and communication technologies. The changes may be long-lived, with the majority of the new adopters saying that they will continue to use the technologies that they started using in response to the pandemic. A generational comparison showed that while baby boomers and the silent generation were less likely than younger adults to have made recent adoptions, the older generations did not significantly differ in terms of interest in using new technologies. This presentation will also report on how COVID-19-related changes in technology experience varied by other demographic and socio-economic characteristics.

### THE INFLUENCE OF COVID-19 ON RETIREMENT AND LONGEVITY PLANNING: A MULTIGENERATIONAL PERSPECTIVE

Julie Miller,[1] Martina Raue,[2] and Lisa D'Ambrosio,[3]
*1. MIT, Cambridge, Massachusetts, United States,*
*2. MIT AgeLab, Cambridge, Massachusetts, United States,*
*3. Massachusetts Institute of Technology, Cambridge, Massachusetts, United States*

For many, the COVID-19 pandemic has painted a new economic picture of longevity. Results from the series demonstrated that younger generations surveyed—Gen Xers, Millennials, and Zoomers— generally reported elevated levels of worry about how the pandemic would affect their long-term finances. Baby Boomers, by comparison, evinced significantly less worry. And, although the pandemic has had a disproportionate economic impact on lower-income individuals, a higher income did not appear to confer freedom from worry. When it comes to planning for longevity, worry can be paralyzing. Zoomers were the generation most likely to agree that the future is too uncertain for them to even think about planning financially for retirement, and this sentiment applied for other generations as well. This presentation will also describe other ways in which the COVID-19 pandemic had influenced respondents' perceived longevity preparedness and attitudes across a variety of demographic and socio-economic characteristics.

### Session 1320 (Symposium)

### UNIQUE OPPORTUNITIES AND CHALLENGES FROM TWO ONLINE PSYCHOSOCIAL RANDOMIZED CLINICAL TRIALS

Chair: Carol Musil Co-Chair: Britney Webster Discussant: Rachel Pruchno

Research aimed at testing readily delivered online psychosocial interventions for addressing the needs of custodial grandfamilies (CGF) has been scarce. This symposium reports on two NIH-funded randomized clinical trials (RCT) involving fully online interventions: Study 1 (S1)-dyadic social Intelligence training for custodial grandmothers and their adolescent grandchildren, and Study 2 (S2)-4 week resourcefulness training with daily journaling intervention for grandmothers only. We presented here on the unique advantages and challenges of online RCTs as they apply to CGFs and similar hard-to-reach populations. First, in a cross-study collaboration, Jeanblanc et al. report data from both studies on how COVID-19 influenced coping habits, grandchild's remote learning, household conflict, uncertainty, and finances. Second, Castro et al. investigate how baseline positive and negative affect were reported in daily diaries completed by both grandmothers and grandchildren across 14 days at pretest in S1. Third, Musil et al. describe the challenges and benefits of using an entirely online design for the distribution and collection of longitudinal data, as exemplified by 4 weeks of qualitative daily journals from S2. Lastly, Webster et al. report on the benefits and challenges of recruitment and retention strategies encountered across S1 and S2. As discussant, Rachel Pruchno considers how the specific methodological advantages and disadvantages of online RCTs covered in the above papers apply to family caregiving research in general.

### COMPARISONS BETWEEN RECRUITMENT AND RETENTION STRATEGIES FOR TWO ONLINE RCTS

Britney Webster,[1] Alexandra Jeanblanc,[2] Gregory Smith,[1] and Frank Infurna,[3] *1. Kent State University, Kent, Ohio, United States, 2. Case Western Reserve University, Cleveland, Ohio, United States, 3. Arizona State University, Tempe, Arizona, United States*

Custodial grandfamilies (CGF) comprise a small, diverse group of the US population which can make samples difficult to recruit and retain. Two online RCT studies (S1 & S2) for CGF used a variety of recruitment strategies with varying success. S1, for grandmothers (GM) only, successfully recruited from Facebook (47.95%) and community flyers (17.73%). S2, dyadic study for GM and adolescent grandchildren (AGC), recruited through emails to high school counselors (43.29%) and community (30.94%) and professional (17.13%) kinship support organizations. The advantages of online RCTs for hard-to-reach populations include expedited administration, buffering against social distancing, nationwide enrollment (S1-42 states; S2-43 states), and generalizability of findings. Challenges of online RCTs are establishing rapport and building trust with participants who are not comfortable with technology and designing screenings to identify false participants. Overall, these studies highlight the advantages of an online RCT, especially for hard-to-reach populations like custodial grandfamilies.

### MANAGEMENT AND ANALYSIS OF LARGE QUALITATIVE DATA SETS: LESSONS LEARNED FROM DAILY JOURNALS FOR A BEHAVIORAL RCT

Carol Musil,[1] and Alexandra Jeanblanc,[2] *1. CWRU School of Nursing, Cleveland, Ohio, United States, 2. Case Western Reserve University, Cleveland, Ohio, United States*

As part of a national RCT of a resourcefulness skills training intervention, 342 grandmother caregivers, completed daily online journals for 4 weeks, reporting “challenges or difficulties you faced today with your grandchildren or other family members and how you handled them.” In this paper, we describe the challenges and benefits of using an entirely online design for the distribution and collection of daily journals. We used NVIVO-12 Plus to perform directed content analysis to assess intervention enactment fidelity and compare content between intervention (trained in and prompted to discuss skills) and control groups. Over 92%(n=317) of participants completed daily journals. There was variation in reporting of skill use between groups: 36% of controls spontaneously used the skill “seek professional help” whereas only 4% of the control group reported the skill “change from usual reaction”. With careful management, qualitative data from large samples can be obtained and effectively analyzed for fidelity.

### DAILY EMOTIONAL DYNAMICS OF CUSTODIAL GRANDFAMILIES: CROSS-DYAD CORRELATIONS OF AFFECT MEANS AND VARIABILITY

Saul Castro,[1] Frank Infurna,[2] Britney Webster,[3] Gregory Smith,[3] Max Crowley,[4] Megan Dolbin-MacNab,[5] and Carol Musil,[6] *1. Arizona State University, Arizona State University, Arizona, United States, 2. Arizona State University, Tempe, Arizona, United States, 3. Kent State University, Kent, Ohio, United States, 4. Pennsylvania State University, University Park, Pennsylvania, United States, 5. Virginia Polytechnic University, Blackburg, Virginia, United States, 6. CWRU School of Nursing, Cleveland, Ohio, United States*

Evidence indicates that daily emotional dynamics are associated with mental and physical health. However, these processes have not been examined among custodial grandmothers taking care of adolescent grandchildren. This daily diary study examined correlations between grandmothers and adolescents’ mean levels and variability in negative (NA) and positive affect (PA). Custodial dyads (M = 214) across the nation completed two weeks of daily surveys. For both grandmothers and adolescents, their own PA means were negatively correlated with NA means, PA variability, and NA variability; NA means were positively correlated with variability in PA and NA (ps <.01). Across dyads, grandmothers’ PA means were positively correlated with adolescents’ PA means and negatively correlated with adolescents’ NA means, PA variability and NA variability. Grandmothers’ NA means were positively correlated with adolescents’ variability in PA and NA (ps <.01). Our findings demonstrate how daily emotional dynamics are correlated within and between family members.

### PREDICTORS OF COVID-19 RELATED STRESS AMONG CUSTODIAL GRANDMOTHERS

Alexandra Jeanblanc,[1] Megan Dolbin-MacNab,[2] Carol Musil,[3] and Gregory Smith,[4] *1. Case Western Reserve University, Cleveland, Ohio, United States, 2. Virginia Polytechnic University, Blackburg, Virginia, United States, 3. CWRU School of Nursing, Cleveland, Ohio, United States, 4. Kent State University, Kent, Ohio, United States*

This paper examined predictors of COVID-19 stressors among 316 custodial grandmothers raising school-aged grandchildren using regression. Grandmothers, who were participants in two nationwide behavioral RCTs, completed an online questionnaire in Spring 2020. Predictors included grandmother demographics, depressive symptoms, perceived caregiving stress and reward, stress management strategies, and grandchild factors. Outcomes included grandmothers’ stress related to using bad coping habits (r2=.24), grandchildren’s remote learning(r2=.39), household conflict (r2=.29), COVID-19 fear and uncertainty (r2=.28), and finances(r2=.24). Regression results indicated that grandmothers’ pre-existing depressive symptoms predicted all outcomes except remote learning stress. Higher caregiving stress was associated with all outcomes, except concerns about using bad coping habits. Grandmothers with less perceived access to care reported greater concern about bad coping habits and remote learning stress, while minority grandmothers reported more financial stress and COVID-19 fear and uncertainty. Findings suggest that the COVID-19

pandemic has compounded the stress experienced by already burdened custodial grandmothers.

### Session 1325 (Symposium)

### UNPACKING DATA FROM ADULT DAY CENTERS IN ORDER TO REALIZE THEIR UNTAPPED POTENTIAL IN DEMENTIA CARE

Chair: Tina Sadarangani Discussant: Lauren Parker

Adult day centers (ADCs) in the United States represent a vital, but commonly overlooked, resource for dementia care among community-dwelling older adults. However, the severity of dementia in ADC users, their medical complexity, the supports offered to them, and health outcomes associated with adult day services among persons living with dementia is poorly understood. This is in part due to a lack of standardized data collection in this industry. In this symposium, we present the most current research on these issues, as well as strategies to improve data collection across ADCs to strengthen care. The symposium begins with analysis of data from the state of California that identifies patterns of chronic conditions in ADC users with dementia that are associated with emergency department visits and hospitalizations. We then examine data from the Centers for Disease Control, comparing dementia specialized ADCs and their participants to non-specializing ADCs. We compare the extent to which states with ADC programs require collection of patient centered reported outcomes on persons with dementia. Finally, we explore an innovative collaboration between researchers and community partners to simplify data collection in these centers. Our findings suggest that persons with dementia in ADCs are an extremely complex population and that some ADCs are better suited than others to meet their extensive needs. Additional patient-centered data collection can be supported with widely available software, and has the potential to demonstrate the effectiveness of ADCs, aid in program development, and help leverage funding opportunities.

### EXPLORING THE INTERSECTION OF CHRONIC DISEASE, FUNCTION, AND SOCIAL CARE IN ADULT DAY CARE CLIENTS WITH DEMENTIA

Tina Sadarangani,[1] Jonelle Boafo,[2] Bei Wu,[3] Abraham Brody,[4] and Gary Yu,[5] *1. New York University Rory Meyers College of Nursing, New York University Rory Meyers College of Nursing, New York, United States, 2. Rory Meyers College of Nursing, New York, New York, United States, 3. New York University, New York, New York, United States, 4. NYU Hartford Institute for Geriatric Nursing, New York, New York, United States, 5. NYU Rory Meyers College of nursing, New York, New York, United States*

The interrelationships among dementia, concomitant disease, and social determinants of health are poorly understood and have critical implications for disease course, treatments, and caregiving needs. The aim of this study was to identify patterns of co-occurring chronic conditions among persons with dementia and the relationship of these patterns with clinical characteristics, demographic predictors and functional status. A latent class analysis (LCA) was conducted using data from 53 California adult day centers (n=3,053). Four distinct groups emerged: "dementia only"; "dementia +: > 2; + > 3; + >5 chronic conditions. Having dementia + >5 was associated (p <.001) with greater risk of falls, isolation, medication mismanagement, and reduced likelihood of using an adaptive device. Dementia in day center clients is complicated by clinical conditions, functional decline, and a need for supports that may be lacking. Center staff must be trained and resourced to manage the complex needs of persons with dementia.

### DEMENTIA SPECIALIZATION AMONG ADULT DAY SERVICES CENTERS: NATIONAL STUDY OF LONG-TERM CARE PROVIDERS

Jessica Lendon,[1] Manisha Sengupta,[2] and Amanuel Melekin,[1] *1. National Center for Health Statistics, Hyattsville, Maryland, United States, 2. CDC/NCHS, Hyattsville, Maryland, United States*

Adult day services centers (ADSC) are a source of community-based care for persons with Alzheimer's disease/ other dementias. This study compares dementia specialized ADSCs (DSADSC) and their participants to other ADSCs that do not specialize in dementia care using the 2016-2018 National Study of Long-term Care Providers. DSADSCs account for 10% of all ADSCs and serve 15% of all ADSC participants with dementia. About half of DSADSC participants have dementia, compared to 30% in other ADSCs. A higher percentage of DSADSCs, compared to other ADSCs, were in the Midwest, were nonprofit, had a social model, and employed nursing aides. Fewer DSADSCs, compared to other ADSCs, provided nursing, mental health, and transportation services. More DSADSC participants were 75 years of age or older and needed assistance with eating and toileting. Findings may help identify how ADSCs, particularly, DSADSCs, meet the unique care needs of older adults with dementia.

### USING EXISTING SOFTWARE SYSTEMS TO COLLECT OUTCOME DATA IN ADULT DAY SERVICES

Keith Anderson,[1] Lisa Peters-Beumer,[2] and Laurie Duff,[3] *1. University of Texas at Arlington, Arlington, Texas, United States, 2. Concordia University Chicago, River Forest, Illinois, United States, 3. Easterseals New Hampshire, Manchester, New Hampshire, United States*

Recently, there has been a resounding call for standardized outcome data collection in adult day services (ADS). Outcome data have the potential to demonstrate the effectiveness of ADS, aid in program development, and help leverage funding opportunities. Unfortunately, many ADS centers do not collect outcome data for several reasons, including the cost of data collection software and systems. In this presentation, we present one effort to utilize an existing multiuse 'off the shelf' software solution to collect ADS outcome data for a network of ADS providers. The researchers collaborated with software developers and ADS providers to adapt the software to incorporate outcome measures and reporting functionality on both the individual and program levels. Adaptation and adoption required attention to HIPAA compliance, workflow integration, measurement fidelity, and data management processes. Despite these challenges, adapting existing software systems may be a cost-effective way to enable expanded outcome data collection in ADS.

### DOCUMENTING THE NEED FOR PATIENT-CENTERED RELEVANT OUTCOMES IN ADULT DAY SERVICES

Jonelle Boafo,[1] Keith Anderson,[2] Abraham Brody,[3] and Tina Sadarangani,[4] *1. Rory Meyers College of Nursing, New York, New York, United States, 2. University of Texas at Arlington, Arlington, Texas, United States, 3. NYU Hartford Institute for Geriatric Nursing, New York, New York, United States, 4. New York University Rory Meyers College of Nursing, New York University Rory Meyers College of Nursing, New York, United States*

ADCs are not uniformly regulated at the federal or state level, resulting in the absence of uniform data collection. The lack of large-scale data has resulted in a dearth of evidence on the role ADC services play in the health and well-being of their clients, particularly persons living with dementia (PLWD). The purpose of this study was to compare data being collected across states and evaluate the degree to which patient centered relevant outcomes (PCROs) are being collected. A review of ADC regulations in 50 states found that <10 states, required standardized reporting on ADC participants. Regulatory forms relied on clinical judgment as opposed to validated tools, and focused on eligibility for services as opposed to independence, engagement, or clinical interventions in the ADC. Emphasizing collection of PCROs in ADCs, beginning at the state level, is an essential step in documenting the value and effectiveness of ADCs, particularly for PLWD.

### Session 1327 (Symposium)

### SENSORY HEALTH AND DEMENTIA CARE: UNDERSTANDING AND ADDRESSING UNMET NEEDS IN ADRD

Chair: Carrie Nieman Co-Chair: Heather Whitson
Discussant: Laura Gitlin

Sensory health in dementia stands at the intersection of two major public health challenges. Hearing and vision impairments are among the most common and disabling comorbidities in dementia and may worsen the trajectory of decline yet frequently go unrecognized and unaddressed. Improving sensory function may be an accessible and cost-effective nonpharmacological intervention to aid in the management of neuropsychiatric symptoms, improve quality of life for persons with dementia, and reduce burden for care partners. This symposium presents the latest evidence on the impact of sensory impairment in dementia and efforts to integrate sensory health into the care of persons with dementia. This symposium will cover emerging evidence of the impact of hearing loss and vision impairment on persons living with dementia, specifically around neuropsychiatric symptoms, disability, and cost. In moving toward solutions, we will discuss new approaches to provide vision and hearing care for persons with dementia in diverse settings, from audiology to specialized memory clinics to home-based care. This discussion will include findings from a systematic review of telehealth in dementia care, which highlights the limitations of existing literature on accounting for the sensory needs of persons with dementia and their care partners. Finally, we will share new international practice recommendations on vision and hearing impairment among persons living with dementia. The symposium highlights the large, yet often unrecognized, sensory health needs of persons with dementia and the multi-prong approach required to identify and support sensory health and, ultimately, healthy aging among persons with dementia.

### NEUROPSYCHIATRIC SYMPTOMS AND HEARING LOSS IN DEMENTIA: UNMET NEED AND OPPORTUNITY FOR INTERVENTION

Carrie Nieman,[1] Alexander Kim,[1] Emmanuel Garcia Morales,[2] Constantine Lyketsos,[3] Nicholas Reed,[2] Valerie Cotter,[4] Sara Mamo,[5] and Esther Oh,[1] *1. Johns Hopkins University School of Medicine, Baltimore, Maryland, United States, 2. Johns Hopkins Bloomberg School of Public Health, Baltimore, Maryland, United States, 3. Johns Hopkins Bayview, School of Medicine, Baltimore, Maryland, United States, 4. Johns Hopkins School of Nursing and School of Medicine, Baltimore, Maryland, United States, 5. University of Massachusetts Amherst, Amherst, Massachusetts, United States*

Hearing loss is one of the most common comorbidities among persons with dementia, with prevalence of 60->90%. Most go untreated and disparities exist. However, sensory impairment may influence the health of individuals and care partners. We will share findings from a clinic-based cohort of persons with dementia (n=101). Controlling for demographic and clinical factors, we found that every 10 decibel increase in hearing loss was associated with nearly an additional neuropsychiatric symptom ($b = 0.7$ per 10 dB; $p = 0.01$) and 1.3-point increase in severity ($b = 1.3$ per 10 dB; $p = 0.04$). These findings provide the first estimates that utilize objective audiometry. Furthermore, hearing aid use appeared to be protective. Hearing care may represent an additional, but underutilized, non-pharmacological intervention. We will discuss these findings in the context of the epidemiology of hearing loss in dementia and highlight new opportunities for managing hearing loss through community-based approaches.

### VISION IMPAIRMENT IN DEMENTIA CARE

Heather Whitson, *Duke University School of Medicine, Durham, North Carolina, United States*

Epidemiological evidence indicates that 3-4% of community-dwelling adults over age 65 years old have functionally limiting deficits in both vision and cognition. The comorbidity prevalence is higher in older age strata and in long-term care. Seniors with co-occurrence of vision impairment and dementia have six times higher odds of disability and higher average annual Medicare fee for service costs ($13,655 [95% confidence interval: $9,931-$18,798], compared to peers with dementia alone ($8,867 [95% confidence interval: $7,360-10,683]) or neither condition ($4,518 [95% confidence interval: $4,360-$4,682]). This talk will review evidence that people with early dementia and vision problems can experience improved function through appropriately tailored vision rehabilitation interventions. The talk will provide recommendations for unbiased cognitive assessment in visually impaired people. The session will outline research opportunities regarding the question of whether preventing or treating vision impairment may improve cognitive trajectories and neuropsychiatric symptoms in people with dementia.

### CONSIDERING THE TECHNOLOGICAL AND SENSORY NEEDS OF PATIENTS WITH COGNITIVE IMPAIRMENT IN THE ERA OF TELEHEALTH

Esther Oh,[1] Julie Yi,[2] Corrine Pittman,[3] Carrie Price,[4] and Carrie Nieman,[1] *1. Johns Hopkins University School of Medicine, Baltimore, Maryland, United States, 2. Johns Hopkins University, Baltimore, Maryland, United States, 3. Howard University College of Medicine, Washington, District of Columbia, United States, 4. Towson University, Towson, Maryland, United States*

During the COVID-19 pandemic, telehealth has become an important means of delivering memory care. Telehealth that is responsive to the technological abilities and preferences as well as the sensory needs of persons living with dementia is critical to advancing access to care. We conducted a systematic review to investigate the use of telehealth among older adults with cognitive impairment. The search yielded 3,551 titles and abstracts that led to 17 full-text articles. Studies showed that telehealth can be used for routine care, cognitive assessment and telerehabilitation with good efficacy and satisfaction. Three studies investigated telemedicine delivery in the home and 16/17 studies relied on support staff and care partners to navigate technology. No studies reported adaptations to account for sensory impairments and 5/17 studies excluded individuals with sensory impairments. This talk will review barriers and facilitators totelehealth for older adults with cognitive impairment and adaptations to address sensory needs.

### CARING FOR PERSONS WITH DEMENTIA IN AUDIOLOGY

Marilyn Reed, *Baycrest Health Sciences, Toronto, Ontario, Canada*

While hearing loss is highly prevalent among patients with dementia, it frequently goes unidentified and unmanaged. It has been a commonly-held belief that older adults with dementia are unable to benefit from hearing rehabilitation, but recent evidence shows that many individuals with dementia can successfully use amplification, helping to improve communication, social interaction and quality of life for these individuals and their caregivers. This presentation will describe how modifications to practice led to successful outcomes for the majority of patients of a geriatric audiology clinic with co-morbid hearing loss and cognitive impairment. In a study of hearing aid use in 67 patients with these comorbidities, over 90% used hearing aids successfully with measurable benefit for both patients and caregivers. Furthermore, we will discuss approaches to improving communication for LTC residents with dementia and hearing loss through the support of audiologists during remote visits with physicians and families during the pandemic.

### DEVELOPMENT OF INTERNATIONAL PRACTICE RECOMMENDATIONS FOR PERSONS WITH HEARING, VISION, AND COGNITIVE IMPAIRMENT

Barbara Weinstein,[1] Jenna Littlejohn,[2] and Iracema Leroi,[3] *1. City University of New York, New York, New York, United States, 2. University of Manchester, Manchester, England, United Kingdom, 3. Global Brain Health Institute, Dublin, Dublin, Ireland*

Many older adults being evaluated for dementia have unrecognized hearing and/or vision problems which can confound results of neuropsychological assessment(s) and can impact care recommendations. International care standards for detection, assessment, and management of people living with dementia (PwD) are rarely addressed yet are critical. We propose a set of recommendations crafted to foster the highest quality health care to enable PwD to live well with these combined impairments. The focus is detection, diagnosis, treatment, and support of PwD who have age-related hearing and/or vision impairments. The guiding principles underlying the recommendations was a focus on promotion of a person-centered approach, but to be pragmatic in considering all contextual levels including professional care pathways and socio-economic/policy factors internationally. The recommendations are inclusive of all stakeholders who work together to promote equity and mutual respect across the domains. The guidelines are designed to be pragmatic, implementable, resource sparing, and sustainable.

### Session 1330 (Symposium)

### ABUSE OF AND INTERVENTION FOR OLDER ADULTS DURING THE COVID-19 PANDEMIC

Chair: Pamela Teaster Discussant: Cory Bolkan

Beginning in the United States in 2020, SARS-CoV-2 lead to unprecedented changes in the lives of both younger and older people. Efforts to mitigate the spread of the novel coronavirus, which included physical distancing and self-quarantine not only upended the lives of many people but also created natural laboratory conditions for the mistreatment of older adults. Exploring the mistreatment of older adults during the pandemic presented an unprecedented opportunity to examine perspectives of service providers and affected older adults. This symposium offers four perspectives on this subject. Dr. Karen Roberto and colleagues will present changes and challenges that COVID-19 brought for Adult Protectives Service staff and the vulnerable adults whom they serve. Ms. Lori Smetanka and colleagues will present changes and challenges that COVID-19 created for state and local Long-Term Care Ombudsman. Dr. Holly Ramsey-Klawsnik and Ms. Tammy Seaver will report on how the pandemic affected Nevada Adult Protective Services clients, casework, and staff. Finally, Dr. Pamela Teaster and colleagues will discuss how older adults experienced exploitation attempts during the early months of the pandemic. Dr. Cory Bolkan will begin the discussion, highlighting how conditions brought about by COVID-19 both enabled and thwarted efforts to address elder abuse.

### ADULT PROTECTIVE SERVICES STUDY ON THE IMPACT OF COVID-19

Karen Roberto,[1] Pamela Teaster,[2] Jyoti Savla,[1] Imogen Fua,[3] Karl Urban,[4] and Emily Hoyt,[1] *1. Virginia Tech, Blacksburg, Virginia, United States, 2. Virginia Tech, BLACKSBURG, West Virginia, United States, 3. WRMA, Inc., Rockville, Maryland, United States, 4. WRMA, Inc., Arlington, Virginia, United States*

The purpose of study was to explore changes in implemented of APS programs in response to the COVID-19 pandemic. This three-phase study began with telephone interviews with eight state-level APS administrators of early phase "hot-spots" that were used to inform questions for the Phase II national survey of state APS programs. Forty-seven states completed an on-line survey on the effects of the pandemic on work and workload, policy and practice, staff, partnerships, and preparedness. Phase III interviews with 7 local APS programs provided an "on-the-ground" view of challenges of meeting clients' needs. Although most APS programs reported fewer reports of adult maltreatment than before the pandemic, the stress and disruption of COVID-19 required new work arrangements, to which programs adapted technological and managerial support to care for the needs of both APS staff and the clients they served. Finding provide insight for policy and planning requirements for future catastrophic events.

### COVID-19 AND THE LONG-TERM CARE OMBUDSMAN PROGRAM: FINDINGS FROM A NATIONAL STUDY

Lori Smetanka,[1] Chenguang Du,[2] Pamela Teaster,[3] and Kathryn Ratliff,[2] *1. The National Consumer Voice for Quality Long-Term Care, Washington, District of Columbia, United States, 2. Center for Gerontology, Virginia Tech, BLACKSBURG, Virginia, United States, 3. Virginia Tech, BLACKSBURG, West Virginia, United States*

The purpose of our study was to explore changes for long-term care ombudsman programs across the country in response to the COVID-19 pandemic. The study team explored the effect of COVID-19 on programs: cases, resident engagement, complaint Investigation and resolution, services, complaints, changes in visitation, and preparedness plans. The research team developed survey items and beta-tested them with state and local LTCOs prior to distributing the survey nationally to State Long-Term Care Ombudsman and Local Long-Term Care Ombudsman in order to characterize experiences of the participants. From 62 state LLTC respondents we learned there were 81.0% fewer cases received, 97.36% were less able to engage with residents, 78.95% were less involved in the engagement of residents in complaint investigation and resolution and there were 71.05% fewer activities involved in investigations. Not surprisingly, there was an 80.0% increase in information that the LTCO provided to the media.

### COVID PANDEMIC IMPACT ON NEVADA ADULT PROTECTIVE SERVICES

Holly Ramsey-Klawsnik, *Klawsnik & Klawsnik Associates & Consultant, Nevada Adult Protective Services Program, Canton, Massachusetts, United States*

Empirical data regarding Covid pandemic impact on the Nevada Adult Protective Services (APS) Program clients, casework, and staff was gathered and analyzed as part of a multi-faceted program evaluation. Key findings include: 66% of the staff agreed or strongly agreed that the pandemic made their jobs more challenging. Respondents reported Covid-related challenges faced by clients, the program, and themselves as social workers serving older and vulnerable adults. Adverse client impacts observed included increased social and emotional isolation, loss of housing, exacerbation of symptoms of mental illness, necessary services being cut from clients subsequent to testing Covid positive, and fear and reluctance to allow needed visiting service providers, such as home health aides, into their homes. We will discuss the implications of the findings on APS services and clients, and on related health and human services designed to promote the wellness and independence of older and vulnerable adults.

### FINANCIAL EXPLOITATION OF OLDER ADULTS DURING THE EARLY MONTHS OF THE COVID-19 PANDEMIC

Pamela Teaster,[1] Karen Roberto,[2] Jyoti Savla,[2] Chenguang Du,[3] Emily Hoyt,[2] Scott Beach,[4] Neil Charness,[5] and Peter Lichtenberg,[6], *1. Virginia Tech, BLACKSBURG, West Virginia, United States, 2. Virginia Tech, Blacksburg, Virginia, United States, 3. Center for Gerontology, Virginia Tech, BLACKSBURG, Virginia, United States, 4. University of Pittsburgh, University of Pittsburgh, Pennsylvania, United States, 5. Florida State University, Tallahassee, Florida, United States, 6. Wayne State University, Detroit, Michigan, United States*

COVID-19 created a "perfect storm" for financial exploitation directed at older adults. We invited adults aged 60 and older enrolled in gerontology research registries at Virginia Tech, Florida State University, Wayne State University, and University of Pittsburgh to complete an on-line survey about experiences with financial exploitation by strangers. The 997 respondents ranged in age from 60 to 98 (M = 71.3; SD = 6.8); most identified as White (93.4%), female (64.2%) and living with a spouse/partner (58%). Approximately one-half of respondents (56.87%) reported experiencing a scam attempt about COVID-19 issues. Most contact by scammers was electronic (49%) and frequently occurred two or more times (40%). Most respondents ignored the request (i.e., hung up phone, deleted text/email, threw away mail). However, 9% sent the requested payment, and 4% gave the scammer their personal information. Confidence in financial matters and having attended financial educational programs protected older adults from being scammed.

### Session 1335 (Paper)

### ADULT PROTECTION AND ELDER ABUSE

### CHANGING FAMILY RELATIONSHIPS DURING THE COVID-19 PANDEMIC: THE CASE OF ELDER ABUSE

Elsie Yan,[1] Daniel Lai,[2] and Vincent Lee,[3] *1. The Hong Kong Polytechnic University, Hong Kong, Not Applicable, Hong Kong, 2. Hong Kong Baptist University, Hong Kong Baptist University, Not Applicable, Hong Kong, 3. The Hong Kong Polytechnic University, The Hong Kong Polytechnic University, Not Applicable, Hong Kong*

Since the first confirmed case being identified in January 2020, authorities in Hong Kong have implemented various measures in an attempt to control the spread of the disease. These measures include compulsory quarantining of infected persons and those suspected of exposure, temporary closure of high-risk premises, and suspension of public activities and services, encouraging work-from-home arrangement etc. These measures, however, may exacerbate the impact of known risk factors and create new avenues for elder mistreatment. Life stress, financial strains and work-from-home arrangements increase chances of family conflicts, cessation of public services increases burden in the already stressed caregivers. This study examines the changing intergenerational family relations in the midst of the pandemic. A total of 1200 community dwelling senior citizens participated through responding to a telephone survey. Information was collected on participants' demographic characteristics, perceived disruptions brought about by COVID-19, family relations, physical and mental health, etc. Family conflicts and abuse were commonly reported: 27.8% reported family conflicts, 14.5% psychological abuse, 3.1% physical abuse, 3.9% financial abuse. A large proportion of participants (41.8%), however, also reported improved family relations during the pandemic. Results of logistic regression indicate that advanced age, female gender, poor financial situation were significant predictors for family conflicts and abuse. Contrary to our expectations, pandemic related disruptions in daily lives and perceived safety in the community were not associated in family conflicts and abuse in the present sample.

### INCIDENCE AND RISK FACTORS OF ELDER MISTREATMENT IN THE COMMUNITY: A LONGITUDINAL POPULATION-BASED STUDY

David Burnes,[1] David Hancock,[2] John Eckenrode,[3] Mark Lachs,[2] and Karl Pillemer,[3], *1. University of Toronto, Toronto, Ontario, Canada, 2. Weill Cornell Medicine, New York City, New York, United States, 3. Cornell University, Ithaca, New York, United States*

Prior population-based elder mistreatment (EM) risk factor research has focused on problem prevalence using cross-sectional designs, which cannot make causal inferences between proposed risk factors and EM or discern existing cases from new cases entering the population. This study sought to estimate the incidence of EM and identify risk factors for new cases. It is a ten-year prospective, population-based cohort study with data collected between 2009 (Wave 1) and 2019 (Wave 2). Based on Wave 1 random, stratified sampling to recruit English/Spanish-speaking, cognitively intact, community-dwelling older adults (age ≥ 60) across New York State, this study conducted computer assisted telephone interviews (CATI) with 628 respondents participating in both Wave 1 and Wave 2 interviews (response rate=60.7%). Ten-year EM incidence was regressed on factors related to physical vulnerability, living arrangement, and socio-cultural characteristics using logistic regression. Ten-year incidence rates included overall EM (11.4%), financial abuse (8.5%), emotional abuse (4.1%), physical abuse (2.3%), and neglect (1.0%). Poor self-rated health at Wave 1 significantly predicted increased risk of new Wave 2 overall EM (odds ratio [OR]=2.8), emotional abuse (OR=3.67), physical abuse (OR=4.21), and financial abuse (OR=2.8). Black older adults were at significantly heightened risk of overall EM (OR=2.61), specifically financial abuse (OR=2.8). Change from co-residence (Wave 1) toward living alone (Wave 2) significantly predicted financial abuse (OR=2.74). Healthcare visits represent important opportunities to detect at-risk older adults. Race is highlighted as an important social determinant for EM requiring urgent attention. This study represents the first longitudinal, population-based EM incidence study.

### PERCEIVED FINANCIAL VULNERABILITY IS RELATED TO PERCEIVED COGNITIVE IMPAIRMENT AND LIVING ALONE

Peter Lichtenberg,[1] Maggie Tocco,[2] and Juno Moray,[2]
*1. Wayne State University, Detroit, Michigan, United States, 2. Wayne State University, Wayne State University/Detroit, Michigan, United States*

**Objective:** Perceived financial vulnerability is linked to physical and mental health and also to risk for financial exploitation (Lichtenberg et al., 2020a,b). This study examined the relationship of risk scores for financial exploitation to demographic variables, perceived memory loss and living alone.

**Methods:** The 17-item self-report Financial Exploitation Vulnerability Scale (FEVS) posted on our website https://olderadultnestegg.com was completed by a convenience sample of 258 older adults. Correlational, multiple regression and Chi Square analyses were used.

**Results:** Thirty percent of the sample scored at an elevated risk for financial exploitation due to perceived financial vulnerability. Although this was a convenience sample the results were similar to what was found in a sub-study of the HRS. Thirty eight percent of participants were living alone and 38% reported that their memory was less reliable than a year ago. Financial vulnerability risk score was significantly related to decreased education (r=-.12), living alone (r=.21) and perceived memory loss (r=.35). Eighteen percent of the variance was accounted for in a multiple regression ($F(5,250)=10.73$, $p<0.001$, $r2=0.18$) with all three measures predicting FEVS risk score independently. The combination of perceived memory loss and living alone was significantly associated with the highest percentage of elevated risk scores.

**Discussion:** Perceived financial vulnerability and its relationship to health (e.g. memory loss) and financial exploitation, continues to be under-appreciated in gerontology and geriatrics research. Our findings, consistent with GSA's Longevity Fitness report further highlights this important dimension.

### RISK FACTORS OF FINANCIAL EXPLOITATION VERSUS SCAM

Pi-Ju Liu,[1] Stacey Wood,[2] Aining Wang,[1] Yaniv Hanoch,[3] David Hengerer,[4] and Mary Muskat,[5], *1. Purdue University, West Lafayette, Indiana, United States, 2. Scripps College, Claremont, California, United States, 3. University of Southampton, Southampton, England, United Kingdom, 4. Claremont Graduate University, Claremont, California, United States, 5. Purdue University, West Lafayette, California, United States*

Financial exploitation (FE) perpetrators are usually seen in a position of trust, such as family members or friends, whereas perpetrators of scam tend to be unknown individuals. Few empirical studies have examined victim risk factors, and this study aimed to systematically compare risk factors of FE versus scam. One-hundred-and-ninety-five adults (ages 18-89) were recruited to complete a 60-minute survey and interview at Purdue University in Indiana (n1=97) and Scripps College in California (n2=98). Risk factors assessed included cognitive tasks (overall cognition, memory, and executive decision), socio-emotional questionnaires (depression, resilience, ostracism, and social integration), financial measures (numeracy, objective financial knowledge, retirement worries, and financial well-being), physical health and demographics (age, gender, education level, marital status, ethnicity). Additionally, participants reported experiences of FE and scam, including (1) the 11-item short-form Older Adult Financial Exploitation Measure, (2) seven questions on scam from the Health and Retirement Study, and (3) likelihood to contact a scammer after reviewing lottery scam materials. The three dependent variables were log-transformed before OLS regression models were built. Each dependent variable was associated with different risk factors. Lower standard of living (p=.02) and ostracism (p<.05) independently predicted FE. Lower physical health (b=-.02, p=.003) was the strongest predictor of scam, with lower level of financial well-being (p=.02) serving as an independent predictor. For lottery scams contact likelihood, ostracism (b=.04, p=.005) and being male (b=-.23, p=.04) were the strongest predictors. Since risk factors differed between FE and scam, prevention and intervention programs should target the unique profiles of risk factors for each.

### STRUCTURAL AND INDIVIDUAL AGEISM PREDICTS ELDER ABUSE PROCLIVITY AND PERPETRATION

E-Shien Chang,[1] Joan Monin,[2] Daniel Zelterman,[2] and Becca Levy,[3], *1. Weill Cornell Medicine, New York City, New York, United States, 2. Yale School of Public Health, New Haven, Connecticut, United States, 3. Yale University, Woodbridge, Connecticut, United States*

Considering that elder abuse affects one in six older persons worldwide, a need exists to identify factors that predict this abuse. Previous studies have found that ageism operates at both structural (i.e., societal-level stigmatizing views toward older persons) and individual levels (i.e., negative age beliefs) to affect health. However, it was not known whether and if so, how these two levels work together to impact perpetrators committing elder abuse. Thus, examining the mechanism between ageism and elder abuse was the aim of the current study. We hypothesized that structural and individual ageism would simultaneously predict elder abuse. In addition, following Stereotype Embodiment Theory, the impact of structural ageism on elder abuse would be mediated by individual ageism. In Sample 1, participants described their proclivity to abuse older people if they could do so without punishment (n=1,580). In Sample 2, family caregivers described actual abuse of their older care recipients (n=400). Overall, elder abuse proclivity (33% in Sample 1) and perpetration (56% in Sample 2) were prevalent. As hypothesized, structural ageism and individual ageism simultaneously predicted elder abuse proclivity and perpetration. Also as predicted, individual ageism significantly mediated the association between structural ageism and elder abuse in both samples. This the first study that examined the mechanistic pathways between structural and individual levels of ageism in the context of elder abuse. Effective solutions to prevent elder abuse should incorporate upstream interventions to mitigate the adverse effects of ageism.

### Session 1340 (Symposium)

### BUILDING MOMENTUM FOR DIVERSITY, EQUITY, AND INCLUSION IN GERIATRICS AND GERONTOLOGY EDUCATION

Chair: Jennifer Severance Co-Chair: Barbara Gordon
Discussant: Brian Lindberg

With an increasingly multicultural and diverse older adult population, health care professionals must be prepared to serve older adults from varied backgrounds and marginalized communities; address health determinants and disparities; and promote diversity, equity, inclusion, and empathy within systems of care. The National Association for Geriatrics Education (NAGE) is a non-profit organization representing geriatric and gerontology education and training programs, including Health Services and Resource Administration (HRSA) funded Geriatric Workforce Enhancement Programs (GWEPs), and Geriatric Academic Career Awardees (GACAs). The 44 GWEPs focus on improving health outcomes for older adults by enhancing geriatrics and primary care training of the healthcare workforce. The 26 GACA awards support leaders in Age-Friendly health care transformation and interprofessional clinical geriatrics training. This symposium examines the role both programs have in reducing racial health disparities in older adults by promoting increased diversity of the geriatrics/gerontology workforce and advancing public policies for racial equity and inclusion. First, presenters will introduce the NAGE Diversity and Racial Equity Workgroup that supports a broader and unified effort across GWEPs and GACAs for equity and inclusion in geriatrics and gerontology education. Presenters will then share strategies to mobilize system-level changes within their institutions. Finally, examples of progress showcase individual GWEP and GACA projects and partnerships aimed at reducing racial health disparities within a multidimensional and local context. Presenters discuss strategies and opportunities to disrupt and transform health professions education at multiple levels and implications for policies supporting optimal aging for all older adults.

### ANSWERING THE CALL FOR DIVERSITY AND RACIAL EQUITY: THE NATIONAL ASSOCIATION FOR GERIATRIC EDUCATION

Katherine Bennett,[1] Rosellen Rosich,[2] Linda Edelman,[3] Barbara Gordon,[4] Anna Goroncy,[5] and Jennifer Severance,[6] *1. University of Washington, Seattle, Washington, United States, 2. University of Alaska Anchorage, Anchorage, Alaska, United States, 3. University of Utah College of Nursing, Salt Lake City, Utah, United States, 4. University of Louisville, Louisville, Kentucky, United States, 5. University of Cincinnati Department of Family and Community Medicine , Cincinnati, Ohio, United States, 6. University of North Texas Health Science Center - Ft. Worth, TX, Fort Worth, Texas, United States*

The National Association for Geriatric Education (NAGE) is a non-profit organization representing geriatric and gerontology programs, including Health Services and Resource Administration funded Geriatric Workforce

Enhancement Programs (GWEPs), and Geriatric Academic Career Awardees (GACAs). NAGE responded to the renewed call to address systemic racism and racial inequities by forming a Diversity and Racial Equity Workgroup. The Workgroup explored ways to disseminate educational resources, support members to address racial inequities among older adults, promote increased diversity of the geriatrics/gerontology workforce, and support public policy initiatives that address racism and health disparities. Initial outputs include creating a Diversity and Racial Equity resource page, identifying liaisons to the Workgroup from each NAGE Committee to ensure impact across the organization, and organizing collaborations across GWEPs and GACAs to share successful initiatives. Future plans include education and advocacy with members and collaborating organizations to address systemic racism and racial health inequities impacting older adults.

### REFLECTION LEADING TO ACTION ON DIVERSITY, EQUITY, AND INCLUSION AT THE VIRGINIA GWEP

Charles Alexander,[1] Patricia Slattum,[2] Ishan Williams,[3] and Leland Waters,[1] *1. Virginia Commonwealth University, Richmond, Virginia, United States, 2. Department of Pharmaceutics, Richmond, Virginia, United States, 3. University of Virginia, Charlottesville, Virginia, United States*

Last year's Black Lives Matter protests inspired the Virginia Geriatric Education Center (VGEC) GWEP's plenary to engage in reflection and discussion on diversity, equity, and inclusion (DEI) in our work together. During each bi-monthly meeting, we dedicate time to generate ideas to improve our programming, how we work together, and how we partner and recruit for our programs. Champions for DEI on our plenary led an effort to develop a DEI newsletter clarifying DEI concepts and introducing resources thematically related to the monthly VGEC faculty development program curriculum. By incorporating these resources into our monthly curriculum, facilitators have a new access point to incorporate content on health equity and policy into our curriculum. The intentional focus on DEI is opening the door to deeper reflection and conversation with a goal of improving all our programming, cultivating a new social awareness, and bringing new voices and perspectives to the table.

### PARTNERING WITH AFRICAN AMERICAN FAITH-BASED COMMUNITIES TO SUPPORT DEMENTIA CAREGIVERS

Monica Long,[1] Shellie Williams,[2] Katherine Thompson,[1] Jason Molony,[3] and Jeff Graupner,[2] *1. University of Chicago, Chicago, Illinois, United States, 2. University of Chicago Medicine, Chicago, Illinois, United States, 3. The University of Chicago Medicine, Chicago, Illinois, United States*

African Americans (AA) are twice as likely to develop Alzheimer's Disease as Caucasians. Historically, houses of faith have been a center of the AA community and a trusted source of information and support. Based on these facts, as well as community needs, the SHARE Network (a Geriatrics Workforce Enhancement Program on the South Side of Chicago) in partnership with faith-based community leaders, created an opportunity for community members to train to become resource experts on Alzheimer's Disease & Related Dementias (ADRD) and create sustainable caregiver support groups (CSGs). The resulting initiative, Dementia Resource Champions, is a train-the-trainer style health education initiative piloted in 2018, and subsequently expanded and modified due to COVID. Participants receive instruction on stress reduction, ADRD, and community resources. They discuss how to structure CSGs to meet community needs. Results of this initiative include development of five brand-new CSGs with faith communities on Chicago's South Side.

### GWEP TAG-TEAM RESPONSE: COVID-19 HEALTH DISPARITIES IN ORANGE COUNTY, CALIFORNIA

Jung-Ah Lee,[1] Julie Rousseau,[2] Neika Saville,[3] Sonia Sehgal,[2] and Lisa Gibbs,[4] *1. University of California, Irvine, Irvine, California, United States, 2. University of California, Irvine, Orange, California, United States, 3. Division of Geriatric Medicine and Gerontology, Orange, California, United States, 4. UC Irvine Health, UC Irvine Health, California, United States*

Health disparities follow zip codes, and in Orange County, CA, both COVID-19 cases and deaths are highly concentrated in our diverse geriatric populations in Santa Ana (44,075) and Anaheim (40,984) where our two UCI Federally Qualified Health Centers (FQHCs) are located, and Garden Grove (16,174) and Buena Park (7,581), where University of California Irvine (UCI) TAG-TEAM GWEP community partner FQHCs are located. Collectively, our FQHCs serve diverse populations, with 83-88% of patients identifying as Hispanic/Latino or Asian. As we support these clinics in becoming Age-Friendly Health Systems, UCI's GWEP pivoted to provide COVID-19 education in the form of multi-lingual materials and videos available in Spanish, Vietnamese, Korean, Mandarin, and Farsi. Additionally, through our Age-Friendly Geriatrics Tele-ECHO Series we are working to build Mental Health care competencies among these FQHC providers since the pandemic morbidity and mortality disparities have resulted in a profound mental health crisis in our communities.

### MITIGATING IMPLICIT RACIAL BIAS IN THE CLINICAL SETTING: A QUALITATIVE STUDY OF FAMILY MEDICINE RESIDENTS

Anna Goroncy, *University of Cincinnati Department of Family and Community Medicine, Cincinnati, Ohio, United States*

Implicit racial bias (IB) in physicians contributes to racial health inequities. Health profession trainees are not consistently trained to address IB. This phenomenological study explored Family Medicine (FM) residents' experience of applying strategies to mitigate IB during home visits (HVs) to homebound older adults. FM residents completed pre-work related to IB, applied strategies to mitigate IB during HVs, then completed written reflections and commitments-to-change (CTC). A two-month survey assessed completion of targeted changes and barriers faced. Researchers completed a thematic analysis identifying five themes: Response to IAT, barriers, strategies, value of HVs and mindfulness. All residents' stated level of CTC remained the same (9/9, 100%) and 8/9 residents (89%) had partially or fully implemented their intended change at 2 months. Residents continued applying newly-learned strategies two months after training with transference to other clinical settings and bias types. These findings can facilitate development of clinically-based IB curricula with lasting impacts.

### Session 1345 (Paper)

### CARE TRANSITIONS

### ANTIPSYCHOTIC AND PSYCHOPHARMACOLOGICAL MEDICATIONS: CARE TRANSITIONS AMONG NURSING HOME RESIDENTS

Sarah Holmes,[1] Aida Kuzucan,[2] Abisola Olopoenia,[2] Nicole Brandt,[3] Becky Briesacher,[4] Danya Qato,[2] Barbara Zarowitz,[2] and Linda Wastila,[2], *1. University of Maryland School of Nursing, Baltimore, Maryland, United States, 2. University of Maryland School of Pharmacy, Baltimore, Maryland, United States, 3. University of Maryland School of Pharmacy, University of Maryland School of Pharmacy, Maryland, United States, 4. Northeastern University, Boston, Massachusetts, United States*

Nursing home residents are frequently prescribed antipsychotic and other psychopharmacologic medications (AP/PPM) to manage behavioral and psychological symptoms. Residents also experience care transitions between nursing homes and other healthcare institutions. Limited research exists on the relationship between AP/PPM use and care transitions in this population. The purpose of this study is to compare odds of care transitions among those with and without AP/PPM use, controlling for resident characteristics. This cross-sectional study used data from a 5% random sample of Medicare beneficiaries between 2011-2015 who resided in a nursing home and were continuously enrolled in Medicare Parts A, B, and D. Chi-square tests compared resident characteristics and AP/PPM use between those with <2 and ≥2 transitions. Multivariate logistic regression evaluated the odds of transitions in those with and without AP/PPM use during a one-year follow-up, including interaction effects among AP and PPM use. Among 132,263 eligible residents, the majority were female(68%), white(85%), and >75 years old(63%). Of residents who experienced ≥2 transitions, 17% received AP and 82% received PPM, compared to those with <2 transitions for which 9% and 66% received AP/PPM, respectively (p<.001). Logistic results showed that for residents with PPM use, those who take AP have 1.33 the odds of experiencing ≥2 transitions than PPM users who do not take AP (p<.001). Findings demonstrate a significant association between AP/PPM use and care transitions in nursing home residents. Prudent use of AP/PPM should thus be optimized with a goal of reducing care transitions and improving quality of care.

### HOW CAN PROVIDERS BETTER ADDRESS RECOVERY NEEDS FOR GERIATRIC TBI SURVIVORS?

Mia Vogel,[1] Wonkyung Jung,[2] and Hilaire Thompson,[2] *1. University of Washington, St. Louis, Missouri, United States, 2. University of Washington, Seattle, Washington, United States*

Older adults are at increased risk for poorer recovery from traumatic brain injury (TBI), incidence of pre-injury frailty, and comorbidity compared to younger adults. In this study, a longitudinal multiple case study approach was taken to describe gaps in health service delivery identified by older adults following mild-moderate TBI. Participants were interviewed at 1 week, and 1, 3, 6, and 12 months post injury (5 times). In total, 57 interviews were conducted with 13 participants and transcribed verbatim. Codes were identified inductively to develop a codebook for guiding thematic analysis in NVIVO. Two independent investigators double coded 16 transcripts (28%) and reached consensus; remaining transcripts were allocated to the two investigators for independent coding and verified and reconciled with the other coder until consensus was reached. Many participants were happy with the care they received. However, ongoing health issues and TBI symptoms sometimes remained unaddressed. Follow-up and care planning would have been helpful for most patients as well as ways to address common balance and dizziness issues. Among some, the unwillingness to go to a provider for follow-up or adhere to medication and physical therapy exercises were issues. In some cases, providers were unable to help or understand the reason for patients' symptoms (e.g., smell/taste changes, ear issues, etc.). Sometimes, providers did not address the issues that were of most concern to patients, or they would prescribe treatments that were not acceptable to patients. Empathetic, tailored, and patient-centered approaches are needed to improve care delivery and outcomes.

### I'M THE ONLY SON SHE GOT LEFT: MALE CAREGIVERS' EXPERIENCES MANAGING OLDER ADULTS' POST-ACUTE CARE

Michael Bueno,[1] David Russell,[2] and Jo-Ana Chase,[3] *1. University of Missouri - Columbia / University of California, Irvine, Placentia, California, United States, 2. Appalachian State University, Boone, North Carolina, United States, 3. University of Missouri, Columbia, Missouri, United States*

Family caregivers (FCGs) play an integral role supporting older adults transitioning to post-acute care following a hospitalization. FCGs function as advocates, information agents, and most importantly, care managers and providers. Although, caregiving has been traditionally seen as a female role, men are increasingly undertaking these roles and responsibilities. This research addresses a gap in the existing literature by exploring the subjective experiences of male FCGs of older adults in the post-acute setting. Using data from two parent qualitative studies on caregiving in the post-acute setting (N=40), we conducted a qualitative secondary analysis using conventional content analysis of male caregiver participants' interview data (n=11). Interviews explored the subjective experiences of male caregivers' interactions with home health care supportive personnel and conducting medical/nursing tasks for older adults. Five themes emerged: areas of abandonment, financial needs, masculinity, organization of care, and preparation. These themes highlighted areas of both confidence and struggle for male FCGs and captured their unique experiences managing the care of an older adult in the post-acute setting. Furthermore, the themes illustrate male FCGs' feelings of guilt, financial impact, work disruptions, and the perceived effect of masculinity on their caregiving role. Findings can inform clinicians' provision of focused and tailored resources to meet the specific needs of male FCGs. Future research should explore the evolving experiences of male FCGs over time, particularly those FCGs of older adults with chronic illnesses.

### RECEIPT OF TRAINING AMONG FAMILY CAREGIVERS ASSISTING WITH A POST-HOSPITAL CARE TRANSITION

Julia Burgdorf,[1] Chanee Fabius,[1] Catherine Riffin,[2] and Jennifer Wolff,[1] *1. Johns Hopkins Bloomberg School of Public Health, Baltimore, Maryland, United States, 2. Weill Cornell Medicine, New York, New York, United States*

Medicare Conditions of Participation require hospitals to provide training to family and unpaid caregivers when their support is necessary to enact the post-discharge care plan. However, caregivers often report feeling unprepared for this role. We perform a cross-sectional analysis of the 2017 National Health and Aging Trends Study and its linked National Study of Caregiving (nationally representative surveys of older adults and their family and unpaid caregivers, respectively) to assess the prevalence of, and factors associated with, caregiver receipt of adequate transitional care training. Our analytic sample includes 795 (weighted n=7,083,222) family caregivers who assisted an older adult during a post-hospital care transition in the past year. The outcome of interest caregiver-reported receipt of the training needed to manage this transition ("adequate transitional care training") from hospital staff. Six in ten (59.1%) caregivers who assisted during a post-hospital care transition reported receiving adequate transitional care training. In weighted, multivariable logistic regression models, caregivers were half as likely to report receiving adequate transitional care training if they were black compared to white (Adjusted Odds Ratio (aOR): 0.52; 95% CI: 0.31-0.89) or experienced financial difficulty (aOR: 0.50; 95% CI: 0.31-0.81). Findings suggest that socially vulnerable family caregivers of older adults are less likely to report receiving adequate transitional care training. Changes to the discharge process, such as using standardized caregiver assessments, may be necessary to ensure equitable support of family caregivers.

### TOLERABILITY OF LITHIUM: A NATURALISTIC DISCONTINUATION STUDY IN OLDER ADULTS (≥60 YEARS)

Marinke Flapper,[1] Els van Melick,[2] Jos van Campen,[3] Natasja Schutter,[4] and Rob Kok,[5] *1. Onze Lieve Vrouwe Gasthuis Amsterdam, Amsterdam, Drenthe, Netherlands, 2. Hospital Reinier de Graeff, Delft, Zuid-Holland, Netherlands, 3. Hospital OLVG, Hospital OLVG, Noord-Holland, Netherlands, 4. Arkin Ouderen, Amsterdam, Noord-Holland, Netherlands, 5. Parnassia, Den Haag, Zuid-Holland, Netherlands*

Lithium is one of the most effective treatment options in both bipolar disorder and treatment-resistant depression. The use of lithium in older adults declined during the last decades, probably resulting in undertreatment of older adults. To investigate how well lithium is tolerated in old age, we aimed to determine the frequency, reasons and possible predictors of discontinuation due to adverse effects in a cohort of hospitalized adults aged 60 years or older who had started with lithium.

We performed a retrospective cohort study based on chart reviews. Participants were in treatment at Parnassia Group at The Hague, the Netherlands. After inclusion (between January 2010 and December 2016), participants were followed until April 2017, when we performed data extraction and analysis.

In our sample of 135 patients (median age 69 years, median follow-up duration 18 months), 49 (36.3%) participants discontinued lithium. Only a minority (11 (8.1%)) of the participants discontinued solely due to adverse effects. The majority discontinued lithium due to psychiatric (18,5%) reasons, (most commonly mentioned within this subgroup: lack of effectiveness and non-compliance) or a combination of reasons (7.4%). None of the factors we studied (age, gender, Charlson Comorbidity Index (CCI), polypharmacy, renal function and neurological history) were significantly associated with discontinuation due to adverse effects.

The frequency of lithium discontinuation in our cohort was in range with frequencies reported in younger patients. Older age itself should not be a reason to withhold lithium treatment.

### Session 1350 (Paper)

### CAREGIVING AND WELL-BEING

### A CASE FOR TRAUMA-INFORMED DEMENTIA CARE: THE EMANCIPATORY POWER OF PSYCHOLOGICAL RESILIENCE AND TRAUMA FROM COVID-19

Adrienne Ione, *Silver Linings Integrative Health, LLC, Tacoma, WA, Washington, United States*

The dominant nonpharmacological therapeutic approach in dementia care is "person-centered." The racial disparities in disease and diagnosis affliction, represents an urge to incorporate trauma sensitivity. Antipsychotic use rates among this population being substantially higher than two years ago, as well as increasing evidence of ACE scores being a significant risk factor in dementia development, we need to consider the emancipatory power of trauma-informed dementia care. Data were collected from 103 peer-reviewed journal articles. Person-centered care practices were analyzed for adherence to SAMHSA's principles that guide a trauma-informed approach. Studies were rated on a Likert-scale in six areas: safety; trustworthiness and transparency; peer support; collaboration and mutuality; empowerment and choice; and cultural, historical & gender issues. It was observed that of the 103 search returns, 38 met study criteria. Within these data, it was observed that the majority of studies scored low on incorporation and adherence to SAMSHA's trauma-informed approach. While a few studies mentioned the magnifying effects of a current traumatic event on preexisting vulnerabilities, no study highlighted the growth or flourishing potential in triggering past trauma and the current traumatic event offering an opportunity of mind-somatic integration. This paper proposes trauma-informed dementia practices consider: 1) intake and assessment to include a screening of ACE score; 2) evaluation of (re)integrating current losses into current developmental and lifespan phase; and 3) offering therapeutic support of current traumatic event (COVID-19) to metabolize past traumatic events in the context of current developmental and lifespan phase.

### ADULT SIBLING TENSION AND OLDER MOTHERS' PSYCHOLOGICAL WELL-BEING: THE MODERATING ROLE OF CAREGIVING

Megan Gilligan,[1] J. Jill Suitor,[2] Yifei Hou,[3]
Barbra Brottman,[4] Jeenkyoung Lee,[4] and Eunbea Kim,[4]
*1. Iowa State University, Iowa State University, Iowa, United States, 2. Purdue University, Purdue University, Indiana, United States, 3. Purdue University, West Lafayette, Indiana, United States, 4. Iowa State University, Ames, Iowa, United States*

The life course perspective concept of "linked lives" suggests that the lives of adult children and older parents are interconnected and consequential for the well-being of members of both generations. In this work, we consider the association between tension among adult siblings and older mothers' psychological well-being. We focus specifically on tension in the adult sibling relationship because research has shown that negative relationship quality is especially consequential for well-being. We consider this association in the context of caregiving because this is a time when offspring are often required to coordinate with each other to provide assistance. We utilized data from 304 older mothers (average age = 78) and 736 of their adult children (average age = 49) from the Within-Family Difference Study (WFDS) II. First, we examined the direct association between adult sibling tension and mothers' reports of depressive symptoms. Second, we examined whether the association between sibling tension and mothers' depressive symptoms was moderated by mothers' need for care. Preliminary results indicated no direct effect of sibling tension on mothers' depressive symptoms. However, moderation analysis revealed that sibling tension was associated with an increase in mothers' depressive symptoms among mothers who reported needing assistance. These findings highlight the importance of understanding the interconnected nature of adult family relationships especially in the context of later-life family caregiving. In particular, the findings reveal that older mothers in need of care are especially vulnerable to tension in the relationships among their adult children.

### HOW GENDER SHAPES THE EFFECTS OF IMMEDIATE FAMILY MEMBERS' DEATHS ON ADULTS' PSYCHOLOGICAL WELL-BEING

J. Jill Suitor,[1] Megan Gilligan,[2] Catherine Stepniak,[3]
Yifei Hou,[3] and Robert Frase,[3] *1. Purdue University, Purdue University, Indiana, United States, 2. Iowa State University, Iowa State University, Iowa, United States, 3. Purdue University, West Lafayette, Indiana, United States*

The deaths of family members constitute one of the most serious negative life events experienced in adulthood. The impact of these losses on psychological well-being may differ considerably by the structural relationship between the deceased and the survivors, and by the genders of both family members; however, few studies have been able to explore these variations by generation, gender, and time since death. In this paper, we use mixed-methods data to explore how depressive symptoms are affected differentially in adulthood by the deaths of mothers, fathers, and siblings, as well as by the gender of survivors. We address these questions using data collected from approximately 600 adult children nested within 250 later-life families, in which approximately 55% experienced the death of at least one parent and 15% experienced the death of a sibling in the previous decade. Preliminary multilevel regression analyses showed that deaths of siblings predicted sisters' but not brothers' depressive symptoms. In the case of parents, only mothers' deaths were found to predict daughters' depressive symptoms, whereas neither parents' deaths predicted sons' well-being. Further, these patterns differed little by time since death. Qualitative data revealed that women were more likely to report that both their mothers' and siblings' deaths had led to higher conflict within the sibling network, which previous research has shown predicts psychological well-being. Taken together, these findings demonstrate the salient role of gender in shaping well-being in the face of events of deaths of parents and siblings in adulthood.

### SANDWICH GENERATION CAREGIVING AMONG BABY BOOMER AND GENERATION X CAREGIVERS IN THE UNITED STATES

Christina Miyawaki,[1] Erin Bouldin,[2] Eva Jeffers,[3] and
Lisa McGuire,[4] *1. University of Houston, Houston, Texas, United States, 2. Appalachian State University, Boone, North Carolina, United States, 3. Centers for Disease Control and Prevention (CDC), Atlanta, Georgia, United States, 4. CDC, Atlanta, Georgia, United States*

Sandwich generation caregivers are generally those who care for both a child and an older adult. Baby Boomer and Generation X belong to this age cohort. Using data from the 2015-2018 Behavioral Risk Factor Surveillance System Caregiver Module, we compared the prevalence and characteristics of sandwich caregivers across these two generations. Data represent adults from 44 jurisdictions. We categorized caregivers into generations using their age at the time of the survey (N=34,777). Sandwich caregivers were classified as those who lived with a child (≤18 years) and provided care/assistance to a parent/grandparent with a long-term illness/disability during the past 30 days. Prevalence ratios (PR) from log-binomial regression models that included generation, sandwich caregiver status, sex, and race/ethnicity were used to compare weighted estimates. Six percent of Baby Boomers and 31% of Generation X were sandwich caregivers ($p<0.001$). In adjusted models, sandwich caregivers had a lower prevalence of any chronic health condition (PR=0.77, $p=0.01$) and fair/poor health (PR=0.87, $p=0.003$) than other caregivers, but similar frequent mental and physical distress prevalence. Baby Boomer caregivers were more likely to report a chronic health condition, fair/poor health, and frequent physical distress than their Generation X counterparts, but less likely to report frequent mental distress. Sandwich caregivers in these generations appear to be in better health than other caregivers. Nonetheless, it is critical to support the needs of sandwich caregivers as they age, given their important role in meeting the needs of both children and older adults and the additional challenges created by the pandemic.

### THE EFFECT OF NURSE AIDE RETENTION ON OHIO'S NURSING HOME RESIDENT CARE EXPERIENCE SCORES: A FACILITY-LEVEL ANALYSIS

Katherine Kennedy, *Miami University, Oxford, Ohio, United States*

The objective of the study was to analyze whether higher nurse aide retention was related to better resident care experiences using an overall score and seven domain scores among a sample of Ohio nursing homes. The 2017 Ohio Biennial Survey of Long-Term Care Facilities was used in combination with the 2017 Ohio Nursing Home Resident Satisfaction Survey. These data were merged with the Ohio Medicaid Cost Report, Certification and Survey Provider Enhanced Reports, LTC Focus, Area Health Resource File, Rural Urban Commuting Area data, and Payroll-based Journal Public Use Files. The analytic sample (N=690) represents freestanding facilities with a full-year cost report. The analysis included means and frequencies, ANOVAs with Tukey adjustments, and linear regressions that controlled for heteroskedasticity. Quartiles of the CNA retention rate were used to define four groups: low, medium, high, and extremely high. After controlling for facility and county characteristics, facilities with high CNA retention (61-72%) had significantly higher overall resident care experience scores by 1.27 percentage points and better environment scores by 1.35 percentage points compared to those with low CNA retention (0-48%). Medium retention (49-60%) also had significantly better environment scores than low retention. Compared to the high retention group, facilities with extremely high retention (73%+) had significantly lower scores for the overall resident care experience, facility culture, caregivers, and spending time. Maintaining a high retention rate of CNAs is important, but there were surprising negative effects from having extremely high retention potentially due to high burnout or poor person-job fit.

### Session 1355 (Paper)

### COVID-19 AND WELL-BEING

### GROWTH MINDSET AND OLDER ADULTS' WELL-BEING DURING THE COVID-19 PANDEMIC

Yena Kyeong,[1] Pamela Sheffler,[1] Esra Kürüm,[1] Leah Ferguson,[1] Elizabeth Davis,[1] Carla Strickland-Hughes,[2] and Rachel Wu,[3] *1. University of California, Riverside, Riverside, California, United States, 2. University of the Pacific, University of the Pacific, California, United States, 3. University Of California - Riverside, Riverside, California, United States*

Growth mindset, the belief that abilities and attributes are improvable, may help buffer against older adults' feelings of social isolation during the COVID-19 pandemic, as it may foster effective self-regulation and resilience. This study examined the effects of growth mindset on older adults' well-being and adjustment, compared to younger and middle-aged adults. Participants self-reported on their growth mindset, depression, well-being, and daily habits amid the pandemic. For older adults (N = 178, 82% female, M age = 70.42, SD age = 6.50, range 60-90), regression analyses (controlling for gender, education, income, and age) revealed that growth mindset was associated with decreased depression ($\beta = -.29$, $p = .001$) and increased well-being ($\beta = .38$, $p < .001$). In addition, a logistic regression showed that older adults with a higher growth mindset were more likely to adjust their daily tasks during the pandemic (e.g., using technology to remotely socialize; OR = 1.77, p = .012). The same set of analyses in samples of younger (N = 235, 72% female, M age = 29.84, SD age = 5.89, range 18-39) and middle-aged adults (N = 188, 74% female, M age = 50.02, SD age = 6.10, range 40-59) revealed that growth mindset was associated with decreased depression and increased well-being. However, in these groups, growth mindset did not predict the likelihood of adjusting daily tasks. Findings suggest that while growth mindset is linked to enhanced well-being during the pandemic, its effect on adjusting to new circumstances might be salient in older adulthood.

### IT WAS HEARTBREAKING . . . IT WAS HORRIBLE: THE EXPERIENCES OF NURSING HOME RESIDENTS' CHILDREN DURING COVID-19

Vivian Miller,[1] Nancy Kusmaul,[2] Jacquelyn Burns,[1] and Ji Hyang Cheon,[3] *1. Bowling Green State University, Bowling Green, Ohio, United States, 2. University of Maryland, Baltimore County, Baltimore , Maryland, United States, 3. University of Maryland, Baltimore County, University of Maryland Baltimore County, Maryland, United States*

As a result of COVID-19, the Centers for Medicare and Medicaid Services (CMS) suspended all outside visitors from entering nursing homes on March 13, 2020. For more than six months, care partners were only permitted in compassionate care situations, so adult children of residents were only able to contact their parents via phone calls, video chats, window visits, and in some cases, limited outdoor visits. Experts have written on the adverse, detrimental impact of this lack of connection and isolation has had on residents. However, the lived experiences from the perspectives of residents' adult children remain largely absent from the literature. To uncover the experiences of these care partners, semi-structured interviews were conducted (N=12) from December 2020 to February 2021. Adult children shared witnessing their parent' physical and cognitive decline which they attributed to the lack of visitors. Care partners expressed feeling frustrated that they were unable to observe their parent's health condition, and could not provide support. Also, many rightfully worried they would never see their loved one again. Findings from this study reveal implications for nursing home leaders and policymakers, such as building infrastructure and systems that both ensure safety and allow care partners to regularly see their residents in long-term care to avoid the unintended adverse consequences of these policies. Further, findings from this research indicate the need for future programs to mitigate and lessen the long-term consequences this isolation has had on both residents and their adult children.

### MENTAL HEALTH EFFECTS OF COVID-19 IN OLDER ADULTS ARE MODERATED BY EXISTING MENTAL HEALTH NEEDS AND EMOTIONAL SUPPORT

Tianyin Liu, Shiyu Lu, Terry YS Lum, Walker Siu Hong Au, Man-Man Peng, Dara KY Leung, and Gloria HY Wong, *The University of Hong Kong, Hong Kong, Not Applicable, Hong Kong*

Mitigating mental health consequences is one of the priorities for the society to advance, and the aim of this study is to investigate the mental health effects of COVID-19 in older adults and to explore risk and protective factors. Social workers recruited 722 older adults living in the community (mean age 73.6±8.4) from January 2020 to February 2021 in Hong Kong, and interviewed them for basic demographics (age, gender, education, and living status), mental health service use in recent three months (proxy for existing needs), number of people to talk to when feeling down (proxy for emotional support network), and assessed their depression using Patient Health Questionnaire-9 (PHQ-9). Impacts of COVID-19 were indicated by local daily effective reproductive number (Rt) and Nth wave since the start of the pandemic. Generalized linear models (GLM) were applied. Basic demographics were not associated with depression, existing mental health needs ($\beta$=2.99, p<.001), Rt ($\beta$=1.08, p<.05) and Nth wave ($\beta$=0.49, p<.05) were positively associated with depression, while emotional support network was negatively associated with depression ($\beta$=-2.47, p<.001). There was also a significant interaction between Rt and Nth wave on depression ($\beta$=0.69, p<.05), suggesting ongoing COVID-19 took a toll on older adults' mental health. Three-way interactions between COVID-19 Rt, Nth wave and existing mental health needs ($\beta$=0.25, p<.05) and emotional support network ($\beta$=-0.12, p=.07) on depression further indicated that older adults with existing mental health needs warrant more attention, and wider emotional support network may buffer the impact of the pandemic on mental health.

### RACIAL DIFFERENCES IN THE EROSION OF PSYCHOLOGICAL RESILIENCE FOLLOWING COVID-19 RELATED FINANCIAL HARDSHIP

Miles Taylor,[1] Dawn Carr,[1] and Kendra Jason,[2] *1. Florida State University, Tallahassee, Florida, United States, 2. UNC Charlotte, Charlotte, North Carolina, United States*

**Objectives:** Research on the impact of COVID-19 among older adults has primarily focused on virus outcomes, but it is also possible the pandemic's hardships have eroded the adaptive capacity of older adults. It is also likely these impacts vary by race and ethnicity. We examine changes in psychological resilience (PR) among older adults pre and post-pandemic to determine whether financial and social hardships have altered this resource for White, Black, and Hispanic older adults.

**Method:** Using the COVID module recently released by the HRS (n=735), we examined changes in PR between 2016 and 2020 related to specific COVID experiences. We tested interactions to determine whether the effects of these experiences were conditioned by race and ethnicity.

**Results:** Consistent with previous literature, resilience was relatively stable during this time on average. Financial hardship due to COVID-19 diminished resilience, but this effect was concentrated primarily among White Americans. PR was unchanged related to financial hardship among Black Americans.

**Discussion:** The results suggest that PR is a relatively stable resource in later life, even during a pandemic. However, this resource may be impacted in the face of specific and especially new challenges in later life. Policies and interventions related to job loss and financial hardship during the pandemic should be seen as supporting the capacity for older adults to adapt to current as well as future challenges.

### Session 1360 (Paper)

### COVID-19 PANDEMIC I

### CHANGES IN HEALTH AND WELL-BEING IN COVID-19 CLINICALLY VULNERABLE OLDER ENGLISH PEOPLE DURING THE PANDEMIC

Giorgio Di Gessa,[1] and Debora Price,[2] *1. University College London, London, England, United Kingdom, 2. University of Manchester, Manchester, England, United Kingdom*

People with specific health profiles and diseases (such as diabetes, lung and heart conditions) have been classified as 'clinically vulnerable' (CV) to Covid-19, i.e. at higher risk of severe illness and mortality from Covid-19, and were targeted for shielding. However, there is as yet little evidence on how the pandemic and shielding impacted the health and social well-being of CV older people. Using data from Wave 9 (2018/19) and the first Covid-19 sub-study (June/July 2020) of the English Longitudinal Study of Ageing, we investigated changes in health and well-being during the pandemic by clinical vulnerability. We also explored the interactions between CV and age-group (50s, 60s, 70s, 80+), and between CV and shielding. Results suggest that CV older people (~39% of the sample) were more likely to report worse health and social well-being outcomes during the pandemic compared to non-CV participants, even considering pre-pandemic levels of health and well-being. However, changes in health were not uniform across age groups, with those in their 50s and 60s more likely to report greater deterioration in mental health than those in their 70s and over 80. Moreover, older adults who were shielding and were CV reported the most substantial rises in anxiety, depression, receipt of formal care as well as decreases in well-being and physical activity. While policies focussing on shielding CV older people reduce rates of hospitalisation and death from Covid-19, policymakers should address the wider needs of this group if their long-term health and social well-being are not to be compromised.

### COMPARISON OF EMBEDDED SENSOR DATA FOR LONG-TERM CARE RESIDENTS BEFORE AND AFTER ONSET OF THE COVID-19 PANDEMIC

Erin Robinson, Wenlong Wu, Geunhye Park, Gashaye M. Tefera, Kari Lane, Marjorie Skubic, James Keller, and Mihail Popescu, *University of Missouri, Columbia, Missouri, United States*

Older adults have experienced greater isolation and mental health concerns during the COVID-19 pandemic. In long-term care (LTC) settings, residents have been particularly impacted due to strict lockdown policies. Little is known about how these policies have impacted older adults. This study leveraged existing research with embedded sensors installed in LTC settings, and analyzed sensor data of residents (N=30) two months pre/post the onset of the U.S. COVID-19 pandemic (1/13/20 to 3/13/20, 03/14/20 to 5/13/20). Data from three sensors (bed sensors, depth sensors, and motion sensors) were analyzed for each resident using paired t-tests, which generated information on the resident's pulse, respiration, sleep, gait, and motion in entering/exiting their front door, living rooms, bedrooms, and bathrooms. A 14.4% decrease was observed in front door motion in the two months post-onset of the pandemic, as well as a 2.4% increase in average nighttime respiration, and a 7.6% increase in nighttime bed restlessness. Over half of our sample (68%) had significant differences ($p<0.05$) in restlessness. These results highlight the potential impact of the COVID-19 pandemic and social distancing policies on older adults living in LTC. While it is not surprising that significant differences were found in the front door motion sensor, the bed sensor data can potentially shed light on how sleep was impacted during this time. As older adults experienced additional mental health concerns during this time, their normal sleep patterns could have been affected. Implications could help inform LTC staff, healthcare providers, and self-management of health approaches among older adults.

### CROSS-NATIONAL ANALYSIS OF BEREAVEMENT FROM COVID-19 AND DEPRESSIVE SYMPTOMS AMONG OLDER ADULTS IN EUROPE

Haowei Wang,[1] Ashton Verdery,[1] Rachel Margolis,[2] and Emily Smith-Greenaway,[3] *1. The Pennsylvania State University, University Park, Pennsylvania, United States, 2. University of Western Ontario, London, Ontario, Canada, 3. University of Southern California, Los Angeles, California, United States*

The COVID-19 pandemic has left older adults around the globe grieving the sudden death of relatives and friends. We examine if COVID-19 bereavement corresponds with older adults' depressive symptoms in 27 countries, and test for variation by gender and country context. We analyzed the Survey of Health, Ageing and Retirement in Europe (SHARE) COVID-19 data collected from N=51,383 older adults (age 50–104) living in 27 countries between June-August 2020, of whom 1,363 reported the death of a relative or friend from COVID-19. We estimated pooled-multilevel logistic regression models to examine if COVID-19 bereavement was associated with depressive symptoms and worsening depressive symptoms for older men and women, and we tested whether the national COVID-19 mortality rate in their country had an additive, or multiplicative, influence. COVID-19 bereavement from the death of a relative or friend is associated with significantly higher odds of reporting depressive symptoms, and reporting that these symptoms have recently worsened since the outbreak of COVID-19. Net of personal loss, living in a country with the highest COVID-19 mortality rate corresponds further with women's depressive symptoms; however, living in the midst of more COVID-19 deaths does not alter the implications of personal loss for depressive symptoms. COVID-19 deaths have lingering mental health implications for surviving older adults. Even as the collective toll of the crisis is apparent, bereaved older adults are in particular need of mental health support.

### FINANCIAL HARDSHIP AND THE PAIN OF SOCIAL DISCONNECTION DURING THE COVID-19 PANDEMIC IN THE UNITED STATES

Tirth Bhatta,[1] Nirmala Lekhak,[2] Timothy Goler,[3] Eva Kahana,[4] and Sfurti Maheshwari,[5] *1. University of Nevada, Las Vegas, Nevada, United States, 2. University of Nevada Las Vegas, Las vegas, Nevada, United States, 3. Norfolk State University, Newport News, Virginia, United States, 4. Case Western University, Case Western reserve University, Ohio, United States, 5. University of Nevada, Las Vegas, Las Vegas, Nevada, United States*

Considerable scholarly attention has been directed at increasing social isolation and loneliness during the COVID-19 pandemic, and their adverse impact on later life psychological well-being. Notably absent is the focus on financial hardship in the context of overlapping unprecedented economic and public health crisis. It is unclear whether loneliness continues to differ across different levels of financial hardship even amidst immense uncertainty, social isolation, and anxiety induced by the pandemic. Based on our nationwide web-based survey of adults aged 50 years and older (n=1861), we used ordinal logistic regression to examine the influence of financial hardship on loneliness and assessed the role of socioeconomic status (SES), emotional support, and health status in contributing to such influence. We found significantly higher odds of greater loneliness ($\beta = .28$, $p < .001$) among individuals who reported experiencing greater financial hardship. Among two measures of SES, only household income contributed substantially to the influence of financial hardship on loneliness. We documented significantly lower emotional support and greater health disadvantage among individuals experiencing greater financial hardship. Consideration of emotional support and health status explained the remaining influence of financial hardship, due to their association with both financial hardship and loneliness. Despite a sense of shared vulnerability and social isolation across the general population, our findings suggest that SES inequalities in later life loneliness are maintained even in the midst of the pandemic.

### REPRESENTING DUTCH OLDER ADULTS DURING COVID-19: WHAT CAN WE LEARN?

Jolanda Lindenberg,[1] and Miriam Verhage,[2] *1. Leyden Academy on Vitality and Ageing, Leiden, Zuid-Holland, Netherlands, 2. Leyden Academy on Vitality and Ageing, Leyden Academy on Vitality and Ageing, Zuid-Holland, Netherlands*

From the very beginning of the COVID-19 pandemic older adults have been at the heart of public debate. Early articles argued that public representation of older persons displayed a a resurgence of ageist stereotypes and beliefs in (inter)national media (e.g. Ayalon et al. 2020, Fraser et al 2020, Lichtenstein 2020, Sotomayer et al. 2020). Yet studies confirming this are absent up to now. In this paper, we present findings on the representation of Dutch older adults during the first six months of the COVID-19 crisis in The Netherlands. We analysed 1141 articles about older adults of the five largest newspapers using quantitative content analyses and discourse analysis to systematically explore patterns, sentiments and meaning in the articles. We show that the majority of these articles were published in general news and that older adults were rarely (2%) cited in these articles. Most prominent adjectives were vulnerable and weak. Most prominent substantives were attention, long-term care facility and loneliness. The sentiment was largely negative. Additionally, we find three discursive frames predominate: 'an older people's disease', 'vulnerability' and 'solidarity'. This evidences that the Dutch reporting on older adults during COVID-19 reproduced a discourse of dependency highlighting and further emphasizing the sociopolitical context before COVID-19 while drawing out earlier ageist tendencies. On the basis of our findings and drawing on advisory experiences, we discuss implications for policy, education and practice and how we can reframe and differently address older adults specifically in terms of language and their more (un)conscious positioning in (public) debate.

### Session 1365 (Symposium)

### CREATING VIRTUAL COMMUNITY FOR OLDER ADULTS: DURING THE COVID-19 PANDEMIC AND BEYOND

Chair: Neil Dsouza Co-Chair: Alexis Travis Discussant: Erica Solway

Combining data on health and well-being from the University of Michigan National Poll on Healthy Aging (NPHA) with case studies and data from GetSetUp, a virtual online learning community, and the Michigan Department of Health and Human Services (MDHHS), this symposium will highlight how virtual community can be created and supported during the COVID-19 pandemic and beyond. Polling data on loneliness and physical environments demonstrate the need for opportunities for connection before and during the pandemic. Other polling data from the NPHA shows telehealth visits increased significantly as did the use of video chat technology. These findings suggest that comfort with technology may help support aging in place. GetSetUp helps to make this possible with customized learning to help older adults overcome hurdles to tech adoption and use. GetSetUp classes focus on supporting social connection and providing information on resources and services. Beyond the pandemic, these services will remain critical for many older adults, including those facing mobility limitations, those with limited community, and those looking to diversify their networks. The Senior Deputy Director of Aging and Adult Services Agency will highlight how Michigan combines data and technology to support Michigan's aging network. The GetSetUp and MDHHS virtual community allowed for a statewide connection to health and aging services, including programs such as vaccine information sessions. The data and case studies described will highlight the need for connection during the COVID-19 pandemic and how a startup and State worked together to address this need.

### LESSONS IN CUSTOMIZING VIRTUAL COMMUNITIES TO MEET THE NEEDS OF OLDER ADULTS

Neil Dsouza, *GetSetup, Midvale, Utah, United States*

GetSetUp started as a technology learning platform for older adults. During Covid-19 we realized that many adults not only wanted to learn technology but were eager for lifelong learning opportunities. Our specially designed technology platform offered a safe, welcoming space that fosters growth, learning, and community for older adults over 60,000 Michiganders over 60 joined in just 4 months. Case studies show how older adults can benefit from technology to feel empowered, reduce social isolation, and improve wellbeing through a job or volunteer opportunities and new friendships. Reports highlight this ongoing need for those with limited community, mobility issues, or those looking to diversify their communities. A trusted virtual community helps connect the physical world digitally to assure older adults understand processes like vaccine enrollment procedures. Virtual communities will not replace physical communities but offer research opportunities, reach more remote communities, and allow for collaboration with state and aging networks.

### LESSONS ON USING HEALTH RESEARCH & TECHNOLOGY TO BROADEN RESOURCES FOR OLDER ADULTS ACROSS THE STATE

Alexis Travis, *Michigan Department of Health and Human Services, Lansing, Michigan, United States*

The vision of the Michigan state unit on aging is for residents to live well and thrive as they age. The COVID-19 pandemic exacerbated the existing problem of older adult social isolation. Social engagement and community involvement are keys to healthy aging. Combining state resources with the GetSetUp virtual community allowed for statewide connections and extended resources, creating an almost around-the-clock virtual senior center. Through customized courses the state was able to offer vaccine navigation sign-up classes, among other classes, to help older adults interact with essential health and aging services. As Michigan continues to work to address health equity and social determinants of health beyond the pandemic, technology designed specifically for older adults is an important component of programmatic offerings. It also allows for a public-private partnership opportunity to support older adults as they age.

### POLL FINDINGS ON SOCIAL CONNECTION AND TECHNOLOGY USE DURING THE COVID-19 PANDEMIC

Erica Solway, *University of Michigan, Ann Arbor, Michigan, United States*

The National Poll on Healthy Aging conducted an online survey of a nationally representative sample of adults age 50-80 (n=2,074) in June 2020 about experiences related to loneliness, their physical environments, and telehealth and technology use. 41% felt a lack of companionship, and 46% reported infrequent social contact. Feelings of loneliness were more likely among those who lived alone or who did not have access to features in their neighborhood and community which may offer opportunities for safe interactions. The poll also found that 26% of adults age 50-80 had a telehealth visit March through June 2020 and 64% overall reported being comfortable with video conferencing technology, with notable differences by demographic subgroup. These results highlight the need for new opportunities for older adults, especially those with the greatest social and economic need, to feel connected and to be confident using technology, both during and after the pandemic.

### Session 1370 (Symposium)

### CURRENT AND FUTURE DIRECTIONS IN MINORITY AGING: EMBRACING INTERDISCIPLINARY MODELS

Chair: Heather Farmer Co-Chair: Amy Thierry Discussant: Keith Whitfield

Racial/ethnic disparities in health among older adults are well-documented. More research is needed to clarify the complex and multifactorial mechanisms underlying these associations. This symposium will feature research that employs innovative theoretical and methodological approaches to understand the biopsychosocial mechanisms that underlie racial/ethnic disparities in older adults' health and determine sources of within-group heterogeneity in minority aging. Dr. Forrester will integrate stress biology and intersectionality to demonstrate the importance of stress and resilience (e.g., John Henryism) with biological aging within Black adults participating in the Coronary Artery Risk Development in Young Adults (CARDIA) Study. Dr. Brown Hughes will present innovative research using data from the African American United Memory and Aging Project (AA-UMAP) on the importance of Alzheimer's disease-specific knowledge and perceptions among Black older adults. Dr. Gamaldo will employ a within-race approach to understand how knowledge and perceptions of Alzheimer's disease and related dementias (ADRD) shape cognitive performance among Black older adults in the AA-UMAP study. Dr. Mitchell will use Health and Retirement Study data to explore the role of midlife stress exposure in accounting for racial disparities in trajectories of cognitive functioning. Drs. Thierry and Farmer will use HRS data to examine how psychosocial resilience (e.g., mastery) affects the relationship between perceived neighborhood conditions (e.g., disorder) and cognition among Black older adults. This work highlights the importance of applying an interdisciplinary lens to move the study of minority aging forward and ultimately, to reduce the unnecessary burden of morbidity and mortality among minoritized groups.

### EXPLORING HOW NEIGHBORHOOD CHARACTERISTICS AND PSYCHOSOCIAL RESILIENCE AFFECT COGNITION AMONG OLDER BLACK ADULTS

Heather Farmer,[1] Amy Thierry,[2] Marina Armendariz,[3] Sydney Kirven,[4] and Kyler Sherman-Wilkins,[5] *1. University of Delaware, Newark, Delaware, United States, 2. Xavier University of Louisiana, Xavier University of Louisiana, Louisiana, United States, 3. The Pennsylvania State University, University Park, Pennsylvania, United States, 4. Xavier University of Louisiana, New Orleans, Louisiana, United States, 5. Missouri State University, Springfield, Missouri, United States*

Black older adults are at greater risk for poor cognitive health than Whites, and adverse neighborhood conditions may contribute to this disparity. Moreover, limited research examines how resilience is implicated in the relationship between neighborhoods and cognition among Blacks. Using 2006-2016 waves of the Health and Retirement Study, we examine how perceived neighborhood characteristics (physical disorder and social cohesion) and psychosocial resilience (social support, mastery, and sense of purpose) contribute to cognitive functioning among 1,655 Black adults ages 65+. Results from multilevel linear regression models show that greater physical disorder was associated with worse cognitive functioning, and this was attenuated after adjustment for socioeconomic status. We found a positive association between purpose and mastery with cognitive functioning, even after accounting for socioeconomic, psychosocial, and health-related characteristics. Thus, high levels of purpose and mastery may be protective for cognitive functioning among Black older adults in spite of experiencing negative neighborhood contexts.

### NAVIGATING BLACK AGING: THE IMPACT OF STRESS AND THE POWER OF RESILIENCE IN PROMOTING HEALTH

Sarah Forrester,[1] Keith Whitfield,[2] Catarina Kiefe,[1] and Roland Thorpe, Jr.,[3] *1. University of Massachusetts Medical School, Worcester, Massachusetts, United States, 2. University of Nevada Las Vegas, Las Vegas, Nevada, United States, 3. Johns Hopkins Bloomberg School of Public Health, baltimore, Maryland, United States*

The health profile of African Americans clearly shows that stress works to worsen chronic conditions. To improve the health of aging African Americans, interventions need to address how effects of stress are reduced by individual resilience factors and exacerbated by anxiety or other traits. We will characterize the effects of stress by measuring rate of biological aging (RBA) over thirty years in a Black cohort (aged 18-30 at baseline) of approximately 2,000 individuals from the longitudinal CARDIA study. Biological aging (BA) captures premature physiological aging beyond that predicted by an individual's chronological age. RBA will be characterized by within person change in BA over 30 years. We will measure the association between RBA and anxiety and will further measure the extent to which various forms of individual resilience factors mitigate the effects of anxiety on BA. We will also explore how intersectionality is evinced in sex differences in RBA.

### THE AFRICAN AMERICAN UNITED MEMORY AND AGING PROJECT: AD KNOWLEDGE AND FAMILY HISTORY AS IT RELATES TO COGNITION

Alyssa Gamaldo,[1] Allison Caban-Holt,[2] and Travonia Brown-Hughes,[3] *1. The Pennsylvania State University, University Park, Pennsylvania, United States, 2. Maya Angelou Center for Health Equity, Winston-Salem, North Carolina, United States, 3. School of Pharmacy, Hampton, Virginia, United States*

This study explores the influence of Black adults' Alzheimer's disease (AD) knowledge and family history of AD on cognition. A sample of Black adults (n=66, age range=45-84) completed a computerized cognitive (CogState Brief) battery and surveys of AD knowledge, family history of AD diagnosis, and health. On the 14-item AD knowledge survey, participants correctly answered a mean of 10.80 (SD=1.50) items. Approximately, 56% reported a biological family member diagnosed with AD, of these 30% reported this being a mother or father. Linear regression models suggested no significant association between AD knowledge and cognitive performance. However, adults with a family member diagnosed with AD had worse visual learning accuracy even after adjusting for age, education, and income. Increased age was associated with worse processing speed, particularly in adults with a mother diagnosed with AD. These findings demonstrate the importance of examining the influence of family history on Black adults' cognitive health.

### THE EFFECTS OF MID-LIFE STRESS EXPOSURE ON BLACK-WHITE DIFFERENCES IN COGNITIVE DECLINE

Uchechi Mitchell, *University of Illinois Chicago, School of Public Health, Chicago, Illinois, United States*

Cognitive decline is a precursor to cognitive impairment and dementia. Recent research suggests that cognitive decline may begin earlier in the life course for Blacks and that Black-white disparities in cognitive function emerge in midlife. Disproportionate exposure to chronic and acute stressors during mid-life may explain Black-white differences in trajectories of cognitive function over time. In this study we use data from approximately 3,700 Black and white respondents age 51-64 from the Health and Retirement Study to examine race differences in cognitive decline and the role mid-life stress exposure play in these differences. Initial findings suggest that mid-life Blacks have lower levels of cognitive function, but their rates of cognitive decline do not differ significantly from mid-life whites. Financial strain and everyday experiences of discrimination are inversely associated with cognitive decline and only partially explain differences in cognitive decline between mid-life Blacks and whites.

### REDEFINING THE USE OF DIGITAL COMMUNITIES: AD KNOWLEDGE IN AN ONLINE EDUCATED COHORT OF MIDLIFE AND OLDER BLACKS

Travonia Brown-Hughes,[1] Alyssa Gamaldo,[2] Corinne Pettigrew,[3] Allison Caban-Holt,[4] Nihal Mohamed,[5] and Roland Thorp, Jr,[6] *1. School of Pharmacy, Hampton, Virginia, United States, 2. Penn State University, University Park, Pennsylvania, United States, 3. Johns Hopkins University, Baltimore, Maryland, United States, 4. Maya Angelou Center for Health Equity, Winston-Salem, North Carolina, United States, 5. Icahn School of Medicine at Mount Sinai Department of Oncological Sciences, New York, New York, United States, 6. Johns Hopkins Bloomberg School of Public Health, Baltimore, Maryland, United States*

The normalization of memory loss continues to contribute to diagnostic delays among older adult African Americans with dementia. We utilized an innovative recruitment method to establish a solely online study to examine perceptions and knowledge levels of Alzheimer's Disease in a highly educated geographically diverse cohort of 223 African Americans aged 50-84. Participants were recruited through largely electronic communications. Sample participants were primarily female (n=196), with 51.1% having completed a master's degree, and 58.2% of participants with household incomes of $90,000 or higher. Study findings revealed that although highly educated, 42% of sample participants believed significant memory loss was a normal part of aging and 59.6% felt that God's Will was a possible cause of AD. A sizable majority of participants, 86.5%, felt most family physicians were not trained to diagnose AD. Findings underscore the need for physician and community education within diverse populations, regardless of education and SES status.

### Session 1375 (Symposium)

### DEVELOPING SYNERGISTIC RELATIONSHIPS BETWEEN AGE-FRIENDLY UNIVERSITIES AND AGE-FRIENDLY COMMUNITIES

Chair: Joann Montepare Co-Chair: Wendy Rogers

The Age-Friendly University (AFU) initiative was designed to support the Global Network for Age-Friendly Cities and Communities (WHO, 2018) and offers a range of opportunities for institutions of higher education to help communities adapt to their new age-diverse social structures as a result of shifting age demographics. In turn, age-friendly community partnerships are helping to fuel campus efforts to advance age-inclusivity through education, research, and community engagement. At present over 70 institutions have joined the AFU global network, as more campuses prepare to become age-friendly partners. In this collaborative symposium (Directors of Aging Centers and AFU Interest Groups), campus leaders will describe synergistic relationships between their age-friendly campus efforts and the age-friendly efforts of their neighboring communities. Montepare (Lasell University) will provide an overview of the AFU initiative and its set of 10 principles, and make the case that campuses and communities are necessary partners for creating and developing age-friendly efforts. Demonstrating this assertion, Pastor and Rogers (University of Illinois Urbana-Champaign) will describe linkages between their community and campus initiatives, including developing a Panel of Elders, television programming for older adults, and hosting joint events. Black and Andel (University of South Florida) will discuss the intersection between the AFU principles and the processes undertaken by age-friendly communities. Revell and Viveiros (University of Massachusetts Dartmouth) will show how campus collaborations with nearby communities are instrumental in sustaining age-friendly efforts, especially during a pandemic.

### AGE-FRIENDLY UNIVERSITIES (AFU): NECESSARY PARTNERS FOR AGE-FRIENDLY COMMUNITIES

Joann Montepare, *Lasell University, Newton, Massachusetts, United States*

The pioneering Age-Friendly University (AFU) initiative, endorsed in 2016 by GSA's Academy for Gerontology in Higher Education (AGHE), calls for institutions of higher education to respond to shifting demographics and the needs of aging populations through more age-friendly campus programs, practices, and partnerships. The case will be made that AFU institutions can also play vital roles in helping neighboring communities develop, launch, assess, and sustain their age-friendly efforts through research and related endeavors that engage students and faculty. In addition, AFU campus-community partnerships can play a critical role in breaking down age-segregation that fuels ageism, building intergenerational connections, and increasing aging literacy among rising community members - all of which are necessary steps for building age-friendly communities.

### ENHANCING COLLABORATIONS BETWEEN OUR AGE-FRIENDLY COMMUNITY AND UNIVERSITY INITIATIVES

Brian Pastor, and Wendy Rogers, *University of Illinois Urbana-Champaign, Champaign, Illinois, United States*

Community involvement and synergistic partnerships are key to fostering a holistic approach to programming and outreach that assess and meet the needs of the older adults in our communities. The University of Illinois Urbana-Champaign has created an Age-Friendly network to facilitate these partnerships featuring our designation of Age-Friendly City and Age-Friendly University as well as partnerships with our state and local governments, community aging services providers, and continuing care retirement communities. Through these partnerships, we have identified the landscape of the community, assessed the unique needs older adults, and identified novel solutions. We will discuss our plans for activities that will empower older adults in our community and at our university by promotion connection, collaboration, and inclusion.

### LEVERAGING THE INTERSECTION BETWEEN AGE-FRIENDLY UNIVERSITIES AND AGE-FRIENDLY COMMUNITIES

Kathy Black, *University of South Florida, Sarasota-Manatee, Florida, United States*

Age-friendly Universities represent a growing contribution to the worldwide age-friendly movement. For universities, the international effort aims to highlight the role higher education plays in responding to the opportunities associated with an aging population. The initiative outlines ten principles to engage older adults via collegiate mission pertaining to research, education and service. Shared practices suggest diverse and unique application of the guiding tenets across participating colleges and universities. However Age-friendly Universities are also part of a broader ecosystem, situated in geographic locales reflecting actual or prospective age-friendly community status. The global Age-friendly Community movement is a decade-old effort to improve the environments in which we age via a cyclical process. This paper identifies the intersection between Age-friendly University principles and Age-Friendly Community processes and discusses reciprocal considerations for mutual advancement of the broader movement.

### CREATING AN AGE-FRIENDLY MODEL SYSTEM THROUGH A COMMUNITY AND UNIVERSITY PARTNERSHIP

Andrew Revell,[1] and Jennifer Viveiros,[2] *1. University of Massachusetts Dartmouth, University of Massachusetts Dartmouth, Massachusetts, United States, 2. University of Massachusetts Dartmouth, North Dartmouth, Massachusetts, United States*

The University of Massachusetts 5-campus system was the first university system to receive the Age-Friendly University designation in the AFU Global Network (Business West, 2019). Simultaneously, the town of Dartmouth and city of New Bedford became Age-Friendly Communities. This allowed for dynamic collaboration between our university and communities. This presentation highlights several examples. The Ora M. DeJesus Gerontology Center faculty and student researchers developed the original age-friendly survey items for New Bedford's initial community assessment; and the College of Nursing and Health Sciences faculty and student researchers compiled data for Dartmouth's survey. Community service during the pandemic has flourished. The Community Companions program, which matches students with community members in social need, went virtual. Nursing students and faculty have been on the frontline in the vaccination efforts in the town of Dartmouth. These partnerships will be presented as examples of potential opportunities for other age-friendly communities. Community-university partnerships are encouraged.

### Session 1380 (Symposium)

### DISPARITIES RESEARCH AT THE DEEP SOUTH ALZHEIMER'S DISEASE CENTER OF THE UNIVERSITY OF ALABAMA AT BIRMINGHAM

Chair: Maria Pisu Discussant: David Geldmacher

Residents of the US Deep South (Alabama, Georgia, Louisiana, Mississippi, and South Carolina) have a 20–30% higher risk of developing Alzheimer's disease or related dementia (ADRD). Moreover, >20% of African Americans, who are at higher ADRD risk than whites, live in this region. Therefore, one important goals of the Deep South Alzheimer's Disease Center (DS-ADC) of the University of Alabama at Birmingham is to spearhead research to address these disparities. This panel presents current DS-ADC research, with two presentations focusing on the local patient population and the last two on the Deep South population compared to the rest of the nation. Addressing the challenge of recruiting representative samples in clinical research, the first paper is part of a research program to understand difference that may exist between African American and white research participants. The second paper examines patients with multiple conditions, in particular dementia and cancer, showing a marked disadvantage in cognition outcomes for African Americans. The next two papers take a broader perspective to better understand the population of older adults with ADRD in the Deep South and in the rest of the US. The third paper examines socioeconomic and medical contexts of African American and white older Medicare beneficiaries with ADRD, and the fourth paper examines differences in utilization of specialists, ADRD drugs, and hospitalizations in the two regions taking these contexts into account. The discussant will close the session by placing these studies in the larger context of the disparities research at the DS-ADC.

### HEALTH CARE UTILIZATION IN DIVERSE OLDER ADULTS IN THE DEEP SOUTH AND THE REST OF THE UNITED STATES

Maria Pisu,[1] Roy Martin,[1] Liang Shan,[2] Giovanna Pilonieta,[3] Richard Kennedy,[1] Gabriela Oates,[1] and David Geldmacher,[4] *1. University of Alabama at Birmingham, Birmingham, Alabama, United States, 2. University of Alabama at Birmingham, BIrmingham, Alabama, United States, 3. University of Alabama At Birmingham, Birmingham, Alabama, United States, 4. University of Alabama at Birmingham, University of Alabama at Birmingham, Alabama, United States*

We examined racial/ethnic (R/E) differences in health care utilization among older adults with Alzheimer's disease and related dementia (ADRD) from US Deep South [DS] and non-DS, and individual or context-level factors that affect this utilization. Data were 2013-2015 claims for Medicare beneficiaries with ADRD; county-level data were used to define context-level covariates; adjusted analyses were conducted separately for DS and non-DS. Across R/E groups, 33%-43% in DS, 43%-50% in non-DS used ADRD specialists; 47%-55% in DS, 41%-48% in non-DS used ADRD drugs; 42.9%-53.4% in DS, 42%-51.8% in non-DS had hospitalizations in a one-year follow-up. R/E differences were not significant, with few exceptions. Comorbidities, poverty, and medical resources availability were associated with specialist use and hospitalizations; comorbidities and specialist use were associated with drug use. In non-DS only, other individual, context-level covariates were associated with health care outcomes. Research should further examine determinants of health care utilization in these populations.

### THE CHALLENGE OF IDENTIFYING REPRESENTATIVE SAMPLES IN RESEARCH INVOLVING MINORITY PARTICIPANTS

Giovanna Pilonieta,[1] and David Geldmacher,[2] *1. University of Alabama At Birmingham, Birmingham, Alabama, United States, 2. University of Alabama at Birmingham, University of Alabama at Birmingham, Alabama, United States*

Determining participants' demographics, cognition, and functional performance by race is crucial to understanding disparities in clinical research on Alzheimer's disease. We compared demographic and performance variables between Black/African American (B/AA; N=30; 41%) and White participants (N=43, 59%) in the UAB Alzheimer's Disease Center. Among 73 participants, 38 (52%) were women, mean age was 65.7 (SD 9.47), and mean education was 16 (2.31) years. Significant differences in gender proportions across race groups were observed. B/AA women represented 70 % of their race group, white women represented 39.5 %. There were no statistically significant differences in age, education, cognitive or functional severity, reasons to participate in research, referral source, objective measures of cognition, or informant-rated daily function by race group. In conclusion, despite 50% oversampling of B/AA participants compared to the State population, no differences in cognitive and functional performance at the time of enrollment were associated with race.

### SOCIAL DETERMINANTS OF ALZHEIMER'S DISEASE AND RELATED DEMENTIAS: RACIAL AND REGIONAL VARIATIONS

Roy Martin,[1] Liang Shan,[2] David Geldmacher,[3] Giovanna Pilonieta,[4] Richard Kennedy,[1] Gabriela Oates,[1] and Maria Pisu,[1] *1. University of Alabama at Birmingham, Birmingham, Alabama, United States, 2. University of Alabama at Birmingham, BIrmingham, Alabama, United States, 3. University of Alabama at Birmingham, University of Alabama at Birmingham, Alabama, United States, 4. University of Alabama At Birmingham, Birmingham, Alabama, United States*

To examine whether racial and regional social determinants of health disparities exist for older adults with Alzheimer's disease and related dementias (ADRD). We identified 115,879 African American (AA) and White older adults with ADRD (10% from the Deep South) from administrative claims data for a 5% random sample of Medicare beneficiaries (2013-2015). We examined racial and regional differences across sociodemographic characteristics, county-level linked poverty indicators, medical resource availability categories, insurances quality indicators. Social context differences were found between regions including Deep South older adults with ADRD having higher economic impoverishment and lower access rates to specialty medical care services. Older Deep South AA had higher Medicare/Medicaid eligibility rates and less medical access. Significant socioeconomic disparities exist between Deep South and other US regions across several social determinant factors in older adults with ADRD. Social context differences were especially prominent for older Deep South AA with ADRD.

### THE RELATIONSHIP BETWEEN PRIOR CANCER DIAGNOSIS AND ALL-CAUSE DEMENTIA PROGRESSION AMONG U.S. ADULTS

Mackenzie Fowler, Nicole Wright, Kristen Triebel, Gabrielle Rocque, Ryan Irvin, and Richard Kennedy, *University of Alabama at Birmingham, Birmingham, Alabama, United States*

Cancer-related cognitive impairment is a common effect of cancer that shares symptoms with dementia. Only one study examined cancer's longitudinal association with dementia. This analysis expands to a larger clinical sample. Electronic health record data were extracted from July 2003-February 2020. Baseline cognition/progression on the Alabama Brief Cognitive Screener (ABCs) by cancer history were assessed using linear mixed effects models, with interaction by race. After adjustment for demographics/socioeconomics, those with cancer history had higher baseline cognition (□: 1.49 [0.91-2.07]), and declined slower (□: 0.40 [0.08-0.71]) than those without. Health behaviors/comorbidities attenuated this association. Non-Hispanic Blacks with cancer history demonstrated lower cognition throughout follow-up compared to non-Hispanic Whites / other race/ethnicities with cancer history and participants without cancer history. Health behaviors/comorbidities confound and race modifies the relationship between cancer and dementia. Exploring the role of health behaviors/comorbidities on this association and causes of racial disparities is needed.

### Session 1385 (Symposium)

### EMERGING SCHOLARS AND JUNIOR FACULTY PRESENT NEW STRATEGIES FOR STUDENT ENGAGEMENT IN GERONTOLOGY

Chair: Sarah Hahn Discussant: Jennifer Kinney

With the rapid aging of the population, the need for gerontological educators to identify pedagogical strategies to increase interest and prepare students continues to grow. Innovative approaches and educational practices contribute greatly to student success in the gerontological classroom. Literature on gerontological pedagogy has shed light on the success of high-impact practices, creative assignments, pedagogical interventions, and even different course modalities when it comes to effectively delivering gerontological content and engaging students. Additionally, the Academy for Gerontology in Higher Education (AGHE) provides a wealth of suggestions for creating and implementing effective gerontology courses and assignments. However, while we are familiar with these practices, we are not familiar with how specific groups of academics, such as emerging scholars and junior faculty, are utilizing them. Emerging scholars and junior faculty experience several major transitions as they prepare for life in academia. To ensure that emerging scholars and junior faculty are well prepared, we need to continue to empower these individuals to foster growth. This can be done by highlighting how emerging scholars and junior faculty have met the goals of maximizing and optimizing student learning. As such, the purpose of this symposium is to examine innovative approaches used by emerging scholars and junior academics in the gerontological classroom that have optimized student learning. This includes presentations on strategies for team-based learning, using intersectionality as a theoretical lens, and two creative written assignments, The Gerontological Movie Database Review and Interview an Elder.

### INTERVIEWING AN ELDER: STUDENTS' PERCEPTIONS OF CHANGE IN AN INTRODUCTION TO GERONTOLOGY CLASSROOM

Sarah Hahn, *Mercy College, Dobbs Ferry, New York, United States*

Within the gerontological classroom, high-impact practices and creative assignments have consistently shown to help engage students, optimize learning, and increase positive attitudes toward older adults and aging (e.g., Chonody, 2015; Yamashita, et al., 2018). One such creative assignment, interviewing an older adult, has been cited as both an influential and valuable experience to gerontology students (e.g., O'Hanlon & Brookover, 2002). Although this assignment has been popular in and out of gerontology courses, more data regarding this and student's understanding is needed. As such, this presentation aims to 1) introduce and establish the value of using the written assignment, "Interview an Elder" in the gerontology classroom and 2) present preliminary qualitative data on how students' perceptions of older adults changed after the assignment. Using thematic analysis, results suggest that students are not only surprised by what they learned, but have increased positive perceptions of older adults overall.

### FLIPPING THE SCRIPT ABOUT AGING THROUGH FILMS: THE GERONTOLOGICAL MOVIE DATABASE REVIEW

Sara Hackett, *University of South Florida , Wesley Chapel, Florida, United States*

Education about the heterogeneity of the older adult population is an important step for reducing ageist attitudes. As many undergraduate students view gerontology as an unrelatable discipline, educators are tasked with identifying innovative strategies to make course content engaging. The purpose of this presentation is to share an emerging educator's experience with creating a novel essay assignment. Based off the International Movie Database (IMDb), the Gerontological Movie Database (GMDb) Review encourages students to use their knowledge to evaluate how older adults are portrayed in films. Explicitly, students must 1) choose a film that focuses on older adult characters and 2) apply key gerontological concepts (e.g., the life course perspective) to critique the film's representation of aging. Though movie reviews are not a typical genre of writing, this assignment increases students' understanding of how their perception of aging, coupled with master narratives embedded within today's culture, influences the construction of age.

### USING TEAM-BASED LEARNING IN AN ONLINE SYNCHRONOUS GERONTOLOGY COURSE

Leah Janssen, *Scripps Gerontology Center, Oxford, Ohio, United States*

Team-based learning (TBL) was chosen for its learner-centered approach to intentional engagement and purposeful application of course material in a cross-listed, upper-level gerontology class (i.e., Social Forces in Aging). Intedashboard, an online TBL platform, was utilized to support the online synchronous course, which is especially useful for its integration of class material, module assessments, peer/course evaluations, and dashboard display of live team activity. From the perspective of an emerging scholar, this symposia session will explore the application of TBL as a tool for developing teams, helping students personally connect with course material, and support inclusive teaching initiatives. More specifically, this presentation examines how a scaffolded TBL exercise on cumulative advantage/disadvantage, intersectionality, and social identities led to increased identification and awareness of students' social location, and the perceived impacts on their later lives.

### USING INTERSECTIONALITY THEORY AS A LENS IN A PERSPECTIVES ON AGING CLASS

Kelly Munly, *Penn State Altoona, Altoona, Pennsylvania, United States*

The presenter will discuss strategies for using intersectionality as a theoretical lens in her Perspectives on Aging class in order to support students to understand the relevance of aging studies—including health and social disparities in aging—for their contemporary lived lives, as well as for prior generations. With this relevance established, the class also examines the significance and justification for the development of policy, such as Social Security legislation, as well as the need for aging-related career areas. The presenter will discuss the application of key course resources, including research that looks at aging in historic contexts, as well as content highlighting the importance of Age Friendliness and the diversity of career areas to support Age Friendliness and more optimal aging experiences overall. Examining historic roots of aging-related experiences in social contexts creates an informative platform for understanding experiences of aging in society today.

### Session 1390 (Symposium)

### ESPO AND HEALTH SCIENCES SECTION SYMPOSIUM: CONDUCTING CLINICAL TRIAL RESEARCH DURING THE COVID-19 PANDEMIC: TRANSFORMING RESEARCH PRACTICE FOR THE FUTURE

Chair: Brianna Morgan Co-Chair: An Nguyen Discussant: An Nguyen

The COVID-19 pandemic caused significant disruptions for people and institutions across healthcare settings. Clinical trials are an important research tool to test interventions in real-world healthcare settings and provide high quality evidence that supports older adults' longevity and wellness. Clinical trialists must consider how to account for unpredictable and ever-changing environmental contexts. The COVID-19 pandemic is a specific example of a changing context that impacted all stages of the clinical trials process from planning, to administration, and outcomes. Reflecting on ways clinical trialists navigated their studies during the COVID-19 pandemic may unlock opportunities to design flexible clinical trials that meet the needs of older adults in real-world environments. This symposium highlights five clinical trials for older adults that occurred during the COVID-19 pandemic. Dr. Carpenter will discuss lessons learned in implementing a palliative care intervention in nursing homes. Brianna Morgan will describe the pivots needed to complete a clinical trial testing an advance care planning website for nursing home residents with dementia. Dr. Nuckols will describe obstacles and opportunities to implementing a randomized controlled trial on hospital nursing units, including implications for medication safety. Dr. Pevnick will highlight barriers and facilitators to implementing a pharmacist-led intervention to reduce hospital readmissions. Dr. Stark will share novel procedures for conducting clinical trials in the community that reduce burden for older adult participants while maintaining fidelity. Presenters will address practice transformations that researchers can bring forward to design flexible clinical trials that meet the needs of older adults in different healthcare contexts.

### ADAPTING AN ADVANCE CARE PLANNING WEBSITE FOR PERSONS WITH DEMENTIA IN NURSING HOMES DURING THE COVID-19 PANDEMIC

Brianna Morgan,[1] Liza Behrens,[2] Sonia Talwar,[3] Emily Summerhayes,[4] Mary Ersek,[4] and Nancy Hodgson,[5]
*1. University of Pennsylvania, Philadelphia, Pennsylvania, United States, 2. University of Pennsylvania School of Nursing, Philadelphia, Pennsylvania, United States, 3. University of Pennsylvania, School of Nursing, Philadelphia, Pennsylvania, United States, 4. University of Pennsylvania school of nursing, Philadelphia, Pennsylvania, United States, 5. University of Pennsylvania, School of Nursing, philadelphia, Pennsylvania, United States*

We partnered with a national for-profit nursing home (NH) organization to test the acceptability and use of an advance care planning (ACP) website for people living with dementia using a randomized controlled trial (RCT) design. Concurrently, the COVID-19 pandemic disproportionately impacted NHs and halted in-person research. We will present challenges, opportunities, and adaptations in site engagement, recruitment, and data collection. Initially, NHs were overwhelmed by pandemic efforts and research staff were unable to enter sites. We capitalized on time and available resources by beta-testing the website in a comparable population and designing surveys to elicit COVID-19's impact on ACP. Once able, NH staff took on recruitment and data collection efforts intended for research staff. We supported NHs by pivoting to remote data collection, providing technology on site, and offering flexible communication. Flexibility is key in supporting site engagement, recruitment, and data collection and has implications for designing pragmatic RCTs.

### INNOVATION IN TIMES OF UNCERTAINTY: CLINICAL TRIALS IN NURSING HOMES DURING SARS-COV-2

Joan Carpenter, *University of Maryland School of Nursing, Baltimore, Maryland, United States*

In March 2020 the Centers for Medicare & Medicaid Services (CMS) announced restrictions on visitors and nonessential personnel in nursing homes to protect residents and facilities from SARS-CoV-2 (severe acute respiratory syndrome coronavirus 2) outbreaks. At the time, these measures were "temporary" but they continued well into 2021 resulting in a prolonged pause on in-person study activities in a palliative care clinical trial in 12 nursing homes. This session will address the impact of this pause and decisions made to overcome the potential failure of the trial. Of utmost importance was respecting nursing homes rapidly changing context, continued communication with the site leadership, transitioning to phone and video-conference study activities, and designing a retrospective study using existing data to answer a different but similar research question. As clinical researchers move forward implementing trials and complex interventions in nursing homes, we must use the lessons learned to design flexible trial protocols.

### CONDUCTING A CLUSTER RCT ON MEDICATION SAFETY IN NURSING UNITS OVERTAXED BY THE COVID-19 PANDEMIC

Teryl Nuckols,[1] Ed Seferian,[1] Bernice Coleman,[1] Carl Berdahl,[1] Tara Cohen,[1] and Andrew Henreid,[2]
*1. Cedars-Sinai Medical Center, Los Angeles, California, United States, 2. Cedars-sinai Medical Center, Los Angeles, California, United States*

Medication errors continue to harm many hospitalized patients. In other high-risk industries, voluntary incident reporting is widely used to improve safety. Reporting is widely used in hospitals, but not as effectively. This AHRQ-funded cluster RCT will assess the effects of the SAFE Loop, which includes five enhancements in incident reporting implemented on hospital nursing units. Analyses will compare changes in nurses' attitudes toward reporting, event reporting rates, report quality, and medication event rates between intervention and control arms. The COVID-19 pandemic has created both obstacles and opportunities. The intervention requires study staff to engage nursing unit directors, attend daily nursing "huddles", and train overtaxed front-line nurses in a geographic area greatly impacted by COVID-19 surges. This created uncertainty around the best time to start the trial.

Conversely, we have collected unique data on the implications of COVID-19 for medication safety while testing our instruments during the trial preparation phase.

### ADDRESSING COVID-19 BARRIERS TO CLINICAL TRIAL ENROLLMENT AND IMPLEMENTATION IN THE PHARM-DC STUDY

Joshua Pevnick,[1] Michelle Keller,[2] Korey Kennelty,[3] Michelle Ko,[4] Logan Murry,[3] An Nguyen,[1] Andrew Henreid,[5] and Jeffrey Schnipper,[4] *1. Cedars-Sinai Medical Center, Los Angeles, California, United States, 2. Cedars Sinai, Cedars Sinai, California, United States, 3. University of Iowa, Iowa City, Iowa, United States, 4. Brigham and Women's Hospital, Boston, Massachusetts, United States, 5. Cedars-sinai Medical Center, Los Angeles, California, United States*

Recent hospitalization puts older adults at higher risk of experiencing adverse drug events (ADEs) that are a common cause of hospital readmission. Yet, most ADEs are preventable. The PHARMacist Discharge Care (PHARM-DC) study is a multi-site randomized controlled trial that seeks to evaluate the effect of pharmacist-led peri- and post-discharge interventions on 30-day hospital readmissions among older adults taking ≥10 medications or ≥3 high-risk medications. The PHARM-DC intervention includes pharmacist-led patient counseling, medication reconciliation at discharge, and a follow-up phone call post-discharge. We will highlight study protocol adaptations undertaken during the COVID-19 pandemic to address challenges to enrollment and to minimize risk of COVID-19 exposure for study participants and research personnel. Additionally, we will share insights from focus groups and semi-structured interviews with pharmacist interventionists and pharmacy leaders on barriers and facilitators to implementation due to the pandemic and strategies for future clinical trials to overcome barriers.

### CAN WE DO BETTER FOR OLDER ADULT RESEARCH PARTICIPANTS? CLINICAL TRIAL IMPROVEMENTS PROMPTED BY A GLOBAL PANDEMIC

Susy Stark, *Washington Unversity, St Louis, Missouri, United States*

Retaining older adults in clinical trials has often been a challenge for researchers. Clinical trial procedures, aimed at improving fidelity, often offer barriers to frail older adults who have challenges traveling to medical centers and enduring long clinical assessment visits. During the COVID-19 pandemic, we modified the procedures of two randomized controlled trials. COMPASS: A novel transition program to reduce disability after stroke is a clinical trial examining the efficacy of a transition home program that provides home modifications and self-management strategies compared to stroke education. HARP: Removing home hazards for older adults living in affordable housing is a pragmatic trial examining the effectiveness of a home hazard removal program for residents of low-income housing. Modifications to the trials were designed to reduce human contact but in some cases reduced the burden on trial participants. Modified procedures addressed retention, assessment of endpoints and intervention methods.

### Session 1395 (Paper)

### FAMILY CAREGIVERS

### DAUGHTERS-IN-LAW IN NEGOTIATING THE INTERGENERATIONAL CONTRACT IN RURAL CHINA: A QUALITATIVE CASE STUDY

Yong-Zhen Li, and Crystal Kwan, *The Hong Kong Polytechnic University, Kowloon, Not Applicable, Hong Kong*

Daughters-in-law play a key role in intergenerational relationships, especially in Rural China. Albeit, their voices are less heard and examined. This study explores how daughters-in-law in Rural China view and negotiate intergenerational contracts with their older adult parents-in-law. A qualitative case study design was used, and multiple data collection methods (including semi-structured interviews, observation and document review) with thematic analysis were employed. Findings highlight that daughters-in-law play a key role in shaping the intergenerational contracts between their spouse and their spouse's parents. In particular, the daughters-in-law provided instrumental support to their parents-in-law who were without self-care abilities or at risk when their adult child (the daughter-in-law's spouse) went to the city/county for work. There were also unique findings highlighting diverse negotiations of the intergenerational contract between daughters-in-law and their older adult parents-in-law. In the context of growing austerity and the current pandemic, whereby informal social supports and networks become key to older adults' wellbeing, identifying strengths and barriers of intergenerational support from daughters-in-law, is significant to support both the individual members and family wellbeing.

### DIVERSE CAREGIVERS IN THE UNITED STATES: IMPLICATIONS FOR CAREGIVING ACROSS THE LIFESPAN

C. Grace Whiting,[1] Rita Choula,[2] Yanira Cruz,[3] Lauren Pongan,[4] and Feylyn Lewis,[1], *1. National Alliance for Caregiving, Washington, District of Columbia, United States, 2. AARP Public Policy Institute, Washington D.C., District of Columbia, United States, 3. National Hispanic Council on Aging, Washington, D.C., District of Columbia, United States, 4. Diverse Elders Coalition, Diverse Elders Coalition, New York, United States*

Caregivers with diverse backgrounds make up an important part of the landscape of caregiving in the US. Their unique experiences have been traditionally under-researched in the field of social sciences and underrecognized by society. To further understand the impact of race, ethnicity, class, gender and sexuality onto caregiving, the National Alliance for Caregiving (NAC) and the AARP Public Policy Institute commissioned an in-depth analysis of the dataset collected from the Caregiving in the US 2020 study, which segments populations based on ethnic and sexual identity, geographic location, and income level. Utilizing survey interviews with 1,392 caregivers in the US, this study found differences amongst the African-American, Latinx, and Asian American-Pacific Islander populations in relation to age, time spent caregiving, ADL/IADLs, caregiving strain and intensity, receipt of formal and informal support, and financial impact. In consideration of the distinct challenges presented by diverse caregiving throughout the lifespan, this presentation will also feature results from a 2020-2021 Diverse Elders Coalition and NAC commissioned study on the unmet caregiving needs in diverse communities. 11 virtual listening sessions were held with 400 caregivers of color, including American Indian/Alaska Native caregivers, and LGBTQ caregivers across the nation. Presentation attendees can expect to learn new insights into the experiences of diverse caregivers, while also gaining a fresh understanding of informal and formal support preferences with a multicultural lens. Finally, this presentation will provide recommendations to further prioritize the needs of historically marginalized caregivers in policy and practice.

### HEALTH OF AGING FAMILIES: COMPARING COMPOUND AND NONCOMPOUND CAREGIVERS

Fei Wang,[1] Christina Marsack-Topolewski,[2] Rosanne DiZazzo-Miller,[3] and Preethy Samuel,[3] *1. Case Western Reserve University, Cleveland Heights, Ohio, United States, 2. Eastern Michigan University, Ypsilanti, Michigan, United States, 3. Wayne State University, Detroit, Michigan, United States*

Providing care to a family member with disabilities takes a toll on the caregiver and the whole family's health. Among aging caregivers, compound caregiving (i.e., caring for additional family members) has become an increasingly common scenario. However, few research studies have focused on compound caregivers. Extant literature describes individual-level outcomes, with sparse knowledge on family-level outcomes. The present study examines the differences in the individual and family health of aging compound and noncompound caregivers, using the family quality of life framework. Web-based cross-sectional data collected from 112 aging caregivers (i.e., over 50 years) was analyzed using chi-square and independent sample t-tests to examine differences between caregivers. Compound caregivers had worse perceptions of personal health ($t = -2.96$, $p = .004$, $d = -.61$) than noncompound caregivers. In terms of family health, although all caregivers shared similar perceptions on the importance, opportunities, initiative, and stability, compound caregivers had lower attainment ($t = -2.64$, $p = .009$, $d = -.44$) and satisfaction ($t = -3.90$, $p < .001$, $d = -.73$) than noncompound caregivers. Findings have practice implications for identifying caregivers' multiple responsibilities. It is necessary to develop individual and family level programs focused on health promotion and caregiving training.

### STATE MEDICAID POLICY AND LTC: A MULTI-STATE QUALITATIVE STUDY OF POLICY INFLUENCE ON CAREGIVER EXPERIENCES

Tonie Sadler,[1] Kevin Yan,[1] Daniel Brauner,[2] Harold Pollack,[3] and R. Tamara Konetzka,[3] *1. The University of Chicago, Chicago, Illinois, United States, 2. Western Michigan University, Kalamazoo, Michigan, United States, 3. University of Chicago, Chicago, Illinois, United States*

This study focuses on long-term care (LTC) state Medicaid policy and its impact on caregiver decisions and experiences. It examines respondents' general knowledge of LTC state policies and services, challenges with navigating LTC policies and services, and decision-making pathways based on these factors. Using purposive sampling, 63 family caregivers across eight states participated in open-ended qualitative interviews (2019-2020) until thematic saturation was reached. Questions broadly examined caregivers' experiences and decisions, focusing on decisions made around type of care setting and experiences with LTC state policy. States were selected to represent variation in Home and Community Based Service (HCBS) expenditures as a percentage of total Medicaid long-term services and support expenditures. While LTC policies and services vary significantly by state, we identified many parallels in caregiver experiences and perceptions across states, as respondents often lacked specific knowledge about LTC policies and services and how to access them. Overarching themes include LTC policy navigation challenges, distrust in state-funded LTC services and supports, and moral expectations of caregiving. To manage these challenges, caregivers employed coping strategies such utilizing informal support networks, hiring care coordination assistance, and "stretching things thin" to fill the policy and service gaps. Study findings highlight potential strategies to improve LTC services across states. There is a need to improve community trust with state services by employing transparent regulatory and evaluation procedures for LTC. Wider access to case management may improve communication and knowledge of available services to maximize benefit from HCBS expansions.

### TIMING AND SUPPORT MATTER: CAREGIVING DEMANDS AT THE INTERSECTION OF STRESS PROCESS AND LIFE COURSE PERSPECTIVES

Yeonjung Lee,[1] Alex Bierman,[2] and Margaret Penning,[3] *1. University of Calgary, Calgary, Alberta, Canada, 2. University of Calgary, University of Calgary, Alberta, Canada, 3. University of Victoria, Victoria, British Columbia, Canada*

Extensive research documents the outcomes of family caregiving. However, perspectives differ, with some suggesting that caregiving provides psychological rewards and others suggesting that the stress of caregiving carries psychological costs. We argue that both of these perspectives are correct, but their applicability will differ based on the timing of caregiving and the availability of social support. A life course perspective suggests that the timing of a stressor in the life course will create variations in its mental health impacts, whereas a stress process perspective suggests that the consequences of a given stressor for mental health will vary based on the availability of social support. A synthesis of these two perspectives then suggests that social support will act as stress buffer differently depending on the age of caregiver. To examine these questions, we use a subsample of respondents who reported caregiving (N=20,441) in the 1st wave of the Canadian Longitudinal Study on Aging. Analyses provide evidence of different outcomes of caregiving, according to both the timing of caregiving and the availability of support. In particular, a high level of caregiving demands are associated with greater depression and lower life satisfaction. Social support inhibits both associations, and the association between high demands and life satisfaction is stronger in older caregivers. Social support does not buffer high caregiving demands more strongly at older ages, though, showing two distinct process. Demanding caregiving appears particularly detrimental for psychological well-being as people age, and the efficacy of social support resources do not increase to compensate.

### Session 1400 (Symposium)

### FOUR APPROACHES FOR IMPLEMENTING INTERPROFESSIONAL EDUCATION

Using Microsoft Teams, the students from Psychology and marketing at Upper Iowa University, create a marketing plan focused on proposing a product or service targeting older adults. The Michigan LEND program, engages a minimum of 4 disciplines in practice online simulation approaches to respond to a case study. At Wayne State University and University of Detroit Mercy, during a zoom visit with community dwelling 50+ old adults, students from 9 disciplines collaborate on recommendations, referrals, and resources to improve health and/or quality of life. Marquette University students from 10 health professions participate in a series of four half-day workshops, designed in alignment with the Interprofessional Education Collaborative (IPEC) core competencies.

### MARKETING AND PSYCHOLOGY: AN INTERDISCIPLINARY PARTNERSHIP

Melinda Heinz, and Summer Zwanziger Elsinger, *Upper Iowa University, Fayette, Iowa, United States*

Students enrolled in Psychology of Aging and Consumer Behavior combined efforts during an 8-week course to create marketing plans focused on proposing a product or service targeting older adults. The goal of the project was to 1) infuse aging content into the curriculum 2) increase awareness of older adult consumers and 3) decrease aging stereotypes. Student teams were engaged in this project one day each week over 8 weeks. Instructors created weekly tasks to break down components of the project and each student group was required to turn in evidence of their completed task. During the 2020-2021 academic year, participants used Microsoft Teams and recorded their tasks for instructors to grade. A rubric was used to facilitate grading of weekly team tasks and similar weights/points were used in both classes to create similar levels of student "buy in." Suggested implementation tips for both face-to-face and online modalities will be discussed.

### SIMULATION APPROACH TO INTERPROFESSIONAL EDUCATION

Margaret Greenwald, *Wayne State University, Wayne State University, Michigan, United States*

A team practice simulation approach to interprofessional education is presented. Participants (79 trainees over 4 years) were assigned to one of six teams representing clinical services for a client with complex clinical needs (medical care, outpatient therapy, dental, nutrition, speech and hearing, leadership). Each student within the team was assigned a specific role (e.g., primary care, policy maker, family member). A critical component of this activity is that each participant adopted the role and perspective of an individual in a different clinical area than their own. In preparation for a live discussion by all participants, each team met to study their assigned clinical roles and to prepare a one-page slide addressing specific questions given only to their team. At the live session, the overall goal was to develop a coherent clinical plan for the client. This is an effective approach for IPE in care of clients across the lifespan.

### USING CANVAS FOR AN INTERPROFESSIONAL VIRTUAL TEAM VISIT

Ashley Reed, *Wayne State University, Detroit, Michigan, United States*

The is session will demonstrate how to use the Canvas learning management system (LMS) to organize and facilitate interprofessional education experiences (IPE) amongst students and faculty. Emphasis will be placed on the use modules as a way to organize content and facilitate requirements associated with IPE. In addition, the session will include demonstration on how to assign disciplines to sections to aide in faculty abilities to review of student submissions.

### CURRICULAR APPROACH TO IPE: PREPARING HEALTH PROFESSIONS STUDENTS TO DELIVER TEAM-BASED CARE

Stacy Barnes, and Kelly Horton, *Marquette University, Milwaukee, Wisconsin, United States*

Interprofessional education (IPE) is essential to prepare students for future healthcare careers and to meet accreditation requirements for health profession schools. After surveying successful IPE programs across the country, Marquette University developed a curricular approach. Over 1,500 students from 10 health professions (Athletic Training, Medical Laboratory Science, Counseling Psychology, Dentistry, Medicine, Nursing, Occupational Therapy, Physical Therapy, Physician Assistant Studies, Speech-Language Pathology) currently participate in a series of four interactive, half-day courses which are aligned with the Interprofessional Education Collaborative (IPEC) core competencies. Courses were moved online in response to the pandemic and are currently delivered using Microsoft Teams. Feedback from learners and faculty is gathered using post-event surveys and has been overwhelmingly positive. Learner outcomes are measured using the Interprofessional Collaborative Competencies Attainment Survey. Overall, this approach has proven to be an effective and efficient model for delivering IPE to large numbers of students.

### Session 1405 (Symposium)

### GOING VIRTUAL WHEN THE DOORS CLOSE: ADDRESSING GERIATRIC WORKFORCE TRAINING NEEDS DURING A PANDEMIC

Chair: Linda Edelman Co-Chair: Gail Towsley Discussant: Timothy Farrell

The focus of our Geriatric Workforce Enhancement Program (GWEP) is to enhance long-term services and support (LTSS) and primary care healthcare workforce capacity through interprofessional education (IPE) and to increase patient, family, and caregiver engagement. When it became evident that LTSS settings, schools, and communities were going to be adversely impacted by the COVID-19 pandemic for the unforeseeable future, our GWEP quickly pivoted to address new challenges and initiate technology to continue our programs. In this symposium, we describe four programs implemented or revised during the COVID-19 pandemic. We utilized CARES (Coronavirus Aid, Relief and Economic Security) funding to develop a 3-part Project ECHO on utilizing telehealth in LTSS settings. We pivoted quarterly Fireside Chats – community-based educational programs held at partnering LTSS settings for older adults and caregivers – to bi-weekly and now monthly webinars addressing topics relevant to COVID-19 and combatting social isolation. Because students could no longer attend an in-person IPE course introducing them to long-term care, we revised the course to be online with a partnering nursing home participating in an interactive mock care conference. Finally, a 2-semester undergraduate Honors College project-based course introducing students to successful aging utilized virtual activities to expose students to the challenges of hospice care during a pandemic. With these adaptations, as well as activities that advocated for, and supported, LTSS settings and older adults, our GWEP program was able to continue to provide education and support to the setting and individuals most impacted by COVID-19.

### VIRTUAL TRANSITIONS AND OPPORTUNITIES IN LTSS EDUCATION POST-PANDEMIC

Gail Towsley,[1] Jacqueline Telonidis,[2] Cherie Brunker,[3] and Linda Edelman,[1] *1. University of Utah College of Nursing, Salt Lake City, Utah, United States, 2. University of Utah College of Nursing, Salt lake City, Utah, United States, 3. University of Utah School of medicine, Salt Lake City, Utah, United States*

The Utah Geriatric Education Consortium Learning Community transitioned to the Age-Friendly Long-Term Services and Support (LTSS) ECHO with support from Comagine Health, our local QIN-QIO. ECHO sessions utilize case-based learning and mentorship to help community providers gain the expertise required to provide needed care and/or services to older adults. Since March 2020, and in response to the needs of our partners, four ECHO sessions (average of 47 attendees per session) have focused on COVID-19 training including COVID-19 briefings, infection prevention, positive thinking and coping with stress. With our partners, we also co-created a 3-part LTSS telehealth ECHO series to illustrate how telehealth can address the unique challenges of COVID-19. We will discuss 1) how we met the educational needs of our partners during a health crisis 2) the process we took to develop the LTSS telehealth ECHO series, and 3) opportunities for continued virtual education application.

### PIVOTING COMMUNITY-BASED EDUCATIONAL PROGRAMMING FOR FORMAL AND INFORMAL CAREGIVERS DURING COVID-19

Kara Dassel,[1] Jacqueline Telonidis,[2] Catherine Witt,[1] and Linda Edelman,[3] *1. University of Utah, Salt Lake City, Utah, United States, 2. University of Utah College of Nursing, Salt lake City, Utah, United States, 3. University of Utah College of Nursing, Salt Lake City, Utah, United States*

The Utah Geriatric Education Consortium provides community-based education about Age-Friendly Health Care and Dementia-Friendly Communities targeted towards informal and professional caregivers. As such, we have collaborated with our community partners to host a series of "Fireside Chats". Since March of 2020, we have hosted 17 Fireside Chats. Our attendance has exceeded our expectations, with over 500 attendees (average of 32 attendees per session). The professional attendees come from a variety of interdisciplinary backgrounds including nursing, medicine, public health, allied health, aging services, and health and long-term care administration. Our non-professional attendees include family caregivers, students, and older adults in the community. This session will address: a) the logistical steps we took (and lessons learned) as we "pivoted" our Fireside Chats into a virtual video-conference format, b) how we redesigned the curriculum to address topics related to COVID-19, and c) will review our evaluation feedback.

### ADAPTING A PROJECT-BASED AGING LABORATORY DURING A PANDEMIC

Troy Andersen,[1] and Linda Edelman,[2] *1. University of Utah, University of Utah College of Social Work, Utah, United States, 2. University of Utah College of Nursing, Salt Lake City, Utah, United States*

Aging Well in Utah is a competitive 2-semester project-based Honor's College Praxis Laboratory for 9 students from different degree programs dedicated to deepening understanding of the aging process through a broad gerontological lens. This session will address how the course was adapted during the COVID-19 pandemic, including: 1) scheduling virtual class times with guest lecturers; 2) conducting older adult interviews via Zoom to provide students experience in communicating "what matters most", one of the 4Ms of Age Friendly HealthCare; 3) adapting a student-designed medical narrative project highlighting stories of transition and healing through the dying process for previously unsheltered residents of a hospice program to focus on the lived experience of hospice patients during COVID-19; and 4) utilizing virtual technology to interview hospice patients, family members and hospice staff. In spite of challenges, student evaluations were above average and reported increased interest in incorporating age-friendly concepts into future careers.

### IPE IN LTC IMMERSION EXPERIENCE: CREATING A BRIDGE TO CAREERS IN LONG-TERM CARE

Megan Thomas Hebdon,[1] Christina Wilson,[2] Catherine Bernier-Carney,[3] Jacqueline Telonidis,[4] and susan Chase-Cantarini,[5], *1. University of Texas at Austin, University of Texas at Austin, Texas, United States, 2. University of Utah College of Nursing, salt lake City, Utah, United States, 3. University of Utah College of Nursing, salt Lake City, Utah, United States, 4. University of Utah College of Nursing, Salt lake City, Utah, United States, 5. University of Utah College of Nursing, Salt Lake City, Utah, United States*

The Utah Geriatric Education Consortium seeks to enhance healthcare provider workforce capacity. The purpose of our interprofessional education (IPE) in Long Term Care

(LTC) course is to enhance students' understanding of team-based care for older adults, presenting avenues for career opportunities. The course was offered via distance learning technology to adapt to the COVID-19 pandemic. Health sciences students (n=55) enrolled in the course and completed pre- and post-course work asynchronously. The course also included a two-hour virtual session in partnership with a LTC interprofessional team with a resident case study and discussion using the 4M's Framework. We will discuss the a) COVID-19 course adaptations, b) IPE case study using the 4M's Framework to help students conceptualize older adult care, and c) student pre- and post-course discussion responses demonstrating their prior experience with older adults and course impact on their views about caring for older adults in LTC settings.

### Session 1410 (Paper)

### HEALTH PROMOTION (BSS PAPER)

### DIABETES DIAGNOSIS, SOCIAL SUPPORT, AND HEALTH BEHAVIOR CHANGES IN OLDER ADULTS

Weidi Qin, *Case Western Reserve University, Cleveland, Ohio, United States*

The growing disease burden of diabetes in older adults highlights the importance of health-promoting behaviors in this population. A new diagnosis of diabetes can be a teachable moment that motivates older adults to engage in health behavior changes. Guided by the convoy model of social relations, social support from family and friends may influence health behaviors, and moderate the effects of a diabetes diagnosis on health behaviors. The current study investigates health behavior changes in drinking, smoking, and physical activity before and after a diabetes diagnosis, and whether social support moderates the relationships. A sample of 13,143 older adults without diabetes at baseline were selected from the Health and Retirement Study, and followed up for six waves. Social support from family and friends were measured separately. Mixed-effects regression models were performed. Sampling weights were adjusted to generate population estimates. After a diabetes diagnosis, older adults reduced alcohol consumption and were more likely to quit smoking. More social support from family was associated with decreased alcohol consumption, and more social support from friends was associated with increased physical activity. Significant interaction between social support from family and diabetes diagnosis was found. Specifically, among older adults with a diabetes diagnosis, more social support from family was associated with less drinking and smoking cessation. The study findings suggest that health practitioners can consider the timing of diabetes diagnosis to facilitate health behavior changes. Furthermore, diabetes educators can help older adults mobilize support from family and friends to better engage in health-promoting behaviors.

### DOES MONEY MATTER? CHARACTERISTICS ASSOCIATED WITH JOINT PAIN MEDICATION ACCESS AMONG OLDER ADULTS

Aviad Tur-Sinai,[1] Netta Bentur,[2] and Jennifer Shuldiner,[3] *1. Yezreel Valley College, Mevasseret Zion, Yerushalayim, Israel, 2. Tel Aviv University, Tel Aviv University, Tel Aviv, Israel, 3. University of Toronto, University of Toronto, Ontario, Canada*

The experience of pain is a widespread phenomenon among adults, especially older adults, and entails high costs to both individuals and society. The objective of the current research is to determine if the ability to pay and supplementary insurance are factors associated with pain medication among individuals over 50. Data came from Survey of Health, Aging and Retirement in Europe (SHARE). The sample included 64,281 individuals 50+ from nineteen European countries and Israel. Joint pain was common with one out of three reporting joint pain. Prevalence of pain was similar among different age groups, and more women reported joint pain. Among those in pain, about 21.5% of the individuals reported mild pain, 52.9% moderate and 26% severe pain. In the multivariate logistic regression, we found that men and those older than 60 suffered more from joint pain, while controlling for education and subjective assessment of the ability to cope economically (Able to make ends meet). A large percentage of those with pain were not taking medication to manage their pain, and there were significant demographic differences between those that did and did not take medication. Those that took medication were younger, male, had more education, were able to cope economically and had supplementary insurance. Our study showed that about half of the individuals with pain were not taking medication to manage their pain. Our results demonstrate that among individuals over 50 in Europe income is strongly associated with taking pain medication and that there is economic inequity in medication access.

### HOME AND COMMUNITY BASED SERVICE USE AMONG VETERANS WITH DEMENTIA LIVING IN RURAL VIRGINIA

Jyoti Savla,[1] Mamta Sapra,[2] Lauren Hagemann,[3] and Katherine Luci,[4] *1. Virginia Tech, Blacksburg, Virginia, United States, 2. Salem Veteran Affairs Medical Center, Salem VAMC, Virginia, United States, 3. Salem VAMC, salem, Virginia, United States, 4. Salem VA Medical Center, Salem, Virginia, United States*

Despite the overall expansion of rural Veteran health care facilities, older Veterans in these areas are still underserved and face challenges and barriers to access services. Using data from 60 family caregivers of persons with dementia (PwD; Mean Age = 67 years, Range = 39-84; 92% White; 71% Spouse) we examined the types of home-based and community services they utilized. We also examined reasons that family caregivers provided for not using these services. Next, we applied Andersen's Behavioral Model of Health Services to examine how predisposing factors such as demographics, available resources, and PwD's needs were associated with the use of services. We found that Veterans living in rural counties had lower access to caregiver support groups, homemaker services, adult day centers, and home-based respite services. The top three reasons for not using services were that the family caregiver chose to do it themselves, the PwD did not want the service or the service provider to help, or it was too far from the caregiver's home. Regression analysis further showed that caregivers caring for PwDs with greater ADL challenges and memory and behavior problems were more likely to need and utilize paid services. Disparities based on gender, age, and race were also explored. Findings suggest the need to develop effective service promotion strategies and destigmatizing the use of paid services among Veteran families to reduce health disparities in rural regions.

### REFLECTIONS ON IDENTITY IN MEMOIRS OF WRITERS WITH APHASIA: LESSONS LEARNED ON THE PATH TOWARD RECOVERY

Hanna Ulatowska,[1] and Gloria Olness,[2] *1. University of Texas at Dallas, Dallas, Texas, United States, 2. University of North Texas, Denton, Texas, United States*

Personal stories provide insight into the experience of illness as it intersects with one's identity. Prior studies by the first author examined identity as manifested in personal accounts of U.S. World War II veterans with and without dementia. The current study examines identity as revealed through written memoirs of middle-aged and older adults who have aphasia, from a cross-section of North American, European, and Australian cultures. The abrupt onset of stroke and associated aphasia, and the subsequent path toward re-engagement in life with an often-chronic communicative impairment, provide a unique window into the nature and evolution of the identity of the writer. The written modality offers an opportunity for reflective formulation that is not afforded to the memoir-writers in their verbal expression. Nineteen memoirs and biographical accounts of individuals with aphasia from a range of primarily individualistic cultures were examined for content reflective of the identity of the author, focused on post-stroke phases of restitution and quest. Primary authors were people with aphasia or rarely their close family member. Some were professional editors, poets or authors. Gender and life backgrounds were varietal. Manifestations of personal identity, its reinforcement, and its evolution were evidenced in: the provision of lessons learned from living with aphasia; content of letters exchanged with friends; engagement with family in life and recovery; fictional and poetic expression; spiritual insight; renewed or altered occupational pursuits; and comments on facing one's mortality. Findings hold implications for the cross-cultural practice of narrative medicine with the older adult population.

### THE PERKS OF DOING HOUSEWORK: ITS IMPACTS ON PHYSICAL HEALTH, MENTAL WELL-BEING, COGNITIVE PERFORMANCE, AND SURVIVAL

Li Chu,[1] Xianmin Gong,[2] Jennifer Lay,[3] Fan Zhang,[4] Timothy Kwok,[5] and Helene Fung,[5] *1. Stanford University, Stanford University, California, United States, 2. The Chinese University of Hong Kong, Hong Kong, Not Applicable, Hong Kong, 3. University of Exeter, Exeter, England, United Kingdom, 4. Jinan University, Jinan University, Guangzhou, Guangdong, China (People's Republic), 5. The Chinese University of Hong Kong, Shatin, N.T., Hong Kong, Not Applicable, Hong Kong*

Previous research has shown mixed results regarding the effects of doing housework. While some earlier studies have found no association between performing heavy housework and health, other studies have found various benefits of doing housework, including body leanness and lower mortality rate. This study examined the effects of housework on older adults' survival over a period of 14 years, and investigated the underlying mechanisms. A total of 2,768 older adults in Hong Kong (female: 47.29%; age: 65-98) from a longitudinal survey study were included in the current analyses. Linear regression analysis revealed that doing more housework was significantly associated with surviving more days ($\beta = 45.36$, SE = 6.40, $p < .001$). We then examined whether the association between housework and survival was mediated by physical health, mental health and/or cognitive functioning using a parallel mediation model with multiple mediators. Results showed a significant partial mediating effect of physical health ($\beta = 1.20$, SE = .53, $p = .003$), a marginally significant partial mediating effect of cognitive functioning ($\beta = 1.35$, SE = .70, $p = .054$) and no mediating effect of mental health. All the analyses remained consistent after controlling for sex, education, marital status, subjective social status and living arrangement. These results suggest that doing housework may benefit survival by improving physical and cognitive functioning. Our findings have implications for better understanding factors that influence mortality, developing accessible physical activity interventions for older adults, and supporting aging in place.

### Session 1415 (Symposium)

### HETEROGENEITY IN VULNERABILITY AND RESILIENCE AMONG CENTENARIANS

Chair: Daniela Jopp Co-Chair: Charikleia Lampraki
Discussant: Dario Spini

Given their exceptional longevity, centenarians have long been considered as examples of successful aging. Yet, with increases in empirical studies, findings suggest that they may show vulnerability and resilience at the same time. This symposium offers a more in-depth perspective on both constructs in centenarians. Zaccaria and colleagues investigated the link between social isolation and loneliness within the Fordham Centenarian Study. Results indicate the existence of four subgroups combining expressions of isolation and loneliness, suggesting different vulnerability patterns in centenarians. Uittenhove and colleagues analyzed patterns of coping strategies in the Second Heidelberg Centenarian Study. Cluster analysis identified two coping profiles, one characterized by a wide coping repertoire including problem-directed and internal strategies, while the other showed low problem-solving. Lampraki and Jopp examined the effects of (lacking) resources and psychological strengths (optimism) on depressive symptoms in the Fordham Centenarian Study. Findings suggest that the effect of resources is mediated by psychological strengths, demonstrating their beneficial value in very old age. Jopp and colleagues report findings from the ongoing SWISS100 Study. Based on telephone interviews conducted during the COVID-19 pandemic, they found that centenarians did not feel vulnerable. While half of the centenarians and their proxies reported no changes in everyday life, the other half experienced substantial challenges due to lack of activities and absence of social contacts due to governmental regulations. In sum, centenarians are vulnerable and resilient at the same time, highlighting the future research needs on its predictors, and the application of this knowledge within the context of crisis.

### LONELINESS AND SOCIAL ISOLATION AMONG CENTENARIANS AND NEAR-CENTENARIANS: RESULTS FROM THE FORDHAM CENTENARIAN STUDY

Daniele Zaccaria,[1] Stefano Cavalli,[2] Barbara Masotti,[1] and Daniela Jopp,[3] *1. University of Applied Sciences and Arts of Southern Switzerland, Manno, Ticino, Switzerland, 2. University of Applied Sciences and arts of southern switzerland, Manno, Ticino, Switzerland, 3. University of Lausanne, Lausanne, Vaud, Switzerland*

Although loneliness and social isolation are often discussed together, they are mainly examined separately. The few studies examining both concepts simultaneously focus usually on the wider category of older people (65+), with no or little attention to very old age. Our main aim was to investigate loneliness and social isolation in combination among near-centenarians and centenarians. Analyzing data from the Fordham Centenarian Study (N=94; MAge=99.2; range=95-107), we found no or very weak associations between loneliness and social isolation. Combining measures of loneliness (UCLA Loneliness scale) and social isolation (Lubben Scale) we built a typology with four different groups (Not lonely or isolated; Lonely and isolated; Lonely but not isolated; Isolated but not lonely). The factors that most strongly predicted the distribution among these four groups were gender, widowhood, education, and self-rated health. Findings highlight the importance of jointly studying both concepts to better understand social risks in very old age.

### COPING IN CENTENARIANS: PATTERNS AND CORRELATES

Kim Uittenhove,[1] Daniela Jopp,[2] and Kathrin Boerner,[3] *1. UNIL, Lausanne, Vaud, Switzerland, 2. University of Lausanne, Lausanne, Vaud, Switzerland, 3. University of Massachusetts Boston, Boston, Massachusetts, United States*

Coping strategies are a source of resilience, yet little is known about their use in centenarians. We examined patterns in coping strategy use and determined how these patterns were associated with characteristics such as personality, cognitive status, quality of life, and health. We analyzed data from the Fordham Centenarian Study (N = 119), where centenarians responded to 40 items covering 10 coping dimensions (e.g., active problem-solving, support seeking, reappraisal). Findings revealed two clusters which differed in amount and strategy types: One was characterized by high use of many strategies which addressed the problem and its appraisal. The other was characterized by a smaller strategy repertoire, with very limited use of problem-focused strategies. The more varied and problem-focused coping pattern was associated with other characteristics, such as personality (e.g., extraversion) and quality of life (e.g., well-being). Findings suggest variation in coping profiles associated with resilience in centenarians.

### BASIC RESOURCES AND PSYCHOLOGICAL STRENGTHS AS PREDICTORS OF DEPRESSIVE SYMPTOMS

Charikleia Lampraki, and Daniela Jopp, *University of Lausanne, Lausanne, Vaud, Switzerland*

Loss of personal resources is expected to have a negative effect on well-being in all ages, however, in very old age, this effect may be exacerbated. Centenarians, who are confronted with accumulated age-related losses, may be at higher risk of experiencing depressive symptoms. This study investigated the link between basic resources (i.e., health, social network) and depressive symptoms and whether it was mediated by psychological strengths (i.e., meaning, optimism) in 119 centenarians and near-centenarians (Mage = 99.7 years). Results indicated that meaning in life fully mediated the link between health and depressive symptoms, and the link between social network size and depressive symptoms. Similarly, optimism mediated the link between network and depression, but no mediation effect was found when considering health as basic resource. In sum, basic resources are only indirectly associated to depressive feelings, with psychological strengths playing an important intervening role in very old age.

### CENTENARIANS' EXPERIENCE OF THE COVID PANDEMIC IN SWITZERLAND

Daniela Jopp,[1] Stefano Cavalli,[2] Armin von Gunten,[3] François Herrmann,[4] Carla Gomes Da Rocha,[5] Garnelle Ziade,[6] and Kim Uittenhove,[7] *1. University of Lausanne, Lausanne, Vaud, Switzerland, 2. University of Applied Sciences and arts of southern switzerland, Manno, Ticino, Switzerland, 3. CHUV - University Hospital of Vaud, Lausanne, Vaud, Switzerland, 4. University of Geneva, Geneva, Geneve, Switzerland, 5. HES-SO Valais Wallis, Sion, Valais, Switzerland, 6. University of Lausanne, lausanne, Vaud, Switzerland, 7. UNIL, Lausanne, Vaud, Switzerland*

Being considered as individuals with elevated risk of severe health reactions to the COVID19 infections, governments around the world have put in place wide-ranging measures to protect very old individuals from the virus. In the present study, we investigated centenarians' experience of the COVID19 pandemic, to reach a better understanding of their vulnerability and resilience. As part of the SWISS100 study, we conducted telephone interviews with 30 centenarians and 40 family members. While almost all centenarians felt not, qualitative data suggested the existence of two groups: One included centenarians lived rather withdrawn and isolated before the crisis and therefore did not experience major changes. The other group included centenarians who suffered substantially from no longer being able to see family and friends and missed valued activities. Family members reported challenges, including centenarians' decline in mental and physical health. Findings highlight the importance of different vulnerability profiles and lock-down side effects.

### Session 1420 (Symposium)

### IMPLICATIONS OF THE COVID-19 PANDEMIC ON ADULT DAY SERVICES

Chair: Katherine Marx Co-Chair: Laura Gitlin Discussant: Tina Sadarangani

Adult day service (ADS) centers serve an important role in care provision for people living with dementia (PLWD). These programs provide socialization, activities, and access to many therapies for PLWD. Additionally, they offer respite to family caregivers so they can work, run errands, and recharge. In March 2020, like much of the nation, ADS sites across the nation were shut down. This left many PLWD and their caregivers without access to the services they depended on to manage day to day care. It also left many sites without a revenue source to pay employees and maintain buildings. Almost a year later, many states have still not reopened ADS and sites that have reopened have done so with a lower census, increased costs, and the lingering fear of a second closure. Much focus has been on the care of older adults in nursing homes or other residential long-term care settings but the challenges of ADS and the people they serve has been mostly ignored. The purpose of this symposium is to highlight the implications of the COVID-19 pandemic on ADS centers. Holly Dabelko-Schoeny will present data gathered from ADS Centers across Ohio. Lauren Parker, will then present data from ADS sites across the United States that examines the effect of COVID-19 on closures and programming during the closures. Katherine Marx will present the effect of ADS closures on family caregivers of persons living with dementia. Finally, Joseph Gaugler will explore this from a policy perspective and provide recommendations moving forward.

### CARING FOR A FAMILY MEMBER LIVING WITH DEMENTIA WHEN ADULT DAY SERVICES CLOSE

Katherine Marx,[1] Lauren Parker,[2] Joseph Gaugler,[3] Holly Dabelko-Schoeny,[4] and Laura Gitlin,[5], *1. Johns Hopkins School of Nursing, Baltimore, Maryland, United States, 2. Johns Hopkins Bloomberg School of Public Health, Baltimore, Maryland, United States, 3. University of Minnesota, Minneapolis, Minnesota, United States, 4. The Ohio State University, The Ohio State University, Ohio, United States, 5. Drexel University, College of Nursing and Health Professions, Drexel University, Pennsylvania, United States*

Adult Day Service (ADS) centers play an important role in community services that help families keep a person living with dementia (PLWD) at home. We interviewed 33 family caregivers about their experience during the COVID-19 Pandemic and the shutdown of the ADS centers where the PLWD attends. All 33 (100%) reported that the ADS center was shut for a period of time (range: 2 weeks – remain closed). Caregivers reported a decline in their physical health (33%,n=11) and mental health (52%,n=17) and an increase in feelings of loneliness (48%,n=16). For the PLWD, the caregivers noted, a decline in physical (48%,n=16) and mental (55%,n=18) health and an increase in behaviors (39%,n=13). The shutdown of most ADS centers across the country due to the COVID-19 pandemic has had implications not only for the ADS sites but for the families that entrust them with the care for a family member.

### THE IMPACT OF COVID-19 ON ADULT DAY SERVICES' CLOSURES AND PROGRAMMING

Lauren Parker,[1] Katherine Marx,[2] Joseph Gaugler,[3] and Laura Gitlin,[4] *1. Johns Hopkins Bloomberg School of Public Health, Baltimore, Maryland, United States, 2. Johns Hopkins School of Nursing, Baltimore, Maryland, United States, 3. University of Minnesota, Minneapolis, Minnesota, United States, 4. Drexel University, College of Nursing and Health Professions, Drexel University, Pennsylvania, United States*

Nationally, adult day services (ADS) were forced to closed due to the COVID-19 pandemic. The forced closure of ADS programming consequentially impacted the services provided to clients. Many ADS continued to provide telephonic/remote services to clients, despite limited reimbursement from national and state sources for these services. Using data from ADS sites participating in the ADS-Plus Program (n= 22), this presentation examines the effects of COVID-19 on ADS closures and programming provided during the closure. About 86% (n=19) of the centers reported having to closed due to COVID-19. One-hundred percent of the sites reported offering telephone support to clients. Nearly 45% (n=10) of the centers reported not being reimbursed for this service. As ADS is a vital community-based resource for many families, it is important to demonstrate the crucial services provided by ADS to inform policymakers of the essentiality of day centers.

### ADULT DAY SERVICES AND COVID: A CRISIS IN OHIO

Holly Dabelko-Schoeny,[1] Susan Wallace,[2] and Salli Bolin,[3] *1. The Ohio State University, The Ohio State University, Ohio, United States, 2. LeadingAge Ohio, Columbus, Ohio, United States, 3. MemoryLane Care Services, Toledo, Ohio, United States*

An Ohio Executive Order forced adult day service providers across the state to close from March 24, 2020 until September 21, 2020 due to COVID, resulting in significant hardship for providers and families. In fact, 65% of programs reported laying off or reducing staff and 83% of directors reported participants had to move to higher and more expensive levels of care such as nursing homes and assisted living. Programs reported that 74% of caregivers had to choose between working and taking care of their family members. Ninety-one percent of ADS program directors in Ohio reported their caregivers were experiencing an increase in stress and anxiety. This paper explores the experiences of Ohio adult day providers during the COVID epidemic, and identifies the challenges and opportunities to coalition building to educate policy makers about day services and the crucial care centers provide.

### ADULT DAY SERVICES AS AN ESSENTIAL SERVICE AND SUPPORT

Joseph Gaugler,[1] Katherine Marx,[2] Holly Dabelko-Schoeny,[3] Lauren Parker,[4] Keith Anderson,[5] Elizabeth Albers,[1] and Laura Gitlin,[6], *1. University of Minnesota, Minneapolis, Minnesota, United States, 2. Johns Hopkins School of Nursing, Baltimore, Maryland, United States, 3. The Ohio State University, The Ohio State University, Ohio, United States, 4. Johns Hopkins Bloomberg School of Public Health, Baltimore, Maryland, United States, 5. University of Texas at Arlington, Arlington, Texas, United States, 6. Drexel University, College of Nursing and Health Professions, Drexel University, Pennsylvania, United States*

Throughout the COVID-19 pandemic, the significant challenges and gaps related to the care of older people in the U.S. were made distressingly apparent. This summary presentation will consider the effects of COVID-19 and associated shutdowns on older persons who use ADS programs, their family caregivers, and programs/staff themselves. Among recommendations to consider are the classification of adult day services and similar community-based long-term care providers as essential (and clarifying their difference from senior centers). In addition, considering new financing approaches and utilizing ADS or similar community-based programs as incubators of evidence-based innovation are options to consider to better align ADS with optimal dementia care.

### Session 1425 (Symposium)

### INNOVATIVE POPULATION AND INTERVENTION RESEARCH FOR LGBTQ+ OLDER ADULTS WITH DEMENTIA IN A COVID WORLD

Chair: Charles Emlet Co-Chair: Karen Fredriksen Goldsen

This past year, the lives of vulnerable older adults, including those within the older LGBTQ+ community have been disrupted dramatically, as has the research agendas designed to improve their lives. Older people, including LGBTQ+ older adults with dementia, have been placed at increased risk for social isolation and mental health issues during COVID, making viable interventions even more crucial. Additionally, how research is conducted within these communities needed to be adjusted in order to preserve viability. This symposium draws upon data from the National Health Aging and Sexuality/Gender study, the first longitudinal study of LGBTQ+ older adults in the United States, as well as data from Aging with Pride: IDEA (Innovations in Dementia Empowerment and Action), the first randomized controlled trial (RCT) designed to improve quality of life of LGBTQ+ adults living with dementia and their care partners. (1) Kim and Fredriksen Goldsen examine modifiable behavioral and social factors that can improve quality of life among LGBTQ+ older adults with cognitive impairment. (2) Fredriksen Goldsen, Teri, Emlet and colleagues present initial efficacy findings from the IDEA study and how the intervention needed to be altered to be viable in a COVID world. (3) The importance of Motivational Interviewing (MI) as part of a LGBTQ+ sensitive intervention designed for LGBTQ+ older adults with dementia and their care partners is discussed by Petros, Fredriksen Goldsen and Teri. As COVID continues to impact vulnerable populations as well as research and service delivery, identifying new and innovative strategies will become increasingly important.

### THE FIRST INTERVENTION STUDY FOR LGBTQ+ OLDER ADULTS WITH DEMENTIA AND CAREGIVERS: COVID-19 LESSONS LEARNED

Karen Fredriksen Goldsen,[1] Linda Teri,[1] Hyun-Jun Kim,[2] Charles Emlet,[3] Ryan Petros,[1] Charlotte Brown,[1] Glenise McKenzie,[4] and David La Fazia,[1] *1. University of Washington, Seattle, Washington, United States, 2. School of Socal Work, Seattle, Washington, United States, 3. University of Washington, Tacoma, Washington, United States, 4. Oregon Health & Science University, Portland, Oregon, United States*

LGBTQ+ older adults face significant health disparities with higher rates of cognitive impairment and social isolation. Yet, the cognitive health needs of LGBTQ+ adults and caregivers have not been adequately addressed in clinical trials and services. In this presentation, we will share findings from Aging with Pride: IDEA (Innovations in Dementia Empowerment and Action), the first randomly controlled trial (RCT) intervention study designed to improve quality of life of LGBTQ+ adults living with dementia and caregivers, and to reduce institutionalization. In this presentation, we will share preliminary efficacy findings, the effectiveness of culturally responsive approaches, and Covid-19 adaptations, including delivery by virtual chat rather than in-home, technology training, ensuring safety of virtual intervention components, and providing on-going technology support. Preliminary findings suggest a higher intervention retention rate in the virtual delivery as compared to in-home. This study illustrates innovative ways to serve disadvantaged communities in dementia care and aging services.

### MODIFIABLE FACTORS OF HEALTH-RELATED QUALITY OF LIFE AMONG LGBTQ OLDER ADULTS LIVING WITH COGNITIVE IMPAIRMENT

Hyun-Jun Kim,[1] and Karen Fredriksen Goldsen,[2]
*1. School of Socal Work, Seattle, Washington, United States, 2. University of Washington, Seattle, Washington, United States*

Cognitive impairment can lead to significant decline in health-related quality of life (HRQOL) in later life especially among those who are socially marginalized. While Lesbian, gay, bisexual, transgender, and queer (LGBTQ) older adults are documented to be at heightened risks of cognitive impairment, they may face unique challenges due to discrimination, social isolation, and other LGBTQ-related risks. This study examined factors associated with psychological and physical HRQOL among LGBTQ adults aged 50 and older analyzing a sub-set of longitudinal data (N = 646) from National Health, Aging, and Sexuality/Gender Study: Aging with Pride. Lifetime LGBTQ discrimination and victimization and insufficient food intake were negatively, and physical and leisure activities were positively associated with both HRQOL dimensions. Community engagement, social support, and social activities were positively associated with psychological HRQOL. Culturally responsive interventions addressing these modifiable factors are needed to improve HRQOL of this socially marginalized but resilient population.

### AGING WITH PRIDE: CULTURAL RELEVANCE OF MOTIVATIONAL INTERVIEWING FOR LGBTQ ADULTS WITH DEMENTIA

Ryan Petros, Karen Fredriksen Goldsen, and Linda Teri,
*University of Washington, Seattle, Washington, United States*

LGBTQ adults disproportionately experience dementia and are more likely to rely on informal social support to meet care needs in the community compared to cisgender, heterosexual peers. Culturally responsive interventions will accommodate the unique strengths and independence of this population and support self-determination as they navigate reduced capacity for self-care, increased need for support, and changes to autonomous, independent decision-making.

Motivational Interviewing (MI) is a culturally responsive approach to treatment, amenable to integration with other evidence-based practices (EBP), and is especially relevant for the LGBTQ community for its compatibility with self-determination theory. This paper describes the unique contributions of MI that resulted in culturally relevant adaptations to a leading EBP for dementia and culminated in an efficacious intervention (Aging with Pride: IDEA) that is compatible with self-determination theory, designed for individuals and care providers in the LGBTQ community affected by dementia, and adapted for online delivery during the COVID pandemic.

### Session 1430 (Symposium)

### MANY FACES OF SLEEP: DIVERSE SLEEP CHARACTERISTICS AND THEIR JOINT ASSOCIATIONS WITH STRESS ON HEALTH IN ADULTHOOD

Chair: Hye Won Chai Co-Chair: Soomi Lee Discussant: Nancy Sin

Two separate bodies of literature point to the significant roles of sleep and stress and their associations with health outcomes in adulthood. To further extend the field's knowledge on sleep, stress, and health, it is essential to consider the multi-dimensional aspects of sleep and diverse stress contexts and identify ways in which the three factors are interrelated to each other. Different sleep characteristics may have varying implications for stress processes that, in turn, shape health outcomes. Therefore, this symposium integrates diverse characteristics of sleep (duration, quality, and pileup) in conjunction with various stress processes and experiences (bereavement, stressor exposure and appraisals, rumination), and examines their associations with cognitive, emotional, and physiological health outcomes. The first paper by Vigoureux and colleagues investigates the interaction between daily sleep and stressor frequency and severity on daily rumination. The second paper by Slavish and colleagues examines the bidirectional associations between daily stress and sleep duration and efficiency. The third paper by Mu and colleagues explores the mediated associations of sleep quality and sufficiency with work impairment through perceived cognitive abilities and rumination. The fourth paper by Lee uses the stress concept of pileup and tests how pileup of insufficient sleep is associated with day-to-day trajectories of affective and physical well-being. The final paper by Chai and colleagues examines whether sleep quality moderates the association between family bereavement and heart rate variability. The discussant, Dr. Nancy Sin, will integrate key points and discuss considerations for mechanisms and long-term implications of sleep, stress, and health.

### SLEEPING IT OFF: SLEEP QUALITY MODERATES THE ASSOCIATION BETWEEN FAMILY BEREAVEMENT AND HEART RATE VARIABILITY

Hye Won Chai,[1] Dylan Jester,[2] Soomi Lee,[3] Susanna Joo,[4] Debra Umberson,[1] and David Almeida,[5] *1. The University of Texas at Austin, Austin, Texas, United States, 2. School of Aging Studies, University of South Florida, Tampa, Florida, United States, 3. University of South Florida, Tampa, Florida, United States, 4. Yonsei University, Seodaemun-gu, Seoul-t'ukpyolsi, Republic of Korea, 5. Pennsylvania State University, University Park, Pennsylvania, United States*

While previous studies evince a strong link between family bereavement and worse cardiovascular functioning, factors that may influence the association remain unexplored. This study examined the relation between experiencing the death of an immediate family member and heart rate variability (HRV) and whether the associations differed by sleep quality. The sample included respondents from the Midlife in the United States (MIDUS) Biomarker Project who reported losing an immediate family member – parents, spouse, siblings, or children – within a year before project (n = 94) and those who did not experience any deaths (n = 872). Results showed that the death of a family member was associated with worse HRV only among those who reported having a poor sleep quality and not for those with good sleep quality. These findings suggest that poor sleep quality may indicate psychophysiological vulnerability for those who experienced the death of an immediate family member.

### PILEUP OF INSUFFICIENT SLEEP AND DAY-TO-DAY TRAJECTORIES OF AFFECTIVE AND PHYSICAL WELL-BEING

Soomi Lee, *University of South Florida, Tampa, Florida, United States*

This study examined whether and how pileup of insufficient sleep is associated with day-to-day trajectories of affective and physical well-being. Participants from the Midlife in the United States Study (N=1,795) provided diary data for eight days. Pileup of insufficient sleep was operationalized as the number of consecutive nights with <6 hours of sleep. Multilevel models evaluated the linear, quadratic, and cubic effects of pileup of insufficient sleep on daily well-being, adjusting for sociodemographic covariates. Daily negative affect increased and positive affect decreased in curvilinear fashion as the pileup of insufficient sleep increased. For example, daily negative affect increased, but the rate of increase decelerated as the pileup of insufficient sleep increased. In the days most distal to baseline, the rate of increase in negative affect accelerated again. Results were consistent for physical symptoms. Findings suggest that making efforts to break the vicious cycle of insufficient sleep may protect daily well-being.

### ASSOCIATIONS OF DAILY SLEEP AND STRESS WITH RUMINATION: AN ECOLOGICAL MOMENTARY ASSESSMENT AND ACTIGRAPHY STUDY

Taylor Vigoureux, and Soomi Lee, *University of South Florida, Tampa, Florida, United States*

Unconstructive repetitive thoughts are indicative of rumination about daily experiences. Given that poor sleep is associated with greater reactivity to daily stressors, we examined joint associations of daily sleep and stress with daily rumination. 143 nurses completed 14 days of ecological momentary assessments (EMA; assessments of daily sleep, stress, and rumination) and actigraphy. After controlling for age, sex, education, income, inpatient vs. outpatient nurse, workday, and day vs. night shift, there were significant joint associations of sleep and stress with daily rumination. Daily rumination was lowest when lower (-1SD) stressor severity or stressor frequency were paired with longer (+1SD) EMA or actigraphy sleep duration. Daily rumination was highest when higher stressor frequency was paired with higher actigraphy wake after sleep onset (i.e., poorer sleep quality). Future studies should assess whether rumination about daily experiences is associated with quality of patient care provided by nurses in a hospital setting.

### DAILY STRESS AND SLEEP ASSOCIATIONS VARY BY WORK SCHEDULE: A BETWEEN- AND WITHIN-PERSON ANALYSIS IN NURSES

Danica Slavish,[1] Jessica Dietch,[2] Heidi Kane,[3] Joshua Wiley,[4] Yang Yap,[4] Kimberly Kelly,[1] Camilo Ruggero,[1] and Daniel Taylor,[5] *1. University of North Texas, Denton, Texas, United States, 2. Oregon State University, Corvallis, Oregon, United States, 3. University of Texas at Dallas, Richardson, Texas, United States, 4. Monash University, Clayton, Victoria, Australia, 5. University of Arizona, Tucson, Arizona, United States*

Nurses experience poor sleep and high stress due to demanding work environments. Night shift work may exacerbate stress-sleep associations. We examined bidirectional associations between daily stress and sleep, and moderation by shift worker status and daily work schedule. 392 nurses (92% female, mean age = 39.54) completed 14 days of sleep diaries and actigraphy, plus daily assessments of stress and work schedule upon awakening. Nurses were classified as recent night shift workers if they worked 1+ night during the past 14 days. Greater daily stress predicted shorter diary sleep duration and lower diary sleep efficiency. Shorter diary and actigraphy sleep duration and lower diary sleep efficiency predicted higher next-day stress. Compared to recent night workers, day workers had higher stress after nights with shorter sleep. Associations did not vary by daily work schedule. Sleep disturbances and stress may unfold in a toxic cycle and are prime intervention targets among nurses.

### SUBJECTIVE AND PERSEVERATIVE COGNITION MEDIATE THE RELATIONSHIP BETWEEN SLEEP AND WORK IMPAIRMENT

Christina Mu, Brent Small, and Soomi Lee, *University of South Florida, Tampa, Florida, United States*

The study examined the mediating role of subjective and perseverative cognition on sleep and work impairment. Sixty nurses completed a background survey and 14-days of ecological momentary assessments (EMA) and sleep actigraphy. Each day, participants evaluated their subjective cognition (mental sharpness, memory, processing speed), perseverative cognition (rumination) and work impairment (how much did you cut back on normal paid work, how much did the quality of your work suffer). Multiple sleep characteristics were measured by EMA and actigraphy. Multilevel mediation models adjusted for sociodemographics and work shift. At the between-person and within-person levels, there were mediated associations of sleep quality and sufficiency (but not actigraphy-measured sleep) with work impairment through subjective and perseverative cognition. Better sleep quality or higher sleep sufficiency were associated with better subjective and perseverative cognition, which, in turn, were associated with less work impairment.

### Session 1435 (Paper)

### MILD COGNITIVE IMPAIRMENT

### ACCEPTANCE OF TELE-TECHNOLOGY-BASED MIND-BODY CLASSES FOR INDIVIDUALS WITH MILD COGNITIVE IMPAIRMENT

Kara Cohen,[1] Patricia Griffiths,[2] and Tracy Mitzner,[3] *1. Georgia Institute of Technology, Atlanta, Georgia, United States, 2. GA Tech, Atlanta, Georgia, United States, 3. Georgia Institute of technology, Atlanta, Georgia, United States*

Individuals with Mild Cognitive Impairment (MCI) face many challenges, including cognitive declines and reduced independence which are associated with poor health outcomes. Although there is no cure for MCI, mind-body exercise classes may improve cognitive function and reduce risk of falls (Wayne, Yeh, & Mehta, 2018). However, such classes are often not accessible for individuals with MCI due to lack of transportation, fear of being stigmatized, or inability to find instructors who have experience working with individuals with MCI (Hobson & Middleton, 2008; Rimmer, 2005). Tele-technology, such as video-conferencing software, has the potential to remove barriers to participation by allowing individuals to attend classes from home. The goal of this study was to assess the feasibility of using tele-technology to deliver mind-body classes to individuals with MCI. We evaluated technology acceptance and usability for OneClick.chat, a web-based video-conferencing platform designed for older adults. Stakeholders (4 subject matter experts, 2 individuals with MCI, and 2 care partners) participated in a user study that included questionnaires and a short interview. The technology acceptance data indicate that OneClick.chat was perceived as easy to use. Some individuals expressed privacy and security concerns which could be addressed with additional education and support. These findings have implications for interface design, education, and training for deployment of tele-technology delivered mind-body classes for those with MCI.

### BODY MASS INDEX TRAJECTORY AND INCIDENT MILD COGNITIVE IMPAIRMENT AMONG AFRICAN AMERICAN OLDER ADULTS

Adrienne Aiken Morgan,[1] Ana Capuano,[2] Robert Wilson,[2] and Lisa Barnes,[3] *1. North Carolina A&T State University, Greensboro, North Carolina, United States, 2. Rush, Chicago, Illinois, United States, 3. Rush University Medical Center, Chicago, Illinois, United States*

Previous research suggests a decline in body mass index (BMI) among older adults is associated with negative health outcomes, including mild cognitive impairment (MCI) and incident dementia (Gao et al., 2011). However, few studies have examined BMI longitudinal trajectories and how they change after MCI diagnosis among older African Americans. To characterize trajectories of change in BMI among older African American participants with no cognitive impairment at baseline we used data from the Minority Aging Research Study, MARS (N=408, 76.5% women, mean age = 73.5, mean education = 15.0). We constructed piecewise linear mixed-effects models that included a random intercept and two random slopes. The first slope began at baseline. The second slope began at MCI diagnosis allowing for acceleration in the rate of decline after the diagnosis. The results showed BMI declined over time (B=-0.19, SE=0.04, p<.001), and there was a faster decline after MCI (additional decline, B=-0.18, SE=0.068, p=.007). In a second model controlling for age, higher education was associated with a lower BMI at baseline (B=-0.36, SE=0.092, p<.001) but slower decline before MCI (B=0.02, SE=0.006, p=.001). However, after MCI the decline of participants with higher education was faster (B=-0.06, SE=0.022, p=.003). These results suggest an accelerated decline in BMI following MCI diagnosis, with higher education related to an even faster BMI decline, possibly a consequence of cognitive reserve.

### EARLY VERSUS LATE MILD COGNITIVE IMPAIRMENT: NEURAL EVENT-RELATED OSCILLATIONS DURING A GO/NO GO TASK

Elizabeth Lydon,[1] Lydia Nguyen,[2] Shraddha Shende,[1] Hsueh-Sheng Chiang,[3] and Raksha Mudar,[4] *1. University of Illinois Urbana-Champaign, Champaign, Illinois, United States, 2. iN2L, Denver, Colorado, United States, 3. UT Southwestern Medical Center, UT Southwestern Medical Center, Texas, United States, 4. University of Illinois-Urbana Champaign, Champaign, Illinois, United States*

Amnestic mild cognitive impairment (aMCI) is marked by episodic memory deficits, which is used to classify individuals into early MCI (EMCI) and late MCI (LMCI). Growing evidence suggests that individuals with EMCI and LMCI differ in other cognitive functions including cognitive control, but these are less frequently studied. Using a semantic Go/NoGo task, we examined differences in cognitive control between EMCI and LMCI on behavioral (accuracy and reaction time) and neural (scalp-recorded event-related oscillations in theta and alpha band) measures. Although no behavioral differences were observed between the groups, EMCI and LMCI groups differed in patterns of neural oscillations for Go compared to NoGo trials. The EMCI group showed differences in theta power at central electrodes and alpha power at central and centro-parietal electrodes between Go and NoGo trials, while the LMCI group did not exhibit such differences. Furthermore, the LMCI group had higher theta synchronization on Go trials at central electrodes compared to the EMCI group. These findings suggest that while behavioral differences may not be observable, neural changes underlying cognitive control processes may differentiate EMCI and LMCI stages and may be useful to understand the trajectory of aMCI.

### PAIN PERSISTENCE IS ASSOCIATED WITH INCREASED ODDS OF MCI IN LATE MIDLIFE AND EARLY OLDER ADULTHOOD

Tyler Bell, Jeremy Elman, Carol Franz, and William Kremen, *University of California San Diego, La Jolla, California, United States*

Twenty percent of older adults will experience persistent pain, the sensation of bodily harm lasting three or more months. Persistent pain doubles the risk of dementia, but we know less about the impact on earlier stages, such as mild cognitive impairment (MCI). As a step for clarification, this study leveraged data from the Vietnam Era Twin Study of Aging (VETSA) to understand how pain persistence relates to MCI in late midlife to early older adulthood. Participants (n=1,465, 100% male) were recruited across three waves at average ages 56, 62, and 68. At each wave, participants completed the SF-36 and were asked to rate their pain intensity from none (1) to very severe (6). Clinical pain was coded as pain intensity rated more than mild (>3/6). As a time-varying predictor, pain persistence was then calculated as a running frequency of the total waves reporting clinical pain. MCI diagnosis was based on Jak-Bondi criteria. Age, depressive symptoms, comorbidities, and opioid use were included as time-varying covariates. Age and education were included as time-invariant covariates. General estimating equations showed that pain persistence over two waves, reported in 35% of the sample, increased MCI odds by 57% (OR=1.57, 95%CI: 1.28 to 1.94). Pain persistence over three waves, reported in 17% of the sample, increased MCI odds by 98% (OR=1.98, 95%CI: 1.44 to 2.70). The findings emphasize the role of pain in earlier stages of dementia and the potential importance of pain management in offsetting cognitive decline.

### Session 1440 (Symposium)

### NEW DIRECTIONS IN DIETARY RESTRICTION: REMEMBERING EDWARD MASORO

Chair: Arlan Richardson

In 1935, Clive McCay reported that severe restriction of food increased the lifespan of male rats. In the following four decades, several laboratories replicated this observation with less sever restrictions, which will be referred to as dietary restriction (DR). However, there were concerns even in the aging community in the 1970s as to whether DR increased lifespan by retarding aging. It was the research of two former Kleemeier Awardees, Edward Masoro and Roy Walford, that conclusively demonstrated in the 1980s that DR retarded aging resulting in improved healthspan and reduced pathology. Ed Masoro's research was focused on lipid metabolism when he was invited to attend a workshop on metabolism and aging in 1969. His interest in aging was piqued such that the more he learned about aging, the more interested he became. In a subsequent workshop in 1973, Ed heard Morris Ross describe his research on restricting food intake on cancer and longevity. Ed was impressed that a relatively simple manipulation had such dramatic effects, and he decided to focus his research on DR. After an extensive review of the DR literature up to the 1970s, Ed established the 40% restriction paradigm, which is used in almost all DR studies to date. Ed's group was the first to study aging and DR under barrier conditions which he established at San Antonio. Over the next two decades, Ed would direct a Program Project that showed DR had a dramatic effect on most age-related pathologies and improved many physiological functions. Studying the restriction of fat, protein, micronutrients, Ed came to the conclusion that total calories consumed was a key factor in the effect of DR on longevity. His group was the first to show that DR significantly reduced circulating levels of glucose and insulin, which was subsequently shown to occur because of increased insulin sensitivity and is now recognized as a hallmark of DR and potentially important in the anti-aging action of DR. Ed was chair of the Biological Sciences Section of GSA in 1979 and President in 1995. This session is dedicated to Edward Masoro who passed away on July 11, 2020 at the age or 95.

Dr. Masoro was president in 1995 and BS chair in 1979, Clive McCay was President in 1949.

### INTERMITTENT FASTING: FROM CALORIES TO TIME RESTRICTION

Rafael de Cabo, *NIA, Baltimore, Maryland, United States*

Classic implementation of calorie restriction (CR) in laboratory animals increases health and longevity in most model organisms. Traditionally, chronic CR is the reduction of daily energy intake without malnutrition. Recently, paradigms have emerged that recapitulate some of the beneficial aspects of this intervention, avoiding some of its challenges. The length of daily fasting length and periodicity have emerged as potential drivers behind CR's beneficial health effects. Numerous strategies and eating patterns, including prolonged periods of fasting, have been successfully developed to mimic many of CR's benefits without its austerity. These new feeding protocols range from short mealtimes designed to interact with our circadian system (daily time-restricted feeding) to more extended fasting regimens known as intermittent fasting. We will discuss the current status of knowledge on different strategies to reap the benefits of CR on metabolic health in rodent models and humans without the rigor of chronic reductions in caloric intake.

### IMPACT OF CALORIC RESTRICTION ON MOLECULAR AND FUNCTIONAL NETWORKS IN RHESUS MONKEYS

Rozalyn Anderson, *University of Wisconsin College of Medicine, University of Wisconsin, Madison, Wisconsin, United States*

Caloric restriction (CR) delays aging and the onset of age-related disease in diverse species. Several diseases of aging including diabetes, cancer, and neurodegeneration, have an established metabolic component. Although the mechanisms of CR remain unknown, numerous factors implicated in longevity regulation by CR converge on regulation of metabolism. The reprograming of metabolism with CR is tissue specific, but mitochondrial activation and changes in redox metabolism are among the shared features. Changes in non-coding miRNA and in processing of transcripts are contributing mechanisms in integrating metabolic and growth pathways. Our studies in simple cell culture shows that small changes in metabolic status can precipitate large-scale multi-modal functional changes across cellular processes. We propose that modest failures in metabolic integrity with age broadly impact homeostasis and adaptation, creating shared vulnerability to diseases and conditions despite differences in their etiology, and that CR harnesses this same axis to promote health and enhanced longevity.

### CIRCADIAN ALIGNMENT OF FEEDING REGULATES LIFESPAN EXTENSION BY CALORIC RESTRICTION

Filipa Rijo-Ferreira[1], Mariko Izumo[1], Pin Xu[2], Carla B. Green[1], Joseph S. Takahashi[1], *1. UT Southwestern Medical Center, Dallas, Texas, United States 2. Icahn School of Medicine at Mount Sinai, New York, New York, United States*

Caloric restriction (CR) promotes longevity in several species. Classic CR protocols often lead to chronic cycles of 2h-feeding/22h-fasting, raising the question whether calories, fasting or time of day are causal. To address this, we tested an AL control group and five CR protocols with different timing and duration of feeding/fasting cycles. C57BL/6J male mice were subjected to 30% CR as one single meal a day at the beginning of the day or night (classical protocols with < 2h feeding, CR-day and CR-night), or smaller meals distributed for 12h (CR-day-12h and CR-night-12h), or evenly spread out throughout 24h (CR-spread) to abolish the otherwise daily feeding pattern adopted by nocturnal animals. We found that CR alone is sufficient to extend lifespan without fasting. However, the benefits are enhanced if feeding/fasting cycles are present and match their normal nocturnal activity. Circadian alignment of feeding with at least 12h fasting boosts CR-mediated increase on survival in mice, independently body weight. Aging leads to widespread upregulation of inflammation-related genes and downregulation of metabolic pathways in liver from ad lib fed mice; whereas CR at night ameliorates these aging-related changes and preserves circadian oscillations in gene expression. Overall, our results demonstrate that circadian interventions promote longevity and provide a novel perspective for elucidating mechanisms of aging.

### THE IMPACT OF SHORT-TERM DIETARY RESTRICTION ON STEM CELL FUNCTION

Archana Unnikrishnan, *University of Oklahoma, Oklahoma City, Oklahoma, United States*

Stem cells play a critical role in the maintenance of tissue function and their proliferative/regenerative capacity is essential to this role. Because stem cells persist over the lifespan of an animal, they are susceptible to gradual accumulation of age-associated damage, resulting in the loss of regenerative function that can impair organ function. Understanding the mechanism(s) that regulates stem cell function is essential for retarding the aging process, and stem cells are attractive targets for aging interventions. Dietary restriction (DR), the most robust anti-aging intervention to-date, has been shown to enhance the activity and integrity of stem cells in a variety of tissues (e.g., muscle, bone marrow, and intestine), and it is believed that effect of DR on stem cells plays an important role in the anti-aging action of DR. For example, DR has been shown to preserve and increase the number of intestinal stem cells (ISCs) and enhance their regenerative capacity in young animals. Data from my lab shows that ISCs from old mice have limited proliferation activity and form few if any organoids in vitro (a surrogate for a fully functional crypt) and that ISCs isolated from old mice on life-long DR show an improved ability to form organoids. While it is well accepted that life-long DR increases lifespan and has anti-aging effects an important aspect of DR that has been largely overlooked is that DR implemented only for a short time early in life can increase lifespan of rodents even when rodents are fed ad libitum the remainder of their life. In line with this, we recently found that ISCs from old mice fed DR for only a short-period resulted in a dramatic increase in ability of the ISCs to form organoids. This is the first evidence that short-term DR administrated late in life can rescue the loss in ISC function that occurs with age.

### Session 1445 (Symposium)

### NURSING HOME SOCIAL WORK WITH RESIDENTS WITH SEVERE MENTAL ILLNESS, THOUGHTS OF SUICIDE, OR DEMENTIA

Chair: Mercedes Bern-Klug
Discussant: Amy Restorick Roberts

Many of the close to 3 million persons who receive care in a U.S. nursing home in any given year face mental-health-related challenges that range from minor to severe. One of the core professionals involved with care planning for the psychosocial needs of nursing home residents with mental health concerns is the social worker. Reporting data from a 2019 nationally representative survey of nursing home social services directors, this session provides information about the training needs of nursing home social workers in terms of their work with residents diagnosed with a severe mental illness such as schizophrenia or severe depression, residents who are suicidal, and residents with dementia.

### DEMENTIA CARE INVOLVEMENT AND TRAINING NEEDS OF SOCIAL SERVICES DIRECTORS IN U.S. NURSING HOMES

Jung Kwak,[1] Kevin Smith,[2] and Mercedes Bern-Klug,[2]
*1. The University of Texas at Austin, Austin, Texas, United States, 2. University of Iowa, Iowa City, Iowa, United States*

This study describes social services directors' involvement in dementia care in U.S. nursing homes, focusing on interest in and needs for dementia care training. Respondents were 841 social service directors from U.S. nursing homes. We found that 87% of social service departments engaged in cognitive assessment; 59% of social services directors were strongly interested in dementia care training, and 23% would need up to 10 hours of preparation time or would not be able to train staff on dementia-related care. Racial minority background, fewer years of experience in nursing homes, and barriers to staffing predicted strong interest in dementia care training. These findings demonstrate social services directors' active involvement in dementia care and need for training.

### NURSING HOME SOCIAL SERVICES DIRECTORS CARING FOR RESIDENTS WITH SERIOUS MENTAL ILLNESS

Denise Gammonley,[1] Xiaochuan Wang,[1] Kelsey Simons,[2] Kevin Smith,[3] and Mercedes Bern-Klug,[3] *1. University of Central Florida, Orlando, Florida, United States, 2. University of Rochester School of Medicine & Dentistry, Department of Psychiatry, Rochester, New York, United States, 3. University of Iowa, Iowa City, Iowa, United States*

Psychosocial care for residents with serious mental illness (SMI) requires understanding of co-morbidities and careful attention to needs, rights, and preferences. Analyses of social services directors (SSDs) responses (n=924) to the National Nursing Home Social Service Director Survey considered perceived roles and competence to provide care stratified by the percentage of NH residents with SMI. Depression screenings and biopsychosocial assessments were common roles regardless of the percentage of residents with SMI. About one-quarter lacked confidence to train colleagues in recognizing distinctions between depression, delirium and depression (23.4% unable) or to develop care plans for residents with SMI (26% unable). A bachelor's degree (OR=0.64, 95% CI:0.43, 0.97) or less (OR= 0.47, 95% CI:0.25, 0.89) was associated with less perceived competence in care planning compared to those with a master's degree. SSDs reported less involvement in referrals or interventions for resident aggression in homes with a high proportion of residents with SMI.

### NURSING HOME SOCIAL SERVICES DIRECTORS' ROLES AND SELF-EFFICACY IN SUICIDE RISK MANAGEMENT

Xiaochuan Wang,[1] Kelsey Simons,[2] Denise Gammonley,[1] Amy Restorick Roberts,[3] and Mercedes Bern-Klug,[4]
*1. University of Central Florida, Orlando, Florida, United States, 2. University of Rochester School of Medicine & Dentistry, Department of Psychiatry, Rochester, New York, United States, 3. Miami University, Oxford, Ohio, United States, 4. University of Iowa, Iowa City, Iowa, United States*

Nursing home (NH) residents face many risk factors for late life suicide, and transitions into and out of NHs represent risk periods for suicide. Based on data from the 2019 National Nursing Home Social Services Directors survey (n = 924), this presentation describes NH social services directors (SSDs) roles in managing suicide risk and factors that influence self-efficacy in this area. Nearly one-fifth (19.7%) of SSDs lack of self-efficacy in suicide risk management, reporting needing significant preparation time or being not able to train others on this topic. Results of ordinal logistic regression indicate that SSDs who consider insufficient social services staffing as a minor barrier (comparing with a major barrier) to psychosocial care, those who report greater involvement in safety planning for suicide risk, and those with Master's degree, are more likely to perceive greater self-efficacy in suicide risk management. Implications for training and staffing will be discussed.

### Session 1450 (Symposium)

### OPPORTUNITIES TO IMPACT DISABILITY: TRENDS, COSTS, AND EVIDENCE-BASED INTERVENTIONS

Chair: Sarah Szanton Discussant: David Grabowski

As healthcare shifts to a focus on social determinants and population health, and older adults increasingly seek to "age in community," it is vital to understand the functional capabilities and related costs for older adults with disability. This symposium will present data on five major areas related to older adult disability. The 1st presenter will describe recent national disability trends. The 2nd will present Medicare costs by disability, dementia, and community-dwelling status in order to illustrate how these different demographic groups vary in Medicare expenditures over time. This information is critical to policymakers and health systems leaders to plan for these populations. They will then describe a 3rd project, which employs a novel longitudinal modeling approach, Group Based Trajectory Modeling, to identify and describe the distinct trajectories of Emergency Department use after incident disability. This work assesses the heterogeneity in health care use after disability, which may be shaped by available supports. The 4th presentation will describe a combined analysis of the 11 sites that have published data from implementations of the CAPABLE program. This program is a 10 session, home-based interprofessional program that provides an occupational therapist, a nurse, and a handyworker to addresses older adults' self-identified functional goals by enhancing individual capacity and the home environment. Taken together, these presentations can inform interventions and policies that improve the health and quality of life of older adults with disabilities.

### CAPABLE PROGRAM IMPROVES DISABILITY IN RESEARCH AND IMPLEMENTATION SETTINGS

Sarah Szanton,[1] Qiwei Li,[2] and Laura Gitlin,[3] *1. Johns Hopkins University, Baltimore, Maryland, United States, 2. Johns Hopkins School of Nursing, Baltimore, Maryland, United States, 3. Drexel University, College of Nursing and Health Professions, Drexel University, Pennsylvania, United States*

Interventions to reduce disability are crucial for older adults with disabilities to avert unnecessary hospitalizations or nursing home placements and improve daily life. Developed and tested at one research site, multiple health systems and community based organizations have since implemented CAPABLE. All published or peer reviewed tests of CAPABLE were reviewed (six studies, 11 sites) with a total of 1087 low-income community-dwelling older adults with disabilities. Participants were an average age of 74-79, cognitively intact, and self-reported difficulty with one or more activities of daily living (ADL). These trials were reviewed by extracting the participants' scores on main outcomes, ADLs and IADLs, and when available, fall efficacy, depression, pain and cost savings. All studies yielded improvements in ADL and IADL limitations, with small to strong effect sizes. Studies with the complete dose of CAPABLE showed more improvement in ADLs and cost savings than the studies that implemented a decreased dose.

### MEDICARE COSTS BY DISABILITY AND COMMUNITY-DWELLING STATUS

Claire Ankuda, and Katherine Ornstein, *Icahn School of Medicine at Mount Sinai, New York, New York, United States*

Understanding population-level Medicare expenditure patterns for older adults with functional disability is critical to focus supports to reduce costly and potentially burdensome health care use. We used the National Health and Aging Trends Study (NHATS) to assess quarterly Medicare expenditures over the 12 months following NHATS interview. We examine Medicare expenditure patterns for older adults in nursing homes (N=386), in the community and without disability (N=20,103), with disability and dementia (N=2,008), and with disability but not dementia (N=2,945). One-year mortality ranged from 2.0% for those without disability in the community to 25.9% for those residing in nursing homes. Among those surviving 1 year, Medicare expenditures the first quarter after NHATS survey ranged from $1,794 (95% CI $1,690-$1,898) for those with no disability to $5,177 (95% CI $4,535-$5,818) for those with disability and dementia. We assess trends over the following two years, and find that trajectories vary by clinical grouping.

### DISABILITY TRENDS AMONG COMMUNITY-DWELLING OLDER ADULTS AND RELATED DETERMINANTS OF DISABILITY

Qiwei Li,[1] and Sarah Szanton,[2] *1. Johns Hopkins School of Nursing, Baltimore, Maryland, United States, 2. Johns Hopkins University, Baltimore, Maryland, United States*

The growing aging population with disabilities poses challenges to caregiving and health care services but there is little recent data on disability trends. Some studies have shown that disability is decreasing while others have shown it increasing. Understanding these trends among community-dwelling older adults is critical for communities to allocate resources and develop policies. This study updates disability trend data among community-dwelling older adults using nationally representative National Health & Aging Trends Study data. Results revealed that about 30% of Medicare beneficiaries had at least one limitation of the activity of daily living (ADL) from 2011 to 2019. Age 75-79 (IRR=1.55), 80-84 (IRR= to 4.60), 85-89 (IRR=2.99), 90+ (IRR=4.60), female, (IRR=1.18), not a house owner (IRR=1.51), financial strain (IRR=1.71), and receiving Medicaid (IRR=1.84) are associated with a higher likelihood of becoming ADL limited or having more ADL limitations. We will discuss potential policy, intervention, and research implications.

### TRAJECTORIES OF EMERGENCY DEPARTMENT USE AFTER INCIDENT FUNCTIONAL DISABILITY

Katherine Ornstein, and Claire Ankuda, *Icahn School of Medicine at Mount Sinai, New York, New York, United States*

Emergency department (ED) visits for older adults with functional disability may represent unmet needs and are often burdensome to patients and families. While it is known that older adults with functional disability use the ED at high rates, this does not capture the heterogeneity of experience after the onset of disability. Using NHATS, we identified a cohort of older adults with incident disability, or who reported they began to receive help with self-care and/or mobility in the prior year. Using the month that they report first receiving help, we linked to Medicare data to assess quarterly patterns of ED use. We used Group Based Trajectory Modeling to assess the trajectories of ED use after disability. We identified three distinct trajectories of ED use: persistently high, declining, and persistently low. We describe the clinical, household, and sociodemographic characteristics associated with likely membership in each trajectory group.

### Session 1455 (Symposium)

### PROVIDING SPECIALTY TELEHEALTH CARE TO OLDER, RURAL PATIENTS: VOICES FROM FIELD

Chair: Eileen Dryden Co-Chair: Lauren Moo

Older, rural adults have limited access to quality geriatric specialty care for several reasons including relatively few geriatric specialists in rural areas and lack of transportation options or patient ability to travel to more urban centers.

GRECC Connect is a promising telehealth-hub and spoke model that provides rural patients access to teams of multidisciplinary geriatric specialists in more urban medical centers primarily by video connection with affiliated community-based outpatient clinics (CBOCs). This model provides a viable option for increasing access to geriatric specialty care for rural patients but is not used to the extent it could be. To date, much of our understanding of this model has come from the experts at the hub medical centers. To learn more about the experience of this model from the field we interviewed CBOC staff and providers as well as Veterans and their caregivers about geriatric specialty telehealth services. In this symposium we will discuss facilitators and barriers to implementing this model from the perspective of the field and then explore more deeply both the context of the CBOC environment and the older patient population served by rural CBOCs to further understand the challenges that are faced in attempting to connect older patients with telehealth services. Finally, we will share the perceived value of the service and alignment with local needs. This deeper understanding of the experience of the 'spoke' may help enhance access to much needed geriatric specialty care for rural veterans.

### QUALITATIVE EVALUATION: GRECC CONNECT AS A METHOD OF DELIVERING HEALTH CARE TO RURAL OLDER VETERANS

Lauren Moo,[1] William Hung,[2] Eileen Dryden,[3] Camilla Pimentel,[4] Laura Kernan,[3] and Kathryn Nearing,[5] *1. VA Bedford Health Care System, Bedford, Massachusetts, United States, 2. Veterans Health Administration, New York City, New York, United States, 3. Veterans Health Administration, Bedford, Massachusetts, United States, 4. VA Bedford Healthcare System, Bedford, Massachusetts, United States, 5. Veterans Health Administration, Aurora, Colorado, United States*

The VA Office of Rural Health-funded GRECC Connect program uses telehealth modalities to provide geriatric specialty care to rural older veterans and education to clinicians in VA Community-based outpatient clinics (CBOCs). Qualitative evaluation of GRECC Connect has included interviews with three stakeholder groups: geriatrics specialty teams at 15 hub medical centers, rural CBOC staff, and patients/family caregivers. CBOC staff interviews included 50 individuals from 13 different CBOCs. Staff roles included clinic managers, social workers, psychologists, physicians, nurses, and telehealth technicians. Older veterans who had recently been involved in a GRECC Connect video visit were also invited to share their views on the visit. By including multiple perspectives on the program, we are better positioned to increase reach, access, and improve care for older rural veterans.

### THE INTEGRAL ROLE OF CBOCS IN RURAL HEALTHCARE: PROMISES AND CHALLENGES

Camilla Pimentel,[1] Kathryn Nearing,[2] Laura Kernan,[3] Eileen Dryden,[3] and Lauren Moo,[4] *1. VA Bedford Healthcare System, Bedford, Massachusetts, United States, 2. Veterans Health Administration, Aurora, Colorado, United States, 3. Veterans Health Administration, Bedford, Massachusetts, United States, 4. VA Bedford Health Care System, Bedford, Massachusetts, United States*

Community-based outpatient clinics are critical to extending the geographic reach of VA's healthcare delivery system. Nationwide, 733 CBOCs provide outpatient care to nearly half of the VA's patient population. The 13 rural CBOCs in the study sample provide outpatient primary care, mental health care, and a limited number of specialty care services. Located 1–3.5 hours away from their closest VA Medical Center, these CBOCs have a wide—sometimes interstate—service catchment area. To effectively serve increasingly older and medically complex patient populations, they rely heavily on partnerships with larger VA Medical Centers and local community providers for inpatient, residential, and additional outpatient services. CBOCs experience myriad staffing challenges, including staff turnover, "access providers" working at multiple CBOCs, and highly variable training in rural health and geriatrics. While some CBOCs have robust telehealth offerings, others cannot currently grow their telehealth capacity owing to constraints in clinic space and provider schedules.

### ANOTHER SET OF EYES: RECIPIENTS' VIEWS OF THE BENEFITS OF GERIATRIC SPECIALTY TELEHEALTH SERVICES

Eileen Dryden,[1] Laura Kernan,[1] Kathryn Nearing,[2] Camilla Pimentel,[3] and Lauren Moo,[4] *1. Veterans Health Administration, Bedford, Massachusetts, United States, 2. Veterans Health Administration, Aurora, Colorado, United States, 3. VA Bedford Healthcare System, Bedford, Massachusetts, United States, 4. VA Bedford Health Care System, Bedford, Massachusetts, United States*

The aim of GRECC Connect is to increase access to specialty care for medically complex, older, rural patients through e-consultations and telehealth visits. We interviewed 50 outpatient clinic staff and providers as well as 30 patients and caregivers about these services. Overall, the services were considered beneficial. For patients and caregivers, services alleviated the stress and cost of travel, they improved quality of life by increasing their understanding of the progression of an illness and providing treatment and guidance to increase patient functioning and reduce disruptive behaviors, and they eased anxiety associated with not receiving needed care. Having 'another set of eyes' on the patients reduced stress and anxiety for providers. Concerns included alignment of telehealth modality with the capabilities of older patients with cognitive problems, hearing loss and/or limited technological abilities and, for some providers, that the referral for and recommendations resulting from the service added to their workload.

### DIGITAL DIVIDE MAGNIFIED FOR OLDER VETERANS LIVING OFF THE GRID

Kathryn Nearing,[1] Camilla Pimentel,[2] Eileen Dryden,[3] Laura Kernan,[3] and Lauren Moo,[4], *1. Veterans Health Administration, Aurora, Colorado, United States, 2. VA Bedford Healthcare System, Bedford, Massachusetts, United States, 3. Veterans Health Administration, Bedford, Massachusetts, United States, 4. VA Bedford Health Care System, Bedford, Massachusetts, United States*

Compared to urban Veterans, rural Veterans are more likely to be older (55-74), not employed, have less education, more service-related disabilities and unmet healthcare needs.

Interviews with a national sample of community-based outpatient clinic providers described highly-rural Veterans who are "off the grid." These Veterans, by choice and/or circumstance, do not have access to reliable internet, associated devices or knowledge/skills. Providers described the difficulties of connecting with these Veterans even by phone. The healthcare shift to virtual telehealth modalities in response to COVID-19 highlights the digital divide as a social determinant of health. For "off-the-grid" Veterans, past experiences and present-day circumstances converge to perpetuate and exacerbate inequalities in accessing healthcare. Their situation underscores that telehealth is not a panacea for increasing access to care and confronts us with the moral imperative to reach those with whom it may be most difficult to connect to span social, geographic and digital divides.

### A STRATEGY MATCHING TOOL FOR BOOSTING IMPLEMENTATION OF GERIATRIC TELEHEALTH SERVICES IN RURAL CBOCS

Laura Kernan,[1] Eileen Dryden,[1] Camilla Pimentel,[2] Kathryn Nearing,[3] and Lauren Moo,[4] *1. Veterans Health Administration, Bedford, Massachusetts, United States, 2. VA Bedford Healthcare System, Bedford, Massachusetts, United States, 3. Veterans Health Administration, Aurora, Colorado, United States, 4. VA Bedford Health Care System, Bedford, Massachusetts, United States*

Fifteen Veterans Administration Medical Centers (VAMCs) offer geriatric specialty care telehealth services through a hub and spoke model to patients at affiliated community-based outpatient clinics (CBOCs). These services are not used to the extent they could be. Through interviews with 50 staff and providers at rural CBOCs we identified several implementation facilitators and barriers. CBOC-level barriers included space constraints, low staffing, internet connection issues, and limited knowledge of services available and referral processes. Patient-level barriers included discomfort with technology, cognitive decline, and inability to travel to the CBOC. We found that champions within the CBOC and iterative, targeted outreach from the hub helped facilitate uptake of services. We entered the identified barriers into the CFIR-ERIC (Consolidated Framework for Implementation Research-Expert Recommendations for Implementing Change) Implementation Strategy Matching Tool to help generate targeted strategies that will be used to refine each hub's implementation approach.

### Session 1460 (Symposium)

### SLEEP, LIFESTYLE, AND SOCIOECONOMIC MARKERS OF MENTAL AGING AND WELL-BEING: LESSONS FROM ENGLAND, CHINA, AND JAPAN

Chair: Andrew Steptoe

Healthy ageing has become a popular topic worldwide. We investigated the role of sleep, leisure activities, and socioeconomic inequalities in relation to cognitive decline, wellbeing, and quality of life in data from the English Longitudinal Study of Ageing (ELSA), Chinese Health and Retirement Longitudinal Study (CHARLS), and Japanese Study of Aging and Retirement (JSTAR), national representative samples of England, China and Japan, respectively. We found an inverted U-shaped association between sleep quality and memory in English adults and a positive dose-response association in Chinese older adults (Brocklebank). In another examination, we found that younger English individuals playing games had lower quality-of-life than older participants who game, and this association is more pronounced for widowed individuals than others (Almeida-Meza). Cognitive impairment and dementia represent significant challenges worldwide. In a cross-country investigation, we found that the prevalence of MCI was twice as great in England compared with Japan, but that the two nations differ slightly across socioeconomic correlates (Gireesh). In another cross-country comparison between England and China, we found that the rate of memory change appeared socioeconomically patterned, primarily by education and area-based characteristics (urban vs. rural), with a more substantial impact on rural China inequalities compared to England (Cadar). Our results indicate more robust educational and geographical disparities in China and increased occupational impact among English and Japanese participants. Our findings highlight the imperative need for policy interventions and tailored strategies to protect those particularly disadvantaged in England and China.

### SLEEP QUALITY AND COGNITIVE DECLINE: A CROSS-COUNTRY COMPARISON BETWEEN ENGLAND AND CHINA

Laura Brocklebank,[1] Dorina Cadar,[1] Li Yan,[2] Yaohui Zhao,[2] and Andrew Steptoe,[1] *1. University College London, London, England, United Kingdom, 2. Peking University, Guangzhou, Guangdong, China (People's Republic)*

Too little or too much sleep is associated with accelerated cognitive decline in older adults. However, sleep duration does not capture other sleep problems prevalent in older adults, such as difficulties with falling or staying asleep. Less is known about the impact of sleep quality on cognitive ageing, and if this relationship differs between England and China. Therefore, the aim of this study is to examine the relationship of self-reported sleep quality with cognitive performance and rate of change over 6-7 years follow-up in two nationally-representative samples of English and Chinese older adults. The primary outcome was a memory score (range 0-20), which was assessed using immediate and delayed 10-word recall tests in both cohorts. The results of bivariate descriptive analyses at baseline suggest there may be an inverted U-shaped association between sleep quality and memory in English older adults, and a positive dose-response association in Chinese older adults.

### ONLINE GAMING AND WELL-BEING IN THE ENGLISH LONGITUDINAL STUDY OF AGEING

Pamela Almeida-Meza, Dorina Cadar, Andrew Steptoe, and Carrie Ryan, *University College London, London, England, United Kingdom*

Play is considered an important contributor to healthy ageing. Using data from 3,067 participants aged 50+ from the English Longitudinal Study of Ageing, we explored online gaming assessed at wave 6 (2012/13) and quality-of-life, loneliness, and depression at wave 9 (2018/19). Covariates were age, sex, marital status, education, work status, depression, self-rated health, physical activity, smoking and alcohol consumption. We found that 22% of respondents engaged in gaming. Interaction analyses indicated that for younger individuals (<65 years), gaming predicted lower scores in the self-realization sub-scale of the quality-of-life scale in comparison to older gamers. Furthermore, there was a significant association between gaming and lower quality-of-life for widowed individuals only, particularly in terms of autonomy, self-realization, and pleasure. There were non-significant associations between gaming and loneliness and depression. Online gaming might be independently associated with lower levels of quality of life, especially for younger and widowed adults.

### SOCIOECONOMIC INEQUALITIES AND MILD COGNITIVE IMPAIRMENT: EVIDENCE FROM ENGLAND AND JAPAN

Aswathikutty Gireesh,[1] Pamela Almeida-Meza,[1] Hashimoto Hideki,[2] Andrew Steptoe,[1] and Dorina Cadar,[1] *1. University College London, London, England, United Kingdom, 2. University Of Tokyo, Tokyo, Tokyo, Japan*

Japan is the world's fastest ageing population, with a higher prevalence of dementia than in the UK. Less clear is the role of socioeconomic inequalities in neurocognitive disorders between these countries. This study aims to assess comparatively the relationship between education, a marker of cognitive reserve, and income in relation to mild cognitive impairment (MCI) and dementia in England and Japan. We ascertained MCI using a validated algorithm based on one standard deviation below the mean on two standardised cognitive tests. Multinomial logistic regression models were used to study the associations between socioeconomic markers and MCI/dementia. The prevalence of MCI was almost twice as high among English adults compared to Japanese. Results suggest that nations are similar in overall socioeconomic inequalities of MCI/dementia, but this might differ across socioeconomic markers. Considerable variability in the health inequalities could be attributed to the country-specific socio-cultural-political factors, which remains to be further explored.

### SOCIOECONOMIC DETERMINANTS OF COGNITIVE AGING: A CROSS-COUNTRY COMPARISON BETWEEN ENGLAND AND CHINA

Dorina Cadar,[1] Yaohui Zhao,[2] Li Yan,[2] Laura Brocklebank,[1] and Andrew Steptoe,[1] *1. University College London, London, England, United Kingdom, 2. Peking University, Guangzhou, Guangdong, China (People's Republic)*

Lower educational attainment is associated with a higher risk of dementia and a steeper cognitive decline in older adults. However, less clear is how other socioeconomic markers contribute to cognitive ageing and if these socioeconomic influences on cognitive ageing differ between England and China. We examined the relationship of education, household wealth, and urbanicity with cognitive performance and rate of change over 7-8 years follow up in the English Longitudinal Study of Ageing and Chinese Health and Retirement Longitudinal Study, national representative samples of England and China. We found that the rate of cognitive change appears to be socioeconomically patterned, primarily by education and area-based characteristics (urban vs rural), with a stronger impact of inequalities seen in rural China. Public health strategies for preventing cognitive decline and dementia should target socioeconomic gaps to reduce health disparities and protect those particularly disadvantaged in England and China.

### Session 1465 (Paper)

### SOCIAL ISOLATION AND MENTAL HEALTH

### CATASTROPHIC HEALTH EXPENDITURES AND MENTAL HEALTH IN OLDER CHINESE PEOPLE: THE ROLE OF SOCIAL HEALTH INSURANCE

Wei Yang,[1] and Bo Hu,[2], *1. King's College London, London, England, United Kingdom, 2. London School of Economics and Political Science, London, England, United Kingdom*

Catastrophic health expenditure (CHE) has considerable effects on household living standards, but little is known regarding the impacts of CHE on people's mental health. Using China as an example, this study examines the association between CHE and mental health and investigates whether and to what extent social health insurance (SHI) can lessen the impacts of CHE on mental health among older people aged over 60 in China. The data come from three waves of the China Health and Retirement Longitudinal Study (CHARLS 2011, 2013, and 2015, N = 13,166). We built fixed-effects quantile regression models to analyse the data. We found that incurring CHE has significantly detrimental effects on older people's mental health, whereas the SHI demonstrates a protective effect. The observed protective effects of SHI are the strongest among those with relatively mild mental health problems, i.e., people whose CES-D scores are below the 50th percentile. Our findings provide empirical evidence that encourages the integration of psychologically informed approaches in health services. We also urge governments in low- and middle-income countries to consider more generous health financing mechanisms for those with higher healthcare needs.

### LIVING ARRANGEMENTS AND PSYCHOSOCIAL WELL-BEING AMONG OLDER ADULTS: EVIDENCE FROM THE HEALTH AND RETIREMENT STUDY

Rashmita Basu,[1] Huabin Luo,[1] Adrienne Steiner,[2] and Alan Stevens,[3], *1. East Carolina University, Greenville, North Carolina, United States, 2. East Carolina University, East Carolina University, North Carolina, United States, 3. Baylor Scott & White Health Research Institute, Temple, Texas, United States*

Despite growing attention to the association between living arrangements and health outcomes, less is known about how emotional well-being and life satisfaction vary by living arrangements. Using data from the 2014 and 2016 Leave Behind Questionnaires from the Health and Retirement Survey (N=13,275), we estimated generalized linear regression models comparing emotional well-being (a ratio of positive to negative emotion) and life satisfaction (the satisfaction with life scale, SWLS) by living alone versus living with others, controlling for socioeconomic and other health-related characteristics. Overall, individuals who lived alone had lower emotional well-being (β=-0.11; p<0.01), and SWLS score (β=-0.42; p<0.001), compared to those living with others. The direction of these relationships stratified by the cognitive status was the same. Policies and programs designed to support the growing population of older adults living alone should focus on improvement in these positive outcomes to enhance the quality of life.

### LONELINESS, STRESS, AND DEPRESSIVE SYMPTOMS AMONG OLDER IMMIGRANTS: A MODERATING ROLE OF FAMILIAL RELATIONSHIPS

Heejung Jang, *University of Michigan, Ann Arbor, Michigan, United States*

**Objectives:** Immigration is a stressful life event, and immigrants commonly experience loneliness, a risk factor for depression. However, little is known about how and whether older immigrants' perceived stress exposure/appraisals mediate the association between loneliness and depressive symptoms. Further, this study explores whether familial relationships moderate the indirect or direct effects of the mediation models.

**Method:** This study uses the 2012 Health and Retirement Study from a sample of 719 immigrants age 57 and older. A series of moderated mediation analyses were conducted across the total number of stress exposure and eight stress appraisal domains.

**Results:** The findings indicate that the total number of stress exposure and five domains of stress appraisals (health problems in self, physical/emotional problems in spouse/child, financial strain, housing problems, and close relationships in others) mediate the association between loneliness and depressive symptoms. In addition, the perceived negative strain from family moderated the mediating effect of health problems and housing problems in the relationship between loneliness and depressive symptoms.

**Discussion:** This study suggests that negative relationships with family may increase upsetting in stress appraisals on health and housing problems, which turn in increased depressive symptoms for lonely older immigrants. Practitioners need to assess older immigrants' stressors and family relationships to understand their loneliness and depressive symptoms.

### SOCIAL ISOLATION AS A RISK FACTOR FOR CIGARETTE SMOKING IN OLDER ADULTS

Gilbert Gimm,[1] Mary Lou Pomeroy,[2] and Thomas Cudjoe,[3] *1. George Mason University, Vienna, Virginia, United States, 2. George Mason University, George Mason University, Virginia, United States, 3. Johns Hopkins University School of Medicine, Baltimore, Maryland, United States*

**Objective:** This study examined the prevalence of social isolation and cigarette smoking in a national sample of community-dwelling older adults, and assessed the role of social isolation on the risk of cigarette smoking.

**Methods:** Using data from 8,044 participants (age 65+ years) across two waves of the National Health and Aging Trends Study (NHATS), we analyzed the prevalence of social isolation in older adults and as a risk factor for cigarette smoking. Social isolation was measured across 4 relationship domains (Cudjoe, 2018) on a scale of 0 to 4, using objective measures of social interactions. Descriptive and logistic regression analyses were conducted to assess how social isolation is associated with smoking.

**Results:** Preliminary results showed that 18.2% of older adults were socially isolated (3.5% severely isolated) and 7.1% of participants reported current smoking. We found that both social isolation (OR = 2.5, p<.001) and severe isolation (OR = 5.9, p<.001) increased the odds of smoking. Also, older adults with depression (OR = 1.6, p<.01) and dual-eligible beneficiaries (Medicare and Medicaid) with TRICARE coverage (OR = 4.6, p<.05) had greater odds of smoking. However, we did not find evidence that the odds of smoking varied significantly by the number of chronic conditions.

**Conclusion:** Social isolation is associated with an increased risk of cigarette smoking among older adults. Smoking may be an important behavior in the pathway between social isolation and its association with morbidity and mortality.

### THERE IS NO COMMUNITY HERE: LIVING ALONE, PLACE, AND OLDER PEOPLES' RISK OF SOCIAL ISOLATION

Rachel Weldrick, *Simon Fraser University, Vancouver, British Columbia, Canada*

Existing research has identified significant risk factors for experiencing social isolation in later life including chronic health conditions, mobility impairments, and living alone among others. Although many older people who live alone maintain active social lives, living alone remains a top predictor of social isolation. Less is known about other types of risk factors, such as place-based risks and social exclusion. Despite calls to examine the role of place and social exclusion in social isolation risk, few studies have investigated the links. Models of isolation risk have often omitted place-based factors and social exclusion and focused largely on individual-level risks. In order to address these gaps, this paper presents the findings of 17 in-depth, qualitative interviews with community-dwelling older people who live alone (aged 65-93). Participants were recruited using a theoretical sampling strategy to ensure that a diverse range of neighbourhood types were represented among the participants (e.g., walkable vs. car-dependent neighbourhoods). Interview transcripts were analyzed using a constructivist grounded approach resulting in several major themes. Participants described aspects of their local environments as shaping their risk of isolation including infrastructure and amenities delivered in place, and neighbourhood makeup, among others. These themes are further examined through the lens of place-based exclusion and used to conceptualize how dimensions of both place and social exclusion fit into the model of known isolation risk factors. An adapted model of risk is presented to guide future research and intervention planning.

### Session 1470 (Symposium)

### TEACHING ANTI-RACISM IN GERONTOLOGY: AN INTERACTIVE PROGRAM OF RECOGNITION, SELF-WORK, PEDAGOGY, AND ACTION

Chair: Althea Pestine-Stevens Discussant: Tina K. Newsham

Older adults with intersecting identities as persons of color experience disparities in health and well-being due to racism in individual and structural spheres, which have been amplified by health, economic, and social consequences of COVID-19. We can begin the work to reduce these inequities by training scholars and practitioners to disrupt the systems within which we work that relegate advantages and disadvantages throughout the life course and in later life by racial groups. This interactive symposium presents resources on anti-racist gerontological education and provides an opportunity to engage critically with peers in all stages of their careers and anti-racism journeys who are interested in integrating anti-racism into their teaching. The first presenter introduces conversations to begin anti-racist pedagogy and assumptions to dismantle. The second presenter describes cultural humility as an essential step towards self-awareness and critical self-reflection for educators and practitioners. The third presenter presents how anti-racist pedagogy, a teaching approach that combines racial content, pedagogy, and organizing, may be applied to gerontology education. Fourth, an example will be presented from an online course module developed to guide Master of Social Work students toward recognizing racial disparities in aging services systems and identifying concrete suggestions for improvement. Finally, strategies for curriculum design will be presented with examples from Public Health education. This symposium is designed to include ample time for group discussion on this critical and under-addressed area of teaching in gerontology across disciplines, such that participants can better connect with others to build awareness, competency, and resources.

### TEACHING ANTI-RACISM IN AN AGING SERVICES COURSE: LESSONS DEVELOPED AND LESSONS LEARNED

Althea Pestine-Stevens,[1] and Emily Greenfield,[2] *1. Rutgers Social Work, New Brunswick, New Jersey, United States, 2. Rutgers, The State University of New Jersey, New Brunswick, New Jersey, United States*

Despite high levels of racial disparities in health and well-being among older adults, curricula addressing how aging services systems contribute to or work to ameliorate these disparities are scarce. This paper introduces a module on inequalities and anti-racism in aging developed for an online course on aging services within a Master of Social Work program. First, materials that help students identify and understand racial inequalities in aging and in the programs that serve older adults are presented. Next, students are introduced to the applied context of how COVID-19 has exacerbated these inequalities. Finally, students critically engage in reflections and assessments of the available resources within aging services and advocacy organizations, providing recommendations for how these systems may better incorporate anti-racist practices. Challenges and opportunities will be discussed, including piloting this module in a virtual, asynchronous environment.

### THE MASTER'S TOOLS WILL NEVER DISMANTLE THE MASTER'S HOUSE: THE ROLE OF DIVERSITY IN ANTI-RACIST EDUCATION

Karen Lincoln, *University of Southern California, Los Angeles, California, United States*

Diversity is a strange fruit that requires critical analysis to understand its meaning, value and impact on education. Depending on the era, diversity has been defined in a number of different ways and has a variety of meanings across a range of contexts. The lack of shared meaning and understanding of diversity and who controls the diversity narrative have significant implications for the development of anti-racist pedagogy in gerontological education. This presentation will discuss the history and evolution of the "diversity discourse" and how mainstream notions of diversity impact diversity initiatives, curriculum design and anti-racist pedagogy. Strategies for engaging in an historical analysis of diversity and how this process relates to the design, leadership and ownership of anti-racist curriculum will be discussed, as well as the role of gerontology in leading these efforts.

### CULTURAL HUMILITY: THE BIAS CHECKER

Natalie Moore-Bembry, *Rutgers, The State University of New Jersey, Camden, New Jersey, United States*

Historically we have been taught to understand and embrace cultural competency, however, this focus has often led to a superficial understanding of others and seldom required one to better understand themselves. Cultural humility is based on one's ability to engage in individual accountability and institutional accountability. Individual accountability is based on critical self-reflection and critique, lifelong learning, and the challenging of power imbalances. Institutional accountability requires one to challenge structural power. This session will: (1) explore ways to engage in critical self-reflection and critique; (2) describe how values and beliefs impact the interactions of our personal and professional lives; and (3) strategize ways to collectively model and practice in cultural humility in one's personal and professional life.

### REFLECTIONS ON APPLYING ANTI-RACIST PEDAGOGY TO TEACHING ABOUT AGING

Rona Karasik, and Kyoko Kishimoto, *Saint Cloud State University, SAINT CLOUD, Minnesota, United States*

Racial disparities are well documented in all facets of aging (e.g., health, housing, retirement). Helping gerontology students to recognize racial inequity, however, is only the first step toward effecting change. Empowering students to investigate root causes of these disparities (e.g., power relations) is essential in order to begin to identify ways to dismantle institutional racism and create systems that are more equitable. With its attention to historical/political context and fostering skills for critical analyses and social change, anti-racist pedagogy offers educators a framework from which to introduce and explore these issues (Karasik & Kishimoto, 2020). This session will consider how anti-racist pedagogy may be applied within the gerontological curriculum using examples of teaching about (1) retirement and (2) housing for older adults. Associated challenges such as finding and preparing appropriate material, as well as engaging students and faculty in work they are unfamiliar and/or uncomfortable with will also be discussed.

### CONSIDERATIONS IN CURRICULUM DESIGN FOR TEACHING ABOUT RACE AND RACISM IN GERONTOLOGY

Darren Liu,[1] and Betty Burston,[2] *1. Des Moines University, Des Moines, Iowa, United States, 2. University of Nevada, Las Vegas, Las Vegas, Nevada, United States*

The recognition of structural racism as a public health crisis has enlarged the breadth and depth of discussions of the unique "Isms" in higher education classrooms. A significant number of undergraduate or graduate level gerontology programs participate in the delivery of training through instruction on the topics of aging, gerontology, or long-term care administration to future labor market participants. Absent from these classroom conversations, however, has been an analysis of how syntax, the analytical vocabulary, and other framing of such conversations can impact learners. While these educational programs provide highly critical information on disease, illness and injury prevention, self-care, population health, and other topics, professors can also introduce perspectives on race, racism, and how they may be related to the delivering of care to the aging population. Specifically, this session will introduce an example of curricular design for the identification of structural racism in the operation of long-term care institutions.

### Session 1475 (Symposium)

### TECHNOLOGY TO SUPPORT SELF-REGULATION IN OLDER ADULTHOOD: INSIGHTS FROM DESIGN TO IMPLEMENTATION

Chair: Shannon Meija Discussant: Hans-Werner Wahl

Across the lifespan, individuals adapt to change through the careful monitoring and adjustment of goals, demands, and performance—processes of self-regulation. Technology in support of self-regulatory processes may compensate for deficiencies in the ability to set, monitor, and work toward goals. Our purpose in this symposium is to forward the discourse on how health technology—from design to implementation—can assist older adults in their efforts to support their health and well-being in daily life. Our symposium begins with design considerations for technologies that support processes of information seeking, reflection, and action. Chin presents a process for designing conversation agents that guide dialogues with older adults to support informal self-regulated learning of health information. Nie and colleagues synthesize the literature on visual feedback to provide a framework that illustrates how visual design elements can link feedback to action. The symposium continues with papers that speak to older adults' experiences using technology to accomplish their goals. Mejía and colleagues use insight from older adults who had self-assessed their balance for 30 consecutive days to explore themes of self-monitoring accuracy and feedback preferences. Francis and colleagues use data from the Detroit-based Social Relations Study to illustrate how technology use and its implications vary when older adults engage with their weaker social ties. The symposium will conclude with a discussion led by Wahl, who will situate the papers, and the discourse on health technology design and application, within lifespan developmental and action perspectives on aging.

### DESIGNING CONVERSATIONAL AGENTS TO SUPPORT SELF-REGULATED LEARNING FOR OLDER ADULTS

Jessie Chin,[1] and Smit Desai,[2], *1. University of Illinois at Urbana-Champaign, University of Illinois at Urbana-Champaign, Illinois, United States, 2. University of Illinois, Urbana Champaign, Champaign, Illinois, United States*

The rapid growth of the off-the-shelf smart speakers (such as Amazon Alexa and Google Home), also called Conversational Agents (CAs), creates potential to deliver everyday life support to users at home (such as checking weather, listening to news, scheduling events). Literature demonstrated the technology acceptance of CAs among older adults (including novice users) given the low barriers to use CAs. The natural conversations among CAs and users enable the opportunities to build deeper understandings about a topic through theory-driven guided dialogues. Our study has designed the metacognition strategies in the guided dialogues of CAs to support informal self-regulated learning of health information among older adults. The study has shown the feasibility and acceptance of CAs to help older adults learn new health information on their own through these guided dialogues. Additional analyses on the feasibilities to implement different metacognitive strategies in guided dialogues in the off-the-shelf CAs were also conducted.

### DESIGNING BEHAVIORAL FEEDBACK VISUALIZATIONS TO SUPPORT HEALTH BEHAVIOR CHANGE

Qiong Nie,[1] Daniel Morrow,[2] Maurita Harris,[1] and Wendy Rogers,[2] *1. University of Illinois at Urbana Champaign, Champaign, Illinois, United States, 2. University of Illinois Urbana-Champaign, Champaign, Illinois, United States*

Health technology has the potential to support behavior change by measuring performance and providing users with visualizations of this performance as feedback. Such visual feedback has had limited success in changing health behaviors, but it is not clear why. We conducted a systematic review of the visual feedback literature to develop an organizational framework representing the visual feedback-action process. We identified the components that have been investigated in the context of visual feedback. These components are classified into four categories: visualization types (e.g., bar graph) and variables (e.g., color); feedback characteristics (e.g., social comparison); psychological processes (e.g., motivation) and action (e.g., exercise). The insights will inform the design of feedback visualizations in a smartphone application to support medication adherence for older adults. More broadly, this integrative perspective will yield principles of feedback visualization techniques and components that influence the behavior change process and develop a roadmap to facilitate the design.

### PERSPECTIVES ON HOW FALL PREVENTION TECHNOLOGIES CAN SUPPORT OLDER ADULTS' SELF-MONITORING PROCESSES

Shannon Meija,[1] Sungjae Hong,[1] Aileen Griffin,[2] Tai-Te Su,[3] and Jacob Sosnoff,[4], *1. University of Illinois, Champaign, Illinois, United States, 2. University of Illinois at Urbana-Champaign, Aurora, Illinois, United States, 3. University of Illinois at Urbana-Champaign, Champaign, Illinois, United States, 4. School of Health Professions, University of Kansas Medical Center, Kansas City, Kansas, United States*

Fall risk increases as older adults lose the functional resources necessary to maintain balance while completing everyday activities. As functional resources often decline gradually with age, momentary deficits may not be apparent until after a fall occurs. Mobile fall prevention technologies could support older adults in self-monitoring their ability to safely navigate their environments. In this paper we present perspectives on self-monitoring and feedback in a sample of older adults (n = 20, 50% female, age 65+) who had self-assessed their balance via a smartphone for 30 consecutive days. Thematic analysis of semi-structured interviews showed that fall history differentiated a) participants' awareness of day-to-day variation in functional ability; b) trust in the accuracy of self-monitoring; and c) imaginations of what types of feedback a mobile fall prevention technology should provide. Insight on older adults' internal self-monitoring processes and guidelines for feedback design are discussed.

### WEAK TIES THAT BIND: ICT USE, SOCIAL RELATIONS, AND DEPRESSIVE SYMPTOMS AMONG OLDER ADULTS

Jess Francis, Noah Webster, and Nour Fakhoury, *University of Michigan, Ann Arbor, Michigan, United States*

Information and communication technology (ICT) use has been associated with well-being among older adults. This link is often attributed to the fact that technology use facilitates connecting with social relations generally. What is less known, however, is the extent to which distinct dimensions of social relations uniquely influence how ICT use affects health. Thus, the importance of weak ties is receiving increased attention. Using data from the Detroit-based Social Relations Study collected in 2015, we examine the extent to which separate dimensions of weak ties (contact frequency and network size) mediate and moderate the link between technology use and depressive symptoms among adults age 65+ (n=213). A greater number of less close relations mediated the link as it was associated with technology use and fewer depressive symptoms. A moderating effect was also found as technology use was associated with fewer depressive symptoms only among those with lower contact frequency.

### Session 1480 (Symposium)

### THE STUDY OF MUSCLE, MOBILITY, AND AGING (SOMMA): AN OVERVIEW

Chair: Steve Cummings Co-Chair: Peggy Cawthon
Discussant: Russell Hepple

SOMMA is an NIA-funded cohort study to identify biological determinants of mobility and fitness. The overall aim of SOMMA is to use biopsies, novel biomarkers, advanced imaging, and intensive physical and cognitive assessments to elucidate the biological processes that contribute to changes in mobility and physical fitness with aging. SOMMA will recruit 875 people age 70+ (of whom about 200 have been enrolled.) We take biopsies of the vastus lateralis muscle to quantify mitochondrial content and function of the electron transport chain. We use 31PMR spectroscopy to quantify mitochondrial capacity to generate ATP in quadriceps muscle (ATPmax). We will quantify other biological properties in biopsies including denervation, autophagy and accumulated biochemical damage and use gene expression to discover pathways that contribute to mobility and fitness. SOMMA uses MR for quadriceps volume and D3Cr dilution for total skeletal muscle mass, cardiopulmonary exercise testing to measure fitness (VO2 peak). We are also making many other intensive assessments of physical and cognitive function. Mobility endpoints include baseline and three year change in 400 m and 4 meter gait speed and fitness. SOMMA is building a large biobank of muscle, adipose blood, and urine specimens that will be available for ancillary studies. In this Symposium, we will present results from analyses of associations between muscle mitochondrial function and strength, muscle mass, cognitive performance, gait speed, and fitness. The symposium will also preview opportunities for collaborations and ancillary studies with SOMMA.

### INITIAL RESULTS FROM SOMMA: CONTRIBUTION OF MITOCHONDRIAL FUNCTION TO WALKING AND FITNESS

Steve Cummings,[1] Peggy Cawthon,[1] Bret Goodpaster,[2] Russell Hepple,[3] Nancy W. Glynn,[4] Stephen Kritchevsky,[5] Anne Newman,[6] and Paul Coen,[2] *1. California Pacific Medical Center, San Francisco, California, United States, 2. AdventHealth, Orlando, Florida, United States, 3. University of Florida, Gainsville, Florida, United States, 4. University of Pittsburgh Graduate School of Public Health, Pittsburgh, Pennsylvania, United States, 5. Wake Forest School of Medicine, Winston Salem, North Carolina, United States, 6. University of Pittsburgh, Pittsburgh, Pennsylvania, United States*

We hypothesize that the capacity of mitochondria in quadriceps skeletal muscle to generate ATP energy by respirometry (OXPHOS) in biopsies from the vastus lateralis, and in whole quadriceps muscle by 31PMRS (ATPmax) would contribute to 4 and 400m gait speed and to peak oxygen consumption on treadmill testing (VO2peak). In analyses from the first SOMMA participants recruited (N=122), OXPHOS was similarly associated with 4m (r=0.21) and 400 m (r=0.21) walking speed (P<0.01). However, ATPmax was not associated with either 4m or 400m walking speed (r=-0.02 and -0.07 respectively). In contrast both OXPHOS (r=0.43) and ATP max (r=0.35) were more strongly correlated with fitness (VO2 peak). These findings suggest that in older people, the mitochondrial capacity to generate ATP plays an important role walking speed and may be even more important to fitness.

### MUSCLE QUALITY REVISITED: BIOPSY ENERGETICS, MR SPECTROSCOPY, AND MUSCLE POWER AND STRENGTH

Anne Newman,[1] Adam Santanasto,[2] Elsa Strotmeyer,[1] Barbara Nicklas,[3] Paul Coen,[4] Bret Goodpaster,[4] and

Steve Cummings,[5] *1. University of Pittsburgh, Pittsburgh, Pennsylvania, United States, 2. University of Pittsburgh, Pittsburgh, Pittsburgh, Pennsylvania, United States, 3. Wake Forest School of Medicine, Winston-Salem, North Carolina, United States, 4. AdventHealth, Orlando, Florida, United States, 5. California Pacific Medical Center, San Francisco, California, United States*

With age, strength may decline faster than muscle mass pointing to a deterioration in muscle quality. Aspects of muscle quality and function are being measured in SOMMA; we hypothesized that in vitro and in vivo bioenergetics capacity of muscle would be related to muscle strength and power. Associations differed between men and women. In men (n=48, ATPMAX, 70, max OXPHOS and max ETS) but not women (n=68, ATPMAX, n=103, max OXPHOS and max ETS), muscle ATP regeneration by 31P MR spectroscopy was correlated with leg power (r = 0.27, p= 0.05). Energy production in tissue was similarly more strongly correlated with power in men than women, though not statistically significant. Correlations between the tissue measures and strength were also stronger in men than women. In ongoing follow-up, we will be able to determine what role that muscle tissue energetics plays in explaining the loss of strength and power with aging.

### MUSCLE BIOENERGETICS AND COGNITIVE EXECUTIVE FUNCTION: THE SOMMA STUDY BASELINE

Stephen Krtichevsky,[1] Stephen Kritchevsky,[2] Steve Cummings,[3] Anne Newman,[4] Paul Coen,[5] Anthony Molina,[6] Russell Hepple,[7] and Peggy Cawthon,[3] *1. Wake Forest School of Medicine, Wake Forest School of Medicine, North Carolina, United States, 2. Wake Forest School of Medicine, Winston Salem, North Carolina, United States, 3. California Pacific Medical Center, San Francisco, California, United States, 4. University of Pittsburgh, Pittsburgh, Pennsylvania, United States, 5. AdventHealth, Orlando, Florida, United States, 6. UCSD, La Jolla, California, United States, 7. University of Florida, Gainsville, Florida, United States*

Better executive function has been associated with faster walking speed, but the basis for this association is unclear. Systemic factors appear to contribute mitochondrial function across multiple tissues including muscle and brain. We hypothesized that muscle-based measures of bioenergetics capacity would be associated with cognitive function at SOMMA's baseline. MRI-based ATPMAX and muscle fiber respirometry-based max OXPHOS were correlated with scores on the MoCA (mean: 24.0; SD: 3.2); Trails B (mean: 138 seconds; SD: 73) and the Digit Symbol Coding Tests (mean: 50.8; SD: 14.9). The spearman correlations between ATPmax and the three measures were: 0.10 (p=0.29); -0.20 (p=0.03) and 0.16 (p=0.09), respectively. The association between max OXPHOS were: 0.18 (p=0.02); -0.20 (p<0.01) and 0.11 (p=0.13), respectively. Some associations appeared stronger in men than women. Gender interactions and whether energetics mediate some of the association between cognitive function and gait speed will be explored in the full baseline sample.

### ASSESSING MITOCHONDRIAL ENERGETICS OF SKELETAL MUSCLE IN THE STUDY OF MUSCLE MOBILITY AND AGING (SOMMA)

Bret Goodpaster,[1] Peggy Cawthon,[2] Stephen Kritchevsky,[3] Anne Newman,[4] Russell Hepple,[5] Steve Cummings,[2] and Paul Coen,[1], *1. AdventHealth, Orlando, Florida, United States, 2. California Pacific Medical Center, San Francisco, California, United States, 3. Wake Forest School of Medicine, Winston Salem, North Carolina, United States, 4. University of Pittsburgh, Pittsburgh, Pennsylvania, United States, 5. University of Florida, Gainsville, Florida, United States*

Mitochondria produce energy as ATP that essential for muscle contraction and movement. We hypothesize that age-related decreases in the capacity to generate ATP in muscle plays a major role in loss of mobility with aging. In SOMMA, we use high-resolution respirometry to measure the activity of electron transport system (ETS) in permeabilized muscle fibers from muscle biopsies. This allows us to assay ETS function in a highly controlled ex vivo experiment at the myocellular level, removed from other potentially limiting physiological factors including supplies of substrates and oxygen. We are also measuring the maximal capacity to generate ATP (ATPmax) in vivo by 31PMRS. ATPmax reflects the rate of phosphocreatine replenishment via oxidative phosphorylation. Analysis from the first 113 participants indicates that ATPmax correlates with Maximal OXPHOS (r=0.27, P=0.005), and Maximal ETS capacity (r=0.17, P=0.08). This suggests that these approaches provide complementary information on skeletal muscle energetics.

### ASSOCIATIONS OF BODY COMPOSITION AND MITOCHONDRIAL FUNCTION IN OLDER ADULTS

Peggy Cawthon,[1] Paul Coen,[2] Bret Goodpaster,[2] Russell Hepple,[3] Stephen Kritchevsky,[4] Anne Newman,[5] and Steve Cummings,[1], *1. California Pacific Medical Center, San Francisco, California, United States, 2. AdventHealth, Orlando, Florida, United States, 3. University of Florida, Gainsville, Florida, United States, 4. Wake Forest School of Medicine, Winston Salem, North Carolina, United States, 5. University of Pittsburgh, Pittsburgh, Pennsylvania, United States*

SOMMA assessed body composition by whole body magnetic resonance imaging (MRI) scans analyzed by AMRA (fat-to-weight ratio, mean: 57% ± 0.1; thigh muscle volume, mean: 8.9L ± 2.4; weight-to-muscle volume ratio, mean: 9.0 kg/L ± 1.6). Deuterated creatine dilution (D3Cr muscle mass, mean: 21.8kg ± 6.8) was used to measure muscle mass. Mitochondrial function was measured in fresh muscle tissue by respirometry (maximum OXPHOS and maximum ETS) and non-invasively by 31PMRS (ATPmax). Maximum OXPHOS and maximum ETS capacity were positively correlated with thigh muscle volume (r=0.42 and 0.44, respectively; p<.001) and D3Cr muscle mass (r=0.37 and 0.33, respectively; p<.001) and negatively correlated with fat-to-weight ratio (r=-0.36 and 0.26, respectively; p<.001) and weight-to-muscle volume ratio (r=-0.42 and -0.37, respectively; p<.001). Stratified analyses suggest that some associations may differ by sex. Associations between ATPmax and these body composition measures were more modest.

### Session 1485 (Paper)

### TRANSPORTATION ISSUES AT OLDER AGES

### ALCOHOL- AND DRUG-ASSOCIATED INJURY OUTCOMES AMONG OLDER ADULTS INVOLVED IN CAR CRASHES

Oluwaseun Adeyemi, *University of North Carolina at Charlotte, Mankato, Minnesota, United States*

Understanding how recent alcohol or drug use among older adults involved in car crashes can inform emergency care team on the morbidity and mortality risks. This study aimed to assess the odds of worsened health outcomes among older adults with evidence of alcohol or drug ingestion. This cross-sectional analysis used crash census data from the National EMS Information System. The outcome variable was the health outcome after EMS care, measured on a four-point ordinal scale: lower acuity, emergent, critical, and dead. The predictor variable was alcohol/drug use (present/not present). Age, race, gender, part of the body injured, and the revised trauma score of the patients were used as confounders. Odds ratio were calculated using proportional ordinal logistic regression. A total of 42,992 individuals, aged 65 years and older, were involved in car crash events, which required EMS activation. About 22.9% needed emergent care, 4.4% were classified as critical, and 0.4% died without resuscitation efforts. At the time of crash, 3.8% of the older population had evidence of alcohol or drug use. After adjusting for age, gender, race, injury location and revised trauma score of the crash patients, alcohol/drugs were associated with 54% increased odds of worse clinical outcome (AOR:1.54; 95% CI: 1.32-1.80). The adjusted odds remained elevated in urban (AOR: 1.69; 95% CI: 1.42-2.02) and suburban (AOR: 2.21; 95% CI: 1.12-4.35) and not significantly elevated in rural areas. Study findings can inform EMS service and emergency room care as well as policies that strengthen the urban and suburban EMS.

### CAREGIVING IS DRIVING MY TIME: CAREGIVER AND CARE RECIPIENT DRIVING BEHAVIORS AND HOURS OF CARE

Athena Koumoutzis,[1] Jonathon Vivoda,[1] and Jiawei Cao,[2] *1. Miami University, Oxford, Ohio, United States, 2. Miami University, Miami University, Ohio, United States*

Informal caregivers often provide transportation assistance as older adult care recipients (CRs) begin regulating their driving (e.g., avoid certain driving situations, decrease/cease driving). This study examined how caregiver and CR driving frequency and CR's driving avoidance behaviors impact caregiving intensity. Using data from Round 7 of the National Health and Aging Trends Study and the linked National Survey of Caregiving (n=1048 dyads), results indicated that caregiving intensity was highest among caregivers who drove everyday (5.38 hours) and for CRs who had not driven in the last month/did not drive (4.65 hours). Negative binomial regression techniques were used to assess and compare driving-related predictors. Compared to CRs who reported no avoidance of nighttime driving, caregivers of CRs who do not drive at all can expect to provide about 36% more hours of caregiving per day. Caregiving intensity was not significantly related to CR's driving alone, on the highway, or in bad weather avoidance behaviors. CRs who drove every day, most days, and rarely required between 33% and 40% fewer expected hours per day of caregiving compared to CRs who had not driven in the past month. The expected number of hours spent providing care per day was 36% higher among caregivers who drove the care recipient every day, 28% higher among most-day drivers, and 30% higher among those who never drove as opposed to caregivers who drove some days per week. Results suggest that caregiving intensity is related more to caregiver and CR driving frequency than CR driving avoidance behaviors.

### CHANGES IN TRANSPORTATION DURING THE COVID-19 PANDEMIC: RESULTS FROM A SURVEY OF MIDDLE-AGED AND OLDER FLORIDIANS

Anne Barrett, Cherish Michael, and Jessica Noblitt, *Florida State University, Tallahassee, Florida, United States*

The pandemic's numerous effects on everyday life include reductions in driving and changes in the use of other transportation modes, like getting rides from family and friends, walking, and biking. Aside from broad patterns, however, little is known about these changes, including how they affected different groups of the population and how they felt about them. Our study addresses these issues using data collected from an online survey of over 4,000 Floridians aged 50 or older, conducted between December 2020 and April 2021 and funded by the Florida Department of Transportation to support its aging road user program, Safe Mobility for Life. Changes in driving and in rides from family and friends were more striking than those in other transportation modes. Nearly 30 percent of respondents decreased their driving during the pandemic, while 20 percent got fewer rides from family and 25 percent got fewer rides from friends. In contrast, only 11 percent decreased their walking, and the same percentage increased it. Less common were changes in biking, with percent 7 decreasing and only 4 percent increasing it. Multivariate analyses revealed that these changes were influenced by gender, race, age, socioeconomic status, and health. Further insight was gained from analysis of an open-ended item, revealing positive and negative assessments of the changes. Positive assessments centered on feeling satisfied with working at home, spending more time outdoors, having more free time, and saving money. Negative assessments centered on social isolation, dissatisfaction with government responses to the pandemic, and reduced transportation options.

### DRIVER SPEED PROFILES INDEX WARNING SIGNS OF MCI

Jennifer Merickel,[1] Ruiqian Wu,[2] Matthew Rizzo,[2] and Ying Zhang,[2] *1. University Of Nebraska Medical Center, Omaha, Nebraska, United States, 2. University of Nebraska Medical Center, Omaha, Nebraska, United States*

Goal: Use driver behavior profiles to screen and index early warnings of cognitive decline and Alzheimer's disease (AD). Hypothesis: Real-world driver speed behavior profiles discriminate mild cognitive impairment (MCI).

**Methods:**Sensors were installed in personal vehicles of 74 legally-licensed, active drivers (age: 65-90 years, $\mu = 75.85$)

who completed 2, 3-month real-world driving assessments, including demographic and cognitive assessments, 1 year apart (244,564 miles driven). MCI status was indexed using 8 neuropsychological tests (spanning executive function, visuospatial skills, processing speed, and memory), relevant to MCI and driving. Driving environment was indexed from state speed limit (SL; roadway type: residential, commercial, interstate) and sunrise-sunset databases (time of day: day vs. night). Models: Data were randomly split into training (66%) and validation (33%) sets. An optimal mixed effects logistic regression model was determined from validation data AUC values.

**Results:**MCI drivers drove slower with optimal discrimination (estimated for every 5 mph decrease in speeding) in 1) residential roads (SL 25-35 mph; MCI odds increased by 6% [95% CI: 2-11%]), 2) interstate roads (SL >55 mph; MCI odds increased by 14% [95% CI: 8-20%]), and 3) night environments (MCI odds increased by 7% [95% CI: 2-12%]).

**Conclusion:** Quantitative indices of real-world driver data provide "ground truth" for screening and indexing phenotypes of cognitive decline, in line with ongoing efforts to link driver behavior with age-related cognitive decline and AD biomarkers. Behavioral biomarkers for diagnosing early warnings of dementia could ultimately bolster our ability to detect and intervene in early AD.

### MODES OF TRANSPORTATION TO MEDICAL AND PRIMARY CARE AMONG OLDER ADULTS

Zainab Suntai, Kefentse Kubanga, Emmanuel Adanu, and Abhay Lidbe, *University of Alabama, Tuscaloosa, Alabama, United States*

Transportation is an increasingly meaningful concern for older adults as physical, cognitive, and psychological changes in older adulthood impact mobility and accessibility. While several studies have examined the modes of transportation used among older adults, few have explored specifically how older adults are accessing primary care/medical care services. As such, this study aimed to determine the specific modes of transportation used among older adults for primary care visits. Data were derived from the 2018 National Health and Aging Trends Study (NHATS), an annual longitudinal panel survey of older adults aged 65 and older living in the United States. Descriptive analyses were conducted to examine the prevalence of several modes of access and logistic regression models were used to predict the likelihood of using the two most prevalent transportation modes, based on sociodemographic and socioeconomic factors. Results showed that 70% of older adults drive themselves to their doctor, 34.8% rely on a family member, friend, or paid person, 2.4% have a home visit, 2.1% use public transportation, 1.5% walk to their doctor and 1.1% use a taxi. Additionally, having higher income, being of younger age, being White, and having post-secondary education was associated with driving oneself to the doctor. These results indicate that while most older adults are still self-reliant on transportation to medical providers, those with lower socioeconomic status are particularly at risk of losing driving independence. Transportation-related interventions should therefore consider targeting individuals with lower economic capital by proving financial assistance, ride-share programs, and other innovative approaches.

### Session 1490 (Symposium)

### USING CLINICAL TRIALS AS AGENTS OF TRANSFORMATION IN POPULATIONS BURDENED BY DISPARITIES

Chair: Daniel Jimenez Discussant: Giyeon Kim

Older adults from racial/ethnic backgrounds as well as those from rural areas experience a disproportionate burden of physical and mental health risk factors. Given the prevalence of comorbid physical and mental health conditions in later life, the inadequacies of current treatment approaches for averting years living with disability, the disparities in access to the health care delivery system (including mental health care), and the workforce shortages to meet the mental and physical health needs of racial/ethnic and rural populations, development and testing of innovative strategies to address these disparities are of great public health significance and have the potential to change practice. This session will illustrate how four different interventions are being used to address mental and physical health needs in Latino and rural-dwelling older adults with the goal of reducing and ultimately eliminating disparities in these populations. Particular attention will be paid to the use of non-traditional interventions (e.g. social support, health promotion, technology). Results of clinical research studies will be presented alongside clinical case presentations. This integrated focus highlights the importance of adapting research interventions to real-world clinical settings.

### OVERCOMING HEALTH PROMOTION BARRIERS IN RURAL HEALTH: A QUALITATIVE ANALYSIS

John Batsis, *University of North Carolina at Chapel Hill, Chapel Hill, North Carolina, United States*

Weight loss interventions are fraught with difficulties for older adults in rural areas due to transportation difficulties, reduced availability of staff, and lack of programs. Telemedicine can overcome these barriers. A qualitative analysis of data from 44 exit-interviews from a rural-based, older adult weight loss study, informed by thematic analysis, was conducted. Participant's age was 73 years (73% female) and BMI was 36.5kg/m2. Distance to the site was 24 miles (31 min). Key themes included: a) telemedicine can help improve one's health, is more practical than in-person visits, is less costly, and time efficient; b) the majority (60%) were initially apprehensive about using telemedicine, a fear that resolved quickly; c) setting up telemedicine was easy and acceptable, despite a quick learning curve; d) having a team member for troubleshooting was important. Using telemedicine in older adults with obesity residing in rural areas should be considered in health promotion interventions.

### CERTIFIED OLDER ADULT PEER SUPPORT SPECIALISTS' USE OF TECHNOLOGY TO SUPPORT OLDER ADULTS IN THE COMMUNITY

Karen Fortuna, *Dartmouth College, Lebanon, New Hampshire, United States*

Middle-aged and older adults with mental health conditions have a high likelihood of experiencing comorbid physical health conditions, premature nursing home admissions, and early death compared with the general population of middle-aged and older adults. An emerging workforce of certified older adult peer support specialists aged 50 years or above is one of the fastest growing mental health workforces and may be a suitable community-based workforce to simultaneously support the mental health, physical health, and aging needs of middle-aged and older adults with a serious mental illness. Older adult peer support specialists are people with a lived experience of aging into middle age and older adulthood with a mental health condition. This presentation will present three single-arm pilot studies examining how certified older adult peer support specialists' incorporate technology, including text messaging, ecological momentary assessments, and smartphone applications into practice and clinical outcomes among older adults with serious mental illness.

### ENGAGING ISOLATED HISPANIC AND LATINO OLDER ADULT CAREGIVERS IN REMOTE BEHAVIORAL INTERVENTIONS

Caroline Silva, *University of Rochester School of Medicine, Rochester, New York, United States*

Recruiting Hispanic/Latino older adults for behavioral clinical trials is challenging, particularly those who are isolated. This presentation describes the recruitment process and feasibility/acceptability outcomes for the adaptation of two interventions (Connect for Caregivers and Engage Coaching) aimed at improving the social relationships and supports of older adult Hispanic/Latino caregivers of a family member with dementia. We compare online and community-based recruitment methods for English and Spanish-speaking Hispanic/Latino caregivers across the United States and present recruitment challenges during COVID-19. Of eligible participants, 82% were identified via online (e.g., research registry) and 18% via community-based (including snowball sampling) methods. Of participants recruited via online methods, 22% were Spanish-speaking, versus 100% of those recruited by community-based methods. Overall, 91% of all eligible/interested participants enrolled in at least one of the interventions. We discuss further the feasibility/acceptability of study procedures and the interventions, as the study was conducted remotely (via phone/Zoom) due to COVID-19.

### Session 1495 (Symposium)

### VOICES IN MOTION: HOW SOCIAL SINGING CAN FACILITATE PSYCHOLOGICAL HEALTH FOR PERSONS WITH DEMENTIA AND CAREGIVERS

Chair: Stuart MacDonald Co-Chair: Debra Sheets
Discussant: Theresa Allison

Dementia is a global public health priority that exerts significant impact on individuals, families, healthcare systems, and society. Worldwide, over 35 million individuals are estimated to have some subtype of dementia – a projection expected to triple by 2050. Despite progress, a cure for dementia remains elusive, and approved pharmacotherapies are selectively effective for but a limited duration. Increasingly, arts-based interventions for persons with dementia (PwD) and their caregivers are being recognized as inexpensive, non-invasive, and non-pharmacological lifestyle interventions with the potential to improve psychological function as well as reduce healthcare costs. This symposium overviews the Voices in Motion (ViM) study – a sociocognitive intervention exploring the impact of participation in an intergenerational choir on psychological, social, and cognitive function for PwD and their caregivers (n=32 dyads). PwD, caregivers, and local high school students sang in a professionally-conducted choir for as many as three seasons (each ~12 weeks long), spanning up to 18 months of follow-up. Employing an intensive repeated measures design, psychosocial, physiological, and cognitive function was measured every four to six weeks (up to 9 total assessments). The symposium papers provide a representative overview of the broad impact that this novel, non-pharmacological lifestyle intervention can offer vis-à-vis mitigating dementia symptoms and facilitating the psychological health of caregivers. Choir participation has important and significant impacts on psychosocial well-being and quality of life. Discussion focuses on policy implications and the need for community-based programs that reflect a social model for dementia and support living well through engaging and meaningful activities.

### THE EMERGENCE AND BENEFITS OF SOCIAL RELATIONSHIPS IN TWO COMMUNITY-BASED DEMENTIA CHOIRS

Andre Smith, Debra Sheets, Mary Kennedy, Tara Erb, Ruth Kampen, Chandra Berkan Hozempa, and Stuart MacDonald, *University of Victoria, Victoria, British Columbia, Canada*

Community choir participation for persons with dementia (PwD) confers benefits to health and well-being, including the benefit of socializing which can reduce feelings of loneliness and social isolation. Using the concept of social capital, this study examines the degree to which two intergenerational Voices in Motion choirs facilitate the development of social relationships between PwD, caregivers, and high school students. Data collection involved interviews with 17 dyads of PwD and caregivers, completion of a social relationship questionnaire, and focus groups with a total of 29 high school students. The results show a gradual increase in the level of interactions between all participants, with students in particular interacting more frequently with PwD. Over time, trust and reciprocity emerged within the choirs as more people shared information about themselves. Students' understanding of dementia changed over time as they learned to appreciate PwD as unique human beings with rich life stories and experiences.

### THE PROMISING IMPACT OF A VIRTUAL MUSIC INTERVENTION FOR PERSONS WITH DEMENTIA

Cynthia McDowell,[1] Jannell Walton,[2] Debra Sheets,[2] Andre Smith,[2] Robert Stawski,[3] and Stuart MacDonald,[2] *1. University of Victoria, University of Victoria, British Columbia, Canada, 2. University of Victoria, Victoria, British Columbia, Canada, 3. Oregon State University, Corvallis, Oregon, United States*

This study examines the within-person association between negative affect and global cognitive function for persons with dementia. Participants (n=33) engaged weekly in the Voices in Motion (ViM) sociocognitive choral intervention spanning up to 18-months and 9 individual assessments. Results revealed a significant time-varying association whereby within-person improvements in negative affect dynamically covaried with improvements on the Mini Mental State Examination (p<.05) across months of participation. These findings imply that, within-persons, reducing comorbidities associated with dementia (e.g., elevated negative affect) through participation in a lifestyle intervention, may facilitate increases in cognitive function. During the current pandemic, ViM transitioned to an online choir allowing for individuals to continue participating in the intervention and to maintain necessary social connections. The discussion focuses on the implications of this virtual choral intervention and the importance of modifiable risk factors such as negative affect and social isolation on the maintenance of cognitive health.

### INVESTIGATING THE CORTICAL CORRELATES OF SINGING: POTENTIAL NEURAL BENEFITS OF CHOIR FOR PERSONS WITH DEMENTIA

Nicholas Tamburri, Debra Sheets, Drew Halliday, Andre Smith, and Stuart MacDonald, *University of Victoria, Victoria, British Columbia, Canada*

Through leveraging the known advantages of musical engagement and socialization, choir interventions are known to facilitate psychological and cognitive benefits for persons with dementia (PwD). Surprisingly, no research has explored whether social singing may also confer neurological advantages. In this study, we employed functional near infrared spectroscopy (fNIRS) to investigate the cortical correlates of both social and solo singing in PwD (n=13). Paired-sample t-tests were used to evaluate within-person differences in frontal cortical activation between the social vs solo singing. Results showed significant activation differences in three frontal channels, with social singing requiring comparatively less frontocortical activation. These findings indicate potential neural benefits of social singing – with less frontal activation being a proxy for greater reliance on intact proceduralized systems – and serve to highlight the utility of fNIRS in better understanding the neural correlates underlying the benefits of social singing interventions for PwD.

### MODULATORS OF CAREGIVER DISTRESS AND CORRESPONDING IMPACTS ON PERSONS WITH DEMENTIA

Michaella Trites,[1] Sebastian Santana,[1] Debra Sheets,[1] Andre Smith,[1] Robert Stawski,[2] and Stuart MacDonald,[1] *1. University of Victoria, Victoria, British Columbia, Canada, 2. Oregon State University, Corvallis, Oregon, United States*

Extensive literature documents the detrimental effects of caregiver distress (CD) for caregivers. Less is known about the impact that CD exerts upon their care recipients, particularly persons with dementia (PwD). Using multilevel modeling, this study employed dyadic data from the Voices in Motion study to examine time-varying within-person associations between key caregiver and care recipient indicators of psychosocial function. An initial dyadic coupling model indicated that cognitive functioning for PwD and caregiver well-being significantly predicted shifts in CD. A second time-varying dyadic model found that, within dyads, high levels of CD predicted lower positive affect and increased depression scores in PwD. Most sociocognitive interventions target a sole member of a dyad; our results suggest that both dyad members are impacted simultaneously. Caregiver well-being impacts CD, which in turn, impacts well-being in PwD. The symbiotic relationship between care partners and PwD is nuanced, with further research required to understand the interdependencies.

### Session 1500 (Symposium)

### VULNERABILITY AND RESILIENCE AMONG OLDER ADULTS DURING THE COVID PANDEMIC

Chair: Carolyn Aldwin

The COVID-19 pandemic is particularly challenging for many older adults. They are strongly encouraged to practice social distancing and sheltering in place to decrease morbidity and mortality risks. However, social isolation and loneliness increase the risk of mental and physical health problems. Nonetheless, there are indications that older adults may be more resilient than originally thought. Park et al. present longitudinal findings that younger adults fared worse than middle-aged or older ones, reporting greater distress and less social support, mindfulness, and emotion regulation skills. For older participants, acceptance of negative emotions and social support predicted lower distress. Choun et al. also present longitudinal data, showing that depressive symptoms decreased among older adults during the lockdown phase of the pandemic, but reflected changes in loneliness and physical health symptoms. Stellman et al. found that moderate levels of combat experience were helpful for some older Vietnam veterans coping with the pandemic, although a few found that it made coping more difficult. Turner et al. found that positive, non-familial intergenerational contacts were associated with a higher number of positive pandemic-related changes, such as finding new hobbies and meaning in work. Finally, Aldwin et al. present a social ecological approach to vulnerability and resilience. Using qualitative data, they found that stressors and resources were reported at personal, interpersonal and societal levels. Further, older adults both took comfort from and contributed to community resources during this pandemic. In summary, this symposium identified factors that contribute to older adults' resilience during this pandemic.

### EMOTION REGULATION RESOURCES EXPLAIN MIDDLE-AGED AND OLDER ADULTS' COVID-19-RELATED DISTRESS

Cystal Park,[1] Beth Russell,[2] and Michael Fendrich,[2] *1. University of Connecticut, Storrs Mansfield, Connecticut, United States, 2. UCONN, Storrs Manfield, Connecticut, United States*

As the pandemic caused widespread disruption across the world, studies suggested younger adults were faring more poorly than other adults. We hypothesized that younger adults might possess fewer emotion regulation resources and skills, accounting for their greater distress. In a national sample of 1528 adults, we examined how baseline resources

(in mid-April, during initial peak infections) predicted distress (depression, anxiety, PTSD symptoms) five weeks later, when states began initial reopenings. Younger adults reported greater distress and less social support, mindfulness, and emotion regulation skills than did middle aged and older adults.. Controlling for stress exposure, younger adults' distress was predicted by impulsivity and lack of perceived strategies while middle-aged and older adults' distress was predicted by acceptance of negative emotions; perceived social support was related to lower distress for both groups but mindfulness was unrelated. Results suggest that emotion regulation skills are a promising prevention and intervention focus.

### LONELINESS, SOCIAL CONTACT, AND HEALTH AMONG OLDER ADULTS DURING THE COVID-19 PANDEMIC

Soyoung Choun, Carolyn Aldwin, and Dylan Lee, *Oregon State University, Corvallis, Oregon, United States*

The COVID-19 pandemic is a challenging situation for many older adults at elevated risk for mortality. Social distancing and lockdown to prevent contagion may result in social isolation and feelings of loneliness, which can have adverse effects on health. We examined how depressive symptoms were associated with between-person differences and within-person variations in loneliness, social contacts, and daily physical problems during 8 weeks. We sampled 247 older adults (Mage = 71.1, SD = 7.3, range = 51 - 95), who participated at micro-longitudinal online surveys (baseline and 7 weekly follow-ups) from April 28 to June 23. Multilevel modeling analysis controlling age, gender, marital status, and education showed that depressive symptoms were significantly decreased during 8 weeks. Further, depressive symptoms were positively coupled with both loneliness and physical problems for both the within-and between-person levels. Increase in social contact was related to decreases in depressive symptoms only at the between-person level.

### COPING WITH COVID: DOES PRIOR MILITARY SERVICE PLAY A ROLE FOR VIETNAM VETERANS?

Jeanne Stellman,[1] Steven Stellman,[2] Anica Kaiser,[3] Avron Spiro,[4] and Brian Smith,[5] *1. Mailman School of Public Health, Columbia University, Mailman School of Public Health, Columbia University New York, New York, United States, 2. Department of Epidemiology, Mailman School of Public Health Columbia University New York, New York, United States, 3. National Center for PTSD at VA Boston Healthcare System, Boston University School of Medicine, VA Boston Healthcare System Boston, Massachusetts, United States, 4. Boston University, Boston, Massachusetts, United States, 5. Women's Health Sciences Division, National Center for PTSD, VA Boston Healthcare System Boston, Massachusetts, United States*

We investigated the impact of earlier military combat on ability to cope with the COVID-19 pandemic in 379 male Vietnam veterans who responded to surveys in 1984, 1998, and 2020. Combat exposure was scored with a validated scale, contrasting lowest tertile (8-15) vs. medium/high (16-40). About one-fourth of veterans (26%) reported that their military experience made it easier to cope with the pandemic, while over half (59%) said it had no effect. Medium/high-combat veterans were more likely to report that their military experience made coping easier (OR = 1.8, p = 0.03), but were less likely to report no effect of service on their coping than low-combat veterans (OR = 0.40, p<0.001). All 19 respondents (5%) who said military experience made coping more difficult were medium/high combat veterans. Military experience, and combat particularly, affected many of these veterans' ability to cope with the pandemic decades after their service.

### INTERGENERATIONAL RELATIONSHIPS AS A RESOURCE FOR RESILIENCE DURING THE COVID-19 PANDEMIC

Shelbie Turner,[1] Karen Hooker,[2] Shannon Jarrott,[3] and John Geldhof,[1] *1. Oregon State University, Oregon State University, Oregon, United States, 2. Oregon State University, Corvallis, Oregon, United States, 3. The Ohio State University, Columbus, Ohio, United States*

The intergenerational ties that offer support to older adults are likely useful for resilience during the COVID-19 pandemic. We analyzed whether positive and negative intergenerational contact was associated with positive pandemic-related personal change. We utilized data collected from 566 adults aged 50 and older between August 2020 and January 2021 via MTurk and a statewide research registry. Participants reported the quality of their contact with younger adults, and whether they experienced positive changes (i.e. new hobbies, healthier behavior, greater meaning in work) as a result of the pandemic. Higher positive, but not lower negative, non-familial intergenerational contact was associated with a higher number of positive pandemic-related changes (estimate = 0.07, SE = 0.03, p=0.02). The quality of familial intergenerational relationships were not associated with positive pandemic-related changes. Non-familial intergenerational relationships may be especially important for resilience, and should be supported during a time when they may be difficult to maintain.

### A SOCIAL ECOLOGICAL MODEL OF VULNERABILITY AND RESILIENCE IN OLDER ADULTS DURING THE COVID-19 PANDEMIC

Carolyn Aldwin,[1] Heidi Igarashi,[2] Maria Kurth,[1] Hye Soo Lee,[1] Soyoung Choun,[1] and Dylan Lee,[2] *1. Oregon State University, Corvallis, Oregon, United States, 2. Oregon State University, Oregon State University, Oregon, United States*

**Objectives:** We used a social ecological model to examine vulnerability and resilience among older adults during the COVID-19 pandemic.

**Methods:** We analyzed two open-ended questions included in a survey of 235 respondents, 51–95 years old (M = 71.35; SD = 7.39; 74% female), which asked about COVID-19-related difficulties and positive experiences during the past week. We identified three different levels for difficulties and positives at the personal, interpersonal, and/or societal levels.

**Results:** Fewer than half of the respondents reported on difficulties (41%) and positives (40%) just at the personal level. In terms of crossing levels, people were most likely to report events spanning the personal and interpersonal levels (14% and 18%, respectively). A few individuals reported difficulties and positives at the societal level.

**Discussion:** Older individuals were acutely aware of challenges and positives existing at all three levels, and contributed to resources at the interpersonal and community levels.

### Session 1505 (Symposium)

### THE DIVERSE SPIRIT: SPIRITUAL CARE PERSPECTIVES AND THE IMPLICATIONS FOR OLDER ADULTS IN VARIOUS SETTINGS

Chair: Lydia Manning Co-Chair: Chad Federwitz
Discussant: Julie Hicks Patrick

Religiosity and spirituality are commonly supported and viewed as essential elements of well-being in old and very old age, particularly at end of life. These essential elements often include the exploration of the meaning in life, inner peace, belonging, contentment, and near-end-of-life-completion. The positive outcomes of religious and spiritual beliefs and practices have been well established. However, these experiences and related positive outcomes may not always include a diverse array of older adults. The spiritual care of older adults is becoming more culturally diverse and includes differing perspectives on what constitutes spiritual care, both in approach and practice. This symposium will explore the current state of spiritual care for older adults through a lens of cultural diversity and inter-religious/spiritual perspectives. A focus on the current practices of spiritual care for older adults and future implications will also be considered. Recommendations pertaining to future gerontological inquiry in the importance of spiritual care, as well as diverse approaches within gerontological practice will be highlighted and discussed.

### SELF-TRANSCENDENT WISDOM MEDIATES THE ASSOCIATION BETWEEN SPIRITUALITY AND WELL-BEING IN SIX NATIONS

Monika Ardelt,[1] Juensung Kim,[2] and Michel Ferrari,[2], *1. University of Florida, Gainesville, Florida, United States, 2. University of Toronto, Toronto, Ontario, Canada*

Distraught individuals sometimes turn to religion for solace, particularly in old age, so spirituality is not necessarily positively related to well-being. However, spirituality might lead to greater well-being if it promotes self-transcendent wisdom. Using a sample of 307 respondents from six nations (USA, Canada, Serbia, Ukraine, Iran, and China), ranging in age from 59 to 99 years (M=73.00, SD=8.13), this study tested the generalizability of the hypothesized mediated pathway. Results showed only weak correlations between spirituality and well-being measures in the whole sample. Yet, as predicted, spirituality, mediated by self-transcendent wisdom, was indirectly related to greater well-being in all six nations, despite significant differences by nation in variable means. Spirituality had additional direct positive effects on life satisfaction in Canada, Iran, and China and on general well-being in Iran and China. These findings suggest that spirituality likely results in greater well-being when it transcends egocentric concerns.

### SPIRITUALITY TRAINING AS AN ESSENTIAL ELEMENT OF PERSON-CENTERED CARE

Raya Kheirbek, *University of Maryland School of Medicine, University of Maryland School of Medicine, Maryland, United States*

Palliative Medicine, built on the biopsychosocial-spiritual model of care, has long recognized the critical role of spirituality in the care of patients with complex, serious, and chronic illnesses. We conducted focus groups to arrive at a consensus definition of “spiritual care.” Additionally, we collected and compared frameworks and models that recognize that providers cannot be made compassionate simply through the imposition of rules; methods were needed to achieve behavior change. The created curricula covered the definitions of spiritual care, self-awareness, cultural sensitivity, assessment, and skills. As part of ongoing curriculum development processes, training included evaluation tools to accompany competency standards. Results demonstrated improvements in self- reported abilities to (a) establish appropriate boundaries with patients; (b) apply the concept of compassionate presence to clinical care; (c) understand the role of spirituality in professional life; (d) identify ethical issues in inter-professional spiritual care. Clinicians need to address patients’ spiritual needs.

### COPING WITH UNCERTAINTY: USE OF CONTEMPLATIVE PRACTICES AMID A PANDEMIC

Nirmala Lekhak,[1] Tirth Bhatta,[2] Tim Goler,[3] and Eva Kahana,[4] *1. University of Nevada Las Vegas, Las vegas, Nevada, United States, 2. University of Nevada, Las Vegas, Nevada, United States, 3. Norfolk State University, Norfolk, Virginia, United States, 4. Case Western University, Case Western reserve University, Ohio, United States*

Contemplative practices have been used as coping resources to reduce the negative influences of adverse life situations on mental health. The COVID-19 pandemic has disproportionately impacted older adults, causing immense uncertainty, stress, and anxiety. By using data from our “Coping with Pandemic” nationwide web-based survey (n=1861), we examine the utilization of practices such as meditation, prayer, and yoga across social, economic, and health status during the pandemic. Consistent with studies conducted before the pandemic, we find significantly greater utilization of meditation and yoga among women and higher educated individuals. Findings showed significantly greater usage of prayer among women and Blacks. Unlike previous studies, we documented greater usage of meditative practices among Blacks than Whites. Older adults with higher anxiety were significantly more likely to practice meditation and yoga. Our study offers much needed guidance for future intervention studies aimed at improving mental health among diverse groups of older adults.

### AN EVOLVING INQUIRY OF MONASTIC SPIRITUAL CARE FOR AGING INMATES

Alex Bishop,[1] and Kevin Randall,[2] *1. Oklahoma State University, Oklahoma State University, Oklahoma, United States, 2. Sam Houston State University, Sam Houston State University, Texas, United States*

This investigation involved focus-group inquiry of the Oblates in Prison Program, a faith-based ministry founded on monastic principles in the Rule of St. Benedict. Data from a Benedictine Order monk and program coordinator, ordained prison minister, and lay ministry volunteer were collected. Participants were asked a series of questions regarding the spiritual care of aging prisoners. Responses were coded and cross-compared for thematic content. Of central thematic importance was implementation of a spiritual care model using traditional monastic rules for daily living. A second theme centered on purposeful rebuilding of self-renewal through stability and obedience. A final emergent theme encompassed institutional acceptance in the provision of religious sacraments, sacred texts, and artifacts. Results highlight the broader implications of providing spiritual care and outreach to aging prisoners. The role of restorative justice for successful delivery of faith-based spiritual care for improved rehabilitation of aging inmates will be further addressed.

### Session 2000 (Symposium)

### ADVANCE CARE PLANNING IN THE CONTEXT OF COVID-19: COMPLEXITIES ACROSS A RANGE OF CONSTITUENTS

Chair: Brian Carpenter Discussant: Karen Hirschman

The COVID-19 pandemic brought serious illness and death into close proximity for a large number of people, whether through personal experience, infection in family members or friends, or unremitting media coverage on the effects of the virus and widespread mortality. Because of a collective vulnerability to illness and the heightened possibility of death, more people began to contemplate what kinds of medical care they would want if they ever became seriously ill. In other words, more people began the process of advance care planning (ACP). This symposium explores how the COVID-19 pandemic shifted interest in and execution of ACP across a range of groups. The first presentation reviews survey data from a large, community-based sample of older adults about their ACP conversations before and after the start of the pandemic. Shifting to the experience of clinicians, the second presentation summarizes a survey with multidisciplinary healthcare professionals about ACP conversations in their personal lives during the pandemic and how their observations of patients influenced their own plans. The third presentation describes the reactions of undergraduate students to an ACP class exercise, including COVID-19 as a motivating factor for pursuing ACP. The final presentation concludes with a review of two clinical cases that illustrate how COVID-19 has upended traditional ACP and highlighted the need for new policies and processes, with a particular focus on ethics and equity. Together, these presentations offer diverse insights into how ACP may shift in a post-pandemic world.

### HAS THE COVID-19 PANDEMIC INCREASED ADVANCE CARE PLANNING DISCUSSIONS HELD BY OLDER ADULTS?

Gloria Gutman,[1] Brian deVries,[2] Robert Beringer,[3] Paneet Gill,[4] and Helena Daudt,[5] *1. Simon Fraser University, Vancouver, British Columbia, Canada, 2. San Francisco State University, San Francisco, California, United States, 3. University of Victoria, Victoria, British Columbia, Canada, 4. Simon Fraser University, Surrey, British Columbia, Canada, 5. Victoria Hospice, Victoria, British Columbia, Canada*

In an online survey exploring older Canadians' experiences during the COVID-19 pandemic, 3989 respondents aged 55-99 indicated whether they had discussed their future care preferences and with whom, prior to and since the outbreak. Pre-pandemic, 62% had held such discussions; since the pandemic 43% did so, 11% for the first time. Rates were significantly higher among white respondents than among persons of color, women than men, and those 65+ than younger respondents. Respondents were most likely to have talked, respectively, with their spouse (58% before; 40% since), family (35%; 22%), and friends (12%; 10%)—with higher rates for white, women and older respondents. Surprisingly, only 4% before and 2% since the pandemic had discussed their care preferences with a doctor. Initiation of some new discussions was encouraging but there were fewer than expected, perhaps due to denial, superstition, or disbelief of pandemic severity. Advance care planning remains an under-utilized resource.

### WHEN THE PROFESSIONAL TURNS PERSONAL: HEALTHCARE PROVIDERS AND THEIR OWN ADVANCE CARE PLANNING DURING THE PANDEMIC

Brian Carpenter, *Washington University in St. Louis, Saint Louis, Missouri, United States*

As healthcare professionals counseled patients and care partners during the pandemic about treatment options, clinical probabilities, and preparations for death, they had opportunities to reflect on how they themselves would want to be treated if they fell ill. We conducted a survey with healthcare professionals who were caring for patients during the pandemic and asked their work had affected their own advance care planning. Based on their clinical observations, 28% revised their personal interest in life-prolonging medical interventions. Substantial proportions had initiated conversations with partners (45%), parents (46%), and their primary care physician (29%) about their medical preferences. Similarly high percentages had encouraged family members and friends to update or complete their advance care plans, and 26% intended to initiate planning in the near future. Interest in advance care planning is high among healthcare professionals, who may benefit from tailored resources that acknowledge their clinical experience.

### COVID-19 AND DEATH ANXIETY: THE IMPACT ON STUDENTS' APPRAISALS TO COMPLETING ADVANCE DIRECTIVES

Carlyn Vogel,[1] Debra Dobbs,[2] Maureen Templeman,[1] Victoria Marino,[1] and William Haley,[1] *1. University of South Florida, Tampa, Florida, United States, 2. University of South Florida, School of Aging Studies, University of South Florida, Florida, United States*

This study examined possible effects of COVID-19 on students' appraisals, coping, and responses to completing advance directives (ADs). We used the transactional model of stress and coping to explore 93 undergraduate students' responses to an AD assignment completed in an undergraduate course during COVID-19. Students watched a recorded lecture, read content related to ADs, and examined a sample copy of a 5 Wishes document. Students completed an assignment reflecting on reactions to completing ADs. Content analysis of 65 responses indicated almost 10% of students mentioned COVID-19 or the pandemic as a reason to complete ADs. Approximately 18% mentioned their youth and 40% mentioned sudden or serious illness as reasons to complete ADs. Nearly 30% mentioned death anxiety as a reason for being unprepared to complete ADs. Instructors should consider ways to inform and help students process their emotions given contextual factors (e.g. the pandemic) when teaching about ADs.

### ADVANCE CARE PLANNING IN THE TIME OF COVID: REAL-WORLD CASE DISCUSSIONS

Erica Frechman,[1] and Harleah Buck,[2] *1. Nursing, Nashville, Tennessee, United States, 2. University of Iowa, Iowa City, Iowa, United States*

COVID has accelerated the science and practice of palliative care while creating a sense of urgency in advance care planning (ACP). Past palliative care principles and practices have been stressed to the breaking point, causing the need for new ACP approaches to elicit patient-and-family goals/values/preferences. In this presentation, two cases will be used to discuss pragmatic implications and real-world implementation strategies in the trenches of the major health care system disruptor of this generation. We will discuss access to documentation, immediacy and complexity of the decision-making process, provider impacts, role of the family, and even an upside to this crisis. This will include questions of ethics and equity. The first case involves an older man whose family disregarded his wishes, while the second case discusses the pitfalls and pearls when family cannot visit. These two cases highlight COVID's impact on ACP and cause us to re-think its process and delivery.

### Session 2005 (Paper)

### AGEISM (SRPP PAPER)

### AGEISM AND PERCEPTIONS OF VULNERABILITY: FRAMING OF AGE DURING THE COVID-19 PANDEMIC

Jasmin Tahmaseb McConatha, jordan Broussard, and Jacki magnerelli, *West Chester University, West Chester, Pennsylvania, United States*

Media representations of the Covid-19 pandemic and its devastating consequences have shaped people's fears, anxiety, and perceptions of vulnerability. Social scientists have examined the consequences of how information is "framed." Framing theory asserts that issues can be portrayed differently by emphasizing or de-emphasizing aspects and information. According to Lakoff (2004) the impact of a message is not based on what is said but how it is said. Theories of framing focus on how the media frames issues, which then structure and shape attitudes and policies. A news article serves as a frame for an intended message. The purpose of this project is to analyze the ways that "age" has been framed during the Covid-19 pandemic. One of the most dominant frames in terms of COVID-19 coverage is how the pandemic has been analyzed through the lens of age and framed in terms of age discrimination. Method: A thematic analysis of New York Times and Washington Post news articles addressing older adults and illness vulnerability was conducted. The results of news articles appearing in these prominent newspapers indicated that the perceptions of older men and women tended to focus on the relationship between age and vulnerabilities to severe consequences from Covid-19. The frames in which these new articles were presented indicated ageist tones and messages that had the potential to either reinforce or lead to age stereotyping and discrimination.

### AGEIST POLICIES THAT FAVOR OLDER PEOPLE: WHAT DO OLDER PEOPLE THINK?

Hakan Jonson,[1] and Tove Harnett,[2] *1. Lund University, Lund University, Skane Lan, Sweden, 2. Lund University, Lund, Skane Lan, Sweden*

Policies on supportive services have frequently used chronological age to determine rights and needs of people within the adult population. Such policies have been described as ageist, but could also be regarded as favoring older people in cases where chronological age is used as a proxy for needs. In Sweden, municipalities have recently been allowed to grant people above a certain age some home care services without individual needs testing, and several political parties have suggested that a nursing home guarantee at the age of 85 should be introduced. The aim of the study that this presentation reports on was to investigate views among older people on age as an organizing principle for distributing eldercare services. Data was collected through an online surveys to members of pensioners' organisations (N=1540). Respondents were asked about their views on a number of age-related policies that are used or proposed as part of the eldercare system in Sweden. The analysis revealed a general support for the use of chronological age as a proxy for needs. This suggest that respondents used an interest groups perspective and supported stereotypical arrangements that favored older people. When free-text answers were included in the analysis, it became evident that the use of chronological age was not related to the problem of ageism. In the presentation we will discuss the potential gap between anti-ageism and views of older people and what a framework on ageism brings into the moral economy of eldercare.

### EMPLOYERS' SUPPORT OF OLDER ADULTS FACING AGEISM IN THE WORKPLACE: A SCOPING REVIEW OF THE LITERATURE

Amanda Bull, Raza Mirza, Andrea Gardiola, Christopher Klinger, and Jessica Hsieh, *University of Toronto, Toronto, Ontario, Canada*

As the Canadian population continues to age rapidly, addressing the social structures that negatively impact older adults is of increasing importance. The most prominent of these social structures is the workplace, which can be a potential source of age discrimination. The goal of this scoping review was to analyze the literature that addresses strategies for employers to support older workers experiencing ageism, in order to answer the research question: How can employers support older adults (50+) facing ageism in the workplace? Following Arksey and O'Malley's five-step framework, an electronic database and grey literature search was conducted between September and December 2020. Thematic content analysis was performed to establish key themes. The search revealed 3,635 peer-reviewed and grey literature sources that were evaluated by three investigators. Thirty-six articles, published between 2006 and 2020, met inclusion criteria and examined various support strategies for employers. Five major emerging themes were identified from the literature: (1) Recruitment practices, (2) Training opportunities, (3) Education for managers, leaders, and employees in the workplace, (4) Flexible employment opportunities, and (5) Methods to change the psycho-social environment of the workplace. Implementation of these interventions is required to support older adults who may be experiencing workplace ageism. Longitudinal research of these interventions is required to determine the lasting effects of these strategies; however, the existing literature supports the implementation of these supportive actions, which is vital to ensuring that older adults are able to attain and maintain valuable work, in healthy environments, now and into the future.

### THE OTHERING OF OLDER PEOPLE IN DISABILITY POLICIES

Hakan Jonson, *Lund University, Lund University, Skane Lan, Sweden*

Disability policies in Sweden rest on the idea that all humans have equal value and a goal of policies has been to enable persons with disabilities to be able to live like other members of society, but older people do not seem to be included as part of this goal. The presentation concerns the Swedish support system's legal discourse, and investigates the rationale for excluding people over the age of 65 from services that younger people with disabilities may obtain. Data consists of government texts and court decisions under the Severe Disability Act about services for people over the age of 65. It was found that little in the legal discourse concerns the needs and rights of older people, and the general belief is that the Severe Disability Act is primarily intended for children, young people, and adults of working age. The legal discourse contained a type of "residual ageism" that was justified through the indirect construction of older people as different. Othering of older people was present in assumptions about differences in categorizations (people with disabilities vs older people with support needs), needs (active age vs not active age), and comparisons (with people without disabilities of the same age vs with others receiving eldercare). The presentation outlines potential changes of these policies.

### Session 2010 (Symposium)

### AGING IN PLACE: SOCIAL CONNECTEDNESS AND THE HEALTH OF OLDER SUBSIDIZED HOUSING RESIDENTS

Chair: Thomas Cudjoe Co-Chair: Judith Gonyea
Discussant: Robyn Stone

Findings on the prevalence and impacts of loneliness and social isolation on the lives of older adults have led to the lack of social connectedness being viewed as a major public health issue. Although an understudied population, emerging evidence suggests that for the nearly 3 million older low-income adults living in subsidized housing the impacts of lack of social connection may be especially profound. From a life course perspective, "cumulative economic disadvantage," if combined with a "cumulative connectedness disadvantage," may heighten exposure to health risk factors and negative health outcomes. Addressing social risk factors in this population thus may have the potential to reduce health disparities as well as improve quality of life. Decades of epidemiologic evidence suggest that social isolation is a key factor in disparate outcomes. Evidence-based interventions are needed to improve aging in place for this population, but little is known about the individual or contextual factors that influence social connectedness and health among this high-risk population of older adults. This symposium includes interdisciplinary perspectives and methods as well as present results from four studies that examine 1) perspectives on aging in place in subsidized housing, 2) relationship between social connection and food insecurity 3) relationship between social isolation and healthcare utilization and 4) between resident ties and links to health outcomes. Papers in this symposium will highlight the implications of study findings for informing future interventions to improve aging in place for older adults living in subsidized housing.

### RESOURCES WITHIN: BETWEEN RESIDENT TIES AND LINKS TO HEALTH AMONG OLDER SUBSIDIZED HOUSING RESIDENTS

Noah Webster, *University of Michigan, Ann Arbor, Michigan, United States*

Increasing evidence points to the importance of non-family ties in promoting health among older adults. Less is known though about these ties within the context of subsidized housing. In this study we examine prevalence of social ties between residents and examine links to health. Data were collected through interviews conducted with 39 residents age 62 and older living in a subsidized housing community in Southeast Michigan. Residents reported knowing on average 10 (SD=6.5) other residents, and nominated three (SD=4.2) residents into their close social networks. Residents who reported getting out of the community less often and those with one or more health limitations nominated significantly more residents into their network. Also, getting out of one's apartment more often was associated with knowing more residents in the community. Findings highlight between resident ties may serve as an important resource for those geographically restricted and may be useful to integrate into interventions.

### AGING IN PLACE FOR OLDER SUBSIDIZED HOUSING RESIDENTS: THE INFLUENCE OF SOCIAL CONNECTION AND SOCIAL ENVIRONMENT

Rebecca Brown,[1] David Reyes-Farias,[2] Erin Finucane,[2] Amanda Watson,[2] Momana Jahan,[2] Sonia Pandit,[2] Carolina Reid,[3] and Barbara Resnick,[4], *1. Perelman School of Medicine of the University of Pennsylvania, Wynnewood, Pennsylvania, United States, 2. University of Pennsylvania, Philadelphia, Pennsylvania, United States, 3. University of California, Berkeley, Berkeley, California, United States, 4. University of Maryland School of Nursing, Baltimore, Maryland, United States*

Older adults living in subsidized housing experience health disparities including disproportionate rates of social isolation and nursing home admission. Little is known about how social relationships and social environment influence aging in place for this population. We interviewed 58 residents aged 62 or older. Qualitative thematic analyses revealed that social relationships both inside and outside the building contributed to residents' experience of aging in place. Relationships with other residents and staff members provided social support, while connections to family and friends outside the building "opened up" the residents' world and provided a sense of connection to the larger community. Social and physical environment also contributed, with residents' ability to move between private and public spaces leading to feelings of freedom and independence. Discussion focuses on expanding definitions of aging in place to encompass residents' experiences and implications for improving aging in place for this population.

### SOCIAL ISOLATION AND HEALTHCARE UTILIZATION AMONG OLDER ADULTS LIVING IN SUBSIDIZED HOUSING

Thomas Cudjoe,[1] Laura Prichett,[2] Katherine Runge,[3] Laura Andes,[3] carl latkin,[4] and Cynthia Boyd,[5] *1. Johns Hopkins University School of Medicine, Baltimore, Maryland, United States, 2. Johns Hopkins BEAD, Baltimore, Maryland, United States, 3. Mercy Housing, Denver, Colorado, United States, 4. Johns Hopkins Bloomberg School of Public Health, Baltimore, Maryland, United States, 5. Johns Hopkins University, Baltimore, Maryland, United States*

Older adults living in subsidized housing are often at high risk for having multiple chronic conditions and nursing home placement. Previous studies in this population have not examined the relationship between social isolation and healthcare utilization. We examine this using Lubben Social Network Scale-6 and self-reported healthcare utilization. Utilizing data from a multi-state non-profit subsidized housing provider, we performed descriptive and multivariate analyses on a sample of older adults (N=3,822). Overall, 95 % reported having a checkup within the last 12 months and an average of less than one emergency room visits (mean= 0.58) or hospitalizations (mean= 0.34). In adjusted models, Socially isolated older adults had lower levels of routine checkup (OR=0.50, CI 0.36,0.70) and higher levels of hospitalizations (IRR=1.30, CI 1.10,1.54) compared to older adults who were not socially isolated. Efforts to address healthcare utilization should identify social isolation and explore strategies to promote social connectedness to improve health.

### FOOD INSECURITY AMONG OLDER URBAN SUBSIDIZED HOUSING RESIDENTS: THE IMPORTANCE OF SOCIAL CONNECTEDNESS

Judith Gonyea,[1] Arden O'Donnell,[1] and Alexaandra Curley,[2] *1. Boston University, Boston, Massachusetts, United States, 2. Case Western Reserve University, Cleveland, Ohio, United States*

Poverty and food insecurity are associated with poor health in later life. Although housing is recognized as a social determinant of health; relatively little research has explored food insecurity in the marginalized population of older subsidized housing residents. In this study, we examined factors associated with food insecurity and particularly how social connectedness was associated with food insecurity. We hypothesized that social connection measures (i.e., loneliness, sense of belonging) independent of sociodemographic, health and food program variables would contribute to food insecurity. Our data are from interviews with 216 residents ages 55-plus (50% Black, 45% LatinX). The 6-item USDA Household Food Security Survey found high rates of food insecurity, 40% for ages 55-69 and 20% for ages 70-plus. Multivariate logistic regression models revealed that loneliness was significantly related to food insecurity even after other factors were controlled. Discussion centers on strategies for addressing social risk factors to ameliorate food insecurity.

### Session 2015 (Symposium)

### ALZHEIMER'S DISEASE RESEARCH AND OUTREACH IN THE NEW NORMAL: TRANSITIONING TO THE VIRTUAL WORLD

Chair: Stacy Andersen Co-Chair: Patricia Heyn

Following disruptions to research, clinical trials, and support for individuals with Alzheimer's disease and related dementias (ADRD), 2020 taught us important lessons about the need for creativity, flexibility, and resourcefulness during an urgent and global public health crisis. The COVID-19 pandemic showed that we have knowledge and technology that can be rapidly implemented, adopted, and utilized by many sectors to allow the continued care and research of our older adult population with ADRD. Thus, this symposium will address virtual methods that are transforming ADRD research and support. First, Dr. Rhodus will discuss the implementation of online assessments in clinical trials at an Alzheimer's Disease Research Center and the effects of sociodemographic disparities in online accessibility. Next, Dr. Bazzano will describe methods of remote collection of brain health data through tablets, smartphones, and wearables in the Bogalusa Heart Study. Then, Dr. Andersen will report on the transition from in-person to virtual assessments of cognitive and physical function in centenarian studies and address strategies for inclusivity of individuals with limited technology experience. Next, Dr. Fazio will introduce Project VITAL which aims to impact social isolation by increasing accessibility to virtual education and support for care community staff, family caregivers, and individuals with dementia. Finally, Dr. Penfold will report on the translation of a paper-based, face-to-face intervention for reducing caregiver burden into a self-directed online learning program. Overall, these presentations highlight successes and challenges in incorporating virtual-based methods to maintain engagement with participants, individuals with ADRD, and caregivers during the pandemic and beyond.

### LONGEVITY STUDIES IN THE NEW NORMAL: THE MOVE TO VIRTUAL ASSESSMENT

Stacy Andersen,[1] Sandra Rizer,[2] Lance San Souci,[1] Melissa Berlin,[1] Emily Harris,[1] Stephanie Cosentino,[3] Paola Sebastiani,[4] and Thomas Perls,[5] *1. Boston University*

School of Medicine, Boston, Massachusetts, United States, 2. Columbia University Irving Medical Center, New York, New York, United States, 3. Columbia University Medical Center, New York, New York, United States, 4. Tufts Medical Center, Physician Organization, BOSTON, Massachusetts, United States, 5. Boston University, Boston, Massachusetts, United States

Extreme longevity is associated with resilience to Alzheimer's disease. A major goal of centenarian studies is therefore to identify factors associated with maintaining cognitive function throughout life. Over the past year, two studies of centenarians and their offspring (age 60-110+ years) have pivoted from in-home assessments of cognitive and physical function to hybridized, Zoom-based assessments including comprehensive cognitive testing, blood pressure, grip strength, and accelerometry and biological sample collections. Protocols were optimized for accessibility for individuals with limited technology experience (e.g., investigator remotely controls all functions of the participant's tablet) and sensory impairments (e.g., integration of wireless headphones) and include high-sensitivity data collection (e.g., sensor-based wearables and digital recording of cognitive test responses). Advantages of virtual administration included the ability to accommodate fatigue through multi-day assessment and to include geographically-isolated individuals. Disadvantages included participant burden due to equipment setup and inability to collect certain measures virtually (e.g., carotid ultrasounds).

### A NEW NORMAL? CONTINUED IMPACT OF COVID19 ON ALZHEIMER'S CLINICAL TRIALS RESEARCH

Elizabeth Rhodus,[1] Allison Gibson,[2] Shoshana Bardach,[3] Erin Abner,[1] and Gregory Jicha,[1] *1. University of Kentucky, Lexington, Kentucky, United States, 2. University of Kentucky, University of Kentucky, Kentucky, United States, 3. Dartmouth University, Dartmouth University, New Hampshire, United States*

The COVID-19 pandemic has led to unprecedented challenges in Alzheimer's disease and related dementias (ADRD) clinical trials research. Scientists continue to grapple with the potential and multifaceted consequences of COVID-19. This presentation will discuss strategies used at a U.S. Alzheimer's Disease Research Center to implement virtual methods to counter COVID-19's impact on safety for continued research engagement; address the disparate impact by age, race, and ethnicity for online accessibility; and plans for virtual engagement in future research. As scientists navigate lasting implications of COVID-19, future study planning, design, and management will likely be altered. Specifically, increased awareness of participant-centered approaches, inclusion of psychosocial implications, and focus on ways to meet older adults' unique needs of virtual accessibility will be needed. We must be intentional to counter COVID-19's lasting impact on ADRD clinical trials research while maintaining rigor and reproducibility to uphold and progress advances toward treatment and cures for ADRD.

### BUILDING ON EXISTING TECHNOLOGY TO ENHANCE REMOTE ASSESSMENTS OF BRAIN HEALTH

Lydia Bazzano,[1] Camilo Fernandez,[1] Philip Hwang,[2] Ileana DeAnda-Duran,[1] Enejda Senko,[2] Honghuang Lin,[2] Vijaya Kolachalama,[2] and Rhoda Au,[2] *1. Tulane University, New Orleans, Louisiana, United States, 2. Boston University, Boston, Massachusetts, United States*

Tablets, smartphones, linked devices have features such as high-fidelity microphones, accelerometers, GPS locators, and gyroscopes can be used to capture brain health-related data. Collection of data remotely is especially important given the vulnerability of older adults to COVID and the need to protect from such exposure. As part of an American Heart Association/Gates Venture Strategically Funded Network, a number of remote assessments are being deployed to capture information related to brain health in a subset of the Bogalusa Heart Study cohort (mean age 51.4, SD 5.3). The Linus Health Platform includes applications that measure cognitive abilities, and collect digital voice features and phone sensor data that can be derived into surrogate measures of cognitive function and mood. A readily available suite of games (Lumosity) is also being used to assess cognitive health. These devices and applications offer a largely unexplored opportunity for acquiring and assessing data related to brain health.

### PROJECT VITAL: A STATEWIDE INITIATIVE USING TECHNOLOGY TO IMPACT SOCIAL ISOLATION

Sam Fazio, *Alzheimer's Association, Chicago, Illinois, United States*

Project VITAL leverages customized technology to enhance connection, engagement, education and support to individuals with dementia and their caregivers. The goal was to positively impact social isolation, stress, and well-being and help mitigate the effects of isolation experienced during the pandemic and beyond. Phase One included three components designed to impact connection, engagement, education and support. It's Never Too Late provided 300 customized tablets to 150 care communities to facilitate connections to family members and increase individualized, person-centered engagement. Project ECHO, a video-based learning platform, educated and supported care community staff. Virtual education and support were offered to family caregivers at home. Phase Two provided the program to an additional 150 care communities and added online professional training/certification. Phase Three focuses on 150 family caregivers and individuals with dementia living at home. In addition to connection and individualized engagement, family caregivers now had direct access to virtual programs and services.

### STAR CAREGIVERS: VIRTUAL TRAINING AND FOLLOWUP: IMPLEMENTATION

Robert Penfold,[1] Magaly Ramirez,[2] Susan McCurry,[3] Linda Terry,[4] James Ralston,[1] and Kelly Hansen,[1] *1. Kaiser Permanente Washington Health Research Institute, Seattle, Washington, United States, 2. University of Washington School of Public Health, Seattle, Washington, United States,*

*3. University of Washington School of Nursing, Seattle, Washington, United States, 4. university of Washington School of Nursing, Seattle, Washington, United States*

STAR Caregivers is an evidence-based intervention designed to reduce caregiver burden for caregivers of people living with dementia. This study translated the paper-based, face-to-face intervention into a 6-session, self-directed online learning program supported by 6, 30-minute telephone calls with a clinically trained coach. Our approach is designed to overcome issues of access to training. Eligible caregiver-patient dyads at Kaiser Permanente Washington were identified automatically via electronic health records. Qualitative interviews were conducted with a sample of patients to elicit information about their needs and preferences for training. We developed a "learning management system" (analogous to compliance training modules) complete with slides, voice-over narration, and testing. The training sessions are mounted on the KP Learn site and accessible to anyone. Baseline and outcomes data on standardized instruments are collected remotely via REDCap. Recruitment for the study is ongoing and initial participant feedback on the program is very positive.

### Session 2020 (Paper)

### BIOLOGY AND ENVIRONMENT OF AGING

### AIR POLLUTION EXPOSURE IS ASSOCIATED WITH COGNITIVE PERFORMANCE: RESULTS FROM THE EINSTEIN AGING STUDY

Nelson Roque,[1] Charles Hall,[2] Mindy Katz,[2] and Martin Sliwinski,[3] *1. University of Central Florida, Orlando, Florida, United States, 2. Albert Einstein College of Medicine, Bronx, New York, United States, 3. The Pennsylvania State University, University Park, Pennsylvania, United States*

Prior research has established that those exposed to higher levels of fine particulate matter (PM1, PM2.5) air pollution have higher levels of accumulated amyloid-beta (Aβ) and tau in frontal cortex at autopsy, higher error rates on cognitive function assessments, and lower scores on memory and both verbal and non-verbal intelligence assessments. We explored the relationship between regional air quality monitoring measures (EPA AirData) and baseline cognitive performance of 312 older adults, from the Einstein Aging Study (EAS, NIA P01AG003949). Participants completed neuropsychological assessments at baseline and each followup wave (i.e., delayed free recall and total recall; Trails A & B, Digit Symbol substitution task (DSST), MoCA). For each participant, based on their zipcode, we computed average PM2.5 exposure at various exposure windows (1-15, 30-60, 60-90, 90-120 days prior to baseline). Adjusting for age, education, and gender across all models, mean of daily particulate matter exposure at various exposure windows (30-60, 60-90 days) was significantly related to performance on the MoCA and Trails A & B, in expected directions (i.e., higher pollution, worse cognitive performance - more error, slower speed). Models with memory performance as the outcome indicated that only distant time horizons were related to memory performance (i.e., 60-90, 90-120 days prior). These findings suggest that particulate matter air pollution likely affects different cognitive domains at different timescales. This methodology cannot address contributions from indoor air quality and mobility - an exposure misclassification likely resulting in significant biases towards the null in the estimation of the effects of air pollution.

### BLOOD PRESSURE VARIABILITY AND COMPLEXITY IN NURSING HOME RESIDENTS BEFORE DEATH

Laura A. Graham,[1] Sei Lee,[2] Michael Steinman,[2] Carmen Peralta,[3] Anna Rubinsky,[3] Bocheng Jing,[4] Kathy Fung,[4] and Michelle Odden,[5] *1. VA Palo Alto Health Care System, Menlo Park, California, United States, 2. University of California San Francisco, San Francisco, California, United States, 3. University of California, San Francisco, San Francisco, California, United States, 4. San Francisco VA Medical Center, San Francisco, California, United States, 5. Stanford University, Stanford, California, United States*

Blood pressure (BP) is a complex dynamic system in the human body and an important determinant of healthy aging. Exploring BP as a dynamic data system may provide important insights into how BP patterns can provide complementary information to the static, one-time BP measurements that are more commonly used for clinical decision making. Thus, we sought to describe BP as a dynamic data system in older adults nearing death. Using a prospective cohort study design, we assessed BP measures 6 months before death in Veterans Health Administrative nursing home residents between 10/1/2006 and 9/30/2017. Variability was characterized using standard deviation and mean square error after adjusting for diurnal variations. Complexity (i.e., amount of novel information vs. redundancy) was examined using Shannon's entropy (bits). Generalized linear models were used to examine factors associated with overall BP variability. We identified 17,953 patients (98.0% male, 82.5% White, mean age 80.2 years, and mean BP 125.7/68.6 mmHg). In the last 6 months of life, systolic BP decreased slightly (□-7.2mmHg). Variability was stable until the last month of life, at which point variability increased by as much as 30%. In contrast, complexity did not change in the 6 months before death (□0.02 bits). Factors associated with BP variability before death include hospitalizations, hospice care, and medication changes. Systolic BP decreases in the last 6 months before death, and BP variability increases in the last month of life. Further, the increase in BP variability may be driven by increasingly complex care patterns as one approaches death.

### NOVEL POSITIONAL AND BIOLOGICAL CANDIDATE GENE FOR GRIP STRENGTH IN OLDER ADULTS: THE LONG LIFE FAMILY STUDY

Adam Santanasto,[1] Mary Wojczynski,[2] Ryan Cvejkus,[3] Lihua Wang,[4] Bharat Thyagarajan,[5] Kaare Christensen,[6] E. Warwick Daw,[7] and Joseph Zmuda,[3] *1. University of Pittsburgh, Pittsburgh, Pittsburgh, Pennsylvania, United States, 2. Washington University School of Medicine, Washington University School of Medicine, Missouri, United States, 3. University of Pittsburgh, Pittsburgh, Pennsylvania, United States, 4. Washington University School of Medicine, St. Louis, Missouri, United States,*

*5. University of Minnesota, Minneapolis, Minnesota, United States, 6. Department of Public Health, University of Southern Denmark, Odense, Syddanmark, Denmark, 7. Washington University in St. Louis, St. Louis, Missouri, United States*

Grip strength is a robust indicator of overall health, is moderately heritable and predicts longevity in older adults. Using genome-wide linkage analysis, we identified a novel locus on chromosome 18p linked to grip strength in 4534 individuals from 582 families (age 70.0 ± 15.8, range 24–110 years; 54% women). DNA sequencing was completed to identify single nucleotide variants (SNVs) in the 3.44 – 4.04 mega-basepair region on chromosome 18p. Using the sequencing data, we performed association analyses between the 7312 SNVs in the region and grip strength in families exhibiting evidence for linkage. Models were adjusted for age, age2, sex, height, field center and population substructure. There were 23 families (263 individuals) that contributed to the linkage peak (cumulative logarithm of the odds [LOD] score = 12.4). Six families (112 individuals) accounted for most of the linkage signal (LOD = 6.4). In these 6 families, we found highly significant associations between SNVs in the Disks Large-associated Protein 1 (DLGAP1) gene and grip strength (lead SNV: β= -0.75kg ± 0.15, p-value= 4.3*10-6). Correcting for the top SNV in DLGAP1 reduces the LOD by 72% in these families. Further, the effect allele frequency is much higher in these 6 families (39.7%) compared with both the NHLBI's Trans-OMICs for Precision Medicine (23.5%) and 1000Genomes (28.0%) references panels. The DLGAP1 gene plays an important role in post-synaptic density of neurons; thus, it is a novel positional and biological candidate gene for follow-up studies aimed at uncovering genetic determinants of muscle strength.

### PLEIOTROPIC GENES FOR PULMONARY FUNCTION AND AGING-RELATED TRAITS: THE LONG LIFE FAMILY STUDY (LLFS)

Mary Feitosa,[1] Mary Wojczynski,[2] Jason Anema,[3] E. Warwick Daw,[4] Lihua Wang,[5] Marianne Nygaard,[6] Adam Santanasto,[7] and Michael Province,[2] *1. Washington University School of Medicine in St. Louis, St. Louis, Missouri, United States, 2. Washington University School of Medicine, Washington University School of Medicine, Missouri, United States, 3. Washington University School of Medicine, St Louis, Missouri, United States, 4. Washington University in St. Louis, St. Louis, Missouri, United States, 5. Washington University School of Medicine, St. Louis, Missouri, United States, 6. Department of Public Health, University of Southern Denmark, Odense, Syddanmark, Denmark, 7. University of Pittsburgh, Pittsburgh, Pittsburgh, Pennsylvania, United States*

Pulmonary function progressively declines with aging. Forced expiratory volume 1-second (FEV1) and forced vital capacity (FVC) are predictors of morbidity of cardiovascular diseases and all-cause mortality. Reduced pulmonary function has shown association with elevated chronic low-grade systemic inflammation and low muscle strength, and glycosylated hemoglobin (HbA1c). We evaluated whether FEV1 and FVC share common genetic factors with interleukin-6 (IL-6), high-sensitivity C-reactive protein (hsCRP), body mass index (BMI), handgrip strength, plasma glucose, and HbA1c employing correlated meta-analysis (CMA) in up to 3888 individuals (age range: 26-106). CMA tests whether combining genome-wide association (GWA) P-values from two or more traits enhances the ability to detect variants concomitantly influencing such traits. We considered a variant pleiotropic if the univariate GWA P≤1.0x10-02 and CMA P≤5.0x10-08. We identified pleiotropic loci for FEV1 (or FVC), IL-6 and hsCRP within CYCS, CALN1, TRIM9, AXIN2, MICAL3, and CMIP; FEV1 (or FVC) and BMI within CYP2U1, LINC00871-RPL10L-MDGA2, and LOC105372472; FEV1 (or FVC) and grip strength within XXYLT1 and MAL2; FEV1 and FPG within MAF; and FVC and HbA1c within HDHD3. Some loci were reported as GWAS suggestive (P<10-6) for pulmonary function. Additionally, the identified loci harbor genes with roles in pro-inflammatory cytokine production, immune and inflammatory pathway, T-helper signaling pathway, transforming growth factor-beta, and T-cell receptor-alpha enhancer. Our findings suggest that inflammation is a feature of reduced pulmonary function with IL-6, hsCRP, BMI, low muscle strength, glucose, and HbA1c. Pleiotropic genetic associations across these traits may explain part of the correlated genetic architecture between pulmonary function and aging-related traits.

### THE POSITIVE IMPACT OF A NOVEL AIR PURIFICATION TECHNOLOGY ON ENVIRONMENTAL AND CLINICAL OUTCOMES

Susan Schlener,[1] and Kathryn Worrilow,[2],*1. Phoebe Ministries, Allentown, Pennsylvania, United States, 2. LifeAire Systems, Allentown, Pennsylvania, United States*

Reducing sources of illness improves resident care. An advanced air purification technology (AAPT) was designed to destroy the DNA and RNA of all bacteria, fungi and viruses, rendering them non-infectious and to remediate volatile organic compounds (VOCs). This study compares the biological, fungal and VOC loading using the AAPT to standard high efficiency particulate air (HEPA) filtration. It was hypothesized that the AAPT would be associated with reductions in airborne and surface pathogens, VOCs and improved clinical metrics. A control floor with HEPA filtration and study floor with AAPT remediation were studied. Measurements of total VOCs and airborne and surface bacteria and fungi were measured in five locations on each floor. The facility acquired infection (FAI) rate, the number of infections divided by total patient days, showed a 57% difference between the control floor (2.33 FAIs/month) and the study floor (1.00 FAIs/month) and a decrease of 39.75% pre-installation (1.66 FAIs/month) to post-installation (1.00 FAIs/month). The viable pathogen loading measured on the study floor was reduced from an average of 483.8 colony forming units (CFU)/m3 pre-installation to an average of 56 CFU/m3 post-installation. VOCs were reduced from an average of 641.66 parts per billion (PPB) to 64.96 PPB and viable surface bacteria from an average of 110.6 CFU/m3 to 97.2 CFU/m3. The AAPT significantly reduced levels of infectious airborne and surface pathogens and VOC levels. As a result, residents on the AAPT floor demonstrated significant improvements in FAI rates. The findings support the hypothesis that environmental factors impact resident wellness.

### Session 2025 (Paper)

### CHALLENGES OF COVID-19

### CHALLENGES FOR CHILDREN 65+ CARING FOR PARENTS 90+ WITH DEMENTIA DURING COVID-19

Elizabeth Gallagher,[1] Kathrin Boerner,[2] Yijung Kim,[3] Kyungmin Kim,[4] and Daniela Jopp,[5] *1. University of Massachusetts Boston, Boston, MA, Massachusetts, United States, 2. University of Massachusetts Boston, Boston, Massachusetts, United States, 3. The University of Texas at Austin, Austin, Texas, United States, 4. Seoul National University, Seoul, Seoul-t'ukpyolsi, Republic of Korea, 5. University of Lausanne, Lausanne, Vaud, Switzerland*

With the rise of the novel coronavirus, family caregivers of persons with dementia have been tasked with adapting to an entirely new caregiving landscape. Adult children caring for parents in the 'oldest old' age group bear an additional burden. Namely, children that are older adults themselves are navigating the joint vulnerability of both their own and their parents' aging-related issues (e.g., health problems). The aim of this study was to explore the experiences of dementia caregivers during COVID-19 from the unique perspective of children aged 65 and older caring for parents aged 90 and older. Participants were 30 caregivers from the Boston Aging Together Study with whom we conducted in-depth interviews between March 2020 and February 2021. Thematic analysis revealed key challenges related to COVID-19. Children were worried about the prospect of their parent contracting the virus and took steps to minimize their parent's exposure, such as discontinuing use of formal supports (e.g., home health aides) or assistance from other family and friends. Forgoing these supports often created greater responsibilities for caregivers as well as contributed to greater social isolation for both child and parent. In situations where parents resided in institutional settings, children were often unable to provide necessary help and support to parents due to restrictions. Caregivers also faced difficulties due to their parent not understanding or practicing COVID-19 regulations and in utilizing alternative means of communication with their parent (e.g., video conferencing). Supports and services should be designed in light of the unique challenges of this group.

### COVID-19 AND EMANCIPATORY GERONTOLOGY: PERSPECTIVES OF POLITICAL ECONOMY AND THE AMERICAN RESCUE PLAN

carroll Estes,[1] Nicholas DiCarlo,[2] and Jarmin Yeh,[3] *1. UCSF, University of California San Francisco, California, United States, 2. University of California, San Francisco, institute for Health and Aging, UCSF, California, United States, 3. University of California, San Francisco, San Francisco, California, United States*

The present historic moment – a pandemic worsened by far-right extremism – reveals how mounting individual and collective precarity across the lifecourse and in old age resides within societal institutions of colonialism, white supremacy, patriarchy, and capitalism. Contradictions between systems of democracy and capitalism construct an ageist society aligned with neoliberal ideologies attempting to dismantle and privatize Social Security, Medicare, and Medicaid. These issues confront the call for a critical inquiry that matters in the lives of those who daily experience social injustices (Denzin, 2017). This paper presents emancipatory gerontology (Estes & DiCarlo, 2019) as a critical praxis to challenge assumptions, frameworks, and delirium writ large in American society as it relates to how we conceive of age, aging, and generations. We elucidate how the $1.9 trillion 2021 American Rescue Plan represents a paradigm shift that aims to supplant austerity economics with human, public, and community benefit. This knock on the hegemonic commitment to austerity and its mantra is an opportunity to interrogate the effects of, and advance, emancipatory policies and practices. Gerontology is inadequate without a lens for examining how critical analysis and social action might inform one another. To shift from disruption to transformation in the "new normal," scholars must bring the past and future into the present to engage realistic utopian pedagogies of hope. Emancipatory gerontology offers a theoretical framework and vocabulary for interrogating individual and social consequences of major policy and institutional forces in relation to aging and generations across the lifecourse.

### HOW STATES SUPPORTED OLDER ADULTS AND PERSONS WITH DISABILITIES DURING COVID-19 THROUGH THE MEDICAID PROGRAM

Edward Miller,[1] Lisa Beauregard,[2] Pamela Nadash,[3] Elizabeth Simpson,[3] Molly Wylie,[4] and Michael Gusmano,[5] *1. University of Massachusetts Boston, Boston, Massachusetts, United States, 2. Executive Offce of Elder affairs, Executive Office of Elder Affairs, Massachusetts, United States, 3. University of Massachusetts Boston, University of Massachusetts Boston, Massachusetts, United States, 4. University of Massachusetts Boston, University of Massachusetts boston, Massachusetts, United States, 5. Rutgers University School of Public Health, Rutgers University School of Public Health, New Jersey, United States*

COVID-19 has presented challenges for older adults who receive Medicaid home and community-based services. The federal government has allowed states to seek approval for certain flexibilities to better serve this population, including increasing provider payment rates, allowing family members to be caregivers, and permitting case management entities to provide direct services. This study uses cross-sectional data to identify factors associated with states' adopting these Medicaid flexibilities using multivariate methods. The results indicate that the factors associated with state adoption varied depending on the flexibility. The findings suggest that states increased provider payment rates in response to prevalence of COVID-19 within their state. As cases increase, states may come under pressure to increase provider rates further which may not be feasible because of budget constraints. The results also suggest that demand for and supply of services may be a factor in whether states allowed family members to be paid caregivers. States with a higher proportion of individuals aged 85 years and older were more likely to permit caregivers to be paid which may suggest that these states may not have enough providers to care for the population. Lastly, the results suggest that provider supply was associated with whether a state allowed case management entities to provide direct care services. States with fewer home health agencies were more likely to allow this flexibility. Based on these results, states may be pursuing available Medicaid flexibilities to address provider and workforce shortages which existed prior to COVID-19 but have been exacerbated by the pandemic.

### MODERATING EFFECTS OF LONELINESS ON COVID-19-RELATED STRESS AND ANXIETY IN MIDDLE AND LATER LIFE IN SOUTH KOREA

Sukyung Yoon,[1] and Soo Chan Choi,[2] *1. University of Wyoming, Laramie, Wyoming, United States, 2. Yonsei University, Seoul, Seoul-t'ukpyolsi, Republic of Korea*

Many people have suffered from psychological distress in the form of stress, loneliness, and anxiety resulting from the COVID-19 epidemic (Havnen et al., 2020; Luchetti et al., 2020). Along with these factors, physical health (hereafter health), resilience, and living arrangements as protective factors were examined. The research aims were to investigate 1) factors affecting the association between COVID-19-related stress (hereafter stress) and anxiety, and 2) moderating effects of loneliness on this association. Data was collected on 450 middle-aged and older adults (ages 45 through 76) living in South Korea during COVID-19. A multi-group path analysis was employed. Measurement invariance was examined by comparing unconstrained and fully constrained models. Both models fit. Moderating effects of loneliness existed. Stress was negatively associated with health and living arrangements for people with both higher and lower levels of loneliness. Health was positively associated with resilience for both groups. Resilience was negatively associated with anxiety for both groups. For people with higher levels of loneliness only, stress and health were negatively associated with resilience and anxiety, respectively. The association between stress and anxiety was significant for both groups. However, the impact of stress on anxiety was significantly larger for people with higher levels of loneliness than for people with lower levels of loneliness. Health practitioners and service providers should develop programs to maintain and promote resilience, social support, and good health among middle-aged and older adults in South Korea to mitigate negative mental health consequences during the pandemic.

### THE IMPACT OF COVID RESTRICTIONS AMONG RATES OF FALLING IN ASSISTED LIVING RESIDENTS

Kamil Hester,[1] Melanie Fong,[2] and Margaret Danilovich,[3]
*1. Loyola University, Chicago, Illinois, United States,*
*2. University of Chicago, Chicago, Illinois, United States,*
*3. CJE SeniorLife, Evanston, Illinois, United States*

As a result of the COVID-19 pandemic, assisted living group activities and congregate dining stopped and residents were confined to their rooms. While this may have kept residents safer from contracting the virus, it also reduced their physical activity levels. The aim of this study was to investigate if rates of falls in one assisted living community varied as a result of COVID-19 restrictions. We analyzed fall incident reports from n=155 residents from October 2019 to October 2020. Results showed a total of n=802 falls in the year-long period (range of 1-30 falls per resident; mean = 5.17; SD=5.6 in the 12 month period). The majority (65%) of falls occurred in resident rooms. 55% of falls occurred between 6am and 6pm. The primary cause of these falls was loss of balance (30%). Comparing falls that occurred 5 months pre-restriction (Oct 2019-Feb 2020) with 5 months post-restriction (April 2020-August 2021) showed non-significant differences between time periods (p=.59). However, analyzing rates of falls by month showed a range of 46 - 88 falls by month with the lowest number occurring in winter months and the peak number of falls occurring in both Oct 2019 and 2020. Despite the majority of falls occurring in resident rooms, Covid restrictions of room confinement did not appear to impact the prevalence of falls in this sample. However, the seasonal variation warrants further research and those in assisted living should consider seasonal variations and proactively implement policies to prevent falls during these times.

### Session 2030 (Symposium)

### CHANGES IN MENTAL HEALTH, SOCIAL ENGAGEMENT, AND PHYSICAL ACTIVITIES DURING THE COVID-19 PANDEMIC

Chair: Wenjun Li Discussant: Su-I Hou

The current COVID-19 pandemic has profoundly changed our behaviors and health, especially vulnerable community-dwelling older adults. This symposium includes three presentations that evaluated the pandemic's impacts on mental health, social engagement and physical activity in healthy community-living older adults and those with dementia. Dr. Wenjun Li and his team examined the pandemic impact on mental health and social engagement among relatively healthy older adults residing in suburban and rural neighborhoods in Central Massachusetts, USA. The study reported significant variations in pandemic impacts by sex, age, race, income, living arrangement, and neighborhood housing density, suggesting the pandemic has had disproportionally negative impacts on socially and economically disadvantaged vulnerable older adults. Dr. W. Quin Yow and her team evaluated the impacts of government mandated social distancing and lockdowns on older adults with dementia and their caregivers in Singapore. The study found significant increases in irritability, aggression and hallucinations among older adults with dementia, and possible deterioration of health conditions and heightened moderate level of stress. The results suggest that social distancing may have resulted in negative outcomes in this vulnerable population with dementia and their caregivers. Dr. Ladda Thiamwong reported her team's efforts on forming an international aging research collaborative to mitigate heath consequences of COVID-19 crisis from the international perspective. The team consists of ten scholars from five countries, including Hong Kong, Nepal, Singapore, Thailand, and the United States. They collect data using combinations of online and face-to-face surveys. Their important findings will be discussed in detail in this symposium.

### CHANGES IN SOCIAL ISOLATION, ANXIETY AND DEPRESSION AMONG OLDER ADULTS DURING THE COVID-19 PANDEMIC

Linda Churchill, Hannah Siden, Annabella Aquirre, Elizabeth Procter-Gray, and Wenjun Li, *University of Massachusetts Lowell, Lowell, Massachusetts, United States*

Social distancing and business lockdowns may have severe negative impact on daily living, mental and physical health of community-living older adults. Our Healthy Aging and Neighborhood Study surveyed 370 older adults in Central Massachusetts in 2020 and 2021. Participants were queried about pre-post pandemic changes in social and physical activities, mental and physical health, and lifestyle factors including food purchasing, diet and physical exercise; and attitude towards and receiving of vaccination. The study is ongoing and data are being accumulated. Preliminary analysis suggested that social distancing and lockdowns have negative impacted social engagement, communications with close friends, relatives and family members, food purchasing, frequency of outdoor exercises, especially group activities. The impact appeared to differ by sex, advancing age, and living arrangement. In summary, social distancing and business lockdowns may have negative impacts on most older adults while the impacts were more severe in those older and socioeconomically disadvantaged.

### COVID-19 LOCKDOWN MEASURES: IMPACT ON OLDER ADULTS WITH DEMENTIA AND THEIR CAREGIVERS IN SINGAPORE

Tharshini Lokanathan,[1] Hui-Ching Chen,[1] and W. Quin Yow,[2] *1. Singapore University of Technology and Design, Singapore University of Technology and Design, Not Applicable, Singapore, 2. Singapore University of Technology & Design, Singapore, Not Applicable, Singapore*

Family caregivers typically rely on community-based services and social support networks to supplement their caregiving of older adults with dementia (OAwD). In April 2020, the Singapore government implemented a partial lockdown to contain the spread of COVID-19. We assessed the policy's impact on the physical and mental health of caregivers and their OAwD. As part of a larger study, 30 caregivers were interviewed and rated the stress they experienced when caring for an OAwD during the partial lockdown. Qualitative analyses found significant behavioral changes in OAwD such as irritability, aggression and hallucinations, which led some caregivers to believe their dependents' condition had deteriorated, as well as heightened levels of caregiving stress. Overall, our preliminary results suggested that although social distancing measures may be effective in containing the spread of COVID-19, these measures could lead to negative outcomes on vulnerable populations such as OAwD and their caregivers.

### AN INTERNATIONAL AGING RESEARCH COLLABORATION DURING THE COVID-19 CRISIS: MITIGATING GLOBAL HEALTH CONSEQUENCES

Wayne Chong,[1] Rick Kwan,[2] Inthira Roopsawang,[3] Ramraj Gautam,[4] Vivian, W. Q. Lou,[5] and Ladda Thiamwong,[6] *1. Nanyang Technological University, Singapore, Not Applicable, Singapore, 2. The Hong Kong Polytechnic University, Hong Kong, Hong Kong, 3. Mahidol University, Bangkok, Thailand, 4. UMass Lowell, Lowell, Massachusetts, United States, 5. The University of Hong Kong, Hong Kong, Not Applicable, Hong Kong, 6. College of Nursing, University of Central Florida, Orlando, Florida, United States*

There are several reasons for forming an aging international research collaboration; however, creating a successful and productive research team during the global crisis may require extensive planning and efforts. Our team consists of ten scholars from five countries, including Hong Kong, Nepal, Singapore, Thailand, and the United States. To accomplish this initiative, we employ ten simple rules for establishing international research collaborations proposed by R. de Grijs (2015). We aim to examine impacts of the pandemic on physical activity, frailty, falls, depression and social networks in diverse older adults. We collect data by online survey and/or face-to –face survey using questionnaires including fear of the COVID, face mask use, Social Network, Rapid Assessment of Physical Activity, a simple frailty questionnaire, CDC fall risk checklist, short Fall-Efficacy Scale International and Patient Health Questionnaire-9. Topics of discussion included: research progression, lessons learned and barriers to international collaboration during the COVID-19 crisis.

### Session 2035 (Symposium)

### CLARK TIBBITTS AWARD AND HIRAM J. FRIEDSAM MENTORSHIP AWARD LECTURES

Chair: Kara Dassel

The Clark Tibbitts Award lecture will feature an address by Debra Dobbs, PhD, FGSA in memory of the 2021 award recipient, Kathryn Hyer, PhD, FGSA. AGHE's Clark Tibbitts Award was established in 1980 and named for an architect of the field of gerontological education. The award is given each year to an individual or organization that has made an outstanding contribution to the advancement of gerontology and geriatrics education. The Hiram J. Friedsam Award lecture will feature an address by the 2021 award recipient, Pamela Elfenbein, MSW, PhD, FAGHE, HS-BCP. Hiram J. Friedsam was the professor, co-founder, and director of the Center for Studies in Aging and dean of the School of Community Service at the University of Northern Texas. Dr. Friedsam was an outstanding teacher, researcher, colleague, and mentor to students, faculty, and administrators, as well as a past president of AGHE. The purpose of this award is to recognize those who emulate Dr. Friedsam's excellence in mentorship.

### KATHRYN HYER'S LASTING LEGACY IN GERONTOLOGY EDUCATION AND AGING POLICY RESEARCH

Debra Dobbs, *University of South Florida, Tampa, Florida, United States*

Kathy Hyer, our dear friend, colleague, former Gerontological Society of America President and Professor and Director of the Florida Policy Exchange Center on Aging, University of South Florida posthumously has been awarded the Clark Tibbett's Award for her achievements in the advancement of the field of gerontological education in higher education. This lecture will reflect on some of her decades of accomplishments including her dedication to AGHE's mission to train and educate students in gerontology. Her greatest achievement is in the area of training and education of long-term care administration and aging studies undergraduate students to be nursing home and assisted living administrators. This lecture will also highlight several initiatives of Dr. Hyer's that involved undergraduate, graduate students, and junior faculty who she mentored including the Geriatric Workforce Enhancement Project, NIA SAFEHAVEN and COVID research on disasters, the Dementia Training Academy, Age Friendly Initiatives both nationally and internationally and student mentorship for graduate assistantships through the USF Center for End of Life Care.

### HIRAM J. FRIEDSAM AWARD: REFLECTIONS OF MENTORS AND MENTORSHIP

Pamela Elfenbein, *University of North Georgia, Gainesville, Georgia, United States*

Being honored with the AGHE Hiram J. Friedsam Mentorship Award brought into sharp focus the mentors who have guided my journey and a desire to share this moment with them. Preparation for this session provided the opportunity to reach out to those who have shaped my path and share memories of our journeys. Lesa Huber, when awarded the Hiram Friedsam Mentorship Award in 2013, wrote “those of us who have had great mentors know it is not so much what is said, but how it is said that is at the core of effective mentorship.” This presentation reflects on relationships built, wisdom shared, guidance offered, lessons learned by design and unintentionally, and passed on in my own way to the next generation.

### Session 2040 (Paper)

### COGNITIVE IMPAIRMENT PROGRAMS

### CHILDHOOD ADVERSITY ACROSS THE LIFESPAN: ASSESSING THE RELATIONSHIP BETWEEN ACES AND SUBJECTIVE COGNITIVE DECLINE

Elizabeth Avent,[1] Jeanine Yonashiro-Cho,[2] Roberta Peterson,[2] Laura Mosqueda,[3] and Zachary Gassoumis,[3] *1. University of Southern California, Los Angeles, California, United States, 2. Keck School of Medicine of USC, Los Angeles, California, United States, 3. Keck School of Medicine of USC, Alhambra, California, United States*

Adverse childhood experiences (ACEs) have long-term impacts on health throughout the life course. Emerging research found that 3+ ACEs are associated with increased risk of cognitive impairment, nearly 11 times more than those who have not experienced childhood adversity. This study further investigates the ACEs-SCD relationship using data from the 2011 Behavioral Risk Factor Surveillance System (BRFSS). Seven ACE questions were asked of respondents in California, Washington, and Wisconsin (n=5,898, aged 55+); SCD was measured as experiencing progressive confusion or memory loss in the last 12 months. A series of logistic regressions were run to separately model the presence of ACEs and ACE score on SCD. Fourteen percent reported SCD, with 65.4% of those reporting 1+ ACE. More SCD respondents reported 4+ ACEs (10.8%) than non-SCD respondents (4.8%). The most frequently reported ACEs among those with SCD were psychological abuse (34.9%) and substance abuse in the household (30.5%). Regression results showed greater SCD risk with increased ACE scores, up to 2.90 odds of SCD for 4+ ACEs compared to 0 ACEs (p<.0001). Those reporting physical abuse and sexual abuse had the greatest odds (1.75 & 1.70, p<.0001) of SCD. Findings demonstrate a strong association between childhood adversity and SCD, with physical and sexual abuse placing individuals at greatest risk. Results show possible pathways to which ACEs can lead to cognitive impairment. Findings implicate the importance of considering a lifespan perspective in childhood adversity and family violence work and the importance of considering early-life adversity when assessing risk for cognitive impairment.

### COMPARATIVE ANALYSIS OF DEMENTIA CARE PROGRAMS AND DELIVERY MODELS

Allie Peckham,[1] Marianne Saragosa,[2] Madeline King,[3] Monika Roerig,[3] and Gregory Marchildon,[3] *1. Arizona State University, Pheonix, Arizona, United States, 2. University of Torono, Toronto, Ontario, Canada, 3. University of Toronto, Toronto, Ontario, Canada*

Dementia has significant social and economic impacts for those living with dementia and their caregivers. Despite an increase in prevalence of complex chronic conditions and dementia, long-term care services are continuously pushed out of institutional settings and into the home and community. The majority of people living with dementia in Canada and the United States (U.S.) live at home with support provided by family, friends, or other unpaid caregivers. Ten dementia care programs and service delivery models across five different North American jurisdictions in Canada and the U.S. are compared using a deductive analytical approach using a comparative policy framework developed by Richard Rose. The policy efforts included in this research attempt to improve health system flow and access for vulnerable populations. One common theme among all jurisdictions are long-standing institutional barriers that can make change difficult. These barriers can prevent the ability for systems to be flexible and adapt to meet the changing needs of populations. Incrementalism is often considered an appropriate approach to health system reform. Yet, incremental change efforts lead to policy layers and these layers can lead to tension between different policy mixes and unintended consequences. These programs were introduced in a manner that did not fully consider how to patch current structures and risk creating further system redundancies. One approach to reduce this risk is to combine evaluative efforts that assess ‘goodness of fit’. The degree to which these programs have embedded these efforts successfully is low, with the possible exception of DSRIP from NY.

### EDUCATION, OCCUPATION, AND THE COGNITIVE PERFORMANCE DISTRIBUTION OF SOUTH KOREAN OLDER ADULTS

Katherine Ford,[1] and Anja Leist,[2] *1. University of Luxembourg, Esch-sur-Alzette, Not Applicable, Luxembourg, 2. University of Luxembourg, Esch-sur-Alzette, Diekirch, Luxembourg*

Earlier research suggests that educational attainment up to early adulthood are crucial for the development of cognitive reserve, while intellectually stimulating activities later in the life course are of limited impact. We sought to explore the effects of educational attainment and occupational factors (occupation type and currently having work) across the distribution of cognitive performance for adults aged 45-65 years in South Korea. We analysed scores from the Korean Mini Mental State Exam (MMSE) provided in the 2006 wave of the Korean Longitudinal Study of Aging. We used quantile regressions to both investigate relationships across the distribution and to reduce bias for measures of the central tendency as the MMSE is known for its ceiling effects. The quantile function at the lowest conditional decile of MMSE scores suggested that education level was the dominant significant factor for adult performance on the MMSE (lowest MMSE decile, primary education: $\beta = 6.11$ points, $p < 0.001$; secondary education $\beta = 9.56$ points, $p < 0.001$). All occupational factors were non-significant. Further factors with a significant association with the MMSE were hearing loss, the log-transformed household income, and age squared. With the conditional median function, occupational factors became significant in the middle of the distribution but remained much less important than education levels. In summary, educational levels were more important to explain variation in cognitive functioning than occupational factors, echoing studies with Western samples. We discuss the findings with regard to the historically gender unequal educational and occupational opportunities in Korea.

### RETURN HOME INTERVIEWS FOR MISSING OLDER ADULTS WITH DEMENTIA: A SCOPING REVIEW

Noelannah Neubauer,[1] Elyse Letts,[2] Christine Daum,[2] Antonio Miguel-Cruz,[3] Lauren McLennan,[2] and Lili Liu,[2] *1. University of Waterloo, University of Waterloo, Ontario, Canada, 2. University of Waterloo, Waterloo, Ontario, Canada, 3. University of Alberta, Edmonton, Alberta, Canada*

**Introduction:** Persons living with dementia are at risk of becoming lost. When a person is returned home safely after a missing incident, an interview with the person or care partner may identify ways to prevent repeat incidents. It is not known if these interviews are being conducted for this population.

**Objectives:** The purpose of this review was to understand return home interviews and whether they are being used with persons who have dementia.

**Methods:** Scholarly and grey literature were searched in 20 databases. Articles were included from any language, year, study design if they included terms resembling “return home interview”, “missing,” “lost,” or “runaway”.

**Results:** Eleven articles in scholarly, and 94 in grey literature sources were included, most from the United Kingdom. The majority of academic (55%) and grey (61%) articles were related to missing children, and none were specifically about persons living with dementia. Interviews were typically conducted within 72 hours after a missing person was returned, and by police or charitable organizations. The main reasons were to understand the causes of the incident and confirm the missing person’s safety, identify support needs, and to provide support to reduce repeat missing incidents.

**Conclusion:** Existing reasons for interviews can also apply to persons with dementia. This review informs future research on return home interviews. It also informs community organizations, and police services interested in adopting this practice with persons living with dementia. Evaluations would confirm if these interviews can reduce repeat incidents and help keep people with dementia safe.

### THE STRONGERMEMORY PROGRAM: A BRAIN HEALTH INTERVENTION FOR MILD COGNITIVE IMPAIRMENT

Catherine Tompkins,[1] Emily Ihara,[1] Rob Liebreich,[2] Jessica Fredericksen,[2] and Lauren Bradley,[2] *1. George Mason University, Fairfax, Virginia, United States, 2. Goodwin House, Falls Church, Virginia, United States*

The StrongerMemory program is a brain health intervention targeting older adults with mild cognitive impairment (MCI). Approximately 20% of individuals over 65 years old have MCI and 38% will potentially develop dementia after 5 years of MCI onset. Participants in the 16-week StrongerMemory program practice exercises involving math, writing, and reading aloud for 20-30 minutes a day, stimulating the prefrontal cortex of the brain, which governs the brain’s ability to retrieve memories. The current research exploring participants’ experiences with the StrongerMemory program was approved by the University’s Institutional Review Board. A semi-structured interview was implemented with each participant and the interviews took about 15 to 30 minutes to complete. There was a total of 24 respondents associated with a local continuing care retirement community, each providing their verbal consent to be interviewed and recorded over Zoom. The respondents were mostly female ranging in age from 65 to 90. Most respondents reported having advanced degrees and were participating in the program on their own, with a spouse or friend. Each interview transcript was coded by at least two members of the Mason research team. Participants reported being motivated to participate in the program due to fearing memory loss because of experiences of family members and friends and others wanted to remain “cognitively fit.” Codes and themes representing participants’ perceptions and reactions to exercises and their challenges and lessons learned from their participation will be discussed, including participants feelings of being “less foggy” and better able to remember information.

### Session 2045 (Symposium)

### COMMON DATA ELEMENTS FOR WORKFORCE AND STAFFING MEASUREMENTS IN LONG-TERM CARE

Chair: Charlene Chu Co-Chair: Franziska Zúñiga
Discussant: Kirsten Corazzini

The workforce in residential long-term care (LTC) is key in providing high-quality, person-centered care for residents.

However, low staffing and adverse staffing outcomes such as turnover or job dissatisfaction hinder the provision of high-quality care. International research can add valuable insights for policy and practice by learning from different settings and cultures. The initiative "To Harmonize Research In long-term care liVing Environments (WE-THRIVE)", is led by an international group of LTC researchers to identify common data elements (CDE) for cross-comparative research that support older adults thriving in LTC. In this symposium, we will present an overview of the WE-THRIVE initiative with a specific focus on CDEs and measurement. The first talk will provide the context for the WE-THRIVE initiative, and discuss the collaborative and iterative processes required to develop the initial CDEs in the area of workforce and staffing. In the second talk, we will discuss which staff should be "in the house" to meet the needs of residents during and after a pandemic, and what type of workforce data system should be available to ensure the best quality outcomes for residents and carers. Next, current issues in the measurement of staffing in LTC based on a review of reviews of staffing's relationship to quality of care will be discussed. Finally, we extend the debate to consider theoretical and empirical explanations for the relationship between staffing and quality in LTC and the promotion of person-centred care outcomes.

### LAYING DOWN THE GROUNDWORK FOR AN INTERNATIONAL MEASUREMENT INFRASTRUCTURE

Katherine McGilton,[1] Franziska Zuniga,[2] Michael Lepore,[3] Kirsten Corazzini,[4] and Charlene Chu,[5] *1. KITE-Toronto Rehabilitation, University Health Network, Toronto, Ontario, Canada, 2. Basel University, Ostermundingen, Basel-Stadt, Switzerland, 3. LiveWell Alliance, Southington, Connecticut, United States, 4. University of Maryland School of Nursing, Baltimore, Maryland, United States, 5. University of Toronto, Toronto, Ontario, Canada*

The COVID-19 epidemic has brought to light the significant problems in the long-term care (LTC) sector, specifically the lack of an infrastructure to collect and aggregate data between LTC sectors in different countries. This talk will briefly describe goals of the WE-THRIVE initiative, and focus on exploring the development of "workforce and staffing" common data elements for LTC. We will describe how the subgroup is "laying down the groundwork" within this domain with various methodologies to develop CDEs related to workforce and staffing. The CDEs aim to measure staff retention and turnover, evaluating nursing supervisor effectiveness, and staff training in LTC. Anticipated challenges of this international work will also be highlighted. International research on LTC can valuably inform LTC policy and practice, and the proposed CDEs can facilitate data sharing and aggregation internationally, including low-, middle-, and high-income countries.

### CHALLENGES IN THE MEASUREMENT OF STAFFING: A REVIEW OF REVIEWS

Magdalena Osinska, Catherine Blatter, Michael Simon, and Franziska Zúñiga, *University of Basel, Basel, Basel-Stadt, Switzerland*

The question concerning the relationship of staffing and quality of care of residents in residential long-term care (LTC) has been explored extensively; however, no consistent evidence has been brought forth so far. Inconsistent measurement of staffing might hinder this research field to move forward. We assessed its measurement in a narrative review of reviews that explore the staff – quality of care relationship. We identified 12 systematic reviews, covering 1960 to May 2018. Most studies included had a cross-sectional design, were performed in the USA and worked with secondary, administrative data (e.g., OSCAR). Comparability of studies was limited by diverse definitions and measurement methods for staffing, including data about grade-mix, number of staff, and staff-resident ratios. We suggest performing international multi-case studies to compare and contrast LTC staffing and develop appropriate international common data elements. Logic models support the description of the expected relationship between staffing aspects and quality.

### WHO'S IN THE HOUSE: STAFFING IN LONG-TERM CARE HOMES DURING THE COVID-19 PANDEMIC

Shirin Vellani,[1] Charlene Chu,[2] Annica Backman,[3] Astrid Escrig-Pinol,[4] José Tomás Mateos,[5] Franziska Zúñiga,[6] karen spilsbury,[7] and Katherine McGilton,[8] *1. Toronto Rehabilitation Institute-University Health Network, Toronto, Ontario, Canada, 2. University of Toronto, Toronto, Ontario, Canada, 3. Umea University, Umea, Vasterbottens Lan, Sweden, 4. The Mar Nursing School, Universitat Pompeu Fabra, Barcelona, Catalonia, Spain, 5. University of Lleida, Lleida, Catalonia, Spain, 6. University of Basel, Basel, Basel-Stadt, Switzerland, 7. University of Leeds, University of Leeds, Leeds, England, United Kingdom, 8. KITE-Toronto Rehabilitation, University Health Network, Toronto, Ontario, Canada*

There is an absence of high-quality workforce data that could be used globally for comparative research on workforce planning in the residential long-term care (LTC) sector. We know that older adults residing in the LTC settings have multimorbidities resulting in complex care needs, yet the workforce is insufficiently able to meet their needs. A further reduction in LTC workforce was noted during the COVID-19 pandemic which increased the risk of adverse outcomes for residents. Survey results focused on the workforce in LTC homes collected from several countries during the current pandemic, highlighted that several members of the workforce were either absent or worked virtually (e.g., physicians, social workers). A better understanding of who is/or should be in the house to meet the needs of residents during or after future pandemics requires a workforce data system that routinely collects this information to ensure best quality outcomes for residents and their carers.

### UNDERSTANDING AND EXPLAINING HOW STAFF PROMOTE QUALITY FOR OLDER PEOPLE LIVING IN LONG-TERM CARE FACILITIES

Julienne Meyer,[1] Kirsty Haunch,[2] Carl Thompson,[2] and karen spilsbury,[2] *1. City, University of London, Guildford, England, United Kingdom, 2. University of Leeds, University of Leeds, Leeds, England, United Kingdom*

Little is known about how the workforce influences quality in long term care facilities for older people. Conceptually, quality is complex, often contested, and dynamic, has overlapping physical, social, psychological and emotional dimensions and can refer to both quality of life and quality of care. Assuming 'more staff equates to better quality' is intuitively appealing but research suggests that a more nuanced, non-linear, relationship exists. A programme of research in the UK is developing theoretical and empirical explanations of how staff promote quality for older people living in long-term care facilities. It shifts the debate from numbers of staff and their relationship to quality indicators toward recognising the ways in which staff more broadly influence quality. Our work will be useful for people and organisations making policy and delivering services on the best ways to deploy and support quality in long term care through the most valuable resource: its staff.

### Session 2050 (Symposium)

### COMMUNITY COLLEGE GERONTOLOGY PROGRAMS: REVISITING AND RE-VISIONING WORKFORCE, CAREER PATHWAYS, AND TRANSFER

Chair: Jennifer (Jenny) Sasser Co-Chair: Roger Anunsen Discussant: Michael Faber

The session will focus on new and existing innovative ways that Community Colleges are effectively addressing workforce needs resulting from a rapidly aging population; the ever-expanding career pathways available to students in the field of gerontology; as well as the continuing higher education needs of students transferring to bachelor's and graduate-level gerontology programs. A panel of Community College and University gerontology professionals, representing both the GSA Community College and Aging Workforce Interest Groups, will share the innovative ways that they are working to address the three focus areas of this symposium. We will also include opportunities for discussion with participants about their experiences with and ideas for addressing these issues.

### INNOVATIONS IN GERONTOLOGY EDUCATION

Kimberly McDonald,[1] and Jennifer Ellis,[2], *1. Northwood Technical College, New Richmond, Wisconsin, United States, 2. Northwood Technical College, Superior, Wisconsin, United States*

This session examines innovative approaches to effectively address the aging services workforce needs in rural, Midwestern settings. Presenters will explore career pathways and transfer opportunities in gerontology education, as well as best practices for addressing the educational, professional and personal needs of diverse student populations.

### THE IMPACT OF DECLINING ENROLLMENT AND RE-CAREERING ON COMMUNITY COLLEGE GERONTOLOGY PROGRAMS

Judith Robertson Phillips, *California State University San Marcos, California State University San Marcos, California, United States*

According to Inside Higher Ed (2020), community college enrollment declined by 10.1% during the fall 2020 semester and this decline in enrollment continued into the spring 2021 semester for many community colleges. Gerontology programs within community colleges were among curriculums impacted by this downswing in enrollment. This presentation will discuss what this means for community college Gerontology programs across the United States in a time when the older adult population is rapidly increasing, and a qualified workforce is needed to fill the multiple fields serving this aging population. One area of discussion will be the number of returning adults who are re-careering into Gerontology, some because of the impact of a family caregiving situation while others are motivated to change careers because of their interest in the numerous career pathways available in the field of aging. Suggestions for encouraging and supporting these newly returning adults will be discussed.

### ADULT LEARNING AND EMPLOYMENT OPPORTUNITIES FOR OLDER WORKERS IN AUSTRALIA AND THE UNITED STATES: LESSONS FOR ADULT EDUCATION

Philip Taylor,[1] Takashi Yamashita,[2] Leah Janssen,[3] and Phyllis Cummins,[4] *1. Federation University Australia, Berwick, Victoria, Australia, 2. University of Maryland, Baltimore County, Baltimore, Maryland, United States, 3. Scripps Gerontology Center, Oxford, Ohio, United States, 4. Miami University, Oxford, Ohio, United States*

This study examined the role community colleges (U.S.) and Technical and Further Education (TAFE; Australia) institutes play in providing educational opportunities to older workers in the U.S. and Australia. Employment for adults of all ages has been impacted by job automation in recent decades. We analyzed national level data to estimate the impacts of job automation by age group. In both the U.S. and Australia, about 65% of older workers in sales occupations are at risk for job loss due to automation. Additionally, we reviewed occupational projection data and employment opportunities for workers who may be displaced by automation. Needs for health care support occupations, such as nursing assistants and occupational and physical therapy assistants are expected to grow rapidly. We will provide several recommendations based on the integration of our findings related to education/training programs and the aging workforce in the context of community colleges and TAFEs.

### ADDRESSING THE CALIFORNIA MASTER PLAN ON AGING THROUGH INNOVATIVE GERONTOLOGY SPECIALIZATIONS

Pamela Abbott-Enz, *Chaffey College, Rancho Cucamonga, California, United States*

The 2021 Master Plan for Aging outlines five bold goals to pursue over the next ten years, including addressing the issues of housing, health, and quality of life, finance, and caregiving, the plan also addresses public safety and emergency services, community programs and public spaces, access, inclusion, and equity. In order to prepare the workforce to meet these goals, California Community Colleges have the unique opportunity to collaborate and develop Aging Specialists in fields not traditionally age-focused. This presentation explores the possible scope and path toward curriculum development of five innovative collaborations that will prepare Gerontology specialists for the workforce that will be created through the execution of the California Master Plan.

### Session 2055 (Paper)

### COVID-19 VACCINATIONS, EDUCATION, AND AWARENESS

### AN INTERNATIONAL PERSPECTIVE ON THE IMPACTS OF COVID-19 ON ADULT EDUCATION AND TRAINING

Oksana Dikhtyar,[1] Abigail Helsinger,[1] Phyllis Cummins,[2] and Nytasia Hicks,[3] *1. Scripps Gerontology Center, Miami University, Oxford, Ohio, United States, 2. Miami University, Oxford, Ohio, United States, 3. Department of Veterans Affairs, San Antonio, Texas, United States*

The COVID-19 pandemic has caused one of the worst economic crises since the Great Depression and the current recession has been more detrimental to older workers compared to other age groups. Not only has it forced more older workers out of their jobs, but it has also made it much harder for jobless older workers to find a new job. Furthermore, due to increased automation and digitalization in the workplace, older workers will likely need upskilling or reskilling to improve their employment prospects in the changed labor market. This situation brings the importance of offering training and continuous education programs that target older workers to the forefront of adult education policy and practice. This qualitative study examines measures taken in response to COVID-19 in adult education and training (AET) in seven countries including Sweden, Norway, the Netherlands, Australia, Singapore, Canada, and the United States. The findings are based on key informant interviews with international policy experts and scholars in the field of AET in addition to information gathered from written materials (e.g., government and organizational reports). To expedite their economic recovery and improve labor market outcomes for their workers, some countries have increased government funding for vocational and continuing education or offered financial support for post-secondary students while others have provided funds to employers to offer training and retraining for their employees. Some of these measures have the potential to expand adult educational opportunities in the post-pandemic world. Implications for policy and practiced are discussed.

### COVID-19 VACCINATION IN HOME HEALTH AND HOSPICE: BARRIERS TO VACCINATION AND RESULTS FROM A HOME VACCINATION PROGRAM

Robert Rosati, Steven Landers, and Tami Videon, *VNA Health Group, Holmdel, New Jersey, United States*

Little is known about vaccination rates in home health and hospice populations. Results draw upon two separate data sources from The Visiting Nurse Association Health Group (VNAHG). Among VNAHG patients surveyed between February 2 and March 1, 202, 24% had received at least one COVID-19 vaccine. Among vaccinated patients, roughly one quarter did not travel to get the vaccine (received inpatient vaccination). They mostly traveled by car (88%), and 70% received help from a family member. Of patients who had not received a vaccine (76%), 81% were pursuing or planning to pursue obtaining a vaccine. Additionally, of those not pursuing a vaccine, 30% indicated it was because they could not get to a vaccine site. 44% of patients in the VNAHG "in home" vaccination pilot were bedbound, and 100% of patients had ambulation difficulties that make it impossible for them to leave home. All (100%) had a health care provider(s) recommended they get the vaccine. Only 38% have internet access. A quarter tried to call to schedule a vaccine, but only one was able to speak to someone. 40% of the patients attempted to get a COVID-19 vaccine prior to enrollment in the program. Most patients (81%) did not have someone available to assist with their transportation to get vaccinated, and most indicated difficulty securing an appointment. Many indicated severe traveling difficulties (requiring oxygen, needing ambulance transport). These findings highlight the high barriers for homebound patients, and the need and value of clinicians traveling to provide in-home vaccines.

### COVID-19 VACCINE HESITANCY AMONG OLDER ADULTS: EVIDENCE FROM THE MEDICARE CURRENT BENEFICIARY SURVEY

Divya Bhagianadh,[1] and Kanika Arora,[2] *1. University of Iowa, IOWA CITY, Iowa, United States, 2. University of Iowa, Iowa city, Iowa, United States*

**Objective:** Older adults have been the most enthusiastic cohort about the COVID-19 vaccine since its rollout. However, there is limited evidence on vaccine hesitancy, particularly among community-dwelling older adults. In this study, we examine the prevalence and predictors (especially information sources) of vaccine hesitancy in this group.

**Methods:** We use the Medicare Current Beneficiary Survey (MCBS)- Fall 2020 supplement data and employ multivariable logistic regression models to explore this association. We study heterogeneous effects by gender, metro/non-metro residence status, race, and age.

**Results:** Depending on healthcare providers (HCP), social media, the internet, and family/friends as the main COVID-19 information source was associated with higher odds of negative vaccine intent when compared to those who rely on regular news. We did not find any association of 'unsure' vaccine intent and different information sources.

**Discussion:** Recommendation from an HCP is a strong predictor of any vaccine acceptance and higher negative intent for COVID-19 vaccine among those who depend on HCP for information is concerning. This could be due to vaccine hesitancy among HCPs themselves or due to other mechanisms like infrequent interactions with the health system.

### COVID-19 VACCINE INTENTIONS WITHIN THE MEDICARE POPULATION

Tami Swenson, *Des Moines University, Des Moines, Iowa, United States*

COVID-19 vaccine intentions by older adults reflect individual care seeking behavior and medical system trust and broader systemic cultural shifts related to vaccine hesitancy. The purpose of this paper is to examine the October wave of the rapid response panel survey fielded by the Centers for Medicare and Medicaid Services (CMS) to track and monitor the effects of the pandemic within the Medicare population. With a sample size of 9686 Medicare beneficiaries, the calculated statistics use replicate weights to adjust for the complex survey sample design and balanced repeated replication using Fay's adjustment of 0.3 for variance estimation. When asked about the likelihood of getting the COVID-19 vaccine if one were available, 58 percent of the Medicare population definitely or probably intended to get the vaccine, 16 percent expressed they would probably or definitely not, and 26 percent were not sure. Black or Hispanic Medicare beneficiaries were significantly more likely to express they would probably not or definitely not get the vaccine than White, non-Hispanic Medicare beneficiaries. Distrust of what government says about the vaccine and concern about the safety or side effects were the most common reasons for not intending to get the vaccine. Those expressing intentions to not get the COVID-19 vaccine in the October 2020 survey wave were more likely to lack access to the internet, which is a potential systematic barrier if they changed their intentions following the FDA approvals of the COVID-19 vaccines and more information became available in the winter and spring of 2021.

### PERCEPTIONS OF THE GOVERNMENT'S RESPONSE TO THE PANDEMIC: VOICES FROM THE COVID-19 COPING STUDY

Haley Gallo,[1] Lindsay Kobayashi,[2] and Jessica Finlay,[2]
*1. USC, Big Bear Lake, California, United States,*
*2. University of Michigan, Ann Arbor, Michigan, United States*

The COVID-19 pandemic was met with conflicting government strategies in the handling of the virus. Older adults were disproportionately impacted by the pandemic, yet little is known about their perspectives of the government response. Using data collected in September and October, 2020 from the online, nation-wide COVID-19 Coping Study, we conducted qualitative thematic analysis on a subsample of respondents (N=500) proportionate to the age, gender, race/ethnicity, and education of the U.S. population age 55+. Two researchers individually coded a random subsample of 50 open-ended responses to the question "How do you feel about federal government responses to and handling of the COVID-19 pandemic?" Using NVivo qualitative software, the researchers compared codes and reconciled differences to achieve a Kappa score of >0.8. The first author coded the remaining responses using the established coding strategy. Analyses identified themes related to President Trump's leadership, Congress, the broader federal government, and science. Some participants indicated that the federal government's response to the pandemic was "inadequate," "too political," and "lacking coordination." Others expressed that the president "did the best he could" or that "it's not the federal government's responsibility." While some praised vaccine development efforts and expressed their appreciation for Dr. Fauci, others expressed scientific distrust. Participants' perspectives were divergent, reflective of the country's polarization surrounding COVID-19 policies and practices. Differences in perspectives exist by race/ethnicity, gender, geographic region, and age. Study results can help identify groups of older adults who may need targeted programs and policy support.

### Session 2060 (Paper)

### DISPARITIES AND HEALTH

### HEALTH DISPARITIES IN DELIRIUM

Robert Dicks,[1] Jimmy Choi,[2] Christine Waszynski,[3]
Kadesha Collins-Fletcher,[3] Beth Taylor,[4]
Catherine Martinez,[5] and David O'Sullivan,[6] *1. Hartford Hospital, West Simsbury, Connecticut, United States, 2. Institute of Living, Institute of Living, Hartford, Connecticut, United States, 3. Hartford Hospital, Hartford, Connecticut, United States, 4. Evernorth, Saint Louis, Missouri, United States, 5. Vanderbilt University, Nashville, Tennessee, United States, 6. Hartford Healthcare, Hartford, Connecticut, United States*

Racial and ethnic minority populations in the US experience greater cumulative disease burden, as well as social and economic barriers, stressors, and limited advocacy/access to culturally informed healthcare. This increased risk burden is expected to be associated with an increased risk for delirium during acute care encounters. Previous studies on health disparity and delirium are limited and report equivocal findings regarding delirium incidence, possibly related to sample bias or non-validated measures. Risk for delirium during acute care in health disparity populations (HDP) that include Black African Americans (BAA) and Hispanic-Latinx (HL) has not been systematically studied using validated measures. We conducted a retrospective analysis utilizing our delirium program (ADAPT) registry that systematically assessed all hospitalized patients through their entire hospital stay for the years 2018-2019 (36K patients, 80% NHW, 11% HL, 9% BAA). The Confusion Assessment Method (CAM and CAM-ICU) and Richmond Agitation Sedation Scale (RASS) were used as screening assessments to identify delirium. We know from previous studies that negative CAM results in our environment have high specificity. The incidence of delirium between populations was compared using a chi-square test. Delirium incidence was higher in HDP (BAA combined with HL) compared to NHW in 71-80yo (16.0% vs 12.6%, p=0.003). Delirium incidence was not different in all other age groups compared; <65yo (p=0.191), 61-70yo (p=0.223), 81-90yo (p=0.644). Understanding the association, or lack thereof, between health disparities, ethnic and race-based risks for delirium is expected to provide important insights into more focused delirium assessment, prevention and mitigation strategies in these populations.

### HEALTH RISKS, PERCEPTIONS, AND SELF-CARE PATTERNS: A COMPARATIVE STUDY BETWEEN OLDER AND YOUNGER FILIPINOS

Julienne Ivan Soberano,[1] Marysol Caciata,[2]
Jo Leah Flores,[3] Erwin William Leyva,[3]
Mary Abigail Hernandez,[3] Josefina Tuazon,[3] and
Lorraine Evangelista,[4] *1. University of the Philippines, Mandaluyong City, National Capital Region, Philippines, 2. VA Long Beach Healthcare System, VA Long Beach Healthcare System, California, United States, 3. University of the Philippines, University of the Philippines, National Capital Region, Philippines, 4. University of Texas Medical Branch School of Nursing, University of Texas Medical Branch School of Nursing, Texas, United States*

Worldwide trends in health risks, lifestyle behaviors, health perceptions, and health-seeking patterns suggest alarming disparities among individuals from low- and middle-income countries; particularly for older individuals (≥ 60 years). This study aims to compare health risks, perceptions, lifestyle behaviors, and health-seeking patterns between younger (< 60 years) and older (≥ 60 years) Filipinos from rural communities in the Philippines; and assess relationships between demographic, health risks and perceptions, and lifestyle behaviors to bolster health promotion efforts. A comparative cross-sectional study was employed with 863 younger and 427 older Filipinos. Results show that older participants were more likely to be single/widowed and had ≤ high school education. Older participants had higher rates of hypertension, dyslipidemia, diabetes, and depression but were more likely to report higher quality of life, ≥ 150 minutes of physical activity per week, ≥ 5 servings of fruits and vegetable per day, more difficulty falling asleep, report seeing a physician regularly, going to the community health center when sick, and attend stress management classes compared to their younger counterparts (all p's < .001). There were no differences in rates of obesity, self-medication, and use of integrative health. Older age was associated with higher risks, improved health perceptions, healthier lifestyle behaviors, and better health-seeking patterns. Our data suggest that health risks are higher in older individuals but risky lifestyle behaviors were higher in younger individuals and suggest the need to design separate health promotion interventions that target the unique needs of older and younger Filipinos from rural communities.

### IMMIGRATION-RELATED FACTORS AND DEPRESSION HELP-SEEKING AMONG OLDER CHINESE AMERICANS

Dexia Kong,[1] Man Guo,[2] Melissa Simon,[3] and XinQi Dong,[4] *1. Rutgers University, New Brunswick, New Jersey, United States, 2. The University of Iowa, Iowa City, Iowa, United States, 3. Northwestern University Feinberg School of Medicine, Chicago, Illinois, United States, 4. Rutgers University, Rutgers Institute for Health, New Jersey, United States*

Asian Americans have the lowest mental health service utilization rate among all racial/ethnic groups. One important yet understudied aspect of this group's mental health service use is its potential associations with immigration-related factors such as migration reasons, years in U.S., acculturation, and ethnic enclave residence. Using data from the Population-based Study of Chinese Elderly in Chicago (collected 2013-2015, N=3,123), this study investigates whether and how immigration-related factors shape mental health service utilization. Four categories of help-seeking behaviors for depressive symptoms were examined, including not seeking help (23.5%), seeking help from informal source(s) only (40%), seeking help from both informal and formal sources (28.7%), and seeking help from formal source(s) only (8.8%). Results of logistic regressions showed that U.S. Chinese older adults who migrated for family reasons were less likely to seek help from informal sources only than those who migrated for other reasons [Odds Ratio (OR)=0.64, 95% Confidence Interval (CI)=0.42-0.99). Less acculturated older immigrants (OR = 0.88, 95% CI = 0.79-0.97) and those who lived in Chinatown (OR = 2.34, 95% CI = 1.21-4.52) were more likely to seek help from formal sources only (relative to not seeking any help). Our findings showed that majority of the older Chinese Americans with depressive symptoms either did not seek help or sought help from informal sources only. Their help-seeking behaviors were shaped by their migration and acculturation experiences. Leveraging informal support networks and ethnicity-specific resources in Chinatown represent a culturally appropriate approach to facilitate mental health help-seeking among U.S. Chinese older adults.

### ROLE OF DISEASE-SPECIFIC INCIDENCE AND SURVIVAL IN DISPARITIES IN LIFE EXPECTANCY IN THE UNITED STATES

Julia Kravchenko,[1] Bin Yu,[2] and Igor Akushevich,[1] *1. Duke University, Durham, North Carolina, United States, 2. Duke University, Duke University/Durham, North Carolina, United States*

There are persisting geographic and racial disparities in life expectancy (LE) across the United States (US). We used 5% Medicare Claims data (2000-2017) to investigate how disease incidence and survival contribute to such disparities. Disease-specific hazard ratios (HRs) were calculated for Medicare beneficiaries living in the US states with the lowest LE (the states with the highest LE were used as a reference group), in gender- and race-/ethnicity-specific populations. Analysis of incidence showed that the greatest contribution to between-the-state disparities in LE was due to higher incidence (HRs≥1.30) of atherosclerosis, heart failure, influenza/pneumonia, Alzheimer's disease, and lung cancer among older adults living in the states with the lowest LE. The list of diseases that contributed most to LE through the differences in their survival substantially differed from the above listed diseases: namely, diabetes, chronic ischemic heart disease, and cerebrovascular disease had HRs≥1.28 for their respective survival rates, with the highest HRs for lung cancer (HR=1.37, in females) and prostate cancer (HR=1.30). Respective race-/ethnicity-specific patterns of incidence and survival HRs were investigated and diseases contributed most to racial disparities in LE were identified. Study showed that when planning the strategies targeting between-the-state differences in LE in the US, it is important to address both 1) primary and secondary prevention for diseases demonstrating substantial differences in contributions of incidence, and 2) treatment choice, adherence to treatment, and comorbidities for diseases contributing to LE disparities predominantly through the differences in survival. Such strategies can be disease-, race-/ethnicity-, and geographic area-specific.

### SLOWDOWN IN LIFE EXPECTANCY IMPROVEMENTS FOR EUROPEAN COUNTRIES FROM 2000 TO 2019

Nicholas Steel,[1] John Ford,[2] and Jurgen Schmidt,[3] *1. University of East Anglia, Norwich Norfolk, England, United Kingdom, 2. Cambridge University, Cambridge, England, United Kingdom, 3. Public Health England, London, England, United Kingdom*

Life expectancy improvements have slowed across Europe since around 2010 for unknown reasons. We aimed to assess the contribution of specific conditions and risk factors to changes in life expectancy. We compared Global Burden of Disease (GBD) 2019 estimates for life expectancy at birth, years of life lost to premature mortality (YLLs) and population attributable fractions (PAFs) for risk factors, for 17 European Economic Area (EEA) countries from 2000 to 2010 and from 2010 to 2019. All 17 countries experienced a slowdown in life expectancy improvements after 2010, after decades of improvement. Denmark experienced the smallest drop in improvement from 2000 to 2010 compared to 2010 to 2019 (0.75 years drop), followed by Norway (0.79), Iceland (0.86), Finland and Sweden (both 0.89). The 5 countries with the largest drop in improvement were Spain (1.6 years drop), the Netherlands (1.88), Portugal (1.92), the United Kingdom (UK) (2.13), and Ireland (2.77). Ischaemic heart disease and stroke made the biggest contribution to the slowdown in life expectancy. Important risk factors for mortality varied by country and included tobacco, drug and alcohol use, and high fasting plasma glucose. The Nordic countries have maintained improvements in life expectancy substantially better than other European countries. The different patterns in different countries suggest multiple factors are contributing to the changes, including specific conditions, risks and behaviours, and broader societal determinants of health. Large scale, international, co-ordinated research is needed to better understand these changes and inform policy actions, particularly as the COVID-19 pandemic will increase international differences.

### Session 2065 (Symposium)

### DISRUPTION TO TRANSFORMATION: ENGAGED RESEARCH

Chair: Carol Geary Co-Chair: Katherine Abbott Discussant: Erin McGaffigan

With changes in funders' requirements, engagement of persons with "lived experience" in the planning, conduct, and dissemination of research is increasingly common. Although patient and stakeholder engagement is expected, the body of literature describing necessary structures and processes is severely limited. Therefore, the purpose of this symposium is to aid in the movement of engaged research from disruption to translation. To do so, we will describe gaps in researcher knowledge and skills associated with patient and stakeholder engagement; structures and processes in current use with older adults, and outcomes associated with engagement. We will begin by sharing findings within advisory board models of engagement. Dr. Lessem will describe the Sage Resource Project researcher needs assessment. Researchers (N=103) shared both their engagement interests and their perceived knowledge and capability gaps. Dr. Berman will describe training developed within the same project to overcome researchers' perceived gaps. Then, Dr. Roes will describe a qualitative evaluation of persons with dementia perspectives on rewards and benefits of advisory board involvement. Our final two presenters will describe engagement using a variety of engagement approaches. Dr. Douglas will share experiences with adapting Montessori philosophies and processes to engage CNAs in development of innovative programming for dementia care within a long term care environment. Finally, Dr. Geary will share her team's findings from interviews completed using appreciative inquiry with sites with over five-years' experience engaging patients in research. Each site has developed unique infrastructures and processes to most effectively achieve desired outcomes.

### STRUCTURE, PROCESS AND OUTCOMES IN PATIENT ENGAGEMENT

June Eilers,[1] Cheryl Jernigan,[2] Kim Kimminau,[3] and Carol Geary,[4] *1. UNMC, Omaha, Nebraska, United States, 2. Retired, Univeristy of Kansas Medical Center, Kansas, United States, 3. MU, MU, Missouri, United States, 4. University of Nebraska Medical Center, University of Nebraska Medical Center, Nebraska, United States*

Although patient engagement in research is gaining acceptance by researchers and funding bodies, descriptions of implementation options and associated outcomes are limited. In this appreciative inquiry of the 12 institutions involved in the Great Plains Collaborative of the Patient-Centered Outcomes Research Network (PCORnet), we interviewed patient engagement officers and patient partners to enhance understanding of approaches to organizational structure, research engagement processes, and associated outcomes of the engaged research. Multiple structures have been identified including operational affiliations in both hospital and multiple university departments. Professional affiliations of patient engagement officers vary widely, including nurses, social workers, and public health professionals, among others. Patient engagement processes also vary, but with the majority using forms of advisory boards. All sites reported outcomes of their work including completed and/or ongoing research and co-authored publications.

### THE SAGE RESOURCE PROJECT: READYING RESEARCHERS TO TRANSFORM RESEARCH THROUGH ENGAGEMENT

Rebecca Berman,[1] Jesse Bella,[2] Margaret Danilovich,[3] and Rachel Lessem,[3] *1. Leonard Schanfield Research Institute, CJE SeniorLife, Illinois, United States, 2. Gerontology Department, Northeastern Illinois University, Illinois, United States, 3. CJE SeniorLife, Chicago, Illinois, United States*

The Sage Model enables engagement of older adults receiving Long Term Services and Supports (LTSS), a group typically excluded in research. This presentation focuses on lessons learned from The Sage Resource Project, a Patient Centered Outcomes Research Institute funded project. We collaborated with RCMAR and Roybal centers to encourage NIH-affiliated researchers to embrace stakeholder engagement through promotion of the Sage Model. Few studies include an assessment of researcher needs when it comes to stakeholder engagement. We conducted a needs assessment (n=103) finding <50% of researchers presented work to older adults and only 41% interacted with older adults receiving LTSS. However, >90% were likely to attend webinars to learn more. Additionally, 70% of respondents were interested in setting up their own Sage Model research advisory boards. We identify opportunities for transforming LTSS research by including older adults as well as directions for future research on engagement, based on researchers' identified needs.

### BUILDING RESEARCHERS' CAPACITY FOR EMBRACING THE ENGAGEMENT OF OLDER ADULTS IN RESEARCH

Rachel Lessem,[1] Margaret Danilovich,[1] and Rebecca Berman,[2], *1. CJE SeniorLife, Chicago, Illinois, United States, 2. Leonard Schanfield Research Institute, CJE SeniorLife, Illinois, United States*

The Sage Resource Project aimed to broaden the pool of researchers who include the voice of older adults using long-term services and supports (LTSS) in research processes. We developed training to build researcher capacity to engage older adults through the development of Sage Model research advisory boards. Methods included training strategies for learning mode, design, duration, and emphasis of content that were informed by results of a researcher needs assessment and input from 2 older adult research advisory boards. Over 100 researchers registered for a 4-webinar series. All respondents to webinar evaluations (22) reported learning about topics that aligned with webinar objectives and had interest in engaging older adult stakeholders and/or developing an older adult research advisory board in the future. Representatives from five universities expressed interest attending online interactive workshops to build advisory boards. Lessons learned identify directions for research on best practices for developing older adult advisory groups.

### MOTIVATION FOR PEOPLE LIVING WITH DEMENTIA TO ENGAGE IN RESEARCH ADVISORY BOARDS

Dianne Gove,[1] Ana Diaz,[1] and Martina Roes,[2]
*1. Alzheimer Europe, Luxembourg, Diekirch, Luxembourg, 2. German Center for Neurodegenerative Diseases (DZNE), Witten, Nordrhein-Westfalen, Germany*

The importance of Public Involvement (PI) is increasingly being recognized in the field of dementia research. In 2012, Alzheimer Europe set up the European Working Group of People with Dementia (EWGPWD) which provides advice and input for all activities of the organization including several large European-funded research projects. The German Center for Neurodegenerative Diseases (DZNE) created a research advisory patient board in 2020 with the intention of supporting the board in strategic research decisions. Both groups are composed of people with dementia and act independently. With the aim of finding out whether PI in research is mutually rewarding and beneficial, members of both groups were asked about their motivation to be involved in PI research activities and the value this had for them. This was collected either through narrative interviews or during meetings. People with dementia described several reasons for being involved in PI activities in dementia research.

### ENGAGING DIRECT CARE PROVIDERS IN THE IMPLEMENTATION OF MONTESSORI PROGRAMMING FOR DEMENTIA

Natalie Douglas, *Central Michigan University, Mt Pleasant, Michigan, United States*

There is a need to engage direct care providers such as certified nursing assistants (CNAs) explicitly in efforts to implement innovative programming in long-term care environments. This presentation will outline engagement strategies that supported the implementation of Montessori programming in a community of 20 individuals living with severe dementia. Examples about positioning the CNA at the center of decision making, negotiating and building trust, cultivating opportunities for mutual consultation, creating spaces for new ideas to emerge, and synthesizing diverse perspectives will be highlighted. Although the program achieved positive outcomes on a number of measures including decreased responsive behaviors from people living with dementia, decreased negative qualities of relationships between CNAs and persons with dementia, and increased positive qualities of relationships between CNAs and persons with dementia, this presentation will focus on the “how” of engagement between the project team and the CNAs by highlighting qualitative data.

### Session 2070 (Paper)

### ELDER ABUSE SCREENING AND PREVALENCE

### EMERGENCY DEPARTMENT UTILIZATION PATTERNS AMONG PHYSICAL ELDER ABUSE VICTIMS IN COMPARISON TO OTHER OLDER ADULTS

Tony Rosen,[1] Katherine Wen,[2] Sunday Clark,[3] Alyssa Elman,[4] Philip Jeng,[5] Yiye Zhang,[5] Karl Pillemer,[2] and Yuhua Bao,[5] *1. Weill Cornell Medical College / NewYork-Presbyterian Hospital, PELHAM, New York, United States, 2. Cornell University, Ithaca, New York, United States, 3. Boston University School of Medicine, Boston, Massachusetts, United States, 4. Weill Cornell Medical College / NewYork-Presbyterian Hospital, New York, New York, United States, 5. Weill Cornell Medical College, New York, New York, United States*

**Background:** Physical elder abuse is common and has serious health consequences. Little is known, however, about the patterns of health care utilization among these victims, including whether opportunities may exist for earlier identification and intervention. Our goal was to describe Emergency Department (ED) utilization known physical elder abuse victims compared with non-victims.

**Methods:** We used Medicare insurance claims to examine ED utilization patterns among a well-characterized cohort of 139 known physical elder abuse victims in the year before abuse was identified and compared this to control subjects matched on age, sex, race, and residential zip code.

**Results:** Physical elder abuse victims were significantly more likely than control subjects to visit the ED (47.5% vs. 35.9%, p=0.01) during the year before identification and to have at least one visit for an injury-related complaint (14.4% vs. 8.3%, p=0.03). Victims were also more likely to have multiple visits (18.7% vs. 14.6%, p=0.24), visit multiple EDs (7.9% vs. 6.7%, p=0.63), or be high frequency utilizers (≥4 visits, 3.6% vs. 2.7%, p=0.58), but differences did not reach statistical significance. The most common diagnoses in ED visits among victims were: open wound of knee/ankle, exacerbation of chronic bronchitis, pneumonia, and chest pain.

**Conclusion:** This work provides preliminary evidence that physical elder abuse victims use the ED more frequently and potentially have different patterns of utilization than other older adults. We plan to further characterize these different patterns to potentially to use them to develop tools for earlier identification.

### PATIENT-CENTERED DIGITAL SCREENER FOR ELDER ABUSE IN THE EMERGENCY DEPARTMENT

Fuad Abujarad,[1] Esther Choo,[2] James Dziura,[3] Chelsea Edwards,[1] Michael Pantalon,[1] Karen Jubanyik,[4] Gail D'Onofrio,[5] and Thomas Gill,[1] *1. Yale University, New Haven, Connecticut, United States, 2. Oregon Health & Science University, Portland, Oregon, United States, 3. Yale University, New H, Connecticut, United States, 4. Yale University School of Medicine, New Haven, Connecticut, United States, 5. Yale University School of Medicine, Yale University School of Medicine, Connecticut, United States*

Elder abuse is a growing problem where many cases are left unidentified by professionals. For some older adults, the emergency department may be the sole point of care where they have an opportunity to be identified as victims of abuse. However, current methods of screening tend to miss less obvious forms of abuse and may deter older adults from self-reporting due to either a lack of understanding of abuse or fear of potential consequences. VOICES is an innovative, self-administrated, and automated tablet-based tool that combines screening, educational content, and brief motivational interviewing to enhance and improve identification of elder abuse cases. Combining an elder abuse screener and digital coach designed to guide the older adult through a customized pathway to encourage self-identification and self-reporting of abuse, VOICES is a robust tool engineered to place the screening process in the hands of the older adults, rather than the providers. We will discuss preliminary results of the ongoing feasibility study currently being conducted in the ED, which has successfully enrolled over 500 older adults. Current data indicate that 93% of patients find the tool to be satisfying, engaging, and easy to use. Preliminary findings also suggest that older adults who come in with “Little to none” knowledge of elder abuse increase knowledge of abuse after using the tool. In summary, VOICES appears to be a feasible tablet-based screening tool in the emergency department.

### SELF-ADMINISTRATED ELDER ABUSE SCREENING TOOL FOR OLDER ADULTS WITH VISUAL AND HEARING DISABILITIES

Sarah Swierenga,[1] Fuad Abujarad,[2] Jennifer Ismirle,[1] and Chelsea Edwards,[2], *1. Michigan State University, Michigan State University, Michigan, United States, 2. Yale University, New Haven, Connecticut, United States*

Older adults age 60+ with disabilities are at greater risk of elder abuse compared to those without disabilities. We will describe results from our study to evaluate the usability and feasibility of the VOICES tablet-based elder abuse screening tool with older adults who have visual and hearing disabilities. VOICES is a digital health tool that screens, educates, and motivates older adults to self-report elder abuse. The VOICES tool has been developed and tested to be used with older adults without disabilities. We conducted a usability study with (n=14) older adults who were blind, had low vision, or were hard of hearing. Our evaluation method included both quantitative and qualitative measures to evaluate the ease of use and usefulness of the VOICES tool. Usability was measured as the percentage of tasks completed successfully, the average time to perform a task and the issues observed during performance of the tasks. Usability satisfaction was measured by written or verbal feedback on the questionnaires, and verbal comments from each session. Six participants completed the tasks successfully on their own; seven participants (mostly blind participants) completed the tasks with some intervention or help from the moderator. The majority of participants had System Usability Scale (SUS) scores 80 or above. Of all the participants, twelve (92%) stated that they would recommend the VOICES tool to others. Our findings generated universal considerations for more inclusive digital health interventions that accounts for the needs, wants and limitations for older adults with disabilities.

### SOCIAL PARTICIPATION AND ELDER MISTREATMENT IN A NATIONAL SAMPLE OF OLDER ADULTS

Emmy Yang,[1] Nadra Lisha,[2] Ashwin Kotwal,[3] Jaclyn Wong,[4] and Alison Huang,[2] *1. Icahn School of Medicine at Mount Sinai, New York, New York, United States, 2. University of California San Francisco, San Francisco, California, United States, 3. University of California San Francisco, University of California San Francisco, California, United States, 4. University of South Carolina, Columbia, South Carolina, United States*

Little is known about how social participation influences older adults' susceptibility to elder mistreatment. We conducted a cross-sectional analysis of a national probability sample of community-dwelling U.S. adults from 2015-2016 (1,268 women and 973 men; mean age 75 and 76 years, respectively; 82% non-Hispanic white). Frequency of participation in formal activities (community meetings, religious services, and volunteering) and informal social activities (socializing with friends and family) was assessed by questionnaire. Additional measures assessed emotional, physical, and financial mistreatment since age 60. Multivariable logistic regression examined associations between social participation and elder mistreatment, adjusting for age, race/ethnicity, education, and comorbidity. Forty percent of women and 22% of men reported at least one form of mistreatment (emotional, physical, or financial). Women reporting at least monthly formal social participation were more likely to report emotional mistreatment (adjusted odds ratio (AOR) 1.57, 95% confidence interval (CI) 1.08-2.29) and financial mistreatment (AOR 1.56, 95% CI 1.02-2.38) than women with less frequent engagement. Older women who socialized at least weekly were more likely to report emotional mistreatment (AOR 0.59, 95% CI 0.44-0.78) and financial mistreatment (AOR 0.59, 95% CI 0.42-0.85). These associations were not seen among older men. Frequent social engagement in the community does not preclude risk for elder mistreatment, and informal socializing may be associated with decreased exposure to certain forms of mistreatment. Assessment of older adults' social activities may help guide strategies for detecting and mitigating elder mistreatment in the community.

### Session 2075 (Paper)

### FAMILY CAREGIVING AND PERSONS WITH DEMENTIA

### CAREGIVING INTENSITY AND DEPRESSIVE SYMPTOMS AMONG OLDER ADULTS AFTER PARTNERS' ONSET OF DEMENTIA

Melissa Harris,[1] Jinkyung Ha,[2] and Geoffrey Hoffman,[2] *1. Duke University, Durham, North Carolina, United States, 2. University of Michigan, Ann Arbor, Michigan, United States*

Caring for a partner with Alzheimer's disease or related dementia (ADRD) can create tremendous care burdens. However, the extent to which onset of ADRD in a partner impacts caregiving intensity and emotional health, the relationship of increased care intensity to emotional health, and whether relationships vary across the older adult population, are less clear. We used 9 waves (years 2000-2016) of the nationally representative Health and Retirement Study dataset to examine the number of weekly caregiving hours provided and depressive symptoms for older (ages ≥51) individuals after partners' ADRD onset (measured with the Telephone Cognitive Interview Survey). We compared changes in outcomes from before to after partners' ADRD onset using zero-inflated negative binomial regression models, overall and among sub-populations with potential vulnerability to excess care burdens – women and racial/ethnic minorities. In our sample of 2,186 older Americans with 10,120 unique observations, we observed a 215% increase ($p<0.001$) in weekly caregiving hours provided and 21% increase ($p<0.001$) in depressive symptoms reported by older respondents after partners' ADRD onset. Even larger impacts were observed for women and for non-Hispanic whites. Increased amounts of caregiving were associated with increases in depressive symptoms after a partner's ADRD onset. In all, ADRD has substantial impacts on family. Improved support mechanisms, including enriched community resources, clinician focus on dyadic needs, respite care, and policy efforts such as tax credits for caregivers, will be needed to meet the needs of couples increasingly affected by ADRD.

### FEASIBILITY OF THE ACTPLAN PROGRAM FOR AFRICAN AMERICAN DEMENTIA CAREGIVERS: A SELF-DIRECTED MULTIMEDIA DELIVERY

Yashika Watkins,[1] William Collinge,[2] Alysha Hart,[3] Rita Tharpe,[4] Neelum Aggarwal,[5] and Gloria Bonner,[6] *1. Chicago State University, Cliffside Park, New Jersey, United States, 2. Collinge and Associates, Inc., Eugene, Oregon, United States, 3. University of Illinois at Chicago College of Nursing, Chiavgo, Illinois, United States, 4. University of Illinois at Chicago College of Nursing, Chicago, Illinois, United States, 5. Rush University, Chicago, Illinois, United States, 6. Unversity of Illinois at Chicago College of Nursing, Chicago, Illinois, United States*

African Americans (AA) are less likely than White Americans to complete advance care plans or end-of-life treatment documents. They face significantly greater risk of Alzheimer's Disease, a silent epidemic for this population, and other dementias. The healthcare system's lack of dementia support for AAs contributes to disparate care. A four-session caregiver group education program was conducted on advance care planning for AA dementia family providers. The program was based on Kolb's Experiential Learning Model and initially found effective in an R01 study using in-person delivery by a professional. The present pilot assessed feasibility of delivering the program in a self-directed multimedia format without professional facilitation, using Session 1 on tube feeding decisions as the test session. Twenty-six AA dementia caregivers completed the session in groups of 5 to 8 at a church equipped with a large TV screen. On-screen prompts guided navigation through the program which included recorded lecture, slides, short videos on decision-making, and group discussions. Using quantitative and qualitative methods, pre-and post-survey instruments were administered and interviews conducted. Usability ratings averaged 84%. Knowledge and self-efficacy gains exceeded those of the R01, with a 35% increase in correct responses on knowledge items, versus 18% for the R01 subjects; and increase in perceived decisional self-efficacy of 31% versus 30% for the R01 subjects. Qualitative feedback was universally positive. These findings confirm the feasibility of the self-guided multimedia approach to delivery of the program. A large RCT is planned which, if successful, will support wide dissemination to AA caregivers in need.

### HOME-VISIT INTERVENTION TO REDUCE STRESS OF UNDERSERVED FAMILY CAREGIVERS FOR PERSONS WITH DEMENTIA

Jung-Ah Lee,[1] Seyed Amir Hossein Aqajari,[2] Eunae Ju,[2] Priscilla Kehoe,[2] Lisa Gibbs,[3] and Amir Rahmani,[2]
*1. University of California, Irvine, Irvine, California, United States, 2. University of California Irvine, Irvine, California, United States, 3. UC Irvine Health, UC Irvine Health, California, United States*

Immigrant family caregivers for persons living with dementia (PWD) have constant stress due to the 24/7 responsibility. These family caregivers of PWD often have high morbidity and mortality. We provided a cultural and language specific home-visit intervention for these vulnerable family caregivers. There is a lack of an objective measure of stress for caregivers. We assessed caregivers' stress by measuring heart rate variability (HRV), a physiological measure of stress, using a smartwatch for a one-month intervention. Weekly home visits for a month were provided to dementia family caregivers by trained community health workers with stress reduction techniques: mindful breathing and compassionate listening. Linear mixed-effect models were used to analyze the trends for the daily stress levels as measured by HRV from the smartwatch. We had 22 participants who completed the 4-week intervention (8 Latinos, 8 Koreans, 6 Vietnamese). The models showed a significant decrease in the stress level of all participants for 3 weeks (all Ps<0.01). At 28 days (4 weeks) all three groups showed a decrease in stress: Korean group (Beta= -0.405, P<0.001), Vietnamese group (Beta = -0.150, P=0.028), Latino group (Beta= -0.154, P=0.073) and all caregivers (Beta = -0.235, P< 0.001). The findings demonstrated a reduction of immigrant family caregiver stress with a home-visit weekly intervention for one month using mindful breathing and compassionate listening by culturally/linguistically appropriate community health workers. Large-scale studies to determine long-term outcomes of family dementia caregivers are necessary and should be carried out.

### NEGATIVE IMPACT AND POSITIVE VALUE OF CAREGIVING IN SPOUSE CARERS OF PERSONS WITH DEMENTIA IN SWEDEN

Marcus Johansson,[1] Kevin J McKee,[1] Lena Dahlberg,[1] Martina Summer-Meranius,[2] Christine Williams,[3] and Lena Hammar,[4] *1. Dalarna University, Falun, Dalarnas Lan, Sweden, 2. Mälardalen University, Västerås, Vastmanlands Lan, Sweden, 3. Florida Atlantic University, Boca Raton, Florida, United States, 4. Mälardalen University / Dalarna University, Västerås, Vastmanlands Lan, Sweden*

As welfare providers struggle to meet the care needs of persons with dementia (PwDs), most of their needs are being met by a family carers, most often a spouse. The situation for spouse carers is unique, e.g., with grief, loneliness and loss of intimacy combining with stress and poor health. Research is needed to develop adequate support for spouse carers based on evidence of what influences negative and positive outcomes of care. The present study investigated psychosocial correlates of spouse carers' (i) negative impact and (ii) positive value of caring. Data from a cross-sectional survey of 165 spouse carers community-resident in Sweden was analysed in two hierarchical regression models to predict negative impact and positive value of caring. Results found that negative impact and positive value were explained by different variables, significant predictors for negative impact included carer stress, health, and emotional loneliness, and change in intimacy with the care-recipient, while positive value was predicted by mutuality, change in closeness to the care-recipient and quality of support. Negative impact and positive value shared variance of only 17.2%. Thus, negative impact and positive value represent different aspects of the carer situation. Consequently, support needs to target several aspects in carers' life, aiming to; facilitate for spouses to manage PwD's impairment, increase emotional support while also strengthening the relationship between carer and PwD to reduce negative impact while increasing positive value.

### PERCEPTIONS OF FAMILY CAREGIVERS OF PEOPLE WITH DEMENTIA REGARDING SYMPTOM MANAGEMENT AND THE COVID-19 PANDEMIC

Melissa Harris,[1] and Marita Titler,[2] *1. Duke University, Durham, North Carolina, United States, 2. University of Michigan, Ann Arbor, Michigan, United States*

Nearly 98% of older adults with Alzheimer's disease and related dementias (ADRD) experience behavioral and psychological symptoms of dementia (BPSD). Although BPSD are linked to caregiver burden, perceptions of family caregivers on the impact of BPSD and their experiences addressing them in the home are unclear, and little is known about the impact of the pandemic on these experiences. Study aims were to explore: 1) the experiences of family caregivers of community dwelling older adults with ADRD regarding BPSD and how they manage BPSD in the home, and 2) how the pandemic impacted family caregivers' experiences, BPSD of their relatives, and BPSD management. A qualitative, exploratory approach was used; 21 family caregivers were interviewed virtually. Content analysis and constant comparative methods were used. Ten major themes emerged: 1) Emotional and psychological responses of caregiver, 2) Loss, 3) Anticipation, 4) Reliance, 5) Learning to caregive, 6) Rewarding, 7) Emotional and psychological responses of care recipient 8) Cognition of care recipient, 9) Care strategies, 10) Caregiver perspectives. Caregivers did not use terms "behaviors" or "symptoms", instead they described their relatives' and their own experiences interdependently. Caregiving challenges presented before the pandemic (e.g. equivocal effects of medications, increasing care demands), many of which were compounded by the pandemic. Future research should explore the experiences of caregivers from a range of backgrounds. Findings illustrate communication barriers exist between clinicians, community services, people with ADRD and their families which may be addressed through clinician education, family-centered care planning, and policies to expand support service access.

### Session 2080 (Symposium)

### FINDINGS FROM IMPLEMENTATION OF THE EIT-4-BPSD TRIAL

Chair: Barbara Resnick Co-Chair: Marie Boltz Discussant: Ann Kolanowski

Behavioral and psychological symptoms of dementia (BPSD) include aggression, agitation, depression, anxiety, apathy and hallucinations and are exhibited by up to 90% of nursing home (NH) residents with dementia. BPSD result in negative health outcomes, functional decline, high care costs, increased risk for inappropriate use of antipsychotic medications and social isolation. Behavioral approaches are endorsed as the first line of treatment for BPSD. Despite regulatory requirements, less than 2% of nursing homes consistently implement these approaches. The EIT-4-BPSD Trial was done to test a novel implementation approach to assure that staff in nursing homes use non-pharmacologic, behavioral approaches for the management of BPSD. EIT-4-BPSD is a theoretically-based four-step approach that includes: 1. Assessment of the environment and policies; 2. Education of staff; 3. Establishing person-centered care plans; and 4. Mentoring and motivating staff. Implementation of the four-step approach was guided by the Evidence Integration Triangle (EIT). The EIT brings together evidence and key stakeholders from the NH and uses a participatory implementation processes, practical evidence-based interventions, and pragmatic measures of progress toward goals. A total of 55 nursing homes from two states and 553 residents were included in this study. Nursing homes were randomized to EIT-4-BPSD or education only. This symposium will describe the utility of the EIT as an implementation framework based on the Reach, Effectiveness, Adoption, Implementation, and Maintenance model, report detailed effectiveness outcomes of EIT-4-BPSD at the setting and resident levels, and address recruitment and measurement challenges and future solutions to these challenges.

### TESTING THE USEFULNESS OF THE EVIDENCE INTEGRATION TRIANGLE FOR IMPLEMENTATION OF EIT-4-BPSD

Barbara Resnick, *University of Maryland School of Nursing, Baltimore, Maryland, United States*

The effectiveness of evidence-based practices, such as use of behavioral interventions, can be improved when delivered under conditions of an implementation framework. This pragmatic trial used the Evidence Integration Triangle (EIT) which is a parsimonious, community-engaged participatory framework that brings evidence and facility stakeholders together. Active engagement empowers key stakeholders to integrate evidence into practice using a simple three-pronged framework: (1) A participatory implementation process which was done via monthly meetings and weekly emails between stakeholders and a clinical expert as they worked on facility based goals; (2) Implementation of the four steps delineated in the EIT-4-BPSD; and (3) evaluation using practical progress measures. There was some evidence of implementation of the EIT-4-BPSD based on participation in Stakeholder team meetings, settings working towards goal achievement, and increased use of behavioral interventions among staff. The EIT approach is a useful implementation framework to help change staff behavior in long term care.

### EFFECTIVENESS OF THE EIT-4-BPSD INTERVENTION ON RESIDENT AND SETTING OUTCOMES

Elizabeth Galik, *University of Maryland, Baltimore, Maryland, United States*

The effectiveness of EIT-4-BPSD was based on testing the following hypotheses: (1) Settings exposed to EIT-4-BPSD will demonstrate improvements in Environment and Policy assessments, better quality of care interactions, and more person-centered care approaches for management of behavioral symptoms in care plans compared to Education Only settings; and (2) Residents in EIT-4-BPSD settings will have fewer behavioral symptoms and less pain, maintain or improve function, use fewer psychotropic medications, and have improved quality of life compared to residents in Education Only settings. There was not a significant treatment effect at the setting or resident level. Reasons for lack of effectiveness include limited evidence of behavioral symptoms at baseline, nationally based environment and policy requirements related to behavioral management, and measurement challenges in identifying behaviors and other outcomes. Future work should focus more on process and changing how staff approach care which was demonstrated in this trial.

### FACILITATORS AND BARRIERS TO IMPLEMENTATION OF THE EIT-4-BPSD INTERVENTION

Kiernan Riley,[1] Marie Boltz,[2] Ann Kolanowski,[3] Kimberly Van Haitsma,[4] and Liza Behrens,[5] *1. Penn State University, college Station, Pennsylvania, United States, 2. Pennsylvania State University, University Park, Pennsylvania, United States, 3. Penn State, University Park, Pennsylvania, United States, 4. The Pennsylvania State University, University Park, Pennsylvania, United States, 5. University of Pennsylvania School of Nursing, Philadelphia, Pennsylvania, United States*

This study aimed to explore the perceptions of stakeholders (site champions, administrators, and front-line, social service, and activity staff) regarding the EIT-4-BPSD implementation strategy, including its utility, and the barriers and facilitators to implementation in real-world settings. A process evaluation included qualitative data from focus groups conducted with 93 stakeholders of 21 nursing homes (NHs) that implemented the EIT-4-BPSD strategy. Data were analyzed using a conventional content analysis. Emerging codes were sorted into categories then organized in meaningful clusters based on the domains of the RE-AIM framework. Challenges, facilitators, and contextual factors explain variability in implementation of EIT-4-BPSD strategy among NHs in six key categories: multi-stakeholder engagement, multi-level outcomes, process adaptations, uptake and utility of EIT resources, adoption barriers and facilitators, and future planning. Overall, stakeholders reported that the EIT-4-BPSD strategy can be successfully implemented in NHs and is helpful in improving staffs' approach to BPSD.

### RECRUITMENT AND MEASUREMENT CHALLENGES AND FUTURE SOLUTIONS

Kimberly Van Haitsma, *The Pennsylvania State University, University Park, Pennsylvania, United States*

Recruitment of residents in the EIT-4-BPSD study required that residents have evidence of at least one behavioral symptom noted by staff in the past month. Even with this inclusion criteria 25% of the sample had no behavioral symptoms at baseline based on the Cornell Scale for Depression in Dementia, the Cohen-Mansfield Agitation Inventory, the Resistance to Care Scale, Pain in Advanced Dementia Scale, and Quality of Life in Late-stage Dementia Scale. Further, settings had good quality of care. Challenges to recruitment included the lack of willingness of residents with significant behavioral symptoms to assent to participate and legally authorized representatives to consent. Challenges to measurement included recall by staff and assumptions that behavioral symptoms were normal, short observation periods, and resident inability to provide input. Future solutions include some revisions in measures that will be described, longer assessment periods, and elimination of requirements that make recruitment of this population difficult.

### Session 2085 (Symposium)

### HEARING AND VISION IMPAIRMENT IN OLDER ADULTS: IMPLICATIONS FOR COGNITIVE, MENTAL, AND BRAIN HEALTH

Chair: Jennifer Deal Co-Chair: Frank Lin Discussant: Bonnielin Swenor

Sensory impairment impacts over 55% of Americans aged 60 years or older and may have important downstream consequences for the cognitive health of older adults. This session will present evidence for a relationship between sensory impairment and accelerated cognitive decline, increased risk of incident dementia, and increased mental and physical fatigability from two observational cohort studies. Additionally, this session will investigate the possible nature of these relationships. It may be that sensory impairment is a marker of dementia-related pathological changes in the brain, with potential ramifications for risk prediction and stratification. Alternatively, sensory impairment may have a direct impact on the aging brain, a potential causal mechanism liking sensory impairment and brain health, with implications for disease prevention. As part of this session, we will present evidence for associations between central auditory processing, a potential risk marker, and brain volumes measured with structural magnetic resonance imaging (MRI), and retinal vasculature density, as measured with optical coherence tomography. We will conclude by describing associations between age-related macular degeneration, a leading cause of blindness, and neurocognitive test performance and regional changes in brain atrophy and connectivity.

### VISION, COGNITIVE IMPAIRMENT, AND DEMENTIA IN THE AGING, DEMOGRAPHICS, AND MEMORY STUDY

Bonnielin Swenor,[1] Yunshu Zhou,[2] Kenneth Langa,[2] and Joshua Ehrlich,[3] *1. Johns Hopkins School of Medicine, Baltimore, Maryland, United States, 2. University of Michigan, Ann Arbor, Michigan, United States, 3. University of Michigan, University of Michigan, Michigan, United States*

Vision impairment (VI) is common in late-life and may be a modifiable risk factor for cognitive decline and dementia. In this study, using data from the population-based Aging, Demographics and Memory Study (ADAMS), we analyzed the association of VI with cognitive impairment no dementia (CIND) and dementia. We found that VI (binocular presenting acuity <20/40) was significantly associated with incident CIND (OR=3.5, 95% CI=1.4-8.9, p=0.008) and dementia (OR=1.8, 95% CI=1.0-3.1, p=0.040) after adjusting for age. However, among those with CIND, VI was not associated with dementia (OR=0.9, 95% CI=0.4-1.8, p=0.733). The association between VI and CIND remained significant in models fully adjusted for demographic and health factors (OR=2.7, 95% CI=1.0-7.5, p=0.049). We conclude that VI is associated with development of CIND but not with subsequent onset of dementia. These findings suggest that the association between VI and dementia is driven by the elevated risk of dementia among those with CIND.

### WORSE SELF-REPORTED HEARING ABILITY IS ASSOCIATED WITH GREATER PERCEIVED PHYSICAL AND MENTAL FATIGABILITY

Kyle Moored,[1] Frank Lin,[2] Justin Golub,[3] Mary Wojczynski,[4] Robert Boudreau,[1] Angeline Galvin,[5] Nancy W. Glynn,[6] and Theresa Gmelin,[7] *1. University of Pittsburgh, Pittsburgh, Pennsylvania, United States, 2. Johns Hopkins University, Johns Hopkins University, Maryland, United States, 3. Columbia University, New york, New York, United States, 4. Washington University School of Medicine, Washington University School of Medicine, Missouri, United States, 5. University of Southern Denmark, Odense, Hovedstaden, Denmark, 6. University of Pittsburgh Graduate School of Public Health, Pittsburgh, Pennsylvania, United States, 7. University of Pittsburgh Graduate School of Public Health, Pittsburgh, Pennsylvania, United States*

Older adults with hearing loss often report higher fatigue due to effortful listening. We evaluated whether self-reported hearing ability is associated with perceived physical and mental fatigability (a more sensitive measure than fatigue) using the Pittsburgh Fatigability Scale (PFS). Older adults (N=2,558) from the Long Life Family Study Visit 2 (71.5±11.4 years; 54.8% women) completed PFS and self-reported hearing ability (worse=[fair,poor,very poor,deaf] or better=[good, excellent]). Age-adjusted PFS Physical and Mental scores were 2.3 and 2.5 lower, respectively, for worse vs. better hearing (p<.0001). Generalized estimating equations adjusted for family-relatedness, site, age, sex, cognitive function (Mini-Mental State Examination), education, and self-reported health. Compared to individuals with better hearing, those with worse hearing had a 42% and 44% greater odds of physical (≥15) (CI:1.12-1.80,p=0.0042) and mental(≥13) (CI:1.13-1.84,p=0.0034) fatigability, respectively. These observed associations may potentially be explained via complex psychosocial and cognitive aging pathways (e.g. effortful listening) to be examined in future work.

### RETINAL VASCULAR DENSITY ON OCT-A AND AGE-RELATED CENTRAL AND PERIPHERAL HEARING LOSS IN AN ITALIAN OLDER POPULATION

Ilaria Bortone,[1] Francesco Panza,[2] Giancarlo Sborgia,[3] Gianluigi Giuliani,[3] Valentina Pastore,[3] Francesco Boscia,[3] Nicola Quaranta,[4] and Rodolfo Sardone,[5], *1. Institute of clinical Physiology - National Research Council, Pisa, Toscana, Italy, 2. Gerontology Unit, National Institute of Gastroenterology "Saverio de Bellis", Research Hospital, Castellana Grotte, Bari, Italy., Castellana Grotte, Puglia, Italy, 3. Eye Clinic, Department of Basic Medical Sciences, Neuroscience and Sense Organs, University of Bari "Aldo Moro", Bari, Italy., Bari, Puglia, Italy, 4. Otolaryngology Unit, Department of Basic Medical Science, Neuroscience and Sense Organs, University of Bari Aldo Moro, Bari, Italy., Bari, Puglia, Italy, 5. Population Health - Sensorial Frailty Laboratory - NATIONAL INSTITUTE IRCCS "S DE BELLIS" Research Hospital, Castellana Grotte, Puglia, Italy*

Age-related hearing loss (ARHL) and retinal vessel changes have both been associated to neurodegeneration/dementia, suggesting a possible link between these two conditions in older age. We analyzed data on 886 older participants (65 years+, age range: 65-92 years) in the cross-sectional population-based Salus in Apulia Study. OCT-A scan was used to measure SVD and DVD of the capillary plexi of the macula in different retinal quadrants. Peripheral ARHL was defined as >40 dB HL of PTA (0.5,1,2, and 4KHz) in the worst ear, and age-related CAPD as <50% at the SSI-ICM test in at least one ear. DVD at the whole retina and at the parafoveal quadrant were inversely associated only with age-related CAPD [OR:0.93; 95%CI: 0.88-0.96 and OR:0.94; 95 CI:0.90-0.99, respectively]. The association of retinal vascular density with age-related CAPD may bring us a further step forward in understanding the biological mechanisms underlying the links between neurodegeneration/dementia and ARHL.

### ASSOCIATIONS OF CENTRAL AUDITORY PROCESSING WITH BRAIN VOLUMES

Josef Coresh,[1] Kathleen Hayden,[2] Clifford Jack,[3] Thomas Mosley,[4] James Pankow,[5] Frank Lin,[6] Jennifer Deal,[7] and Kening Jiang,[8] *1. Department of Epidemiology, Johns Hopkins Bloomberg School of Public Health, Baltimore, Maryland, United States, 2. Wake Forest School of Medicine, Winston-Salem, North Carolina, United States, 3. Department of Radiology, Mayo Clinic, Rochester, Minnesota, United States, 4. The University of Mississippi Medical Center, Jackson, Mississippi, United States, 5. Division of Epidemiology and Community Health, University of Minnesota School of Public Health, Minneapolis, Minnesota, United States, 6. Johns Hopkins University, Johns Hopkins University, Maryland, United States, 7. Johns Hopkins University, Baltimore, Maryland, United States, 8. Johns Hopkins Cochlear Center for Hearing and Public Health, Baltimore, Maryland, United States*

We investigated the cross-sectional associations of speech-in-noise performance with magnetic resonance imaging brain volumes among 588 cognitively normal participants (77±4 years, 56% female) from the Aging and Cognitive Health Evaluation in Elders Study (randomized trial embedded in the Atherosclerosis Risk in Communities (ARIC) Study) baseline in 2018-19 (N=427, with hearing loss) and ARIC (N=161, normal hearing) Visit 6/7 in 2016-17/2018-19. Central auditory processing was measured by Quick Speech-in-Noise (QuickSIN) test; range: 0 to 30, lower scores=worse performance. In models adjusted for demographic and disease covariates, every 5-point decrease in QuickSIN score was associated with smaller volumes of the temporal lobe overall (-0.07SD, 95% CI:-0.13,-0.01) as well as subregions including but not limited to those important for auditory processing (amygdala:-0.13SD, 95% CI:-0.21,-0.04; middle temporal gyrus:-0.08SD, 95% CI:-0.15,-0.00; superior temporal gyrus:-0.08SD, 95% CI:-0.15,-0.01). Further research is needed to understand the mechanisms underlying these observed associations.

### AGE-RELATED MACULAR DEGENERATION AND THE AGING BRAIN

Alexandra Badea,[1] Jacques Stout,[1] Simon Davis,[1] Jie Zhuang,[2] David Madden,[1] and Heather Whitson,[3] *1. DUKE UNIVERSITY, Durham, North Carolina, United States, 2. Shanghai University of Sport, Durham, North Carolina, United States, 3. Duke University School of Medicine, Durham, North Carolina, United States*

Age-related macular degeneration (AMD), a leading cause of vision loss in older Americans, is associated with cognitive decline and, particularly, worse performance on verbal fluency tasks. To determine whether AMD is associated with changes in brain structure that may underlie decline in cognition, we conducted a longitudinal, observational study of 39 visually impaired AMD patients and 33 age-matched peers with healthy eyes. Participants (mean age 74.3) underwent cognitive assessments and 3T magnetic resonance imaging (MRI) at baseline and two years. At baseline, AMD patients exhibited lower cortical volume and worse white matter tract integrity, especially in inter-hemispheric connections (FDR <0.05). Principal components analyses revealed faster white matter decline in the AMD group, especially in visual cortex and left hemisphere, which is implicated in language tasks. Understanding patterns of regional brain atrophy in AMD sheds light on mechanisms for the AMD-cognition link and opens windows of opportunity for intervention.

### Session 2090 (Symposium)

### IMPLICATIONS OF DISABILITY FOR FUNCTIONAL TRAJECTORY, SERVICE USE, AND EXPENDITURES

Chair: Wayne Anderson Discussant: Gretchen Alkema

People with disabilities face a diverse array of health care and support needs. These needs can vary by disability type, degree, and timing of the advent of functional limitations. These differences have implications for needed health care service use and related expenditures. The symposium will open with a Centers for Disease Control and Prevention-sponsored analysis of adult disability-associated health care expenditures, both nationally and by U.S. state, in total, by per adult, by per adult with disability, and by payer, to illustrate the contribution and variation of these expenditures to individual states and the health care system. We will next present a U.S. Department of Health and Human Services'

Office of the Assistant Secretary for Planning and Evaluation effort to identify onset and patterns of reduced functional ability at end of life for older adults with and without dementia as related to other comorbidities. The last paper will present a Commonwealth Foundation study on older adults with functional disabilities and multiple chronic conditions, comparing those with high health care needs versus the subset of those people who are also high cost. Patterns of utilization differed between these two groups, and by state. These findings have implications for the development of care models that might best meet people's needs. Our discussant will respond to the studies' findings and discuss the important role that efforts to understand the nature of disability and functional status and the scale and scope of service use and costs have for people with disabilities.

### STATE-LEVEL HEALTH CARE EXPENDITURES ASSOCIATED WITH DISABILITY

Olga Khavjou,[1] Amanda Honeycutt,[1] Laurel Bates,[1] NaTasha Hollis,[2] Scott Grosse,[2] Hilda Razzaghi,[2] and Wayne Anderson,[1] *1. RTI International, Research Triangle Park, North Carolina, United States, 2. Centers for Disease Control and Prevention (CDC), Atlanta, Georgia, United States*

This study updated prior (2003) state-level estimates of disability-associated health care expenditures (DAHE). We combined 2013-2015 data from three national data sets to estimate using multivariate regression all state-level DAHE for US adults in total, by payer, and per adult and per (adult) person with disability (PWD). In 2015, DAHE were $868 billion nationally (State range, $1.4 billion to $102.8 billion) accounting for 36% of total health care expenditures (range, 29%-41%). From over a decade ago, total DAHE increased by 65% (range, 35%-125%). DAHE per PWD was $17,431 (range $12,603 to $27,839). From over a decade ago, per-PWD DAHE increased by 13% (range, –20% to 61%). In 2015, Medicare DAHE per PWD ranged from $10,067 to $18,768. Medicaid DAHE per PWD ranged from $9,825 to $43,365. DAHE are substantial and vary by state and payer. Stakeholders can use these results to develop public health programs to support people with disabilities.

### UTILIZATION AND EXPENDITURES OF HIGH-NEED, HIGH-COST OLDER ADULTS WITH DISABILITIES

Cleanthe Kordomenos,[1] Molly Knowles,[1] Micah Segelman,[1] and Sarita Karon,[2], *1. RTI International, Research Triangle Park, North Carolina, United States, 2. RTI International, Waltham, Massachusetts, United States*

The factors that lead people to have high needs for care can vary greatly, with implications for the best approaches to serving their needs. One high need group of interest is older adults with disabilities and multiple comorbidities. There is variation in need within this group. Of particular interest is the subset that is both high need and high cost (HNHC). We present work describing Medicare and Medicaid utilization and expenditures for this high need group and the HNHC subset. Over 7.6 million people were identified as high need; 13.6% of them also were defined as HNHC. Patterns of utilization differed between these groups, with the HNHC group more likely to use inpatient care and nursing home care, but less likely to use community-based long-term services and supports. These findings have implications for the development of care models that might best meet the needs of this population.

### FUNCTIONAL TRAJECTORIES FOR PEOPLE WITH DEMENTIA AND OTHER COMORBIDITIES IN THE LAST YEARS OF LIFE

Lauren Palmer,[1] Emily Graf,[2] Qinghua Li,[3] Zhanlian Feng,[3] Helen Lamont,[4] Judith Dey,[4] Iara Oliveira,[4] and Ila Broyles,[5] *1. RTI International, RTI International, Massachusetts, United States, 2. UC Berkeley, Berkeley, California, United States, 3. RTI International, Research Triangle Park, North Carolina, United States, 4. Office of the Assistant Secretary for Planning and Evaluation, Washington, District of Columbia, United States, 5. RTI International, RTI International, North Carolina, United States*

A hallmark of end-of-life (EOL) is reduced functional ability. However, the impact of dementia and other comorbidities on EOL decline for older adults is less well understood. We estimated the effect of having dementia and comorbidities on activity of daily living (ADL) scores using the 2000-2012 Health and Retirement Study. We identified 5,853 people over age 65 who died and predicted monthly ADL impairments in the last 4 years of life controlling for dementia and other characteristics. Stroke and obesity were associated with significantly higher ADL scores, regardless of dementia status. However, if both dementia and either stroke or obesity were present, dementia was associated with significantly higher ADL scores approximately 1-4 years before death. Functional decline occurred closer to death if they had these conditions and no dementia. Differences in function when patients have dementia and comorbidities may affect understanding of survival time and access to appropriate care.

### Session 2095 (Paper)

### LONG-TERM CARE AND COVID-19

### BREATHING UNEASY: FIT-TESTED N95 RESPIRATOR ACCESS IN WASHINGTON STATE LONG-TERM CARE FACILITIES

Carolyn Ham, and Mikiko Nakamura, *Washington State Department of Health, Shoreline, Washington, United States*

Long-term care facilities (LTCF) have been disproportionately impacted by illness and death from COVID-19. Shortages of respirators for staff, especially Particulate Filtering Facepiece Respirators (N95), have limited LTCFs ability to follow public health recommendations for preventing COVID-19 transmission. Use of N95 respirators was infrequent in Washington State (WA) LTCFs prior to May 2020. N95 respirators must be individually fit tested to provide intended protection; a fit test is a procedure that tests the seal between the N95 respirator and the wearer's face. The WA Department of Health (WA DOH), collaborated with stakeholders to survey LTCFs in November 2020 regarding needs for fit tested respirators and analyzed responses (n=384). Responses by facility type: 8.3% nursing homes, 17.7% assisted living, 62.8% adult family home, 11.2% other. In WA, adult family homes (AFH) are licensed for six or fewer residents. 23.70% of LTCFs indicated they did not have any N95 respirators in stock at their facility; 96.7% of these were AFH. In August 2020 WA DOH surveyed AFH owners and received 110 responses; 9.76% reported having at least one staff member fit tested for respirators. Smaller facilities may experience increased burden in accessing N95 respirators and fit testing due to lack of established relationships with suppliers and small volumes being purchased. WA DOH used federal COVID funding to contract with mobile fit testing providers and prioritized AFHs for this service. Between December 1, 2020-February 28, 2021, staff at 290 LTCFs were fit tested. The project will continue throughout 2021.

### LTSS DIRECT CARE WORKER EMPLOYMENT DURING COVID-19

Janette Dill,[1] and Bianca Frogner,[2] *1. University of Minnesota, Minneapolis, Minnesota, United States, 2. University of Washington, Seattle, Washington, United States*

The crisis of COVID-19 in long-term care services and supports (LTSS) has brought attention to challenges in staffing long-term care organizations, as shortages of direct care workers led to a dramatic inability to provide needed care for many residents in nursing homes and other residential care settings. In this study, we examine unemployment among LTSS direct care workers during the crisis and recovery. This study uses monthly data from January 2019 to December 2020 from the Current Population Survey, a monthly household survey collected by the Bureau of Labor Statistics, and we compare an individual's 2019 monthly employment patterns to their 2020 monthly employment. Long-term care workers had an unemployment rate of 2.8% in April 2020, when unemployment rates in the US reached a peak; however, new unemployment among long-term care workers has not declined as consistently as in other settings. Female health care workers were significantly more likely to be unemployed compared to their male counterparts, a trend that is consistent with the overall economy, and workers who earned the lowest wages were more likely to have transitioned to unemployment. COVID-19 has added significant complexity to the provision of direct care services, making LTSS a hazardous place to work. Concerns remain about unemployment in long-term care where demand for workers remains high; additional measures need to be taken to ensure that direct care workers have the resources they need to remain employed.

### STATE ADOPTION OF NURSING HOME IMMUNITY FROM LEGAL LIABILITY DURING THE COVID-19 PANDEMIC

Pamela Nadash,[1] Edward Miller,[2] Elizabeth Simpson,[1] Michael Gusmano,[3] and Lisa Beauregard,[4] *1. University of Massachusetts Boston, University of Massachusetts Boston, Massachusetts, United States, 2. University of Massachusetts Boston, Boston, Massachusetts, United States, 3. Rutgers University School of Public Health, Rutgers University School of Public Health, New Jersey, United States, 4. Executive Offce of Elder affairs, Executive Office of Elder Affairs, Massachusetts, United States*

Twenty-eight states have provided nursing homes (NHs) with immunity from legal liability related to COVID-19. This study places these provisions in the context of prior actions protecting NHs from legal action and explores factors influencing the adoption of such immunity provisions across states. It uses cross-sectional data to examine patterns of policy adoption and to assess states' likelihood of adopting immunity provisions using multivariate methods. Variables of interest include information on state political, socioeconomic, programmatic, and COVID-19-related characteristics as well as data on campaign contributions and lobbying activity at the state level. Factors significantly related to NH immunity provision adoption included measures of state fiscal health (unemployment), ideology (percent legislators Democrat), governing capacity (unified government), and NH characteristics (percent not-for-profit, hospital-based, and chain). Population density and Medicaid as a percentage of state general fund expenditures proved significant as well. Against these complex influences, organizations lobbying on behalf of NH residents and their families have found themselves ineffectual in creating avenues for accountability. Results indicate that enforcing accountability for NH deaths during the COVID-19 pandemic is a complex process, constrained by available policy tools and made more complicated by factors external to the NH environment that contributed to high death rates. Historically, the NH industry has been successful in avoiding consequences for poor quality care, a pattern that has persisted in that NHs have generally been successful in avoiding liability for negligence during the COVID-19 pandemic.

### STAYING IN TOUCH DURING COVID-19: LONG-TERM CARE FACILITY COMMUNICATION STRATEGIES AND FAMILY PERCEPTIONS

Jane Straker,[1] and Mi Sun Choi,[2] *1. Miami University, Oxford, Ohio, United States, 2. Silla Universiy, Silla University, Pusan-jikhalsi, Republic of Korea*

During the COVID-19 pandemic, family concerns regarding residents in long-term care facilities (LTCFs) increased dramatically due to the higher proportion of deaths in LTCFs and an inability to visit, observe care, or easily communicate with residents. However, little is known about how these facilities communicated with families and how communications were related to family perceptions about the facility. To address these knowledge gaps, we implemented an online survey of family members or friends of residents in LTCFs from April 28 to June 19, 2020. A total of 174 responses nationwide reported the types of communications used, frequency of communication and alternative visits, and whether the families had peace of mind, would recommend the facility and whether they were considering removing the resident from the facility. We performed chi-square and t-tests to identify differences in perception among families. Results showed that respondents had more negative perspectives of a facility when they were not informed about confirmed COVID cases. There were no differences in family members' perceptions of a facility based on the frequency of alternative visits. When respondents could communicate with their family members in an LTCF by telephone, email, mail, and window visits, they had more peace of mind. Respondents were more likely to recommend the facility to others when they were able to communicate via mail with the facility. Our findings suggest multiple communications and transparency about COVID status were most effective in keeping positive family perceptions about the facility. Our results can inform future facility communication protocols.

### THE ETHICAL DILEMMA OF SAFETY VERSUS SELF-DETERMINATION IN LONG-TERM CARE COMMUNITY RESIDENTS DURING COVID-19

Rebecca Davis,[1] Cheryl Monturo,[2] Maria O'Reilly,[3] and Diana Sturdevant,[4] *1. Grand Valley State University, Grand Rapids, Michigan, United States, 2. West Chester University, West Chester, Pennsylvania, United States, 3. Central Queensland University, Bundaberg, Queensland, Australia, 4. University of Oklahoma HSC, Oklahoma City, Oklahoma, United States*

The pandemic profoundly affected the care of older adults in long term care communities (LTCC) across the world. More than one third of pandemic deaths were linked to nursing homes. Most nations and states had strict guidelines on visitation, with many, especially in the United States, totally prohibiting visitation for over an entire year. Well-intentioned measures to protect through isolation caused a profound ethical tension between safety and self-determination. The aim of the project was to examine this dilemma using a case study and the Madison Collaborative Ethical Reasoning in Action Framework. Eight key questions of fairness, outcomes, rights, responsibilities, character, liberty, empathy, and authority were applied in the context of federal and state mandates in the US and Australia. Results highlighted issues of ageism, paternalism vs empathy, regulatory vs family authority, a focus on short-term outcomes while forfeiting long-term outcomes, community responsibilities to the resident trumped individual resident rights, the potential loss of community character in lieu of basic care provision, a loss of personal freedoms, and the emphasis of physical well-being over holistic well-being. The results of this analysis can inform future policy and provide lessons learned for the future.

### Session 2100 (Symposium)

### MEASURING WHAT MATTERS IN LONG-TERM CARE: COMMON DATA ELEMENTS IN BRAZIL, CHINA, AND THE UNITED STATES

Chair: Michael Lepore Co-Chair: Kirsten Corazzini
Discussant: Sheryl Zimmerman

Internationally sharable common data elements on residential long-term care (LTC) settings, such as nursing homes and assisted living facilities, can facilitate comparisons across diverse LTC settings for valuable insights on LTC regulation and oversight, practice and operations, infrastructure development, human resources issues, and quality and safety. However, such insights are predicated on the premise that data elements capture information that matters to the full LTC community, including residents, relatives and staff, and are able to be collected across diverse care settings, including low-resource contexts. A critique of much current LTC measurement is its focus on deficits and loss, rather than thriving, person-centered care, and healthy aging, which have been established as important to LTC communities internationally. Further, measurement burden, cultural differences in perceptions of data sharing, and data infrastructure differences are key issues for international data. An international collaborative of LTC researchers—Worldwide Elements to Harmonize Research in Long-Term Care Living Environments (WE-THRIVE)—has developed a set of common data elements that are recommended for parsimoniously assessing key outcomes, workforce and staffing, person-centered care, and the contexts within which LTC settings operate. The studies in this symposium provide insights into the validation and implementation of WE-THRIVE recommended measures in diverse, low-resource LTC contexts, including LTC settings in Brazil, China, and rural Midwest US. Study findings validate WE-THRIVE measures, and provide new knowledge to inform capacity-building for the measurement of person-centered care and healthy aging outcomes in diverse, low-resource, LTC settings.

### MEASURING WHAT MATTERS ACROSS INTERNATIONAL LONG-TERM CARE SETTINGS

Kirsten Corazzini,[1] and Michael Lepore,[2] *1. University of Maryland School of Nursing, Baltimore, Maryland, United States, 2. LiveWell Alliance, Southington, Connecticut, United States*

Measuring what matters most to residents, relatives and staff in residential long-term care settings is critical, yet underdeveloped in our predominantly frailty and deficits-focused measurement frameworks. The Worldwide Elements to Harmonize Research in Long-Term Care Living Environments (WE-THRIVE) consortium has previously prioritized measurement concepts in the areas of care outcomes, workforce and staffing, person-centered care, and care context. These concepts include knowing the resident and what matters most to the resident, and outcomes such as quality of life, and personhood. We present findings of our currently recommended measures, including both general population and dementia-specific measures, such as the Person-Centered Care Assessment Tool (PCAT), the Personhood in Dementia Questionnaire (PDQ), and the ICEpop CAPability Measure for Older People (ICECAP-O). We also describe remaining gaps in existing measures that will need to be addressed to fully specify common data elements focused on measuring what matters most to residents, relatives and staff.

### PERSON-CENTERED CARE POST-PANDEMIC IN RURAL U.S. SETTINGS: USE OF WE-THRIVE MEASURES

Michael Lepore,[1] Nancy Kusmaul,[2] Sarah Holmes,[3] Deanna Myer,[3] Zahra Rahmaty,[3] and Kirsten Corazzini,[3] *1. LiveWell Alliance, Southington, Connecticut, United States, 2. University of Maryland, Baltimore County, Baltimore , Maryland, United States, 3. University of Maryland School of Nursing, Baltimore, Maryland, United States*

Frontier communities are rural counties that are sparsely populated with limited resources to provide residential long-term care; COVID-19 has heightened these barriers and adversely affected residents, their families and staff. This study describes the feasibility of implementing recommended

WE-THRIVE measures in one residential long-term care setting in a frontier community in the rural Midwest, and the capacity for administrative leadership to draw upon results of measures to implement person-centered care post-pandemic. During the COVID-19 pandemic, early efforts to advance person-centered care in the setting were displaced by a focus on infection control and containment. WE-THRIVE measures assess person-centered care following immunization distribution, and are discussed in terms of how to develop a dashboard to 'de-escalate' a COVID-19 focus and rebuild momentum towards person-centered care. Particular attention is given to the context of measurement, including data sharing and measurement burden, to inform advancing person-centered long-term care in other frontier communities.

### PERSPECTIVES TOWARD LONG-TERM CARE MEASUREMENT FROM FRONTLINE WORKERS IN BRAZIL

Patrick Wachholz,[1] Paulo José Fortes Villas Boas,[2] Vivian Schutz,[3] Michael Lepore,[4] Deanna Myer,[5] Ester Villalonga Olives,[6] Kirsten Corazzini,[5] and Ruth Caldeira de Mello,[2], *1. São Paulo State University, São Paulo State University - Unesp, Sao Paulo, Brazil, 2. University of São Paulo, Universidade de São Paulo, Sao Paulo, Brazil, 3. University of Maryland Baltimore, school of Nursing, Baltimore, Maryland, United States, 4. LiveWell Alliance, Southington, Connecticut, United States, 5. University of Maryland School of Nursing, Baltimore, Maryland, United States, 6. University of Maryland, School of Pharmacy, Baltimore, Maryland, United States*

The Brazilian long-term care (LTC) sector remains poorly structured and underdeveloped. COVID-19 did not bring unprecedented focus to the sector just because of the high mortality; it also affected the quality of care. In this pilot study, we evaluated the perspectives toward WE-THRIVE LTC measurements from Brazilian frontline workers in five long-term care facilities. For the four WE-THRIVE domains of LTC measurement (workforce and staffing, person-centered care, organizational context, and care outcomes), respondents used a 4-point Likert scale to rate their importance and answered open-ended questions about how these aspects of care changed since COVID-19. With few exceptions, respondents rated these aspects of LTC as extremely important or very important. Qualitative results highlighted concerns about and impacts of COVID-19, such as challenges related to the isolation of residents. The assessed measurement domains are confirmed to be important by frontline staff in Brazil. Measurement adoption must account for current issues.

### PERSPECTIVES TOWARD PERSON-CENTERED LONG-TERM CARE IN CHINA

Kirsten Corazzini,[1] Bei Wu,[2] and Jing Wang,[3] *1. University of Maryland School of Nursing, Baltimore, Maryland, United States, 2. New York University, New York, New York, United States, 3. Fudan University, Chapel Hill, North Carolina, United States*

Health care aides provide direct care for older residents with advanced dementia in long-term care facilities. This study aims to understand care aides' perceptions of what is 'good' care, what is person-centered care, and how to provide person-centered care for older residents with advanced dementia, as preparatory work of the WE-THRIVE consortium's efforts to develop internationally-relevant common data elements of person-centered dementia care and launch comparative research in LMICs. Semi-structured interviews were conducted with health care aides (N=35) from 2 government-owned and 2 private long-term care facilities in urban China. Directed and conventional content analysis were used, drawing upon core constructs of person-centered dementia care and Nolan's (2006) senses framework. We found that although care aides were not trained in person-centered care, they did incorporate person-centeredness in their work by tailoring their care to the needs of older residents and facilitating interactions with residents and their peers through communication cues.

### Session 2105 (Symposium)

### MOBILITY PERFORMANCE IN OLD AGE: A WINDOW INTO BRAIN INTEGRITY

Chair: Qu Tian Co-Chair: Andrea Rosso Discussant: Caterina Rosano

Over two decades of research has established brain aging as a critical component of mobility decline. Studies consistently report that motor slowing predicts cognitive decline and neurodegenerative diseases, but reported associations are often modest. Both mobility and brain aging are complex processes and steady-state assessments are typically used (usual pace gait and structural MRI). We aim to elucidate the complex relations between brain aging and mobility by considering (a) strategies to maintain function such as interlacing periods of activity and rest (fractionation), (b) interventions that target brain and body (motor skill training), (c) multi-modal neuroimaging (functional connectivity and cerebral small vessel disease (cSVD)), (d) challenged walking (dual-tasks, uneven surfaces), and (e) reduced resources (hearing loss). This symposium focuses on community-dwelling older adults from observational and intervention studies using state-of-the-art and real-life assessments of gait (quality and fragmentation by tri-axial accelerometry) and brain (near-infrared spectroscopy (fNIRS), resting-state functional MRI). First, we examine activity strategies that modify the relation between slow gait and AD risk (Tian). Second, using fNIRS, we investigate the extent to which motor skill training increases automaticity of gait (Chen). Third, we examine how functional connectivity may compensate for the detrimental effects of cSVD on mild parkinsonian signs (Hengenius). Fourth, we investigate the effects of challenged walking on gait quality and the relation with cognitive function (Suri). Finally, we demonstrate relations of hearing and cognition with mobility (Pupo). We seek to generate discussions on shared pathways underlying motor slowing and the aging brain and future prevention and intervention strategies.

### ACTIVITY FRACTIONATION MODERATES THE RELATIONSHIP OF GAIT SPEED WITH ALZHEIMER'S DISEASE RISK

Yang An,[1] Jennifer Schrack,[2] Pei-Lun Kuo,[3] Amal Wanigatunga,[2] Eleanor Simonsick,[4] Susan Resnick,[5]

Luigi Ferrucci,[5] and Qu Tian,*1. NIA, Baltimore, Maryland, United States, 2. Johns Hopkins Bloomberg School of Public Health, Baltimore, Maryland, United States, 3. National Institute on Aging, National Institute on Aging, Maryland, United States, 4. National Instute on Aging/NIH, Baltimore, Maryland, United States, 5. National Institute on Aging, Baltimore, Maryland, United States*

Older adults with slow gait have a modestly elevated risk of Alzheimer's disease (AD). Whether strategies to maintain function, such as interlacing periods of activity and rest, modify this relationship is unknown. We analyzed 577 initially cognitively normal participants aged 50+(53%women,26%Black) who had baseline data on gait speed and fractionation via ActiHeart. Diagnoses of mild cognitive impairment (MCI)/AD were adjudicated during an average 7.3years follow-up. We examined gait speed, fractionation, and their interaction with MCI/AD risk using Cox proportional-hazards models, adjusted for demographics and APOE-e4. Each 0.2m/sec faster gait speed was associated with 24% lower risk of MCI/AD(p=0.04). Fractionation was not associated with MCI/AD risk(p>0.05). There was a significant gait*fractionation interaction(p=0.013). At high fractionation, gait was not predictive of MCI/AD. Slow gait speed is less predictive of future MCI/AD in individuals who fractionate their activity to maintain function, possibly indicating brain function that drives such compensatory strategy is still conserved.

### MILD PARKINSONIAN SIGNS ARE RELATED TO LOWER CORTICO-STRIATAL CONNECTIVITY IN EXECUTIVE NETWORKS

Theodore Huppert, Andrea Rosso, Caterina Rosano, and James Hengenius, *University of Pittsburgh, Pittsburgh, Pennsylvania, United States*

Mild Parkinsonian signs (MPS) affect up to 24% of community-dwelling older adults. We hypothesize that MPS are associated with Parkinson's-like alterations of functional connectivity (FC) in sensorimotor, executive, and reward cortico-striatal networks. Participants (N=266; mean age=83; 57% female) without Parkinson's completed resting-state fMRI and Unified Parkinsonian Disease Rating Scale (UPDRS). FC between striatum and cortex was measured within each network. Logistic regression tested associations of each network's FC with MPS (UPDRS>0), adjusted for MPS risk factors, then including white matter hyperintensities (WMH). MPS was associated with lower cortical-striatal FC in the left executive cortico-striatal network (OR [95%CI]: 0.188 [0.043,0.824]). Association survived adjusting for risk factors (0.162 [0.030,0.874]) but was attenuated after including WMH (0.209 [0.036, 1.200]). In stratified analyses, left executive cortico-striatal FC was associated with MPS only for those with higher WMH (0.077 [0.010,0.599]). Future work should examine whether higher FC protects against the influence of WMH on MPS.

### MOTOR SKILL TRAINING EFFECT ON REAL-TIME PREFRONTAL CORTEX ACTIVATION DURING WALKING

Subashan Perera,[1] Theodore Huppert,[1] Jennifer Brach,[2] Andrea Rosso,[1] and Nemin Chen,[1], *1. University of Pittsburgh, Pittsburgh, Pennsylvania, United States, 2. University of pittsburgh, Pittsburgh, Pennsylvania, United States*

We aimed to test the effects of motor skill training (MST) on gait automaticity measured by changes in prefrontal cortex (PFC) activation during actual walking. We used data from a 12-week trial of older adults (mean age=75.5, 60.5% women) randomized to standard physical therapy and standard+MST in a 1:1 ratio. Functional near infrared spectroscopy (fNIRS) measured PFC activation during simple and dual task walking. We will apply linear mixed models to assess effects of task, time, and MST on PFC activation. We will compare the PFC activation 1) during dual task walking compared to simple walking; 2) across visits after intervention; and 3) between participants receiving MST compared to standard physical therapy. These results will demonstrate whether gait automaticity, as evidenced by PFC activation during walking, is affected by MST.

### ASSOCIATION BETWEEN DUAL-TASK GAIT AND COGNITIVE FUNCTION IN OLDER ADULTS

Jessie VanSwearingen,[1] Mark Redfern,[2] Ervin Sejdic,[2] Andrea Rosso,[3] and Anisha Suri,[3] *1. School of Health and Rehabilitation Sciences, Pittsburgh, Pennsylvania, United States, 2. Swanson School of Engineering, University of Pittsburgh, Pittsburgh, Pennsylvania, United States, 3. University of Pittsburgh, Pittsburgh, Pennsylvania, United States*

Community mobility involves walking with physical and cognitive challenges. In older adults (N=116; results here from initial analyses: N=29, Age=75±5 years, 51% females), we assessed gait speed and smoothness (harmonic-ratio) while walking on even and uneven surfaces, with or without an alternate alphabeting dual-task (ABC). ANOVA assessed surface and dual-task effects; Pearson correlations compared gait with global cognition and executive function composite z-scores. The four conditions (even, uneven, even-ABC and uneven-ABC) affected speed(m/s) (0.97±0.14 vs 0.90±0.15 vs 0.83±0.17 vs 0.79±0.16). Smoothness (2.19±0.48 vs 1.89±0.38 vs 1.92±0.53 vs 1.7±0.43) was affected by only surface (controlled for speed). Greater speed was associated with better global cognition(ρ=0.47 to 0.49, p<0.05) for all conditions and with better executive function for even-ABC(ρ=0.39, p=0.04) and uneven-ABC(ρ=0.40, p=0.03). Executive function was associated with smoothness during even(ρp=-0.42, p=0.03) and uneven(ρp=-0.39, p=0.04) walking. Type of walking challenge differentially affects gait quality and associations with cognitive function.

### COGNITION MODERATES THE RELATIONSHIP BETWEEN HEARING AND MOBILITY IN COGNITIVELY NORMAL OLDER ADULTS

Brent Small,[1] Jennifer Deal,[2] Nicole Armstrong,[3] Susan Resnick,[4] Frank Lin,[5] Luigi Ferrucci,[4] Qu Tian,[4] and Daniel Pupo,[6]*1. University of South Florida, Tampa, Florida, United States, 2. Johns Hopkins University, Baltimore, Maryland, United States, 3. Warren Alpert Medical School of Brown University, Providence, Rhode Island, United States, 4. National Institute on Aging, Baltimore, Maryland, United States, 5. Johns Hopkins University, Johns Hopkins University, Maryland, United States, 6. University of South Florida, University of South Florida, Florida, United States*

Recent data has shown a consistent but modest association between hearing impairment and poor mobility; both are strongly associated with cognition. Cognitive function may moderate the relationship between hearing and mobility. We analyzed 601 cognitively normal older participants from the Baltimore Longitudinal Study of Aging who had concurrent data on cognition (attention, executive function, sensorimotor function), hearing (pure-tone average, PTA), and mobility (6-meter gait speed, 400-meter time). We performed multivariable-adjusted linear regression to test two-way interactions between each cognitive measure and PTA. There were significant PTA interactions with all cognitive measures on 400-meter time. There was a significant interaction between PTA and sensorimotor function on 6-meter gait speed. Among cognitively normal older adults, poorer hearing is more strongly associated with poor mobility in those with low cognition, especially sensorimotor function. Future studies are needed to understand how cognition may moderate the relationship of hearing impairment with mobility decline over time.

### Session 2110 (Symposium)

### MOLECULAR RESILIENCY AND AGING

Chair: Adam Salmon

Resilience is described as the ability to respond to acute forms of stress and recover to normal homeostasis. There is growing evidence that biology of resilience is entwined with the biology of aging. With increasing age, resilience decreases and is a likely contributor to increased morbidity, frailty and susceptibility to death with age. Conversely, increased resilience across numerous physiological markers of function is associated with longevity and healthy aging. The variation in resilience in populations suggests biological and molecular regulatory mechanisms that might provide insight into interventions to improve resilience, healthy aging and longevity. In this session, speakers will provide insight regarding short-term assays of resilience in animal models that prove useful both in delineating these biological mechanisms as well as inform on potential translational models to better understand biological resilience in human populations. The sessions focus is on defining these assays and discussion of the biological relevance each resilience assay in terms of the regulation of aging. The goals of these studies range from identifying potential predictors of individual lifespan within markers of functional resilience to leveraging geroscience to define whether markers of resilience can be modified through interventions to the aging process. Moreover, better understanding of the biology of resilience could assist in defining novel interventions that improve resilience and thereby enhance longevity.

### CELLULAR RESILIENCE AS A POTENTIAL PREDICTOR OF LIFESPAN

Adam Salmon, *University of Texas Health San Antonio, San Antonio, Texas, United States*

The progressive decline of resilience during the aging process across multiple functional systems suggests basic biological mechanisms of regulation. We exploited a primary cell model to identify markers of cellular resilience or the ability of cells in culture to respond and return to homeostasis following acute challenge including metabolic, oxidative, or proteostatic stress. Using primary fibroblasts from minimally-invasive skin biopsies of genetically heterogeneous mice, we are able to determine individual cellular resilience as well as the normal lifespan and healthspan of each donor. Our studies suggest donor age and sex affect cellular resilience and that this measure of resilience can predict functional outcomes in some interventional studies. While longevity studies continue, these studies point to a potential highly important marker of healthspan and longevity as well as a model to delineate the biology of resilience in animal and translational models.

### RESILIENCE AS A DETERMINANT OF HEALTHSPAN AND LIFESPAN IN MICE

Nathan LeBrasseur, *Mayo Clinic, Rochester, Minnesota, United States*

Dynamic measures of physical resilience—the ability to resist and recover from a challenge—may be informative of biological age far prior to overt manifestations such as age-related diseases and geriatric syndromes (i.e., frailty). If true, physical resilience at younger or middle ages may be predictive of future healthspan and lifespan, and provide a unique paradigm in which interventions targeting the fundamental biology of aging can be tested. This seminar will discuss research on the development of clinically relevant measures of physical resilience in mice, including anesthesia, surgery, and cytotoxic drugs. It will further highlight how these measures compare between young, middle-aged, and older mice, and how mid-life resilience relates to later-life healthspan and even lifespan. Finally, it will provide insight into whether interventions targeting the biology of aging can modify physical resilience in mice.

### ROLE OF PHYSIOLOGICAL RESILIENCE IN AGING: CHALLENGES AND OPPORTUNITIES

Derek Huffman, *Albert Einstein College of Medicine, Bronx, New York, United States*

Lifespan and healthspan remain a cornerstone of documenting efficacy in aging research. However, it is becoming increasingly appreciated that housing rodents in conventional, unprovoked conditions, rather than exposed to the same variety of stressors normally encountered by free-living humans, has limited our understanding of how these strategies can be translated. Resilience can be defined as the ability of an organism to respond to a physical challenge or stress and return to homeostasis. Indeed, physiologic resilience is recognized to decline with age from a weakening of interactions among multiple physiologic regulatory functions. Here, we have attempted to optimize stress assays as a means of measuring physiologic resilience in mice. Our data demonstrate that these assays can readily detect age-related deficits in recovery, are amendable to geroprotector strategies, including rapamycin, while acute exposure to a stress can accelerate aging and mortality, thereby serving as a potentially useful paradigm for testing age-delaying interventions.

### GENETIC VARIANTS CORRELATE WITH BETTER PROCESSING SPEED

Anastasia Gurinovich,[1] Kaare Christensen,[2] Marianne Nygaard,[2] Jonas Mengel-From,[2] Stacy Andersen,[3] Thomas Perls,[1] Paola Sebastiani,[4] and

Zeyuan Song,[5] *1. Boston University, Boston, Massachusetts, United States, 2. Department of Public Health, University of Southern Denmark, Odense, Syddanmark, Denmark, 3. Boston University School of Medicine, Boston, Massachusetts, United States, 4. Tufts Medical Center, Physician Organization, BOSTON, Massachusetts, United States, 5. Boston University School of Public Health, Boston, Massachusetts, United States*

Some cognitive abilities, such as vocabulary, are resilient to brain aging, while others such as conceptual reasoning, memory, and processing speed, decline with age and their rate of decline is genetically regulated. Despite the strong genetic heritability of processing speed assessed by the digit symbol substitution test (DSST), previous studies have failed to identify robust common genetic variants associated with this test. The Long Life Family Study (LLFS) includes long lived individuals and their family members who maintain good DSST scores as they age and who may carry variants associated with better DSST. We therefore conducted a genome-wide association study (GWAS) of DSST in LLFS using ~15M genetic variants imputed to the HRC panel of 64,940 haplotypes with 39,635,008 sites and replicated the findings using genetic data imputed to the 1000 Genomes phase 3 reference panel combining two Danish cohorts: the Middle Aged Danish Twins and the Longitudinal Study of Aging Danish Twins. The GWAS in LLFS discovered 20 rare genetic variants reaching genome-wide significance (p-value < 5x10-8), including 18 variants associated with better processing speed with large effect size. The genetic associations of rs7623455, rs9821776, rs9821587, rs78704059 in chromosome 3 were replicated in the combined Danish cohort. These genetic variants tagged two hormone receptor related genes, THRB and RARB, both related to cognitive aging. Further gene-based tests in LLFS confirmed that these two genes have protective variants associated with better processing speed.

### Session 2115 (Paper)

### MORBIDITY, MORTALITY, AND AGING

### CREATION AND VALIDATION OF A POLYSOCIAL SCORE FOR MORTALITY AMONG COMMUNITY-DWELLING OLDER ADULTS IN THE UNITED STATES

Yongjing Ping,[1] Chenkai Wu,[1] Michelle Odden,[2] Robert Stawski,[3] and Hoda Magid,[4] *1. Duke Kunshan University, Kunshan, Jiangsu, China (People's Republic), 2. Stanford University, Stanford, California, United States, 3. Oregon State University, Corvallis, Oregon, United States, 4. Stanford University, Stanford University, California, United States*

The interrelatedness between social determinants of health impedes researchers to identify important social factors for health investment. Since the older population had highly diverse social backgrounds, a new approach is needed to quantify the aggregate effect of social factors and develop person-centered social interventions. Participants ([n = 7383], 54.5% female) were aged 65 years or above who complete an additional psychosocial questionnaire in the Health and Retirement Study (HRS) at study entry in 2006 or 2008. Social determinants of health encompassing five social domains: economic stability, neighborhood and physical environment, education, community and social context, and health care system. Five-year mortality was calculated as the number of years from the interview date to the death date. We used the forward stepwise logistic regression to construct the polysocial score and multivariate logistic regressions to assess the associations between polysocial score and five-year mortality. Polysocial score (range: 7 to 59, mean±SD: 35.5±7.5) was created using 15 social determinants of health. Of the 7383 participants, 491 (30.8%), 599 (17.2%), and 166 (7.8%) deaths occurred over five years among participants with a low (0-29), intermediate (30-39), and high (40+) polysocial score, respectively. Participants with an intermediate (Odds Ratio [OR]=0.76; 95% CI, 0.65-0.89) or high (OR=0.46; 95% CI, 0.36-0.59) polysocial score had higher odds of death than those in the low category in the fully adjusted model, respectively. The polysocial approach may offer possible solutions to monitor social environments and suggestions for older adults to improve their social status for specific health outcomes.

### EPIDEMIOLOGIC DETERMINANTS OF DYNAMICS IN HEART FAILURE PREVALENCE AND MORTALITY IN OLDER U.S. ADULTS

Bin Yu,[1] Igor Akushevich,[2] Arseniy Yashkin,[3] and Julia Kravchenko,[2] *1. Duke University, Duke University/ Durham, North Carolina, United States, 2. Duke University, Durham, North Carolina, United States, 3. Duke University, Morrisville, North Carolina, United States*

Recent declines in heart failure (HF) prevalence and increases in mortality among older adults in the US suggest the need for research to investigate the relative contribution of the epidemiological determinants of these two processes to their historical and current trends. Study data were derived from a 5% sample of Medicare beneficiaries, 1991-2017. Partitioning analysis was used to decompose age-adjusted prevalence and incidence-based mortality (IBM) into their constituent components. HF prevalence trend decomposition demonstrated three phases: (a) Decelerated Increasing Prevalence (1994-2006) mainly driven by decreasing incidence, overpowering increasing survival, (b) Accelerated Declining Prevalence (2007-2014) and (c) Decelerated Declining Prevalence (2015-2017), mainly driven by declining incidence, overpowering declining survival. For HF IBM four phases were identified: (a) Decelerated Increasing Mortality (1994-2001) with declining incidence and increasing survival driving deceleration, (b) Accelerated Declining Mortality (2002-2012), (c) Decelerated Declining Mortality (2013-2016), mainly driven by declining incidence, overpowering declining survival, and (d) Accelerated Increasing Mortality (2017) mainly driven by declining survival, overpowering declining incidence. Study findings suggest that the recent decade-long decline in HF prevalence and 15-year decline in HF mortality mainly reflected decreasing incidence, while the most recent increase in mortality was due to declining survival, which may be associated with the Hospital Readmission Reduction Program. If current trends of incidence and survival persist, HF prevalence and mortality are forecasted to grow, suggesting that actions to reduce HF risk factors and improve treatment and management of HF after diagnosis are warranted.

### PREDICTORS OF MULTIMORBIDITY AMONG KOREAN OLDER ADULTS: LONGITUDINAL SECONDARY DATA ANALYSIS

Eunkyung Kim, TaeWha Lee, and Yoonjung Ji, *Yonsei University, Seoul, Seoul-t'ukpyolsi, Republic of Korea*

Multimorbidity has become a global concern for an aging society. It has been reported to be associated with increased health service utilization, leading to poor health outcomes including quality of life. However, the incidence of multimorbidity and its related factors are poorly understood. The aim of this study was to determine the socioeconomic and health-related factors predicting the incidence of multimorbidity in Korean older adults using longitudinal secondary data from the Korean Longitudinal Study of Aging (KLoSA) dataset from 2008 to 2018. The KLoSA aimed to collect basic data to be used for developing socioeconomic policy for the aging society in Korea. The sample included 3,019 older adults aged 65 years and over who had 0-2 chronic diseases at baseline in 2008. Multimorbidity was measured with the incidence of co-existence of three or more chronic diseases using Cox's proportional-hazards model. Among 3,019 respondents (female 57.6%, mean age 73.07±6.30 years), 586 (19.4%) incidents of multimorbidity were reported after 10 years of follow-up. Low participation in social activities, being overweight or obesity, more depressive symptoms, current or past drinkers, and lower life satisfaction were identified as significant predictors of multimorbidity among Korean older adults. This study identified high risk groups with overlapping senility and multimorbidity, who require more attention from health care providers in the course of chronic disease monitoring and management. This longitudinal approach will contribute to the development of preventive strategies to reduce the incidence of multimorbidity among older adults.

### SEX DIFFERENCE IN ALL-CAUSE AND INFECTION-SPECIFIC MORTALITY OVER 10 YEARS POST HIP FRACTURE

Rashmita Bajracharya,[1] Jack Guralnik,[2] Jay Magaziner,[3] Michelle Shardell,[4] Alan Rathbun,[3] Takashi Yamashita,[5] and Denise Orwig,[2] *1. University of Maryland, School of Medicine, Catonsville, Maryland, United States, 2. University of Maryland, Baltimore, Baltimore, Maryland, United States, 3. University of Maryland Baltimore, Baltimore, Maryland, United States, 4. University of Maryland School of Medicine, Baltimore, Maryland, United States, 5. University of Maryland, Baltimore County, Baltimore, Maryland, United States*

Men die at a twice higher rate than women in the first two years after fracture and also experience higher infection-related mortality. Most research has only looked at differences in short-term mortality after hip fracture. The objective was to determine if cumulative incidence of all-cause mortality and infection-specific mortality is higher in men compared to women over ten years. Data came from Baltimore Hip Studies7th cohort. Women were frequency-matched (1:1) to men on timing of fracture to ensure equal numbers of men and women. The association of sex and all-cause mortality was analyzed using Cox proportional hazard model and a cause-specific hazard model for infection-specific mortality. Both models controlled for age, cognition, comorbidity, depressive symptoms, BMI, and pre-fracture ADL limitations. Complete-case sample size was 300 (men=145, women=155). By the end of ten years from the date of admission for a hip fracture, there were 237 (men=132, women=105) all-cause deaths and 38 (men=25, women=13) infection-specific deaths. Men had significantly higher all-cause mortality risk [73.7% vs 59.3%; HR=2.31(2.02-2.59)] and infection-specific mortality [17.2% vs 8.3%; HR=4.43(2.07-9.51)] compared to women. In addition to sex, older age, cognition, and comorbidities were associated with all-cause mortality whereas only BMI was associated with infection-specific mortality in adjusted models. Men had a higher risk of mortality over 10 years compared to women, specifically two-fold higher risk of infection-specific mortality compared to all-cause mortality. Findings imply that interventions to prevent/treat infection, tailored by sex, may be needed to narrow significant differences in long-term mortality rates between men and women.

### VALIDATION OF SELF-REPORTED CANCER DIAGNOSIS BY COGNITIVE STATUS IN THE HEALTH AND RETIREMENT STUDY

Megan Mullins, Jasdeep Kler, Marissa Eastman, Mohammed Kabeto, Lauren Wallner, and Lindsay Kobayashi, *University of Michigan, Ann Arbor, Michigan, United States*

Exploring the relationship between cognition and cancer is increasingly important as the number of older adults in the US grows. The Health and Retirement Study (HRS) has longitudinal data on cognitive status and self-reported cancer diagnoses, but these self-reports have not been validated. Using HRS linked to Medicare Fee for Service (FFS) claims (1998-2016), we evaluated the validity of self-reported cancer diagnoses (excluding non-melanoma skin) against Medicare claims by respondent cognitive status. We included 8,280 Medicare-eligible HRS participants aged ≥67 with at least 90% FFS coverage. Cognitive status was ascertained from the HRS interview following the date of cancer diagnosis (or reference claim date) using the Langa-Weir method and was classified as normal, cognitive impairment no dementia (CIND), or dementia. We calculated the sensitivity, specificity, and Cohen's kappa for first incident malignant cancer diagnosis by cognitive status group. The majority (76.4%) of participants scored as cognitively normal, 9.6% had CIND, 14.0% had dementia and, overall, 1,478 had an incident cancer diagnosis. Among participants with normal cognition, sensitivity of self-reported cancer diagnosis was 70.2% and specificity was 99.8% (kappa=0.79). Among participants with CIND, sensitivity was 56.7% and specificity was 99.8% (kappa=0.66). Among participants with dementia, sensitivity was 53.0% and specificity was 99.6% (kappa=0.64). Results indicate poor validity of self-reported cancer diagnoses for older adults with CIND or dementia. These findings suggest researchers interested in cancer and cognition should use the HRS-Medicare linkage to ascertain cancer diagnosis from claims, and they highlight the importance of cognitive status in research among older adults.

### Session 2120 (Paper)

### PAIN ASSESSMENT AND MANAGEMENT

### BARRIERS AND FACILITATORS OF PAIN MANAGEMENT IN PERSONS WITH DEMENTIA IN LONG-TERM CARE: A SCOPING REVIEW

Yo-Jen Liao,[1] Ying-Ling Jao,[2] Diane Berish,[2] and Lisa Kitko,[3] *1. Penn State University, University Park, Pennsylvania, United States, 2. Pennsylvania State University, University Park, Pennsylvania, United States, 3. Pennsylvania State University, Pennsylvania State University, Pennsylvania, United States*

Approximately 50% of individuals with dementia regularly experience moderate to severe pain, which is largely undermanaged. Several studies have explored the barriers and facilitators of pain management for persons with dementia; yet the evidence has not been systematically reviewed. This review aimed to synthesize current evidence on the barriers and facilitators of pain management in persons with dementia in long-term care. A PRISMA guided literature search was conducted in PubMed, CINAHL, and PsycINFO. Titles, abstracts, and full texts were screened. Included articles were original research examining the barriers or facilitators of pain assessment and treatment in individuals with dementia in long-term care. Quality assessment was conducted using the Risk of Bias tool and Johns Hopkins Level of Evidence. Ten studies were identified, including four quantitative studies, five qualitative studies, and one with both quantitative and qualitative research. Barriers of pain management identified include residents' ability to self-report pain, pain medication side effects, need discrepancy among residents and their families, reluctance in administering analgesics, lack of pain assessment tools, lack of guidance in providing nonpharmacological interventions, and lack of clinical guidelines. Facilitators of pain management include clinicians with caring and enthusiastic characteristics, clinicians' knowledge of residents, positive relationships among clinicians, good communication skills, using validated pain assessment tools, understanding pain indicators, clinical experience, and need-driven continuing education. These results can guide clinical practice in long-term care. Interventions should be developed to target these barriers and facilitators and improve pain management in persons with dementia.

### PAIN ASSESSMENT IN IMPAIRED COGNITION (PAIC15) INSTRUMENT: CUTOFFS AGAINST THREE STANDARDS

Jenny van der Steen, Margot de Waal, and Wilco Achterberg, *Leiden University Medical Center, Leiden, Zuid-Holland, Netherlands*

Observational pain scales can help identify pain in persons with impaired cognition including dementia who may have difficulty expressing pain verbally. The Pain Assessment in Impaired Cognition-15 (PAIC15) observational pain scale covers 15 important items that are indicative of pain, but it is unclear how likely pain is for persons with each summed score (theoretical range 0-45). The goal of our study was to determine sensitivity and specificity of cut offs for probable pain on the PAIC15 against three possible standards. We determined cut offs against (1) self report when able, (2) the established Pain Assessment in Advanced Dementia (PAINAD) cut off of 2, and (3) observer's overall estimate based on a series of systematic observations. We used data of 238 nursing home residents with dementia who were observed by their physician in training or nursing staff in the context of an evidence-based medicine (EBM) training study, with 137 residents assessed twice. The area under the ROC curve was excellent against the PAINAD cut off (□ 0.8) at both assessments, but acceptable or less than acceptable for the other two standards. Across standards and criteria for optimal sensitivity and specificity, cut offs at the PAIC15 could be 3 or 4. Guided by self report we recommend PAIC15 scores of 3 and higher to represent probable pain with sensitivity and specificity in the 0.5 to 0.7 range.

### RELATIONSHIPS OF PAIN TREATMENT WITH DEMENTIA AND FUNCTIONAL OUTCOME IN MEDICARE HOME HEALTH CARE

Jinjiao Wang,[1] Kenrick Cato,[2] Yeates Conwell,[3] Kathi Heffner,[1] Fang Yu,[4] Thomas Caprio,[3] and Yue Li,[1] *1. University of Rochester, Rochester, New York, United States, 2. Columbia University, New York, New York, United States, 3. University of Rochester Medical Center, Rochester, New York, United States, 4. Arizona State University, Phoenix, Arizona, United States*

Adequate pain management is important to post-acute care functional recovery, yet persons with Alzheimer's disease and related dementias (ADRD) are often undertreated for pain. The objectives of this study were to examine in Medicare post-acute home health (HH) recipients with daily interfering pain 1) if analgesic use at home is related to functional outcome, and 2) if ADRD is related to the likelihood of analgesic use at home. We analyzed data from the Outcome and Assessment Information Set, Medicare claims, and electronic medical records of 6,039 Medicare beneficiaries ≥ 65 years who received care from a large HH agency in New York in 2019 and reported daily interfering pain. Analgesic use was identified in medication reconciliation of HH visits and categorized into any analgesics or opioid(s). ADRD was identified from ICD-10 codes and significant cognitive impairment. Functional outcome was measured as change in the composite score of Activity of Daily Living (ADL) limitations from HH admission to HH discharge. Use of any analgesics at home was associated with greater ADL improvement from HH admission to HH discharge ($\beta$= -0.20 [greater improvement by 0.2 ADLs], 95% Confidence Interval [CI]: -0.37, -0.04; p=0.017). Compared with patients without ADRD, those with ADRD were less likely to use any analgesics (Odds Ratio [OR] = 0.66, 95% CI: 0.49, 0.90, p=0.008) or opioids (OR=0.53, 95% CI: 0.47, 0.62, p<0.001) at home. Adequate pain management is essential to functional improvement in post-acute HH care. Patients with ADRD may be under-treated for pain in post-acute HH care.

### REMOTELY SUPERVISED CRANIAL ELECTRICAL STIMULATION AND CLINICAL PAIN FOR OLDER ADULTS WITH KNEE OSTEOARTHRITIS

Hyochol Ahn,[1] Hongyu Miao,[2] and Yumna Ali,[2] *1. University of Texas Health Science Center School of Nursing, Houston, Texas, United States, 2. The University of Texas Health Science Center at Houston, Houston, Texas, United States*

Knee osteoarthritis (KOA) is one of the most prominent causes of chronic pain, functional impairment, and disability in older adults. The current standards of care for KOA are aimed toward reducing pain and are largely comprised of analgesic medications, but existing pharmacologic approaches often produce significant adverse effects. Moreover, recent evidence suggests that KOA pain is characterized by alterations in pain-related brain mechanisms. Cranial electrical stimulation (CES), which delivers a low-amplitude alternating electric current to the brain, can facilitate the reversal of maladaptive brain function. Portable CES devices can be used at home with real-time monitoring through a secure videoconferencing platform to facilitate high adherence. Thus, the purpose of this pilot clinical study was to examine the preliminary efficacy of remotely supervised CES on clinical pain severity in older adults with KOA. Thirty participants with KOA were randomly assigned to receive 10 daily sessions of remotely supervised CES with 0.1 mA at a frequency of 0.5 Hz for 60 minutes (n=15) or sham CES (n=15). We measured clinical pain severity using the numeric rating scale (NRS; range, 0 – 100). Participants (67% female) had a mean age of 59 years. Active CES significantly reduced scores on the NRS (Cohen's d = 1.43, P < 0.01). Participants tolerated CES well without any adverse events. Our findings demonstrate the promising clinical efficacy of remotely supervised CES for older adults with KOA. Future studies with larger-scale randomized controlled trials with follow-up assessments are needed to validate and extend our findings.

### Session 2125 (Symposium)

### PATIENT, CAREGIVER, AND PHYSICIAN BARRIERS TO HOME-BASED PALLIATIVE CARE: FINDINGS FROM A TERMINATED STUDY

Chair: Susan Enguidanos
Discussant: Stephanie Wladkowski

Despite two decades of palliative care services, there remains numerous barriers to patient and caregiver use of palliative care. For many years, policymakers believed lack of funding for palliative care was the primary obstacle to accessing palliative care services. In 2017, we undertook a randomized controlled trial to test the effectiveness of a home-based palliative care (HBPC) program within accountable care organizations and in partnership with an insurance company that covered the cost of HBPC. After 20 months, we had recruited just 28 patients. This symposium will: (1) describe outcomes from various approaches undertaken to engage primary care physicians and recruit patients and their caregivers into this trial; (2) present barriers to HBPC referral identified from a qualitative study of primary care physicians; (3) present findings from a qualitative study of patient- and caregiver-identified barriers to HBPC; (4) describe physician and patient barriers to research participation; and (5) discuss implications of these findings for researchers and healthcare providers. Information presented in this symposium will inform researchers and policy makers about challenges and facilitators to recruiting patients, caregivers, and physicians to participate in research studies as well as inform healthcare practitioners of potential obstacles to increasing patient access to HBPC.

### TRIALS AND TRIBULATIONS: PALLIATIVE CARE TRIAL RECRUITMENT APPROACHES AND CHALLENGES

Anna Rahman, Sindy Lomeli, and Susan Enguidanos, *University of Southern California, Los Angeles, California, United States*

In 2017, we received funding form the Patient-Centered Outcomes Research Institute to conduct a large, state-wide, randomized controlled trial to test the effectiveness of a home-based palliative care (HBPC) program within accountable care organizations. Participants were randomized to either HBPC or enhanced usual care, where physicians were provided added training and support in core palliative care practices. Originally, we planned to obtain patient referrals to the trial from primary care physicians, however we were unable to engage primary care physicians in patient identification processes. In this session we will describe the numerous trial modifications made to our trial recruitment methods and the success of each approach. Ultimately, after 20 months of trial recruitment, we had recruited just 28 patients and 10 of their caregivers. Findings from this terminated trial may inform other researchers in development of participant recruitment methods.

### IT'S YET ANOTHER THING: BARRIERS TO AND RECOMMENDATIONS FOR PHYSICIAN REFERRALS TO HOME-BASED PALLIATIVE CARE

Valeria Cardenas,[1] YuJun Zhu,[2] Jenna Giulioni,[3] Anna Rahman,[2] Susan Enguidanos,[2] and Alexis Coulourides Kogan,[4] *1. University of Southern California, University of Southern California, California, United States, 2. University of Southern California, Los Angeles, California, United States, 3. USC Division of Biokinesiology and Physical Therapy, Los Angeles, California, United States, 4. USC Keck School Of Medicine, Los Angeles, California, United States*

To understand primary care providers' (PCPs) experiences with referring patients to home-based palliative care (HBPC), we conducted individual, key-informant interviews with 31 PCPs. About half participants were male (54.8%), White (42.5%), US-born (58.1%), and were 57 years old (SD=9.17), on average. About one-third of participants (32.3%) indicated they refer 10+ patients annually to HBPC, while most (80.7%) reported "strong" comfort discussing palliative care with patients. Qualitative analysis revealed three prominent thematic categories, each related to barriers PCP experienced when referring patients to palliative care: (1) PCP-level (lack of knowledge and comfort); (2) perceived patient-level (culture, family disagreement, need, home-based aspect); and (3) HBPC program-level (need to close the loop with PCP, insurance coverage, program availability, and eligibility). PCP recommendations for overcoming identified barriers will be discussed. Findings hold important implications for timely patient-referrals to palliative care by PCPs and for sustaining palliative programs that rely on these referrals.

### RELUCTANCE TO ACCEPT PALLIATIVE CARE AND RECOMMENDATIONS FOR IMPROVEMENT

Anna Rahman,[1] YuJun Zhu,[1] Susan Enguidanos,[1] and Valeria Cardenas,[2] *1. University of Southern California, Los Angeles, California, United States, 2. University of Southern California, University of Southern California, California, United States*

Although insurance companies are increasingly paying for home-based palliative care (HBPC), enrollment remains low. To identify patient and caregiver perceived barriers to HBPC and their recommendations for overcoming these barriers, we conducted semi-structured individual interviews. Our interview protocol elicited participants' perspectives on HBPC services; positive and negative aspects of the palliative program explanation; and suggestions for improving HBPC messaging. Seventeen patients and eight caregivers who were eligible for a randomized controlled trial of HBPC were interviewed. Themes related to HBPC referral barriers included reluctance to have home visits, enrollment timing, lack of palliative care knowledge, and patients' self-perceived health condition. Themes related to recommendations for overcoming these obstacles included ensuring that HBPC referrals come from healthcare providers or insurance companies and presenting HBPC more clearly. Findings reinforce the need for palliative care education among seriously ill patients and the importance of delivering palliative care information and referrals from trusted sources.

### BARRIERS TO AND RECOMMENDATIONS FOR RESEARCH RECRUITMENT OF INDIVIDUALS AFFECTED BY SERIOUS ILLNESS

Anna Rahman,[1] Jenna Giulioni,[2] Alexis Coulourides Kogan,[3] Susan Enguidanos,[1] and Valeria Cardenas,[4] *1. University of Southern California, Los Angeles, California, United States, 2. USC Division of Biokinesiology and Physical Therapy, Los Angeles, California, United States, 3. USC Keck School Of Medicine, Los Angeles, California, United States, 4. University of Southern California, University of Southern California, California, United States*

Researchers are encountering increasing challenges in recruiting participants for healthcare research. We conducted semi-structured individual interviews to identify participant barriers to research and recommendations for overcoming these challenges. We recruited 17 patients and eight caregivers who were approached to participate in a randomized control trial. We also recruited 31 primary care physicians. Using grounded theory, three researchers independently coded the transcripts and then met to compare codes and reconcile discrepancies. Themes from patient and caregiver interviews included time constraints, privacy concerns, lack of research familiarity, disconnect with research institution, self-perceived health status, and concerns with study randomization and repetitive questions. Physician-identified barriers focused on time constraints and study randomization. Patient and caregiver recommendations for study recruitment included various recruitment techniques. Physician recommendations were related to incentives. Although patients and caregivers prefer that their physicians recruit them for health-related research studies, physicians identified time constraints as a barrier to research involvement.

### Session 2130 (Paper)

### PHYSICAL FUNCTIONING AND ACTIVITY MEASUREMENT

### EFFECTS OF AGE ON DUAL TASK WALKING PERFORMANCE AS MEASURED USING A SMARTPHONE APPLICATION IN MIDDLE-AGED ADULTS

Junhong Zhou,[1] Gabriele Cattaneo,[2] Wanting Yu,[3] Jose Tormos,[4] Lewis Lipsitz,[3] David Bartres-Faz,[4] Alvaro Pascual-Leone,[5] and Brad Manor,[6] *1. Harvard Medical School/Hebrew SeniorLife, Roslindale, Massachusetts, United States, 2. Universitat Autònoma de Barcelona, Barcelona, Catalonia, Spain, 3. Hebrew SeniorLife, Boston, Massachusetts, United States, 4. University of Barcelona, Barcelona, Catalonia, Spain, 5. Harvard Medical School, Boston, Massachusetts, United States, 6. Hinda and Arthur Marcus Institute for Aging Research, Harvard Medical School, Boston, Massachusetts, United States*

After the age of 65, one's ability to walk while performing an additional cognitive task (i.e., dual-tasking) is predictive of both future falls and cognitive decline. However, while it is well-known that older adults exhibit diminished dual-task performance, the time course of age-related dual-task decline has not been established. We thus conducted an analysis of data collected within the ongoing Barcelona Brain Health Initiative, a prospective population-based study characterizing the determinants of brain health maintenance in middle-aged adults. Cognitively-unimpaired participants (n=655) aged 40-65 years without neuro-psychiatric disease completed laboratory-based trials of walking normally (single-task) and walking while performing a verbalized serial subtraction task (dual-task). A smartphone-based gait assessment application was used to capture data and derive both the mean stride time (ST) and stride time variability (STV, defined as the coefficient of variation about the mean stride time) of each trial. The dual-task costs (DTC) to each gait metric were obtained by calculating the percent change from single- to dual-task conditions. We categorized participants into five groups according to age (e.g. Group 1: 40-45 years; Group 5: 60-65 years). Age group did not have an effect on single-task gait outcomes ($p>0.51$). However, the oldest age group, as compared to each of the other groups, exhibited greater DTC to both ST and STV ($p<0.03$). These results indicate that dual-task walking performance in particular may begin to diminish in late middle age even in the absence of detectable cognitive issues, DTC may offer a sensitive metric to age-related change in cognitive function.

### OBJECTIVELY MEASURED PHYSICAL ACTIVITY, SEDENTARY BEHAVIOR, AND INCIDENT FRACTURE IN OLDER WOMEN: THE OPACH STUDY

Steve Nguyen,[1] John Bellettiere,[1] Michael LaMonte,[2] Carolyn Crandall,[3] and Andrea LaCroix,[1], *1. University of California, San Diego Herbert Wertheim School of Public Health and Human Longevity Science, La Jolla, California, United States, 2. University at Buffalo, Buffalo, New York, United States, 3. University of California, Los Angeles, Los Angeles, California, United States*

Women aged 65 and older experience nearly three-fourths of the 2 million osteoporotic fractures annually in the US, yet whether accelerometer-measured volumes and intensities of physical activity and sedentary behavior (SB) are associated with reduced fracture risk is understudied. We investigated associations of accelerometer-measured light physical activity (LPA), moderate-to-vigorous physical activity (MVPA), sedentary time (ST), and mean sedentary bout duration (MBD) with incident clinical fractures (hip, vertebral, pelvis, lower leg, upper arm, forearm, and wrist) in the WHI OPACH cohort. Participants (N=6248; mean±SD age=78.6±6.7; 34% Black, 17% Hispanic) without prior hip fracture wore the ActiGraph GT3X+ for 7 days between May 2012-April 2014 and were followed through March 2020 for incident clinical fracture (N=711). Cox models estimated hazard ratios (HR) and 95% confidence intervals (CI), adjusting for age, race-ethnicity, education, alcohol, smoking, height, weight, falls history, RAND-36 physical function, diabetes, thiazide use, prescription osteoporotic therapy, and age at menopause. The HR(95% CI) across MVPA quartiles was 1.00(reference), 1.15(0.93-1.41), 0.90(0.72-1.13), and 0.79(0.61-1.02); p-trend=0.01. The HR(95% CI) for a one-interquartile range increment in MVPA (42 minutes/day) was 0.86(0.76-0.97). Associations were modified by prescription osteoporotic therapy [no: HR=0.77(0.66-0.89), yes: HR=1.03(0.85-1.25); p-interaction=0.01] and varied in magnitude by age[<80: HR=0.78(0.64-0.96), ≥80: HR=0.92(0.79-1.07); p-interaction=0.09], BMI [<30 kg/m2: HR=0.85(0.75-0.97), ≥30 kg/m2: HR=0.90(0.67-1.19); p-interaction=0.08], and race-ethnicity [Black: HR =0.63(0.44-0.89), Hispanic: HR =0.78(0.56-1.09), White: HR =0.92(0.80-1.06); p-interaction=0.16]. LPA, ST, or MBD were not associated with incident fractures. These data suggest that MVPA may reduce and not increase fracture risk and that LPA and SB do not increase fracture risk.

### PREMENOPAUSAL BILATERAL OOPHORECTOMY EFFECTS ON CLINICAL AND REAL-WORLD PHYSICAL FUNCTION MEASURES

Emma Fortune, Omid Jahanian, Melissa Morrow, Virginia Miller, and Michelle Mielke, *Mayo Clinic, Rochester, Minnesota, United States*

Women with premenopausal bilateral oophorectomy (PBO) are at increased risk for physical function (PF) declines. This study investigated the relationships of field-based physical activity measures with clinical PF and strength parameters in post-menopausal women with and without PBO. Women with (n=21; age=64±4 years; BMI=32±8 kg.m-2) and without (n=15; age=67±6 years; BMI=28±6 kg.m-2) PBO performed PF and strength tests (walking speed, distance walked, short physical performance battery (SPBB), leg and chest strength), and wore ankle accelerometers for 7 days (daily step count and loading index [the cumulative sum of each step's skeletal loading]). Age, BMI, step count and loading index were entered into stepwise multiple regression to identify significant predictors of PF and strength parameters. Step count was a predictor of SPBB score in both groups. In women without PBO, step count was a predictor of walking speed; loading index was a predictor of leg strength; step count and loading index were predictors of distance; and step count and age were predictors of chest strength. For PBO women, loading index and BMI were predictors of walking speed and distance; BMI was a predictor of leg strength; and there were no predictors of chest strength. These data suggest while field-based physical activity was strongly and positively associated with clinical PF and strength measures for women without PBO, BMI was a dominant negative factor for PF in women with PBO. Future work will include a larger sample size and additional confounders to further elucidate underlying factors of reduced PF and mobility after PBO.

### THE RELATIONSHIP BETWEEN KNEE PAIN AND HEART FAILURE IN OLDER BLACK AND LATINO WOMEN

Raya Kheirbek,[1] Bernadette Siaton,[2] Brock Beamer,[3] Jacob Blumenthal,[2] Les Katzel,[2] John Sorkin,[4] and Beth Hogans,[5] *1. University of Maryland School of Medicine, University of Maryland School of Medicine, Maryland, United States, 2. University of Maryland School of Medicine, Baltimore, Maryland, United States, 3. Baltimore Veterans Affairs Medical Center, BALTIMORE, Maryland, United States, 4. University of Maryland, Baltimore VA Medical Center, Maryland, United States, 5. Johns Hopkins University, Johns Hopkins University, Maryland, United States*

**Background:** Knee pain is the second-most prevalent and disabling common pain condition globally, having deleterious effects on daily function including mobility and exercise capacity; chronic knee pain is especially prevalent in older adults. There is substantial evidence to indicate that physically inactive individuals have higher rates of cardiovascular disease. Nonetheless, studies investigating cardiovascular risks with osteoarthritis have had mixed results.

**Objective:** This study explores the relationship between knee pain and heart failure especially examining the factors of age, gender, race in U.S. older adults.

**Methods:** Retrospective secondary analysis of Medicare claims data for 1.478 million adults over age 65. The standard analytical file for 2017 was segmented according to the presence of any of several ICD-10 codes for heart failure (HF). Medicare beneficiaries with and without HF diagnoses were evaluated for knee pain and other common pain-associated conditions; pain condition data was stratified by age, gender and race codes.

**Results:** Knee pain was markedly increased in women with HF in the 65-70- and 70–75-year-old age-cohorts and relatively less increased in older age-cohorts and males. Knee pain in women was especially elevated in those with Medicare race codes indicating Black and Hispanic status.

**Conclusion:** in a large cohort of Medicare beneficiaries, knee pain was noted to be markedly increased in younger cohorts of older women with HF, and more prevalent in Black and Hispanic women. Further studies should evaluate lifestyle, biomechanics, and inflammatory factors that may be contributing to this relationship.

### THE SEX DIFFERENCE IN GAIT SPEED: HOW DO SOCIODEMOGRAPHIC, LIFESTYLE, SOCIAL, AND HEALTH DETERMINANTS CONTRIBUTE?

Lena Sialino,[1] Laura Schaap,[1] Sandra van Oostrom,[2] Susan Picavet,[3] Johannes Twisk,[4] Monique Verschuren,[3]

Marjolein Visser,[1] and Hanneke Wijnhoven,[1] *1. Vrije Universiteit Amsterdam, Amsterdam, Noord-Holland, Netherlands, 2. National Institute for Public Health and the Environment, Amsterdam, Noord-Holland, Netherlands, 3. National Institute for Public Health and the Environment, Bilthoven, Utrecht, Netherlands, 4. Amsterdam University Medical Centre, Amsterdam, Noord-Holland, Netherlands*

This study explores whether a sex difference in sensitivity to (strength of the association) and/or in exposure to (prevalence) determinants of gait speed contributes to the observed lower gait speed among women compared to men. Data from the Longitudinal Aging Study Amsterdam (LASA) were used. In total 2407 men and women aged 55-81 years were included, with baseline measurements in 1992/2002 and follow-up measurements every 3-4 years for 15/25 years. Multivariable mixed model analysis was used to investigate sex differences in sensitivity (interaction term with sex) and in exposure to (change of the sex difference when adjusted) for socio-demographic, lifestyle, social and health determinants of gait speed. On average, women had a 0.054 m/s (95%CI:0.076-0.033, adjusted for height and age) lower mean gait speed compared to men. Higher BMI and lower physical activity were more strongly associated with lower gait speed in women compared to men (i.e. higher sensitivity). Lower educational level, living alone and having more chronic diseases, pain and depressive symptoms among women compared to men also contributed to observed lower gait speed in women (i.e. higher exposure). In conclusion, both a higher sensitivity and higher exposure to determinants of gait speed among women compared to men contributes to the observed lower gait speed among women aged 55 years and older. The identified (modifiable) contributing factors should be taking into account when developing prevention and/or treatment strategies aimed to enhance healthy physical aging. This might require a sex-specific approach in both research and clinical practice.

### Session 2135 (Symposium)

### PRESIDENTIAL SYMPOSIUM: SOCIAL ENGAGEMENT, ISOLATION, AND LOSS DURING THE COVID-19 PANDEMIC

Chair: Toni Antonucci Co-Chair: Debra Umberson
Discussant: Deborah Carr

The COVID-19 pandemic has transformed social life across the globe and had a particularly profound impact on older people. In this BSS Presidential Symposium, we address the annual meeting's theme—Disruption to Transformation: Aging in the "New Normal"—by inviting noted experts to address implications of the COVID-19 pandemic for social engagement and isolation. The speakers address this theme from sociological, psychological, demographic, and public health perspectives with attention to racial disparities and the impact of the pandemic on patterns of isolation and loneliness in the population, methods for conducting research with older populations, patterns of bereavement and loss of family ties, and caregiver mental health. The panel will shed light on these issues through individual presentations and dialogue.

### INTERGENERATIONAL RELATIONSHIPS DURING THE COVID-19 PANDEMIC: CHALLENGES AND OPPORTUNITIES OF DATA COLLECTION

Megan Gilligan,[1] and J. Jill Suitor,[2] *1. Iowa State University, Iowa State University, Iowa, United States, 2. Purdue University, Purdue University, Indiana, United States*

Family scholars experienced numerous unique challenges and opportunities when studying intergenerational relationships during the COVID-19 pandemic. In this presentation, we draw examples from the Within-Family Differences Study to highlight some of the ways in which respondents' patterns of study participation, reports of their relationships with their late-life parents and their own young adult children, and their psychological well-being, subjective physical health, and health behaviors reflected the fluctuating waves of the pandemic. Among the patterns we discuss are systematic variations in the intensity of respondents' answers to both open and closed-ended items, respondents' expressions of concern regarding choosing the "right" answers to questions, and their attempts to negotiate their responses with interviewers. Our observations led us to conclude that measures family gerontologists use to capture many constructs central to the field are subject to different "meanings" by respondents in the face of disaster.

### ISOLATION AND LONELINESS: IMPLICATIONS FOR HEALTH AND WELL-BEING OF OLDER POPULATIONS

julianne Holt-Lunstad, *Brigham Young University, Provo, Utah, United States*

Evidence suggests social isolation and loneliness are prevalent within the population and may potentially be exacerbated due to the pandemic. Social connections have powerful influences on health and longevity, and lacking social connection qualifies as a risk factor for premature mortality. Evidence from the recent National Academy of Science consensus report on social isolation and loneliness among older adults will be summarized, providing the scope of the health effects, potential mechanisms and risk factors, as well as current gaps in the evidence. Importantly, this evidence points to several implications for solutions across sectors, including medical and healthcare practice and policy.

### CARING DURING COVID-19: INEQUALITIES IN RESOURCE- AND INFORMATION-SHARING BY RACE, ETHNICITY, AND NATIVITY

Sung Park, *Harvard University, Cambridge, Massachusetts, United States*

Americans experienced significant challenges as a result of the pandemic, further magnifying the weak U.S. social safety net. With few institutional supports available, individuals turned to each other for assistance. Relying on multiple nationally representative surveys, this study examines resource-sharing by race, ethnicity, and nativity over a one-year period during COVID-19. Furthermore, this study examines knowledge-related behaviors, such as information-seeking and information-sharing, which were also important tools utilized during the pandemic. Differences in both resource- and information-sharing contributed to disparities in the perceptions of risk, the reported levels of need, and concomitant behaviors linked to well-being. This research emphasizes the importance of personal relationships during times of crisis, and the role of social connections in shaping health and economic inequalities between minority- and non-minority populations.

### Session 2140 (Symposium)

### RESEARCH APPLYING ADAPTIVE LEADERSHIP FRAMEWORK FOR CHRONIC ILLNESS ACROSS SETTINGS OF CARE

Chair: Ashley Leak Bryant Co-Chair: Anna Beeber
Discussant: Bei Wu

The carepartner is an essential member of the team to facilitate and assist in maximizing the independence of the older adult. The four papers in this symposium applies The Adaptive Leadership Framework for Chronic Illness (ALFCI) at the point of design, as well as an analytic framework for literature synthesis, intervention design, and analysis of existing data. In the first paper, a qualitative metasummary of a scoping review synthesizes qualitative findings about fatigue adaptation and challenges for stroke survivors, care partners, and healthcare professionals. The second paper describes the use of the ALFCI in an intervention study to manage symptom challenges in older adults with acute myeloid leukemia. The third paper shares staff's experiences of providing direct care for older residents with advanced dementia in long-term care facilities. The fourth paper describes use of the carepartner–assisted intervention to improve oral hygiene of older adults with cognitive impairment. The ALFCI is a useful framework for intervention design 1) this framework provides a comprehensive way to examine the context of symptoms/behaviors (not just the symptom/behavior in isolation), 2) the framework guides "collaborative work", 3) analytically it can help guide development of shared meaning of communication and "collaborative work" of dyads (family caregivers, long-term care staff and older adults), and 4) helps understand process of staff utilizing their strengths and doing adaptive work to facilitate interactions and communication, managing older residents' behavioral and psychological symptoms of dementia, and improving their care provision and work life.

### USING THE ADAPTIVE LEADERSHIP FRAMEWORK FOR CHRONIC ILLNESS TO DESIGN A PALLIATIVE AND COLLABORATIVE INTERVENTION

stephanie Becantur,[1] Ya-Ning Chan,[2] Rachel Hirschey,[1] Marianne Baernholdt,[3] Ruth Anderson,[4] and Ashley Leak Bryant,[5] *1. UNC School of Nursing, Chapel Hill, North Carolina, United States, 2. UNC Chapel Hill, Chapel Hill, North Carolina, United States, 3. University of North Carolina at Chapel Hill School of Nursing, Chapel Hill, North Carolina, United States, 4. University of North Carolina at Chapel Hill, UNC Chapel Hill, North Carolina, United States, 5. School of Nursing, Durham, North Carolina, United States*

Our study addresses symptoms and functional management early in acute myeloid leukemia treatment using a PAlliative and Collaborative Care inTervention (PACT) delivered by clinical staff. PACT is an interdisciplinary nurse-led intervention of nursing [RN], occupational therapy [OT], physical therapy [PT] for adults ≥ 60 years of age at time of initial treatment. Finding from our preliminary qualitative work led to use of the Adaptive Leadership Framework for Chronic Illness to guide optimal engagement of the patient in self-management of symptoms and functional activities to address QOL outcomes. We share lessons learned of how the ALFCI was applied to the PACT intervention to empower patients with self-management skills to reduce symptoms and optimize function. In summary, the team and patient identify and address emotional, motivational, attitudinal barriers so that the patient can do the work required for self- management to reduce symptoms and avoid functional decline.

### USING A THEORETICAL FRAMEWORK IN A QUALITATIVE METASUMMARY ABOUT FATIGUE ADAPTATION AFTER STROKE

Ruth Anderson,[1] Leslie Davis,[2] and Chiao-Hsin Teng,[3] *1. University of North Carolina at Chapel Hill, UNC Chapel Hill, North Carolina, United States, 2. The University of North Carolina at Chapel Hill, Chapel Hill, North Carolina, United States, 3. UNC Chapel Hill, Chapel Hill, North Carolina, United States*

We describe how we used a theoretical framework, Adaptive Leadership Framework for Chronic Illness (ALFCI), to complete a qualitative metasummary in a scoping review of 26 articles. We abstracted and grouped qualitative findings relevant to fatigue adaptation in stroke survivors using constructs of the ALFCI as 4 main themes: 1) adaptive challenges, 2) adaptive work, 3) adaptive leadership and collaborative work, and 4) technical challenges and technical work. We found that stroke survivors encountered different aspects of challenges (e.g., physical dysfunction vs. mental distress) and utilized various adaptive work (e.g., conserving energy vs. restructuring normality) as well as what stroke survivors needed from healthcare professionals (e.g., basic knowledge about fatigue). The ALFCI provides a useful lens to synthesize qualitative findings regarding fatigue adaptation and therefore researchers can target different problems that need to be tackled for stroke survivors, care partners, or healthcare professionals, respectively.

### PROVIDING CARE FOR OLDER RESIDENTS WITH DEMENTIA IN NURSING HOMES IN CHINA: THROUGH THE LENS OF ADAPTIVE LEADERSHIP

Xueli Bian,[1] Ruth Anderson,[2] Anna Beeber,[2] Junqiao Wang,[3] and Jing Wang,[4] *1. Fudan University, Shanghai, Shanghai, China (People's Republic), 2. University of North Carolina at Chapel Hill, UNC Chapel Hill, North Carolina, United States, 3. Fudan University, shanghai, Shanghai, China (People's Republic), 4. UNC Chapel HIll, Shanghai, Shanghai, China (People's Republic)*

This study aims to understand staff's experiences of providing direct care for older residents with advanced dementia in long-term care facilities through the lens of Adaptive Leadership Framework for Chronic Illness (ALFCI). Semi-structured interviews were conducted with health care aides (N=35) from 2 government-owned and 2 private long-term care facilities in urban China. Directed and conventional content analysis were used, drawing upon core constructs of ALFCI. We found that health care aides are confronted with multiple challenges such as high intensity of work, stress from managing older residents' behavioral and psychological symptoms of dementia (BPSD), a lack of access to on-the-job dementia-specific training, and a lack of support from nurses and managing team. Some of the health care aides demonstrated use of their strengths and doing adaptive work to improve work life and care for older residents by using communication cues, enhancing person-centeredness in their care, and facilitate peer interactions.

### QUALITATIVE ANALYSIS OF COACHING WITH CARE PARTNERS OF PEOPLE WITH COGNITIVE IMPAIRMENT

Chiao-Hsin Teng,[1] Ya-Ning Chan,[1] Youngmin Choi,[1] Cass Dictus,[1] Janelle Perez,[2] Jing Wang,[3] Victoria Bartoldus,[4] and Bianca Shieu,[5] *1. UNC Chapel Hill, Chapel Hill, North Carolina, United States, 2. University of North Carolina at Chapel Hill School of Nursing, Chapel Hill, North Carolina, United States, 3. Fudan University, Chapel Hill, North Carolina, United States, 4. University of North Carolina at Chapel Hill, Chapel Hill, North Carolina, United States, 5. University of Pittsburgh/ School of Medicine, Pittsburgh, Pennsylvania, United States*

We apply the adaptive leadership framework for chronic Illness in a care partner–assisted intervention to improve oral hygiene of older adults with cognitive impairment. Care partners receive four coaching sessions which we recorded and transcribed verbatim. We will describe how our team of seven investigators codes the data using a priori codes from the framework. The data from 17 care-partners contributes 68 individual sessions for coding. We have two subgroups of 7 individuals with mild dementia (MD) and 10 with mild cognitive impairment (MCI). We will discuss the plan for multiple comparisons such as (a) longitudinal across 3 months of intervention, b) within MD and within MCI and c) between MD and MCI. To illustrate, we will discuss our approaches to reaching coding consensus and rigor and will present results of the within group analyses. Finally, we will discuss next steps and the end products we aim to achieve.

### Session 2145 (Paper)

### SENSORY IMPAIRMENT AND LONELINESS

### HEARING LOSS AND PREVENTABLE HOSPITALIZATIONS

Nicholas Reed, and Emmanuel Garcia Morales, *Johns Hopkins Bloomberg School of Public Health, Baltimore, Maryland, United States*

Nearly half of all adults over the age of 60 years have hearing loss. Recent research suggests adults with hearing loss experience increased health care expenditures and hospitalization. However, little is known about whether these are preventable hospitalizations which may indicate poorer healthcare system engagement. In this cross-sectional analysis, we examined data from combined 2016-2018 Medicare Current Beneficiary Survey (MCBS) datasets. Participants are asked to describe their self-perceived trouble hearing. Preventable hospitalizations were defined and generated from administrative claims files based on the Agency for Healthcare Research and Quality identified conditions that should be manageable in ambulatory care settings. Multivariate regression models adjusted for demographic/socioeconomic characteristics and general health determinants were used to explore the association between trouble hearing and outcomes. The combined 2016-2018 MCBS administrative claims files included 18,814 participant-years, 49.8% reported no trouble hearing, 43.4% reported a little trouble and 6.8% a lot of trouble hearing, respectively. A higher proportion of those with a lot of trouble hearing (6.8%) experienced at least one preventable hospitalization compared to those with a little trouble hearing (3.4%) and no trouble hearing (2.5%). In a fully adjusted logistic regression model, hearing loss was associated with 1.35 times the odds of experiencing at least one preventable hospitalization per year (OR=1.35; 95% CI=1.03-1.77). Medicare beneficiaries with hearing loss experience higher rates of preventable hospitalizations. This may be due to avoidance of care due to communication barriers. Further work is needed to understand underlying reasons and whether addressing hearing loss modifies the observed association.

### IMPACT OF VISION AND HEARING IMPAIRMENT ON COGNITION AND LONELINESS: EVIDENCE FROM THE MEXICAN HEALTH AND AGING STUDY

Kimberly Hreha,[1] Rafael Samper-Ternent,[2] Brian Downer,[3] Joshua Ehrlich,[4] Paige Downer,[3] Timothy Reistetter,[5] and Heather Whitson,[6] *1. Duke University, Durham, North Carolina, United States, 2. The University of Texas Medical Branch, Galveston, Texas, United States, 3. University of Texas Medical Branch, Galveston, Texas, United States, 4. University of Michigan, University of Michigan, Michigan, United States, 5. University of Texas Health Science Center at San Antonio, Dickinson, Texas, United States, 6. Duke University School of Medicine, Durham, North Carolina, United States*

Poor vision and hearing have been associated with lower cognitive function and greater social isolation (i.e., loneliness) among older adults. However, this evidence is based largely on data from non-Hispanic populations. Therefore, we investigated whether self-reported vision and hearing was associated with cognitive function and loneliness in a nationally-representative study of Mexican adults aged 50 and older in Wave 3 of the Mexican Health and Aging Study. The final sample included 12,426 participants. The majority were female (58%), and the mean age was 67. Self-reported vision and hearing status were categorized as excellent-very good [ref], good, and fair-poor. Measures for global cognition, memory, and non-memory cognition were calculated using z-scores based on nine cognitive tests. Participants who reported frequently feeling a lack of companionship, left out, or isolated were categorized as feeling lonely. All analyses controlled for age, sex, and years of education. Participants with fair-poor vision had lower global ($\beta$= -0.06, p <.01), memory ($\beta$= -0.07, p <.01), and non-memory cognition ($\beta$= -0.06, p <.01) than participants with excellent-very good vision. In addition, participants with fair-poor hearing had higher non-memory cognition ($\beta$= 0.03, p <.05) but not global cognition ($\beta$=0.02) or memory ($\beta$=0.001). Fair-poor vision (OR=1.53, 95% CI=1.25-1.87) but not fair-poor hearing (OR=1.16, 95% CI=0.97-1.38) was associated with higher odds of being lonely. Poor vision may be a potentially modifiable risk factor for lower cognition and loneliness among Mexican adults. Future research should incorporate robust measures of sensory health and investigate the longitudinal association between vision, cognition, and loneliness.

### INTERVENTIONS FOR QOL AND OTHER OUTCOMES AMONG CAREGIVERS OF OLDER ADULTS WITH VISUAL IMPAIRMENTS

Afeez Hazzan,[1] Pamela Haibach-Beach,[2] Lauren Lieberman,[3] and Jamia Williams,[3] *1. State University of New York at Brockport, Hilton, New York, United States, 2. SUNY Brockport, State University of New York, Brockport, New York, United States, 3. State University of New York, Brockport, State University of New York, Brockport, New York, United States*

Unpaid family caregivers play a critical role in the care of older adults with visual impairments (VI). Caring for older adults with VI requires much time and energy, often resulting in psychological stress and reduced quality of life (QoL). However, there is a paucity of data on the impact of caregiving on QoL and related outcomes among these caregivers. The purpose of this study was to conduct a scoping review examining issues of QoL, health, stress, burden, and barriers among unpaid caregivers of older adults (i.e. aged 60 years or more) with VI. The study aimed to summarize interventions for addressing these issues. This study followed the Arksey and O'Malley (2005) five stage approach for scoping reviews. We performed a search of published peer-reviewed articles available in PubMed, CINAHL Complete, and PsycINFO to identify relevant studies. Two reviewers conducted the screening of titles, abstracts, and full-texts. Of the 452 records identified, 24 were eligible for full-text screening and five articles met the final inclusion criteria. The following four themes were identified: (1) prevalence of QoL-related barriers among unpaid caregivers of older adults with VI; (2) adverse events among unpaid caregivers of older adults with VI; (3) interventions for unpaid caregivers of older adults with VI; and (4) potential impacts of intervention on unpaid caregivers of older adults with VI. These findings reveal a lack of interventions for unpaid caregivers of older adults with VI, despite the prevalence of QoL-related barriers and adverse events. Research addressing these issues are urgently needed.

### LONELINESS, SLEEP QUALITY, AND COGNITIVE FUNCTION IN COMMUNITY-DWELLING OLDER ADULTS

Kexin Yu,[1] and Bernadette Fausto,[2] *1. USC, LA, California, United States, 2. Rutgers University–Newark, Newark, New Jersey, United States*

Loneliness is a risk factor for cognitive decline in older adults, however, the underlying mechanisms are less understood. Individuals who experience frequent loneliness tend to have poorer sleep quality. Empirical evidence supports the influence of sleep on cognitive health. This study examined the possible mediating effect of sleep characteristics on the relationship between loneliness and cognition. The study sample included 557 participants from wave 2 of the National Social Life, Health, and Aging Project who had actigraphy sleep measures (mean age = 73.17, 52.6% female). Loneliness was assessed with the 3-item UCLA Loneliness Scale. Cognitive function was measured with the Montreal Cognitive Assessment. Five sleep quality indicators were objectively recorded with wearable devices: assumed sleep time; actigraphy sleep time; time spent awake after sleep onset (WASO); sleep fragmentation; and sleep percentage (actigraphy sleep/(assumed sleep + WASO)). Path analysis model results show that WASO, fragmentation, and sleep percentage mediate the link between loneliness and cognitive function. Loneliness was positively related to WASO, and WASO was negatively associated with cognition. Loneliness correlated with increased sleep fragmentation which was associated with worse cognitive function. Individuals who had more frequent loneliness had a lower sleep percentage, and sleep percentage was positively associated with cognitive function. Nonetheless, the path from loneliness to these three sleep characteristics became insignificant after controlling for depressive symposiums. Depressive symptoms and fragmentation were found to double mediate the association between loneliness and cognitive function. Sleep and depression could be underlying pathways for the association between loneliness and cognition.

### THE LONGITUDINAL RELATIONSHIPS BETWEEN SOCIAL ISOLATION, FRAILTY, AND HEALTH OUTCOMES AMONG CANADIAN OLDER ADULTS

Fereshteh Mehrabi,[1] and François Béland,[2], *1. School of Public Health (ESPUM), Université de Montréal, Montreal, Quebec, Canada, 2. Université de Montréal, Montreal, Quebec, Canada*

Social isolation and frailty are global public health issues that may lead to poor health outcomes. We tested the two following hypotheses: 1) changes in social isolation and frailty are associated with adverse health outcomes over two years, 2) the associations between social isolation and health vary across different levels of frailty. We estimated a series of latent growth models to test our hypotheses using data from the FRéLE longitudinal study among 1643 Canadian community-dwelling older adults aged 65 years and over. Missing data were handled by pattern mixture models with the assumption of missing not at random. We measured social isolation through social participation, social networks, and social support from different social ties. We assessed frailty using Fried's criteria. Our results revealed that higher frailty at baseline was associated with a higher rate of comorbidity, depression, and cognitive decline over two years. Less social participation at baseline was associated with comorbidity, depression, and changes in cognitive decline. Less social support from friends, children, partner, and family at baseline was associated with comorbidity, cognitive decline, and changes in depression. Fewer contacts with grandchildren were related to cognitive decline over time. The associations of receiving less support from partner with depression and participating less in social activities with comorbidity, depression, and cognitive decline were higher among frail or prefrail than robust older adults over time. This longitudinal study suggests that intimate connectedness and social participation may ameliorate health status in frail older populations, highlighting the importance of age-friendly city policies.

### Session 2150 (Paper)

### SEXUALITY

### AGING LGBT ADULTS' ACCESS TO SOCIAL RESOURCES ACCORDING TO LGBT IDENTITY AND SOCIODEMOGRAPHICS

Krystal Kittle,[1] Kathrin Boerner,[2] and Kyungmin Kim,[3]
*1. University of Nevada, Las Vegas, Las Vegas, Nevada, United States, 2. University of Massachusetts Boston, Boston, Massachusetts, United States, 3. Seoul National University, Seoul, Seoul-t'ukpyolsi, Republic of Korea*

Research suggests that social resources positively influence the health and well-being of lesbian, gay, bisexual, and transgender (LGBT) aging adults, but their access to social resources may vary according to LGBT identity. Using data from Aging with Pride: National Health, Aging, and Sexuality/Gender Study (N=2,536), multivariate models tested how access to social resources varied by LGBT identity and whether the effect of LGBT identity showed additional variations by sociodemographic characteristics (i.e., age and education) among aging LGBT adults. Lesbian respondents had larger social networks than gay respondents, while gay respondents had smaller networks than transgender respondents. Lesbian respondents had more social support and community belonging than other identity groups. Bisexual male respondents and transgender respondents had less support than gay respondents and bisexual male respondents reported less community belonging than gay respondents. Education and age moderated the association between LGBT identity and social support. Findings highlight the importance of considering social support separately from social network size with the understanding that large social networks do not necessarily provide ample social support and this distinction was particularly relevant for transgender respondents who had larger social networks, but less social support than gay respondents. Results also suggest that feelings of LGBT community belonging vary among LGBT identity groups. Health and human service professionals should not only consider the sexual and gender identity of their aging LGBT clients, but also consider the clients' additional sociodemographic characteristics when assessing their access to social resources.

### COMPARISON OF TWO SURVEYS USING THE SEXUALITY ASSESSMENT TOOL (SEXAT) IN FLANDERS

Els Messelis,[1] Michael Bauer,[2] Elisabeth Vander Stichele,[3] and els Elaut,[4], *1. LACHESIS, Ghent, Oost-Vlaanderen, Belgium, 2. La Trobe University, Melbourn, Victoria, Australia, 3. AZ Sint Jan, Brugge, West-Vlaanderen, Belgium, 4. UGent, Gent, Oost-Vlaanderen, Belgium*

From 2015 it is mandatory in Flanders, Belgium, to develop a policy to deal with sexual abuse in elderly care. Residential Aged Care Facilities (RACF'S) try to focus on this mandatory, but should also pay attention to implement an overall Sex and Intimacy Policy. This study contains a Comparison of two surveys (Messelis & Bauer, 2020 and Vander Stichele, e.a. 2020) in Flanders, Belgium, both using the Sexual Assessment Tool (SeAT, Messelis & Bauer, 2017). Both studies aimed to assess how supportive residential aged care facilities are of residents' sexual expression. In the survey of Messelis & Bauer 750 aged care facilities were contacted in 2017-2018 and 69 (9,2%) completed the SexAT survey after three reminders. Vander Stichele e.a. contacted 100 aged care facilities managers in 2019. Twenty of them (20% response rate) completed the SexAT after three reminders. Findings of the Messelis & Bauer survey indicate that 70% of the facilities rated 'very good' to 'good' (score between 21-59/69), while Vander Stichele e.a. found a prevalence of 76% of this score. Both found no facilities were rated 'excellent' (score greater than 60/69). In the category 'improvement needed' (score less than 20/69), percentages were 30% and 23%; a difference of 7% (CI95% of difference in percentage includes zero, not significant). There is room for improvement in residential aged care facilities for the support of sexual expression of residents. The more recent study confirms results of the previous one, and no significant evolution was observed in two consecutive cross-sectional surveys.

### SEXUAL EXPRESSION, POLICY, AND PRACTICES IN SKILLED NURSING FACILITIES: AN UPDATED ASSESSMENT IN THE STATE OF KANSAS

Sarah Jen,[1] and Mijin Jeong,[2] *1. University of Kansas, Lawrence, Kansas, United States, 2. University of Kansas, LAWRENCE, Kansas, United States*

Prior studies have reported barriers to meeting the sexual needs of older adults within skilled-nursing facilities, such as a lack of privacy, lack of supportive practices and policies, and judgement or discomfort on the part of the staff (Doll, 2013; Hajjar & Kamel, 2003). In 2008, Doll and colleagues assessed the scope of sexual behaviors, staff perceptions of and responses to such behaviors, and whether facilities had a sexual policy in place in SNFs in the state of Kansas (Doll, 2013). In the present study, an online survey was distributed to the same population to provide an updated assessment of sexual behaviors, policies, and practices. Of 60 survey respondents, 62.7% reported knowledge of individual sexual acts (e.g., masturbation) within the past year and 34.5% reported interactional (between two or more residents) sexual acts. When encountering a sexual event, staff were most likely to report the incident to an administrator (76.7%) and treat residents with respect (70.0%), while 35.0% and 41.7% were expected to respond with embarrassment and discomfort, respectively. Only 40% of administrators reported having a policy related to sexual expression. Findings indicate that staff are likely to respond differently to LGBTQ residents due to discomfort and those living with cognitive impairment due to concerns related to consent. The proportion of facilities in Kansas with policies related to sexual expression has increased from 26% to 40% in the past 12 years, but there remains a need for greater specificity of sexuality-related policies and trainings.

### THE ROLE OF MINORITY STRESS AND SOCIAL RESOURCES IN THE HEALTHCARE UTILIZATION OF AGING LGBT ADULTS

Krystal Kittle,[1] Kathrin Boerner,[2] and Kyungmin Kim,[3],
*1. University of Nevada, Las Vegas, Las Vegas, Nevada, United States, 2. University of Massachusetts Boston, Boston, Massachusetts, United States, 3. Seoul National University, Seoul, Seoul-t'ukpyolsi, Republic of Korea*

Research suggests that minority stress can influence the healthcare utilization of aging LGBT adults, and that social resources can buffer the effect of stress on healthcare utilization. Using data from Aging with Pride: National Health, Aging, and Sexuality/Gender Study (N = 2,560), multiple logistic regression assessed the associations between minority stress (i.e., internalized stigma and LGBT identity disclosure) and healthcare utilization (i.e., health screenings, emergency room use, routine checkups, and regular provider). We also examined the moderating effect of social resources, including social network size, social support, and LGBT community belonging, in these associations. Internalized stigma was negatively associated with having a routine checkup in the previous year (OR = 0.82, p = .038). Disclosure was positively associated with having a health screening within the past 3 years (OR = 1.52, p = .000) and having a regular provider (OR = 1.33, p = .021). Further, we found that social support moderated the association between disclosure and health screenings (OR = 1.52, $p < .001$); thus, having higher levels of social support and disclosure in tandem increased the likelihood of getting a health screening in the last three years. Health and human service professionals should provide information about internalized stigma and LGBT identity disclosure to educate their clients about the ways in which these minority stressors can impact their healthcare experiences. Providers should assess the social support of their aging LGBT clients and inform them about the added benefit that social support can have in their healthcare experiences.

### Session 2155 (Symposium)

### TRANSFORMING DEMENTIA CARE: IMPLEMENTATION CHALLENGES MOVING EVIDENCE-BASED PROGRAMS TO HEALTH CARE

Chair: Laura Gitlin Co-Chair: Kenneth Hepburn
Discussant: Sara Czaja

Although evidence for dementia care programs continues to grow, families and health providers do not have ready access to programs, nor have they been widely disseminated and routinized in healthcare. Understanding implementation considerations when embedding evidence-based programs in healthcare systems can inform ways to effectively transform dementia care. This symposium will examine similarities and differences in implementation challenges encountered and strategies used when implementing four evidence-based programs being tested in different healthcare environments using distinct study designs. Dr. Gaugler et al., will discuss implementation challenges encountered with a staff-delivered intervention (ADS Plus) to support caregivers in adult day services that is being tested using a mixed methods hybrid trial design in >50 sites nationally. Dr. Hodgson et al., will discuss adaptions and their measurement to COPE, a home-based dyadic support program being embedded in 10 PACEs of a large healthcare system using a noninferiority trial testing staff training strategies. Dr. Forester et al., will examine implementation of the Care Ecosystem for dementia patients in a high-risk, integrated care management program using a pilot embedded pragmatic trial. Dr. Hepburn et al., will explore tactical challenges of implementing Tele-Savvy, an online caregiver psychoeducation program, within the context of a pilot pragmatic clinical trial. Drawing upon implementation science, themes discussed include balancing adaptations and fidelity, measurement of implementation outcomes and organizational readiness, and staff training implications. Also highlighted are research design considerations. Dr. Czaja, an expert in the design and implementation of dementia care interventions from in-person to technology-based will be the discussant.

### ADDRESSING TACTICAL CHALLENGES IN EMBEDDING TELE-SAVVY IN A PILOT PRAGMATIC TRIAL

Carolyn Clevenger,[1] Karina Berg,[2] Richard Fortinsky,[3] and Kenneth Hepburn,[4] *1. Emory University, Emory University, Georgia, United States, 2. University of connecticut, farmington, Connecticut, United States, 3. University of Connecticut School of Medicine, Farmington, Connecticut, United States, 4. Emory University, Atlanta, Georgia, United States*

This presentation describes how we plan to address the challenges of testing an evidence-based caregiver program in a real-world setting without the infrastructure and personal contact of a typical RCT. Instead of screening participants for eligibility, clinic staff will pre-identify participants whom clinicians then confirm. Each clinic will include Tele-Savvy as standard of care; we will thus be able to obtain IRB approval for a waiver of consent. By securing agreement from each clinic to incorporate a small set of standard instruments (e.g., Pearlin Caregiver Competence scale) into their standard operating procedure of routinely collecting caregiver data gathering, we will be able to eliminate the need for a central research staff to conduct baseline and follow-up interviews and will, instead, collect key outcome data through the electronic health record. Instead of in person training of Tele-Savvy facilitators, we will rely on an online training program and facilitator manuals.

### CHALLENGES IN IMPLEMENTING EVIDENCE-BASED DEMENTIA CARE PROGRAMS IN COMMUNITY-BASED SETTINGS: ADS PLUS

Katherine Marx,[1] Lauren Parker,[2] Keith Anderson,[3] Holly Dabelko-Schoeny,[4] Elma Johnson,[5] Elizabeth Albers,[6] Laura Gitlin,[7] and Joseph Gaugler,[6] *1. Johns Hopkins School of Nursing, Baltimore, Maryland, United States, 2. Johns Hopkins Bloomberg School of Public Health, Baltimore, Maryland, United States, 3. University of Texas at Arlington, Arlington, Texas, United States, 4. The Ohio State University, The Ohio State University, Ohio, United States, 5. University of Minnesota School of Public Health, University of Minnesota/ Minneapolis, Minnesota, United States, 6. University of Minnesota, Minneapolis, Minnesota, United States, 7. Drexel University, College of Nursing and Health Professions, Drexel University, Pennsylvania, United States*

The Adult Day Service Plus Program (ADS Plus) augments the usual care provided by ADS programs by integrating education, referrals, and problem-solving strategies for family caregivers of persons with dementia. Utilizing a mixed-methods, hybrid effectiveness design, we were in the process of conducting a national evaluation of ADS Plus across xx geographically and culturally diverse programs across the

U.S. when the COVID-19 pandemic resulted in the shutdown of almost all of the programs participating in ADS Plus. Qualitative and quantitative data collected during the evaluation suggested that a more robust incorporation of implementation domains and measures (e.g., organizational readiness to change) may have helped avoid some of the challenges related to staff training, fidelity, and other critical intervention delivery aspects. Incorporating implementation science frameworks and measures as early as possible in intervention design may have helped to overcome some of the challenges experienced in ADS Plus.

### TRACKING ADAPTATION AND FIDELITY WHEN EMBEDDING COPE, EVIDENCE-BASED DEMENTIA CARE, IN PACE SITES

Laura Gitlin,[1] and Nancy Hodgson,[2] *1. Drexel University, College of Nursing and Health Professions, Drexel University, Pennsylvania, United States, 2. University of Pennsylvania, School of Nursing, philadelphia, Pennsylvania, United States*

One essential question in moving dementia care interventions to practice is, "What is the optimal balance between fidelity to, and adaptation of, a proven program in "real world" settings?" We present a protocol for measuring the adaptation/fidelity and implementation of an evidence-based dementia care program (Care of Persons in their Environment, COPE) in PACE settings. During pre-implementation, science-based elements of COPE were documented including the theory of change, logic model and core components. Possible adaptations to COPE in its delivery were identified and included program structure (sequence of sessions), content (assessments), and delivery methods (online). During implementation, documentation of implementation strategies is captured using an evidence-informed checklist derived from the Expert Recommendations for Implementing Change (ERIC) workgroup. Ongoing documentation of fidelity/adaptation aspects of program implementation is conducted using the FRAME framework. Understanding methods and measures deployed in adaptation and implementation of evidence-based dementia programs can help guide future translation efforts.

### ADAPTATION OF THE CARE ECOSYSTEM INTERVENTION FOR INDIVIDUALS WITH DEMENTIA IN A HIGH-RISK, CARE MANAGEMENT PROGRAM

Karen Donelan,[1] Christine Vogeli,[2] Christine Ritchie,[3] and Brent Forester,[4] *1. Heller School for Social Policy Management, Brandeis University, Waltham, Massachusetts, United States, 2. Mass General Brigham, Somerville, Massachusetts, United States, 3. Massachusetts General Hospital, Boston, Massachusetts, United States, 4. McLean Hospital, McLean Hospital, Massachusetts, United States*

The Care Ecosystem (CareEco) model is a telephone-based dementia care program providing standardized, personalized and scalable support and education for caregivers and persons living with dementia (PLWD), medication guidance, and promotion of proactive decision-making. It has demonstrated improvement in quality of life for PLWD and reduced unnecessary healthcare expenditures. We initiated a pragmatic, embedded randomized pilot trial of an adapted CareEco model for nurses who provide high-risk care management and are embedded in primary care practices within a large healthcare system. Outcomes include feasibility of collecting emergency department visits, usability and acceptability of the intervention by nurse care managers, caregiver strain, behavioral symptoms of dementia and healthcare expenditures. Challenges of implementation include engaging key care management leaders, adaptation of the CareEco training modules for nurses, identification of primary caregivers, training and reinforcing knowledge and skills of the nurses, embedding clinical assessments into care manager workflows and integration with the EMR.

### Session 2160 (Paper)

### TRENDS AND ISSUES OF OLDER ADULTS LIVING WITH HIV/AIDS

### MEDIATING ROLE OF LONELINESS ON STIGMA AND DEPRESSIVE SYMPTOMS AMONG OLDER PERSONS LIVING WITH HIV

Moka Yoo-Jeong, *Northeastern University, Brookline, Massachusetts, United States*

Studies have shown associations among stigma, loneliness, and depressive symptoms in older persons living with HIV (OPLWH) but research assessing the mediating pathway among these variables is lacking. As such, the aims of this study were to assess the association between stigma and depressive symptoms and to test the mediating effects of loneliness. A sample of 146 OPLWH (50 years of age and older) recruited from an outpatient HIV clinic in Atlanta, GA, completed a cross-sectional survey. Mediation analysis, guided by Baron and Kenny's (1986) criteria, was conducted using Stata v14.2 to assess the direct and indirect effects of loneliness on the association between stigma and depressive symptoms while controlling for covariates (self-rated health [0=poor to fair, 1=good to excellent]; past unstable housing [0=No, 1=Yes]; and HIV disclosure status [0=to none; 1=to someone]). Loneliness mediated the association between stigma and depressive symptoms ($\beta$=0.79, SE=0.23, $p < .001$). The model reflected a very good fit ($\chi 2$=0.09, p=.765; CFI=1.00, TLI=1.09, RMSEA $< 0.001$) and explained 27% of the variance in loneliness and 33% of the variance in depressive symptoms. Stigma predicted higher loneliness, which in turn predicted more depressive symptoms. Findings suggest that addressing depressive symptoms in OPLWH may require multifaceted interventions targeting psychosocial and interpersonal factors including stigma and loneliness.

### OVERWEIGHT, OBESITY, AND NEUROPSYCHOLOGICAL PERFORMANCE: RESULTS FROM THE WOMEN'S INTERAGENCY HIV STUDY

Elizabeth Vasquez,[1] Mark Kuniholm,[2] Leah Rubin,[3] Anjali Sharma,[4] Kathleen Weber,[5] Margaret A. Fischl,[6] Michael Plankey,[7] and Deborah Gustafson,[8] *1. University of Albany, University of Albany, New York, United States, 2. University at Albany (SUNY), Rensselaer, New York, United States, 3. Johns Hopkins University School of*

Medicine, Baltimore, Maryland, United States, 4. Albert Einstein College of Medicine, Bronx, New York, United States, 5. Cook County Health, Chicago, Illinois, United States, 6. Sylvester comprehensive cancer center, Miami, Florida, United States, 7. Georgetown University, Washington, District of Columbia, United States, 8. SUNY Downstate Health Sciences University, Brooklyn, New York, United States

Conflicting associations of body mass index (BMI) and waist circumference (WC) with neuropsychological performance (NP) are observed in the general population and among people living with HIV. We examined BMI and WC in middle-aged women living with HIV (WLWH) and without HIV (HIV-) in relation to 10-year trajectories of NP in the Women's Interagency HIV Study (WIHS). NP assessments occurred biennially from 2009-2019. Demographically-adjusted T-scores were calculated for six NP domains: learning, memory, executive function, processing speed, attention and working memory, and motor function. Multivariable linear models stratified by HIV serostatus examined whether baseline (2009) BMI and WC were associated with NP domains - 1) cross-sectionally and 2) longitudinally over 10 years. The sample included 432 WLWH and 367 HIV- women, >40 years old. Most women (73%) were overweight (BMI=25-29.9kg/m2) or obese (BMI=>30kg/m2). Among WLWH, 28% were overweight, 45% obese; among HIV- women, 26% were overweight; 56% obese. Cross-sectionally at baseline, WLWH who were overweight versus normal weight (BMI=18.5-24.9kg/m2), performed worse on executive function, processing speed, and motor function (all p<0.05). HIV- women who were overweight versus normal weight performed worse on memory, learning, executive function, processing speed and motor function (all p<0.05). Baseline BMI and WC were not associated with worsening NP domains in this younger, primarily overweight and obese sample of WLWH or HIV- women (all p>0.05).Future follow-up of these women will enhance understanding of the age when total and/or central obesity may influence NP trajectories and health of the aging brain.

### TRENDS IN ANTIRETROVIRAL REGIMEN COMPLEXITY AMONG MEDICARE BENEFICIARIES WITH HIV, 2014-2018

Sean Fleming,[1] and Linda Wastila,[2] *1. University of Maryland Baltimore, University of Maryland Baltimore, Maryland, United States, 2. University of Maryland School of Pharmacy, Baltimore, Maryland, United States*

Little is known about antiretroviral therapy (ART) patterns among Medicare beneficiaries with Human Immunodeficiency Virus (HIV). ART has significant implications for spending in Medicare Part D as use of single-tablet regimens (STR) grows, generic availability remains low, and price increases for branded therapies consistently exceed inflation. The objective of this study is to detail patterns of STR utilization among Medicare beneficiaries with HIV. We conducted a retrospective trend analysis using a 5% sample of Medicare Chronic Conditions Data Warehouse, 2014-2018. We included each person-month that fee-for-service beneficiaries with HIV had Parts A, B, and D coverage. Trends in annual prevalence of STR overall, by ART class, and by age, sex, and race subgroups were estimated. The study included 9,509 beneficiaries who contributed 345,708 person-months to the analysis. The prevalence of STR increased from 21.8% (95%CI, 21.5-22.1) in 2014 to 44.6% (95%CI, 44.3-45.0) in 2018 (p <0.0001), an increase of 104.6%. Integrase strand transfer inhibitors (INSTI) saw the largest increase in utilization between 2014 (4.4% [95%CI 4.2-4.5]) and 2018 (35.1% [95%CI 34.8-35.4]) (p<0.0001), a 701.8% increase. All sociodemographic subgroups experienced similar growth in STR use between 2014 and 2018. STR and INSTI utilization increased significantly over the study period, suggesting increased ART spending under Part D. Although increasing availability of generic multi-tablet ART regimens (MTR) may offer cost-savings, further research is needed comparing generic MTR to branded STR with regards to patient preferences, adherence, healthcare resource utilization, and total costs in the growing population of Medicare beneficiaries with HIV.

### VISION DIFFICULTY AND ENGAGEMENT IN CARE AMONG AGING MEN LIVING WITH HIV

Alison Abraham,[1] Weiqun Tong,[2] Valentina Stosor,[3] Mackey R. Friedman,[4] Roger Detels,[5] and Michael Plankey,[6] *1. University of Colorado--Denver, Aurora, Colorado, United States, 2. Johns Hopkins University, Baltimore, Maryland, United States, 3. Northwestern University Feinberg School of Medicine, Chicago, Illinois, United States, 4. University of Pittsburgh, Pittsburgh, Pennsylvania, United States, 5. UCLA School of Public Health, Los Angelas, California, United States, 6. Georgetown University, Washington, District of Columbia, United States*

For aging adults living with HIV (AALH) who have complex medical care needs, vision impairment may be an added burden that may lead individuals to disengage from their own medical care. We examined the relationships of self-reported vision difficulty with indicators of care engagement: 1) adherence to HIV antiretroviral therapy (ART; defined as taking ≥95% of medications); 2) self-reported avoidance of medical care; 3) self-reported tendency to ask a doctor questions about care (> 2 questions at a medical visit). A modified version of the National Eye Institute vision function questionnaire was administered at three semi-annual visits (from October 2017 to April 2018) to assess difficulty performing vision-dependent tasks (no, a little, moderate to extreme difficulty). We included 1063 AALH participants (median age 60 years, 24% Black). Data were analyzed using repeated measures logistic regression with generalized estimating equations adjusted for fixed race, and at visit values for age, education level, depressive symptoms, alcohol use, and smoking status. Compared to no vision difficulty, those reporting moderate to extreme vision difficulty on at least one task (18%) had 1.95 times higher odds (95% CI: 1.36, 2.79) of having less than optimal ART adherence and 1.92 times higher odds [95% CI: 1.06, 3.47]) of avoiding necessary medical care, but 1.6 times higher odds [95%CI: 0.93, 2.72] of asking more questions. These findings suggest that vision impairment plays a role in medical care engagement among older adults living with HIV, and may contribute to poorer management of HIV and chronic comorbidities.

### Session 2165 (Symposium)

### UPDATE ON POLLING AND POLICY EFFORTS ON LONELINESS, TELEHEALTH, CAREGIVING, AND ADVANCE CARE PLANNING

Chair: Erica Solway Discussant: Brian Lindberg

Older adults and their caregivers experienced dramatic changes in many aspects of their lives during the COVID-19 pandemic which resulted in important shifts in organizational and federal priorities and policies. To explore older adults' changing experiences and perspectives amidst the pandemic, the University of Michigan National Poll on Healthy Aging (NPHA), a recurring, nationally representative household survey, polled over 2,000 adults age 50-80 at multiple timepoints through January 2021 about their feelings of loneliness and use of telehealth. In June 2020, the NPHA also surveyed adults age 50-80 about advance care planning before and during the COVID-19 pandemic and asked family caregivers about their care challenges in the three months since the pandemic. This session will start with a presentation of results from these polls, first exploring change over time in loneliness and telehealth use and then focusing on experiences related to advance care planning and caregiving challenges. Next, presenters from diverse national coalitions and organizations, including the Coalition to End Social Isolation and Loneliness, the National Academy for State Health Policy, the National Alliance for Caregiving, and the Coalition to Transform Advanced Care will describe their organizations' efforts, including their work with research and advocacy partners, state and federal agencies, and the Biden administration to facilitate dialogue and advance activities and policies related to these timely topics.

### FINDINGS ON LONELINESS, TELEHEALTH, AND ADVANCE CARE PLANNING FROM THE NATIONAL POLL ON HEALTHY AGING

Matthias Kirch,[1] Dianne Singer,[1] Jeffrey Kullgren,[1] Cheryl Lampkin,[2] Teresa Keenan,[2] Alison Bryant,[2] Preeti Malani,[3] and Erica Solway,[1], *1. University of Michigan, Ann Arbor, Michigan, United States, 2. AARP, Washington, District of Columbia, United States, 3. University of MIchigan, Ann Arbor, Michigan, United States*

The University of Michigan National Poll on Healthy Aging (NPHA) taps into the perspectives of older adults to inform health care policy and practice using a nationally representative sample of more than 2,000 adults age 50-80. Questions about lack of companionship and feelings of loneliness were tracked over three time points; 34% expressed feelings of loneliness in October 2018, 41% in June 2020, and 37% in January 2021. The NPHA also found that use of telehealth increased from 4% in May 2019 to 30% in June 2020 to 43% in January 2021. Finally, the NPHA found that 37% have completed both medical durable power of attorney and advance directive with 7% completing at least one of these documents in the first three months of the COVID-19 pandemic. These poll results can be used to inform actions by coalitions and organizations to advance state and federal policy.

### CAREGIVING CHALLENGES DURING THE COVID-19 PANDEMIC: FINDINGS FROM THE NATIONAL POLL ON HEALTHY AGING

Alicia Carmichael,[1] Natalie Leonard,[1] Jeannette Jackson,[1] Erica Solway,[1] Matthias Kirch,[1] Dianne Singer,[1] RIchard Gonzalez,[1] and Amanda Leggett,[2] *1. University of Michigan, Ann Arbor, Michigan, United States, 2. University of Michigan, Ypsilanti, Michigan, United States*

The COVID-19 pandemic posed new challenges for caregivers. This study examines the prevalence of pandemic care challenges (e.g., decreasing care to reduce virus spread, difficulty accessing medical care) and their associations with caregiver mental health and interpersonal well-being in a nationally representative sample of 311 caregivers who participated in the June 2020 National Poll on Healthy Aging. We consider seven care challenges and supports as key predictors of caregiver mental health (care-related stress, self-reported mental health, three depressive symptoms) and interpersonal well-being (lack of companionship, isolation) in bivariate tests and ordinary least squares regressions. Each care challenge/support was endorsed by between 13-23% of caregivers. Difficulty getting needed medical care was the most predictive challenge associated with increased caregiver stress, depressive symptoms, and worsened interpersonal well-being. All care challenges predicted an increase in caregiver stress. Effective caregiver tools and supports must consider changing policies and care needs, especially during a pandemic.

### EFFORTS BY THE COALITION TO END SOCIAL ISOLATION AND LONELINESS

Andrew MacPherson, *Healthsperien, Washington, District of Columbia, United States*

Established in the Fall 2018 and based in Washington, D.C., the Coalition to End Social Isolation & Loneliness brings together dozens of national organizations including consumer groups, community-based organizations, health plans, mental and behavioral health organizations, health care innovators, and many others to lead a multi-stakeholder dialogue to address the crisis of social isolation and loneliness in America. The Coalition focuses on three major areas to achieve this goal: Disseminating research findings, developing and advocating for federal and state legislative and regulatory policy interventions, and leading public awareness events in Washington, D.C. and across the nation. The COVID-19 pandemic has greatly accelerated efforts to engage Congress and the Executive Branch on a range of federal policy priorities, including leveraging and advancing social services and supports, supporting health care delivery to support those who are socially isolated and/or lonely, and advancing federally-funded research initiatives.

### EFFORTS BY THE NATIONAL ACADEMY FOR STATE HEALTH POLICY

Wendy Fox-Grage, *NASHP, Washington, DC, District of Columbia, United States*

The National Academy for State Health Policy hosts both the RAISE Act Family Caregiving Resource and Dissemination Center and the Hub for State Strategies to Build and Support Palliative Care, with generous funding from The John A. Hartford Foundation. The value of supporting individuals with serious illness and complex conditions as well as their family caregivers through telehealth, care management, advance care planning, and other added family caregiver supports has been especially evident during the COVID-19 pandemic. Policymakers are now grappling with how to restructure hard-hit health care and long-term services and supports systems to better support these individuals and their family caregivers. The State Hub provides concrete resources for states working to implement and expand high-quality palliative care, and the RAISE Center is assisting the Family Caregiving Advisory Council with creating the country's first national Family Caregiver Strategy.

### EFFORTS BY THE NATIONAL ALLIANCE FOR CAREGIVING

C. Grace Whiting, *National Alliance for Caregiving, Washington, District of Columbia, United States*

The National Alliance for Caregiving (NAC) conducts research, does policy analysis, develops national best-practice programs, and works to increase public awareness of family caregiving issues. In addition to national research and advocacy, NAC provides technical assistance to a national network of caregiving coalitions representing nearly 30 states. NAC recognizes that family caregivers provide important societal and financial contributions toward maintaining the well-being of those in their care. The need to support the nation's nearly 53 million family caregivers and sustain them as the backbone of our chronic and long-term care system is a central issue in national and state efforts to reform healthcare, especially in light of the challenges presented by the COVID-19 pandemic. This presentation will provide information on the current status of these national and state efforts.

### Session 2166 (Symposium)

### CAREGIVER STRESS: BIOMARKERS LINKED TO DISEASE RISK AND THE PSYCHOBIOLOGY OF STRESS REDUCTION

Chair: Janelle Beadle Co-Chair: Felipe Jain

Caregivers to older adults with chronic diseases frequently experience chronic stress which can negatively affect caregivers' physical and mental health, and increase disease risk. This interdisciplinary symposium will highlight critical factors influencing caregiver stress, and the role of biomarkers in detecting caregiver disease risk. First, we will discuss the effects of stress and emotional experiences on risk for cardiovascular disease in caregivers of persons with dementia (PWD). In the first talk, Dr. Mausbach will examine relationships among perceived stress, blood glucose and risk of diabetes and cardiovascular disease in caregivers of PWD. Next, Dr. Losada-Baltar will discuss the degree to which caregivers' ambivalent feelings towards providing care are associated with inflammatory markers of cardiovascular risk. Following this, two talks will investigate critical links between stress and caregiver emotional well-being. Dr. Liu will report relationships among the stress-related hormone cortisol, sleep, and anxiety in the context of adult day services. Dr. Beadle will examine the degree to which caregivers' affiliative, empathetic interactions with others relate to their experience of stress through cortisol assessments and neuroimaging. The final talk by Dr. Jain will investigate the effects of a Mentalizing Imagery Therapy intervention for family PWD caregivers on stress, evidence for mindfulness as a causal mediator of stress reduction, and the relationship to brain networks associated with emotion regulation. Taken together, this symposium will identify relevant psychosocial and biological factors that contribute to caregiver stress, as well as discuss the psychobiology of amelioration of caregiver stress.

### CAREGIVER STRESS AND EMPATHY: NEURAL AND HORMONAL CORRELATES

Shalmali Mirajkar,[1] David Warren,[2] and Janelle Beadle,[3]
*1. Saint Louis University, St. Louis, Missouri, United States, 2. Department of Neurological Sciences, University of Nebraska Medical Center, Omaha, Nebraska, United States, 3. University of Nebraska at Omaha, Omaha, Nebraska, United States*

Providing care to older adults with chronic conditions can be emotionally meaningful and stressful. The tend-and-befriend theory highlights the role of affiliation/empathy in stress reduction, but it has not been established whether this theory extends to caregivers for older adults. Addressing this gap, we assessed caregiver empathy and stress through behavioral, hormone, and neuroimaging measures. In Experiment 1, we compared 19 caregivers (Mage=67.1) to 24 non-caregivers (Mage=72.6), and found that caregivers with a greater reduction in cortisol to an empathic context showed greater prosocial behavior ($r2=0.3$). In experiment 2 (N=32), we examined differences between caregivers and non-caregivers in whole brain resting-state functional connectivity (RSFC) with seed regions of interest (posterior cingulate cortex (PCC); amygdala), and covariation of RSFC with empathy ($\alpha=0.05$). For emotional empathy, caregivers had stronger connectivity between the PCC seed, medial prefrontal cortex, and right supramarginal gyrus, and between the amygdala seed and the right middle frontal gyrus.

### CAUSAL MEDIATION OF STRESS REDUCTION IN FAMILY DEMENTIA CAREGIVERS: A FOCUS ON MINDFULNESS

Liliana Ramirez Gomez, and Felipe Jain, *Massachusetts General Hospital, Harvard Medical School, Boston, Massachusetts, United States*

Although mindfulness therapies have demonstrated benefits for reducing stress and improving psychological symptoms in family dementia caregivers, the mechanisms underlying these salutary effects are unknown. We report a causal mediation pathway to improvement of stress symptoms in family dementia caregivers with Mentalizing Imagery Therapy (MIT), which employs mindfulness and guided imagery tools to reduce stress and improve understanding of self and others. In a randomized controlled trial of short-term 4-week MIT (N=24) versus a psychosocial support group (N=22), MIT demonstrated superior benefit for reducing perceived stress ($p=.006$). Increased trait mindfulness was a causal mediator of this effect ($p=.02$). Neuroimaging pre and post intervention found that increased mindfulness was associated with strengthened connectivity of the dorsolateral prefrontal cortex with an emotion regulation network (p<.001). The results are discussed in light of theories of cognitive control and may inform the design of future studies aimed at reducing family caregiver stress.

### DEMENTIA FAMILY CAREGIVERS' AMBIVALENT FEELINGS AND CARDIOVASCULAR RISK: LONGITUDINAL CORRELATES

Rosa Romero-Moreno,[1] Carlos Vara-García,[2] Samara Barrera-Caballero,[3] Javier olazarán,[4] Brent Mausbach,[5] Roland von Känel,[6] Ricardo Olmos,[7] and Andrés Losada-Baltar,[2] *1. Universidad Rey Juan Carlos de Madrid, Madrid, Madrid, Spain, 2. Universidad Rey Juan Carlos, Madrid, Madrid, Spain, 3. Rey Juan Carlos University, Alcorcón, Madrid, Madrid, Spain, 4. HGU Gregorio Marañón,, Madrid, Madrid, Spain, 5. University of California San Diego, La Jolla, California, United States, 6. University Hospital Zurich, Zurich, Zurich, Switzerland, 7. Universidad Autónoma de Madrid, Madrid, Madrid, Spain*

Cross-sectional data show that caregivers' ambivalent feelings are associated with psychological distress. The association of ambivalent feelings with caregivers' cardiovascular risk has not been studied. For this purpose we analyzed preliminary data from the Spanish Longitudinal Caregiving Spanish Longitudinal Study (CUIDA-LONG). One-year follow-up data were available for 96 dementia family caregivers. The following variables were assessed: sociodemographics, body mass index (BMI), disruptive behaviors, ambivalence, depressive symptomatology and cardiovascular risk with the inflammatory biomarker C-reactive protein (CRP). A hierarchical regression model was tested. Sociodemographic variables and change over time in stressors, ambivalence and depression were entered as predictors of change in CRP. 27% of the variance in CRP was explained through the model. More time since being a caregiver, higher BMI and greater increase in ambivalence contributed significantly to an increase in CRP. Ambivalent feelings contribute significantly to the cardiovascular risk of those who care for a relative with dementia.

### AN INTENSIVE LONGITUDINAL STUDY OF THE ASSOCIATION OF STRESS WITH HYPERGLYCEMIA USING REAL-TIME DATA COLLECTION

Brent Mausbach, *University of California San Diego, La Jolla, California, United States*

Caregivers of persons with dementia (PWD) are at significantly elevated risk for cardiovascular disease (CVD) s. A higher risk for diabetes is one potential mechanism of morbidity in caregivers. Diabetes has been associated with dyslipidemia, hypertension, oxidative stress, increased low-grade inflammation, and endothelial dysfunction, which all place individuals at risk for CVD. Elevated blood glucose, even in the nondiabetic range, is a significant risk marker for the development of CVD. The current study examined the semi-continuous association between stress and glucose. Participants wore a continuous glucose monitor that measured blood glucose every 5 minutes for a period of 10 days (n = 2,880/participant). Ecological Momentary Assessment (EMA) was used to measure stress, positive affect, negative affect, and dietary intake 3x/day over the 10-day period. Hierarchical linear models indicated significant within-person associations between stress and blood glucose levels (t = 3.88, df = 3.92, p = .018; $R2$ = 26.2%).

### DAILY CORTISOL TOTAL OUTPUT MEDIATED SLEEP AND AFFECT AMONG DEMENTIA FAMILY CAREGIVERS

Daniel Fleming, Elizabeth Fauth, and Yin Liu, *Utah State University, Logan, Utah, United States*

Cortisol is a primary stress hormone associated with sleep. We examined daily cortisol as the potential mechanism linking prior night's sleep and daily mood among 173 dementia family caregivers (M (SD) age = 61.97 (10.66)) who used adult day services (ADS) at least two days a week. Caregivers self-reported sleep characteristics (bed and wake time, sleep quality, care receiver's night-time problems) and affect (anxiety, depressive symptoms) across eight consecutive ADS/non-ADS days. Salivary cortisol was collected five times each day. Multilevel mediation analysis suggested that daily cortisol total output (assessed as "area under the curve") mediated prior nights' total time in bed and daily anxiety, but only on high-stress (non-ADS) days. Mediation was non-significant on low-stress (ADS) days, and at the between-person level. ADS use is respite from a chronically stressful role. Reducing exposure to stress via respite may protect against harmful processes related to sleep, cortisol reactivity, and daily anxiety.

### Session 2170 (Paper)

### AGEISM (BSS PAPER)

### AGE DISCRIMINATION DURING THE COVID-19 PANDEMIC: ASSOCIATIONS WITH DAILY WELL-BEING

Lydia Ong, Patrick Klaiber, Anita DeLongis, and Nancy Sin, *University of British Columbia, Vancouver, British Columbia, Canada*

During the COVID-19 pandemic, ageist attitudes have been pervasive in public discourse, interpersonal relationships, and medical decision-making. For example, older adults have been portrayed as vulnerable while younger adults have been portrayed as reckless. The current study examined age discrimination during COVID-19 and associations with daily affect and physical symptoms. Positive events and age were examined as moderators. From March to August 2020, 1493 participants aged 18-91 (mean=40) in the U.S. and Canada completed surveys for seven consecutive evenings about discrimination, positive events, affect, and physical symptoms. Multilevel models controlled for age, race, income, education, sample (university students vs. community), and country of residence. Results indicated that individuals who reported more age discrimination had higher negative affect (b=36.44, SE=3.97), lower positive affect (b=-19.07, SE=4.10), and increased physical symptoms (b=3.85, SE=0.49; p<0.001 for all), compared to those with fewer reports of age discrimination. Within-persons, days with age discrimination were associated with higher negative affect (b=3.66, SE=1.36, p=0.008), lower positive affect (b=-2.60, SE=1.23, p=0.037), and increased physical symptoms (b=0.26, SE=0.11, p=0.02), compared to days on which age discrimination was not reported. Positive events moderated the between-person association of age discrimination with physical symptoms such that individuals with more age discrimination and more frequent positive events reported fewer daily physical symptoms than those with more age discrimination and less frequent positive events. Age did not moderate the associations. Age discrimination was associated with poorer daily well-being during the COVID-19 pandemic and may have long-term impacts on intergenerational solidarity and attitudes toward aging.

### IMPACT OF STRUCTURAL AGEISM ON GREATER VIOLENCE AGAINST OLDER PERSONS: A CROSS-NATIONAL STUDY OF 56 COUNTRIES

E-Shien Chang,[1] Joan Monin,[2] Daniel Zelterman,[2] and Becca Levy,[3] *1. Weill Cornell Medicine, New York City, New York, United States, 2. Yale School of Public Health, New Haven, Connecticut, United States, 3. Yale University, Woodbridge, Connecticut, United States*

Violence directed against older persons is recognized as a global health problem. However, structural drivers for violence remain under-studied. This country-level ecological study aimed to examine a previously unexplored link between structural ageism and violence against older persons. Following extensive structural stigma literature, structural ageism consisted of two components: (1) discriminatory national policies related to older persons' economic, social, civil, and political rights, gathered from global databases including UN, WHO, and others; and (2) societal-level prejudicial social norms against older persons, measured by negative attitudes toward older persons by the World Values Survey. Two components were z scored and combined such that higher score indicated greater structural ageism. Prevalence rates of violence per 100,000 persons aged 70 and over in each country were drawn from the Global Burden of Diseases Study. Final analysis included 56 countries, representing 63% of the world's aging population aged 60 and over across all six WHO regions. As predicted, structural ageism was significantly associated with the prevalence rates of violence in multivariate models ($\beta$ =205.7, SE=96.3, P=.03), after adjusting for country-level sociodemographic and health covariates. Three sets of sensitivity analyses supported the robustness of our findings. That is, structural ageism did not predict other types of violence and other types of prejudice did not predict violence against older persons. Public health and population-based violence prevention policies may benefit from a targeted approach that tackles the harmful effects of structural ageism.

### INTERGENERATIONAL CONTACT EXPERIENCES AND THEIR RELATION TO AGEISM AS A MULTIDIMENSIONAL CONSTRUCT

Tim Kuball,[1] Georg Jahn,[2] and Claas Pollmanns,[2] *1. Chemnitz Technical University, Leipzig, Sachsen, Germany, 2. Chemnitz Technical University, Chemnitz, Sachsen, Germany*

Research on intergroup contact suggests that negative contact experiences affect cognitive representations such as stereotypes more strongly than positive contact experiences. To comprehensively examine the full effect of intergroup contact, the valence of the contact experience as well as the affective and cognitive dimensions of prejudice should be assessed. In ageism research, previous studies typically focused only on contact of positive valence and were limited to the perspectives of younger individuals on older adults. Primary objective of this study is to examine both positive and negative contact frequency and their relation to affective and cognitive dimensions of ageism from the perspectives of younger adults between the age of 18 and 25 (study 1) and older adults between the age of 60 and 92 (study 2). Consistent with previous research on intergroup contact, our results confirm that both types of contact were similarly predictive of affective facets of prejudice. However, only in study 2 that assessed older adults' agreement with contemporary stereotypes about young men and women, negative compared to positive contact frequency proved to be a stronger predictor of the cognitive dimension of ageism. Our findings emphasize the importance of focusing on all dimensions of prejudice and highlight the need to consider the perspectives of young and old in ageism research.

### OLDER PEOPLE WITH DEMENTIA RUNNING A POP-UP RESTAURANT: HOW TO USE REALITY SHOWS TO REDUCE THE STIGMA OF DEMENTIA

Wenqian Xu, *Linköping University, Norrköping, Ostergotlands Lan, Sweden*

The present study focused on a Chinese reality show, Forget Me Not Café, which brought together five older people (aged 65 and older) living with dementia to run a pop-up restaurant and intended to reduce the stigma of dementia. The study aims to explore how the reality-show participants describe dementia in older people and how their views relate to the macrosocial context of dementia and older people. This study performed a thematic discourse analysis on the written and spoken content about dementia in older people (or later life) presented in the reality show. Four discursive themes were identified including: (1) age is a risk factor for dementia; (2) early signs and symptoms of dementia in older people deserve attention; (3) putting pressure on family caregivers of people with dementia; (4) expectations to maintain social engagement and slow down the development of dementia. This study also found that the views of the reality-show participants highlight the capability of older people with dementia to communicate effectively and live with the condition, their personal goals of sustaining a happy, meaningful and sociable life, as well as their actions to positively influence personal circumstances. The results of this study indicate that this reality show might help reduce the stigma of dementia and empower older people living with dementia, while it also tends to stress the responsibility for care on family carers and shift the responsibility of managing the dementia-related challenges to older people living with dementia.

### THE SOCIAL EFFECTS OF AN INTERGENERATIONAL E-MAIL PAL PROGRAM

Kristi Lise,[1] Jillian Minahan,[2] and Karen Siedlecki,[3] *1. CUNY Queens College, New Rochelle, New York, United States, 2. Fordham University, New Providence, New Jersey, United States, 3. Fordham University, Bronx, New York, United States*

Ageist attitudes and loneliness negatively impact both younger and older adults (e.g., Sun et al., 2019). This study utilized a randomized waitlist-control design to investigate the effects of a six-week intergenerational e-mail pen pal program on loneliness in younger and older adults and ageism in younger adults. Thirty-three younger adults (18-30 years) and 28 older adults (over age 65) completed an online survey assessing ageist attitudes, loneliness, well-being, and other individual differences. One week after completing a baseline survey, 17 email pen pal pairs began the six-week e-mail intervention. Participants repeated the survey one week after the completion of the intervention (which was eight weeks after the baseline for the control participants). Analyses showed that at baseline, younger adults (M=2.41, SD=.76) reported higher levels of loneliness compared to older adults (M=1.65, SD=.77), $t(59) = 3.85$, $p < .001$. Repeated measures ANOVAs showed that the intervention did not have a significant effect on ageism or loneliness in either younger or older adults. However, the effect size of the intervention for loneliness among older adults was moderate to large ($\eta 2= .07$). Descriptive statistics indicated that older adults in both the intervention and control groups experienced an increase of loneliness during the post-test. However, the older adults in the intervention group experienced less of an increase compared to older adults in the control group. This suggests that the intervention may have buffered the increase in loneliness that older adults may experience during the winter months and during the onset of the COVID-19 pandemic.

### Session 2175 (Symposium)

### AGING STRONG: PROMOTING RESILIENCE THROUGH OPTIMISM, PURPOSE, AND SOCIAL CONNECTIONS

Chair: Ellen Wicker Co-Chair: James Schaeffer

Resilience is defined as the ability to adapt and cope with circumstances in a way that empowers one to emerge stronger, thrive, and incorporate lessons learned. Resilience as a trait can be learned and modified and have a significant impact on healthy aging. UnitedHealthcare (UHC) and AARP Services, Inc. (ASI) are committed to the health and well-being of participants in UHC's Medicare Supplement insurance plans, recognizing that health and wellness need to be promoted on a holistic level to ensure successful aging. In this effort, an initiative titled Aging Strong 2020 was developed to promote health, well-being, and increase resilience by focusing on the key individual pillars of enhanced purpose in life, social connectedness, and optimism. To accomplish this goal, a series of eight interventions over three years were created and delivered, with a focus on the key pillars in order to improve clinical and psychological health outcomes and participants' satisfaction with health care. This symposium will specifically discuss efforts related to the Aging Strong 2020 program. First, we will describe the prevalence and outcomes of the pillars in a large national survey. Next, key challenges and successes in recruitment and retention for the various interventions will be highlighted, followed by overall findings from the eight interventions targeting the pillars. Finally, qualitative findings on participant experience as a result of participation will be discussed. Results from these initiatives demonstrate that interventions designed to improve well-being among older adults contribute to the holistic model of health.

### RESILIENCE, PURPOSE IN LIFE, LONELINESS, AND ASSOCIATED MEDICAL COSTS IN OLDER ADULTS

Rifky Tkatch,[1] Gandhi Bhattarai,[2] Sandra Kraemer,[3] James Schaeffer,[1] Charlotte Yeh,[4] and Timothy Barnes,[1] *1. UnitedHealth Group, Minnetonka, Minnesota, United States, 2. OptumLabs, Minnetonka, Minnesota, United States, 3. UnitedHealthcare, Minnetonka, Minnesota, United States, 4. AARP Services, Inc., Washington, District of Columbia, United States*

Resilience, purpose in life (PIL), and loneliness have been linked, and used to characterize the health and well-being of older adults. Studies demonstrate that higher resilience, PIL, and minimal loneliness are associated with better late-life outcomes. However, research on how these constructs negatively impact medical costs is limited. Using survey and claims data from a large sample of older adults age 65+ (N=4,496), resilience, PIL, and loneliness were examined to determine associations with medical costs. Among study participants, 11% exhibited low resilience, 19% severe loneliness, and 35% low PIL. Low resilience was associated with 24% higher medical costs compared to participants with high resilience, severe loneliness with 20% higher costs compared to participants with no loneliness, and low PIL marginally associated with 12% higher costs compared to participants with high PIL. Interventions targeting resilience, PIL, and loneliness could be beneficial to promoting successful aging and lowering medical costs.

### RECRUITING OLDER ADULTS IN A MEDICARE SUPPLEMENT POPULATION FOR WELLNESS INTERVENTION PILOT STUDIES

Rifky Tkatch,[1] Lizi Wu,[1] Laurie Albright,[2] Michael McGinn,[3] Charlotte Yeh,[4] and Catherine Zaidel,[3] *1. UnitedHealth Group, Minnetonka, Minnesota, United States, 2. UnitedHealth Group, Minneapolis, Minnesota, United States, 3. OptumLabs, Minnetonka, Minnesota, United States, 4. AARP Services, Inc., Washington, District of Columbia, United States*

Older adults are often underrepresented in the health promotion literature, in part due to challenges in recruiting older adults for such studies. Aging Strong 2020 was specifically designed to address the health needs of older adults. A subset of adults aged 65 and older with an AARP Medicare Supplement plan insured by UnitedHealthcare were recruited for participation in one of eight interventions. Recruitment lists for each program were drawn from a pool previously screened for loneliness, purpose in life, optimism, and resilience, administered by an interactive voice response (IVR) telephone survey. Recruitment efforts were multifaceted and included emails, direct mailers, and phone calls. Incentives ranging from $25-$100 for completing surveys did not correspond with higher recruitment rates. Overall, recruitment phone calls reached 28,058(32%) individuals on the recruitment lists; a total of 1,766 participated, demonstrating that targeted efforts to recruit older adults for research opportunities can be successful.

### IMPROVING RESILIENCE, OPTIMISM, PURPOSE, AND LONELINESS AMONG OLDER ADULTS

Lizi Wu,[1] Laurie Albright,[2] Rachel Ungar,[1] Catherine Zaidel,[3] Yan Cheng,[1] James Schaeffer,[1] Charlotte Yeh,[4] and Rifky Tkatch,[1] *1. UnitedHealth Group, Minnetonka, Minnesota, United States, 2. UnitedHealth Group, Minneapolis, Minnesota, United States, 3. OptumLabs, Minnetonka, Minnesota, United States, 4. AARP Services, Inc., Washington, District of Columbia, United States*

Aging Strong 2020 was developed to promote health and well-being and increase resilience by focusing on the pillars of enhanced purpose in life, social connectedness, and optimism. A series of eight interventions over three years tested the feasibility of enhancing these pillars. Interventions included: 1) An expressive writing program, 2) Animatronic pets, 3) A telephonic reminiscent program, 4) An online self-compassion mindfulness program, 5) A technology-based behavior change tool, 6) An online and workbook tool for purpose, 7) An online happiness program, and 8) A peer-to-peer support program. Each program demonstrated efficacy dependent on the pillar targeted and the population sampled. Overall, some improvement was found among participants in resilience (47%), purpose (49%), optimism (44%), and loneliness (48%). Further, participant satisfaction improved in each program with Net Promoter Scores increasing between 7-19 points. Results demonstrate that Aging Strong 2020 was successful, contributing to a holistic model of healthy aging.

### OLDER ADULTS' EXPERIENCES PARTICIPATING IN AGING STRONG 2020 PROGRAMS

Rachel Ungar,[1] Laurie Albright,[2] James Schaeffer,[1] Ellen Wicker,[3] and Janella Hudson,[4] *1. UnitedHealth Group, Minnetonka, Minnesota, United States, 2. UnitedHealth Group, Minneapolis, Minnesota, United States, 3. AARP Services, Inc., Washington, District of Columbia, United States, 4. Optum/United Healthcare, Tampa, Florida, United States*

User satisfaction assessments are integral to demonstrating intervention efficacy. Towards that end, older participants across the Aging Strong 2020 suite of offerings participated in semi-structured interviews (n = 248) to provide feedback about their experiences in the program and resulting satisfaction. Overall, most participants were satisfied with the Aging Strong 2020 interventions and reported gaining new skills, tools, or coping strategies. Participants endorsed program features that facilitated social interaction, community building, and social support. Program content specifically adapted for older adults and appropriate life stage concerns and/or areas of interest were considered especially helpful. Results demonstrate that the current test and learn model offers an opportunity for participant feedback to refine and improve future iterations of project offerings. Participant feedback led to key improvements in subsequent versions of the Aging Strong 2020 programs and their contributions to successful aging among older adults.

### Session 2180 (Paper)

### ALZHEIMER'S DISEASE I (HS PAPER)

### IMPROVING USEFULNESS OF COGNITIVE DECLINE POPULATION MEASURES IN PREDICTING FUTURE DEMENTIA BURDEN

Benjamin Olivari,[1] Christopher Taylor,[1] and Lisa McGuire,[2] *1. Centers for Disease Control and Prevention (CDC), Atlanta, Georgia, United States, 2. CDC, Atlanta, Georgia, United States*

Alzheimer's disease and related dementias begin with mild early symptoms of memory loss, progressing to more severe cognitive and functional impairment. Reports of worsening memory and subjective cognitive decline (SCD) are often the earliest possible signs of dementia onset. The trajectory of certain types of dementia may require early detection of worsening memory in the disease progression for successful interventions. However, the predictive value of subjective measures of cognitive decline is limited; the majority of those who report subjective symptoms do not progress to diagnosed cognitive impairment or dementia. These two realities create a significant challenge in confronting the growing dementia crisis. Population-level data can be beneficial in tracking trends in SCD. Data from the Behavioral Risk Factor Surveillance System (BRFSS) core questions related to chronic diseases and from the SCD optional module from survey years 2015-2019 were aggregated across the participating 50 states, D.C., and Puerto Rico for this analysis. Among 181,097 U.S. respondents aged ≥45 years, 11.3% (95% CI=10.9-11.6) reported SCD; among 20,424 with SCD symptoms, 39.4% (37.6-40.6) reported functional difficulties associated with SCD symptoms and 33.9% (32.4-35.5) needed assistance with day-to-day activities resulting from symptoms. Studies suggest persons experiencing SCD symptoms and associated functional difficulties are at increased risk for dementia compared with those with SCD without functional difficulties. Combining responses about SCD with associated functional difficulties, anxiety, and other measures might help to better inform the future burden of more severe cognitive impairment than SCD status alone.

### MENTAL DISORDERS FORECAST ALZHEIMER'S DISEASE AND RELATED DEMENTIAS IN 1.7 MILLION NEW ZEALANDERS

Leah Richmond-Rakerd,[1] Stephanie D'Souza,[2] and Barry Milne,[2], *1. University of Michigan, Ann Arbor, Michigan, United States, 2. University of Auckland, Auckland, Auckland, New Zealand*

Neurodegenerative conditions, including Alzheimer's disease and related dementias (ADRD), have an outsized impact on disability and loss of independence in older adults. As such, there is a growing need to identify modifiable risk factors for ADRD at the population level. We conducted a nationwide administrative-register study to investigate mental disorders as a potential preventable risk factor for later-life ADRD. Data were drawn from the New Zealand Integrated Data Infrastructure, a collection of whole-of-population administrative data sources linked at the individual level by a common spine. We identified all individuals born in New Zealand between 1928-1967 and followed them for three decades (N = 1,711,386; observation period = 1988-2018; age at baseline = 21-60 years). Diagnoses of mental disorders were ascertained from public-hospital records. Diagnoses of ADRD were ascertained from public-hospital records, mortality records, and pharmaceutical records. Individuals with a mental disorder were at elevated risk for developing Alzheimer's disease and related dementias relative to those without a mental disorder. This prospective association was evident in both men and women, across age, and after accounting for pre-existing physical diseases. If associations are causal, ameliorating mental disorders could extend population healthspan and reduce the societal burden of neurodegenerative diseases.

### PREVALENCE AND IMPACT OF COMORBID CANCER AND DEMENTIA ON HEALTH OUTCOMES IN OLDER ADULTS: A LONGITUDINAL STUDY

Jyotsana Parajuli,[1] Diane Berish,[2] Ying-Ling Jao,[2] Yo-Jen Liao,[3] and Lee Ann Johnson,[4] *1. Univerisity of North Carolina at Charlotte, Charlotte, North Carolina, United States, 2. Pennsylvania State University, University Park, Pennsylvania, United States, 3. Penn State University, University Park, Pennsylvania, United States, 4. Univeristy of Virginia, University of Virginia, Virginia, United States*

Dementia and cancer are two common chronic conditions in older adults. However, there are few studies examining the prevalence of comorbid cancer and dementia and the longitudinal impact of these comorbid conditions on health outcomes. This study investigated the prevalence and longitudinal impact on health outcomes in older adults with comorbid cancer and dementia. This is a secondary analysis, using data from the 2010 and 2016 waves of the Health and Retirement Study (HRS). The health outcomes of the study included nursing home stay, hospital stay, home care use, activities of daily living (ADL) limitations, instrumental activities of daily living (IADL), self-rated health status, mortality, and the out-of-pocket medical expenditure in older adults with cancer and dementia. Data were analyzed using descriptive statistics, logistic regression, and linear regression analyses. The results revealed that the prevalence of comorbid cancer and dementia ranged from 2.6% to 2.8% over the 6-year period. Older adults with comorbid cancer and dementia demonstrated higher likelihood of nursing home stay, ADL and IADL limitations, and mortality; but a decreased likelihood of homecare use and hospital stay compared to older adults with cancer only or dementia only (some outcomes were not significant for dementia only group). Findings point out the risk of increased functional decline and mortality in older adults with comorbid cancer and dementia. Future research is needed to explore the contributing factors of the risk and identify interventions to promote physical function and reduce mortality for this population.

### SAFETY CONSIDERATIONS OF ADAPTIVE HORSEBACK RIDING PROGRAM FOR ADULTS WITH DEMENTIA AND THEIR FAMILIES

Alicia Oestreich, and Beth Fields, *University of Wisconsin-Madison, Madison, Wisconsin, United States*

Human-animal interactions, including equine-assisted services, are becoming increasingly popular to enhance the quality of life of adults with dementia and their families. However, there is a lack of knowledge on safety considerations when serving this population. The purpose of this qualitative descriptive study was to explore the safety perspectives of key stakeholders involved in an adaptive horseback riding program for adults with dementia and their families. Ten, 30-minute semi-structured interviews and two, 60-minute focus groups were conducted with horseback riding program instructors and staff, dementia specialists, and adults with dementia and their families. Thematic analysis of data were guided by the Professional Association of Therapeutic Horsemanship International's Core Safety Standards and completed using NVivo 12. Stakeholders described two central themes to consider when offering equine-assisted services at therapeutic horseback riding centers to adults with dementia and their families 1) dementia and horse training parameters, and 2) enrollment procedures. Stakeholders recommended that training should encompass "how to interact and communicate in a positive way with the adult with dementia…redirect if a behavior comes up", and horses should be specially selected to "tolerate standing in a ramp during a difficult mount". Stakeholders also shared that enrollment in the program should include learning the adult's health and prior horse experience, precautions and contraindications, horseback riding readiness, and availability of and support from family. Information gleaned from this study may help researchers, instructors, and staff develop policies that demonstrate optimal safety practices when delivering equine-assisted services to adults with dementia and their families.

### VALIDITY OF THE CHINESE VERSION OF THE PERSON-ENVIRONMENT APATHY RATING (PEAR-C) FOR PERSONS WITH DEMENTIA

Ying-Ling Jao,[1] Ying-Yu Chao,[2] Yo-Jen Liao,[3] Diane Berish,[1] An-Yun Yeh,[4] and Shang-Ti Chen,[5] *1. Pennsylvania State University, University Park, Pennsylvania, United States, 2. Rutgers University, Newark, New Jersey, United States, 3. Penn State University, University Park, Pennsylvania, United States, 4. Hunter College of CUNY, New York, New York, United States, 5. National Dong-Hwa University, Shoufeng, Hualien, Taiwan (Republic of China)*

Apathy is a prevalent neurobehavioral symptom in dementia. Despite that environmental stimulation plays a key role in apathy, it is often overlooked in assessment. The Person-Environment Apathy Rating (PEAR) scale is currently the only validated apathy scale for persons with dementia that addresses environmental stimulation and is only available in English. This project translated the PEAR scale into Mandarin Chinese and evaluated its content validity. The PEAR scale includes two subscales: PEAR-Environment and PEAR-Apathy. Each subscale includes six items. The PEAR scale translation and validation were conducted through a four-step process. First, the PEAR scale was translated from English into Chinese by two bilingual PhD-prepared researchers. Second, the two Chinese versions of PEAR (PEAR-C) were back-translated into English by another two bilingual PhD-prepared researchers. Third, three content experts reviewed the two translated scales and reconciled a final PEAR-C scale. Finally, these three experts individually rated the PEAR-C and evaluated its content validity item-by-item in two aspects: 1) content equivalence: appropriateness to use this scale in Chinese cultural setting, and 2) semantic equivalence: the scale remains the same meaning after translation. The content validity index (CVI) was calculated to sum the ratings across experts. The CVI of content equivalence for all items was 1.0 for both subscales. The CVI of semantic equivalence was 0.98 for the PEAR-Environment and 0.97 for the PEAR-Apathy. The PEAR-C shows substantial content validity. Its reliability and construct validity need further evaluation. This scale is promising to assess apathy for individuals with dementia in the Chinese-speaking community.

### Session 2190 (Symposium)

### CAREGIVING THROUGH TURBULENT TIMES: FINDINGS FROM THE MIT AGELAB'S LONGITUDINAL STUDY OF FAMILY CAREGIVERS

Chair: Julie Miller Discussant: Julie Miller

Nearly one in five Americans is an unpaid family caregiver, and the need for family caregivers is projected to grow over the next several decades in the face of longer lifespans (AARP 2020). Yet the increasing centrality of family caregivers for providing care to an aging population highlights two knowledge gaps: first, the degree and experience of burden and stress caregivers manage around balancing care with other family and work responsibilities; and second, a lack of knowledge about the caregiver journey and the microtasks of care, including how caregivers leverage – or not – different tools, technologies and resources to support the care they provide. To develop a deeper understanding of these questions and others, the MIT AgeLab has built a research panel of over 1200 caregivers providing care to another adult family member. This symposium will present findings from the MIT AgeLab Caregiver Panel, including: 1) an examination of the extent to which family caregivers identify as such and how they feel about their roles; 2) how family caregivers experienced the COVID-19 pandemic both personally and around the care they provide; 3) caregivers' use of and attitudes toward technology to support the care they provide; and 4) what caregivers identify as their key unmet needs. The session will include a facilitated discussion around the intersection of COVID-19 with caregivers' technology use, experience of caregiving, and future needs, as well as to identify additional research questions and directions for future research with the MIT AgeLab Caregiver Panel.

### CAREGIVERS' LANGUAGE AND EMOTIONS AROUND CARE

Lisa D'Ambrosio, *Massachusetts Institute of Technology, Cambridge, Massachusetts, United States*

Caregiving encompasses a range of roles and activities, but not all people providing care identify as "caregivers." Understanding the vocabulary and emotions that caregivers have should first, contribute to an understanding of caregiving and caregivers per se, and second, aid in communicating with them more effectively. Analysis of survey data from members of the MIT AgeLab Caregiver Panel shows variance in self-identification as caregivers and in language and emotions around caregiving, reflecting diversity in the care experience, but consensus around the core concept of a caregiver. This presentation will report on how caregivers' relationships, gender and care tasks affect their language and identify a caregiver experience-identity gap: a space between what caregivers do and what they report. We highlight how an understanding of caregivers' experiences of what they do – as opposed to a catalog of tasks they do – may be more important for understanding their experience of strain.

### TECHNOLOGIES AND SERVICES TO SUPPORT CARE: CAREGIVERS' EXPERIENCES AND PERSPECTIVES

Chaiwoo Lee, *Massachusetts Institute of Technology, Cambridge, Massachusetts, United States*

Technologies developed to make life easier for the general population – including smart home products, internet-enabled services, communication platforms, and health management systems – also have the potential to assist individuals who provide care to loved ones. While caregivers may be eager users of technology to support their responsibilities, some technologies remain untapped resources. An in-depth survey conducted with the MIT AgeLab Caregiver Panel around attitudes toward and use of technology for themselves and for caregiving showed that while caregivers use a wide range of technologies for themselves, their use for caregiving is limited. However, while caregivers did not universally use technologies or services to support the care they provided, those who did so generally reported positive feelings about their use. This presentation will report on technology experiences – including perceived usefulness, ease of use and integration, impacts, and overall satisfaction – among caregivers of various characteristics and conditions.

### CAREGIVING THROUGH THE PANDEMIC: EXPLORING THE IMPACTS OF COVID-19 ON CAREGIVERS AND THEIR CAREGIVING EXPERIENCES

Alexa Balmuth, *MIT AgeLab, Cambridge, Massachusetts, United States*

In the midst of the COVID-19 pandemic, many caregivers have been tasked with a unique role; not only to keep themselves safe and healthy, but also to protect and find new ways to aid their care recipients, many of whom are older adults at relatively high risk for severe complications from COVID-19. These challenging circumstances have driven caregivers to quickly adapt as they continue to manage their personal lives and caregiving responsibilities. Utilizing three waves of survey data from the MIT AgeLab Caregiver Panel, this presentation will examine the attitudes, experiences and worries of family caregivers at several time points along the course of the COVID-19 pandemic, as well as caregivers' preparations and coping behaviors along the way. Differences between caregiving situations will also be discussed.

### Session 2195 (Paper)

### CHRONIC DISEASE MANAGEMENT (HS PAPER)

### ENERGY DECLINE MAY PREDICT MILD PARKINSONIAN SIGNS IN COMMUNITY-DWELLING OLDER ADULTS

Rebecca Ehrenkranz,[1] Qu Tian,[2] Andrea Rosso,[1] Nancy W. Glynn,[3] Lana Chahine,[1] James Hengenius,[1] Xiaonan Zhu,[1] and Caterina Rosano,[1] *1. University of Pittsburgh, Pittsburgh, Pennsylvania, United States, 2. National Institute on Aging, Baltimore, Maryland, United States, 3. University of Pittsburgh Graduate School of Public Health, Pittsburgh, Pennsylvania, United States*

Mild Parkinsonian Signs (MPS) are common in older adults without overt neurological disease. MPS are often progressive and predict disability and dementia, yet little is known about predictors for MPS. Low self-reported energy is associated with mobility impairment, which is a hallmark of MPS. Yet whether self-reported energy relates to MPS is unknown. We explored the association of changes in self-reported energy with MPS in 293 participants (aged 83 ± 2.8 years, 58% women, 61% White) free of dementia and Parkinson's Disease in the Health, Aging and Body Composition Study. Self-reported energy was assessed on a 0-10 scale annually between Year 2 and Year 10 (mean follow-up: 8 years) and its slope was estimated via linear mixed effects models. MPS were evaluated at Year 10 based on the Unified Parkinson Disease Rating Scale motor component. On average, self-reported energy declined 0.06 points per year. In a linear regression model adjusted for age, fatigue, and comorbidities, those with MPS had steeper SEL decline (β [Standard Error] = -0.358 [0.119]) in the prior eight years than those without MPS. Thus, declining self-reported energy may be a risk factor for MPS. Self-reported energy is easily evaluated in routine clinic visits, and may be a modifiable risk factor that can be targeted to reduce the incidence of MPS.

### HEALTHY LIFESTYLE AND SOCIAL NETWORK PROLONG DISABILITY-FREE SURVIVAL IN OLDER ADULTS WITH DIABETES

Ying Shang,[1] Wei Wu,[2] Abigail Dove,[1] Jie Guo,[1] Anna-Karin Welmer,[3] Debora Rizzuto,[1] Laura Fratiglioni,[3] and Weili Xu,[1] *1. Karolinska Institutet, Solna, Stockholms Lan, Sweden, 2. Hubei University of Chinese Medicine, Hubei University of Chinese Medicine, Hubei, China (People's Republic), 3. Karolinska Institutet, 17175, Stockholms Lan, Sweden*

**Aim:** We aimed to estimate the extent to which diabetes shortens disability-free survival, and identify which factors may prolong disability-free survival in older adults with diabetes.

**Methods:** A total of 2,216 disability-free participants aged ≥60 were followed up to 15 years. Diabetes was ascertained through antidiabetic drug use, medical records, or HbA1c ≥ 6.5%. Disability-free survival was defined as the survival until the occurrence of disability. Data on behaviours (healthy vs. unhealthy), leisure activities (active vs. inactive), and social network (moderate-to-rich vs. poor) were collected at baseline. A favourable (vs. unfavourable) lifestyle profile was defined as the presence of at least one of healthy behaviours, active engagement in leisure activities, and/or moderate-to-rich social network. Data were analysed with Cox regression and Laplace regression.

**Results:** During the follow-up, 1,345 (60.7%) participants developed disability/death. Diabetes was related to the outcome (HR 1.29, 95% CI 1.06–1.57), and shortened 2.15 (1.02–3.27) years of median disability-free survival. Additionally, disability-free survival (95% CI) was shortened by 3.29 (1.21–5.36), 3.92 (2.08–5.76) and 1.66 (0.06–3.28) years for participants with diabetes plus unhealthy behaviours, inactive leisure activities, or poor social network, respectively (reference: no diabetes plus healthy behaviours, leisure activities, or moderate-to-rich social network). Among participants with diabetes, a favourable profile led to a non-significant HR of 1.19 (0.93–1.56) for disability/death and prolonged disability-free survival by 3.26 (2.33–4.18) years than those with unfavourable profile.

**Conclusions:** Healthy lifestyle and/or moderate-to-rich social network attenuates the risk of diabetes on disability/death and prolongs disability-free survival in people with diabetes by 3 years.

### IMPLEMENTATION OF A SHARED DECISION-MAKING TOOL FOR OSTEOARTHRITIS TREATMENT TO REDUCE DECISIONAL CONFLICT

Yashika Watkins,[1] Rose Gonzalez,[2] Charla Johnson,[3] and Ravneet Kaur,[4] *1. Chicago State University, Cliffside Park, New Jersey, United States, 2. Movement is Life, Lake Ridge, Virginia, United States, 3. Franciscan Missionaries of Our Lady Health System, Baton Rouge, Louisiana, United States, 4. University of Illinois at Chicago, Crown Point, Indiana, United States*

Shared decision making is a key component of patient centered care where clinical evidence and the patient's preference and values are considered. Physical activity and weight loss are often recommendations in the treatment plan, especially in mild to moderate stage of osteoarthritis (OA). Movement is Life (MIL) created an innovative SDM tool to provide a framework for patient-centered discussions. The tool leverages an underlying Markov Model and represents the likely pain, activity levels, and lost productivity at three future time points. By comparing the patient's likely progression depending on treatment choices compared to doing nothing, the patient has an illustration of future state. A pilot of N=108 women, ages 45-64, with chronic knee pain for at least three months and at least one co-morbidity (obesity, hypertension, diabetes) were randomized to a control (n=54) or intervention (n=54) arm of the study at eight centers across the United States. Results showed the demographic profiles were similar between the groups. At one-month, n=47 control and n=50 intervention patients returned for evaluation. Self-reported level of physical activity increased in the intervention group (56% vs 34%, p = 0.0229). Qualitative feedback from the intervention group indicated high satisfaction with use of the tool. Both groups reported a high likeliness to recommend the provider to a friend or family member. Inclusion of the SDM tool added an average of one minute to the patient counseling time over the control group. The quasi script provides a consistent communication pathway and may reduce disparities by addressing unconscious bias.

### MULTIPLE CHRONIC CONDITION COMBINATIONS, AFFECT, AND MEMORY AMONG BLACK AND WHITE OLDER ADULTS

Aaron Ogletree,[1] Steph Cooke,[2] and Benjamin Katz,[2] *1. American Institutes for Research, Washington, District of Columbia, United States, 2. Virginia Tech, Blacksburg, Virginia, United States*

Research demonstrates the adverse effects of coexisting multiple chronic conditions (MCCs) on older adults' health and wellbeing. While most research relies on total counts of chronic conditions, little work explores how specific MCC combinations may have compounding effects on depression and memory. Furthermore, no published research explores differences in the prevalence and correlates of MCC

combinations between Black and White older adults. The current study assesses within- and between-group heterogeneity in the prevalence and correlates of MCC combinations to advance health equity research. We utilize a sample of 16,757 Black and White older adults drawn from the 2014 wave of the Health and Retirement Study. Respondents were categorized into one of 32 MCC combination groups. Depressive symptoms and self-rated memory were calculated separately for Black and White respondents across each of the 32 groups. Chi-square tests, t-tests, and ANCOVAs were used to compare differences. Black and White respondents differed significantly in the prevalence of 14 out of 32 MCC combinations. Within-group differences were found such that 45% of Black respondents experiencing only Lung Disease met criteria for clinical depression; this rate is similar to Black respondents experiencing Diabetes + Heart Condition + Hypertension + Lung Disease (44.5%). Between-group differences revealed that Black respondents experiencing Arthritis + Diabetes + Hypertension had worse self-rated memory than White counterparts (MB = 3.24, MW = 3.13; two sample t[1139]= -2.04, $p < .05$; Cohen's d = 0.13). Additional findings are presented, and theoretical and practical implications for this work are discussed.

### PREDICTING SYMPTOM SEVERITIES IN MIDDLE-AGED AND OLDER ADULTS WITH ARTHRITIS AND MULTIMORBIDITY

Wenhui Zhang, *Emory University, Atlanta, Georgia, United States*

**Introduction:** Uncertainties increase with disease guideline-driven decision-making for older adults as their numbers of chronic conditions and functional limitations increase. A national study found that people with arthritis plus ≥ one other chronic condition have reported significantly higher social participation restriction, serious psychological distress, and work limitation than those with ≥two non-arthritis chronic conditions. However, how arthritis comorbidities contribute to the symptoms such as pain, fatigue, sleep, depression, anxiety, and cognitive abilities that chronically impair people's daily functioning remain unexplored.

**Purpose:** To explore how arthritis comorbidities predict the symptom severities of pain interference, fatigue, sleep disturbance, depression, anxiety, and cognitive abilities among community-dwelling middle-aged and older adults. Method: 140 community people aged over 50 with arthritis and multimorbidity were recruited. Stepwise regressions predicted the PROMIS symptoms of pain interference, fatigue, sleep disturbance, depression, anxiety, and cognitive abilities with arthritis type and 18 comorbidities measured by the Functional Comorbidity Index checklist after controlling for demographics.

**Results:** Obesity, chronic obstructive pulmonary disease, diabetes, and income significantly predicted pain interference (adjusted R2=35%). Marital status, obesity, and peripheral vascular disease significantly predicted fatigue (adjusted R2=17%). Depression diagnosis and income adequacy significantly predicted depressive symptoms (adjusted R2=23%). Depression, income adequacy, and anxiety diagnosis significantly predicted anxiety (adjusted R2=23%). Age significantly predicted cognitive abilities (adjusted R2=12%).

**Discussion:** Comorbidities and socio-demographics, especially income, impact symptom experiences of people aging with arthritis and multimorbidity. Future studies should explore the pathogenesis among arthritis, comorbidities, and symptoms for tailored intervention while disclosing health disparities associated with the arthritis multimorbidity.

### Session 2200 (Paper)

### COGNITIVE IMPAIRMENT AND DECLINE

### CANNABIS USE AND SUBJECTIVE COGNITIVE DECLINE AMONG MIDDLE-AGED AND OLDER ADULTS

Maritza Dowling, *George Washington University - School of Nursing, Washington, District of Columbia, United States*

Cannabis is the most commonly-used drug in the US, with older adults being the fastest-growing group of users. National surveys among 50+ adults found poorer executive function among current or past cannabis users, but better performance with daily users. However, there is little evidence linking levels of cannabis use and subjective cognitive decline (SCD) among middle-age and older adults. This study sought to examine the association between levels of cannabis use in the past 30 days and SCD during the past 12 months using Behavioral Risk Factor Surveillance System (BRFSS) data (2016-2019) from 45+ individuals while controlling for demographics, chronic conditions, exercise, general and mental health. Logit models with SCD as outcome were estimated using complex survey weights. Multiple group analyses examined differences across age groups: 45-64 vs. 65+. Levels of cannabis use in the past 30 days were categorized as: no use, 1-4 days, 5-20 days; and 21-30 days. Adjusted results indicated that those who reported no cannabis use were from 35% to 47% less likely to report SCD compared to cannabis users at all levels: 1-4 days (OR=0.65, 95%CI=0.46, 0.92); 5-20 days (OR=0.53, 95%CI=0.36,0.78); 21-30 days (OR=.61, 95%CI=0.45,0.83). In multi-group analyses, levels of cannabis use effects on SCD remained statistically significant in the 45-64 age group, but not in the 65+ group. Further research targeting SCD is needed to design interventions particularly for middle-age cannabis users whose health has been compromised by disease or age-related vulnerabilities and are at greater risk for adverse cognitive outcomes from cannabis use.

### CLINICAL RECOMMENDATIONS FOR REDUCING THE RISK OF COGNITIVE DECLINE

G. Adriana Perez,[1] Kelly O'Brien,[2] Marwan Sabbagh,[3] and Michelle Bruno,[4] *1. University of Pennsylvania School of Nursing, Phildelphia, Pennsylvania, United States, 2. UsAgainstAlzheimer's, Chicago, Illinois, United States, 3. Cleveland Clinic Nevada, Las Vegas, Nevada, United States, 4. Avalere Health, Washington, District of Columbia, United States*

As much as 40% of dementia cases can be attributed to modifiable risk factors (Livingston et al., 2020). Much of that risk-reduction can be accomplished by changing behavior in midlife. In light of the emerging evidence that dementia may be preventable, UsAgainstAlzheimer's convened a workgroup of national experts to develop new recommendations that primary care clinicians and general neurologists can use to initiate primary prevention conversations with their patients about cognitive decline. Few resources address steps that clinicians can take in their routine care to help patients reduce risk. Some relevant resources provide excellent guidance but tend to be more focused on early detection or slowing disease progression rather than primary prevention. The Risk Reduction Workgroup (RRWG) was convened to help address the need for clinicians to know how to discuss cognitive decline with their patients. The workgroup aligned on 11 recommendations for primary care clinicians and general neurologists. In addition the RRWG provide considerations for implementing the recommendations in clinical practice. The recommendations are mindful of social determinants of health, account for cultural differences, and are designed for general accessibility. This effort is part of a broader initiative by UsAgainstAlzheimer's to address risk-reduction for cognitive decline and early interventions. Under the guidance of a multidisciplinary Provider Leadership Group consisting of representatives from some of the nation's largest health provider serving organizations, three independent workgroups are developing guidance and tools to assist providers in their clinical practice and improve health outcomes for patients at-risk for Alzheimer's and related dementias.

### PATIENT COGNITIVE IMPAIRMENT ASSOCIATED WITH GREATER CARE INTENSITY DURING MEDICARE HOME HEALTH CARE

Julia Burgdorf,[1] and Kathy Bowles,[2] *1. Johns Hopkins Bloomberg School of Public Health, Baltimore, Maryland, United States, 2. University of Pennsylvania School of Nursing, Philadelphia, Pennsylvania, United States*

Medicare beneficiaries with cognitive impairment are more likely to access home health care than those without such impairment, and an estimated 1 in 3 Medicare home health patients has diagnosed dementia. However, recent changes to the Medicare home health payment system do not adjust for patients' cognitive impairment. To the extent that cognitive impairment prompts higher intensity care, this could create a financial disincentive for providers serving this patient population. We draw on a nationally representative sample of 1,214 (weighted n=5,856,333) community-living Medicare beneficiaries who received home health care between 2011-2016. We measure care intensity by the number and type of visits received during an index home health care episode. We model care intensity as a function of patient cognitive impairment during the episode, measured via clinician reports in standardized patient assessment data. In propensity score adjusted, multivariable models holding all covariates at their means, home health patients with identified cognitive impairment received a significantly greater number of visits. During the index home health episode, cognitively impaired patients received an additional 2.82 total visits (95% CI: 1.32-4.31; p<0.001), 1.39 nursing visits (95% CI: 0.49-2.29; p=0.003), 0.72 physical therapy visits (95% CI: 0.06-1.39; p=0.03), and 0.60 occupational therapy visits (95% CI: 0.15-1.05; p=0.01). Findings suggest that recent changes to Medicare home health care reimbursement do not reflect the more intensive care needs of patients with cognitive impairment, and may threaten access to care for these individuals.

### RISK FACTORS FOR COGNITIVE DECLINE IN OLDER ADULTS IN PUERTO RICO: ASSESSING BIAS FROM SAMPLE ATTRITION

Brian Downer,[1] Caitlin Pope,[2] Tyler Bell,[3] Sadaf Milani,[1] Ross Andel,[4] and Michael Crowe,[5], *1. University of Texas Medical Branch, Galveston, Texas, United States, 2. University of Kentucky, Lexington, Kentucky, United States, 3. University of California San Diego, La Jolla, California, United States, 4. University of South Florida, Tampa, Florida, United States, 5. University of Alabama at Birmingham, Birmingham, Alabama, United States*

Many risk factors for cognitive decline are associated with mortality and are common among older adults who cannot complete a survey interview. Our objective was to compare analyses of risk factors for cognitive decline among older adults in Puerto Rico with and without accounting for sample attrition. Data came from the Puerto Rican Elderly: Health Conditions Study. Our sample included 3,437 participants interviewed in 2002/03. Cognitive function was measured using the Mini-Mental Caban (MMC). The outcome was the change in MMC score between 2002/03 and 2006/07. Logistic regression was used to estimate inverse probability weights for being interviewed in 2006/07 (n=3,028) and completing the MMC at follow-up (n=2,601). Linear regression models were used to assess the association between stroke, hypertension, diabetes, smoking status, and cognitive decline with and without the IPWs. In the unweighted analysis, stroke was associated with a significantly greater decline in cognition (b=-0.62, standard error [SE]=0.30, p=0.04). Hypertension (b=-0.02, SE=0.12, p=0.84), diabetes (b=-0.22, SE=0.13, p=0.10) and being a current (b=0.05, SE=0.22, p=0.84) or former smoker (b=0.05, SE=0.14, 0.74) were not associated with cognitive decline in the unweighted analysis. The results were similar when including the IPW for mortality (stroke b=-0.63; hypertension b=-0.03; diabetes: b=-0.20; current smoker: b=0.08; former smoker: b=0.07) and having completed the MMC at follow-up (stroke b=-0.58; hypertension b=-0.03; diabetes: b=-0.20; current smoker: b=0.03; former smoker: b=0.09). These findings indicate that stroke is a risk factor for cognitive decline among older Puerto Rican adults even after accounting for selective attrition.

### THE UTILITY OF THE WHO INTRINSIC CAPACITY SCREENING TOOL TO IDENTIFY PHYSICAL AND MENTAL FUNCTION DECLINES

Lina Ma, Yaxin Zhang, Pan Liu, and Yun Li, *Xuanwu Hospital, Capital Medical University, National Research Center for Geriatric Medicine, Beijing, Beijing, China (People's Republic)*

**Background:** The disease concept is increasingly being replaced by a functional approach to address the healthcare needs of the older people. WHO proposed the Integrated Care for Older People (ICOPE) screening tool to identify older people with priority conditions associated with declines in intrinsic capacity (IC). Very few evidence on the clinical utility of the ICOPE tool is available.

**Objectives:** To determine if the tool can identify adults with poor physical and mental function. Method: 376 participants aged 50–97 years were included. IC was assessed with the WHO ICOPE screening tool, covering the following five domains: cognitive decline, limited mobility, malnutrition, sensory loss, and depressive symptoms. We assessed the activities of daily living, the Fried frailty phenotype, FRAIL scale, SARC-F scale, MMSE, GDS, social frailty, and quality of life. Peak expiratory flow, bones mineral density, body composition were obtained.

**Results:** 69.1% of the participants showed declines in IC. Participants with declines in IC were older, had more chronic diseases, worse general health, worse physical function as indicated by lower Barthel index, walk speed, grip strength, and physical fatigue, worse mental function indicated by lower MMSE scores, higher GDS scores, more mental fatigue, and worse social function. After adjusting for age, IC was positively correlated with walking speed, resilience score, and MMSE score and negatively correlated with frailty, SARC-F score, IADL score, GDS score, and physical and mental fatigue.

**Conclusion:** The WHO ICOPE screening tool is useful to identify adults with poor physical and mental function in Chinese older adults.

### Session 2205 (Symposium)

### COVID VACCINE ROLLOUT FOR OLDER PEOPLE: EAST MEETS WEST

Chair: Nengliang Yao Co-Chair: Tom Cornwell Discussant: Cheryl Camillo

Older adults should be one of the first groups to receive COVID-19 vaccines, because the risk of dying from COVID-19 increases with age. However, it takes time to distribute the vaccines to different countries, and the challenges in administering vaccines may differ by health system characteristics and local culture. This international symposium will discuss the vaccine rollout issues in eight countries (Isreal, Japan, South Korea, China, France, United Kingdom, Canada, and United States). We will use an interview and dialog format, instead of presentations. We will cover extensive topics including: Availability - What vaccines? Access, Acceptance, Caregivers – How are providers responding/handling caregivers wanting to be vaccinated?Cost/Financing Issues, Distribution Logistics/Transport/Safety, Lessons Learned, Mutations/Variants, Partnerships needed to vaccinate homebound patients (community partners; home health agencies, etc.), Who can/should provide vaccination? The situation with COVID-19 is still very fluid. Countries are at different stages of vaccinating older people. The chair didn't ask the speakers to write an abstract now, instead, the speakers will collect more information during the next few months and plan to have a prep meeting one month before the Annual Meeting.

### COVID VACCINE ROLLOUT FOR OLDER ADULTS IN JAPAN

Tadashi Wada, *Irahara Primary Care Hospital, Chiba, Chiba, Japan*

It has just started in Japan. We will provide detailed information later. The symposium has experts from 8 countries. We will use an interview and dialog format, instead of presentations.

### COVID VACCINE ROLLOUT FOR OLDER ADULTS IN SOUTH KOREA

Chan Mi Park, *Asan Medical Center, Songpa-gu, Seoul-t'ukpyolsi, Republic of Korea*

It has just started in South Korea. We will provide detailed information later. The symposium has experts from 8 countries. We will use an interview and dialog format, instead of presentations.

### COVID VACCINE ROLLOUT FOR OLDER ADULTS IN ISRAEL

Naim Mahroum, *Sabar Health, Even Yehuda, Tel Aviv, Israel*

The COVID vaccine rollout in Israel has prioritized older adults. It led to a substantial decline in the incidence of COVID-19 in older adults. The new variants are threats to the current achievements. We will provide detailed information later. The symposium has experts from 8 countries. We will use an interview and dialog format, instead of presentations.

### COVID VACCINE ROLLOUT FOR OLDER ADULTS IN FRANCE

Matthieu De Stampa, *Assistance Publique - Hôpitaux de Paris, Paris, Ile-de-France, France*

Only about three million people in France have so far received at least one dose of a Covid-19 vaccine. Those aged over 75 are offered either Pfizer or Moderna vaccines in a vaccination center. Older people with pre-existing conditions can now get AstraZeneca's Covid-19 vaccine. We will provide detailed information later. The symposium has experts from 8 countries. We will use an interview and dialog format, instead of presentations (please refer to the program overview).

### COVID VACCINE ROLLOUT FOR OLDER ADULTS IN THE UNITED KINGDOM

Huajie Jin, *King's College London, London, England, United Kingdom*

As of early March, at least 22 million adults had received one dose of a Covid vaccine in the UK, with 1.2 million of those fully vaccinated with two shots. Anybody aged 56 and over can book an appointment to get the Covid-19 vaccine. We will provide detailed information later. The symposium has experts from 8 countries. We will use an interview and dialog format, instead of presentations.

### Session 2210 (Symposium)

### DISCRIMINATION, STRESS, AND HEALTH ACROSS THE LIFE COURSE

Chair: Roland Thorpe, Jr. Discussant: Carl V. Hill

There is a paucity of research that seeks to understand why race disparities in health across the life course remain elusive. Two such explanations that have been garnering attention is stress and discrimination. This symposium contains papers seeking to address the impact of discrimination or stress on African American health or health disparities across the life course. Brown and colleagues examine the differential effects of chronic stress exposure by means of latent class analysis on mental and physical health in the HRS. Analysis revealed four subgroups, each demonstrated a typological response pattern with the most pronounced health consequences for high stress exposure, appraisal and few or no coping mechanisms. This suggests an alternative approach to examining the stress-health link by using a combined person- and variable-centered approach. Thomas Tobin and colleagues evaluate the life course processes through which early life racial discrimination (ELRD) and racial centrality shape adult allostatic load (AL) among older Blacks in the Nashville Stress and Health Study. Findings indicate that racial centrality is protective against adult high AL for those who experienced racial discrimination as children or adolescents. Cobb and colleagues examine how multiple attributed reasons for everyday discrimination relates to all-cause mortality risk among older Blacks in HRS. The authors report the 3 or more attributed reasons for everyday discrimination is a particularly salient risk factor for mortality in later life. This collection of papers provides insights into how discrimination or stress impacts African American health or health disparities in middle to late life.

### STRESS IS A LATENT CONSTRUCT: EXPLORING THE DIFFERENTIAL EXPERIENCE OF STRESS AMONG BLACK OLDER ADULTS

Catherine Garcia,[1] Alexis Reeves,[2] John Pamplin,[3] Uchechi Mitchell,[4] and Lauren Brown,[5] *1. University of Nebraska - Lincoln, Lincoln, Nebraska, United States, 2. University of Michigan, Ann Arbor, Michigan, United States, 3. NYU Center for Urban Science and Progress, Brooklyn, New York, United States, 4. University of Illinois Chicago, School of Public Health, Chicago, Illinois, United States, 5. San Diego State University School of Public Health, San Diego State University School of Public Health, California, United States*

While evidence highlights the detrimental health consequences of stress exposure for Black Americans, the impact of stress exposure on health varies by the stressor, individual appraisal and coping mechanisms examined. In this study, we aim to explore the differential effects of chronic stress exposure by means of latent class analysis on mental and physical health. Data come from 800 Black older adults ages 52+ from the 2006 Health and Retirement Study. A set of items that include stress exposure, appraisal and coping were used to assess chronic stress burden on anxiety, depressive symptoms and chronic conditions to identify stress and health clusters. Analysis revealed four subgroups, each demonstrated a typological response pattern with the most pronounced health consequences for high stress exposure, appraisal and few or no coping mechanisms. Results show an alternative approach to examining the stress-health link by using a combined person- and variable-centered approach.

### EARLY-LIFE RACIAL DISCRIMINATION, RACIAL CENTRALITY, AND ADULT ALLOSTATIC LOAD AMONG AFRICAN AMERICAN OLDER ADULTS

Angela Gutierrez,[1] Roland Thorpe, Jr.,[2] and Courtney Thomas Tobin,[3] *1. Edward R. Roybal Institute on Aging, Los Angeles, California, United States, 2. Johns Hopkins Bloomberg School of Public Health, baltimore, Maryland, United States, 3. Fielding School of Public Health, University of California Los Angeles, Fielding School of Public Health UCLA, California, United States*

This study evaluated the life course processes through which early life racial discrimination (ELRD) and racial centrality (i.e., the importance of Black identity to one's sense of self) interact to shape allostatic load (AL) among African American (AA) adults aged 50+ in the Nashville Stress and Health Study (N=260). Adolescent ELRD was associated with greater racial centrality in adulthood and conferred 35% greater risk of high adult AL; greater centrality was also linked to high adult AL. Centrality accounts for 24% of the association between ELRD and AL. ELRD and centrality interact to shape adult AL, such that racial centrality is protective against high AL for adults who experienced racial discrimination as children or adolescents. Findings highlight the multiple pathways through which race-related stressors and psychosocial resources interact to shape physiological dysregulation in later life and underscore the health significance of racial identity for older AAs.

### NUMBER OF ATTRIBUTED REASONS FOR EVERYDAY DISCRIMINATION AND MORTALITY AMONG OLDER BLACKS

Ryon Cobb, *University of Georgia, Athens, Georgia, United States*

To date, little is known about the significance of the number of attributions for everyday discrimination on all-cause mortality risk among older Blacks. Data are from a subsample of older Black respondents in the Health and Retirement Study (HRS), a nationally representative panel study of adults above the age of 50 in the 2006/2008 HRS waves, respondents completed a battery of questions on experience with psychosocial stressors, which included the number of attributed reasons for everyday discrimination. Vital status was obtained from the National Death Index and reports from key household informants (spanning 2006–2016). Cox proportional hazard models were used to estimate the risk of mortality. During the 10-year observation period, 450 deaths occurred. A higher number of attributed reasons for everyday discrimination was associated with a higher likelihood of death after adjusting for demographic characteristics and remained significant after further adjustments for other psychosocial, health, behavioral, and economic covariates.

### Session 2215 (Paper)

### EDUCATION AND TRAINING IN TOPICS ON AGING

### DEPLOYING IMPLEMENTATION STRATEGIES TO FACILITATE PROFESSIONALS' USE OF THE HOME CARE FRAILTY SCALE

Lisa Juckett,[1] Haley Oliver,[1] Leah Bunck,[2] Crystal Kurzen,[2] Andrea Devier,[2] and Fannisha Page,[2] *1. The Ohio State University, Columbus, Ohio, United States, 2. LifeCare Alliance, Columbus, Ohio, United States*

Home- and community-based service (HCBS) organizations play an instrumental role in maximizing the independence of older adults, ages 60 and over. HCBS clients typically have multiple health complications, placing them at great risk of frailty—a complex condition associated with health decline and institutionalization. However, despite their frequent contact with older adults, HCBS professionals are not required to assess the frailty levels of their clients, creating a missed opportunity to monitor the needs of this at-risk population. The purpose of this quality improvement study was to test a package of five implementation strategies designed to support HCBS professionals' use of the evidence-based Home Care Frailty Scale (i.e., Frailty Scale) with all new clients at one large HCBS organization. Implementation strategies included (a) selecting one professional to serve as the organization's Frailty Scale "champion," (b) holding three training sessions with 25 HCBS professionals, (c) modifying client charts to allow professionals to document Frailty Scale results, (d) pilot testing the Frailty Scale with a small group of clients, and (e) completing monthly chart audits to monitor rates of Frailty Scale implementation. During the first three months of Frailty Scale use, HCBS professionals administered the Frailty Scale to 414 out of 467 eligible clients (88.6%). For Month 1, 87.4% of eligible clients were administered the Frailty Scale, followed by 90.8% in Month 2, and 85.6% in Month 3. This quality improvement study suggests that a multifaceted package of implementation strategies can support professionals' use of an evidence-based frailty instrument in the HCBS setting.

### LESSONS ON LEARNING IN LATER LIFE WITH THE MIT AGELAB 85+ LIFESTYLE LEADERS

Taylor Patskanick,[1] Julie Miller,[2] Lucas Yoquinto,[2] Lisa D'Ambrosio,[3] and Joseph Coughlin,[2] *1. MIT, Somerville, Massachusetts, United States, 2. MIT, Cambridge, Massachusetts, United States, 3. Massachusetts Institute of Technology, Cambridge, Massachusetts, United States*

Previous research has established the role of lifelong learning in promoting psychological wellbeing and active aging. Population aging necessitates an understanding of the unique opportunities and challenges around formal and informal learning in later life. This paper will share findings from a mixed methods study with the MIT AgeLab 85+ Lifestyle Leaders, a panel comprised of octogenarians and nonagenarians from across the United States. Drawing on an online survey and virtual focus groups with 29 Lifestyle Leaders from January 2021, findings suggest the Lifestyle Leaders most often learned new things from talking with others (46%) and reading print (54%) or online (54%) sources. The majority were familiar with attending in-person lectures or classes (89.7%) and were now using videoconferencing to do these (78.6%). A majority (56.7%) had or are currently participating in a lifelong learning program. Most consider themselves lifelong learners and described this around remaining curious and engaged with life, choices around what one learns, and greater enjoyment of learning. In the survey, a plurality of Lifestyle Leaders indicated the top two challenges affecting their ability to learn were sensory burdens (e.g., hearing loss, declining eyesight) (35%) and their energy level (32.4%); focus group data revealed that recall also is a barrier. Focus group data further highlighted generational experiences around early life learning and career paths, specifically how gender roles, diagnoses of learning disabilities, and evolving digital technology have affected these and changed over the course of their lifetimes.

### RESOLVING DILEMMAS IN ICT-ENHANCED INTERVENTIONS: A CROSS-PLATFORM-MEDIATED STRATEGY

Dara KY Leung, Frankie HC Wong, Gloria HY Wong, and Terry YS Lum, *The University of Hong Kong, Hong Kong, Not Applicable, Hong Kong*

Face-to-face interventions in social care settings are severely disrupted under COVID-19. Previous studies support Information and communication technology (ICT) enhanced intervention as an effective alternative. While difficulties older adults experienced in using ICT were examined extensively, there are fewer discussions on how innate medium characteristics of the delivery mode influence therapeutic interactions. This study explored these embedded challenges in ICT-enhanced psychosocial interventions and possible solutions. We conducted on-site observations and focus groups with 12 participants from two teleconferencing-aided intervention groups for chronic pain with exercise and psychotherapy elements. Observation notes and transcriptions of focus groups recordings were analyzed using thematic analysis. We identified three overarching themes: empowerment, dilemmas, and cross-platform mediated strategy. ICT empowered participants by promoting autonomy and self-management, yet two dilemmas that stemmed from technological affordances undermined the quality of communication. A screen-camera dilemma occurred when participants tried to observe instructions from interventionists while demonstrating their posture in front of the camera for guidance. The blurring boundary between therapy and home settings presented another dilemma. Although teleconferencing increased flexibility and comfort in participation, interruptions from the background environment and intersections of family living spaces disrupted audio-visual communication and jeopardized the sense of security. As a solution, interventionists adopted a cross-platform mediated strategy to bypass the dilemmas. They communicated and delivered supplementary materials through different media, including printed materials and video streamings. Interventionists could consider the unique structural features in different media and the potential impact of participants' sociodemographic factors, especially those associated with digital literacy.

### RETURN MIGRATION AS AN ASSET MAXIMIZATION STRATEGY IN RETIREMENT

Mara Sheftel, *Penn State University, Brooklyn, New York, United States*

Mexican immigrants make up an increasing proportion of the US population 65 and older. Estimating outcomes for this population is complicated by return migration. Due to data limitations, theoretical frameworks and empirical evidence fail to provide clear indication of the economic selection mechanism of return migration, especially at older ages making it difficult to estimate economic determinants of return. Here two waves of data from the US based Health and Retirement Study and the Mexican Health and Aging Study are combined to create a novel dataset that enables a comparison of assets at older ages for those who stay in the US, those who return before age 50 and those who return at 50 and older. Unadjusted results show no difference in total net wealth at older ages between the three groups, with higher business assets among returnees and higher concentration of wealth in home equity among stayers. With evidence of higher inequality among stayers, lower median wealth in Mexico, and asset advantages operating through citizenship, older age return can be interpreted as a means to acquire a higher standard of living in retirement for non-citizen immigrants. Comparing assets between 2000 and 2012 reveal the vulnerability of stayers during the US housing crisis. These findings are novel because they point to return migration as a retirement strategy and expose a source of vulnerability among those Mexican immigrants who remain in the US into older ages.

### USING DOCUMENTARY SHORT FILM FOR HEALTH IMPACT: AN EXAMPLE FOR SUPPORTING FAMILY CAREGIVERS

Janice Bell,[1] and Jessica Zitter,[2] *1. University of California, Davis, Sacramento, California, United States, 2. Highland Hospital, Oakland, California, United States*

Storytelling through film is a powerful tool with potential to improve understanding, spark discussion, shape perceptions of health and illness, and influence related behavior. We developed a film discussion guide for the documentary short film Caregiver: A Love Story. The 24-minute film follows the experience of a man who leaves his job to become the primary caregiver of his 59 year-old wife, who opts out of non-beneficial chemotherapy, choosing instead to remain at home with hospice support. The 2-hour program was facilitated by an experienced social worker and offered on five different days/times using Zoom video (n=60 total attendees; 9-15/ session). At each session, we showed the film, discussed self-care and caregiver resources, and fielded a survey to assess satisfaction, format acceptability and session impact (response rates 67-100%/session). Attendees liked the session format (90%); found the film relevant to their situations (80%); intended to look for new professional resources (79%); were motivated to do something different(71%); learned something new (64%); and intended to ask for more help from family or friends (64%). Many commented that they preferred the video meeting format over in-person meetings. The film viewing and discussion format is acceptable and accessible to family caregivers who may otherwise not be able to attend given competing demands. This format also has potential to improve support access to resources. Extensions to this work are planned to tailor the film discussion guide for health care providers and students working with family caregivers across inpatient, outpatient and hospice settings.

### Session 2220 (Symposium)

### EFFECTS OF AGING ATTITUDES ON DEVELOPMENT IN OLD AGE

Chair: Maria Clara P de Paula Couto Co-Chair: Klaus Rothermund

This session will focus on aging attitudes and their effects on different aspects of development in old age (e.g., preparation, age stereotypes, age discrimination, and well-being). Cultural differences and how they shape individual aging are also explored. The first two presentations focus on cross-cultural differences in preparation for old-age. Nikitin et al. examine financial preparation and how expectations about support from the state influence it. People's beliefs about the utility and the risk of aging preparation and their role in preparatory activities is investigated by Kim-Knauss et al. Tsang et al. explore age differences in pursuing autonomy and independence during the COVID-19 pandemic and the role of perceived social obligation. Cultural differences in the accuracy between perceived retrospective changes in well-being and actual changes is explored by Park et al. The last presentation (de Paula Couto et al.) focuses on country- and age-related differences in personal experiences of age discrimination in different life domains. Taken together, findings suggest that attitudes toward, and preparation for aging, are not static. Situational contexts and personal assessments of the contexts can shape such attitudes and behavior.

### PERSONAL EXPERIENCES OF AGE DISCRIMINATION IN DIFFERENT LIFE DOMAINS: DETERMINANTS AND CONSEQUENCES

Jana Nikitin,[1] Sylvie Graf,[2] Klaus Rothermund,[3] and Maria Clara P de Paula Couto,[3] *1. University of Vienna, Vienna, Wien, Austria, 2. University of Bern, Bern, Bern, Switzerland, 3. Friedrich-Schiller University Jena, Jena, Thuringen, Germany*

Age discrimination is pervasive in society which bears far-reaching consequences for individuals in terms of decreased psychological and physical health. Age discrimination can be experienced in different life-domains and perceived as a social (others' experiences) or as a personal phenomenon (own experiences). Our first goal was to examine country- and age-related differences in personal experiences of age discrimination in distinct life domains, reported by 2,817 participants aged 40 to 90 years from the US, China, Germany, the Czech Republic, and Taiwan. As another goal, we investigated the impact of age discrimination on life satisfaction. Personal age discrimination was domain-specific, with more experiences reported in the family, work, and personality domains. Personal age discrimination increased with age and was higher in China and Taiwan. Age discrimination negatively predicted life satisfaction. This negative effect was more pronounced if age discrimination was experienced in domains with high subjective importance.

### COUNTRY DIFFERENCES IN FINANCIAL PREPARATION: WHAT DO PEOPLE EXPECT FROM THE STATE?

Maria Clara P de Paula Couto,[1] Sylvie Graf,[2] Klaus Rothermund,[1] and Jana Nikitin,[3] *1. Friedrich-Schiller University Jena, Jena, Thuringen, Germany, 2. University of Bern, Bern, Bern, Switzerland, 3. University of Vienna, Vienna, Wien, Austria*

A considerable gap between one's pension and living expenses in old age exists in almost all developed countries, making savings and financial preparation for old age inevitable. Nevertheless, financial preparation for old age substantially differs across countries. Using the data from the AAF project, we investigated what motivates people in different countries (USA, Germany, Czech Republic, Hong Kong, and Taiwan) to financially prepare for old age. Financial preparation was the highest in the USA, followed by Germany and the Czech Republic. The lowest levels of financial preparation were found in Hong Kong and Taiwan. These differences were explained by age-related expectations on a "paternalistic" role of the state: Greater endorsement of the idea that the state should provide financial support to older citizens led to less preparation. These findings are in line with the idea that individuals' beliefs and expectations regarding the role of institutions shape personal actions.

### ACTION-RELATED THOUGHTS AND BELIEFS REGULATE THE EFFECT OF AGE STEREOTYPES ON AGING PREPARATION

Frieder Lang,[1] Helene Fung,[2] Dwight Tse,[3] and Yaeji Kim-Knauss,[4] *1. Institute of psychogerontology, University of Erlangen-Nuremberg, Nuremberg, Bayern, Germany, 2. The Chinese University of Hong Kong, Shatin, N.T., Hong Kong, Not Applicable, Hong Kong, 3. School of Psychological Sciences and Health, University of Strathclyde, Glasgow, Scotland, United Kingdom, 4. University of Erlangen-Nuremberg, Nuremberg, Bayern, Germany*

Thinking about old age stereotypically affects one's engagement in age-related behaviors and developmental regulation. We hypothesized that positive or negative aging stereotype (AS) would be associated with more or less aging preparation, while action-related thoughts and beliefs might exert influence thereon. We used the AAF online-study dataset consisting of 591 German, 348 Chinese, and 139 American adults (aged 18–93 and 55% female). Using a count measure of 15-preparatory-activities, we first explored the role of AS measured by a bipolar scale and how perceived utility and risk of aging preparation differentiate this association. Findings revealed that perceiving more utility buffered the impact of negative AS, which suggests that one's action-related thoughts are more proximal and self-relevant predictor of aging preparation. Besides, Chinese and Americans were more susceptible to the presence of AS than Germans, implying that cultural background or societal conditions might also shape one's belief system and thereby regulate behaviors.

### OLDER ADULTS PURSUE MORE AUTONOMY DURING PANDEMIC: AN EXPLANATION BY SOCIAL OBLIGATION

Chunyan Mai,[1] Helene Fung,[2] and Hiu Ling Vivian Tsang,[3] *1. The Chinese University of Hong Kong, Hong Kong, Not Applicable, Hong Kong, 2. The Chinese University of Hong Kong, Shatin, N.T., Hong Kong, Not Applicable, Hong Kong, 3. Hong Kong Metropolitan University, The Chinese University of Hong Kong, Hong Kong*

Older adults are considered more vulnerable under the COVID-19 pandemic. Nevertheless, the pandemic also highlights the social obligation of all individuals, young and old. We investigated whether older adults pursued more autonomy during the pandemic than did middle-aged adults, and the moderating effect of perceived social obligation. One hundred and twenty-three Hong Kong citizens (62 females, Mage=60.59±13.28 years old) participated in this study in 2018 (before pandemic) and 2020 (during pandemic). Comparing these two waves, the results showed a larger increase of perceived importance of independence and autonomy among older adults than among middle-aged adults. Moreover, the age difference became stronger with a higher increase in expectation on social obligation, suggesting that the pandemic might make older adults feel more socially obligated to be independent and autonomous, so as not to be a burden on others. Future ageism-related studies should take the social obligation of older adults into consideration.

### RETROSPECTIVE PERCEPTIONS OF CHANGES IN WELL-BEING: THE IMPACT OF AGE, CULTURE, AND AGING ATTITUDES ON ACCURACY

Allura Lothary,[1] Thomas Hess,[2] and Jeongsoo Park,[3] *1. University of Illinois Urbana-Champaign, Urbana, Illinois, United States, 2. North Carolina State University, Raleigh, North Carolina, United States, 3. Ajou University, Suwon-si, Kyonggi-do, Republic of Korea*

Aging attitudes have important consequences on functioning in later-life. A critical question concerns whether such attitudes may bias perceptions of one's own aging, with potentially negative effects on important outcomes. Using data from adults aged 30 – 85 in the US (n=315), Hong Kong (n=317), and Germany (n=623), we examined the impact of age and aging attitudes on accuracy of perceptions of change in well-being over five years in different domains of functioning. Across contexts, comparisons revealed good correspondence between retrospective reports and actual change. However, older adults and those with negative attitudes retrospectively reported less positive change over this period. Accuracy of perceived change was affected by aging attitudes, with positive attitudes being associated with greater accuracy across most domains, although culture moderated these effects. The results highlight the complex relationship between culture and perceptions of well-being, as well as the potentially insidious effects of attitudes on their accuracy.

### Session 2225 (Symposium)

### ENHANCE: THE CENTER FOR ENHANCING NEUROCOGNITIVE HEALTH, ABILITIES, NETWORKS, & COMMUNITY ENGAGEMENT

Chair: Sara Czaja Co-Chair: Walter Boot Discussant: Michelle Bourgeois

Approximately 9 % of those aged 65 and over have a cognitive impairment due to a variety of causes including Alzheimer's disease and other forms of dementia, Mild Cognitive Impairment (MCI), Parkinson's disease, traumatic brain injury (TBI), and stroke. Few technology solutions have been directed towards supporting older adults with cognitive impairments and the literature regarding the efficacy of these solutions is sparse. In this symposium, we describe our new Center called ENHANCE (Enhancing Neurocognitive Health, Abilities, Networks, and Community Engagement), which is focused on developing technology support for aging adults with a cognitive impairment due to MCI, TBI, and Stroke. Sara Czaja will provide an overview of the conceptual framework, goals, and structure of ENHANCE, and describe the STRUMM project, that focuses on the design and evaluation of an innovative intelligent adaptive software package aimed at providing cognitive and social support to aging adults with cognitive impairments. Wendy Rogers will discuss the ENACT project, a longitudinal needs assessment focusing on understanding the needs, challenges, and support preferences of our target population and informal caregivers. Neil Charness will describe the AUGMENT development project, which is concerned with developing an instructional support tool for mobility activities, such as wayfinding, locating, and using transportation services. Finally, Walter Boot will discuss the DREAM development project, which is focused on developing a technology-based cognitive aid to support prospective memory activities. Michelle Bourgeois will serve as the discussant for the symposium and highlight the unique opportunities and challenges associated with ENHANCE.

### STRUMM: AN INTELLIGENT, ADAPTIVE SOFTWARE PACKAGE FOR OLDER ADULTS WITH A COGNITIVE IMPAIRMENT

Sara Czaja,[1] Marco Ceruso,[2] Walter Boot,[3] Neil Charness,[3] and Wendy Rogers,[4] *1. Weill Cornell Medicine/Center on Aging and Behavioral Research, New York, New York, United States, 2. Weill Cornell Med, New York, New York, United States, 3. Florida State University, Tallahassee, Florida, United States, 4. University of Illinois Urbana-Champaign, Champaign, Illinois, United States*

Many older adults have a cognitive impairment (CI), which negatively impacts on their quality of life and threatens their independence. In this presentation, we provide an overview of the conceptual framework, structure, and processes of our multi-site Center, ENHANCE, which is focused on developing technology support for aging adults with a CI. ENHANCE has two cross-site research projects, two cross-site development projects, training, and dissemination components. A core battery of measures is collected across all projects. We also discuss the Supportive Technology Resources through Usability & Machine-learning Methods (STRUMM) research project, which focuses on an innovative intelligent adaptive software package aimed at providing cognitive and social support, and support for resource access to aging adults with a CI. STRUMM is designed to meet the user's varying cognitive needs. Finally, we present preliminary data regarding the perceived usability and value of STRUMM from our clinical partners and potential user groups.

### EVERYDAY NEEDS ASSESSMENT FOR COGNITIVE TASKS: CHALLENGES FOR PERSONS WITH COGNITIVE IMPAIRMENT

Raksha Mudar,[1] Maurita Harris,[2] Elizabeth Lydon,[3] Widya Ramadhani,[3] Sara Czaja,[4] Walter Boot,[5] Neil Charness,[5] and Wendy Rogers,[3], *1. University of Illinois-Urbana Champaign, Champaign, Illinois, United States, 2. University of Illinois at Urbana Champaign, Champaign, Illinois, United States, 3. University of Illinois Urbana-Champaign, Champaign, Illinois, United States, 4. Weill Cornell Medicine/Center on Aging and Behavioral Research, New York, New York, United States, 5. Florida State University, Tallahassee, Florida, United States*

ENACT (Everyday Needs Assessment for Cognitive Tasks) is an exploration and discovery project to gather information on challenges in daily and community living experienced by individuals aging with compromised cognition due to mild cognitive impairment, traumatic brain injury, or post-stroke. We are exploring their challenges through a longitudinal needs assessment study involving interviews with older adults with cognitive impairment and their care partners. We will describe the study development process wherein we interviewed subject matter experts, including persons with professional (neurology, rehabilitation, gerontology) or personal experience with individuals who have cognitive impairment. Based on their collective insights, we selected the following categories of activities for the ENACT in-depth interviews: health, social engagement, transportation, domestic life, and leisure/recreation. The ENACT longitudinal data will provide insights to guide development of adaptive, context-sensitive technology-based supports for the AUGMENT, DREAM, and STRUMM projects described in this symposium, as well as other initiatives.

### AUGMENT: NAVIGATION APPS INSTRUCTION FOR OLDER ADULTS WITH COGNITIVE IMPAIRMENT

Walter Boot,[1] Sara Czaja,[2] Wendy Rogers,[3] Nicholas Gray,[4] Dorota Kossowska-Kuhn,[5] Michael Pervratil,[5] and Neil Charness,[1] *1. Florida State University, Tallahassee, Florida, United States, 2. Weill Cornell Medicine/Center on Aging and Behavioral Research, New York, New York, United States, 3. University of Illinois Urbana-Champaign, Champaign, Illinois, United States, 4. Psychology Department, Florida State University, Florida, United States, 5. Psychology Department, Tallahassee, Florida, United States*

Augmenting User Geocordinates and Mobility by ENhanced Tutorials (AUGMENT) is a development project in the ENHANCE Rehabilitation Engineering Research Center aiming to promote community engagement for aging adults with cognitive impairment (CI) from stroke, traumatic brain injury, and mild cognitive impairment. AUGMENT aims include 1) providing proof of concept that a robust instructional package can support successful use of existing, complex navigation apps, Google maps and rideshare app Uber, by a diverse set of people with CI; and 2) providing proof of product by testing performance with and without instruction. We discuss the needs assessment phase and development of new tests to assess wayfinding abilities and reported difficulties with navigation, using a control sample of 384 community-dwelling older adults. We found that self-reported navigation difficulties are predicted ($R$-square = .28) by gender, a spatial orientation test, self-reported memory ability, and severity of memory difficulty.

### DEVELOPMENT OF THE DREAM SYSTEM: DIGITAL REMINDERS FOR EVERYDAY ACTIVITY MEMORY

Neil Charness,[1] Sara Czaja,[2] Wendy Rogers,[3] Edie Sanders,[1] Robin Stuart,[1] Ronald Andringa,[1] and Walter Boot,[1] *1. Florida State University, Tallahassee, Florida, United States, 2. Weill Cornell Medicine/Center*

*on Aging and Behavioral Research, New York, New York, United States, 3. University of Illinois Urbana-Champaign, Champaign, Illinois, United States*

Prospective memory, the ability to remember to execute an intention in the future, is crucial for the performance of many everyday tasks important for independent living. Prospective memory abilities decline with age, and older adults living with mild cognitive impairment (MCI), cognitive impairment due to traumatic brain injury (TBI), and cognitive impairment due to stroke are especially susceptible to prospective memory failures. The goal of the Digital Reminders for Everyday Activity Memory (DREAM) project is first to establish proof of concept for an adaptive cognitive aid to support the prospective memory of older adults with various cognitive impairments, and then establish proof of product in studies examining the use of a working prototype within the lab and within participants' homes. Data will be presented from initial work verifying product requirements through engagement with stakeholders, including subject matter experts, older adults with cognitive impairments, and their care partners.

### Session 2230 (Symposium)

### ESPO AND ACADEMY FOR GERONTOLOGY IN HIGHER EDUCATION SECTION SYMPOSIUM: A NEW NORMAL IN TEACHING? INCORPORATING UNCONVENTIONAL AND CREATIVE IDEAS INTO GERONTOLOGY CURRICULUM

Chair: Lauren Bouchard Co-Chair: Yan-Jhu Su Discussant: Marilyn Gugliucci

This symposium is intended to highlight novel, applied examples and classroom activities in gerontology curriculum. In accordance with the AGHE gerontological education competencies, these authors will provide insightful and fun connections to arts/humanities, popular culture, technology, and current events to inspire conversation, interest, self-reflection, and empathy in the classroom. The first author will discuss social media (e.g., TikTok) as a segue to difficult classroom conversations regarding negative stereotypes and ageism in society. Presenter two will discuss cross-field educational connections between music education and gerontology. Next, presenter three will put present a unique activity regarding technology, homeownership, and retirement with a competitive flair. Presenter four utilizes documentary to encourage empathy in nursing. Finally, presenter five will present a timely class debate regarding United States political office and ageism that is sure to create lively and relevant conversation.

### DECODING TIKTOK: UTILIZING SOCIAL MEDIA FOR DIFFICULT CONVERSATIONS ABOUT AGEISM

Lauren Bouchard, *Concordia University Chicago, Chicago, Illinois, United States*

Understanding ageism is a key aspect of gerontological curriculum. Media examples (e.g., television and movies) can be effective tools, and yet gerontological educators should stay updated on new media trends to encourage student interest. This presentation will explore a new social media application (i.e., TikTok) to help students recognize and dismantle their own ageist beliefs. The presenter will describe and explain the classroom activity, instructions for finding and downloading content, as well as the social media application itself. In this activity, students brainstorm their preconceived notion of older adults to catalyze open discussion regarding societal beliefs. Next, a few video examples, with both positive and negative portrayals of older adults are presented for discussion. Students may also bring other examples for participation credit to this class. This symposium presentation will include an interview activity guide, additional breakout group instructions, and other tips for creating impactful class discussion on ageism.

### INTERDISCIPLINARY EDUCATION MODEL IN THE STUDY OF AGING: EXAMPLE AND COURSE DESIGN

Yan-Jhu Su, *University of Massachusetts Boston, Boston, Massachusetts, United States*

Collaboration among various disciplines is essential to the gerontology curriculum because it is a a new and comprehensive subject. This presentation will discuss the design of interdisciplinary courses to include practical applications in the study of aging. The presenter will share examples based on personal experience to illustrate how music and psychology may be applied to the study of aging. In addition, the presentation will include analysis of actual course designs to show how different fields can be integrated in the classroom setting. This symposium presentation intends to improve cross-discipline applications as well as help students contribute to and benefit from the study of aging.

### ASSESSING KNOWLEDGE, ATTITUDES, AND BELIEFS OF NURSES ABOUT LGBTQ OLDER ADULTS USING A DOCUMENTARY VIDEO

Suzanne Dutton, *Sibley Memorial Hospital/Johns Hopkins, washington, District of Columbia, United States*

Statistics reveal that lesbian, gay, bisexual, transgender, and queer (LGBTQ) older adults experience health disparities and barriers to accessing healthcare because of discrimination and fear of disclosing sexual orientation. Nurses receive limited education regarding care for LGBTQ older adults. This study exposed nurses to the documentary, Gen Silent, which details LGBTQ older adult experiences. The objective of the study was to increase participants' understanding of LGBTQ health disparities. A one-group pre-/post-test design was conducted to test the effect of the documentary on knowledge and attitudes about LGBTQ health. A total of 379 nurses participated in the study. A questionnaire including a 16-item standardized scale and an open-ended question asking how participants would change their practice was administered before and after the intervention. Findings revealed statistically significant increases in LGBTQ knowledge and inclusive attitudes. This research supports the use of a documentary as an educational method related to LGBTQ older adults.

### AGE LIMITATIONS FOR PRESIDENTIAL CANDIDATES? EXPLORING STUDENT PERSPECTIVES USING CLASSROOM DEBATES

Jason Garbarino, *University of Vermont, Burlington, Vermont, United States*

Ahead of the 2020 Presidential Election, Donald Trump (age 73) and his primary opponent, Joseph Biden (age 76) received extensive criticism regarding the aptness of their candidacies based upon their current ages. While the United States Constitution requires candidates to have "attained the age of thirty-five years", no age cap for presidential candidates exists. In response to timely public discussion, undergraduate interprofessional gerontology students worked in assigned groups to prepare to debate either in favor of, or in opposition to a constitutional amendment capping the age of presidential candidates. Following classroom debates, course faculty moderated in-depth conversation examining cogent arguments made throughout the debates. After attending this session, participants will understand the logistics of planning in-class debates, moderating post-debate student discussions, and evaluation methods of student debate performance and on a corresponding reflective writing assignment. Student and faculty takeaways and prospective classroom debate ideas will be provided.

### HOUSE HUNTERS, GERONTOLOGY STYLE: A UNIQUE CLASSROOM ACTIVITY FOR UNDERGRADUATES

Meghan McDarby, *Washington University, St. Louis, Missouri, United States*

Small group discussion activities that capitalize on students' interest in technology may generate enthusiasm for course content in gerontology. We describe a unique classroom activity that supports discussion about retirement issues in older adulthood by leveraging student dexterity in utilizing web applications. In this activity, students act as real estate agents for a retired older adult couple who is relocating to be closer to family. Students are presented with details about the couple, including demographic information (e.g., age, functional limitations, hobbies) and the couple's "wish list" for features and amenities of their future home. Then, students use these details to choose a home for the couple on Zillow and prepare a "pitch" of the home that is presented to the class and judged by the course instructor. Feedback from students suggests that this activity offers a "real world application to course material" and facilitates enthusiasm about course content.

### Session 2235 (Symposium)

### FACTORS AND FUNCTIONS ASSOCIATED WITH HEALTH AND WELL-BEING AMONG OLDER ADULTS: EVIDENCE FROM NHATS

Chair: Loretta Anderson Co-Chair: Alexandra Wennberg
Discussant: Allison Gibson

The National Health and Aging Trends Study (NHATS) is a nationally representative sample of Medicare beneficiaries aged 65 and older. From 2011 through 2020, annual in-person interviews have collected data in many areas, including health, environment, wellbeing, cognition, and function. With a decade of follow-up, including replenishment samples, NHATS is an ideal setting to investigate trends and trajectories of aging. Aging is heterogeneous and understanding the myriad of factors and functions that impact health and wellbeing is critical to developing interventions and care to promote health and wellbeing. Considering a multifactorial, wholistic approach to aging will provide a deeper understanding to create an impact. This symposium features pivotal research conducted using NHATS data, while highlighting overall strengths of the dataset for future research. The first presentation of this symposium investigates the factors that define cognitive profiles associated with dementia diagnosis over a period of five years. The second presentation investigates the role engagement in personally meaningful activities play in cognitive, emotional, functional, and health-related outcomes in older adults. The third presentation investigates the association between sleep medication use and fall risk among older adults with and without dementia. The session concludes with an investigation of end-of-life communication in persons with dementia and hearing impairment.

### COGNITIVE DECLINE OVER A 5-YEAR PERIOD: THE NATIONAL HEALTH AND AGING TRENDS STUDY

Sarah Holmes,[1] Virgina Richardson,[2] and Allison Gibson,[3] *1. University of Maryland School of Nursing, Baltimore, Maryland, United States, 2. The Ohio State University College of Social Work, Columbus, Ohio, United States, 3. University of Kentucky, University of Kentucky, Kentucky, United States*

The classification of Alzheimer's disease and related dementia (ADRD) is important for understanding the progression of cognitive decline. This longitudinal study used data from the National Health and Aging Trends Study (NHATS). A sample of 3,287 eligible Medicare beneficiaries were included in the study. Nine cognitive profiles were examined from Waves 1 to 5 (2011-2015). Discriminant factor analysis was used to identify factors that differentiated across the cognitive profiles. Results showed that 1,076 had some measure of "possible" or "probable" dementia over the 5 years. In Wave 1, there were 104 self-reported ADRD diagnoses, and in Wave 5, there were 327 self-reported ADRD diagnoses. Social participation was an important factor in those that impairment reversed from probable to possible ADRD. Findings support previous evidence that certain activities may slow or reverse cognitive decline and can inform future studies exploring the causality of dementia onset.

### FAVORITE ACTIVITY AND IMPLICATIONS FOR COGNITION, MENTAL HEALTH, AND FUNCTION IN PERSONS WITH AND WITHOUT DEMENTIA

Jeanine Parisi,[1] Nancy Perrin,[2] Laura Gitlin,[3] and Natalie Regier,[4] *1. Johns Hopkins Bloomberg School of Public Health, Baltimore, Maryland, United States, 2. Johns Hopkins University School of Nursing, Baltimore, Maryland, United States, 3. Drexel University, College of Nursing and Health Professions, Drexel University, Pennsylvania, United States, 4. School of Nursing, Johns Hopkins School of Nursing, Maryland, United States*

Little is known about the impact of engagement in personally meaningful activities for older adults. This study examines the impact of engagement in one's favorite activity on cognitive, emotional, functional, and health-related outcomes in older adults with and without dementia. Data were obtained from 1,397 persons living with dementia (PLWD) and 4,719 cognitively healthy persons (CHP) participating in wave 2 of the National Health and Aging Trends Study (NHATS). Sociodemographic characteristics were examined by cognitive status. A multivariate analysis of variance indicated that, for PLWD, engagement in favorite activity was associated with greater functional independence and decreased depression ($F(6,1201)=3.01$, $p<.01$, Wilks's $\Lambda=.985$, partial $\eta2=.015$). For CHP, engagement in favorite activity was associated with greater functional independence, decreased depression and anxiety, and better performance on memory measures ($F(6,4107)=11.46$, $p<.001$, partial $\eta2=.016$,). Findings suggest that engagement in personally meaningful activities may have significant and distinct benefits for persons with and without dementia.

### FALLS AMONG HIGH- AND LOW- FREQUENCY SLEEP MEDICATION USERS WITH AND WITHOUT DEMENTIA

Alexandra Wennberg,[1] and Loretta Anderson,[2]
*1. Karolinska Institutet, Stockholm, Stockholms Lan, Sweden, 2. University of Maryland School of Medicine, Reisterstown, Maryland, United States*

Difficulty with sleep and falls are prevalent among older adults. Sleep medication use is associated with falls in older adults, but little is known about its impact in older adults with dementia. We used data from the 2011 National Health and Aging Trends Study to assess the association of low- versus high- frequency sleep medication use with falls in older adults with self-reported dementia. In our fully adjusted model, among those with dementia, high-frequency sleep medication users were more likely to fall than low-frequency sleep medication users (OR=3.86, 95% CI: 1.31, 11.37). Among those without dementia, high-frequency sleep medication users were more likely to fall than low-frequency sleep medication users (OR=1.40, 95% CI: 1.11, 1.77). Reducing sleep medication use in older adults with and without dementia may help reduce the risk of falls and fall-related outcomes in older adults.

### HEARING LOSS AND NEUROPSYCHIATRIC SYMPTOMS IN PERSONS WITH DEMENTIA AT THE END OF LIFE

Natalie Regier,[1] and Carrie Nieman,[2] *1. School of Nursing, Johns Hopkins School of Nursing, Maryland, United States, 2. Johns Hopkins University School of Medicine, Baltimore, Maryland, United States*

Access to effective communication is critical to the conversations that occur at end-of-life and represents an unaddressed need within palliative care. These challenges may disproportionately affect persons with dementia (PWD). Hearing loss is one of the most common comorbidities among PWD and is independently associated with neuropsychiatric symptoms. However, relatively little is known about the potential impact of hearing loss on PWD at end-of-life. We examined last month of life (LML) data from 971 proxies of deceased PWD from the National Health and Aging Trends Study (2011-2020). Hearing difficulty was associated with increased anxiety/sadness in PWD, $\chi2(1)=4.596$, $p=.032$, such that 65.6% of persons with hearing difficulty reported anxiety/sadness in the LML. Binary logistic regression found that hearing difficulty was significantly associated with increased anxiety/sadness (OR=1.40, 95% C.I. 1.00 – 1.80, $p < 0.05$). Interventions that optimize communication for PWD may be a meaningful approach to improving the end-of-life experience.

### Session 2240 (Symposium)

### HEALTH DISPARITIES IN COVID-19: IMPLICATIONS FOR RESEARCH, POLICY, AND PRACTICE

Chair: Chivon Mingo Co-Chair: Ronica Rooks

The rapid transmission of COVID-19 has resulted in more than 100 million confirmed cases in over 200 countries and continues to have wide-community spread. Consistently, it has been reported that older adults are at a greater risk for requiring hospitalization or dying from the virus compared to younger adults and children. In fact, compared to those age 18-29, age 65-74 are five times more likely to be hospitalized and 90 times more likely to experience death. The risk increases exponentially with age. Individuals 85 and older are 13 times more likely to require hospitalization and 630 times more likely to die from the disease. The physical health-age correlation has permeated the media and many discussions concerning the pandemic. However, fewer discussions have centered on the interaction of age and social variables that further exacerbate COVID-19 related burden or mortality such as race/ethnicity, socioeconomic status, and limited access to healthcare. Therefore, this symposium will bring direct attention to COVID-19 related health disparities that compromise public health, discuss implications on future research, policy, and practice, and discuss opportunities to reduce the burden and mitigate health inequities. The symposium presenters will specifically address the impact of social support during COVID-19, disparities in the effects of social distancing on health status, the economic impact on health, cognitive decline among low-income older adults navigating a global pandemic, and factors associated with higher rates of hospitalizations among racial/ethnic diverse older adults.

### RACIAL AND AGE GROUP DISPARITIES IN THE EFFECT OF SOCIAL DISTANCING ON HEALTH STATUS

Karen Lincoln, *University of Southern California, Los Angeles, California, United States*

African Americans are dying from COVID-19 at younger ages than Whites. Social distancing (SD) prevents the spread of the virus, but because of work demands, transportation needs, and living arrangements SD may be difficult for many African Americans, many of whom are experiencing higher unemployment, poverty, food insufficiency, and social isolation. This study will determine if the health of African Americans and Whites are differentially impacted by SD measures. SD rules can increase or decrease health disparities by: (a) directly impacting the symptoms and progression of chronic health conditions; (b) influencing availability of protective factors and exposure to risk factors; and (c) mitigating or exacerbating the effects of sources of disparities. These hypotheses will be tested using data from the Understanding America's COVID Study. Findings can advance understanding of how public health requirements can reduce or increase health disparities and identify protective factors to facilitate adherence to public health guidelines.

### SOCIAL SUPPORT, COPING BEHAVIORS, AND RESILIENCE AMONG AFRICAN AMERICANS DURING COVID-19

Sung Park, *Harvard University, Cambridge, Massachusetts, United States*

Enduring structural inequalities in the United States by race have only become more apparent during COVID-19, as African Americans experienced significant health and economic challenges that far exceeded those observed among other racial and ethnic groups. Relying on multiple nationally representative surveys, this study examines the diversity of ways in which middle-aged and older African Americans' managed the stress and pressures associated with the pandemic. I summarize the inequities faced by African Americans before and during COVID-19, as well as trends in the utilization of social support, coping behaviors, and degree of resilience. Furthermore, this study investigates the relationship between social support and coping strategies to multiple health outcomes over time. When appropriate, comparisons to other racial and ethnic groups are made. This research underscores the importance of considering social relationships and modifiable coping behaviors when studying African American aging and well-being during times of crisis.

### MEMORY PROBLEMS DURING COVID IN LOW-INCOME OLDER ADULTS

Faika Zanjani, *Virginia Commonwealth University, Richmond, Virginia, United States*

Prevention, with widespread lifestyle risk reduction at the community-level, is considered an effective method to decrease Alzheimer's disease (AD). Diverse low-income older adults in Virginia managing either diabetes/cardiovascular symptoms, were offered weekly lifestyle telephone-health coaching for 12-weeks, providing education, motivations, self-efficacy, and referral services for AD lifestyle risk. Participants provided positive anecdotal feedback and the need for continued health coaching during COVID-19. Thirty participants (predominantly African American/Black female) consented for continued health coaching during the pandemic with 47% reporting memory problems. Findings indicated poorer health status associated with reporting memory problems for poor physical health days, poor mental health days, total mental/physical health poor days, sad days, worried days, tired days, feelings of emptiness, feelings of rejection, feelings of failure, little interest/pleasure, and feeling down. This preliminary work creates the impetus for future large-scale AD prevention investigations to improve the lives of AD-risk, low-income, diverse older adults reporting memory problems.

### THE DEVASTATING ECONOMIC IMPACT OF COVID-19 ON OLDER BLACK AND LATINX ADULTS: IMPLICATIONS FOR HEALTH AND WELL-BEING

Catherine Garcia, *University of Nebraska - Lincoln, Lincoln, Nebraska, United States*

The ongoing COVID-19 pandemic and subsequent economic recession have wreaked havoc on the United States' economy and brought to the forefront stark racial and ethnic inequalities in our society. Older Black and Latinx adults are particularly hard hit by the pandemic as they have relatively lower levels of income and wealth to protect against crises. This study used data from the 2020 COVID-19 module of the Health and Retirement Study, to highlight how the COVID-19 pandemic has economically impacted older Black, U.S.-born Latinx and foreign-born Latinx adults. Results show the pandemic has economically devastated older Black and Latinx adults across a host of economic factors, with foreign-born Latinx experiencing greater economic hardships relative to other groups. Our findings document stark inequalities that are being exacerbated by the pandemic. We discuss the implications of the economic shocks of the pandemic for the health and well-being of older Black and Latinx adults.

### Session 2245 (Symposium)

### HEALTHY BRAIN AGING

Chair: Saul Villeda

### SYSTEMIC MECHANISMS OF BRAIN REJUVENATION

Saul Villeda, *University of California San Francisco*

Aging drives cellular and cognitive impairments in the adult brain. It is imperative to gain mechanistic insight into what drives aging phenotypes in the brain in order to maintain, and even restore, functional integrity in the elderly. We, and others, have shown that systemic manipulations - such as heterochronic parabiosis (in which a young and old circulatory system are joined) and administration of young blood or exercise induced blood factors - can reverse age-related impairments in regenerative, synaptic and inflammatory processes, as well as rescue cognitive faculties in the aged brain. These studies have revealed an age-dependent bi-directionality in the influence of the systemic environment indicating pro-youthful factors in young blood elicit rejuvenation while pro-aging factors in old blood drive aging. It has been proposed that introducing pro-youthful factors or mitigating the effect of pro-aging factors may provide effective strategies to rejuvenate aging phenotypes in the brain. Despite this potential, much is unknown as to the systemic and molecular mechanisms regulating pro-youthful and pro-aging effects of blood-borne factors. I will discuss work from my research group that begins to provide mechanistic insight into the systemic and molecular drivers promoting rejuvenation in the aging brain.

### INSIGHTS INTO HIPPOCAMPAL BIOLOGY FROM YOUTH-ASSOCIATED PLASMA FACTORS

Joseph Castellano, *Icahn School of Medicine at Mount Sinai, New York, New York, United States*

### FEMALE RESILIENCE IN BRAIN AGING

Dena Dubal, *University of California San Fransisco, San Francisco, California, United States*

### TARGETING ANTICIPATORY NEUROGENESIS TO MAINTAIN COGNITIVE RESERVE

Amar Sahay, *Massachusetts General Hospital Harvard Medical School Lexington, Massachusetts, United States*

Memory imprecision is a hallmark of age-related cognitive decline and mild-cognitive impairment (MCI) and is characterized by increased memory interference and decreased stability of memory representations. Evidence from humans, non-human primates and rodents demonstrate reduced hippocampal neurogenesis, excitation-inhibition imbalance and inflexible hippocampal remapping during age-related cognitive decline and MCI. Developing strategies to reverse cognitive decline during aging and Mild Cognitive Impairment necessitates an understanding of molecular, cellular, circuit and network mechanisms that support memory functions of the hippocampus. Over the last decade we have built a multifaceted program grounded in basic neuroscience that is aimed at improving memory in aging and MCI. We have demonstrated how we can Rejuvenate the aged hippocampus by selectively increasing neurogenesis and how we can Re-engineer connectivity of aged inhibitory microcircuits to improve memory precision in aging. Ongoing efforts include strategies to Repairing neurogenic niche fitness by targeting intercellular communication in the aging hippocampus. In today's talk I will present a fourth approach catalyzed by our discovery of the first transcriptional regulator of neural stem cell expansion in the adult hippocampus. We will present data in support of this claim and convey how this discovery may guide strategies to maintain cognitive reserve embodied in the pool of neural stem cells in the adult hippocampus.

### Session 2250 (Symposium)

### HOW TO PUBLISH: GUIDANCE FROM GSA'S JOURNAL EDITORS

Chair: Suzanne Meeks

Each year he GSA publications team sponsors a symposium to assist authors who wish to publish in GSA's high impact and influential journals. The first part of the session will include five brief presentations from the editors of The Gerontologist, Innovation and Aging, and the Journals of Gerontology Series A and B plus GSA's managing editors. We will integrate practical tips with principles of publication ethics and scholarly integrity. The topics will be as follows: (1) Preparing your manuscript: strong and ethical scholarly writing for multidisciplinary audiences, (2) common problems that affect peer review, (3) addressing translational significance and fit to journal expectations, (4) transparency, documentation, and Open Science; and (5) working with Scholar One. Following these presentations, we will hold round table discussions with editors from the GSA journals portfolio. At these round tables, editors will answer questions related to the podium presentations and other questions specific to each journal. Intended audiences include emerging and international scholars, and authors interested in learning more about best practices and tips for getting their scholarly work published.

### PREPARING YOUR MANUSCRIPT: STRONG AND ETHICAL SCHOLARLY WRITING FOR MULTIDISCIPLINARY AUDIENCES

Suzanne Meeks, *University of Louisville, Louisville, Kentucky, United States*

This presentation will emphasize the importance of plain, good writing. Editors read 10 or more manuscripts per week with pressure to reject 80-90% of them. If the point and contribution are not clear in a quick scan of the paper, it will not be reviewed favorably. I will provide tips for writing that are commonly violated in submissions, provide references for additional writing support, cover expectations for language consistent with GSA's Reframing Aging initiative, and discuss some common publication ethics issues that arise during the review process, including author contributions and embedding your scholarship in the context of prior work.

### COMMON PROBLEMS THAT IMPACT PEER REVIEW

Rozalyn Anderson, *University of Wisconsin College of Medicine, University of Wisconsin, Madison, Wisconsin, United States*

This presentation will review the most common issues that affect how reviewers see a manuscript submission. These include clarity, use of figures, and attention to existing research, especially establishing the significance and novelty of the work, and how to frame a narrative. I will also address responding to peer review. The focus will be on the biological science perspective (Journals of Gerontology Series A), but these issues are relevant to all submissions to GSA journals.

### DEFINING TRANSLATIONAL SIGNIFICANCE IN GERONTOLOGY

Steven Albert, *University of Pittsburgh, Pittsburgh, Pennsylvania, United States*

Innovation in Aging requires a statement from authors on translational significance. This requirement forces authors to consider the implications of their research for changing some component of aging. How does the research address a challenge posed by aging bodies, minds, relationships, or societies? The editorial board has developed criteria for assessing translational significance. Translational research must meet at least one of three criteria. It (i) must predict or explain a health or behavioral outcome, (ii) be advanced enough in deployment or development to assess these effects, and (iii) have a clear pathway to large-scale program delivery or change in clinical practice. The criteria rule out some kinds of submissions, such as scale development, single-case studies, or reviews of literature. We use these criteria to structure each article's required translational significance statement. Rethinking translation may help focus research across the full set of GSA journals.

### TRANSPARENCY, DOCUMENTATION, AND OPEN SCIENCE

Derek Isaacowitz, *Northeastern University, Northeastern University, Massachusetts, United States*

Some GSA journals are especially interested in promoting transparency and open science practices, reflecting how some subdisciplines in aging are moving toward open science practices faster than others. In this talk, I will consider the transparency and open science practices that seem most relevant to aging researchers, such as preregistration, open data, open materials and code, sample size justification and analytic tools for considering null effects. I will also discuss potential challenges to implementing these practices as well as reasons why it is important to do so despite these challenges. The focus will be on pragmatic suggestions for researchers planning and conducting studies now that they hope to publish later.

### GSA MANAGING EDITORS' PERSPECTIVE ON SUBMISSION DOS AND DON'TS

Kathleen Jackson, *The Gerontological Society of America, Washington, District of Columbia, United States*

In this presentation, the managing editors of GSA's peer-reviewed journals will discuss how the editorial offices operate and their roles in the publishing process. The topics will include how to navigate the ScholarOne submission system, why it is important to read the Instructions to Authors, and how authors can work with the editorial offices to increase the visibility and impact of their published articles.

### Session 2255 (Symposium)

### LEVERAGING A POPULATION-BASED DYADIC DATA SET TO PROMOTE HEALTH EQUITY AMONG CHINESE AMERICANS

Chair: XinQi Dong Co-Chair: Dexia Kong

Recognizing the central role of family-oriented values in Chinese culture, developing a family-based understanding of health and wellbeing in Chinese Americans is imperative. By linking two unique population-based datasets (one on Chinese older adults, and another on their corresponding adult children caregivers), the purpose of this symposium is to present interactive analyses of dyad-level data to achieve an interpersonal understanding of health outcomes of Chinese older adults and their adult children within the family context. Data were obtained from 807 Chinese older adults-adult children dyads by merging data from two epidemiological studies, namely the Population Study of ChINese Elderly in Chicago (the PINE study) and the PIETY study of corresponding adult children caregivers of PINE participants. Specifically, this symposium presents findings from five interconnected research projects. Session 1 provides an overview of study design and sample characteristics of the dyadic dataset. Session 2 examines the relationship between adult children's endorsement of the filial piety value and older parents' mental health outcomes. Session 3 investigates the level of congruence between older parents' self-perceived mental health and adult children's evaluation of their parents' mental health. Session 4 investigates the extent to which depressive symptoms among older parents were associated with those of their adult children. Session 5 explores the relationship between older parents' physical function and adult children's perceived caregiving burden. Taken together, this symposium presents potential contributions of dyad-level analyses in advancing minority population health. Study findings have the potential to inform the development of family-centered intervention strategies targeting Chinese Americans.

### DYADIC DATA ON U.S. CHINESE OLDER ADULTS AND THEIR ADULT CHILDREN: STUDY DESIGN AND SAMPLE OVERVIEW

XinQi Dong,[1] and Dexia Kong,[2], *1. Rutgers University, Rutgers Institute for Health, New Jersey, United States, 2. Rutgers University, New Brunswick, New Jersey, United States*

This paper aims to describe study design of the unique dyadic older Chinese American-adult children dataset, and present sample characteristics of the dyads. A total of 807 older parents were matched with their adult children (characteristics of matched versus not matched participants will be compared). On average, adult children were 48 years old, had 12 years of education, lived with 3 persons in household, had 2 children, and lived in U.S. for 17 years. Approximately 65% of the adult children sample were female, 82% married, 93% preferred to speak Chinese dialects, and over 97% foreign-born immigrants. On the other hand, older parents were 74 years old, had 7 years of education, lived with 3 persons in household, had 3 children, and lived in U.S. for 17 years on average. About 60% of the older parent sample were female, 73% married, over 99% foreign-born immigrants who preferred to speak Chinese dialects.

### CHILDREN'S FILIAL PIETY AND PARENTS' DEPRESSIVE SYMPTOMS: FINDINGS FROM A DYADIC STUDY

Qun Le,[1] XinQi Dong,[2] and Stephanie Bergren,[1] *1. Rutgers University, New Brunswick, New Jersey, United States, 2. Rutgers University, Rutgers Institute for Health, New Jersey, United States*

Filial piety is an important Chinese cultural value that prescribes child behavior towards their parents, but little is known about its relationship to the parents' psychological wellbeing. This study utilizes dyad data from the PINE and PIETY Studies. Filial piety was measured by asking how much the participant thought children should 1) care; 2) respect; 3) greet; 4) please and make happy; 5) obey; and 6) provide financial support to their parents. Depressive symptoms were measured by Patient Health Questionaire-9 with a cutoff of 5 indicating the presence of depressive symptoms. Logistic regressions were used to examine the associations controlling for both children's and parents' sociodemographic characteristics. Higher filial piety in happy (OR:0.80, (0.65-0.99)) or obey (OR:0.83, (0.68-1.00)) was associated with a lower likelihood of depressive symptoms among parents. Future research should explore the potential causal relationships between children's filial piety and parents' mental health.

### PARENTS' SELF-REPORTED VERSUS CHILD EVALUATION OF PARENTS' MENTAL HEALTH OUTCOMES: IS THERE A DIFFERENCE

Dexia Kong,[1] Qun Le,[1] XinQi Dong,[2] and Yingxiao Hua,[1] *1. Rutgers University, New Brunswick, New Jersey, United States, 2. Rutgers University, Rutgers Institute for Health, New Jersey, United States*

Older Chinese-Americans are more likely to experience depressive symptoms compared to the general U.S. aging population. This paper aims to examine the level of congruence between parents' self-reported mental health and children's evaluation of their parents' mental health. Dyad-level understanding is particularly relevant considering the family-based medical decision-making preference in the Chinese-community. Older parents' depressive symptoms were measured by PHQ-9 with a cutoff of 5 indicating the presence of depressive symptoms. Adult children were asked whether their parents informed them of their depressive symptoms or if they suspected that their parents were depressed. Logistic regressions were conducted. Parents' self-reported depressive symptoms were associated with both adult children's awareness (OR:3.28 (2.00-5.39)) and suspicion (OR:3.10 (2.02-4.77)) of their parents' depressive symptoms. Results remained consistent among mother-child and father-child dyads. Study findings underscore the importance of incorporating adult children's' perspective in mental health research in the Chinese community.

### PROFILE OF PARENT-CHILD WELL-BEING IN IMMIGRANT FAMILIES

Qun Le,[1] XinQi Dong,[2] and Mengting Li,[3] *1. Rutgers University, New Brunswick, New Jersey, United States, 2. Rutgers University, Rutgers Institute for Health, New Jersey, United States, 3. Rutgers, The State University of New Jersey, New Brunswick, New Jersey, United States*

Earlier caregiving research focused on psychological well-being of either caregivers or care recipients, while less is known about the caregiving pattern with optimal outcome for both caregivers and care recipients. Data were from the PINE and PIETY studies, with 804 parent-child dyads. Depressive symptoms were measured by PHQ-9 with a cutoff of 5 distinguishing happy or depressed. Parent-child dyads were divided into four groups: happy-parent-happy-child (HPHC, n=572, 71.1%), depressed-parent-happy-child (DPHC, n=139, 17.3%), happy-parent-depressed-child (HPDC, n=65, 8.1%), and depressed-parent-depressed-child (DPDC, n=28, 3.5%). Multinomial logistic regression was used to compare the sociodemographic differences among the groups. Compared to the HPHC group, the DPHC group had older parents, more mother-child dyads and lower-income children, the HPDC group had more female children. However, there was no significant difference between the HPHC and the DPDC group. Future research could explore the predictors of parent-child well-being to inform intervention strategies.

### THE ASSOCIATION BETWEEN PARENTS' PHYSICAL FUNCTION AND ADULT CHILDREN'S CAREGIVING BURDEN

Dexia Kong,[1] XinQi Dong,[2] and Qun Le,[1], *1. Rutgers University, New Brunswick, New Jersey, United States, 2. Rutgers University, Rutgers Institute for Health, New Jersey, United States*

Using data from 544 older parents-adult children Chinese American dyads, this study aims to understand the association between older parents' physical function and their adult children's perceived caregiving burden. Parents' physical function was assessed by the Katz Index of Activities of Daily Living (ADL) and the Lawton Instrumental ADL (IADL), with higher scores indicating more functional limitations. Adult children's caregiving burden was assessed in five dimensions, including time dependence, developmental, physical, social, and emotion burden. Logistic regression was used to examine the association. More ADL limitations were associated with a higher likelihood of developmental burden (OR:1.14 (1.06-1.23)) and physical burden (OR:1.14 (1.06-1.23)) burden. More IADL limitations was associated with a higher likelihood of time dependence burden (OR:1.08 (1.03-1.12)), developmental burden (OR:1.06 (1.03-1.09)), and physical burden (OR:1.08 (1.04-1.12)). Parents' physical function was not related to children's social and emotional burdens. Practice and research implications will be discussed.

### Session 2260 (Symposium)

### MAJOR DISASTERS' IMPACTS ON LONG-TERM CARE SETTINGS, VULNERABLE OLDER ADULTS, AND CARE PROVIDERS

Chair: Leah Haverhals Co-Chair: Katie Cherry

The COVID-19 pandemic has disproportionately negatively affected older adults, and has specifically devasted older adults who are minorities and those who reside in long-term care (LTC) facilities. For professionals working in LTC facilities, major stressors and challenges due to the pandemic must be navigated, sometimes in parallel with the effect that major disasters like hurricanes can have on LTC facilities. This symposium will focus on the impact major disasters, including the COVID-19 pandemic and Hurricane Irma, had on LTC settings and those who live and work there, as well as older adults who are minorities and their communities. First, Dr. Roma Hanks will present findings from a study of community members and leaders in a majority African-American community in the United States (US) about their experiences with and challenges faced related to the pandemic. Second, Dr. Lisa Brown will share experiences and perceptions of mental health clinicians from across the US who worked in LTC settings before and during the pandemic. Third, Dr. Ella Cohn-Schwartz will describe how the pandemic impacted Holocaust survivors ages 75+ in Israel compared to older adults who did not experience the Holocaust. Fourth, Dr. Lindsay Peterson will present findings from interviews with nursing home and assisted living community representatives in the US regarding vulnerabilities LTC facilities experienced related to Hurricane Irma in 2017. As a whole, these presenters will provide insights into experiences of older adults, care providers, LTC facilities, and communities as they navigated challenges associated with the COVID-19 pandemic and a major hurricane.

### JUST ANOTHER STORM: CONCEPTUALIZING OLDER ADULTS' PERCEPTIONS OF THE COVID-19 PANDEMIC

Christopher Freed,[1] Shoon Lio,[2] Martha Arrieta,[1] and Roma Hanks,[1] *1. University of South Alabama, Mobile, Alabama, United States, 2. Spring Hill College, Mobile, Alabama, United States*

Older adults of color who experience health disparities are especially vulnerable to health and economic adversity related to COVID-19. This study focuses on nine zip codes wherein 70.2% of residents are of African-American descent and an estimated 31.5% of residents live in poverty. To understand the lived experience of the COVID-19 pandemic, perceived challenges of COVID-19, and the dissemination of information related to COVID-19, we collected interview and focus group data in Spring 2020 from fifteen community members, leaders, or advocates. Analyses reveal that older individuals approach the COVID-19 pandemic with familiar disaster mitigation strategies. Other persons perceive the pandemic as another community challenge that African-Americans must confront. Older adults report generational differences in perceptions of the risk of COVID-19 and compliance with health guidelines. Overall, analyses reveal a deeply cultural context for intergenerational responses associated with COVID-19 and a sense of agency among older community leaders as health advocates.

### PERCEPTIONS OF MENTAL HEALTH CLINICIANS WORKING IN LONG-TERM CARE FACILITIES DURING THE COVID-19 PANDEMIC

Rachel Ward,[1] Savannah Rose,[1] Lisa Lind,[2] Roma Hanks,[3] and Lisa Brown,[4] *1. Palo Alto University, Palo Alto, California, United States, 2. Deer Oaks Behavioral Health, San Antonio, Texas, United States, 3. University of South Alabama, Mobile, Alabama, United States, 4. Palo Alto University, Moss Beach, California, United States*

During the COVID-19 pandemic, mental health clinicians were initially not considered essential workers, and most were prevented from entering long-term care (LTC) facilities. This study investigated the perceptions and experiences of licensed clinicians who were providing services in LTC settings before and during the pandemic. Respondents included 126 clinicians from 31 states who completed a 90-item survey to assess the impact of COVID-19. Visitor restrictions were perceived to have had a negative effect on patients' emotional, behavioral, and cognitive status. The pandemic adversely impacted clinicians financially, personally, and emotionally, with more than half (67%) reporting that they experienced burnout. This study found that the COVID-19 pandemic adversely impacted clinicians working in LTC settings, their patients' wellbeing, and the delivery of mental health services. Understanding the impact that the COVID-19 pandemic has had on LTC patients and clinicians alike has implications for the provision of services during future pandemics.

### MENTAL HEALTH OF HOLOCAUST SURVIVORS AND OTHER OLDER ADULTS DURING THE COVID-19 PANDEMIC IN ISRAEL

Yaacov Bachner, Sara Carmel, and Ella Cohn-Schwartz, *Ben-Gurion University, Beer-Sheva, HaDarom, Israel*

Holocaust survivors could be especially vulnerable to the negative effects of the COVID-19 pandemic due to their early life traumas. Thus, the current study examines the effects of the pandemic on the mental health of Holocaust survivors in Israel, compared to adults who did not experience the Holocaust. We collected quantitative data from 305 adults aged 75+ (38% Holocaust survivors) in Israel during the COVID-19 pandemic. The results indicate that Holocaust survivors were worried to a greater extent from COVID-19 and reported greater depression which became worse during the pandemic. On the other hand, despite these differences, the two groups were similar in their will to live. In conclusion, Holocaust survivors seem to be more vulnerable to the COVID-19 pandemic, strengthening the vulnerability hypothesis, while also showing resilience in their will to live. Policy makers and practitioners should pay special attention to this particularly vulnerable population during these difficult times.

### DISASTER VULNERABILITY IN LONG-TERM CARE: THE IMPORTANCE OF SOCIAL AND ORGANIZATIONAL CONNECTIONS

Debra Dobbs,[1] Joseph June,[2] David Dosa,[3] Kathryn Hyer,[4] and Lindsay Peterson,[4] *1. University of South Florida, School of Aging Studies, University of South Florida, Florida, United States, 2. University of South Florida, Tampa, Florida, United States, 3. Brown University, Providence, Rhode Island, United States, 4. University of South Florida, University of South Florida, Florida, United States*

The risks to older adults in nursing homes (NHs) and assisted living communities (ALCs) exposed to disasters are evident in prior research. However, little research has been conducted to understand the factors related to facilities' vulnerability. This research examined NH and ALC experiences during Hurricane Irma in 2017. Qualitative interviews were conducted with representatives of facilities (N=100), transcripts were analyzed using Atlas.ti version 8. Team members met to reach consensus on codes and major themes and subthemes, which they analyzed using a conceptual model designed to identify factors related to the disaster vulnerability in long-term care (LTC). We found physical factors (e.g. location, physical characteristics) are important, but physical strength is not enough. Multiple social/organizational factors are critical. Results indicate managing a major disaster and protecting LTC residents involve social and organizational connections across a range of groups from staff and family members to emergency mangers and neighborhood associations.

### Session 2265 (Paper)

### MOBILITY, DISABILITY, AND SOCIAL CONTEXTS

### FORMAL AND INFORMAL CARE USE OVER THE COURSE OF COGNITIVE DETERIORATION AMONG ADULTS WITH A DISABILITY

HwaJung Choi,[1] Kenneth Langa,[1] Edward Norton,[1] Tsai-Chin Choi,[1] and Cathleen Connell,[2] *1. University of Michigan, Ann Arbor, Michigan, United States, 2. University of Michigan School of Public Health, Ann Arbor, Michigan, United States*

The dynamics between formal and informal care among persons with a disability may substantially differ over the course of their cognitive decline.

Based on a nationally representative study of older adults, the analysis sample included 3,685 individuals who had at least one activity of daily living (ADL) limitation. We estimated probabilities of using formal care and informal care in the years before and over the course of dementia after controlling for sociodemographic factors, survey mode, and proxy interview status.

The adjusted probability of receiving care from an informal helper increased before the onset of dementia: 36% in 4 years prior to the onset (T=-4); 46% at T=-2. In contrast, the increase in the probability of using formal care was pronounced primarily at the onset of dementia; for example, the probability of overnight nursing home stay was 12% at T=-2 vs. 31% at T=0, which continued to increase over the subsequent years (39% at T=6). The probability of using nursing home care at the onset was significantly greater for women vs. men (Adjusted risk ratio (ARR)=1.21; p=0.010); non-Hispanic white vs. Hispanic (ARR=1.62; p=0.004); those with low vs. high wealth (ARR=1.60; $p < 0.001$); those without a spouse vs. with a spouse prior to the onset (ARR=1.39; $p < 0.001$); and those with all adult children living far vs. at least one coresident adult child prior to the onset (ARR=1.51; p= < 0.001).

Public policies and interventions aimed at providing for the needs of people with dementia should consider disparities in care use across racial/ethnic and socioeconomic groups.

### GENDER DIFFERENCES IN AVOIDING LATER-LIFE DISABILITY: A LIFE COURSE PERSPECTIVE

Patricia Morton, *Wayne State University, Detroit, Michigan, United States*

Identifying the early origins of adult health has underscored how experiences in the earliest stages of life can have lasting consequences. Whereas most research on the early origins of adult health has linked childhood conditions to worse health in adulthood, this study considered whether childhood conditions are associated with healthy aging. Guided by the World Health Organization's emphasis on functional ability as a core component of healthy aging, the present study investigated the association between childhood social conditions and avoiding later-life limitations in basic and instrumental activities of daily living, referred to as disability-free status. This study also tested potential health-related and socioeconomic mediators and examined whether these life course antecedents of healthy aging vary by gender. Analyzing a sample of 9,376 adults over age 50 from the Health and Retirement Study over 10 years (2006-2016) revealed that childhood socioeconomic disadvantage reduced the odds of avoiding disability over time. For women, adult health lifestyles mediated this relationship whereas adult socioeconomic status (SES) mediated this relationship for men. Conditional indirect effects indicated that the mediational effects of body mass and education differed between men and women (i.e., moderated mediation). The direct effects of childhood and adult SES also varied by gender. These results demonstrate that the life course antecedents, especially SES, of healthy aging are distinct for men and women. Interventions should prioritize reducing early-life exposure to socioeconomic disadvantage, especially for women. Given the gendered differences in the mediating effects, midlife interventions can be tailored for men and women.

### OLDER AMERICANS WITH DISABILITY ARE VULNERABLE TO ECONOMIC AND FOOD INSECURITY DURING COVID-19

Shinae Choi,[1] Eun Ha Namkung,[2] and Deborah Carr,[3]
*1. The University of Alabama, Tuscaloosa, Alabama, United States, 2. Korea Institute for Health and Social Affairs, Sejong, Ch'ungch'ong-namdo, Republic of Korea, 3. Boston University, Boston, Massachusetts, United States*

This study investigated whether older Americans with physical disability were vulnerable to three types of economic insecurity (difficulty paying regular bills, difficulty paying medical bills, income loss) and two types of food insecurity (economic obstacles, logistical obstacles) during the early months of the COVID-19 pandemic. We evaluated the extent to which associations are moderated by three personal characteristics (age, sex, race/ethnicity) and two pandemic-specific risk factors (job loss, COVID-19 diagnosis). Data were from a random 25 percent subsample of the Health and Retirement Study participants who completed a COVID-19 module administered in 2020. Our analytic sample included 3,166 adults aged 51 and older. We estimated logistic regression models to document the odds of experiencing each hardship. Persons with three or more functional limitations reported significantly higher odds of both types of food insecurity, and difficulty paying regular and medical bills, relative to those with no limitations. After controlling for health conditions, effects were no longer significant for paying medical bills, and attenuated yet remained statistically significant for other outcomes. Patterns did not differ significantly on the basis of the moderator variables. Older adults with more functional limitations are vulnerable to economic and food insecurity during the pandemic, potentially exacerbating the physical and emotional health threats imposed by the pandemic. Our findings reveal an urgent need to promote policies and procedures to protect older adults with disability from economic and food insecurity. Supports for older adults with disability should focus on logistical as well as financial support for ensuring food security.

### TOWARD A MULTIDIMENSIONAL UNDERSTANDING OF LATER LIFE DISABILITY: A LATENT PROFILE ANALYSIS

Natasha Peterson,[1] Jeongeun Lee,[2] and Eva Kahana,[3]
*1. Iowa State University, Iowa State University, Iowa, United States, 2. Iowa State University, Ames, Iowa, United States, 3. Case Western University, Case Western reserve University, Ohio, United States*

Disability is difficult to define succinctly. Current literature on disability has primarily focused on physical functional limitations. However, relying on a single dimension or index cannot accurately represent disability as the experience of disability is nuanced and complex. To address these gaps, this study aims to understand the multidimensional nature of disability among retired, community-dwelling older adults. Using a sample of 414 older adults between the ages of 72 and 106 years (M=84.84, SD=4.56), latent profile analysis was employed to identify classes based on five indicators of disability across three domains. The five indicators of disability included difficulties with activities of daily living (ADLs), cognitive impairment, physical impairment, sensory impairment, and participation restrictions. Three classes were found to represent the data best. The most favorable and highly functioning group comprised the highest number of participants (n=242, 59.5%). The next group, class 2 (n=157, 37.9%), was characterized by high physical impairment and ADL-difficulty. The smallest group, class 3 (n=15, 3.6%), had the highest ADL-difficulty and participation restrictions but drastically lower cognitive and sensory impairment. Multinomial logistic regression revealed that class membership was related to sociodemographic characteristics. Finally, class membership predicted several mental health outcomes such as depressive symptoms, positive affect, and life satisfaction in the expected direction. If supported by future work, these findings could inform practitioners in developing more specific interventions relevant to older adults based on their disability profiles. Understanding various combinations of disablement has potential implications for services and interventions to be tailored to individuals' distinct disability-related needs.

### Session 2270 (Symposium)

### NEW BRAIN AGING CENTER

Chair: Feng Lin Co-Chair: Yeates Conwell Discussant: Janine Simmons

Evidence indicates an association between emotional well-being (EWB) and underlying brain processes, and that those processes change with both normal and pathological brain aging. However, the nature of these associations, the mechanisms by which EWB and its component domains change with brain aging, and how those changes may be associated with common neuropathologies like Alzheimer's disease and related dementias (ADRD), are largely unexplored. The NIA-funded Network for Emotional Well-being and Brain Aging (NEW Brain Aging) has the goal of developing a nationwide community of investigators dedicated to research that identifies and tests mechanisms by which brain aging influences EWB and how EWB may impact risk for and progression of ADRD. Synthesizing human and animal literature, our premise is that relationships between EWB and ADRD are bidirectional – normal and pathological changes in aging brain influence EWB and EWB contributes to brain health and illness, such as ADRD. NEW Brain Aging will identify and coalesce resources for interested investigators and provide pilot funding opportunities to stimulate research and development of the field. Component presentations of this symposium will include (1) an overview by Dr. Robert Kaplan of the current state of research on EWB; (2) the role of animal studies (Kuan Hong Wang) and (3) human subjects research (Feng Vankee Lin) in EWB and aging; and (4) design of NEW Brain Aging and resources it will provide (Yeates Conwell). Janine Simmons will explain NIA's vision for EWB research and lead open discussion.

### EMOTIONAL WELL-BEING HUMAN STUDIES

Feng Lin, *University of Rochester Medical Center, Rochester, New York, United States*

Early evidence indicates an association between EWB and underlying brain processes, and that those processes change with both normal and pathological brain aging. However, the nature of these associations, the mechanisms by which EWB and its component domains change with brain aging, and how those changes may be associated with common neuropathologies in ADRD, are largely unexplored. We propose an appraisal-adaptation model in understanding relationships between EWB and ADRD. For human models, we encourage the use of well-established measures that directly assess eudaimonic and hedonic EWB, including abnormal scenarios (e.g., neuropsychiatric symptoms, anhedonia, loneliness, etc.), as well as older adults with exceptional cognition (i.e., superagers or supernormals). Dr. Lin will review premises associated with the appraisal-adaptation model in conducting human research on EWB, aging, and ADRD.

### ACTIVITIES OF NEW BRAIN AGING

Yeates Conwell, *University of Rochester Medical Center, Rochester, New York, United States*

The Network for Emotional Well-being and Brain Aging (NEW Brain Aging) was funded by NIA with the goal of forming a national, transdisciplinary collaborative that includes investigators with research expertise in emotional well-being (EWB), Alzheimer's disease and related dementias (ADRD), human and animal neuroimaging, stress regulation, and computational/quantitative methods. Our objective is to stimulate mechanistic research identifying and testing mechanisms by which brain aging influences EWB and how EWB may impact risk for and progression of ADRD. This presentation will explain the structure and functions of the network that serve as a resource for investigators interested in EWB and aging research, and how to access them: a transdisciplinary community of scholars interested in brain, aging, and EWB research from both human and animal fields; webinars; workgroups to establish priorities for NEW Brain Aging activities; a resource repository; and pilot project funding opportunities to which network members can apply.

### EMOTIONAL WELL-BEING ANIMAL MODELS

Kuan Wang, *University of Rochester, Rochester, New York, United States*

Clinical studies suggest an association between EWB and the risk or progression of AD. However, the mechanistic link and causal relationship between EWB and AD remain unknown, due to limited experimental access and control of the underlying human brain processes. Animal models offer genetic control of AD mutations and neural circuit analysis tools, but subjective feelings of EWB cannot be assessed through self-report. To study EWB across species, we adopt a theoretical framework that views emotions as central brain states that respond to exteroceptive or interoceptive stimuli and cause multiple cognitive, somatic and behavioral changes. Recent neuroanatomical and functional imaging studies have identified evolutionarily related brain circuits in the encoding and regulation of central emotional states in animals. Dr. Wang will review progress in elucidating the functional activities of these circuits and discuss the challenges and opportunities to link these neural representations to EWB and AD related pathological progression.

### OVERVIEW OF EWB AND AGING

Robert Kaplan, *Stanford University, Stanford University, California, United States*

The accumulation of scientific knowledge has been hampered by inconsistent usage of terms and categories. Ontology is the study of categories, their properties, and the relations between them. This presentation considers the definition and measurement of emotional well-being (EWB), a term that has been used inconsistently in research and clinical practice. The category contains eudaimonic and hedonic well-being that represent interrelated but conceptually distinct aspects of mental health. This presentation will review the definition and measurement of EWB and evidence for the validity of the construct. Evidence suggests EWB increases after age 50 and is important for maintenance of cognitive function in old age. Further, low in EWB may be a risk factor for incident ADRD, and is likely to impair cognitive functioning.

### NIA PRIORITIES ON EMOTIONAL WELL-BEING

Janine Simmons, *NIA, NIH, District of Columbia, United States*

In 2021, NIH funded six high-priority research networks designed to develop resources to support and advance the study of emotional well-being (EWB) and its core components. These research networks aim to advance the field by facilitating transdisciplinary research in the social, behavioral, psychological, biological, and neurobiological sciences. The National Institute on Aging (NIA) co-sponsored the RFA, and provided funding for NEW Brain Aging, because of the central importance of EWB to health trajectories across the adult lifespan. In this presentation, Dr. Simmons, Chief of the Individual Behavioral Processes Branch within the NIA Division of Behavioral and Social Research (BSR), will discuss how EWB research fits within NIA priorities. She will then facilitate open discussion about NIA and BSR's vision for the EWB 'network of networks,' the synergy of NEW Brain Aging with other members of the larger network, and the opportunities these networks will provide for investigators interested in EWB.

### Session 2275 (Symposium)

### NOVEL STRATEGIES TO REACH AND ENGAGE OLDER VETERANS DURING COVID-19

Chair: Amanda Peeples Discussant: Kim Van Orden

The COVID-19 pandemic and associated public health measures to prevent its spread have important implications for the health and wellbeing of older Veterans. Prior to the pandemic, social isolation was already recognized as a risk for older adults, contributing to increased risk of depression, physical inactivity, and mortality. Stay-at-home orders, social distancing, and transitions to new ways of delivering care have meant that many of the ways in which older Veterans connect with VA and others have changed. Older Veterans and Veterans with serious mental illness (SMI) are especially vulnerable to experience negative impacts from social isolation and loneliness. This symposium will present on four novel and adapted strategies for engaging with older Veterans during the COVID-19 pandemic and beyond: 1) VA Connection Plans, a whole health intervention to promote social connections for older Veterans with and without SMI (Peeples); 2) telehealth adaptations to PEER, an in-person, peer-delivered exercise intervention for older Veterans with SMI (Muralidharan); 3) VA Compassionate Contact Corps, a VA Voluntary Service program to connect older Veterans with friendly volunteers via telephone (Sullivan); and 4) group telehealth interventions to foster social connection among older Veterans and their families (Weiskittle). Kim Van Orden, geropsychologist and director of the Hope Lab (Helping Older People Engage) at the University of Rochester Medical Center, will serve as discussant.

### VA CONNECTION PLANS: A WHOLE HEALTH INTERVENTION TO PROMOTE SOCIAL CONNECTIONS FOR OLDER VETERANS

Samantha Hack,[1] Anjana Muralidharan,[2] and Amanda Peeples,[3] *1. VA Maryland Health Care System, Baltimore, Maryland, United States, 2. Veterans Affairs Capitol Healthcare Network, Baltimore, Maryland, United States, 3. VISN 5 MIRECC, Baltimore, Maryland, United States*

The Connection Plan intervention was created as a brief intervention to assist older adults experiencing social isolation during COVID-19. Based in Cognitive Behavioral Therapy (CBT), it is designed to help older adults create a "Connection Plan" to cope with distress related to social isolation. In 1-2 sessions, interventionists work with the older adult to create a Connection Plan with three parts: Mind (ways to change negative thoughts), Body (ways to change unpleasant body sensations), and Connections (ways to increase social engagement). Through soliciting feedback from key stakeholders (Veterans and VA clinicians), the Connection Plan intervention was adapted for the VA context. This paper will present this process of creating the VA Connection Plans manual, as well as associated efforts to disseminate the intervention to 900 VA staff and deliver it to 600 older Veterans with (age 50+) and without (age 65+) serious mental illness.

### ENGAGING OLDER VETERANS WITH SERIOUS MENTAL ILLNESS IN PHYSICAL ACTIVITY: IN-PERSON, REMOTE, AND HYBRID MODELS

Sera Havrilla,[1] Alicia Lucksted,[1] Deborah Medoff,[2] Karen Fortuna,[3] Amanda Peeples,[1] and Anjana Muralidharan,[4] *1. VISN 5 MIRECC, Baltimore, Maryland, United States, 2. University of Maryland School of Medicine, Baltimore, Maryland, United States, 3. Dartmouth College, Lebanon, New Hampshire, United States, 4. Veterans Affairs Capitol Healthcare Network, Baltimore, Maryland, United States*

Older adults with serious mental illness (SMI) have complex care needs across medical, psychiatric, cognitive, and social domains. This growing population exhibits high levels of medical comorbidity and sedentariness. Innovative interventions that promote holistic recovery for this group are needed, especially in the context of the COVID-19 pandemic. Peer Education on Exercise for Recovery (PEER) is a peer coaching intervention, delivered by VA Peer Specialists (Veterans with lived experience of mental illness), to promote exercise and physical activity among older adults with SMI. This paper will present on three different models of PEER: fully in-person, fully remote, and a hybrid model with both in-person and remote elements. Preliminary data indicates that PEER is (1) engaging and well-liked, (2) associated with greater sustained increases in physical activity compared to an active control, and (3) can lead to sustained physical activity increases that are resilient to situational constraints such as physical distancing.

### VA COMPASSIONATE CONTACT CORPS: A PHONE-BASED INTERVENTION FOR VETERANS INTERESTED IN SPEAKING WITH PEERS

Lisa Gualtieri,[1] Maura Campbell,[2] Heather Davila,[3] Jacquelyn Pendergast,[4] Prince Taylor,[5] and Jennifer Sullivan,[6] *1. VA, Syracuse, New York, United States, 2. Department of Veterans Affairs, St. Lois, Missouri, United States, 3. VA Boston Healthcare System, VA Boston Healthcare System, Massachusetts, United States, 4. CHOIR, VA Boston healthcare System, Boston, Massachusetts, United States, 5. Department of Veterans Affairs, Washington, District of Columbia, United States, 6. VA Boston Healthcare System, Boston, Massachusetts, United States*

The VA Voluntary Service has developed and implemented a new social prescription program called Compassionate Contact Corps which was created during the COVID-19 pandemic when in-home volunteers could no longer enter Veterans' homes. The program targets Veterans who are lonely, socially isolated or seeking additional social connection. Volunteers and Veterans are matched based on common interests. Trained volunteers provide support by making periodic phone calls. Program referrals are made from VA providers in several clinical programs (e.g. Home-based Primary Care). To date, CCC has been implemented in more than 80 sites in the VA, with 310 volunteers, 5,320 visits, and 4,757 hours spend with Veterans.

### GROUP TELEHEALTH INTERVENTIONS FOSTERING SOCIAL CONNECTION AMONG OLDER VETERANS AND THEIR FAMILIES

Michelle Mlinac,[1] and Rachel Weiskittle,[2], *1. VA Boston Healthcare System, Boston, Massachusetts, United States, 2. VA Boston Healthcare System, Jamaica Plain, Massachusetts, United States*

During the early months of the COVID-19 pandemic, virtual and telephone visits rapidly replaced most in-person care within the Veterans Health Administration (VA) to reduce virus spread. To address the emerging mental health needs of older Veterans (e.g., isolation, loneliness), we developed an 8-week group treatment manual, deliverable over telephone or videoconference, to foster social connection and address pandemic anxieties. The manual was disseminated in March 2020 as a rapid response to emergent COVID-19 pandemic realities, during which many locations in the United States called for immediate self-quarantine measures for unknown durations. This talk will present the user-centered design of the manual, preliminary feasibility and acceptability findings from provider surveys, and introduce versions of the manual targeting specific populations (e.g., caregivers, Spanish speakers) currently in development or in pilot testing.

### Session 2280 (Symposium)

### ORAL HEALTH, COGNITIVE FUNCTION, AND MORTALITY: FINDINGS FROM NATIONAL SURVEYS

Chair: Bei Wu Co-Chair: Susie Keepper
Discussant: Michèle Saunders

Poor oral health, diabetes mellitus (DM), and cognitive impairment are common problems in older adults. Using national surveys, this symposium aims to present new findings regarding the impact of the co-occurrence of DM and poor oral health on cognitive function, cognitive decline, and mortality. This symposium will also cover the topic of dental care use among adult populations in the U.S. Using data from the Health and Retirement Study (HRS) (2006- 2018), the first study shows that adults with both DM and edentulism had the worst cognitive function, followed by those with edentulism alone, and those with DM alone. Using the same HRS data, the second study found that co-occurrence of DM and edentulism had a higher risk of more rapid cognitive decline with advancing age than the presence of each condition alone. The third study used data from the 2006-2016 HRS linked with mortality files, and revealed that the risk of diabetes and edentulism on mortality may vary across racial/ethnic groups. Using the Behavioral Risk Factor Surveillance System survey (2002-2018), the fourth study examined disparities of dental service utilization among racial/ethnic groups (Whites, Hispanics, Blacks, Asians, American Indians or Alaska Natives, and Native Hawaiian or other Pacific Islanders). Age differences in dental services were also compared between older adults and other younger and middle-aged populations. This symposium highlights the role of oral health in improving cognitive health. Policies and programs are needed to increase dental care access, a critical way to help maintain good oral health.

### DIABETES MELLITUS, EDENTULISM, AND TRAJECTORY OF COGNITIVE DECLINE AMONG OLDER ADULTS

Chenxin Tan,[1] Brenda Plassman,[2] Frank Sloan,[2] Mark Schwartz,[3] Samrachana Adhikari,[3] Angela Kamer,[4] Huabin Luo,[5] and Bei Wu,[4], *1. New York University, Jersey City, New Jersey, United States, 2. Duke University, Durham, North Carolina, United States, 3. New York University, NYC, New York, United States, 4. New York University, New York, New York, United States, 5. East Carolina University, Greenville, North Carolina, United States*

We examined the impact of diabetes mellitus (DM) and edentulism on the trajectory of cognitive decline, using the Health and Retirement Study. We analyzed self-reported DM and edentulism collected in 2006 and cognition data from 2006 and its follow up waves through 2018. Among 15,709 eligible participants age 50+ in 2006, 65.96% had neither DM nor edentulism (Group 1), 15.12% had DM alone (Group 2), 13.79% had edentulism alone (Group 3), and 5.12% had both conditions (Group 4). Results from linear mixed-effects models show that in comparison to Group 1, individuals in Group 4 had the lowest level of cognitive function, followed by those in Group 3 and Group 2. Group 4 had a modestly faster rate of cognitive decline ($p=0.052$). This study illustrates that co-occurrence of DM and edentulism has a higher risk of more rapid cognitive decline with advancing age than the presence of each condition alone.

### THE IMPACT OF DIABETES AND EDENTULISM ON ALL-CAUSE MORTALITY: RACIAL AND ETHNIC DISPARITIES

Huabin Luo,[1] Frank Sloan,[2] Brenda Plassman,[2] Samrachana Adhikari,[3] Mark Schwartz,[3] Xiang Qi,[4] Bei Wu,[5] and Chenxin Tan,[6] *1. East Carolina University,*

*Greenville, North Carolina, United States, 2. Duke University, Durham, North Carolina, United States, 3. New York University, NYC, New York, United States, 4. Rory Meyers College of nursing, New York, New York, United States, 5. New York University, New York, New York, United States, 6. New York University, Jersey City, New Jersey, United States*

This study examined the relationships between the concomitance of diabetes mellitus (DM) and edentulism and mortality among Black, Hispanic, and White older adults in the US. We used data from the 2006-2016 Health and Retirement Study with 2,108 Black, 1,331 Hispanic, and 11,544 White respondents aged 50+. Results of weighted Cox proportional hazards models showed that the concomitance of DM and edentulism was associated with a higher mortality risk for Blacks (Hazard Ratio [HR] = 1.58, p < 0.01), Hispanics (HR = 2.16, p < 0.001) and Whites (HR = 1.61, p < 0.001). Findings also indicated that DM was a risk factor for mortality across all racial/ethnic groups, but edentulism was a risk factor only for Whites (HR = 1.30, p < 0.001). This study revealed that the risk of DM and edentulism on mortality varied among racial/ethnic groups. Our study gives alternative explanations for the observed findings.

### EFFECTS OF THE CO-OCCURRENCE OF DIABETES AND TOOTH LOSS ON COGNITIVE FUNCTION

Chenxin Tan,[1] Brenda Plassman,[2] Frank Sloan,[2] Mark Schwartz,[3] Samrachana Adhikari,[3] Xiang Qi,[4] Bei Wu,[5] and Huabin Luo,[6] *1. New York University, Jersey City, New Jersey, United States, 2. Duke University, Durham, North Carolina, United States, 3. New York University, NYC, New York, United States, 4. Rory Meyers College of nursing, New York, New York, United States, 5. New York University, New York, New York, United States, 6. East Carolina University, Greenville, North Carolina, United States*

Using data from the 2006, 2012, and 2018 waves of the Health and Retirement Study, we estimated effects of co-occurrence of diabetes mellitus (DM) and complete tooth loss (CTL), both self-reported, on cognitive function among 10,816 adults age 50+. Cognitive function was measured using a shortened version of the Telephone Interview for Cognitive Status. Results from the fixed effects linear regression model show that in comparison to those with neither condition, adults having both DM and CTL had the worst cognitive function (b = 1.49, p < 0.001), followed by having CTL alone (b = 0.78, p < 0.001), and having DM alone (b = 0.42, p < 0.001). Our study suggests that CTL is a stronger risk factor for lower cognitive function than DM, and the co-occurrence of DM and CTL poses additive risk. Further research is needed to investigate the pathway from DM and CTL to poor cognition.

### DISPARITIES IN DENTAL SERVICE USE AMONG ADULT POPULATIONS IN THE UNITED STATES

Wei Zhang,[1] Bei Wu,[2] and Yan Yan Wu,[3] *1. University of Hawaii at manoa, Honolulu, Hawaii, United States, 2. New York University, New York, New York, United States, 3. University of Hawaii at Manoa, University of Hawaii at Manoa, Hawaii, United States*

This paper aimed to examine disparities of dental service utilization for younger (20-49), middle-aged (50-64), and older adults (65+), among Whites, Hispanics, Blacks, Asians, American Indians or Alaska Natives (AIAN), and Native Hawaiian or other Pacific Islanders (NHOPI). Weighted logistic regression models were conducted to analyze nine waves of data (2002-2018) from the Behavioral Risk Factor Surveillance System. Results show that the all-wave average prevalence was 71% and racial/ethnic disparities increased with age. Black older adults had the lowest level of dental service utilization (65%), comparing to the two highest groups: White older adults (79%) and Asian older adults (76%). The younger adult populations had low prevalences with the lowest among Asians (65%). The AIAN and NHOPI all age groups tended to have average or below average prevalences. Health policy, federal funding, and community-based programs should address needs of dental service utilization for racial/ethnic minorities including Blacks, AIANs, and NHOPIs.

### Session 2285 (Symposium)

### PERCEIVED PHYSICAL FATIGABILITY: A PROGNOSTIC MARKER OF BIOLOGICAL, ORGAN SYSTEM, AND BRAIN AGING

Chair: Nancy W. Glynn Co-Chair: Eleanor Simonsick
Discussant: Basil Eldadah

Characterizing perceived physical fatigability enables researchers to quantify an individual's susceptibility to experiencing fatigue in the context of a standardized physical task. This approach eliminates self-pacing, and is a less-biased, more sensitive means to measure the degree to which fatigue may limit activity. Our previous work with two validated measures of perceived fatigability, the Pittsburgh Fatigability Scale (PFS) and Borg Rating of Perceived Exertion (RPE) at the end of a standardized 5-minute treadmill walk, are prognostic indicators of phenotypic aging. This symposium will present new directions related to greater fatigability as a marker of biological aging, organ system health and functioning, as well as brain pathology and structure. Specifically, Mr. Katz will explore the relationship between leukocyte telomere length, a marker of biological aging, with PFS fatigability in participants from the Long Life Family Study. The other four papers use data from the Baltimore Longitudinal Study of Aging (BLSA) and RPE fatigability (RPE). Drs. Simonsick and Karikkineth investigate fatigability as an early marker of aging and disease related impacts on key organ systems, specifically diminished renal function as reflected in estimated Glomerular Filtration Rate and cardiovascular health evaluated as vascular stiffness. Ms. Liu and Dr. Schrack will share whether there are associations of perceived fatigability with brain health, specifically Alzheimer's disease-related pathology (PiB) and changes in brain structure. Lastly, our Discussant, Dr. Eldadah, will critically review the presentations in the context of new directions in fatigability research.

### ASSOCIATION OF LEUKOCYTE TELOMERE LENGTH WITH PERCEIVED PHYSICAL FATIGABILITY

Joseph Zmuda,[1] Joseph Lee,[2] Lawrence Honig,[3] Kaare Christensen,[4] Mary Feitosa,[5] Mary Wojczynski,[6]

Nancy W. Glynn,[7] and Ryan Katz,[8] *1. University of Pittsburgh, Pittsburgh, Pennsylvania, United States, 2. Columbia University, New York, New York, United States, 3. Columbia University Irving Medical Center, New York, New York, United States, 4. Department of Public Health, University of Southern Denmark, Odense, Syddanmark, Denmark, 5. Washington University School of Medicine in St. Louis, St. Louis, Missouri, United States, 6. Washington University School of Medicine, Washington University School of Medicine, Missouri, United States, 7. University of Pittsburgh Graduate School of Public Health, Pittsburgh, Pennsylvania, United States, 8. University of Pittsburgh Graduate School of Public Health, Pittsburgh, Pennsylvania, United States*

Leukocyte telomere length (LTL) is a potential marker of biological aging, but its relationship to fatigability, a prognostic indicator of phenotypic aging (e.g., functional decline) is unknown. We hypothesized shorter LTL would predict greater perceived physical fatigability. Two generations of participants (N=1,997; 309 probands, 1,688 offspring) were from the Long Life Family Study (age=73.7±10.4, range 60-108, 54.4% women). LTL was assayed at baseline and 8.0±1.1 years later perceived physical fatigability was measured using the validated, self-administered 10-item Pittsburgh Fatigability Scale (PFS, 0-50, higher scores=greater fatigability). Prevalence of greater physical fatigability (PFS scores≥15) was 41.9%. Using multivariate linear regression, one kilobase pair shorter LTL predicted higher PFS Physical scores (β=0.9, p=0.025), adjusted for family relatedness, generation (indicator for age), field center, follow-up time, sex, and follow-up body mass index, physical activity, health conditions. LTL, a promising marker of future fatigability, may allow for early identification of those at-risk for deleterious aging.

### FATIGABILITY: AN EARLY MARKER OF DIMINISHED RENAL FUNCTION?

Ann Moore,[1] Michelle Shardell,[2] Pei-Lun Kuo,[3] Ajoy Karikkineth,[1] Luigi Ferrucci,[1] and Eleanor Simonsick,[4] *1. National Institute on Aging, Baltimore, Maryland, United States, 2. University of Maryland School of Medicine, Baltimore, Maryland, United States, 3. National Institute on Aging, National Institute on Aging, Maryland, United States, 4. National Instute on Aging/NIH, Baltimore, Maryland, United States*

Renal function declines markedly with age due to normal aging and/or disease processes and impacts multiple systems. Diminished renal function may manifest as low exercise tolerance and fatigue threshold. Using data on 951 well-functioning (usual gait speed >.67m/s and no difficulty walking ¼ mile) men and women (51%) aged 60-89 years in the Baltimore Longitudinal Study of Aging, we evaluated the cross-sectional association between perceived fatigability (Rating Perceived Exertion after 5-minute treadmill walk at 1.5mph) categorized as 6-7, 8-9, 10-11 and 12+ and GFR using Cockcroft-Gault. For each fatigability increment, likelihood of suboptimal (GFR=75-89, 21%), diminished (GFR=60-74, 26%) and poor renal function (GFR=15-59, 30%) relative to GFR≥90 was respectively OR(95%CI) p-value 1.51(1.16-1.96).002, 1.38(1.04-1.83).027 and 1.68(1.22-2.31).002 adjusted for demographics, weight, height, smoking, exercise and anemia. Findings were similar for men and women. Perceived fatigability may facilitate identification of apparently well-functioning older adults on the precipice of suboptimal to poor renal function.

### ASSOCIATION BETWEEN ARTERIAL STIFFNESS AND FATIGABILITY IN WELL-FUNCTIONING OLDER ADULTS

Luigi Ferrucci,[1] Eleanor Simonsick,[2] and Ajoy Karikkineth,[1] *1. National Institute on Aging, Baltimore, Maryland, United States, 2. National Instute on Aging/NIH, Baltimore, Maryland, United States*

The association between vascular health measured by arterial stiffness and fatigability, a marker of future mobility decline, is unknown. We examined 1210 men (47.7%) and women from the Baltimore Longitudinal Study of Aging, mean age 66.6 ± 13.9 years. Perceived fatigability was assessed after a 5-minute, treadmill walk using Borg rating (range 6-20). Arterial stiffness was determined by carotid femoral pulse wave velocity (PWV). In linear regression analyses fatigability and PWV were associated in men (Beta/P-value) (0.160/0.001) and women (0.136/0.008). Adjustment for mean arterial and pulse pressure attenuated the association in women (0.104/0.050) but not men (0.160/0.001). The association was significant among those with slower usual and rapid gait speeds, longer 400m walk time and slower repeated chair stands pace (all p<0.05). Arterial stiffness is associated with a greater proneness to fatigue especially in older adults exhibiting poorer mobility. The underlying mechanisms appear to differ between men and women.

### ASSOCIATIONS BETWEEN PERCEIVED FATIGABILITY AND AMYLOID STATUS IN THE BALTIMORE LONGITUDINAL STUDY OF AGING

Ryan Dougherty,[1] Amal Wanigatunga,[1] Murat Bilgel,[2] Yang An,[3] Eleanor Simonsick,[4] Susan Resnick,[2] Jennifer Schrack,[1] and Fangyu Liu,[5] *1. Johns Hopkins Bloomberg School of Public Health, Baltimore, Maryland, United States, 2. National Institute on Aging, Baltimore, Maryland, United States, 3. NIA, Baltimore, Maryland, United States, 4. National Instute on Aging/NIH, Baltimore, Maryland, United States, 5. Johns Hopkins University, Baltimore, Maryland, United States*

Higher level of and greater longitudinal increase in perceived fatigability are linked to cognitive decline and lower brain volumes in older adults. However, it remains unclear whether perceived fatigability is associated with Alzheimer's disease-related brain pathology. In the BLSA, 163 participants without neurological disease or cognitive impairment (aged 74.7+/-8.4 years, 45% men) were assessed for perceived fatigability using rating of perceived exertion after a 5-minute (0.67 m/s) treadmill walk and Aß burden using 11C-Pittsburgh compound B (PiB) positron emission tomography. Forty-four participants were PiB+ based on a mean cortical distribution volume ratio (DVR) cut point of 1.066. After adjusting for demographics, body composition, comorbidities and ApoE-e4, higher perceived fatigability was not associated with PiB+ status (OR=0.84; 95% CI: 0.69, 1.05). Results suggest perceived fatigability may contribute to cognitive decline through pathways other than Aß

pathology. Future studies should target other mechanisms linking perceived fatigability and cognitive decline.

### LONGITUDINAL ASSOCIATION BETWEEN PERCEIVED FATIGABILITY AND BRAIN VOLUMES IN COMMUNITY-DWELLING OLDER ADULTS

Fangyu Liu,[1] Yang An,[2] Amal Wanigatunga,[3] Alden Gross,[3] Eleanor Simonsick,[4] Luigi Ferrucci,[2] Susan Resnick,[2] and Jennifer Schrack,[3], *1. Johns Hopkins University, Baltimore, Maryland, United States, 2. National Institute on Aging, Baltimore, Maryland, United States, 3. Johns Hopkins Bloomberg School of Public Health, Baltimore, Maryland, United States, 4. National Instute on Aging/NIH, Baltimore, Maryland, United States*

Perceived fatigability is linked to declining physical and cognitive performance, yet whether fatigability reflects early subclinical change in brain structure is unknown. Using mixed effects models, we assessed the longitudinal association of 3T MRI-derived brain volumes with perceived fatigability after a 5-min treadmill walk (0.67 m/s, 0% grade) using the Borg Rating of Perceived Exertion scale (range 6-20) in 802 BLSA participants (age 68.2+/-12.4 years, 45% men 66% White). In models adjusted for intracranial volume, demographics, chronic conditions, and CESD score, declining gray matter volumes in the frontal (β=-0.01) and temporal (β=-0.02) lobes, as well as the hippocampus (β=-0.25), precuneus (β=-0.10) and thalamus (β=-0.19) were associated with higher fatigability. Larger ventricular volumes were also associated with higher fatigability (β=0.02). Brain atrophy, particularly in gray matter and the hippocampal region, is longitudinally associated with increased fatigability in cognitively normal older adults, making it a potential marker of brain atrophy.

### Session 2290 (Symposium)

### PROSPECTIVE MONITORING OF NEWLY MARKETED DRUGS IN FRAIL OLDER ADULTS USING REAL-WORLD DATABASES

Chair: Dae Kim Co-Chair: Elisabetta Patorno

In recent years several new drugs have been approved for treatment of heart failure and type 2 diabetes. Despite their life-prolonging benefits, uptake of new drugs is often slow among older patients with frailty due to under-representation of frail older adults in pivotal clinical trials and concerns for adverse events. To optimize pharmacotherapy, timely evaluation of the drug benefits and risks is urgently needed. We propose a novel drug monitoring framework that prospectively evaluates the effectiveness and safety of newly marketed drugs for frail and non-frail patients in real-world databases. This framework utilizes a validated claims-based frailty index (CFI) (range: 0-1; frail if ≥0.20) to find early signals for effectiveness and safety of new drugs by updating the analysis at regular intervals as new data become available. In this symposium, we present early results of this prospective monitoring framework for 2 new drug classes using Medicare claims data from the approval date until the end of 2017: 1) angiotensin receptor-neprilysin inhibitor (ARNI) (approved in July 2015) for heart failure with reduced ejection fraction (HFrEF) and 2) sodium-glucose cotransporter-2 inhibitors (SGLT2i) (approved in March 2013) for type 2 diabetes. We first show the uptake of ARNI and SGLT2i over time among the eligible Medicare beneficiaries by clinical characteristics, including frailty. Subsequently we present the results of sequential cohort analysis for the effectiveness and safety results of ARNI and SGLT2i. After these presentations, the panel will discuss the strengths, limitations, and challenges of implementing our monitoring framework in real-world databases.

### MONITORING THE EFFECTIVENESS AND SAFETY OF ARNI VS. ANGIOTENSIN RECEPTOR BLOCKER BY FRAILTY STATUS

Elisabetta Patorno,[1] Chandrasekar Gopalakrishnan,[1] and Dae Kim,[2] *1. Brigham and Women's Hospital, Boston, Massachusetts, United States, 2. Hebrew SeniorLife, Boston, Massachusetts, United States*

Using Medicare data 2015-2017, we conducted 5 sequential 1-to-1 propensity score-matched analyses of ARNI initiators and angiotensin receptor blockers (ARB) initiators, mimicking the accrual of new data every 6 months. Primary effectiveness endpoint was a composite of heart failure hospitalization or all-cause mortality and primary safety endpoint was a composite of hospitalization or emergency department visits for hypotension, acute kidney injury, hyperkalemia, and angioedema. Among non-frail patients (n=5,014), the rates (per 100 person-years) for ARNI vs ARB were 12.7 and 9.2 (rate difference: 3.4, 95% CI: 0.8 to 6.1), respectively, for the effectiveness endpoint and 5.2 and 3.6 (rate difference: 1.5, 95% CI: -0.1 to 3.2), respectively, for the safety endpoint. Among frail patients (n=2,694), the corresponding rates were 19.8 and 21.6 (rate difference: -1.8, 95% CI: -7.0 to 3.4) for the effectiveness endpoint and 10.9 and 8.0 (rate difference: 2.9, 95% CI: -0.6 to 6.4) for the safety endpoint.

### MONITORING THE COMPARATIVE SAFETY OF SGLT2I VS GLP-1 RA IN OLDER ADULTS WITH TYPE 2 DIABETES BY FRAILTY STATUS

Alexander Kutz,[1] Chandrasekar Gopalakrishnan,[2] Dae Kim,[3] and Elisabetta Patorno,[2] *1. Brigham and Women's Hospital, Harvard Medical School, Boston, Massachusetts, United States, 2. Brigham and Women's Hospital, Boston, Massachusetts, United States, 3. Hebrew SeniorLife, Boston, Massachusetts, United States*

Using Medicare (4/2013-12/2017), we conducted 9 sequential analyses of patients with type 2 diabetes initiating SGLT2i vs. GLP1-RA mimicking the accrual of new data every 6 months to monitor SGLT2i safety with respect to diabetic ketoacidosis (DKA) since their U.S. approval. For each analysis, we estimated cumulative HRs (95% CIs) after 1:1 propensity score matching on >70 covariates comparing treatments within frail and non-frail patients. By analysis 1, SGLT2i were associated with a higher DKA rate vs. GLP-1RA in both frail and non-frail patients, but results were highly imprecise due to few events. With the accrual of more DKA events, precision of the estimates continued to improve through analysis 9 [HR=2.95 (95% CI, 1.19-7.31)] in frail patients; [HR=1.77 (1.15, 2.75)] in non-frail patients], with sufficiently precise estimates by analysis 6 in frail patients [HR=2.80 (95% CI, 1.03, 7.61)] and by analysis 7 in non-frail patients [HR=1.62 (95% CI, 1.01, 2.57)].

### MONITORING UTILIZATION TRENDS OF GLUCOSE-LOWERING DRUGS FOR TYPE 2 DIABETES IN OLDER ADULTS BY FRAILTY STATUS

Dae Kim,[1] Alexander Kutz,[2] Elisabetta Patorno,[3] and Chandrasekar Gopalakrishnan,[3] *1. Hebrew SeniorLife, Boston, Massachusetts, United States, 2. Brigham and Women's Hospital, Harvard Medical School, Boston, Massachusetts, United States, 3. Brigham and Women's Hospital, Boston, Massachusetts, United States*

Using Medicare fee-for-service data from 2013-17, we identified a cohort of patients with type 2 diabetes (T2D) who initiated a glucose-lowering drug (mean [SD] age, 74.8 (6.9) years). Amongst frail patients (CFI≥0.20), metformin use remained stable from 29.1% to 29.4%, whereas sulfonylureas (25.8% to 22.1%) and insulin (21.2% to 19.0%) use declined. Amongst non-frail patients (CFI <0.20), metformin (35.3% to 33.1%) and sulfonylurea (26.2% to 22.2%) use decreased whereas insulin (11.7% to 10.6%) use remained stable. DPP-4i and glitazones use remained stable whereas the use of newer agents such as SGLT-2i and GLP-1 RA increased steadily over the study period in both frail and non-frail patients, though their use remains low ( <8%). In conclusion, sulfonylureas and insulin accounted for about one-third of initiated glucose-lowering medications and were more frequently used by frail patients, though their use declined steadily over time with the availability of newer agents.

### TRENDS OF ARNI AND OTHER HEART FAILURE MEDICATION USE IN OLDER ADULTS

Elisabetta Patorno,[1] Chandrasekar Gopalakrishnan,[1] Dae Kim,[2] and Yu-Chien Lee,[3] *1. Brigham and Women's Hospital, Boston, Massachusetts, United States, 2. Hebrew SeniorLife, Boston, Massachusetts, United States, 3. Linkou Chang-Gung Memorial Hospital, Linkou Chang-Gung Memorial Hospital, Taoyuan, Taiwan (Republic of China)*

In 2015-2017, we identified 276,679 to 315,788 Medicare beneficiaries with HFrEF (mean age 76.6-76.7 years, 75.0-76.2% male, 82.0-83.4% Whites, and 44.8-50.9% frail). Since its approval in July 2015, ARNI use increased from 0.3% to 5.7%. ARNI uptake was lower in patients with older age (6.6% for 65-74 years vs 3.4% for ≥85 years), non-Hispanic race (7.3% for Hispanic vs 5.6-6.6% for other race), no dual eligibility (6.4% for dual eligibility vs 5.5% for no dual eligibility), frailty (5.1% for frailty vs 6.1% for non-frailty) and dementia (3.8% for dementia vs 6.1% for no dementia). Frail patients were less likely than non-frail patients to receive disease-modifying treatments, such as angiotensin-converting enzyme inhibitors (32.4% vs 38.9%), angiotensin receptor blockers (14.5% vs 17.5%), aldosterone antagonists (20.8% vs 23.4%), and beta-blockers (65.1% vs 68.3%), but more likely to receive symptomatic treatment with loop diuretics (56.4% vs 48.0%).

### Session 2295 (Symposium)

### RECRUITMENT, ENROLLMENT, AND RETENTION: CREATIVE TECHNIQUES TO OVERCOME OBSTACLES

Chair: Justine Sefcik Discussant: Darina Petrovsky

The process of recruiting, enrolling, and retaining older adults in research studies has been challenging, even prior to the COVID-19 pandemic. This symposium presents research conducted and lessons learned on recruiting, enrolling, and retaining older adults, including those with cognitive impairment. Insights are provided on what techniques are most beneficial for improving rates of research participation, spanning time prior to and during the pandemic. The first presentation reports on qualitative perspectives of persons living with dementia and their caregivers as to what helped them decide to enroll into a clinical trial together. The second presentation speaks to how variations in incentive payment allocations played a role in consent decisions of patients with amnestic mild cognitive impairment and their study partners. The third presentation discusses the effectiveness of an adapted framework and strategies to increase the recruitment and retention of older Latinos with Alzheimer's Disease and Related Dementias (ADRD) into a clinical trial. The fourth presentation shares techniques for recruiting older adults for a survey study during the pandemic. The fifth presentation defines challenges during a longitudinal study when the pandemic and other natural disasters occurred and strategies for success. Taken together, these presentations will inform researchers on techniques that could be used to improve recruitment, enrollment, and retention of older adults in clinical research.

### DYADS' PERCEPTIONS: RECRUITING PERSONS LIVING WITH DEMENTIA AND CAREGIVERS IN A CLINICAL TRIAL

Darina Petrovsky,[1] Glenna Brewster,[2] Junxin Li,[3] Nalaka Gooneratne,[4] Nancy Hodgson,[5] Miranda McPhillips,[6] and Justine Sefcik,[7] *1. Rutgers University, Philadelphia, Pennsylvania, United States, 2. Nell Hodgson Woodruff School of Nursing Emory University, Atlanta, Georgia, United States, 3. Johns Hopkins University, Baltimore, Maryland, United States, 4. University of Pennsylvania School of Medicine, Philadelphia, Pennsylvania, United States, 5. University of Pennsylvania, School of Nursing, philadelphia, Pennsylvania, United States, 6. University of Pennsylvania, University of Pennsylvania, Pennsylvania, United States, 7. Drexel University, College of Nursing and Health Professions, Philadelphia, Pennsylvania, United States*

Recruiting persons living with dementia (PLWD) and their caregivers (dyads) into research is challenging and costly. The purpose of this study was to better understand factors that influence dyads decisions to enroll in a clinical trial. We used Ajzen's Theory of Planned Behavior (TPB) to develop a qualitative interview guide and analyze the data with a directed content analysis. We conducted semi-structured telephone interviews with 12 PLWD and 9 caregivers who all enrolled in one clinical trial. Aligning with the TPB we found the following positively influenced enrollment: 1) wanting to learn, in-person meetings with knowledgeable staff, and the money always helps (attitudes toward joining); 2) to support another person (perceived norm); and 3) easy to participate (perceived behavioral control). Flexible scheduling and the study taking place in the home was comfortable and convenient for participants. Findings can inform future recruitment efforts and research studies.

### VARIATIONS IN PAYMENT ALLOCATION TO PERSONS LIVING WITH COGNITIVE IMPAIRMENT AND STUDY PARTNERS

Junxin Li,[1] Justine Sefcik,[2] Darina Petrovsky,[3] Glenna Brewster,[4] Nancy Hodgson,[5] Nalaka Gooneratne,[6] and Miranda McPhillips,[7] *1. Johns Hopkins University, Baltimore, Maryland, United States, 2. Drexel University, College of Nursing and Health Professions, Philadelphia, Pennsylvania, United States, 3. Rutgers University, Philadelphia, Pennsylvania, United States, 4. Nell Hodgson Woodruff School of Nursing Emory University, Atlanta, Georgia, United States, 5. University of Pennsylvania, School of Nursing, philadelphia, Pennsylvania, United States, 6. University of Pennsylvania School of Medicine, Philadelphia, Pennsylvania, United States, 7. University of Pennsylvania, University of Pennsylvania, Pennsylvania, United States*

There is a paucity of research focused on monetary incentives for recruiting dyads (participants with cognitive impairment and study partners) into research. Our objective was to evaluate if two different variations in allocating compensation among dyads changed consent rates in one clinical trial, Memories2. This trial is evaluating cognitive and functional outcomes of obstructive sleep apnea treatment in patients with amnestic mild cognitive impairment (aMCI). Prior to phone screening, participants were randomly assigned to one of two groups (1) $200 to participant with aMCI or (2) $100 to participant with aMCI and $100 to study partner at consent visit. Allocating all the payment to the participant with aMCI yielded a 2.6% consent rate, while splitting the payment yielded at 1.7% consent rate. We will also discuss how demographic factors affected consent decision by group. This study provides insight into novel strategies that may enhance enrollment of dyads into clinical trials.

### RECRUITMENT AND RETENTION FRAMEWORK FOR A TIMED-ACTIVITY INTERVENTION AMONG OLDER LATINOS WITH ADRD

G. Adriana Perez, *University of Pennsylvania School of Nursing, Phildelphia, Pennsylvania, United States*

Latino participation in ADRD research is essential to advance cognitive health equity. We present results of an adapted framework to increase recruitment and retention of older Latinos with ADRD and caregivers (CGs) in a timed-activity intervention. Framework factors include 3 structures with strategies informed by a Latino Community Advisory Board. For Characteristics of Study Processes, we included linguistically equivalent data collection procedures/measures, scheduled at times most convenient for participants/CGs. Participants were called weekly for questions/guidance with procedures. Intervention sessions built-in additional time to embed Latino cultural values: familismo, personalismo, confianza and respeto. Study Team Infrastructure, included bilingual/bicultural members/students; and trusted community partners to assist with participant referrals. For Preferences and Beliefs Toward Research, we conducted a series of focus groups to understand beliefs about "memory health" and perceptions of ADRD risks. Strategies yielded effective results. We reached our recruitment goal; started a wait-list of interested participants; had zero (n=0) attrition.

### RECRUITMENT OF OLDER ADULTS DURING THE COVID-19 PANDEMIC: UTILIZING TWO RECRUITMENT TECHNIQUES

Justine Sefcik,[1] Zachary Hathaway,[2] Rose Ann DiMaria-Ghalili,[1] and Martha Coates,[3], *1. Drexel University, College of Nursing and Health Professions, Philadelphia, Pennsylvania, United States, 2. Drexel University, Philadelphia, Pennsylvania, United States, 3. Drexel University, Bryn Mawr, Pennsylvania, United States*

The COVID-19 pandemic has limited in-person interactions and reduced access to research participants. To recruit older adults for a study on the impact of COVID-19 on physical, mental, and social wellbeing we utilized two recruitment techniques: 1) ResearchMatch, a free recruitment database, and 2) a convenience sample of residents in a retirement community. Messages were sent via ResearchMatch to 1,491 adults age 65 and over. In total, 228 individuals responded over 2 weeks; 194 responded in the first 24 hours. Eighty-four completed the online survey. For the retirement community, recruitment information was shared during a Zoom townhall meeting; 44 expressed interest and 30 completed the study (half over the phone with a research assistant). We will discuss differences between the older adults recruited by each strategy (e.g., the ResearchMatch group was highly educated; more staff needed to interview retirement community participants). Overall, these were effective recruitment techniques during challenging times.

### IMPLEMENTING PHYSICAL ACTIVITY STUDIES DURING COVID-19 AND WINTER STORMS: LESSONS LEARNED

Calliope Murphy,[1] Tony Chao,[2] Charles Morrison,[1] Karen Chapman,[1] Ronald Lindsey,[1] Mary Hastings,[3] and Annalisa Na,[4] *1. University of Texas Medical Branch at Galveston, Galveston, Texas, United States, 2. University Of Texas Medical Branch, Galveston, Texas, United States, 3. Washington University School of Medicine in St. Louis, St. Louis, Missouri, United States, 4. Drexel University, College of Nursing and Health Professions, Drexel University College of Nursing and Health Professions, Pennsylvania, United States*

Patient recruitment and retention are challenging for longitudinal studies. Stay-at-home restrictions for the Galveston and Houston regions in 2020 for COVID-19 and in 2021 for the Winter Storms shut down elective healthcare activities and created additional recruitment barriers during the implementation of a 12-month study examining the physical function of older adults receiving a total knee arthroplasty. This presentation describes recruitment and retention strategies during natural disasters. Ten participants started the study during the pandemic and 6 remained through the winter storms (3 withdrew, 1 no showed). Physical activity monitors were distributed and collected through mail, patient reported outcomes were completed online or over the phone, clinician-initiated measures were only collected when clinics were open, and efforts were made to minimize staff burden and follow evolving hospital guidelines. Most importantly, regular communication and follow-up with participants, research team, and department personnel created a sense of community.

### Session 2300 (Paper)

### SOCIAL DETERMINANTS OF HEALTH I

### ADULT CHILDREN'S EDUCATION AND OLDER MOTHERS' HEALTH: ARE OFFSPRING'S PROBLEMS MEDIATORS OR MODERATORS?

Robert Frase,[1] Shawn Bauldry,[2] J. Jill Suitor,[2] and Megan Gilligan,[3] *1. Purdue University, West Lafayette, Indiana, United States, 2. Purdue University, Purdue University, Indiana, United States, 3. Iowa State University, Iowa State University, Iowa, United States*

Despite the growing body of literature documenting positive effects of adult children's education on older mothers' health outcomes there is limited research exploring the mechanisms that underlie and influence this relationship. This lack of knowledge limits our understanding of how or under what conditions older mothers benefit from their offspring's resources. In this paper, we draw from theories of the life course, cumulative inequality, and the social foreground to explore how adult children's problems (physical and emotional, personal and financial, and deviant behaviors) mediate and moderate the effect of adult children's education on older mothers' self-rated health and depressive symptoms. To address this question we use data collected from 420 mothers aged 75-85 reporting on their 1,514 adult children, as part of the Within Family Differences Study. Theoretically, this project adds to existing scholarship on intergenerational support in later-life families by identifying the conditions under which adult children's resources improve parents' well-being. Preliminary findings reveal that less educated adult children experience more problems, which in turn, negatively impact mothers' health. Additionally, when adult children experience problems in their own lives, mothers receive less care and financial support from their offspring, even from those who are well-educated and would otherwise have been expected to have shared resources. The findings will have implications for practice by increasing health care providers' awareness that older parents may be at risk for unmet needs for care even when adult children have resources that would have been expected to serve as a safety net.

### HEALTH AND SOCIAL DETERMINANTS ASSOCIATED WITH DELAY OF HEALTH CARE AMONG RURAL OLDER ADULTS

Autumn Decker, and Raven Weaver, *Washington State University, Pullman, Washington, United States*

Delaying healthcare has the capacity to increase morbidity and mortality, especially among individuals with chronic and acute health conditions. Older adults in rural areas are more likely to have chronic health conditions and are more likely to delay care due to financial barriers than their urban counterparts. To further investigate these associations, we conducted descriptive, bivariate, and regression analyses using data from a needs assessment designed to identify health needs and service delivery gaps among an economically diverse eight-county region. A random sample of adults responded to the survey, with 1,226 respondents aged 60+ (mean age = 71). The majority of respondents were White, female, and had insurance coverage. Overall, 35% of respondents experienced a delay in healthcare. We used logistic regression to determine the associations of age, gender, number of health conditions, household income, distance from medical facility, and perceived quality of neighborhood with delay of healthcare. Individuals with younger age ($p = .017$), more chronic conditions ($p < .001$), lower income ($p < .001$), and lower perceived quality of neighborhood ($p = .008$) were more likely to experience a delay in healthcare. These findings highlight risk factors that were salient prior to the onset of the COVID-19 pandemic. However, the pandemic has contributed to an increasing trend of delaying healthcare and may have amplified existing challenges. Findings may inform efforts led by healthcare providers and policy makers to facilitate timely and preventive healthcare use. Future research is needed to investigate the compounding long-term health implications of delaying healthcare.

### LIFE COURSE STRESSORS AND FUNCTIONAL LIMITATIONS IN LATER LIFE AMONG WHITE, BLACK, AND HISPANIC ADULTS

Madison Sauerteig,[1] Kenneth Ferraro,[1] and Shawn Bauldry,[2] *1. Purdue University, West Lafayette, Indiana, United States, 2. Purdue University, Purdue University, Indiana, United States*

Although striking racial and ethnic disparities in health are manifest during later life, they may be rooted in early-life exposures. Drawing from cumulative inequality theory, we investigate whether experiencing life course stressors increases the risk of later-life functional limitations and whether this relationship differs by race and ethnicity. This study utilizes longitudinal data from the Health and Retirement Study to test whether six indicators of child stressors and eleven indicators of adult stressors predict trajectories of the onset and severity of functional limitations in later life among a diverse sample of adults. We find that child and adult stressors are associated with earlier onset and greater severity of functional limitations during later life. Mediation analyses reveal the indirect influence of child stressors via adult stressors on onset and severity of functional limitations; however, the indirect effects are slightly stronger for Black and Hispanic adults than their White counterparts (i.e., moderated mediation). In sum, child stressors, in and of themselves, do not increase functional limitations among Black and Hispanic people but are associated with greater adult stress exposure, leading to more functional limitations in later life. Disparities in functional limitations are also partly due to lower education and less wealth among Black and Hispanic adults.

### SOCIAL ENGAGEMENT AND DEPRESSIVE SYMPTOMS AMONG MIDDLE-AGED AND OLDER ADULTS IN INDIA

Kasturi Banerjee,[1] and Tamara Baker,[2] *1. University of Kansas, Lawrence, Kansas, United States, 2. University of North Carolina, Chapel Hill School of Medicine, University of North Carolina at Chapel Hill (School of Medicine), North Carolina, United States*

Social networks and family support are known to have benefits for many older adults globally, and India is no exception. However, the exact nature of the impact of these factors on depression in the Indian population remains largely unexplored. Considering the aging Indian population and changing socio-cultural landscape, it is important to identify the role of social engagement and neighborhood factors in the mental health needs of these older adults. To address this need, this study aimed to identify predictors of depressive symptoms among individuals aged 45+ years from four states in India. Data were taken from the Longitudinal Aging Study in India (LASI) pilot survey in 2010, focusing on the influence social activities, family factors and neighborhood satisfaction variables have on depressive symptomatology. A hierarchical multiple regression analysis was conducted and found that residing in a southern state (Karnataka and Kerala) ($\beta$=.178, $p<0.05$); lower life satisfaction ($\beta$= -.261, $p<0.05$); having more living children ($\beta$=.110, $p<0.05$), less frequently or never visiting friends and relatives ($\beta$=.079, $p<0.05$) and not liking the neighborhood ($\beta$=.072, $p<0.05$) were predictors of depressive symptoms. These findings are consistent with limited extant literature on the importance of family and social engagement as predictors of depressive symptoms in this population. Future research should focus on qualitatively examining the interaction between depressive symptoms and social engagement within this population, thereby helping develop targeted interventions, measure outcomes and long term, community engagement-based prevention programs.

### THE 2008 RECESSION AND BIOLOGICAL HEALTH: PSYCHOLOGICAL WELL-BEING AND SOCIAL DISADVANTAGE MODIFY VULNERABILITY

Julie Kirsch, *University of Wisconsin-Madison, University of Wisconsin-Madison, Wisconsin, United States*

Racial minorities and educationally disadvantaged experienced more housing loss, unemployment, and financial strain during the 2007-2009 Great Recession. These hardships may heighten stress and amplify persistent and growing health inequities, which were further worsened by the recent COVID-19 pandemic. It is therefore essential to identify factors that contribute to individual differences in vulnerability so that more effective interventions can be implemented, especially in older adult populations who may face unique economic hardships tied to age discrimination. According to the reserve capacity model, higher levels of psychosocial resources, including psychological well-being, can protect against the negative health outcomes related to heightened stress exposure. This study tested the intersections between recession hardship, pre-existing vulnerability defined as racial and educational disadvantage, and psychological well-being as predictors of biological indicators of chronic allostatic load. Chronic allostatic load was assessed with cardiovascular reactivity and recovery to acute mental stress and systemic inflammation (basal indicators of C-reactive protein and interleukin 6). Biological data came from a national sample of adults known as the Midlife in the US Study ( MIDUS; age = 25-75, N=863) that completed assessments after the recession. Multiple regression models revealed that more widespread recession hardship predicted greater biological dysregulation. Tests of three-way interactions revealed that the association between recession hardship and biological dysregulation was strongest among respondents with combined disadvantages of low educational status and low levels of psychological well-being. This study connected a major economic event to individual variation in health vulnerability and identified potential biological pathways to future disease outcomes.

### Session 2305 (Symposium)

### SOCIAL RELATIONSHIPS IN OLDER ADULTHOOD AND LINKS WITH PSYCHOLOGICAL AND PHYSICAL WELL-BEING

Chair: Emily Willroth Discussant: Patrick Hill

Positive social relationships are fundamental to psychological and physical well-being across the lifespan. This symposium showcases rigorous daily-diary and longitudinal investigations that (a) examine change in social relationships and loneliness in older adulthood, and (b) investigate links between social relationships and psychological and physical well-being outcomes in older adulthood. First, we present results from a coordinated analysis of three longitudinal studies demonstrating that loneliness tends to increase across the second half of life (Talk 1). Second, we share converging evidence that suggests positive social relationships tend to decline with age. In turn, these longitudinal changes in loneliness and social relationships predict later physical health outcomes (Talk 2). Together, these findings suggest that positive social relationships tend to decrease and loneliness tends to increase with age, resulting in physical health costs. In the second half of the symposium, we turn to research on how positive social relationships may promote psychological well-being, and in turn, better physical health in older adulthood. Using daily diary data, we demonstrate that on days when older adults report more positive social interactions, they also report feeling more sense of purpose (Talk 3). Finally, we show that higher sense of purpose and more positive change in sense of purpose in midlife prospectively predicts better physical health in older adulthood (Talk 4). Together, the research presented in this symposium reveals normative declines in social relationships in late life, while also highlighting the potential health benefits of increasing positive social relationships in older adulthood.

### ADVANCING THE STUDY OF LONELINESS AND HEALTH IN OLDER ADULTS THROUGH THE USE OF INTEGRATIVE DATA ANALYSIS

Eileen Graham,[1] Kathryn Jackson,[2] Emorie Beck,[3] Jing Luo,[1] Olivia Atherton,[1] Emily Willroth,[1] Daniel Mroczek,[1] and Anthony Ong,[4] *1. Northwestern University, Chicago, Illinois, United States, 2. Northwestern university, Chicago, Illinois, United States, 3. northwestern University, Chicago, Illinois, United States, 4. Cornell University, Ithaca, New York, United States*

Recent work has shown the importance of studying loneliness and social isolation across adulthood for understanding healthy aging. This project explored loneliness trajectories across multiple independent samples. Using coordinated IDA, we estimated and meta-analyzed identical multilevel growth models in loneliness using three samples (ELSA, SHARE, HRS). We found u-shaped change, suggesting that loneliness may decline from young adulthood to midlife, then increase after midlife. These trajectories were significant across all three datasets and not fully explained by demographics or depression. We found that divorce, widowhood, social isolation, and functional limitations were associated with higher overall loneliness. Additionally, divorce and functional limitations, and sex (being male) were associated with deeper dips in loneliness in midlife and steeper increases in old age. These findings suggest that loneliness increases across the second half of life and point to the need for evidence-based strategies for addressing social disparities in midlife and later adulthood.

### EARLY TRAUMA AND LATER HEALTH: EXAMINING THE MEDIATION OF SOCIAL RELATIONSHIPS IN ADULTHOOD IN HRS

William Chopik,[1] Amanda Leggett,[2] Jooyoung Kong,[3] Courtney Polenick,[4] and Yin Liu,[5], *1. Michigan State University, East Lansing, Michigan, United States, 2. University of Michigan, Ypsilanti, Michigan, United States, 3. University of Wisconsin-Madison, Madison, Wisconsin, United States, 4. University of Michigan, Ann Arbor, Michigan, United States, 5. Utah State University, Logan, Utah, United States*

Early trauma is associated with compromised health and well-being in later life, but whether social functioning mediates the association is unclear. Participants in the Health and Retirement Study (n = 15,946) had baseline surveys in years 2006 and 2008 (T1), and were followed up twice (T2-3) every 4 years. Health outcomes included depressive symptoms, chronic health conditions, and subjective memory complaints. Social relationships were measured by contacts, relationship strains, and feelings of loneliness. Early trauma was measured by parental physical abuse and alcohol and drug problems in the family before the age of 16. Social contacts decreased over time, while relationship strains and loneliness increased especially for older adults with early trauma, which in turn mediated the associations between early trauma and poorer health in later life. The findings suggested that maintaining positive social relationships are beneficial for better health in late life, especially for individuals with early trauma exposures.

### DAILY SOCIAL INTERACTIONS AS A ROUTE TO PURPOSEFUL DAYS IN OLDER ADULTHOOD

Gabrielle Pfund,[1] Mathias Allemand,[2] and Matthias Hofer,[2] *1. Washington University in St. Louis, Saint Louis, Missouri, United States, 2. University of Zurich, Zurich, Zurich, Switzerland*

Sense of purpose predicts slower cognitive decline, reduced risk for health issues, and greater longevity (Pfund & Lewis, 2020). However, work is limited regarding how we can help older adults maintain purposefulness in daily life. The current study explored positive daily social interactions as a route to daily purposefulness in older adults, using a measurement burst design. Older adults completed surveys for five-day bursts spread six months apart (Mean age = 70.75, SD = 7.23; n = 104). Multilevel models demonstrated that on days when individuals reported more positive social interactions, they reported feeling more purposeful (b = 0.39, 95% CI [0.28, 0.51]) when accounting for health, employment, and relationship status. Employment status moderated this association, as daily social interactions were more strongly associated with daily purpose for unemployed/retired individuals (b = -0.23, 95% CI [-0.38, -0.08]). Positive social interactions thus may help older adults maintain purposefulness, particularly after retirement.

### MAINTAINING SENSE OF PURPOSE IN MIDLIFE PREDICTS BETTER PHYSICAL HEALTH

Daniel Mroczek, and Emily Willroth, *Northwestern University, Chicago, Illinois, United States*

Having a sense of purpose in life is fundamental to psychological and physical well-being. Despite the myriad benefits of purpose, it may be difficult to hold onto purpose as people age and experience fewer future-oriented goals. The present research used reliable change indices to estimate change in sense of purpose during midlife in three diverse samples. On average, sense of purpose declined slightly with age in all three samples. Next, we used linear regression to examine associations between sense of purpose levels and sense of purpose change and later self-reported physical health outcomes. Consistent with our preregistered hypotheses, higher sense of purpose predicted better health in the two larger samples and more positive sense of purpose trajectories better health in all three samples. Together, these findings suggest that both having a sense of purpose and holding onto it may be important for physical health in middle to older adulthood.

### Session 2310 (Symposium)

### STRESS PROCESSES OVER 2 DECADES: FINDINGS FROM THE NATIONAL STUDY OF DAILY EXPERIENCES

Chair: David Almeida Discussant: Daniel Mroczek

Daily experiences of stress and the associated sequelae of affective and physiological changes represent the multiple dimensions of a complex, time-dependent process of how stressors unfold in daily life. Daily diaries capture these time-sensitive processes as they occur under real world conditions. Longitudinal changes in stress processes can then be tracked using a measurement burst design: daily diaries repeated longitudinally. Using this design, the National Study of Daily Experiences (NSDE) has generated more than 35,000 days of data from a national sample of over 2,500 adults assessed repeatedly across 20 years of adulthood. The NSDE features details of more than 10,000 reports daily stress including exposure, appraisal and affective responses from adults ranging in age from 24 to 95 years. The current symposium leverages this unique and influential dataset to examine age differences and aging-related changes in daily stress processes with four presentations from the NSDE. First, Dr. Robert Stawski will discuss longitudinal change and age-related differences in exposure to multiple types of daily stressors. Next, Dr. Susan Charles will examine age differences and change in a key element of the stress process: negative affect. Third, Dr. Eric Cerino will describe longitudinal change in appraisals of daily stressors focusing on stressor control. Finally, Dr. David Almeida will examine changes in negative affect reactivity to daily stressors across the 20 years of the NSDE. Dr. Dan Mroczek will discuss the picture these presentations provide of how aging and age-differences impact the daily stress process and future directions for understanding these trajectories.

### AGE-RELATED CHANGE IN THE DAILY STRESSOR REACTIVITY ACROSS 20 YEARS OF ADULTHOOD

Jacqueline Mogle,[1] Jonathan Rush,[2] and David Almeida,[3]
*1. Penn State University, University Park, Pennsylvania, United States, 2. University of Victoria, Victoria, British Columbia, Canada, 3. Pennsylvania State University, University Park, Pennsylvania, United States*

Affective reactivity to everyday stressful events has been shown to be an important predictor of poor mental and physical health. The purpose of this study was to examine longitudinal changes in daily stress across 30 years of adulthood as a critical first step for understanding aging-related trends in daily stress. We used data from the National Study of Daily Experiences (NSDE) to calculate exposure and reactivity to daily stressors collected during telephone interviews over the course of 8 consecutive days. These daily assessment bursts were conducted in 1997, 2007, and 2018. Data were comprised of 33,931 daily interviews from 2,880 adults ages 25-74 at the first burst. Results indicated decreased stressor reactivity over time but this decrease was greater for younger adults. Discussion will focus on how examining change in daily stress processes is critical for illuminating stress and health.

### AGE DIFFERENCES AND LONGITUDINAL CHANGE IN EXPOSURE TO DAILY STRESSORS: THREE WAVES OF DIARY DATA ACROSS 20 YEARS

Eric Cerino,[1] Jonathan Rush,[2] and Robert Stawski,[3]
*1. Pennyslvania State University, State College, Pennsylvania, United States, 2. University of Victoria, Victoria, British Columbia, Canada, 3. Oregon State University, Corvallis, Oregon, United States*

Exposure to daily stress is an important risk factor for healthy aging. We examined cross-sectional age-related differences and longitudinal aging-related change in stressor exposure across three waves of the National Study of Daily Experiences (N=2,914, M=51.53 years, SD=13.55, 56.35% Female) spanning 20 years. Exposure to six types of stressors (arguments, avoided arguments, work overloads, home overloads, network stressors, other) were obtained from telephone interviews over 8 consecutive days in waves conducted in ~1996, ~2008, and ~2017. Longitudinal analyses revealed declines in stressor exposure across 20 years ($p<.01$), driven by declines in arguments, work overloads, and network stressors specifically. Cross-sectional analyses indicated that older individuals reported stressors less frequently ($p<.01$), driven by decreases in arguments, avoided arguments, work overloads, and home overloads specifically. Rates of longitudinal decline did not depend on age at baseline. Results suggest that aging-related changes and baseline age differences inform daily stress trajectories in mid- and later-life.

### CHANGE IN DAILY NEGATIVE AFFECT ACROSS 20 YEARS

Jennifer Piazza,[1] Jonathan Rush,[2] and Susan Charles,[3]
*1. California State University, Fullerton, California State University, Fullerton, California, United States, 2. University of Victoria, Victoria, British Columbia, Canada, 3. University of California, Irvine, Irvine, California, United States*

The current study examined levels of daily NA among people (N=413) who participated in three waves of the National Study of Daily Experiences (~1996; ~2008; ~2017). At each wave, participants reported how often they had experienced six negative emotional experiences every day for eight consecutive days. Cross-sectional analyses at each time-point show age-related decreases in NA. Trajectories over time, however, were moderated by age (Est = .006, SE = .002, p = .001), revealing a curvilinear pattern. Among people who were 25-50 years-old at the first wave, daily NA decreased over time, with decreases more pronounced among the younger adults. For people at least 50 years-old at the start of the study, daily NA increased over time, with the slopes steepest for older adults. Findings indicate that cross-sectional and longitudinal age-related patterns in NA differ when examining data collected from 1996 to 2017.

### CHARACTERIZING PERCEIVED CONTROL OVER DAILY STRESS: LONGITUDINAL CHANGES AND ASSOCIATIONS WITH AFFECT

Jacqueline Mogle,[1] Robert Stawski,[2] Jonathan Rush,[3] David Almeida,[4] and Eric Cerino,[5] *1. Penn State University, University Park, Pennsylvania, United States, 2. Oregon State University, Corvallis, Oregon, United States, 3. University of Victoria, Victoria, British Columbia, Canada, 4. Pennsylvania State University, University Park, Pennsylvania, United States, 5. Pennyslvania State University, State College, Pennsylvania, United States*

Perceived control is an important psychosocial correlate of healthy aging. Using data from the National Study of Daily Experiences (N=1,047, M=55.82 years, SD=10.35, 57.27% Female), we examined cross-sectional age-related differences and longitudinal aging-related change in perceived control over daily stress across 10 years and explored the influence of stressor control on negative affect (NA) and positive affect (PA). Stressor control, NA, and PA were obtained from telephone interviews over 8 consecutive days in measurement bursts conducted in ~2008 and ~2017. Longitudinal analyses revealed significant declines in stressor control across 10 years ($p<.001$). Cross-sectional analyses revealed marginally lower stressor control among older individuals ($p<.10$). Within-person associations revealed lower NA and higher PA on days when stressor control was higher than usual ($ps<.001$). Results suggest that stressor control declines with age and holds promise as an important component of daily stress processes with relevance for health and well-being outcomes across the lifespan.

### Session 2315 (Paper)

### SUCCESSFUL AGING

### DEVELOPMENT OF A COMPREHENSIVE CHINESE SUCCESSFUL AGING SCALE: INCORPORATING THE VIEWPOINTS OF OLDER ADULTS

Edwin K. H. Chung,[1] and Dannii Yeung,[2] *1. City University of Hong Kong, Hong Kong, Hong Kong, 2. City University of Hong Kong, Hong Kong, Not Applicable, Hong Kong*

Inspiring by Martinson and Berridge's (2015) systematic review, the current definition of successful aging (SA) fails to acknowledge the laypeople's conceptualization of SA. Adopting a mixed-method approach, two studies were conducted with the aim of soliciting older adults'

perceptions of SA and to develop a multidimensional instrument for assessing SA. Study 1 was a qualitative study and 27 community-dwelling older adults (Mage=68.07 years, SD=7.10, range=60–83; 56.3% females) were interviewed. Interview transcripts were analyzed, and seven themes were emerged. An initial item pool for the Successful Aging Scale (SAS) was then established based on these themes as well as those in the SA literature, such as acceptance and independence. Study 2 was a survey study which was conducted among 414 community-dwelling older adults (Mage=64.50 years, SD=4.01, range=60–82; 55.3% females) to identify optimal items for constitution of the SAS. Exploratory factor analysis revealed a 12-factor solution, accounting for 62% of the variance. The 12 factors are adequate health, perceived constraints, flexible attitudes toward life, acceptance of age-related change, life embracement, active engagement, harmonious family, supportive friendship, civic awareness, social contribution, living independently, and adaptive coping strategies. The 12 factors exhibit similar strength of associations with most of the well-being measures, but certain factors show stronger correlation with depressive symptoms and social relationship, suggesting the uniqueness of each factor. Overall, the SAS demonstrates promising psychometric properties. These findings disclose that the older adults' perceptions of SA could cover broader dimensions than those in Rowe and Kahn's model (1997).

### I THINK I AGE GRACEFULLY: A FOCUS GROUP STUDY OF SUCCESSFUL AGING CONCEPTIONS AMONG OLDER WOMEN LIVING WITH HIV

Anna Rubtsova,[1] Tonya Taylor,[2] Gina Wingood,[3] Igho Ofotokun,[4] Deborah Gustafson,[2] and Marcia Holstad,[5] *1. Emory University Rollins School of Public Health, Emory University, Georgia, United States, 2. SUNY Downstate Health Sciences University, Brooklyn, New York, United States, 3. Columbia University Mailman School of Public Health, New York, New York, United States, 4. Emory University School of Medicine, Atlanta, Georgia, United States, 5. Emory University, Atlanta, Georgia, United States*

Our previous quantitative research found high prevalence of self-rated successful aging (SA) among older (age ≥50) women living with HIV (OWLH) enrolled in the Women's Interagency HIV Study (WIHS). However, little is known about how OWLH define SA. Most studies have examined SA among predominantly white men living with HIV. Therefore, the purpose of our qualitative study was to examine subjective understandings of SA among OWLH and, as a comparison group, older HIV-seronegative women at risk of HIV. Four focus group discussions (FGD) were conducted among 23 participants (12 OWLH, 11 HIV-seronegative). These women were recruited from WIHS participants previously enrolled in our quantitative study of SA, "From Surviving to Thriving" (FROST), at two WIHS sites – Atlanta and Brooklyn. At each site, we conducted two FGD – one with OWLH and one with older HIV-seronegative women in February-March of 2019. Participants were, on average, 56 years old (range, 51-70), 78% Black, and 60% with annual income ≤ $12,000. A team of coders conducted thematic coding of fully transcribed FGD using MAXQDA software. Several themes emerged. Both OWLH and older HIV-seronegative women defined SA as "aging gracefully," i.e. accepting and celebrating aging after having survived hardships of earlier life (e.g., HIV diagnosis, drug use). They also emphasized taking care of themselves (e.g., taking their meds) and spirituality in their definitions of SA. In contrast to HIV-seronegative participants, who prioritized sobriety as taking good care of themselves, OWLH emphasized taking care of their HIV (e.g., "staying on top of your numbers").

### LINKAGES BETWEEN INDIGENOUS CULTURAL GENERATIVITY AND SOBRIETY TO PROMOTE SUCCESSFUL AGING AMONG ALASKA NATIVES

Jordan Lewis, *Memory Keepers Medical Discovery Team/ University of Minnesota Medical School, Duluth campus, Duluth, Minnesota, United States*

This article builds on the People Awakening Project, which explored an AlaskaNative understanding of the recovery process from alcohol use disorder and sobriety. The presentation will explore motivating and maintenance factors for sobriety among older AN adult participants (age 50+) from across Alaska. Ten life history narratives of Alaska Native older adults, representing Alutiiq, Athabascan, Tlingit, Yup'ik/Cup'ik Eskimos, from the PA sample were explored using thematic analysis. AN older adults are motivated to abstain from, or to quit drinking alcohol through spirituality, family influence, role socialization and others' role modeling, and a desire to engage in indigenous cultural generative activities with their family and community. A desire to pass on their accumulated wisdom to a younger generation through engagement and sharing of culturally grounded activities and values, or indigenous cultural generativity, is a central unifying motivational and maintenance factor for sobriety. The implications of this research indicates that family, role expectations and socialization, desire for community and culture engagement, and spirituality are central features to both Alaska Native Elders' understanding of sobriety, and more broadly, to their successful aging. Sobriety can put older Alaska Native adults on a pathway to successful aging, in positions to serve as role models for their family and community, where they are provided opportunities to engage in meaningful indigenous cultural generative acts.

### PET OWNERSHIP AND LONGITUDINAL CHANGE IN PHYSICAL FUNCTION: EVIDENCE FROM THE BLSA

Erika Friedmann,[1] Nancy Gee,[2] Eleanor Simonsick,[3] Erik Barr,[4] Barbara Resnick,[5] Emily Werthman,[6] and Ikmat Adesanya,[1] *1. University of Maryland, Baltimore, Maryland, United States, 2. Virginia Commonwealth University, Richmond, Virginia, United States, 3. National Instute on Aging/NIH, Baltimore, Maryland, United States, 4. University of Maryland Baltimore, University of Maryland Baltimore, Maryland, United States, 5. University of Maryland School of Nursing, Baltimore, Maryland, United States, 6. University of Maryland, Baltimore, Baltimore, Maryland, United States*

Successful aging depends on avoiding disease and disability, maintaining high physical and cognitive function, and psychological adaptation. Research examining the relationship of pet ownership (PO) or human-animal interaction (HAI) to human health supports contributions to these successful ag-ing-related outcomes at some point in the life-cycle, mostly in populations with diseases or disabili-ties. We examine the contributions of PO to maintaining physical capacity among generally healthy community-dwelling older participants in the Baltimore Longitudinal Study of Aging (BLSA). Partici-pants' [N=637, mean age=68.3 years (SD=9.6), pet owners N=149] completed a standardized physi-cal function test battery (among other measures) every 1-4 years and a ten-year PO history. Linear mixed, or generalized linear mixed, models with time varying PO were used to examine change in successful aging-related outcomes over up to 13 years [mean=7.5, (SD=3.6)] according to PO. Physi-cal function declined across all domains examined, but was observed to be less severe with PO in overall physical performance (p<0.001), rapid gait speed (p=0.041), 400-meter walk time (p<0.001), and reported physical wellbeing (p=0.032). No differences were observed for grip strength (p=0.56), usual gait speed (p=0.07), and leisure time physical activity (p=0.26) after con-trolling for age. This study provides the first longitudinal evidence that PO may promote successful aging among community-dwelling healthy older adults by moderating age-related declines in physical functional status in late-life.

### THE USE OF CONCEPT-MAPPING TO STRUCTURE THE CULTURAL ADAPTATION OF EDUCATIONAL CURRICULA FOR LATINO OLDER ADULTS

Lissette Piedra,[1] Melissa Howe,[2] Yadira Montoya,[2] and Molly Hofer,[3] *1. University of Illinois at Urbana-Champaign, Urbana, Illinois, United States, 2. NORC at the University of Chicago, Chicago, Illinois, United States, 3. University of Illinois Extension, Chicago, Illinois, United States*

Culture, embedded in language and reflected in colloquial expressions, influences behaviors and cognitive constructs that affect health. To reach Latino older adults, health promotion efforts should include congruent cultural aspects—such as relevant metaphors, values, and proverbs—that will resonate with their cognitive constructs. However, this content should also be situated within a broader social context. For community-dwelling Latino older adults, this means considering their care systems and the multiple stakeholders within. In this paper presentation, we describe an innovative, interdisciplinary collaboration to culturally and linguistically adapt existing Illinois Extension curricula to meet the needs of Latino older adults and their families living in Cook County, which includes Chicago and its neighboring suburbs. We will demonstrate how concept-mapping (CM) studies can be used to structure the cultural adaptation of educational curriculum to a Latino audience. Specifically, we describe these CM studies, which asked how multiple stakeholders and Latino older adults living in the Chicagoland area defined positive aging provided empirically-grounded direction for our 11-member steering committee, composed of investigators, service leaders, and Latino older adults. We also will describe how the current project deepens relationships in the community that facilitate dissemination efforts to Latino older adults.

### UNCOVERING PET ISSUES: A SURVEY OF PROFESSIONALS WORKING WITH OLDER ADULTS AND CARE PARTNERS

Jessica Bibbo,[1] Justin Johnson,[1] Jennifer Drost,[2] and Margaret Sanders,[3] *1. Benjamin Rose Institute on Aging, Cleveland, Ohio, United States, 2. Summa Health System, Akron, Ohio, United States, 3. Northeast Ohio Medical University, ROOTSTOWN, Ohio, United States*

Pets can play an important role in older adults' health behaviors and decisions. However, the degree to which these issues are encountered or addressed by professionals working with this population remains unknown. An interdisciplinary (e.g., healthcare, social services) sample of professionals (N=72, 93.05% female, Mage=48.82, SDage=12.57) completed an online survey focused on the pet ownership issues they have encountered while working with older adults, persons with dementia, and care partners. The professionals (n=66) estimated 42.86% of their clients had been pet owners, and 45.58% regularly asked their clients about pets. Issues raised to the professionals varied by type of client. Older adults most often brought up exercising the pet, routine veterinary care, and the financial aspect of ownership (all 37.50%). Persons with dementia most often discussed accessing pet care items (12.50%), exercising the pet (9.72%), and basic pet care (8.33%). Care partners brought up basic pet care (33.33%), planning for the pet due to their care recipients' housing transition (26.38%), and exercising the pet (25.00%). Professionals reported talking to clients about planning for the pet due to housing transition, concerns about falling, and concerns about the pet's behavior (all 31.94%). The professionals (n=69) were very favorable toward pet ownership in general (M=4.43, SD=0.78) (1=extremely unfavorable, 5=extremely favorable), less favorable about older adult pet ownership (M=4.15, SD=0.72, p=.002), and even less favorable about persons with dementia owning pets (M=3.51, SD=0.93, p<.001). The results provide evidence that pet ownership issues are likely encountered in geriatric service settings and may shape healthy aging.

### Session 2320 (Symposium)

### SUPPORT FROM A DISTANCE: HOW HOME CARE AGENCIES INFLUENCE PAID CAREGIVING IN THE HOME

Chair: Emily Franzosa Discussant: Robyn Stone

Paid caregivers (e.g., home health aides, personal care attendants, and other direct care workers) who care for functionally impaired older adults in the home frequently report that while rewarding, their work is logistically, physically, and emotionally demanding. Unlike direct care workers in institutional settings, paid caregivers work with care recipients one-on-one in private settings and often have limited contact with or support from their employers. These factors contribute to high workforce turnover and may impact the quality of patient care. In this symposium, we explore ways that home care agency policies and practices influence the experience of giving and receiving care in the home. First, Bryant et al. describe the range of agency-based models and the impact of workplace design in creating supportive working environments. Next, Fabius et al. explore characteristics of direct care agencies across Maryland, with implications for worker training and support. Reckrey et al. describe the differing perceptions of aides, caregivers and providers around the role agencies play in defining paid caregivers' roles, and how this may lead to conflict within the caregiving team. Finally, in the context of COVID-19, Franzosa et al. examine communication and coordination between Veterans Affairs-paid agencies and home health aides during the pandemic, while Tsui et al. present a case study of an agency's efforts to support paid caregivers through group support calls. Together, these studies highlight challenges in the structure, organization and perceptions of home care agencies, and identify potential avenues for agencies to support paid caregivers and their clients.

### JOB DESIGN FOR HOME CARE WORK: PERSPECTIVES FROM EMPLOYERS AND HOME CARE AIDES

Robyn Stone,[1] Alex Hennessa,[2] and Natasha Bryant,[2]
*1. LeadingAge, Washington, District of Columbia, United States, 2. LeadingAge LTSS Center @UMass Boston, Washington, District of Columbia, United States*

Home-based care is a rapidly growing sector becoming more important to individuals, families, providers, and payers. The ways in which agencies create the work environment for home care aides who are essentially in their clients' homes is not adequately documented and may be changing rapidly with labor market innovations. This qualitative study describes how different home care business models (e.g., non-profit VNAs, for-profit franchises, uber-style matching, worker-owned coops) address job design and the overall work environment for home care aides. Interviews with employers and focus groups with home care aides examine workplace practices, how work is organized and supported when the workforce is virtual and the workplace is a client's home, and the perceived attributes of a positive workplace environment across business models. This study fills significant knowledge gaps about home care workplace design and the role of agencies in creating a supportive environment.

### CHARACTERISTICS, CHALLENGES, AND SUPPORT OF MARYLAND'S DIRECT CARE WORKERS: FINDINGS FROM A STATEWIDE AGENCY SURVEY

Deirdre Johnston,[1] Jennifer Wolff,[2] and Chanee Fabius,[2],
*1. Johns Hopkins University, Baltimore, Maryland, United States, 2. Johns Hopkins Bloomberg School of Public Health, Baltimore, Maryland, United States*

Direct care workers (e.g., personal care aides) are paid health care professionals who provide hands on assistance with daily activities to persons with disabilities in home, community, and institutional settings. Many workers are employed by direct care agencies, but little is known or understood about the organizational attributes of these agencies. We describe results from a mixed mode (postal mail, electronic, and telephone) survey of n=1112 residential care agency administrators in Maryland to assess organizational (e.g., size, supplemental services) and direct care worker (e.g., training) characteristics. Preliminary findings indicate that half of direct care agencies' revenue comes from Medicaid and roughly 40% of clients are living with dementia. Administrators report challenges managing dementia-related behaviors (70%), communicating with persons living with dementia (63%) and interacting with family caregivers (63%). Findings from this work will inform the development of an organizational level intervention that targets training and support of direct care workers.

### WHOSE DECISION IS IT? PERSPECTIVES ON AGENCY INVOLVEMENT IN DETERMINING PAID CAREGIVERS' ROLES IN DEMENTIA CARE

Deborah Watman,[1] Emma Tsui,[2] and Jennifer Reckrey,[3]
*1. Icahn School of Medicine at Mount Sinai, New York, New York, United States, 2. CUNY Graduate School of Public Health & Health Policy, CUNY Graduate School of Public Health & Health Policy, New York, United States, 3. Icahn School of Medicine at Mount Sinai, Icahn School of Medicine at Mount Sinai, New York, United States*

Individuals living at home with dementia often rely on a team of caregivers and health care providers. Yet little is known about how the role of paid caregivers within this team is determined. We identified patients with moderate to severe dementia (n=9) and conducted individual interviews with their care teams (family caregiver, paid caregiver, physician) (n=27) to explore perspectives on paid caregiver roles. Participants disagreed on who determined the paid caregiver's role. Agencies were perceived to set limitations on the scope of care (particularly by physicians) but agency care plans were often seen as inadequate and failing to capture important nuances of care. Most family caregivers believed they should guide what paid caregivers did in the home, while most paid caregivers reported relying on their own experience and knowledge. Understanding and addressing these differing perceptions is critical to improving the quality of paid care in the home.

### NAVIGATING COVID-19: COMMUNICATION AND COORDINATION BETWEEN HOME CARE AGENCIES AND AIDES CARING FOR OLDER VETERANS

Kimberly Judon,[1] Eve Gottesman,[2] Tessa Runels,[3] Nicholas Koufacos,[1] and Emily Franzosa,[4] *1. James J. Peters VA Medical Center, Bronx, New York, United States, 2. US Department of Veterans Affairs, Bronx, New York, United States, 3. West Haven VAMC, West Haven, Connecticut, United States, 4. Icahn School of Medicine at Mount Sinai, Icahn School of Medicine at Mount Sinai, New York, United States*

Home health aides are essential members of the home care team, but often report limited communication with agency supervisors. To explore the impact of COVID-19 on these dynamics, we conducted semi-structured interviews with providers (n=9), contracted home health agencies (n=6), and aides caring for veterans (n=8) at an urban Veterans Affairs medical center. Data were analyzed through thematic analysis. Agencies relied on aides to observe and report on patients' conditions, including COVID-19 symptoms, but aides were not always aware of follow-up and wanted more information about their patients' health and COVID-19 status. Agencies also reported providing personal protective equipment (PPE) and infection prevention guidance to aides; however, some aides reported purchasing their own PPE and seeking out private COVID-19 testing. Supporting aides by providing needed training and protective resources, and engaging them more collaboratively in medical care, may help improve job satisfaction and quality of care.

### RETHINKING EMPLOYER COMMUNICATION AND SUPPORT FOR HOME CARE AIDES: A CASE STUDY OF PANDEMIC GROUP SUPPORT CALLS

Emma Tsui, *CUNY Graduate School of Public Health & Health Policy, CUNY Graduate School of Public Health & Health Policy, New York, United States*

This case study explores an employer-initiated biweekly group support call for home care aides implemented by a large New York City-based home care agency during the COVID-19 pandemic. Specifically, we investigate how agency staff used information gathered through these calls to intervene into existing agency communication and support systems for aides. Our single-site case study analyzes detailed notes from almost 100 support calls that took place between April 2020 and March 2021, as well as interviews with agency staff from communications, human resources, nursing, and other departments that support aides. We compare and contrast new communication and support mechanisms advanced in conjunction with these calls with agency systems pre-pandemic. Our findings suggest that while calls were initially targeted toward providing emotional and operational support, staff also advocated for more systemic supports. We discuss the sustainability of these new efforts, as well as ongoing barriers and gaps.

### Session 2325 (Paper)

### TECHNOLOGY AND COVID-19

### I WOULD BE MORE AT A LOSS WITHOUT IT: TECHNOLOGY AS A TOOL FOR RESILIENCE FOR OLDER ADULTS DURING THE COVID-19 PANDEMIC

Rebecca Newmark,[1] Theresa Allison,[2] Alexander Smith,[3] Carla Perissinotto,[2] and Ashwin Kotwal,[4], *1. University of California San Francisco, Oakland, California, United States, 2. University of California San Francisco, San Francisco, California, United States, 3. UCSF, UCSF, California, United States, 4. University of California San Francisco, University of California San Francisco, California, United States*

COVID-19 associated shelter-in-place orders led to concerns about worsening social isolation and inadequate access to technology among older adults, yet little is known about technology use in this population during the pandemic. We examined older adults' experiences with technology during shelter-in-place in order to identify lessons learned for a post-pandemic world. We conducted semi-structured in-depth interviews with a purposive sample of 20 community-dwelling older adults in San Francisco. Two independent coders conducted concurrent data analysis using inductive and deductive approaches to identify salient themes. Participants were 78 years on average (range 64-99), 55% female, 25% Black, 75% lived alone, and 60% reported at least one ADL impairment. Technology emerged as core aspect of resilience, indicating whether older adults could navigate pandemic restrictions, with two primary themes identified. First, many participants reported discovery of new technologies to maintain or develop new connections, including Zoom-based community groups and telehealth services ("there's all kinds of virtual programs where you can exercise"). Second, older adults were resourceful in identifying community resources and enlisting family members to learn ("I had to ask one of my granddaughters how to make the chat thing work"). Despite difficulty navigating passwords, software updates and other common obstacles, most participants expressed gratitude for technology and the connectivity made possible. Many indicated an intention to integrate new technology-based social interactions into everyday life even after restrictions ended. The COVID-19 pandemic has highlighted the role technology can play in fostering resilience among older adults in adapting to external stressors.

### INFORMATION AND COMMUNICATION TECHNOLOGY CHANGES IN LONG-TERM CARE DUE TO COVID-19

Amy Schuster, and Shelia Cotten, *Clemson University, Clemson, South Carolina, United States*

Residents of long-term care (LTC) facilities (e.g., nursing homes, assisted living facilities) have historically been vulnerable to feelings of loneliness and social isolation. Due to the COVID-19 pandemic, LTC facilities were required to restrict public access in March 2020. LTC communities were not prepared for the residents' increased socioemotional needs which arose because of the mandated facility lockdown. This study investigated ICT use in LTC facilities and how ICTs are being used by residents since the onset of the COVID19 pandemic. Seventy LTC administrators in South Carolina (12 nursing homes and 58 assisted living facilities) completed an online survey exploring ICT access and use in LTC facilities and whether access and use changed as a result of COVID-19. Administrators from fifty-three percent of LTC facilities reported purchasing ICTs for their residents to use for communicating with family members and telehealth since the onset of COVID-19. LTC administrators reported that using the ICTs helped residents to socialize more frequently and feel more socially connected to their family members, friends and/or other residents. Barriers to ICT use included staff not having time to assist residents with technology, broken technology, and residents not wanting to share technology. LTC facilities were not adequately prepared to support the socioemotional needs of their residents in the event of a federally mandated facility lockdown. Future research should investigate the ICTs available for residents' use in a national sample of LTC facilities and how LTC administrators adapted the ICTs available as a result of their experiences with COVID-19.

### OLDER ADULT TECHNOLOGY USE DURING A GLOBAL PANDEMIC: A STUDY OF MENTAL HEALTH, SOCIAL SUPPORTS, AND RESILIENCY

Geunhye Park, Erin Robinson, and Gashaye M. Tefera, *University of Missouri, Columbia, Missouri, United States*

Older adults have been disproportionately impacted by the COVID-19 pandemic, which has led many to isolate during this time. Technology enables people to remain connected, however little is known about how older adults have used technology and the impact it has had on their mental health and connectedness. This study was to explore how the COVID-19 pandemic has influenced older adult mental health and social connectedness, with a particular emphasis on how technology has played a role. One-on-one interviews (N=29) were conducted with adults aged 65+ (Mean age=71.5; 86% female) via phone/Zoom. Participants were asked open-ended questions about the impact social distancing has had on their quality of life, health, and social connectedness as well as their technology use to remain connected. Findings highlight the mental health stressors experienced by older adults during the pandemic, as well as much resiliency and innovation. In speaking of the isolation and its effect on her mental health, one participant said, "I thought fighting cancer was bad, but this is worse." Nearly all of the participants had used technology in some form to remain connected to others, which the most common being a smart phone to call, text, and video-interface. One participant commented, "You can't beat an iPhone. How in the world could we ever live without an iPhone?" Many participants had learned a new technology during the pandemic, such as Zoom. Our findings raise the possibility that technology may be a good strategy for enhancing well-being of aging population amid the pandemic.

### PREFERRED COMMUNICATION CHANNELS AMONG OLDER ADULTS DURING COVID-19

Cheryl Der Ananian,[1] Brad Doebbeling,[1] G. Mauricio Mejía,[1] Hallie Wine,[1] Haley Harelson,[1] Michelle Houchins,[1] Frank Infurna,[2] and Claire Pishko,[1] *1. Arizona State University, Phoenix, Arizona, United States, 2. Arizona State University, Tempe, Arizona, United States*

Social distancing measures put in place during the COVID-19 pandemic limited in-person interactions and may have increased the risk for social isolation and loneliness in older adults.

**Purpose:** The purpose of this study was to understand the communication channels used by older adults (age 50+) during the COVID-19 pandemic to mitigate social isolation and loneliness.

**Methods:** Older adults (n=22) who were selected from a longitudinal study, 'Aging in the time of COVID,' and who had self-reported they successfully avoided loneliness, participated in a semi-structured online interview. Participants were asked to describe the communication techniques and efforts they used to stay connected to family and friends during the pandemic. All interviews were recorded and transcribed verbatim. A thematic analysis approach was used to identify common approaches.

**Results:** Participants were primarily female and white (100%) with a mean age of 64.7 years. Preliminary findings (n=5) suggest older adults relied heavily on technology to facilitate communication with family and friends including texting, phone calls, email, video calls or conferences (e.g., Zoom), WhatsApp and social media, primarily Facebook. In-person communication strategies, including one-on-one and small group gatherings following social distancing guidelines, were preferred, but used less often than technology-based approaches. Living close to friends and family, and previous experience with technology were facilitators. Competing work and family demands, distance, and technology challenges limited communication.

**Conclusions:** While older adults may adopt technology at a lower pace, they relied on digital communication technology to maintain social connections during COVID.

### USE AND DISUSE OF TECHNOLOGY AMONG PERSONS WITH MEMORY CONCERNS AND THEIR CAREGIVERS DURING THE COVID-19 PANDEMIC

Ashley Millenbah,[1] Elizabeth Albers,[2] Jude Mikal,[2] Jessica Finlay,[3] Eric Jutkowitz,[4] Lauren Mitchell,[5] Brenna Horn,[2] and Joseph Gaugler,[2], *1. University of Minnesota, University of Minnesota, Minnesota, United States, 2. University of Minnesota, Minneapolis, Minnesota, United States, 3. University of Michigan, Ann Arbor, Michigan, United States, 4. Brown University, Brown University, Rhode Island, United States, 5. Emmanuel College, Boston, Massachusetts, United States*

Social distancing and shelter-in-place orders designed to curb the spread of COVID-19 increased isolation among persons with memory concerns (PWMC) and increased the burden on individuals providing their care. Technology, such as smartphones or tablets, has demonstrated potential to improve the social connections and mental health of older adults; however, older adults historically have been reluctant to adopt new technology. We aimed to understand why some PWMC and their caregivers used new technology to adapt to lifestyle changes during the COVID-19 pandemic while others did not. In this study, we used data collected in 20 qualitative interviews from June to August, 2020 with PWMC and their family caregivers to assess changes in and barriers to technology use. Qualitative thematic analysis identified three themes which explained motivations for using new technology during a pandemic: 1) seeking relief from caregiver burden, 2) alleviating boredom, and 3) maintaining social connection. Results further revealed lingering barriers to PWMC and caregiver adoption of technologies, including: 1) PWMC dependence upon caregivers, 2) a lack of familiarity with technology, and 3) difficulties using technology. This in-depth investigation suggests that technology has the ability to provide caregivers relief from caregiving duties and provide PWMC with more independence during periods of pronounced isolation

### Session 2330 (Symposium)

### THE SILENT MAJORITY: AGING WITH HIV

Chair: Paul Nash Discussant: Molly Perkins

Those over the age of 50 represent the majority of people living with HIV (PWH), most of the HIV research, prevention and service retention work is targeted at 'at-risk' communities under age 50. Given this diverse and growing population, intersections of age with HIV need to be prioritized. This focus would actively increase quality of care and life experience for older PWH and the growing numbers transitioning into old age. Using local, national, and international data, this symposium will highlight the unmet social needs of older PWH. Presentations will provide evidence of unmet need, decreased self-esteem, enhanced health burden, and the damaging nature of stigma. Given the impact of COVID-19

globally, the data will further demonstrate the need to support immunocompromised older PWH. Older PWH are a marginalized community and the effects of COVID-19 have been disproportionately severe. With the adverse health outcomes experienced because of COVID-19 and intersectional stigma, it is important to understand the support structures that are and are not in place for older PWH. Advance care directives make up an integral part of future planning, especially for those living with chronic health concerns, yet little research has previously evidenced the steps taken by OPWH. Finally, using data from sub-Saharan Africa, emotional and instrumental social support sufficiency will be described to highlight the unmet needs of these older PWH. Our discussion will focus on the need for policies and programs to support this growing segment of the HIV population with increasingly diverse and unmet needs.

### FAILING TO PLAN IS PLANNING TO FAIL: UTILIZATION OF ADVANCE CARE DIRECTIVES IN OLDER ADULTS LIVING WITH HIV

Annie Nguyen, [1] Anna Egbert, [2] Mark Brennan-Ing, [3] Stephen Karpiak, [4] and Paul Nash, [5] *1. University of Southern California, Alhambra, California, United States, 2. Ronin Institute, Montclair, New Jersey, United States, 3. Brookdale Center for Healthy Aging Hunter College, CUNY, New York, New York, United States, 4. GMHC, National Resource Center on Aging and HIV, New York, New York, United States, 5. University of Southern California, Los Angeles, California, United States*

Advance Care Planning (ACP) makes up an integral part of the care continuum, especially for those living with chronic conditions such as HIV. Little research exists to understand how intersections of race, gender, sexuality and gender identity combine to influence the choices made by older adults living with HIV regarding ACP. The Research on Older Adults with HIV (ROAH) 2 study collected data from across the US and investigated the incidence and range of ACP amongst those 50+ living with HIV. Correlational analysis indicated that being White was significantly related to having at least one directive (R=0.070, p=0.035) where being African American correlated negatively with several forms of ACP. Additionally, there were also significant relationships between being Transgender, being gay, and being a woman as to the engagement with ACP options. Further analysis explored the impact of finance, self-rated health and social support networks.

### LIVING WITH HIV THROUGH THE COVID-19 PANDEMIC: IMPACTS ON OLDER ADULTS IN NEW YORK CITY

Stephan Karpiak,[1] and Annie Nguyen,[2], *1. GHMC, New York, New York, United States, 2. University of Southern California, Alhambra, California, United States*

New York City was among the first to institute physical distancing and shutdowns to curb community spread of COVID-19. The pandemic has amplified issues related to isolation. We investigated the challenges created by the pandemic older adults living with HIV in NYC. 137 participants were recruited Sept-Nov 2020 from the oldest ASO in NYC, to complete surveys. Demographics: mean age=60.4; 58.3% men; 43.1% black/AA, 24.1% white; 48.9% gay, 30.7% straight; mean years living with HIV= 23.0, 92.6% reported undetectable viral loads. About one-third experienced hunger/food insecurity during the pandemic and 48.2% said they were not getting enough financial support from usual sources. Some (43.3%) reported skipping doses of HIV medications and 69.8% felt more isolated compared to before the pandemic. Those who lived alone (77.4% of total) were significantly more likely to report feeling depressed, follow media coverage on COVID-19, skip HIV medications, and experience changes in sleep patterns.

### PERCEPTIONS OF SOCIAL SUPPORT SUFFICIENCY AMONG OLDER ADULTS WITH HIV IN SUB-SAHARAN AFRICA

Jennifer Kaufman,[1] Kristen Porter,[2] Catherine MacPhail,[3] Janet Seeley,[4] Stephen Karpiak,[5] and Mark Brennan-Ing,[1] *1. Brookdale Center for Healthy Aging Hunter College, CUNY, New York, New York, United States, 2. John W. McCormack Graduate School of Policy and Global Studies, Moultonborough, New Hampshire, United States, 3. School of Health & Society, University of Wollongong, Bulli, New South Wales, Australia, 4. London School of Hygiene and Tropical Medicine, London, England, United Kingdom, 5. GMHC, National Resource Center on Aging and HIV, New York, New York, United States*

Globally, the greatest number of older people with HIV (OPWH) are in sub-Saharan Africa (3.7 million). This population will continue to expand with greater access to antiretroviral therapy. Compared to OPWH in high income counties, these OPWH have constrained access to government and community-based services and largely rely on assistance from family, friends, and neighbors for their social support needs. We examined factors related to perceptions of instrumental and emotional support sufficiency (availability and adequacy) among OPWH age 50 and older in Uganda (n = 101) and South Africa (n = 108). Significant covariates of instrumental support sufficiency included not having an AIDS diagnosis, greater support from family, and less support from friends. Significant covariates of emotional support sufficiency were fewer depressive symptoms, greater support from family, and geographic location (Uganda). Explanation of these findings based on social network characteristics and implications for policy and program development will be discussed.

### STIGMA IMPACTS HEALTH DISPARITIES AND INEQUITIES IN LGBTQ AND PEOPLE OF COLOR AGING WITH HIV

Paul Nash,[1] Mark Brennan-Ing,[2] Stephen Karpiak,[3] and Anna Egbert,[4] *1. University of Southern California, Los Angeles, California, United States, 2. Brookdale Center for Healthy Aging Hunter College, CUNY, New York, New York, United States, 3. GMHC, National Resource Center on Aging and HIV, New York, New York, United States, 4. Ronin Institute, Montclair, New Jersey, United States*

The impact of stigmatizing attitudes and discriminatory behaviors on health disparities and inequities in non-heterosexual individuals, people of color (PoC), older adults, and persons living with HIV becomes increasingly recognized. This quartette of stigmatized characteristics elevates the risk of barriers to medical services, burden of disease and unfavorable health outcomes in LGBTQ-PoC aging with HIV. Using data from ROAH 2.0 study (N=723), we explored facets of stigma, barriers to medical services and health status in racial/ethnic minorities of older adults with HIV (OAH) living in California, New York, and Illinois. Stigma was evident in >50% of OAH who expressed reservation to self-disclose HIV status. Importantly, 20%-24% of Asian, Black/African-American, Hispanic/Latinx and Multiracial vs. 7% White OAH withheld this information from at least one health care provider. Over 10% of OAH experienced prejudice/discrimination while accessing service. Non-disclosure and prejudice/discrimination were linked to lower self-rated health status, thus, evidencing stigma-related health burden.

### Session 2335 (Symposium)

### THE WISCONSIN LONGITUDINAL STUDY: NEW COGNITIVE, GENETIC, BIOLOGICAL, AND SOCIAL DATA AND A DIVERSIFYING SAMPLE

Chair: Michal Engelman

The Wisconsin Longitudinal Study (WLS) has followed a sample of one in three Wisconsin high school graduates from the class of 1957 for over 64 years, making it an excellent data source for researchers interested in linking early and midlife characteristics to a wide range of later-life outcomes. The WLS is unique among major studies of aging cohorts for its duration of follow up, the inclusion of siblings, and the combination of rich social and health information. This symposium will provide an overview of the WLS, describe recent data collection and linkages, and introduce ongoing efforts to diversify the educational and racial/ethnic composition of the study sample. WLS data cover nearly every aspect of the participants' lives from early life socioeconomic background, schooling, family, and work, to physical and mental health, social participation, civic engagement, well-being, and cognition. The study is linked to administrative data including Medicare records, Social Security records, mortality records, and resource data on primary and secondary schools attended by participants as well as characteristics of their employers, industries, and communities of residence. Recent data collection efforts have generated a wealth of new biological and cognitive information, including genetic data collected from saliva and blood samples, measures of the gut microbiome, and derived polygenic scores for educational attainment, cognitive performance, depression, and subjective well-being. The currently-fielding ILIAD effort is implementing rigorous AD diagnostic protocols to track the progression of dementia across cognitive phenotypes. The symposium will conclude with practical information on accessing and using the data.

### THE WISCONSIN LONGITUDINAL STUDY: OVERVIEW, DATA LINKAGES, AND FUTURE PLANS

Michal Engelman, *University of Wisconsin-Madison, Madison, Wisconsin, United States*

The WLS is a study of Wisconsin high school class of 1957 graduates, with follow-ups in 1964, 1975, 1993, 2004, 2011, and 2020. The data reflect the life course of the graduates (and their siblings), initially covering education, switching to family, career, and social participation in midlife, and physical and mental health, cognitive status, caregiving, and social support as respondents age. The WLS is linked to multiple administrative data sources including: parent earnings from state tax records (1957-60) and Social Security earnings and benefits for respondents; 1940 Census data; characteristics of high schools and colleges, employers, industries, and communities of residence; voting records from 2000-2018; Medicare claims; and the National Death Index. Efforts are underway to expand the racial/ethnic and educational composition of the WLS by supplementing the original sample with a new cohort of age-matched adults drawn from Wisconsin's Black, Hispanic, Asian-American, and Native American communities.

### WLS-ILIAD: INITIAL LIFETIME'S IMPACT ON ADRD

Pamela Herd, *Georgetown University, Georgetown University, District of Columbia, United States*

Between 2021 and 2025, WLS will collect two new waves of data, which will capture detailed measures of cognitive change and dementia as the cohort reaches their early to mid 80s. In this session, I will provide an overview of the data that we're collecting, as well as opportunities to explore early and mid-life determinants of cognitive change and dementia onset in this unique study. Compared to existing studies, the WLS offers some novel opportunities. First, it will provide one of the only opportunities to study how early and midlife life conditions and experiences, on data gathered prospectively, can shape cognitive trajectories and dementia in later life. Second, its unique sibling design provides significant analytic advantages, improving causal inference. Third, the study includes a large group of rural participants, allowing for closer examinations of how rural conditions may shape risk and resilience against cognitive decline and dementia in later life.

### WLS-ILIAD: NEW LONGITUDINAL RESOURCE FOR COGNITIVE AND DEMENTIA DATA

Pamela Herd,[1] Victoria Williams,[2] and Sanjay Asthana,[3] *1. Georgetown University, Georgetown University, District of Columbia, United States, 2. Division of Geriatrics and Gerontology, UW-Madison,, University of Wisconsin-Madison, Wisconsin, United States, 3. University of Wisconsin, Madison, Wisconsin, United States*

One of the distinctive strengths of WLS is the availability of Henmon-Nelson IQ scores on all participants while in high school, followed by prospective collection of data through cognitive batteries of varying size and sophistication. Launched in 1993, the initial longitudinal cognitive testing included 8 abstract reasoning items followed by the administration of larger cognitive batteries in 2004 and 2011 comprised of a 10-item word recall test, digit ordering task, phonemic and category fluency, as well as repeated and new items from the WAIS-R similarities task first administered in the 1993 survey. In 2018, with R01 funding from NIA, the scope of cognitive testing expanded significantly and includes administration of a phone-based cognitive screening measure, and a comprehensive in-person neuropsychological assessment for individuals identified at risk for dementia targeting a range of cognitive domains, including memory, language, attention, visuospatial abilities, and executive functioning.

### BIOLOGICAL MEASURES IN THE WLS: GENETIC AND MICROBIOME DATA

Kamil Sicinski, *University of Wisconsin-Madison, Madison, Wisconsin, United States*

Ever since releasing genotype data in 2017, the WLS continually expands resources available to users interested in genetic research. Key advantages to the WLS data for genetics research include its sibling sample and nearly full life course longitudinal study design. In 2021, we now have state-of-the-art polygenic scores available in multiple domains, such as health, cognition, fertility, personality, risk behaviors and attitudes, and life satisfaction. The scores cover phenotypes spanning from adventurousness, through educational attainment, to age at which voice deepened. Additionally, the genotype data was re-imputed in 2021 to the superior Haplotype Reference Consortium reference panel and the WLS expects to obtain copy number variants data next year. In addition to genetic data, we have a set of novel microbiome data on a subset of participants that allows researchers to study relationships between environments and gut microbial composition.

### HOW TO ACCESS AND USE DATA FROM THE WISCONSIN LONGITUDINAL STUDY

Carol Roan, *University of Wisconsin - Madison, Madison, Wisconsin, United States*

With over 27,000 analysis variables covering more than 60 years of participants' lives, the WLS data can be overwhelming to new users who are looking for the measures they need to answer their research questions. Core WLS survey data is free and easy to download from our website. As we add new types of measures and new waves of data, we refine our data sharing methods to balance our need to make the data easily available with the need to protect the confidentiality of participants. This presentation will teach users how to access to the data files they need for their research and how to use our online documentation of survey instruments and data files. Symposium attendees will also receive a USB drive with the publicly available data and complete documentation.

### Session 2340 (Symposium)

### TRANSITIONS TO LONG-TERM RESIDENTIAL CARE SETTINGS

Chair: Bram de Boer Co-Chair: Hilde Verbeek Discussant: Joseph Gaugler

During their life course, many older adults encounter a transition between care settings, for example, a permanent move into long-term residential care. This care transition is a complex and often fragmented process, which is associated with an increased risk of negative health outcomes, rehospitalisation, and even mortality. Therefore, care transitions should be avoided where possible and the process for necessary transitions should be optimised to ensure continuity of care. Transitional care is therefore a key research topic. The TRANS-SENIOR European Joint Doctorate (EJD) network builds capacity for tackling a major challenge facing European long-term care systems: the need to improve care for an increasing number of care-dependent older adults by avoiding unnecessary transitions and optimising necessary care transitions. During this symposium, four presenters from the Netherlands and Switzerland will present different aspects of transitions into long-term residential care. The first speaker presents the results of a co-creation approach in developing an intervention aimed at preventing unnecessary care transitions. The second speaker presents an overview of interventions aiming to improve a transition from home to a nursing home, highlighting the clear mismatch between theory and practice. The third speaker presents the impact of the COVID-19 pandemic on transitions into long-term residential care using an ethnographic study in a long-term residential care facility in Switzerland. The final speaker discusses the results of a recent Delphi study on key factors influencing implementing innovations in transitional care. The discussant will relate previous findings on transitional care with a U.S. perspective.

### DEVELOPING A REABLEMENT PROGRAM AIMED AT PREVENTING UNNECESSARY CARE TRANSITIONS AFTER GERIATRIC REHABILITATION

Hilde Verbeek,[1] Gertrudis Kempen,[1] Jolanda van Haastregt,[1] Ellen Vlaeyen,[2] Geert Goderis,[2] Silke Metzelthin,[1] and Ines Mouchaers,[1] *1. Maastricht University, Maastricht, Limburg, Netherlands, 2. KU Leuven, Leuven, Vlaams-Brabant, Belgium*

Patients returning home after geriatric rehabilitation may encounter several challenges related to daily functioning, which only manifest after returned home due to the large difference in environment and amount of support provided in both settings. This study aimed to develop an intervention preventing transitional care. A co-creation design was used, including literature research, observations, interviews, and working groups including a variety of stakeholders (n=13), including care professionals, policymakers of the municipality, client representatives, and an expert in the field of geriatric rehabilitation. Results indicated four main causes for transitional care problems: lack of communication between patients and professionals, coordination and continuity of care, patients' limited self-management skills, and insufficient preparation. To solve these problems, an intervention was developed consisting of six intervention components aiming to increase self-management during meaningful daily activities, narrow the gap between the rehabilitation and home setting, and enhance communication and coordination.

### A MISMATCH BETWEEN THEORY AND PRACTICE IN THE TRANSITION FROM HOME TO A NURSING HOME: A SCOPING REVIEW

Amal Fakha,[1] Bram de Boer,[1] Jan Hamers,[1] Matheus van Achterberg,[2] Erik van Rossum,[3] Hilde Verbeek,[1] and Lindsay Groenvynck,[1] *1. Maastricht University, Maastricht, Limburg, Netherlands, 2. KU Leuven, Leuven, Vlaams-Brabant, Belgium, 3. Zuyd University of Applied Sciences, Heerlen, Limburg, Netherlands*

The transition from home to a nursing home is a complex process, existing of three transition phases (pre-, mid- and post-transition). It is often fragmented, leading to negative outcomes for older persons and informal caregivers. To prevent these negative outcomes, knowledge of existing transitional care interventions is paramount. Therefore, a scoping review was performed, summarizing current interventions aiming to improve transitional care. The review identified 17 studies, describing eight multi- and five single-component interventions. From the multi-component interventions, seven main components were identified: education, relationships/communication, improving emotional well-being, personalized care, continuity of care, support provision, and ad hoc counseling. This review identified a clear mismatch between theory on optimal transitional care and current transitional care interventions. All interventions focused on either a specific phase or target population throughout the transition process. This inhibits a continuous transition process in which a partnership between all stakeholders involved exists.

### TRANSITION INTO LTRC DURING THE COVID-19 PANDEMIC: AN ETHNOGRAPHIC CASE STUDY

Franziska Zúñiga,[1] Hilde Verbeek,[2]
Sandra Staudacher-Preite,[3] and Megan Davies,[1]
*1. University of Basel, Basel, Basel-Stadt, Switzerland,*
*2. Maastricht University, Maastricht, Limburg, Netherlands,*
*3. Nursing Science, Department of Public Health, University of Basel/Basel, Basel-Stadt, Switzerland*

COVID-19 has affected long-term residential care (LTRC) disproportionally due to the high-risk population, lack of resources and insufficient preventative measures. Protective measures, including quarantine and strict visitation restrictions have made transitions into LTRC more challenging. Further insight is needed to understand how residents, relatives and staff have experienced this during the COVID-19 pandemic. During four months of fieldwork in a LTRC facility in Switzerland, a rapid ethnography consisting of interviews, observations, informal conversations and document analysis was conducted. This study included a total of 14 residents, 21 healthcare staff from varying departments and 7 relatives of residents. First results indicate that protective measures interfere with a resident's ability to find meaningful activities and interactions within LTRC as well as the possibility to maintain mobility. This and limited family contact following a move into LTRC prevents a smooth transition from home to LTRC and impacts overall resident quality of life.

### IMPLEMENTATION OF TRANSITIONAL CARE INNOVATIONS: CONSIDERING THE ORGANIZATIONAL CONTEXT AND PROCESS IS KEY

Bram de Boer,[1] Matheus van Achterberg,[2] Jan Hamers,[1] Hilde Verbeek,[1] and Amal Fakha,[1] *1. Maastricht University, Maastricht, Limburg, Netherlands, 2. KU Leuven, Leuven, Vlaams-Brabant, Belgium*

Many transitional care innovations (TCI) are implemented to improve long-term care services for older persons during the transition between various care settings. Nevertheless, multiple contextual factors (barriers; facilitators) influence the implementation of TCI at different levels such as but not limited to the organizational environment, outer setting, or innovation's characteristics. By conducting a modified Delphi study involving 29 international experts from 10 countries, eleven influencing factors were prioritized and agreed upon (with ≥ 85% consensus level) as the most important for implementing TCI. These top factors were linked mostly to the organizational setting (e.g. resources, financing) or the implementation process (e.g. engaging key stakeholders). Moreover, the feasibility to address the majority of these factors with implementation strategies was rated as difficult. Our work concludes a compilation of major factors to be aware of and aim to tackle when preparing to implement a new TCI in any long-term care setting.

### Session 2345 (Symposium)

### A TOOLKIT FOR ADVANCING AGE INCLUSIVITY IN HIGHER EDUCATION

Chair: Joann Montepare Co-Chair: Kimberly Farah

The pioneering Age-Friendly University (AFU) initiative, endorsed by GSA's Academy for Gerontology in Higher Education (AGHE), calls for institutions of higher education to respond to shifting demographics and the needs of our aging populations through more age-friendly programs, practices, and partnerships. Over 70 institutions have joined the AFU global network and adopted the 10 AFU guiding principles. In support of the initiative, a GSA-AGHE-AFU workgroup was organized to develop strategies to help GSA members and their campuses explore how they can be more age-inclusive and create pathways to joining the AFU network. One outcome of the workgroup's efforts was the creation "Tools for Advancing Age Inclusivity in Higher Education", designed with support from AARP. In this symposium, workgroup members describe this suite of tools which can be used by faculty, students, administrators, and other campus leaders. Montepare will introduce the symposium with an overview of the AFU network and the workgroup's goals. Morrow-Howell and Schumacher will discuss tools for "Making the Case" with examples from efforts on their campuses. Porter and Bergman will describe tools for "Getting Started" and how campuses can begin to mobilize age-friendly efforts. Andreoletti and June will share tools for "Gaining Momentum" with tips for creating age-friendly campus connections and collaborations. Silverstein and Gugliucci will describe tools for "Assessing and Tracking Success" that can be used at any stage of the process for exploring a campus's age-friendliness. Information about joining the AFU network will be provided.

### LEARN, ENGAGE, AND ACT TO ADVANCE AGE INCLUSIVITY IN HIGHER EDUCATION

Joann Montepare, *Lasell University, Newton, Massachusetts, United States*

Shifting age demographics are reshaping our social structures with far-reaching implications for higher education. Aging populations mean more older adults are looking to higher education to meet their professional needs and personal interests, and the longevity economy is calling for a trained workforce to provide services to support the health and functioning of individuals as they age. As well, there is a need to improve students' aging literacy, along with developing synergistic age-friendly campus-community partnerships to address aging issues. How can institutions explore, create, develop, and sustain more age-friendly programs, practices, and partnerships? This presentation will introduce the toolkit specially designed by the GSA-AGHE Workgroup for use by faculty, students, administrators, and other campus leaders, and will provide an overview of the

Age-Friendly University (AFU) initiative and its 10 guiding principles for creating more age-inclusive campuses.

### MAKING THE CASE FOR ADVANCING AGE INCLUSIVITY

John Schumacher,[1] and Nancy Morrow-Howell,[2]
*1. University of Maryland, Batlimore County, Baltimore, Maryland, United States, 2. Washington University in St. Louis, Saint Louis, Missouri, United States*

How do you present the most effective case for promoting age-inclusivity to your campus leadership? Educational institutions differ in their missions and resources; and these factors affect their readiness to becoming more age-inclusive. This presentation suggests that the best approaches are tailored to intentionally and robustly advance your institution's values, mission, and strategic plan as demonstrated through your proposed age-inclusivity initiative. We review the persuasive arguments for increasing the focus on age-inclusivity in higher education, including securing increasing external research and development funding, supporting employees and alums exploration of encore careers, attracting more students in light of demographic shifts, contributions to overall campus diversity, etc. Identifying the most compelling arguments for particular institutions, consistent with their missions, is connected to the various resources in the AFU toolkit. Finally, we show examples from a range of institutions who successfully made their cases for embracing age inclusivity and have not looked back.

### GAINING MOMENTUM AROUND ADVANCING AGE INCLUSIVITY

Andrea June,[1] and Carrie Andreoletti,[2] *1. Central Connecticut State University, Central Connecticut State University, Connecticut, United States, 2. Central Connecticut State University, New Britain, Connecticut, United States*

Have you already experienced some success with age friendly initiatives at your institution but are wondering how you might broaden your reach? Fostering connections across disciplines and units on your campus as well as with organizations in your community is the key to gaining momentum and advancing age inclusivity. This presentation will discuss strategies for connecting and engaging faculty, staff, students, and community members in age friendly programs and practices. We will share examples and tips for supporting others to be more age inclusive in their teaching, research, and community engagement. We will share ideas from the AFU toolkit for creating learning groups, collaborative community events, and intergenerational exchange as well as our own experience which has demonstrated that many smaller efforts over time can go a long way toward building momentum and creating a more age inclusive campus.

### WHERE TO START THE JOURNEY TO ADVANCE AGE INCLUSIVITY AT YOUR INSTITUTION

Elizabeth Bergman,[1] and Michelle Porter,[2], *1. Ithaca College Gerontology Institute, Ithaca, New York, United States, 2. University of Manitoba, Centre on Aging, Winnipeg, Manitoba, Canada*

Each institution's journey to becoming more age inclusive will to depend on its unique characteristics, and be dependent on its strengths and existing gaps. A good place to start is to explore how to build connections and leverage existing initiatives, such as research programs, community connections and importantly the institution's strategic plan. At this point, elements to consider include coalition building, identifying strengths and gaps, and reframing aging. Because ageism can be a hindrance in many ways, strategies to address ageism should be included. GSA initiatives and tools such as the Reframing Aging Initiative, Ageism First Aid and AARPs Disrupt Aging will be highlighted in our presentation. Examples of how several universities have charted their course to becoming more age-inclusive and age-friendly will be outlined.

### ASSESSING AND TRACKING SUCCESS OF ADVANCING AGE INCLUSIVITY

Marilyn Gugliucci,[1] and Nina Silverstein,[2]
*1. University of New England College of Osteopathic Medicine, Kennebunk, Maine, United States, 2. University of Massachusetts Boston, Boston, Massachusetts, United States*

Assessment is an important component of advancing age inclusivity on your campus, and the AFU Principles are a useful guiding framework. Assessment helps move the campus from making a commitment to endorse the principles to actually taking stock of current campus practices and movement toward achieving the vision of an age-friendly institution of higher education. To establish a baseline of campus practices, assessment can be done before or after an institution joins the AFU Global Network. Evaluation also follows periodically to assess how well a campus is adhering to the AFU Principles once measurable goals are established and priorities are integrated within an institution's strategic plan. The toolkit contains examples from multiple campuses of mapping the principles, conducting an audit, doing a photovoice evaluation, holding listening tours, and using the newly developed AFU Inventory and Campus Climate Survey (ICCS).

### Session 2350 (Symposium)

### AGING WITH INTELLECTUAL AND DEVELOPMENTAL DISABILITIES: A CRITICAL EXAMINATION OF SUPPORTS FOR HEALTHY AGING

Chair: Lieke van Heumen Co-Chair: Kelly Munly
Discussant: Patricia Heyn

The number of older adults with intellectual and developmental disabilities (IDD) in the U.S. is expected to double and potentially triple by 2030. Despite this demographic urgency, there continues to be a lack of research directly addressing aging of people with IDD. Individuals with IDD have on average twice as many health problems than others without IDD, experience earlier age-related declines in health and function than the general population and are more likely to develop secondary conditions as they age. The increase in the number of people aging with IDD and the challenges experienced by this population have demanded new directions for research, practice and policy that promote social justice and improve this population's health and well-being. This symposium brings together research that critically examines and calls for a "new normal" of supports for healthy aging provided to the population aging with IDD. The first presentation consists of a systematic review of healthy aging interventions for adults with IDD. The authors conclude that such interventions for adults with IDD remain scarce, incipient and sporadic. The second presentation critically reviews an interprofessional education model aimed to address the complex and unique needs of older adults with IDD and dementia. The authors provide recommendations for the future development of interprofessional education in this field. In the third and final presentation the authors offer further transformation toward a new normal as they outline future directions for research on aging with IDD that is informed by positive psychology and disability studies theory.

### POSITIVE PSYCHOLOGY AND DISABILITY STUDIES: DIRECTIONS FOR RESEARCH ON AGING WITH IDD

Tamar Heller,[1] and Lieke van Heumen,[2] *1. University of Illinois at chicago, Department of disability and Human Development, University of Illinois at Chicago, Illinois, United States, 2. University of Illinois at Chicago, Chicago, Illinois, United States*

This presentation outlines future directions for research on aging with intellectual and developmental disabilities (IDD) informed by positive psychology and disability studies theory. Research on aging with IDD often focuses on losses in functioning that come with age and on ways to prevent such decline. When applying a positive psychology lens to the experience of aging with IDD the focus shifts to determinants of positive emotions and strength and on ways of increasing meaningfulness in life as one ages. A positive psychology lens also relies on the assumption that people can self-direct and organize their lives, which is essential for individuals aging with IDD who historically have lacked opportunities to exercise self-determination. Positive psychology as applied to disability aligns with disability studies perspectives that emphasize that different ways of being in the world can be 'sources of knowledge, satisfaction, creativity, and happiness' (Chivers, 2011 p.9).

### ARE PERSONS WITH INTELLECTUAL AND DEVELOPMENTAL DISABILITIES BEING EMBRACED IN HEALTHY AGING INTERVENTIONS?

Johanna Zurek,[1] Matthew Janicki,[2] and Flavia Santos,[3]
*1. Hochschule Doepfer, Cologne, Nordrhein-Westfalen, Germany, 2. University Of Illinois at Chicago, Chicago, Illinois, United States, 3. University College Dublin, Dublin, Dublin, Ireland*

We carried out a systematic review of healthy ageing interventions for adults with IDD. Twenty-three prospective studies including 2,398 men and women [average age: 44.3 years old] were found worldwide. Among them were only five RCTs. The designs usually were within or between subjects involving small sample sizes (ranging from 8 to 379 participants), mostly non-randomised or without follow up. We identified four thematic areas: Physical activity - nutrition and health (n = 10); Health education and health exams (n = 6); Social inclusion and community participation (n = 3); and Multi-components (n = 4). Overall, studies found effective outcomes, such as loss of body weight and improvement in nutritional habits, despite a few negative findings. We conclude that healthy ageing initiatives for people with IDD continue to be scarce, incipient, and sporadic. More research should embrace health promotion in people with IDD as a programme practice and public policy.

### DEVELOPING A NEW MODEL OF INTERPROFESSIONAL EDUCATION IN AGING AND IDD

Faith Helm,[1] Edward Ansello,[2] and Phillip Clark,[1]
*1. University of Rhode Island, Kingston, Rhode Island, United States, 2. Virginia Commonwealth University, Richmond, Virginia, United States*

Health and social care providers are ill-equipped to address the complex needs of individuals growing older with IDD and their families when dementia is suspected or diagnosed. Addressing the growing need for professionals to acquire practical diagnostic, treatment, and management methods requires an interorganizational and interprofessional approach. A consortium of aging and IDD organizations developed a successful Project ECHO (Extension for Community Healthcare Outcomes) model to create a virtual community of practice connecting a hub team and participating spoke sites. This paper reviews reasons for the model's success, including: (1) curriculum providing practical solutions to complex problems, (2) integration of interprofessional team approach, (3) "all teach, all learn" model promoting sharing among participants, and (4) the inclusion of case studies engaging participants in developing solutions and strategies to improve the quality of life of clients and families. Implications of this model and recommendations for future professional educational programs are presented.

### Session 2355 (Paper)

### ALCOHOL, SUBSTANCE USE, AND ADDICTIONS

### BRIEF ALCOHOL INTERVENTIONS WITH OLDER ADULTS: RESULTS OF A SYSTEMATIC REVIEW OF LITERATURE

Catherine Lemieux, and Gregory Purser, *Louisiana State University, Baton Rouge, Louisiana, United States*

Older persons are especially vulnerable to the negative effects of alcohol misuse. National reports show that the older-adult population is the least likely group to perceive a need for treatment and be screened for alcohol-related problems. Little research has examined the impact of brief interventions on different drinking outcomes in at-risk older adults. To address this gap, the current study sought to systematically review empirical literature examining the effectiveness of brief alcohol interventions (BAI) implemented with adults (≥50) engaged in at-risk drinking. The authors developed specific a priori inclusion criteria (e.g., alcohol-related outcome measures, randomized controlled trials, RCT) before beginning the search process. Key terms were entered into 9 databases to yield an initial pool of 5,909 articles, from which 5,572 were excluded. A total of 337 articles remained, from which an additional 89 were excluded. Next, the authors independently reviewed 248 full-text, empirical articles and subsequently excluded 237 that did not satisfy inclusion criteria. Thus, the current systematic review yielded 11 studies representing RCT or experimental designs that employed random assignment. Findings of the review indicated that 7 (63.6%) studies showed a positive effect, with only 1 showing no positive effect of the intervention. For the remaining 3 (27.2%), the positive effect of the intervention was not conclusively determined due to study design issues. Overall findings suggest that BAI are effective in reducing alcohol consumption in the older-adult population. Additional evidence is needed to further knowledge consistent with recent initiatives (e.g., Age-Friendly Health Systems, 4Ms) that promote healthy aging.

### CARE FACILITIES FOR OLDER PEOPLE WITH LONG-TERM SUBSTANCE USE: PROMISING PRACTICES FROM SWEDEN

Tove Harnett,[1] and Hakan Jonson,[2] *1. Lund University, Lund, Skane Lan, Sweden, 2. Lund University, Lund University, Skane Lan, Sweden*

The stigma of alcohol and long-term substance use is well-known and may be even greater for older people. This is a presentation on “wet” eldercare facilities, i.e. care settings designed for older people with long-term substance use problems, where abstinence is abandoned for well-being. Wet eldercare facilities exist in several European countries and the Swedish ones have a hybrid formal organization: They target people over 50 years, but are regarded as nursing homes and residents lease their own flats inside the setting, which makes it correct to describe residents as tenants. Guided by symbolic interactionism, the aim is to analyze how residents in wet eldercare facilities manage to view these places in a positive light. Forty-two residents of four facilities were interviewed, revealing how the hybrid status of these places enabled residents to frame their situation as being “in the right place”, but for different reasons. Some framed the place as a nursing home, others as an ordinary flat. Although wet eldercare facilities are undisputedly linked to stigma and the inability to become sober, the formal hybrid organization enabled residents to construct less stigmatized characterizations of the place and of themselves. The study suggests that it is an (often-neglected) gerontological responsibility to counter stigma and improve the sense of dignity for older people living in stigmatized settings. Based on promising practices in the Swedish system, the study therefore presents strategies that enable older people to ascribe positive characteristics to themselves and to the place where they live.

### EXPLORING KNOWLEDGE, BELIEFS, AND ATTITUDES OF OLDER ADULTS ABOUT PRESCRIPTION OPIOIDS

Noell Rowan,[1] Tamatha Arms,[2] and Susan Glose,[3]
*1. University of North Carolina Wilmington, Wilmington, North Carolina, United States, 2. UNC Wilmington, Wilmington, North Carolina, United States, 3. UNC Wilmington, UNC Wilmington, North Carolina, United States*

Over the past two decades, opioids have been considered important and acceptable in the treatment of pain for older adults, especially for chronic health conditions. Despite the fact that older adults are prescribed opioid medications at high rates, there is little research examining older adults' knowledge, beliefs, and attitudes about opioid medications. The purpose of this study was to explore the knowledge, beliefs, and attitudes surrounding prescription opioid medications of community living older adults in a southeast area of the United States. A cross-sectional, descriptive, anonymous survey design of participants aged 55 or over was used. Study participants (N=119) reported bias in their attitudes and beliefs about the use and misuse of prescription opioid medications. Multiple regression analyses revealed that gender, age, work, marital status, and education level all had significant results in explaining variance in the statistical models. Even though study participants demonstrated high levels of education and understanding of the potential of addiction to opiates, there were a number of misconceptions revealed about prescription pain medications. This urges the necessity of increased awareness via further research, presentations, and creative discourse to assist in the understanding of precursors of addiction and ways to deal with pain that do not automatically rely on prescription opioid medicines. Implications include outreach to a larger and more diverse sample to address knowledge, beliefs, and attitudes surrounding prescription opioid medications of community living older adults.

### THE MEANING OF AGE: IN A CONTEXT OF ELDERCARE AND SUBSTANCE USE

Tove Harnett,[1] and Hakan Jonson,[2], *1. Lund University, Lund, Skane Lan, Sweden, 2. Lund University, Lund University, Skane Lan, Sweden*

Some people age with substance abuse and social problems and several countries provide members of this population with a type of arrangement referred to as “wet” eldercare facilities. These facilities provide care for people who are judged as unable to become sober, in some cases with a lower age-limit at 50 years. The aim of this study was to investigate the meaning of age for judging the fit between the person and the arrangement. The study was based on interviews with 42 residents, 10 case workers and 21 staff members at five facilities in Sweden. Respondents were asked about the relevance of age and if the facility should include younger people as well. Some staff argued that younger people should be excluded since they could not have the history of multiple failures in treatment that was a prerequisite for admission. Regarding the low age-limit, substance abuse was said to accelerate the process of ageing so that a person aged 50 could be considered 20 years older and in need of eldercare. Residents had a tendency to equate age with activity and argued that people below the age of 50 were active and energetic and the inclusion of younger people would lead to disturbance of the calm pace of the facilities. Given that facilities have been described as “end-stations”, it was puzzling that few respondents linked the question of admitting younger person to the matter of giving up ambitions to make the person sober.

### Session 2360 (Paper)

### ALZHEIMER'S DISEASE AND OTHER DEMENTIAS

### FACILITY CHARACTERISTICS ASSOCIATED WITH INTENSITY OF CARE OF NURSING HOME RESIDENTS WITH ADVANCED DEMENTIA

Meghan Hendricksen,[1] Susan Mitchell,[2] Ruth Lopez,[3] Kathleen Mazor,[4] and Ellen McCarthy,[5] *1. Hinda and Arthur Marcus Institute for Aging Research, Boston, Massachusetts, United States, 2. Hinda and Arthur Marcus Institute for Aging Research, Roslindale, Massachusetts, United States, 3. MGH Institute of Health Professions, Boston, Massachusetts, United States, 4. University of Massachusetts Medical School, Worcester, Massachusetts,*

*United States, 5. Marcus Institute for Aging Research, Hebrew SeniorLife, Boston, Massachusetts, United States*

Profound variations in care intensity of nursing home (NH) residents with advanced dementia exist for NHs within and across hospital referral regions (HRRs). Little is known about how these levels of influence relate. Nationwide 2016-2017 Minimum DataSet was used to categorize NHs and HRRs into 4 levels of care intensity based on hospital transfer and tube-feeding rates among residents with advanced dementia: low intensity NH in low intensity HRR; high intensity NH in low intensity HRR; low intensity NH in high intensity HRR; and high intensity NH in high intensity HRR. We used multinomial logistic regression to identify NH characteristics associated with belonging to each of 4-levels of intensity as compared to low intensity NH in low intensity HRRs (reference). We found high intensity NHs in high intensity HRRs were more likely to be in an urbanized area, not have an dementia unit, have an NP/PA on staff, have a higher proportion of residents who were male, age <65, of Black race, and had pressure ulcers, and relatively fewer days on hospice. Whereas in low intensity HRRs, higher proportion of Black residents was the only characteristic associated with being a high intensity NH. These findings suggest potentially modifiable factors within high intensity HRRs that could be targeted to reduce burdensome care, including having a dementia unit, palliative care training for NP/PAs, or increased use of hospice care. This study underscores the critical need to better understand the role race plays in the intensity of care of NH residents with dementia.

### RISK OF ALZHEIMER'S DISEASE AND RELATED DEMENTIA AMONG ADULTS WITH CONGENITAL AND ACQUIRED DISABILITIES

Elham Mahmoudi,[1] Paul Lin,[2] Neil Kamdar,[2] Anam Khan,[3] and Mark Peterson,[4] *1. University of Michigan, Commerce Township, Michigan, United States, 2. University of Michigan, Ann Arbor, Michigan, United States, 3. University of Michigan School of Public Health, Ann Arbor, Michigan, United States, 4. University of Michigan, University of Michigan, Michigan, United States*

**Objective:** Adults with congenital (cerebral palsy or spina bifida (CP/SB)) or acquired disabilities (spinal cord injury (SCI) or multiple sclerosis (MS)) have higher incidence of age-related health conditions. There is a gap in the literature about the risk of dementia among adults living with these disabilities. This study aimed to examine time to incidence of Alzheimer's disease and related dementia (ADRD) among these disability cohorts.

**Method:** Using national private payer claims data from 2007-2017, we identified adults (45+) with diagnosis of CP/SB (n=7,226), SCI (n=6,083), and MS (n=6,025). Adults without disability diagnosis were included as controls. Using age, sex, race/ethnicity, cardiometabolic, psychologic, and musculoskeletal chronic conditions, and socioeconomic variables, we propensity score matched persons with and without disabilities. Incidence of ADRD was compared at 4-years. Cox Regression was used to estimate adjusted hazard ratios (aHR) for incident early and late onset ADRD.

**Results:** Incidence of early and late onset ADRD were substantially higher among people with disabilities compared to their non-disabled counterparts. Adults with CP, SCI, and MS had higher risk for early [CP/SB: aHR= 3.35 (95% CI: 2.18-5.14); SCI: aHR=1.93 (95% CI:1.06-3.51); and MS: aHR=4.49 (95% CI:2.62-7.69)] and late [CP: aHR=1.68 (95% CI:1.38-2.03); SCI: aHR: 1.77 (95% CI:1.55-2.02); and MS: aHR=1.26 (95% CI:1.04, 1.54)] onset ADRD.

**Conclusions:** Risk of ADRD was higher among adults with CP/SB, SCI, and MS compared to their matched cohort without disability. Investment in early screening and use of therapeutic or rehabilitative services that may help preserving cognitive function among these patient cohorts is warranted.

### SYSTEMS BARRIERS TO MEDICATION MANAGEMENT DURING HOSPITAL TO HOME TRANSITIONS OF OLDER ADULTS WITH DEMENTIA

Maningbe Keita-Fakeye,[1] Rhea Sharma,[2] Sylvan Greyson,[3] Quincy Samus,[4] Ayse Gurses,[5] Sara Keller,[6] and Alicia Arbaje,[4] *1. Johns Hopkins Bloomberg School of Public Health, Parkville, Maryland, United States, 2. Virginia Commonwealth University, Virginia Commonwealth University, Virginia, United States, 3. Bayview Medical Center, Baltimore, Maryland, United States, 4. Johns Hopkins University School of Medicine, Baltimore, Maryland, United States, 5. Johns Hopkins School of Medicine, Baltimore, Maryland, United States, 6. Johns Hopkins School of Medicine, Lutherville-Timonium, Maryland, United States*

The hospital-to-home transition is a high-risk period for medication errors and adverse events for older adults living with dementia. Researchers conducted a qualitative study using semi-structured interviews and participant solicited diaries. Caregivers of adults ages 55 and older were recruited to understand barriers to medication management during hospital to skilled home health care transitions. We used a human factors engineering approach to guide our understanding of systems level barriers. At least two researchers independently coded each transcript using content analysis and the ATLAS.ti software. We interviewed 23 caregivers and identified five barrier types stemming from systems breakdowns related to: (1) knowledge and information, (2) access to and use of resources and tools, (3) caregiver burden, (4) pandemic concerns, and (5) health limitations. Caregivers grappled with receiving overwhelming, insufficient, incorrect, or conflicting information, and had difficulty managing information from different sources. Latinx caregivers encountered language barriers that impeded role and task clarity. Caregivers expressed mistrust in health systems elements and inability to access resources. Caregivers were in need of additional caregiving assistance, financial aid, and tools to manage medications. Balancing multiple medications and responsibilities left caregivers burdened. The health limitations of the older adult and COVID-19 concerns related to reduced access to resources and ability to deliver and receive in person care complicated task management. Altogether these barriers reflect systems level breakdowns impeding task understanding, execution, and overall management. These findings will inform the development of interdisciplinary strategies to ensure safer care transitions.

### THE COST-EFFECTIVENESS OF NON-DRUG INTERVENTIONS THAT REDUCE NURSING HOME ADMISSIONS FOR PEOPLE WITH DEMENTIA

Eric Jutkowitz,[1] Fernando Alarid-Escudero,[2] Peter Shewmaker,[3] Joseph Gaugler,[4] and Laura Pizzi,[5] *1. Brown University, Brown University, Rhode Island, United States, 2. Center for Reserach and Teaching in Economics, Aguascalientes, Aguascalientes, Mexico, 3. Brown Universtiy, Brown University, Rhode Island, United States, 4. University of Minnesota, Minneapolis, Minnesota, United States, 5. Health Outcomes, Policy, and Economics (HOPE) Program, Pscataway, New Jersey, United States*

Although people generally want to age in their community, individuals living with dementia are likely to move to a nursing home. In randomized trials, psychosocial interventions reduce the risk of people living with dementia transitioning to a nursing home, but the cost-effectiveness of these interventions is unknown. We used an evidence-based mathematical model to simulate a place of residence (community or nursing home) for people living with dementia. Our model also predicts time caregiving, health care costs, and quality of life. We modeled the reduction in nursing home rate (i.e., hazard ratio (HR) treatment effect) identified from two trials of non-drug interventions for people living with dementia and their caregiver. Using trial data, we account for the disease stage of when interventions are implemented. Specifically, we modeled MIND (HR: 0.63; 18-month effect), an in-home intervention for people with mild-moderate dementia, and the NYU Caregiver Intervention (HR: 0.53; 42-month effect), which is for people with moderate dementia. We evaluated each intervention's cost-effectiveness relative to usual care for the duration of the intervention from a societal perspective. The MIND and NYU Caregiver Intervention resulted in $23,900, and $6,600 costs savings relative to usual care, respectively. The model predicted an improvement in the quality of life for people living with dementia for both interventions. The largest cost saving was attributed to reductions in family nursing home spending. Medicare and Medicaid received modest cost savings but are likely to be tasked with paying for these interventions.

### USE OF TANGIBLE, EDUCATIONAL, AND PSYCHOLOGICAL SUPPORTIVE SERVICES AMONG CHINESE AMERICAN DEMENTIA CAREGIVERS

Jinyu Liu,[1] Yifan Lou,[2] Ethan Siu Leung Cheung,[2] and Bei Wu,[3] *1. Columbia University, Columbia University, New York, United States, 2. Columbia University, New York, New York, United States, 3. New York University, New York, New York, United States*

**Background and Objectives:** Though many studies have examined the service utilization of dementia caregivers, there is limited empirical evidence from Asian Americans and the lack of incorporating community resources and sociocultural factors in this field. Guided by the Andersen's Behavioral Model of Health Services Use (ABM), we aimed to understand whether and how predisposing, enabling and need factors were associated with utilizing multiple types of services among Chinese Americans dementia caregivers.

**Research Design and Methods:** We collected survey data from 134 Chinese dementia caregivers in New York City. Logistic regression models were conducted to test the associations between predisposing, enabling and need factors and the likelihoods of using tangible (home health aide, adult daycare, respite care), educational (lectures and workshops), and psychological (peer support groups and psychological counseling) services.

**Results:** Consistent with prior literature, caregiver's knowledge about services, caring tasks, length of care and burden and care recipient's physical and cognitive deteriorations, were significantly associated with higher possibilities of using multiple types of services among these Chinese American dementia caregivers. Three sociocultural factors, including residing in Chinatowns, availability of alternative family caregivers and diagnosis of cognitive deterioration, were also associated with higher likelihoods of using educational or psychological services.

**Discussion and Implications:** The findings extended the existing literature on service utilization of caregivers by highlighting the importance of distinguishing types of services and the necessity of considering sociocultural factors in future research and practice.

### Session 2365 (Symposium)

### AN INTERDISCIPLINARY COLLABORATION TO IMPROVE HYPERTENSION MEDICATION ADHERENCE FOR OLDER ADULTS

Chair: Jeannie Lee Co-Chair: Wendy Rogers

Hypertension is highly prevalent in older adults (74.5% in ≥60 years) with dire consequences, and adherence to hypertension medications is low (approximately 50%). With increased smartphone use among older adults (81% for 60-69 years, 62% for ≥70 years), technology innovations can improve medication adherence. This symposium highlights the efforts of an innovative interdisciplinary team of experts (clinical, cognitive aging, human factors, health technology) to develop and implement the Medication Education, Decision Support, Reminding, and Monitoring (MEDSReM) system to improve hypertension medication adherence for older adults. MEDSReM is a theory-based, integrated mobile application (app) and companion web portal that educates, supports missed dose decisions, reminds, monitors adherence, and incorporates blood pressure feedback. In this symposium, we describe the interdisciplinary development efforts. Insel et al. will present the theory-based intervention, technology translation, and advancement of the MEDSReM system. Lee et al. will describe the interdisciplinary team and describe the work by the decision support subteam that created the medication formulary and generated an algorithm to guide missed-dose decisions based on pharmacology of aging. Rogers et al. will discuss the education subteam's development of educational information about hypertension, medications, and adherence for the MEDSReM system. Mitzner et al. will illustrate the instructional support subteam's efforts to ensure older adults can interact with both the smartphone app and online portal. Lastly, Hale et al. will describe the user testing subteam's usability processes including the integration of blood pressure self-monitoring.

These efforts will provide insights for other interdisciplinary teams developing technology interventions for older adults.

### FOUNDATIONAL BASIS FOR THE DEVELOPMENT OF MEDSREM

Gilles Einstein,[1] Daniel Morrow,[2] Jeannie Lee,[3] Wendy Rogers,[2] Tracy Mitzner,[4] and Kathie Insel,[5] *1. Furman University, Greenville, South Carolina, United States, 2. University of Illinois Urbana-Champaign, Champaign, Illinois, United States, 3. University of Arizona, Tucson, Arizona, United States, 4. Georgia Institute of technology, Atlanta, Georgia, United States, 5. Behavioral and Social Science, Tucson, Arizona, United States*

Discovering a composite of measures of executive function/working memory predicted everyday medication adherence among older adults, led to the development of a behavioral intervention, the Multifaceted Prospective Memory Intervention (MPMI) to improve hypertension medication adherence. The intervention resulted in a 35% improvement in adherence compared to an active education and attention control condition. However, adherence slowly declined over an additional five months of adherence monitoring without the presence of interventionists in the home. We proposed that the use of technology might help individuals maintain the prospective memory strategies, resulting in sustained adherence. An interdisciplinary team was formed to translate the behavioral intervention to technology, resulting in the first version of the MEDSReM system. In this presentation we describe the evolution of the project, from the components of the successful MPMI to the design and initial testing of MEDSReM. These efforts provide general insights about translating interventions into technology tools.

### INTERDISCIPLINARY TEAM FOR MEDSREM-2 AND DECISION SUPPORT THROUGH PHARMACOLOGY OF AGING PRINCIPLES

Kathie Insel,[1] J. Nicholas,[2] Amani Albadawi,[2] and Jeannie Lee,[3] *1. Behavioral and Social Science, Tucson, Arizona, United States, 2. University of Arizona College of Pharmacy, Tucson, Arizona, United States, 3. University of Arizona, Tucson, Arizona, United States*

The interdisciplinary team members with distinct and complementary expertise working collaboratively to advance MEDSReM to MEDSReM-2 will be introduced. The decision support functionality in MEDSReM-2 application (app) is to guide older users on making decisions about missed doses. MEDSReM-2 medication formulary was created to include safe hypertension medications for older adults. Pharmacology of aging, including Pharmacokinetic and Pharmacodynamic principles, along with published studies and expert peer reviews, were used to create an algorithm for safe window of time to take the missed medications. We will present the processes for developing the decision support algorithm for the MEDSReM-2 App and how this guide will be communicated to the users to inform their decision making about missed doses. Interdisciplinary collaboration including pharmacy, nursing, cognitive aging, and technology development that was crucial for designing and implementing decision support within the MEDSReM-2 app for older users will be shared.

### DEVELOPING EDUCATIONAL MATERIALS TO SUPPORT OLDER ADULTS WITH HYPERTENSION MANAGEMENT

Qiong Nie,[1] Maurita Harris,[1] Stacy Al-Saleh,[2] Ysabel Beatrice Floresca,[3] and Wendy Rogers,[3] *1. University of Illinois at Urbana Champaign, Champaign, Illinois, United States, 2. University of Arizona, Tucson, Arizona, United States, 3. University of Illinois Urbana-Champaign, Champaign, Illinois, United States*

A comprehensive approach to hypertension management requires medication adherence as well as more general health behavior changes. Our primary objective is to provide evidence-based and tailored education about hypertension, medications, and health self-management strategies with consideration for different stages of behavior change, health literacy, education, disease knowledge, and experience. To facilitate health behavior change, enable information seeking, and increase engagement, the educational materials provide different layers of information, including tips and information in the MEDSReM app, as well as more detailed educational content on the web portal. We will present examples of the materials in different formats to show how they are tailored to ease comprehension, support adherence, and influence behavior change. These educational materials will have broad utility outside of the MEDSReM system, and will also serve as the education-only comparison condition for the randomized controlled trial.

### DEVELOPING INSTRUCTIONAL SUPPORT FOR MEDSREM-2 THROUGH HUMAN FACTORS DESIGN PRINCIPLES

Maurita Harris,[1] Kenneth Blocker,[2] Mimi Trinh,[3] and Tracy Mitzner,[4] *1. University of Illinois at Urbana Champaign, Champaign, Illinois, United States, 2. University of Illinois at Urbana-Champaign, Champaign, Illinois, United States, 3. University of Illinois Urbana-Champaign, Champaign, Illinois, United States, 4. Georgia Institute of technology, Atlanta, Georgia, United States*

Appropriate instruction is critical for ensuring the MEDSReM-2 system (i.e., smartphone app, blood pressure monitor, online portal) will be easily and effectively used and will, therefore, be more likely to be adopted. We will present our iterative processes for developing instructional support for MEDSReM 2 using human factors design principles (e.g., task analyses, comparative analyses, expert evaluation of mock-ups with screen flows). The instructional supports include user manuals, videos, as well as instructions within the MEDSReM 2 app. We will also highlight design principles used to empower the user and the benefits of using an interdisciplinary approach (i.e., gerontology, cognitive psychology, educational psychology, design, community health) to develop instructional support for older adult users.

### USABILITY EVALUATION METHODS USED TO ENSURE THE USABILITY OF THE MEDSREM-2 SYSTEM

Carson Smith,[1] Mimi Trinh,[1] Wendy Rogers,[1] and Timothy Hale,[2] *1. University of Illinois Urbana-Champaign, Champaign, Illinois, United States, 2. University of Illinois Urbana-Champaign, University of Illinois Urbana-Champaign, Illinois, United States*

A key objective of the MEDSReM-2 study is to promote medication taking decisions and improve adherence to hypertensive medications for older adults. New functionalities include enhanced decision-support algorithms for missed medications, automated entry of blood pressure measurements, improved data visualizations, and an easy-to-use online web portal. In support of these enhancements, the User Testing subteam is tasked with providing ongoing evaluation and feedback on the usability of early design concepts, prototypes, beta software, wireless blood pressure monitors, and instructional materials. The overall project comprises multiple working teams, whose efforts must be coordinated. We will describe the challenges of working with these interdisciplinary teams and the usability evaluation methods used to support the needs of each team in creating the enhanced MEDSReM-2 system that is easy-to-use and effective in helping older adults improve their hypertension medication adherence. These processes inform the research and design efforts of other technology interventions.

### Session 2370 (Symposium)

### BUILDING AND USING DATA RESOURCES FOR RESEARCH ON JOB CHARACTERISTICS, HEALTH, AND RETIREMENT

Chair: Amanda Sonnega Discussant: Gwen Fisher

A growing literature seeks to understand the relationship between the experience of work and important later-life outcomes. Rich longitudinal measurement of both sides of this equation in datasets such as the Health and Retirement Study (HRS) have made this research possible. These data take the form of self-reported experiences of work (such as physical demands, job flexibility, job satisfaction etc.). Increasingly, researchers are looking to add potentially complementary information on the work environment available in the Occupational Information Network (O*NET) database through a linkage using occupation and industry codes in the survey data. The session talks will describe research conducted using O*NET linked with HRS data as well as ongoing work to create a new data resource that will allow other researchers to undertake research with O*NET-HRS linked data. Each presentation will include some discussion of both the value and limits of using the linkage to O*NET. Carpenter will provide a detailed description a new project linking the 2019 O*NET data to the HRS for public use.This presentation explains the types of variables that will be made available in the O*NET-HRS occupation project and will provide examples for how the measures can be used in longitudinal HRS studies. Using O*NET-HRS linked data, Carr will present on work examining the role of preretirement job complexity in alternative retirement paths and cognitive performance. Helppie-McFall will used the linked data to discuss the role of mismatch between demands of work and workers' ability to meet those demands in retirement decisions.

### CREATING A PUBLIC DATA RESOURCE: THE HRS-O*NET LINKAGE

Dawn Carr,[1] Brooke Helppie-McFall,[2] Julia Beckel,[3] and Rebekah Carpenter,[1] *1. Florida State University, Tallahassee, Florida, United States, 2. University of Michigan, Ann Arbor, Michigan, United States, 3. Colorado State University, Fort Collins, Colorado, United States*

Few longitudinal studies provide detailed information about the characteristics of the jobs older workers engage in, limiting the ability to evaluate the potential consequences of extended working lives. In this session, we introduce a new project linking the 2019 O*NET taxonomy and corresponding data to the Health and Retirement Study for public use. We describe the procedures taken to develop an O*NET linkage to be released to HRS users in the form of a publicly available data file, allowing aging researchers to evaluate detailed aspects of occupations in the 50+ population. We explain the types of variables that will be made available in the O*NET-HRS occupation project, and provide examples for how the measures can be used in longitudinal HRS studies.

### USING O*NET LINKAGES TO ADVANCE RESEARCH: AN EXAMPLE EVALUATING COGNITIVE FUNCTION AND WORK TRANSITIONS

Dawn Carr, *Florida State University, Tallahassee, Florida, United States*

The type of work older adults engage in has potential to play a key role in shaping health and wellbeing. In this presentation, using data drawn from an O*NET crosswalk linked with the Health and Retirement Study, I show how different types of transitions out of the workforce shapes cognitive function differently for individuals retiring from different types of occupations. Based on a factor analysis of 36 job-related abilities, activities, and contexts, this paper shows that retirement has a more significant consequence for cognitive function for those who retire from jobs with low levels of cognitive complexity, but no significant consequences for those who retire from jobs with high levels of cognitive complexity. I discuss these results in the context of the ways in which O*NET classifications of jobs can provide critical insights into the potential influence of changing retirement trajectories on wellbeing in later life.

### USING HRS-ONET LINKED DATA TO STUDY SUBJECTIVE AND OBJECTIVE MISMATCH BETWEEN WORK DEMANDS AND CAPACITY AT OLDER AGES

Amanda Sonnega,[1] Gwen Fisher,[2] and Brooke Helppie-McFall,[1] *1. University of Michigan, Ann Arbor, Michigan, United States, 2. Colorado State University, Fort Collins, Colorado, United States*

Mismatch between demands of work and workers' ability to meet those demands may play an important role in retirement decisions. This presentation extends earlier work using Health and Retirement Study data linked to O*NET to develop measures of discrepancy between individual's own reports of physical and mental abilities and 1) their perceptions of the physical and mental demands of their jobs and 2) O*NET ratings of the physical and mental demands of their jobs. In particular, we utilize newly available linked information using 2010 Census codes and 2019 O*NET ratings that reflect more current jobs. We then examine the impact of each type of mismatch (subjective and objective) on retirement timing. Overall, we find a stronger connection between subjective mismatch relative to objective mismatch. We discuss implications of this finding in terms of the value of the O*NET linkage and potential interventions aimed at extending working lives for positive aging.

### Session 2375 (Paper)

### CHALLENGES OF COVID-19 FOR MINORITY AND DIVERSE POPULATIONS

### ACTIVITY ENGAGEMENT PATTERNS AMONG OLDER CHINESE IMMIGRANTS

Fengyan Tang, and Mary Rauktis, *University of Pittsburgh, Pittsburgh, Pennsylvania, United States*

Activity engagement is a major component of well-being in later life. However, very few studies have focused on older immigrants who are often at risk for social isolation and psychological distress. We aim to map the pattern of activity engagement and examine its variations in relation to immigration-related factors and social aspects of neighborhoods in a representative sample of older Chinese immigrants. We used data from the Population Study of Chinese Elderly in Chicago (PINE), a population-based epidemiological study of US Chinese older adults that were conducted between 2011 and 2013 (N=3,157). Latent class analysis and multinominal regression analysis were conducted to identify activity engagement patterns and examine the associated factors. Four patterns of activity engagement were identified: restricted (15%), diverse (31%), informal social (32%), and community-based social (21%). Acculturation and family-oriented immigration differentiated the restricted from the diverse class membership. Positive attributes of social environment measured by social network size, positive social support, neighborhood cohesion, and sense of community were associated with the probabilities of class membership relative to the restricted class. Findings point to the importance of positive attributes of social environment in enhancing engagement with life among older Chinese immigrants. Efforts are needed to assist the vulnerable restricted group and recent older immigrants while meeting the demands of older immigrants who are less educated and less acculturated. Creating a supportive environment is important to provide information, access, and resources needed for activity engagement in the marginalized minority aging populations

### AGING OF A MODEL MINORITY: A DIACHRONIC ANALYSIS OF TWO QUANTITATIVE RESEARCH STUDIES ON AGING OF JAPANESE IN NEW YORK

Itsuko Toyama,[1] and Taeko Nakashima,[2] *1. St. Andrew's University, Souraku-gun, Kyoto, Japan, 2. Nihon Fukushi University, Handa-city, Aichi, Japan*

This is a diachronic analysis of two quantitative research studies on the aging of Japanese and Japanese Americans living in Greater New York. How have older Japanese individuals, who once have been referred as "model minority," lived and aged in Greater New York? All the data in this paper are based on the first research study conducted in 2006 and the second in 2018 (Ethical approval reference number 6, 2018). This paper reveals both the social transitoriness and the cultural immutability of the Japanese elderly community in Greater New York. The following is a summary of the findings: (1) a growing Japanese American community with US citizenship, higher academic qualification, and better communication competency has been observed. (2) The allowable range of private expense to hire personal caregivers has been widened. (3) Not only the concerns and anxieties for later lives but also the plans and preparations for aging are much the same. (4) The elderly are provided with culturally specific care (with regard to language, food, and concept of care)—even allowed to live with other Japanese people—and the needs of caregivers who can understand Japanese culture are satiated. (5) Almost half of those in the community find it difficult to eliminate the possibility of returning to Japan, and some of them have already chosen to migrate back to Japan.

### COVID-19 AND THE INTERSECTION OF RACE, ETHNICITY, AND SEXUAL MINORITY STATUS.

Robert Beringer,[1] Brian de Vries,[2] Gloria Gutman,[3] Paneet Gill,[4] and Helena Dault,[5] *1. University of Victoria, Victoria, British Columbia, Canada, 2. San Francisco State University, Palm Springs, California, United States, 3. Simon Fraser University, Vancouver, British Columbia, Canada, 4. Simon Fraser University, Surrey, British Columbia, Canada, 5. Victoria Hospice, Victoria, British Columbia, Canada*

The COVID-19 virus has caused millions of deaths and impaired physical and mental health and social disconnection for countless persons around the world; concomitantly, the pandemic has exposed and exacerbated the pervasive effects of racism and stigma experienced by Black, Indigenous, or People of Color (BIPOC) and other marginalized/stigmatized groups. This study adopts an intersectional perspective examining multiple marginalized identities (i.e., the combination of LGBTQ and BIPOC status) and COVID-19 pandemic health stressors. We report on data from an online survey (conducted between Aug 10 and Oct. 10, 2020) focusing on current experiences and future planning during the COVID-19 pandemic in Canada. LGBTQ respondents (n=415) indicated significantly higher levels of depression, loneliness, sadness, and isolation in comparison to heterosexuals (n=3916). Heterosexual white respondents (n=3446) reported significantly higher levels of acceptance in their community and reported greater happiness but also higher rates of feeling of isolation than heterosexual BIPOC heterosexuals (n=470) who reported significantly higher rates of feeling judged/shamed by others than the heterosexual white respondents. In contrast to our expectations, white LGBTQ respondents (n=366) reported significantly more depression, loneliness, anxiety, and sadness than their BIPOC LGBTQ peers (n=49). These findings are interpreted as reflecting a complex mix of the effects of marginalization (as experienced by LGBTQ persons in general), and privilege and relative deprivation (as experienced by heterosexual and LGBTQ white persons) along with resilience and the moderated expectations and experiences of BIPOC LGBTQ persons.

### FACTORS RELATED TO COVID-19 VACCINE UPTAKE IN BLACK AMERICAN COMMUNITIES

Julene Johnson,[1] Orlando Harris,[2] Carl V. Hill,[3] Peter Lichtenberg,[4] Sahru Keiser,[2] Thi Tran,[5] Tam Perry,[4] and Elena Portacolone,[6] *1. University California San Francisco, San Francisco, California, United States, 2. University of California, San Francisco, San Francisco,*

*California, United States, 3. Alzheimer's Association, Chicago, Illinois, United States, 4. Wayne State University, Detroit, Michigan, United States, 5. UCSF/ San Francisco, UCSF Institute of Health and Aging/ San Francisco, California, United States, 6. University of California, Berkeley, California, United States*

Black/African Americans represent 13% of the population, yet account for more than 24% of COVID-19 deaths. Emerging evidence indicates that Black Americans are receiving COVID-19 vaccines at lower rates than whites. However, there is minimal information about why vaccination rates are lower. To address this gap, we examined the effects of the COVID-19 pandemic among Black Americans, with an emphasis on understanding trust and vaccine uptake. Data were collected between July and September 2020 using 8 virtual focus groups in Detroit, MI and San Francisco Bay Area, CA with 33 older Black Americans and 11 caregivers of older Black Americans with cognitive impairment. Inductive/deductive content analysis was used to identify themes. The first theme pointed to a sense of feeling abandoned by healthcare providers and the government at local and state levels, which exacerbated uncertainty and fear about the vaccine and in general. The second theme emphasized a sense of deep distrust towards healthcare providers and the government, especially during the pandemic. The third theme pointed to a reluctance in receiving the vaccine because of distrust of pharmaceutical companies and the government, as well as misinformation and the rapid speed of vaccine development. These findings suggest that underlying systemic issues need to be addressed immediately to accelerate vaccine uptake among older Black Americans. New initiatives are needed to foster trust and address abandonment by healthcare and government systems. In addition, public health campaigns with reliable information about the COVID-19 vaccine are needed.

### MEETING NEEDS OF DIVERSE OLDER ADULTS AND CAREGIVERS DURING PUBLIC HEALTH EMERGENCIES: WHAT CAN WE LEARN FROM COVID-19?

Alycia Bayne,[1] Rachel Singer,[1] Candace Spradley,[2] Lauren Isaacs,[3] Eva Jeffers,[4] Benjamin Olivari,[4] Kevin Matthews,[5] and Alaina Whitton,[6], *1. NORC at the University of Chicago, Bethesda, Maryland, United States, 2. CDC Foundation, Atlanta, Georgia, United States, 3. NORC at the University of Chicago, Chicago, Illinois, United States, 4. Centers for Disease Control and Prevention (CDC), Atlanta, Georgia, United States, 5. NCCDPHP, Centers for Disease Control, Georgia, United States, 6. The CDC Foundation, Atlanta, Georgia, United States*

With support from the CDC Foundation and technical assistance from the Centers for Disease Control and Prevention, NORC at the University of Chicago conducted studies to examine the needs and concerns of older adults and unpaid caregivers during COVID-19, including their trusted sources of COVID-19 information and available public health interventions. Methods included a nationally representative survey of 1,030 adults aged 50+ years using computer-assisted telephone and web interviewing; online focus groups with older adults and caregivers in Spanish and English; a survey and interviews with stakeholder organizations; secondary analysis of U.S. caregiver surveys; analysis of public social media posts; and searches of peer-reviewed and grey literature in Spanish and English to identify interventions. Results suggest that needs and concerns differed among older adult subpopulations, including racial and ethnic minority populations, people with lower incomes, rural and tribal populations, people with limited English proficiency, and people with disabilities as well as caregivers. Older adults perceived news media, the internet, and healthcare providers as important resources for COVID-19 information, although trusted sources varied by race and ethnicity, urbanicity, and income. Findings suggested the need to increase awareness of existing public health interventions and resources to support older adults and caregivers during public health emergencies like COVID-19. Strategies for tailoring communication for diverse older adults and caregivers include partnering with national organizations, leveraging community-level infrastructure, and disseminating information through trusted sources. Studying the needs of older adults and caregivers during COVID-19 can inform future public health emergency response priorities.

### Session 2380 (Symposium)

### COMMUNITY-ENGAGED RESEARCH WITH INDIGENOUS COMMUNITIES TO IMPROVE ELDER HEALTH AND WELL-BEING

Chair: Jordan Lewis

Much of the past research conducted with tribal communities was coined "helicopter research," because researchers would enter the community, gather data, and leave the community, never to inform communities how the data was used or published, creating mistrust. Community Based Participatory Research (CBPR) is a research approach conducted as an equal partnership between community members, organizational representatives, and researchers that serve as guidelines for researchers working collaboratively with communities. This symposium will offer a panel of presentations highlighting research studies with tribal communities that honor and respect tribal sovereignty in addressing health and wellbeing among their older adults. The panel presentations will consist of presentations on dementia caregiving, generativity and successful aging, social support and diabetes management, elder-centered research methods.

### SOCIAL SUPPORT AND DIABETES MANAGEMENT AMONG OLDER AMERICAN INDIANS

Molly Grant,[1] Kathleen Conte,[2] Lisa Lefler,[3] and R. Turner Goins,[4] *1. Phoenix Counseling and Crisis Center, Gastonia, North Carolina, United States, 2. DePaul University, Chicago, Illinois, United States, 3. Western Carolina University, Cullowhee, North Carolina, United States, 4. Western Carolina University, Western Carolina University, North Carolina, United States*

We examined social support among older American Indians in relation to their diabetes management. In-depth interviews were conducted with 28 participants aged ≥ 60 years who were members of a federally-recognized tribe. We examined professionally transcribed audio recordings with a systematic text analysis approach. Main sources of social support were family/friends, clinicians/formal services, community/culture, and spiritual/God. Most of the support was instrumental in nature, including food shopping, meal preparation, and medication management. Social support had both positive and negative influences diabetes management while there were some participants who lacked support. The four main social support types were present, including instrumental, emotional, informational, appraisal support. Value orientations among American Indian families command lateral-group relational behavior rather than autonomy and independence with extended social systems fostering interdependence. A deeper understanding is needed of how social relationships can be better leveraged to aid in the effective diabetes management among older American Indians.

### ALASKA NATIVE ELDER-CENTERED RESEARCH METHODOLOGY

Maria Crouch,[1] Jordan Lewis,[2] and Rosellen Rosich,[3] *1. Yale School of Medicine Substance Abuse Treatment Unit & The Consultation Center, Yale university, New Haven, Connecticut, United States, 2. Memory Keepers Medical Discovery Team/ University of Minnesota Medical School, Duluth campus, Duluth, Minnesota, United States, 3. University of Alaska Anchorage, Anchorage, Alaska, United States*

Alaska Native (AN) Elders have historically been underrepresented in research. Innovative AN research posits that practice-based evidence is fundamental to culturally grounded, multifaceted methods. AN Elders is a cultural convention distinguishing Elders who continue to serve as an integral part of their family and community and recognized by their community as role models. Several studies will be discussed which employed Elders at every level of the research, ensuring cultural relevancy, outcomes, and dissemination activities. The findings lay the foundation for an Elder-centered research methodology that can be adapted and applied in other studies to encourage engagement of older adults. This methodology has potential to impact research for underrepresented groups and to rethink and reshape Western-centric practices. Findings from this research provides best practices for capacity building and sustainability, strategies for empowerment and prevention, and a framework for supporting the AN community in all phases of research.

### CULTURAL CONTINUITY IN A RESERVATION NURSING HOME

Pamela Monaghan-Geernaert, *Northern State University, Aberdeen, South Dakota, United States*

Throughout our lifespan we experience the culture of our families and communities. Our cultural selves guide our understanding of health and illness. However the health care system often ignores our culture in the delivery of care. This can have devastating effects on individuals and particularly the elderly. This presentation reviews a case study of a tribally owned and operated nursing home. The emphasis on maintaining cultural activities, feasting on traditional foods, offering sacred practices led to high satisfaction of the health care experience by residents and staff. Creating this environment was difficult and barriers in culturally responsive care delivery will also be discussed.

### COMMUNITY-BASED PARTICIPATORY RESEARCH ON DIET AND ACTIVITY WITH AN INDIGENOUS PUEBLO COMMUNITY

Karen Kopera-Frye, *University of New Mexico, Las Cruces, New Mexico, United States*

Tribal Critical Race Theory (Brayboy, 2005) supports the use of decolonizing methodologies such as Community-Based Participatory Research when collaborating with Indigenous communities. This paper highlights the underlying processes in working with a Pueblo community on an intergenerational health project. Indigenous participants included 16 Piro Pueblo individuals who collaborated on a project examining healthy diets and activity in their community. The project involved providing information on the importance of activity and healthy eating of traditional foods to promote healthy living. Thematic analysis of open-ended questions exploring the role of culture in food and activity yielded important themes of gathering, resilience, history, honoring ancestors at mealtime, cultural ways, and activities such as dancing and drumming. The results suggest that projects addressing diet and activity collaborate to ensure cultural values, e.g., connectedness, cultural ways, e.g., gathering, dancing, and Indigenous knowledge are represented in the project and viewed through an Indigenous lens.

### CPBR PROCESS IN URBAN SETTINGS AND THE UNIQUE CHALLENGES AND SUCCESSES

Steffi Kim, *University of Alaska Anchorage, Anchorage, Alaska, United States*

CBPR is a framework that allows for the collaboration of researchers and communities as co-partners and is a supported approach for Indigenous communities. The community engagement and co-partnership in this study allowed for the researcher's flexibility to be responsive to culturally appropriate practices and priorities of the communities and participants. CBPR principles, including the Elder Advisory Committee (EAC), were utilized in this urban-based project. Challenges presented in many ways, including the processes of a) entering communities, b) relationship building, c) time involvement, and d) recruitment. Successes represented the unique opportunity to enter communities at an interpersonal level, b) close community engagement, c) gathering information beneficial for the research team and the community, and d) extended community engagement. While challenges exist, this approach's benefits are far-reaching promoting trust, support, and interest in future research endeavors. The presenter will discuss strategies and processes helpful in engagement, recruitment, and data collection.

### Session 2385 (Symposium)

### CREATIVE ARTS-BASED APPROACHES TO IMPROVE THE WELL-BEING OF OLDER ADULTS

Chair: Darina Petrovsky Discussant: Justine Sefcik

As older adults age they may face cognitive impairment, disruption in their sleep, and a decrease in mood and overall well-being. Given the negative consequences of the COVID-19 pandemic, they may experience a disruption in their access to health care services. Creative arts-based approaches have shown promise in improving the well-being of older adults and may be helpful in augmenting health care services. In this symposium, we will present research results of creative arts-based interventions aimed at improving the well-being of older adults, including those with dementia. We will also discuss ways to successfully engage with organizations that serve older adults using arts-based interventions. The first presentation will focus on the results from a feasibility randomized controlled trial examining the effects of a tailored music listening intervention on sleep outcomes in older adults living with dementia and their caregivers. The second presentation will report findings from the Mason Music & Memory Initiative aiming to improve mood and behavioral outcomes in persons living with dementia in nursing homes. The third presentation will focus on the preliminary results of a pilot study that integrated the delivery of music therapy telehealth with remote social work support and service linkage for rural older adults from low-income areas. The fourth presentation will report findings from the capacity-building program for teaching artists, health/aging organizations, and arts organizations committed to strengthening Creative Aging efforts through research. Implications for future research and creative arts-based intervention development for older adults will be discussed.

### TAILORED MUSIC LISTENING TO IMPROVE SLEEP IN OLDER ADULTS WITH DEMENTIA: A RANDOMIZED CLINICAL TRIAL

Nalaka Gooneratne,[1] Joke Bradt,[2]
Miranda Varrasse McPhillips,[3] Ime Etokebe,[3]
Laura Gitlin,[4] Nancy Hodgson,[5] and Darina Petrovsky,[6]
*1. University of Pennsylvania School of Medicine, Philadelphia, Pennsylvania, United States, 2. Drexel University, Phialdelphia, Pennsylvania, United States, 3. University of Pennsylvania, Philadelphia, Pennsylvania, United States, 4. Drexel University, College of Nursing and Health Professions, Drexel University, Pennsylvania, United States, 5. University of Pennsylvania, School of Nursing, philadelphia, Pennsylvania, United States, 6. Rutgers University, Philadelphia, Pennsylvania, United States*

Sleep disturbances in persons living with dementia (PLWD) contribute to reduced well-being. Music has shown promise to improve sleep among older adults, but there is limited evidence of music interventions improving sleep specifically in PLWD. The purpose of this wait-list RCT was to examine the i) feasibility; ii) acceptability and iii) preliminary efficacy of tailored music listening intervention in community-dwelling PLWD and their caregivers (dyads). Thirty consented dyads out of 33 (91%) completed the RCT. Tailored music for sleep was feasible based on screening (26%), enrollment (89%), and recruitment (3 dyads/month) rates. The intervention was found acceptable, as evidenced by post-intervention interviews. Compared to controls, PLWD in the intervention group reported greater global sleep quality improvement post-intervention (PSQI mean change -0.08 vs -1.65; p=0.06). The results from this feasibility RCT have informed the development of a music mobile application that will be tested in a future clinical trial.

### IMPLEMENTING A PERSONALIZED MUSIC INTERVENTION FOR PERSONS LIVING WITH DEMENTIA IN NURSING FACILITIES

Meng-Hao Li,[1] Emily Ihara,[2] Catherine Tompkins,[2]
Christi Clark,[3] Shannon Layman,[2] and Megumi Inoue,[2],
*1. George Mason University, Arlington, Virginia, United States, 2. George Mason University, Fairfax, Virginia, United States, 3. Harpeth Consultant Advisory Group, Franklin, Tennessee, United States*

The Mason Music & Memory Initiative (M3I) team has implemented a personalized music intervention in nursing facilities across Virginia aiming to improve behavioral and psychological symptoms of persons living with dementia. This person-centered intervention uses a unique music playlist comprising songs, artists, and preferred musical genres. The preliminary findings from a randomized controlled trial will be reported, the purpose of which was to examine the intervention impact on nursing home residents' mood and behavior. Based on the findings from 16 facilities with 158 residents who have completed the study, both quantitative and qualitative data indicate the positive effects on residents, including improved sleep and mood, as well as reduced agitation. The challenges in implementing intervention research in nursing facilities during the COVID-19 pandemic and the principles of telehealth and virtual support for facilities that were used to address those challenges will also be discussed.

### Session 2390 (Paper)

### DISASTERS AND AGING

### CHALLENGES AND STRATEGIES TO MAINTAIN FIDELITY TO THE MIRROR-TCM INTERVENTION DURING THE COVID-19 PANDEMIC

Mary Naylor,[1] Karen Hirschman,[1] Brianna Morgan,[1]
Molly McHugh,[1] Elizabeth Shaid,[1] Kathleen McCauley,[1]
Christina Whitehouse,[2] and Mark Pauly,[1] *1. University of Pennsylvania, Philadelphia, Pennsylvania, United States, 2. Villanova University, Villanova Univesity, Pennsylvania, United States*

Randomized clinical trials (RCTs) have demonstrated that the multicomponent Transitional Care Model (TCM), an advanced practice registered nurse-led, team-based, care management strategy improves outcomes for older adults transitioning from hospital to home. However, healthcare systems' adoption of the model has been limited. A multisystem, replication RCT (MIRROR-TCM) enrolling older adults hospitalized with heart failure, chronic obstructive pulmonary disease or pneumonia began in February 2020 just as the outbreak of COVID-19 in the U.S. dramatically changed the healthcare and research landscape. The goal of this qualitative descriptive study is to explore the impact of COVID-19 on fidelity to the TCM intervention during this clinical trial. Using directed content analysis, recorded monthly meetings with health system leaders and staff were coded to identify challenges and strategies to maintaining fidelity to the intervention in the context of the pandemic. Analyses showed that COVID-19 impacted all 10 TCM components. The components with the most challenges were delivering services from hospital-to-home due to quarantining, restrictive facility policies, lack of personal protective equipment and limited telehealth availability; coordinating care due reduced availability of services, and screening at risk individuals because of fewer eligible patients. Strategies for addressing challenges included: exploring alternatives (e.g., increasing reliance on telehealth, expanding study eligibility), building and engaging networks (e.g., direct outreach to skilled nursing facility staff) and anticipating needs (e.g., preparing for shorter hospital stays). Findings highlight the importance of monitoring the contextual challenges to implementing an evidence-based intervention and actively engaging partners in identifying strategies to achieve fidelity.

### CHANGES IN MODIFIABLE HEALTH BEHAVIORS DURING THE PANDEMIC AND EFFECTS ON MENTAL HEALTH: EVIDENCE FROM ENGLAND

Giorgio Di Gessa, and Paola Zaninotto, *University College London, London, England, United Kingdom*

COVID-19 mitigation efforts (including lockdowns and advice to stay at home as much as possible) are likely to have resulted in changes in health behaviours such as the amount of sleep, physical exercise, alcohol use, and eating. To date, little is known about how and to what extent these changes in health behaviours since the beginning of the pandemic are related to mental health. Using pre-pandemic data from Wave 9 (2018/19) and from two Covid-19 sub-studies (with data collection in June/July and November/December 2020) of the English Longitudinal Study of Ageing, we investigate how changes in health behaviour during the initial months of the pandemic are associated with subsequent mental health among older people. In our regression analyses, we considered depression and anxiety and controlled for pre-pandemic measures of mental health. Between March and June/July 2020, about a third of older people reported less physical activity; one in five less sleep; and one in ten eating less food and drinking more. Compared to respondents who did not change their behaviours, those who reported sleeping and eating both more and less, and who mentioned less physical activity were more likely to report depression and anxiety, even taking into account pre-pandemic mental health. An increase in drinking was also marginally associated with higher depression. Policymakers should encourage older people who have engaged in unhealthier behaviours to modify them to reduce the negative long-term effects on their mental health.

### IMPACT OF COVID-19 MORTALITY ON U.S. LIFE EXPECTANCY BY SOCIOECONOMIC RANK OF COUNTY OF RESIDENCE

Denys Dukhovnov, and Magali Barbieri, *University of California, Berkeley, Berkeley, California, United States*

Mortality disparities due to COVID-19 pandemic in the US accentuated the gap in the targeted public health and education response among disadvantaged communities. We use county data from John Hopkins University of Medicine in conjunction with county socioeconomic decile rankings, and weekly national data from the Centers for Disease Control to uncover the impact of county-level socioeconomic deprivation on the spatio-temporal dynamic of COVID-19 mortality. We estimate that over the course of 2020, the pandemic reduced the life expectancy at birth by 1.33 years (95% CI 1.0-1.56), and by 0.84 years (95% CI 0.59-1.0) by age 85 across all county SES decile groups, relative to the previous year's projection. The highest losses occurred in counties at the ends of the deprivation spectrum, and least affecting those in its middle. Decomposition of the impact of the COVID-19 mortality by seasonal time periods of 2020 reveals that coastal urban and high-SES counties endured a heavy death toll in the initial stages of the pandemic, though managed to cut it by more than a half by the end of 2020. The least affluent, inland, and rural counties have experienced a dramatic and lasting increase in deaths toward the second half of the year. We find that preexisting socioeconomic disparities before COVID-19 remained in force during the pandemic, leaving populations at all ages residing in underserved communities at a greater risk. This both calls into question and further instructs the ongoing public health interventions enabling more effective and equitable infectious disease mitigation strategies.

### IMPACT OF HURRICANE SANDY ON HOSPITAL ADMISSIONS 2 YEARS LATER

Laura Sands,[1] Pang Du,[2] Quyen Do,[1] Yunnan Xu,[1] and Rachel Pruchno,[3] *1. Virginia Tech, Blacksburg, Virginia, United States, 2. Virginia Tech, Virginia Tech, Virginia, United States, 3. Rowan University, Stratford, New Jersey, United States*

Disaster exposure is often followed by acute illness and injuries requiring hospital admission in the weeks after the disaster. It is not known whether disaster exposure is associated with hospitalization in the years after the disaster. We examined the extent to which disaster exposure is associated with hospitalization two years after Hurricane Sandy. The analyses fill a gap in our understanding of long-term physical health consequences of disaster exposure by identifying older adults at greatest risk for hospitalization two years after disaster exposure. Older adults (n=909) who participated in a longitudinal panel study provided data before and after Hurricane Sandy. These data were linked with Medicare inpatient files to assess the impact of Hurricane Sandy on hospital admissions after the post-hurricane interview. Those who reported experiencing a lot of fear and distress in the midst of Hurricane Sandy were at an increased risk of being hospitalized in the second or third years after the hurricane [Hazard Ratio=1.81 (1.15 – 2.85)]. Findings held after controlling for pre-hurricane demographics, social risks, chronic conditions, and decline in physical functioning after the hurricane. These findings are the first to show that disaster exposure increases risk for hospital admissions two years after a disaster, and that older adults' appraisal of their emotional distress during the disaster has prognostic significance that is not explained by known risks for hospital admissions. The findings suggest that interventions during the storm and after the storm, may reduce long-term health consequences of disaster exposure among older adults.

### SOCIOCULTURAL FACTORS INFLUENCING THE MANAGEMENT OF MENINGITIS AMONG OLDER ADULTS IN KWARA STATE, NIGERIA

Kafayat Mahmoud,[1] Moshood Issah,[2] and Hameed Bakare,[3] *1. University of Kansas, Lawrence, Kansas, United States, 2. University of Ilorin, Ilorin, Kwara, Nigeria, 3. University of Ilorin, University of Ilorin, Kwara, Nigeria*

During epidemic and non-epidemic seasons, the Kwara North, Nigeria, has consistently reported high incidence rates for meningitis, a disease which mostly affects older members of the community. Limited studies have investigated the nexus between climate change-meningitis and socio-cultural factors influencing the management and control of meningitis among the older adults. This study explored the lived experiences of older individuals with meningitis and relationships with their caregivers in Kaiama Local Government area of Kwara state, Nigeria. 15 participants, 6 men and 9 women, aged 65+ years were purposively selected, and in-depth interviews were conducted. Results indicated that most of the older adults believed that the disease is caused by spiritual or supernatural forces (such as witchcrafts, demons, evil spirits among others), and the treatment and management, using orthodox medicine of it has been hampered by certain socio-cultural beliefs. Due to beliefs about contagion, older adults are mostly left on their own, with adult children occasionally visiting their parents to provide care only to return to their own homes. Also, children visit with traditional healers who perform rites of purification and give older adults concoctions to use. The study concluded that meningitis is one of the leading causes of untimely death of some older adults in the study communities and it has been worsened by some socio-cultural practices. Based on this conclusion, the study recommended massive enlightenment of the general public about best practices in the treatment and management of the disease among the older members of the study communities.

### Session 2395 (Symposium)

### DIVERSITY, EQUITY, AND INCLUSION IN GERONTOLOGICAL RESEARCH MENTORING AND METHODOLOGIES

Chair: Ronica Rooks Co-Chair: Chivon Mingo Discussant: Chivon Mingo

With a rapid increase in our nation's diversity and in particular the diversity of the aging population, research focused on the well-being and quality of life for all older adults is imperative. Within GSA, The Minority Issue in Gerontology Advisory Panel is charged with providing support to the membership that ultimately will yield an increase in the quantity and quality of research related to minority aging issues. Therefore, understanding best practices for minority-focused gerontological research and gerontological education curriculum is warranted. The advancement of the field is predicated on the ability to have trained professionals with skills and competencies that effectively meet the needs of a diverse aging population. This symposium will include a presentation highlighting practical strategies for strengthening gerontology research by intentionally incorporating anti-racist methodological approaches. The second presentation will consist of recommendations on how to support, promote, and advance gerontology education in a manner that increases the diversity of those pursuing a research or an applied career in this area of study. Presenters will share an overview of the literature, findings from program implementation focus groups, and recommendations for tailoring strategies to fit your intended audience. This session will prove beneficial as we make strides to ensure that diversity, equity, and inclusion remain a core value and an inherent practice of all gerontology professionals.

### THE GSA NIA R13 DIVERSITY MENTORING AND CAREER DEVELOPMENT WORKSHOP: WHAT DID WE LEARN AFTER 3 YEARS?

Patricia D'Antonio,[1] Keith Whitfield,[2] and Patricia Heyn,[3] *1. The Gerontological Society of America, Washington, District of Columbia, United States, 2. University of Nevada Las Vegas, Las Vegas, Nevada, United States, 3. University of Colorado Anschutz Medical Campus, Arvada, CO, Colorado, United States*

The goal of the GSA NIA R13 Diversity Mentoring and Career Development Technical Assistance Workshop (GSA Diversity TAW) is to support, promote, and advance the training of diverse students in aging research. The program's main aim is to increase the number of early career scientists who are historically underrepresented in gerontological research. Thus, from 2018 to 2020, more than 60 trainees and 16 faculty from diverse backgrounds participated in this unique gerontological training that included peer mentoring opportunities and engagements at the GSA Annual Meeting. The workshop curriculum included scientific presentations, networking, NIH grant preparation, career planning, and effective professional communication. Trainees and faculty were involved in the evaluation of the workshop, which included electronic surveys and focus groups that informed the design and curriculum of subsequent workshops. This paper will discuss the curricular design and objectives of the GSA Diversity TAW and present a summary of the trainees' feedback results about the program and the iterative changes made based on that data.

### STRENGTHENING ANTI-RACIST GERONTOLOGY RESEARCH: DOES ONE SIZE FIT ALL?

Lyn Holley, *University of Nebraska at Omaha, Omaha, Nebraska, United States*

This paper is based on a scoping review and conceptual analysis of research literature about incorporating anti-racism into social science research practices. In his examination of how anti-racist research can effectively borrow key concepts such as "validation and "reliability" from traditional social science research, Dei concludes that these concepts must be reconsidered to addresses the main issues of anti-racism. (2005). A further critique of these concepts is that they do not account for differences among racism as it is applied to different minoritized groups. Public Health Nurses and other practitioners have long recognized the importance of understanding and taking these differences into account in their "culturally competent" practice. (Lipscomb, Culture Care) Although there is some literature about de-centering whiteness in research (e.g., https://libguides.umn.edu/antiracismlens ), little is available to guide research that acknowledges and addresses overlapping yet differing contours of racism as experienced by different "races", e.g., Black-Americans, Native American Indians".

### Session 2400 (Paper)

### DIVERSITY, RACIAL MINORITIES, AND AGING I

### AGE-FRIENDLY AS TRANQUILO AMBIENTE: HOW SOCIO-CULTURAL PERSPECTIVES SHAPE OLDER LATINOS' LIVED ENVIRONMENT

Melanie Plasencia, *University of California, Berkeley, Albany, California, United States*

Researchers have increasingly considered the importance of age-friendly communities to improve the health and well-being of older adults. Studies have primarily focused on the built environment, such as community infrastructure, older adult behavior, and environmental expectations. Less attention, however, has been given to the role of cultural characteristics in shaping perceptions of age-friendly environments for Latinos. Using an ethnographic methodological approach, including participant observation in a Latino community near New York City and 72 semi-structured interviews, this study provides empirical insights into how older Latinos characterize age-friendly communities. Latino older adults described their community as age-friendly using Tranquilo Ambiente (TA), which translates to a calm or peaceful environment. According to older adults, a TA possesses the following: 1) a sense of personal safety, including protection of their body, 2) ethnic, social connectedness, including networks with other Latinos and important social and cultural events; and 3) a comparative understanding of their communities treatment of seniors versus other geographical and spatial locations. While much has been written on the role of the built and social environment in developing and implementing age-friendly communities, more research on the cultural significance and understanding of place among marginalized older adults is necessary. TA and its characteristics demonstrate that cultivating an age-friendly setting requires adapting structures and services to promote Latino older adults' social and cultural support and engagement.

### AGING OUT OF PLACE: FACTORS RELATED TO QUALITY OF LIFE AMONG OLDER REFUGEES IN THE UNITED STATES

Jonix Owino,[1] and Heather Fuller,[2], *1. North Dakota State University, Horace, North Dakota, United States, 2. North Dakota State university, Fargo, North Dakota, United States*

Refugees flee their home countries, migrating to countries such as the US for safety. The psychological distress they experience may compromise their adaptation and well-being. However, little is known about quality of life among aging refugees who migrate to the US as adults, and in particular whether quality of life varies among refugees by sociodemographic factors such as age, sex, country of origin, and length of residence. Moreover, limited research exists examining the role of social connectedness for aging refugees's quality of life. The current study explores sociodemographic and social connection factors associated with quality of life among aging refugees (N = 108; aged 50+). Refugees from Bhutan, Burundi, and Somalia were recruited from a Midwestern small city to complete an in-depth survey assessing social factors and well-being. Hierarchical regression analyses showed that females, older individuals, and African refugees reported lower quality of life, while length of residence was not associated with quality of life. When controlling for sociodemographic factors, greater social integration and lower loneliness were significantly associated with higher quality of life. There was also a significant interaction between loneliness and sex in predicting quality of life, indicating that greater loneliness was associated with reduced quality of life for women but not men. Study findings will be discussed in light of cultural variations within refugee groups and with the goal of highlighting ways to best support aging refugees' well-being and develop social programs that can effectively cater to issues of aging among refugees.

### IMPACT OF A PHARMACIST-LED DIABETES MANAGEMENT INTERVENTION TO IMPROVE HEALTH EQUITY

Renae Smith-Ray,[1] Tanya Singh,[1] Evie Makris,[2] Jaime Horan,[3] and Michael Taitel,[1] *1. Walgreen Co, Deerfield, Illinois, United States, 2. Walgreens Co., Deerfield, Illinois, United States, 3. Walgreens, Deerfield, Illinois, United States*

The COVID-19 pandemic and Black Lives Matter movement brought increased recognition to the need for health equity. Diabetes, the 7th leading cause of death, is one of many conditions where health inequities are evident. A higher percentage of Black (11.7%) and Hispanic (12.5%) U.S. adults are diagnosed with diabetes compared to non-Hispanic Whites (7.5%). To address this health inequity, a nationwide pharmacy chain implemented telephonic 'Advanced Care' (AC) outreach for patients with diabetes. During the AC call, pharmacists used motivational interviewing techniques to counsel patients on the importance of closing gaps in care and reducing barriers to medication adherence. Gaps included timely A1C testing, exams (eye, foot, kidney), immunizations (influenza, pneumonia, Hepatitis B), and recommendation of additional therapies for patients with multiple chronic conditions (ACE/ARB, statins). Medication fill gaps were compared between the Intervention period (8/1/20-1/31/-21) and a pre-intervention period (2/1/20-7/31/20). The AC pilot occurred in 8 Chicago Walgreens locations that primarily serve Black and Hispanic patients. Eight control stores were matched on census block-level household income and race/ethnicity, patient volume, and insurance mix. A pre/post-test vs. control difference-in-difference (DID) analysis was conducted to compare on-time refill rates. Of the 1,009 older patients (age≥50) called, 59.9% were reached. The DID analysis showed that patients in pilot stores had improved pre-post on-time refill rates compared to controls ($p<0.0001$). Diabetes self-management is key to reducing diabetes-related complications. Early findings from this pilot demonstrate that the Walgreens AC intervention improves medication adherence - an important step toward improving health equity.

### INSTRUMENTAL ACTIVITIES OF DAILY LIVING DIFFICULTIES PREDICT COGNITIVE DECLINE IN OLDER PUERTO RICAN ADULTS

Caitlin Pope,[1] Tyler Bell,[2] Brian Downer,[3] Sadaf Milani,[3] Lauren Roach,[1] and Michael Crowe,[4], *1. University of Kentucky, Lexington, Kentucky, United States, 2. University of California San Diego, La Jolla, California, United States, 3. University of Texas Medical Branch, Galveston, Texas, United States, 4. University of Alabama at Birmingham, Birmingham, Alabama, United States*

Given the hypothesized bidirectional association between functional and cognitive decline, further characterization of the temporal association between the two is needed, especially in Latinx samples as they are the most rapidly growing demographic in the United States and at greater risk for Alzheimer's disease. This study assessed bidirectional associations between instrumental activities of daily living (IADL) difficulty and cognition in older Puerto Rican adults. Participants included 2,840 community-dwelling adults (60+ years) without cognitive impairment who completed baseline and a four-year follow-up in the Puerto Rican Elderly: Health Conditions (PREHCO) project. At each wave, cognition (using the Mini-Mental Cabán) and self-reported IADL difficulty (a sum score of 10 everyday tasks) were measured. Covariates included age, gender, education, comorbidities, and depressive symptoms. Bidirectional associations were tested using a path model with concurrent and cross-lagged paths between cognition and IADL difficulty adjusting for covariates. Lower baseline cognition related to more baseline IADL difficulty (B=-0.08, SE=0.02, p<.001). Cognitive decline at follow-up related to greater IADL difficulty at follow-up (B=-0.06, SE=0.02, p=.012). Looking at cross-lagged associations, greater baseline IADL difficulty associated with more cognitive decline at follow-up (B=-0.10, SE=0.04, p=.012). However, baseline cognition was not significantly associated with change in IADL difficulty at follow-up (B=-0.003, SE=0.02, p=.869). Findings support the growing body of literature that IADL difficulties can predict future cognitive decline in samples of community-dwelling older adults. More research into both functional and cognitive decline in Latinx samples will provide a more generalizable view of aging.

### POPULATION AGING AND MULTICULTURAL DIVERSITY IN THE UNITED STATES: IMPLICATIONS FOR OLDER CONSUMERS' NEEDS AND EXPECTATIONS

Julie Miller,[1] Taylor Patskanick,[2] Lisa D'Ambrosio,[3] and Joseph Coughlin,[1], *1. MIT, Cambridge, Massachusetts, United States, 2. MIT, Somerville, Massachusetts, United States, 3. Massachusetts Institute of Technology, Cambridge, Massachusetts, United States*

Looking ahead to 2030 and beyond, the United States will be both older and more multicultural than presently. To explore the impacts and characteristics of an increasingly diverse population beginning to age, the MIT AgeLab conducted online focus groups in August 2020 (n=92) with ethnically diverse participants ages 40-69 on topics related to household composition, use of technology and digital engagement. Regarding household composition, Black and Latinx participants were more likely to report living with grandchild(ren), and Asian, Latinx, and White participants were more likely to report living with a parent(s) or parent(s)-in- law. Latinx participants often described ways in which caregiving for aging parents was a cultural value, but many participants who had raised children in the United States but who were not born in the United States themselves described cultural gaps in family attitudes that had sometimes widened across the generations. While all participants were using some technology, due to the coronavirus pandemic, digital tools were being used more widely than ever before. Racial/ethnic identity groups were more similar than different in terms of their responses to questions around consumer digital engagement. There were notable differences in overall trust in technology across racial/ethnic groups, with Asian participants reporting the highest average overall level of trust in technology and Multiracial participants reporting the lowest. Looking ahead, the intersection of aging and growing racial/ethnic diversity in the United States will yield a wider array of consumer needs and expectations.

### Session 2405 (Symposium)

### EFFECTS OF LONELINESS AND SOCIAL ISOLATION ON COGNITIVE HEALTH: LATEST PERSPECTIVES AND FUTURE DIRECTIONS

Chair: Kheng Siang (Ted) Ng Co-Chair: Kexin Yu
Discussant: James Lubben

Loneliness and social isolation as antecedents of cognitive decline have received substantial attention in recent research. This symposium addresses this year's conference theme of aging in the "new normal". The COVID-19 pandemic has highlighted the negative impacts of loneliness and social isolation on older adults' health and wellbeing. This symposium includes studies that shed light on the relationships between loneliness, social isolation and cognitive health using a multidisciplinary approach, and provide recommendations and future directions for advancing this research area. The first presentation examines cardiovascular biomarkers as potential mechanisms that mediate the longitudinal relationship between loneliness and cognitive decline with the HRS dataset. The second presentation examines several social isolation indicators and their effects on cognitive decline in a Canadian longitudinal study. Using the US ADRC longitudinal study of aging, the third study shows the effect of loneliness on cognitive health in older adults pre- and post-onset of the COVID-19 pandemic. The symposium concludes with a literature review of the different measures employed to operationalize the constructs of loneliness, isolation, which resulted in heterogeneous study findings on their influences on the risk of developing dementia. This review calls for consistent measures to produce comparable evidence on the health consequences of loneliness and isolation. In all, this symposium reports and reviews the latest evidence on the association between social isolation, loneliness and cognitive health amidst the ongoing COVID-19 pandemic. It also echoes the conference theme of transforming disruption to opportunities in aging health service and research.

### BIOLOGICAL PATHWAYS UNDERLYING THE LONGITUDINAL ASSOCIATION BETWEEN LONELINESS AND COGNITIVE DECLINE

Kheng Siang (Ted) Ng,[1] and Kexin Yu,[2] *1. University of Colorado Anschutz Medical Campus, Aurora, CO, Colorado, United States, 2. USC, LA, California, United States*

Loneliness has been recognized as a risk factor for cognitive decline (CD) in older adults. However, how loneliness "gets under the skin" to influence CD has been conceptually proposed but rarely empirically examined. The purpose of this study is to investigate whether cardiovascular and kidney biomarkers mediate the longitudinal association between loneliness and CD. We used the cross-lagged panel model (CLPM) to examine the hypothesized relationships with 2006, 2010 and 2014 waves of data from the Health and Retirement Study (HRS). Loneliness is measured with 3-item UCLA loneliness scale. Cognitive health was assessed using the total cognition score. Biomarkers considered including HbA1C, total cholesterol, HDL cholesterol, CRP, and Cystatin C. Among all five biomarkers examined, HbA1c significantly mediated the longitudinal association between loneliness and CD. The other biomarkers examined did not mediate the relationship between loneliness and CD. The study findings show loneliness might affect CD through elevating HbA1C levels.

### A PUBLIC HEALTH ANALYSIS OF THE RELATIONSHIP BETWEEN LONELINESS, ISOLATION, AND DEMENTIA

Christina Victor, *Brunel University London, Uxbridge, England, United Kingdom*

Loneliness and isolation are now characterised as major public health problems largely because of reported associations with negative health outcomes including dementia. We adopt a public health perspective and review the relationship between loneliness/isolation and dementia focussing on how these concepts are defined, measured, and reported. We identified community based longitudinal studies which measured loneliness/isolation at baseline and dementia at follow up (minimum 12 months) published up to February 2021. We identified 12 papers for loneliness and 15 for isolation which demonstrated substantial heterogeneity in how exposure (loneliness/ isolation) and outcome (dementia) were measured and reported. For example, dementia was measured in 5 different ways: death, hospitalisation, clinical diagnosis, dementia screening tools or cognitive function. Evidence to support a relationship between loneliness/isolation and dementia is inconclusive largely because of this methodological heterogeneity. Using consistent exposure and outcome measures is a prerequisite for determining the health consequences of loneliness and isolation.

### THE RELATIONSHIP BETWEEN LONELINESS AND THE COVID-19 PANDEMIC ON COGNITION AND WELL-BEING IN OLDER ADULTS

Justin Barber,[1] Allison Gibson,[2] Shoshana Bardach,[3] Kelly Parsons,[1] Julia Johnson,[1] Gregory Jicha,[1] and Elizabeth Rhodus,[1], *1. University of Kentucky, Lexington, Kentucky, United States, 2. University of Kentucky, University of Kentucky, Kentucky, United States, 3. Dartmouth University, Dartmouth University, New Hampshire, United States*

Social distancing is necessary to limit the spread of Covid-19. However, many older adults are predisposed to isolation and loneliness despite calls to socially distance. The current study examined loneliness during Covid-19 in relation to cognition and wellbeing in older adults. Data were extracted from a U.S. ADRC longitudinal study of aging database. Cognition was assessed using the NACC UDS 3.0 battery. Measures of well-being include: Short Form Health Survey, Subjective Memory Assessment, and Geriatric Depression Scale. Measurement of loneliness was selected from the NIH ADRC Covid-19 questionnaire. Data were from 115 older adults with normal cognition or MCI with a visit ≤18 months before research stoppage in March 2020 and after resumption in late-June 2020. Cognition and wellbeing are compared before and after onset of pandemic. Isolation due to Covid-19 may have long-term implications. Results of this study will highlight the need for acute assessments and psychosocial interventions.

### THE LONGITUDINAL ASSOCIATION BETWEEN SOCIAL ISOLATION AND COGNITIVE DECLINE AMONG CANADIAN OLDER ADULTS

François Béland,[1] and Fereshteh Mehrabi,[2] *1. Université de Montréal, Montreal, Quebec, Canada, 2. School of Public Health (ESPUM), Université de Montréal, Montreal, Quebec, Canada*

Social isolation is an emerging public health concern with an emphasis on its potential preventive impact on cognitive impairment. We hypothesize that change in social isolation is associated with cognitive decline over two years. Latent growth models were used to test the hypothesis using data from the FRéLE longitudinal study among 1643 Canadian community-dwelling older adults aged 65 years and over. We assessed social isolation using social participation, social networks, and support from various social ties. We found that less contact with friends and children, less social support from friends, children, and partner, and the absence of partner were significantly associated with cognitive decline. Higher baseline social participation was related to the lower rate of cognitive decline over time. Fewer contacts with grandchildren were associated with cognitive decline over time. Our study suggests that public health policies in age-friendly cities and intergenerational contacts may promote cognitive health in older adults.

### Session 2410 (Symposium)

### ESPO AND BUTLER-WILLIAMS SCIENTIFIC SYMPOSIUM: DISRUPTING HEALTH DISPARITIES AND TRANSFORMING CARE OF OLDER ADULTS

Chair: Patricia Jones Co-Chair: Jamie Justice Discussant: Roland Thorpe, Jr.

The NIA's Butler-Williams Scholars Program and GSA's ESPO Section are united in providing career development opportunities in a manner that promotes leadership, diversity, and inclusivity. Our emerging scholars are called to examine health inequity and overcome barriers by continuous and incremental improvements (disruption) or dramatic change (transformation) to improve health and access to care for our diverse aging population. Among the chief concerns of our aging population are disparities in health associated with race/ethnicity, experience, sociocultural and socioeconomic factors. GSA's early career professionals and 2020 alumni of the prestigious NIA Butler-Williams Scholars Program address these issues. Dr. Karen Moss will discuss new findings from a study of African American caregivers of persons living with dementia. Dr. Jasmine Travers will present on mitigating disparities in access to long-term services and supports among older adults. Dr. Maricruz Rivera-Hernandez, will present current work on aging and care delivery or health of Hispanic older adults, including impact of COVID19 in Hispanic groups. Dr. Marc Garcia will present on interaction of race/ethnicity, nativity, and gender with key social and economic factors on health, COVID19, and aging. Finally, Dr. Shana Stites will present on social challenges in advancing diagnosis and treatment of Alzheimer's disease dementia. The featured talks by rising stars deepen our understanding of the influence of health disparity so we can disrupt inequity and transform systems of care across our diverse aging populations.

### EXPERIENCED FAMILY CAREGIVER PERSPECTIVES ON ADVANCE CARE PLANNING FOR AFRICAN AMERICANS LIVING WITH DEMENTIA

Kathy Wright,[1] Laurel Myers Hurst,[1] Abigail Grieff,[1] Karen Rose,[1] Todd Monroe,[2] Celia Willis,[1] and Karen Moss,[1] *1. The Ohio State University, Columbus, Ohio, United States, 2. Ohio State University, Columbus, Ohio, United States*

Most existing advance care planning (ACP) programs do not meet the needs of lower socioeconomic status (SES) African American (AA) older adults living with dementia. The perspectives of experienced family caregivers are integral to achieving appropriate ACP tailoring. The purpose of this study is to describe experienced family caregiver perceptions about needs and preferences for tailoring ACP for family caregivers of lower SES AA older adults living with dementia. This qualitative, descriptive, cross-sectional study is embedded within a larger community-based participatory study aimed at intervention development. Caregivers are completing up to two interviews. Preliminary data describes themes involving Caregiver Stress and suggestions for Service Improvements addressing grief and loss pre- and post-death. Caregiver findings and other stakeholder data from healthcare providers and community leaders will guide the design of a “new normal” enhanced, preference-consistent ACP intervention to improve end-of-life care during a global pandemic that is amplifying pre-existing healthcare disparities.

### A PROFILE OF BLACK AND LATINX OLDER ADULTS RECEIVING CARE IN NURSING HOMES: 2011-2017

Andrew Dick,[1] Bei Wu,[2] David Grabowski,[3] Mansi Agarwal,[4] Gayani Perera,[5] Patricia Stone,[6] and Jasmine Travers,[2] *1. Rand Corporation, Boston, Massachusetts, United States, 2. New York University, New York, New York, United States, 3. Harvard Medicaid School, Boston, Massachusetts, United States, 4. Division of Biostatistics, Washington University School of Medicine, St Louis, Missouri, United States, 5. Center for Health Policy Columbia University School of Nursing, New York, New York, United States, 6. Columbia University School of Nursing, New York, New York, United States*

Between the years 1999-2008, a substantial increase in nursing home use occurred among Black and Latinx older adults, while white older adults’ use of nursing homes decreased. These disparate trends suggested potential racial and ethnic disparities in options for preferred long-term services and supports (LTSS) settings. Over the last decade, several initiatives have been put in place to support LTSS needs in the community. However, it is unclear whether Black and Latinx older adults are continuing to use nursing home services at disproportionate rates. We used LTCfocus data for 2011-2017 to explore current trends in nursing home use and access among Black and Latinx older adults in light of these current initiatives. Our findings reveal a continued rise in Black and Latinx older adults’ use of nursing homes while white older adults’ use continues to decline. More notably, there has been a decline in nursing homes servicing these minority groups.

### THE IMPACT OF COVID-19 ACROSS NURSING HOMES THAT DISPROPORTIONALLY SERVE MINORITY RESIDENTS

Amit Kumar,[1] Indrakshi Roy,[1] Amol Karmarkar,[2] KImberly Erler,[3] James Rudolph,[4] Julie Baldwin,[1] and Maricruz Rivera-Hernandez,[4],*1. Northern Arizona University, Flagstaff, Arizona, United States, 2. Sheltering Arms Institute, Richmond, Virginia, United States, 3. MGH Institute of Health Professions, Boston, Massachusetts, United States, 4. Brown University School of Public Health, Providence, Rhode Island, United States*

The Coronavirus-2019 (COVID-19) pandemic has disproportionally affected communities of color and older adults in the United States. Nursing homes (NHs) have reported over 130,000 COVID-19 deaths (or one-fourth of all US deaths) circa March 2021, a high share of the nation’s total death count (CMS COVID-19 NH Data). These inequities partially driven by barriers to care, segregation and structural racism have resulted in the unequal impact of COVID-19 across NHs (Li et al., 2020). In this presentation, I will describe NHs that disproportionally care for minority residents and the effect of NH composition on COVID-19-related mortality and outcomes. In 2020, minority older adults were less likely to have access to high quality facilities. From June – August, NHs with a high proportion of minority residents reported higher COVID-19 mortality rates per 1000 residents. Equal access to high quality of care across the life-course among racial and ethnic groups is needed.

### THE INTERSECTION OF RACE AND ETHNICITY, NATIVITY, AND SEX ON COGNITIVE TRAJECTORIES OF OLDER ADULTS IN THE UNITED STATES

Wassim Tarraf,[1] and Marc Garcia,[2] *1. Institute of Gerontology & Department of Healthcare Sciences, Wayne State University, Detroit, Michigan, United States, 2. Syracuse University, Syracuse, New York, United States*

We used longitudinal data from the Health and Retirement Study (1998-2016) to estimate sex-specific age-graded changes in global cognition and memory among White, Black, and U.S.- and foreign-born Latino adults 51 years and older. Among males, racial/ethnic and nativity differences in cognitive function were mainly evident at younger ages, particularly for Blacks compared to Whites. We found no evidence to support male racial/ethnic or nativity differentials in trajectories of cognitive aging. For women, older Blacks and U.S.-Born Latinas, and to a lesser degree foreign-born Latinas, had lower cognitive function at younger ages. However, White women showed more pronounced cognitive aging in comparison to U.S.- and foreign-born Latinas. Results applied to both global and memory outcomes. Our findings support calls for nuanced considerations of racial/ethnic and nativity effects on cognitive aging and ADRDs. Continued monitoring of differential cognitive aging trends is warranted as the vascular and neurologic sequelae of COVID-19 manifests.

### HOW BIOMARKER-BASED DIAGNOSIS AND TREATMENT AFFECT ALZHEIMER'S STIGMA: RESULTS OF A RANDOMIZED TRIAL

Jeanine Gill,[1] Emily Largent,[1] Kristin Harkins,[1] Abba Krieger,[2] Jason Karlawish,[1] and Shana Stites,[1]
*1. University of Pennsylvania, Philadelphia, Pennsylvania, United States, 2. Wharton School of Business, Philadelphia, Pennsylvania, United States*

Alzheimer's disease (AD) causes progressive disability and, ultimately, death. Currently no therapy can delay or slow cognitive and functional decline. This prognosis contributes to the general public's negative reactions—discrimination, pity, and social distance—toward individuals with AD and their families. But what if, using AD biomarker tests, diagnosis was made earlier and treatment was available? Stigma of AD might change. This project aimed to discover how diagnosis and treatment of AD before the onset of cognitive impairment would change public stigma, and how these effects might differ in ethnoracial populations. Comparisons of 12 experimental conditions (i.e., 2 (biomarker test result) x 2 (treatment availability) x 3 (cognitive impairment: none, mild, moderate)) are conducted in two independent samples of self-identified White (N=800) and Black (N=800) Americans. Findings anticipate the translation of the preclinical AD construct into care and will inform public policies and interventions to mitigate public stigma of AD.

### Session 2415 (Symposium)

### GENDER, FAMILY HISTORIES, AND LATE-LIFE ECONOMIC WELL-BEING

Chair: Deborah Carr Co-Chair: Pamela Smock
Discussant: Teresa Ghilarducci

Text TK.

### MARITAL HISTORIES AND LATE-LIFE ECONOMIC SECURITY: DO SOCIAL SECURITY BENEFITS RULES PERPETUATE DISPARITIES?

Deborah Carr, *Boston University, Boston, Massachusetts, United States*

Disparities in late-life economic security persist along the lines of gender, marital status, race, and educational attainment. We propose that these disparities are partly due to the fact that Social Security benefits are structured such that never-married, divorced, and cohabiting persons, those who were widowed prematurely, or were in a dual-earner couple face benefit penalties. Drawing on data from the Wisconsin Longitudinal Study (WLS), a study that has followed men and women from age 18 (in 1957) through age 72 (in 2011), we examine disparities in Social Security earnings and poverty risk on the basis of gender and marital histories. Our results reveal a large disadvantage for divorced and never-married persons (relative to their married counterparts), with women and those divorced two or more times experiencing the largest toll. We discuss the implications of our results for revamping Social Security to better meet the needs of 21st century families.

### HOW MARITAL STATUS SHAPES GRANDPARENTING CHILDREN WITH DISABILITIES

Madonna Harrington Meyer, *Sociology, Syracuse, New York, United States*

How does marital status shape grandparent care work when grandchildren have disabilities? Based on 50 in-depth interviews with grandparents who provide various types of care for grandchildren with disabilities, we find that marital status shapes care work in three distinct ways: (1) Many who are married describe both grandparents working as a team to provide vital care; (2) Some who are married describe spouses, primarily grandfathers, who are either unable or unwilling to provide care; and (3) Many who are not married, primarily grandmothers, describe providing relatively high levels of care and support despite relatively low resources. While nearly all report a great deal of joy and satisfaction with their care work, those who are single, have greater care responsibilities, and fewer resources are more likely to report adverse social, emotional, physical, and financial impacts. More robust social policies could alleviate the impact of marital status on grandparent care work.

### THE EFFECTS OF GENDER AND MARITAL STATUS ON ACCRUED DEBT IN RETIREMENT PLANNING

Zibei Chen,[1] and Karen Zurlo,[2] *1. University of Southern Mississippi, Hattiesburg, Mississippi, United States, 2. Rutgers University, Rutgers University, New Jersey, United States*

The effects of gender and marital status on accrued debt in retirement planning becomes an urgent concern because unmarried women face greater financial challenges in retirement than their counterparts. This study used data from the National Financial Capability Study (NFCS), designed by FINRA. We identified debt that influences retirement planning among a sample of pre-retirees, aged 51 to 61 years, and consider the associations of gender, marital status, debt, and retirement planning. Our results indicated that mortgage debt and credit card debt were negatively associated with retirement planning for women. Having a retirement account is positively associated with retirement planning and it also mediates the relationship between credit card debt and retirement planning. We urge women and financial planning executives to take time during the pre-retirement years to assess their various forms of debt and determine how it affects retirement planning objectives given current marital status.

### Session 2420 (Symposium)

### JOSEPH T. FREEMAN AND EXCELLENCE IN REHABILITATION AWARD LECTURE

Chair: Elsa Strotmeyer

The Joseph T. Freeman Award lecture will feature an address by the 2021 Freeman Award recipient Pamela Cacchione, PhD, CRNP, BC, FGSA, FAAN, of the University of Pennsylvania. The Joseph T. Freeman Award is a lectureship in geriatrics awarded to a prominent physician in the field of aging, both in research and practice. The award was established in 1977 through a bequest from a patient's estate as a tribute to Dr. Joseph T. Freeman. The Excellence in Rehabilitation of Aging Persons Award lecture will feature an address by the 2021 Excellence in Rehabilitation Award recipient Gregory Hicks, MPT, PhD of the University of Delaware. The Excellence in Rehabilitation of Aging Persons Award is designed to acknowledge outstanding contributions in the field of the rehabilitation of aging individuals.

### EXCELLENCE IN REHABILITATION OF AGING PERSONS AWARD: DEVELOPING A FUNCTION-FOCUSED APPROACH TO PAIN MANAGEMENT FOR OLDER ADULTS WITH CHRONIC LOW BACK PAIN

Gregory Hicks, *University of Delaware, Newark, Delaware, United States*

The overarching goal of our research program is to improve physical function and participation in older adults with chronic musculoskeletal conditions, particularly low back pain (LBP). Despite the fact that LBP is a common problem for older adults and is associated with poor outcomes in this vulnerable age group, little research has focused on LBP in people over age 65. With minimal research available, clinicians do not have clear evidence-based guidance regarding management of older adults with LBP. This presentation will focus on our pathway towards developing a focused treatment algorithm designed to prevent and address the pain-related disability found in older adults with LBP. Our work has largely focused on the identification of potentially modifiable "rehabilitation risk factors" that are associated with pain and functional limitations, as well as the development of interventions to address these risk factors. Specifically, we will discuss our findings relative to trunk muscle impairments and clinical hip impairments in the context of the older adult with low back pain.

### JOSEPH T. FREEMAN AWARD: CREATING SYNERGY IN INTERDISCIPLINARY AGING RESEARCH AND PRACTICE

Pamela Cacchione, *University of Pennsylvania, University of Pennsylvania School of Nursing, Pennsylvania, United States*

Over 30 years of interdisciplinary practice stimulated many research questions. Early intervention research in sensory impairment, specifically vision and/or hearing impairment was heavily supported by interdisciplinary colleagues from Geriatric Medicine, Nursing, Occupational Therapy, Optometry and Audiology. Challenges and opportunities from this research created a growing interest in developing and designing technologies for older adults. Creating the need for partnerships with engineering. My expertise in aging and access to willing research participants made me an ideal research partner. Effectively expanding my focus beyond sensory impairment interventions to designing and testing robots with older adults. Currently we are testing low cost mobile robots in acute care and the community and are developing and testing a soft robot to assist persons out of a chair as well as turn and lift persons up in bed. The synergy of interdisciplinary practice and research is helping us innovate to improve the lives of older adults.

### Session 2425 (Paper)

### LONG-TERM CARE II (SRPP PAPER)

### HOME CARE SERVICES FOR SENIORS: A TYPOLOGY OF INSTRUMENTS AND ADMINISTRATIVE BURDENS IN INDUSTRIALIZED COUNTRIES

Patrik Marier,[1] Daniel Dickson,[1] and Kyuho Lee,[2]
*1. Concordia University, Montreal, Quebec, Canada, 2. Daegu University, Daegu University, Kyongsang-bukto, Republic of Korea*

This contribution has two key objectives. First, inspired by earlier studies in comparative welfare state and in (social) gerontology, we develop a conceptualization of autonomy that is rooted in its social dimensions. This concept is then deployed to assess its policy considerations within the field of home care, both with regards to access and generosity in 21 industrialized countries. Second, this contribution performs a comparative assessment of the key factors resulting in a prioritization of the social dimensions of home care and social services in long term care. This study involves an-depth analysis of policy instruments deployed by public authorities to enhance the (social) autonomy of older adults, complemented with interviews with policy makers in diverse home care policy settings (Canada, France, South Korea, Sweden, and the United States). As such, this study features an evaluation of the presence of social elements in the definition and supply of care needs across 21 countries. It leads to the construct of a social dimensions of autonomy index based upon these instruments and the budgetary prioritization of home care within long term care policies. Among core findings, one discovers broader access and more generous funding when home care responsibilities are firmly embedded at the local level.

### NURSING HOME POST-ACUTE CARE SPECIALIZATION GROUPS AND FINANCIAL OUTCOMES

Xiao (Joyce) Wang,[1] Jeffrey Burr,[2]
Robert Weech-Maldonado,[3] Jennifer Hefele,[4] and
Kathrin Boerner,[5] *1. University of Massachusetts, Boston, Massachusetts, United States, 2. McCormack Graduate School, Boston, Massachusetts, United States, 3. University of Alabama at Birmingham, Birmingham, Alabama, United States, 4. Booz Allen Hamilton, McLean, Virginia, United States, 5. University of Massachusetts Boston, Boston, Massachusetts, United States*

Nursing homes (NHs) have increasingly specialized in post-acute care (PAC). However, it remains unclear as to why some NHs engage in more specialization than the others. Furthermore, the relationship between financial outcomes and PAC specialization has not been examined using more accurate financial indicators. This study developed a NH PAC specialization typology and examined financial outcomes (i.e. total revenue per inpatient day, operating margin) of different specialization groups. We employed NH-level panel data from 2011 through 2017 and focused on over 9,000 urban NHs per year. Multiple data sources were utilized like the Certification and Survey Provider Enhanced Reporting data; Medicare Cost Reports; and Brown University's LTCfocUS. We employed Latent Profile Analysis to develop distinct NH care specialization groups based on PAC staffing levels. This analysis revealed heterogeneous and clustered patterns of PAC staffing utilization and identified a four-group typology: "low specialization," "mixed specialization," "moderate PAC specialization," and "intensive PAC specialization." Using fixed-effects modeling, we then examined financial outcomes of the four PAC specialization groups. Although being in a group with higher level of commitment to PAC specialization was associated with higher revenues, it was not necessarily associated with higher operating margins. Further, in stratified analyses, for-profit and not-for-profit NHs showed different patterns in these associations. This suggested that although NHs compete for patients paid at higher reimbursement policies, increased costs may offset higher revenues as a result of specialization. Future studies should track financial outcome trajectories of NHs by care specialization groups in light of various payment innovations.

### THE BENEFIT OF AUTONOMY AND CONTROL: A POSITIVE CHARACTERISTIC OF THE HOME HEALTH AIDE POSITION

Hayley Gleason,[1] and Edward Miller,[2] *1. Colorado Department of Health Care Policy & Financing, Denver, Colorado, United States, 2. University of Massachusetts Boston, Boston, Massachusetts, United States*

Home Health Aides' (HHAs) are one of the fastest growing workforces in the country, yet the industry struggles to recruit new aides into the field and retain current workers. This study explored HHAs' experiences with the level of autonomy and control granted to them within their day-to-day work. Findings from six focus groups with 37 HHAs showed that many aides select home care because of the control and independence the positions offer. Interacting one-on-one with clients and being able to self-structure their daily tasks were major benefits that drew HHAs to the field. Additionally, the HHAs highlighted the control they have over their schedule and the flexibility the position offers to enable them to accommodate other responsibilities, like childcare or other jobs. Being able to decline a client because of travel distance, the hours required, or not feeling that it is a "good fit" was also a welcomed aspect of the position. Despite complaints about the job, such as low pay, lack of benefits, and limited support, many of the HHAs admitted staying on in their positions because of the flexibility, autonomy, and control provided. Findings highlight the value that HHAs place on autonomy and control and the potential benefit that these job qualities have for promoting greater recruitment and retention of the home care workforce. Amplifying opportunities for these aspects of the job may thus entice new individuals to pursue a career as an HHA, as well as help to maintain those individuals currently in the position.

### UNDERGIRDING CNAS IN LTC: THE EXPERIENCE OF COLLABORATIVE LN-CNA CAREGIVING PAIRS

Cynthia Beynon,[1] Katherine Supiano,[2] Elena Siegel,[3] Linda Edelman,[4] Connie Madden,[5] and Sara Hart,[6]
*1. Weber State University, Ogden, Utah, United States, 2. University of Utah, Salt Lke City, Utah, United States, 3. University of California, Davis, Sacramento, California, United States, 4. University of Utah College of Nursing, Salt Lake City, Utah, United States, 5. University of Utah, Salt Lake city, Utah, United States, 6. University of Utah, SALT LAKE CITY, Utah, United States*

This research explores support provided by licensed nurses (LNs) to certified nurse aide (CNA) coworkers in the nursing home (NH). Using purposive sampling, we interviewed 12 LN and 12 CNA participants individually and as part of an LN/CNA caregiving pair. Semi-structured interviews were recorded, transcribed verbatim, and coded for meaning using NVivo software. LN and CNA participants described anticipated and unanticipated holistic support for CNAs. We applied the term undergirding to this phenomenon , and we present descriptions and examples of undergirding in nine categories: listen and respond, show respect, help with resident care and answer call lights, protect the CNA, support physical needs, and provide emotional support. Undergirding promotes work success for the CNA and the LN, as the LN job includes oversight of CNA responsibilities. Most notably, participants report undergirding facilitates high-quality resident care. These findings may be helpful for educators and administrators, but perhaps are most important for policymakers. CNAs need additional support to decrease turnover, improve retention, and elevate NH residents' quality of care. The study design identified and explored optimal collaboration as it is possible in the current NH setting. It does not represent all LN/CNA caregiving pairs.

### Session 2430 (Symposium)

### MENTAL HEALTH AMONG INFORMAL CAREGIVERS OF OLDER ADULTS IN ASIA

Chair: Xiang Gao Co-Chair: Kaipeng Wang
Discussant: Fei Sun

The purpose of this symposium is to highlight the mental health needs and factors associated with mental health among informal caregivers of older adults in Asia. The symposium consists of five papers. The first paper explores the perceived role, needs, and rewards of informal caregiving among caregivers of residents in independent long-term care facilities in South India. The second paper presents a systematic review and meta-analysis on the association between long-term care service use and informal caregiver burden, depression, and health status. The third paper examines the association between caregivers' characteristics and quality of life among informal caregivers of older adults with cognitive impairment in China. The fourth paper examines the association between coping strategies and caregiver burden and depression among Chinese caregivers of older adults with cognitive impairment. The last paper examines the association between cohort, meaning making, and depression among adult caregivers during the COVID-19 pandemic in Hong Kong. Taken together, these five papers underscore of the mental health needs and protective and risk factors of mental well-being among caregivers in Asia. Findings of those papers inform the development and adaptation of culturally sensitive interventions to improve mental health outcomes among informal caregivers in Asia. The disccuant will comment on the strengths and limitations of these papers in terms of their contributions to the theory, research, and practice on mental health among informal caregivers in Asia.

### INFORMAL CAREGIVING BETWEEN RESIDENTS IN LTC FACILITIES IN INDIA: DEMANDING OR REWARDING ROLE

Shantha Balaswamy, *The Ohio State University, Columbus, Ohio, United States*

Research on caring for older adult with health problems by Informal caregivers (IC) in the community in developing countries like India is increasing. However, IC in institutions is largely unacknowledged. This exploratory study examines the perceived role, demands, and rewards of informal caring for residents in independent LTC facilities in South India. A total of 187 residents were interviewed in Tamil and Kannada using structured and open-ended questions on demographics, health, mental health, residents' interactions, tasks performed and personal experiences. About 50% reported assisting other residents with ADLs, 30% helped with IADLs, and 75% provided emotional support. The caregivers' appraisals as residents and their relationship with care-recipient was both positive and negative. In addition to socialization, personal accomplishment, caregivers reported emotional exhaustion, stress, and burnout. Implications related to paid and unpaid labor policy in LTC and reducing IC stress are discussed.

### LONG-TERM CARE SERVICE USE AND CAREGIVER BURDEN, DEPRESSION, AND HEALTH: A SYSTEMATIC REVIEW AND META-ANALYSIS

Cindy Ng,[1] Ringo Ho,[2] and Wayne Chong,[1] *1. GeroPsych Consultants Pte Ltd, Singapore, Not Applicable, Singapore, 2. Nanyang Technological University, Singapore, Singapore, Singapore*

This study examined whether long-term care service use (LTCSU) is associated with informal caregivers' burden, depression, and health status. Eligible articles collected data directly from caregivers, written in English, and allowed for extraction or computation of effect sizes. MEDLINE, PsycINFO and ProQuest Dissertations & Theses Global databases were searched between September 2017 and January 2018. The risk of bias of individual studies was assessed regarding confounding, study power, and other biases. This unfunded study was registered with PROSPERO: CRD42018108827. Of the 419, 209 and 346 articles identified, 24, 14 and 15 articles that involved 12,530, 6,687 and 7,331 informal caregivers respectively, were eligible for analyses regarding the above associations. With unadjusted effect sizes, omnibus tests found statistically non-significant overall effect estimates in the association of LTCSU with caregiver burden, depression, and health status. Subgroup analyses, however, revealed that the above associations differed by service type, caregiver sex, and country, respectively.

### ASSOCIATES OF PERCEIVED QUALITY OF LIFE IN CHINESE OLDER ADULTS LIVING WITH COGNITIVE IMPAIRMENT

Xiang Gao, *Huazhong University of Science and Technology, Wuhan, Hubei, China (People's Republic)*

This study examined perceived quality of life in Chinese older adults living with cognitive impairment in a group of urban Chinese older adults and explore its associations with caregivers' characteristics. Questionnaires were administered in person to 300 caregiver-care recipient dyads from three urban communities in mainland China in 2019. The 40-item Alzheimer's Disease-related Quality of Life tool asked caregiver respondents to indicate care recipients' life conditions. Higher levels of caregiving burden ($\beta$ = -0.19, $p < 0.01$) and more depressive symptoms ($\beta$ = -0.19, $p < 0.01$) amongst caregivers were significantly associated with lower quality of life of care recipients. The results suggested that reducing caregivers' burden and depressive symptoms are essential to promote quality of life of care recipients. Formal support from health professionals, service organizations, and communities are urgently called for to promote the wellbeing of Chinese families affected by cognitive impairment.

### COPING AND CAREGIVER BURDEN AND DEPRESSION AMONG CHINESE CAREGIVERS OF OLDER ADULTS WITH COGNITIVE IMPAIRMENT

Xiang Gao,[1] and Kaipeng Wang,[2] *1. Huazhong University of Science and Technology, Wuhan, Hubei, China (People's Republic), 2. University of Denver, Denver, Colorado, United States*

Coping strategies are important factors that influence caregivers' mental health outcomes. The purpose of this study is to examine the association between coping strategies and caregiver burden and depression among Chinese caregivers of older adults with cognitive impairment. Data came from structured interviews with 300 primary family caregiver-care recipient dyads in Wuhan, China. We used OLS to examine the association between coping strategies and caregiver burden and depression. More positive reframing and acceptance were associated with lower caregiver burden, whereas more self-distraction was associated with higher caregiver burden. More positive reframing was associated with lower caregiver depression, whereas higher self-distraction and religion were associated with higher caregiver depression. Findings of this study suggest that a psychosocial intervention package that emphasizes on enhancing positive reframing skills and affirming acceptance may be effective in reducing caregiver burden and depression among Chinese caregivers of older adults with cognitive impairment.

### IMPACT OF COVID-19 ON MEANING MAKING OF DEMENTIA CAREGIVERS IN HONG KONG: FROM THE GENERATIONAL PERSPECTIVES

Reynold Leung,[1] and Vivian, W. Q. Lou,[2] *1. The University of Hong Kong, Pokfulam, Not Applicable, Hong Kong, 2. The University of Hong Kong, Hong Kong, Not Applicable, Hong Kong*

This study examined the impact of COVID-19 on meaning making among adult children dementia caregivers and the association with caregiver mental well-being. Adult caregivers (n=601) from two generations, 1946-1964 (Baby Boomers) and 1965-1980 (Generation X), were recruited in Hong Kong between October 2019 and June 2020. Participants were assessed on depressive symptoms (PHQ-9) and meaning making (Finding Meaning Through Caregiving Scale-FMTC). Generation X scored higher on sense of loss ($p = 0.04$) and lower on provisional meaning of FMTC ($p=0.017$). Moreover, an interaction effect ($p=0.003$) between generation and COVID-19 were found. During the pandemic, Generation X caregivers were more likely to suffer from higher losses, higher depressive symptoms (>23.2% moderate to severe) and lower provisional meaning ($p=0.03$) compared to their boomer counterparts. The level of meaning making is more important to Generation X caregivers, especially in COVID-19 situation. Government should consider generation-responsive services and education support in guiding service implementation.

### Session 2435 (Paper)

### NEIGHBORHOOD ENVIRONMENTS AND AGING

### NEIGHBORHOOD CHARACTERISTICS AND ACCELERATED AGING: EVIDENCE FROM THE HEALTH AND RETIREMENT STUDY

Haena Lee, Jennifer Ailshire, and Eileen Crimmins, *University of Southern California, Los Angeles, California, United States*

An individual's rate of aging directly impacts one's functioning, morbidity and mortality. Identifying factors related to accelerated or delayed aging may provide important information for potential areas of intervention. While race/ethnicity, socioeconomic status and behavior characteristics have been linked to biological aging, it is unclear whether neighborhood characteristics are associated with one's rate of aging. We use a novel aging measure, Expanded Biological Age, from the 2016 Health and Retirement Study Venous Blood Study (HRS-VBS) to investigate whether individuals living with unfavorable neighborhood conditions are experiencing accelerated aging compared to those living in more favorable conditions. We constructed a summary measure of expanded biological age using 22 novel biomarkers in the HRS-VBS; we then regressed the summary measure on age and used the residuals as indicators of accelerated or delayed aging. We measured neighborhood physical disorder, presence of green space, and perceived social cohesion using the 2016 HRS Interviewer Observation data and Self-Administered Questionnaire. We find that individuals living with higher levels of neighborhood physical disorder appeared 1.05 years older biologically than the average for those of the same chronological age. Individuals living near green space including parks were 1.5 years younger biologically than expected based on their chronological age though this association was marginally significant. We did not find an association between neighborhood social cohesion and accelerated aging. This implies that living with severe neighborhood disorder, characterized by presence of disrepair, trash/litter, and abandoned structures, and living near green space, play an important role in who lives longer.

### NEIGHBORHOOD COHESION AND THE MENTAL HEALTH OF MULTIMORBID OLDER ADULTS: CLSA PATH ANALYSIS THROUGH LONELINESS

Daniel R. Y. Gan, Andrew Wister, and John Best, *Simon Fraser University, Vancouver, British Columbia, Canada*

More older adults with multimorbidity are aging in place than ever before. Their mental health may be affected by housing and neighborhood factors. In this paper, we use structural equation modelling (SEM) to examine how the physical environment influences life satisfaction and depressive symptoms in two separate models. We included social environment (i.e., social support, social participation, walking) and loneliness as intermediate variables. Data were drawn from baseline and the first follow-up (after 3-4 years) of the Canadian Longitudinal Study on Aging (CLSA). Participants were N=14,301 adults aged □65 with □2 chronic illnesses. Good model fit were found after controlling for age, sex, education and baseline values (TFI=1.00; CFI=1.00; RMSEA<0.001; SRMR<0.001). The total effects of housing quality (Btotal=0.08,-0.07) and neighborhood cohesion (Btotal=0.03,-0.06) were weak but statistically significant in the expected direction. Together, the intermediate variables explained 21-31% of the total effects of housing quality and 67-100% of the total effects of neighborhood cohesion. Loneliness explains 27-29% of the total effects of physical environment on mental health, whereas walking explained a mere 0.4-0.9% of their total effects. Walking did not mediate between housing quality and mental health outcomes. Overall, the results support our path analysis framework: physical environment -> social environment -> loneliness -> mental health. Our model provided excellent explanations of the effects of neighborhood cohesion, especially on life satisfaction. If these associations reflect causal effects, community-based age-friendly interventions should focus on neighborhood cohesion and loneliness to promote the well-being of older adults who are aging in place with multimorbidity.

### NEIGHBORHOOD ENVIRONMENTS AND COGNITIVE DECLINE IN MIDDLE AND OLD AGE IN CHINA: GENDER AND AGE VARIATIONS

Ye Luo,[1] Xi Pan,[2] and Lingling Zhang,[3] *1. Clemson University, Central, South Carolina, United States, 2. Texas State University, San Marcos, Texas, United States, 3. University of Massachusetts Boston, Boston, Massachusetts, United States*

Older adults are more vulnerable to neighborhood physical and social conditions due to longer exposure, increased vulnerability, changing spatial use, and a greater reliance on access to community sources of integration. Previous research has demonstrated an association between neighborhood environments and cognitive function in older adults. However, most studies were cross-sectional, focused on western countries, and did not examine potential moderating factors. This study examined gender and age variations in the relationship between neighborhood environments and cognitive decline in middle and old age in a developing country that is experiencing rapid population aging and rising prevalence of Alzheimer's disease and related dementias. Using data from a nationally representative sample of adults aged 45 years and older from the three waves of China Health and Retirement Longitudinal Study (CHARLS 2011-2015), this study estimated multilevel growth curve models for the effects of neighborhood environments on cognitive decline separately for men and women and for those aged 45 to 64 and those aged 65 and above. It showed that the cross-sectional effect of outdoor facility and longitudinal effect of handicapped access were more significant for men, but the cross-sectional effect of community social participation and longitudinal effects of raining days, number of disasters, employment service, and community SES were more significant for women. The cross-sectional effect of infrastructure advantages and longitudinal effects of employment service and old age income support were more significant for adults aged 65 and over. These findings suggest that community-level interventions may be more beneficial for older women.

### SOCIAL ISOLATION AND SUICIDAL IDEATION OF OLDER PEOPLE: THE BUFFERING EFFECTS OF NEIGHBORHOOD SOCIAL COHESION

Choi Bomi,[1] Hey Jung Jun,[1] and Susanna Joo,[2] *1. Yonsei University, Seoul, Seoul-t'ukpyolsi, Republic of Korea, 2. Yonsei University, Seodaemun-gu, Seoul-t'ukpyolsi, Republic of Korea*

This study aimed to examine the buffering effect of neighborhood social cohesion on the association of social isolation and suicidal ideation among Korean older people. The sample was older adults who were 65 years old or older and participated in the Korea Health Survey 2017 collected by the Center for Disease and Prevention (N=67,835). Social isolation was measured with three indicators: living-alone, contact isolation (less than weekly contact with family, friends, or neighbors), and participation isolation (less than monthly social organization attendance). Neighborhood social cohesion was measured with two indicators: trust in neighbors and the welfare budget ratio to represent social capital and social inclusion capabilities, respectively. Multilevel logistic regression analyses were performed to estimate the dynamic relationships between social isolation, neighborhood social cohesion, and suicidal ideation. Results of the main effect indicated that social isolation is a significant risk factor for suicidal ideation and neighborhood social cohesion works as a protective factor against suicidal ideation. Results of cross-level interactions showed that the welfare budget ratio moderated the association between participation isolation and suicidal ideation (OR=0.960, p<.001). The negative effect of participation isolation was reduced as the welfare budget ratio of the neighborhood increased. Neighborhood social cohesion indicators did not moderate the association between the remaining types of social isolation and suicidal ideation. The evidence from this study highlights the importance of social welfare expenditures when building suicide prevention interventions and age-friendly communities.

### TWIN SIMILARITY FOR NEIGHBORHOOD, GEOGRAPHIC MOBILITY, AND HEALTH OUTCOMES IN LATE ADULTHOOD

Deborah Finkel,[1] Ida Karlsson,[2] Malin Ericsson,[2] Tom Russ,[3] Anna Dahl Aslan,[4] and Nancy L. Pedersen,[2] *1. Indiana University Southeast, New Albany, Indiana, United States, 2. Karolinska Institutet, Stockholm, Stockholms Lan, Sweden, 3. University of Edinburgh, Edinburgh, Scotland, United Kingdom, 4. Hälsohögskolan Skövde, Skövde, Vastra Gotaland, Sweden*

Socioeconomic status (SES) is one of the most robust predictors of health. The source of SES-health associations is heavily debated; one approach is investigating neighborhood-level environmental characteristics. Challenges include selection effects and the possibility of reverse causation: people choose their neighborhoods. Longitudinal twin research can overcome these issues by assessing location choice over time as well as twin similarity; however, few existing twin studies have incorporated neighborhood-level data, and none of those focus on aging. Using longitudinal data from the Swedish Adoption/Twin Study of Aging, the current study examined the impact of location at various points in life. Location at birth and in 1993 were available for 972 participants. Birth years ranged from 1926 to 1948; mean age in 1993 was 54.55 (range = 35-67). Thirty-nine percent of the sample had moved to a different county between birth and midlife: individuals who moved had significantly higher parental SES and had achieved significantly higher education. Moreover, identical twin concordance for geographic mobility (77%) was significantly higher than fraternal twin concordance (65%), indicating a modest but significant genetic contribution. Geographic mobility did not impact identical twin similarity on a functional aging factor (corrected for age and education), but fraternal twins concordant for mobility were more similar than discordant twins, suggesting genetic contributions to mobility may also impact health. Ongoing retrieval of location information for twins born 1900-1925 and geocoding of location information available at 9 waves of data collection will allow for expanded investigation of the SES-health relationship at the neighborhood level.

### Session 2440 (Symposium)

### NEW FRONTIERS ON CELLULAR SENESCENCE

Chair: Laura Niedernhofer

### IDENTIFYING SENESCENT CELLS THAT DRIVE AGING

Laura Niedernhofer, *University of Minnesota, Minneapolis, Minnesota, United States*

Cellular senescence is a potent tumor suppressor mechanism. However, the untoward effect is that the accumulation of senescent cells promotes loss of resilience, aging and age-related diseases. One approach to maintaining the benefits of senescence while preventing the negative consequences is senolytic therapies: drugs that do not prevent senescence, but selectively kill senescent cells. Since virtually any type of cell can become senescent, it is important to identify the lineages of senescent cells that are most potent at driving loss of tissue homeostasis and aging. This will enable honing development of senolytics. We used a genetic approach to drive increased genotoxic stress, a potent inducer of senescence, in a tissue specific manner. The impact of this targeted senescence on different organs and cell types will be discussed, identifying a lead target for senolytics.

### SENOLYTICS REDUCE CORONAVIRUS-RELATED MORTALITY IN OLD MICE

Christina Camell, *Institute on the Biology of Aging and Metabolism, University of Minnesota, Province, Minnesota, United States*

The elderly and chronically ill are among groups at the highest risk for morbidity and mortality to several infections, including SARs-CoV-2. Cellular senescence contributes to inflammation, multiple chronic diseases, and age-related dysfunction, but effects on responses to viral infection are unclear. Old mice acutely infected with pathogens that included a SARS-CoV-2-related mouse β-coronavirus experienced increased senescence and inflammation with nearly 100% mortality. Targeting SCs using senolytic drugs before or after pathogen exposure significantly reduced mortality, cellular senescence, and inflammatory markers and increased antiviral antibodies. Thus, reducing the SC burden in diseased or aged individuals should enhance resilience and reduce mortality following viral infection, including SARS-CoV-2.

### IMMUNE SURVEILLANCE OF SENESCENT CELLS

Scott Lowe, *Memorial Sloan Kettering Cancer Center & Howard Hughes Medical Institute, New York, New York, United States*

Cellular senescence involves a stable cell cycle arrest and a secretory program that modulates the tissue environment. In cancer, senescence acts as a potent barrier to tumorigenesis and, though many cancers evade senescence during the course of tumor evolution, ionizing radiation and conventional chemotherapy can, to varying degrees, induce senescence in tumor cells leading to potent anticancer effects. Conversely, the aberrant accumulation of senescent cells can reduce regenerative capacity and lead to tissue decline, contributing to tissue pathologies associated with age or the debilitating side-effects of cancer therapy. Our laboratory studies mechanisms of cellular senescence with the ultimate goal of developing strategies to modulate senescence for therapeutic benefit. We have focused on how senescent cells trigger immune surveillance to facilitate their own elimination or, when that fails, how synthetic immune cells (i.e. CAR T cells) can be directed to eliminate senescent cells. Recent advances in understanding senescent cell surveillance by the immune system will be discussed.

### CELL SENESCENCE AS A MEDIATOR OF AGE-DEPENDENT BRAIN INFLAMMATION

Xu Zhang,[1] Vesselina Pearsall,[1] Nathan LeBrasseur,[1] and Marissa Schafer,[2] *1. Mayo Clinic, Rochester, Minnesota, United States, 2. Physiology and Biomedical Engineering, Rochester, Minnesota, United States*

Cellular senescence and inflammation are interconnected causes and consequences of tissue aging. Here, we implemented orthogonal approaches to study their interaction in steady-state mature and aged mouse brain. Using single cell sequencing, we identified a putative senescent microglial population, which increased in abundance with age and was characterized by increased expression of p16 and chemotactic senescence associated secretory phenotype (SASP) factors. Using p16-INK-ATTAC transgenic mice to eliminate p16ink4a-positive senescent cells and mass cytometry, we show that p16ink4a-positive cell targeting reduced the abundance of activated inflammatory cells in the aged female brain. Age-dependent declines in executive cognitive function were improved following transgenic p16ink4a-positive cell targeting, and executive function robustly correlated with inflammatory brain cell composition in females. Collectively, our findings demonstrate fundamental differences in the age- and sex-dependent brain inflammatory landscape and implicate p16ink4a-positive senescent cell targeting as a therapeutic strategy to attenuate age-related inflammation and cognitive decline.

### Session 2445 (Symposium)

### NURSING HOMES AND COVID-19: STAFF EXPERIENCES

Chair: Verena Cimarolli Co-Chair: Joann Reinhardt
Discussant: Sheryl Zimmerman

Nursing homes (NHs) faced an unprecedented crisis during the rapid spread of COVID-19. This pandemic has had a devastating impact on both NH residents and workers who are often on the frontlines providing hands-on care. These workers are vulnerable to the health risks of COVID-19 due to daily exposure to residents with COVID-19, residence in areas with high infection rates, and challenges specific to low-income workers (e.g. reliance on mass transportation). Research has highlighted the experiences of NH workers during the pandemic to learn how to better support them now and during future pandemics. This symposium will add to this research and present new findings from studies conducted in the United States to capture the unique experiences of NH employees. First, Bryant illustrates specific COVID-19-related challenges that NH frontline workers faced and how these workers' experiences compare to workers in other long-term services and support settings. Reinhardt reports findings from a qualitative study examining the multi-level challenges experienced by nursing assistants during the pandemic. Cimarolli examines if quality of employer communication and workers' perceived COVID-19-related preparedness mitigate the impact of work-related stress on NH workers' decision to resign. Franzosa shares recommendations based on priorities identified by nursing assistants and administrators to build future resilience based on lessons learned. Finally, Simpson identifies factors associated with states' decisions to adopt COVID-19 testing mandates for workers in NHs. Dr. Zimmerman discusses study findings and their contributions for creating supportive NH work environments to ensure most optimal NH worker and resident quality of life.

### CHALLENGES FACED BY NURSING HOME DIRECT CARE WORKERS DURING THE COVID-19 PANDEMIC: A COMPARISON ACROSS CARE SETTINGS

Verena Cimarolli,[1] Robyn Stone,[1] and Natasha Bryant,[2] *1. LeadingAge, Washington, District of Columbia, United States, 2. LeadingAge LTSS Center @UMass Boston, Washington, District of Columbia, United States*

The COVID-19 pandemic has generated awareness of the value of the direct care workforce to provide care in settings serving those most at risk from the disease. However, few studies have gauged the impact of COVID-19 on this workforce and their pandemic-related challenges. The purpose of this study was to examine the challenges and stress experienced by direct care workers (N=1,414) and their perceptions of preparation and quality of employer communication during this health crisis. Nursing home (NH) workers reported separation from family members and understaffing as the top external and work-related challenges. They felt adequately prepared and gave their employers high marks for communicating with them during the pandemic. NH direct care workers were more likely to report increased workload and understaffing as a challenge compared to workers in home and community-based settings. They also experienced a significantly higher number of work-related challenges compared to workers in assisted living.

### JOB RESIGNATION IN NURSING HOMES DURING COVID-19: THE ROLE OF EMPLOYER COMMUNICATION AND WORKER PREPAREDNESS

Natasha Bryant,[1] Robyn Stone,[2] Francesca Falzarano,[3] and Verena Cimarolli,[2] *1. LeadingAge LTSS Center @UMass Boston, Washington, District of Columbia, United States,*

*2. LeadingAge, Washington, District of Columbia, United States, 3. Weill Cornell Medicine, Douglaston, New York, United States*

Although research on factors mitigating the negative impact of strain/stress experienced by nursing home (NH) workers during the pandemic is emerging, there is no research on how COVID-19-related work stress and employer supports influence NH workers decision to resign. The purpose of this study was to investigate if high quality communication related to COVID-19 by the employer – a form of job support - can mitigate the impact of work stress on NH employees (N=1,730) decision to resign by optimizing employees' preparedness to care for residents with COVID-19. Guided by the Job-Demands-Control-Support Model and employing path analyses, results indicate that higher stress was associated with greater likelihood of resigning, which operated through the paths of communication quality and preparedness. While higher stress was associated with less optimal quality of communication, good quality of communication was associated with more optimal preparedness which was associated with reduced likelihood of leaving one's job.

### CHALLENGES REPORTED BY CERTIFIED NURSING ASSISTANTS DURING COVID-19: A QUALITATIVE STUDY

Emily Franzosa,[1] Wingyun Mak,[2] Orah Burack,[2] and Joann Reinhardt,[2] *1. Icahn School of Medicine at Mount Sinai, Icahn School of Medicine at Mount Sinai, New York, United States, 2. The New Jewish Home, New York, New York, United States*

Certified nursing assistants (CNAs) who care for vulnerable nursing home residents faced unprecedented circumstances due to the COVID-19 pandemic. While staff and PPE shortages were ubiquitous and widely known, the focus of this qualitative work was to gain a broader understanding of the numerous challenges they faced. We conducted 10 remote focus groups with CNAs at 5 nursing homes (N=56) in downstate New York. Content analysis was conducted, and emerging themes identified. Results showed a myriad of emotional challenges including helplessness, fear and anxiety. Operational challenges focused on lack of COVID testing capacity, information, and consistent guidance and support, in addition to staff and equipment. Individual challenges included personal experience of COVID illness and that of colleagues, and balancing high concurrent demands of work and family. These results are discussed in the context of developing recommendations to promote future safety, skill refinement and enhanced resilience in the workforce moving forward.

### IT WAS TRIAL BY FIRE: RECOMMENDATIONS FOR BUILDING A STRONGER NURSING HOME WORKFORCE AFTER COVID-19

Wingyun Mak,[1] Orah Burack,[1] Kenneth Boockvar,[1] Joann Reinhardt,[1] and Emily Franzosa,[2] *1. The New Jewish Home, New York, New York, United States, 2. Icahn School of Medicine at Mount Sinai, Icahn School of Medicine at Mount Sinai, New York, United States*

The COVID-19 crisis showed the urgent need for a unified, well-supported nursing home workforce. The objective of this qualitative study was to examine the lived experience of certified nursing assistants (CNAs) and administrators during COVID-19 to identify best practices moving forward. Six administrator interviews and 10 remote focus groups with CNAs at 5 nursing homes (N=56) were examined through directed content analysis. Based on priorities identified by CNAs and administrators, the following practices may be most impactful: 1) ongoing and responsive staff training; 2) transparent, direct, and two-way communication channels; 3) prioritizing hiring permanent staff to avoid shortages and reliance on agency staff; 4) building collaborative staff-management relationships; 5) providing flexible job benefits; 6) providing staff-centered emotional support resources; and 7) appraising COVID-19 innovations. Our results suggest that rather than returning to "business as usual," nursing homes can draw on these lessons to build a more sustainable workforce and industry.

### FACTORS ASSOCIATED WITH STATE MANDATE OF NURSING HOME STAFF TESTING DURING COVID-19

Edward Miller,[1] Molly Wylie,[2] Lisa Beauregard,[3] Pamela Nadash,[4] Michael Gusmano,[5] and Elizabeth Simpson,[4] *1. University of Massachusetts Boston, Boston, Massachusetts, United States, 2. University of Massachusetts Boston, University of Massachusetts boston, Massachusetts, United States, 3. Executive Offce of Elder affairs, Executive Office of Elder Affairs, Massachusetts, United States, 4. University of Massachusetts Boston, University of Massachusetts Boston, Massachusetts, United States, 5. Rutgers University School of Public Health, Rutgers University School of Public Health, New Jersey, United States*

COVID-19 has presented challenges for nursing homes (NHs) and other congregate living settings which serve older adults at high risk for morbidity and death from the virus. This study identified factors associated with states' adopting a mandate for regular staff testing for COVID-19 in NHs. Potential correlates included state government ideology and capacity, NH supply and demand, prevailing economic conditions, existing state policies, and NH characteristics. Findings indicate that percent for profit NHs is most strongly associated with adoption of a state staff testing mandate. Governing capacity (average legislative salary), percent population at risk for COVID-19, and existing public policy (percent Medicaid spending devoted to long-term services and supports (LTSS) were also associated with the probability of adoption. Based on these results, states with more proprietary facilities and greater capacities for government action, investment in Medicaid LTSS, and at-risk populations were more likely to mandate regular staff testing in NHs.

### Session 2450 (Symposium)

### OBSERVING PERSONALITY IN EVERYDAY LIFE: HOW PERSONALITY INFLUENCES DAILY ACTIVITIES AND MOOD

Chair: Shiyang Zhang

Personality reflects the influence of older adults' past experiences on their tendencies to engage in certain behaviors and generate emotions. An increasing number of studies have focused on the expression of personality in older adults' everyday life. Specifically, personality features have been associated with daily activities, affect reactivity, and mood. This symposium draws on Ecological Momentary Assessments and longitudinal analysis to understand how personality is manifested in daily life, and how daily emotional experiences accumulate over time to influence physical health. Lee and colleagues examine how personality is associated with daily activities and find that extraversion is positively associated with activity diversity in two adult samples. Pasquini and colleagues consider the within-person fluctuations in personality traits and demonstrate the possibility of using daily behaviors and affect as markers of extraversion and neuroticism. Zhang and Fingerman assess how positive and negative moods concurrently change in reaction to daily social contacts and confirm that narcissism moderates such associations. Finally, this symposium also focuses on the cumulative effect of daily emotions on physical health. Leger and colleagues address the long-term association between personality (e.g., neuroticism, conscientiousness) and physical health and identify the mediating role of negative reactivity to daily stressors. Collectively, the presentations provide an in-depth analysis of personality's impact on concrete daily behaviors and emotions, as well as their profound long-term effect on physical health. Our discussion outlines future research directions and highlights how inter-individual differences, simultaneous life events, and social interactions intertwine to influence individuals' behaviors that occur in natural settings.

### SOCIAL ENCOUNTERS AND MOOD: THE MODERATING ROLE OF NARCISSISM

Karen Fingerman, and Shiyang Zhang, *The University of Texas at Austin, Austin, Texas, United States*

Social contacts may lead to more positive and less negative emotions in late life, yet we know little about how narcissism influences such associations, and whether contacts with close and not-close social partners impact mood differently. This study examined associations between social contacts, narcissism, and mood on the within- and between- person level. Older adults aged 65 + (N = 303) completed ecological momentary assessments in which they reported social contacts and mood every 3 hours for 5 to 6 days. Older adults had higher positive mood after contacting either close or not-close social partners, but only not-close social partners reduced negative mood. Multilevel models found positive associations between average social contacts number and positive mood among people scored lower on narcissism, and positive associations between social contacts and negative mood for those who scored higher on narcissism. Findings suggest the necessity of considering interpersonal differences in interventions targeting well-being.

### WHO HAS ACTIVE LIFESTYLES? PERSONALITY AND ACTIVITY DIVERSITY IN ADULTHOOD

Soomi Lee,[1] Susan Charles,[2] Karen Fingerman,[3] and David Almeida,[4], *1. University of South Florida, Tampa, Florida, United States, 2. University of California, Irvine, Irvine, California, United States, 3. The University of Texas at Austin, Austin, Texas, United States, 4. Pennsylvania State University, University Park, Pennsylvania, United States*

Broad and even participation across daily activities ("activity diversity") has been found to be associated with better health. Less is known about who has greater activity diversity. We examined whether personality traits are associated with activity diversity in two independent samples of adults. Data came from the Midlife in the United States Study II and Refresher (n=2623, Mage=54yrs) and Daily Experiences and Well-being in Late Life Study (n=308, Mage=74yrs) who responded to daily activity questions. We constructed activity diversity scores in each sample using Shannon's entropy. We focused on three personality traits — conscientiousness, extraversion, neuroticism — often associated with health. Higher extraversion was associated with greater activity diversity, replicated across the two samples. The associations were independent of conscientiousness and neuroticism (both were not significant), total activity time/frequency, age, gender, race, education, and self-rated health. Results suggest that future activity interventions may need to target those with lower extraversion.

### CAN ECOLOGICAL MOMENTARY ASSESSMENTS BE USED AS DAILY MARKERS OF PERSONALITY TRAITS?

Andreas Neubauer,[1] Nicholas Eaton,[2] Sean Clouston,[3] Eileen Graham,[4] Daniel Mroczek,[4] Stacey Scott,[2] and Giancarlo Pasquini,[2], *1. Leibniz Institute for Research and Information in Education, Frankfurt, Mecklenburg-Vorpommern, Germany, 2. Stony Brook University, Stony Brook, New York, United States, 3. Renaissance School of Medicine, Stony Brook University, Renaissance School of Medicine, Stony Brook University, New York, United States, 4. Northwestern University, Chicago, Illinois, United States*

This study hypothesized that select ecological momentary assessment (EMA) survey items are sensitive to day-to-day fluctuations in personality traits Extraversion (E) and Neuroticism (N). As part of the Einstein Aging Study, 312 older adults (Mage=76.96 years, SD=4.85 years, range=70-90 years) completed up to 5 EMA surveys per day for 16 days and a Big Five trait personality measure. Parallel two-factor multilevel confirmatory factor analyses were conducted for E (Daily-E; Trait-E) and N (Daily-N; Trait-N). The E model showed good fit (CFI=.95; TLI=.94; RMSEA=.02) and a significant correlation of .20 between Daily-E and Trait-E factors. The N model showed poor fit (CFI=.68; TLI=.61; RMSEA=.06). Results suggest EMA items can be used as daily markers of Extraversion, yet results are unclear for Neuroticism due to poor model fit. Daily markers of Extraversion can be used to detect fluctuations in personality traits across days that may predict long-term personality change.

### AFFECT REACTIVITY TO DAILY STRESSORS MEDIATES THE RELATIONSHIP BETWEEN PERSONALITY TRAITS AND PHYSICAL HEALTH

Nicholas Turiano,[1] William Bowling,[2] Jessica Burris,[2] David Almeida,[3] and Kate Leger,[2], *1. West Virginia University, Morgantown, West Virginia, United States, 2. University of Kentucky, Lexington, Kentucky, United States, 3. Pennsylvania State University, University Park, Pennsylvania, United States*

Researchers hypothesize that how people react to daily stressful events partly explains the personality-health relationship, yet no study has examined longitudinal associations between these factors. The current study examined the role of negative affect reactivity to daily stressors as a mediating pathway between personality and physical health outcomes using three waves of data spanning 20-years from a nationwide probability sample of 1,176 adults. Results indicate that Wave 1 neuroticism was associated with greater negative affect reactivity at Wave 2, which then predicted the development of chronic conditions and functional limitations at Wave 3. Higher conscientiousness was associated with less negative affect reactivity, which in turn predicted better physical health at Wave 3. Negative affect reactivity partially mediated both personality traits and physical. These findings highlight the usefulness of using a daily stress framework for understanding how personality impacts health over time, which has important implications for disease prevention.

### Session 2455 (Symposium)

### ORAL HEALTH'S TRANSFORMATIVE IMPACT ON DIET AND SYSTEMIC HEALTH OUTCOMES

Chair: Michèle Saunders

The 2020-25 Dietary Guidelines for Americans identified dental caries as a major diet-related chronic disease of public health concern and suggested in the section for adults over 60, "Good dental health is critical to overall health, as well as the ability to chew foods properly." Poor oral health can lead to chronic diseases and impede one's ability to chew fruits, vegetables, whole grains, and other nutrient-rich foods across the life span. Almost 90 percent of adults ages 20 to 64 years and 96 percent of those over 65 years of age have dental caries. The overall prevalence of complete tooth loss is 2.2 percent among adults ages 20 to 64 years and jumps to 17.3 percent for those over age 65. As a result of COVID-19, some seniors are not seeking regular oral health services, which increases the need for preventive oral health practices and consuming a healthy dietary pattern recommended in the new Dietary Guidelines. Recent research will underscore the importance of saliva and oral health in cancer patients on radiation and in other chronic diseases. Saliva has also been shown to reduce specific infections that are related to influenza and HIV. Participants in this session will gain understanding of factors linking poor oral health and nutrition practices to chronic diseases and guidance on critical preventive oral health practices to increase saliva flow and decrease dental caries through all stages of the life cycle. Promoting oral health is the responsibility of the interdisciplinary team overseeing older adults.

### MINIMIZING COMPLICATIONS OF AGING THAT LEAD TO DRY MOUTH AND POOR ORAL HEALTH

Athena Papas, *Tufts University School of Dental Medicine, Boston,, Massachusetts, United States*

Poor oral health causes severe pain and untreated infections to spread throughout the body. For older adults, the prevalence of root decay exceeds that of any other medical condition. Our research shows tooth loss and edentulousness were associated with increased mortality and inversely associated with BMI, waist circumference, blood pressure, and fasting blood glucose. Our Stop-it study found people who lost bone density had fewer teeth, problems chewing, and involuntary weight loss and frailty. 88% of the elderly take medications that cause loss of saliva. Sjögren's and radiation therapy for head and neck cancer patients heighten risk. Without saliva, patients have increased tooth decay, periodontal disease and fungal infections, salivary gland blockage, and problems swallowing and speaking. Dry mouth leads people to suck on candy that further increase caries. Substituting sugarfree gum for candy increases salivary flow and reduces dental caries. Brushing, flossing, and limiting sugar also lessen tooth decay.

### NUTRITION, ORAL HEALTH, AND CHRONIC DISEASES INEXTRICABLY LINKED

Teresa Marshall, *University of Iowa, Iowa City, Iowa, United States*

The 2020-25 Dietary Guidelines identified dental caries as a diet-related chronic disease of major importance. Preventing dental caries and other oral infectious diseases is critical to maintaining an individual's capacity to chew food, consume nutrient-rich diets, and sustain optimal nutrition status. Pain and infection from dental caries complicates consumption of adequate amounts of fruits, vegetables, dairy, and lean protein recommended in the Dietary Guidelines. Nutrition and dietary intake can affect the development and integrity of oral cavity and progression of oral diseases. Increased snacking throughout the day in place of three-meals daily raises the risk of obesity and dental caries throughout the life cycle. Older adults who make routine oral health preventive practices, such as brushing, cleaning between teeth, drinking fluoridated water, and chewing sugarfree gum to increase saliva flow can reduce dental caries and oral infectious diseases. Professionals must also consider the impact of sugar-sweetened beverages and sugar intake.

### RESEARCH CONFIRMS EFFECTIVENESS OF ORAL HEALTH PREVENTIVE PRACTICES

Michael Dodds, *Mars Wrigley, Chicago, Illinois, United States*

Lack of insurance or funds for dental services, lack of access to dental offices, fear of dentists, and avoidance of dental offices during COVID can lead to oral health problems in older adults. Brushing, flossing, and drinking fluoridated water can protect teeth when dentists are unavailable. Limiting intake frequency of carbohydrates and chewing sugarfree gum after eating add protection. A recent systematic review and meta-analysis confirmed the effectiveness of sugarfree gum in reducing caries, in children and adults who chewed sugarfree gum compared with those who did not chew. Chewing sugarfree gum significantly reduced caries increment, with a prevented fraction of 28 percent, roughly equivalent to the prevented fractions for fluoride toothpastes and supplements. A follow-up systematic review provides further evidence that chewing sugarfree gum reduces the numbers of Streptococcus mutans in the oral cavity. Finally, chewing sugarfree gum could alleviate symptoms of xerostomia and may reduce caries.

### Session 2460 (Symposium)

### PRESIDENTIAL SYMPOSIUM: FROM DISRUPTION TO TRANSFORMATION: CHALLENGING AND CHANGING THE NEW NORMAL

Chair: Deborah Waldrop Co-Chair: Philip Rozario
Discussant: Emily Greenfield

While the refrain “We’re all in this together” is meant to describe a sense of universality of our exposure and adaption to the Covid-19 pandemic life, the deeply rooted racial and economic injustices and ongoing health crises continue to expose the inequities experienced by many older adults. In this symposium, we focus on existing disparities and possibilities for transformation. The first paper discusses systemic racism as a structural driver of practices and policies that influence poverty, poor housing and neighborhood conditions, worse health profiles, relationship loss and social isolation among older Black adults. The second paper illuminates the importance of health equity and collaboration between aging and healthcare systems to improve the well-being outcomes and address disparities of older adults from racial-ly/ethnically diverse backgrounds. The third paper illustrates how the privatization of Medicare has created bureaucratic complexities that increase cost and burdens for beneficiaries. The fourth paper presents the ways that the pandemic has exposed the challenges of a nonexistent Long Term Services and Support system; specifically, in refocusing our attention on the working conditions of in-home and residential workers, such as poor compensation, and high turnover and mounting demands on families. The fifth paper addresses the importance of collaboration between nursing homes and assisted living communities with governmental emergency operations in times of disasters and public health crises. Each paper addresses pressing issues that have created the “new normal” for older adults; together the presenters explore the disruptions and offer solutions for renewed transformation.

### RACISM AND THE LIFE COURSE: SOCIAL AND HEALTH EQUITY FOR OLDER BLACK AMERICANS

Harry Taylor,[1] Robert Taylor,[2] and Linda Chatters,[3]
*1. Center for the study of Aging and Human Development, Durham, North Carolina, United States, 2. University of Michigan, Ann Arbor, Michigan, United States, 3. University of Michigan, ANN ARBOR, Michigan, United States*

Racism and the Life Course: Social and Health Equity for Older Black Americans examines the impacts of systemic racism on adult development and the aging trajectories of Black Americans. Using the life course perspective (e.g., socio-historical events, linked lives), we discuss systemic racism as a structural driver of practices and policies (e.g., racial residential segregation) that have shaped the social and health circumstances of older Black Americans. These life circumstances include high rates of poverty, poor housing and neighborhood conditions, worse health profiles, and relationship loss and social isolation—conditions that, for too many older Black adults, represent the ‘normal’ state of affairs. Creating a ‘new normal’ of social and health equity for older Black Americans requires recognizing and disrupting the operation of systemic racism in our policies and practices. Selected recommendations and actions for achieving health and social equity for older Black Americans are discussed.

### MAKING MEDICARE COMPLICATED: THE CONSEQUENCES OF PRIVATIZATION

Pamela Herd, *Georgetown University, Georgetown University, District of Columbia, United States*

Starting with policy changes in the 1980s, Medicare has largely become privatized, with nearly 40 percent of beneficiaries enrolled in private Medicare Advantage plans and another 30 percent with private supplemental coverage, including for prescription drug coverage. As a result, Medicare has become laden with administrative burdens and barriers. Beneficiaries are faced with a confusing array of plans and coverage options when they enroll, and are expected to choose a new plan every year. The choice they make has large implications for their health care costs, as well as their actual access to health care. While we typically think that targeted policies are burdensome and social insurance programs are accessible, Medicare contradicts this easy categorization. Instead, it demonstrates how private sector involvement in public programs can increase complexity and increase burdens for beneficiaries.

### PROMOTING HEALTH EQUITY THROUGH PARTNERSHIPS

Karon Phillips, *Trust for America's Health, Trust for America's Health, District of Columbia, United States*

Funded by The John A. Hartford Foundation, Trust for America’s Health’s (TFAH) Healthy Aging initiative has supported states as they develop Age-Friendly Public Health Systems (AFPHS). The goal of this national initiative is to make healthy aging a core function of state and local public health departments. Through this initiative, TFAH is working directly with states as they work to improve the health of older adults, with a particular focus on health equity. Given the increased prevalence of health disparities, prioritizing health equity has become important for many organizations. Through new partnerships and collaboration with aging services providers and health care systems, public health departments have developed innovative ways to improve the health and well-being of older adults from racial/ethnically diverse backgrounds. Areas of collaboration between the public health and aging sectors include sharing data on older adult health and working together to address social isolation.

### REIMAGINING LONG-TERM SERVICES AND SUPPORTS IN A POST-PANDEMIC WORLD

Katherine Abbott,[1] Gretchen Alkema,[2] and Robert Applebaum,[1], *1. Miami University, Oxford, Ohio, United States, 2. The SCAN Foundation, Long Beach, California, United States*

Prior to the global pandemic, the United States struggled to coordinate, deliver, and finance quality, person-centered long-term services and supports (LTSS) through the default primary payer, Medicaid. The pandemic highlights the challenges of not having a LTSS system. LTSS workers are underpaid, overworked, and turning over at alarming rates. Families face mounting pressures of caring for a growing number of loved ones, some with very complex care. Costs continue to climb, and quality indicators are not improving. While our approach to LTSS has improved, costs and quality challenges still dominate the landscape. We are at juncture when we need to reimagine the LTSS system, one that genuinely puts the care recipients and their caregivers at the heart of the system. The pandemic has provided some lessons about how to think differently about what long-term services can look like. Now is the time to embrace innovative opportunities building on this adversity.

### THE DIFFERENCE LTC CORPORATE OWNERSHIP MAKES IN COLLABORATIONS WITH EMERGENCY OPERATION CENTERS DURING DISASTERS

Joseph June,[1] David Dosa,[2] Lindsay Peterson,[3] Kathryn Hyer,[3] and Debra Dobbs,[4], *1. University of South Florida, Tampa, Florida, United States, 2. Brown University, Providence, Rhode Island, United States, 3. University of South Florida, University of South Florida, Florida, United States, 4. University of South Florida, School of Aging Studies, University of South Florida, Florida, United States*

Collaboration between nursing homes (NHs) and assisted living communities (ALCs) with state and local entities (e.g., emergency operation centers (EOCs)) is critical during a disaster. The corporate structure of NHs and ALCs can make a difference in their ability to collaborate with these entities during a disaster. This mixed-method study examines differences in satisfaction with collaboration with state and local entities during Hurricane Irma in Florida in 2017 between corporate-owned NHs (N=24), larger (25+ beds) ALCs (N=38) and smaller ALCs (N=30). We also explore collaboration in Florida NHs (N=35) and ALCs (N=123) specific to COVID19. Scaled 1-5 survey data results indicate that small ALCs are the least satisfied (M=2.90) with EOC collaboration, compared to NHs (M=3.04) and larger ALCs (M=3.33) during Irma. Smaller ALCs were more dissatisfied with COVID19 mandates compared to larger ALCs and NHs. Ways to improve collaboration during a disaster, especially for smaller ALCs, will be discussed.

### Session 2465 (Paper)

### PSYCHOSOCIAL WELL-BEING

### ARE ALL DOMAINS OF LIFE SATISFACTION EQUAL? DIFFERENTIAL ASSOCIATIONS WITH HEALTH AND WELL-BEING IN OLDER ADULTS

Julia Nakamura,[1] Scott Delaney,[2] Ed Diener,[3] Tyler VanderWeele,[4] and Eric Kim,[1], *1. University of British Columbia, Vancouver, British Columbia, Canada, 2. Harvard T. H. Chan School of Public Health, Boston, Massachusetts, United States, 3. University of Virginia, Charlottesville, Virginia, United States, 4. Harvard T.H. Chan School of Public Health, Boston, Massachusetts, United States*

Growing evidence documents strong associations between overall life satisfaction and favorable health and well-being outcomes. However, because most previous studies have assessed satisfaction with one's life as a whole, we know little about whether specific domains of life satisfaction (e.g., satisfaction with income) might be driving better health and well-being outcomes. Data were from 13,752 participants in the Health and Retirement Study—a nationally representative cohort of US adults aged >or=50. We evaluated if positive changes in seven domains of life satisfaction (between t0;2008/2010 and t1;2012/2014) were associated with 35 indicators of physical, behavioral, and psychosocial health and well-being (at t2;2016/2018). Satisfaction with family life and non-work activities showed the largest associations with subsequent psychological factors, followed by satisfaction with financial situation and income. Effect estimates were double in magnitude for certain domains of life satisfaction (e.g., the association between satisfaction with family life and purpose in life ($\beta$=0.22, 95% CI:0.16,0.27) was more than twice as large as the association between satisfaction with housing and purpose in life ($\beta$=0.09, 95% CI:0.02,0.16). Further, some domains showed associations with physical health outcomes (e.g., participants with the highest satisfaction with health had a 21% decreased mortality risk (95% CI: 0.66,0.95)), health behaviors (e.g., higher satisfaction with income decreased risk of sleep problems by 11% (95% CI:0.80,0.99)), and social factors (e.g., loneliness ($\beta$: -0.16 to -0.42)). Individual domains of life satisfaction might be novel targets for interventions and policies seeking to enhance specific facets of health and well-being in our rapidly aging population.

### ATTACHMENT, EMOTION, AND PHYSIOLOGICAL COREGULATION AMONG ELDERLY MOTHERS AND THEIR ADULT DAUGHTERS

Elizabeth Jain,[1] Gisela Labouvie-Vief,[2] and Mark Lumley,[3] *1. AKSA Consulting, Saint Charles, Illinois, United States, 2. University of Geneva, University of Geneva, Geneve, Switzerland, 3. Wayne State University, Wayne State University, Michigan, United States*

Examination of physiological coregulation among marital partners suggests a dynamic interplay between partner physiology. Further, attachment dimensions of anxiety and avoidance mediate this coregulation during conflict. This study examined the role of attachment and race in predicting physiological coregulation for mothers and their adult daughters during emotional discussions. A sample of 23 African American and 17 Caucasian mother-daughter pairs (aged 26 to 83) completed interview sessions and Relationships Questionnaires. Pairs engaged in discussions (neutral, conflict, happy), while monitoring heart rate. HR difference scores were computed between pairs (bps; 0 meant no difference). Multiple Regressions revealed attachment anxiety and avoidance predicted HR variation between pairs for the neutral and happy discussions, differently by racial group ($F(7,33)=3.297$, $p < 0.01$). For African American women, increased anxiety predicted increased HR variation during neutral and happy discussions, whereas for Caucasian women, increased avoidance predicted increased HR variation. However, during conflict anxiety singularly predicted increased HR covariation ($b = 5.03$, $p = 0.01$), for both groups. Low anxiety and low avoidance predicted physiological coregulation (lower HR variance between pairs). Increased anxious attachment predicted partner dysregulation (increased HR variation between pairs) across all 3 discussions, moderated by avoidance for the Caucasian women. Results suggest attachment plays a role in regulating physiology under emotional stress, and that there may be important cultural differences in this relationship. Further examination will explore the dynamic interplay between attachment and physiological coregulation across adulthood and later life.

### LONGITUDINAL AND AGE-RELATED IMPLICATIONS OF PRIMARY AND SECONDARY CONTROL FOR HAPPINESS

Masahiro Toyama, *University of the Ozarks, Clarksville, Arkansas, United States*

While previous research addressed two distinct types of happiness, including hedonia and eudaimonia, the longitudinal associations of primary and secondary control with these happiness constructs had not been fully studied. The present study aimed to contribute to the literature by examining these associations and their age differences. Using data from the second and third waves of the Midlife in the United States (MIDUS; N = 4,963, aged 28 to 84 at baseline), the present study conducted structural equation modeling analyses to examine whether primary and secondary control predicted residualized changes over around a decade in the latent constructs of hedonia and eudaimonia and whether there were age differences in these associations. The results indicate that while only primary control predicted change in eudaimonia overall, the associations of primary and secondary control with changes in hedonia and eudaimonia differed by age. Particularly, in comparing these effects for younger and older individuals, primary control predicted increases in eudaimonia only for younger individuals, whereas secondary control predicted decreases in hedonia for younger individuals but predicted increases in eudaimonia for older individuals. Considering these findings, the importance of primary and secondary control for happiness may vary between adults of different ages, which is possibly due to their life priorities that may change with age. The present study suggested potential directions of future research further examining the role of primary and secondary control for happiness and exploring potential interventions to promote happiness, for example, by modifying primary and/or secondary control for adults of different ages.

### POSITIVE ASPECTS OF CAREGIVING IN INCIDENT AND LONG-TERM CAREGIVERS: ROLE OF SOCIAL ENGAGEMENT AND DISTRESS

Chelsea Liu,[1] Victoria Marino,[2] Virginia Howard,[3] William Haley,[2] and David Roth,[4], *1. Harvard School of Public Health, Brookline, Massachusetts, United States, 2. University of South Florida, Tampa, Florida, United States, 3. University of Alabama at Birmingham, Birmingham, Alabama, United States, 4. Johns Hopkins University, Baltimore, Maryland, United States*

Positive aspects of caregiving (PAC) are positive appraisals that caregivers report about their role such as feeling appreciated or important, and may increase with caregiver adaptation over time. We aimed to examine differences in PAC by caregiving duration and social engagement, controlling for measures of distress. A total of 283 African American or White caregivers from the Caregiving Transitions Study with a wide range of caregiving durations were included in our analysis. We used multivariable linear regressions to model total PAC score on years of caregiving and social engagement (social network, monthly social contact), adjusting for age, sex, race, marital status, relationship to care recipient, dementia status of care recipient and measures of distress (depressive symptoms, perceived stress, caregiving strain). Caregivers with higher social engagement reported significantly higher PAC while caregivers with longer duration of care reported marginally higher PAC in most analytic models. African American caregivers reported higher PAC compared to White caregivers. Dementia caregivers reported lower PAC than non-dementia caregivers in models that adjusted for demographic variables and social network size, but the association was attenuated with the addition of caregiving strain. In summary, higher social engagement and longer care duration were associated with higher PAC after adjusting for demographic variables and measures of distress. Future studies should aim to understand how caregivers shift appraisal to positive aspects of their role and explore implementation of interventions targeting PAC in order to improve the caregiving experience.

### THE EFFECTS OF STIGMA ON THE CAREGIVERS OF ELDERLY PATIENTS WITH PSYCHIATRIC ISSUES IN A CHINESE COMMUNITY

Joanne Siu Ping So,[1] and Kai Nin Joseph Chan,[2]
*1. University of Victoria, Victoria, British Columbia, Canada, 2. Asia Pacific Career Development Association, 20850, Maryland, United States*

World Health Organization in 2017 indicates the proportion of persons over the age of 60 years will exponentially grow from 12% in 2015 to 22% in 2050. Advanced age is a common risk factor for multiple conditions, including psychosis, depression, and other mental illnesses linked to cognitive and neurologic disorders. The majority of the studies identify ethnicity and socioeconomic status as primary determinants of mental health care access. Recent studies show that up to 12% of elderly Chinese have had a history of mental problems. However, over 50% of Chinese with mental disorders have failed to obtain professional help. Lack of access to health care for mental disorders has been linked to multiple underlying socioeconomic and cultural factors. These Chinese Americans lack an in-depth understanding of their psychosis, and psychiatric conditions are often a minority in nature. This study will systematically review the existing situations relating the factors to the stigma on caregivers. The result shows that the leading cause of psychiatric disorders, physical and emotional components of the elderly population, needs to be incorporated in the care plan in nursing homes and hospitals. In North America, the constant perception of discrimination and the inherent feeling of isolation and stigma among families with elderly members remains challenging. This review could contribute to the policy reform, which can help design effective control strategies to manage gaps of most mental disorders that continue to disproportionately affect different ethnic groups across the U. S. and Canada.

### Session 2470 (Paper)

### PURPOSE IN LIFE

### MEANINGFUL ENGAGEMENT OF RESIDENTS WITH DEMENTIA IN ASSISTED LIVING: THE ROLE OF DIRECT CARE WORKERS

Jennifer Craft Morgan,[1] Joy Ciofi,[2] Candace Kemp,[3] Andrea Hill,[4] and Alexis Bender,[5] *1. Georgia State University, Georgia State University, Georgia, United States, 2. Georgia State University, Avondale Estates, Georgia, United States, 3. Gerontology Institute, Georgia State University, Atlanta, Georgia, United States, 4. Georgia State University, Atlanta, Georgia, United States, 5. Emory University, Atlanta, Georgia, United States*

Meaningful engagement has important implications for quality of care for persons living with dementia. Yet, little research has focused on direct care workers' (DCWs) role in facilitating engagement opportunities for residents in assisted living. Using data from our ongoing NIA-funded study, "Meaningful Engagement and Quality of Life among Assisted Living Residents with Dementia," we describe DCW approaches to engaging residents and the factors that influence the use and successful application of these approaches. Focal residents (N=33) were followed at four diverse assisted living communities for one year. Data includes care partner interviews (N=100), including 28 DCWs and 1560 hours of field observation data. DCWs interviewed had between 2 months and 12 years' experience in their current position and were mostly African American and/or immigrant women of color. Findings suggest that DCW-resident interactions are key opportunities to engage residents in a meaningful way and can facilitate positive, trust-based relationships. This analysis elaborates on our previous work identifying four approaches: knowing the person, connecting with and meeting people where they are, being in the moment, and viewing all encounters as opportunities. We identified factors affecting opportunities for, and experiences with, meaningful engagement between residents and DCWs, including community staffing, consistent assignment, scheduling, DCW training and tenure, and community resources. We conclude with implications for practice emphasizing how elaboration of these approaches can inform the development of DCW training, opportunities for career advancement, and integration of approaches consistently into daily practice in an effort to support meaningful engagement of residents living with dementia.

### PURPOSE IN LIFE: A RECONCEPTUALIZATION FOR VERY LATE LIFE

Keith Anderson,[1] Noelle Fields,[1] Jessica Cassidy,[1] and Lisa Peters-Beumer,[2], *1. University of Texas at Arlington, Arlington, Texas, United States, 2. Concordia University Chicago, River Forest, Illinois, United States*

Across disciplines, we have long sought to understand the factors that contribute to purpose in life. Theorists have posited that having life goals, feeling productive, and remaining active are essential contributing elements to purpose in life (Crumbaugh & Maholick, 1969; Rowe & Kahn, 1997; Ryff, 1989). While these factors can undoubtedly contribute to purpose in life, they may not fully explain purpose in life for older adults in very late life (85 years old and older) who have long past and short future time horizons. In this presentation, we explore the concept of purpose in life for older adults in very late life and how current measures may not fully or accurately apply to this group. We examine the two most commonly used measures of purpose in life, the Purpose in Life Test (Crumbaugh & Maholick, 1964, 1969) and the Ryff Purpose Subscale (Ryff, 1989; Ryff & Keyes, 1995), and identify specific items that should be reconsidered for use with older adults in very late life. We then reconceptualize purpose in life for the oldest old based on several foundational theories, including Socioemotional Selectivity Theory, the Theory of Gerotranscendence, and Terror Management Theory. Stemming from this analysis, we posit that purpose in life in very life consists of three domains – the very long past, the very near future, and the transcendental post-mortem. Based upon this reconceptualization, we recommend the development of new measures of purpose of life in very late life that capture these three domains.

### SENSE OF PURPOSE IN LIFE PROMOTES RESILIENCE TO MEMORY DEFICITS RELATED TO DEPRESSIVE SYMPTOMATOLOGY

Nathan Lewis,[1] and Patrick Hill,[2], *1. University of Victoria, Victoria, British Columbia, Canada, 2. Washington University in St. Louis, St. Louis, Missouri, United States*

Individuals higher in depressive symptoms commonly present with neuropsychological deficits including poorer memory performance. Sense of purpose in life, a component of psychological well-being, has been shown to promote resilience to cognitive impairment in older adulthood, but it is unclear whether it may also protect against cognitive deficits associated with higher depressive symptoms. This study examined whether purpose in life moderated the effect of depressive symptoms on cognitive functioning in a large longitudinal study of 4599 American older adults (Mage = 74.33 years, range = 65–104 years, 56.84% female) across a 12-year follow-up period. Depressive symptomatology was assessed at each wave using the 8-item Center for Epidemiologic Studies Depression Scale. Multilevel models assessed the influence of depressive symptoms and the interaction with sense of purpose in life on changes in word recall and mental status. Higher depressive symptoms were associated with poorer recall at baseline, but not rate of change over time. A negative interaction was observed between sense of purpose in life and depressive symptoms such that individuals higher in purpose experienced a less negative impact of depressive symptoms on word recall. No significant interaction of sense of purpose and depressive symptoms was observed on mental status. Having a sense of purpose in life may help protect older adults from memory deficits associated with higher depressive symptoms. The present findings underscore the potential for sense of purpose to promote cognitive reserve in older adulthood, allowing individuals to maintain cognitive performance in the face of accruing neuropsychological challenges.

### Session 2475 (Paper)

### SOCIAL CONNECTION AND RESEARCH CHALLENGES DURING THE COVID-19 PANDEMIC

### FEASIBILITY AND CONSIDERATIONS FOR REMOTE INTERVIEWING OF PERSONS LIVING WITH DEMENTIA: ADAPTATIONS FOR COVID-19

Jeanine Yonashiro Cho,[1] Elizabeth Avent,[2] Laura Mosqueda,[3] and Zachary Gassoumis,[3] *1. University of Southern California, Alhambra, California, United States, 2. University of Southern California, Los Angeles, California, United States, 3. Keck School of Medicine of USC, Alhambra, California, United States*

The continuing COVID-19 pandemic has necessitated changes to research protocols and approaches to mitigate health risks to both study participants and researchers. This is particularly true of studies exploring the biopsychosocial well-being and personal perspectives of older adults and those at elevated risk of COVID-19 complications. While videoconferencing platforms have enabled remote work and social activities, reliance on them may potentially exclude some individuals (e.g., those without digital devices, access to high speed internet or proficiency with technology). Persons living with dementia (PLWD) may experience difficulties navigating videoconferencing systems and building rapport with interviewers, though the inclusion of PLWD in research is necessary to ensuring their equitable representation. This presentation disseminates promising practices and lessons learned from a longitudinal study conducting remote interviews on sensitive topics with PLWD and their care partners (CP). Findings are drawn from a case study of the Better Together Dementia Care Study, an 18-month longitudinal study of PLWD (N=8) and their CPs (N=13), which implemented remote interviewing in Summer 2020 to gather data on the quality-of-life, resilience, relationship quality, adverse childhood experiences, mistreatment, and health status of PLWD. Researchers were able to interview most enrolled PLWD (n=7) via videoconferencing. Paper surveys were mailed to phone-interviewed participants, enabling them to view questions and answer choices in concordance with verbal queries. Researchers also tested a protocol asking CPs to leave the room while PLWD answered questions on sensitive topics. Findings support the use of remote interviewing with PLWD and provide insights to guide replication of these approaches.

### FEASIBILITY STUDY OF A TABLET-BASED INTERVENTION TO ENHANCE THE MEANING IN LIFE AMONG OLDER ADULTS

Seok In Nam, and Sangyoon Han, *Yonsei University, Seoul, Seoul-t'ukpyolsi, Republic of Korea*

Recommendations to improve older adults' health and well-being focus on enhancing meaning in life through social interaction. Research studies have suggested that a tablet-based intervention can create opportunities to enhance meaning in life, thus reducing social isolation and loneliness. The purpose of this study was to examine the feasibility of using a tablet-based intervention to enhance meaning in life among older adults. Senior Meaning in Life Enhancement (SMiLE) is a tablet-based application developed and implemented based on person-centered counseling, logotherapy, and Acceptance Commitment Therapy (ACT). Thirty-one participants (adults aged over 65 years) were randomized for intervention (n = 15) or waitlist control (n = 16). The intervention group received a tablet with our embedded app. Participants were invited to participate in the app-based 2-month program for 30-minutes each day with the tablet. We evaluated pre-and post-semi-structured interviews, meaning in life scales, and usability tests. Data were analyzed using thematic analyses, descriptive statistics, and Mann–Whitney and Wilcoxon tests. Findings confirmed that at two months after the intervention, there was a statistically significant difference in the gap between pre-and post-meaning in life scores between the two groups ($Z = -2.08$, $p < .05$). Furthermore, qualitative findings included positive changes in behavior, relationships, and usability. This pilot study suggests the feasibility of a tablet-based intervention in older adults and demonstrates its potential benefit for meaning in life. These findings are valuable to researchers, practitioners, and designers interested in technological interventions for older adults.

### PARTICIPATORY RESEARCH WITH OLDER WORKERS IN A PANDEMIC: INNOVATIONS AND LESSONS LEARNED

Cal Halvorsen, Kelsey Werner, and Elizabeth McColloch, *Boston College, Chestnut Hill, Massachusetts, United States*

In the spring of 2020, and as the implications of the COVID-19 pandemic became increasingly dire, in-person studies halted throughout the world. This included our planned study to examine the role of the Senior Community Service Employment Program (SCSEP)—the sole federal workforce training program for low-income older adults—in influencing participant financial, physical, and mental well-being. While our original plans were to hold a series of in-person workshops with SCSEP participants and case managers using a form of participatory research called community-based system dynamics (CBSD), we paused the launch of our study to determine the safest path forward. This presentation will describe how we responded as well as innovations and implications for future research with harder to reach populations. First, we met with the Massachusetts state SCSEP director to assess the feasibility of moving our sessions online with this particular population. After determining that virtual and telephone sessions would both be needed to increase accessibility, we identified virtual whiteboard software rigorous enough to utilize CBSD-specific activities, user-friendly enough for populations less familiar with virtual environments, and with security features that would be approved by our university, as well as discussed what types of activities to conduct on the telephone for such a visual research method. Our CBSD study was one of the first to utilize virtual and telephone formats in the history of this method, and our results indicate that it is possible—and sometimes beneficial—to move in-person participatory methods to these environments to increase inclusion and efficiency.

### THE IMPACT OF THE CARING CALLERS PROGRAM ON SENIOR COMPANION VOLUNTEERS AND CLIENTS DURING THE COVID-19 PANDEMIC

Kathy Lee,[1] Noelle Fields,[2] Gretchen Feinhals,[3] and Melanie Calhoun,[3] *1. University of Texas at Arlington, Coppell, Texas, United States, 2. University of Texas at Arlington, Arlington, Texas, United States, 3. The Senior Source, Dallas, Texas, United States*

**Purpose of the Study:** The purpose of this study was to examine the impact of the Caring Callers Program on older adults and volunteers. Our research team piloted this telephone reassurance program during the COVID-19 pandemic. In the Caring Callers Program, socially isolated older adults were paired with older adult volunteers from the Senior Companion Program (20 pairs).

**Methods:** In the Caring Callers Program, Senior Companion volunteers provided the clients with emotional support through a weekly phone call over the 12 weeks period (May through July 2020). Prior to the intervention implementation, the volunteers received a two-hour group-based training through a teleconference platform. Program outcomes were measured through quantitative and qualitative approaches.

**Results:**The clients (mean age=73.5) showed significantly increased overall self-rated health at post-test, compared to pre-test and they discussed social and emotional benefits. The clients were very satisfied with the program and indicated that the program met their expectations. Our individual, in-depth interviews with the volunteers (mean age=73.2) also revealed that the volunteers were able to develop their skills that are helpful for their Senior Companion volunteer activities and experience mutual benefits by spending their time more purposefully. Overall, our participants shared that they wanted to continue participating in the Caring Callers Program.

**Discussion:** We learned the importance of training not only for the volunteers but also for the clients, prior information on their pair, making sure of the volunteer-client fit, and benefits of using telephone particularly in this group of vulnerable older adults.

### Session 2480 (Paper)

### SOCIAL DETERMINANTS OF HEALTH II

### BIRTH COHORT DIFFERENCES IN MULTIMORBIDITY BURDEN AMONG AGING U.S. ADULTS

Nicholas Bishop,[1] Steven Haas,[2] and Ana Quiñones,[3]
*1. Texas State University San Marcos, San Marcos, Texas, United States, 2. Penn State, University Park, Pennsylvania, United States, 3. Oregon Health & Science University, Portland, Oregon, United States*

Multimorbidity is the co-occurrence of two or more chronic health conditions and affects more than half of the US population aged 65 and older. Recent trends suggest increased risk of poor self-reported health, physical disability, cognitive impairment, and mortality among later born birth cohorts, yet we are unaware of work examining cohort trends in multimorbidity among aging US adults. Observations were drawn from the Health and Retirement Study (2000–2018) and included adults aged 51 and older across 7 birth cohorts (1923 and earlier, 1924–1930, 1931–1941, 1942–1947, 1948–1953, 1954–1959, and 1960–1965). Multimorbidity was measured as a count of 9 chronic conditions including heart disease, hypertension, stroke, diabetes, arthritis, lung disease, cancer (excluding skin cancer), depression, and cognitive impairment. General linear models adjusting for repeated measures and covariates including age, sex, race/ethnicity, and education were used to identify whether trends in multimorbidity varied across birth cohort. 31,923 adults contributed 153,940 total observations, grand mean age was 68.0 (SD=10.09), and mean multimorbidity was 2.19 (SD=1.49). In analyses adjusted for age and other covariates, adults born 1948–1953 reported .34 more chronic conditions (SE=.03, p<.001), adults born 1954–1959 reported .42 more chronic conditions (SE=.03, p<.001), and adults born 1960–1965 reported .55 more chronic conditions (SE=.03, p<.001), than those born 1931–1941, respectively. Our preliminary results confirm increasing multimorbidity among later birth cohorts of older Americans and should help guide policy to manage impending health declines among older Americans.

### PERSONAL NETWORK BRIDGING POTENTIAL AMONG RURAL AND OLDER POPULATIONS

Adam Roth, Siyun Peng, and Brea Perry, *Indiana University, Bloomington, Indiana, United States*

Personal social networks play a fundamental role in the daily lives of older adults. Although many studies examine how life course factors and personal preferences shape network formation, fewer consider how the places in which older adults live present opportunities and obstacles to cultivate social relationships. In the present study, we explore how geographic context is associated with the ability to interact with non-overlapping social groups within one's personal network (i.e., network bridging). This unique network formation offers older adults access to diverse social stimuli, non-redundant information, and social autonomy. By analyzing data from the Person-to-Person Health Interview Survey (N=709), we found that a minority of respondents reported the ability to bridge social groups within their networks. Respondents residing in rural and semi-rural counties engaged in fewer non-overlapping social groups compared to those residing in urban counties. These findings suggest that the communities in which older adults live condition opportunities for accessing unique social resources. Identifying the link between geographic residence and personal network structure has important implications for how individuals navigate the uncertainty and elevated support needs of later life. Additional research adopting a social network perspective is needed to provide insight into geographic health disparities occurring among the older population.

### RACE DIFFERENCES IN TRAJECTORIES OF HOPELESSNESS AMONG U.S. OLDER ADULTS: DO SOCIAL CONDITIONS MATTER?

Uchechi Mitchell,[1] Elena Graetz,[2] and Jing Wang,[3], *1. University of Illinois Chicago, School of Public Health, Chicago, Illinois, United States, 2. University of Illinois Chicago, College of Liberal Arts and Science, University of Illinois, Chicago, Illinois, United States, 3. University of Illinois, Chicago, University of Illinois Chicago, Illinois, United States*

Despite being a risk factor for cardiovascular disease, suicidal ideation, and mortality among U.S. older adults, research on hopelessness and how it changes over time are lacking. Although hopelessness generally increases with age, levels of hopelessness may be influenced by race/ethnicity and social or economic factors. This study uses longitudinal data from 8,359 individuals from the Health and Retirement Study to examine race differences in trajectories of hopelessness from 2006 to 2018. We used linear mixed models to estimate trajectories of hopelessness for blacks, whites and Hispanics age 51 and older. The model was fit with a natural spline cubic function to model changes in time trends of hopelessness and the interaction between time and race. Models controlled for demographic characteristics, socioeconomic status, health status, and psychosocial factors that influence hopelessness. We found that older Hispanics have the highest levels of hopelessness, followed by non-Hispanic blacks and non-Hispanic whites. Trajectories of hopelessness were non-linear and differed by race. For older whites, hopelessness increased from 2006-2010 and then decreased until 2018. For older blacks, it decreased the entire time period but did so at a decreasing rate; and, for older Hispanics, hopelessness decreased from 2006-2012 and then increased thereafter. Our study shows that hopelessness generally decreased over time among older adults between 2006 and 2018 in race-specific ways, despite generally increasing with age. These findings suggest that race, age and period effects differentially influence trajectories of hopelessness. Factors contributing to these differences may be related to concurrent social and economic conditions.

### TEMPORAL TRENDS IN THE PREVALENCE OF DEMENTIA IN SOUTH KOREA

Jung Hyun Kim,[1] and Anja Leist,[2] *1. University of Luxembourg, University of Luxembourg, Diekirch, Luxembourg, 2. University of Luxembourg, Esch-sur-Alzette, Diekirch, Luxembourg*

Background. Secular decreases in the prevalence of cognitive impairment and dementia have been observed in several Western countries, however, few systematic investigations of temporal trends in dementia have been conducted in South Korea. Method. Data came from N=8,006 individuals (N=2,110 assessed twice) aged 65 years and older participating in the Korean Longitudinal Study of Aging 2008 and 2018. Dementia was indicated by a score ≤ 17 on the Korean Mini-Mental State Examination (K-MMSE). Dementia was regressed on the year of survey, adjusting for multiple demographic and socio-economic confounders, and, in additional models, also chronic diseases and lifestyle factors related to health, social, and religious activities. Results. Across waves, the share of individuals with low socio-economic status decreased. The prevalence of chronic diseases, including diabetes, heart diseases, stroke, and psychiatric diseases, increased over time. Alcohol consumption increased, whereas smoking rates, religious affiliation, and participation in religious activities decreased. Controlling for all covariates and compared to 2008, we observe decreases in dementia prevalence in 2018 by 52% (2018: OR 0.48, CI 0.42, 0.56). Women's MMSE scores were more than two times as likely as men's to indicate dementia (OR 2.59, CI 2.15, 3.14). Discussion. Decreases in dementia prevalence in Korea are partly attributable to improved socio-economic conditions and can be observed despite the increased prevalence of chronic conditions. However, secular trends were not fully explained by these and lifestyle factors. We discuss further individual-level and contextual-level mechanisms that may have contributed to these findings.

### THE INTERACTION OF LIFE COURSE SOCIOECONOMIC STATUS AND LEISURE ACTIVITIES ON COGNITIVE PERFORMANCE IN OLD AGE

Rongjun Sun,[1] and Zhenmei Zhang,[2] *1. Cleveland State University, Cleveland, Ohio, United States, 2. Michigan State University, Department of Sociology, Michigan, United States*

While the separate effects of socioeconomic status and engaging in leisure activities on cognition have been well documented, their interaction effect has rarely been examined. After examining life course socioeconomic status (SES) on cognitive impairment in old age, this paper is focused on exploring the interaction effects between life course SES and leisure activities. We use data from the Chinese Longitudinal Healthy Longevity Survey, which covers five waves of interviews of adults aged 65 or older between 2002 and 2014. Cognitive impairment is measured by the Chinese version of Mini-Mental Status Examination. Two sets of variables are used to reflect an older person's life course SES in childhood and adulthood, respectively. Seven leisure activities are included in this analysis. We adopt the lagged independent variable approach and a Generalized Linear Mixed Model to examine the association between leisure activity and cognitive impairment over time. Results show that there is an independent impact of SES in both childhood and adulthood on cognitive decline in Chinese older population. Furthermore, as the focus of this study, there are substantial interactions between life course SES and engaging in leisure activities with a consistent pattern: those of higher life course SES enjoy extra benefits from engaging in leisure activities. The interactions between life course SES and leisure activities promise a competing approach accounting for cognitive health inequality among older adults.

### Session 2485 (Paper)

### TECHNOLOGY AND OLDER ADULTS

### DETERMINANTS OF INTENTION TO USE DIGITAL TECHNOLOGY FOR OLDER ADULTS BY ENVIRONMENTAL DIMENSIONS

Sangyoon Han, and Seok In Nam, *Yonsei University, Seoul, Seoul-t'ukpyolsi, Republic of Korea*

Due to the COVID-19 pandemic, it is common to hear news of older adults being socially isolated due to difficulties in purchasing or accessing online services and in interacting with family or friends through video calling apps. Despite an increasing ease of access to digital devices, such access far from universal. Thus, digital inequality has become a serious problem for older adults. To understand why digital inequality issues are so relevant for older adults, we must understand older adults' entire life contexts and the potential of digital technologies in their lives. With these understandings, the purpose of this study was to explore the technology acceptance process and identify key precursors to acceptance of digital technology using the Technology Acceptance Model (TAM) 3 as a framework. This study used data from the 2018 Digital Divide Survey of the Ministry of Science and ICT. A total of 1,662 older adults (aged 55+) were analyzed using structural equation modeling with bootstrap sampling. Model fit indices (CFI = .928; SRMR = .074; RMSEA = .044) suggested an acceptable fit. Results indicated that two environmental dimensions, personal environment (self-efficacy and value recognition) and social environment (social norms and social support systems), had a significant impact on the intention to use technology both directly and indirectly. Furthermore, perceived usefulness and perceived ease of use mediated between environmental domains and the intention. This study indicates that providing appropriate digital support for older adults is important to achieve greater digital inclusion.

### DIGITAL DEVELOPMENTS IN SOCIETY THAT PERSONS 75 YEARS AND OLDER HAVE BEEN PART OF: A SCOPING REVIEW

Moonika Raja,[1] Jorunn Bjerkan,[2] Ingjerd Kymre,[1] Kathleen Galvin,[3] and Lisbeth Uhrenfeldt,[1], *1. Nord University, Nord University, Bodø, Nordland, Norway,*

*2. Nord University, Nord University, Levanger, Nord-Trondelag, Norway, 3. University of Brighton, University of Brighton, England, United Kingdom*

The population in Europe is ageing and people are becoming more than ever dependent on digital technologies. The present study aims to map relevant evidence about digital developments in society involving people aged 75 and over in European countries. It focuses on their experiences and the main barriers to, and facilitators of, societal digital demands. Scoping reviews can be used when the purpose is to identify types of available evidence and clarify concepts, this process was guided by a framework proposed by Arksey and O`Malley. The studies included in the review covered digital technology, digital devices and telehealth, and the context covered participants` own home or surroundings. A comprehensive search was made on CINAHL, Embase, Pubmed/MEDLINE, Scopus and Open Grey. Out of 727 identified citations, 13 sources which met the inclusion criteria (9 original study articles, 2 theses, 1 letter about a product and 1 project report). The studies included varied in their focus, design and location. Older European citizens have experienced technology making life easier and the opposite. The outstanding facilitator found was that technology should be easy to use. Interestingly, both social support and lack of social support were found as facilitators of using new technology and difficulty in remembering the instructions was seen as an important barrier. As technology develops rapidly, there is a need for new and additional research among older European citizens. Future research should cover participants` access to the devices, social support and the technical solutions most relevant to older people today.

### PRIVACY AND SMART SPEAKERS IN RESEARCH WITH OLDER ADULTS

Kelly Quinn,[1] Jessie Chin,[2] Smit Desai,[3] Carrie O'Connell,[4] David Marquez,[5] and Naoko Muramatsu,[6], *1. University of Illinois at Chicago, Chicago, Illinois, United States, 2. University of Illinois at Urbana-Champaign, University of Illinois at Urbana-Champaign, Illinois, United States, 3. University of Illinois, Urbana Champaign, Champaign, Illinois, United States, 4. University of Illinois at Chicago, University of Illinois at Chicago, Illinois, United States, 5. Rush University, University of Illinois at Chicago, Illinois, United States, 6. University of Illinois Chicago, Chicago, Illinois, United States*

Advances in artificial intelligence and computational linguistics have made smart speakers, such as Amazon Alexa^TM^ and Google Home^TM^, economical and widely available. For older adults particularly, devices with voice interfaces can help to overcome accessibility challenges that often accompany interaction with today's technologies. However, voice-activation also requires devices to be in a continuous state of ambient listening, which can create a significant privacy risk for the user, one that is often amplified as smart speakers are placed in highly personal home spaces to facilitate their utility. Deployment of these devices in research settings poses additional risk, as traces of data filter through research teams, app developers, and third-party services that support research efforts. This presentation addresses the privacy aspects of deploying Google Home Mini^TM^ speakers in research that examined their feasibility for enhancing physical activity among sedentary older adults. Interviews with participants were conducted in two studies: the first included a demonstration of the device and physical activity program (n=15); and the second included in-home use of devices and a physical activity program (n=15). Content analysis of study documentation, field notes, and interviews revealed specific areas that require additional attention when utilizing smart speakers in research, including the capture of identifying information, protocols for data handling, and requirements for informed consent. These findings are discussed in context with extant literature on individual privacy concerns and behaviors related to smart household devices. Results from this study can inform future research efforts incorporating smart speakers, to mitigate potential risks of privacy violation.

### THE FINAL MILE: EVALUATING EHEALTH AND MHEALTH UTILIZATION AMONG OLDER ADULTS

Mary Krebs, *University of Massachusetts Boston, Warren, Maine, United States*

While health endures as a term to describe looking after oneself, looking after loved ones, and receiving care, the added component of electronic technology has emerged to affect all levels of health care delivery. Despite the prevalence of digital health and empirical evidence strongly supporting improved outcomes--the final mile--as it is sometimes called, is the sustained patient engagement with eHealth and mHealth we have yet to achieve. This research identifies a gap in the literature for understudied characteristics of digital health adoption, use affecting the aging population in the U.S., and contributes a deeper understanding of the key barriers to use of health-related technology. A mixed-methods research approach explores prevalent barriers to digital health utilization by older adults through a pre-post data collection strategy to empirically test an educational health-related intervention rooted in the Technology Acceptance Model. This validated analytic framework represents a decision 'core' as a user pathway for actual use. Evaluation of score data utilized a quantitative test of group means while thematic coding was employed for qualitative analysis. The results from this study are two-fold. The work strongly suggests specific barriers to adoption and use, confirming a distrust and reluctance to engage. However, additional evidence, both quantitative and qualitative illuminates substantive skills, positive perceptions, hopeful attitudes, as well as the rationale for use of available digital resources. Findings suggest future research would benefit from expanded use of the two-pronged approach to foster health-related technology engagement.

### THE ROLE OF INTERSECTIONALITY IN ACCESS TO TECHNOLOGY AMONG OLDER ADULTS

Zainab Suntai,[1] and Susanny Beltran,[2] *1. University of Alabama, Tuscaloosa, Alabama, United States, 2. University of Central Florida, Orlando, Florida, United States*

In the era of COVID-19, technology has become a primary means of connecting with the world while maintaining physical distance, which is crucial for older adults who are at disproportionately high risk of infection and death. Throughout the pandemic, there has been increased emphasis on using telehealth to access medical and mental health care, and technology (e.g., apps, social media, video calls) for social interactions/communication to mitigate loneliness/isolation. Thus, COVID-19 has increased the need for older adults to access technology, and widened disparities experienced by those with limited access. This study used data from the 2018 National Health and Aging Trends Study, an annual longitudinal panel survey of Medicare beneficiaries aged 65+ in the U.S, to explore the association between the interaction of race/ethnicity and sex, and access to both a working cell phone and laptop/computer. Chi-square tests and multivariable logistic regressions were conducted. The sample (N=2,442) was 83.7% white, 8.5% Black, and 7.8% Hispanic. After accounting for other explanatory variables, logistic regression analysis indicated significantly higher odds of not having both a working cell phone or computer/laptop among White women (OR=1.518, CI=.1.510-1.527), Black men (OR=.1.741, CI= 1.720-1.763), Black women (OR=2.567, CI= 2.545-2.589), Hispanic men (OR=1.036, CI=1.022-1.050), and Hispanic women (OR=2.265, CI=.2.243-2.287) compared to White men. Overall, Hispanic and Black women were the least likely to have access to technology compared to other groups. Addressing technological equity remains a need. Future research should consider how the provision of devices along with technological literary programs can improve well-being among BIPOC women.

### Session 2495 (Symposium)

### THE IMPORTANCE OF PSYCHOSOCIAL FACTORS IN AGING: THE MINORITY AGING RESEARCH STUDY

Chair: Lisa Barnes Co-Chair: Brittney Lange-Maia
Discussant: Carlos Mendes de Leon

Psychosocial factors can provide crucial insight into lived experiences that influence healthy aging. Though psychosocial factors are often used to explain health disparities seen between different racial/ethnic groups, within-group investigations can be particularly powerful for identifying culturally specific psychosocial factors that impact heterogeneity in aging among minority populations. The Minority Aging Research Study (MARS) is an ongoing, longitudinal epidemiologic cohort of 797 older African Americans from the Chicago, IL metropolitan area. Participants are on average 73.4 (standard deviation [SD]=6.6) years of age, 78.2% are women, and mean years of education is 14.8 (SD=3.7). At baseline, 75.3% of participants were without cognitive impairment, 20.8% had mild cognitive impairment, and 3.9% had mild dementia. Participants were recruited starting in 2004 and complete annual visits including a clinical evaluation, cognitive and motor testing, and assessment of risk factors related to Alzheimer's Disease risk, including those hypothesized to be associated with a higher burden of cognitive impairment among older African Americans. This symposium will discuss the longitudinal association between John Henryism and cognitive function and decline (McSorley), participation in social activities and risk of all-cause mortality (Lamar), and the predictive relationship between experiences of everyday discrimination and incident disability (Lange-Maia). Finally, we will examine multilevel correlates—including environmental, sociocultural, behavioral, and biological factors—related to perceived stress (Glover). Mendes de Leon will critically consider what appear to be the most potent psychosocial factors for minority aging and possible implications of integrating these factors into interventions focused on promoting healthy aging among older African Americans.

### DISCRIMINATION AND RISK OF INCIDENT DISABILITY IN OLDER AFRICAN AMERICANS

Melissa Lamar,[1] Sue Leurgans,[2] Aron Buchman,[3] Lisa Barnes,[2] and Brittney Lange-Maia,[2] *1. Rush Alzheimer's Disease Center, Rush University Medical Center, Rush Alzheimer's Disease Center, Illinois, United States, 2. Rush University Medical Center, Chicago, Illinois, United States, 3. Rush University, Chicago, Illinois, United States*

Discrimination is linked to poor health outcomes, but most studies examine young or midlife populations. We assessed associations between discrimination and disability in African Americans. The Detroit Areas Study Everyday Discrimination Scale quantified experiences of interpersonal mistreatment. Separate Cox-proportional hazards models tested the associations between baseline discrimination and incident mobility, activities of daily living (ADLs), and instrumental activities of daily living (IADLs) disability, adjusting for age, sex, education, BMI, smoking, depressive symptoms, and vascular diseases. At baseline, 441, 674, and 469, participants were initially free of mobility, ADL, and IADL disability, respectively, and 257, 185, and 269 new cases of mobility, ADL, and IADL disability were observed over approximately 8.5 years. Discrimination was associated with higher risk of ADL disability (hazard ratio: 1.03 per 1-point higher discrimination score, 95% confidence interval: 1.00-1.06) but no other disability type. Everyday discrimination is associated with risk of ADL disability.

### JOHN HENRYISM AND COGNITIVE FUNCTION AND DECLINE

Christopher Howard,[1] Bryan James,[2] Raj Shah,[3] Patricia Boyle,[4] Lisa Barnes,[5] and V. Eloesa McSorley,[5], *1. The Utah State Hospital, Provo, Utah, United States, 2. Rush University, Chicago, Illinois, United States, 3. Rush, Rush University Medical Center, Illinois, United States, 4. Rush Alzheimer's Disease Center, Chicago, Illinois, United States, 5. Rush University Medical Center, Chicago, Illinois, United States*

Older Black adults in the US have higher prevalence and incidence of dementia and perform lower on cognitive tests than whites. Some of these differences have been attributed to facets of structural racism such as access to and quality of education and fewer socioeconomic resources. Here, we consider whether John Henryism (JH), a measure of self-perceived high-effort coping in the face of chronic environmental and psychosocial stressors, is associated with cognitive function and decline. JH has been associated with adverse cardiovascular health outcomes among African-Americans, especially those with fewer socioeconomic resources. Using data from MARS, we assessed whether JH, measured with an 8-item questionnaire (mean=16.9, sd=4.8, range: 4-27), was associated with level of cognitive function and rate of cognitive decline. We found one standard deviation increase in JH was associated with lower average cognitive function (□=-0.05, 95% CI: -0.09, -0.01). Higher JH was not associated with rate of cognitive decline.

### SOCIAL ACTIVITY AND ALL-CAUSE MORTALITY IN OLDER BLACK ADULTS OF THE MINORITY AGING RESEARCH STUDY

Bryan James,[1] Ana Capuano,[2] Robert Wilson,[2] Lisa Barnes,[3] and Melissa Lamar,[4], *1. Rush University, Chicago, Illinois, United States, 2. Rush, Chicago, Illinois, United States, 3. Rush University Medical Center, Chicago, Illinois, United States, 4. Rush Alzheimer's Disease Center, Rush University Medical Center, Rush Alzheimer's Disease Center, Illinois, United States*

Negative social stressors (e.g., perceived loneliness and/or social isolation) predict mortality in older adults; less is known about the role of positive social activities. What research does exist focuses on White adults, leaving gaps in knowledge regarding specific activities that may decrease mortality risk in Black adults. We investigated whether self-reported late-life social activity, generally and by type, predicted mortality risk in 768 Black adults (age~73yrs; non-demented at baseline) participating in MARS. Over ~6.5 follow-up years, 25% of participants died (n=192; age-at-death~83yrs). In fully-adjusted Cox models including demographic, health, and relevant psychosocial covariates, mortality risk decreased by 32% (HR=0.68,95%CI=0.49,0.93) in those with higher compared to lower social activity generally, and with higher volunteer-, church- and group-related activities specifically. Engaging in social activity, especially altruistic or faith-based activities, reduces mortality risk in older Blacks regardless of overall health or social stressors pointing toward community-based approaches to increase longevity in this population.

### CORRELATES OF PERCEIVED STRESS AMONG COMMUNITY-DWELLING OLDER AFRICAN AMERICANS WITHOUT DEMENTIA

Ana Capuano,[1] Robert Wilson,[1] David Bennett,[2] Lisa Barnes,[3] and Crystal Glover,[4], *1. Rush, Chicago, Illinois, United States, 2. Rush University, Chicago, Illinois, United States, 3. Rush University Medical Center, Chicago, Illinois, United States, 4. Rush University Medical Center, Rush Alzheimer's Disease Center, Illinois, United States*

The purpose of this study was to identify correlates of perceived stress among older African Americans without dementia. We grouped correlates into four levels – environmental, sociocultural, behavioral, and biological – guided by the National Institute on Aging's (NIA) Health Disparities Research Framework. We performed a cross-sectional data analysis with the Minority Aging Research Study using ordinal logistic regression analyses. Participants were 722 African Americans without dementia [mean age = 73.61 years (SD=6.33)]. Correlates from environmental (e.g., larger life space), sociocultural (e.g., larger social network size), behavioral (e.g., more purpose in life), and biological (e.g., higher global cognition) levels were associated with lower odds of having greater levels of perceived stress. Perceived stress was associated with correlates from every level. Future research is needed to examine how changes in these correlates may be related to perceived stress in older African Americans.

### Session 2500 (Symposium)

### THE IMPORTANCE OF SOCIAL SUPPORT NETWORKS ON MENTAL HEALTH STATUS OF CUSTODIAL GRANDPARENTS

Chair: Deborah Whitley Co-Chair: Youjung Lee Discussant: Yanfeng Xu

This symposium presents a collection of papers that examine the concept of social support and its effect on custodial grandparents' (CG) mental health state. Each paper explores a different perspective about grandparents' access to and/or use of social support networks and mental health outcomes; several papers view social support within the context of the COVID-19 pandemic. Nadorff and colleagues explore social support by middle-generation family members and its effects on grandparents' stress and depressive symptoms. Musil and colleagues report on psychosocial and social support predictors of self-appraised healthcare and financial security by CG during the Covid-19 pandemic. Whitley and Kelley describe current social networks relied upon by a preliminary sample of CG while managing the daily stresses and strains associated with COVID-19 and its restrictive mandates. The final two papers report the use of specialized technology and support services delivered to homebound CG during the COVID-19 pandemic. Lee and colleagues describe a telemental health model using Solution-Focused Brief Therapy to serve socially isolated grandparents experiencing mental health distress as during the pandemic. Mendoza and Park report on program challenges and outcomes of implementing a support service for grandparents living under COVID-19 restrictions. The highlights of the papers will be discussed by Yanfeng Xu and give attention to the ways scholars and practitioners can build upon these works to maximize the mental health outcomes of CG, while managing to live in socially restrictive and challenging environments.

### LIVING WITH COVID-19: DESCRIPTIONS OF CUSTODIAL GRANDPARENTS' MENTAL HEALTH STRESS AND SOCIAL SUPPORT NETWORKS

Susan Kelley, and Deborah Whitley, *Georgia State University, Atlanta, Georgia, United States*

Research suggests custodial grandparents (CG) with chronic health conditions, limited economic resources, and restricted social connections are at risk for adverse mental health outcomes. The growing uncertainty surrounding COVID-19 seems to accentuate these findings. This paper presents preliminary descriptions of mental stress by a small sample of CG (n=26) surveyed after the onset of COVID-19. They described the social groups comprising their networks, and the methods used to engage with them. A majority of the sample (96.2%) reported experiencing mental stress since the onset of the virus; based on Brief Symptom Inventory results, five CG scored in the clinical range for stress. Food access, fear of getting sick, and grandchildren's school requirements are leading sources of stress. Despite such challenges, CG report varying levels of social engagement with their support networks. The reported descriptions give preliminary insight how CG can maximize their social networks to build/sustain positive mental health well-being.

### CONNECTING GRANDPARENT CAREGIVERS THROUGH TELEMENTAL HEALTH DURING COVID-19

Laura Bronstein,[1] Kelley Cook,[2] and Youjung Lee,[3], *1. Binghamton University, Binghamton University/ Binghamton, New York, United States, 2. Community Research and action, Binghamton University/ Binghamton, New York, United States, 3. Binghamton University, Department of Social Work, Binghamton, New York, United States*

Since the COVID-19 outbreak, children and their caregivers throughout the world are experiencing unprecedented long-term social isolation. For too many, especially grandparent-headed families, underrepresented minorities, and those living in poverty, this precipitates and exacerbates mental health conditions including anxiety and depression. Despite these families' increased needs for mental health services during the pandemic, professionals often lack experience and expertise in telemental health, which is a safe and effective way to provide these services. In this symposium, we will present a telemental health model for working with grandparent-headed families that draws upon Solution-Focused Brief Therapy (SFBT), an evidence-based approach focusing on strengths. This SFBT-based telemental health training program prepares mental health professionals to implement this safe and innovative intervention, enabling them to effectively serve isolated and marginalized grandparent caregivers and their families when providing in-person services is not possible.

### THE MODERATION OF MIDDLE-GENERATION SUPPORT ON THE RELATION OF STRESS AND DEPRESSION IN CORESIDENT GRANDPARENTS

Melissa Barnett,[1] Loriena Yancura,[2] and Danielle Nadorff,[3], *1. University of Arizona, Tucson, Arizona, United States, 2. University of Hawaii at Manoa, Honolulu, Hawaii, United States, 3. Mississippi State University, Mississippi State, Mississippi, United States*

Consistent with Cohen & Wills' Buffering Hypothesis, social support has been found to moderate the relation between stress and depressive symptoms but has yet to be examined among coresident grandparents (CGPs), a population at risk of increased stress and depression. The current study sought to extend the model to this highly prevalent, vulnerable population. Participants were 180 grandparents across the USA living with their grandchildren. Measures included depression, stress, and satisfaction with support provided by the middle generation (MG) parent of the grandchild. After controlling for age, gender, income, and household type (skipped or multigen), MG support moderated the relation between perceived stress and depressive symptoms, accounting for 49% of variance. For CGPs least satisfied with support provided by the MG, the more stress, the higher their depressive symptoms. These findings indicate that improving relationships with grandchildren's parents is an important avenue for interventions focused on grandparent caregivers' mental health.

### GRANDMOTHERS RAISING GRANDCHILDREN: PREDICTORS OF HEALTHCARE ACCESS AND FINANCIAL SECURITY DURING COVID-19

Alexandra Jeanblanc,[1] Chris Burant,[1] and Carol Musil,[2] *1. Case Western Reserve University, Cleveland, Ohio, United States, 2. CWRU School of Nursing, Cleveland, Ohio, United States*

Grandmothers living with or raising grandchildren who had just completed the final data point of an NIH-funded, national, behavioral RCT were asked to complete an additional data collection point to capture the effects of the Covid-19 pandemic on their families' access to healthcare and financial security. In Spring 2020, 258 grandmothers completed measures of access to healthcare and financial security (3 and 4 item composite scales), family strain, family functioning, and psychosocial and demographic variables. Financial security (Adj. R2=.52) was explained by knowing other grandfamilies; better family functioning; and fewer financial worries, unmet service needs, and depressive symptoms. Access to healthcare (Adj. R2=.24) was explained by being married, employed and having fewer financial worries and unmet service needs. Findings that family functioning, knowing other grandfamilies and depressive symptoms contributed to financial security, and that marital and employment status affect access to healthcare show the importance of support.

### IMPLEMENTING AN INTERVENTION PROGRAM DURING THE COVID-19 PANDEMIC: CHALLENGES AND SUCCESSES

Nancy Mendoza, *The Ohio State University, The Ohio State University, Ohio, United States*

During the COVID-19 pandemic, the implementation of intervention programs for grandfamilies are facing multiple challenges. In this paper, we will present some of the challenges and successes of introducing the GRANDcares Plus Project (GRANDc+) during the COVID-19 pandemic. As an intervention program, GRANDc+ has demonstrated positive outcomes for grandfamilies, such as increased satisfaction with life, knowledge of services, self-care practices, and supportive social networks. Due to the pandemic, the implementation of GRANDc+ has been met with many challenges including, training of facilitators, following CDC's COVID-19 guidelines/recommendations, and considering grandfamilies needs, concerns and safety. The pandemic has and continues to have detrimental effects on grandfamilies; this makes it more vital than ever to support grandfamilies through interventions like GRANDc+, despite what challenges we may face. Our presentation will provide insights into identifying, managing, and overcoming the challenges of implementing interventions during the COVID-19 pandemic.

### Session 2505 (Symposium)

### THE NEED FOR PERSON-CENTERED MEASURES FOR DEMENTIA RESEARCH AND CARE

Chair: Sam Fazio Co-Chair: Sheryl Zimmerman
Discussant: Laura Gitlin

The importance of person-centered medical and psychosocial care has become widely recognized, but there is abundant evidence that care is not always person-centered. In 2018, the Alzheimer's Association published their evidence-informed Dementia Care Practice Recommendations, which address nine domains all grounded in a person-centered perspective. Following that work, the Association launched LINC-AD -- Leveraging an Interdisciplinary Consortium to Improve Care and Outcomes for Persons Living with Alzheimer's and Dementia. An early effort of LINC-AD, and the focus of this symposium, examined what measures are available to guide care and assess outcomes, and the extent to which they embrace person-centeredness. The results have been disappointing. This session will highlight the importance of person-centered measures in five domains of the Dementia Care Practice Recommendations, based on comprehensive reviews of literature. Each paper, presented by LINC-AD research advisors, will examine available measures and raise questions about gaps using a person-centered lens. Katie Maslow will describe frequently used measures and identify person-centered measures that could be added to studies of alternate procedures intended to increase detection and diagnosis. Drs. Mast and Molony will discuss a person-centered approach to item development and testing for assessment. Emilee Ertle will discuss the need to measure interpersonal and contextual factors associated with behavioral expressions. Drs. Prizer and Zimmerman will compare measures of dressing ability and their person-centered components. Dr. Calkins will examine the strengths and limitations of environmental assessment tools. As Discussant, Dr. Gitlin will integrate the findings from all five presentations, suggesting directions for the future.

### ADDING PERSON-CENTERED MEASURES TO RESEARCH ON DETECTION AND DIAGNOSIS OF DEMENTIA

Katie Maslow, *Gerontological Society of America, Washington, District of Columbia, United States*

In the United States, numerous studies on detection and diagnosis of dementia show that large proportions of subjects refuse initial screening tests. Moreover, among those who accept the tests, score poorly, and are therefore referred for a diagnostic evaluation, large proportions do not follow up to get the evaluation. Available data on characteristics of subjects who refuse initial screening and follow-up evaluation suggest that incorporating procedures based on person-centered concepts and practices, such as procedures that acknowledge individuals' unique characteristics and attempt to involve, enable, and empower them, could lead to more effective detection and diagnosis. Based on results of an analysis of measures used in studies conducted in the U.S. and elsewhere, this presentation will describe frequently used measures and identify person-centered measures that could be added to studies of alternate procedures intended to increase detection and diagnosis.

### PERSON-CENTERED ASSESSMENT: EVALUATING POSITIVE PSYCHOSOCIAL MEASURES IN DEMENTIA RESEARCH

Sheila Molony,[1] and Benjamin Mast,[2] *1. Quinnipiac University, Hamden, Connecticut, United States, 2. University of louisville, louisville, Kentucky, United States*

Person-centered principles continue to redefine the nature of dementia care, but less attention has been given to integration of person-centered principles into clinical assessment and dementia research. As a result, identification of deficits and cognitive impairment tends to dominate clinical and research efforts, whereas strengths and positive characteristics need more research. This paper examines existing positive psychosocial measures of psychological wellbeing, hope, spirituality, resilience, social relationship, dignity, and at-homeness. Many of these measures demonstrate strong psychometric properties and have been identified as promising outcome measures for strengths-based studies and approaches to care. This paper will evaluate the extent to which these measures used a person-centered approach to item development and testing, and whether item content is consistent with person-centered principles. Future directions for instrument development require greater inclusion of people living with dementia and family caregivers.

### PERSON-CENTERED ASSESSMENT OF BEHAVIOR CHANGES IN PEOPLE WITH DEMENTIA

Benjamin Mast,[1] Gail Mountain,[2] Ann Kolanowski,[3] Esme Moniz-Cook,[4] Margareta Halek,[5] and Emilee Ertle,[6],*1. University of louisville, louisville, Kentucky, United States, 2. University of Bradford, West Yorkshire, England, United Kingdom, 3. Penn State, University Park, Pennsylvania, United States, 4. University of Hull, Hull, England, United Kingdom, 5. Witten/Herdecke University, Witten, Nordrhein-Westfalen, Germany, 6. University of Louisville, Louisville, Kentucky, United States*

Behavioral and psychological symptoms of dementia are increasingly being reconceptualized as expressions of distress and unmet needs. Measures that evaluate context are needed to increase our understanding of factors that influence these expressions. This review evaluated measures for two common behavioral states that are experienced as challenging for caregivers: apathy and resistance to care. A systematic literature search identified measures of apathy or resistance to care for people living with dementia. Eight measures of apathy and three measures of resistance to care were identified. Reliability and validity of these measures were evaluated using the COSMIN framework, as well as reported contextual factors within which the behavior occurs. The identified measures had fair to good reliability and validity in people living with dementia. However, available measures need to move beyond symptomatic constructs for this complex paradigm, and toward the varied interpersonal and contextual factors associated with behavioral expression.

### MEASUREMENT FOR SOMETHING AS PERSONAL AS DRESSING IS NOT PERSONALIZED

Sheryl Zimmerman,[1] and Lindsay Prizer,[2], *1. Cecil G. Sheps Center for Health Services Research, Chapel Hill, North Carolina, United States, 2. Emory University, Atlanta, Georgia, United States*

In 2018, the Alzheimer's Association set forth Dementia Care Practice Recommendations in nine domains, one being support for activities for daily living (e.g., dressing, toileting, eating/nutrition). For example, preservation of dressing independence is important for dignity, autonomy, and to decrease caregiver burden. Measurement is necessary to guide care and assess outcomes related to dressing, but availability of related measures to assess processes, structures, and outcomes of care has not been examined; more so, the extent to which the related measures are person-centered is completely unexplored territory. This session will present a critical assessment of available measures grounded in the Donabedian Model. Of 21 identified measures, 4 assessed dressing alone,

16 included dressing as part of a larger scale, and 1 included dressing as a part of a scale to screen for dementia; none were person-centered. This session will suggest modifications to and need for new measures for person-centered dressing.

### ENVIRONMENTAL ASSESSMENT TOOLS FOR SHARED RESIDENTIAL SETTINGS FOR INDIVIDUALS LIVING WITH DEMENTIA

Migette Kaup,[1] Addie Abushousheh,[2] and Margaret Calkins,[3] *1. Kansas State University, Manhattan, Kansas, United States, 2. Center for Health Design, West Bend, Wisconsin, United States, 3. IDEAS Institute, Cleveland Hts, Ohio, United States*

The overarching goal of conducting research in and on settings for individuals living with dementia is to be able to identify associations between features, or set(s) of features, and outcomes of interest. A major challenge to this goal is the disconnect between the rapidly changing arena of LTC design over the past 20 years toward more residential-styled settings and existing assessment tools, many of which were developed over 20 years ago, and which are based on and reflect more traditional models of care, service delivery and design. This session examines the strengths and limitations of both existing and emerging assessment tools from the US, Canada, UK and Australia. More recently developed tools are more directly focused on the values of person-centered care than older tools.

### Session 2510 (Symposium)

### VARIETY OF IDENTIFYING AND ASSESSING PREFERENCES OF EVERYDAY LIVING OF OLDER ADULTS

Chair: Martina Roes Discussant: Kimberly Van Haitsma

Identifying preference of older adults supports person-centred care. The most sophisticated instrument is the preference for everyday living inventory (PELI). The PELI has been translated into German language and tested in different care settings. For people who experience difficulties communicating their preference the PELI has been combined with photographs. The voice of older immigrants could lead to an enhancement of the PELI as well other preference tools. Thus, our symposium title: Variety of identifying and assessing preferences of everyday living of older adults. Our symposium includes four presentations: Dr. Bergmann will present data from a preference study in three different care settings (long-term care, nursing homes, adult day care) in Germany. The results indicate that the importance of certain preferences distinguishes between the care settings. Dr. Vanessa Burshnic will present data from her content validity study of photographs used to supplement the Preferences for Everyday Living Inventory-Nursing Home (PELI-NH) from the perspective of older adults. Content analysis revealed thematic codes describing participants' photograph preferences including image quality, context, subject diversity, and relevance to long-term care. Mike Rommerskirch-Manietta will present results from a review to identify Instruments which can be used to assess preferences for everyday living of older adults. Interestingly instruments either represent multiple or only one domain. The study from Viktoria Peters-Nehrenheim does focus on preferences of older immigrants. She will present results how older immigrants (first generation) define preferences and how they can be assessed. Prof. Van Haitsma will be our discussant.

### TESTING A PREFERENCE TOOL IN DIFFERENT CARE SETTINGS IN GERMANY: DESCRIPTIVE RESULTS

Tobias Stacke,[1] Mike Rommerskirch-Manietta,[2] Daniel Purwins,[1] Christina Manietta,[1] Armin Stroebel,[3] Martina Roes,[4] and Johannes Bergmann,[1] *1. DZNE, Witten, Nordrhein-Westfalen, Germany, 2. DZNE, German Center for Neurodegenerative Diseases, Nordrhein-Westfalen, Germany, 3. Center for Clinical Studies, Erlangen, Bayern, Germany, 4. German Center for Neurodegenerative Diseases (DZNE), Witten, Nordrhein-Westfalen, Germany*

**Background:** The tool "Preferences for Everyday Living Inventory" (PELI) for Nursing Homes (NH) was developed in the USA. In our project PELI-D, the PELI was translated from English into German and piloted in three care settings: Nursing Home (NH), Home Care (HC) and Adult Day Care (AD).

**Objective:** The objective is to provide insights in preferences of importance of older adults in need of care in Germany.

**Methods:** Data collection was carried out in 2019 on multiple measurement points: n=48 baseline (T0) and n=41 two-week follow-up (T1).

**Results:** The results indicate that the importance of certain preferences distinguishes between the care settings: In NH preferences for body care and aspects of professional care are important. Additionally, in HC the aspects of social contact and eating/drinking are perceived as important. Comparing T0 and T1, importance of the item's daily routines, social contact and aspects of privacy seem to be reliable.

### COGNITIVE INTERVIEWING TO EVALUATE THE CONTENT VALIDITY OF A PHOTO-SUPPORTED PREFERENCE ASSESSMENT

Kelly Knollman-Porter,[1] Rachel Topper,[1] Eleanor McConnell,[2] Katherine Abbott,[1] Kimberly Van Haitsma,[3] and Vanessa Burshnic-Neal,[4], *1. Miami University, Oxford, Ohio, United States, 2. Duke University School of Nursing, Durham, North Carolina, United States, 3. The Pennsylvania State University, University Park, Pennsylvania, United States, 4. Department of Veterans Affairs, Durham VA Health Care System, North Carolina, United States*

Photo-supported verbal assessments have shown to improve comprehension and expression of choices by older adults living with cognitive-communication challenges. The purpose of this study was to assess content validity (CV) of photographs used to supplement the Preferences for Everyday Living Inventory-Nursing Home (PELI-NH) from the perspective of older adults, using cognitive interviewing methods. Participants (N=21) were average age 75 (SD=5.67), mostly male (62%) and white (90%), living in residential communities (86%), with no known cognitive or communication deficits. Interview data was used to iteratively assess and revise photographs. A total of 46 photographs demonstrated CV; 26 demonstrated CV after revisions; 3 did not demonstrate CV after revisions. Content analysis revealed thematic codes describing participants' photograph preferences including image quality, context, subject diversity, and relevance to long-term care. Discussion will include implications for clinicians and researchers on how to evaluate and improve CV of photo-supported verbal assessments.

### INSTRUMENTS TO ASSESS PREFERENCES FOR EVERYDAY LIVING OF OLDER ADULTS WITH VARIOUS CARE NEEDS: AN EVIDENCE MAP

Daniel Purwins,[1] Kimberly Van Haitsma,[2] Katherine Abbott,[3] Martina Roes,[4] and Mike Rommerskirch-Manietta,[5], *1. DZNE, Witten, Nordrhein-Westfalen, Germany, 2. The Pennsylvania State University, University Park, Pennsylvania, United States, 3. Miami University, Oxford, Ohio, United States, 4. German Center for Neurodegenerative Diseases (DZNE), Witten, Nordrhein-Westfalen, Germany, 5. DZNE, German Center for Neurodegenerative Diseases, Nordrhein-Westfalen, Germany*

**Background:** Instruments to identify and assess preferences for everyday living are important tools for health professionals. For research purposes, they appear equally essential, for example, to develop new care approaches based on the preferences of the older adults. So far, it seemed unknown which instruments already exist to identify and assess preferences for everyday living.

**Method:** We conducted an evidence map to identify instruments, to understand how preferences are assessed and which instruments are focusing everyday living.

**Results:** We plotted our results in the form of a bubble plot. We identified instruments that map multiple domains (e. g. function and leisure activities) or only one topic (e. g. food, personal hygiene or brightness). Preferences are assessed using direct questions, frequencies, sorting, stimuli, or even scores. Our results show the variety of how preferences are defined and the range of instruments to assess preferences for everyday living of older adults.

### PREFERENCES OF OLDER IMMIGRANTS IN NURSING CARE: A SCOPING REVIEW

Huerrem Tezcan-Guentekin,[1] Martina Roes,[2] and Viktoria Peters-Nehrenheim,[3] *1. ASH, Berlin, Berlin, Germany, 2. German Center for Neurodegenerative Diseases (DZNE), Witten, Nordrhein-Westfalen, Germany, 3. DZNE, German Center for Neurodegenerative Diseases, Nordrhein-Westfalen, Germany*

**Background:** Worldwide, our societies are characterized by an increasing diversity, greatly contributed by immigrants. When in need of care older immigrants face various barriers and serious challenges in terms of unmet preferences. To provide person-centered care, health care professionals need to consider the personal background of immigrants to identify and assess their individual preferences.

**Objective:** To understand how preferences of older immigrants in nursing care are defined and how they can be assessed.

**Methods:** A scoping review will be conducted to identify and analyze preferences of older immigrants across health care settings.

**Preliminary results:** The literature search revealed that older immigrants in need of care define their preferences in terms of expectations and priorities. Differences among immigrants are related to the age of the person at the time of immigration, on cultural differences and/or on how the concepts of preferences in the country of origin is understood.

### Session 2515 (Paper)

### WELL-BEING IN OLDER ADULTS

### COMPARING NIGHTTIME TO RUSH HOUR AVOIDANCE AS INITIAL DRIVING SELF-REGULATION BEHAVIORS

Jonathon Vivoda,[1] Lisa Molnar,[2] David Eby,[2] Carolyn DiGuiseppi,[3] Vanya Jones,[4] Guohua Li,[5] Thelma Mielenz,[5] and David Strogatz,[6], *1. Miami University, Oxford, Ohio, United States, 2. University of Michigan Transportation Research Institute, Ann Arbor, Michigan, United States, 3. University of Colorado Denver, Aurora, Colorado, United States, 4. Johns Hopkins University, Baltimore, Maryland, United States, 5. Columbia University, New York, New York, United States, 6. Bassett Research Institute, Cooperstown, New York, United States*

Aging is associated with an increase in avoidance of challenging driving situations (e.g., driving at night, during rush hour, on freeways, and in unfamiliar areas). Such avoidance behavior may be due to driving self-regulation (SR), an intentional response to perceived declining abilities, or it may be due to other factors such as lifestyle changes or preferences. Most previous research has not studied SR as the reason for avoidance, and has treated avoidance behaviors interchangeably. In addition, previous research has not differentiated one's first SR behavior from those reported later in the process. This study included 1,557 participants from the AAA Longitudinal Research on Aging Drivers (LongROAD) to assess older adults' initial self-regulatory behavior by comparing the frequency of nighttime, rush hour, freeway, and unfamiliar area avoidance among those who reported only one SR behavior. Nighttime SR was most common (58.8%), followed by rush hour (25.5%), unfamiliar areas (11.0%), and freeways (4.8%). Binary logistic regression was used to assess how demographics, function, and self-reported driving variables were related to different odds of reporting nighttime vs. rush hour avoidance (the two most common) as one's initial SR behavior. Higher odds of reporting nighttime avoidance (compared to rush hour) as one's initial SR behavior were related to female gender, low income, impaired visual acuity, better self-reported ability to see during the day, worse self-reported ability to see at night, less comfort driving at night, and more comfort driving during rush hour, and in unfamiliar areas.

### COMPARING YOUNG AND OLD ADULTS' NIGHT HAZARD DETECTION WITH DRIVING SIMULATION AND ON ROAD

Anne Dickerson, and Juliette Leonardo, *East Carolina University, Greenville, North Carolina, United States*

While there is validity of using driving simulation as a proxy for on-road performance, few studies have examined hazard detection at night. Night driving is a self-restricting practice with little evidence demonstrating the need with healthy older adults. This study's objective was to analyze night driving using eye-tracking technology examining differences between on-road/simulated drives and older/younger adults. A 2 (old, young) x 2 (simulator, on-road) repeated-measures design measured three roadway "hazards" of pedestrains looking at their cell phone while posed to cross the roadway. Pupil glances were recorded using outcome measures of total fixation duration, number of fixations, and time-to-first fixation for the pedestrains on-road and on a specifically designed scenario matching the on-road route. Thirty-three healthy, community-living drivers age 65+ years (N=16) and drivers age 20-40 years (N=17) completed both drives. Using non-parametric statistics, results demonstrated that night hazard detection was similar across driving conditions except for time-to-first fixation, which was faster on-road for both age groups ($p<.001$). At some hazard locations, there were significant differences between the two age groups, with older adults taking longer to initially see hazards. Results suggest, older adults detected hazards similarly to younger adults, especially during on-road performance, suggesting avoidance of night driving may not be necessary. Results also support using driving simulation as a proxy for on-road with night driving needing to be incorporated. Additionally, eye-tracking has the potential for research in hazard detection with emphasis on the time-to-first fixation outcomes when considering driving analysis.

### FACTORS AFFECTING COGNITIVE DYSFUNCTION SCREENING FOR LATINX ADULTS WITH TYPE 2 DIABETES

Heather Cuevas,[1] Julie Zuñiga,[1] and Stephanie Morgan,[2], *1. The University of Texas at Austin, The University of Texas at Austin/Austin, Texas, United States, 2. The University of Texas at Austin, Austin, Texas, United States*

Before development of overt type 2 diabetes (T2DM), changes in brain structure and activation patterns are found in insulin resistance, indicating many with T2DM may already have alterations in cognitive function. How best practices are met for screening for cognitive dysfunction, specifically Latinx adults with T2DM who are at higher risk, remains unclear. The purpose of this study was to examine aspects influencing screening Latinx adults with T2DM for cognitive problems by identifying patient-, clinician- and clinic- level factors. This was a mixed methods study which used semi-structured interviews with Latinx adults with T2DM (n=30; mean age: 68; 57% Mexican American); surveys and interviews with health care providers (n = 15); and inventories of four outpatient clinics to identify factors (e.g. time, clinic policies) influencing screening. Data were analyzed via thematic analysis (interviews) and descriptive statistics (surveys and inventories). For patients, screening was important, but inability to work related to a possible diagnosis of dementia was a concern. Providers and patients agreed other health issues (e.g. hyperglycemia) took precedence to screening. Providers (96.7%) were expected to screen but did not have support/time from clinics and relied on patients for initial prompts. Only one clinic reported staff education on cognitive screening with an emphasis on potential cultural differences in test results and adequate resources related to dementia for Latinx adults. Clinics serving Latinx adults have a responsibility to deliver appropriate care. Leadership should consider innovative practices such creation, with patients, of educational materials for screening -a need highlighted by most participants.

### Session 3000 (Symposium)

### BENEFITS AND BARRIERS TO EMERGING TECHNOLOGIES TO PROMOTE HEALTH, WELL-BEING, AND INDEPENDENCE OF OLDER ADULTS

Chair: Walter Boot

Emerging technologies, such as voice assistant systems and artificial companion robots, hold a great deal of promise for improving the health, wellbeing, and independence of older adults. However, these solutions will likely be ineffective in the absence of research to understand barriers to the adoption and use of these technologies and without an exploration of the needs and preferences of older adults. This symposium focuses on both the potential of such technologies and factors that may affect their success. H. Spangler will present a detailed analysis of privacy concerns of older adults, with and without cognitive impairment, related to the use of Voice Assistant Systems (VAS). R. Nicholson will discuss the potential of a VAS app for promoting exercise among older adults and their caregivers to enhance mobility independence, with a focus on perceived benefits and dislikes about the app that may impact use. Finally, C. Berridge will present an exploration of perceptions of and attitudes toward artificial companion (AC) robots across the lifespan, before and after the start of the COVID-19 pandemic, including concerns about privacy. Together, these talks will highlight novel methods through which emerging technologies can support older adults and issues to consider if these methods are to produce meaningful change.

### PRIVACY CONCERNS AMONG OLDER ADULTS USING VOICE ASSISTANT SYSTEMS

Tiffany Driesse,[1] Robert Roth,[2] Xiaohui Liang,[3] David Kotz,[4] John Batsis,[5] and Hillary Spangler,[6] *1. UNC School of Medicine, Chapel Hill, North Carolina, United States, 2. Dartmouth-Hitchcock, Lebanon, New Hampshire, United States, 3. University of Massachusetts Boston, Boston, Massachusetts, United States, 4. Dartmouth College, Hanover, New Hampshire, United States, 5. University of North Carolina at Chapel Hill, Chapel Hill, North Carolina, United States, 6. UNC Hospitals, Chapel Hill, North Carolina, United States*

Voice Assistant Systems (VAS) are software platforms that complete various tasks using voice commands (e.g., Amazon Alexa), with increasing usage by older adults. It is unknown whether older adults have significant privacy concerns with VAS. 55 participants were evaluated from ambulatory practice sites for a study on VAS detection of early cognitive decline. The mean age was 73.3±5.6 years, 58% female, 93% white, and 53% had mild cognitive impairment. Privacy concerns were assessed via Likert-based surveys. Participants believed data was used with consent (71%) and stored properly (67%); however, 71% wanted new privacy regulations, 43% were comfortable with daily activity monitoring, and 85% thought the data needs to be highly protected. Qualitative themes included "listening-in", "tracking", and unwanted sharing of information. Findings suggest that older adults do not have significant privacy concerns for VAS use, but requested additional regulations. Future research can compare VAS privacy concerns between age groups.

### OLDER ADULT USER FEEDBACK ON DESIGN AND FUNCTIONALITY OF ENGAGE, A VOICE-ACTIVATED EXERCISE PROGRAM

Yadira Montoya,[1] Saira Shervani,,[2] Chelsea Smith,[2] Louise Hawkley,[1] Megan Huisingh-Scheetz,[3] and Roscoe Nicholson,[4] *1. NORC at the University of Chicago, Chicago, Illinois, United States, 2. University of Chicago, chicago, Illinois, United States, 3. University of Chicago, Chicago, Illinois, United States, 4. University of Chicago, Evanston, Illinois, United States*

The EngAGE Alexa app is a socially motivated exercise program targeting older adult-caregiver dyads to promote mobility independence. EngAGE provides exercise routines that older adults can perform in the home in conjunction with a messaging component to facilitate motivation from caregivers and a tracking component to monitor progress. This presentation will describe the qualitative results that have informed the app's design and evaluation of its feasibility and functionality following a 14-week feasibility study in 10 dyads of older adult exercisers and their caregivers. The presentation will cover the perceived benefits of EngAGE's older adult users (including "real world" clinically relevant improvements, the comprehensiveness of the exercises, and exercise knowledge gained), as well as likes and dislikes that contributed to our assessment of the app's functionality. Finally, we will discuss how the feedback contributes to future directions in the development of the app's features, supporting materials, design and content.

### COMFORT AND DATA SHARING WITH ARTIFICIAL COMPANION ROBOTS AMONG AN ONLINE COHORT

Yuanjin Zhou,[1] Julie Robillard,[2] Nora Mattek,[3] Sarah Gothard,[3] Jeffrey Kaye,[3] and Clara Berridge,[1] *1. University of Washington, Seattle, Washington, United States, 2. University of British Columbia, Vancouver, British Columbia, Canada, 3. Oregon Health & Science University, Portland, Oregon, United States*

Results from a June 2020 survey on comfort with two forms of artificial companion (AC) robots in normal compared with pandemic times will be presented. 1,082 adults age 21-92 (mean 64) completed the online survey for a response rate of 45%. Significantly greater comfort is reported with small AC robots relative to larger human-shaped robots in both normal and pandemic times. In bivariate and adjusted models, younger age and male gender were most commonly associated with greater comfort with AC robots. Most participants (68.7%) did not think an AC robot would make them feel less lonely. About half (52.8 %) of the participants reported that they probably or definitely would want their facial expressions to be read, while a minority (15.0%) were at least somewhat comfortable with AC robots recording their conversations. The most common person participants wanted these data types shared with is themselves, a spouse/partner, and medical provider.

### Session 3005 (Symposium)

### BRIDGING AGE-FRIENDLY HEALTH SYSTEMS INTO THE COMMUNITY

Chair: Erin Emery-Tiburcio Discussant: Rani Snyder

As the Age-Friendly Health Systems initiative is implemented across the country, building bridges into the community assures that older adults live safely, enjoy good health and stay involved in their communities. In this symposium, we present innovative approaches that bridge the Age-Friendly Health Systems initiative into the community. Each presentation explores how the 4Ms concepts are integrated into their programs and enhance community with older adults. CATCH-ON Connect provides free cellular-enabled tablets and individual training to older adults to help them do What Matters to them by using their tablets. Rush@Home is a home-based primary care program that addresses the 4Ms in the comfort of an older adult's home. Social Connections was co-designed with older adults to decrease loneliness by connecting older adults to each other. The Caregiver Initiative identifies and supports caregivers of older adults by meeting the 4Ms health needs of both caregiver and care recipient. Dementia Friendly Communities engage community stakeholders in a process to become educated, creating safe and respectful environments for individuals with dementia. By exploring these approaches, we can bridge the Age-Friendly Health Systems initiative into the community to support older adults outside the four walls of the health system.

### CO-DESIGNING A SOCIAL CONNECTIONS PROGRAM WITH OLDER ADULTS

Jasmine Chandy,[1] Padraic Stanley,[1] Grisel Rodriguez-Morales,[1] and Erin Emery-Tiburcio,[2] *1. Rush University Medical Center, Chicago, Illinois, United States, 2. Rush University, Chicago, Illinois, United States*

Loneliness presents a higher risk for mortality than smoking 15 cigarettes per day. COVID-19 has exacerbated loneliness for many older adults, without access to family, friends, and community. Friendly caller programs utilizing volunteers to talk with older adults who are lonely can be helpful, providing much-needed contact. However, few lasting connections have formed in these programs. To enhance our social connections program, we systematically engaged a group of older adults who struggle with social isolation to co-design a program to meet their needs. This group met virtually twice for two hours to (1) identify contributors to their isolation, generate ideas for ideal program components, and how best to connect older adults to each other; and (2) to refine the multi-component program created by staff based on the first discussion. Group process and themes will be presented, along with a discussion of key issues in program co-design with older adults.

### CATCH-ON CONNECT: A PROGRAM TO INCREASE TECHNOLOGY ACCESS AMONG OLDER ADULTS DURING COVID-19

Erin Emery-Tiburcio,[1] Robyn Golden,[2] Salvador Castaneda,[2] Michelle Newman,[2] and Janis Sayer,[2]

*1. Rush University, Chicago, Illinois, United States, 2. Rush University Medical Center, Chicago, Illinois, United States*

During the COVID-19 pandemic, technology became an essential tool to maintain connections to social support, health professionals, and services. However, many older adults do not have access to technology or do not feel comfortable using it. CATCH-ON Connect provides cellular-enabled tablets and individual, personalized technical assistance to older adults. Adults age 65+ in project partner primary care clinics who do not have an internet-ready device or who lack digital literacy are eligible to participate. Older adults learn how to access their electronic health record portal, use pre-installed apps (e.g., Lyft, Zoom), and receive education about COVID-19 and the 4Ms. Of the 40 participants enrolled to date, 46% have never accessed the internet with a tablet or smartphone. Initial qualitative outcomes indicate high satisfaction and increased electronic socialization. Quantitative results of participant technology challenges, loneliness, and utilization of telehealth services will be discussed.

### DEMENTIA-FRIENDLY AMERICA RECOGNITION: STRATEGIES FOR URBAN, AFRICAN AMERICAN COMMUNITIES

Susan Frick,[1] Raj Shah,[2] and Tarisha Washington,[1] *1. Rush University Medical Center, Chicago, Illinois, United States, 2. Rush, Rush University Medical Center, Illinois, United States*

Dementia-Friendly America is a network of communities across the United States who have committed to a process to support people living with dementia and their caregivers. Through technical support from Dementia Friendly Illinois, CATCH-ON, a HRSA Geriatric Workforce Engagement Program, has identified key characteristics for the 17 communities in Illinois achieving national recognition and for communities that have engaged but not yet achieved national recognition. In addition to communities in rural regions, urban communities with a large number of African Americans residents have necessitated more grassroots engagement than other communities. Partnerships are vital for providing information and education about the movement and for supporting multi-sectoral engagement. This presentation highlights barriers and facilitators in diverse communities, particularly urban African American communities, becoming recognized by Dementia Friendly America.

### THE CAREGIVER INITIATIVE: A MODEL FOR CAREGIVER HEALTH AND WELLNESS IN AGE-FRIENDLY HEALTH SYSTEMS

Robyn Golden,[1] Leslie Pelton,[2] and Ellen Carbonell,[1]
*1. Rush University Medical Center, Chicago, Illinois, United States, 2. Institute for Healthcare Improvement, Boston, Massachusetts, United States*

Health care systems have historically relied on family caregivers to provide long-term care for older adults, often including medically complex care for which they have no medical training. Yet there are few interventions in place to assist them within health care systems. The Rush Caregiver Initiative (Rush-CGI) provides system-level and caregiver interventions. Rush-CGI's system-level interventions focus on culture change, including modifying workflows, training, and electronic health record data. Rush-CGI caregiver interventions begin with assessment of needs and provision of resources for caregivers, offering interprofessional family-based interventions. Interventions include a Teach-Back Clinic, Family Care Planning sessions, and Goals of Medical Care meetings, all held on an outpatient basis either in person or virtually. Outcomes include decreased caregiver depression and anxiety, and increased caregiving self-efficacy. This presentation will discuss creating system level change and providing customized caregiver interventions, including how the Rush-CGI can be modified to fit a variety of patient populations.

### RUSH@HOME: HOME-BASED PRIMARY CARE FOCUSED ON UNDERSERVED COMMUNITIES AND HEALTH EQUITY

Alexander Rackman, Elizabeth Davis, Leticia Santana, Robyn Golden, and Walter Rosenberg, *Rush University Medical Center, Chicago, Illinois, United States*

Homebound patients are often medically complex and are among those in greatest need of care and services. This is especially true for those that reside in underserved communities, where they face the added risk stemming from scarce community resources. Often these patients are only able to access health care for emergencies, which is ineffective and high cost. Rush@Home is a home-based primary care program that exemplifies the Age-Friendly Health System mission with a focus on the 4Ms, incorporating navigation and social work. Patients reflect the West Side of Chicago, with 80% of patients identifying as Black and/or Latino. During the first two years, Rush@Home demonstrated better care at a lower cost with readmission rates decreased by 11.8%, hospitalizations by 17.5%, length of stay by 8.7%, ED visits by 17.9%, and missed appointments by 72%. This presentation will highlight outcomes and discuss key issues in home-based primary care.

### Session 3010 (Symposium)

### BRIDGING THE FAMILY CARE GAP

Chair: Joseph Gaugler Discussant: Richard Schulz

This symposium aims to create a scientific and policy roadmap to offset the impending shortage of family caregivers available to assist older adults in the U.S. (i.e., the "family care gap"). Drawing on public health, cultural frameworks, family care science, and policy analysis, this symposium will orient future research, intervention development, dissemination and implementation, and policy innovation to more effectively address the family care gap. The selected presentations will include the need to apply and understand cultural adaptation and humility to support a rapidly diversifying older population (Drs. Nkimbeng and Parker). In addition, systematic review methodology will be applied to obtain insights as to what intervention models/strategies actually reduce caregiving time (Drs. Baker, Jutkowitz, and Gaugler). The next presentation will leverage the existing evidence base of translational efforts that aim to disseminate and implement dementia caregiver interventions into practice (Drs. Hodgson and Gitlin). The final presentation of our symposium will focus in-depth on a potential solution to the family care gap: more systematic approaches to identifying and assessing family caregivers in healthcare systems (Drs. Riffin and Wolff). Our discussant, Dr. Richard Schulz, will bring his extensive and renowned experience in caregiving to summarize the public health and policy implications of the family care gap.

### DIVERSE, CULTURALLY RICH APPROACHES TO FAMILY CARE IN THE UNITED STATES

Lauren Parker,[1] and Manka Nkimbeng,[2] *1. Johns Hopkins Bloomberg School of Public Health, Baltimore, Maryland, United States, 2. University of Minnesota, Minneapolis, Minnesota, United States*

Despite the projected rise in the diversity of caregivers and caregiving in the US, the health system is not prepared to accommodate this growth. Interventions and supports often are not adequately tailored to meet the cultural needs of older adults. Additionally, the limited interventions available for racial/ethnic minority populations frequently fail to capture and report culturally tailored perspectives. Therefore, the purpose of this presentation is to describe how culture influences caregiving in the US. Specifically, it will: (1) provide a contemporary definition of culture; (2) identify cultural domains that impact caregiving; (3) offer examples of how caregiving is influenced by different cultural/demographic backgrounds; (4) provide examples of culturally tailored caregiving programs, and (5) discuss how to approach cultural needs that may not be addressed by current interventions.

### A SYSTEMATIC REVIEW OF INTERVENTIONS THAT REDUCE FAMILY CAREGIVING TIME

Eric Jutkowitz,[1] Joseph Gaugler,[2] and Zachary Baker,[3], *1. Brown University, Brown University, Rhode Island, United States, 2. University of Minnesota, Minneapolis, Minnesota, United States, 3. University of Minnesota, University of Minnesota/Minneapolis, Minnesota, United States*

Due to multiple long-term sociodemographic and health trends contributing to the impending family care gap, there likely is no single policy or intervention that could increase the number of family caregivers in the U.S. to the levels required to fill such a gap. However, the amount of time that a family caregiver spends providing assistance is potentially mutable. Given the pressing concerns of the family care gap, identifying interventions or approaches that could reduce existing caregiving time is of considerable importance. This presentation provides the results of a systematic review of published research to identify the effects of interventions on the amount of time family caregivers spend on their caregiving tasks. Pharmaceutical approaches directed to care recipients, technology interventions, case management, multicomponent interventions, and care settings all appeared to reduce caregiving time. Improved operationalization, study design, and similar factors will help guide future intervention research to reduce caregiving time.

### IMPLEMENTING AND SUSTAINING FAMILY CARE PROGRAMS IN REAL-WORLD SETTINGS: BARRIERS AND FACILITATORS

Nancy Hodgson, *University of Pennsylvania, School of Nursing, philadelphia, Pennsylvania, United States*

This presentation will summarize the extant published studies on the translation of proven family care programs for dementia in different care settings. This review is the first to our knowledge to examine the specific implementation efforts deployed in care settings for different family caregiver programs. In this review, we sought to answer three basic questions: (1) What theory base(s) or conceptual framework(s) guided the implementation of evidence-based family care programs?; (2) What implementation strategies were used to support translation into practice?; and (3) What were the identified drivers of and barriers to organizational change required for adoption of an evidence-based program? Understanding the frameworks and strategies deployed in translational studies published to date can help guide future translation efforts, inform the design of new family caregiver support programs that optimize their implementation potential, and ultimately help to minimize the "family care gap."

### IDENTIFYING, ASSESSING, AND SUPPORTING FAMILY CAREGIVERS IN HEALTH AND LONG-TERM CARE: PROGRESS AND OPPORTUNITIES

Jennifer Wolff,[1] and Catherine Riffin,[2], *1. Johns Hopkins Bloomberg School of Public Health, Baltimore, Maryland, United States, 2. Weill Cornell Medicine, New York, New York, United States*

Family caregivers are a largely hidden but vital workforce within medical and long-term care settings. Family caregivers are actively involved throughout care delivery systems and provide crucial assistance to people with chronic conditions. Building on the person- and family-centered care approach and recent recommendations from national organizations, this presentation sets forth a roadmap for research, policy, and practice that outlines practical solutions and opportunities to address existing barriers to systematic assessment and support of family caregivers in clinical practice. With the impending family care gap and projections for a steep decline in the availability of family caregivers in the coming decades, it is more important than ever to prepare health care systems for this shift. If put into action, the recommendations of this presentation can help to bridge the care gap by promoting sustainable solutions and infrastructure to ensure that families are recognized and adequately supported in care delivery settings.

### Session 3015 (Symposium)

### DELIVERING PERSON-CENTERED CARE DURING A PANDEMIC: STAKEHOLDER PERSPECTIVES

Chair: Katherine Abbott Discussant: Kirsten Corazzini

Person-centered care (PCC) is an approach to care that both nursing homes (NH) and assisted living (AL) communities strive to provide. PCC is a philosophy that recognizes knowing the person and honoring individual preferences. However, when COVID-19 emerged, the NH and AL environments were ground zero for infection spread and disproportionate numbers of deaths among residents. As a result, many practices changed dramatically in efforts to reduce the transmission of COVID-19 in these communities. The purpose of this symposium is to discuss several projects that can speak to the impact of the pandemic on stakeholder efforts to provide PCC. First, Dr. Roberts presents feedback from residents and family members on the challenges COVID-19 created for family involvement in care conferences. In the second study, Dr. Behrens examines focus group data from direct-care nurses on their perceptions of delivering PCC related to risk of harm to staff and residents. The third study presents the voices of activities professionals who were implementing a PCC quality improvement project to communicate resident preferences, which illustrates both the importance of PCC during the pandemic, but also the challenges implementing during the pandemic. Fourth, the Kansas PEAK 2.0 program used provider feedback to direct and inform program responses through components such as consistent staffing. Finally, Dr. Zimmerman presents qualitative data from over 100 AL administrators, medical, and mental health care providers on their experiences pivoting during COVID-19. Our discussant will explore the implications of these studies in terms of the future of PCC in residential settings.

### WE'RE STILL VERY MUCH IN LIMBO: PROVIDERS' PERSPECTIVES ON IMPLEMENTING A PERSON-CENTERED PROJECT

Megan Kelley,[1] Alex Heppner,[2] Kimberly Van Haitsma,[3] Katherine Abbott,[1] and Miranda Corpora,[1], *1. Miami University, Oxford, Ohio, United States, 2. Scripps Gerontology Center, Oxford, Ohio, United States, 3. The Pennsylvania State University, University Park, Pennsylvania, United States*

**Background:** The Preferences for Everyday Living Inventory assists nursing home (NH) providers in assessing residents' preferences and can be used to make Preferences for Activity and Leisure (PAL) Cards, which are personalized 5x7 laminated cards that reflect a resident's recreation and leisure preferences. We sought to understand the barriers and facilitators to implementing PAL Cards in NH communities during COVID-19.

**Methods:** NH providers from five states (n=29) were recruited to create PAL Cards in their communities. Monthly coaching calls with project champions assessed the implementation process. Calls were recorded, transcribed verbatim, checked for accuracy, and coded via thematic analysis.

**Results:** Four themes emerged: Adapting to COVID, Preoccupied with COVID, Future Thinking, and PAL Cards Filling the Gaps.

**Conclusion:** Some providers were successful adapting to COVID, while others struggled to implement a new program during the pandemic. Those that were successful expressed how PAL Cards helped promote person-centered care.

### NURSING STAFF PERCEPTIONS OF RISK OUTCOMES IN DELIVERING PREFERENCE-BASED PERSON-CENTERED CARE

Marie Boltz,[1] Ann Kolanowski,[2] Mark Sciegaj,[3] Katherine Abbott,[4] Caroline Madrigal,[5] Kimberly Van Haitsma,[6] and Liza Behrens,[7], *1. Pennsylvania State University, University Park, Pennsylvania, United States, 2. Penn State, University Park, Pennsylvania, United States, 3. Penn State University, University Park, Pennsylvania, United States, 4. Miami University, Oxford, Ohio, United States, 5. Providence VA Medical Center, Providence VA Medical Center, Rhode Island, United States, 6. The Pennsylvania State University, University Park, Pennsylvania, United States, 7. University of Pennsylvania School of Nursing, Philadelphia, Pennsylvania, United States*

Effective management of the perceived risks associated with delivering preference-based person-centered care (PBPCC) is historically challenging for nursing home staff. Existing research lacks the granularity needed to guide clinicians who fear negative health and safety outcomes for residents. This study examined direct-care nursing staff perceptions of outcomes associated with delivering PBPCC. Participants (N=27) worked in NHs experiencing 6-12 health citations, were mostly female (85%), and represented diverse ages, race, education, and collective work experience in NHs. Content analysis of verbatim transcripts from 12 focus groups identified an overarching theme of: "person-centered outcomes related to risk engagement"; and sub-themes of: harms to staff (e.g. fear, frustration, guilt); harms to residents (e.g. negative moods and behaviors, physical discomfort); and positive shared outcomes (e.g. building nurse-resident relationships, positive care environment). Implications for risk management that improves quality of care and life outcomes in a post-COVID era will be discussed.

### RESIDENT AND FAMILY ENGAGEMENT IN CARE CONFERENCES: IMPORTANT PROCESSES AND SUPPORTING STRATEGIES

Elizabeth Cox,[1] Thuy Dan Tran,[1] Hannah Tepsa,[1] and Tonya Roberts,[2], *1. University of Wisconsin, Madison, Wisconsin, United States, 2. University of Wisconsin - Madison, University of Wisconsin - Madison, Wisconsin, United States*

Self-determination is a core value of person-centered care. Research has shown residents and families want to be involved in decisions about care. Care conferences are one existing structure where residents and families can engage in decision-making about care goals. However, there are few tools to support effective engagement. To inform future tool development, this study sought to understand what resident and family stakeholders value about engaging in care conferences. In virtual meetings, 16 stakeholders identified 3 key areas of engagement: being informed about health/well-being, influencing care goals, and advocating for needs. They indicated current approaches do not achieve these engagement goals, which is particularly problematic during COVID when families cannot engage in person. Stakeholders offered ideas for supporting engagement such as provision of data before the conference. The study has implications for individualizing care conferences and encouraging resident and family engagement in decision-making both during and beyond COVID.

### COVID-19 AND PERSON-CENTERED CARE: LESSONS LEARNED THROUGH A STATEWIDE PROGRAM FOR NURSING HOMES

Migette Kaup, and Laci Cornelison, *Kansas State University, Manhattan, Kansas, United States*

Frail elders in nursing homes are the highest risk group for developing complications of COVID-19. This lead to a response from CMS and state regulators that was heavily focused on protection and safety through segregation and infection control. The purpose of this study was to gather the narrative of this pandemic response and understand the impact on person-centered care and be able to address provider needs in real-time. This qualitative method focused on nursing home providers who are a part of PEAK 2.0, a Medicaid pay-for-performance program in Kansas. Interviews with nursing home staff (n=168) revealed two critical themes of need; mandated responses disregarded elders' autonomy and self-determination in decision making, and infection control strategies required new approaches to facets of resident care that still maintained dignity. This data, along with COVID-19 guidance were then used to inform feasible resource development and education to maintain PCC practices during the pandemic.

### COVID-19 PERSPECTIVES OF ASSISTED LIVING AND HEALTH CARE PROVIDERS IN SEVEN STATES

Philip Sloane,[1] Johanna Hickey,[2] Kali Thomas,[3] Julia Thorp,[2] Meredith Poole,[2] Aisha Chaudhry,[2] Paula Carder,[4] and Sheryl Zimmerman,[5] *1. UNC Medical School, Sheps Center, Chapel Hill, North Carolina, United States, 2. University of North Carolina at Chapel Hill School, Chapel Hill, North Carolina, United States, 3. Brown University, Brown University/Providence, Rhode Island, United States, 4. OHSU-PSU School of Public Health, Portland, Oregon, United States, 5. Cecil G. Sheps Center for Health Services Research, Chapel Hill, North Carolina, United States*

Thirty percent of COVID-19 deaths in long-term care were in assisted living (AL), indicating challenges providing care. This project recruited AL administrators and medical and mental health care providers in a seven-state stratified random sample of 250 communities; it asked what was most challenging responding to COVID-19, what was successful, how to have better dealt with COVID-19, and how others could have helped. The most common challenge was addressing residents' psychosocial needs, explained as "No contact - no hugging. The seniors require touch. It's something we've always done, and we can't do; we're required not to do it." Successes included infection prevention, and in hindsight, administrators discussed staffing. Related to external entities, one commented, "Come in the building and see what we're doing. Don't sit behind a freaking screen and act like you know what we're doing." Providers stressed patient access to care and social isolation. Implications will be discussed.

### Session 3020 (Symposium)

### DISRUPTION TO TRANSFORMATION: AGING IN THE NEW NORMAL: A CHAT WITH NIA SENIOR LEADERSHIP

Chair: Melinda Kelley Discussant: Melinda Kelley

The National Institute on Aging (NIA) at the National Institutes of Health, Department of Health and Human Services, is the federally designated lead agency on aging research and supports significant research on aging as a lifelong process. In the last six years, NIA has experienced a tripling of its budget. Although much of this funding is targeted to Alzheimer's disease (AD) and AD-related dementias research, there has been an increase in funds allocated to non-AD research in keeping with the overall growth of NIH. This symposium will provide a forum for exploration of the implications of the budget increases for the general research community. NIA's senior staff will discuss research priorities and programs supported by the Institute. A question-and-answer session will follow these remarks on current funding and future priorities and research directions of NIA.

### NIA DIVISION OF EXTRAMURAL ACTIVITIES

Kenneth Santora, *National Institute on Aging, Bethesda, Maryland, United States*

Dr. Santora will discuss the work of the NIA Division of Extramural Activities. Dr. Santora will also be available for small group discussion.

### NIA OFFICE OF SPECIAL POPULATIONS

Patricia Jones, *National Institute on Aging, Bethesda, Maryland, United States*

Dr. Jones will discuss the work of the NIA Office of Special Populations. Dr. Jones will also be available for small group discussion.

### OVERVIEW OF NIA BUDGET

Richard Hodes, *National Institute on Aging, Bethesda, Maryland, United States*

Dr. Hodes will discuss budget and overall research priorities for the National Institute on Aging.

### NIA DIVISION OF AGING BIOLOGY

Ronald Kohanski, *National Institute on Aging, Bethesda, Maryland, United States*

Dr. Kohanski will discuss research priorities for the Division of Aging Biology. Dr. Kohanski will also be available for small group discussion.

### NIA DIVISION OF GERIATRICS AND CLINICAL GERONTOLOGY

Evan Hadley, *National Institute on Aging, Bethesda, Maryland, United States*

Dr. Hadley will discuss research priorities for the Division of Geriatrics and Clinical Gerontology. Dr. Hadley will also be available for small group discussion.

### NIA DIVISION OF NEUROSCIENCE

Eliezer Masliah, *National Institute on Aging, Bethesda, Maryland, United States*

Dr. Masliah will discuss research priorities for the Division of Neuroscience. Dr. Masliah will also be available for small group discussion.

### NIA DIVISION OF BEHAVIORAL AND SOCIAL RESEARCH

Lisbeth Nielsen, *National Institute on Aging, Bethesda, Maryland, United States*

Dr. Nielsen will discuss research priorities for the Division of Behavioral and Social Research. Dr. Nielsen will also be available for small group discussion.

### RISING STAR EARLY-CAREER FACULTY AWARD LECTURE AND CYBER-PEDAGOGY PANEL DISCUSSION

Kara Dassel,[1] and Candace Brown,[2] *1. University of Utah, Salt Lake City, Utah, United States, 2. Duke University, Durham, North Carolina, United States*

The Rising Star Early-Career Faculty Award lecture will feature an address by 2021 recipient Candace S. Brown, PhD,

MA, MEd, of the University of North Carolina, Charlotte. The Rising Star Early-Career Faculty Award acknowledges new faculty whose teaching and leadership stand out as influential and innovative. This event will also feature a panel discussion led by the AGHE Awards Review Panel titled, "Cyber-Pedagogy to the Rescue: Creating Effective Online Programming for Students and Trainees During the Pandemic."

### CYBER-PEDAGOGY TO THE RESCUE: CREATING EFFECTIVE ONLINE PROGRAMMING FOR STUDENTS AND TRAINEES DURING THE PANDEMIC

Kara Dassel, *University of Utah, Salt Lake City, Utah, United States*

COVID-19 upended in-person educational programming in areas such as classroom instruction within academic institutions, engagement of adult learners, and training of direct care workers. In-person educational offerings were forced, as a result of health restrictions, to pivot into either asynchronous or synchronous web-based instruction. This panel discussion will discuss lessons-learned in cyber-pedagogy in three areas: 1) Faculty Consultation: Faculty who teach online regularly and have completed training in quality online educational practices are experts who were called upon to assist others with transitioning courses to an online format. This presentation outlines the ways in which certified online instructors, at one academic center, tutored and assisted faculty in the health sciences with online instruction. 2) Adult Learning: The rapid transition to online learning has implications for adult learners pertaining to accessibility, diverse learning and technology abilities, and course and peer engagement. This presentation will explore strategies that faculty can utilize to offer adult learners differentiated learning and engagement opportunities. This discussion will also highlight the nexus among these pedagogical strategies and the Age-Friendly University Global Network, providing guidance for how universities can connect online learning methods to the Age-Friendly University principles. 3) Workforce Training: The direct care workforce employed in community-based services and support programs and long-term care settings tend to receive little or no training in geriatric care. This presentation will discuss how educational trainings offered through a Geriatric Workforce Enhancement Program transitioned onto web-based platforms in order to accommodate these ongoing educational needs throughout the pandemic.

### CREATING 'THIS IS IT' MOMENTS IN GERONTOLOGICAL EDUCATION

Candace Brown, *Duke University, Durham, North Carolina, United States*

We all have had "this is it" moments. And while many gerontologists will say they "fell" into the field, I solicit that educational paths are set before us and we have choice to which path that we take. Paths provide opportunities for discoveries about ourselves and in the case of gerontology, we have a breadth of choices. This is how I view teaching and why developing new courses in the field are a necessity. Change is constant; creating new paths for opportunities is optional. This lecture will provide how, as an educator, I accept my 'this is it' moments so I can effectively foster students to recognize their own 'this is it' moment(s) within gerontological education.

### Session 3030 (Paper)

### DIVERSITY, RACIAL MINORITIES, AND AGING II

### BINGE DRINKING AND HEAVY DRINKING AMONG OLDER MILITARY VETERANS: APPLYING THE THEORY OF INTERSECTIONALITY

David Albright,[1] Justin McDaniel,[2] Zainab Suntai,[1] and Julianne Wallace,[2] *1. University of Alabama, Tuscaloosa, Alabama, United States, 2. Southern Illinois University, Carbondale, Illinois, United States*

The post-service impact of military experiences include post-traumatic stress disorder, depression, substance misuse and several other adverse outcomes that persist well into older adulthood. As such, older military veterans are at risk of developing alcohol dependency and those with existing stressors from other identities are at the highest risk of engaging in binge drinking or heavy drinking. This study used the theory of intersectionality to examine alcohol misuse by veteran status and age, veteran status and race and veteran status and sex. Data were derived from the 2016, 2017 and 2018 Brief Risk Factor Surveillance System (BRFSS) from the Centers for Disease Control and Prevention (CDC). The BRFSS is an annual survey conducted over the phone in all 50 states and territories. Survey-weighted logistic regression models were used to examine alcohol misuse among adults aged 65+ by veteran status and the intersection between age, race, and sex. Results showed no interaction between veteran status and age, and no interaction between veteran status and sex. However, there was a significant interaction between veteran status and race, in that Black/Other race veterans were more likely to engage in both binge drinking and heavy drinking compared to White veterans, White nonveterans and nonveterans of the same race. Interventions geared towards this population should therefore engage culturally sensitive approaches that consider the historical and systemic factors that contribute to these disparities in rates of alcohol misuse among older military veterans.

### DIAGNOSIS OF BEHAVIORAL SYMPTOMS OF DEMENTIA AND CNS-ACTIVE DRUG USE AMONG DIVERSE PERSONS LIVING WITH DEMENTIA

Johanna Thunell,[1] Geoffrey Joyce,[1] Dima Qato,[1] Jenny Guadamuz,[2] and Julie Zissimopoulos,[1] *1. University of Southern California, Los Angeles, California, United States, 2. University of Southern California, University of Southern California, California, United States*

Approximately 90% of persons living with dementia (PLWD) experience behavioral and psychological symptoms of dementia (BPSD). Studies demonstrated high use of central nervous system (CNS) active drugs in nursing homes; one recent study documented high use among community-dwelling PLWD. Racial/ethnic disparities in BPSD diagnosis and CNS-active drug use, however, are unknown. We quantified disparities in BPSD diagnoses and CNS-active drug use using 100% Medicare Part A and B claims, 2017-2019, and Part D, 2018-2019. Beneficiaries were ages 65 and older in 2017, community-dwelling, and had a dementia diagnosis (n=801,597). We estimated models of CNS-active drug use to quantify racial/ethnic differences adjusting for confounders. Among PLWD, 66% had a BPSD diagnosis and 65% were taking a CNS-acting drug. Asians/Pacific Islanders were less likely to have a BPSD diagnosis (55%) than other groups, particularly affective diagnoses (40%). Whites were most likely to have any diagnosis (67%). Blacks were most likely to have hyperactivity diagnoses (7%). Antidepressants were most commonly used drug class (44%). Thirteen percent used an antipsychotic. Models adjusted for age, sex, comorbid conditions, dual-eligibility and BPSD diagnoses, showed non-Whites were less likely to use any CNS-active drug than Whites, but Blacks and Hispanics were slightly more likely to use antipsychotics. We found racial/ethnic differences in BPSD diagnoses and CNS-active drug use. Whether these disparities are due to differences in BPSD symptoms, health-care access or care-seeking remains an important question. Further study of disparity in outcomes associated with use will inform risk and benefit of CNS-active drugs use among PLWD.

### RACE AND ETHNIC GROUP DIFFERENCES IN SOCIAL ENGAGEMENT AMONG OLDER ADULTS

Lien Quach,[1] Uyen-Sa Nguyen,[2] Van Pham,[3] and Jeffrey Burr,[4], *1. University of Massachusetts Boston, Newton, Massachusetts, United States, 2. University of North Texas Health Science Center (UNTHSC), School of Public Health (SPH), Fort Worth, Texas, United States, 3. Center for Promotion of Advancement of Society, Hanoi, Ha Noi, Vietnam, 4. McCormack Graduate School, Boston, Massachusetts, United States*

Social engagement is considered crucial for older adults' well-being, generating social capital, connecting them to information about healthy lifestyles, and providing coping strategies for addressing daily challenges. Little is known about race and ethnic disparities regarding social engagement. This study examines the relationship between race, Hispanic ethnicity, and social engagement among community-dwelling adults age 65 or older. Data are taken from the Health and Retirement Study (2014) (n=6,221). Race and ethnic status are measured as: non-Hispanic white, non-Hispanic black, non-Hispanic "Asians and other race," and Hispanic (any race). Social engagement includes frequency of contact with friends and family and participation in social activities (e.g. volunteering and attending religious services). Covariates included age, sex, education, number of co-morbidities, and alcohol consumption. Linear regression analyses were performed using SAS 9.4. The mean age was 74.6, and sixty percent of the sample was female. Race and ethnic distribution were 78.6% non-Hispanic white, 11.9% non-Hispanic black, 7.89% Hispanics, and 1.7% non-Hispanic "Asians and other race." The mean score for our social engagement index was 3.3 (range 0-6). Hispanic persons, Asian persons, and persons from other race groups had lower social engagement compared with non-Hispanic white persons [β:-0.29, p<.0001; β:-0.27, p=0.04 respectively), after adjusting for covariates. These race and ethnic group differences in social engagement likely contribute to well-document health disparities in later life. Understanding racial and ethnic disparities in social engagement and the factors that create these differences can help identify appropriate social intervention programs regarding improving the well-being of all older adults.

### Session 3035 (Paper)

### ELDER ABUSE AND NEGLECT

### EVALUATING A MULTIDISCIPLINARY TEAM APPROACH TO ELDER ABUSE

Carmen Morano,[1] and Erin Berical,[2], *1. SUNY-Albany, Albany, New York, United States, 2. University at Albany, Albany, New York, United States*

This paper presents findings from a University and Community-based Agency collaboration to design and implement a preliminary evaluation of the Elder Abuse Multidisciplinary Team (E-MDT) Intervention. This intervention brings professionals from a variety of fields to investigate and respond to elder abuse. Data from 22 Interviews with staff along with anonymous survey data from E-MDT team members/staff (n=312) sought to establish team successes, challenges in implementation, and ongoing functioning. Themes that emerged in creating successful teams include: Establishing Buy-In and Trust of the team members, The Benefit of sharing experience and practical knowledge with other program sites; and Recognizing the Differences related to Onboarding and Sustaining New programs versus Sustaining Existing Programs. Themes related to responding during COVID revealed challenges such as Adapting to Technology and Inconsistent Access to the Internet. It was noted that remote meetings were easier to attend than face-to-face meetings. Data from the survey found the vast majority of respondents view the E-MDTs as having a positive impact on Clients (93%); while 93% of respondents indicated a positive impact on their Approach to Practice and the service area of their agency. Approximately 80% of the respondents indicated their multidisciplinary teams were Effective. Responses to 3-Open Ended questions included in the survey echoed similar themes from the interviews, as well as comments about their Professional Development and the complexity of responding to elder abuse. The paper will close with a discussion of the strategies used to facilitate the collaboration and complete the evaluation during the COVID-19 pandemic.

### EVALUATING RISK OF NEGLECT AMONG OLDER ADULTS USING NSHAP ROUND 3 (2015-2016)

Melissa Howe,[1] Grey Pierce,[2] Selena Zhong,[2] Lissette Piedra,[3] and Won Choi,[2] *1. NORC at the University of Chicago, Chicago, Illinois, United States, 2. University of Chicago, Chicago, Illinois, United States, 3. University of Illinois at Urbana-Champaign, Urbana, Illinois, United States*

As functional health declines, dependency on others increases along with the risk of neglect and its harmful consequences. In this paper, we use data collected during 2015-16 (Round 3) of the National Social Life, Health, and Aging Project (NSHAP) to identify older adults at risk of neglect and to test the hypothesis that high neglect risk predicts poorer health. Specifically, we use NSHAP's functional health survey module and follow-up care receiving "loop" to categorize respondents as having either "high" or "low" neglect risk. NSHAP's functional health module assesses respondents' difficulties with Activities of Daily Living (ADLs)

and Instrumental Activities of Daily Living (IADLs). Because ADLs and IADLs are integral to the maintenance of physical health, hygiene, and well-being, the unrequited desire for help with such activities could signal neglect. Accordingly, we assign “high neglect risk” to respondents who report either that they: (a) want but are not getting help with an ADL or IADL; or (b) are getting help with an ADL or IADL, but from a helper who is not very reliable. Motivated by current research that documents higher rates of morbidity and mortality among neglected older adults, we examine associations between neglect risk and other key NSHAP measures, including indicators of physical health, mental health, cognition, social support, social strain, and field interviewer assessed respondent hygiene. Results suggest that this method of risk assessment can be useful in identifying vulnerable populations of older adults. Follow-up interviews are needed to further confirm its utility as a risk assessment tool.

### INVESTIGATING ELDER ABUSE AND NEGLECT IN DIVERSE REFUGEE COMMUNITIES IN GREENSBORO, NC

S. Sudha,[1] and Narayan Khadka,[2], *1. University of North Carolina Greensboro, Greensboro, North Carolina, United States, 2. Institute for Peace and Harmony, Albany, California, United States*

Elder abuse and neglect (EAN) comprises multiple dimensions, is experienced by about 10% of older adults in the U.S in diverse communities, and is severely detrimental to older adults' (OA) health and wellbeing. However, documentation of EAN among refugee OA is greatly lacking as are services for these communities. Refugee OA are overall underserved members of marginalized communities. This paper reports on a community-engaged study to collect information and raise awareness of EAN among OA in 2 North Carolina refugee communities - Nepali-speaking Bhutanese and Congolese. Research partners included University researchers and community refugee-serving organizations. Surveys and focus group interviews were conducted. 17 Nepali-speaking Bhutanese and 13 Congolese filled out survey questions, including the Elder Abuse Suspicion Index. They participated in focus group discussions (FGDs), separately for men and women of each community. Survey results indicated EAN more among Congolese than Nepali-speaking Bhutanese. FGD results showed both communities prefer to depend on family members, and experience difficulties with language, transportation, and economic insecurity. No EAN was reported in the FGDs. In line with principles of community-engaged approaches, a capacity-building event to increase awareness of EAN was held, attended by 25 persons from the two communities. This study adds documentation on an under-researched area and marginalized communities. Action recommendations include disseminating culturally appropriate EAN information, strengthening English language and job skills and transportation options, encouraging cooperation across state, nonprofit, educational, and service organizations to address needs of older refugee adults.

### OLDER ADULTS AND FAMILY DISCORD OR VIOLENCE DURING THE COVID-19 PANDEMIC: RESULTS OF A CANADA-WIDE SURVEY

Gloria Gutman,[1] Brian de Vries,[2] Robert Beringer,[3] Paneet Gill,[4] Helena Dault,[5] and Mojgan Karbakhsh,[1], *1. Simon Fraser University, Vancouver, British Columbia, Canada, 2. San Francisco State University, Palm Springs, California, United States, 3. University of Victoria, Victoria, British Columbia, Canada, 4. Simon Fraser University, Surrey, British Columbia, Canada, 5. Victoria Hospice, Victoria, British Columbia, Canada*

Child abuse and intimate partner violence rates are known to increase during and in the aftermath of disasters. Research on elder abuse during disasters, including the current pandemic, is limited. As part of an online survey that explored older Canadians' current experiences and future care plans during the COVID-19 pandemic, we aimed to determine the prevalence, contributing factors and potential outcomes of frequent family discord involving physical violence (FFD/PV) as a proxy for elder abuse. The survey was conducted between Aug 10 and Oct 10, 2021. Respondents (n=4380) were recruited using social media, direct email, Facebook advertising and with the assistance of 85 local community, regional and national organizations. The sub-sample reporting FFD/PV (n=76, 1.8%) was compared with other survey respondents regarding socio-demographic characteristics, negative and positive emotions, difficulty accessing basic needs, food, health care and support. Respondents experiencing FFD/PV were found to be significantly younger and less educated and were more likely to be non-white and not working than other respondents. The subgroup sustaining FFD/PV reported significantly higher rates of feeling depressed, lonely, isolated, anxious, sad, and judged/shamed and felt less happy, relaxed and accepted in their community. They also reported higher rates of challenges in accessing basic material needs such as food, support, medical care, mental health treatment and experienced more changes in life routines. Although only a small percentage reported FFD/PV, our results highlight a disturbing pattern that merits serious attention of adult protection agencies, seniors' advocates and disaster response organizations.

### PREDICTORS OF DEPRESSION IN HEALTHCARE PATIENTS AT RISK OF SELF-NEGLECT

Miriam Rose,[1] Farida Ejaz,[1] and Courtney Reynolds,[2], *1. Benjamin Rose Institute on Aging, Cleveland, Ohio, United States, 2. Benjamin Rose Institute on Aging, Benjamin Rose Institute on Aging, Ohio, United States*

More than half of reports to Adult Protective Services agencies nationwide involve allegations of self-neglect. An intensive case management intervention for preventing self-neglect was evaluated in a longitudinal study conducted collaboratively by a large healthcare system, Adult Protective Services, and a gerontological research institute. Patients (444) who were older (60+ years) and/or disabled (18+ years) were randomly selected for participation from 19 primary-care clinics if they had risk factors for self-neglect, including depression, substance abuse, dementia, and/or impairment in activities of daily living. Average age was 68 years (SD=12.5), 68% were Hispanic, 68% had monthly income of less than $1,361, and 67% were female. Clinics were randomized into intervention and control groups. Intervention clinic patients received intensive case management services; control clinic patients received usual care, including social work services. Subjects were interviewed at baseline and four months later. The Stress Process Model guided a multiple regression analysis. Domains of background characteristics, primary and secondary stressors, and support (patients in intervention or control group) were entered in blocks to predict depression levels at post-test. While no significant differences were found in post-test depression levels between intervention and controls, the final model was statistically significant (adjusted R2=.452). Significant predictors of depression were: younger age (disabled adults), poorer self-rated physical and emotional health, greater loneliness, and less social support. Future analyses will examine effects of moderating variables on post-test depression levels. Practice implications of preliminary analyses include addressing disabled adults' mental health needs, especially if they are isolated and lack social support.

### Session 3040 (Symposium)

### EXPANDING THE SCOPE OF ADMINISTRATIVE HEALTH RECORDS THROUGH ADVANCED STATISTICAL METHODS

Chair: Igor Akushevich Co-Chair: Carl V. Hill
Discussant: Konstantin Arbeev

The objective of the Symposium is to expand familiarity of the application of advanced methods of modern statistical modeling and data management, to administrative health data by combining methodological innovations with practical hands-on demonstrations. Topics will cover a range of methodological and substantive topics including: i) decomposition and partitioning approaches in analysis of disparities and time trends in AD/ADRD; ii) new artificial intelligence technologies that allow us to enrich electronic health record datasets with self-report scores in geriatrics; iii) using administrative data to model adherence to disease management and health-related behavior; iv) the use of longitudinal extension of the average attributable fraction to study health disparities and multimorbidity; and v) the geographic and racial disparities in total and remaining life expectancies after diagnoses of AD/ADRD and other chronic conditions. The increasing availability of large-scale datasets based on electronic health records and administrative claims records provide an unprecedented opportunity for obtaining nationally representative results based on individual-level measures that reflect the real care-related and epidemiological processes. This makes the reduction of barriers to entry to the use of such data of vital importance to the community of geriatrics and health researchers.

### DECOMPOSITION OF DISPARITIES IN ALZHEIMER'S DISEASE AND RELATED DEMENTIA

Igor Akushevich, *Duke University, Durham, North Carolina, United States*

This study uses Medicare data to non-parametrically evaluate race- and place-of-residence-related disparities in AD/ADRD prevalence and incidence-based mortality, separate them out into the epidemiological causal components including race-related disparities in incidence and survival, and finally explain these in terms of health-care-related factors using causal methods of group variable effects (propensity scores and the rank-and-replace method) and regression-based analyses (extended Fairlie's model and generalized Oaxaca-Blinder approach for censoring outcomes). Partitioning analysis showed that the incidence rate is the main predictor for temporal changes and racial disparities in AD/ADRD prevalence and mortality, though survival began to play a role after 2010. Arterial hypertension is the leading predictor responsible for racial disparities in AD/ADRD risks. This study demonstrated that Medicare data has sufficient statistical power and potential for studying disparities in AD/ADRD in three interacting directions: multi-ethnic structure of population, place of residence, and time period.

### NEW AI TECHNOLOGIES TO ENRICH ELECTRONIC HEALTH RECORD DATA SETS WITH SELF-REPORT SCORES IN GERIATRICS

Ricardo Pietrobon, *Spore Data, SporeData, North Carolina, United States*

Although electronic health record data present a rich data source for health service researchers, for the most part, they lack self-report information. Although recent CMS projects have provided hospitals with incentives to collect patient-reported outcomes for select procedures, the process often leads to a substantial percentage of missing data, also being expensive as it requires the assistance of research coordinators. In this presentation, we will cover Artificial Intelligence-based based technologies to reduce the burden of data collection, allowing for its expansion across clinics and conditions. The technology involves the use of algorithms to predict self-report scores based on widely available claims data. Following previous work predicting frailty scores from existing variables, we expand its use with scores related to quality of life, i.e. mental health and physical function, and cognition. Accuracy metrics are presented both in cross-validation as well as external samples.

### USING ADMINISTRATIVE CLAIMS TO MODEL HEALTH-RELATED BEHAVIORS: MEASURES OF SCREENING AND MEDICATION ADHERENCE

Arseniy Yashkin, *Duke University, Morrisville, North Carolina, United States*

We demonstrate how administrative claims records can be used to model certain behavioral patterns and associated health effects. The inability of administrative claims, which are in essence a billing record, to account for differences in behavior is a major limitation of such data which usually requires an externally linked source to overcome. However, for certain diseases, for which well-defined and accepted guidelines on screening and medication use exist, the claims themselves can provide a way for modeling health-related behavior. A practical application to screening and medication adherence for type ii diabetes mellitus is presented. Diverse methods of the calculation of such indexes with their pros, cons and variation in identified effects are discussed and demonstrated using results based on administrative claims drawn from a 5% sample of Medicare beneficiaries.

### DIFFERENCES IN THE RACIAL CONTRIBUTION OF DEMENTIA AND CHRONIC CONDITIONS TO HOSPITALIZATION, SNF ADMISSION

Heather Allore, *Yale School of Medicine, New Haven, Connecticut, United States*

We estimate the contribution for experiencing hospitalization, skilled nursing facility admission and mortality using a measure of attributable fraction that incorporates both the prevalence, incidence and risk called Longitudinal Extension of the Average Attributable Fraction (LE-AAF). We estimate the LE-AAF for Non-Hispanic whites and Non-Hispanic Blacks for dementia and 10 chronic conditions, for three outcomes. This approach analyses the temporal relationships among conditions to estimate their population-level average attributable fractions. Unlike standard measures of attributable fraction, the sum of the contribution of each condition based on the LE-AAF will not exceed 100 percent, enabling us to compute the contribution of pairs, triads or any combination of conditions. Furthermore, in studying multimorbidity, the LE-AAF has the desirable feature of being based on all combinations of the risk factors and covariates present in the data with final values for the individual LE-AAFs obtained by averaging across these observed combinations of predictors.

### DISPARITIES IN DISEASE-SPECIFIC REMAINING LIFE EXPECTANCY AMONG MEDICARE BENEFICIARIES IN THE UNITED STATES

Bin Yu,[1] and Julia Kravchenko,[2] *1. Duke University, Duke University/Durham, North Carolina, United States, 2. Duke University, Durham, North Carolina, United States*

Racial and geographic disparities in life expectancy (LE) in the US are a persistent problem. We used 5% Medicare Claims for 2000-2017 to investigate the patterns of remaining LE (RLE) in the U.S. with the highest and the lowest LE. RLEs in race-/ethnicity specific populations aged 65+ were calculated in patients with specific diseases and in the total population using the area under the Kaplan-Meier estimator. The Cox model was used to investigate the effect of state-specific residence on total LE and RLE. Between-the-states differences in RLE were most pronounced for cerebrovascular disease, atherosclerotic heart disease, breast and prostate cancer. RLE was the lowest for lung cancer and sepsis, followed by Alzheimer's disease, dementia, pneumonia, and heart failure. RLE for myocardial infarction and cerebrovascular disease decreased over time, while for renal failure, diabetes, atherosclerotic heart disease, and cancers of breast and prostate RLE increased.

### Session 3045 (Symposium)

### EXPERIENCING THE COVID-19 PANDEMIC AT AGE 85 AND OVER: AN MIT AGELAB STUDY WITH THE 85+ LIFESTYLE LEADERS PANEL

Chair: Taylor Patskanick Discussant: Lisa D'Ambrosio

The oldest of older adults remain at the highest risk of developing severe illness, requiring hospitalization, or dying if infected with COVID-19. As a result, the discourse about the COVID-19 pandemic has centered on short-term sacrifices to "protect" older adults. Yet much remains to be known about the prolonged impact of the pandemic on the over-85 age demographic. This symposium shares findings from a longitudinal, mixed methods study with the MIT AgeLab's 85+ Lifestyle Leaders panel, a panel of octogenarians and nonagenarians convened since 2015. This symposium offers an update to a 2020 GSA session shared regarding the initial, cross-sectional work conducted in March 2020 with this panel. Findings will be drawn from a series of 85 interviews with 15 participants, 14 focus groups ($\bar{x}$ participants=19.3), and three surveys (March 2020, N=28; August 2020, N=18; November 2020, N=16) conducted with the panel regarding the impact of the pandemic on this group over the past year. The first presentation covers the Lifestyle Leaders' adoption and use of technology throughout the pandemic, with a focus on telehealth. The second takes an in-depth look at the unique lived experiences of Lifestyle Leaders living in senior housing communities during the pandemic. The third explores the Lifestyle Leaders' perspectives on cultivating resilience and caring for their mental health while in a pandemic. Finally, the fourth presentation shares the Lifestyle Leaders' experiences with social isolation and loneliness during the pandemic, with a focus on how family relationships and engagement in intergenerational programming have changed.

### PERSPECTIVES FROM THE 85+ LIFESTYLE LEADERS ON SOCIAL ISOLATION AND MULTIGENERATIONAL RELATIONSHIPS

Taylor Patskanick, *MIT, Somerville, Massachusetts, United States*

The oldest of older adults are especially impacted by many of the measures recommended to slow the spread of COVID-19. This presentation explores changes in Lifestyle Leaders' experiences with loneliness and their beliefs about the impact of COVID-19 on multigenerational relationships and intergenerational programming. For example, 55.6% strongly agreed or agreed with the statement, "The pandemic will have been more socially impactful on younger generations than older generations." Lifestyle Leaders remain interested (68%) in virtual or socially distanced intergenerational programming. Particular activities of interest included technology tutoring, pen pals, and outdoor or virtual socializing. Additionally, this presentation will highlight how the Lifestyle Leaders have been impacted by a loss in weak ties and the extent to which the pandemic has prompted them to take on new roles in their families, including "accepting" paid and unpaid caregiving and experiences living with children and grandchildren during the pandemic.

### MENTAL HEALTH AND RESILIENCE AMONG THE 85+ LIFESTYLE LEADERS THROUGHOUT THE COVID-19 PANDEMIC

Julie Miller, *MIT, Cambridge, Massachusetts, United States*

Recent research suggests older adults may be uniquely able to cope and cultivate psychological resilience during the COVID-19 pandemic. This presentation will describe how the Lifestyle Leaders' overall mental health (including worries and experiences of social isolation and loneliness) and thoughts about the future have changed throughout the pandemic, as well as the ways in which they remained resilient throughout. For example, a survey in March 2020 indicated that 68% of Lifestyle Leaders were very or extremely worried about COVID-19, compared to only 33% in November 2020. Interviews with Lifestyle Leaders revealed that the pandemic led many to engage in more focused thinking about their own mortality and, for some, presented or compounded challenges of older age (e.g., widowhood, downsizing, etc.). The presentation will also highlight ways in which Lifestyle Leaders' past experiences and current activities have contributed to their mental health and fortitude during the ongoing pandemic.

### UNDERSTANDING TECHNOLOGY ADOPTION, TROUBLESHOOTING, AND TELEHEALTH AMONG THE LIFESTYLE LEADERS

John Rudnik, *MIT, Cambridge, Massachusetts, United States*

The COVID-19 pandemic has prompted widespread adoption of and greater reliance on digital technologies across the generations. In interviews and focus groups, the Lifestyle Leaders reported increased use of teleconferencing services, especially for virtual programming, socializing, and telehealth. Approximately 61% of Lifestyle Leaders had attended between one and five virtual or phone-based healthcare appointments since March 2020. Beliefs about telehealth across interviews varied: appointments were convenient but lacked the "in-person feedback and understanding" of an in-person visit. Half of the Lifestyle Leaders (50%) agreed with the statement, "Telehealth is the future of personalized medicine." This presentation will also report on the Lifestyle Leaders' challenges associated with using technology during the pandemic. These included concerns about: having devices able to keep up with the demands of the pandemic; using technology-enabled grocery services; and troubleshooting technology. Many were concerned about their growing dependence on technology because of the pandemic.

### LIFESTYLE LEADERS' EXPERIENCES IN SENIOR HOUSING DURING THE COVID-19 PANDEMIC

Taylor Patskanick,[1] Julie Miller,[2] and Kathryn Chan,[3] *1. MIT, Somerville, Massachusetts, United States, 2. MIT, Cambridge, Massachusetts, United States, 3. Massachusetts Institute of Technology AgeLab, Cambridge, Massachusetts, United States*

Millions of older adults living in close communal contact in senior housing communities remain vulnerable during the COVID-19 pandemic. Approximately 30% of Lifestyle Leaders currently live in senior housing. This presentation will cover the unique challenges these participants have encountered, including experiences with and the impact of changing norms and pandemic-related policies within communities over time. In March 2020, 75% of Lifestyle Leaders rated the response of their senior housing community to COVID-19 as "Excellent" or "Very Good." In August 2020, they reported they believed they were less likely to contract COVID-19 living in senior housing compared to people not living in senior housing (80%). Interview data revealed Lifestyle Leaders in these environments held favorable views toward their communities. This presentation will further discuss how the Lifestyle Leaders who do not live in senior housing perceive senior living and how these perceptions have shifted during the pandemic.

### Session 3050 (Paper)

### FAMILY CAREGIVING (BSS PAPER)

### CONTINUITY OF CAREGIVERS' ENSURING MEDICAL CARE AND CARE-TRANSITION PREPAREDNESS: A MEDIATION MODEL

Nosaiba Rayan-Gharra,[1] Orly Tonkikh,[1] and Nurit Gur-Yaish,[2] *1. University of Haifa, Haifa, Hefa, Israel, 2. Oranim Academic College of Education, Haifa, HaZafon, Israel*

Studies show that informal support provided during hospitalization is essential for communicating with the healthcare team and explaining medical care. Less is known about factors explaining family caregivers' Ensuring and Explaining Medical Care (EEMC) during hospitalization and its impact on care-transition-preparedness of patients in terms of their understanding of the explanations and instructions for continued care. This study examined whether EEMC during the current hospitalization mediates the association between involvement of the caregiver in ensuring and explaining medical care prior the current hospitalization and patients' care-transition-preparedness for discharge. A prospective cohort study includes 456 internal-medicine-patients at a tertiary medical center in Israel, who were accompanied by an informal caregiver. Involvement in EEMC prior and during the hospitalization, covariates such as health literacy (HL) levels, demographic, health, and functional status were reported by the patients during the hospitalization; and care-transition-preparedness was reported by the patients in a week after discharge. After controlling for covariates, only high HL levels of patients and their caregivers were positively associated with EEMC during hospitalization and care-transition-preparedness ($P<0.05$). Moreover, mediation analysis indicated significant direct (B(unstandardized)=1.69; $p=0.003$) and indirect effect (Mediated effect (ME)=1.28; CI= 0.81 to 1.87) of prior involvement in EEMC on care-transition-preparedness through high EEMC during the current hospitalization, controlling for baseline characteristics of patients and their caregivers (total effect: B=2.95; $p<0.001$). These findings suggest that caregivers' experience and involvement prior the hospitalization may be an essential factor in improving EEMC during the current hospitalization, and in turn improve transition outcomes.

### DOES PERSONAL GOAL PURSUIT ALLEVIATE FAMILY CAREGIVER STRESS?

Shelbie Turner,[1] and Karen Hooker,[2], *1. Oregon State University, Oregon State University, Oregon, United States, 2. Oregon State University, Corvallis, Oregon, United States*

Family caregivers may experience reduced stress by maintaining their sense of self throughout their time in a caregiving role. Working towards personal goals is helpful for maintaining a sense of self, but pursuing one's own goals amidst caregiving responsibilities may be challenging. In this study, we analyze the processes by which caregivers pursue their own personal goals – and how those processes impact daily stress – in an effort to develop a deeper understanding of goal-pursuit as a potential caregiver stress-reducing strategy. We utilized daily data from spousal (N=256 days) and adult-child (N=400 days) caregivers who participated in the PULSE (Personal Understandings of Life and Social Experiences) Project, a 100-day microlongitudinal study on goal pursuit amongst people 50 and older (Hooker et al., 2013). In daily surveys, caregivers reported progress made towards a personally-identified health and social goal, along with a 4-item measure of daily stress. We ran multi-level models to assess how daily goal progress was associated with same-day stress. Spousal caregivers' daily stress was lower on days when their health goal (Estimate = -1.07, SE = 0.20, $p<.0001$)

and social goal (Estimate = -0.97, SE = 0.15, p<.0001) progress was higher. Similarly, adult-child caregivers' daily stress was lower on days when their health goal (Estimate = -0.67, SE = 0.19, p<.001) and social goal (Estimate = -0.52, SE 0.24, p=0.03) progress was higher. Results support the hypothesis that maintaining personally-meaningful goals can alleviate caregiver stress, and is a promising tool for caregiver health promotion.

### ENHANCING ACTIVE ENGAGEMENT FOR DEMENTIA CAREGIVERS: A SYNTHESIS OF INTERVENTIONS

Jacqueline Eaton, *University of Utah, University of Utah, Utah, United States*

In a recent meta-analysis of interventions for dementia caregivers, psychoeducational interventions were found to be effective only if they required caregivers to apply knowledge and skills through active engagement. This emphasizes the importance of understanding which intervention components enhance application in order to improve caregiving interventions and the mechanisms by which they work. The purpose of this presentation is to identify and assess elements of active engagement within dementia caregiving interventions. Articles included in this review were published between 2009 and 2018 and identified as psychoeducational dementia caregiving interventions. Each intervention was assessed to describe: 1) how active engagement was defined, 2) the logistics for implementing the active engagement techniques, 3) and the process for evaluating active engagement components. Of 36 articles meeting inclusion criteria, 25 mentioned active engagement components of the intervention. Active components included discussion, problem-solving, practice, role-play, action plans, and homework. Only five articles provided partial descriptions of the active components, five mentioned assessing active engagement, and only one study examined the efficacy of an engagement technique. This demonstrates a significant gap in our understanding of interventions for dementia caregivers. Active engagement enhances outcomes, yet to our knowledge, the specific steps taken to engage caregivers actively and the mechanisms by which these work are unclear. This is a barrier to optimizing active engagement within intervention delivery. Clarifying processes and methods for testing mechanisms of action can further enhance caregiver engagement with interventions.

### LONGITUDINAL EFFECTS OF STRESS AND COGNITIVE FUSION IN ANXIETY AND DEPRESSIVE SYMPTOMS OF FAMILY CAREGIVERS

Samara Barrera-Caballero,[1]
Rosa Romero-Moreno,[2] Carlos Vara-García,[3]
Javier olazarán,[4] María del Sequeros Chaparro,[5]
Lucía Jiménez-Gonzalo,[6] José Adrián Fernandes-Pires,[6]
and Andrés Losada-Baltar,[3], *1. Rey Juan Carlos University, Alcorcón, Madrid, Madrid, Spain, 2. Universidad Rey Juan Carlos de Madrid, Madrid, Madrid, Spain, 3. Universidad Rey Juan Carlos, Madrid, Madrid, Spain, 4. HGU Gregorio Marañón,, Madrid, Madrid, Spain, 5. Universidad Autónoma de Madrid, Madrid, Madrid, Spain, 6. Rey Juan Carlos University, Alcorcón, Madrid, Spain*

Dementia caregiving has been commonly associated with negative psychological consequences in caregivers. Cognitive fusion, that is, the tendency for been overly influenced by cognition, has been linked to psychological distress in caregivers in cross-sectional studies. Female caregivers and those who are exposed to more stressors such as behavioral and psychological symptoms of dementia report higher levels of distress. However, longitudinal analysis of predictors of caregivers levels of distress are sparse, with no available study analyzing the longitudinal effect of cognitive fusion. The aim of this study is to analyze the longitudinal effect of cognitive fusion in depressive and anxiety symptoms of family dementia caregivers, after controlling for other relevant variables. Face to face interviews were conducted each year through a two-year period (three assessments) with 143 caregivers. Linear mixed models analysis were used to analyze the associations between time-varying values for cognitive fusion, frequency and reaction to care-recipient behavioral problems and depressive and anxiety symptoms, after controlling for caregivers' age and gender, daily hours and time caring, care-recipient functional capacity and caregivers' transitions (cessation of caregiving). Results suggest that increases in cognitive fusion and in reaction to behavioral problems, being a female caregiver and being younger, significantly predicted increases in anxiety symptoms over time. Also, increases in cognitive fusion and in reaction to behavioral problems, decreases in care-recipient's functional capacity and ending of the caregiving role significantly predicted increases in depressive symptoms. Psychological strategies aimed at reducing cognitive fusion and stress levels may be especially helpful for reducing caregivers' distress.

### MET AND UNMET NEEDS OF COGNITIVELY IMPAIRED OLDER ADULTS AND BURDEN AND BENEFITS OF THEIR CAREGIVERS

Pildoo Sung, Johan Suen, Nawal Hashim,
Rahul Malhotra, and Angelique Chan, *Duke-NUS Medical School, Singapore, Not Applicable, Singapore*

Previous studies typically assess caregiver needs when trying to interpret caregiver burden. We propose that both met and unmet needs of care recipients translate into different caregiving experiences with varying levels of benefits and burden combined. We use data on 263 caregivers of community-dwelling Singaporean older adults with cognitive impairment who participated in a community-based dementia care study conducted in 2018-2020. Our analysis produces three major findings. First, latent class analysis identifies three distinct types of caregiving experience based on caregiver-reported burden and benefits of caregiving: intensive (high burden and high benefits, 11% of caregivers), satisfied (low burden and high benefits; 54%), and dissatisfied (low burden and low benefits; 35%). Second, multinomial logistic regression shows that both met and unmet needs of care recipients are positively associated with the intensive caregiving experience, while only met needs are positively associated with the satisfied caregiving experience, in comparison to dissatisfied caregiving experience. Third, met needs in the areas of daytime activities, memory assistance, and mobility are positively related to the satisfied caregiving experience, compared to the dissatisfied caregiving experience. In other words, caregivers are more likely to be satisfied in their caregiving experiences (i.e., low burden and high benefits) if their care recipients' problems with memory, mobility, and finding suitable and adequate daytime activities are properly managed. Our findings thus call for interventions to fulfill care recipients' needs in a more tailored manner in order to increase satisfaction among caregivers.

### Session 3055 (Symposium)

### HEALTH DISPARITIES IN ALZHEIMER'S DISEASE: A WAKEUP CALL FOR TRANSFORMATION

Chair: Allison Gibson Co-Chair: Lenora Smith
Discussant: Robert Turner

It is well documented in the scientific literature that health disparities exist within the Alzheimer's disease and related dementias (ADRD) population, particularly among socially disadvantaged individuals experiencing limited opportunities to achieve optimal health. In this symposium, presenters will introduce some of the significant health disparities observed across varying ADRD research. The first presentation, by Robinson-Lane and colleagues, examines caregiving coping and health among Black ADRD families. Findings suggest in addition to traditional stress and coping strategies, additional interventions are needed that improve physical health for family caregivers. Next, Yu and colleagues will discuss the higher levels of emotional distress reported among individuals diagnosed with mild cognitive impairment, compared to their cognitively normal counterparts. In the third presentation, Lin and colleagues share their work on changes in dementia-related behavioral symptoms observed by hospice staff during COVID-19. The pandemic has affected nearly every aspect of healthcare delivery, and many hospice staff are reporting patients diagnosed with dementia have also felt the effects. Next, Xu et al identified that non-Hispanic Black older adults in their help-seeking behaviors and diagnosis process of ADRD, and often were not seen in healthcare settings by an ADRD specialist compared to their White counterparts. In the final session, Agboji will speak on the issue on findings from a systematic examination of the literature that demonstrates apathy being underrecognized and undertreated in healthcare settings. This symposium will conclude with a discussion on how researchers can disrupt these disparities by promoting health equity across ADRD healthcare and social services.

### PRELIMINARY FINDINGS OF A NATIONAL SURVEY OF BLACK ADRD FAMILY CAREGIVERS

Florence Johnson,[1] Nicholas Mazzara,[1] Kayla DeMarco,[1] Ivaylo Dinov,[2] and Sheria Robinson-Lane,[2], *1. University Of Michigan School of Nursing, Ann Arbor, Michigan, United States, 2. University of Michigan School of Nursing, Ann Arbor, Michigan, United States*

The National Caregiver Survey aims to capture a representative sample of Black Alzheimer's disease and/or related dementias (ADRD) family caregivers who are 55+ to better understand the relationships between adaptation to caregiving, coping, and health. Following targeted social media marketing, ADRD family caregivers (n=60) completed an electronic survey capturing over 200 data elements. Analysis was completed using Spearman correlation coefficients. Preliminary results suggest that 55% of participants were hypertensive (n=33) and 27% (n=16) had diabetes. Participants were generally overweight with an average BMI of 29. 28% (n=17) of the sample were smokers. A negative correlation was identified between the level of care needs of the recipients (IADLs) and alcohol use (p=0.037). There was also a correlation between identifying positive aspects of caregiving and adaptive coping (p=0.045). Caregiver support programs should facilitate development of effective coping strategies for new family caregivers, with particular attention on smoking cessation, brain-healthy diet, and exercise.

### COMPARING THE EMOTION STATUS OF INDIVIDUALS WITH MCI TO THEIR COGNITIVELY NORMAL COUNTERPARTS: I-CONECT PROJECT

Katherine Wild,[1] Lisa Silbert,[1] Jeffrey Kaye,[1] Hiroko Dodge,[1] and Kexin Yu,[2], *1. Oregon Health & Science University, Portland, Oregon, United States, 2. USC, LA, California, United States*

Socially isolated older adults with MCI are at greater risk of developing ADRD. This study compares the emotional status of older adults with MCI to their cognitively normal counterparts within a socially isolated sample. We used baseline data from the Internet-based Conversational Engagement Clinical Trial (NCT02871921). MCI status was determined according to clinical diagnosis. Three emotion domain scores were calculated: negative affect, social satisfaction, and psychological wellbeing. Linear regressions were conducted for all 17 Emotion Battery measures and 3 domain scores. The 127 participants' mean age was 81.1 (SD=4.6). About 54% were diagnosed with MCI. Older adults with MCI had more negative affect, yet no difference was observed in social satisfaction and psychological wellbeing. Individuals with MCI had higher levels of fear, perceived hostility, perceived stress, sadness, and lower self-efficacy. Better understanding the emotional status could inform the development of behavioral health interventions and early detection of MCI.

### CHANGES IN DEMENTIA-RELATED BEHAVIORAL SYMPTOMS OBSERVED BY HOSPICE STAFF DURING COVID-19

Kimberly Convery,[1] Tessa Jones,[2] Aditi Durga,[3] Abraham Brody,[4] and Shih-Yin Lin,[5] *1. New York University Rory Meyers College of Nursing, New York, New York, United States, 2. New York University Silver School of Social Work, New York, New York, United States, 3. NYU Rory Meyers College of Nursing, New York, New York, United States, 4. NYU Hartford Institute for Geriatric Nursing, New York, New York, United States, 5. New York University, New York, New York, United States*

COVID-19 infection control precautions (e.g., social distancing) and associated isolation and changes to routines can worsen dementia-related behavioral symptoms. A cross-sectional online survey was administered to 101 hospice staff (95% female; mean age 49) to investigate what dementia-related behavioral symptoms in their care recipients had changed from before to after the COVID-19 outbreak. Of the 101 participants, 47 (46.5%) reported changes in symptoms, three (3%) had not been able to physically observe/assess their care recipients, two (2%) reported changes in routines, and 49 (48.5%) reported no changes. The most common changes in symptoms were increased agitation (N=19), depression (N=16), confusion (N=10), and anxiety (N=6). Some participants (N=14) also commented on potential causes, including not having visitors and inability to go outside for normal activities while not understanding why. Interventions to normalize social environments for hospice recipients with dementia are likely imperative to alleviate behavioral symptoms exacerbated by COVID-19-related precautions.

### RACIAL DIFFERENCES IN THE PATHWAY TO DIAGNOSIS OF ALZHEIMER'S DISEASE AND RELATED DEMENTIAS

James burke,[1] Matthew Dupre,[1] Se Hee Min,[2] Ruth Anderson,[3] David Page,[1] Truls Ostbye,[4] and HANZHANG XU,[5] *1. Duke University School of Medicine, Durham, North Carolina, United States, 2. Duke University School of Nursing, Durham, North Carolina, United States, 3. University of North Carolina at Chapel Hill, UNC Chapel Hill, North Carolina, United States, 4. Duke University, Durham, North Carolina, United States, 5. Duke University School of Medicine, Duke University, North Carolina, United States*

This study examined differences in the pathway to diagnosis of Alzheimer's disease and related dementias (ADRD) between Black and White older adults. Using electronic health records from a large health system, we included 2,085 non-Hispanic Black and 6,269 non-Hispanic White older adults with a final/primary diagnosis of ADRD between 2014 and 2020. Black older adults were more likely to receive the ADRD diagnosis from a primary care provider (35.4% vs. 29.8%), during a hospital admission (19.5% vs. 13.6%), or during an emergency department visit (4.2% vs. 2.0%); but were less likely to be diagnosed by an ADRD specialist (31.6% vs. 45.2%). Black older adults had nearly twice as many clinical encounters in the two years prior to the ADRD diagnosis than their White counterparts (43 vs. 26). Despite having more clinical encounters, Black older adults were more likely to be at a later stage when diagnosed than White older adults.

### BARRIERS TO AND FACILITATORS FOR OPTIMAL INTERVENTION FOR APATHY IN OLDER ADULTS WITH DEMENTIA

Shannon Freeman,[1] and Aderonke Agboji,[2] *1. University of Northern British Columbia, Prince George, British Columbia, Canada, 2. University of Northern British Columbia, Quesnel, British Columbia, Canada*

Apathy is a persistent symptom in brain disorders. It affects 84% of people with brain disorders. Those affected are more than two times likely to die early than those without. Yet it is often ignored and undertreated. An integrative review guided by Whittemore and Knafl's (2005) framework was carried out to identify the factors that inhibit or facilitate the diagnosis and management of apathy among older adults with dementia. The result of the findings revealed five barriers and three facilitators. Barriers included inconsistencies in the definition and diagnostic criteria, lack of awareness, overlap with other neuropsychiatric disorders, a paucity of evidence-based information, and lack of familiarity. Facilitators were standardized definition and assessment tools, good communication among the interdisciplinary team, and adequate training, education, and experience. In conclusion, efforts should be geared towards raising awareness and developing a practice guideline to aid healthcare professionals in detecting and managing apathy optimally.

### Session 3060 (Symposium)

### INNOVATIVE MULTIDISCIPLINARY CARE MODELS PROVIDED VIA TELEHEALTH DURING THE COVID PANDEMIC

Chair: Shahla Baharlou Discussant: Lee Lindquist

Integrated and collaborative care lead to better care. Addressing the behavioral and mental health care needs of patients results in better health outcomes. Interdisciplinary and multi-disciplinary approaches to health care delivery yield more effective health care planning. A holistic approach to healthcare sees the individual as more than the sum of diseases. Research studies have supported these assertions and yet, in actual practice, they are often more aspirational than actualized. The COVID-19 pandemic has made it even more difficult to implement collaborative care delivered by varied professional disciplines. This symposium describes efforts to provide more holistic and multidisciplinary care in the primary care geriatrics practice of the Dept. of Geriatrics & Palliative Medicine, Icahn School of Medicine. This New York City practice has 4,500 patients with diverse backgrounds and a median age of 85. In the first paper, Baharlou and her colleagues describe the establishment of an IMPACT collaborative care depression model in the middle of the COVID-19 pandemic. It was adapted to be provided by telephone and uses a different psychosocial intervention than is usually implemented. Hinrichsen and Leipzig outline the successful integration of Cognitive Behavioral Therapy for Insomnia into geriatrics primary care to improve insomnia in older adults and deprescribe sleep medications. Munoz and her colleagues describe the ALIGN program which is an interdisciplinary team effort, informed by the social determinants of health framework, to facilitate access to an array of services delivered virtually because of the pandemic.

### IMPROVING MOOD PROMOTING ACCESS TO COLLABORATIVE TREATMENT (IMPACT) PROGRAM IN GERIATRICS PRIMARY CARE

Gregory Hinrichsen,[1] Lizette Munoz,[2] Katherine Currey,[2] sheila Barton,[2] Fay Kahan,[2] Blair MacKenzie,[2] Laili Soleimani,[1] and Shahla Baharlou,[3], *1. Icahn School of Medicine at Mount Sinai, New York, New York, United States, 2. Mount Sinai Health System, New York, New York, United States, 3. Icahn School of Medicine at Mount Sinai, Icahn School of Medicine at Mount Sinai, New York, United States*

Improving Mood Promoting Access to Collaborative Treatment (IMPACT) is a well-established model for the treatment of depression in primary care. The COVID pandemic has caused increased distress and depression among the older patients in our New York City geriatric practice. This paper describes the establishment of a virtual IMPACT model during the pandemic in which IMPACT services have been provided via telephone. This effort was a multidisciplinary collaboration among geriatric medicine, geriatric psychiatry, social work, and geropsychology. Our IMPACT program uses a brief form of Interpersonal Psychotherapy (IPT) for depression as the psychosocial component instead of Problem Solving Treatment. Delivery of IMPACT by telephone appears to have enhanced engagement and sustained involvement in the program compared with prior efforts to deliver it by in-person meetings. IPT as a psychosocial modality was well-received by patients. To date, treatment outcomes have been favorable and will be reported in this presentation.

### COGNITIVE BEHAVIORAL THERAPY FOR INSOMNIA IN GERIATRIC PRIMARY CARE

Rosanne Leipzig, and Gregory Hinrichsen, *Icahn School of Medicine at Mount Sinai, New York, New York, United States*

Insomnia is common in older adults and may have adverse cognitive, emotional, and physical consequences. Some older people are prescribed sleep medications for insomnia despite longstanding concerns about their use with older people (i.e., BEERS criteria). Cognitive Behavioral Therapy for Insomnia (CBT-I) is highly effective in the treatment for insomnia in adults and older adults. However, most studies of CBT-I in late life have been conducted with individuals younger than 70. This paper discusses four years of experience of providing CBT-I to older people in geriatric primary care, two-thirds of whom were older than 75 years of age. Among the subgroup of 29 individuals who completed a full course of CBT-I, almost all of those who had been on sleep medications discontinued them. Treatment outcomes were large and clinically meaningful. This paper will also describe our experience in providing CBT-I via telehealth because of the COVID pandemic.

### ALIGNING TO A NEW NORMAL DURING COVID-19

Lizette Munoz, *Mount Sinai Health System, New York, New York, United States*

The Acute Life interventions Goals and Needs Program (ALIGN) is an inter-professional team of medical and social work providers dedicated to offering time-limited intensive ambulatory care to the most complex, high cost, high needs older patient population at Mount Sinai Hospital in NYC. During the 2020 COVID19 pandemic, ALIGN pivoted to focus on emergency planning actions. Such actions included language and culturally concordant goals of care discussions with patients and family, completion of electronic Medical Orders for Life Sustaining Treatment, reassessment of patient's social determinants of health, determination of adequate access to food, medication, and emotional support to those alone and isolated, and assistance with video telemedicine. ALIGN's model of care has shown how adaptable this program and others were during the height of the pandemic.

### Session 3065 (Symposium)

### LESSONS LEARNED IN THE DELIVERY OF LIVE REMOTE GROUP WEIGHT LOSS AND PHYSICAL ACTIVITY INTERVENTIONS FOR OLDER ADULTS

Chair: Jason Fanning Discussant: Barbara Nicklas

Social connection lies at the root of lasting health behavior change, and as such most effective interventions are built around social tools. Group leaders and peers provide education, and act as models of successful change and collaborators in addressing common barriers to behavioral adoption and maintenance. Unfortunately, many older adults do not have access to high quality group programs due to factors such as limited transport options, lack of local availability, or worries over personal safety. Importantly, developing effective, synchronous remote group programming is not as simple as delivering an in-person session via teleconference software. Instead, careful consideration must be paid to technology selection, fostering effective group communication, and developing confidence for use of remote intervention tools. This symposium provides key lessons learned from three group-based activity and weight loss interventions for older adults that focused on live, remote interaction. Jason Fanning will share lessons from the MORPH study, which paired remote group-mediated behavioral counseling with dietary weight loss and the accumulation of aerobic activity across the day. Christina Hugenschmidt will share her experiences adapting a group program involving improvisational dance or social gaming for remote delivery. Kushang Patel will present results from a mixed-methods study on the feasibility and acceptability of a remotely-delivered exercise program for older adults with knee osteoarthritis. Finally, Barbara Nicklas will place these experiences in the context of the development of exercise interventions for older adults over time, and highlighting vital next steps for ensuring more older adults have access to this important behavioral medicine.

### DELIVERING A GROUP-MEDIATED WEIGHT LOSS AND ACTIVITY PROGRAM TO OLDER ADULTS WITH CHRONIC PAIN IN THE MORPH STUDY

Amber Brooks,[1] Barbara Nicklas,[2] W. Jack Rejeski,[3] and Jason Fanning,[4] *1. Department of Anesthesiology, Wake Forest School of Medicine, North Carolina, United States, 2. Wake Forest School of Medicine, Winston-Salem, North Carolina, United States, 3. Department of Health and Exercise Science, Wake Forest University, North Carolina, United States, 4. Wake Forest University, Winston Salem, North Carolina, United States*

Chronic pain in aging is a potent cause and consequence of obesity, inactivity, and prolonged sedentary behavior, making these especially important targets for behavioral intervention. This study aimed to refine a theory-based group-mediated diet and sedentary behavior intervention for older adults with chronic pain. Participants (N=28) attended 12 weekly group meetings generally in home via WebEx and used an mHealth self-monitoring app as they attempted to move more often and reduce caloric intake. Relative to a control condition, the program produced improvements in physical function ($\eta^2$=.08), pain intensity ($\eta^2$=.12), sedentary time ($\eta^2$=.07), and weight loss ($\eta^2$=.21). Key findings related to effective remote group intervention delivery included: (1) the importance of a self-efficacy-enhancing technology orientation; (2) the value of small group bonding activities to seed communication; and (3) the impact of software choice on interpersonal communication. We will discuss the value of these findings for future remote intervention design.

### THE VIRTUALIZATION OF A MOVEMENT AND SOCIAL GROUP-ACTIVITY INTERVENTION FOR OLDER ADULTS AND THEIR CAREGIVERS

Deepthi Thumuluri,[1] Christina Soriano,[2] Rebecca Barnstaple,[3] Jason Fanning,[4] Jessie Laurita-Spanglet,[5] Edward Ip,[6] and Christina Hugenschmidt,[6], *1. Wake Forest School of Medicine, winston-Salem, North Carolina, United States, 2. wake forest university, winston-Salem, North Carolina, United States, 3. York University, Toronto, Ontario, Canada, 4. Wake Forest University, Winston Salem, North Carolina, United States, 5. University of Southern Maine, Portland, Maine, United States, 6. Wake Forest School of Medicine, Winston-Salem, North Carolina, United States*

COVID-related safety concerns mandated suspension of our ongoing trial testing the effects of movement and social engagement in older adults with early-stage dementia and their caregivers (dyads). Participant vulnerability and the requirement for group social interaction complicated intervention resumption. We present results from a successful pilot to rapidly and iteratively optimize study interventions for remote delivery targeting intervention mediators (social connection, movement) based on participant feedback. Three-dyad groups (n=6 individuals) completed cycles of intervention via Zoom immediately followed by an interview with open-ended and quantitative feedback. Cycles were repeated until no new information was solicited, then repeated with new participants. Optimization revealed needs for technological support, more intensive movement, and social connection. Specifically, the inability to make eye contact, see others' full body, and technology-associated timing asynchronies impeded social connection in the movement group. We will present practical tips for crafting remote group interventions for caregiver/person living with dementia dyads.

### FEASIBILITY AND ACCEPTABILITY OF TELE-ENHANCE FITNESS IN OLDER ADULTS WITH KNEE OSTEOARTHRITIS

Elise Hoffman,[1] Neta Simon,[1] Nancy Gell,[2] and Kushang Patel,[1], *1. University of Washington, Seattle, Washington, United States, 2. University of Vermont, Burlington, Vermont, United States*

Enhance Fitness (EF) is an evidence-based, group exercise program for older adults. When COVID-19 halted in-person EF classes nationally, we adapted EF for remote delivery (tele-EF) by engaging key stakeholders. To determine feasibility and acceptability of tele-EF, we conducted a mixed methods study among 42 older adults (≥65 years) with knee osteoarthritis. Participants attended EF classes for 1-hour, 3 days/week for 4-5 months (1-3 months in-person EF and 2-4 months in tele-EF). Attendance for in-person EF was 80.0% versus 91.0% for tele-EF. Nearly all participants (95.2%) reported that they were satisfied or very satisfied with tele-EF. Qualitative exit interview data mapped well onto Social Cognitive Theory constructs. With tele-EF, participants found that livestream classes facilitated accountability and self-efficacy to participate in exercise and that interactive instruction provided encouragement and support to exercise. Thus, tele-EF is a viable remotely-delivered exercise program for older adults that retains many features of in-person EF.

### Session 3070 (Paper)

### LOVE, ROMANCE, AND AGING

### DOES RELATIONSHIP END PRECEDE COGNITIVE DECLINE? AN ANALYSIS OF THE HEALTH AND RETIREMENT STUDY

Douglas Hanes,[1] and Sean Clouston,[2] *1. Stony Brook University, Stony Brook, New York, United States, 2. Renaissance School of Medicine, Stony Brook University, Renaissance School of Medicine, Stony Brook University, New York, United States*

Relationship status is thought to be associated with cognitive health in older adults, with married persons performing better on memory assessments than unmarried-cohabitating, single, divorced, and widowed persons. However, questions remain about whether relationship termination causes cognitive decline, is a result of it, or whether they share a cause; and the mechanisms by which such a relationship might operate. To address this gap in the literature, we hypothesized that relationship termination could affect cognition via the following five pathways: (1) post-termination depression; (2) loss of distributed-cognition partner; (3) cognitive depletion from caring for partner in declining and ultimately terminal health; (4) divorce to preserve assets to qualify for Medicaid to cover healthcare for cognitive decline; and (5) post-termination changes in neuropsychiatric symptoms alongside a pre-existing neurodegenerative condition that also causes cognitive decline. Using data from the 2000–2016 waves of the Health and Retirement Study (HRS; N = 23,393), we found that relationship termination, whether due to divorce or widowhood, was associated with cognitive decline. Using mixed-effects regression we found that the rate of cognitive decline increased after relationship termination (widowhood: □ = -0.587, p <0.001; divorce: □ = -0.221, p <0.001), supporting mechanism (5). Using HRS data for respondents and their spouses' mental and physical health, health insurance, and activities of daily living, we also find support for mechanisms (1) and (3). Relationship termination is a critical juncture in a person's life course that has multiple implications and may, ultimately, worsen patients' conditions.

### PERCEPTIONS OF PARTNER AFFECT MEDIATE AFFECT CONTAGION IN OLDER COUPLES' DAILY LIFE: AN EXPERIENCE-SAMPLING STUDY

Elisa Weber,[1] and Gizem Hueluer,[2] *1. University of Zurich, University of Zurich, Zurich, Switzerland, 2. University of South Florida, Tampa, Florida, United States*

Intimate relationship partners dynamically covary in their affective states. One mechanism through which intimate relationship partners experience and shape each other's affective states is affect contagion, i.e., the spread of affective states from one person to another. The degree to which social-cognitive processes are involved in affect contagion in daily life remains unclear. The majority of older adults live together with a spouse/partner, and intimate relationships are one of the most important social contexts in their daily lives. Expanding on previous research, we focused on contagion of positive and negative affect between older relationship partners, and examined whether processes of affect contagion were mediated by perceptions of partner affect, i.e., how individuals thought their partners felt at previous moments. We used data from an experience sampling study with 152 older heterosexual couples (304 participants; 65+ years old) who reported on their positive and negative affect, perceptions of their partner's positive and negative affect, and presence or absence of partners 6 times a day for 14 days. Dyadic multilevel mediation models were used to evaluate our hypotheses. We observed strong evidence that processes of positive affect contagion between partners were mediated by perceptions of partner's affective states. Negative affect contagion was directed from men to women, but not vice versa, and mediated by perceptions of partner's affective states. Partner presence was unrelated to processes of affect contagion. Our findings help identify underlying mechanisms of affect contagion and support the notion that perceptions of close others' emotions might shape our own feelings.

### ROMANTIC ATTACHMENT, STRESS, AND COGNITIVE FUNCTIONING IN A LARGE SAMPLE OF MIDDLE-AGED AND OLDER COUPLES

Rebekka Weidmann, and William Chopik, *Michigan State University, East Lansing, Michigan, United States*

Romantic relationships are a key factor contributing to health across the lifespan. Within this research line, attachment theory has been a useful framework to understand how relationships impact health. One primary health concern in late adulthood is reduced cognitive functioning: Alzheimer's disease and related neurodegenerative disorders become increasingly prevalent with age affecting millions of people. Even though much research has identified various sociodemographic, medical, and behavioral risk factors, little knowledge exists on romantic attachment's psychosocial role for cognitive decline. The purpose of this study was to examine the link between insecure attachment, stress, and cognitive functioning in a large sample of middle-aged and older couples. In particular, we wanted to investigate how insecure attachment is linked to both partners' cognitive functioning and whether stress mediates these associations. To that aim, we used data of 1,043 romantic couples (Mage = 64.7 years; 38.5% same-sex couples) who reported on their attachment anxiety and avoidance, their stress levels, their cognitive decline, and their and their partners' dementia symptoms. Couple members also participated in a memory performance task. The results suggest that anxiety is linked to participants' cognitive decline, while avoidance was linked to partners' cognitive decline and poorer memory performance. We also detected significant mediational effects for stress in the association between insecure attachment and cognitive functioning. We conclude that potentially malleable psychosocial factors, such as insecure attachment and stress, are important research objects when understanding cognitive functioning in middle and late adulthood.

### THE CRUSH: A PROTO-ROMANTIC RELATIONSHIP ACROSS THE LIFE COURSE

Joseph Kotarba,[1] and Amanda Couve,[2], *1. TxSt University, San Marcos, Texas, United States, 2. Encompass Health, San Antonio, Texas, United States*

This presentation describes the "crush" experience as it occurs among older adults. A basic definition of a crush is a one-sided, proto-romantic relationship. The scholarly and commonsense understanding in American culture focuses on the crush as most commonly occurring during the developmental phases of adolescence and pre-adolescence. Symbolic interactionists view life course as a somewhat fluid process of adapting to changing situations in life. Experiences like the crush can potentially occur at almost any age at which romantic thoughts and feelings are possible. Our ethnographic research on older adults residing either in group facilities or in domiciliary locations indicates that crushes are fairly common. These crushes follow the same general narrative as crushes among younger people: a beginning, a middle and an end. There are two narrative styles among older adults: face-to-face and mediated. The crush in a group facility is encouraged by interaction during social hours, meals, entertainment, and religious/spiritual activities. Crushes are more observable among women who do not have to delve into their past for objects of their affection. Available paramours from the mass media include young celebrities such as Michael Buble and Josh Groban. These crushes differ from those among younger women in the denouement, to the degree affection generally fades away from memory rather than comes to a distinct end. Factors such as increased access to electronic media and music, and increased sociality in the community and in residential environments will create situations in which the security, excitement and rewards of a crush are plausible.

### Session 3075 (Paper)

### MOOD, EMOTIONS, AND HEALTH

### AGE-RELATED DIFFERENCES IN CLINICAL AND PSYCHOSOCIAL PREDICTORS OF UNMET NEEDS IN BLADDER CANCER SURVIVORS

Nihal Mohamed,[1] Tung Ming Leung,[2] Katherine Ornstein,[3] Naomi Alpert,[3] Travonia Brown-Hughes,[4] Emanuela Taioli,[3] and Natasha Kyprianou,[3] *1. Icahn School of Medicine at Mount Sinai Department of Oncological Sciences, New York, New York, United States, 2. Northwell Health, Northwell Health, New York, United States, 3. Icahn School of Medicine at Mount Sinai, New York, New York, United States, 4. School of Pharmacy, Hampton, Virginia, United States*

Understanding of unmet needs and their predictors among bladder cancer (BC) survivors is critical to optimize health care planning for patients. This study compares between younger (<65 Years) and older (≥65 Years) BC patients across seven domains of unmet needs (e.g., informational, psychological, supportive care, daily living, communication, logistic, and sexuality needs) and their demographic, clinical, and psychosocial predictors. BC survivors (N=159; 47% women) were recruited from the Bladder Cancer Advocacy Network and completed a questionnaire that included the needs assessment survey (BCNAS-32), hospital anxiety and depression scale (HADS), coping (BRIEF COPE), social provisions scale (SPS), and self-efficacy beliefs (GSE) scale. Although no significant group differences in all reported needs emerged, both groups reported more communication (IQR = 50 (62.5) and less sexuality needs

(IQR =13 (52.1). Older patients reported higher depression and anxiety (IQR = 32 (11.5); N = 68) than younger patients (IQR = 28 (11.0); p < .01; N = 88). Multivariable analyses stratified by age showed significant effects of gender among older patients with women experiencing more psychological, care, communication, and sexuality needs than men. Multivariable analyses also showed age-related differences (p < .05) in the predictors of needs controlling for covariates (e.g., gender). Among older patients both higher depression and anxiety and lower self-efficacy beliefs were associated with more psychological, care, and communication needs. Among younger patients, higher depression and anxiety were associated with more psychological, logistic, daily living, and communication needs. Results emphasize the importance of tailoring care planning for patients based on age.

### LINKING RELIGIOUS IDENTITY, PARTICIPATION, AND FAITH TO DOMAINS OF MENTAL HEALTH IN LATE LIFE

Eva Kahana,[1] Poshan Dahal,[2] Tirth Bhatta,[3] and Polina Ermoshkina,[4], *1. Case Western University, Case Western reserve University, Ohio, United States, 2. Case Western Reserve University, Case Western Reserve University, Ohio, United States, 3. University of Nevada, Las Vegas, Nevada, United States, 4. Case Western Reserve University, Cleveland, Ohio, United States*

Religiosity in late life has been linked to psychological well-being outcomes. However, there has been insufficient attention to complex associations between different domains of religiosity and domains of psychological wellbeing. We explored associations between religious identity, religious participation, religious coping (trust in God), and mental health indicators of depressive symptoms, life satisfaction, and positive/negative affect among 797 independent, retirement community-dwelling older adults. At baseline, religious identity (expressed as self- concept) and religious participation (church attendance) each were associated with fewer depressive symptoms (b=-0.47, p<0.05; b=-0.19, p<0.05). Religious identity, however, was significantly associated with both life satisfaction and positive affects but not with negative affect. Religious coping was associated with greater life satisfaction and positive affect. Our longitudinal analysis documented a statistically significant decline in depressive symptoms, and increase in life satisfaction and positive affect, with corresponding increase in religious identity over time. However, changes in religious identity did not lead to significant changes in negative affect over time. Religious coping and church attendance fully explained the influence of religious identity on changes in life satisfaction. Although the influence of religious identity on depressive symptoms and positive affect was weakened, its significant influence was maintained even after the consideration of religious coping and church attendance. Beyond religious identity, we also observed a significant increase in positive affect with a corresponding increase in religious coping. Overall, our findings support expectations that religious identification and practices are associated with greater psychological well-being among community dwelling old- old adults.

### THE ASSOCIATION BETWEEN MIDLIFE EDUCATION AND DEPRESSIVE SYMPTOMS IN LATE LIFE: THE AGES-REYKJAVIK STUDY

Milan Chang,[1] Chiharu Nishizuka,[2] Hrafnhildur Eymundsdottir,[1] Sigurveig Sigurdardottir,[3] Alfons Ramel,[3] Vilmundur Gudnasson,[3] and Palmi Jonsson,[4], *1. The Icelandic Gerontological Research Institute, Reykjavik, Hofuoborgarsvaoio, Iceland, 2. Faculty of Social Science, Reykjavik, Hofuoborgarsvaoio, Iceland, 3. University of Iceland, Reykjavik, Hofuoborgarsvaoio, Iceland, 4. University hospital Landspitali, Reykjavik, Hofuoborgarsvaoio, Iceland*

**Background:** Disability and depression are associated with cumulative health adversities such as socioeconomic status (SES), nutrition, medical care, and education among older adults. However, there is little evidence on the longitudinal association between mid-life education level with a disability and depressive symptoms in older adults in Iceland. The aim of the study was to examine the association between mid-life education and prevalence of activity of daily living (ADL) dependency and high depressive symptoms in late-life.

**Methods:** A large community-based population residing in Reykjavik, Iceland (n=4991, 57.3% women, 76.9±5.8 yrs) participated in a longitudinal study with an average of 25 years of follow-up. Mid-life education was categorized into 4 groups (primary, secondary, college, and university). ADL dependency and high depressive symptoms were assessed on average 25 (±4) years later. The 5-item ADL dependency score ranged between 0 (no difficulty) and 18. Depressive symptoms were assessed by the 15-item Geriatric Depression Scale (GDS).

**Results:** After controlling for demographic and health-related risk factors, those with higher education at mid-life were significantly less likely to have high depressive symptomatology (6 or higher GDS scores, Odds Ratio (OR) = 0.65, 95% Confidence Interval (CI): 0.52 ~ 0.82, P < 0.0001). However, mid-life education was not associated with ADL dependency in later life.

**Conclusion:** Our study shows that mid-life education is associated with depressive symptoms 25 years later, while no association found with ADL dependency among Icelandic older adults.

### TRANSITION TO WIDOWHOOD: TRAJECTORIES OF DEPRESSIVE SYMPTOMATOLOGY AMONG JAPANESE OLDER ADULTS

Masumi Iida,[1] Shohei Okamoto,[2] Ikuko Sugawara,[3] and Erika Kobayashi,[4] *1. Arizona State University, Tempe, Arizona, United States, 2. Tokyo Metropolitan Institute of Gerontology, Itabashi, Tokyo, Japan, 3. Bunri University of Hospitality, Suginami-ku, Tokyo, Japan, 4. Tokyo Metropolitan Institute of Gerontology, Itabashi-ku, Tokyo, Japan*

Spousal loss is one of the most consequential negative life events for the surviving partners. While there is abundant research on mental health and well-being of widows, most of these studies rely on the post-bereavement data. In this study, we use the data from the National Survey of Japanese Elderly (NSJE), which is a publicly available longitudinal data set collected from Japanese adults aged 60 years and older. The current study uses the first seven waves of data from 1987 to

2006, where participants were followed every three to four years. Using the NSJE advances our understanding of the bereavement process as it allows us to observe the levels and trajectories of depressive symptom before, during, and after the loss of their spouses. In our analyses, we selected 522 participants (average age at bereavement: 75.0 years; 27% male) who experienced spousal loss at some point during the seven waves. We examined the trajectories of depressive symptoms assessed using CES-D as these participants transition to widowhood. The results showed a small significant increase in depressive symptoms leading up to the time of the loss. There was also a significant increase in symptoms at the time of the loss, but we did not observe any decline in symptoms after the loss. In addition, we found that their age at bereavement significantly moderated the pattern, such that the increase in depressive symptoms at the time of the loss was attenuated for older participants. The implications of these findings will be discussed.

### Session 3080 (Symposium)

### MULTIPLE PERSPECTIVES ON DISASTER PREPAREDNESS IN LONG-TERM CARE: FROM HEAT TO HOSPICE

Chair: David Dosa Co-Chair: Ross Andel
Discussant: Lisa Brown

Preparedness of residents in long-term care (LTC) exposed to disasters continues to warrant concern. Prior work by our research team highlights explicit evidence of the profound vulnerability of Florida nursing home (NH) residents exposed to Hurricane Irma in 2017. This research adds to our knowledge of the profound effect of disasters on long term care residents. This symposium will utilize mixed methodologies to discuss the varied effects of Hurricane Irma on vulnerable older adults residing in Florida NHs and Assisted Living communities (ALCs). Using a novel methodology for identifying a cohort of ALC residents, the first presentation will present the morbidity and mortality effects of Hurricane Irma on Florida ALC residents and identify high risk groups by health condition. The second presentation will document the effect of Hurricane Irma on NH Residents previously enrolled in Hospice and expound on the effect of the disaster on hospice enrollment after the storm. The third presentation will present qualitative results of interviews with ALC administrators highlighting the effect of the storm on both large and small (<25 beds) facilities. The fourth presentation will address the issue of heat exposure in the days after Hurricane Irma and consider the preventative effect of generators on morbidity and mortality. Finally, a fifth presentation will examine NH staffing level variation in the days leading to the hurricane. To conclude, this symposium offers a multi-faceted view of a disaster's effects on LTC residents across Florida, including novel data from the NH environment and lesser-examined ALCs.

### RESOURCES AND RELATIONSHIPS IN DISASTERS: DIFFERENCES AMONG SMALL AND LARGE ASSISTED LIVING COMMUNITIES

Debra Dobbs,[1] Joseph June,[2] David Dosa,[3] Kathryn Hyer,[4] and Lindsay Peterson,[4] *1. University of South Florida, School of Aging Studies, University of South Florida, Florida, United States, 2. University of South Florida, Tampa, Florida, United States, 3. Brown University, Providence, Rhode Island, United States, 4. University of South Florida, University of South Florida, Florida, United States*

Disaster preparedness among assisted living communities (ALCs) has not been widely researched, despite the growth of ALCs and evidence of disability in this population. An additional issue of concern is the way in which ALCs vary, including variation by size. The purpose of this paper was to explore the experiences of ALCs in Florida that experienced Hurricane Irma in 2017 and how experiences varied by ALC size. Qualitative interviews and focus groups were conducted with representatives of small ALCs (<25 beds; n=32) and large ALCs (25+; n=38). Transcripts were analyzed using Atlas.ti version 8, and research team members collaborated to reach consensus on codes and further analyze differences based on ALC size. Results suggest there are differences among ALCs in their disaster preparedness and response, and these differences are related to size (e.g., access to resources, organizational characteristics). Implications for ALC resident wellbeing and future disaster planning will be discussed.

### THE ROLE OF HOSPICE IN FLORIDA NURSING HOMES POST-HURRICANE IRMA

Julianne Skarha,[1] Lily Gordon,[2] Dylan Jester,[3] Lindsay Peterson,[4] David Dosa,[1] and Debra Dobbs,[5] *1. Brown University, Providence, Rhode Island, United States, 2. Health Services, Policy and Practice, Brown University, Providence, Rhode Island, United States, 3. School of Aging Studies, University of South Florida, Tampa, Florida, United States, 4. University of South Florida, University of South Florida, Florida, United States, 5. University of South Florida, School of Aging Studies, University of South Florida, Florida, United States*

There is little known about the effect of hospice post-disaster. This study utilized exposure to Hurricane Irma (2017) to evaluate the differential mortality effect of the disaster on Florida NH residents (N=45,882) compared to a control group of residents in the same NHs in 2015 (N=47,690) by hospice status. We also examine the difference in hospice utilization rates post-storm for short- and long-stay (LS) residents. There was an increase in mortality for those in the cohort not on hospice within 90 days in 2017 compared to 2015 (OR= 1.06, 95% CI: 1.01, 1.11). For the rate of hospice enrollment post-storm among residents previously not on hospice, there was an increase among LS residents within 30 days (OR =1.15, 95% CI: 1.02, 1.23) and 90 days (OR= 1.12, 95% CI: 1.05, 1.20). It is important to further examine the increase in the rate of hospice enrollment in LS NH residents post-storm.

### NURSE STAFFING IN NURSING HOMES DURING HURRICANE IRMA

Kali Thomas,[1] Lindsay Peterson,[2] David Dosa,[3] Ross Andel,[4] Kathryn Hyer,[2] and Dylan Jester,[5] *1. Brown University, Brown University/Providence, Rhode Island, United States, 2. University of South Florida, University of South Florida, Florida, United States, 3. Brown University, Providence, Rhode Island, United States, 4. University of South Florida, Tampa, Florida, United States, 5. School of*

*Aging Studies, University of South Florida, Tampa, Florida, United States*

Little is known about the effect of hurricanes on nurse staffing in nursing homes. Hurricane Irma made landfall on September 10th, 2017 in Florida. This study examined daily nurse staffing levels from September 3rd-24th, 2017 in 653 nursing homes; 81 facilities evacuated and 572 facilities sheltered-in-place. Data from Payroll-Based Journaling (PBJ), Certification and Survey Provider Enhanced Reports (CASPER), and Florida's health providers' emergency reporting system were used. Among all facilities, we found significant increases in staffing for licensed practical nurses (p=.02) and certified nursing assistants (p<.001), but not for registered nurses (p=.10) before Hurricane Irma made landfall. In comparison to facilities that sheltered-in-place, evacuating facilities increased staffing levels of all nurse types (all p<.001). From one week before landfall to two weeks after landfall, an additional estimated $2.41 million was spent on nurse staffing. Policymakers attempting to reduce the burden of natural disasters on nursing homes should reimburse staffing-related expenses.

### EXCESS MORTALITY ATTRIBUTED TO HURRICANE IRMA AMONG ASSISTED LIVING RESIDENTS WITH CHRONIC CONDITIONS IN FLORIDA

Kali Thomas,[1] Lindsay Peterson,[2] Debra Dobbs,[3] Ross Andel,[4] David Dosa,[5] and Cassandra Hua,[5], *1. Brown University, Brown University/Providence, Rhode Island, United States, 2. University of South Florida, University of South Florida, Florida, United States, 3. University of South Florida, School of Aging Studies, University of South Florida, Florida, United States, 4. University of South Florida, Tampa, Florida, United States, 5. Brown University, Providence, Rhode Island, United States*

Little is known about the impact of hurricanes on residents in assisted living communities (ALs), especially among individuals with chronic conditions that increase their risk of death after storms. We examined how the association between exposure to Hurricane Irma in 2017 and mortality differed by select chronic conditions. With Medicare data, we identified cohorts of AL residents in 2015 (n= 30,712) and 2017 (n= 29,842 ) and compared their rates of 30-day and 90-day and mortality. We adjusted rates for demographic characteristics and other comorbidities. AL residents with diabetes were at highest risk of death after the storm; between 2015 and 2017 they experienced a 50% increase in their 30-day mortality rates (0.6% in 2015, 0.9% in 2017) and a 43% increase in their 90-day mortality rates (2.1% in 2015, 3.0% in 2017). Policy makers should consider strategies to ensure that diabetic residents maintain continuity of medical care during disasters.

### HEALTH EFFECTS OF POWER LOSS AFTER HURRICANE IRMA ON NURSING HOME RESIDENTS IN FLORIDA

Lily Gordon,[1] Julianne Skarha,[1] Nazmus Sakib,[2] Joseph June,[3] Dylan Jester,[4] Lindsay Peterson,[5] and David Dosa,[1] *1. Brown University, Providence, Rhode Island, United States, 2. Department of Industrial and Management Systems, University of South Florida, Tampa, Florida, United States, 3. University of South Florida, Tampa, Florida, United States, 4. School of Aging Studies, University of South Florida, Tampa, Florida, United States, 5. University of South Florida, University of South Florida, Florida, United States*

Previous research establishes that hurricanes adversely affect nursing home (NH) resident health but specific causal pathways are still unclear. We combined power outage data with Medicare claims to determine the effects of power loss from Hurricane Irma(2017) among NH residents in Florida. Out of 580 facilities, 289 reported power loss. These facilities had higher star ratings; higher beds counts, and were preferentially in the Southeast region of Florida compared to facilities without outages. There were 27,767 residents living in a NH without power. They were comparable in characteristics to residents that did not lose power (N=26,383). We ran adjusted generalized linear models with robust standard errors, clustering for NH. We found power loss was associated with a trend towards increased odds of mortality within 7-days (OR:1.12, 95% CI:0.96, 1.30) and 30-days (OR:1.10, 95% CI:1.00, 1.21) post-storm, but not with hospitalization. Future research should investigate the time-specific effects of power outages.

### Session 3085 (Symposium)

### NEW DEVELOPMENTS IN VIEWS ON AGING RESEARCH: VARIABILITY, INNOVATIVE CONCEPTS, AND CONTEXTUAL PERSPECTIVES

Chair: Anna Kornadt Co-Chair: Hans-Werner Wahl
Discussant: Susanne Wurm

Views on aging (VoA) such as attitudes toward own aging, awareness of aging or subjective age, have a large impact on outcomes related to positive development in later life. Recent research in this domain has focused on complex research designs and inter-systemic linkages at different levels. Indicators of short-term variability of VoA have increasingly been investigated, linking the respective findings with performance indicators, biomarkers, and trait-like data. In addition, bidirectional relationships of VoA and outcomes over time as well as data contextualizing VoA across historical time may offer new insights on the plasticity of VoA seen in bio-cultural co-construction. The symposium will showcase these recent trends with studies from the U.S. and Germany. First, Zhu and Neupert extend previous studies by linking established VoA indicators with future time perspective, all assessed by means of a daily diary study with 60-90 year-old adults. Kornadt et al. examined the variability of subjective age within a day and the relationship with trait subjective age and cortisol levels. Mejia et al. extend VoA to the area of subjective awareness of fall risks in daily life and links them with physical performance. Wettstein et al. investigate the bidirectional relationship of VoA indicators and perceived stress over time. Finally, we move from the micro to a macro-micro design in Wahl et al.'s presentation addressing historical change in VoA across 20 years in the Berlin Aging Study and in MIDUS. Susanne Wurm will discuss how different levels of VoA analysis will find better interlinkage in the future.

### PERCEIVING MORE AGE-RELATED LOSSES? THE ROLE OF GENERAL CONTROL BELIEFS AND DAILY DISTURBANCE TO PLANS

Xianghe Zhu,[1] and Shevaun Neupert,[2], *1. North Carolina State University, North Carolina State University, North*

*Carolina, United States, 2. North Carolina State University, Raleigh, North Carolina, United States*

We examined daily fluctuations in future time perspective within the daily stress and awareness of aging processes. Awareness of age-related change (AARC) focuses on everyday experiences that highlight changes in behavior and functioning as a result of growing older. We integrated individual differences in control beliefs because those with higher control tend to be more resilient to stressors. We conducted a daily diary study of 112 older adults (aged 60-90) who completed measures of control beliefs at baseline and then daily measures of stressor exposure, appraisal (e.g., threats to future plans), and AARC for eight consecutive days. Increases in threats to future plans were associated with increases in AARC losses, and those with low control were especially vulnerable to increases in threats to future plans. With a constricted future time perspective, any threats to future plans may be especially harmful for older adults who are low in control beliefs.

### SHORT-TERM FLUCTUATION OF SUBJECTIVE AGE AND ITS CORRELATES: AN ECOLOGICAL MOMENTARY ASSESSMENT OF OLDER ADULTS

Theresa Pauly,[1] Denis Gerstorf,[2] Ute Kunzmann,[3] Oliver Schilling,[4] David Weiss,[3] Anna Luecke,[5] Hans-Werner Wahl,[5] and Anna Kornadt,[6], *1. University of Zurich, University of Zurich, Zurich, Switzerland, 2. Humboldt University of Berlin, Humboldt University of Berlin, Brandenburg, Germany, 3. Leipzig University, Leipzig, Sachsen, Germany, 4. University of Heidelberg, Heidelberg, Baden-Wurttemberg, Germany, 5. Heidelberg University, Heidelberg, Baden-Wurttemberg, Germany, 6. University of Luxembourg, Esch-sur-Alzette, Diekirch, Luxembourg*

We examined short-term fluctuations of subjective age with data obtained from 123 young-old (Mage = 67.19 years) and 47 old-old adults (Mage = 86.59 years) who reported their momentary subjective age six times a day over seven consecutive days as they were going about their everyday lives. Participants felt younger on a large majority of occasions, and 25% of the total variability in subjective age could be attributed to within-person variation. Those with younger trait subjective ages exhibited larger moment-to-moment variation, while chronological age did not impact variability. Furthermore, we investigated relationships between within-day fluctuations of subjective age and daily cortisol fluctuations. Our findings extend the literature on subjective age by showing that how old people feel can vary on a momentary basis, that state and trait components of subjective age are related, and that fluctuations in subjective age are related to biomarkers of stress.

### AWARENESS OF BALANCE AS AN INTRAINDIVIDUAL DYNAMIC OF OBJECTIVE AND SUBJECTIVE EXPERIENCES OF FALL RISK IN DAILY LIFE

Tai-Te Su,[1] Aileen Griffin,[2] Faith Washington,[3] Jason Fanning,[4] Jacob Sosnoff,[5] and Shannon Meija,[3] *1. University of Illinois at Urbana-Champaign, Champaign, Illinois, United States, 2. University of Illinois at Urbana-Champaign, Aurora, Illinois, United States, 3. University of Illinois, Champaign, Illinois, United States, 4. Wake Forest University, Winston Salem, North Carolina, United States, 5. School of Health Professions, University of Kansas Medical Center, Kansas City, Kansas, United States*

Falls are life-changing events in older adulthood. With an accurate understanding of balance, older adults can adapt to age-related changes in physical ability without prematurely restricting physical activity. The Daily Balance Project examines the implications of older adults' awareness of fall risk in daily life. For 30-consecutive days, following a fall-risk assessment, 40 older adults used a smartphone to report balance confidence and then perform four balance assessment and a 30-second sit-to-stand task to measure postural sway and fall-risk. Measures of postural sway showed greater intraindividual variability than balance confidence and fall risk. Multilevel models showed that awareness of balance fluctuated during the study and varied across individual differences in baseline fall-risk. Baseline fall risk also differentiated how balance confidence and postural sway were linked to subsequent momentary fall risk assessments. The findings are discussed within the framework of action-perspectives of adult development and awareness of aging.

### LONGITUDINAL RELATIONS BETWEEN VIEWS ON AGING AND PERCEIVED STRESS: EVIDENCE FOR MUTUAL ASSOCIATIONS

Hans-Werner Wahl,[1] Anna Kornadt,[2] and Markus Wettstein,[3], *1. Heidelberg University, Heidelberg, Baden-Wurttemberg, Germany, 2. University of Luxembourg, Esch-sur-Alzette, Diekirch, Luxembourg, 3. German Centre of Gerontology, Berlin, German Centre of Gerontology, Berlin, Berlin, Germany*

We investigated the reciprocal longitudinal relation between perceived stress and three established domains of views on aging (VoA): (1) subjective age; (2) attitude toward own aging [ATOA]; and (3) aging-related cognitions including social loss, physical decline, and continuous growth. We also examined the potentially moderating role of chronological age. Data of the German Ageing Survey, comprising two measurement occasions (2014 and 2017) and a sample of 4,588 individuals aged between 40 and 95 years, were analyzed. Controlling for socio-demographic and health-related indicators, cross-lagged models indicated mutual longitudinal relations between VoA and stress. Whether the pathway from stress to VoA or the opposite pathway was stronger varied depending on the VoA considered. With increasing age, most VoA domains were less strongly associated with subsequent perceived stress. Our findings suggest that less favorable VoA predict higher perceived subsequent stress, but they are also preceded and predicted by higher levels of perceived stress.

### NO HISTORICAL CHANGE IN VIEWS ON AGING AND THEIR CORRELATES: EMERGING EVIDENCE FROM GERMANY AND THE UNITED STATES

Johanna Drewelies,[1] Sandra Duezel,[2] Margie Lachman,[3] Jacqui Smith,[4] Nilam Ram,[5] Ulman Lindenberger,[2] Denis Gerstorf,[6] and Hans-Werner Wahl,[7], *1. Humboldt University, Berlin, Berlin, Germany, 2. Max-Planck-Institute for Human Development, Berlin, Berlin, Germany, 3. Brandeis University, Brandeis University, Massachusetts, United States, 4. University of Michigan, University of*

*Michigan, Michigan, United States, 5. Stanford University, Stanford, California, United States, 6. Humboldt University of Berlin, Humboldt University of Berlin, Brandenburg, Germany, 7. Heidelberg University, Heidelberg, Baden-Wurttemberg, Germany*

To examine historical changes in views on aging, we compared matched cohorts of older adults within two independent studies that assessed differences across a two-decade interval, the Berlin Aging Studies (BASE, 1990/93 vs. 2017/18, each n = 256, Mage = 77) and the Midlife in the United States Study (MIDUS, 1995/96 vs. 2013/14, each n = 848, Mage = 67). Consistent across four different dimensions of individuals' subjective views on aging (age felt, age appeared, desired age, attitudes towards own aging) in the Berlin Aging Studies and corroborated with subjective age felt in the MIDUS, there was no evidence whatsoever that older adults of today have more favorable views on how they age than older adults did two decades ago. We discuss reasons for our findings, including the possibility that individual age views may have become increasingly decoupled from societal age views.

### Session 3090 (Symposium)

### OLDER ADULT SUBSTANCE USE, SUPPORT, HEALTH, AND WELL-BEING: UNDERSTANDING A COMPLEX PICTURE

Chair: Bethany Bareham Co-Chair: Verena Cimarolli
Discussant: Alexis Kuerbis

Substance Use Disorders (SUDs), particularly hazardous alcohol use and Opioid Use Disorder (OUD), are increasingly common in older populations. Both are associated with poor mental and physical wellbeing. Alcohol use can also contribute positively to older people's social and emotional wellbeing, and may be used by older people in managing pain. Health concerns can make older people more aware of the impact of their lifestyle, including substance use, for their health. The relationship between substance use, health and wellbeing in old age is complex, raising questions for how older people with SUDs are best supported. We aim to give insight into this complexity, drawing on five recent studies. Our first presentation examines the impact of health concerns in old age on alcohol use. Our second presentation explores the complicated relationship between experiences of pain, and alcohol use with age. Our third presentation considers wellbeing in older people with OUD, and the impact of Adverse Childhood Experiences, psychosocial, health and sociodemographic factors; and what this means for how wellbeing is promoted in this population. Our fourth presentation explores how those supporting people with dementia navigate alcohol use amongst older residents, in a care context where autonomy is prioritised, but health risk behaviours must be managed. Our final presentation considers alcohol use in 'the new normal'; examining the impact of the COVID-19 pandemic on older people's alcohol use, and motivations to engage in healthier/unhealthy use. We consider implications of these complex considerations around addressing substance use amongst older people for practice.

### COVID-19, OLDER PEOPLE'S ALCOHOL USE AND ENGAGEMENT WITH SUPPORT: A RAPID EVIDENCE SYNTHESIS

Eileen Kaner,[1] Liam Spencer,[1] Hannah O'Keefe,[1] Barbara Hanratty,[1] and Bethany Bareham,[2] *1. Newcastle University Population Health Sciences Institute, Newcastle upon Tyne, England, United Kingdom, 2. Newcastle University, Newcastle upon Tyne, England, United Kingdom*

COVID-19, and associated restrictions, have likely impacted older people's alcohol use and related support needs, given disrupted routines and stress increase alcohol use in older populations. This rapid evidence synthesis aimed to examine older people's (aged 50+) alcohol use, and engagement with alcohol support services during COVID-19. Seventy-six articles were identified through systematic database searches, reporting 63 survey, five qualitative, three pilot, and five hospital admission studies; of general and service-user populations of older drinkers. Data were drawn together through narrative synthesis. Alcohol use increased for up to 32% of older people, including service-users; particularly older women. Increased use was linked to anxiety, depression and emotional distress. Decreased use was more common in some older populations; particularly Mediterranean. Barriers such as web access and safe transport affected older service-users' engagement with support. Support to address hazardous alcohol use amongst older people must be prioritised in wake of the pandemic.

### OLDER ADULT HEALTH AND REMISSION OF RISKY DRINKING AND ALCOHOL USE DISORDER: A LIFESPAN-DEVELOPMENTAL STUDY

Ellen Yeung,[1] Thomas Kwan,[2] Kenneth Sher,[3] and Matthew Lee,[2], *1. George Washington University, Washington, District of Columbia, United States, 2. Rutgers University, Piscataway, New Jersey, United States, 3. University of Missouri, Columbia, Columbia, Missouri, United States*

Older-adult drinking is a growing public-health concern. As part of a larger project investigating older adulthood by contrasting this with other adult developmental periods, this study used longitudinal U.S.-representative data to test bidirectional associations between drinking and health, emphasizing aging-related health concerns as potential mechanisms of remission from risky/problem drinking. In multiple-group cross-lag models, we found that effects of poor self-reported health on drinking reductions increased with age, reached significance around midlife, and were strongest in older adulthood. However, a caveat revealed by additional Markov transition models was that these effects did not extend to relatively severe older-adult drinkers (indexed by DSM-5 AUD). In some instances, poor health even predicted less older-adult AUD remission. Altogether, findings support the notion of aging-related health concerns as important mechanisms of older-adult drinking reduction; but highlight a need to understand barriers to these mechanisms among severe older-adult drinkers, in part toward guiding lifespan-developmentally-informed interventions.

### AGE EFFECT ON THE BIDIRECTIONAL RELATIONS BETWEEN ALCOHOL CONSUMPTION AND PAIN

Matthew Lee,[1] Kenneth Sher,[2] and Ellen Yeung,[3], *1. Rutgers University, Piscataway, New Jersey, United States, 2. University of Missouri, Columbia, Columbia, Missouri, United States, 3. George Washington University, Washington, District of Columbia, United States*

Alcohol consumption reduces but pain rises over the life course. Thus, we hypothesized that developmental variability in the bidirectional association between alcohol consumption and pain would vary as a function of age. This hypothesis was tested across three age groups – younger (<29), middle (29-65), and older (>65) using NESARC wave 1 and 2 data (N=34,653). The effect of pain interference at baseline on alcohol consumption at follow-up was non-significant across the age groups, indicating that self-medication theory was unsupported. The effect of alcohol consumption at baseline on pain interference at follow-up was significant among the middle (Estimate -.007, p=.002) and older (Estimate -.019, p<.001) groups, but non-significant among the younger group. This latter effect differed significantly between the younger and older groups (p =.005) and the middle and older groups (p=.041). Results show that alcohol consumption reduces pain interference, especially later in life.

### GENDER DIFFERENCES IN WELL–BEING AMONG PEOPLE AGE ≥ 50 IN MEDICATION-ASSISTED TREATMENT FOR OPIOID USE DISORDER

Mary Mitchell,[1] Molly Perkins,[2] Alexis Bender,[3] and Sahil Angelo,[4] *1. Friends Research Institute, Baltimore, Maryland, United States, 2. Emory University of Medicine, Atlanta, Georgia, United States, 3. Emory University, Atlanta, Georgia, United States, 4. Medstar Georgetown University Hospital, Medstar Georgetown University Hospital, District of Columbia, United States*

The impact of Adverse Childhood Experiences (ACEs) on the physical and mental well–being of older adults with Opioid Use Disorder (OUD) is unclear, and we know even less about gender differences. This study explores this association and investigates additional factors (e.g., pain, depression) that may affect physical and mental well–being in this population with a focus on gender. The sample for the present analysis includes 90 adults aged 50 and older from a larger study focused on aging with OUD across eight opioid treatment programs in Georgia. We performed multivariable linear regression analyses by gender. There was a small, but significant, association between ACEs and mental well–being for men only. Other significant predictors of physical and mental well-being (e.g., insurance status, pain, satisfaction with social role, stigma) varied by gender. We discuss the importance of these gender differences in identifying appropriate areas for intervention to improve physical and mental well–being.

### WHERE'S THE BAR? ALCOHOL AND MEANINGFUL ENGAGEMENT AMONG ASSISTED LIVING RESIDENTS WITH DEMENTIA

Candace Kemp,[1] Elisabeth Burgess,[2] and Alexis Bender,[3] , *1. Gerontology Institute, Georgia State University, Atlanta, Georgia, United States, 2. Georgia State University, Georgia State University - Atlanta, Georgia, United States, 3. Emory University, Atlanta, Georgia, United States*

Alcohol use across the life course provides some physical and psychological benefits when used in moderation. As a social model of care, assisted living (AL) communities emphasize autonomy; yet, we do not know how this philosophy extends to drinking. Using ethnographic and interview data from a larger 5-year NIA-funded study in four diverse AL communities designed to identify best practices for the meaningful engagement of AL residents with dementia, we examine how residents, families, and staff interpret residents' rights about alcohol use and how staff and families facilitate or limit alcohol use of residents with dementia. Findings indicate staff and families frequently rely on a narrative of "watchful oversight" to limit or restrict alcohol consumption while simultaneously affirming the social connection of drinking (e.g., alcohol-free socials). We discuss the implications of our findings for research and practice aimed at promoting meaningful engagement and quality of life among persons with dementia.

### Session 3095 (Paper)

### PAIN MANAGEMENT

### ACCEPTANCE AND COMMITMENT THERAPY AND EXERCISE: CAN THEY HELP OLDER ADULTS MANAGE CHRONIC PAIN?

Dara KY Leung,[1] Annabelle PC Fong,[2] Wai-Wai Kwok,[2] Angie KY Shum,[2] Tianyin Liu,[1] Gloria HY Wong,[1] and Terry YS Lum,[1], *1. The University of Hong Kong, Hong Kong, Not Applicable, Hong Kong, 2. The University of Hong Kong, Pokfulam, Not Applicable, Hong Kong*

Chronic pain is common among older adults and affects their physical and psychological well-being. While exercise can reduce pain and promote physical functions, psychological interventions may enhance pain management by addressing the psychosocial contributors to the prolonged pain. Acceptance and Commitment Therapy (ACT) is a psychological intervention that emphasizes on psychological flexibility, values, and mindfulness. This approach may be particularly helpful in dealing with chronic pain, where symptoms can be beyond one's control. This single group pre-post study investigated the feasibility and efficacy of an intervention combining ACT and exercise for chronic pain management in older adults. The intervention consisted of 16 sessions delivered over eight weeks. ACT and exercises were modified according to the individual's capability when needed. Clinical outcomes regarding pain severity and interference, pain acceptance, value of life, depression, anxiety, and physical functioning were assessed. Twenty-four older adults attended all sessions and completed the assessments. Preliminary results showed that, while participants experienced similar level of pain after the intervention, they reported less pain interference on mood and enjoyment of life, and improved chronic pain acceptance, pain self-efficacy, success at living their values, committed action, depressive symptoms, physical functioning in the lower body strength, aerobic and endurance, agility and dynamic balance, and upper body strength (all p<.050). This study lends support to the feasibility of a combined ACT and exercise intervention for chronic pain management in older adults. The efficacy of

ACT may not be directly on reducing pain, but on increased psychological flexibility to co-live with pain.

### CAREGIVER-ASSISTED PAIN COPING SKILLS TRAINING FOR PATIENTS WITH DEMENTIA: A PILOT STUDY

Laura Porter,[1] Francis Keefe,[2] Deborah Barnes,[3] Lisa Gwyther,[4] Kenneth Schmader,[5] and Christine Ritchie,[6] *1. Duke University Medical Center, Durham, North Carolina, United States, 2. Duke University School of Medicine, Durham, North Carolina, United States, 3. University of California, San Francisco, San Francisco, California, United States, 4. Duke Center for Aging, Chapel Hill, North Carolina, United States, 5. Duke, Durham, North Carolina, United States, 6. Massachusetts General Hospital, Boston, Massachusetts, United States*

Pain is common and undertreated in patients with dementia, and contributes to disability, psychological distress, neuropsychiatric symptoms and caregiver stress. The goal of this study was to develop a caregiver-assisted pain coping skills training protocol tailored for community-dwelling adults with mild-moderate dementia and their family caregivers. We conducted interviews with patients and caregivers to develop the protocol. We then conducted a single arm pilot test of the intervention's feasibility and acceptability. Patients were recruited from an outpatient memory care clinic and screened for pain using the validated Pain, Enjoyment, General Activity (PEG) scale. The intervention included five sessions of training in pain assessment, relaxation, pleasant activity scheduling, and integrative movement. Initially sessions were conducted in person or by videoconference according to the dyad's preference; during COVID-19 (latter half of study) all sessions were conducted remotely. Eleven dyads consented and provided baseline data [patients: mean age=77.7 years (SD=4.8), 70% non-Hispanic white; caregivers: mean age=69.6 years (SD=13.3); 91% non-Hispanic white; 73% spouses]. Nine dyads (82%) completed all five sessions. Caregivers reported high levels of satisfaction with the intervention (mean=3.4 on 1-4 scale) and frequent use of pain coping skills (mean=3-4 days/week). On average, patients reported pre-post decreases in pain severity (mean=-1.2, SD=1.8) and pain interference (mean=-0.64, SD=0.67) on the Brief Pain Inventory. Overall these findings suggest that a behavioral pain coping intervention for patients with mild-moderate dementia and their caregivers is feasible, acceptable, and potentially helpful for managing pain.

### DEMENTIA CARE: ADDRESSING PAIN AND MAXIMIZING COMFORT

Deepa Vinoo, *NYC Health Hospitals/Coler, Roosevelt Island, New York, United States*

Pain is common in older people who have Dementia, and is associated with a number of chronic and acute conditions. There is evidence that as many as 83% of nursing home residents experience pain that often goes unrecognized or inappropriately treated. Pain has a powerful effect on mood, sleep quality, functional ability, and overall quality of life. Rejecting care due to pain is very common among patients with Dementia. An association between pain and increased agitation has been noted, Significant reduction of agitation and psychotropic usage have been demonstrated by pain treatment in patients with moderate to severe dementia. This project was conducted in six memory care units with 150 residents at 815 bedded long-term geriatric care facility. All residents in memory care units from May 2018 to December 2019 were individually assessed for pain management, rejection of care, usage of psychotropics, falls and physical altercations. Trained interdisciplinary staff to evaluate pain by using PAIN AD. Educated interdisciplinary team on pharmacological and non-pharmacological pain management, and Pain management has improved from 40% to 90%, Rejection of care reduced from 80% to 30%. Usage of antipsychotics reduced by 12%. Falls reduced from 12% to 2%. Physical altercations reduced to zero. Staff call out due to work related injury significantly reduced. Staff verbalized improved job satisfaction and increased morale.

### PSYCHOLOGICAL RESILIENCE, STRESS, AND PAIN: THE MODERATING ROLE OF POSITIVE AFFECT AND WELL-BEING

Calia Morais,[1] Michael Robinson,[1] Roger Fillingim,[2] and Emily Bartley,[3] *1. University of Florida, University of Florida, Florida, United States, 2. University of Florida, gainesville, Florida, United States, 3. University of Florida, Gainesville, Florida, United States*

Chronic low back pain is the leading cause of disability among older adults. The impact of psychological factors, including high levels of stress, are associated with increased risk for pain. Despite the growing evidence suggesting that psychological well-being is associated with better health outcomes, limited research has examined positive psychological factors in the context of pain among older adults. In this secondary data analyses of we examined the association of perceived stress on pain and physical functioning, and the moderating role of positive affect and well-being (PAW) on these relationships. A total of 60 adults over the age of 60 completed completed questionnaires assessing perceived stress (Perceived Stress Scale) and positive affect and well-being (Neuro-QOL PAW). The Back Performance Scale measured back-related physical functioning and movement-evoked pain. We hypothesized that PAW would be inversely associated with pain outcomes and would moderate the relationship between perceived stress and pain. Bivariate correlations assessed the association between study variables, while the interaction of PAW and perceived stress was examined via linear regression. Age (r=.30), income (r=.28), and being married (r=.32) were associated with higher PAW scores, while there was an inverse association with movement-evoked pain (r=-.28). After controlling for demographic covariates, moderation analysis revealed that higher levels of perceived stress were associated with poorer physical functioning, but only among those with lower positive affect and well-being (b=0.14). As seen, examining the influence of positive psychological functioning on pain-related outcomes has important clinical implications that may promote positive pain adaptation in this population.

### RURAL OLDER ADULTS' EXPERIENCES WITH PAIN FROM CHRONIC ILLNESSES AND ITS TREATMENT

Hyunjin Noh,[1] Zainab Suntai,[1] and Cho Rong Won,[2], *1. University of Alabama, Tuscaloosa, Alabama, United States, 2. University of Alabma, Tuscaloosa, Alabama, United States*

Although pain control is an essential factor in promoting quality of life, pain is undertreated among certain sub-populations, such as older adults and rural residents. The purpose of this study was to explore pain experiences and its treatment among rural older adults. A qualitative research design was adopted to capture the common essence of participants' experiences through a phenomenological method. Purposeful sampling was used, and the participant criteria was: age 55+, have good thinking skills, resident of Alabama, have chronic/serious health conditions, and experienced pain or discomfort in the last 3 months. Twenty-three participants were recruited from rural counties of West and South Alabama through the local Area Agency on Aging and health and senior service centers. Individual semi-structured interviews were conducted via phone and were recorded and transcribed verbatim. Thematic analysis was conducted to identify emerging themes and repeated patterns from the data. Our results revealed themes in four categories: 1) impact of pain: physical limitations and coping strategies, 2) Impact of Covid-19: physical health, social, and mental health impact, 3) challenges in pain treatment: transportation (driver/time/cost/Covid-19 exposure) and non-transportation related problems (lacking resources/mistrust/limited health insurance coverage), and 4) suggestions: transportation-related (more transportation options/financial assistance) and non-transportation-related support (improved insurance coverage/non-pharmacological care) . Findings of this study highlight rural older adults' unique needs in access to pain treatment, further amplified during the Covid-19 pandemic. Increase in sustainable, funded transportation programs and the supply of local pain specialists is critical to meet such needs and improve their quality of life.

### Session 3100 (Symposium)

### PRESIDENTIAL SYMPOSIUM: DISRUPTION TO TRANSFORMATION OF COMMUNITIES OF CARE: KEY ISSUES AND OPPORTUNITIES IN HEALTH SCIENCES

Chair: Christine Mueller Co-Chair: Kirsten Corazzini
Discussant: Jennifer Wolff

Consistent with the theme of the conference this year, this symposium explores the impact of the pandemic and our commitment to redress structural racism and health inequities on health of older adults, and our collective capacity to transform and innovate through our gerontological health sciences lens. Each presenter will focus on one sector of care: (1) the health system and healthcare workforce; (2) older persons; and (3) families and care partners. The first presenter provides a systems-level perspective on key disruptors in healthcare systems for care of older adults and the workforce, and emerging innovations to address increasingly transparent care inequities. Emerging research implications as a result of these disruptions will be highlighted. The second presenter highlights how the predominant features of the pandemic in older adults, loneliness and isolation, are co-occurring with significant resilience and innovation, and the resultant potential to create a paradigm shift in how we design and advance communities of care. The third presenter provides the perspective of family members and care partners of older adults during the time of the pandemic, focusing on disruptions that have informed changes going forward, innovations that have emerged, and implications for research to be addressed. Our discussant situates the presentations within the larger context of 'the new normal', with a particular focus on considering the global, inter-connected context of communities of care, a commitment to reducing inequities for older adults, and implications for health sciences education and policy.

### LEVERAGING DISRUPTIONS TO CREATE AN EQUITABLE, AGE-FRIENDLY, LEARNING HEALTH SYSTEM

Eric Lenze,[1] Brian Carpenter,[2] Nancy Morrow-Howell,[2] and Beth Prusaczyk,[1] *1. Washington University School of Medicine in St. Louis, Saint Louis, Missouri, United States, 2. Washington University in St. Louis, Saint Louis, Missouri, United States*

In a learning health system, the system's own data and the experiences of its workforce are integrated with external evidence to provide better care. In an age-friendly health system, core principles of age-friendly care are integrated into every point in the system. Disruptions caused by the COVID-19 pandemic, and the innovations that addressed them, present an opportunity to discuss how these two frameworks may be combined and leveraged to transform care for older adults. We will present examples of pandemic-related disruptions, including rapid changes in how patients and providers move within and between facilities and the significant toll on healthcare workers' mental health. We will also highlight innovative solutions to these disruptions that could transform healthcare systems. Critical to these points is a discussion of how these disruptions have disproportionately impacted healthcare workers and patients of color and how the innovations must be implemented using an anti-racist, health equity lens.

### INNOVATION DURING DISRUPTION: SUPPORTING WHAT MATTERS MOST TO OLDER ADULTS THROUGH PERSON-ENVIRONMENT FIT

Sarah Szanton, *Johns Hopkins University, Baltimore, Maryland, United States*

The fragile and improvised systems of care for older adults have been decimated by isolation and fragmented care during the pandemic. However, innovations are increasingly being offered to older adults to improve the fit between them and their environment. This includes fit within the home, the social environment, the policy environment, and with clinicians. Advancing these "fits" requires evidence-based solutions like CAPABLE, a 4 month self-efficacy and function program that provides an occupational therapist, nurse and handyworker to assess and address older adults' functional goals. The older adult identifies what matters most and experiences a tailored program that taps into their purpose in life and supports engaging in meaningful activities. Starting in research sites, CAPABLE is now offered in 34 sites in 17 States and expanding through policy and insurers. Such efforts to leverage the strength of older adults and their families, builds capacity to evolve our communities of care.

### LESSONS FROM DISRUPTION: A NEW NORMAL FOR FAMILIES AND CARE PARTNERS

Beth Fields, *University of Wisconsin-Madison, Madison, Wisconsin, United States*

COVID-19 has forced community-dwelling older adults to rely on family members and care partners more than ever before for support. Often at the expense of their own health and well-being, family members and care partners help older adults manage physical and psychosocial needs, navigate a complex, ever-changing healthcare system, and follow public health guidelines. Given the increasing demands and poor outcomes, there is no better time than now to develop policies and practices that better recognize and support family members and care partners of older adults. To inform policy and practice development, this symposium will present findings from a literature review of peer-reviewed studies published from 2019 through 2021 that identifies and addresses challenges and opportunities related to caregiving for an older adult in a pandemic. The experiences of the past year demonstrate that the new normal needs to recognize and support family members and care partners.

### Session 3105 (Symposium)

### RE-ENVISIONING AGING IN THE RIGHT PLACE TO DISRUPT HOMELESSNESS FOR OLDER PEOPLE

Chair: Rachel Weldrick Co-Chair: Sarah Canham
Discussant: Joyce Weil

Recent developments in the aging-in-place literature have recognized the significance of aging-in-the-right-place. That is, aging in a place that supports an individual's unique values, vulnerabilities, and lifestyles. This symposium will build upon existing research by critically examining the potential for older persons with experiences of homelessness (OPEH) and/or housing insecurity to age-in-the-right-place. Presenters will include interdisciplinary researchers with a diversity of perspectives stemming from gerontology, social work, and environmental design. The symposium will begin with Weldrick and Canham presenting a conceptual framework for aging-in-the-right-place that has been developed to outline indicators relevant to OPEH and housing-insecure older people. Elkes and Mahmood will then discuss findings from a study of service providers working with OPEH to consider the relative benefits and challenges of temporary housing programs. Following, Brais and colleagues will present findings from an environmental audit, developed as a novel assessment tool to evaluate the accessibility and physical design of housing programs for OPEH. A final presentation by Kaushik and Walsh will highlight findings from a photovoice study on perspectives of aging-in-the-right place among OPEH during the Covid-19 pandemic. Joyce Weil, an expert in measurement of person-place fit and life course inequalities, will discuss the implications of these papers and reflect on the potential for the aging-in-the-right-place framework to address the diverse needs of the growing population of OPEH through policy and practice. Together, the participants of the symposium will advance this emerging scholarship using a wide range of methods and perspectives.

### AGING IN THE RIGHT PLACE: A CONCEPTUAL FRAMEWORK FOR HOUSING INSECURE OLDER PEOPLE

Sarah Canham,[1] Atiya Mahmood,[2] Tamara Sussman,[3] Christine Walsh,[4] and Rachel Weldrick,[2], *1. University of Utah, Salt Lake City, Utah, United States, 2. Simon Fraser University, Vancouver, British Columbia, Canada, 3. McGill University, Montreal, Quebec, Canada, 4. University of Calgary, Calgary, Alberta, Canada*

Emerging research has highlighted the significance of aging in the right place (AIRP) by recognizing that secure and optimal housing should support an individual's unique vulnerabilities and lifestyles. Existing literature, however, has yet to consider what it means for older people experiencing homelessness and/or housing insecurity to age-in-the-right-place. In order to address this knowledge gap, a review of person-environment fit models for older people and other relevant literature was conducted to determine critical identifiers of AIRP for housing insecure older people. Findings from this literature review were then refined in collaboration with interdisciplinary scholars and community partners to establish a conceptual framework. This paper presents the resulting conceptual framework and outlines the key indicators of AIRP relevant to housing insecure older people. The proposed framework provides a practical and meaningful contribution to the literature which can be used to promote housing security among individuals often excluded from existing aging-in-place models.

### AGING IN THE RIGHT PLACE? PHOTOVOICE WITH OLDER ADULTS RESIDING IN SHELTERS DURING COVID-19

Jill Hoselton Christine Walsh, and Vibha Kaushik, *University of Calgary, Calgary, Alberta, Canada*

Aging in the right place (AIRP) involves supporting older adults to live as long as possible in their homes and communities, recognizing that where an older person lives impacts their ability to age optimally and must match their unique lifestyles and vulnerabilities. Photovoice, a participatory action research strategy, allows people to document their experiences through photography, promoting critical dialogue about issues such as AIRP and rights-based housing. This presentation highlights the concept of AIRP from the perspectives of a diverse group of older adults living in promising practices shelters in Vancouver, Montreal, and Calgary, Canada using photovoice. Findings indicate that the process promoted a sense of empowerment among participants. Insights about older adults' perceptions of AIRP residing in shelters to best meet their intersectional identities, housing, and support needs will be shared. Findings inform policy initiatives that promote AIRP and the right to adequate housing for older adults experiencing homelessness.

### EVALUATING SPACES FOR OLDER ADULTS EXPERIENCING HOMELESSNESS: FINDINGS FROM AN ENVIRONMENTAL AUDIT

Émilie Cormier,[1] Diandra Serrano,[2] Atiya Mahmood,[3] Tamara Sussman,[2] Valérie Bourgeois-Guerin,[1] and Hannah Brais,[4], *1. Université du Québec à Montréal, Montreal, Quebec, Canada, 2. McGill University, Montreal, Quebec, Canada, 3. Simon Fraser University, Vancouver, British Columbia, Canada, 4. Old Brewery Mission, Old Brewery Mission, Quebec, Canada*

Homeless populations require spaces and services that take into account their life trajectories. The Aging in the Right Place - Environmental Checklist (AIRP-ENV) is an environmental audit tool developed by our team to evaluate the accessibility and overall design features of housing targeted for aging individuals experiencing homelessness. Researchers in Vancouver, Calgary and Montreal employed this tool in 2021 to evaluate environmental features in selected promising practices to identify built environment factors that promote aging in the right place. Preliminary findings reveal the following themes across sites: access to communal and recreational spaces encourage social inclusion and meaningful recreation opportunities; barrier-free built environment features foster independence and safety; and access to services and amenities encourage community mobility. Findings demonstrate a need to employ a broader evaluative lens that incorporates psycho-social factors to gain a nuanced understanding of aging in the right place for older adults who have experienced homelessness.

### TEMPORARY HOUSING FOR OLDER PEOPLE: ADDRESSING HOUSING INSECURITY TO PROMOTE AGING IN THE RIGHT PLACE

Atiya Mahmood,[1] Holly Lemme,[1] Gelareh Modara,[1] Emily Lam,[1] Maria Juanita Mora,[1] Sarah Canham,[2] and Shelby Elkes,[1], *1. Simon Fraser University, Vancouver, British Columbia, Canada, 2. University of Utah, Salt Lake City, Utah, United States*

The Aging in the Right Place (AIRP) project is a multi-year, multi-city partnership grant on aging, housing insecurity and homelessness. This paper presents findings from provider/staff interviews (N=5) at a Temporary Housing Program (THP) serving older people experiencing (or at risk of) homelessness (OPEH) in Vancouver, Canada. The researchers sought to understand the strengths and weaknesses of the program, scale-up (i.e., policies) and/or scale out impacts (i.e., on people and communities), as well as how the program promotes housing security and stability for OPEH. Narrative data reveals the program provided housing stability to OPEH by offering increased access to resources (food, pharmaceutical, transportation, social support and engagement). Additionally, through the promotion of client autonomy, privacy and security in their housing unit, the organization and staff work to support and foster AIRP among their clients and help to transform a temporary housing space into a secure home-type setting.

### Session 3110 (Symposium)

### SOCIAL ENGAGEMENT AND WELL-BEING AMONG CHINESE OLDER ADULTS

Chair: Wei Zhang Co-Chair: Bei Wu Discussant: Yan Yan Wu

Social engagement is increasingly recognized as a protective factor to promote healthy aging. This symposium provides new findings on social participation and social isolation in relation to individuals' health and well-being among the Chinese populations. Using the 2002–2018 Chinese Longitudinal Healthy Longevity Survey, the first study examined the trends of leisure activity engagement among young-old adults aged 65–74 in China over a 16-year period. Findings revealed a general downward trend of engaging in any form of social leisure activity and upward trends for engaging in home-bound and solitary leisure activities. Similarly, the second study examined social participation patterns and individual factors associated with these patterns using three nationally representative data in China, UK, and US. Their findings highlighted several underlying participation patterns across these nations as well as differences in how socio-demographics were associated with these patterns. Using data collected among Chinese older adults in Hawaii, the third study examined the associations of social isolation with psychological well-being. Results showed that social isolation was positively related to psychological distress, and negatively related to life satisfaction and happiness. These associations were partially mediated by resilience. Their findings revealed the detrimental health effects of social isolation. Using the same dataset in Hawaii, the last study examined the associations between neighbourhood conditions and psychological well-being for Chinese older adults. Their findings revealed that both physical and social neighbourhood conditions were associated with psychological well-being, particularly for foreign-born older adults, and psychological resources such as self-management abilities could mediate the associations.

### TRENDS IN LEISURE-TIME ACTIVITY PARTICIPATION AMONG YOUNG-OLD ADULTS IN CHINA

Qiushi Feng,[1] Wei Zhang,[2] Huashuai Chen,[3] and Joelle Fong,[1], *1. National University of Singapore, Singapore, Not Applicable, Singapore, 2. University of Hawaii at manoa, Honolulu, Hawaii, United States, 3. Xiangtan University, Durham, North Carolina, United States*

Engagement of leisure activities is highly associated with health and wellbeing in later life. In this study, we examined the trends of leisure activity engagement in young-old adults aged 65–74 in China for a 16-year period. Panel data for a nationally representative sample of young-old adults were obtained from the 2002–2018 Chinese Longitudinal Healthy Longevity Survey. Findings revealed that, compared with 2002, young-old adults in subsequent years were less likely to engage in any form of social leisure activity. The odds of participating in social events, regular exercise, and outdoor activities decreased most prominently over time, while downward trends in tourism and joining outdoor activities showed signs of reversal post-2014. In contrast, trends for engaging in home-bound and solitary leisure activities generally increased. The future elderly in China have generally tended towards solitary leisure activities over time and public health interventions are required to reverse such trends.

### SOCIAL PARTICIPATION PATTERNS AND WELL-BEING AMONG OLDER ADULTS: FINDINGS FROM THE UNITED STATES, THE UK, AND CHINA

Yun Zhou,[1] Bei Wu,[2] and Chenxin Tan,[3] *1. Department of Sociology, Peking University, Beijing, Beijing, China (People's Republic), 2. New York University, New York, New York, United States, 3. New York University, Jersey City, New Jersey, United States*

This study used Latent Class Analysis to examine patterns of social participation among older adults in the US, the UK, and China, from the three nationally representative surveys conducted in 2018-2019: The Health and Retirement Study, the English Longitudinal Study of Ageing, and the China Health and Retirement Longitudinal Study. Although the profiles of social participation were distinctively different, several common patterns were found: Comprehensive Participants, Occasional Participants, and Deficient Participants. It was estimated that less than 10% of older adults from these countries were extensively engaged in social participation. Seventy-seven percent of Chinese older adults were shown being "Deficient Participants", and the percentages were 29% and 20% in the US and the UK, respectively. The findings showed positive associations of levels of participation with socioeconomic status and health. The magnitudes of these associations varied across the nations. Actions are needed to promote levels of participation for Chinese older adults.

### SOCIAL ISOLATION, RESILIENCE, AND PSYCHOLOGICAL WELL-BEING IN OLDER CHINESE AMERICANS

Wei Zhang,[1] Katherine Wang,[2] Yaolin Pei,[3] Bei Wu,[3] and Xiang Qi,[4], *1. University of Hawaii at manoa, Honolulu, Hawaii, United States, 2. Duke University, Durham, North Carolina, United States, 3. New York University, New York, New York, United States, 4. Rory Meyers College of nursing, New York, New York, United States*

Using data collected in 2018 on 398 older Chinese Americans aged 55+ residing in Hawaii, we examined the associations of social isolation with psychological well-being and the mediating role of resilience. Social isolation was measured by their marital status, living arrangement, contact with children/family/friends, and participation in social activities. Psychological well-being was measured by psychological distress, life satisfaction, and happiness. Results from multivariate linear regressions and ordered logistic regressions showed social isolation was positively associated with psychological distress ($\beta$=0.017, $p<0.05$), and negatively associated with life satisfaction ($\beta$=-0.220, $p<0.001$) and happiness ($\beta$=-0.086, $p<0.05$) . By contrast, resilience was associated with lower psychological distress and higher life satisfaction and happiness. Moreover, mediation analysis showed that resilience contributed to 32% of the association between social isolation and psychological distress, 24.9% of the association between social isolation and life satisfaction, and 16.3% of the association between social isolation and happiness.

### NEIGHBORHOOD CONDITIONS, SELF-MANAGEMENT ABILITIES, AND PSYCHOLOGICAL WELL-BEING AMONG CHINESE OLDER ADULTS

Bei Wu,[1] Wei Zhang,[2] and Keqing Zhang,[3], *1. New York University, New York, New York, United States, 2. University of Hawaii at manoa, Honolulu, Hawaii, United States, 3. University of Hawaii at Manoa, Honolulu, Hawaii, United States*

Abstract Few studies have examined the associations between neighborhood conditions and psychological well-being for Chinese older adults in the U.S. This study examined how neighborhood conditions were associated with psychological well-being through self-management abilities as a pathway among Chinese older adults in Hawai'i. Survey data were collected in 2018 and ordinary Least Square regressions and mediation analysis were conducted. For the whole sample, both neighborhood physical conditions and social cohesion were significantly associated with psychological well-being, and the associations were significantly mediated by self-management abilities. The foreign-born subsample shared similar results with the whole sample. For the U.S.-born subsample, psychological well-being was only significantly associated with neighborhood physical conditions, and the association was mediated by self-management abilities. Our findings suggest that both physical and social neighborhood conditions are associated with psychological well-being, particularly for foreign-born older adults, and psychological resources such as self-management abilities could mediate the associations.

### Session 3115 (Paper)

### SOCIAL SUPPORT DURING THE COVID-19 PANDEMIC

### CORRELATES OF SOCIAL ISOLATION AMONG MINORITY OLDER ADULTS DURING THE COVID-19 PANDEMIC

Omolola Adepoju,[1] Daniel Howard,[2] Kendra Smith,[3] Luz Herrera,[4] Daikwon Han,[5] and LeChauncy Woodard,[6] *1. University of Houston, Houston, Texas, United States, 2. Texas A&M University at College Station, Texas A&M University at College Station, Texas, United States, 3. Smith Research & Consulting LLC, Spring, Texas, United States, 4. Texas A&M University, Fort Worth, Texas, United States, 5. Texas A&M University, Texas A&M University, Texas, United States, 6. University of Houston College of Medicine, Houston, Texas, United States*

**Background:** Over the past year, engagement with older adults has been severely curtailed given the high rates of COVID-19 morbidity and mortality in this population. This study examined the correlates of social isolation among African American and LatinX older adults during the COVID-19 pandemic.

**Methods:** Working with community-based organizations and senior living centers, we administered a survey to older adults 55+, in the Houston metroplex, between 11/2020 and 01/2021 (n=575). The survey assessed COVID-19 prevention behaviors and health-related social needs. Responses to "How often do you feel lonely or isolated from those around you?" were used to create a dichotomous social isolation dependent variable. The main independent variable, family/community support, was based on responses to the validated question "If for any reason you need help with day-to-day activities such as bathing, preparing meals, shopping, managing finances, etc., do you get the help you need?" Multivariate logistic regression adjusting for socioeconomic status, medical conditions, positive COVID test (for self or family), COVID-19 prevention behaviors, and emergency preparedness levels was used.

**Results:** Limited family/community support was strongly associated with social isolation (OR=6.2; $p<0.01$), as was having any chronic condition (OR=2.9, $p=0.02$). Females and seniors who reported daily social distancing were more likely to report being socially isolated (OR=2.4, $p=0.04$; OR=1.09; $p=0.09$, respectively). Of all chronic conditions examined, diabetes was the single strongest predictor of social isolation (OR=2.49, p=0.02).

**Conclusion:** Being female, having diabetes and limited family/community supports are associated with COVID-19-induced social isolation in African American and Latinx communities.

### MODERATING EFFECT OF SOCIAL SUPPORT BETWEEN LONELINESS AND DEPRESSION: YOUNG-OLD AND OLD-OLD ADULTS

Sunghwan Cho,[1] and Kyuhyung Chung,[2], *1. Virginia Commonwealth University, Richmond, Virginia, United States, 2. Semyung University, Semyung University, Ch'ungch'ong-bukto, Republic of Korea*

Depression increases suicidal risk lowers quality of life in older adults. However, it is unknown how loneliness and depression are associated with young-old and old-older adults. This study examined association of loneliness and depression from the National Social Life, Health, and Aging Project (NSHAP) (2015-2016), estimating moderating effects of social support. The sample of this study was community dwelling Medicare beneficiaries aged 65+ (n=1,532): young-older adults (n=903) and old-older adults (n=629). Loneliness was measured by the Revised University of California, Los Angeles Loneliness Scale short form (3 items; young, M=.86, SD=.73; old, M=.87, SD=.67; range 0-3). Social support consists of two variables each measured by 4 items, spouse/partner support (young, M=2.29, SD=.50; old, M=2.26, SD=.51; range 0-3) and family support (young-old, M=2.19, SD=.52; old-old, M=2.23, SD=.52; range 0-3). Depression was measured by Center for Epidemiological Studies Depression scale (11 items, young, M=1.41, SD=.42; old, M=1.45, SD=.42; range 1-4). Multiple linear regression was used in this study, including relevant covariates. Findings indicated loneliness in both groups (young, p<.001; old, p<.001), spouse support in both groups (young, p<.001; old, p<.001) had statistical significance in depression. Family support in young-older adults (p<.05) had a statistical significance for depression. Interaction of loneliness and spouse support moderated the relationship between loneliness and depression in old-older adults (p<.05). Findings suggest old-older adults' loneliness and depression could be soothed by spousal support. Spousal support could be important in that the informal caregiver is likely to focus on older adults with fragility at the end of their lives.

### SENIOR CENTERS AND LGBT PARTICIPANTS: ENGAGING OLDER ADULTS VIRTUALLY IN A PANDEMIC

Suzanne Marmo,[1] Manoj Pardasani,[2] and David Vincent,[3], *1. Sacred Heart University, Fairfield, Connecticut, United States, 2. Adelphi University, Garden City, New York, United States, 3. SAGE, NEW YORK, New York, United States*

Upon the outbreak of COVID-19, recommendations to cease all non-essential in-person social services were mandated across the United States to prevent transmission to non-infected individuals. As a result, approximately 96% of all senior centers in the United States were closed to in-person programming (National Council on Aging, 2020). LGBT older adults in particular were at higher risk of isolation and declines in overall health as they were more likely to live alone, experience loneliness or have less immediate family support systems when compared to non-LGBT older adults (Yang, Chu & Salmon, 2017). The purpose of this presentation is to explore how LGBT older adult participants in senior centers transitioned to virtual programming during the pandemic. Using a risk-resiliency theory framework, the purpose of this presentation is to share the impact of virtual programming on the health and well-being of LGBT community-dwelling older adults. An exploratory, cross-sectional study was conducted utilizing an online survey to understand their needs, concerns and experiences. Participants reported a relatively easy adaptation to technology, steady participation in programs and services, satisfaction with virtual senior center programming and a consistent sense of engagement with their peers. Higher levels of engagement with senior center programs were associated with greater perceptions of social support. Additionally, stronger perceptions of social support and participation in exercise and fitness programming were associated with higher life satisfaction and lesser symptoms of depression and anxiety. Strategies for outreach, engagement and service provision will be presented.

### STRESS RELATED TO COVID-19 AND LONELINESS AMONG MIDDLE-AGED AND OLDER ADULTS IN THE UNITED STATES

Sukyung Yoon, and Neely Mahapatra, *University of Wyoming, Laramie, Wyoming, United States*

Intensified levels of stress and loneliness have been attributed to the COVID-19 pandemic (Havnen et al., 2020; Luchetti et al., 2020). Moreover, loneliness has been reported to exacerbate psychological and physical health issues (Holt-Lunstad et al., 2015). The current research aims to investigate the impact of stress-related to COVID-19 on loneliness. The roles of age, sex, living arrangements, health, and resilience were also investigated. Data was collected on 267 middle-aged and older adults (ages 45 through 88) living in the U.S during COVID-19. A path analysis was employed. For both the direct and indirect effects, 95% confidence intervals were estimated using bootstrapping (a bootstrap sample of 1,000 was specified). Model fit was acceptable. X2 (5) = 7.913, p > 0.05, CFI=0.972, RMSEA =0.047. Regarding direct effects, the results indicate that COVID-19 related stress (hereafter stress) was negatively associated with perceived good health (hereafter health) ($\beta$ = -.213, p<0.001). It was also found that health was positively associated with resilience ($\beta$ = .324, p<0.001). Being male was positively associated with resilience ($\beta$= .144, p<0.05), and resilience was negatively associated with loneliness ($\beta$= .230, p<0.001). Meanwhile, stress had negative indirect effects on resilience, whereas stress had positive indirect effects on loneliness. Finally, health and being male had negative indirect effects on loneliness. The findings indicate that health practitioners and service providers should develop programs to improve and maintain good health, resilience, and social support among middle-aged and older adults during the COVID-19 pandemic. Moreover, gender-based services are also needed.

### THE ASSOCIATION BETWEEN ONLINE SOCIAL NETWORK SUPPORT AND FEAR OF COVID-19 AMONG OLDER ADULTS

Andrew Steward,[1] Matthew Schilz,[2] Kaipeng Wang,[2] M. Pilar Ingle,[2] Carson De Fries,[2] and Leslie Hasche,[2]
*1. University of Denver, Lone Tree, Colorado, United States, 2. University of Denver, Denver, Colorado, United States*

Public health concerns related to the COVID-19 health crisis are particularly salient among older adults. Fear surrounding COVID-19 has also been associated with increased spread, morbidity, and mortality of the disease. Prior to the pandemic, loneliness and social isolation were already a concern for older adults, and the pandemic further constrained how older adults may socially connect with others because of public health safety precautions. Online social networks are a valuable form of support for older adults, and usage of online social networks during the pandemic may have expanded. Thus, the purpose of this study is to examine the association between online social networks and fear of COVID-19 among older adults. A convenience sample (n = 239) of adults 60+ years of age in the U.S. completed a 20-minute, online survey. The independent variable utilized the Lubben Social Network Scale (four items), focusing on online support. The dependent variable was measured by the Fear of COVID-19 scale (eight items). Results of ordinary least squares regression show that increased online social network support was significantly associated with decreased fear of COVID-19 ($p < 0.05$), while holding constant age, sex, race, marital status, education, whether a respondent lives alone, and self-rated health. Findings highlight the importance of online social networks for older adults during the COVID-19 crisis. Existing online networks which engage older adults should be expanded, and efforts should be made to provide older adults with online forms of social support who may experience barriers or inequities related to accessing technology.

### Session 3120 (Symposium)

### STEP-HI STUDY: A MULTIMODAL INTERVENTION OF EXERCISE AND TESTOSTERONE THERAPY: DESIGN AND REAL-WORLD CHALLENGES

Chair: Ellen Binder Discussant: Jay Magaziner

Hip fractures are common among older women and can have a devastating impact on their ability to remain independent. Many women who were high functioning before the hip fracture do not return to their pre-fracture level of function, have persistent weakness and mobility impairments, and may require ongoing supportive services. Age-associated androgen deficiency may contribute to deficits in muscle mass, strength and power that are common in older female hip fracture patients. The Starting a Testosterone and Exercise Program after Hip Injury (STEP-HI ) Study is a three-group, randomized, double-blinded, placebo-controlled Phase III clinical trial designed to evaluate a multi-modal intervention aimed at improving functional outcomes in older female hip fracture patients. 168 female hip fracture patients, age 65 yrs. and older are being recruited from 6 clinical sites in the USA. Participants are being assigned to one of three groups: supervised exercise (EX) plus 1% testosterone topical gel; EX plus placebo gel; or enhanced usual care. The primary outcome is six-minute walk distance. This symposium will present information related to key aspects of the design of the STEP-HI trial, including recruitment of a frail patient population during a period of injury recovery, the testosterone and exercise interventions and related fidelity procedures, and implementation challenges for this multi-modal intervention prior to and during the COVID-19 pandemic. In addition, the underlying mechanisms by which testosterone and exercise are expected to have a synergistic effect on muscle strength and function will be discussed.

### THE STEP-HI TRIAL: PRAGMATIC CHALLENGES AND DESIGN ISSUES

Ellen Binder, *Washington University in St. Louis School of Medicine, Saint Louis, Missouri, United States*

Multi-modal interventions present many implementation challenges, especially for studies of frail older adults. The STEP-HI study is an ongoing multi-center, randomized, controlled, double-blinded clinical trial that is evaluating whether six months of topical testosterone therapy combined with a supervised center-based exercise-training program can improve mobility, functional performance, and quality of life after hip fracture, compared to exercise training alone or Enhanced Usual Care. Female hip fracture patients ≥ 65 yrs. old who are living in the community or assisted living are being randomized within 26 weeks of surgical repair for the fracture, and re-evaluated 24 weeks later. This presentation discusses the rationale and study design, and modifications to the protocol in response to challenges, including the COVID-19 pandemic.

### RECRUITMENT CHALLENGES AND STRATEGIES FOR MULTIMODAL INTERVENTION TRIALS

Jenna Bartley, *Center on Aging, Farmington, Connecticut, United States*

Determining ways to improve hip fracture recovery in older adults is important, however recruitment of this target population into clinical trials is challenging. Multimodal interventions that target multiple mechanisms of recovery may improve outcomes, but each component presents unique recruitment barriers. While exercise interventions have been shown to be beneficial for hip fracture recovery, offering exercise following completion of conventional physical therapy can be viewed as a burdensome time commitment. Hormone replacement therapy may hold promise for overcoming anabolic resistance, but concern about adverse side effects can also deter participation. STEP-HI is a multisite trial testing whether exercise and testosterone can improve hip fracture recovery in older women. In this talk, recruitment barriers experienced in STEP-HI and strategies employed to overcome these barriers will be discussed. Strategies include: partnering with hospitals, skilled nursing facilities and orthopedic surgeons. providing talks and education materials; and featuring past participant testimonials in recruitment materials.

### IMPLEMENTATION OF A REMOTE FIDELITY OVERSIGHT PROGRAM FOR A MULTI-SITE EXERCISE INTERVENTION

Jennifer Stevens-Lapsley, *University of Colorado School of Medicine, Aurora, Colorado, United States*

The STEP-HI exercise protocol is a supervised, 2 phase, multimodal, high-intensity exercise program that emphasizes resistance training. Exercise sessions are conducted at an exercise facility and occur on two non-consecutive days/week for 6 months. During specified exercises, the exercise interventionist targets the participant's eight-repetition maximum (8-RM), defined as the greatest resistance that can be moved 8 times through full range of motion with good form. A rigorous, remote fidelity monitoring program maximizes consistency of the intervention across sites. This fidelity oversight program is a model for future exercise studies because of its unique remote, hierarchical structure. All exercise interventionists are initially certified by written examination and direct observations. Some exercise sessions are also video recorded and reviewed using fidelity checklists. After initial certification, repeated direct observation and video-based verification of fidelity are repeated at prescribed intervals for each interventionist to ensure sustained consistency of implementation across sites.

### HOW DOES TESTOSTERONE AUGMENT THE ANABOLIC RESPONSE TO EXERCISE IN FRAIL OLDER ADULTS?

Shalendar Bhasin, *Brigham and Women's Hospital, Harvard Medical School, Boston, Massachusetts, United States*

Testosterone treatment increases muscle mass, strength, and leg power in menopausal women, hypogonadal men, older men with mobility limitation, COPD and ESRD. Testosterone's effects on muscle mass and strength are augmented by exercise training and growth hormone. Testosterone treatment improves some measures of physical performance, such as stair climbing power and aerobic capacity; the improvements in gait speed have been modest. Testosterone increases muscle mass by inducing the hypertrophy of type 1 and 2 muscle fibers, and by increasing satellite cell number. Testosterone promotes the differentiation of mesenchymal progenitor cells into myogenic lineage and inhibits their differentiation into adipogenic lineage by activating Wnt-target genes, including follistatin that plays an important role in mediating testosterone's effects on the muscle. Testosterone also increases polyamine synthesis in the muscle. Combined administration of testosterone plus multi-component exercise intervention that includes functional training may be needed to improve function and mobility in older adults.

### Session 3125 (Symposium)

### THE IMPLICATIONS OF AGING COUPLES' LINKED LIVES: DYADIC ASSOCIATIONS IN HEALTH AND WELL-BEING

Chair: Stephanie Wilson Co-Chair: Christina Marini
Discussant: Amy Rauer

Older adults age in the context of their intimate partnerships. Partners' lives—their emotions, behaviors, and health—are intricately linked as they navigate the challenges associated with aging. This symposium presents research that illuminates ways partners influence one another later in life. The talks are diverse with regard to their timescale (e.g., years vs. hours) and context (e.g., dementia vs. pain). Dr. Martire will examine associations between declines in one spouse's physical health over 5 years and the other's mental health. This talk will further consider whether discussing health concerns exclusively with one's spouse intensifies such associations. Ms. Nah will show how the pain of both partners (care providers and recipients) contributes to escalating marital conflict over 2 years. Dr. Wilson will demonstrate that emotional reactivity to spousal distress in the lab is associated with increased proinflammatory gene expression up to 80 minutes later, a risky pattern for health if repeated over time. Dr. Monin will examine actor and partner associations of affect and depressive symptoms among people with early-stage dementia and their spouses; the absence of partner associations suggests that emotional spillover may operate differently in early-stage dementia dyads. Dr. Novak will identify correlates of four latent profiles derived from couples' physical, psychological, and relationship well-being: happy, healthy couples; unhappy, unhealthy couples; and two groups with blissful marriages despite individual problems. Dr. Amy Rauer, an internationally recognized scholar of relationships and health, will discuss ways in which this research advances our understanding of couples' linked lives.

### EXPRESSION OF EMOTIONS AND GENES: PROINFLAMMATORY GENE EXPRESSION RISES WITH SPOUSAL DISTRESS

Steven Cole,[1] M. Shrout,[2] Janice Kiecolt-Glaser,[2] and Stephanie Wilson,[3] *1. UCLA School of Medicine, Los Angeles, California, United States, 2. The Ohio State University College Of Medicine, columbus, Ohio, United States, 3. Southern Methodist University, DALLAS, Texas, United States*

Marital quality shares ties to inflammation-related conditions like cardiovascular disease and diabetes. Lab-based studies implicate hostility during marital conflict as a mechanism via inflammatory reactivity. However, developmental theories suggest that conflict declines with age. Spousal distress is an important but overlooked context for aging couples as networks shrink and assistance needs increase. To examine the effects of spousal distress on changes in proinflammatory gene expression, 38 adults ages 40-81 witnessed their spouse relive an upsetting personal memory aloud, rated their mood before and after, and provided blood samples at baseline and twice post-task. Those whose negative mood increased more in response to spousal disclosure showed larger elevations in proinflammatory gene expression 40 ($p=.022$) and 80 minutes ($p<.0001$) after the task. Effects were robust to race, gender, age, alcohol, smoking, and body mass index. These novel findings identify spousal distress as a key marital context that may escalate inflammation-related health risks.

### EFFECTS OF LATE-LIFE HEALTH TRANSITIONS ON SPOUSES' PSYCHOLOGICAL WELL-BEING

Ruixue Zhaoyang,[1] Christina Marini,[2] and Lynn Martire,[1] *1. The Pennsylvania State University, University Park, Pennsylvania, United States, 2. Adelphi University, Garden City, New York, United States*

Declining physical health likely affects not only older adults' own well-being, but also that of their spouse. Using two waves of data from 610 couples in the National Social Life, Health and Aging Project, we examined effects of health declines over five years on change in self and spousal psychological well-being. Actor-Partner Interdependence Model findings showed that declines in spouses' physical health (i.e., increased pain and decreased physical and cognitive function) predicted increases in older adults' anxiety. Given the increasing importance of later-life social ties outside of marriage, we further considered the role of non-spousal health confidants. Preliminary findings suggest that effects of health declines on both partners' well-being depend on the availability of these confidants. When older adults have people in addition to their spouse with whom they can talk about their health, detrimental effects of spouses' declining health on older adults' well-being are weakened for some health outcomes

### A DYADIC LATENT PROFILE ANALYSIS OF OLDER COUPLES' PSYCHOLOGICAL, RELATIONAL, AND PHYSICAL HEALTH

Ashley Ermer,[1] Stephanie Wilson,[2] and Josh Novak,[3]
*1. Montclair State University, Montclair, New Jersey, United States, 2. Southern Methodist University, Dallas, Texas, United States, 3. Auburn University, Auburn University, Alabama, United States*

The present study explored the heterogeneity of older couples' psychological, relational, and physical health using a sample of 535 couples above the age of 62. A dyadic latent profile analysis was conducted to identify and predict unique clusters of couples' relative psychological (depressive symptoms and daily hassles), relational (problematic affective communication and marital satisfaction), and physical health (number of health problems and self-reported health satisfaction). Predictors of class membership included relationship length, age, income, and hours worked outside the home. Results revealed 4 distinct classes: Happy & Healthy Together (63.5%), Individually & Relationally Strained (14.7%), Relationally Happy with Strained Wives (12.3%), and Relationally Happy with Strained Husbands (9.3%). Typology descriptions and predictors of class membership will be discussed. These findings highlight that health promotion efforts should be tailored to the specific psychological, relational, and physical health concerns of both partners rather than a one-size-fits-all approach.

### EFFECTS OF OWN AND PARTNER PAIN ON SPOUSAL CAREGIVERS' MARITAL QUALITY

Lynn Martire, and Suyoung Nah, *The Pennsylvania State University, University Park, Pennsylvania, United States*

Little is known about whether care recipients' and their spousal caregivers' own pain influence the marital quality perceived by caregivers. Considering that experiencing and witnessing pain may be related to marital distress, we hypothesized that care recipient and caregiver pain would be associated with caregivers' greater increases in marital conflict over time. We focused on 264 spousal caregivers of older adults with chronic illnesses or disability from the 2015 and 2017 National Study of Caregiving. Sixty-nine percent of care recipients and 54% of caregivers in this study were bothered by pain at baseline. Findings revealed that caregiver ($b = 0.25$, $p = .02$) and care recipient pain ($b = 0.34$, $p < .01$) at baseline were both associated with caregivers' higher marital conflict at follow-up. These findings suggest the importance of accounting for not only care recipients' pain but also spousal caregivers' own pain when examining caregivers' marital quality.

### INTERPERSONAL ASSOCIATIONS BETWEEN AFFECT AND DEPRESSIVE SYMPTOMS IN PERSONS WITH EARLY DEMENTIA AND THEIR SPOUSES

Amanda Piechota,[1] Sumaiyah Syed,[1] and Joan Monin,[2],
*1. Yale School of Public health, New Haven, Connecticut, United States, 2. Yale School of Public Health, New Haven, Connecticut, United States*

Positive and negative affect have independent effects on health and occur frequently in close relationships. No research to our knowledge has examined self-reported affective experiences of persons with dementia (PWD) and their spouses and interpersonal associations with their psychological health. Secondary analysis of baseline interviews from a randomized clinical trial testing a stress reduction intervention in 45 couples (n=90) examined whether individuals' positive and negative affect were associated with their own depressive symptoms (actor effects) as well as their partner's depressive symptoms (partner effects) and whether these associations differed for PWD and spouses. Actor partner interdependence model results showed that for PWDs and spouses, one's own positive affect was related to one's own lower depressive symptoms ($B=-3.10$, $SE=.59$, $df=58.70$, $p<.001$), and one's own negative affect was associated with one's own greater depressive symptoms ($B=6.62$, $SE=.60$, $df=65.67$, $p<.001$). These effects were independent from each other. Partner effects were not significant.

### Session 3130 (Symposium)

### THE INTERSECTION OF CANCER, GERIATRICS, AND GERONTOLOGY: A MULTIDISCIPLINARY APPROACH ACROSS THE CANCER CONTINUUM

Chair: Jessica Krok-Schoen

Despite the majority of cancer survivors being older adults, the connection between oncology, geriatrics, and gerontology remains unexplored. Our symposium will provide insights across the cancer continuum from prevention through survivorship as well as a comprehensive view of the connection between gerontological and geriatric factors in oncology. Specifically, we will discuss the biopsychosocial and behavioral factors among older adults with cancer, their effect on health outcomes, and how researchers and clinicians can intervene to improve health outcomes. The first abstract by Dr. Cadet found that despite a lack of knowledge of options and harms of cancer screening among older adults with low health literacy, there was a desire to understand more to better their health. The second abstract by Dr. Bhattacharyya found that older patients with cancer experience high levels of social isolation, loneliness, and fear that are heightened by individual and technology-based barriers to telehealth. The third abstract by Dr. Carroll found that breast cancer survivors with good sleep quality had less accelerated biological aging than those with sleep problems. The fourth abstract by Dr. Krok-Schoen utilized one of the largest datasets of older women, the Women's Health Initiative, and found multiple gerontological and geriatric factors associated with physical activity among older female cancer survivors. Closing this symposium is Dr. Guida, a Program Director at the National Cancer Institute (NCI), who will profile the current research efforts, programmatic priorities, and current funding in aging and cancer. These multidisciplinary researchers and clinicians will provide a comprehensive symposium regarding geriatric oncology.

### SHARED DECISION-MAKING AND COLORECTAL CANCER SCREENING BEHAVIORS AMONG OLDER ADULTS WITH LOW HEALTH LITERACY

Rebekah Halmo,[1] Siobhan McDonold,[1] Mara Schonberg,[2] and Tamara Cadet,[1], *1. Simmons University, Boston, Massachusetts, United States, 2. Division of General Medicine, Beth Israel Deaconess Medical Center, Harvard Medical School, Boston, Massachusetts, United States*

National guidelines recommend adults >75 engage in shared decision making (SDM) around colorectal cancer (CRC) screening because of the uncertain benefit to risk ratio. There are no decision tools to support CRC decision making for adults >75 years with low health literacy (LHL). The purpose of this mixed-methods study was to better understand the perspectives of adults >75 with LHL on SDM around CRC screening and to obtain their feedback on an existing higher literacy CRC decision aid. Utilizing the Brief Health Literacy Screening Tool to identify participants with LHL, semi-structured interviews were conducted with 30 adults. Findings indicate that 80% of participants were non-Hispanic Black and 42% had < high school degree. 76% felt they would benefit from CRC screening despite their age. Themes related to CRC screening included lack of knowledge of options and harms, but a desire to understand more to better take care of their health.

### BIOLOGICAL AGING IN BREAST CANCER SURVIVORS AND THE ROLE OF SLEEP

Judith Carroll, *University of California, Los Angeles, Los Angeles, California, United States*

Although cancer treatments can prolong life, they may lead to long-term changes in physical health and well-being. The lasting symptoms experienced after cancer treatment include greater fatigue, pain, cognitive complaints, and functional decline. Cancer and its related cytotoxic treatments are proposed to directly altering biological aging pathways. Our recent findings support this hypothesis, suggesting that women with breast cancer exposed to therapy have alterations in indicators of biological aging, including elevated DNA damage, reduced telomerase activity, and more rapid epigenetic aging. There was variability in risk for signs of biological aging, and given the high prevalence of sleep problems among breast cancer survivors, we sought to examine whether healthy sleep might be protective. Results suggest that those with good sleep quality had less accelerated biological aging than those with sleep problems. Results point to healthy sleep as a modifiable target to protect women with breast cancer from experiencing biological aging.

### PREDICTORS OF PHYSICAL ACTIVITY AMONG OLDER BREAST CANCER SURVIVORS: FINDINGS FROM THE WHI LILAC STUDY

Michael Pennell,[1] Michelle Naughton,[2] Xiaochen Zhang,[2] Aladdin Shadyab,[3] Candyce Kroenke,[4] Nazmus Saquib,[5] Electra Paskett,[6] and Jessica Krok-Schoen,[7], *1. College of Public Health, The Ohio State University, Columbus, Ohio, United States, 2. Comprehensive Cancer Center, Columbus, Ohio, United States, 3. University of california, San Diego, San Diego, California, United States, 4. Kaiser Permanente Northern California Division of Research, Oakland, California, United States, 5. College of Medicine, Sulaiman Al Rajhi University, Bukairyah, Al Qasim, Saudi Arabia, 6. Department of Internal Medicine, College of Medicine, The ohio State University, Columbus, Ohio, United States, 7. The Ohio State University, Columbus, Ohio, United States*

We examined the factors associated with physical activity following cancer treatment among older breast cancer survivors from the WHI LILAC study. The majority of participants (n=3,710, mean age=78.8±5.9) were white (86%), and had in situ/localized breast cancer (79%). Women who received radiation therapy, were underweight/normal weight, had fewer reported cancer-related symptoms, no lymphedema, higher self-rated health, higher physical functioning, no pain, no depressive symptoms, and higher social support had significantly greater participation and duration of physical activity (all $p<0.05$). Women aged <75 who received radiation had longer duration of total minutes of physical activity ($\beta=19.7$, $p<0.05$), while women aged 75-85 who received radiation had shorter duration of total minutes of physical activity ($\beta=-3.2$, $p<0.05$). These results indicate that multiple health and social factors are associated with physical activity in this cohort. Interventions to facilitate physical activity among this group should consider body weight, symptom burden, comorbidity status, and social support.

### NATIONAL CANCER INSTITUTE'S CANCER AND AGING PRIORITIES

Jennifer Guida, *National Cancer Institute, Bethesda, Maryland, United States*

Modern improvements in cancer detection and treatment coupled with the implementation of population-based cancer prevention and control strategies have contributed to a sustained decline in overall cancer mortality rates. Although this trend is promising, challenges at the nexus of cancer and aging are, in turn, becoming more prominent. Older adults (age 65 years and older) are the largest growing segment of the U.S. population, and aging into older adulthood is disproportionally associated with the incidence of common cancers. Many survivors of childhood cancer will live for decades after cancer treatment and mature into older age. Strategic investments in aging research will contribute to population health by preserving or improving healthspan and ensuring equitable access to – and benefit from – advances in cancer prevention, control, and population science. This presentation will describe ongoing cancer and aging efforts at the National Cancer Institute, including programmatic priorities and current funding opportunities.

### Session 3135 (Symposium)

### THE NEW FACES AND NEW PLACES OF GEROSCIENCE

Chair: Felipe Sierra

The field of geroscience is rapidly evolving, as well as expanding worldwide. The Program will highlight new approaches and players in the field. Notably, geroscience was initiated as an effort to improve recognition of the role played by basic aging biology in our efforts to improve the health of older adults. Substantial recognition by multiple players of that role of basic aging biology have resulted in significant interest on the part of clinicians and translational biology practitioners. The program will highlight examples of hand-picked efforts in industry and academia, both in the US and in Europe, and will bring into the same stage researchers interested in the various facets of geroscience, from basic biology, translation, clinical and, ultimately, industry viewpoints.

### INSPIRE: A NEW EFFORT ON GEROSCIENCE IN TOULOUSE, FRANCE

Bruno Vellas,[1] and Felipe Sierra,[2], *1. CHU Toulouse: Centre Hospitalier Universitaire de Toulouse, Gérontopôle de Toulouse, Institut du Vieillissement, Midi-Pyrenees, France, 2. Toulouse, Midi-Pyrenees, France*

The Inspire project of the Toulouse Hospital System is a comprehensive approach to health care in older adults, focused on maintenance of health and physical function. At the core of the project are human, mouse and killifish cohorts, which in the case of humans, is comprised of 1,000 subjects of ages 20 and above, which are followed for a total of 10 years, both via visits to the clinic, and electronic follow-up via the ICOPE app. At recruitment they are stratified as robust, pre-frail or frail according to Fried's criteria, and then followed for loss of Intrinsic Capacities, as defined by WHO. A parallel cohort of Swiss mice with enhanced (exercise) and decreased (high fat diet) health will be used to measure concordant parameters. The project is generating a significant biorepository that is being used to pursue research in several areas where Toulouse has a significant research strength.

### THE RISE AND THE DEATH OF SENESCENT CELLS: FROM MECHANISMS TO INTERVENTIONS

Marco Demaria, *Medical Faculty, Groningen, Groningen, Netherlands*

Aging is at the root of age-related diseases and therapies targeting basic age-associated mechanisms have the potential to extend healthy lifespan. A common feature of older organisms is the accumulation of senescent cells – cells that have irreversibly lost the capacity to undergo replication. Senescent cells are characterized by an irreversible cell cycle arrest and by the Senescence-Associated Secretory Phenotype (SASP), which include many tissue remodeling and pro-inflammatory factors. Senescent cells are intermittently present during embryogenesis and in young organisms. On the contrary senescent cells accumulate and persist in aging tissues. Significantly, these persistent senescent cells can drive low-grade chronic inflammation, and their genetic or pharmacological elimination is sufficient to delay a number of diseases and to improve health span. Here, I will discuss the mechanisms by which senescent cells can promote tissue aging and dysfunction and the potential of targeting senescent cells to delay human aging.

### THE GROWING GLOBAL HEALTHY LONGEVITY ECOSYSTEM

Thomas Seoh, *Kitalys Institute, Charlottesville, Virginia, United States*

The geroscience field has started to grow exponentially in recent decades. This in turn has led to a rapidly emerging global ecosystem of players and nodes that has radiated out into fields from clinical investigation to medical practice to capital markets and startup activity to consumer-facing goods and services, regulations, laws and policies and the general wellness-conscious public. These are still early days, and there is uncertainty and a lack of awareness about the shape and activities of this rapidly growing and evolving community. This presentation will attempt a high-level survey of the current landscape in the hope of promoting awareness and collaborations among diverse, multiple initiatives that can accelerate the field.

### TRANSLATIONAL GEROSCIENCE: HUMAN MODELS OF HEALTHY AGING AND LONGEVITY

Sofiya Milman, *Albert Einstein College of Medicine, Bronx, New York, United States*

While insulin like growth factor-1 (IGF-1) is a well-established modulator of aging and longevity in model organisms, its role in humans is less well understood. Previous ambiguities in part have been attributed to cohort characteristics and unawareness of interactions between age and IGF-1. Centenarians have emerged as an ideal model of healthy aging because they delay the onset of age-related diseases and often remain disease free for the duration of their lifespan. In cohorts of centenarians and generally healthy older adults, we demonstrated that reduced IGF-1 is associated with extended lifespan and health-span. Additionally, we confirmed that IGF-1 interacts with age to modify risk in a manner consistent with antagonistic pleiotropy: younger individuals with high IGF-1 are protected from dementia, vascular disease, diabetes, cancer, and osteoporosis, while older individuals do not exhibit IGF-1-associated protection from disease. These findings offer evidence for IGF-1 modulating health-span and lifespan in humans.

### Session 3140 (Symposium)

### THE OLDER AMERICANS ACT, THE AGING NETWORK, AND THE PANDEMIC

Chair: Brian Lindberg

This session provides insights into how the pandemic challenged the capabilities and ingenuity of the Older Americans Act (OAA) programs and the aging network and what it means for in-home and community aging services now and in the future. Speakers will include key aging network stakeholders, who will discuss the overnight evolution of programs serving often isolated older adults.

### OLDER AMERICANS ACT MEALS PROGRAMS: RESPONDING TO THE PANDEMIC

Katie Jantzi, *Meals on Wheels America, Arlington, Virginia, United States*

This session provides insights into how the pandemic challenged the capabilities and ingenuity of the Older Americans Act (OAA) programs and the aging network. Speakers will include key aging network stakeholders, who will discuss the overnight evolution of programs serving often isolated older adults.

### OLDER AMERICANS ACT SUPPORTS AND SERVICES: ADAPTING TO THE PANDEMIC

Amy Gotwals, *National Association of Area Agencies on Aging, Washington, District of Columbia, United States*

This session provides insights into how the pandemic challenged the capabilities and ingenuity of the Older Americans Act (OAA) programs and the aging network. Speakers will include key aging network stakeholders, who will discuss the overnight evolution of programs serving often isolated older adults.

### Session 3145 (Symposium)

### THE TIES THAT BIND: HOW ONLINE AND OFFLINE INTERACTIONS AFFECT SOCIAL SUPPORT AND QUALITY OF LIFE FOR OLDER ADULTS

Chair: Shelia Cotten

Though a digital divide still exists, older adults are increasingly using a range of information and communication technologies (ICTs) – smartphones, apps, tablets, and computers – to communicate and engage with social ties. This symposium focuses on modalities of interaction – whether online or offline – that older adults use to interact with social ties. The research projects detailed examine the frequency of different interaction modalities, as well as impacts of these interaction modalities on older adults' perceptions of social support and quality of life. Kadylak and colleagues focus on social robots and how older adults may engage with this evolving technology to improve social engagement and aging in place. Kim and Fingerman investigate whether daily social media use is associated with same-day negative or positive mood in later life. Xie and colleagues examine older adults' patterns of both online and offline social interaction during COVID-19, and how older adults perceive these interactions. Schuster and Cotten, using a national sample of individuals aged 65 and older, examine whether social media use may be related to a range of quality of life indicators. Each of these studies provides additional insights into the ways through which older adults interact and communicate with social ties, and potential impacts of the different ways through which they interact, which may provide insights into groups seeking to increase social engagement among older adults in general and during times when social isolation may be exacerbated due to societal stressors, such as pandemics.

### OLDER ADULTS' ONLINE AND OFFLINE SOCIAL INTERACTIONS DURING THE COVID-19 PANDEMIC

Kristina Shiroma,[1] Atami De Main,[2] Nathan Davis,[3] and Bo Xie,[3], *1. The University of Texas at Austin, The University of Texas at Austin, Texas, United States, 2. School of nursing, the university of texas at Austin, Austin, Texas, United States, 3. The University of Texas at Austin, Austin, Texas, United States*

During the COVID-19 pandemic, much of our social interaction has transitioned from in-person to online. This study examined older adults' social interaction during COVID-19, online and offline. Participants were recruited from community-dwelling older adults in Central Texas. Data collection took place via the telephone during June-August 2020 (N = 200; age range: 65-92 years; Mean: 73.6; SD: 6.33). Participants used a variety of communication modes, including phone or texting (used by 99% of the participants); email (44%); in person (35%); video chat (31%); social media (24%); and postal mail (4%). Most participants (77%) used more than one communication mode. Participants discussed their preferences for and challenges of technology (i.e., smart phones) and its applications (i.e., video chat, telehealth, and social media). Participants' self-reported experiences ranged from positive (50%), mixed (35%), to negative (15%). These findings will inform policy and community interventions to promote older adults' social interactions during the pandemic.

### WELL-BEING FACTORS THAT RELATE TO FACEBOOK-USING OLDER ADULTS' PERCEIVED SOCIAL SUPPORT ON FACEBOOK

Travis Kadylak,[1] Shelia Cotten,[2] and Amy Schuster,[2] *1. University of Illinois Urbana Champaign, Champaign, Illinois, United States, 2. Clemson University, Clemson, South Carolina, United States*

The majority of literature on Facebook use and well-being focuses on younger demographics. The number older adults using Facebook continues to increase. Facebook use by older adults has been found to increase well-being and decrease feelings of depression. This study investigates the effect that perceived social support on Facebook may have on loneliness, depression, social support (offline), and fear of missing out (FOMO) for older adult Facebook users. Older adults aged 65 and older in the U.S. completed a Qualtrics survey (N=798). Participants were, on average, 74 years old. Perceived social support on Facebook had a positive association with social support, depression, and FOMO. The results suggest that among Facebook using older adults, higher levels of perceived social support on Facebook were associated with higher levels of social support, feelings of depression, and FOMO. Future research should investigate the possibility that depression could be driving perceived social support on Facebook.

### DAILY SOCIAL MEDIA USE, SOCIAL TIES, AND EMOTIONAL WELL-BEING IN LATER LIFE

Karen Fingerman, and Yijung Kim, *The University of Texas at Austin, Austin, Texas, United States*

Research has seldom explored older adults' daily social media use and its interface with 'offline' social ties. Using data from the Daily Experiences and Well-being Study (N = 310; Mage = 73.96), we investigated whether more daily social media use was associated with the same-day negative or positive mood in later life, and how these associations varied with older adults' daily social encounters and social network structure. More daily social media use was associated with less same-day negative mood. Additionally, more daily social media use was associated with less negative mood on days with more in-person encounters, compared to the days with fewer in-person encounters. More daily social media use was also associated with more positive mood for individuals with a relatively small social network, but not for their counterparts. Post-hoc analyses supported a compensatory function of social media for those older adults lacking social connections in their daily lives.

### EXPLORING SOCIAL AND ASSISTIVE DOMESTIC ROBOTS FOR OLDER ADULTS: ROBOT SOCIABILITY, TRUST, AND ACCEPTANCE

Megan Bayles,[1] Wendy Rogers,[1] and Travis Kadylak,[2]
*1. University of Illinois Urbana-Champaign, Champaign, Illinois, United States, 2. University of Illinois Urbana Champaign, Champaign, Illinois, United States*

Older adults prefer to age in place, to live independently while maintaining social connection and engagement with the community. Though older adults can encounter barriers to these goals, social and assistive domestic robots hold promise for promoting independence and online/offline social engagement. However, social robots must be designed to meet their needs and preferences. Open questions remain regarding how to facilitate the development of trust and acceptance in robot support. We investigated the associations between robot social characteristics, sociability, trust, and acceptance for instrumental activities of daily living. We used an online survey to assess older adults' perceptions towards social and assistive robots. Robots with more social abilities were rated as more acceptable and trustworthy across different task types. We discuss design implications that may promote the development of robot trust and acceptance by older adults, and ultimately help enable aging in place and social engagement.

### Session 3150 (Symposium)

### THE VERTICAL AND HORIZONTAL RELATIONS OF KOREAN AND KOREAN AMERICAN OLDER ADULTS AND THEIR WELL-BEING

Chair: Meeryoung Kim Co-Chair: Nan Sook Park
Discussant: Michin Hong

Various relationships are important for the well-being of older adults. This session focuses on the vertical and horizontal relations of Korean and Korean American older adults and their well-being. The purpose of this session is to highlight the importance of intergenerational relations and social involvement of Korean and Korean American older adults. For vertical relations, two studies focus on intergenerational relationships and solidarity. The first study investigated whether intergenerational relationships and social support mediate the distressing consequences of life events, and how this improved the psychological well-being of Korean older adults. The second study developed a standardized measurement tool for intergenerational solidarity because intergenerational conflicts caused by rapid socioeconomic changes have highlighted the importance of strengthening intergenerational solidarity. The third and fourth studies focus on horizontal relations involving social isolation and social involvement. Guided by the double jeopardy hypothesis, the third study examined the health risks posed by the coexistence of social and linguistic isolation in older Korean Americans. As the opposite of social isolation, social involvement was an important factor of social integration of older adults. The fourth study examined volunteering as an example of social involvement by focusing on older adults' volunteering on the social integration and role identity. Implications of this study suggest not only the importance of social involvement but also the intergenerational relationships on older adults' well-being.

### INTERGENERATIONAL RELATIONSHIPS, SOCIAL SUPPORT, AND PSYCHOLOGICAL WELL-BEING AMONG KOREAN OLDER ADULTS

Janet Wilmoth, and Yooumi Lee, *Syracuse University, Syracuse, New York, United States*

Using longitudinal data from the 2006 to 2018 Korean Longitudinal Study of Aging, this study explores depression trajectories among individuals who are 60 or older with at least one living adult child at baseline. We estimated linear growth curve models of depression trajectories separately for married, unmarried and widowed using the Center for Epidemiologic Studies Depression Scale (CES-D). Results indicate that declining health and recent widowhood are positively related to depressive symptoms. Satisfactory intergenerational relationships and social support in the form of caregiving decrease depressive symptoms of older parents, especially among the widowed. Having at least one son and a first-born daughter positively impact psychological well-being of older parents. A son was particularly important for those who are widowed. We conclude that the psychological benefits of intergenerational relationships and social support are contingent upon the vulnerability of Korean older adults and discuss the implications for public policy.

### A STUDY ON THE DEVELOPMENT AND VALIDATION OF AN INTERGENERATIONAL SOLIDARITY MEASURING TOOL USING MIXED METHODS

Seok In Nam, and Joosuk Chae, *Yonsei University, Seoul, Seoul-t'ukpyolsi, Republic of Korea*

Intergenerational conflicts caused by rapid socioeconomic changes have highlighted the importance of strengthening intergenerational solidarity, emphasizing the necessity of tool designed to measure intergenerational solidarity. This study developed a standardized intergenerational solidarity measurement tool using mixed methods. In the qualitative research stage, 27 main survey questions were derived through literature research, in-depth interviews, and content validity verification. In the quantitative research stage, based on the results of a survey of 1,109 adults, both exploratory and confirmatory factor analyses of the questions were conducted, and the validity of the questions was confirmed. The analysis results were used to develop a 10-item measurement tool consisting of two factors: "recognition of intergenerational solidarity in the family" and "recognition of social intergenerational solidarity." This study is the first attempt to develop a standardized measure of intergenerational solidarity, and it can be used for nationwide panel surveys in academic and policy research.

### THE IMPACT OF VOLUNTEERING MOTIVATION FOR OLDER KOREAN ADULTS ON SOCIAL INTEGRATION AND ROLE IDENTITY

Meeryoung Kim, *Daegu University, Daegu, Kyongsang-bukto, Republic of Korea*

As life expectancy increases, older Korean adults need more activities for the next 20 to 30 years after their retirement. Rowe and Kahn indicate active social participation as an area of successful aging. After retirement, older adults uphold a desire to be part of society. This study examines the motivation effects for volunteering on social integration, role identity and volunteer satisfaction. Subjects for this study are 303 older volunteers belonging to the Korean Senior Citizens' Association throughout South Korea. According to the results, the skills obtained through volunteering had a significant effect on social integration, role identity, and volunteer satisfaction. Value motivation also had a significant effect on social integration, and reinforcement motivation significantly affected role identity. Implications of this study were found to have various effects according to the motivation for volunteering. Therefore, it will be important to understand the older adults' motives so that they can volunteer accordingly.

### HEALTH RISKS POSED BY SOCIAL AND LINGUISTIC ISOLATION IN OLDER KOREAN AMERICANS

Yuri Jang,[1] Hyunwoo Yoon,[2] Nan Sook Park,[3] David Chiriboga,[3] Miyong Kim,[4] and Juyoung Park,[1]
*1. University of Southern California, Los Angeles, California, United States, 2. School of Social Work, Portland State University, Portland, Oregon, United States, 3. University of South Florida, Tampa, Florida, United States, 4. School of Nursing, The University of Texas at Austin, Austin, Texas, United States*

Guided by the double jeopardy hypothesis, the present study examined the health risks posed by the coexistence of social and linguistic isolation in older Korean Americans. Using data from the Study of Older Korean Americans (SOKA, n = 2,032), comparisons of four isolation typologies (no isolation, social isolation only, linguistic isolation only, and dual isolation) were made, and their impacts on physical (self-rated health), mental (mental distress), and cognitive health (cognitive performance) were examined. The 'dual isolation' group exhibited greater sociodemographic and health disadvantages. The odds of having fair/poor health, mental distress, and cognitive impairment were 2.21-3.17 times higher in the 'dual isolation' group than those in the group with no isolation. Our findings confirm that both social relationships and language proficiency are key elements for older immigrants' social connectedness and integration, deprivation of which puts them at risk in multidimensions of health.

### Session 3155 (Symposium)

### TRAINING IN INTERDISCIPLINARY, PRACTICE-ORIENTED MINORITY AGING RESEARCH: HONORING THE WORK OF DR. JAMES JACKSON

Chair: Briana Mezuk Co-Chair: Robert Taylor
Discussant: Roland Thorpe, Jr.

Few scientists had the breadth and depth of scholarship, the keen interest in interdisciplinary scientific collaboration, and the commitment to mentoring the next generation of scientists as Dr. James Jackson. His passing remains a tremendous loss for the field. This symposium, organized by members of the Michigan Center for Urban African American Aging Research (MCUAAAR), which was founded by James over 20 years ago, reflects on the impact of transdisciplinary team science, of the importance of research networks and resource sharing, of the need to center research within practice and community, and of the scientific innovation that comes from integrating conceptual models, data sources, and methodological approaches from seemingly disparate fields. The session is co-chaired by Dr. Robert Taylor, longtime faculty member and current PI of MCUAAAR. The talk by session chair Dr. Briana Mezuk will discuss the ways in which the training approach of Analysis Core has inspired new training programs on integrative methods focused on minority health and disparities. The talk by Dr. Tam Perry will describe the innovations of the Community Liaison and Recruitment Core, including how COVID-19 impacted the activities of the Healthier Black Elder Center. The third talk by Dr. Rodlescia Sneed, a MCUAAAR early-career scientist, provides an example of how this Center supports interdisciplinary minority aging research through her project focused on older adults who have a history of incarceration. Finally, Discussant Dr. Roland Thorpe, a member of the MCUAAAR Advisory Board, will reflect on Dr. Jackson's legacy of mentorship and collaboration.

### METHODS TRAINING TO ADVANCE INTEGRATIVE SCIENCE ON MINORITY AGING

Monica Firestone,[1] Wassim Tarraf,[2] and Briana Mezuk,[3] *1. Department of Epidemiology, University of Michigan School of Public Health, Michigan, United States, 2. Institute of Gerontology & Department of Healthcare Sciences, Wayne State University, Detroit, Michigan, United States, 3. University of Michigan, Ann Arbor, Michigan, United States*

Minority aging is an inherently interdisciplinary field. However, it can be difficult for early-career investigators to develop skills on how to integrate data sources, study designs, measurement approaches, and analytic tools from disparate fields into their research programs. This session will illustrate how the biopsychosocial framework has been used to structure the content and delivery of methods training related to minority health/aging research in two NIH-funded exemplar programs: the MCUAAAR Analysis Core, and the Michigan Integrative Well-Being and Inequality (MIWI) Training Program. This talk will illustrate how the 20-year history of MCUAAAR informed the development of MIWI, and how both initiatives approach early-career scientist training through: i) centering learning within a mentorship structure to model team science, ii) avoiding false dichotomizes and hierarchies in study designs and data sources, and iii) attending to the unique challenges faced by scientists working in minority health through knowledge sharing.

### PROMOTING CONNECTIONS THROUGH CREATIVE APPROACHES TO RESEARCH ENGAGEMENT FOR OLDER AFRICAN AMERICANS

Jamie Mitchell,[1] Kent Key,[2] Vanessa Rorai,[3] Sean Knurek,[4] Peter Lichtenberg,[5] and Tam Perry,[5], *1. University of Michigan School of Social Work, Ann Arbor, Michigan, United States, 2. Michigan State University, Flint, Michigan, United States, 3. Wayne State University Institute of Gerontology, Detroit, Michigan, United States, 4. Michigan State University, Corunna, Michigan, United States, 5. Wayne State University, Detroit, Michigan, United States*

This presentation will feature innovative retention approaches that contributed to sustaining connections to older Black participants in the long-standing Healthier Black Elders Center (HBEC). The HBEC aims to address and reduce health disparities through research and education. In 2020, this outreach has included a telephone outreach program and a weekly social group, "The Party Line," to promote connections and collect data on mental health, coping mechanisms and newly acquired skills, as well as health care access including access to masks, testing and tele-health. The presentation will also describe tailored approaches to initiating a Community Advisory Board and programming in Flint, MI and creative efforts to retain participants in Detroit, MI, thus ensuring the relationships between researchers and older community members are sustained despite program modifications.

### SOCIAL RELATIONSHIPS AND SOCIAL ENGAGEMENT AMONG AFRICAN AMERICAN ADULTS WITH A HISTORY OF INCARCERATION

Bridget Farmer, Jennifer Johnson, and Rodlescia Sneed, *Michigan State University, Flint, Michigan, United States*

Strong social relationships and social engagement are crucial for both successful aging and successful community re-entry after incarceration. Here, we utilized a mixed methods approach to understand the impact of incarceration on social relationships and social engagement among formerly incarcerated community-dwelling African-American adults aged >50. Participants in the 2012 or 2014 waves of the Health and Retirement Study answered questions regarding prior incarceration, social relationships, and participation in social activities. Additionally, we utilized key informant interviews to further explore how incarceration might impact relationships and social engagement. This presentation will describe quantitative associations between prior incarceration and social relationship structure & function. Further, we will use our qualitative interview data to further explore possible explanations for our findings. Finally, we will describe how MCUAAAR Scientist/Faculty interactions facilitated this work.

### Session 3160 (Paper)

### ALZHEIMER'S DISEASE II (HS PAPER)

### ASSOCIATION BETWEEN LATE-LIFE HYPERTENSION AND RESILIENCE TO ALZHEIMER DEMENTIA AMONG OLDER ADULTS

Mo-kyung Sin,[1] and Yan Cheng,[2] *1. Seattle University, Seattle, Washington, United States, 2. George Washington University, Washington, District of Columbia, United States*

While midlife hypertension is known as one contributing factor for cognitive impairment and Alzheimer dementia in late-life older adults, less is known about the role of late-life hypertension in resilience to Alzheimer dementia. We examined the relationship between late-life hypertension and Alzheimer dementia resilience among older adults using the National Alzheimer's Coordinating Center data from 2005-2020 (n=3,170). Hypertension, captured within 5 years prior to death, was defined as blood pressure (BP) ≥ 140/90 mmHg in at least two visits and/or ever treated with anti-hypertensive agents. Resilience was defined as positive Alzheimer disease (AD) pathology (CERAD score moderate or severe and BRAAK stage V or VI) from autopsy and Clinical Dementia Rating (CDR) - Sum of Boxes (SOB): 0.5-2.5 or CDR global (0-0.5) from last data point before autopsy. Student's t-tests and Chi-square tests were conducted to compare patients with and without resilience. A multivariate logistic regression was conducted to estimate the association between late-life hypertension and resilience, adjusting for covariates of demographics and neuropathological characteristics. We had 55 resilient cases among 1,195 positive AD pathology cases. Those resilient were older (88±6.7) and had higher systolic BP (136 ± 18.2 mmHg) than non-resilient (82±7.9 years old, 130±20 mmHg. Untreated hypertension had a protective effect on resilience (adjusted OR: 3.69 (1.10-13.5, p=0.05). Patients with a systolic BP in the range of 135-145 mmHg and a diastolic BP in the range of 65-75 mmHg had the highest resilience possibility. Unlike midlife hypertension, late-life hypertension may have different effect on dementia, prompting further studies.

### LINKS OF AUTOIMMUNE THYROID DISORDERS TO ALZHEIMER'S DISEASE FOR MEDICARE BENEFICIARIES AGES 65+

Stanislav Kolpakov Nikitin,[1] Arseniy Yashkin,[2] and Igor Akushevich,[1], *1. Duke University, Durham, North Carolina, United States, 2. Duke University, Morrisville, North Carolina, United States*

To explore racial disparities in the interaction between Hashimoto thyroiditis (HT), Graves disease (GD), and Alzheimer's disease (AD)we used age-adjusted rates for Medicare beneficiaries aged 65+. We investigated race/ethnicity/gender-specific trends of age-adjusted prevalence of HT and GD over 1991-2017 and used the Cox proportional hazards model with propensity score matching to explore the associations between AD and these thyroid disorders. The total age-adjusted prevalence of GD was increasing over the study period reaching a maximum of 0.35% in 2014 followed by a gradual decline. Except for relatively high prevalence rates in Native American Males, no statistically significant sex/race-related differences were identified. GD was found to be associated with an increased risk of AD onset [HR: 1.129; CI: 1.050-1.215]. The mechanism of interaction between thyroiditis and AD could follow several alternative pathways. The primary mechanism involves changes in blood vessel function, mostly arterial wall stiffness, which leads to increased pulse wave velocity and consequently to the higher amplitude of oscillation of the peripheral microcapillary system in the brain leading to cerebral leukoaraiosis and damage in kidney tissue. This, in turn, leads to kidney diseases (themselves associated with AD/ADRD), increased for HT and decreased for GD LDL cholesterol levels and an increased for HT and decreased for GD total/HDL cholesterol ratio, which has an important role in increased common carotid intima-media thickness (CIMT) in hypothyroid patients and is linked to arteriosclerosis. Additionally, both, endothelial dysfunction and arterial stiffness are risk factors for coronary or artery disease and consequently increase risks for AD/ADRD.

### NEIGHBORHOOD GREENSPACE AND COGNITION: THE CARDIOVASCULAR HEALTH STUDY

Sara Godina,[1] Andrea Rosso,[1] Gina Lovasi,[2] Lilah Besser,[3] Jana Hirsch,[4] Jonathan Platt,[5] Annette Fitzpatrick,[6] and Yvonne Michael,[7] *1. University of Pittsburgh, Pittsburgh, Pennsylvania, United States, 2. Drexel Dornsife School of Public Health, Philadelphia, Pennsylvania, United States, 3. Florida Atlantic University, Boca Raton, Florida, United States, 4. Drexel University, Philadelphia, Pennsylvania, United States, 5. Columbia University Mailman School of Public Health, New York, New York, United States, 6. University of Washington, Seattle, Washington, United States, 7. Drexel University, PHILADELPHIA, Pennsylvania, United States*

Access to greenspace has been positively associated with cognition among older adults, however prior research has been limited in temporal and geographic scope. We evaluated associations between neighborhood greenspace and incidence of dementia and change in cognitive functioning using a longitudinal sample of non-demented adults (n=2,465) from the Cardiovascular Health and Cognition Study. Percent greenness (1-km radial buffers) was derived from the National Land Cover Dataset. Cognitive function was measured using the Mini-Mental State Examination (3MSE) and dementia status was clinically adjudicated. Cox proportional hazard and logistics regression analyses were used to examine associations of baseline greenness with risk of incident dementia and risk of mild cognitive impairment, respectively. Generalized linear mixed models accounting for within-subject correlations were used to examine the association between greenspace in the neighborhood at baseline and 3MSE score (1991-1999). Ongoing results will be presented, along with modifiers and mediators of associations.

### PREVENTABLE HOSPITALIZATIONS IN ADULTS WITH ALZHEIMER'S DISEASE AND RELATED DEMENTIAS: UNITED STATES, 2016–2018

Christopher Taylor,[1] Benjamin Olivari,[1] Roshni Patel,[2] Raza Lamb,[3] Matthew Baumgart,[3] and Lisa McGuire,[4] *1. Centers for Disease Control and Prevention (CDC), Atlanta, Georgia, United States, 2. Center for Disease Control and Prevention, Atlanta, Georgia, United States, 3. Alzheimer's Association, Chicago, Illinois, United States, 4. CDC, Atlanta, Georgia, United States*

Alzheimer's disease and related dementias (ADRD) are a significant public health burden. Preventing hospitalizations in adults with ADRD is a public health priority. Data from the 2016–2018 Healthcare Cost Utilization Project National Inpatient Sample, an all-payer representative sample of US hospitalizations, were used to describe potentially preventable hospitalizations in adults ≥45 years with ADRD using International Classification of Disease, Tenth Edition, Clinical Modification (ICD-10-CM) codes. Definitions for principal or any-listed ICD-10-CM codes from the Agency for Healthcare Research and Quality defined potentially preventable hospitalizations where admissions might have been avoided by appropriate outpatient primary care management. Of discharges in adults ≥45 years with a potentially preventable hospitalization diagnosis, 11.4% (N=389,155) had a diagnosis of ADRD listed in any position. Of those discharges with ADRD, a significantly higher proportion (82.6%) with diagnosis related to potentially preventable hospitalizations were aged ≥75 years compared to 78.9% without potentially preventable hospitalizations. Additionally, of those with ADRD and potentially preventable hospitalization diagnoses, a higher proportion died in the hospital (5.7%) compared to those without potentially preventable hospitalization diagnoses (3.4%). The most common potentially preventable hospitalization diagnoses among adults with ADRD were related to sepsis (34.0%), injuries (20.8%), urinary tract infections (14.2%), and heart failure (12.7%). Measures focusing on preventing injuries as well as identifying early signs and symptoms of potentially preventable hospitalizations like urinary tract infections and sepsis in adults with ADRD could reduce the number of preventable hospitalizations in this population.

### THE EFFECT OF TRAUMATIC BRAIN INJURY ON ALZHEIMER'S DISEASE AND COGNITIVE DECLINE IN VETERANS AND NON-VETERANS

Igor Akushevich,[1] Arseniy Yashkin,[2] Stanislav Kolpakov Nikitin,[1] and Julia Kravchenko,[1], *1. Duke University, Durham, North Carolina, United States, 2. Duke University, Morrisville, North Carolina, United States*

We assess the differences in the effect of traumatic brain injury (TBI) on the decline in cognitive status and the risk of Alzheimer's disease and related dementia (AD/ADRD) between veteran and non-veteran respondents of the Health and Retirement Study (HRS) and measure the sensitivity of these differences to the incremental introduction of controls for associated risk factors. Three groups of AD/ADRD risk-related variables were used: i) demographic/socioeconomic factors, including gender, race, marital status, education, income, and the number of limitations in activities of daily living; ii) comorbidities, including co-existing depression/post-traumatic stress syndrome (PTSD), substance (alcohol, tobacco and/or prescription drug) abuse, diabetes mellitus, stroke, and heart failure; and iii) genetic factors, including the presence of at least one pair of the APOE4 allele and a series of polygenic risk scores associated with AD hallmarks. The dynamics of changes in cognitive impairment in response to TBI, PTSD, and mild cognitive impairment were validated against respective measures estimated using the Department of Defense Alzheimer's Disease Neuroimaging Initiative (DoD-ADNI) data. The results of the analyses showed that TBI and PTSD were strongly associated with cognitive decline and the risks of AD/ADRD in both veteran and non-veteran subpopulations in HRS data and the difference between them was not statistically significant. Effect magnitude decreased with the addition of risk-related control variables but remained associated with the increased risks. Prevalence of mild cognitive impairment was associated with TBI at baseline in DoD-ADNI data, but no cognitive decline was observed during one year of follow-up.

### Session 3165 (Symposium)

### BREAKING NEW GROUND: INCENTIVIZING INNOVATIVE CAREGIVING PROGRAMS

Chair: C. Grace Whiting Discussant:
Dolores Gallagher-Thompson

Caring for someone with chronic illness is a demanding job, and as a result 46% of caregivers caring for adults with chronic illnesses report a significant level of burden (AARP and National Alliance of Caregiving, 2015). Recent reviews note a prevalence rate of 31.2% for depression (Collins & Kishita, 2019) and 32.1% for anxiety (Kaddour & Kishita, 2020). In addition, most caregivers also report high levels of negative emotions including frustration, guilt, and a sense of hopelessness regarding the future (Schulz & Eden, 2016). This symposium will focus on innovative programming to address caregiver needs and concerns. The first presenter will set the foundation as she explores her caregiver journey and the issues she experienced as a caregiver. Using her personal experience, this healthcare professional will explore her interactions with the medical system as a caregiver, including the unique issues experienced during the pandemic. The second presenter will examine why are caregiver program needed, what benefits can be expected, and what is considered best practices when addressing the unmet needs of family caregivers in caregiver programs. The third and fourth presenters will discuss two exemplar caregiving programs - Caregiver Clinic at the Memorial Sloan Kettering Cancer Center and the Caregiver Initiative from the Rush University Medical Center. They will describe the program, the process of creation, funding, barriers experienced, and working solutions. Pertinent data regarding the integration of the programs within the medical systems, the programs scope, and the effects of the pandemic on the programs will be shared.

### THE LONG AND WINDING ROAD: A CAREGIVER'S JOURNEY

Theresa Harvath, *University of California, Davis, UC Davis, California, United States*

In a discussion about innovative caregiving programs, it is important to hear the voice of the caregiver. In this section, using her personal experience, this healthcare professional will explore her interactions with the medical system as a caregiver for her partner. She will discuss her caregiver journey from the cancer diagnosis in 2014 through her partner's final days in hospice, which occurred during the pandemic. She will discuss pertinent issues regarding in what ways the healthcare system response was helpful, which responses were problematic, and where it outright failed to address caregiver needs. Issues, such as caregiver perceived invisibility during the hospital stay, how ageism affected policy decisions and what effect those policies had on her as the caregiver, and other important issues will be explored, including the how the pandemic affected her as a caregiver.

### CAREGIVER SERVICES: NEEDS, BENEFITS, AND BEST PRACTICES

Susan Reinhard, *AARP, Washington, District of Columbia, United States*

Just under 1 in 5 Americans (19.2%) are caregivers for adults with chronic illnesses. Caregiving is the great equalizer as caregiving remains an activity that occurs among all generations, racial/ethnic groups, income or educational levels, family types, gender identities, and sexual orientations. This presentation will provide a snapshot of the current status of caregiving in the United States. It will explore why caregiver services are needed and will highlight the impacts many caregivers face as a result of their stepping up to help family and friends. In addition, this presentation will discuss what is considered best practice in caregiver services and how the public and private sectors can work together to develop solutions to support family caregivers and those under their care.

### CAREGIVERS CLINIC AT MEMORIAL SLOAN KETTERING CANCER CENTER

Allison Applebaum, *Memorial Sloan Kettering Cancer Center, New York, New York, United States*

The mission of the Caregivers Clinic at Memorial Sloan Kettering Cancer Center (MSK) is to assure that no caregiver of an MSK patient experiencing significant distress as a result of their caregiving responsibilities goes unidentified and deprived of necessary psychosocial services. This presentation will cover the steps taken and barriers faced in the development of the Clinic, including advocating for caregivers to receive their own unique medical records. Data regarding the number of caregivers seen for psychotherapy and for medication management will be presented, as will data regarding presenting complaints and average length of care. Also included is a discussion of the challenges faced in expanding and maintaining the capacity of the Clinic, especially in the setting of the pandemic during which caregivers' use of psychosocial care at MSK is notably higher than in years past. Several current adjunct approaches to address capacity needs currently being piloted will be discussed.

### THE RUSH CAREGIVER INITIATIVE: A MODEL FOR CAREGIVER HEALTH AND WELLNESS IN AGE-FRIENDLY HEALTH SYSTEMS

Leslie Pelton,[1] Ellen Carbonell,[2] and Robyn Golden,[2]
*1. Institute for Healthcare Improvement, Boston, Massachusetts, United States, 2. Rush University Medical Center, Chicago, Illinois, United States*

The Rush Caregiver Health and Well-Being Initiative (Caregiver Initiative) draws together evidence-based practices into a single framework to improve care for older adults and caregivers. The Caregiver Initiative has two components: system-level and caregiver level interventions. The complexities of system change take place within leadership, data management, and provider teams throughout the health care system, and solutions to resistance have been developed. Caregiver-level interventions start with an assessment using evidence-based tools, and offer an opportunity to participate in a Teach-Back Clinic, Family Care Planning sessions, and/or Goals of Medical Care meetings, all connected to the 4Ms of an Age-Friendly Health System. Contact and follow-up issues were addressed, and as of February 2021, 191 caregivers have enrolled. Outcomes to date show statistically and clinically significant reductions in depression, anxiety, and caregiver burden. This presentation will highlight lessons learned in the development of the model and caregiver outcomes to date.

### Session 3170 (Symposium)

### CHALLENGES OF IMPLEMENTING THE PRISM 2.0 TRIAL FOR SUPPORTING SOCIAL CONNECTIVITY THROUGH TECHNOLOGY

Chair: Walter Boot Co-Chair: Sara Czaja
Discussant: Dana Plude

Following the success of the Personal Reminder Information and Social Management (PRISM) trial, which found that a specially designed computer system for older adults can enhance social connectivity and reduce loneliness among older adults at risk for social isolation, the PRISM 2.0 trial sought to replicate and extend these results to a new technology platform (tablet rather than desktop) with expanded social features and diverse populations of older adults, including older adults living in rural areas, assisted living communities, and senior housing. This symposium discusses the aims of the trial conducted by the Center for Research and Education on Aging and Technology Enhancement (CREATE), challenges encountered (including challenges related to the COVID-19 pandemic), and solutions to those challenges. S. Czaja will begin with an overview of the PRISM 2.0 system and the trial. J. Sharit will discuss challenges encountered working within the context of assisted living facilities and with impaired participants. This will be followed by a discussion of technical challenges encountered during the course of the trial presented by N. Charness. W. Rogers will present training issues involved (both with respect to participants and assessors). Finally, W. Boot will describe challenges encountered with measuring and quantifying technology use during the trial. Lessons learned are applicable to many types of technology interventions administered in diverse contexts. D. Plude, Deputy Director in the Division of Behavioral and Social Research of NIA, will serve as discussant.

### PRISM 2.0: A TECHNOLOGY SYSTEM TO SUPPORT RESOURCE ACCESS AND SOCIAL AND COGNITIVE ENGAGEMENT

Sara Czaja,[1] Walter Boot,[2] Neil Charness,[2]
Wendy Rogers,[3] and Joseph Sharit,[4], *1. Weill Cornell Medicine/Center on Aging and Behavioral Research, New York, New York, United States, 2. Florida State University, Tallahassee, Florida, United States, 3. University of Illinois Urbana-Champaign, Champaign, Illinois, United States, 4. Department of Industrial Engineering, University of Miami, Coral Gables, Florida, United States*

Social isolation and lack of engagement are common among older adults and present a risk for emotional, physical and cognitive decline. Technology offers the potential of remediating these risks and enhancing opportunities for connectivity. In this paper we present an overview of the PRISM 2.0 multi-site RCT, which evaluated a simple to use Personalized Reminder Information and Social Management System (PRISM) among a sample of two hundred and forty-eight adults age 65+ in diverse contexts (Rural Locations, Assisted Living Communities and Senior Housing). PRISM 2.0 is a tablet-based system, intended to provide support for access to resources and information, new learning, social and cognitive engagement, and memory. We describe the goals and content of PRISM, the user-centered design process, and measurement strategies. We also discuss the challenges of conducting the trial during the COVID-19 pandemic and the strategies used to adapt the trial protocol within the three contexts.

### CHALLENGES OF IMPLEMENTING THE PRISM 2.0 CLINICAL TRIAL WITH ASSISTED LIVING AND IMPAIRED PARTICIPANTS

Sara Czaja,[1] Jerad Moxley,[2] Carlos Almirola,[3] and
Joseph Sharit,[4], *1. Weill Cornell Medicine/Center on Aging and Behavioral Research, New York, New York, United States, 2. Weill Cornell Medicine, New York, New York, United States, 3. University of Miami Miller School of Medicine, Miami, Florida, United States, 4. Department of Industrial Engineering, University of Miami, Coral Gables, Florida, United States*

The PRISM 2.0 clinical trial examined the benefits of a software system, implemented on a computer tablet, which was designed to support access to information, engagement, and social connectivity among older people. Participants across three sites were recruited from rural locations, senior living housing facilities, and assisted living facilities (ALFs) and correspondingly randomized into either the Prism or control (tablet computer without the PRISM system) conditions. In this talk, we focus on the challenges associated with including ALF participants at key stages of the trial. These stages included telephone prescreening, baseline assessment, training on the system, and 6-, 9-, and 12-month follow-up assessments. Inability to meet inclusion criteria related to cognitive and sensory-motor considerations was a common problem, as was the ability to sustain attention during the training sessions. Recommendations for recruitment and retaining older adults in ALFs for these types of studies will be offered.

### PRISM 2.0: TECHNICAL CHALLENGES

Walter Boot,[1] Sara Czaja,[2] Wendy Rogers,[3] Joseph Sharit,[4] and Neil Charness,[1], *1. Florida State University, Tallahassee, Florida, United States, 2. Weill Cornell Medicine/Center on Aging and Behavioral Research, New York, New York, United States, 3. University of Illinois Urbana-Champaign, Champaign, Illinois, United States, 4. Department of Industrial Engineering, University of Miami, Coral Gables, Florida, United States*

PRISM 2.0 was designed to run on Android tablets and made use of both customized apps that relied on Google's browser and e-mail functionality as well as commercial apps, such as Microsoft's Skype for videoconferencing. We also made use of functionality provided by our partner AT&T, such as their sim cards to provide cell-based internet connectivity to participants who did not have access to Wi-Fi internet services to their home (cable, DSL), as well as tablet management software to deploy updates. The Miami site provided central management and tablet deployment and redeployment services and support as well as coordinating locally provided tech support at the three sites. We discuss some of the technical challenges associated with these arrangements. We focus on how changes to the operating system broke some of our apps necessitating substitution of other apps and provision of new training, and how Covid-19 affected technical support.

### TRAINING CHALLENGES FOR EFFECTIVELY IMPLEMENTING A TECHNOLOGY CLINICAL TRIAL: A SNAPSHOT FROM PRISM 2.0

Tracy Mitzner,[1] Kara Cohen,[2] Jerad Moxley,[3] and Wendy Rogers,[4], *1. Georgia Institute of technology, Atlanta, Georgia, United States, 2. Georgia Institute of Technology, Atlanta, Georgia, United States, 3. Weill Cornell Medicine, New York, New York, United States, 4. University of Illinois Urbana-Champaign, Champaign, Illinois, United States*

Technology interventions can only be adequately assessed for efficacy if participants are adequately trained to use the technology. Only then can an evaluation be made about whether the technology intervention affects the outcome of interest. In the PRISM study, our goal was to teach inexperienced older adults to use either a tablet computer (control) or the PRISM 2.0 system. In this presentation we will discuss the training processes we used for both groups (e.g., segmenting sessions, providing homework, observations), to enable us to evaluate the relative benefits of PRISM for social connectedness. We will describe the training challenges and the need for assessors to be able to troubleshoot technology issues. We will evaluate individual differences in training success and drop-outs to provide insights for other technology intervention studies. Understanding these individual differences can provide guidance for the deployment of new technologies that may benefit health, social interaction, or cognitive engagement.

### CHALLENGES OF QUANTIFYING PRISM 2.0 AND TABLET USE

Neil Charness,[1] Jerad Moxley,[2] and Walter Boot,[1], *1. Florida State University, Tallahassee, Florida, United States, 2. Weill Cornell Medicine, New York, New York, United States*

As with the PRISM 1.0 trial, an important outcome of the PRISM 2.0 trial is use of the PRISM system and use of the PRISM system compared to the control condition (a standard tablet without the PRISM software). Frequent use over time is an important measure of system success. Further, use data provide key measures of system usefulness and usability. What features do participants use most and how often? Within those features, what activities do they engage in? What are the patterns of use throughout the trial, and how does PRISM system use compare to the control condition? However, quantifying use is not an easy task. This talk presents the challenges of quantifying use of a complex, multi-faceted system, and of making meaningful comparisons in use between two very different systems. Analysis approaches and solutions are discussed.

### Session 3175 (Paper)

### COGNITIVE AGING I

### COGNITION-MORTALITY ASSOCIATIONS ARE STRONGER WHEN ESTIMATED JOINTLY IN LONGITUDINAL AND TIME-TO-EVENT MODELS

Stephen Aichele,[1] Sezen Cekic,[2] Patrick Rabbitt,[3] and Paolo Ghisletta,[2], *1. Colorado State University, Fort Collins, Colorado, United States, 2. University of Geneva, University of Geneva, Geneve, Switzerland, 3. University of Oxford, University of Oxford, England, United Kingdom*

**Objectives:** With aging populations worldwide, there is growing interest in links between cognitive decline and elevated mortality risk—and, by extension, analytic approaches to further clarify these associations. Toward this end, some researchers have compared cognitive trajectories of survivors vs. decedents while others have examined longitudinal changes in cognition as predictive of mortality risk. A two-stage modeling framework is typically used in this latter approach; however, several recent studies have used joint longitudinal-survival modeling (i.e., estimating longitudinal change in cognition conditionally on mortality risk, and vice versa). Methodological differences inherent to these approaches may influence estimates of cognitive decline and cognition-mortality associations. These effects may vary across cognitive domains insofar as changes in broad fluid and crystallized abilities are differentially sensitive to aging and mortality risk.

**Methods:** We applied each of the above analytic approaches to data from a large-sample repeated-measures study of older adults (N = 5,954, of whom 4,453 deceased; ages 50–87 years at assessment).

**Results:** Cognitive trajectories indicated worse performance in decedents and when estimated jointly with mortality risk, but this was attenuated after adjustment for health-related covariates. Better cognitive performance predicted lower mortality risk, and, importantly, cognition-mortality associations were stronger when estimated in joint models.

Associations between mortality risk and crystallized abilities only emerged under joint estimation, confirming the greater power of this statistical approach.

**Discussion:** These results suggest that joint estimation of cognition-mortality associations may be beneficial for research in cognitive epidemiology and cognitive reserve in adult development.

### EFFECTS OF HEALTH-PROMOTING LIFESTYLES IN MIDLIFE ON COGNITIVE FUNCTIONING IN LATER LIFE

Eunsaem Kim,[1] Yunhwan Lee,[1] Jonggak Shin,[2] Gyeonghui Kim,[2] and Jihye Yoon,[1], *1. Ajou University School of Medicine, Suwon, Kyonggi-do, Republic of Korea, 2. Korea Employment Information Service, EUMSEONG-GUN, Ch'ungch'ong-bukto, Republic of Korea*

Maintaining cognitive function in later life is key to healthy aging because cognitive impairments compromise everyday functional abilities, impeding independent living. Numerous studies have discovered early life experiences and lifestyle behaviors over the lifespan to have substantial influences on cognitive functioning with age. Especially, subtle brain changes related to dementia occur as early as midlife, and lifestyle factors in midlife influence neuropathological development, suggesting that midlife is a critical period for preserving cognitive health in later life. This study investigated the association between lifestyle behaviors in midlife and cognitive performance in later life using 12-year follow-up data from the Korean Longitudinal Study on Aging (KLoSA). Cognitive function was assessed with the Harmonized Cognitive Assessment Protocol (HCAP) for KLoSA. Eight thousand respondents from the KLoSA sample were administered HCAP neuropsychological tests. Hierarchical multiple regression analyses were used to examine whether health-promoting lifestyles at baseline (2006) predicted cognitive function in 2018 after controlling for health-related covariates. We identified a positive influence of health-protective behaviors (non-smoking, moderate drinking, regular exercise, weight management, and health screening) at baseline on language abilities in 2018 ($\beta = .05$, $p < .05$). In addition, health-promoting behaviors covering interpersonal relationships, social engagement, optimistic outlook, and positive attitudes at baseline were predictive of language abilities ($\beta = .08$, $p < .01$), executive function ($\beta = .06$, $p < .01$), and the visuospatial ability ($\beta = .06$, $p < .05$) in 2018. This study highlights the importance of midlife health-promoting lifestyles in maintaining cognitive health in later life.

### HAPLOGROUPS OF MITOCHONDRIAL DNA DIFFERENTIATE PATTERNS OF COGNITIVE CHANGE OVER 7 YEARS

Amber Watts,[1] Elias Michaelis,[1] and Russell Swerdlow,[2] *1. University of Kansas, Lawrence, Kansas, United States, 2. University of Kansas Alzheimer's Disease Center, Fairway, Kansas, United States*

Mitochondrial DNA (mtDNA) may play an important role in Alzheimer's disease (AD) and cognitive decline. A particular haplogroup of mtDNA (haplogroup J), has been observed more commonly in patients with AD than in cognitively normal controls. We used mtDNA haplogroups to predict change in cognitive performance over seven years. We hypothesized that haplogroup J would predict poorer cognitive function and steeper cognitive decline. We analyzed data from 140 cognitively normal older adults (age 65+) who participated in the University of Kansas Alzheimer's Disease Center annual registry. We used factor analysis to create three composite scores (verbal memory, attention, executive function) from 11 individual cognitive tests. We performed latent growth curve modeling to describe trajectories of cognitive performance and change. We compared haplogroup H, the most common, to haplogroup J, the potential risk group. Results indicated haplogroup J carriers had significantly lower baseline performance (B=-.049, $p< .01$) and slower rates of improvement (B=-.046, $p < .05$) on tests of verbal memory compared to haplogroup H. For executive function, groups did not differ at baseline (B=.065, $p>.10$), but haplogroup J had slower rates of improvement (B=-.097, $p < .01$). There were no differences in attention across groups in performance (B=.135, $p>.10$) or change (B=-.01, $p>.10$). Our results reinforce the important role of mtDNA in changes to cognitive function with aging and imply that the effects of haplogroup J may vary across cognitive domains. Future research should investigate the mechanisms by which mtDNA might affect performance on specific cognitive domains across haplogroups.

### OLDER ADULTS' ENGAGEMENT IN COGNITIVELY STIMULATING ACTIVITIES PRIOR TO THE PANDEMIC PREDICTS LONELINESS

Lilian Azer,[1] Isaac Quintanilla Salinas,[1] Esra Kürüm,[1] Leah Ferguson,[1] Elizabeth Davis,[1] Weiwei Zhang,[1] Carla Strickland-Hughes,[2] and Rachel Wu,[3], *1. University of California, Riverside, Riverside, California, United States, 2. University of the Pacific, University of the Pacific, California, United States, 3. University Of California - Riverside, Riverside, California, United States*

Loneliness, which may be more prevalent in older than younger adults, may lead to increased subjective cognitive decline and cognitive impairment may in turn predict perceived loneliness. COVID-19 physical distancing restrictions may exacerbate perceived loneliness, especially that experienced by older adults. The present study investigated whether self-reported cognitive abilities (i.e., executive functions) would predict loneliness during the COVID-19 pandemic. Younger (YA; n = 136, 18-35 years), middle-aged (MA; n = 126, 36-54 years), and older (OA; n = 171, 55-88 years) adults completed questionnaires assessing self-reported executive functions (EF) and perceived loneliness using the BRIEF-A and UCLA Loneliness scale respectively. Forty-nine of the 171 older participants partially completed a cognitive learning intervention, which has previously been found to increase EF. In the current study, age group did not significantly predict perceived loneliness. However, OA who participated in the prior intervention reported less loneliness than those who did not participate in the intervention. Additionally, OA who participated in the intervention and self-reported worse EF during the current study, also reported feeling lonelier than adults who did not participate in the intervention. Although results from our prior research found most OA who participated in the intervention improved their EF, the results from the current study suggest that it left them more susceptible to the negative effects of physical distancing restrictions if they had lower self-reported EF during the pandemic. Decreased engagement, real or perceived, in cognitively stimulating activities due to the pandemic strengthens the relationship between lower self-reported EF and perceived loneliness.

### THE IMPACT OF GROWTH MINDSET ON OLDER ADULTS' COGNITIVE FUNCTIONING IN A MULTI-SKILL LEARNING INTERVENTION

Pamela Sheffler,[1] Esra Kürüm,[1] Angelica Sheen,[2] Leah Ferguson,[1] Diamond Bravo,[1] George Rebok,[3] Carla Strickland-Hughes,[4] and Rachel Wu,[5] *1. University of California, Riverside, Riverside, California, United States, 2. University of California, Irvine, Irvine, California, United States, 3. Johns Hopkins University, Baltimore, Maryland, United States, 4. University of the Pacific, University of the Pacific, California, United States, 5. University Of California - Riverside, Riverside, California, United States*

Motivational factors, such as perceived control and self-efficacy, have been shown to affect older adults' cognitive functioning. Growth mindset, the belief in the malleability of intelligence and abilities, represents a related but distinct factor that has been widely studied in children and young adults' learning but less applied to the older adult population. Two studies investigated growth mindset, motivation, and cognitive functioning in a 3-month multi-skill learning intervention that incorporated weekly discussions on growth mindset and successful aging. Participants reported on their growth mindset, general pursuit of novel skill learning, and intrinsic motivation to learn, and completed a cognitive battery before, during, and after the intervention. Study 1 (n = 15, 67% female, M age = 68.67 years, SD age = 8.68, range 58-86) included both an experimental and control group and indicated that from pretest to post-test, intervention participants increased their growth mindset, while control participants did not. Study 2, which included a larger, all experimental sample (n = 28, 68% female, M age = 69.36 years, SD age = 7.00, range 58-86) revealed strong positive associations between growth mindset, pursuit of novel skill learning and intrinsic motivation. Further, participants showed a significant increase in growth mindset from pretest to post-test. Participants with higher pre-existing growth mindset showed larger cognitive gains at post-test, although growth mindset change did not affect post-test change in cognitive functioning. These findings suggest that growth mindset may facilitate older adults' continued learning and cognitive gains, and they may complement older adult learning interventions.

### Session 3180 (Symposium)

### CONDUCTING RESEARCH IN PERSONS WITH DEMENTIA DURING THE COVID-19 PANDEMIC: CHALLENGES AND STRATEGIES

Chair: Ying-Ling Jao Co-Chair: Diane Berish
Discussant: Ann Kolanowski

The COVID-19 pandemic has caused a health crisis for vulnerable older adults, physically and psychologically. Despite the urgent demand for clinical research for people with dementia, research activities are restricted due to the pandemic. This symposium will share the experiences of researchers conducting studies with older persons with dementia during the COVID-19 pandemic. The presenters share their strategies to overcome challenges at different stages of the study process during the pandemic. The research projects include work conducted in acute care, assisted living, nursing homes, and the community. The presentations include perspectives from different geographic areas and across countries in North America. The first presenter reports the challenges in continuing an ongoing research project, and shares strategies to engage stakeholders and plan a new protocol for recruitment and in-person data collection with residents with dementia in nursing homes. The second presenter reports on the barriers and facilitators of conducting an ongoing clinical trial with older adults with dementia across hospital and community settings and discusses strategies to meet project goals which include modifications to the protocol and analytic plan. The third presenter describes adaptations made to a study intervention designed to promote quality resident-staff interactions in assisted living and alterations to stakeholder engagement. The fourth presenter describes challenges and strategies to engage older adults with dementia via technology. The discussant will synthesize the findings across studies and highlight policy and research implications for the COVID-19 pandemic as well as other emergency situations.

### EXAMINING CAREGIVER-RESIDENT COMMUNICATION AND APATHY IN DEMENTIA IN NURSING HOMES DURING THE COVID-19 PANDEMIC

Diane Berish,[1] Yo-Jen Liao,[2] Marie Boltz,[1] and Ying-Ling Jao,[1], *1. Pennsylvania State University, University Park, Pennsylvania, United States, 2. Penn State University, University Park, Pennsylvania, United States*

This presentation shares lessons learned from conducting a study examining the impact of staff caregivers' communication approach on apathy in residents with dementia in nursing homes. Due to COVID-19 restrictions, this study had to be paused and required major revisions to continue, which resulted in significant delays and increased expenses. Additionally, this study required in-person data collection and video recordings to capture staff caregivers' communication with residents with dementia during caregiving activities. However, due to the pandemic, nursing home residents' daily routines have been significantly changed, making it challenging to capture the nature of caregiver-resident interactions. Furthermore, using masks created unforeseen barriers for capturing communication between staff caregivers and residents including difficulties in identifying residents' facial expressions, which are a vital component of assessing apathy. The presentation describes approaches to communication with founders, collaborators, and clinical sites and discusses strategies to recruit participants and conduct data collection.

### PILOT TESTING OF THE PROMOTING POSITIVE CARE INTERACTIONS (PPCI) IN ASSISTED LIVING STUDY DURING A PANDEMIC

Elizabeth Galik,[1] Barbara Resnick,[2] Kelly Doran,[2] Marie Boltz,[3] Shijun Zhu,[1] and Anju Paudel,[4] *1. University of Maryland, Baltimore, Maryland, United States,*

2. University of Maryland School of Nursing, Baltimore, Maryland, United States, 3. Pennsylvania State University, University Park, Pennsylvania, United States, 4. University of Maryland, University of Marland, Maryland, United States

The purpose of this study was to test the feasibility and preliminary efficacy of Promoting Positive Care Interactions (PPCI)—a four step intervention designed to establish positive care interactions between staff and residents with cognitive impairment or dementia in Assisted Living (AL). Initially designed as a traditional on-site intervention, PPCI was later transformed to be conducted remotely through webinar and virtual meetings due to challenges related to onsite engagement in AL during the COVID-19 pandemic. Additionally, the study adopted shorter timeline, a single group pretest-posttest design, and limited recruitment to staff only; 17 care staff were recruited, and data was collected via online surveys and interviews. PPCI was successfully implemented as intended with considerable stakeholder engagement. Findings demonstrated feasibility and promising staff adoption of PPCI. Continued research is needed to optimize the quality of care interactions in AL and evaluate whether online approach to staff training can change staff behavior.

### CLINICAL RESEARCH IN THE HOSPITAL DURING THE PANDEMIC: WHAT'S WORKED AND NOT WORKED?

Ashley Kuzmik,[1] Irene Best,[1] Jacqueline Mogle,[2] and Marie Boltz,[1], *1. Pennsylvania State University, University Park, Pennsylvania, United States, 2. Penn State University, University Park, Pennsylvania, United States*

Under normal conditions, the hospital setting presents multiple challenges to research with persons with dementia and their care partners. This presentation describes the additional barriers posed by the COVID-19 pandemic, as well as the strategies to meet those challenges, in a cluster randomized controlled trial that examines the efficacy of a nurse-family partnership to promote functional recovery of persons with dementia. In response to research restrictions, the research team altered their plan for recruitment, implementation of the intervention, data collection, and analytic approach. This presentation describes these alterations and discusses the plan to meet the aims of the project while meeting the requirements of the Institutional Review Board, accountability to the funder, and university regulations. Modifications in staffing patterns, staff training, and procedures will also be discussed, as well as the study timeline. Finally, strategies to maintain a positive attitude and productivity within the team will be discussed.

### SUPPORTING PATIENT ENGAGEMENT IN DEMENTIA RESEARCH VIA TECHNOLOGY DURING THE COVID-19 PANDEMIC

Sophie yang,[1] Mario Gregorio,[2] Alison Phinney,[1] and Lillian Hung,[1], *1. University of British Columbia, Vancouver, British Columbia, Canada, 2. Vancouver, British Columbia, Canada*

The COVID-19 pandemic brings challenges to patient partnerships in research. In-person research meetings with patient partners were prohibited. In this presentation, we outline specific issues we encountered in a patient-led dementia research project, which involved a literature review study and gathering community stakeholders to identify the top 10 local priorities in the development of a dementia-friendly community. We will describe how we found shared solutions to complete the project. In response to COVID, computers and training were provided for patient partners to maintain team connection, plan project activities, conduct team analysis, and host a community workshop in the lockdown time. The drastic shift to virtual research methods created barriers and opportunities for co-research with older people with dementia. Virtual meetings can generate inequities for those who do not have a computer and knowledge in videoconferencing. Practical strategies to overcome barriers to using virtual technologies will be explored.

### Session 3185 (Symposium)

### COPING WITH COVID-19: CHALLENGES AND RESILIENCE

Chair: Lauren Mitchell Co-Chair: Lauren Mitchell
Discussant: Daniel Mroczek

COVID-19 has introduced unprecedented challenges for older adults. At the same time, older adults have adapted to meet the challenges of the pandemic. In this symposium, we explore a number of difficulties brought about by COVID-19, while also investigating the ways in which individual, social, and community resources and strengths have bolstered older adults' resilience through the pandemic. Paper 1 investigates family caregivers of older adults with dementia living in long-term residential care facilities, a group that has been especially heavily affected by the pandemic. Using longitudinal data spanning Fall 2017-Spring 2021, the authors estimate caregivers' trajectories of well-being pre-and-post pandemic. With an exceptionally large qualitative data sample, Paper 2 examines the influence of COVID-19 on older adults' neighborhood engagement. Thematic analysis has revealed diverse patterns of response to the pandemic, as well as community and personal characteristics that have facilitated older adults' coping and resilience. Papers 3 and 4 examine how older adults' personality traits may influence their responses and adjustment to the pandemic, each using assessments of personality taken before the pandemic. Specifically, Paper 3 investigates the relationship between Big Five personality traits and older adults' concerns, precautions, and preparatory behaviors. Paper 4 explores how optimism predicts older adults' emotional responses, including worry, loneliness, and benefit-finding. Using a variety of methods and populations, these studies illustrate the challenges that older adults and their families have faced over the past year, as well key factors at the level of individuals, relationships, and communities that have contributed to their resilience.

### DEMENTIA CAREGIVERS' TRAJECTORIES OF WELL-BEING BEFORE AND DURING THE COVID-19 PANDEMIC

Elizabeth Albers,[1] Robyn Birkeland,[2] Colleen Peterson,[2] Anna Drake,[3] Joseph Gaugler,[1] and Lauren Mitchell,[3] *1. University of Minnesota, Minneapolis, Minnesota, United States, 2. University of Minnesota, University of Minnesota, Minnesota, United States, 3. Emmanuel College, Boston, Massachusetts, United States*

Residents of long-term care settings and their family caregivers have been severely impacted by the COVID-19 pandemic. The present mixed-methods study examines trajectories of well-being pre- and post-pandemic onset for caregivers of persons with dementia living in residential long-term care. Participants were taking part in, or had recently completed, an ongoing intervention trial to support families transitioning a relative into long-term care. Beginning in summer 2020, we started assessing caregivers' COVID-19-related experiences and added three surveys spanning 4-months beyond the 12-month parent study period to capture caregivers' adjustment throughout the pandemic. Using latent growth curve models, we estimated caregivers' (N = 104) trajectories of depressive symptoms, burden, and self-efficacy before and during the pandemic. We also tested whether the counseling intervention had protective effects for participants in the treatment group, and examined moderators including long-term care facility size, care recipient's dementia and health status, and quality of staff interactions.

### OPTIMISM IS PROSPECTIVELY ASSOCIATED WITH RESILIENCE DURING THE COVID-19 PANDEMIC

Jeewon Oh,[1] Mariah Purol,[1] Eric Kim,[2] and William Chopik,[1], *1. Michigan State University, East Lansing, Michigan, United States, 2. University of British Columbia, Vancouver, British Columbia, Canada*

Emerging research has identified how protective factors—like optimism—are associated with resilience to stress during the COVID-19 pandemic. However, the majority of research is cross-sectional, which creates ambiguity around the causal direction because these very protective factors might have also changed due to the pandemic. In the current study, we used longitudinal data from the Health and Retirement Study (N = 921; Mage = 64.54, SD = 10.71; 59.6% female; 57.5% White) to examine how optimism measured in 2016 predicted adjustment during the pandemic (in 2020). Higher baseline levels of optimism were subsequently associated with less worrying and stress resulting from changes in social contacts ($\beta s > |.10|$), less loneliness and not feeling overwhelmed ($\beta s > |.16|$), and greater COVID-related resilience and benefit-finding ($\beta = .21$). The findings will be discussed in the context of mechanisms that facilitate the protective functions of optimism and other psychological characteristics.

### AGING IN PLACE SINCE THE COVID-19 PANDEMIC ONSET: A QUALITATIVE STUDY OF NEIGHBORHOOD ENGAGEMENT

Lindsay Kobayashi,[1] Melissa Cannon,[2] Gabriella Meltzer,[3] and Jessica Finlay,[1], *1. University of Michigan, Ann Arbor, Michigan, United States, 2. Western Oregon University, Independence, Oregon, United States, 3. Department of Social and Behavioral Sciences, New York University, New York, United States*

The COVID-19 pandemic may fundamentally change neighborhood environments and ways of aging in place. This research aimed to investigate perceptions of and engagement in neighborhoods since the pandemic onset among online survey respondents of the COVID-19 Coping Study. We analyzed a random stratified sample of 500 open-ended responses collected July-September 2020 with quotas for age, gender, race/ethnicity, and education to match the US population aged 55+. Qualitative thematic analysis identified both increased and decreased local activity and varying levels of social interaction, support, and civic engagement. Community characteristics including age structure, socioeconomic diversity, density, housing patterns, weather, and social infrastructure were related to neighborhood perceptions. These interacted with personal characteristics such as duration of residence, living arrangements, family closeness, health status, and preferred lifestyle. Results highlight coping strategies among aging adults and their neighbors, sources of individual and community vulnerability, and opportunities to strengthen social infrastructure and resiliency within neighborhoods.

### DOES PERSONALITY SHAPE COVID-19 RESPONSES IN OLDER ADULTS?

Damaris Aschwanden,[1] Angelina Sutin,[2] Amanda Sesker,[2] Ji Hyun Lee,[2] Martina Luchetti,[2] Yannick Stephan,[3] and Antonio Terracciano,[4] *1. Florida State University, Tallahassee, Florida, United States, 2. Florida State University College of Medicine, Tallahassee, Florida, United States, 3. University of Montpellier, Montpellier, Languedoc-Roussillon, France, 4. FLORIDA STATE UNIVERSITY, Florida State University, Florida, United States*

Knowing how personality plays out in a pandemic can provide guidance to improve public health messaging. In a sample of 2066 participants (Mage = 51.42; 48.5% female), we examined whether personality is associated with concerns, precautions, preparations, and duration estimates of the COVID-19 pandemic. Personality traits were measured before the pandemic; responses were assessed in late March 2020. We investigated whether age moderates the trait-response associations because older adults are at higher risk for severe complications of COVID-19. Among the 65-96-year-olds, higher conscientiousness was associated with more preparations, higher openness was associated with greater concerns, and both higher openness and agreeableness were related to more preparations and longer duration estimates. This pattern has implications: If all older adults took COVID-19 seriously, individual differences in personality should not matter; however, our findings indicate that they do matter and could be considered in the development of personality-tailored communication to older adults.

### Session 3190 (Paper)

### DEVELOPMENTAL CHANGE OVER THE LIFE COURSE

### CHILD'S DEVELOPMENTAL DISABILITIES AND PARENTAL HEALTH IN LATER LIFE: DO PARENTAL RACE AND GENDER MATTER?

Juha Lee,[1] Manjing Gao,[2] and Chioun Lee,[2], *1. University of California, Riverside, Anaheim, California, United States, 2. University of California, Riverside, Riverside, California, United States*

Parents, particularly mothers, who experienced early life adversities (ELAs) are more likely to have a child with developmental disabilities (DD). We have little knowledge about how parental health varies across race-gender groups among those with a DD child and the role of ELAs in the associations. Using Black and White adults (n = 8,778; 25% Blacks) from the Midlife in the United States (MIDUS) study, we examine racial disparities in the impact of having a child with DD (vs. having healthy children) on parental health outcomes. This study questions (1) the extent to which parents' ELAs (e.g., poverty and abuse) are associated with having a child with DD and (2) how considering early-life factors reveals racial and gender disparities in the impact of having a child with DD. We found that as the number of ELAs increases, the probability of having a healthy child decreases for all race-gender groups, but most dramatically for Black women. Having a DD has adverse effects on chronic illnesses and functional limitations more for mothers than fathers. Black women are most adversely affected, with no effect on Black men. There is no gender difference in the impact of having a DD child on depressive symptoms, yet White parents are more vulnerable than Black parents. After controlling for ELAs, the adverse effects of having a DD child on both physical and mental health remain significant. Future research should identify life-course circumstances that reveal why the impact of having a DD child varies by race and gender.

### CHILDHOOD LEAD EXPOSURE AND COGNITIVE FUNCTIONING AMONG OLDER ADULTS: EVIDENCE FROM THE HEALTH AND RETIREMENT STUDY

Haena Lee,[1] Mark Lee,[2] and John Robert Warren,[2], *1. University of Southern California, Los Angeles, California, United States, 2. University of Minnesota, Minneapolis, Minnesota, United States*

Many children born in the early 20th century were exposed to water-borne lead, a neurotoxin that negatively impacts brain development. While lead exposure has been linked to poor cognition among children and young adults, no population-level research has examined the long-term implications of lead exposure for cognitive functioning in later life. Our study is the first to address this gap by utilizing novel data linkages between the 1940 U.S. Census and the Health and Retirement Study (HRS). Our sample includes respondents who were under age 17 (born 1924-1940) by the time of the decennial enumeration on April 1, 1940. Given that the dominant source of lead exposure was water during this period, we assessed lead exposure by using water chemistry and piping material data for each HRS respondent's city of residence in 1940. Late-life cognitive functioning for HRS participants (observed 1998-2016) was measured using the Telephone Interview for Cognitive Status. We find that lead exposure during childhood is significantly and negatively associated with cognitive functioning in later life. HRS participants who lived in cities with lead pipes and acidic or alkaline water—the conditions required for lead to leech into municipal water—showed lower levels of cognitive functioning decades later as compared to other participants. This association persisted net of race, gender, childhood socioeconomic status and childhood health. However, the association was largely accounted for by adjusting for educational attainment. This implies that childhood lead exposure impacts later-life cognition via its effect on educational attainment.

### CHILDHOOD PHYSICAL ABUSE CASTS A VERY LONG SHADOW: PHYSICAL AND MENTAL ILLNESS AMONG OLDER ADULTS

Esme Fuller-Thomson,[1] and Anna S. Buhrmann,[2]
*1. University of Toronto, Toronto, Ontario, Canada, 2. McMaster University, McMaster University, Hamilton, Ontario, Canada*

A burgeoning literature indicates adverse childhood experiences (ACEs) are associated with chronic illness. Most research, to date, has not focused on health outcomes among older adults. The objectives of the current study were to identify the prevalence and adjusted odds of two mental health and six physical health conditions among survivors of childhood physical abuse (CPA) who were aged 60 and older (n=409) in comparison to their peers who had not been physically abused (n=4,659). Data were drawn from a representative sample of older British Columbians in the Canadian Community Health Survey. Logistic regression analyses took into account sex, race, age, immigration status, marital status, education, income, smoking, obesity, binge drinking and number of other ACEs. For 3 health outcomes, CPA survivors had adjusted odds ratio more than twice that of their peers (Anxiety OR=2.22; 95% CI=1.46, 3.38; Depression OR=2.17; 95% CI=1.57, 3.01; COPD OR=2.03; 95% CI=1.40, 2.94). For CPA survivors, the adjusted odds ratios were more than 50% higher for cancer (OR=1.71; 95% CI=1.31, 2.24), migraine (OR=1.67; 95% CI=1.15, 2.45) and debilitating chronic pain (OR=1.58; 95% CI=1.22, 2.03), and 33% higher for arthritis (OR=1.33; 95% CI=1.05, 1.69). CPA was not significantly associated with either heart disease or diabetes (p>.05). The association between CPA and two mental health and four physical health outcomes remained significant, even after controlling for sociodemographic characteristics, health behaviors and other ACEs. Further research is needed to investigate potential pathways through which childhood physical abuse is linked to a wide range of chronic later-life health problems.

### LINKS BETWEEN EARLY-LIFE CONTEXTUAL FACTORS AND LATER-LIFE COGNITION AND THE ROLE OF EDUCATIONAL ATTAINMENT

Jordan Palms,[1] and Laura Zahodne,[2], *1. University of Michigan, Ann Arbor, Michigan, United States, 2. Clinical Science, Ann Arbor, Michigan, United States*

Educational attainment is a well-documented predictor of later-life cognition, but less is known about upstream contextual factors. This study aimed to identify which early-life contextual factors uniquely predict later-life global cognition and whether educational attainment mediates these relationships. Participants were drawn from the Michigan Cognitive Aging Project (N=461; Mage=63.51; SDage=3.13; 50% non-Hispanic Black). School-level contextual factors included U.S. region during elementary school (Midwest, South, Northeast), racial diversity of school (mostly White, mostly Black, diverse), self-reported education quality, and school type (public versus private). Household-level contextual factors included mother's and/or father's education, number of adults (1, 2, 3+), and number of children. Later-life global cognition was operationalized with a composite score derived from a comprehensive neuropsychological battery. A mediation model controlling for sociodemographics estimated total, direct, and indirect effects of contextual factors through educational attainment (years). Lower education quality, attending a mostly Black or diverse school, attending a public school, and reporting three or more adults in the household were each associated with lower cognition. After accounting for educational attainment, associations remained for education quality, school type, and reporting three or more adults in the household. Indirect effects through educational attainment were observed for elementary school region, education quality, racial diversity of school, and mother's education. School context appears to more consistently predict later-life cognition than household context, highlighting the potential long-term benefits of school-level interventions for cognitive aging. Future research should consider causal relationships among household-level and school-level contextual factors, as well as additional mediators beyond educational attainment.

### Session 3195 (Symposium)

### DISRUPTION TO TRANSFORMATION: AGING IN THE NEW NORMAL: NIA SESSION FOR EARLY-CAREER RESEARCHERS

Chair: Melinda Kelley Discussant: Melinda Kelley

The National Institute on Aging (NIA) at the National Institutes of Health, Department of Health and Human Services, supports biomedical and behavioral research with a lifespan focus. NIA research seeks to understand the basic processes of aging, improve prevention and treatment of diseases in later life, improve the health of older persons, in addition to a focus on Alzheimer's disease and related dementias. The NIA also supports the training and career development of scientists focusing on aging research and the development of research resources. This symposium, meant for junior faculty and emerging scholars, will provide an update on the latest research findings from the NIA followed by a segment on funding mechanisms and strategies. An opportunity will be provided to meet and consult with NIA extramural staff.

### OVERVIEW OF NIA RESEARCH AND PRIORITIES

Richard Hodes, *National Institute on Aging, Bethesda, Maryland, United States*

Dr. Hodes will provide an overview of NIA's structure and mission, in addition to discussing research foci from across the Institute's scientific divisions.

### HOW TO GET AN NIA GRANT

Kenneth Santora, *National Institute on Aging, Bethesda, Maryland, United States*

Dr. Santora will provide an overview of the NIA application process and will share information on relevant policy changes.

### Session 3200 (Symposium)

### EMERGING CONCEPTS IN DYADIC RESEARCH

Chair: Karen Lyons Discussant: Amy Rauer

This session includes four papers that explore and expand upon emerging concepts in dyadic research in health, illness, and end-of-life. First, Dr. Karen Lyons and colleagues examine the concept of dyadic mental health in mid-late life couples living with lung cancer. The paper examines the impact of having optimal versus poor dyadic mental health at diagnosis on the physical health of the couple over time, but also explores the ways we examine the concept of dyadic health in research and potential implications of these methods. Second, Dr. Lyndsey Miller and colleagues take a dyadic approach to understanding the roles of social activity and connectedness on depressive symptoms in a sample of community-dwelling older couples. The paper not only highlights important gender differences, but also the salient role of incongruent dyadic physical health. Third, Dr. Ranak Trivedi and colleagues describe the findings of a novel pilot intervention targeted at improving dyadic self-management in care dyads where one member is a Veteran with chronic conditions. The paper draws upon concepts of collaboration and dyadic coping to conceptualize self-management as a dyadic phenomenon. Finally, Dr. Buck and colleagues explore the novel concept of dyadic dissolution in a sample of family caregivers after the death of their care partner. The paper explores the concept as a cognitive and affective process with implications for how the surviving partner adapts over time. Speakers and Discussant, Dr. Amy Rauer, will focus on implications of these concepts for advancing dyadic science of health and illness across the lifespan.

### THE ROLE OF OPTIMAL DYADIC MENTAL HEALTH IN COUPLES LIVING WITH LUNG CANCER: INTERDEPENDENCE, CONGRUENCE, OR BALANCE?

Lyndsey Miller,[1] and Karen Lyons,[2], *1. School of Nursing, Oregon Health & Science University, Oregon, United States, 2. Boston College, Chestnut Hill, Massachusetts, United States*

Optimizing dyadic health is a central goal of dyadic frameworks. Yet, research has focused on interdependent individual health or the transactional nature of health within dyads. Emerging research has explored dyadic health through the lens of congruence and balance. This longitudinal study examined dyadic mental health in 76 couples ($M = 67.88 \pm 11.54$) during the first year of lung cancer. As expected, multilevel modeling found mental and physical health of couples were significantly associated at baseline ($p < .05$). Congruence in mental health was significantly associated with changes in physical health over time for survivors ($p < .05$) but not partners, whereas balanced mental health had differential effects on the physical health of survivors and partners ($p < .01$). Discussion will focus on the implications of congruent versus balanced dyadic health for the couple, evaluation of interventions, and propose ways to define optimal dyadic health.

### DISTINCT INFLUENCES OF SOCIAL ACTIVITY AND SOCIAL CONNECTEDNESS ON DEPRESSIVE SYMPTOMS IN OLDER ADULT COUPLES

Joel Steele,[1] Chao-Yi Wu,[2] Hiroko Dodge,[2] Jeffrey Kaye,[2] Karen Lyons,[3] and Lyndsey Miller,[2], *1. Portland State University, Portland, Oregon, United States, 2. Oregon Health & Science University, Portland, Oregon, United States, 3. Boston College, Chestnut Hill, Massachusetts, United States*

This study aimed to simultaneously examine the associations between social activity and connectedness and depressive symptoms in older adult couples. Using SEM and data from 116 community-dwelling couples (age $76.18 \pm 8.49$), we found that engagement in social activities was associated with lower depressive symptoms in men ($p = 0.014$), whereas more close friendships were associated with lower depressive symptoms in women (p = 0.018), controlling for partner effects, age, education, and cognitive function (CFI: 1.00, TLI: 1.35, RMSEA: 0.00 [0.00, 0.08]). Unexpectedly, we also found better female physical health to be associated with greater depressive symptoms in males (p = 0.029). When examined as dyadic physical health, more incongruence between the physical health of partners was associated with greater depressive symptoms in men (p = 0.007). Discussion will focus on distinct influences of social activity and connectedness on mental health, and the context of gender, marriage, and dyadic health.

### A WEB-BASED SELF-MANAGEMENT INTERVENTION FOR VETERANS WITH CHRONIC CONDITIONS AND THEIR CAREGIVERS: A PILOT STUDY

Katherine Plummer,[1] Madhuvanthi Suresh,[2] Rashmi Risbud,[3] Marika Humber,[4] Donna Zulman,[5] Christine Timko,[5] John Piette,[6] and Ranak Trivedi,[1]
*1. Stanford University, Menlo Park, California, United States, 2. Palo Alto University, Atlanta, Georgia, United States, 3. VA Palo Alto Health Care System, Palo Alto, California, United States, 4. VA Palo Alto Healthcare System, Menlo Park, California, United States, 5. VA Palo Alto Health Care System, Menlo Park, California, United States, 6. University of Michigan, Menlo Park, California, United States*

Web-based Self-management Using Collaborative Coping Enhancement in Diseases (Web-SUCCEED) is a dyadic intervention for patients and their caregivers designed to improve self-management through improving dyadic stress coping, dyadic relationships, and positive emotions. Veterans Affairs (VA) patients with one or more chronic conditions and positive screen for self-management distress were recruited with their informal caregiver from VA Palo Alto. Of the 17 patients and 16 caregivers recruited (62.3% of eligible), 8 patients and 8 caregivers (48.5%) completed the intervention and assessments. Twelve participants withdrew mostly citing the stress of the pandemic as their reason; 5 did not respond to multiple outreach efforts. Veterans were 66□18 y and caregivers were 58□16 y. Veterans and caregivers who completed the program rated it high on usability and acceptability. Pre-post t-tests across a psychosocial battery did not reveal significant differences; results were limited by incomplete post-intervention data. Further testing with modified retention strategies is recommended.

### CAREGIVERS' LOSS OF THE DYADIC EXPERIENCE AFTER THEIR CARE PARTNER'S DEATH

Harleah Buck, *University of Iowa, Iowa City, Iowa, United States*

One emerging dyadic concept is the experience of family caregivers when their care partner dies and their dyadic relationship comes to an end. This study qualitatively examined and characterized the loss of the dyadic experience for the caregiver after the death of their care partner. Data was accrued as part of a randomized clinical trial in 29 older hospice caregivers. Iterative thematic analysis focused on dyadic processes before, during and post death. Using two relational parameters from Relational Turbulence Theory resulted in a preliminary characterization of a new concept - dyadic dissolution as a cognitive and affective process whereby a remaining member of a dyad experiences relational uncertainty and partner interference while adapting (or not) to the death of their care partner. Findings suggest that asking several open-ended questions about the dyadic relationship will enable assessment for any continuing impact of relational uncertainty and partner interference on bereaved caregivers.

### Session 3205 (Symposium)

### ENGAGING ISOLATED AND UNDERSERVED OLDER ADULTS IN 4MS CARE: AGE-FRIENDLY CARE, PA

Chair: Diane Berish Discussant: Terry Fulmer

Older adults, the largest segment of the US rural population, face significant disparities in health and healthcare compared to their non-rural peers, including more chronic health conditions, financial challenges, and social isolation. They have limited access to healthcare and social services for prevention, management and treatment of chronic conditions. Age-Friendly Care-PA, a partnership between Primary Health Network and Penn State College of Nursing, aims to reduce these disparities in care and services for rural older adults through co-designing their Geriatric Workforce Enhancement Program. Age-Friendly Health Systems, an initiative of the John A Hartford Foundation and the Institute for Healthcare Improvement, in partnership with the American Hospital Association and the Catholic Health Association of the United States, equips providers, older adults, and their care partners with the support necessary to address What Matters, Medication, Mentation, and Mobility. This symposium describes how the 4Ms are integrated into clinician training and competencies, older adult education, operations, care delivery, and quality improvement. Year two outcome data will be shared. Drs. Hupcey and Fick will provide an overview of the project and its reach. Dr. Berish will describe the process of engaging stakeholders in co-developing our 4M metrics and the data generated. Jenny Knecht, CRNP, will describe a pilot study to extend the reach and acceptability of telehealth to hard-to-reach older persons. Finally, Dr. Garrow will detail a new initiative focused on equity in care. Our discussant, Dr. Terry Fulmer will lead a discussion of this work as well as next steps and policy implications.

### BUT HOW WILL WE MEASURE IT? CO-CREATING ASSESSMENTS OF OUTCOMES IN AGE-FRIENDLY CARE, PA

Diane Berish, *Pennsylvania State University, University Park, Pennsylvania, United States*

Moving from concept to quantitative measurement can be complex. There were several challenges in co-designing measures to assess the impact of Age-Friendly Care, PA, a geriatric workforce enhancement program. First as a FQHC, our clinical partner had not captured the metrics of interest. Second, the co-developed operational definitions for our metrics should be feasible, relevant, and useful for all project members. Third, funder reporting requirements must also be addressed. Working within this context, we co-created 11 outcome indicators structured around the 4Ms (IHI) now with 9 months of data. EMR changes to make data reportable included measuring opioid misuse mitigation, high-risk medication elimination, cognitive assessment and dementia care management, advanced care planning, care partner presence, annual wellness visit completion, pneumonia vaccination rates, colorectal screening rates, mobility goal tracking, and presence of a caregiver. Work continues around formulating themes to create a reportable mechanism for assessing What Matters.

### AGE-FRIENDLY CARE, PA: A GERIATRIC WORKFORCE ENHANCEMENT PROGRAM

Judith Hupcey,[1] Marie Boltz,[1] Lisa Kitko,[2] and Donna Fick,[1], *1. Pennsylvania State University, University Park, Pennsylvania, United States, 2. Pennsylvania State University, Pennsylvania State University, Pennsylvania, United States*

Age-Friendly Care, PA is co-led by Primary Health Network, the largest Federally Qualified Health Center in Pennsylvania, and Penn State College of Nursing that aims to bring reliable, high-quality, age-friendly care to all older adults living in rural PA. Sponsored by HRSA through its Geriatric Workforce Enhancement Program, Age-Friendly Care, PA utilizes the ECHO, all-teach-all-learn, platform to engage isolated rural providers in incorporating the 4Ms (IHI) into their practice. Age-Friendly Care, PA reaches out directly to rural older adults and their care partners to co-design education and support. We have hosted 28+ events and reached 450+ individuals. Results include tracking and improvement in quality indicators assessed including support for individuals living with dementia and their care partners (NA-66.7%), risk for opioid misuse (NA-78%), high-risk medication management (NA-47.8%), fall-risk management (NA-9.4%), and advanced care planning (NA-8.9%). We will discuss the creation, co-development, implementation, lessons learned, and future of Age-Friendly Care, PA.

### REACHING OUT TO OLDER ADULTS IN RURAL COMMUNITIES BY UTILIZING COMMUNITY HEALTH WORKERS DURING A PANDEMIC

Jenny Knecht, *Pennsylvania State University, University Park, Pennsylvania, United States*

Older adults in rural communities have access, isolation, and technology barriers to healthcare that are exacerbated by the COVID- 19 pandemic. A shortage of healthcare professionals combined with limited resources and poor broadband access have limited their ability to benefit from telehealth. The pandemic has further worsened isolation in rural communities. This Age-Friendly Care, PA pilot study uses community health workers (CHW) as a bridge to connect isolated and underserved older adults with their healthcare team. The CHWs facilitate access to telehealth provided by a Federally Qualified Health Center (FQHC), and also provide "check-ins' to housebound patients. The focus of the intervention is CHW delivered facilitation of telehealth and other supports to better manage their healthcare needs. We will describe the co-design of the project and discuss lessons learned in attempting to bridge the digital divide for rural older adults during and after the pandemic.

### ONE POSSIBLE CONSEQUENCE OF COVID-19 VACCINE: INEQUITABLE DISTRIBUTION

George Garrow, *Primary Health Network, Sharon, Pennsylvania, United States*

Primary Health Network (PHN) is the largest Federally Qualified Health Center (FQHC) in Pennsylvania expanding over 17 counties. Getting Pennsylvanians vaccinated is a critical step in reducing the spread and impact of COVID-19, although research suggests that the inequitable distribution of the COVID-19 vaccine may be a critical barrier. Although concerns regarding vaccine hesitancy are prevalent, experts also suggest that disparities in vaccination rates are in part due to the lack of accessible scheduling; adversely affecting underserved, such as rural communities, and minority populations. To address these obstacles, Primary Health Network is creating a COVID-19 Vaccination/Health Equity Team. Their objectives include: creating tools to provide comprehensive information on vaccine supply, identifying potential challenges and proactively planning for ways to mitigate likely disparities, identifying people who wish to be vaccinated but lack the means to do so, and connecting them in an equitable way, to vaccinations.

### Session 3210 (Symposium)

### ENVIRONMENTAL GERONTOLOGY DURING COVID-19: AGING IN PLACE SINCE THE PANDEMIC ONSET

Chair: Melissa Cannon Co-Chair: Jessica Finlay
Discussant: Graham Rowles

The COVID-19 pandemic is fundamentally changing neighborhood landscapes as we shelter in place and adjust our lifestyles. To age-in-place is to live in one's home and/or community "safely, independently, and comfortably." The ability to age-in-place is a public health priority for all, regardless of income or health status, and requires a variety of community resources to be sustainable. Since the pandemic onset, access to neighborhood resources was limited to reduce transmission risks. Changes to economic arrangements and socio-spatial norms have profoundly impacted daily life, though how these influence health and well-being is largely unknown. It is likely that these effects may vary in different communities and contexts; for example, neighborhoods that are able to self-organize to safely provide social support and resources may fare better. This symposium brings together cutting-edge studies in urban and rural U.S. places to explicate how the pandemic is transforming aging-in-place experiences and perspectives. The first presentation shows how rapidly community-based services have adjusted operations to meet the needs of their communities. The second presentation explores strategies to provide social support in rural communities. The third presentation highlights the social health needs of a subset of older adults who had not formed friendships with their neighbors. Together, these studies suggest that close examinations of aging-in-place conditions and mechanisms from organizational, socio-spatial, and social network perspectives are evermore important amid a pandemic. We discuss the implications of these empirical findings in relation to emerging theories within environmental gerontology.

### UNSUNG HEROES: HOW SENIOR CENTERS HAVE ADAPTED TO PROVIDE ESSENTIAL SERVICES IN THE FACE OF COVID-19

Jan Mutchler, Caitlin Coyle, and Ceara Somerville, *University of Massachusetts Boston, Boston, Massachusetts, United States*

This presentation will describe the ways in which senior centers in Massachusetts have adapted during the COVID-19

pandemic. Three surveys (distributed in April, August, and November, 2020) were conducted with 342 senior centers in the state to learn about current operations through the pandemic, challenges faced, and steps taken to solve those challenges. Results suggest that almost all senior centers (91%) continued to provide limited programming or essential services during the pandemic. Senior centers are prioritizing socialization and nutritional needs as critical services, but are changing the way they operate to continue to meet those needs. Despite facing uncertainty about the future, senior centers continue to adapt to changing conditions as they seek to meet their core mission. This presentation will discuss effects of COVID-19 on how senior centers will continue to operate through and post-pandemic times as well as local and state policy implications.

### COMMUNITY LEADERS' PERSPECTIVE OF STRATEGIES TO ENHANCE SOCIAL CONNECTEDNESS IN RURAL COMMUNITIES

Len Kaye,[1] Lori Parham,[2] and Patricia Oh,[3], *1. University of Maine, Orono, Maine, United States, 2. AARP Maine, Portland, Maine, United States, 3. UMaine Center on Aging, Bangor, Maine, United States*

Age-Friendly communities are charged with fostering a social environment where social connections are available, accessible, and meaningful. Thematic content analysis of 67 interviews (representing 73 communities) conducted between 12/09/2019 and 01/24/2020) and 59 interviews (representing 71 communities) conducted between 12/14/2020 and 1/19/2021 with age-friendly leaders in rural Maine suggested the importance of expanding multi-sectoral collaborations and developing flexible strategies that allow older people to create and maintain social connections, even during COVID. Prior to the pandemic, common strategies were: (1) local partners and volunteers; (2) in-person activities; (3) accessibility; (4) reciprocity; and, (5) neighborhood-specific solutions. During the pandemic, there was an increased reliance on regional partners and funders to develop low or no-tech and tech-enabled social opportunities. Additional adaptive strategies included: (1) intergenerational ties; (2) emphasizing fun; and, (3) flexibility. The study has implications for understanding how rural age-friendly communities develop and grow multi-sectoral collaborations to enhance social connections.

### SOCIAL CONTACT PRIOR TO COVID-19 AND LONGITUDINAL MENTAL HEALTH TRAJECTORIES DURING COVID-19 AMONG ADULTS AGES ≥55

John Best,[1] Jessica Finlay,[2] and Daniel R. Y. Gan,[1], *1. Simon Fraser University, Vancouver, British Columbia, Canada, 2. University of Michigan, Ann Arbor, Michigan, United States*

Social support protects mental health during a crisis. We examined whether prior contact with social organizations and friends/neighbors was associated with better trajectories of loneliness, depression and self-rated memory during the COVID-19 pandemic. We conducted latent class analysis and regression analysis on longitudinal data from the COVID-19 Coping Study of US adults aged ≥55 from April-October 2020 (n=3105). Overall, prior contact with friends(B=-.075,p<.001), neighbors(B=-.048,p=.007), and social organizations(B=-.073,p<.001) predicted better mental health amid COVID-19. Three classes were identified: Class1 had the best outcomes, whereas Class3 had the worst outcomes and were most likely to live alone(B=.149,p<.001). For Class1, prior contact with social organizations(B=-.052,p=.044) predicted decreasing loneliness. For Class2, prior contact with friends(B=-.075,p<.001) predicted decreasing loneliness and better memory(B=-.130,p=.011). Conversely, prior contact with neighbors(B=-.165,p=.010) predicted worsening loneliness among Class3. Our findings pose new questions on the role of neighborhood networks to mitigate poor mental health outcomes among older adults during a crisis.

### Session 3215 (Symposium)

### ESPO AND BEHAVIORAL AND SOCIAL SCIENCES SECTION SYMPOSIUM: ADDRESSING THE NEEDS OF BLACK, INDIGENOUS, AND PEOPLE OF COLOR (BIPOC) COMMUNITIES THROUGHOUT THE STAGE MODEL

Chair: Briana Sprague Co-Chair: Kylie Meyer
Discussant: Chivon Mingo

Behavioral interventions have been successfully deployed to prevent and manage chronic conditions among older persons, improve mental health, and support caregivers' ability to cope with care stressors. However, intervention effects may not be equally distributed among populations, nor equally acceptable or accessible among Black, Indigenous, and People of Color (BIPOC) communities. In this symposium, we will discuss how behavioral interventions can better meet the needs of BIPOC older adults and caregivers, who may not equally benefit from advancements in behavioral interventions due to issues such as a reliance on non-diverse study samples and lack of cultural tailoring. This symposium will be structured in accordance with the National Institutes of Health Stage Model of Behavioral, and will feature researchers whose work address BIPOC needs across the trajectory of intervention development. Representing Stage 1 research, Fayron Epps, PhD, RN, will describe her use of a community advisory council to develop a faith-based toolkit to support African Americans living with dementia and their caregivers. Next, Laura Gitlin, PhD, MA, will describe her experiences testing a Stage 3 intervention to lower depression among African Americans, including challenges advancing the culturally-tailored program to Stage 4. Lastly, Shanae Rhodes, BSN, RN will describe her Stage 2 evaluation of a conversation group created and attended by women of color to socially connect in response to COVID-19. Although speakers will describe research projects that represent specific research Stages, this symposium will have a large discussion-based component and will cover all parts of the Stage Model of Behavioral Intervention.

### USING ADVISORY BOARDS TO DEVELOP A FAITH-BASED TOOLBOX TO SUPPORT AFRICAN AMERICAN FAMILIES FACING DEMENTIA

Fayron Epps, *Emory University, Atlanta, Georgia, United States*

For this project, we are designing and testing the feasibility of employing components of a Faith-based Home Activity Toolbox (Faith-HAT). The goal of this NIH stage

1 intervention development project is to go beyond the four walls of the church to find ways to meet the spiritual needs of persons living with moderate and severe dementia "where they are" to help them remain religiously and spiritually engaged. This mixed-methods project is designed in 2 phases: (a) developing a prototype Faith-HAT and (b) testing the feasibility and exploring preliminary effectiveness. To successfully conduct this project, we have included a community advisory board of church leaders, caregivers, and persons living with dementia as members of the research design team to advise on the design and implementation of the Faith-HAT. Brainstorming workshops with the board are used to ensure the research is meeting the needs of the African American families affected by dementia.

### STUCK IN STAGE 3: THE CASE OF AN EFFECTIVE DEPRESSION INTERVENTION FOR AFRICAN AMERICAN OLDER ADULTS

Laura Gitlin, *Drexel University, College of Nursing and Health Professions, Drexel University, Pennsylvania, United States*

Beat the Blues (BTB) is a culturally tailored depression program for older African Americans. Tested in an NIA Stage 3 efficacy trial, findings showed statistically and clinically significant benefits, including decreased depressive symptoms, improved depression knowledge and symptom recognition, and behavioral activation. The multi-component intervention was co-constructed in partnership with a large senior center. Drawn from previously tested depression programs and tailored to preferences/needs of the targeted population, its five components included care management, depression education and symptom recognition, resources/referrals, and stress reduction and behavioral activation techniques. Despite significant findings, strong effect sizes and high acceptability, moving BTB to NIA Stage 4 (effectiveness) or 5 (dissemination) has been challenging. Challenges that will be discussed include lack of senior center funding to support training and delivery and infrastructure to embed BTB in community-based programs, and reluctance of health systems to adopt BTB because of its focus on one racial group.

### BONDING IN SISTERHOOD: A QUALITATIVE STUDY OF A VIRTUAL, HEALTH-RELATED PROGRAM FOR WOMEN OF COLOR AMID COVID-19

Shanae Rhodes, *UT Health San Antonio, San Antonio, Texas, United States*

My Sister's Keeper is an online education and support group created by women of color in response to disproportionate stresses related to COVID-19 experienced by women of color. The current study aims to examine the Stage 2 evidence that an online support group formed by members of the community may help mediate inequity-related stressors and increase receptiveness to health-related recommendations. To begin to develop this evidence, a thematic analysis of 8 in-depth individual interviews was performed. Resulting themes included: 1) feeling empowered; 2) solidarity in sisterhood (e.g., shared ownership of a virtual community); 3) being focused (on women of color) yet being inclusive; 4) currency of knowledge (e.g., responsibility to share knowledge with others); and 5) preferring virtual accessibility to stay connected. Preliminary data suggest that social support offered through an online platform dedicated to women of color can promote health during the pandemic and possibly beyond.

### Session 3220 (Symposium)

### ESPO AND SOCIAL RESEARCH, POLICY, AND PRACTICE SECTION SYMPOSIUM: IT'S A PRACTICE, NOT AN END STATE: CENTERING EQUITY IN GERONTOLOGICAL RESEARCH AND POLICY

Chair: Sarah Dys Co-Chair: Claire Pendergrast

Social, economic, and health inequities shape the experience of aging, reflecting a landscape of unequal resources, opportunities, and stressors that accumulate over the life-course. These inequities are not accidental, but rather reflect systems of power that act through institutions, policies, and people to simultaneously privilege some groups and disadvantage others based on socially constructed categories. These systems include, but are not limited to, racism, ageism, and capitalism. The unequal and unjust distribution of resources and opportunities over the lifespan results in health, social, and economic disparities in older adulthood. For example, Black older adults are at higher risk of experiencing chronic disease burden and shorter life expectancy than white older adults due to greater economic disinvestment, interpersonal and systemic racial discrimination, and lower health services access over the life course. This symposium features three leading scholars whose work centers racial and health equity in later life. The symposium will engage with issues related to long-term services and supports infrastructure, community-engaged and culturally relevant programs and education, and research activities (e.g., recruitment, study design, grant writing, dissemination). Panelists will also discuss their research agendas and recent scholarship, career trajectories, insights, and practices. We hope symposium attendees will identify opportunities and strategies for focusing on elimination of health disparities across the life-course in their own work. We believe this symposium can serve as an opportunity for SRPP members and emerging scholars and practitioners to center equity, highlight intersectionality, and amplify our colleagues at the forefront of addressing inequity through their work.

### ENGAGING RURAL AND RACIAL-ETHNIC POPULATIONS IN GERONTOLOGICAL RESEARCH

Ishan Williams, *University of Virginia, Charlottesville, Virginia, United States*

Older adults from racial/ethnic populations, as well as rural-dwelling older adults, are often at heightened risk for experiencing health disparities. Reasons for these disparities may include access issues, language barriers, distrust, lack of awareness, and of culturally appropriate materials. Racial/ethnic populations and rural-dwelling populations are also less likely to be included in research to help minimize the impact of these disparities. Shifting from reducing disparities to eliminating disparities will require attentiveness to designing programs and research that focus on increasing representation of racial/ethnic groups in research, integrating diverse populations (particularly rural and other marginalized groups) into the development of ideas and projects, and finally a commitment to culturally appropriate and inclusive approaches to research and education. Applying these strategies can provide guidance on how to best facilitate inclusive and equitable research, collaborative partnerships, and equitable healthcare for everyone, especially those from populations often underrepresented.

### FROM CARE MANAGER TO RESEARCHER: ADDRESSING HEALTH DISPARITIES IN LONG-TERM SERVICES AND SUPPORTS

Chanee Fabius, *Johns Hopkins Bloomberg School of Public Health, Baltimore, Maryland, United States*

Racial and socioeconomic disparities are prevalent in long-term services and supports (LTSS). There is a need for innovative research with practical application informing aging and disability policies to reduce health care disparities for older adults and people with disabilities using LTSS. This presentation will provide an overview of the career trajectory of Dr. Chanee Fabius, whose research agenda is informed by applied care management experience, where she helped older adults remain at home and delay the need for nursing home care. She will also present findings from work that (1) examines networks of care used by older adults (e.g., paid care and/or support from family and unpaid caregivers) and how they vary by race and socioeconomic status and (2) describes the effect of LTSS utilization on quality of life and health service utilization across diverse groups of older adults.

### ANTI-RACISM AND HEALTH EQUITY AS MISSING VALUES TO PRODUCTIVE AGING: IMPLICATIONS FOR EMERGING PROFESSIONALS

Ernest Gonzales, *New York University, New York, New York, United States*

Productive aging scholarship has grown in scope and rigor over the last four decades, yet anti-racism and health equity have not been formally integrated into the conceptual framework. Furthermore, there is a dearth of research that explicates heterogeneity among a growing diverse older adult population. This presentation will integrate anti-racism and health equity as core values to productive aging scholarship in order to explore risk and protective factors to employment, volunteering, and caregiving among a growing diverse older adult population. Part of this presentation will include major findings from longitudinal population-based studies as well as key findings from a Consensus Statement by the National Academies of Sciences, Engineering, and Medicine (NASEM) on work and retirement trajectories. Dr. Gonzales will also share professional strategies (e.g., grant submissions, publishing, teaching) with ESPO members who want to center anti-racism, health equity, and social justice in their scholarship.

### Session 3225 (Paper)

### FAMILY AND INTERGENERATIONAL RELATIONSHIPS I

### CHANGES IN GRANDPARENTING DURING THE PANDEMIC AND EFFECTS ON MENTAL HEALTH: EVIDENCE FROM ENGLAND

Giorgio Di Gessa,[1] Valeria Bordone,[2] and Bruno Arpino,[3] *1. University College London, London, England, United Kingdom, 2. University of Vienna, Vienna, Wien, Austria, 3. University of Florence, Florence, Toscana, Italy*

Policies aiming at reducing rates of hospitalisation and death from Covid-19 encouraged older people to reduce their physical contacts. For grandparents in England, this meant that provision of care for grandchildren was allowed only under very limited circumstances. To date, evidence on changes in grandparenting during the pandemic is scarce and little is known about whether and to what extent reduction in grandchild care provision impacted grandparents' mental health. Using pre-pandemic data from Wave 9 (2018/19) and the second Covid-19 sub-study (November/December 2020) of the English Longitudinal Study of Ageing, we first described changes in grandparenting since the start of the pandemic. Then, using regression models, we investigated associations between changes in grandparenting and mental health (depression, quality of life, life satisfaction, and anxiety) during the pandemic, while controlling for pre-pandemic levels of the outcome variables. Almost a third of grandparents reported that the amount of grandchild care during the pandemic reduced or stopped altogether, whereas 10% provided as much or more care compared to pre-pandemic levels, mostly to help parents while working. Compared to grandparents who provided grandchild care at some point during the pandemic, those who stopped altogether were more likely to report poorer mental health, even taking into account pre-pandemic health. A reduction in grandparenting was only marginally associated with higher depression. Although policies to limit physical contacts and shield older people reduced their risks of getting ill from Covid-19, our study shows the consequences of stopping childcare provision in terms of poorer mental health among grandparents.

### CHILDHOOD ABUSE AND CAREGIVING FOR PERPETRATING PARENTS: IMPACTS ON ADULT CHILD WELL-BEING

Jaime Goldberg,[1] Jooyoung Kong,[1] and Sara Moorman,[2] *1. University of Wisconsin-Madison, Madison, Wisconsin, United States, 2. Boston College, Chestnut Hill, Massachusetts, United States*

Combining the stress process model of caregiving and life course perspective, this study examined the long-term influences of childhood abuse on perpetrating parent-adult child relationships and adult child well-being in the context of caregiving. Using a sample of family caregivers from the Wisconsin Longitudinal Study (969 caregivers of mothers; 280 caregivers of fathers), we investigated whether contact frequency and emotional closeness with an abusive parent mediate the longitudinal effects of parental childhood abuse on adult child caregivers' depressive symptoms and the moderating effects of self-acceptance and mastery on this mediational association. Key findings indicate that maternal childhood abuse may negatively affect emotional closeness between an adult child caregiver and perpetrating mother ($b = -0.24$, $p < .001$). This could lead the adult child caregiver to experience increased depressive symptoms ($b = 0.02$, $p < .05$). Although the mediation paths for the effect of maternal childhood abuse on depressive symptoms via emotional closeness with mothers did not differ by caregivers' level of psychological resources, we found that psychological resources significantly moderated the association between maternal childhood abuse and depressive symptoms ($b = -0.08$, $p < .05$). Further research may explore this phenomenon in light of the heterogeneity of contemporary families. Practitioners working with adults with a history of parental childhood abuse who are caregiving for their perpetrator are encouraged to employ a trauma-informed approach to maximize the caregivers' health and well-being.

### EARLY INTERGENERATIONAL RELATIONSHIPS AND LATER SUPPORT PROVIDED TO OLDER PARENTS: TIME-TO-DEATH AS A CONTINGENCY

Merril Silverstein,[1] Wencheng Zhang,[2] Douglas Wolf,[3] and Maria Brown,[1], *1. Syracuse University, Syracuse, New York, New York, United States, 2. Syracuse University, Syracuse University, New York, United States, 3. Syracuse University, Syracuse, New York, United States*

This paper focuses on whether stronger relationships with parents early in the family lifecycle results in adult children providing more support to them 45 years later, and whether this association is contingent on parents' remaining years of life. We test time-to-death of parents as an indicator of vulnerability, an easy to ascertain and potentially powerful predictor of support. Data derived from the Longitudinal Study of Generations, a panel of three-generation families, originally fielded in 1971 and continuing to 2016. Focusing on the youngest generation (mean age = 19 in 1971), the analytic sample consists of 356 child-father relationships 473 child-mother relationships. We examined trajectories of instrumental support provided to parents over four waves between 1997 and 2016 as a function of each parent's remaining years of life (mortality data from the National Death Index). We also examined variation in those trajectories based on frequency of shared activities and intensity of emotional closeness in 1971. Ordinal multi-level growth curve analysis revealed that proximity to death was a significant predictor of instrumental support provided over time. Only in child-father relationships did greater emotional closeness, as expressed in 1971, produce stronger associations between remaining years of life and provision of instrumental support. Findings are discussed in terms of understanding intergenerational dynamics that unfold over many decades and the utility of time-to-death as an alternative metric for assessing vulnerability. This research is timely in light of growing uncertainty about the family as a reliable source of care in later life, particularly for older men.

### EFFECTS OF INTERGENERATIONAL RELATIONSHIP AND SUPPORT ON MHEALTH APP ADOPTION AMONG OLDER ADULTS

Yi-Hsuan Tung,[1] Shinyi Wu,[2] and Iris Chi,[3] *1. University of Southern California, PASADENA, California, United States, 2. University of Southern California, SANTA MONICA, California, United States, 3. University of Southern California, University of Southern California, California, United States*

As a growing body of literature examining the effects of mHealth for older adults' diabetes self-management, how relational factors affect seniors adopting mHealth is still unclear. Guided by the transactional approach of intergenerational relations and the technology acceptance model, this study aims at investigating the perceived ease-of-use, perceived usefulness, and intention-to-use of a mHealth app among older adults with Type-2 diabetes in relation to familial (parent-child) relationship and to e-learning support from child/ren or from external youth volunteering tutors. Using data from the Intergenerational Mobile Technology Opportunities Program (IMTOP), 304 Taiwanese participants (an average age of 64.6 years, 43% female, and 62.5% received at least a high school degree) who had at least a child were included for analysis using structural equation modeling. Results showed that perceived ease-of-use ($\beta = .58$, $p < .001$) and perceived usefulness ($\beta = .27$, $p < .001$) are significant predictors of intention-to-use. Positive associations are found only between external intergenerational, but not familial, e-learning support and perceived ease-of-use ($\beta = .45$, $p < .001$) and perceived usefulness ($\beta = .42$, $p < .001$). Parent-child relationship is positively associated with both familial ($\beta = .73$, $p < .001$) and external intergenerational support for e-learning ($\beta = .36$, $p < .001$), as well as directly ($\beta = .12$, $p = .030$) and indirectly related to intention-to-use. Our findings suggest the importance of intergenerational relationship and appreciation of both familial and external support to facilitate and sustain older adults' adoption for mHealth programs.

### INTERGENERATIONAL EXCHANGES IN AGING SOUTH ASIAN MUSLIM FAMILIES: AN INTERSECTIONAL LIFECOURSE PERSPECTIVE

Mushira Khan,[1] Karen Kobayashi,[2] and Andre Smith,[2]
*1. University of Victoria, Plainfield, Illinois, United States,*
*2. University of Victoria, Victoria, British Columbia, Canada*

International migration flows are increasing at a rapid pace and are often accompanied by emergent global realities, (re)negotiation of identities and familial bonds, anticipated challenges, and unforeseen exigencies. Concomitantly, advances in public health have resulted in longer lives with an increasing proportion of the global population now 65 years and older. While these demographic shifts have received considerable research attention, little is known about aging South Asian Muslim families in the US and the ways in which they adjust and adapt to shifting social realities. To address this gap, this qualitative study explores the intersections of faith, culture, gender, age, and immigrant status, and how these seminal life course events shape intergenerational care and support exchanges in South Asian Muslim families. Building on findings from 30 in-depth narrative interviews with three generations of immigrant South Asian Muslim women, and using an intersectional lifecourse perspective, this study explores the (re)negotiation of familial bonds and the enactment of religious beliefs and practices such as those around filial expectations in a transnational Islamic context. It shows how, for the grandmothers, daughters, and granddaughters in the study, their Islamic faith was a part of both the public sphere and a collective ideology, as well as a deeply personal and intimate attachment that provided structure and continuity in their everyday lives. Finally, the implications of these findings in the broader context of Islamophobia and salient structural barriers to accessing available health and social support services for aging South Asian Muslim families are discussed.

### Session 3230 (Paper)

### FAMILY CAREGIVER IMPACT OF THE COVID-19 PANDEMIC

### COVID-19'S IMPACT ON BURDEN AND NUTRITION FOR FAMILY CAREGIVERS OF PEOPLE WITH PARKINSON'S DISEASE

Dara LoBuono,[1] Rachel Robin,[2] and Mehmet Uygur,[2], *1. University of Rhode Island, North Haledon, New Jersey, United States, 2. Rowan University, Glassboro, New Jersey, United States*

The COVID-19 pandemic has worsened Parkinson's disease (PD) symptoms; however how COVID-19 has impacted family caregivers of people with PD (PwPD) is unknown. A 38-item open-and closed-ended online survey that explored caregiver burden and nutrition behaviors during COVID-19 was completed by 34 caregivers. Quantitative variables related to how COVID-19 has impacted caregiver burden are reported as percentages. Responses to open-ended questions related to COVID-19's impact on caregiver burden and dietary behaviors were double-coded by two researchers, differences in codes were discussed until consensus was reached, and themes were finalized. The mean age of caregivers was 67.2±8.7 (47-82 years of age) and the majority were female (64.7%). Since the COVID-19 pandemic, 61.7% of caregivers felt their relationship with their PwPD stayed the same or slightly improved, 41% reported having to make a slight or increased number of adjustments to their schedules to provide care and experienced a slight or increased physical strain because of providing care. 58.8% reported a slight or increased number of times they felt sad/hopeless and 76.5% reported a slight or increased number of times they felt anxious/worried. Themes related to COVID-19's impacts on caregiver burden included: fear, stress, and isolation; increased caregiver responsibilities; no change in caregiving. Themes highlighting COVID-19's impact on dietary behaviors included: healthier dietary patterns; increase in snack foods and boredom eating; no change in dietary patterns. Results suggest COVID-19 has negatively impacted caregiver well-being and further exploration in changes in dietary intake are warranted.

### DETERMINING THE IMPACT OF COVID-19 ON END-OF-LIFE EXPERIENCES OF FAMILY CAREGIVERS FOR PEOPLE LIVING WITH DEMENTIA

Sameer Mithani, Gwen McGhan, Deirdre McCaughey, and Kristin Flemons, *University of Calgary, Calgary, Alberta, Canada*

COVID-19 has impacted all of our lives, but the population most at risk are older adults. Family caregivers (FCGs) for people living with dementia (PLWD) face challenges in providing care, which are compounded with the introduction of COVID-19 public health policies. The purpose of this study was to examine the experiences of FCGs where the PLWD died during the COVID-19 pandemic. FCGs were invited to participate in an online survey to examine their caregiving experiences during the COVID-19 pandemic, with the option of participating in a follow-up focus group. Sixteen FCGs whose family members with dementia died during the pandemic participated in the survey. A follow-up focus group was conducted to further examine how COVID-19 policies impacted their role as a caregiver in long-term care (LTC) and affected their ability to grieve. The results of the survey and focus group suggest that a lack of role clarity and inadequate communication channels between the FCG and LTC due to COVID-19 increased the strain FCGs faced during end-of-life care. At the end of life, public policies, such as reduced or no visitation, led to feelings of inadequacy and regret. Several participants also expressed appreciation for completing Advanced Care Planning documentation prior to COVID-19. Based on these results, policymakers can help ease the increased turmoil faced by FCGs during end-of-life care in future public health emergencies by involving FCGs of PLWD in the decision-making process. The completion of Advanced Care Planning documentation can also ease the burden FCGs may experience during end-of-life care.

### RESOURCES AND SERVICES FOR FAMILY CAREGIVERS IN THE TIME OF COVID-19

Gwen McGhan, Deirdre McCaughey, Kristin Flemons, and Whitney Hindmarch, *University of Calgary, Calgary, Alberta, Canada*

COVID-19 has led to increased burden on family caregivers (FCGs) for people living with dementia (PLWD), while simultaneously limiting the resources available to them. Our study surveyed Alberta, Canada FCGs to assess their needs and generate recommendations to inform policies about care access, resources, and agency supports. We conducted a mixed methods study using a sequential triangulation design (QUANTITATIVE + qualitative). Our Community Advisory Committee was involved in all stages of study planning, execution, and dissemination. Survey results informed the qualitative data collected from focus groups with FCGs. A total of 230 FCGs participated in the survey, with an average age of 59. The average age of PLWD was 75. The majority were women (77%), 46% were spouses and 41% were adult children. Respondents reported feeling more isolated (69%), more strain (66%) and decreased quality of life (55%) compared to pre-pandemic. Resource use by FCGs decreased from an average of 5 resources pre-pandemic to 1.6 during COVID-19. Services including day programs and home care were no longer available or reconfigured, leading to greater strain and heightened need for respite, which was also unavailable. Focus groups highlighted that system navigation and accessing services during COVID-19 was overly burdensome, leaving FCGs feeling abandoned by the system. FCGs reported an increase in caregiving responsibility and less access to services resulting in PLWD experiencing a decline in wellness and function. As such: 1) resources should be consistently available for FCGs and 2) FCGs require clear, correct, and concise information about COVID-19.

### THE EXPERIENCE OF FAMILY MEMBERS IN LONG-TERM CARE FACILITIES DURING THE SARS COV2 (COVID-19) PANDEMIC

Raeann LeBlanc,[1] and Hyeyoung Park,[2], *1. University of Massachusetts, Amherst, Royalston, Massachusetts, United States, 2. University of Massachusetts Amherst, Amherst, Massachusetts, United States*

In-person visits are one form of care support offered by family members for residents in a Long-term Care Facility

(LTCF). Family member visitation may extend to close social relationships with the residential care staff, which can be important in managing care. The long-term care population has been significantly impacted by a high number of SARS-COV2 (Covid-19) cases in morbidity and mortality but, in-person visits were limited due the public health concern. This study aimed to describe the experience of family members of persons in LTCFs during the Covid-19 pandemic. We used an online survey of 34 questions. Forty-six family members were recruited through online caregiver support platforms, and 22 completed the survey. Average participant age was 57. Majority were female with high-moderate (M=3.48) Kessler psychological distress scores. Participants reported less frequent communication with their family members in LTCFs. Difficult to reach nursing staff, who were the primary contact, was a concern. Their preferred means of communication was the telephone followed by window visits; residents preference remained for in-person visits followed by telephone. Participants described a decrease in relationship closeness with staff and a decrease in confidence in the quality of care. These results, limited by sample size, offer a beginning insight into the importance of communication between the family member and nursing staff, including the contact frequency. Structural disparities such as LTCF nursing staff levels may partly explain these deficits in supporting families during the Covid-19 pandemic. Opportunities to support family members remain a needed focus of long-term care reforms.

### Session 3235 (Paper)

### FAMILY CAREGIVING II (BSS PAPER)

### CROSS-CULTURAL DIFFERENCES IN CAREGIVING: INVESTIGATING THE ROLE OF FAMILISM AND SOCIAL SUPPORT

Francesca Falzarano,[1] Jerad Moxley,[2] Karl Pillemer,[3] and Sara Czaja,[4], *1. Weill Cornell Medicine, Douglaston, New York, United States, 2. Weill Cornell Medicine, New York, New York, United States, 3. Cornell University, Ithaca, New York, United States, 4. Weill Cornell Medicine/Center on Aging and Behavioral Research, New York, New York, United States*

Cultural diversity in the United States (US) reflects a demographic shift, with a growing population of minority older adults and a subsequent increase in minority family caregivers providing care to aging adults. Research has demonstrated heterogeneity in the caregiving experience, with increasing focus placed on examining the impact of cultural values on caregiver (CG) outcomes. Familism has been investigated as a driving mechanism of cross-cultural differences in caregiving outcomes, yet prior work examining this relationship has yielded mixed findings. Using the sociocultural stress and coping model as a guiding framework, we examined, in a sample of 243 CGs who participated in the Caring for the Caregiver Network Study, a randomized controlled trial examining a culturally-tailored technology-based psychosocial intervention, the influence of familism and social support on positive aspects of caregiving, depressive symptoms, and caregiver burden. We also examined how these relationships vary as a function of race/ethnicity, the CG's relationship to the care-recipient, other sociodemographic characteristics (e.g., SES status), and acculturation. Results showed that African American and Hispanic CGs exhibited higher levels of familism compared to Whites. In African Americans, familism predicted higher positive caregiving appraisals, and social support significantly predicted lower burden and depression. In Hispanics, levels of familism varied as a function of acculturation, with lower levels of familism identified among US Hispanic natives. Our findings highlight that cultural beliefs, such as familism, as well as social support may be adaptive in protecting against adverse CG outcomes and point to directions for future culturally congruent, family-centered intervention approaches.

### DEHUMANIZATION OF OLDER FAMILY MEMBERS: NOVEL DETERMINANTS OF ELDER ABUSE PROCLIVITY BY CAREGIVERS

E-Shien Chang,[1] Joan Monin,[2] Daniel Zelterman,[2] Naomi Isenberg,[3] and Becca Levy,[4], *1. Weill Cornell Medicine, New York City, New York, United States, 2. Yale School of Public Health, New Haven, Connecticut, United States, 3. University of Wisconsin, Madison, Madison, Wisconsin, United States, 4. Yale University, Woodbridge, Connecticut, United States*

Elder abuse affects one in six older persons globally. Three limitations converge to impede progress in prevention: most research is victim- rather than perpetrator-based; the reliance on explicit, self-reported factors; and failure to account for psychological factors that motivate abuse in the first place. The current study will be the first to address these gaps by examining whether family caregivers' dehumanization of older persons, or the denial of humanness to older persons as one of the most hateful age stereotypes, could explain elder abuse proclivity. Implicit dehumanization of older persons was measured by a novel implicit-association-test developed for this study. Explicit dehumanization was measured by a semantic differential question widely used in the literature. We used the reliable and validated 8-item Caregiver Abuse Screen to measure elder abuse proclivity. In the final survey of 585 caregivers, dehumanization was found to be prevalent with 51% of the caregivers implicitly and 31% explicitly dehumanizing older persons. As predicted, implicit and explicit dehumanization uniquely contributed to elder abuse proclivity (OR=1.23, 95% CI=1.02-1.50, p=.03) and (OR=1.26, 95% CI=1.05-1.51, p=.01), respectively, after adjusting for relevant covariates including caregiver burden, and caregivers' and care-recipients' health. Also as predicted, implicit dehumanization improved the prediction of abuse proclivity above and beyond the explicit dehumanization of older persons and caregiver burden. Socio-etiological models of elder abuse perpetration and corresponding prevention design should consider the inclusion of dehumanization as a key risk factor for abuse proclivity in family caregivers.

### DURATION OF FAMILY CAREGIVING AND ITS EFFECTS ON INFLAMMATION IN THE CAREGIVING TRANSITIONS STUDY

David Roth, *Johns Hopkins University, Baltimore, Maryland, United States*

Sustained caregiving for older adult family members with disabilities can be a chronically stressful experience that may adversely affect the health of caregivers. Systemic inflammation is thought to be one mechanism by which caregiving stress might impact health, but previous studies of inflammation in caregivers have generally found inconsistent or very small effects with questionable clinical significance when comparing caregiving and non-caregiving control samples. The Caregiving Transitions Study (CTS) enrolled 283 family caregivers and 283 carefully-matched controls from an ongoing national epidemiologic study. This population-based sample of caregivers included an unusual subsample of 32 long-term caregivers who had been providing care to the same care recipients for over 9 consecutive years. Analyses of covariance indicated that these 32 long-term caregivers had statistically significant ($p < 0.05$) elevations on three circulating biomarkers of inflammation – C-reactive protein, Interleukin-6, and D-dimer – compared 1) to their 32 individually-matched non-caregiving controls, and 2) to the 248 caregivers who had been providing care for less than 9 years. Covariates in the analytic models included age, sex, race, and body mass index. Similar effects were observed for caregivers of persons with or without dementia. Polynomial regression models across all caregivers revealed significant curvilinear associations of inflammation with caregiving duration. Inflammation was not markedly elevated throughout the first several years of caregiving but then begin to increase more dramatically at around 10 years of caregiving. These findings suggest that long-term caregiving, in particular, may be associated with specific physical health risks through chronically elevated systemic inflammation.

### PREVALENCE AND CHARACTERISTICS OF SUBJECTIVE COGNITIVE DECLINE AMONG CAREGIVERS, 2015-2019

Eva Jeffers,[1] Roshni Patel,[2] Erin Bouldin,[3] Kenneth Knapp,[4] Dana Guglielmo,[1] Christopher Taylor,[1] and Lisa McGuire,[5], *1. Centers for Disease Control and Prevention (CDC), Atlanta, Georgia, United States, 2. Center for Disease Control and Prevention, Atlanta, Georgia, United States, 3. Appalachian State University, Boone, North Carolina, United States, 4. New York Medical College, Valhalla, New York, United States, 5. CDC, Atlanta, Georgia, United States*

Approximately 20% of U.S. adults provide unpaid care to family members and friends with a health condition or disability, and 20% of caregivers reported being in fair or poor health themselves. Much of the assistance caregivers provide have cognitive components, such as medication or financial management, yet little is known about caregivers' cognitive functioning. Subjective cognitive decline (SCD), or self-reported worsening of memory over the past year, among caregivers could impact the quality of care they provide. This study assessed prevalence of SCD by caregiving status and, among caregivers, the distribution of sociodemographic and other characteristics by SCD status. The study included 93,851 community-dwelling adults aged ≥45 years in 22 states who completed both the Behavioral Risk Factor Surveillance System Cognitive Decline and Caregiving modules during 2015-2019. All data were weighted; comparisons are based on modified Rao-Scott chi-square tests ($\alpha=0.05$). Among caregivers (n=21,238), 12.6% (95%CI:11.7-13.5) reported SCD, compared with 10.2% (95%CI:9.7-10.7) of non-caregivers ($p<0.0001$). Caregivers with SCD had more chronic health conditions, lower educational attainment, and were less likely to be married or employed than caregivers without SCD, despite a similar age distribution. Caregivers with SCD were also more likely than caregivers without SCD to report fair or poor health, frequent mental distress, a history of depression, and frequent activity limitations. SCD may negatively impact caregivers' health, function, and ability to provide care. With the anticipated increases in the need for caregiving, it is critical to understand the cognitive health of caregivers to better support caregivers and care recipients.

### PSYCHOMETRIC TESTING OF THE BRI UNMET NEED INSTRUMENT: A COMPREHENSIVE MEASURE OF DEMENTIA CAREGIVERS' NEEDS

Morgan Minyo,[1] David Bass,[2] Kate McCarthy,[3] and Katherine Judge,[1], *1. Cleveland State University, Cleveland, Ohio, United States, 2. Benjamin Rose Institute on Aging, Cleveland, Ohio, United States, 3. Benjamin Rose Institute on Aging, Benjamin Rose Institute on Aging, Ohio, United States*

Compared to non-dementia caregivers, family/friend caregivers of individuals with dementia experience more negative caregiving consequences. One reason is the myriad of negatively impacted life domains including: managing symptoms; family communication; financial and legal matters; and finding and coordinating services. Few psychometrically tested measures exist for assessing the range of potential unmet needs of dementia caregivers. Such a measure would describe the frequency and correlates of unmet needs and provide a key outcome for intervention research. This study tested the psychometric properties of a comprehensive measure of unmet needs, the BRI Unmet Need Instrument. Data from 192 family/friend dementia caregivers was used to test reliability and four validity types. Results showed total unmet needs, as well as its nine subscales, had good reliability (Cronbach's alpha .70 - .95). Discriminant validity was confirmed through factor analyses of the 45 unmet needs and items in measures of depression and care-related strain. Unmet need items loaded on separate factors that were deemed acceptable (.72-.38). Predictive validity was assessed by the association with depression, which was significant and an acceptable range ($r = .22$, $p < .01$). Convergent validity was confirmed by significant associations with three caregiver strain measures, mastery ($r = .40$, $p <.01$), emotional strain ($r = .19$, $p < .01$), and relationship strain ($r = .15$, $p <.05$). Good structural validity for nine predetermined unmet needs subscales was found using principal component analysis (loadings = .82-.39). Results suggest the BRI Unmet Needs Instrument is a ready-to-use, reliable and valid comprehensive measure.

### Session 3240 (Paper)

### FINANCIAL EXPLOITATION AND ADULT PROTECTION

### A SCOPING REVIEW OF FINANCIAL ELDER EXPLOITATION INTERVENTIONS

Stephanie Skees, *Washington University in St. Louis, Saint Louis, Missouri, United States*

Elder financial exploitation (EFE), defined by the National Center for Elder Abuse (2021) as “the misappropriation of an older person’s money or property,” is a continuing public health crisis shown to cost individuals at least $2.9 billion a year (MetLife Mature Market Institute, 2011). Many believe this impact will increase exponentially due to the effects of COVID-19. In fact, a recent study conducted by Chang & Levy (2021) found that the prevalence of elder abuse as a whole increased from 1 in 10 older adults to 1 in 5 in the past year. Although increased collaboration between state attorneys general, Adult Protective Services, and financial institutions has driven progress in the field; there is still little known regarding EFE interventions. To address this issue, this study conducts a scoping review of the EFE intervention literature. This approach was chosen over a systematic review primarily due to the lack of a universal definition of EFE, as well as the limited number of studies available delineating between EFE and elder abuse as a whole. The main findings of the review reveal that current EFE intervention practices are focused on preventing abuse before it occurs by addressing risk factors for abuse in older adults; and are largely reliant on Adult Protective Services and the legal system. This finding is significant because state policies differ in their qualifications of EFE, thus leaving many older adults vulnerable and unprotected. Further interventions that address EFE while it is occurring and alignment across governing bodies are needed.

### I MIND MY OWN BUSINESS AND EXPECT OTHERS TO DO THE SAME: CONVERSATIONS ABOUT DECLINING FINANCIAL CAPACITY

Marguerite DeLiema,[1] and Mingyang Zheng,[2] *1. University of Minnesota, Twin Cities, SAINT PAUL, Minnesota, United States, 2. University of Minnesota, Minneapolis, Minnesota, United States*

**Introduction:** One of the smartest ways to prepare for declines in financial decision making capacity is to appoint an agent under power of attorney for finances and to share important financial information and preferences with trusted family or friends. Yet only 12% of older Americans with children think that they’ll need help with their finances as they age, and more than half are uncomfortable talking about their finances with children.

**Method:** We conducted four in-depth interviews with older adults and four focus groups with Black, Latino, low income, and low-middle income adults aged 65 and older. An average of 9 participants were in each 2-hour focus group.

**Results:** Barriers included lack of awareness, denial of future changes in capacity, lack of trustworthy surrogate decision-makers, shame about one’s financial situation, desire for privacy, fear of being a burden, and resistance to overtures by children. Barriers differed by ethnicity and socioeconomic status, with lower income older adults having less knowledge of advance planning and Powers of Attorney.

**Implications:** Significant education is needed around Powers of Attorney and how to begin the advance planning process. Study findings informed the Thinking Ahead Roadmap, a guide to facilitate planning and communication around future money management. The Roadmap uses an empowerment framework to motivate individuals to appoint trusted financial advocates and prepare them for a smooth transition in money management, thereby reducing risk of exploitation, costly mistakes, and family conflict.

### IDENTITY THEFT AND OLDER ADULTS: HOW MINORITIES AND THE POOR SUFFER THE WORST CONSEQUENCES

Marguerite DeLiema,[1] David Burnes,[2] and Lynn Langton,[3] *1. University of Minnesota, Twin Cities, SAINT PAUL, Minnesota, United States, 2. University of Toronto, Toronto, Ontario, Canada, 3. RTI International, RTI International, District of Columbia, United States*

Society’s growing reliance on technology to transfer and store private information has created more opportunities for identity thieves to access personal data. Prior work using data from the National Crime Victimization Survey (NCVS) Identity Theft Supplement (ITS) showed that baby boomers were significantly more likely than Millennials to be victims of identity theft and that older people and minorities experience more severe economic and psychological consequences. This study examines how socioeconomic status, demographic characteristics, and incident-specific factors relate to how much money is stolen during identity theft, the likelihood of experiencing out-of-pocket costs, and emotional distress among identity theft victims age 65 and older. Using combined data from the 2014 and 2016 NCVS-ITS, this study examines the correlates of financial and psychological consequences of identity theft among 2,307 victims age 65 and older. Older Black victims are more likely to have greater amounts of money stolen and are more likely feel distressed than older non-Latino white identity theft victims. The most disadvantaged older adults living at or below the federal poverty level are nearly five times as likely to suffer out-of-pocket costs. The length of time information is misused and the hours spent resolving identity theft are significantly associated with emotional distress. More than one-third of older victims experience moderate to severe emotional distress following identity theft, and those who can least afford it suffer out-of-pocket costs. Greater advocacy and psychological support are needed to help older adults recover, in addition to tools to protect their personal information from misuse.

### OLDER VICTIMS OF MASS MARKETING SCAMS: AN ANALYSIS OF DATA SEIZED FROM SCAMMERS

Marguerite DeLiema,[1] and Lynn Langton,[2] *1. University of Minnesota, Twin Cities, SAINT PAUL, Minnesota, United States, 2. RTI International, RTI International, District of Columbia, United States*

Mass marketing scams are some of the most common frauds in America, and include scams perpetrated through the mail. A growing body of research indicates that older adults face a greater risk of victimization due to age-related changes in cognitive functioning and social isolation, and may be more likely to fall victim repeatedly. The aim of this study is to determine the frequency of repeat mass marketing fraud (revictimization) among older adults and patterns of victimization associated with age, scam type, seasonality, and geography. We use two decades of non-public administrative data from the United States Postal Inspection Service (USPIS). These databases were seized during law enforcement investigations of mass mailing scam organizations and contain more than 2 million unique U.S. victims and their transactions with four different fraud organizations. Victims were matched across datasets using name, address, and a change of address file. We find that revictimization rates increase with age in psychic scams. The 10,000 victims who responded the most times (between 82 and 562 times) were 78 years old on average and suffered $4,700 in total losses per person. Other significant trends emerged for lottery and sweepstakes scams. Unlike prior fraud victimization studies, inferences on victim characteristics are based on actual victim experiences with fraud rather than hypothetical scenarios or surveys where victims must self-report fraud. Findings provide valuable policy-relevant information regarding older victims and the patterns of chronic victimization.

### USING VOICE AND TOUCHSCREEN TECHNOLOGY TO PROTECT VULNERABLE CLIENTS IN RESIDENTIAL CARE FACILITIES

Jocelyn Brown,[1] Joan Davitt,[2] Pam Luby,[3] and Dorinda Adams,[3] *1. University of Maryland Baltimore, Martinsburg, West Virginia, United States, 2. University of Maryland, Baltimore, Maryland, United States, 3. Social Services Administration, Baltimore, Maryland, United States*

Due to the COVID-19 pandemic, the Centers for Medicare and Medicaid Services (CMS) issued a memorandum for long term care facilities to restrict visitation by all nonessential personnel, including guardianship case managers. To enhance caseworker access to their guardianship clients, an eastern seaboard state agency distributed Amazon Echo Show devices to clients in long term care facilities. These touchscreen and voice-activated devices have both video and audio capabilities. This study reports the results of the first phase of a comprehensive evaluation, pilot testing the devices via a group of "superuser" case managers to understand the potential challenges and benefits of using these devices. Sixteen case managers participated in two virtual focus groups before and after the installation of an Echo device with one of their guardianship clients. Participants were asked to discuss experiences in accessing clients and client engagement before and after device installation. The focus groups were audio-recorded and transcribed verbatim and two researchers independently identified themes using open and axial coding. Major themes identified included: challenges to device installation and use, strategies to overcome challenges, benefits to using Echo devices, and ethical concerns. These findings suggest that touchscreen or voice-activated devices with video capability can assist case managers in protecting vulnerable clients and ensuring their well-being when in-person access is restricted. Additionally, the devices can be used to enable isolated residents to connect to the outside world, including family, friends, and case managers through technology. Strategies for replication of this innovative program will be discussed.

### Session 3245 (Paper)

### GERIATRIC CARE MANAGEMENT

### ADDRESSING THE NEW NORMAL IN HEALTH CARE USING AN INTERPROFESSIONAL MODEL OF CARE COORDINATION

Sam Cotton,[1] Anna Faul,[2] and Pamela Yankeelov,[2] *1. University of Louisville Trager Institute, University of Louisville Trager Institute, Louisville KY, Kentucky, United States, 2. University of Louisville, University of Louisville, Kentucky, United States*

There has been significant discourse surrounding the widespread system failures within healthcare during COVID-19. Older, frailer, and poorer persons across the United States have been the most impacted by the pandemic. Given this, our FlourishCare team, received funding through the COVID Cares Act, as part of our Geriatric Workforce Enhancement Program (GWEP) grant, to create innovative programming for individuals that were the most impacted by the pandemic. Remote patient monitoring (RPM) is one intervention been shown as an effective way to assist persons in managing their conditions. Patients from our Optimal Aging Clinic were identified as struggling with hypertension, diabetes and/or COPD. Interprofessional teams of nursing and social work learners were assigned to work with patients. The kits contain all of the necessary technology and a virtual app platform that allows a patient to check their heart rate, blood pressure, oxygen levels, and glucose levels. This information was then disseminated to the team coordinating the patient's care. The sample was mostly female (88%), African American (64%) and retired (70%). The mean age was 60 (SD=4), and 40% had less than a high school diploma. After 3 months in the program, we saw a significant improvement across all determinants of health, with a particular overall change in access to health services and individual health behaviors. This study showed the importance of providing patients with access to technology and the support of an interprofessional team can improve patient outcomes, lead to improvements in individual health behaviors and improve health literacy.

### COMPARATIVE PERSPECTIVES ON GERIATRICS-SURGERY CO-MANAGEMENT PROGRAM BY SPECIALTY AND STAFF ROLE

Sonia Pandit,[1] Mark Simone,[1] Alyson Michener,[1] Lisa Walke,[2] and Ingrid Nembhard,[3], *1. University of Pennsylvania, Philadelphia, Pennsylvania, United States, 2. University of Pennsylvania, University of Pennsylvania, Pennsylvania, United States, 3. The Wharton School, University of Pennsylvania, Philadelphia, Pennsylvania, United States*

Co-management programs between geriatrics and surgical specialties have gained popularity in the last few years. Little is known about how these programs are perceived across surgical specialties and staff roles. We conducted a mixed methods study to assess perspectives on a geriatrics-surgery co-management program (GSCP) at a hospital where geriatricians co-manage patients 65 or older admitted to Orthopedic Trauma, General Trauma, and Neurosurgery. We used semi-structured interviews (n=13) and online surveys (n=45) to explore program value, facilitators, use, understanding, and impact by specialty and staff roles (physicians, advanced practice providers, nurses, case managers, social workers). Interview transcripts were analyzed using qualitative thematic analysis, and survey data were analyzed using Kruskal-Wallis, ANOVA, and Fisher's exact tests. Interviews revealed three themes: 1) GSCP is valued because of geriatricians' expertise in older adults, relationship with patients and families, and skill in addressing social determinants of health; 2) GSCP facilitators include consistent availability of geriatricians, clear communication, and collaboration via shared data-driven goals; and 3) GSCP use varies by surgical specialty and role depending on expertise and patient complexity. Survey data analysis affirmed interview themes and showed significant differences (p-values<0.05) between perspectives of surgical specialties and roles on GSCP use, understanding, impact, and which specialty should manage specific clinical issues. Findings suggest that while there are similarities across surgical specialties and roles regarding the value of, and facilitators for, a GSCP, specialties and roles differ in use, understanding, and perceived program impact on care. These findings suggest strategies for optimizing this intervention across groups.

### GERIATRIC COMANAGEMENT REDUCES HOSPITAL-ACQUIRED GERIATRIC SYNDROMES IN OLDER VASCULAR SURGERY INPATIENTS

Janani Thillainadesan,[1] Sarah Aitken,[2] Sue Monaro,[3] John Cullen,[3] Richard Kerdic,[3] Sarah Hilmer,[4] and Vasi Naganathan,[2] *1. Concord Hospital, NSW, New South Wales, Australia, 2. University of Sydney, Concord, New South Wales, Australia, 3. Concord Hospital, Concord, New South Wales, Australia, 4. The University of Sydney, St Leonards, New South Wales, Australia*

Aims Based on our meta-analysis, surveys and qualitative studies of geriatricians in Australia and New Zealand, we designed and implemented a novel inpatient model to co-manage older vascular surgical inpatients at a tertiary academic hospital in Sydney. This model, called Geriatrics co-management of older vascular surgery patients (Gerico-V), embedded a geriatrician into the vascular surgery unit who introduced a range of interventions targeting older people. Here we evaluated this model of care. Methods We undertook a prospective before-and-after study of consecutive patients aged ≥65 years admitted under vascular surgery. One hundred and fifty-two GeriCO-V patients were compared with 150 patients in the pre- GeriCO-V group. The primary outcomes were hospital-acquired geriatric syndromes, delirium, and length of stay. Results The GeriCO-V group had more frail (43% vs 30%), urgently admitted (47% vs 37%), and non-operative patients (34% vs 22%). These differences were attributed to COVID-19. GeriCO-V patients had fewer hospital-acquired geriatric syndromes (49% vs 65%; P =.005) and incident delirium (3% vs 10%; P = .02), in unadjusted and adjusted analyses. Cardiac (5% vs 20%; P <.001) and infective complications (3% vs 8%]; P = .04) were fewer in the GeriCO-V group. LOS was unchanged. Frail patients in the GeriCO-V group experienced significantly less geriatric syndromes and delirium. Conclusions The Gerico-V model of care led to reductions in hospital-acquired geriatric syndromes, delirium, and cardiac and infective complications. These benefits were seen in frail patients. The intervention requires close collaboration between surgeons and geriatricians, and may be translated to other surgical specialties.

### REFERRAL PROCESSES AND DISPOSITIONS IN A MULTIDISCIPLINARY TEAM FOR OLDER ADULTS WITH COGNITIVE VULNERABILITY

Richard Fortinsky,[1] Kristen Annis-Brayne,[2] Marie Smith,[3] Kathleen Obuchon,[4] Julie Robison,[3] George Kuchel,[5] and Shawn Ladda,[4], *1. University of Connecticut School of Medicine, Farmington, Connecticut, United States, 2. UConn Health, UConn Health, Connecticut, United States, 3. University of Connecticut, University of Connecticut, Connecticut, United States, 4. UConn Health, Farmington, Connecticut, United States, 5. University of Connecticut Health, Farmington, Connecticut, United States*

Multidisciplinary team care for community-dwelling older adults with multiple chronic conditions has proven value. Older adults receiving team care experience better outcomes than by solo practitioners alone, and teams are being established as outgrowths of primary care and other clinical settings. Yet little is known about the inner workings of multidisciplinary teams, both in terms of how referral patterns among team members are established and the extent to which older adults and their families accept referrals from team leaders to other clinical disciplines within teams. In this presentation, we provide details about referral patterns and rates of acceptance by study participants in an ongoing clinical trial testing a multidisciplinary team designed to provide care management to older adults (age >65) with cognitive vulnerability due to dementia, depression, and/or delirium (3D Team). Nurse practitioners lead the 3D Team, conduct in-home clinical assessments and make referrals to other team members based on study protocols specifying participants' eligibility for each 3D Team member. Results are based on the first 209 older adults randomized to the 3D Team. Pharmacist: all 209 members accepted having their medications reviewed and reconciled. Registered Dietician: of 134 referrals, 52 (38.8%) accepted. Occupational Therapist, of 117 referrals, 65 (55.6%) accepted. Physical Therapist: of 109 referrals, 92 (84.4%) accepted. Community Health Educator: of 106 referrals, 101 (95%) accepted. LCSW for depression-related problem solving therapy: of 76 referrals, 55 (72.4%) accepted. Criteria for referrals and interpretations of variations in referral acceptance rates by older adults and their families will be discussed.

### STAFF PERSPECTIVES ON LESSONS LEARNED FROM AN INTERDISCIPLINARY MEMORY CARE COORDINATION PROGRAM

Inga Antonsdottir,[1] Quincy Samus,[2] Melissa Reuland,[3] Deirdre Johnston,[3] Morgan Spliedt,[4] and Danetta Sloan,[5] *1. Johns Hopkins School of Nursing, Baltimore, Maryland, United States, 2. Johns Hopkins University School of Medicine, Baltimore, Maryland, United States, 3. Johns Hopkins University, Baltimore, Maryland, United States, 4. The Johns Hopkins University, Baltimore, Maryland, United States, 5. Johns Hopkins Bloomberg School of Public Health, Baltimore, Maryland, United States*

MIND at Home is a home-based care coordination program for persons living with dementia (PLWD) and their informal care partners (CP). Assessments, care planning and coordination is delivered by trained non-clinical Memory Care Coordinators (MCCs), working together on an interdisciplinary team with nurses and geriatric psychiatrists. We report qualitative results from program staff (two nurses and eight MCCs) who implemented the program in the context of two clinical trials. Care team respondents answered open-ended questions covering 5 domains pertaining to: helpful skillsets; positive and challenging factors aspects of care coordination; barriers to care coordination for clients; and improvements suggestions/resources to strengthen the program.

Compassion, finding common ground, listening, organization, and time management were reported as critical skills. Staff enjoyed team collaboration, being in and learning about the community, increasing CP confidence and mastery when caring for a PLWD. Reported challenges included documentation in EHR, accessing/navigating resources, driving long distances, unsafe neighborhoods, ambiguous assessment tools, and working with low engagement clients. Common barriers faced by clients (as reported by staff) were financial struggles/poverty, and lack of insurance coverage for needed services. Staff suggested several improvements: better communication strategies, integration with LTSS services and medical providers, 24-hour program hotline, continuous education for staff, simplified data collection and care delivery tracking process. This presentation on the experience of MIND at Home trained nurses and MCCs provides deep insight on how this and similar care coordination programs might be successfully implemented or strengthened.

### Session 3250 (Paper)

### GERIATRIC EDUCATION FOR HEALTH PROFESSIONALS

### INTERGENERATIONAL LEARNING: AN OPPORTUNITY TO TRANSFORM NURSING STUDENTS' PERSPECTIVES OF OLDER ADULTS

Marleen Thornton,[1] Kathryn Burns,[2] and Lara Street,[2], *1. Notre Dame of Maryland University, Baltimore, Maryland, United States, 2. Notre Dame of Maryland, Baltimore, Maryland, United States*

The purpose of this pilot project was to explore the experience of an intergenerational learning environment focused on healthy aging for nursing students and older adults. Intergenerational learning experiences provide opportunities for individuals from different age groups to communicate and participate in learning activities together. The growing population of older adults calls for increased geriatric nursing expertise. Nursing students' attitudes toward older adults are often negative though, and result in decreased interest in geriatric nursing. The opportunity to transform nursing students' perspectives on older adults has the potential to improve nursing care for older adults, and the number of nurses focused on geriatric nursing care. This qualitative inquiry used a convenience sample of 10 participants from a cross-listed university course on healthy aging for baccalaureate nursing students and older adult members of a lifelong learning institute. Semi- structured focus group interviews were conducted. Narrative transcripts were analyzed using an inductive approach. Analysis illustrated improved nursing students' perspectives of older adults and aging. A similar theme was noted for older adults' perspectives of younger adults. The importance of social interaction within an intergenerational learning environment and the need for opportunities to challenge ageist perspectives was illustrated. Increased exposure to healthy older adults, personally and professionally, may increase nursing students' interest in geriatric nursing and improve nursing care for older adults. Future research should examine more specifically how intergenerational learning experiences can decrease ageism, improve nursing students' and nurses' perspectives on older adults, and improve nursing practice for older adults.

### PERSON-CENTERED CARE FOR FAMILY CAREGIVERS: CO-DESIGNING AN EDUCATION PROGRAM FOR THE HEALTHCARE WORKFORCE

Jasneet Parmar,[1] Sharon Anderson,[2] Cheryl Pollard,[3] Lesley Charles,[4] Bonnie Dobbs,[4] Myles Leslie,[5] Cecelia Marion,[6] and Gwen McGhan,[5], *1. Department of Family Medicine, University of Alberta, Edmonton, Alberta, Canada, 2. Faculty of Medicine & Dentistry University of Alberta, Edmonton, Alberta, Canada, 3. McEwan University, Edmonton, Alberta, Canada, 4. University of Alberta, Edmonton, Alberta, Canada, 5. University of Calgary, Calgary, Alberta, Canada, 6. Covenant Health, St. Albert, AB, Alberta, Canada*

**Background:** Research recommends the healthcare workforce receive competency-based education to support family-caregivers [FCGs}. typically, education has been directed at FCG's to increase their care skills rather that at healthcare providers to provide person-centered care to FCGs.

**Objectives:** We present the co-design process used to create a competency-based education program for the healthcare workforce that ensures a person-centered focus on FCGs and introduce our Health Workforce Caregiver-Centered Care Education.

**Approach:** Co-design is the act of creating with stakeholders to ensure useable results that meet stakeholder's needs. We began by coining the concept "caregiver-centered care," defined as a collaborative working relationship between families and healthcare providers aimed at supporting FCGs in their caregiving role, decisions about care management, and advocacy. From this definition we co-designed, then validated the Caregiver-Centered Care Competency Framework in a Delphi Process. Stakeholders (n= 101) including FCGs, providers, policy makers, community organizations, researchers, and educational designers then used effective practices for health workforce education to co-design the 'foundational' level of a Caregiver Centered Care education.

**Results:** Teaching and learning resources include six competency-aligned educational modules with videos and interactive exercises that encourage reflection. With the COVID-19 pandemic, we moved the education online (caregivercare.ca). In the first four months online, 815healthcare providers completed the education. We continue to use mixed methods to evaluate the Caregiver-Centered Care Education, for acceptability and effectiveness, in five care contexts (primary, acute, home, supportive living, long-term care).

**Conclusion:** We expect that our education will support caregiver-centered care in all healthcare settings.

### TEACHING END-OF-LIFE DECISION MAKING TO UNDERGRADUATE NURSING STUDENTS

Sherry Greenberg,[1] Nancy Innella,[2] and Bryan Pilkington,[3] *1. Seton Hall University College of Nursing, Seton Hall University College of Nursing, New Jersey, United States, 2. Seton Hall University College of Nursing, Nutley, New Jersey, United States, 3. Seton Hall University, Nutley, New Jersey, United States*

This presentation highlights the development, implementation, and results of an educational session with undergraduate nursing students about end-of-life decision making. The purpose of this qualitative thematic analysis study was to explore student perspectives of end-of-life decision making following an education session. The aims were to 1) develop themes from student feedback on end-of-life decision making and 2) refine educational strategies to teach end-of-life decision making to nursing students. The study was conducted with 72 junior level baccalaureate nursing students enrolled in an undergraduate gerontological nursing course. An interactive lecture was developed, following short philosophical ethics readings, which brought the students up to date on the history of end-of-life discussions, key cases, and different frameworks to approach a cluster of ethical issues associated with end-of-life care. A debate pedagogical model was employed as an engaging activity in which students directly applied recently learned concepts. In the debate activity, students were divided into two teams. Each team was assigned a position, which was a specific response to the case question: Should practitioners assist in their patient's committing suicide? Should practitioners offer medical aid in dying? Each team conferred, presented their position, responded to the arguments or reasons from the opposing position. The session ended with a debrief by the course instructors. In the first semester, 31 nursing students completed four open-ended questions following the class. Results included increased student confidence discussing end-of-life issues and identification of two concepts commonly referred to in end-of-life care discussions and in bioethics, autonomy and dignity.

### VIRTUAL GERIATRIC EDUCATION PREFERENCES OF RURAL SOCIAL WORKERS

Joan Ilardo,[1] Raza Haque,[2] and Angela Zell,[1] *1. Michigan State University College of Human Medicine, East Lansing, Michigan, United States, 2. Michigan State University, East Lansing, Michigan, United States*

Older adults in rural communities need access to comprehensive healthcare services provided by practitioners equipped with geriatric knowledge and skills. The Geriatric Rural Extension of Expertise through Telegeriatric Service (GREETS) project goal is to use telemedicine and telehealth to expand geriatric service options to underserved Michigan regions. GREETS educational programs train health practitioners to provide geriatric care for vulnerable older adults. To determine gaps in geriatric competencies, the team conducted an online survey of health professionals including behavioral health practitioners. Respondents identified educational topics and preferred virtual delivery methods. Demographic information included respondent's professional position, practice setting, and county. The respondents were asked to indicate level of educational need using a scale ranging from a low, medium, or high need. Fifty (47%) of 106 total responses were from social workers. We compared the percent of social workers to other practitioners' responses in our analysis. Four topics emerged for both groups as medium or high educational needs: 1) transitional care when changing residential settings or post-hospitalization; 2) assisting family caregivers cope with caregiving responsibilities; 3) incorporating community-based services into care plans; and 4) and managing frail older adults. Social workers noted higher need than the other respondents for: 1) managing chronic pain; 2) managing care of patients with multiple chronic conditions; 3) having serious illness conversations; 4) diagnosing dementia; and 5) discussing advance care planning. Both social worker and other respondents indicated interactive case-based webinars; published tools, toolkits, tip sheets; and didactic webinars as their top three learning formats.

### Session 3255 (Symposium)

### IMPROVING QUALITY AND ACCESS TO CARE IN LONG-TERM CARE SETTINGS

Chair: Nancy Dudley Discussant: Nancy Dudley

Innovative delivery models that assure access to quality care in long-term care settings are needed for the diverse high-risk aging U.S. population. The 2008 National Academy of Medicine report, "Retooling for an Aging America: Building the Health Care Workforce" highlighted the need for changing the roles of health care providers in order to provide high-quality and cost-effective care to older Americans. Moreover, from the providers' perspective, workplace violence in health care institutions, such as nursing homes, negatively affect the delivery, quality, and accessibility of health care. In this symposium, we identify needs and care provisions in the context of older adults aging in long-term care settings and discuss the implications for policy and health care transformation. This symposium comprises three distinct presentations: (1) identifying and addressing the needs of diverse older adults aging in low-income independent living facilities in community health practice; and based on pilot survey results from nurses in the State of New Hampshire, (2) reframing residents' violence directed toward providers as self-protection and (3) proposing legislative and policy changes designed to meet the needs of staff and residents of long-term care facilities. These presentations represent efforts across long-term care settings to improve access and quality of care in the context of diverse older adults aging in the U.S.

### ADDRESSING NEEDS OF OLDER ADULTS IN LOW-INCOME INDEPENDENT LIVING FACILITIES IN COMMUNITY HEALTH

Lisa Rauch,[1] Toby Adelman,[1] Daryl Canham,[1] and Nancy Dudley,[2], *1. San Jose State University, San Jose, California, United States, 2. San Jose State University, Los Altos, California, United States*

Access to quality care in long-term care settings including independent living facilities is needed for a diverse high-risk aging U.S. population. There is an urgent need to assess and address complex care needs of older adults living longer with chronic conditions and serious illness. However, a system to assess and identify health problems, intervene, and evaluate outcomes is lacking. This session presents learnings from a pilot study developed in collaboration with Nurse Managed Centers at low-income independent living facilities for older adults and undergraduate nursing students in community health practice. We will discuss the adaptation of the Omaha System for provision of care in independent living facilities to address complex care needs. Finally, we will discuss the impact of this project and its potential for healthcare transformation in independent living facilities and transformation of education in undergraduate nursing programs.

### REFRAMING OLDER ADULTS' VIOLENCE TOWARD STAFF AS SELF-PROTECTION

Rosemary Taylor,[1] Lisa Mistler,[2] Pamela DiNapoli,[3] Karla Armenti,[3] and Raelene Shippee-Rice,[4] *1. University of Massachusetts Medical School, Worcester, Massachusetts, United States, 2. Geisel School of Medicine; Dartmouth-Hitchcock Health; New Hampshire Hospital, Concord, New Hampshire, United States, 3. University of New Hampshire, Durham, New Hampshire, United States, 4. University of New Hampshire, Nottingham, New Hampshire, United States*

One of the first studies on workplace violence in nursing homes was published in 1985. Forty-five (45) years later, resident violence against staff continues to increase in incidence and severity. At the request of a state senator, a New Hampshire psychiatrist formed a research group to conduct the first New Hampshire survey on staff experience of workplace violence. Study questions focused on experiences of workplace violence and incident reporting, and the availability and benefit of workplace violence training programs. Results were consistent with recently published literature: violence is an expected, normalized element when providing care; potential repercussions and perceived resident lack of intent were major reasons for incident non-reporting. Analysis of study results and review of the literature led to the question: Are older residents' violent behaviors towards staff an act of self-protection?

### IMPROVING POLICY AND LEGISLATION RELATED TO HEALTHCARE WORKPLACE VIOLENCE

Rosemary Taylor,[1] Raelene Shippee-Rice,[2] Pamela DiNapoli,[3] Karla Armenti,[3] and Lisa Mistler,[4] *1. University of Massachusetts Medical School, Worcester, Massachusetts, United States, 2. University of New Hampshire, Nottingham, New Hampshire, United States, 3. University of New Hampshire, Durham, New Hampshire, United States, 4. Geisel School of Medicine; Dartmouth-Hitchcock Health; New Hampshire Hospital, Concord, New Hampshire, United States*

Our group conducted the first known New Hampshire survey on healthcare staff experience of workplace violence. Study questions focused on experiences of workplace violence, incident reporting mechanisms, and the availability and benefit of workplace violence training programs. Results were consistent with recently published literature: violence against healthcare workers remains a serious public health problem that is under-reported, understudied, "tolerated and largely ignored." We will discuss the aspects of policy and legislative responses to workplace violence that have been insufficient to address this problem. We also will present some strategies that, if implemented, could lead to improved policy and legislation that may be more proactive in reducing such violence.

### Session 3260 (Symposium)

### INEQUITIES IN PALLIATIVE AND DEMENTIA CARE AND HEALTH IN LONG-TERM SUPPORTIVE SERVICES SETTINGS

Chair: Abraham Brody

Most older adults with serious illness, including Alzheimer's Disease and Related Dementias (ADRD) reside in community-based settings. These individuals and their care partners rely on Long Term Supportive Services (LTSS) including nursing home, home health, hospice, and adult day centers to provide support. LTSS are often under-resourced and reimbursed however, with significant regulatory restrictions on the care they can provide. These issues combined with other systemic factors in our healthcare system and society, including racism and poverty, lead to substantial inequities. Even preceding the use of LTSS, ADRD is diagnosed later in non-white individuals and access to high-quality services, including palliative care is severely limited. Moreover, few palliative care interventions address ADRD and even fewer have been specifically tailored to address the needs of our multi-cultural, racially and ethnically diverse society. This symposium will therefore utilize data from several nationwide data sets collected as part of routine care for clinical, billing, and/or regulatory purposes to assess inequities that exist across LTSS sites related to ADRD and palliative care. The individual abstracts show a clear pattern of inequities that stem from endemic systems failures towards people of color in the United States that must be addressed through a multipronged approach. This research shows that policies must be changed to require adequate collection of social determinants of health, to target policies that allow substandard or limited access to care, and research and clinical reform to produce a more culturally sensitive approach to care for those with ADRD and other serious illnesses.

### ASSOCIATIONS BETWEEN DEMENTIA, RACE-ETHNICITY, AND INTENSIVE AND PATIENT-CENTERED END-OF-LIFE CARE

Amanda Reich,[1] Robert Semco,[1] Holly Prigerson,[2] Joel Weissman,[1] Adoma Manful,[1] and Elizabeth Luth,[2], *1. Harvard Medical School/Brigham and Women's Hospital, Boston, Massachusetts, United States, 2. Weill Cornell Medicine, New York, New York, United States*

A retrospective cohort analysis of Medicare administrative claims data from 2016-2018 compared intensive and patient-centered end-of-life care measures in persons with and without dementia, including the moderating effects of race/ethnicity. Over half (53%) of 485,209 Medicare decedents had a dementia diagnosis. Decedents with dementia were 31-34% less likely to receive intensive end-of-life care (hospital death 95%CI: 0.64-0.67; hospitalization in last 30 days 95%CI: 0.68-0.70) and 50% more likely to receive timely hospice care (95%CI: 1.48-1.52). The association between dementia and end-of-life care varied by decedent race/ethnicity. Compared to non-Hispanic white decedents without dementia, non-Hispanic Black, Hispanic and Asian decedents with dementia were significantly more likely to receive intensive end-of-life care. Non-Hispanic Black decedents with dementia were 23% more likely to receive timely hospice care (95%CI: 1.11-1.36). Additional research is needed to understand why persons with dementia receive less intensive end-of-life care and why differences exist based on racial/ethnic status.

### NATIONWIDE INEQUITIES IN NURSING HOME PALLIATIVE CARE SERVICES

Jordan Harrison,[1] Andrew Dick,[2] Patricia Stone,[3] and Leah Estrada,[3], *1. RAND Corporation, Pittsburgh,*

*Pennsylvania, United States, 2. Rand Corporation, Boston, Massachusetts, United States, 3. Columbia University School of Nursing, New York, New York, United States*

Inequities exist in nursing home (NH) quality of care for racial/ethnic minorities, but the extent of palliative care (PC) disparities in unknown. We used cross-sectional national survey data (2017-18) from 869 NHs to measure PC services (summative score: 0-100). Survey linked to Minimum Data Set and Area Health Resources Files. Descriptive statistics and NH-level, multivariable regressions examined regional differences in NH PC services by varying concentrations of Black and Latino residents. Substantial regional differences were recorded in mean PC score and by concentration. Mean PC services were highest in the Northeast and lowest in the South: Northeast ($\bar{x}$=50.45, SE=1.50); West ($\bar{x}$=49.96, SE=1.74); Midwest ($\bar{x}$=48.18, SE=1.17); South ($\bar{x}$=44.71, SE=1.30). After adjusting for urbanicity and county level poverty, NHs in the Northeast and West with increasing concentrations of Black and Hispanic residents offered significantly fewer PC services. Overall, NHs serving predominantly serving minority populations offer fewer PC services.

### INEQUITY IN HEALTH AMONG PEOPLE LIVING WITH ALZHEIMER'S DISEASE AND RELATED DEMENTIAS IN ADULT DAY CENTERS

Gary Yu,[1] Bei Wu,[2] Abraham Brody,[3] Tina Sadarangani,[4] and Jonelle Boafo,[5], *1. NYU Rory Meyers College of nursing, New York, New York, United States, 2. New York University, New York, New York, United States, 3. NYU Hartford Institute for Geriatric Nursing, New York, New York, United States, 4. New York University Rory Meyers College of Nursing, New York University Rory Meyers College of Nursing, New York, United States, 5. Rory Meyers College of Nursing, New York, New York, United States*

In adult day centers (ADCs), 58% of clients identify as racial/ethnic minorities, and at least 30% have Alzheimer's Disease and related dementias (ADRD). ADCs offer culturally and linguistically congruent care to clients, making them well-positioned to address potential health disparities affecting persons with ADRD. We used data from 53 California ADCs (n=3,053) to identify differences in clinical characteristics among ADC clients' with ADRD based on demographics such as race and English proficiency. We found that, when compared to their respective counterparts, a significantly greater proportion of racial/ethnic minorities and non-English speakers (p<.001) had 5 or more chronic conditions in addition to ADRD. We noted considerable missing data on race, likely because ADCs in California are not mandated to report data on race/ethnicity. In order to identify inequities in care within this complex population, social determinants of health, including race, must be a standard component of client assessment.

### INEQUITIES IN ACCESS TO HIGH-QUALITY HOME HEALTH AGENCIES AMONG RACIAL AND ETHNIC MINORITIES WITH AND WITHOUT DEMENTIA

Bei Wu,[1] Abraham Brody,[2] and Chenjuan Ma,[3] *1. New York University, New York, New York, United States, 2. NYU Hartford Institute for Geriatric Nursing, New York, New York, United States, 3. NYU, NYU Rory Meyers College of Nursing, New York, United States*

There are rising concerns of inequities in access to high-quality home health agencies (HHA). Using multiple national data sources that included 574,682 individuals from 8,634 HHA, we examined access to high-quality HHA care among racial and ethnic minorities with and without dementia. Approximately 9.9% of the individuals were Black, 6.2% Hispanic, and 3.3% other race/ethnicity. Over one-third (36.3%) had been diagnosed with dementia. Black and Hispanic individuals were 5.5 percentage points (95% CI, 5.2% - 5.9%) and 7.4 percentage points (95% CI, 7.0% - 7.8%) respectively more likely to receive care from agencies defined as having low-quality compared to White counterparts. Persons living with dementia were 1.3% less likely to receive care from high-quality agencies. Having dementia increased the inequity in accessing high-quality HHA between Black and White individuals. Racial and ethnic minorities, particularly those with dementia were at a disadvantaged position to receive care from high-quality HHA.

### HOSPICE CARE INEQUITIES IN INDIVIDUALS WITH ALZHEIMER'S DISEASE AND RELATED DEMENTIAS

Leah Estrada,[1] Aditi Durga,[2] Shih-Yin Lin,[3] Ariel Ford,[2] and Abraham Brody,[4], *1. Columbia University School of Nursing, New York, New York, United States, 2. NYU Rory Meyers College of Nursing, New York, New York, United States, 3. New York University, New York, New York, United States, 4. NYU Hartford Institute for Geriatric Nursing, New York, New York, United States*

Despite known benefits of hospice, inequities exist. Using data from a multi-site pragmatic trial in a representative groups of hospices, we examined inequities in length of stay (LOS) and general inpatient use (GIU) for 12,153 patients with dementia (primary and secondary diagnosis) using descriptive statistics and association tests. There were significant associations between race/ethnicity and GIU and LOS (p< 0.001). In those with primary diagnosis of dementia, Asian (31%) and Black/AA (24%) individuals had significantly greater utilization of GIU than Hispanic (19%) and white individuals (21%). Greater inequities were found in those with a secondary diagnosis. LOS amongst Asians were shortest with 78% having an LOS ≦14 vs 50-59% in other groups. Differences in long-stay >60 days (7%) vs 14-22% in other groups were found. There were similar differences examining by primary vs. secondary diagnosis. These inequities point to cultural and systems factors that require further study and intervention.

### Session 3265 (Symposium)

### INTEGRATING STUDY DESIGNS ON EMOTIONAL REACTIVITY AND REGULATION IN OLD AGE: NEW EVIDENCE FROM THE EMIL STUDY

Chair: Oliver Schilling Discussant: Gloria Luong

Key insights into emotional reactivity and regulation have been gained by studying how these dynamics evolve as older people are confronted with controlled stressors in the lab, go about their everyday routines, or develop across adulthood and old age. Yet, we are only beginning to understand how the dynamics on the different time scales observed in these study designs interact . Aiming for a comprehensive picture of the predictors, correlates, and consequences of emotional reactivity and regulation, the EMIL study integrates a lab-based study with ambulatory in-vivo assessments and a classic long-term longitudinal study. 130 young-old (65-69 years) and 59 very-old adults (83-89 years) from the ILSE study, contributing four waves of health, cognitive, and psycho-social data over almost 25 years, were tested in the lab and assessed six times a day over seven consecutive days. We provide an overview of and first across-design results from EMIL: Katzorreck et al. examined whether the frequency of exposure to daily stressors affects emotion regulation capacity as tested in the lab. Lücke et al. analyzed daily working memory performance, sleep, and its association with long-term change in cognitive functioning. Wieck et al. present differential effects of discrete negative emotions as induced in the lab and reported in daily life on social cognitive performance as indicated by empathic accuracy. Gerstorf et al. examined how long-term cognitive aging affects positive feelings and stressor reactivity in daily life. Gloria Luong will discuss the presentations, considering challenges and opportunities of integrating lab-based, ambulatory, and longitudinal study designs.

### LINKING EMOTION REGULATION CAPACITY AND THE FREQUENCY OF DAILY STRESSORS IN OLD AND VERY OLD AGE

Denis Gerstorf,[1] Anna Lücke,[2] Hans-Werner Wahl,[2] Oliver Schilling,[3] Ute Kunzmann,[4] and Martin Katzorreck,[5], *1. Humboldt University of Berlin, Humboldt University of Berlin, Brandenburg, Germany, 2. Heidelberg University, Heidelberg, Baden-Wurttemberg, Germany, 3. University of Heidelberg, Heidelberg, Baden-Wurttemberg, Germany, 4. Leipzig University, Leipzig, Sachsen, Germany, 5. Humboldt University of Berlin, Berlin, Berlin, Germany*

Lifespan theories and lab-based research both suggest that the ability to downregulate negative emotions is often well preserved into old age, but becomes increasingly fragile in very old age. However, little is known about factors that may alleviate such age differences. Here, we ask whether exposure to daily stressors helps very old adults to maintain effective emotion regulation skills. We used data from 130 young-old (65-69 years, 48% women) and 59 very-old adults (83-89 years, 58% women) who watched negative emotion evoking film clips in the lab under emotion regulation instructions and also reported stress situations they experienced in everyday life (42 occasions across seven days). Initial results indicate that very-old adults were indeed less successful in regulating sadness than young-old adults, but those very-old adults who reported many daily stressful situations were as capable of emotion regulation as young-old adults. We discuss possible factors contributing to our age-differential findings.

### SLEEP AND WORKING MEMORY: SHORT-TERM LINKS IN DAILY LIFE AND LONG-TERM ASSOCIATIONS

Cornelia Wrzus,[1] Denis Gerstorf,[2] Ute Kunzmann,[3] Martin Katzorreck,[4] Oliver Schilling,[5] and Anna Lücke,[1] *1. Heidelberg University, Heidelberg, Baden-Wurttemberg, Germany, 2. Humboldt University of Berlin, Humboldt University of Berlin, Brandenburg, Germany, 3. Leipzig University, Leipzig, Sachsen, Germany, 4. Humboldt University of Berlin, Berlin, Berlin, Germany, 5. University of Heidelberg, Heidelberg, Baden-Wurttemberg, Germany*

Sufficient sleep is relevant for both momentary cognitive functioning and long-term cognitive developments. However, which factors make people particularly vulnerable to the cognitive consequences of sleep loss remains an open question. Here, we obtained data from 122 young-old (66-69 years) and 35 very old (85-89 years) adults who provided six daily ambulatory assessments of working memory performance and daily sleep over one week, and long-term trajectories in processing speed and working memory performance. Our results add to current knowledge in three ways: First, results from multilevel structural equation models showed both too little and too much daily sleep was associated with poorer working memory in everyday life. Secondly, this association was independent of cognitive aging over the preceding four years. Thirdly, average sleep duration did not predict cognitive changes over the next year. Participants' age and health as well as emotional functioning are discussed as further influences on the associations.

### UNDERSTANDING THE LINK BETWEEN DISCRETE NEGATIVE EMOTIONS AND EMPATHIC ACCURACY

Denis Gerstorf,[1] Oliver Schilling,[2] Martin Katzorreck,[3] Anna Lücke,[4] Ute Kunzmann,[5] and Cornelia Wieck,[5] *1. Humboldt University of Berlin, Humboldt University of Berlin, Brandenburg, Germany, 2. University of Heidelberg, Heidelberg, Baden-Wurttemberg, Germany, 3. Humboldt University of Berlin, Berlin, Berlin, Germany, 4. Heidelberg University, Heidelberg, Baden-Wurttemberg, Germany, 5. Leipzig University, Leipzig, Sachsen, Germany*

Extant theories have suggested that negative emotions generally harm cognitive processes. However, adopting a discrete emotion perspective, in this study, we predicted that only anger and fear but not sadness should be negatively associated with empathic accuracy, a process that has been shown to be cognitively highly demanding. Over 100 participants (Mage = 66.66 years, SDage = 1.00) reported their emotional reactions in response to a negative film in the laboratory, documented their everyday momentary emotions six times a day over seven consecutive days, and completed a film-based empathic accuracy test. Initial findings suggest that only fear but not anger or sadness was related to empathic accuracy. More specifically, high levels of fear both in the laboratory and in everyday life predicted low empathic accuracy. This pattern of findings will be discussed in the context of discrete emotions theories.

### LONG-TERM COGNITIVE AGING TRAJECTORIES AS PREDICTORS OF DAILY AFFECT

Oliver Schilling,[1] Ute Kunzmann,[2] Martin Katzorreck,[3] Anna Lücke,[4] Hans-Werner Wahl,[4] Nilam Ram,[5] and Denis Gerstorf,[6], *1. University of Heidelberg, Heidelberg, Baden-Wurttemberg, Germany, 2. Leipzig University, Leipzig, Sachsen, Germany, 3. Humboldt University of Berlin, Berlin, Berlin, Germany, 4. Heidelberg University, Heidelberg, Baden-Wurttemberg, Germany, 5. Stanford University, Stanford, California, United States, 6. Humboldt University of Berlin, Humboldt University of Berlin, Brandenburg, Germany*

Multiple-time scale studies provide new opportunities to examine how developmental processes evolving on different cadences are intertwined. Theories about age-related accumulation of stress suggest that long-term progressive loss of cognitive resources should manifest in and shape short-term daily affective experiences. Applying growth modeling and intraindividual variability methods to data obtained from 123 young-old (65-69 years, 51% women) and 47 very-old adults (85-88 years, 49% women) who provided 20+ year longitudinal data on Digit Symbol performance and 42-occasion momentary data about the emotions and stressors they experienced during everyday life (6 reports per day), we found that shallower long-term loss of cognitive performance was associated with less fluctuation in momentary positive affect, as well as less "spikiness" of and reactivity to stress. We discuss and present further results highlighting how mid-term processes surrounding age, gender roles, and health additionally contribute to and shape links between long-term and short-term dynamics of aging.

### Session 3270 (Symposium)

### LATE BREAKING SESSION: EMERGING COVID-19 RESEARCH: NEW UNDERSTANDING OF THE EFFECTS OF COVID-19 ON HEALTH AND LONGEVITY

### GENETIC AND PHENOTYPIC EVIDENCE FOR THE CAUSAL RELATIONSHIP BETWEEN AGING AND COVID-19

Ranran Zhai,[1] Timothy Pyrkov,[2] Anastasia Shindyapina,[3] Marco Mariotti,[4] Peter Fedichev,[2] Xia Shen,[1] Vadim Gladyshev,[4] and Kejun Ying,[5], *1. Sun Yat-Sen University, Guangzhou, Guangdong, China (People's Republic), 2. Gero, Singapore, Not Applicable, Singapore, 3. Brigham and Women's Hospital, Harvard Medical School, Brigham and Women's Hospital, Harvard Medical School, Massachusetts, United States, 4. Brigham and Women's Hospital, Boston, Massachusetts, United States, 5. Harvard University, Boston, Massachusetts, United States*

Epidemiological studies revealed that the elderly and those with comorbidities are most susceptible to COVID-19. To understand how genetics affects the risk of COVID-19, we conducted a multi-instrument Mendelian Randomization (MR) analysis and found that the genetic variation that supports a longer life is significantly associated with the lower risk of COVID-19 infection, as well as being hospitalized after infected. The odds ratio is 0.31 (P = 9.7e-6) and 0.46 (P = 3.3e-4), respectively, per additional 10 years of life. We further applied aging clock models and detected an association between biological age acceleration and future incidence and severity of COVID-19 infection for all subjects and individuals free of chronic disease. Biological age acceleration was also significantly associated with the risk of death in COVID-19 patients. A bivariate genomic scan for age-related COVID-19 infection identified a key contribution of the Notch signaling pathway and immune system. Finally, we performed MR using 389 immune cell traits as exposure and observed a significant negative correlation between their effect on lifespan and COVID-19 risk, especially for B cell-related traits. More specifically, we discovered the lower CD19 level on B cells indicates an increased risk of COVID-19 and potentially decreases the lifespan expectancy, which is further validated in clinical data from COVID-19 patients. Our analysis suggests that the factors that accelerate aging and limit lifespan cause an increased COVID-19 risk. Thus, the interventions target these factors (e.g., reduce biological age), after further validation, may have the opportunity to reduce the risk of COVID-19.

### METFORMIN SUPPRESSES MONOCYTE INFLAMMATION AND METABOLIC REPROGRAMMING BY SARS-COV-2 SPIKE PROTEIN

Theodore Cory,[1] Russell Emmons,[2] Johnathan Yarbro,[1] and Brandt Pence,[2], *1. University of Tennessee Health Science Center, Memphis, Tennessee, United States, 2. University of Memphis, Memphis, Tennessee, United States*

COVID-19 disproportionately affects older adults, and a hallmark of the disease is a hyperinflammatory state that is associated with severity. Various anti-inflammatory therapeutics have shown mixed efficacy in treating COVID-19, and the mechanisms by which hyperinflammation occurs are not well understood. Previous research indicated that monocytes, a key innate immune cell, undergo metabolic reprogramming and produce inflammatory cytokines when stimulated with SARS-CoV-2. We hypothesized that binding by the viral spike protein mediates this effect, and that drugs which regulate immunometabolism – including the geroprotector drug metformin – could inhibit the inflammatory response in monocytes. Monocytes stimulated with recombinant SARS-CoV-2 spike protein subunit 1 showed a dose-dependent increase in glycolytic metabolism that was associated with production of pro-inflammatory cytokines including interleukin-6 and tumor necrosis factor-alpha. This response was dependent on hypoxia-inducible factor-1alpha, as chetomin inhibited glycolysis and cytokine production. Inhibition of glycolytic metabolism by 2-deoxyglucose (2-DG) or glucose deprivation also inhibited the glycolytic response, and 2-DG strongly suppressed cytokine production. Glucose-deprived monocytes rescued cytokine production by upregulating oxidative phosphorylation, an effect which was not present in 2-DG-treated monocytes due to the known effect of 2-DG on suppressing mitochondrial metabolism. Finally, pretreatment of monocytes with metformin strongly suppressed spike protein-mediated cytokine production in monocytes, and abrogated glycolytic and mitochondrial metabolism. In summary, the SARS-CoV-2 spike protein induces a pro-inflammatory immunometabolic response in monocytes that can be suppressed by treatments that interfere with glycolytic activation, including metformin. This has potential implications for the treatment of hyperinflammation during COVID-19, which disproportionately affects older adults.

### BIOLOGICAL AGING PREDICTS VULNERABILITY TO COVID-19 SEVERITY IN UK BIOBANK PARTICIPANTS

Chia-Ling Kuo,[1] Luke Pilling,[2] Janice Atkins,[3] Jane Masoli,[2] Joao Delgado,[4] George Kuchel,[1] David Melzer,[2] and Morgan Levine,[5], *1. University of Connecticut Health, Farmington, Connecticut, United States, 2. University of Exeter, Exeter, England, United Kingdom, 3. University of Exeter Medical School, University of Exeter, England, United Kingdom,*

*4. University of Exeter Medical School, Exeter, England, United Kingdom, 5. Yale University, New Haven, Connecticut, United States*

Age and disease prevalence are the two biggest risk factors for COVID-19 symptom severity and death. We therefore hypothesized that increased biological age, beyond chronological age, may be driving disease-related trends in COVID-19 severity. Using the UK Biobank England data, we tested whether a biological age estimate (PhenoAge) measured more than a decade prior to the COVID-19 pandemic was predictive of two COVID-19 severity outcomes (inpatient test positivity and COVID-19 related mortality with inpatient test-confirmed COVID-19). Logistic regression models were used with adjustment for age at the pandemic, sex, ethnicity, baseline assessment centers, and pre-existing diseases/conditions. 613 participants tested positive at inpatient settings between March 16 and April 27, 2020, 154 of whom succumbed to COVID-19. PhenoAge was associated with increased risks of inpatient test positivity and COVID-19 related mortality (ORMortality=1.63 per 5 years, 95% CI: 1.43-1.86, p=4.7x10E-13) adjusting for demographics including age at the pandemic. Further adjustment for pre-existing disease s/conditions at baseline (OR_M=1.50, 95% CI: 1.30-1.73 per 5 years, p=3.1x10E-8) and at the early pandemic (OR_M=1.21, 95% CI: 1.04-1.40 per 5 years, p=0.011) decreased the association. PhenoAge measured in 2006-2010 was associated with COVID-19 severity outcomes more than 10 years later. These associations were partly accounted for by prevalent chronic diseases proximate to COVID-19 infection. Overall, our results suggest that aging biomarkers, like PhenoAge may capture long-term vulnerability to diseases like COVID-19, even before the accumulation of age-related comorbid conditions.

### Session 3275 (Paper)

### LONG-TERM CARE III (SRPP PAPER)

### ANTIPSYCHOTIC PRESCRIBING IN VA-CONTRACTED COMMUNITY NURSING HOMES AND INCIDENT USE AMONG VETERANS

Patience Moyo,[1] Emily Corneau,[2] Portia Cornell,[2] Amy Mochel,[2] Kate Magid,[3] Cari Levy,[3] and Vincent Mor,[4], *1. Brown University School of Public Health, Providence, Rhode Island, United States, 2. Providence VA Medical Center, Providence, Rhode Island, United States, 3. Rocky Mountain VA Medical Center, Aurora, Colorado, United States, 4. Brown University, Providence, Rhode Island, United States*

The Veterans Health Administration (VA) is increasingly purchasing long-term care for eligible Veterans from non-VA, community nursing homes (CNHs). Antipsychotics present safety risks for older adults, but it is unknown how the prevalent use of antipsychotics at CNHs influences whether newly admitted Veterans will initiate antipsychotic therapy. This study used 2013-2016 VA data, Medicare claims, Nursing Home Compare, and Minimum Data Set (MDS) assessments. We identified 10,531 long-stay CNH episodes for Veterans not prescribed antipsychotics 6 months before CNH admission. We categorized Veterans by whether, 12 months before admission, they were diagnosed with FDA-approved indications (including schizophrenia, Tourette's syndrome, Huntington's disease) for antipsychotic use. The exposure was the proportion of all CNH residents prescribed antipsychotics in the quarter preceding a Veteran's admission. Using adjusted logistic regression, we analyzed two outcomes measured using MDS assessments collected ~100 days after CNH admission: 1) new antipsychotic use, and 2) new diagnosis for an FDA-approved indication among Veterans without these conditions at admission. Among antipsychotic-naïve Veterans admitted to CNHs, 7,924 (75.2%) lacked an antipsychotic indication. Prevalent antipsychotic use in CNHs ranged 0%-10.9% (quintile 1) and 25.7%-91.4% (quintile 5). The odds of initiating antipsychotic use increased with higher CNH antipsychotic use rates (OR=2.52, 95% CI:2.05-3.10, quintile 5 vs. 1), as did the odds of acquiring a new indication (OR=2.08, 95% CI:1.27-3.40, quintile 5 vs. 1). Provider practices may be influencing new diagnoses indicating antipsychotic use at CNHs with high antipsychotic use. It may be important for VA to consider antipsychotic use when contracting with CNHs.

### CASE MANAGEMENT IN PRIMARY CARE ASSOCIATED WITH SERVICE USE BY ADULTS WITH DEMENTIA AND COMORBIDITIES

Emma Quach,[1] Lauren Moo,[2] Christine Hartmann,[3] Shibei Zhao,[4] and Pengsheng Ni,[5], *1. Center for Healthcare Organization and Implementation Research, VA Bedford Healthcare System, Massachusetts, United States, 2. VA Bedford Health Care System, Bedford, Massachusetts, United States, 3. VA Bedford Healthcare System, VA Bedford Healthcare System, Massachusetts, United States, 4. VA Bedford Healthcare System, Bedford, Massachusetts, United States, 5. Boston University School of Public Health, Boston University School of Public Health, Massachusetts, United States*

Community-dwelling adults with dementia are at higher risks than counterpart without dementia of poor health outcomes, and those with dementia and co-occurring conditions face even greater risks. Optimal treatment for dementia includes functional and psychosocial support through long-term services and supports (LTSS), but use remains low. Our study investigated whether case management provided in primary care and in dementia care settings facilitated LTSS use for Veterans with dementia and comorbidities. We performed a cross-sectional analysis of 2019 VA-paid health care on a cohort of Veterans with dementia, defined by clinical diagnoses (International Classification of Disease, Tenth Revision). Receipt of case management was measured by whether or not a Veteran enrolled in a VA (1) home-based primary care, (2) geriatric primary care, or (3) dementia clinic. Comorbidities were measured by an adapted Elixhauser comorbidities index and dichotomized as ≤ 3 or ≥ 4 comorbidities. LTSS use was measured by whether or not Veterans used home health, home respite, adult day care, hospice, or veteran-directed care. Multivariate logistic regressions showed that LTSS use was higher for enrollees in each case management program compared to Veterans not enrolled in any. LTSS use was also higher for enrollees in each primary care program with more comorbidities than program counterparts with fewer comorbidities. Case management in primary care settings may facilitate functional and psychosocial support to meet dementia and non-dementia related needs for adults who have dementia with comorbidities.

### IMPACT OF INFORMAL CARE ON HEALTH CARE UTILIZATION AMONG OLDER PEOPLE IN CHINA

Yixiao Wang, *King's College London, London, England, United Kingdom*

Population aging has become a challenge to long-term care and health care for the society. Using China as a case study, this paper assesses allocative efficiency of resources in informal care and health care, to explore the effectiveness of the policy, i.e., encouraging informal care as a more cost-effective way to reduce public health care spending. Drawing data from the 2011, 2014, and 2018 waves of the Chinese Longitudinal Healthy Longevity Survey, this study examines the impact of informal care on utilization of health care as well as amount of health care expenditures among older people with functional limitations in China. Using random effects model with instrumental variable approach, our findings suggest that informal care significantly reduces the utilization of health care, primarily by reducing the utilization of outpatient care. However, informal care significantly increases the amount of inpatient care expenditures for inpatient care users. We do not observe significant association between informal care and amount of outpatient care expenditures for outpatient care users. This study highlights a pressing need for the Chinese government to support informal caregivers by taking economic values of informal caregiving into consideration, and to improve efficiency in inpatient care by a more integrated resource allocation mechanism

### NURSING FACILITY AND HEALTH CARE SERVICE USE AND COSTS UNDER THE CMS FINANCIAL ALIGNMENT INITIATIVE

Lauren Palmer,[1] Matt Toth,[2] Joyce Wang,[3] Emily Schneider,[2] Allison Dorneo,[2] Giuseppina Chiri,[3] and Edith Walsh,[1], *1. RTI International, RTI International, Massachusetts, United States, 2. RTI International, Research Triangle Park, North Carolina, United States, 3. RTI International, Waltham, Massachusetts, United States*

The Centers for Medicare & Medicaid Services created the Financial Alignment Initiative (FAI) to test the impact of integrated care and financing models for dually eligible Medicare-Medicaid beneficiaries. Using Medicare claims, the Minimum Data Set 3.0, and state-provided enrollment files, we evaluated demonstration impacts on long-stay nursing facility (NF) use, other health care service utilization, and costs for the overall eligible population in two FAI demonstration States with managed fee-for-service models, Colorado and Washington. We used quasi-experimental, difference-in-differences regression models for the impact analyses. In Colorado, there was a 7.2 percent decrease ($p<0.001$) in long-stay NF use, relative to the comparison group. Otherwise, the demonstration showed unfavorable service utilization results—increases in preventable emergency department (ED) visits and declines in 30-day follow-up after mental health discharge (MHFU)—and no impact on Medicare costs. In Washington, there was also a decrease in long-stay NF use (12.4 percent, $p<0.001$) and skilled NF admissions (21.7 percent, $p<0.001$). However, the demonstration resulted in decreases in physician visits and 30-day MHFU. There was a favorable decrease in Medicare costs. The impact of the FAI demonstrations on NF use was favorable for both States, while the impact on service utilization and Medicare costs was mixed and more favorable in Washington. Washington's care coordination model was intensive and targeted to high-cost individuals while Colorado provided minimal care coordination. Coordinated care and integrated long-term services and support may help postpone NF institutionalization, but there is no evidence these activities reduced preventable hospitalizations or ED visits.

### Session 3280 (Symposium)

### MAXWELL A. POLLACK AWARD LECTURE

Chair: Bob Harootyan

The lecture will feature an address by the 2020 Pollack Award recipient, Karl Pillmer, PhD, FGSA of Cornell University. The 2021 Pollack Award recipient is Namkee G. Choi, PhD, FGSA, of the University of Texas at Austin. The Maxwell A. Pollack Award for Contributions to Healthy Aging Award recognizes instances of practice informed by research and analysis, research that has directly improved policy or practice, and distinction in bridging the worlds of research and practice.

### MAXWELL A. POLLACK AWARD LECTURE

Karl Pillemer, *Cornell University, Ithaca, New York, United States*

The lecture will feature an address by the 2020 Pollack Award recipient, Karl Pillmer, PhD, FGSA of Cornell University. The 2021 Pollack Award recipient is Namkee G. Choi, PhD, FGSA, of the University of Texas at Austin. The Maxwell A. Pollack Award for Contributions to Healthy Aging Award recognizes instances of practice informed by research and analysis, research that has directly improved policy or practice, and distinction in bridging the worlds of research and practice.

### Session 3285 (Paper)

### MEDICATIONS AND PRESCRIBING

### DEPRESCRIBING BLOOD PRESSURE TREATMENT IN VA LONG-TERM CARE RESIDENTS

Michelle Odden,[1] Sei Lee,[2] Michael Steinman,[2] Anna Rubinsky,[3] Bocheng Jing,[4] Kathy Fung,[4] Laura A. Graham,[5] and Carmen Peralta,[3] *1. Stanford University, Stanford, California, United States, 2. University of California San Francisco, San Francisco, California, United States, 3. University of California, San Francisco, San Francisco, California, United States, 4. San Francisco VA Medical Center, San Francisco, California, United States, 5. VA Palo Alto Health Care System, Menlo Park, California, United States*

There is growing interest in deprescribing of antihypertensive medications in response to adverse effects, or when a patient's situation evolves such that the benefits are outweighed by the harms. We conducted a retrospective cohort study to evaluate the incidence and predictors of deprescribing of antihypertensive medication among VA long-term care residents ≥ 65 years admitted between 2006 and 2017. Data were extracted from the VA electronic health record, CMS Minimum Data Set, and Bar Code Medication Administration. Deprescribing was defined as a reduction in the number of antihypertensive medications, sustained for 2 weeks. Potentially triggering events for deprescribing included low blood pressure (<90/60 mmHg), acute renal impairment (creatinine increase of 50%), electrolyte imbalance (potassium below 3.5 mEq/L, sodium decrease by 5 mEq/L), and fall in the past 30 days. Among 22,826 VA nursing home residents on antihypertensive medication, 57% had describing event during their stay (median length of stay = 6 months). Deprescribing events were most common in the first 4 weeks after admission and the last 4 weeks of life. Among potentially triggering events, acute renal impairment was associated with greatest increase in the likelihood of deprescribing over the subsequent 4 weeks: among residents with this event, 32.7% were described compared to 7.3% in those without (risk difference = 25.5%, p<0.001). Falls were associated with the smallest increased risk of deprescribing (risk difference = 2.1%, p<0.001) of the events considered. Deprescribing of antihypertensive medications is common among VA nursing home residents, especially after a potential renal adverse event.

### OPIOID USE AMONG RURAL MEDICARE BENEFICIARIES

Yvonne Jonk,[1] Heidi O'Connor,[2] Karen Pearson,[3] Zachariah Croll,[2] and John Gale,[3], *1. University of Southern Maine, Muskie School, Portland, Maine, United States, 2. University of Southern Maine, Portland, Maine, United States, 3. University Of Southern Maine, Portland, Maine, United States*

This study examines differences in opioid prescribing rates among a nationally representative sample of Medicare beneficiaries across rural and urban areas, as well as among beneficiaries with chronic overlapping pain conditions (COPCs). We assess whether prescribing patterns exceed the Centers for Disease Control and Prevention guidelines for dose and duration, and identify socioeconomic and health risk factors associated with opioid prescribing using logistic regression analyses. Data were from the 2010-2017 Medicare Current Beneficiary Survey files. Rural-Urban Commuting Area codes were used to identify patients' residential location. The Area Health Resource Files were used to identify market characteristics such as primary care and mental health shortage areas. With the exception of 2010, over years 2011-2017, higher percentages of community-dwelling rural beneficiaries received opioid prescriptions (21.8-25.4%) compared to their urban counterparts (19.1-23.7%). During the same time period, facility-dwelling rural beneficiaries were more likely to receive opioid prescriptions (39.8-47.2%) compared to their urban counterparts (28.8-35.0%). Higher percentages (18.8%) of the community dwelling population in rural had COPCs compared to urban (15.2%), and a higher percentage of rural beneficiaries with COPCs (31.4%) received an opioid prescription than their urban counterparts (22.2%). Previous research points to other factors contributing to a lack of alternatives to opioids for pain management in rural areas, including greater reliance on primary care providers, lack of access to chronic pain specialists and alternative therapies, and travel barriers. Improving the capacity of rural primary care to deal with COPCs and expanding access to specialists via telehealth warrants further attention from policymakers.

### PHARMACIST-LED INTERVENTIONS TO IMPROVE MEDICATION ADHERENCE IN OLDER ADULTS: A META-ANALYSIS

Zachary Marcum,[1] Shangqing Jiang,[1] Jennifer Bacci,[1] and Todd Ruppar,[2], *1. University of Washington, Seattle, Washington, United States, 2. Rush University College of Nursing, Chicago, Illinois, United States*

As pharmacists work to ensure reimbursement for chronic disease management services on the national (e.g., Medicare) level, summative evidence of their impact on important health metrics, such as medication adherence, is needed. The objective of this study was to assess the effectiveness of pharmacist-led interventions on medication adherence in older adults. In April 2020, a comprehensive search was conducted in six databases for publications of randomized clinical trials of pharmacist-led interventions to improve medication adherence in older adults. English-language studies with codable data on medication adherence and diverse adherence-promoting interventions targeting older adults (age 65+) were eligible. A standardized mean difference effect size (intervention vs. control) was calculated for the medication adherence outcome in each study. Study effect sizes were pooled using a random-effects meta-analysis model. Moderator analyses were then conducted to explore for differences in effect size due to intervention, sample, and study characteristics. The primary outcome was medication adherence using any method of measurement. This meta-analysis included 40 unique randomized trials of pharmacist-led interventions with data from 8,822 unique patients (mean age, range: 65 to 85 years). The mean effect size was 0.57 (95% Confidence Interval [CI]: 0.38-0.76). When two outlier studies were excluded from the analysis, the mean effect size decreased to 0.41 (95% CI: 0.27-0.54). Moderator analyses showed larger effect sizes for interventions containing medication education and when interventions had components delivered at least partly in patients' homes. In conclusion, this meta-analysis found a significant improvement in medication adherence among older adults receiving pharmacist-led interventions.

### SEX DIFFERENCES IN POTENTIALLY INAPPROPRIATE PRESCRIBING AMONG OLDER ADULTS WITH MULTIMORBIDITY

Maria Ukhanova,[1] Sheila Markwardt,[1] Jon Furuno,[1] Laura Davis,[2] Brie Noble,[1] and Ana Quiñones,[1] *1. Oregon Health & Science University, Portland, Oregon, United States, 2. Neighborhood Health Center, Hillsboro, Oregon, United States*

Sex differences in prescribing potentially inappropriate medications (PIMs) for various multimorbidity patterns are not well understood. This study sought to identify sex specific risk of PIMs in older adults with cardiovascular-metabolic patterns. Secondary analysis of the Health and Retirement Study interview data (2004-2014; n=6,341,

≥65 y/o) linked to Medicare claims data was conducted. Four multimorbidity patterns were identified based on the list of 20 chronic conditions and included: 'cardiovascular-metabolic only', 'cardiovascular-metabolic plus other physical conditions', 'cardiovascular-metabolic plus mental conditions', and 'no cardiovascular-metabolic disease' patterns. Presence of PIM prescribing was identified using the 2015 American Geriatrics Society Beers Criteria, limited to the list of medications to avoid in older adults. Chi-square tests and logistic regressions were used to identify sex differences in prescribing PIMs across multimorbidity patterns: (1) for PIMs overall and (2) for each PIM drug class. Results indicate that on average women were prescribed PIMs more often than men (39.4% and 32.8%, respectively). Women with cardiovascular-metabolic plus other physical patterns (Adj.OR=1.25, 95% CI: 1.07-1.45) and cardiovascular-metabolic plus mental patterns (Adj.OR=1.25, 95% CI: 1.06-1.48) had higher odds of PIM compared to men, however, there were no sex differences in PIM prescribing in the cardiovascular-metabolic only patterns (Adj.OR=1.13, 95% CI: 0.79-1.62). There was variation by sex across different PIM drug classes. Our study emphasizes the need to further reduce PIM prescribing among older adults, and identifies target populations for potential interventions to improve medication prescribing practices.

### THYME AND OREGANO TERPENOIDS ACTIVATE AUTOPHAGY AND PROTECT AGAINST HEPATIC STEATOSIS

Gabriele Civiletto, Guillaume Eric Jacot, Federico Sizzano, Kamila Muller, Aurélie Hermant, Umberto De Marchi, Jerome Feige, and Philipp Gut, *Nestlé Research, Lausanne, Vaud, Switzerland*

Caloric restriction has been shown to reduce chronic illness in aging and increase life expectancy in most living organisms including mammals. Autophagy, a ubiquitous catabolic pathway of cellular quality control, is a key mechanism mediating the benefits of caloric restriction. In addition, mutations in genes involved in autophagy have been associated with the early onset of age-related diseases such as neurodegeneration, highlighting autophagy as a potential therapeutic target. Here, we aimed to discover autophagy inducers from a library of edible molecules for potential use in food applications. To this end, we developed a novel in vivo high-content screening strategy using fluorescent reporter zebrafish that monitor autophagy flux in skeletal muscle. We identify the thyme and oregano constituent thymol as a novel potent autophagy inducer in zebrafish, human cells and mouse tissues. Mechanistically, thymol triggers an hormetic effect on mitochondria in synergism with a calcium-dependent autophagy response which, in turn, leads to mobilization of intracellular lipid stores. We tested the effects of chronic thymol supplementation in mice fed a high-fat diet and showed that thymol mobilizes fatty acids, reduces liver triglycerides and improves markers of liver damage. In sum, we validate the use of zebrafish screening as a discovery model for autophagy-based therapeutics and demonstrate that thymol is an autophagy inducer with potential for the prevention of chronic metabolic diseases and other age-related conditions.

### Session 3290 (Symposium)

### NOVEL APPLICATIONS OF ACCELEROMETRY DATA FOR HEALTH OUTCOMES IN OLDER ADULTS: THINKING BEYOND MVPA

Chair: Jennifer Schrack Co-Chair: Jacek Urbanek
Discussant: Manini Manini

Physical activity is a well-established predictor of health and longevity. Wearable accelerometers produce high-frequency, time series data that capture multiple aspects of daily physical activity across the spectrum of intensity. Historically, the majority of accelerometry-based physical activity research has employed summary threshold metrics such as moderate-to-vigorous physical activity, or "MVPA." Although these measures are important for understanding compliance with physical activity guidelines, they underutilize the potential of this data. To advance the science of physical activity in older adults, more sensitive, clinically translatable measures are needed. This symposium will examine the associations between novel measures of accelerometry-derived physical activity and various aging-related health outcomes. Dr. Wanigatunga will discuss the association of physical activity volume and fragmentation with the frailty phenotype in the Study to Understand Vitamin D and Fall Reduction in You (STURDY). Dr. Cai will present evidence on the association of physical activity quantities and patterns with measures of visual impairment in the Baltimore Longitudinal Study of Aging. Ms. Qiao will present a novel accelerometry-derived measure of performance fatigability in the Developmental Epidemiologic Cohort Study. Finally, Dr. Urbanek will discuss the role of accelerometry-derived free-living gait cadence in defining fall risk in STURDY. Collectively, these presentations highlight critical associations between objective measures of physical activity and health outcomes in older adults and illuminate the need for thinking beyond MVPA to improve prevention and intervention efforts.

### ACCELEROMETER-DERIVED PATTERNS OF PHYSICAL ACTIVITY AND INCIDENT FRAILTY

Yurun Cai,[1] Jacek Urbanek,[2] David Roth,[3] Jeremy D. Walston,[4] Karen Bandeen-Roche,[1] Lawrence Appel,[2] Jennifer Schrack,[1] and Amal Wanigatunga,[1] *1. Johns Hopkins Bloomberg School of Public Health, Baltimore, Maryland, United States, 2. Johns Hopkins School of Medicine, Baltimore, Maryland, United States, 3. Johns Hopkins University, Baltimore, Maryland, United States, 4. Johns Hopkins University School of Medicine, Baltimore, Maryland, United States*

Low physical activity (PA) is a common phenotype of frailty, but whether disengagement of daily lifestyle PA signals impending frailty remains unexplored. Using STURDY (Study to Understand Fall Reduction and Vitamin D in You) data from 499 robust/prefrail adults (mean age=76 + 5 years; 42% women), we examined whether accelerometer patterns (activity counts/day, active minutes/day, and activity fragmentation) were prospectively associated with incident frailty over 2 years of follow-up; 48 (10%) participants developed frailty. In Discrete-Cox hazard models adjusted for demographics, medical conditions, and device wear days, every 30 min/day higher baseline active time, 100,000 more activity counts/day, and 1% lower activity fragmentation was associated with a 13% (p=0.003), 10% (p=0.001), and 8% (p<0.001) lower risk of frailty, respectively. Our results show that both reduced amounts and fragmented patterns of daily PA captured from accelerometry are associated with phenotypic frailty and might signal frailty onset.

### VISUAL IMPAIRMENT AND OBJECTIVELY MEASURED PHYSICAL ACTIVITY IN MIDDLE-AGED AND OLDER ADULTS

Jennifer Schrack,[1] Jian-Yu E,[2] Amal Wanigatunga,[1] Jacek Urbanek,[3] Eleanor Simonsick,[4] Bonnielin Swenor,[3] and Yurun Cai,[1] *1. Johns Hopkins Bloomberg School of Public Health, Baltimore, Maryland, United States, 2. Johns hopkins Bloomberg School of Medicine, Baltimore, Maryland, United States, 3. Johns Hopkins School of Medicine, Baltimore, Maryland, United States, 4. National Instute on Aging/NIH, Baltimore, Maryland, United States*

Vision loss is associated with restricted physical activity (PA), yet the relationship between multiple domains of vision measures and objectively measured PA, especially activity patterns, in mid-to-late life remains unclear. In 603 BLSA participants (mean age=73.5±11 years; 56% women; 69% white), best-corrected and presenting visual acuity (VA), contrast sensitivity, visual fields (VF), stereo acuity were assessed from 2015 to 2019. Free-living PA was assessed using a wrist-worn ActiGraph accelerometer for 7 days. Linear regression models showed that participants with vs. without best-corrected VA impairment had 29.3 fewer active minutes/day (p=0.03) and trended towards fewer activity counts (p=0.05), adjusting for sociodemographic and health characteristics. VF impairment was associated with 268,636 fewer activity counts (p=0.02), 46.2 fewer active minutes/day (p=0.02), and a 3% greater activity fragmentation (p=0.009). Older adults with visual impairment have restricted and more fragmented activity patterns. Longitudinal studies are warranted to examine causality between visual impairment and PA decline.

### DETECTING A NOVEL WALKING-BASED PERFORMANCE FATIGABILITY MARKER WITH ACCELEROMETRY IN OLDER ADULTS

Jaroslaw Harezlak,[1] Robert Boudreau,[2] Jacek Urbanek,[3] Kyle Moored,[2] Jennifer Schrack,[4] Eleanor Simonsick,[5] Nancy W. Glynn,[6] and Yujia (Susanna) Qiao,[7] *1. Indiana University, Bloomington, Indiana, United States, 2. University of Pittsburgh, Pittsburgh, Pennsylvania, United States, 3. Johns Hopkins School of Medicine, Baltimore, Maryland, United States, 4. Johns Hopkins Bloomberg School of Public Health, Baltimore, Maryland, United States, 5. National Instute on Aging/NIH, Baltimore, Maryland, United States, 6. University of Pittsburgh Graduate School of Public Health, Pittsburgh, Pennsylvania, United States, 7. University of Pittsburgh, University of Pittsburgh, Pennsylvania, United States*

Walking-based performance fatigability measures (e.g., lap-time difference) may not adequately capture performance deterioration as self-pacing is a common compensatory strategy in those with low activity tolerance. To overcome this limitation, we developed a new approach with accelerometry (ActiGraph GT3X+, sampling=80 Hz, non-dominant wrist) during fast-paced 400m-walk (N=57, age=78.7±5.7 years, women=53%). Cadence (steps/second) was estimated using raw accelerometer data (R “ADEPT”). Penalized regression splines (R “mgcv”) were used to estimate the individual-level smoothed cadence trajectories. “Time-to-slow-down” was defined as first time-point where the full confidence interval of change in cadence<0. Five participants were censored at stopping time (not slowdown or complete walk). Median “time-to-slow-down” was 1.86 minutes (IQR=0.98-2.73, range=0.57-6.25). Participants with longer “time-to-slow-down” had slower starting cadence, longer 400m-walk time, and greater perceived fatigability (Pittsburgh Fatigability Scale), p’s<0.05 (linear regression). Our preliminary findings revealed that detecting accelerometry-based performance fatigability/deterioration in older adults is feasible and needs to account for initial pace.

### FREE-LIVING GAIT CADENCE MEASURED BY WEARABLE ACCELEROMETERS FOR ASSESSING FALL RISK

David Roth,[1] Marta Karas,[1] Amal Wanigatunga,[2] Stephen Juraschek,[3] Lawrence Appel,[4] Jennifer Schrack,[2] and Jacek Urbanek,[4], *1. Johns Hopkins University, Baltimore, Maryland, United States, 2. Johns Hopkins Bloomberg School of Public Health, Baltimore, Maryland, United States, 3. Harvard Medical School, Boston, Massachusetts, United States, 4. Johns Hopkins School of Medicine, Baltimore, Maryland, United States*

Accelerometers are widespread in research applications, yet whether they are superior to structured clinic-based assessments is unknown. Using negative binomial regression, we compared traditional in-clinic measures of mobility (6-minute gait cadence, speed, and distance, and 4-meter gait speed) with free-living gait cadence from wrist accelerometers (Actigraph GT9X) in predicting fall rates in 432 older adults (age 77.29±5.46 yrs, 59.1% men, 80.2% White) participating in the Study to Understand Fall Reduction and Vitamin D in You (STURDY). Accelerometry-based gait cadence was estimated with the Adaptive Empirical Pattern Transformation algorithm. Across all participants, every 10 steps/min higher cadence was associated with a 13.2% lower fall rate (p=0.036). Mobility measures were not related to falls (p>0.05). Among higher-functioning participants (cadence ≥100 steps/min), every 10 steps/min higher free-living cadence (p=0.01) was associated with a 27.7% lower fall rate. Data collected from accelerometers may provide a more sensitive indicator of fall risk than in-clinic tests.

### Session 3295 (Symposium)

### NOVEL APPROACHES EXAMINING SLEEP HEALTH AS A MARKER OF SUCCESSFUL AGING

Chair: Soomi Lee Discussant: Meredith Wallace

Sleep is a modifiable determinant of health. It changes with advancing age and in response to diverse contexts (e.g., related to work or one’s health). Previous studies have often used single measures of sleep duration or sleep quality. However, a recent paradigmatic shift towards multidimensional sleep health emphasizes the importance of examining how multiple sleep measures are simultaneously associated with health. This approach presents many opportunities for understanding sleep phenotypes and their potential contributions to health. Yet it also presents methodological challenges in analyzing multidimensional sleep data. This symposium showcases the most recent approaches and novel ideas examining the role of sleep health in successful aging. Paper 1 examines sleep profiles (i.e., latent groups with varying sleep characteristics) in middle-aged adults and their linkages to psychological well-being and chronic conditions with differences by age groups. Paper 2 investigates 24-hour patterns of sleep-activity rhythms and their associations with physical functioning performance in older men and women. Paper 3 showcases the utility of a sleep health composite score in examining sleep disparities and their drivers in middle- and later-adulthood. Paper 4 examines whether and how a composite sleep health measure based on actigraphy data is associated with specific characteristics of adult bipolar disorder patients. These papers use different cohorts, such as the Midlife in the United States Study, Osteoporotic Fractures in Men study, and Multi-Ethnic Study of Atherosclerosis. At the end, Dr. Wallace will discuss key findings from these studies, their methodological contributions and implications for aging, and directions for future research.

### IDENTIFICATION OF LATENT SLEEP PROFILES IN MIDDLE-AGED ADULTS AND CONNECTIONS TO WELL-BEING

Soomi Lee,[1] and Claire Smith,[2], *1. University of South Florida, Tampa, Florida, United States, 2. University of South Florida, University of South Florida, Florida, United States*

For middle-aged adults, achieving adequate sleep is a challenge but essential for long-term health. The present study identified latent sleep profiles to clarify how multiple sleep variables (i.e., regularity, satisfaction, alertness, timing, efficiency, and duration) cooccur within middle-aged adults and the implications these holistic sleep experiences have for well-being. Three profiles emerged within the Midlife in the United States II dataset (MIDUS; N=4030, Mage=56.23 years): (i) good sleepers, (ii) nappers/poor night sleepers, and (iii) sufficient but irregular sleepers. Generally, good sleepers reported the best well-being, sufficient/irregular sleepers reported comparatively moderate well-being, and nappers/poor night sleepers reported the worst well-being across a variety of indicators (i.e., chronic health conditions, life satisfaction, positive affect, negative affect, and psychological well-being) after adjusting for sociodemographic characteristics. Age moderated these associations. Our findings advance understanding of sleep health as a multifaceted construct and of its connection to well-being in middle-aged adults.

### REST-ACTIVITY RHYTHM PATTERNS AND PHYSICAL FUNCTIONAL PERFORMANCE IN COMMUNITY-DWELLING OLDER MEN

Gregory Tranah,[1] Patrick Bradshaw,[2] Terri Blackwell-Hoge,[1] Jamie Zeitzer,[3] Sonia Ancoli-Israel,[4] Kristine Yaffe,[5] Katie Stone,[6] and Dorothy Chen,[7], *1. Sutter Health, San Francisco, California, United States, 2. UC Berkeley, Berkeley, California, United States, 3. Stanford University, Palo Alto, California, United States, 4. UC San Diego, La Jolla, California, United States, 5. UCSF, UCSF, California, United States, 6. University of California, Berkley, San Francisco, California, United States, 7. Sutter Health, SAN FRANCISCO, California, United States*

Sleep and activity patterns have been linked to physical performance in older adults. Traditional parametric models of 24-hour activity rhythms fail to adequately capture specific diurnal sleep and wake patterns; functional principal components analysis (fPCA) is a non-parametric approach that addresses this limitation. Using fPCA, we modeled accelerometry data from n = 2,960 participants in the Osteoporotic Fractures in Men (MrOS) ancillary sleep study (mean age 77y) and examined cross-sectional associations with gait speed and grip strength measurements. Lower daytime activity (expected difference = -0.049 [-0.072, -0.028] m/s), increased sleep duration and a reduced midday dip in activity (-0.015 [-0.035, 0.006] m/s) were modestly associated with worsening gait speed. A modest association between both later sleep and wake times and increased sleep duration with worsening grip strength outcomes was observed (-1.11 [-1.90, -0.32] kg). Specific daily activity patterns may serve as predictive biomarkers for changing physical function in aging populations.

### MULTIDIMENSIONAL SLEEP HEALTH: CONCEPTS, ADVANCES, AND IMPLICATIONS FOR RESEARCH AND INTERVENTION

Joon Chung,[1] Matthew Goodman,[1] Tianyi Huang,[2] Suzanne Bertisch,[1] and Susan Redline,[3] *1. Brigham and Women's Hospital, Harvard Medical School, Boston, Massachusetts, United States, 2. Brigham and Women's Hospital, Boston, Massachusetts, United States, 3. Harvard Medical School, Boston, Massachusetts, United States*

Sleep is a complex process, sensitive to aging, with theoretical and evidentiary basis for influence on multiple health outcomes. Recent scholarship has argued for a 'multi-dimensional' approach to sleep health, that is, a recognition that healthy sleep consists of more than its quantity (duration) and is more than the absence of sleep disorders. This new conception of sleep health acknowledges sleep's complexity yet presents challenges for methodological treatment. How do we operationalize/analyze multiple dimensions of sleep, some of which are correlated due to physiological reasons, common measurement tools, or sensitivity to common stressors? Is it sensible to talk about 'sleep health' as a single, composite entity with multiple components, akin to a dietary pattern rather than a collection of individual nutrients? Exemplar data from a racial-ethnic disparities project in aging adults suggest the utility of a composite approach, and the value of considering inter-correlations among sleep metrics.

### FINDING A COMPOSITE MEASURE FOR DATA FROM WRIST ACTIGRAPHY IN BIPOLAR DISORDER

Ellen Lee,[1] David Wing,[2] Sonia Ancoli-Israel,[3] Colin Depp,[1] Ho-Kyoung Yoon,[4] Lisa Eyler,[1] and Christopher Kaufmann,[5] *1. Department of Psychiatry, University of California San Diego, La Jolla, California, United States, 2. Center for Wireless and Population Health Systems, University of California San Diego, La Jolla, California, United States, 3. UC San Diego, La Jolla,*

California, United States, 4. Department of Psychiatry, Korea University College of Medicine, Seoul, Seoul-t'ukpyolsi, Republic of Korea, 5. University of California San Diego, La Jolla, California, United States

Actigraphy can objectively measure sleep in studies on Bipolar Disorder (BD) where subjective sleep ratings might be influenced by affect. Actigraphy data are complex necessitating data reduction approaches. We created a composite score of actigraphy sleep metrics (total sleep time [TST], wake after sleep onset [WASO], and percent sleep [PS]) in BD. We computed z-scores of sleep measures for n=51 BD vs. n=80 healthy subjects and averaged scores. We examined associations with participant characteristics and used LASSO to identify metrics best explaining composite variability. Higher composite scores (better sleep) were seen in employed vs. unemployed (t=2.40, df=34, p=0.02), and correlated with higher medication load (r=0.41, p=0.004), lower mania symptomatology (r=-0.33, p=0.04) and lower interleukin (IL)-6 levels (r=-0.32, p=0.02). TST best explained variability in medication load and PS best explained employment, mania symptoms and IL-6. Given observed specificity of associations, selecting theory-driven sleep metrics may be more appropriate than a composite.

### Session 3300 (Symposium)

### PHYSICAL ACTIVITY AND SEDENTARY BEHAVIOR PATTERNS PRIOR TO AND DURING THE COVID-19 PANDEMIC

Chair: Nancy Gell Co-Chair: Dori Rosenberg
Discussant: John Bellettiere

Understanding patterns in the types of activities older adults engage in during physical activity and sedentary time could help shape intervention designs. Few studies have adequately described the physical activity and sedentary pursuits older adults undertake, including during the COVID-19 pandemic. To answer these questions, this symposium uses data from three recent studies: Adult Changes in Thought (ACT),an epidemiologic study with self-reported and device-based measures of physical activity and sedentary time including time spent in various domains of activity; Objective Physical Activity and Cardiovascular Disease Health in Older Women (OPACH), an epidemiologic study with device and self-report measures of sedentary behavior; and an ongoing clinical trial, the Healthy Aging Resources to Thrive (HART) study with device and self-reported data on sitting time and patterns as well as physical activity. The first session in this symposium will present a description of the rates of meeting the aerobic, strength, and balance recommendations among older adults in the ACT study. Next, we will have a presentation describing sedentary activities in older adults by age, sex and device-based sitting patterns in the ACT study. In the third presentation we will use OPACH data to examine patterns and context of sedentary in relation to aging-related outcomes. Finally, we will describe changes in physical activity and sedentary time in the HART trial in the cohort enrolled prior to the COVID-19 pandemic vs. those enrolled during the pandemic. Our Discussant will provide new insights on the roles of sedentary behavior and physical activity in aging and health.

### DEVICE-MEASURED SEDENTARY PATTERNS AND PHYSICAL ACTIVITY BEFORE AND DURING THE COVID-19 PANDEMIC

Mikael Anne Greenwood-Hickman,[1] Jing Zhou,[2] Julie Cooper,[3] David Arterburn,[2] Andrea Cook,[2] and Dori Rosenberg,[2], *1. Kaiser Permanente Washington, Seattle, Washington, United States, 2. Kaiser Permanente Washington Health Research Institute, Seattle, Washington, United States, 3. kaiser Permanente Washington Health Research Institute, Seattle, Washington, United States*

Little is known about objective levels of sitting time (ST), patterns of ST, and physical activity (PA) among older adults before compared to during the COVID -19 pandemic. We used data from the Healthy Aging Resources to Thrive Trial to examine differences in activPAL-assessed ST, standing time, breaks from sitting, and steps in study enrollees prior to March 2020 (N = 97, % female = 60.8, % white = 81.4; Mean BMI = 35.2) compared to post-March 2020 (N = 47, % female = 70.2, % white = 72.3; Mean BMI = 36.1). During the pandemic, participants had higher sitting time (Mean = 11.5 vs. 10.7 hours/day), fewer breaks from sitting (Mean = 40 vs. 44 breaks/day), and fewer steps (Mean = 4441 vs. 5931 steps/day) than prior to the pandemic. Interventions may be needed to support older adults with obesity in recovering losses in time spent physically active.

### AEROBIC, STRENGTHENING, AND BALANCE ACTIVITIES PERFORMED BY COMMUNITY-DWELLING OLDER ADULTS

Bonnell Levi,[1] Nancy Gell,[1] and Mariana Wingood,[2] *1. University of Vermont, Burlington, Vermont, United States, 2. University of Vermont, University of Vermont, Vermont, United States*

Little is known about whether older adults meet the recommended physical activity (PA) guidelines, including aerobic, strength, and balance components. Given this gap, we examined self-report PA data from 1,352 older adult participants of the Adult Changes in Thought (ACT) study. We classified participants as meeting some components, meeting the full guidelines, or being insufficiently active. Multinomial regression was used to identify factors associated with meeting PA guidelines. Despite performing 9.5 hours of weekly PA, only 11% met the full guidelines, 13% met the aerobic, and 26% met the balance or strength recommendations. Increasing age and body mass index, needing assistance with instrumental daily activities, heart disease, and low income were associated with decreased odds of meeting PA guidelines. Older adults primarily perform aerobic PA and lower intensity PA with fewer participating in strength and balance activities. Interventions targeting strength, balance, and higher intensity PA should be developed.

### CHARACTERIZING COMPONENT ACTIVITIES OF OLDER ADULT SEDENTARY TIME BY AGE, GENDER, AND DEVICE-BASED SITTING PATTERNS

Rod Walker,[1] John Bellettiere,[2] Andrea LaCroix,[2] Dori Rosenberg,[1] and Mikael Anne Greenwood-Hickman,[3] *1. Kaiser Permanente Washington Health Research Institute, Seattle, Washington, United States, 2. University of California, San Diego Herbert Wertheim School of Public Health and Human Longevity Science, La Jolla, California,*

*United States, 3. Kaiser Permanente Washington, Seattle, Washington, United States*

The activities that compose older adults' considerable sedentary time are not well characterized. We described daily time spent in self-reported sedentary activities and explored differences by age, gender, and activPAL sitting patterns. Participants self-reported a total of 10.7 hours of sitting time and spent the most time watching TV (2.6 hrs/day), using the computer (1.7 hrs/day), and reading (1.6 hrs/day). Women spent more time watching TV, engaged in hobbies, and socializing and less time on the computer compared to men. Older participants spent more time watching TV, reading, and participating in group activities and less time on the computer than younger participants. Those with low activPAL sitting time and frequent activPAL sitting breaks (low mean bout duration) ~1 hr /day less watching TV than those with high activPAL sitting time. These findings help illuminate future intervention targets and lay the path to explore associations between different sedentary activities and health.

### ACCELEROMETER-MEASURED PATTERNS OF SEDENTARY BEHAVIOR IN OLDER WOMEN: THE OPACH STUDY

Eric Hyde,[1] John Bellettiere,[2] Blake Anuskiewicz,[3] Andrea LaCroix,[2] and Benjamin Schumacher,[4], *1. UC San Diego, San Diego, California, United States, 2. University of California, San Diego Herbert Wertheim School of Public Health and Human Longevity Science, La Jolla, California, United States, 3. San Diego State University, San Diego, California, United States, 4. University of California, San Diego, San Diego, California, United States*

Excessive sedentary behavior (SB) is related to deleterious health outcomes. Understanding the patterns and contexts in which SB accumulates can promote healthy aging. Daily sitting time and mean sitting bout duration (MBD) were measured by triaxial accelerometers. Participants self-reported how much time they spent sitting while: watching TV, reading, using the computer, driving, working, or taking phone calls. Data were compared across aging-related characteristics. Age-adjusted sitting time (minutes/day) for 5,838 diverse (33.2% Black, 16.9% Hispanic), older women (mean age 78.7±6.7) were 577.2 for Hispanic women, 630.3 for Black women, and 632.0 for White women. Those in the lowest vs. highest physical function category had the longest MBD (16.1 vs. 11.7 minutes/bout). Watching television was the most common self-reported sedentary activity. The highest vs. lowest quartile of MBD spent, on average, 30.6 and 22.3 minutes/day watching television, respectively. This presentation will illuminate critical factors associated with sitting patterns in older adults.

### Session 3305 (Symposium)

### PRESIDENTIAL SYMPOSIUM: FROM GLOBAL TO LOCAL: AN AGE-INCLUSIVE AND RIGHTS-BASED APPROACH TO GERONTOLOGICAL EDUCATION

Chair: Dana Bradley Co-Chair: Judith Howe

In June 2020, the Gerontological Society of America (GSA) joined in solidarity in the movement to condemn the entrenched racism undermining American society and build upon a gero-rich international historical base of supporting human rights. However, as gerontological educators, we need to expand on the conversation of racism to the broader global discussion of inclusivity and elimination of discrimination. A global focus on human rights of older persons, which began in 1982 at the World Assembly on Aging and has led to the current discussion of the proposed UN Convention on the Rights of Older Persons. The Academy of Gerontology in Higher Education (AGHE) is GSA's education group of colleges and universities that offers education, training, curricular innovations, and research programs in the field of aging. The work of this group is grounded in an age-inclusive and rights-based perspective, and members are committed to an international view demonstrated through AGHE's tagline Global Leaders in Advancing Education on Aging; This symposium explores the role of age-inclusivity and a rights-based perspective in gerontology and geriatrics education and offers both challenges and best practices for moving forward. The first presentation explores the meaning of age-inclusivity in aging education in a global context and asks how do we build upon our international roots? Our second presenter shares a proposed framework for a rights-based approach to gerontology education. The third presentation explores an example of a rights-based training program. We conclude with a lively discussion focusing on how to take action through education.

### A PROPOSED FRAMEWORK FOR IMPLEMENTING A RIGHTS-BASED APPROACH TO GERONTOLOGICAL EDUCATION AND TRAINING

Laura Allen,[1] and Dana Bradley,[2], *1. Bar-Ilan University, Ramat Gat, Tel Aviv, Israel, 2. UMBC Erickson School of Aging Studies, Baltimore, Maryland, United States*

This paper uses the nine general principles that underpin human rights (Non-discrimination, Respect, Dignity, Autonomy, Equality, Self-fulfillment and Personal development, Full and effective participation, Intergenerational solidarity, and Recognition of intrinsic value and worth as a human being) to frame a right's based approach. This framework looks beyond the older person and the issues they are facing to the structure and culture of the society itself and the ways in which it is contributing to challenges. Using this lens, we will discuss how to develop definitions and standards of right's- based education that are culturally and contextually appropriate, define right's based competencies and recognize, that despite the universal rights of older persons, the implementation may need to be adjusted for unique sociocultural environments. . Lastly we will outline a strategy to identify and train multidisciplinary teaching and research teams using this proposed framework.

### PAST, PRESENT, AND FUTURE OF HUMAN RIGHTS IN GERONTOLOGICAL EDUCATION

Rona Karasik,[1] and Judith Howe,[2], *1. Saint Cloud State University, SAINT CLOUD, Minnesota, United States, 2. Icahn School of Medicine at Mount Sinai, New York, New York, United States*

The rights of older persons, essential to our work as gerontologists, were discussed in the World Assembly on Aging (1982) and adopted through the United Nations Principles of Older Persons and followed by the Madrid International Plan of Action on Aging (MIPPA) in 2002. Although it has been endorsed by the General Assembly of the United Nations, in contrast to conventions, it is not binding on member states. This paper discusses the rights of older persons and our obligations as educators and researchers to focus on core issues associated with the rights and quality of life of older people. We will review the role of education in meeting this call to action through examples like the UN Decade of Healthy Ageing where education is a required element to accomplish the action areas and the Age-Friendly University movement. Both have involved multiple university communities on a global scale.

### BRINGING RIGHTS OF OLDER PERSONS TO THE REAL WORLD: LESSONS LEARNED FROM A RIGHTS-BASED TRAINING

Kelly Fitzgerald,[1] and Kim Stoeckel,[2] *1. University of Maryland Baltimore County, Baltimore, Maryland, United States, 2. UMBC, Baltimore, Maryland, United States*

When gerontological education and training are grounded in a rights-based approach, this framing provides a tool to ensure the rights of older people are advocated for and experienced. A "train the trainer" program was given to non-governmental organizations (NGOs) providing services to refugees in Jordan. The goal of the training was to educate NGOs on how to use a rights-based approach when responding to and supporting older refugees. The training covered a range of topics in ensuring the rights of older people in the provision of care, protection, and inclusion. Training outcomes revealed an increased awareness of the rights of older people. Skills and knowledge gained as a result of this training empowered participants to further develop their own work, within their cultural context, to reflect a rights-based approach to services and programs.

### Session 3310 (Paper)

### SOCIAL-ENVIRONMENTAL CONTEXTS AND WELL-BEING

### AIR QUALITY IMPROVEMENT IS ASSOCIATED WITH DECREASING DEPRESSIVE SYMPTOMS IN OLDER WOMEN

Andrew Petkus,[1] Xinhui Wang,[1] Diana Younan,[2] Daniel Beavers,[3] Mark Espeland,[3] Joshua Millstein,[2] Margaret Gatz,[2] and Jiu-Chiuan Chen,[2], *1. University of Southern California, LOS ANGELES, California, United States, 2. University of Southern California, Los Angeles, California, United States, 3. Wake Forest School of Medicine, Winston-Salem, North Carolina, United States*

Air pollution exposure is an environmental risk factor in brain aging and may also be associated with late-life depressive symptoms (DS). It is unknown if air quality (AQ) improvement is associated with reductions in DS in later life. Longitudinal data from 917 cognitively intact women with no prior history of depression (baseline age 66.4 ± 1.6 years old) participating in the Women's Health Initiative Memory Study of Younger Women (WHIMSY; 2008-2016) were analyzed to examine whether AQ improvement over the 5-years prior to WHIMSY baseline was associated with trajectories of DS (measured by 15-item Geriatric Depression Scale). Annual PM2.5 (fine particulate matter of aerodynamic diameter <2.5) and NO2 were estimated at the participants' residence using regionalized universal kriging models. Estimates were aggregated to the 3-year average at 5 years (remote) and immediately (recent) before WHIMSY baseline. Associations between AQ improvement (difference between remote to recent exposure) and trajectories of DS were estimated using linear mixed effect models, adjusting for sociodemographic, lifestyle, and clinical covariates. AQ improved prior to baseline (PM2.5: 1.62 ± 1.45 μg/m3 and NO2: 3.70 ± 2.81 ppb). Women residing in locations with greater improvement in NO2 (per IQR = 4.34 ppb) or PM2.5 (per IQR = 2.30 μg/m3) reported significant annual reductions in DS (βNO2=3.1%, p=.046; βPM25=1.6%, p=.046), similar to the effect of engaging in moderate to vigorous physical activity four times or more a week. These findings suggest that improving air quality may reduce depressive symptoms in older women.

### AMBIENT AIR POLLUTION AND INCREASING DEPRESSIVE SYMPTOMS IN OLDER WOMEN: THE MEDIATING ROLE OF THE PREFRONTAL CORTEX

Andrew Petkus,[1] Xinhui Wang,[1] Diana Younan,[2] Daniel Beavers,[3] Mark Espeland,[3] Joshua Millstein,[2] Margaret Gatz,[2] and Jiu-Chiuan Chen,[2], *1. University of Southern California, LOS ANGELES, California, United States, 2. University of Southern California, Los Angeles, California, United States, 3. Wake Forest School of Medicine, Winston-Salem, North Carolina, United States*

Exposure to air pollution may accelerate brain aging and increase risk of late-life depressive symptoms (DS). Brain structures underlying these associations are unknown. Longitudinal data from 829 community-dwelling women without dementia (baseline age 81.6 ± 3.6 years old) who participated in both the Women's Health Initiative Memory Study Magnetic Resonance Imaging study (WHIMS-MRI; 2005-06) and the WHIMS-Epidemiology of Cognitive Health Outcomes (2008-16) were analyzed to examine whether volumetric measures of brain structures mediated associations between long-term exposure to ambient air pollutants and annual increases in DS (as measured by annually assessed 15-item Geriatric Depression Scale). Annual PM2.5 (fine particulate matter of aerodynamic diameter <2.5) and NO2 were estimated at the participants' residence using regionalized universal kriging models and aggregated to the 3-year average prior to the WHIMS-MRI. Structural equation models were constructed to estimate associations between exposure, structural brain variables, and trajectories of DS (standardized on baseline mean and SD). Living in locations with higher NO2 (standardized β = 0.023; 95% Confidence Interval (CI) = 0.004, 0.042) or PM2.5 (standardized β = 0.021; 95% CI = 0.004, 0.038) was associated with larger annual increases in DS (~60% larger annual increase in DS). Higher NO2, but not PM2.5, was associated with smaller prefrontal cortical volumes (standardized β = -0.431; 99% CI = -0.518; -0.344). Prefrontal cortical volume explained 30.4% of the total association between annual DS increase and NO2. These findings underscore the importance of the prefrontal cortex in associations between NO2 exposure and increasing DS in later-life.

### CRIME AND PERCEIVED NEIGHBORHOOD SAFETY ACROSS RACIAL-ETHNIC GROUPS

Alfredo Velasquez,[1] Fangqi Guo,[2] and Jennifer Robinette,[1] *1. Chapman University, Orange, California, United States, 2. Chapman University, Irvine, California, United States*

Crime often increases safety concerns for residents, and safety concerns are generally associated with worse health. Despite that marginalized racial/ethnic groups are more likely than non-Hispanic Whites to live in areas with more crime, prior studies have documented that these groups differentially view crime as a threat to safety. Furthermore, older adults are more likely to report safety concerns than younger adults, despite a lesser chance of being victimized. Using multiple waves of data from the Health and Retirement Study, a representative sample of US adults aged 51 years and older (n= 11,161, mean age of 66 years), we conducted weighted repeated cross-sectional linear regressions to examine whether the association between crime and perceived neighborhood safety varies by racial/ethnic group, by age, or by wave of data collection. Study results indicated that higher crime rates consistently predicted more safety concerns among non-Hispanic Whites, Hispanics, and "Others," but were inconsistently associated with safety concerns among non-Hispanic Blacks, adjusting for age, household wealth, and census tract-level concentrated disadvantage, population density, and racial/ethnic heterogeneity. Furthermore, among non-Hispanic Whites, greater crime predicted more safety concerns before, but not after including a measure of racial/ethnic heterogeneity. These patterns persisted across the full age span. Racial/ethnic differences in the crime-safety link could be explained by additional sociopolitical and environmental variables including diversity that vary over time. Follow-up analysis is needed to determine if the racial/ethnic differences in crime-safety links extend to health.

### SPACE, CONTEXT, HUMAN CAPITAL: A MACRO-MICRO PERSPECTIVE ON SOCIAL ENVIRONMENT AND FINANCIAL LITERACY IN LATER LIFE

Yang Li,[1] Jan Mutchler,[2] Edward Miller,[2] Jing Jian Xiao,[3] and Reginald Tucker-Seeley,[4], *1. University of Massachusetts, Boston, Massachusetts, United States, 2. University of Massachusetts Boston, Boston, Massachusetts, United States, 3. University of Rhode Island, Kingston, Rhode Island, United States, 4. University of Southern California, Los Angeles, California, United States*

Despite a large body of literature documenting the association between individual characteristics and financial literacy, our understanding of the impact of macro-environmental conditions on individual financial literacy remains limited, particularly in later life. Drawing from a micro-macro perspective on the social environment and individual processes, we examined the extent to which three state-level contextual characteristics were associated with individual later-life financial literacy in the United States: tertiary educational attainment, poverty prevalence, and Internet penetration. We utilized data from the 2019 Understanding America Study (UAS) for adults aged 50 years or older to assess financial literacy (n=2,930), and data from the American Community Survey to evaluate contextual conditions. The UAS is a nationally representative survey panel supported by the Social Security Administration and the National Institute on Aging. Cross-sectional multilevel regression models were used to examine the hypothesized effects. We found that state-level poverty prevalence was negatively associated with individual financial literacy while state-level Internet penetration was positively associated with individual financial literacy, over and above individual characteristics known to impact financial literacy. No association was found between state-level educational attainment and individual financial literacy after controlling for respondents' own education. Findings suggest that the social environment may condition financial literacy in later life through exposure to opportunities that promote knowledge acquisition. Interventions to enhance later-life financial literacy may benefit from targeted approaches that take into account the environmental characteristics of their locations of residence.

### UNDERSTANDING THE PATHWAYS TO THE HEALTHY AGING FRAMEWORK: EMPIRICAL EVIDENCE AND FUTURE DIRECTIONS

Terry YS Lum, Shiyu Lu, Yingqi Guo, Yuqi Liu, and On Fung Chan, *The University of Hong Kong, Hong Kong, Not Applicable, Hong Kong*

The WHO's healthy ageing framework has highlighted the impact of the environment on health among older adults. However, their pathways are still understudied. This study investigates whether processes of functioning as an agency and that of developing belonging can help better understand the pathways to healthy ageing. We used two existing data, including community-dwellers aged 65 years and above (N=2,081 and N=1,553) in Hong Kong, by linking environmental data (population density, urban greenness, land-use diversity, and service destinations). Mental health variables were measured by the 15-item Geriatric Depression Scale (Study 1) and Mental Component Summary scale of the 12-item Short-form Health Survey (Study 2). The agency process was measured by social activities (SA) and functional ability (FA) (Study 1). The belonging process was measured by the 8-item Brief Sense of Community Scale (Study 2). Multilevel structural equation modeling was used in Study 1-2. Study 1 found that SA mediated the relationship between community facilities and depressive symptoms, while FA mediated the relationship between urban greenness and commercial facilities and depressive symptoms, providing support to the agency process. Study 2 found that land use mix and recreational services had positive total effects on both mental health, partially mediated by sense of community, providing support to the belonging process. Our findings enhance the theoretical understanding of healthy ageing by investigating two potential pathways and have implications for urban planning policy and health promotion. We discussed how to move forward by incorporating the ambulatory assessment and ecological momentary assessment into healthy ageing research.

### Session 3315 (Symposium)

### TESTING IMPLEMENTATION OF FFC-AL-EIT

Chair: Barbara Resnick Co-Chair: Marie Boltz

Residents in assisted living settings engage in limited amounts of physical activity and decline functionally more rapidly than peers in nursing homes. To address the persistent functional decline and increased time spent in sedentary activity Function Focused Care was developed. Function Focused Care involves teaching caregivers to evaluate residents' underlying functional capability and physical activity and engage them in physical activity during all care interactions. Prior research has demonstrated that implementing function focused care improves or maintains function and increases physical activity, improves mood and decreases behavioral symptoms among residents. To optimize implementation of Function Focused Care a theoretically based implementation strategy, Function Focused Care for Assisted Living Using the Evidence Integration Triangle (FFC-AL-EIT), was developed. FFC-AL-EIT combines the social ecological model, social cognitive theory and the Evidence Integration Triangle. The social ecological model includes intrapersonal, interpersonal, environmental, and policy factors that influence behavior. Social cognitive theory guides the interpersonal interactions that motivate caregivers and residents to engage in function focused care. Lastly, the Evidence Integration Triangle facilitates systemic implementation of function focused care. A total of 85 facilities from three states were randomized (FFC-AL-EIT versus Education Only) and 794 residents consented. The Reach, Effectiveness, Adoption, Implementation, and Maintenance (RE-AIM) model was used to evaluate outcomes. This symposium will provide the implementation outcomes and value of the Evidence Integration Triangle, the effectiveness of FFC-AL-EIT on function and physical activity and the effectiveness on psychosocial outcomes and care interactions.

### THE VALUE OF THE EVIDENCE INTEGRATION TRIANGLE

Barbara Resnick, *University of Maryland School of Nursing, Baltimore, Maryland, United States*

The Evidence Integration Triangle involved engaging stakeholders in the 12 month FFC-AL-EIT activities including identifying community specific goals, supporting the staff implementing the intervention, and intervening when champions or staff were not engaged in intervention activities. Ongoing participation of the stakeholder team occurred through monthly meetings. Evaluation of implementation was based on the Reach Effectiveness Adoption Implementation and Maintenance (RE-AIM) Model. Reach was based on 85 of 90 communities participating and 794 residents recruited. Effectiveness was supported based on less functional decline and more function focused care performed by residents. Adoption was based on evidence that monthly meetings were held, 77% of settings engaged as, or more than expected, and caregivers increased the amount of function focused care provided. The intervention was implemented as intended, knowledge was received, and environments and policies supporting function focused care were maintained. The Evidence Integration Triangle is an effective implementation approach for assisted living.

### THE IMPACT OF FFC-AL-EIT ON RESIDENT AND SETTING OUTCOMES

Marie Boltz, *Pennsylvania State University, University Park, Pennsylvania, United States*

FFC-AL-EIT was implemented by a Research Nurse Facilitator working with a community champion and stakeholder team for 12 months to increase function and physical activity among residents. FFC-AL-EIT included four steps: (Step I) Environment and Policy Assessments; (Step II) Education; (Step III) Establishing Resident Function Focused Care Service Plans; and (Step IV) Mentoring and Motivating. A total of 85 communities and 794 residents were included. The age of participants was 89.48 (SD=7.43), the majority was female (N=561, 71%) and white (N=771, 97%). Resident measures, obtained at baseline, four and 12 months, included function, physical activity, and performance of function focused care. Setting outcomes, obtained at baseline and 12 months, included environment and policy assessments and service plans. Effectiveness was based on less decline in function ($p<.001$), more function focused care ($p=.012$) and better environment ($p=.032$) and policy ($p=.003$) support for function focused care in treatment sites.

### THE EFFECTIVENESS OF FFC-AL-EIT ON PSYCHOSOCIAL OUTCOMES AND CARE INTERACTIONS

Elizabeth Galik, *University of Maryland, Baltimore, Maryland, United States*

This study included a subset of 59 communities and 550 residents from the full FFC-AL-EIT study. Participants were mostly white (98%), female (69%) and had a mean age of 89.30 (SD=7.63). Sites were randomized to the four step FFC-AL-EIT intervention implemented by a function focused care nurse facilitator working with a facility champion over 12 months versus education only. Resident measures included depression, agitation, resistiveness to care and the quality of care interactions and were obtained at baseline, 4 and 12 months. There was a significant positive treatment effect related to depression, agitation, resistiveness to care and quality of care interactions with either less decline or some improvement in these behaviors and symptoms and improvement in the quality of care provided between the treatment versus control group. The study suggests there is some benefit to implementing FFC-AL-EIT for psychosocial outcomes and care interactions among residents in assisted living communities.

### Session 3320 (Symposium)

### 2020 EDITOR'S CHOICE ARTICLES FROM JGMS: FOCUS ON VULNERABLE POPULATIONS

Chair: Lewis Lipsitz Discussant: Tamara Baker

This symposium will present four 2020 "Editor's Choice" articles from the Journal of Gerontology Medical Sciences that focus on issues relevant to vulnerable older populations. Justin Golub and colleagues, in their article "Audiometric Age-Related Hearing Loss and Cognition in the Hispanic Community Health Study", broaden the scope of age-related studies on audiometric hearing loss by using a large Hispanic cohort, a community largely excluded from previous hearing loss studies. By examining audiometrically-defined hearing loss and cognitive measures, Golub found links between hearing loss and lower neurocognition. Janice Atkins and colleagues, in "Preexisting Comorbidities Predicting COVID-19 and Mortality in the UK Biobank Community Cohort", challenge the practice of simple age-based targeting of older adults to prevent severe COVID-19 infections, and show that specific high-risk comorbidities are better indicators of hospitalization and mortality. "Comparison of Recruitment Strategies for Engaging Older Minority Adults: Results from Take Heart", by Jessica Ramsay and colleagues, examines methods used to recruit older adults of color from primarily low socio-economic households for behavioral and clinical health research. Ryon Cobb and coauthors, in their article "Self-reported Instances of Major Discrimination, Race/Ethnicity, and Inflammation among Older Adults: Evidence from the Health and Retirement Study", investigate whether self-reported lifetime discrimination is a psychosocial factor influencing inflammation in older adults. Tamara Baker, the discussant, will highlight commonalities and lessons learned from these studies, including links between racial, socio-economic, or disease-related vulnerabilities of older adults and their health status, as well as best practices to account for these factors in future clinical trials.

### MAJOR DISCRIMINATION, RACE-ETHNICITY, AND INFLAMMATION AMONG OLDER ADULTS

Lauren Parker,[1] Roland Thorpe, Jr.,[2] and Ryon Cobb,[3]
*1. Johns Hopkins Bloomberg School of Public Health, Baltimore, Maryland, United States, 2. Johns Hopkins Bloomberg School of Public Health, baltimore, Maryland, United States, 3. University of Georgia, Athens, Georgia, United States*

This study examines the relationship between self-reported instances of major discrimination and inflammation among older adults, and explores whether this relationship varies in accordance with race/ethnicity. Data from 2006/2008 Health and Retirement Study was used to collect measures of self-reported instances of major discrimination and high-risk C-reactive protein (CRP), which was assayed from blood samples. Modified Poisson regression with robust standard errors was applied to estimate the prevalence ratios of self-reported instances of major discrimination, as it relates to high-risk CRP (CRP ≥ 22 kg/m2), and test whether this relationship varies by race/ethnicity. Respondents who experienced any instances of major discrimination had a higher likelihood of high-risk CRP (prevalence ratio [PR]: 1.14, 95% confidence interval [CI] = 1.07–1.22) than those who did not report experiencing any instances of major discrimination. This relationship was weaker for blacks than whites (PR: 0.81, 95% CI = 0.69–0.95).

### AUDIOMETRIC AGE-RELATED HEARING LOSS AND COGNITION IN THE HISPANIC COMMUNITY HEALTH STUDY

Adam Brickman,[1] Adam Ciarleglio,[2] Nicole Schupf,[3] José Luchsinger,[1] and Justin Golub,[4] *1. Columbia University, New York, New York, United States, 2. George Washington University, Washington, DC, District of Columbia, United States, 3. Columbia University, Mailman School of Public Health, New York, New York, United States, 4. Columbia University, New york, New York, United States*

Studies associating age-related hearing loss (HL) with cognition have been limited by non-Hispanic cohorts, small samples, or limited confounding control. We overcome these limitations in the largest study of formal, audiometric HL and cognition to date using the multicentered Hispanic Community Health Study (n=5,277, mean age=58.4 [SD=6.2]). The main exposure was audiometric HL. The main outcome was neurocognitive performance. Adjusting for demographics, hearing aid use, and cardiovascular disease, a 20-dB increase (one-category worsening) in HL was cross-sectionally associated with worse performance in multiple neurocognitive measures: -1.53 (95% CI = -2.11, -0.94) raw score point difference on Digit Symbol Substitution Test, -0.86 (-1.23, -0.49) on Word Frequency Test, -0.76 (-1.04, -0.47) on Spanish-English Verbal Learning Test (SEVLT) 3 trials, -0.45 (-0.60, -0.29) on SELVT recall, -0.07 (-0.12, -0.02) on Six-Item Screener. Because HL is common and potentially treatable, it should be investigated as a modifiable risk factor for neurocognitive decline/dementia.

### COMPARISON OF RECRUITMENT STRATEGIES FOR ENGAGING OLDER MINORITY ADULTS: RESULTS FROM TAKE HEART

Cainnear Hogan,[1] Mary Janevic,[2] Rebecca Courser,[2] Kristi Allgood,[2] Cathleen Connell,[2] and Jessica Ramsay,[3]
*1. VA Ann Arbor Healthcare System, Ann Arbor, Michigan, United States, 2. University of Michigan School of Public Health, Ann Arbor, Michigan, United States, 3. University of Michigan School of Public Health, Detroit, Michigan, United States*

Few studies report best practices for recruiting older adults from minority, low SES communities for behavioral interventions. In this presentation, we describe recruitment processes and numbers for Take Heart, a randomized controlled trial testing the effectiveness of an adapted heart disease self-management program for primarily African American, low SES adults 50 years or older in Detroit. Community-based (CB), electronic medical record (EMR), and in-person hospital clinic (HC) recruitment methods were implemented. Within 22 months, 453 participants were enrolled, with an overall recruitment yield of 37%. The CB method had the highest yield (49%), followed by HC (36%) and EMR (16%). The average cost of recruiting and enrolling one participant was $142. Face-to-face interactions and employing a community health worker were particularly useful in engaging this population. Further research is needed to confirm these findings in other minority and low SES populations and share lessons learned about recruitment challenges and successes.

### PREEXISTING COMORBIDITIES PREDICTING COVID-19 AND MORTALITY IN THE UK BIOBANK COMMUNITY COHORT

Jane Masoli,[1] Joao Delgado,[2] Luke Pilling,[1] Chia-Ling Kuo,[3] George Kuchel,[3] David Melzer,[1] and Janice Atkins,[4], *1. University of Exeter, Exeter, England, United Kingdom, 2. University of Exeter Medical School, Exeter, England, United Kingdom, 3. University of Connecticut Health, Farmington, Connecticut, United States, 4. University of Exeter Medical School, University of Exeter, England, United Kingdom*

Hospitalized COVID-19 patients tend to be older and frequently have hypertension, diabetes or CHD, but whether these co-morbidities are more common than in the general older population is unclear. We estimated associations between pre-existing diagnoses and hospitalized COVID-19

alone or with mortality (during the first COVID-19 outbreak, tests performed between March 16 and April 26, 2020). In 269,070 UK Biobank participants aged 65+, 507 (0.2%) became COVID-19 hospital inpatients, of which 141 (27.8%) died. Common preexisting co-morbidities in hospitalized inpatients were hypertension (59.6%), history of falls/fragility fractures (29.4%), CHD (21.5%), T2 diabetes (19. 9%) and asthma (17.6%). However, in adjusted models, pre-existing diagnoses of dementia, T2 diabetes, COPD, pneumonia, depression, atrial fibrillation and hypertension emerged as independent risk factors for COVID-19 hospitalization, the first five remaining statistically significant for related mortality. There are specific high risk pre-existing co-morbidities for COVID-19 hospitalization and deaths in community based older men and women.

### Session 3325 (Symposium)

### A STAKEHOLDER-BASED STUDY IMPROVING RESIDENT AND FAMILY ENGAGEMENT IN THE SAFETY OF ASSISTED LIVING

Chair: Anna Beeber Co-Chair: Ruth Anderson
Discussant: Lindsay Schwartz

Assisted living (AL), is a long-term care service that provides housing and care for over 800,000 older adults in 30,000 residences. AL culture and operations have been transforming to enhance resident personhood and increase autonomy, however, these practices are balanced with the need to minimize safety issues (e.g., medication errors, infections, falls, and in cases of dementia, elopement and injuries). In this stakeholder-based study, we are translating existing strategies for improving patient safety to AL residences and developing an evidence-based tool for implementing these engagement strategies in AL. This symposium presents the methods and findings from a federally-funded mixed methods study including qualitative interviews with 105 AL residents, staff and family caregivers, and a series of focus groups with an AL stakeholder group to develop a toolkit to improve resident and family engagement in AL safety. The first paper outlines our methodological approach, including our efforts to work with stakeholders throughout the research process. The second paper reports findings from a scoping review of existing tools to support resident and family engagement in the safety of AL. The third paper presents the findings from our interviews with AL residents, families and staff exploring their safety priorities, and how they differ across stakeholder groups. The fourth paper presents the findings from our qualitative interviews exploring the challenges and promising practice to resident and family engagement in AL safety during the COVID-19 pandemic. All four presentations in this symposium illustrate important issues for future practice, policy, and research.

### STAKEHOLDER-BASED METHODS TO DEVELOP A TOOLKIT TO PROMOTE ENGAGEMENT IN ASSISTED LIVING SAFETY

Ruth Anderson,[1] Matthias Hoben,[2] Stephanie Chamberlain,[3] Victoria Bartoldus,[4] Stephanie Palmertree,[5] and Anna Beeber,[1] *1. University of North Carolina at Chapel Hill, UNC Chapel Hill, North Carolina, United States, 2. University of Alberta at Edmonton, Edmonton, Alberta, Canada, 3. University of Alberta, University of Alberta, Alberta, Canada, 4. University of North Carolina at Chapel Hill, Chapel Hill, North Carolina, United States, 5. University of North Carolina at Chapel Hill School of Nursing, Chapel Hill, North Carolina, United States*

This presentation provides and overview of a mixed-methods stakeholder engaged study to develop a toolkit to encourage resident and family engagement in the safety of assisted living (AL). This study uses stakeholder-based data and stakeholder engaged processes to adapt existing tools and strategies from other settings to encourage resident and family engagement in the safety of AL. We will improve resident safety in AL by developing an evidence-based tool to implement these engagement tools/strategies in AL. The presentation will outline the theoretical base, the approach for this study, including efforts to recruit and retain stakeholders throughout the study, and stakeholder engaged process to develop the toolkit. The presentation will include challenges and strategies to encourage participation of AL staff, residents, and family caregivers in the study. The presentation will conclude with a discussion of implications for future design and research efforts aiming to impact AL care, policy, and research implementation.

### TOOLS TO ENHANCE RESIDENT AND FAMILY ENGAGEMENT IN ASSISTED LIVING SAFETY: A SCOPING REVIEW.

Jennifer Leeman,[1] Marianne Baernholdt,[1] Christine Kistler,[2] Elizabeth Moreton,[3] Terri Ottosen,[3] Pam Dardess,[4] Anna Beeber,[5] and Thi Vu,[6] *1. University of North Carolina at Chapel Hill School of Nursing, Chapel Hill, North Carolina, United States, 2. University of North Carolina at Chapel Hill Division of Geriatric Medicine and Dept. of Family medicine, Chapel Hill, North Carolina, United States, 3. University of North Carolina at Chapel Hill Health Sciences Library, Chapel Hill, North Carolina, United States, 4. Institute for Patient- and Family-Centered Care, McLean, Virginia, United States, 5. University of North Carolina at Chapel Hill, UNC Chapel Hill, North Carolina, United States, 6. Yale University, New Haven, Connecticut, United States*

This presentation reports the results of a scoping review which identified and evaluated existing engagement strategies, tools, and interventions for their fit with assisted living (AL). Using the PRISMA criteria, we evaluated 54 empirical studies in assisted living/residential care or nursing homes (NH) for how they engaged families and residents, promoted person-centered and/or safety in AL/NH care, and assessed relevant outcomes (safety, experience, service use, satisfaction with care, health behaviors, and quality of life). The strategies, tools, and interventions aimed to improve residents' activities of daily living, function, and quality of life. Studies also targeted staff and family caregivers to increase knowledge, improve relationships, and decrease caregiving stress. Overall, the studies reported statistically significant changes in resident quality of life, agitation, antipsychotic use, staff knowledge and job satisfaction. Results from this systematic review will inform the development of a testable toolkit to increase engagement and improve safety in AL.

### UNDERSTANDING RESIDENT, FAMILY, AND STAFF SAFETY PRIORITIES TO GUIDE DEVELOPMENT OF AN ENGAGEMENT TOOLKIT

Victoria Bartoldus,[1] Cloie Chiong,[2] Tabitha Linville,[3] Stephanie Palmertree,[3] Anna Beeber,[4] and Youngmin Cho,[1], *1. University of North Carolina at Chapel Hill, Chapel Hill, North Carolina, United States, 2. University of North Carolina at Chapel Hill Gillings school of Global Public Health, Chapel Hill, North Carolina, United States, 3. University of North Carolina at Chapel Hill School of Nursing, Chapel Hill, North Carolina, United States, 4. University of North Carolina at Chapel Hill, UNC Chapel Hill, North Carolina, United States*

Resident and family engagement (the desire, ability, and activation as a partner in care) is a necessary component of keeping assisted living (AL) residents safe. Barriers to engagement include differing priorities between the resident/family and staff. This presentation outlines the results of a content analysis of qualitative interviews with 105 AL staff, residents, and family members, in which we examined AL stakeholder priorities for safety. Qualitative interviews were analyzed to first identify safety priorities by stakeholder type (staff, resident, and families), and then compared across stakeholder group. Stakeholder-specific safety priorities were identified, including infection management (COVID-19 and others), medications errors, falls, elopement, lack of AL resources/staffing, conflict, adverse events, nutrition, physical hazards, building security, chemical agents, fire/natural disasters, and abuse/neglect – the importance of these priorities vary by stakeholder type. Presentation discussion will include implications for future intervention to address the top safety problems in AL.

### ENGAGEMENT IN ASSISTED LIVING DURING THE COVID-19 PANDEMIC: CHALLENGES AND PROMISING PRACTICES.

Youngmin Cho,[1] Janelle Perez,[2] Jing Wang,[3] Stephanie Palmertree,[2] Anna Beeber,[4] and Victoria Bartoldus,[1], *1. University of North Carolina at Chapel Hill, Chapel Hill, North Carolina, United States, 2. University of North Carolina at Chapel Hill School of Nursing, Chapel Hill, North Carolina, United States, 3. Fudan University, Chapel Hill, North Carolina, United States, 4. University of North Carolina at Chapel Hill, UNC Chapel Hill, North Carolina, United States*

The "lockdown" in assisted living (AL) from the COVID-19 pandemic has physically isolated residents from the outside world and affected resident and family engagement in care. This presentation outlines a content analysis of qualitative semi-structured telephone interviews conducted from April 2020 with 105 AL staff, residents, and family members exploring COVID-19 experience/restrictions and engagement during the pandemic. Analysis revealed AL families and residents expressed difficulties with COVID-19 visiting and distancing restrictions, reduced family visitations, discontinuity of care, and worries about COVID-19 infection. Staff/administrators expressed uncertainty about lack of knowledge about COVID-19, worries about transmission, and if staff will get exposed outside of work. Promising factors include enhanced communication between staff and families regarding care, improved virtual communication, creative strategies to socially engage residents, and improved infection control practices and staff training. The presentation discusses the implications of the findings for future research, policy, and practice.

### Session 3330 (Symposium)

### A SYSTEMS-WIDE APPROACH FOR EARLY DETECTION AND MANAGEMENT OF DEMENTIA IN PRIMARY CARE

Chair: Annette Fitzpatrick Discussant: Basia Belza

Dementia is frequently unrecognized and under-reported by health care providers. The needs of an aging population increase the burden on an already over-worked primary care system that is often without the appropriate training, resources, and reimbursement to address the growing number of people with cognitive decline in the US. In this symposium we present a systems-wide approach within University of Washington (UW) Primary Care to increase awareness of early signs and symptoms, detection of cognitive impairment, and support of providers, patients and caregivers that will ultimately improve outcomes of care. This quality improvement (QI) program integrates stakeholder-selected components of the GSA KAER (Kickstart-Assess-Evaluate and Refer) Model and Toolkit (2020 Edition), developed by the Gerontological Society of America (GSA), into primary care practice. We describe content and logistics of a continuing education intervention for primary care providers and clinical staff to increase skills for evaluation and management of dementia. Working with UW clinic managers and information technology (IT), we have developed a pragmatic system for streamlining operations and documenting care utilizing newly developed interdisciplinary workflows and electronic health record order sets. Using input from our Community Advisory Board, we explain development of a web-based resource directory to be used in-clinic and at home to support providers, staff, patients, families, and caregivers across cognitive changes. Strategies presented here are aimed to help other health care systems initiate steps to integrate KAER and other tools into a practical QI program for improving detection and management of dementia through support of primary care.

### INTEGRATING THE GSA KAER TOOLKIT INTO PRACTICAL TRAINING FOR PRIMARY CARE CLINICS

Jaqueline Raetz,[1] Basia Belza,[1] Monica Zigman Suchsland,[2] Judit Illes,[3] Benjamin Olivari,[4] Lisa McGuire,[5] Annette Fitzpatrick,[1] and Barak Gaster,[1], *1. University of Washington, Seattle, Washington, United States, 2. University of Washington, Seattle, University of Washington, Washington, United States, 3. The Gerontological Society of America, Washington, District of Columbia, United States, 4. Centers for Disease Control and Prevention (CDC), Atlanta, Georgia, United States, 5. CDC, Atlanta, Georgia, United States*

Education is central to driving change in clinical practice. First, primary care providers and their clinic team members need to understand why detecting cognitive impairment is important, how it can be done efficiently, and what the next steps in referral and management are. To engage primary care clinics in this change process, we developed a continuing education intervention, based on the KAER Model, using a live video format. Four evidence-based, 45-minute training modules presented core knowledge skills, including how to have difficult conversations, which are essential to diagnosing cognitive impairment. To overcome the obstacles to doing so in primary care, our team relied on a deep understanding of busy primary care practice. With a combined 35 years of direct experience in primary care, our collaborative interdisciplinary team was able to use the KAER Model to develop a highly acceptable intervention for primary care.

### IMPLEMENTATION OF THE GSA KAER TOOLKIT IN A LARGE CLINIC SYSTEM: WORKFLOW MODIFICATIONS AND EMR TOOLS

Barak Gaster,[1] Basia Belza,[1] Monica Zigman Suchsland,[2] Judit Illes,[3] Benjamin Olivari,[4] Lisa McGuire,[5] Annette Fitzpatrick,[1] and Jaqueline Raetz,[1]
*1. University of Washington, Seattle, Washington, United States, 2. University of Washington, Seattle, University of Washington, Washington, United States, 3. The Gerontological Society of America, Washington, District of Columbia, United States, 4. Centers for Disease Control and Prevention (CDC), Atlanta, Georgia, United States, 5. CDC, Atlanta, Georgia, United States*

We implemented the KAER toolkit in the University of Washington primary care clinics. In this session we share the workflows implemented to promote the KAER model and share the tools we developed within EPIC, the system's electronic medical record (EMR). We collaborated with clinic staff to develop interdisciplinary workflows including: training patient service representatives, social workers, nurses, and medical assistants (MAs) about 'red flags;' training medical assistants to complete the Patient Health Questionnaire (PHQ-9) and Montreal Cognitive Assessment (MoCA); and assuring they are appropriately entered into flowsheets in EPIC. We created a checklist (EPIC 'SmartPhrase') and educated the clinics' interdisciplinary teams to utilize it within their scope of practice. Additionally, we created an order set (EPIC 'SmartSet') of commonly ordered tests and referrals to expedite evaluation of patients with suspected cognitive impairment. Lastly, we created a direct link from our EMR to our website containing community resources.

### COGNITION IN PRIMARY CARE COMMUNITY RESOURCE DIRECTORY FOR INDIVIDUALS, CAREGIVERS, AND PROVIDERS

Annette Fitzpatrick,[1] Barak Gaster,[1] Jaqueline Raetz,[1] Judit Illes,[2] Benjamin Olivari,[3] Lisa McGuire,[4] Basia Belza,[1] and Monica Zigman Suchsland,[5] *1. University of Washington, Seattle, Washington, United States, 2. The Gerontological Society of America, Washington, District of Columbia, United States, 3. Centers for Disease Control and Prevention (CDC), Atlanta, Georgia, United States, 4. CDC, Atlanta, Georgia, United States, 5. University of Washington, Seattle, University of Washington, Washington, United States*

A KAER Model recommendation is to refer individuals diagnosed with dementia to resources that help them prepare for the future and services that provide ongoing support. The purpose of this project was to locate local quality services and develop a resource directory for persons with cognitive impairment for use by providers, staff, individuals, families, and caregivers. We worked with a Community Advisory Board and interviewed individuals and caregivers to understand what resources are useful and important to include in the resource directory. We built a web-based resource directory that allows users to query resources based on specific needs. We integrated the resource directory within the electronic health record for providers to include after visit summaries. A resource directory was deployed for community use, with goals of sustainability and longevity after this project is completed.

### Session 3335 (Symposium)

### APPROACHING MULTIMORBIDITY FROM A TRANSLATIONAL GEROSCIENCE PERSPECTIVE

Chair: Anne Newman

Multimorbidity describes the accumulated burden of chronic disease. Multimorbidity erodes physiologic reserve, increasing the risk of frailty, disability and death. Most older adults have at least one chronic health condition by age 65. Once established, many age-related conditions progress and accumulate with age. Geroscience holds that there are key biologic pathways that explain the increase with age in multimorbidity, frailty and disability Translation of geroscience principles to human studies requires careful assessment of biomarkers of these pathways and multisystem outcomes. In this symposium, translational researchers in geriatric medicine and gerontology will present current work to elucidate biologic underpinnings of aging and potential intervention targets. We will address whether blood biomarkers of aging processes are prognostic using combinatorial techniques and explore the potential for proteomics to identify novel pathways for health aging. New insights into the role of inflammation will be discussed with an emphasis on its relationship to multimorbidity. Brain aging will be considered with respect to the interactions between external stressors and resilience evaluating the role of ketone bodies which have immunomodulatory effects particularly on innate immune cells. Finally, the role of multimorbidity as an intervention target and potential intermediate outcomes including biomarkers will be presented with discussion of next steps needed to realize the potential for translational geroscience clinical trials to improve health span.

### OPERATIONALIZING HEALTHSPAN AS AN OUTCOME FOR CLINICAL TRIALS IN GEROSCIENCE

Jamie Justice, *Wake Forest School of Medicine, Wake Forest School of Medicine, North Carolina, United States*

Efforts targeting biological aging pathways are advancing interventions which could extend healthy lifespan. Design of clinical trials to test such interventions necessitates an operational definition of healthspan, such as slowed accumulation or progression of multiple chronic diseases, functional decline, and disability. In this talk we explore these composite measures of healthspan proposed as outcomes for clinical trials in aging. This will be examined in example cases including multimorbidity and deficit accumulation frailty indices in an 8-Year intensive lifestyle intervention trial, and an update on multimordbity, functional, and biomarker endpoints in the trial Targeting Aging with MEtformin (TAME). Through these examples we will explore issues related to effect sizes and statistical challenges related to composite endpoints. Finally, we will discuss the role existing and emerging biomarkers of aging in clinical trials in geroscience and summarize evidence linking biomarkers to clinically meaningful outcomes.

### UNBIASED PROTEOMICS AND TARGETED BIOMARKERS ASSOCIATED WITH EXCEPTIONAL LONGEVITY AND MULTIMORBIDITY IN HUMANS

Jason Sanders, *Brigham and Women's Hospital, Boston, Massachusetts, United States*

Biomarkers ideal for geroscience trials could be those simultaneously identified using targeted and discovery assays and which strongly associate with complementary disease (multimorbidity) and longevity (exceptional survival) outcomes. To identify a tractable set of biomarkers for use in geroscience trials, we used the Cardiovascular Health Study (CHS), whose participant makeup closely aligns with the Targeting Aging with MEtformin (TAME) trial. In ~4800 CHS participants, quantitative assays of nine a priori-identified biomarkers were used to construct a biomarker index which strongly associated with the TAME primary outcome of mortality and multimorbidity over 6 and 10 years of follow-up. In ~3000 CHS participants, 1300 proteins were measured with unbiased aptamer proteomics and associated with survival to age 90 over 25 years of follow-up. Proteins in the biomarker index were identified as some of the strongest associated with survival to 90. This convergent evidence suggests these biomarkers may be well-suited for geroscience trials.

### CHRONIC INFLAMMATION AND THE ACCELERATION OF CHRONIC DISEASE STATES

Jeremy D. Walston, *Johns Hopkins University School of Medicine, Baltimore, Maryland, United States*

The chronic activation of the immune system is commonly observed in older adults, and is highly associated with multiple chronic disease states and Geriatric syndromes including physical frailty, sarcopenia and mild cognitive impairment. Chronic inflammation is multifactorial, and the individual inflammatory mediators that drive the development and propagation of disease states impact normal tissue homeostasis as well as stem cell vitality. This session will discuss age-related etiologies of chronic inflammation and specific inflammatory mediators and their measurement, including Tumor Necrosis Factor (TNF) alpha and its receptors. Inflammation-driven molecular pathways that most impact relevant chronic disease states such as the tryptophan degradation pathway, and its relationship to pathophysiological changes, will also be considered. Finally, discussion of potential treatment modalities, including several emerging from Geroscience research, will be described as will their impact on chronic disease states.

### IMMUNOMETABOLIC STRESS AND KETONE BODIES IN DISORDERS OF THE AGING BRAIN

John Newman, *Buck Institute for Research on Aging, Novato, California, United States*

Delirium is an acute confusional state that is a common complication of acute illness in older adults, and is associated with increased risk of death, disability, and dementia. Delirium in older adults is an example of a geriatric syndrome, with multifactorial, multi-system causes that include existing aging-related physiological changes as well as external acute stressors. Its pathophysiology delirium is not well understood but may include glycolytic energy deficits associated with acute inflammation in the brain. The endogenous ketogenic system provides ketone bodies as a lipid-derived alternative to glucose for cellular energy, and ketone bodies are increasingly understood to have immunomodulatory effects particularly on innate immune cells. We used a mouse model of acute inflammation-associated behavioral change to investigate how age-related differences in energy utilization in the brain affect delirium-like phenotypes, focusing on energy metabolism and innate immune activation in the brain as an example of immunometabolic approaches to geriatric syndromes.

### Session 3340 (Symposium)

### BEHAVIORAL HEALTH, SOCIAL ENGAGEMENT, AND LONG-TERM CARE SERVICES USE AMONG COMMUNITY OLDER ADULTS: USA VS. TAIWAN

Chair: Su-I Hou Co-Chair: Chien-Ching Li
Discussant: Darren Liu

As healthcare advances, older adults are living longer. While 90% of older adults prefer aging in their own homes and communities, it is important to examine key factors influencing healthy aging-in-community and community-based long-term care (LTC) services available in different countries. This symposium examines behavioral health, social engagement, and LTC services utilization among community-dwelling older adults in the USA and Taiwan. Lessons learned from older adults across countries will provide insights for tailored community-based LTC services and program development. Dr. Hou from The University of Central Florida (UCF) will highlight similarities and differences in behavioral health profiles and the topics that most interest community-dwelling older Americans participating in three aging-in-community programs in Central Florida. Dr. Wang from Case Western Reserve University will examine the impact of neighborhood social cohesion on mobility among community-dwelling older Americans aged 65 and older from the national Health and Retirement Study. Dr. Liu from National Cheng-Kung University in Taiwan will share results of healthy lifestyle on quality of life among community-dwelling older adults in southern Taiwan. Dr. Young from State University of New York at Albany will compare long-term care use among community-dwelling older adults with and without dementia in Central Taiwan. Finally, Drs. Cao and Hou from UCF will analyze home and community-based services in the USA versus Taiwan. This symposium will further discuss similarities and differences of key factors related to healthy aging-in-community, along with practical recommendations and lessons learned across countries and cultural environments to improve community-based long-term care services and programs.

### BEHAVIORAL HEALTH AND MOST-INTERESTED TOPICS AMONG OLDER ADULTS IN THREE AGING-IN-COMMUNITY PROGRAMS IN FLORIDA

Su-I Hou, *School of Global Health Management & Informatics, University of Central Florida, University of Central Florida, Florida, United States*

This study compares behavioral health and most-interested topics among older adults in three aging-in-community (AIC) programs: a university-based lifelong learning program (LLP; 38%), a county neighborhood lunch program (NLP; 29%), and a village program sample (33%) (total n=289). Mean age was 72.4 (SD=8.68) years. Although perceived health was similar (mean=3.76), LLP and village members reported higher quality of life than NLP participants (p=.004). Two-thirds of the participants indicated at least half of their daily plates filled with fruits and vegetables, and at least 10 min. walking in 4.5 days during a typical week. The duration of each walking was lower among NLP members (23 min.), compared with village (31 min.) or LLP members (35 min.) (p=.002). The top three most interested topics were brain health, giving back, and keep community healthy. Older adults in AIC programs were overall healthy and active. Results have implication on tailored program development.

### NEIGHBORHOOD COHESION AND MOBILITY LIMITATIONS AMONG OLDER AMERICANS: THE ROLES OF DEPRESSIVE SYMPTOMS AND MASTERY

Weidi Qin,[1] Jiao Yu,[2] and Fei Wang,[3], *1. Case Western Reserve University, Cleveland, Ohio, United States, 2. Department of Sociology, College of Arts and Sciences, Case Western Reserve University, Cleveland, Ohio, United States, 3. Case Western Reserve University, Cleveland Heights, Ohio, United States*

Neighborhood environment has been playing an important role in late-life health; yet, the social aspect of neighborhood environment and its impact on mobility limitations have been rarely examined. This study examines the relationship between neighborhood social cohesion and mobility limitations and the potential mediators (i.e., depressive symptoms, mastery) of this relationship. A total of 8,317 Americans aged 65 and older were selected from the Health and Retirement Study. Using ordinary least squares (OLS) regressions, this study shows that neighborhood social cohesion is negatively associated with mobility limitations, and this association is mediated by depressive symptoms and mastery respectively. Specifically, neighborhood social cohesion can reduce mobility limitations through mitigating depressive symptoms and increasing mastery. The findings have implications for developing community measures to promote neighborhood social cohesion and applying psychosocial interventions to reduce depressive symptoms and improve mastery among older adults.

### HEALTHY LIFESTYLE DOES MATTER FOR COMMUNITY-DWELLING ADULTS PURSUING QUALITY OF LIFE

Li-Fan Liu, *National Cheng Kung University, National Cheng Kung University, Tainan, Taiwan (Republic of China)*

It has been well documented that socioeconomic factors influence lifestyle behaviors and all the physical and mental status at the individual level do matters for elderly people experiencing healthy aging. This study aimed to explore to what extent the healthy lifestyle including exercise and social participation influence on the health status of the community dwellings and their quality of life in Taiwan. Using a cross-sectional survey design, 1032 adults, aged ≥ 50 years, were interviewed with complete data from four communities in southern Taiwan. The results showed that for older community dwellings adults, doing exercise was significantly associated with feeling less stress, less depress, higher life satisfaction and higher quality of life (p<0.001). Being volunteers was found to be significantly associated with better quality of life (p<0.001). In the aging society, it is necessary to apply multifaceted approaches extending from individual solutions to public policy efforts in promoting healthier lifestyles.

### A COMPARISON OF LONG-TERM CARE USE BY COMMUNITY-DWELLING OLDER ADULTS WITH AND WITHOUT DEMENTIA

Ya-Mei Chen,[1] Kuo-Piao Chung,[1] HSIU-HSI CHEN,[2] Yen-Po Yeh,[3] and Yuchi Young,[4], *1. National Taiwan University, School of Public Health, Taipei, Taipei, Taiwan (Republic of China), 2. National Taiwan University, National Taiwan University, Taipei, Taiwan (Republic of China), 3. Changhua County Public Health Bureau, Changhua City, Changhua, Taiwan (Republic of China), 4. SUNY at Albany, Rensselaer, New York, United States*

Introduction. This study compares long-term care (LTC) use among community-dwelling older adults with and without dementia. Methods. Participants (n=14,483) were aged 65+ residents of Changhua County, Taiwan who qualified for LTC services. Data were collected (4/1/2017-10/26/2018) through health assessments. Multivariate logistic regression quantifies the study aim. Results. Preliminary results show that on average participants with dementia are older than people without dementia (81.1 vs. 80.5; p<.001), more females (13.4% vs. 8.0%; p<.001), higher mean ADL (12,4 vs. 9.8; p< .001) and IADL (21.4 vs. 17.8; p<.001), and lower mean comorbidity (2.5 vs. 2.8; p<.001). Multivariate regression results indicate people with dementia use twice the health-related LTC services than their counterpart (OR= 2.0; 95% CI 1.90–2.14). Discussion. People with dementia use more health-related LTC services. Future dementia studies should examine the pattern of non-health-related LTC services concomitant with health-related services, so that person-centered care can be tailored to foster aging-in-community.

### HOME AND COMMUNITY-BASED SERVICES BETWEEN THE USA AND TAIWAN

Su-I Hou,[1] and Xian Cao,[2], *1. School of Global Health Management & Informatics, University of Central Florida, University of Central Florida, Florida, United States, 2. University of Central Florida, University of Central Florida, Florida, United States*

Home and community-based services (HCBS) are critical to support our rapidly growing and aging population around the world. This paper takes initial steps to compare HCBS between the USA and Taiwan from four perspectives: funding sources, service types, challenges, and recommendations. Peer-reviewed articles and governmental reports (both

English and Chinese) in the U.S.A. and Taiwan were reviewed. Analyses showed both countries mainly use tax dollars to fund HCBS. Although both countries have similar service categories, USA lack a clear organization scheme whereas Taiwan has detailed and clear services provisions. Workforce quality and shortage were common challenges for both countries, especially from culture perceptive. Recommendations for USA include expanded funding pool, better coordination between agencies, and rebalancing HCBS and institutional care with limited budget. Recommendations for Taiwan include expanded service coverage and quality to reduce disparity in rural areas, and providing more support for informal caregivers.

### Session 3345 (Paper)

### BROADENING THE VIEW OF GERONTOLOGICAL EDUCATION AND PRACTICE

### ALZHEIMER'S DISEASE AMONG AMERICAN MINORITY POPULATIONS: AN ECOLOGICAL EXPLORATORY STUDY

Maria LaQuaglia,
Marina Celly Martins Ribeiro de Souza, and
Carolina Marques Borges, *The College of New Jersey, The College of New Jersey, New Jersey, United States*

A significant public health concern with regards to increasing rates of Alzheimer's is that it disproportionately affects minority groups in the United States. The present ecological exploratory study uses secondary aggregate data from the fifty United States in the year of 2019. The purpose of this study was to address the disparities in Alzheimer's in minority populations in the US and explore associated factors. The "minority" populations considered were African American and Latino populations, and the "majority" population was referred to as "white". The data was extracted from the United States Census Bureau, the CDC National Center for Health Statistics, and the Behavioral Risk Factor Surveillance System (BRFSS) Dataset. The prevalence rates of Alzheimer's disease are greatest in both older Latinos (12.2%) and African Americans (13.8%), compared to older whites (10.3%) in the investigated time period. Our results showed that being over 65 years old (p=.009), with a below-average ($62,843) median household income (p=.024), history of stroke (p=.029), and being a part of the Latino population (p=.036), were significantly associated with Alzheimer's mortality rates in the United States. By identifying disparities in access to Alzheimer's healthcare and at-risk communities, more comprehensive intervention strategies can be developed to promote change and advocate for more Alzheimer's education and resource allocation for minority populations.

### DIFFERENCES IN GERIATRIC-FOCUSED EDUCATION PREPARATION AND BELIEFS ABOUT PROVIDING CARE TO OLDER PATIENTS IN GHANA

Grace Karikari,[1] and Samuel Asante,[2] *1. University of North Dakota, Grand Forks, North Dakota, United States, 2. Northeastern State University, Tahlequah, Oklahoma, United States*

With the increasing growth rate of older persons and a rise in related illnesses in Ghana, it is vital that health practitioners are equipped with geriatric-oriented knowledge and expertise to provide the needed services to geriatric patients. The purpose of this study was to examine (i) the differences in the level of geriatric-focused educational preparation between graduating medical and nursing students in a Ghanaian public institution, and (ii) the association between educational preparation and students' beliefs about providing care to geriatric patients. The study hypothesized that students with more positive beliefs will be linked to higher educational preparation. Descriptive and inferential statistics were conducted (n=136 students). Findings show significant differences in the educational preparation of medical and nursing students (t [134] = -3.790, p < .001). Graduating nursing students, comparatively, had higher educational preparation (M=23.14, SDe3.30) than medical students (M=21.14, SD=2.76). However, there was no significant association between students' educational preparation and their beliefs about geriatric care. The findings underscore the need for extensive or more focused training. Further, the association between beliefs and educational preparation warrants further exploration. The need to examine the social cultural environment within which the research participants reside are discussed.

### ONLINE GERONTOLOGY COURSES: ISSUES OF EQUITY

Anita Sharma, *University of Louisiana at Monroe, Monroe, Louisiana, United States*

The COVID-19 Pandemic changed higher education in several significant ways. The most significant impact was on methods of course delivery. In March 2020, all educational institutions changed their methods of instruction to fully online instruction. It happened almost overnight and left the students as well as the instructors unprepared for the unanticipated metamorphosis . The sudden and unanticipated change in the method of instruction and delivery of course contents also highlighted issues of equity. There appeared to be high levels of inequity in the use of technology across school and college campuses. A survey of students conducted by the author at the University of Louisiana at Monroe revealed different types of inequity such as, lack of finances to buy equipment, lack of training in the use of technology, and lack of personal space to study from home. A significant percentage of student population at ULM consists of first-generation college students. These students were impacted the most by the new methods of course delivery. Additionally, the author looked up similar surveys at other educational institutions and conducted a meta-analysis of published studies. This paper presents these findings.

### THE STATE OF GERONTOLOGICAL TRAINING IN GHANA: IMPLICATIONS FOR A SPECIALIZED WORKFORCE AND THE AGING POPULATION

Samuel Asante,[1] and Grace Karikari,[2], *1. Northeastern State University, Tahlequah, Oklahoma, United States, 2. University of North Dakota, Grand Forks, North Dakota, United States*

The rise in older population in Ghana is accompanied by several challenges that may require trained professionals with specialized knowledge in geriatrics and gerontology to help address. Extensive review of existing literature, however, indicates a lack of comprehensive geriatric focused training in Ghana's education system. Unfortunately, the scope of this training deficiency, potential impact on the geriatric workforce, as well as the health and wellbeing of the aging population on the national level have not been extensively examined. With the older adult population set to increase over the next few years, the need for geriatric-trained human service professionals in diverse disciplines, including medicine, nursing, psychology, social work and public health has become more pronounced. This paper is a review of the current state of gerontological education in higher institutions in Ghana. Specifically, authors examined (1) current geriatric-focused training programs available to students in all public/government owned institutions, (2) existing national level programs, and policies addressing training deficiencies, and (3) the implications of findings for future geriatric workforce as well as the health and wellbeing of older Ghanaians. The findings call attention to the need for a nationwide geriatric-focused training system and policies aimed at prioritizing specialized care for the older population. Culturally appropriate recommendations for integrating gerontological training and education in tertiary institutions are discussed. Guidelines and standards based on the Academy for Gerontology in Higher Education (AGHE) Competencies for Undergraduate and Graduate Education are proposed.

### WHAT DO ISRAELI GERONTOLOGY STUDENTS THINK ABOUT MEDICAL MARIJUANA USE FOR ALZHEIMER'S AND PARKINSON'S DISEASE?

Offer Edelstein,[1] Richard Isralowitz,[2] Oren Wacht,[2] Alexander Reznik,[2] and Yaacov Bachner,[3], *1. Ben-Gurion University of the Negev, Beer-Sheva, HaDarom, Israel, 2. Ben-Gurion University of the Negev, Be'er-Sheva, HaDarom, Israel, 3. Ben-Gurion University, Beer-Sheva, HaDarom, Israel*

**Aims:** The aims of the current study were as follows: 1) to assess gerontology graduate students' beliefs about medical marijuana's (MMJ) effectiveness for two common age-related conditions - Alzheimer's (AD) and Parkinson's disease (PD); 2) to assess students' beliefs and attitudes toward MMJ; 3) to explore associations linking background characteristics, MMJ-related attitudes and beliefs, and beliefs about the MMJ effectiveness for AD and PD.

**Method:** A sample of 104 (84 women and 20 men) gerontology graduate students voluntarily participated in an anonymous online survey.

**Results:** The vast majority (95%) of the participants indicated they had no formal education about MMJ and reported being unprepared to answer clients' MMJ-related questions (84.6%). Most of the participants believed that MMJ is effective for use with AD (70.2%) and PD (80.8%) patients. Participants reported favorable beliefs about MMJ benefits, concerns about risks, the need for training, and positive attitudes toward recreational marijuana use legalization. Prior marijuana use (e.g., self-use, friends or family) was found to be associated with more positive beliefs about MMJ benefits, risks, and its legalization for recreational purposes. Prior marijuana use was the only factor associated with the belief that MMJ is an effective therapy for use with individuals diagnosed with AD or PD.

**Conclusions:** The study findings stress the need for students' MMJ education in order to provide future gerontology service providers with the necessary knowledge and ability to address clients' questions about MMJ use. Efforts to develop curricula and training programs need to be promoted.

### Session 3350 (Symposium)

### CONGRESSIONAL UPDATE

Chair: Brian Lindberg

This popular annual session will provide cutting-edge information on what the 117th Congress has and has not accomplished to date, and what may be left for end of the First Session. Speakers will discuss key issues such as pandemic relief, Social Security, Medicare, Medicaid, and the Older Americans Act.

### Session 3355 (Symposium)

### DISASTER PREPAREDNESS AND COPING AMONG OLDER ADULTS: EMPIRICAL ANALYSIS AND VIRTUAL REALITY PLATFORM DEVELOPMENT

Chair: Zhen Cong

This symposium included 4 studies that use national and regional data to examine older adults' disaster preparedness and coping. The first study examined age differences in preparedness for the continuation of COVID-19 with a sample of 443 residents in Dallas, TX. The findings highlight older adults' resilience and special needs for different types of support during the pandemic. The second study examined the association of having COVID-19 and intergenerational relationships using the COVID-19 module of the Health and Retirement Study with a sample of 3266 respondents. Using a national sample of 1,467 respondents from the 2017 U.S. National Household Survey, the third study examined age differences relationships among the type of disasters (i.e., disasters with different lead-time), response efficacy, and disaster preparedness. The findings highlighted older adults' unique vulnerability and resilience in different types of disasters. The fourth study discussed a pilot virtual reality platform under development to assist older adults to develop tailored household emergency preparedness plans and practice those plans with simulated extreme weather conditions and warnings for older adults to practice disaster response and develop relevant knowledge and skills as well as test and revise their emergency preparedness plans. Overall, this symposium emphasizes the uniqueness of older adults' needs, vulnerability, and resilience to disasters.

### AGE DIFFERENCES IN PREPAREDNESS FOR THE CONTINUATION OF COVID-19: IMPORTANT ROLES OF SOCIAL SUPPORT

Zhen Cong,[1] Daan Liang,[2] Guofeng Cao,[3] and Zhirui Chen,[1], *1. University of Texas at Arlington, Arlington, Texas, United States, 2. University of Alabama, Tuscloosa, Alabama, United States, 3. University of Colorado Boulder, Boulder, Colorado, United States*

This study examined the association between age and preparedness for the continuation of COVID-19. The moderation effects of three types of social support, namely, emotional, financial, and instrumental assistance were also tested. Using a sample of 443 adults in Dallas county which has the most confirmed cases in Texas, results of multiple linear regressions showed that compared to those aged between 18 and 64, older adults aged 65 and reported better preparedness for the continuation of COVID-19. Receiving emotional, financial, and instrumental assistance were respectively more important for older people to get better prepared than for younger adults, which is consistent with the socioemotional selectivity theory. Our findings directed attention to the strengths and resilience of older adults during COVID-19 from a life course perspective and highlighted the importance of social support and social relationship in their post-disaster recovery and ongoing preparedness.

### THE COVID-19 PANDEMIC AND INTERGENERATIONAL SUPPORT: RESULTS BASED ON THE HEALTH AND RETIREMENT STUDY COVID-19 MODULE

Zhen Cong,[1] and Guanggang Feng,[2] *1. University of Texas at Arlington, Arlington, Texas, United States, 2. University of Texas at Arlington, School of Social Work, Texas, United States*

This study examined relationships between COVID-19 exposure and intergenerational support patterns. The data was from the 2020 Health and Retirement Study (HRS) COVID-19 Module (N=3266). The latent class analysis (LCA) was used to identify the types of intergenerational support based on respondents' reports on whether they provided and received financial and instrumental support from either coresident children or non-coresident children. Two classes were identified, namely, the high interaction group and the low interaction group. Logistic regression showed that respondents who had COVID-19 and had increased spending as a result of COVID-19 were more likely to be in the high interaction group. Other types of COVID-19 exposure, i.e., knowing someone being diagnosed or knowing someone who died from COVID-19 were not significant.

### RESPONSE EFFICACY AND ACTION TO PREPARE FOR DISASTERS WITH DIFFERENT LEAD TIME: AGE DIFFERENCES

Zhen Cong, and Zhirui Chen, *University of Texas at Arlington, Arlington, Texas, United States*

This study investigated how disaster types, namely those with short and longer warning lead time, contextualized individuals' preparatory action, especially as associated with their response efficacy and age. The working sample included 1,467 respondents from the 2017 U.S. National Household Survey. Logistic regressions showed that individuals with higher levels of response efficacy were more likely to prepare after learning information about how to prepare. Respondents in areas prone to short lead-time disasters were less likely to prepare than those in longer lead-time disasters areas. Response efficacy was more important for action taking for short lead-time disasters, which was observed only among older adults when older and younger adults were examined separately. These findings revealed the impacts of disaster types and response efficacy on disaster preparedness and older adults' unique vulnerability and resilience, which could guide policymaking and interventions to promote national disaster preparedness tailored to regional peculiarities.

### DEVELOPMENT OF A VIRTUAL REALITY PLATFORM TO PROMOTE DISASTER PREPAREDNESS AMONG OLDER ADULTS

Chen Kan, Aaron Hagedorn, Sam Thomas, Reid Yeager, and Zhen Cong, *University of Texas at Arlington, Arlington, Texas, United States*

This project develops a tailored and adaptive virtual reality platform to innovatively promote older adults' disaster preparation in a socially engaging environment. The platform serves the following purposes: 1) assist older adults to develop tailored household emergency preparedness plans, 2) simulate extreme weather conditions and warnings for older adults to practice disaster response and develop relevant knowledge and skills as well as test and revise their emergency preparedness plans, 3) use the process as a social engagement tool to reduce social isolation and promote a sense of community. The virtual environments are designed in Unity to simulate extreme weather conditions/natural disasters and older adults are guided to use the HTC VR headset and experience the selected disaster scenario. The pilot VR platform will be tested among community-dwelling older adults in the Dallas-Fort Worth Metropolitan Area.

### Session 3360 (Symposium)

### DISRUPTING OTHERING: A CONTINUUM OF AGE-INTEGRATED STRATEGIES FOR GERONTOLOGY EDUCATION

Chair: Lisa Borrero Co-Chair: Lisa Borrero

Given the extensive negative impacts of ageism, it is incumbent upon institutions of higher education to lead the charge in promoting age inclusivity and dismantling ageism, particularly in the classroom. Traditional teaching approaches are insufficient to meet the needs of our increasingly diverse student population or our aging post-COVID society. Gerontology classrooms require new, innovative, and purposeful ways of engaging students in course content. This symposium brings together faculty who intentionally promote age inclusivity by immersing students in age-integrated experiences to varying degrees and in different ways. These approaches fundamentally reject the idea of older adults as "other" by promoting an age-inclusive reality in which individuals of all ages are equally valued. Dr. Lyn Holley will describe a new course which requires projection of a future, older self and two systematic life interviews – one of a real US elder and one of an imagined counterpart in a different country; comparative analysis reveals how aging is shaped by society. Dr. Skye Leedahl will discuss challenging ageism by examining existing age-focused policies and programs and having students engage in six intergenerational discussions and reflective writing. Dr. Tina Newsham will describe a newly revised practicum in which immersive, real-world experiences with older adults are incorporated, along with meaningful reflection and the development of an e-portfolio. Finally, Drs. Laura Donorfio and Lisa Borrero will explain the ways "othering" is disrupted when students imagine themselves as older adults, and use creative approaches that demonstrate how to negotiate key aspects of the aging process.

### USING VISUAL DEMONSTRATIONS OF LEARNING TO PROMOTE AGE-INTEGRATED PROBLEM SOLVING

Lisa Borrero, *University of Indianapolis, Indianapolis, Indiana, United States*

Working to dismantle the "othering" of older adults requires active effort to reverse deeply ingrained cultural perceptions and attitudes. As gerontology educators, we are uniquely positioned to "move the needle" on this issue by providing students with the opportunity to engage with older adults in meaningful ways and to see the world from their perspective. In this presentation, visual demonstrations of student learning will be shared that allow students to demonstrate their mastery of course concepts in a creative way and to problem-solve a particular issue by engaging with their own future selves. This approach also allows for a deviation from the routine of written demonstrations of learning by appealing to a different set of student skills. Approaches discussed will include concept maps to deconstruct community collaboration; book bentos to explore optimal aging; multimedia presentations on meaning-making in older adulthood; and a photo elicitation project to address outdoor fall hazards.

### VISUALIZING INCLUSIVITY: AGING LIFELONG AND WORLDWIDE THROUGH THE LENS OF GERONTOLOGY AS SOCIAL SCIENCE

Lyn Holley, *University of Nebraska at Omaha, Omaha, Nebraska, United States*

A new course actively engages students in applying social science to examine their own prospective aging and the aging of others. This Grand Project begins with self-assessment of their expectations for the "Future Self" at age 67, then conducting two structured interviews each followed by writing a "life story" – one of an US older adult and the other an imagined older adult from a different county. Comparative analysis of these three stories highlights the impacts of society, heredity, and choice on shaping the experience of old age. Each project is presented to the class. Seeing life from this personal "tour" of the "other" informs beliefs about differences. Students gain knowledge about gerontology as a social science and develop personal understanding of their own and others' aging, a good step toward becoming comfortable with diversity and inclusivity. Ageism is the only "ism" guaranteed to include all who survive long enough.

### EMBRACING AGE-INCLUSIVITY THROUGH MULTIMODAL, ACTIVE STUDENT LEARNING IN A HEALTH AND AGING POLICY COURSE

Skye Leedahl, *University of Rhode Island, Kingston, Rhode Island, United States*

One of the goals for an upper-level undergraduate seminar course is for students to identify and discuss existing policies, programs, and resources for meeting the needs of the aging population. To encourage active student learning, the course provides a mix of readings and assignments, six intergenerational discussions, and reflective writing. The idea is that these varying experiences help foster a deep understanding of how these policies and related issues pertain and matter to everyone, not only older individuals, and how their future career and family experiences will benefit from the knowledge gained in the course regardless of their next career steps. This course model has been effective for challenging ageism, and has been taught four times, each time with modifications based on student numbers, current issues, and the pandemic. The presenter will discuss assessment methods, identify best practices, and offer suggestions for others interested in utilizing a similar model.

### DISRUPTING OTHERING BY FOSTERING LIGHT-BULB MOMENTS IN IMMERSIVE PRACTICAL EXPERIENCES

Tina K. Newsham, *University of North Carolina Wilmington, University of North Carolina Wilmington, North Carolina, United States*

Fully immersive experiences (i.e., practica, internships, clinicals) with older adults are one successful strategy to reduce ageist beliefs among students, as long as the experiences are high quality and students are given an opportunity to reflect on their observations, feelings, and application of classroom knowledge. Representing a more intensive level of engagement than single-session interviews or multisession intergenerational activities, such experiences are not without challenges. Instructors are not present on-site continually (or at all) to observe what the student is seeing or doing, therefore providing limited opportunity to recognize and reframe situations that may reinforce the "othering" of older adults. Through creating carefully curated reflection prompts and assessment strategies (such as eportfolios), this presenter will discuss how instructors can ensure students completing practica, internships, and clinicals recognize the breadth and diversity of aging experiences, engage ethically and appropriately with older adults in non-ageist ways, and avoid reinforcing "othering" and ageism.

### GETTING TO KNOW YOURSELF ACROSS THE LIFESPAN

Laura Donorfio, *University of Connecticut, Waterbury, Connecticut, United States*

New teaching and learning methods must be developed to accommodate exclusive online environments. The utilization of experiential learning in an aging course is a key teaching method that helps tap into the affective domain of learning (i.e., feelings, values, and attitudes). In a society where ageist attitudes abound and are often internalized, purposeful challenges to the "othering" of older adults is needed to disrupt this cycle. This presentation will share an experiential/age inclusivity activity that can be utilized in any teaching environment. Over a semester, students are required to write themselves four separate letters from their older selves at varying ages to their younger selves. They must research and provide a snapshot of what their life will be like at these ages in predetermined areas (i.e., health, mobility, family/friends, policy, and cultural variables, etc.) Worksheets will be provided capturing this "Getting to Know Yourself Across the Lifespan" experiential learning activity.

### Session 3365 (Symposium)

### DISRUPTING THE STATUS QUO: THE NEW NORMAL OF FAMILY CAREGIVERS' SERVICE USE

Chair: Karen Moss Co-Chair: Verena Cimarolli
Discussant: Karen Rose

Family caregivers are essential partners in chronic disease management for older adults. However, being a family caregiver can have negative mental and physical health consequences, making it important for some caregivers to rely on supportive services, either for themselves (e.g. support groups) or to get help with caregiving tasks (e.g. home care). Supportive service use by family caregivers is well documented; yet, this research has often not included specific subgroups of caregivers (e.g. the racially/ethnically, or geographically diverse). Hence, the purpose of this symposium is to share new findings from research on supportive service use in understudied caregiving populations. First, Dr. Cimarolli presents findings from a study on the types of supportive services long-distance caregivers use for themselves and the factors associated with supportive service use in this caregiver population. Then, Dr. Wyman reports findings from a survey on the use of home and community-based resources by family caregivers in a Native American community. Dr. Wright will share the results of a systematic review of self-care interventions designed for caregivers of African Americans living with dementia. Finally, Dr. Mavandadi presents the results of a study examining the effectiveness of a telephone-based, collaborative dementia care program for improving outcomes in caregivers of military veterans living with dementia. Dr. Karen Rose will discuss the implications of each of these study findings for the development and evaluation of supportive interventions for these specialized family caregiver groups.

### CARING FROM AFAR: LONG-DISTANCE CAREGIVERS' USE OF SUPPORTIVE SERVICES FOR THEMSELVES

Molly Wylie,[1] Jillian Minahan Zucchetto,[2] Francesca Falzarano,[3] Amy Horowitz,[4] and Verena Cimarolli,[5], *1. University of Massachusetts Boston, University of Massachusetts boston, Massachusetts, United States, 2. Fordham University, Psychology Department, Bronx, New York, United States, 3. Weill Cornell Medicine, Douglaston, New York, United States, 4. Fordham University, Graduate School of Social Service, New York, New York, United States, 5. LeadingAge, Washington, District of Columbia, United States*

Although long distance caregivers (LDCs) are starting to be recognized as a subgroup of care partners experiencing unique challenges and stresses, it is unknown 1) what types of supportive services LDCs use for themselves and 2) what factors are associated with supportive service use in this understudied caregiving population. In our sample of 304 LDCs (Mage=56.9), the most frequently utilized service was video phone/webcam systems to monitor the care recipient (CR). Guided by Andersen's Model of Health Care Utilization and using multiple hierarchical regression analysis, younger age of the LDC (a predisposing factor) and need-related characteristics (greater caregiving burden and depressive symptoms, more time spent helping the CR, and worse CR functional status) were associated with greater use of supportive services. Enabling factors were not associated with service use. These study findings can help inform how to engage LDCs in supportive service utilization.

### A SYSTEMATIC REVIEW OF SELF-CARE INTERVENTIONS FOR AFRICAN AMERICAN FAMILY CAREGIVERS

Eunjung Ko,[1] Karen Moss,[1] Tara O'Brien,[2] Loren Wold,[3] Karen Rose,[1] and Kathy Wright,[1] *1. The Ohio State University, Columbus, Ohio, United States, 2. The Ohio State University, Center for Healthy Aging, Self-Management, and Complex Care College of Nursing, Columbus, Ohio, United States, 3. The Ohio State Univesity, College of Nursing andDepartment of Physiology and Cell Biology, Columbus, Ohio, United States*

African Americans (AA) family caregivers provide the bulk of unpaid care to persons living with dementia (PLWD). This role leaves little time for the adoption of self-care behaviors—critical to the prevention and management of chronic diseases. In this systematic review, we appraise caregiver self-care interventions that include AA caregivers of PLWD. PubMed, CINAHL, PsycINFO, Cochrane, and Embase databases were searched. Terms included AA/Black, self-care, caregiving, lifestyle, intervention, psychological stress, and faith/community. We initially found 250 references and after application of exclusion criteria and removal of redundant references, 18 articles were analyzed. Most interventions focused on impacting levels of caregiver burden, depression, physical activity, anxiety, or wellbeing. Psychoeducation, physical activity, and spiritually-focused interventions were most effective in improving outcomes in caregivers. Future studies should examine the impact of interventions on the prevention and management of chronic disease in AA caregivers of PLWD.

### UTILIZATION OF HOME AND COMMUNITY-BASED RESOURCES BY FAMILY CAREGIVERS IN A NATIVE AMERICAN COMMUNITY

Debra Miller,[1] Sunshine Wheelock,[2] Florence Petri,[2] Elijah Metoxen,[3] Nickolas Lambrou,[4] Carey Gleason,[5] and Mary Wyman,[6], *1. Oneida Comprehensive Health Division, Oneida, Wisconsin, United States, 2. Oneida Alzheimer's Community Advisory Board, Oneida, Wisconsin, United States, 3. Oneida Aging & Disability Services, Oneida, Wisconsin, United States, 4. University of Wisconsin, Madison, Madison, Wisconsin, United States, 5. University of Wisconsin, Madison, Wisconsin, United States, 6. UNIVERSITY OF WISCONSIN, Madison, Wisconsin, United States*

Family caregiving is uniquely significant for elder care within American Indian/Alaska Native (AI/AN) communities. Compared to other populations, AI/AN older adults are disproportionately impacted by chronic conditions and AI/AN are more likely to be family caregivers. However, AI/AN are underrepresented in aging research. We describe a successful research partnership with the Oneida Nation of Wisconsin and report results of a recent survey of tribal members and affiliates (N=405), covering demographics of caregiving, awareness and use of home and community-based resources, and perceptions of factors impacting service use. Approximately 42% of respondents were current caregivers; of these, roughly one-third knew how to access various resources. Most common sources of knowledge were a health care/social worker or finding information on their own. Traditional cultural values were viewed as variably supportive of resource utilization, depending on service type. Implications for efforts to address disparities for AI/AN aging and support caregivers will be discussed.

### THE EFFECTIVENESS OF A CAREGIVER SUPPORT AND EDUCATION INTERVENTION DESIGNED FOR CAREGIVERS OF VETERANS

April Eaker,[1] Katherine Van Treese,[1] Brenda Jeffries-Silmon,[1] Laura Wray,[2] and Shahrzad Mavandadi,[3], *1. VA Center for Integrated Healthcare, VA Western New York Healthcare System, Buffalo, New York, United States, 2. VA Center for Integrated Healthcare, Buffalo, New York, United States, 3. Corporal Michael J Crescenz VA Medical Center, Corporal Michael J Crescenz VA Medical Center, Pennsylvania, United States*

Veterans are at increased risk for dementia and multiple comorbid conditions, often making the family caregiving experience particularly challenging. The objective of this study was to examine the effectiveness of a telephone-based, collaborative dementia care program for improving caregiver (CG) outcomes in CGs of veterans with dementia. All CGs (n=107) received individual care management and were randomized to either individual intervention alone or individual plus group education and social support. CGs were on average 72.5 (+/-11.0) years old, and the majority were female, spouses/partners of the care recipient (CR), and providing care for ≥1 year. Pre-post analyses indicate that CGs experienced a significant reduction in both the frequency of CRs' dementia-related symptoms (e.g. memory difficulties, disruptive behaviors, depressive affect) and their own distress in response to these symptoms. Our study adds to the literature on the development and evaluation of understudied CG populations, such as CGs of veterans with dementia.

### Session 3370 (Paper)

### ECONOMICS AND FINANCIAL WELL-BEING IN AGING

### DISPARITIES IN FINANCIAL STRAIN FOR OLDER ADULTS AND PEOPLE WITH DISABILITIES IN CALIFORNIA

Lei Chen,[1] Kathryn Kietzman,[2] and Rebecca Allen,[3], *1. University of California, Los Angeles, Los Angeles, California, United States, 2. University of California, Los Angeles, UCLA Center for Health Policy Research, California, United States, 3. University of Alabama, Tuscaloosa, Alabama, United States*

This study examines disparities in the experience of financial strain among older adults and people with disabilities by age, gender, race/ethnicity, poverty, and disability type. People with disabilities refer to those who report cognitive impairment, difficulties performing activities of daily living (ADLs) and/or instrumental activities of daily living (IADLs). Financial strain includes challenges that participants incurred during the last 12 months in acquiring food, housing, health care, or income. This study uses the data from the 2019 California Long-Term Services and Supports (LTSS) survey that was merged with data from the omnibus California Health Interview Survey (CHIS) (N=1097). This is the most comprehensive population-level dataset to examine LTSS needs, unmet needs, and uses of LTSS in California. Initial findings show that 50% of participants report spending less on food, while 40% report cutting down on saving for retirement, receiving and borrowing money from others, and experiencing a decline in household income. More than 20% note that they could not make rent or mortgage payments, had debt due to medical bills, and had to spend less on prescription medications or medical care. We also find significant disparities in financial strain by age, gender, poverty, and disability type; however, no significant disparities by race/ethnicity. This study is among the first to examine disparities in various financial strain types for people who need LTSS in California. The findings have policy implications for the Master Plan for Aging (MPA), which serves as a blueprint to build environments that promote an age-friendly California.

### DOES THE HEALTH INSURANCE PROGRAM REDUCE HEALTH-INDUCED POVERTY?: NEW EVIDENCE FROM THE CHINA HOUSEHOLD INCOME PROJECT

Yalu Zhang,[1] and Qin Gao,[2], *1. Peking University, Beijing, Beijing, China (People's Republic), 2. Columbia University, New York, New York, United States*

The growing cost of healthcare services has been a concern for many countries in the world. In China, medical expenditures can account for as much as 65% of per capita income in some low-GDP counties in 2011. One of the primary goals of the New Rural Cooperative Medical Insurance (NRCMI) is to provide financial protection and alleviate the financial burdens of rural residents in China. This paper examined whether NRCMI participation impacted the incidence of catastrophic health expenditure (CHE) among middle-aged and older adults (45 years old and above) using the China Household Income Project 2007 rural data and an instrumental variable estimation method in two provinces where there was heterogeneity in NRCMI implementation schedule. The results show that NRCMI enrollment could not impact the likelihood of experiencing CHE among middle-aged and older adults. However, NRCMI participation increased the actual amount of medical expenses in one province but not in the other. Although none of the prior studies have used instruments and village fixed effects or take endogeneity issues into account to investigate the impact of NRCMI on relative financial burden among recipients, the results found in this study are generally aligned with the prior findings, especially with those using quasi-experimental studies. Findings from this study provide empirical evidence to the policymakers that the effect of NRCMI participation on financial protections is limited despite its broad population coverage. The limited effects are probably due to the low reimbursement rate and more utilization of expensive healthcare services.

### HEALTH, QUALITY OF LIFE, AND ECONOMIC IMPACTS OF HOME CARE VOUCHERS FOR MIDDLE-INCOME ADULTS

Joanne Spetz, Laura Wagner, Jacqueline Miller, Susan Chapman, and Connie Kwong, *University of California, San Francisco, San Francisco, California, United States*

The Support at Home pilot program provided financial support for home care services by middle-income adults with disabilities in San Francisco to support aging in place. This presentation reports the results of the mixed-methods evaluation of the program, which incorporated administrative records, surveys of clients and comparison group members, surveys of informal caregivers of clients, surveys of the care providers hired by clients, and focus groups with clients and with informal caregivers. Outcome measures included the Older People's Quality of Life Questionnaire, Patient Health Questionnaire-2, an adapted Burden Scale for Family Caregivers, and self-reported falls, emergency department visits, and hospitalizations. Analyses included pre-post chi-squared and t-test comparisons and comparisons of changes between the client and comparison groups. Multivariate regression analyses were conducted to control for demographic differences between the groups. An economic analysis was conducted to learn whether changes in costs associated with medical appointments, emergency department visits, and hospitalizations were greater than the costs of the program, including both voucher and administrative costs. Results indicated statistically significant positive changes in personal stress and financial stress, but not in the composite quality of life score. There also were statistically significant reductions in attendance at medical appointments, falls, emergency department visits, and hospitalizations. The focus group data supported the findings regarding personal and financial stress, and also indicated that clients and their caregivers perceived positive quality of life benefits. The economic analysis indicated substantial cost savings from the program due to reduced use of medical services.

### THE INFLUENCE OF MARITAL STATUS AND GENDER ON FINANCIAL WELL-BEING

Jing Geng, *Virginia Tech, Blacksburg, Virginia, United States*

Research consistently documents gender differences in financial status in later life, and some also examine marital status in this regard. However, the subjective aspects of financial well-being are less well-explored, especially as this relates to both gender and marital status in the U.S. Using a gender-sensitive approach, this study examines the extent to which gender and marital status affect the financial well-being of older American adults. Different from previous studies that use only objective measures of financial well-being, this study also takes a subjective assessment in terms of financial satisfaction into account so that the role of marital status and gender in both objective measures and subjective assessments can be identified. This study uses the 2014 Health and Retirement Study and employs ordinary least squares regressions and ordinal logistic regression analyses. Examining those aged 65 and over, the sample varies from N=10,325 (financial well-being) to 4,280 (financial satisfaction). Differences in gender and marital statuses across all objective measures of financial well-being show up, with women being disadvantaged while the married (regardless of gender) being advantaged. Concerning financial satisfaction, being divorced and separated were negatively related to financial satisfaction for both men and women. These findings indicate that both marital status and gender are important indicators of financial well-being in later life.

### WHOM CAN I RELY ON? THE IMPACT OF CHINA'S PUBLIC PENSION PROGRAM EXPANSION ON THE EXPECTATIONS FOR OLD-AGE SUPPORT

Qian Zhang, *Beijing Normal University, Beijing, Beijing, China (People's Republic)*

Aging is a global trend and China is no exception. Older people in China mostly rely on their adult children for old-age support. This traditional provision pattern of old-age support, however, is challenged by hundreds of millions of internal migrant workers. They relocate from rural to urban regions for better employment and are no longer able to provide old-age support to their older parents in rural areas. The aim of this study was to determine the impacts of China's public pension program expansion in rural areas on older people's expectations for old-age support. Utilizing the natural experiment of program expansion, this study identified an instrumental variable as the county adoption of the pension program. In addition, the study analyzed a nationally representative longitudinal dataset CHARLS with fixed effects model. Results from the statistical model showed that given the participation in the pension program, older adults reported more reliance on pension for old-age support financially and less reliance on children. Heterogeneous effects were found for older adults living together with children and older adults living independently. These important findings suggest that the government partially assumes the responsibility for the old-age support of adult children in the traditional sense. The potential benefits of this study provide a policy implication for developing countries to alleviate old-age support problems and enable internal migration for economic development.

### Session 3375 (Paper)

### END OF LIFE

### ASSOCIATION BETWEEN ADVANCE CARE PLANNING FOR OLDER ADULTS AND FAMILY CAREGIVERS' SENSE OF SECURITY

Masumi Shinohara,[1] Mariko Sakka,[2] Asa Inagaki,[2] Taisuke Yasaka,[2] Chie Fukui,[2] Maiko Noguchi-Watanabe,[2] Ayumi Igarashi,[2] and Noriko Yamamoto-Mitani,[2],
*1. University of Tokyo, Bunkyo, Tokyo, Japan,*
*2. The University of Tokyo, Bunkyo, Tokyo, Japan*

Family caregivers' (FCs') sense of security benefits older adults who receive homecare. Advance care planning (ACP) is reported to have positive effects on FC's experience, but it might differ depending on FCs' kin relationships with the older adults. We examined whether ACP for older adults in homecare settings is associated with FCs' sense of security. Further, we assessed whether such an association depends on their status as spouses or as adult children. We conducted a secondary analysis of data from a prospective cohort study in Japan. The participants were older adults who used home-visit nursing services, their FCs, and the nurses who cared for them. The FCs were asked to answer a sense of security questionnaire, and nurses were asked whether ACP was conducted. Multivariate logistic regression analyses were performed to examine the association between ACP implementation and positive changes in the sense of security scores after three months. Data from 169 cases were analyzed. Of the FCs, 28.1% were men and 55.6% were spouses. ACP was performed in 53.8% of the cases. The results of the multivariate analyses showed an interactive effect between ACP implementation and FC kin relationships. For spouses, ACP was significantly associated with a positive change in their sense of security. For adult children, such an association was not found. ACP might have a positive effect on caregiving spouses' sense of security. Adult child caregivers, who often have multiple responsibilities and have difficulties facing their parents' physical decline, may need support, in addition to ACP.

### PREFERENCES AND DETERMINANTS OF END-OF-LIFE CARE AMONG OLDER CHINESE AMERICANS

Dexia Kong,[1] Elissa Kozlov,[1] and XinQi Dong,[2], *1. Rutgers University, New Brunswick, New Jersey, United States, 2. Rutgers University, Rutgers Institute for Health, New Jersey, United States*

End-of-life (EOL) care awareness and practice remain particularly low among older Chinese Americans. More empirical evidence regarding EOL is needed to develop culturally-relevant interventions to promote EOL engagement in this minority population. Using population-specific data, this study investigates preferences and associated sociodemographic and health determinants related to EOL among older Chinese Americans. Data were from the Population-based Study of Chinese Elderly in Chicago (collected 2017-2019, N=3,124). Linear and logistic regressions were conducted. Of the sample, 46.1% considered EOL care planning as important or somewhat import. Nearly 22% had EOL discussions with families. The most preferred EOL locations were home (43.7%), hospital (35.5%), nursing home (10.1%), and hospice (4.3%). Overall, 47.1% perceived EOL care as family decisions, 39.6% regarded EOL care as personal decisions, 7.5% preferred children to make EOL decisions, and 3.3% preferred a spouse to make EOL decisions. Chinese older adults who were female (B=0.10, p<0.01), married (B=0.11, p<0.01), had higher education (B=0.02, p<0.001), acculturation level (B=0.02, p<0.001), and religiosity (B=0.12, p<0.001), and more chronic conditions (B=0.05, p<0.001) were more likely to consider EOL as important. Those with older age [Odds Ratio (OR)=1.02, 95% Confidence Interval (CI)=1.01-1.03], female gender (OR=1.44, 95% CI=1.18-1.77), higher levels of education (OR=1.02, 95% CI=1.01-1.04), acculturation (OR=1.04, 95% CI=1.01-1.06), and religiosity (OR=1.11, 95% CI=1.02-1.21), longer U.S. residence (OR=1.02, 95% CI=1.01-1.03), and more chronic conditions (OR=1.13, 95% CI=1.06-1.21) were more likely to have discussed EOL preferences with their families. Study findings underscore low engagement in EOL planning in this population and the need for culturally-appropriate interventions.

### THE BURDEN OF HAVING TO WONDER: MINORITY STRESS EXPERIENCES OF LGBTQ+ HOSPICE FAMILY CAREGIVERS

Kristin Cloyes,[1] Miranda Jones,[2] Marilisa Vega,[2] Megan Hebdon,[3] Casidee Thompson,[2] Susan J. Rosenkranz,[2] Maija Reblin,[4] and Lee Ellington,[2], *1. University of Utah, Portland, Oregon, United States, 2. University of Utah, Salt Lake City, Utah, United States, 3. University of Utah, University of Utah, Utah, United States, 4. Moffitt Cancer Center, Tampa, Florida, United States*

Home hospice care relies heavily on informal caregivers, often patients' family and close others. Hospice family caregivers report stress, burden, and unmet support needs associated with poor health and bereavement outcomes. These outcomes are sensitive to the quality of interactions with professional hospice providers, especially for historically marginalized groups, yet little research examines experiences of LGBTQ+ hospice family caregivers. Informed by minority stress theory, we conducted in-depth interviews with LGBTQ+ home hospice family caregivers across the U.S. (N=20). Participants reported demographics and described their caregiving experiences including interactions with hospice providers. Interviews were audio-recorded, transcribed, and content-analyzed. Participants were mostly white (n=15, 75%), non-Hispanic (n=19, 95%), cisgender (n=19, 95%), gender binary (n=19, 95%), lesbian (n=10, 50%), women (n=12, 60%); average age was 52.3 (range 25-67, SD=13.84). Along with known end-of-life caregiving stressors, participants experienced minority stress that complicated caregiver-provider communication. Distal stressors included lack of LGBTQ+ competent resources, inadequate legal protections, providers' assumptions about relationships, and difficult dynamics with unaccepting relatives. Proximal stressors included perceived risks of disclosure, expectation of poor treatment, feeling the need to modify presentation of self or home, and wondering whether negative provider interactions were due to being LGBTQ+. This generated a background level of uncertainty, caution, and concern that was particularly distressing in the home setting. Minority stress affects LGBTQ+ people across the lifespan and generates added burdens and support needs for hospice family caregivers. Providers who understand these effects are better positioned to deliver safe, effective care to all families at end of life.

### UNMET FAMILY NEEDS IN HOSPICE AND RESEARCH PRIORITIES: PERSPECTIVES FROM A NATIONAL SAMPLE OF HOSPICE AGENCIES

Todd Becker, and John Cagle, *University of Maryland, Baltimore, Baltimore, Maryland, United States*

Although hospice cares for nearly 1.5 million patients and families annually, little is known about practitioners' opinions of current gaps in care and research. To this end, we posed two open-ended questions to hospice representatives to identify practice-relevant research priorities. Data stem from two optional questions (Q1: N = 72; Q2: N = 73) appended to Cagle et al.'s (2020) national survey of 600 randomly selected hospices, stratified by state and profit status. Most participants provided the majority of care in-home (84.7%; 79.5%) and worked at a medium-sized hospice (50.0%; 49.3%). Responses to Q1 ("What is the biggest unmet need for hospice patients and families?") and Q2 ("In your opinion, what is the most pressing topic that hospice researchers need to study?") were analyzed for content and then synthesized. Analyst triangulation and peer debriefing improved trustworthiness. Emerging domains included: access to hospice, hospice services and workforce issues, and education. The access to hospice domain contained a subtheme regarding the need for earlier referrals. Participants noted that short lengths of stay undermine the clinical benefits to patients and families, and that hospice enrollment criteria may contribute to inadequate lengths of stay. The hospice services and workforce issues domain largely focused on burnout prevention. Participants acknowledged that provider self-care was linked to the quality of patient care. The education domain contained subthemes related to improving physician knowledge regarding prognostication and referral, and to patients and families regarding misconceptions about hospice care. Findings highlight critical needs for future hospice research and policy change.

### Session 3380 (Paper)

### FAMILY CAREGIVING I (SRPP PAPER)

### CARING AGAIN: PARENT CAREGIVERS FOR THEIR WOUNDED ADULT CHILDREN VETERANS

Linda Nichols,[1] Jeffrey Zuber,[2] Robert Burns,[3] and Jennifer Martindale-Adams,[2], *1. VA Medical Center 11H, Memphis, Tennessee, United States, 2. University of Tennessee Health Science Center, Memphis, Tennessee, United States, 3. University of Tennessee Health Science Center, University of Tennessee Health Science Center, Tennessee, United States*

With military personnel in Iraq and Afghanistan surviving what were previously fatal injuries, there is ongoing discussion about how to provide care for them and support their families. Parents frequently provide care for their unmarried, injured adult children, especially those returning with polytraumatic injuries, PTSD, or Traumatic Brain Injury (TBI). Parents (n=160) of combat injured adult children who participated in a DoD-funded behavioral intervention study are described. Parents were mainly mothers, average age 60.2 years, with ages ranging from 45 to 79. The veterans had functional limitations, and only 9.2% were employed. Parents, on average, had been caregivers for 6.6 years and daily spent 7.7 hours providing care and 17.2 hours on duty, primarily focused on supervision and daily life management rather than physical care. Average caregiver burden score approached high and was related to veteran TBI diagnosis, aggressive behavior toward others, and functional limitations. Few parents (22.7%) worked full-time; 85.3% had decreased personal spending, 84.0% dipped into personal savings, and 58.9% reduced retirement saving. These findings are similar to those of aging parent caregivers of adult children with serious mental illness or developmental disabilities in amount of care provided to their adult children, their level of burden, financial and career cost to themselves, and concern about their future and their children's future. As these parents and their adult children age, providing care and resources will present greater challenges for them, for the military and veteran care systems they rely on for support, and for society.

### THE MODERATING EFFECT OF CAREGIVER ENGAGEMENT IN TRANSITIONAL CARE INTERVENTION OUTCOMES: A META-ANALYSIS

Kristin Levoy,[1] Eleanor Rivera,[2] Molly McHugh,[1] Alexandra Hanlon,[3] Karen Hirschman,[1] and Mary Naylor,[1], *1. University of Pennsylvania, Philadelphia, Pennsylvania, United States, 2. University of Illinois Chicago, Chicago, Illinois, United States, 3. Virginia Tech, Roanoke, Virginia, United States*

As chronically ill adults age, increased fluctuations in health status result in frequent care transitions. Caregiver engagement is often a core component of evidence-based transitional care interventions, yet little is known about the relative contribution of this element to observed outcomes. This meta-analysis aimed to synthesize evidence of caregiver engagement in randomized control trials (RCT's) of transitional care interventions, estimate the overall intervention effects on all-cause hospital readmissions, and test caregiver engagement as a moderator of interventions' effects. Relative risk was the effect size, and the overall effect was estimated using inverse variance weighting. Fifty-four studies met criteria, representing 31,399 participants and 65 effect sizes. The weighted sample mean age was 64 years. The majority (64%) of interventions targeted participants with specific diagnoses, such as heart disease, but more than half (54%) lacked caregiver engagement components. Among all reviewed studies of transitional care interventions, the overall effect on all-cause readmissions at 1 month was nonsignificant (p=.123, k=28). However, intervention effects at 2 or more months were significant (RR=0.89, 95% CI: 0.82, 0.97, p=.007, k=26), indicating a 12% reduction in the relative risk of all-cause readmissions among intervention participants compared to controls. Caregiver engagement was found to moderate intervention effects (p=.05). Specifically, interventions that included caregiver engagement produced more robust effects (RR=0.83, 95% CI: 0.75, 0.92, p=.001), than those without such engagement (RR=0.97, 95% CI: 0.87, 1.08, p=.550). Findings suggest that transitional care interventions need to more explicitly engage caregivers as active partners in order to optimize patient outcomes.

### VIETNAMESE FAMILIES' STRENGTH AND RESILIENCE AND HEALTHCARE PROFESSIONALS' ROLE DURING THE PANDEMIC

Christina Miyawaki,[1] Minhui Liu,[2] and Kyriakos Markides,[3], *1. University of Houston, Houston, Texas, United States, 2. Central South University, Changsha, Hunan, China (People's Republic), 3. University of Texas Medical Branch, Galveston, Texas, United States*

Traumatic escape from Vietnam in 1975 brought 1.3 million Vietnamese refugees to the U.S. Today, Vietnamese are the largest Asian subethnic group in Houston, Texas (81,000+), making Houston the 3rd largest Vietnamese-populated city in the U.S. Despite these numbers, health research on Vietnamese population is limited. To address this gap, we developed the Vietnamese Aging and Care Survey and collected data on Vietnamese older adults (≥65 years) and their caregivers (N=199). The purpose of this study was to examine the association between caregivers' caregiving characteristics and care recipients' mental health (N=58 dyads). Descriptive statistics and logistic regression models were used. Caregivers were on average 53 years-old, Vietnam-born (97%), and working (66%). The majority (84%) lived with their care recipients and provided care for 20+ hours/week (69%) in good/excellent health (76%). Care recipients were on average 75 years-old, Vietnam-born (100%) in fair/poor health (81%). Regression results showed stressed caregivers with more-depressed care recipients (OR=1.47, 95%CI:1.02, 2.13) but positive caregiving experiences (OR=0.85, 95%CI:0.74, 0.97) and burdened caregivers (OR=0.79, 95%CI:0.65, 0.96) with less-depressed care recipients. We found the association between stressed caregivers and depressed care recipients (Life Stress Paradigm), but care recipients becoming a "helpful company" reduces caregiver burden and care recipients' depression (Social Exchange Theory). Vietnamese families live in multigenerational households within ethnic enclaves and remain a tightly-knit family unit showing resilience to their low socioeconomic status (≤25K, 91%). Leveraging a family as their strength, healthcare professionals should take a caregiver-care recipient dyad approach when planning COVID-19 pandemic interventions in Vietnamese communities.

### WORKING FAMILY CAREGIVER WELL-BEING: WORK IMPACT, WORKPLACE SUPPORTS, AND FAMILY DISCORD

Jiayun Xu,[1] Pi-Ju Liu,[1] Yisheng Peng,[2] and Scott Beach,[3], *1. Purdue University, West Lafayette, Indiana, United States, 2. George Washington University, Washington, District of Columbia, United States, 3. University of Pittsburgh, University of Pittsburgh, Pennsylvania, United States*

A recent study by our team showed that family discord about care provision among multiple family caregivers was a significant predictor of caregiver burden, anxiety, depression, and activity restrictions. This study examined the combined effects of family discord, work impacts (missed work, caregiving affects work), and workplace supports (flexible hours, caregiver benefits) on caregiver well-being. We conducted a secondary analysis using cross-sectional survey data from the Western Pennsylvania Family Caregiving Project 2017-2018. Participants were family caregivers who worked outside the home and shared older adult (i.e. over age 50) caregiving responsibilities with family (n=364, mean age: 52.59 years, female: 71.7%, White: 79.7%, cared for a parent: 69.5%, mean work hours/week: 37.30). Hierarchical regression analyses were conducted testing for main effects of family discord, work impacts, and workplace supports; and interactions between discord and work impacts/workplace supports. The presence of family discord negatively impacted all caregiver well-being outcomes ($p<0.05$). Having more work impacts increased the risk for anxiety ($p<0.04$), activity restrictions ($p<0.01$) and burden ($p<0.01$). No main effects were found for workplace support. Moderating effects were found, such that anxiety was high when family discord and work impacts were higher ($p=0.025$). Additionally, more activity restrictions occurred when caregivers had low workplace support and higher family discord ($p=0.020$). Results suggest having less family discord, more workplace support, and less negative work impacts may improve caregiver well-being. Future work is needed to determine which work supports are most beneficial to this population and how family discord and negative work impacts can be reduced.

### Session 3385 (Symposium)

### FRIENDSHIP AND LONELINESS AMONG PEOPLE LIVING WITH DEMENTIA: SOCIAL PRACTICES AND IDENTITY

Chair: Pamela Saunders Co-Chair: Daniel R. Y. Gan Discussant: John Swinton

More people living with dementia (PLWD) are aging in place in the community. The number of PLWD aging in community is estimated to comprise 61-81% of the total number of PLWD in North America. Since most PLWD do not drive (Foley et al., 2000), many may (or may not) spend much of their time closer to home, barring occasional visits out of town. Yet, one's everyday environment may not always provide "ways of being in the world that are more accepting and embracing" (Hillman & Latimer, 2017) and kind, to the varied socio-cognitive struggles of PLWD. Meaningful relationships are required to support continued social participation and citizenship (Bartlett & O'Connor, 2007; Swinton, 2020). In addition to dementia diagnosis, these everyday experiences in community may significantly alter PLWD's self-perception and confidence. PLWD may feel more or less comfortable forging relationships depending on their past experiences. In other words, the identity of PLWDs are often challenged and (re)constructed (Saunders et al., 2011). Amid persistent power imbalances, malignant social practices may reshape one's identity such that social isolation, whether self-imposed and/or due to restrictions from others, appear the best way to tide over overwhelming loneliness. This symposium explores how community and friendships may intercept the formation of such lonely self-identity among PLWD. We use advanced qualitative methods to elucidate the varied experiences and challenges of PLWD in community. Findings from three perspectives, namely sociolinguistics, sociology, and social work, will be discussed identify new social practices to undo stigma and support PLWD in community.

### COMMUNICATIVE COPING AND FRIENDSHIP AMONG PERSONS LIVING WITH DEMENTIA: FINDINGS FROM LONG-TERM CARE

Pamela Saunders, *Georgetown University, WASHINGTON, District of Columbia, United States*

The study of identity is central to many disciplines, however there is a special link that connects language and discourse to identities. The way people speak reveals a lot about who they are. Through discourse and communication individuals convey and negotiate their sense of self (de Fina, 2020). Regardless of cognitive status, persons living with dementia (PLWD) use language to construct for themselves a social identity of being included in friendship networks (de Medeiros et al., 2011). This paper uses data from the Friendship Study to examine the use of such communicative coping behavior (CCB) for friendship formation. Ethnographic observations of PLWD were conducted in a Long-Term Care residential setting. Sociolinguistic discourse analysis of verbatim transcripts with reference to the CCB Checklist (Saunders et al., 2016) reveal evidence of CCB use. Results suggest that different types of CCBs were used to construct identity and negotiate friendship challenges in different contexts.

### UNSPOILED IDENTITIES OF PEOPLE LIVING ALONE WITH DEMENTIA: RESISTING STIGMA BY HELPING OTHERS

Laura Girling,[1] and Kate de Medeiros,[2] *1. University of Maryland, Baltimore County, Baltimore, Maryland, United States, 2. Miami University, Oxford, Ohio, United States*

Goffman (1963) described stigma as the shift from being viewed as a whole and usual person to one with a spoiled identity. People living with dementia (PLWD) often report feeling stigmatized. Many dementia stereotypes highlight losses (e.g., loss of self) and negatively position the person as a passive, dependent care recipient. Here, we present findings from a qualitative study of people living alone with dementia (N=10) in the community that challenge these stereotypes. Analysis of in-depth interviews revealed that many participants resisted the spoiled identity label through active engagement in the community such as participating in paid employment, providing care for neighbors and family members, and volunteering. Overall, findings underscore the need to rethink and challenge common perceptions of PLWD that are focused solely on care, to recognize their active and valuable role in the lives of others. How PLWD negotiate these identities should inform policies of dementia in community.

### ADDRESSING STIGMA IN THE COMMUNITY: A RIGHTS-BASED APPROACH TO COMMUNITY-BUILDING

Deborah O'Connor, *University of British Columbia, Vancouver, British Columbia, Canada*

Article 12 of the United Nations Convention of Rights of Persons with Disabilities (CRPD) affirms the rights of persons with physical and mental disabilities to be treated as equal, and deserving of state support to realize their full human potential. This focus on a 'positive' right to support (as opposed to the 'negative' right to non-interference) has established an important set of expectations around societal responses to people living with dementia(PLWD). This presentation examines the contributions of a rights-based approach to build community with and for PLWD. Data is drawn from Participatory Action Research (PAR) and bi-weekly online action groups with N=10 PLWD in urban and rural British Columbia. Two thematic targets were identified. First, it is important to bring together PLWD in ways that create a sense of solidarity and inclusion. Second, fostering community requires addressing the stigma and discrimination which often leave PLWD feeling isolated, excluded, and marginalized.

### Session 3390 (Symposium)

### GLOBAL PUBLISHING OPPORTUNITIES IN GERONTOLOGY: A VIEW FROM THE EDITOR'S DESK

Chair: Edward Miller Co-Chair: Elizabeth Simpson

Global aging has proceeded at an unprecedented and accelerating rate. The aging of the population creates both opportunities and challenges for elders, their families, and society in general. Importantly, there is substantial variation in the effects of and response to global aging both within and across nations depending, in part, on prevailing cultural expectations and values, political and economic imperatives, and social and demographic characteristics. Thus, while some regions and countries have responded with innovative policies and programs to better enable the growing cohort of older adults to remain active and engaged in the community, other regions and countries have struggled with their response or barely begun to plan for the rising population of elders. This symposium assembles editors at five leading gerontological journals to demonstrate the role that peer-reviewed scholarship can play in disseminating knowledge that informs gerontological research, policy, and practice internationally. Editors include: Kyungmin Kim, PhD, Research on Aging; Jessica Kelley, PhD, Journal of Gerontology: Social Sciences; Edward Alan Miller, PhD, Journal of Aging & Social Policy; Julie Hicks Patrick, PhD, International Journal of Aging & Human Development; and Julie Robison, PhD, The Journal of Applied Gerontology. Each presenter will review the scope, content, and focus of their journals and the role and opportunities for international scholarship.

### THE ROLE OF GLOBAL SCHOLARSHIP IN THE JOURNAL OF AGING & SOCIAL POLICY

Elizabeth Simpson,[1] Michael Gusmano,[2] Pamela Nadash,[1] and Edward Miller,[3] *1. University of Massachusetts Boston, University of Massachusetts Boston, Massachusetts, United States, 2. Rutgers University School of Public Health, Rutgers University School of Public Health, New Jersey, United States, 3. University of Massachusetts Boston, Boston, Massachusetts, United States*

Policymakers, practitioners, and researchers need a balanced, thoughtful, and analytical resource to meet the challenge of global aging at a rate that's historically unprecedented. The Journal of Aging & Social Policy (JASP), which was founded in 1989, serves this role by drawing contributions from an international panel of policy analysts and scholars who assume an interdisciplinary perspective in examining and analyzing critical phenomena that affect aging and the development and implementation of programs for elders from a global perspective. Study settings extend beyond the United States to include Europe, the Middle East, Australia, Latin America, Asia, and the Asia-Pacific rim. This presentation will document the scope, content, and focus of JASP, including the rise of international submissions, which now account for approximately half of articles published. Opportunities for publishing in JASP will be discussed; so too will strategies for navigating the peer-review process successfully.

### RESEARCH ON AGING: THE INTERNATIONAL VIEW FROM THE EDITORS' DESKS

Jeffrey Burr,[1] Changmin Peng,[2] and Kyungmin Kim,[3] *1. McCormack Graduate School, Boston, Massachusetts, United States, 2. University of Massachusetts Boston, Boston, Massachusetts, United States, 3. Seoul National University, Seoul, Seoul-t'ukpyolsi, Republic of Korea*

We review the scope, content, and focus of the peer-reviewed journal, Research on Aging (SAGE), publishing its 422nd volume this year. We will discuss how scholarship produced from researchers around the globe has changed over the years. Data on submissions, acceptance rates, and the important role of an international editorial board will be presented. The review process will be described, along with suggestions on how to increase chances of success when submitting original research. Although Research on Aging is sometimes considered to focus primarily on social gerontology, the scope in recent years has widened considerably, with manuscripts in aging studies published from such fields as economics, psychology, demography, public health, and public policy, as well as from sociology, and social work, among others. One of several special issues forthcoming in the journal will be described to demonstrate the possibilities for international impact.

### JOURNAL OF GERONTOLOGY: SOCIAL SCIENCES—GLOBAL SCHOLARSHIP CHALLENGES AND OPPORTUNITIES

Jessica Kelley, *Case Western Reserve University, Celeveland, Ohio, United States*

Journal of Gerontology: Social Sciences aims to publish the highest quality social scientific research on aging and the life course in the U.S. and worldwide. The disciplinary scope is broad, encompassing scholarship from demography, economics, psychology, public health, and sociology. A key substantive focus is identifying the social, economic, and cultural contexts that shape aging experiences worldwide. In the coming decade, social gerontology research is poised to present many opportunities for cross-national and cross-cultural scholarship – driven in part by the proliferation of large parallel data sets from many nations in Europe, Latin America, and Asia. I will discuss the role that peer-reviewed cross-national scholarship can play in disseminating knowledge that informs gerontological research, policy, and practice internationally. I will also identify under-researched areas that will be of great interest to scholars in the coming decade, including LGBT older adults, aging in the Global South, reconfigured families, and centenarians.

### THE JOURNAL OF APPLIED GERONTOLOGY: AN INTERNATIONAL FORUM FOR INFORMATION

Julie Robison, *University of Connecticut, University of Connecticut, Connecticut, United States*

The mission of applied gerontology is to bridge science and practice to benefit the health and well-being of older persons, their families, their communities, and other contexts. This presentation will provide insights from the Journal of Applied Gerontology and its attempts to publish and disseminate scholarship that has international application. Following an overview of the growing internationalization of peer-reviewed submissions to the Journal of Applied Gerontology on a variety of topics and from a range of perspectives, the presentation will highlight key achievements as well as ongoing concerns and opportunities to better achieve the goals of applying gerontological scholarship to aging contexts worldwide. Concluding comments will examine how outlets for dissemination and authors themselves can better position their work to enhance their influence on aging in an international context.

### TIPS FOR TIMELY (AND POSITIVE) REVIEWS: THE INTERNATIONAL JOURNAL OF AGING & HUMAN DEVELOPMENT

Julie Patrick, *West Virginia University, Morgantown, West Virginia, United States*

For more than 40 years, under the leadership of four editors and two publishers, The International Journal of Aging and Human Development (IJAHD) has featured multidisciplinary scholarship related to aging processes and older adults. With the publication of eight issues a year, with over 800 pages of scientific content, the IJAHD places emphasis upon psychological and social studies of aging and the aged. However, the Journal also publishes research that integrates observations from other disciplines that illuminate the "human" side of gerontology. A more recent focus includes midlife development, as well. About half (47%) of the publications in the IJAHD are from international colleagues. This presentation will discuss tips for both international and US-based scholars for ensuring timely reviews and positive decisions for manuscript submissions, including such areas as key words, suggesting unbiased reviewers, formatting, writing mechanics, clearly-articulated methods, and a sound theoretical basis.

### Session 3395 (Paper)

### HEALTH CARE AND AGING IN PLACE

### CHRONIC DISEASE AMONG MEDICAID BENEFICIARIES OVER 50: THE POTENTIAL IMPACT OF MEDICAID WORK REQUIREMENTS

Rodlescia Sneed,[1] Graham Gardner,[2] Alexander Stubblefield,[3] and Briana Mezuk,[4], *1. Michigan State University, Flint, Michigan, United States, 2. Michigan State University, Michigan State University, Michigan, United States, 3. Michigan State University, East Lansing, Michigan, United States, 4. University of Michigan, Ann Arbor, Michigan, United States*

Since 2018, several states have proposed requiring work or other community engagement activities as a condition of receiving Medicaid; however, there has been little inquiry into the impact of such policies on Medicaid recipients over 50. Here, we describe the prevalence and burden of chronic disease among Medicaid beneficiaries over 50 who might be impacted by Medicaid work requirements. We used data from the 2016 wave of the Health and Retirement Study, a large-scale population-based study of adults aged >50. Our sample included individuals over 50 who were not Medicare-eligible (<65 years old) and not receiving Social Security Income. We used logistic regression models to compare those working <20 hours per week (the minimal community engagement/work cutoff) to those working >=20 hours per week, adjusting for age, race/ethnicity, sex, education, and marital status. Individuals working <20 hours per week had greater prevalence of chronic health conditions, including greater odds of diabetes (OR: 2.06; 95% CI: 1.37-2.46), hypertension (OR:2.56; 95% CI: 1.69-3.89), arthritis (OR: 2.96; 95% CI: 2.06-4.62, and lung disease (OR: 4.11; 95% CI: 2.31-7.32). Further, among those with chronic health conditions, those working <20 hours per week reported more, medication use, more worsening of their conditions in the past 2 years, and more hospitalization than their counterparts. Taken together, these finding suggest that Medicaid work requirements in this population would have the most impact on the most medically vulnerable individuals. Policymakers should consider this as they propose policies impacting Medicaid coverage in this population.

### CONNECTICUT'S VETERANS-DIRECTED CARE PROGRAM OUTCOMES COMPARED TO HCBS WAIVER PARTICIPANTS: HCBS CAHPS RESULTS

Kathy Kellett,[1] Martha Porter,[1] Dorothy Wakefield,[1] and Julie Robison,[2], *1. UConn Health, Farmington, Connecticut, United States, 2. University of Connecticut, University of Connecticut, Connecticut, United States*

Connecticut (CT) Veterans Directed Home and Community Based Services Program (VDC) is an innovative

Veterans Administration (VA) services option providing veterans at risk of institutionalization with person-centered consumer-directed long-term services and supports at home. Funded by an Administration for Community Living grant, the CT Department of Aging and Disability Services partnered with the VA, the five CT Area Agencies on Aging, and UConn Health Center on Aging (UConn). UConn researchers conducted the Consumer Assessment of Healthcare Providers and Systems in Home and Community Based Services (HCBS CAHPS) survey with VDC participants (n=36) from October 2019 through March 2020. The standardized, validated HCBS CAHPS survey, which Connecticut administers to individuals in most CT Medicaid HCBS programs, is a universal, cross-disability tool to assess/improve the quality of HCBS programs. Analyses compared VDC participants' program experiences to survey results from individuals in the Connecticut Home Care Program (CHCP) (for older adults) (n=629), Personal Care Assistance (PCA) (n=282), and Acquired Brain Injury (ABI) (n=327) waiver programs. Notably, more VDC participants (91%) knew who their support broker was, compared to CHCP, ABI, and PCA (82%, 79%, and 72%, respectively) who knew their case manager; 91% of VDC participants gave their support broker the highest rating, compared to 66% to 74% of participants in other programs who rated their case manager. This study provides strong evidence that the CT VDC program is positively impacting veterans and that the AAAs and support brokers are effectively helping them receive the HCBS they need in a consumer-directed way.

### OLDER BREAST CANCER SURVIVORS: PERSPECTIVES ON HEALTHCARE ENCOUNTERS AND UNMET NEEDS

Victoria Raveis,[1] Anita Nirenberg,[2] Yumeng Liu,[3] and Simona Kwon,[4], *1. New York University, New York University, New York, United States, 2. Hunter College, Hunter College, CUNY, New York, United States, 3. New York University, New York, New York, United States, 4. NYU School of Medicine, New York University School of Medicine, New York, United States*

Breast cancer treatment advances have lengthened the survivorship period. Limited attention has focused on the myriad issues breast cancer survivors experience related to their cancer and other health conditions as they age. Focus groups, conducted Fall 2019 – Spring 2020 with a diverse sample of breast cancer survivors from the New York metropolitan region (N=28) explored survivors' healthcare encounters and goals, quality of life, survivorship lifestyle, other health conditions and risks, e.g. emergence of COVID-19. Participants were 40-82 years old (57% were 56 or older); racially diverse (57% White, 18% Black, 14% Hispanic, 11% Bi-racial); 32% were married/partnered and 57% were parents. Mean diagnosis age was 51. Treatments received included lumpectomy (64%), chemotherapy (57%), radiation (46%), hormonal therapy (39%), and single/bilateral mastectomy (36%). Survivors expressed the importance of restoring normality in their life and the necessity to be pro-active in ensuring their health issues were addressed in medical encounters. Person-centered care and clinician engagement was valued, but not routinely experienced. Survivors evaluated treatment options not just on being cancer-free, but on how it would impact their whole life. They expressed concerns about the future and anxiety over long-term survival. Long term survivors, recipients of early experimental and/or extensive treatments, worried about an emergence of long-delayed adverse health consequences and complications managing other health issues in the future, particularly as they grew older. COVID-19 raised additional health concerns, particularly among those with high risk health conditions due to prior cancer treatments; various self-mandated protective activities were integrated into their self-care practices.

### THE COSTS AND BENEFITS OF CHOICE: FAMILY MANAGERS IN DIRECTLY FUNDED HOME CARE

Lisette Dansereau,[1] Christine Kelly,[1] Katie Aubrecht,[2] Amanda Grenier,[3] and Allison Williams,[4] *1. University of Manitoba, Winnipeg, Manitoba, Canada, 2. St. Francis Xavier University, St. Francis Xavier University, Nova Scotia, Canada, 3. University of Toronto and Baycrest Hospital, Toronto, Ontario, Canada, 4. McMaster University, Hamilton, Ontario, Canada*

Directly funded (DF) home care, or consumer directed home care, gives program users a budget to choose their own services. Set in the Canadian province of Manitoba, our study examines the local DF program "Self and Family Managed Care", which does not allow program users to hire and pay a family member. Incorporating a disability lens into care and aging studies, we share findings from a qualitative study based on 24 semi-structured interviews with DF users. We focus on the experiences of family managers, that is, representatives acting as a decision maker for an older adult. About half of the family managers in this study care for people living with dementia or cognitive decline. We identify two main themes: 1) service flexibility in DF reduces caregiver strain, 2) family managers tend to hire agencies rather than individuals to avoid administrative burden. Our discussion highlights the costs of DF from the perspective of caregivers as administrative burden (financial paperwork, finding workers, choosing a 'good' agency), and the benefits as flexibility (choosing workers, trusting workers, setting schedules, assigning work). We also consider the goals of family managers to enhance quality of life and avoid long-term residential care, in contrast to younger self-managers who desire control and autonomy. We recommend that DF programs need to reduce administrative work for users, support users in making informed choices, and find better ways to support, acknowledge and value the work of family managers and substitute decision makers.

### THEY HAVE THE ABILITY TO SAY YES OR NO: PROVIDERS' PERSPECTIVES ON VETERANS' SERVICE REFUSAL AS A CAUSE OF READMISSIONS

Nytasia Hicks,[1] Polly Noel,[2] Lauren Penney,[3] and Jacqueline Pugh,[4], *1. Department of Veterans Affairs, San Antonio, Texas, United States, 2. South Texas Veterans Health Care system, South Texas Veterans Health System, Texas, United States, 3. South Texas Veterans Health Care System, San Antonio, Texas, United States, 4. South Texas Veterans Health Care system, San Antonio, Texas, United States*

sService refusal, where patients actively refuse clinicians' recommendations for additional services needed to achieve safe and full recovery after discharge, is a key but often overlooked cause of readmissions. There is a dearth of literature on the extent of service refusal and providers' (e.g. clinicians, nurses, social workers) observations regarding how to deal with these situations. As part of a larger, 10 VA site organizational case study of readmission, semi-structured interviews exploring causes of readmission were conducted with 21-41 staff members at each site (n=314). 41 providers identified Veteran service refusal and decision-making as causes of readmission. Providers acknowledged the need to honor patient autonomy/self-determination in decisions while at the same time worrying about potential adverse outcomes. Incongruence between Veterans' and providers' perceptions (especially for capacity for self-care), goals, and discharge plans was also cited as a factor in service refusal. Frustration was also raised about initial acceptance of service followed by refusal at time of service delivery. Providers also felt readmissions increased even further when combined with lack of or inadequate caregiving arrangements/family support, lack of cognitive capacity, homelessness, or home care affordability. Findings point to the need for interventions to evaluate congruence between provider and patient assessment of self-care capabilities and provide more in-depth goal setting and motivational interviewing techniques to help patients reach more realistic post-discharge care goals.

### Session 3400 (Symposium)

### HEALTH DISPARITIES RESEARCH WITH THE NATIONAL INSTITUTE ON AGING (NIA)

Chair: Cerise Elliott Co-Chair: Patricia Jones
Discussant: Patricia Jones

The National Institute on Aging has taken special efforts to support research endeavors that explore ways to address health disparities. For example, the NIA Health Disparities Research Framework was developed in 2015 to provide a visualization of priority areas in Aging Research. The Framework can help researchers assess advances and potential opportunities for stimulating and supporting rigorous methods to address health disparities in Aging Research among the phases of research. The goal of this symposium is to highlight the different resources and research opportunities that NIA offers to support aging researchers, centers, and institutions for health disparities-related research or programs. Dr. Ron Kohanski will present a concept piece on how laboratory animals might be leveraged to mimic the impact of early life disparities on aging over the life-course, based on research in the hallmarks of aging support by NIA's Extramural Division of Aging Biology. Dr. Damali Martin will identify the different resources focused on health disparities related research within the Division of Neuroscience. Dr. Lyndon Joseph will discuss the different health disparities research opportunities that are available from the Division of Geriatrics and Clinical Geriatrics. Dr. Frank Bandiera will highlight the different resources and research opportunities that are available to address health disparities within the Division of Behavior and Social Research. These presentations, taken together, will provide important information that bolsters knowledge of resources and research opportunities to address health disparities over the lifecourse and in late life.

### CRITICAL THOUGHTS ABOUT HEALTH DISPARITIES WITH THE NIA DIVISION OF NEUROSCIENCE

Damali Martin,[1] and Cerise Elliott,[2], *1. NIA, NIH, Bethesda, Maryland, United States, 2. National Institute on Aging, Bethesda, Maryland, United States*

Population-based health disparities studies requires improved research design and appropriate research questions for investigation that will inform evidenced-based interventions and prevention strategies. The NIA Division of Neuroscience is committed to supporting new studies that 1) invests in health priorities as reflected by needs of minoritized populations (e.g. Race/Ethnic minorities; Rural or Sexual Gender Minorities); 2) examines Alzheimer's Disease and cognitive changes across the individual lifespan; and 3) understands intersectionality of cohorts and minimize potential biases in participant selection. This brief session will outline updated steps to permit critical thought about the use of the NIA's Health Disparities Framework to examine relevant biological, sociocultural, behavioral and environmental across multiple levels of influence.

### THE DIVISION OF AGING BIOLOGY ON BASIC RESEARCH IN HEALTH DISPARITIES

Ronald Kohanski, *National Institute on Aging, Bethesda, Maryland, United States*

The core mission of the Division of Aging Biology is to explore the molecular and cellular mechanisms of aging. Work supported by the Division is best known for research using laboratory animals, but we have a less well-recognized program of research engaging human participants. With a goal expanding our presence in clinical research and the hope that we can have an impact on health disparities, this talk will provide an overview of how our grantees approach basic mechanistic questions of aging in human communities. In addition, a forward-looking but speculative presentation will be made on ways in which laboratory animals might be used to study health disparities from the perspective of hallmarks of aging.

### HEALTH DISPARITIES SCIENTIFIC RESEARCH IN THE DIVISION OF GERIATRICS AND CLINICAL GERONTOLOGY

Lyndon Joseph, *NIA, Bethesda, Maryland, United States*

The Division of Geriatrics and Clinical Gerontology (DGCG) supports clinical and translational research on health and disease in the aged, and research on aging over the human lifespan, including its relationships to health outcomes. Key areas include development of new interventions for age-related conditions and pathologies, prevention and treatment of multiple chronic conditions, geriatric palliative care, factors influencing the progression of chronic diseases over the life span, and predictive markers of aging that may inform potential interventions for extension of health span. Population diversity and health disparities are critical aspects of science that cut across DGCG research areas. This presentation will highlight several examples of DGCG-supported studies related to health disparities and discuss potential future research directions. One potential upcoming research area of interest involves leveraging large data sets to examine disparities in risks and benefits of long-term osteoporosis drug therapy and drug holidays according to racial and ethnic groupings

### NIA DIVISION OF BEHAVIORAL AND SOCIAL RESEARCH PRIORITIES IN HEALTH DISPARITIES

Frank Bandiera, *National Institute on Aging, Bethesda, Maryland, United States*

This presentation will include priority research areas in health disparities in the NIA Division of Behavioral and Social Research (BSR). It will include a portfolio analysis and description of BSR program and research in health disparities. Specifically, the 2019 BSR National Advisory Committee on Aging (NACA) recommended BSR's number one research priority in health disparities. In 2020 BSR held a workshop on structural racism. BSR supports funding announcements in health disparities in Alzheimer's Disease and Related Dementias, sleep, Native American Health, and immigration among others. Key themes for BSR include racial disparities that often center on context and resources, that is, factors such as residential segregation, rurality, individual and neighborhood SES, and access to health care; persistent racial differences in chronic health conditions; disparities in health systems; immigration and nativity closely linked with race and health outcomes; and racial discrimination linked to poorer mental health and psychological stress.

### Session 3405 (Paper)

### HEALTHY AGING AND HEALTH PROMOTION

### DYNAMIC ASSOCIATIONS BETWEEN LIFESTYLE FACTORS AND POSITIVE AND NEGATIVE AFFECT IN MIDDLE-AGED AFRICAN AMERICANS

Raheem Paxton,[1] Chuong Bui,[2] Rebecca Allen,[1] and Edward Sazonov,[2], *1. University of Alabama, Tuscaloosa, Alabama, United States, 2. University of Alabama, University of Alabama, Alabama, United States*

**Purpose:** The purpose of this study was to examine the dynamic association between lifestyle factors and both positive and negative effect in middle-aged African Americans.

**Methods:** Study participants (N = 69, Mean age=51 years, 80% female) were recruited from two African American churches in the Deep South. Participants completed daily surveys on positive and negative effect, physical activity, sedentary behavior, diet quality, and sleep quality daily for up to 10-days. Mixed-effect models were used to examine associations between the variables of interest.

**Results:** On days that participants were more active, they experienced higher mean positive effect (P = .015) and lower mean negative effect (P = .028) scores. Conversely, more time spent sitting in lagged models (i.e., T-1) was associated with higher mean negative effect (P = .001) and lower mean positive effect (P = .040) scores. In lagged models, better sleep quality was associated with higher positive effects (P = .007) scores but reported lower negative effects (P < .0001) scores on the same day. Lastly, on days where diet quality was higher, positive effect scores were higher (P < 0.05). Association between diet quality and positive effect was moderated by age (P = .025).

**Conclusion:** The data suggest that same and previous day health behaviors may have a significant impact on the health and well-being of middle-aged African Americans. More research is needed to determine whether these behaviors can be targeted in real-time as a means of improving mental health outcomes in this population.

### INTRINSIC CAPACITY AND ITS RELATIONSHIP WITH LIFE SPACE AREA (INCREASE) IN COMMUNITY-DWELLING OLDER ADULTS

Jia Qi Lee,[1] Yew Yoong Ding,[2] Laura Tay,[3] Aisyah Latib,[3] and Yee Sien Ng,[4], *1. Duke-NUS Graduate Medical School, Singapore, Singapore, 2. Geriatric Education and Research Institute, Singapore, Not Applicable, Singapore, 3. Sengkang General Hospital, Singapore, Not Applicable, Singapore, 4. Singapore General Hospital, Singapore, Not Applicable, Singapore*

Intrinsic capacity (IC), defined as 'the composite of all physical and mental capacities of an individual', is of increasing interest in geriatrics as a potential multidimensional measure of health in older adults. According to the International Classification of Functioning, Disability and Health (ICF) framework, IC, through its interactions with environmental factors, determines a person's participation in the community. However, there is lack of empirical evidence demonstrating this association. The primary aim of this study was to examine the association of IC with Life Space Area (LSA; a measure of participation) among community-dwelling older adults. The secondary aim was to determine whether age and gender modify this relationship. Cross sectional analysis was performed on data from the Individual Physical Proficiency Test for Seniors (IPPT-S) study conducted in the Northeastern region of Singapore. Standardized IC factor scores were calculated through confirmatory factor analysis using variables that represented the 5 IC domains. Association of IC with LSA and its effect modification by age and gender were examined with regression analyses. The study included 751 participants with mean age of 67.6 and mean LSA score of 88.6. IC showed a positive and significant association with LSA (B=6.33, P<0.001) and the effect remained significant even after controlling for potential confounders (B=4.76, P<0.001). Age and gender did not show significant modification on this relationship. Our findings support the empirical rigour of the ICF framework and provide guidance for healthcare providers who aim to enhance life space mobility and promote healthy aging in older adults.

### PERCEPTIONS OF HEALTHY AGING AMONG COMMUNITY-DWELLING OLDER PERSONS IN AN URBAN SLUM IN IBADAN, NIGERIA

Olubukola Omobowale, *University of Ibadan, Nigeria, Ibadan, Oyo, Nigeria*

**Background:** Healthy Ageing is the process of the development and maintenance of functional capacity which allows well-being at an older age. This understanding is comprehensive and relevant for all older persons. Despite the global attention being given to healthy ageing, there is limited information on the perceptions of this concept among older persons in low resource settings like Nigeria, hence the need for this study.

**Methods:** In depth interviews were conducted among older persons aged 60 years and above residing in Idikan

Community, an urban slum area in Ibadan, Nigeria. Using an interview guide, perceptions of older persons on meanings of healthy ageing, factors related to healthy ageing and experience of ageing were explored.

**Results:** A total number of 24 interviews were conducted. Slightly more than half of the respondents were females. The majority of the respondents were of the opinion that healthy ageing is about being " strong" and able to move around, without being dependent on anyone for mobility and activities of daily living. In their opinion, healthy ageing is related to different health dimensions: biological (adoption of healthy habits and behaviors with self-responsibility, psychological (feelings of optimism and happiness), spiritual (faith and religiosity) and family and social support (healthy and well children, friends and family).

**Conclusion:** Urban Community dwelling older persons' perception of healthy ageing was positive and incorporating their opinions on healthy aging from the perspective of the older persons can support the activities of professionals who work with this population group.

### REDUCING ASPIRATION PNEUMONIA RISK FOR OLDER PEOPLE: EFFECT OF EVIDENCE-BASED ORAL CARE

Lynette Goldberg,[1] Leonard Crocombe,[2] Silvana Bettiol,[3] Anna King,[4] and Sangeeta Khadka,[3], *1. University of Tasmania, Hobart, Tasmania, Australia, 2. Latrobe University, Latrobe University Melbourne, Victoria, Australia, 3. University of Tasmania, University of Tasmania, Hobart, Tasmania, Australia, 4. University of Tasmania, University of Tasmania, Tasmania, Australia*

Poor oral health increases the risk of aspiration pneumonia for older people. This is due primarily to six pathogens found in the mouth: five bacteria and one fungus. With a cohort of older people who were dependent on others for their oral care, we analyzed the load and type of bacteria and fungi from swabs of cheek, gum, and tongue mucosa. There were no significant differences between the three sites for load of bacteria (H (2) = .89; p = .64); there were significant differences between the sites for type of bacteria (F (2,78) = 11.97; p <.001) with the tongue showing the greatest diversity. There were no significant differences between the three sites for load (H (2) = 2.94; p = .23) or type (F (2,77) = .46; p = .63) of fungi. We then investigated the effect of regular compared to evidence-based oral care over a six-week period, and whether evidence-based oral care could significantly reduce the absolute count of the six oral pathogens specifically related to aspiration pneumonia. Participants self-selected into Regular Care (n = 10) and Evidence-based Care (n = 17) Groups. Evidence-based oral care resulted in significant decreases (p = .02 to p < .001) in the load of four potentially pathogenic bacterial species, including E. coli, gut-based bacteria, and in an increased load of Lactobacillus reuteri, a host-protective normal flora in the mouth, compared to baseline. There were no significant differences between groups for the abundance and type of fungi.

### USING IMPLEMENTATION SCIENCE STRATEGY MAPPING ON THE AGE-FRIENDLY HEALTH SYSTEM'S 4MS JOURNEY

Mary Dolansky,[1] Anne pohnert,[2] and Sherry Greenberg,[3], *1. Case Western Reserve University, Cleveland, Ohio, United States, 2. CVS Minute Clinic, Woonsocket,, Rhode Island, United States, 3. Seton Hall University College of Nursing, Seton Hall University College of Nursing, New Jersey, United States*

**Background:** Implementation science is the study of methods to promote the adoption and integration of evidence-based practices into routine health care to improve the quality of care. The purpose of this study was to use Implementation Mapping to guide the implementation of The John A. Hartford evidence-based Age-Friendly Health Systems (AFHS) 4Ms Framework: What Matters, Medications, Mentation, and Mobility.

**Methods:** Implementation Mapping, a systematic process for planning implementation strategies, guided the 9-month integration of the 4Ms Framework in the 1,100 MinuteClinics across the US. Implementation Mapping includes five tasks: (1) conduct an implementation needs assessment and identify program adopters and implementers; (2) state adoption and implementation outcomes and performance objectives, identify determinants, and create matrices of change objectives; (3) choose theoretical methods (mechanisms of change) and select or design implementation strategies; (4) produce implementation protocols and materials; and (5) evaluate implementation outcomes.

**Results:** The implementation plan, developed by the implementation mapping method, was carried out over 9-months. Seven implementation strategies were identified from the Expert Recommendations for Implementing Change (ERIC) project including the provision of education, electronic health record integration, internal champion facilitation, cues to action, and a dashboard to monitor progress. To date, the implementation mapping has resulted in the adoption of the 4Ms by 1145 providers (37%). Monitoring of the adoption of the 4Ms Framework and consideration of future implementation strategies is ongoing.

**Conclusions:** Implementation Mapping provided a systematic process to develop strategies to improve the adoption, implementation, sustainment, and scale-up of the evidence-based 4Ms Framework.

### Session 3410 (Paper)

### INTERGENERATIONAL TEACHING, LEARNING, AND ENGAGEMENT

### EFFECTIVENESS OF AN INTERGENERATIONAL SERVICE-LEARNING PROGRAM TO CHANGE ATTITUDES ON AGING

Sara Bartlett,[1] Sky Bergman,[2] Phyllis Solomon,[3] and Zvi Gellis,[3], *1. Cal Poly, San Luis Obispo, Los Osos, California, United States, 2. Cal Poly, San Luis Obispo, San Luis Obispo, California, United States, 3. University of Pennsylvania, Philadelphia, Pennsylvania, United States*

This study evaluated the efficacy of a 10-hour intergenerational service-learning program administered to undergraduates to determine if it would increase knowledge about aging, improve attitudes about older adults, and reduce ageism more than a course with less service-learning activity. Making maximum impact on students in these areas in a short amount of time is particularly relevant in short, quarter-based university programs. A quasi-experimental design using a convenience sample compared pre-test and post-test scores between an experimental intervention (N=68) and a comparison (N=71) group on The Facts on Aging Quiz Multiple Choice version, Aging Semantic Differential, and Fabroni Scale on Ageism. Qualitative data via open-ended survey questions was also collected. The experimental intervention, the Lives Well Lived project, was based on a documentary film by the same name, which incorporates themes of successful aging. During the project students and older adults interviewed one another about living a life well lived, participated in a photo shoot, and created a Memoir for the older adult. The comparison group included two social visits to a congregate meal program. Results from multiple regression analysis showed that students in the intervention group had less ageist stereotypes and less negative bias about aging at post-test and qualitative data indicated they were more inclined to participate in intergenerational relationships in the future. Programs like this one that are longer and more relational may be useful for consideration in undergraduate gerontology courses in reducing ageism and promoting intergenerational relationships which benefit those of all ages.

### FACULTY PERCEPTIONS OF ENGAGING OLDER ADULTS IN HIGHER EDUCATION: THE NEED FOR INTERGENERATIONAL PEDAGOGY

Jason Dauenhauer,[1] Afeez Hazzan,[2] and Kristin Heffernan,[3], *1. State University of New York, Brockport, Brockport, New York, United States, 2. State University of New York at Brockport, Hilton, New York, United States, 3. State University of New York, Brockport, State University of New York, Brockport, New York, United States*

Institutions of higher education need to become more age friendly. Creating an on-campus lifelong learning program can offer older adults opportunities to audit classes and engage in multigenerational classrooms, but can also promote intergenerational learning when instructors consciously use pedagogy that fosters engagement between learners from various generations. Promoting intergenerational learning to facilitate reciprocal sharing of expertise between generations is also the fourth principle of the Age Friendly University framework. This qualitative interview study examines the perspectives of 27 faculty members who have opened their face to face classrooms to older adult auditors to 1) Explore perceived benefits and challenges associated with having older adults in the college classroom and to 2) Determine what levels of intergenerational learning may be taking place. Compared to lecture-based courses, faculty whose pedagogy promotes discussion, sharing, and small group work reported detailed examples of older adult learners and traditionally-aged college students engaging in course-related discussion. The unique, historical and diverse perspectives of older adults improved the quality of education for students, and fostered in-depth learning. Challenges related to older adult auditors included poor/limited attendance, sharing of strong opinions/dominating class discussion, sensory/mobility and technology accessibility. Recommendations include training to promote intergenerational engagement in college classrooms.

### GOVERNMENT AND UNIVERSITY PARTNER FOR VIRTUAL SERVICE-LEARNING TO SUPPORT INTERGENERATIONAL SOCIAL INTERACTION

Martha Kemeny,[1] Adelle Williams,[1] and Stephanie Cole,[2] *1. Slippery Rock University, Slippery Rock, Pennsylvania, United States, 2. State of Pennsylvania, Harrisburg, Pennsylvania, United States*

Pre-pandemic, evidence existed that intergenerational service-learning programs support knowledge of aging and positive attitudes and perceptions (Monahan et al., 2020). As spring 2020 COVID-19 lock downs and public health warnings urged physical distancing of community dwelling older adults, growing concern about the unintended consequences of increased social isolation on mental and physical health prompted the Secretary's Office of Pennsylvania Department of Aging to design a pilot project with university faculty for virtual intergenerational social interaction. The Department identified older adults at the highest risk for social isolation (live alone, in poverty, with a disability). The resulting pilot project is fully integrated as a high impact practice into eight sections of recreational therapy and gerontology courses with participation by 210 undergraduate students and 210 older adults for 9 weeks of both the fall and spring semesters. Students, who received extensive classroom instruction aimed at avoiding negative stereotypes of older adults as helpless and dependent, called their assigned partner several times a week for at least an hour of communication. Using the UCLA loneliness scale, community-dwelling older adults reported frustration with isolation due to the pandemic. Those with low and moderate loneliness reported positive feelings about program and looking forward to interactions with students. Students gained virtual communication skills that may contribute to telehealth competencies, intervention skills such as assessment, life review/reminiscence, mindfulness techniques, and leisure education. Moreover, an analysis of student reflections revealed positive changes in attitudes toward older adults and the ability to enjoy common interests despite age differences.

### PROMOTING INTERGENERATIONAL ENGAGEMENT WITHIN THE COLLEGE CLASSROOM: FACULTY TRAINING NEEDS

Afeez Hazzan,[1] Kristin Heffernan,[2] and Jason Dauenhauer,[3], *1. State University of New York at Brockport, Hilton, New York, United States, 2. State University of New York, Brockport, State University of New York, Brockport, New York, United States, 3. State University of New York, Brockport, Brockport, New York, United States*

As population aging accelerates worldwide, institutions of higher education are increasing efforts to focus on ways to meet the growing needs of older adult learners. Many institutions are addressing these needs by joining the Age-Friendly University (AFU) Global Network. Affiliated institutions are required to promote intergenerational learning to facilitate the reciprocal sharing of expertise between learners of all ages, including older adults. However, these institutions will need to provide instructors with the training to ensure that intergenerational engagement is being actively fostered in the classrooms. In this study, we examine the perspectives of faculty members who have opened their classrooms to older adult auditors. The research question was: What types of training do faculty recommend to promote intergenerational engagement in the classroom? In-depth face to face interviews were conducted with 27 faculty members. Qualitative content analysis of the data yielded the following four themes: 1) Provide accessible training to teach faculty their role 2) Educate faculty about the importance of becoming aware of generational time periods/context 3) Learn to approach auditors with a mindset that they are adults and have had careers/experiences, and 4) Train faculty on how to foster discussion. Overall, these findings point to a need for training that focus on intergenerational curricular design and multigenerational classroom management.

### STUDENTS' ATTITUDE CHANGE: VIRTUAL VS. IN-PERSON INTERGENERATIONAL, ARTS-BASED COURSES

Meghan Young,[1] Elizabeth Lokon,[2] and Yue Li,[2], *1. Scripps Gerontology Center, Miami University, Oxford, Ohio, United States, 2. Miami University, Oxford, Ohio, United States*

When higher education classes went virtual at the start of the COVID-19 pandemic, converting an in-person, arts-based, service-learning course into a meaningful, virtual experience seemed impossible. However, the Opening Minds through Art (OMA) program developed online courses where students met older adults weekly over Zoom to create and discuss art. Undergraduate and graduate students at Miami and Marian Universities (n=47) came from more than 20 different areas of study and had varying knowledge of gerontology and dementia. Pre- and post-assessments were administered at the start and end of the academic semester. Paired-samples t-tests were conducted to examine pre-post changes in students' attitudes toward people living with dementia (PLWD) using the Dementia Attitude Scale (DAS) (O'Connor & McFadden, 2010) and the extent students actually like PLWD using the Allophilia scale (Pittinsky et al, 2011). Students in virtual OMA courses showed significant improvement in overall DAS and Allophilia scores and all subdomain scores (i.e., general knowledge about dementia, affection, social comfort level, kinship, and engagement and enthusiasm when interacting with PLWD), with moderate to high effect sizes (Cohen's d range between 0.39 and 1.10). The magnitudes of these effect sizes for virtual OMA are comparable to previous studies examining students' participation in face-to-face OMA sessions, where Cohen's d on DAS and Allophilia scales ranged between 0.48 and 1.07 (Lokon et al, 2017, 2018). Overall, we found that it is possible to design virtual service-learning courses that improve students' attitudes toward PLWD as effectively as face-to-face courses.

### Session 3415 (Symposium)

### KUAKINI HHP CENTER FOR TRANSLATIONAL RESEARCH ON AGING: AIMS AND PRELIMINARY FINDINGS

Chair: Bradley Willcox Co-Chair: Richard Allsopp
Discussant: Peter Martin

Kuakini Medical Center (Kuakini) is creating an interdisciplinary Hawai'i-based Center for translational research on aging. This Center will build upon Kuakini's five-decades of NIH-funded research, its 420,000-specimen biorepository, and existing strengths in aging research, notably, the 56-year ongoing Kuakini Honolulu Heart Program cohort study (Kuakini HHP), Kuakini Honolulu-Asia Aging Study (Kuakini HAAS), and Kuakini HHP Offspring Study. The overall goal is to find practical means to enhance healthy human lifespan (healthspan). Four research project leaders (RPLs) have been selected from various disciplines for mentorship in translational aging research. The first RPL presentation will introduce a novel mouse model, enabling controlled expression of the pro-longevity gene FoxO3, and assess the impact on lifespan and healthspan phenotypes in mice. These phenotypes will be compared to similar phenotypes in humans with/without the FOXO3 longevity genotype. The second RPL presentation will assess the relation between leukocyte telomere attrition rates (from banked blood collected at three time points over 20-plus years) in older Kuakini HHP men with/without the FOXO3 longevity genotype. The third RPL presentation will assess whether FOXO3 genotype, peripheral leukocyte telomere dynamics (attrition rate, telomerase activity) and inflammatory cytokines mediate the human brain integrity and function with age. This project will utilize structural and functional MRI data from male and female Kuakini HHP Offspring Study participants. The fourth RPL presentation will assess whether APOE e2, e4, and FOXO3 longevity-associated alleles impact 34-year incidence of intracerebral hemorrhage. We will summarize the findings, address the healthspan implications and provide future directions. Supported by NIH 5P20GM125526.

### KUAKINI HHP CENTER FOR TRANSLATIONAL RESEARCH ON AGING: AN INTRODUCTION

Kamal Masaki,[1] Tim Donlon,[2] Donald Willcox,[3] Brian Morris,[4] Richard Allsopp,[1] and Bradley Willcox,[5], *1. Kuakini Medical Center, Honolulu, Hawaii, United States, 2. JABSOM, Honolulu, Hawaii, United States, 3. Department Of Gerontology And Public Health, Okinawa International University, Okinawa, Japan, 4. University of Sydney, Sydney, New South Wales, Australia, 5. Kuakini Medical Center, Kuakini Medical Center/Honolulu, Hawaii, United States*

sKuakini Medical Center (Kuakini) is establishing an interdisciplinary Hawai'i-based Center of Biomedical Research Excellence (COBRE) for clinical and translational research on aging. - Specific Aim 1: Create a world class, innovative, interdisciplinary Center for Translational Research on Aging by building on the existing rare and valuable Kuakini Honolulu Heart Program (Kuakini HHP) and its related studies and research infrastructure. - Specific Aim 2: Advance the genomic, biological and clinical science of aging, and enhance the world class character of our Center, by enlisting accomplished senior scientists to mentor promising Hawaii-based new investigators in interdisciplinary aging research and career development. - Specific Aim 3: Elevate the capacity of our Center by: i) creating a state-of-the-art Clinical and Translational Core, and ii) collaborating with other Centers of Biomedical Research Excellence. This presentation will briefly introduce the Center and discuss its aims, research strategy, and future plans.

### ASSESSING LIFESPAN AND AGING PHENOTYPES RESULTING FROM FOXO3 INDUCTION USING MOUSE MODELS

Brian Hew,[1] Christopher Tran,[1] Kristal Xie,[1] Bradley Willcox,[2] and Jesse Owens,[1], *1. Kuakini Medical Center, Honolulu, Hawaii, United States, 2. Kuakini Medical Center, Kuakini Medical Center/Honolulu, Hawaii, United States*

Environmental signals, including caloric restriction and oxidative stress, trigger FoxO3 to upregulate genes involved in stress resistance, metabolism, cell cycle arrest, and apoptosis that may help mitigate age-related diseases. Activation of FoxO3 has been shown to have a profound life-extending effect on model organisms. Protective SNPs in FOXO3 are strongly associated with exceptional longevity in humans. The objective of this study is test the relation between FoxO3 and longevity using mouse models. We generated a mouse line containing an extra copy of FoxO3 that can be induced at any age. In our model, FoxO3 remains driven by its natural promoter to avoid mis-expression in inappropriate cells and to maintain the gene's ability to respond to signals such as stress. We are utilizing this new model to assess survival endpoints and test a panel of aging phenotypes reflecting healthspan throughout the mouse lifespan and compare these to similar human phenotypes.

### ANALYSIS OF CELL AND MOLECULAR PHENOTYPES OF A HIGHLY REPLICATED LONGEVITY-ASSOCIATED FOXO3 VARIANT IN OLDER ADULTS

Randi Chen,[1] Richard Allsopp,[1] Bradley Willcox,[2] and Philip Davy,[1], *1. Kuakini Medical Center, Honolulu, Hawaii, United States, 2. Kuakini Medical Center, Kuakini Medical Center/Honolulu, Hawaii, United States*

Aging demographics in the US, and other industrialized nations, are resulting in rapidly increasing health care costs from age-related diseases. New therapeutic interventions to extend healthspan in older adults requires understanding connections between basic aging biology and human longevity factors. Using clinical samples from the Kuakini Honolulu Heart Program (HHP) and their Offspring, we are examining potential links between molecular and cellular mechanisms of aging and the longevity associated FOXO3 genotype (carriage of SNP rs2802292 "G" allele). Telomere dynamics in leucocytes (LTL) have shown strong correlation with multiple lifestyle and health factors. We previously demonstrated a significant protective relation between FOXO3 longevity genotype and LTL in a cross-sectional study. Now we are assessing a longitudinal relation, at three time points over 20+ years, in older men. We are also exploring stem cell frequency and differentiation capacity in neurological and peripheral blood samples to assess FOXO3 genotype and human cell dynamics.

### FOXO3, TELOMERE DYNAMICS, AND HEALTHY BRAIN AGING: A COBRE STUDY

Bradley Willcox,[1] Kamal Masaki,[2] Richard Allsopp,[2] and Kalpana Kallianpur,[2], *1. Kuakini Medical Center, Kuakini Medical Center/Honolulu, Hawaii, United States, 2. Kuakini Medical Center, Honolulu, Hawaii, United States*

Human longevity is linked to genetic, cellular, and other complex biological and psychosocial traits. Aging is typically accompanied by gradual brain atrophy and cognitive decline, but the mechanisms are unclear. Cellular aging, characterized by telomere shortening and altered telomerase activity, is related to mortality and brain aging. Decelerated brain aging is associated with greater peripheral blood leukocyte telomere length (LTL) and, we hypothesize, may be linked to FOXO3 genotype. We will use MRI to assess brain structure and function cross-sectionally in 100 Kuakini Honolulu Heart Program Offspring. Atrophy and disrupted functional connectivity, markers of brain aging, will be examined in relation to FOXO3 and LTL. Associations between brain structural and functional differences, FOXO3 genotype and LTL will be investigated over a wide range of ages, controlling for other biological and psychosocial factors. Results may provide insight into mechanisms influencing the rate of brain aging, and may eventually extend human healthspan.

### THE IMPACT OF APOE AND FOXO3 GENOTYPE ON THE RISK OF INTRACEREBRAL HEMORRHAGE AMONG AMERICAN MEN OF JAPANESE ANCESTRY

Randi Chen,[1] Steven Greenberg,[2] G. Ross,[3] Bradley Willcox,[4] Kamal Masaki,[1] and Kazuma Nakagawa,[5], *1. Kuakini Medical Center, Honolulu, Hawaii, United States, 2. Massachusetts General Hospital, Boston, Massachusetts, United States, 3. VA Pacific Islands Health Care System, Honolulu, Hawaii, United States, 4. Kuakini Medical Center, Kuakini Medical Center/Honolulu, Hawaii, United States, 5. University of Hawaii and Kuakini Medical Center, Honolulu, Hawaii, United States*

This study assessed the impact of APOE e2, e4 minor alleles and the FOXO3 longevity-associated genotype (carrier of SNP rs2802292 "G" allele) on 34-year incidence of intracerebral hemorrhage (ICH). Cox regression models were performed to assess the impact of the APOE e2, e4 and FOXO3 G alleles on the incidence of ICH. A total of 6483 participants were eligible for the analyses. 213 participants developed ICH. Cox-regression model showed neither APOE minor allele vs. common genotype (APOE e3/e3: RR 0.89, 95% CI: 0.64-1.22, p=0.46) nor FOXO3 G carrier status (RR 0.97, 95% CI: 0.72-1.29, p=0.82) was associated with incident ICH. Conversely, both hypertension (RR: 1.46, 95% CI: 1.07-2.00, p=0.02) and low cholesterol level (RR: 0.99, 95% CI: 0.99-1.00, p=0.001) were associated with incident ICH. Carriage of APOE e2 or E4 alleles and the FOXO3 G allele do not appear to impact risk of ICH over 34 years in this cohort.

### Session 3420 (Symposium)

### LEVERAGING ACL FUNDING TO IMPLEMENT AN EVIDENCE-BASED FALLS PREVENTION PROGRAM IN THREE GWEPS

Chair: Ellen Flaherty Discussant: Nina Tumosa

Primary care practices have a robust capacity to screen older adults for falls risk and refer them to evidence-based falls prevention programs delivered by Community Based Organizations (CBOs). However, due to a difference in the culture and nature of the work done in these two systems of care, there is often a lack of coordination and communication. Dartmouth has worked to bridge this gap for the past five years through our Health Resources and Services Administration (HRSA)-funded Geriatric Workforce Enhancement Program (GWEP). GWEP goals include the promotion of Age-Friendly Health Systems by focusing on the 4 Ms: What Matters Most, Medication, Mentation and Mobility. GWEPs commonly operationalize the Mobility component via falls risk screening and prevention programs. Though CBOs are well suited to deliver falls prevention programs, implementing, disseminating and sustaining community-based falls prevention programs in an environment of cost containment, limited funds for community-based services and workforce issues is challenging. Previous Administration for Community Living (ACL) grant funding enabled us to develop the Dartmouth Falls Prevention Training Center (D-TC) using our expertise in training and community-based implementation of evidence-based interventions. The D-TC offers training and implementation support to primary care and CBOs on screening, referring and capacity-building for falls prevention programs. We will discuss challenges and successes implementing the Dartmouth falls prevention model with two additional GWEP grantees, Baystate and the University of Rhode Island. Benefits of leveraging ACL and HRSA funding to achieve synergistic goals to reduce falls in older adults will be explored.

### THE DARTMOUTH FALLS PREVENTION TRAINING PROGRAM: PRIMARY CARE AND COMMUNITY-BASED ORGANIZATION COLLABORATION

Dawna Pidgeon, *Dartmouth Hitchcock Medical Center, Lebanon, New Hampshire, United States*

Falls are a leading cause of fatal and non-fatal injuries in older adults. Older adult participation in community-based falls prevention programs can significantly reduce falls risk, however, identifying and referring individuals to appropriate programs can be challenging. Through Administration for Community Living (ACL) funding, we have developed a comprehensive Dartmouth Falls Prevention Training Program for healthcare and community based organizations that includes (1) Falls screening in primary care; (2) “Balance Days”, a community-based education and balance screening event encompassing falls risk stratification and coaching into programs; (3) Instructor Training for Tai Ji Quan: Moving for Better Balance®, a highly effective falls prevention program; and (4) Implementation Training, a research informed workshop shown to enhance community-based program sustainability through participant retention. We will share strategies for sustainable collaborations between primary care and CBOs to reach at-risk individuals and improve lives and decrease costs associated with falls.

### ADOPTING AND ADAPTING A FALLS PREVENTION PROGRAM: LESSONS LEARNED FROM IMPLEMENTING A MODEL FROM A DIFFERENT CONTEXT

Carol Petrie, Christine Ferrone, Phillip Clark, and Alexandra Morelli, *University of Rhode Island, Kingston, Rhode Island, United States*

Geriatric Workforce Enhancement Programs (GWEPs) are ideally suited to develop and implement educational programs to transform the geriatric care system. They link academic programs, clinical partners, and community-based organizations to bridge care system gaps to improve the health and social care of older adults. Such a collaboration is especially important in falls prevention, where primary care assessments generate referrals to community programs that enroll older adults to reduce their risk of falling. However, exporting an evidence-based model developed in one context for implementation in another is not without its perils and pitfalls. This paper explores the challenges of applying a model developed elsewhere to the Rhode Island context, including the need to understand how structural differences in academic, primary care, and community-based systems require flexibility, innovation, and persistence in overcoming the networking challenges in these different settings. Recommendations for implementing program models in a variety of settings are explored.

### ADAPTING TO CREATE INNOVATIVE VIRTUAL FALLS PREVENTION PROGRAMS FOR AT RISK OLDER ADULTS DURING A GLOBAL PANDEMIC

Maura Brennan, and Rebecca Dobert, *Baystate Health, Springfield, Massachusetts, United States*

Baystate Health’s Geriatrics Workforce Enhancement Program (GWEP) postponed implementation of Group Medical Visits focused on falls reduction for older adults in Springfield, Massachusetts due to COVID-19 and quickly shifted efforts to participate in Dartmouth’s Falls Prevention Training Program. Long standing GWEP Community Based Organizations (CBOs) were consulted, and all believed that the virtual Tai Ji Quan Moving for Better Balance® (TJQMBB) program would combat social isolation and improve older adults’ comfort with technology in addition to reducing falls during the COVID-19 pandemic. Baystate’s GWEP was able to reallocate grant dollars to support the purchase of equipment for CBOs to deliver TJQMBB virtually. While many challenges continue to arise, the innovative and collaborative approach between the two GWEPs and Baystate’s CBOs leveraging Administration for Community Living falls prevention funding has led to high level engagement and rapid implementation. Dartmouth’s model capitalizes on and strengthens existing GWEP partnerships with its CBOs.

### Session 3425 (Symposium)

### LEVERAGING PROMISING POLICIES TO SUPPORT LONG-TERM CARE RESIDENTS' QUALITY OF LIFE POST-PANDEMIC

Chair: Deanne Taylor Co-Chair: Janice Keefe
Discussant: Heather Cook

Long-term care (LTC) is highly regulated and often the policy language is complex and in tension with residents’ quality of life goals. Prior to COVID-19, LTC policy levers prioritized safety over other quality domains such as privacy, dignity, spirituality, and comfort. During the pandemic, this focus on safety regulations, while important, intensified in ways that often negatively impacted residents’ overall quality of life. In this symposium, we share findings from a five- year research project where we conducted a unique and expansive review of regulatory policy across four Canadian jurisdictions. We highlight how 11 different quality of life domains are supported and which texts offering promising policy language to enhance a well-rounded quality of life for residents. These are timely insights to offer as policy-makers look to the future and consider the lessons learned from the pandemic. We contend that creating more LTC policy is not a timely pathway forward to LTC reform. Instead, we suggest that existing policy can be leveraged when applied within a resident-centred quality of life lens. We will guide attendees through examples of existing promising policies highlighting how they might leveraged in planning for a better LTC system. The discussion will be rooted in our unique resident-centred approach to policy analysis using specific domains of quality of life and then applied to four different perspectives: residents, families, staff and volunteers. Our discussant a Ministry of Health decision-maker will address the implications of our research for post-pandemic planning to improve resident quality of life

### FAMILIES AS VALUED CONTRIBUTORS TO LTC RESIDENTS' QUALITY OF LIFE: POLICY PERSPECTIVES

Janice Keefe, *Mount Saint Vincent University, Halifax, Nova Scotia, Canada*

Family members are essential contributors to Q0L of LTC residents. This paper analyzes how the system views family's role in residents' QoL and enables or inhibits family involvement. Our analysis of 21 policies that regulate LTC in four Canadian Provinces reveal differences in their portrayal of residents' families. In many policies, family roles are characterized procedurally (task-oriented) or relationally (interactive) by policy type. Operational standards (regulatory policies) linked to licensing employ more formal terminology, while LTC program guidelines use facilitative language to engage families and build relationships through voluntary means. Specific examples of orientation and admission procedures, care protocols including use of restraints, right to live at risk, and end-of-life care are presented to reveal inter-provincial variations. We argue there are opportunities to further engage families within the current regulatory framework.

### PROMISING LONG-TERM RESIDENTIAL CARE POLICY GUIDANCE FOR STAFF TO SUPPORT RESIDENT QUALITY OF LIFE

Mary Jean Hande, *Mount Saint Vincent University, Halifax, Nova Scotia, Canada*

This paper reviews 63 policy documents in four Canadian jurisdictions that guide long term residential care staff on how to enhance 11 resident quality of life in Canada. We found guidance in each jurisdiction that provide clear language to support staff discretion and flexibility to navigate regulatory tensions and enhance resident quality of life. Newer policies tend to reflect more interpretive approaches to staff flexibility and broader quality of life concepts. We argue that if interpreted through a resident quality of life lens and with the right structural supports, these promising texts offer important counters to the rigidity of long term residential care policy landscape and can be leveraged to effectively broaden and enhance quality of life for residents in long term residential care.

### TRACING THE EXPRESSION OF RESIDENT QUALITY OF LIFE POLICIES IN CANADIAN LONG-TERM CARE SETTINGS

Pamela Irwin, and Janice Keefe, *Mount Saint Vincent University, Halifax, Nova Scotia, Canada*

Policies favouring safety, security, and order are expressed in preference to those oriented towards person-centred resident quality of life in Canadian long-term care settings. Factors impacting the expression of these latent (under-utilised) rules were uncovered through an analysis of long-term care related policies in four provinces. 84 policies relating to resident quality of life in long-term care were analysed in three sequences, incorporating jurisdictions, policy types, and quality of life domains, over time. The analysis revealed three policy levers: situations–providing explicit and implicit examples of resident oriented quality of life policy suppression in each jurisdiction; structures–identifying which types of policy and quality of life expressions are more vulnerable to dominance by others; and trajectories–confirming the cultural shift towards more person-centredness in Canadian long-term care related policies over time. Although these policies exist, their potentiality remains dormant in the dominant policy discourse, thereby signaling a positive post-pandemic possibility.

### DOES LONG-TERM CARE POLICY ENABLE OR LIMIT VOLUNTEERS' ROLES IN ENHANCING RESIDENT QUALITY OF LIFE?

Emily Hubley, and Mary Jean Hande, *Mount Saint Vincent University, Halifax, Nova Scotia, Canada*

This paper examines how volunteer roles are represented in Canadian long term care (LTC) policy in four Canadian jurisdictions, attending to how these regulated roles might impact resident quality of life. Overall, we found that policies define volunteer roles narrowly, which may limit residents' quality of life. This happens through (1) omitting volunteers from most regulatory policy, (2) likening volunteers to supplementary staff rather than caregivers with unique roles, and (3) over-emphasizing residents' safety, security and order. We offer insights into promising provincial policy directions for LTC volunteers, yet we caution against further regulating volunteers. Instead, we argue, addressing the cultural, social and structural changes required for volunteers to enhance LTC residents' quality of life effectively.

### Session 3430 (Symposium)

### MAINTAINING ENERGY: A POTENTIAL TRANSFORMATIVE POWER TO ADAPT TO THE CHALLENGES OF OLDER AGE?

Chair: Rebecca Ehrenkranz

Reduced energy is a hallmark feature of aging. Maintaining higher energy late in life may be a key adaptive strategy to the challenges that accompany older age and ultimately promote resilience. Perceived lack of energy is often construed as synonymous with fatigue, and energy and fatigue are frequently considered opposite aspects of the same phenomenon. However, evidence suggests that energy and fatigue have distinct underlying neurobiology. Further exploration of the energy/fatigue dichotomy is needed in community-dwelling older adults free of neuropathologies and clinically overt conditions. This symposium will first present clinical and epidemiologic justifications for operationalizing energy as a separate construct from fatigue and then will provide evidence on the underlying neurobiological correlates. Taken together, our results suggest perceived energy: a) overlaps with but is distinct from lower fatigability (Katz); b) may signal resilience against age-related declining mood and gait speed despite self-reported tiredness (Ehrenkranz); c) appear negatively influenced by Alzheimer's neuropathology (Dougherty); and d) may reflect a distinct spatial distribution of brain functional connectivity (Hengenius). Thus, this symposium will explore energy as a mechanism related to yet distinct from fatigue and its implications for both healthy aging and neuropathological processes.

### ENERGY AND FATIGUE PREDICT GAIT SPEED AND MOOD DECLINE: RESULTS FROM THE HEALTH, AGING AND BODY COMPOSITION STUDY

Xiaonan Zhu,[1] Nancy W. Glynn,[2] Caterina Rosano,[1] and Rebecca Ehrenkranz,[1], *1. University of Pittsburgh, Pittsburgh, Pennsylvania, United States, 2. University of Pittsburgh Graduate School of Public Health, Pittsburgh, Pennsylvania, United States*

Older adults may report high energy alongside tiredness or vice versa; little is known about whether discordant self-reported energy (SEL) and tiredness predict trajectories of mood, cognition, or gait speed. SEL (0-10 scale dichotomized at median) and tiredness (present/absent) were obtained in 2,613 older adults (aged 74.6± 2.87 years) and used to create four groups (energized/not tired, low energy/tired, energized/tired, low energy/not tired). Center for Epidemiologic Studies Depression Scale (CES-D) and gait speed were measured over 10 years; mixed effect models compared trajectories in these domains across each group with low energy/tired group as referent. Each group was significantly associated with CES-D and gait. Adjusting for demographics, the high SEL/not tired group showed the least decline in mood (ß = -0.17, p<0.01); the high SEL/tired group showed the least decline in rapid gait (ß = 0.008, p = 0.02). High SEL may indicate resilience for mood and gait speed decline.

### ENERGY AND EXHAUSTION MAY EXPLAIN DIFFERENT SUBDOMAINS OF PERCEIVED FATIGABILITY

Rebecca Cohen,[1] Theresa Gmelin,[1] Kyle Moored,[2] Yujia (Susanna) Qiao,[3] Maggie Slavin,[1] Nancy W. Glynn,[4] and Ryan Katz,[1], *1. University of Pittsburgh Graduate School of Public Health, Pittsburgh, Pennsylvania, United States, 2. University of Pittsburgh, Pittsburgh, Pennsylvania, United States, 3. University of Pittsburgh, University of Pittsburgh, Pennsylvania, United States, 4. University of Pittsburgh Graduate School of Public Health, Pittsburgh, Pennsylvania, United States*

Fatigability is a more sensitive measure of one's perception of fatigue. To identify an appropriate fatigue question when a fatigability measure is unavailable, we examined associations between widely used global fatigue questions and perceived physical and mental fatigability. Participants (N=896, age=74.7±6.6, 58.1% women) from two aging research registries completed the valid Pittsburgh Fatigability Scale (PFS, 0-50) and five global fatigue questions: energy level (0-10), running out of energy (0-5), feeling energetic (0-6), feeling tired (0-6), and feeling exhausted (0-6) over past four weeks. All fatigue measures were correlated (p<0.0001) with physical (|r| range=0.48-0.57) and mental fatigability (|r| range=0.31-0.39). "Energy level" and "feeling exhausted" had strongest associations with physical and mental fatigability, respectively, in age, sex, BMI-adjusted regression models (p's<0.001), suggesting older adults can distinguish between physical and mental domains. Future work will explore how these constructs are distinct but related, and confirm the optimal proxy for the two fatigability subdomains.

### ASSOCIATION OF WALKING ENERGETICS WITH AMYLOID STATUS: FINDINGS FROM THE BALTIMORE LONGITUDINAL STUDY OF AGING

Fangyu Li,[1] Amal Wanigatunga,[1] Qu Tian,[2] Eleanor Simonsick,[3] Murat Bilgel,[2] Jennifer Schrack,[1] and Ryan Dougherty,[1], *1. Johns Hopkins Bloomberg School of Public Health, Baltimore, Maryland, United States, 2. National Institute on Aging, Baltimore, Maryland, United States, 3. National Instute on Aging/NIH, Baltimore, Maryland, United States*

Higher energetic costs for mobility are associated with slow and declining gait speed. Slow gait is linked to cognitive decline and Alzheimer's disease (AD), but the physiological underpinnings are note well-understood. We investigated the cross-sectional association between the energetic cost of walking and amyloid status (+/-) in 174 cognitively unimpaired men and women (52%) aged 78.5±8.6 years. The energetic cost of walking was assessed as the average oxygen consumption (VO2) during 2.5 minutes of customary-paced overground walking. Amyloid status was determined from 11C-Pittsburgh compound B (PiB) positron emission tomography (PET) imaging. Average energetic cost of walking was .169±.0379 ml/kg/m and 30% of the sample was PiB+. In logistic regression adjusted for demographics, APOE-e4, body composition and comorbidities, each 0.01ml/kg/m higher energy cost was associated with 12% increased odds of being PiB+ (OR=1.12; 95% CI:1.01-1.24). Inefficient walking may be a clinically meaningful physiological indicator of emerging AD-related pathology.

### CORTICO-STRIATAL FUNCTIONAL CONNECTIVITY REFLECTS CHANGES IN SUBJECTIVE ENERGY AND TIREDNESS

Rebecca Ehrenkranz, Theodore Huppert, Caterina Rosano, and James Hengenius, *University of Pittsburgh, Pittsburgh, Pennsylvania, United States*

Subjective feelings of energy and tiredness may reflect different neural processes. Functional connectivity (FC) was measured in 272 HealthABC participants via resting state functional MRI in striatal-associative, striatal-limbic and striatal-sensorimotor networks. Subjective energy level (scored 1-10) and tiredness (tired/not-tired) during the prior month were collected via self-report from year 2 to year 10 (mean energy follow-up=8 years, tiredness follow-up=7 years). Participants who never reported being tired during follow-up (N=119) had significantly lower FC in the striatal-limbic network (mean difference [95%CI]: -0.055 [-0.1020,-0.00879], p=0.02). Participants with stable energy level over time (N=94, defined as decline <1.0 SD below the mean) had significantly higher FC in the striatal-associative network (mean difference [95% CI]: 0.041 [0.00192,0.0807], p=0.04). Associations were similar when adjusted for brain atrophy, demographics, and education. Although based on subjective measures, the distinct spatial patterns of these associations support our hypothesis that neural basis of energy and fatigue may differ.

### Session 3435 (Symposium)

### MULTISCALE BRAIN AGING IN THE CONTEXT OF NEURODEGENERATION AND ALZHEIMER'S DISEASE

Chair: Kyra Thrush Co-Chair: Yaroslav Markov

The brain, with a diverse array of specialized cells, regional substructures, and a relatively isolated microenvironment, represents a uniquely challenging organ system for aging research. The brain can experience physical trauma, interact with the periphery, and is responsible for cognitive and behavioral modifications that can feed back into the molecular processes of aging both within and external to the brain. Advances to our understanding and ability to intervene in the complexity that personifies brain aging and associated neurodegeneration will require integrated, multiscale approaches operating in tandem. Therefore, we have organized this symposium to highlight promising new approaches to study brain aging through the lens of multiple biological levels of organization. We will provide insight not only into normal brain aging, but will also suggest key spurious processes that may drive neurodegeneration and functional decline.

### DEEP LEARNING METHODS CAPTURE NON-LINEAR BRAIN AGING PATTERNS UNDERLYING ALZHEIMER'S DISEASE AND RESILIENCE

Albert Higgins-Chen, Yaroslav Markov, Raghav Sehgal, Morgan Levine, and Kyra Thrush, *Yale University, New Haven, Connecticut, United States*

The current era of multi-omics data collection has enabled researchers to obtain exceptionally comprehensive profiling of disease subjects. However, exceptionally high dimensionality can ultimately be an obstacle to biological insight. Previously, we presented a method in which penalized regression of methylation principal components reduces noise and improves prediction of age, disease, and Alzheimer's Disease (AD) pathophysiology. However, strictly linear methods may overly simplify the complex epigenetic aging landscape. We hypothesized that non-linear deep learning methods could identify molecular signatures that better reflect individual resilience to AD. Through the use of an autoencoder to represent high dimensional methylation array data, and supplemental machine learning methods, we connect latent nonlinear representations of the brain to aging, resilience, and indications of AD. In particular, resultant age-predicting representations of methylation were correlated with enrichment of methylation regions and biological pathways. Contextualized within AD pathology, this work provides valuable, ongoing insight into resilience in AD.

### THE ARC OF ASTROCYTE AGING: INSIGHTS FROM SCRNASEQ

Christopher Minteer,[1] Morgan Levine,[2] and Margarita Meer,[3], *1. Yale University, Yale University, Connecticut, United States, 2. Yale University, New Haven, Connecticut, United States, 3. Yale School of Medicine, New Haven, Connecticut, United States*

There is an urgent need to increase our understanding of brain aging and its role in neurodegeneration. While, evidence suggest that many hallmarks of aging, including epigenetic alterations and cellular senescence may be implicated in dementia, studying these and other progressive molecular changes in the brain remains extremely challenging. We asked whether something as simple as artificially aging cells in culture could recapitulate the changes that occur during organismal aging. To test this, we passaged human primary astrocytes and performed single-cell RNA sequencing (scRNAseq) of cells at passages 2-10. We observe that the sequential passaging—that terminates with a cluster of senescent cells—can be captured by manifolds and used to quantify a pseudo-time measure of progressive transcriptional changes. We identify genes underlying this transition and apply this signature of in vitro astrocyte passaging to scRNAseq from human and mouse brain aging studies, demonstrating associations with aging and neuropathology.

### NEURONAL EXCITATORY STATE IS LINKED TO STRESS RESILIENCE

Bruce Yankner, and Joseph Zullo, *Harvard Medical School, Boston, Massachusetts, United States*

The aging human brain is a study in both the importance and limitations of human stress response factors. Individual neurons can maintain functionality for 80 or more years, testifying to the potency of their stress response pathways. However, failure of these pathways during aging drastically increases the risk of neurodegenerative diseases. The transcriptional repressor REST is induced in the brains of long-lived humans but is lost in neurodegenerative disease. Here, we explore one modality of REST's protective effects: regulation of neuronal excitability. We show that excitatory capacity and stress response are inversely correlated in the human brain. We find that REST and its C. elegans orthologs repress neuronal excitation in response to stressful conditions. Further, exogenously suppressing neuronal excitation restores stress resistance to REST-deficient animals, while enhancing stress response in wildtype ones. Thus, regulation of neuronal activity is an important aspect of neuronal stress response and a potential therapeutic modality.

### EARLY PREDICTION OF COGNITIVE DEFICITS AFTER TRAUMATIC BRAIN INJURY BASED ON AD-LIKE PATTERNS OF NEURODEGENERATION

Alexander Maher,[1] Kenneth Rostowsky,[2] Nikhil Chaudhari,[1] Nahian Chowdhury,[1] Elliot Jacobs,[1] David Robles,[1] Ammar Dharani,[1] and Andrei Irimia,[2], *1. University of Southern California, Los Angeles, California, United States, 2. University of Southern California, University of Southern California, California, United States*

Traumatic brain injuries (TBIs) are frequently followed by persistent brain alterations and by cognitive sequalae, especially in older adults. Although mild TBI (mTBI) is a risk factor for Alzheimer's disease (AD), the extent to which the two conditions are related remains largely unexplored. Using structural, functional and diffusion magnetic resonance imaging (MRI), we have identified AD-like post-traumatic neurodegeneration patterns that accurately prognosticate cognitive decline after geriatric mTBI. Our results indicate that these features involve cortical regions and circuitry mediating memory and executive function, and that AD neurodegeneration has key structural and functional similarities to post-traumatic neurodegradation. Using machine learning of such similarities, we have accurately forecast the severity of chronic cognitive deficits after geriatric mTBI based on acute neuroimaging measures. Our findings demonstrate that AD-like alterations in brain structure and function observed early after injury can predict post-traumatic mild cognitive impairment, which is itself strongly associated with AD risk.

### EXERCISE-ASSOCIATED PATHWAYS AS NOVEL NEUROPROTECTANTS AGAINST CNS AGING AND ALZHEIMER'S DISEASE

Constanza Cortes, *University of Alabama at Birmingham, University of Alabama at Birmingham, Alabama, United States*

Skeletal muscle has recently arisen as a novel regulators of Central Nervous System (CNS) function and aging, secreting bioactive molecules known as myokines with proteostasis and metabolism-modifying functions in targeted tissues. We have recently generated a novel transgenic mouse with enhanced muscle proteostasis via moderate overexpression of Transcription Factor E-B (TFEB), a powerful master regulator of cellular clearance and proteostasis. We have discovered that the resulting enhanced skeletal muscle proteostasis function can significantly ameliorate proteotoxicity in the aging CNS and improve cognition and memory in aging mice. These neuroprotective benefits are markedly reminiscent of those observed in the aging CNS post-exercise, suggesting enhancing muscle proteostasis may be sufficient to replicate the local and systemic effects of exercise. Identification of pathways regulating crosstalk between skeletal muscle and CNS may yield targets with high therapeutic potential for diseases of the aging CNS.

### Session 3440 (Paper)

### NURSING HOME STAFF

### COVID-19 IMPACT ON ALBERTA NURSING HOME WORKERS: AN INTERPRETIVE DESCRIPTIVE STUDY WITH DIRECT CARE PROVIDERS

Jude Spiers,[1] Heather Titley,[1] Amber Savage,[1] Trina Thorne,[1] Sandra Young,[2] Neda Asadi,[3] Corinne Schalm,[4] and Carole Estabrooks,[1] *1. University of Alberta, Edmonton, Alberta, Canada, 2. University of Alberta, University of Alberta, Alberta, Canada, 3. University of Alberta, University of Alberta/ Edmonton, Alberta, Canada, 4. Ablerta Health, Alberta Health, EDMONTON, Alberta, Canada*

COVID-19 has devastated the LTC sector, but we lack systematic information on the impact on frontline staff. Our research, a partnership with the continuing care branches of Alberta Health and Alberta Health Services, was aimed at assessing COVID-19 impacts on staffs' well-being and quality of work-life and quality of care and life among residents. Here we report on staff. Using an interpretive descriptive approach, we interviewed 140 staff from January through April 2021, in 34 nursing homes. Facilities selected varied in ownership (public/private) and COVID-19 status (high, moderate, or low incidence). Virtual interviews focused on three key areas of impact: (a) staff mental and physical health, well-being, and work-life, (b) the facility, and (c) on residents. Interviews were analyzed using inductive content analysis. Dominant themes included a commitment of staff to resident wellbeing; a norm of stoicism in which accumulative stress of COVID-19 is recognized in participants' private lives but not their work; the critical role of teamwork in managing extra workload associated with COVID-19 protocols; role flexibility, particularly managers', enables workers to minimize interruptions to care activities; governmental wage subsidies and the restriction of workers to only one facility benefits residents and workers in terms of time and familiarity, but some health care aides faced a wage reduction of 30-40%. Alongside the research component, we regularly met with stakeholders and end-users to discuss emerging findings and potential areas needing urgent intervention, as well as longer-term programming as the impact of COVID-19 will persist for many years.

### FACTORS INFLUENCING RESIDENT RESPONSIVE BEHAVIORS TOWARD STAFF IN NURSING HOMES: A SYSTEMATIC REVIEW

Lori Weeks,[1] Abubakar Mohamed Nassur,[2] Fajr Haq,[3] Viraji Rupasinghe,[4] Carole Estabrooks,[5] and Yuting Song,[5], *1. Dalhousie University, Dalhousie University, Nova Scotia, Canada, 2. University of Alberta, University of Alberta, Alberta, Canada, 3. University of Calgary, Calgary, Alberta, Canada, 4. Dalhousie University, Halifax, Nova Scotia, Canada, 5. University of Alberta, Edmonton, Alberta, Canada*

When staff experience various types of resident responsive behaviors, this can lead to decreased quality of work-life and lower quality of care. We synthesized empirical quantitative and qualitative evidence on factors associated with resident responsive behaviors directed towards staff in nursing homes. We searched 12 bibliographic databases and "grey" literature with two key words: long-term care and responsive behaviors resulting in 7671 sources. Pairs of reviewers independently completed screening, data extraction, and risk of bias assessment. Based on extracted data, we developed a coding scheme of factors utilizing the ecological model as an organizational structure. We then applied the coding scheme to quantitative and qualitative articles and prepared narrative summaries for each factor. From 86 included studies (57 quantitative, 28 qualitative, 1 mixed methods), multiple factors emerged, such as staff training about responsive behaviors (individual level); staff approaches to care (interpersonal level); leadership, staffing resources, and physical environment (institutional level); and racism and patriarchy (societal level). Quantitative and qualitative results each provided key insights, such as qualitative results pertaining to leadership responses to reports of responsive behaviors, and quantitative findings on the impact of staff approaches to care on responsive behaviors. By synthesizing both quantitative and qualitative evidence, this review provides a comprehensive overview of factors associated with resident responsive behaviors towards staff. Our findings offer insights into promising factors for long-term care system and nursing home managers to address to strive to reduce responsive behaviors of residents toward staff in nursing homes.

### LONGITUDINAL CHANGES IN LEADERSHIP AND PERSON-CENTERED CARE OVER 5 YEARS IN SWEDISH NURSING HOMES

Annica Backman,[1] karin sjögren,[1] Hugo Lövheim,[1] Marie Lindkvist,[1] and David Edvardsson,[2], *1. Umea University, Umea, Vasterbottens Lan, Sweden, 2. La Trobe University, La Trobe University Melbourne, Victoria, Australia*

Nursing home leadership has been described as crucial for person-centred care and psychosocial climate, but longitudinal data are lacking. The significance of manager educational qualifications and operational model of nursing homes for perceived leadership, person-centred care and psychosocial climate also needs further exploration. This study aimed to explore changes in nursing home managers' leadership, person-centred care and psychosocial climate comparing matched units in a five-year follow-up. Also, to explore changes in leadership characteristics' and the significance of manager qualifications for perceived leadership, person-centred care and climate. Repeated cross-sectional, valid and reliable, measures of leadership, person-centred care, psychosocial climate and demographic variables were collected from managers and staff n=3605 in 2014 and n=2985 staff in 2019. Descriptive and regression analyses were used. Leadership remained significantly associated to person-centred care in a five-year follow-up, but no changes in strength of associations were seen. Leadership also remained significantly associated to psychosocial climate, with stronger associations at follow-up. Also, certain leadership characteristics significantly increased over time, thus, partly confirms previous findings. It was also shown that a targeted education for managers was significantly associated to person-centred care.

### SUSTAINABILITY OF INFORM: A COMPLEX TEAM-BASED IMPROVEMENT INTERVENTION IN LONG-TERM CARE

Matthias Hoben,[1] Liane Ginsburg,[2] Whitney Berta,[3] James Dearing,[4] Peter Norton,[5] Malcolm Doupe,[6] Janice Keefe,[7] and Jude Spiers,[8], *1. University of Alberta at Edmonton, Edmonton, Alberta, Canada, 2. York University, York University, Alberta, Canada, 3. University of Toronto, University of Toronto, Ontario, Canada, 4. Michigan State University, Michigan State University, Michigan, United States, 5. University of Calgary, University of Calgary, Alberta, Canada, 6. University of Manitoba, Winnipeg, Manitoba, Canada, 7. Mount Saint Vincent University, Halifax, Nova Scotia, Canada, 8. University of Alberta, Edmonton, Alberta, Canada*

Improving Nursing Home Care Through Feedback On perfoRMance Data (INFORM) was a complex, theory-based, three-arm, parallel cluster-randomized trial. In 2015–2016, we successfully implemented two theory-based feedback strategies (compared to a standard approach to feedback) to increase nursing home (NH) care aides' involvement in formal communications about resident care (formal interactions [FI], the primary outcome). Here, we report the extent to which FI was sustained 2.5 years following withdrawal of intervention supports. We also report on several determinants of sustainability. We analyzed data from 18 NHs (46 units, 529 care aides) in the control group, 19 NHs (60 units, 731 care aides) in the basic assisted feedback group (BAF), and 14 homes (41 units, 537 care aides) in the enhanced assisted feedback group (EAF). We assessed sustainability of FI, using repeated measures, hierarchical mixed models, adjusted for care aide, care unit and facility variables. In EAF, FI scores increased from T1 (baseline) to T2 (end of intervention) (1.30–1.42, p=0.010), remaining stable at T3 (long-term follow-up) (1.39 p=0.065). FI scores in BAF increased from T1 to T2 (1.33–1.44, p=0.003) and continued to increase at T3 (1.49, p<0.001). In the control group, FI did not change from T1 to T2 (1.25–1.24, p=0.909), but increased at T3 (1.38, p=0.003). Better culture, evaluation and fidelity enactment significantly increased FI at long-term follow-up. Theory-informed feedback provides long lasting benefits in care aides' involvement in FI. Greater intervention intensity neither implies greater effectiveness nor sustainability. Modifiable context elements and fidelity enactment may facilitate sustained improvement.

### THE RELATIONSHIP BETWEEN LEADERSHIP STYLES, JOB SATISFACTION, AND TENURE AMONG NURSING HOME ADMINISTRATORS

Carey Peerman, *Radford University, Elliston, Virginia, United States*

Long-term care is considered a subset of health care administration as the characteristics and leadership skills needed differ from other areas of health care. Leadership style directly relates to organizational development, success, and effectiveness. For study purposes, specific focus was placed upon determining the degree to which nursing home administrators (NHAs) perceived styles of leadership determined job satisfaction with tenure as an NHA. Perceptions of leadership style and levels of job satisfaction were determined using a non-experimental, quantitative design, specifically employing a survey research approach. The research instrument in this study, the MLQ, provided the data essential to addressing the research questions and accompanying hypotheses. The effect of study participant response to items on the MLQ associated with the research questions was assessed using univariate analysis of descriptive factors and inferential statistical techniques for statistical significance testing purposes. A total of 87% of study participants indicated that they perceived their leadership style as Nursing Home Administrators (NHA's) was reflective of Transformational Leadership. However, findings indicated that participants who had a Transactional Leadership style were more likely than other types of leadership styles to select an NHA as a career path if given the opportunity to choose this line of work in the future.

### Session 3445 (Symposium)

### PARTNERSHIP WITH FAMILIES DURING HOSPITALIZATION OF PERSONS WITH DEMENTIA: INTERVENTION AND MEASUREMENT STRATEGIES

Chair: Marie Boltz Co-Chair: Barbara Resnick
Discussant: JUDITH TATE

Persons with dementia have high rates of hospitalization and are at risk for complications including psychological distress, and functional and cognitive decline. In turn, their family caregivers often face increased stress related to lack of preparedness to meet the complex needs of the patient during hospitalization and in the post-acute period. Hospitalization provides an opportunity to reframe the role of family caregivers from the traditional passive one to that of partners with the hospital team. The aim of the Family-centered, Function-focused Care (Fam-FFC) clinical trial is to test a nurse-family partnership model that incorporates a four step approach to optimize behavioral, functional, and cognitive outcomes in hospitalized persons with dementia and increase preparedness of caregivers to continue to optimize these outcomes in in the acute and post-acute recovery period. In this symposium we provide a description of the intervention with regard to theoretical support, four step process, and cultural appropriateness of the process. Two presentations describe, among Black and white dyads, evidence to support the psychometric properties of major outcome measures, caregiving preparedness and neuropsychiatric symptoms, in hospitalized dyads living with dementia. The final presentation describes a strategy to engage the dyad in goal development and evaluation, and its effect upon hospital readmissions. Findings from this symposium will help to identify intervention and measurement resources for those working with hospitalized persons with dementia and their family caregivers, and guide ongoing research needs in this area. Our discussant will synthesize the research findings and discuss implication for research, policy, and practice.

### ENGAGEMENT OF FAMILY IN A GOAL SETTING STRATEGY: IMPACT UPON 30-DAY HOSPITAL READMISSIONS

Marie Boltz,[1] Ashley Kuzmik,[1] Barbara Resnick,[2] Irene Best,[1] and Jacqueline Mogle,[3], *1. Pennsylvania State University, University Park, Pennsylvania, United States, 2. University of Maryland School of Nursing, Baltimore, Maryland, United States, 3. Penn State University, University Park, Pennsylvania, United States*

Family-centered Function-focused Care (Fam-FFC) works with family caregivers as care partners in the assessment, function-promoting goal setting, implementation, and evaluation of goal attainment during hospitalization and immediate post-acute period. ANCOVA technique examined the preliminary impact of Fam-FFC upon 30-day hospital readmissions and logistic regression tested the association of goal attainment, measured with the Goal Attainment Scale (GAS) with 30-day hospital readmissions. The majority of the patients were Black (50%), female (62%), had a mean age of 81.6 (SD=8.4), mean Barthel Index of 60.29 (SD=27.7), and mean MoCA of 10.67 (SD=7.0). Goals represented six main categories: mobility, cognition, self-care, toileting, sleep, and pain management. Patients in the intervention group had less 30-day hospitalizations ($F= 4.6$, $p=.033$) and goal attainment was significantly associated with less recidivism ($B=.179$, Wald= 2.8 (1), $p= .045$). FamFFC shows promise in reducing 30-day hospital readmissions; results support the contribution of family engagement and use of GAS

### CULTURAL APPROPRIATENESS OF AN INTERVENTION TO PROMOTE FUNCTIONAL RECOVERY FROM HOSPITALIZATION: CAREGIVER VIEWS

Marie Boltz,[1] and Rhonda BeLue,[2] *1. Pennsylvania State University, University Park, Pennsylvania, United States, 2. St. Louis University College for Public Health and Social Justice (CPHSJ), St. Louis University, Missouri, United States*

The Fam-FFC model includes caregiver education and care pathway to promote physical function, wellbeing, and cognition. The Ecological Model (EM) provided a framework to assess the cultural appropriateness of the Fam-FFC intervention, through interviews with family caregivers, patients, and nurse champions, and focus groups with staff. Findings are described within the eight dimensions of the EM: 1 ) language: perceptions of the dyads' comfort level with intervention information; (2) persons: representation of dyads' ethnic /racial group within the nurse champions' ethnicity/race; (3) metaphors: use of cultural terms equivalent to those used by participants; (4) content: integration of participants' values, customs, and traditions in the intervention; (5) concepts: congruence of caregiving concepts with cultural norms; (6) goals: congruence of the intervention goals with participants' cultural norms and goals; 7) methods: the culturally appropriateness of the delivery of the intervention; and (8) context: alignment of the intervention with the participant's socio-community context.

### THE PREPAREDNESS FOR CAREGIVING SCALE IN AFRICAN AMERICAN AND WHITE CAREGIVERS OF HOSPITALIZED PERSONS WITH DEMENTIA

Ashley Kuzmik, *Pennsylvania State University, University Park, Pennsylvania, United States*

This study evaluated the Preparedness for Caregiving Scale (PCS) upon discharge from the hospital. The caregivers reported a mean age of 60.5 years (SD=13.9). The majority of caregivers were female (72%), married (59%), non-Hispanic/Latino (98%) and either white (52%) or African American (48%). Fifty percent were employed outside of the home and averaged 40.7 (SD= 14.4) hours of outside work per week. The average PCS was 24.4 (SD=6.9, 0-32). One-factor structure of the PCS and measurement invariance by race was fully supported. Predicative validity revealed significant association between the PCS and anxiety ($\beta =-.41$, $t = -7.61(287)$, $p <.001$), depression ($\beta =-.44$, $t =-8.39$ (287), $p <.001$), and strain ($\beta =-.48$, $t =-9.29(287)$, $p <.001$). The PCS is a valid and meaningful tool to measure preparedness in African American and white family caregivers of persons with dementia during post- hospitalization transition.

### RELIABILITY AND VALIDITY OF THE NEUROPSYCHIATRIC INVENTORY QUESTIONNAIRE IN DYADS WITH DEMENTIA AT HOSPITALIZATION

Barbara Resnick, *University of Maryland School of Nursing, Baltimore, Maryland, United States*

This study expanded on the limited psychometric testing of the Neuropsychiatric Inventory-Questionnaire (NPI-Q), and extended testing to include hospitalized persons with dementia upon admission to the hospital, with reports from family caregivers. Using data from 318 dyads in the ongoing Fam-FFC trial, a Rasch analysis was conducted. Most patients were female (62%), non-Hispanic (98%), and Black (50%) with a mean age of 81.62 (SD=8.43). There was evidence of internal consistency for all subscales (behavior, severity, caregiver distress); a DIF analysis showed invariance across race and gender. The items on the NPI-Q fit with each subscale. Hypothesis testing showed a significant association between the AD8 (F=30.04, p=.001) and MoCA (F= 5.05, p=.03) with behaviors; the AD8 (F =27.91, p=.001) and MoCA (F = 6.65, p=.01) with severity; and the AD8 (F = 29.23, p=.001) with caregiver distress. Findings provide support for the NPI-Q use in persons with dementia during acute illness.

### Session 3450 (Symposium)

### PROMOTING SELF-CARE IN CAREGIVERS OF OLDER ADULTS LIVING WITH CHRONIC ILLNESS: THE ICARE4ME STUDY

Chair: Lauren Massimo Co-Chair: Karen Hirschman
Discussant: Harleah Buck

Informal caregivers provide a substantial amount of social support to older adults which can be stressful and lead to poor self-care. When stressed, caregivers of persons living with chronic illness are less vigilant and less motivated to engage in self-care behaviors that are important for maintaining their own physical and emotional health. Support interventions can encourage self-care by helping caregivers to focus on values, solve problems, and transform their goals into action. In this symposium, we will discuss the iCareMe study, a randomized controlled trial (RCT) (NCT03988621) that tests a virtual support intervention which utilizes health coaching to increase self-care behaviors in caregivers of older adults living with chronic illnesses, such as heart failure and dementia. The first session will discuss the translation of self-care theory to the basis for the "Virtual Caregiver Coach for You" (ViCCY) intervention and will provide an overview of the iCare4Me randomized control trial designed for caregivers of persons living with advanced heart failure. The second session will describe the adaptation of the iCare4Me RCT to caregivers of persons living with dementia. The third session will highlight findings from a qualitative descriptive study examining the characteristics of effective health coaching used in these two RCTs. Finally, the last session will share findings from a cross-sectional analysis examining moderators of self-care in heart failure caregivers. Together, these presentations will illustrate the unique and innovative approach that iCare4Me has taken to improve self-care in caregivers of older adults living with chronic illness.

### SELF-CARE THEORY AND TRANSLATION TO INTERVENTION

Barbara Riegel, and Karen Hirschman, *University of Pennsylvania, Philadelphia, Pennsylvania, United States*

Self-care is defined as a process of maintaining health through health promoting practices and managing illness when it occurs. Self-care is integral in the management of chronic conditions, but even those without illness engage in some level of self-care daily. In our on-going study we promote self-care as a means to control the stress associated with caregiving. We acknowledge the burden of caregiving for a loved one experiencing a serious chronic illness. That responsibility is typically associated with significant stress for the caregiver. We use stress theory to address the caregivers' appraisal of events and coping responses. Three experienced health coaches were hired to provide 10 sessions of coaching over a 6-month period to each of the caregivers randomized to the intervention group. The emphasis of the iCare4Me coaching sessions is to address primary and secondary appraisal and coping as a means to improve self-care and thereby decrease stress.

### IMPROVING SELF-CARE OF INFORMAL CAREGIVERS OF ADULTS WITH FRONTOTEMPORAL DEGENERATION

Michelle Sharkey,[1] Lauren Fisher,[2] and Lauren Massimo,[2]
*1. University of Pennaylvania, Philadelphia, Pennsylvania, United States, 2. University of Pennsylvania, Philadelphia, Pennsylvania, United States*

Frontotemporal degeneration (FTD) is a common cause of young-onset dementia that results in progressive deterioration in executive functioning and social comportment. A tremendous burden is placed on young caregivers, typically spouses, who often sacrifice their own self-care needs in order to manage the cognitive decline and subsequent functional impairments of their loved one, contributing to extraordinarily high levels of stress and depression in caregivers of individuals with FTD. Very few interventions have been tested specifically in FTD caregivers, and those that exist have generally focused on education around patient behavior management. In this session, we will discuss how we adapted the iCare4Me study, originally designed for heart failure caregivers, for caregivers of persons with FTD and we will share initial findings from iCare4Me for FTD, a randomized controlled trial which evaluates the efficacy of a virtual health coach intervention aimed at increasing self-care behaviors and reducing stress in FTD caregivers.

### A QUALITATIVE STUDY OF CHARACTERISTICS OF AN EFFECTIVE HEALTH COACH: PERSONAL, PROFESSIONAL, AND PROGRAM BASED

Frances Barg,[1] Barbara Riegel,[2] and Caitlin Clason,[2]
*1. University of Pennsylvania Perelman School of Medicine, Philadelphia, Pennsylvania, United States, 2. University of Pennsylvania, Philadelphia, Pennsylvania, United States*

Health coaching continues to grow in popularity as an effective intervention to empower and engage patients and their caregivers. However, little is known about what characteristics contribute to the success of health coaches in implementing evidence-based interventions. This study examines the characteristics that contribute to effective health coaches. Semi-structured interviews were conducted with health coaches and an interdisciplinary research team of an ongoing study examining a virtual health coaching intervention. Interviewees identified three discrete themes of characteristics that contribute to the success of health coaches: personal (e.g. compassion), professional (e.g. transferability of soft skills) and program based (e.g. training regimen). We conclude that it is not just innate personality attributes that make a health coach effective in their role, but training and program design intended to support health coaches are also important in implementing interventions.

### SUPPORT QUALITY MODERATES THE IMPACT OF TOTAL HOUSEHOLD OCCUPANCY ON SELF-CARE NEGLECT IN INFORMAL CAREGIVERS

Barbara Riegel, and Austin Matus, *University of Pennsylvania, Philadelphia, Pennsylvania, United States*

Caregiver self-care may be impacted by the household environment. We evaluated the impact of support quality (e.g., ratings of quality of emotional support, information, material help, errands performed by others) and total household occupancy on a validated measure of self-care neglect in caregivers of patients with heart failure. Multivariate regression modeling was used to examine predictors of self-care neglect and we introduced an interaction term between support quality and household occupancy. The main effects model included terms for years of caregiving experience, hours caregiving daily, support quality, and total household occupancy (R2: 0.31; p <0.05). The interaction term between support quality and household occupancy contributed significantly (p < .05) to the respecified model (R2: 0.41; p <0.05). We suggest that the potential benefit of total household occupancy on caregiver self-care depends on perceived support quality. Clinicians should assess quality of household resources with caregivers during interactions.

### Session 3455 (Symposium)

### REINVENTING HOUSING CARE: ENVIRONMENTAL NEGOTIATIONS MADE IN CONGREGATE SETTINGS DURING COVID-19

Chair: Ian Johnson Discussant: Terri Lewinson

The COVID-19 pandemic prompted an urgent reconsideration of space and place within congregate housing. Research has only underscored the need for health-promoting physical alterations to residential environments (Peters & Halleran, 2020), but also generated lasting questions about the relationships between congregate environments and their residents, visitors, and workforce —among them, what ways can environments be negotiated to reduce risk (Dosa et al., 2020)? How can environments enact care for formal caregivers (Chen & Chavalier, 2021)? Who might be challenged by this care which may question the dangers associated with proximity (Lynn, 2020)? This symposium focuses on the ways stakeholders within congregate housing observed, related to, and negotiated changes to space and place during the pandemic. Paper 1 presents an organizational case study investigating provider perspectives of how housing and healthcare responses to COVID have shaped palliative care with unhoused patients during the pandemic. Paper 2 highlights the collaborative work of a multi-sector coalition working to address timely needs of residents in low-income senior buildings. Paper 3 reflects on the formation of a cross-national senior housing network and the interdisciplinary exchange of best practices and policy recommendations that emerged. The collective findings of these papers challenge previous notions of care in congregate environments, illuminate how provider networks respond to crises and share emergent knowledge, and consider how institutional decisions about the pandemic have re-placed and re-spaced provider and patient experiences. This symposium offers observations and strategies that may assist in envisioning successful congregate care during COVID-19 and beyond.

### EFFECTS OF THE COVID-19 PANDEMIC ON PLACE IN END-OF-LIFE CARE: INSIGHTS FROM A HOMELESS MOBILE PALLIATIVE CARE TEAM

Ian Johnson, *University of Washington, SEATTLE, Washington, United States*

The effects of the COVID-19 pandemic on both those experiencing homelessness (Tsai & Wilson, 2020) and those with life-limiting illnesses (Abbott et al., 2020) is of great public health concern. This presentation details the findings from an organizational case study (Yin, 2014) aimed at investigating COVID-related changes to the service environments in which unhoused palliative care patients receive care. Through ethnographic field observation (Phillippi & Lauderdale, 2017) and interviews with a homeless palliative care team and their community partners (Turner, 2005), findings included 1) decreased staff capacity due to de-congregated care; 2) efforts to extend care in community settings due to relocation barriers; 3) conflict between reducing viral risk and honoring unique population needs; and 4) provider perceptions of COVID-19 as an "equalizer." Findings illustrate the impact of emergency response within housing and healthcare systems on unhoused patients' care and offer potential pathways to quality end-of-life care for homeless populations.

### THE ENACTMENT OF CARE: LESSONS LEARNED FROM A MULTISECTOR COALITION ADVOCATING FOR THOSE LIVING IN SENIOR HOUSING

Claudia Sanford,[1] Dennis Archambault,[2] Michele Waktins,[3] Zach Kilgore,[4] Michael Appel,[5] and Tam Perry,[6], *1. United Community Housing Coalition, Detroit, Michigan, United States, 2. Authority Health, Detroit, Michigan, United States, 3. Volunteers of America-Michigan, Southfield, Michigan, United States, 4. CSI Support & Development, Warren, Michigan, United States, 5. Develop Detroit, Detroit, Michigan, United States, 6. Wayne State University, Detroit, Michigan, United States*

This presentation explores how a coalition, Senior Housing Preservation-Detroit, considered and planned for "care" in senior buildings in Detroit, Michigan. Detroit was one of the American cities affected in the early days of the pandemic; the coalition pivoted its work in creative, collaborative ways which included understanding the rapidly changing context for those living in low-income senior buildings. Older minority adults have been shown to be disproportionally affected by COVID-19; the coalition successfully advocated for testing to be brought to senior buildings (and now vaccine distribution) and addressed mask distribution and food insecurity in several senior buildings (see Archambault, Sanford and Perry, 2020). Without the long-established partnerships, "care" could not have been as coordinated, multi-sector and trusted. The presentation will discuss lessons learned that can be applied to future challenges in supporting the well-being of residents as they negotiate their residential spaces.

### PLANNING FOR SENIORS HOUSING IN CHANGING CITIES: LESSONS LEARNED FROM A CROSS-NATIONAL EXCHANGE

Tam Perry,[1] Karen Kobayashi,[2] Denise Cloutier,[2] Yasir Mehmood,[3] Emma Helfand-Green,[4] Sander Hitzig,[5] and Christine Sheppard,[5], *1. Wayne State University, Detroit, Michigan, United States, 2. University of Victoria, Victoria, British Columbia, Canada, 3. Wayne State University, Wayne State, Michigan, United States, 4. City of Toronto, Toronto, Ontario, Canada, 5. Sunnybrook Research Institute, Toronto, Ontario, Canada*

Across North America, a growing number of older adults have a core housing need and lack access to affordable, suitable or adequate housing. Although federal, state/provincial and local backdrops vary across Canadian and American contexts, seniors' housing providers in both countries face similar challenges and must develop innovative policy and program responses to help older adults age in place. We hosted an international seniors' housing conference to create a platform for cross-national collaboration among multidisciplinary seniors housing experts. This event offered an opportunity to exchange best practices, emerging research, and policy solutions, and establish a set of shared priorities for advancing seniors housing that were applicable to two nations with different social systems. This paper will reflect on the exchange of knowledge and best practices related to housing preservation, eviction prevention, and access to supports during COVID-19, and the lessons learned fostering a cross-national collaborative network of seniors housing experts.

### Session 3460 (Paper)

### SLEEP: OLDER ADULTS AND CAREGIVERS

### DAY AND NIGHT, NIGHT AND DAY: ASSOCIATIONS OF SLEEP AND ACTIVITY VARIABILITY AND MORTALITY

Jade Benson,[1] Elena Jauregui,[2] and Diane Lauderdale,[2]
*1. University of Chicago, Chicago, Illinois, United States,*
*2. The University of Chicago, Chicago, Illinois, United States*

Self-reported sleep duration has been repeatedly found to predict mortality. Actigraphy has recently been added to population-based studies to provide more accurate sleep measures. Actigraphy sleep duration has not consistently predicted mortality, but actigraphy measures of sleep disruption measures are generally found to be predictive of mortality for older adults. A few studies have more fully used actigraphy data and constructed variables to summarize 24-hour activity patterns, which have also predicted mortality. In this study, we use a nationally representative study of Americans aged 61 – 91 to examine the associations between mortality and actigraphy-derived measures of variability, for both sleep and 24-hour activity patterns. We use 72-hour wrist actigraphy data from a substudy of the 2010/11 round of the National Social Life, Health and Aging Project (NSHAP) linked to the National Death Index (NDI) to establish 5-year mortality. Sleep variability was represented by sleep fragmentation and the standard deviation of wake and bed times. Intraday variability and between day (interday) variability described the 24-hour activity patterns. Cox proportional hazards models were adjusted for sociodemographic confounders and average daytime activity. In general, more variability was associated with increased death hazard for all measures. Fragmentation (HR: 1.04, 95% CI: [1.01, 1.07], p = 0.01), standard deviation of bedtimes (HR: 1.16, 95% CI: [1.02, 1.31], p = 0.02), and intraday variability (HR: 1.19, 95% CI: [0.98, 1.43], p = 0.07) showed the strongest associations. This study suggests that both consistent sleep and 24-hour activity patterns are associated with better prospective health.

### OBJECTIVE SLEEP QUALITY AND THE UNDERLYING FUNCTIONAL NEURAL CORRELATES AMONG OLDER ADULTS WITH PROBABLE MCI

Chun Liang Hsu,[1] Ryan Falck,[2] Daniel Backhouse,[2] Patrick Chan,[2] Elizabeth Dao,[2] Lisanne ten Brinke,[2] Brad Manor,[3] and Teresa Liu-Ambrose,[2] *1. Hinda and Arthur Marcus Institute for Aging Research, Harvard Medical School, Burnaby, British Columbia, Canada, 2. University of British Columbia, Vancouver, British Columbia, Canada, 3. Hinda and Arthur Marcus Institute for Aging Research, Harvard Medical School, Boston, Massachusetts, United States*

Poor sleep is a strong risk factor for dementia and is commonly reported among older adults with mild cognitive impairment (MCI). However, the neural underpinnings of poor sleep among older adults with MCI remains equivocal. The goal of this cross-sectional analysis was to explore the relationship between resting-state functional connectivity in the brain and sleep quality as measured by actigraphy. We hypothesize lower sleep efficiency and higher sleep fragmentation may be associated with aberrant functional connectivity of brain regions involved in somatosensory, somatomotor, and attentional processing. Thirty-six community-dwelling older adults with probable MCI between 65-85 years (mean=71.8 years) were assessed for sleep quality using a motion watch to quantify sleep efficiency and fragmentation over 14 days. All participants completed resting-state functional magnetic resonance imaging (fMRI) within 14 days of sleep monitoring. Independent associations between network connectivity and sleep quality were determined using general linear models. Examined networks included the somatosensory network (SMN), dorsal attention network (DAN), ventral attention network (VAN), frontoparietal network (FPN), and default mode network (DMN). Mean Montreal Cognitive Assessment score was 22.5 (SD=2.7) and Mini-Mental State Examination score was 28.3 (SD=1.5). Mean sleep efficiency and fragmentation index was 80.1% and 31.8 respectively. Higher sleep fragmentation correlated with increased connectivity between the SMN and insula, the SMN and posterior cingulate, as well as FPN and primary motor area (Z=3.1; p<0.05). These results suggest aberrant functional connectivity between brain regions involved in attentional and somatosensory processes may be associated with disrupted sleep mechanisms in older adults with MCI.

### SLEEP DISTURBANCES AND THE SPEED OF MULTIMORBIDITY DEVELOPMENT IN OLD AGE

Amaia Calderón-Larrañaga,[1] Laura Pérez,[2] Davide Vetrano,[1] Federico Triolo,[1] Linnea Sjöberg,[1] Alexander Darin-Mattsson,[1] Marco Inzitari,[2] and Shireen Sindi,[1], *1. Karolinska Institutet, Solna, Stockholms Lan, Sweden, 2. Parc Sanitari Pere Virgili, Barcelona, Catalonia, Spain*

Sleep disturbances are prevalent among older adults and are associated with various individual diseases. The goal of this study was to investigate whether sleep disturbances are associated with the speed of multimorbidity development among older adults. Data were gathered from the Swedish National study of Aging and Care in Kungsholmen (SNAC-K), an ongoing population-based study of subjects aged 60+ (N=3363). The study included a subsample (n=1189) without multimorbidity at baseline (<2 chronic diseases). Baseline sleep disturbances were assessed using the Comprehensive Psychiatric Rating Scale, and categorized as none, mild, moderate-severe. The number of chronic conditions throughout the nine-year follow-up was obtained from clinical examinations. Linear mixed models were used to study the association between sleep disturbances and the speed of chronic diseases accumulation, adjusting for sex, age, education, physical activity, smoking, alcohol consumption, depression, pain, and psychotropic drug use. We repeated the analyses including only cardiovascular, neuropsychiatric, and musculoskeletal diseases as the outcome. Moderate-severe sleep disturbances were associated with a higher speed of chronic disease accumulation (ß/year=0.142, p=0.008), regardless of potential confounders. Significant positive associations were also found between moderate-severe sleep disturbances and neuropsychiatric (ß/year=0.041, p=0.016) and musculoskeletal (ß/year=0.038, p=0.025) disease accumulation, but not with cardiovascular diseases. Results remained stable when participants with baseline dementia, cognitive impairment, or depression were excluded. The finding that sleep disturbances are associated with faster chronic disease accumulation points towards the importance of early detection and treatment of sleep disturbances as a possible strategy to reduce chronic multimorbidity among older adults.

### SLEEP PHENOTYPES OF CAREGIVERS FOR PERSONS LIVING WITH DEMENTIA

Glenna Brewster,[1] Christina Pierpaoli,[2] Fayron Epps,[3] Kalisha Bonds Johnson,[3] and Kate Yeager,[3] *1. Emory University, Stonecrest, Georgia, United States, 2. University of Alabama, Tuscaloosa, Alabama, United States, 3. Emory University, Atlanta, Georgia, United States*

Sleep disturbance is prevalent among caregivers of people living with dementia. Gaps exist regarding when caregivers begin to experience sleep disturbance along their caregiving trajectory. This study aimed to identify and describe phenotypes of current caregivers' sleep patterns before and during caregiving and describe caregivers' perception of their current sleep quality relative to their pre-caregiving sleep. We conducted semi-structured interviews with 19 caregivers participating in a larger, randomized controlled trial. Interviews were about caregivers' sleep patterns and were conducted after caregivers completed the first 6 months of the study. Interviews were audio-recorded using a video-conferencing platform and ranged from 20 to 45 minutes. We conducted thematic analysis of the interview transcripts. Four distinct caregiver-sleep phenotypes emerged from the qualitative data: Changed and Dissatisfied, Changed and Satisfied, Unchanged and Dissatisfied, and Unchanged and Satisfied. Caregivers whose sleep was categorized as Changed experienced a difference in their pre-caregiving sleep, usually from good to poor sleep. Caregivers whose sleep was Unchanged had poor sleep pre-caregiving and continued to have poor sleep during caregiving. Caregivers also reported being Satisfied or Dissatisfied with their current sleep pattern, defined in terms of daily distress and impairment. These 4 subtypes highlight the heterogeneity of caregivers'

sleep experiences and debut a useful clinical framework with which to identify, categorize, and target caregivers at risk for sleep disturbance. Identifying caregivers' sleep phenotypes will enable healthcare providers to determine caregivers' needs and readiness for interventions.

### Session 3465 (Symposium)

### SOCIAL DETERMINANTS OF COGNITIVE HEALTH: STUDIES ON PHYSICAL AND SOCIAL ENVIRONMENTS AND COGNITION

Chair: Kexin Yu Co-Chair: Kheng Siang (Ted) Ng
Discussant: Patricia Heyn

Living environments profoundly influence the aging process. This symposium presents research on two main aspects of the living environment and their relationships with cognitive health. The living environment is broadly defined, including both physical and social aspects. The physical environment is the characteristics of the built environment, such as tripping hazard in the home, cleanness of the community streets, and presence of deserted buildings, etc. The social environment is the cohesiveness with other people living in the neighborhood. Living environments have multiple layers; the physical environments encompass both in-home and in-community domains, whereas the social environment can be categorized as domestic versus community cohesiveness. This symposium includes studies with investigation scopes spanning from the micro to mezzo levels. The first presentation scrutinizes the buffering effect of marital relationships, as a form of domestic social environments, on cognition among older adults with vision and hearing impairments. Using the NHATS dataset, the second presentation examines social isolation as a potential mediator for the association between physical, social environments and global cognitive functioning. The third presentation evaluates the impact of living environments on cognition among Canadian older adults with multimorbidity. The last presentation examines how the physical environment affects sleep quality and thus influences older adults' cognition. All four presentations are closely linked to the overarching theme of evaluating the environmental impact on cognition and provide possible explanations mediating the association observed. This symposium contributes to advancing gerontological knowledge by offering new perspectives on the social determinants of cognitive health.

### EXAMINING SOCIAL ISOLATION AS A MEDIATOR FOR THE ENVIRONMENTAL INFLUENCE ON COGNITIVE FUNCTIONING

Kexin Yu, *USC, LA, California, United States*

Environments serve as one of the most pervasive stimuli to the brain, shaping cognitive functionality through neuroplasticity. Both the physical and social environments of living could affect the cognitive functioning of older adults. Nonetheless, the mechanisms on how the environments "get under the skin" are unknown. This study examines the potential mediating effect of social isolation on the relationships between environment and cognitive health. Wave 9 data from the National Healthy Aging Trend Study was employed. The working sample includes 2,313 older adults. Path analysis results showed that in-home disorder was positively related to social isolation. A more cohesive social environment was negatively related to social isolation. Higher social isolation scores were associated with worsen global cognitive functioning. In-home disorder, community disorder, and social environment had significant direct effects on cognitive health after adjusting for the mediating effect. Social Isolation partially mediates the environmental influence on cognitive functioning.

### AT-HOMENESS INFLUENCES THE COGNITION OF MULTIMORBID OLDER ADULTS: LONGITUDINAL PATH ANALYSIS THROUGH LONELINESS

John Best,[1] Andrew Wister,[1] and Daniel Gan,[2], *1. Simon Fraser University, Vancouver, British Columbia, Canada, 2. simon fraser university, Burnaby, British Columbia, Canada*

Approximately two-thirds of older adults' experience multimorbidity in North America. Challenges of symptoms management and reduced mobility often coincide with late-life depression which is associated with a 2 to 5-fold increased dementia risk. Loneliness and depression are connected in the prodromal phases. We examine the effects of physical environment (e.g., housing and neighborhood factors) and social environment (e.g., social support) on loneliness, depression, and cognition using path analysis, controlling for baseline. Data(n=15,087) was drawn from the Canadian Longitudinal Study on Aging. Measures of housing, neighborhood and life satisfaction were used to construct an index of "at-homeness" based on theory. We found good model fit (TLI=.989; CFI=.999; RMSEA=.026; SRMR=.006). At-homeness(B=-.20, p<.001) rivaled the effect of social environment(B=-.19, p<.001) on loneliness. Together, physical environment and loneliness had as much effect on cognition as depression. If causality is supported, modifying older adults' satisfaction with their home environment may reduce loneliness and cognitive decline.

### WAKE AFTER SLEEP ONSET MEDIATES THE LINK BETWEEN NEIGHBORHOOD SOCIAL ENVIRONMENT AND COGNITION IN OLDER ADULTS

Kexin Yu,[1] Paul Duberstein,[2] and Bernadette Fausto,[3]
*1. USC, LA, California, United States, 2. Rutgers University School of Public Health, Piscataway, New Jersey, United States, 3. Rutgers University–Newark, Newark, New Jersey, United States*

Cognition is influenced by the neighborhood social and physical environment, but the underlying mechanisms by which neighborhood environment affects cognition are unclear. We tested the hypothesis that sleep mediates the effects between environmental exposures and cognition. We employed structural equation modeling to examine interrelationships among neighborhood social and physical environment, actigraphic sleep characteristics, and global cognition in a sample of older adults (N=3,196) from Round 2 of the National Social Life, Health, and Aging Project. Results indicated that participants with better cognition lived in salutary (e.g., cohesive, safe) social environments (est.=0.03, p<.001) and less disruptive (e.g., noisy, polluted) physical environments (est.=-0.04, p<.001). The mediation hypothesis was partially supported. Time spent awake after sleep onset mediated the social environment-cognition relationship, but sleep characteristics did not mediate the physical environment-cognition relationship. Future work should identify other environmental influences on sleep and cognition in aging to inform public health intervention priorities.

### MARITAL QUALITY AS A MODERATOR OF THE ASSOCIATION BETWEEN SENSORY IMPAIRMENTS AND COGNITIVE FUNCTIONING

Corinna Tanner,[1] Avalon White,[2] Stephanie Richardson,[2] Melanie Hill,[2] Shaylee Bench,[1] Brian Stagg,[3] Joshua Ehrlich,[4] and Jeremy Yorgason,[5] *1. College of Nursing, Brigham Young University/Provo, Utah, United States, 2. School of Family Life, Brigham Young University/ Provo, Utah, United States, 3. University of Utah Moran Eye Center, University of Utah/Salt Lake City, Utah, United States, 4. University of Michigan, University of Michigan, Michigan, United States, 5. Brigham Young University, Provo, Utah, United States*

Research suggests that marital quality may buffer the impact of sensory impairments in later life, and that marital quality relates to cognitive functioning. This study explored how marital quality moderated links between sensory impairments and cognitive functioning. We used data from 723 paired marital dyads from two cohorts in the NHATS and NSOC studies across three-year periods (n=340 dyads from waves 1, 2, 3; n=383 dyads from waves 5, 6, 7). Growth curve models of executive functioning indicated that marital quality moderated effects of both hearing and vision impairment on changes in cognitive functioning longitudinally. Specifically, higher marital quality was associated with higher executive functioning across time. Results suggested no improvement in executive functioning among those with average or lower marital quality. Although cognition declines with advanced age and with sensory impairments, results suggest that older adults with higher marital quality may improve in some aspects of cognition longitudinally.

### Session 3470 (Paper)

### SOCIAL DETERMINANTS OF MENTAL HEALTH

### A NEW AND WORRISOME PREDICTOR OF ATTITUDES TOWARD SUICIDE: SELF-RATED HEALTH

Julene Cooney, *Syracuse University, Manlius, New York, United States*

Do people have the right to end their own lives? The General Social Survey has monitored the attitudes of Americans towards suicide since 1977 using four questions: Do you think a person has the right to end his or her own life if this person has an incurable disease, has gone bankrupt, has dishonored his or her family, or is tired of living and ready to die? These four responses can be combined into a reliable index representing an individual's attitude toward suicide. As average population education levels have increased and religiosity has fallen, attitudes favoring the right to suicide have increased across the population. This research project introduces a previously understudied predictor of attitudes toward suicide: self-rated health. Using logistic and ordinal logistic regression, and controlling for age, education level, religiosity, marital status, survey year, race, and sex, I find that, over time, self-rated health has become a significant predictor of attitudes toward suicide. Since 2002, respondents who perceived themselves to be in poor health are significantly more likely to favor the right to end one's life, especially if the individual has an incurable disease. After stratifying by age and race, I find that the relationship between self-rated health and attitudes toward suicide is strongest among individuals in the mid-life and is equally significant as a predictor for White and Black Americans after 2010. These findings provide further evidence that mental health screening is an increasingly vital component of physician/patient interactions and highlight the importance of continuity of care.

### COVID-19 OUTBREAKS AND CONTROL MEASURES ARE ASSOCIATED WITH DEPRESSION RISK AMONG OLDER ADULTS

Man-Man Peng, Tianyin Liu, Walker Siu Hong Au, Terry YS Lum, and Gloria HY Wong, *The University of Hong Kong, Hong Kong, Not Applicable, Hong Kong*

Local COVID-19 outbreaks and infection control measures may affect mental health in older persons. This study aims to investigate the effects of COVID-19 outbreaks and control measures on depression risk in community-dwelling older adults in Hong Kong. With rolling cross-sectional design, telephone screenings for depressive risk were conducted among 8163 older people using Patient Health Questionnaire-2 (PHQ-2) from February to September 2020. COVID-19 outbreaks across thirty weeks were measured using real-time effective reproductive number (Rt), infected new cases, and change of infected new cases by week. Infection control measures were assessed using four policy indices, including government response, government stringency, containment and health, and economic support. Linear regressions were used to test the associations of depression risk with COVID-19 outbreaks and control measures. We found that being female and higher Rt were associated with higher depression risk in the overall sample. In older adults without pre-existing mental health issues, higher depression risk were related to older age ($t=-1.974$, 95%CI[-0.006, 0.000], $p<0.05$), a higher level of government stringency ($t=2.954$, 95%CI[0.007, 0.033], $p<0.01$), and less stringent containment and health-related policy ($t=-2.599$, 95%CI[-0.041, -0.006], $p<0.01$). In older adults with pre-existing mental health issues, greater changes in newly infected cases were related to higher depression risk ($t=2.813$, 95%CI[0.002, 0.010], $p<0.01$). In conclusion, the effects of COVID-19 infection risk and control measures on depression risk differ among older Chinese by pre-existing mental health issues. Future public health communication could build on resilience to balance awareness of infection risks and mental health risks in older persons.

### EXPLAINING GENDER DIFFERENCES IN DEPRESSIVE SYMPTOMS AMONG CAREGIVERS OF OLDER PATIENTS WITH CRITICAL ILLNESS

Wenhao Fu, and Jiajia Li, *Shandong University, Jinan, Shandong, China (People's Republic)*

The aging of baby boomers makes caring for the elderly an increasingly important topic. As rising cost of health care, the care for seriously ill patients has gradually shifted from hospitals to families, particularly in the countryside. Along with growing demand for informal care, informal caregivers are at increased risk of depression. The aim of this study was to explore the potential protective factors or risk factors associated with depressive symptoms of caregivers for patients with critical illness (45 to 93 years of age) across gender groups, explain their different pathways of influence, and elucidate targeted measures to improve their outcomes (N=518). Results from the statistical model showed that the paths of effect from care needs to caregiver depressive symptoms differed between male and female informal caregivers. Care needs were not significantly associated with depression symptoms among informal caregivers, for either men or women. Care hours of more than 12 hours per day and financial difficulties at home are risk factors for depressive symptoms in caregivers, with significance of OR=3.42; 95%CI,1.97 to 5.94; P=0.000 and OR=2.98; 95%CI, 1.46 to 6.05; p=0.003, respectively. For male caregivers, years of caregiver education and the feel relied upon by relative's were both protective factors, whereas Job-Caregiving conflict, was a risk factor ($P<0.05$). For female caregivers, caregiver burden and higher caregiver age were its risk factors ($P<0.05$). These important findings demonstrate that to be effective in reducing depressive symptoms among informal caregivers, both cointerventions and triage interventions by gender are warranted.

### Session 3475 (Symposium)

### THE EXPERIENCE OF HEALTH CARE WORKERS CARING FOR OLDER ADULTS DURING THE COVID-19 PANDEMIC

Chair: Tiffany Washington Co-Chair: Terri Lewinson
Discussant: Jennifer Craft Morgan

Older adults are at increased risk for COVID-19 illness, hospitalization, and mortality. Essential health care workers became the backbone of their care during the pandemic, and their experiences are worthy of discussion. This symposium will highlight the emotional impact of COVID-19 on health care workers, and their scope of practice in various health settings. Using data from a cross-sectional survey, presenter one will describe concerns and coping strategies among nursing home social workers during COVID-19. Next, presenter two will present findings on the emotional health and wellbeing of home care workers. Then, presenter three will describe the experience of the VA's strike teams sent into 82 Florida nursing homes most impacted by COVID. Presenter four will describe findings from a qualitative study on emotional distress experienced by hospital-based medical directors, nurse practitioners, and other health care workers. Finally, presenter five will describe findings from a scope of practice among health care social workers study, with an emphasis on how policies in their respective settings impacted their work. Taken together, the presenters' findings in this symposium have implications for practice and policy recommendations to improve the experience of health care workers during pandemics.

### POLICY CONSIDERATIONS FOR GERIATRIC SOCIAL WORKERS IN HEALTHCARE SETTINGS DURING COVID-19

Terri Lewinson,[1] and Tiffany Washington,[2] *1. Georgia State University, Atlanta, Georgia, United States, 2. University of Georgia, Athens, Georgia, United States*

Social workers are essential to the delivery of health care with older adults the during COVID-19 pandemic. This paper focuses on the impact of policies in health care systems that affect geriatric social work practice. Semi-structured interviews were conducted with 55 social workers from a variety of health care settings. Data were analyzed to identify the scope of social work practice in health care settings during the COVID-19 pandemic, and how policies in their respective settings impacted their work. Conditions that impeded participants' ability to provide quality care and work within their scope of practice included inconsistent expectations of interdisciplinary team members, disparate access to resources, restriction of opportunities to address emotional distress experienced by workers. Recommendations for policy enhancements in health care settings include interprofessional education on effective team communication, protocol development for the equitable distribution of resources among essential workers, and trauma-informed in-service trainings for health care administrators.

### WORK DOES NOT END WHEN YOU LEAVE: CONCERNS AND COPING STRATEGIES AMONG NURSING HOME SOCIAL WORKERS DURING COVID-19

Vivian Miller,[1] Keith Anderson,[2] Nancy Kusmaul,[3] and Noelle Fields,[2], *1. Bowling Green State University, Bowling Green, Ohio, United States, 2. University of Texas at Arlington, Arlington, Texas, United States, 3. University of Maryland, Baltimore County, Baltimore, Maryland, United States*

Nursing home social workers (NH SW) at the frontline during COVID-19 are faced with many challenges in meeting the psychosocial needs of residents while balancing their own well-being needs. In order to explore the experiences of NH SW during COVID-19, the study utilized a cross-sectional survey distributed to social media sites (e.g., Reddit, Facebook) and professional networks. The survey asked participants (N = 63) open-ended questions which were analyzed using the rigorous and accelerated data reduction (RADaR) method. Themes suggested that fear for self, lack of administrative support, and overall stress were notable concerns among NH SW. Findings also suggested that support from family/friends and self-care were most personally helpful to NH SW. Lastly, themes related to coping strategies included talking with co-workers, mindfulness, and boundary setting. Findings suggest the need for increased supports for NH SW. Implications related to stress and coping during COVID-19 are offered.

### GET IN AND GET OUT: THE IMPACT OF COVID-19 ON THE EMOTIONAL HEALTH OF HOME CARE WORKERS

Leah Janssen, *Scripps Gerontology Center, Oxford, Ohio, United States*

This research explores the emotional health of home care workers (HCWs) during the coronavirus pandemic. In-depth, qualitative interviews were conducted with 17 home care workers in the fall of 2020. Thematic analysis revealed important connections between emotional health and success on the job. Perception of time and appreciation emerged as key elements that impacted emotional health. HCWs expressed the pressure to perform as usual while simultaneously taking on extra tasks as distracting from direct client care and reinforcing a task-oriented care approach. As a result of these tensions, HCWs experienced a loss of appreciation by the client, who prioritized personal safety and a "get in and get out" attitude, leaving the HCW feeling less fulfilled in their work. Implications of this research highlight the importance of HCW emotional health needs when retaining HCWs as valuable members of the long-term care workforce is paramount.

### THE VA'S LONG-TERM CARE STRIKE TEAM SUPPORTING FLORIDA'S NURSING HOMES WORKFORCE

Adam Golden, *Orlando VA Healthcare System, Orlando, Florida, United States*

In coordination with the Florida Department of Health, the VA Sunshine Healthcare Network (VISN 8) established Long-Term Care Strike Teams to provide services to the LTC facilities most affected by the COVID-19 pandemic across the state of Florida. Between April 2020 through September 2020, the Strike Teams provided direct patient care to community residents, infection control/ prevention education, and patient/ staff COVID-19 swabbing. We encountered facilities with large numbers of staff infected with COVID-19 and agency staff that were refusing to come to "COVID-infected" facilities. Remaining staff, including the administrators, were under much psychological distress. However, our experience supporting the long-term care facilities also had a major impact on our own perceptions of nursing home care. The bravery, dedication, and caring that we witnessed reinforced that the health care workers in long-term care facilities are true heroes.

### THE PSYCHOLOGICAL IMPACT OF COVID-19 ON HOME BASED PRIMARY CARE PROVIDERS IN NEW YORK: A QUALITATIVE STUDY

Emily Franzosa,[1] Sybil Masse,[2] Abraham Brody,[3] Jonathan Ripp,[4] Katherine Ornstein,[2] Alex Federman,[2] and Ksenia Gorbenko,[2], *1. Icahn School of Medicine at Mount Sinai, Icahn School of Medicine at Mount Sinai, New York, United States, 2. Icahn School of Medicine at Mount Sinai, New York, New York, United States, 3. NYU Hartford Institute for Geriatric Nursing, New York, New York, United States, 4. Mount Sinai Health System, New York, New York, United States*

Research on professional burnout during the pandemic has focused on hospital-based health care workers. This study examined the psychological impact of the pandemic on home-based primary care (HBPC) providers. We interviewed 13 participants from six HBPC practices in the New York including medical/clinical directors, program managers, nurse practitioners, and social workers and analyzed the transcripts using inductive qualitative analysis approach. HBPC providers experienced emotional exhaustion and a sense of reduced personal accomplishment. They reported experiencing grief of losing many patients at once and pressure to adapt to changing circumstances quickly. They also reported feeling guilty for failing to protect their patients and reduced confidence in their professional expertise. Strategies to combat burnout included shorter on-call, regular condolence meetings to acknowledge patient deaths, and peer support calls. Our study identifies potential resources to improve the well-being and reduce the risk of burnout among HBPC providers.

### Session 3485 (Symposium)

### UNDERSTANDING AND MEASURING FRAILTY: INSIGHTS FROM THE CANADIAN NUAGE AND CLSA COHORTS

Chair: Nancy Presse Co-Chair: Alan Cohen

Frailty is one of the most central concepts in geriatrics; nonetheless, multiple definitions and operationalizations abound, and the underlying biology remains a topic of much discussion. Here, we bring together four talks that join questions of understanding with questions of measurement, in order to explore how answering each is necessary to make progress on the other. We cannot measure frailty if we have not understood and defined it, but we cannot understand if we cannot measure it and study it. Turcot et al. present work on operationalizing frailty in the NuAge cohort. Mayo et al. establish a scale to test the extent to which frailty can be operationalized as a ladder rather than a condition, again using the NuAge cohort. Mendo et al. use mediation analyses to understand how grip strength and other aspects of frailty may play a role in the relationship between diabetes and atherosclerosis. Ghachem et al. test the relationship between physiological dysregulation of different systems and different criteria of the Fried model, in order to assess the evidence for frailty as an emergent physiological state. Together, these talks will push the boundaries of how we think about frailty at levels ranging from biological to clinical to operational.

### COMPARISON OF DIFFERENT APPROACHES TO OPERATIONALIZE FRIED'S PHENOTYPIC FRAILTY IN THE NUAGE COHORT

Alan Cohen,[1] Pierrette Gaudreau,[2] Véronique Legault,[3] José Morais,[4] Nancy Presse,[3] Stéphanie Chevalier,[5] and Valérie Turcot,[6] *1. Universite de Sherbrooke, Sherbrooke, Quebec, Canada, 2. Université de Montréal, Montreal, Quebec, Canada, 3. Université de Sherbrooke, Sherbrooke, Quebec, Canada, 4. McGill University, Montreal, Quebec, Canada, 5. McGill University, Ste-Anne-de-Bellevue, Quebec, Canada, 6. CIUSSS de l'Estrie-CHUS, Sherbrooke, Quebec, Canada*

Many operationalization approaches were proposed to identify frailty in older adults. The common use of Fried's original criteria or other cut-offs based on cohort distribution may not apply in every cohort leading to potential bias in the identification of frail individuals. We thus aimed to apply different Fried's phenotypic frailty operationalization approaches in the Quebec NuAge cohort of generally healthy community-dwelling older adults (n=1,753; aged 67-84 years), and longitudinally compare prevalence, incidence and predictive strength on outcomes, such as functional autonomy, falls, hospitalization and mortality. Significant variability in prevalence, classification agreement and predictive strengths were observed between approaches, notably using different types of distribution cut-offs, variables, or ways to handle missing data. This strategy helped us to prioritize a specific Fried's phenotypic frailty operationalization in NuAge, which could then be used in secondary research projects aiming to study determinants of Fried's phenotypic frailty and its role in health outcomes.

### VALIDATION OF A FRAILTY LADDER USING RASCH ANALYSIS: IF THE SHOE FITS

Mylène Aubertin-Leheudre,[1] Kedar Mate,[2] Sabrina Figueiredo,[2] Julio Fiore,[2] Mohammad Auais,[3] Susan Scott,[4] José Morais,[2] and Nancy Mayo,[2] *1. Universite du Quebec à Montreal, Montreal, Quebec, Canada, 2. McGill University, Montreal, Quebec, Canada, 3. Queen's University, kingston, Ontario, Canada, 4. McGill University Health Centre Research Institute, Montreal, Quebec, Canada*

The current measurement approach to frailty is to classify people on frailty status, rather than measure the degree to which they are frail. Here, we test the extent to which a set of items identified within the frailty concept fits a hierarchical linear model (Rasch model) and form a true measure reflective of the frailty construct and confirm the model using the NuAge dataset. The development sample included 234 individuals (aged 57 to 97) drawn from three sources: at-risk seniors (n=141); post-colorectal surgery (n=47); and post-rehabilitation hip fracture (n=46). We defined our frailty construct based on items commonly used in frailty indices, self-report measures, and performance tests. Of the 68 items, 29 fit the Rasch Model: 19 self-report items on physical function and 10 performance tests including one for cognition. Items typically identified as reflecting the frailty concept fit the Rasch model. The Frailty Ladder would facilitate personalized intervention.

### CARDIOVASCULAR RISK FACTORS AND CAROTID INTIMA MEDIA THICKNESS: MEDIATION AND INTERACTION BY GRIP STRENGTH

Mark Keezer,[1] Marie-Pierre Sylvestre,[2] and Christian Mendo,[3], *1. McGill University, Montreal, Quebec, Canada, 2. Université de Montréal, Montreal, Quebec, Canada, 3. Universite de Montreal, Montreal, Quebec, Canada*

Frailty is often described as being an increased vulnerability to the effects of stressors. There is little research investing how frailty may act as either a mediator or participate in interactions in the associations between risk factors and chronic disease. We will present novel analyses of the Canadian Longitudinal Study on Aging, focusing on the 30,000 study participants who underwent serial physical evaluations at one of 11 data collection sites between 2011 and 2018. Using the 4- way decomposition method elaborated by Vanderweele, we investigate the role of grip strength, as a component of physical frailty, in the effect of cardiovascular risk factors on the atherosclerotic burden of individuals (measured using carotid intima media thickness). Our findings clarify the mechanisms underlying of grip strength in the associations between cardiovascular risk factors and carotid intima media thickness.

### THE FRAILTY SYNDROME: AN EMERGENT PROPERTY OF PARALLEL DYSREGULATION IN MULTIPLE PHYSIOLOGICAL SYSTEMS?

Linda Fried,[1] Véronique Legault,[2] Karen Bandeen-Roche,[3] Nancy Presse,[2] Pierrette Gaudreau,[4] Alan Cohen,[5] and Ahmed Ghachem,[5], *1. Mailman School of Public Health, New York, New York, United States, 2. Université de Sherbrooke, Sherbrooke, Quebec, Canada, 3. Johns Hopkins Bloomberg School of Public Health, Baltimore, Maryland, United States, 4. Université de Montréal, Montreal, Quebec, Canada, 5. Universite de Sherbrooke, Sherbrooke, Quebec, Canada*

Despite its widespread presence in older adults, frailty etiology is still unclear, being associated with dysregulation in diverse physiological systems. Here, we show evidence that frailty emerges from broad loss of homeostasis integrated through complex systems dynamics. Using the NuAge and WHAS cohorts, we calculated Mahalanobis distance-based physiological dysregulation in six systems and tested the breadth, diffuseness, and nonlinearity of associations between frailty and system-specific dysregulation. We found clear support for breadth of associations, but only partial support for diffuseness and nonlinearity: 1) physiological dysregulation is positively associated with frailty in many or all systems, depending on analyses; 2) the number of dysregulated systems or the total amount of dysregulation are more predictive than individual systems, but results only partially replicated across cohorts; 3) dysregulation trends are exponential, but not always significant. These results suggest, but do not fully prove, that frailty is an emergent property of complex systems dynamics.

### Session 3490 (Symposium)

### EAST MEETS WEST: HOME AND COMMUNITY BASED CARE TO ENHANCE AGING IN PLACE

Chair: Takashi Amano Co-Chair: Megumi Inoue

Although the magnitude and rate of aging in Japan and the United States differ, the drastic change in population structure has resulted in common challenges in both countries. One challenge is assisting older people in staying in the community. Enhancement of home- and community-based care allows older people to remain in their homes or spaces of their choice without moving into an institution to receive necessary care. This symposium includes four presentations (two from Japan and two from the U.S.) examining various efforts surrounding home- and community-based care designed to strengthen older people's abilities to stay in the community. The presenters will cover a wide range of strategies that have been implemented in both countries. The first presenter will describe the development and delivery of a project to expand Arizona's dementia capable system. The second presenter will describe initiatives of a professional association of geriatrics to promote the concept of aging in place. The third presenter will discuss the Home Hazard Removal Program (HARP), a new home hazard removal and fall risk self-management program delivered in the home by occupational therapists. The fourth presenter will discuss Japan's national policy priority of promoting the use of home health care within the community-based integrated care system. The symposium will conclude with a review of similarities and differences of various efforts, summarize common goals and challenges, and identify best practices.

### EXPANDING ARIZONA'S DEMENTIA CAPABLE SYSTEM

DAVID Coon, *Arizona State University, Phoenix, Arizona, United States*

Currently, 5.8 million US adults live with Alzheimer's disease (ADRD); the number is expected to double by 2050. Arizona will experience the greatest percent increase in ADRD by 2025. This project targeted three underserved groups in order to expand Arizona's dementia capable system: people living alone with ADRD; people with Down Syndrome or another intellectual/developmental disability (DS/IDD) aging with ADRD and their family caregivers; and people with ADRD and their caregivers in the Latino community. This presentation describes the development and delivery of the project's educational workshops, case management services, and evidence-based programs. Over 2,220 participants have participated in workshops to date with the largest percentage being case managers, care coordinators, and discharge planners. Evaluations have been extremely positive with 86.1% being "very likely" to recommend the project to others. The presentation concludes with findings and lessons learned regarding the delivery of the project's evidence-based programs and case management services.

### GERIATRICS PERSPECTIVES FROM JAPAN

Satoru Mochizuki,[1] and Masahiro Akishita,[2], *1. Hino-Nozomi Clinic, Hino, Tokyo, Japan, 2. The University of Tokyo, Bunkyo-ku, Tokyo, Japan*

In 2025, Japan's baby boomers will cross the threshold of 75 years of age; a phenomenon that has been referred to as "the 2025 crisis", resulting in a significant burden on the healthcare system. To address this issue, the Japanese government is establishing the Integrated Community Care System, to provide comprehensive medical and long-term care services in each community. In cooperation with government and affiliated organizations, the Japan Geriatrics Society (JGS) has been working to develop the Integrated Community Care System. As a result of this effort, geriatric medicine is being integrated into the health care system through incentives for practitioners. For instance, medical facilities can be reimbursed if they perform comprehensive geriatric assessments (CGA) and CGA-based management/care. Additionally, home care medicine and polypharmacy are emerging issues of interest to the government. In this symposium, I will discuss how JGS has been trying to achieve "Aging in Place" in Japan.

### A HOME HAZARD REMOVAL PROGRAM TO REDUCE FALLS IN COMMUNITY-DWELLING OLDER ADULTS

Susy Stark, *Washington Unversity, St Louis, Missouri, United States*

The majority of falls experienced by older adults occur in the home with home hazards associated with an increased risk of falling. Low-income older adults, who have more disability and live in substandard housing, need feasible interventions to help them safely age in place. The Home Hazard Removal Program (HARP) is a new home hazard removal and fall risk self-management program delivered in the home by occupational therapists to prevent falls. To evaluate the program, a randomized control trial was conducted with 310 community-dwelling older adults receiving aging services in the community. HARP had high acceptability with older adults and was feasible to deliver in the community. Adjusted for fall risk, individuals in the HARP group fell 1.4 times versus 2.2 times in the control group over 12 months. This low-cost home hazard removal program demonstrated acceptability, feasibility, and a significant reduction in falls for at-risk community-dwelling older adults.

### JAPAN'S NEW FRAMEWORK ON DEMENTIA CARE

Kenji Toba, *Tokyo University, Tokyo Metropolitan Institute for Gerontology, Tokyo, Japan*

The number of people with dementia in Japan is ever-increasing. In 2020, 6 million people lived with dementia. The number is expected to increase to 9 million in 2040. This means that a person with dementia will be supported by three working people. To prepare for the big wave of dementia, Japan released the New Orange Plan in January 2015. In 2019, the Framework for Promoting Dementia Care was issued by the Japanese cabinet in which prevention and the opportunity for persons with dementia to age in place were set as the main goals. This framework requires all ministries to promote people's awareness about individuals with cognitive impairment. The educational targets include taxi drivers, retail shop clerks, bankers, police, and people working in the criminal justice system. I will discuss the New Framework which has potential to assist the country in supporting people living with dementia.

### Session 3495 (Symposium)

### IRVING S. WRIGHT AWARD OF DISTINCTION LECTURE, VINCENT CRISTAFALO AWARD LECTURE, AND TERRIE FOX WETLE AWARD LECTURE

Chair: Steven Austad Co-Chair: Terrie Wetle

The Irving S. Wright Award of Distinction Lecture will feature an address by the 2021 recipient Malene Hansen, PhD of the Buck Institue for Research on Aging. The Vincent Cristofalo Rising Star Award in Aging Research lecture will feature an address by the 2021 recipient, Morgan Levine, PhD, of Yale University. This award is given by the American Federation for Aging Research, Inc. The Terrie Fox Wetle Award lecture will feature an address by the 2020 recipient, Kali Thomas, PhD, FGSA of Brown University and an address by the 2021 recpient, Andrea Gilmore-Bykovskyi of the University of Wisconsin, Madison. These awards are given by the American Federation for Aging Research, Inc.

### TERRIE FOX WETLE AWARD (2021): ADVANCING MULTIDISCIPLINARY HEALTH SERVICES SCIENCE: DEVELOPMENTS IN A DEMENTIA-FOCUSED PROGRAM OF RESEARCH

Andrea Gilmore Bykovskyi, *University of Wisconsin-Madison, Madison, Wisconsin, United States*

The Terrie Fox Wetle Rising Star Award in health Services and Aging Research is an award named in honor of Fox Wetle, PhD, who is internationally recognized for her contributions to aging, public health, and health care research. The award recognizes health services researchers in early or middle-career phases who have made significant contributions that embody the value of multidisciplinary health services science and are likely to have a sustained, high impact on practice and research. This aware lecture will be presented by the 2021 Award Recipient, Andrea Gilmore-Bykovskyi, PhD, RN, and will highlight emergent findings and foci in her dementia-focused health services research program. In particular, the award lecture will discuss progress in investigating social and behavioral communication patterns among individuals with moderate to advanced dementia; and the role of temporally situated observational measures and inclusion of persons with dementia and their caregivers in this line of research. The lecture will conclude with a discussion of next steps for this area of investigation surrounding assessment of episodes of lucidity in advanced dementia; and considerations for strengthening progress in outcome evaluation among persons living with dementia through multidisciplinary and community-informed health services research.

### IRVING S. WRIGHT AWARD: CELLULAR RECYCLING IN AGING AND DISEASE: THE IMPORTANCE OF TAKING OUT THE TRASH

Malene Hansen, *Sanford Burnham Prebys Medical Discovery Institute, La Jolla, California, United States*

Aging is greatly influenced by quality-control processes that keep the materials inside our cells in proper shape and function. One of these processes is called autophagy, which means "self-eating". This cellular recycling process can digest damaged components to provide new and better parts for the cell. Autophagy plays important roles in many age-related diseases and has been directly linked to aging. In our laboratory, we use the microscopic soil-dwelling round worm C. elegans to understand how autophagy is linked to aging and disease. In this Wright Award seminar, I will discuss our progress on understanding how autophagy is regulated during normal aging and how it may promote a long and healthy lifespan.

### TERRIE FOX WETLE AWARD (2020): THE ROLE OF HOME-DELIVERED MEALS PROGRAMS IN IMPROVING HEALTH AND PROMOTING COMMUNITY INDEPENDENCE FOR OLDER ADULTS

Kali Thomas, *School of Public Health, Providence, Rhode Island, United States*

Dr. Terrie "Fox" Wetle is internationally recognized as a leader who conducts and advocates for multi-disciplinary and multi-method investigations centered on aging, public health and health care with direct implications for shaping policy and practice. This award lecture, given in Dr. Wetle's name, will be presented by the 2020 award recipient, Kali Thomas, PhD. Dr. Thomas will present a line of multi-disciplinary and multi-method research focused on the impact of home-delivered meals as it relates to the health outcomes of homebound, food insecure older adults. Findings will include results from observational and intervention studies conducted at both the local and national levels. Examples of how this evidence has influenced policy and practice, including greater integration with healthcare, will be provided. The lecture will conclude with discussion about future opportunities for collaboration with community partners to measure and understand the impact of these vital social services on the lives of older adults.

### VINCENT CRISTOFALO "RISING STAR"AWARD: DNA METHYLATION LANDSCAPES IN AGING

Morgan Levine, *Yale University, New Haven, Connecticut, United States*

The epigenetic code can be thought of as the operating system of the cell. It controls the most basic and critical cellular processes including differentiation, replication, metabolism, and signaling. Yet, with age, the epigenetic landscape is remodeled, bringing about widespread consequences for cellular and tissue identity, integrity, and functioning. But, what if like computer programmers, we could discover how to recode or restore the original program? The revolutionary discoveries by Yamanaka and Takahashi suggests this may be possible. While early experiments showed that Yamanaka factors could be used to convert somatic cells into induced pluripotent stem cells, more recent work by us and others have shown that signatures of epigenetic aging are also wiped clean during this process. What's more, epigenetic age reversal appears to take place early in the process and thus can be achieved without the cell _needing to dedifferentiate. Building off of this discovery, our lab is combining novel experiments and advanced bioinformatic techniques to decipher the epigenetic code and determine how it is remodeled during aging, development, and reprogramming. In our recent work, we have made advancements in mapping the epigenetic alterations observed in aging and linking them to both cellular processes and disease etiology. We have identified specific age changes in mouse and human cells that reflect mitotic history, cellular senescence, oxidative damage, and mitochondrial dysfunction. We have also demonstrated that these changes inform differences in organismal lifespan and/or disease etiology at the tissue level. Overall, this work has sweeping implications for our basic understanding of epigenetic aging and reprogramming, and will help provide the foundation for potent therapeutics that extend healthspan and lifespan.

### Session 3500 (Symposium)

### ADVANCING AGE INCLUSIVITY IN A PANDEMIC: AGE-FRIENDLY UNIVERSITY (AFU) CAMPUSES TAKE ACTION

Chair: Joann Montepare Co-Chair: Kimberly Farah

The COVID-19 pandemic presented extraordinary challenges for professionals in the aging field across campuses and communities, calling for rethinking and redesigning how their work was structured, their programs were delivered, and their connections were sustained. The pandemic also made clear the value of being an age-friendly institution of higher education, especially as we experience historic changes in age demographics. This symposium features campus leaders representing institutional partners of the Age-Friendly University (AFU) global initiative (endorsed by GSA's Academy for Gerontology in Higher Education) who will discuss how their age-friendly programs were adapted during the pandemic to continue to advance age inclusivity. These diverse responses exemplify the vast potential of age-friendly opportunities. June and Andreoletti (Central Connecticut State University) will discuss how the Scholars for Life! program supported the engagement of older learners in the neighboring community through the engagement of faculty. Elfenbein (University of North Georgia) will describe how learning experiences for older learners and intergenerational exchange were created beyond the classroom through the Personal Enrichment, Action and Knowledge (PEAK) program. Terhune (Northern Kentucky University) will describe how student support practices and services were adapted to provide working adult students with guidance for navigating their educational needs during the pandemic. Kheirbek (University of Maryland, Baltimore) will describe how age-friendly collaborations with the institution's medical school leveraged intergenerational connections and technology to foster social connection for hospitalized older adults. Gautam and Melillo (UMass Lowell) discuss how a campus partnership with the Learning in Retirement Association (LIRA) adapted efforts around healthy aging.

### SCHOLARS FOR LIFE! BUILDING FACULTY AND COMMUNITY CONNECTIONS ON AN AGE-FRIENDLY UNIVERSITY CAMPUS

Carrie Andreoletti,[1] and Andrea June,[2], *1. Central Connecticut State University, New Britain, Connecticut, United States, 2. Central Connecticut State University, Central Connecticut State University, Connecticut, United States*

Central Connecticut State University's Scholars for Life! supports the engagement of older learners in the community through faculty guest lectures. During the COVID-19 pandemic, participation in the virtual format frequently swelled to over 100 attendees, which is five times the number participating pre-pandemic. Moreover, faculty engagement increased. This presentation will share results of a study that used an Age-Friendly University (AFU) lens to explore this expanded connection to community members with the intention to build on its successful faculty-community engagement. 132 participants responded to the survey (M age = 69), mostly identifying as local retired alumni and community members. Participants reported high satisfaction with the lectures, connection to the university, interest in joining future travel abroad experiences, and utilizing campus resources when safe. Indeed, 84% are now aware of CCSU's AFU status and 61% expressed interest in the 62+ course tuition waiver. Implications and future directions will be addressed.

### TAKING AGE-FRIENDLY CAMPUS EFFORTS TO NEW PEAKS

Pamela Elfenbein, *University of North Georgia, Gainesville, Georgia, United States*

The University of North Georgia's Personal Enrichment, Action, Knowledge Series (PEAKS) was developed as a monthly series of engaging and seasonally appropriate presentations and activities designed to expressly meet the needs of the region's large and quickly growing older adult population during the Covid-19 pandemic and resultant isolation. The PEAKS programs are available virtually to allow for broad participation throughout the 30 county UNG service region. While we developed PEAKS to specifically reach-out to older adults in our region, we have found that our audience is actually multi-generational, actively engaging with our speakers and one another. AFU underpinnings for PEAKS include Principle #8 - To enhance access for older adults to the university's range of health and wellness programs and its arts and cultural activities; and #9 - To engage actively with the university's own retired community.

### SUPPORTING EDUCATIONAL NEEDS OF OLDER ADULT LEARNERS: STRATEGIES FOR VIRTUAL TRANSITIONING AND STUDENT ENGAGEMENT

Sara Conwell,[1] Amy Danzo,[1] Allyson Graf,[1] Suk-Hee Kim,[1] and Katherina Terhune,[2], *1. Northern Kentucky University, Highland Heights, Kentucky, United States, 2. Northern Kentucky University, Lexington, Kentucky, United States*

The pandemic has revealed a multitude of challenges disproportionately impacting older adults, including older adult learners. Institutions of higher education are uniquely positioned to respond to various challenges using the guiding framework of the Age-Friendly University global initiative. This presentation highlights how preexisting university student support practices and services were adapted to provide older adult learners with guidance for navigating their educational needs during the pandemic. Specifically, it expands on strategies utilized by Adult Learner Programs and Services to effectively pivot to virtual services to support the advising and programming needs of older adult learners. Survey data identifying areas of interest for virtual programming for older adult students will be explored. Recommendations will be discussed for promoting effective transitioning to virtual support systems, preserving student engagement and intergenerational learning, and advocating for aging to remain central to university diversity and inclusion initiatives.

### AN AGE- FRIENDLY CAMPUS PARTNERSHIP FOR HOSPITALIZED OLDER ADULTS IN THE COVID ERA AND BEYOND

Heavner Mojdeh,[1] Marjorie Fass,[2] Christina Cafeo,[1] Giora Netzer,[1] Mangla Gulati,[3] Nicole Brandt,[4] Barbara Resnick,[5] and Raya Kheirbek,[6], *1. University of Maryland, Baltimore, Maryland, United States, 2. University of Maryland School of Nursing , Baltimore, Baltimore, Maryland, United States, 3. University of Maryland Medical Center, Baltimore, Maryland, United States, 4. University of Maryland School of Pharmacy, University of Maryland School of Pharmacy, Maryland, United States, 5. University of Maryland School of Nursing, Baltimore, Maryland, United States, 6. University of Maryland School of Medicine, University of Maryland School of Medicine, Maryland, United States*

As hospitals isolate COVID-19 patients to prevent the spread of this highly contagious disease, patients and family are separated during times of critical illness. For many older adults inflicted with coronavirus it is not the fear of dying that matters the most, it is the fear of dying alone. Utilizing the 4Ms approach, University of Maryland, Baltimore (UMB) and University of Maryland Medical Center (UMMC) responded with several initiatives including intergenerational programs designed to shape and inform the development of future healthcare clinicians in addressing what matters the most to patients and leveraging technology to connect them with families, provide mobility opportunities, monitor medications, and reduce errors.

### A STEP BACKWARD AND FORWARD IN AN AGE-FRIENDLY UNIVERSITY INITIATIVE: ADAPTING A CAMPUS LEARNING PARTNERSHIP

Karen Devereaux Melillo, Carol McDonough, and Ramraj Gautam, *UMass Lowell, Lowell, Massachusetts, United States*

The 5-campus UMass system received designation as an Age-Friendly University (AFU) in 2019. AFU Principle 1 highlights the importance of involving older adults in University activities. UMass Lowell's Center for Gerontology Research and Partnerships collaborated with the Learning in Retirement

Association (LIRA) in Spring 2020 to offer aging-related courses around healthy aging. However, due to COVID-19, these were canceled and are re-scheduled for Spring 2021 via Zoom. The paper will describe the process of selecting course offerings with LIRA and the subsequent cancellation/rescheduling process and adaptation needed. A course will focus on AFU initiative and the opportunities and challenges at UMass Lowell. Likewise, the other course will offer a session on technology and aging where age-based digital divide and strategies for reducing it will be discussed. This paper will reflect on how the collaboration with LIRA and course selection process relates to the AFU principles 1, 5 and 9.

### Session 3505 (Symposium)

### COVID-19: HOW IT SHAPED NURSING HOME CARE AND ELDER JUSTICE

Chair: Brian Lindberg

This session will provide updates on how the pandemic led to horrific situations in long-term care facilities and how the pandemic influenced major federal efforts to address elder abuse, neglect, and exploitation.

### THE ELDER JUSTICE ACT AND THE PANDEMIC

Robert Blancato, *Elder Justice Coalition, Washington, District of Columbia, United States*

This session will provide updates on how the pandemic led to horrific situations in long-term care facilities and how the pandemic influenced major federal efforts to address elder abuse, neglect, and exploitation.

### THE PANDEMIC AND NURSING HOME CARE

Lori Smetanka, *The National Consumer Voice for Quality Long-Term Care, Washington, District of Columbia, United States*

This session will provide updates on how the pandemic led to horrific situations in long-term care facilities and how the pandemic influenced major federal efforts to address elder abuse, neglect, and exploitation.

### PANDEMIC LESSONS FROM A LONG-TERM CARE OMBUDSMAN

Mairead Painter, *Long-Term Care Ombudsman Program, Hartford, Connecticut, United States*

This session will provide updates on how the pandemic led to horrific situations in long-term care facilities and how the pandemic influenced major federal efforts to address elder abuse, neglect, and exploitation.

### Session 3510 (Symposium)

### DOING AGING RESEARCH TOGETHER: INNOVATIVE PERSPECTIVES ON PARTICIPATORY APPROACHES

Chair: Anna Wanka Co-Chair: Anna Urbaniak

The symposium aims to take a closer look at what it means to involve older participants in ageing research - beyond the role of research subjects. By discussing projects that deploy different participatory approaches we investigate the manifold ways in which older adults can become co-creators of the research process. We do so comparing such approaches in different domains, with different outcomes and in different stages of the research process. Consequently, this symposium (1) looks at the research process through the lens of benefits and challenges resulting from involving older adults as co-creators; (2) showcases projects across different domains and different jurisdictions that applied participatory approach in ageing research to discuss benefits and challenges, and (3) advances scientific insights into participatory approaches involving older adults. After an introductory contribution outlining theories, concepts and developments of participatory approaches in ageing research, we present insights from three empirical studies in different cultural and thematic settings. In our first presentation, Anna Wanka and Anna Urbaniak open the symposium by presenting an overview of participatory approaches that involve older adults. In the first empirical presentation, Julia Nolte and Hamid Turker discuss the process of involving older adults in data analysis and therefor present data from the US. In the third presentation, Lillian Hunn highlights how the recent COVID-19 pandemic impacted patient involvement in research in Canada. Finally, Anna Urbaniak discusses the process of planning participatory research with hard to reach population among older adults in Austria, namely those who are socially excluded.

### OLDER ADULTS AS CO-CREATORS IN THE RESEARCH PROCESS: AN OVERVIEW OF CONCEPTS, METHODS, AND APPROACHES

Anna Wanka, *Goethe University Frankfurt, Frankfurt, Hessen, Germany*

Participatory approaches have a long-standing tradition in the social sciences and approaches have diversified across multiple research domains. Also in ageing, there is a growing interest in involving older adults, particularly in fields like gerontechnology development, environmental gerontology or patient involvement. In this contribution, ask what participatory approaches and co-creation means in the context of ageing research. What are the benefits and challenges of involving older adults in different research domains, stages of the research process and deploying various participatory methods and approaches? To approach this question, we present preliminary results of a comprehensive scoping review of the literature and provide examples of how older adults can be involved in developing research questions together with researchers, collecting and analysing the data, as well as validating and disseminating study results.

### DESIGNING PARTICIPATORY AGING RESEARCH ON EXCLUSION FROM SOCIAL RELATIONS: A CITIZEN SCIENCE PROJECT

Anna Urbaniak, *University of Vienna, Wien, Wien, Austria*

Many policies and initiatives aim at enhancing the social participation of older adults. Despite this growing interest in increasing social inclusion and combating social exclusion in older age, the voices of socially-excluded older adults and their experiences remain underrepresented in research. Based on data from the Austrian research project “Socially Excluded Older Adults: Voices and Experiences” (SEVEN), I reflect on what it means to co-create research with the hard-to-reach populations of socially excluded older adults. Data discussed is derived By inviting this group to participate in each stage of the research, the project develops an innovative approach that, on the one hand, facilitates and advances ways for socially-excluded older adults to express their voices, thereby empowering them and their self-advocacy, and, on the other hand, creates research insights that are able to grasp the life worlds of older, socially excluded adults more accurately.

### INVOLVING OLDER ADULTS IN THE DATA ANALYSIS PROCESS

Hamid Turker, and Julia Nolte, *Cornell University, Ithaca, New York, United States*

Because involvement of older adults in the research process continues to be low, we review existing approaches to engaging older adults in qualitative and quantitative data analysis. In doing so, we examine in which contexts older adults have or could serve as analysis consultants, collaborators, or even leaders. We also discuss the benefits and challenges associated with inviting older adults into the analysis process: On the one hand, this age group can contribute unique viewpoints and draw on a lifetime of experience and expertise. On the other hand, involving older adults in data analyses typically requires resources and time while bearing the risks of health-related attrition, tokenistic treatment, and power imbalances between researchers and older adults. As a result, we close with recommendations on how to navigate this process effectively and respectfully (e.g., building trust, reducing hierarchies, being mindful of older adults' needs, and assessing their satisfaction throughout the collaboration).

### RELATIONAL APPROACHES IN PATIENT-ORIENTED RESEARCH DURING THE COVID-19 PANDEMIC

Lillian Hung, *University of British Columbia, Vancouver, British Columbia, Canada*

The COVID-19 pandemic has exposed the fragile state of patient involvement in research. The involvement of the most vulnerable population (older people with dementia) in research was even more challenging. This presentation outlines challenges my research team encountered in patient-led projects (older people with dementia) and describes how we found creative strategies to set up and complete research during the time of pandemic. I will describe how the team applied collaborative participatory principles to engage a team with diverse backgrounds in the lockdown time to maintain research progress. Patient partners in my research team actively led recruitment, research planning and decision-making, team analysis and knowledge exchange. University students in our research team helped to make technology easy to use for our patient partners. The friendly, flexible and accessible exchange between students and patient partners reinforced the importance of a respectful relational approach in patient-oriented research.

### Session 3515 (Paper)

### EDUCATION AND TRAINING (SRPP PAPER)

### CHALLENGES TO ENGAGE LOW-SKILLED ADULTS IN EDUCATION AND TRAINING: AN INTERNATIONAL PERSPECTIVE

Sydney Shadovitz,[1] Abigail Helsinger,[2] and Phyllis Cummins,[1], *1. Miami University, Oxford, Ohio, United States, 2. Scripps Gerontology Center, Miami University, Oxford, Ohio, United States*

The demand for adult education and training (AET) opportunities throughout the life course is substantial as labor markets often require workers to obtain advanced skills. AET opportunities are more often pursued by high-income and high-skilled workers than low-skilled or low-income workers. With the increased prominence of job automation and technological advances in the workforce, low-skilled workers are at risk for fewer opportunities within the labor market. These factors emphasize the importance of providing learning opportunities throughout the life course. In this mixed-methods study, we analyzed 2012/2014 data from the Program for the International Assessment of Adult Competencies (PIAAC) for the U.S., Canada, the Netherlands, Norway, and Sweden to compare participation rates in non-formal education (NFE) by high and low-skilled adults. Countries were selected based on qualitative findings that inform best practices. Additionally, to gain insights of policies and programs that promote NFE, international key informant interviews (n = 33) were conducted. AET policies and programs, along with barriers such as cost, motivation, and time, were explored with key informants. Findings include (1) aging and skills are negatively correlated in all nations of interest; (2) low-skilled adults are less likely to participate in NFE than their high-skilled counterparts; (3) low-skilled workers in Norway and the Netherlands are more likely to participate in NFE than their U.S. counterparts; and (4) NFE is often more acceptable to low-skilled adults due to previous negative experiences with formal education. Using these findings, we discuss successful AET programs in Nordic countries for overcoming barriers.

### DOMESTIC AND INTERNATIONAL PERSPECTIVES ON FINANCING ADULT EDUCATION AND TRAINING

Abigail Helsinger,[1] Oksana Dikhtyar,[1] Phyllis Cummins,[2] and Nytasia Hicks,[3], *1. Scripps Gerontology Center, Miami University, Oxford, Ohio, United States, 2. Miami University, Oxford, Ohio, United States, 3. Department of Veterans Affairs, San Antonio, Texas, United States*

Adult education and training (AET) over the life-course is necessary to participate in economic, social, and political activities in the time of globalization and technological advancement. However, little research has been done to identify mechanisms to fund AET opportunities among middle-aged and older adults from a comparative international perspective. Our study aimed to identify strategies to finance AET opportunities for middle-aged and older adults through an international lens, to help identify barriers and facilitators in effort to best support adult learners regardless of education background or socioeconomic characteristics. We carried out a descriptive qualitative study to facilitate an in-depth understanding of funding mechanisms available to adult learners in the selected countries, from the perspective of adult education and policy experts. Data were collected using semi-structured interviews with 61 international adult education experts from government agencies, non-governmental organizations, and education institutions. Our informants represented 10 countries including Australia, Canada, Germany, Italy, the Netherlands, Norway, Singapore, Sweden, the

United Kingdom, and the United States. Data included at least one in-depth phone or web-based qualitative interview per informant in addition to information gathered from written materials (e.g., peer-reviewed publications and organizational reports). We identified three financing options that arose as themes: government-sponsored funding; employer-sponsored funding; and self-funding. We found that government-sponsored funding is especially important for low-skilled, low-income older adults for whom employer-sponsored or self-funding is not available. Our results have implications for lifelong AET policy changes, such as adaptations of successful AET funding programs across global communities.

### REDUCING PESSIMISM ABOUT SOCIAL SECURITY'S FUTURE THROUGH GERONTOLOGICAL EDUCATION

Catheryn Koss, *California State University, Sacramento, Sacramento, California, United States*

A recent Pew Research Center poll found less than half of non-retired U.S. adults believe they will receive Social Security benefits when they retire. Pessimism about Social Security's future is even stronger among people under 50, raising concerns about political support for the program. The current study examined to what extent education about how Social Security functions and strategies to address program funding shortfalls could increase optimism about Social Security's future. In Fall 2020, twenty-two undergraduate students enrolled in an on-line aging and social policy course were asked how likely they would receive Social Security income when they retired. Consistent with national polling data, only 38% believed they would probably or definitely receive Social Security retirement income. Students were also asked to briefly explain why they answered the way they did. Common reasons given for pessimism about receiving Social Security retirement income included having heard Social Security will run out of money and believing only U.S. citizens are eligible for Social Security benefits. Over a two-week period, students completed asynchronous on-line activities covering Social Security retirement eligibility, worker and spousal benefits, current funding, and proposed changes to address funding shortfalls. Upon completion, 67% of students stated they would probably or definitely receive Social Security retirement income when they retired (p = .07). These preliminary results suggest that education about the program can reduce pessimism about Social Security's future. Additional data will be collected from 45 students enrolled in the same course in Spring 2021.

### SOCIAL WORK GRADUATES ENTERING THE FIELD OF GERONTOLOGY DURING COVID-19: EXPLORING PERCEIVED READINESS AND NEEDS

Susanny Beltran,[1] and Vivian Miller,[2], *1. University of Central Florida, Orlando, Florida, United States, 2. Bowling Green State University, Bowling Green, Ohio, United States*

Gerontological social workers (GSW) are on the frontlines supporting the biopsychosocial needs of older adults in hospitals, communities, and long-term care settings. However, it is unclear whether social workers (SW) are trained to meet the emerging needs of older adults during COVID-19. This study describes training received, perceived readiness, and training needs of GSWs new to the field during 2020. A cross-sectional online survey was conducted with recent graduates from U.S. SW programs. Survey questions explored training received and ongoing needs, perceived self-efficacy (adapted from the Geriatric Social Work Competency Scale), demographics, and confidence in ability to work with populations 55+. A total of 15 recent SW graduates specializing in gerontology completed the survey. Fifty-three percent of the sample held MSWs and over half (53.3%) were licensed social workers or registered interns. Nearly all participants (73.3%) reported taking an introductory aging course, and almost half (46.7%) completed coursework in aging and diversity, aging policy, and end-of-life care/bereavement; 80% completed fieldwork in aging. Participants report moderate skill in assessing issues related to losses or transitions (46.7%), and physical functioning (53.3%), and advanced skill in assessing cognitive functioning (60.0%), and caregiver stress/needs (53.3%). Nearly half of respondents who rated their training as good-excellent indicate being very-extremely confident (42.8%) in their ability to practice with older adults. Training needs among participants include disaster preparedness, telehealth, and coordination of scarce resources. Curriculum development and continuing education are necessary to support emerging gerontological social workers in their practice during COVID-19 and other emergencies.

### TRAINING SAVVY CAREGIVER PROGRAM GROUP LEADERS THROUGH AN ONLINE COURSE

Kenneth Hepburn,[1] Carey Sherman,[2] John Hobday,[3] and Lai Reed,[1], *1. Emory University, Atlanta, Georgia, United States, 2. Family Care Strategies, LLC, Ann Arbor, Michigan, United States, 3. Healthcare Interactive, inc, St. Louis Park, Minnesota, United States*

A significant factor limiting organizations' implementation of the Savvy Caregiver program, a widely disseminated dementia caregiver psychoeducation course, is the need to provide training to program leaders to ensure their understanding of Savvy core principles and strengthen their teaching and coaching skills. Such training has typically been provided through in-person group sessions led by the Savvy developers. To facilitate broader availability, we have embarked on an NIA-supported program to develop a fully online self-paced Savvy train-the-trainer course. The course, delivered individually on a widely used teaching platform, is in seven sections: the first introduces Savvy principles and the trainer role; the next six cover the content and teaching strategies of each of Savvy's six sessions. In the first development phase, 33 individuals from 13 organizations across the country took part in training (average age 49.5; almost all college level or professional women). Qualitative interviews with 11 trainees and debriefing sessions with others yielded consistently positive responses: the training enhanced their own appreciation for caregiving; they endorsed the self-paced learning and; and it established expectations for positive benefits of Savvy for caregivers. Trainees' feedback has led to several improvements, including resolving reported technical glitches (e.g., navigating the course). New videos illustrating group delivery methods have been added. Fidelity monitoring strategies are supported as organizations have been encouraged to augment the online training by convening meetings of trainees while in training to enable role playing, and greater personalization is achieved via post-training Zoom meetings with trainees and the Savvy training team staff.

### Session 3520 (Symposium)

### EVALUATING HOW AND WHY THE ENVIRONMENTAL CONTEXT SHAPES THE COURSE OF DEVELOPMENT ACROSS THE ADULT LIFESPAN

Chair: Omar Staben Co-Chair: Frank Infurna Discussant: Eileen Crimmins

There is a long-standing literature that has documented the importance of both the immediate and distal context in impacting mental and physical health across the adult lifespan. The goal of this symposium is to bring together a collection of papers that target the extent to which the immediate and distal context as measured through objective and subjective indicators relate to pertinent outcomes of mental and physical health. Staben and colleagues use an intensive longitudinal design in middle-aged adults to show that objective and subjective indicators of the neighborhood are associated with higher levels of and are protective against the impact of monthly adversity on mental health and well-being. Munoz and colleagues evaluate associations between objective and subjective early-life neighborhood contexts and whether they play a role in cognitive function at midlife. They find that poorer age-five self-report conditions were associated with lower working memory. Osuna and colleagues examine how both neighborhood and housing conditions play a role on psychological well-being. They find that housing and neighborhood safety conditions are associated with depressive symptoms over time. Piazza and colleagues examine associations between daily financial thoughts, SES, and indices of emotional and physical health. They find that individuals who reported more daily financial thoughts also reported more negative affect and physical symptoms. The discussion by Crimmins will integrate the four papers by highlighting the importance of how different forms of context can impact development in adulthood and old age, particularly in relation to health and well-being and consider future routes of inquiry.

### DO OBJECTIVE OR SUBJECTIVE NEIGHBORHOOD INDICATORS PROTECT AGAINST ADVERSITY ON MENTAL HEALTH AND WELL-BEING?

Frank Infurna, Kevin Grimm, Suniya Luthar, and Omar Staben, *Arizona State University, Tempe, Arizona, United States*

The neighborhood context through which individuals interact is shown to be associated with mental and physical health across adulthood. Much less is known regarding potential underlying reasons why, such as protecting against the deleterious effects of stress. This study explores whether objective and subjective neighborhood factors are associated with maintenance of mental health and well-being in the context of monthly adversity. We use longitudinal data from a sample of midlife (N =362) who completed monthly questionnaires for two years. Results show that experiencing a monthly adversity was associated with poorer mental health and well-being. Living in a neighborhood with more disorder was associated with stronger declines in mental health and well-being when a monthly adversity was reported. Our discussion focuses on why the neighborhood context is relevant for middle-aged adults and the various ways through which neighborhood context has the potential to shape the course of development in adulthood.

### THE EFFECT OF EARLY NEIGHBORHOOD CONTEXTS ON COGNITIVE FUNCTION IN MIDLIFE

Heewon Yoon,[1] Jean Choi,[2] Giancarlo Pasquini,[3] Alexa Allan,[4] Martin Sliwinski,[5] Stacey Scott,[3] and Elizabeth Munoz,[6], *1. University of Texas at Austin, Austin, Texas, United States, 2. The University of Texas at Austin, Austin, Texas, United States, 3. Stony Brook University, Stony Brook, New York, United States, 4. Pennsylvania State University, University Park, Pennsylvania, United States, 5. The Pennsylvania State University, University Park, Pennsylvania, United States, 6. The University of Texas at Austin, The University of Texas at Austin, Texas, United States*

We evaluated associations between objective and subjective early-life neighborhood contexts and cognitive function at midlife. Study participants grew up in different addresses but resided in the same urban zip code at the time of data collection thus controlling for concurrent neighborhood contexts. Participants provided their home address when they were five-years-old and recalled their age-five neighborhood conditions (Mage= 40.59 (7.91); n = 130). Age-five addresses were geocoded and linked with harmonized longitudinal Census tract boundaries and variables. Predictive models with a self-reported neighborhood conditions score, an objective neighborhood deprivation indicator, and other sociodemographic covariates indicated that poorer age-five self-reported neighborhood conditions were significantly associated with lower baseline (Cohen's d = -.24) and average daily (d = -.21) working memory performance. There were no associations with objective age-five neighborhoods. Results contribute to a growing literature on the role of psychosocial neighborhood contexts on cognition that may extend back to childhood neighborhoods.

### DAILY FINANCIAL THOUGHTS AND INDICES OF MENTAL AND PHYSICAL HEALTH: THE IMPORTANCE OF SOCIOECONOMIC STATUS

Jonathan Rush,[1] Eric Cerino,[2] Jacqueline Mogle,[3] Robert Stawski,[4] Susan Charles,[5] David Almeida,[6] and Jennifer Piazza,[7], *1. University of Victoria, Victoria, British Columbia, Canada, 2. Pennyslvania State University, State College, Pennsylvania, United States, 3. Penn State University, University Park, Pennsylvania, United States, 4. Oregon State University, Corvallis, Oregon, United States, 5. University of California, Irvine, Irvine, California, United States, 6. Pennsylvania State University, University Park, Pennsylvania, United States, 7. California State University, Fullerton, California State University, Fullerton, California, United States*

The current study examined the associations between daily financial thoughts, socioeconomic status (SES), and indices of emotional (positive and negative affect (PA/NA)) and physical health (physical symptoms and cortisol). Participants (N = 782) from the National Study of Daily Experiences, a subsample of the Midlife in the United States Refresher survey, completed daily diary interviews and provided saliva samples, from which cortisol was assayed. Participants who, on average, reported more daily financial thoughts also reported more NA, less PA, more physical symptoms, and had higher cortisol AUCg (all p's < .05). These effects were more pronounced among people reporting lower SES. Daily fluctuations in financial thoughts also predicted daily fluctuations in NA, PA, and physical symptoms (all p's <. 01). Again, these associations were more pronounced among people reporting lower SES. Results indicate that intrusive, daily financial thoughts may be one pathway explaining the link between SES and health outcomes.

### IMPACT OF HOUSING AND NEIGHBORHOOD ON DEPRESSION AMONG OLDER ADULTS IN THE HEALTH AND RETIREMENT STUDY

Rachel Wilkie,[1] Jennifer Ailshire,[2] and Margarita Osuna,[3]
*1. Spatial Sciences Institute, USC, University of Southern California, California, United States, 2. University of Southern California, Los Angeles, California, United States, 3. USC, University of Southern California, California, United States*

Prior research has suggested that poor neighborhood and housing conditions can lead to worse psychological wellbeing. Most studies examine either neighborhood or housing conditions, but not both. Since neighborhood and housing conditions may be correlated it raises the question of whether one is a proxy for the other. We use data from the 2006 and 2008 waves of the Health and Retirement Study to examine associations between perceived neighborhood and housing conditions in 2006 and depressive symptoms (CES-D 8) score in 2008. We find that worse housing conditions and neighborhood safety are associated with more depressive symptoms two years later, even when controlling for prior depressive symptoms. Furthermore, housing and neighborhood conditions are independently related to increased depression symptoms over time. Our research contributes to a deeper understanding of the relationship between home and neighborhood environments and psychological wellbeing in older adults.

### Session 3525 (Symposium)

### FAMILY AND AGING IN CROSS-NATIONAL CONTEXTS

Chair: Jennifer Ailshire Discussant: Jennifer Ailshire

The global aging of the population, combined with shifts in the structure and composition of families, has led to increased attention to the role of family and social relationships in the aging experience. The importance of family in determining healthy aging, however, may largely depend on the social, political, and economic context in which individuals are embedded. Cross-national investigations offer a unique opportunity to understand how family relationships and family caregiving influence health and well-being among older adults by comparing family dynamics across different sociocultural contexts. The HRS-family of surveys, which have been harmonized within the Gateway to Global Aging, provides remarkable opportunities for cross-national comparative analysis. The papers in this session use harmonized data from the Gateway to compare the influence of family on health across different social dimensions in multiple countries from around the world, including examinations of: 1) the impact of grandparenting on health of older adults in Europe and China; 2) psychological well-being among European older adults whose partners receive formal care; 3) the influence of parent-child relationships on health and well-being of older adults in China and the U.S.; and 4) how loneliness among older adults is patterned according to their living arrangements and relationship quality. The discussion will highlight the promises and challenges of cross-national research on families and aging and how harmonized aging data facilitates international comparisons.

### GRANDCHILD CARING AND LATE-LIFE DEPRESSION: A COMPARATIVE LONGITUDINAL STUDY IN CHINA AND EUROPE

Yazhen Yang, *University of Southampton, Southampton, England, United Kingdom*

The impact of grandparenting on the grandparents' health has been relatively under-studied, and international comparisons can provide useful lessons for grandparents and policymakers. This study examined country differences in the effects of grandchild care provision on the grandparents' depression in Italy, Spain, China, Denmark and Sweden using the longitudinal Harmonised CHARLS and SHARE data collected between 2010-5. Controlling for the grandparents' depression in 2011, grandparents providing non-intensive grandparental care in China, Sweden and Denmark in 2013 were less likely to report depression in 2015 compared to those who did not provide any care in 2013. Such effects were more pronounced among grandmothers compared to grandfathers. The results indicate that the effects of grandchild caring on the grandparents' depression in China was comparable to Denmark and Sweden. Future research can focus on identifying the causal pathways between grandparenting and wellbeing, and the implications of such pathways for older persons' wellbeing worldwide.

### PARTNER CARE ARRANGEMENTS AND OLDER EUROPEANS' WELL-BEING: VARIATIONS BY GENDER AND CONTEXT

Nekehia Quashie,[1] Karen Glaser,[2] Martina Brandt,[1] and Ginevra Floridi,[3], *1. Technische Universitat Dortmund, Dortmund, Nordrhein-Westfalen, Germany, 2. King's College London, London, England, United Kingdom, 3. Nuffield College, University of Oxford, Oxford, England, United Kingdom*

Across Europe, partners are often primary caregivers to older adults with care needs. Yet, a variety of partner care arrangements may arise. Little is known about the interrelations between partners' care arrangements, (potential) caregivers' gender, and the context in which care is embedded. We use 2015 SHARE data from 17 countries on 3,465 couples aged 50+ where one partner receives care. We examine how life satisfaction and depressive symptoms of (potential) caregivers vary across five care arrangements: solo-care; shared formal; shared informal; outsourced formal; and outsourced informal. We explore heterogeneity by gender and across four contexts: Northern, Western, Southern, and Eastern Europe. Outsourcing partners'

care to formal or informal providers is linked with higher well-being among Northern and Western European women, but with lower well-being among women in Southern Europe, where traditional female caregiving responsibilities are stronger. Among men, outsourcing partner care is linked to higher well-being regardless of context.

### CHILD-PARENT RELATION AND OLDER ADULTS' HEALTH: A CROSS-CULTURAL COMPARISON BETWEEN CHINA AND THE UNITED STATES

Dexia Kong,[1] Mack Shelley,[2] and Peiyi Lu,[2], *1. Rutgers University, New Brunswick, New Jersey, United States, 2. Iowa State University, Ames, Iowa, United States*

Western culture emphasizes independence in the child-parent relationship while Chinese culture values interdependence between adult children and older parents. This study compared the association of child-parent relationships with older adults' multidimensional health over time in the U.S. and China. Two waves of data (2012-2015) from HRS and CHARLS were used (n=6,641, aged ≥65). Linear regression models were estimated. Results showed that, compared to Chinese older adults, fewer older Americans co-resided with or lived nearby their children, had less weekly contact, and fewer financial transfers from/to their children. Most child-parent relationship variables were nonsignificant predictors of older Americans' health. However, a closer child-parent relationship was linked to fewer depressive symptoms and better cognition among older Chinese. Co-residence was associated with poorer health among Chinese parents. The associations of child-parent relationships with older adults' health exhibited cross-cultural differences. A cultural perspective is recommended in understanding how family relations affect older adults' health.

### OLDER ADULTS' SOCIAL STRESS PROFILES: A CROSS-COUNTRY COMPARISON OF THE UNITED STATES AND MEXICO

Christina Marini,[1] and Stephanie Wilson,[2], *1. Adelphi University, Garden City, New York, United States, 2. Southern Methodist University, DALLAS, Texas, United States*

Older adults face heightened risks for loneliness due to social isolation. Low-quality relationships also fuel loneliness. Because living arrangements and family norms differ between countries, cultural differences may arise in the stress of isolation, loneliness, and difficult relationships. To examine social stress profiles in the US and Mexico, HRS (N=17,878) and MHAS (N=15,001) participants rated their loneliness, whether they lived alone, and relationship quality with their spouse, children, and friends. Five latent classes emerged in both samples: lonely and isolated; lonely with poor relationships; moderately lonely with ambivalent relationships; moderately lonely and unhappily married; and low social stress. Lonely isolation was most common among Americans (23.4%), but least common among Mexicans (14.0%). The highest risks for loneliness coincided with living alone in the US, but with low-quality relationships in Mexico. Results reveal undercurrents of older adults' social stress that were common to both countries, as well as important cultural differences.

### Session 3530 (Paper)

### FAMILY AND INTERGENERATIONAL RELATIONSHIPS II

### ALL IN THE FAMILY? AGE, PERIOD, AND COHORT DIFFERENCES IN KINSHIP TIES AMONG OLDER U.S. ADULTS

Ellen Compernolle,[1] Khoa Phan Howard,[2] and Eric Hedberg,[3], *1. NORC at the University of Chicago, Chicago, Illinois, United States, 2. Northwestern University, Evanston, Illinois, United States, 3. Abt Associates, Chicago, Illinois, United States*

In general, older adults' social networks—characteristics of which (e.g., size, type, frequency) have been linked to important health and well-being outcomes--tend to be kin-centered, although this has changed over time. Disentangling these changes, however, is difficult given typical mobility decline and shrinking networks in old age (age), the rapid social and demographic changes that occurred during the 20th century (cohort), and, in recent decades, the 2008 Recession and technological advances (period). This study uses data from the National Social Life, Health, and Aging Project (NSHAP), a nationally representative sample of older adults (ages 57-85; 2005-2016), to examine patterns in older adults' social networks, with particular emphasis on the role that family plays. Specifically, we ask: 1) Have older adults' social networks become less kin-centered over the past decade (2005-2016)? 2) Are they less kin-centered among younger cohorts? And 3) Does the recession explain part of these period effects? We find that, between 2005 and 2016, family still comprises the majority of older adults' social networks, although their network size and range have grown larger and become less family-centric. They also report fewer close family members and friends, living with fewer family members, and less frequent interaction with network ties. Results from multi-level regression models suggest that age, and to a much lesser extent, cohort, plays a key role in many of these changes, although this varies between the first and second 5-year intervals of data collection, underscoring older adults' adaptivity to current social and economic circumstances.

### COSTS OF RAISING GRANDCHILDREN ON GRANDMOTHER-ADULT CHILD RELATIONS IN BLACK AND WHITE FAMILIES

Yifei Hou,[1] J. Jill Suitor,[2] Megan Gilligan,[3] Destiny Ogle,[2] Catherine Stepniak,[1] and Yufu Jiang,[1], *1. Purdue University, West Lafayette, Indiana, United States, 2. Purdue University, Purdue University, Indiana, United States, 3. Iowa State University, Iowa State University, Iowa, United States*

The cost of raising grandchildren on grandmothers' mental and physical health has been well-documented; however, little is known about whether raising grandchildren also has a cost on grandmothers' relationships with the adult children whose children the grandmothers have raised. Drawing from theories of exchange and affect, stress process model, and racial differences in intergenerational solidarity, we tested how raising grandchildren affects grandmother-adult child relations. Further, we explored the extent to which these patterns differed by race. To address this question, we used mixed-methods data collected from 553 older mothers regarding their relationships with their 2,016 adult children; approximately 10% of the mothers had raised one or more of their grandchildren "as their own." Data were provided by the Within-Family Differences Study-I. Multilevel analyses showed that raising grandchildren was associated with greater closeness in grandmother-adult child relationship in Black families; however, in White families, raising grandchildren was associated with greater conflict in the grandmother-adult child relationship. Further, the differences by race in the effects of raising grandchildren on closeness and conflict were statistically significant. Qualitative analyses revealed that race differences in the association between raising grandchildren and relationship quality could be explained by mothers' reports of greater family solidarity in Black than White families. Our findings highlight the ways in which race and family solidarity interact to produce differences in the impact of raising grandchildren on Black and White mothers' assessment of the quality of their relationships with their adult children, consistent with broader patterns of racial differences in intergenerational cohesion.

### DOES RELIGIOSITY IN THE TRANSITION TO ADULTHOOD PREDICT FILIAL NORMS IN MIDLIFE?

Jeung Hyun Kim,[1] Woosang Hwang,[2] Maria Brown,[2] and Merril Silverstein,[2], *1. Syracuse University, Syracuse, New York, United States, 2. Syracuse University, Syracuse, New York, New York, United States*

**Objective:** This study aims to identify multiple dimensions of religiosity among young adults at the beginning and end of the transition to adulthood, and describe how transition patterns of religiosity in early adulthood are associated with filial elder-care norms in midlife.

**Background:** There is a broad consensus that religiosity is multidimensional in nature, but less is known regarding transitions in multiple dimensions of religiosity from early to middle adulthood and predicted filial eldercare norms as a function of those religiosity transitions.

**Methods:** The sample consisted of 368 young adults participating in the Longitudinal Study of Generations in 2000 (mean age = 23 years) and 2016 waves. We conducted a latent class and latent transition analyses to address our aims.

**Results:** We identified three religious latent classes among young adults in both 2000 and 2016 waves: strongly religious, weakly religious, and doctrinally religious. Staying strongly religious young adults between 2000 to 2016 waves reported higher filial elder-care norms in the 2016 Wave than those who were in staying weakly religious, staying doctrinally religious, and decreasing religiosity transition patterns between 2000 to 2016 waves.

**Conclusion:** Our findings suggest that religiosity is still an important value for young adults shaping their intergenerational relationships with their aging parents.

**Keywords:** religiosity, filial eldercare norms, young adults, transition to adulthood

### STRESSFUL FAMILY CONTEXTS AND HEALTH IN DIVORCED AND MARRIED MOTHERS

Kandauda Wickrama,[1] and Eric Klopack,[2] *1. University of Georgia, Athens, Georgia, United States, 2. University of Southern California, Los Angeles, California, United States*

Using prospective data over 25 years from a sample of 416 women, the first objective of the current study was to investigate the biopsychosocial process over the second-half of the life course comparing mothers with diferent marital histories. The second objective was to investigate this biopsychosocial process for 296 maried mothers focusing on their marital quality over middle years. The results suggested that, compared to being married, divorcing in early-midlife launched an adverse biopsychosocial process for women leading to physical pain, physical limitations, and depressive symptoms over their mid-later years, largely through early-midlife financial stress, regardless of later recoupling. However, subsequent financial stress did not influence divorced mothers' later-life health problems, suggesting their development of resilience. For consistently married mothers, both marital stress and financial stress uniquely influenced all three health problems throughout their mid-later years. For all mothers, these health problems progressed over mid-later years, as indicated through their stabilities and mutual influences, and these health problems also selected mothers into further escalating financial and marital stress over their mid-later years. Elucidating differential short- and long-term health influences of marital and financial stressors for divorced and married mothers provides a potentially useful information for targeted early preventive intervention efforts and policy formation. Such interventions can promote and develop resiliency factors, thereby aiding middle-aged mothers to prevent from adverse biopsychosocial processes.

### WHY DO PEOPLE DIVORCE LATE IN LIFE? SWEDISH GRAY DIVORCE NARRATIVES

Peter Öberg,[1] and Torbjorn Bildtgard,[2], *1. University of Gävle, Gävle, Gavleborgs Lan, Sweden, 2. Stockholms University, Stockholm, Stockholms Lan, Sweden*

Divorce rates for people 60+ has increased in many parts of the Western world in what has been described as a "grey divorce revolution". In Sweden these divorce rates have more than doubled since the millennium. But why do people choose to divorce late in life and what is the impact of life phase typical transitions? Qualitative interviews with 37 Swedish men and women (aged 62-81) divorced after the age of 60 were collected, covering themes regarding the divorce process: motives for and experiences of divorce, and life as grey divorcee. The results by thematic analysis show that motives for divorcing earlier in the life-course, such as abuse, unfaithfulness and addiction are prevalent also among older people. However, they tend to be framed differently in later life and be integrated into divorce narratives informed by age. We identified four life phase typical narratives for divorce: 1) Lack of a common project for the third age. 2) Partners personality change due to age related disease. 3) Increased freedom after empty-nest allowing emancipation from a dominant partner. 4) A final romantic adventure as a form of rejuvenation. All these life-phase typical narratives are related to the third age as a time of self-fulfillment, where the partner can either be part of or an obstacle to that project. The results will be used to discuss current older cohorts' views of family norms and later life from the perspective that current older cohorts participated in the divorce revolution in the 1970s as young adults.

### Session 3535 (Paper)

### FAMILY CAREGIVING INTERVENTIONS

### A SYSTEMATIC REVIEW OF EFFECTS OF PHYSICAL ACTIVITY OF FAMILY CAREGIVERS OF OLDER ADULTS WITH CHRONIC DISEASE

Dawon Baik,[1] Jiyoun Song,[2] Aluem Tark,[3] Heather Coats,[4] and Catherine Jankowski,[4], *1. University of Colorado, Aurora, Colorado, United States, 2. Columbia University, Columbia University, New York, United States, 3. University of Iowa, University of Iowa, Iowa, United States, 4. University of Colorado, University of Colorado, Colorado, United States*

More than 17 million family caregivers (FCGs) provide care for older adults with chronic illness in the US. Caregiving for older adults with chronic disease places a considerable burden on FCGs and they tend to neglect their personal health. Generally, physical activity (PA) programs benefit the physical and psychological health of FCGs. However, no review of PA randomized clinical trials (RCTs) focused on FCGs of older adults with chronic disease. In this systematic review, we analyzed the most recent trends (2010-2020) in RCTs identifying the effects of PA in this population. This review followed the Preferred Reporting Items for Systematic Reviews and Meta-Analyses guidelines. Electronic databases (PubMed, CINAHL, Embase, PsycInfo, Cochrane Library) were searched for publications dated from 2010 to 2020. All studies included were appraised for quality using the Cochrane Collaboration Risk of Bias Tool. Of the resulting 16 studies, most studies (n=11) targeted FCGs of older adults with dementia or cancer. Most FCGs were non-Hispanic white. PA interventions with mixed modes (e.g., aerobic and resistance exercise), mixed delivery methods (e.g., in-person and telephone) and mixed settings (e.g., supervised gym- and unsupervised home sessions) were used most frequently. PA interventions significantly improved psychological health but had inconsistent effects on physical health. Tailored PA programs, designed based on FCGs' goals, preferences and limitations, may improve upon physical health outcomes. Future PA studies should include samples of racially and ethnically diverse FCGs of older adults representing a broader range of chronic diseases.

### CHARACTERIZING SUPPORTIVE SERVICES USE BY CAREGIVING RELATIONSHIP STATUS

Jasmine Travers,[1] and Chanee Fabius,[2], *1. New York University, New York, New York, United States, 2. Johns Hopkins Bloomberg School of Public Health, Baltimore, Maryland, United States*

Informal caregivers of aging older adults experience a high degree of burden and strain. These emotional experiences often stem from stressful tasks associated with caregiving. Caregiving supportive services that target the provision of support for stressful tasks are instrumental in alleviating caregiving burden and strain. Research is limited on what types of caregiving supportive services caregivers are accessing by relationship status and their source of information. We sought to characterize caregiving supportive services use by caregiving relationship status. We analyzed cross-sectional data from the 2015 National Study of Caregiving limited to caregivers of older adults □65 years. Caregiver relationship status (i.e., spouse, child, other relative/non-relative) was the independent variable. Bivariate analyses were performed to examine the association with caregiver relationship status and 1) any use of supportive services, 2) type of supportive service used among users, and 3) source of information about supportive services. Our sample consisted of 1,871 informal caregivers, 30.7% reported using supportive services. By caregiver relationship status, children had the greatest use of supportive services compared to spouses and other relatives/non-relatives (33.3% vs. 22.5% vs. 22.1%, p=.02, respectively). Among users of services, there were no differences in type of services used. Spouses primarily received their information about services from a medical provider or social worker (73.8%, p=.004). Our findings highlight the need to ensure that other caregiving groups such as spouses have access to important supportive services such as financial support. Medical providers and/or social workers should be better leveraged and equipped to provide this information.

### DESIGN THINKING AS A METHOD FOR DEVELOPING CAREGIVER (AND OTHER) INTERVENTIONS

Leila Aflatoony,[1] Molly Perkins,[2] Drenna Waldrop,[3] and Kenneth Hepburn,[3] *1. Georgia Tech, Atlanta, Georgia, United States, 2. Emory University of Medicine, Atlanta, Georgia, United States, 3. Emory University, Atlanta, Georgia, United States*

"Design Thinking," an innovative, human-centric approach to problem-solving, seeks to ensure that design efforts "solve the right problem." This presentation describes the Design Thinking process and illustrates its use in the context of three design studio sessions with of family caregivers of patients at the Integrated Memory Care Clinic (IMCC), a comprehensive medical home for persons living with dementia. The Design Thinking process entails five steps – Empathize, Define, Ideate, Prototype, Test – that engage consumers/end-users to identify, as precisely as possible, the issues or concerns that are most important to them and to further identify the possible solutions that seem to most fully address these concerns. The process can be described as one of divergent and convergent thinking. In the first session, the Empathize phase, IMCC caregivers were asked to think as broadly as possible about needs not being met by IMCC. These topics were reviewed more convergently in the second session, the Define phase; here the participants agreed on a shorter, prioritized list of needs to be addressed. In the third session (that combined the Ideate and pre-Prototype stages), participants identified 14 topics (interventions) they felt should be included in this program. Finally, in the Test phase, they assessed the topics and agreed that the most important need IMCC could address would be to provide a comprehensive orientation program for new caregivers. IMCC clinicians concurred with the salience of the problem to be solved and saw addressing it as contributing substantially to the improvement of IMCC clinical care.

### ETHICAL AND METHODOLOGICAL CONSIDERATIONS INTERVIEWING DEMENTIA CAREGIVERS

Quinton Cotton,[1] Laura Block,[2] Clark Benson,[2] Amanda Friz,[3] Britta Chelgren,[4] Brady Stroik,[2] Emily Ploch,[4] and Andrea Gilmore Bykovskyi,[2]
*1. University of Wisconsin - Medicine & Public Health, Fitchburg, Wisconsin, United States, 2. University of Wisconsin-Madison, Madison, Wisconsin, United States, 3. University of Washington, University of Washington, Washington, United States, 4. University of Wisconsin-Madison, University of Wisconsin-Madison, Wisconsin, United States*

Greater inclusion of people living with dementia (PLWD) and their caregivers in research is a global research priority and an expressed priority of dementia advocacy organizations. Absent inclusion of PLWD and caregivers, our understanding of dementia-related experiences and optimization of care and caregiving interventions is stymied. Qualitative interviewing techniques constitutes a primary method for obtaining PLWD and caregivers' perspectives. Yet, there is little guidance on use of qualitative interviewing techniques among PLWD and caregivers or discussion of potential challenges encountered, despite unique vulnerabilities faced throughout the research process, which may be further heightened among historically excluded groups. Meaningful progress toward inclusion of PLWD and their caregivers in dementia research necessitates broader examination of associated methodological and ethical considerations that arise in the conduct of interviews. Drawing from a large multi-site qualitative study of dementia caregivers with exposure to high levels of social disadvantage, we used a multiple-triangulation qualitative approach across interview transcripts, memos, and interviewer discussions to identify methodological and ethical challenges that arose during the interviewing process. Challenges were identified across all phases of research, and included relational concerns with PLWD and family members due to disclosure of sensitive information, risk of re-traumatization in discussing past experiences, multiple roles of caregivers with conflicting perspectives, variable recall capacity, limited prior appraisal of caregiving, and request of interviewers for medical advice or selecting services . We outline events evidencing these challenges and proposed strategies (i.e. use of research consults, interview debriefing) to strengthen research capacity to anticipate and respond to them.

### PREDICTORS OF PARTICIPATION RESTRICTION IN MIDLIFE CAREGIVERS: AN EXPLORATORY STUDY

Tara Klinedinst,[1] Scott Beach,[2] Heidi Donovan,[1] and Grace Campbell,[1], *1. University of Pittsburgh, Pittsburgh, Pennsylvania, United States, 2. University of Pittsburgh, University of Pittsburgh, Pennsylvania, United States*

Mid-life family caregivers (CGs) are at risk for participation restrictions (reduced engagement in valued roles and activities) due to competing demands of work, parenting, and family caregiving responsibilities. When CGs experience participation restrictions, quality of care for care recipients (CR) decreases, yet CG burden and risk for poor health increases. The purpose of this study was to identify the factors contributing to decreased participation in midlife CGs. Participants were CGs aged 45-64 years ($n = 677$) from the National Study of Caregiving/National Health and Aging Trends Study. We used multivariate logistic regression to determine attributes of CGs, CRs, and the care situation that independently contribute to participation restrictions. We found that negative aspects of caregiving (OR = 1.51, 95% CI = 1.33, 1.71) and CR depression and anxiety (OR = 0.90, 95% CI = 0.83, 0.99) significantly predicted participation restrictions ($p < 0.05$). Positive aspects of care (OR = 0.87, 95% CI = 0.74, 1.01), frequency of helping with chores (OR = 1.30, 95% CI = 0.98, 1.70), frequency of providing personal care (OR = 1.24, 95% CI = 0.97, 1.59), and frequency of providing help getting around the home (OR = 1.30, 95% CI = 0.97, 1.75) showed trends for association with participation restrictions ($p < 0.10$). We identified factors that are related to participation restriction in midlife CGs. Some of these factors (e.g., positive and negative aspects of caregiving, frequency of assistance provided) are potentially modifiable intervention targets that could bolster participation in this at-risk group.

### Session 3540 (Symposium)

### FRIENDSHIP AND LONELINESS AMONG PEOPLE LIVING WITH DEMENTIA: TOWARD COMMUNITY AND SHARED HUMANITY

Chair: Daniel R. Y. Gan Co-Chair: Habib Chaudhury
Discussant: Jim Mann

An increasing number of people living with dementia (PLWD) age in community. In North America, this number ranges from 61-81% of the total number of PLWD. As many as one in three PLWD do not live with a care partner. Since most PLWD do not drive, many may spend a significant proportion of time within half a mile of their homes. Yet, the neighbourhood may or may not provide "ways of being in the world that are more accepting and embracing of the kinds of disruptions that dementia can produce" (Hillman & Latimer, 2017). To support continued social participation, meaningful everyday networks are required. PLWD and care partners may identify more or less strongly with a community depending on their position in the network, its spaces, and activities. According to Nancy (1991), "community" has been conjured as an antidote to the loneliness of the human condition, which explains its allure. In response, Costello (2014) argued that "community" requires one to constantly try and "fall short" in caring for another's changing experiences. The value of a community thus depends on the quality of its friendships – the ability of otherwise lonely individuals to empathize – which may be threatened by challenges to PLWD's personhood. This symposium brings together expertise in community gerontology, philosophy, and neuropsychology to advance current conceptualizations of personhood in community amid cognitive decline. These will be discussed in relation to lived experiences, with the aim to inform future research and practice of dementia care and prevention in community.

### AT-HOMENESS: RETHINKING PERSONHOOD-IN-COMMUNITY THROUGH THE LENS OF SOCIAL IDENTITY

Graham Rowles,[1] Habib Chaudhury,[2] and
Daniel R. Y. Gan,[2], *1. University of Kentucky, Lexington, Kentucky, United States, 2. Simon Fraser University, Vancouver, British Columbia, Canada*

Since Chaudhury's seminal work (2008), spatial ethnographies of the everyday lives of people living with dementia(PLWD) have proliferated. From an experiential perspective, geographies of home (Blunt & Varley, 2004) and geographies of dementia may overlap significantly. We conducted a meta-ethnographic synthesis of n=28 articles to identify points of convergence and divergence in these literatures using comparative thematic analysis with NVivo 12. Whereas geographies of home highlight at-homeness (e.g., ontological safety and daily activities), geographies of dementia underscore communal and civic participation (e.g., social relationships). These themes converge around "social identity" which may be an important construct that helps PLWD feel at home. The quality of life of PLWD in the community may be influenced by prior (and present) experiences of at-homeness. These become more pertinent as older adults shelter in place. We discuss the implications of these findings in relation to relational models of personhood-in-community (Swinton, 2020) and community gerontology.

### MUTUAL RECOGNITION: EMPATHY AS THE FOUNDATION OF COMMUNITY IN DEMENTIA

Peter Costello, *Providence College, Providence, Rhode Island, United States*

This paper explores the challenges of developing a healthy, genuine community as some of its members experience cognitive decline or dementia. I draw upon philosophical discussions on community (Stein, 2000) and Husserlian empathy (1931;1939) to identify these challenges. First, community is organic; it relies on the differentiated roles of individual members to remain healthy. The ability to recognize the contribution of each member is essential for its health. Second, dyadic relationships may similarly be healthy or waning depending on the presence or absence of mutual empathy. Empathy is embodied. Persons living with dementia (PLWD) need to experience being recognized as persons, in person, in order for dyadic relationships and communities to thrive. As such, some communities may become unhealthy in the absence of mutual recognition. In these instances, careful interventions, e.g., through shared experiences and embedded memories, may be required to promote the well-being of the community and its members.

### SHARED HUMANITY: ADVANCING THE PERSONHOOD OF PEOPLE LIVING WITH DEMENTIA

Steven Sabat, *Georgetown University, Georgetown University, District of Columbia, United States*

During the past three decades, the idea of personhood, and the degree to which people living with dementia (PLWD) possess it and ought to be treated as such, has been discussed by a number of important scholars such as Tom Kitwood and John Swinton. Although both asserted that PLWD ought to be treated as persons, their notions of personhood appear to be quite different. Kitwood noted that personhood was a status "bestowed" on another individual, whereas Swinton asserted that personhood was endemic to human beings. Yet, these approaches are complementary. I show, using case studies, how supportive communities are required for PLWD to teach us about our humanity, so long as we remain "finely aware and richly responsible" (Nussbaum, 1990). Our humanity is shared, in the Zulu concept of "Ubuntu" (Tutu, 1981;1989), when each individual's humanity is ideally expressed in relationship with others. This has important implications for clinical practice.

### Session 3545 (Symposium)

### FROM MARGIN TO CENTER: INTERSECTIONALITY AND THE POLITICS OF ENVIRONMENTAL GERONTOLOGY

Chair: Austin Oswald Co-Chair: Jarmin Yeh
Discussant: Jarmin Yeh

As the population becomes increasingly older and culturally diverse, so too does the need for critical scholarship that examines the complex lives of differently positioned older adults in relation to their social and physical ecologies. This innovative symposium reflects the importance of putting intersectional frameworks (Collins & Bilge, 2016) in conversation with environmental gerontology to critically examine structures of power in assessing who matters and who benefits from place-based initiatives that intend to support healthy aging (Phillipson, 2004). Perry et al.'s paper addresses the politics of responsibility, asking who is responsible for keeping older people safe in light of Covid-19 though a citywide senior housing coalition in Detroit. The second paper, authored by Johnson, speaks to the politics of access and structural inequalities that create disparities in end-of-life care for unhoused older adults. The third and fourth papers, by Stinchcombe and colleagues and Oswald, critically examine dominant paradigms of age-friendliness in Canada and the United States though a politics of representation that highlights who is (in)visible in these initiatives. The final paper by Reyes, on the civic participation of Latinx and African American older adults, illustrates how structural change cannot happen without engaging these populations in the political process. Together, these papers exemplify the politics of environmental gerontology and demonstrate that without acknowledgment of multi-layered identities and the structures contributing to their inequities, environmental gerontology is inadequate, as it may overlook important social and environmental factors that connect older people to the places where they live and die.

### CRITICAL AGE-FRIENDLY RESEARCH AND REPRESENTATIONAL ETHICS

Austin Oswald, *CUNY Graduate Center, New York, New York, United States*

As the efforts of the Global Age-friendly Cities and Communities movement mature and continue to grapple with society's shifting dynamics, blind spots and knowledge gaps are exposed. This research applies critical discourse analysis to examine the evolution of Age-friendly NYC using an intersectional lens committed to an ethics of representation. Over 1,000 pages of public records were analyzed to trace the history of this movement in relation to age, race, sexuality, gender, ability, and class. Findings suggest that Age-friendly NYC is a global leader of the age-friendly movement, yet social identities are represented neither equally nor universally in its initiatives. Discussions of race, sexuality, and gender are subtle. They also overlook how these identities may intersect and shape the aging experience for differently positioned older adults. A comprehensive understanding of the aging experiences of those with multiple intersecting identities is needed to inform future age-friendly policies and programs.

### A CRITICAL EXAMINATION OF SENIOR HOUSING: PROMOTING EQUITY THROUGH ENSURING SAFE SPACES

Zach Kilgore,[1] Michael Appel,[2] Michele Waktins,[3] Claudia Sanford,[4] Dennis Archambault,[5] and Tam Perry,[6], *1. CSI Support & Development, Warren, Michigan, United States, 2. Develop Detroit, Detroit, Michigan, United States, 3. Volunteers of America-Michigan, Southfield, Michigan, United States, 4. United Community Housing Coalition, Detroit, Michigan, United States, 5. Authority Health, Detroit, Michigan, United States, 6. Wayne State University, Detroit, Michigan, United States*

As affordable senior housing communities aimed to address the health and well-being concerns of residents in the COVID-19 pandemic, special attention to safety during renovation had to be addressed. This paper offers case studies from members of a city-wide advocacy group, Senior Housing Preservation-Detroit. Eighty one percent of covid deaths in the City of Detroit are those 60 and above; 81.2% of deaths have been among African Americans (Detroit Health Department, 2021). With the grief and challenge in a city hit early on in the 2020 pandemic, these case studies will highlight how Covid-19 affected planned projects in senior buildings, how stakeholders such as developers, staff and residents responded and key considerations for future emergencies affecting senior housing communities. This paper offers critical perspectives applicable to many urban landscapes in order to raise awareness to policy makers, and practitioners.

### INVESTIGATING BARRIERS AND FACILITATORS TO CONGRUENCE IN PLACE OF CARE AMONG UNHOUSED PALLIATIVE CARE PATIENTS

Michael Light,[1] and Ian Johnson,[2], *1. Harborview Medical Center, Seattle, Washington, United States, 2. University of Washington, SEATTLE, Washington, United States*

An important quality marker for end-of-life services is congruence with patient's preferred place of care, but this congruence in place of care is less likely for those facing structural inequalities (Grunier et al., 2007). As homelessness among older adults in the United States grows (Culhane et al., 2013), the urgency in understanding place of care in palliative care with unhoused patients grows. This presentation illustrates results from an organizational case study (Yin, 2014) of a novel homeless palliative care team and focuses on a qualitative content analysis of charts of patients over 50 receiving care both before and during the COVID-19 pandemic (n=27). Findings highlight (1) the interplay between environmental factors, psychosocial resources and constraints, and medical acuity in determining where care can take place, (2) facilitators for care in marginal settings such as emergency shelters and encampments, and (3) where opportunities for more equitable age-friendly healthcare system interventions exist.

### CIVIC PARTICIPATION AMONG LATINX AND AFRICAN AMERICAN OLDER ADULTS, AN INTERSECTIONALITY LIFE-COURSE PERSPECTIVE

Laurent Reyes, *Rutgers University, Rutgers University, New Jersey, United States*

Older adults' civic participation has received considerable attention, but most scholarship has focused on formal volunteerism and voting. The literature shows that rates of voting and volunteering have been consistently lower among African Americans and Latinx older adults compared to their White counterparts. However, little research has explored civic participation in the context of historical structures of inequality that exclude these populations from participating in formal civic activities and continue to do so today. In addition, other civic activities are going unrecognized. To understand civic participation through the lens of Latinx and African American older adults I draw from intersectional life course perspective to contextualize participants' lived experiences across the life course and within historical and current socio-political space in which they live and participate. Study's findings could improve conceptualizations and measurements of civic participation for future studies, and inform efforts to support civic participation among these populations.

### AGE-FRIENDLY AND LGBTQ2+ FRIENDLY COMMUNITY INITIATIVES IN CANADA: PRELIMINARY FINDINGS

Emma Whitehouse,[1] Kimberley Wilson,[1] and Arne Stinchcombe,[2], *1. University of Guelph, Guelph, Ontario, Canada, 2. Brock University, St. Catharines, Ontario, Canada*

Age-Friendly Communities (AFC) initiatives are gaining momentum in Canada and around the globe with many communities making commitments to becoming age-friendly. Aging lesbian, gay, bisexual, transgender, queer, and two-spirit (LGBTQ2+) Canadians are a diverse subpopulation whose social histories and contexts may not have been considered in such initiatives. In response, many community-level organizations have created programs and supports for older LGBTQ2+ persons. Through a survey and environmental scan, we sought to identify and profile such initiatives. In addition, in-depth interviews were held with representatives from community groups to ascertain how community leaders construct and define communities that are both age and LGBTQ2+-inclusive. Participants were also asked to reflect on how their sense of community and support was impacted by the Covid-19 pandemic. The findings indicated that many supports for LGBTQ2+ older adults emerged outside of formal AFC initiatives and in response to a perceived normativity among services for older adults.

### Session 3550 (Paper)

### HEALTH OF OLDER BLACK ADULTS

### DISCRIMINATION, CHURCH SUPPORT, PERSONAL MASTERY, AND PSYCHOLOGICAL DISTRESS AMONG BLACK PEOPLE IN THE UNITED STATES

James Muruthi,[1] Bertranna Muruthi,[2] Reid Thompson Cañas,[3] Lindsey Romero,[2] Abiola Taiwo,[2] and Peter Ehlinger,[4], *1. University of Oregon, Eugene, Oregon, United States, 2. University of Oregon, University of Oregon, Oregon, United States, 3. university of oregon, University of Oregon, Oregon, United States, 4. University of Oregon, 97402, Oregon, United States*

**Objective:** This study used the stress process model to test the mediating effects of personal mastery and moderating effects of church-based social support on the relationship between everyday discrimination and psychological distress across three age groups of African American and Afro-Caribbean adults.

**Methods:** Using a national sample of 5008 African Americans and Afro-Caribbean adults from the National Survey of American Life Study, this study employs structural equation modeling to investigate the relationships between everyday discrimination, personal mastery, church-based social support, and psychological disorders.

**Results:** Everyday discrimination was an independent predictor of psychiatric disorders across all groups. Group- and age-specific comparisons revealed significant differences in the experience of everyday discrimination and psychiatric disorders. Mastery was a partial mediator of the relationship between discimination and psychiatric disorder among Afro-Caribbeans while church support was a significant moderator only among the young and older African Americans.

**Implications:** Together, our study findings provide useful first steps towards developing interventions to reduce the adverse psychological impacts of everyday discrimination on African Americans and Afro-Caribbeans. Intervention efforts such as individual psychotherapy aimed to improve Afro-Caribbean individuals' sense of mastery would be a partial solution to alleviating the adverse effects of discrimination on their psychological health.

### EDUCATIONAL MOBILITY AND AGE-RELATED DECREMENTS IN KIDNEY FUNCTION ACROSS ADULTHOOD AMONG BLACK AND WHITE ADULTS

Agus Surachman,[1] Alexis Santos,[2] Jonathan Daw,[3] Lacy Alexander,[3] Christopher Coe,[4] and David Almeida,[2], *1. University of California San Francisco, San Francisco, California, United States, 2. Pennsylvania State University, University Park, Pennsylvania, United States, 3. The Pennsylvania State University, University Park, Pennsylvania, United States, 4. University of Wisconsin - Madison, Madison, Wisconsin, United States*

This paper examines the association between educational mobility and age-related decrements in kidney function. Data from the main survey and the Biomarker Project of the Midlife in the United States (MIDUS) Wave 2 and Refresher samples were combined, resulting in 1,861 adults (54.5% female; age 25-84, Mage=53.37) who self-identified as non-Hispanic Black (n=326) and non-Hispanic white (n=1,535). The estimated glomerular filtration rate (eGFR) was based on serum creatinine, calculated using the CKD-EPI formula. Intergenerational educational mobility was based on the comparison between parental education (no high school/HS degree versus HS degree or higher) and participant's education level (HS degree or lower versus some college versus bachelor's degree or higher). Results from regression analysis indicated that Black participants in the moderate upward mobility group (parental education = no HS degree, participant's education = some college) showed significantly steeper age-related decrements in eGFR across adulthood compared to Black adults with higher stable high status (parental education = HS degree or higher, participant's education = bachelor's degree or higher), B=-0.70, SE=0.26, p=.008, or white adults with higher stable high status, B= 0.58, SE=0.29, p=.044. A steeper age-related decrement in eGFR is known as a reliable risk factor for chronic kidney disease and cardiovascular disease. These findings support the notion of skin-deep resilience among Black adults who experience upward socioeconomic mobility. We explored multiple psychosocial factors that may explain these findings, including lifetime and daily discrimination, social status and financial strains, and perceived stress and depressive symptoms.

### LOOKING BEYOND FAMILY FOR SUPPORT AND INTIMACY: HOW OLDER SINGLE AND WIDOWED BLACK WOMEN COPE WITH LONELINESS

Margaret Salisu, *SUNY@Downstate, Brooklyn, New York, United States*

Older adults are at risk of depression and higher rates of suicide due to loneliness. Loneliness is even more pronounced for single and bereaved older adults. Although loneliness is one of the benchmarks for measuring well-being, little is known about how older Black women understand and cope with loneliness. The aim of this qualitative phenomenological study is to explore how older Black women understand and cope with loneliness. Fourteen older single and/or widowed Black women in New York City participated in this study. The application of the Black feminist standpoint theory helped to understand the loneliness of the participants in the context of their Blackness. Three themes emerged from the study: loneliness increasing with age, looking beyond the family for intimacy, and balance. All the participants expressed feeling some degree of loneliness, regardless of whether they lived alone or with family. Although they had robust social circles, they experienced loneliness, feelings of isolation, and a loss of emotional connection and intimacy. However, these losses went unexpressed, as the participants struggled to balance their position as Black matriarchs—which they considered an important familial role due to their identity as older Black women—with their emotional needs. These two roles did not converge for the women, as the role of Black matriarch posed an expectation they would not experience emotional loss in old age. The implications of this study for policy and practice pertain to the intersection of race, age, gender, and sexuality in assessing the risk of loneliness.

### SELF-MANAGEMENT OF DIABETES IN BLACK MEN: THE FLINT MANUP INTERVENTION STUDY

Dana Carthron,[1] Wayne McCullough,[2] Samir Chatterjee,[3] Kent Key,[2] Kelsey Lemke,[4] David Gordon,[5] Gretchen Piatt,[6] and Harold Neighbors,[2], *1. North Carolina Central University, North Carolina Central University,*

*North Carolina, United States, 2. Michigan State University, Flint, Michigan, United States, 3. Claremont Graduate University, Claremont, California, United States, 4. University of Michigan-Flint, Flint, Michigan, United States, 5. University of Michigan-Flint, Ann Arbor, Michigan, United States, 6. University of Michigan, University of Michigan, Michigan, United States*

The story of John Henry, the "steel-drivin' man", is well known to Black men in the United States. John Henry is considered a hero because he demonstrated tremendous strength and self-determination. The MANUP diabetes program used the John Henryism, defined as high-effort active coping in the face of adversity, as the basis of a diabetes intervention for Black men. MANUP conducted four community-based focus groups identifying topics of concern to Black men with type 2 diabetes (T2D). Interestingly, the men reported that high-effort active coping was crucial for successful diabetes self-management. MANUP then developed and implemented a longitudinal culturally targeted self-management program for 33 Black men with T2D in Flint, Michigan. MANUP included discussion groups, physical activity, and an app incorporating text-messaging, group-chat, and a blood glucose monitoring dashboard to improve glycemic control (A1c). This single-group, repeated measures intervention assessed A1c three times over a six-month period. Improvements in A1c were observed at: baseline – time 2: 8.9% vs 8.6%, p=0.14; time 2 – time 3: 8.6% vs 8.1%, p=0.21; and baseline – time 3: 8.9% vs 8.1%, p=0.005. After controlling for age and insulin use, the significant reduction in A1c over 6 months remained (p=0.01). These findings demonstrate that combining mobile health technology and moderate physical activity with culturally targeted discussion topics can improve T2D self-management and reduce A1c in Black men. More community-driven longitudinal intervention studies that improve diabetes self-management among Black men are needed to achieve gender and racial health equity.

### TESTING THE JOHN HENRYISM HYPOTHESIS ON CARDIOMETABOLIC HEALTH AMONG OLDER AFRICAN AMERICANS

Yanping Jiang,[1] Jennifer Gómez,[2] Jacqueline Rodriguez-Stanley,[1] and Samuele Zilioli,[3], *1. Wayne State University, Detroit, Michigan, United States, 2. Wayne State University, Wayne State University, Michigan, United States, 3. Wayne state university, Detroit, Michigan, United States*

In the context of racism, the John Henryism Hypothesis posits that prolonged high-effort coping, which is referred to as John Henryism, may take a toll on physical health among individuals from low socioeconomic status (SES) backgrounds, particularly low SES African Americans. This study aimed to test the John Henryism Hypothesis among older African Americans by examining the combined effect of John Henryism and childhood SES on cardiometabolic health indexed by metabolic syndrome and systemic inflammation. Data were drawn from a sample of 170 urban older African Americans (Mage = 67.4 years, 75.9% female), who completed questionnaires assessing John Henryism and childhood SES (i.e., parental education). Blood pressure, waist circumference, and fasting blood were also collected to assess metabolic syndrome and systemic inflammation. Results indicated that John Henryism was significantly associated with elevated metabolic syndrome symptoms among older African Americans reporting low childhood SES (b = 0.42, 95%CI = [0.02, 0.83]), but not among those with high childhood SES (b = -0.33, 95%CI = [-0.78, 0.13]). This result was robust to a variety of demographic variables, lifestyle behavioral factors, and health conditions that are known to be associated with metabolic syndrome. A similar pattern of results, however, did not emerge for systemic inflammation. Our findings highlight the importance of considering the joint impact of early childhood socioeconomic backgrounds and individual psychological proclivities in explaining the elevated cardiovascular disease risk among older African Americans.

### Session 3555 (Paper)

### INNOVATIONS IN GERONTOLOGICIAL PEDOGOGY AND PRACTICE

### AN INTEGRATIVE APPROACH TO PROMOTING WELLNESS IN OLDER ADULTS: AN ONLINE UNDERGRADUATE COURSE

Robin Majeski, *University of Maryland, Baltimore County, Catonsville, Maryland, United States*

As gerontology and geriatric programs look to grow their connections with health and allied health areas, examining emerging health care areas is important. Thus, this session will present key concepts and pedagogical strategies from an innovative online undergraduate course, An Integrative Approach to Promoting Wellness in Older Adults, that is taught in the Erickson School at the University of Maryland at Baltimore County. The course brings together a conventional western allopathic approach and a holistic, patient-centered, integrative approach which includes complementary therapies, to promote wellness in older adults. Specifically, the session will present a comparison of the philosophical premises of conventional western and integrative approaches to health promotion. It will present a holistic model of health, an overview of the effectiveness and safety of different complementary therapies, and examples of how an integrative approach with conventional western and complementary therapies can be used in to promote health in older adults with particular chronic conditions. Also, a discussion of the incorporation of diversity content in the course, an integrative approach to health promotion for underserved populations, and specific pedagogical strategies used to facilitate learning in synchronous and asynchronous online learning environments are included. Session presentation techniques include a PowerPoint presentation, demonstration of examples of conventional western and integrative approaches to health promotion in older adults through case studies, and audience participation.

### CREATIVE ARTS INTERVENTIONS AS A WAY TO COMBAT AGEISM AND INCREASE STUDENT INTEREST IN GERONTOLOGY

Emily Ihara, Catherine Tompkins, Megumi Inoue, and Kendall Barrett, *George Mason University, Fairfax, Virginia, United States*

Despite the continuous, growing need for health professionals who are trained to work with older adults living with Alzheimer's disease and related disorders (ADRD), research shows that recruitment and retention of gerontological health care professionals remains low. Ageism plays an important role in this resistance and continues to have societal impact, even proliferating in disturbing ways during the COVID-19 pandemic via stereotypes, discrimination, and framing in the media. Gerontologists in various health professional educational settings continue to address the need to infuse aging content in creative ways and increase the competency of all health professionals to combat ageism and understand the importance of specialized care for this population. Our gerontological research team has engaged students in various ways to increase interest in aging issues and ADRD. Current research projects involve the implementation of non-pharmacological, creative arts interventions, including Mason's Music & Memory Initiative (M3I) and TimeSlips, both which have the potential to appeal to intergenerational partnerships and provide students with tools to communicate better with those living with ADRD. We examined attitudes about aging among undergraduate and graduate students (N=78) who have completed our training modules and/or participated in these two projects. The asynchronous trainings provide content on ADRD and the implementation of non-pharmacological, creative arts interventions. We examined students' attitudes about aging and ADRD and analyzed their open-ended responses regarding their experiences with someone living with ADRD. Various levels of education, relationships with older adults, and life experience influenced their responses regarding their attitudes about aging.

### DESIGNING SOCIALLY ASSISTIVE ROBOTS FOR PEOPLE WITH ALZHEIMER'S AND RELATED DEMENTIA

Kimberly Mitchell,[1] Xiaopeng Zhao,[2] Jinxiao Yu,[3] and Luke MacDougall,[4], *1. University of Tennessee, Knoxville, Tennessee, United States, 2. University of Tennessee, University of Tennessee, Tennessee, United States, 3. University of Tennessee, Knoxville, Knoxville, Tennessee, United States, 4. University of Tennessee - Knoxville, Knoxville, TN, Tennessee, United States*

Alzheimer's disease (AD) is the most common form of dementia and is associated with memory loss and cognitive impairments that affect daily life. Approximately 5.8 million older adults in the U.S. are living with AD. People with AD often require high levels of care and assistance to maintain daily activities. The majority of care provided to a person living with AD or other forms of dementia is from a family caregiver, representing 18.6 billion hours of unpaid care valued at $244 billion. The long duration, time-intensive nature of caregiving imposes high burdens on caregivers. We designed a socially assistive robot to engage in conversation with people with AD, by engaging in conversation, helping them conduct simple daily tasks, and relieving caregivers of some of their responsibilities. Using design-thinking methodology, a prototype social robot has been created using 3-d printing technology and a single board computer based on raspberry Pi. Interaction between human and robot is implemented using the Mycroft open source voice assistant. The authors demonstrated that the robot is able to have natural conversations with human users. The overall cost of the robot is estimated to be less than $300, rendering it possible for wide distribution among the public. Future research includes further implementation of various cognitive assessment and cognitive training programs using the social robot to improve the quality of life for people living with AD.

### GENERATIONAL VIEWS OF LIFE CHALLENGES AND THE AGING PROCESS: INFLUENCE OF RELATIONSHIP TYPE, FREQUENCY, EXPERIENCE

Mary Pagan, *SUNY Oswego, Baldwinsville, New York, United States*

This study, conducted within a higher education setting, examines and compares views of younger adults (age 20-24) and older adults (age 65-97) in two major areas; life challenges and aging processes. Are they complimentary, neutral, or opposing? We investigate views and the potential association to relationship type (family relation, close contact, casual), frequency (times interacting per month) including mode of communication (face-to-face, phone, visual technology), and narrative of overall experience. A mixed-method approach incorporated convenience survey data and extensive in-depth interviews. Data collection instruments were designed and conducted by students in an upper-division wellness and aging course at SUNY Oswego. Students partnered with older adult study participants. Participant (n=80) inquiries centered on life challenges (COVID, adversity, loss, discrimination/bias, regrets) and aging processes (views on aging, life expectancy, changes in health). The study also examines the impact of the 3-credit course on aging views; specifically did they change their life expectancy choice (years) from the week one by the end of the course.

### INNOVATIONS IN WORKFORCE EDUCATION FOR FAMILY CAREGIVING

Kathryn Sexson,[1] Jennifer Mongoven,[2] Lisa Badovinac,[1] and Theresa Harvath,[1], *1. University of California, Davis, UC Davis, California, United States, 2. University of California, Davis, Sacramento, California, United States*

The number of adults providing care to a family member in the US is estimated at more than 50 million, with nearly half of those individuals providing complex care. National organizations, such as AARP, and federal programs, such as the Geriatric Workforce Enhancement Program, have identified the family caregiver as an integral member of the health care team, yet there is a paucity of clinical workforce education programs for how best to partner and support family caregivers. A virtual summit was held in September 2020 to highlight existing educational programs designed to prepare undergraduate and graduate health professional students or practicing clinicians in their efforts to support family caregivers. The meeting consisted of a keynote, 6 podium presentations and 12 poster presentations. Primary themes emerged around target learners, curricula topics, and outcomes. Programs targeted learners from across the workforce, from undergraduate students to continuing professional education programs, with the majority targeting graduate (masters and doctoral) learners. Several programs were interprofessional in development, delivery and target learner. Curricula topics varied across programs and included caregiver assessments, multicultural considerations, communication, care plan development and risk screening. Education outcomes primarily focused on assessment of participants' confidence and knowledge. The summit highlighted that the topic of family caregiving is included in clinical education inconsistently, if at all. The summit helped identify gaps in education, curriculum development, and the need for common learning outcomes to strengthen a clinician's ability to support family caregivers as part of the care team.

### Session 3560 (Symposium)

### INTERGENERATIONAL PROGRAMMING DURING THE PANDEMIC: TRANSFORMATION DURING (CONSTANTLY) CHANGING TIMES

Chair: Shannon Jarrott Co-Chair: Skye Leedahl
Discussant: Donna Butts

Implementing intergenerational programming amidst the COVID-19 pandemic has required creativity, partnership, and dedication to the work. Most intergenerational programs involving in-person meetings or events are accompanied by guidelines to protect participant health and safety. Programming is routinely cancelled or postponed due to poor weather or contagious illness, particularly when a vulnerable population is involved. The needs for safety precautions and continued intergenerational contact were both amplified during the pandemic, leading many to modify or innovate ways to engage generations rather than eliminate contact for extended periods. Technology has afforded new approaches to engage young people and older people with each other; non-technological ways have also proven effective. This symposium will address strategies used to implement intergenerational programs during the pandemic. Authors will highlight lessons learned and strategies they expect to retain in the future. The first paper describes a pivot in nutrition programming designed for a shared site with preschool children and frail older adults. In paper two, authors discuss their partnership-based approach shifting to remote offerings of Cyber-Seniors programming. Paper three addresses how MentorUp Service-Learning expanded its reach by adaptations to virtual programming for older adults in retirement communities. The final paper presents evaluation data comparing arts programming delivered in-person pre-pandemic and again virtually during the pandemic. In each case, researchers and community partners learned techniques to maintain their programmatic foci. Some projects developed strategies they plan to maintain post-pandemic. Donna Butts, Executive Director of Generations United serves as the symposium discussant.

### RESPONDING TO INTERGENERATIONAL FOOD SECURITY AND NUTRITION EDUCATION NEEDS WITH REMOTE PROGRAMMING

Rachel Scrivano,[1] Shannon Jarrott,[1] and Jill Juris Naar,[2] *1. The Ohio State University, Columbus, Ohio, United States, 2. Appalachian State University, Boone, North Carolina, United States*

In-person intergenerational programming focused on nutrition education and healthy food access among older adults and preschool children in care settings was abandoned last year when COVID forced center closures. Food for a Long Life (FFLL), a 5-year community-based participatory research (CBPR) project, re-oriented programming in response to heightened community food insecurity and social isolation during COVID. With county Extension agents, FFLL modified and initiated new partnerships to expand food pantry services for several hundred families and deliver nutrition programming to youth (n=28) and older adult (n=130) participants in two states. In this presentation we share how the CBPR method supported adaptive programming and evaluation while continuing to advance project goals, including to promote the sustainability of an intergenerational food pantry and nutrition programming delivery after funding ends in summer 2021.

### KEEPING INTERGENERATIONAL PROGRAMMING ALIVE DURING THE PANDEMIC THROUGH COLLABORATION AND TECHNOLOGY

Erica Estus,[1] Catherine Taylor,[2] and Skye Leedahl,[1], *1. University of Rhode Island, Kingston, Rhode Island, United States, 2. Age-Friendly Rhode Island, Providence, Rhode Island, United States*

The University of Rhode Island Cyber-Seniors' in-person intergenerational programming was quickly shut down during the Spring 2020 semester due to the pandemic. Since then, we have worked diligently and collaboratively with partners to offer creative intergenerational options for university students and older adults living in the community. We partnered with Age-Friendly RI and the Census Outreach to provide phone-based wellness checks to 11,500 older adults, and this evolved into a statewide weekly call with partners (n=34 calls) focused on reducing social isolation for older adults. Our students moved to offering phone or Zoom-based appointments with 21 community organizations across Rhode Island and became mentors for the new Cyber-Seniors ® organization digital offerings (n=90 students). In this presentation, we will share our experiences with the pivot from in-person to mostly technology-based interactions. We will discuss challenges and lessons learned, some of which will be retained regardless of the pandemic situation.

### INTERGENERATIONAL SERVICE-LEARNING DURING COVID-19

Tamar Shovali, *Eckerd College, Eckerd College, Florida, United States*

Mentor Up is a technology training program designed to reduce loneliness through technology training and intergenerational relationships. The program, which has similarities to Cyber Seniors, has been held at Eckerd College for four years and has traditionally been held in-person at a local Continuing Care Retirement Community (CCRC). During the pandemic we partnered with AARP to re-think the program and offer a modified version of Mentor Up on Zoom. Five one-hour one-on-one virtual technology training sessions led by 16 students were scheduled. Participants joined to ask questions about how to use features on their smartphones or how to navigate the Zoom virtual platform more effectively. The remote nature of programming allows for expanded 2021 participation, including AARP members across Florida and all three Westminster Communities of Florida in the area (CCRC, ALF, SNF). Participation rates, strategies to implement intergenerational programs on a virtual platform, and lessons learned will be highlighted.

### THE PHOTOGRAPHY AND MEMORY PROJECT: ADAPTING AN INTERGENERATIONAL PROGRAM DURING A GLOBAL PANDEMIC

Roddy MacInnes,[1] Anne Walker,[1] Amy DelPo,[2] Leslie Hasche,[1] Andrew Steward,[3] Matthew Schilz,[1] and Carson De Fries,[1], *1. University of Denver, Denver, Colorado, United States, 2. Denver Public Library, Denver, Colorado, United States, 3. University of Denver, Lone Tree, Colorado, United States*

Since 2019, a university-community partnership has connected undergraduate students with older adults from independent living and community-based settings (i.e., library, art museum) for a photography-based intergenerational program. This study compares the implementation and impacts of this photography-based intergenerational program both in an in-person format before the COVID-19 pandemic (n=34) and an online format during the COVID-19 pandemic (n=25). Pre- and post- results from older adult and undergraduate student participants regarding social connection and evaluation comments from program participants and staff were compared between the pre-pandemic, in-person format, and the same program's offering the next year in a virtual format. Results indicate that the benefits of this intergenerational photography program were experienced at a similar level during and before COVID-19. Findings demonstrate that intergenerational programs could effectively continue in remote formats, while also identifying potential challenges in implementation for staff regarding managing logistics and maintaining engagement among participants.

### Session 3565 (Paper)

### INTERVENTIONS WITH PERSONS WITH DEMENTIA AND THEIR CAREGIVERS

### INTERVENTION DESIGN WITH COGNITIVELY IMPAIRED POPULATIONS: THE OPTIMIZE DEPRESCRIBING INTERVENTION

Orla Sheehan,[1] Elizabeth Bayliss,[2] Ariel Green,[3] Melanie Drace,[2] Jonathan Norton,[4] Emily Reeve,[5] Kathy Gleason,[6] and Cynthia Boyd,[4], *1. Johns Hopkins University School of Medicine, Johns Hopkins University, Maryland, United States, 2. Kaiser Permanente Colorado, Denver, Colorado, United States, 3. Johns Hopkins University School of Medicine, Baltimore, Maryland, United States, 4. Johns Hopkins University, Baltimore, Maryland, United States, 5. University of South Australia, Adelaide, South Australia, Australia, 6. Kaiser Permanente, Kaiser Permanente Institute for Health Research, Colorado, United States*

Older adults with cognitive impairment and multiple other chronic conditions often have polypharmacy which increases their risks of medication related cognitive effects, adverse drug events, hospitalization and death and leads to higher health care costs. Deprescribing, the process of reducing or stopping potentially inappropriate medications may improve outcomes for those older adults with cognitive impairment and multiple chronic conditions. The OPTIMIZE trial examined whether a primary care-based, patient- and family-centered intervention educating and activating patients, family members, and clinicians about deprescribing reduces numbers of chronic medications and potentially inappropriate medications for older adults with dementia or mild cognitive impairment and multiple chronic conditions. We explored the mechanisms of intervention effectiveness through post hoc qualitative stakeholder interviews and surveys with 15 patients, 7 family caregivers, and 28 clinicians. All stakeholder groups endorsed the acceptability of the intervention. Success of the intervention was affected by contextual factors including prior knowledge and openness to deprescribing, cognition and prognosis. Positive outcomes included patients and care partners scheduling specific appointments to discuss deprescribing and providers remembering to consider deprescribing in cognitively impaired older adults. Recollection of intervention materials was inconsistent over time but highest shortly after intervention delivery. The time required to mail intervention materials to patients prior to a scheduled appointment limited the reach of the intervention by excluding persons with rapidly scheduled appointments. Our work identifies key learnings in intervention roll out which can guide future translation of our intervention to other settings and other pragmatic intervention studies in this vulnerable population.

### PERSON- AND TASK-CENTERED MEALTIME CARE: IMPACT ON POSITIVE, NEUTRAL, AND CHALLENGING BEHAVIORS IN PEOPLE WITH DEMENTIA

WEN LIU,[1] Kristine Williams,[2] Melissa Batchelor,[3] Yelena Perkhounkova,[4] and Maria Hein,[4], *1. The University of Iowa College of Nursing, Iowa City, Iowa, United States, 2. University of Kansas School of Nursing, Kansas City, Kansas, United States, 3. George Washington University, Washington, D.C., District of Columbia, United States, 4. University of Iowa, Iowa City, Iowa, United States*

Mealtime is an important daily activity to ensure intake. Person-centered and task-centered care may influence individual positive, neutral, and challenging mealtime behaviors. Yet, little work has fully examined their relationships. This study aimed to examine the association between person-centered and task-centered care approaches and individuals' positive, neutral, and challenging mealtime behaviors. This secondary analysis of 110 videotaped mealtime observations involved 29 staff and 25 residents with dementia (42 unique staff-resident dyads) in 9 nursing homes. Videos were coded using the refined Cue Utilization and Engagement in Dementia mealtime video-coding scheme. Logistic regression models were fit to four dependent variables representing resident mealtime behaviors: 1) positive/neutral behaviors (nonverbal), 2) positive utterances (verbal), 3) functional impairments (nonverbal), and 4) resistive behaviors (verbal and nonverbal). Independent variables were staff person-centered care modifications (nonverbal), person-centered utterances (verbal), and task-centered behaviors (verbal and nonverbal). Covariates included resident age, gender, eating function, and video duration. Resident positive utterances were associated with staff person-centered care utterances (OR =1.38, 95% CI = 1.09,1.76). Resident functional impairments were associated with staff person-centered care modifications (OR=1.33, 95% CI=1.02, 1.74) and fewer staff person-centered utterances (OR=0.81, 95% CI=0.66, 1.00). Resident resistive behaviors were associated with more staff person-centered utterances (OR=1.65, 95% CI=1.18, 2.31). Findings provided preliminary information supporting the role of staff person-centered care on resident positive and challenging mealtime behaviors. Findings inform use of verbal and nonverbal person-centered care strategies to improve positive communication and reduce challenging behaviors during mealtime in people with dementia.

### RESULTS OF OPTIMIZE: A CLUSTER RANDOMIZED TRIAL OF PATIENT, FAMILY, AND PROVIDER EDUCATION IN PRIMARY CARE

Ariel Green,[1] Elizabeth Bayliss,[2] Susan Shetterly,[2] Melanie Drace,[2] Jonathan Norton,[3] Emily Reeve,[4] Orla Sheehan,[5] and Cynthia Boyd,[3], *1. Johns Hopkins University School of Medicine, Baltimore, Maryland, United States, 2. Kaiser Permanente Colorado, Denver, Colorado, United States, 3. Johns Hopkins University, Baltimore, Maryland, United States, 4. University of South Australia, Adelaide, South Australia, Australia, 5. Johns Hopkins University School of Medicine, Johns Hopkins University, Maryland, United States*

Individuals with cognitive impairment frequently have multiple chronic conditions (MCC), increasing their risk for polypharmacy and associated adverse outcomes. Optimizing medications through deprescribing (reducing or stopping the use of inappropriate medications or medications unlikely to be beneficial) may improve outcomes for this population. Optimize was a pragmatic, 12-month cluster-randomized trial of deprescribing in primary care within a not-for-profit integrated delivery system. Participants were age 65+ with dementia or mild cognitive impairment (MCI), 2+ chronic conditions, and 5+ chronic medications. The intervention consisted of a deprescribing educational brochure for patients/caregivers, and Tip Sheets for primary care clinicians. Outcomes were the number of chronic medications and presence of potentially inappropriate medications (PIM). In total, 1,433 patients received, and 1,579 control clinic patients would have been eligible to receive, the intervention (N=3,012). After 6 months, mean estimates of chronic medications were 6.23 in the intervention group and 6.33 in the control group adjusting for baseline counts, age, and gender (p=0.13). Excluding those without complete 90 days follow-up increased the adjusted effect size to 0.14 (p=0.08). In sub-analyses of individuals with 7+ medications at baseline (N= 1,434), the adjusted effect size was 0.19 (p=0.07) at 6 months and 0.21 (p=0.045) when excluding those without complete 90 days' follow-up. Change in proportions of PIM did not differ between intervention and control groups. An educational intervention for patients, caregivers and clinicians may prompt reductions in chronic medications. The relatively small effect size highlights the complexity of medication management for individuals with dementia or MCI and MCC.

### VOICE YOUR VALUES, TAILORED ADVANCE CARE PLANNING IN PERSONS LIVING WITH MILD DEMENTIA: A FEASIBILITY STUDY

Shirin Vellani,[1] Martine Puts,[2] Katherine McGilton,[3] and Andrea Iaboni,[4], *1. Toronto Rehabilitation Institute-University Health Network, Toronto, Ontario, Canada, 2. University of Toronto, University of Toronto/Toronto, Ontario, Canada, 3. KITE-Toronto Rehabilitation, University Health Network, Toronto, Ontario, Canada, 4. Toronto Rehabilitation Institute, University Health Network, Toronto, Ontario, Canada*

Older adults diagnosed with mild dementia can identify their wishes, values and goals of care with a high degree of accuracy and reliability. However, there is a paucity of research to guide best practices on how to incorporate Advance Care Planning (ACP) in the care of older adults living with mild dementia. Thus, only a minority of them participate in any ACP discussions. We developed an intervention called Voice Your Values (VYV) that healthcare professionals can implement to identify and document values of older adults. This single group pretest and posttest design aimed to determine the feasibility, acceptability and preliminary efficacy of the intervention. A convenience sample of 20 dyads of older adults and their trusted individuals were recruited from 4 geriatric clinics. Tailored VYV intervention was delivered to dyads on a one-on-one basis over two sessions using videoconferencing. Feasibility was determined through recruitment and retention rates, and intervention fidelity. Acceptability was assessed using modified Treatment Evaluation Inventory. Primary outcome was the Surrogate Decision-Making Confidence Scale. Secondary outcomes included an ACP engagement survey to assess older adults' engagement in ACP; Dementia Knowledge Assessment Tool for trusted individuals; and the Kessler Psychological Distress Scale for all participants. The recruitment rate was 45%, retention rate was 100% and 92% participants rated VYV as highly acceptable. Trusted individuals showed statistically significant improvement in decision-making confidence (p=.02) and psychological distress (p=.02); but no improvement in dementia knowledge (p=.47). Older adults demonstrated statistically significant improvement in ACP engagement (p=<.01). Initial feasibility of VYV was demonstrated.

### Session 3570 (Symposium)

### LGBTQIA+ CAREGIVING AND CARE NEEDS OF PERSONS LIVING WITH ALZHEIMER'S DISEASE AND RELATED DEMENTIAS

Chair: Jason Flatt Co-Chair: Whitney Wharton
Discussant: Joel Anderson

Lesbian, gay, bisexual, transgender, queer, intersex, and/or asexual (LGBTQIA+) older adults are a growing population. LGBTQIA+ persons living with Alzheimer's disease and related dementias (ADRD) face unique challenges in terms of accessing care and support compared with their non-LGBTQIA+ counterparts. The care challenges faced by LGBTQIA+ people living with ADRD may be compounded by the fact they are more likely to be single, more likely to live alone, and less likely to have children. Several studies have started to explore the unique needs of LGBTQIA+ caregivers and persons living with ADRD. In this symposium, we highlight current research addressing the psychosocial and health-related needs of LGBTQIA+ caregivers and persons living with ADRD. Two presentations address psychosocial factors and health among LGBTQIA+ caregivers of persons with ADRD. Krystal Kittle will present analyses using population-based data on LGBTQIA+ caregivers in terms of caregiving burden and mental health. Shana Stites will present results from the Health and Retirement Study highlighting differences among same-sex spouses in terms of caregiving patterns and research participation. Next, Ethan Cicero will present prevalence estimates of care needs and challenges among diverse transgender adults living with memory problems. Finally, we will highlight a promising intervention for LGBTQIA+ caregivers of persons with ADRD. Jason Flatt will describe the adaptation and feasibility of the Savvy Caregiver program for LGBTQIA+ caregivers. Joel Anderson, an expert in LGBTQIA+ caregiving for persons with ADRD, will facilitate a conversation about these results and place them in the context of current LGBTQIA+ and ADRD research.

### HEALTH CONCERNS FOR LGBTQ+ CAREGIVERS OF PEOPLE WITH ALZHEIMER'S DISEASE AND RELATED DEMENTIAS

Joel Anderson,[1] Jennifer Pharr,[2] Sheniz Moonie,[2] Jason Flatt,[2] and Krystal Kittle,[3], *1. University of Tennessee at Knoxville, Knoxville, Tennessee, United States, 2. University of Nevada Las Vegas, Las Vegas, Nevada, United States, 3. University of Nevada, Las Vegas, Las Vegas, Nevada, United States*

We examined four states with data on LGBTQ+ identity and the ADRD caregiving modules from the 2019 Behavioral Risk Factor Surveillance System. Multivariable regression models examined the associations between LGBTQ+ identity and health outcomes. Among the ADRD caregivers, 55,920 (4.7%) identified as LGBTQ+. Compared with non-LGBTQ+ caregivers, LGBTQ+ caregivers were younger and more likely to live in rural counties. Half of LGBTQ+ caregivers spent 20+ hours per week providing care, and nearly 72% reported helping with personal care. LGBTQ+ caregivers reported more days when their mental health was not good than non-LGBTQ+ caregivers (B = 8.01;95% CI= 2.32-13.75). Female caregivers overall were twice as likely than males to experience depression (OR = 2.11;95% CI=1.29-3.45). These findings provide insight into characteristics of LGBTQ+ caregivers and their health concerns. Interventions that promote mental health and reach diverse LGBTQ+ caregivers in rural communities are crucial in supporting LGBTQ+ caregivers of people with ADRD.

### PATTERNS AMONG SAME-SEX SPOUSAL COUPLES: DIVERSE SOCIOCULTURAL REPRESENTATION IN ALZHEIMER'S RESEARCH

Shana Stites, *University of Pennsylvania, Philadelphia, Pennsylvania, United States*

Emerging evidence shows that understanding characteristic patterns between study partners (SP) and subjects can inform initiatives to diversify representation of sociocultural groups in ADRD research. This study examined same-sex spousal dyads with the goal of identifying bellwethers of opportunities to build diversity in ADRD research. Descriptive analysis of The Aging, Demographics and Memory Study (ADAMS), which enrolled a subset of subjects from the Health and Retirement Study and a SP for each subject. Eight same-sex spousal couples were among 718 SP-subject dyads (1.1%). Gay men were 3 times as likely to be spousal SPs (n=6) than lesbians (n=2), even though women far outnumber men overall. Patterns in caregiving and other characteristics also differed. Same-sex couples are underrepresented in ADRD research. Patterns among those enrolled suggest masculine and feminine norms may drive research engagement. This is discussed in the context of increasing sociocultural diversity in ADRD research across key social groups.

### MEMORY-RELATED LIMITATIONS AND CARE NEEDS OF GENDER MINORITY BIPOC ADULTS

Nickolas Lambrou,[1] Whitney Wharton,[2] Jason Flatt,[3] and Ethan Cicero,[4], *1. University of Wisconsin, Madison, Madison, Wisconsin, United States, 2. Emory University, Atlanta, Georgia, United States, 3. University of Nevada Las Vegas, Las Vegas, Nevada, United States, 4. Emory University School of Nursing, Atlanta, Georgia, United States*

Limited research exists investigating cognitive impairment and Alzheimer's disease and related dementias (ADRD) among gender minority (GM) adults (transgender/non-binary/, including examining memory-related challenges among GMs who also identify as Black, Indigenous, or People of Color (BIPOC). 2015-2019 Behavioral Risk Factor Surveillance System data were used to explore care needs of GM and cisgender (CG) adults with subjective cognitive decline (SCD, N=441), which may be the first clinical manifestations of ADRD. Regression models examined SCD-associated functional limitations and care needs among GM-BIPOC, GM-White, CG-BIPOC, and CG-White adults. GM-BIPOC and GM-White were 2-4x more likely to have SCD-related limitations, require assistance with daily tasks, be unable to do day-to-day or social activities when compared to CG-White. GM-BIPOC were 2-5x more likely to be uninsured and experience cost-related healthcare barriers compared to GM-White and CG-White/BIPOC. Additional research is needed to improve care and well-being for this understudied population.

### EVALUATION OF THE SAVVY CAREGIVER PROGRAM FOR LGBTQIA ADULTS LIVING WITH ALZHEIMER'S DISEASE AND RELATED DEMENTIAS

Kiera Pollock,[1] Yeonsu Song,[2] Whitney Wharton,[3] Joel Anderson,[4] and Jason Flatt,[5], *1. Los Angeles LGBT Center, Los Angeles, California, United States, 2. UCLA, Los Angeles, California, United States, 3. Emory University, Atlanta, Georgia, United States, 4. University of Tennessee at Knoxville, Knoxville, Tennessee, United States, 5. University of Nevada Las Vegas, Las Vegas, Nevada, United States*

Approximately 350,000 LGBTQIA+ older adults in the U.S. currently have Alzheimer's disease and related dementias (ADRD), with projections nearing 1 million by 2030. LGBTQIA+ older adults face challenges in receiving adequate and inclusive care and caregiving support due to the inability to rely on traditional family networks, greater disability, and discrimination when seeking aging services. Working with the Los Angeles LGBT Center Aging in Community Initiative, we evaluated the: 1) Adaptation of the Savvy caregiver training program for care providers of LGBTQIA+ persons living with ADRD; and 2) Feasibility and acceptability of the program. Care providers were very satisfied with the program, strategies, information, and activities of the tailored Savvy program. For psychosocial outcomes, there were trends in greater care planning, increases in asking friends/family for support, and decreased loneliness. Additional research is needed on culturally-relevant aging services and behavioral interventions for care providers of LGBTQIA+ persons living with ADRD.

### Session 3575 (Symposium)

### MARGRET M. AND PAUL B. BALTES AWARD LECTURE: OPTIMISM AND HEALTH: RESOURCE OR DELUSION?

Chair: Eileen Crimmins

The lecture will be given by the 2020 Baltes Award recipient, William Chopik, PhD, of Michigan State University. The recipient of the 2021 Baltes Award is Laura B. Zahodne, PhD, of the University of Michigan. The Margret M. and Paul B. Baltes Foundation Award in Behavioral and Social Gerontology recognizes outstanding early-career contributions in behavioral and social gerontology. The award is generously funded by the Margret M. and Paul B. Baltes Foundation.

### OPTIMISM AND HEALTH: RESOURCE OR DELUSION

William Chopik, *Michigan State University, East Lansing, Michigan, United States*

There is a general, widely-held belief that optimism is always a good thing. While there is much previous research suggesting that optimists enjoy several health and wellness benefits, there is also a large body of research suggesting that optimism is not always advantageous. Examining how optimism develops and changes across the lifespan may give us insight into how we use optimism and allow us to determine if and when optimism is helpful or maladaptive. In this talk, I will review evidence debating the efficacy of optimism, as well as examine how optimism develops across the lifespan. I also discuss how life events may or may not impact the developmental trajectory of optimism. Lastly, I address currently unanswered questions and emphasize the contextual nature of optimism's advantages. Ultimately, being persistently optimistic about the future is a nearly universal human trait. But the exact contexts in which this positive thinking is helpful--if ever--is an intriguing question that speaks to how we think about ourselves, how we think about others, and how we think about our many possible futures.

### Session 3580 (Paper)

### MARRIAGE AND HEALTH

### MARITAL QUALITY AND HEAVY ALCOHOL USE AMONG OLDER COUPLES

Angela Curl, Jennifer Bulanda, and
Amy Restorick Roberts, *Miami University, Oxford, Ohio, United States*

Supportive marital relationships may reduce partners' problematic health behaviors, whereas unhappy relationships may lack efficacious spousal monitoring of health and increase the likelihood of using maladaptive coping strategies, such as heavy alcohol use, to deal with relationship problems. We used pooled data from the 2014 and 2016 waves of the Health and Retirement Study to examine how both partners' perceptions of marital quality were associated with heavy drinking. Our analytic sample included married couples in which both spouses were over age 50, completed the leave-behind psychosocial questionnaire, and provided non-missing data on marital quality and alcohol use (n=2,095 couples). Measures included both positive and negative dimensions of marital quality and controls for sociodemographic, economic, health, household and marital characteristics. Using Proc Glimmix, we estimated a dual-intercept Actor-Partner Interdependence Model (APIM), in which separate equations were computed simultaneously for husbands and wives. For husbands, higher negative marital quality was associated with an increase in the odds of their own heavy drinking (OR=1.27), but there was no significant association between wives' marital quality and husbands' heavy drinking behavior. For wives, marital quality was not significantly associated with their own heavy drinking, but husbands' higher ratings of both negative and positive marital quality increased the risk of wives' heavy drinking (OR=1.60 and OR=1.75, respectively). Results suggest that marital quality is associated with heavy drinking in later life: self-ratings of marital quality matter for men, whereas spousal perceptions of marital quality are more important for women.

### MARITAL STATUS, MARITAL TRANSITIONS, AND SLEEP QUALITY IN MID TO LATE LIFE

Kristin August, *Rutgers University, Camden, New Jersey, United States*

Sleep is an important behavior in the prevention and management of chronic conditions in later life. Marital status may account for variability in sleep quality, but little is known about this association in the later part of life or how transitions into and out of marriage are related to changes in sleep quality. This study used the resource model and crisis model as frameworks to understand how marital status and marital transitions were related to sleep quality in mid to late life and whether these findings differed by gender. Interview data from 2,872 participants 50-74 years old (M=59.77 years; 63.7% women) from the ORANJ BOWL, a longitudinal panel study in New Jersey, were used. Marital status and sleep quality were examined in two waves approximately 10 years apart. All analyses controlled for health and sociodemographic characteristics. Weighted regressions revealed that individuals in committed romantic relationships and women had worse sleep quality than those in other marital status groups and men ($p<.005$). Weighted fixed effects regressions revealed that compared to individuals who remained married, individuals who remained divorced or widowed or who became widowed had better sleep quality, whereas those who became divorced had worse sleep quality ($ps<.05$); individuals who transitioned into marriage had better sleep quality than those who remained divorced or widowed ($ps<.03$). Findings differed depending on the index of sleep quality examined. Efforts to understand which middle-aged and older adults are most vulnerable to sleep disturbances can inform the design of interventions to promote better sleep quality.

### MARITAL STRAIN AND SUPPORT AND SUBJECTIVE WELL-BEING IN LATER LIFE: ASCRIBING A ROLE TO EARLY-LIFE CONDITIONS

Laura Upenieks,[1] and Yingling Liu,[2] *1. University of Texas at San Antonio, San Antonio, Texas, United States, 2. Baylor University, Waco, Texas, United States*

Decades of research have the beneficial effects of marital support and the detrimental consequences of marital strain on health and well-being. However, we know relatively less about how circumstances in childhood—a key developmental period of the life course—influence the relational structure in which later life is embedded and any implications this may hold for well-being. We integrate the life course perspective with the stress process model to offer a framework for how childhood conditions (childhood happiness, family structure, and financial strain) moderate the relationship between marital support/strain and subjective well-being in older adulthood in potentially different ways for men and women. The consequences of marital strain may be more severe and the benefits of marital support may not be as strongly felt for those adults who experienced greater adversity during childhood. Drawing on longitudinal data from Waves 2 (2010-2011) and 3 (2015-2016) of the NSHAP project (N = 1,376), results from lagged dependent variable models suggest that marital support buffers the effect of not living with both parents in childhood on subjective well-being for men. Meanwhile, women raised in families that experienced financial hardship reported lower subjective well-being in the context of marital strain in later life. No significant interaction effects were obtained for childhood happiness. Taken together, our findings suggest that adverse experiences in childhood can be scarring, particularly in the context of strained intimate relationships. However, a supportive marriage can, in some cases, offset the effects of childhood hardship on subjective well-being in later life.

### VALIDATION STUDY OF THE SPANISH VERSION OF THE MARITAL SATISFACTION QUESTIONNAIRE FOR OLDER PERSONS

José Adrián Fernandes-Pires,[1] Andrés Losada-Baltar,[2] María del Sequeros Pedroso-Chaparro,[1] Laura Gallego-Alberto,[3] Isabel Cabrera,[3] Lucía Jiménez-Gonzalo,[1] María Márquez-González,[3] and Jorge González Guardia,[1], *1. Rey Juan Carlos University, Alcorcón, Madrid, Spain, 2. Universidad Rey Juan Carlos, Madrid, Madrid, Spain, 3. Universidad Autónoma de Madrid, Madrid, Madrid, Spain*

Interpersonal relationship quality is relevant for older adults´ well-being and mental health. Studies focused on methods to evaluate marital satisfaction in older adults and the relationship of this variable with psychological correlates are scarce. This study examined the psychometric properties of the Spanish version (Castro-Díaz et al., 2012) of the Marital Satisfaction Questionnaire For Older Persons (MSQFOP; Haynes et al., 1992) in a sample of middle-aged and older adults from Spain. Participants were 130 individuals (60.8% women) 40 years or older (M= 60.31, SD=11) involved in a marital/partner relationship. The assessed variables were marital satisfaction (MSQFOP), marital warmth, positive emotions, frequency of arguments, perceived stress associated with the COVID-19 pandemic, and anxiety and depressive symptoms. The results from the exploratory factor analysis yielded a three factor structure (compatibility, communication, and sex) explaining 77.8% of the variance. Even though the factor structure was the same as that of the original version, some items loaded on other factors. The internal consistency (Cronbach's alpha) was 0.97. The results revealed significant ($p < .001$) positive associations between marital satisfaction, marital warmth, and positive emotions. In addition, significant negative associations were found between marital satisfaction and frequency of arguments, stress associated with the COVID-19 pandemic, and anxious and depressive symptomatology ($p < .05$). The findings suggest that Spanish version MSQFOP has good psychometric properties that recommend its use with middle-aged and older adults. Marital satisfaction seems to be a relevant construct for understanding stress, well-being, and mental health in middle-aged and older adults.

### Session 3585 (Symposium)

### MEETING THE CHALLENGES OF COVID-19 IN LONG-TERM CARE: LESSONS LEARNED FROM AN INTERNATIONAL PERSPECTIVE

Chair: Karin Wolf-Ostermann Discussant: Marie Boltz

The outbreak of COVID-19 is a major challenge for health care systems all over the world. Older residents of long-term-care facilities (LTCF) such as nursing homes (NH) are among those at highest risk for COVID-19 and comprise a population with dramatically higher rates of morbidity and mortality than the general community. NH staff are also affected by the pandemic as they are challenged by increased workloads, emotional burden caused by the loss of resident life, and the fear of becoming infecting themselves or infecting family members. Finally, the pandemic places emotional and practical demands upon informal carers who are involved in the life of the NH resident. Therefore, research should investigate different perspectives on LTCF during the pandemic and discuss major challenges and possible support structures and strategies. Such an understanding is necessary to optimize care, support post-pandemic recovery, and prepare for future public health challenges. This international symposium will therefore provide four presentations to address these issues. The first presentation will report on global mortality data associated with COVID-19 in LTCF. The second presentation reports on the situation in German NHs addressing the complex situation of morbidity, care dependency, and social isolation. The third presentation will describe the effects of the pandemic upon NH staff in Poland. The final presentation examines the impact and guidelines of allowing visitors in NHs in the Netherlands for residents, family caregivers and staff. Our discussant, Marie Boltz, will synthesize the research findings and lead a discussion of future directions for policy and practice.

### COVID-19 IN GERMAN NURSING HOMES: A PANEL STUDY ON MORBIDITY, BURDEN OF CARE, AND SOCIAL ISOLATION

Kathrin Seibert,[1] Dominik Domhoff,[2] Franziska Heinze,[3] Benedikt Preuss,[4] Heinz Rothgang,[4] and Karin Wolf-Ostermann,[5], *1. Institute for Public Health and Nursing Research, Universtity of Bremen, Bremen, Germany, 2. Institute for Public Health and Nursing Research, University of Bremen, Bremen, Germany, 3. Research Center on Inequality and Social Policy (SOCIUM),*

*university of Bremen, Bremen, Germany, 4. Research Center on Inequality and Social Policy (SOCIUM), University of Bremen, Bremen, Germany, 5. University of Bremen, University of Bremen, Bremen, Germany*

Germany was hit by the second wave of the pandemic much harder than by the first wave. The study aims to describe the extent to which nursing homes (NH) are affected by COVID-19. About 8,000 NHs were invited to participate in two waves of an online survey, with a share of 5-10% participating. The share of all deceased NH-residents with COVID-19 is about 50% (04/2020-02/2021). Factors that increase the risk of an outbreak in NH are the spread of the virus in the population, the size of the institution and staff-resident-ratio. The initial lack of protective equipment has decreased during the second wave, but the facilities have to cope with massive additional care needs with reduced staff. NHs have partly banned contacts between residents and relatives. As a conclusion the support of NH in their attempt to fight the impact of this and further pandemic situations requires highest attention.

### THE IMPACT OF VISITATION GUIDELINES DURING COVID-19 ON WELL-BEING AND DAILY LIFE IN NURSING HOMES

Debby Gerritsen,[1] Ramona Backhaus,[2] Bram de Boer,[2] Judith Urlings,[3] Raymond Koopmans,[1] Jan Hamers,[2] and Hilde Verbeek,[2], *1. Radboud University Medical Centre, Nijmegen, Gelderland, Netherlands, 2. Maastricht University, Maastricht, Limburg, Netherlands, 3. Living Lab in Ageing and Long-Term Care, Maastricht, Limburg, Netherlands*

Nursing homes across the world have taken very restrictive measures, including a ban for visitors, to prevent and control COVID-19 infections. This study reports on findings of a study investigating guidelines on allowing visitors in nursing homes and the impact on residents' well-being, family caregivers and staff. In total, 76 nursing homes in the Netherlands were followed using a survey study, including three waves of data collection in (May 2020, September 2020, March 2021. Results indicated a negative impact of a visitation ban for residents' overall well-being. There was a variety in guidelines of allowing visitors in nursing homes, and showed that safe visiting was possible during the COVID-19 pandemic. Staff perceived a fragile balance between infection prevention and the impact of restriction on residents. In conclusion, a general ban for visitors is not necessary and may do more harm than good for residents living in nursing homes.

### MENTAL HEALTH CONSEQUENCES OF SARS-COV-2 PANDEMIC ON LONG-TERM CARE FACILITY SOCIAL CARE PROVIDER IN POLAND

Dorota Szcześniak,[1] Adrianna Senczyszyn,[1] Maria Maćkowiak,[1] Marta Ciułkowicz,[1] Katarzyna Lion,[2] and Joanna Rymaszewska,[1], *1. Department of Psychiatry, Wroclaw Medical University, Poland, Wroclaw Medical Univeristy, Dolnoslaskie, Poland, 2. Menzies Health Institute Queensland, Griffith University, Australia, Menzies Health Institute Queensland, Queensland, Australia*

During the pandemic long-term care facilities (LTCF) social health providers constantly remain in a dilemma between loyalty to people with dementia and concerns for their own families. All of these factors could contribute to the mental burden, burnout, and increased chance of depression, anxiety and post-traumatic symptoms. In our study we aimed to provide a window on psychopathological consequences (somatic symptoms, anxiety and insomnia, social dysfunction, and depression) associated with the exposure of LTCF employees to the risk of the SARS-CoV-2 contagion in Poland. Moreover, we investigated if institutional factors, such as personal protection equipment availability, safety guidelines or access to psychiatric and psychological support at the workplace, contribute to the decrease of psychological distress of the LTCF personnel. The results can serve as ready-made guidelines for mitigating the SARS-CoV-2 impact on dementia care and constitute the basis for further analysis of long-term consequences of this precedential situation.

### INTERNATIONAL EVIDENCE ON THE COVID-19 DEATHS OF PEOPLE WHO LIVE IN LONG-TERM CARE FACILITIES

Joseba Zalakain,[1] Elizabeth Lemmon,[2] David Henderson,[3] Amy Hsu,[4] Andrea Scmidt,[5] Greg Arling,[6] Florien Kruse,[7] and Adelina Comas-Herrera,[8], *1. SIIS Centro De Documentación Y Estudios, Donostia San Sebastián, Pais Vasco, Spain, 2. University of Edinburgh, Edinburgh, Scotland, United Kingdom, 3. University of Edinburgh, NINE Edinburgh BioQuarter, Scotland, United Kingdom, 4. Bruyère Research Institute, Ottawa, Ontario, Canada, 5. Austrian National Public Health Institute (Gesundheit Österreich GmbH, GÖG), Vienna, Wien, Austria, 6. Purdue University, West Lafayette, Indiana, United States, 7. Radboud University Medical Center, Nijmegen, Gelderland, Netherlands, 8. London School of Economics and Political Science, London School of Economics and Political Science, England, United Kingdom*

The COVID-19 pandemic has had a disproportionate impact, in terms of mortality, on people who live in Long-Term Care Facilities (LTCFs). This study involved compiling data on number of deaths of people who live in LTCFs and analyzing the extent to which differences between countries could be attributed to measures taken to control the spread of COVID-19 to LTCFs or to other factors. The study found that differences in how the data is collected make international comparisons difficult but that there is a clear correlation between number of COVID-19 deaths of residents in LTCFs and number of COVID-19 deaths of people living in the community. The study also found that countries that experienced a particularly high number of deaths in LTCFs during the first COVID-19 wave tended to have lower relative mortality in LTCFs in the subsequent waves, which potentially could be attributed to learning from the initial shock.

### Session 3590 (Paper)

### MENTAL HEALTH IMPACT OF THE COVID-19 PANDEMIC

### FOOD INSECURITY AND MENTAL HEALTH TRAJECTORIES DURING THE COVID-19 PANDEMIC: LONGITUDINAL EVIDENCE

Dexia Kong,[1] Peiyi Lu,[2] Elissa Kozlov,[1] and Mack Shelley,[2] *1. Rutgers University, New Brunswick, New Jersey, United States, 2. Iowa State University, Ames, Iowa, United States*

The extent to which food insecurity impacts changes in mental health outcomes over time in the context of Covid-19 remains unknown. Using longitudinal data from a nationally representative survey, the objectives of the present study were to: (1) assess the prevalence of food insecurity among U.S. adults amid the Covid-19 pandemic; and (2) investigate the relationships between food insecurity statuses and changes in mental health outcomes over time as the pandemic unfolds. Longitudinal data from the Internet-based Understanding Coronavirus in America survey collected bi-weekly between April and December 2020 were used (n=4,068, 15 repeated measures). Adult respondents (aged ≥18) were asked about their food insecurity experiences and stress/anxiety/depressive symptoms. Linear mixed-effect models examined changes in mental health outcomes over time among groups with various food insecurity statuses. Overall prevalence of food insecurity was 8%. Food insecurity was consistently associated with higher levels of stress/anxiety/depressive symptoms ($p<0.001$). Stress/anxiety/depressive symptoms declined over time among food-secured U.S adults. However, mental health trajectories of respondents with various food insecurity categories, including food insecurity status, persistent food insecurity, and food insecurity of higher severity and longer duration, remained stable or worsened over time. Moreover, the mental health gap between food-secured and food-unsecured participants widened over time. Food insecurity represents a pressing public health problem during the Covid-19 pandemic with substantial mental health implications. Persistent and severe food insecurity may contribute to mental health disparity in the long term. Food insecurity reduction interventions may alleviate the estimated alarming mental health burden as the pandemic unfolds.

### GLOBAL RISE OF DEPRESSION PREVALENCE AMID THE COVID-19 PANDEMIC

Anna Egbert,[1] Stephen Karpiak,[2] Richard Havlik,[3] Sadiye Cankurtaran,[4] and Sirinnaz Ozturk,[5], *1. Ronin Institute, Montclair, New Jersey, United States, 2. GMHC, National Resource Center on Aging and HIV, New York, New York, United States, 3. National Institutes of Health, National Institute of Aging, New York, New York, United States, 4. Jagiellonian University, Kraków, Malopolskie, Poland, 5. University of Warsaw, Warsaw, Mazowieckie, Poland*

The immense burden of depressive disorders is on the rise, with global prevalence estimates in 2017 ranging from 4% to 13%. The novel coronavirus SARS-CoV-2 is likely to impact the established risk factors for depressive disorders. Thus, a rapid increase in depression prevalence can be expected amid the COVID-19 pandemic. Using epidemiologic data (N=111,225) derived from an extant online survey "Measuring Worldwide COVID-19 Attitudes and Beliefs" (launched by Fetzer and colleagues, March-April 2020) in 178 countries, we examined age-dependent global prevalence of depression and assessed the impact of social factors caused by the COVID-19 pandemic on depressive symptomatology. Point prevalence of depression was measured using the PHQ8 standard cut-off score (i.e., ≥10). Correlates of depressive symptoms were analyzed with hierarchical regression modeling separately in three age groups, i.e., 18-34, 35-54 and 55+ years. We found that nearly 20% of individuals globally revealed significant symptoms of depression, including 27% of young, 15% middle-aged, 9% adults aged 55+. These data suggest that the prevalence of depression is 2-5 times higher than global estimates preceding the COVID-19 pandemic. Regression modeling explained approx. 50% variability in depressive symptoms across the three age groups. Increased risk of depression was found in females, single or divorced individuals, and those who presented poorer health and higher anxiety. Social restrictions amid the COVID-19 pandemic were marginal risks for depression. Together, this study highlights the impact of the COVID-19 pandemic on the mental health of people of different ages and urges the development of increased access to psychological interventions.

### MENTAL HEALTH AND WELL-BEING AMONG INDIVIDUALS WITH A SENSORY LOSS DURING COVID-19 LOCKDOWN MEASURES

Natascha Merten,[1] Amy Schultz,[2] Matthew Walsh,[2] Suzanne van Landingham,[2] Paul Peppard,[2] Carol Ryff,[2] and Kristen Malecki,[2], *1. University of Wisconsin - Madison, Madison, Wisconsin, United States, 2. University of Wisconsin-Madison, Madison, Wisconsin, United States*

Hearing and vision impairment are highly prevalent chronic conditions and are associated with poorer mental health and well-being. Mental health problems may be exacerbated by COVID-19-related lockdown measures and limitations of in-person contacts may affect those with sensory impairments more severely. We aimed to determine whether hearing and/or visual impairment were associated with worse mental health and psychological well-being during lockdown measures in Spring/Summer 2020 in Wisconsin. We included 1341 (64% women, aged 20-92 years) Survey of the Health of Wisconsin participants of a COVID-19 survey (May-June, 2020). We assessed self-reported current mental health and psychological well-being and vision and hearing impairment. Logistic regression models with vision and hearing impairments as determinants and multiple mental health and well-being outcomes were used and adjusted for age, gender, race, education, heart disease, hypertension, hyperlipidemia and diabetes. In preliminary analyses, we found associations of vision impairment with increased odds of generalized anxiety disorder (odds ratio=2.10; 95% confidence interval=1.32-3.29) and depression (2.57; 1.58-4.11). Individuals with a vision impairment were more likely to be taking medication for depression (1.75; 1.13-2.68), report being lonely (1.65; 1.00-2.64) and report hopelessness (1.45; 1.01-2.08). Individuals with a hearing impairment were more likely to be taking depression medications (1.72; 1.07-2.73) and to report being lonely (1.80; 1.05-2.98). Sensory impairment was not associated with stress levels or sense of purpose in life. Individuals with sensory impairment may represent a particularly vulnerable population during the COVID-19 pandemic. Future research should determine underlying reasons and interventions to mitigate this populations' disadvantages.

### PREDICTORS OF MENTAL HEALTH DURING THE COVID PANDEMIC

Helen Lach,[1] Devita Stallings,[2] Rebecca Lorenz,[3] John Taylor,[4] and Janice Palmer,[5], *1. Saint Louis University, Saint Louis, Missouri, United States, 2. Saint Louis University, St. Louis, Missouri, United States, 3. University*

*at Buffalo, Buffalo, New York, United States, 4. Saint Louis University, Hillsboro, Missouri, United States, 5. Webster University, St. Louis, Missouri, United States*

Health professionals have been concerned about mental health of older adults during the COVID pandemic. To explore their experiences, we conducted an online survey of community-dwelling older people to examine their mental health related to stress, based on Pearlin's Stress Process Model. A snowball approach was used; we sent recruitment e-mails through senior organizations and contacts with e-mail lists of potential participants; there were 504 respondents. We used regression analysis to explore predictors of mental health based on Pearlin's model. Background characteristics included age (m = 75.7, SD 4.95), gender (77.4% female) and race (White = 93.4%). The CESD-10 provided a measure of mental health. Scores indicated 62.3% of the sample scored in the low range for depressive symptoms and 37.7% in the moderate to high range. Stressors were measured using the Perceived Stress Scale that includes subscales of perceived helplessness and perceived self-efficacy. We also measured perceived social Isolation, and current life space as predictor variables. Results of regressing the CESD-10 onto the set of theoretical predictors revealed that the inclusion both subscales of the Perceived Stress Scale, social isolation, and current life space jointly accounted for approximately 63.0% of the variability in the outcome beyond the baseline model (FChange[4, 449] = 211.15, $p < .01$), which included age, race, and gender. The model overall, accounted for approximately 66.5% (R2adjusted = 66.0%) of the variability in CESD-10 scores, (F[7, 449] = 127.473, $p < .01$). Addressing stress among older adults is important to help them maintain positive mental health.

### SOCIAL CONNECTIVITY IN THE CONTEXT OF COVID-19 AND LONG-TERM CARE

Anna Garnett,[1] Hannah Pollock,[1] Natalie Floriancic,[2] Lorie Donelle,[1] Yolanda Babenko-Mould,[1] Abe Oudshoorn,[1] Carri Hand,[1] and Cheryl Forchuk,[1],
*1. Western University, London, Ontario, Canada,*
*2. Western University, Western University, Ontario, Canada*

The COVID-19 pandemic has disproportionately impacted older adults, particularly those residing in long-term care homes (LTCHs), causing immense loss of life and resulting in overall health declines in LTCH residents. These vulnerable older adults have also experienced extreme loneliness, anxiety and depression. Social connectedness is an important contributor to well-being and quality of life of older adults in LTCHs and family members are an essential component to this. However, restrictions driven by policies to protect resident safety, have constrained family members' access to long-term care homes and limited in-person contact between residents and their families. In their absence, health providers have been integral to supporting connections between residents and their families within LTCHs. This study aimed to understand the experiences of social connectedness between residents and family members who have been physically separated due to the current pandemic and, to examine LTCH health providers' experiences and responses to support social connectedness. Using a qualitative descriptive design, in-depth semi-structured interviews were conducted with 21 family members and 11 healthcare providers. Emergent themes from qualitative content analysis are: (a) all-encompassing impacts of separation; (b) advocacy became my life; (c) the emotional toll of the unknown; 4) the burden of information translation; 5) precarious balance between safety and mistrust for the healthcare system; and (d) a formulaic approach impedes connectivity. A more comprehensive understanding of the experiences and support needs of LTCH residents and their family members within the context of a pandemic can inform practice approaches to support social connections going forwards.

### Session 3595 (Paper)

### MENTAL HEALTH OF OLDER ADULTS

### ASSOCIATION BETWEEN POST-TRAUMATIC STRESS AND DEPRESSION AMONG OLDER PUERTO RICANS: HOW DOES LONELINESS MATTER?

Kaipeng Wang,[1] Adolfo Cuevas,[2] Anao Zhang,[3] Carson De Fries,[1] Ladson Hinton,[4] and Luis Falcón,[5]
*1. University of Denver, Denver, Colorado, United States,*
*2. Tufts University, Medford, Massachusetts, United States,*
*3. University of Michigan, Ann Arbor, Michigan, United States, 4. University of California, Davis, Sacramento, California, United States, 5. University of Massachusetts Lowell, Lowell, Massachusetts, United States*

Puerto Ricans have a significantly higher prevalence of post-traumatic stress disorder (PTSD) than other racial and ethnic groups. PTSD can lead to co-morbid depression, which exacerbates the risk for compromised mental and physical health among older adults. Loneliness, a robust indicator of poor social support, is likely to worsen the dysphoric symptoms of PTSD and increase the risks of depression. However, few studies have focused on the moderating role of loneliness on the association between post-traumatic stress and depression. This study examines the association between post-traumatic stress and depression and whether such an association differs by loneliness among older Puerto Ricans. Data were collected from 304 Puerto Ricans aged 60-81 living in the Greater Boston area who responded to questionnaires in a survey. We used ordinary least squares regression to examine the association between post-traumatic stress, loneliness, and depression. We found that post-traumatic stress was significantly associated with higher levels of depression; however, the association was contingent on experiences of loneliness. The association between post-traumatic stress and depression was stronger for those experiencing a higher degree of loneliness. Findings underscored the role that loneliness plays in exacerbating the risk of developing co-morbid PTSD and depression among older Puerto Ricans. In working with older Puerto Ricans experiencing post-traumatic stress, it is important for mental health professionals to incorporate the assessment of loneliness and to prevent and reduce co-morbid depression by addressing loneliness through improving social skills, enhancing social support, and reducing maladaptive social cognition.

### DISPARITIES OF MENTAL HEALTH SERVICE NEEDS AND UTILIZATION AMONG OLDER ADULTS

Yuanyuan Hu,[1] and Qingwen Xu,[2], *1. New York University, Elmhurst, New York, United States, 2. New York University, New York University, New York, United States*

Prior studies have examined mental health disparities, however, without adequate attention to the older adult population. Framed by the Andersen Behavioral Model of Health Service Use, this study was to examine the prevalence of depression and anxiety and the mental health service use among older adults of different race/ethnicity; and to investigate factors associated with mental health services use(counseling and psychotropic medication). Data from the National Health Interview Survey 2019 were analyzed by bivariate tests and logistic regression analyses. Hispanic older adults have the highest rates of depressive and anxious symptoms, followed by Whites, Blacks, and Asians. Non-Hispanic Asians and Blacks reported significantly lower rates of taking medication. The severity of depression and anxiety was consistently associated with mental health service use across all groups. Education was positively associated with counseling use in white and black groups. For older whites, better general health, male and foreign-born were significantly predicting less medication use. Older blacks with better general health were significantly less likely to use medication. For Hispanic older adults, female and being single were associated with anxiety medication use. Results suggest that older adults, despite different perceptions and cultural understandings of mental health, use mental health services for severe conditions. This study also highlights the important role that education and health literacy could have played in the use of counseling services. For the medication use, the result—that general health status was important for both black and white older adults, but not Hispanics—could suggest a few directions for further exploration.

### FEASIBILITY PILOT: PROBLEM ADAPTATION THERAPY FOR EMOTION REGULATION IN COMMUNITY-DWELLING OLDER ADULTS

Julia Sheffler,[1] Melissa Meynadasy,[2] Dimitris Kiosses,[3] and Natalie Sachs-Ericsson,[2], *1. Florida State University College of Medicine, Tallahassee, Florida, United States, 2. Florida State University, Tallahassee, Florida, United States, 3. Weill-Cornell Medicine, White Plains, New York, United States*

Emotion regulation (ER) difficulties in older adults are associated with increased depression and decreased resiliency to stressful life events. In general, maladaptive ER is a transdiagnostic risk factor for a range of psychological and physical problems across the lifespan. Thus, interventions targeting ER may be valuable in reducing risk for a range of late-life pathologies. The present study evaluated and adapted an existing ER-focused treatment (i.e., Problem Adaptation Therapy (PATH)) for community older adults. We completed a small clinical pilot study to assess the feasibility of the adapted protocol and initial signals of effect of the intervention on ER, depression, and resiliency. Participants were recruited using an online survey, which was used to then identify participants scoring in the highest and lowest quartiles for ER. Individuals in the lowest ER quartile (N=27) were randomly assigned to the PATH condition or a physical health education (PHET) control condition. Of the 27 participants in the low ER group, four participants (3 PATH, 1 PHET) dropped out of the intervention. A paired samples t-tests revealed significant decreases in depressive symptoms, significant increases in self-reported ER skill, and improvements in resiliency (all $ps<.05$) for the PATH condition. For the PHET condition, only significant increases in self-reported ER skill ($t(12) = -2.68$, $p = .020$) were observed. In sum, the intervention protocol proved feasibility and demonstrated initial signals of effect in the expected directions. Future studies will examine mechanisms of action and the efficacy of the adapted PATH protocol.

### FUNCTIONAL MOBILITY, AGING, AND PEOPLE WITH MENTAL ILLNESS: ISSUES AND CHALLENGES

Michelle Zechner,[1] Ellen Anderson,[2] and Kenneth Gill,[3], *1. Rutgers, Princeton, New Jersey, United States, 2. Rutgers, Newark, New Jersey, United States, 3. Rutgers, Piscataway, New Jersey, United States*

People with serious mental illness (SMI) are more likely to experience chronic health conditions at younger ages, which increases the risk of premature death. Co-morbid health conditions and risk for premature death are well-studied in the population, however less is understood about the impact of aging and SMI on functional ability. Research suggests that the population walk less and may have lower fitness levels than other populations (Gill et al., 2016). Specific data exploring functional age of people with SMI is sparse. The authors compared published standardized geriatric functional fitness values for people over 65 to baseline values of a community sample of people living with SMI who participated in a community health promotion intervention. The average age of the sample was 50 (SD=11). Three physical functioning measures were used in the comparison to measure physical functioning; the Sit to Stand Test, 6 Minute Walk, and Single Legged Stance. Results indicated significant differences in mean physical functioning values between the sample and standardized geriatric values. The sample performed at levels 20-30 years older than their chronological age. This finding suggests that mental health and aging services may need to adjust interventions, services and methods to improve physical functioning in middle-aged and older adults living with SMI. Premature functional decline impacts community living skills, independent living, housing choice, vocational options, and may impede personal goal attainment. Recommendations for interventions will be offered, as will suggestions for policies targeting services that cross aging and mental health silos.

### TRAUMA, POSTTRAUMATIC STRESS DISORDER, AND TREATMENT AMONG MIDDLE-AGED AND OLDER WOMEN

Laura Sampson,[1] Shaili Jha,[2] Andrew Ratanatharathorn,[1] Andrea L. Roberts,[1] Laura D. Kubzansky,[1] Eric B. Rimm,[1] and Karestan C. Koenen,[1], *1. Harvard T.H. Chan School of Public Health, Boston, Massachusetts, United States, 2. Harvard T.H. Chan School of Public Health, Harvard T.H. Chan School of Public Health, Massachusetts, United States*

Posttraumatic stress disorder (PTSD) is twice as prevalent in women as in men, and is an established risk factor for chronic disease, but few studies have comprehensively assessed lifetime PTSD in middle-aged and older civilian women. We surveyed 33,328 women aged 54-74 from the Nurses' Health Study II from August 2018 to January 2020 to understand trauma exposure, PTSD based on the Diagnostic and Statistical Manual of Mental Disorders Version 5, and trauma-related treatment use. The majority (82.2%) of women reported one or more lifetime traumas.

10.5% of those with trauma had lifetime PTSD and 1.5% had past-month PTSD. The most common trauma types were sudden or unexpected death of a loved one (44.9%) and interpersonal or sexual violence (43.5%). Almost 30% experienced occupational (nursing-related) trauma. Interpersonal or sexual violence event types explained the largest proportion of PTSD cases (33.6%) out of seven categories of events assessed. Only 25% of women with trauma ever accessed trauma-related treatment, but this proportion was higher (66.4%) among those with diagnosable PTSD, and among those with current depression (35.9%). Treatment was most common among women who experienced interpersonal/sexual violence and lowest among those with occupational trauma, but treatment satisfaction did not vary by worst trauma type. Psychotherapy was the most common type of treatment. These results demonstrate that trauma is nearly universal in middle-aged to older women, which has important implications for their long-term health and well-being—particularly in the era of COVID-19 which is likely to produce additional trauma in this population.

### Session 3600 (Symposium)

### MODELS TO STUDY AGING

Chair: Viviana Perez

### UNIVERSAL DNA METHYLATION AGE ACROSS MAMMALIAN TISSUES

Zhe Fei,[1] Ken Raj,[2] Steve Horvath,[1] and Ake Lu,[3], *1. UCLA, Los Angeles, California, United States, 2. Centre for Radiation, Chemical and Environmental Hazards, Public Health England,, Chitlon, England, United Kingdom, 3. UCLA, LOS ANGELES, California, United States*

Aging is often perceived as a degenerative process caused by random accrual of cellular damage over time. In spite of this, age can be accurately estimated by epigenetic clocks based on DNA methylation profiles from almost any tissue of the body. Since such pan-tissue epigenetic clocks have been successfully developed for several different species, it is difficult to ignore the likelihood that a defined and shared mechanism instead, underlies the aging process. To address this, we generated over 10,000 methylation arrays, each profiling up to 37,000 cytosines in highly-conserved stretches of DNA, from over 59 tissue-types derived from 128 mammalian species. From these, we identified and characterized specific cytosines, whose methylation levels change with age across mammalian species. Genes associated with these cytosines are greatly enriched in mammalian developmental processes and implicated in age-associated diseases. From the methylation profiles of these age-related cytosines, we successfully constructed three highly accurate universal mammalian clocks for eutherians, and one universal clock for marsupials. The universal clocks for eutherians are similarly accurate for estimating ages (r>0.96) of any mammalian species and tissue with a single mathematical formula. Collectively, these new observations support the notion that aging is indeed evolutionarily conserved and coupled to developmental processes across all mammalian species - a notion that was long-debated without the benefit of this new and compelling evidence.

### THE COMMON MARMOSET: A HIGHLY TRANSLATABLE SMALL NONHUMAN PRIMATE MODEL OF AGING

Ricki Colman, *University of Wisconsin Madison, Madison, Wisconsin, United States*

The common marmoset (Callithrix jacchus) has been used in biomedical research for many years, but within the last decade its popularity has increased dramatically prompted to a large degree by their realized utility for neuroscience and aging research. Many factors make the marmoset an attractive model system including their genetic and physiological similarity to humans, relatively short lifespan (average of ~13 years, maximum of ~20 years), high fertility (highest of any primate, routine production of 2-3 offspring every 5-6 months), rapid development (reproductively competent by ~1.5 years of age, aged by 7-8 years of age), small size (~400 grams), human-like social structure consisting of cooperative breeding with shared parenting responsibilities, and lack of zoonotic diseases of concern to humans. Marmosets share ~93% sequence identity with the human genome and they develop similar age-related conditions as humans. Marmosets may strike the perfect balance between similarity to humans and abbreviated aging course.

### BATS: SECRETS OF EXTENDED HEALTHSPAN

Emma Teeling, *University College Dublin, Dubling, Ireland*

Of all mammals, bat possess the most unique and peculiar adaptations that render them as excellent models to investigate the mechanisms of extended longevity and potentially halted senescence. Indeed, they are the longest-lived mammals relative to their body size, with the oldest bat caught being >41 years old, living approx. 8 times longer than expected. Bats defy the 'rate-of-living' theories that propose a positive correlation between body size and longevity as they use twice the energy as other species of considerable size, but live far longer. The mechanisms that bats use to avoid the negative physiological effects of their heightened metabolism and deal with an increased production of deleterious Reactive Oxygen Species (ROS) is not known, however it is suggested that they either prevent or repair ROS damage. Bats also appear to have resistance to many viral diseases such as rabies, SARS and Ebola and are the suspected reservoir species for a huge diversity of newly discovered viruses, including Sars-CoV-2 This suggests that their innate immunity is different to other mammals, perhaps playing a role in their unexpected longevity. Here the potential genomic basis for their rare immunity and exceptional longevity is explored across multiple bat genomes and divergent ageing and immune related markers (e.g. microbiome, telomeres, mitochondria, cellular dynamics, cytokine response) studied in wild bat populations. These findings provide a deeper understanding of the causal mechanisms of ageing and tolerant immunity, potentially uncovering the key molecular pathways that could be utilised to benefit society.

### Session 3605 (Paper)

### NURSING HOMES

### A CROSS-SECTIONAL STUDY COMPARING YOUNGER AND OLDER NURSING HOME RESIDENTS IN WESTERN CANADA

Bianca Shieu,[1] Todd Schwartz,[2] Anna Beeber,[3] Matthias Hoben,[4] Mark Toles,[2] and Ruth Anderson,[3]
*1. University of Pittsburgh/ School of Medicine, Pittsburgh, Pennsylvania, United States, 2. UNC Chapel Hill, UNC Chapel Hill, North Carolina, United States, 3. University of North Carolina at Chapel Hill, UNC Chapel Hill, North Carolina, United States, 4. University of Alberta at Edmonton, Edmonton, Alberta, Canada*

Specialized care for younger nursing home (NH) residents may be necessary to meet their unique health and quality of life needs; however, key attributes of younger NH residents are poorly understood and limit the development of effective, tailored interventions. This study described differences in clinical and nonclinical characteristics of younger vs. older nursing NH residents. In a retrospective cohort study, we used SPSS and analyzed comprehensive Resident Assessment Instrument – Minimum Data Set (RAI-MDS 2.0) data from NHs in Western Canada, for the period from January 2016 to December 2017. We included all assessments (full and abbreviated) performed quarterly. These findings indicated that younger (age 18-64) vs. older (age >=65) NH residents differed considerably: younger residents were predominately male, single, more obese, more depressed, had higher prevalence of depression, cerebral vascular accident, and hemi- or quadriplegia, and required more assistance in activities of daily living than older residents. The findings will contribute a better comprehension of the characteristics of the younger NH population and how they differ from other residents. The study provides useful information to policymakers, providers, and researchers to guide them in developing tailored policies, programs, and interventions. Also, findings may guide consumers as they plan for long-term care needs of loved ones. Finally, the findings provide a baseline estimate as researchers continue to track the growth of and changes in, the populations served in nursing homes.

### ACUITY DIFFERENCES AMONG NEWLY ADMITTED MEDICARE RESIDENTS IN RURAL AND URBAN SKILLED NURSING FACILITIES

Yvonne Jonk,[1] Andrew Coburn,[2] Catherine McGuire,[2] Deborah Thayer,[2] and Karen Mauney,[2], *1. University of Southern Maine, Muskie School, Portland, Maine, United States, 2. University of Southern Maine, Portland, Maine, United States*

Using the 2015 national Minimum Data Set Version 3.0, the Area Health Resources Files, the 2015 Provider of Services File, and the Rural-Urban Commuting Area codes, this study assessed rural-urban differences in newly admitted, Medicare skilled nursing facility (SNF) residents' functional status, cognitive performance, and behavioral issues using self-performance, early loss, and late loss Activities of Daily Living (ADLs); the Cognitive Function Scale (CFS); and indicators of aggression, psychosis, or wandering, respectively. The study evaluated 686,881 unique patient assessments for newly admitted Medicare SNF residents across 15,157 facilities in 47 states. Negative binomial and generalized linear models with state fixed effects and clustering by SNFs were used to evaluate rural-urban acuity differences before and after adjusting for socio-economic factors; admission source, and market area characteristics. Compared to urban SNF residents, rural residents were more likely to be cognitively impaired (45% Isolated Small Rural, 44.5% Small Rural, 41% Large Rural, 38.8% Urban), and have behavioral issues (6.7% rural, 4.8% urban). Unadjusted and adjusted regression models confirmed bivariate findings that rural SNF residents were less functionally impaired (IRR range: 0.974-.987), but had more cognitive and behavioral issues in more remote rural locations than urban. The (unadjusted) odds of cognitive impairment were 1.1-1.3 times higher for residents of rural vs urban SNFs; while the odds of having any one of the behavioral issues were 1.2-1.6 times higher in more remote rural locations. The capacity of rural SNFs to manage complex cognitive and behavioral problems deserves further research.

### FACTORS AFFECTING THE SUSTAINMENT, SUSTAINABILITY, AND SPREAD OF PRACTICE CHANGES IN CANADIAN LONG-TERM CARE HOMES

Lauren MacEachern,[1] Yuting Song,[2] Liane Ginsburg,[3] Malcolm Doupe,[4] Adrian Wagg,[2] Jude Spiers,[2] and Whitney Berta,[5], *1. University of Toronto, Toronto, Ontario, Canada, 2. University of Alberta, Edmonton, Alberta, Canada, 3. York University, York University, Alberta, Canada, 4. University of Manitoba, Winnipeg, Manitoba, Canada, 5. University of Toronto, University of Toronto, Ontario, Canada*

Our understanding of the post-implementation sustainment, sustainability, and spread (SSS) of complex quality improvement interventions is limited. We explored factors that influenced the SSS of a care aide-led quality improvement initiative (Safer Care for Older Persons (in residential) Environments [SCOPE]) implemented in 6 Manitoba long-term care homes two years after the conclusion of SCOPE in 2017. We analyzed small group interview data collected from all unit- and facility-level managers who participated in SCOPE and were still working in these facilities. We asked about SCOPE implementation, post-SCOPE quality improvement activities, factors that influenced them, and about inter-unit spread of SCOPE following the project's conclusion. The interviews were audio-recorded, transcribed verbatim, de-identified, and analyzed using thematic analysis. Five of the 6 facilities reported sustained SCOPE quality improvement activities, tools, and facilitative structures. In the same 5 facilities, SCOPE benefits (e.g., increases in care aide empowerment and self-efficacy, manager belief in care aide capacity) continued post-implementation. Spread beyond the original SCOPE units had occurred in 3 facilities. Factors that influenced the SSS of SCOPE were related to the team (e.g., care aides' quality improvement capacity), to the unit and facility (e.g., culture of innovation and change), and to the long-term care system (e.g., competing imperatives). Some factors influencing SSS differ from factors known to influence implementation. The identified factors affecting SSS highlight the influence of social dynamics (i.e., interactions, communication, relationships) among staff on SSS. Further research is warranted to explore interactions among these influencing factors and how they lead to SSS.

### FEASIBILITY OF ROUTINE QUALITY-OF-LIFE ASSESSMENT IN LONG-TERM CARE HOMES

Matthias Hoben,[1] Sube Banerjee,[2] Anna Beeber,[3] Stephanie Chamberlain,[4] Laura Hughes,[5] Hannah O'Rourke,[4] Kelli Stajduhar,[6] and Jude Spiers,[7], *1. University of Alberta at Edmonton, Edmonton, Alberta, Canada, 2. University of Plymouth, University of Plymouth, England, United Kingdom, 3. University of North Carolina at Chapel Hill, UNC Chapel Hill, North Carolina, United States, 4. University of Alberta, University of Alberta, Alberta, Canada, 5. University of Sussex, University of Sussex, England, United Kingdom, 6. University of Victoria, University of Victoria, British Columbia, Canada, 7. University of Alberta, Edmonton, Alberta, Canada*

Maximizing long-term care (LTC) residents' quality of life (QoL) is the primary goal of care. However, most residents have cognitive impairment and care staff time is severely limited, leading to various complexities in measuring QoL. This study developed and assessed the feasibility of an approach to routinely measuring QoL in LTC residents. We used the DEMQOL-CH, a practical, reliable, valid tool, developed in the UK to be completed by care aides to assess QoL in residents with moderate to severe dementia. We recruited 45 care aides in 10 LTC homes in Alberta, Canada who we surveyed on the QoL of 263 residents via video calls. We assessed time to complete; care aide and manager perceived feasibility of completing the DEMQOL-CH; internal consistency and inter-rater reliability of DEMQOL-CH scores; and we conducted cognitive interviews with 7 care aides to assess care aide comprehension of the tool. Time to complete was on average 4 minutes with little variation. Care aides and managers rated using the DEMQOL-CH as highly feasible and valuable. The internal consistency of the DEMQOL-CH score was 0.80. The DEMQOL-CH score inter-rater agreement was 0.73. Cognitive interviews suggested good comprehension overall with some comprehension problems especially in care aides who speak English as a second language. Asking care aides to complete the DEMQOL-CH is highly feasible, requires minor resources, and reliability is high. However, some items caused comprehension and reliability problems. Reasons and possible solutions will be subject to further investigations.

### IMPACT OF RACE ON DECISION MAKING FOR NURSING HOME RESIDENTS WITH ADVANCED DEMENTIA: CAN WE DISRUPT AND TRANSFORM?

Ruth Lopez,[1] Ashley Roach,[2] Meghan Hendricksen,[3] Anita Rogers,[4] Fayron Epps,[5] and Ellen McCarthy,[6], *1. MGH Institute of Health Professions, Boston, Massachusetts, United States, 2. OHSU, Portland, Oregon, United States, 3. Hinda and Arthur Marcus Institute for Aging Research, Boston, Massachusetts, United States, 4. University of Tennessee at Martin, Parsons, Tennessee, United States, 5. Emory University, Atlanta, Georgia, United States, 6. Marcus Institute for Aging Research, Hebrew SeniorLife, Boston, Massachusetts, United States*

Despite 20 years of research and numerous experts and associations advocating a palliative approach to care for nursing home (NH) residents with advanced dementia, research consistently demonstrate striking and persistent racial differences in the use of burdensome interventions such as feeding tubes and hospital transfer. Most notable is that Black NH residents experience more burdensome interventions at the end of life. The reasons for these differences are poorly understood. The purpose of this study was to examine NH staff members' perceptions of advance care planning with proxies of Black and White residents. We conducted thematic analysis of semi-structured interviews with 158 NH staff members gathered as part of the ADVANCE study. This is a large qualitative study in 13 NHs in 4 regions of the country aimed at explaining regional and racial factors influencing feeding tube and hospital transfer rates. We found that NH staff, regardless of region of the country, held several assumptions about Black proxies including: being attached or not wanting to let go; not wanting to talk about death, believing everything must be done; not wanting to play God; having large conflicted families, not trusting; putting on attitude, and tending not to use NHs. We found that these assumptions led some NH staff to feel that rather than engaging in shared decision making, they were engaged in a battle with proxies leading them to pick and choose their battles and at times even giving up trying. Whether these assumptions can be disrupted and transformed will be discussed.

### Session 3610 (Symposium)

### ORAL HEALTH IN LATE LIFE: CHALLENGES AND SOLUTIONS

Chair: Xi Chen Co-Chair: Bei Wu
Discussant: Stephen Shuman

Older adults face a unique challenge in maintaining their oral health due to an increased disease burden, polypharmacy, functional impairment and other reasons. The five papers in this symposium describe the oral health issues in various groups of older adults and discuss different approaches to improve oral health for older adults. Using data from the Population Study of Chinese Elderly in Chicago, the first paper examined the relationship between self-reported discrimination and oral health related quality of life and investigated how resilience mediated such a relationship among foreign-born older Chinese Americans. The second paper described the oral health concerns and related treatment needs in older adults receiving palliative care using a mixed method design. The third paper demonstrated how to use behavior change techniques to improve oral self-care skills of individuals with mild dementia and support their family caregivers. The fourth paper described a project that integrates the age-friendly health system's principles into specialty dental care to address healthy aging and oral health. This initiative helped prevent and change the false belief that aging inevitably involves deterioration in oral health. The fifth paper described the impact of COVID-19 on the management of oral health problems and access to dental care in older adults. Transformative changes in care delivery and the impact of vaccination on access to care was also explored. This symposium helps better understand the oral health needs in older adults and provides new evidence to improve oral health for these individuals.

### ORAL HEALTH IN ADULT PATIENTS RECEIVING PALLIATIVE CARE: A MIXED METHOD STUDY

Violet D'Souza,[1] Timothy Thomsen,[2] Stephanie Gilbertson-White,[3] Jirakate Madiloggovit,[4] Chandler Pendleton,[5] Xianjin Xie,[5] arshi munjal,[5] and Xi Chen,[4], *1. University of Toronto, Toronto, Ontario, Canada, 2. University of Iowa Roy J. and Lucille A. Carver College of Medicine, Iowa City, Iowa, United States, 3. University of Iowa College of Nursing, Iowa City, Iowa, United States, 4. University of Iowa, Iowa City, Iowa, United States, 5. University of Iowa College of Dentistry, Iowa City, Iowa, United States*

Oral disease is highly prevalent in persons receiving palliative care (PRPC). Yet, little is known about how PRPC perceive their oral health status and related treatment needs. Forty-nine PRPC were recruited. They first completed a structured oral symptom review, followed by an oral exam. A nested sample of 11 participants also completed an in-depth interview querying their perceived oral health concerns and related treatment needs. Quantitative and qualitative data was analyzed and integrated for interpretation. Eighty-six percent of participants reported at least one oral symptom, including dry mouth (83.7%), a pain-related symptom (40.8%), or oral function difficulties (51.0%). About 40% of participants reported compromised quality of life due to oral conditions; however, the perceived impacts and treatment needs were modest. Oral disease was highly prevalent in PRPC, yet its overall impact was modest. Except for painful symptoms, most participants reported limited desire to seek treatment for oral health conditions.

### SELF-REPORTED DISCRIMINATION, RESILIENCE, AND ORAL HEALTH-RELATED QUALITY OF LIFE AMONG OLDER CHINESE AMERICANS

Bei Wu,[1] Iris Chi,[2] Wei Yang,[3] XinQi Dong,[4] and Weiyu Mao,[5], *1. New York University, New York, New York, United States, 2. University of Southern California, University of Southern California, California, United States, 3. University of Nevada Reno, Reno, Nevada, United States, 4. Rutgers University, Rutgers Institute for Health, New Jersey, United States, 5. University of Nevada, Reno, Reno, Nevada, United States*

There is a lack of empirical evidence on self-reported discrimination and oral health-related quality of life (OHRQoL). Further, the mechanism linking the two constructs is not well understood. This study aimed to examine the relationship between self-reported discrimination and OHRQoL and investigate resilience as a mediator in such a relationship among foreign-born older Chinese Americans. Data came from the Population Study of Chinese Elderly in Chicago collected between 2017 and 2019. The working sample included 3,054 foreign-born Chinese Americans (60+ years of age). Mediation analysis was conducted to examine the direct and indirect pathways towards OHRQoL. Self-reported discrimination was directly and indirectly associated with poorer OHRQoL. Resilience mediated the relationship between self-reported discrimination and OHRQoL. Specifically, individuals experienced discrimination reported weaker resilience, and subsequently, reported poorer OHRQoL. Findings illustrate the importance of studying self-reported discrimination in relation to OHRQoL and further identify resilience as an intermediary pathway to promote OHRQoL.

### REFINING AN ORAL HEALTH CARE PARTNER INTERVENTION USING BEHAVIOR CHANGE TECHNIQUES

Rachel Hirschey,[1] courtney caiola,[2] Ya-Ning Chan,[3] Brenda Plassman,[4] Bei Wu,[5] Donald BaileyJr.,[6] Ruth Anderson,[7] and Ashley Leak Bryant,[8], *1. UNC School of Nursing, Chapel Hill, North Carolina, United States, 2. East Carolina University, Greenville, North Carolina, United States, 3. UNC Chapel Hill, Chapel Hill, North Carolina, United States, 4. Duke University, Durham, North Carolina, United States, 5. New York University, New York, New York, United States, 6. duke University School of Nursing, Durham, North Carolina, United States, 7. University of North Carolina at Chapel Hill, UNC Chapel Hill, North Carolina, United States, 8. School of Nursing, Durham, North Carolina, United States*

Following a pilot, we refined an oral health carepartner intervention for individuals with mild dementia (IMD). In this intervention, we use behavior change techniques (BCTs) to foster changes by carepartners including using new oral-care techniques and developing skills for using cueing and communications approaches to support behavior changes by IMD (duration and frequency of toothbrushing and oral-hygiene skills); thus, improving plaque and gingival indices. We describe our approach to refining the intervention manual including a) completing the self-paced BCT taxonomy course, b) developing a coding schema, c) coding the original manual for evidence of BCTs, and d) refining the manual to improve use of BCTS in the refined intervention. Our results detail how BCTs can be applied to refine and improve interventions. This research demonstrates the value in using BCTs for interventions to address how carepartners and IMD can collaborate to improve oral hygiene care.

### ADOPTING THE 4MS FRAMEWORK AND AGE-FRIENDLY CONCEPTS INTO DENTAL CARE

Annette Medina-Walpole,[1] Thomas Caprio,[2] and Szilvia Arany,[3], *1. University of Rochester, Rochester, New York, United States, 2. University of Rochester Medical Center, Rochester, New York, United States, 3. Specialty Care, General Dentistry, Eastman Institute for Oral Health, University of Rochester, Rochester, NY, New York, United States*

We report on launching a dental initiative that addresses healthy aging and oral health, focusing on prevention and changing the false belief that aging inevitably involves deterioration in oral health. Our project integrates the age-friendly health system's principles into specialty dental care at Eastman Institute for Oral Health, URMC. We established an oral health-centered framework with project elements of the 4Ms - what matters, medication, mentation, and mobility. We developed pilot 4Ms templates integrated into dental charts to implement healthy-aging key oral health processes, including oral health assessment, treatment planning, and oral hygiene support. We engaged all members of the dental team through educational sessions for providing care for patients affected by Alzheimer's disease and related dementias. We leveraged on locally available interdisciplinary resources and the collaboration reflecting undergoing efforts at the University of Rochester Division of Geriatrics and the Finger Lakes Geriatric Education Center, a Geriatric Workforce Enhancement.

### PANDEMIC-DRIVEN DISRUPTIONS IN ORAL HEALTH: TRANSFORMATIVE TRENDS IN CARE FOR OLDER ADULTS

Samuel Zwetchkenbaum,[1] Mary Fisher,[2] Brooke Fukuoka,[3] Jeffrey Dodge,[4] Michael Helgeson,[5] and Elisa Ghezzi,[6], *1. Rhode Island Department of Health, Providence, Rhode Island, United States, 2. Michigan Geriatric Dental Care, West Bloomfield, Michigan, United States, 3. Your Special Smiles PLLC, Jerome, Idaho, United States, 4. CareLink, Woonsocket, Rhode Island, United States, 5. Apple Tree Dental, Mounds View, Minnesota, United States, 6. University of Michigan School of Dentistry, Ann Arbor, Michigan, United States*

Oral healthcare for the aging was severely disrupted during the pandemic of coronavirus disease 2019 (COVID-19). Transformative changes in care delivery involved teledentistry, mobile/portable dentistry, minimally invasive dentistry, aerosol minimization, and interprofessional oral care. Management of chronic oral health problems evolved through periods of limited to no access to daily and professional oral healthcare. Access to care has been influenced by availability of the oral care workforce, variability in long term care policy, and the lack of funding to cover medically necessary services delivered via asynchronous telehealth technologies. Impacts were identified six and twelve months into the pandemic. These will be compared to the state of oral healthcare for the aging 18 months from the start of the pandemic. The impact of vaccination on access to care will be explored. Variability between states (Idaho/Michigan/Minnesota/Rhode Island) will be addressed. Directions of new and needed research opportunities will be discussed.

### Session 3615 (Symposium)

### POST-RETIREMENT PAID WORK AND INEQUALITIES AT OLDER AGES

Chair: Benjamin Shaw Co-Chair: Kevin Cahill
Discussant: Michael Giandrea

Participation in paid work frequently extends beyond pensionable age, with the Organization for Economic Cooperation and Development observing, in “Pensions at a Glance” (2017, pp. 126–7), that effective retirement ages in high-income countries exceed normal full-pension-eligibility ages by 10 months for men and two months for women. While working after pensionable age is becoming ever more common, not all workers on the cusp of retirement are able to continue in their current position or find a new job. Remarkably, little is known about the implications of unequal access to post-retirement work for social and income inequalities in later life, nor how job quality might change as people work into the years normally set aside for retirement. The four papers in this symposium address the following questions: 1) do bridge employment transitions exacerbate or mitigate income inequality later in life? 2) how does job quality (job satisfaction, physical and psychosocial working conditions) compare before and after pensionable age? 3) which processes lead to changes in working conditions in the late career? and 4) might empirical and theoretical gains be made by considering post-pensionable-age paid work as a specific career stage? The presenters use longitudinal data from the United States (the Health and Retirement Study, HRS), Sweden (Swedish Longitudinal Occupational Survey of Health, SLOSH), and Japan (Japanese Study of Aging and Retirement, JSTAR) complemented by interviews with older workers in Sweden. This symposium will provide insights into the nature and consequences of working after pensionable age in contrasting institutional settings.

### DOES BRIDGE EMPLOYMENT MITIGATE OR EXACERBATE INEQUALITIES LATER IN LIFE?

Michael Giandrea,[1] Joseph Quinn,[2] Lawrence Sacco,[3] Loretta Platts,[3] and Kevin Cahill,[4], *1. U.S. Bureau of Labor Statistics, Washington, District of Columbia, United States, 2. Boston College, Chestnut Hill, Massachusetts, United States, 3. Stockholm University, Stockholm, Stockholms Lan, Sweden, 4. Center on Aging & Work at Boston College, Chestnut Hill, Massachusetts, United States*

This paper explores how gradual retirement impacts inequality later in life, with a focus on transitions from career to bridge employment. We use 26 years of longitudinal data from the Health and Retirement Study to document the various pathways that older Americans take when exiting the labor force, and examine how bridge employment impacts non-housing wealth and total wealth, including the present discounted value of Social Security benefits. We find that gradual retirement in the form of bridge employment neither exacerbates nor mitigates wealth inequalities among Americans who held career jobs later in life. We do find evidence that wealth inequalities grow among the subset of older career workers who transition from career employment to bridge employer at older ages. These findings provide quantitative evidence that bridge employment at older ages is taken by those who need to continue working financially and those who continue working for nonpecuniary reasons.

### HAVING A POST-RETIREMENT JOB: IMPROVISATION AND CONTAINING COMMITMENTS

Agnieszka Ignatowicz,[1] Hugo Westerlund,[2] Dara Rasoal,[3] and Loretta Platts,[4], *1. University of Birmingham, Birmingham, England, United Kingdom, 2. Stockholm University, Stockholm University, Stockholms Lan, Sweden, 3. Dalarna University, Falun, Dalarnas Lan, Sweden, 4. Stockholm University, Stockholm, Stockholms Lan, Sweden*

This qualitative paper focuses on individuals who work after pensionable age, a distinctive period in the late career when workers are supported by the known and reliable income of a pension. Using constant comparative analysis, we analyzed interviews from a purposive sample of 25 Swedish people in their late sixties and early seventies. We examined conditions for being in paid work in terms of enabling factors (self-employment, shift work, shortage occupation), improvisation, and the role of chance. The interviews revealed that post-retirement workers took charge of the aspects of work that mattered most to them, evading the disciplinary aspects of work by controlling scheduling and limiting the duration of their commitment. These constrained commitments had knock-on effects of improving psychosocial working conditions. Women and immigrants—groups facing low pensions—experienced the greatest financial consequences of being unable to work in their retirement years in order to supplement their pension income.

### CHANGES IN JOB QUALITY AS PEOPLE WORK BEYOND PENSIONABLE AGE IN SWEDEN

Kevin Cahill,[1] Hugo Westerlund,[2] Loretta Platts,[3] and Lawrence Sacco,[3], *1. Center on Aging & Work at Boston College, Chestnut Hill, Massachusetts, United States, 2. Stockholm University, Stockholm University, Stockholms Lan, Sweden, 3. Stockholm University, Stockholm, Stockholms Lan, Sweden*

This paper uses data from the biennial Swedish Longitudinal Occupational Survey of Health to examine changes in job quality among older workers, controlling for work intensity and employment characteristics. Job quality outcomes included job satisfaction and physical (dangerous, strenuous or unpleasant work) and psychosocial (job strain, effort-reward imbalance, work time control) working conditions. First difference estimation was used to analyze within-individual changes in job quality, as well as changes in hours, employment characteristics (shifting to a non-permanent contract, the private sector and self-employment) and health. Individuals who worked beyond pensionable age experienced statistically significant improvements in job quality, with larger improvements among those who reduced working hours and shifted from permanent to non-permanent contracts, from the public into the private sector, and from wage-and-salary to self-employment. We conclude that work beyond pensionable age is a distinctive period characterized by employment that becomes more flexible and rewarding and less stressful.

### JOB QUALITY IN THE LATE CAREER IN SWEDEN, JAPAN, AND THE UNITED STATES

Loretta Platts,[1] Lawrence Sacco,[1] Ayako Hiyoshi,[2] Kevin Cahill,[3] Stefanie König,[4] and Hugo Westerlund,[5], *1. Stockholm University, Stockholm, Stockholms Lan, Sweden, 2. Örebro University, Örebro, Orebro Lan, Sweden, 3. Center on Aging & Work at Boston College, Chestnut Hill, Massachusetts, United States, 4. University of Gothenburg, Gothenburg, Gotlands Lan, Sweden, 5. Stockholm University, Stockholm University, Stockholms Lan, Sweden*

This paper examines job satisfaction and psychosocial and physical job quality over the late career in three contrasting national settings: Sweden, Japan and the United States. The data come from an ex-post harmonized dataset of individuals aged 50 to 75 years constructed from the biennial Swedish Longitudinal Occupational Survey of Health (SLOSH, 2006–2018, n=13936 to 15520), Japanese Study of Ageing and Retirement (JSTAR, 2006–2013, n=3704) and the United States Health and Retirement Study (HRS, 2006–2016, n=6239 and 8002). The job quality outcomes were physical labour, psychosocial working conditions (time pressure, discretion, pay satisfaction, job security) and job satisfaction. Random effects modelling was performed with age modelled with spline functions in which two knots were placed at ages indicating eligibility for pensions claiming or mandatory retirement. Interestingly, in each country, post-pensionable-age jobs were generally less stressful, freer, and more satisfying than jobs held by younger workers.

### Session 3620 (Symposium)

### PRESIDENTIAL SYMPOSIUM: STORIES OF STRUGGLE AND RESILIENCE: EXPERT INTERDISCIPLINARY REFLECTIONS OF THE PAST YEAR

Chair: Danielle Waldron Co-Chair: Kalisha Bonds Johnson

Are you an ESPO member curious about what the “new normal” means for your future career in the field of aging? Or are you a GSA member interested in hearing from your colleagues about their experiences over the past year? Welcome to the ESPO Presidential Symposium! During this session, speakers will share honest and candid insights about their careers in the field of aging amidst the pandemic, racial discrimination/social unrest, and economic insecurity. Speakers in the ESPO Presidential symposium include: Dr. Thomas K.M Cudjoe, Dr. Candace S. Brown, and Dr. Marnin J. Heisel. Dr. Cudjoe, a physician, will discuss his clinical experience treating older adults with COVID-19, the shift to tele-health, and his research on the impact of social isolation on older adults. Dr. Brown, an academician, will discuss how the new attention to the longstanding issues of social injustice in the U.S. shaped her teaching pedagogy, research, student mentorship, and provide critical context regarding the impact of COVID-19 on Black and Indigenous People of Color (BIPOC) professors. Dr. Heisel, a clinical psychologist, will share how his intervention research on resiliency and well-being in older adulthood shifted amidst the “new normal,” as well as how older adults in his clinical practice encountered and coped with difficulties over this past year. As our society confronts social injustice, tackles health implications of COVID-19, and adjusts to a new way of life, we must consider how these factors, together, inform the interdisciplinary stories of struggle and resilience in the field of aging.

### A PANDEMIC PARADOX: INNOVATIONS IN PSYCHOLOGICAL RESILIENCY AND SUICIDE PREVENTION IN OLDER ADULTS

Marnin Heisel, *Schulich School of Medicine & Dentistry, The University of Western Ontario, London, Ontario, Canada*

The COVID-19 pandemic has had a substantial negative impact on the health and well-being of older adults, a demographic with the highest proportion of fatalities in North America. Long-term care and retirement homes have been especially hard hit. Sheltering in place can increase social isolation among older adults and contribute to feelings of stigmatization, burden, stress, anxiety, anger, and despair. As older adults also account for high rates of suicide, fear of infection, reduced access to professional and social supports, and growing apathy, hopelessness, and social isolation could amplify suicide risk (see Zortea et al., 2020). The speaker will discuss how his program of research on psychological resiliency and suicide prevention in older adults has pivoted online over the past year, and how the pandemic has paradoxically inspired innovative approaches to research, education, clinical practice, and social advocacy for older adults that will likely continue well beyond the present time.

### SOCIAL CONNECTIONS: TRANSFORMATIVE EXPERIENCES AMID DISRUPTIVE TIMES

Thomas Cudjoe, *Johns Hopkins University School of Medicine, Baltimore, Maryland, United States*

Today many older adults are experiencing intensified social isolation and loneliness as they attempt to "stay safe at home." The notion, is a stark contrast from our understanding of the importance of social connections on health and well-being. This session highlights: first hand experiences caring for older adults during the COVID-19 pandemic and the implications of social isolation on the health of older adults. The speaker will offer perspectives for ESPO members on the role of community engagement in orienting research agendas, both now (amid the pandemic) and into the future.

### BLACK, PROUD, SILENT, AND LOUD: EXPERIENCES OF A JUNIOR FACULTY MEMBER IN 2020

Candace Brown, *University of North Carolina, Charlotte, Charlotte, North Carolina, United States*

Several social injustice issues, well known within the Black community, were brought to light to other ethnic/racial groups in 2020 and could no longer be ignored within the academic community. This led to personal, departmental, and institutional initiatives meant to increase racism awareness and apply change in thought and action. These initiatives often came at a cost of personal time and resources to Black and Indigenous People of Color academics, expected to contribute to these initiatives, redefine classroom syllabi, uphold research agendas, and continue with mentoring activities amidst their home environment (due to COVID-19) while monitoring their own feelings of pride, hurt, anger, anxiousness, and often- fatigue. This presentation will present the perceived triumphs and failed experiences of a junior faculty member, how they navigated this process, and explain the continued importance of institutions' forward movement of initiatives meant to change the social and racial academic atmosphere.

### Session 3625 (Symposium)

### REABLEMENT SERVICES ACROSS THE WORLD: EFFECTIVENESS AND IMPACTING FACTORS

Chair: Stan Vluggen Co-Chair: Lise Buma
Discussant: Barbara Resnick

Due to the ageing of the world population, solutions are necessary to reduce the increasing demand for care. Besides the need for more care, older people often wish to remain as independent as possible and retain as much control as possible. A possible solution are services based on the concept of reablement, which includes working in a more rehabilitative and person-centered manner and has been researched in various forms internationally. Reablement services are promising and use the patient's strengths and, through interdisciplinary cooperation, aims to achieve the goals important for, and set by, the individual to remain/become as independent as possible. During this symposium, five presenters from the US, New-Zealand, Norway and the Netherlands talk about the impact and implementation of reablement services. The first presentation is about the results of a systematic review of the effects of reablement on daily functioning and identifying common features of effective interventions. The second presentation is about a systematic scoping review mapping how physical activity strategies are integrated and explored in reablement research and identifying knowledge gaps. The third presentation is about the significant impact of COVID-19 and its associated restrictions on residents in assisted living communities. The fourth presentation is on combining lessons learned and practical implications from research on reablement services into the SELF-intervention. The fifth presentation describes the implications of funding on practice and outcomes of reablement. This symposium represents the current practice and future directions regarding implementation and research of reablement services across the world.

### THE EFFECTIVENESS AND COMMON FEATURES OF REABLEMENT SERVICES ON CLIENTS' DAILY FUNCTIONING: A SYSTEMATIC REVIEW

Stan Vluggen, Sandra Zwakhalen, Gertrudis Kempen, Silke Metzelthin, and Lise Buma, *Maastricht University, Maastricht, Limburg, Netherlands*

This systematic review, guided by the ReAble-definition, (1) provides an overview of reablement interventions and their effect on Activities of Daily Living (ADL), and (2) identifies common features of effective interventions. A systematic search was conducted from 2002 to 2020, identifying nineteen studies from eight countries with a total of 6,534 participants. Ten studies (with moderate to high quality) revealed improvements in ADL functioning. Three common intervention features were identified within effective interventions: use of multidisciplinary teams; a protocoled or standardized assessment; and using multiple components such as ADL-training, education and exercise programs. This review emphasizes that future studies should provide a more consistent and detailed reporting on the intervention and its components. Furthermore, a uniform approach regarding components, follow-up times and outcome measures can contribute to the comparison of reablement interventions and better determine their effectiveness, independent of the healthcare system or country in which it is used.

### INTEGRATION OF PHYSICAL ACTIVITY IN REABLEMENT FOR COMMUNITY-DWELLING OLDER ADULTS: A SYSTEMATIC SCOPING REVIEW

Cathrine Moe,[1] Elissa Burton,[2] Lisbeth Uhrenfeldt,[3] and Hanne Leirbekk Mjøsund,[1], *1. Nord University, Bodø, Nordland, Norway, 2. Curtin University, Perth, Western Australia, Australia, 3. Nord University, Nord University, Bodø, Nordland, Norway*

The aim of this study was to map evidence of how physical activity (PA) strategies are integrated and explored in research of interdisciplinary, time-limited reablement for community dwelling older adults and to identify knowledge gaps. Following an apriori protocol, we searched eight databases for eligible studies, in addition to citation and reference searches. Study selection and data-extraction was made independently by two reviewers. Fifty-one studies were included, showing that exercises and practice of daily activities were included in the majority of intervention studies, but in most cases little information about exercise components or strategies for increasing PA were provided. There was insufficient evidence for any synthesis of how reablement affects older adults' PA levels, their physical fitness or how PA is experienced in reablement. There is a need to further investigate how the promotion of PA can be adequately implemented in reablement and how it may affect older adults' function.

### THE IMPACT OF COVID-19 AND ASSOCIATED RESTRICTIONS ON PHYSICAL ACTIVITY AMONG ASSISTED LIVING RESIDENTS

Barbara Resnick,[1] Rachel McPherson,[2] and Elizabeth Galik,[3], *1. University of Maryland School of Nursing, Baltimore, Maryland, United States, 2. University of Maryland Baltimore and Baltimore County, Catonsvile, Maryland, United States, 3. University of Maryland, Baltimore, Maryland, United States*

COVID-19 and associated restrictions significantly impacted residents in assisted living (AL) communities. This was a descriptive study of 35 AL communities that were participating in an implementation trial of Function Focused Care for Assisted Living Residents with Dementia during the COVID-19 pandemic. Within twelve months of the COVID-19 pandemic, 18% of the AL communities had at least one resident who was positive for COVID-19. Almost half of the ALs allowed health care providers into the setting. All of the ALs facilitated family visits outside and by telephone and technology, but only 11% allowed visitors inside the community. Over 50% stopped using recreational supplies to encourage physical activity and 28% reported that residents experienced more behavioral and psychological symptoms of dementia. Restrictions designed to prevent the spread of COVID-19 may have negatively impacted resident behavior and the AL staff's engagement of residents in physical and recreational activities during the pandemic.

### FUNDING INFLUENCING PRACTICE AND OUTCOMES IN REABLEMENT

Paul Rouse,[1] and Matthew Parsons,[2], *1. University of Auckland, Auckland, Auckland, New Zealand, 2. University of Waikato, Hamilton, Waikato, New Zealand*

Internationally, Home Care is invariably funded through fee-per-service, e.g., if an hour of care is delivered, the provider receives an associated amount of funding. However, the funding model discourages reductions in packages-of-care when a client's functional capacity improves, and further disincentivises providers to discharge clients. Similarly, staff income is often directly associated to the delivered hours-of-care and if a client's hours are reduced, so is their income; again, discouraging the right behaviour, such as reporting improvements in independence levels. In 2008 in New Zealand, we developed a case-mix funding methodology and have been progressively implementing the new model since that time. This presentation highlights the findings in relation to how Home Care service hour allocations titrate against needs following implementation of the model as well as a number of other key quality outcomes that have been observed as a result of the case-mix model.

### FEASIBILITY OF A GENERIC FFC INTERVENTION FOR LONG-TERM CARE: EVIDENCE FROM INTERVENTIONS IN VARIOUS CARE SETTINGS

Silke Metzelthin,[1] Janneke de Man-van Ginkel,[2] Getty Huisman-de Waal,[3] Sandra Zwakhalen,[1] and Stan Vluggen,[1], *1. Maastricht University, Maastricht, Limburg, Netherlands, 2. UMC Utrecht, Utrecht, Utrecht, Netherlands, 3. Radboud University Medical Center, Nijmegen, Gelderland, Netherlands*

Function Focused Care (FFC) interventions support nurses to adapt their level of care to the capabilities of older people and to optimize their self-reliance. Recently, three FFC-interventions were implemented in various Dutch care settings. Lessons learned and implications were synthesized and an advanced FFC-program 'SELF' was developed for wide application. SELF comprises interactive and multidisciplinary sessions, is theoretically grounded, primarily focuses on behavior change in nurses, and is tailored to the team's needs. It also includes policy and environment review, goal-setting, and coaching-on-the-job. SELF was tested in one Dutch psychogeriatric ward. Afterwards, focus groups were conducted with nurses, trainers, manager and coaches. The interactive content, mutual discussions, and practice-based working methods were highly valued. SELF increased awareness and willingness to practice FFC and was considered feasible in practice. Increased involvement and support of allied health professionals and the manager was preferred. A nationwide effectiveness trial is planned after refining SELF.

### Session 3630 (Symposium)

### RURAL RESILIENCE THROUGH COVID-19

Chair: Shannon Freeman Co-Chair: Raven Weaver
Discussant: Shannon Freeman

The effects of the COVID-19 pandemic have been felt globally affecting everyone, but have disproportionately harmed some of the most vulnerable and marginalized including individuals residing in rural and remote areas. The geographic isolation initially thought to protect rural and remote communities from the pandemic soon became a disadvantage, requiring individuals to navigate long-standing systemic barriers (e.g., lack of transportation issues, limited access to healthcare resources, and fragmented accessibility to vaccines), alongside the new challenges posed by COVID-19 restrictions to mitigate the spread of disease. The purpose of this symposium is to showcase examples of rural resiliency in the face of significant struggle. Taking a strength-based approach, the papers discuss efforts to identify healthy coping and positive aspects of physical distancing (Paper 1; Weaver), explore social support and psychological mindset (Paper 2; Fuller), inform successful strategies to pivot programming to remote coalition engagement for obesity prevention (Paper 3; Buys), implement a peer mentoring program to spur development of new strategies to build community resilience (Paper 4; Oh), and review elements of rurality that empower or exclude older people and the implications for a post-COVID world (Paper 5; Curreri). As we continue to uncover and learn about the short and long-term implications of living through the pandemic, these papers describe ways in which rural communities demonstrate resilience in the face of adversity. Our presenters will showcase a range of US and international perspectives and offer policy and program recommendations for building resilience in the longer term.

### ADAPTATION AND COPING AMONG RURAL OLDER ADULTS THROUGHOUT THE COVID-19 PANDEMIC

Andrea Huseth-Zosel,[1] and Heather Fuller,[2] *1. North Dakota State University, Fargo, North Dakota, United States, 2. North Dakota State university, Fargo, North Dakota, United States*

In the past year, older adults have faced challenges due to COVID-19, yet many have also shown great resilience. This qualitative study explores older adults' experiences and perceptions of adaptation, social connection, and coping across the first six months of the COVID-19 pandemic, with a particular focus on unique resilience factors among rural older adults. A Midwestern sample (35% rural) of 70 older adults aged 70-97 completed three phone interviews (April, June, and October 2020) about their experiences with social distancing due to COVID-19. Thematic analysis of qualitative responses identified themes of resilience including: 1) purposeful and flexible social connections, 2) positive psychological mindset, and 3) hardiness and life experience. Strains related to the loss of community connections were evident, yet older adults demonstrated signs of adaptability and coping as compensation. Implications and future directions will be discussed in the context of change over time and geographic variation.

### EXPERIENCES WITH PHYSICAL DISTANCING: COPING STRATEGIES AND POSITIVE EXPERIENCES

Raven Weaver, *Washington State University, Pullman, Washington, United States*

A representative U.S. sample of adults completed an online survey (N=360) about perceived changes in social health and wellbeing since the implementation of physical distancing restrictions in April. Analyses are conducted on a subsample of adults aged 60+ (n=93; m=65.7 years; SD=4.7). Baseline bivariate descriptive analyses showed no geographic-based differences in self-rated health, resilience, perceived financial wellbeing, or family/friend support measures. Content analysis of rural residents' (n=20) responses about coping strategies and positive experiences across three time points (April/July/November) revealed aspects of resilience. Individuals coped via acceptance and planning; engaging in activities; and keeping with routines. Positive experiences were relatively stable over time, with individuals describing improved health habits and enhanced social connectedness with family/neighbors. Individuals identified societal betterment and saving money as unintended benefits of the efforts to mitigate the spread of COVID-19. When faced with adversity, identifying positive experiences may help individuals cope with challenges in the long-term

### COMMUNITY-ENGAGED OLDER ADULT-LED POLICY, SYSTEMS, AND ENVIRONMENT INTERVENTIONS IN THE MS HIGH OBESITY PROGRAM

Masey Smith,[1] Katie Halfacre,[2] Megan Holmes,[2] and David Buys,[3] *1. Mississippi State University, Starkville, Mississippi, United States, 2. Mississippi State University, MS State, Mississippi, United States, 3. Mississippi State University, STARKVILLE, Mississippi, United States*

Older adults in rural areas are at unique risk for poor outcomes due to social isolation and limited access to resources. The Mississippi High Obesity Program (HOP) aims to enhance access to social connections and resources like community gardens, food pantries, and physical activity as part of its broader objective to prevent and reduce obesity. Through policy, systems and environment strategies, development of Memoranda of Understanding (MOUs) between aforementioned entities, and community based participatory research approaches, Mississippi HOP efforts enhance food systems improvement efforts; grow multi-sectoral collaboration; and evaluate the effectiveness of new policies, and specifically MOUs, in reaching these goals. Older adults represent more than 40% (n=27) of all coalition members and stakeholder leaders (n=61); they are essential for the success of these initiatives. This presentation will highlight the work done during the COVID-19 pandemic and the role of and benefits to older adults, especially ones in rural communities.

### PEER MENTORING TO FOSTER RESILIENT AGE-FRIENDLY RURAL COMMUNITIES IN MAINE

Jennifer Crittenden,[1] Laura Lee,[2] and Patricia Oh,[3] *1. University of Maine, University of Maine, Maine, United States, 2. Maine Community Foundation, Ellsworth, Maine, United States, 3. UMaine Center on Aging, Bangor, Maine, United States*

Maine has a growing number of age-friendly community initiatives (AFCIs); 116 communities are actively working to adapt the social, service, and built environments for aging and 71 have formally joined the AARP Network of Age-Friendly States and Communities. During COVID, rural municipalities were faced with dynamic changes that limited older resident's access to services and social engagement. To overcome these limitations, it is critical for emergent AFCIs to have tools and strategies to maintain and further enhance healthy environments and resilient communities. This study uses group interviews with 6 leaders of established AFCIs and 6 leaders of emergent AFCIs to explore how the Lifelong Fellows Program, a peer mentoring model that matches experienced leaders with newly formed initiatives, was able to spur development of new strategies to build community resilience. Prominent themes were (1) engaging new local and regional partners; (2) intergenerational volunteerism; (3) fun and flexibility; and (4) relationship-building.

### FEATURES OF RURAL COMMUNITIES IN LATIN AMERICA AND SUB-SAHARAN AFRICA THAT INFLUENCE WELL-BEING OF OLDER PERSONS

Andrew Banda,[1] Norah Keating,[2] Jaco Hoffman,[3] Jose Parodi,[4] and Nereide Curreri,[5], *1. University of Zambia, Lusaka, Lusaka, Zambia, 2. Swansea University, Swansea, Wales, United Kingdom, 3. North-West University, Vanderbijlpark, Gauteng, South Africa, 4. San Martin de Porres University, Lima, Lima, Peru, 5. North-West University, South Africa, Vanderbijlpark, North-West, South Africa*

In their recent volume, Critical Rural Gerontology, Skinner et al (2021) challenge us to set aside unidimensional notions of rural communities as bypassed vs very supportive; and to identify the elements of rurality that empower or exclude older people and how these differ across cultures and settings. Covid-19 has highlighted the need for safe and inclusive communities. Given that LMIC will be home to the majority of older adults (Gonzales et al. 2015), we undertook a scoping review of features of rural communities that influence wellbeing of older people in countries across Latin America and Sub-Saharan Africa. The review included literature in English, French, Spanish and Portuguese, using search engines MEDLINE, CINAHL Complete, PsycInfo, SocINDEX, SciELO, AJOL (Africa Journals Online), LILACS, Redalyc, LatinIndex and Clacso. Findings illustrate diversity in how community features including remoteness, infrastructure and belonging influence material, social and subjective wellbeing of older residents.

### Session 3635 (Symposium)

### SHIFTING ISSUES OF SUPPORT EXCHANGE UNDER 20-YEAR IMPLEMENTATION OF JAPANESE LONG-TERM CARE INSURANCE PROGRAM

Chair: Tomoko Wakui

Japan has faced numerous issues in the last twenty years with its mandatory long-term care (LTC) insurance program. This LTC insurance program obviously affected older adults' informal support exchanges, reducing support from family and the community, which became more valuable, subjectively. Furthermore, changes in support have impacted older adults' subjective well-being and children's perceived care motivation. Additionally, a mandatory uniform system challenges the issue of tolerance of diversity, meaning how non-traditional families' opinions be involved LTC situations. This symposium discusses unexpected shifting issues in Japan in the implementation of a public LTC program with a focus on older adults' support exchanges. The first paper examines the long-term impacts of formal and informal support by examining the effects of implementing formal services. The second paper assesses a community's role in relation to family in the presence of a public LTC program. The third paper examines the subjective impacts of older parents, who provided support to adult children and their reciprocal expectations of receiving LTC. The fourth paper, on the other hand, articulates reciprocal impacts on sons' care motivation, which has become more important, since the introduction of the LTC program reinforced men's participation in LTC. Finally, the fifth paper clarifies how a public uniform program accommodates informal support from non-traditional families when the program premises the presence of family in advanced care planning. Our findings have long-term implications for aging societies in relation to formal and informal support exchanges.

### TRENDS IN INFORMAL AND FORMAL LONG-TERM CARE USE AMONG OLDER ADULTS WITH DISABILITIES IN JAPAN

Yoko Sugihara,[1] Erika Kobayashi,[2] Taro Fukaya,[3] Jersey Liang,[4] and Hidehiro Sugisawa,[5] *1. Tokyo Meropolitan University, Tokyo, Tokyo, Japan, 2. Tokyo Metropolitan Institute of Gerontology, Itabashi-ku, Tokyo, Japan, 3. Tokyo Metropolitan Institute of Gerontology, Tokyo, Tokyo, Japan, 4. School of Public Health, University of Michigan, Ann Arbor, Michigan, United States, 5. J.F. Oberlin University, Tokyo, Tokyo, Japan*

Whether increased formal long-term care (LTC) reduces informal LTC use by serving as a substitute or has a complementary role that boosts both informal and formal LTC use has been an important issue for evaluating LTC policy effectiveness. We described trends in in-home LTC use among older adults and LTC availability in relation to changes in LTC policy in Japan. In addition, we examined whether these trends differ by living arrangements, gender, income, and disability levels. We used five waves of repeated cross-sectional data starting in 1999 to 2017. The use of both informal and formal LTC types combined increased until 2006 and then gradually decreased while remaining higher than in 1999. Although implementing the LTC program may have temporarily contributed to the complementary use of both LTC types, eligibility limitations brought about by LTC reform potentially reduced the effects of formal LTC's complementary role.

### NEIGHBORHOOD RELATIONSHIP MATTERS FOR WHOM?: INTERACTION WITH FAMILY STRUCTURE AND FUNCTIONAL CONDITIONS

Erika Kobayashi,[1] and Ikuko Sugawara,[2] *1. Tokyo Metropolitan Institute of Gerontology, Itabashi-ku, Tokyo, Japan, 2. Bunri University of Hospitality, Suginami-ku, Tokyo, Japan*

Living environment is considered to have unignorable effect on our health and well-being, especially when we face shrinkage of mobility as we age. Social interaction with neighbors constitutes our social environment, and it may affect our well-being by interacting with other social resources such as support from family and professional care providers. In this study we examined the effect of social environment in neighborhood on older people's well-being, and how its effect is moderated by their family structure, functional conditions, and LTC service usage. Data was obtained from the survey conducted in 2012 with nationally representative sample of Japanese adults aged 60 years and older. The results showed that for people with functional limitation but were not certified as needing LTC, neighborhood social network was positively associated with well-being. These results suggest the unique function of neighbors for frail people to keep everyday life in the community.

### WHEN DOES SUPPORT TO ADULT CHILDREN NEGATIVELY AFFECT THE SUBJECTIVE WELL-BEING OF OLDER JAPANESE?

Erika Kobayashi, *Tokyo Metropolitan Institute of Gerontology, Itabashi-ku, Tokyo, Japan*

Providing time and money to adult children may enhance perceived usefulness and consequently the subjective well-being (SWB) of older parents. However, non-reciprocal relationships with children and conflicts with leisure activities could negatively affect parents' SWB. It was hypothesized that a substantial amount of support to children would be associated with lower SWB when older parents (a) had a low expectation of receiving long-term care from the children, and (b) were engaged in hobbies/learning activities. Life satisfaction and depressive symptoms measured as SWB were predicted based on the Generalized Estimating Equations, using panel data (2012-2017) with a nationwide representative sample of Japanese adults aged 60 years and older (1,212 parents). Providing child-rearing support (i.e., grandchild care) of 30 hours or more per month was positively associated with SWB regardless of conditions (a) and (b).

Hypothesis (b) was partially supported: providing financial support enhanced depressive symptoms among older adults with hobbies/learning.

### ROLE OF EMOTIONAL ATTACHMENT AND RECIPROCITY IN SONS' PERCEIVED CARE MOTIVATION FOR PARENTAL FIGURES

Ryo Hirayama,[1] Ichiro Kai,[2] and Tomoko Wakui,[3]
*1. Osaka City University, Osaka, Osaka, Japan, 2. The University of Tokyo, Tokyo, Tokyo, Japan, 3. Tokyo Metropolitan Institute of Gerontology, Tokyo, Tokyo, Japan*

The collapse of the traditional Japanese household system and the subsequent social advancement of women has led supporting parents as a family matter, and led more men to assume caregiving roles; however, very few studies have focused on sons' care motivation. This study aimed to understand adult sons' perceived care motivation and to examine the respective related factors of emotional attachment and reciprocity. A total of 1322 men (M [age] = 44.5) participated in a web-based questionnaire survey. Perceived care motivation for providing five types of support (e.g., helping with daily activities and housework) to each parent and parent-in-law was assessed. Regression analyses revealed that emotional attachment with parents and parents-in-law predicted perceived care motivation for all types of support. Furthermore, the role of reciprocity was indicated by the association between rearing by mother-in-law and son-in-law's motivation to provide assistance in financial matters, housework, and visiting a hospital.

### WHO SHOULD MAKE CARE ARRANGEMENT FOR OLDER ADULTS? HETERONORMATIVE FAMILY RESPONSIBILITY IN JAPAN

Ryo Hirayama, *Osaka City University, Osaka, Osaka, Japan*

In Japan, despite the greater availability of public care services upon implementation of national long-term care insurance, families are still considered as primarily responsible to make care arrangement for older adults. My aim in this study was to explore (hetero)normative ideas about families that underlie Japan's institutionalized practices of elder care. In doing so, I focused on care managers, who are certified care practitioners helping families to make care arrangement, and whether they would count older adults' same-sex partners as legitimate family members to participate in such arrangement. Data were collected from 1,580 care managers working for officially designated in-home care providers across the nation. Preliminary analysis revealed that although most care managers believed the voices of same-sex partners should be preferably reflected in the process of care arrangement, they also thought that these partners could not participate in such process without permission from older adult's "blood relatives" (e.g., siblings).

### Session 3640 (Symposium)

### SOCIAL DETERMINANTS OF MEN'S HEALTH ACROSS THE LIFE COURSE

Chair: Roland Thorpe, Jr. Discussant: Keith Whitfield

There is a paucity of research focusing on the complex interaction between social, behavioral, biological, and psychosocial factors, and health outcomes among men. This symposium contains a collection of papers that discuss some key social determinants of health (SDOH) that can provide insights to advance our understanding of men's health and aging across the life course. Dawn will discuss the Stroke Counseling for Risk Reduction (SCORRE) intervention designed to increase awareness, risk perceptions, and health behaviors to reduce stroke risk in African Americans. Findings suggest tailoring the intervention to the needs and preferences of young African American men. Archibald and colleagues seek to determine if race differences in allostatic load (AL) among adult men vary by age. Black men 45-64 had a higher AL score (PR = 1.14, 95% CI 1.02, 1.28) than White men. Skipper and colleagues used a grounded theory approach to examine the negative interactions of 35 religious middle and old age Black men. Analyses reveal that church-related negative interactions broadly fall within the following themes: (1) ageism within intergenerational churches, (2) people are messy, and (3) issues with leadership. Bruce and colleagues examine the association between religious service attendance and mortality among Black men. Participants who attended at least once per week were 18% less likely to die than their peers who did not attend a religious service at all (HR 0.82; 95% CI 0.68-0.99). These presentations collectively will bolster our knowledge on key SDOH among men across the life course.

### TAILORING STROKE COUNSELING FOR RISK REDUCTION INTERVENTION TO AFRICAN AMERICAN MEN

J. Taylor Harden,[1] Laura Salazar,[2] Gayenell Magwood,[3] Patricia Clark,[2] and Dawn Aycock,[2], *1. University of Texas at Austin, Austin, Texas, United States, 2. Georgia State University, Atlanta, Georgia, United States, 3. Medical University of South Carolina, Charleston, South Carolina, United States*

Early life course achievement and maintenance of ideal cardiovascular health is associated with reduced risk of developing stroke later in life. The Stroke Counseling for Risk Reduction (SCORRE) intervention is an age-and-culturally relevant intervention originally designed to correct inaccurate stroke risk perceptions and improve lifestyle behaviors to reduce stroke risk in AAs age 20-35. In a study testing SCORRE, fewer men participated, but most were not at a stage of readiness for behavior change; many did not think they were at risk despite averaging three modifiable risk factors, and while improvements in outcomes were observed in women they were not in men. These differences led to tailoring SCORRE to young AA men. The methods for tailoring SCORRE and resulting strategies for attracting, engaging, and empowering them towards stroke risk reduction, including hypotheses concerning food supply, housing, economic and social relationships, education, and mental health care will be raised for discussion.

### IT'S NOT HEALTHY FELLOWSHIP: NEGATIVE INTERACTIONS AND THE IMPLICATIONS FOR MIDDLE- AND OLD-AGE BLACK MEN

Loren Marks,[1] Cassandra Chaney,[2] and Antonius Skipper,[3],
*1. Brigham Young University, Provo, Utah, United States, 2. Louisiana State University, Baton Rouge, Louisiana, United States, 3. Georgia State University, Atlanta, Georgia, United States*

Despite the benefits of social support on the well-being of Black men across the life course, scholars are more closely examining the potentially negative outcomes associated with some social networks. As one social support system, the Black church frequently serves middle and old age Black men who identify as religiously involved. Yet, higher levels of religious involvement have also been associated with more church-related negative interactions. The present study utilizes a grounded theory approach to examine the negative interactions of religious middle and old age Black men. A semi-structured interview protocol is used to gather data from 35 Black men between the ages of 45 and 76. Analyses reveal that church-related negative interactions broadly fall within the following themes: (1) Ageism Within Intergenerational Churches, (2) People are Messy, and (3) Issues with Leadership. Since negative interactions can be more detrimental than social support is beneficial, health-related implications are discussed.

### RACE DIFFERENCES IN ALLOSTATIC LOAD AMONG BLACK AND WHITE MEN: DOES AGE MATTER?

Sarah Hill,[1] Hossein Zare,[2] Marino Bruce,[3] Keith Norris,[4] Keith Whitfield,[5] Roland Thorpe, Jr.,[6] and Paul Archibald,[7], *1. Johns Hopkins School of Medicine, Baltimore, Maryland, United States, 2. John Hopkins Bloomberg School of Public Health, Baltimore, Maryland, United States, 3. University of Mississippi Medical Center, Jackson, Mississippi, United States, 4. University of California Los Angeles, Los Angeles, California, United States, 5. University of Nevada Las Vegas, Las Vegas, Nevada, United States, 6. Johns Hopkins Bloomberg School of Public Health, baltimore, Maryland, United States, 7. CUNY, Staten Island, New York, United States*

Although Black-White disparities in health and mortality among men persist, there has been a paucity of work focusing on race differences in physiological dysregulation of biological processes resulting from the cumulative impact of stressors among men. The purpose of this study was to assess potential race differences in Allostatic Load (AL) among adult men and if such differences varied by age. Data were drawn from the 1999-2016 NHANES and the study population included 21,529 non-Hispanic Black (NHB) and 34,282 Non-Hispanic White (NHW) born in US. Adjusting for potential confounders, NHB men 25-44 and 45-64 had a higher AL score (OR = 1.19, 95% confidence interval (CI) 1.00, 1.42) and (OR = 1.14, 95% confidence interval (CI) 1.02, 1.28) NHW men. No race differences with respect to AL score were observed among the other age groups. The results suggest that age plays a role in race differences in AL

### RELIGIOUS SERVICE ATTENDANCE, ALLOSTATIC LOAD, AND MORTALITY AMONG BLACK MEN

Bettina Beech,[1] Dulcie Kermah,[2] Gillian Marshall,[3] Paul Archibald,[4] Genee Smith,[5] Roland Thorpe, Jr.,[6] and Marino Bruce,[7], *1. University of houston, Houston, Texas, United States, 2. Charles R. Drew University of Medicine and Science, Los Angeles, California, United States, 3. University of Washington, Tacoma, Washington, United States, 4. CUNY, Staten Island, New York, United States, 5. Johns Hopkins Bloomberg School of Public Health, Baltimore, Maryland, United States, 6. Johns Hopkins Bloomberg School of Public Health, baltimore, Maryland, United States, 7. University of Mississippi Medical Center, Jackson, Mississippi, United States*

Black men experience high levels of social and psychological stress and religion has been a coping strategy. The purpose of this study was to examine the association between religious service attendance and mortality among Black men. Data were drawn from the NHANES III (1988-1994) sample linked to the 2015 public use Mortality File. The analytic sample (n=2300) was restricted to Black men. All-cause mortality was the primary outcome and religious service attendance was the primary independent variable. Findings from Cox proportional hazards models indicated participants who attended at least once per week were 18% less likely to die than their peers who did not attend a religious service at all (fully adjusted HR 0.82; CI 0.68-0.99). The robust association between religious service attendance and mortality among Black men suggest that prospective studies are needed to further examine the influence of religion on health among this population.

### Session 3645 (Symposium)

### SOCIAL DETERMINATES OF HEALTH AMONG OLDER ADULTS LIVING WITH HIV/AIDS

Chair: Erin Robinson Co-Chair: Tonya Taylor

In the United States (U.S.), people aged 55 years and older make up 36% of people living with HIV/AIDS (PLWHA). Nearly 20% of new HIV infections occur among people aged 50+. While medical breakthroughs in HIV treatment have allowed PLWHA to live longer, healthier lives, structural conditions still exist that affect health outcomes of older adults living with HIV/AIDS (OALWHA). These conditions continue to disproportionately burden OALWHA, particularly older adults of color. Therefore, a greater understanding of the social determinates of health (SDH) is essential to continue making progress in HIV treatment, maintenance, and prevention. The U.S. Centers for Disease Control and Prevention (CDC) has highlighted several SDH among OALWHA, including: poverty, education, income, employment status, health insurance coverage, and housing. This symposium will highlight emerging research that examines several of these indicators among OALWHA. Using a variety of research methodologies, the five abstracts included in this symposium aim to address: 1) psychosocial risk factors of quality of life; 2) life instability and mental health; 3) institutional barriers and facilitators of successful aging; 4) determinants of engaging in advance care planning; and 5) a needs assessment of OALWHA, with particular emphasis on SDH. Results from this research identify several priority areas (such as housing instability, mental health, food insecurity, and isolation) for healthcare leaders to consider in targeting future policy, programming, and funding. Future initiatives are essential to help continue the progress in HIV/AIDS treatment and prevention, including addressing SDH among the aging population living with HIV/AIDS.

### PSYCHOSOCIAL RISK FACTORS OF QUALITY OF LIFE OUTCOMES AMONG OLDER ADULTS LIVING WITH HIV

Monique Brown, *University of South Carolina, Columbia, South Carolina, United States*

Antiretroviral therapy, higher education, and HIV disclosure have been linked to improved quality of life (QoL) among people living with HIV. However, research examining psychosocial risk factors of QoL among older adults living with HIV (OALH) is lacking. Therefore, the main aim of this study was to examine the psychosocial risk factors of QoL among OALH. Data were obtained from 156 adults aged 50 and older living with HIV in South Carolina. Multivariable regression models adjusting for sociodemographic characteristics were used to determine the association between psychosocial risk factors and QoL domains among OALH. Stigma was associated with the physical (β=0.058, p=0.023), social (β=-0.149, p=0.006), and spiritual (β=0.124, p=<0.001) domains. Resilience was associated with the psychological (β=0.206, p=<0.001), independence (β=0.100, p=0.010), social (β=0.166, p=0.004), and environmental (β=0.312 p=<0.001) domains. Depression and experiencing trauma were also associated with varying QoL domains. Findings may inform interventions geared towards improving QoL among OALH.

### EXPLORING LIFE INSTABILITY'S RELATIONSHIP TO THE MENTAL HEALTH OF OLDER ADULTS WITH HIV

Audrey Harkness,[1] Gail Ironson,[2] Cho-Hee Shrader,[1] Dustin Duncan,[3] Steven Safren,[2] and Elliott Weinstein,[4], *1. Department of Public Health Sciences, University of Miami, Florida, United States, 2. Department of Psychology, University of Miami, Florida, United States, 3. Mailman School of Public Health, Columbia University, New York, United States, 4. Psychology, Miami, Florida, United States*

The study is one of the first to examine both the prevalence of life instability among older adults with HIV (OAWH) in a community clinic and its relationship to their mental health. OAWH (N=623) from a community medical clinic completed an interviewer-administered assessment (English/Spanish) which included an additive Life Instability Index (LII) composed of indicators at the individual (e.g. education, housing instability, employment status) and community (e.g. poverty, transportation) levels. Participants were a mean age of 60 years (SD = 5.90) with the majority identifying as Black-non-Hispanic (65.9%), cisgender male (60.8%), and heterosexual (80.6%). Participants reported an average of 6.08 destabilizing factors (SD = 1.44). In multiple linear regression analyses LII was significantly related to increased substance use among participants (b= 0.08, p < 0.01), but not with anxiety or depression. An LII is an innovative approach to assess the relationship between OAWH's mental health and social determinants of health.

### PERCEPTIONS OF SOCIAL DETERMINANTS OF SUCCESSFUL AGING AMONG OLDER MEN LIVING WITH HIV

David Moore,[1] Dilip Jeste,[2] Marcia Holstad,[3] and Anna Rubtsova,[4], *1. University of California, San Diego, San Diego, California, United States, 2. University of California San Diego, La Jolla, California, United States, 3. Emory University, Atlanta, Georgia, United States, 4. Emory University Rollins School of Public Health, Emory University, Georgia, United States*

The overall purpose of this qualitative study was to examine barriers and facilitators of successful aging among older men living with HIV (OMLH). Participants were recruited through HIV Neurobehavioral Research Program at the University of California, San Diego. Our sample included 14 OMLH: average age - 62 years old (range: 53 to 72), 79% white, 43% living alone, 79% men who have sex with men, 57% having college education or higher. Semi-structured interviews lasted from 43 to 114 minutes and were fully transcribed. Several themes emerged related to perceived barriers to successful aging stemming from social institutions: i.e., age discrimination and ageism, sexual and HIV-related stigma, social isolation, lack of resources, and food insecurity. Perceived institutional solutions promoting successful aging included mixed-age/inter-generational support groups, computer literacy training, health education, information and resources related to healthy lifestyle on a limited budget, and increased transparency of resources available to older adults.

### BARRIERS AND FACILITATORS TO ADVANCE CARE PANNING AMONG VETERANS AGING WITH HIV

Sean Halpin,[1] Vincent Marconi,[2] Amy Justice,[3] Theodore Johnson II,[4] D. Keith McInnes,[5] Molly Perkins,[6] and Emily Pinto Taylor,[4], *1. University of Georgia, Decatur, Georgia, United States, 2. Division of Infectious Diseases, Emory University School of Medicine, Atlanta, Georgia, United States, 3. West Haven VA Medical Center, West Haven, Connecticut, United States, 4. Emory University School of Medicine, Atlanta, Georgia, United States, 5. Boston University School of Public Health, Boston, Massachusetts, United States, 6. Emory University of Medicine, Atlanta, Georgia, United States*

Advance care planning (ACP) and hospice services are underutilized by patients living with HIV (PWH). Little is known about how older PWH approach ACP; the purpose of this qualitative study was to understand barriers and facilitators to ACP within the context of the patient-clinician relationship. Data are from a larger multimethod study designed to understand social determinants of health (SDH) that shape the lives and healthcare experiences of veterans aging with HIV. The sample includes 25 veterans from the Veterans Aging Cohort Study (VACS) recruited from an urban VA medical center. Semi-structured interviews were performed and analyzed using thematic analysis. Less than half of participants reported engaging in ACP. Key barriers to ACP include: fragile social ties, distrust of the healthcare system, and fear of disclosure and discrimination. We offer several recommendations for clinicians to engage in these conversations successfully and highlight the importance of considering SDH when designing interventions.

### SOCIAL DETERMINANTS OF HEALTH AMONG THOSE WITH AND WITHOUT HIV INFECTION IN NYC, THE EPICENTER OF THE U.S. CRISIS

Tonya Taylor, *SUNY Downstate Health Sciences University, Brooklyn, New York, United States*

The COVID-19 pandemic in NYC, the epicenter of the US crisis, revealed indisputable evidence that social determinants of health (SDoH, e.g., racism, crowded housing, employment risks) and disparities in comorbid health risk factors produce higher burdens of disease and death among racial and ethnic populations. We conducted a needs assessment of SDoH

among 1400 patients in several ambulatory care clinics to explore the impact among older adults, across different clinical populations. Among older adults with HIV (OAH), we found lower rates of food and housing insecurity compared to older adults without HIV. Despite higher levels of COVID knowledge and prevention adherence, we also found significantly higher levels of isolation, loneliness, depressive symptoms, and anxiety among OAHs compared to those without HIV. Access to Ryan White entitlements did buffer some impacts but preexisting high burdens of mental health issues were exacerbated, perhaps due to heightened perceptions of increased vulnerability to COVID-19.

### Session 3650 (Symposium)

### TECHNOLOGY IS THE NEW NORMAL FOR INDIVIDUALS AGING-IN-PLACE WITH DISABILITIES

Chair: Travis Kadylak Discussant: Susy Stark

Technology designers often exclude individuals aging with diverse needs, capabilities, and disabilities from engaging in the design process, which can hinder the usability and usefulness of emerging technologies. In this symposium, investigators report on research and development efforts aimed at understanding the needs of, and developing supportive technologies for, people aging with long-term disabilities. This symposium features projects from the Rehabilitation Engineering Research Center on Technologies to Support Aging-in-Place for People with Long-Term Disabilities (RERC TechSAge), which is an interdisciplinary collaboration between Georgia Tech and the University of Illinois at Urbana-Champaign. First, Bayles et al. will discuss findings from the Aging Concerns, Challenges, and Everyday Solution Strategies II study, focused on Deaf older adults' use of technologies as solution strategies for common everyday challenges. Next, Mitzner et al. will highlight the development of an evidence-based group exercise intervention (Tellewellness Tai Chi for Arthritis) aimed at promoting both physical exercise and social interaction for older adults with long-term mobility disabilities. Kadylak et al. will describe how voice-activated digital assistants can support older adults aging with mobility disabilities by reporting on findings from a longitudinal demonstration project with older adults in assisted and independent living communities. Exploring the potential for smartbathroom technology to promote aging in place, Sanford et al. will discuss how smartbathroom sensor data can be analyzed and vizualized to identify ways to communicate insight from sensor data to improve training of occupational therapy practitioners. Susy Stark from Washington University will serve as the discussant for the symposium.

### TECHNOLOGY SOLUTIONS FOR EVERYDAY BARRIERS AMONG DEAF OLDER ADULTS

Lyndsie Koon,[1] Shraddha Shende,[2] Wendy Rogers,[2] Jenny Singleton,[3] and Megan Bayles,[2], *1. University of Kansas, Lawrence, Kansas, United States, 2. University of Illinois Urbana-Champaign, Champaign, Illinois, United States, 3. University of Texas at Austin, Austin, Texas, United States*

American Sign Language (ASL) is the primary form of communication for approximately 250,000 people in the U.S. (Mitchell et al., 2006). As these individuals age, they may experience challenges in their everyday activities. For example, ASL users rely on visual cues, but have age-related change in vision. Moreover, ASL users may need to utilize technology to communicate with non-ASL users, but the technology may not be suitable/usable for older adults. We explored these issues in the Aging Concerns, Challenges, and Everyday Solution Strategies (ACCESS) study, wherein we interviewed Deaf older adults (N=60) in ASL, who provided insights into unique, everyday challenges they encounter. We will focus on the technology solution strategies they incorporate to address and overcome challenges with daily activities. Understanding how participants think about, adapt, and utilize different technologies can inform future technology design to successfully support diverse, aging populations.

### DELIVERING A TAI CHI INTERVENTION TO ADULTS AGING WITH MOBILITY DISABILITIES USING ZOOM

Elena Remillard,[1] Kara Cohen,[2] Lelah Cochran,[2] and Tracy Mitzner,[3], *1. Georgia Institute Of Technology, Atlanta, Georgia, United States, 2. Georgia Institute of Technology, Atlanta, Georgia, United States, 3. Georgia Institute of technology, Atlanta, Georgia, United States*

Many individuals aging with mobility disabilities experience barriers to participating in physical activity, including transportation challenges and the need for specialized instruction. Since the COVID-19 pandemic began, these participation barriers have been amplified due to lockdowns and restrictions. Tele-technologies, including videoconferencing platforms like Zoom, can facilitate access to exercise classes from one's home. Virtual group exercise classes that incorporate social interaction have particular potential to support the physical and mental health of this population. This session will highlight lessons learned from launching the 'Tele Tai Chi' study, in which we are delivering an evidence-based Tai Chi program (Tai Chi for Arthritis) via Zoom to small group classes of older adults with long-term mobility disabilities. We will describe adaptations made in translating the in-person program to an interactive, online class, and provide an overview of a 'Telewellness' Tool that provides guidelines for using Zoom to deliver exercise classes to older adults.

### EXPLORING USE OF DIGITAL HOME ASSISTANTS FOR OLDER ADULTS: A DEMONSTRATION PROJECT

Maya Malecki,[1] Leonardo Galoso,[1] Saahithya Gowrishankar,[2] Amy Brown,[3] Ramavarapu Sreenivas,[2] Wendy Rogers,[1] and Travis Kadylak,[4], *1. University of Illinois Urbana-Champaign, Champaign, Illinois, United States, 2. University of Illinois at Urbana-Champaign, Champaign, Illinois, United States, 3. CRIS Healthy Aging Center, Danville, Illinois, United States, 4. University of Illinois Urbana Champaign, Champaign, Illinois, United States*

Emerging digital home assistant technology has potential to support older adults in their homes. Voice-activated assistants can be used for entertainment, environmental control, physical activities, health management, and social engagement. However, many older adults have limited experience with these devices, which are not designed with them in mind. We conducted a demonstration project to explore how seven older adult assisted and independent living residents interacted with digital assistants over four months. We conducted monthly semi-structured telephone interviews and pre/post questionnaires. Participants desired to use their devices to communicate with others, and for a range of health activities, including nutrition tracking, medication management, and health information searching. However, numerous usability barriers emerged. Some participants perceived their device as a social companion. These findings indicate that older adults are willing to use digital assistants for various activities that may enhance independence, although instructional and training materials are needed to support their use.

### SMARTBATHROOM DATA VISUALIZATION TOOL TO INFORM OT CLINICAL REASONING

Avinandan Basu,[1] Yangyi Xu,[2] and Jon Sanford,[1] *1. Georgia Tech, Georgia Tech/Atlanta, Georgia, United States, 2. Georgia Tech, GA Tech/Atlanta, Georgia, United States*

Traditionally, Occupational Therapy assessment of an older adult's toilet transfer performance has been based on qualitative observation and client self-report. The purpose of this study was to evaluate the effectiveness of supplementing traditional clinical reasoning with quantitative transfer performance data about body and foot position, balance, hand placement and grasping forces on grab bars. Specifically, we conducted an online survey of occupational therapy practitioners and educators to assess the usefulness and usability of 2D and 3D graphic visualizations representing foot and hand position and forces exerted on the floor, toilet seat and grab bars. These data were captured by sensors located throughout GA Tech's SmartBathroom laboratory during a study of transfer performance. Findings are being used to identify the most useful sensor data and the most effective ways to convey that data to improve training of occupational therapy students.

### Session 3655 (Symposium)

### TECHNOLOGY TO SUPPORT SOCIAL, HEALTH, AND WELL-BEING OUTCOMES AMONG OLDER ADULTS

Chair: Walter Boot

In response to the COVID-19 pandemic, information and communication technologies (ICTs) are primarily how many people communicate, socialize, and receive healthcare. In a recent Pew report, experts in the role of technology in society believe that post-COVID-19 pandemic, society will continue to be far more technology-driven than pre-pandemic. That is, technology will play an even greater role in our lives in the "new normal." However, compared to younger adults, many older adults are less likely to adopt the technologies needed to perform these everyday tasks. Differences in technology proficiency, acceptance, and adoption between groups is often referred to as the "digital divide," and older adults are more likely to be on the disadvantaged side of this digital divide. This session explores the potential of technology to support social, health, and wellbeing outcomes among older adults, and the challenges involved. This session will start with a talk by A. Lothary on the success and challenges of using a simple video chat platform to address loneliness and social isolation. S. Shende will present a video-technology intervention for older adults with and without cognitive impairment, and how this intervention was designed to facilitate engagement. This will be followed by a presentation by X. Lin on the relationship between social media usage and well-being across the lifespan, and mediators of this relationship. The session will conclude with a presentation by W. Qin on predictors of older adults' use of telehealth technology to support health and wellbeing during the COVID-19 pandemic.

### VIDEO CHAT TECHNOLOGY TO SUPPORT HOME AND COMMUNITY-BASED ORGANIZATIONS

Brielle Ross,[1] Allura Lothary,[2] Dillon Myer,[3] Raksha Mudar,[4] Wendy Rogers,[1] and Madina Khamzina,[1], *1. University of Illinois Urbana-Champaign, Champaign, Illinois, United States, 2. University of Illinois Urbana-Champaign, Urbana, Illinois, United States, 3. OneClick. Chat, Philadelphia, Pennsylvania, United States, 4. University of Illinois-Urbana Champaign, Champaign, Illinois, United States*

Concerns about loneliness and social isolation for older adults were already evident but have been exacerbated during the pandemic. Home and Community Based Organizations (HCBOs) provide support for their older clients in the community and need to support their staff, who may be working remotely. We are exploring the potential of video chat technology to connect older adults with their friends, families, and other support. We review the technologies available to older adults in the community and staff working with older adults to promote social engagement. We are collaborating with OneClick.chat to identify the needs of the HCBOs through a literature review and qualitative interviews of staff members from different senior living environments. Their challenges and successes of engaging older adults through video chat technologies will provide guidance for design of an HCBO dashboard for OneClick.chat that will support diverse needs.

### CONTENT DEVELOPMENT FOR A VIRTUAL SOCIAL ENGAGEMENT INTERVENTION

Allura Lothary,[1] Justine King,[2] Sarah Jones,[3] Raksha Mudar,[3] Dillon Myers,[4] Wendy Rogers,[5] and Shraddha Shende,[5] *1. University of Illinois Urbana-Champaign, Urbana, Illinois, United States, 2. iN 2. L, Greenwood Village, Colorado, United States, 3. University of Illinois-Urbana Champaign, Champaign, Illinois, United States, 4. OneClick.chat, Philadelphia, Pennsylvania, United States, 5. University of Illinois Urbana-Champaign, Champaign, Illinois, United States*

Video technology has the potential to provide older adults with socially and cognitively engaging activities for in-home participation. We are exploring use of OneClick.chat, a video technology platform, to present older adults with and without mild cognitive impairment opportunities for engagement. In collaboration with iN2L we have developed events that will facilitate conversations that do not rely on episodic memory, cover a range of topics, and represent different cultures and interests. We selected event topics that were positive, socially and cognitively engaging, and included a range of pictures based on our previous research. Events were carefully controlled for length of presentation, picture type, and readability. Discussion questions related to the events were designed to stimulate engaging conversations through open-ended questions and to not burden memory recall or enforce stereotypes. Our work highlights potential future avenues for researchers and home and community-based organizations to use technology to promote social engagement.

### THE RELATIONSHIP BETWEEN SOCIAL MEDIA USE AND WELL-BEING: THE MEDIATING ROLE OF SOCIAL SUPPORT

Margie Lachman,[1] and Xin Yao Lin,[2] *1. Brandeis University, Brandeis University, Massachusetts, United States, 2. Brandeis University, Waltham, Massachusetts, United States*

Frequent social media usage can have negative effects on well-being, but the mechanisms involved are unclear. This study explored the mediating role of giving and receiving support. Using the Midlife in the United States Refresher eight-day daily diary study (N=782, age 25-75), multilevel structural equation modeling examined the hypothesized relationships at both the within- (intraindividual) and between-person (interindividual) levels. Results showed that at the within-person level, days with more social media use were associated with a larger proportion of time giving support and worse well-being (less positive affect and more stress, negative affect, and loneliness). At the between-person level, more social media use was associated with worse well-being. Giving support, but not receiving support, mediated the relationship between social media use and well-being at the within, but not the between-person level. Discussion focuses on ways to address the negative consequences of social media use related to social connections and well-being.

### ADOPTION OF TELEHEALTH AMONG OLDER ADULTS DURING THE COVID-19 PANDEMIC

Weidi Qin, *Case Western Reserve University, Cleveland, Ohio, United States*

The COVID-19 pandemic has disrupted older adults' in-person healthcare services. Many individuals rely on remote communication with their healthcare providers for non-urgent health or mental health issues. The present study investigated the effects of technology learning and depressive symptoms on new adoption of telehealth (e.g. online messaging, video call) to communicate with healthcare providers during the COVID-19 pandemic. A sample of 1,500 Medicare beneficiaries aged 65 or older was selected from the National Health and Aging Trend Study. A series of logistic regressions were performed. Results showed that older adults who learned a new online technology during the COVID-19 outbreak were more likely to adopt telehealth. Also, older adults with a higher level of depressive symptoms were more likely to start using telehealth. The findings highlight the importance of technology training to help older adults go online. Telehealth can be an important coping tool for depressive symptoms during the pandemic.

### Session 3660 (Symposium)

### TRAUMATIC EVENTS AND HEALTH: AN ECOLOGICAL AND LIFE COURSE PERSPECTIVE

Chair: XinQi Dong

During the past decades, researchers have shown an increasing interest in the study of traumatic events among aging populations. The majority of studies on trauma focus on mental health, which overlooks the possibility that trauma may also have an adverse effect on other health outcomes, such as cognitive function. A number of studies focus on a single traumatic event. However, this approach may underestimate its health impact as many people experience multiple forms of traumatic events. Indeed, the impact of traumatic events on health depends on the event itself (e.g., single or multiple forms, time) as well as ecological factors. This symposium aims to address the above limitations. The first longitudinal study An Ecological Model of Risk Factors in Elder Mistreatment (EM) Victims tested different dimensions of the ecological model to prevent recurrence of EM. The second study Polyvictimization and Cognitive Function in an Ethnic Minority Aging Population explored whether exposure to multiple forms of EM affects cognitive function. The third study Traumatic Events and Cognitive Function: Does Time Matter? examined whether traumatic events happened in childhood, adulthood, or old age will influence late-life cognitive function. The fourth study Face-saving and Help-seeking among Older Adults with EM identified cultural determinants of help-seeking behaviors in EM victims. This symposium will advance knowledge in the health consequences of polyvictimization and exposure to traumatic events in different life stages. It will also inform interventions to stop the recurrence of EM in immigrant families and enhance the help-seeking behaviors of ethnic minority older adults.

### POLYVICTIMIZATION AND COGNITIVE FUNCTION IN AN ETHNIC MINORITY AGING POPULATION

XinQi Dong,[1] and Mengting Li,[2] *1. Rutgers University, Rutgers Institute for Health, New Jersey, United States, 2. Rutgers, The State University of New Jersey, New Brunswick, New Jersey, United States*

Globally, around 1 in 6 older adults experienced some form of elder mistreatment in community settings. However, little is known about the prevalence of polyvictimization, or experience of multiple forms of abuse, which may exacerbate negative outcomes over that of any one form of victimization in isolation. Data were drawn from the PINE study. Polyvictimization was defined as exposure to multiple forms of victimization, including psychological, physical, and sexual mistreatment, financial exploitation, and caregiver neglect. Cognitive function was evaluated by global cognition, episodic memory, executive function, working memory, and MMSE. Regression analyses were performed. Among 3153 participants, 128 experienced two forms of abuse while 12 experienced three or more forms of abuse. Polyvictimization was associated with lower global cognition (b=-0.05, SE=0.02, p<.05), episodic memory (b=-0.06, SE=0.03, p<.05), working memory (b=-0.14, SE=0.07, p<.05), and processing speed (b=-0.68, SE=0.33, p<.05). Interventions could target older adults with polyvictimization and protect their cognitive function.

### AN ECOLOGICAL MODEL OF RISK FACTORS IN OLDER ADULTS WITH REPEATED EXPOSURE TO ELDER MISTREATMENT

Mengting Li,[1] XinQi Dong,[2] and Qun Le,[3] *1. Rutgers, The State University of New Jersey, New Brunswick, New Jersey,*

United States, 2. Rutgers University, Rutgers Institute for Health, New Jersey, United States, 3. Rutgers University, New Brunswick, New Jersey, United States

Limited empirical studies examined the factors related to repeated EM exposures among Chinese older immigrants. Guided by the ecological model, this study aims to explore what are the risk factors leading to recurrence of EM. Data were drawn from the two-wave PINE Study with 725 participants having EM at baseline and 191 reported repeated EM after two years. EM was evaluated by a 66-item instrument, including psychological, physical, and sexual mistreatment, financial exploitation, and caregiver neglect. Logistic regression was used. Increasing financial independence was associated with lower possibility of repeated EM (OR: 0.72, 95%CI 0.56-0.92). Every one unit increase in ADL impairment (OR: 1.10, 95%CI 1.02-1.18), IADL impairment (OR: 1.09, 95%CI 1.05-1.13) and increase frequency of alcohol consumption (OR: 1.33, 95%CI 1.06-1.66) were associated with higher possibility of repeated EM. Social service could improve physical function, provide financial support, and reduce health-risk behavior to prevent the recurrence of EM.

### TRAUMATIC EVENTS AND COGNITIVE FUNCTION: DOES TIME WHEN TRAUMATIC EVENTS HAPPEN MATTER?

Gabriella Dong, *Rutgers, The State University of New Jersey, New Brunswick, New Jersey, United States*

People at different life stage may respond differently to traumatic events and result in different cognitive health. This study aims to examine the relationship between life stage at which one experiences traumatic events and cognitive function. The data were drawn from the 2017-2019 PINE study (N = 3,125). The time of life events happened was evaluated by childhood (<20), adulthood (20-59), and old age (60 and above). Cognition was measured through global cognition, episodic memory, working memory, processing speed, and MMSE. Linear regression was used. Individuals with the latest exposure to traumatic events at adulthood or old age have higher cognitive function than those without traumatic events over the life course. Exposure to traumatic events in middle or later life stimulates cognition, while trauma exposure in earlier life stage does not. Future research to understand the impact of traumatic events on health could consider the time when traumatic events happen

### FACE-SAVING AND HELP-SEEKING AMONG U.S. CHINESE OLDER ADULTS WITH ELDER MISTREATMENT

Dexia Kong,[1] XinQi Dong,[2] and Ying-Yu Chao,[3],
*1. Rutgers University, New Brunswick, New Jersey, United States, 2. Rutgers University, Rutgers Institute for Health, New Jersey, United States, 3. Rutgers University, Newark, New Jersey, United States*

Chinese culture places a high value on saving face and not bringing shame to the family. This study aimed to examine the associations between face-saving and help-seeking among U.S. Chinese older adults who experienced elder mistreatment (EM). Data were retrieved from the PINE study. Regression analyses were performed. Most EM victims sought help from informal sources only (48.21%), followed by no help (26.79%), informal plus formal help (19.64%), and formal help only (5.36%). For EM screening, face-saving was associated with informal help-seeking intentions ($p < .05$). For EM subtypes, face-saving was associated with overall help-seeking intentions for financial exploitation ($p < .05$), but not on physical mistreatment, psychological mistreatment, and caregiver neglect. Face-saving was not associated with help-seeking behaviors. Study findings underscore the significance of a unique cultural value in understanding EM help-seeking intentions among Chinese older adults. Cultural constructs should be considered in future EM research in diverse populations.

### Session 4000 (Symposium)

### ACCESS AND BARRIERS TO USE OF LONG TERM SERVICES: CONTEXTUAL ISSUES

Chair: Allen Glicksman Co-Chair: Lauren Ring
Discussant: Norah Keating

The challenges that some older adults face in accessing both health and social services is a topic of continuing concern. This panel will focus on contextual issues that often shape specific challenges. These contextual issues usually emerge either from issues of diversity among the older persons themselves (for example, minority status or foreign born) and diversity between the ways in which services are offered (usually established at the national or in the case of the United States, at the state level). The intersection of these two forms of diversity often define the specific challenges faced by older persons in accessing health and social services. Further, unexpected events, such as the COVID pandemic, can affect both types of diversity (greater challenges for persons who do not speak the dominant language; inability of services to quickly adapt to radically changed environment). Our panel will address these issues through four presentations, each taking a different look at the ways in which diversity affects access. Our first paper, by Torres, will place this discussion in wider context by presenting results from a scoping review. Our second paper, by Diederich looks at access to services by immigrant generation (that being another source of diversity) in Germany. The third paper, by Thiamwong looks at how the COVID crisis affected older Hispanic women. Finally, Ring will examine how a national policy, here the definition of poverty, affects outcome and access for older person in the United States.

### DEFINING POVERTY AS AN ELIGIBILITY REQUIREMENT FOR SUPPORTIVE SERVICES

Lauren Ring, and Allen Glicksman, *Philadelphia Corporation for Aging, Philadelphia, Pennsylvania, United States*

Deciding which individuals qualify as “poor” often depends on how each country or municipality defines the term ‘poverty’. In the United States, program eligibility is often tied to the Federal Poverty Level (FPL), using 100% of the FPL as a cut-off for receipt of services. However, research has shown that incomes of 200% of the FPL and higher are often needed to establish even minimum levels of economic security. Using data from an omnibus health study conducted in 2018 that included 1,581 persons ages 60+ who were asked about their health and service needs, we compared persons making 100% of the FPL or less to persons making 101%-199% and 200%+, respectively. Results show that poor health status and need for services among persons in the 101%-199% are similar to those with incomes less than 100% FPL, and significantly higher than persons with incomes at 200%+ of the FPL.

### ETHNICITY AND RACE IN ACCESS AND USAGE OF HEALTH AND SOCIAL CARE: RESULTS FROM A SCOPING REVIEW

Sandra Torres, *Department of Sociology, Uppsala, Uppsala Lan, Sweden*

Scholarship on ethnicity and old age is at a crossroad now that increased diversity is a given in older populations. The same holds true for the study of the role that ethnicity and race play in access and usage of health and social care in old age. This presentation relies on a scoping review of scholarship published between 1998 and 2020 that brings attention to the ways in which ethnicity & race – as grounds for stratification and disadvantage - are made sense of in this scholarship. The presentation will describe the topics that the review divulged, whether racism has been acknowledged in this scholarship so far, and how this has been the case. In doing so, this presentation will argue that if we are to address the inequalities that older ethnic minorities face we need not only a diversity-astute research agenda but also an injustice-aware one.

### CULTURAL DIFFERENCES IN OLDER IMMIGRANTS' HEALTH AND SOCIAL SERVICES USE

Freya Diederich, *University of Bremen, Bremen, Bremen, Germany*

Even though Germany has a mandatory health and long-term care insurance with no or only very low co-payments, immigrants and the native population differ in their health and social services use. Differences in cultural traits and a lack of knowledge about the institutional setting are frequently mentioned as contributing factors. Relying on the epidemiological approach in the economic literature, this empirical study shows that both cultural traits that prevail in older immigrants' country of origin and older immigrants' knowledge about the host country's institutional setting affect their health and social services use in Germany. We distinguish foreign-born immigrants and their descendants as both groups differ in their connection to the home and the host country. The results will be used to discuss immigrants' access and potential barriers to the use of health and social services in comparison to the native population.

### HOW THE COVID-19 CRISIS AFFECTED DIVERSE OLDER ADULTS: A MIXED METHODS CASE SERIES

Ladda Thiamwong, *College of Nursing, University of Central Florida, Orlando, Florida, United States*

The coronavirus disease (COVID-19) pandemic has magnified inevitable physical, mental and social health consequences, especially in Hispanic older adults who experience health disparities and ageism. Even though physical distancing has been adopted as a key strategy to help reduce further spread of COVID-19, prolonged periods of physical distancing may worsen existing health problems. This study aims to explore how the COVID crisis affected diverse older adults. An explanatory sequential mixed methods design was utilized. Quantitative data were collected by questionnaires via Qualtrics survey and qualitative data were collected by individual phone interviews with four open-end questions. One in 4 older adults lives alone and one in 20 has no friend to call on. More than half of the participants were afraid of COVID and a fourth of them were afraid of losing their life to COVID. Participants identified keeping themselves busy as key to staying healthy during the pandemic.

### Session 4005 (Symposium)

### ACTIVE AGING FROM THEORY TO PRACTICE: NATIONAL EXPERIENCES OF POLICY MAKING IN EUROPE AND CANADA

Chair: Francesco Barbabella

Born in Europe as a concept aiming to counteract new demographic and societal challenges, active aging has progressively become a key pillar of an extended welfare state for aging populations in many high-income countries. Needs, interests, and preferences of new aging cohorts are changing, becoming more diverse and requiring a better understanding and greater attention by policy makers, beyond mere social welfare programmes for those with social, economic or health needs. Active aging policies aim at improving individuals' quality of life by optimizing opportunities for health, participation, and security (WHO 2002), hence unlocking the potential of older people as active citizens in the community and the society. Since the focus is on a multidimensional concept of quality of life, active aging works at the intersection of labour, social, educational, family, infrastructure, and many other policy areas. However, there may be gaps and discrepancies between the concept in itself and its application at the policy level. The purpose of this symposium is to present and discuss how different post-industrial societies are advancing and implementing active aging policies, in the context of overarching societal challenges and competing needs. In this respect, the symposium focuses on four countries representing different traditional welfare state models: Canada, Italy, Poland, and the United Kingdom. These four case studies bring analyses of active aging policies at national and/or regional level, providing a picture of how such policies have been designed, how they evolved and what they have achieved in recent years.

### HOW ACTIVE AGING POLICIES EVOLVED IN ITALY? A SYSTEMATIC REVIEW OF NATIONAL AND REGIONAL POLICIES

Francesco Barbabella, Davide Lucantoni, Marco Socci, Giovanni Lamura, and Andrea Principi, *National Institute of health and science on ageing (IRCCS INRCA), Ancona, Marche, Italy*

In recent years, active aging became a concept progressively considered by policy makers in Italy. A national project for creating a multilevel and co-managed coordination of active aging policies was launched in 2019 by the Italian Government and the National Institute of Health and Science on Ageing (IRCCS INRCA). A systematic review of active aging policies was conducted at both national and regional level. Results showed that national policies still reflect the general categorical fragmentation of Italian welfare system, although substantial developments were found in some sectors, i.e. prolonging working life, supporting families, social inclusion, and healthy ageing. Ten out of twenty-one regions have approved laws dedicated to active aging, although in most cases these laws are too recent to show impact. National and regional policy makers still need to overcome several cultural, coordination and fragmentation challenges for improving active aging policies and harmonise them with societal needs.

### AGING EQUALLY? EMPLOYING ACTIVE AGING POLICY IN THE UNITED KINGDOM

Liam Foster, *University of Sheffield, Sheffield, England, United Kingdom*

The UK's responses to the challenges of ageing have largely focused on productivist notions of active ageing, with more comprehensive responses tending to be reactive and largely remedial. This presentation will show that productivist policies, often characterised by individual responsibility, including raising the retirement age, restricting access to early retirement, and providing a stronger link between pension benefits and contributions, have incentivised remaining in the labour market. These strategies have been justified in the context of ageing populations and increasing pension costs. However, opportunities to extend working lives have not been experienced equally. In practice most policies are gender blind. Furthermore, a more comprehensive approach to active ageing in the UK needs a collective emphasis to mobilise a wide range of societal resources, underpinned by a commitment to public welfare, which is highly problematic under neo-liberalism. Therefore, a comprehensive approach to ageing in the UK requires a substantial ideological shift.

### POLICY IMPLICATIONS OF THE ACTIVE AGING FRAMEWORK IN POLAND

Jolanta Perek-Białas, and Maria Varlamova, *Jagiellonian University, Krakow, Malopolskie, Poland*

Poland's relatively young population in the past, is aging rapidly, which provokes a growing interest in the realisation of the older population's potential within the framework of the concept of active ageing. From 2012, when the first Governmental Program on Social Participation (ASOS 2012-2013) was introduced, the active aging framework remains one of the dominant strategies in developing and implementing social policies for the older generation. In the current paper, the focus is made on the employment and social participation of older citizens policies, showing the considerable gap in the prioritisation and hence in the outcomes, highlighted both by Active Ageing Index (AAI) indicators and more in-depth analysis. We will discuss the perceived risks and pitfalls of the current long-term ageing policy approach in Poland and provide recommendations for improvement.

### SOCIAL ISOLATION AND AGING OUT OF PLACE AMONG IMMIGRANTS AND REFUGEE SENIORS IN CANADA

Shanthi Johnson,[1] Juanita-Dawne Bacsu,[2] Tom McIntosh,[3] Bonnie Jeffery,[3] and Nuelle Novik,[3], *1. University of Alberta, Edmonton, Alberta, Canada, 2. University of Saskatchewan, Saskatoon, Saskatchewan, Canada, 3. University of Regina, Regina, Saskatchewan, Canada*

Immigrant and refugee seniors experience cultural barriers, discrimination, and limited networks which increase the risk of social isolation and thus hinder their active participation in the society. This paper explores social isolation among immigrant and refugee seniors in Canada based on an environmental scan of federal/provincial/territorial and community-based programs and a systematic scoping review. Findings revealed important gaps and regional disparities in opportuntiies to reduce social isolation and great active participation. Research was limited, often qualitative in nature, typically based on larger urban centres, with measurement issues related to the need for consideration beyond one's living arrangements. The results highlight the need for greater understanding Canada's immigration and refugee system and policies, and collaboration across levels of government. Reducing issues of social isolation and enabling better active aging for vulnerable seniors require a more nuanced and multidimensional conceptualization with prioritization on addressing the unique factors of culture and geographical context.

### Session 4010 (Symposium)

### AGEISM: OUTCOMES, INTERVENTIONS, FUTURE DIRECTIONS

Chair: Kelly Trevino Discussant: Becca Levy

Ageism is stereotyping and discrimination against individuals or groups based on their age. Ageism toward older adults is ubiquitous in American society and takes many forms including prejudicial practices and institutional policies that lead to unfair treatment of older adults. Ageism negatively impacts older adults in numerous domains such as health care and the workplace. Older adults themselves often internalize ageist views with detrimental effects on physical and mental health including increased risk for suicidal ideation and worse memory performance. This symposium addresses ageism from multiple perspectives and describes strategies for detecting and combatting ageism. The first speaker is Patricia D'Antonio, Vice President of Policy and Professional Affairs of The Gerontological Society of America (GSA). Ms. D'Antonio will describe GSA's Reframing Aging Initiative which aims to improve the public's understanding of the meaning of aging in order to counter ageism and support policies and programs that benefit older adults. The second speaker, Dr. Fredriksen Goldsen will use an Age Equity Framework to present her research on the relationship between ageism and mental and physical health and quality of life in LGBTQ older adults. The third speaker, Dr. Gendron will describe a content analysis of an anti-ageism resource that evaluates ageism interventions using an ecological framework. The fourth speaker, Dr. Hinrichsen will discuss ways psychotherapists can help older adults identify and move beyond internalized ageist beliefs. Finally, Dr. Levy, an internationally recognized expert in ageism will discuss themes across speakers and comment on the future of work in this area.

### REFRAMING AGING: A GENERATION'S WORK

Patricia D'Antonio, *The Gerontological Society of America, Washington, District of Columbia, United States*

Changing American culture is challenging and changing attitudes and behaviors around the universal experience of aging especially so. Unless the field of advocates who care about aging issues cultivates a more visible, more informed conversation on older people, it will remain difficult to advance the systemic changes needed to adjust to a society with increased and increasing longevity. Advocates will need to be vigilant to avoid cueing negative attitudes towards aging and aging policies. The Reframing Aging Initiative is a long-term, social change endeavor designed to improve the public's understanding of what aging means and the many contributions older people bring to society. Using evidence-based research, the initiative seeks to teach how to tell an effective story about aging that will promote positive perceptions of aging and reduce ageism.

### AGE EQUITY: A FRAMEWORK FOR ADDRESSING AGEISM, STIGMA, AND BIAS

Charles Emlet,[1] and Karen Fredriksen Goldsen,[2], *1. University of Washington, Tacoma, Washington, United States, 2. University of Washington, Seattle, Washington, United States*

In the Covid-19 context, researchers and policy makers have turned their attention to long-standing disparities in health equity, including by race, ethnicity, poverty, sexuality, and gender. Yet, scholarship to date does not conceptualize age as a critical aspect of difference requiring an equity lens. In this presentation, we utilize an Age Equity Framework to investigate ageism based on research findings from the 2018 National Health, Aging and Sexuality/Gender Study (NHAS): Aging with Pride. Investigating ageism, stigma, and bias, we found nearly half of LGBTQ older adults feel invisible and disrespected. After adjusting for background characteristics, experiences of ageism were associated with higher rates of stigma, lifetime victimization, discrimination, lower support and community engagement, and adverse outcomes (lower mental and physical health and quality of life). The rapidly growing older adult population highlights the pressing need to consider age inequities and the importance of achieving age equity across the life course.

### ANTI-AGEISM INTERVENTIONS: AN ECOLOGICAL APPROACH

Alexa Van Aartjik,[1] Kyrie Carpenter,[2] Ryan Backer,[3] Ashton Applewhite,[4] and Tracey Gendron,[1], *1. Virginia Commonwealth University, Richmond, Virginia, United States, 2. OldSchool Clearinghouse, San Francisco, California, United States, 3. OldSchool Clearinghouse, Mednham, New Jersey, United States, 4. This Chair Rocks, Brooklyn, New York, United States*

Ageism, discrimination based on age, is a systemic problem that occurs at multiple levels of the ecological system – meaning that ageism manifests at the individual, dyadic, institutional and societal levels. The expression of ageism within the levels of the ecological system makes ageism a continually shifting and dynamic force of oppression. Although ageism is a well-documented phenomenon with wide-reaching negative impacts, interventions to mitigate ageism's effects remain understudied. Little is known about the taxonomy of interventions available addressing ageism at the individual, dyadic, subcultural, institutional and societal levels. The current study conducted a deductive content analysis of an anti-ageism resource clearinghouse, OldSchool.info, to evaluate ageism interventions using an ecological framework. Results indicate the majority of ageism interventions are passive-oriented societal-level macrosystem approaches. A gap analysis will be discussed that indicated more active-oriented interventions with engageable content to address ageism at the personal and relational levels are needed.

### AGEISM ON THE THERAPEUTIC COUCH: AGING ATTITUDES AND LATE-LIFE PSYCHOTHERAPY

Gregory Hinrichsen, *Icahn School of Medicine at Mount Sinai, New York, New York, United States*

Ageist stereotypes characterize older adults as depressed, demented, and dependent. A large body of research has documented the adverse physical and emotional impact of ageism on older adults. Mental health professionals, however, often see the minority of older adults who, in fact, are depressed, have cognitive impairments, and/or are increasingly dependent on others. To what degree do pre-existing attitudes about aging come into play in psychotherapy with older people? With all age groups, psychotherapists often help clients better understand and challenge longstanding negative assumptions about self and world (sometimes called "the unconscious" or "underlying schemas"). These assumptions often impede the individual's ability to successfully contend with life problems. This presentation will discuss ways in which psychotherapists can assist older clients in clarifying their underlying (and often self-limiting) negative assumptions about aging, moving beyond them to better contend with late life stressors, and improving emotional well-being.

### Session 4015 (Paper)

### AGING AND TECHNOLOGY INTERVENTIONS I

### EFFICACY OF ONLINE GOAL MANAGEMENT TRAINING FOR AGE-ASSOCIATED EXECUTIVE IMPAIRMENT

Lynn Zhu,[1] Danielle D'Amico,[2] Susan Vandermorris,[1] Yushu Wang,[1] Laryssa Levesque,[1] Jordan Lass,[1] Angela Troyer,[1] and Brian Levine,[1], *1. Baycrest, Toronto, Ontario, Canada, 2. Ryerson University, Toronto, Ontario, Canada*

Goal Management Training® (GMT) is a standardized cognitive rehabilitation program that enhances individuals' awareness of executive function impairments and trains them to regularly monitor and manage their goals. In-person GMT is well-validated among numerous subpopulations, including people experiencing age-related cognitive impairment or acquired brain injury, and people with psychiatric disorders. The goal of this study was to evaluate the efficacy and usability of online GMT relative to computerized "brain training" in a registered randomized controlled trial (protocol NCT03602768 at Trials.gov). Both interventions were administered in a self-paced format, with background therapist support provided for GMT. Primary outcomes were measured as self-reported executive impairment on standardized measures (the Dysexecutive Questionnaire and the Cognitive Failures Questionnaire) at pre-, immediate post-, and 6 weeks post-intervention. 62 older adults without psychiatric or neurological diagnoses completed the trial (online GMT: n = 37, age[mean] = 69 years; computerized brain training: n = 25, age[mean] = 64 years; both groups: 76%

female). Improvements on the primary outcomes were observed post-intervention and were maintained at follow-up. GMT and computerized brain training groups could not be differentiated statistically, possibly due to restriction of range in the outcome measures at baseline. Additionally, the self-paced format prolonged the intervention beyond the recommended duration, which may have diluted efficacy. GMT was well-received, with participants reporting frequent use of the trained metacognitive strategies. Future studies will examine online GMT's effectiveness in samples with documented executive impairment and with additional supports to promote engagement for this virtual program.

### EVALUATION OF AN ECOLOGICAL MOMENTARY INTERVENTION FOR DEPRESSION IN OLDER ADULTS LIVING ALONE

Heejung Kim,[1] Soyun Hong,[2] Sangeun Lee,[3] Kijun Song,[1] Mijung Kim,[4] Yuntae Kim,[4] and Hyein Kim,[1], *1. Yonsei University, Seoul, Seoul-t'ukpyolsi, Republic of Korea, 2. Yonsei university, Seoul, Seoul-t'ukpyolsi, Republic of Korea, 3. University of Illinois, Chicago, Illinois, United States, 4. Mapo Senior Welfare Center, Seoul, Seoul-t'ukpyolsi, Republic of Korea*

Depression is a common but treatable mental health problem among older adults. Daily management and monitoring is very important; thus, diverse approaches have been developed. The objective of this research was to develop and pilot-test a counseling-plus-mobile health (mHealth) intervention using ecological momentary intervention (EMI) to reduce daily depressive mood for older adults living alone. Of 64 older adults living alone in community settings, 44 completed mHealth training and EMI for 7 weeks between October 2018 and October 2019. Study participants were randomized into experimental and control groups. The intervention was based on the protocol developed for an mHealth program for older Korean adults. Participants wore an actiwatch that measured their depressed moods four times a day for 2 weeks. Depressive symptoms were measured using the Hamilton Depression Rating Scale (K-HDRS) and the Korean version of the Short Geriatric Depression Scale (SGDS-K). Sleep quality was assessed using the Korean version of the Pittsburgh Sleep Quality Index (PSQI-K). The mean age of the study sample was 76.05±5.66 years, and the majority of the participants were female (61.0%, 36/59). There were no demographic characteristic differences between intervention and control groups. Based on multi-level modeling, EMI was not associated with significant improvements in depressive symptoms. However, depressive symptoms showed an initial decreasing trend, leveling off toward the end of the intervention period. This study finding could function as preliminary data to develop mHealth-based EMIs for older users in a larger, long-term study.

### EXPANSION OF A WEB-BASED PLATFORM TO SUPPORT CAREGIVERS IN CALIFORNIA USING CARENAV

Heather Young,[1] Janice Bell,[2] Kathleen Kelly,[3] Tina Kilaberia,[2] and Jennifer Mongoven,[2], *1. University of California–Davis, Sacramento, California, United States, 2. University of California, Davis, Sacramento, California, United States, 3. Family Caregiver Alliance, San Francisco, California, United States*

About one in five Americans is engaged in providing care to a family member. Caregivers (unpaid family members or friends) support older adults and persons with disability with a variety of conditions, including challenges in physical, cognitive, and mental health. In California, 4.5 million family caregivers are assisting individuals over the age of 18. The CA Department of Health Care Services funds 11 Caregiver Resource Centers (CRC) to support caregivers and, in 2019, provided support to expand information technology services through adoption of a statewide online assessment platform and client portal, CareNav™, to serve as a client record and referral tool. CareNav™ facilitates collection of consistent state-wide data that can inform program improvement and policy. This study evaluated the implementation process from the perspective of 35 CRC team members in guided focus group discussions. CRC staff identified several potential benefits to adopting CareNav™, including ease of client access and convenience, the ability to aggregate data to inform planning and policy, and a streamlined process for resource sharing. Challenges included customizing site-specific data needs, as well as concerns about equitable access to internet services. Ongoing evaluation will focus on validation and visualization of data, and translation of data into actionable information to improve quality and reach.

### SMARTWATCH TECHNOLOGY FOR PHYSICAL ACTIVITY IN OLDER ADULTS: A QUALITATIVE STUDY

Mengchi Li,[1] Miranda McPhillips,[2] Sarah Szanton,[3] Jennifer Wenzel,[4] and Junxin Li,[3], *1. Johns Hopkins University, BALTIMORE, Maryland, United States, 2. University of Pennsylvania, University of Pennsylvania, Pennsylvania, United States, 3. Johns Hopkins University, Baltimore, Maryland, United States, 4. Johns Hopkins University SON, baltimore, Maryland, United States*

Older adults' experiences using smartwatch technology for physical activity (PA) have not been well studied. We studied older adults' acceptance, capacity, and experience using smartwatches for self-monitoring and promoting PA. We conducted individual interviews using semi-structured interview guides with 15 older adults who participated in two studies. The two studies employed smartwatches in combination with personalized exercise training to promote PA and the interventions were 4 and 24 weeks in length. Interviews were transcribed verbatim. Two researchers conducted inductive content analysis using NVivo V.12 to identify and categorize codes into major themes. Participants reported high overall acceptance, ease of use (i.e., charging, checking steps, reading the screen), and attractive design of smartwatches. Participants' positive descriptions of their smartwatch experience included: increased activity awareness (step counts and heart rate), improved exercise accountability, and enhanced motivation (response to prompts). Most participants expressed interest in long-term smartwatch use. While participants expressed enjoyment of smartwatch technology for self-monitoring and PA promotion, some reported decreased motivation over time. Participants' concerns of smartwatch functionalities included short battery life, inaccurate step-recordings, and touchscreen insensitivity. Some also reported failure to troubleshoot smartwatch syncing/ pairing problems with smartphones and daily smartwatch charging issues. Smartwatch Bluetooth connectivity and battery life can be improved to increase usability and acceptability among older adults. Future research should explore the role of smartwatches for older adults' PA with emphasis on behavior change over time.

### USING TECHNOLOGY FOR PRESCRIPTION AND ADHERENCE IN AN ALZHEIMER'S PREVENTION PROGRAM

Amber Watts,[1] Angela VanSciver,[2] Jon Clutton,[2] Katrina Finley,[2] Erica Flores,[2] Amanda Szabo-Reed,[2] Jeffrey Burns,[2] and James Vacek,[3], *1. University of Kansas, Lawrence, Kansas, United States, 2. University of Kansas Medical Center, Fairway, Kansas, United States, 3. University of Kansas Medical Center, Kansas City, Kansas, United States*

Healthy lifestyle change is difficult to adopt and maintain without support. Often physicians recommend exercise to their patients, but have limited means to support this change. A major goal of our study is to provide physicians with a simple method of referring patients to a program that supports adoption and maintenance of exercise that meets recommended guidelines for older adults. The Lifestyle Empowerment for Alzheimer's Prevention program (LEAP! Rx) is a yearlong intervention to support cognitively normal older adults in adoption and maintenance of moderate to vigorous exercise, a key prevention factor for Alzheimer's disease. The program uses the electronic medical record and builds relationships with physicians to identify patients eligible to participate. It electronically communicates about patients' progress back to referring physicians to facilitate ongoing physician-patient interaction. Participants receive exercise coaching to reach their weekly exercise goals and have access to online lifestyle education classes (e.g., nutrition, sleep, stress management). The study is currently enrolling (n= 121 enrolled; mean age 71.4; 12% non-white, 4% Hispanic/Latino, and 83% female). Physician referrals originate from five clinics represented by 48 physicians. The study design will actively compare the physician referral process to self-referrals from the community (n=20). We have adapted the protocol to the conditions of the pandemic including online exercise coaching and support. This presentation will discuss successes and lessons learned from this novel method of recruitment and adherence to exercise.

### Session 4020 (Symposium)

### AGING WITH INTELLECTUAL AND DEVELOPMENTAL DISABILITIES: WHEN DEMENTIA IS DIAGNOSED OR SUSPECTED

Chair: Phillip Clark Discussant: Kelly Munly

Individuals with lifelong intellectual and developmental disabilities (IDD) have unique needs associated with aging that pose challenges for them and their families. In particular, an increased likelihood for early onset Alzheimer's disease is a major concern that can place individuals at risk for a host of biomedical, psychological, and social challenges. Faced with providers not trained in how to properly screen for, diagnose, and treat conditions, individuals and families are often left with inadequate care, services, and support. To address these concerns, education for professionals is essential in providing accurate information based on clinical best practices. This symposium presents an innovative and interprofessional model developed by a partnership of geriatrics and IDD educational and service organizations based on Project ECHO (Extension for Community Healthcare Outcomes) methodology. A virtual community is created in which participants both teach and learn from each other through a combination of didactic and case presentations. The first paper describes the ECHO model, including the development of the hub and spoke structure, recruitment of providers, and collaborative and multidisciplinary process of curriculum development. The second paper explores educational experiences of participating spoke agencies in the program, including professionals' and clients' outcomes. The third paper presents the implications of creating a foundation based on interprofessional education and networking principles to bridge the gap between health and social care disciplines and parallel service systems. The final paper provides recommendations and implications for developing and refining methods to address the need for provider education in this rapidly expanding field.

### CREATING, RECRUITING, AND DEVELOPING: KEY TASKS AND THEIR CHALLENGES

Faith Helm,[1] and Edward Ansello,[2], *1. University of Rhode Island, Kingston, Rhode Island, United States, 2. Virginia Commonwealth University, Richmond, Virginia, United States*

The ECHO model is uniquely suited to developing education for a wide range of agencies and providers serving the needs of older adults with IDD. The program's structure and its educational philosophy depend on modeling teamwork in both the hub and the spokes. Recruitment of participants included paid caregivers, healthcare practitioners, and direct service providers, focusing on team participation at each site. In developing the curriculum, it was critical to recognize the roles played by each sector, as well as the complementary contributions of others. Consequently, curriculum content needed to be multidisciplinary and multifocal, and recognize both the breadth of contributors and time limits in selecting content for each session. Didactic presentations and case studies embodied these features. Priorities included best practices in person-centered care; differential diagnoses; and physical, social, and environmental factors. The facilitators of, and challenges to, these priorities offer implications for advancing educational programs with similar objectives.

### EDUCATIONAL EXPERIENCES WITHIN THE LEARNING COMMUNITY: ECHO EFFECTS ON PARTICIPANTS AND CLIENTS

Kathleen Bishop,[1] and Yumi Shirai,[2], *1. National Task Group on ID and Dementia Practices, Lee Center, New York, United States, 2. University of Arizona, Tucson, Arizona, United States*

Although Project ECHO is a well-established, effective model to promote quality care in the general healthcare field, this project is the first attempt to implement the model with community care providers of individuals with IDD who are affected by dementia. In order to capture spokes' (community providers and NTG-Affiliated Regional Trainers) experiences and explore potential benefits, we conducted a content analysis of qualitative data gathered from a spoke feedback session and follow-up surveys from the same group (n = 20). Our findings indicated that spokes appreciated (1) didactic sessions by gaining new knowledge; (2) in-depth, mutual discussion about their cases; (3) experts taking time and ensuring their good practices; and (4) the ability to apply this gained knowledge to their day-to-day practices to improve quality of life for individuals with IDD and their families. We will discuss specific examples that inform future practices.

### BRIDGE BUILDERS AND BOUNDARY SPANNERS: INTERSECTORAL, INTERORGANIZATIONAL, AND INTERPROFESSIONAL EDUCATION

Phillip Clark, *University of Rhode Island, Kingston, Rhode Island, United States*

The needs of individuals aging with intellectual and developmental disabilities (IDD) and their families do not fall neatly within defined policies, programs, and professions. They comprise complex challenges based on medical, psychological, social, environmental, economic and familial dimensions. These needs pose a challenge for providers in developing solutions at three levels: (1) different policies and programs create barriers based on different funding sources, eligibility requirements, and administrative restrictions; (2) clinical and community-based programs embody the gap between healthcare and human service providers; and (3) different professions are trained in their own methods of assessment and care plan development that impede the design of integrated approaches to defining and solving problems. This paper proposes an intersectoral, interorganizational, and interprofessional framework for addressing these problems based on networking and collaborative practice principles embodying bridge-building, boundary-spanning, and team-working as a basis for provider education. Implications for expanded education in this field are explored.

### IMPLICATIONS AND RECOMMENDATIONS FROM A PROJECT ECHO SERIES ON AGING WITH IDD

Jenni Mathews,[1] and Faith Helm,[2], *1. Virginia Geriatric Education Center, Richmond, Virginia, United States, 2. University of Rhode Island, Kingston, Rhode Island, United States*

Ongoing evaluations of innovative educational programs provide opportunities for quality improvement. This paper reports results from a Project ECHO series on lifelong IDD and dementia. Participant responses (n = 85) were collected from spoke sites in various settings across the US. Using a 5-point (5 very effective) Likert scale, data were collected from assessment items on 4 didactic presentations and 5 case studies representing essential components of the ECHO model. Overall scores by spoke sites for satisfaction with the didactic and case presentations ranged from 3.94 to 4.94; relevance of case studies to the work setting ranged from 4.0 to 4.75. Knowledge gain questions showed consistently positive directionality. As a result of their participation, spokes rated intent to provide better care for patients (57% to 88%), train staff (62% to 81%), and educate family/caregivers (57% to 88%). Implications of findings for the application of quality improvement methods are discussed.

### Session 4025 (Symposium)

### AGINGPLUS: A RANDOMIZED TRIAL TO INCREASE PHYSICAL ACTIVITY IN MIDDLE-AGED AND OLDER ADULTS

Chair: Manfred Diehl Discussant: Jennifer Schrack

Engagement in physical activity (PA) has well-documented benefits for delaying or preventing age-related diseases. Thus, it is important to study innovative ways to increase PA in the adult population. This symposium describes AgingPLUS, an ongoing trial that addresses three psychological mechanisms to increase adults' PA: Negative views of aging (NVOA), low self-efficacy beliefs, and deficient goal-planning skills. The symposium also presents preliminary findings, based on a pre-pandemic subsample, on changes in explicit NVOA, implicit VOA, and changes in PA. Diehl et al. describe the theoretical background and study design of the ongoing RCT. This also includes the main study hypotheses. Rebok et al. present preliminary findings showing significant effects of the intervention on NVOA and frequency of moderate intensity exercise. Effects on physical function and accelerometry measures were not statistically significant in this subsample. Tseng et al. examined the effects of the intervention on two measures of implicit VOA: a lexical decision-making task (LDMT) and the Brief Implicit Association Test (BIAT). Findings showed that differences in post-intervention latencies on the LDMT were not statistically significant. Differences on post-intervention BIAT d scores also failed to be significant. Finally, Nehrkorn-Bailey et al. tested a multiple mediator model examining the mediational role of self-efficacy (SE) and exercise intention (EI) on PA. Results showed that Week 4 SE significantly mediated the effect of intervention condition to Week 8 anticipated PA engagement. Week 4 EI significantly mediated the effect of intervention condition on Month 6 PA engagement. Anticipated PA effects predicted subsequent involvement in PA.

### AGINGPLUS: THEORETICAL BACKGROUND AND STUDY DESIGN OF THE RANDOMIZED TRIAL

George Rebok,[1] David Roth,[1] Kaigang Li,[2] Abigail Nehrkorn-Bailey,[2] Diana Rodriguez,[2] Katherine Thompson,[2] and Manfred Diehl,[2], *1. Johns Hopkins University, Baltimore, Maryland, United States, 2. Colorado State University, Fort Collins, Colorado, United States*

The AgingPLUS program targets three psychological mechanisms that are known barriers to middle-aged and older adults' engagement in physical activity (PA): Negative views of Aging (NVOA), low self-efficacy beliefs, and poor goal planning skills. These risk factors are addressed in a 4-week intervention program that is compared to a generic health education program as the control group. Middle-aged and older adults (age 45-75 years) are enrolled in the trial for 8 months, with four assessment points: Baseline (pre-test), Week 4 (immediate post-test), Week 8 (delayed post-test), and Month 6 (long-term follow-up). The major outcome variables are participants' engagement in PA as assessed via daily activity logs and actigraphs. Positive changes in NVOA, self-efficacy beliefs, and goal planning are the intervention targets and hypothesized mediating variables leading to increases in PA. This trial adopted the experimental medicine approach to assess the short- and long-term efficacy of the AgingPLUS program.

### EXAMINING THE EFFECTS OF THE AGINGPLUS PROGRAM ON PHYSICAL ACTIVITY: A MULTIPLE MEDIATOR MODEL

George Rebok,[1] David Roth,[1] Shang-En (Michelle) Chung,[1] Kaigang Li,[2] Diana Rodriguez,[2] Katherine Thompson,[2] Manfred Diehl,[2] and Abigail Nehrkorn-Bailey,[2], *1. Johns Hopkins University, Baltimore, Maryland, United States, 2. Colorado State University, Fort Collins, Colorado, United States*

This study examined the effect of the AgingPLUS program on anticipated physical activity (PA) and PA engagement, along with the hypothesized mediator roles of self-efficacy (SE) and exercise intention (EI). Data came from 147 participants (Mage = 60.11 years; SD = 8.28 years) of the ongoing trial. Structural equation modeling tested the effects of the intervention, week 4 EI, and week 4 SE on anticipated PA at week 8 and engagement in PA at 6 months. The pathway from week 8 anticipated PA to month 6 PA was also assessed. Results showed that week 4 SE significantly mediated the pathway of intervention condition to week 8 anticipated PA, whereas week 4 EI significantly mediated the pathway from intervention condition to engagement in PA at 6 months. Furthermore, anticipated PA predicted subsequent engagement in PA. Results from these analyses provide preliminary support for the efficacy of the AgingPLUS program.

### THE AGINGPLUS TRIAL: INTERVENING TO PROMOTE PHYSICAL ACTIVITY IN MIDDLE-AGED AND OLDER ADULTS

David Roth,[1] Shang-En (Michelle) Chung,[1] Kaigang Li,[2] Abigail Nehrkorn-Bailey,[2] Katherine Thompson,[2] Manfred Diehl,[2] and George Rebok,[1], *1. Johns Hopkins University, Baltimore, Maryland, United States, 2. Colorado State University, Fort Collins, Colorado, United States*

This paper investigated whether the AgingPLUS program promotes physical activity in middle-aged and older adults by examining outcomes at weeks 4 and 8 with baseline scores included as covariates. The analyses assessed intervention effects on negative views of aging (NVOA), physical activity (CHAMPS), physical function (SPPB, VO2max), and accelerometry measures (e.g., minutes sedentary). We found significant intervention effects on NVOA ($p < .001$) and frequency of moderate intensity exercise ($p = 0.048$), but no significant effects on physical function, VO2max, or the accelerometry measures. Standardized effect sizes for the significant effects ranged from 0.31 to 1.03 standard deviation units. These findings suggest that AgingPLUS improved motivational factors for engaging in physical activity but did not lead to objective changes in physical activity in the short term. Further research will investigate the mediational role of these motivational factors in enhancing physical activity over the longer term (6 months).

### AGINGPLUS: EXAMINING THE MALLEABILITY OF IMPLICIT VIEWS OF AGING

Garrett Forsyth, Abigail Nehrkorn-Bailey, Diana Rodriguez, Kat Thompson, Manfred Diehl, and Heidi Tseng, *Colorado State University, Fort Collins, Colorado, United States*

AgingPLUS also examines whether the intervention can change participants' implicit VOA. To that end, participants completed a lexical decision-making task (LDMT) and the Brief Implicit Association Test (BIAT) at baseline and post-intervention. One-way ANCOVAs with baseline scores as covariate were used for these analyses. For LDMT, there was no significant difference between the groups regarding their post-intervention latencies for old-positive words, $F(1,181) = 0.01$, $p = .60$, old-negative words, $F(1,181) = 0.43$, $p = .51$, young-positive words, $F(1,181) = 0.19$, $p = .67$, and young-negative words, $F(1,181) = 1.16$, $p = .28$. For BIAT, both groups showed a slight preference for the young at baseline (mean $d = 0.39$), and post-intervention (mean $d = 0.38$). There was no significant difference between the groups regarding post-intervention d scores, $F(1,181) = 0.002$, $p = .97$. These preliminary findings suggest that in the current subsample, AgingPLUS did not significantly change participants' implicit VOA.

### Session 4030 (Symposium)

### ALZHEIMER'S ALPHABET SOUP: ADRD RESEARCH IN NHATS AND NSOC

Chair: Loretta Anderson Co-Chair: Alexandra Wennberg Discussant: Frank Lin

There are 5.8 million Americans living with Alzheimer's disease, but currently there is no cure or effective treatment. Alzheimer's disease and related dementias (ADRD) are characterized by cognitive decline, but patients also have behavioral symptoms and functional decline. Understanding the gamut of risk and prognostic factors for ADRD and those associated with the task of caring for these patients is layered. The National Health and Aging Trends Study (NHATS) and the sister National Study of Caregiving (NSOC) are excellent resources to investigate layers of ADRD incidence, progression, and caregiving in the population. NHATS is a nationally representative sample of Medicare beneficiaries aged 65 and older. Since 2011, annual in-person interviews have collected data in many areas, including health, environment, wellbeing, cognition, and function. NSOC has been conducted at three timepoints corresponding with NHATS rounds and collects detailed data on caregivers, including information on care activities, caregiver burden, and caregiver wellbeing. This symposium illustrates the broad range of ADRD research questions that can be probed using NHATS/NSOC data. The session begins with a presentation the association between caregiver burden and ADRD patient cognitive outcomes. The second presentation examines the role of physical performance as a predictor of developing ADRD. The third presentation investigates the role of dual sensory impairment – both hearing and vision impairment – on dementia incidence. The session concludes with an examination of whether hearing impairment among dementia patients is associated with different caregiving needs.

### CAREGIVER BURDEN IS ASSOCIATED WITH DEMENTIA PATIENT COGNITIVE PERFORMANCE IN NHATS

Loretta Anderson,[1] and Alexandra Wennberg,[2] *1. University of Maryland School of Medicine, Reisterstown, Maryland, United States, 2. Karolinska Institutet, Stockholm, Stockholms Lan, Sweden*

Informal caregivers of dementia patients engage in multicomponent care that is often stressful. In heart failure patients, caregiver burden has been associated with occurrence of cardiovascular events. However, little is known about how caregiver burden affects patient cognition in dementia care dyads. Using data from the National Health and Aging Trends Study and National Study of Caregiving, we examined the association between caregiver burden, assessed on 38 aspects of caring, and patient cognition, assessed with the immediate and delay word recall, Clock Drawing, and self-rated memory. In fully adjusted models at round 7 (2017) higher caregiver burden was cross-sectionally associated with lower immediate (B=-0.02, 95% CI -0.03, -0.01) and delayed (B=-0.03, 95% CI -0.04, -0.02) word recall. Longitudinally, across rounds 7-9 (2017-2019) higher burden was associated with lower patient Clock Draw score (B=-0.01, 95% CI -0.03, -0.001). These findings have implications for economic assistance and interventions in dementia care dyad.

### LINKS OF SHORT PHYSICAL PERFORMANCE BATTERY SCORE WITH INCIDENT DEMENTIA: RESULTS FROM THE NHATS

Emily Smail,[1] Jennifer Schrack,[2] Amal Wanigatunga,[3] Judith Kasper,[4] Adam Spira,[1] and Di Wu,[1] *1. Department of Mental Health, Johns Hopkins Bloomberg School of Public Health, Baltimore, Maryland, United States, 2. Department of Epidemiology, Johns Hopkins Bloomberg School of Public Health, Baltimore, Maryland, United States, 3. Johns Hopkins Bloomberg School of Public Health, Baltimore, Maryland, United States, 4. Department of Health Policy and Management, Johns Hopkins Bloomberg School of Public Health, Baltimore, Maryland, United States*

Physical performance is associated with cognitive function in later life, but few studies have examined the prospective association of physical performance with incident dementia. We studied 4539 community-dwelling National Health and Aging Trends Study (NHATS) participants aged ≥65 years with data on demographics and the Short Physical Performance Battery (SPPB) in 2011, who were followed through 2014. Our outcome was dementia diagnosis from a validated NHATS algorithm. We applied survey weights to make results nationally representative and performed Cox regression analyses. After adjustment for potential confounders, lower baseline SPPB scores were associated with incident dementia (HR=1.68, p < 0.01). Slower gait speed was the SPPB component most strongly associated with incident dementia (HR=1.21, p < 0.01). We found that poorer physical performance was linked to incident dementia in a cohort of older adults. More research is needed to examine the effect of improving physical performance on the prevention of dementia.

### PREVALENCE OF CONCURRENT FUNCTIONAL VISION AND HEARING IMPAIRMENT AND ITS ASSOCIATION WITH DEMENTIA

Pei-Lun Kuo,[1] Alison Huang,[2] Joshua Ehrlich,[3] Judith Kasper,[4] Nicholas Reed,[2] Frank Lin,[5] Bonnielin Swenor,[6] and Jennifer Deal,[7], *1. National Institute on Aging, National Institute on Aging, Maryland, United States, 2. Johns Hopkins Bloomberg School of Public Health, Baltimore, Maryland, United States, 3. University of Michigan, University of Michigan, Michigan, United States, 4. Department of Health Policy and Management, Johns Hopkins Bloomberg School of Public Health, Baltimore, Maryland, United States, 5. Johns Hopkins University, Johns Hopkins University, Maryland, United States, 6. Johns Hopkins School of Medicine, Baltimore, Maryland, United States, 7. Johns Hopkins University, Baltimore, Maryland, United States*

Vision and hearing impairment are common and independently linked to dementia risk. Adults with concurrent vision and hearing impairment (dual sensory impairment, DSI) may be particularly at-risk. Data were from the National Health and Aging Trends Study (NHATS) (2011–2018, N=7,562). Functional sensory impairments were self-reported (no impairment, vision only, hearing only, and DSI). We calculated age-specific prevalence of sensory impairments. Discrete time proportional hazards model with a complementary log-log link were used to assess 7-year dementia risk. Of 7,562 participants, overall prevalence of functional vision, hearing and DSI was 5.4%, 18.9% and 3.1%, respectively. DSI prevalence increased with age, impacting 1 in 7 adults ≥90 years. DSI was associated with a 50% increased 7-year dementia risk (adjusted hazard ratio 1.50; 95% confidence interval, 1.12–2.02) compared to no impairment. Sensory rehabilitative interventions for multiple impairments may be an avenue for consideration in efforts to reduce dementia risk.

### WORKING WHILE CARING FOR OLDER ADULTS WITH HEARING IMPAIRMENT AND DEMENTIA: EVIDENCE FROM THE NSOC

Varshini Varadaraj,[1] Bonnielin Swenor,[2] Nicholas Reed,[3] and Emmanuel Garcia Morales,[3] *1. The Wilmer Eye Institute, Baltimore, Maryland, United States, 2. Johns Hopkins School of Medicine, Baltimore, Maryland, United States, 3. Johns Hopkins Bloomberg School of Public Health, Baltimore, Maryland, United States*

Age-related hearing loss (HL) and dementia are common among older adults. The implications of caregiving for older adults with dementia is documented. Whether the presence of HL modifies these association is unknown. We used data from the 2011 NHATS/NSOC. Hearing loss and dementia were identified among care recipients (CR). Our outcomes included: hours of care provided, and caregiver's work activities. Among 1,013 caregivers, 456 assisted individuals without HL or dementia (HL-/D-), 229 with dementia (D+), 193 with HL, and 135 with HL and dementia (HL+/D+). In fully adjusted models, as compared to caregivers of HL-/D-, caregivers of D+ spent 39.1 hours more (95% CI: 13.6,64.6) in caregiving, caregivers of HL+/D+ spent 56.6 more hours (95% CI: 25.1,88.1). We found no differences in work activities between CR groups. The presence of HL increases the caregiving needs of adults with dementia. The additional time does not affect the labor participation of caregivers.

### Session 4035 (Symposium)

### CHRONOBIOLOGICAL FACTORS RELATED TO SLEEP AND NEUROPSYCHIATRIC SYMPTOMS IN PERSONS LIVING WITH DEMENTIA

Chair: Nancy Hodgson Co-Chair: Fanghong Dong

Circadian rhythm disturbances (CRD) are commonly seen in people living with dementia. A clear understanding of the role of CRD in dementia etiology will be beneficial by exploring the exogenous factors (externally influence the duration of sleep hours, such as light/dark cycles) and endogenous factors (internal biological rhythm, such as diurnal cortisol pattern). This symposium will apply a chronobiological approach to study exogenous and endogenous factors that influence circadian rhythm and their effects on sleep and neuropsychiatric symptoms in persons living with dementia (PLWD). Four paper presentations will use secondary data analysis of data from the Healthy Patterns Clinical Trial (NCT03682185), a randomized controlled trial of a home-based activity intervention designed to improve circadian rhythm disorders in PLWD. We will first describe the circadian rhythm pattern reflected by endogenous factors (salivary cortisol), then examine salivary cortisol (endogenous) and white light intensity (exogenous) and on subjective sleep and neuropsychiatric symptoms (including depression) in PLWD, respectively. In session 1, we will present cortisol diurnal rhythm pattern in PLWD using a cross-sectional design. In session 2, we will discuss the relationship between salivary cortisol indicators and depressive symptoms. In session 3, we focus on the association between diurnal cortisol slope and neuropsychiatric symptoms using the baseline data. In session 4, we describe the association between evening white light exposure and subjective sleep. The discussant will describe how these findings build on our understanding the nature of circadian rhythm disturbance in dementia and inform future research and treatment approaches.

### BEDTIME SALIVARY CORTISOL AND DEPRESSIVE SYMPTOMS IN OLDER ADULTS LIVING WITH DEMENTIA

Miranda McPhillips,[1] Darina Petrovsky,[2] Liming Huang,[3] Adriana Adriana,[3] Nancy Hodgson,[4] and Fanghong Dong,[5], *1. University of Pennsylvania, University of Pennsylvania, Pennsylvania, United States, 2. Rutgers University, Philadelphia, Pennsylvania, United States, 3. University of Pennsylvania, Philadelphia, Pennsylvania, United States, 4. University of Pennsylvania, School of Nursing, philadelphia, Pennsylvania, United States, 5. UPenn, PHILADELPHIA, Pennsylvania, United States*

The dysregulation of cortisol has been associated with depressive symptoms in older adults. To date, no prospective longitudinal studies have examined whether salivary cortisol is a risk factor for depressive symptoms in persons living with dementia (PLWD). With a sample of 123 PLWD, baseline salivary cortisol was collected at awaking, 30 minutes after awaking, and bedtime. Depressive symptoms were assessed at baseline and the four-week follow-up. Cortisol indicator were centered. Baseline bedtime cortisol level was significantly associated with depressive symptoms in a curvature style while controlling age, gender, and baseline depressive symptoms ($\beta$=3.76 for linear term and $\beta$=-1.57 for quadratic term, both ps<0.04). No other baseline cortisol measures were significant prospective predictors. Our results suggest the bedtime cortisol was a significant risk factor for depressive symptoms in PLWD. These findings suggest that bedtime cortisol may play a role in the etiology of depressive symptoms in PLWD.

### SALIVARY CORTISOL PATTERNS IN PEOPLE LIVING WITH DEMENTIA

Fanghong Dong,[1] Liming Huang,[2] Subhash Aryal,[3] G. Adriana Perez,[4] Nancy Hodgson,[5] and Darina Petrovsky,[6], *1. UPenn, PHILADELPHIA, Pennsylvania, United States, 2. University of Pennsylvania, Philadelphia, Pennsylvania, United States, 3. University of Pennsylvania School of Nursing, Philadelphia, Pennsylvania, United States, 4. University of Pennsylvania School of Nursing, Phildelphia, Pennsylvania, United States, 5. University of Pennsylvania, School of Nursing, philadelphia, Pennsylvania, United States, 6. Rutgers University, Philadelphia, Pennsylvania, United States*

Salivary cortisol has a well-documented circadian pattern in older adults. Yet, the pattern of salivary cortisol in persons living with dementia (PLWD) due to circadian rhythm disturbances is unknown. This study examined diurnal salivary cortisol patterns in 176 PLWD (mean age 73.6±8.8, 33.3% male, clinical dementia rating >=0.5) by collecting saliva at waking (AM1), 30 minutes after waking (AM2) and bedtime (PM) over two consecutive days. Cortisol awakening response (CAR) was calculated as the change between AM2 and AM1 cortisol levels. The mean baseline salivary cortisol levels (ug/dl) were 0.35 (SD:0.3) at AM1, 0.40 (SD:0.39) at AM2, and 0.19 (SD:0.4) at PM. On average, cortisol levels decreased from morning to evening, with 58% exhibiting a positive CAR (mean 0.05; SD:0.34). There were no significant associations between cortisol levels with age, sex, obesity, and comorbidities. The findings demonstrated that diurnal cortisol rhythms are maintained in PLWD with a flattened CAR.

### RELATIONSHIPS BETWEEN SALIVARY CORTISOL SLOPE AND NEUROPSYCHIATRIC SYMPTOMS IN PERSONS LIVING WITH DEMENTIA

Fanghong Dong,[1] G. Adriana Perez,[2] Nancy Hodgson,[3] and Yeji Hwang,[4], *1. UPenn, PHILADELPHIA, Pennsylvania, United States, 2. University of Pennsylvania School of Nursing, Phildelphia, Pennsylvania, United States, 3. University of Pennsylvania, School of Nursing, philadelphia, Pennsylvania, United States, 4. University of Pennsylvania, School of Nursing, Philadelphia, Pennsylvania, United States*

While a flatter diurnal cortisol slope has been related to poor health outcomes in healthy populations, little is known about this relationship in persons living with dementia (PLWD). The purpose of this study was to examine the association between diurnalcortisol slope and neuropsychiatric symptoms in PLWD. Secondary data analysis was conducted using baseline data from the Healthy Patterns Study (N=168). Diurnal cortisol slope was calculated using the difference between changes in salivary cortisol from 30 minutes after awakening to bedtime. Spearman rho coefficients were used. Flatter cortisol slope was associated with the presence of symptoms of agitation (r=-0.191, p=0.013) and disinhibition (r=-0.168, p=0.03). Steeper cortisol slope was related to a more severe level of anxiety symptoms (r=0.36, p=0.009) and higher frequency of insomnia (r=0.292, p=0.011). We found that cortisol slope was associated with neuropsychiatric symptoms in PLWD. Future research is needed to examine the mechanisms underlying the relationships.

### ASSOCIATION BETWEEN EVENING LIGHT EXPOSURE AND SUBJECTIVE SLEEP MEASURES AMONG PEOPLE LIVING WITH DEMENTIA

Yeji Hwang,[1] Sonia Talwar,[1] Nancy Hodgson,[2] and Miranda McPhillips,[3], *1. University of Pennsylvania, School of Nursing, Philadelphia, Pennsylvania, United States,*

2. University of Pennsylvania, School of Nursing, philadelphia, Pennsylvania, United States, 3. University of Pennsylvania, University of Pennsylvania, Pennsylvania, United States

Excessive light exposure before bedtime can disrupt one's circadian rhythm and can lead to poor sleep. The purpose of this study was to describe the relationship between evening light exposure and subjective sleep measures in people living with dementia (PLWD). We conducted secondary data analysis using the baseline data from Healthy Patterns Clinical Trial (N=137). We used Actiwatch Spectrum Plus to collect light data over three consecutive days. We defined evening light exposure as the average white light intensity for 4 hours before sleep. Sleep measures included Epworth Sleepiness Scale and PROMIS Sleep-Related Impairment. We used univariate regression analysis. We found that that greater evening intensity of light exposure was associated with higher daytime sleepiness ($\beta$=0.209, p=0.015) and more sleep impairment ($\beta$=0.228, p=0.014). The results of our study suggest that exposure to bright light during evening can disturb nighttime sleep and increase daytime sleepiness in PLWD.

### Session 4040 (Symposium)

### CONTRIBUTION OF SENSORY FUNCTION TO PRECLINICAL INDICATORS OF PHYSICAL AND COGNITIVE FUNCTIONING WITH AGING

Chair: Yuri Agrawal Co-Chair: Jennifer Schrack
Discussant: Bonnielin Swenor

There are well established associations between sensory loss and physical and cognitive deficits with aging, but gaps remain in our understanding of the associations between sensory function and early preclinical indicators of physical and cognitive decline. This symposium will present data from the Baltimore Longitudinal Study of Aging (BLSA) on a series of studies investigating the links among sensory function, motor function, and physical and cognitive outcomes in older adults. In the first study, Dr. Gross will present an operational definition of early cognitive impairment (ECI) based on a combination of two cognitive measures – the Card Rotations test and the California Verbal Learning Test Immediate Recall – to predict progression to MCI/AD. In the second study, Dr. Cai will evaluate the relationship between multisensory impairment (in vision, hearing, olfaction, proprioception and vestibular function) and the algorithmic definition of ECI. In the third study, Dr. Armstrong will evaluate the association between multisensory impairment and another biomarker of ECI or preclinical AD, specifically PET-PiB deposition. In the fourth study, Dr. Schrack, will present the joint contribution of multisensory (hearing and vision) impairment and motor function (gait speed) on risk of incident MCI/AD in longitudinal analyses. Finally, Dr. Martinez Amezcua will present the longitudinal association between hearing and vestibular function and decline in higher level physical function and endurance performance. Taken together, these studies present compelling data about the contribution of sensory function to preclinical indicators of physical and cognitive functioning with aging.

### DERIVATION AND VALIDATION OF AN ALGORITHMIC CLASSIFICATION OF EARLY COGNITIVE IMPAIRMENT

Yang An,[1] Frank Lin,[2] Luigi Ferrucci,[3] Jennifer Schrack,[4] Yuri Agrawal,[5] Susan Resnick,[3] and Alden Gross,[4] *1. NIA, Baltimore, Maryland, United States, 2. Johns Hopkins University, Johns Hopkins University, Maryland, United States, 3. National Institute on Aging, Baltimore, Maryland, United States, 4. Johns Hopkins Bloomberg School of Public Health, Baltimore, Maryland, United States, 5. Otolaryngology, Baltimore, Maryland, United States*

The long prodromal period for dementia pathology demands valid and reliable approaches to detect cases before clinically recognizable symptoms emerge, by which time it may be too late to effectively intervene. We derived and compared several algorithms for early cognitive impairment (ECI) using longitudinal data on 1704 BLSA participants. Algorithms were based on cognitive impairment in various combinations of memory and non-memory tests, and the CDR. The best-performing algorithm was defined based on 1SD below age-and race-specific means in Card Rotations or California Verbal Learning Test immediate recall, two tests that in prior work show the earliest declines prior to dementia onset. While this ECI algorithm showed low concordance with concurrent adjudicated MCI/dementia (AUC: 0.63, sensitivity: 0.54, specificity: 0.73), it was among the best predictors of progression to MCI/dementia (HR: 3.65, 95% CI: 1.69,7.87). This algorithm may be useful in epidemiologic work to evaluate risk factors for early cognitive impairment.

### SENSORY IMPAIRMENT AND ALGORITHMIC CLASSIFICATION OF EARLY COGNITIVE IMPAIRMENT IN MIDDLE-AGED AND OLDER ADULTS

Yuri Agrawal,[1] Jennifer Schrack,[2] Alden Gross,[2] Nicole Armstrong,[3] Eleanor Simonsick,[4] Susan Resnick,[5] and Yurun Cai,[2] *1. Otolaryngology, Baltimore, Maryland, United States, 2. Johns Hopkins Bloomberg School of Public Health, Baltimore, Maryland, United States, 3. Warren Alpert Medical School of Brown University, Providence, Rhode Island, United States, 4. National Instute on Aging/NIH, Baltimore, Maryland, United States, 5. National Institute on Aging, Baltimore, Maryland, United States*

Sensory function has been linked to cognitive impairment and dementia, but the link between multiple sensory impairments and early cognitive impairment (ECI) is unclear. Sensory function (vision, hearing, vestibular, proprioception, and olfaction) was measured in 390 BLSA participants (age=75±8 years; 57% women; 69% white) from 2012 to 2018 over a mean 3.6 years. ECI was defined based on 1 standard deviation below age-and race-specific means in Card Rotations or California Verbal Learning Test immediate recall. Cox proportional hazard models examined the risk of ECI for each sensory impairment and across categories of impairments. Vision impairment (vs. no vision impairment) was associated with a 70% greater risk of ECI (HR=1.70, p=0.05). Participants with 1 or ≥2 sensory impairments had triple the risk of ECI (HR=3.74 and 3.44, p=0.008 and 0.02, respectively) compared to those without impairment. Future studies are needed to examine whether treatment for sensory impairments can modify these risks.

### SENSORY IMPAIRMENT AND BETA-AMYLOID DEPOSITION IN THE BALTIMORE LONGITUDINAL STUDY OF AGING

Yurun Cai,[1] Hang Wang,[2] Jennifer Schrack,[1] Yuri Agrawal,[3] Eleanor Simonsick,[4] Susan Resnick,[5] and

Nicole Armstrong,[6], *1. Johns Hopkins Bloomberg School of Public Health, Baltimore, Maryland, United States, 2. Johns Hopkins University, Baltimore, Maryland, United States, 3. Otolaryngology, Baltimore, Maryland, United States, 4. National Instute on Aging/NIH, Baltimore, Maryland, United States, 5. National Institute on Aging, Baltimore, Maryland, United States, 6. Warren Alpert Medical School of Brown University, Providence, Rhode Island, United States*

Studies have demonstrated a link between sensory impairment and dementia risk, but little is known about the presence of beta-amyloid plaques in individuals with single and multisensory impairments. Sensory function (combinations of vision, hearing, vestibular function, and proprioception) and amyloid PET imaging were measured in 170 BLSA participants (age=78± 9 years; 53% women; 77% white; 28% amyloid positive) from 2012 to 2019. Log-binomial regression models were used to examine the prevalence ratios (PR) of amyloid positivity for individual sensory impairments and across categories of impairments. While crude associations indicate associations of vision impairment (PR=1.72, p=0.04) and impairments in all four senses (PR=2.38, p=0.03) with amyloid positivity, these associations were insignificant after adjusting for age, sex, race, and education. There were no other crude and adjusted associations. These results suggest sensory impairments may be related to dementia independent of AD pathology. Future studies with larger sample sizes are warranted.

### MOTOR AND SENSORY FUNCTION AS PREDICTORS OF MCI AND DEMENTIA IN THE BALTIMORE LONGITUDINAL STUDY OF AGING (BLSA)

Amal Wanigatunga,[1] Yurun Cai,[1] Hang Wang,[2] Yuri Agrawal,[3] Susan Resnick,[4] Luigi Ferrucci,[4] Qu Tian,[4] and Jennifer Schrack,[1], *1. Johns Hopkins Bloomberg School of Public Health, Baltimore, Maryland, United States, 2. Johns Hopkins University, Baltimore, Maryland, United States, 3. Otolaryngology, Baltimore, Maryland, United States, 4. National Institute on Aging, Baltimore, Maryland, United States*

Motor and sensory impairments are linked with dementia risk, but whether there is a joint effect of deficits in motor and sensory function is unknown. We analyzed 649 BLSA participants (aged 72±11 years; 55% women; 68% white) who had concurrent baseline 6-meter usual gait speed and sensory function (vision, hearing) between 2012-2019. Mild cognitive impairment (MCI) and dementia were adjudicated during an average follow-up of 3 years. We examined the association between baseline gait speed, z-scored sensory function, and a gait*sensory interaction with risk of MCI/dementia using Cox proportional hazard models, adjusted for demographics and chronic conditions. Each .01 m/s faster baseline gait was associated with a reduced risk (HR:0.98 (0.96-0.99)) of MCI/dementia, and each 1 SD higher in hearing and vision z-score was associated with an increased risk (HR:1.84 (1.1-3.1)) increased risk. The was no significant interaction, suggesting motor and sensory impairments may be independently associated with MCI/dementia risk.

### ASSOCIATION OF HEARING IMPAIRMENT WITH HIGHER LEVEL PHYSICAL FUNCTIONING AND WALKING ENDURANCE

Pei-Lun Kuo,[1] Nicholas Reed,[2] Eleanor Simonsick,[3] Yuri Agrawal,[4] Frank Lin,[5] Jennifer Deal,[6] Jennifer Schrack,[2] and Pablo Martinez Amezcua,[7], *1. National Institute on Aging, National Institute on Aging, Maryland, United States, 2. Johns Hopkins Bloomberg School of Public Health, Baltimore, Maryland, United States, 3. National Instute on Aging/NIH, Baltimore, Maryland, United States, 4. Otolaryngology, Baltimore, Maryland, United States, 5. Johns Hopkins University, Johns Hopkins University, Maryland, United States, 6. Johns Hopkins University, Baltimore, Maryland, United States, 7. Columbia University Irving Medical Center, New York, New York, United States*

The longitudinal associations between hearing impairment and higher-level functional measures and the potential confounding role of vestibular function have not been assessed. We investigated these associations in 831 participants of the Baltimore Longitudinal Study of Aging (2012–2019). Hearing was measured using pure-tone audiometry and categorized using WHO standards. Physical function was assessed with the Health Aging and Body Composition Physical Performance Battery (HABCPPB, higher=better) and walking endurance with time to walk 400 meters. Multivariable regression models tested the hypotheses that participants with hearing impairment have poorer physical outomes. In a subset, we further adjusted for vestibular function. Hearing impairment was associated with decrements in higher-level physical performance and walking endurance, and faster decline over time, regardless of vestibular function. Among participants with any hearing impairment, hearing aid users were faster in the 400-m walk. Early screening for higher-level functional loss among older adults with hearing loss is warranted.

### Session 4045 (Paper)

### COVID-19 AND COMMUNITY DWELLING ADULTS

### AGING THROUGH THE TIME OF COVID-19: HEALTHCARE ACCESS FOR OLDER ADULTS LIVING WITH CHRONIC CONDITIONS

Allie Peckham,[1] Molly Maxfield,[2] Keenan Pituch,[2] M. Aaron Guest,[2] Shalini Sivanandam,[3] and Brad Doebbeling,[2], *1. Arizona State University, Pheonix, Arizona, United States, 2. Arizona State University, Phoenix, Arizona, United States, 3. Arizona State University, Arizona State University, Arizona, United States*

Chronic conditions require on-going continuous management and preventive treatment. Over 80% of adults aged 65 and older have multiple chronic conditions. Concerns have arisen about how the COVID-19 pandemic is affecting the management of chronic conditions. Delay, avoidance, and poor management of healthcare during the COVID- 19 pandemic may increase the risk of unnecessary hospitalizations and mortality. This study aims to understand the impact of COVID-19 on healthcare access in a U.S. sample of Americans 50 years of age or older. Participants completed an online survey about healthcare access and other risk factors during the COVID-19 pandemic. Multinomial regression analysis examined the results of two key access points: healthcare provider /doctor (n=468) and medication (n=754). One-half (56%) of those who needed access to a provider were able to be seen. Participants who were older, had multiple chronic conditions, and those with a provider were more likely to have access. However, when individuals with more chronic conditions did not have access, they indicated that this lack of access was due to COVID-19. When not receiving access to medications, unemployed participants attributed the lack of access more often to COVID-19 than other reasons. These findings demonstrate an important lack of access to providers and medication among older adults during the pandemic. In multivariate models, this lack of access was most often due to COVID-19, in addition to traditional factors such as insurance, employment, and medical and behavioral comorbidity. Interventions are needed to lower access barriers to care even further during COVID-19.

### CAREGIVING DURING COVID-19: A MULTI-STATE QUALITATIVE STUDY OF FAMILY CAREGIVER EXPERIENCES AND DECISION MAKING

Tonie Sadler,[1] Kevin Yan,[1] Daniel Brauner,[2] Harold Pollack,[3] and R. Tamara Konetzka,[3], *1. The University of Chicago, Chicago, Illinois, United States, 2. Western Michigan University, Kalamazoo, Michigan, United States, 3. University of Chicago, Chicago, Illinois, United States*

COVID-19 poses unique challenges to family caregivers. This study explores how family caregivers for older adults with cognitive impairments experience and make decisions about caregiving during a global pandemic. Using purposive sampling, 63 family caregivers across eight states participated in open-ended qualitative interviews (2019-2020), until thematic saturation was reached. Questions broadly examined caregivers' experiences and decisions, focusing on decisions made around type of care setting. Questions about responses to the Pandemic were added as events unfolded. States were selected to represent variation in Home and Community Based Service (HCBS) expenditures as a percentage of total Medicaid long-term services and supports expenditures. Family caregivers experienced significant concern about COVID-19 itself, and about the indirect consequences of caregiving caused by the pandemic. Caregivers also displayed flexibility and adaptability in ceasing selected services, contingently continuing services, and utilizing telemedicine and other remote healthcare interventions to protect their loved ones. Many family caregivers utilized remote health care tools such telemedicine, no-contact prescription and grocery delivery. Such measures improved service access and reduced caregiver workload. Given the persistent challenges posed by COVID-19, long-term service organizations have an opportunity to enhance their policies to meet the needs of caregivers and those they care for. There is a need to expand telemedicine and other remote healthcare tools, while adapting these technologies to the needs of families. Also, procedures are needed for safe pathways to utilize HCBS and nursing care during a pandemic including communication supports, sufficient PPE, increased staffing, and utilization of evidence-based protocols.

### HOW HAS COVID-19 IMPACTED OLDER ADULTS AND THEIR NEEDS FOR SERVICES?

Alycia Bayne,[1] Mallory Kennedy,[1] Emily Alvarez,[2] Bernadette Wright,[3] Lucy Theilheimer,[4] and Jennifer Benz,[5], *1. NORC at the University of Chicago, Bethesda, Maryland, United States, 2. NORC at the University of Chicago, Chicago, Illinois, United States, 3. Meals on Wheels America, Arlington, Virginia, United States, 4. Meals On Wheels America, Arlington, Virginia, United States, 5. NORC at the University of Chicago, Kamuela, Hawaii, United States*

COVID-19 has had profound effects on older adults and will have lasting impacts on their preferences and needs for services, including those offered by Meals on Wheels and other community organizations. Organizations serving older adults would benefit from insights about how to prioritize resources and services to address older adults' needs during the pandemic and beyond. On behalf of Meals on Wheels America, NORC at the University of Chicago conducted a study to explore COVID-19's impacts on older adults and older adults' needs during the pandemic. We conducted two data collection activities with adults age 60 and older: a nationally representative survey with 1,535 respondents and 24 interviews. Results indicated that COVID-19 has affected older adults' physical and mental health, social connectedness, employment, and use of services and technology. Informal networks of family members and friends are a source of assistance for 50% of older adults during the pandemic. Impacts of COVID-19 differed by income, rurality, disability status, and living situation. Findings documented the extent to which older adults had unmet needs during the pandemic, such as activities to help keep busy at home and affordable food to meet dietary needs. Survey respondents who have a lower income, are 75 and older, live alone, and who are Black were more likely to have unmet needs. Findings suggested a need to strengthen partnerships among organizations that serve older adults to address diverse needs, conduct ongoing assessments of older adults' needs and preferences, and enhance assistance for informal support networks.

### LONGITUDINAL EFFECTS OF COPING STRATEGIES ON MENTAL HEALTH OF OLDER ADULTS LIVING ALONE DURING THE COVID-19 PANDEMIC

Seoyoun Kim,[1] Hyunwoo Yoon,[2] and Yuri Jang,[3], *1. Texas State University, San Marcos, Texas, United States, 2. Gongju National University, Gongju, Ch'ungch'ong-namdo, Republic of Korea, 3. University of Southern California, Los Angeles, California, United States*

The COVID-19 pandemic and related social distancing measures have posed a significant threat to the mental health of older adults, particularly those living alone. Accordingly, the World Health Organization implemented the #HealthyAtHome program, encouraging people to keep in regular contact with loved ones, stay physically active, and keep a regular routine. The current study aims to examine a micro-longitudinal link between positive coping strategies (e.g., exercise, meditation, relaxation, and virtual social contacts) and depressive symptoms among older adults who live alone during the COVID-19 pandemic. We used 21 biweekly waves of longitudinal data from the Understanding America Study (UAS) collected between April 2020 and February 2021 (N=839, observation= 16,256). The multilevel models with correlated random effects were estimated to examine lagged effects of coping strategies (t-1) on depressive symptoms

(t). The analysis used the xthybrid command with clustered standard errors in Stata 15.1. The results show that exercise (b=-.10, p=0.02), relaxation (b=-02, p=0.01), and virtual social contacts (b=-.01, p=0.01) were predictive of lower depressive symptoms even after controlling for time-invariant and time-varying covariates. Meditation, however, was associated with higher depressive symptoms (b=.01, p=0.02). The results show that modifiable lifestyle factors, such as taking time to exercise or relax, may enhance mental health and well-being for older adults living alone. Virtual social contacts such as video calls could be an effective way to keep older adults socially connected and emotionally healthy.

### SOCIAL ISOLATION AND COVID-19 MITIGATION: PERSPECTIVES OF KEY INFORMANTS IN THE UNITED STATES AND JAPAN

Emily Ihara,[1] Megumi Inoue,[1] Cortney Hughes Rinker,[1] and Naoru Koizumi,[2], *1. George Mason University, Fairfax, Virginia, United States, 2. George Mason University, Arlington, Virginia, United States*

The deleterious health effects of social isolation and loneliness among older adults have been well-established and were exacerbated by the forced separation for those at health risk of contracting the COVID-19 virus. Both the United States and Japan are experiencing phenomenal growth of the older adult population; Japan is considered a "super-aged" society, with the highest proportion of people aged 65 and older in the world. This study examined how COVID-19 and mitigation measures may have affected services for older adults. We conducted key informant interviews with specialists in aging and older adult care in both Japan (n=5) and the United States (n=14). All interviews were conducted over Zoom and lasted 30-60 minutes. The research team transcribed and checked the interviews for accuracy and conducted multiple coding sessions to identify, sort, and consolidate the codes using Atlas.ti. Key themes in both countries that emerged included the many cracks in the system of programs and services for older adults, the inaccessibility to technology and the internet, and the particular difficulties of socioeconomic inequities, especially for those living alone. Older adults were motivated to become more technologically proficient and local communities came forward to help provide support. One key informant from the U.S. noted that their organization experienced a 600% increase in interest among volunteers as a result of the pandemic. Despite the many challenges of the pandemic, many silver linings emerged. One participant poetically stated, "I think that's human nature – when you have no other choice, you find a way."

### Session 4050 (Paper)

### DEPRESSION AND OUTCOMES

### ANTIDEPRESSANT USE AND RISK OF SUICIDAL BEHAVIOR IN OLDER PERSONS WITH DEPRESSION: A COHORT STUDY IN HONG KONG

Yi Chai,[1] Hao Luo,[1] Kenneth K.C. Man,[2] Wallis C.Y. lau,[2] and Ian C.K. Wong,[1], *1. The University of Hong Kong, Hong Kong, Not Applicable, Hong Kong, 2. University College London, London, England, United Kingdom*

**Background:** Depression is highly prevalent in older adults and requires treatment. However, debate persists on whether antidepressant use is associated with an elevated risk of suicidal behavior. This study aims to examine the short- and long-term risk of suicidal behavior by various classes of antidepressants in older persons with depression.

**Methods:** Persons aged 40 years and above and received a clinical diagnosis of depression between January 1, 2001, and December 31, 2016 were identified from the Clinical Data Analysis and Reporting System in Hong Kong. The risk of suicidal behavior in persons who were prescribed antidepressants was compared with persons who were not prescribed any antidepressant drugs. Antidepressants were classified as tricyclic and related antidepressant drugs (TCAs), selective serotonin reuptake inhibitors (SSRIs), noradrenergic and specific serotonergic antidepressants (NaSSAs), serotonin–norepinephrine reuptake inhibitors (SNRIs) and others. Incidence and adjusted hazard ratio (aHR) of subsequent self-harm and suicide within one-year and the whole study period were estimated by age groups.

**Results:** A total of 34,927 persons aged 40-64 years, and 19,300 persons aged 65+ years were included. In the younger age group, the highest short-term and long-term risks were found in others (aHR, 2.33; 1.02-5.34) and NaSSAs (2.88; 2.15-3.86), respectively. In the older age group, no significant association was observed between antidepressant use and suicidal behavior across all antidepressant classes.

**Conclusion:** The self-harm and suicide associated risks vary across antidepressant classes and age groups. Cautions are always needed for antidepressant prescriptions.

### ASSOCIATION OF EARLY-LIFE FAMILY AND NIEGHBORHOOD CIRCUMSTANCES WITH DEPRESSIVE SYMPTOMS IN CHINESE OLDER ADULTS

Yan Chen, and Jie Tan, *Duke Kunshan University, Kunshan, Jiangsu, China (People's Republic)*

A growing body of literature suggests that early life circumstances can influence mental health throughout the lifespan. However, how these early life circumstances cumulatively contribute to depression in old age is not completely understood. The present study examined the associations of eight factors representing multifaceted early life experience at individual, family, and community levels with depression among community-dwelling older adults. Data were from the China Health and Retirement Longitudinal Study. We included 8,239 community-dwelling individuals who were ≥60 years, completed the life history questionnaire, and had assessment of depression. Chi-square test was used to examine the unadjusted associations between each of the eight early life risk factors and depression. An early life disadvantage index was established using risk factors that were significantly associated with depression. Logistic regression was used to examine the association of each early life risk factor and the index with depression. Of 8,239 individuals included, 2,055 (24.9%) had depression. In bivariate analysis, each of eight early life risk factors was significantly associated with depression. Except for maternal and paternal education, all risk factors persisted to be associated with depression after multivariable adjustment. In the multivariable-adjusted model, a one-point higher in the early life disadvantage index (range: 0-6) was associated with a 45% (95% CI: 37%, 53%) higher odds of depression. There was a strong association between early life environments and depressive symptoms among Chinese community-dwelling older adults. Adverse early life circumstances could contribute cumulatively to depression in old age.

### COGNITIVE FRAILTY AND ITS LONG-TERM EFFECT ON DEPRESSION IN AN OLDER POPULATION IN KOREA

Yoonjung Ji, TaeWha Lee, and Eunkyung Kim, *Yonsei University, Seoul, Seoul-t'ukpyolsi, Republic of Korea*

Cognitive frailty is a condition where physical frailty and mild cognitive impairment (MCI) co-exist without dementia. It occurs in 1.8%-8.9% of the general older population, and older people with depression have a higher risk of frailty. However, the relationship between cognitive frailty and depression is still unclear. This study aimed to determine the relationship between cognitive frailty and depression of older adults by time using comparative group analysis. A secondary analysis was conducted using the Korean Longitudinal Study of Aging (KLoSA) dataset from 2010 to 2018. A sample was 981 older adults who were 65 years old and without dementia over residing in the community. Cognitive frailty was defined as having a mini-mental state examination score of 18-23 and 3 or more of the Fried frailty indexes. Generalized Estimating Equation model and chi-square test were employed. Of the 981 subjects, the cognitive frailty(CF) was 28.5%, followed by robust (37.7%), physical frailty (PF, 29.4%), mild cognitive impairment (MCI, 4.4%) at baseline. The group differences on depression measured by the Center for Epidemiological Studies Depression (CESD) were statistically significant in the PF ($F=4.70$, $p<.001$) and the CF ($F=4.95$, $p<.001$) group compared to the robust group. The time difference effect ($F=.09$, $p=.05$) and a group-by-time interaction effect were observed ($p<.001$). This study confirmed that cognitive frailty is strongly associated with depression. Effective approaches to managing psychological wellbeing, including dementia, are essential for older adults with cognitive frailty.

### DEPRESSION AND AGE-RELATED CHANGES IN BODY COMPOSITION, CARDIOVASCULAR FUNCTION, GRIP STRENGTH, AND LUNG FUNCTION

Julian Mutz, and Cathryn Lewis, *King's College London, London, England, United Kingdom*

Individuals with mental disorders, on average, die prematurely, have higher levels of physical comorbidities and may experience accelerated ageing. In individuals with lifetime depression and healthy controls, we examined associations between age and multiple physiological measures. The UK Biobank study recruited >500,000 participants, aged 37-73, between 2006–2010. Generalised additive models were used to examine associations between age and grip strength, cardiovascular function, body composition, lung function and bone mineral density. Analyses were conducted separately in males and females with depression compared to healthy controls. Analytical samples included up to 342,393 adults (mean age = 55.87 years; 52.61% females). We found statistically significant differences between individuals with depression and healthy controls for most physiological measures, with standardised mean differences between -0.145 and 0.156. There was some evidence that age-related changes in body composition, cardiovascular function, lung function and heel bone mineral density followed different trajectories in individuals with depression. These differences did not uniformly narrow or widen with age. For example, BMI in female cases was 1.1 kg/m2 higher at age 40 and this difference narrowed to 0.4 kg/m2 at age 70. In males, systolic blood pressure was 1 mmHg lower in cases at age 45 and this difference widened to 2.5 mmHg at age 65. Individuals with depression differed from healthy controls across a broad range of physiological measures. Differences in ageing trajectories differed by sex and were not uniform across physiological measures, with evidence of both age-related narrowing and widening of case-control differences.

### WAYS COMMUNITY-BASED ORGANIZATIONS ENHANCE LATE-LIFE DEPRESSION CARE IN PARTNERSHIP WITH CLINICS

Theresa Hoeft,[1] Melissa Gosdin,[2] Jenny Wagner,[2] Stuart Henderson,[2] Mindy Vredevoogd,[3] Katherine James,[3] Jurgen Unutzer,[3] and Ladson Hinton,[4], *1. University of Washington, Seattle, Washington, United States, 2. University of California Davis, University of California Davis, California, United States, 3. University of Washington, University of Washington, Washington, United States, 4. University of California, Davis, Sacramento, California, United States*

Late-life depression is a serious public health concern in the U.S., especially as the population ages. To improve care coordination and increase the number of providers working to improve depression outcomes, primary care clinics and community-based organizations (CBOs) can partner and improve care. Addressing social determinants of health is one area CBOs can help respond to but there are other ways CBOs can bring value to these partnerships with primary care clinics. As part of a qualitative evaluation of the Care Partners Project, 84 key informant interviews and 20 focus groups were conducted over five years with selected primary care physicians, care managers, administrators and psychiatric consultants. These data were coded and organized using an inductive and deductive thematic analysis approach. CBOs contributed to care through 1) adding new services that focus on clients' social needs (e.g., assistance locating affordable housing, reliable transportation, applying for social security benefits) that were foundational to effective depression care; 2) strengthening core aspects of existing care; 3) incorporating a lay health workforce to enhance care; and/or 4) adding home visits that supported deeper understanding of patient's life context, enhanced trust and improved access to care. CBOs can enhance depression care through increasing access and quality of care. Findings can inform conversations about the value CBOs offer when partnering with health care systems and improve partnership efforts. Such conversations are worth revisiting as organizations deepen their connections and work together over time.

### Session 4055 (Paper)

### DEVELOPMENTAL CHALLENGES IN LATER LIFE

### DIFFERENTIAL TREATMENT OF OLDER WORKERS DUE TO COVID-19: POTENTIAL FOR AGEISM AND AGE DISCRIMINATION AT WORK

Lisa Hollis-Sawyer, *Northeastern Illinois University, Chicago, Illinois, United States*

This paper examines the implications of employers' current COVID-19 protective workplace attendance policies toward older workers, potentially creating the outcomes of increased numbers of involuntary retirees and the discouraged older worker syndrome among otherwise qualified older workforce participants. How potential ageist assumptions and age discrimination under COVID-19 affect workplace decisions in reflection on the Age Discrimination in Employment Act (1967) guidelines is discussed. Older workers may remain in the workforce longer than ever before due to having healthier life expectancies. Workplace policies need to be increasingly sensitive to older employees' rights to sustain their workplace engagement (Cummins, 2014; Cummins, Harootyan, & Kunkel, 2015). The author reviewed current unemployment trends in 2020 and emerging litigation in reflection upon general issues of COVID-19 related age discrimination in the older workers' workplace attendance decisions by employers and the historical framework of the Age Discrimination in Employment Act (1967, with significant amendments in 1978 and 1986). The policy analysis paper presents the implications of employers' COVID-19 protective policies on older workers and how it may affect the "health" of the workplace and older adults and the economy beyond the pandemic. Lastly, strategies to address an "age-friendly" workplace during a pandemic and post-pandemic are discussed.

### EARLY RETIREMENT AND SENSORY IMPAIRMENTS: THE MODIFYING EFFECT OF TOTAL ASSETS

Emmanuel Garcia Morales, and Nicholas Reed, *Johns Hopkins Bloomberg School of Public Health, Baltimore, Maryland, United States*

Sensory impairments are common among older adults. Little is known on the association between sensory impairments, which impact labor productivity, and the effect modification of wealth. We used the 2006-2018 rounds of the Health and Retirement Study. Hearing (HI) and vision (VI) impairments (self-report) at baseline, and working status throughout the study period was observed. Logistic regression models, adjusted for demographic, socioeconomic, and health characteristics, were used to characterize the association of sensory impairment and early retirement (i.e., before age 65). Secondary analysis stratified by assets. Among 1,688 adults ages 53-64, 1,350 had no impairment, 140 had HI only, 141 VI only, and 57 had dual sensory impairment (DSI). Only adults with HI had higher odds of early retirement (Odds Ratio [OR]: 1.6; 95% Confidence Interval [CI]: 1.0,2.5) relative to those without sensory impairment. Among those with large assets, those with HI had higher odds (OR:2.6, 95% CI: 1.4,5.2) and those with VI had lower odds (OR. 0.37; 95% CI: 0.2,0.8) of early retirement. Among the low asset group, we found no differences across impairment groups for the odds of retirement. In sample of older adults, we provide evidence that the presence of hearing impairment is associated early retirement. Secondary analyses suggest wealth may modify this association which highlights the wealth disparities faced by people with sensory impairments.

### ELDERCARE RESPONSIBILITIES AND PHYSICAL HEALTH SYMPTOMS AMONG MANUFACTURING WORKERS

Richard Fortinsky,[1] Janet Barnes-Farrell,[2] Jennifer Garza,[1] Samantha Lacey,[2] Hannah Austin,[2] James Grady,[1] and Martin Cherniack,[1], *1. University of Connecticut School of Medicine, Farmington, Connecticut, United States, 2. University of Connecticut, Storrs, Connecticut, United States*

Working adults responsible for providing care to older relatives at home (eldercare) have reported greater psychological health problems such as depressive symptoms and stress than workers without eldercare responsibilities. Less is known about how eldercare is associated with physical health symptoms such as sleep-related problems and pain. Among manufacturing workers, such physical health symptoms have the capacity to diminish productivity. Therefore, we explored associations between eldercare responsibilities and physical health symptoms that could affect work performance in a sample of 357 adult employees from five manufacturing companies in a northeastern US state. Research questions were: are workers with eldercare responsibilities more likely than those without eldercare responsibilities to report sleep-related and pain-related symptoms, and are the number of eldercare tasks associated with these physical health symptoms? Among sample members, 52 (14.6%) provided eldercare, 62% were male, mean(standard deviation) age=49.8(12.7), and 77% were non-Hispanic White; no demographic differences were found between those with and without eldercare responsibilities. In bivariate analyses, we found that providing eldercare was associated with lower sleep quality (p=.05), fewer hours of sleep during the workweek (p=.04), more pain interference at home and at work (p=.02), and more pain on average in the past week (p=.01). Providing more types of eldercare tasks ranging from personal care to providing transportation was associated with more pain on average in the past week (p = .04). We conclude that eldercare is associated with physical health symptoms that could directly affect job performance among manufacturing workers. Workplace policy implications will be discussed.

### RETIREMENT MIGRATION AND PRECARITY: MATERIAL AND SOCIAL ASPECTS

Marion Repetti,[1] and Toni Calasanti,[2] *1. University of Applied Sciences and Arts Western Switzerland HES·SO // Valais-Wallis, Sierre, Valais, Switzerland, 2. VIrginia Tech, Blacksburg, Virginia, United States*

Discussions of precarity in later life have tended to focus on the uncertainties of material resources, and the feelings of anxiety that this evokes (e.g., Lain et al. 2019) as some older people thus face the risk of being excluded from the broader society. Although scholars often point to inequalities, such as those based on class and gender, as having an influence on the likelihood of older people experiencing such precarity, ageism is considered only to the extent that it can exacerbate the impact of these statuses through, for instance, labor market experiences. Here, we expand upon the impact of ageism on the social aspects of precarity: the loss of recognition and respect as a person that is at the core of social bonds. Drawing on qualitative interviews we have conducted among Swiss, British, and U.S. older people who migrated to cheaper countries in retirement, we demonstrate that ageism can influence precarity regardless of classes. We find that even among wealthier older migrants, who otherwise might fit the image of the retiree seeking an active lifestyle in a sunny location, the attempt to escape the devaluation heaped upon older people in their original country plays an important role. In their new countries, retired migrants of all classes felt that they were valued and part of a community, and this differed from the ageism in their home countries. We thus argue that ageism be considered in future analyses of precarity in later life.

### Session 4060 (Symposium)

### DYNAMIC RELATIONSHIPS BETWEEN PERCEIVED HOUSING AND LIFE TRANSITIONS: A BETTER UNDERSTANDING OF GOOD AGING IN PLACE

Chair: Frank Oswald Co-Chair: Steven Schmidt
Discussant: Malcolm Cutchin

Housing has gained increased relevance as a central factor for health and well-being. Many countries have implemented ageing in place policies, which provide services focused on improving the physical environment. Housing needs change as people grow older and experience different transitions across their life courses. Studies have demonstrated relationships between housing and health and well-being in later life on the one hand and life transitions and health and well-being in later life on the other hand. However, research on life transitions in combination with perceived housing in relation to indicators of good ageing is virtually nonexistent. This symposiums aims to address the dynamic relationship between perceived housing and life transitions and how they impact health, well-being, functioning, and social/neighborhood participation as people age by data from a mixed-method approach in Sweden and Germany. The first contribution by Slaug and colleagues introduces changes in how older adults perceive their housing following the life transition of a fall at home. Second, Eriksson and colleagues present qualitative results on the experience of relationships between perceived housing, several life transitions and well-being among community-dwelling Swedish older adults. Third, Wanka and colleagues present partially different results from a comparable study in German on the same topic but emphasizing the experience of interrelationships between different life course transitions. Fourth, Granbom and colleagues explore how low-income older adults in Sweden reason about their current housing situation and a future life transition of relocation. Finally, Malcolm P. Cutchin will serve as the session's discussant.

### TO FALL OR NOT TO FALL – FALLS AND PERCEPTIONS OF CONTROL AND MEANING OF THE HOME

Susanne Iwarsson, Steven Schmidt, and Björn Slaug, *Lund University, Lund, Skane Lan, Sweden*

Falls are a major public health problem among older people. Even if the outcome of a fall is not fatal, it may be a traumatic experience with both physical and psychological consequences. However, there is a lack of studies examining how falls in the home may impact the perception of the home. To compare perceptions of the home between those who had fallen and those who had not, we utilized data from the Swedish SNAC-GÅS study (N=371; mean age=68; 43% men). Perceptions of control and meaning of the home were captured by established psychological instruments. Excluding falls in other environments than the home (n=99), those who fell in the home the previous year (n=34) scored notably higher on housing control by "powerful others" (p=0.053) and notably lower on perceived "behavioral home bonding" (p=0.056) compared to non-fallers (n=238). These results warrant further research into the impact of falls on perceived housing.

### REASONING ABOUT LIFE TRANSITIONS IN RELATION TO PERCEIVED HOUSING: IN-DEPTH DATA FROM SWEDEN

Steven Schmidt,[1] Maya Kylén,[2] and Erik Eriksson,[1]
*1. Lund University, Lund, Skane Lan, Sweden, 2. CASE Lund University, Lund, Skane Lan, Sweden*

The accessibility and suitability of housing to improve health and well-being for the growing share of older adults in the population has important policy implications. Yet, current housing policies tend to neglect the heterogeneity of older adults housing needs which vary across age, health, and personal preferences. Little is known about how life transitions in combination with perceived housing relates to good aging. This qualitative study aims to explore the relationship between perceived housing, life transitions, well-being, health, and participation in older age. Participants were community-dwelling and aged 65-75 years. All participants reported multiple transitions considered as important for the way they viewed their housing situation and social relationships as well as for the choices they made around their housing. The findings from this project may be used to develop health promotion programs that proactively support housing decisions along the process of ageing and enable full participation in society.

### REASONING ABOUT LIFE TRANSITIONS IN RELATION TO PERCEIVED HOUSING: IN-DEPTH DATA FROM GERMANY

Karla Wazinski,[1] Frank Oswald,[1] and Anna Wanka,[2]
*1. Goethe University Frankfurt, Frankfurt am Main, Hessen, Germany, 2. Goethe University Frankfurt, Frankfurt, Hessen, Germany*

Partially different to the Swedish contribution, this paper analyses the relationships between perceived housing, life course transitions and wellbeing among community-dwelling older adults in Germany. Based on 15 qualitative interviews with persons aged 60-75 years, the contribution focuses on the experience of interrelationships between different life course transitions and perceived housing, and how they contribute to wellbeing in later life. First findings indicate a concourse of different transitions around the retirement age (e.g. illnesses, changes in partnerships) and a temporal as well as causal relationship between the two transitions relocation and retirement (for example, relocation becomes possible only after retiring or people relocate with the retirement phase in mind). The entanglement of life course transitions, in turn, shapes the person-place-relationships and perceived housing in different ways, which will be exemplified and interpreted in the presentation. However, further research is needed to consider the effects of social inequalities in these processes.

### Session 4065 (Symposium)

### EMERGING BIOTECHNOLOGY MARKERS OF COGNITIVE IMPAIRMENT

Chair: Megan Huisingh-Scheetz
Discussant: Jennifer Schrack

The early detection of cognitive impairment is among the National Institute on Aging's (NIA) current research priorities. Sensor-based technologies have exploded in recent years allowing remote, continuous measurement of older adults' free-living activity. This highly granular data has stimulated exciting new research exploring how change in health can be detected remotely using novel "biotechnology" markers. Yet, this area of research is in its infancy as it relates to predicting cognitive function. This symposium will provide an overview of the sensor-cognition research landscape and will feature 5 new studies exploring the relationship between biotechnology markers and cognitive function, each with unique sensors, cognitive measures and samples. The first three presentations will report associations between accelerometry-based activity measures (chest or wrist devices) and cognitive function (assessed by diagnosis, a neurocognitive assessment, or microstructural changes on DTI) in the Baltimore Longitudinal Study on Aging, a large, NIA-funded epidemiologic dataset. The fourth presentation will report the significance of free-living hip accelerometry activity measures beyond clinically-available information in a random forest prediction model of 1-year change in Montreal Cognitive Assessment scores among urban, predominantly African-American older adults without moderate-severe dementia residing in the community. The final presentation will report associations between room-to-room transitions as detected by in-home, infrared motion sensors and mild cognitive impairment using data from a community-dwelling sample of older adults residing alone. This symposium will provide a substantial expansion of current knowledge in this research space and will be relevant to clinicians or researchers with an interest in sensor technology or dementia.

### RELATIVE VIGOROUS-INTENSITY PHYSICAL ACTIVITY PREDICTS BRAIN MICROSTRUCTURAL CHANGES IN OLDER ADULTS

Jennifer Schrack,[1] Bennett Landman,[2] Amal Wanigatunga,[1] Susan Resnick,[3] Luigi Ferrucci,[3] and Qu Tian,[3], *1. Johns Hopkins Bloomberg School of Public Health, Baltimore, Maryland, United States, 2. Vanderbilt University, Nashville, Tennessee, United States, 3. National Institute on Aging, Baltimore, Maryland, United States*

Physical activity especially at moderate-to-vigorous intensity may preserve brain structure in old age. However, current findings are cross-sectional and rely on absolute intensity. This study aimed to examine whether relative or absolute vigorous-intensity physical activity (VPA) predicts brain microstructural changes. We analyzed 260 initially cognitively normal and well-functioning participants(age=70.5yrs) who had VPA data via ActiHeart and longitudinal brain microstructure by DTI(follow-up=3.7yrs). Associations of VPA with microstructural changes were examined using linear mixed-effects models, adjusted for demographics. Each SD higher relative VPA defined by heart rate reserve (i.e. 21 min/day) was significantly associated with less decline in memory-related microstructural integrity, including mean diffusivity of entorhinal cortex and parahippocampal gyrus and fractional anisotropy of uncinate fasciculus and cingulum-hippocampal part, and not executive/motor-related microstructure. Absolute VPA was not associated with microstructural markers. Among well-functioning older adults, participating in VPA defined by heart rate reserve may predict less brain microstructural decline in memory-related areas.

### DAILY PHYSICAL ACTIVITY PATTERNS: A WINDOW ON COGNITIVE DECLINE IN THE BALTIMORE LONGITUDINAL STUDY OF AGING (BLSA)

Fangyu Liu,[1] Hang Wang,[1] Jacek Urbanek,[2] Yang An,[3] Eleanor Simonsick,[4] Susan Resnick,[5] Jennifer Schrack,[6] and Amal Wanigatunga,[6], *1. Johns Hopkins University, Baltimore, Maryland, United States, 2. Johns Hopkins School of Medicine, Baltimore, Maryland, United States, 3. NIA, Baltimore, Maryland, United States, 4. National Instute on Aging/NIH, Baltimore, Maryland, United States, 5. National Institute on Aging, Baltimore, Maryland, United States, 6. Johns Hopkins Bloomberg School of Public Health, Baltimore, Maryland, United States*

Gradual disengagement from essential daily physical activity (PA) necessary for independent living could signal present or emerging mild cognitive impairment (MCI) or Alzheimer's disease (AD). We used BLSA data to examine whether PA patterns including: 1) total activity counts/day, 2) minutes/day spent active, and 3) activity fragmentation (reciprocal of the mean active bout length) differs between participants with adjudicated normal cognition (n=498) and MCI/AD diagnoses (n=32). Linear models were used and adjusted for demographics, APOE-e4 status, morbidity, and gait speed. Compared to those with normal cognition, those with MCI/AD had 3.0% higher activity fragmentation (SE=1.1%, p=0.006) but similar mean total activity counts/day (p=0.08) and minutes/day spent active (p=0.19). Results suggest that activity fragmentation may arise as a compensatory strategy in the absence of reduced activity in MCI and early AD and that activity monitoring may be potentially useful for detecting MCI and AD at an earlier stage.

### CIRCADIAN REST AND ACTIVITY RHYTHMS AND COGNITIVE CHANGE IN THE BALTIMORE LONGITUDINAL STUDY OF AGING

Yang An,[1] Sarah Wanigatunga,[2] Vadim Zipunnikov,[3] Mark Wu,[4] Eleanor Simonsick,[5] Susan Resnick,[6] Adam Spira,[7] and Jill Rabinowitz,[7], *1. NIA, Baltimore, Maryland, United States, 2. Johns Hopkins, Baltimore, Maryland, United States, 3. Department of Biostatistics, Bloomberg School of Public Health, Johns Hopkins, Baltimore, Maryland, United States, 4. Johns Hopkins University School of Medicine, Baltimore, Maryland, United States, 5. National Instute on Aging/NIH, Baltimore,*

*Maryland, United States, 6. National Institute on Aging, Baltimore, Maryland, United States, 7. Johns Hopkins Bloomberg School of Public Health, Baltimore, Maryland, United States*

Alterations in 24-hour movement patterns, or circadian rest/activity rhythms (RARs), commonly occur with aging. Using linear mixed effects (LME) modeling, we examined associations of baseline RARs with longitudinal change in cognition. Participants (N=424; 47% male, baseline age 72.8±10.1 years) were from the Baltimore Longitudinal Study of Aging and completed 5.6±0.8 nights of wrist actigraphy at baseline. Tests of memory, executive function, attention, language, and visuospatial ability were administered at baseline and subsequent visits (3.7±1.7 years of follow-up in those with >1 visit (n=295)). In unadjusted random intercept and slope LME models, greater RAR stability predicted slower memory decline, and higher activity during participants' least active 5 hours (L5) predicted slower decline in visuospatial ability. After covariate adjustment, higher activity in participants' most active 10 hours (M10) and higher L5 predicted slower decline in visuospatial ability (p<.05). Further research is needed on RARs as risk factors for later-life cognitive decline.

### HIP ACCELEROMETRY ACTIVITY PATTERNS IMPROVE MACHINE LEARNING PREDICTION OF 1-YEAR MOCA SCORE CHANGE

Chengjian Shi,[1] Jacek Urbanek,[2] Niser Babiker,[3] Alan Gonzolez,[3] Jovany Soto,[4] Andrey Rzhestsky,[3] and Megan Huisingh-Scheetz,[3], *1. University of Chicago, chicago, Illinois, United States, 2. Johns Hopkins School of Medicine, Baltimore, Maryland, United States, 3. University of Chicago, Chicago, Illinois, United States, 4. Illinois Institute of Technology, Orland Park, Illinois, United States*

We tested whether free-living hip accelerometry measures improved prediction of 1-year change in Montreal Cognitive Assessment (MoCA) scores beyond clinically available information. We analyzed data (n=126) from predominantly African American (78.2%) older adults without moderate-severe dementia residing near our geriatrics clinic. Age (73.6 ±6.1 years), gender, education, comorbidities, income, and MoCA performance were collected at baseline; participants then wore a right hip, triaxial Actigraph accelerometer (30Hz) continuously for 7 days. A MoCA was repeated at 1 year. Six measures were calculated from the daytime (7am-5pm) data: mean/variance of hourly counts per minute, mean/variance of daily percent of time spent in the lowest activity quartile, and mean/variance of daily percent of time spent in the highest activity quartile. In a random forest model containing baseline MoCA, demographics and comorbidities, the accelerometry measures improved prediction of 1-year MoCA performance by ~17.8%. Accelerometry data may be clinically useful for predicting early cognitive decline.

### STABILITY AND VARIABILITY OF INDOOR ROOM TRANSITIONS AND MILD COGNITIVE IMPAIRMENT IN OLDER ADULTS

Christina Reynolds,[1] Lisa Barnes,[2] Lisa Silbert,[1] Hiroko Dodge,[1] Jeffrey Kaye,[1] Zachary Beattie,[1] and Chao-Yi Wu,[1], *1. Oregon Health & Science University, Portland, Oregon, United States, 2. Rush University Medical Center, Chicago, Illinois, United States*

Indoor room transition is an underexplored real-world activity outcome. We estimated the stability and variability of indoor room transitions and their associations with mild cognitive impairment (MCI) in older adults. Older adults living-alone (n=159, age=78.3±8.8 years, 14% MCI) from the Oregon Center for Aging & Technology (ORCATECH) and the Minority Aging Research Study (MARS) were included. Room transitions were detected using passive infrared motion sensors in bathroom, bedroom, kitchen, and living room. The hourly number of room transitions was used to calculate the interdaily stability and intradaily variability of room transitions. MCI was operationalized by the Clinical Dementia Rating equaled 0.5. Generalized estimating equations models adjusted for demographics, health, and environmental factors revealed that older adults with MCI had a lower interdaily stability of room transitions than cognitive healthy peers (z=-2.06,p=0.03). A pervasive-sensing system deployed in homes can obtrusively measure room transition activities to inform cognitive health in older adults.

### Session 4070 (Paper)

### FALL RISK

### ACCELEROMETER-MEASURED DAILY STEPS, PHYSICAL FUNCTION, AND SUBSEQUENT FALL RISK IN OLDER WOMEN: THE OPACH STUDY

Benjamin Schumacher,[1] John Bellettiere,[2] Michael LaMonte,[3] and Andrea LaCroix,[2], *1. University of California, San Diego, San Diego, California, United States, 2. University of California, San Diego Herbert Wertheim School of Public Health and Human Longevity Science, La Jolla, California, United States, 3. University at Buffalo, Buffalo, New York, United States*

We sought to investigate the association between steps per day (steps/d) and incident fall risk while also assessing the role of physical functioning on this association. Steps/d were measured by accelerometer for 7 days in 5,545 women aged 63 to 97 years between 2012 - 2014. Falls were ascertained from daily fall calendars for 13 months. Median steps/d were 3,216. There were 5,473 falls recorded over 61,564 fall calendar months. The adjusted incidence rate ratio comparing women in the highest vs. lowest step quartiles was 0.71 (95% confidence interval, 0.54 - 0.95; P-trend across quartiles of steps/d = 0.01). After further adjustment for physical function using the Short Physical Performance Battery, the rate ratio was 0.86 (0.64-1.16; P-trend = 0.27). Mediation analysis estimated that 66.7% to 70.2% of the association of steps/d and fall risk may be mediated by physical function. In conclusion, higher steps/d were related to lower incident falls primarily through their beneficial association with physical functioning. Interventions that improve physical function, including those that involve stepping, could reduce falls in older adults.

### FINDING STATIC STABILITY LIMITS: COMPARISON OF REACTIVE BALANCE IN OLDER PEOPLE WITH AND WITHOUT A HISTORY OF FALLS

Michel Hackbarth, Jessica Koschate, Sandra Lau, and Tania Zieschang, *Carl von Ossietzky University Oldenburg, Oldenburg, Niedersachsen, Germany*

Reactive balance is a highly relevant fall risk factor, but is rarely considered in clinical practice. Especially medio-lateral perturbations lead to a pronounced instability of the gait pattern. However, there is no consensus on a method for the assessment of individually challenging perturbation intensities to apply during walking. The aim of this study is to determine and compare the static stability-limits in older adults with and without a history of falls. Twelve older adults with (OAF; 75.6 ±3.66,9♀) and 19 older adults without a history of falls (OA; 77.5 ±4.99,12♀) were subjected to progressive-intensifying perturbations while standing on a perturbation treadmill. In addition, functional performance (Mini-BESTest), fear of falling (FES-I), and physical activity (kcal) were assessed Deflection of the treadmill-platform was randomized by timing and direction and was increased until the subject had to compensate with a step (stability-limit). The maximum deflection distance for each direction, as well as the FES-I score, mini-BESTest score, and activity level were evaluated for group differences using the t-test and Mann-Whitney-U test (α≤5%). There were no significant group differences in the mini-BESTest and between the maximum tolerated deflection distances. The OAF-subjects showed an increased FES-I score (median for OA=18.0 and OAF=22.0, p=0.032) and higher activity levels (median for OA=1974 kcal and OAF=3365 kcal, p=0.011). Despite an increased fear of falling, the older adults with a fall history showed a similar stability-limit, but higher activity levels. In future experiments these static stability limits should be tested during walking and evaluated via motion analysis.

### MEAN ARTERIAL PRESSURE AND RISK OF FALLS RESULTING IN HOSPITAL PRESENTATION IN OLDER ADULTS

Sultana Monira Hussain,[1] Michael Ernst,[2] Christopher Reid,[3] Andrew M. Tonkin,[1] Johannes Neumann,[1] Le Thi Phuong Thao,[1] Anna Barker,[4] and John McNeil,[1], *1. Monash University, Melbourne, Victoria, Australia, 2. University of Iowa, Iowa City, Iowa, United States, 3. Curtin University, Perth, Western Australia, Australia, 4. Silver Chain, Melbourne, Victoria, Australia*

Utilising data from the ASPirin in Reducing Events in the Elderly trial participants aged 70-years, we estimated MAP and variation in MAP defined as within-individual SD of MAP from baseline and first 2 annual visits. Falls were confined to those involving presentation to a hospital. Cox proportional hazards regression was used to calculate hazard ratio (HR) and 95% confidence interval (CI) for associations with falls. Amongst 16,703 participants (1,540 falls), MAP was not associated with falls irrespective of antihypertensive medication status (all: HR 1.00, 95% CI 0.99-1.01, not on antihypertensive: HR 1.01, 95% CI 0.99, 1.02, on antihypertensive: HR 1.01, 95% CI 0.99-1.02). Amongst 14,818 participants who remained in the study up to year 2 without falls, 1 unit escalation in MAP variability increased the risk (HR 1.01, 95% CI 1.00-1.03). Compared with those in the lowest tercile of variability, those in the middle or highest tercile of variability experienced an increased risk of falling (middle: HR 1.32, 95% CI 1.06-1.65; highest: HR 1.25, 95% CI 1.01-1.55). When stratified for antihypertensive medication status, those receiving diuretics (HR 1.18, 95% CI 1.00-1.39) or beta-blockers (HR 1.37, 95% CI 1.08-1.73) were at increased risk compared to those receiving renin-angiotensin-system acting agents. All results persisted after adjustment for multiple covariates. The association of diuretics and beta-blockers with falls remained significant even after excluding those with history of heart failure. Older community-dwelling adults with high variability in MAP are at increases risk of falls, particularly amongst those receiving beta-blockers or diuretics.

### MEASUREMENT DISPARITIES IN FRAILTY AMONG KIDNEY TRANSPLANT PATIENTS: IMPACT OF DIFFERENTIAL ITEM FUNCTIONING

Nadia Chu,[1] Alden Gross,[2] Xiaomeng Chen,[1] Qian-Li Xue,[1] Karen Bandeen-Roche,[2] Dorry Segev,[1] and Mara McAdams-DeMarco,[1], *1. Johns Hopkins University School of Medicine, Baltimore, Maryland, United States, 2. Johns Hopkins Bloomberg School of Public Health, Baltimore, Maryland, United States*

Frailty is commonly measured for clinical risk stratification during transplant evaluation and is more prevalent among older, non-White kidney transplant (KT) patients. However, group differences may be partially attributable to misclassification resulting from measurement bias (differential item functioning/DIF). We examined the extent that DIF affects estimates of age, sex, and race differences in frailty (physical frailty phenotype/PFP) prevalence among 4,300 candidates and 1,396 recipients. We used Multiple Indicators Multiple Causes with dichotomous indicators to assess uniform DIF in PFP criteria attributable to age (≥65vs.18-64 years), sex, and race (Black vs.White). Among candidates (mean age=55 years), 41% were female, 46% were Black, and 19% were frail. After controlling for mean frailty level, females were more likely to endorse exhaustion (OR=1.20,p=0.003), but less likely to endorse low activity (OR=0.83,p=0.01). Younger candidates were more likely to endorse weight loss (OR=1.30,p=0.005), exhaustion (OR=1.60,p<0.001), and low activity (OR=1.80,p<0.001). Black candidates were more likely to endorse exhaustion (OR=1.25,p<0.001), but less likely to endorse weakness (OR=0.79,p<0.001). Among recipients (mean age=54 years), 40% were female, 39% were Black, and 15% were frail. Younger recipients were more likely to endorse weight loss (OR=1.55,p=0.005) and low activity (OR=1.61,p=0.02); however, no DIF was detected by sex or race. Results highlight the impact of DIF for specific PFP measures by age, sex, and race among candidates, but only by age for recipients. Further research is needed to ascertain whether candidate- and/or recipient-specific thresholds to correct for DIF could improve risk prediction and equitable access to KT for older, female, and Black candidates.

### PREDICTORS OF FALLS IN OLDER ADULTS WITH AND WITHOUT DEMENTIA

Safiyyah Okoye,[1] Chanee Fabius,[2] and Jennifer Wolff,[2]
*1. Johns Hopkins University, Baltimore, Maryland, United States, 2. Johns Hopkins Bloomberg School of Public Health, Baltimore, Maryland, United States*

Persons living with dementia (PLWD) have up to twice the risk for falling and three-times the risk of serious fall-related injuries as those without dementia. Falls are a leading cause of hospitalizations among PLWD, who are more likely to incur high costs and experience negative health consequences (e.g, delirium, in-hospital falls) than persons without dementia. Few studies have examined risk factors for falls comparing Americans with and without dementia. We used data from the 2015 and 2016 rounds of the National Health and Aging Trends Study (n=5,581) to prospectively identify risk factors for a single fall and recurrent (2+) falls over a 12-month period among community-living older adults ≥65 years with and without dementia in a series of bivariate logistic regressions. Overall, we identified fewer predictors of single or recurrent falls among PLWD compared to persons without dementia. For example, socioeconomic indicators (e.g., income, financial hardship) predicted recurrent falls in persons without dementia, but not in PLWD. Among PLWD, falling in the previous year was associated with both single (odds ratio (OR): 3.38, 95% confidence interval (CI): 1.77, 6.49) and recurrent falls (OR: 6.19, 95% CI: 3.50, 10.93). PLWD who experienced recurrent falls were also more likely to be identified as having a fear of falling (OR: 2.17, 95% CI: 1.33, 3.54), physical function impairments, depression symptoms (OR: 2.23, 95% CI: 1.34, 3.71), and anxiety symptoms (OR: 1.73, 95% CI: 1.14, 2.62). Further study of fall-risk factors could inform screening, caregiver education and support, and prevention strategies for PLWD.

### Session 4075 (Paper)

### FALLS AND MOBILITY

### ASSOCIATION OF IN-LAB AND FREE-LIVING MOBILITY MEASURES WITH FALLS AND FEAR OF FALLING IN UNDERACTIVE OLDER PEOPLE

Stacey Schepens Niemiec,[1] Cheryl Vigen,[1] Jeanine Blanchard,[1] Matthew Niemiec,[2] and Brittany Eng,[1], *1. University of Southern California, Los Angeles, California, United States, 2. Independent contractor, Glendale, California, United States*

Falls in older adults have significant consequences—a single fall can lead to serious injury, psychological trauma, activity restriction, and increased mortality. This study describes differences in mobility-related characteristics of underactive (<150 minutes/week of physical activity), racially diverse, older adults (65–84 years) classified by self-reported fall status (0, 1, 2+ falls in previous 12mo) and fear of falling (yes/no). We analyzed baseline data from 105 individuals (mean age=72.1 years; 73% female; 64% white, 29% Black, 12% Asian) who participated in a trial of a physical activity smartphone intervention for older people. Total minutes of daily stepping and medium-to-brisk (≥75 steps/min) and brisk (≥100) cadence bouts in free-living conditions was gathered over 3 days via ActivPal activity monitor. Gait speed was determined from a 4-meter walk test for those pretested prior to COVID-19 mandates (n=60). Of the median 81.8 minutes spent stepping daily, very few minutes involved moderate-to-brisk (14.0) or brisk cadence (10.1). Groups classified by fall status (non-fallers n=74, 1x fallers n=18, repeat fallers n=13) differed significantly in daily minutes spent in medium-to-brisk (p=0.04) and brisk cadence (p=0.02), but not in 4-meter gait speed or total minutes stepping. Individuals who reported fear of falling versus those with no fear did not differ significantly on any mobility-related parameters. Four-meter gait speed was significantly negatively correlated with both cadence measures (p=0.02) but not total minutes stepping. This study indicates that faster walking behaviors in everyday activity may be a useful target for intervention to prevent falls in underactive older adults.

### SYMPTOMS CONTRIBUTING TO MOBILITY LIMITATIONS AND FEAR OF FALLS IN OLDER ADULTS.

Michelle McKay,[1] Janell Mensinger,[2] Melissa O'Connor,[3] Alexander Costello,[1] and Suzanne Leveille,[4], *1. Villanova University, Villanova University, Pennsylvania, United States, 2. Villanova University, Fitzpatrick College of Nursing, Villanova University, Pennsylvania, United States, 3. Villanova University, M. Louise Fitzpatrick College of Nursing, Villanova, Pennsylvania, United States, 4. University of Massachusetts Boston, University of Massachusetts Boston, Massachusetts, United States*

Mobility limitations in older adults are associated with negative outcomes including fear of falls (FOF) and poorer quality of life. However, self-reported symptoms contributing to mobility difficulty have not been fully explored as an area for intervention. The study aimed to identify the prevalence of self-reported symptom causes of difficulty walking and stair-climbing. In addition, we examined associations between symptoms and FOF in a population-based cohort of community-dwelling older adults in the MOBILIZE Boston Study. Of the 243 older adults who reported difficulty with walking one quarter of a mile or climbing stairs, 67% were women, 72% were white, average age=79.4y (SD=5.7). FOF was measured with the Tinetti Falls Efficacy Scale. Pain was most commonly reported as the primary symptom responsible for mobility difficulty (38.4%) followed by endurance (21.1%), multiple symptoms (15.6%), weakness (13.2%), balance (8.7%), other symptoms (2.9%). Factorial ANCOVA determined gender differences in associations between symptoms and FOF, adjusting for age. In pairwise comparisons, women who identified balance as their primary symptom had higher FOF than women identifying endurance (p=.017), pain (p=.015), other (p=.017), or multiple (p=.050) symptoms. There were no FOF differences for women identifying balance compared to weakness as the primary issue (p=.395). Men who identified balance as their primary symptom had higher FOF than those who identified pain (p=.036); no other FOF differences were noted in men identifying balance compared to other symptoms. Understanding common symptoms experienced by older adults, and symptoms associated with greatest FOF, will assist in developing tailored interventions for mobility improvement.

### TRANSITIONS IN ADHERENCE TRAJECTORIES FROM INTERVENTION TO MAINTENANCE OF A FALLS PREVENTION EXERCISE PROGRAM

Pildoo Sung,[1] May-Ling June Lee,[2] Kok Yang Tan,[1] Rahul Malhotra,[1] and Angelique Chan,[1], *1. Duke-NUS Medical School, Singapore, Not Applicable, Singapore, 2. Duke-NUS Medical School, Duke-NUS Medical School, Not Applicable, Singapore*

The successful implementation of a falls prevention exercise program for older adults hinges on self-maintenance after active intervention. However, little is known about the pattern of adherence from the intervention to the maintenance phase of such programs, and the factors influencing adherence. We investigate transitions in exercise adherence trajectories from the active intervention to the maintenance phase of a falls prevention exercise program in Singapore, and whether exercise self-efficacy is associated with adherence in the maintenance phase. We analyze data of 143 older adults who participated in a 12-week, group-based falls prevention exercise program, followed by a 6-month maintenance phase, in 2018-2019. Sequential process latent class growth modeling identifies the distinct exercise adherence trajectories in the active intervention and the maintenance phase separately and their transition patterns. Multivariable regression examines whether baseline and change in self-efficacy during the active intervention predict adherence during the maintenance phase. The analysis reveals three exercise adherence trajectories— adherent (40% of participants), intermittent (38%), and disengaged (22%)—in the active intervention phase, and two trajectories—adherent (33%) and disengaged (67%)—in the maintenance phase. Those adherent in the maintenance phase comprise participants who were adherent (42%) or intermittent (58%) in the active intervention phase. Baseline and increase in exercise self-efficacy during the active intervention are positively associated with adherence in the maintenance phase. The findings capture the heterogeneity in exercise adherence patterns within and across the active intervention and maintenance phases of falls prevention exercise program, and the importance of exercise self-efficacy in continued adherence to exercise.

### TRANSLATION OF A FALLS PREVENTION BEHAVIORAL INTERVENTION FOR FAMILY CAREGIVER ADMINISTRATION

Victoria Panzer,[1] Veronica Smith,[2] Dorothy Wakefield,[3] and Richard Fortinsky,[4], *1. Brookside Research & Development, Freeland, Washington, United States, 2. Brookside Research & Development, Freeland, WA, Washington, United States, 3. UConn Health, Farmington, Connecticut, United States, 4. University of Connecticut School of Medicine, Farmington, Connecticut, United States*

FallsTalk is a one-month evidence-based falls prevention (FP) program that focuses attention on causes of an individual's falls and encourages new FP behaviors. We translated the program for family caregivers of persons with cognitive impairment or dementia (PwCID) to administer to the PwCID and examined the number of new FP behaviors (#newFPBs) reported during the intervention period by the Caregiver. Thirty-four Dyads (Caregiver+PwCID) were trained to conduct brief daily FP discussions together using paper (FTCGnoTech) or computerized (FTCGTech, n=20) guidance. Dyads had FallsTalk training, daily discussions and weekly check-in calls. FTCGTech included discussion suggestions tailored to Caregiver concerns. To examine the use of technology, Poisson regression models compared #newFPBs between FTCGnoTech and FTCGTech and included covariates age (<80 or >=80), Mini-Mental Status Exam (MMSE >25 or <=25) and interactions. To evaluate the influence of Caregiver participation, #newFPBs reported by 115 non-demented clinical trial participants (no Caregiver-FT-ClTrNoCG) were compared with the Caregiver outcomes. Dyads using technology reported significantly more #newFPBs (MeanFTCGnoTech=5.34(SEM=0.68), MeanFTCGTech=8.46(SEM=0.76); p=.004) during the intervention month. A significant interaction was observed whereby Dyads with MMSE<=25 using technology, reported significantly more #newFPBs than the non-technology group (MeanFTCGnoTech=4.23(SEM=0.72), MeanFTCGTech=9.01(SEM=0.90); p=.047). Caregiver (n=34) involvement substantially increased #newFPBs (MeanFT-ClTrNoCG=1.39(SEM=0.15), MeanCaregiver=7.21(SEM=0.49); p<.0001), independent of technology. Across studies, participants or Caregivers for those with MMSE<=25 and <80yo reported significantly more #newFMBs (Mean=4.38(SEM=0.55) than those 80+yo (Mean=2.06(SEM=0.30); p=.0026). FallsTalk Caregiver provides an effective means to promote new Dyad FP strategies. The influence of Caregiver involvement and technology show promise in encouraging behavioral change to prevent falls.

### WEATHER, BUILT ENVIRONMENT, OR PERSONAL FACTORS: PREDICTORS OF WALKING BY INDEPENDENT LIVING RESIDENTS WITH FRAILTY

Margaret Danilovich,[1] Aura Espinoza,[2] and Christie Norrick,[2], *1. CJE SeniorLife, Evanston, Illinois, United States, 2. CJE SeniorLife, Chicago, Illinois, United States*

Environmental factors influence older adult physical activity. However, the evidence about which factors lead to increased physical activity is mixed and few have studied how these factors affect those with frailty or living in retirement communities. This study investigated how environmental and weather factors influence physical activity among pre-frail and frail older adults residing in independent living retirement communities. We used ActivPal accelerometers to measure 7-day step counts among (n=108) pre-frail and frail residents in 9 independent living residences in metropolitan Chicago. We conducted environmental audits using the MAPS Abbreviated tool and collected National Weather Service Station data (temperature, precipitation, and daylight minutes) during the ActivPal periods. Participants were on average 80.0 years, 74% female, and average daily step count was 3,450 (range 151 - 11,663). Four buildings were in suburban areas and 5 in urban areas and four were private-pay residences while 5 offered subsidized rent. ANOVA results showed private-pay buildings had higher total MAPS scores than subsidized buildings (p=0.001), and urban buildings had higher total MAPS scores than suburban buildings (p < 0.000). Mean step differences were non-significant between different building types: (mean steps = 3,317 private-pay, 3,629 subsidized, 3,536 urban, 3,350 suburban). Pearson product-moment correlations showed a positive association between steps and MAPS positive streetscape features (p=0.011). Multiple regression analysis showed higher temperature days, precipitation, and more minutes of daylight were associated with higher step counts (p=.04). Given the dramatic variation in individual step counts, future research should investigate personal factors that contribute to activity among independent living residents.

### Session 4080 (Symposium)

### FAMILY CAREGIVING DISRUPTED BY COVID-19: OVERCOMING CHALLENGES THROUGH RESILIENCE AND FLEXIBILITY

Chair: Mary Wyman Discussant: Laura Wray

The impacts of the COVID-19 pandemic have been global and pervasive – yet only partially understood. Older adults and their family caregivers have experienced profound and unprecedented challenges as a result of COVID-19. This symposium features research on these disruptions and the creative adaptations undertaken in response. Four presentations present a variety of geographic regions, caregiving settings, and research focal points. Avidor and Ayalon present findings from interviews with family caregivers of residents in long-term care, highlighting the issues faced as a result of dramatic shifts in policy and procedures in support of pandemic infection control. Gum and colleagues focused on how service access has changed for community-dwelling older adults and their families during the pandemic, and how agencies may best leverage the flexibility of caregivers to adapt service provision. Boucher et al. compare the challenges faced by family caregivers during COVID-19 with those faced during a natural disaster, and highlight unique differences in service access for caregivers of military veterans. Finally, Ko and co-authors share their experience with the implementation of a technology intervention to reduce caregiver stress as part of a research trial, and how the protocol adjustments necessitated by COVID-19 revealed the unique potential of such technologies to support caregivers under isolated conditions. The presenters will focus on themes of resilience and lessons learned for health care systems, service agencies, and society to best support family caregivers in challenging circumstances moving forward.

### DISASTER CAREGIVING: CAREGIVERS NEGOTIATING STORMS AND COVID-19 FOR VETERANS AND NON-VETERANS ON THE GULF COAST

Nathan Boucher,[1] Kevin McKenna,[2] Carrie Dombeck,[2] Amy Clark,[2] Ke Wang,[3] Jennifer Olson,[4] and Megan Shepherd-Banigan,[2], *1. Durham VA Health Care System, Durham, North Carolina, United States, 2. Duke University School of Medicine, Durham, North Carolina, United States, 3. Rosalynn Carter Institute for Caregivers, Americus, Georgia, United States, 4. Rosalynn Carter Institute for Caregiving, Americus, Georgia, United States*

Natural disasters and COVID-19 likely add complexity to caregiving efforts, yet little is known about these effects. We will discuss our findings exploring additional needs and challenges experienced by caregivers during hurricanes, floods, and COVID focused on US Gulf Coast states. We interviewed caregivers of both Veterans (n=13) and non-Veterans (n=11). The presentation will include an overview of 1) types of resources needed or used related to storms and to COVID, including social support and access to information for both emergency planning and recovery; 2) caregiver experience before, during, and after the disaster including psychological effects on caregivers and addressing special health needs; 3) comparisons of challenges during storms versus COVID including emotional impact and access to health and specialty care; and 4) additional resources used by caregivers of Veterans. We will also address how these data are informing national caregiver support programs.

### PANDEMIC DEPLOYMENT OF A SMARTHEALTH TECHNOLOGY TO IMPROVE STRESS IN DEMENTIA FAMILY CAREGIVERS

Karen Rose,[1] Kristina Gordon,[2] Emma Schlegel,[3] Matthew McCall,[2] Ye Gao,[4] Jason Jabbour,[5] and Eunjung Ko,[1], *1. The Ohio State University, Columbus, Ohio, United States, 2. The University of Tennessee Department Of Psychology, Knoxville, Tennessee, United States, 3. The Ohio State University College Of Nursing, Columbus, Ohio, United States, 4. University Of Virginia Department of Computer Science, Charlotesville, Virginia, United States, 5. University of Virginia Department of Engineering Systems and Environment, Charlotesville, Virginia, United States*

Caregiving stress from repetitive and heavy caregiving workloads can trigger poor emotional health, such as stress, anxiety, and depression, leading to higher caregiver mortality rates. Interest in technology-based interventions for this population has increased among researchers due to availability, acceptability, and flexibility compared to in-person services, especially now, during an unprecedented pandemic. Our study focuses on in-home SmartHealth technologies for caregivers of persons with Alzheimer's Disease and related dementias, delivered using Ecological Momentary Assessment and a novel acoustic monitoring, mood recognition, and self-learning recommendation system. The system provides mindfulness-based stress management in response to interpersonal conflict in real-time. We will report challenges and solutions of creating and deploying a SmartHealth system for older adults in their home during the COVID-19 pandemic. Potential effects of this system on caregivers' emotional health are also examined. Findings suggest SmartHealth technologies may assist caregiving populations adapt and thrive in a new, more isolated normal.

### THE CHALLENGES FACED BY LONG-TERM CARE RESIDENTS AND THEIR FAMILIES DURING COVID-19

Liat Ayalon,[1] and Sharon Avidor,[2], *1. Bar Ilan University, Ramat Gan, HaMerkaz, Israel, 2. Ruppin Academic Center, Ruppin Academic Center, HaZafon, Israel*

The present research aims to examine the effects of protective measures due to the coronavirus disease (COVID-19) within long-term care (LTC) settings on the residents and their family members. Open-ended qualitative interviews were conducted with 14 family members of older adults who resided in LTC settings during the first wave of the pandemic in Israel. The first theme identified is Rupture, including physical disconnect; the disruption in routine treatment to residents; and decline in the satisfaction with the setting. The second theme is Response, including sharing viewpoints and involvement in decision making, as well as an intense ambivalence shared by family members. Our findings highlight the distress caused to residents and family members by the isolation and restrictions in LTC settings during the pandemic, and underscore values and priorities that are central to them and their family members, including maintaining continuity, transparency, and working in unison with their families.

### Session 4085 (Paper)

### FAMILY CAREGIVING III

### DEMENTIA CARE-RELATED STRESS AND WORKING MEMORY PERFORMANCE: EXAMINING THE MEDIATING ROLE OF SLEEP

Ashley Blasi,[1] and Francesca Falzarano,[2], *1. Fordham University, Bronx, New York, United States, 2. Weill Cornell Medicine, Douglaston, New York, United States*

Dementia Family caregivers often experience significant stress and burden, which has been associated with a myriad of adverse effects on physical and mental health, as well as cognition. The impact of caregiving on health and well-being may have negative implications on the provision of quality and effective care. Specifically, working memory is a key domain of cognition that ultimately underlies logic and decision making processes. Thus, the purpose of the current study is to examine the associations between dementia care-related stress and working memory, as well as potential mediators of this relationship, in a sample of 50 primary caregivers who completed measures examining stress, including burden and overload, and several domains of cognition. Our results showed that higher levels of caregiving overload were associated with worse working memory performance, measured using the N-back task. Additionally, we found that lower sleep quality fully mediated the relationship between overload and working memory performance, such that the negative effects of overload on working memory performance may operate as a result of sleep impairment. By determining the mediating role of sleep while also providing evidence to support the negative relationship between stress and working memory, our results provide support for the development of interventions that target factors such as burden and sleep quality to help mitigate stress in caregivers.

### EXAMINING MENTAL AND PHYSICAL HEALTH STATUS AMONG SANDWICH GENERATION CAREGIVERS

Hansol Kim,[1] Richard Schulz,[1] Scott Beach,[2] and Heidi Donovan,[1], *1. University of Pittsburgh, Pittsburgh, Pennsylvania, United States, 2. University of Pittsburgh, University of Pittsburgh, Pennsylvania, United States*

With a sample of 54,076 caregivers, the Behavioral Risk Factor Surveillance System (BRFSS, 2015-2017) provides an opportunity to assess the impact of caregiving on U.S. adults varying in age, gender, and race. We focused on sandwich generation (SG) caregivers, aged 30-59, with childcare and eldercare responsibilities (n=8,805). In multivariate regression models of mental and physical health, we evaluated the association of age, gender, race and caregiving factors controlling for marital status, education, housing status, income, employment status, and self-rated health status. SG caregivers were predominantly female (65.6%), white (69.6%), black (13%) and were caring for a parent/parent-in-law (50%) or another relative (26.4%). Age, gender, and race were associated with mental health outcomes such that SG caregivers who were younger (aged 30-39), female, and white reported poorer mental health during the past month ($p<.05$). Older (aged 50-59), female, and white SG caregivers reported poorer physical health during the past month ($p<.05$). Caregiving factors were not associated with mental health, but SG caregivers who had been providing care for a longer period of time were more likely to report poor physical health in the past month. Finally, education, income, employment, housing status, and self-rated health status were statistically significant covariates in both models ($p<.05$). These findings demonstrate that young caregivers are at risk for poor mental health; older caregivers for poor physical health. Being female, white, with lower socioeconomic status is associated with poor mental and physical health. Future research should address the unique needs of SG caregivers with dual caregiving responsibilities.

### EXPLORING THE SUBJECTIVE CAREGIVING EXPERIENCE AMONG CHINESE, KOREAN, AND SOUTHEAST ASIAN COMMUNITIES

Sara Powers,[1] Rachel Schaffer,[1] David Bass,[1] Ocean Le,[2] and Lauren Pongan,[2], *1. Benjamin Rose Institute on Aging, Cleveland, Ohio, United States, 2. Diverse Elders Coalition, Diverse Elders Coalition, New York, United States*

Although the Asian American community is one of the fastest growing racial groups in the US, members of this group continue to be underserved and understudied, especially when it comes to the needs of family caregivers. Therefore, through a national initiative to understand the lived experiences of diverse family and friend caregivers, survey data was collected from a variety of Asian American ethnic subgroups including Chinese (n=148), Korean (n=131), and Southeast Asian (i.e., Vietnamese, Hmong, Cambodian, Laotian; n=161). Surveys were distributed in-person and online, and also offered in the translated native languages of the abovementioned groups. Caregivers had to be 18 years and older and providing care to a person aged 55 and older who needed assistance because of ongoing health problems or disabilities. For the overall sample of Asian American caregivers (n=440), participants were on average 51.68 years of age (SD=15.98), identified as female (n=336), were not born in the US (n=348), lived with the care receiver (n=247), and reported less than $10,000 in income per year (n=199). As guided by the Stress Process Model and through a series of ANOVA tests, when compared on all major outcomes, Southeast Asian caregivers significantly reported: 1) more difficulty with care related tasks (e.g., financial/legal decisions), 2) a stronger cultural commitment to caregiving, 3) higher work strain, and 4) more depressive symptomology. Discussion will focus on opportunities for professionals to meet the needs of Asian American caregivers through the use of available trainings and programs aimed to support diverse caregivers.

### FAMILY CAREGIVING FOR PERSONS WITH EMOTIONAL, DEVELOPMENTAL, AND BEHAVIORAL DISORDERS DURING COVID-19

Scott Beach,[1] Richard Schulz,[2] Heidi Donovan,[2] Ann-Marie Rosland,[2] Tara Klinedinst,[2] and Hansol Kim,[2] *1. University of Pittsburgh, University of Pittsburgh,*

*Pennsylvania, United States, 2. University of Pittsburgh, Pittsburgh, Pennsylvania, United States*

COVID-19 has the potential to exacerbate stresses of family caregiving. While studies during COVID-19 have focused on caregivers of those with memory / cognitive issues like Alzheimer's Disease, less attention has been paid to those caring for persons with emotional / developmental / behavioral disorders. This paper compares family caregivers of persons with emotional / developmental / behavioral disorders (EDB); physical conditions (PH); and memory / cognitive problems (MC) during the early phase of the pandemic. We focus on demographics, caregiving context, COVID-related caregiver stressors, and validated physical, psychosocial, and financial well-being outcomes. We conducted a cross-sectional national online survey during April-May, 2020 (n = 556). The sample included 274 PH (50%), 141 MC (25%), and 141 EDB (25%) caregivers. EDB caregivers were younger, with younger care recipients who were more likely to be their child. EDB caregivers reported more COVID-related caregiver stressors than MC or PH caregivers, including increased caregiving duties ($p<.01$); more family disagreements about care provision ($p=.05$); and worsened mental health of the care recipient ($p<.01$). In multivariate regression models, EDB caregivers had significantly higher anxiety; depression; and fatigue (all $p<.01$); more sleep disturbance ($p=.05$); less social participation ($p<.05$); and poorer overall financial well-being ($p<.05$). MC caregivers also reported more negative outcomes, but effects were consistently strongest for EDB caregivers. This study shows that EDB caregivers are at significantly elevated risk for negative impacts due to COVID-19 and should receive increased support and attention during this public health crisis.

### PSYCHOLOGICAL CORRELATES OF (NON)USE OF FORMAL RESOURCES BY FAMILY CAREGIVERS OF PEOPLE WITH DEMENTIA

Laura Gallego-Alberto,[1] Isabel Cabrera,[1] María Márquez-González,[1] María del Sequeros Chaparro,[1] Laura Mérida-Herrera,[1] Cristina Huertas,[2] and Andrés Losada-Baltar,[2]
*1. Universidad Autónoma de Madrid, Madrid, Madrid, Spain, 2. Universidad Rey Juan Carlos, Madrid, Madrid, Spain*

Caring for a relative with dementia is a stressful task characterized by a high number of demands extended in time. Therefore, caregivers frequently report the need for assistance to cope with the situation. However, formal resources use is low among that population. The objective of this study was to explore the correlates of (non)use of formal resources (day care center and home care) by family caregivers of people with dementia. Participants were 225 dementia family caregivers that were individually assessed in a) use of formal resources, b) sociodemographic variables, c) stressors (frequency and reaction to behavioral problems), and d) psychological variables (depression, anxiety, and dysfunctional thoughts about caregiving). A logistic regression was done comparing those who used formal resources with those who did not use them. Caregivers who did not use formal resources were younger (OR = .95; 95% CI [.92 - .98]), devoted more daily hours to caring (OR = 1.07; 95% CI [1.02 - 1.11]), reported higher levels of dysfunctional thoughts about caregiving (OR = 1.07; 95% CI [1.04 – 1.10]) and higher anxiety levels (OR = 1.07; 95% CI [1.00- 1.13]), and their care-recipient had a higher functional autonomy (OR = 1.04; 95% CI [1.02 – 1.05]). Higher levels of anxiety and dysfunctional thoughts in caregivers may be act as barriers to seek for formal support. Targeting these variables may help to increase the use of formal resources by family caregivers of people with dementia.

### Session 4090 (Symposium)

### ISOLATION IN OLDER ADULTS IN THE UNITED STATES AND JAPAN: AN EARLY EXAMINATION OF IOT POSSIBILITIES

Chair: Dana Bradley Co-Chair: carmen Sceppa
Discussant: carmen Sceppa

Isolation in older adults is a growing problem in both the US and Japan. This symposium showcases work funded by NSF and JST (Japan Science Technology Committee) to develop smart technology to create caring, connected communities by integrating gerontology and technology research. The U.S. and Japan are experiencing dramatic population aging and share several similarities: Populous (U.S. 327 million at #3 and Japan 127 million at #11) and economically developed (GDP: the U.S. #1 and Japan #3) and isolation was increasing even before the pandemic. This multi-year project addresses the challenges of isolation by using smart technologies in culturally appropriate ways to support older adults and suggests ways that isolation and loneliness may be managed by older persons, local governments, and NGOs. The first paper examines the experiences of loneliness as characterized by early retirees in both countries. The interdisciplinary research team has used this qualitative set of case studies to identify promising technology support points. Our second paper explores these issues surrounding isolation using data from Study on the Lifestyle and Values of Senior Citizens (Japan). This analysis focuses on longitudinal data from both countries and helps situate our work outside the Covid-19 Pandemic. The third presentation focuses on the intersection between culture and technology and proposes a continued research collaboration model. Our discussion will highlight how community stakeholders in U.S. and Japan have a role in creating evidence-based adaptive environments to detect and mitigate isolation by developing and using gero-centric approaches.

### A QUALITATIVE COMPARISON OF THE POST-COVID RETIREMENT EXPERIENCE BETWEEN JAPANESE AND AMERICAN OLDER ADULTS

Mariko Nishikitani,[1] Mie Ariyoshi,[2] Fumihiko Yokota,[3] Gretchen Tucker,[4] Laura Allen,[5] and Dana Bradley,[4]
*1. Kyushu University Hospital, Medical Information Center, Kyushu University Hospital, Fukuoka, Japan,*
*2. St Cahterines University, Matsuyama, Ehime, Japan,*
*3. Kyushu University, Kyushu, Fukuoka, Japan, 4. UMBC Erickson School of Aging Studies, Baltimore, Maryland, United States, 5. Bar-Ilan University, Ramat Gat, Tel Aviv, Israel*

The purpose of this collaborative study between researchers in Japan and the U.S. was to understand the retirement experience and potential changes in social interactions (amount, type, and mode of communication) among older adults living independently in the community. Specifically, we were interested in individuals' expectations about retirement and the types of social interactions experienced prior to and post-retirement, situated within the context of the COVID-19 pandemic. Both research teams conducted in-depth one-on-one interviews with community-residing retired older adults in early 2021. In the findings we explore similarities and differences between the retirement experiences of Japanese and American older adults, including methodological differences that transpired; specifically, we evaluate the meaning and experience of the COVID-19 pandemic in each of these cultural contexts and how retired older adults experienced its impact in their social interactions.

### A QUANTITATIVE COMPARISON OF SOCIAL INTERACTIONS OF OLDER ADULTS PRE-COVID-19 BETWEEN THE UNITED STATES AND JAPAN

Mariko Nishikitani, *Kyushu University Hospital, Medical Information Center, Kyushu University Hospital, Fukuoka, Japan*

Using the Study on the Lifestyle and Values of Senior Citizens (The Eighth International Study by the Japan Cabinet Office), the social interactions were assessed in the context of health and life satisfaction of the older adults of the U.S and Japan to confirm the relationship between ICT usage and social interactions. The less social interaction was defined as those who answered that they had no "role in the family," "working," or "social activities such as volunteering." The proportion of less-social interaction people and non-use of ICT increased with age, but the proportions of Japanese were higher than that in Americans. The adjusted odds ratio for non-use of ICT to the risk of isolation of the older adults in Japan was 2.43 (95% CI: 1.59-3.73), but no significant relationship was observed in American older adults. Future research will examine the use of ICT by older adults in each country.

### DEVELOPING CULTURALLY COMPETENT TECHNOLOGY FOR OLDER ADULTS IN JAPAN AND THE UNITED STATES

Dana Bradley,[1] Gretchen Tucker,[1] and Laura Allen,[2],
*1. UMBC Erickson School of Aging Studies, Baltimore, Maryland, United States, 2. Bar-Ilan University, Ramat Gat, Tel Aviv, Israel*

The United States and Japan are experiencing an exponential growth in the number of persons age 65 and older. To address certain aging-related issues, assistive technological advancements are being developed. These technologies need to be reliable, safe, secure, and culturally accepted by older adults. In addition, technology must be developed within the unique cultural contexts of each country. One approach currently being used is an interdisciplinary team approach comprised of researchers representing gerontology, information systems, robotics, health sciences, sociology, and computer sciences between two universities in the United States and two Japanese universities. This collaborative project between institutions and countries highlights the need to understand the cultures and traditions of each of these countries. To further develop culturally competent technology, an integrative research plan is being utilized, which incorporates the use of community engagement to examine the influence of the cultural context among older adults.

### Session 4095 (Symposium)

### MANAGED LONG-TERM CARE SERVICES: A PLAYBOOK INNOVATION OR A HAIL MARY?

Chair: Larry Polivka Discussant: Robert Applebaum

The approach to providing long-term services and supports (LTSS) has changed dramatically over the last three decades in both the financing and delivery arenas. In the U.S., long-term strategies have varied by state in organizational structure, scope of delivery and administrative practices. In the past two decades an additional change has emerged with over half the states adopting some form of managed LTSS. This shift has deepened the divide in state approaches to LTSS system design and delivery. The shift to managed LTSS has been largely fueled by ideological expectations and concerns about growing Medicaid costs: Empirical research findings have played a minimal role. For example, the large CMS evaluation conducted in this area did not include Medicaid data or encounter data from the managed care plans as part of the study efforts. However, the managed LTSS experiment does create an opportunity to compare costs and outcomes of these different models of financing and delivery. This symposium will present preliminary evaluation findings from two states, Ohio and Pennsylvania, which are generating data to assess both the implementation and outcomes of these alternative LTSS models. To set the context an initial paper will discuss the expansion of managed LTSS programs across the nation and examine how these efforts compare to the development occurring in the European LTSS systems. The third presentation will discuss the results of the Community Catalyst dual eligibles' managed care demonstration program monitoring project.

### OHIO'S DUAL ELIGIBLE POPULATION: EFFECTS OF PROGRAM DESIGN AND IMPLEMENTATION ON CARE MANAGEMENT

Katherine Abbott, Athena Koumoutzis, and Jennifer Heston-Mullins, *Miami University, Oxford, Ohio, United States*

MyCare Ohio is a prospective blended managed care payment model program tasked to provide comprehensive and coordinated care to Ohio residents who are dully eligible for

Medicare and Medicaid. To understand the administration and day-to-day implementation of care management within MyCare Ohio, n=75 interviews with a total of n=331 personnel from Area Agencies on Aging, Managed Care Plans, and service providers were conducted. Interviews were audio recorded, transcribed, and checked for accuracy. Data were analyzed by iterative reviews and deductive coding in Dedoose. Respondents provided insights on how care management activities are affected by program design features (e.g., ability to opt-out of the Medicare component), transitions between acute and long-term care settings, documentation systems and data-sharing, and high numbers of beneficiaries with behavioral health diagnoses. Implications for practice and policy will be discussed.

### OHIO'S LARGE SCALE EXPERIMENT ON INTEGRATED CARE: RESULTS AND IMPLICATIONS FOR LONG-TERM SERVICES REFORM

Matt Nelson, Robert Applebaum, and John Bowblis, *Miami University, Oxford, Ohio, United States*

Implemented through five health plans, Ohio's MyCare demonstration began in 2014 and was designed to coordinate primary, acute care, behavioral health and long-term services in the major urban areas of the state. Individuals who are dually eligible for both Medicaid and Medicare and who reside in specified geographic regions must enroll into a managed MyCare plan. MyCare beneficiaries are assigned to two primary categories: community well and those needing long-term services and supports (LTSS). Individuals receiving the integrated MyCare intervention were expected to have lower acute care hospitalizations, lower long-term nursing home use, better longevity and lower overall health and long-term care costs. Using a propensity score matching design, the evaluation compared MyCare enrollees to comparison group members in non-MyCare counties of the state, using Medicaid and Medicare claims data. The 120,000 MyCare program participants represented about half of the dual eligible individuals in the state.

### PENNSYLVANIA'S TRANSITION TO MEDICAID MANAGED LONG-TERM SERVICES AND SUPPORTS

Howard Degenholtz, *University of Pennsylvaniz, Pittsburgh, Pennsylvania, United States*

Implemented through five health plans, Ohio's MyCare demonstration began in 2014 and was designed to coordinate primary, acute care, behavioral health and long-term services in the major urban areas of the state. Individuals who are dually eligible for both Medicaid and Medicare and who reside in specified geographic regions must enroll into a managed MyCare plan. MyCare beneficiaries are assigned to two primary categories: community well and those needing long-term services and supports (LTSS). Individuals receiving the integrated MyCare intervention were expected to have lower acute care hospitalizations, lower long-term nursing home use, better longevity and lower overall health and long-term care costs. Using a propensity score matching design, the evaluation compared MyCare enrollees to comparison group members in non-MyCare counties of the state, using Medicaid and Medicare claims data. The 120,000 MyCare program participants represented about half of the dual eligible individuals in the state.

### Session 4100 (Paper)

### MIGRATION AND AGING

### AGE OF MIGRATION AND THE HEALTH STATUS OF OLDER LATINOS: FINDINGS FROM THE HEALTH AND RETIREMENT STUDY

Blakelee Kemp,[1] and Marc Garcia,[2], *1. University of Nebraska, Lincoln, Nebraska, United States, 2. Syracuse University, Syracuse, New York, United States*

Life course research emphasizes the importance of considering how early life experiences set individuals on specific trajectories over time with implications across multiple health domains. Life experiences of older Latinos are shaped by where they were born and, for the foreign-born, when they immigrated to the United States. Prior research examining the extent to which age of migration is associated with health has largely been limited to regional studies. To address this gap in knowledge, we use nationally representative data from the Health and Retirement Study to examine associations between age of migration and multiple physical health outcomes among older Latinos residing in the United States. We examine 2010 prevalence and follow-up incidence to 2016 of cardiovascular issues, diabetes, one or more activities of daily living (ADLs), one or more instrumental activities of daily living (IADLs), cognitive issues, and mortality incidence. Preliminary results indicate similar health profiles across Latinos who migrated in early life (<18), during adulthood (18-34), and during later adulthood (35+). Most health profiles were similar among Latino men and women except for prevalence and incidence of experiencing difficulties with at least one ADL. Latino women who migrated in later-adulthood have higher prevalence of ADLs and women who migrated early in life (>18) have higher ADL incidence than Latino men who migrated during the same life course periods. A greater understanding of the how immigrant experiences influence physical health outcomes offers important insights into the development of actionable and culturally appropriate social and health policies.

### CAN RURAL MIGRANT WORKERS AFFORD TO RETIRE IN CHINA? A STUDY OF CHINESE MIGRANT WORKER'S RETIREMENT SAVINGS

Jing Liu,[1] Heying Zhan,[2] and Fengxian Qiu,[3], *1. Zhejiang University of Finance and Economics, Hangzhou, Zhejiang, China (People's Republic), 2. Georgia State University, Georgia State University, Georgia, United States, 3. Anhui Normal University, Wuhu, Anhui, China (People's Republic)*

This paper makes connections between social policies of retirement, migrant worker's migration experience, and migrant workers' retirement savings. Using insight from the political economy of aging and stress theory, this paper links the macro levels of understanding with the micro levels of work and aging experiences for migrant workers. Using binary logistic regression with a sample of 699 Chinese migrant workers from three emigration provinces (Anhui, Henan, Sichuan), this paper explores four specific aspects of migrant worker's migration experience in relation to their retirement savings: financial status; length of employment; social support, and levels of hopefulness. Findings reveal that migrant workers with better financial status, social support, and higher level of hopefulness towards future are more likely to have retirement savings as compared to their counterparts. Discussions linking the macro and micro levels of social policies were provided. Policy implications were discussed.

### DISABILITY AND OLDER AGE RETURN MIGRATION: EVIDENCE AGAINST THE SALMON BIAS

Mara Sheftel, *Penn State University, Brooklyn, New York, United States*

Mexican immigrants make up an increasing proportion of the US population 65 and older. Whereas this population has among the lowest rates of disability at working ages, there is growing evidence of high rates of disability at older ages, findings which contradict what mechanisms of selection, namely the "salmon bias," would predict. However, largely due to data limitations disability rates between those who stay in the US into older ages and those who return to Mexico are rarely compared. Here two waves of data from the US based Health and Retirement Study and the Mexican Health and Aging Study are combined to create a novel dataset that enables an interrogation of the widely held assumption of negative selection on health among return migrants. Investigating three measures of functional limitation and disability, results show higher prevalence of disability for stayers as compared to both younger and older returnees. These results are robust to controls for childhood background, adult socioeconomic status, and migration related variables and hold for those who immigrated during different immigration policy regimes. These findings are novel not only because they stand in opposition to previous assumptions about the direction of health selective return migration, but also because they mean that those remaining in the United States into older ages are among the most vulnerable.

### THE RELATIONSHIP BETWEEN PLACE OF DEATH AND IMMIGRANT STATUS

Yujin Franco,[1] Margarita Osuna,[2] and Jennifer Ailshire,[3], *1. University of Southern California, University of Southern California, California, United States, 2. USC, University of Southern California, California, United States, 3. University of Southern California, Los Angeles, California, United States*

Increasing attention is being paid to improving care at the end-of-life, including developing a better understanding of where individuals die, and factors related to place of death. The older immigrant population in the United States is increasing rapidly, and while prior research suggests they may differ in their end-of-life experiences, we know relatively little about foreign-born differences in where people die. This study investigates how the place of death (home, hospital, and nursing home) differs between the U.S.-born and foreign-born. We used data on 9,180 U.S.-born and 969 foreign-born respondents from the nationally representative Health and Retirement Study (HRS) for who end-of-life surveys were conducted with a proxy between 2002 and 2016. Approximately one-third of deaths occurred in nursing homes in both groups. Hospital deaths were more common in US-Born decedents (31.9%) than foreign-born decedents (25.2%), while death at home was lower for US-born (35.5%) than foreign-born (40.2%). We used multinominal logistic regression analysis to determine whether sociodemographic characteristics, cause of death, or receipt of family caregiving explained the observed differences in place of death by foreign-born status. Results from fully adjusted multivariate models indicate the foreign-born differences in place of death cannot be explained by socioeconomic, health, or family factors. Our research shows key differences in the end-of-life experience between US-born and foreign-born older adults and highlights the importance of examining end-of-life experiences for this small, but rapidly growing segment of the older U.S. population.

### WHERE TO RETIRE? EXPERIENCES OF OLDER AFRICAN IMMIGRANTS IN THE UNITED STATES

Manka Nkimbeng,[1] Alvine Akumbom,[2] Marianne Granbom,[3] Sarah Szanton,[4] Tetyana Shippee,[5] Roland Thorpe, Jr.,[6] and Joseph Gaugler,[1], *1. University of Minnesota, Minneapolis, Minnesota, United States, 2. Johns Hopkins School of Nursing, Baltimore, Maryland, United States, 3. Centre of Ageing and Supportive Environments (CASE), Lund University, Skane Lan, Sweden, 4. Johns Hopkins University, Baltimore, Maryland, United States, 5. University of Minnesota, University of Minnesota, Minnesota, United States, 6. Johns Hopkins Bloomberg School of Public Health, baltimore, Maryland, United States*

The needs and conceptualization of age-friendliness likely vary for immigrant older adults compared to native-born older adults. For example, Hispanic immigrant older adults often return to their home country following the development of ill health. Doubling in size since the 1970's, the aging needs of African immigrants are not fully understood. This qualitative study examined experiences of aging and retirement planning for African immigrant older adults in the United States (U.S.). Specifically, it explored the factors, processes, and ultimate decision of where these older adults planned to retire. We analyzed semi-structured interviews with 15 older African immigrants in the Baltimore-Washington Metropolitan area. Data were analyzed using thematic analyses in NVivo. The majority of participants were women, with a mean age of 64. We identified three overarching themes with ten subthemes. The themes included: 1) cultural identity: indicating participant's comfort with the U.S. society and culture; 2) decision making: factors that impact participants' choice of retirement location, and 3) decision made: the final choice of where participants would like to retire. Age-friendliness for immigrant older adults in the U.S. is complex and it includes the traditional domains such as physical and sociocultural environment (e.g. housing, transportation, and income). However, immigrant age-friendliness also needs to include wider contextual aspects such as political climate in their country of origin, immigrant status, family responsibilities, and acculturation in the U.S. More research is needed understand and facilitate age-friendly environments for transnational immigrant older adults.

### Session 4105 (Symposium)

### NEW ASPECTS IN METABOLISM OF AGING

Chair: Rozalyn Anderson

In recent years there has been a renewed emphasis on metabolism as a key contributor to a host of chronic non-communicable conditions: cancer, neurodegeneration, frailty, and functional declines in immune and inflammatory processes. All share a common connection in metabolic dysfunction. Furthermore, aging itself is associated with changes in metabolism, although the underlying drivers for these changes are unknown. Here we introduce speakers working at the cutting edge in metabolism research, and whose studies are of direct relevance to aging. Dr. Chandel will focus on mitochondrial biology, describing recent advances in understanding the mechanisms of the beneficial effects of metformin. Dr. Haigis takes the mitochondrial theme to cancer biology, the area of research that revived metabolic perspectives in biomedical research. Dr. Najt's talk describes a less well studied organelle, the lipid droplet, and its role in a rapidly expanding area of research on lipid metabolic regulation specifically in the context of aging. Dr. Brown-Borg will present data on nutritional and genetic modulation of metabolism and how pathways converge to influence chromatin and epigenetic regulation of gene expression. Together our speakers explore new concepts in metabolism research that are of particular relevance to aging. This session aligns with the concept of GeroScience, the more we know of aging biology the better we understand diseases and disorders of aging. This session will demonstrate that metabolism, its regulation, and its influence on key processes linked to health and longevity, place it in a central position as we seek to discover targets and interventions to improve human aging.

### THE ROLE OF MITOCHONDRIA IN AGING AND CANCER

Marcia Haigis, *Harvard Medical School Boston, Massachusetts, United States*

### METHIONINE METABOLISM IN AGING REGULATION

Holly M. Brown-Borg, *University of North Dakota School of Medicine & Health Sciences, Grand Forks, North Dakota, United States*

Aging is the major risk factor for many diseases but the mechanisms are poorly understood. The risk of developing hepatic steatosis increases with age and the health impact of this disease is negative and high. When challenged with high fat diets, long living Ames mice withstand the detrimental metabolic effects that occur in normal mice. We examined transcriptomic and epigenomic profiles of Ames and wild type hepatocytes in the presence or absence of fat to demonstrate that the epigenomic profile drives transcription factor and downstream gene expression resulting in susceptibility or resistance to fatty liver disease. We found that markers of steatosis are related to gene expression in wild type and Ames mice, and dwarf mice retain fewer lipid droplets compared to wild type mice. These studies will provide data to guide our understanding of mechanisms leading to hepatic disease and define factors that provide protection from age-related metabolic disorders.

### METFORMIN INHIBITS MITOCHONDRIAL COMPLEX I TO PROMOTE HEALTH

Navdeep Chandel, *Northwestern University, Illinois, United States*

The major function of mitochondria in cellular homeostasis has been the generation of ATP through oxidative phosphorylation. However, we have previously demonstrated that mitochondria can serve as signaling organelles by releasing low levels of reactive oxygen species (ROS) and TCA cycle metabolites that are essential for hypoxic activation of HIF, antigen activation of T cells, cellular differentiation and proliferation of cancer cells. The anti-diabetic drug metformin has been proposed to inhibit mitochondrial complex I. We will present data indicating that metformin inhibits mitochondrial complex I to exert it's biological effects through controlling ROS, ATP, and NAD+.

### LIPID DROPLET SIGNALING IN METABOLIC HEALTH AND AGING

Charles Najt, Douglas G. Mashek, *University of Minnesota, Minneapolis, Minnesota, United States*

Lipid droplets (LDs) are neutral lipid rich organelles involved in lipid storage, fatty acid trafficking, and signaling. Emerging evidence from our laboratory and others suggests that the specific LD resident proteins couple/uncouple cells and tissues from inflammation and metabolic dysfunction. However, the mechanism by which LD proteins influences these critical pathways remains unknown. We will present data delving into the role of LD proteins Perilipin (PLIN) 2 and 5 in balancing cellular energy metabolism, mitochondrial function, and inflammation. Data will be presented defining novel mechanisms through which PLIN2 orchestrates eicosanoid production as a means to promote inflammation. We will contrast these findings to PLIN5, which uncouples LD accumulation from metabolic dysfunction and inflammation, in part due to its promotion of SIRT1 signaling. Overall, these studies will highlight a crucial role of LD metabolism and signaling in regulating cellular energy homeostatic processes known to be key players in governing healthspan.

### Session 4110 (Paper)

### PHYSICAL ACTIVITY AND WELL-BEING

### DELAYING HEALTH CARE DUE TO THE COVID-19 PANDEMIC: ASSOCIATIONS WITH PHYSICAL AND MENTAL HEALTH AND PREVENTIVE CARE

Felicia Wheaton,[1] Terika Scatliffe,[2] and Matilda Johnson,[2], *1. Xavier University of Louisiana, New Orleans, Louisiana, United States, 2. Bethune-Cookman University, Daytona Beach, Florida, United States*

Health care is important for maintaining optimal physical and mental health. However, due to the COVID-19 pandemic, many older adults have delayed or postponed care. Data from the special midterm release of the 2020 Health and Retirement Study (HRS) were used to examine the relationship between chronic conditions and delayed care, as well as between delayed care and mental health outcomes and preventative care among Americans aged 50+ (N=3,266). Approximately 30% of respondents said yes when asked "Since March 2020, was there any time when you needed medical or dental care, but delayed getting or did not get it at all?" Of those, 55% said their provider cancelled, closed or suggested rescheduling, 28.5% decided it could wait, and 20.8% were afraid to go. Results from OLS and logistic regression, controlling for sociodemographic characteristics, indicate that those with lung disease and those with a heart condition had significantly higher odds of delaying care. Delaying care was associated with significantly higher odds of poor self-rated health and feeling depressed, as well as significantly higher average hopelessness, loneliness and negative affect and significantly lower average positive affect. Surprisingly, delaying care was not associated with receiving a flu shot, cholesterol test, colonoscopy, mammogram or prostate exam in the previous two years. It is likely that the full effects of delaying health care during the pandemic have yet to be felt and there is a need to study the implications of such delays.

### LIMITATIONS IN PHYSICAL ACTIVITIES AND LONELINESS: THE ROLE OF GUILT FOR PERCEIVING ONESELF AS A BURDEN

María del Sequeros Chaparro,[1] Isabel Cabrera,[1] Carlos Vara-García,[2] José Adrián Fernandes-Pires,[3] Samara Barrera-Caballero,[4] Laura Mérida-Herrera,[1] María Márquez-González,[1] and Andrés Losada-Baltar,[2], *1. Universidad Autónoma de Madrid, Madrid, Madrid, Spain, 2. Universidad Rey Juan Carlos, Madrid, Madrid, Spain, 3. Rey Juan Carlos University, Alcorcón, Madrid, Spain, 4. Rey Juan Carlos University, Alcorcón, Madrid, Madrid, Spain*

Loneliness is a prevalent problem associated with negative health consequences for older adults, such as greater cognitive decline. Limitations to perform physical activities have been associated with greater loneliness in older adults. This association could be moderated by maladaptive social cognition or feelings, such as guilt associated with perceiving oneself as a burden. The objective of this study was to analyze the moderating effect of guilt associated with perceiving oneself as a burden in the relationship between limitations in physical activities and loneliness. Participants were 195 community-dwelling people 60 years or older not showing explicit cognitive or functional limitations that prevent activities of daily life, but who may present limitations in some physical activities (e.g., walking a kilometer or more). A linear regression analysis was conducted for testing the interaction between limitations in physical activities and guilt for perceiving oneself as a burden in loneliness, controlling for gender and age. The interaction between limitations in physical activities and guilt for perceiving oneself as a burden was the only significant predictor of loneliness and the model explained 18.30% of the variance. Limitations in physical activities influenced loneliness when people have high levels of guilt for perceiving oneself as a burden. This study suggests that guilt for perceiving oneself as a burden may play an important role in the association between limitations in some physical activities and loneliness.

### LUNG FUNCTION IN OLD AGE AND PHYSICAL ACTIVITY FROM MIDLIFE TO OLD AGE: LONGITUDINAL STUDY WITH 24-30 YEARS' FOLLOW-UP

Ingemar Kåreholt,[1] Liberta Mehmedi,[2] and Charlotta Nilsen,[2], *1. Jönköping University, Jönköping, Jonkopings Lan, Sweden, 2. Jönköping University, Aging Research Network Jönköping (ARN-J), Jonkopings Lan, Sweden*

The study investigated the role of physical activity, from midlife to older age, in relation to lung function in older age. In order to increase the understanding of the relationship between physical activity and lung function, the relationship between physical activity in midlife and physical activity in older age was also studied. Two Swedish studies based on nationally representative samples were used in this study, the Level of Living Survey, LNU, and the Swedish Panel Study of Living Conditions of the Oldest Old, SWEOLD. The participants were interviewed on three occasions; at the mean ages of 53 years, 61 years, and 81 years. The average follow-up time from the last to the first interview was 24-30 years. The results show that more physical activity in late midlife (mean age of 61 years) was associated with better lung function in older age. The association persists but attenuates when physical activity in older age was included in the analyses. There was also a strong association between physical activity in older age and better lung function in older age. Physical activity in late midlife had a positive association with physical activity in older age. This study shows the importance of physical activity in late midlife and in older ages to maintain good lung function in older ages. To invest in preventive actions in the form of physical activity are vital to be able to promote healthy aging, and should include the oldest old (76+).

### PSYCHOSOCIAL AND PHYSICAL ACTIVITY OUTCOMES AMONG GROUP LIFESTYLE BALANCE PROGRAM PARTICIPANTS WITH ARTHRITIS

Taylor Hudzinski,[1] Ferdinand Delgado,[2] and Cheryl Der Ananian,[1], *1. Arizona State University, Phoenix, Arizona, United States, 2. Arizona State University, Arizona State University, Arizona, United States*

**Background:** Weight loss and physical activity (PA) are recommended for arthritis management. The Group Lifestyle Balance (GLB) Program(TM) is an evidence-based, lifestyle change program for weight loss in individuals with prediabetes, but hasn't been evaluated in people with arthritis.

**Purpose:** The purpose of this study was to evaluate the effectiveness of an adapted version of the GLB program on PA and psychosocial outcomes related to weight loss among overweight (Body Mass Index >27) individuals with arthritis.

**Methods:** A single-group, quasi-experimental design was used to examine the effects of the adapted GLB program on measures of PA and psychosocial outcomes. All participants (N=15) received the GLB program and completed the following surveys: CHAMPS PA, Self-Efficacy for PA (SE), Social Support for PA (SS), Weight Loss Efficacy (WEL) and Barriers to Healthy Eating (BHE) at baseline, 12-weeks, 6 months, and 12 months. Repeated measures ANOVA and the Friedman Test were used to examine changes over time.

**Results:** Participants (aged 53-79 years) were primarily female (82%), white (94%), and college educated (94%). Significant improvements were found in BHE subsections of self-control and motivation (p=0.002), daily mechanics (p=0.042), and WEL subsections of availability (p=0.049), social pressure (p=0.010), physical discomfort (p=0.011), and positive activities (p=0.007). Weekly caloric expenditure

(p=0.004), metabolic equivalent minutes (p=0.022) for all activities, and moderate-intensity activities (p=0.019) also showed significant improvements. However, most improvements were seen in the short-term.

**Conclusions:** The GLB program should be further evaluated for its effectiveness in people with arthritis.

### THE IMPACT OF LOW BACK PAIN AND PHYSICAL ACTIVITY ON MENTAL AND PHYSICAL HEALTH OUTCOMES IN OLDER ADULTS WITH ARTHRITIS

Janiece Taylor,[1] Natalie Regier,[2] Minhui Liu,[3] Sarah Szanton,[1] and Richard Skolasky,[4], *1. Johns Hopkins University, Baltimore, Maryland, United States, 2. School of Nursing, Johns Hopkins School of Nursing, Maryland, United States, 3. Central South University, Changsha, Hunan, China (People's Republic), 4. Johns Hopkins University School of Medicine, Baltimore, Maryland, United States*

Nearly 50% of Americans aged 65 and above have been diagnosed with arthritis at any point in time, and an estimated 80% of adults experience low back pain (LBP) at some point in their lives. However, little is known about the experience of LBP in older adults with arthritis and whether or not it is related to adverse mental and physical health outcomes over and above those linked to arthritis. This study examined the relationship between LBP and four associated physical and mental health conditions (depression, insomnia, mobility limitations, and self-rated health) in older adults with arthritis. We also examined whether physical activity mediated the relationships between LBP and these four conditions. A cross-sectional analysis was conducted using data from the National Health and Aging Trends Study. Descriptive analyses and chi-square tests assessed whether there were demographic differences between persons with and without LBP. Binary logistic regressions found that participants with LBP were 30% more likely to endorse insomnia (95% confidence interval (CI) =1.1 to 1.5, p<.001), had 40% higher odds of depression (95% CI=1.1 to 1.6, p<.001), and 70% higher odds of poor self-rated health (95% CI=1.5 to 1.9, p<.001) than those without LBP. Activity mediated the relationship between LBP and the four health outcomes in unadjusted models. Findings indicate that LBP may be associated with adverse mental and physical health in older adults with arthritis, and treatment for older adults with arthritis and comorbid LBP should include interventions targeting LBP and routine assessment of mental and physical health.

### Session 4115 (Paper)

### PHYSICAL ACTIVITY INTERVENTIONS

### EFFECTS OF A MOVEMENT-BASED MIND-BODY INTERVENTION IN MANAGING OSTEOARTHRITIS SYMPTOMS IN OLDER ADULTS

Juyoung Park,[1] and Carson Herron,[2], *1. Florida Atlantic University, Boca Raton, Florida, United States, 2. Florida Atlantic University, Florida Atlantic University, Florida, United States*

In a secondary analysis, this study examined differences in age (younger vs. older geriatric groups), gender, and living arrangement (living alone vs. living with others) in elderly patients with osteoarthritis (OA) who utilized chair yoga (CY) as an type of movement-based mind-body intervention (MMBI) for symptom management. A two-arm, assessor-blinded, randomized control trial was used to examine effects of CY (twice-weekly 45-minute sessions for 8 weeks) on pain interference, physical function, and psychosocial outcomes by gender, age, and living arrangement in older adults with OA who could not participate in traditional exercise. A total of 112 older adults completed CY or a health education program (HEP) and participated in five data collection points. Older women in the CY group showed greater reduction in pain interference during the CY intervention than those in HEP, $F(4, 86) = 3.255$, $p = .016$, $\eta 2 = .131$. The younger group (ages 61 to 74) had decreased depression scores during the intervention, $F(4, 87) = 2.598$, $p = .042$, $\eta 2 = .107$. Regardless of the intervention (CY or HEP), depression scores in older adults who were living alone decreased substantially during the intervention. Group-based and supervised CY interventions are recommended for older adults with OA to reduce pain interference, reduce depressive symptoms, and develop social networks. Online-based synchronous CY sessions may address physical activity needs and improve mental well-being in this population in light of physical distancing practices due to COVID-19.

### EPISODIC MEMORY IN OLDER ADULTS WITHOUT DEMENTIA: A META-ANALYSIS OF AEROBIC EXERCISE INTERVENTIONS

Sarah Aghjayan,[1] Themistokles Bournias,[2] Chelsea Stillman,[3] Shannon Donofry,[3] Anna Marsland,[3] and Kirk Erickson,[3], *1. University of Pittsburgh, Pittsburgh, Pennsylvania, United States, 2. Carnegie Mellon University, Carnegie Mellon University, Pennsylvania, United States, 3. University of Pittsburgh, University of Pittsburgh, Pennsylvania, United States*

The effect of aerobic exercise interventions on episodic memory performance among older adults without dementia remains a matter of intense debate. Prior meta-analyses examining this association have reported minimal improvements in episodic memory performance following exercise training but have also been plagued by several limitations, including restrictive inclusion criteria, combined sample populations, and infrequent examination of the effect of exercise parameters (e.g., volume). To address these gaps, we conducted a meta-analysis of randomized controlled trials (RCTs) to determine if aerobic exercise interventions influence episodic memory performance in older adults without dementia and to examine potential moderators of these effects (e.g., sample and intervention characteristics). Included studies met the following criteria: (1)Studies of adults (M≥55 years) with normal cognition, subjective cognitive decline, or mild cognitive impairment; (2)Aerobic exercise RCTs; and (3) Assessment of episodic memory. Intervention effects were represented by Hedges' g and combined into pooled effect sizes using random- and mixed-effects models. Thirty-three studies met inclusion criteria, representing data from 2,488 participants. The primary analysis yielded a significant positive effect of aerobic exercise on episodic memory (Hedges' g[CI]=0.28[0.10-0.47]; p=0.003). Mixed-effects analyses demonstrated a positive effect on episodic memory among studies with a high percentage of females (>66%), participants with normal cognition, studies reporting intensity, studies with a no-contact or nonaerobic physical activity control group, and studies prescribing 2,100–3,900 total minutes of activity (range 540–8,190 minutes). These results suggest that aerobic exercise may act as an accessible, nonpharmaceutical intervention to improve episodic memory in late adulthood before changes in cognition are detected.

### HIGH-INTENSITY INTERVAL TRAINING IN OLDER ADULTS WITH TREATMENT NAIVE CHRONIC LYMPHOCYTIC LEUKEMIA

David Bartlett,[1] Grace MacDonald,[1] Mike Deal,[1] Erik Hanson,[2] Carl Pieper,[1] J. Brice Weinberg,[1] Danielle Brander,[1] and Andrea Sitlinger,[1] *1. Duke University, Durham, North Carolina, United States, 2. University of North Carolina, Chapel Hill, North Carolina, United States*

Chronic lymphocytic leukemia (CLL) is the most common leukemia, affecting predominantly older adults. Treatment naïve patients (CLLtn) with low physical fitness have poor survival following commencement of treatment. CLLtn is characterized by inadequate immune functions, increased risk of secondary malignancies and infections. The aims of this study were to determine the feasibility and preliminary effects of 12-weeks of high-intensity interval training (HIIT) on CLLtn patients. We enrolled eighteen CLLtn patients (64.9±9.1yrs.). Eleven (5M/6F) were allocated to HIIT and seven (4M/3F) to the control group (CON). HIIT consisted of three 30-minute treadmill sessions/week plus two 30-minute strength training sessions/week. Feasibility was confirmed if >70% of HIIT participants completed >75% of prescribed sessions and prescribed minutes, and if >80% of high-intensity intervals were at a heart rate corresponding to 80% of aerobic capacity (139±19 bpm). Results are presented as mean±SD and effect sizes (d), with 0.2, 0.5 and 0.8 representing small, medium and large effect sizes, respectively. Feasibility was achieved, with HIIT completing 5.0±0.2 sessions/week and 99±3.6% of prescribed minutes/week at 142±19 bpm. No adverse safety events were observed. Compared to CON, HIIT increased leg (d=2.602), chest (d=1.285), and seated row (d=3.323) strength, while aerobic capacity difference between groups was d=0.431. Compared to CON, HIIT increased in vitro natural killer immune cell cytolytic activity against K562 (d=1.586) and OSU-CLL (d=0.917) cancer cell lines, and autologous CLL cells (d=1.362). HIIT is safe and feasible in older adults with CLLtn. Preliminary effects suggest that HIIT increases muscle strength and important components of immune function.

### NON-INFERIORITY OF A GROUP LIFE VERSION COMPARED TO THE ORIGINAL, INDIVIDUAL LIFE TO PREVENT FALLS

Carl-Philipp Jansen,[1] Corinna Nerz,[2] Sarah Labudek,[3] Sophie Gottschalk,[4] Judith Dams,[4] Jochen Klenk,[5] Clemens Becker,[2] and Michael Schwenk,[3] *1. Robert-Bosch-Hospital Stuttgart, Karlsruhe, Baden-Wurttemberg, Germany, 2. Robert-Bosch-Hospital, Stuttgart, Baden-Wurttemberg, Germany, 3. Heidelberg University, Heidelberg, Baden-Wurttemberg, Germany, 4. University Medical Center Hamburg-Eppendorf, Hamburg, Hamburg, Germany, 5. IB University of Applied Health and Social Sciences, Stuttgart, Baden-Wurttemberg, Germany*

The 'Lifestyle-integrated Functional Exercise' (LiFE) program has been shown to reduce risk of falling via improvements in balance and strength while increasing physical activity in older adults. Its one-to-one delivery comes with considerable costs hampering large scale implementability. To potentially reduce costs, a group format (gLiFE) was developed and analyzed for its non-inferiority to LiFE in reducing activity-adjusted fall incidence after 6 months. Further, intervention costs and physical activity were analyzed. Older adults (70+ years) at risk of falling were included in this multi-centre, single-blinded, randomized non-inferiority trial. LiFE was delivered in nine intervention sessions to increase balance, strength, and physical activity, either in a group (gLiFE) or at the participant's home (LiFE). 309 persons were randomized into gLiFE (n=153) and LiFE (n=156). Non-inferiority for activity-adjusted falls was inconclusive; the incidence risk ratio (IRR) of gLiFE was 1.350 (95% CI: 0.856; 2.128) at 6 months. Falls were largely reduced in both groups. Physical activity was superior in the gLiFE group (gLiFE +880 steps; CI 252, 1,509) which also had a cost advantage under study conditions as well as real world estimations. GLiFE was associated with lower intervention costs, making it a cost-efficient alternative to the individually delivered LiFE. The added value of gLiFE is the greater effect on physical activity, making it particularly attractive for large scale PA promotion in public health concepts. Depending on individual needs and preferences, both formats could be offered to individuals, with a greater focus on either fall prevention (LiFE) or physical activity promotion (gLiFE).

### RECRUITING AND SCREENING OLDER ADULTS WITH ALZHEIMER'S DISEASE FOR THE FIT-AD TRIAL

Fang Yu,[1] Jean Wyman,[2] Susan Greimel,[2] and Lin Zhang,[3], *1. Arizona State University, Phoenix, Arizona, United States, 2. University of Minnesota, Minneapolis, Minnesota, United States, 3. University of Minnesota, University of Minnesota, Minnesota, United States*

Recruiting older adults with Alzheimer's disease (AD) into clinical trials has been very challenging even for resource-rich trials. This presentation will discuss the recruitment rate, screening ratio, and recruitment yield and costs in the FIT-AD Trial. The FIT-AD Trial was a single-site, pilot randomized controlled trial testing the effects of 6-month aerobic exercise on cognition and hippocampal volume in community-dwelling older adults with mild-to-moderate AD dementia. Ten recruitment strategies and a 4-step screening process were used to ensure a homogenous sample and exercise safety. The target sample size was 90. During the 48-month recruitment period, 396 individuals responded to our recruitment, 301 were reached, and 103 were tentatively qualified at step 4. Of these 103, 67 (69.8%) completed the optional magnetic resonance imaging (MRI) component of the trial and 7 were excluded due to abnormal MRIs. In year 4, our sample size was increased to allow individuals in the screening process a chance to enroll, resulting in a final sample size of 96. Per enrolled participant, the recruitment rate was 2.15, the screen ratio was 2.92, and the recruitment yield was 31.9%. Over 49% of the enrolled participants were yielded through referrals (28.1%) and Alzheimer's Association events/services (21.9%). The total recruitment cost was $38,246 ($398 per randomized participant). The results indicate that a multi-prong, extensive community outreach-based approach is essential in recruiting older adults with AD dementia into an exercise trial. Referral was the most cost-effective strategy. Two individuals needed to be screened to enroll one participant.

### Session 4120 (Paper)

### PHYSICAL HEALTH IMPACT OF THE COVID-19 PANDEMIC

### CHANGES IN PHYSICAL FITNESS OF OLDER PEOPLE DURING THE COVID-19 PANDEMIC IN GERMANY: OBJECTIVE TRAINING DATA

Jessica Koschate, Michel Hackbarth, Sandra Lau, and Tania Zieschang, *Carl von Ossietzky University Oldenburg, Oldenburg, Niedersachsen, Germany*

The purpose of this study was to analyze objective training data on changes in leg muscle training before and after the COVID-19 lockdown during spring 2020 in Germany. Overall, the training data of 4435 individuals in the age group (AG) 45-64 years (55±5 years, 66% ♀) and of 2853 in the AG 65-95 years (72±6 years, 54% ♀) were exported from chip-controlled exercise circuits. Training weight and number of repetitions performed on the leg extensor were used to calculate a leg score (LS), considering the last three training sessions before the lockdown (baseline) and the first ten individual sessions as well as the averaged sessions for August, September and October after individual training resumption. Based on the baseline LS, three training intensity groups (TG_low, medium, high) were defined, and analyzed for differences (ANOVA). The LS in TG_low remained stable after the lockdown, but increased compared to baseline in both AGs after the first ten sessions ($p<0.05$). In TG_medium, LS was reduced at the first post training session ($p<0.05$) and returned to baseline levels at training session eight in the younger and session two in the older adults. In both AGs, LS was reduced in the TG_high ($p<0.001$), and did not reach baseline levels by October. Hence, the LS of TG_high was identified as being particularly affected by the training interruption, irrespective of age. More individually tailored training recommendations should be made for these individuals to be able to regain their initial training levels and avoid long-term adverse health effects.

### FACTORS PREDICTING DELAY OF MEDICAL AND DENTAL CARE AMONG AMERICAN ADULTS OVER 50 DURING THE COVID-19 PANDEMIC

Athena Chung Yin Chan,[1] and Rodlescia Sneed,[2], *1. University of Minnesota, St Paul, Minnesota, United States, 2. Michigan State University, Flint, Michigan, United States*

Delaying or avoiding medical care is associated with a range of poor health-related outcomes. Due to concerns about the coronavirus (COVID) pandemic, many older adults delayed or avoided seeking medical and dental care in 2020. The purpose of this study was to examine factors predicting delay or avoidance of care among older adults. Participants were U.S. adults aged ≥ 50 who participated in the Health and Retirement Study, a population-based study of community-dwelling adults. Delays in seeking or obtaining medical and dental care were assessed via self-report in June 2020. Hierarchical logistic regression models were used to predict the influence of demographic variables (e.g. age, marital status, race and ethnicity, gender, education, work status), health insurance status, health status, and COVID-related experiences (self or household history of COVID diagnosis, knowing anyone who died from COVID, willingness to take risks, and pandemic concerns) on delay or avoidance of medical or dental care. Overall, 30% of our sample reported delaying or avoiding medical or dental care. Delayed care was lower among younger age (0.97; 0.96-0.99); non-Hispanic Blacks (0.59; 0.43-0.80), Hispanics (0.63; 0.45-0.87) and women (0.78; 0.63-0.97). Moreover, care avoidance was significantly higher among persons with disability or on sick leave 1.56; 1.04-2.33), those with chronic diseases (1.41; 1.00-2.01), those with fair/poor self-rated health (1.33; 1.03-1.73), and those with high COVID-related concerns (1.34; 1.07-1.68). Understanding factors associated with medical care will inform targeted care delivery and health promotion encouraging persons in need to safely seek timely healthcare services.

### HOW COVID-19 IMPACTED OLDER ADULT WALKING GROUP MEMBERS IN SCOTLAND: A MIXED-METHODS STUDY

Kathryn Martin,[1] Kevin Stelfox,[1] Wei Lynn Khor,[1] Amudha Poobalan,[1] Daniel Powell,[1] Rute Vieira,[1] Marjory D'Arcy,[2] and Peter Murchie,[1], *1. University of Aberdeen, Aberdeen, Scotland, United Kingdom, 2. Grampian 50. + Network, Aberdeen, Scotland, United Kingdom*

Scotland has enacted strict social distancing and stay-at-home policies during the COVID-19 pandemic, at times prohibiting outdoor group-based physical activity. This mixed-method study examined the changing role of older adult walking groups in North East Scotland around the first lockdown and how restrictions impacted members' well-being. Three consecutive surveys were posted or emailed to members of the Grampian 50+ Network over summer 2020, with questions about social contact, loneliness, well-being, physical activity, public health messages, help-seeking behavior, and socio-demographics. 346 members completed the June survey, with 268 (83%) returning the follow-up survey in July, and 258 (80%) in August. Twenty participants (selection criteria - gender and geographic location) participated in repeated semi-structured interviews. Participants were, on average, 72±7 years old (range: 58-90), retired (94%), and women (80%). Participants reported missing in-person interaction from not regularly meeting with their walking group. Groups adapted to stay-at-home measures by using technology (i.e. videoconferencing/text/email/telephone) to maintain relationships. Easing restrictions required groups to modify format, location and size. Concerns about safe transport, mask-wearing, maintaining social distance (2m/6ft), and potential lack of socialisation emerged as barriers for future engagement. While, participants generally expressed confidence in the Scottish Government's pandemic response and public health messaging, they expressed dissatisfaction that 'over-70s' were grouped together. Findings suggest that these walking group members fared well and were adaptive in response to the pandemic. Promoting group-based opportunities for physical activity and social interaction remain vital for the health and well-being of older adults in the near and long term.

### IMPACT OF THE COVID-19 STAY-AT-HOME ORDER ON DIET AND HEALTH IN COMMUNITY-DWELLING OLDER ADULTS WITH OBESITY

Kathryn Porter Starr,[1] Michael Borack,[2] Marshall Miller,[2] Heather Hutchins-Wiese,[3] Alyssa King,[2] Kenlyn Young,[2] and Connie Bales,[2], *1. Duke University School of Medicine, Hillsborough, North Carolina, United States, 2. Duke University School of Medicine, Durham, North Carolina, United States, 3. Eastern Michigan University, Ypsilanti, Michigan, United States*

Profound restrictions were placed on previously free-living older adults due to mandatory stay-at-home orders for Covid-19. Recognizing the potential for worsening health and heightened risk of Covid-19 complications with older age and obesity, we conducted a survey to assess the impact of stay-at-home requirements on diet, health/social behaviors, and food security in 58 older adults (age=70.8±6.2, 55% Black, 93% female) who had participated in past obesity-reduction trials. A 71-item questionnaire was administered by phone and included demographics, health, lifestyle and dietary habits, food attitudes, and food security questions. Results showed indicators of heightened health risk, including health care appointments either delayed/cancelled (69%) and self-reported weight gain (62%). Of those with weight gain, 22% reported a gain of >10 pounds (33% gained 5-10 pounds and 7% < 5 pounds). Increased food intake was reported by 67% and 45% felt their eating patterns were less healthy due to increased snacking (71%) and consumption of sweets (41%). Food access and isolation were also an issue, as 51% were concerned about leaving the house for food and 81% reported eating alone. While some positive behaviors were reported (new ways to access food and health information, more reported cooking at home), the majority of findings indicated increased risk of obesity and its complications. The findings emphasize the need for effective lifestyle interventions that can be delivered remotely to high-risk older adults; this would benefit those presently self-restricted for Covid-19 concerns as well as other isolated older adults who need better access to individualized interventions.

### PRIOR PHYSICAL PERFORMANCE IMPACTS SOCIAL CONTACT AND SOCIAL PARTICIPATION OF U.S. OLDER PEOPLE DURING COVID-19

Yun Zhang,[1] Wei Hou,[2] and Sean Clouston,[2] *1. Renaissance School of Medicine, Stony Brook University, Stony Brook, New York, United States, 2. Renaissance School of Medicine, Stony Brook University, Renaissance School of Medicine, Stony Brook University, New York, United States*

**Objective:** To assess what roles the prior physical and cognitive performances of US older people played in changes of social contact and social participation due to the COVID-19 pandemic.

**Methods:** We used the 2020 National Health and Aging Trends Study (NHATS) COVID-19 questionnaire (n=3,188, response rate=80.5%), linked to demographic information from the 2019 NHATS wave. We excluded participants who reported COVID-19 diagnosis and/or symptoms (n=288), and those represented by a proxy (n=414). We compared older people's social contact with family and friends, social contact with health care providers, and social participation, prior to and during the COVID-19. We used paired t-test for the summed scores, Wilcoxon signed-rank for paired categorical variables, and McNamara test for paired binary variables. We further used weighted linear and logistic regressions adjusted for multiple covariates to investigate the effects of prior physical and cognitive performances on social contact and social participation, prior to, during, and changes in the COVID-19.

**Results:** The sample included 2,486 participants that were predominantly females (56.20%) and non-Hispanic whites (88.43%), with participants averaging 78.24 years old. The COVID-19 pandemic resulted in significantly increased social contact with family and friends but decreased social participation of the US older people. Better prior physical performance was associated with significantly increased video calls and volunteering work during the COVID-19, while prior cognitive ability was not a significant risk factor.

**Conclusion:** Prior physical performance, rather than prior cognitive ability, significantly affected the social way old people responded to a pandemic crisis.

### Session 4125 (Paper)

### SOCIAL ISOLATION AND LONELINESS (BSS PAPER)

### DO SOCIAL CONNECTIONS BUFFER LONELINESS ASSOCIATED WITH LIVING ALONE?

Markus Schafer, Haosen Sun, and Jin Lee, *University of Toronto, Toronto, Ontario, Canada*

The growth of solo living has important implications for the rising "loneliness epidemic" among older adults. This study considers whether two forms of social connectedness—extra-household core discussion networks and formal social participation—buffer the loneliness associated with living alone. Our study uses data from two surveys (National Social Life, Health, and Aging Project; Survey of Health, Ageing and Retirement in Europe) encompassing 20 developed Western countries in 2009/2010 and 2015/2016 (n = 110,817). Harmonizing measures across data sets, we estimate survey-specific and pooled linear regression models with interaction terms. Results indicated that high levels of social connectedness only moderately buffer the loneliness associated with living alone in later life. Findings were largely consistent across regions of Europe and the United States, though the buffering patterns were most robustly identified for widowed solo dwellers. Taken together, the results suggest that extra-household connections are partial compensators, but do not seem to fully replace the ready companionship afforded by residential co-presence in later life. Future research is needed to understand whether the efficacy of compensatory connections differs by gender, race/ethnicity, and across more diverse global regions.

### HOW IS DAILY SOCIAL INTERACTION RELATED TO LONELINESS IN OLDER ADULTS? THE ROLES OF TRAIT LONELINESS AND PERSONALITY

Ruixue Zhaoyang,[1] Stacey Scott,[2] Karra Harrington,[1] and Martin Sliwinski,[1], *1. The Pennsylvania State University,*

*University Park, Pennsylvania, United States, 2. Stony Brook University, Stony Brook, New York, United States*

Loneliness is prevalent among older adults and is associated with increased risks for morbidity and mortality. This study examined what types of social interactions could reduce loneliness for older adults and who would benefit the most from social interactions. We used data from 312 community-dwelling older adults (aged 70 to 90 years) who completed ecological momentary assessments (EMA) five times a day for 16 consecutive days using smartphones (n=20,507 reports), as part of the ongoing Einstein Aging Study (EAS). At each EMA, participants reported their social interactions in the past 3 to 4 hours and their current feelings of loneliness. Results from multilevel models revealed that older adults reported lower levels of loneliness on occasions when they had pleasant social interactions (p<.000) or interactions with family (p=.001) in the past few hours, compared with occasions when they had no social interaction. In contrast, they reported higher levels of loneliness if they had unpleasant social interactions in the past few hours (p=.004). These within-person (WP) effects of social interactions on momentary loneliness were significantly moderated by participants' trait levels of loneliness and neuroticism; and were significantly stronger among those with higher (vs. lower) trait loneliness (ps <.001) or neuroticism (ps <.042). Other personality traits (Extraversion, Openness, Conscientiousness, Agreeableness) did not moderate any WP association. These results highlight the importance of having pleasant social interactions and frequent interactions with family for reducing older adults' loneliness in daily life, especially for those higher in trait loneliness and neuroticism.

### LONELINESS AT OLDER AGES IN JAPAN: VARIATION IN LONELY LIFE EXPECTANCY AND THE ROLE OF SOCIAL ISOLATION

Shiro Furuya,[1] and James Raymo,[2], *1. University of Wisconsin--Madison, Madison, Wisconsin, United States, 2. Princeton University, Princeton University, New Jersey, United States*

Despite growing media, policy, and research attention to loneliness, it remains an understudied dimension of inequality in demography. Additionally, research on loneliness often fails, both methodologically and conceptually, to distinguish loneliness from social isolation. This is an important limitation given the positive correlation between measures of these two distinct concepts, a relationship that may be particularly relevant in collectivistic societies. This study focuses on Japan, describing the synthetic cohort duration of exposure to loneliness at older ages, with and without adjusting for the correlation between loneliness and social isolation. Combining life tables from the Human Mortality Database with individual data from the National Survey of Japanese Elderly, we calculated isolation-adjusted lonely life expectancies. We also evaluated regional and educational differences in isolation-adjusted lonely life expectancies. Results showed significant differences in lonely life expectancy before and after adjusting for social isolation; however, the attention to social isolation did little to alter our general understandings of trends and differentials in lonely life expectancy. In contrast to public perceptions of growing loneliness, we find that lonely life expectancy is short among older Japanese and has not increased over time. Additionally, we found no clear regional nor educational differences in isolation-adjusted lonely life expectancy.

### PERCEIVED DISCRIMINATION, SOCIAL ENVIRONMENT, AND OLDER ADULTS' PSYCHOLOGICAL WELL-BEING

Min-Kyoung Park,[1] and Christine Mair,[2], *1. University of Maryland, Baltimore & Baltimore County, Ellicott City, Maryland, United States, 2. University of Maryland, Baltimore County (UMBC), Baltimore, Maryland, United States*

Experiencing discrimination can have detrimental effects on psychological well-being. For older adults in the U.S., discrimination on the basis of country of origin may be a particularly alienating experience. A positive social environment, however, has been shown to buffer associations between discrimination and poorer psychological well-being. However, this hypothesis has not been tested in a sample of older Americans who perceive discrimination because of country of origin. As the United States continues to diversify and politically polarize, understanding older adults' experiences with discrimination and identifying potential buffers to these negative effects is increasingly important. We analyze 942 older Americans (aged 50+) from the Psychosocial Module of the most recent wave of the Health and Retirement Study (HRS, 2020). Specifically, we analyze associations between perceived discrimination on the basis of country of origin and three psychological well-being outcomes: loneliness, anxiety, and life satisfaction. We further test if the social environment buffers negative effects by examining interactions between discrimination and social support as well as discrimination and neighborhood environment. Our results reveal clear and consistent associations between older adults' perceived discrimination and increased loneliness and decreased life satisfaction. These negative associations, however, appear to be buffered by social support and positive neighborhood environment, respectively. The potential buffering effect of positive social environments on psychological well-being is particularly pronounced for older adults under the age of 65. We discuss these findings in light of the prevalence of discrimination in the U.S. and consider potential mechanisms for improving the social environment of older adults.

### SOCIAL ISLANDS: EXAMINING THE INTERSECTION OF ISOLATION AND TECHNOLOGY USE

Joseph Svec,[1] and Megan Gilligan,[2], *1. Iowa State University, Ames, Iowa, United States, 2. Iowa State University, Iowa State University, Iowa, United States*

Life course theory suggests that social relationships are tied to overall well-being. In the extant literature social isolation negatively impacts physical and mental health outcomes in later life. In this study, we focus specifically on the association between social isolation and older adult's self-rated health status. We also examine whether and the extent to which technology use augments negative health consequences attributed to isolation. Using data on 3,758 older adults (ages 65+) from the 2018 wave of the Health and Retirement Study, we contribute to current scholarly examinations at the intersection of technology and isolation. We conduct a series of ordinal logistic regressions to estimate the odds of respondents' higher self-rated health (from poor = 1 to excellent = 5) on subjective measures of social isolation (i.e. feel left out, lack of companionship and isolated from others) in addition to whether respondents live with a partner and have an adult child who lives in close geographic proximity. Preliminary results show that individuals who perceived higher levels of social isolation evaluated their own health status as poorer. We also find that the use of computers for virtual communications corresponds with higher self-rated health statuses, regardless of the proximity of children or other family members. However, a negative interaction between computer use and isolation indicates the positive effects of technology are limited for those who are highly isolated. These findings suggest that technology impacts on health are nuanced, where an overreliance on technology as a substitute may not consistently yield positive outcomes.

### Session 4130 (Symposium)

### SPEAKING OF DEMENTIA: HOW TO REFER TO DEMENTIA IN RACIAL-ETHNIC MINORITY COMMUNITY-FACING COMMUNICATIONS

Chair: Zachary Baker Co-Chair: Tetyana Shippee
Discussant: Joseph Gaugler

What do you call "dementia"? In academic writing, researchers often chose the inclusive, "Alzheimer's Disease and Alzheimer's Disease Related Dementias (AD/ADRD)". When referring to the people experiencing dementia, the person-centered language: "persons living with dementia (PLWD)" is preferred. This is a welcome departure from the antiquated disease-centered language of "dementia patients" or "the demented". Still, AD/ADRD and PLWD may be less fitting in community-facing education or participant recruitment. For instance, community-facing materials may benefit from choosing terms like "memory loss", "issues related to memory or aging", or "changes in ability, behavior, or judgment". In this symposium we present a range of viewpoints focused on how to refer to "dementia" in community-facing materials/conversations. These viewpoints include those of several racial and ethnic groups (i.e., African Americans, African Immigrants, American Indians, Asians, Hispanics/Latinos/as/x/e, and Whites). We also include viewpoints from people interfacing with many different diseases that cause dementia (i.e., Alzheimer's disease, dementia with Lewy bodies, Early-onset Alzheimer's disease, and Parkinson's disease dementia) because of the different manifestations of dementia that can arise from those diseases. Viewpoints were gathered through 1) a nation-wide community advisory board, 2) community conversations with African Immigrants, 3) a national effort to increase the representation of Hispanics/Latinos/as/x/e PLWD in AD/ADRD research, and 4) eight community projects exploring the African American AD/ADRD experience. These talks will present possible terms to use within groups, considerations to increase inclusiveness, issues with translation into native languages, considerations surrounding symptoms that may be most recognizable to community members, and stigmatized terminology.

### GOING INTO A COMMUNITY, WE NEED TO START FROM WHERE THEY'RE AT: CAREGIVER AND ADVOCATE PERSPECTIVES

Zachary Baker, *University of Minnesota, University of Minnesota/Minneapolis, Minnesota, United States*

We recorded and inductively coded an open-ended discussion of jargon surrounding "dementia" with the "Supporting Dementia Caregivers After Death" community advisory board (CAB). CAB-members included current and former caregivers of PLWD due to early- and normal-onset Alzheimer's, Lewy body, and Parkinson's, a co-president of the Alzheimer's Association (ALZ) Young Champions, a dementia trainer/consultant and member of a Catholic church that preserves American Indian spiritual traditions, a senior program manager at ALZ who was entrusted by American Indian reservation elders to provide dementia education, a care partner support group leader, and an Alzheimer's Ambassador chosen by multiple US senators. Themes identified included differential inclusiveness of terms like "memory loss" versus "dementia", misuse and misunderstanding of "dementia" versus "Alzheimer's," and the difficulty of translating "dementia" into the American Indian Ojibwe (i.e., Anishinaabemowin) language where suggested translations directly translated to "slow memory loss", "brain deterioration", "absent mindedness", or even "craziness".

### I DIDN'T REALLY FULLY UNDERSTAND UNTIL I CAME INTO THE STATES: AFRICAN IMMIGRANTS' ADRD INTRODUCTION AND AWARENESS

Kwame Akosah,[1] Tetyana Shippee,[2] Christina Rosebush,[3] Wynfred Russell,[4] Joseph Gaugler,[1] and Manka Nkimbeng,[1], *1. University of Minnesota, Minneapolis, Minnesota, United States, 2. University of Minnesota, University of Minnesota, Minnesota, United States, 3. University of Minnesota, Twin Cities, Minneapolis, Minnesota, United States, 4. The African, Career Resources and Education Inc, Brooklyn Park, Minnesota, United States*

Most African immigrants report that they had never heard about dementia until their arrival in the United States. Conversations and insights from project advisory board meetings of the African Immigrant Memory Loss and Dementia Education projects (5 conversations and 8 meetings in the Minneapolis area) reveal unique cultural and immigrant characteristics surrounding dementia terminology and awareness. Dementia is often lumped together with mental illness which is associated with stigma. In addition to the fear of bad news and death, mental health issues are often considered a result of witchcraft, spiritual attack or punishment. Additionally, there are no traditional or cultural words for dementia in many African languages and current terms used are related to mental illness and all have negative connotations. There is a need to identify appropriate words for dementia in many tribal and immigrant dialects that can facilitate dementia awareness and education programs in African communities.

### FROM DEMENTIA TO DEMENCIA: WAY MORE THAN A SINGLE LETTER

Maggie Britton, *University of Houston, University of Houston/Houston, Texas, United States*

Hispanic or Latino/a/x/e (H/L) individuals are at 1.5x risk for Alzheimer's Disease and Related Dementias (ADRD) compared to non-H/L White individuals. Although H/L individuals make up roughly 18% of the U.S. population, they are vastly underrepresented in ADRD research. For example, less than 9% of individuals in the National Alzheimer's Coordinating Center (NACC) data set are H/L. Collaborative efforts like the ECHAR Network are working to increase the representation of H/L individuals in ADRD research across the U.S. A non-exhaustive list of barriers to H/L participation include limited health literacy, perspectives on aging, and preferences for family-centered care that aligns with H/L cultural values (e.g., familismo). For example, H/L individuals are less likely to have a medical conceptualization of the Spanish translation for dementia. The cognate "demencia" is more likely to be conflated with alternative meanings like insanity, which may create barriers when developing community-facing recruitment and study materials.

### LESSONS LEARNED FROM FOCUS GROUPS AND COMMUNITY EDUCATION PROVISION IN THE AFRICAN AMERICAN COMMUNITY

Robbin Frazier, *University of Minnesota, University of Minnesota/Minneapolis, Minnesota, United States*

The present symposium will synthesize themes derived from eight different projects designed to better understand dementia in Minnesota (MN) within the African American community (where individuals are disproportionately susceptible to dementia and the tolls of dementia care). These projects included focus groups, community outreach, community education, networking with aging service providers, and community forums. Projects were funded by and conducted in partnership with the MN Department of Human Services, the Alzheimer's Association, the MN Board on Aging, and the MN Leadership Council on Aging's Diverse Elders Coalition. Themes included the unique ways that African American elders share their viewpoints and the importance of faith-based outreach. Another major theme, which connects to the other symposium talks, was the importance of three S's: Stigma, Shame, and Silence as cultural considerations in the African American, West African, and Latino/a/x/e communities as they apply to access to information and training to better understand AD/ADRD.

### Session 4135 (Paper)

### SPIRITUALITY AND HEALTH

### CARING FOR A FAMILY MEMBER WITH MILD DEMENTIA: PERCEPTIONS, CONNECTIONS, AND RELATIONAL DYNAMICS WITH THE SACRED

Jocelyn McGee,[1] Dennis Myers,[1] Rebecca Meraz,[2] and Davie Morgan,[1], *1. Baylor University, Waco, Texas, United States, 2. Baylor University, Dallas, Texas, United States*

Researchers define spirituality as the search for or connection with the "sacred", which is transcendent and considered blessed, holy, or revered. For some, the sacred is connection with a divinity (e.g., God, gods) and for others, a close relationship with something else bigger than themselves (e.g., the Universe, Nature, a life philosophy). Current research reports that family caregivers with a strong connection to the sacred, as compared with those who do not, have fewer symptoms of depression, more positive perceptions of the caregiving experience, improved coping, and bolstered resilience. However, there is limited research on the impact of spirituality on the perceptions of familial caregivers whose loved ones have recently been diagnosed with dementia. In this study, 27 family caregivers of persons with mild dementia (CDR=1) were interviewed using the Dimensions of Caregiving Interview (DCI, McGee & Carlson, 2013). The DCI identified positive psychological aspects of the caregiving experience, including spirituality. Three heuristic themes emerged from Directed Content Analysis: perceptions about the sacred reflect variability in the early part of the caregiving journey; specific characteristics, traits, and functions of the sacred shape caregiver coping and adjustment; and the relational dynamics between caregivers and the sacred inform adaptation. Recommendations for clinical practice and additional research are provided.

### EITHER SENT BY GOD OR USED BY GOD: IMPACT OF COVID-19 ON THE RELIGIOUS LIVES OF BLACK FAMILIES WITH DEMENTIA

Fayron Epps, Yiran Ge, Mayra Sainz, and Janelle Gore, *Emory University, Atlanta, Georgia, United States*

The COVID-19 pandemic has underscored systemic disparities and laid its effects on the Black community. Often overlooked is how health disparities heighten stress and affect the emotional well-being of Black American caregivers. The purpose of this study is to explore the impact of COVID-19 on church engagement for Black families affected by dementia. A qualitative design was employed to collect data from current caregivers, faith/church leaders, and persons with cognitive impairment. Participants (n = 17) were predominantly female, all identified as Black. During semi-structured interviews, participants were asked how COVID-19 has impacted their participation in faith practices. The following themes emerged: (a) ability to continue faith practices, (b) increased church engagement, (c) new normal, (d) importance of fellowship, and (e) role of technology. Participants believed COVID-19 did not impact their faith practice partly due to the ability to continue with faith traditions in a virtual format. Online worship services enabled more families affected by dementia to participate. Many church leaders expressed the intent of continuing to provide online worship services post-pandemic. Families highlighted their need to have fellowship with other parishioners. Technology was perceived as a double-edged sword that serves as both a motivator and a barrier to religious engagement. These findings will support faith leaders and churches in understanding the needs of their congregation during the COVID-19 pandemic, specifically, it will allow families living with dementia to continue engaging in religious activity and living in meaningful ways.

### GIVE THANKS IN ALL CIRCUMSTANCES? GRATITUDE TOWARD GOD AND HEALTH IN LATER LIFE AFTER MAJOR LIFE STRESSORS

Laura Upenieks,[1] and Joanne Ford-Robertson,[2]
*1. University of Texas at San Antonio, San Antonio, Texas, United States, 2. University of Texas at San Antonio, University of Texas at San Antonio, Texas, United States*

Gratitude is foundational to well-being throughout the life course, and an emerging body of work suggests that older adults may be more inclined to attribute gratitude to a non-human target (God). Drawing on life course theory and Erikson's lifespan development framework, we use data from a national sample of Christian older adults from the United States (N = 1,005) to examine whether gratitude towards God buffers the noxious health effects of the death of a loved one or personal illness. Results suggest that gratitude towards God tends to predict better age-comparative and global self-rated physical health in the aftermath of stress, a moderation effect which is partially mediated by stronger beliefs in God-mediated control (that God is a collaborative partner in dealing with problems). We conclude by proposing some interventions for clinicians and counselors centered around gratitude and religiosity that may assist older adults in coping with major life stressors.

### HOLY AND HELPING: THE ROLE OF SANCTIFICATION IN THE COMMUNAL COPING OF OLDER AFRICAN AMERICAN COUPLES

Antonius Skipper,[1] Andrew Rose,[2] Jhazzmyn Joiner,[1] Ethan Jones,[2] and Alex Reeves,[1], *1. Georgia State University, Atlanta, Georgia, United States, 2. Texas Tech University, Lubbock, Texas, United States*

Disproportionately affected by numerous relational stressors (e.g., financial strain, morbidity), older African American couples frequently find solace in religion and each other. Research notes that both married and cohabiting couples effectively respond to difficult situations by sharing the ownership of a stressor and organizing a collaborative, collective response. However, little is known about the influence of religion on shared coping experiences, particularly among older African American couples. This study examined dyadic data from the Strong African American Couples Project to capture the influence of relational sanctification on the communal coping practices of married and cohabiting older African American couples. The sample included 194 African American couples (146 married and 48 cohabiting) between the ages of 50 and 86 years. With the use of Actor Partner Independence Models, this study found that men's sanctification predicted both their own communal coping and their partner's communal coping. However, there were no significant effects when women's sanctification was used as a predictor of communal coping among older African American couples. These findings are both important and novel, because these relationships had never before been examined within the United States, much less among older African American couples. Similar to existing research among majority White couples, this research finds that men's religiosity may be a more influential predictor of relational outcomes than women's religiosity. Such findings offer a valuable foundation for future studies seeking to consider how relational sanctification and communal coping may impact other outcomes associated with the romantic relationships of older African Americans.

### SPIRITUAL EXPERIENCE IN DEMENTIA FROM THE HEALTH CARE PROVIDER PERSPECTIVE: IMPLICATIONS FOR INTERVENTION

Jennifer Palmer,[1] Michelle Hilgeman,[2] Tracy Balboni,[3] Sara Paasche-Orlow,[4] and Jennifer Sullivan,[5], *1. VA Boston Healthcare System, Newton, Massachusetts, United States, 2. Tuscaloosa VA Medical Center, Tuscaloosa, Alabama, United States, 3. Harvard Medical School, Boston, Massachusetts, United States, 4. Hebrew SeniorLife, Roslindale, Massachusetts, United States, 5. VA Boston Healthcare System, Boston, Massachusetts, United States*

Spiritual care seeks to counter negative outcomes from spiritual distress and is notably needed in dementia. Such care needs disease-appropriate customization. Employing "cognitive apprenticeship" theory's focus on learning from contrast, we explored spiritual needs salient within dementia as related to other disease states; we aimed to inform future dementia-focused spiritual care design. Accordingly, we conducted semi-structured qualitative interviews with 24 providers who serve older adults inclusive of persons with dementia. We sampled participants purposively by discipline (chaplains, nursing staff, social workers, activities professionals) and religious tradition (for chaplains). Our interview guide inquired about the nature of spiritual needs in dementia and stakeholders' roles in addressing them. Hybrid inductive/deductive thematic analysis was employed. A thematic structure emerged with two themes: 1) spiritual experience in dementia compared to other medical conditions (sub-themes: the salience of (a) fear; (b) loss of self; (c) dementia's progressive and incurable nature; (d) dementia's impact on accessing faith); and 2) the need for spiritual intervention at the mild stage of dementia (sub-themes: (a) awareness in mild dementia and its influence on spiritual distress; (b) a window of opportunity). These findings pointed to possibilities for the "what" of spiritual needs and the "who" and "when" of implementing spiritual care. Implications included the imperative for dementia-specific spiritual assessment tools, interventions targeting fear and loss early in the disease, and stakeholder training. Researchers should study the "how" of dementia-appropriate spiritual care given recipients' cognitive and linguistic challenges. Conjointly, these efforts could promote the spiritual well-being of persons with dementia worldwide.

### Session 4140 (Symposium)

### SUSTAINABILITY OF THE AGING NETWORK'S COVID-19 RESPONSES: ISOLATED IMPACT OR TRANSFORMATIVE CHANGE?

Chair: Suzanne Kunkel Discussant: Suzanne Kunkel

The COVID-19 pandemic mobilized individuals, organizations, and communities. Area Agencies and Aging (AAAs) and Title VI Native American Programs, core organizations of the network of community-based organizations (CBOs) that serve older adults and their families, pivoted their service delivery methods to provide life-sustaining services. Their long-standing expertise in community needs assessment, pre-existing cross-sectoral partnerships, and an infusion of emergency federal funding, enabled this rapid response. Recently concluded studies using key-informant interviews and national surveys of AAAs and Title VI programs highlight these service adaptations, from expansion of home-delivered meal programs to new partnerships in telehealth. These organizations also reported expansion of services offered and people served, and the emergence or strengthening of partnerships with other CBOs, businesses, and governmental organizations such as public health entities. For example, 78% of the respondents to the recent survey of AAAs reported that they have a role in vaccination outreach, scheduling support, or delivery. The papers in this symposium will use these new studies to describe the nature, origins, and potential sustainability of new and expanded services and partnerships. The Collective Impact Model for community change (introduced in the Stanford Social Innovation Review) will provide a framework for the discussion. Built on the importance of cross-sector coordination, the five pillars of success for collective rather than isolated impact are: a common agenda, mutually reinforcing activities, continuous communication, shared measurement, and a backbone organization. Each of these five pillars is relevant to the heightened community response during the pandemic, and to the likelihood of sustainability.

### DELIVERY OF HOME AND COMMUNITY BASED SERVICES DURING A PANDEMIC: UNEXPECTED CONSEQUENCES

Adeola Ayedun,[1] Jane Straker,[2] Traci Wilson,[3] Amanda Brewster,[4] Chris Rubeo,[4] Leslie Curry,[1] and Emily Cherlin,[5], *1. Yale School of Public Health, New Haven, Connecticut, United States, 2. Miami University, Oxford, Ohio, United States, 3. National Association of Area Agencies on Aging, Washington, District of Columbia, United States, 4. University of California, Berkeley School of Public Health, Berkeley, California, United States, 5. School of Public Health, New Haven, Connecticut, United States*

The COVID-19 pandemic required AAAs to pause essential services, serving as a catalyst for innovation. We examined such innovations as part of an explanatory mixed-methods, positive deviance study of AAA partnerships with health and social service organizations. We identified 8 AAAs with many partners serving areas with lower levels of health care use, and 3 AAAs with few partners serving areas with higher levels of health care use. We interviewed AAA and partners, (total = 123). Using the constant comparative method, we identified recurrent themes: 1) AAAs adapted to increased demand for services by developing new ways to deliver services, 2) the pandemic raised awareness of unmet needs such as social connection, 3) changes in delivery of services included embracing technology, and 4) AAAs and their partners identified resources to rapidly pivot services. AAAs and partners demonstrated resiliency to not only to sustain programs, but to innovate throughout the pandemic.

### THE EVOLVING ROLE OF AREA AGENCIES ON AGING THROUGH THE COVID-19 PANDEMIC AND BEYOND

Elizabeth Blair,[1] and Traci Wilson,[2] *1. n 4. a, Washington, District of Columbia, United States, 2. National Association of Area Agencies on Aging, Washington, District of Columbia, United States*

Area Agencies on Aging (AAAs) have played an important and well-documented role in meeting the nutritional and wellness needs of older adults during COVID-19. To better understand the continued impact of COVID-19 pandemic on AAA services, partnerships, and clients, the National Association of Area Agencies on Aging surveyed the nation's 618 AAAs in February 2021, with a 27% response rate. As a result of COVID-19, most AAAs reported both increased numbers of new clients and needs of existing clients; three-quarters of AAAs developed new external partnerships; and over half are implementing strategies to address equity and inclusion regarding their services and clients. Nearly 80% of AAAs are involved with COVID-19 vaccine outreach and delivery, from scheduling appointments to administering the vaccine to homebound clients. We will describe these and other transformations of services, partnerships, and client needs; discuss challenges and opportunities; and provide examples and video vignettes from AAA directors.

### CASE STUDY ON THE COMMUNITY RESILIENCE WITHIN TITLE VI PROGRAMS DURING COVID-19: A QUALITATIVE ANALYSIS

Abigail Bailey, *Scripps Gerontology Center, Oxford, Ohio, United States*

Health inequalities increased for Native Americans during the COVID-19 pandemic due to poor infrastructure, lack of electricity, health disparities, limited transportation, and rural location (Yellow Horse, 2021). Title VI programs--aging network organizations that serve tribal elders--had to be resourceful to meet increased needs and restrictions on service delivery options. Qualitative data from the national 2020 Title VI Native American Aging Programs Survey illustrated the challenges faced and the resiliency of these organizations and their communities. Two rounds of thematic coding of 479 open-ended responses to the survey revealed that communication across organizations, a sense of shared mission, and sharing of resources allowed these agencies to provide more services in innovative ways. Challenges included limited funding, regulatory barriers, and staff burnout. A video presentation by a Title VI program director will provide context for the results of the survey.

### Session 4145 (Symposium)

### TELEHEALTH AS A RESOURCE FOR CONTINUITY OF CARE IN THE FACE OF DISASTER

Chair: Walter Boot Co-Chair: Judith Robertson Phillips

This symposium co-sponsored by the Disasters and Older Adults and the Technology and Aging special interest groups of GSA aims to highlight the promise of, and barriers to, the use of telehealth to support continuity of care in the face of disasters and crises, such as the ongoing COVID-19 pandemic. M. Mattos will showcase a home-based medical care (HBPC) program to address chronically ill and homebound persons living with dementia and caregivers' needs during the pandemic. T. Wyte-Lake will present the results of a national survey on how changes were made to the Department of Veterans Affairs (VA) HBPC programs in response to the pandemic. G. Demiris describes a large caregiver study in which problem solving therapy and positive appraisal theory interventions designed specifically to support family caregivers of hospice patients during the COVID-19 pandemic were implemented via telehealth. D. Lindeman will specifically discuss challenges and implementation strategies for telehealth solutions applied to low-income older adults living in affordable housing communities. Finally, H. Xu will present the results of an analysis examining the effectiveness of telehealth in reducing readmissions among heart failure patients during the COVID-19 pandemic. While the COVID-19 pandemic has especially impacted older adults and those who care for them, these talks highlight the potential of telehealth services and interventions to provide support and facilitate the continuity of care during times of crisis.

### AN INTERDISCIPLINARY HOME-BASED MEDICAL CARE PROGRAM TO REACH HOMEBOUND ADULTS LIVING WITH DEMENTIA

Karen Duffy,[1] Tuula Ranta,[2] Justin Mutter,[2] and Meghan Mattos,[3], *1. UVA Health, Charlottesville, Virginia, United States, 2. University of Virginia, School of Medicine, Charlottesville, Virginia, United States, 3. University of Virginia, School of Nursing, Charlottesville, Virginia, United States*

The COVID pandemic has impacted access to care, particularly for older, homebound persons living with dementia (PwD). At the beginning of the pandemic, our interdisciplinary team introduced a home-based medical care program (HBMC) to address chronically ill and homebound PwD and caregivers' needs to promote aging in place. The purpose of this presentation is to describe PwD and caregiver service use and experiences with Virginia at Home (VaH) HBMC during the pandemic. All PwD participating in VaH program are offered home telehealth access with necessary devices. We will discuss telehealth access and use and dyad-care provider communication across up to 20 dyads to facilitate continuity of care. These data are supplemented by qualitative interviews with dyads presenting needs, preferences, and experiences accessing and using services across the first six months of program launch. We will conclude with a discussion of participant-informed program alterations to facilitate optimal overall care and outcomes.

### ADAPTATIONS TO IN-HOME HEALTH CARE DUE TO COVID-19: THE VA'S HOME-BASED PRIMARY CARE PROGRAM

Leah Haverhals,[1] Chelsea Manheim,[1] Nelly Solorzano,[1] Suzanne Gillespie,[2] and Tamar Wyte-Lake,[3], *1. Denver Center of Innovation for Veteran Centered and Value Driven Care (COIN), Denver, Colorado, United States, 2. Finger Lakes VA Healthcare System, Canandaigua, New York, United States, 3. US Department of Veterans Affairs, U.S. Department of Veterans Affairs (VA), California, United States*

The COVID-19 pandemic disrupted traditional Home Based Primary Care (HBPC) care processes, including changes to provision of face-to-face care in-home for older adults. Our study describes and explains care delivery changes Department of Veterans Affairs (VA) HBPC programs made in response to the pandemic. We fielded a national survey to all 140 VA HBPC programs, targeting interdisciplinary care teams and HBPC leadership. We structured survey questions using a mixed method approach with both closed and open-ended questions, applying a qualitative content analysis approach to open-ended responses complemented by analysis of descriptive quantitative data. Preliminary findings highlight the value and consideration of different telehealth modalities when caring for an older, homebound population, as well as creative adaptations HBPC teams made to deliver care during the pandemic. Implications include nascent development of decision-making paradigms beyond the pandemic particularly for appropriate use of telehealth modalities for older homebound adults.

### USE OF TELEHEALTH TO SUPPORT FAMILY CAREGIVERS OF HOSPICE PATIENTS DURING THE COVID-19 PANDEMIC

Debra Parker Oliver,[1] Karla Washington,[1] and George Demiris,[2], *1. Washington University in St. Louis, St. Louis, Missouri, United States, 2. School of Nursing, University of Pennsylvania, University of Pennsylvania, Pennsylvania, United States*

Family caregivers of hospice patients faced additional challenges in the context of the COVID-19 pandemic where social isolation and loneliness that are often observed among those taking care of a loved one at the end of life, were exacerbated by social distancing rules and workflow changes introduced by hospice agencies. The use of telehealth technologies has the potential to facilitate the delivery of supportive services for family caregivers. We conducted a study examining the use of telehealth for the delivery of a supportive intervention based on problem solving therapy and positive appraisal theory designed specifically to support family caregivers of hospice patients during the COVID-19 pandemic. We recruited 248 caregivers who each participated in three telehealth sessions over a month; caregivers reported higher levels of quality of life and lower levels of anxiety post intervention. Specific recommendations for inclusive telehealth design are discussed based on lessons learned.

### LESSONS FROM LIGHTHOUSE: OPERATIONALIZING TECHNOLOGY TO SUPPORT OLDER ADULTS IN AFFORDABLE HOUSING COMMUNITIES

David Lindeman, *UC Berkeley, Oakland, California, United States*

Lighthouse for Older Adults, an innovative public-private partnership, was developed in response to COVID-19 as a means of advancing telehealth for low-income older adults living in affordable housing communities. Residents of these communities often don't have reliable access to devices, sufficient bandwidth for telehealth, or adequate social services, further complicated by the need for multi-lingual and culturally sensitive programs. This presentation will share program implementation strategies and outcomes, including the essential role telehealth services play in the care and wellbeing of older adults during and beyond COVID-19. This session will review evidence-based components of a telehealth intervention, including digital literacy training and technology support. Key drivers for successful implementation (e.g., peer led training, user input into technology selection) as well as barriers to implementation (e.g., broad band installation, internet service availability/cost, tech support) will be reviewed. Lessons learned through program replication and scaling of Lighthouse telehealth services will be discussed.

### TELEHEALTH AND 30-DAY READMISSIONS AMONG HEART FAILURE PATIENTS DURING THE COVID-19 PANDEMIC

Bradi Granger,[1] Eric Peterson,[2] Matthew Dupre,[3] and HANZHANG XU,[4], *1. Duke university School of Nursing, Durham, North Carolina, United States, 2. University of Texas Southwestern Medical Center, Dallas, Texas, United States, 3. Duke University School of Medicine, Durham, North Carolina, United States, 4. Duke University School of Medicine, Duke University, North Carolina, United States*

This study examined whether outpatient follow-up within 14 days of discharge via telehealth visits are as effective as in-person visits for reducing 30-day readmission in heart failure (HF) patients. Using electronic health records from a large health system, we included HF patients (n=1,722) who were hospitalized during the period of March 15-July 15, 2020. Overall, 28.1% of patients received an early outpatient follow-up visit. Patients who received telehealth visits (n=119) were more likely to be older and live in areas with higher median household incomes than those with in-person visits (n=365). Thirty-day readmission rates were 20.5% during the COVID-19 period. Multivariate models showed that patients who received a telehealth (OR=0.36, 95%CI [0.23-0.56]) or an in-person (OR=0.42, 95%CI [0.31-0.57]) visit were less likely to be readmitted within 30 days compared with patients without an early outpatient follow-up. Telehealth visits were just as effective as in-person visits at reducing 30-day readmissions.

### Session 4150 (Paper)

### TELEHEALTH INTERVENTIONS FOR OLDER ADULTS: COVID-19

### RURAL-URBAN DIFFERENCES IN AVAILABILITY OF TELEMEDICINE SERVICES AMONG MEDICARE BENEFICIARIES DURING COVID-19

Yvonne Jonk,[1] Erika Ziller,[2] and Heidi O'Connor,[2], *1. University of Southern Maine, Muskie School, Portland, Maine, United States, 2. University of Southern Maine, Portland, Maine, United States*

The COVID-19 pandemic has created substantial disruptions to all aspects of rural and urban U.S. life. At the same time, it has provided opportunities for shifts in health service delivery, including policy innovations to increase telehealth availability and use for diagnosis and treatment of health concerns. However, it is unclear whether rural residents, particularly older adults, have the same access to telehealth services as their urban counterparts. Rural providers may face unique barriers to delivering telehealth services, and rural patients may have more difficulty accessing those services from their homes. This study used the Fall and Summer 2020 Medicare Current Beneficiary Survey COVID-19 Supplement Public Use Files to examine rural-urban differences in the telemedicine services available to Medicare beneficiaries from their primary care providers, as well as their ability to access those services. Preliminary findings suggest that rural beneficiaries are less likely to have access to telehealth services during the pandemic, they were more likely to exhibit hesitancy towards receiving the COVID-19 vaccine, they were less likely to engage in preventive behaviors such as hand washing and sterilizing surfaces, and more likely to have missed diagnostic or medical screening tests (37%) compared to urban (27%) beneficiaries. Finally, rural beneficiaries were less likely to have a smartphone, computer, or tablet at home and less likely to have access to the internet (78% rural; 84% urban). Policy implications include the need for outreach efforts to better inform the provider community, and efforts to improve rural health system infrastructure available to support telehealth.

### TECHNOLOGY IS OUT THERE FOR THE BETTERMENT OF US: AFRICAN AMERICAN FAMILY CAREGIVERS AND COVID-19

Afeez Hazzan,[1] Carol D'Agostino,[2] and Phyllis Jackson,[3], *1. State University of New York at Brockport, Hilton, New York, United States, 2. Geriatric Mental Health Specialist, Geriatric Mental Health Specialist, New York, United States, 3. Common Ground Health, Common Ground Health, New York, United States*

Unpaid family caregivers are mostly responsible for bearing the costs associated with caring for older adults with dementia. Importantly, the ongoing COVID-19 pandemic has created unforeseen challenges for many family caregivers. Specifically, the restrictions put in place to limit the spread of the coronavirus may be exacerbating the challenges faced by these caregivers as they try to navigate the system. Further, studies have shown that family caregivers who are members of a racial or ethnic minority group such as African-Americans or Hispanics face unique challenges when caring for their loved ones. Additional challenges may include socioeconomic disadvantages, health disparities, and language barriers that make it more difficult to access healthcare and social services. In this study, we examined the perspectives of African-American family caregivers of older adults on the feasibility of utilizing technology as a coping strategy (including for research participation) during the ongoing COVID-19 pandemic. The research question was: What are the perspectives of African-American family caregivers of people with dementia on the feasibility, opportunities, and challenges of technology as a means to engage family caregivers during a pandemic? In-depth one-on-one interviews were conducted with 12 African-American/black family caregivers. Thematic analysis of the qualitative data yielded the following three themes: (1) Acceptance that technology will play a greater role in the world going forward, and family caregivers need to adapt; (2) Opportunities to avoid social isolation while maintaining links with critical community resources; and (3) Challenges due to possible loss of privacy and lack of physical interactions

### TELEHEALTH: A RESOURCE FOR VULNERABLE POPULATIONS TO ACCESS ORAL HEALTHCARE DURING THE COVID-19 PANDEMIC

Victoria Raveis,[1] David Glotzer,[2] and Andre Ritter,[3], *1. New York University, New York University, New York, United States, 2. New York University, New York University,*

*New York, United States, 3. New York University, New York, New York, United States*

Dental care and adherence to daily oral hygiene practices are particularly important for healthy aging. For socially disadvantaged or impoverished, older community residents, populations who are at risk for long-standing oral disease, public dental clinics are safety nets. In March 2020, when COVID-19 cases surged in the New York metropolitan area, a months-long suspension of the area's community dental services occurred, including clinical operations at the NYU College of Dentistry. To ameliorate the impact of this widespread service suspension, NYU Dentistry implemented an interim Dental Telehealth Service, open to the community, with telehealth consultations delivered by NYU faculty. This consultative service served a diverse population, adhering to guidelines the American Dental Association (ADA) issued on "urgent" and "emergency" care, with the goal of treating with a minimally invasive approach, to relieve the burden on hospital emergency rooms. Older adults, experiencing dental issues and fearing they were particularly vulnerable to the virus, called into this service, as they desperately wanted to avoid the overwhelmed public hospital ERs. A range of significant dental issues, i.e. pain, swelling, tooth fractures, were addressed. Implementing this community resource was a creative strategy to address a serious health services gap during this public health crisis. It also yielded important insights regarding the feasibility, acceptability and utility of telehealth, as a routine component of dental practice, when treating older adults, who often have serious co-morbidities and limited mobility. Certainly, the conversational nature of telehealth is a less stressful and anxiety-provoking clinical encounter.

### TELEMEDICINE USE AMONG OLDER ADULTS DURING THE COVID-19 PANDEMIC

Anita Szerszen,[1] Yulia Kogan,[2] and Edith Burns,[2]
*1. Northwell Health, Staten Island, New York, United States, 2. Northwell Health, New Hyde Park, New York, United States*

**Objective:** Although technology adoption among older adults is improving, ethnic minorities and those with socioeconomic disadvantages may have lower utilization of telemedicine. Here, we evaluate telemedicine uptake amongst community-based older adults.

**Materials and Methods:** Using a retrospective cohort design, we examined electronic medical records (EMR) for documentation of telemedicine use among patients > 65 years old at Geriatric practices in the New York metropolitan area from January-November 2020. Demographic details and insurance payer were captured for telemedicine visits and compared to in-person encounters. Multivariable regression was used to evaluate the association of demographic, socioeconomic factors and visit type.

**Results:** A total of 712 patients (32.3%) engaged in 1,085 telemedicine visits. Telemedicine represented 80% and 66% of all encounters during April and May, respectively and averaged 11.8% between June and November. Use was similar across age groups, gender, race and insurance payer status between telemedicine versus in-person encounters. Patients with greater number of comorbidities were more likely to use telemedicine-. Medicaid recipients had preference for video visits. 47.5% of patients who engaged in video visits had another person/family member present during an encounter.

**Conclusions:** Telemedicine augmented access to health care for older individuals during the peak of the COVID pandemic and continues to be utilized to improve access to care for older Americans. Given the distinct preference for video visits among patients with multiple medical conditions and those who have Medicaid, telemedicine has potential to serve as a tool to reduce enduring health care disparities beyond the pandemic.

### Session 4155 (Symposium)

### THE PATHWAYS TO HEALTHY AGEING: EVIDENCE FROM LONGITUDINAL STUDIES AND REAL-TIME DATA

Chair: Terry YS Lum

The WHO has replaced its active ageing policy framework developed in 2002 with the new Healthy Ageing framework developed in 2015 and declared the decade between 2020 and 2030 as the Decade of Healthy Ageing. Healthy Ageing framework emphasizes the pivotal role of functional ability (FA) among older adults and conceptualizes that FA can be determined by intrinsic capacity (IC), environments (EN), and their interaction. WHO calls for global research to advance theoretical understanding of Healthy Ageing framework and translate the evidence into policy actions. This symposium provides the latest findings on Healthy Ageing from multi-country studies using real-time data and longitudinal study design. Dr. Röcke explored daily time-out-of-home and place visit diversity with daily emotional and stress processes in Zurich, using sensor-based and self-reported mobility and activity indicators to capture FA. Dr. Lu investigated the EN and 4-year trajectories of IC and their impact on FA trajectories among older adults in Hong Kong. Dr. Liu explored the longitudinal associations between neighborhood physical EN and depressive symptoms of older adults in Hong Kong and the moderating effects of terrain slope and declining daily activity of living. Dr. Guo investigated the relationship between perceived EN (environmental cognition) and mental health and the mediating roles of physical activity and place attachment. Dr. Chan explored neighborhood physical EN and cognition among older people and identified whether this association varies among different older age groups. Based on these findings, this symposium will discuss the future research direction on Healthy Ageing and its policy implication.

### DAY-TO-DAY MOBILITY, AFFECT, AND STRESS COUPLINGS IN SWISS OLDER ADULTS

Eun-Kyeong Kim,[1] Pascal Griffel,[2] Robert Moulder,[2] Cheng Fu,[1] Minxia Luo,[1] Mike Martin,[1] Robert Weibel,[1] and Christina Roecke,[2], *1. University of zurich, Zurich, Zurich, Switzerland, 2. University of Zurich, Zurich, Zurich, Switzerland*

The Mobility, Activity, and Social Interactions Study (MOASIS) is part of a global effort to more closely examine indicators of functional ability in relation to person characteristics and life contexts as proposed by the WHO's healthy aging definition. In MOASIS, sensor-based and self-reported mobility and activity indicators were used to capture functional ability in 153 community-dwelling older adults aged

65-91 over 30 days. The present study examines daily time out-of-home and place diversity and its within-person associations with positive and negative affect and stress. Initial between-person analyses indicate that mobility is only weakly related to indicators of physical and mental health. We propose that the health- and well-being implications of mobility more strongly play out in daily life and at the within-person level, and will examine general health, cognitive ability, and marital status as intrinsic capacity moderators accounting for some of the expected interindividual heterogeneity.

### NEIGHBORHOOD ENVIRONMENT, INTRINSIC CAPACITY AND 4-YEAR LATE-LIFE FUNCTIONAL ABILITY TRAJECTORIES

Yuqi Liu, Yingqi Guo, Hung Chak Ho, Hiu Kwan Chui, Chris Webster, Lai Har Chiu, Terry YS Lum, and Shiyu Lu, *The University of Hong Kong, Hong Kong, Not Applicable, Hong Kong*

Knowledge on how intrinsic capacity (IC) and neighbourhood physical environment shape functional ability (FA) trajectories in later life remains understudied. We investigated the 4-year trajectories of IC and their impact on FA trajectories, and the associations between neighbourhood physical environments and FA trajectories over time among older adults. We conducted a four-wave longitudinal study from 2014-2017 in Hong Kong with 2,081 adults aged 65 and above. FA was assessed by The Chinese Lawton Instrumental Activities of Daily Living. We used cognition, affect, locomotion, sensory capacity, and vitality to capture multi-domains of IC. Neighbourhood physical environment attributes included green space, land use diversity, and facilities availability, assessed within 200- and 500-meter buffers of respondents' homes. IC and FA each decreased significantly over time. Individuals with declines in IC experienced faster declines in FA over time. Green space, the number of leisure facilities and public transport slowed the decreasing FA rate.

### ASSOCIATIONS BETWEEN NEIGHBORHOOD ENVIRONMENTS AND DEPRESSIVE SYMPTOMS OF OLDER ADULTS

Shiyu Lu, Yingqi Guo, Hung Chak Ho, Hiu Kwan Chui, Chris Webster, Lai Har Chiu, Terry YS Lum, and Yuqi Liu, *The University of Hong Kong, Hong Kong, Not Applicable, Hong Kong*

Little is known about the accumulative impacts of neighbourhood physical environments on depression among older adults. Based on a cohort study of 2,081 older adults in Hong Kong, this study examined longitudinal relationships between neighbourhood physical environments and depressive symptoms among older adults and the moderating effects of the slope of terrain and individual functional ability using latent growth curve modelling. Results indicated that the availability of community centres and passive leisure facilities reduced depressive symptoms over time. The protective effects of residential surrounding greenness on depressive symptoms among older adults differed by the slope of terrain. Longitudinal associations between neighbourhood physical environments and depressive symptoms varied between older adults with and without functional limitations. Identifying environmental barriers and applying targeted residential environment interventions are essential.

### ENVIRONMENTAL COGNITION AND MENTAL HEALTH: PHYSICAL ACTIVITY AND PLACE ATTACHMENT AS PATHWAYS

Shiyu Lu, Yuqi Liu, On Fung Chan, Hiu Kwan Chui, Terry YS Lum, and Yingqi Guo, *The University of Hong Kong, Hong Kong, Not Applicable, Hong Kong*

Few studies have explored the underlying pathways between environment cognition (i.e., perception of environment) and mental health in older adults. We tested the mediation effects of physical activity and place attachment in the relationship between environmental cognition and mental health, based on a survey study of 1,553 older adults in Hong Kong using structural equation model. The results showed that significant relationship between negative environmental cognition (i.e., residing in higher accessible area but perceive lower) on access to convenient stores, leisure facilities, clinics, community centers, religious places and lower mental health can be explained by lower daily average physical activity time. Place attachment can significantly mediate the positive effect of positive environmental cognition (i.e., residing in lower accessible area but perceive higher) towards all types of services on mental health. Findings from this study have policy implication for urban planning and age-friendly community design

### NEIGHBORHOOD PHYSICAL ENVIRONMENT AND COGNITION IN LATER LIFE: A CROSS-SECTIONAL STUDY IN HONG KONG

Yuqi Liu, Yingqi Guo, Shiyu Lu, Hiu Kwan Chui, Lai Har Chiu, Chris Webster, Terry YS Lum, and On Fung Chan, *The University of Hong Kong, Hong Kong, Not Applicable, Hong Kong*

This study examined the association between neighbourhood physical environment and cognition among community-dwelling older people and identifies whether this association varies among different older age groups. Data came from a cross-sectional survey data with 2,081 older people living in 12 public housing estates in Hong Kong. We merged individual data with neighbourhood physical environment data from community audit and GIS. Multivariable linear regression model was used. Young-old who resided in neighbourhoods with a higher land use mix and more public transport terminals, were associated with better cognition. Only the number of community centres was positively associated with the cognition for old-old. A curvilinear association was found between cognition and the number of active leisure facilities in the overall sample and young-old. Our findings could inform urban planners and policymakers on planning community facilities and physical environments based on the needs of older people in different age groups.

### Session 4160 (Symposium)

### THE RATIONAL USE OF HORMONE REPLACEMENT THERAPY IN OLDER ADULTS

Co-Chair: Jennifer Mammen Co-Chair: Eleanor Simonsick
Discussant: Eleanor Simonsick

Multiple hormone levels are lower in older compared to younger adults, but the clinical implications are controversial. Lower thyroid hormone, testosterone, estrogen and vitamin D are variously associated with metabolic syndrome, sarcopenia, low bone density and cognitive decline, for which treatment has been recommended in the past. However, evidence from the T Trials of testosterone therapy failed to show benefit for endpoints other than sexual function. In the Women's Health Initiative, a large RCT of estrogen replacement in older women, found evidence of an interaction between estrogen therapy and metabolic risk factors such as diabetes that actually exacerbates risk of cognitive decline. Longitudinal observation of thyroid hormone and thyrotropin patterns in the Baltimore Longitudinal Study of Aging demonstrate heterogeneity that might account for a lack of benefit in studies of treatment for subclinical hypothyroidism in older adults. At the same time, new data suggest the need for a more aggressive threshold for vitamin D in older adults, with a lower threshold associated with a drop in physical function compared to younger adults. Complexity in the regulation of hormonal pathways and the downstream effects on target tissues means multiple individuals with similar hormone levels may have different underlying physiology, with divergent clinical needs. Changes in activity and diet common during aging, and exacerbated by the pandemic, lead to physical and mood changes associated with hormonal dysfunction in popular culture and patient requests for evaluation. The ultimate goal should be personalized treatment decisions based on comprehensive evaluation and pathophysiology.

### THE MANY FACES OF ELEVATED TSH: WHEN TO AVOID THYROID HORMONE THERAPY IN OLDER ADULTS

Enoch Abbey,[1] John McGready,[2] Luigi Ferrucci,[3] Eleanor Simonsick,[4] and Jennifer Mammen,[1], *1. Johns Hopkins University School of Medicine, Baltimore, Maryland, United States, 2. Johns Hopkins University Bloomberg School Of Public Health,, Baltimore, Maryland, United States, 3. National Institute on Aging, Baltimore, Maryland, United States, 4. National Instute on Aging/NIH, Baltimore, Maryland, United States*

We have previously demonstrated that hypothalamic-pituitary-thyroid axis aging is characterized by several distinct patterns. An elevated thyrotropin (TSH) level (mean 5.6mIU/L) with normal free thyroxine (FT4) was present in 75 BLSA participants with at least 3 visits. Twenty-one percent had an historical pattern consistent with primary gland failure, while 13% had a pattern consistent with an HPT response to stressors (aging-adaptation). The remainder had intermediate patterns of change. FT4 >0.92pg/ml identified those in whom TSH elevations occurred with aging-adaptation with a 90.0% sensitivity and 93.8% specificity, indicating no need for therapy. In addition, among 597 participants with stable TSH levels in the reference range, being on thyroid hormone therapy increased mortality risk (IRR=1.8; 95% CI 0.9-2.1). Thus, including FT4 in the diagnostic criteria for hypothyroidism in older adults could target therapy to avoid the potential harm of reversing the aging adaptations in those who do not have true early hypothyroidism.

### SEX-SPECIFIC 25-HYDROXYVITAMIN D THRESHOLD CONCENTRATIONS FOR FUNCTIONAL OUTCOMES IN OLDER ADULTS

Jack Guralnik,[1] Eleanor Simonsick,[2] Stephen Kritchevsky,[3] Peggy Cawthon,[4] and Michelle Shardell,[5], *1. University of Maryland, Baltimore, Baltimore, Maryland, United States, 2. National Instute on Aging/NIH, Baltimore, Maryland, United States, 3. Wake Forest School of Medicine, Winston Salem, North Carolina, United States, 4. California Pacific Medical Center, San Francisco, California, United States, 5. University of Maryland School of Medicine, Baltimore, Maryland, United States*

25-Hydroxyvitamin D [25(OH)D] has extra-skeletal effects, but it is not known whether the minimum sufficient serum levels for such targets, like muscle, differ from those for bone health (typically recommended at 20 or 30 ng/dL). Therefore, we derived and validated sex-specific thresholds for serum 25(OH)D predictive of poor physical function using 5 cohorts comprising 16,388 community-dwelling older adults (60.9% women). Using a cohort-stratified random two-thirds sample, we found incident slow gait was best discriminated by 25(OH)D<24.0 versus 25(OH)D>=24.0 ng/mL among women (Relative Risk=1.29; 95% CI 1.10-1.50) and 25(OH)D<21.0 versus 25(OH)D >=21.0 ng/mL among men (RR=1.43; 95% CI 1.01-2.02). Estimates from the remaining one-third validation sample were similar. Empirically identified and validated sex-specific 25(OH)D thresholds from multiple well-characterized cohorts of older adults may yield more biologically meaningful definitions in important sub-populations. Such thresholds may serve as candidate reference concentrations or inform design of vitamin D intervention trials in older adults.

### TESTOSTERONE THERAPY FOR MEN WITH AGE-RELATED LOW TESTOSTERONE: TEMPEST IN A TEACUP

Shehzad Basaria, *HARVARD MEDICAL SCHOOL, BOSTON, Massachusetts, United States*

Serum testosterone concentrations decrease in men with age, but benefits and risks of raising testosterone levels in older men remain controversial. In the T-Trials, a total of 790 men, age 65 and older, with a serum testosterone concentration of < 275 ng/dL and symptoms of sexual dysfunction, fatigue or physical dysfunction were randomized to either testosterone gel or placebo gel for 1 year. Treatment in the testosterone arm increased serum testosterone levels to the mid-normal range for young men. Testosterone replacement was associated with a significant increase in sexual activity (p<0.001), libido and erectile function. In contrast, there was no improvement in vitality or physical function. Adverse findings included increases in non-calcified plaque formation and a higher rate of prostate events. In sum, testosterone treatment in older men was associated with modest benefits, while the risk on prostate and cardiovascular health remain unclear.

### ASSOCIATIONS BETWEEN ENDOGENOUS ESTROGEN, POSTMENOPAUSAL HORMONE THERAPY, AND COGNITIVE CHANGES IN OLDER WOMEN

Christina Hugenschmidt, and Mark Espeland, *Wake Forest School of Medicine, Winston-Salem, North Carolina, United States*

How markers of brain health are associated with endogenous estrogen and use of postmenopausal hormone therapy (HT) varies depending on women's years from menopause and metabolic health status, ranging from potential benefit to harm. The Women's Health Initiative (WHI) included 7,233 women age 65-80 who underwent a randomized clinical trial of various HT preparations for an average of 5.9 years. Over up to 18 years of post-trial follow-up, diabetes (DM2) increased the risk of dementia (hazard ratio [HR] 1.54 [95% CI 1.16–2.06]). Having DM2 and also treatment with unopposed conjugated equine estrogens increased the risk to HR=2.12 [1.47-3.06]. We hypothesize that the metabolic effects of estrogen in the brain drives this interaction. In support of this, the metabolic transition following menopause may alter the impact of other treatments on cognition, for example behavioral weight loss therapy to treat obesity in women with type 2 diabetes (interaction p=0.02 for executive function).

### Session 4165 (Symposium)

### WELL-BEING AMONG AGING WOMEN: OBSERVATIONS FROM THE WOMEN'S HEALTH INITIATIVE STUDY (WHI)

Chair: Barbara Cochrane Discussant: Barbara Cochrane

Previous efforts to assess well-being in relation to health have relied on descriptive analyses of hedonic or eudaemonic well-being indicators. Factor scores from principal components analysis offer a summary measure of well-being, but limited interpretability in epidemiologic analyses, e.g. estimated risk ratios. Use of latent class analysis to identify groups differing by levels of both hedonic and eudaemonic indicators preserves information about both dimensions while supporting interpretation of well-being effects on health indicators important for studying older women's health. For this symposium, we report on analyses to: (1) Develop a multidimensional profile of well-being that would preserve the individual dimensions of well-being (hedonic, eudaemonic) using latent class analysis; and (2) Support epidemiologic analyses of well-being as both an outcome and a predictor of health outcomes, enhancing interpretability of levels of well-being across various dimensions. Data on well-being in over 80,000 older women in the Women's Health Initiative Study were obtained in 2011-2012, along with their baseline demographic characteristics in 1993-1998. All-cause mortality included death from any cause occurring from 2012-2020. Four classes of well-being were identified: (1) lowest hedonic and eudaemonic well-being scores; (2) higher hedonic/lower eudaemonic scores; (3) lower hedonic/higher eudaemonic scores; and (4) highest hedonic and eudaemonic scores. Results will be presented in three papers addressing the development of the well-being profile; relationships of the profile with predictors of well-being; and the relationship of the profile to all-cause mortality. The discussion will focus on future research questions, such as likely factors mediating well-being and health outcomes.

### ASSESSING WELL-BEING AMONG AGING WOMEN: OBSERVATIONS FROM THE WOMEN'S HEALTH INITIATIVE

Eileen Rillamas-Sun,[1] Barbara Cochrane,[2] Nancy Woods,[2] and Kenneth Pike,[2], *1. Fred Hutchinson Cancer Research Center, Seattle, Washington, United States, 2. University of Washington, Seattle, Washington, United States*

Our aim was to develop a profile of well-being preserving the ability to estimate differential effects of both hedonic and eudaemonic dimensions of well-being on health outcomes. Numerous indicators of well-being from over 80,000 aging women included hedonic (enjoyment of life, happiness, satisfaction with life, quality of life) and eudaemonic (personal growth, purpose in life, environmental mastery, control, self mastery) dimensions. Using latent class analysis, we identified groups of women with distinct profiles of well-being. A four-class solution had both good statistical fit and made conceptual sense. Class 1 (n=9,146, 11%) had the lowest scores on hedonic and eudaemonic indicators, while Class 4 (n=38,246 47%) had the highest levels of all well-being indicators. Class 2 (n=7,106, 9%) had higher hedonic and lower eudaemonic scores and Class 3 (n=26,650, 33%) had lower hedonic and higher eudaemonic scores. These classes form a well-being profile useful for estimating differential effects on health outcomes.

### PREDICTORS OF CLASSES OF WELL-BEING AMONG AGING WOMEN

Eileen Rillamas-Sun,[1] Barbara Cochrane,[2] Kenneth Pike,[2] and Nancy Woods,[2], *1. Fred Hutchinson Cancer Research Center, Seattle, Washington, United States, 2. University of Washington, Seattle, Washington, United States*

Our aim was to examine the relationship of predictors of well-being from prior studies to the well-being profile developed from data from aging WHI participants. Class 1 included women with both low hedonic and eudaemonic well-being scores, class 4 with the highest scores. Classes 2 and 3 had moderate scores, with class 2 having higher hedonic and lower eudaemonic scores and class 3 having lower hedonic and higher eudaemonic scores. We examined associations between predictors and well-being classes. Youngest women were in Class 4 (mean=60.2 years) and oldest in Class 3 (mean=63.2). African American women had higher proportions in in Classes 2 and 3, Latinas in Classes 1 and 3, and Asian/Pacific Islanders in Class 3. College graduates, married women and those with household incomes >$50,000 were most likely in Class 4. Associations with age, race/ethnicity, education, marital status and income were consistent with prior analyses incorporating individual well-being indicators.

### WELL-BEING AND ALL-CAUSE MORTALITY IN AGING WOMEN IN THE WOMEN'S HEALTH INITIATIVE (WHI)

Kenneth Pike,[1] Barbara Cochrane,[1] Nancy Woods,[1] and Eileen Rillamas-Sun,[2], *1. University of Washington, Seattle, Washington, United States, 2. Fred Hutchinson Cancer Research Center, Seattle, Washington, United States*

To study the relationship between well-being and all-cause mortality, we estimated mortality among women in four classes of well-being using the well-being profile from the Women's Health Initiative Study (WHI). Demographic characteristics were self-reported at enrollment (1993-98). All-cause mortality included death from any cause between 2012-2020. We used logistic regression to examine all-cause mortality risk across the classes, using Class 4 (highest hedonic and eudaemonic well-being scores) as the referent, adjusting for age and race. Compared to Class 4, all other classes had higher age- and race-adjusted odds of death.

Highest risks were in Class 1 women (OR=2.61; 95% CI: 2.46-2.76) and Class 3 women (OR=1.62; 95% CI: 1.55-1.68). Women in Class 4 had the lowest risk of all-cause mortality over an 18-year follow-up. These results confirm the utility of a profile of well-being for predicting all-cause mortality while preserving ability to identify the differences among well-being indicators across classes.

### Session 4167 (Symposium)

### RECRUITMENT AND RETENTION IN DIVERSE COHORTS: LESSONS FROM COMMUNITY-ENGAGED EFFORTS

Chair: Carrie Nieman Co-Chair: Haera Han
Discussant: George Rebok

Effective behavioral interventions and associated trials reflect the complexity and context of the communities with which they are tailored and the behaviors they seek to address. Community-engaged methodology can serve to capture these complexities, particularly when focusing on health inequities. Significant health and healthcare disparities persist among racial/ethnic minorities and representation of racial/ethnic minorities is lacking within trials that reflects the diversity of the U.S. population. Novel approaches are needed to increase the diversity of participants within behavioral intervention research. This symposium covers the unique barriers and facilitators related to recruitment and retention across a range of populations, including African American and Hispanic/LatinX older adults with hearing loss to diverse dementia family caregivers and community-dwelling Korean American older adults. Beyond the challenges and opportunities, the symposium will focus on effective recruitment strategies. The discussion will include 1) findings from 10 years of recruiting older Korean Americans into community-based trials, 2) lessons in tailoring recruitment efforts to dementia family caregivers, 3) the integration of human-centered design into a community-engaged hearing care intervention targeting low-income and African American older adults, 4) successful recruitment and retention efforts in a community-based participatory research trial in a borderlands community, and 5) the deployment of strategies to recruit Latino, Asian, and African American older adults with depression and anxiety in the setting of the COVID-19 pandemic. This symposium seeks to build the evidence related to recruitment of older racial/ethnic minorities in diverse settings, which is fundamental to addressing health inequities through behavioral intervention research.

### COMMUNITY ENGAGEMENT AND HUMAN-CENTERED DESIGN: LESSONS FROM HEARS IN INCLUSIVE RECRUITMENT OF OLDER ADULTS

Hae-Ra Han,[1] Nicole Marrone,[2] Jonathan Suen,[3] Sarah Szanton,[4] Jami Trumbo,[5] Frank Lin,[6] and Carrie Nieman,[7], *1. Johns Hopkins School of Nursing, Baltimore, Maryland, United States, 2. University of Arizona, Tucson, Arizona, United States, 3. Johns Hopkins University School of Nursing, Baltimore, Maryland, United States, 4. Johns Hopkins University, Baltimore, Maryland, United States, 5. Cochlear Center for Hearing & Public Health, Baltimore, Maryland, United States, 6. Johns Hopkins University, Johns Hopkins University, Maryland, United States, 7. Johns Hopkins University School of Medicine, Baltimore, Maryland, United States*

Within hearing care, significant disparities persist despite the highly prevalent nature of age-related hearing loss and relatively few trials include representation of racial/ethnic minorities. HEARS is an affordable, accessible hearing care intervention delivered by older adult peer mentors. The HEARS randomized controlled trial (NCT03442296) is a community-engaged RCT with an embedded human-centered design practitioner. Recruitment efforts occurred over 18 months in partnership with 13 affordable housing and social centers. The cohort (n=151) includes 43% (n=65) who self-identify as African American and 63.6% (n=96) with <$25,000 annual household income. The cohort represents the largest to-date of African American and low-income older adults with hearing loss. Recruitment efforts entailed 470.5 staff hours and $4,917.26 in supplies, equating to 1.4 hours and $14.13 per 1 individual screened and 3.1 hours and $32.56 for 1 participant randomized. Community-engaged research, partnered with human-centered design, may offer critical approaches to increasing representation within behavioral intervention trials.

### COMMUNITY-ENGAGED STRATEGIES FOR RECRUITMENT OF KOREAN AMERICANS IN COMMUNITY-BASED RESEARCH STUDIES

Hochang Lee,[1] Miyong Kim,[2] and Haera Han,[3], *1. University of Rochester, Rochester, New York, United States, 2. School of Nursing, The University of Texas at Austin, Austin, Texas, United States, 3. Nursing, Baltimore, Maryland, United States*

With increasing numbers of researchers targeting ethnic minorities to address health disparities, it is important to address the unique needs of Korean American (KA) older adults—a "hard to reach" yet one of the most rapidly increasing ethnic and age groups in the nation. The purpose of this paper is to describe the main barriers to research participation and to identify facilitators for recruitment of older KAs. We have analyzed recruitment data pertaining to more than 10 community-based KA research studies we have conducted for the last ten years. There were a number of unique recruitment challenges in regard to the culture, language, and sociodemographic characteristics of the participants. Examples of effective recruitment strategies included: aligning the research agenda with the priorities of the community; establishing collaboration with ethnic churches and ethnic media; recruiting and training bilingual volunteers and community health workers; and placing liaison research staff in the community.

### PRIORITIZING COMMUNITY IN RESEARCH DECISION-MAKING THROUGH PARTNERSHIP

Maia Ingram,[1] Aileen Wong,[1] Rosie Piper,[2] Sonia Colina,[1] Scott Carvajal,[1] Laura Coco,[1] and Nicole Marrone,[1], *1. University of Arizona, Tucson, Arizona, United States, 2. Mariposa Community Health Center, Nogales, Arizona, United States*

In behavioral intervention research, taking a community-based participatory research approach enhances recruitment and retention while facilitating the transfer of research findings into social change. Successes with recruitment and retention are secondary to enacting fundamental principles of trust, reciprocity, cultural humility, empowerment, and respect. This presentation will describe a longitudinal clinical trial in a Southwest borderlands community, Oyendo Bien. The study was co-developed and implemented with community partnership throughout the research process. Dyads were recruited to participate in a community-delivered group education and support program addressing hearing loss for Spanish-speakers age 50+ years (n=132 participants randomized). We highlight the critical role that community health workers (promotoras) held as members of the research team. Furthermore, we describe an innovative approach for language mediation that integrates and empowers community participation. This presentation will include examples of lessons learned from the community in collaborating to conduct research in a way that truly serves.

### RECRUITING DIVERSE DEMENTIA FAMILY CAREGIVERS: WHAT WORKS FOR WHICH GROUPS?

Valerie Cotter,[1] Hae-Ra Han,[2] and Kyra Mendez,[2], *1. Johns Hopkins School of Nursing and School of Medicine, Baltimore, Maryland, United States, 2. Johns Hopkins School of Nursing, Baltimore, Maryland, United States*

The purpose of this presentation is to compare success of recruitment methods by race/ethnicity, age, and kinship of dementia family caregivers. We conducted a cross-sectional study and recruited a convenience sample of dementia family caregivers using community-based and online methods. Recruitment success was tracked through survey questions, direct referrals, and community event sign-ups. Using chi-squared statistics, we examined the success of each method by caregiver race/ethnicity, age, and relationship to person with dementia (kinship). There were significant differences in recruitment source based on race/ethnicity, age, and kinship (P<.001). Specifically, referrals and newspaper advertisements were most successful for recruiting older (54 years+), White, non-Hispanic, and spousal or child caregivers; community events and reputable websites for recruiting older, minority, child caregivers; ResearchMatch for recruiting younger, minority, child/grandchild caregivers; and social media for recruiting younger, White, non-Hispanic, and child caregivers. Findings support the importance of implementing tailored methods to reach diverse dementia caregivers.

### COMMUNITY RECRUITMENT OF ASIAN, LATINO AND AFRICAN AMERICAN OLDER ADULTS WITH DEPRESSION SYMPTOMS DURING COVID-19

Ravali Mukthineni,[1] Sahnah Lim,[2] Aida Jimenez,[3] Caroline Ferreira,[1] sheri Lapatin Markle,[1] Margarita Alegria,[1] and Irene Falgas Bague,[4], *1. Disparities Research Unit. Massachusetts General Hospital, Boston, Massachusetts, United States, 2. New York University, New York, Massachusetts, United States, 3. Department of Psychology, University Of Puerto Rico - Rio Piedras/ San Juan, Massachusetts, United States, 4. Massachusetts General Hospital, Harvard Medical School, Boston, Massachusetts, United States*

Recruitment and engagement of racial/ethnic minority older adults in clinical trials is crucial to expand implementation of evidence-based interventions for disability prevention. Public Health measures to counteract COVID-19 pandemic have increased the challenges on reaching this population. This study seeks to comprehensively evaluate a set of recruitment strategies to enroll Latino, Asian and African American older adults with symptoms of depression and anxiety during the first year of a randomized clinical trial. A partnership of three academic sites across the U.S. (NYC, MA and PR) involving several collaborations with community agencies recruited racial/ethnic minority older adults using different strategies involving bilingual interviewers calling from hospital research dataset and community agencies' list of clients, referrals from primary care providers or psychotherapy waitlist. In this presentation we will report various recruitment and retention data including individual and organizational predictors of successful recruitment as well as challenges across all three sites.

### Session 4170 (Symposium)

### KENT AND KLEEMEIER AWARD LECTURE AND PRESENTATIONS

Chair: Debra Dobbs

The Robert W. Kleemeier Award lecture will feature an address by the 2020 Kleemeier Award recipient, Matt Kaeberlein, PhD, FGSA, of the University of Washington. The Kleemeier Award is given annually to a member of The Gerontological Society of America in recognition for outstanding research in the field of gerontology. The Donald P. Kent Award lecture will feature an address by the 2020 Kent Award recipient, David Ekerdt, Phd, FGSA, of the University of Kansas. The Kent Award is given annually to a member of The Gerontological Society of America who best exemplifies the highest standards of professional leadership in gerontology through teaching, service, and interpretation of gerontology to the larger society.

### TARGETING BIOLOGICAL AGING: A NEW PARADIGM FOR 21ST CENTURY MEDICINE

Matt Kaeberleien, *University of Washington, North Bend, Washington, United States*

Biological age is the greatest risk factor for nearly every major cause of death and disability, including COVID-19. Yet, traditional biomedical research and clinical approaches have focused on waiting until people are sick and treating individual diseases one at a time. Attempts to "cure" age-related diseases have proven unsuccessful, and the impact of "disease-first" approaches continue to be incremental. Recent advances in understanding them mechanisms linking biological aging to disease, or geroscience, have identified interventions that directly target the molecular hallmarks of aging. Unlike disease-specific approaches, such interventions have the potential to prevent multiple diseases of aging simultaneously, thereby greatly enhancing healthspan and lifespan for most individuals. Here I will provide an overview of translational geroscience, which I believe will become the paradigm for the practice of medicine in the 21st century. I will also discuss recent work with one such intervention, the drug rapamycin, and our efforts to eventually delay or reverse biological aging in companion dogs and people.

### AGING AS READINESS AND WARINESS

David Ekerdt, *University of Kansas, Lawrence, Kansas, United States*

Gerontology concerns itself with events in time, either things that have happened or things that may happen. In the former, our work is to describe and explain. In the latter, the occurrence of events is unknowable, but we can nonetheless study people's imagination of them (how it arises) and how that imagination shapes behavior and attitudes in the present (how it matters). The subjective experience of aging, thus, is one of looking ever forward—welcoming, waiting for, or hoping to avoid what the future may hold. This personal experience of aging toggles between readiness and wariness of the time ahead, one stance incurring or else eclipsing the other. Transitions are fruitful opportunities to study people's readiness and wariness toward the time ahead, for example, widowhood, the prospect of retirement, and residential relocation. This is when people are more likely to conjure, in their minds, whom they may become. Arguably, the fundamental transition that looms and occupies aging minds (and the minds of loved ones) is not death but rather the potential passage into the "fourth age" of frailty and vulnerability. This prospect hovers above all else: its occurrence increasingly likely but its timing uncertain. About this prospect, gerontology has the capacity, nay the obligation, to promote narratives about later life that shape wariness and readiness for the practical future (e.g., financial matters, bodily care, living arrangements) as well as for the emotional reception of an old age coming ever closer.

### Session 4175 (Symposium)

### ADAPTIVE CARE AND REMOTE TRAINING: MODELS OF RESILIENCE FOR GWEPS DURING A PANDEMIC

Chair: Max Zubatsky

Service and training are interconnected for GWEP's, whose dual missions are to advance training as well as service delivery to improve the care of older adults. The COVID-19 pandemic has necessitated a pivoting from in-person to remote delivery of program content and services. As a result, older adults and their families require the continuity of services with their providers due to the barriers that the pandemic has created. Additionally, universities and organizations have adapted virtually to teach learners how to work with older adults around different health initiatives. The pandemic required these programs to develop immediate services that provided an alternative to remote delivery services. This collection of GWEP programs utilized students and trainees in their older adult services and initiatives. The goal of this symposium is to demonstrate new models of educational and program delivery to enhance and extend training and service to new audiences. The symposium centers on best practices including technological tools to promote GWEP aims and will allow a discussion of challenges and outcomes faced. The session will be comprised of presentations from five university-based, Geriatric Workforce Enhancement Programs (approximately 12 minutes each), a 20 minute discussion and sharing of best practices, and a 10 minute question/answer session. Individual presentations will address areas that include: 1.) group interventions for dementia and caregivers, 2.) teaching interdisciplinary interns in conducting telehealth visits, 3.) adapting geriatrics regional conferences to reach older adults virtually, and 4.) Developing new programs and services for underserved and underrepresented older adult populations.

### SAINT LOUIS UNIVERSITY GWEP PIVOTS TO VIRTUAL SERVICE DELIVERY: LESSONS LEARNED

Max Zubatsky,[1] John Morley,[2] and Marla Berg-Weger,[3], *1. Medical Family Therapy Program, Saint Louis University, Missouri, United States, 2. Division of Geriatric Medicine, Saint Louis University School of Medicine, Missouri, United States, 3. Saint Louis University, Saint Louis, Missouri, United States*

In response to the COVID-19 pandemic, Saint Louis University GWEP quickly pivoted service initiatives to online formats. Despite challenges of technology literacy and access, GWEP faculty, staff, and students creatively adapted in-person programming to online delivery and developed new virtually-delivered services. These service delivery adaptations provided opportunities for educating students, residents, faculty, community partners, and older adults and their caregivers to gain new knowledge and skills while continuing to participate in programming. This presentation will highlight innovations in the area of services to persons with dementia through Cognitive Stimulation Therapy, caregivers through education and support programs, older adults experiencing loneliness and social isolation through Circle of Friends, and older adults and caregivers through a virtual geriatric assessment clinic. We share highlights here of our efforts to pivot programming, access new funding streams, and, in some cases, create online delivery, including valuable lessons learned.

### SUCCESSES AND LESSONS LEARNED IN MOVING ALL EDUCATION AND SERVICE ACTIVITIES TO VIRTUAL LEARNING AND SERVICES

Anna Faul,[1] Pamela Yankeelov,[1] Barbara Gordon,[2] Joseph D'Ambrosio,[1] and Sam Cotton,[3], *1. University of Louisville, University of Louisville, Kentucky, United States, 2. University of Louisville, Louisville, Kentucky, United States, 3. University of Louisville Trager Institute, University of Louisville Trager Institute, Louisville KY, Kentucky, United States*

Despite technology challenges, the UofL GWEP was able to provide access to quality care during the pandemic by pivoting all health and behavioral health patient appointments to telehealth. We found being flexible and using technology patients were most familiar with, was the most successful way easing into telehealth. We trained providers in a variety of technology tools and modalities to support this flexibility. We implemented a remote patient monitoring program and a virtual friendly visitors' program for our most vulnerable patients. Our workforce development focus supported us in teaching our 60+ interdisciplinary interns how to conduct telehealth visits, how to collaborate as an interdisciplinary team in managing the remote patient monitoring program, and how to do virtual case conceptualization and care planning meetings. To re-create the dynamic atmosphere of an interprofessional learning experience, we have paired students together in teams, and provide them with regular opportunities to meet and engage.

### REIMAGINING TRAINING DURING A PANDEMIC: THE EXPERIENCE OF MIDDLE TENNESSEE GWEP

James Powers,[1] and Shana Rhodes,[2], *1. Vanderbilt University, Nashville, Tennessee, United States, 2. Vanderbilt University Medical Center, Nashville, Tennessee, United States*

A major component of The Middle Tennessee GWEP involves delivery of an annual regional geriatrics update conference. Formerly in-person, the planning committee transformed the 34th Annual Update Conference to a virtual platform within a six-month period. The University partner provided a Zoom platform with licensing and training of program staff. National marketing was achieved through professional societies and purchased e-mailings. Participants numbered 79, including 8 disciplines. Presenters were instructed on platform techniques including screen sharing, polling function, and breakout rooms to enhance audience participation. REDCap registration captured demographic information and facilitated evaluations and post-attendance intention-to-change surveys. Lessons learned were shared with community partners and advisory board members who demonstrated changes in service delivery models and training of new staff to support care to greater numbers of clients and participants. Virtual platforms can extend outreach for valuable learning and service outcomes and maintain high levels of satisfaction among target audiences.

### NEW NETWORKS, NEW CONNECTIONS: GERIATRIC STUDENT SCHOLARS EMBRACE ADAPTIVE LEARNING

Robin McAtee, *University of Arkansas for Medical Sciences, Little Rock, Arkansas, United States*

The Arkansas Geriatric Education Collaborative (AR's GWEP) embraces, nurtures, and encourages students with a passion for caring for older adults. Each year five geriatric scholars are chosen from across the spectrum of health services schools (MD, RN, PT, PA, Pharm D, dental hygiene, etc.) to enhance their geriatric knowledge and experience. Requirements focus on geriatric academic and community-based opportunities. However, these opportunities drastically changed with the pandemic. Therefore, the students became very innovative as they trudged forward to meet and exceed the scholar objectives. They participated in various virtual events to fulfil their academic and community participation requirements. They worked together to develop and implement an interdisciplinary final project that marketed to and engaged rural isolated older adults in a fun educational event aimed at preventing social isolation in older adults and caregivers. Students learned how to connect to and bridge the digital divide with isolated rural older adults.

### BRIDGING DIGITAL DIVIDES: GWEP PIVOTS TO SUPPORT TELEHEALTH FOR CLINICAL CARE AND EDUCATION

Lisa Gibbs,[1] Julie Rousseau,[2] Sonia Sehgal,[2] Neika Saville,[3] and Jung-Ah Lee,[4], *1. UC Irvine Health, UC Irvine Health, California, United States, 2. University of California, Irvine, Orange, California, United States, 3. Division of Geriatric Medicine and Gerontology, Orange, California, United States, 4. University of California, Irvine, Irvine, California, United States*

Early in the pandemic, the University of California, Irvine (UCI), GWEP pivoted to focus on building telehealth and remote patient monitoring, while supporting team-based interdisciplinary learners. Our Health Assessment Program for Seniors (HAPS) adapted to provide hybrid remote/in-person evaluations with our Geriatric Fellows and Doctor of Nurse Practitioner (DNP) students working alongside our multidisciplinary team. Learner teams innovatively bridged the digital divide through weekly DNP support phone calls, and the Fellows delivered family conferences through Zoom. In ASSIST, medical students and nursing students gained digital competencies through a phone support system for isolated older adults with friendly weekly check-ins providing referrals to community resources. Another IRB-approved pilot, Healing at Home, diverted patients from the Emergency Room and In-Patient care with a team of ED, Hospitalists, Geriatricians teaching DNP and Fellows telehealth management. GWEP successfully piloted symbiotic learning for both older adults and health profession students through new virtual formats.

### Session 4180 (Paper)

### AGING IN THE COMMUNITY

### FACTORS ASSOCIATED WITH DEFICIENT HOUSING AMONG COMMUNITY-LIVING OLDER ADULTS IN THE UNITED STATES

Safiyyah Okoye,[1] Laura Samuel,[1] Sarah Szanton,[1] and Jennifer Wolff,[2], *1. Johns Hopkins University, Baltimore, Maryland, United States, 2. Johns Hopkins Bloomberg School of Public Health, Baltimore, Maryland, United States*

Housing quality is a recognized social determinant of health. Qualitative evidence suggests the ability of older adults to maintain their homes is affected by the domains of financial resources, social environment, and functional abilities, but this conceptualization has not been tested quantitatively. This cross-sectional study examined associations between financial resources (indicated by socioeconomic characteristics: education, racial-status, annual income, financial hardship, Medicaid eligibility), social environment (living arrangement, social integration), and functional abilities (lower extremity performance, self-care disability, independent-living disability, homebound-status, dementia, depression) with deficient housing among 6,489 community-living adults ≥ 65 years participating in the nationally representative 2015 National Health and Aging Trends Study. Sampling weights accounted for study design and non-response. An estimated 9.2% (3.2 million) older Americans lived in housing with ≥1 deficiency (any peeling paint, evidence of pests, flooring in disrepair, broken windows, crumbling foundation, missing siding, or roof problems). In bivariate logistic regressions, factors from all three domains were associated with deficient housing. In a multivariable model that included all variables above and adjusted for age and sex, indicators of financial resources and social environment remained associated with deficient housing (including financial hardship, adjusted odds ratio (aOR)=1.48, 95% confidence interval (CI): 1.10,1.98; and living with non-spousal others versus alone, aOR=1.48; 95% CI:1.09, 2.03), whereas indicators of functional abilities did not. To ensure quality housing for all community-dwelling older adults, efforts that increase financial resources and further examine the role of social environment in deficient housing are needed.

### NEIGHBORHOOD ENVIRONMENT AND CARDIOMETABOLIC DISEASE IN INDIVIDUALS AGING WITH PHYSICAL DISABILITY

Anam Khan,[1] Paul Lin,[2] Neil Kamdar,[2] Elham Mahmoudi,[3] and Philippa Clarke,[4], *1. University of Michigan School of Public Health, Ann Arbor, Michigan, United States, 2. University of Michigan, Ann Arbor, Michigan, United States, 3. University of Michigan, Commerce Township, Michigan, United States, 4. Institute for Social Research, Ann Arbor, Michigan, United States*

The environment may be particularly important for facilitating participation and health for individuals aging with physical disability. However, little is known about which features of the neighborhood are particularly pertinent for this population. This study aims to address this gap by examining the type(s) of neighborhood environments associated with cardiometabolic disease. We identified ~26,000 individuals with a diagnosis of physical disability using a national private health insurance claims database in the U.S. Geocoded information for individuals was used to assign them to features of their neighborhood from the National Neighborhood Data Archive. An adapted typology was used to classify neighborhoods into the following based on density of health-promoting and harming features: 1) High health-promoting/harming (service-dense), 2) High health-promoting/low harming, 3) Low health-promoting/high harming, 4) Low health-promoting/harming, and 5) Average. We used time-varying Cox models to estimate adjusted hazard ratios (HR) and 95% confidence intervals (CI) for time-to incident cardiometabolic conditions. High neighborhood-level affluence, and low disadvantage scores characterized service-dense neighborhoods. They had more than 2x higher density of health-promoting resources (e.g., transit) compared to other neighborhood types. Individuals residing in service-dense neighborhoods had an 8% lower risk of any cardiometabolic disease (HR 0.92, 95% CI: 0.85-0.99) compared to those in average neighborhoods. Similar effects were observed for Hypertension and Type 2 Diabetes, with effects most pronounced for the latter (HR 0.82, 95% CI: 0.71-0.94). For individuals aging with physical disabilities, service-dense neighborhoods may be protective against cardiometabolic morbidity. Findings can inform community design that support cardiometabolic health in this population.

### NEIGHBORHOOD ENVIRONMENT AND SOCIAL SUPPORT RECEIVED: AN EXAMINATION OF RACE AND GENDER IN BALTIMORE CITY

Sol Baik,[1] Christine Mair,[2] Amanda Lehning,[1] Ji Hyang Cheon,[3] Shari Waldstein,[4] Michele Evans,[5] and Alan Zonderman,[6], *1. University of Maryland, Baltimore, Baltimore, Maryland, United States, 2. University of Maryland, Baltimore County (UMBC), Baltimore, Maryland, United States, 3. University of Maryland, Baltimore County, University of Maryland Baltimore County, Maryland, United States, 4. University of Maryland, Baltimore County, Baltimore, Maryland, United States, 5. NIA, Baltimore, Maryland, United States, 6. HANDLS, Baltimore, Maryland, United States*

Social support in urban settings is likely shaped by the context of the neighborhood environment. Patterns of support may also differ by the type of support received as well as characteristics of the person receiving support. For example, women and Black residents may have stronger support networks compared to men and white individuals, and variation by gender and race in social support may have important implications for promoting well-being in disadvantaged neighborhoods. To investigate the presence of these potential patterns in a disadvantaged urban environment, we analyzed 2,553 Baltimore City residents (ages 30-64) from the baseline wave (2004-2009) of the Healthy Aging in Neighborhoods of Diversity across the Life Span (HANDLS) study. We tested associations between self-assessed neighborhood environment (disorder, cohesion, and control) and social support (from partners, children, and/or friends) and further explored variation by intersections of race and gender using multi-group structural equation modeling. Our results suggest that individuals are more likely to receive support when they perceive their neighborhood to have higher social control and cohesion, particularly in terms of support from friends. Although interactions by race and sex were not statistically significant, a descriptive pattern emerged wherein Black women are particularly likely to receive support from multiple sources when they report more social control in their neighborhood. On the other hand, there is almost no association between neighborhood environment and social support for Black men. We discuss these findings in light of potential neighborhood inequities in informal support access in Baltimore City and similar urban settings.

### RESIDENCE IN HUD HOUSING ASSOCIATED WITH GREATER BENEFIT FROM HCBS SERVICES FOR MEDICAID ENROLLEES IN PENNSYLVANIA

Damian Da Costa,[1] and Howard Degenholtz,[2], *1. University of Pittsburgh, Pittsburgh, Pennsylvania, United States, 2. University of Pennsylvaniz, Pittsburgh, Pennsylvania, United States*

State Medicaid programs seek to shift the delivery of long-term care services away from institutional settings and toward community-based settings by expanding access to home-and-community-based services (HCBS). HCBS are hypothesized to prevent or delay the need for protracted nursing home stays. This study explores the question of which types of community residence maximize this protective effect of HCBS. We used a probabilistic matching technique to identify whether waiver-eligible Medicaid enrollees were likely to reside in project-based HUD housing in 2013. We applied multinomial logistic regression to observe the risk of long-stay nursing home admission (>100 days) relative to persistent community residence in the subsequent four years. Our model controlled for age, race, gender, urban status, and receipt of home-and-community based services. Our predictor of interest was the interaction between receipt of home and community based services (HCBS) and residence in HUD housing. The eligible baseline population included 152,632 community-residing Pennsylvania Medicaid enrollees in 2013. The analytic sample excluded individuals who died during 2013 or who were no longer waiver-eligible after 2013. Residence in HUD project-based housing while receiving HCBS is independently associated with a 27% percent reduction in risk of long-stay nursing home admission (p = .01) when controlling for individual-level demographics. No significant association was observed between the predictor of interest and risk of death during the follow-up period, suggesting that this finding is not likely confounded by individual health status. Further research should test whether this association is causal and specify possible mechanisms.

### THE INTERSECTION OF DEMENTIA-FRIENDLY INITIATIVES AND AGE-FRIENDLY ENVIRONMENTS: THE INTEGRATION MODELS

Fei Sun,[1] Ha Neul Kim,[1] Lucas Prieto,[1] Stéfanie Fréel,[2] katrin Seeher,[2] and Huali Wang,[3], *1. Michigan State University, East Lansing, Michigan, United States, 2. World Health Organization, Geneva, Geneve, Switzerland, 3. Peking University Institute of Mental Health (Sixth Hospital), Beijing, Beijing, China (People's Republic)*

While age friendly city (AFC) initiatives aim to build supportive physical and social environments for older adults, dementia-friendly initiatives (DFI) see the critical need of persons living with dementia (PWD) to be included in society. Given the close relationship between advanced age and dementia risk, communities facing challenges of aging and dementia will benefit from the integration of DFI and AFC. This study aims to summarize the differences between AFC and DFI practice and to identify integrative models for DFI and AFC based upon cases in the U.S.A and China. Qualitative interviews with 11 stakeholders from Massachusetts and Michigan of the U.S.A. and Beijing and Shanghai in mainland China were recorded via Zoom and transcribed for analyses in order to identify different integration models. A summary of differences and commonalities between AFC and DFI core values, key players, major activities, and outcomes is reported. Four practice models of AFC and DFI based upon case analyses were described as sequential integration, concurrent integration, sequential separation, and concurrent separation. Massachusetts' model is unique in the support from the state government to integrate both from the beginning, and Michigan witnessed separate efforts between grassroots-based agencies and the state government. Shanghai model represents a sequential integration that includes DFI in local government's long-term aging policy plan, while AFC and DFI in Beijing have a loose connection despite progress made for each initiative. Communities need to develop a practice model considering its local community needs, policy support, and sustainable resources available.

### Session 4185 (Symposium)

### CARE IN CONTEXT: DEMENTIA SUPPORT IN MEXICO AND THE UNITED STATES

Chair: Sunshine Rote Co-Chair: Jacqueline Angel
Discussant: William Vega

Due to rapid demographic transitions, the number of people with dementia is rising in the Americas, and is expected to double in the coming decades, increasing from14.8 million in 2030 to over 27 million by 2050. The burden of dementia is especially pronounced for the Mexican-origin population in Mexico and the U.S. For Mexico, financial support for older low-income citizens and medical care are universal rights, but limited fiscal resources and the needs of a large low-income population create inevitable competition for limited resources among the old and the young. Although the United States has a more developed economy and well-developed Social Security and health care financing systems for older adults, Mexican-origin individuals in the U.S. do not necessarily benefit fully from these programs. The institutional and financial problems are compounded in both countries by longer life spans, smaller families, as well as changing gender roles and cultural norms. Such changes affect the Mexican-origin population in particular because of a higher prevalence rates of cognitive impairment than other racial and ethnic groups, and the lower access to resources to provide care. In this GSA Symposium, the authors of four papers deal with the following topics as they relate to dementia care in Mexico and the United States: (1) living alone in late life; (2) living arrangements and dementia care; (3) the role of non-governmental organizations in care; (4) next steps to address dementia care needs in the U.S. and Mexico.

### WHEN STRANGERS BECOME FAMILY: THE ROLE OF CIVIL SOCIETY ORGANIZATIONS IN THE CARE OF OLDER PEOPLE

Veronica Montes De Oca,[1] and Ronald Angel,[2] *1. UNAM, Mexico City, Distrito Federal, Mexico, 2. UT Austin, UT Austin, Texas, United States*

As in other nations, the aging of the population of Mexico presents many challenges specially in dependence. These social and political changes occur in the context of a series of interacting political, social and demographic transformations. At the end of the 20th and beginning of the 21st Centuries civil society organizations have begun to define a third sector. A growing desire of individuals to exercise more direct democracy, has accompanied the growth of identity politics and the rise of groups representing women, indigenous populations, racial and religious minorities, environmental interests, older persons, and others. These groups have changed public discourse and today give individuals greater capacity to demand their basic human and social rights. This paper reviews the impact of these changes on older people and multidimensional care.

### DEMENTIA AND LIVING ARRANGEMENTS AMONG MEXICANS AND MEXICAN AMERICANS

Jiwon Kim,[1] Sunshine Rote,[2] Silvia Mejia-Arango,[3] Mariana López-Ortega,[4] Jacqueline Angel,[5] and Phillip Cantu,[6], *1. UT Austin, Austin, Texas, United States, 2. University of Louisville, Louisville, Kentucky, United States, 3. Department of Population Studies El Colegio de la Frontera Norte, Tijuana, Baja California, Mexico, 4. National Institute of Geriatrics, National Institutes of Health, Mexico CIty, Distrito Federal, Mexico, 5. UT Austin, UT Austin, Texas, United States, 6. Sealy Center on Aging, University of Texas Medical Branch, Texas, United States*

This study compares the living arrangements of adults 85 and older in Mexican-origin adults in Mexico and the United States. The study uses 475 Mexican-Americans in five southwestern states (Hispanic Established Population for the Epidemiologic Studies of the Elderly, H-EPESE) and 1,710 Mexicans from 32 states in Mexico (Mexican Health and Aging Study, MHAS) to examine living alone vs living with others based on the presence of dementia controlling for demographic, health, and financial correlates. In both countries, more than 20% of respondents living alone have dementia. Dementia is associated with household extension in both countries. Homeownership increases household extension in Mexico but not the U.S. The findings show that individuals with dementia are more likely to live alone in the U.S. than in Mexico. Older individuals with dementia may be at elevated risk of isolation in later life if families or formal organizations cannot provide on-going assistance.

### LIVING ALONE IN LATE LIFE

Jacqueline Torres, *University California San Francisco, San Francisco, California, United States*

Approximately one third of older adults in the United States (US) and 13% of older adults in Mexico live alone. In both countries, the prevalence of living alone is higher for women and increases with advanced age; in the US, an estimated 4.3 million older adults continue to live alone with cognitive impairment or dementia. We will present research from the US and Mexico on the receipt of long-term services and supports and unmet needs for care among older adults living alone, including with cognitive impairment, as well as factors that may modify these outcomes. For the US, we will describe recent findings about the health, health care, and caregiving outcomes of older adults living alone vs. living with others during the COVID-19 pandemic.

### FRAMING BINATIONAL CHALLENGES OF MENTAL AND COGNITIVE HEALTH CARE IN MEXICAN-ORIGIN OLDER ADULTS: CONSENSUS FINDINGS

Flavia Andrade,[1] Mariana López-Ortega,[2] Emma Aguila,[3] and Stephanie Grasso,[4], *1. University of Illinois at Urbana-Champaign, University Of Illinois At Urbana-Champaign, Illinois, United States, 2. National Institute of Geriatrics, National Institutes of Health, National Institute Of Geriatrics, National Institutes Of Health, Distrito Federal, Mexico, 3. Sol Price School of Public Policy, University of Southern California, California, United States, 4. University of Texas at Austin, The University of Texas at Austin, Texas, United States*

Growth in older populations, and hence in the number of persons living with dementia, is particularly rapid for individuals of Mexican origin living in the U.S. and Mexico. In order to identify influences on cognitive health in this diverse population, the University Texas at Austin and Mexican National Institute of Geriatrics (INGER) organized their second Bridging Conference titled: "Framing Challenges of Cognitive and Mental Health Care in Mexican-origin Older Adults in Mexico and the U.S". In this presentation, we highlight the results of a consensus-building session, during which bi-national expert opinions were generated and synthesized addressing gaps in research, knowledge, and policy, as well as the setting of priorities for immediate action and future research. Reducing barriers to adequate care for those aging-in-place with dementia was a central theme of the identified priorities. Critical areas of identified need, more specifically, included reducing social isolation, caregiver burden, and diminishing retirement income.

### LIVING BEYOND 80: A LONGITUDINAL STUDY OF COGNITIVE AND IADL DISABLEMENT AMONG THE OLDEST MEXICAN AMERICANS

Jacqueline Angel,[1] Sunshine Rote,[2] and Jiwon Kim,[3], *1. UT Austin, UT Austin, Texas, United States, 2. University of Louisville, Louisville, Kentucky, United States, 3. UT Austin, Austin, Texas, United States*

Mexican Americans live longer on average than other ethnic groups, but often with protracted cognitive and physical disability. Little is known, however, about the role of cognitive decline for transitions in IADL disability and tertiary outcomes of the IADL disablement for the oldest old. We employ the Hispanic Established Populations for the Epidemiologic Study of the Elderly (2010-2011, 2012-2013, 2016, N=1,078) to investigate the longitudinal patterns of IADL decline using Latent Transition Analysis. Three IADL groups were identified: independent (developing mobility limitations), emerging dependence (limited mobility and community activities), and dependent (limited mobility and household and community activities). Declines in cognitive function were a consistent predictor of greater IADL disablement, and loneliness was a particularly salient distal outcome for emerging dependence. These results highlight the social consequences of cognitive decline and dependency as well as underscore important areas of intervention at each stage of the disablement process.

### Session 4190 (Paper)

### COGNITION, SENSORY IMPAIRMENT, AND COMMUNICATION

### BILINGUALISM AND HEALTHY AGING: ONSET AGE OF BILINGUALISM AS A PREDICTOR OF OLDER ADULT THEORY OF MIND

W.Quin Yow,[1] Xiaoqian Li,[2] and Jia Wen Lee,[3]
*1. Singapore University of Technology & Design, Singapore, Not Applicable, Singapore, 2. Singapore University of Technology and Design, Singapore, Not Applicable, Singapore, 3. Singapore University of Technology & Design, Singapore University of Technology & Design, Not Applicable, Singapore*

The ability to understand and speak more than one language (i.e., bilingualism) may protect against age-related cognitive deterioration (Abutalebi et al., 2015). While there is mounting evidence suggesting that bilingualism confers advantages in domain-general cognitive abilities in late adulthood (see Bialystok, 2017, for a review), few studies have investigated the influences of bilingualism on socio-cognitive abilities such as theory of mind (ToM) in the normal aging process. Thus, in this study, we examine how bilingualism factors (i.e., onset age of bilingualism, language balance, and diversity in language use) are associated with individual differences in ToM in healthy older adult bilinguals aged 58-79 (N=44). ToM abilities were assessed using the Theory-of-Mind Task Battery (Hutchins et al., 2008), where participants viewed vignettes and answered questions about the protagonists' cognitive and affective mental states. All participants completed a self-report language background questionnaire and the Montreal Cognitive Assessment (MoCA) test as a measure of general cognitive ability. Results revealed that better ToM was negatively correlated with participants' chronological age (r=-.43, p=.004) and the onset age of second-language acquisition (r=-.41, p=.006), but not language balance and diversity (ps>.40). Partial regression analyses showed that earlier onset age of bilingualism predicted better ToM performance (β=-.40, p=.009), even after controlling for age, education, and general cognitive ability. These findings suggest that bilingual language experience, particularly earlier exposure to a second language, may provide benefits to older adults in preserving their ability to understand others' mental states, acting as a cognitive reserve against age-related declines in socio-cognitive functions.

### EXAMINING THE COMBINED ESTIMATED EFFECTS OF HEARING IMPAIRMENT AND DEPRESSION ON COGNITIVE DECLINE AND DEMENTIA

Danielle Powell,[1] Willa Brenowitz,[2] Kristine Yaffe,[2] Frank Lin,[3] Alden Gross,[4] and Jennifer Deal,[1], *1. Johns Hopkins University, Baltimore, Maryland, United States, 2. UCSF, UCSF, California, United States, 3. Johns Hopkins University, Johns Hopkins University, Maryland, United States, 4. Johns Hopkins Bloomberg School of Public Health, Baltimore, Maryland, United States*

Late-life depression is a comorbidity which may co-occur in older adults with hearing loss- each as prevalent and independent modifiable risk factors for dementia. We used data from 1,820 participants (74 ± 2.8 years, 38% Black race) from the Health Aging and Body Composition Study to test if the hearing loss-dementia/cognitive decline (Modified Mini Mental State Exam[3MS] and Digit Symbol Substitution[DSST]) relationship differed in hearing impaired participants who also had depressive symptoms. Depressive symptoms were defined as CES-D 10 ≥10) at one or more visits from years 1-5. Algorithmic incident dementia defined using medication use, hospitalizations and cognitive test scores. Audiometric hearing loss was measured at year 5 and categorized as normal/mild vs ≥moderate loss. In linear mixed models adjusted for demographic and clinical covariates, presence of both hearing loss and depressive symptoms (vs. having neither) was associated with faster rates of decline in 3MS (-0.30, 95% CI:-0.78, -0.19) and DSST (-0.35,95% CI:-0.67, -0.03) over 10 years of follow-up. Both hearing loss and depressive symptoms (vs. neither) was associated with increased risk (hazard ratio (HR):2.91, 95%CI: 1.59, 5.33) of incident dementia in multivariable-adjusted Cox proportional hazards models. Comorbid conditions among hearing impaired older adults should be considered and may aid in dementia prevention and management strategies.

### INHIBITORY CONTROL IN AGE-RELATED HEARING LOSS

Shraddha Shende,[1] Lydia Nguyen,[2] Grace Rochford,[3] and Raksha Mudar,[3], *1. University of Illinois Urbana-Champaign, Champaign, Illinois, United States, 2. iN 2L, Denver, Colorado, United States, 3. University of Illinois-Urbana Champaign, Champaign, Illinois, United States*

Inhibitory control involves suppression of goal irrelevant information and responses. Emerging evidence suggests alterations in inhibitory control in individuals with age-related hearing loss (ARHL), however, few have specifically studied individuals with mild ARHL. We examined behavioral and event related potential (ERP) differences between 14 older adults with mild ARHL (mean age: 69.43 ± 7.73 years) and 14 age- and education-matched normal hearing (NH, mean age: 66.57 ± 5.70 years) controls on two Go/NoGo tasks: a simpler, basic categorization task (Single Car; SC) and a more difficult, superordinate categorization task (Object Animal; OA). The SC task consisted of exemplars of a single car and dog, and the OA task consisted of exemplars of multiple objects and animals. Participants were required to respond to Go trials (e.g., cars in SC) with a button press, and withhold responses on NoGo trials (e.g., dogs in SC task). Behavioral results revealed that ARHL group had worse accuracy on NoGo trials on the OA task, but not on the SC task. ARHL group had longer N2 latency for NoGo compared to Go trials in the simpler SC Task, but no differences were observed on the OA task between Go and NoGo trials. These findings suggest that more prolonged neural effort in the ARHL group on the SC task NoGo trials may have contributed to their ability to successfully suppress false alarms comparable to the NH group. Overall, these findings provide evidence for behavioral and neural changes in inhibitory control in ARHL.

### NONVERBAL COMMUNICATION IN DEMENTIA FAMILY CAREGIVING: USING THE VNVIS-CG SCALE FOR IN-HOME VIDEO OBSERVATIONS.

Carissa Coleman,[1] Kristine Williams,[2] Kacie Inderhees,[1] and Michaela Richardson,[1], *1. University of Kansas, Kansas City, Kansas, United States, 2. University of Kansas School of Nursing, Kansas City, Kansas, United States*

Communication is fundamental for dementia care and identifying communication behaviors is key to identifying strategies that facilitate or impede communication. To measure caregiver nonverbal communication, we adapted the Verbal and Nonverbal Interaction Scale for Caregivers (VNVIS-CG) for second-by-second behavioral coding of video observations. The VNVIS-CG was adapted for computer-assisted Noldus Observer coding of video interactions captured at home by family caregivers from the FamTechCare clinical trial. Operational definitions for nonverbal communication behaviors were developed and inter-rater reliability was excellent (Kappa = .88) using two independent coders. Videos N=232 were coded featuring 51 dyads; caregivers who were primarily female (80%) spouses (69%) of men (55%) diagnosed with moderate to severe dementia (64.7%). Mean caregiver age was 65 years. Emotional tone conveyed by caregivers was primarily respectful, occurring 68.1% of the time, followed by overly nurturing (9%), bossy, harsh, or antagonistic (6.2%), and silence occurred 16.7 % of the time. Caregiver gestures and positive postures (i.e., animated facial expressions, head nodding, or caregiver body movements) were the most commonly occurring overt behaviors (46.5%), followed by changing the environment to help the PWD (19.9%), and expressing laughter/joy (18.9%). The least common nonverbal behaviors were negative posture, aggression, compassion, and rejecting. The adapted behavioral coding scheme provides a reliable measure that characterizes dementia caregiver nonverbal communication behaviors for analysis of video observations. Ongoing research will identify strategies that facilitate communication as well as determine how strategies vary by dementia stage, diagnosis, and dyad characteristics.

### VERBAL COMMUNICATION IN DEMENTIA FAMILY CAREGIVING: USING THE VNVIS-CG SCALE FOR IN-HOME VIDEO OBSERVATIONS.

Kristine Williams,[1] Carissa Coleman,[2] Iman Aly,[2] and Paige Wilson,[2], *1. University of Kansas School of Nursing, Kansas City, Kansas, United States, 2. University of Kansas, Kansas City, Kansas, United States*

Communication is fundamental for dementia care and identifying communication behaviors is key to identifying strategies that facilitate or impede communication. To measure caregiver verbal communication, we adapted the Verbal and Nonverbal Interaction Scale for Caregivers (VNVIS-CG) for second-by-second behavioral coding of video observations. The VNVIS-CG was adapted for computer-assisted Noldus Observer coding of video interactions captured at home by family caregivers from the FamTechCare clinical trial. Operational definitions for verbal communication behaviors were developed and inter-rater reliability was excellent (Kappa = .86) using two independent coders. Videos (N=232) were coded featuring 51 dyads; caregivers were primarily female (80%) spouses (69%) of men (55%) diagnosed with moderate to severe dementia (64.7%). Mean caregiver age was 65 years. Silence occurred most frequently (44.9% of the time), followed by caregiver direction or instruction (22.6%), and the person with dementia (PWD) verbalizing (22.8%). Caregiver communication also included asking questions (14.2%), verbalizing understanding (7.9%), repeating information (2.1%), affirmations (1.0%), acknowledging emotions (0.3%), and ignoring (0.0%). Questions most commonly requested clarification, showed interest, or repetitive quizzing; few questions sought to engage PWD input (ex. offers choices, encourages emotional expression, or ask permission). Tone was overwhelmingly neutral rather than humorous, aggressive, or patronizing. The adapted behavioral coding scheme provides a reliable measure that characterizes dementia caregiver verbal communication behaviors for analysis of video observations. Ongoing research will identify strategies that facilitate communication as well as determine how strategies vary by dementia stage, diagnosis, and dyad characteristics.

### Session 4195 (Paper)

### COGNITIVE IMPAIRMENT

### EXAMINING THE GENDER DISPARITIES IN HEALTH SERVICE UTILIZATION AMONG OLDER ADULTS MANAGING COGNITIVE IMPAIRMENTS

Camille McKenzie,[1] and Patricia Findley,[2], *1. Rutgers University--New Brunswick, New Jersey, United States, 2. Rutgers University--New Brunswick, Rutgers University--New Brunswick, New Jersey, United States*

Comorbid conditions can complicate healthcare access, yet little is known about the impact of cognition on accessing those services. This study aims to examine the effect of gender on health service utilization among the aging population. Data from the National Health Interview (2019) Survey (n=3,058) were utilized to assess the impact of gender on the use of physician and dental services among older adults managing cognitive impairments. We estimated logistic regression models controlling for demographic, health/physical functioning, and psychological predictors. Findings indicated older male respondents managing cognitive impairments were less inclined to utilize both physician ($p<.05$) and dental ($p<.01$) health services on a yearly basis as compared to their female counterparts. Household income (aOR = 0.62 [-0.91, -0.06]; aOR = 0.34 [-1.25, -0.90]) and health insurance coverage (aOR = 0.18 [-2.31, -1.13]; aOR = 0.32 [-1.68, -0.62]) respectively were the strongest structural factors limiting access to both physician and dental services, with lower levels of educational attainment (aOR = 0.46 [-1.00, -0.55]) being an additional barrier for dental service utilization. Respondents with low self-rated health were inclined to utilize physician more than dental services (aOR = 2.31 [0.38, 1.29]; aOR = 0.73 [-0.50, -0.14]). It is imperative geriatric practitioners adopt a more integrative approach to health, especially among the aging population managing chronic disabilities, where additional community supports are warranted to promote and maintain physical and oral health.

### GREATER ADOLESCENT COGNITIVE ABILITY IS LINKED TO LOWER RISK OF COGNITIVE IMPAIRMENT IN LATER LIFE

Tara Gruenewald,[1] Catalina Zavala,[2] Molli Grossman,[2] Thalida Arpawong,[2] Jennifer Manly,[3] Susan Lapham,[4] Margaret Gatz,[2] and Carol Prescott,[2], *1. Chapman University, Orange, California, United States, 2. University of Southern California, Los Angeles, California, United States, 3. Columbia University, Columbia University, New York, United States, 4. American Institutes for Research, American Institutes for Research, Virginia, United States*

There have been few investigations of the role that adolescent cognitive ability plays in predicting later-life cognitive impairment, and the mechanisms, such greater life course educational exposure, that might underlie these connections. This knowledge gap is due, in part, to a lack of cohorts with early-life cognitive assessment who are followed to later adulthood. We capitalized on data from the 1960 Project Talent (PT) high school cohort (n>360,000) and two recent follow-ups, the Project Talent Twin & Sibling (PTTS; n=2,491 in 2014) Study and the Project Talent Aging Study (PTAS; n=6,421 in 2018), to examine these potential links. In 1960, ability was assessed in multiple cognitive domains (e.g., general aptitude, quantitative, reasoning). Participants/proxies reporting 2 or more symptoms of cognitive impairment in 2018 on the AD8 Dementia Screener were classified as having a positive screen. Binary logistic generalized estimating equations with race, sex, and adolescent family SES covariates, indicated that in multiple cognitive domains, higher ability in adolescence predicted lower odds of a positive AD8 screen in later life (ORs of 0.80 - 0.85). The effects were only slightly attenuated with inclusion of life course educational attainment. Sibling models found a similar pattern of associations and effect sizes, indicating that the association is not attributable to shared family and genetic background. These findings indicate that higher cognitive ability as indicated by better performance in multiple cognitive domains in adolescence may be protective against cognitive impairment five decades later and life course educational attainment only partially mediates this association.

### INTENSITY OF WALKING TRAINING IMPACTS COGNITION AMONG ASSISTED LIVING RESIDENTS WITH FRAILTY

Margaret Danilovich,[1] and Christie Norrick,[2],
*1. CJE SeniorLife, Evanston, Illinois, United States,*
*2. CJE SeniorLife, Chicago, Illinois, United States*

Exercise has many proven cognitive benefits but the precise intensity to modify cognition is unclear. This pilot study investigated the role of exercise intensity on cognitive outcomes among assisted living residents. We enrolled n=33 frail or pre-frail residents who completed an 8 week, twice-weekly walking intervention. Participants were 66% female, and on average were 88 years old with a MMSE score=25.6, and low cognitive scores (Category Fluency Animals=10.45, Category Fluency Vegetables=7.67, Trail Making Test A=60.82 seconds, Trail Making Test B=155.18 seconds). Walking sessions used 5-minute intervals focused on maximizing steps, fast speeds, and multi-directions for 45 minutes per session. Participants in the high intensity group walked at >70% heart rate maximum or RPE 15-17 and those randomized to the casual intensity group walked at <60% heart rate maximum or RPE <13. Results showed the casual-intensity group improved more on Category Fluency tests (increase of 0.88 animals and 1.06 vegetables) compared to the high-intensity group (increase of 0.12 animals and increase of 0.35 vegetables). On Trail Making Test A, high-intensity participants had a 7.47 second decrease in time to complete compared to the casual-intensity group (2.00 seconds increase). On Trail Making Test B, high-intensity participants decreased time to complete by 27.13 seconds compared to a 26.19 decrease in the casual-intensity group. Results show promising trends in the role of exercise intensity in impacting different elements of cognition among assisted living residents.

### PREVALENCE OF DEMENTIA AMONG THE ELDERLY IN LATIN AMERICA AND THE CARIBBEAN: A SYSTEMATIC REVIEW AND META-ANALYSIS

Fabiana Ribeiro, Ana Carolina Teixeira-Santos, and Anja Leist, *University of Luxembourg, Esch-sur-Alzette, Diekirch, Luxembourg*

**Background:** Over the last decades, life expectancy in Latin America and the Caribbean showed a rapid increase, which led to a significant increase in the number of people with dementia. Moreover, 9% of the population in this part of the world are aged 65 or older, and by 2050 this percentage is projected to at least double. For this reason, it is essential to estimate the prevalence of dementia in LAC countries with the aim to determine suitable actions to enhance the quality of life of those affected.

**Methods:** Database searches for articles were conducted September 2020 throughout Pubmed, Web of knowledge, Scopus, Lilacs, and SciELO. The inclusion criteria comprised population- or community-based studies, published in English, Spanish, or Portuguese, reporting data on the prevalence of dementia collected in LAC countries. The complete data search retrieved 1719 non-duplicates.

**Results:** A total of 58 studies met the high-quality inclusion criteria, published 1991-2020, including participants in the following countries: Brazil, Mexico, Argentina, Colombia, Peru, Cuba, Dominican Republic, Venezuela, Ecuador, Trinidad and Tobago, and Jamaica. The most common form of dementia studied was Alzheimer's disease with prevalence ranging from 5.9% to 23.4%. Estimates differed by age, gender, and education, with oldest, women, and lower-educated adults living in rural areas presenting higher dementia prevalence.

**Conclusion:** This is the first study giving a comprehensive overview of dementia prevalence in LAC countries, which is relevant to estimate care needs and economic costs related to dementia treatment and care.

### TRANSFORMING AGING: INCREASED ACCESS TO CARE MINIMIZES RURAL AND URBAN DIFFERENCES IN COGNITIVE CHANGE

Timothy Ly,[1] Rebecca Allen,[2] Barbara Jackson,[3]
Amy Albright,[4] John Bell,[3] Dana Carroll,[5] and
Anne Halli-Tierney,[3], *1. University of Alabama, University of Alabama, Alabama, United States, 2. University of Alabama, Tuscaloosa, Alabama, United States, 3. The University of Alabama, Tuscaloosa, Alabama, United States, 4. VA Maine HCS, Gorham, Maine, United States, 5. Auburn University Harrison School of Pharmacy, Tuscaloosa, Alabama, United States*

Rural-urban disparities in cognitive health outcomes, such as greater prevalence of cognitive decline among rural-dwelling older adults, have been linked to inequity in access to care. However, few studies have demonstrated whether longitudinal increased access to care may mitigate such disparities. This paper presents data from ongoing systematically collected behavioral health data on new and returning patients at an interdisciplinary geriatrics clinic at the University of Alabama Medical Center. The aim of this study was to determine baseline predictors of cognitive change across three annual visits (n = 42, mean age of 75.63 years (SD = 9.15)). Adjusting for baseline cognitive status, baseline subjective health literacy, and baseline depression and anxiety, results from a univariate ANCOVA showed that age at first visit (B = -.024, 95% CI [-.041, -.008], t(35) = -2.990, p = .005) and rural-urban status (B = .555, 95% CI [.123, .988], t(35) = 2.608, p = .013) predicted cognitive change at timepoint three (T3). Specifically, individuals from rural areas were less likely to experience cognitive decline and scored .555 points better than individuals from urban areas on cognitive screeners at T3 compared with baseline cognitive status. These results suggest that increased access to and utilization of care may ameliorate traditional disparate rates of cognitive decline between rural- and urban-dwelling older adults. Moreover, behavioral health screenings in primary geriatrics clinic care may help identify patient cognitive needs and facilitate integrated care through combined medical, pharmacological, and behavioral interventions to promote positive cognitive health outcomes.

### Session 4200 (Paper)

### COVID-19 AND MENTAL HEALTH

### COVID-19 PANDEMIC-RELATED DISTRESS AND SELF-EFFICACY TO MANAGE DEMENTIA CARE AMONG FAMILY CAREGIVERS

Lena Thompson,[1] Haley Schneider,[1] Maria Donohoe,[1] Elizabeth Saathoff,[1] Lubna Hossain,[1] and Sato Ashida,[2], *1. University of Iowa College of Public Health, Iowa City, Iowa, United States, 2. University of Iowa, Iowa City, Iowa, United States*

The COVID-19 pandemic posed numerous challenges to persons with dementia (PWD) and their caregivers. To better understand these challenges, we conducted a mixed methods study analyzing data from interviews with family caregivers participating in an ongoing intervention study. Telephone interviews were conducted with 58 family caregivers of PWD diagnosed within the past two years. Participants reported self-efficacy (SE) using a 5-item scale (e.g. handle problems with memory, keep PWD at home) and rated pandemic-related distress on a 1-10 scale. They also qualitatively described effects of the pandemic on care recipients and themselves. Qualitative data were coded and organized by concepts from the Stress Process Model. Distress level ranged from 1-9 and was negatively associated with SE to manage dementia care (r=-316, p=0.036). Caregivers described distress related to primary stressors such as loss of services (respite care, assistance with daily tasks) and resistance to mask wearing by PWD due to behavioral symptoms. Secondary stressors included managing work or supervising children's schoolwork at home while providing care. Caregivers most often expressed distress related to inability to access coping resources such as family or friends, and worried that PWD were not able to rely on their support systems. At all reported levels of distress, inability to interact with members of support networks (e.g., family, friends, service providers) was identified as most distressing. This was compounded by lower self-efficacy to manage dementia care. Efforts to decrease pandemic impacts must consider strategies to safely keep PWD and caregivers connected with family, friends, and service providers.

### DISCRIMINATION AND MENTAL HEALTH DURING THE BLACK LIVES MATTER MOVEMENT AND THE COVID-19 PANDEMIC

Peiyi Lu,[1] Dexia Kong,[2] and Mack Shelley,[1] *1. Iowa State University, Ames, Iowa, United States, 2. Rutgers University, New Brunswick, New Jersey, United States*

Discrimination has been more prevalent since the pandemic. Black Lives Matter (BLM) movement flourished in the summer of 2020 as protests against police brutality and racial injustice. However, the extent to which individuals' discrimination experiences and associated mental health outcomes change amid a global pandemic and a dramatic societal movement in American society remains unknown. This study examines the dynamic relationship of racism and/or Covid-19-related discrimination with changes in mental health in the context of BLM and Covid-19. Data were from U.S. adults (age ≥18) who completed the online Understanding Coronavirus in America survey in June and September of 2020 (n=3,502). Respondents were asked to attribute their discrimination experience to 8 main reasons: (1) Covid-19; (2) national origin/race/skin color; (3) gender/sexual orientation; (4) age; (5) physical feature; (6) education/income; (7) health condition; and (8) religion/other. Quasi-Poisson regression models examined the associations between discrimination and anxiety/depression/stress. Results indicated about 33% of respondents reported discrimination in June compared to 21% in September. Racism was significantly associated with more anxiety/depression/stress in June, but not in September or in the longitudinal trend. Covid-19-related discrimination was associated with elevated levels of anxiety/depression/stress in September and in the longitudinal trend, but not in June 2020. We concluded that individuals' discrimination experiences are shaped by societal contexts. Specifically, racism was the dominant discrimination attribution in June 2020 when BLM was at its peak. However, Covid-19 discrimination overtook racism as the primary attribution and showed a significant relationship with poorer mental health over time.

### DISPARITIES IN COVID-19 RELATED WORRY AMONG OLDER ADULTS IN THE UNITED STATES

Felicia Wheaton,[1] Terika Scatliffe,[2] and Matilda Johnson,[2], *1. Xavier University of Louisiana, New Orleans, Louisiana, United States, 2. Bethune-Cookman University, Daytona Beach, Florida, United States*

Racial disparities in COVID-19 exposure, illness, hospitalization and mortality have been well-documented, however, less is known about whether African Americans and other minorities experience greater worry related to the COVID-19 pandemic. Data from the special midterm release of the 2020 Health and Retirement Study (HRS) were used to examine the relationship between race (white, African American, and other) and ethnicity (Hispanic/Non-Hispanic) and COVID-19 related worry among older Americans (N=2,069). Participants were asked, "because of the coronavirus pandemic how worried are you about 1) your own health, 2) the health of others in your family? 3) Your financial situation? 4) Being able to get help if you needed it from family, friends, or others? 5) What will happen in the future?" (0=not at all worried and 10=very worried). Results from OLS regression controlling for age, gender and education showed that compared with whites, African Americans had significantly higher average worry for all items except the last (other race did not differ). On the other hand, Hispanics had significantly lower worry, on average, for each of the five items. In addition, women had significantly higher average worry, while age was negatively associated with all items except the first. These findings indicate that in addition to the previously documented disparities in COVID-19, older African Americans experienced more worry. This has important implications for long-term physical and mental health.

### EFFECTS OF ISOLATION AMONG OLDER ADULTS DUE TO CORONAVIRUS RESTRICTIONS

Cynthia Thomas, *International Network for the Prevention of Elder Abuse, Rockville, Maryland, United States*

Semi-structured interviews were conducted with 35 residents in a Maryland condominium, four to six months after the presence of the epidemic in the US was recognized in mid-March. The objective was to determine to what extent the restrictions resulting from the presence of a new disease was affecting older adults in their daily lives, and in their plans for the future. All respondents were over the age of 60 and half were more than 80 years old. Two-thirds lived by themselves; most others lived with a husband or wife. Respondents for the most part were following guidelines to wear masks, practice social distancing and avoid close contact with persons outside their homes, including other family members. Over half had already made dramatic changes in their daily activities. Some found an opportunity to develop new skills, had connected with people from the past, or had become more introspective. Others, while exhibiting some of the same characteristics, were more focused on the restrictions they faced, and were more aware than ever of the limited amount of time left in their lives. Differences between respondents in the emphasis of their perspectives are explored, by age, gender, and other characteristics.

### WORSENING MENTAL HEALTH AND SELF-RATED HEALTH IN OLDER INCARCERATED PERSONS DURING THE COVID-19 PANDEMIC

Lisa Barry,[1] Deborah Noujaim,[2] Alexandra DePalma,[2] Emil Coman,[2] and Dorothy Wakefield,[2], *1. University of Connecticut Center on Aging, Farmington, Connecticut, United States, 2. UConn Health, Farmington, Connecticut, United States*

Incarcerated persons age 50 and older comprise one of society's most vulnerable groups given high rates of chronic illness, estrangement from family/friends, and suicide. Consequently, the mental health impact of COVID-19 on this population may be especially salient. Using data from the ongoing Aging Inmates' Suicidal Ideation and Depression study (Aging INSIDE), we determined change in older incarcerated persons' mental health (anxiety and depression symptoms) and change in self-rated health (SRH) from before to during the COVID-19 pandemic, and evaluated how these variables were related. Of the 202 still-incarcerated Aging INSIDE participants, 157 (77%) completed Check-In Surveys between August-September 2020. Participants were 96% male, racially diverse (41% White, 41% Black, 18% Hispanic/Other) and average age was 56.0(±5.8) years. From before to during the COVID-19 pandemic, average anxiety symptom scores, assessed by the GAD-7, increased (worsened) (from 6.4±5.7 to 7.8±6.6; $p<0.001$), average depressive symptoms scores, measured by the PHQ-8, increased (worsened) (from 5.5±6.0 to 8.1±6.5; $p<0.001$), and average SRH decreased (worsened) (from 3.0±0.2 to 2.6±0.2; $p<0.001$). Worsening anxiety led to worsening depressive symptoms (direct effect = 0.339; $p<0.05$). A mediation model controlling for age, race, chronic conditions, years until release, and change in social support score found a total effect of change in anxiety on SRH change of -0.04 ($p<0.001$), of which 34.2% flows indirectly through change in depression ($p<0.001$). Older incarcerated persons experienced worsening mental health and SRH during the COVID-19 pandemic. Future research will determine if mental health and SRH improve following vaccination and return to "normal" procedures.

### Session 4205 (Symposium)

### CROSS-NATIONAL PERSPECTIVES ON HEALTH AND WELL-BEING IN LATER LIFE

Chair: Jennifer Ailshire Discussant: Yuan Zhang

Cross-national comparisons are increasingly being used to re-evaluate whether our theories of aging are dependent on sociocultural context and to develop new insights into both long-standing and emerging issues in aging. The papers in this symposium use harmonized data from the Gateway to Global Aging to examine a range of topics in aging and provide new insights into key questions in aging research. Liu et al., determine whether social relationships mediate the association between early life adversity and later life health and well-being using longitudinal data from the U.S. and England. Considering multiple domains of the social determinants of health, such as socioeconomic status, social relationships, and health care access, Cho et al. identify which are related to self-rated health in China, Japan, and South Korea. Cheng et al. test the age-as-leveler hypothesis for explaining how socioeconomic differentials in health change in older ages in China and 19 European countries to determine if the protective effect of socioeconomic status on multi-morbidity changes with age. Harmonized data facilitate international comparisons papers, but additional efforts are sometimes needed to calibrate specific measures across international surveys. A major challenge in cross-national aging research, for instance, has been the limited ability to compare cognition across countries. Nichols and colleagues present a novel method for cocalibration of cognitive function across the U.S., England, and India that could be used for additional cross-national comparative research. The papers in this symposium demonstrate both the potential and challenges of cross-national studies of health and well-being among older adults.

### EARLY ADVERSITY AND LATER HEALTH: THE PATHWAY OF SOCIAL RELATIONSHIPS IN ADULTHOOD IN HRS AND ELSA

Andrew Steptoe,[1] William Chopik,[2] Amanda Leggett,[3] Jooyoung Kong,[4] Courtney Polenick,[5] and Yin Liu,[6], *1. University College London, London, England, United Kingdom, 2. Michigan State University, East Lansing, Michigan, United States, 3. University of Michigan, Ypsilanti, Michigan, United States, 4. University of Wisconsin-Madison, Madison, Wisconsin, United States, 5. University of Michigan, Ann Arbor, Michigan, United States, 6. Utah State University, Logan, Utah, United States*

Early adversity is associated with compromised health and well-being in later life, but whether social functioning mediate the association is unclear. We examined 2 longitudinal samples of older adults (>= 50 years) whose baseline surveys were between 2006 and 2008 with follow-up until 2016 in the Health and Retirement Study (HRS, n = 15,946) and its sister study in England (ELSA, n = 9,692). Health outcomes included depressive symptoms, chronic health conditions, and subjective memory complaints. Social relationships were measured by contacts, relationship strains, and feelings of loneliness. Early adversity was measured by parental physical abuse and alcohol and drug problems in the family before the age of 16. Patterns of association were similar in these 2 samples, where social contacts decreased over time, while relationship strains and loneliness increased especially for older adults with early trauma, which in turn mediated the associations between early adversity and poorer later health.

### SOCIOECONOMIC STATUS AND LATER-LIFE HEALTH: LONGITUDINAL EVIDENCE FROM EUROPE AND CHINA

Nicolas Sommet,[1] Daniela Jopp,[2] Dario Spini,[2] and Mengling Cheng,[2], *1. University of Lausanne Faculty of Social and Political Sciences, Lausanne, Vaud, Switzerland, 2. University of Lausanne, Lausanne, Vaud, Switzerland*

Scholars are divided as to how the protective effect of SES on health (the SES-health gradient) varies over the later-life course: The age-as-leveler perspective suggests that the SES-health gradient weakens with age, whereas the cumulative (dis)advantages perspective suggests that it strengthens with age. To clarify this, we used SHARE 2004-2017 (73,407 respondents from 19 European countries) and CHARLS 2011-2018 (8,370 Chinese respondents). Congruent with the age-as-leveler perspective, growth curve models revealed that the overall protective effect of SES on multimorbidity was weaker for older than younger adults (the country-specific effects were significant in two thirds of the case). We interpret this as a selection effect. However, the within-participant protective effect of SES on multimorbidity did not vary over the later-life course (the country-specific effects were nonsignificant in the majority of the case). Findings suggest that extant cross-sectional studies should be interpreted with caution and that longitudinal, cross-national studies are needed.

### INFLUENCE OF SOCIAL DETERMINANTS ON SELF-RATED HEALTH IN THREE COUNTRIES OF EAST ASIA

Takeshi Nakagawa,[1] Dannii Yeung,[2] and Jinmyoung Cho,[3], *1. National Center for Geriatrics and Gerontology, Obu, Aichi, Japan, 2. City University of Hong Kong, Hong Kong, Not Applicable, Hong Kong, 3. Baylor Scott & White Health Research Institute, Temple, Texas, United States*

This study aims to compare five domains of social determinants and their associations with self-rated health (SRH) among older adults in China, Japan, and Korea, where they share some cultural values but the development and conditions of economic status, health care system, and education system vary. A total of 10,111 participants aged 65 years and older were included from three harmonized datasets at baseline CHARLS, KLoSA, and JSTAR. Guided by the Healthy People 2020 Framework, five domains of social determinants were included: education levels, economic stability (total income, working for pay), social and community context (social engagement, living with children), health care access (medical center utilization), and neighborhood (rural vs. urban). Regression models showed that working for pay, social engagement, and medical center utilization were significant predictors for SRH in three countries. However, unique predictors have also been observed in China and Korea, which will be discussed in the presentation.

### CO-CALIBRATION OF COGNITIVE DOMAINS ACROSS DIFFERENT COUNTRIES WITHOUT COMMON LINKING ITEMS

Richard Jones,[1] Alden Gross,[2] and Emma Nichols,[3] *1. Brown University, Providence, Rhode Island, United States, 2. Johns Hopkins Bloomberg School of Public Health, Baltimore, Maryland, United States, 3. Johns Hopkins University School of Public Health, Baltimore, Maryland, United States*

Modern psychometric methods allow for cocalibration of cognition across cross-national surveys, given the presence of common tests across studies. For narrow cognitive domains, there may not be common tests due to cultural and linguistic differences in testing. We developed a novel method to facilitate cocalibration that entails (1) identifying a common score across studies highly correlated with the focal domain; (2) deriving scores separately in each study for the domain of interest; and (3) applying stratified equipercentile equating to equate domain scores in (2) to the distribution of the common metric in (1). We tested this method by equating executive functioning in the Harmonized Cognitive Assessment Protocols (HCAP) in the US (N=3496), India (N=4096), and England (N=1273). The method preserves the rank order of executive functioning derived separately (r>0.99 in England; r>0.99 in India), while preserving between-study differences observed in general cognitive functioning. We discuss limitations and future directions.

### Session 4210 (Symposium)

### CULTURAL ADAPTATIONS TO RECRUITMENT STRATEGIES AND COMMUNITY-BASED INTERVENTIONS FOR DEMENTIA CAREGIVERS

Chair: Lauren Parker Co-Chair: Katherine Marx
Discussant: Maria Aranda

Nearly 30 years after the 1993 National Institute of Health Revitalization Act, minority groups' low participation in research remains (which required the inclusion of women and racial/ethnic minority groups into government-funded clinical trials). This is particularly the case for participation in research on Alzheimer's Disease and related dementias (ADRD). Deeply rooted historical race-based mistreatment in research and in the health care system at large persist as barriers to low-participation of minorities (i.e. Black/African American, Hispanic/Latino) and immigrants in research studies, who remain at disparate risk for adverse ADRD health outcomes and expedited mortality. The use of culturally adapted approaches in recruitment strategies and community-based interventions might be helpful to encourage the participation of underrepresented groups into research. As such, this presentation highlights three studies that seek to use cultural adaptation to inform recruitment strategies and community-based interventions. First, Dr. Parker will present how tenets from Critical Race Theory can be used to inform culturally-adapted recruitment strategies of Black/African American caregivers into community-based research by drawing upon two ongoing studies: a randomized trial providing caregiver support through Adult Day Services (ADS) and the evaluation of impact of ADS on stress levels of Black/African American using biomarker measures.

Next, Ms. Johnson will present results on cultural adaptions to the ADS-Plus Program for Spanish-speaking populations. Finally, Dr. Nkimbeng will present on the process of culturally-tailoring dementia education for African immigrants in Minnesota. Findings from this presentation identify opportunities for researchers to use cultural adaptations to encourage participation of underrepresented populations into ADRD research.

### THE USE OF CRITICAL RACE THEORY TO INFORM CULTURALLY ADAPTED RECRUITMENT STRATEGIES FOR COMMUNITY-BASED RESEARCH

Joseph Gaugler,[1] Laura Gitlin,[2] and Lauren Parker,[3], *1. University of Minnesota, Minneapolis, Minnesota, United States, 2. Drexel University, College of Nursing and Health Professions, Drexel University, Pennsylvania, United States, 3. Johns Hopkins Bloomberg School of Public Health, Baltimore, Maryland, United States*

Black/African American participation in non-pharmacological randomized trials and community-based research on Alzheimer's Disease and related dementias (ADRD) remains low. This presentation introduces Critical Race Theory (CRT), its tenets and how it can be used in tandem with national strategies to encourage participation of Black/African Americans in ADRD research. CRT is a transdisciplinary methodology that draws on anti-racist tenets to study and transform the relationships among race, racism, and power. We describe how CRT constructs (e.g., race consciousness, counter-storytelling) can be used to inform recruitment strategies to enroll Black/African American ADRD caregivers into community-based research by drawing upon two ongoing studies: a randomized trial providing caregiver support through Adult Day Services (ADS) and the evaluation of impact of ADS on stress levels of Black/African American using biomarker measures. In the spirit of CRT this presentation is a call for action and transformation of dementia care research to actively include Black/African Americans.

### THE AFRICAN IMMIGRANT DEMENTIA EDUCATION PROJECT: A COMMUNITY-UNIVERSITY PARTNERSHIP

Wynfred Russell,[1] Joseph Gaugler,[2] and Manka Nkimbeng,[2], *1. African Career, Education & Resource Inc, Brooklyn Park, Minnesota, United States, 2. University of Minnesota, Minneapolis, Minnesota, United States*

The African Immigrant Dementia Education project is a community-university partnership with the goal of developing a culturally tailored dementia education program with African immigrants in Minnesota. In collaboration with our community partner (African Career, Education & Resource, Inc.), a project advisory board that features professionals and family members from the African immigrant community was assembled and its first meeting was held in February 2021. Preliminary discussions about content, mode of delivery and cultural considerations of an eventual dementia education intervention have begun. This presentation will offer details on the process of working with an advisory board and community partner to identify and culturally tailor an evidenced-based dementia education curriculum for a unique cultural group. Also, we will present challenges encountered during this process and offer suggestions and strategies to promote successful researcher-community partnerships.

### ADAPTING THE ADS PLUS PROGRAM AND STUDY FOR A SPANISH SPEAKING POPULATION

Katherine Marx,[1] Manka Nkimbeng,[2] Joseph Gaugler,[2] Laura Gitlin,[3] Lauren Parker,[4] and Elma Johnson,[5], *1. Johns Hopkins School of Nursing, Baltimore, Maryland, United States, 2. University of Minnesota, Minneapolis, Minnesota, United States, 3. Drexel University, College of Nursing and Health Professions, Drexel University, Pennsylvania, United States, 4. Johns Hopkins Bloomberg School of Public Health, Baltimore, Maryland, United States, 5. University of Minnesota School of Public Health, University of Minnesota/ Minneapolis, Minnesota, United States*

While Hispanic/Latinos are at increased risk for Alzheimer's Disease, they are often cited as a "difficult-to-reach population" to engage in community-based research or clinical trials. One reason may be that many community-based supportive interventions for dementia caregivers are not adapted for Spanish-speaking populations. The purpose of this presentation is to describe the process of adapting the Adult Day Services Plus (ADS Plus) program for this population. In addition to translating ADS Plus into Spanish, staff, familiar with the program from four sites, which serve a predominantly Hispanic population, participated in a set of three focus groups that reviewed recruitment and intervention materials. Emerging themes included, Hispanic caregivers do not refer to themselves as caregivers but as the familial relationship (e.g. daughter, son, wife), and Hispanics often view research as a waste of resources. Future studies should consider these cultural elements towards caregiving in developing programs for Spanish-speaking dementia caregivers.

### Session 4215 (Symposium)

### EAST MEETS WEST SYMPOSIUM: COMMUNITY-BASED PROGRAMS FOR DEMENTIA PREVENTION AND SUPPORT

Chair: Fei Sun Discussant: Melissa Batchelor

This East Meets West Symposium presents cross-cultural evidence of community-based programs developed to address cognitive health needs of older adults or to support dementia family caregivers. It includes five studies from mainland China, Taiwan, and the U.S.A. The first study explored the adherence to computerized cognitive training to promote cognitive health among Chinese older adults using a qualitative approach. External encouragement, and self-awareness of performance improvement were found influential to older adults' adherence. The second study spearheaded by American Association of Retired Persons (AARP) in the U.S.A shed further light on technology use in promoting brain health by comparing in-person versus online cognitive training to older Americans during the COVID-19 pandemic. The third study tested the effectiveness of a cognitive health management model for community-dwelling older adults including healthy and mild cognitively impaired (MCI) ones in Shanghai, China. Older adults with MCI who received the management service showed better cognition outcomes compared to their counterparts in the control group. The fourth study focused on a community program named "Carer Café", developed to support dementia family caregiver in Taiwan. Participants (n=375) reported reduced stress and increased access to service referrals. The last study examined the stakeholders' perceptions of dementia friendly initiatives (DFI) being implemented in U.S.A, mainland China, and Taiwan to support persons with dementia and family caregivers. The impacts of DFI made, challenges DFI faced, and strategies DFI used during COVID-19 pandemic were discussed. The discussant will comment on each article's contributions and limitations in the context of literature.

### EXPLORING FACTORS CONTRIBUTE TO ADHERING IN COMPUTERIZED COGNITIVE TRAINING AMONG HEALTHY OLDER ADULTS IN CHINA

Siman Lv,[1] Cuiping Ni,[1] and Yu Liu,[2], *1. China Medical University School of Nursing, Shenyang, Liaoning, China (People's Republic), 2. China Medical University, shengyang, Liaoning, China (People's Republic)*

Computerized cognitive intervention has the potential to enhance cognition among healthy older adults. However, little is known of the factors associated with adherence in computerized cognitive training among healthy older adults in China. This study was designed to explore these factors utilizing a descriptive qualitative method. A semi-structured interview was used to interview 13 informants. The analysis suggested that factors associated with adherence to the computerized cognitive intervention, included 3 core themes:(1) individual characteristics, with three subthemes of "having free time", "emotion", and "persistence characteristics"; (2) encouragement, with three subthemes of "peer group support", "support from healthcare professional", and "supervision from facilitators"; and (3) self-recognized improvement related to training, with two subthemes of "better brain function" and "emotion improved". The results revealed multifactors promote adherence including personal and social aspects.

### A COMPREHENSIVE COGNITIVE HEALTH MANAGEMENT MODEL FOR COMMUNITY-DWELLING OLDER ADULTS IN SHANGHAI

Jing Nie,[1] yuan fang,[1] Xia Li,[1] and Jiayuan Qiu,[2], *1. Shanghai Jiaotong University school of medicine, Shanghai, Shanghai, China (People's Republic), 2. shanghai mental health center, shanghai mental health center, Shanghai, China (People's Republic)*

To investigate whether a comprehensive cognitive health management model improves cognitive function among community-dwelling older adults in Shanghai, China. The comprehensive cognitive health management model included brain health screening, individualized brain health consultation and referrals, and annual follow-up monitoring. We compared 161 older adults (43 with MCI, 118 with normal aging) who received the health management model, to an equal size of control group who did not receive the model on their cognition and emotion using Montreal Cognitive Assessment Scale (MoCA) and Geriatric Depression Scale (GDS). Over one-year, participants with MCI in the intervention group showed a significant increase of MoCA score than their MCI counterparts in the control group. Participants with normal aging in the intervention group showed no differences in MoCA and GDS with those in the control group. The individualized cognitive health management model is promising to assist community-dwelling older adults with MCI to maintain brain health.

### CARER CAFÉ IN TAIWAN: AN INNOVATIVE MODEL PROVIDING CAREGIVER SUPPORT FOR OLDER ADULTS

Tsuann Kuo,[1] and Su-I Hou,[2], *1. Chung Shan Medical University, Taichung, Taichung, Taiwan (Republic of China), 2. School of Global Health Management & Informatics, University of Central Florida, University of Central Florida, Florida, United States*

Physical places and environments play critical roles in shaping how people interact. This paper introduces an innovative Carer Café model in Taiwan with social infrastructure support and services for family caregivers caring for older adults. Carer cafés are community-based initiatives of Taiwan Association of Family Caregivers (TAFC), aiming to help carers recognizing their own needs and increase awareness of long-term care resources available. Through partnerships with local coffee shops, respite-focused services are provided. The "free coffee for two" campaign encourages family members and friends taking a caregiver out for a "respite coffee". Carer Café has also become an important "third place" hosting respite programs and the "a shop within a shop" style creating designated space to build a sense of community. Pilot survey (n=375) showed 77% perceived reduced stress, 83% appreciated services provided, and 250% increased referrals within one year. Implications on impact and future opportunities will be discussed.

### COMMUNITY-BASED BRAIN HEALTH PROMOTION DURING THE COVID-19 PANDEMIC: EFFORTS OF AARP IN THE USA

Lindsay Chura,[1] Rachel Lazarus,[2] and Sarah Lock,[1], *1. AARP, Washington, District of Columbia, United States, 2. Staying Sharp, AARP, Washington, District of Columbia, United States*

The pandemic has created new barriers for the delivery of healthcare resources and information, as well as in-person delivery of health care, caregiving, and social engagement. AARP created trainings designed for volunteer-led community-based brain health promotion. Due to the COVID, we have had to convert them to virtual presentations, distributed through technology. Staying Sharp is AARP's online platform that educates users about integrated, holistic, lifestyle-based approaches for maintaining brain health as we age. This platform was created for convenience and scalability – we currently have over 800,000 users. During a period of necessary isolation, this platform has performed as an ideal way to get helpful information about maintaining brain health to our consumers, who are now stuck at home without access to in-person (e.g., community-based) alternatives. We will discuss lessons learned from these two different approaches along with preliminary data on behavior change based on these engagements.

### IMPLEMENTING DEMENTIA FRIENDLY INITIATIVES IN THE COMMUNITY: PERCEPTIONS OF STAKEHOLDERS IN THE USA AND CHINA

Ha Neul Kim,[1] Lucas Prieto,[1] Christian Conyers,[2] Fredrika Opur,[3] and Fei Sun,[1], *1. Michigan State University, East Lansing, Michigan, United States, 2. University of Michigan, University of Michigan, Michigan, United States, 3. MSU, East Lansing, Michigan, United States*

To address the exclusion of persons living with dementia (PWD), Dementia Friendly Initiatives (DFI) are being launched to build a friendly and supportive environment for PWD in the U.S.A, mainland China, and Taiwan. This study aims to identify the impact of DFI, the challenges DFI encountered, and strategies used to address such challenges within the COVID-19 context in American and Chinese societies. Individual interviews via Zoom with 9 stakeholders from the U.S.A, 8 from mainland China and one from Taiwan were transcribed for analyses. DFI have shown effectiveness in raising the public's dementia awareness and engaging PWD in the community. COVID-19 pandemic posed the challenges of serving isolated PWD due to resources and attention shifted to COVID-19 prevention. Person-centered and technology-based means were used to deliver services for PWD during the pandemic. DFI in American and Chinese societies experienced similar sustainability challenges but showed resilience during the COVID-19 pandemic.

### Session 4220 (Paper)

### EMPLOYMENT AND SOCIAL ENGAGEMENT IN LATER LIFE

### ACTIVE AGING AND THE MATTHEUS EFFECT

Per Jensen, *Aalborg University, Aalborg, Nordjylland, Denmark*

While active ageing has been discursivized in international organizations and among researchers as a major means to combat the challenges of demographic ageing, this study aims to make a critical-theoretical and empirical assessment of the active ageing concept. It falls into three parts, the first showing how the conceptual framework of active ageing is undertheorized, lacks conceptual and analytical clarity, and that the theoretical framework does not hold clear ideas regarding the factors conditioning active ageing. The second part investigates the main patterns and structuring mechanisms of active ageing in an outcome perspective using Danish data subject to a correspondence analysis. Here, a Matthew Effect of accumulated advantage is found; that is, older adults who are blessed in one sphere of life are also blessed in others, and such inequalities in old age are the outcomes of social life biographies (i.e., cumulative advantages/disadvantages over the life course). Although nursed by the political system, EU ideas about active ageing are only weakly translated into policies and programs. Part three discusses some of the reasons for this, one obviously being that active ageing is elusive and lacks well-defined cause-and-effect descriptions. Another reason is that the concept has been developed in global elite networks that are quite distant from policymakers; at least in a decentralized political system like the Danish welfare state.

### MIGRATION AND HEALTH: FREEDOM OF MOVEMENT AND SOCIAL BENEFITS FOR CHINESE MIGRANT WORKERS

Fengxian Qiu,[1] Heying Zhan,[2] and Jing Liu,[3], *1. Anhui Normal University, Wuhu, Anhui, China (People's Republic), 2. Georgia State University, Georgia State University, Georgia, United States, 3. Zhejiang University of Finance and Economics, Hangzhou, Zhejiang, China (People's Republic)*

Nearly 30% of China's workforce consists of China's rural-to-urban migrant workers, accounting for nearly 300 million of China's population. Even though they have gained freedom of movement since the 1980s, they still have no access to healthcare in urban areas where they work. This study utilizes a mixed method of a survey with a sample of migrant workers from three Chinese emigration provinces (n=817) in 2018 and follow-up interviews with 30 migrant workers in 2020 to examine factors of migration experience affecting migrant worker's health and healthcare. Using binary logistic regression, we found that migrant workers' longer work experience is correlated with poorer self-rated health, their better financial status and level of hopefulness towards the future are positively correlated to self-rated health. Qualitative findings shed light on the cumulative effect of the length of work experience and fear of medical cost on migrant workers' declining health. The lack of portability in health insurance and different reimbursement rates in health care access are structural barriers in health-seeking behaviors among migrant workers. Policy implications are presented in the global context of social rights and freedom of movement.

### REVIEW OF RESEARCH INTO EMPLOYER APPROACHES TO YOUNGER ONSET DEMENTIA AND MILD COGNITIVE IMPAIRMENT IN THE WORKPLACE

James Carino,[1] Philip Taylor,[1] and Damian Morgan,[2], *1. Federation University Australia, Berwick, Victoria, Australia, 2. Federation University Australia, Churchill, Victoria, Australia*

Younger Onset Dementia (YOD) and Mild Cognitive Impairment (MCI) are relatively prevalent conditions globally which can affect the job performance of individuals in their working lives. This presentation considers the existing research documenting actual, stated or intended approaches taken by employers to managing and supporting employees with these conditions. Nine relevant research projects were identified based on an extensive exploration of the peer reviewed literature. These show that employers have some knowledge of dementia, but do not recognise this as a possible explanation when performance changes occur in the workplace. Employees typically leave or are removed from the workplace before a formal diagnosis or soon after. The literature shows both supportive and unsupportive behaviours toward employees. Drawing from this literature recommendations for increasing the quality of support provided to employees are offered: awareness training to foster earlier detection, clarification of these conditions as a disability, and application of methods to support employees to continue to contribute to the workplace while this is feasible and desirable from both the employer and the employee's perspective.

### Session 4225 (Paper)

### ENHANCING CARE SUPPORT, COMPETENCIES, AND OUTCOMES

### EFFECTS OF SIMULATION-BASED LEARNING ON NURSING STUDENTS' LEARNING OUTCOMES OF A GERONTOLOGY COURSE

Hsueh-Fen Kao,[1] Minzhi Ye,[2] and Lin Chen,[3], *1. The University of Texas at El Paso, El Paso, Texas, United States, 2. Benjamin Rose Institue on Aging, Benjamin Rose Institue on Aging, Ohio, United States, 3. Fudan University, Shanghai, Shanghai, China (People's Republic)*

Simulation-Based Learning (SBL) is beneficial to nursing education. Nevertheless, recent studies have shown a side effect of being overwhelmed by repeated exposures to simulation. Thus, how many times simulation scenarios should be provided to students remains a question. The objectives of this study were to (1) explore the changes in nursing students' perceived competence, self-efficacy, and learning satisfaction after repeated exposures to simulations, and (2) determine the acceptable frequency of SBL in the 'Care of Older Adults' course. A one-group repeated measurement experimental design with self-administered questionnaires in a convenient sample of 84 senior nursing undergraduate students was used at a university in southern Taiwan, and 79 students completed all measurements. After taking the baseline measurements (T0), students were exposed to 75-mininute simulation scenarios from Time 1 (T1) to Time 3 (T3) three weeks apart throughout the semester. Students' perceived nursing competence, self-efficacy, and learning satisfaction were measured immediately after each exposure. There were statistically significant improvements from T0 to T3 ($p < .001$) in all three areas; however, no significant difference when comparing scores from T1 to T2 and from T2 to T3. To conclude, SBL is effective in improving nursing students' perceived competence, self-efficacy, and learning satisfaction. While the primary changes occur at the first simulation effort, it is the accumulated multiple exposures collectively improve students' learning outcomes. Multiple instructional strategies are recommended to maintain students' learning interests to achieve optimal learning outcomes of the course across a semester.

### ENHANCING BEHAVIORAL HEALTH COMPETENCIES: INTERPROFESSIONAL COLLABORATION BETWEEN SOCIAL WORK AND MEDICINE

Bronwyn Keefe, *Boston University, Boston, Massachusetts, United States*

This presentation will describe the creation and findings from an interprofessional curriculum in behavioral health developed by social work faculty for medical students. Training in behavioral health is needed more than ever during a time of increased isolation and fear during the COVID pandemic. Older adults with untreated behavioral health concerns are a vulnerable population, which can result in negative effects, including emotional distress, reduced physical health, increased mortality, and suicide (IOM, 2012). Healthcare is increasingly complex with a need to focus on the physical, social, and behavioral aspects of daily living, and providers are realizing the importance of interprofessional collaboration. Towards that aim, I created a module for 4th year medical students in mental health and older adults, which is now part of their medical education curriculum. I will present outcomes in: (1) satisfaction; (2) acquired knowledge and skills (post-test); (3) application of knowledge and skills (pre-post competency assessment and comfort around asking about depression); and (4) patient outcomes (frequency of depression screening and number of referrals to social worker). Feedback from the 143 medical students is positive with 95% strongly agreeing or agreeing that this expanded their knowledge and understanding in mental health issues among older adults. At baseline, 17% of medical students were moderately to very comfortable in asking questions on the GDS compared to 42% at post-assessment. After completing the course, almost 25% of medical students made a referral to social work during their rotation. This collaboration resulted in curriculum that is both rigorous and impactful.

### EXAMINING THE EFFICACY AND RESULTS OF A SHORT-FORM ALZHEIMER'S SURVEY WITH COLLEGE STUDENTS

Debra Valencia-Laver,[1] Brooke Buchanan,[1] Chelsea McPheron,[1] Anna Rogers,[1] Alex DeTurck,[1] Jasmine Shapiro,[1] and Gary Laver,[2], *1. California Polytechnic State University, San Luis Obispo, Cal Poly/ San Luis Obispo, California, United States, 2. California Polytechnic State University, San Luis Obispo, Cal Poly, San Luis Obispo, California, United States*

College students are important stakeholders in addressing the significant costs of Alzheimer's disease in their future roles as caretakers, health care consumers, taxpayers, and as individuals in the workforce whose careers may interact with and impact those with Alzheimer's and their caregivers. To assess their knowledge of Alzheimer's, a 10-item True/False on-line quiz was presented to 912 students in Introductory Psychology classes. Participants were 61% white, 13% Asian/Asian American, and 10% Latinx, with 14% reporting other racial and ethnic groups, including that of mixed heritage; 59% of the sample self-reported as female. The quiz was counterbalanced such that items appearing in one format (e.g., True) appeared in the other format (e.g., False) across the two forms of the quiz. A significant difference was found for percent correct in Form A (61.4%) versus Form B (59.3%). In order to prompt participants to consider the ways the disease may impact their own lives, additional questions examined students' own experience with Alzheimer's, their interest and willingness to take action towards supporting Alzheimer's research, and their perceptions about how Alzheimer's would impact their lives personally, financially, and in their career pursuits. The research extends the findings of earlier research on student knowledge of Alzheimer's (e.g., Bailey, 2000; Eshbaugh, 2014) by allowing the results to be broken down by gender, race/ethnicity, and student major. It also expands upon those findings by identifying how college students project the societal effects and costs of Alzheimer's to their own lives and livelihoods.

### ONLINE TRAINING THAT CHANGES FAMILY CAREGIVER BEHAVIOR AND ATTITUDES

Vicki de Klerk-Rubin, *Validation Training Institute, Pleasant Hill, Oregon, United States*

The Family Caregiver Course (FCC) is an 18-week Validation training to sensitize family carers to the psychosocial needs of their relatives, integrate new behaviors that build relationships and specific verbal and non-verbal techniques to increase communication. Validation Training Institute partnered with the Alzheimer's Association of Colorado in 2019 and 2020 in delivering this course. Due to COVID-19 limitations, the 2020 iteration was completely digital, using principles of online learning. To replace the two-day in-person component of this course, we developed four, 4-hour Zoom sessions that allowed participants to: practice specific Validation techniques, exercise, process and apply what was learned, role play and receive coaching to anchor skills. Important online rules were maintained, such as, offer opportunities for participant engagement every 10 minutes and create a community of inquiry. Pre- and Post-Surveys of the 2019 and 2020 iterations of FCC showed that after taking this course, family carers: reported that they knew what to do when faced with challenging behaviors from their relative; understood that lying or pretending to agree with a disoriented person was not an effective strategy for communication; gained knowledge of the different forms of dementia and that the differences are significant; were clear about the differences between Validation and other methods.

### PERSON-CENTERED CARE FOR FAMILY CAREGIVERS: EVALUATING AN EDUCATION PROGRAM FOR THE HEALTHCARE WORKFORCE

Sharon Anderson,[1] Jasneet Parmar,[2] Cheryl Pollard,[3] Bonnie Dobbs,[4] Myles Leslie,[5] and Gwen McGhan,[5], *1. Faculty of Medicine & Dentistry University of Alberta, Edmonton, Alberta, Canada, 2. Department of Family Medicine, University of Alberta, Edmonton, Alberta, Canada, 3. McEwan University, Edmonton, Alberta, Canada, 4. University of Alberta, Edmonton, Alberta, Canada, 5. University of Calgary, Calgary, Alberta, Canada*

**Background:** While family caregivers [FCGs] provide 75-90% of care for people living in the community, most healthcare providers are not trained to provide person-centered care to FCGs. We followed research recommendations that the healthcare workforce receive competency-based education to identify, assess, support and partner with FCGs.

**Objective:** Mixed methods evaluation healthcare workforce education program. Approach: We began by coining the concept "caregiver-centered care," defining it as a collaborative working relationship between families and healthcare providers aimed at person-centered support for FCGs. From this definition, interdisciplinary stakeholders including FCGs (n=101) co-designed the Foundational Caregiver-Centered Care education. Learning resources included six competency-aligned educational modules with videos and interactive exercises that encourage reflection. Kirkpatrick Barr's healthcare training evaluation framework underpinned our mixed methods evaluation. We measured participant's reaction to the education (Level 1) and changes in learner's knowledge and confidence to work with FCGs (Level 2).

**Results:** 352 healthcare providers completed the education online (caregivercare.ca). Learners were satisfied with quality of education (Mean 4.75/5; SD=.5) and the education increased their motivation to learn more about caregiver-centered care (Mean 4.75/5; SD .5). Student's paired samples T-test indicates pre-post education changes in knowledge and confidence to work with FCGs were significant [Pre (M=37.8, Sd=7.6) to post (M=47.2, SD=3.5) t (125) = -14.39, p<.0005 (two-tailed)]. Qualitative results derived from open responses mirrored the quantitative results.

**Conclusion:** The Caregiver-Centered Care education provides a foundation for educating healthcare providers working with FCGs to provide care to FCGs to maintain their wellbeing and sustain care.

### Session 4230 (Paper)

### ENVIRONMENT AND AGING

### ADVANCING STATE AND LOCAL HOME MODIFICATION PRACTICE AND POLICY: FINDINGS FROM AGING NETWORK SURVEYS

Julie Overton,[1] Jon Pynoos,[2] Emily Nabors,[3] Damon Terzaghi,[4] Elizabeth Blair,[5] Traci Wilson,[6] Bernard Steinman,[7] and Suzanne Kunkel,[8], *1. University of Southern California, Los Angeles, California, United States, 2. University of Southern California, University of Southern California, California, United States, 3. USC Leonard Davis School of Gerontology, Los Angeles, California, United States, 4. ADvancing States, Arlington, Virginia, United States, 5. n4a, Washington, District of Columbia, United States, 6. National Association of Area Agencies on Aging, Washington, District of Columbia, United States, 7. University of Wyoming, University of Wyoming, Laramie, Wyoming, United States, 8. Miami University, Oxford, Ohio, United States*

Home modification (HM) can promote older adults' functioning as their needs change, reduce fall risks, and support caregivers. A supportive home environment is increasingly important as homes become healthcare delivery sites for home and community-based services (HCBS). HM is funded and administered by disparate agencies, often hindering access to HM services for at-risk older adults who need them the most. The Aging Network (State Units on Aging (SUAs), Area Agencies on Aging (AAAs), and Title VI organizations serving Native American older adults) plays an important but not well understood role in HM. To address this lack of research, the USC Leonard Davis School of Gerontology, ADvancing States, and the National Association of Area Agencies on Aging in cooperation with Scripps Gerontology Center conducted three national surveys, with support from the Administration for Community Living: 1) directors of the 56 SUAs with an 89% response rate; 2) directors of the 618 AAAs with a 79% response rate; and 3) directors of 276 Title VI programs with an 84% response rate. Exemplary practices included HM advocacy through interagency coalitions; state and local plan priority setting; creative HM financing with housing, disability, and health care sectors, including partnerships with Medicaid agencies; and integration of HMs into state and local HCBS, including nursing home transition and caregiver support programs. Findings on the types of HM activities, service delivery barriers, funding sources, collaborations, and targeted populations will inform HM policy and practice for the Aging Network's critical state and local agencies serving low-income older adults.

### DETROIT NEEDS ASSESSMENT STUDY: A COMMUNITY ACADEMIC PARTNERSHIP WITH THE DETROIT AREA AGENCY ON AGING

Faith Hopp,[1] Fay Keys,[1] Elizabeth Chapleski,[1] La Cheryl Wicker,[1] Patricia Rencher,[2] Shirley Thomas,[3] Anne Davis,[4] and Ronald Taylor,[4], *1. Wayne State University, Detroit, Michigan, United States, 2. Urban Aging L3C, Detroit, Michigan, United States, 3. Wayne State University, Ypsilanti, Michigan, United States, 4. Detroit Area Agency on Aging, Detroit, Michigan, United States*

This presentation discusses a comprehensive needs assessment to inform long-term strategic planning for the Detroit Area Agency on Aging. The goal was to provide in-depth input from the older population (age 60+) and key agency stakeholders, using surveys (413 community participants), listening sessions (132 participants), 23 interviews with homebound older adults, and online surveys (94) targeting medical, church, government, academic, media and HSO stakeholders. Findings indicate that many participants were not aware of available community services. For example, one-third (33.3%) had not heard of Medicaid waivers providing services outside of nursing homes, while nearly one in five (22.0%) had not heard about senior employment services. The most common areas of unmet need were for caregiver workshops (16.3%) and diabetes management classes (15.7%). Community services most often noted as 'extremely important' included health and wellness programs (68.8%), services to help access health and supportive services (71%), easy to find service information (67.7%), home care and housekeeping services (66.4%), and caregiver support (63.7%). Stakeholder findings provide insight regarding this lack of awareness. Asked "How familiar do you think the general public is with DAAA?" , 10.8% answered 'very familiar' and 33% 'unfamiliar'. Findings related to an "Age-Friendly City" suggest the importance of access to supportive community services, transportation, safety, housing, and healthcare. Engagement of older adults in needs assessments plays a vital role in Area Agencies on Aging meeting the needs of emerging aging cohorts by developing 'age friendly' strategies to address increasing racial, ethnic, socioeconomic, and cultural diversity.

### DEVELOPING A SHARED LANGUAGE TO DESCRIBE THE AGE-FRIENDLY ECOSYSTEM

Kim Dash,[1] Jody Shue,[2] Tim Driver,[2] Alice Bonner,[3] Leslie Pelton,[3] Rani Snyder,[4] and Terry Fulmer,[5], *1. Education Development Center, Waltham, Massachusetts, United States, 2. Age Friendly Foundation, Waltham, Massachusetts, United States, 3. Institute for Healthcare Improvement, Boston, Massachusetts, United States, 4. The John A. Hartford Foundation, New York, New York, United States, 5. The John A. Hartford Foundation, New York City, New York, United States*

While multiple sectors—cities and communities, education, employment, health, and public health—have identified and implemented strategies to promote age-friendly systems, their efforts have mainly advanced in siloes. Each sector has met goals specific to its constituents, however, the major transformations required to realize systemic inclusivity and well-being among diverse groups of older adults remains indefinable. To begin to address this gap, we have engaged age-friendly sectors in a process of coordinated planning to define and operationalize an age-friendly ecosystem (AFE) that advances cross-sector and age-friendly solutions to meet the needs of all older adults. Our process borrows from Kania and Kramer (2011) who describe conditions to achieve substantial collective impact when coordinating efforts across sectors: a common agenda, shared measurement systems, mutually reinforcing activities, and continuous communication. In this presentation, we describe our stepwise process to set a common agenda, by engaging older adults and working with experts across sectors, to agree on a series of characteristics that define an AFE. Specifically, we surveyed older adults about their perceptions of an age-friendly ecosystem as well as conducted a review and analysis of relevant activities (i.e., policies, programs, and practices) associated with five age-friendly sectors. Next, activities were organized by common and defining characteristics. We then convened more than 40 international experts representing diverse age-friendly sectors to review and revise the AFE characteristics. Through structured and facilitated group processes, we worked with experts to identify and define six critical AFE characteristics as well as examples of corresponding activities.

### SPILLOVER BENEFITS AND ACHIEVING SUSTAINABILITY OF AGE-FRIENDLY COMMUNITIES

Shayna Gleason,[1] Caitlin Coyle,[2] and Jan Mutchler,[2], *1. University of Massachusetts, Boston, Somerville, Massachusetts, United States, 2. University of Massachusetts Boston, Boston, Massachusetts, United States*

As the age-friendly movement grows in its second decade, urgent questions of initiative sustainability have taken hold in the academic literature and among advocates implementing age-friendly changes. The creation of authentically age-inclusive environments requires not just the continuation of the initiative itself, but that its principles become embedded in formal and informal organizational processes, across sectors and beyond familiar networks focused on older populations. This paper argues that sustainability of age-friendly initiatives is enhanced by "spillover" effects, in which a behavioral or policy change in one environment spurs change in another environment. Evidence for such spillover and an understanding of how and when it occurs is currently limited in the context of age-friendly environments. This paper draws on the experiences of Age-Friendly Boston, which has been working towards age-friendly goals for more than five years. Based on Boston's experience, we identify, describe, and exemplify three pathways to positive spillover across environments: 1) branding positively; 2) publicizing successes; and 3) embarking on new relationships, while strengthening existing ones, in pursuit of shared goals. We also draw conclusions about what positive spillover means for the sustainability of age-friendly environments.

### Session 4235 (Paper)

### GENDER, CONTROL, AND CAREGIVING

### CORPORATE POLICY, MALE BREADWINNERS, AND THEIR FAMILY CARE IN AGING JAPAN

Hiroko Umegaki-Costantini,[1] and Glenda Roberts,[2], *1. SciencesPo, Paris, Ile-de-France, France, 2. Waseda University, Tokyo, Tokyo, Japan*

A rapidly emerging set of carers are men who combine care for older relatives with employment. In Japan, a 2015 government initiative aimed at reforming work to make employment and care compatible by 2020 failed to reduce the approximately 100,000 annually quitting employment mainly due to care for older relatives. This paper aims to evaluate the initiative's limited impact through a multilevel understanding of the roll-out of the family care policy. Stakeholder views, based on 32 interviews including with employers, the Japanese Business Federation, local care providers and NPOs, are juxtaposed with the perspectives of employed male family carers drawn from 37 qualitative in-depth narrative interviews complemented by participant observation in the Tokyo area in 2019. The ethnographic fieldwork evidences informants' diverse engagement with care for older relatives underpinned by strongly held cultural views of care provision being a 'private' issue, which contrasts with government attempts to make family care a 'social' issue by broadening stakeholder participation. Further, corporates tend to have tacit reluctance to transform working practices to accommodate care. Thus, employed men's devotion to work competes with the culturally embedded notion that carers should be committed to care provision. In conclusion, such a disjuncture is a major factor in the government initiative's failure. Although cultural values and meanings in policy evaluation theories are often neglected, this research points to the significance of ongoing (re)construction of the socio-cultural notions congruent with social policy enablement.

### DOING EVERYTHING: INTERSECTIONS OF GENDER AND SEXUAL ORIENTATION IN CAREGIVING FOR A SPOUSE WITH DEMENTIA

Toni Calasanti,[1] and Brian de Vries,[2], *1. VIrginia Tech, Blacksburg, Virginia, United States, 2. San Francisco State University, Palm Springs, California, United States*

Gender inequalities are rooted in and drive the division of labor over the life course, which result in heterosexual men and women acquiring different resources, skills, and identities. Gendered differences in caregiving reflect these varying gender repertoires. Whether and how these repertoires vary by sexual orientation is lesser understood. Our qualitative study seeks to explore the ways that sexual orientation and gender, and the related division of both paid and unpaid labor, shapes caregiving for a spouse or partner with Alzheimer's disease and related disorders (AD). Our data, obtained from in-depth interviews conducted among lesbian (n=9), gay (n=6), and heterosexual spousal and partner (23 women and 14 men) caregivers of those with AD, reveal that, although all the caregivers spoke about "having to do everything," with a particular focus on decision-making, they interpret this experience differently based on the intersections of gender and sexuality. The heterosexual women reported they were used to managing daily household life, yet they described having to make decisions as quite stressful: "I don't like to be the boss." Heterosexual husbands also lamented that they "had to do everything," but commenting that they hadn't realized what it took to "manage a household." The concerns reported by lesbian and gay spouses and partners were similarly situated but more varied, as each group tended to report their previous divisions of labor as "less well-defined." Our findings reflect both the influence of gender inequalities on how respondents experience "doing everything," and their potential modification in same-sex relationships.

### FACTORS ASSOCIATED WITH ENGAGING IN REGULAR PHYSICAL ACTIVITY AMONG WOMEN FAMILY CAREGIVERS

Abiola Keller, *Marquette University, Milwaukee, Wisconsin, United States*

Regular physical activity is important for promoting the health of family caregivers. In this study, we used data from the 2015 and 2017 Behavioral Risk Factor Surveillance System Questionnaire-Caregiver module to examine factors associated with meeting physical activity guidelines among women caregivers. Meeting physical activity guidelines was defined as participating in 150 minutes (or vigorous equivalent minutes) of physical activity weekly. We used survey-weighted multivariate regression analyses to examine relationships between sociodemographic, caregiving, and health characteristics and meeting physical activity guidelines. All variables were entered into the model simultaneously. The Wald test was used to test the significance of interactions between race and ethnicity and other covariates. 50.7% of 10,542 women caregivers met physical activity guidelines. The amount of time spent caregiving each week was not associated with the odds of meeting guidelines. Caregivers in the paid workforce had decreased odds (OR=0.73, 95%CI [0.62-0.87]) of meeting guidelines. Compared to women caregiving for <6months, women caregiving for 6 months to 2 years had increased odds of meeting guidelines (OR =1.33, 95%CI [1.08-1.64]). Increasing education was associated with an increased odds of meeting guidelines, but being college educated had a more positive effect for Hispanic than white caregivers (pinteraction=0.03). Having children did not affect the odds of meeting guidelines for white caregivers, but for black caregivers having two or more children decreased the odds (pinteraction=0.03). Understanding how sociodemographic, caregiving, and health characteristics impact engagement in regular physical activity is critical to designing effective interventions and ultimately improving the health of caregivers.

### PERCEIVED CONTROL AND ICD CONCERNS IN OLDER ICD RECIPIENTS: SEX AS A MODERATOR

Abigail Latimer,[1] Jennifer Miller,[2] Misook Lee Chung,[2] Muna Hammash,[3] and Debra Moser,[2], *1. University of Kentucky, Lexington, Kentucky, United States, 2. University of Kentucky College of Nursing, Lexington, Kentucky, United States, 3. University of Louisville, Louisville, Kentucky, United States*

Implantable cardioverter defibrillators (ICDs) reduce the risk of sudden cardiac death for those with a history of or high risk for lethal arrhythmias. In 2016, 105,000 ICDs were implanted in older adults (age ≥ 60) in the US. Approximately 25% of ICD recipients report significant ICD concerns with women reporting higher levels than men. Little is known about the experience of older adults living with life-saving/extending medical technologies, particularly related to sex differences in ICD concerns. Perceived control may decrease ICD concerns, but sex differences have not been explored. The aim of this cross-sectional study was to determine the moderation effect of sex on the association between perceived control and ICD concerns in older adults (age≥ 60). Participants completed a questionnaire on ICD Concerns (ICDC-8) and the Control Attitudes Scale-Revised, a measure of perceived control. We conducted a moderation analysis using Hayes' PROCESS for SPSS with 5,000 bootstrap samples. Of the 99 participants (73.7% male; age 70 + 7 years; education 13 + 3 years), most participants were white (79.8%) and married (69.7%). There were no differences in participant characteristics, perceived control, or ICD concerns, by sex. We found an interaction between sex and perceived control (b= -.5715, p= 02), indicating that for women (-.5801, p =.007), as perceived control increased, ICD concerns decreased. For men (-.0089, p =.9439), ICD concerns remained the same despite level of perceived control. Future clinical and research interventions to decrease ICD concerns should include ways to increase perceived control particularly for older women living with ICDs.

### THE ROLE OF SECONDARY CARE NETWORKS, GENDER, AND RACE ON PRIMARY CAREGIVER BURDEN

Jiaming Liang, and Maria Aranda, *University of Southern California, Los Angeles, California, United States*

In addition to primary caregivers, many older adults receive care from secondary care networks (SCN), which include family members and friends. Literature rarely considers support that SCN provided to primary caregivers. This study examines: (a) the association between SCN support and primary caregiver burden, and (b) the intersectional effects of gender (male/female)-race (White/Black) identities of primary caregivers on the association. A cross-sectional study using data from 2015 National Health and Aging Trend Study (NHATS) and National Study of Caregiving (NSOC) was conducted. A total of 967 older adults, 967 primary caregivers, and 2253 secondary caregivers were selected. SCN support was measured by (a) care domain overlap, and (b) proportion of caregiving by SCN. Negative binomial regressions on overall and split samples estimated main effects of SCN support and the intersectional effects of gender and race. Both SCN-related variables were associated with primary caregiver burden, but significant three-way interaction was only found between gender, race, and proportion of caregiving by SCN. Black female caregivers reported heaviest burden and having SCN support was associated with lower risk of being burdened. Whereas Black male caregivers reported lightest burden and SCN support was not associated with their perceived burden. Our findings support the positive role of SCN in reducing stress of primary caregivers, and demonstrate that positive impacts of SCN support vary across gender-race groups. The results indicate a strong need for support programs aimed at promoting cooperation among family caregivers for burden reduction, especially families with female and Black primary caregivers.

### Session 4240 (Symposium)

### GERIATRIC WORKFORCE ENHANCEMENT PROGRAMS' PARTICIPATION IN THE NURSING HOME COVID ACTION NETWORK

Chair: Leland Waters Discussant: Nina Tumosa

In late September, 2020, the Geriatric Workforce Enhancement Program's (GWEP) Program Officer, at the Health Resources Services Administration (HRSA), alerted the 48 GWEPs about a nationwide initiative focusing specifically on the pandemic's effect in nursing facilities. The ECHO Institute at the University of New Mexico negotiated a national contract with the Agency for Healthcare Research and Quality (AHRQ) to provide a nationwide educational intervention via the CARES Act Provider Relief Fund. The ECHO Institute recruited over 100 Training Centers as educational coordinators for the Project ECHO Nursing Home National COVID Action Network. Our Project Officer suggested that individual GWEPs participate in this effort and take the lead or provide geriatric educators for these Training Centers. Project ECHO (Extension for Community Healthcare Outcomes) is an innovative telementoring program that creates virtual learning communities, bringing together healthcare providers and subject matter experts using videoconference technology for brief presentations, and case-based learning, fostering an "all learn, all teach" approach. This symposium will describe the journeys that five GWEPs experienced becoming Training Centers, rapidly deploying a nursing home ECHO project, to support nursing home staff on best practices for protecting patients, staff, and visitors from coronavirus infection and spread. GWEPs from The University of Louisville, the University of North Carolina, the University of North Texas, the University of Rochester and The Virginia Geriatric Education Center's two ECHO Hubs, joined the National COVID Action Network. This presentation will provide an overview of why GWEPs are well positioned to address emergent needs with short notice.

### LESSONS LEARNED IN EXECUTING THE NURSING HOME COVID ACTION NETWORK

Samantha Cotton,[1] Pamela Yankeelov,[2] Barbara Gordon,[1] and Anna Faul,[2], *1. University of Louisville, Louisville, Kentucky, United States, 2. University of Louisville, University of Louisville, Kentucky, United States*

The University of Louisville ECHO Hub for the Nursing Home COVID Action Network put together a hub of experts that could effectively address the diverse needs of the 240 nursing homes in the 7 cohorts launched. We included an infectious disease expert, a geriatrician, and a behavioral health specialist who adjusted the curriculum to be more in line with the needs of the nursing homes. Our nursing homes were diverse in terms of geography, size and location. We created space for our cohorts to feel comfortable with each other, despite their differences. To foster this sense of togetherness, our facilitators used anonymous opinion polls and incorporated the use of virtual breakout rooms to encourage small group discussions. These strategies assisted in developing a sense of community within the Project ECHO sessions, that will continue to evolve in the post COVID world.

### NC CAN COVID-19 ACTION NETWORK: PROJECT ECHO IN NORTH CAROLINA

Cristine Henage,[1] Jennifer Hubbard,[2] J. Marvin McBride,[2] and Ben Blomberg,[2], *1. The University of North Carolina at Chapel Hill, UNC, North Carolina, United States, 2. UNC Chapel Hill School of Medicine, Chapel Hill, North Carolina, United States*

Experts in geriatrics, infection control and nursing home administration joined the ECHO Hub team led by The Carolina Geriatrics Workforce Enhancement Program (CGWEP) at the University of North Carolina at Chapel Hill (UNC). Ninety-two of North Carolina's 423 nursing homes enrolled in a 16-week videoconference series designed to address clinical, logistical, and leadership issues related to COVID-19. The CGWEP coordinated recruitment with two other Training Centers at UNC Family Medicine and the Mountain Area Health Education Center, reaching 58% of all NC nursing homes (N=245). Faculty used curriculum and pre-recorded videos provided by the Institute for Healthcare Improvement (IHI). Discussions demonstrated real-world problem solving as participants applied what they learned to local conditions. Quality Improvement (QI) experts from IHI mentored participants in gathering data and completing Plan, Do, Study, Act cycles to better respond to the challenges of COVID-19 among a critically vulnerable population.

### LEVERAGING PARTNERSHIPS FOR A NURSING HOME COVID LEARNING COMMUNITY

Jennifer Severance, Janice Knebl, Susanna Luk-Jones, and Sarah Ross, *University of North Texas Health Science Center - Ft. Worth, TX, Fort Worth, Texas, United States*

The rapid and uncertain trajectory of community spread in nursing homes statewide spurred action by the University of North Texas Health Science Center to create a nursing home (NH) COVID learning community. As an existing ECHO hub, we assembled an interdisciplinary team leveraging local NH partnerships, a regional Quality Improvement Organization (QIO), and a regional emergency response task force to rapidly scale up delivery. Specialist teams include a geriatrician and NH medical director, administrator, nursing administration, infection control expert, and a QIO specialist. With the IHI curriculum as a road map for essential training elements, we adapt each week's agenda based on the interests and concerns of the participating nursing facilities and the incidence rate in our community. At this time, we have two more sessions before completion of phase 1. The three cohorts are engaging 151 participants from 68 nursing facilities with a total attendance of 747.

### NURSING HOME COVID-19 ACTION NETWORK: RESPONDING WITH STRESS FIRST AID AND CARING FOR RESIDENTS WITH DEMENTIA

Barbara Ganzel,[1] Adam Simning,[2] and Thomas Caprio,[3], *1. Ithaca College Gerontology Institute, Ithaca, New York, United States, 2. University of Rochester, Rochester, New York, United States, 3. University of Rochester Medical Center, Rochester, New York, United States*

The GWEP at the University of Rochester (New York) has an established network of nursing homes participating in Project ECHO. This ECHO hub includes geriatric medicine, psychiatry, pharmacy, aging services network and the Alzheimer's Association focusing on best practices in geriatric mental health and dementia care. With the COVID-19 pandemic, this infrastructure quickly pivoted to expansion of 80 facilities and the addition of expertise in medical direction, trauma informed care, and infectious disease. A stress first aid training module was developed in partnership with Ithaca College and the National Center for PTSD to support front line nursing home workers. Dementia care experts contributed to practical problem-solving in addressing social isolation and mental health. Work now is focusing on vaccination and how to best support trauma-informed needs of residents with dementia.

### VIRGINIA'S RESPONSE TO THE NURSING HOME COVID ACTION NETWORK

Anne Rhodes,[1] Shannon Arnette,[1] Dan Bluestein,[1] Emily Ihara,[2] Megumi Inoue,[2] Catherine Tompkins,[2] and Leland Waters,[1], *1. Virginia Commonwealth University, Richmond, Virginia, United States, 2. George Mason University, Fairfax, Virginia, United States*

The Virginia Geriatric Education Center's GWEP recruited 195 of Virginia's 273 eligible nursing homes, using two Project ECHO Nursing Home Training Centers located at George Mason University and Virginia Commonwealth University. These sessions promoted collaboration, allowed for sharing of successes and challenges, and nurtured quality improvement projects. Our next steps are to survey Virginia's nursing homes to see if they are interested in future ECHO sessions with other topics. We plan to share these results with the Institute for Healthcare Improvement so that we may be able to continue to enhance this national network of Training Centers with faculty and staffing dedicated to quality assurance and performance improvement. The program has initiated new collaborations with nursing homes across many healthcare disciplines, strengthened connections between nursing homes and research institutions, and will help foster innovative ways to collaborate in this post-pandemic virtually connected world.

### Session 4245 (Paper)

### GRANDPARENTING

### INTENSITY OF GRANDPARENT CAREGIVING AND WELL-BEING IN A CULTURAL CONTEXT: A SYSTEMATIC REVIEW

Athena Chung Yin Chan,[1] Sun-Kyung Lee,[2] Jingchen Zhang,[2] Jasmine Banegas,[3] Scott Marsalis,[4] and Abigail Gewirtz,[2], *1. University of Minnesota, St Paul, Minnesota, United States, 2. University of Minnesota -- Twin Cities, St. Paul, Minnesota, United States, 3. University of Minnesota -- Twin Cities, Minneapolis, Minnesota, United States, 4. University of Minnesota -- Twin Cities, St Paul., Minnesota, United States*

With improved longevity and changes in family structure, grandparents are key resources in providing care for grandchildren. However, mixed findings indicate that multiple role engagement may enhance well-being or bring demands on grandparents raising grandchildren. Little is known about how the intensity of grandparent caregiving is associated with their well-being in different family contexts (i.e., structures, cultures/regions, and reasons of care). This systematic review examines the association between the intensity of grandparent caregiving and their well-being. Peer-reviewed articles published after 1990 were identified in five electronic databases. A keyword search was performed for keywords associated with: (a) grandparent caregivers raising grandchildren, and (2) well-being (i.e., physical, mental, cognitive, and life satisfaction). Only quantitative studies were included. Fifty-six articles from 28 countries/regions were included. Findings suggested that the well-being of grandparents is optimal when they provide caregiving of moderate intensity, with optimal amounts varying across sociocultural contexts. In Europe and Australia, providing supplementary care seems beneficial for grandparents' well-being, especially supporting dual-earner families. In Asia, economic resources buffer the adverse effect of primary care on grandparents' well-being. In the U.S., findings vary across ethnicity/race. White grandparents enjoy health benefits providing supplementary care with support from adult children. However, Hispanic grandparent caregivers in multigenerational households have better well-being than those in skipped-generation households, whereas Black custodial caregivers have better well-being than supplemental caregivers. Collectively, the intensity of grandparent caregiving and well-being is complicated by their roles in the family and cultural differences. This systematic review calls for culturally-tailored family interventions.

### LIVING ARRANGEMENTS AND GENDERED WORK PROSPECTS AMONG CHINESE GRANDPARENTS

Jing Ye,[1] and Feinian Chen,[2], *1. University of Maryland College Park, University Park, Maryland, United States, 2. University of Maryland College Park, University of Maryland, Maryland, United States*

Recent literature on grandparenthood in China overwhelmingly focuses on the role of grandparents as caregivers for grandchildren. However, many become grandparents at an age when they are still active in the labor force. Using data from the China Health and Retirement Longitudinal Study (2011-2015), this study examines the extent to which coresidence with grandchildren affects grandparents' labor force participation and work hours. Results from our fixed-effect models show that, living with grandchildren has a positive effect on men's work participation and hours worked, especially for those with flexible jobs. For women with inflexible jobs, coresidence with grandchildren has a negative impact on their work prospect. Furthermore, grandparents in skipped generational households are less likely to scale back in work than those in multigenerational households, indicating a high level of double burden from both work and caregiving responsibilities. Our study extends prior work by emphasizing grandparents' role as active workers and highlights the importance to understand work and caregiving demands in a gendered and dynamic household context.

### MATERIAL HARDSHIP AMONG CUSTODIAL GRANDPARENTS AND GRANDCHILDREN'S PHYSICAL AND MENTAL HEALTH IN COVID-19

Yanfeng Xu,[1] Qianwei Zhao,[2] Brittany Schuler,[3] and Sue Levkoff,[1], *1. University of South Carolina, Columbia, South Carolina, United States, 2. Baylor University, Waco, Texas, United States, 3. Temple University, Temple University, Pennsylvania, United States*

COVID-19 has increased economic hardship for many families, including custodial grandparent-headed families. We aim to examine latent classes of material hardship among custodial grandparent-headed families, to assess predictors associated with identified classes, and to investigate associations with grandchildren's physical and mental health outcomes during COVID-19. Data was collected from a cross-sectional survey in June 2020. The sample comprised of 362 grandparents. Latent class analysis and logistic regression were conducted. Three latent classes of material hardship were identified: Class 1 (n = 232; 64.1%) low overall hardship with high medical hardship, class 2 (n = 52; 14.4%) moderate overall hardship with high utility hardship, and class 3 (n = 78; 21.5%) severe overall hardship. Factors, such as race, household income, labor force status, financial assistance status, and trigger events to raise grandchildren, were associated with class membership. Class 2 (OR = 0.19, $p < 0.05$) compared to Class 1 was significantly associated with grandchildren's physical health. Our findings suggest that material hardship is heterogeneous among custodial grandparents during COVID-19, and children in households experiencing utility hardship have a higher risk for poorer physical health outcomes. Results highlight the needs to meet grandparents' material needs and call for future research to examine the mechanism that explains the link between material hardship and grandchildren's outcomes.

### RESOURCEFULNESS SKILLS USE BY GRANDMOTHERS RAISING GRANDCHILDREN: A LONGITUDINAL CASE STUDY APPROACH

Alexandra Jeanblanc,[1] Carol Musil,[2] Elizabeth Tracy,[1] and Jaclene Zauszniewski,[1], *1. Case Western Reserve University, Cleveland, Ohio, United States, 2. CWRU School of Nursing, Cleveland, Ohio, United States*

In the U.S., over 2.7 million grandparents are primary caregivers to grandchildren. It is critical to understand the experiences of grandparent caregivers to design tailored, supportive programs. Our aim was to analyze 4 weeks of daily online journals of 129 grandmothers with respect to their use of a set of Resourcefulness Skills© following web-based skills training. Using a thematic analysis approach, coding was completed by a three person team using NVIVO 12. Percent agreement among coders was over 90% (Kappa = .956). Twelve cases were randomly selected for case study development. Comparative case study analysis was used to look within and across cases for instances where skills were used and how skill use changed over time. The pattern of skill use showed that grandmother caregivers used resourcefulness skills to deal with the grandchild's behavior and developmental issues as well as within the entire family system to manage conflicted relationships with the grandchild's parents, balance relationships with their spouse/partner, and maintain relationships with other relatives. Case studies will be presented to show skill use over the four weeks of journaling in the context of the family system, as well as the strategies used by participants who improved skill use over time and those who faced barriers to skill use. Findings highlight the use of journals as a means to assess enactment fidelity of treatment interventions and the importance of the family network in skills training program implementation and ways to help grandmothers make use of skills training in the family setting.

### SUPPORTING ONE'S OWN? GRANDPARENTS' HELP TO GRANDCHILDREN WHO LIVE WITH OTHER UNRELATED CHILDREN

Teresa Cooney, *University of Colorado Denver, Denver, Colorado, United States*

The structures of young families today are becoming increasingly complex, which may impact grandparents' involvement. I examine whether grandparents' support to adult children's households differs for those with biological grandchildren only, versus households with both biological and non-biological (step, unrelated) grandchildren. The resource dilution hypothesis and sociobiology theory suggest that grandparents will be less supportive of grandchildren when other unrelated children co-reside in their households. Grandparents (mean age 62.23) in the Add Health Parent Study (2015-2017) reported on instrumental and financial help given to each of their adult children's families in the past year. These data were merged with information from their adult children (mean age 36.76) who participated in Add Health Wave V (2016-2018). Adult children's household structures—biological children only (n=400) or biological + other children (n=51)—were determined using their fertility histories and household rosters. No significant differences were found in the likelihood that grandparents offered any instrumental or financial support to these two household types (controlling for grandparent resources and adult child characteristics). Nor was the level of grandparents' financial support significantly different for the two groups. However, grandparents gave significantly fewer hours of help to adult children heading households including both biological grandchildren and unrelated children. Grandparents appear less willing to devote time to assisting their grandchildren's families when their investment is diluted by the presence of unrelated children. Perhaps time with grandchildren is less pleasing or comfortable when unrelated children are present. This same issue does not impact financial giving, which need not involve contact.

### Session 4250 (Symposium)

### GWEP SUCCESSES AND LESSONS LEARNED FROM MAKING COMMUNITIES AGE FRIENDLY

Chair: Katherine Thompson Co-Chair: Jennifer Ouellet

Geriatrics Workforce Enhancement Programs (GWEPs), funded by the Health Resources and Services Administration have a strong focus on age friendly care and community engagement. With a wide range of populations, locales, and health systems served, GWEPs have significant experience working with a wide variety of communities to implement age friendly care. In this symposium, we present successes and lessons learned from GWEP projects representing diverse populations and approaches to achieving age friendly communities. For instance, one GWEP is utilizing Patient Priorities Care to lay the framework for What Matters in clinical decision-making. Another GWEP is focusing on What Matters by uniquely embedding Area Agencies on Aging care coordinators within primary care settings to invite the participation of aging patients in advance care planning, among other health interventions. A third GWEP is using the 4Ms to educate patients and caregivers in geriatric psychiatry clinics in a population of veterans. Another GWEP is pairing Age Friendly Health System efforts within a health system with community-based efforts to become an age friendly and dementia friendly city. A final GWEP is using multiple educational modalities to create Age-Friendly Communities and assure that health systems, community-based organizations, and older adults and families are educated about the 4Ms. By exploring successes and lessons learned in making communities age friendly, we can improve existing and future programs centered on age friendly care for older adults.

### EDUCATION OF CAREGIVERS AND VETERANS TO IMPROVE THE CARE OF THE GERIATRIC PSYCHIATRIC PATIENT

Maritza Buenaver, *Oklahoma City VA Health Care Systems, Oklahoma City, Oklahoma, United States*

The geriatric psychiatry outpatient clinic provides assessment of the elderly Veteran with mental illness and behavioral and psychological symptoms of dementia. I will describe strategies developed and implemented in this setting to provide education to the caregiver (family) to improve early identification of delirium, depression and cognitive impairment. This education proved to reduce the number of pharmacological treatment and increase the use of nonpharmacological interventions based on "what matters to the patient" and following the BEERS criteria guidelines. One of the most important outcomes of the education and evaluation in the geriatric psychiatric clinic was a decrease in number of emergency room visits of elderly, specifically those with dementia.

### SUCCESSES AND LESSONS LEARNED FROM AGE-FRIENDLY COMMUNITY COLLABORATIONS: BAYSTATE HEALTH GWEP

Rebecca Dobert, and Maura Brennan, *Baystate Health, Springfield, Massachusetts, United States*

Baystate is the largest health system in Western Massachusetts with 4 hospitals, 3 Community Health Centers (CHCs) and a large primary care network. Baystate Medical Center (BMC) is in Springfield, Massachusetts. BMC and the CHCs were the first health care sites nationally to be recognized by the Institute for Healthcare Improvement as "Committed to Care Excellence" in the age friendly movement. Collaboration with a city-wide coalition of community-based organizations led to simultaneous recognition of Baystate as "age friendly" and recognition of the city as both dementia and age friendly. The 3 awards were presented at a Springfield senior center with media coverage and the participation of the mayor and other political leaders. This collaboration persists and the GWEP and coalition partners continue to participate in multiple joint educational and community outreach projects. As a result, the city coalition has added health care to its initial focus on housing and transportation.

### WHAT MATTERS MOST: ACP EVOLVING IN PRACTICE

Kevin Valadares, *University of Southern Indiana, Evansville, Indiana, United States*

The University of Southern Indiana (USI) GWEP uniquely embeds Area Agencies on Aging (AAA) care coordinators within primary care settings to invite the participation of aging patients in advance care planning (ACP), among other health interventions. Two subsequently developed features of the USI GWEP's ACP initiative emerged to address the What Matters metric of the 4Ms: 1) Patients are invited to engage in What Matters Most conversations through multiple touchpoints that frame Medicare Wellness Visits with a Deaconess provider and introduce a free, online ACP platform, Prepare for Your Care. 2) Provider, patients and families are supported in having ACP conversations with the dedication of a new Advance Care Planning facilitator position. Certified in Respecting Choices and jointly funded by the GWEP and Deaconess, the ACP facilitator supports individuals in navigating these essential healthcare conversations about balancing quality care with quality of life.

### CATCH-ON EDUCATIONAL INTERVENTIONS FOR PROVIDERS, OLDER ADULTS, AND CAREGIVERS

Erin Emery-Tiburcio,[1] Robyn Golden,[2] and Michelle Newman,[2], *1. Rush University, Chicago, Illinois, United States, 2. Rush University Medical Center, Chicago, Illinois, United States*

CATCH-ON, the collaborative GWEP led by Rush University Medical Center, is working to create Age-Friendly Communities by assuring that health systems, community-based organizations, and older adults and families are educated about the 4Ms. For providers, CATCH-ON offers a monthly Learning Community that focuses on one of the 4Ms each quarter. Each session provides practical recommendations for 4Ms implementation and opportunities to share experiences in small groups. CATCH-ON also partnered with Community Catalyst, older adults, and caregivers to develop a 4Ms educational brochure. The brochure is available electronically and by paper to educate older adults and caregivers about the 4Ms and discussing them with their healthcare team. Additionally, CATCH-ON created 4M online modules for older adults and families. This session will explore the success and lessons learned in developing educational interventions for diverse audiences and how this approach strengthens Age-Friendly Communities.

### Session 4255 (Paper)

### HEALTH PROMOTION (SRPP PAPER)

### AGE-TASTIC: AN EVIDENCE-BASED INTERVENTION TO IMPROVE HEALTH, SAFETY, AND WELL-BEING IN OLDER ADULTS

Manoj Pardasani,[1] Jacquelin Berman,[2] Mebane Powell,[3] and Madison Gates,[4], *1. Adelphi University, Garden City, New York, United States, 2. NYC Department for the Aging, NYC Department for the Aging, New York, United States, 3. NYC Department for the Aging, New York, New York, United States, 4. NYC Department for the Aging, NYC, New York, United States*

Age-Tastic! is a holistic intervention that enhances the well-being, health and safety of older adults. Most evidence-based interventions aimed at older adults have focused on singular aspects of health such as cognitive health, falls prevention, depression, advanced care planning, etc. There are few interventions that encompass a holistic approach to health and safety. Age-tastic! is one such intervention that encompasses various aspects of health – social support, financial well-being, physical safety, mental health, health care management, and nutrition. Designed as a competitive board game to entice older adults, this intervention integrates concepts of cognitive restructuring, behavioral activation and game theory to educate, motivate and encourage healthful behaviors. At the core of this intervention is a focus on increasing awareness of health and safety issues, improving health literacy and changing harmful behaviors. A randomized control trial was conducted with 98 older adults assigned to an experimental and control group. Interviews were conducted at baseline, right after the intervention ended (8 weeks) and again after a short time (8 weeks after intervention ended). The results showed significant increases among experimental group participants in knowledge of healthy behaviors ($p=0.05$), perception of self-efficacy for engaging in healthful behaviors ($p<0.001$) and engagement in health behaviors ($p=0.001$). Regression analyses demonstrated that greater knowledge and self-efficacy were associated with behavioral change within the intervention group ($p<0.001$). Knowledge about the importance of preventing falls and communication with medical providers was positively correlated with the corresponding behavioral change ($p<0.05$). Implications for health literacy among older adults will be shared.

### CHILDHOOD DISADVANTAGE AND LATER-LIFE MULTIMORBIDITY: DEMONSTRATING ROBUSTNESS UNDER IMPERFECT IDENTIFICATION

Brayan Seixas, and James Macinko, *UCLA, Los Angeles, California, United States*

This study assesses the relationship between indicators of early childhood disadvantage and later-life multimorbidity within a nationally representative sample of Brazilians aged 50 and over ($n = 9,412$). Data come from the baseline assessment (2015/2016) of the Brazilian Longitudinal Study of Aging (ELSI). We employed survey-weighted Ordinary Least Squares regression to estimate the effects of individual and combined measures of childhood disadvantage on the total number of chronic conditions in later life. Mediation analysis assessed whether adult socioeconomic status (SES) mediated the relationship between childhood disadvantage and chronic conditions. We found that individual and combined measures of childhood disadvantage were associated with the total number of chronic conditions, even after controlling for potential confounders. Mediation analysis suggested that part of the effect of childhood adversity is mediated by higher SES in adulthood (~10%). A formal strategy of sensitivity analysis showed that omitted variable bias is extremely unlikely. To rule out the observed effect, an unobserved hypothetical confounder would need the explanatory power of the residual variance of both the independent and the dependent variables that is at least 30 times larger than that of BMI or 5 times larger than the explanatory power of age. Results should inform efforts to strengthen interventions targeting early childhood development and to improve other key inputs (such as education) to enhance adult SES and lessen the impact of early life stressors on health in older adulthood.

### FOOD INSECURITY, FOOD ENVIRONMENTS, AND DISPARITIES IN DIET QUALITY AND OBESITY

Yeon Jin Choi, *University of Southern California, Los Angeles, California, United States*

Food insecurity is a public health concern that is associated with poor diet and obesity. Poor food environments with low access to healthy, affordable food may amplify the negative impact of food insecurity on diet and obesity. This study aims to investigate whether food insecurity and food environments are jointly associated with an increased risk of poor diet quality and obesity. We used data from a nationally representative sample of 6,395 older adults in the Health and Retirement Study Health Care and Nutrition Survey and the National Neighborhood Data Archive. Weighted regression models were estimated to examine the relationship between food insecurity and food environments with diet quality and obesity. Both food insecurity and poor food environment were associated with lower healthy eating index scores, indicating poorer quality diet. Food insecure older adults were more likely to be obese than food secure older adults and poor food environments exacerbate the negative impact of food insecurity on obesity risk. However, there was no statistical difference in obesity risk by food environment among food secure respondents. Findings from this study highlight the negative impact of limited access to healthy food due to financial difficulties and/or poor food environments on diet quality and obesity risk. Providing financial or nutritional supports along with efforts to promote healthy food environment may reduce disparities in diet quality and obesity. Special support should be provided to food insecure older adults with poor food environment, those at the greatest risk of poor diet quality and obesity.

### GENDER, TOBACCO CONTROL POLICIES, AND RECALCITRANT SMOKING AMONG OLDER ADULTS IN 20 EUROPEAN COUNTRIES

Manjing Gao, Chioun Lee, and Soojin Park, *University of California, Riverside, Riverside, California, United States*

Little is known about sociodemographic and macro-level predictors of “recalcitrant smoking,” defined as persistent smoking when one has developed a health condition that is likely caused by smoking. We aim to investigate the impact of gender, education, and tobacco control policies on recalcitrant smoking among older adults in Europe from 2006 through 2013. Data from 33,839 respondents—aged 50 years and older with a smoking history and at least one smoking-related health condition—were pooled from the 2006–07, 2011, and 2013 waves of three harmonized longitudinal studies on ageing (SHARE, ELSA, and TILDA). We fitted gender-specific logistic regression models with two-way fixed effects and tested interaction terms between gender, tobacco control policies, and education, adjusting for age, marital status, GDP per capita, smoking prevalence, country, and year-fixed effects. Compared to men and individuals with higher levels of education, women and less educated individuals were more likely to be recalcitrant smokers. The association between education and recalcitrant smoking was stronger for women than men. The inverse association between the TCS and recalcitrant smoking was stronger for those having upper secondary education (for men: OR = 0.905, CI = 0.849–0.965; for women: OR = 0.897, CI = 0.834–0.964) and tertiary education (for men: OR = 0.802, CI = 0.717–0.898; for women: OR = 0.739, CI = 0.603–0.907), compared to those having less than upper secondary. As women and less educated individuals are vulnerable to recalcitrant smoking, future policies targeting these marginalized groups are needed to prevent recalcitrant smoking in old age.

### THE ASSOCIATION BETWEEN THE USE OF DIGITAL HEALTH MANAGEMENT TOOLS AND SUBJECTIVE WELL-BEING AMONG OLDER ADULTS

Mai See Yang,[1] Haowei Wang,[2] and Yong Kyung Choi,[3], *1. UC Davis, UC Davis, California, United States, 2. The Pennsylvania State University, University Park, Pennsylvania, United States, 3. University of California Davis School of Medicine, Sacramento, California, United States*

This study aims to examine the association between the use of digital health management tools and subjective well-being in later life. Research is limited about technology use (e.g., participation in online wellness program, finding medical information, using devices to monitor health) among community dwelling older adults. This study used data from the Health and Retirement study 2012 Module “Technology Use: Barriers and Benefits” (N = 1,416). We used multiple regression methods to test the association between technology use and subjective well-being (i.e., self-rated health, life satisfaction, and depressive symptoms). Over half of the participants reported using technology (58%). The mean age for this group was 68.7 (SD 9.6). Majority of the respondents were female (55%). About 18% were non-Hispanic Blacks, 2% were non-Hispanic other, 11% were Hispanic, and 68% were non-Hispanic Whites. For this sample of technology users, the usage of digital health management included online exercise programs (16%), online wellness programs or health monitoring programs (7%), searching for medical and health information online (43%), digital devices to monitor health (31%), and physical activity-based video game such as Wii Fit (7%). Over 88% of the sample have used at least one of these formats to monitor their health. Results from regression models suggested that the use of any digital health management tools was related to fewer depressive symptoms and better self-reported health. Findings from this study provide insight into how digital health management can protect older adults from poor subjective well-being in later life.

### Session 4260 (Symposium)

### INTERPRETING THE EMERGING DISCOURSE AROUND ELDERHOOD: LIFE STAGE, ANTI-AGEISM STRATEGY, OR SOMETHING ELSE?

Chair: Jennifer Inker

The emerging discourse around elderhood hints at the possibility of a later life stage characterized by a focus on balancing development and decline, with potential to empower elders. However, little agreement exists about whether elderhood is a valid and useful construct. The first presenter questions the aging "mystique" through an analysis of the concepts of elderhood, sageing, croning, and eldering in popular and academic literature, underscoring the importance of avoiding othering and critically thinking beyond labels, even if positive. The second presenter explores the concept of agency in later life through a feminist philosophical lens, arguing that confrontations with one's existential vulnerability need not be an obstacle to agency in elderhood, but rather can inspire alternative conceptualizations of it. The third presenter contrasts his personal and professional experiences of studying cultural aspects of aging, concluding that elderhood is neither a stage of a life nor a rite of passage but rather an individual, voluntaristic process. The fourth presenter explores 943 texts written by Finnish older adults, finding that the writers creatively position themselves as a group of older persons with a special contribution to make to society, even where elderhood is not explicitly mentioned, and potentially offer an alternative view to countering ageism. The fifth and final presenter explores a novel elderhood video intervention among first-year medical students (N = 585). Thematic findings of neutrality, elderhood as development, elderhood as othering, and elderhood as an opportunity to reframe stigma suggest that elderhood may be a viable and productive anti-ageism strategy.

### THE ELDERHOOD MYSTIQUE

Kate de Medeiros, *Miami University, Oxford, Ohio, United States*

While elderhood recognizes untapped potential and continued growth and creativity in later life, it also risks becoming a dismissive label that positions older people as different, otherworldly, and mysterious. By analyzing the concept of elderhood and similar movements (e.g., sageing, croning, eldering) in popular and academic literature, paying close attention to how elderhood is defined and framed, I found that elderhood has a role in some religious and cultural practices. However, newer approaches to elderhood have emerged from middle aged writers who imagine an idealized role in later life – the elderhood mystique. Often grounded in introspective passivity and selflessness, elderhood parallels Kathleen Woodward's depiction of wisdom as a disempowering label that discourages activism and resistance by older people. Subsequently, elderhood and wisdom risk becoming new forms of othering or exclusion. Overall, findings underscore the importance of critical analysis of age-related terms, regardless of how positive they seem.

### ELDERHOOD, AGENCY AND EXISTENTIAL VULNERABILITY

H.E. Laceulle, *University of Humanistic Studies, Utrecht, Netherlands, Utrecht, Netherlands*

Popular conceptualizations of elderhood often use a spiritually inspired language of personal growth and wisdom. These conceptualizations are rightly critical of the language of activity and productivity that abounds in dominant successful aging discourses. Instead, the emphasis is placed on embracing our diminishing strength and increasing dependence with an attitude of resignation and gracious acceptance. Problematically, however, this can reinforce the ageist cultural assumption that old age lacks agency. If the emerging discourse about elderhood is truly to serve as a more inspiring cultural image of late life, it requires a reconceptualization of agency in the face of existential vulnerabilities. This paper aims to present a possible philosophical outlook for such a reconceptualization. It will draw on sources from feminist philosophy to argue how confrontations with vulnerability need not be an obstacle, but rather inspire alternative conceptualizations of agency that are a welcome addition to gerontological thinking.

### ELDERS OR OLD MEN?

Thomas Cole, *McGovern Medical School, Houston, Texas, United States*

Thomas R. Cole, GSA Abstract, 3.9.2021 Elders or Old Men? My book Old Man Country is about 12 successful, respected older men who think back on their lives and current aging. When starting my research, I first questioned my own aspirations for aging: What would my aging be like? Who would I become? What would be my purpose as an old man? Although I expected that strength and resilience would be the common thread of elderhood, it was actually their vulnerabilities that defined them (accepting losses, acknowledging dependency.) More so, these vulnerabilities did not demarcate a descent but rather a continuous uphill struggle that differentiates elderhood from growing old. Ultimately, I argue that elderhood is not a life stage or a right of passage but rather an individual process to be worked through, if one so chooses.

### ELDERHOOD IN PROTESTANT RELIGIOUS CONTEXTS: STEPPING STONES IN RELIGIOUS LANGUAGE

Jenni Spännäri, *University of Eastern Finland, Joensuu, Pohjois-Karjala, Finland*

Elderhood is an emerging concept for making meaning in older age, often contextualized in spiritual but not religious traditions. But what kinds of frameworks for elderhood are woven into protestant religious contexts? This paper explores 943 texts written by Finnish older adults in study groups organized by a pensioners' organization. A key finding is that religious language – known through religious songs and prayers learned by heart at school – offers a medium to explore and express their elderhood. The writers creatively use the rhythm and wordings of these textual patterns to position themselves as a group of older persons with a special contribution to make to society. These results will aid examining elderhood and its potential in various contexts where the concept might not be explicitly used. This examination potentially leads to new ways to support experiences of elderhood and thus to offer an alternative view to countering ageism.

### ELDERHOOD AS AN ANTI-AGEISM INTERVENTION

Shannon Arnette, Jennifer Inker, Sarah Marrs, Maddie McIntyre, Waters Bert, and Tracey Gendron, *Virginia Commonwealth University, Richmond, Virginia, United States*

Ageism is a complex, multi-layered phenomenon impacting feelings, thoughts and behaviors toward self and others. Due to the complexity of ageism, evidence-based anti-ageism interventions have proved challenging and costly. To date, using the concept of elderhood as a mechanism to mitigate the negative impacts of ageism has not been explored. As an anti-ageism strategy, elderhood reframes later life as a stage that encompasses growth and development and expected loss and decline. The current study evaluated a brief video intervention among first-year medical students before participating in a year-long senior mentoring program. First-year medical students (N = 585) from 2018-2021 responded to open-ended questions after viewing the video. Thematic analysis revelated four themes: neutrality, elderhood as development, reframing stigma and elderhood as othering. Findings suggest that elderhood may be a viable and productive anti-ageism strategy.

### Session 4265 (Symposium)

### LEVERAGING FREE PUBLIC USE DATA FOR AGING AND LIFE COURSE RESEARCH

Chair: Phyllis Moen Discussant: Kathleen Cagney

This symposium will showcase life course and aging research that is possible using freely available integrated census and survey data available via IPUMS. This session is organized by the Network for Data-Intensive Research on Aging (NDIRA) initiative at the University of Minnesota's Life Course Center. NDIRA seeks to build and support an interdisciplinary community of scientists leveraging powerful data resources in innovative ways to understand health outcomes at older ages, as well as the demography and economics of aging. The session features papers that illustrate how to examine aging-related topics including health at older ages, work and socioeconomic conditions, and living conditions with a common thread of examining heterogeneity within groups. These papers all leverage freely available census and nationally-representative survey data, highlighting the potential value of these data for studying aging and the life course. By combining papers on an array of topics from a variety of data sources, this symposium highlights exemplar papers that demonstrate the types of novel research possible using public use census and survey data that NDIRA seeks to foster.

### DERAILED BY THE COVID-19 ECONOMY? OLDER ADULTS' PAID WORK BY INTERSECTIONS OF AGE, GENDER, RACE-ETHNICITY, AND CLASS

Phyllis Moen,[1] Joseph Pedtke,[2] and Sarah Flood,[1], *1. University of Minnesota, Minneapolis, Minnesota, United States, 2. University of Minnesota (Life Course Center), Minneapolis, Minnesota, United States*

This paper addresses the uneven employment effects on older Americans (Boomers and Genxers, ages 50-75) of the COVID-19 pandemic. Drawing on monthly CPS data from January through December 2020 (IPUMS) with an intersectional approach, we first chart shifts in employment and non-employment for population subgroups defined by age, gender and race/ethnicity, including explanations for not working (unemployment, retired, disabled, not in the workforce for other reasons – NILF-other). We then examine uneven transitions --monthly individual-level shifts out of and into paid work for population subgroups, considering also disparities by educational level. We find increases in proportions unemployed, especially for women in their 50s, as well as increases in the proportions reporting they are NILF-Other, especially for Asian and Hispanic women, with small increases for Asian and Hispanic men as well. There is little change in age-graded reports of being retired, regardless of gender or race/ethnicity, though there are education-level effects.

### LONG-TERM EFFECTS OF SOCIAL INSURANCE ON ADULT MORTALITY: EVIDENCE FROM THREE SOCIAL PROGRAMS IN MEXICO

William Dow,[1] Susan Parker,[2] and Emma Aguila,[3], *1. UC Berkeley, Berkeley, California, United States, 2. University of Maryland, College Park, Maryland, United States, 3. Sol Price School of Public Policy, University of Southern California, California, United States*

Research on the mortality effects of social insurance programs for older adults has generated conflicting results. Some studies suggest important health benefits, others find no effects, and still others find unintended adverse effects potentially linked to pathways such as increased obesity. Evidence has focused predominantly on short-run effects rather than net long-run mortality effects and their effects on the health of older adults has been particularly understudied. Mexico offers a unique opportunity for studying the long-run effects of social programs on adult mortality. Within a ten-year period, Mexico introduced the following influential social insurance programs: Progresa conditional cash transfer (CCT) program in 1997, 70 y más unconditional cash transfer (UCT) program for older persons in 2007, and Seguro Popular, a public health insurance program (PHI) for the uninsured, in 2004. In this paper we analyze effects on mortality for middle-age and older adults, by gender, 10-20 years after program implementation.

### CHANGES IN SOCIODEMOGRAPHIC AND DISEASE PREVALENCE AMONG FIVE BIRTH COHORTS OF OLDER LATINOS

Jennifer Ailshire,[1] and Catherine Garcia,[2], *1. University of Southern California, Los Angeles, California, United States, 2. University of Nebraska - Lincoln, Lincoln, Nebraska, United States*

Latinos are often treated as an amalgamated group without respect to Latinos' composition included in sampling designs in different periods. This matters because the Latino population is continuously changing over time with respect to migration patterns, socioeconomic status, sociocultural characteristics, and geographic dispersion across the U.S., which may influence disease patterns in later life. We use data from the Health and Retirement Study and the National Health Interview Survey to investigate changes in older Latinos' composition by examining five birth cohorts. Results indicate that there have been significant demographic and health changes over time among older Latinos, with later-born cohorts more racially and ethnically diverse, more educated, and exhibiting a higher prevalence of hypertension, diabetes, and obesity. Understanding these shifting dynamics is imperative for crafting strategies and public policies that meet this group's health needs, reduce the cost of health care, and increase the quality of life for older Latinos.

### INFLUENCE OF LABOR MARKET DISPARITIES ON SEX AND GENDER INEQUALITIES IN COGNITIVE DECLINE

Precious Esie,[1] Jennifer Manly,[2] and Justina Avila-Rieger,[3], *1. Columbia University, New York, New York, United States, 2. Columbia University, Columbia University, New York, United States, 3. Taub Institute for Research on Alzheimer's Disease and the Aging Brain, New York, New York, United States*

State-level labor market disparities have been linked to health outcomes. The current study examines how labor market disparities may shape different patterns of sex/gender inequalities in cognition across race/ethnicity, place, and time. We leverage cognitive outcome data from multiple cohort and nationally representative longitudinal studies, as well as historical data on labor force participation and occupational status from IPUMS CPS. Multilevel modeling analyses was used to examine heterogeneity in sex/gender inequalities in cognitive trajectories within and between race/ethnicity and U.S. state of birth and determine whether such variability is explained by a state-level labor market opportunity composite. We expect women to demonstrate an advantage over men on cognitive measures. Women's advantage will be more pronounced in states with a small sex/gender gap in labor market opportunities and less pronounced in states with a large gap. The magnitude of this advantage will be greater for White women compared with Black women.

### HOUSEHOLD STRUCTURE AND OLDER PERSONS

Sandile Simelane,[1] Tapiwa Jhamba,[1] Rachel Snow,[1] and Sainan Zhang,[2], *1. United Nations Population Fund, New York, New York, United States, 2. UNFPA, New York, New York, United States*

This research explores the life circumstances of older persons (aged 60 years and above), focusing on the sociodemographic and socioeconomic conditions of those who live alone. We situate the living arrangements of older persons within the global context of changing household structures in 76 countries from all regions of the world. Older persons who live alone are among those most likely to need governmental and other forms of social support. The analysis presented here is crucial for supporting policy responses to the needs of older persons, including the special attention they require during the current COVID-19 crisis. It also supports the operationalization of the Madrid International Plan of Action on Ageing (MIPAA)(United Nations, 2002), the realization of United Nations Principles for Older Persons (United Nations, 1991), and the broader framework of the Programme of Action of the International Conference on Population and Development(ICPA-POA).

### Session 4270 (Paper)

### LOCAL, STATE, AND FEDERAL POLICIES

### COMMUNITY OPTIONS TO FUND AGING SERVICES: A NATIONAL STUDY TO TRACK LOCAL INITIATIVES

Athena Koumoutzis, Jennifer Heston-Mullins, Pamela Mayberry, and Robert Applebaum, *Miami University, Oxford, Ohio, United States*

The majority of federal support for older people needing in-home services and supports comes from the Medicaid program. However, less than 10% of older people are eligible for Medicaid and to receive long-term services, a person must have a severe disability. Many older people with moderate levels of disability or those who are not impoverished are not eligible. In response to these system limitations, some counties across the nation have developed alternative funding strategies, such as property tax levies, to better serve older members of their communities. After identifying 15 states with such initiatives, a survey was distributed to 414 contacts within these states, with a response rate of 55%. Respondents included organizations such as area agencies on aging, councils on aging, and county departments on aging. Local funding varied within and across states, with annual funding ranging from $8,000-$47 million. Most commonly provided services with local funds include home-delivered (81%) and congregate (73%) meals, transportation (61%), and homemaker services (49%). A majority of programs (63%) indicated that local funds are used to provide at least one family or friend caregiver service. This study is the first compilation and description of locally-funded elder service initiatives in the U.S. Locally-funded initiatives can help older people with long-term services needs continue to live in their own homes and communities. On the other hand, some have raised questions about whether this is a good approach to funding aging services, raising concerns that this will lead to further inequities across states and communities.

### DISTINGUISHING FEATURES OF AAA-HEALTHCARE PARTNERSHIPS: BUSINESS ACUMEN IS NECESSARY BUT NOT SUFFICIENT

Leslie Curry,[1] Emily Cherlin,[2] Adeola Ayedun,[1] Chris Rubeo,[3] Traci Wilson,[4] Amanda Brewster,[3] and Jane Straker,[5], *1. Yale School of Public Health, New Haven, Connecticut, United States, 2. School of Public Health, New Haven, Connecticut, United States, 3. University of California, Berkeley School of Public Health, Berkeley, California, United States, 4. National Association of Area Agencies on Aging, Washington, District of Columbia, United States, 5. Miami University, Oxford, Ohio, United States*

Stronger relationships among service providers in the health care and social service sectors may contribute to positive outcomes such as lower health care use and spending. Such partnerships have grown in recent years, including Area Agencies on Aging (AAAs) contracting with health care organizations, and their impact on health care utilization has been demonstrated. Nevertheless, knowledge about how AAAs establish and manage successful collaborations is limited. This study was designed to understand how AAAs in regions with low levels of avoidable health care utilization develop and sustain partnerships with health care organizations. We conducted an explanatory sequential mixed-methods, positive deviance study. In the quantitative phase, we identified 8 AAAs with multiple health care partners serving areas with little utilization of nursing homes by residents with low-care needs, and 3 with few partners and high utilization for comparison. In the qualitative phase, we identified key informants within AAAs and their partners for in-depth interviews (total n = 123). We used the constant comparative method of analysis to identify 5 factors that characterized partnerships in the highly-partnered, low-utilization sites: 1) Regional context (e.g., breadth of health care provider market, cross-sectoral coalitions), 2) AAA human resource assets (e.g., community expertise, business acumen), 3) AAA organizational culture (e.g., visionary leadership, risk taking), 4) Interdependence among organizations (e.g., mutual benefit, alignment), and 5) Interpersonal dynamics (e.g., trust, relationships). The importance of these regional, organizational, and relational factors suggests that AAA business acumen is necessary but not sufficient to build and sustain robust cross-sectoral partnerships.

### FEDERAL INTERVENTIONS TARGETING SOCIAL ISOLATION AND LONELINESS: AN EXPLORATORY REVIEW

Lauren Palmer,[1] Jennifer Howard,[2] Helena Voltmer,[3] Abigail Ferrell,[4] Natalie Mulmule,[5] Abbie Levinson,[3] and Sarita Karon,[3], *1. RTI International, RTI International, Massachusetts, United States, 2. RTI International, Durham, North Carolina, United States, 3. RTI International, Waltham, Massachusetts, United States, 4. Lake Erie College of Osteopathic Medicine, Erie, Pennsylvania, United States, 5. RTI International, RTI International, North Carolina, United States*

Even prior to the COVID-19 Public Health and Medical Emergency, the experiences of chronic social isolation and loneliness (SIL) were growing among older adults. Countries began increasing national visibility for these issues and implementing programs and services focused on addressing them. In the United States (US), however, little is known about successful national interventions or their effectiveness in tackling SIL among older Americans. We conducted a rapid review of the peer-reviewed and grey literature from 2009-2019, focusing on existing federal programs, health systems, and health care models in the US that address SIL among older adults. Of the 110 articles identified, 36 met the inclusion criteria and were synthesized. Our review found few federal interventions that directly address SIL; several may be addressing SIL as an auxiliary outcome to addressing social determinants of health, such as group exercise, transportation support, or food insecurity. While these interventions may provide a promising opportunity, implementation and evaluation challenges were identified. Thus, federal and state agencies face significant obstacles to understanding the impact of existing interventions and their effectiveness in addressing SIL, hampering progress toward large scale implementation. As SIL receives increasing attention, we add another voice to existing literature that indicates significant heterogeneity among existing programs; we found that few evidence-based, scalable federal initiatives exist in the US that target SIL. Without resources from federal and state agencies, the ability of health entities, community-based organizations, and direct care providers to implement effective interventions is significantly diminished.

### THE EFFECT OF INCREASING STATE MINIMUM WAGE ON FAMILY CAREGIVING

Eric Jutkowitz,[1] Derek Lake,[1] and Peter Shewmaker,[2], *1. Brown University, Brown University, Rhode Island, United States, 2. Brown Universtiy, Brown University, Rhode Island, United States*

Paid long-term care workers, such as personal care aides, compliment family caregivers in the delivery of care for people with long-term care needs. Nearly 12% of paid long-term care workers live in poverty. There is a call to raise the federal minimum wage from $7.25/hr to $15/hr. Long-term care workers may financially benefit from an increase in minimum wage, but families that rely on paid long-term care may be unable to afford higher wages. We obtained Health and Retirement Study (HRS; 2006-2014) respondents' state of residence which we linked with state minimum wage data. Between 2006 and 2010 the federal minimum wage increased from $5.15 to $7.25. We identified 25 states in which the 2006 to 2010 (pre period) increases in federal minimum wage increased the state's effective minimum wage (higher of state and federal minimum wage). Seven of these states continued to increase their minimum wage from 2010 to 2014 (post period). The remaining 18 matching control states did not increase their minimum wage after 2010. We used a difference-in-differences design and ordinary least squares regression to compare hours of unpaid and paid caregiving HRS respondents received in treatment and control. There was no statistically significant change in unpaid (-2.15; 95%CI: -8.53, 4.23) or paid (2.42; 95%CI: -1.33, 6.20) caregiving hours received between HRS respondents that lived in states that did and did not increase their minimum wage. Increasing state minimum wage may improve the economic wellbeing of long-term care workers without adversely affecting people with long-term care needs.

### THE EFFECT OF MEDICAID ENROLLMENT ON FAMILY CAREGIVING

Eric Jutkowitz,[1] Peter Shewmaker,[2] Derek Lake,[1] and Momotazur Rahman,[2], *1. Brown University, Brown University, Rhode Island, United States, 2. Brown Universtiy, Brown University, Rhode Island, United States*

Most people with long-term care needs rely on family caregivers. People with long-term care needs are also more likely to be eligible for Medicaid, which is the largest public payer of home and community based long-term care services. Whether enrolling in Medicaid compliments or substitutes for family caregiving is unknown. We linked Health and Retirement Study (HRS) respondents with their Medicaid enrollment data (2002-2012), to determine the effect of enrolling in Medicaid on family caregiving hours. We identified 130 people that participated in the HRS interview prior to enrolling in Medicaid in the same year (i.e., untreated) and 142 people that participated in the HRS interview after recently enrolling in Medicaid (i.e., treated). Untreated and treated respondents had similar demographic characteristics (age, sex, race). We estimated a series of inverse probability weighted linear regression adjusted models to determine the difference in monthly family caregiving hours between individuals that newly enrolled in Medicaid compared to people that had yet to enroll. We controlled for HRS respondents' demographics, health care utilization, and nursing home utilization. HRS respondents interviewed after enrolling in Medicaid received 5.98 (95%CI: -27.60, 39.57) fewer monthly hours of family caregiving than respondents that had yet to enroll in Medicaid. HRS respondents interviewed after enrolling in Medicaid were not statistically more likely to receive any family caregiving (risk difference 0.05% 95%CI: -0.16, 0.06) than HRS respondents that had yet to enroll in Medicaid. Initial enrollment in Medicaid does not substitute for family caregiving.

### Session 4275 (Paper)

### LONG-TERM CARE POLICY

### DISCRIMINATION IN LONG-TERM CARE FACILITIES: LEGAL CONSCIOUSNESS AND PROBLEM-SOLVING AMONG STAFF

Angela Perone, *University of Michigan, Ypsilanti, Michigan, United States*

Over half of direct care workers in long-term care facilities are women of color. Building on legal consciousness theory–which explains how individuals invoke legal principles to define everyday experiences–this study examines how staff understand and resolve discrimination between residents and staff and among staff. This study employs a multi-method qualitative extended comparative case approach. Data includes in-depth semi-structured interviews (n=80) and participant and non-participant observation (n=8 months) at two facilities that vary in staff racial composition. Findings reveal rampant unreported instances of race and sex discrimination toward Black staff by white staff and residents. Black staff at all levels did not invoke rights or discrimination rhetoric when they experienced overt race discrimination by residents but engaged in significant emotional labor to respond to race discrimination by residents. Black staff, however, perceived microaggressions and unequal treatment by white staff as discrimination. At both facilities, floor staff and management adopted diverse team approaches across race and staff hierarchy for responding to race discrimination by residents toward Black female staff. These findings suggest the need for new and targeted policy and practice approaches that recognize extensive emotional labor expended by staff of color when addressing discrimination by residents and challenges from white staff when addressing race discrimination by staff. These findings have theoretical implications by extending legal consciousness theory to multi-level staff understandings of discrimination. Findings also provide useful tools and case examples for policymakers and practitioners interested in racial justice, particularly given how COVID has exacerbated racial inequities in long-term care.

### HOW LONG-TERM CARE QUALITY ASSURANCE MEASURES ADDRESS DEMENTIA IN AUSTRALIA, ENGLAND, JAPAN, AND THE UNITED STATES

Michael Lepore,[1] David Edvardsson,[2] Ayumi Igarashi,[3] and Julienne Meyer,[4], *1. LiveWell Alliance, Southington, Connecticut, United States, 2. La Trobe University, La Trobe University Melbourne, Victoria, Australia, 3. The University of Tokyo, Bunkyo, Tokyo, Japan, 4. City, University of London, Guildford, England, United Kingdom*

The prevalence of people with dementia living in long-term care (LTC) is high and rising internationally, and the need to improve LTC for people with dementia is widely recognized. In some countries, LTC quality assurance programs use quantitative measures of LTC quality, and international bodies emphasize the importance of person-centered care and healthy ageing outcomes. To better understand how LTC quality assurance programs address dementia, programs were reviewed in four countries—Australia, England, Japan, and the United States. Quality measures from each program were identified (n = 38) and examined to determine how they address dementia. Most measures did not address dementia, but four risk-adjusted for dementia (antipsychotic use, fractures, falls, mobility), one was dementia-specific (dementia/delirium hospitalizations), and one excluded people with dementia (losing bowel/bladder control). The other 32 measures were calculated equally regardless of the prevalence of dementia among LTC residents. Overall, LTC quality measurement differs internationally, but few measures address dementia. When dementia is addressed in quality measure calculations, it is most often as a risk-adjustor. Risk adjustment can help with attributing performance on these measures to the LTC setting rather than to the types of residents that the setting serves, but risk adjustment factors also are highly amenable to fraud, and thus require ongoing monitoring. Although LTC quality assessment programs and measures can help ensure people with dementia have access to quality LTC, adoption of measures that are meaningful to people with dementia—including measures of person-centered care and healthy ageing outcomes—remains needed.

### LONG-TERM CARE INSURANCE POLICY INTENSITY AND THE POLICY MATCHING DEGREE OF PILOT CITIES IN CHINA

Rong Peng,[1] and Bei Wu,[2], *1. School of Economics, Guangzhou, Guangdong, China (People's Republic), 2. New York University, New York, New York, United States*

Since China launched long-term care insurance (LTCI) pilot in 15 cities in 2016, the LTCI policy schemes have been developed gradually. Based on the 61 LTCI policy documents, this study evaluated the LTCI policies intensity in each pilot area by building a PMC index model with 10 primary variables and 44 two-level variables. Using the coupled coordination model, the coordination development indexes were calculated to evaluate the level of matching between LTCI policies and economy development and population structure. The results showed that the PMC index valued between 0.646 and 0.922. The three cities having the highest level of policy strength (PMC>0.8) were as follows: Qingdao, Nantong, and Jingmen. The three cities having the lowest level (PMC<0.7) were Ningbo, Qiqihar and Chongqing. The indexes of the coordination degree varied from 0.263 to 0.594. Shanghai, Qingdao and Guangzhou had the highest level of coordination degree, while Anqing, Chende and Qiqihaer are the lowest .The difference of financing mechanism of pilot cities was one of the main determinants of policy intensity. The matching degree was relatively low. Qingdao was a unique city having both high policy intensity and matching degree. It was suggested that the intensity and matching degree of LTCI policies should be improved to develop a national LTCI system in China.

### PROVIDING NURSING HOME CARE IN AN ERA OF INCREASING SCARCITY: THE CASE OF PENNSYLVANIA

Edward Miller,[1] Molly Wylie,[2] Elizabeth Simpson,[3] and Marc Cohen,[3], *1. University of Massachusetts Boston, Boston, Massachusetts, United States, 2. University of Massachusetts Boston, University of Massachusetts boston, Massachusetts, United States, 3. University of Massachusetts Boston, University of Massachusetts Boston, Massachusetts, United States*

Medicaid financing of nursing home (NH) care provides the strongest safety net for low income older adults, persons who have high-intensity long-term care (LTC) needs, and consumers with exorbitant LTC costs. Yet, NHs currently face serious threats to their financial viability, particularly in the context of the COVID-19 pandemic, where the costs of caring for residents in a safe way have increased significantly, even as the ability to recoup these costs from the Medicaid program has been constrained. The purpose of this study is to assess key demand and supply factors affecting the performance of the NH industry in Pennsylvania over time. It draws from several large, national data sources, including NH Compare, LTCFocus.org, the U.S. Bureaus of the Census and Labor Statistics, and Certification and Survey Provider Enhanced Reports, as well as state-level population projections and Departments of Health and Human Services data. An aggregate database was constructed with historical data points at the facility, regional, and state level. Annual total and regional trends were examined from 2010 to 2020. Findings suggest a growing gap between what NHs require to meet the needs of residents and the level of reimbursement paid by the largest funder: Medicaid. Considering demographic trends, this gap will only grow over time in the absence of policy change. The pandemic has further highlighted the existing challenges resulting from an underfunded service infrastructure and the need for additional investment if NHs are to provide high quality care to a growing cohort of older adults requiring support.

### USING POPULATION-LEVEL DATA TO ASSESS NEED FOR AND USE OF LONG-TERM SERVICES AND SUPPORTS IN CALIFORNIA

Kathryn Kietzman,[1] Lei Chen,[2] and Rebecca Allen,[3], *1. University of California, Los Angeles, UCLA Center for Health Policy Research, California, United States, 2. University of California, Los Angeles, Los Angeles, California, United States, 3. University of Alabama, Tuscaloosa, Alabama, United States*

In response to aging and disability stakeholder advocacy in California, the state's 2018-19 budget included support for the development of a study of Californians with needs for long-term services and supports (LTSS). Existing data on LTSS most typically represents those who already use specific programs or services. Yet many programs do not uniformly collect and report data, or have little capacity to share data across different delivery systems. In response to these gaps, we developed a 15-minute follow-on survey to the 2019-2020 California Health Interview Survey (CHIS), gathering statewide population-level data to assess LTSS needs and use by Californians 18 years of age and older. This paper reports on preliminary findings from the 2019 CHIS-LTSS survey conducted with a sample of 1097 respondents. Screening questions identified respondents reporting difficulties with concentrating, remembering, or making decisions (60%), performing basic daily activities such as dressing or bathing (26%), or getting out of the house to shop or to see the doctor (52%). Nearly half of respondents (45%) reported needing help with routine care needs while 16% needed help with personal care needs. Additional findings illustrate specific LTSS needs, service use, consequences of unmet needs, financial concerns, and consumer experiences. At a time when California policy makers, program planners, and advocates are engaged in implementing a 10-year Master Plan for Aging, these findings can be used to identify and address gaps in the types of services and supports that are essential to meet the LTSS needs of older adults and people with disabilities.

### Session 4280 (Paper)

### MENTAL HEALTH AND AGING

### AN INNOVATIVE MENTAL HEALTH MODEL FOR TREATING CULTURALLY DIVERSE OLDER ADULTS

Tobi Abramson,[1] Jacquelin Berman,[2] and Madison Gates,[1] *1. NYC Department for the Aging, NYC, New York, United States, 2. NYC Department for the Aging, NYC Department for the Aging, New York, United States*

The mental health needs of older adults are largely unmet, a finding even more prevalent within culturally diverse older adult populations. Added to this is the high rate of social isolation. Research has indicated increased connection to mental health services when services are embedded within physical health care settings. For those attending community centers, 85% indicate that they are socially isolated, 68% indicate they are lonely, and 53% have a mental health need (compared to 20% nationally). The need for innovative programming is evident. When examining the needs of diverse older adults, it is increasingly important that new and innovative approaches address social isolation, loneliness, and mental health problems experienced by this cohort. Utilizing this knowledge an innovative model of embedding and integrating mental health services, provided by bilingual and bicultural clinicians, into congregate sites (older adult centers) was implemented. Those that participated were mainly female (72.1%), 68.5% English-speaking, 14.5% Spanish-speaking, 13.6% Chinese-speaking and 3.4% other. Spanish-speakers had more depression than English-speakers and both had more depression than Chinese-speakers. English and Spanish-speakers reported more social isolation and Chinese-speakers compared were more likely to participate in engagement. Chinese-speakers were less likely to be in clinical services with a positive screen compared to English-speakers. Overall, 75% engaged in treatment; 37.3% and 41% showed a 3-month improvement of depression and anxiety, respectively. This presentation focuses on the innovative components of this model, how to engage diverse older adults to utilize treatment, steps needed for replication, and policy implications around integrated mental health treatment.

### ASSOCIATION OF SOCIAL RELATIONSHIPS AND DEPRESSION AMONG OLDER MEXICAN ADULTS: DOES A RURAL OR URBAN CONTEXT MATTER?

Paige Downer, and Rebeca Wong, *University of Texas Medical Branch, Galveston, Texas, United States*

Social characteristics such as strong community and family ties have been associated with positive mental health outcomes in older adults. However, this evidence is based primarily on non-Hispanic White populations and may vary according to living in a rural versus urban community. We hypothesize that the positive impact of available social networks, perceived support, and social participation on older Mexican adults' likelihood for high depressive symptoms (i.e., depression) will be greater for those living in rural (community < 2,500 people) than urban communities. Data came from the 2012 Wave of the Mexican Health and Aging Study. Depressive symptoms were measured using a 9-item version of the Center for Epidemiologic Studies Depression Scale. Social participation is the respondent's self-reported participation in hobbies, religious activities, volunteering, and visits with neighbors. Available social network is measured as having relatives and/or good friends living in the neighborhood. Perceived support is the respondent's perception of friends/family's willingness to help with finances and personal care. The final sample of 6,266 respondents was majority (62.4%) female, mean age of 69 years, 17.8% lived in a rural community, and 34.5% with depression. Logistic regression models stratified by rural/urban indicated that available social network and perceived social support were not associated with depression in rural or urban communities. In general, the social participation activities were associated with significantly lower odds of depression for older adults living in urban but not rural communities. This research highlights the influence of older adults' community on their social relationships and mental health.

### THE EFFECTS OF ASSETS AND DEBT ON THE DEPRESSION TRAJECTORY OF LOW-INCOME OLDER ADULTS IN SOUTH KOREA

Jihee Woo,[1] and Hyojin Choi,[2], *1. University of Pittsburgh, Pittsburgh, Pennsylvania, United States, 2. Sungkyunkwan University, Seoul, Seoul-t'ukpyolsi, Republic of Korea*

Evidence suggests that assets and debt are among the most significant factors affecting older adults' mental health. This study focuses specifically on South Korea, where the poverty rate of older adults is the highest among all OECD countries. Given that low-income older adults with fewer assets and more debt may be at greater risk for depression, we investigated how assets and debt affected the depression trajectory of low-income older adults in South Korea. We used the six most recent waves of data from the Korean Welfare Panel Study (2014-2019) to estimate the longitudinal effects of assets and debt on depression in low-income older adults. Our sample was restricted to low-income Korean heads of household aged 55 and above (N=2,832). Using latent growth curve modeling, the unconditional model revealed decreasing trends in depression over time, while the conditional model, controlling for sociodemographic variables (i.e., age, gender, education, general health, marital status, employment status, income), suggested that assets and debt had contrasting impacts on depression. Specifically, although it did not impact the depression trajectory, debt did have a positive impact on depression at baseline. Most notably, assets negatively affected both depression at baseline (B=-1.911, SE=0.284, p<.001) and its trajectory (B=-0.235, SE=0.081, p<.01). These findings highlight the importance of holding assets over time as a protective factor against depression and thus the need for interventions such as savings programs and financial education for low-income older adults.

### THE LONG ARM OF CHILD MALTREATMENT AND MENTAL HEALTH IN LATER LIFE: THE EFFECTS OF MALTREATMENT AND FAMILY CONTEXT?

Chengming Han,[1] Tirth Bhatta,[2] Eva Kahana,[3] and Brian Gran,[1], *1. Case Western Reserve University, Cleveland, Ohio, United States, 2. University of Nevada, Las Vegas, Nevada, United States, 3. Case Western University, Case Western reserve University, Ohio, United States*

Purpose. This article examines the role of family context in shaping the influence of childhood maltreatment on later life psychological well-being in the cultural context of Chinese society. Method. Data were drawn from the China Health and Retirement Longitudinal Study (CHARLS) baseline. Maltreatment was measured by corporal punishment by either mother or father in childhood. We used family violence, parents' family socioeconomic status (SES) and mental health to represent family context. Result. Our ordinary least square regression analysis shows that corporal punishment administered by a mother was associated with higher depressive symptoms (b=0.308, p<0.05) in later life while being hit by father did not result in higher depressive symptoms. Family contexts had residual ("long arm") influence on respondents' mental health: violence in the family, including being hit by siblings (b=0.657, p<0.001) and witnessing violence between parents (b=0.658, p<0.001) contributed significantly to higher depressive symptoms. Conclusion. Corporal punishment by parents had long term effects on mental health of their children in later life. Cultural values, such as filial piety did not eliminate the negative impacts of being hit in childhood on mental health in later life. Family contexts including violence between parents also played important roles in shaping the relationship between child maltreatment and mental health in later life. Implication. Our study offers important insights about the complex matrix of cultural traditions, social circumstances and diversity in dealing with child rearing stress and their consequences for later life mental health.

### USE OF COMMUNITY-BASED PARTICIPATORY RESEARCH IN MENTAL HEALTH STUDIES WITH OLDER ADULTS: A SYSTEMATIC REVIEW

Jessie Ho-Yin Yau,[1] Walker Siu Hong Au,[1] Tianyin Liu,[1] Anna Y Zhang,[2] Gloria HY Wong,[1] and Terry YS Lum,[1], *1. The University of Hong Kong, Hong Kong, Not Applicable, Hong Kong, 2. The University of Hong Kong, The University of Hong Kong, Not Applicable, Hong Kong*

Community-based participatory research (CBPR), a bottom-up approach that community stakeholders and academics are involved equitably, is an effective approach for enhancing relevance and value in public health research and has gained popularity in recent decades. However, little is known about how CBPR can be used in mental health studies with older adults. This systematic review examined the current state of knowledge about how CBPR approach has been adopted in mental health research among older adults in different societies. According to the PRISMA guidelines, we searched five major databases and screened the literature using these criteria: 1) journal articles reporting use of CBPR in mental health research among older adults, 2) articles published in English language, 3) studies conducted in any settings with any mental health research. Initial search found 3,227 articles and preliminary screening identified 23 eligible articles. We found that around 90% of studies were conducted in the West. Most studies adopted CBPR to develop community-based mental health interventions or to revise current interventions or models while addressing the cultural needs of their studied population. Few studies adopted CBPR to evaluate existing mental health workshops or programmes. The extent of involvement of older adults in the CBPR approach varied across studies, from questionnaire design to programme evaluation. Our review uncovered ways of CBPR implementation across different societies and elements of successful implementation in CBPR practices in mental health research among older adults.

### Session 4285 (Symposium)

### PODCASTS AND YOUTUBE VIDEOS: INNOVATIVE TOOLS FOR DISSEMINATING MENTAL HEALTH AND DEMENTIA EDUCATION TO THE PUBLIC

Chair: Regina Koepp Co-Chair: Natali Edmonds

This symposium will discuss two examples of innovative public education tools used to disseminate evidence-based information to the general public about mental health and aging and Alzheimer's Disease and related dementias. The first is the Psychology of Aging Podcast hosted by Regina Koepp, Clinical Geropsychologist. Since it's launch in April 2020, there have been 50 weekly episodes and more than total 25,000 downloads. The goal of the Psychology of Aging podcast is to facilitate access to information and education about mental health and brain health among older adults with the hope of de-stigmatizing mental health care for older adults, reducing ageism, and promoting access to mental health and dementia care for older adults and their families. The second is Dementia Careblazers, created and hosted by Dr. Natali Edmonds, board certified Geropsychologist. The goal of Dementia Careblazers videos is to offer easy to access information to family members who care for someone with dementia. In her weekly videos, Dr. Edmonds provides actionable, evidence-based information and resources focused on dementia caregiving in brief videos. Since it's launch on YouTube November 2016, Dementia Careblazers, has 65,000 subscribers, has posted 231 videos, and has had more than 4 million views nationally and internationally. Drs. Koepp and Edmonds will discuss the role podcasts and YouTube videos play in public education and share tips for professionals wanting to start an evidence-based program of their own. This virtual modality may be of increased interest considering recent health risks through face to face interactions and advancements in technology.

### PSYCHOLOGY OF AGING PODCAST: PUBLIC EDUCATION TOOL FOR SHARING EVIDENCE-BASED MENTAL HEALTH AND AGING INFORMATION

Regina Koepp, *Center for Mental Health & Aging, Brookhaven, Georgia, United States*

Psychology of Aging Podcast, created and hosted by Dr. Regina Koepp, Clinical Geropsychologist, is the first podcast of its kind devoted solely to mental health and aging. The goal of the Psychology of Aging podcast is to facilitate access to information and education about mental health and brain health among older adults with the hope of de-stigmatizing mental health care for older adults, reducing ageism, highlighting diversity, and promoting access to mental health and dementia care. The format includes a combination of expert interviews and "solo-casts". Topics range from depression and suicide prevention to Alzheimer's Disease and related dementias to the unique needs of LGTBQ older adults and caregivers to health disparities experienced by African American and Latin-X communities related to dementia and the COVID-19 pandemic. During this session, Dr. Koepp will discuss the role podcasts play in public education and share tips for starting an evidence-based podcast.

### DEMENTIA CAREBLAZERS YOUTUBE: PUBLIC EDUCATION TOOL FOR SHARING EVIDENCE-BASED DEMENTIA CARE INFORMATION

Natali Edmonds, *Owner and Founder, Phoenix, Arizona, United States*

Dementia Careblazers, created and hosted by Dr. Natali Edmonds, board certified Geropsychologist, offers weekly YouTube videos to family caregivers of people living with dementia. These free brief videos provide actionable, evidence-based information and resources focused on dementia caregiving. This virtual modality is particularly relevant for caregivers of people living with dementia given the difficulty family caregivers have in finding supervision and care for the person with dementia in their absence and considering recent health risks through face to face interactions. Furthermore, the free archive of Dementia Careblazers videos allows for access to evidence-based dementia care information at any time, regardless of geographic location or time zone. During this session, Dr. Edmonds will discuss the role YouTube videos play in public education and share tips for starting an evidence-based YouTube channel.

### Session 4295 (Paper)

### RELIGION AND SPIRITUALITY

### A MULTIDIMENSIONAL CONSTRUCT OF RELIGIOSITY AMONG BABY BOOMERS AND TRAJECTORIES OF SOCIAL ATTITUDES

Joonsik Yoon,[1] Woosang Hwang,[2] Maria Brown,[2] and Merril Silverstein,[2], *1. Syracuse University, Syracuse, New York, United States, 2. Syracuse University, Syracuse, New York, New York, United States*

Although a number of studies have examined relationships between religiosity and social attitudes, less is known about how these relationships change over the life course using a multidimensional construct of religiosity among Baby Boomers. A multidimensional construct of religion allowed us to take a more person-centered approach to religiosity, whereby we examine the association between Baby Boomers with different types of religiosity and the trajectories of their political and gender role attitudes over a period of transition from early to later adulthood. We selected 798 young-adult Baby Boomers from the 1971 wave (mean age: 19 years) of the Longitudinal Study of Generations (LOSG) and tracked their political and gender role attitudes through until the 2016 wave (mean age: 64 years). Using latent class analysis, we identified four latent religious typologies: strongly religious, weakly religious, liberally religious, and privately religious. We found that Baby Boomers in the strongly religious class reported the most conservative political and gender role attitudes among the four classes over this period of transition. Baby Boomers in the privately religious class were conservative in their political and gender role attitudes than those in the weakly religious class. The liberally religious group generally reported the second most conservative political attitudes among the four identified groups, but reported the least conservative gender role attitudes of the four groups. Findings suggest that early religiosity may serve as a significant predictor affecting political and gender role attitudes throughout the adult life course.

### FAMILY CAREGIVERS OF PERSONS WITH MILD DEMENTIA SHARE THEIR SPIRITUAL STRUGGLES

Jocelyn McGee, Davie Morgan, and Dennis Myers, *Baylor University, Waco, Texas, United States*

The lives of family caregivers of persons with Alzheimer's disease and related dementias (ADRD) may change dramatically with disease progression in their loved one. Many rely on spirituality as a resource for coping. There is evidence that persons experiencing transition/losses, as a consequence of disease/illness, can experience spiritual struggles or a crises in meaning. However, there is limited research related to spiritual struggles among family caregivers of persons with ADRD, particularly in the beginning stages of the disease process. In this study, three domains of spiritual struggle were identified after analyzing 27 caregiver interviews using the constant comparative method: 1) changes in relationship with their higher power (e.g., feelings of anger towards, feeling punished by, feeling disconnected from, and questioning); 2) changes in spiritual practices (e.g., decreased participation as a consequence of feeling unsupported, judged, or misunderstood by spiritual communities); and 3) dissonance between previously held core beliefs and current life circumstances (e.g., feelings of shame, doubt, and guilt as well as cessation of self-care activities due to the belief that they must sacrifice everything for their loved one). Notably, 74% experienced spiritual struggle in one domain; 33% in two domains, and 11% in three domains. The majority of participants had come to resolution of these spiritual struggles by the time they were interviewed. However, 40.7% were experiencing ongoing spiritual struggles, at the time of interview, suggesting the importance of identifying and addressing spiritual struggles in this population over time in order to enhance coping and adaptation.

### HMONG OLDER ADULTS' END-OF-LIFE CARE PREFERENCES: PHYSICAL, PSYCHOSOCIAL, CULTURAL, RELIGIOUS, AND SPIRITUAL

Youhung Her-Xiong, *William S. Middleton Memorial Veterans Hospital, Madison, Wisconsin, United States*

As the Hmong community continues to grow and age in the United States (US), mainstream healthcare providers may encounter Hmong older adults who prefer their cultural end-of-life (EOL) care. The challenge for these providers is to offer culturally sensitive EOL care to Hmong older adults within the realm of the Western healthcare system. One factor contributing to this challenge may be the lack of knowledge regarding Hmong older adults' EOL care preferences. Another is Hmong EOL care is interwoven with care from domains such as culture, religion, and spirituality. The purpose of this study is to garner an understanding of the care preferences of Hmong older adults during the dying process. A qualitative study using inductive content analysis was conducted. Thirty Hmong older adults who reside in Wisconsin participated in semi-structured interviews that were audio recorded and transcribed. Data was analyzed using inductive content analysis by Elo & Kyngäs (2008). The findings revealed that participants preferred care at EOL in the domains: physical, psychosocial, cultural, religious, and spiritual. Physical care included ADL's while psychosocial care related to communication and companionship. Cultural care included children as caregivers and decision-makers. Religious and spiritual care surrounded Animism and Christian beliefs such as soul calling and prayers. Findings also suggest Hmong older adults' care preferences as heterogenous and holistic. The findings have implications for the Hmong community and formal care services to collaborate on how culturally sensitive care can be provided to Hmong older adults at end of life.

### RELIGIOSITY AMONG YOUNG-ADULT BABY BOOMERS: ASSOCIATIONS WITH PSYCHOLOGICAL WELL-BEING OVER 45 YEARS

Kent Jason Cheng,[1] Maria Brown,[2] Woosang Hwang,[2] and Merril Silverstein,[2], *1. Syracuse University, Syracuse, New York, United States, 2. Syracuse University, Syracuse, New York, New York, United States*

Past studies on the influence of religiosity on psychological well-being tended to be cross-sectional in nature and neglected generational differences. In this study, we assess how religiosity in early adulthood (mean age = 19) affects baby-boomers' psychological well-being over the life course. We used waves 1 to 9 or 45 years of survey data from the Longitudinal Study of Generations (LSOG) (N=798), a sample of Southern Californians. First, we used latent class analyses on five domains to identify three typologies of baby boomers' religiosity in early adulthood. We call these typologies "strongly religious," "weakly religious," and "personally religious." Then, we used latent growth curve modelling to ascertain the influence of these religiosity typologies on psychological wellbeing from waves 1 to 9, controlling for time-invariant (religious affiliation, age, sex, race, parental income)

and varying (religious salience, education, marital status, and annual income) factors. We found that the strongly religious have a consistently upward psychological wellbeing trend throughout the study period whereas wellbeing started to decline for the weakly religious and personally religious at around wave 6, on when they were about mid-40s to almost 50. We provide evidence that religiosity in early adulthood – a period in life characterized by the exploration of various options for the future brought about by greater personal freedom – positively influences baby boomer's psychological wellbeing over the life course.

### Session 4300 (Symposium)

### STRUCTURE AND CHANGE: NEW DEVELOPMENTS IN RESEARCH ON CUMULATIVE DIS/ADVANTAGE

Chair: William Dannefer Discussant: carroll Estes

In a time of heightened social inequality and concern to reckon with its sources and consequences, the relevance of cumulative dis/advantage (CDA) to understanding patterns of aging has become even clearer, and CDA research has continued to expand in several fresh directions. Papers in this symposium will review the current state of knowledge regarding CDA and will present new analyses addressing key questions of its intersections with social change and its structural patterning. We will begin with a review of knowledge on comparative evidence regarding cumulative dis/advantage and its cross-national patterning. With regard to change, will examine the compare the effect of the 2008 recession and subsequent recovery across generational cohorts through a comparative examination of trajectories of income inequality. We will also present evidence on the impact of gender, focusing on women's late-life health.

### CUMULATIVE DIS/ADVANTEGE, STRUCTURE, AND CHANGE: THREE NEW DIRECTIONS FOR CDA RESEARCH

Chengming Han,[1] and William Dannefer,[2], *1. Case Western Reserve University, Cleveland, Ohio, United States, 2. Case Western Reserve University, cleveland, Ohio, United States*

Over the past several decades, evidence for cumulative dis/advantage as a regular feature of cohort aging has continued to cumulate, while new questions concerning the underlying dynamics continue to emerge. This paper reviews the accumulated knowledge base, and focused on three recently emerging lines of inquiry that hold great promise for expanding more fully our understanding of CDA processes: 1) the intersection of class stratification and race in the operation of CDA processes, 2) factors accounting for cross-national variations, and 3) the intersection of robust intracohort processed that generate cda with intercohort processes and the impact of historical and social change. These three new directions are briefly discussed.

### CUMULATIVE DIS/ADVANTAGE, STRUCTURE, AND CHANGE: THREE NEW DIRECTIONS IN CDA RESEARCH

William Dannefer, *Case Western Reserve University, cleveland, Ohio, United States*

Over the past several decades, evidence for cumulative dis/advantage as a regular feature of cohort aging has continued to cumulate, while new questions concerning the underlying dynamics continue to emerge. This paper reviews the accumulated knowledge base, and focused on three recently emerging lines of inquiry that hold great promise for expanding more fully our understanding of CDA processes: 1) the intersection of class stratification and race in the operation of CDA processes, 2) factors accounting for cross-national variations, and 3) the intersection of robust intracohort processed that generate cda with intercohort processes and the impact of historical and social change. These three new directions are briefly discussed.

### IMPACT OF ECONOMIC SHOCKS ON CUMULATIVE ADVANTAGE PROCESSES: RECESSION, RECOVERY, AND TRAJECTORIES OF INEQUALITY

Stephen Crystal, *Rutgers University, Princeton, New Jersey, United States*

This study compares the effect of the 2008 recession and subsequent recovery across generational cohorts by evaluating age-cohort trajectories of income inequality. Using data from the 2007 to 2016 waves of the Survey of Consumer Finances, we examine the trajectory of inequality for the overall population and by cohort in years spanning the Great Recession and subsequent recovery. We find that increases in per-capita income and wealth observed at the population-level during the recovery were not reflected among households below the median, leading to increasing inequality. Within cohorts, we observe growing inequality within cohorts in their primary working years. Findings are consistent with a model of integrative cumulative dis/ advantage, which predicts increasing within-cohort inequality over the life course influenced both by persistent micro- and macro-level processes of increasing heterogeneity. Our analyses highlight the potential role of extreme business cycle fluctuations, booms and busts, to exacerbate this underlying process.

### TRAJECTORIES, TURNING POINTS, AND INTERRUPTIONS: HOW SOCIAL INEQUALITY SHAPES WOMEN'S LATER-LIFE HEALTH

Marissa Gilbert,[1] and Jessica Kelley,[2], *1. Case Western Reserve University, cleveland, Ohio, United States, 2. Case Western Reserve University, Celeveland, Ohio, United States*

We explore women's health in midlife and later life at the nexus of structural sexism and the life course perspective, applying Dannefer's (2018) concept of life course reflexivity, which emphasizes social dynamism with potential health-changing 'input' at all ages. We present three types of reflexive changes in the gendered life course that shape women's health as they age: (1) trajectories of lifetime labor market disadvantage leading to limited health-protective resources in later life; (2) turning points in family structure and need, with draining caregiving demands; (3) interruptions in midlife, such as divorce, erasing the social and economic benefits of marriage. We provide support for critical arguments that theoretical work on the life course has too-often utilized the 'privileged' or the 'male' life course with insufficient attention to structural sexism as a fundamental cause of women's health disparities in later life.

### Session 4305 (Paper)

### SUCCESSFUL AGING IN PLACE

### FUNCTIONAL MRI TO ASSESS DECISION-MAKING CAPACITY OF OLDER ADULTS WITH DEMENTIA: A PROOF OF CONCEPT STUDY

Thomas TANNOU, *Besançon University Hospital, BESANCON, Franche-Comte, France*

Assessment of decision-making capacity is essential to respect older adult dignity, particularly concerning major decision such as ageing in place. To date, it is the clinician's assessment, based on a global analysis of his clinical evaluation and neuropsychological tasks, which enables decision-making assessment. Given the difficulty it represents, and the ethical and societal issues raised, the research question concerns the contribution of neuro-imaging technologies as an aid to the evaluation of decision-making capacity. We included in our proof-of-concept study 4 healthy older patients and 2 older patients with dementia (mild stage) followed in a memory clinic. Each of the participants completed neuropsychological tests with a focus on executive functions, anosognosia and judgemental skills. Next, they performed a decision-making task, the Balloon Assessment Risk Task (BART) in functional MRI, and, finally, they participated in a semi-structured interview completed with interview of their caregiver. For both patients, their referring geriatrician was questioned a priori on his assessment of their decision-making capacity. The results showed a common activation pattern in functional MRI between the patient considered competent in decision-making and the healthy subjects, unlike the patient who was not clinically competent. The qualitative analysis highlighted major anosognosia in both pathological situations, but decision-making in everyday life situations differed between the 2 patients. This study shows the feasibility, on a sensitive topic, to explore the potential contribution of functional neuroimaging and semi-directed interviews as tools. It also demonstrates the value of conducting mixed research, combining neurosciences and social science to explore complex clinical issues.

### I WOULDN'T SEARCH THAT WITH MY MOBILE PHONE: CREDIBILITY AND TRUST IN OHIRS AMONG LOWER-INCOME BLACK OLDER ADULTS

Christina Harrington,[1] and Amanda Woodward,[2], *1. DePaul University, Chicago, Illinois, United States, 2. Michigan State University, East Lansing, Michigan, United States*

Online health information resources (OHIRs) such as conversational assistants and smart devices that provide access to consumer health information in the home are promoted as viable options for older adults to independently manage health. However, there is question as to how well these devices are perceived to meet the needs of marginalized populations such as lower-income Black older adults who often experience lower digital literacy or technology proficiency. We examined the experiences of 34 lower-income Black older adults aged 65-83 from Chicago and Detroit with various OHIRs and explored whether conversational resources were perceived to better support health information seeking compared to traditional online web searching. In a three-phase study, participants tracked their experiences with various OHIRs and documented health-related questions in a health diary. Participants were then interviewed about their diaries in focus groups and semi-structured interviews, followed by a technology critique and co-design session to re-envision a more usable and engaging conversational device. We present preliminary results of the themes that emerged from our analysis: cultural variables in health information seeking practices, perceptions of credibility, likelihood of use, and system accessibility. Participants indicated that their trust of different resources depended on the type of information sought, and that conversational assistants would be a useful resource that require less technology proficiency, even among those with lower e-health literacy. Although our findings indicate that familiarity and trust were salient constructs associated with perceptions of OHIRs, these devices may address digital literacy and technology familiarity with certain design considerations.

### MIGRATION AND AGING IN THE RIGHT PLACE: OLDER PUERTO RICAN ADULTS' NARRATIVES

Brooke Jespersen, *Case Western Reserve University, Cleveland, Ohio, United States*

This research examines meanings of aging in the "right" place (Golant, 2015) among older adults who have grown up and grown older in migratory contexts. This qualitative research is based on semi-structured and life history interviews with 30 low socio-economic status Puerto Rican adults over the age of 60 who reside in Cuyahoga County, Ohio and have engaged in Puerto Rico-US migration throughout the life course. Inductive thematic analysis of interviews revealed fraught, multi-scalar narratives of aging in the "right" place. At the level of residence type, older adults' narratives exhibited a tension between interdependence and independence. That is to say, they struggled to reconcile cultural preferences for family-based living arrangements with fears of becoming a burden. At the level of nation, a similar tension manifested. Older adults reported navigating differential citizenship rights, access to healthcare and social services, natural disasters, and experiences of social inclusion and exclusion via migration between Puerto Rico and the US mainland. Thus, aging in the "right" place was complex, if not altogether elusive, as inequitable circumstances obliged older adults to make tradeoffs regardless of where they lived. These findings extend scholarship on aging in the "right" place, which has focused on residence type, by considering how older adults negotiate aging within and across households, communities, and nations. Moreover, these findings highlight how challenging aging in the "right" place can be for migrating and disadvantaged populations.

### MOVING THE NEEDLE ON OUTCOME MEASUREMENT: A LONGITUDINAL ANALYSIS OF FRAILTY AMONG HOME-DELIVERED MEAL RECIPIENTS

Lisa Juckett,[1] Haley Oliver,[1] Leah Bunck,[2] Crystal Kurzen,[2] Andrea Devier,[2] and Fannisha Page,[2], *1. The Ohio State University, Columbus, Ohio, United States, 2. LifeCare Alliance, Columbus, Ohio, United States*

Frailty is a complex condition highly associated with malnutrition and can lead to the devastating loss of independence among older adults. Home-delivered meals (HDMs) aim to combat frailty and malnutrition and provide nutritional support to nearly 10 million older adults each year. Though self-reported metrics indicate that HDMs help older adults maintain their independence, few studies have systematically collected longitudinal data that objectively represent the health benefits of HDMs. The present study implemented two evidence-based instruments designed to measure frailty levels of HDM recipients (age 60 to 99 years) at two time points. HDM staff at one organization underwent multifaceted training to implement The Home Care Frailty Scale and the Clinical Frailty Scale with HDM recipients at the start of HDM enrollment and at three-month follow-up. Activity of daily living impairments ($B = .46$, $p < .001$) and instrumental activity of daily living impairments ($B = .28$, $p < .001$) were significant predictors of higher frailty levels at baseline ($N = 245$). Sixty-two recipients were analyzed at 3-month follow-up. Clinical Frailty Scale scores indicated stable frailty levels from baseline to follow-up (4.08 vs. 4.08). Home Care Frailty Scale scores indicated a slight increase in frailty levels (7.4 vs 7.63) though not statistically significant, $t(61) = -.34$, $p = .74$. These stable frailty metrics suggest that HDMs contribute to older adults' ability to remain living in their own homes and communities and can support the importance of increased financial investments in HDM programs at the state and national levels.

### WHAT KEEPS OLDER PEOPLE OUT OF NURSING HOMES? A META-ANALYSIS

Joseph Gaugler,[1] Rachel Zmora,[1] Colleen Peterson,[2] Lauren Mitchell,[3] Robyn Birkeland,[2] Eric Jutkowitz,[4] and Sue Duval,[1], *1. University of Minnesota, Minneapolis, Minnesota, United States, 2. University of Minnesota, University of Minnesota, Minnesota, United States, 3. Emmanuel College, Boston, Massachusetts, United States, 4. Brown University, Brown University, Rhode Island, United States*

Perhaps one of the most examined, and costly, health transitions older people experience is nursing home admission. In addition to the financial costs nursing home admission poses to older people, their families, and other payers (e.g., the public), institutionalization is linked with a range of negative outcomes and represents a loss of independence and quality of life to many older persons. The current meta-analysis attempted to synthesize all available randomized controlled trials available to ascertain which intervention approaches appeared to prevent nursing home entry for older adults. The MEDLINE, PsycInfo, CINAHL, Cochrane, and EMBASE databases were searched to August, 2020. Abstracts were screened ($N = 28{,}120$) to identify randomized controlled trials of interventions to prevent or delay nursing home admission as well as systematic reviews. Identified studies were cross-referenced until the point of saturation, resulting in 1,786 studies for additional inclusion/exclusion screening. Following a consensus-based review among the authors that included risk of bias, 323 randomized controlled trials were included in the meta analysis. Although several intervention modalities appeared protective against nursing home admission and approached statistical significance, preliminary results suggest that comprehensive geriatrics assessment (pooled OR = .69, 95% CI: .50, .95) and specialized, inpatient geriatrics care (pooled OR: .77, 95% CI: .59, .99) were most consistent in helping to prevent institutionalization among older persons. The findings emphasize the importance of geriatrics when delivering optimal care to older persons. Integrating such approaches more effectively into a largely fee-for-service healthcare paradigm remain a critical challenge.

### Session 4310 (Symposium)

### THE CANNABIS AND OLDER PERSONS STUDY: HAS SCIENCE CAUGHT UP WITH PRACTICE YET?

Chair: Brian Kaskie

Since 2016, the Cannabis and Older Persons Study has examined the increasing use of cannabis among Americans over 60 years old. Our current work dives into particular groups of cannabis users and explores outcomes related to medical conditions and symptoms. This symposium also features a range of methodological approaches from an analysis of the BRFSS caregiving and cannabis modules, a convenience sample of more than 4,000 older cannabis users enrolled in the Illinois Medical Cannabis Program and qualitative interviews conducted with aging veterans. Kanika Arora examines the association between informal caregiving and marijuana use and whether this association varies by age. Julie Bobitt shares findings from 32 interviews with older Veteran cannabis users. Alton Croker examines cannabis use as a complement or alternative to palliative care. HyoJung Kang clusters negative outcomes experienced by older persons who use cannabis. Brian Kaskie compares cannabis use among persons with Multiple Sclerosis (N=135) and persons diagnosed with arthritis (N=582) or cancer (N=622). While we certainly find reason to remain concerned that cannabis use alone and co-occurring use with prescription opioids may contribute to increased rates of substance misuse and other undesirable outcomes among older adults, we find it increasingly difficult to overlook the benefits many persons derive when taking cannabis as a method to manage pain or address other medical conditions. At this point, public policy officials and program administrator should strive to strike a balance between addressing cannabis harms relative to promoting benefits such as opioid reduction and diversion.

### COMPARING CANNABIS USE ACROSS DIAGNOSED CONDITIONS: APPLES AND ORANGES?

Brian Kaskie, *University of Iowa, Iowa City, Iowa, United States*

Although researchers have identified medications that relieve symptoms of Multiple Sclerosis (MS), none are entirely effective and some persons with multiple sclerosis (PwMS) use alternatives. Our study compared cannabis use among PwMS (N=135) and persons diagnosed with arthritis (N=582) or cancer (N=622) who participated in the Illinois medical cannabis program. We tested for significant differences across psychological well-being, quality of life and three behavioral outcomes, and also considered effects of co-occurring prescription opioid use. A majority of all individuals used cannabis to address pain and improve quality of sleep. PwMS reported lower levels of productivity, exercise and social activity, and cannabis was less helpful with improving these particular outcomes. Most persons used cannabis for sleep or digestive problems and we found no differences across groups in terms of well-being and quality of life. This comparative evaluation suggests cannabis mechanisms are not specific as much as they impact common processes.

### CAREGIVERS' CANNABIS USE: DOES BURDEN LEAD TO BLUNTS?

Kanika Arora, *University of Iowa, Iowa city, Iowa, United States*

Research on risky health behaviors among caregivers is limited. In this paper, we examine the association between informal caregiving and marijuana use and whether this association varies by age. Using data from Behavioral Risk Factor Surveillance System (2016-2019), a multivariable logistic regression model compared marijuana use in “caregivers” and “expectant caregivers.” We stratified the analyses by age and also assessed the association between caregiving intensity and marijuana use. Among younger individuals (18-49 years), informal caregiving was associated with higher odds of marijuana use. In this group, higher prevalence of marijuana use was positively associated with care intensity. There was no detectable association between caregiving and marijuana use among older individuals (50 years or older). Health behaviors among caregivers differ by age. Combined exposure to informal caregiving and marijuana in young adulthood may lead to adverse long-term health consequences. Immediate effects of marijuana use may negatively influence care recipient outcomes.

### CANNABIS USE AMONG VETERANS: IT SHOULD BE EASIER TO GET SOME

Julie Bobitt, *Center for Dissemination and Implementation Science, University of Illinois at Chicago, Illinois, United States*

and PTSD. From December 2020 – February 2021 we conducted 32 semi-structured interviews with Veterans who responded to our initial and follow-up surveys and agreed to discuss their cannabis use. We coded and themed the interviews using inductive thematic analysis. We found that many Veterans are using cannabis in place of other medications such as opioids and benzodiazepines and often do so to avoid the negative side effects. However, barriers such as Veterans Administration policies and cost of medical cannabis affect Veterans ability to obtain medical cannabis. Our results inform clinicians and policy makers on the use of cannabis as an alternative to prescription medications for treating chronic pain and other conditions in older Veterans.

### CANNABIS AT THE END OF LIFE: A ROAD MORE TRAVELED

J Alton Croker, *University of Iowa, University of Iowa, Iowa, United States*

This study examines medical cannabis as a complement or alternative to palliative care (PC). Using cross-sectional survey data from 708 terminal patients in the Illinois Medical Cannabis Program, we compare those in PC (n = 115) to those who are not (n = 593). Increased odds of PC utilization were observed for prior military service, cancer diagnosis, low psychological wellbeing, and medical complexity. PC was positively associated with improvement scores for pain, and ability to manage health status. Higher pain levels were also observed for PC patients who indicated concurrent use of cannabis and opioids, compared to those not using opioids. While most terminal patients use cannabis as an alternative to PC, medical cannabis does operate as a therapeutic complement for individuals in PC to help manage pain and overall health status, and is used at higher levels of pain when patients are also using opioids.

### NEGATIVE CLUSTERS ASSOCIATED WITH CANNABIS USE: TANGLED UP IN BLUES

Hyojung Kang, *University of Illinois, University of Illinois, Illinois, United States*

Previous studies concerning older adults have focused on whether cannabis use leads to positive or negative outcomes. In this study, we identified clusters of negative health outcomes associated with medical cannabis use. In total, we examined eight health outcomes: pain, sleep, falls, memory, digestive issues, mental health conditions, exercise, and general productivity reported by 2,968 persons over 60 who participated in the Illinois Medical Cannabis Program. We used association analysis to simultaneously identify groups of negative outcomes reported by participants. The distribution of non-positive outcomes shows a bell-shaped curve: 1.4% of participants responded that cannabis use improved all outcomes, while 4.1% of participants answered that cannabis use did not. When looking at negative outcomes, 86% of participants reported none worsened, and 11% reported one of the outcomes was affected. Only a small fraction of the participants (3%) claimed more than one negative outcomes after cannabis use.

### Session 4315 (Symposium)

### THE HALLMARKS OF AGING: LEVERAGING ON THEIR INTERACTIONS

Chair: Ana Maria Cuervo

### SELECTIVE AUTOPHAGY: A LINK ACROSS THE HALLMARKS OF AGING

Ana Maria Cuervo, *Albert Einstein College of Medicine, Bronx, New York, United States*

Autophagy function has been closely linked with the loss of proteostasis that characterizes most old organisms and tissues. However, the cellular functions of selective types of autophagy such as chaperone-mediated autophagy (CMA) go beyond cellular quality control. CMA can degrade fully functional proteins to terminate their function and thus contribute to regulation of multiple cellular processes. To fully understand the consequences of loss of CMA function with age, we have developed genetic and pharmacological ways to modulate this pathway in old mice. Our data supports involvement of CMA in other hallmarks of aging such as metabolism, senescence, cellular response to stress, epigenetics and cellular stemness. This interconnection among the cellular processes that drive aging highlights the potential of acting on only some of them with geroprotective effects.

### ANTI-AGING INTERVENTIONS TARGETING THE HALLMARKS OF AGING

Brian Kennedy, *National University of Singapore, Singapore*

### EFFECTS OF CALORIC RESTRICTION ON THE EPIGENETIC LANDSCAPE OF HEMATOPOIETIC STEM CELLS

Isabel Beerman, *NIA, Baltimore, Maryland, United States*

During aging, alterations of hematopoietic stem cells are associated with functional decline of the blood system. Caloric restriction (CR) interventions have been reported to improve adult stem cells in other tissue types during aging so we sought to evaluate the effects of CR on the aged HSC compartment. We find significant epigenetic alterations in HSCs isolated from aged mice after life-long CR compared to ad libitum fed aged mice. We further evaluated the epigenetic landscapes and functional potential of aged HSCs shortly after allowing life-long CR mice access to ad libitum food. We uncover epigenetic modification associated with functional alterations of the HSCs, defining potential mechanisms by which restrictions in food consumption affect the aging hematopoietic compartment.

### CALORIC RESTRICTION MIMETICS ATTENUATE THE HALLMARKS OF AGING

Guido Kroemer, *University of Paris, Villejuif/Paris, France*

Nutrient depletion, which is one of the physiological triggers of autophagy, results in the depletion of intracellular acetyl coenzyme A (AcCoA) coupled to the deacetylation of cellular proteins. We found that there are at least 4 possibilities to mimic these effects, namely (i) the depletion of cytosolic AcCoA by interfering with its biosynthesis, (ii) the stimulation cytosolic AcCoA consumption, (iii) the inhibition of protein acetyltransferases, or (iii) the stimulation of protein deacetylases. Thus, AcCoA depleting agents, AcCoA-consuming agents, acetyltransferase inhibitors or deacetylase activators are highly efficient inducers of autophagy and reduce aging-associated diseases including diabetes, obesity, cardiac failure and failing cancer immunosurveillance. Hence, we classify them as “caloric restriction mimetics” (CRM). We have initiated the systematic search for CRMs based on their cellular effects in vitro. We built screening assays amenable to high-throughput technology for the identification of CRMs. These results will be discussed.

### Session 4320 (Symposium)

### WELL-BEING DURING THE COVID-19 PANDEMIC: THE ROLES OF AGE, RACE, AND GENDER

Chair: Nicky Newton Discussant: Jennifer Lodi-Smith

In the early months of COVID-19, behavioral modifications (i.e., social distancing) were the only means available to ameliorate contagion. These had widespread ramifications for well-being, although older adults showed relatively less disruption and high resilience than their younger counterparts (Carney et al., 2021). Early findings highlight the need for a life course perspective when examining reactions to COVID-19, based on social structure, personal agency, and individual differences such as age, gender, and personality (Settersten et al., 2020). The presentations in this symposium contribute to a developing body of research that delves deeper into individual lived experiences during COVID-19. Using data from the Health and Retirement Study, Ryan examines cohort and age differences in pandemic-related social contact, communication, loneliness, and well-being for women in the US, revealing that the impact of pandemic-attributed psychosocial experiences on well-being differed by age group. Newton et al. examine associations between perceptions of future time, COVID-19 disruption, and psychological well-being among older Canadian women, finding that COVID-19 disruption moderated the relationship between constrained time horizons and well-being. Birditt and colleagues assessed racial disparities in relationships between COVID-related stress, social isolation, and depression among adults aged 18-97 from the Survey of Consumers, and found ethnic/racial minorities reported greater pandemic-related stress and that stress and social isolation had detrimental effects on well-being. A discussion by Lodi-Smith will emphasize the necessity to include individual differences – age, race, gender, cohort, cultural context –when examining pandemic-related well-being in order to provide a more nuanced body of research.

### COVID-RELATED PERCEPTIONS OF THE FUTURE AND WELL-BEING AMONG OLDER CANADIAN WOMEN

Hua (Poppy) Huo,[1] Lauren Hytman,[1] Cara Ryan,[1] and Nicky Newton,[2], *1. Wilfrid Laurier University, Waterloo, Ontario, Canada, 2. Wilfried Laurier University, Waterloo, Ontario, Canada*

Socioemotional Selectivity Theory (SST; Carstensen, 1993) posits that time horizons - or Future Time Perspective (FTP) - change with age and/or the priming of endings. Fung and Carstensen (2006) found that SARS-CoV in 2003 naturalistically primed fragility, with consequences for both FTP and well-being. The current SARS-CoV-2 (COVID-19) pandemic provides a similar context: During the early months of COVID-19, age and time horizon were related to greater emotional well-being for American adults (Carstensen et al., 2020); Dozois (2020) found that, for Canadian adults, anxiety and depression rose. The current study examines relationships between FTP, COVID-19 impact, and psychological well-being in older Canadian women (N = 190; Mage = 70.38). We found that COVID-19 impact and FTP were both related to well-being; additionally, COVID-19 impact moderated the relationship between FTP and well-being. The complexity of what remains or becomes increasingly important for older women during a global health crisis is discussed.

### THE PANDEMIC AND OLDER WOMEN IN THE UNITED STATES: IMPACTS ON SOCIAL NETWORKS AND WELL-BEING

Lindsay Ryan, *University of Michigan, Ann Arbor, Michigan, United States*

The current study examines the unique impacts of the ongoing COVID-19 Pandemic on the well-being of middle aged to older women from the 2020 Health and Retirement Study (n = 1252) and how their reports of social contact during the pandemic compare to age-matched women from 2018 (n = 2063). Although up to a third of women across age categories reported changes in social contact due to the pandemic, their rates of communication with friends and family were not significantly different from their counterparts in 2018. Results find expected age patterns in satisfaction with life during the pandemic), where the young-old report the highest levels. However, the association of life satisfaction with the extent to which women reported more loneliness during the pandemic was only significant among the young old and oldest old. Age differences in pandemic-specific experiences in relation to well-being are discussed within a life course developmental framework.

### RACE DIFFERENCES IN COVID-19 STRESS AND SOCIAL ISOLATION: IMPLICATIONS FOR DEPRESSIVE SYMPTOMS

Akari Oya,[1] Angela Turkelson,[1] Courtney Polenick,[1] Karen Fingerman,[2] and Kira Birditt,[1], *1. University of Michigan, Ann Arbor, Michigan, United States, 2. The University of Texas at Austin, Austin, Texas, United States*

The experience of the COVID-19 pandemic may vary widely by race. This study examined race differences in pandemic-related stress, social isolation and the implications for well-being. Participants included 1260 adults (45% women) ages 18 to 97 from the May and June 2020 nationally representative Survey of Consumers and 562 who completed a 6 month follow up in November/December. A total of 76% were White, 10% were Black, 3% were Asian, and 11% were Hispanic. Participants reported experiences of pandemic-related stress, social isolation and depressive symptoms in the last month. Analyses showed that minority groups reported greater pandemic related stress that had negative implications for depressive symptoms over time. The implication of social isolation for the stress-depressive symptoms link also varied by race. Overall this study showed racial inequities in the implications of COVID-19 pandemic and that reducing social isolation may only be beneficial for certain racial/ethnic groups.

### Session 4325 (Symposium)

### A MODEL TO TRANSFORM COMMUNITIES TOWARD BECOMING DEMENTIA INCLUSIVE

Chair: Jennifer Drost Discussant: Margaret Sanders

As the US population ages, the prevalence of people living with dementia will also increase. It is estimated that by 2050, 13.8 million American's 65 and older will be diagnosed with dementia, and currently only 40% of those living with dementia receive an official diagnosis. 70% of people living with dementia live in the community. In order to optimize quality of life and extend each person's ability to remain living in their homes for as long as possible, it is important for communities to educate consumers and providers alike about Alzheimer's Disease and related dementias, focusing on behaviors and interventions. This education must cross multiple sectors to effectively increase awareness, decrease stigma, and enable participation in community living for people living with dementia and their caregivers. Dementia Friends USA offers a framework for implementation of dementia friendly inclusive community initiatives that spans professions and incorporates patient and caregiver perspectives. The four symposia will 1) lead us through the evidence that supports the Dementia Friends USA approach, 2) demonstrate how this approach can be operationalized in a truly integrated fashion at the community level using HRSA's Geriatric Workforce Enhancement Program (GWEP), 3) provide step-by-step instructions for implementing Dementia Friends Community sessions, focusing on one sector at a time (in this case the Developmental Disability population), and 4) discuss the individual and community level outcomes of Dementia Friends implementation.

### CREATING DEMENTIA-INCLUSIVE COMMUNITIES USING A GERIATRIC WORKFORCE ENHANCEMENT PROGRAM FRAMEWORK

Margaret Sanders,[1] and Jennifer Drost,[2], *1. Northeast Ohio Medical University, ROOTSTOWN, Ohio, United States, 2. Summa Health System, Akron, Ohio, United States*

The Geriatric Workforce Enhancement Program (GWEP) sponsored by HRSA provides an organizing framework around which dementia inclusive community initiatives can be successfully implemented and sustained. The overarching goal of all GWEPs is to improve outcomes for older adults by promoting evidence-based education that spans the continuum of care. This includes integration of academic, clinical, and community-based providers. By their very nature, all GWEPs partner across agencies throughout the state to deliver interprofessional education that will impact people living with dementia and their caregivers at the community, primary care, and acute care levels. Dementia inclusive community initiatives must have this kind of high-level interagency coordination. Our GWEP has successfully implemented Dementia Friends sessions across multiple sectors (Veterans, EMS, clergy, libraries, developmentally disabled, living alone) both in-person and virtually due to COVID-19. This symposium will share the methods to organize at the community level to deliver a unified message community-wide.

### FROM AWARENESS TO SOCIAL ACTION: THE ROLE OF DEMENTIA FRIENDS IN SUSTAINING DEMENTIA INCLUSIVENESS

Martha Williman, and Bonnie Burman, *Ohio Council for Cognitive Health, New Albany, Ohio, United States*

According to the World Dementia Council, three components are important to effectively engage a community to become dementia inclusive, 1) raising awareness and consequently decreasing stigma, 2) enabling participation, and 3) providing support—including in health and care settings. Too many times these components are separate initiatives thus limiting their effectiveness and sustainability. By applying the collective impact model and utilizing the Dementia Friends program as the link between the three, all dementia inclusive efforts can be enhanced and sustained regardless of the range of activities and approaches a community chooses to adopt. This symposium provides both evidence and examples of how to personalize and employ the Dementia Friends program to optimize the process, outcome, and impact of dementia inclusive initiatives. By engaging the entire community, awareness is raised, the structure is in place to enable action, and cross-sector collaboration will ensure continuation and sustainability of these important efforts.

### IMPROVING CARE FOR INDIVIDUALS LIVING WITH INTELLECTUAL DEVELOPMENTAL DISABILITIES AND DEMENTIA

Donna Barrett, *Summit County Public Health, Akron, Ohio, United States*

Ultimately, transformation of communities can only occur through educational efforts delivered to specific community sectors. Although the portion of people with Intellectual Developmental Disabilities who develop dementia as they age is equal to that of the general population, individuals with Down syndrome are at a much higher risk. This symposium will describe how a county health department partnered with the local County Board of Developmental Disabilities to systematically incorporate Dementia Friends for Intellectual Developmental Disabilities with Alzheimer's disease training to their staff and provider network. We will describe who to get on board with the idea, how to organize, and how to deliver specific trainings. Outcomes related to increase in participant knowledge, increases in service provision and outcomes related to staff mentoring will be discussed.

### ESTABLISHING A DEMENTIA-INCLUSIVE COMMUNITY: THE EVALUATION OF DEMENTIA FRIENDS IN CLEVELAND HEIGHTS

Jennifer Cardellini, Sarah Nicolay, and Jessica Bibbo, *Benjamin Rose Institute on Aging, Cleveland, Ohio, United States*

Cleveland Heights, in northeast Ohio, is currently working towards becoming a member of the Dementia Friendly America National Network. Utilizing the Dementia Friends curriculum to raise community members' awareness of issues related to dementia is a key component of this initiative. Our initial efforts toward this goal targeted two sectors, namely community member and libraries. Participants completed on-line surveys at the beginning and end of each session. The surveys include the Brief Tool for Dementia-Friendly Education and Training Sessions developed by the Administration for Community Living. Of the 22 participants, nine had not previously attended a Dementia Friends session and completed both pre- and post-session surveys. Results indicated participants felt more confident interacting with people living with dementia at post-session compared to pre-session ($t = -2.83$, $p=.022$). Changes at the individual level may create more inclusive communities for people living with dementia and those who care for and about them.

### Session 4330 (Paper)

### AGING AND HEALTH AND SOCIAL SERVICES

### COMPARISON OF ASSOCIATION BETWEEN DIVORCE AND ACCESS TO HEALTHCARE SERVICES AMONG MARRIED IMMIGRANTS

Suyeong Bae,[1] James Graham,[2] Sanghun Nam,[1] and Ickpyo Hong,[1], *1. Yonsei University, Wonju, Kangwon-do, Republic of Korea, 2. Colorado State University, Colorado State University, Colorado, United States*

The number of married immigrants is increasing in Korea, and family dissolution is also growing in this population. Although divorce could impact immigrants' health status, it is unclear whether they have difficulties accessing healthcare and medical services. Thus, we examined whether divorce in married immigrants is independently associated with access to healthcare services. A retrospective analysis of 11,778 adults who participated in the 2018 National Multicultural Family Survey. We used three different covariate adjustment methods (multivariate logistic regression, inverse probability of treatment weighting, 1:1 greedy propensity score matching) to examine the association between divorce and access to healthcare services after accounting for various demographic and clinical characteristics. Overall, 5.8% ($n = 691$) of married immigrants reported a history of divorce. The divorce group included 107 (15.5%) males and 584 (84.5%) females, with an average age of 45.17 years ($SD = 10.9$). The non-divorced group included 1992 males (18.0%) and 9095 (82.0%) females, with an average age of 39.1 years ($SD = 10.5$). After propensity score matching, all variables were balanced (all $p>0.05$). Individuals who experienced divorce were more likely to have difficulties in healthcare service access than those who did not experience divorce (adjusted odds ratio 1.423, 95% CI [1.075, 1.882]). Our findings revealed that divorce increased the risk of limited healthcare services among immigrants in Korea. Healthcare policymakers should be aware of the healthcare access issues in this minority population. In addition, to improve the lifestyles of minority populations, it is necessary to study their overall lives.

### FACTORS IMPACTING TREATMENT DECISION MAKING IN OLDER ADULTS WITH INDOLENT NON-HODGKIN LYMPHOMA

Kelly Trevino,[1] Peter Martin,[2] and John Leonard,[2], *1. Memorial Sloan Kettering Cancer Center, New York, New York, United States, 2. Weill Cornell Medicine, New York, New York, United States*

Indolent non-Hodgkin lymphomas (NHL) are among the most common lymphomas and up to two-thirds of diagnoses are made in older adults (age≥65 years). Initial treatment options include cancer-directed therapy or active monitoring by the oncologist for disease progression. Despite the disparate nature of these treatments, the factors impacting older adults' treatment decisions are unknown. This study examines the reasons older adults chose their initial treatment, factors influencing this decision, shared decision-making preferences, and differences in these factors relative to younger adults (age<65 years). Adult patients (≥21 years) with a new diagnosis of indolent NHL in the past six months completed electronic self-report measures. The final sample consisted of 86 patients; 43.0% (n=37) were older adults. Over two-thirds of older adults (n=25, 67.6%) were being monitored by their oncologist with no age differences in current treatment (p=.55). Most older adults chose their treatment plan to "maximize my long-term health" (n=24, 64.9%) which did not differ from younger adults (p=.77). The primary factors impacting older adults' treatment decisions were their doctor's recommendation (M=3.92, SD=.28, Range=0-4) and their personal preference (M=2.88, SD=1.68, Range=0-4). Factors impacting treatment decisions did not differ by age (all p's>.05). Most older adults (n=25, 69.4%) expressed a preference for shared decision-making with their oncologist which did not differ from younger adults (p=.17). Treatment planning for older adults should consider long-term health, consistent with older adults' values. Older adults may view treatment decision-making similarly to younger adults; assumptions about patients' values and decision-making preferences based on age are likely inappropriate.

### HEART FAILURE AND HOSPITAL UTILIZATION TRAJECTORIES BEFORE AND AFTER HIP FRACTURE SURGERY

Sijia Wei,[1] Wei Pan,[2] Chiyoung Lee,[3] Hideyo Tsumura,[4] Tingzhong (Michelle) Xue,[4] and Eleanor McConnell,[4], *1. Duke University School of Nursing, Apex, North Carolina, United States, 2. Duke University, Duke University School of Nursing, North Carolina, United States, 3. University of Washington, Bothell, Bothell, Washington, United States, 4. Duke University School of Nursing, Durham, North Carolina, United States*

Long-term hospital utilization trajectories in the context of surgery are understudied. Heart Failure (HF) is associated with an increased risk for rehospitalization after hip fracture surgery. This study aimed to examine whether older adults (>= 65 years old) have distinct patterns of long-term hospital utilization trajectories and whether HF influences these trajectories before and after hip fracture surgery. An initial cohort of 1,172 older adults hospitalized for hip fracture surgery between October 2015 and December 2018 was extracted from electronic health records. To adjust selection bias in baseline characteristics, we used propensity score 1:1 ratio matching to identify a final cohort of older adults with (n = 288) and without (n = 288) HF. Monthly frequencies of emergency department (ED) and inpatient encounters 1-year before and after the hip fracture surgery were used to identify distinct utilization trajectories from group-based trajectory analysis. Logistic regression models were used to compare the differences in ED and inpatient trajectories among patients with and without HF. High ED users (9.5%) had constant high ED use, and high inpatient users (20.1%) had significantly higher inpatient usage around the index hip fracture surgery hospitalization. Both low ED (90.5%) and inpatient (79.9%) users had low but slightly increased use around the index hospitalization. Compared with older adults without HF, older adults with HF were more likely to be long-term high inpatient user (OR = 1.94, 95% CI 1.25-3.01, p = 0.003), but not significantly different in long-term ED utilization (OR=1.87, 95% CI 0.97-3.59, p = 0.62).

### PSYCHOMETRIC PROPERTIES OF THE HEALTH LITERACY SURVEY EUROPEAN QUESTIONNAIRE-12

Suyeong Bae,[1] Sung-Ji Park,[2] Hee-Soon Woo,[2] and Ickpyo Hong,[1], *1. Yonsei University, Wonju, Kangwon-do, Republic of Korea, 2. Wonkwang university, Wonkwang University/Iksan, Cholla-bukto, Republic of Korea*

The importance of health literacy has increased as the public awareness of health has increased. Health Literacy Survey European Questionnaire-47 (HLS-EU-Q47) is a representative assessment tool for evaluating health literacy, and its psychometric properties have been examined in various countries. This study analyzed the item-level psychometric properties of a short version of the HLS-EU-Q47 using a Rasch measurement model. We collected 254 Korean adults who completed the 12 items of the HLS-EU-Q47 in hospital settings. We used confirmation factor analysis (CFA) to examine the unidimensionality assumption of the HLS-EU-Q12. We analyzed item fit, precision, and differential item functioning (DIF) across sex, age and education groups. The CFA model confirmed that HLS-EU-Q12 satisfies the unidimensionality assumption (CFI = 0.96, TLI = 0.96, RMSEA = 0.09) and no local independence in the 12 test items (residual correlations ranged from -0.16 to 0.19). The HLS-EU-Q12 demonstrated high reliability (Cronbach's $\alpha = 0.90$) and no DIF across sex, age and education groups ($p > 0.05$). The person strata by the instrument were 3.80, which is equivalent to a traditional reliability value of 0.87. In short, the study findings indicate that the HLS-EU-Q12 has good psychometric properties with the 254 Korean adults. Since the HLS-EU-Q12 can accurately and precisely evaluate the health literacy of Korean adults, this instrument could be used in clinical settings.

### THE OCCUPATIONAL NARRATIVES OF OLDER ADULTS PARTICIPATING IN MUSIC-BASED OCCUPATIONS

Penelope Moyers Cleveland, Kassidy Beckstein, Allie Gartner, Lexy Hay, Mackenzie King, Sammy McLeish, and Courtney Romatz, *University of Indianapolis, Indianapolis, Indiana, United States*

The purpose of this study was to implement an occupational therapy intervention that could be used for telehealth services with an emphasis on participants learning ways to independently choose and sustain engagement in meaningful music activities, known as occupations. The researchers' aim was to examine how music occupation interventions lower risks of occupational deprivation (i.e., prolonged restriction from participation in necessary or meaningful activities) that could occur due to the COVID-19 pandemic. Eight adults participated who were 65 years or older, lived in the community, and enjoyed music. The researchers used narrative qualitative methodology to analyze pre- and post-intervention focus group data. The participants completed seven intervention sessions designed to increase and sustain music engagement outside of the sessions. The pre-intervention focus group data resulted in an occupational pattern analysis and a single occupational narrative. Triangulation of data post-intervention included the two focus groups and their pattern analyses and narratives, field notes from each intervention session, and documents produced through group completion. The final analysis produced an occupational change pattern analysis and narrative. The focus of the change narrative was on the participant's management or prevention of occupational deprivation. The researchers identified several common themes involving change in routines and habits to include regular engagement in meaningful music activities, skills for using occupational participation as an important method of coping with COVID-19, and developing new technological skills to access music to replace in-person participation of attending live concerts and shows when deemed unsafe because of potential for virus transmission.

### Session 4335 (Paper)

### AGING AND TECHNOLOGY INTERVENTIONS II

### ACTIVITY SPACE AND FUNCTIONAL OUTCOMES IN FRAIL OLDER PERSONS USING GPS ANALYSIS

Sandra Lau,[1] Frerk Mueller-von Aschwege,[2] Tania Zieschang,[1] Juergen Bauer,[3] Andreas Hein,[1] and Rebecca Diekmann,[1], *1. Carl von Ossietzky University Oldenburg, Oldenburg, Niedersachsen, Germany, 2. OFFIS e.V. - Institute for Information Technology, Oldenburg, Niedersachsen, Germany, 3. Heidelberg University, Heidelberg, Baden-Wurttemberg, Germany*

With increasing age, walking becomes a main functional ability to participate in activities of daily living and supports independence and mobility. Frailty in older, multimorbid patients has a negative impact on physical activity and may reduce the personal activity space (AS). In this pilot study, GPS data were used to identify walking tracks to define individual AS and to compare functional performance in frail older persons. GPS data of 20 community-dwelling adults (84.5(±5.2) years, 85% women, mean frailty phenotype 1.9 (70% ≥2) points) were analyzed using a customized software to assess individual AS over a ten-months period. A geriatric home assessment including Short Physical Performance Battery (SPPB), gait speed (GS) and Timed-up-and-Go (TUG) was conducted monthly. GPS analysis revealed three different walking types presenting AS similarities: Type A walkers prefer smaller short walks nearby the home while Type B can be characterized by taking larger regular walks. Type C presents the widest AS using different transportation modes, but only a moderate number of walks. Mean group difference in functional performance of Type A walkers showed significantly reduced GS (0.45(±0.1) m/s), TUG (23.4s(±4.9)) and SPPB scores (3.8(±0.8) points; $p<0.05$) compared to Type C (0.82(±0.1)m/s (GS); 13.2(±1.4) s (TUG); 7.0(±1.3) points (SPPB)). Functional performance of Type B walkers (0.63(±0.2)m/s (GS); 17.1(±4.4)s (TUG); 6.5(±2.4)points (SPPB)) revealed significantly higher SPPB scores compared to Type A ($p<0.05$). Walks and individual AS can be mapped via GPS under everyday conditions. High heterogeneity within frail older people was observed. Persons with lower functional performance showed a reduced AS and physical activity.

### DEVELOPING, TESTING, AND IMPLEMENTING A FALLS PREVENTION AND HEALTHY AGING APP (KEEP-ON-KEEP-UP) FOR OLDER ADULTS

Emma Stanmore, *University of Manchester, Manchester, England, United Kingdom*

Falls are a common and costly concern for older adults. Digital technologies can offer new, inexpensive approaches to increase access and engagement with falls prevention programmes. Keep-On-Keep-Up is a personalised, falls prevention App with strength and balance exercises plus health literacy games. This study reports on the user-centred design, usability testing and implementation of the KOKU App. Older adults aged 55 years and older in the UK were invited to take part in the study. Data collection included focus groups; baseline and 6 week questionnaires and assessments; semi-structured interviews and one focus group with falls prevention therapists to explore App usability. Thirty older adults were invited to use KOKU unsupervised, 3 times a week for 6 weeks. Data were analysed using thematic content analysis. Focus groups (n=11) with 66 older users and 11 therapists informed development. Thirty older adults (mean age = 75) were recruited for the in-depth testing. Mean SUS score was 71 indicating high usability. Qualitative themes included: ease of use (app usability; iPad properties; exercise presentation), usefulness (physical/psychological benefits; falls education), attitude towards the App and intention to use (technological barriers; flexibility of use; exercise class versus App). Therapists (n=6) viewed the KOKU platform positively and suggested extensions for further progression. No adverse events were reported during the study. This research demonstrates that KOKU is an acceptable and easy to use falls prevention intervention that facilitates older adults' ability to access falls prevention training at a time, and in a location, that suits them.

### FEASIBILITY AND ACCEPTABILITY OF AN MHEALTH ACP TOOL IN PRIMARY CARE

Desh Mohan,[1] Katelin Cherry,[1] Tatiana Fofanova,[1] Taylor Huffman,[1] Glenn Davis,[2] Anthony Comito,[3] and Elissa Kozlov,[4], *1. Koda Health, Houston, Texas, United States, 2. Cypress Physicians Association, Spring, Texas, United States, 3. FOUNDRY41, Houston, Texas, United States, 4. Rutgers University, New Brunswick, New Jersey, United States*

With only 7% of Medicare beneficiaries having completed Advance Care Planning with their physicians, engagement in Advance Care Planning in the clinical setting has been historically low. This study investigated the feasibility of introducing the Koda Health Advance Care Planning software platform in the primary care setting, and whether patients would engage in advance care planning through this medium. The Koda platform is a video-driven, web application that guides patients through advance care planning concepts, including values and quality of life exploration, surrogate decision maker selection, life-support treatments, and advance directive completion. The study was completed over a six-month period in two primary care clinics in the Houston, Texas area. Inclusion criteria were age 55 or older, English-speaking, and capacity for medical decision making. 339 patients met eligibility criteria and had a median age of 73 (range 59-89). All participants were offered the platform, and 262 (77%) created an account and began planning for their care. Of the patients that created an account, 87% completed all ACP steps on the platform and 72% identified a surrogate decision maker. The median time spent on the platform was 18 minutes. The Koda platform appears to be a useful tool for patients and providers to improve engagement in advance care planning and improve surrogate decision maker identification. Further research is needed to understand whether the Koda platform aids in providing goal-concordant care.

### IMPROVING STAFF-FAMILY END-OF-LIFE COMMUNICATION AT ISRAELI GERIATRIC FACILITIES BY USING A MOBILE APP

Rinat Cohen,[1] Gal Maydan,[2] Shai Brill,[3] and Jiska Cohen-Mansfield,[4], *1. Tel-Aviv University, Hadera, Hefa, Israel, 2. Beit Rivka, Petah-Tikva, HaMerkaz, Israel, 3. Clalit Health Services, Tel-Aviv, Tel Aviv, Israel, 4. Tel-Aviv University, Tel-Aviv, Tel Aviv, Israel*

Family caregivers (FCs) of persons institutionalized at geriatric facilities present significant unmet communication needs regarding receiving regular updates about their loved one's condition and having available healthcare professionals (HPs) to approach when needed. We developed and tested a mobile-app for staff-family communication with both parties having active roles in app planning to tailor it to their needs and abilities. The app includes a daily-update module for FCs and a chat option for FCs and HPs. App use was piloted at one geriatric-medical-center for 15 months (unit-level randomization resulted in one complex-care and one assisted-ventilation unit in each group- intervention and control) and one single-unit nursing-home for three months. Personal interviews were conducted with 55 FCs (28 from intervention-group and 27 FCs from control-group) before-and-after app use (with mean duration of use 1.6[S.D.=.6] months. Most participants were women and the children of the patients; their mean age was 55.9 years (S.D.=12.4). Repeated-measures Analysis-of-Variance for the end-of-life communication sub-scale on the Quality-of-communication questionnaire yielded a main effect for time (F(1,53)=8.31, p=.006) with both groups' ratings increasing over time and an interaction effect (F(1,53)=4.78, p=.033) with a greater increase for intervention-group compared to control-group. Intervention-group participants rated the app as convenient to use. Qualitative data revealed that FCs perceived app use as improving quality of communication with the HPs who used it and improving their own well-being. The app offers a feasible and an effective mode of communication that incorporates technology in daily communication between FCs and HPs while addressing FCs' unmet needs.

### TEXT-MINING IN LONG-TERM CARE: EXPLORING THE USEFULNESS OF COMPUTER-AIDED ANALYZING METHODS

Sil Aarts,[1] Coen Hacking,[2] Hilde Verbeek,[2] Jan Hamers,[2] and Katya Sion,[2], *1. Maastricht university, Maastricht, Limburg, Netherlands, 2. Maastricht University, Maastricht, Limburg, Netherlands*

In nursing homes, narrative data are collected to evaluate quality of care as perceived by residents or their family members. This results in a large amount of textual data which exceeds the capability of humans to analyse it. This study aims to explore the usefulness of text-mining approaches regarding narrative data gathered in a nursing home setting. Data has been collected as part of the project 'Connecting Conversations': assessing experienced quality of care by conducting individual interviews (n=125) with residents of nursing homes, family members and care professionals. Several pre-processing steps were applied to the textual data. Finally, a variety of text-mining analyses were conducted: individual and bigram word frequencies, correlation analysis and sentiment analysis. A survey was conducted to establish a sentiment analysis model tailored to text collected in long-term care for older adults. Residents, family members and care professionals uttered respectively 285, 362 and 549 words per interview. Word frequency analysis showed that words that occurred most frequently in the interviews are often positive. Although there are some differences in wording such as the use of 'mother' and 'breakfast', correlation analysis displayed that similar words are used by all three groups to describe quality of care. The majority of interviews displayed a neutral sentiment. Care professionals are more diverse in their sentiment than residents and family members: while some express a more positive sentiment, others express more negativity. This study demonstrates the usefulness of text-mining to extend our knowledge regarding quality of care in a nursing home setting.

### Session 4340 (Paper)

### BLACK CAREGIVERS' HEALTH

### MASTERY GOALS FOR BLACK AMERICAN DEMENTIA CAREGIVERS

Kalisha Bonds Johnson,[1] Fayron Epps,[1] Glenna Brewster,[2] Carolyn Clevenger,[3] Gaea Daniel,[3] Victoria Pak,[3] Sudeshna Paul,[3] and Kenneth Hepburn,[1], *1. Emory University, Atlanta, Georgia, United States, 2. Emory University, Stonecrest, Georgia, United States, 3. Emory University, Emory University, Georgia, United States*

About 5.8 million older American adults live with Alzheimer's disease and related dementias; Black American older adults' prevalence is more than twice that of non-Hispanic white older adults. The Black American dementia caregiving experience can be pictured within the Black Family Social-Ecological Context Model, which provides a conceptual basis for examining social determinants of health at individual, family, community, and societal levels with careful consideration for how the intersecting identities of race, gender, and class of Black American caregivers influence the multiple dimensions of their caregiving experiences. Family dynamics, community setting, and healthcare systems have a potentially bidirectional influence on these caregivers, which is informed by the larger historical reality of systemic racism and general disenfranchisement. This paper outlines how Stress Process and Perceived Control frameworks offer ways for Black American dementia caregivers to achieve a sense of mastery within the complicated and fraught ecology within which their caregiving occurs. We propose a research and development agenda to create a program for enhancing a sense of mastery among Black American dementia caregivers. Two concepts in particular, "constraints" and "efficacy expectations," provide ways to develop a systematic approach to developing successful coping strategies for the constraints perceived by individuals as they undertake and function in the caregiving role. The recognition of the complexity of the caregiving ecosystem and intersectionality of caregivers' experience emphasize the importance of individualization: each caregiver's experience of this ecosystem– and therefore each Black American caregiver's way to mastery within it– will be uniquely shaped and experienced.

### MY FAITH GIVES ME STRENGTH: BLACK CHURCHES SUPPORTING DEMENTIA CARE PARTNERS

Danetta Sloan,[1] Valerie Cotter,[2] Quincy Samus,[3] Morgan Spliedt,[4] Samuel Means,[5] Janice Bowie,[1] and Darrell Gaskin,[1], *1. Johns Hopkins Bloomberg School of Public Health, Baltimore, Maryland, United States, 2. Johns Hopkins School of Nursing and School of Medicine, Baltimore, Maryland, United States, 3. Johns Hopkins University School of Medicine, Baltimore, Maryland, United States, 4. The Johns Hopkins University, Baltimore, Maryland, United States, 5. Baltimore, Maryland, United States*

Inequities in dementia care require an in depth understanding of dementia care partner life experiences, care needs, sources of support and ways to improve those experiences at the community level. In partnership with Black Churches in Baltimore, MD and rural Pennsylvania, we facilitated three focus groups (n=30) with Black dementia care partners to understand: 1) unique dementia care-related needs and challenges, and 2) how the church can be a supportive care partner. Participants noted three overarching themes related to challenges and needs: 1) unique challenges in caregiving - assuming various roles to provide comprehensive care, working within policies of support services, environmental challenges (neighborhood safety), and family conflicts; 2) lack of dementia education and educational materials; and 3) unmet needs of the care recipient – lack of dementia friendly neighborhoods, lack of meaningful activities, and undiagnosed dementia. Regarding the supportive role of the church, the theme of perceived role of the church included providing emotional and social support for care partners and recipients, reducing burden through providing respite, support groups, networking/ connections to resources, and promoting health events.

### PAIN AMONG AFRICAN AMERICAN FAMILY CAREGIVERS: ASSOCIATION WITH HEALTH OUTCOMES

Janiece Taylor,[1] Lyndsay DeGroot,[2] Thomas Travison,[3] Richard Skolasky,[4] Sarah Szanton,[1] Haera Han,[5] and Martha Abshire Saylor,[6], *1. Johns Hopkins University, Baltimore, Maryland, United States, 2. Johns Hopkins University, Johns Hopkins University School of Nursing, Maryland, United States, 3. Harvard Medical School, Boston, Massachusetts, United States, 4. Johns Hopkins University School of Medicine, Baltimore, Maryland, United States, 5. Nursing, Baltimore, Maryland, United States, 6. Johns Hopkins School of Nursing, Baltimore, Maryland, United States*

African Americans experience high rates of undermanaged pain as they age. African Americans often become family or informal caregivers, and undermanaged pain may have implications for their overall health and ability to perform caregiving tasks. The purpose of this study was to examine if pain was related to overall health and number of caregiving activities among African American caregivers. We used data from round 7 of the National Study of Caregiving (N= 646 African American caregivers, mean age= 57 years [SD=15]). Pain over the prior month was participant-assessed. Of this sample, 451 (69.7%) were women, 324 (50.1%) had pain, and 309 (47.8%) had multiple comorbidities. The number of caregiving activities ranged from 8 to 14 with mean of 11.6 (SD= 1.4). In a logistic regression model, controlling for income, education, and comorbidities, we found having pain in the last month was associated with lower likelihood of very good or excellent health (Adjusted OR 0.48; 95% CI 0.33 to 0.69) and greater likelihood of having depressive symptoms (Adjusted OR 2.04; 95% CI 1.34 to 3.10). In a linear regression model, however, pain was not significantly related to the number of caregiving activities (R2 = 0.04). Findings suggest that while pain is related to poorer physical and mental health outcomes among African American caregivers, even pain does not deter them from providing care for care recipients. Management of pain among African American caregivers may be beneficial for improving their own overall health and mental health.

### TRANSCENDING INEQUITIES IN DEMENTIA CARE IN BLACK COMMUNITIES: LESSONS FROM THE MIND CARE COORDINATION PROGRAM

Danetta Sloan,[1] Deirdre Johnston,[2] Chanee Fabius,[1] Inga Antonsdottir,[3] Morgan Spliedt,[4] Tabitha Pyatt,[2] Melissa Reuland,[2] and Quincy Samus,[5], *1. Johns Hopkins Bloomberg School of Public Health, Baltimore, Maryland, United States, 2. Johns Hopkins University, Baltimore, Maryland, United States, 3. Johns Hopkins School of Nursing, Baltimore, Maryland, United States, 4. The Johns Hopkins University, Baltimore, Maryland, United States, 5. Johns Hopkins University School of Medicine, Baltimore, Maryland, United States*

Resolution of ongoing inequities in dementia care requires careful examination of how care is delivered to ensure we are aware of, and meeting needs for all people affected, especially those most vulnerable and in under-resourced communities. Maximizing Independence at Home (MIND) is a multicomponent, home-based dementia care program designed to provide high quality, wholistic care coordination for to persons and families living with dementia. Program goals are to delay transition from home, improve life quality, and reduce unmet care needs. We completed three focus groups (n = 25) with Black (e.g., African American) dementia caregivers who received the intervention to understand (1) the unique dementia related needs of Black dementia caregivers and barriers and challenges to caregiving experienced within the Black community , (2) perceived benefits of MIND, and (3) ways to improve the MIND intervention. Participants noted three overarching themes related to needs and challenges in dementia care in the Black community: difficulty finding and accessing dementia information, help, and related services; familial conflict/lack of sibling and familial support; and lack of effective communication about dementia within Black Communities. Regarding benefits of the program, four themes emerged including that the program helped find resources (formal and informal); provided caregivers an opportunity for socialization and interaction; included comprehensive assessments and helpful linked information; and resulted in a "much needed break." Increased diversity of MIND personnel and greater clarity and consistency in MIND program promotion and communications were themes for how the program could be improved.

### WORSHIP EXPERIENCES OF BLACK FAMILIES AT DEMENTIA-FRIENDLY CHURCHES: A MULTIPLE-CASE STUDY DESIGN

Fayron Epps,[1] Janelle Gore,[1] Mayra Sainz,[1] and Glenna Brewster,[2], *1. Emory University, Atlanta, Georgia, United States, 2. Nell Hodgson Woodruff School of Nursing Emory University, Atlanta, Georgia, United States*

Literature supports the significance of religiosity as a protective factor to promote well-being for Black families facing dementia; However, minimum reports exist on the worship experiences of these families. The purpose of this multiple-case study was to report the experiences of Black families facing dementia who attended worship services at dementia-friendly churches. Data were collected through participant observations of family caregivers (n = 4) and persons living with dementia ( n = 4) during worship services and semi-structured interviews with the family caregivers over six months. Data were analyzed case by case followed by a cross-case analysis. Four overarching themes emerged: welcoming church culture, the significance of support with differences in perceived and actual support, engagement during worship service, and connectedness between the caregiver and their family member living with dementia. Family caregivers reported that their family member with dementia was attentive and expressed moments of clarity during and immediately after worship services. Religious worship can play an essential role in the quality of life among Black families affected by dementia. Health practitioners are encouraged to acknowledge the influences of religion among dementia families and integrate religious practices within interdisciplinary care plans and programs.

### Session 4345 (Paper)

### COGNITIVE AGING II

### CHILDHOOD SOCIOECONOMIC STATUS AND COGNITIVE AGING: AN INTERSECTIONAL LIFE COURSE PERSPECTIVE

Addam Reynolds,[1] Emily Greenfield,[2] Sara Moorman,[3] and Laurent Reyes,[4], *1. Rutgers, the State University of New Jersey, Brick, New Jersey, United States, 2. Rutgers, The State University of New Jersey, New Brunswick, New Jersey, United States, 3. Boston College, Chestnut Hill, Massachusetts, United States, 4. Rutgers University, Rutgers University, New Jersey, United States*

Greater childhood socioeconomic status (cSES) is associated with better later life cognition. Largely absent from this literature is how structural racism potentially influences this relationship. Guided by intersectional life course theory, we examined if the influence of cSES and region of schooling on later life cognitive outcomes differs among non-Hispanic White (NHW) and Black older adults. We used data from the 2010-2016 waves of the Health and Retirement Study for participants ages 65 and older in 2010. Using growth mixture modeling, we estimated the associations between race, cSES (parental education, social, and financial capital), and region of schooling at age 10 (southern versus not) on cognitive performance. Consistent with prior research, there was a main effect of race on cognitive performance levels (but not with decline over time), with lower scores among older Black adults, on average. Among NHWs, higher cSES was protective for later life cognition, especially for NHW participants from the South. Although Black older adults who attended school outside of the South had higher levels of cognitive performance than their counterparts who attended school at age 10 in the South, Black older adults who attended school outside of the South--regardless of cSES--still had lower average scores on cognition at baseline than the most disadvantaged NHW participants. This paper implicates the effects of structural racism on cognitive performance among older Black adults, indicating the need for heightened attention to structural racism within interventions for optimizing brain health and promoting equitable cognitive aging across the life course.

### CUMULATIVE STRESS BURDEN AND COGNITIVE FUNCTION IN AFRICAN AMERICAN ADULTS LIVING IN LOW-INCOME NEIGHBORHOODS

Erica Fan,[1] Tamara Dubowitz,[2] Wendy Troxel,[2] Andrea Weinstein,[1] Tiffany Gary-Webb,[3] Meryl Butters,[1] Bonnie Ghosh-Dastidar,[4] and Andrea Rosso,[1], *1. University of Pittsburgh, Pittsburgh, Pennsylvania, United States, 2. RAND Corporation, Pittsburgh, Pennsylvania, United States, 3. University of Pittsburgh, Graduate School of Public Health, Pittsburgh, Pennsylvania, United States, 4. RAND Corporation, Arlington, Virginia, United States*

African Americans (AA) are more likely to experience stressors due to racial discrimination and segregated neighborhoods, potentially contributing to higher risk for dementia. We investigated the association between stressors and cognitive function in older AA adults through cumulative stress burden (CSB) indices. Stressors and cognitive domains were measured in 253 participants >50 years, recruited from primarily AA neighborhoods in Pittsburgh, PA. CSB included perceived and psychological distress, unfair treatment, post-traumatic stress disorder, and neighborhood-level stressors such as walkability, safety, satisfaction, and social cohesion. Indices were formed by dichotomizing stressor scores and summing. Cognitive domains were z-scores adjusted for age, sex, and education. Adjusted generalized linear models assessed the relation between CSB indices and cognition, and between specific stressors and cognition. Interactions with age were tested. Greater individual-level CSB index was associated with lower language (□= -0.11, p= 0.03) and executive function (□= -0.087, p=0.04). The neighborhood-level CSB index was not associated with any cognitive domain. The combined index was marginally associated with language in adjusted models (□= -0.07, p= 0.05). There were no significant associations between specific stressors and cognition, except for neighborhood safety with 3MS (□= -0.28, p= 0.001) and language (□= -0.16, p=

0.02). Age interactions indicate that findings were stronger for younger participants. Greater cumulative stress is associated with poorer cognitive function in some domains in older AA. A comprehensive assessment of cumulative stress is vital in understanding the dimensionality of racialized stress for older adults potentially experiencing cognitive decline.

### GREATER ADOLESCENT COGNITIVE ABILITY LINKED TO LOWER RISK OF EARLIER MORTALITY

Tara Gruenewald,[1] Molli Grossman,[2] Catalina Zavala,[2] Thalida Arpawong,[2] Gwen Fisher,[3] Kenneth Shultz,[4] Margaret Gatz,[2] and Carol Prescott,[2], *1. Chapman University, Orange, California, United States, 2. University of Southern California, Los Angeles, California, United States, 3. Colorado State University, Fort Collins, Colorado, United States, 4. California State University, San Bernardino, San Bernadino, California, United States*

There have been few investigations of the role that adolescent cognitive ability might play in predicting physical resilience across the life course, including decreased risk of early mortality. Our limited knowledge of how multiple cognitive ability domains shape trajectories of longevity is due, in part, to a lack of aging cohorts with early life cognitive assessments, and family data that allow for examination of shared family and genetic characteristics that may play a role in cognitive ability-health links. We capitalized on data from the 1960 Project Talent high school cohort (n>360,000, born 1942-1946) and mortality data (n=22,584; 5,497 deceased) collected as part of two recent follow-ups, the Project Talent Twin & Sibling Study and the Project Talent Aging Study, to examine these potential associations. In 1960, ability was assessed in multiple cognitive domains (e.g., general aptitude, quantitative, reasoning). Mortality status was ascertained through 2016. Binary logistic generalized estimating equations with race, age, sex, and adolescent family SES covariates, indicated that each 1 standard deviation higher ability in multiple cognitive domains in adolescence predicted lower odds of earlier mortality (ORs of 0.79 - 0.87). Co-sibling control models indicated a similar pattern, suggesting that benefits associated with higher cognitive performance do not simply reflect shared environmental and genetic background, but may represent a direct protective effect. These findings indicate that better performance in multiple cognitive domains in adolescence, above and beyond the influence of genetic and family environmental factors, may be or point to modifiable protective factors against risk of early mortality.

### LONG-TERM COGNITIVE EFFECTS FROM A REAL-WORLD MULTI-SKILL LEARNING INTERVENTION IN OLDER ADULTS

Leah Ferguson,[1] Debaleena Sain,[1] Esra Kürüm,[1] Carla Strickland-Hughes,[2] George Rebok,[3] and Rachel Wu,[4], *1. University of California, Riverside, Riverside, California, United States, 2. University of the Pacific, University of the Pacific, California, United States, 3. Johns Hopkins University, Baltimore, Maryland, United States, 4. University Of California - Riverside, Riverside, California, United States*

Previous cognitive learning interventions have focused primarily on learning one or two novel real-world skills at a time, or utilizing computer-based programs to enhance specific cognitive skills (Ball et. al 2002; Park et. al, 2014). While these studies yielded immediate cognitive improvements in participants, the long-term benefits of continuing to learn several real-world skills in older adulthood is unclear. In the present two studies, the long-term (1-year post-intervention) benefits of a multi-skill learning intervention were investigated with older adult participants. Study 1 (a pilot sample) included 6 participants (67% female, M = 66.33 years, SD = 6.41, range = 58–74 years old) and Study 2 included 27 participants (67% female, M = 69.44 years, SD= 7.12, range = 58–86 years old). Following a three month intervention which entailed simultaneously learning at least three real-world skills, such as photography, drawing, and Spanish, participants' cognitive abilities were assessed using four tasks (Flanker, Set-Shifting, Dot Counting, and N-Back), as well as RAVLT and Digit Span. Follow-up assessments were completed at three-, six-, and 12-month follow-ups after the interventions. Linear mixed-effects regression models revealed significant cognitive improvements across time points up to one year following the intervention compared to baseline assessments. These promising results support the idea that intense learning experiences may lead to considerable cognitive growth in older adulthood, as they do earlier in the lifespan.

### THE DUAL-LANGUAGE SEMANTIC COMPUTERIZED PROGRAM (DISC) MAINTAINED LOCAL SWITCH COSTS IN MCI OLDER ADULTS

W.Quin Yow,[1] Hui-Ching Chen,[2] and Tharshini Lokanathan,[2], *1. Singapore University of Technology & Design, Singapore, Not Applicable, Singapore, 2. Singapore University of Technology and Design, Singapore University of Technology and Design, Not Applicable, Singapore*

It has been proposed that switching cost deficit in executive control (Velichkovsky et al., 2020) could be used as an early marker for abnormal aging processes. Although research with technology-based intervention has shown benefits in improving cognitive performance with older adults, the overall results are mixed (Ge et al, 2018). This study aims to investigate whether computerized intervention program (e.g., DISC) would help to reduce the switching costs deficits in mild-to-moderate cognitively-impaired older adults (MCI-OA). Fourteen MCI-OA (79.75±6,94) and 9 cognitively-healthy OA (age 77,25±6,9) were randomly assigned to an experimental group or a control group (a final sample size of 30 MCI and 40 cognitive-healthy older adults would be ready by conference time). All participants first completed a set of cognitive tasks as part of a larger study (i.e., pre-tests) (e.g., MMSE, Ravens, cued-base Task Switching Task). The experimental group then played cognitive games on a touch-screen tablet for about 30-40 minutes per session with a total of 24 sessions over 8-12 weeks. The control group continued their daily activity as per usual for 8-12 weeks. Participants were then asked to complete the same set of cognitive tasks again post-test. Control group MCI-OA performed worse for the local costs in the cued Task Switching task ($p<.05$), whereas experimental group MCI-OA maintained their performance ($p=.40$) post-test compared to pre-test. All cognitively-healthy OA did not show any difference in performance irrespective of condition. This suggests that the DISC program could be an effective tool in slowing down the abnormal accelerated aging process.

### Session 4350 (Paper)

### COGNITIVE IMPAIRMENT AND CAREGIVING

### DYADIC RELATIONSHIPS BETWEEN SELF-RATED HEALTH AND COGNITION AMONG OLDER ADULTS AND THEIR SPOUSAL CAREGIVERS

Ruotong Liu,[1] Iris Chi,[2] and Shinyi Wu,[3], *1. University of Southern California, Marina del Rey, California, United States, 2. University of Southern California, University of Southern California, California, United States, 3. University of Southern California, SANTA MONICA, California, United States*

This study examines spouses who are in a caregiving situation to discern how they influence each other's health. Previous studies reported health concordance and cross-domain effects among caregiver and care-recipient dyads. However, it is less understood of the health dyadic relationships among spouses who are in a caregiving situation. No studies have specifically looked into the relationship between self-rated health (SRH) and cognitive functioning among spousal caregiving dyads over time. In this study we analyzed the longitudinal reciprocal relationships between SRH and cognitive functioning measured by the Telephone Interview for Cognitive Status among older adults and their spousal caregivers, and whether the relationship differed by whether husband or wife was the caregiver. Longitudinal data from the Health and Retirement Study (2010-2016) on 540 dyads were pooled and analyzed using structural equation modeling under an actor-partner interdependence model. Results revealed cognitive concordance among older spouses in which caregivers' cognition is associated with care-recipients' cognition subsequently ($\beta$=0.05, p<.05). SRH concordance was not significant. Cross-domain results showed only one significant direction, that is, care-recipients' cognition in the subsequent time was significantly correlated with caregivers' SRH, regardless of whether husband ($\beta$=0.09, p<.05) or wife ($\beta$=0.08, p<.05) was the caregiver. Our study found that married couples in a spousal caregiving situation displayed cognitive but not overall health concordance, and cross-domain effects of caregiver's SRH on spousal care recipient's cognition subsequently. The reciprocal associations suggest that addressing and improving either partner's physical health and cognition may benefit both dyad members.

### EXPLORING THE EXPERIENCES OF PERSONS LIVING WITH DEMENTIA AND THEIR CARE PARTNERS DURING THE COVID-19 PANDEMIC

Jeanine Yonashiro Cho,[1] Elizabeth Avent,[2] Laura Mosqueda,[3] and Zachary Gassoumis,[3], *1. University of Southern California, Alhambra, California, United States, 2. University of Southern California, Los Angeles, California, United States, 3. Keck School of Medicine of USC, Alhambra, California, United States*

Public health measures implemented to mitigate the spread of COVID-19 have transformed the physical and social environments in which we live. The effects of these policies on persons living with dementia (PLWD) and their care partners (CPs) are not fully understood. This study explores the experiences, attitudes, and perceptions of caregiving dyads during the COVID-19 pandemic. Cross-sectional survey data were drawn from a larger longitudinal study examining the relationship between PLWD aged 65+ and their CPs being conducted in a metropolitan city significantly affected by COVID-19. Interviews with were conducted remotely via videoconferencing and telephone. Data on sources and types of care provided for the PLWD, relationship quality and conflict, and caregiver stress were collected and analyzed using descriptive statistics and tests of independence. Preliminary results from PLWD (n=8) and CPs (n=13) confirmed a reduction in social interaction with family members and friends. CPs reported they (n=5) or other family members (n=2) changed their schedules to provide care for the PLWD. CPs reported increased conflict with the PLWD regarding care provision, going out or welcoming visitors, and home management. In contrast, PLWD reported a lack of conflict among household members (n=6) and the perception of good changes (n=2) and increased quality time with CPs. Preliminary findings provide empirical evidence of the effects of pandemic public health policies on dyads enrolled in this study and reveal differences in perceived relational conflict between PLWD and their CPs. Further research is needed to better understand the experiences of dyads and develop supportive interventions.

### GAPS IN THE SYSTEM: SUPPORTING PEOPLE LIVING WITH DEMENTIA AND THEIR CAREGIVERS

Madeline King,[1] Allie Peckham,[2] Monika Roerig,[1] Husayn Marani,[1] and Gregory Marchildon,[1], *1. University of Toronto, Toronto, Ontario, Canada, 2. Arizona State University, Pheonix, Arizona, United States*

As individuals are living longer, the prevalence of older adults living with dementia and other complex health and social care needs is on the rise (Alzheimer's Association, 2020; CIHI, 2020). Correspondingly, efforts to develop supportive programming and policies for persons living with dementia (PLWDs) are of paramount importance (CIHR, 2019). The challenges faced by PLWDs and other complex health and social needs are widely known (CIHR, 2019), however, a systematic understanding of how and if current and long-standing efforts are adequately meeting the needs of these individuals remains elusive. This research sought to understand how program administrators, decision makers, PLWD, and caregivers across five North American jurisdictions (British Columbia, Ontario, Newfoundland and Labrador, New York State, and Vermont) perceived specific dementia care programs and support services within their respective jurisdictions. We performed an inductive analysis of semi-structured interviews (N=37) and identified on-going care gaps experienced by participants. We present three main gaps: 1) disconnected and uncoordinated system infrastructure, 2) lack of comprehensive services to meet the diverse needs of PLWD and their caregivers, and 3) inconsistency in how dementia is understood; with associated perceived remedies. The results suggest that even when attempts to address the needs of PLWD and their caregivers are put in place there remains significant limitations of systems. The perspectives of decision makers, program administrators and individuals with lived experience offer unique insight into how these experiences may be improved to better support the complex needs of PLWD and their caregivers.

### REFINING CARE TO PLAN: DELIVERING PERSONALIZED RECOMMENDATIONS TO SUPPORT DEMENTIA CAREGIVERS

Jinhee Cha,[1] Colleen Peterson,[1] Ashley Millenbah,[1] Katie Louwagie,[1] Zachary Baker,[2] Christine Jensen,[3] and Joseph Gaugler,[4], *1. University of Minnesota, University of Minnesota, Minnesota, United States, 2. University of Minnesota, University of Minnesota/Minneapolis, Minnesota, United States, 3. Riverside Center for Excellence in Aging & Lifelong Health, Williamsburg, Virginia, United States, 4. University of Minnesota, Minneapolis, Minnesota, United States*

Caregivers of persons living with Alzheimer's disease and Alzheimer's disease-related dementias (PLWD; AD/ADRD) benefit from unique interventions to address their different needs. While information on which interventions best meet specific needs exists, less is known about how to match caregivers with those interventions. To address this research gap, we tested Care to Plan (CtP) within a large healthcare system. After care navigators guided caregivers through twenty CtP tailoring questions to identify caregivers' greatest needs, the online tool provided the intervention type best suited to meet their needs, along with region-specific information on available programs. This mixed methods analysis evaluated the utility of the CtP tool with 20 family caregivers of PLWD after a 1 month follow-up. Most caregivers agreed that the CtP tool was helpful (85%) and would recommend the tool to other caregivers (90%). Caregivers also said they valued being able to discuss the CtP recommendations with the care navigator (95%). However, only 65% said they found services that met their needs or planned on using services recommended by CtP. Interview data indicate time constraints and restricted availability of resources due to COVID-19 precautions reduced caregivers' abilities to pursue some recommendations. In addition, the stage of dementia experienced by their care recipient may explain why others found CtP less useful. However, these caregivers noted the potential utility of the resources for their future care planning needs. A larger evaluation of the CtP tool within the healthcare system is ongoing.

### THE EDUCATION OF FAMILY MEMBERS AND OLDER ADULT COGNITIVE HEALTH: DIFFERENCES ACROSS RACIAL GROUPS

Sindhu Vasireddy,[1] Jenjira Yahirun,[2] and Mark Hayward,[3], *1. University of St Andrews, UK, St. Andrews, Fife, Scotland, United Kingdom, 2. Bowling Green State University, Bowling Green State University, Ohio, United States, 3. University of Texas at Austin, University of Texas at Austin, Texas, United States*

Education is a strong predictor of cognitive health among older adults, and recent research indicates that apart from one's own education, the educational resources of family members also play a crucial role in shaping cognitive health over the life course. We add to this literature by investigating whether the advantages of highly educated family members matter for both Blacks and Whites in the U.S. Specifically, we ask whether the resources of family members-parents and offspring-partially explain the racial gap in both the prevalence and incidence of cognitive health across both groups. For this, we employ logistic regression models to examine the prevalence of cognitive impairment at baseline, and discrete-time event history models to assess the incidence of cognitive impairment, using data from the Health and Retirement Study (HRS) for the years ranging from 2000 to 2012. Preliminary results indicate that at the baseline, respondent's own education is predictive of cognitive impairment among Whites, but not Blacks. Once respondent-level health conditions and behaviors are taken into consideration, parental or offspring education is not associated with the prevalence of cognitive impairment. For respondents who are not impaired at the baseline, our results from the incidence models align with the "adjacent generations" literature suggesting that both parental and offspring education has a salient positive effect on later-life cognitive health. However, we find notable differences across groups to suggest that the education of parents and offspring may play a larger role in mitigating cognitive decline among African Americans, compared to Whites.

### Session 4355 (Paper)

### COVID-19 AND MORTALITY IN NURSING HOMES

### COVID-19 DEATH IN HIGH-MEDICAID NURSING HOMES: THE ROLE OF EMPLOYMENT EMPOWERMENT

Ganisher Davlyatov,[1] Justin Lord,[2] Akbar Ghiasi,[3] and Robert Weech-Maldonado,[4], *1. University of Oklahoma Health Sciences Center, Oklahoma City, Oklahoma, United States, 2. Louisiana State University at Shreveport, Bossier City, Louisiana, United States, 3. University of the Incarnate Word, San Antonio, Texas, United States, 4. University of Alabama at Birmingham, Birmingham, Alabama, United States*

This study examines the association between COVID-19 death and employee empowerment in under-resourced nursing homes (70% or higher Medicaid census). Employee empowerment captures elements of participative decision making, autonomy, responsibility, open communication, decentralization, and decision-making flexibility within an organization. Survey data from 391 Directors of Nursing (response rate of 37%) from 2017-2018, were merged with secondary data from CMS Nursing Home COVID-19 Public File, LTCFocus, Area Health Resource File, and Nursing Home Compare. A Poisson regression was used to examine reported COVID-19 death and employee empowerment. The independent variable employee empowerment was the mean score of summated Likert scale questions. Control variables included organizational (size, location, ownership, chain affiliation, quality, payer mix, acuity, occupancy, and race/ethnicity, staffing mix), and county factors (Medicare Advantage penetration, per capita income, poverty, unemployment, education, 65+ population, and competition). Employee empowerment was associated with a lower number of COVID-19 cases ($p < 0.05$). Rural, not-for-profit, and payer-mix were also significantly associated with a lower number of COVID-19 deaths. Employee empowerment captures the decentralization of authority and an employee's ability to make decisions without approval. In light of this crisis, empowerment may have helped under-resourced nursing homes be more agile and faster in their response. High-Medicaid nursing homes may need to consider different decision-making practices when faced with a crisis, such as, COVID-19.

### COVID-19 MORTALITY IN ADULT CONGREGATE LIVING SETTINGS ACROSS FIVE U.S. STATES

David Rein,[1] Lindsey Shapiro,[2] Mairin Mancino,[3] and Caroline Pearson,[3], *1. NORC, Atlanta, Georgia, United States, 2. NORC, NORC, Chicago, Illinois, United States, 3. NORC, NORC, Maryland, United States*

The magnitude of COVID-19 mortality in adult congregate living settings other than nursing homes (NH) is unknown. To address this, we created an individual property level dataset for five U.S. states (Colorado, Connecticut, Florida, Georgia, and Pennsylvania) using multiple public and private sources. The data included information on each observation's state and county, level of care (LOC), the estimated number of residents, COVID-19 deaths through December 31, 2020, and county-level cases of COVID-19 per 100,000. We restricted our sample to market grade properties with 25+ residents, for which we able to estimate resident and LOC information. We defined LOC as County, non-congregate (CN), Independent Living (IL), Assisted Living (AL), Memory Care (MC), and Nursing Home (NH). We used multilevel, multivariable logistic regression models to estimate the expected death rate for each LOC controlling for differences in reported COVID-19 infections and county and state reporting differences. We identified 3,059 properties that met our inclusion criteria (69 CN, 477 IL, 1,118 AL, 179 MC, and 1,216 NH). We estimated deaths per 1,000 persons of 4.4 (95% CI: 4.0-4.8) for CN, 4.4 (3.9-4.9) for IL, 16.2 (14.7-17.9) for AL, 50.3 (44.4-56.8) for MC, and 32.0 (29.3-34.9) for NH. The order of death rate severity was the same in each state, except MC in Georgia. Additional research is needed to evaluate whether death rate differences resulted from congregate living risks, from COVID mortality risk factors at each LOC, or a combination of factors.

### COVID-19 NURSING HOME DEATHS IN COOK COUNTY: PREDICTORS AND MODIFIABLE RISKS

Andrew DeMott,[1] Susan Hughes,[2] Michael Gelder,[2] and Sage Kim,[2], *1. UIC, Chicago, Illinois, United States, 2. University of Illinois at Chicago, Chicago, Illinois, United States*

Investigators at the University of Illinois Chicago, the Illinois Department on Aging (IDOA), the Illinois Department of Public Health, and the Health and Medicine Policy Research Group are collaborating to examine comparative rates of Covid-19-related deaths among older adults who reside in nursing homes vs. the community in Illinois. As a first step, we have examined data from the Cook County Medical Examiner's office to compare nursing home resident fatalities to those who died in the community. Deaths with Covid-19 listed as primary or secondary cause of death that occurred between January 1, 2020 to September 30, 2020 among older adults ages 60 and over were identified from the Cook County Medical Examiner's Office case archive file. Location at death and race/ethnicity were obtained from the same source. Location at death was matched with data in the Center for Medicare and Medicaid Services (CMS) Covid-19 Nursing Home Data to identify persons who died in skilled nursing facilities (SNFs) as well as facility and staff characteristics. We found that the 3,937 deaths among persons over the age of 60 comprised 75% of total deaths in Cook County. Of the total older adult deaths, 2,090 (53%) died in the community and 1,837 (47%) died in SNFs. Regression analyses that controlled for CMS quality ratings found that larger, for-profit nursing homes, with high levels of staff infected with Covid-19 were associated with higher mortality. The policy implications of these findings will be discussed.

### DISPARITIES IN COVID-19 MORTALITY IN HIGH-MINORITY NURSING HOMES

Robert Weech-Maldonado,[1] Justin Lord,[2] Ganisher Davlyatov,[3] Akbar Ghiasi,[4] and Gregory Orewa,[1], *1. University of Alabama at Birmingham, Birmingham, Alabama, United States, 2. Louisiana State University at Shreveport, Bossier City, Louisiana, United States, 3. University of Oklahoma Health Sciences Center, Oklahoma City, Oklahoma, United States, 4. University of the Incarnate Word, San Antonio, Texas, United States*

Racial/ethnic disparities in healthcare have been highlighted by the recent COVID-19 pandemic. Minorities continue to utilize nursing home services at a higher rate than White residents, contributing to existing health inequity concerns. This study examined the relationship between nursing home racial/ethnic mix and COVID-19 resident mortality using the CMS Nursing Home COVID-19 Public File. As of October 25, 2020, high minority nursing homes reported 6.5 COVID-19 deaths as compared to 2.6 deaths for nursing homes that had no racial/ethnic minorities. Four nested sequential negative binomial regressions were used to model the relationship between racial/ethnic disparities in COVID-19 deaths and the separate contributions of facility-level resident characteristics (percent of females, percent of residents 65 years and older, percent of residents with congestive heart failure, hypertension, and obesity, and the average level of residents' acuity), resource availability (nursing homes' payer-mix, occupancy rate, county-level Social Deprivation Index, and nursing home location), and other organizational characteristics (nursing home for-profit status, chain affiliation, and self-reported nursing, clinical, aides, and other staff shortages). After controlling for interstate differences, facility-level resident characteristics, resource availability, and organizational characteristics, high-minority nursing homes had 61% more COVID-19 deaths (Incidence Rate

Ratio [IRR] = 1.61; $p < 0.001$) as compared to nursing facilities with no minorities. From a policy perspective, nursing homes, that serve primarily minority populations, may need additional resources, such as, funding for staffing and equipment in the face of the pandemic. The COVID-19 pandemic has sharpened the focus on healthcare disparities and societal inequalities in the long-term care.

### Session 4360 (Paper)

### CULTIVATING MORE AGE-FRIENDLY CAMPUSES

### BRINGING TRANSDISCIPLINARY AGING RESEARCH FROM THEORY TO PRACTICE

Lana Sargent,[1] Tracey Gendron,[2] Marissa Mackiewicz,[3] Ana Diallo,[4] Faika Zanjani,[2] Elvin Price,[5] Pamela Parsons,[2] and Gregory Ford,[6], *1. VCU School of Nursing, Ashland, Virginia, United States, 2. Virginia Commonwealth University, Richmond, Virginia, United States, 3. VCU Institute for Inclusion, Inquiry, and Innovation (iCubed): Health and Wellness in Aging Populations, Richmond, Virginia, United States, 4. Virginia Commonwealth University School of Nursing, Richmond, Virginia, United States, 5. Virginia Commonwealth University/ School of Pharmacy, Richmond, Virginia, United States, 6. Beacon Housing Communities, Richmond, Virginia, United States*

There is a growing emphasis to use a transdisciplinary team approach to accelerate innovations in science to solve complex conditions associated with aging. However, the optimal organizational structure and process for how to accomplish transdisciplinary team science are unclear. In this study, we illustrate our team's experience using transdisciplinary approaches to solve challenging and persistent problems for older adults living in urban communities. We describe our challenges and successes using the National Institutes of Health four-phase model of transdisciplinary team-based research. Using a de-identified survey, the team conducted an internal evaluation to identify features that created challenges including structural incongruities, interprofessional blind spots, group function, and group dynamics. The team then identified responses to address the features that created challenges and determined indicators for success. Indicators for success were identified by the team as a place for continued evaluation of the teams' collaborative effectiveness, transdisciplinary integration, and impact on the university and aging community. This work resulted in the creation of the team's Transdisciplinary Conceptual Model. This model became essential to understanding the complex interplay between societal factors, community partners, and academic partners. Conducting internal evaluations of transdisciplinary team processes is integral for teams to move beyond the multi- and interdisciplinary niche and to reach true transdisciplinary success. More research is needed to develop measures that assess team transdisciplinary integration. Once the process of transdisciplinary integration can be reliably assessed, the next step would be to determine the impact of transdisciplinary team science initiatives on aging communities.

### IMPLEMENTING AGE-FRIENDLY UNIVERSITY PRINCIPLES: INSTITUTIONAL SUPPORTS, BARRIERS, AND OUTCOMES

Katarina Felsted,[1] and Jacqueline Eaton,[2], *1. University of Utah, Salt Lake City, Utah, United States, 2. University of Utah, University of Utah, Utah, United States*

The University of Utah Gerontology Interdisciplinary Program received an Age-Friendly University (AFU) seed grant through GSA's Academy of Gerontology in Higher Education, funded by AARP, to develop a model for promoting lifelong learning in partnership with university and community stakeholders. We designed and instituted a targeting marketing campaign that supported our goals: 1) to implement AFU principles; 2) to promote awareness of HB60, a legislative bill allowing people 62+ to audit courses at public universities for a minimal cost; 3) to enhance HB 60 enrollment through increased communication of online course options and tuition waiver support; and 4) to improve university and community stakeholder engagement. This presentation describes project benefits, including increased awareness of AFU initiatives, promoting age diversity, safe participation through online coursework, and enhanced community partnerships. The initiative garnered strong departmental support for marketing, communications, and structure for the post-award process. Barriers occurred due to a lack of HB60 infrastructure at the university level, which inadvertently obstructs organizational engagement. This initiative targeted AFU principles while supporting the university's strategic goal of engaging communities and preparing to pursue membership in the AFU Global Network. Future AFU goals include developing advocacy channels within the university to improve organizational support.

### OLDER STUDENT EXPERIENCES: A CRITICAL EXAMINATION OF AGING ON CAMPUS

Cassandra Barragan,[1] and Sarah Walsh,[2], *1. Eastern Michigan University, Ypsilanti, Michigan, United States, 2. Eastern Michigan University, Eastern Michigan University, Michigan, United States*

There is overwhelming evidence that the number of older learners on college campuses has been steadily increasing since the 1970s. The needs of older learners differ from traditional students, and many services and resources available at higher education institutions are geared towards students aged 18-25 (Silverstein, Choi, & Bulot, 2001). Age Friendly University (AFU) principles highlight the need to consider older learners at a university and provide structure to evaluate programs and practices and to enhance inclusion and diversity based upon age. This study examined how an AFU designated university is working to better understand their older students.

**Methods:** A web-based pilot survey of older learners (N=248) asked all students ages 40 and older a series of questions regarding motivation to attend school, barriers and supports, campus environment, and connection with AFU principles.

**Analysis:** A regression analysis found that older learners who felt more welcomed by faculty (p=.001), administration (p=.002),and student organizations (p=.026) were more likely to feel connected to campus, and younger-older students (p=.031) and those who did not feel their job was a barrier to attending school (p=.037) were more likely to feel satisfied with their level of engagement on campus. Additionally, older learners felt the AFU principles were demonstrated by their university.

**Discussion:** The experiences of older learners are important as we continue to see higher numbers of students over the age of 40. Our results demonstrate the need to engage older learners as part of diversity and inclusion efforts to facilitate connection to the campus community.

### THE INTERSECTION OF OLDER STUDENTS AND DISABILITY: HOW AGE-FRIENDLY UNIVERSITIES CAN BOOST VISIBILITY AND ACCESSIBILITY

Cassandra Barragan,[1] and Sarah Walsh,[2], *1. Eastern Michigan University, Ypsilanti, Michigan, United States, 2. Eastern Michigan University, Eastern Michigan University, Michigan, United States*

The needs of older learners differ from traditional students, and many services and resources available at higher education institutions are geared towards students aged 18-25 (Silverstein, Choi, & Bulot, 2001). Age Friendly University (AFU) principles highlight the need to consider older learners at a university. Older learners face various barriers to education including balancing schoolwork with responsibilities and accessibility of campus resources (Silverstein et al., 2001). This study examined how an AFU designated university is working to better understand their older students.

**Methods:** A web-based pilot survey of older learners (N=248) asked all students ages 40 and older a series of questions regarding motivation to attend school, barriers and supports, campus environment, and connection with AFU principles.

**Analysis:** A t-test analysis explored differences in motivation, barriers and challenges, and connection to campus between students who identified as having a disability and those who did not.

**Findings:** We found there were significant differences between the groups in how health impacted their education (p=.001), being able to physically access campus (p=.014), the availability of online classes (p=.047), and the hours of operation of student support services (p=.045). There were also differences between groups in how connected they felt to campus based on feeling welcomed by faculty (p = .033) and feeling satisfied with their level of engagement at the university (p = .002).

**Discussion:** Our results demonstrate the need to fully engage older learners with a disability as part of diversity and inclusion efforts to facilitate connection to the campus community.

### TOWARD AGE-FRIENDLY HIGH EDUCATION: AN INTERGENERATIONAL PARTICIPATORY CO-DESIGN APPROACH

Vivian, W. Q. Lou,[1] Esther Woo,[2] Nicol Pan,[2] Peter J. Cobb,[2] Xiao Hu,[2] and Michael Cheng,[2] *1. The University of Hong Kong, Hong Kong, Not Applicable, Hong Kong, 2. The University of Hong Kong, The University of Hong Kong, Not Applicable, Hong Kong*

**Objective:** When aging becomes a global challenging, we believe it is timely important to equip aging knowledge among university students regardless of their disciplinary study subjects. This study aims to describe principles and process of development an aging-related curriculum in high education entitled "Intergenerational Participatory Co-design Project (IPCP)" and evaluate its impacts.

**Methodology:** Guided by a key principle of involving participants of any learning context as co-creators of both the learning process and learning outcomes, IPCP went through four stages of development including capacity building, co-creation on learning objectives, deliberated content learning, and learning outcome dissemination. Mixed methodology including qualitative in-depth interview and quantitative questionnaire were applied in evaluation. A total of 26 participants, from three generations recruited from one university, one secondary school, and a pool of senior champions under a geron-infusion initiative participated.

**Findings:** after attaining capacity building workshops applying Optimal Quality Intergeneration Interaction Framework, three learning groups formulated. A common theme "preserving cultural heritage" emerged, while each group has identified a specified focus (e.g., food, Tai Ji, and historic sites guide). Quotes collected and survey data revealed positive impacts in reducing stereotype and enhancing learning experiences.

**Conclusion:** IPCP demonstrated good practices in role models in multi-disciplinary collaboration in pedagogy innovation. It also paved solid way towards a learning community interwoven with continuous innovation: IPCP becomes a pioneer contributor of library's digital data hub solution; common core office starts to develop a human lifespan cluster; two research team members started new collaboration on geron-infusion in Faculty of Education.

### Session 4365 (Symposium)

### CURRENT RESEARCH ON SUICIDE IN OLDER ADULTS

Chair: Montgomery Owsiany Discussant: Yeates Conwell

Rates of suicide are elevated in middle- and late-life, yet studies focusing on suicidal ideation and behavior in older adults are limited compared to research in younger adults. The studies included in the present symposium offer valuable findings on suicide in older adults across the span of late-life. Owsiany et al. focus on age differences between older and younger adults in the association between anxiety symptoms and suicide risk. In Heisel et al., an online intervention is assessed for improving the outcomes of psychological well-being and suicide risk in older adult men who are transitioning into retirement during the COVID-19 pandemic. Crnek-Georgeson and Wilson reviewed the link between retirement patterns and psychological effects, including suicidal behaviors, among older adults. Additionally, this review includes recommendations for policy makers and employers in an effort to assist older adults with the transition into retirement. Utilizing baseline data from the Helping Older

Adults Engage study, Fenstermacher et al. research the association between volunteering and suicidal ideation in a predominantly lonely older adult sample across the span of late-life. Together, these studies provide foundation for future research on suicide in late-life to build upon. Future studies should continue to focus on risk and protective factors for suicide in older adults and aim to improve screening and intervention for suicidal thoughts and behaviors in this population. Yeates Conwell, M.D., Director of Geriatric Psychiatry and Co-Director of the Center for the Study and Prevention of Suicide at the University of Rochester Medical Center, will serve as discussant.

### AGE DIFFERENCES IN THE ASSOCIATION BETWEEN ANXIETY SYMPTOMS AND SUICIDE RISK

Ruifeng Cui, Amy Fiske, and Montgomery Owsiany, *West Virginia University, Morgantown, West Virginia, United States*

Suicide rates increase over the life-span, necessitating concern in older adults. Recent studies suggest that anxiety disorders are associated with suicidal thoughts and behavior. The present study examined the association between anxiety symptoms (General Anxiety Disorder-7) and suicide risk (Suicide Behaviors Questionnaire-Revised), testing whether the association differs between younger and older adults. Depression symptoms (Patient Health Questionnaire-8) were controlled for in the analyses. In a sample of 944 participants (46% 60+ years), anxiety symptoms, depression symptoms, and suicide risk were lower among older adults (60+ years) than younger adults (all $p < .01$). Age moderated the significant association between anxiety symptoms and suicide risk ($\Delta R2 = .008$, $p < .01$). Results indicate that an increase in anxiety is associated with a smaller increase in suicide risk for older adults than younger adults. The need for suicide risk screening among individuals with elevated anxiety symptoms is critical, especially for younger adults.

### RELATION BETWEEN VOLUNTEERING AND SUICIDAL IDEATION AMONG OLDER ADULTS

Montgomery Owsiany,[1] Amy Fiske,[1] Kim Van Orden,[2] and Erika Fenstermacher,[1], *1. West Virginia University, Morgantown, West Virginia, United States, 2. University of Rochester School of Medicine & Dentistry, Rochester, New York, United States*

Suicide disproportionally affects older adults, necessitating further investigation into potential protective factors for suicide in late life. Volunteering is associated with psychological well-being in older adults. However, there is scant literature examining the association between volunteering and suicidal ideation or behavior in this age group. The present study involved a cross-sectional examination of volunteering and suicidal ideation using baseline data from the Helping Older People Engage study. This sample (N = 245, 88% White, 68% female, 60-92 years old) was recruited from primary care practices. Inclusion criteria included clinically significant loneliness (UCLA loneliness scale M = 49.5; SD = 5.7). Forty-three percent of the sample reported engaging in recent volunteer activity. Passive and active suicidal ideation in the past month were endorsed, respectively, by 20% and 9% of the sample. Results of hierarchical linear regression analyses examining the association between volunteering and suicidal ideation, controlling for covariates, will be discussed.

### RETIREMENT AND SUICIDAL BEHAVIOURS: A REVIEW OF THE INTERNATIONAL LITERATURE

Leigh Wilson,[1] and Kylie Crnek-Georgeson,[2], *1. The University of Sydney, Camperdown, New South Wales, Australia, 2. The University of Sydney, Camperdown/ Darlington, New South Wales, Australia*

This scoping review maps published literature on retirement patterns and the effect retirement has on individuals. Recommendations are provided for future research, including changes to retirement policies, to decrease the prevalence of suicidal behaviours for older adults. A literature search (2014 - 2020) was conducted in Medline, PubMed, Cinahl and Scopus, using the terms retirement, transition, redundancy, and pathways. A rapid review of global literature was undertaken, identifying 204 articles, with 27 papers chosen for full review. Search terms included retirement, transition, redundancy and pathways to retirement. Themes arising from the data were experience of retirement, planning, health (physical and psychological), and levels of social participation. This review provides information for policy makers, health workers and employers to assist individuals with retirement, emphasising the need to maintain competency in a complex set of skills to improve health literacy and decrease psychological stress/ suicidal behaviours in older adults.

### TESTING ONLINE MEN'S GROUPS TO PROMOTE PSYCHOLOGICAL WELL-BEING AND REDUCE DESPAIR DURING THE COVID-19 PANDEMIC

Paul Links,[1] Sisira Sarma,[2] Gordon Flett,[3] Kimberly Wilson,[4] Simon Hatcher,[5] Sylvie Lapierre,[6] David Conn,[7] and Marnin Heisel,[8], *1. McMaster University, Hamilton, Ontario, Canada, 2. The University of Western Ontario, London, Ontario, Canada, 3. York University, Toronto, Ontario, Canada, 4. University of Guelph, Guelph, Ontario, Canada, 5. University of Ottawa, Ottawa, Ontario, Canada, 6. Universite du Quebec a Trois-Rivieres, Trois-Rivieres, Quebec, Canada, 7. Baycrest; University of Toronto, Toronto, Ontario, Canada, 8. Schulich School of Medicine & Dentistry, The University of Western Ontario, London, Ontario, Canada*

Suicide prevention is a healthcare and social justice priority. Older adults have the highest rates of suicide and the highest COVID-19 fatality rates in North America. The combined impacts of social isolation, fear of infection, apathy, and hopelessness could amplify suicide risk among older adults, as appears to have been the case during the 2003 SARS epidemic in Hong Kong. Innovative interventions are thus needed to promote social interaction and reduce risk for suicide in these challenging times. We are currently testing an online version of our Meaning-Centered Men's Group (MCMG; Heisel et al., 2020), an upstream psychological intervention designed to promote psychological well-being and reduce suicide risk among men struggling with the transition to retirement, in the context of pandemic-related public health restrictions. This presentation will focus on adaptations to MCMG for online delivery, and share participant experiences and findings on positive and negative psychological outcomes.

### Session 4370 (Symposium)

### DISRUPTION TO SUBSTANCE AND OPIOID USE DISORDER: THE DEEP SOUTH SUBSTANCE AND OPIOID USE RURAL TRAINING GRANT

Chair: Rebecca Allen Co-Chair: Lindsey Jacobs
Discussant: Bruce Rybarczyk

The primary objective of this symposium is to describe our integrated, interprofessional behavioral health training program in substance use and opioid use disorders (SUD/OUD) across the adult lifespan (19 to 80) within our clinical psychology graduate program in the Deep South. Due to the COVID-19 pandemic, our assessment, treatment, and prevention delivery has occurred via telehealth. The first paper describes our Clinical Training Model in two federally qualified health centers (one peri-urban and one rural) and one residential drug and alcohol rehabilitation program. Graduate and undergraduate students provide prevention, assessment, and treatment with an emphasis on 1) mindfulness-based relapse prevention, 2) literacy-adapted treatment for chronic pain, and 3) trauma and recovery. The second paper describes the participant population. Specifically, participants (N = 105) receiving prevention, assessment and treatment services report high levels of substance and opioid use and are underserved, impoverished, and have low levels of education and health/mental health literacy. The third paper explores the relation of age, adverse childhood experiences, and PTSD symptoms within the context of substantial or severe SUD/OUD. The final paper describes issues surrounding telehealth delivery in the rural south with underserved populations. The discussant, an expert in integrated, interprofessional telehealth delivery across the adult lifespan, will provide insight on program sustainability and dissemination. Given the pronounced need for SUD/OUD treatment in underserved populations with attention to the intersection of age and urban/rural residence, this project is poised to make a substantive impact across the adult lifespan.

### INTEGRATING PSYCHOLOGICAL SERVICES INTO COMMUNITY-BASED CLINICS

Lindsey Jacobs, *The University of Alabama, The University of Alabama, Alabama, United States*

In Alabama, where mental health stigma is a critical barrier to care, integrated behavioral health services are vital to address the mental health needs that underlie substance use disorder (SUD) and opioid use disorder (OUD). Since October 2019, our team has developed partnerships with one rural and two peri-urban primary care clinics to offer behavioral health services with an emphasis on SUD/OUD prevention, screening, and treatment. The patient populations receiving services at these three facilities are under-resourced with multiple disadvantages placing them at risk for morbidity, mortality, SUD/OUD, and poor behavioral and mental health outcomes. Behavioral health services have been delivered primarily via telehealth due to the COVID-19 pandemic. This presentation will describe the process, current status, and future goals for implementing integrated behavioral health care, with a focus on identifying the barriers and facilitators during the COVID-19 pandemic era.

### CHARACTERISTICS OF OUR PATIENT POPULATION

Kyrsten Hill, *The University of Alabama, Tuscaloosa, Alabama, United States*

To date, 106 patients have completed behavioral health assessments across three sites: a rural primary care clinic (n = 32), urban federally qualified health center (n = 33), and state-certified residential rehabilitation facility (n = 41). Patients ranged from 18 to 65 years of age (M = 38.6, SD = 11.4). Approximately 51% were female and 75% were non-Hispanic White (followed by 22% African American). Over 60% had a high school degree or less and found it at least somewhat difficult to pay for basic needs. Most patients endorsed substantial (44%) or severe (39%) drug use, with 40% endorsing opioid use. There were no significant differences in substance use by age group. Moderate to severe symptoms of depression (43%) and anxiety (49%) were common. Approximately 70% endorsed adverse childhood experiences, and 44% reported clinically significant post-traumatic stress symptoms. Measures of cognitive functioning and objective health literacy are currently being collected.

### AGE, CHILDHOOD TRAUMA, POST-TRAUMATIC STRESS DISORDER, AND SUBSTANCE USE DISORDER IN THE DEEP SOUTH

Rebecca Allen,[1] and Candice Reel,[2], *1. University of Alabama, Tuscaloosa, Alabama, United States, 2. The University of Alabama, Tuscaloosa, Alabama, United States*

We examined the relationship of reported childhood trauma and PTSD symptoms in a sample (N = 105) of individuals aged 19 to 80 receiving treatment for substance use and opioid use disorders in federally qualified health centers. Fifty-two percent of the sample was age 39 or younger, 30% were in their 40s and 18% were aged 50 and older. Thirty-two percent did not graduate high school and 36% had a high school education or equivalent. Seventy percent reported experiencing adverse childhood experiences (ACES). Although harmful alcohol use was low, 83% of the sample reported substantial or severe substance use, with 41% of the total reporting opioid use. ACES predicted current PTSD symptoms. Telehealth treatment considerations include: 1) internet access, 2) health and mental health literacy, and 3) monitoring for dissociation when using mindfulness-based relapse prevention treatment.

### Session 4375 (Symposium)

### DIVERSITY AND VARIATION IN ASSISTED LIVING CARE, NATIONALLY AND OVER TIME

Chair: Portia Cornell Discussant: Tetyana Shippee

Assisted living is generally understood to offer a greater degree of privacy and independence than a nursing home; most residents pay privately, with some receiving support from state subsidies and Medicaid; regulation and oversight are the purview of state agencies. Within these broad parameters, however, one assisted living community may look quite different from another across the country, or down the street, in its resident population and the regulations that govern its operating license. The purpose of this symposium is to explore that variation. The papers leverage an in-depth review of changes in assisted-living regulation from 2007 to 2019 and a methodology to identify Medicare beneficiaries in assisted living using ZIP codes. To set the stage, the first paper examines variation across assisted living licenses to identify six regulatory types and compare their populations' characteristics and health-care use. The second paper analyzes trends over time in the clinical acuity of assisted living residents associated with changes in nursing home populations. The third paper investigates racial disparities in assisted living associated with memory-care designations and proportions of Medicaid recipients. The fourth investigates how regulation of hospice providers in assisted living affect end-of-life care and place of death. The final paper describes requirements related to care for the residents with mental illness in seven states. The symposium concludes with an expert in long-term care disparities and quality discussing the implications for policymakers, providers, and the population needing long-term care in assisted living.

### A TYPOLOGY OF HEALTH SERVICES REGULATED IN U.S. ASSISTED LIVING COMMUNITIES

Lindsey Smith,[1] Wenhan Zhang,[2] Sheryl Zimmerman,[3] Philip Sloane,[4] Kali Thomas,[5] and Paula Carder,[1],
*1. OHSU-PSU School of Public Health, Portland, Oregon, United States, 2. Brown University, Providence, Rhode Island, United States, 3. Cecil G. Sheps Center for Health Services Research, Chapel Hill, North Carolina, United States, 4. UNC Medical School, Sheps Center, Chapel Hill, North Carolina, United States, 5. Brown University, Brown University/Providence, Rhode Island, United States*

State agencies regulate assisted living (AL) with varying approaches across and within states. The implications of this variation for resident case mix, health service use, and policy, are not well described. We collected health services-relevant AL regulatory requirements for all 50 states and DC and used a mixed-methods approach (thematic analysis; k-means cluster analysis) to identify six types: Housing, Affordable, Hybrid, Hospitality, Healthcare, and Hybrid-Healthcare. We stratified Medicare claims data by regulatory type, identifying variation in resident case mix and health service use. Housing and Affordable clusters have larger proportions of dual-eligible beneficiaries, Black residents, and residents of Affordable had more long-term nursing home use compared to other clusters. Dual-eligible beneficiaries account for 26.6% of Housing cluster residents compared to 8.1% of Hybrid Healthcare cluster residents. We provide other examples and explain the implications in terms of sampling AL for single and multi-state studies, racial disparities, and health-related policies.

### RACIAL DISPARITIES IN USE OF MEMORY CARE AND PREVALENCE OF DUALS IN ASSISTED LIVING

Portia Cornell, *Providence VA Medical Center, Providence, Rhode Island, United States*

Assisted living (AL) communities with memory care licenses are disproportionately located in affluent and predominantly White communities and Black older adults are underrepresented in AL. But little is known about characteristics of AL that care for Black residents. We estimated the association of facility-level characteristics as proxy measures for AL resources, such as memory care designations and percentage of dual-eligible residents, across low (0-5%), medium (5-10%) and high (>10%) percentages of Black residents. We found broad differences among communities in the three levels of Black-resident prevalence. High percentage of Black residents was associated with large differences in the percentage of Medicaid-enrolled residents (high 54% duals [s.d.=34], med 28% [31], low=13% [22], p<0.001). ALs with high Black populations were less likely to have a memory-care designation than ALs with medium and low percentages of Black residents (high 4.7% memory care, med 11%, low 17%).

### TRENDS IN ACUITY OF RESIDENTS IN ASSISTED LIVING

Cassandra Hua, *Brown University, Providence, Rhode Island, United States*

Assisted living serves as a substitute for nursing home residents with low care needs, especially in markets with a high proportion of dually eligible Medicare beneficiaries. This study examines trends in the acuity of residents in assisted living communities over time in comparison to nursing homes to characterize how substitution has affected the resident compositions of both settings. We also examine how trends in acuity are shaped by dual eligibility. Using Medicare claims data, we identify cross-sectional samples of beneficiaries in each setting from 2007-2017. The proportion of residents in assisted living with high care needs has increased 18% in assisted living communities compared to 8.7% in nursing homes. Acuity levels are higher among dually eligible assisted living residents compared to assisted living residents who are not dually eligible. Policy makers and administrators should examine whether assisted living is prepared to provide care for an increasingly acute population.

### PLACE OF DEATH AMONG ASSISTED LIVING RESIDENTS AS A FACTOR OF HOSPICE REGULATIONS

Joan Teno,[1] David Dosa,[2] Wenhan Zhang,[2] Pedro Gozalo,[2] Kali Thomas,[3] and Emmanuelle Belanger,[4], *1. Oregon Health and Science University, Portland, Oregon, United States, 2. Brown University, Providence, Rhode Island, United States, 3. Brown University, Brown University/Providence, Rhode Island, United States, 4. Brown University School of Public Health, Providence, Rhode Island, United States*

Our objective was to examine the likelihood of dying in RC/AL among a national cohort of fee-for-service Medicare beneficiaries who died in 2018 (N=31,414) as a factor regulations allowing hospice care. We estimated multivariable logistic regression models to examine the association between RC/AL as place of death and supportive hospice regulations, controlling for demographic characteristics, dual Medicare/Medicaid eligibility, years in AL, and hospital referral region (HRR) to control for hospice practice patterns. A majority of beneficiaries in our cohort died in RC/AL; more than half while receiving hospice services. In unadjusted models, the odds of remaining in RC/AL communities until death were significantly higher in the presence of regulations supportive of hospice care. This relationship was no longer significant once adjusting for covariates and an HRR fixed effect, suggesting important variation in end-of-life experiences for AL residents not explained by hospice regulations.

### INTER- AND INTRASTATE VARIATION OF MENTAL HEALTH REQUIREMENTS FOR ASSISTED LIVING IN SEVEN STATES

Sarah Dys, *Portland State University, Portland, Oregon, United States*

Little is known about states' approaches to regulating mental health (MH) services in assisted living (AL) settings. Yet, one in nine AL residents are diagnosed with serious mental illness (Hua et al, 2020). This study describes the MH regulatory requirements in AL regulations within Arkansas, Louisiana, New Jersey, New York, Oklahoma, Pennsylvania, and Texas. Using health services regulatory analysis (Smith et al, 2021), we reviewed 2018 regulations for the 45 identified AL licenses within these states sourced from Nexis Uni. We summarize 16 MH requirements related to admission, care transitions, resident assessment, third-party services access, and staff training. Each state explicitly addressed at least one of the identified MH requirements, though few states have consistency across all AL types within a state. The most commonly addressed requirements related to admission limitations, assessment, and transfer to psychiatric units. Understanding these requirements promotes a holistic approach to practices that meet residents' needs.

### Session 4380 (Paper)

### DYADIC RESEARCH (BSS PAPER)

### FUTURE ELDERCARE PLANNING AMONG CHINESE AGING FAMILIES IN HONG KONG

Xue Bai, Joanne Luk, Ranran He, and Yanyee Kwong, *The Hong Kong Polytechnic University, Hong Kong, Not Applicable, Hong Kong*

Increasing attention has been paid to the potential role of care planning in buffering future eldercare challenges. However, little is known about the characteristics of care planning among Chinese ageing families. It is also of interest to reflect how recent events such as COVID-19 pandemic may affect their views of the future care planning. From a family systems perspective, this study explored the extent, processes, and contents of intergenerational care planning of Chinese ageing families in Hong Kong. Dyadic interviews were conducted with 60 adult child-older parent pairs, and individual interviews were conducted with another 33 adult children. Intergenerational discrepancies in extent and processes of care planning, intergenerational congruence of care expectations and struggles, facilitating role of family capital and hindering role of cultural capital in care planning were primary themes. Although both generations demonstrated strong awareness of future eldercare needs, they were found engaged in different levels and processes of care planning. Adult children's level of engagement in planning activities may influence parents' extent and contents of care planning. Intergenerational transmission of eldercare values contributed to intergenerational congruence of care expectations but also led to similar struggles and ambivalent attitudes toward future care. Moreover, family capital was found to facilitate family care planning while Chinese cultural values that emphasize family care may hinder both generations' efforts in care planning. The findings will deepen our understanding on characteristics of intergenerational care planning in Asian Chinese communities and inform services to improve adult children and ageing parents' preparation for future eldercare.

### MEASURING DYADIC INTERACTIONS AND DINING ENVIRONMENT IN DEMENTIA MEALTIME CARE: A SYSTEMATIC REVIEW OF INSTRUMENTS

WEN LIU,[1] and Sohyun Kim,[2], *1. The University of Iowa College of Nursing, Iowa City, Iowa, United States, 2. University of Iowa, Iowa City, Iowa, United States*

It is critical to use validated instruments to assess mealtime dyadic interactions and dining environment for people with dementia to evaluate the process and efficacy of mealtime interventions. However, the quantity and psychometric quality of such instruments are unknown. This systematic review aimed to synthesize the quantity and quality of instruments that assess dyadic interactions, physical environment, and/or social environment during dementia mealtime care. We searched Pubmed, CINAHL, AgeLine, PsychINFO, and Cochrane Library for records published between 1980-2020. Records were eligible if they included any instrument assessing concepts of interest (i.e., mealtime dyadic interactions, physical and/or social dining environment). From eligible records, eligible instruments originally developed or later modified to measure concepts of interest were identified, and instruments' characteristics were extracted: 1) development process, 2) concept/construct assessed, 3) sample/setting, 4) administration method, 5) item description, 6) scoring format/interpretation, 7) reliability, and 8) validity. A newly developed tool was used to evaluate instruments' psychometric quality. In total, 26 eligible instruments were identified. Seventeen instruments assessed dyadic interactions, 1 assessed only physical environment, and 8 assessed physical & social environment. All instruments were observational tools and scored as having low psychometric quality. Reasons for low psychometric quality included use of small sample size compared to the number of items, limited psychometric testing, and inadequate estimates. A number of instruments were developed and/or used to assess dyadic interactions, physical and/or social environment in dementia mealtime care. All instruments warrant further testing to accumulate psychometric evidence in larger diverse samples in different care settings.

### PATTERNS OF INTERGENERATIONAL CARE PLANNING AMONG CHINESE AGING FAMILIES IN HONG KONG

Xue Bai, Chang Liu, and Tongling Xu, *The Hong Kong Polytechnic University, Hong Kong, Not Applicable, Hong Kong*

Care planning before the onset of intensive care needs can increase families' ability to manage caregiving crises and cope with care transitions. However, future care planning has not been substantially examined in a family context. Drawing on the model of Preparation for Future Care Needs and a family systems perspective, this study investigated patterns of intergenerational care planning across multiple planning domains (awareness, avoidance, information gathering, decision making, and concrete planning) among Chinese intergenerational pairs. Quantitative data of 213 pairs of aging parents and adult children were collected in Hong Kong. Latent Profile Analysis was conducted to examine typological structure underlying care planning patterns. Three patterns were discovered: filial-maximal, dyadic-moderate, and filial-minimal. Profile 1 contained approximately 9.9% of pairs, which demonstrated a relatively higher level of avoidance on considering the need of care preparation and engaged less in concrete planning activities. Profile 2 contained 68.5% of intergenerational pairs that had a moderate preparation level. Profile 3 contained 21.6% of intergenerational pairs that were comparatively active in care planning. The findings also indicated that although older adults across three groups demonstrated a similar level of awareness to prepare for future care, their engagement in the concrete planning activities may be driven by their children's awareness and preparation toward future care. The findings will enhance professionals' and service providers' awareness of diverse care planning patterns among Asian aging families, and inform targeted policies and programmes to alleviate unpreparedness for eldercare through intergenerational care planning which can be more effective than unilateral preparation.

### SPILLOVER EFFECTS OF RETIREMENT ON PHYSICAL AND MENTAL HEALTH OF A SPOUSE OR PARTNER: DO GENDER AND SEXUALITY MATTER?

Jingwen Liu, *University of Maryland, college park, college park, Maryland, United States*

While the health implication of retirement on retirees has been widely discussed in social sciences, less is known about its spillover effects on one's spouse or partner. Indeed, large quantities of studies have shown that retirement is a joint decision of couples that may influence the time use of spouses and the incidence of divorce, and so leaving the cross-spouse effects unexplored may underestimate the influence of retirement and social security policies. Using ten waves of Health and Retirement Survey (HRS 1996-2014, N = 85039 observations), this research adopts fixed effects models and instrumental variable methods to examine the causal effects of a retiree's retirement on the physical and mental health outcomes of his/her spouse or partner, with particular attention paid to the gender and sexuality differentials. Regression results suggest that transitioning into retirement is associated with enhanced self-reported health and cognitive function and decreased depressive symptoms, physical limitation, and BMI among the retirees' spouses or partners. However, the timing when retirement occurs also matters that retirement at the culturally expected retirement age leads to beneficial spillover effects, while late retirement brings about detrimental effects. Further comparison analysis indicates that retirement's spillover effects are more prominent among females and heterosexual couples than among their male and gay and lesbian counterparts. These findings inform policymakers of the cross-couple spillover effects of postponing retirement ages and the increasing physical and psychological disparities of females and gay and lesbian couples within families in middle and later life.

### WE SHOULD HAVE TALKED ABOUT HOSPICE: TRANSFORMING COMMUNICATION WITH BEREAVED LGB AGING WOMEN

Korijna Valenti,[1] and Leah Janssen,[2], *1. University of Colorado--Denver, Denver, Colorado, United States, 2. Scripps Gerontology Center, Oxford, Ohio, United States*

Because of historical discrimination, discomfort disclosing information, and differing definitions of family, lesbian, gay, bisexual, and transgender (LGBT) older adults with serious illness need both improved palliative and end-of-life (EOL) care communication with clinicians and recognized inclusion of spouses/partners. Communicating about palliative and EOL care may improve the care goals and emotional trajectory for patients and significant others. Using a descriptive qualitative approach, this study's aim was to analyze the communication experiences during a spouse's/partner's EOL care for bereaved LGB women (n=16) 60 and older. Drawing on queer gerontology, issues relating to access to resources and information and the systemic silencing of older LGB women illuminate areas where policy and practice may be improved. Semi-structured, one-on-one interviews were used to provide deep and meaningful information about palliative and EOL care communication between participants, their spouse or partner, and clinicians. While results reflect certain outcomes found in prior studies with non-LGBT adults, thematic analysis revealed three main findings with evidence specific to this population: 1) avoiding deep discussions about EOL; 2) lack of understanding about palliative or EOL care; and 3) limited communication with clinicians. Findings illuminate the need for better understanding among clinicians regarding palliative and EOL communication with LGBT dyads as well as communication strategies based on recognition and acceptance. Further dyadic communication research may improve care goals for LGBT older adults. Understanding couples' interactions and examining different communication behaviors may lead to improved palliative and EOL care goals for older LGBT adults with serious illness and their spouses/partners.

### Session 4385 (Symposium)

### ENVIRONMENT STILL MATTERS: EXAMINING PERSON-PLACE-RELATIONSHIPS IN THE OLD AND THE NEW NORMAL ACROSS SETTINGS

Chair: Frank Oswald Co-Chair: Habib Chaudhury
Discussant: Amanda Grenier

In environmental gerontology, the home and the neighborhood have always been of particular interest for empirical research. Issues such as orientation and safety, place attachment and biographical bonding, have proven to be important for community dwellings older adults and for those living in care homes. However, with Covid-19, the seemingly stable person-place-relationships have been challenged. This symposium provides a set of applied research contributions that demonstrate the persistent salience of the environment by examining person-place-relationships in the old and the new normal in private homes and care homes. Contributions draw from ideas of "precarious ageing" (Grenier & Phillipson) and "pandemic precarity", for instance to understand housing insecurity, while concepts from environmental gerontology are used to explain processes of environmental agency and belonging. The first contribution by Mahmood and colleagues introduces an environmental audit tool for people at risk of homelessness to assess built environmental features of housing and neighborhood that support housing stability in the face of insecurity. Second, Wanka provides data from people framed as 'risk-groups' through the Covid-19 pandemic and how they dealt with contact restrictions, showing the role of intergenerational neighborhood relations to mediate risks of pandemic precariousness. Third, Elkes examined mobility and wayfinding challenges for residents in a long-term care home and subsequent environmental interventions to improve orientation. Forth, Leontowitsch and colleagues present findings from long-term care home residents during the pandemic to gain understanding of their experiences of social isolation and a biographical sense of resilience. Finally, Amanda Grenier will serve as the session's discussant.

### ENVIRONMENTAL ASSESSMENT OF HOUSING FOR OLDER ADULTS FACING HOUSING INSECURITY

Atiya Mahmood,[1] Muhammad Qureshi,[1]
Habib Chaudhury,[1] Sarah Canham,[2] Rachel Weldrick,[1]
and Shelby Elkes,[1], *1. Simon Fraser University, Vancouver, British Columbia, Canada, 2. University of Utah, Salt Lake City, Utah, United States*

The Aging in the Right Place Environmental Audit (AIRP-ENV) and Secondary Observation (AIRP-ENV-SO) tools were developed to conduct observation-based audit of the built environment in shelters, transitional housing, independent housing with offsite/onsite supports, and permanent supportive housing with onsite medical and/or specialized services for older adults experiencing (or at risk of) homelessness. The 241 item AIRP-ENV tool is used to audit the presence/absence of exterior and interior built environmental features that support housing stability. The seven open-ended questions in the AIRP-ENV-SO tool is used to collect contextual data on function, safety and land-use of surrounding neighborhood. Data were collected at four sites of a transitional housing program in Vancouver, Canada as part of a multi-year, multi-city partnership project on aging and homelessness. Preliminary results demonstrate that built environment and urban design features (e.g., access, privacy, flexible and supportive spaces) contribute towards tenants' residential resiliency and aging in place.

### THE ROLE OF INTERGENERATIONAL NEIGHBORHOOD RELATIONS FOR PRECARIOUS AGING DURING THE COVID-19 PANDEMIC IN GERMANY

Anna Wanka, *Goethe University Frankfurt, Frankfurt, Hessen, Germany*

Throughout the Covid-19 pandemic, the immediate living environment has significantly gained importance - particularly for people framed as 'risk-groups', such as older adults. Effects of contact restrictions to contain the spread of the virus have affected inequalities, uncertainties and loneliness in later life differently depending on the intergenerational relations, informal infrastructures of provisioning and networks of solidarity given in a certain neighborhood. The paper presents findings from a recent mixed-methods study in Frankfurt, Germany, combining a quantitative survey (n=1.000) with a longitudinal qualitative study (n=60). Results show how intergenerational neighborhood relations can play a crucial role in mediating risks of pandemic precariousness in later life, but also how older adults themselves significantly contributing to neighborhood networks of provisioning. Strengthening such very local relations is key to protecting all age groups from the effects of crises beyond the pandemic, and, in conclusion, ways to do so are being discussed.

### RESIDENTS' WAYFINDING CHALLENGES AND ENVIRONMENTAL INTERVENTIONS IN A CARE HOME

Habib Chaudhury, and Shelby Elkes, *Simon Fraser University, Vancouver, British Columbia, Canada*

This study evaluated the role of the built environment on residents' wayfinding behaviours at Louis Brier Home in Vancouver, British Columbia, Canada. The goal of this study was to explore baseline mobility challenges for the residents traveling between their bedrooms and social spaces. In response to this, low-cost environmental interventions were proposed and implemented to support safe and independent wayfinding for the residents. The project consisted of three phases. First phase involved a mixed methods approach using behavior mapping and spatial observations of the residents interacting with their physical environment, combined with one focus group with the staff members. In the second phase, researchers presented actionable environmental interventions for the care home administration to consider and implement. The final phase involved post-implementation behaviour mapping, spatial observations and a focus group session. The implemented environmental interventions influenced in improved resident wayfinding and orientation in the long-term care home.

### DOING TIME: EXPERIENCES OF CARE HOME RESIDENTS IN GERMANY DURING THE EARLY PHASE OF THE COVID-19 PANDEMIC

Frank Oswald,[1] Arthur Schall,[2] Johannes Pantel,[2] and Miranda Leontowitsch,[3], *1. Goethe University Frankfurt, Frankfurt am Main, Hessen, Germany, 2. Goethe University Frankfurt, Frankfurt, Hessen, Germany, 3. Goethe University Frankfurt, Frankfurt / Main, Hessen, Germany*

Residents of care homes across the globe are affected by the spread of SARS-CoV-2 as they have been identified as a high-risk group and because they experienced strict social isolation regulations during the first wave of the pandemic. Social isolation of frail older people is strongly associated with negative health outcomes. The aim of this research project was to investigate how residents in care homes experienced social isolation during the first phases of contact ban in Germany. This paper draws on structured interview data collected from 22 residents in two care homes during early June 2020 in Frankfurt/Main. The findings show that their experiences were shaped by three factors: care home staffs' approach to handling the contact ban; biographical sense of resilience; and a hierarchy of life issues. The findings highlight the importance of locally specific response mechanisms in care homes, and the need to contextualize residents' experiences.

### Session 4390 (Paper)

### ESPO AND BIOLOGICAL SCIENCES SECTION SYMPOSIUM: BEDSIDE TO BENCH: CLINICALLY RELEVANT MODELS OF AGING

### CHARACTERIZATION OF A MOUSE MODEL OF INDUCIBLE FRAILTY: THE HUMANIZED IL-6 MOUSE

Lolita Nidadavolu,[1] Peter M. Abadir,[2] Jeremy D. Walston,[2] Anne Le,[1] Gayane Yenokyan,[3] Liliana Florea,[1] Corina Antonescu,[1] and D. Brian Foster,[1], *1. Johns Hopkins University, Baltimore, Maryland, United States, 2. Johns Hopkins University School of Medicine, Baltimore, Maryland, United States, 3. Johns Hopkins Bloomberg School of Public Health, Baltimore, Maryland, United States*

The cytokine interleukin-6 (IL-6) has pleiotropic effects in aging and is elevated in frail older adults. We have developed a conditional mouse model to better characterize the role of IL-6 in promoting frailty and age-related mitochondrial dysregulation. The human IL-6 (hIL-6) knock-in mouse (TetO-hIL6) was developed utilizing CRISPR/Cas9 technology with transgene donor vector containing a tetracycline response element promoter driving expression of hIL-6 cDNA. Male TetO-hIL6 mice were treated with doxycycline-containing water for six weeks starting at 8 months old. RNAseq analysis of whole blood demonstrated significant upregulation of pro-inflammatory related markers at 6 weeks compared to baseline and upregulated cell proliferation and metabolism pathways. Physical testing of TetO-hIL6 mice before and after hIL-6 induction demonstrated decreased grip strength ($p = 0.003$), decreased running capacity ($p = 0.02$), and 40% increase in falls off of the treadmill ($p = 0.001$). Induced mice also demonstrated decreased basal body temperature ($p < 0.001$). Given the significant dysregulation of metabolism-related genes in RNAseq analysis and changes in basal body temperature following hIL-6 induction, we next performed untargeted metabolomics on plasma from mice at baseline and 6 weeks post-induction to better evaluate metabolic changes associated with hIL-6 elevation. We found changes in key serum metabolites, including circulating adenosine triphosphate (56% reduction, $p = 0.02$), pyruvate (35% reduction, $p = 0.0006$), alpha-ketoglutarate (47% reduction, $p = 0.04$), and succinate (306% increase, $p = 0.001$). The TetO-hIL6 mouse model allows for induction of hIL-6 at various timepoints across the lifespan and demonstrates features of a frailty phenotype.

### EFFECTS OF AGE AND SOCIAL ADVERSITY ON IMMUNE CELL POPULATIONS IN A NON-HUMAN PRIMATE MODEL OF HUMAN AGING

Mitchell Sanchez-Rosado,[1] Noah Snyder-Mackler,[2] James Higham,[3] Lauren Brent,[4] Nicole Marzan-Rivera,[5] Melissa Pavez-Fox,[4] Marina Watowich,[6] and Carlos A. Sariol,[1], *1. University of Puerto Rico Medical Sciences Campus, San Juan, Puerto Rico, United States, 2. Arizona State University, Tempe, Arizona, United States, 3. NYU, New York University, New York, United States, 4. University of Exeter, Exeter, England, United Kingdom, 5. UPR-Medical Sciences Campus, UPR-Medical Sciences Campus, Puerto Rico, United States, 6. University of Washington, Tempe, Arizona, United States*

Significant hallmarks of aging are immune function decline and rising cumulative inflammation. These immunosenescent signatures are also found in individuals who experience chronic social adversity, independently of age. However, no studies to date have examined how social adversity alters immune function across the lifespan –data that are essential to identify the molecular routes through which social adversity might lead to increased aging-related disease. Over a two-year period, we investigated how age and social adversity (quantified by low social status) affected immunity. We measured immune cell proportions at baseline and their gene regulation after in vitro stimulation with pathogen molecules that stimulated both Th1 and Th2 immune responses in a population of free-ranging rhesus macaques. We first performed flow cytometry on peripheral whole blood to quantify changes on immune cell proportions across the lifespan (n=235) and in animals of different social statuses (n=141). We found significant decreases in CD20+ B cells and CD3+/CD4+ T cell proportions with age, suggesting diminished antibody production and adaptive immune responses in older individuals. Age-associated increases in CD3+/CD8+, CD3+/CD4+/CD25+ T regulatory cells and CD14-/CD16+/HLA-DR+ non-classical monocytes indicated heightened baseline inflammation in older animals. Social adversity recapitulated the effects of aging in CD14+/CD16-/HLA-DR+ classical monocytes, indicating immune deficits in phagocytosis and pathogen clearance in older and lower status individuals. Using RNA-seq, our stimulations (n=1,320) will allow us to identify molecular immune pathways that are disrupted by age and social adversity, similarities in response between age and adversity, and how the effect of adversity varies across the lifespan.

### LOSS OF AWARENESS OR URINARY DYSFUNCTION? INVESTIGATING AMYLOIDOSIS AND URINARY PHYSIOLOGY IN A TRANSGENIC MOUSE

Cara Hardy,[1] Ramalakshmi Ramasamy,[1] Dawn Rosenberg,[1] Philip Scarpa,[1] Xiangyou Hu,[1] George Kuchel,[2] and Phillip Smith,[1], *1. UConn Health, Farmington, Connecticut, United States, 2. University of Connecticut Health, Farmington, Connecticut, United States*

Alzheimer's disease (AD) is a devastating disorder primarily affecting older adults and is the most common neurodegenerative disease in the US. More than one in three AD patients experience AD-associated urinary dysfunction (ADUD), which directly contributes to their institutionalization. While ADUD has been clinically regarded as a result of poor cognitive control over urinary function, the physiology underlying loss of urinary control remains unknown. We hypothesize that amyloidosis in the CNS results in pathologic changes in urinary structure and function. Tg-APP/PS1DE9 mice were used before plaque deposition (4-6 months) and after plaque accumulation (8-10 months) and compared to WT littermates. Behavioral assays (open field testing and voiding spot assays) were performed to assess cortical function. Pressure-flow cystometry was conducted under urethane anesthesia to assess autonomic control of urinary function without cortical influence. Pharmacomyography of bladder strips was used to determine tissue-level changes in the absence of CNS input. In Tg-APP/PS1DE9 mice, plaque accumulation resulted in significant cystometric changes to voiding phase parameters, but not storage phase parameters. Pharmacologic studies showed decreased sensitivity to adrenergic stimulation without change in muscarinic sensitivity. Behavioral assays demonstrated significant differences between transgenic animals and WT in locomotion and voiding spot sizes. We interpret our data to support AD-related pathology of Aβ accumulation results in a distinct urinary phenotype in our model, analogous to the ADUD observed in AD patients. Establishing and verifying models of ADUD may improve the efficacy of treating ADUD and increase quality of life for patients and their caregivers.

### METABOLIC ADAPTATIONS TO AEROBIC EXERCISE IN AGED MICE

Tyler Marx, Anastasiia Vasileva, Stephen Hutchison, and Jennifer Stern, *University of Arizona, Tucson, Arizona, United States*

Aerobic exercise training is a potent intervention for the treatment and prevention of age-related disease, such as heart disease, obesity, and Type 2 Diabetes. Insulin resistance, a hallmark of Type 2 Diabetes, is reversed in response to aerobic exercise training. However, the effect of aerobic exercise training on glucagon sensitivity is unclear. Glucagon signaling at the liver promotes fatty acid oxidation, inhibits De novo lipogenesis, and activates AMP Kinase, a key mediator of healthy aging. Like humans, aging in mice age leads to a decline in physical and metabolic function. To understand the role of glucagon signaling in exercise-induced improvements in physical and metabolic function in the mouse, we implemented a 16-week aerobic exercise training protocol in young and aged mice. 16 weeks of exercise training initiated at 6 months of age increased markers of physical function ($P<0.01$) and attenuated age-related weight gain ($P<0.05$) and fat mass ($P<0.0001$). Additionally, exercise training improved glucose clearance ($P<0.01$), enhanced glucose-stimulated insulin secretion ($P<0.01$) and decreased hepatic lipid accumulation ($P<0.05$). Importantly, exercise training decreased hypoglycemia stimulated glucagon secretion ($P<0.01$), with no effect on hepatic glucagon receptor mRNA expression or serum glucagon. Thus, we propose that aerobic exercise training enhances glucagon sensitivity at the liver, implicating glucagon as a potential mediator of exercise-induced improvements in aging. Studies initiating the same aerobic exercise training intervention at 18 months of age in the mouse are currently underway to establish the role of glucagon receptor signaling in exercise-induced improvements in aging.

### Session 4395 (Paper)

### FRAILTY MEASUREMENT

### CONSTRUCTION AND VALIDATION OF A FRAILTY INDEX IN PRIMARY CARE IN ITALY: THE HEALTH-SEARCH FRAILTY INDEX

Davide Vetrano,[1] Alberto Zucchelli,[2] Graziano Onder,[3] Roberto Bernabei,[4] Laura Fratiglioni,[5] Alessandra Marengoni,[6] and Francesco Lapi,[7], *1. Karolinska Institutet, Solna, Stockholms Lan, Sweden, 2. Università degli Studi di Brescia, Karolinska Institutet, Stockholms Lan, Sweden, 3. Italian National Institute of Health, Rome, Lazio, Italy, 4. Catholic University of Rome, Rome, Lazio, Italy, 5. Karolinska Institutet, 17175, Stockholms Lan, Sweden, 6. University of Brescia, Brescia, Lombardia, Italy, 7. Health Search, Florence, Toscana, Italy*

Recognizing frailty in primary care is important to implement personalized care pathways and for prognostication. The aim of this study was to build and validate a frailty index based on routinely collected primary care data in Italy. We used clinical data from 308,280 Italian primary care patients 60+ with at least 5 years of follow-up, part of the Health Search Database. A heuristic algorithm was used to select the deficits to be included in a highly performant frailty index. The fitness of the index was assessed through the c-statistics derived by survival models. Results were externally validated using the Swedish National Study on Aging and Care in Kungsholmen (SNAC-K). After testing 3.4 million of deficits combinations, 25 deficits were selected to be included in the Health Search Frailty Index (HS-FI). After adjusting by sex, age and geographical area, the HS-FI was associated with 5-year mortality (HR per 0.1 increase 1.99; 95%CI 1.95-2.02) and hospitalization rate (HR per 0.1 increase 1.25; 95%CI 1.23-1.27). In the external validation cohort, HS-FI independently predicted mortality, hospitalization, incident disability, incident dementia, and incident falls. This is the first frailty index built following a data-driven approach, using national representative primary care data. The implementation of such tool – derived by routinely collected data – in primary care software will ease the prompt, comparable and reliable recognition of frailty at the population level.

### METABOLOMICS-BASED BIOMARKERS FOR FRAILTY IN CHINESE OLDER ADULTS

Yiming Pan,[1] Pan Liu,[2] Yun Li,[2] and Lina Ma,[2], *1. Capital Medical University Xuanwu Hospital, Beijing, Beijing, China (People's Republic), 2. Xuanwu Hospital, Capital Medical University, National Research Center for Geriatric Medicine, Beijing, Beijing, China (People's Republic)*

**Background:** Frailty is a clinical state characterized by decline in physiological function, and increased vulnerability to adverse outcomes. The biological mechanisms underlying frailty have been extensively studied in recent years. Advances in the multi-omics platforms have provided new information on the molecular mechanisms of frailty. Thus, identifying omics-based biomarkers is helpful for both exploring the physiological mechanisms of frailty and evaluating the risk of frailty development and progression.

**Objective:** To identify metabolomics biomarkers and possible pathogenic mechanisms for frailty with untargeted-metabolomics profiling.

**Methods:** LC-MS-based untargeted metabolomics analysis was performed on serum samples of 25 frail older inpatients and 49 non-frail older controls. The metabolomics profiling was compared between the two groups.

**Results:** We identified 349 metabolites belonging to 46 classes, in which 2 were increased and 3 were decreased in frail older adults. Citrate cycle (with up-regulated cis-Aconitic acid, Fumaric acid, L-Malic acid, and Isocitric acid), fatty acid metabolism (with up-regulated Palmitic acid and L-Palmitoylcarnitine) and tryptophan metabolism (with up-regulated 5-Hydroxy-L-tryptophan, L-Kynurenine, Kynurenic acid, and 5-Hydroxyindoleacetic acid) were significantly associated with frailty phenotype.

**Conclusions:** Our results revealed characteristics of metabolites of frailty in Chinese older adults. The citrate cycle related metabolites (Isocitrate, (s)-Malate, Fumarate and cis-Aconitate), saturated fat (Palmitic acid), unsaturated fatty acid (Arachidonate and Linoleic acid), and some essential amino acid (Tryptophan) might be candidate biomarkers for early diagnosis of frailty. Disorders of energy metabolism, lipotoxicity of saturated fatty acids, disturbances of unsaturated fatty acid metabolism, and increased degradation of tryptophan were potential mechanisms and therapeutic targets of frailty.

### THE ASSOCIATION BETWEEN METABOLIC SYNDROME AND FRAILTY IN HEALTHY COMMUNITY-DWELLING OLDER ADULTS

A R M Saifuddin Ekram,[1] Sara Espinoza,[2] Michael Ernst,[3] Lawrence Beilin,[4] Nigel P. Stocks,[5] Joanne Ryan,[6] and Robyn Woods,[6], *1. Monash University, Monash University, Melbourne, Victoria, Australia, 2. University of Texas Health Science Center San Antonio, San Antonio, Texas, United States, 3. University of Iowa, Iowa City, Iowa, United States, 4. The University of Western Australia (M570), Perth, Western Australia, Australia, 5. University of Adelaide, Adelaide, South Australia, Australia, 6. Monash University, Melbourne, Victoria, Australia*

This study examined the association between metabolic syndrome (MetS) and frailty status in relatively healthy community-dwelling older adults. Participants included 19,114 individuals from the "ASPirin in Reducing Events in the Elderly" (ASPREE) trial. The diagnostic criteria for MetS were according to the International Diabetes Federation Task Force on Epidemiology and Prevention and the American Heart Association/National Heart, Lung, and Blood Institute (2009); and comprised any three of five parameters: waist circumference, triglycerides, fasting blood glucose, high-density lipoprotein cholesterol or hypertension. Frailty and prefrailty were defined using a modified Fried phenotype (FP) comprising exhaustion, body mass index, grip strength, gait speed and physical activity and a deficit accumulation frailty index (FI) of 66 items. The association between MetS and frailty was examined using multinomial logistic regression. At baseline, 51.1% of participants met the criteria of MetS; of those, 41.8% and 2.5 % were prefrail and frail, respectively, according to Fried phenotype, while 49.6% and 11.8 % were prefrail and frail, respectively, according to FI. MetS at baseline was associated with an increased likelihood of prefrailty (RRR: 1.25; 95% CI: 1.17, 1.33) and frailty (RRR: 1.60; 95% CI: 1.28, 2.01) compared to no frailty after adjustment for potential confounders according to Fried phenotype, while the association was stronger for prefrailty (RRR: 2.74; 95% CI: 2.55, 2.94) and frailty (RRR: 5.30; 95% CI: 4.60, 6.11) according to FI. Overall, at baseline, more than half of the participants had MetS, and the presence of MetS was significantly associated with pre-frailty and frailty.

### TRANSPLANT CENTERS THAT MEASURE FRAILTY AS PART OF CLINICAL PRACTICE HAVE BETTER OUTCOMES

Xiaomeng Chen, Yi Liu, Nadia Chu, Jeremy D. Walston, Dorry Segev, and Mara McAdams-DeMarco, *Johns Hopkins University School of Medicine, Baltimore, Maryland, United States*

Frailty predicts adverse outcomes for kidney transplant (KT) patients; yet the impact of clinical assessments of frailty on center-level outcomes remains unclear. We sought to test whether KT centers that measure frailty as part of clinical practice have better pre- and post-KT outcomes. We conducted a survey of US transplant centers (11/2017-4/2018), 132 KT centers (response rate=65.3%) reported frequencies of frailty assessment at candidacy evaluation and KT admission. Center characteristics and clinical outcomes were gleaned from the national registry (2017-2019). Poisson regression was used to estimate incidence rate ratios (IRRs) of waitlist mortality rate and transplantation rate in candidates and graft loss rates in recipients by frequency of frailty assessment. All models were adjusted for case mix and center characteristics. Given similar center characteristics, centers assessing frailty at evaluation had a lower waitlist mortality rate (always=3.5, sometimes=3.2, never=4.1 deaths per 100 person-years). After adjustment, centers assessing frailty at evaluation had a lower rates of waitlist mortality (always IRR=0.91, 95% CI:0.84-0.99; sometimes=0.89, 95% CI:0.83-0.96) and transplantation (always IRR=0.94, 95% CI:0.91-0.97; sometimes=0.88, 95% CI:0.85-0.90) than those never assessing frailty. Centers that always assessed frailty at KT admission had 0.71 (95% CI:0.54-0.92) times the rate of death-censored graft loss than their counterparts never assessing frailty. Assessing frailty at evaluation is associated with lower transplantation rate but better waitlist survival; centers always assessing frailty at admission are likely to have better graft survival. Research is needed to explore how routine assessment of frailty in other clinical practices benefits broader patient populations.

### VALIDATION OF PERCEIVED MENTAL FATIGABILITY USING THE CHINESE VERSION OF THE PITTSBURGH FATIGABILITY SCALE

Yixin Hu,[1] Hangming Zhang,[2] Weihao Xu,[1] siyue wang,[3] Woei-Nan Bair,[4] Li Fan,[1] Yujia (Susanna) Qiao,[5] and Nancy W. Glynn,[6], *1. Chinese PLA General Hospital, Beijing, Beijing, China (People's Republic), 2. Peking university shougang hospital, ., Beijing, China (People's Republic), 3. Peking university School of Public Health, ., Beijing, China (People's Republic), 4. University of the Sciences in Philadelphia, Philadelphia, Pennsylvania, United States, 5. University of Pittsburgh, University of Pittsburgh, Pennsylvania, United States, 6. University of Pittsburgh Graduate School of Public Health, Pittsburgh, Pennsylvania, United States*

**Background:** Recently we validated the simplified-Chinese version of the Pittsburgh Fatigability Scale (PFS) Physical subscale. Next step is to validate the PFS Mental subscale in order to introduce a reliable measure of perceived mental fatigability among Chinese community-dwelling older adults.

**Methods:** This cross-sectional study was conducted in an urban community in Beijing. Internal consistency of the PFS Mental subscale was evaluated by Cronbach's alpha. The participants were divided in half to evaluate the factor structure validity by exploratory factor analyses and confirmatory factor analysis. Convergent validity and discriminant validity were evaluated against cognitive function (assessed by MOCA) and global fatigue from FRAIL Scale.

**Results:** Our study included 370 participants (mean=83.8 years). The simplified-Chinese version of PFS Mental subscale showed strong internal consistency (total Cronbach's alpha=0.82, each items Cronbach's alpha ranged from 0.78 – 0.83). The results of exploratory factor analysis showed all 10 items loaded on two factors: moderate to high and low intensity activities, which explained 60.8% of the total variance. Confirmatory factor analysis showed fit indices: SRMSR = 0.090, RMSEA = 0.120, CFI = 0.89. PFS Mental scores demonstrated moderate concurrent and construct validity against cognitive function ($r = -0.24$, $P<.001$). Additionally, the PFS Mental subscale had strong convergent validity, discriminating according to established cognitive impairment or FRAIL Scale fatigue testing cut points, with differences in PFS Mental scores ranging from 3.2 to 8.4 points.

**Conclusions:** The PFS Mental subscale simplified-Chinese version is a valid tool to assess perceived mental fatigability in Chinese-speaking older adults.

### Session 4405 (Paper)

### HOME HEALTH CARE

### FAMILY CAREGIVER TRAINING DURING MEDICARE HOME HEALTH CARE: CLINICIAN PERSPECTIVES

Julia Burgdorf,[1] and Alicia Arbaje,[2], *1. Johns Hopkins Bloomberg School of Public Health, Baltimore, Maryland, United States, 2. Johns Hopkins University School of Medicine, Baltimore, Maryland, United States*

During Medicare home health care, providers often rely on family caregivers to help meet patients' care needs. Beginning in 2018, CMS requires home health agencies to provide training to family caregivers. This qualitative study is the first research to examine current patterns of family caregiver training, and related facilitators and barriers, during Medicare-funded home health care. We conducted semi-structured key informant interviews with home health nurses and physical therapists (n=19) from 4 diverse agencies, then performed thematic analysis of interview transcripts using a hybrid inductive and deductive coding approach. Clinicians described family caregiver education as a dynamic and cyclical process: simultaneously providing patient care, training family caregivers, and gathering additional information about patient needs and caregiver capabilities, then adjusting the care plan accordingly. We present a model of this cyclic process and describe its four major stages: Initial Assessment, Education, Reassessment, and Adjustment. Additionally, clinicians identified a range of structural, individual, and interpersonal factors which impact their ability to successfully train family caregivers. We define each factor and, using illustrative quotes from our interviews, elucidate its role as a facilitator and/or barrier to clinicians' educational efforts. Findings provide the first model of caregiver training during home health care and highlight policy and practice changes to better support clinicians in these efforts; including greater visit flexibility, access to more experienced clinical mentors, and standardized caregiver assessment tools designed for this unique care setting.

### HOME HEALTH AGENCIES WITH MORE SOCIALLY VULNERABLE PATIENTS HAVE LOWER EXPERIENCE-OF-CARE RATINGS

Jinjiao Wang, Meiling Ying, and Yue Li, *University of Rochester, Rochester, New York, United States*

Little is known about the disparities in patient experience of home health (HH) care related to social vulnerability. This study examined the relationships of patient Medicare-Medicaid dual eligible status and race and ethnicity with patient experience of HH care. We analyzed national data from the Home Health Care Consumer Assessment of Healthcare Providers and Systems (HHCAHPS), Outcome and Assessment Information Set, Medicare claims and Area Health Resources File for 11,137 Medicare-certified HH agencies (HHA) that provided care for Medicare beneficiaries in 2017. Patient-reported experience of care star ratings (1-5) in HHCAHPS included 3 domains (professional care delivery, effective communication, and specific issues in direct patient care) with each dichotomized into high (4-5) and low (1-3) experience of care. The proportion of patients with dual eligibility and the proportion of racial/ethnic minorities were summarized at the HHA level. HHA with higher proportion of dual eligible patients were less likely to have high experience of care rating in professional care delivery (smallest Odds Ratio [OR]=0.514; 95% CI: 0.397, 0.665; p<0.001), effective communication (smallest OR=0.442, 95% CI: 0.336, 0.583; p<0.001), and specific direct care issues (smallest OR=0.697, 95% CI: 0.540, 0.899; p=0.006). HHA with higher proportion of racial/ethnic minorities were also less likely to have high patient experience of care rating across all three domains (smallest OR=0.265, 95% CI: 0.189, 0.370; p<0.001). Disparities in patient experience of HH care exist and they are associated with low income and racial/ethnic minority status, indicating substantial unmet needs among these socially vulnerable patients.

### PATIENT-RATED PERFORMANCE OF HOME HEALTH AGENCIES AND HOSPITALIZATION RISK: A PROPENSITY-SCORE MATCHED ANALYSIS

Chenjuan Ma,[1] Lisa Groom,[2] Shih-Yin Lin,[3] Daniel David,[2] and Abraham Brody,[4], *1. NYU, NYU Rory Meyers College of Nursing, New York, United States, 2. NYU, New York, New York, United States, 3. New York University, New York, New York, United States, 4. NYU Hartford Institute for Geriatric Nursing, New York, New York, United States*

Home health care is the most commonly used home- and community-based service to older adults "Aging in Place". Patient experience of healthcare services is a critical aspect of patient-centered care. Indeed, policymakers have linked patient-rated quality of care to payment to healthcare providers. This study aimed to examine the association between patient-rated care performance of home health agencies and risk for hospitalization among Medicare beneficiaries. This study used several national datasets from 2016 and included 491,718 individuals from 8,459 home health agencies. Home health agencies' performance was measured using patient experience star rating from the Home Health Consumer Assessment of Healthcare Providers and Systems (HHCAHPS). Propensity score matching was used to balance the differences in patient characteristics at baseline between those receiving care from high-performing home health agencies and those in lower-performing agencies. On average, patients were 80.5 years old, 65% female, 81% White, 10% Black, and 6% Hispanic, with 90% taking 5 or more medications. Patients had a mean score of 1.73 (SD=1.69) on the Charlson Index. Respectively, 10% and 16% of patients were hospitalized within 30 and 60 days of home health care initiation. Estimates of logistic regression after propensity score matching found that patients receiving care from lower-performing agencies were at similar risk for both 30-day (OR=0.99, p=0.817) and 60-day (OR=1.02, p=0.616) hospitalization following the start of home health care, compared to those in high-performing agencies. Our findings suggest discrepancies (or no relationship) between patient experience and objective outcomes of home health care.

### Session 4410 (Symposium)

### HOME-BASED CARE IN THE CONTEXT OF COVID-19: DISRUPTIONS, INNOVATIONS, AND LESSONS FOR THE FUTURE

Chair: Emily Franzosa Discussant: Katherine Ornstein

During the initial surge of the COVID-19 pandemic, home-based primary and palliative care (HBPC) practices played a valuable role in maintaining access to health-related services and keeping older, medically complex patients out of hospitals and congregate settings that could heighten their risk for COVID-19. At the same time, these practices faced unique challenges in adapting a traditionally hands-on model of care to accommodate restrictions on in-person contact. In this symposium, we present innovative research highlighting the challenges faced by HBPC practices and patients during spring 2020, as well as their rapid innovations and adaptations. First, Ritchie et al. provide national context with findings from a survey of U.S. home-based primary care practices that highlights the field's most pressing challenges and successful strategies. Shifting to the initial epicenter of the pandemic in New York City, Reckrey et al. present a qualitative study of the perspectives of paid and unpaid caregivers of dementia patients served by an HBPC practice, while Franzosa et al. describe care disruptions among individuals with dementia who died during the initial surge, using a novel chart-based abstraction technique. Finally, two studies (Gorbenko et al. and Kalicki et al.) explore HBPC practices' experience of rapidly transitioning to telehealth through qualitative interviews with NYC-based practices and a provider survey exploring telehealth adoption and readiness in the homebound population. Together, these studies yield important insights into the challenges of providing community-based care for at-risk populations during a pandemic, and practical strategies for home-based models of care moving forward.

### DISRUPTIONS AND ADAPTATIONS IN END-OF-LIFE CARE DELIVERY FOR HOMEBOUND PATIENTS DURING COVID-19

Patricia Kim,[1] Meng Zhang,[1] Jennifer Reckrey,[2] Sara Lubetsky,[3] Emily Xu,[1] Katherine Ornstein,[1] and Emily Franzosa,[2], *1. Icahn School of Medicine at Mount Sinai, New York, New York, United States, 2. Icahn School of Medicine at Mount Sinai, Icahn School of Medicine at Mount Sinai, New York, United States, 3. Icahn School of medicine at Mount Sinai, New York, New York, United States*

The initial COVID-19 pandemic surge in New York City caused widespread delays and disruption in end-of-life services. This study examined the impact of disruptions among homebound adults in an HBPC practice who died between March-June 2020. Through an in-depth mixed-methods chart review, we identified 113 patient deaths (mean age: 87, 73% female, 67% with dementia). Forty-nine (43%) of deaths occurred in April 2020. Through a content analysis of clinician notes, we identified key COVID-related themes, including a shift to intensive phone-based care and to a lesser degree, telehealth; delays in hospice referrals and admissions; and an increase in treatment for behavioral symptoms. Our analysis also demonstrated the central role of family and paid caregivers in coordinating care, and efforts by patients, caregivers and providers to avoid hospital admissions. These findings demonstrate the importance of care coordination across medical, home and community partners to support end-of-life care in emergencies and beyond.

### COVID CHALLENGES AND ADAPTATIONS AMONG HOME-BASED MEDICAL PRACTICES: LESSONS FOR AN ONGOING PANDEMIC

Orla Sheehan,[1] Naomi Gallopyn,[2] Shanaz Sharieff,[3] Abraham Brody,[4] Bruce Leff,[5] and Christine Ritchie,[2] *1. Johns Hopkins University School of Medicine, Johns Hopkins University, Maryland, United States,*

*2. Massachusetts General Hospital, Boston, Massachusetts, United States, 3. Massachusetts General Hospital, Boton, Massachusetts, United States, 4. NYU Hartford Institute for Geriatric Nursing, New York, New York, United States, 5. The Center For Transformative Geriatric Research, Johns Hopkins School of Medicine, Baltimore, Maryland, United States*

Home-based primary care (HBPC) practices rapidly adapted to maintain care during the COVID-19 pandemic. This mixed-methods national online survey of HBPC practices probed responses to COVID-19 surges, COVID-19 testing, the use of telemedicine, practice challenges due to COVID-19, and adaptations to address these challenges. Seventy-nine practices across 29 states were included in the analyses. Eighty-five percent of practices continued to provide in-person care and nearly half cared for COVID-19 patients. Most practices also pivoted to concurrent use of video visits. The top five practice challenges were: patient familiarity with telemedicine, patient and clinician anxiety, technical difficulties reaching patients, and supply shortages. Practices also described creative strategies to physically support the needs of patients. These findings illustrate the need to balance in-person and virtual care for this population, and attend to the emotional needs of patients and staff.

### WHO PROVIDES CARE IN A CRISIS? FAMILY CAREGIVERS' EXPERIENCES OF HOME CARE DURING THE COVID-19 PANDEMIC

Sasha Perez,[1] Katherine Ornstein,[2] and Jennifer Reckrey,[3], *1. Icahn School of Medicine at Mount Sinai Hospital, Icahn School of Medicine at Mount Sinai, New York, United States, 2. Icahn School of Medicine at Mount Sinai, New York, New York, United States, 3. Icahn School of Medicine at Mount Sinai, Icahn School of Medicine at Mount Sinai, New York, United States*

Many homebound individuals with dementia rely on both paid caregivers (e.g., home health aides, home attendants, other homecare workers) and family caregivers to live safely at home. We conducted semi-structured interviews with 15 family caregivers of individuals with severe dementia receiving home-based primary care in NYC to explore how caregiving changed during the COVID pandemic. Most individuals with long-standing paid caregivers experienced infrequent home care disruptions. In fact, paid caregivers were often the primary and sometimes only individuals to provide direct care; family caregivers themselves often stayed away and managed care from a distance. While most family caregivers described heightened attention to infection control, guidance about COVID prevention and safety rarely came from home-based primary care providers or home care agencies and instead was considered "common sense." These findings confirm the essential role paid caregivers play in home-based dementia care teams.

### PROVIDER PERCEPTIONS OF VIDEO TELEHEALTH IN HOME-BASED PRIMARY CARE DURING COVID-19

Emily Franzosa,[1] Abraham Brody,[2] Bruce Leff,[3] Christine Ritchie,[4] Bruce Kinosian,[5] Alex Federman,[6] Katherine Ornstein,[6] and Ksenia Gorbenko,[6], *1. Icahn School of Medicine at Mount Sinai, Icahn School of Medicine at Mount Sinai, New York, United States, 2. NYU Hartford Institute for Geriatric Nursing, New York, New York, United States, 3. The Center For Transformative Geriatric Research, Johns Hopkins School of Medicine, Baltimore, Maryland, United States, 4. Massachusetts General Hospital, Boston, Massachusetts, United States, 5. University of Pennsylvania Perelman School of Medicine, Philadelphia, Pennsylvania, United States, 6. Icahn School of Medicine at Mount Sinai, New York, New York, United States*

The COVID-19 pandemic accelerated the adoption of virtual care. In this qualitative study, we sought to determine provider perceptions of video telehealth during the first wave of COVID-19 in NYC to inform practice for home-based primary care providers nationwide. We conducted semi-structured interviews with clinical directors, program managers, nurse practitioners, nurse managers, and social workers at 6 NYC practices (N=13) in spring 2020. We used combined open and focused coding to identify themes. Participants employed both hospital-supported and commercial technological platforms to maintain care during COVID-19. Benefits of video telehealth included improved efficiency, capacity and collaboration between providers. Barriers included patients' physical, cognitive or technological abilities, dependence on caregivers and aides to facilitate video visits, challenges establishing trust with new patients and addressing sensitive topics over video, and concerns over missing important patient information. Considering patient, clinical, and technological conditions can help optimize telehealth implementation among older homebound adults.

### PROVIDERS' PERCEPTIONS OF TELEHEALTH BARRIERS AMONG HOMEBOUND ADULTS IN IN A HOME-BASED PRIMARY CARE PRACTICE

Alex Kalicki,[1] Peter Gliatto,[1] Emily Franzosa,[2] Katherine Ornstein,[1] and Kate Moody,[1], *1. Icahn School of Medicine at Mount Sinai, New York, New York, United States, 2. Icahn School of Medicine at Mount Sinai, Icahn School of Medicine at Mount Sinai, New York, United States*

The COVID-19 pandemic resulted in a dramatic shift to video-based telehealth use in home-based primary care. We conducted an online 11-item survey exploring provider perceptions of patients' experience with and barriers to telehealth in a large HBPC program in New York City. More than one-third (35%) of patients (mean age of 82.7; 46.6% with dementia; mean of 4 comorbidities/patient) engaged in first-time video-based telehealth encounters between April and June 2020. The majority (82%) required assistance from a family member and/or paid caregiver. Among patients who had not used telehealth, providers deemed 27% (n=153) "unable to interact over video" for reasons including cognitive or sensory ability. Fourteen percent lacked caregivers. Physicians were not knowledgeable about patients' internet connectivity, ability to pay for cellular plans, and video-capable device access. These findings highlight the need for novel approaches to facilitating telehealth and systematic data collection before targeted interventions to increase video-based telehealth use.

### Session 4415 (Symposium)

### IMPLEMENTATION SCIENCE TRANSLATION: PROGRAM SUSTAINMENT FOR MANAGING DISTRESS BEHAVIOR IN DEMENTIA

Chair: Kim Curyto Discussant: Ann Kolanowski

Distress behaviors in dementia (DBD) are common in nursing home settings, are distressing, and result in poorer outcomes for residents and staff. We present on the implementation of STAR-VA, an interdisciplinary intervention for effective management of DBD in Veterans Health Administration (VA) nursing home settings, called Community Living Centers (CLCs). A primary focus of this symposium is the use of implementation science concepts to improve and sustain evidence-based programs through tailored implementation strategies and key partnerships. Key implementation science concepts from conceptual frameworks, including the Consolidated Framework for Implementation Research (CFIR) and the use of organizational Knowledge Reservoirs (KR) for sustaining new clinical practices, formed the basis of this work. Their application in health care practice will be discussed using STAR-VA as an exemplar. Interdisciplinary CLC staff feedback during STAR-VA implementation and sustainment is presented, including feedback regarding barriers to integrating new program interventions into usual care processes. Mapping key implementation strategies onto reported barriers informed development of implementation tools and strategies designed to guide adaptions tailored to the needs of the residents and frontline staff, increasing the chances of successful sustainment. Finally, we highlight the importance of key leadership partnerships in implementation of evidence-based programs to improve care of residents with DBD and present strategies for developing these partnerships. Discussion will include the importance of using implementation science to implement evidence-based interventions for effective management of DBD and strategies for sustainment of these effective practices into usual care.

### TRANSLATING SCIENCE INTO PRACTICE AND MAKING IT STICK: SYSTEM-LEVEL APPROACHES

Kim Curyto,[1] Jennifer Sullivan,[2] and Laura Wray,[3], *1. VA Western New York Healthcare System, Batavia, New York, United States, 2. VA Boston Healthcare System, Boston, Massachusetts, United States, 3. VA Center for Integrated Healthcare, Buffalo, New York, United States*

The delay between establishing evidence-based practice and implementing this evidence base is well documented. This presentation will focus on the application of implementation science principles to real-world clinical programs. A VA priority is to implement evidence-based practice for management of DBD in CLCs. Key implementation science concepts will be introduced, along with a description of how these conceptual models facilitate application of roll-out and sustainment of complex evidence-based interventions. Conceptual frameworks that contributed to intervention selection and facilitation of STAR-VA implementation, including the Consolidated Framework for Implementation Research (CFIR) and Knowledge Reservoirs (KR) framework, and their application in health care practice, will be discussed. The CFIR Expert Recommendation for Implementing Change (ERIC) Mapping Tool will be introduced as useful to identify strategies that address barriers to sustaining implementation. Attendees will be provided with resources to support implementation and sustainment efforts.

### BARRIERS TO SUSTAINED IMPLEMENTATION OF STAR-VA AND STRATEGIES TO OVERCOME THEM

Omonyele Adjognon,[1] Jacquelyn Pendergast,[2] Laura Wray,[3] Michele Karel,[4] Kimberly Curyto,[5] and Jennifer Sullivan,[6], *1. CHOIR, VA Boston Healthcare System, Boston, Massachusetts, United States, 2. CHOIR, VA Boston healthcare System, Boston, Massachusetts, United States, 3. VA Center for Integrated Healthcare, Buffalo, New York, United States, 4. Veterans Health Administration, Washington, District of Columbia, United States, 5. Center for Integrated Healthcare, VA Western NY Healthcare System, Batavia, New York, United States, 6. VA Boston Healthcare System, Boston, Massachusetts, United States*

STAR-VA is an evidence-based, interdisciplinary program helping CLC teams effectively manage DBD. We conducted interviews with 42 key informants involved with STAR-VA implementation in 20 CLCs, guided by a sustainment framework, to understand facilitators and barriers to sustained implementation. We used directed content analysis to identify barriers and mapped them to the CFIR-ERIC Mapping Tool to identify associated implementation strategies. We identified six barriers: 1) staffing issues, 2) lack of written policies, 3) staff buy-in, 4) limited leadership support, 5) exclusion of STAR-VA criteria in performance evaluations, and 6) service line silos. We identified six strategies to overcome these barriers, three strategies most frequently mapped to reported barriers to STAR-VA sustainment: 1) assessing local CLC readiness, facilitators and addressable barriers; 2) identifying and preparing new champions; and 3) altering incentive/allowance structures. The identified strategies can be packaged to further integrate STAR-VA into usual CLC care processes to optimize program sustainability.

### LISTENING TO CLINICAL TEAMS: DEVELOPING STRATEGIES TO SUPPORT SUSTAINED STAR-VA IMPLEMENTATION

Kyle Page,[1] Karen Benson,[2] Laura Wray,[3] Michele Karel,[4] and Kim Curyto,[5], *1. Edward Hines Jr VA Hospital, Hines, Illinois, United States, 2. Home Based Primary Care - Kernersville, W.G. Bill Hefner VA Medical Center, Salisbury, North Carolina, United States, 3. VA Center for Integrated Healthcare, Buffalo, New York, United States, 4. Veterans Health Administration, Washington, District of Columbia, United States, 5. VA Western New York Healthcare System, Batavia, New York, United States*

Feedback obtained from program evaluations and interviews with CLC team members who participated in STAR-VA helped to inform the development of sustained implementation strategies guided by the CFIR-ERIC Mapping Tool. A CLC readiness assessment was developed to guide selection of new champions and assess for local team readiness to implement STAR-VA. Virtual training materials were developed along with a champion training checklist to prepare additional champions and support team training. We identified key implementation steps and optional strategies to support sustained implementation, developed a sustained implementation guide, associated sustained implementation checklist, and sustainability toolkit. We are piloting a regional community of practice model, encouraging development of and building on relationship networks to promote use of program tools, collaborative problem-solving, feedback, and a shared vision for implementation. We will discuss the importance of tailored strategies for integrating new practices into usual care.

### SUPPORT FROM THE TOP: DIVERSE LEADERSHIP PARTNERS FOR SUSTAINED STAR-VA IMPLEMENTATION

A. Lynn Snow,[1] Christine Hartmann,[2] Jenefer Jedele,[3] Lisa Minor,[4] and Michele Karel,[5], *1. Tuscaloosa VA Medical Center, Tuscaloosa, Alabama, United States, 2. VA Bedford Healthcare System, VA Bedford Healthcare System, Massachusetts, United States, 3. Veterans Health Administration, Ann Arbor, Michigan, United States, 4. Geriatrics and Extended Care, Washington, District of Columbia, United States, 5. Veterans Health Administration, Washington, District of Columbia, United States*

The STAR-VA program was an initiative out of what is now called the VA Office of Mental Health and Suicide Prevention, partnering with the national Offices of Geriatrics and Extended Care and Nursing Services. Ongoing collaboration with these national, as well as regional and medical-center-level leaders, has been critical for informing program implementation and dissemination strategies. We will discuss several key partnered strategies, including (1) linking STAR-VA to national CLC systematic quality improvement efforts; (2) engaging national inter-office program leaders in decisions about outreach to and inclusion of facilities in STAR-VA training and implementation; (3) training local STAR-VA champions on strategies for engaging local leadership support; (4) briefing leaders across the system with program updates; and (5) using national VA data to inform STAR-VA sustained implementation. Discussion will address challenges and opportunities for engaging leadership stakeholders in facilitating sustained implementation of evidence-based programs.

### Session 4420 (Paper)

### LONELINESS AND ISOLATION

### CHANGES IN MODES OF SOCIAL CONTACT AND THEIR LINKS WITH MENTAL HEALTH DURING THE COVID-19 PANDEMIC

Laura Finch,[1] and Louise Hawkley,[2], *1. NORC at The University of Chicago, Chicago, Illinois, United States, 2. NORC at the University of Chicago, Chicago, Illinois, United States*

Amid the COVID-19 pandemic, social distancing has been emphasized for older adults because of their greater physical health risks. Using data from the National Social Life, Health and Aging Project (NSHAP), we examined how older adults may have changed their frequency of contact with others via various modes (i.e., in-person, phone calls, messages, and video calls) since the pandemic started, and how these choices may be impacting their mental health. From September 2020 through January 2021, NSHAP respondents (N=2,554 age 50-94 with data from 2015-16) completed a survey via web, phone, or paper-and-pencil. Although some older adults reported reducing their in-person contact with out-of-household family (38%) and friends (40%) since the pandemic started, some also increased contact with them via remote modes such as phone calls (25% and 16% with family and friends respectively); emails, texts, or social media messages (26 and 21%); and video calls (24 and 18%). Net of demographics, living alone, survey mode, and 2015-16 levels of the respective mental health variables, those who decreased in-person contact with family were less happy (B=-0.12, SE=0.06, p=.035), had higher loneliness scores (B=0.23, SE=0.09, p=.011), and more frequently felt depressed (B=0.10, SE=0.05, p=.055). In the presence of decreased in-person contact, increases in remote modes of contact had no net remediating impact—a pattern also found when analyzing contact with friends. Results indicate a persistent adverse effect of reduced in-person contact on mental health despite increased contact with family and friends via remote means.

### LONGITUDINAL ANALYSES INDICATE BIDIRECTIONAL ASSOCIATIONS BETWEEN LONELINESS AND SELF-RATED HEALTH IN ADULTHOOD

Deborah Finkel,[1] Dianna Phillips,[2] and Chandra Reynolds,[2], *1. Indiana University Southeast, New Albany, Indiana, United States, 2. University of California Riverside, Riverside, California, United States*

Loneliness is a potent stressor that increases in prevalence with age in late life and has been linked with numerous adverse physical health outcomes and lower scores on measures of self-rated health (SRH). The association between loneliness and SRH is likely bidirectional—for example, experiencing loneliness may result in physiological changes that alter how individuals perceive their health, and worsening perceptions of one's own health or mobility may act in an increasingly restrictive manner with respect to social interaction. Despite this, limited longitudinal work has examined temporal dynamics between loneliness and SRH. Recently completed harmonization of 9 loneliness items across three longitudinal twin studies of aging in Sweden resulted in sample of 1939 participants aged 40 to 98 at intake (mean age = 74.64) with up to 25 years of follow-up (mean = 7.63) across up to 8 waves (mean = 3.29). Univariate analysis indicated that SRH decreased with age up to age 82 and then leveled off, whereas loneliness continued to increase across the age span. Bivariate dual change score models were used to examine lead-lag relationships across time: which variable contributes to subsequent changes in the other variable. Results indicated a bi-directional relationship: loneliness does not increase after age 82 when SRH is included in the model, and SRH does not level off after age 70 when loneliness is included in the model. Thus, declining SRH may lead to reduced participation in social activities and also feelings of loneliness may intensify perceptions of poor health.

### SOCIAL CONNECTEDNESS RISK AMONG MIDDLE-AGED AND OLDER BLACK MEN WITH CHRONIC CONDITIONS

Matthew Smith,[1] Ledric Sherman,[2] Kirby Goidel,[2] and Thomas Cudjoe,[3], *1. Texas A&M School of Public*

*Health, College Station, Texas, United States, 2. Texas A&M University, College Station, Texas, United States, 3. Johns Hopkins University School of Medicine, Baltimore, Maryland, United States*

Social connectedness is a multi-factorial concept encompassing structural, functional, and quality aspects. For those with chronic conditions, threats to social connectedness can exacerbate illness symptomology and impose barriers to disease self-management. This study identified factors associated social connectedness risk among Black men ages 40+ years with one or more chronic conditions. Data from 2019 were analyzed from a national sample of 1,200 Black males collected with an internet-delivered questionnaire. Three logistic regression models were fitted to assess factors associated with not having enough people to call for help (structural), feeling isolated from others (functional), and not being content with friendships/relationships (quality). All regression models were adjusted for age, education, marital status, number of chronic conditions, self-reported barriers to disease self-management, and having cut-down or skipped social activities because of health problems. On average, participants were age 56.7 (±9.7) years and self-reported 4.0 (±2.9) chronic conditions. Approximately 23% were ages 65+ years, 45.4% cut-down or skipped social activities because of health problems, 25.2% did not have enough people to call for help, 26.0% felt isolated from others, and 23.8% were not content with friendships/relationships. Across the three regression models, men who were middle-aged ($P<0.05$), never married ($P<0.01$), cut-down or skipped social activities because of health problems ($P<0.001$), and reported more barriers to disease self-management ($P<0.001$) were significantly more likely to report social connectedness risk. Findings suggest that efforts to improve the self-management of illness symptomology may mitigate threats to structural, functional, and quality aspects of social connectedness among this male population.

### SOCIAL ISOLATION AMONG OLDER MILITARY VETERANS

Kirsten Laha-Walsh, and Zainab Suntai, *University of Alabama, Tuscaloosa, Alabama, United States*

Social isolation is an increasingly critical issue among older adults and has been found to affect several domains of well-being, including physical, psychological, and cognitive health. Research has found that military veterans often experience hardships in the transition back to civilian life including emotional trauma, depression, substance misuse and pain from combat-related injuries, which have been shown to persist well into older adulthood. As such, this study aimed to examine the prevalence of social isolation among older military veterans and determine which veterans are most at-risk of experiencing social isolation, using the Berkman-Syme Social Network Index as a framework. Data were derived from Round 1 of the National Health and Aging Trends Study (NHATS), an annual longitudinal panel survey of adults aged 65 and older living in the United States. Results showed that about 4.5% of veterans in the NHATS are severely socially isolated while another 20.9% are socially isolated. After controlling for other explanatory variables, being White, being 85 and older, having lower educational attainment, being unmarried/unpartnered and having lower income were associated with an increased risk of experiencing social isolation. Interventions aiming to improve the well-being of older veterans should consider employing both preventative and amendatory measures. These may include the creation and administration of a standardized social isolation scale during visits to veterans' affairs (VA) medical centers and a general effort to address stressors from military service by destigmatizing and improving access to mental health services.

### THE IMPACT OF STRUCTURAL AND FUNCTIONAL SOCIAL RESOURCES ON LONELINESS AMONG AMERICANS AGE 50 YEARS AND OLDER

Jillian Minahan, *Fordham University, New Providence, New Jersey, United States*

Social isolation and loneliness have many negative consequences (e.g., Cacioppo et al., 2006; Griffin et al., 2018; Uchino, 2006), especially among older adults (Perissinotto et al., 2012). According to the cognitive discrepancy theory (CDT), loneliness is the negative psychological state resulting from the perceived discrepancy between one's desired level of social resources and one's actual level of social resources (Peplau & Perlman, 1982; Perlman & Peplau, 1998). Social resources have both structural (e.g., objective) and functional (e.g., perceptions of the quality) aspects (Holt-Lunstad, 2017). The relationship between structural and functional social resources has been described as a filtration process in which functional aspects mediate the association between structural aspects and loneliness (Cacioppo et al., 2016; Hawkley et al., 2008, Hawkley & Kocherginsky, 2018). However, this filtration model has not been empirically tested within the CDT. This study examined the relationship among structural social resources (SSR), functional social resources (FSR), and loneliness cross-sectionally and longitudinally using a sample of 3,345 Americans aged 50 years and older from the 2008 and 2012 waves of the Health and Retirement Study. Results showed that there was a significant indirect effect both cross-sectionally ($\beta = -.07$) and longitudinally ($\beta = -.06$) such that FSR mediated the relationship between SSR and loneliness. Ultimately, the CDT is useful in explaining the complex relationship between structural and functional aspects of one's social resources with loneliness, and interventions may seek to target the functional aspects of one's social network to improve loneliness, rather than focusing solely on structural aspects.

### Session 4425 (Paper)

### LONG-TERM CARE POLICY AND UNMET NEEDS

### A PILOT OF A SUSTAINABILITY-EXTENDING INTERVENTION IN CANADIAN NURSING HOMES

Lauren MacEachern,[1] Yuting Song,[2] Liane Ginsburg,[3] Adrian Wagg,[2] Matthias Hoben,[4] Malcolm Doupe,[5] Carole Estabrooks,[2] and Whitney Berta,[6], *1. University of Toronto, Toronto, Ontario, Canada, 2. University of Alberta, Edmonton, Alberta, Canada, 3. York University, York University, Alberta, Canada, 4. University of Alberta at Edmonton, Edmonton, Alberta, Canada, 5. University of Manitoba, Winnipeg, Manitoba, Canada, 6. University of Toronto, University of Toronto, Ontario, Canada*

Understanding of intervention sustainability processes is limited. Failure to sustain evidence-based innovations means that intended improvements are short-lived, scale-up and spread are unlikely, and real losses are incurred on research investments. We explored the sustainability of a health care aide (HCA)-led quality improvement (QI) initiative, Safer Care for Older Persons (in residential) Environments (SCOPE), that was implemented in long-term care homes (LTCHs) in Manitoba, Canada. Based on our understanding of factors influencing post-implementation sustainability processes, we developed and piloted a "low-dose" and "high-dose" "Booster" intervention to extend the two-year post-implementation period over which SCOPE was naturally sustained. Both versions of the "Booster" involved the following components: a HCA-led team with management support, a workshop to review SCOPE QI approaches and tools, a binder of QI resources, and supports from an experienced Quality Advisor (QA). We collected data from various sources to depict the most accurate account of QI sustainability and conducted thematic analysis to understand each team's experience with sustainability processes. We used a qualitative assessment rubric to evaluate the impact of the "Booster" conditions on the teams' performance against core SCOPE components. Our results suggest that the "Booster" served to establish more relaxed expectations and generally renew interest in LTCH QI initiatives. The calibre of management support was associated with teams' performance and management support varied with the level of QA support. These pilot results will inform the next study phase, which examines longer-term sustainability of QI initiatives in LTCHs beyond the initial 2-year post-implementation period.

### AGING INTO DISABILITY: A CONCEPTUAL CHALLENGE FOR GERONTOLOGY

Jeffrey Kahana,[1] Timothy Goler,[2] and Lawrence Force,[3], *1. Mount Saint Mary College, Newburgh, New York, United States, 2. Norfolk State University, Newport News, Virginia, United States, 3. Mount Saint Mary College / C enter on Aging and Disability Policy, Mount Saint Mary College, New York, United States*

One of the most fraught subjects facing a fast growing aging population is the subject of aging into disability. This paper examines the processes of aging into disability as a distinct challenge for not only older persons, but also for the field of gerontology, and public policy-makers. Disability in youth and in middle age has largely defined the disability rights agenda, and elders aging into disability have not been the subject of much attention from scholars in the field of disability. Surprisingly, however, scholars and policy-makers in gerontology have also by and large avoided the subject of older persons aging into disability—a complex process that involves impairment, environmental disablement, and changes in social relationships. This process accelerates with advancing age, and disproportionately affects women. Moreover, when older adults develop mobility limitations, experience falls, become hard of hearing, or experience other such impairments of age related disability, they do not think of themselves as aging into disability, or being disabled. This lack of disability identity may protect them from stigma and from low self-esteem. At the same time, it stands in the way of seeking accommodations and from developing a bond with other older adults who are aging into disability. This paper explores the dynamics of disability avoidance as an ideal that can harm older adults and their caregivers. It aims to bring disability more fully into the normal life-course, and to suggest lines of inquiry for gerontological research, to broaden the field, and to make service communities more inclusive .

### EVALUATION OF MINNESOTA'S LONG-STAY NURSING HOME QUALITY INDICATORS

Dongjuan Xu,[1] Teresa Lewis,[2] Marissa Rurka,[1] and Greg Arling,[1], *1. Purdue University, West Lafayette, Indiana, United States, 2. Minnesota Department of Human Services, Saint Paul, Minnesota, United States*

The Minnesota Nursing Home Report Card provides 19 clinical quality indictor (QI) ratings. Currently, face validity and expert opinions are employed to group the 19 long-stay QIs into 10 different domains. However, we do not know whether these domains are supported by the data. Under the current scoring program, some QIs may not discriminate very well between facilities. The objective was to evaluate the dimensionality of the QIs and the current scoring approach used to assign points to the domain and total QI scores. Risk-adjusted facility-level rates for the 19 QIs over the 2012-2019 period were used. Our findings indicate it is reasonable to categorize these QIs into 4 domains. Moreover, the current scoring approach is best suited for a facility QI distribution that is approximately normal. However, 11 QIs display a skewed distribution with facilities tightly grouped at the very bottom (floor) or top (ceiling) of the QI distribution. Our findings suggest that the current scoring approach may distort or exaggerate the differences in the QI rates with skewed distributions, assigning widely varying points to facilities that vary little in their QI rates. We recommend a zero-error approach for highly skewed QIs where the QI outcome is achievable and it reflects a serious quality problem. Our study of the QI scoring system is part of a package of recommendations to improve the Minnesota Nursing Home Report Card and value-based reimbursement system. Lessons learned from the study are readily applicable to Medicare's Nursing Home Compare report.

### PREFERABLE USE OF INFORMATION AND COMMUNICATION TECHNOLOGIES IN LONG-TERM CARE SETTINGS: A VIGNETTE SURVEY OF JAPAN

Tomoko Wakui,[1] Tomoko Ikeuchi,[1] Sakiko Itoh,[2] Hiroyasu Miwa,[3] and Kentaro Watanabe,[3], *1. Tokyo Metropolitan Institute of Gerontology, Tokyo, Tokyo, Japan, 2. Osaka University, Osaka, Osaka, Japan, 3. National Institute of Advanced Industrial Science and Technology, Chiba, Chiba, Japan*

The remarkable development of information and communication technologies (ICT), considering the rapidly aging global population, could eliminate the physical and mental burdens involved in caregiving and enhance the perceived dignity and autonomy of older adults. However, the introduction of ICT in long-term care (LTC) in Japan has not yielded good results yet, and the social acceptance of ICT remains understudied. This study aimed to understand and examine people's views on ICT use in LTC settings. An online vignette survey was conducted in August 2020, among community-dwelling persons between the ages of

40–89 years, throughout Japan. A set of four vignettes of different physical and cognitive functional situations, at either a home or nursing home, with or without ICT use, was presented to select a preferred care setting. Multinomial regression analyses were used to examine the relationships between participants' choices and individual characteristics. A total of 4,457 participants (52.8% of whom were women) were analyzed (mean age = 60.8 years). Participants were more likely to choose nursing homes in cognitively dependent situations. Participants who were women, relatively younger, and had higher education were more likely to choose care settings employing ICT for physically and cognitively dependent situations. Those who experienced either informal or formal caregiving were more likely to choose care settings that used ICT. This study revealed that individuals preferred different levels of ICT use based on physical and cognitive situations. Barriers to introducing ICT in LTC settings will be discussed.

### Session 4430 (Paper)

### MENTAL HEALTH INTERVENTIONS

### ANALYSIS OF 29 ITEMS OF THE MEANINGFUL ACTIVITY PARTICIPATION ASSESSMENT-MEANINGFUL SCALE THROUGH RASCH ANALYSIS

Sanghun Nam, Suyeong Bae, and Ickpyo Hong, *Yonsei University, Wonju, Kangwon-do, Republic of Korea*

Individuals find meaning in their personal activities. Meaningful activities can improve an individual's emotional and physical health and quality of life. The Meaningful Activity Participation Assessment-Meaningful Scale (MAPA-M), which can measure these meaningful activities, is measured in 29 items. In this study, the psychometric properties of 29 items of MAPA-M were investigated through Rasch analysis. The data used in this study was the Well Elderly Study 2 data among public data provided by the Inter-university Consortium for Political and Social Research (ICPSR). We used 480 randomized samples from the Well Elderly Study 2 data. Before proceeding with the Rasch analysis, as a result of checking the unidimensionality assumption of 29 items, 19 items satisfied the unidimensionality assumption. As a result of Rasch analysis of 19 items, the Driving item was removed as misfit (infit mean-square = 2.04, infit z-standardized fit statistics = 9.90, outfit mean-square = 1.86, outfit z-standardized fit statistics = 8.99). The 18 items with the misfit items removed show a conceptual item-difficulty hierarchy, and there was no differential item functioning that worked for sex and age groups. The person strata value is 3.97, which corresponds to the confidence value of 0.88. These results indicate that the 18 items in MAPA-M show appropriate item-level psychometric properties. In other words, the modified MAPA-M 18 indicates that meaningful activities can be accurately and stably measured.

### DEVELOPING MULTIMODAL IOMT MONITORING SYSTEM OF GERIATRIC DEPRESSION: A FEASIBILITY STUDY

Heejung Kim,[1] Youngshin Cho,[1] Kyuhee Lim,[1] Sunghee Lee,[2] Yuntae Kim,[3] and Mijung Kim,[3], *1. Yonsei University, Seoul, Seoul-t'ukpyolsi, Republic of Korea, 2. BRFrame Inc., Seoul, Seoul-t'ukpyolsi, Republic of Korea, 3. Mapo Senior Welfare Center, Seoul, Seoul-t'ukpyolsi, Republic of Korea*

The Internet of Medical Things (IoMT) is a promising tool to monitor depression and relevant symptoms. However, the multimodal IoMT monitoring system has been rarely developed considering the characteristics of older adults, particularly living in the community. Therefore, it is necessary to know how to develop multimodal IoMT monitoring systems tailored for older adults and evaluate the feasibility for research and practice. We developed a multimodal IoMT monitoring system that included a smartphone for facial and verbal expressions, smartwatch for activity and heart rates, and ecological momentary assessment (EMA) application. A convenience sample of 21 older Korean adults aged over 65 years was recruited from a community center, and 19 participants completed it. The data were collected in four weeks using self-report questionnaires, IoMT devices, and semi-structured interviews between July and December 2020 and were analyzed in mixed methods. Based on the Geriatric Depression Scale-Short Form scores, eight participants were classified in the depressive group (38.1%) and 13 in the non-depressive group (61.9%). A total of 1,505 (70.72%) EMA data were collected, and 1,277 (60.00%) were analyzed. Furthermore, 1,421 (66.78%) facial expression data were collected and labeled, including anger, happiness, neutral, sadness, surprise, and exception. Voice dialogues were transformed into 5,264 scripts. The depressive group showed lower user acceptance relative to the non-depressive group. However, both groups experienced positive emotions, had regular life patterns, and increased their self-interest. Thus, our multimodal IoMT monitoring system is a feasible and useful measure for acquiring mental health information in older adults' depression.

### IMPLEMENTING BEHAVIORAL HEALTH SOCIAL WORKERS' INCREASED MENTAL HEALTH CARE AMONG OLDER ADULTS

Elizabeth Pfoh, Jessica Hohman, Kathleen Alcorn, Nirav Vakharia, and Michael Rothberg, *Cleveland Clinic, Cleveland, Ohio, United States*

Depression is underdiagnosed and undertreated among older adults. Health systems can screen patients to identify depression, but systemic linkages to treatment are required to ensure care. We used a retrospective stepped-wedge study to identify the impact of implementing behavioral health social workers (BHSWs) on receipt of treatment after a new depression diagnosis. We included adults over 65 years of age with a primary care visit between 2016 and 2019 at a large integrated health system. We excluded patients who were diagnosed with or treated for depression in 2015. Patients were categorized into control (diagnosed before implementation) and intervention (diagnosed after implementation) groups. From our electronic health record, we collected prescriptions for pharmacotherapy and behavioral health visits. Patients were considered treated if they received pharmacotherapy or had a behavioral health visit within 30 days of diagnosis. We used multilevel logistic regression models to identify the association between implementation period (pre versus post) and treatment, adjusted for demographic variables and clustering within site. Our population included 4,475 people. The percent of patients that received treatment increased from 47% to 54% after implementation and the percent of patients with ≥1 behavioral health visit within 30 days increased from 3% to 8% (p<0.01, respectively). The adjusted odds ratio of receiving treatment (AOR: 4.13, 95%CI: 2.84-6.01) and having a behavioral health visit (AOR: 3.12, 95%CI: 2.31-4.24) was significantly higher in the post-implementation period. In conclusion, implementation of BHSWs was associated with increased treatment for older patients with depression.

### LITHIUM RECEIPT AND RISK OF DEMENTIA IN OLDER VETERANS WITH BIPOLAR DISORDER IN THE VA HEALTH SYSTEM

Benjamin Szymanski,[1] Eileen Ahearn,[2] Eric Smith,[3] Jenefer Jedele,[4] John McCarthy,[1] and Ira Katz,[5], *1. Department of Veterans Affairs, Ann Arbor, Michigan, United States, 2. William S. Middleton Memorial Veterans Hospital, Madison, Wisconsin, United States, 3. Edith Nourse Rogers Memorial Veterans Hospital, Bedford, Massachusetts, United States, 4. Veterans Health Administration, Ann Arbor, Michigan, United States, 5. Department of Veterans Affairs, Philadelphia, Pennsylvania, United States*

Older adults with bipolar disorder are at increased risk of developing dementia. The literature suggests lithium treatment may reduce the incidence of dementia. This study sought to inform clinical practice in the Veterans Affairs (VA) health system by estimating the effect of past year lithium receipt on dementia incidence among Veterans with bipolar disorder. Divalproex receipt was used as a comparison. Using VA medical records, 121,094 Veterans aged 50 and older with a diagnosis of bipolar disorder but no dementia diagnosis were identified in fiscal years 2005-2019. Follow-up continued until dementia diagnosis, 36 months from the index date, death, or the end of fiscal year 2020, whichever came first. 4347 (3.6%) were diagnosed with dementia during follow-up. Time-varying indicators of receipt of lithium and divalproex in the prior 365 days were calculated for each day, categorized as 301-365, 61-300, 1-60, or 0 days of receipt. Unadjusted Cox proportional hazards regression analyses indicated reduced dementia incidence with 301-365 (HR=0.86, 95% Confidence Interval [95%CI] 0.75-0.99) and 61-300 (HR=0.75, 95%CI 0.65-0.87) days of lithium receipt, compared to 0 days. For divalproex, 301-365 (HR=1.34, 95%CI 1.23-1.47) and 61-300 (HR=1.13, 95%CI 1.03-1.23) days of receipt were each associated with increased dementia incidence. Lithium effects were not statistically significant after adjusting for age, sex, race, ethnicity, medical comorbidities, and antidepressant, antipsychotic, and anxiolytic medication receipt. Divalproex effects remained statistically significant. Past year divalproex, but not lithium, receipt was significantly associated with dementia incidence among VA patients with bipolar disorder when adjusting for demographics and medical comorbidities.

### MENTAL AND PHYSICAL HEALTH IN SWISS OLDER SURVIVORS OF ENFORCED CHILD WELFARE PRACTICES

Myriam Thoma,[1] Florence Bernays,[2] Carla Eising,[3] Andreas Maercker,[4] and Shauna Rohner,[5], *1. University of Zurich, Zurich, Zurich, Switzerland, 2. University of Zürich, University of Zurich, Zurich, Switzerland, 3. University of Zürich, Zurich, Zurich, Switzerland, 4. University of Zurich, University of Zürich, Zurich, Switzerland, 5. University of Zurich, Zürich, Zurich, Switzerland*

It is the purpose of child welfare practices to provide a protective environment for minors. However, welfare practices for children and adolescents have also been linked to a higher risk for maltreatment, trauma, and deprivation. Due to such early-life adversity, affected individuals often report a life course depicted by further trauma, socio-economic disadvantage, mental and physical ill-health. Examination of the long-term health correlates of enforced child welfare practices, as well as potential mediators, have previously been neglected in later life. It was therefore the purpose of these studies to examine the long-term correlates of enforced child welfare practices; the associated maltreatment, trauma, and deprivation; and the physical and mental health outcomes in Swiss older survivors (n=132, MAGE=71 years) and an age-matched control group (n=125). These studies further examined the mediating role of socio-economic factors (e.g., education, income), self-esteem, and self-compassion. Mental health was assessed with a structured clinical interview; physical health, self-esteem, and self-compassion with psychometric instruments. Survivors reported significantly more types and severity of childhood maltreatment, trauma, and deprivation than the control group. They also reported significantly more lifetime and current mental health disorders and more physical illnesses. Socio-economic factors and self-esteem, but not self-compassion, acted as significant mediators. Exposure to maltreatment, trauma, and deprivation in childhood and adolescence is linked to poorer mental and physical health in later life. Potential targets for intervention and health-protective measures include socio-economic factors and self-esteem, which were found to diminish the detrimental long-term impact of early-life adversity and disadvantage into later life.

### Session 4435 (Paper)

### PHYSICAL FUNCTIONING

### ASSOCIATIONS OF PHYSICAL FUNCTION AND PHYSICAL ACTIVITY WITH COVID-19-LIKE SYMPTOMS

Marguerita Saadeh,[1] Amaia Calderón-Larrañaga,[2] Davide Vetrano,[2] Philip Von Rosen,[3] and Anna-Karin Welmer,[4], *1. Aging Research Center, Karolinska Institutet, Stockholm, Stockholms Lan, Sweden, 2. Karolinska Institutet, Solna, Stockholms Lan, Sweden, 3. Physiotherapy, Stockholm, Stockholms Lan, Sweden, 4. Karolinska Institutet, 17175, Stockholms Lan, Sweden*

Physical function and physical activity have been associated with health outcomes related to the cardiopulmonary and immune systems, but the extent to which they are related to the risk of developing COVID-19-like symptoms remains unclear. We aimed to explore these associations among Swedish older adults. We analyzed data from 904 individuals aged ≥68 years from the population-based Swedish National study on Aging and Care in Kungsholmen. COVID-19-like symptoms were assessed by phone interview (March-June

2020) and included fever, cough, sore throat and/or a cold, headache, pain in muscles, legs and joints, loss of taste and/or odour, breathing difficulties, chest pain, gastrointestinal symptoms and eye inflammation. Muscle strength, mobility, and physical activity were objectively examined in 2016-2018. Data were analyzed using logistic regression models and stratifying by age. During the first outbreak of the pandemic, 325 (36%) individuals from our sample developed COVID-19-like symptoms. Those with longer time to perform the chair stand test had an odds ratio (OR) of 1.5 (95% confidence interval [CI] 1.1-2.1) for presenting with COVID-19-like symptoms compared to those with a faster time to perform the test, after adjusting for potential confounders. The risk was even higher among people aged ≥80 years (OR: 2.6; 95% CI 1.5-4.7). No significant associations were found for walking speed or moderate-to-vigorous physical activity. A weaker muscle strength, especially among the oldest-old adults, may contribute to higher odds of developing COVID-19-like symptoms, emphasizing the need to maintain sufficient levels of muscle strength in old age.

### BACK TO LIFE, BACK TO REALITY: PARTICIPATION AND FUNCTION AFTER ACUTE HOSPITALIZATION IN OLDER ADULTS

Maya Arieli,[1] Racheli Kizony,[1] Efrat Gil,[2] and Maayan Agmon,[3], *1. Department of Occupational Therapy, University of Haifa, Israel, Haifa, HaZafon, Israel, 2. Clalit health services, Haifa and West Galilee, Israel, Haifa, HaZafon, Israel, 3. The Cheryl Spencer Department of Nursing, University of Haifa, Israel, Haifa, HaZafon, Israel*

This study aims to describe and compare functional trajectories (i.e., participation versus basic daily function) from pre-hospitalization period to one and three-months post-discharge, among older adults hospitalized for acute medical illness, of two age groups: ages 65-75, n=39, >75, n=38. A Prospective longitudinal study was conducted, starting during hospitalization in internal ward and followed by home visits (1 month) and telephone interviews (3 months). Participation was measured by the Activity Card Sort (ACS) that queries about instrumental (e.g. shopping), leisure (e.g. physical activity), and social activities. Basic daily function was measured by the Modified Barthel Index (mBI). Wilcoxon test was used to compare between the ACS and mBI total retained scores within age groups. A mixed model repeated measures ANCOVA was used to compare time by group effects in ACS total scores. The results showed that basic function in both age groups was preserved, and both groups experienced a significantly greater decrease in participation level compared with basic function at one month (z=-4.1, p=.001, z=-4.5, p=.001) and at three months (z=-4.1, p=.001, z=-4.1, p=.001) in the "younger" and "older" groups, respectively. Participation trajectories were similar among age groups, however, the "older" group experienced a significantly greater decrease in participation (F(1)=(4.3), p=.042, $\eta 2= .056$). Findings indicate that the traditional measure of basic function does not capture the broad spectrum of older adults' full life and overshadows the reduced participation in meaningful activities. Health care professionals should adopt a comprehensive approach toward functional assessment to encompass participation beyond basic daily function.

### COMPARISONS OF DIFFERENT MULTIMORBIDITY MEASURES TO PREDICT PHYSICAL FUNCTION AMONG MIDDLE-AGED AND OLDER CHINESE

Hui-wen xu,[1] Yan Luo,[1] Zi-Ting Huang,[2] zi-shuo Chen,[1] He-Xuan Su,[1] and Beibei Xu,[3], *1. Peking University School of Public Health, Beijing, Beijing, China (People's Republic), 2. Peking university School of Public Health, Beijing, Beijing, China (People's Republic), 3. Peking University Medical Informatics Center, Beijing, Beijing, China (People's Republic)*

Multimorbidity has been associated with declined physical function. Several methods have been used to measure multimorbidity. However, few studies have compared the associations between different multimorbidity measures and physical function. We aimed to examine and compare the associations between different multimorbidity measures and physical function. We included 16,117 participants aged ≥45 from China Health and Retirement Longitudinal Survey (CHARLS) 2011-2018. Multimorbidity was defined as the co-existence of two or more of fifteen chronic conditions in an individual and measured by condition count at penultimate, multimorbidity patterns at penultimate (examined by exploratory factor analysis) and multimorbidity trajectories from baseline to penultimate living interview (examined by the group-based trajectory model). Physical function was assessed by activities of daily living or instrumental activities of daily living at the last follow-up. Logistic regression models were conducted for establishing prediction models in the training set. We used c-statistic, Integrated Discrimination Improvements (IDI) and Net Reclassification Index (NRI) to compare the performance of different models in the testing set. After adjusting for age and gender, compared with those without any conditions, participants with multimorbidity measured by three methods all had higher risks of poor physical function in the training set. Compared with the model using condition count (c-statistic=0.749), models using multimorbidity trajectory (c-statistic=0.712, IDI=-0.03, NRI=-0.31) and the multimorbidity pattern (c-statistic=0.739, IDI=-0.01, NRI=-0.16) showed poor predictive power (all p<.05). In our study, condition count has the best predictive performance for poor physical function over short time period. It is a simple and useful tool to assess multimorbidity.

### GAIT SPEED MAINTENANCE IS ASSOCIATED WITH SENSORIMOTOR AND FRONTOPARIETAL NETWORK CONNECTIVITY AMONG OLDER ADULTS

Chun Liang Hsu,[1] Brad Manor,[2] and Lewis Lipsitz,[3], *1. Hinda and Arthur Marcus Institute for Aging Research, Harvard Medical School, Burnaby, British Columbia, Canada, 2. Hinda and Arthur Marcus Institute for Aging Research, Harvard Medical School, Boston, Massachusetts, United States, 3. Hebrew SeniorLife, Boston, Massachusetts, United States*

Mobility impairment is a geriatric giant. Particularly, slow gait is associated with elevated risk for cognitive decline, disabilities and dementia. Gait is the product of complex neural network interactions and changes in their connectivity pattern may negatively impact gait speed. However, mechanistic neural correlates for gait speed maintenance and decline remained unclear. As such, the aim of this study is to investigate differences in neural network connectivity in older adults with and without gait speed decline over 24 months. This sub-analysis included 35 community-dwelling older adults age >70 years from the MOBILIZE Boston Study. Baseline assessments included four-meter gait speed test and resting-state fMRI. Gait speed was reassessed at a 24-month follow-up. Participants were stratified to "Maintainer" and "Decliner" groups based upon a cut-off of >0.05 m/s decline in gait speed from baseline to follow-up. A priori selected functional network included sensori-motor network (SMN) and frontoparietal network (FPN). Multivariate analysis of variance was performed to determine between group differences in network connectivity. Discriminant analysis was conducted to identify relative contribution of network connectivity to group classification. Between the 14 Maintainers and 21 Decliners (mean age 83.9 years), Maintainers were younger (p=0.088). After adjusting for age, Maintainers exhibited lower SMN premotor-precentral gyrus connectivity (p=0.023), greater FPN ventral visual-supramarginal gyrus connectivity (p=0.025), and trend level greater SMN-FPN cerebellum-occipital connectivity (p=0.053). Premotor-precentral gyrus connectivity showed greatest contribution to discriminant function. These preliminary findings suggest aberrant connectivity patterns of the SMN and FPN may be predictive of older adults' ability to maintain gait speed.

### THE SEX DIFFERENCE IN PHYSICAL FUNCTIONING: HOW DO RISK FACTORS CONTRIBUTE?

Lena Sialino,[1] Susan Picavet,[2] Hanneke Wijnhoven,[1] Anne Loyen,[2] Monique Verschuren,[2] Marjolein Visser,[1] Laura Schaap,[1] and Sandra van Oostrom,[3], *1. Vrije Universiteit Amsterdam, Amsterdam, Noord-Holland, Netherlands, 2. National Institute for Public Health and the Environment, Bilthoven, Utrecht, Netherlands, 3. National Institute for Public Health and the Environment, Amsterdam, Noord-Holland, Netherlands*

This study explores whether sex differences in the sensitivity to risk factors (strength of the association) and/or in the exposure to risk factors (prevalence) contributes to the sex difference in physical functioning, with women reporting more limitations. Data of the Doetinchem Cohort Study was used (n=5971, initial ages 26-70 years), with follow-up measurements every 5 years (up to 20). Physical functioning (subscale SF-36, range:0-100) and a number of socio-demographic, lifestyle and health-related risk factors were assessed. Mixed-model multivariable analysis was used to investigate sex differences in sensitivity (interaction term with sex) and in exposure (change of the sex difference when adjusting) to risk factors. The physical functioning score among women was 6.75 (95%CL:5.65,7.85,age-adjusted) points lower than among men. In general, men and women had similar risk factors, but pain was more strongly associated with physical functioning (higher sensitivity), and also more prevalent among women (higher exposure). The higher exposure to low educational level and not having a paid job also contributed to the lower physical functioning score among women. In contrast, smoking, mental health problems and a low educational level were more strongly associated with a lower physical functioning score among men and lower physical activity and higher BMI were more prevalent. So, few risk factors seem to contribute the more reported limitations in physical functioning among women aged 26 to 90 years. Our findings provide no indications for reducing this sex difference by promoting a healthy lifestyle but stress the importance of sex differences in pain, work and education.

### Session 4440 (Symposium)

### PSYCHOLOGICAL IMPACTS AND IMPLICATIONS OF AGING DURING A PANDEMIC

Chair: Joseph Mikels Co-Chair: Laura Carstensen
Discussant: Susan Charles

Despite numerous losses associated with advanced age, older adults typically fare better than their younger counterparts in terms of psychological well-being. However, the COVID-19 pandemic has disproportionately threatened the physical and mental well-being of older adults. How have older versus younger adults been doing? The goal of our symposium is to shed light on this question though presentations of intriguing research findings regarding the psychological impacts of the pandemic on older adults. Stone and Mak will describe their work examining momentary changes in affect, activities, locations, and social interactions over time during the first several months of the pandemic for older individuals. Mikels and colleagues will report on completed and ongoing work illuminating the complex ways in which certain older adults have been faring well during the pandemic, whereas others not so much, with attention to underlying factors. Jeste will discuss a diverse line of research that has examined the relationships between loneliness, social isolation, and compassion in older adults before and during the pandemic. Chi and Carstensen will report on completed and ongoing research that links work and prosocial behavior to wellbeing with consideration of associated age differences. Collectively, these presentations will describe the complex and multifaceted psychological impact that the COVID-19 pandemic has had on older individuals, revealing the multiple ways in which they are resilient as well as vulnerable.

### CHANGES IN MOMENTARY EXPERIENCE WERE ASSOCIATED WITH THE ONSET OF THE COVID PANDEMIC

Hio Mak,[1] and Arthur Stone,[2], *1. University of Southern California, University Of Southern California, California, United States, 2. University of Southern California, University of Southern California/Los Angeles, California, United States*

We explored the COVID-19 pandemic's effects on daily experience using momentary recordings of affect, activities, locations, and social interactions, documenting changes in the pandemic's early stages. 123 individuals 50 years or older from an ongoing panel study completed 1-week bursts of Ecological Momentary Assessment (6/day) in March, May, and July. A pronounced spike in negative affect and decrease in positive affect was observed in late March compared with early March, which in May and June returned to early March levels. Levels of fatigue, however, did not follow this pattern. Being with one's spouse/significant other and family also increased, then decreased. Working and interacting with others dropped from early to late March and then remained steady; doing chores had the opposite pattern. Regarding location, being at the workplace dropped from early to late March and remained steady, and being at home had the opposite pattern. Additional analyses explored these patterns.

### AGE DIFFERENCES IN EMOTION AND COPING DURING THE COVID-19 PANDEMIC

Nathaniel Young, Alyssa Minton, and Joseph Mikels, *DePaul University, Chicago, Illinois, United States*

The COVID-19 pandemic unleashed a relentless stressor on the human species with numerous deadly risks. These risks have been disproportionately threatening to the health and wellbeing of older adults. Since April 2020, we have been studying how the pandemic has affected the emotional experiences of older and younger adults broadly in several studies. For instance, in one study, we found that older adults (N=176) experienced fewer negative emotions and coped with greater levels of agency than younger adults (N=181). In additional work, we have been examining how these age differences differ for older workers versus retirees as well as in minority populations. This work broadly supports and illuminates our recent theoretical framework that focuses on how evaluative appraisal processes underlie and contribute to age differences in emotional experience generally, but especially in the context of the stress experienced during a global pandemic.

### LONELINESS AND ISOLATION VERSUS WISDOM AND COMPASSION DURING THE PANDEMIC

Dilip Jeste, *University of California San Diego, La Jolla, California, United States*

Our studies of US national-level samples across adult lifespan as well as older adults in California and in Italy's Cilento region have found a consistently strong inverse correlation between loneliness and wisdom, especially its compassion component. Loneliness and social isolation are associated with worse physical and mental health while the reverse is true for wisdom and compassion. Follow-up of older adults in San Diego during the Covid-19 pandemic showed no change in this pattern. While the effects of the pandemic and the necessary social distancing were heterogeneous, older adults generally handled these stresses better than younger adults, with less loneliness and greater compassion. Our recent studies assessing EEG responses to emotional stimuli as well as alpha and beta diversity in gut microbiome showed opposing biological patterns characterizing loneliness and wisdom. I will also present preliminary data from a compassion training intervention to reduce loneliness among older adults.

### WORKPLACE PROSOCIAL ACTIVITIES AND DAILY WELL-BEING DURING COVID-19

Laura Carstensen,[1] and Kevin Chi,[2], *1. Stanford University, Stanford University, Stanford, California, United States, 2. Stanford University, Stanford, California, United States*

Workplace prosocial activities, such as providing unpaid assistance to colleagues, has been linked to better well-being. However, little is known about how these associations unfold in daily life. This study examines how prosocial activities at work are associated with daily well-being during the COVID-19 pandemic. A sample of 22 employees (aged 22-69 years) from a wealth management firm reported their daily activities and well-being on 10 consecutive workdays. On days when individuals provided help to someone they work with, they experienced higher positive affect, and greater enjoyment and interest at work, compared to days when they did not provide help. Individuals who provided more help reported greater meaning at work. Initial findings suggest that workplace prosocial activities have positive implications for daily well-being during the pandemic. Subsequent analyses will examine whether these findings replicate in a separate sample of working adults. Age differences in helping and meaning will be discussed.

### Session 4445 (Symposium)

### SLEEP, ACTIVITY, AND WELL-BEING IN PERSONS WITH DEMENTIA: FINDINGS FROM THE HEALTHY PATTERNS TRIAL

Chair: Nancy Hodgson Discussant: Darina Petrovsky

Irregular sleep-wake patterns are common in persons living with dementia (PLWD), pose a great burden to caregivers, and are the principal causes of distress and institutionalization of PLWD. A growing body of research supports the importance of activity-based interventions to reduce the frequency and intensity of sleep wake disruption, reduce neuropsychiatric symptoms, and improve quality of life. To date, there are no studies linking sleep disruption and well-being with the nature and timing of activity. This session focuses on lessons learned from the Healthy Patterns Study - a randomized trial of a home-based activity intervention in 200 dyads of PLWD and their caregivers (NCT03682185). Session 1 focuses on the main findings from the clinical trial. Session 2 focuses on the cultural adaptation of the timed activity protocol to improve quality of life (QOL), improve sleep and reduce neuropsychiatric symptoms in older Latinos Session 3 describes the community outreach efforts used over a one-year period to recruit a diverse sample of PLWD and their caregivers for the Healthy Patterns trial. Session 4 examine the relationship between caregiver mastery and neuropsychiatric symptoms in PLWD. Together these findings highlight the complex role of sleep and wake activity in promoting well-being in persons with dementia.

### EFFICACY OF A TIMED ACTIVITY INTERVENTION TO IMPROVE SLEEP: FINDINGS FROM THE HEALTHY PATTERNS STUDY

Miranda McPhillips,[1] Darina Petrovsky,[2] Subhash Aryal,[3] and Nancy Hodgson,[4], *1. University of Pennsylvania, University of Pennsylvania, Pennsylvania, United States, 2. Rutgers University, Philadelphia, Pennsylvania, United States, 3. University of Pennsylvania School of Nursing, Philadelphia, Pennsylvania, United States, 4. University of Pennsylvania, School of Nursing, philadelphia, Pennsylvania, United States*

We conducted a two-arm RCT with dyads of 200 persons living at home with dementia (PLWD) who reported sleep disruption and family caregivers. Components of the Healthy Patterns intervention included: 1) assessing PLWD functional status, preferences and interests; 2) educating caregivers on environmental cues to promote activity and sleep; and 3) training caregivers in timed morning, afternoon, and evening activities. Outcomes included: PLWD quality of life, sleep, and neuropsychiatric symptoms. Sleep-wake patterns were assessed using wrist actigraphy and proxy-reported measures. The main intervention effects were tested using ANCOVA. The average age of participants was 73.4 years, 67% were female, 80% were African American/Black). At 4 weeks, the intervention group demonstrated less sleep-related impairment (p = 0.0031) and reported higher quality of life than the control group (p = 0.0074). These results provide new fundamental knowledge regarding the effects of timing activity on sleep and well-being.

### A CULTURALLY ADAPTED TIMED-ACTIVITY RCT FOR LATINOS WITH ADRD AND CAREGIVERS: FEASIBILITY, ACCEPTABILITY, AND EFFECTS

Liming Huang,[1] and G. Adriana Perez,[2], *1. University of Pennsylvania, Philadelphia, Pennsylvania, United States, 2. University of Pennsylvania School of Nursing, Phildelphia, Pennsylvania, United States*

Latinos are twice as likely to develop Alzheimer's disease (AD) compared to non-Latino whites, yet, account for <2% of clinical trial participants in AD research. This randomized controlled trial examined the feasibility, acceptability and effects of a culturally-adapted timed-activity intervention designed to promote quality of life (QOL) and reduce behavioral symptoms in older Latinos with AD and their caregivers. Healthy Patterns [Pautas Saludables] was implemented among 40 Spanish-speaking dyads. Measures assessed at baseline and 4 weeks post-intervention, indicate improvements in sleep efficiency (p=.06) and QOL (p=.01) among intervention participants. Pautas Saludables was found to be feasible and acceptable. Intervention attendance rate was >90% with low attrition (n=0); no adverse events. Most (74%) rated timed-activity sessions as helpful and appropriate; 58% recommended refreshers. Results provide evidence that Latinos with AD will participate in clinical trials and can improve on key health outcomes, when interventions are adapted to meet their cultural needs.

### EFFECTIVE RECRUITMENT STRATEGIES FOR COMMUNITY-DWELLING PERSONS LIVING WITH DEMENTIA AND THEIR CAREGIVERS

LaShauna Connell,[1] Augustine Boateng,[1] Darina Petrovsky,[2] Justine Sefcik,[3] Nancy Hodgson,[4] and Miranda McPhillips,[5], *1. University of Pennsylvania, Philadelphia, Pennsylvania, United States, 2. Rutgers University, Philadelphia, Pennsylvania, United States, 3. Drexel University, College of Nursing and Health Professions, Philadelphia, Pennsylvania, United States, 4. University of Pennsylvania, School of Nursing, philadelphia, Pennsylvania, United States, 5. University of Pennsylvania, University of Pennsylvania, Pennsylvania, United States*

Recruitment of diverse community-dwelling persons living with dementia (PLWD) and their caregivers (dyads) into randomized controlled trials (RCT) is challenging, time consuming and expensive. This presentation will describe community outreach efforts used over a one-year period to recruit dyads of PLWD and their caregivers in Healthy Patterns RCT. Community outreach yielded 296 inquiries, such that people expressed interest in joining the study. Of the 296 inquiries, almost all (95.6%) identified as African American, and 91(30.7%) consented to join the study. Presentations at senior centers yielded the highest number of inquiries (n=148), followed by staff presence at various community events such as health fairs and senior galas (n=145) and referrals (n=3). We found that community outreach was an effective recruitment strategy to generate inquiries among diverse PLWD and their caregivers to enroll in Healthy Patterns. We will discuss these strategies and provide suggestions for recruiting diverse dyads into clinical trials.

### THE ROLE OF CAREGIVER MASTERY IN ANXIETY SYMPTOMS IN PEOPLE LIVING WITH DEMENTIA

Nancy Hodgson,[1] and Yeji Hwang,[2], *1. University of Pennsylvania, School of Nursing, philadelphia, Pennsylvania, United States, 2. University of Pennsylvania, School of Nursing, Philadelphia, Pennsylvania, United States*

Anxiety symptoms in people living with dementia (PLWD) are the most distressing symptoms for caregivers. While caregiving is bidirectional relationship, little is known how caregivers can influence anxiety in PLWD. The purpose of this study was to examine the relationship between caregiver mastery and anxiety symptoms in PLWD. Secondary data analysis was conducted using baseline data from Healthy Patterns Study. The conceptual model of Factors Associated with Behavioral and Psychological Symptoms of Dementia guided this study. Among the 169 study PLWD, 23.1% (n=39) reported having anxiety symptoms. In a multivariate logistic regression, adjusting for age, dementia stage, sleep, and depression, better caregiver mastery was significantly related to lower odds of having anxiety in PLWD (OR=0.87, p=0.046). These results suggest that interventions aimed at improving caregiver mastery may improve anxiety symptoms in PLWD.

### Session 4450 (Paper)

### SOCIAL AND HEALTH EQUITY

### ADDRESSING SYSTEMIC RACISM BY CREATING A DIVERSITY, EQUITY, AND INCLUSION DEPARTMENT INITIATIVE

Stephanie Chow,[1] Katherine Brown,[2] Martine Sanon,[2] Sasha Perez,[2] Amy Kelley,[1] and Noelle Marie Javier,[2] *1. Icahn School of Medicine at Mount Sinai, New York, New York, United States, 2. Icahn School of Medicine at Mount Sinai Hospital, Icahn School of Medicine at Mount Sinai, New York, United States*

**Background:** Catalyzed by social injustice and worsening racial inequities highlighted by the COVID-19 pandemic, a diverse academic geriatrics and palliative medicine department in NYC launched a DEI initiative. This report presents key program components and lessons learned in launching this initiative in the interprofessional academic medicine setting.

**Methods:** First, DEI core and departmental administration met 2-4 times/month to plan and review program activities, vision, and mission. The team conducted confidential roundtable discussions about DEI issues and 1:1 interviews to assess needs. A monthly Humanities, Arts, and Books (HAB) Initiative provided a safe space for discussion and learning. The HAB platform supported a longitudinal curriculum emphasizing (1) group discussion and self-reflection on DEI concerns, (2) knowledge dissemination including a "Learning Pathway" series, and (3) skill-based workshops. With each event, we collected anonymous feedback. Comments were systematically recorded and an engagement evaluation was conducted to iteratively shape future sessions. Departmental administration was engaged to track DEI-focused measures of recruitment, career advancement, and retention. Finally, we centralized DEI activities on a departmental website, including an anonymous online feedback box.

**Results:** Quantitative and qualitative assessment of DEI initiatives are forthcoming. Metrics include DEI and professional development surveys, departmental demographic and diversity measures, increase in DEI-related projects and grants, and individual participation in DEI programs.

**Conclusions:** Creating a strong and sustainable DEI initiative within an academic medical setting requires a passionate and diverse core team, deliberate backing by administration, and thoughtful dissemination of sensitive content in the midst of a highly charged social justice landscape.

### OLDER VIETNAMESE HAVE THE HIGHEST PREVALENCE OF DISABILITY COMPARED TO WHITE AND OTHER ASIAN GROUPS

Hoang Nguyen,[1] Christina Miyawaki,[2] and Kyriakos Markides,[3] *1. University of Texas Medical Branch at Galveston, Galveston, Texas, United States, 2. University of Houston, Houston, Texas, United States, 3. University of Texas Medical Branch, Galveston, Texas, United States*

The COVID-19 pandemic has highlighted the vulnerability of older adults with pre-existing health conditions and disabilities. A 2011 study reported that Asian older adults had lower prevalence of disability compared to non-Hispanic white. We revisited the estimate a decade later using the recently released 2015-2019 Public Use Microdata Sample (PUMS) from the American Community Survey (ACS). We estimated the prevalence of six types of disability in adults aged 60 years and older who self-identified as Vietnamese, Chinese, Filipino, Japanese, Korean, Asian Indian, or non-Hispanic White. We also compared the risk for each disability type between Vietnamese and non-Hispanic White (reference group) using the adjusted (age, sex, marital status, education and poverty level) odds ratios. All analyses used survey weights for point estimate and the jackknife method for standard error. Significantly higher prevalence of limitations in independent living, self-care, cognitive function, and blindness were reported by Vietnamese than by non-Hispanic White. Vietnamese also had the highest prevalence in all six types of disability of the Asian groups examined. The adjusted odds ratio of limitations in independent living, self-care, and cognitive function was significantly higher for Vietnamese than non-Hispanic White. These findings suggest a possible negative outcome trend with the aging of the Vietnamese population. We discuss the historical accounts of Vietnamese in the United States as war refugees and family reunion migrants, provide possible explanations for these new findings including changing demographic structures, and make recommendations for policy and practice that incorporate existing social and cultural resources in the Vietnamese community.

### RACIAL-ETHNIC DISPARITIES IN ACCESS TO PREVENTIVE SERVICES AMONG PRIVATELY INSURED ADULTS WITH DISABILITIES

Elham Mahmoudi,[1] lauren Groskaufmanis,[2] Neil Kamdar,[2] Anam Khan,[3] and Mark Peterson,[4], *1. University of Michigan, Commerce Township, Michigan, United States, 2. University of Michigan, Ann Arbor, Michigan, United States, 3. University of Michigan School of Public Health, Ann Arbor, Michigan, United States, 4. University of Michigan, University of Michigan, Michigan, United States*

**Introduction:** Cerebral palsy (CP) and spina bifida (SB) are congenital disabilities. Due to life-long disability, adults with CP/SB are with greater needs for preventative care. Little is known about racial/ethnic disparities in use of preventative services in this population. Our objective was to examine racial/ethnic disparities in use of preventative care.

**Methods:** Using 2007-2017 private claims data, we identified White, Black, and Hispanic adults (18+) with CP/SB [n=11,635; White=8,935; Black=1,457; Hispanic=1,243)]. We quantified the National Institute of Medicine (NAM) definition of disparity by matching health related variables (age, sex, comorbid conditions, and Elixhauser index) between Whites and each minority subpopulation. Generalized estimating equations were used and all models were adjusted for age, sex, comorbidities, income, education, and U.S. Census divisions. Outcomes of interest were: (1) any office visit; (2) any physical therapy/ occupational therapy (PT/OT); (3) annual wellness visit; (4) bone density screening; (5) cholesterol screening; (6) diabetes screening.

**Results:** Rate of recommended services for all adults with CP/SB were low and no significant results were found for most preventative services across race/ethnicity. Compared with Whites, Hispanics had lower odds of annual wellness visit (OR: 0.71; 95% CI: 0.53, 0.96) but higher odds of diabetes screening (OR: 1.48; 95% CI: 1.13, 1.93). Blacks had lower odds of bone density screening (OR: 0.54; 95% CI: 0.31-0.95), and annual wellness visit (OR: 0.50; 95% CI: 0.24-1.00).

**Conclusions:** There were no substantial racial/ethnic disparities in use of preventive services among privately insured adults with CP/SB who had a higher-than-average income and education level.

### TRACKING PROGRESS ON PERSON-CENTERED CARE FOR OLDER ADULTS: ARE WE DOING RIGHT BY RACIAL AND ETHNIC MINORITIES?

Jane Tavares,[1] Marc Cohen,[2] and Ann Hwang,[3] *1. LeadingAge LTSS Center @UMass Boston, Boston, Massachusetts, United States, 2. University of Massachusetts Boston, University of Massachusetts Boston, Massachusetts, United States, 3. Commonwealth Care Alliance, Boston, Massachusetts, United States*

Person-Centered care is integral and necessary to high-quality systems of care, providing a holistic approach and addressing the needs and preferences of individuals. Analyzing the 2014 and 2016 Health and Retirement Survey we measure the extent to which the health care system provides person-centered care, to whom and how its receipt affects satisfaction levels and service utilization. About one-third of individuals' report that their preferences were only rarely or sometimes takes account. Results vary greatly by race, highlighting great disparities in person-centered care. One in four Hispanics and one in six Blacks report never having their preferences taken into account compared to roughly one in ten Whites. When people report that their preferences are ignored, they are more likely to forgo medical care and report lower satisfaction with the system. Strategies exist to strengthen and assure advancements in person-centered care, something particularly needed for people of color and low-income populations.

### VIRTUAL INTERDISCIPLINARY COLLABORATION IN STATEWIDE IMPLEMENTATION OF THE MIND AT HOME DEMENTIA CARE PROGRAM

Deirdre Johnston,[1] Jennifer Bourquin,[2] Morgan Spliedt,[3] Inga Antonsdottir,[4] Cody Stringer,[5] Noemi Smithroat,[6] Melissa Reuland,[1] and Quincy Samus,[7], *1. Johns Hopkins University, Baltimore, Maryland, United States, 2. Superior HealthPlan, Austin, TX, Texas, United States, 3. The Johns Hopkins University, Baltimore, Maryland, United States, 4. Johns Hopkins School of Nursing, Baltimore, Maryland, United States, 5. Centene Corporation, Clayton, Missouri, United States, 6. Superior HealthPlan, San Antonio, Texas, United States, 7. Johns Hopkins University School of Medicine, Baltimore, Maryland, United States*

MIND at Home, a well-researched holistic, family-centered dementia care coordination program, provides collaborative support to community-dwelling persons living with dementia (PLWD) and their informal care partners (CP). Through comprehensive home-based assessment of 13 memory-care domains covering PLWD and CPs, individualized care plans are created, implemented, monitored, and revised over the course of the illness. Non-clinical Memory Care Coordinators (MCCs) working with an interdisciplinary team provide education and coaching to PLWD and their identified CP, and serve as a critical liaison and resource and between families, medical professional, and formal and informal community resources. This paper will describe a statewide pilot implementation of the program within a health plan across diverse sites in Texas and will present qualitative and quantitative descriptions of a key component of the program's effective translation to practice, the virtual collaborative case-based learning sessions. Health plan teams completed online interactive training modules and an intensive in-person case-based training with the Johns Hopkins team prior to program launch, and then engaged in weekly, hour-long virtual collaborative sessions that included health plan teams (site-based field teams, health plan clinical supervisory and specialty personnel [RNs, pharmacists, a geriatric psychiatrist, behavioral health specialists] and Johns Hopkins MIND program experts and geriatric psychiatrists. To date, the program has enrolled 350 health plan members, conducted 65 virtual collaborative sessions, and provided 423 CME/CEU units to team members. We will provide an overview of virtual collaborative session structure, participant contributions and discussion topics, case complexity, as well as didactic learning topics covered.

### Session 4455 (Symposium)

### STAKEHOLDER-DRIVEN METHODS CAN ENHANCE CARE DELIVERY FOR NURSING HOME RESIDENTS WITH DEMENTIA

Chair: Natalie Leland Co-Chair: Felicia Chew
Discussant: Jenny Martínez

The ongoing COVID-19 pandemic has underscored the need to optimize care for one of the most affected sectors: older adults in nursing homes and more specifically highly vulnerable populations such as residents with dementia. Research developed in collaboration with stakeholders can optimize impact, relevance, and trustworthiness of study findings thereby informing advances in care. Yet, evidence on stakeholder driven research for enhancing dementia care is limited. This symposium will provide examples of stakeholder-driven research questions that were addressed with stakeholder engagement. First, we will present current evidence about the perspectives of caregivers, including those from communities of color. The second presentation will discuss the perspective of clinical training stakeholders responsible for supporting system-wide clinical program implementation and their experiences with early and later adopter nursing homes within the context of a clinical trial. The third presentation will address the perspective of policy makers and payers via the effect of state-mandated dementia training on resident outcomes. The fourth and final present the findings from a study that examined how nursing home stakeholders responded to a payor requirements for pharmacy services and the relationship between that response and patient outcomes. We will conclude the session with a discussion of stakeholder-engagement methods and recommendations for future nursing home research, which champions stakeholder collaboration.

### EXPLORING INFORMAL CAREGIVERS' PRIORITIES OF NURSING HOME DEMENTIA CARE FROM COMMUNITIES OF COLOR

Marybeth Moscirella,[1] Alexandra Harper,[1] Cara Lekovitch,[1] Rose Turner,[2] Catherine Piersol,[3] Natalie Leland,[1] and Stephanie Rouch,[1], *1. University of Pittsburgh, Pittsburgh, Pennsylvania, United States, 2. University of Pittburgh, Pittsburgh, Pennsylvania, United States, 3. Thomas Jefferson University, Philadelphia, Pennsylvania, United States*

Informal caregivers are critical stakeholders in nursing home (NH) care for individuals with dementia. Given racial and ethnic disparities in United States NHs, there is a need to understand informal caregivers' perspectives, particularly among those that identify as members of a community of color. We conducted a scoping review of informal caregiver priorities of nursing home dementia care. Included studies exclusively examined priorities of informal caregivers identifying as Black, Indigenous, or people of color. The final sample (n=12) included two United States studies representing African American and Korean informal caregivers. The remaining studies were conducted in other countries. Informal caregivers expressed a desire for professional support during the nursing home transition, increased staff knowledge of dementia, and improved resident engagement.

These findings highlight the paucity of informal caregivers identifying as Black, Indigenous, or people of color represented in US nursing home dementia research. Future efforts must include communities of color.

### IMPLEMENTATION IN NURSING HOMES: DESCRIBING EARLY AND LATE ADOPTERS OF AN EVIDENCE-BASED DEMENTIA CARE PROGRAM

Pamela Toto,[1] Felicia Chew,[2] Dawn Bieber,[2] Natalie Leland,[1] and Cara Lekovitch,[1], *1. University of Pittsburgh, Pittsburgh, Pennsylvania, United States, 2. Genesis HealthCare, Kennett Square, Pennsylvania, United States*

Despite national efforts to improve nursing home (NH) quality, care remains variable. Health system efforts to drive improvement often begin with a sub-group of NHs before scaling up across the organization. Yet, there is limited evidence on who to target for the first group. This study addressed this gap by examining facility characteristics of early and late adopters within a multi-site pragmatic clinical trial. Data were obtained from the Organizational Readiness to Change Assessment (ORCA), which was completed by expert trainers, and Nursing Home Compare. Early and late adoption was operationalized according to Roger's Diffusion of Innovations. Sixty-percent of NHs (n=12) were late adopters and 40% (n=8) were early adopters. Between group differences (p<.01) were found in number of health inspection citations and context domain within the ORCA. These findings equip health systems with evidence on how to strategically target partners for initial quality improvement efforts prior to system-wide implementation.

### THE IMPACT OF STATE DEMENTIA TRAINING REQUIREMENTS FOR NURSING HOMES ON RESIDENT OUTCOMES

Yuna Bae-Shaaw,[1] Cara Lekovitch,[2] Felicia Chew,[3] Natalie Leland,[2] Neeraj Sood,[4] and Victoria Shier,[4], *1. University of Southern California, Placentia, California, United States, 2. University of Pittsburgh, Pittsburgh, Pennsylvania, United States, 3. Genesis HealthCare, Kennett Square, Pennsylvania, United States, 4. University of Southern California, Los Angeles, California, United States*

Stakeholders, including policymakers, have prioritized the need to educate nursing home (NH) staff about Alzheimer's disease and dementia. Despite this prioritization and the relationship between staff training and outcomes, dementia-specific knowledge is variable. This study examined state-level training policies between 2011-2016. During this time 12 states (regulators and payers) implemented NH dementia training requirements, creating an opportunity for a natural experiment between states with and without new requirements. We estimated difference-in-differences models to determine the effect of state requirements on outcomes. Data from Nursing Home Compare and LTCFocus.org were linked to data on state policies. Training requirements were associated with 0.39 and 0.17 percentage point reductions in antipsychotics use and restraint use, respectively, and no impact on falls or need for help with daily activities. State requirements for dementia training in NHs are associated with a small, but significant reduction in the use of antipsychotic medication and physical restraints.

### THE IMPORTANCE OF THE PHARMACIST'S ROLE AND THE QUALITY OF PHARMACY SERVICES IN NURSING HOME CARE

Geoffrey Joyce,[1] Seth Seabury,[1] Victoria Shier,[1] Neeraj Sood,[1] and Yuna Bae-Shaaw,[2], *1. University of Southern California, Los Angeles, California, United States, 2. University of Southern California, Placentia, California, United States*

The Centers for Medicare & Medicaid Services requires nursing homes (NHs) to provide pharmacy services to ensure the safety of medication use, such as minimizing off-label medication use for residents with dementia. This study examined NH's response to this requirement and its relationship to medication-related outcomes. The contemporaneous relationship between the quality of pharmacy services and outcome measures were modeled using facility-level longitudinal data from 2011-2017 and facility fixed-effects. The results revealed that deficiency in pharmacy services increased medication-related issues by: 11% in inappropriate medication regimen, 5% in medication error rate >5%, and 3% in any serious medication errors. Additionally, deficiency in pharmacy services was associated with small but statistically significant increases in antipsychotic use, residents with daily pain, number of hospitalizations and rehospitalization rate. The results suggest that pharmacy services have a direct and immediate impact on medication outcomes. The results underscore the importance of pharmacy services in NHs.

### Session 4460 (Paper)

### SUBJECTIVE AGING AND HEALTH

### DAILY PERCEPTIONS OF AGING AND IMPLICATIONS FOR STRESS REACTIVITY

Bethany Wilton-Harding, and Tim Windsor, *Flinders University, Adelaide, South Australia, Australia*

Awareness of one's own aging has received increasing attention in the field of gerontology over the last decade. This study examines the role awareness of age-related change (AARC) may play in the association between daily stressors and well-being. Recently, individuals' awareness of both age-related gains and age-related losses has been shown to vary on a day-to-day basis (Neupert & Bellingtier, 2017). We expected that increases in daily AARC-losses may be associated with increased emotional reactivity to daily stressors, whereas increases in AARC-gains may be associated with decreased reactivity. Data were collected in a daily diary study from a community-based sample of 152 Australian participants aged 53 to 86 (M = 69.18, SD = 5.73). Participants completed daily assessments of AARC, stressors and emotional affect (positive and negative) on their smartphones for 10 consecutive days. Analysis of within-person coupling using multilevel models indicated that daily increases in AARC-losses were associated with increased reactivity to daily stressors (represented by high negative affect and low positive affect). On the other hand, daily increases in AARC-gains were associated with decreased reactivity to daily stressors (represented by low negative affect). Results indicate that even short-term fluctuations in perceptions of aging may be an important factor to consider when investigating associations between daily stressors and well-being in older adulthood. Specifically, greater daily AARC-losses may contribute to lower emotional well-being, whereas an appreciation of positive age-related changes (AARC-gains) may play a role in mitigating emotional reactivity to daily stress experiences in older adulthood.

### DIFFERENCES IN SELF-RATED MEMORY BY RACE/ETHNICITY

Yujin Franco,[1] Joseph Saenz,[2] Yuri Jang,[2] and Jessica Ho,[2], *1. University of Southern California, University of Southern California, California, United States, 2. University of Southern California, Los Angeles, California, United States*

Self-rated memory is an important dimension of well-being among older adults that has also been linked to cognitive impairment over the long term. However, few studies based on nationally-representative samples have examined differences in self-rated memory by race/ethnicity. This study explores differences in self-rated memory across non-Hispanic White, non-Hispanic Black, and Hispanic older adults in the United States. Data were drawn from the 2011 wave of the National Health and Aging Trends Study (NHATS). The sample consisted of older adults aged 65 and older (N=4,753 non-Hispanic Whites, N=1,442 non-Hispanic Blacks, and N=388 Hispanics). Logistic regression was used to examine the association between having poor/fair self-rated memory and race/ethnicity, controlling for socio-demographic characteristics (age, gender, education level, income, and marital status), chronic conditions (heart attack, hypertension, diabetes, stroke, and depressive symptoms), objective memory status, functional limitations (activities of daily living and instrumental activities of daily living), and other social and cultural factors (economic vulnerability, religious practice, and limited English proficiency). I find that non-Hispanic Blacks and Hispanics have significantly higher odds of reporting poor/fair self-rated memory than non-Hispanic Whites. Compared to non-Hispanic Whites, Blacks and Hispanics had 33% and 56% higher odds of reporting poor/fair self-rated memory, respectively, controlling for sociodemographic characteristics, chronic conditions, objective memory status, functional limitations, and social and cultural factors. These results provide evidence that understanding differences in self-rated memory across racial/ethnic groups may have important implications for health professionals, particularly in relation to conducting and interpreting cognitive screening assessments.

### INTERGENERATIONAL AMBIVALENCE, LONELINESS, AND WELL-BEING AMONG OLDER ADULTS IN THE UNITED STATES

Xiaoyan Zhang,[1] and Merril Silverstein,[2], *1. Syracuse University, Syracuse, New York, United States, 2. Syracuse University, Syracuse, New York, New York, United States*

Intergenerational relationships are important sources of informal social support for older people to maintain their emotional well-being. Previous research has extensively investigated the relationship between intergenerational support and older adult's psychological well-being. However, the existing research has not adequately examined intergenerational ambivalence – mixed or contradictory feelings toward a family member in another generation or explored the mechanism that links intergenerational ambivalence and psychological well-being. Further, most studies are cross-sectional, which prevents us from establishing causality. This study utilized data from 2006, 2008, 2010, 2012, 2014, and 2016 waves of Health and Retirement Study (HRS), a national representative sample of U.S. adults aged 50 and more (N= 8,017). Structural equation models were used to examine the longitudinal relationship between intergenerational ambivalence, loneliness, depression, and life satisfaction. The final model indicated very good fit ($\chi 2 = 113.31$, $p < .0001$, CFI = 0.97, RMSEA = .05). The results revealed that ambivalence in older parent-adult child relationships predicted higher subsequent loneliness ($\beta = 0.21$, $p < .0001$), which in turn predicted depressive symptoms ($\beta = 0.25$, $p <.0001$) and life satisfaction ($\beta = -0.30$, $p < .0001$). The results demonstrated that loneliness mediated the relationship between intergenerational ambivalence and depression, and life satisfaction. Multiple group analysis was performed to test whether the study relationships varied by gender. Gender differences were found. Findings have implications for prevention and intervention initiatives targeting improving relationships between parents and children, thereby protecting against mental problems.

### SOCIOCULTURAL DIFFERENCES IN ACCEPTING TECHNOLOGY FOR OLDER ADULTS BETWEEN SOUTH KOREA AND THE UNITED STATES

Jeungkun Kim,[1] and Suk-Young Kang,[2], *1. Kangnam University, Yongin-si, Kyonggi-do, Republic of Korea, 2. Binghamton University, State University of New York, Binghamton, New York, United States*

In recent years, Western-originated technology products for older adults are rapidly spreading in Korea, but discussions on technology acceptance taking into account the socio-cultural characteristics of older adults in Korea are relatively insufficient. The purpose of this study is to analyze the influence of the socio-cultural characteristics of Korean older adults on their intention to use technology compared to the United States. Due to Covid-19, a telephone and non-face-to-face survey was conducted for older adults aged 65-95 residing in New York State in the U.S. and the metropolitan area of South Korea from September 2020 to January 2021(N=155 in South Korea, N=180 in the U.S.).In this study, the expanded technology acceptance model for older adults was conceptualized, and socio-cultural factors were used as mediators or modulators. Results show that Korean older adults had higher expectations that technology use would have a positive impact on their lives, and their product purchase intentions were higher than those in the United States(p<0.001). The main reason was that Korean older adults were less anxious about the leakage of personal information, had higher national trust and were relatively less resistant to robots than American older adults. In addition, Korean older adults were more confident that they could receive help in case of problems with technology products than their counterparts in the United States. This study suggests practical and policy alternatives for securing technology acceptance of older adults, taking into account the social and cultural factors of Korea and the United States.

### VARIATION IN SUBJECTIVE AGING AMONG MIDDLE-AGED AND OLDER LGBTQ+ PEOPLE: AN EXPLORATORY STUDY

Harry Barbee, and Tara McKay, *Vanderbilt University, Nashville, Tennessee, United States*

Studies suggest that women and men have different experiences of subjective aging—including interpretations of age norms, timing of life course stages, and aging anxieties—but few have addressed variation within sexual and gender diverse communities. Drawing on a sample of middle-age and older LGBTQ+ people from Alabama, North Carolina, and Tennessee (n=923), we analyze how four dimensions of subjective aging (age-related self-perceptions, generalized views of aging, aging bodies, and aging anxieties) vary within the LGBTQ+ population by comparing gay and bisexual cisgender men (GBCM), lesbian and bisexual cisgender women (LBCW), and transgender, gender nonconforming, and non-binary (T/GNC/NB) people. Using multivariate regression models, we find that LBCW reported younger ideal ages, more elongated perceptions of the life course, more negative predictions of prospective health, and less aging anxiety compared to GBCM. Regarding self-perceptions and generalized views of aging, T/GNC/NB people reported younger ideal ages and more condensed perceptions of the life course compared to GBCM. Regarding perceptions of aging bodies, T/GNC/NB people reported more positive views of physical changes within the last five years and more negative predictions of prospective health compared to GBCM. Finally, T/GNC/NB people were less anxious than GBCM about future sex lives but were more anxious about not being able to support other people in the future. Overall, while some results align with studies of the general population, we find that sexual and gender diverse people may have different subjective aging trajectories, and thus experience differential physical and mental health outcomes, compared with cisgender heterosexual adults.

### Session 4465 (Symposium)

### THE CHALLENGES OF BEING A FAMILY CAREGIVER DURING THE COVID-19 PANDEMIC IN CANADA

Chair: Gwen McGhan Co-Chair: Deirdre McCaughey

The COVID-19 pandemic has impacted all of our lives, but the population most at risk are older adults. Canadians over the age of 60 account for 36% of all COVID-19 cases but 95% of the deaths, and over two-thirds of ICU admissions. Older adults with chronic health conditions are especially at risk. Prior to COVID-19, family caregivers (FCGs) for older adults were managing their caregiving duties at the limits of their emotional, physical and financial capacity. As such, FCGs need special consideration during these times of uncertainty to support them in their role and enable the continuation of care for their older adult family members. This symposium will report on independently conducted studies from across Canada that have examined how the pandemic and associated public health measures have influenced resource utilization by FCGs and the older adults for whom they provide care. McAiney et al's study examines the deleterious effect of reduced services on community dwelling FCGs and the wellbeing of their family member with dementia. Parmar & Anderson examined the effect of pandemic restrictions on FCGs of frail older adults and found they were experiencing increased distress and decreased wellbeing. Flemons et al report on the experiences of FCGs managing caregiving without critical services and the effect of restrictive visiting policies and the well-being of the caregiving dyad (FCGs and family member with dementia). Finally, McGhan et al will share how FCGs evaluated the efficacy of public health measures and the public health messaging about the pandemic.

### PUBLIC HEALTH MESSAGING AND MEASURES DURING COVID-19: THE EXPERIENCES OF FAMILY CAREGIVERS

Deirdre McCaughey, Kristin Flemons, Whitney Hindmarch, and Gwen McGhan, *University of Calgary, Calgary, Alberta, Canada*

To mitigate the effects of COVID-19, Health Ministries across Canada have enacted numerous public health measures. Our mixed methods study examined the effect of COVID-19 related public health messaging and measures for family caregivers (FCGs) of people living with dementia (PLWD). Of the 230 FCGs completing the survey, most frequently used information sources were television, family/friends, and websites. FCGs over 60 more often used television, newspaper and radio versus websites and social media. FCGs rated public health messaging as good-excellent (64%) especially messaging around the disease spread, symptoms, and finding information. 46% believe the restrictions in long-term care facilities went beyond necessary with 97% reporting restrictions have negatively impacted them. 84% were willing to undertake personal protective equipment and infection control training to ensure continued access to PLWD. Focus groups highlighted concerns about continued access to PLWD, quality of care provision, and increased social isolation's impact on dementia progression.

### EXPLORING THE IMPACT OF COVID-19 ON FAMILY CAREGIVERS OF PERSONS LIVING WITH DEMENTIA IN THE COMMUNITY

Emma Conway,[1] Melissa Koch,[1] Laura Middleton,[1] Heather Keller,[1] Sherry Dupuis,[1] Kate Dupuis,[2] Jennifer Boger,[3] and Carrie McAiney,[1], *1. University of Waterloo, Waterloo, Ontario, Canada, 2. Schlegel-UW Research Institute for Aging, Waterloo, Ontario, Canada, 3. Waterloo University, Waterloo, Ontario, Canada*

COVID-19 public health measures have significantly impacted persons living with dementia (PLWD) and family caregivers (FCGs). Given the restrictions on in-person services, many PLWD were not able to access their usual supports and activities, resulting in FCGs stepping in to support exercise, leisure, socialization, spirituality, and activities of daily living. At the same time, FCGs' own support networks were significantly reduced or no longer available. We conducted in-depth qualitative interviews with 20 FCGs of PLWD in the community to explore the impact of COVID-19 on their well-being. Data were analyzed using thematic analysis. Caregiving during COVID-19 was described as 'draining' and 'stressful', with the support needs of PLWD increasing at a time when fewer supports were available. Reaching out to others, using technology, and setting boundaries were strategies FCGs used to cope. Despite the considerable impacts of COVID-19, FCGs of PLWD demonstrated their resilience in supporting themselves and their PLWD.

### THE IMPACTS OF THE COVID-19 PANDEMIC ON FAMILY CAREGIVERS' CARE WORK, ANXIETY, AND LONELINESS

Sharon Anderson,[1] and Jasneet Parmar,[2] *1. Faculty of Medicine & Dentistry University of Alberta, Edmonton, Alberta, Canada, 2. Department of Family Medicine, University of Alberta, Edmonton, Alberta, Canada*

Our study examined the effects of COVID-19 pandemic and public health measures on family caregivers (FCGs) of frail older adults; specifically, their care work, anxiety, and loneliness all of which are associated with decreased wellbeing. Approximately 604 FCGs completed the survey and findings evidenced COVID-19 creating two solitudes. First, 73% of FCGs for individuals living with them were providing significantly more care during COVID-19. Second, those caring for residents in congregate settings were unable to care. Both situations, community-dwelling and congregate care, increased FCG distress and decreased wellbeing. Anxiety significantly increased from 36% pre COVID-19 to 54% during COVID-19. Loneliness increased from 46% to 85%. FCGs report their mental (58%) and physical (48%) health deteriorated. The detrimental impact of the pandemic and public health measures on FCGs caring at home and in congregate care, and their related needs, need immediate attention from both the health and social systems of care.

### BEYOND THE LOCKDOWN BINARY: FAMILY CAREGIVER NEEDS FOR CREATIVE SOLUTIONS DURING A GLOBAL PANDEMIC

Gwen McGhan, Deirdre McCaughey, Whitney Hindmarch, and Kristin Flemons, *University of Calgary, Calgary, Alberta, Canada*

As COVID-19 lockdowns began in Canada last spring, family caregivers (FCGs) of people living with dementia (PLWD) found themselves facing a catch-22: they and their family members were often most at risk of severe outcomes should they contract the virus, yet the public health measures put in place also detrimentally affected their ability to continue providing care. To understand the nuances of caregiver experiences during the pandemic, we conducted 9 focus groups with 19 FCGs of PLWD in the Calgary region in summer 2020. Caregivers reported negative outcomes resulting from decreased services for both themselves and the PLWD, including increased isolation, poor mental health, and accelerated dementia progression. Caregivers also emphasized the importance thinking beyond the binary of either locking down or opening up; rather, we must find creative solutions to safely continue providing supports to caregivers. This presentation explores FCG suggestions for balancing COVID-19 risk against caregiver needs.

### Session 4470 (Symposium)

### THE DEVELOPMENT OF A SMART REMINDER SYSTEM TO PROMOTE ADHERENCE TO TECHNOLOGY-BASED INTERVENTION AND ASSESSMENT

Chair: Walter Boot Co-Chair: Neil Charness

The overarching aim of the National Institute on Aging funded Adherence Promotion with Person-centered Technology (APPT) Project is to promote adherence to technology-based solutions designed to enhance the early detection and treatment of cognitive decline. The goal is to build and evaluate adaptive, tailored, and integrated technology-based adherence support systems for mobile software platforms that assess and train cognitive skill. The symposium describes the various steps of the development process of the APPT smart adherence support system. N. Charness will present an overview of the APPT project, its aims, and the clinical trials designed to assess the effectiveness of the APPT smart reminder system compared to typical reminder systems. S. Chakraborty will present detailed analyses of past cognitive intervention data to inform understanding of who is likely at risk for poor adherence and how adherence lapses might be predicted in advance to provide just-in-time adherence support. D. Carr will present an exploration of motivating factors for participants to engage in research, and these motivations will be tapped to help develop motivational messages for the APPT adherence support system to be used in the two planned clinical trials. M. Dieciuc will provide additional insights into motivations for engaging in home-based cognitive assessment and training derived from a focus group study. Finally, S. Zhang will describe the results of an initial pilot study examining the effectiveness of motivational reminder messages that match vs. mismatch participants' own motivations. All results inform the design of the APPT system to maximize adherence.

### AIMS OF THE ADHERENCE PROMOTION WITH PERSON-CENTERED TECHNOLOGY (APPT) PROJECT

Walter Boot,[1] Dawn Carr,[1] Shayok Chakraborty,[1] Zhe He,[1] Mia Lustria,[2] Antonio Terracciano,[3] and Neil Charness,[1], *1. Florida State University, Tallahassee, Florida, United States, 2. School Of Information, Florida State University, Florida, United States, 3. FLORIDA STATE UNIVERSITY, Florida State University, Florida, United States*

The APPT project supports the early detection and treatment of age-related cognitive decline and dementia by 1) enhancing adherence to cognitive intervention and assessment protocols, 2) improving understanding of barriers to long-term adherence, and 3) developing algorithms for predicting and preventing adherence failures. Two randomized controlled trials will test an adaptive technology support system predicted to boost adherence to cognitive protocols over a period of six months within samples of older adults with and without cognitive impairment. These studies will provide insight into the benefits of adherence support, and individual difference factors that should shape the adherence protocol, informing the process of identifying individuals who would benefit from additional support and predicting and preventing extended adherence failures before they happen. These studies should improve early detection and treatment of cognitive decline, extend functional independence, and improve lives of those with cognitive impairment as well as the lives of their families.

### MACHINE LEARNING APPROACHES TO UNDERSTANDING AND PREDICTING PATTERNS OF ADHERENCE

Aditya Bhattacharya,[1] Shubo Tian,[1] Nelson Roque,[2] Zhe He,[1] Walter Boot,[1] and Shayok Chakraborty,[1],

*1. Florida State University, Tallahassee, Florida, United States, 2. University of Central Florida, Orlando, Florida, United States*

In cognitive training of older adults, adherence is a major challenge, but appropriate just-in-time adaptive interventions can improve adherence. To understand adherence patterns and predictors of adherence lapses, we aggregated data from two previous trials (N > 230) involving home-based cognitive interventions. This dataset, detailing 40,000 intervention interactions, contains information about intervention engagement and measures of objective and subjective cognitive performance, demographics, technology proficiency, and attitudes. Exploratory analyses were conducted to understand patterns and predictors of faltering adherence, using classification models, together with feature selection to remove redundant variables. Adherence behaviors in a week were predictive of quitting the following week. Game parameters such as the time of play were weak indicators of future playing patterns, whereas game success was a strong predictor of adherence. These and other useful observations will be incorporated in the design and development of the smart reminder system to be deployed in the APPT project.

### FACTORS THAT MOTIVATE OLDER ADULTS TO PARTICIPATE IN RESEARCH: TYPOLOGIES AND IMPLICATIONS

Zhe He,[1] Mia Lustria,[2] Shubo Tian,[1] Maedeh Agharazidermani,[1] Walter Boot,[1] and Dawn Carr,[1], *1. Florida State University, Tallahassee, Florida, United States, 2. School Of Information, Florida State University, Florida, United States*

A key challenge for scholars who study aging is identifying a pool of research volunteers willing to participate. Toolkits and strategies acknowledge the differences in recruitment needed for older adults relative to younger adults, but there is little information about variations among older adult research volunteers. Based on a community sample of older adults age 60+, this study evaluates differences across seven specific motivators across three broad categories: values/altruism, personal growth/improvement, and immediate gratification. We then identify and evaluate four typologies of older adult volunteers based on the combinations of motivations the older adults in our sample identify as important to participation in research studies. Based on these analyses, we describe how our results might inform recruitment and retention practices in aging studies. Further, we will discuss how these results will help shape our technology-based reminder system with a greater understanding of motivations.

### A QUALITATIVE UNDERSTANDING OF MOTIVATIONS, PREFERENCES, AND ATTITUDES TOWARD ADHERENCE-BASED TECHNOLOGY

Shenghao Zhang,[1] Nicholas Gray,[2] Andrew Dilanchian,[3] Dawn Carr,[3] Mia Lustria,[4] Walter Boot,[3] and Michael Dieciuc,[5], *1. Florida State University, TALLAHASSEE, Florida, United States, 2. Psychology Department, Florida State University, Florida, United States, 3. Florida State University, Tallahassee, Florida, United States, 4. School Of Information, Florida State University, Florida, United States, 5. Florida State University, Florida State University, Florida, United States*

The future of cognitive assessments and brain-training programs is very likely to involve mobile applications for phones and tablets. However, adherence to these programs over the long haul is notoriously low. In an effort to countervail this trend, we ran online focus groups with both older and younger adults to understand from a user-centered perspective how to better design apps to increase adherence. Using thematic content analysis (Braun & Clarke, 2006) with an inductive bottom-up approach (Frith & Gleeson, 2004), we found a surprising number of common themes across older and younger adults that superseded many of their superficial differences. For instance, both younger and older adults were reluctant to engage in the program unless it had some obvious perceived benefit; both wanted the program personalized to their individual preferences; both wanted the ability to customize features and reminders; and both generally agreed that the tasks had to be fun.

### THE EFFECT OF REMINDER MESSAGE TAILORING ON COGNITIVE INTERVENTION ADHERENCE

Michael Dieciuc,[1] Andrew Dilanchian,[2] Walter Boot,[2] and Shenghao Zhang,[3], *1. Florida State University, Florida State University, Florida, United States, 2. Florida State University, Tallahassee, Florida, United States, 3. Florida State University, TALLAHASSEE, Florida, United States*

To examine the potential impact of tailored messaging on adherence and attitudes toward text message reminders, a pilot study conducted in advance of the APPT randomized controlled trial systematically manipulated the match between text message content and participants' self-reported motivations to participate in a cognitive intervention study. Older adults (n=40) were asked to engage in cognitive training, in the form of gamified neuropsychological tests, 30 minutes a day for 10 consecutive days, and adherence was tracked remotely over time. Critically, each day text message reminders alternated between messages consistent or inconsistent with participants' previously reported motivations for entering the study. This talk presents results, derived from multilevel modeling, that explore the effectiveness of this simple and cost-effective message tailoring approach for facilitating adherence and engendering positive attitudes toward the reminder system, and implications for programs requiring long-term adherence.

### Session 4475 (Symposium)

### THE IMPORTANCE OF REDEFINING GERIATRIC EXPERTISE IN NURSING HOMES TO REDUCE UNNECESSARY HOSPITALIZATIONS

Chair: Franziska Zuniga Co-Chair: Lori Popejoy
Discussant: Amy Vogelsmeier

Unplanned transfers from nursing homes (NHs) are burdensome, associated with adverse outcomes for residents and costly for health care systems. Internationally, NHs are facing similar issues whereby a lack of geriatric expertise combined with a shortage of NH general practitioners require innovative and adaptable models of care tailored to the organizational context. In this symposium, we will present studies from the MOQI project from the United States, which successfully reduced unnecessary hospitalizations by embedding advanced practice registered nurses (APRN) in 16 US NHs over a 6-year period. We will discuss the influence of race on multiple hospital transfers and present possible interventions to reduce transfers. Next, we will present finding from a study with MOQI APRNs that highlighted their contributions to the COVID-19 pandemic response in NHs and discuss the broader implication or infection control practices. In addition, we will present the INTERCARE project which successfully reduced unplanned hospitalizations in 11 Swiss NHs, by implementing a registered nurse with an expanded role, to compensate for the very limited access to APRNs; which is the case for many European countries. Both MOQI and INTERCARE pinpoint the importance of strategies to support the introduction of a new role in NHs. Both projects will give examples of different models of care which can be feasibly implemented to sustainably decrease unnecessary hospitalizations, in different contexts and with different resources. Finally, data from the INTERCARE study will address the issue of potentially avoidable fall-related transfers and which resources are deemed appropriate to mitigate these.

### POSITIVE EFFECT OF A SUCCESSFULLY IMPLEMENTED MODEL OF CARE ON UNPLANNED TRANSFERS TO HOSPITAL

Raphaëlle Guerbaai,[1] Michael Simon,[2] and Franziska Zuniga,[3], *1. Institute of Nursing Science, Department of Public Health, University of Basel, Institute of Nursing Science, Department of Public Health, University of Basel, Basel-Stadt, Switzerland, 2. University of Basel, Basel, Basel-Stadt, Switzerland, 3. Basel University, Ostermundingen, Basel-Stadt, Switzerland*

Models of care have shown effectiveness in reducing unplanned transfers in nursing homes (NHs) from 11.7% to 6.1%. These include coordination of care and access to skilled medical providers such as geriatricians, specialist nurses or registered nurses with additional training. A hybrid type-2 effectiveness-implementation project (INTERCARE) was developed to improve intervention uptake and to understand the mechanisms behind results. INTERCARE consisted of six core elements and was rolled-out to 11 Swiss NHs with a stepped-wedge design allowing all NHs to receive the intervention. 942 residents were recruited (June 2018 -January 2020). INTERCARE showed a significant reduction of unplanned transfers during the intervention period compared with baseline. The successful implementation of INTERCARE relied on the use of implementation science, building on stakeholder input and careful theory-driven contextual adaptations. INTERCARE's success was driven by registered nurses with expanded roles, on-site coaching, and the use of tools for clinical decision making.

### DEFINING APPROPRIATE RESOURCES FOR NURSING HOMES TO REDUCE POTENTIALLY AVOIDABLE TRANSFERS AFTER A FALL

Michael Simon,[1] Franziska Zúñiga,[1] and Raphaëlle Guerbaai,[2], *1. University of Basel, Basel, Basel-Stadt, Switzerland, 2. Institute of Nursing Science, Department of Public Health, University of Basel, Institute of Nursing Science, Department of Public Health, University of Basel, Basel-Stadt, Switzerland*

Falls are common in nursing home (NH) residents and are the predominant reason for an emergency department (ED) transfer. Falls are responsible for 25% - 87% of ED transfers, a proportion of which are potentially avoidable. INTERCARE – an implementation science study reducing unplanned hospitalizations (2018 – 2020) – involved experts to identify potentially avoidable fall-related transfers. Focus group and stakeholder survey enabled identification of resources to safely manage some falls in NHs. 25.9% of fall-related transfers were potentially avoidable based on using root-cause analysis and discharge reports. Avoidability was associated to ED visit, compared to hospitalizations. Appropriate resources identified by stakeholders included timely access to outpatient services for diagnostic imaging (e.g., X-Ray) and clinical skills' training in suturing and wound care for registered or specialist nurses. Although NHs are striving for a home-like environment, better access to basic diagnostic and treatment services within NHs should be possible.

### MULTIPLE HOSPITAL TRANSFERS AMONG MOQI NURSING HOME RESIDENTS: THE INFLUENCE OF RACE

Elizabeth Fritz,[1] Amy Vogelsmeier,[2] Marilyn Rantz,[2] Lori Popejoy,[2] and Kelli Canada,[3], *1. Sinclair School of Nursing, Columbia, Missouri, United States, 2. University of Missouri - Columbia, Columbia, Missouri, United States, 3. University of Missouri, Columbia, Missouri, United States*

Missouri Quality Initiative (MOQI) was a CMS-funded enhanced care and coordination provider demonstration project (2012-2020) that successfully reduced avoidable hospitalizations and improved nursing home (NH) care quality. Little is known about the influence of race in multiple hospital transfers from NHs. Using a mixed-methods approach we analyzed hospitalization root cause analysis data from 2017-2019 for 1410 residents in 16 MOQI NHs. There were 113 residents who were transferred 609 times. Those with multiple transfers (four or more transfers/year) were compared by race and key characteristics (e.g., code status, diagnosis). A subset of residents with multiple transfers were examined qualitatively to identify and describe key cases. Findings suggest that Black residents have a higher probability for multiple transfers. Findings highlight the need for transfer prevention efforts for Black residents including early assessment and intervention, early/frequent discussion about goals of care, advance directives, resuscitation status, and family/resident understanding of treatment effectiveness.

### THE INFLUENCE OF MOQI APRNS ON THE COVID-19 RESPONSE IN NURSING HOMES

Megan Hiltz, Marilyn Rantz, Amy Vogelsmeier, and Lori Popejoy, *University of Missouri - Columbia, Columbia, Missouri, United States*

During the COVID-19 pandemic Missouri Quality Initiative APRNs worked in 16 nursing homes (NHs) providing clinical expertise and support. To understand their influence on the NH COVID-19 response, we conducted four group interviews with APRNs from 13 of the 16 NHs. Using thematic analysis, we identified similarities and differences between NH groups and then compared groups by COVID-19 infection rates. Leaders from NHs with high COVID-19 rates were unwilling to report infections and were resistant to resident/staff testing. In contrast, leaders from NHs with low COVID-19 rates were strategic about acquiring supplies, held daily huddles, and initiated CDC recommendations almost immediately. All reported residents lost weight, and experienced mood and physical decline resulting from quarantine/isolation. APRNs worked with providers to identify potentially ill residents/staff, improve isolation/quarantine procedures, manage ill residents, and supported efforts to mitigate viral spread. We will discuss implications for broader infection prevention in NHs.

### Session 4480 (Symposium)

### THE M. POWELL LAWTON AWARD LECTURE: THE PERSON-ENVIRONMENT FIT FRAMEWORK, OLDER ADULTS, AND TECHNOLOGY INTERACTIONS

Chair: Debra Dobbs

The lecture will be given by the 2020 recipient, Sara Czaja, PhD, FGSA of Weill Cornell Medicine. The 2020 M. Powell Lawton Award recipient is David Roth, Phd, FGSA, of Johns Hopkins University. The M. Powell Lawton Award is presented annually to an individual who has made outstanding contributions from applied research that has benefited older people and their care. The Lawton Award is generously funded by the Polisher Research Institute of the Madlyn and Leonard Abramson Center for Jewish Life.

### THE M. POWELL LAWTON AWARD LECTURE: THE PERSON-ENVIRONMENT FIT FRAMEWORK, OLDER ADULTS AND TECHNOLOGY INTERACTIONS

Sara Czaja, *Weill Cornell Medicine/Center on Aging and Behavioral Research, New York, New York, United States*

M. Powell Lawton made significant contributions throughout his illustrious career to improve the quality of life of older adults. His landmark theory of person-environment fit (P-E Fit) recognized the importance of understanding the dynamic interactions between older adults and their physical and social environments and the subsequent impact of these interactions on independent living. In today's living environments, technology is ubiquitous and can serve as both a barrier and facilitator to the ability of older people to live independently. This presentation will discuss how the P-F Fit Model can be used to clarify potential mismatches between technology systems and the characteristics, abilities, and preferences of older adult and how it can be used to guide design and training interventions to maximize the ability of aging adults to interact successfully with technology systems. Examples will be drawn from the Center for Research and Education on Aging and Technology Enhancement (CREATE) in the domains of social engagement, work, and health from technology design and intervention perspectives. The CREATE conceptual framework, consistent with the P-E Fit Model posits that users have varying needs, abilities, and attitudes; technology systems and tasks vary in demands; social, physical, and policy environments influence a person's access to and support for technology transactions; and human-technology interactions are dynamic. A focus of the presentation will be on how a user-centered design approach is compatible with the P-E Fit model and can optimize the fit between older adults and technology systems.

### Session 4485 (Paper)

### WORK AND RETIREMENT

### IQ IN YOUNG ADULTHOOD AND DEPRESSIVE SYMPTOMS OVER THE RETIREMENT TRANSITION

Linn Elena Zulka, Valgeir Thorvaldsson, and Linda B. Hassing, *University of Gothenburg, Gothenburg, Vastra Gotaland, Sweden*

Retirement can be a challenging life transition for mental health. Higher levels of IQ in young adulthood have been shown to be advantageous for different outcomes later in life such as quality of life and well-being. However, it remains unclear whether possessing higher cognitive abilities in early life also favors individuals' mental health when facing challenges related to the retirement transition. In this study, we therefore investigated the relationship between IQ in young adulthood and depressive symptoms over the retirement transition. We used data of six waves from the longitudinal population-based HEalth, Aging and Retirement in Sweden (HEARTS) study, as well as data on IQ in young adulthood from conscription. In a piecewise structural equation model, we modelled trajectories of depressive symptoms (measured by the CES-D scale) before and after retirement and in relation to young adulthood IQ (n = 1722 men). Results indicated an average decrease in depressive symptoms over the retirement transition for this sample of men. Higher childhood IQ was associated with further reduction in post-retirement depressive symptoms while controlling for education, retirement age, and memory ability and cardiovascular health at baseline. Our findings support the conclusion that higher IQ in young adulthood may act as a protective factor for mental health in the retirement transition. Individuals with higher IQ in young adulthood may have acquired coping strategies throughout their life-course, which they can apply when handling challenges related to retiring.

### LEARNING TO WORK WITH CHRONIC HEALTH CONDITIONS: HOW TIME SINCE DIAGNOSIS AFFECTS OLDER WORKERS' VITALITY AND WORRIES

Anushiya Vanajan,[1] Ute Bültmann,[2] and Kène Henkens,[3], *1. Netherlands Interdisciplinary Demographic Institute (NIDI), The Hague, Netherlands, Den Haag, Zuid-Holland, Netherlands, 2. University Medical Center Groningen (UMCG), Groningen, Groningen, Netherlands, 3. Netherlands Interdisciplinary Demographic Institute (NIDI), The Hague, Zuid-Holland, Netherlands*

**Background.** Chronic health conditions (CHCs) pose stark detrimental effects on the health and abilities of older workers. The extent of these effects depend on the CHC, the time since its diagnosis and the type of health measure: a rarely explored combination of heterogeneities. Objective. This study examined how four existing and newly diagnosed CHCs influences older workers' vitality and worries about enduring physically and mentally until retirement age.

**Method:** Data from two waves of the NIDI Pension Panel survey conducted in the Netherlands in 2015 and 2018 were used. We analyzed a sample of 1,894 older workers between the ages of 60-62 years at wave 1 using conditional change ordinal least square regression models.

**Results:** Having a CHC at wave 1 was associated with lower levels of vitality and higher levels of worries at wave 2. These effects of CHCs on vitality and worries were much larger for older workers who were newly diagnosed with CHCs compared to those who experienced CHCs for longer. Intriguingly, the new diagnosis of physically disabling conditions increased worries about physical endurance at wave 2, while the new diagnosis of mentally disabling conditions increased worries about mental endurance at wave 2.

**Conclusion:** By distinguishing the effects of four existing and newly diagnosed CHCs on vitality and worries, this study allows the identification of vulnerable groups of older workers. The findings may inform work accommodations and interventions which could improve both the quality and sustainability of work lives, while promoting healthy ageing of older workers.

### WORK EXPECTATIONS AND TRAJECTORIES OF DEPRESSIVE SYMPTOMS AND PASSIVE SUICIDAL IDEATION AMONG BABY BOOMERS

Linh Dang,[1] and Briana Mezuk,[2], *1. University of Michigan School of Public Health, Ann Arbor, Michigan, United States, 2. University of Michigan, Ann Arbor, Michigan, United States*

Expectations regarding work (e.g., probability of retiring at a certain age), whether realized or not, may influence mental health, however there is limited quantitative research on this question. This study examined the longitudinal relationship between expectations of full-time work after age 62 and depressive symptoms and passive suicidal ideation among Baby Boomers, a generation that experienced the Great Recession as they neared retirement. Data came from the Health and Retirement Study, 2008 - 2016 (N = 8,954, mean age = 55.3, 52.2% female, 77.8% non-Hispanic White). Clinically-relevant depressive symptoms were indexed by the Composite International Diagnostic Interview (CIDI). Expectation (probability) of working after age 62 was modeled continuously (range: 0 to 1). Multivariate mixed-effects logistic regression models of screening positive on the CIDI and passive suicide ideation were fit, separately, adjusting for demographics, household income and wealth, and health characteristics. Respondents working at baseline were less likely to screen positive on the CIDI longitudinally (OR: 0.36, 95% CI: 0.26 - 0.51), and while expectations were inversely associated with screening positive on the CIDI this was not significant after accounting for work status (OR: 0.68, 95% CI: 0.43 - 1.09, p=0.104). Longitudinally, higher expectations of working were inversely associated with passive suicidal ideation (OR: 0.54, 95% CI: 0.32 - 0.92) even after accounting for working status. Future research will examine variation in these relationships by contextual factors like wealth, sex, and race/ethnicity to clarify how these features shape the association between work and mental health for this generation of older adults.

### Session 4490 (Symposium)

### (RE)INTRODUCING THE GERONTOLOGY AND GERIATRICS CURRICULAR STANDARDS AND GUIDELINES IN HIGHER EDUCATION

Chair: Tamar Shovali Co-Chair: Marilyn Gugliucci
Discussant: Nina Silverstein

The Academy for Gerontology in Higher Education (AGHE), the education member group of GSA, is the only national institutional membership organization devoted primarily to gerontology and geriatrics education. Its mission provides for development and sponsorship of initiatives to advance the field of aging through its focus on education in gerontology/geriatrics. AGHE first published the Gerontology and Geriatrics Curricular Standards over three decades ago – a document that has been an integral resource for implementing/revising programs in liberal arts, the sciences, and more recently, health professions. To meet the needs in the field for increased breadth and depth of content, the new 7th edition of the educational guidelines fully embraces competency-based education for gerontology, as the health professions programs have for years. Our first presenter will provide an overview of the new edition. The second presenter will focus on associate degree programs in gerontology and their unique contribution to higher education. The third will present on undergraduate programs in gerontology explaining how these programs give students an edge in today's job market. The fourth presenter will address graduate programs in gerontology, describing master's degree programs and doctoral degree programs in gerontology and aging studies. The fifth presenter will discuss health professions programs including geriatrics curricula for osteopathic medical education, gerontology/geriatrics curricula for health-related programs and the doctor of pharmacy degree programs. Presentations will provide expert recommendations for program development through mapping AGHE's Gerontology Competencies for Undergraduate and Graduate Education to programs in higher education. Nina Silverstein will serve as discussant.

### STANDARDS AND GUIDELINES FOR A NEW DECADE

Tamar Shovali, *Eckerd College, Eckerd College, Florida, United States*

As the field evolved, so too, has the Gerontology and Geriatrics Curricular Standards and Guidelines in Higher Education. The 7th edition is focused on the integration of the highly vetted AGHE Gerontology Competencies for Undergraduate and Graduate Education. Through this work we establish a set of standards and guidelines that provides the foundation for excellence in gerontology and health professions education at various levels and across a range of programs in the US and globally. The document provide a model for institutions of higher education in the development of new programs, a basis for program assessment and evaluation, guidance for academic or institutional reviews in existing programs, and a platform for what constitutes a program of study in gerontology/geriatrics or health professions programs for students, the public, and employers. An overview of the development of the 7th edition and suggestions for its use will be provided.

### STANDARDS AND GUIDELINES FOR ASSOCIATE'S DEGREE PROGRAMS IN GERONTOLOGY

Michael Faber,[1] and Suzie Macaluso,[2], *1. Portland Community College, Portland, Oregon, United States, 2. Abilene Christian University, Abilene, Texas, United States*

Associate degree programs in gerontology occupy a unique space in higher education. They must prepare students for a wide variety of careers and opportunities from technical and vocational training to preparation for further gerontology education at a four-year college. It is widely known that there is great variability among the numerous associate degree programs in gerontology; this presentation gives an overview of the revised standards and guidelines for associate degree programs. Associate degree programs will vary based on the faculty, the leadership, the program, and institutional support, this presentation discusses best practices for a variety of program types paying particular attention to competency-based educational strategies.

### STANDARDS AND GUIDELINES FOR UNDERGRADUATE PROGRAMS IN GERONTOLOGY

Laura Donorfio,[1] Karen Kopera-Frye,[2] Robert Maiden,[3] and Carrie Andreoletti,[4], *1. University of Connecticut, Waterbury, Connecticut, United States, 2. University of New Mexico, Las Cruces, New Mexico, United States, 3. Alfred University, Alfred, New York, United States, 4. Central Connecticut State University, New Britain, Connecticut, United States*

Undergraduate programs (majors, minors, certificates) and continuing education programs in gerontology prepare students for entry-level careers in aging and increase competitiveness for graduate work in a variety of fields. Job growth in the field of gerontology is high, especially for positions requiring a bachelor's degree and less. Gerontology education at this level is essential for meeting the growing demand for workers in social services and health services who understand the opportunities and challenges that come with increased longevity and global aging. This presentation will highlight the new recommendations for competency-based gerontology education for undergraduate and continuing education credentials outlined in the latest edition of AGHE Standards and Guidelines. Whether you are developing a new curriculum or revising an old one, we will offer suggestions for using the AGHE competencies and guidelines to ensure that your program adequately prepares students and offers them a competitive edge in today's job market.

### STANDARDS AND GUIDELINES FOR GRADUATE PROGRAMS IN GERONTOLOGY AND GERIATRICS

Rona Karasik,[1] and Julie Masters,[2], *1. Saint Cloud State University, SAINT CLOUD, Minnesota, United States, 2. University of Nebraska, Lincoln, Nebraska, United States*

Graduate programs in gerontology prepare students for advanced academic and/or applied careers in aging. Programs at this level offer greater depth, breadth, and increased emphasis on theory and research. Persons completing a master's and/or doctoral degree in gerontology or aging studies have reached the level of "gerontologist" whereby they have completed the necessary coursework in the physical, psychological, social and built environment in order to understand the unique opportunities and challenges of aging in a scholarly manner. While the depth of treatment of each topic will vary across programs, each of the AGHE Core Competencies should be mastered at a level of "analyzation and evaluation" or higher on Bloom's Taxonomy of Educational Objectives. The current presentation addresses how the AGHE Standards and Guidelines for graduate programs in gerontology were updated as a competency-based curriculum that includes coursework, practicum, and a culminating project (e.g., thesis, comprehensive exam, and/or dissertation).

### QUALITY HEALTH PROFESSIONS PROGRAMS: PATH TO PROGRAM OF MERIT DESIGNATION

Marilyn Gugliucci, *University of New England College of Osteopathic Medicine, Kennebunk, Maine, United States*

In 2015, the AGHE Program of Merit was expanded to implement a voluntary evaluation process for health professions programs that integrate gerontology/geriatrics competencies within their curriculum. These competencies augment students' competence and confidence in the field of aging, preparing them to work with older adults and their care partners. All health professions and medical education programs are eligible to apply for the Program of Merit designation. This international process of evaluation: (1) Verifies program credibility and quality; (2) Informs campus administrators of global guidelines, expectations, and practice in aging for their health professions programs; (3) Assures quality graduates from POM designated programs; and (4) Clarifies for employers the knowledge and skills imparted to student graduates of POM designated health professions programs. Additionally, graduates from POM programs receive a certificate from AGHE stating the health profession program is a designated AGHE Program of Merit.

### Session 4495 (Symposium)

### AGE-FRIENDLY COMMUNITIES: RESEARCH ON PROMISING AND INNOVATIVE PRACTICES

Chair: Patricia Oh Co-Chair: Holly Dabelko-Schoeny
Discussant: William Armbruster

Age-friendly community (AFC) leaders engage with community residents, groups, and organizations to enhance social, service, and built environments for aging. Despite the growing number of AFCs, academic research remains in its nascence. During this symposium, U.S. scholars will present research on AFC implementation. The first two papers address promising practices among AFC leaders. The first paper presents results from a content analysis of American AFC progress reports. Results indicate strong evidence of progress achieved and opportunities for improving the evaluation, documentation, and dissemination of accomplishments. The second paper presents findings from a qualitative descriptive study on community events by AFC initiatives in northern New Jersey. Results indicate how age-friendly leaders use community events to enhance older adults' social participation and inclusion, and as a tactic toward longer-term, multi-domain community change. The third and fourth papers describe the development and testing of innovative technologies to guide AFC implementation. The third paper provides an overview of Safe Routes to Age in Place, a community-engaged mixed methods study using the app MyAmble to examine the needs and utilization of alternative transportation in Franklin County, Ohio. The fourth paper provides a case overview of a university-community partnership to develop a geographical information system (GIS) analytical mapping tool to evaluate the age-friendliness of public amenities in San Antonio, Texas. The symposium will conclude with remarks from AARP Livable Communities leadership about how these studies contribute to building knowledge about AFC implementation and avenues for academic and private partnerships to translate these findings to practice.

### AGE-FRIENDLY COMMUNITY PRACTICE: UNDERSTANDING THE ROLE OF COMMUNITY EVENTS

Natalie Pope,[1] Althea Pestine-Stevens,[1] Clara Scher,[1] and Emily Greenfield,[2], *1. Rutgers Social Work, New Brunswick, New Jersey, United States, 2. Rutgers, The State University of New Jersey, New Brunswick, New Jersey, United States*

To advance an emerging empirical knowledge base for age-friendly community initiatives (AFCIs), we conducted a qualitative descriptive study to explore one manifestation of age-friendly practice: community events (CEs). We aimed to illuminate how AFCI core teams describe CEs as part of their practice and how they perceive the value of CEs for age-friendly progress. Using inductive coding, we analyzed data from semi-structured interviews (n=24) with eight core teams across three time points spanning the early to mid-implementation phases of the AFCIs. Two predominant themes emerged. First, CEs were described as important for working toward age-friendly goals concerning older residents' social participation and inclusion. Second, core teams described the longer-term strategic value of CEs, such as building interorganizational partnerships; providing deeper insight on aging in community; and fostering older adults' leadership as part of the initiative. We discuss implications for advancing transdisciplinary program theory to guide more sustainable and effective AFCIs.

### SAFE ROUTES TO AGE IN PLACE: A FOCUS ON ALTERNATIVE TRANSPORTATION

Noelle Fields,[1] Katie White,[2] Christine Highfill,[3] Qiuchang Cao,[4] Ian Murphy,[4] and Holly Dabelko-Schoeny,[5], *1. University of Texas at Arlington, Arlington, Texas, United States, 2. Ohio State University, Age-Friendly Columbus and Franklin County, Ohio, United States, 3. University of Texas - Arlington, Arlington, Texas, United States, 4. The Ohio State University, Columbus, Ohio, United States, 5. The Ohio State University, The Ohio State University, Ohio, United States*

Informed by social cognitive theory, Age-Friendly Columbus and Franklin County conducted a community-engaged mixed methods study that examined the needs and utilization of alternative transportation by older residents in three pilot neighborhoods in Franklin County, Ohio (n = 32). Participants were provided tablets and used an app (MyAmble) developed at the University of Texas-Arlington to document their traveling experiences. During a 14-day period, 1,190 trips were recorded by older adults and 71.3% of these trips were completed through driving their own personal vehicles. Participants designated 84.5% of trips as important and 72% of the trips improved their mood. Individual (physical and cognitive functioning, cost, time), environmental (lighting, sidewalk conditions, traffic, location of bus stops, weather), and behavioral (no history of bus use, peer to peer information sharing, tracking led to future planning) barriers and facilitators to alternative transportation use such as riding the bus, walking and biking were identified.

### ASSESSING AGE-FRIENDLY COMMUNITY PROGRESS: WHAT HAVE WE LEARNED?

Patricia Oh,[1] and Kathy Black,[2], *1. UMaine Center on Aging, Bangor, Maine, United States, 2. University of South Florida, Sarasota-Manatee, Florida, United States*

The Global Network of Age-friendly Cities and Communities has grown steadily over the past decade across the United States, however surprisingly little is known regarding their accomplishments to date. We utilized content analysis to assess the progress reported by American age-friendly communities (n = 30) that joined by end of year 2015 using the Age-Friendly Community Evidence-based Tool with expanded program evaluation measures including health equity as defined by the World Health Organization. We employed deductive analytic techniques to assess reported community performance in eleven thematic areas across the range of structures and processes that characterize age-friendly efforts. We found strong evidence in the areas of leadership and governance, harnessed resources, application of age-friendly framework, and in multisector collaboration as well as reported provisions. All of the communities reported health equity aims, particularly in promoting accessible physical environments and social inclusion efforts. Our analysis further revealed areas for continued improvement.

### Session 4500 (Paper)

### AGING IN PLACE (SRPP PAPER)

### AGE-FRIENDLY COMMUNITIES AND AGING IN PLACE: FINDINGS FROM LATENT PROFILE ANALYSIS

Seon Kim,[1] Kyeongmo Kim,[2] and junpyo kim,[3], *1. Virginia commonwealth university, Richmond, Virginia, United States, 2. Virginia Commonwealth University, Henrico, Virginia, United States, 3. Gyeongsang National University, Jinju-si, Kyongsang-namdo, Republic of Korea*

Older adults prefer to live in their current home or community and 'Aging in place' has been shown to reduce the cost of caring for older adults and help their successful aging. Although age-friendly communities (AFC) initiatives have been helpful to aging in place, little has been known about the relationship between the types of AFC and aging in place. Using the 2017 AARP Age-Friendly Community Survey, we included 1,079 adults aged 65 or older. We measured aging in place as 'move to a different community', 'move into a different residence within your current community', and 'stay in your current residence', and included eight AFC constructs. We identified the type of AFC using Latent Profile Analysis: low-friendly, mid-friendly, and high-friendly. We also ran multinomial logistic regression to examine whether the types of AFC were associated with aging in place. Of the total participants, 26.0% lived in the low-friendly community, 23.7% in the mid-friendly community, and 50.3% in the high-friendly community. Older adults living in the high-friendly community were more likely to stay in the current residence (64.7%) than those in the low-friendly (47.1%) ($\chi 2$=28.680, p<.001). Also, older adults living in the low-friendly community (OR=3.05, p<.001) and the mid-friendly community (OR=1.42, p<.10) were more likely to move to a different community compared to those living in the high-friendly community. This result suggests that it is important to build an AFC to promote aging in place. For the growing number of older adults' lives, policymakers should consider expanding the AFC initiatives.

### HOME AND COMMUNITY FEATURES, PERCEIVED AGE-FRIENDLINESS, AND INTENTION TOWARD AGING IN PLACE

Yeon Jin Choi, *University of Southern California, Los Angeles, California, United States*

Promoting age-friendliness of communities and supporting aging in place (AIP) are of great importance in aging societies. However, little is known about the mechanism linking home and neighborhood features, older adults' global assessment of community, and their willingness to age-in-place despite the importance in developing policies and interventions. This study used the 2015 AARP Age-Friendly Community Survey, which includes 66 home and neighborhood features under the eight domains specified by the WHO's Age-Friendly Cities Guidelines. A series of linear regression models were estimated to examine the interrelationship between the availability of age-friendly features in eight domains, perceived age-friendliness of community, and intention toward AIP. Overall, a greater availability of age-friendly features was positively associated with perceived age-friendliness of community and AIP intention. The relationship between age-friendly features and AIP intention was mediated by perceived age-friendliness of community (50.3% to 96% of the total effects). When perceived age-friendliness of community was introduced to models, the direct effects of housing, outdoor spaces and buildings, and transportation domains remained significant. Findings suggest that a greater availability of age-friendly features influence older adults' perception on their community, leading to the development of a desire to age-in-place. Domains of housing, outdoor spaces and buildings, and transportation may be the most importance features in promoting age-friendliness of community and the key determinants of aging-in-place. Policy makers and practitioners may need to prioritize promoting age-friendly built environment before social environment in building age-friendly communities.

### OLDER ADULTS' PERCEPTIONS OF SMART CITY INITIATIVES TO AGE IN COMMUNITY

Jongwoong Kim, *West Chester University of Pennsylvania, West Chester, Pennsylvania, United States*

This project explores older American adults' perceptions of smart city initiatives for them to "age in community" particularly in the northeast region. As the U.S. population is aging, it is imperative that the American cities can support their citizens to live in their preferred community environments for as long as they want. While there are many definitions of a smart city, some exemplary smarty city initiatives can be characterized as actively utilizing information and sensor technologies to promote efficiency and sustainability of city-wide systems, ultimately enhancing the quality of citizens' life. This project examines, in particular, seven smart city initiatives that are implemented globally: smart streetlights, health and fall monitoring system, community ridesharing, enhanced CCTVs, "age-friendly map," contact tracing app, and smart traffic system. By surveying those age 55 and older, with a representative sampling from the nine states in the northeast region, this project found that the vast majority of older Americans in this region would prefer to age in rural and suburban communities, and depending on where they prefer to age in (rural-exurban-suburban communities vs. urban-urban center communities) and gender (female vs. male), they perceive particular sets of smart city initiatives as more important for them to age in community. Furthermore, regardless of the community/location preference and demographic (gender, income level, and age) differences, 40% of the respondents expressed no concern of data or information privacy issues from these initiatives, opening some doors for the municipalities that plan to adopt some of these initiatives in the near future.

### THE COST EFFICIENCY OF HOME MODIFICATIONS TO REDUCE HEALTH CARE COSTS

Jesse Abraham, *HomesRenewed, Washington, District of Columbia, United States*

The existing quantity of housing dedicated for older adults is not sufficient to meet the needs of this growing population. And even as the Centers for Medicare and Medicaid Services reimbursement structures are shifting from traditional inpatient and outpatient settings to care in the home, it is a commonplace that most homes were not designed or built to support the needs of aging residents or the provision of healthcare. It is time for America's 100 million existing houses to be made as safe and accessible as possible for aging in place. Falls cost over $50 billion a year in medical expenses. This paper distills current knowledge regarding healthcare cost reductions from home modifications, and then calculates the cost efficiency to society and to the federal government of providing government subsidies for home modifications for older adults at the ages of 50, 65 and 75. Cost sharing among insurers, government and the beneficiary is one way to achieve the positive social returns.

### TYPES OF LATE-LIFE CHALLENGES TO AGING IN PLACE

Kyeongmo Kim, *Virginia Commonwealth University, Henrico, Virginia, United States*

Many older adults prefer to live within their community because they have built strong relationships with their neighbors and neighborhood. Although housing-related factors promote aging in place, findings on the relationships of late-life challenges to aging in place (e.g., cost of living, autonomy) and relocation are mixed. Less is known about the types of challenges to aging in place and about the relationship between the types of challenges and relocation. Using data from the AARP 2015 Age-Friendly Community Surveys (N=3,190 adults aged 65 and older), this study examined the intersection of challenges to aging in place (e.g., home size, cost, safety, independence, family, transportation) and relocation (i.e., move to a different home outside of their community). Using latent class analysis (LCA), we identified five subgroups of late-life challenges to aging in place: multifaceted challenges, cost of living, independence, social connection, no concern. Findings from LCA with a distal outcome showed that older adults with multifaceted challenges (b=0.77, p<.001), were more likely to move out of their community, compared to those with lower levels of challenges, even after adjusting for age, sex, education, income, and chronic diseases. Also, those with challenges regarding the cost of living (b=0.84, p<.001), independence (b=0.64, p<.001), and family connection (b=0.45, p<.001) were more likely to expect to move out of their community. The findings highlight that older adults have different types of challenges to aging in place. Practitioners and policymakers should provide more individualized supportive services, considering the types of challenges to promote aging in place.

### Session 4505 (Symposium)

### AMBULATORY ASSESSMENT OF SOCIO-AFFECTIVE AND COGNITIVE AGING: ZOOMING INTO DAILY PROCESSES

Chair: Tabea Meier Co-Chair: Andrea Horn
Discussant: Christina Roecke

Ambulatory assessment methods offer new possibilities to study cognitive, emotional, and social processes in the setting in which they naturally occur, namely in the daily life of individuals and couples. This allows to zoom into processes with great relevance for healthy aging and well-being over the lifespan. Research on daily psychological processes opens the door for investigating the interplay with contextual (daily stress, social resources) and stable factors (relationship quality status, accumulated discrimination) that are known to shape these dynamic processes. This symposium will present and discuss innovative contributions investigating daily emotional and cognitive functioning and their interplay with social and individual characteristics over the adult lifespan. The first study by Haas and colleagues will present experience sampling data on daily prospective memory performance in couples. Haas et al. will offer a dyadic perspective on cognitive functioning by examining how prospective memory performance is co-regulated in the daily lives of younger and older couples. Meier et al. will present another study from the same couples project; here the focus is on age differences in couples' "we-ness" and its relationship with daily positive emotional experiences and how they are shared with the partner. The series of talks will be completed by Zavala et al. who examined associations between stress and emotional health in daily life and its interplay with prior discrimination experiences of an age-diverse sample of BIPOC adults. After these individual contributions, Christina Roecke will discuss the presented studies and provide her reflections on the results and their implications.

### SHARED POSITIVITY IN DAILY LIFE OF YOUNGER AND OLDER COUPLES: LINKS BETWEEN WE-NESS AND POSITIVE EMOTIONS

Zilla Huber,[1] Olenka Dworakowski,[2] Claudia Haase,[3] Mike Martin,[4] Andrea Horn,[1] and Tabea Meier,[1], *1. University Research Priority Program (URPP), Dynamics of Healthy Aging & Department of Psychology, University of Zurich, University of Zurich, Zurich, Switzerland, 2. University Research Priority Program (URPP), Dynamics of Healthy Aging, & Department of Psychology, University of Zurich, University of Zurich, Zurich, Switzerland, 3. School of Education and Social Policy and (by courtesy) Department of Psychology, Northwestern University, Northwestern University, Illinois, United States, 4. University of zurich, Zurich, Zurich, Switzerland*

Positivity resonance, the shared experience of emotional positivity, may contribute to the quality of romantic relationships and foster couples' sense of "we-ness". The present study examined age differences in couples' positive experiences in daily life, how they are shared with the partner, and how they are related with "we-ness". In a 21-day experience sampling study, 62 younger (18-33 years) and 54 older (57-87 years) couples reported their momentary positive emotional experiences and disclosure. We-ness was operationalized as the rate of we-pronouns in couples' audio-sensed daily conversations. As hypothesized, daily positivity was associated with we-ness, and older couples experienced more positive affect than younger couples. Older couples also showed higher manifestations of we-ness, and these age differences in we-ness were partially explained by more positive disclosure in daily life. Our results contribute to the literature on socio-emotional positivity and aging by showing how these processes manifest in daily life.

### ASSOCIATIONS BETWEEN STRESS EXPOSURE AND EMOTIONAL HEALTH: THE MODERATING ROLE OF DISCRIMINATION

Elizabeth Muñoz,[1] Martin Sliwinski,[2] Stacey Scott,[3] and Daisy Zavala,[4], *1. University of Texas at Austin, University Of Texas At Austin, Texas, United States, 2. The Pennsylvania State University, University Park, Pennsylvania, United States, 3. Stony Brook University, Stony Brook, New York, United States, 4. Stony Brook University, Middle Island, New York, United States*

The Weathering Hypothesis states BIPOC face more stressors, by which over a lifetime they are subjected to the negative consequences of stress (e.g., poorer emotional health). Using ecological momentary assessments, we examined whether subtle discrimination moderated the within-person stressor slope on positive and negative affect. We predicted emotional wellbeing would be worse at stressor moments, and those with greater discrimination experience would be more impacted by stressors. Participants were 334 diverse adults (25-65 years, Mage = 47, 63% Female) from Bronx, New York. Positive affect decreased and negative affect increased significantly at stressor moments (p<.0001). Unexpectedly, subtle discrimination was not a significant moderator for the within-person stressor slope on positive affect and negative affect. Unlike the predictions of the Weathering Hypothesis, these results show that prior discrimination experiences may not exacerbate responses to stressors and entail additional risk in daily life.

### INVESTIGATING EVERYDAY PROSPECTIVE MEMORY IN YOUNGER AND OLDER COUPLES

Tabea Meier,[1] Zilla Huber,[1] Matthias Kliegel,[2] Andrea Horn,[1] and Maximilian Haas,[3], *1. University Research Priority Program (URPP), Dynamics of Healthy*

*Aging & Department of Psychology, University of Zurich, University of Zurich, Zurich, Switzerland, 2. Faculty of Psychology and Educational Sciences & Centre for the Interdisciplinary Study of Gerontology and Vulnerability, University of Geneva, University of Geneva, Geneve, Switzerland, 3. University of Geneva, University of Geneva, Geneve, Switzerland*

Daily events are not simply individual concepts, but shared by the social environment we live in. The present study investigates the role of romantic partners in the co-regulation of cognitive functioning by the example of prospective memory (PM), the ability to remember and correctly execute future intentions. In this context, we examined the impact of social proximity (i.e., physical closeness and psychological intimacy) on interpersonal regulation. Over the course of 21 days, 64 younger couples (18-33 years) and 52 couples of older adults (57-87 years) completed an ambulatory assessment comprising a daily pseudo-randomized PM task. Results reveal that couples' PM performance was higher for younger than for older participants. Further, dyadic PM was correlated with psychological intimacy for both age groups, but the impact of time spent together on intimacy and PM performance, respectively, was stronger in older adults. Possible moderating factors and explanations for these findings will be discussed.

### Session 4510 (Symposium)

### BS GSA AND AAA JOINT SYMPOSIUM

### RIBOSOMAL PROTEIN 6 PHOSPHORYLATION REGULATES TRANSLATIONAL RESPONSES TO DIETARY RESTRICTION

Jarod Rollins, *MDI Biological Laboratory, Salbury Cove, Maine, United States*

Forms of dietary restriction like intermittent fasting (IF) and caloric restriction (CR) promote health and longevity through changes in gene expression. While the transcriptional changes that occur in response to DR have been well described across several species, the role of translational regulation has lagged. Using polysome profiling and mRNA-seq, we quantified changes in actively translated mRNAs that occur in C. elegans under CR compared to well-fed conditions. The analysis revealed hundreds of transcripts regulated on the translational level that would have been missed using conventual transcriptomics. Among the translationally down-regulated genes that where pro-longevity when knocked down were regulators of the cell-cycle: fbxb-24, sdz-33, kbp-1, and cdk-2. In search of the mechanisms regulating selective translation under CR we investigated a role for ribosomal protein 6 (RPS-6) as its phosphorylation status is thought to regulate cell cycle and selective translation of mRNA transcripts. Using RPS-6 phospho-null and phospho-mimetic mutants, we show that phosphorylation and de-phosphorylation of RPS-6 is necessary for the pro-longevity effects of CR and IF. Furthermore, we show that IF is more beneficial for retaining locomotion with age than CR and that endogenously tagged RPS-6 ::mCherry accumulates in body wall muscle under fasting. However, the benefit of IF on locomotion is lost in RPS-6 phospho-mimetic mutants. Together, results suggest that protein translation is enhanced in the muscle under IF to prevent sarcopenia in a way dependent on RPS-6. Translatome analysis of the phosphomutant suggested a role for RPS-6 in selective translation of p38 mitogen-activated protein kinases.

### AGE-RELATED NEUROPROTECTION BY DIETARY RESTRICTION REQUIRES OXR1-MEDIATED RETROMER FUNCTION

Sudipta Bar,[1] George Brownridge,[2] Jennifer Beck,[2] Rachel Brem,[3] Hugo Bellen,[4] Lisa Ellerby,[2] Pankaj Kapahi,[2] and Kenneth Wilson,[2], *1. Buck Institute for Research on Aging, Buck Institute for Research on Aging, California, United States, 2. Buck Institute for Research on Aging, Novato, California, United States, 3. University of California, Berkeley, Berkeley, California, United States, 4. Baylor College of Medicine, Houston, Texas, United States*

Dietary restriction (DR) is the most robust method to delay aging and the onset of neurogenerative disorders across multiple species, though the mechanisms behind this phenomenon remain unknown. To elucidate how DR mediates lifespan extension, we analyzed natural genetic variants that associate with increased longevity under DR conditions in the Drosophila Genetic Reference Panel. We found that neuronal expression of the fly homolog of human Oxidation Resistance 1 (OXR1) is necessary for DR-mediated lifespan extension. Neuronal knockdown of OXR1 also accelerated visual decline but not physical decline, arguing for a specific role of OXR1 in neuronal signaling. Further, we find that overexpression of the TLDc domain from human OXR1 is sufficient for lifespan extension in a diet-dependent manner. Studies from the Accelerating Medicines Partnership - Alzheimer's Disease network show that patients with reduced OXR1 protein levels are more prone to Alzheimer's disease diagnosis, and we find that overexpression of human OXR1 is protective in animal and cell Alzheimer's models. In seeking the mechanism by which OXR1 protects against age-related neuronal decline, we discovered that it provides a necessary function in regulating the neuronal retromer complex, which is essential for the recycling of transmembrane receptors and for maintenance of autophagy. We further discovered that OXR1 deficiency can be rescued by genetic or pharmacological enhancement of retromer function, and that this enhancement extends lifespan and healthspan. Understanding how OXR1 operates could help uncover novel mechanisms to slow neurodegeneration including Alzheimer's disease.

### AGING PREDISPOSES B CELLS TO MALIGNANCY BY ACTIVATING C-MYC AND PERTURBING THE GENOME AND EPIGENOME

Jose Castro,[1] Alessandro Barbieri,[2] João Paulo,[3] Olga Strelkova,[3] John Sedivy,[4] John Manis,[3] Vadim Gladyshev,[5] and Anastasia Shindyapina,[6], *1. I 3. S, Porto, Porto, Portugal, 2. Boston Children's Hospital, Boston, Massachusetts, United States, 3. Harvard Medical School, Boston, Massachusetts, United States, 4. Brown University, Providence, Rhode Island, United States, 5. Brigham and Women's Hospital, Boston, Massachusetts, United States, 6. Brigham and Women's Hospital, Harvard*

*Medical School, Brigham and Women's Hospital, Harvard Medical School, Massachusetts, United States*

Age is the single major risk factor for human cancer, but naturally occurring cancers are rarely studied in aging models. Like humans, mice spontaneously develop cancer with age, and standard laboratory strains are predisposed for B-cell lymphoma. Here, we uncover how B-cell lymphoma develops as a consequence of the aging immune system. We found that aged B cells acquire somatic mutations in tumor suppressors and oncogenes (e.g. Trp53, Pim1, and Myh11) and undergo monoclonal expansions, with some clones representing 86% of splenic B cells. Clonal B cells had hypermethylated promoters and globally silenced expression, suggesting a role of DNA methylation in clonal selection of premalignant B cells. B-cell size, spleen weight, and a novel population of B cells, which we named Myc+ cells, emerged as convenient markers of malignancy. High-throughput analyses of clonal B cells and the use of genetic mouse models revealed that c-Myc drives B-cell size increase and clonal expansion with age. Phoshoproteome and co-culture experiments revealed that c-Myc is activated by signals from the aging microenvironment. Moreover, single-cell RNA-seq suggested that clonal B cells originate from age-associated B cells, further underlying the importance of aging environment in cancer transformation. Longitudinal analyses demonstrated a negative impact of premalignant B cells on mouse lifespan and linked it to age-related myeloid bias. Together, our study revealed cell-autonomous changes that cooperate with the aging microenvironment to give rise to preneoplastic B cells. This stidy established a novel model to study how aging predisposes cells to cancer transformation.

### AN EVOLVING ROLE FOR THE LONG NON-CODING RNA H19 IN AGING AND SENESCENCE

Christian Sell,[1] and Manali Potnis,[2], *1. Drexel University, Philadelphia, Pennsylvania, United States, 2. Drexel University, PHILADELPHIA, Pennsylvania, United States*

The long non-coding RNA (lncRNA) H19 is a maternally imprinted gene transcript that, in conjunction with the neighboring Igf2 gene, is critical in controlling embryonic growth. Loss of H19 results in fetal overgrowth associated with Beckwith Weidemann syndrome, while elevated H19 occurs in human cancers. In the adult, H19 functions in cancer cells where it promotes migration and is correlated with poor prognosis, and in adult stem cells where it is a key regulator of cell fate decisions during differentiation. While the function of H19 in primary somatic cells has not been defined, a reduction in the abundance of H19 has been reported during senescence in endothelial cells. Given the critical importance of H19 in cell fate decisions, it is likely that understanding the precise function of H19 in somatic cells in general and why reduced levels occur with cellular senescence will provide novel insights into both somatic cell maintenance and the senescence program. Towards this end, we examined the role of H19 in somatic cell growth using cardiac interstitial fibroblasts. Our results indicate that H19 is not only vital for somatic cell proliferation and survival, but that depletion of H19 leads to cell cycle arrest and the formation of abnormal nuclei resulting in senescent cells. We are defining both the upstream regulators of H19 and the downstream mediators of senescence following H19 depletion. Overall, these results indicate an essential role for H19 in cell cycle progression, chromatin structure, and possibly proper mitotic division.

### Session 4515 (Symposium)

### CHANGE, CORRELATES, AND STRUCTURE OF PERSONALITY ACROSS ADULTHOOD

Chair: Olivia Atherton Co-Chair: Emorie Beck

Personality is both stable and changing across the lifespan. However, many questions remain about the factors that account for individual differences in change, the consequences of personality for life outcomes, and how best to assess personality at different points in the lifespan. First, Olivia Atherton will discuss research on the development of the Big Five personality traits from young adulthood to midlife with a sample of Mexican-origin individuals, as well as sociodemographic and cultural predictors of personality change in this population. Second, Bill Chopik uses data from 90 countries to examine the consistency of age differences in positive personality traits in the second half of life, from midlife to old age, as well as how cultural characteristics moderate the terminal decline in positive personality traits. Third, Emorie Beck will present research demonstrating that personality traits from the Big Five to beyond are robustly associated with a number of key life events across countries, decades, sociodemographic moderators, and even when controlling for selection bias. Finally, Josh Jackson uses network psychometric techniques to examine coherence and differentiation among indicators of the Big Five from 14 to 85 in a large multinational sample, tracking age differences with consequences for the assessment of personality traits in older adulthood. We will conclude with a panel discussion of emerging issues in personality change, prediction, and assessment across adulthood, with each speaker providing unique experience and insight into the study of each area.

### BIG FIVE DEVELOPMENT FROM YOUNG ADULTHOOD TO MIDLIFE: FINDINGS FROM A LONGITUDINAL STUDY OF MEXICAN-ORIGIN ADULTS

Angelina Sutin,[1] Antonio Terracciano,[2] Richard Robins,[3] and Olivia Atherton,[4], *1. Florida State University College of Medicine, Tallahassee, Florida, United States, 2. FLORIDA STATE UNIVERSITY, Florida State University, Florida, United States, 3. University of California, Davis, Davis, California, United States, 4. Northwestern University, Chicago, Illinois, United States*

A large body of research has documented how personality develops across adulthood, yet very little longitudinal work has examined whether these findings generalize beyond predominantly middle-class, highly-educated White American or Western European individuals. This pre-registered study uses longitudinal data from 1,110 Mexican-origin adults who completed a well-validated personality measure, the Big Five Inventory, up to 6 times across 12 years (median age at Wave 1 = 37.7; range = 26 to 65). Individuals generally maintained their rank ordering on the Big Five over time (rs=.66-.80), and all of the Big Five traits showed small, mean-level decreases across adulthood. These trajectories had few associations with sociodemographic factors (sex, education level,

IQ) and cultural factors (generational status, age at immigration, Spanish/English language preference, Mexican cultural values, American cultural values, ethnic discrimination). Divergences between the present findings and previous research highlight the need to study personality development across diverse aging samples.

### CULTURAL CONSISTENCY IN LATE-LIFE DECLINES IN POSITIVE PERSONALITY TRAITS

William Chopik, *Michigan State University, East Lansing, Michigan, United States*

Personality has elements of both stability and change across the adult lifespan. There has also been evidence for terminal decline—late-life decreases in positive psychological characteristics. However, many of these studies have examined these patterns in primarily Western populations. The current study examined the consistency of age differences in positive personality traits (i.e., character strengths) across cultures. I examined 2,895,051 participants ranging in age from 13 to 100 (Mage = 34.31; 65.3% women) from 90 different countries. I reproduced patterns of terminal decline across cultures. In addition to mean differences between cultures (e.g., focusing on the present is associated with more positive traits [Mr = .45]), cultural characteristics often moderated the effects of age on positive personality traits. For example, terminal decline was more dramatic among people from collectivistic cultures and flatter among people from individualistic cultures. Results will be discussed in the context of cultural variation developmental processes.

### A MEGA-ANALYSIS OF PERSONALITY PREDICTION: ROBUSTNESS AND BOUNDARY CONDITIONS

Joshua Jackson,[1] and Emorie Beck,[2], *1. Washington University in St. Louis, St. Louis, Missouri, United States, 2. northwestern University, Chicago, Illinois, United States*

Decades of studies identify prospective associations between personality characteristics and life outcomes. However, previous investigations of personality characteristic-outcome associations have not taken a principled approach to sampling strategies to ensure the robustness of personality-outcome associations. In a preregistered study, we test whether and for whom personality-outcome associations are robust against selection bias using prospective associations between 14 personality characteristics and 14 health, social, education/work, and societal outcomes across eight different person- and study-level moderators using individual participant data from 171,395 individuals across 10 longitudinal panel studies in a mega-analytic framework with propensity score matching. Two findings emerged: First, personality characteristics remain robustly associated with later life outcomes. Second, the effects generalize, as there are few moderators of personality-outcome associations. In sum, personality characteristics are robustly associated with later life outcomes with few moderated associations. We discuss how these findings can inform studies of personality-outcome associations.

### AGE DIFFERENCES IN PERSONALITY STRUCTURE

David Condon,[1] Emorie Beck,[2] and Joshua Jackson,[3], *1. University of Oregon, Eugene, Oregon, United States, 2. northwestern University, Chicago, Illinois, United States, 3. Washington University in St. Louis, St. Louis, Missouri, United States*

Most investigations in the structure of personality traits do not adequately address age, as few studies look at the structure of personality traits a-theoretically, instead presupposing a theoretical structure e.g., Big Five. As a result, the relationship among indicators within a trait (coherence) are often highlighted but relationships across traits (differentiation) are not thoroughly examined. Using a large-scale sample of 369,151 individuals ranging in age from 14 to 90, the present study examines whether personality indicators show differential relationships as a function of age. Results indicate that coherence shows few changes across the lifespan, while differentiation weakens across adulthood into old age. These finding suggest that Big Five indicators only parallel the Big Five structure among young but not older adults. Thus, using standard Big Five personality trait assessments in older adults may, at best, not reflect reality and, at worse, undermine the predictive utility of personality traits.

### Session 4520 (Symposium)

### COMMUNITY MOBILITY IN OLDER ADULTS: NOVEL METHODOLOGIES, RISK FACTORS, AND INTERVENTIONS

Chair: Andrea Rosso Co-Chair: Michelle Carlson
Discussant: Jana Hirsch

Community mobility is an individual's movement outside the home. It is essential for the completion of many instrumental activities of daily living, such as shopping and healthcare, and promotes physical function, social engagement, independent living, and quality of life. Mobility research often focuses on gait speed measured in clinical settings, a critical but not sufficient determinant of community mobility. Here we present four talks that assess community mobility and its determinants using novel methodologies to enhance our understanding of how to maintain independence in older ages. First, Andrea Rosso presents characteristics of individuals with the strongest associations between environmental walkability, as assessed by virtual audits, and walking. Second, Kyle Moored demonstrates associations of self-reported fatigability with life space among older men, independent of their physical functioning. Breanna Crane introduces GPS-based objective measures of community mobility and their associations with cognitive and physical function of older adults. Finally, Pam Dunlap presents results of a randomized clinical trial of a physical therapy intervention to improve walking in older adults on subjective and objective measures of life space. These talks will provide a better understanding of the factors related to community mobility, introduce attendees to novel methodologies in the assessment of both community mobility and risk factors associated with the loss of community mobility, and demonstrate approaches to improve community mobility in at-risk older adults. The discussant, Jana Hirsch, will provide perspectives on how these data inform our current view of community mobility and will lead a discussion with the audience.

### WHAT DETERMINES VULNERABILITY TO NEIGHBORHOOD WALKABILITY IN OLDER ADULTS?

Caterina Rosano,[1] Alyson Harding,[2] Stephanie Studenski,[1] Philippa Clarke,[3] and Andrea Rosso,[1], *1. University of Pittsburgh, Pittsburgh, Pennsylvania, United States,*

*2. University of Minnesota, Minneapolis, Minnesota, United States, 3. Institute for Social Research, Ann Arbor, Michigan, United States*

Environmental influences are recognized as important predictors of walking behaviors in older adults. However, individuals may differ in vulnerability to low environmental walkability. We determined associations of a walkability index (factor analysis of 16 variables; range -1.65 to 2.23) from audits of online images with self-reported walking behaviors in 406 adults mean age=82 (44% male, 39% Black). Effect modification by 12 variables representing sociodemographics, physical and mental health, and neighborhood characteristics was tested in general linear models. Effect modification was evident for knee pain, marital status, and neighborhood socioeconomic status (nSES) (all p-interaction<0.05); associations were present only in those with knee pain, those who were unmarried, and those in the highest race-specific tertile of nSES. For example, a 1 point higher walkability score was associated with 1.06 (CI: 0.78, 1.44) higher odds of walking in those without knee pain compared to 1.91 (CI: 1.25, 2.90) in those with knee pain.

### ASSOCIATIONS BETWEEN PERCEIVED PHYSICAL AND MENTAL FATIGABILITY AND LIFE SPACE MOBILITY IN OLDER MEN: THE MROS STUDY

Andrea Rosso,[1] Theresa Gmelin,[2] Yujia (Susanna) Qiao,[3] Michelle Carlson,[4] Peggy Cawthon,[5] Jane Cauley,[1] Nancy W. Glynn,[6] and Kyle Moored,[1], *1. University of Pittsburgh, Pittsburgh, Pennsylvania, United States, 2. University of Pittsburgh Graduate School of Public Health, Pittsburgh, Pennsylvania, United States, 3. University of Pittsburgh, University of Pittsburgh, Pennsylvania, United States, 4. Johns Hopkins University, Baltimore, Maryland, United States, 5. California Pacific Medical Center, San Francisco, California, United States, 6. University of Pittsburgh Graduate School of Public Health, Pittsburgh, Pennsylvania, United States*

Physical performance and fatigue can limit mobility within the larger environment (life-space mobility). It is unknown whether perceived fatigability, fatigue anchored to activity intensity and duration, is independently associated with life-space. We assessed this cross-sectionally in the Osteoporotic Fractures in Men Study (MrOS; N=1,681, Meanage=85±4.1). The Pittsburgh Fatigability Scale (PFS, range: 0-50) measured physical (Mean=16.2±9.5) and mental fatigability (Mean=7.5±8.0). Life Space Assessment scores (range: 0-120, higher=greater life-space) incorporated level, frequency, and assistance used for life-space mobility (Mean=84.3±22.0). Compared to the lowest fatigability strata (Physical: PFS 0-4; Mental: PFS 0-3, modeled separately), men in the two highest physical strata (PFS 20-24: B=-4.10±1.67; PFS≥25: B=-6.23±1.72; p's≤.05) and men in the three highest mental strata reported significantly lower life-space mobility (PFS 13-15: B=-3.42±1.74; PFS 16-19: B=-5.38±1.83; PFS≥20: B=-7.96±1.66, p's≤.05), adjusted for physical performance and health covariates. Our results provide evidence linking fatigability and real-world mobility, independent of physical health, in older men.

### METHODS AND RATIONALE FOR USING GPS-DERIVED OBJECTIVE TECHNOLOGIES TO EXAMINE COMMUNITY MOBILITY IN OLDER ADULTS

Kyle Moored,[1] Andrea Rosso,[1] Michelle Carlson,[2] and Breanna Crane,[3], *1. University of Pittsburgh, Pittsburgh, Pennsylvania, United States, 2. Johns Hopkins University, Baltimore, Maryland, United States, 3. Johns Hopkins Bloomberg School of Public Health, Baltimore, Maryland, United States*

Objective measures of community mobility are advantageous for capturing life-space activity. In contrast to subjective, self-reported approaches, GPS-derived objective measures leverage passive, real-time data collection techniques to mitigate recall bias and minimize participant burden. We present methods to quantify community mobility among a sample of 164 community-dwelling older adults (Mean age=77.3±6.5) from a physical therapy intervention aimed at improving walking ability. We characterized community mobility using activity space metrics (e.g., standard deviation ellipse (SDE) area), timing (e.g., time outside home), and shape (e.g., SDE compactness). We will discuss challenges and solutions to generating these metrics as well as their associations with physical and cognitive performance. Time outside of home and SDE area, but not SDE compactness, were correlated with better performance on the 6-Minute Walking Test and Trail-Making Test (Part B) (ρ=.20-.23, p's<.05). These findings will aid in understanding which community mobility measures are associated with functional capacity.

### CHANGES IN GPS-DERIVED COMMUNITY MOBILITY AFTER MOTOR SKILL TRAINING IN OLDER ADULTS

Breanna Crane,[1] Kyle Moored,[2] Michelle Carlson,[3] Subashan Perera,[2] Jennifer Brach,[4] Brooke Klatt,[2] Andrea Rosso,[2] and Pamela Dunlap,[2], *1. Johns Hopkins Bloomberg School of Public Health, Baltimore, Maryland, United States, 2. University of Pittsburgh, Pittsburgh, Pennsylvania, United States, 3. Johns Hopkins University, Baltimore, Maryland, United States, 4. University of pittsburgh, Pittsburgh, Pennsylvania, United States*

The study purpose was to identify the effects of a motor skill training intervention to improve gait speed on community mobility among community-dwelling older adults. The study included 249 participants randomized to standard physical therapy or a standard plus motor skill training program. Community mobility was measured using the Life Space Assessment (LSA) and GPS at baseline, 12 (post-intervention), 24 and 36 weeks. There were 124 participants (M age=77.4±6.7; 68.6% female; LSA: 76.2±17.6) randomized to the standard plus and 125 (M age=77.4±6.4; 62.4% female; LSA: 74.3±18.2) to the standard group. There was no significant between-group difference in pre- or post-intervention LSA scores and no significant pre- to post-intervention change over time in either group. GPS results are pending. While there were no differences in self-reported LSA, we anticipate objective GPS measurement of community mobility will better capture post-intervention changes and differences between groups.

### Session 4525 (Symposium)

### COVID-19 AND PSYCHOSOCIAL CHANGES: RESULTS FROM THE NATIONAL HEALTH AND AGING TRENDS STUDY (NHATS)

Chair: Laura Samuel Discussant: Anthony Ong

The COVID-19 pandemic likely altered many aspects of daily life for older adults, including social connectedness, technology use, financial resources and hopefulness. This symposium examines these exposures and changes during the COVID-19 pandemic and tests their associations with health and related factors. Analyses are all conducted among a nationally representative sample of U.S. adults aged ≥65 years who participated in the NHATS COVID-19 supplement, which was a mail-in survey with participant and proxy respondents conducted between June and October of 2020. Additional NHATS participant data collected between 2011 and 2019 was used to account for individual characteristics before COVID-19, including demographic, socioeconomic and relevant health characteristics. Sampling weights were applied to analyses to account for study design and non-response so that inferences can be drawn to the US population of adults aged ≥65 years. This symposium will present results from five COVID-19 pandemic focused studies that examine the associations between 1) financial changes and health, 2) loneliness and behavioral changes, 3) hopefulness with function, sleep and loneliness, 4) technology use and mental health, and 5) predictors of technology use. These results offer insights into the mechanisms that influence health during the COVID-19 pandemic. Results have clinical, policy and public health implications because they can inform the development of interventions, programs and policies with potential to improve health and health care and advance health equity for older adults.

### FINANCIAL CHANGES AND HEALTH DURING COVID-19 IN THE NATIONAL HEALTH AND AGING TRENDS STUDY

Melissa Hladek,[1] Thomas Cudjoe,[2] Brittany Drazich,[3] Qiwei Li,[1] Sarah Szanton,[4] and Laura Samuel,[4], *1. Johns Hopkins School of Nursing, Baltimore, Maryland, United States, 2. Johns Hopkins University School of Medicine, Baltimore, Maryland, United States, 3. Johns Hopkins University School of Nursing, Baltimore, Maryland, United States, 4. Johns Hopkins University, Baltimore, Maryland, United States*

This study tested associations between income decline and financial difficulty with mental health (lack of feeling anxious/depressed, recurring thoughts/nightmares, avoiding activities/thoughts, feeling jumpy/on guard) and sleep quality during COVID-19 among a national sample of 3,188 older adults. Approximately 8% of US older adults reported income decline and 6% reported financial difficulty. Although income decline and financial difficulty rates were both statistically significantly higher among those financially strained before COVID-19 (19% and 34%, respectively), income decline was more common among those with incomes ≥200% of the poverty threshold (9%) whereas financial difficulty was more common among those with incomes <200% poverty (10%). Adjusting for sociodemographic, health and depressive symptoms before COVID-19, financial difficulty was associated with worse mental health (b= -2.39, p<0.001) and sleep quality (b=-0.820, p<0.001), but income loss was not (b= -0.685, p=0.092 and b= -0.405, p=0.082, respectively). Timely interventions are needed for older adults reporting COVID-19 financial difficulty.

### LONELINESS AND BEHAVIORAL CHANGES DURING THE COVID-19 PANDEMIC

Qiwei Li,[1] Brittany Drazich,[2] Melissa Hladek,[1] Laura Samuel,[3] carl latkin,[4] Cynthia Boyd,[3] and Thomas Cudjoe,[5], *1. Johns Hopkins School of Nursing, Baltimore, Maryland, United States, 2. Johns Hopkins University School of Nursing, Baltimore, Maryland, United States, 3. Johns Hopkins University, Baltimore, Maryland, United States, 4. Johns Hopkins Bloomberg School of Public Health, Baltimore, Maryland, United States, 5. Johns Hopkins University School of Medicine, Baltimore, Maryland, United States*

Concerns for the health impact of loneliness, a risk factor for morbidity and mortality, have risen amid the COVID-19 pandemic. However, relationships between loneliness and behavioral changes remains unclear. Utilizing data from the National Health and Aging Trends Study COVID-19 Supplement, we examine the cross-sectional relationship between loneliness and self-reported increase in each of the following behaviors during the pandemic (n=2,924): walking, vigorous activity, eating, use of alcohol and tobacco, watching television and sleeping. Adjusting for age, race, education, activity of daily living limitations, and chronic conditions, loneliness was significantly associated with a higher odds of more eating (odds ratio- OR: 1.42, confidence intervals-CI: 1.24,1.62), sleeping (OR: 1.35, CI: 1.18,1.56), and watching television (OR: 1.45, CI: 1.30,1.61). These results indicate that during stressful times like our current pandemic, loneliness may lead to morbidity and mortality through sedentary behaviors.

### HOPEFULNESS DURING COVID-19: ASSOCIATIONS WITH FUNCTION, SLEEP, AND LONELINESS IN THE NHATS

Qiwei Li,[1] Laura Samuel,[2] Brittany Drazich,[3] Thomas Cudjoe,[4] Sarah Szanton,[2] and Melissa Hladek,[1], *1. Johns Hopkins School of Nursing, Baltimore, Maryland, United States, 2. Johns Hopkins University, Baltimore, Maryland, United States, 3. Johns Hopkins University School of Nursing, Baltimore, Maryland, United States, 4. Johns Hopkins University School of Medicine, Baltimore, Maryland, United States*

The associations between hopefulness and function, loneliness, and sleep have not been explored in a nationally representative sample of older adults. Additionally, COVID19 dramatically increased stress burden, potentially influencing these relationships. This study used National Health and Aging Trends COVID19 Supplement data (N=2,894 adults aged ≥ 65 years) to evaluate cross-sectional associations between hopefulness about the future during COVID19 with limitations in activities of daily living (ADLs) using a negative binomial model and one-item sleep and loneliness measures using ordered logistic models. Adjusting for age, race/ethnicity, and education and applying sampling weights, increased hopefulness was associated with better ADLs (b= -0.11, p-value=0.021), less loneliness (b=-0.32, p-value=<0.001), and better sleep (b= -0.27, p-value= <0.001). In the midst of a world-wide stressor, hopefulness was associated with better function and symptoms. This relationship is likely bidirectional and further longitudinal research is needed.

### THE ASSOCIATION BETWEEN OLDER ADULT TECHNOLOGY USE AND MENTAL HEALTH DURING THE COVID-19 PANDEMIC

Brittany Drazich,[1] Nancy Perrin,[1] Laura Samuel,[2] Melissa diCardi Hladek,[1] Sarah Szanton,[2] Thomas Cudjoe,[3] Janiece Taylor,[2] and Qiwei Li,[4], *1. Johns Hopkins University School of Nursing, Baltimore, Maryland, United States, 2. Johns Hopkins University, Baltimore, Maryland, United States, 3. Johns Hopkins University School of Medicine, Baltimore, Maryland, United States, 4. Johns Hopkins School of Nursing, Baltimore, Maryland, United States*

Physical distancing during the COVID-19 pandemic may impact the mental health of older adults, but technology use may buffer this impact. This study aimed to 1) examine changes in older adult technology use during the COVID-19 pandemic and 2) determine if technology use moderates the relationships between decreased in-person communication/activity and the mental health of older adults during the pandemic. Data were taken from the NHATS COVID-19 Round 10 (n= 3,188). Older adults engaged in more technology-based activity (b= .237, p<0.001), technology-based healthcare communication (b=.112, p<0.001), and technology-based food acquisition (b= .214, p<0.001) during the COVID-19 pandemic, compared to before. Technology use did not moderate the relationship between decreased in person personal communication (b= .021, p= 0.662)/activity (b= .045, p= 0.749) and mental health during the pandemic. Although older adults are utilizing technology more during the pandemic, it might not be protective against negative mental health outcomes from physical distancing.

### PREDICTING TECHNOLOGY USE AMONG OLDER ADULTS DURING THE COVID-19 PANDEMIC WITH COMMUNITY PARTICIPATION

Brittany Drazich,[1] Laura Samuel,[2] Thomas Cudjoe,[3] Melissa Hladek,[4] Sarah Szanton,[2] and Qiwei Li,[4], *1. Johns Hopkins University School of Nursing, Baltimore, Maryland, United States, 2. Johns Hopkins University, Baltimore, Maryland, United States, 3. Johns Hopkins University School of Medicine, Baltimore, Maryland, United States, 4. Johns Hopkins School of Nursing, Baltimore, Maryland, United States*

Technology use is important for older adults, particularly in the pandemic. The pattern of technology use among older adults varies significantly. We hypothesized that limitations of activities of daily living (ADL), wellbeing, and community participation of community-dwelling older adults before the pandemic would predict technology use during the pandemic. National Health and Aging Trends Study data on 2924 older adults were utilized. Adjusted for age, gender, race, education, marital status, and chronic conditions, previous well-being predicted more online social activities (OR=1.03, p =.03); previous ADL limitations predicted more telehealth use (OR=1.11, p=.014); and previous community participation predicted: learning new technologies (OR=1.46, p <.001), more telecommunication (OR=1.12, p=.007), more online social activity (OR=1.58, p<.001), and more telehealth use (OR=1.09, p= .04). The results of this study imply that high community participation promotes older adults' transition to technology use. Older adults with low participation may need extra attention for such a transition.

### Session 4530 (Symposium)

### CREATIVE ARTS THERAPIES TO ENHANCE MENTAL HEALTH OVER THE COURSE OF AGING: RESEARCH AND IMPLICATIONS

Chair: Shoshi Keisari Co-Chair: Nisha Sajnani
Discussant: Dovrat Harel

The creative arts therapies (CATs) are health care professions that involve the intentional and systematic use of the creative and expressive process of art making to optimize health and well-being. Visual arts, music, dance-movement, drama and poetry provide means of expression to help individuals understand, make sense of, and cope with life challenges within a therapeutic relationship. Older adults develop a better attitude toward CATs, as the creative processes position them as active productive contributors in their own communities, instead of "patients" or "clients". In this sense, CATs encourage participation, and address the negative attitudes and stigma that are sometimes associated with mental health services. This symposium aims to present a diverse picture of studies on CATs for the aging population. Dr. Nisha Sajnani will present a systematic review of studies on CATs for older adults experiencing depression; Dr. Dovrat Harel will present findings from a qualitative study that explored poems written by poetry groups of men in residential care facilities; Dr. Shoshi Keisari will present an evidence-based model that integrates life-review and drama therapy; Silvia Piol, Talia Elkarif and Giada Mola will present a cross-cultural study that explored the experiences of Italian and Israeli participants during an online intervention that focused on the creation of digital photo-collages during COVID-19; Finally, Racheli Lital Gvili will present a study focused on intergenerational music intervention, as a vehicle to bridge the gap between grandparents and grandchildren, which was conducted at the onset of the COVID-19 pandemic.

### CREATIVE ARTS INTERVENTIONS IN ADDRESSING DEPRESSION IN OLDER ADULTS: A SYSTEMATIC REVIEW

Kim Dunphy,[1] Felicity Baker,[1] Ella Dumaresq,[1] Katrina Caroll-Haskins,[2] Jasmin Eickholt,[3] Maya Ercole,[1] Girija Kaimal,[4] and Nisha Sajnani,[5], *1. University of melbourne, University of Melbourne, Victoria, Australia, 2. Holy Family University, Philadelphia, Pennsylvania, United States, 3. University of Applied Sciences, Würzburg, Bayern, Germany, 4. Drexel University, Philadelphia, Pennsylvania, United States, 5. New York University, New York City, New York, United States*

Depression experienced by older adults is proving an increasing global health burden, with rates as high as 27% in the USA. This is likely to increase in coming years as the number and proportion of older adults in the global population rises. Therefore, it is imperative that the effectiveness of approaches to the prevention and treatment of depression are understood. Creative arts interventions, including art, dance movement, drama, and music, are utilized internationally to reduce depressive symptoms in older adults and promote wellbeing. This includes interventions led by trained arts therapists as well as other health and arts professionals. This presentation will include a report of findings from a recent systematic review of the outcomes of four creative arts modalities (art, dance movement, drama, and music) with particular attention paid to processes of change documented in each modality.

### AGING AND MASCULINITY IN BIBLIOTHERAPY AND POETRY GROUPS FOR MEN IN LATE LIFE

Dovrat Harel, *Tel Hai College, Kidron, HaMerkaz, Israel*

The transition to residential care facility may symbolize the joining into the 'community of older people'. This may influence the ways men in late life construct their identities and the intersection of aging and masculinity. Due to some barriers, this experience may be difficult to express explicitly. Bibliotherapy aims to bridge this gap by using literature to address diverse issues in the form of reading and writing activities. In this presentation we will present "The Literary Parliament" project, in which bibliotherapy and poetry groups of men in late life were conducted in residential care facilities in Israel. We will present findings of a qualitative research which explored poems written by group participants, and the way these helped participants to express their perceptions and feelings of ageing and masculinity in the context of residential care. The use of bibliotherapy to encourage psychological discourse among men in late life will be discussed.

### INTEGRATING LIFE-REVIEW AND DRAMA THERAPY FOR COMMUNITY-DWELLING OLDER ADULTS: AN EVIDENCE-BASED MODEL

Shoshi Keisari, *University of Haifa, Department of Gerontology, Faculty of Social Welfare and Health sciences, Mount Carmel, HaZafon, Israel*

Drama therapy is a widely acknowledged way to explore life-stories in late life. This presentation will describe a new model for creative interventions, based on the results of four studies that provide multiple perspectives on the integration of life-review and drama therapy for community dwelling older adults. The results of two quantitative studies (n=55, aged 62-93; n=78, aged 63-96) suggest that the drama therapy interventions have robust therapeutic potential to enhance mental health while aging. The findings of two qualitative studies with therapists (n=8), participants (n=27; aged 63-96) and staff (n=13) provide a better understanding of the process, and support the mechanisms that lead to positive effects on mental health. Combining the results yielded a multidimensional model which points to three potential transformative routes: the evolution of the life-story, the evolution of improvised dramatic expression, and the expansion of social engagement.

### ONLINE DIGNITY THERAPY AND PHOTO COLLAGES: THE NARRATIVES OF ISRAELI AND ITALIAN OLDER ADULTS IN THE COVID-19 ERA

Shoshi Keisari,[1] Talia Elkarif,[2] Giada Mola,[3] Ines Testoni,[4] and Silvia Piol,[5], *1. University of Haifa, Department of Gerontology, Faculty of Social Welfare and Health sciences, Mount Carmel, HaZafon, Israel, 2. University of Haifa, School of Creative Arts Therapies, Faculty of Social Welfare and Health Sciences, Mount Carmel, HaZafon, Israel, 3. Università degli studi di padova, DPSS Department, padova, Veneto, Italy, 4. Università degli studi di padova, FISPPA Department, Section of Applied Psychology, Padova, Veneto, Italy, 5. Università degli Studi di Padova, Padova, Veneto, Italy*

The social isolation imposed by the Covid-19 pandemic has significantly affected older adults, and has impacted both their physical and mental health. The pandemic has led to an increase in ageism associated with poorer mental health and a lower sense of dignity, self-esteem and contribution to society. This cross-cultural study involved 24 participants from Italy and Israel aged 79 to 92. The aim was to develop a brief art-based online intervention to enhance the participants' sense of dignity and sense of meaning in life during this time of crisis. The process focused on the creation of digital photo-collages that captured the participants' values through three perspectives: their past experiences, legacy, and future perspectives. It employed an arts-based research methodology to explore the participants' experiences by analysing their relationship with the artistic expression, the photo collage, and its creative process.

### AN INTERGENERATIONAL ZOOM MUSIC THERAPY GROUP DURING COVID-19

Racheli Lital Gvili, *Bar-Ilan University, Bar-Ilan, University, Ramat Gan, HaMerkaz, Israel*

The COVID-19 pandemic has led to an increase in ageist attitudes and psychological distress and loneliness among older people. The social isolation exacerbated the intergenerational segregation between young and older adults, and has also been expressed within families, since grandparents could not meet their grandchildren in person. The present study involved an intergenerational music intervention, as a vehicle to bridge the gap between grandparents and grandchildren at the COVID-19 pandemic. 41 grandparents aged 56-80, and 45 grandchildren aged 9.9-11.8 took part. Of these, 21 pairs of grandparents and grandchildren participated in a weekly online intergenerational zoom music therapy group for eight weeks, and the rest constituted a waitlist-controlled group. All participants completed the same questionnaires during the same time periods before and after the intervention. The results point to the effectiveness of participation in the sessions in improving intergenerational connections and psychological well-being, and in reducing ageist attitudes and loneliness.

### Session 4535 (Symposium)

### EMERGING TELEHEALTH ADVANCES FOR MENTAL AND COGNITIVE HEALTH IN LATE LIFE

Chair: Cindy Woolverton Co-Chair: Patricia Bamonti
Discussant: Lauren Moo

Over the last year, mental health services offered virtually have increased significantly in response to COVID-19. The rapid adoption of telehealth practices has raised many questions about how to develop and deliver effective interventions for older adults targeting their mental and cognitive health. In this symposium, we present on the feasibility of mental and cognitive health interventions for older adults using telehealth methods with particular focus on how adoption of these telehealth tools have been impacted by the current pandemic. Dr. Touchett and colleagues will present data on the telehealth utilization disparities among older veterans with comorbid disabilities and discuss ethical considerations when providing care for older adults. Dr. Kornblith and colleagues will present pilot data on the feasibility of GOALS, a video telehealth cognitive remediation group intervention for older adults with cognitive and emotional dysfunction related to traumatic brain injuries. Dr. Gould and colleagues will present pilot data on the feasibility and preliminary efficacy of a brief video-delivered self-management intervention BREATHE for older veterans with anxiety disorders. Dr. Weiskittle and colleagues will present their work on the development and dissemination of a brief 8-week telephone group intervention for homebound older adults targeting social isolation. Dr. Jacobs and colleagues will share their findings of a telephone delivered mindfulness intervention for caregivers and persons with dementia. Finally, the discussant, Lauren Moo, MD, an expert in assessing the efficacy of telehealth interventions will tie findings together and provide directions for future research and innovation.

### TELEHEALTH DELIVERY OF GROUP FORMAT COGNITIVE REHABILITATION TO OLDER VETERANS WITH TBI

Sara Schweizer,[1] Kristine Yaffe,[2] Tatjana Novakovic-Agopain,[3] and Erica Kornblith,[3], *1. SFVA, San Francisco, California, United States, 2. UCSF, UCSF, California, United States, 3. SFVA/UCSF, San Francisco, California, United States*

Traumatic brain injury (TBI) is common among older adults, with significant public health costs, and advanced age is a risk factor for poor outcomes after TBI. Older Veterans with TBI-related cognitive and emotional dysfunction without dementia may benefit from cognitive rehabilitation, particularly executive function training, and technology may promote optimal functioning for these patients by increasing access to such treatments. Dr. Kornblith will present pilot data on one such promising group intervention, Goal-Oriented Attentional Self-Regulation (GOALS), administered via in-home video telehealth. Themes gleaned from qualitative feedback collected throughout the intervention and post-treatment feedback questionnaires include the importance of communication and a smooth process with clear instructions for joining study sessions. Preliminary data suggest that only minor adaptions to the existing GOALS protocol are required for telehealth delivery and that delivering group-based executive function training to older TBI-exposed older Veterans with cognitive complaints via telehealth is feasible and acceptable.

### BRIEF VIDEO-DELIVERED INTERVENTIONS TO REDUCE ANXIETY IN OLDER VETERANS: A PILOT RCT

Chalise Carlson,[1] Lauren Anker,[1] Ruth O'Hara,[2] Julie Wetherell,[3] Mary Goldstein,[4] Sherry Beaudreau,[5] and Christine Gould,[1], *1. VA Palo Alto Health Care System, Palo Alto, California, United States, 2. Stanford University School of Medicine, Palo Alto, California, United States, 3. VA San Diego / UCSD, La Jolla Village, California, United States, 4. VA Central Office, Palo Alto, California, United States, 5. VA Palo Alto / Stanford University School of Medicine, VA Palo Alto, California, United States*

Older Veterans with anxiety disorders encounter barriers to receiving mental health services that may be overcome by using brief technology-delivered interventions. To address this, we conducted a pilot randomized controlled trial (RCT) comparing the effects of a guided self-management intervention called BREATHE, a 4-week video-delivered (DVD/internet) intervention and a psychoeducation control (Healthy Living; HL) on anxiety symptom severity. Older Veterans with anxiety disorders (N = 48; 87.5% men; Mean age = 71.77 ± 6.2 years) were randomized to BREATHE or HL. Regarding intervention delivery modality, 67% used DVDs, 23% used the internet, 4% used both to access their assigned intervention. Both groups experienced significant declines in affective anxiety from baseline to 8 weeks followed by an increase in symptoms (i.e., quadratic pattern). HL had significant declines in somatic anxiety, whereas BREATHE did not experience such declines. The longitudinal effects and Veteran satisfaction will be further described in the presentation.

### ADJUNCTIVE TELEHEALTH MINDFULNESS THERAPY FOR PERSONS WITH DEMENTIA AND THEIR CAREGIVERS IN THE RURAL DEEP SOUTH

Daniel Durkin,[1] Michelle Hilgeman,[2] and Lindsey Jacobs,[3], *1. The University of Mississippi, University, Mississippi, United States, 2. Tuscaloosa VA Medical Center, Tuscaloosa, Alabama, United States, 3. The University of Alabama, The University of Alabama, Alabama, United States*

The emotional care needs of persons with dementia (PwD) and their caregivers are multitudinous. Multicomponent interventions may be necessary to meet their multiple needs. Mindfulness interventions have a positive impact on well-being but are often only offered as a stand-alone treatment and typically are available only to the caregiver. This presentation will describe a telephone-delivered adjunctive mindfulness intervention that was offered to caregivers and dyads in conjunction with care consultation. Participants were 26 caregivers and 22 PwD living in the Deep South. The adjunctive mindfulness therapy included four core sessions and an additional five sessions that were optional. Mindfulness was deemed to be a "good fit" for almost 75% of the sample. Duration of mindfulness sessions ranged from 30 to 65 minutes. Participants attended more sessions as a dyad (M=10.10) compared to caregivers alone (M=6.5). Information regarding attendance and treatment engagement will be presented.

### USHERING IN THE SILVER AGE OF TELEHEALTH: ADDRESSING TELEHEALTH DISPARITIES FOR OLDER ADULTS WITH DISABILITIES

Hilary Touchett, *VA, Houston, Texas, United States*

One silver lining of COVID-19 has been the ushering in of 'the golden age of telehealth'. However, this unplanned rapid conversion to telehealth left many providers and clinics unprepared to address systemic barriers that adversely affect older adults, particularly those with disabilities. Data from the VA Corporate Data Warehouse suggest that the rapid adoption of telehealth in mental health clinics during COVID-19 widened telehealth utilization disparities for older Veterans (65+) with disabilities. With 4.5 million Veterans 55+ who have at least one disability more attention to addressing this widening gap is needed. For those with hearing, vision, and complex mobility impairments, there are unique challenges to initiating telehealth services. Dr. Touchett will present preliminary findings while discussing ethical and contextual considerations when using telehealth with older Veterans who have disabilities, while discussing ways to facilitate robust clinical encounters for this population.

### DISSEMINATION OF A COVID-19 RAPID RESPONSE TELEHEALTH GROUP ADDRESSING WORRY AND SOCIAL ISOLATION AMONG OLDER ADULTS

Rachel Weiskittle, *VA Boston Healthcare System, Jamaica Plain, Massachusetts, United States*

In response to the urgent need for virtual mental health treatments during the COVID-19 pandemic, an 8-week group intervention deliverable over video or telephone was developed and disseminated in March 2020. Manual content addressed social isolation and information related to COVID-19. In August 2020, a national web-based provider feedback survey was disseminated to evaluate feasibility of the manual. Respondents (n = 21) across a variety of geriatric mental health clinics reported this intervention to be effective and clinically useful with their patients in providing social support and in mitigating COVID-19 anxieties. The majority of respondents delivered the group in multiple cohorts and found the manual adaptable beyond the early pandemic period.

### Session 4540 (Paper)

### EMPLOYMENT, RETIREMENT, AND AGING

### HEALTH AND WORKING BEYOND RETIREMENT AGE: EXPLORING RACIAL AND GENDER INTERSECTIONALITY

Ronica Rooks,[1] and Allison Leanage,[2], *1. University of Colorado Denver, Denver, Colorado, United States, 2. McMaster University, Hamilton, Ontario, Canada*

Little longitudinal research exists on health and working among older racial and ethnic minority adults. Following previous cross-sectional research, we examine the Health, Aging, and Body Composition (HABC) study comparing working vs. not working overtime among older adults. We hypothesize: 1) Black vs. White adults are more likely to work; 2) Black vs. White differences in working are greater among women than men; and 3) Working relates to fewer prevalent health problems than not working. We used gender-stratified descriptive statistics and generalized mixed-effects logistic regression with covariate adjustments to analyze the HABC cohort study, with community-dwelling, well-functioning Black (42%) and White older adults aged 70-79 in year 1 (n=3,069) to year 6 (n=2,091). We found support for all three hypotheses. Black vs. White adults were more likely to work overtime. Women were less likely to work overtime compared to men. White women were less likely to keep working compared to men and Black women. Lastly, older adults with fewer chronic conditions were more likely to continue working. Our study finds racial and gender differences among older adults working overtime. Intersectionality plays a role in older adults' health and work disparities, leading us to explore the needs and/or benefits of working past retirement in specific groups. Our policy implication is for society to pro-actively invest in older adults' health and productive activities, which may act as social determinants of health solutions to reduce disparities and growing social safety net program costs.

### LOST WORK OPPORTUNITY SCORE PREDICTS HEALTH IN RETIREMENT

Maren Wright Voss,[1] Man Hung,[2] lorie Richards,[3] Wei Li,[3] Pollie Price,[3] Alexandra Terrill,[3] and Lori Wadsworth,[4], *1. Utah State University, Salt Lake City, Utah, United States, 2. Roseman University, South Jordan, Utah, United States, 3. University of Utah, Salt Lake City, Utah, United States, 4. Brigham Young University, Provo, Utah, United States*

**Objectives:** Under-reporting of unemployment or forced retirement has consequences for measuring the impacts of job changes on health at retirement. We analyzed a comprehensive three-part measure of lost work opportunity for evidence of impact on health.

**Methods:** We combined variables from the Health and Retirement Study for 2,576 respondents assessing unemployment, forced retirement, and earlier than planned retirement into a single lost work opportunity score (LOS). We evaluated the reliability and unidimensionality of the LOS. We conducted multivariate regression to assess health impacts controlling for age, gender, education, race, ethnicity, and prior health status.

**Results:** The Cronbach's Alpha for the LOS was a = 0.76 and the LOS variables primarily loaded onto a single component demonstrating undimensionality. The LOS significantly predicted self-reported health (□ = .16; p < .001) with higher lost work associated with negative health outcomes (Cox and Snell R2 = 0.07). The LOS score significantly predicted mental health declines (□ = .07; p = .002)(Cox and Snell R2 = 0.07).

**Discussion:** Population-level data indicates that health declines following both unemployment and retirement, but there is ample evidence that early or planned retirements do not show the same negative health impacts. We examined the health impact of retirement using the construct of lost work opportunity rather than voluntary or involuntary retirement, per se. Our findings indicate that as much as 7% of negative health changes in the early retirement years could be attributable to employment changes that were unplanned or experienced as outside the retiree's control.

### PROFIT STATUS AND EMPLOYEE TURNOVER IN IOWA NURSING HOMES

Hari Sharma,[1] and Lili Xu,[2], *1. The University of Iowa, Iowa City, Iowa, United States, 2. University of Iowa, Iowa City, Iowa, United States*

Employee turnover is a huge concern for nursing homes that care for millions of older individuals whose physical and cognitive impairments make them vulnerable, especially in the middle of a pandemic like COVID-19. Existing research has shown that high turnover of employees can lead to poorer quality of care. Low pay is often cited as one of the key reasons for high turnover of employees in nursing homes. For-profit nursing homes may try to maximize profits by limiting wages paid to their employees. In this study, we examine whether profit-status of a facility is associated with high turnover of its employees. We obtain data on 415 nursing homes operating in Iowa between 2013-2017. We descriptively examine the turnover trends in nurse employees and all employees over time by profit status. We evaluate whether profit status is associated with high turnover using pooled linear regressions controlling for nursing home and resident characteristics. Descriptive results show that for-profit facilities had higher turnover of nurse employees (61.1% vs. 49.6%) and all employees (56.6% vs. 45.4%). Results from multivariate regressions show that, compared to non-profit facilities, for-profit facilities had 6.93 percentage points higher (p<0.01) turnover of all employees, and 7.76 percentage points higher (p<0.01) turnover of nurse employees after controlling for facility and resident characteristics. Given existing evidence on the adverse impact of high employee turnover on nursing home quality, we need policies aimed at lowering employee turnover, targeting for-profit nursing homes.

### Session 4545 (Paper)

### END-OF-LIFE PLANNING AND BEREAVEMENT

### ADVANCE DIRECTIVE COMPLETION AND HOSPITAL OUT-OF-POCKET EXPENDITURES

YuJun Zhu, and Susan Enguidanos, *University of Southern California, Los Angeles, California, United States*

Healthcare costs remain high at end of life. Although advance directives (AD) have been shown to improve care congruence with patients' preferences and lower cost of healthcare services, little is known about the relationship between AD completion and hospital out-of-pocket costs. This study examined whether AD completion was associated with lower hospital out-of-pocket spending at end of life. We used the Health and Retirement Study participants who died between 2000 and 2014 (N=9,228) to examine the association through the use of a two-part analytic model that has been widely used in health economics. We controlled for socioeconomic status, death-related characteristics, and health insurance coverage and imputed missing data using multiple imputation by chained equations. Of the 43.9% of decedents who completed an AD, 90.7% chose to limit care or to be kept comfortable; 78.8% indicated that they wanted to withhold treatment, and 5.6% wanted to prolong life. Having an AD was significantly associated with $632 (95% CI: [-$1,116.47, -$146.71]) lower hospital out-of-pocket costs, with greater savings among younger decedents, dropping from $1,560 (95% CI: [-$2,652, -$268]) at age 50 to $230 (95% CI: [-$445, -$14]) at age 110. Decedents who completed an AD 12 months or less before death had higher out-of-pocket spending ($1,591 on average) than those who completed more than a year before death ($1,001 on average). Our findings have policy implications for physician-patient communication about costs of care and may provide an opportunity for physicians to involve cost-sharing discussions when completing ADs with patients.

### BEREAVEMENT SUPPORT SERVICES IN A NATIONAL SAMPLE OF HOSPICES: A CONTENT ANALYSIS

Todd Becker, and John Cagle, *University of Maryland, Baltimore, Baltimore, Maryland, United States*

Although the Medicare Hospice Benefit mandates that hospices provide bereavement services to families for 1 year following death, it does not stipulate what services should be offered or how. Thus, this study aimed to explore the range of hospice bereavement services. This study stems from Cagle et al.'s (2020) prior study surveying 600 randomly selected agencies, stratified by state and profit status. Most participants (N = 76) worked as clinical supervisors or directors of patient services (41.6%) for medium-sized (53.2%), for-profit hospices (50.6%). Responses to "What types of bereavement support does your hospice provide to families?" were content analyzed. Analyst triangulation and peer debriefing enhanced trustworthiness. Four domains emerged: timing of support, providers of support, targets of support, and formats of support. Each domain reflected substantial variability. All hospices offered postdeath bereavement support. A minority described offering predeath support, often through bereavement risk assessment and supportive services targeting those at risk. Providers frequently included trained bereavement counselors, social workers, and chaplains. Less often, hospices leveraged familiar members of the decedents' care team to encourage family participation. Although bereavement services predominantly targeted surviving adult family members of deceased hospice patients, services tailored to children and hospice-unaffiliated community members also emerged. The format of bereavement services demonstrated the widest variability. Commonly reported formats included written materials, support groups, and phone calls. Most hospices employed multiple formats. Although findings are consistent with prior research, the variability in each domain complicates rigorous investigation of which aspects offer the greatest benefit to bereaved family members.

### EXPLORING THE CONCEPT OF THE ETHICAL WILL AS A WAY TO LEAVE A LEGACY OF VALUES: A SCOPING REVIEW

Sarah Neller,[1] Gail Towsley,[2] and Mary McFarland,[1], *1. University of Utah, Salt Lake City, Utah, United States, 2. University of Utah College of Nursing, Salt Lake City, Utah, United States*

Ethical wills communicate a legacy of values through non-legal emotional and supportive instruction to others and are distinct from legal or living wills. Employed for centuries, little is known about how and why ethical wills are used. We conducted the first scoping review on ethical wills to survey the breadth of published information and identify how they are defined and utilized. We followed the Joanna Briggs Institute methodology for scoping reviews employing an a priori protocol and PRISMA-ScR reporting guidelines . We searched 14 databases in November 2019 and January 2021 without filtering publication date or type. Our final extraction form included frequently used terms describing content, purpose, and outcomes. Two reviewers independently screened 1,568 results. Final extraction included 51 documents from 1997-2020, which were primarily published in lay or peer-reviewed journals within law, estate and financial planning, and religion; only 6 research articles were identified. Most frequently, descriptors characterized ethical wills as a non-material legacy of values, beliefs, wisdom, and life lessons learned written to family or future generations. Ethical wills were utilized most to be remembered, address mortality, clarify life's meaning, and communicate what matters most. They provided opportunity to learn about self, were considered a gift to both writer and recipient and fostered intergenerational interaction and transcendence. Our findings highlight interdisciplinary utilization and dearth of research on ethical wills. Gerontological research is needed to explore ways ethical wills can be used to enhance generativity and intentional living as individuals age and prepare for the end of life.

### FAMILY CONFLICT AND OLDER CHINESE AMERICANS' SELF-EFFICACY IN END-OF-LIFE CARE PLANNING: THE ROLE OF ACCULTURATION

Kaipeng Wang,[1] Fei Sun,[2] Yanqin Liu,[3] and Carson De Fries,[1], *1. University of Denver, Denver, Colorado, United States, 2. Michigan State University, East Lansing, Michigan, United States, 3. Mayo Clinic Arizona, Scottsdale, Arizona, United States*

Family involvement in end-of-life (EOL) care is critical to ensure older adults' health and quality of life. Older adults' self-efficacy in discussing EOL care plans with family members can facilitate family involvement in EOL care planning. Research shows that family relationships are associated with self-efficacy in discussing EOL care with family members among older Chinese Americans. However, the roles of family conflict and acculturation remain unknown. This study examines the association between family conflict and self-efficacy in discussing EOL care with family members and whether such an association differs by acculturation levels among older Chinese Americans. Data were collected from 207 Chinese Americans aged 65-102 in two metropolitan areas in 2017. Ordinary least squares regression was conducted to examine the association between family conflict, acculturation, and self-efficacy in discussing EOL care with family. Family conflict was negatively associated with older adults' self-efficacy in discussing EOL care with family. More specifically, the negative association between family conflict and self-efficacy in discussing EOL care with family members was more pronounced for those with higher levels of acculturation. Findings highlighted differential effects of family conflict on self-efficacy of EOL care plan discussion for older adults with different acculturation levels. Those with higher acculturation may be more independent in their EOL care planning and aware of the possible negative effects of family conflict in their EOL care planning discussions. Acculturation needs to be considered by geriatric health providers to develop family-centered interventions in improving end-of-life care planning for this population.

### THE EFFECTS OF RELIGIOSITY ON DEPRESSION TRAJECTORIES BEFORE AND AFTER WIDOWHOOD

Frances Hawes,[1] Jane Tavares,[2] Corina Ronneberg,[3] and Edward Miller,[4], *1. University of Wisconsin-Eau Claire, eau claire, Wisconsin, United States, 2. LeadingAge LTSS Center @UMass Boston, Boston, Massachusetts, United States, 3. University of Illinois Chicago, Chicago, Illinois, United States, 4. University of Massachusetts Boston, Boston, Massachusetts, United States*

Widowhood is associated with decreased emotional well-being, particularly increased depression. Religiosity may help improve mental health among widowed individuals. However, longitudinal studies exploring the role of religiosity on emotional well-being among widowed older adults is lacking, as are studies which examine this relationship using different dimensions of religiosity. This study analyzed data from the 2006-2016 waves of the nationally representative Health and Retirement Study (HRS). Trajectories of depression among older adults >50 years (N=5,486) were examined to explore patterns of depression among those entering widowhood and the potential impact of religiosity on depressive symptoms during widowhood. Ordinary least squares (OLS) regression analysis was used to examine the association between widowhood and depression as well as the role of religiosity as a moderator of this association. Older adults experienced an increase in depressive symptomology after the onset of widowhood, and although the levels of depressive symptomology decrease post-widowhood, they do not return to their pre-widowhood levels. Additionally, high religious service attendance and higher intrinsic religiosity were both associated with lower depressive symptomology. High religious service attendance moderated the relationship between widowhood and depression. The relationship between high religious service attendance and depression was stronger among widowed older adults living alone. This study highlights the long-term effects of widowhood on depressive symptomology among older adults. The findings also suggest that higher religious service attendance can lessen the effects of widowhood on depressive symptoms, especially for those living alone. These findings may inform intervention development around increased screening and treatment for depression.

### Session 4550 (Paper)

### FRAILTY TRAJECTORIES

### 8-YEAR CHANGES IN FRAILTY IN ADULTS: LINKS TO COGNITIVE AND PHYSICAL FUNCTION AND MORTALITY

Felicia Simpson,[1] Jamie Justice,[2] Judy Bahnson,[3] Joni Evans,[3] Kathleen Hayden,[3] Stephen Krtichevsky,[2] Karen Johnson,[4] and Mark Espeland,[3], *1. Department of Mathematics, Winston-Salem State University, Winston-Salem, North Carolina, United States, 2. Wake Forest School of Medicine, Wake Forest School of Medicine, North Carolina, United States, 3. Wake Forest School of Medicine, Winston-Salem, North Carolina, United States, 4. University of Tennessee Health Science Center, Memphis, Tennessee, United States*

Deficit accumulation frailty indices are being evaluated as clinical markers of biological aging. In this context, it is to be expected that changes over time in such indices should be predictive of downstream changes in cognition, physical function, and mortality. We derived a frailty index (FI) based on deficit accumulation in 38 functional, behavioral, and clinical characteristics and examined associations between 8-year changes in FI and subsequent standardized measures of cognitive and physical function and mortality collected over years 8-18. We drew data from the Look AHEAD clinical trial of a multidomain intensive lifestyle intervention (ILI) in 3841 adults, aged 45-76 years at baseline with overweight/obesity and type 2 diabetes mellitus. Greater FI increases tended to occur among individuals who were older, non-Hispanic White, heavier, and who had greater baseline multimorbidity. Greater increases in FI were associated with subsequently worse levels of composite cognitive function and 400m walk speed (all p<0.001). Additionally, compared with the lowest tertile of FI change, hazard ratios [95% confidence intervals] for 10-year mortality for the middle and highest tertiles of FI change were 1.28 [1.03.1.58] and 1.56 [1.24,1.96], respectively. While assignment to ILI was associated with smaller 8-year increases in FI, this did not translate overall to better cognitive functioning compared to the Diabetes Support and Education control condition across years 8-18. Increase in FI over 8 years predicts subsequent reduced function and greater mortality. However, whether interventions generally targeting FI reduce risks for downstream outcomes remains to be seen.

### ASSOCIATION OF FRAILTY AND SUICIDE IN ADULTS 65 YEARS AND OLDER

Randall Kuffel,[1] Ruth Morin,[2] Kenneth Covinsky,[3] John Boscardin,[3] Yixia Li,[4] and Amy Byers,[3], *1. San Francisco Veterans Affairs Health Care System, San Francisco, California, United States, 2. John D. Dingell VA Medical Center, Detroit, Michigan, United States, 3. University of California, San Francisco, San Francisco, California, United States, 4. San Francisco VA Health Care System, San Francisco, California, United States*

Nearly 10,000 adults aged 65 years and older die by suicide in US annually. Although prior studies have linked individual diagnostic factors to late-life suicide risk, to our knowledge none have examined how accumulated health burden affects suicide risk. Such a metric could be studied utilizing a frailty index (FI). Our primary study objective was to determine the relationship of FI to risk of suicide. We examined a longitudinal cohort of 2,858,876 veterans 65 years and older from fiscal year 2012-2013 (baseline) through 12/31/2017, linking the VA's suicide and mortality databases with medical record data. FI was defined by 31 variables, including morbidity, function, cognition, mood, sensory loss, chronic pain, and failure to thrive. We used Fine-Gray proportional hazards regression to examine time to suicide attempt (fatal and non-fatal). Our sample's average age was 75 (SD 8), 88% White, 9% Black, and 98% male. Thirty-seven percent of veterans were non-frail, 30% were pre-frail, 17% mildly frail, 9% moderately frail, and 7% severely frail. Over the course of the study, 9,043 veterans had a documented suicide attempt with >60% fatal. After adjusting for race, gender, region, substance use disorder, and PTSD, risk of suicide attempt increased across frailty categories: Hazard ratios increased from 1.37 (95%CI: 1.30-1.45) for pre-frail individuals to 1.57 (1.43-1.72) for severely frail individuals. We found similar results after further adjustment for the Charlson Comorbidity Index, suggesting cumulative deficit FI may be a strong prognostic marker for risk of suicide in adults over 65; informing late-life suicide prevention efforts.

### FRAILTY AND MORTALITY IN A COMMUNITY-DWELLING RELATIVELY HEALTHY OLDER POPULATION

A R M Saifuddin Ekram,[1] Joanne Ryan,[2] Sara Espinoza,[3] Michael Ernst,[4] Anne Murray,[5] and Robyn Woods,[2], *1. Monash University, Monash University, Melbourne, Victoria, Australia, 2. Monash University, Melbourne, Victoria, Australia, 3. University of Texas Health Science Center San Antonio, San Antonio, Texas, United States, 4. University of Iowa, Iowa City, Iowa, United States, 5. Hennepin HealthCare Research Institute, Hennepin HealthCare, Minneapolis, Minnesota, United States*

This study examined factors associated with frailty and studied the association between frailty status and mortality in healthy community-dwelling older persons. Participants included 19,114 individuals from the "ASPirin in Reducing Events in the Elderly" (ASPREE) trial. Frailty was defined using modified Fried phenotype comprising exhaustion, body mass index, grip strength, gait speed and physical activity. A deficit accumulation frailty index (FI) using 66 items was also developed. Correlates of frailty were examined using multinomial logistic regression. The association between frailty status at baseline and mortality was analyzed using Cox regression. At baseline, 39.0% (95% CI: 38.3, 39.7) of participants were prefrail, and 2.2% (95% CI: 2.0, 2.4) were frail according to Fried phenotype, while 40.6% (95% CI: 40.0, 41.3) of participants were pre-frail and 8.1% (95% CI: 7.7, 8.5) were frail according to FI. Older age, female sex, lower education, African-American and Hispanic ethno-racial status, smoking, alcohol use, comorbidities, and polypharmacy were associated with frailty status. Pre-frailty increased risk of all-cause mortality significantly (Fried HR: 1.48; 95% CI: 1.28, 1.71; FI HR: 1.54; 95% CI: 1.31, 1.81); and the risk was even higher for frailty (Fried HR: 2.24; 95% CI: 1.67, 3.00; FI HR: 2.34; 95% CI: 1.83, 2.99) after adjustment for covariates. Cardiovascular disease (CVD) and non-CVD-related mortality showed similar trends. These results highlight a considerable burden of pre-frailty among a large group of community-dwelling, initially healthy older adults. Both Fried phenotype and deficit accumulation FI similarly predicted all-cause, CVD and non-CVD-related mortality in relatively healthy older adults.

### IMPACT OF PHYSICAL AND SOCIAL FRAILTY ON THE UTILIZATION OF NURSING CARE SERVICES IN VERY OLD ADULTS

Jaroslava Zimmermann, *University of Cologne, Cologne, Nordrhein-Westfalen, Germany*

Frailty, characterized by increased vulnerability to external stressors, has been found to increase the risk of healthcare utilization and nursing home admission. As the age group of 80 years or older remains frequently underrepresented in previous research, this study examined the impact of physical and social frailty on the utilization of nursing care services in very old population of North Rhine-Westphalia. Using data from a representative cross-sectional study, 1,577 community-dwelling and institutionalized individuals aged ≥80 years were included. Physical frailty was defined according to Fried's criteria (exhaustion, weight loss, low handgrip strength, low physical activity). Social frailty was measured with self-reported loneliness, social isolation, and time spent with others.

The use of outpatient care services, day care, informal and inpatient care were considered. Multinomial regression was applied to investigate the impact of physical and social frailty on the use of outpatient and inpatient care services, controlling for relevant sociodemographic and health related characteristics. Compared to very old adults who did not use any care services, no association was found between frailty and the use of outpatient or informal care. Comparing nonusers of care services with institutionalized individuals, nursing home residents were less likely to experience physical frailty and pre-frailty, but were more likely to be socially isolated and to feel lonely. These findings suggest that physical frailty might have been successfully prevented in the context of institutional inpatient care. However, early identification and intervention focused on social inclusion of the institutionalized very old individuals are needed to reverse social frailty.

### Session 4555 (Paper)

### FRIENDSHIP IN LATER LIFE

### AN EVALUATION OF SOCIAL BRIDGING AND BONDING MECHANISMS IN THE ASSOCIATION OF SOCIAL NETWORKS AND COGNITIVE FUNCTION

Brea Perry,[1] Siyun Peng,[1] Adam Roth,[1] Max Coleman,[1] and Hope Sheean,[2], *1. Indiana University, Bloomington, Indiana, United States, 2. Indiana University, Indianapolis, Indiana, United States*

**Background and Objectives:** Substantial evidence links social connectedness prospectively to cognitive aging outcomes, but there is little agreement about the social processes or mechanisms that drive this relationship. This study evaluated nine measures of social connectedness, focusing on two distinct forms of social enrichment – access to an expansive and diverse set of loosely connected individuals (i.e., social bridging) and integration in a supportive network of close ties (i.e., social bonding). Research Design and

**Methods:** This study used egocentric social network and clinical cognitive data from 311 older adults in the first wave of the Social Networks in Alzheimer Disease (SNAD) study. Linear regressions adjusting for gender, age, education, and depression symptoms were used to estimate the association between nine measures of social connectedness and global cognitive function, verbal memory, and attention.

**Results:** Measures indicative of social bridging (larger network size, lower density, presence of weak ties, and proportion non-kin) were consistently associated with better cognitive outcomes, while measures of social bonding largely produced null effects.

**Discussion and Implications:** These findings suggest that the protective benefits of social connectedness for cognitive function and memory may operate primarily through a cognitive reserve mechanism that is driven by irregular contact with a larger and more diverse group of peripheral others. Population-level interventions that promote the cultivation of social bridging relationships and activities may have benefits for cognition later in life.

### BIG AND MINI: A PROMISING PROGRAM TO LINK GENERATIONS TO COPE WITH SOCIAL ISOLATION

Zhirui Chen,[1] Ling Xu,[1] Noelle Fields,[1] Allen Zhou,[2] Anthony Zhou,[3] and Aditi Merchant,[2], *1. University of Texas at Arlington, Arlington, Texas, United States, 2. The University of Texas at Austin, Austin, Texas, United States, 3. The University of North Texas, Denton, Texas, United States*

**Introduction:** Social isolation disproportionately affected older adults prior to and especially during the COVID-19 pandemic. To help older adults cope with social isolation, a new program "Big and Mini" was created in April 2020 to link young and older adults together (matched through a custom website developed for this program) and to help increase social connectivity through weekly phone calls. Using a survey with both closed and open questions, this study evaluated participant feedback three months after the program was launched.

**Methods:** 63 Bigs (age 50+) and 53 Minis (age 18+) completed the survey. Stress compared to before COVID-19, social isolation, life satisfaction, intergenerational solidarity, and satisfaction with the program were measured for both the Big and Mini participants. Descriptive, bivariate correlation, group comparison and conventional content analyses were conducted.

**Results:** Results showed that 38.1% of Bigs and 37.7% of Minis felt higher levels of stress than before COVID-19. Both Bigs and Minis had medium levels of social isolation. They also reported high levels of satisfaction with life, satisfaction with the program, and intergenerational solidarity. Content analysis suggested that the reasons to join or expectations of the program were curiosity, friendship, mutually beneficial intergenerational connections, and coping with loneliness. Both Bigs and Minis reported benefits from the learning and sharing opportunities that the program offered.

**Conclusions:** The Big and Mini program offers a promising approach with mutual benefits for both Bigs and Minis. Strategies to improve the program and implications for other phone-based intergenerational programs are presented.

### BUILDING FRIENDSHIPS THROUGH VOLUNTEERING IN LATE LIFE: DOES GENDER MODERATE THE RELATIONSHIP?

Emily Lim,[1] Changmin Peng,[1] and Jeffrey Burr,[2], *1. University of Massachusetts Boston, Boston, Massachusetts, United States, 2. McCormack Graduate School, Boston, Massachusetts, United States*

Friendship plays a crucial role in maintaining social connectedness in late life. Volunteering helps older adults to stay socially engaged and often times provides the opportunity to meet and make new friends. A small literature suggests that volunteering may be associated with friendship, but many studies are limited by reliance on small, non-probability samples and simplistic analytic approaches. The literature is also unclear on how volunteering behaviors relate to specific characteristics of friendships and whether there are gender differences that condition these relationships. Using the 2014 and 2018 waves of the Health and Retirement Study (N=1,638 ), we investigate whether volunteer status and hours volunteered in 2014 are associated with friendship characteristics in 2018 (i.e., number of close friends, friendship quality, and contact frequency) among community-dwelling adults aged 50 years and above (M=65.60 years old, SD=8.31). We also examine whether gender moderated these relationships. Volunteer status and hours in 2014 were positively associated with the number of close friends and contact frequency in 2018. Only those who volunteered 200 hours or more in

2014 were positively associated with friendship quality in 2018. Regarding gender differences, men who volunteered 200 hours or more in 2014 had higher friendship quality in 2018 than women, while women who volunteered 100-199 hours in 2014 had greater contact frequency in 2018 than men. Hence, our results suggest volunteering is integral in shaping late-life friendships and volunteering might be more critical for understanding friendship characteristics among older men and women.

### SOCIAL ISOLATION AND SLEEP QUALITY OF OLDER ADULTS IN CHINA: DO FAMILY AND FRIENDSHIP ISOLATION DIFFER?

Dan Zhang,[1] Zhiyong Lin,[2] Feinian Chen,[3] and Shuzhuo Li,[4], *1. Xi'an Jiaotong University, Xi'an, Shaanxi, China (People's Republic), 2. The University of Texas at Austin, Austin, Texas, United States, 3. University of Maryland College Park, University of Maryland, Maryland, United States, 4. Xi'an Jiaotong University, Xi'an, Shaanxi, China (People's Republic)*

This study provides one of the first population-based studies investigating associations between social isolation, especially its two sub-dimensions (family isolation and friendship isolation), and sleep quality among older adults in China. We address three major research questions: 1) Does the risk of poor sleep quality vary by social isolation status? 2) Are the associations between social isolation and sleep quality mediated by mental disorders (depressive symptoms and loneliness) and physical impairments (pain and comorbidity)? and 3) Does the isolation from family members and friends differ in explaining sleep quality? We analyzed data from the 2014 wave of the China Longitudinal Aging Social Survey (CLASS), in which 7,597 respondents (aged 60-98) had complete information on measures of sleep quality (self-rated sleep difficulty), social isolation (using the Lubben Social Network Scale), and other analytical variables. Logistic regression models were estimated to predict the risk of sleep difficulty and Karlson-Holm-Breen (KHB) decomposition method was employed to test potential mediating effects. Results showed that social isolation, both family and friendship isolation, was significantly associated with higher risks of having sleep difficulty. The adverse effect of family isolation was found to be stronger than that of friendship isolation. Although both mental disorders and physical impairments mediated significant shares of associations between social isolation and sleep quality, physical impairments explained a lesser extent of them than mental disorders. These findings will be helpful for health policymakers and practitioners to design effective intervention strategies to help older adults with sleep problems.

### Session 4560 (Symposium)

### FROM DAY TO DAY TO YEAR TO YEAR: DEVELOPMENTAL ANTECEDENTS AND OUTCOMES OF CHANGES IN EMOTION

Chair: Jeremy Hamm Co-Chair: Meaghan Barlow

Research shows that emotions play an important role in successful aging. However, less is known about how day-to-day fluctuations and multi-year changes in positive and negative emotions are implicated in adaptive development. Thus, the present studies address the developmental antecedents and outcomes of micro- and macro-longitudinal changes in different positive and negative emotions. Blöchl, Oertzen, and Kunzmann use 12-year data from the Health and Retirement Study to examine whether socioeconomic resources influence trajectories of positive emotion and physical functioning and their interrelations. Hamm, Wrosch, Barlow, and Kunzmann investigate psychosocial and health-related resources that predict two-year stability and change in adaptive and maladaptive daily patterns of calmness, excitement, sadness, and anger. Pauly et al. examine the extent to which health status moderates the association between daily fluctuations in seven affective states and corresponding changes in stress-related cortisol secretion. Turner, Mogle, Hill, Bhargava, and Rabin study how positive and negative emotions experienced in response to daily challenges (memory lapses) mediate the association between age-related challenges and life satisfaction in a coordinated analysis of two datasets. Finally, Barlow addresses the extent to which variations in daily experiences of positive and negative emotions exhibit age-differential associations with daily satisfaction with life (i.e., emotion globalizing). This symposium thus integrates new research on emotional aging and contributes to a deeper understanding of how adaptive development shapes and is shaped by day-to-day fluctuations and long-term changes in different emotions.

### ASSOCIATIONS BETWEEN AGE-RELATED CHANGES IN POSITIVE AFFECT AND PHYSICAL FUNCTIONING: THE ROLE OF EDUCATION

Timo von Oertzen,[1] Ute Kunzmann,[2] and Maria Blöchl,[3], *1. Universität der Bundeswehr, Neubiberg, Bayern, Germany, 2. Leipzig University, Leipzig, Sachsen, Germany, 3. Leipzig University, Leipzig University, Sachsen, Germany*

Increasing research points to the relevance of educational attainment for positive emotional experiences and physical functioning across adulthood. However, little is known about how age-related developments in positive affect and physical functioning differ by educational attainment. This study used longitudinal data of 10,893 individuals (60–80 years) from the Health and Retirement Study to examine whether educational attainment moderates trajectories of positive affect and physical functioning and their interrelations over 12 years. Initial results from multiple-group bivariate growth models revealed that individuals with less formal education have lower positive affect and poorer physical functioning at baseline. There was, however, no evidence that longitudinal changes in positive affect, longitudinal changes in physical health, and coupled changes between both variables varied with educational attainment. These initial findings suggest that lower educational attainment is primarily related to lower levels of positive affect and physical functioning, but not to greater age-related declines or their interrelations.

### DISCRETE EMOTION PROFILES IN OLD AGE: STABILITY AND CHANGE FOR BETTER OR WORSE

Carsten Wrosch,[1] Meaghan Barlow,[2] Ute Kunzmann,[3] and Jeremy Hamm,[4], *1. Concordia University, Montreal, Quebec, Canada, 2. University of California, Berkeley, University of California, Berkeley, California, United States,*

*3. Leipzig University, Leipzig, Sachsen, Germany, 4. North Dakota State University, Fargo, North Dakota, United States*

Although discrete emotions can change in salience across adulthood, little is known about developmental shifts in the co-occurrence of multiple discrete emotions. The present study (n=389, Mage=73) adopted a person-centered approach to identify stability and change in commonly-occurring profiles of calmness, excitement, sadness, and anger. Daily emotions were assessed over 1-week periods at baseline and two years later. Latent class analyses yielded consistent 3-profile solutions at both waves: a positive emotion (high calmness-moderate excitement-low sadness and anger), a mixed emotion (moderate/high calmness-moderate excitement, sadness, and anger), and an apathetic emotion profile (low calmness, excitement, sadness, and anger). Latent transition analyses revealed both stability (82% remained in the same profile) and change (18% changed profiles) in profile membership. Higher baseline optimism and fewer chronic conditions were associated with adaptive changes in profile membership. Findings point to the importance of considering the co-occurrence of distinct emotions in studying emotional aging.

### DISCRETE AFFECTIVE STATES, CORTISOL, AND SELF-RATED HEALTH IN OLD AGE

Oliver Schilling,[1] Ute Kunzmann,[2] Hans-Werner Wahl,[3] Nilam Ram,[4] Denis Gerstorf,[5] Christiane Hoppmann,[6] and Theresa Pauly,[7], *1. University of Heidelberg, Heidelberg, Baden-Wurttemberg, Germany, 2. Leipzig University, Leipzig, Sachsen, Germany, 3. Heidelberg University, Heidelberg, Baden-Wurttemberg, Germany, 4. Stanford University, Stanford, California, United States, 5. Humboldt University of Berlin, Humboldt University of Berlin, Brandenburg, Germany, 6. University of British Columbia, University of British Columbia, British Columbia, Canada, 7. University of Zurich, University of Zurich, Zurich, Switzerland*

Old age is a developmental phase in which physical vulnerability increases and discrete affective states are uniquely important. The current project combines data from four studies (total N = 476 participants) to investigate within-person fluctuations in salivary cortisol (a marker of physiological arousal), seven discrete affective states, and the moderating role of self-rated health. Each participant provided affect reports and collected salivary cortisol 5-7 times a day for a 7-day period, and rated their health status. Multilevel models showed that cortisol levels were decreased in moments when participants felt happier, more relaxed, and more interested than usual and increased in moments when participants felt angrier, more nervous, more overwhelmed, and sadder than usual. Associations of happy, nervous, overwhelmed, and sad with cortisol were more pronounced in participants of better as compared to those of worse self-rated health. Findings suggest that higher HPA reactivity may indicate preserved health in older adults.

### POSITIVE AND NEGATIVE AFFECT MEDIATE THE RELATIONSHIP BETWEEN MEMORY LAPSES AND LIFE SATISFACTION

Jacqueline Mogle,[1] Nikki Hill,[1] Sakshi Bharhava,[2] Laura Rabin,[3] and Jennifer Turner,[4], *1. Penn State University, University Park, Pennsylvania, United States, 2. Pennsylvania State University, University Park, Pennsylvania, United States, 3. Brooklyn College, The City University of New York, Brooklyn, New York, United States, 4. Pennsylvania State University, State College, Pennsylvania, United States*

Aging is associated with declines and challenges, yet better subjective well-being. Life satisfaction is one aspect of well-being that may be sensitive to daily challenges. Daily memory lapses (e.g., forgetting words or meetings) are common and relevant for many adults. How individuals emotionally respond to challenges like memory lapses is a factor that could determine whether these experiences affect well-being. In a coordinated analysis of two datasets (N=561; ages 25-93 years) using multilevel modeling, we examined whether affective changes related to memory lapses mediated the relationship between memory lapses and life satisfaction. Results were similar across datasets: memory lapses were associated with reduced positive affect and increased negative affect. These associated affective changes also mediated the relationship between lapses and life satisfaction. We discuss the potential implications of our findings for linking proximal events and distal outcomes, and potentially intervening and identifying common challenges to mitigate broad reductions in well-being.

### AGE DIFFERENCES IN EMOTION GLOBALIZING ACROSS THE ADULT LIFESPAN

Meaghan Barlow, *University of California, Berkeley, University of California, Berkeley, California, United States*

Emotion globalizing, the extent to which current emotions impact satisfaction with life, is associated with poorer psychological well-being. Given extant aging theories and research highlighting age-related changes in emotional experiences and emotion regulation, the present study examined age differences in positive and negative emotion globalizing across the adult lifespan. Participants (N = 145 females; aged 23-79) completed assessments of positive emotion (i.e., amused, energetic, calm, happy, interested, excited, and content), negative emotion (i.e., anxious, lonely, sad, annoyed, angry, and distressed), and life satisfaction for 16 days. Multilevel model analyses revealed age differences in negative, but not positive, emotion globalizing. More specifically, older individuals reported lower levels of negative emotion globalizing, as compared to younger individuals. These findings highlight the need to explore downstream consequences of emotion globalizing across the lifespan, as this could unveil novel pathways towards successful aging.

### Session 4565 (Paper)

### HEALTH AND SOCIAL SERVICES

### A NURSE-LED CONCEPTUAL MODEL TO INFORM PATIENT-CENTERED TYPE 2 DIABETES MANAGEMENT IN PUBLIC CLINICAL SETTINGS

Stella Bosun-Arije,[1] and Candidus Nwakasi,[2], *1. Manchester Metropolitan University, Manchester Metropolitan University, England, United Kingdom, 2. Providence College, Providence, Rhode Island, United States*

Globally, there is an increased need to provide patient-centered care for people diagnosed with Type 2 diabetes mellitus (T2DM). In Nigeria, a poorly financed health system worsens the difficulties associated with managing T2DM in clinical settings, resulting in a detrimental effect on patient-centered care. We aimed to develop a conceptual model to promote patient-centered T2DM care in clinical settings. We explored nurses' contextual perceptions of clinical practices and operations that are relevant to T2DM management across public hospitals in Lagos, Nigeria. Identifying a nurse-led intervention is critical to care optimization for people diagnosed with T2DM. Using a qualitative research, we used semi-structured questions and the Constant Comparison Method to interview 17 practice (registered) nurses with over 1-year experience working in public hospitals across Lagos, Nigeria. The Framework Method was used for data analysis. The nurses provided insight into four areas of patient-centered T2DM management in clinical settings. They are: empowering collaboration, empowering flexibility, empowering approach, and empowering practice. The nurses discussed an empowering pathway through which health settings could provide patient-centered care to individuals diagnosed with T2DM. The pathway includes the integration of macro, meso, and micro levels for patient management. Their views informed the development of a conceptual model for the optimization of patient care. The model developed from this research sits within the Patient-Centered Care model of healthcare delivery. The model has the potential to inform patient-centered care in countries with poorly financed healthcare systems, and developed countries with comparatively better healthcare.

### EXAMINING THE RISK OF POTENTIALLY PREVENTABLE HOSPITALIZATION IN ADULTS WITH CONGENITAL AND ACQUIRED DISABILITIES

Elham Mahmoudi,[1] Paul Lin,[2] Anam Khan,[3] and Neil Kamdar,[2], *1. University of Michigan, Commerce Township, Michigan, United States, 2. University of Michigan, Ann Arbor, Michigan, United States, 3. University of Michigan School of Public Health, Ann Arbor, Michigan, United States*

**Introduction:** Adults with congenital (cerebral palsy/spina bifida (CP/SB)) or acquired disabilities (spinal cord injury (SCI) or multiple sclerosis (MS)) are more likely than those without disability to develop medical complications. Little is known about potentially preventable hospitalizations (PPH) among adults with disabilities. PPHs are preventable if a patient had timely access to care. Our objective was to estimate PPH risk for each of the aforementioned disabilities.

**Methods:** We used private payer claims data from 2007-2017 to identify adults (18+) with diagnoses of CP/SB (n=10,617), SCI (n=5,173), and MS (n=6,198). Adults without these disabilities were included as controls. We propensity score matched individuals for age and sex. Logistic regression models with repeated measures were subsequently applied, adjusting for age, sex, race/ethnicity, health indicators, U.S. census divisions, and socioeconomic variables. Odd ratios (OR) were compared over 4-years of follow up.

**Results:** Adults with CP/SB, SCI, and MS had higher odds of any PPH compared with adults without disability [CP/SB: (OR=4.10; 95% CI: 2.31-7.31); SCI: (OR=1.67; 95% CI: 1.21-2.32); and MS: (OR=1.48; 95% CI: 1.00-2.25)]. Use of preventative services reduced the PPH risk. For example, wellness visit reduced the odds of PPH by almost half [CP/SB: (OR=0.52; 95% CI: 0.41-0.67); SCI: (OR=0.57; 95% CI: 0.45-0.71); and MS: (OR=0.53; 95% CI: 0.40-0.66)].

**Conclusions:** Adults with disabilities are at greater odds of PPH compared to adults without disabilities. Clinical guidelines for use of preventative care for adults living with disabilities need to be accordingly updated.

### MEDICATION MANAGEMENT AND THE ROLE OF THE GERIATRIC PHARMACIST IN INTERDISCIPLINARY CARE TEAMS

Christine Jensen,[1] Lauren Burnette,[1] and Faika Zanjani,[2], *1. Riverside Center for Excellence in Aging & Lifelong Health, Williamsburg, Virginia, United States, 2. Virginia Commonwealth University, Richmond, Virginia, United States*

Among adults 65 and older, 30% are taking at least five medications to treat acute and chronic health conditions (Gavin, 2020). As the number of medications increases, the more complex the regimen tends to be, which increases risks with proper management and unwanted side effects. Our interdisciplinary geriatric assessment team has been conducting medication reviews for individuals living with dementia, where geriatric pharmacists meet with these individuals and their family caregiver. These sessions build a trusting relationship, where older adults are able to receive education about their prescription and over-the-counter medications, address any concerns and reach shared goals. Pharmacists routinely recommend deprescribing, and all recommendations are sent to the older adults' primary care provider. After the initial appointment, a follow-up takes place six months later to re-examine adherence to recommendations and assess outcomes. Since April 2019, our pharmacists have served over 300 individuals, where nearly 90% would recommend this review to others. Pharmacists have recommended over 250 medication changes, averaging 2.53 per person. A statistically significant decrease in prescription medications (from 12.48 to 12.16) has been identified, in addition to a trending decline for over-the-counter medications (3.91 to 3.79). Medication reviews have successfully reduced the overall number of medications, as 36% of recommendations have been accepted by the patient and their healthcare provider. Comprehensive medication review programs, where pharmacists are integrated into an interdisciplinary care team, offer high quality, best practice healthcare, where safety and quality of life is improved for older adults.

### PREDICTORS OF CARE MANAGEMENT UTILIZATION

Sharmila Prabhu,[1] and Margaret Danilovich,[2], *1. Loyola University, West Bloomfield, Michigan, United States, 2. CJE SeniorLife, Evanston, Illinois, United States*

Care management is the process of planning and coordinating care to assist individuals or families in managing their health. This may involve managing inpatient or outpatient medical care or helping with other household, legal, or financial needs. Care management service providers are challenged in knowing how best to allocate limited resources (i.e. care manager time) to best meet client needs. Research shows predictors for increased care management needs include advanced age, multiple comorbidities, frequent care transitions, and private insurance coverage, but the association of objectively measured functional assessments and care management hour utilization is unknown. This secondary data analysis aimed to identify factors that predict the amount of care management service among low-income older adults enrolled in a care management program. We used de-identified care management data from the electronic health record at 1 social service agency. We used multivariate regression to predict the number of hours of care management utilization from demographics, comorbidities, intake ADLs/IADLs, physical health, and self-reported quality of life. We found moderate to strong correlations between physical health and quality of life (r=0.58) and activities of daily living and instrumental activities of daily living (r=0.81). Baseline self-reported quality of life predicted the number of hours of care management utilization (p=.03; beta = 6.75). Quality of life can be useful in predicting the number of service hours that a particular client may require from a care management program and should be considered as an intake question to assist social service providers in allocating hours adequately to clients.

### STRENGTHS-BASED INTERPROFESSIONAL PRACTICE AND EDUCATION: TRANSFORMING CARE THROUGH DISRUPTION

Teri Kennedy, *The University of Kansas Medical Center, Kansas City, Kansas, United States*

This is a conceptual paper proposing a new model of Strengths-Based Interprofessional Practice and Education (SB-IPE), incorporating appreciative inquiry and narrative, and its application to improve health and social care practice and policy for older adults. Within people, families, communities, and teams are people who understand their assets and culture, hold a collective wisdom derived from their individual biographies and shared history, and are deeply invested in their success. This wisdom and experience can be mined for strengths and best practices to improve health and social care for older adults and their families. The conceptual framework of the model and relationship between concepts are explained, reviewing and synthesizing relevant literature on the strengths perspective, interprofessional practice and education, evolution of the patient voice, appreciative inquiry, and narrative to leverage the voices and experiences of older adults, their families, and interprofessional teams. Providing person-, family-, and community-centered health and social care through SB-IPE involves eliciting, listening to, and processing stories and narratives, then coalescing and co-creating person/family/team narratives throughout the trajectory of care. Appreciate inquiry and narrative can be harnessed to imagine an improved experience of care for older adults and their families. Incorporating the potential disruption of the voices and perspectives of older adults and their families offers value for health and social care delivery and policy innovation. Application of the SB-IPE model holds promise for harnessing these voices and collective experiences leading from disruption to transformation of health and social care practice, health professions education, policy, and research.

### Session 4570 (Symposium)

### HOW DOES AGING IN PLACE HELP US UNDERSTAND LONELINESS DURING THE COVID-19 PANDEMIC?

Chair: Judith Robertson Phillips Co-Chair: Cassandra Ford
Discussant: Thomas Prohaska

Co-sponsored by the Disasters and Older Adults, Loneliness and Social Isolation, and Rural Aging Interest Groups, five presenters will highlight multiple circumstances regarding the intersection of social isolation or loneliness and the impact of COVID-19. Haverhals and colleagues interviewed veterans and their caregivers to identify the impact of changes in care delivery and social isolation as a result of the pandemic. Findings indicated differences in feelings of isolation among individuals living in their own home or assisted living facilities. Hua et al. examined whether individuals in long-term care communities were lonelier than individuals in the community during the pandemic using data from the NHATS COVID-19 module with higher levels of loneliness reported from individuals living in more restricted communities. Henning-Smith and colleagues explored differences in social activities among rural and urban participants in the COVID-19 Coping Study. Their study provides awareness into the ways rural and urban older adults stayed connected during the pandemic. Peterson et al. examined the effect of COVID-19 on care in Florida nursing homes and assisted living communities and on residents' anxiety with higher levels of anxiety reported by residents in nursing homes. Using the Coping with Loneliness, Isolation and COVID19 Global Survey, O'Sullivan and colleagues utilized the lens of 'place' to examine factors associated with those experiencing loneliness and/or social isolation during the pandemic with insights from a public health perspective. Collectively, these presenters will provide evidence of the challenges associated with older adults' social isolation and loneliness throughout the COVID-19 pandemic.

### DOES PLACE MATTER WHEN UNDERSTANDING LONELINESS AND SOCIAL ISOLATION?

Annette Burns,[1] Christina Victor,[2] Thomas Prohaska,[3] Brian Lawlor,[4] Gerry Leavey,[5] and Roger O'Sullivan,[6], *1. Institute of Public Health in Ireland, Dublin, Dublin, Ireland, 2. Brunel University London, Uxbridge, England, United Kingdom, 3. RRF Foundation for Aging, Fairfax, Virginia, United States, 4. Trinity College Dublin, Dublin, Dublin, Ireland, 5. Ulster University, Belfast, Northern Ireland, United Kingdom, 6. Institute of Public Health in Ireland, Belfast/Dublin, Dublin, Ireland*

Physical distancing and restriction of movements as measures to prevent the spread of Covid-19 required people to change their work, home and social lives. Loneliness and social isolation have emerged as key public health issues during the pandemic. Traditionally when considering loneliness the focus is often on individual factors rather than within the context of structural and environmental dimensions. This paper will utilise data from the Coping with Loneliness, Isolation and Covid-19 global online survey which had over 20, 000 global responses from people aged 18+ in 2020. Analysis will use the lens of 'place' and the 5-item UCLA scale and 6-item Lubben social network scale to understand the social and demographic characteristics and structural and environmental factors associated with those experiencing loneliness and/or social isolation in rural and urban areas both before and during the pandemic. The paper will conclude with key messages from a public health perspective.

### LONELINESS AND SOCIAL CONNECTEDNESS AMONG RURAL OLDER ADULTS SINCE THE COVID-19 PANDEMIC ONSET

Gabriella Meltzer,[1] Lindsay Kobayashi,[2] Jessica Finlay,[2] and Carrie Henning-Smith,[3], *1. Department of Social and Behavioral Sciences, New York University, New York, United States, 2. University of Michigan, Ann Arbor, Michigan, United States, 3. University of Minnesota School of Public Health, Minneapolis, Minnesota, United States*

Rural areas have a higher proportion of older adults aging in place. Rural areas also face structural barriers to supporting social connectedness among older adults, including transportation barriers, greater geographic distances, and access to technological connectivity. This research aims to discuss rural-specific risks of loneliness and social isolation among older adults, as well as rural/urban differences in loneliness and social isolation among older adults using the national COVID-19 Coping Study. Cross-sectional bivariate analyses highlight rural/urban differences in social activities during the pandemic. For example, rural older adults were more likely to use social media daily, compared with urban older adults (67% vs. 61%, $p<0.05$), but were less likely to have phone or video calls with others daily (21% vs. 26%, $p<0.001$). We will also share results of differences within rural older adults in loneliness, isolation, and social activities by socio-demographic characteristics in order to design targeted interventions to improve connectedness.

### SOCIAL ISOLATION IN LONG-TERM CARE FACILITIES RELATED TO COVID-19: EFFECT ON RESIDENT ANXIETY AND CARE

Nazmus Sakib,[1] Joseph June,[2] and Lindsay Peterson,[3], *1. Department of Industrial and Management Systems, University of South Florida, Tampa, Florida, United States, 2. University of South Florida, Tampa, Florida, United States, 3. University of South Florida, University of South Florida, Florida, United States*

Loneliness is a common problem in long-term care. It has been associated with a higher risk of depression, aggressive behaviors, and anxiety and may be a risk factor for cognitive decline. Loneliness can exacerbate social isolation. The COVID-19 emergency brought on measures in Florida, beginning in March 2020, to separate nursing home (NH) and assisted living community (ALC) residents from each other and family members to limit virus spread. This study examines results of a survey with Florida NH (N=59) and ALC (N=117) administrators concerning effects of these measures. Scaled (1-5, lowest to highest) data indicate that resident anxiety was higher in NHs (M=3.40) than ALCs (M=3.17). Care disruptions related to limited resident-to-resident contact also were worse in NHs (M=3.74) than in ALCs (M=3.21), while care disruptions related to loss of family support were higher among ALCs (M=3.19) than in NHs (M=2.86). Implications of these findings will be discussed.

### IMPACT OF SOCIAL ISOLATION DUE TO COVID-19 ON VA HOME-BASED PRIMARY CARE VETERANS AND CAREGIVERS

Chelsea Manheim,[1] Nelly Solorzano,[1] Juli Barnard,[2] Tamar Wyte-Lake,[3] and Leah Haverhals,[1], *1. Denver Center of Innovation for Veteran Centered and Value Driven Care (COIN), Denver, Colorado, United States, 2. Department of Veterans Affairs, Aurora, Colorado, United States, 3. US Department of Veterans Affairs, U.S. Department of Veterans Affairs (VA), California, United States*

In December 2020 we began conducting phone interviews with Veterans, and their caregivers, receiving care through the United Sates (US) Department of Veterans Affairs (VA) Home Based Primary Care (HBPC) program. Our goal was to describe experiences of Veterans and caregivers managing changes in care delivery related to the COVID-19 pandemic and navigating increased social isolation due to social distancing. We interviewed 38 Veterans (average age 78) and caregivers (average age 62) across seven VA HBPC programs. Findings showed those living in their own homes found increased isolation more manageable than those living in assisted living facilities, which restricted visitors. Caregivers had a harder time managing isolation than Veterans, as Veterans were used to being primarily homebound. Veterans and caregivers relied on increased phone communication with their HBPC teams, with some began participating in virtual visits. Implications include insights into better supporting older, homebound adults and their caregivers during disasters.

### COMPARING LONELINESS AMONG INDIVIDUALS IN LONG-TERM CARE SETTINGS AND THE COMMUNITY

Cassandra Hua, *Brown University, Providence, Rhode Island, United States*

We used the NHATS COVID-19 module to examine whether individuals in long-term care communities were lonelier than individuals in the community during the pandemic. Additionally, we examined whether individuals in long-term care communities with more restrictive policies concerning visitors and communal activities were more likely to experience loneliness than individuals in communities with less restrictive policies. Approximately 45% of individuals in long-term care communities (n=134) felt at least a moderate amount of loneliness during COVID-19 when compared to 34% of individuals in the community (n= 2,666) ($p<.05$). However, the association was no longer statistically significant after adjusting for age, race, and sex. Among individuals in long-term care communities with the most restrictive policies, 48% experienced loneliness compared to 44% individuals in less restrictive communities. However, this finding was not statistically significant. Discussion will focus on similarities and differences within these populations that could have led to these results.

### Session 4575 (Symposium)

### INNOVATIVE GERONTOLOGY IN HIGHER EDUCATION: TRANSFORMATIVE EFFECTS OF THE PANDEMIC

Chair: Pamela Saunders Co-Chair: Yoon Chung Kim
Discussant: Debra Dobbs

Gerontology in higher education is experiencing an exciting inflection point rising from the COVID pandemic pushing us to adapt our teaching modalities. Many educators have developed innovative learning experiences making use of creativity, virtual reality, online discussion boards, virtual tours, Jam Boards, videos, and breakout rooms. This symposium will bring together gerontologists and educators to discuss their educational innovations. Dr. Saunders will discuss the use of virtual reality in a Geriatrics clerkship experience to enhance knowledge, empathy, and attitudes towards older adults. Dr. Hanna and Ms. Kim will present the use of a virtual avatar to explore aging identity. Professor Barsness will discuss the participation of older adults from the community as subject matter experts. Ms. Redlich will share her virtual internship experience of exploring the intellectual and social benefits of adult study abroad. Although the pandemic was challenging to gerontological education, substantial transformations have been accomplished. The innovations described in this session broadened engagement of students with older adults to identify their strengths and challenges to flourish in the "New Normal."

### TRANSFORMING EDUCATION USING VIRTUAL REALITY: GERIATRICS CLERKSHIP BEFORE AND DURING PANDEMIC

Pamela Saunders, *Georgetown University, WASHINGTON, District of Columbia, United States*

Since 2006, the Georgetown University School of Medicine has offered a two-week elective in Geriatrics for third-year medical students. Students rotate through diverse clinical experiences, including general geriatrics, geriatric neurology, physical medicine & rehabilitation, memory disorders, Parkinson's and dementia, and palliative care. In addition, students learn about arts, humanities & ethics, communication skills, and taking the patient's perspective. In Fall 2019, pre-pandemic, we added virtual reality (VR) experiences focused on hearing & vision loss, Alzheimer's disease, and end-of-life conversations created by Embodied Labs. Curricular goals included increasing students' empathy and sensitivity, decreasing ageism & stereotyping, and increasing clinical knowledge. Findings suggest regardless of pandemic (pre vs. during) or modality (in-person vs. Zoom) that after participating in the VR labs, students are slightly more comfortable taking care of older adult patients with dementia as well as hearing & vision loss, and participating in end-of-life conversations.

### LEARNING HUMANITIES AND ETHICS OF AGING THROUGH THE LENS OF AN AVATAR CREATION

Yoon Chung Kim, and Gay Hanna, *Georgetown University, Washington DC, District of Columbia, United States*

The main goal of teaching the humanities and ethics of aging is to understand the perspectives of older individuals as they address the challenges and opportunities presented across the aging spectrum. To encourage understanding of this humanistic and ethical process, students were given an assignment to select a profile of an older person with preselected characteristics that they then develop into their avatar, a virtual companion, to accompany them through the course. This assignment included three iterations of the avatar narrative related to what is studied in class around major life transition points related to work, housing, and end of life. These assignments included the creation of Mind Maps which illustrate their avatar's ongoing concerns related to their environment including their social determinants of health. The avatar's formative development throughout the course brought forward discussions around identity, safety, autonomy, and person-centeredness in terms of gerontological practice and policy.

### OLDER ADULTS AS VIRTUAL SUBJECT MATTER EXPERTS IN COVID GRADUATE EDUCATION

Sonya Barsness, *Georgetown University, Washington DC, District of Columbia, United States*

COVID-19 has further illuminated the need for educational approaches in gerontology that are person-centered and experiential. Ideally, this includes in-person experiences with students and older adults. Through their classroom participation as subject matter experts in aging, older adults share their personal experiences, and react to gerontological theories and ideas. Shared learning offers a platform for exploration of shared humanity, so that older adults are not seen as the "other", but "us". This prepares a generation of gerontologists to identify and reject ongoing ageism, again highlighted by the pandemic. COVID-19 has also challenged educators to offer these experiential opportunities. In this presentation we will outline how older adults from a Continuing Care Retirement Community participated virtually in a graduate course. We will discuss how their virtual involvement was structured, how their real-time COVID experiences were integrated, and share feedback from older adult participants and students on their shared learning experiences.

### STUDENT PERSPECTIVE: INTERNSHIP EXPLORING INTELLECTUAL AND SOCIAL BENEFITS OF ADULT STUDY ABROAD

Kim Redlich, *Georgetown University, Washington DC, District of Columbia, United States*

Older adult participation in lifelong learning programs – such as university continuing education opportunities and the Age-Friendly University global network – has grown steadily over the last few years. Many of these programs are characterized by mixed-age classrooms in which undergraduate students share space and learning, remotely during Covid, with older adult participants who pay a nominal fee. Survey findings will be presented from older students involved in two university programs in this category: Temple University's "senior scholars" program and Georgetown University's "senior auditors" program, specifically related to the concept of adult study abroad. Adult study abroad is a new offering that combines the intellectual and social benefits of stimulating coursework with the transformative power of travel, and how the merging of these pursuits can produce purpose, meaning and community, especially for older adults. It is typically residential, academic, intergenerational, and of longer duration than a typical tourist experience.

### Session 4580 (Symposium)

### LINKING OLDER ADULTS' DAILY ACTIVITIES WITH WELL-BEING AND COGNITION: EXAMINING MODERATORS AND MEDIATORS

Chair: Christina Roecke Co-Chair: Minxia Luo
Discussant: Thomas M. Hess

Increasingly more studies are showing that daily activities can be beneficial to wellbeing and cognitive abilities of older adults, but discussions about through which psychological mechanisms daily activities are associated with wellbeing and cognitive health have been scarce. This symposium, including three ambulatory assessment studies and one cross-sectional study, presents emerging theoretical hypotheses and recent empirical findings on this matter. Specifically, with 5-6 days of observations from 313 older adults, Brown and colleagues show that greater daily activity diversity is related to older adults' higher overall cognitive functioning (executive functioning, memory, and crystallized intelligence). Hueluer and colleagues examine the moderating role of interaction modality on the relation of daily social interactions with wellbeing using data from 116 older adults over 21 days. Their results show that more face-to-face interactions — but not telephone or digital interactions — are associated with higher positive affect and lower loneliness. With data from 153 older adults over 15 days, Luo and colleagues show the mediating effect of positive affect in the association between momentary working memory performance and subsequent social activity engagement. Sharifian and colleagues show the mediating effects of solitary-cognitive activities in the association between depressive symptoms and global cognition, using cross-sectional data from 453 older adults, and also examine the moderating role of race. Finally, Tom Hess will serve as a discussant and provide an integrative discussion of the papers, informed by his extensive work on daily activities, motivation, and aging.

### MOMENTARY WORKING MEMORY AND MOMENTARY ACTIVITIES IN HEALTHY OLDER ADULTS: THE MEDIATING ROLE OF AFFECTIVE STATES

Minxia Luo, Robert Moulder, and Christina Röcke, *University of Zurich, Zurich, Zurich, Switzerland*

Research has shown cognitive ability in older age is associated with activity engagement, but little is known about what psychological mechanisms are linking the two constructs. This study investigates an emotional pathway, in which affective states mediate the temporal associations between momentary working memory and momentary activities in older age. We examined data from 153 healthy older adults aged 65 to 91 who completed a smartphone-based ambulatory assessment survey seven times a day over 15 days. In each assessment point, participants reported their momentary activities (e.g., social activities, mentally stimulating activities) and affective states (i.e., positive affect, negative affect) and took a working memory task. Initial results suggest that during an approximate time period of six hours (i.e., across three assessment points), working memory performance influences subsequent likelihood of social activity engagement. Moreover, positive affect mediates this temporal association. Results will be discussed in the context of cognitive aging research.

### SWITCHING UP HOW YOU GET IN YOUR STEPS: DAILY ACTIVITY DIVERSITY AND COGNITIVE FUNCTIONING

Sangha Jeon,[1] Yee To Ng,[2] Soomi Lee,[3] Susan Charles,[1] Karen Fingerman,[4] and Colette Brown,[1], *1. University of California, Irvine, Irvine, California, United States, 2. University of Texas at Austin, Austin, Texas, United States, 3. University of South Florida, Tampa, Florida, United States, 4. The University of Texas at Austin, Austin, Texas, United States*

Active lifestyles are related to better cognitive health. More work is needed, however, to examine whether participating in a variety of daily activities (i.e., activity diversity) has unique importance beyond amount of activity. The current study examined associations between daily activity diversity and cognitive functioning among community-dwelling older adults (N = 313, ages 65-90). Participants completed a cognitive battery, then responded to ecological momentary assessments of their participation in 10 common activity types (e.g., exercise, chores, social visits, volunteering) every 3 hours for 5-6 days, and wore accelerometers to track daily step counts and duration of activity. Multiple regression models revealed that greater daily activity diversity related to higher overall cognitive functioning, executive functioning, memory, and crystallized intelligence. These associations remained significant after adjusting for step count and duration of activity. Findings suggest daily activity diversity has unique importance beyond sheer amount of activity for cognitive health in later adulthood.

### DAILY SOCIAL INTERACTIONS AND WELL-BEING IN OLDER ADULTS: THE ROLE OF INTERACTION MODALITY

Birthe Macdonald,[1] Minxia Luo,[2] and Gizem Hueluer,[3], *1. University of Zurich, Zurich, Zurich, Switzerland, 2. University of zurich, Zurich, Zurich, Switzerland, 3. University of South Florida, Tampa, Florida, United States*

Older adults increasingly use digital communication technologies to stay connected to others. In the present study, we examine the role of social interactions for older adults' daily well-being focusing on three interaction modalities (face-to-face, telephone, and digital). We use data from 116 participants (age: M = 72 years, SD = 5, range = 65 to 94; 41% women), who reported on their social interactions and well-being over 21 days. Our findings show that frequency of face-to-face interactions is more consistently related to well-being than telephone or digital interactions. On days where participants report more face-to-face social interactions than their own average, they report higher positive affect and lower loneliness than usual. Similar effects are not found for telephone or digital interactions. In summary, our findings suggest that face-to-face social interactions are uniquely relevant to older adults' daily well-being. We discuss implications of these findings for future research.

### DEPRESSIVE SYMPTOMS, LEISURE ACTIVITY ENGAGEMENT, AND GLOBAL COGNITION IN NON-HISPANIC WHITE AND BLACK OLDER ADULTS

A. Zarina Kraal,[1] Laura Zahodne,[2] and Neika Sharifian,[1], *1. University of Michigan, Ann Arbor, Michigan, United States, 2. Clinical Science, Ann Arbor, Michigan, United States*

Prior research has linked more depressive symptoms to worse global cognition in older adulthood through lower leisure activity engagement. Less is known regarding which types of activities drive these associations. Additionally, depressive symptoms disproportionately affect cognition in Non-Hispanic Blacks (NHB) versus Non-Hispanic Whites (NHW). This cross-sectional study used data from the Michigan Cognitive Aging Project (n=453, 52% NHB, Mage=63.60 years) to examine whether distinct leisure activities (solitary-cognitive, solitary-creative, community-social, physical, intergenerational-social, cognitive-games) mediated the association between depressive symptoms and global cognition and whether race moderated these associations. Lower engagement in solitary-cognitive activities partially mediated the negative association between depressive symptoms and global cognition. In multi-group models, this indirect effect was only evident in NHBs, who showed a stronger negative association between depressive symptoms and activity engagement than NHWs. While cross-sectional, findings indicate that depressive symptoms may negatively impact cognition by reducing engagement in activities that promote cognitive reserve.

### Session 4585 (Symposium)

### NOVEL GENETIC AND COGNITIVE FINDINGS FROM THE LONG LIFE FAMILY STUDY

Chair: Mary Wojczynski Co-Chair: Nancy W. Glynn
Discussant: Evan Hadley

The Long Life Family Study (LLFS), funded by the National Institute on Aging, is an international collaborative study of the genetics and familial components of exceptional longevity and healthy aging. We phenotyped 4,953 individuals from 539 two-generational families (1,727 proband; 3,226 offspring) at baseline (2006-2009). A second visit (2014-2017) was conducted for 2,904 (478 proband; 2,426 offspring) participants. The longitudinal, comprehensive in-person visits measured domains of healthy aging, including physical performance, cognition, and blood markers. Extensive genetic analyses were performed using the baseline blood draw, including genotyping with the Illumina 2.5M Human Omni array, linkage analyses with the families, whole genome sequencing using the TopMED protocol, and metabolomic assays. Collectively, this symposium will present novel findings that elucidate new potential genes and genetic interactions, investigate metabolomics profiles among APOE4 carriers, and refine phenotypic cognition definitions. Specifically, Dr. Santanasto will share results flanking the mesothelin gene associated with longitudinal gait speed. Then, Dr. An will share his findings of a new intronic variant in PKD1L2 gene associated with adiponectin, a marker of insulin resistance. Next, Dr. Arbeev will discuss genetic interactions between candidate genes for physiologic dysregulation and candidate genes for Alzheimer's disease (AD, UNC5C gene). Ms. Yao will examine metabolomic profile differences between APOE4 carriers who develop AD and those who do not. Lastly, Mr. Xiang will introduce a method for combining multiple cognitive tests into a more sensitive overall cognitive score. As Discussant, Dr. Evan Hadley will share insights and propose future directions for LLFS.

### NOVEL POSITIONAL CANDIDATE GENE FOR GAIT SPEED CHANGE IN OLDER ADULTS: THE LONG LIFE FAMILY STUDY

Mary Wojczynski,[1] Ryan Cvejkus,[2] Bharat Thyagarajan,[3] Kaare Christensen,[4] Nicole Schupf,[5] E. Warwick Daw,[6] Joseph Zmuda,[2] and Adam Santanasto,[7], *1. Washington University School of Medicine, Washington University School of Medicine, Missouri, United States, 2. University of Pittsburgh, Pittsburgh, Pennsylvania, United States, 3. University of Minnesota, Minneapolis, Minnesota, United States, 4. Department of Public Health, University of Southern Denmark, Odense, Syddanmark, Denmark, 5. Columbia University, Mailman School of Public Health, New York, New York, United States, 6. Washington University in St. Louis, St. Louis, Missouri, United States, 7. University of Pittsburgh, Pittsburgh, Pittsburgh, Pennsylvania, United States*

Gait speed is a heritable, robust predictor of longevity in older adults. Using genome-wide linkage analysis in 2379 individuals from 509 families (64±12 years; 45% men), we identified a locus on chromosome 16p linked to gait speed change over 7±1 years (logarithm of the odds score [LOD]=4.2). Gait speed change was calculated using a two-stage growth curve mixed-model. DNA sequencing was completed to identify single nucleotide variants (SNVs) in the linkage region. Association analyses between the 24039 SNVs in the ~1.6mBP region (3.7-5.3mBP) and gait speed change were performed adjusting for age, age2, sex, height, field center, familial relatedness and population substructure. Eleven families (188 individuals) accounted for most of the linkage signal (LOD=6.06). Associations between SNVs flanking the Mesothelin (MSLN) gene and gait speed change were identified (lead SNV rs56850119: $\beta = -0.5 \pm 0.1$, p = 6.4*10-7). Thus, MSLN is a potential positional candidate gene for mobility decline with aging.

### LINKAGE GUIDED SEQUENCE ANALYSIS REVEALED A NOVEL GENE PKD1L2 FOR ADIPONECTIN: THE LONG LIFE FAMILY STUDY (LLFS)

Joseph Lee,[1] Jason Anema,[2] Lihua Wang,[3] Warwick Daw,[4] Kaare Christensen,[5] Joseph Zmuda,[6] Michael Province,[7] and Ping An,[3], *1. Columbia University, New York, New York, United States, 2. Washington University School of Medicine, St Louis, Missouri, United States, 3. Washington University School of Medicine, St. Louis, Missouri, United States, 4. Washington University, St. Louis, Missouri, United States, 5. Department of Public Health, University of Southern Denmark, Odense, Syddanmark, Denmark, 6. University of Pittsburgh, Pittsburgh, Pennsylvania, United States, 7. Washington University School of Medicine, Washington University School of Medicine, Missouri, United States*

Adiponectin is involved in regulating insulin resistance (IR) and is a potential regulator of healthy aging and lifespan. To identify novel variants associated with adiponectin, we further assessed our previously identified linkage peak on 16q23.2 (LODs=3.8). We used sequence data of 632 participants (age, 24-110 years) from 47 families of European ancestry in the Long Life Family Study, a study with familial clustering of exceptional longevity in the US and Denmark. Adiponectin levels were log-transformed, and adjusted for age, sex, sites, and PCs for ancestry. We found a variant in the PKD1L2 (rs527459046, p=2e-8, MAF=3%, r2=1.5%, accounting for linkage=28%). The PKD1L2, 1.4 Mb upstream of the CDH13 (adiponectin receptor gene) is expressed in heart, liver, and adipocytes, known to function as an ion-channel regulator or a GPCR regulator for aging-related lipolysis, IR, and adiponectin/leptin secretion. Haplotyping, epistatic and bioinformatic analyses will be engaged to capture additional/functional variants and regulatory networks.

### GENES INVOLVED IN PHYSIOLOGICAL DYSREGULATION AND DECLINE IN RESILIENCE: ROLE IN ALZHEIMER'S DISEASE

Svetlana Ukraintseva,[1] Olivia Bagley,[1] Hongzhe Duan,[2] Deqing Wu,[1] Igor Akushevich,[1] Alexander Kulminski,[2] Anatoliy Yashin,[1] and Konstantin Arbeev,[1], *1. Duke University, Durham, North Carolina, United States, 2. DUKE UNIVERSITY, Durham, North Carolina, United States*

Our recent GWAS of a composite measure of physiological dysregulation (PD) in the Long Life Family Study (LLFS) found that the top genes associated with age-related changes in PD are involved in biological pathways relevant to maintaining neural networks and brain resilience. In our prior work, PD itself was linked to resilience-related traits. Alzheimer's disease (AD) is a heterogeneous trait and it may involve an accelerated decline in resilience with age as a contributing factor. We proposed that genes associated with aging-changes in PD and brain resilience may contribute to AD risk. We investigated interactions between SNPs in such candidate genes with AD in LLFS and Health and Retirement Study (HRS). Our analysis revealed significant interactions between SNPs in UNC5C and other genes with AD, in both LLFS and HRS. These findings support roles of genetic interactions with UNC5C gene (implemented in axon growth and neuronal apoptosis) in AD.

### METABOLOMIC PROFILE DIFFERENCES BETWEEN DEMENTED AND NON-DEMENTED APOE4 CARRIERS IN THE LONG LIFE FAMILY STUDY

Mohit Jain,[1] Joanne Murabito,[2] Joseph Zmuda,[3] Kaare Christensen,[4] Michael Province,[5] Zhezhen Jin,[6] Joseph Lee,[6] and Yujing Yao,[7], *1. University of California at San Diego, La Jolla, California, United States, 2. Boston University, Framingham, Massachusetts, United States, 3. University of Pittsburgh, Pittsburgh, Pennsylvania, United States, 4. Department of Public Health, University of Southern Denmark, Odense, Syddanmark, Denmark, 5. Washington University School of Medicine, Washington University School of Medicine, Missouri, United States, 6. Columbia University, New York, New York, United States, 7. Columbia University Mailman School of Public Health, New York, New York, United States*

The apolipoprotein ε4 (APOE4) is the most prevalent genetic risk factor for late-onset Alzheimer's Disease (AD). Here we assessed the metabolomic profile differences between APOE4 carriers who develop AD vs. who do not in a sample of 142 participants, aged 65-99 years in the Long Life Family Study (LLFS). Of 7,321 metabolites, we applied a generalized estimating equation model and identified 137 metabolites significantly associated with AD. Subsequent multivariate analyses were performed for prediction and clustering recognition. Among annotated metabolites, 8 metabolites in the eicosanoids and docosanoids group, 3 metabolites in the fatty acids group, and arabitol were associated with elevated risks of AD (OR: 1.6-2.3). On the other hand, a different set of metabolites were associated with reduced risks of AD (OR: 0.34-0.64). These metabolomic profile differences can be used to help with early diagnosis in the population of older APOE4 carriers in the pre-clinical stage.

### DISCOVERING MODALITY OF COGNITIVE FUNCTION USING CLUSTERING ANALYSIS

Stacy Anderson,[1] Thomas Perls,[2] Marianne Nygaard,[3] Paola Sebastiani,[4] and Qingyan Xiang,[2], *1. Boston University School of Medicine, Boston, Massachusetts, United States, 2. Boston University, Boston, Massachusetts, United States, 3. Department of Public Health, University of Southern Denmark, Odense, Syddanmark, Denmark, 4. Tufts Medical Center, Physician Organization, BOSTON, Massachusetts, United States*

In this study with Long Life Family Study (LLFS) participants, we aimed to identify patterns of performance on cognitive function assessments as specific cognitive signatures. We hypothesize that such signatures can be correlated with biomarkers and clinical outcomes. More than 4,700 LLFS participants were administered, at enrollment, a series of neuropsychological tests that measure various cognitive domains. We performed a cluster analysis to group LLFS subjects into clusters characterized by combinations of six neuropsychological test scores. The analysis resulted in 10 clusters of varying size with different cognitive signatures that (1) significantly correlated with physical and pulmonary function, and 31 blood biomarkers and (2) predicted mortality and incident medical events such as dementia, cardiovascular diseases, etc. We conclude that cluster analysis of multiple neuropsychological tests discovers cognitive signatures that are more specific than individual cognitive domains and that these can be correlated with blood biomarkers, incident medical outcomes and mortality.

### Session 4590 (Paper)

### OLDER ADULTS AND HOSPITALIZATION

### EMERGENCY DEPARTMENT ADMISSIONS AMONG OLDER ADULTS LIVING ALONE WITH MULTIMORBIDITY

Jon Barrenetxea,[1] Cynthia Chen,[2] Woon-Puay Koh,[2] Feng Qiushi,[2] Kelvin Bryan Tan,[3] Kevin Chua,[2] and Rachel Tong,[3], *1. Duke-NUS Medical School, Singapore, Not Applicable, Singapore, 2. National University of Singapore, National University of Singapore, Not Applicable, Singapore, 3. Ministry of Health, Singapore, Singapore, Not Applicable, Singapore*

Older adults living alone are at higher risk of mortality, morbidity and healthcare utilization. As more older adults live alone, Emergency Department (ED) admissions could rapidly increase, particularly among those with multimorbidity. We studied the association of living alone on ED admissions among older adults with multimorbidity. We used data from 16,785 older adults of the population-based Singapore Chinese Health Study (mean age: 73 years, range: 61-96 years) who were interviewed in 2014-2016 for living arrangements and medical history. Participants were followed-up for one year on ED admission outcomes (number of admissions, inpatient days and hospitalization costs). We used multivariable logistic regression to study the association between living alone and ED admission, and ran two-part models (probit & generalised linear model) to estimate the association of living alone on inpatient days and hospitalization cost. We found that compared to living with others, living alone was associated with a higher odds of ED admissions [Odds Ratio (OR) 1.28, 95% Confidence Interval (CI) 1.08-1.51)], longer inpatient days (+0.61, 95% CI 0.25-0.97) and higher hospitalization costs (+322 USD, 95% CI 54-591). Compared to those living with others without multimorbidity, living alone with multimorbidity was associated with higher odds of ED admission (OR 1.64 95% CI 1.33-2.03), longer inpatient days (+0.73, 95% CI 0.29-1.17) and higher hospitalization costs (+567 USD, 95% CI 230-906). In conclusion, living alone is associated with higher odds of ED admission, longer inpatient days and higher hospitalization costs among older adults, particularly among those with multimorbidity.

### HOSPITAL-ASSOCIATED DISABILITY ASSOCIATED WITH DELIRIUM AMONG OLDER ADULTS

Richard Kennedy,[1] Hyun Freeman,[2] Roy Martin,[1] Caroline Whittington,[2] John Osborne,[2] Alayne Markland,[2] Thomas Buford,[1] and Cynthia Brown,[3] *1. University of Alabama at Birmingham, Birmingham, Alabama, United States, 2. University of Alabama at Birmingham, University of Alabama at Birmingham, Alabama, United States, 3. Louisiana State University Health Sciences Center - New Orleans, New Orleans, Louisiana, United States*

Hospital-associated disability (HAD), defined as a loss of activities of daily living (ADLs) occurring during hospitalization, is a common complication among older adults. Delirium is also a common complication during hospitalization and is associated with multiple long-term sequelae. We sought to determine the effect of delirium and known covariates on the risk of incident HAD in hospitalized older adults. We examined electronic health record (EHR) data for 35,201 older adults ≥ 65 years of age admitted to the general inpatient (non-ICU) units of UAB Hospital from January 1, 2015 to December 31, 2019. Delirium was defined as a score ≥ 2 on the Nursing Delirium Screening Scale (NuDESC) during hospital admission, and HAD defined as a decline on the Katz ADL scale from hospital admission to discharge. Generalized linear mixed models were used to examine the association between delirium and HAD, adjusting for covariates and repeated observations for individuals with multiple admissions. We found that 21.2% of older adults developed HAD during their hospitalization and experienced higher delirium rates as compared to those not developing HAD (25.2% vs. 16.3%). Presence of delirium, medical comorbidity score, baseline cognitive status, and baseline ADL function were associated (all p <0.001) with incident HAD. Mediation analyses also showed that 8% of the effect of comorbidity on incident HAD was due to delirium (p < 0.001). Reducing rates of delirium can be one component of a comprehensive approach to reduce rates of HAD in older adults.

### PREDICTING UNSCHEDULED EMERGENCY DEPARTMENT REVISITS LEADING TO ACUTE HOSPITAL ADMISSIONS AMONG OLDER ADULTS

Tony Rosen,[1] Yufang Huang,[2] Matthew McCarty,[3] Michael Stern,[3] Yiye Zhang,[4] Daniel Barchi,[5] Rahul Sharma,[3] and Peter Steel,[3], *1. New York, New York, United States, 2. Weill Cornell Medicine, New York, New York, United States, 3. NewYork-Presbyterian Hospital Weill Cornell Medical Center, New York, New York, United States, 4. Weill Cornell Medical College, New York, New York, United States, 5. NewYork-Presbyterian Hospital, New York, New York, United States*

**Background:** Unscheduled emergency department (ED) revisits leading to acute hospital admission (RVA) are tantamount to a failed discharge, associated with physician error, mis-prognosis, and inadequate care planning. Previous research has shown RVA to be associated with adverse outcomes such as ICU admissions, long hospitalizations and mortality. Given the limited impact of pre-existing screening tools for older adults, we developed and validated a machine learning model to predict individual patient risk of RVA within 72 hours and 9 days of index ED visits.

**Method:** A machine learning model was applied to retrospective electronic health record (EHR) data of patients presenting to 2 geographically and demographically divergent urban EDs in 2019. 478 clinically meaningful EHR data variables were included: socio-demographics, ED and comorbidity diagnoses, therapeutics, laboratory test orders and test results, diagnostic imaging test orders, vital signs, and utilization and operational data. Multiple machine learning algorithms were constructed; models were compared against a pre-existing adult ED-RVA risk score as a baseline.

**Results:** A total of 62,154 patients were included in the analysis, with 508 (0.82%) and 889 (1.4%) having 72-hour and 9-day RVA. The best-performing model, combining deep significance clustering (DICE) and regularized logistic regression, achieved AUC of 0.86 and 0.79 for 72-hour and 9-day ED-RVA for older adult patients, respectively, outperforming the pre-existing RVA risk score (0.704 and 0.694).

**Discussion:** Machine learning models to screen for and predict older adults at high-risk for ED-RVA may be useful in directing interventions to reduce adverse events in older adults discharged from the ED.

### REJECTION OF CARE IN HOSPITALIZED PERSONS LIVING WITH DEMENTIA: THE IMPACT OF NURSE COMMUNICATION

Clarissa Shaw,[1] Caitlin Ward,[1] Jean Gordon,[1] Kristine Williams,[2] and Keela Herr,[1], *1. University of Iowa, Iowa City, Iowa, United States, 2. University of Kansas School of Nursing, Kansas City, Kansas, United States*

Rejection of care (RoC) by persons living with dementia (PLWD) has yet to be measured in the hospital setting. Elderspeak communication (i.e., baby talk or infantilization) is an established antecedent to RoC in nursing home dementia care. The purpose of this study was to determine the impact of elderspeak communication by nursing staff on RoC by hospitalized PLWD. Eighty-eight care encounters between 16 PLWD and 53 nursing staff were observed for RoC using the Resistiveness to Care scale in one Midwestern hospital. Audio-recordings of the care encounters were transcribed verbatim and coded for semantic, pragmatic, and prosodic features of elderspeak. Over one-quarter (28.7%) of the duration of nursing staff speech towards PLWD constituted elderspeak and nearly all (96.6%) of the 88 care encounters included some elderspeak. Almost half of the observations (48.9%) included RoC behaviors by PLWD. Rejection of care was modeled as present or absent using a GEE method. Characteristics of the PLWD (e.g., pain, delirium) and the observation (e.g., environmental simulation) were evaluated as potential covariates. After adjusting for pain, length of stay, and gender, a 15-percentage point decrease in the proportion of elderspeak communication by nursing staff reduced the odds of RoC by 62% (OR=0.38, 95% CI=0.21-0.71, p=.002,) and a one unit decrease in pain reduced the odds of RoC by 63% (OR=0.37, 95% CI=0.22-0.63, p<.001). This study identified that pain and elderspeak are two modifiable factors of RoC. Person-centered interventions are needed that address communication practices and approaches to pain management for hospitalized PLWD.

### UNDERSTANDING THE ROLE AND VALUE OF PROCESS QUALITY INDICATORS IN HOSPITALIZED OLDER SURGICAL PATIENTS

Janani Thillainadesan,[1] Sarah Hilmer,[2] Alison Mudge,[3] Sarah Aitken,[4] and Vasi Naganathan,[4], *1. Concord Hospital, NSW, New South Wales, Australia, 2. The University of Sydney, St Leonards, New South Wales, Australia, 3. Royal Brisbane and Women's Hospital, Royal Brisbane and Women's Hospital, Queensland, Australia, 4. University of Sydney, Concord, New South Wales, Australia*

Background Despite the development of geriatrics surgery process quality indicators (QIs), few studies have reported on these QIs in routine surgical practice. Even less is known about the links between these QIs and clinical outcomes, and patient characteristics. We aimed to measure geriatrics surgery process QIs, and investigate the association between process QIs and outcomes, and QIs and patient characteristics, in hospitalized older vascular surgery patients. Methods This was a prospective cohort study of 150 consecutive patients aged ≥ 65 years admitted to a tertiary vascular surgery unit. Occurrence of geriatrics surgery process QIs as part of routine vascular surgery care was measured. Associations between QIs and high-risk patient characteristics, and QIs and clinical outcomes were assessed using clustered heatmaps. Results QI occurrence rate varied substantially from 2% to 93%. Some QIs, such as cognition and delirium screening, documented treatment preferences, and geriatrician consultation were infrequent and clustered with high-risk patients. There were two major process-outcome clusters: (a) multidisciplinary consultations, communication and screening-based process QIs with multiple adverse outcomes, and (b) documentation and prescribing-related QIs with fewer adverse outcomes. Conclusions Clustering patterns of process QIs with clinical outcomes are complex, and there is a differential occurrence of QIs within older vascular surgery patients, suggesting process QIs alone may be unreliable targets for quality improvement. Prospective intervention studies are needed to understand the causal pathways between process QIs and outcomes to help prioritize care processes that are most clearly linked to improved outcomes.

### Session 4595 (Symposium)

### PREPARING FOR THE NEW NORMAL: CHRONICLING THE IMPACT OF COVID-19 ON OLDER ADULTS AND PROVIDERS

Chair: Rose Ann DiMaria-Ghalili Discussant: Justine Sefcik

COVID-19 and social distancing heralded an unprecedented change in the way older adults and health care providers live, work, socialize and manage their health. Early "calls-to-action" included the call for researchers to chronicle the impact of the COVID-19 pandemic on care of older adults to inform models of care and best practices in the new normal. This symposium explores the impact of COVID-19 on the health of older adults across the care continuum and healthcare delivery augmented by technology. The perspectives of older adults living in the community and providers who care for this population are highlighted. Additionally, there is a focus on the most vulnerable, those living in skilled care facilities and continuing care retirement communities. Fisher analyzes the key themes in 37 COVID-19 video communiques over 11 months at a continuing care retirement community. Sefcik explores coping strategies including outdoor activities among community-dwelling older adults. DiMaria-Ghalili examined patterns of physical and mental health, technology usage and loneliness in older adults, including those living in the community and a continuing care retirement community. Using longitudinal data and COVID-19 supplemental survey data from the National Health and Aging Trends Study, Huh-Yoo discusses disparities in online patient-provider communication and implications for the Post-COVID era. Coates discusses the facilitators and barriers perceived by interdisciplinary providers deploying telehealth during the COVID-19 pandemic and implications for healthcare delivery in older adults. The symposium will conclude with a discussion by Dr. Sefcik on the implications for research, practice and policy in the post COVID-19 era.

### PHYSICAL AND MENTAL HEALTH, TECHNOLOGY USE, AND LONELINESS IN OLDER ADULTS DURING THE COVID-19 PANDEMIC

Martha Coates,[1] Zachary Hathaway,[2] Katelyn Moore,[2] Yaegin Park,[2] Jenny Tsui,[2] Justine Sefcik,[3] and Rose Ann DiMaria-Ghalili,[3], *1. Drexel University, Bryn Mawr, Pennsylvania, United States, 2. Drexel University, Philadelphia, Pennsylvania, United States, 3. Drexel University, College of Nursing and Health Professions, Philadelphia, Pennsylvania, United States*

Social isolation is a negative outcome of COVID-19. This study examined patterns of physical and mental health and technology use in older adults, and loneliness during the COVID-19 pandemic. We recruited 115 community-dwelling older adults 65 and older (72% female) from the Pennsylvania region via Research Match (N=84) or from a retirement community (N=31). A significant association between loneliness and worsening of health during the pandemic was observed, Fisher's Exact Test 6.90, p=.03. Those who were lonely demonstrated significantly lower Mental Component Summary Scores (M = 42.75, SD = 11.55) compared to those who were not lonely (M= 55.34, SD= 7.66), t(49) = 5.84, p <.01. Those reporting loneliness were more likely to use a new electronic device to communicate with family during COVID-19 pandemic, X2, (1, N= 107) = 6.24, p =.01. These findings suggest the important role of technology to decrease loneliness in older adults during a pandemic.

### ASSESSING COPING STRATEGIES AND OUTDOOR ACTIVITIES AMONG OLDER ADULTS DURING THE COVID-19 PANDEMIC

Martha Coates,[1] Sarah Wetzel,[2] Janvi Patel,[2] Keyanna Bynum,[2] K. Linh Pham,[2]

Rose Ann DiMaria-Ghalili,[3] and Justine Sefcik,[3], *1. Drexel University, Bryn Mawr, Pennsylvania, United States, 2. Drexel University, Philadelphia, Pennsylvania, United States, 3. Drexel University, College of Nursing and Health Professions, Philadelphia, Pennsylvania, United States*

Information is lacking on how older adults are coping during the pandemic. We explored coping strategies including outdoor activities among community-dwelling older adults (N = 115) 65 and older (mean age 76.45, 71.3% female). Using conventional content analysis, we analyzed responses to: 1) How are you coping with COVID-19? and 2) How often are you going outside during the pandemic and for what reasons? Most common activities are connecting with family and friends (some in person, others on the phone or virtually), reading, tv, game playing, and learning something new (e.g. webinars, online classes). The majority are going outside every day, with walking being the most common activity. Only a few are restricting their out of home activities to essential tasks (e.g. going to the doctors, pharmacy, getting groceries). Findings suggest that many older adults are engaging in positive coping activities. Assessing coping strategies can give insight into wellbeing.

### EXPERIENCES OF INTERDISCIPLINARY PROVIDERS DEPLOYING TELEHEALTH DURING THE COVID-19 PANDEMIC

Katelyn Moore,[1] Zachary Hathaway,[1] Gloria Gonzalez-Kruger,[1] Kymberlee Montgomery,[1] Rose Ann DiMaria-Ghalili,[2] and Martha Coates,[3], *1. Drexel University, Philadelphia, Pennsylvania, United States, 2. Drexel University, College of Nursing and Health Professions, Philadelphia, Pennsylvania, United States, 3. Drexel University, Bryn Mawr, Pennsylvania, United States*

The expansion of telehealth services during COVID-19 is critical for healthcare delivery. This study describes the facilitators and barriers experienced by providers integrating telehealth during COVID-19. The sample consisted of 441 interdisciplinary providers (RNs, APNs, PAs, DPTs, RDs, mental health counselors) who were faculty or alumni of a college of nursing and health professions and completed the online telehealth provider survey. 53% of respondents were nurses/APNs, 59% implemented telehealth within the first week of federal legislation, and 48% received telehealth training once the pandemic started. Respondents reported telehealth changed several services provided during the pandemic (e.g., increased prescription of longer-term medication refills, increased counseling sessions). The greatest reported barrier to utilizing telehealth during the pandemic was the older adults' ability to utilize technology. Understanding the facilitators and barriers experienced by providers during COVID-19 will lead to more robust healthcare delivery models to enhance health outcomes in older adults.

### UNCERTAIN TIMES: A CONTINUING CARE RETIREMENT COMMUNITY'S COVID-19 COMMUNICATION RESPONSE

Justine Sefcik,[1] Martha Coates,[2] Minjung Shim,[3] Don McEachron,[3] Rose Ann DiMaria-Ghalili,[1] and Kathleen Fisher,[3], *1. Drexel University, College of Nursing and Health Professions, Philadelphia, Pennsylvania, United States, 2. Drexel University, Bryn Mawr, Pennsylvania, United States, 3. Drexel University, Philadelphia, Pennsylvania, United States*

The purpose of this qualitative inquiry was to explore conversational video recordings by top administrators from a faith based Continuing Care Retirement Community (CCRC) to help residents, staff, and family members manage the associated uncertainties of the pandemic. Six interdisciplinary researchers explored 37 video communiques from March 2020 to February 2021. Data was independently coded using latent content analysis with the team building consensus on major themes. Themes identified were: Building Trust through Transparency, We're in this Together, Power of One/Individual Responsibility, Converting Challenges into Teaching Moments, and Gratitude/Resilience. Findings suggest attempts to inform, reassure, and encourage maintaining a safe environment (e.g., using masks, restricting visitors, vaccine promotion) for residents, staff, and family members, was met through these conversational videos. Leadership of this CCRC exemplified the mission to provide transparent information during the pandemic, serving as a model to inform other CCRCs' communication during this pandemic and other crisis situations.

### DISPARITY ON ONLINE PATIENT-PROVIDER COMMUNICATION AND IMPLICATIONS FOR A POST-COVID ERA

Michael LeVasseur,[1] Martha Coates,[2] Justine Sefcik,[3] Rose Ann DiMaria-Ghalili,[3] and Jina Huh-Yoo,[1], *1. Drexel University, Philadelphia, Pennsylvania, United States, 2. Drexel University, Bryn Mawr, Pennsylvania, United States, 3. Drexel University, College of Nursing and Health Professions, Philadelphia, Pennsylvania, United States*

Online patient-provider communication (OPPC) increases access to health-related information, enhances self-care, and reduces healthcare expenditures. We investigated OPPC and technology use changes during 6 years. This descriptive cohort analysis involved data collected from the National Health and Aging Trends Study (NHATS) between 2011-2016 and during the pandemic (2020). NHATS consist of a nationally representative sample of Medicare beneficiaries. From a total of N=2,389, we assigned cohorts as those who: (1) own computers (High-Tech, 56.1%), (2) own cell phone only or don't own computer nor cell phones (Low-Tech, 20.2%), and (3) just adopted cell phone or computers (Transition, 23.7%). Only the High-Tech users increased OPPC by 75% over 2011-2016; others remained the same. During the pandemic more participants used mobile technology and internet. Newly adopting technology on its own does not automatically increase OPPC. It is important for Transition users to access training and support to benefit from OPPC.

### Session 4600 (Symposium)

### QUALITATIVE RESEARCH WITH LGBTQ OLDER ADULTS AND OLDER ADULTS LIVING WITH HIV/AIDS DURING COVID-19

Chair: Sara Bybee Co-Chair: Austin Oswald

In this symposium, researchers will discuss conceptual and practical challenges to conducting qualitative research with older LGBTQ+ adults and older adults living with HIV/AIDS during the COVID-19 pandemic. Researchers who conduct studies with LGBTQ+ populations and with older adults living with HIV/AIDS already face challenges in recruiting and retaining large enough sample sizes. Social distancing guidelines during the pandemic prohibited many researchers from conducting in-person studies. Conducting health and social science research entirely online may have its challenges when working with these older adult populations, but it may also present new opportunities for reaching hidden or hard-to-reach groups. Presenters draw on their recent experiences conducting semi-structured interviews, online surveys, and qualitative analyses of public records to illuminate the complex considerations of conducting research remotely with LGBTQ+ older adults and older adults living with HIV/AIDS. Presenters also discuss how conducting remote research with these populations may require methodological changes in study design in order to collect the most meaningful and reliable data from research participants. Ultimately, COVID-19 has required researchers to alter or change their methods of conducting research. As the internet becomes more heavily relied upon for conducting research, it is imperative that scholars in LGBTQ+ aging and HIV/AIDS consider the potential challenges and opportunities that online research presents for their studies, and for research participants who are living with HIV/AIDS or who identify as LGBTQ+.

### RECRUITING AND CONDUCTING ONLINE DYADIC SEMI-STRUCTURED INTERVIEWS WITH LGBTQ+ COUPLES FACING ADVANCED CANCER

Kristin Cloyes,[1] Lee Ellington,[2] Brian Baucom,[3] Katherine Supiano,[4] Kathi Mooney,[2] and Sara Bybee,[2], *1. University of Utah, Portland, Oregon, United States, 2. University of Utah, Salt Lake City, Utah, United States, 3. University of Utah Department of Psychology, Salt Lake City, Utah, United States, 4. University of Utah, Salt Lke City, Utah, United States*

In this study, LGBTQ+ adult couples facing advanced cancer were recruited online. Eligible couples were sent a direct link to electronic consent and surveys in REDCap®. Participants were then invited to complete a 45-minute dyadic semi-structured interview regarding their experience of coping with cancer as a couple. This study faced difficulties in recruiting LGBTQ+ couples, and also faced the challenge of identifying and managing online responses from individuals misrepresenting themselves, and from automated accounts or "bots". LGBTQ+ aging scholars must acknowledge how conducting research remotely with LGBTQ+ adults may necessitate changes in study design, such as changes to recruitment and more comprehensive eligibility screening designed to prevent and detect the collection of untrustworthy data. Ultimately, protecting the integrity of participant data in online research supports research accessibility and inclusion for LGBTQ+ older adults, and is the first step in conducting research that promotes health equity.

### CONDUCTING COMMUNITY-BASED QUALITATIVE RESEARCH WITH LGBTQ OLDER ADULTS OF COLOR DURING COVID-19

Aundaray Guess,[1] and Austin Oswald,[2], *1. GRIOT Circle, Brooklyn, New York, United States, 2. CUNY Graduate Center, New York, New York, United States*

COVID-19 continues to transform the way scientists conduct research with study participants, particularly older adults who are at high risk of becoming seriously ill from the virus. For older adults who may be negatively affected by the digital divide, inclusive data collection practices become even more nuanced. Qualitative researchers moving their research into digital spaces must think critically about their use of technology, and how it affects the quality of data as well as the participant experience. This presentation highlights ethical and methodological considerations from a completely digital, community-based, qualitative research study conducted alongside LGBTQ older adults of color during COVID-19. Strategies to build and strengthen community partnerships are discussed along with challenges and opportunities for collecting data in the current digital landscape. Publicly available records are identified as a potential data source to understand the lives of LGBTQ older adults of color when in-person research is not feasible.

### LGBTQ OLDER ADULT RECRUITMENT IN THE MIDST OF A COVID-19 LOCKDOWN: REMINISCENCES OF A POST-DOCTORAL FELLOW

Robert Beringer, *University of Victoria, Victoria, British Columbia, Canada*

Within days of obtaining ethics approval for a qualitative study "Optimizing LGBTQ Engagement with Hospice and Palliative Care in the Island Health Region" our local Covid-19 lockdown began. It took several months to have new Covid-19 research protocols (Zoom Town Hall meetings/Zoom or telephone interviews) approved. Being impatient, I teamed with another group of researchers to launch "Covid-19: Your Current Experiences and Planning for the Future," an online survey with a large qualitative component where we planned to oversample LGBTQ respondents. In time both projects were approved, and here I reflect on recruitment lessons learned. These include my perceptions how Zoom Town Hall meetings and interviews differ from those I've conducted in-person, reflections on how to use social media (including targeted Facebook advertising) to recruit participants, and sadly, how to manage anti-LGBTQ sentiment that resulted from even the most targeted advertising.

### QUALITATIVE RESEARCH WITH HIV+ OLDER ADULTS LIVING IN RURAL AREAS: METHODOLOGICAL CONSIDERATIONS DURING COVID-19

Erin Robinson, *University of Missouri, Columbia, Missouri, United States*

Older adults living with HIV/AIDS (OALWHA) in rural areas of the U.S. are a highly marginalized community. Intersectional stigma related to age, HIV status, geography, sexual orientation, gender identity, and race oftentimes create a complex lived experience for this population group. While there is a significant need for qualitative research that highlights the intersecting stigmas experienced by OALWHA in rural areas, recruitment challenges exist. Fear of being outed in their rural communities, due to their HIV status and LGBTQ+ identities, makes many OALWHA reluctant to participate in research. However, there is much resiliency in the population as well, especially during the COVID-19 pandemic. In fact, as research approaches have pivoted to phone/virtual data collection during the pandemic, this can help promote anonymity among this population group. This presentation will detail methodological considerations for recruitment, data collection, and analysis for qualitative research with OALWHA in rural areas of the U.S.

### Session 4605 (Symposium)

### SOCIAL RELATIONS, STRESS, AND RACIAL HEALTH DISPARITIES

Chair: Kristine Ajrouch Co-Chair: Noah Webster
Discussant: Toni Antonucci

This symposium brings together four papers that address racial health disparities by investigating stressful aspects of social relations at different points in the life course. Cleary and colleagues focus on racial disparities in psychological health by testing cross-sectional effects of intergenerational stress over time. In particular, they investigate effects of network composition on the relationship between mothers' stressors and their children's depressive symptoms at three time points over 23 years. Camacho and colleagues use longitudinal data from the National Social Life, Health and Aging Project to examine cognitive decline among U.S. African-American, Latino, and White adults aged 60 and above. Results indicate loneliness predicted greater global cognitive decline over time in all groups. However, race differences in this association were found across cognitive function domains. Turner and colleagues consider dementia caregiving challenges among non-Hispanic Blacks. Data from five focus groups were analyzed to reveal distinctive challenges to caregiver health during the COVID-19 pandemic including increased burden and barriers to service access. Finally, Sol and colleagues examined the bidirectional association between loneliness and self-rated health over time among a racially diverse sample. Findings illustrate racial patterns in how loneliness at midlife influences health in later life. Antonucci will discuss the role of stress from social relations as a means to fully understand racial disparities in health across the life course.

### MATERNAL STRESS, FAMILIAL TIES, AND CHILDREN'S WELL-BEING ACROSS 23 YEARS

Jasmine Manalel,[1] Simon Brauer,[2] and Jennifer Cleary,[2], *1. National Human Genome Research Institute, Bethesda, Maryland, United States, 2. University of Michigan, Ann Arbor, Michigan, United States*

According to the family stress model, parental stress impacts child well-being through several mechanisms, which may be amplified in ethnic/racial minority families given increased experiences of stress. We extend this model to examine associations between maternal stress and child well-being at three points spanning 23 years, beginning when children were aged 8-12 years and mothers were aged 24-59 (n=193 dyads). Preliminary results indicate that maternal stressors are associated with increased depressive symptoms in childhood (B=3.56, $p<0.001$), and this association was stronger among Black children compared to White (B=4.12, $p<0.001$). Effects of maternal stress on children's depressive symptoms strengthened among White children with proportionally larger kin networks (B=0.05, $p<0.001$). However, this association weakens as children enter adulthood. Future work will focus on identifying social resources that account for changes in the intergenerational effects of stress.

### CHALLENGES IN RESOURCE UTILIZATION FOR CAREGIVERS OF PERSONS WITH DEMENTIA: A QUALITATIVE STUDY

Jen Weaver,[1] Eric Owens,[1] Meredith Boe,[1] Jessica Bride,[1] Maritza Dowling,[2] Christina Prather,[3] Melinda Power,[1] and Robert Turner,[1], *1. The George Washington University, Washington, District of Columbia, United States, 2. George Washington University - School of Nursing, Washington, District of Columbia, United States, 3. The GW Medical Faculty Associates, Washington, District of Columbia, United States*

This study highlights primary caregivers' experiences with health department policies designed to support people with cognitive impairment/Alzheimer's Disease and Related Dementias (ADRD). Caregivers were defined as individuals aged 45-85 that provide at least 10 hours of unpaid care. Five, 90-minute focus groups were conducted virtually with 24 caregivers of individuals with cognitive impairment/ADRD. Transcripts were analyzed thematically. Caregivers were primarily Black females (75%) with at least a high school education (42%). Care recipients were likely to be community-dwelling parents (71%), with moderate or advanced (79%) dementia. Caregivers described challenges with accessing resources intended for care recipients, especially as cognitive impairment worsened. Caregivers reported providing care 24/7 as traumatizing. Home-based personal aides and companionship services did not reduce this burden. COVID-19 impacted caregivers and care recipient's access to resources increasing burden. Policies need to be flexible for ever-changing needs of individuals with ADRD and support the overall well-being of the caregivers.

### LONGITUDINAL ASSOCIATIONS BETWEEN LONELINESS AND SELF-RATED HEALTH

Simon Brauer, Toni Antonucci, and Ketlyne Sol, *University of Michigan, Ann Arbor, Michigan, United States*

Cross-lagged structural equation models examined the bidirectional association between loneliness and self rated health over three time points. We adjusted for age, gender, network size, and depressive symptoms at baseline. At baseline, the sample was 28% Black and 40% male. Average age at time 1 was 46 years, 56 years at time 2, and 63 years at time 3. Results indicated that loneliness at time 1 was associated with loneliness at time 2; loneliness at time 2 was associated with loneliness at time 3. We had similar findings for associations among self rated health. However, only one of the cross-lagged paths was significant. Specifically, more loneliness at time 2 was associated with worse self rated health at time 3. These associations did not vary across black and white race. Findings indicate that loneliness at later midlife may be detrimental to later life health, regardless of race.

### LONELINESS AND CHANGES IN COGNITIVE FUNCTIONING AMONG RACIALLY DIVERSE OLDER ADULTS IN THE UNITED STATES

Kelly Pacheco,[1] Charles Henderson,[2] M. Cary Reid,[3] Elaine Wethington,[4] and David Camacho,[5], *1. New York University, New York University, New York, United States, 2. Cornell University, Cornell University, New York, United States, 3. Weill Cornell Medicine, Weill Cornell Medicine,*

*New York, United States, 4. Cornell University, Ithaca, New York, United States, 5. Weill Cornell Medicine, New York, New York, United States*

Longitudinal studies examining the association of loneliness with cognitive decline rarely include older members of racial minorities. Guided by a Minority Stress Framework, we used Waves 2 and 3 from the National Social Life, Health, and Aging Project to assess whether loneliness (UCLA-3-items) at W2 predicts cognitive decline (Chicago Cognitive Functioning Measure) among US African-American, Latino, and white older adults (ages≥60). We included interactions of W2 loneliness with race in examining changes in cognitive functioning. Estimates were (N=1,950) adjusted for demographics, chronic disease, depression, and social connectedness. In all groups, loneliness was positively associated with greater global cognitive decline over the 5-year interval. However, analyses of different domains of cognitive functioning (e.g., executive functioning, memory) suggested that this association differs by cognitive domain and race. Future research on interventions to prevent cognitive decline should consider targeting loneliness, include diverse older adults, and examine global and specific domains of cognitive functioning.

### Session 4610 (Symposium)

### STRATEGIES TO DESIGN TECHNOLOGY PROMOTING SOCIAL PARTICIPATION OF PEOPLE WITH DEMENTIA AND THEIR CAREGIVERS

Chair: Karin Wolf-Ostermann Discussant: Jane Flanagan

Community-dwelling people with dementia and their caregivers face increasing challenges to active social participation as the condition progresses. Potential difficulties include disclosing the condition, navigating through available support and sustaining interpersonal relationships. Dementia-friendly support services and interventions targeting caregiving dyads can promote social participation. Interventions serve as a communication channel for the dyads to engage, interact and partake in their community. Technology as a facilitator is gaining momentum; increasing evidence suggests that technological solutions contribute to promoting social health for people with dementia and family caregivers. Patient and public involvement and rigorous evaluations of solutions are needed to ensure successful implementation of dementia-friendly technologies. This symposium, presented as a part of the Marie-Curie Innovative-Training-Network action, H2020-MSCA-ITN, grant agreement number 813196, comprises four pertinent presentations. The first presentation outlines the effectiveness of technological interventions to improve social participation of older adults with and without dementia, and barriers and facilitators these interventions present. The second presentation describes disclosure decisions faced by dyads and Patient and public involvement findings on how an existing empowerment intervention supporting disclosure decision-making can transfer to an online environment. The third presentation reports on findings from a study evaluating a tablet-based activation system designed to engage caregiving dyads in social sessions. The final presentation lifts the focus towards how existing online environments can be adapted through dementia-friendly privacy policy agreements, and thereby support social participation of this user group online. Our discussant, Jane Flanagan, synthesizes the presentations and leads a discussion of future directions for policy and practice.

### TECHNOLOGY-DRIVEN DYADIC INTERACTION SUPPORT FOR COMMUNITY-DWELLING PEOPLE WITH DEMENTIA AND FAMILY CAREGIVERS

Karin Wolf-Ostermann,[1] Lars Steinert,[2] Tanja Shultz,[2] and Viktoria Hoel,[2], *1. University of Bremen, University of Bremen, Bremen, Germany, 2. University of Bremen, Bremen, Bremen, Germany*

People with dementia and their family caregivers struggling with the impacts of the condition on cognitive abilities, experience deterred social interactions and strained relationships. Technology can potentially sustain the relationship by engaging dyads in joint activities and supporting their interaction. This study aimed to evaluate the impact of a tablet-based activation system, I-CARE, specifically designed to engage people with dementia in meaningful activities. In this intervention, community-dwelling people with dementia and their family caregiver engaged in joint activities supported by the I-CARE system. Quantitative measures on quality of life, relationship quality and caregiver burden are collected, while semi-structured interviews explore the impact of Covid-19, as well as what motivates the participants to invite technology into their dyadic interactions. Our findings provide important insight in how technology can support social health and relationship sustenance of dyads living with dementia, and what implications Covid-19 has for their social participation in society.

### INDIVIDUALS' DECISION TO DISCLOSE A DIAGNOSIS OF DEMENTIA AND THE DEVELOPMENT OF AN ONLINE EMPOWERMENT INTERVENTION

Mauricio Molinari Ulate,[1] Jem Bhatt,[2] Jennifer Lynch,[3] Katrina Scior,[2] Georgina Charlesworth,[2] and Gianna Kohl,[4], *1. University of Salamanca, Salamanca, Castilla y Leon, Spain, 2. University College London, London, England, United Kingdom, 3. University of Hertfordshire, Hatfield, England, United Kingdom, 4. University College London, University College London, England, United Kingdom*

Learning to live with a diagnosis of dementia is a complex process. Many people affected by dementia choose not to disclose the diagnosis to others and avoid social activities due to fear of others' adverse reactions. This in turn can limit their social participation and negatively affect their psychosocial health. A systematic review explored factors influencing the decision to disclose or conceal a dementia diagnosis to one's social network, including individuals' attitudes and experiences regarding this decision. The sixteen studies included reveal the complexity of this decision. Findings highlight the role of stigma and individuals' wishes to remain 'normal', but also the need of explaining what has changed. Results were further discussed with people with dementia and informal caregivers as part of patient and public involvement. End users expressed their attitudes, needs, and wishes towards the design of an online empowerment intervention supporting disclosure decision-making in people affected by dementia.

### TECHNOLOGICAL INTERVENTIONS AND SOCIAL PARTICIPATION IN COMMUNITY-DWELLING OLDER ADULTS WITH OR WITHOUT DEMENTIA

Lizzy Boots,[1] Wei Qi Koh,[2] An Neven,[3] Frans Verhey,[1] Marjolein de Vugt,[1] and Pascale Heins,[4], *1. Alzheimer Centrum Limburg, Maastricht University, Maastricht, Limburg, Netherlands, 2. National University of Ireland Galway, Galway, Galway, Ireland, 3. UHasselt - Hasselt University, Transportation Research Institute (IMOB), Diepenbeek, Limburg, Belgium, 4. Maastricht University, Maastricht, Limburg, Netherlands*

Social isolation is a growing health issue in community-dwelling older adults with and without dementia as it can negatively affect their health and well-being. Consequently, psychosocial interventions targeting their social participation are increasingly gaining importance. So far, however, little is known about the potential of technological interventions in this population. Therefore, this systematic review explored the effectiveness of technological interventions in improving social participation of community-dwelling older adults with and without dementia. Records identified through five scientific databases were independently screened by two reviewers. A total of 36 studies published between 2005 and 2020 were included in a narrative synthesis. Studies differed widely in study design, type of technology, used outcome measures, and methodological quality. However, the findings highlight the potential role of technological interventions in improving different dimensions of social participation. At the same time, barriers and facilitators of these interventions to social participation were identified.

### USER-CENTERED DESIGN OF A DEMENTIA-FRIENDLY PRIVACY POLICY FOR THE FINDMYAPPS INTERVENTION

Yvonne Kerkhof,[1] Teake Ettema,[2] Karin Dijkstra,[1] Rose-Marie Dröes,[2] and David Neal,[2], *1. Saxion University of Applied Sciences, Deventer, Overijssel, Netherlands, 2. Amsterdam UMC, Amsterdam, Noord-Holland, Netherlands*

The ability of people with dementia and their caregivers to successfully navigate online environments is increasingly important to their social health. However, uncertainty about privacy online is an important barrier. Theoretically, access to published privacy policies should allow users of websites or software applications to make informed decisions. In practice, such documents are often complicated texts, and consequently even less accessible to people with cognitive impairment than to the general population. We present results from a multi-stakeholder, user-centred design process, towards an accessible alternative: a ‘dementia-friendly privacy policy’. Three design sprints took place in 2021, led by participants of the ‘Smart Solutions Semester’ at Saxion University of Applied Sciences in the Netherlands, in collaboration with cognitively unimpaired laypeople, people with dementia, informal caregivers, and expert stakeholders. Outputs were specifications for the solution, low-fidelity prototypes and high-fidelity prototypes, respectively. The dementia-friendly privacy policy is now ready for implementation and further evaluation.

### Session 4615 (Symposium)

### THE BLUES AND OLDER MINORITY MUSICIANS XXVIII: MORE THAN JUST MUSIC

The Blues has been a ubiquitous music genre for over 150 years throughout the United States. It's not Mississippi, but Phoenix shares a thriving Blues scene and many GeroBlues performers. This year, the “Bo Diddley Track” celebrates 28 years of continued performance of the Blues and Older Minority Musicians: More Than Just Music at GSA, a legacy of and memorial to former Executive Director Paul Kirschner. Join with your colleagues and local notable musicians for a rousing story of challenge, resilience, and some great music worthy of the true spirit of GSA and these great Blues performers to survive, thrive, and grow in the face of challenge and adversity. Enjoy the lecture, mini-performance and then a special musical event at an outstanding local music venue later in the evening. Enjoy one of the best parties at GSA!

### Session 4620 (Symposium)

### THE IMPACT AND RESPONSE TO CLIMATE CHANGE AS LIVED BY OLDER ADULTS

Chair: Robin Fenley Co-Chair: Toni Antonucci
Discussant: Robin Fenley

A growing body of literature documents the domino effects of climate change on the planet and all life. Chief among these changes is the rise in global temperatures, triggering record numbers of heatwaves, and stronger, more dangerous hydrologic events. While climate change looms as a preeminent threat to our planet and future, the public health and human rights ramifications are already apparent. As with many issues in this realm, the effects are felt to a greater degree by our aging populations. As disasters grow in frequency, the more vulnerable populations are at greater risk for more serious outcomes – and will suffer disproportionately from the effects of climate change – resulting in greater inequalities. With the consequences of climate change growing more drastic, these outcomes can be expected to climb unless sufficient measures are enacted to combat global warming. In this symposium we will highlight the link between climate change and its impact on the human rights of older adults, and how climate change threatens progress across the Sustainable Development Goals (SDG) - a blueprint for a more equitable and healthier planet - if decisive actions are not taken. This symposium will demonstrate what valuable opportunities exist to accelerate progress by leveraging the links between SDGs to combat inequalities and climate change. Panelists will discuss the adverse effects of climate change, the human rights and psychological impacts on older adults, and potential action steps and strategies for older persons to become empowered as advocates for climate change reform.

### PSYCHOLOGICAL RISK AND RESILIENCE OF OLDER PEOPLE’S EXPOSURE TO CLIMATE CHANGE

Toni Antonucci, *University of Michigan, Ann Arbor, Michigan, United States*

Climate change places older people at physical and psychological risk. Even small changes in temperature (+/- 1 degree) results in increased morbidity and mortality. Further, the burden of climate change is not borne equally. The stress and mental ill health associated with disasters are especially borne by women, older adults, persons of color, low-income populations, those with preexisting conditions, poor support networks, and those residing in sub-standard housing. Older people are disproportionately represented in these groups, but interventions can ameliorate these risks. At the same time, older people, with the wisdom of experience, can be important assets for families and communities struggling with the effects of climate change. Both addressing the vulnerabilities and enhancing the strengths of older people can serve to meet the immediate needs and the long-term SDGs goals of (3) health and well-being (5) gender equality (10) reduced inequalities (11) sustainable cities and communities (13) climate action.

### ADDRESSING THE ADVERSE EFFECTS OF CLIMATE CHANGE ON THE HUMAN RIGHTS OF OLDER PERSONS

Erin Burk-Leaver, *International Association of Gerontology and Geriatrics, NEW YORK, New York, United States*

The common consequences of climate change events include: displacement, loss of sustainable shelter and housing, and limited access to medical care and other resources such as food, clean water, and sanitation services. These adverse effects coincide to an alarming degree with the human rights most essential to those in vulnerable or marginalized groups, including older populations. Whether through displacement or disruption of supply, the stressors of climate change events greatly exacerbate older populations' vulnerability, especially when compounded by negative social determinants of health, such as existing social, political, and economic barriers to successful aging. Using the SDGs as a framework to develop policies around (13) climate action and the use of improved (9) industry, innovation, and infrastructure to create (11) sustainable cities and communities, it is possible to establish (10) reduced inequalities to promote overall (3) good health and well-being in our older populations.

### ACTION STEPS AND STRATEGIES TAKEN BY OLDER PERSONS TO PREVENT AND MITIGATE EFFECTS OF CLIMATE CHANGE

Martha Bial, *Fordham University, West Harrison, New York, United States*

While the vulnerability of older persons to climate change is recognized by many scholars, there has been less attention to contributions older adults make to the fight against climate change, and their motivations to engage in that fight. Motivations include concern for the environment they will leave to their descendants. Contributions include freed up time, and skills gained in personal or work history to educate others on the issues and to organize and advocate for policy change. This presentation will highlight several national and international organizations of older people devoted to educating community groups, monitoring water quality and changes in wildlife habitats, and testifying before legislative bodies in campaigns for increased environmental regulation. Some of these organizations are intergenerational, providing additional benefits in cross-generational social exchange. Such activities are in line with SDGs 13 (action on climate change), 11 (sustainable cities), 12 (sustainable consumption) and 17 (expanding multi-stakeholder partnerships).

### EMPOWERING CAREGIVERS IN THE MIDST OF CLIMATE CHANGE

Carole Cox, *Fordham University, Fordham University, New York, United States*

More than 2.7 million children in the United States are raised in kinship families, with the majority of these caregivers, grandparents. Nationally, 1 in 11 children is raised in a kinship family, among Black children, the ratio is 1 in 5. Many of these families struggle economically, welcoming their young relatives into small, often substandard public housing where nonexistent or inadequate heating and cooling exacerbate attempts to moderate extreme temperatures in crowded apartments. For others, responsibility for the children follows the loss of life or permanent disruption of family composition due to weather events such as hurricanes or tornadoes. Grandparent resilience is reflected in their commitment to the well-being of these children, especially observed throughout COVID-19. This presentation will demonstrate that a Grandparent Empowerment Program is an effective strategy to tap into their strength as advocates for adequate health care, educational opportunity, resources, and a world free from climate disruption.

### Session 4625 (Symposium)

### TRANSCENDING THE CORPOREAL IN LATER OLD AGE?

Chair: Paul Higgs

This symposium addresses the older body and later life. It focusses on the cultural and social implications of the corporeality of the ageing body. Specifically it seeks to explore the degree to which it is possible to transcend the constraints brought about by the body in later old age. Drawing the distinction between the third and fourth ages for understanding contemporary ageing the papers address three important dimensions of later old age. The first presentation by Gilleard directly addresses the corporeality of late old age noting its seeming undesirability and limitation. Gilleard posits that not only does the ageing body impact on the lived experiences of those in later old age but also acts as a cultural reference point for the representation of this period of the life course. Eliopoulos presents preliminary results from her qualitative study on social exclusion of individuals aged over 80 living in remote island environments of the Pacific Northwest. The research considers how such environments might, even in the absence of high levels of health and social care resources, mitigate some of the constraints associated with the ageing body. The chair, Paul Higgs will discuss the issue of ageism and how it is abstractly inscribed on the ageing body; often with little reference to the lived experiences of older people themselves. He will call for a more reflexive approach to ageism. Overall, the symposium seeks to draw gerontological attention to the complexities and possibilities surrounding the ageing body at later ages.

### THE 9TH DECADE AND BEYOND: THE PERILS OF THE NARRATIVE OF DECLINE

Elaine Eliopoulos, *University College London, University College London, England, United Kingdom*

Twenty-three participants ranging in age from 80-102 years living on remote islands in the Pacific Northwest, USA reported the privileges of their current years. The aim of the study was to explore lived bodily experience and its impact on social exclusion. Participants utilized a unique visual methodology by photographing their experiences which highlighted daily life. While acknowledging that their years 'before' were different, and that life going forward may present unwelcome challenges, life in the now brought new joys and self-determination, despite various bodily compromises. Their perceptions of their bodies defied the dominant narrative of decline. These important findings warrant further investigation of the ways in which this emerging cohort views the challenges of aging bodies and their ability to remain socially connected. The role the dominant narrative of decline plays in their lives may prove to misdirect supports.

### THE CORPOREALITY OF LATE AGE

chris Gilleard, *University College London, UCL, England, United Kingdom*

In a study of over a thousand Germans, Paul Baltes and his colleagues observed that most respondents saw age 80-84 as the preferred age to reach before dying. Living beyond 85 was only desired by a minority. Perhaps this is because this age seems to many the point when bodily disease and physical weakness render life not just unpleasant but actively burdensome. Such views underpin the social imaginary of an undesirable fourth age. This paper discusses the significance of corporeality as both representation and lived experience, raising the question of whether the disparity between real and imagined corporealities resides as much from an 'other' within as without.

### Session 4630 (Symposium)

### VIEWS OF AGING DURING THE CORONA PANDEMIC

Chair: Jennifer Bellingtier Co-Chair: Anna Kornadt

In the Covid-19 pandemic, media stories and government reports have emphasized the heightened risk of being "old" and placed a spotlight on the way we think and talk about older adults and aging. In this symposium we investigate how the pandemic and the public discourse about older adults has shaped views of aging in different countries. Bellingtier et al. report on German children's views of older adults before and during the pandemic. Children placed greater distance between themselves and older adults both before and after the pandemic, suggesting early ageism in children that predates the pandemic. Levy et al. provide experimental evidence that media stereotypes about aging and Covid-19 influence the mental health of older American adults, both in positive and negative. Schwartz and Ayalon found that greater perceptions of age-based discrimination in the healthcare system by Israeli adults 50+ were significantly related to greater Covid-19 worries. Greater worry can motivate older adults to take precautions, but be detrimental if it becomes too high. In line with this finding, Tingvold et al. found in a study with older adults from Luxembourg that more Covid-19 worry predicted feeling older four months later, but only for those in worse health. Finally, Terracciano examined longitudinal change in subjective age and found that American adults reported feeling younger after the emergence of Covid-19 than before, suggesting that perceptions of aging partly reflect a coping process to counter the negativity in the media.

### A MULTI-METHOD EXAMINATION OF AGEISM IN CHILDREN BEFORE AND DURING THE PANDEMIC

Jenny Bauer, Lena-Emilia Schenker, and Jennifer Bellingtier, *Friedrich Schiller University Jena, Jena, Thuringen, Germany*

The pandemic has made age more salient. Access to vaccines, mandates to wear masks, and recommendations for contact restrictions have all varied by age. Developmental intergroup theory proposes that greater salience of a feature can lead to greater stereotyping and prejudice. We investigated this with a multi-method assessment of ageism in children (N = 57, ages 4-8), where data collection occurred both before and during the pandemic. In simulated behavioral measures, children preferred to sit closer to younger adults (mean distance = 1.8 seats) versus older adults (mean distance = 2.8 seats), and, for a simulated treasure hunt, they chose 3.36 younger, versus 1.63 older, teammates. Explicit (picture ratings) and implicit (IAT) ratings also significantly favored younger adults. These preferences were not exacerbated by the pandemic. Although ageism is present at young ages, we found no evidence that this has thus far worsened in the pandemic.

### SELF- AND OTHER-DIRECTED AGEISM AND WORRIES CONCERNING COVID-19 HEALTH CONSEQUENCES

Liat Ayalon,[1] and Ella Cohn-Schwartz,[2], *1. Bar Ilan University, Ramat Gan, HaMerkaz, Israel, 2. Ben-Gurion University, Beer-Sheva, HaDarom, Israel*

Worries associated with COVID-19 health consequences are well-justified. They may motivate people to take safety precautions, but may hinder if they become too intense. Current research examined mainly age and gender as potentially associated with worries. This study instead, focuses on self-perceptions of ageing (SPA) and perceived age discrimination as potential predictors of worry, in light of the ageism pandemic which co-occurred with the COVID-19 outbreak. The study is based on a national representative sample of 1,092 adults aged 50+ in Israel. Phone interviews were conducted between March – May 2020, when Israel gradually moved from strict to partial lockdown. Our findings show that SPA and age-based discrimination in the healthcare system were significant predictors of worries. The findings point to the potentially negative impact of the ageism pandemic in relation to worries. Interventions that address ageism directed by self or others might alleviate people's worries in the COVID-19 pandemic.

### IMPACT OF MEDIA-BASED AGE STEREOTYPES ON OLDER INDIVIDUALS' MENTAL HEALTH DURING THE COVID-19 PANDEMIC

E-Shien Chang,[1] Sarah Lowe,[2] Natalia Provolo,[2] Martin Slade,[2] and Becca Levy,[3], *1. Weill Cornell Medicine,*

*New York City, New York, United States, 2. Yale University, New Haven, Connecticut, United States, 3. Yale University, Woodbridge, Connecticut, United States*

During the COVID-19 pandemic, stigmatization of older persons has increased in traditional and social media. It was unknown whether this negative messaging could be detrimental to the mental health of older individuals, and whether the relatively uncommon positive messaging about older individuals could benefit their mental health. To address these gaps, we designed age-stereotype interventions based on actual news stories that appeared during the pandemic. As expected, the exposure of older individuals to the negative-age-stereotype-messaging interventions led to significantly worse mental health (more anxiety and less peacefulness), compared to a neutral condition; in contrast, the positive-age-stereotype-messaging interventions led to significantly better mental health (less anxiety and more peacefulness), compared to a neutral condition. The results demonstrate the need for media messaging aimed at empowering older individuals during the pandemic and beyond.

### SUBJECTIVE AGE CHANGES DURING THE COVID-19 PANDEMIC

Antonio Terracciano, *FLORIDA STATE UNIVERSITY, Florida State University, Florida, United States*

Aging is associated with an increased risk of COVID-19 morbidity and mortality. In this study, we tested whether the pandemic influenced how old individuals felt by examining longitudinal within-person changes in subjective age. We tested two alternative hypotheses: (a) people felt increasingly older in response to the stress generated by COVID-19; (b) people felt increasingly younger due to psychological distancing from older age. We tested these hypotheses in a large US sample of adults assessed once before and twice during the COVID-19 pandemic. Multilevel analyses indicated that people reported feeling younger with the emergence of COVID-19. We further tested demographic, health, and psychosocial predictors of changes in subjective age. Overall, the findings supported the hypothesis that subjective age partly reflects a coping process of psychological distancing from older age, a process that parallels physical and social distancing.

### EXPLORING THE RELATIONSHIP BETWEEN SUBJECTIVE AGE AND WORRY FOR OLDER ADULTS IN TIMES OF A PANDEMIC

Anna Kornadt,[1] Isabelle Albert,[1] Elke Murdock,[1] Martine Hoffmann,[2] Josepha Nell,[3] and Maiken Tingvold,[4], *1. University of Luxembourg, Esch-sur-Alzette, Diekirch, Luxembourg, 2. RBS, Itzig, Diekirch, Luxembourg, 3. University of Luxembourg, Esch-sur-Alzette, Grevenmacher, Luxembourg, 4. University of Luxembourg, University of Luxembourg, Grevenmacher, Luxembourg*

Given the role of age as a risk factor in the covid pandemic, we examined the longitudinal cross-lagged relationship between subjective age and Covid-related worry, and possible moderators of this relationship. Data were obtained at two-time points (June and October 2020) by a phone/online survey, from N = 611 older participants (Mage = 69.92 years). Participants felt on average 10 and 8.5 years younger than their chronological ages at the two-time points, respectively. Younger subjective age at T1 increased the level of worry at T2 irrespective of age, perceived control and subjective health. Higher worry increased subjective age at T2, but only for those with worse subjective health. Our results show that subjective age and Covid-related worry interact over time. This relation needs to be explored further in order to understand the relationship between subjective age and well-being especially, but not only in the pandemic context.

### Session 4635 (Symposium)

### HEALTH AND AGING DISPARITIES AMONG LATIN AMERICAN OLDER ADULTS: FINDINGS FROM STUDIES IN BRAZIL, COLOMBIA, AND MEXICO

Chair: Margarita Osuna

Aging in Latin America is occurring rapidly, in a context of high levels of poverty and inequality. This symposium is focused on population health and the heath-disparities found in some of Latin America's largest middle-income countries, Mexico, Brazil, and Colombia. This symposium contains presentations on different health-related issues affecting older adults, which can have further implications for wellbeing, health, and disease risk. The papers in this symposium examine a variety of health-related dimensions and disparities among older Latinos that include physiological functioning, cognition, and oral health. Using the Mexican Health and Aging Study (MHAS), Sheehan investigates the associations between personal and familial educational attainment on sleep quality. Also using MHAS, Milani. Using data from the Brazilian Longitudinal Study of Aging, Farina examines the relationship between race and cognition. Garcia uses data from the Colombian Survey of Health, Well-Being, and Aging to study the relationship between Motor Cognitive Risk Syndrome (MCRS) and cognition and frailty. Using the same dataset, Osuna examines variation in oral health in Colombian older adults and the impact this has on their wellbeing. Results indicate which population subgroups in Latin American have increased risk for poorer health and which dimensions of health have gender, race and socioeconomic disparities. The findings highlight the importance of understanding the conditions under which Latin American older adults are aging and the implications this can have in the future.

### ORAL HEALTH-RELATED QUALITY OF LIFE IN COLOMBIAN OLDER ADULTS

Piedad Suarez,[1] Jennifer Ailshire,[2] and Margarita Osuna,[3], *1. University of Southern California, University of Southern California, California, United States, 2. University of Southern California, Los Angeles, California, United States, 3. USC, University of Southern California, California, United States*

Despite its importance for health and wellbeing, oral health quality of life (OHQoL) has received little attention in lower-income countries, such as Colombia. This study describes the prevalence of older adults' OHQoL and variability by socioeconomic status. We use data from the 2015 SABE-Colombia (N=18,700), a nationally representative survey of community-dwelling Colombians ages 60

and older. We used the Simple Count Geriatric Oral Health Assessment Index (SC-GOHAI), a self-reported measure of frequent oral health problems such as chewing, swallowing, and speaking designed to assess OHQoL. The scale ranges from 0-12; higher scores indicate worse OHQoL. About 69% of older Colombians reported at least one OHQoL problem. The most common issues were difficulty chewing hard food and speaking. High education and income were associated with better OHQoL and smoking were associated with worse OHQoL. Oral health may therefore reflect another dimension of social and health inequality for older Colombians.

### THE INFLUENCE OF GENDERED SOCIAL POSITION ON COHORT DIFFERENCES IN SMOKING AMONG OLDER MEXICAN ADULTS

Jaqueline Avila,[1] Rebeca Wong,[2] and Sadaf Milani,[2], *1. Brown University, Providence, Rhode Island, United States, 2. University of Texas Medical Branch, Galveston, Texas, United States*

Disparities throughout the life course, including social position, result in gendered pathways to health, which ultimately result in gender disparities in late life. Using data from the Mexican Health and Aging Study, we explore the concept of gendered social position over the life course (educational achievement, marital history, employment history) and its association with smoking status in old age (current, former, never). We compare two cohorts by gender, those aged 60 to 71 in 2001 (n=4,383) and 60-71 in 2012 (n=5,970), as these cohorts experienced vastly different life courses. Overall, current smoking decreased from 2001 to 2012, but men consistently report higher rates of current and past smoking, compared to women. This presentation will widen the lens of gender to consider the influence of social position established over the life course, on gender differences in smoking, a risk factor for poor health and function in old age.

### THE LIFE COURSE ORIGINS OF THE RACE DISPARITY IN COGNITIVE FUNCTIONING IN BRAZIL

Mateo Farina, *University of Southern California, Los Angeles, California, United States*

**Background:** Cognitive health is a major concern for understanding population health in Brazil. Race inequalities have been found for several health outcomes but less is known about older adult cognitive health. Health inequalities have been tied to several life course factors, but less is known about how the racial stratification in Brazil may contribute to race disparities in cognitive health.

**Method:** Data come from the Brazilian Longitudinal Study of Aging. We used nested regression models to examine the life course origins of the race differences in cognitive functioning.

**Results:** Whites had better cognitive functioning than non-Whites. Education reduced these differences by about half. Health behaviors and cardiometabolic conditions had little to no impact.

**Discussion:** Race differences in cognitive functioning in Brazil are in large part attributable to educational opportunities. These finding point to the importance of cognitive development in childhood to understand racial disparities in later life cognitive health.

### DOES FAMILIAL EDUCATIONAL ATTAINMENT PREDICT SLEEP QUALITY TRAJECTORIES FOR OLDER ADULTS IN MEXICO?

Margarita Osuna,[1] and Connor Sheehan,[2], *1. USC, University of Southern California, California, United States, 2. ASU, Tempe, Arizona, United States*

Researchers have stressed the importance of sleep for healthy aging and longevity. However, there are few population-level studies of sleep quality focusing on older adults in Latin America and Mexico in particular. The objective of this study is to examine the associations between personal and familial educational attainment on sleep quality. We utilized data from the 2001-2015 Mexican Health and Aging Study (N=4,164; MHAS). Our sample consisted of older adults (aged 50+), married with children. We predicted longitudinal reports of restless sleep across four waves of MHAS using mixed-effects logistic regression. We found that lower levels of respondents' education, their spouses' education, and their children's' education were associated with lower levels of sleep quality. When the measures of education were included in the same model, one's own education and children's education remained significantly associated with quality sleep. Our results stress the importance of familial educational attainment for sleep in Mexico.

### MOTORIC COGNITIVE RISK SYNDROME: PREVALENCE AND COGNITIVE PERFORMANCE

Isabela Marquez, Carlos Cano, and Elkin Garcia-Cifuentes, *Pontificia Universidad Javeriana, Pontificia Universidad Javeriana, Distrito Capital de Bogota, Colombia*

Cognitive decline and dementia have a significant impact older adult. Motor Cognitive Risk Syndrome (MCRS) is a pre-dementia stage where slow gait speed and subjective memory complaints are present. MCRS increases the risk of frailty, dementia, disability, falls and overall mortality. We used data from the SABE Colombia study (Health, Well-Being, and Aging) conducted in 2015 in adults aged 60 years and older. After adjusting for confounding variables MCRS was associated with MMSE (OR 0.90, CI 0.82-0.99), pre-frail (OR 9.1, CI 3.26-25.47) and frail (OR 21.38, CI 6.30-72.57). This study found a prevalence of 5.45% of MCRS in Colombian older adults. We found an associations between cognitive performance (MMSE), frailty and MCRS. Our results increase the awareness of a pre-dementia stages different to Mild Cognitive Impairment (MCI), as these individuals are at greater risk than those with MCI to develop dementia.

### Session 9000 (Poster)

### ACUTE CARE AND HOSPITALIZATION

### 65 & THRIVE: IMPROVING PATIENT LENGTH OF STAY, READMISSION, AND QUALITY OF CARE BY BECOMING AN AGE-FRIENDLY HOSPITAL

Tracey Vien,[1] Stella Bobroff,[2] and Ricardo De Ocampo,[2], *1. Kaiser Permanente Med Group/Southern California, Los Angeles, California, United States, 2. Kaiser Permanente, Los Angeles, California, United States*

Data indicates that older persons will increase in numbers along with having an increase of life expectancy in the United States. Kaiser Permanente Los Angeles Medical Center's Utilization Department developed "65 & Thrive"—an age-specialized initiative to provide holistic care that preserves independence, quality of life, prevents functional and cognitive decline, and promotes both patients and their families to continue thriving. The initiative's focus is guided by the 5 M's model on mobility, medication, mentation, multi-morbidity, and what matters. Case management staff were given age-sensitivity trainings, improved workflows and made assessments that identified, addressed, and secured resources for patients throughout their hospitalization. Silver Angel volunteers were specially trained to prevent physical and mental decline and focused on activities to prevent delirium, depression and falls. The volunteers visited with patients daily for these interactions. The initiative was piloted in April 2020 on a stroke telemetry unit and since then the hospital has seen a significant decrease in the overall annual readmission rates by 3.1% when compared to 2019. The average length of stay for older adult patients; however, increased from 4.05 to 4.83 days unfortunately due to COVID-19. This initiative demonstrates the necessity to expand "65 & Thrive" throughout the hospital and ultimately to other Kaiser Permanente medical centers to best provide holistic care to older adults.

### A DELIRIUM RISK STRATIFICATION TOOL AND INTERDISCIPLINARY ROUNDS TO PREVENT DELIRIUM IN HOSPITALIZED OLDER ADULTS

Jennifer Woodward, and Tru Byrnes, *Atrium Health, Charlotte, North Carolina, United States*

Delirium is a disturbance of attention accompanied by a change in baseline cognition that is commonly seen in acute care settings, and effects up to 80% of ICU patients. The development of delirium has adverse effects on patient outcomes and high health care costs. Of patients aged 65+ admitted to our hospital in 2019, non-delirious patients had a five-day length of stay (LOS) compared to a 10-14 days LOS in delirious patients. A five days LOS increase adds an additional $ 8,325 per patient for an extra annual cost of 15 million dollars. Additionally, delirium is often not recognized. A prior retrospective study showed that 31% of older adults seen by a Geriatrics provider were diagnosed with delirium, while only 11% were detected by nurse's CAM screen. Given the need to improve delirium detection and management, a QI project was undertaken with a goal to recruit an interdisciplinary team, create a risk stratification tool to identify patients at substantial risk for developing delirium, and develop a delirium prevention protocol. Patients with a score of ≥ 4 were initiated on a nurse driven delirium protocol that included a delirium precaution sign and caregiver education. 6 months data has shown increased delirium detection of 33%, a reduction in 7.7 days LOS, reduced SNF discharge by 27%, and a significant LOS saving of 231 days. The results were statistically significant, $p < 0.04$ for LOS reduction. The cost avoidance in LOS alone were $384,615 for delirium patients.

### ARE STAKEHOLDER PRIORITIZED POST-ACUTE CARE PRACTICES DOCUMENTED AND DO THEY IMPROVE OUTCOMES?

Natalie Leland, Stephanie Rouch, and Elizabeth Skidmore, *University of Pittsburgh, Pittsburgh, Pennsylvania, United States*

The receipt and intensity of rehabilitation services, such as occupational and physical therapy, have been associated with lower risk of readmissions. Yet, little is known about the care. This study quantified the frequency of documented post-acute care (PAC) stakeholder-prioritized practices and their associations with hospital readmissions. A PAC stakeholder advisory board (e.g., physicians, rehabilitation providers across settings) prioritized key practices to evaluate. Medicare claims and electronic medical records were used to construct an episode of care for patients age 65 or older. Eligible patients were discharged from one of nine acute hospitals to a PAC setting (i.e., inpatient rehabilitation, skilled nursing, home health) within one large health system between August 2016 and August 2018. Descriptive statistics characterized the cohort and frequency of documented practices. Logistic regression examined associations among the practices and readmissions, by setting. Stakeholders prioritized (a) education, (b) cognition assessment and treatment, and (c) medication management. Among these PAC patients (n=3,227) there was variation in documentation for each practice by setting. Documentation of medication management at any point during the stay ranged from less than 1% to 54% of patient stays among settings. There was a significant relationship between the practices and readmissions. Within inpatient rehabilitation, every additional day patient and caregiver education was documented by occupational therapy was associated with 21% lower odds of readmission ($p<0.05$). This study highlights the variability in documentation of stakeholder-prioritized practices across PAC and their associations with readmissions. Future work is needed to enhance the systematic delivery and documentation of these practices.

### CHANGES IN FUNCTIONAL STATUS AMONG CLUSTERS OF OLDER ADULTS AFTER HOSPITALIZATION FOR PNEUMONIA

Chan Mi Park,[1] Hye Chang Rhim,[2] Eun Sik Lee,[3] Wonsock Kim,[4] Jong Hun Kim,[5] and Dae Hyun Kim,[6], *1. Asan Medical Center, Songpa-gu, Seoul-t'ukpyolsi, Republic of Korea, 2. Harvard University, Orlando, Florida, United States, 3. Korea University Anam Hospital, Korea University Anam Hospital, Seoul-t'ukpyolsi, Republic of Korea, 4. Eulji Medical Center, Eulji Medical Center, Kyonggi-do, Republic of Korea, 5. CHA Bundang Medical Center, CHA Bundang Medical Center, Kyonggi-do, Republic of Korea, 6. Hebrew SeniorLife, roslindale, Massachusetts, United States*

Little is known about how social determinants, comorbidity, and disability status are associated with functional recovery after an acute illness. A prospective cohort study was conducted between 2019-2020 at a university hospital in Korea, to investigate functional recovery after hospitalization for pneumonia in older adults with different degrees of social deprivation, disabilities, and comorbidities.

K-means cluster analysis was used to identify groups of patients based on social deprivation score, activities of daily living, instrumental activities of daily living, physical limitation score, and Gagne comorbidity index. Four groups were identified: Group A: non-disabled group with limited social support (n=61 [30.3%]); Group B: multimorbid but non-disabled group with social support (n=45 [22.4%]); Group C: multimorbid and disabled group with social support (n=38 [18.9%]); Group D: multimorbid and disabled group with limited social support (n=57 [28.4%]). Functional status, defined as ability to perform 21 activities and physical tasks independently, was measured via telephone interviews at 1, 3, and 6 months after discharge. Group-based trajectory model identified four functional status trajectories: excellent (n=29 [14.4%]), good (n=51 [25.4%]), fair (n=58 [28.9%]) and poor (n=63 [31.3%]). The most common functional trajectory by four groups was good trajectory (59%) in Group A, excellent trajectory (48.9%) in Group B, fair (50%) and poor trajectory (50%) in Group C, and poor trajectory (77.2%) in Group D. Our results suggest that most patients without disability recover functional status after pneumonia, despite multimorbidity or limited social support. Social support seems to be more important for those with multimorbidity and disability.

### NATIONAL NORMS FOR THE ELIXHAUSER AND CHARLSON COMORBIDITY INDEXES AMONG HOSPITALIZED ADULTS

Christine Loyd,[1] Garner Boogaerts,[2] Yue Zhang,[1] Richard Kennedy,[1] and Cynthia Brown,[3], *1. University of Alabama at Birmingham, Birmingham, Alabama, United States, 2. University of Alabama at Birmingham, University of Alabama at Birmingham, Alabama, United States, 3. Louisiana State University Health Sciences Center - New Orleans, New Orleans, Louisiana, United States*

Multimorbidity has become the defining focus of in-patient geriatric clinical practice and research. Comorbidity assessment burden is often completed using the Elixhauser (ECI) and Charlson comorbidity indexes (CCI), which can predict mortality risk, hospital length of stay and readmission, and healthcare utilization. Yet, the national norms for ECI and CCI have not been reported. Therefore, this study aimed to report comorbidity score national norms of hospitalized patients based on age, race, and sex. Using the 2017 US National Inpatient Sample, ICD-10 coding data from 7,159,694 adult patient's (≥18years) was abstracted to calculate ECI and CCI scores. Scores were stratified into 5-year age increments from age 45-89. Adults aged<45 and >89 were included in the analysis, however not age-stratified. Overall mean comorbidity score for the population using the ECI was 2.76 (95%CI 2.76, 2.76) and CCI was 1.22 (95% CI 1.22, 1.22). Mean scores for both indexes increased with age until age 90, and this increase was independent of race and sex (all p-values<0.001). Some individual comorbidities increased with age including congestive heart failure and dementia, while others including diabetes and chronic obstructive pulmonary disease increased with age but peaked between 60-74 years and declined in older age. Importantly, a report of US national norms for comorbidity burden among hospitalized adults can provide a reference for determining if clinical and research populations have greater or lesser comorbidity than typical hospitalized adults for their age, race, and sex.

### OLDER ADULTS' PERCEPTIONS OF DISPOSITION DECISIONS FROM THE EMERGENCY DEPARTMENT

Karen valcheff,[1] and Hoffart Nancy,[2], *1. University of North Carolina -Chapel Hill, Greensboro, North Carolina, United States, 2. University of Notrht Carolina, Greensboro, Greensboro, North Carolina, United States*

Patient-centered care strives to improve older adult outcomes from the emergency department (ED). Appropriate disposition decisions from the ED for older adults are becoming increasingly complex and challenging. The purpose of this study was to explore the perceptions of older adults as to their disposition from the emergency department, the decision making process, and their engagement in that process. The Three-Talk Shared Decision Making (SDM) model guided the study. A qualitative approach was used to interview seven older adults two days after being treated in the ED. Transcribed data were thematically analyzed using MAXQDA to identify codes, patterns, and themes. Analysis revealed that the Three-Talk SDM model was not being used. Participants identified only one option regarding their disposition from the ED and perceived they had little voice in decision making. They reported a variety of emotional reactions, feelings of helplessness and empathy regarding the decision making process. Three factors that participants perceived as vital to them before making a disposition decision were safety, pain relief, and a definitive diagnosis. The findings of this small sample are clinically meaningful. These older adults wanted to be heard regarding their treatment and disposition decisions. Findings indicate the need for provider education about the use of a model such as the Three -Talk SDM. Further research is needed to look at both the older adult and provider's perception of the ED disposition decision. Additional strategies and skills are warranted to enhance shared decision making in the ED with the growing aging population.

### PAIN ASSESSMENT AND DOCUMENTATION FOR OLDER ADULTS PRESENTING WITH NON-SURGICAL CONDITIONS IN EMERGENCY ROOM

Raza Haque,[1] Mara Bezerko,[2] Lauren Tibbits,[1] and Karen Tate,[1], *1. Michigan State University, East Lansing, Michigan, United States, 2. Michigan State University, College of Human Medicine, Michigan, United States*

Pain is one of the most common reasons for Emergency Department (ED) visits among older adults. However, timely pain assessment and management in this population in ED is a challenging task due to many factors ranging from; sensory, cognitive impairments, chronic pain, reliability of assessment tools, multimorbidity and system factors such as triage-based dynamic ED workflow. Where the implementation of the EMR was anticipated to improve patientcare, literature has indicated the barriers in effective utilization of the EMR for this purpose. We posit that pain assessment and documentation could be variable among older adults presenting with non-surgical conditions.

**Objectives:**1. To examine the proportion of documented initial pain assessment of nonsurgical older adults visiting emergency department 2. To examine the number of initial pain assessments documented in the chart by the five major categories of ICD-10 diagnoses upon discharge.

**Methods:** A retrospective exploratory chart review of 4613 emergency room visits for first pain assessment in the EMR conducted for all adults 65 years or older, presenting with non-surgical conditions, who were discharged same day at an urban teaching hospital.

**Results:** In our study 75.72% of encounters reviewed had a documented pain assessment. Completed pain assessments for the corresponding five most common non-surgical diagnostic categories presenting to our ED: Abdominal pain (92.59%), MSK (92.11 %), chest pain (83.92%), dyspnea ( 80%) and falls (79.46%).

**Conclusion:** Frequency of pain assessment and the management process of older adults presenting with non-surgical conditions in the institution studied was variable and differed based on presenting conditions.

### PREDICTING FACTORS OF CLINICAL OUTCOMES IN TRAUMATIZED ADULTS AND OLDER ADULTS

Pornthip Suyasith,[1] Prangtip Chayaput,[2]
Orapan Thosingha,[3] Suzanne G. Leveille,[4] and
Jatuporn Sirikun,[5], *1. University of Massuchesetts Boston, Bangkok, Samut Sakhon, Thailand, 2. Faculty of Nursing, Mahidol University, Bangkoknoi, Bangkok, Samut Sakhon, Thailand, 3. Faculty of Nursing, Chulabhorn Royal Academy, Laksi, Bangkok, Samut Sakhon, Thailand, 4. CNHS, UMass Boston, CNHS, UMass Boston, Massachusetts, United States, 5. Faculty of Medicine Siriraj Hospital, Bangkoknoi, Bangkok, Samut Sakhon, Thailand*

To investigate factors predicting hospital mortality and hospital length of stay (LOS) in traumatized adults and older adults, we conducted a three-year retrospective study at an academic hospital, Bangkok, Thailand. We reviewed medical records of 627 trauma patients admitted to the ED. Subjects were classified into 2 groups: adults (□55y), and older adults (□55y). Data were collected for demographic and clinical characteristics, physiologic deterioration using the Modified Early Warning Score (MEWS), severity of injury using the Circulation Respiration Abdomen Motor and Speech Score (CRAMS), and outcomes of hospital mortality and LOS. Multivariable logistic and linear regression models were performed. For hospital mortality, an elevated MEWS (Older adults [n= 267]: MEWS≥3, OR=4.80, 95%CI, 1.02-22.56 vs Adults [n = 360]: MEWS≥4, OR=11.63, 95%CI, 1.94-69.82) and CRAMS (Older adults: CRAMS≤9, OR=19.21, 95%CI, 2.78-132.98 vs Adults: CRAMS≤6, OR=18.58, 95%CI, 3.40-101.65) were strongly predictive, adjusted for demographic and clinical data. For LOS, road traffic accident (RTA) (Older adults: $\beta$=0.80, 95%CI, 0.31-1.29, $p < .01$ vs Adults: $\beta$=0.44, 95%CI, 0.21-0.67, $p < .001$) and falls (Older adults: $\beta$=0.88, 95%CI, 0.44-1.32, $p < .001$ vs Adults: $\beta$=0.33, 95%CI, 0.02-0.65, $p < .05$) were associated with LOS, adjusted for demographic and clinical data. MEWS and CRAMS predicted hospital mortality, and RTA and falls predicted LOS in both age groups. Results support the need for interventions for close monitoring and medical management for older traumatized patients based on CRAMS and MEWS assessment to decrease the risk of death, and targeting those sustaining falls and RTA to reduce prolonged LOS.

### Session 9005 (Poster)

### ADULT DEVELOPMENT

### A SCOPING STUDY OF LOW-WAGE WORKERS' RETIREMENT IN THE U.S. AFTER THE GREAT RECESSION

Hyesu Yeo, *University of Georgia, Athens, Georgia, United States*

Background Retirement comes with high risks for low-wage workers because of their cumulative disadvantages, and the Great Recession aggravated this population's working lives. However, there has been a lack of research about this vulnerable population's retirement. The purpose of this scoping study is to offer a comprehensive understanding of low-wage workers' retirement after the Great Recession. Methods Based on the rigorous method of scoping review by Arskey and O'Mally, the researcher systematically searched, selected, and synthesized literature. The articles were collected from eight databases and were published in January 2008 - February 2019. The search terms included terms related to retirement and low-wage. After systemically reviewing titles, abstracts, and full-texts from 5,268 articles, the final chart contains sample characteristics, definitions of low-wage workers, policy/programs, etc., from 23 peer-reviewed empirical studies. Results The results indicated that: 1) Most of the retirement studies covered middle-wage class workers, excluding low-wage workers because of their lack of retirement affordability; 2) There was no common definition of low-wage workers among scholars; 3) 11 studies assumed Social Security is the only retirement income for low-wage workers, and 12 studies investigated how to improve the workers' participation in other retirement programs; 4) Most studies were economic-centered; 5) Low-wage workers had different socioeconomic and labor market characteristics. Conclusion and Implications First, a consensus on the definition of low-wage is required to improve policies and programs associated with this population's retirement. Second, the life-course perspective approach from various disciplines is necessary to improve low-wage workers' retirement, considering diverse backgrounds.

### A STUDY OF SOCIAL ISOLATION, MULTIMORBIDITY AND MULTIPLE ROLE DEMANDS AMONG MIDDLE-AGE ADULTS BASED ON THE CLSA

Lun Li,[1] Andrew Wister,[2] and Barbara Mitchell,[2], *1. University of Simon Fraser, Vancouver, British Columbia, Canada, 2. Simon Fraser University, Vancouver, British Columbia, Canada*

Our understanding of the influence of concurrent multiple social and family roles on social isolation among the middle-aged generation remains limited. Given the increasing complexity of parenting, caregiving and working patterns over recent decades in many countries, and greater concern of multimorbidity in mid-life, this study examines the longitudinal effects of these contexts on social isolation among middle-aged persons. We apply Linear Mixed Models to analyze a sub-sample of 29,847 middle-aged (aged 45 to 64) participants drawn from the Baseline and Follow-up 1 waves of the Canadian Longitudinal Study on Aging. Separated analyses were conducted for participants with or without multimorbidity in order to identify patterns across these groups. Both middle-aged participants with and without multimorbidity experienced greater social isolation over time. Among participants without multimorbidity, holding multiple roles serves as a protective function to prevent social isolation over time. Among participants with multimorbidity, the parenting role remain as a protective factor; however, the caregiving role increases the risk of social isolation over time. This study confirms several life-course transitions from middle age to older age, including increased risk of social isolation and caregiving demand, and decreased parenting and working involvement. Different associations were uncovered among middle-aged persons occupying multiple roles on social isolation with and without multimorbidity over time. The findings emphasize the necessity to study multimorbidity as a salient contextual factor, and to provide enhanced support to multimorbid middle-aged individuals with increasing family caregiving demands.

### ASSOCIATION BETWEEN MUSIC ENGAGEMENT AND MORTALITY IN MIDDLE-AGED AND OLDER ADULTS IN THE US

Andrew Fiscella, Britney Veal, Ming Ji, and Hongdao Meng, *University of South Florida, Tampa, Florida, United States*

Music engagement is a universal human activity that transcends cultural and geographic boundaries. Current evidence suggests that music engages many diverse brain networks with wide-ranging effects on physiological, cognitive, and affective processes. As a result, music activity engagement may be associated with enhanced cognitive reserves and reduced stress. However, it remains unclear whether music activity engagement is associated with any survival advantage in the general population. This study tested this hypothesis in a nationally-representative sample of middle-aged and older adults in the U.S. A cohort of 3,540 respondents from the Health and Retirement Study was followed from 2002 to 2018. Music engagement was measured by self-reported participation in passive and/or active music activities. Potential confounders included socio-demographics, health and functional status, and health-related behaviors. We plotted the Kaplan-Meier survival curves by music engagement level and used Cox proportional hazards model to examine the independent effect of music engagement on mortality. Musical engagement levels were significantly associated with mortality in both the unadjusted and adjusted analyses. Respondents who reported engaging with music at a moderate or high level had lower mortality risk as compared to those who did not (HR=.83, p=0.015; HR=.78, p=0.003, respectively). These findings suggest that music engagement in the middle to late life may have an independent beneficial effect in promoting longevity. Future research should examine whether this observed effect was causal and existed in other populations. If confirmed, interventions should be designed to promote music engagement among middle-aged and older adults.

### IDENTITY, INTEGRITY, AND RELIGIOUS DOUBT IN LATER LIFE

Phoebe Clark,[1] Michiko Iwasaki,[2] Edward Thompson,[3] and Andrew Futterman,[4], *1. Loyola University MD, Gaithersburg, Maryland, United States, 2. Loyola University MD, Department Of Psychology, Maryland, United States, 3. College of the Holy Cross, Broadview Heights, Ohio, United States, 4. Loyola University MD, Loyola University MD, Maryland, United States*

Erik Erikson argued that for religiously involved individuals, the resolution of two stages – Identity vs. Role Diffusion and Integrity vs. Despair – are of critical importance in adult development. Adults typically confront their parents' religious affiliation in adolescence and young adulthood as they establish themselves as independent actors in the world, and in later life, older adults confront their own lived lives, and evaluate in light of their religious commitments whether they have lived meaningfully and with integrity. To examine Erikson's views of religious development, we completed open-ended interviews of 278 community-dwelling older adults (55-101 years). In these interviews, participants describe the development of their religious faith, the nature and development of their religious questioning and doubt, and the relationship between their faith and doubt over their life span. Participants were from Northeast U.S., and were denominationally, racially, and ethnically diverse. More extensive religious doubts were reported during young adulthood regarding the meaningfulness of religion in their family of origin. By contrast, religious doubts are reported less during later life, and more emphasis is placed on the value of religious involvement for themselves and their families. This pattern varies between young-old (under 75 years) and old-old (over 76 years): whereas the young-old seem to be working through their religious doubts, the old-old are more focused on the value of their religious faith. These findings are discussed in terms of Erikson's developmental theory and with respect to cohort differences in religious belief and practice.

### LINKAGES COLORADO: EVALUATION OF INTERGENERATIONAL PROGRAMS TO IMPROVE CONNECTIONS ACROSS ALL AGES

Carson De Fries,[1] Leslie Hasche,[1] Rachel Cohen,[2] Andrew Steward,[3] and Matthew Schilz,[1], *1. University of Denver, Denver, Colorado, United States, 2. Aging Dynamics, Pine, Colorado, United States, 3. University of Denver, Lone Tree, Colorado, United States*

LinkAGES: Colorado is a collaborative group of multisector organizations (e.g., libraries, non-profit service organizations, nursing homes) that uses a capacity-building approach to support the offering of intergenerational programs and evaluation of outcomes over time and across programs. Since 2018, LinkAGES has implemented 20 intergenerational programs involving various activities (e.g., music and art therapy, sharing cultural traditions), across settings, and across modalities (in-person and online). Ages of program participants ranged from 5 months to 96 years old. This study evaluated change in connectedness between generations over time. Participants (n=118) completed pre- and post-program ratings on social connection (i.e., level of intergenerational engagement, self-perception of extent of feeling connected, and self-perception of impact on someone from another generation) on a 4-point Likert scale. Paired sample t-test results indicated that programs significantly improved engagement and perceived impact. Using multiple regression analyses, we tested change over time for each outcome controlling for participant age group, program host setting, and program modality. A greater positive change in level of engagement occurred for older adults and in-person programs. Additionally, feelings of connection and perceived impact significantly improved over time when controlling for age group, program modality, and program host setting, with age group as a significant covariate. This study demonstrates the impact of intergenerational programs on social connectedness across a wider network of organizations than much of the extant literature. While the positive outcomes are promising and consistent across LinkAGES programs and existing literature, further exploration of age group differences should be considered.

### RETIREMENT FINANCIAL PLANING AND LIFE SATISFACTION

Mengya Wang,[1] Suzanne Bartholomae,[2] and Jonathan Fox,[1], *1. Iowa State University, ames, Iowa, United States, 2. Iowa State University, Ames, Iowa, United States*

Retirement has been considered as a major transition in one's life. Financial security in retirement is a major concern for many Americans. Evidence has shown that being financially prepared for retirement could has a significant, positive impact on one's life satisfaction. Employing data from the 2012 and 2018 National Financial Capability Study (N=1023), this study analyzes the relationship between participants' retirement planning in 2012 and their financial satisfaction in 2018. An Ordinary Least Squares regression is used in the current study. This study found relatively low retirement preparedness levels (retirement planning, retirement saving, retirement plan: employer-based or individually held, investment) among the participants in 2012. Based on the descriptive results, adults closest to retirement (ages 55 to 64) are more likely to be planning compared to the other groups, as are adults who were married, highly educated, males, and white. According to the liner regression results, this study found that adults who had a retirement savings goal, had a retirement plan (employer-based or individually held), made regular contributions to retirement plans, and owned investments in 2012 are more likely to be satisfied with their personal financial condition in 2018. As expected, individuals with higher incomes, larger net worth, and those who are older are significantly more likely to be financial satisfied. However, even after controlling for these variables, results show that planning does indeed impact the level of financial satisfaction. Our findings highlight the importance of policies and programs to support Americans with retirement planning.

### Session 9010 (Poster)

### AGEISM AND ATTITUDES TOWARD AGING

### AN AGING NURSING WORKFORCE: THEMATIC ANALYSIS FROM THE NURSTORY PROJECT

Raeann LeBlanc, *University of Massachusetts, Amherst, Royalston, Massachusetts, United States*

**Background:** Nursing in the United States of America is an aging workforce. This study sought to better understand the lived experience of aging nurses. Because nurses work in systems where other forms of interpersonal power dynamics may influence internalized and external stereotype an approach based on intersectional theory was applied.

**Methods:** A qualitative thematic narrative analysis of an existing data set of first-person digital stories in the Nurstory project, authored by a group of nurses, was the data source. An emergent coding method was applied. The collection of five digital stories were analyzed.

**Results:** All stories were first person accounts of experiences that represented their internalized reflections and elements of ageism in how their age interacted with their work environment. Dominant themes included: 1) Role constriction 2) Strength 3) Tired and (re)Tired 4) Age perceived and 5)Loneliness.

**Conclusions:** These aging nursing stories add to the contextual layers of the aging healthcare workplace and aging nursing workforce. These individual experiences offer a nuanced understanding of the internalized responses to aging and ageism. These stories highlight socially constructed and socially reinforced attitudes that are complicated by the personal and occupational expectations of nurse's work, their role and embedded hierarchies in healthcare. Stories such as these are important individual and collective indicators of lived experiences that offer a deeper understanding into the intersections of social identity and aging, that when listened to, can offer insight and a way forward in addressing the stereotype, discrimination and social inequities of ageism.

### ASSESSING FRONTLINE PERSONNEL'S RECOGNITION OF AND RESPONSE TO CASES OF ABUSE IN LATER LIFE

Sarah Marrs, Courtney O'Hara, Ruth Anne Young, Miranda Yelvington, Deijah Patterson, Annie Rhodes, and Edward Ansello, *Virginia Commonwealth University, Richmond, Virginia, United States*

Abuse in later life is experienced by 10% of adults over age 60 (Acierno et al., 2010). Unfortunately, it is estimated that for every one reported case, around 20 remain unreported (APA, 2012). A primary reason for this disparity is the absence of training provided to professionals working at the frontline of elder abuse, such as law enforcement professionals, health care professionals, and aging and victim service providers (e.g., Rose et al., 2016). This leaves the workforce best positioned to intervene in cases of abuse in later life lacking knowledge around what constitutes the different types of abuse and what they should do if they suspect abuse (Rosen et al., 2018). A critical first step to developing the evidence-based training needed to reconcile this gap is to gain a better understanding of the current landscape within this workforce. This qualitative study explored the knowledge and attitudes towards abuse in later life as well as current practices and policies for reporting abuse among law enforcement professionals (n = 1), health care professionals (n = 2), and aging (n = 5) and victim (n = 4) service providers. Participants (N = 12) represented urban (n = 4), suburban (n = 5), and rural settings (n = 2). Themes emerging from the focus groups highlight a number of barriers to identifying and reporting abuse for professionals in each discipline. Our findings also provide strong evidence of the need to intervene and diminish the impact ageist attitudes and behaviors can have on older adults.

### COLLEGE STUDENTS LIFE COURSE DRAWINGS AND THEIR DESCRIPTIONS

Amy Schuster,[1] Katherina Terhune,[2] Tina K. Newsham,[3] M. Aaron Guest,[4] Renee DuMont,[5] Lauren Hackett,[5] J.R. Patton,[5] and Kebafe Segosebe,[4], *1. Clemson University, Clemson, South Carolina, United States, 2. Northern Kentucky University, Lexington, Kentucky, United States, 3. University of North Carolina Wilmington, University of North Carolina Wilmington, North Carolina, United States, 4. Arizona State University, Phoenix, Arizona, United States, 5. University of North Carolina Wilmington, Wilmington, North Carolina, United States*

Drawing as a qualitative method has been employed to elicit views on aging. The subject matter of the drawings, without an explanation from participants, can be misinterpreted. Therefore, in this research, we explored college students' drawings of the life course and the extent to which the content of these drawings corresponded to their written descriptions. A content analysis was performed on 524 college students' life course drawings and their descriptions. Participants drew, on average, five life stages. The majority (75%) of the human beings represented were alone in each life stage. Twelve percent of the drawings were non-human representations of the life course (e.g., flower, tree). The majority of the images (85%) included in the drawings were not mentioned in the written descriptions, for example, hair changes (e.g., from long and straight to curly and short for women). Some physical characteristics (e.g., wrinkles [29%], hunched back [22%]) and some contexts (e.g., tombstones [37%], nursing home [100%]) were present in both the drawing and descriptions. Findings highlight which ideas associated with aging participants thought needed an explanation and which they might have seen as intrinsic to aging, warranting no explanation, emphasizing the importance of examining both drawn and written content when using drawing as a method in aging research. A more thorough and precise examination of the beliefs and perceptions of college students, who will serve as future professionals working with older adults, allows for the development of educational and engagement strategies that accurately target commonly held misperceptions regarding aging.

### NARRATIVE IMAGINATION: DISMANTLING OLD AGE THROUGH THE LITERARY FOODSCAPE IN LORE SEGAL'S "THE ARBUS FACTOR"

Eva-Maria Trinkaus, *University of Graz, Graz, Steiermark, Austria*

Being able to take another person's perspective and understanding the Other is a crucial element of reading, understanding, and processing literature. Especially in the context of old age, many literary texts play into the culturally constructed (cf. Gullette 2004) and biased understanding of old age as decline narrative, rather than reading an old person's story as a narrative of possibility. In her short story "The Arbus Factor" which was first published in The New Yorker in 2007, Lore Segal offers a different perspective on aging. Through creating a space, coming into existence through foodways and food practices, which in my dissertation I will refer to as 'literary foodscape,' she offers a setting and backdrop for the characters to construct a discourse of possibility, creation, and new opportunities at a later stage in life. Segal wittily dismantles age-related stereotypes and opens up a discourse that goes beyond an easy categorization. This paper is going to analyze the ways in which a literary text, through the 'literary foodscape' is able to rewrite a culturally engrained perspective, and offers a different and more accurate understanding of what it means to be old. Gullette, Margaret Morganroth. Aged By Culture. The University of Chicago Press. 2004.

### TOWARD INTERVENTIONS TO REDUCE INTERNALIZED AGEISM

Andrew Steward, *University of Denver, Lone Tree, Colorado, United States*

Ageism is an insidious form of injustice that is internalized from an early age with accumulating negative health impacts across the lifespan. Internalized ageism is associated with numerous public health outcomes, including physical and mental health, functional impairment, cognition, cardiovascular stress, hospitalizations, and longevity. Research has begun to document how ageism negatively impacts health through psychological, behavioral, and physiological pathways. Yet, limited research has addressed interventions to reduce internalized ageism. This study integrates stereotype embodiment theory, theories of successful and productive aging, and recent scholarly literature to present a conceptual model with potential downstream, midstream, and upstream interventions at micro, meso, and macro levels. Micro interventions include: social, physical, and cognitive engagement, as well as stress management. Meso interventions include: education, intergenerational contact, and narrative reframing. Macro interventions include anti-ageism policy, such as amendments to the Age Discrimination in Employment Act (ADEA). The conceptual model is described in detail, and implications for practitioners are discussed. The need to examine how policy influences health through the three pathways in stereotype embodiment theory is discussed. This study provides a working model for scholars and practitioners to use when considering paths toward reducing internalized ageism and optimizing well-being for aging adults.

### Session 9015 (Poster)

### AGEISM AND COVID-19

### "ESPECIALLY FOR PEOPLE OUR AGE": EXPLORING THE CORRELATES OF AGEISM DURING THE COVID-19 PANDEMIC

Jennifer Sublett,[1] Michael Vale,[2] and Toni Bisconti,[1], *1. The University of Akron, Akron, Ohio, United States, 2. University of Akron, Akron, Ohio, United States*

The COVID-19 pandemic has presented an unprecedented context for older adults where they may feel patronized, isolated, and fearful because of their greater risk of getting COVID-19 and being targets of ageism. Previous researchers have linked ageism negatively with health and well-being; although, the majority of this research has highlighted the negative, or hostile, aspects of ageism, and excluded the overaccommodative and patronizing qualities of benevolent ageism. Since the start of the pandemic, both forms of ageism have been noted to be more salient with claims of an ageism outbreak (Ayalon et al., 2020). The correlates of ageism during the COVID-19 pandemic are widely unknown, and the goal of this study was to explore whether experiences of ageism were related to different affective and health-related responses to the pandemic. In a sample of older adults (N=65) collected in September 2020, we found that benevolent ageism positively correlated with pandemic specific experiences of pity (r=.27, p<.05), loneliness (r=.30, p<.05), worry (r=.40, p<.01), and negatively related to self-reported physical health (r=-.31, p<.05) and emotional well-being (r=-.26, p<.05). Hostile ageism did not relate to pity, but positively correlated with loneliness (r=.25, p<.05) and worry (r=.37, p<.01), and negatively related to physical health (r=-.27, p<.05) and emotional well-being (r=-.38, p<.01). This work provides preliminary evidence of how the lives of older adults have been influenced by COVID-19 and the resulting ageism outbreak. Future research should continue this avenue of study with more expansive and inclusive samples and approaches as the pandemic is not over.

### "IT ISN'T GOING TO LAST FOREVER!" THE EFFECTS OF COVID-19 PANDEMIC ON OLDER ADULTS

Chih-ling Liou, *Kent State University, North Canton, Ohio, United States*

The media puts a spotlight on older adults' vulnerability to COVID-19 with limited consideration on how they view and cope with this crisis. This study is to give older adults voices to share their experiences of this pandemic. Data were collected using semi-structured interviews with 46 adults between the ages of 66 and 97 from the midwestern United States. Participants were asked to share how the pandemic affects them, their vision for the future, and how they cope during the pandemic. Although they worried about their health, felt isolated and missed seeing family and friends, most participants shared an optimistic view for the future. Some said that they are looking forward to receiving the vaccine, some believed that the effects of the pandemic are just temporary, and others compared that of the pandemic to the wars and other types of hardships which were much worse. Strategies for coping during the pandemic vary from spiritual practices to positive thinking, from exercises to new hobbies, and from calling family members to cutting down on the news. The results also show that the oldest-old and old-old participants seem to be better at regulating their negative effects compared to the young-old. One female participant in her 90s shared that she does not worry about the pandemic because there is nothing that she can do about it. Older adults' adaptability during the COVID-19 pandemic should be better understood to reverse the image of their vulnerabilities and promote late-life coping during crises.

### AGE AND AGEISM IN THE COVID-19 PANDEMIC: WHAT DOES THE DATA SHOW?

Usha Dhakal, and Suzanne Kunkel, *Miami University, Oxford, Ohio, United States*

Gerontologists were quick to call out the resurgence of ageism that was reflected in the paternalistic, overgeneralized, and deficit views of aging that dominated discussions about age-associated risks of the disease and its consequences. One manifestation of the blunt and potentially ageism-promoting use of age in data about the virus is the failure to routinely distinguish the independent role of age alone, separate from its association with comorbidities. A related problem is the use of broad age categories, which can also mask the role of specific comorbidities. To address that gap, this study uses data from Centers for Disease Control and Prevention, as of Feb 21, 2021 to calculate age-specific COVID-19 death rates (ASDR) and compare the extent to which comorbid conditions potentially associated with COVID-19 deaths were listed on death certificates. Findings showed that the ASDR was significantly higher for those 85 years and over (2249.96 per 100,000); the rate was 802.66 for 75-84 and 312.78 per 100,000 for 65-74. Death certificate information revealed that influenza and pneumonia was the major contributing comorbidity to COVID-19 deaths across all three age groups; (listed on 49% of the death certificates for those 65-74 who died with COVID-19, 46% of those 75-84, and 38% of those 85 and over). Future studies should be more precise about the use of age/age groups, about the rationale for those designations, and about the impact of age separate from comorbidities. Broad use of an arbitrary age as a proxy for frailty and illness contributes to ageism.

### AGEISM AND UNDERGRADUATE ATTITUDES TOWARDS OLDER ADULTS DURING COVID-19

Adam Shea,[1] Aiping Yu,[1] and Jessica Strong,[2], *1. University of Prince Edward Island, University of Prince Edward Island, Prince Edward Island, Canada, 2. University of Prince Edward Island, Charlottetown, Prince Edward Island, Canada*

The COVID-19 pandemic has shed light on the far reaches of ageism in our society. The current study sought to better understand ageist beliefs in Canadian undergraduate students during the pandemic. As part of a larger survey on ageism, we conducted a thematic analysis on open-ended responses to the following questions: 1) "Has your relationship with older adults in your life changed as a result of the COVID-19 pandemic?" and 2) "Have you noticed that attitudes or opinions towards older adults in your community have changed as a result of the COVID-19 pandemic?" Students felt that older adults should be treated differently during the pandemic because they are seen as “high risk” or “vulnerable.” Furthermore, students felt that they needed to be more cautious around older adults because older adults need to be taken care of. Students expressed fear about transmitting the virus to older adults in their lives so chose to isolate from grandparents or avoid older adults in the community in an effort to keep them safe. Finally, examples of negative and positive ageism were present in responses. Negative ageism was seen in comments about how older adults were going to die anyway, the assumption that older adults need more help, and the belief that older adults should be staying home during the pandemic. Positive ageism was present where students realized the importance of their relationships with the older adults in their lives. Results are discussed using the framework of implicit and explicit ageism.

### AGEISM PREDICTS PRIORITIZING COVID-19 VACCINES FOR OLDER ADULTS AND LTC RESIDENTS

Carly Pullen,[1] Jenessa Steele,[2] and Julie Hicks Patrick,[3], *1. Radford University, Radford University, Virginia, United States, 2. Radford University, Radford, Virginia, United States, 3. West Virginia University, Morgantown, West Virginia, United States*

Ageism and ageistic stereotypes regarding older adults have become widespread and influence many policies and practices. Benevolent ageism includes attitudes or behaviors that appear overtly positive but are actually patronizing (Cary et al., 2017). Hostile ageism, usually expressed through negative stereotypes, highlights older adults' poor physical and mental functioning (Cary et al., 2017). The aim of the current study was to examine the role of benevolent and hostile ageism on perceptions of vaccination priority during the COVID-19 pandemic. As part of a larger longitudinal study on perceptions of aging, measures of benevolent and hostile ageism were collected in January 2020, before the pandemic began in the US. In March 2021, the same participants were asked to identify top vaccine priorities by ranking groups (e.g., adults 65 and older, school teachers, etc.). Participants who put older adults in the lowest priority group expressed more benevolent ageism before the pandemic than those who put older adults in the top priority group, $F(2, 88) = 3.93$, $p < .05$. Participants who put older adults in the lowest priority group expressed more hostile ageism prior to the pandemic, compared to those who put older adults in the top priority group, $F(2, 88) = -3.34$, $p < .05$. Similar to Vale and colleague (2020) findings, our results suggest that ageism influences people's ideas about triage for the vaccine. Notably, neither form of ageism related to prioritization for other high-risk groups, including members of racial/ethnic minority groups or health care workers.

### EXAMINING ATTITUDES AND EXPERIENCES OF AGEISM DURING COVID-19: ARE THEY ON THE RISE?

Michael Vale,[1] Jennifer Sublett,[2] and Toni Bisconti,[2], *1. University of Akron, Akron, Ohio, United States, 2. The University of Akron, Akron, Ohio, United States*

Gerontologists have warned of rising ageism amidst the COVID-19 pandemic. Older adults have been portrayed as a homogenous group given their health vulnerabilities and have been viewed with mixed perceptions. For instance, the pandemic has been viewed as an “old person’s” disease and older adults have been inherently linked to imposing health and safety lifestyle changes. Others have responded with acts of overaccommodative care that have minimized older adults’ autonomy. Taken together, there have been inferences of increased hostile and benevolent ageism. Currently, these claims lack empirical data, and the goal of this study was to examine if attitudes and experiences of ageism are on the rise. Across two studies, we examined young adults’ (N=268) attitudes of older adults and older adults’ (N=65) experiences of ageism before and after the start of the pandemic. In study 1, we examined ageist attitudes at 3 time points (2017, 2019, 2020) from separate, but equitable, college samples and found that hostile ageism was higher during the pandemic ($F(2,265)=5.48$, $p<.001$) and benevolent ageism demonstrated no differences. In study 2, we explored older adults’ experiences of ageism pre-and post-pandemic onset (2019, 2020) and, found that they reported experiencing less hostile ageism ($t(64)=2.45$, $p<.05$), with no differences in experienced benevolent ageism. Our findings suggest an increase in hostile ageist views, but a decrease in experiences, partially supporting the alleged claims of rising ageism. Nevertheless, the last year of the pandemic is dynamically contextualized and research should elaborate on the extent and consequences of this rise.

### MEDIA PORTRAYALS OF EMERGING AND OLDER ADULTS DURING COVID-19: PERCEPTIONS OF BIAS AMONG COLLEGE STUDENTS

Taylor Pestritto,[1] Katherine King,[2] Mikala Mikrut,[3] and Kirsten Graham,[3], *1. William James, Newton, Massachusetts, United States, 2. William James College, William James College, Massachusetts, United States, 3. Southern Utah University, Cedar City, Utah, United States*

This study explores media consumption and perceptions of media bias against both older adults and emerging adults during the COVID-19 pandemic. As part of a larger study, 99 students with a mean age of 20.54 (SD = 2.97) completed an online survey in early 2020. Individuals whose media consumption had increased were significantly more likely to report that young adults have been portrayed worse, and older adults better, since the start of COVID-19. Qualitative responses demonstrated broad awareness of ageist and adultist themes in media portrayals of both age groups, e.g., that young adults are careless and reckless whereas older adults are vulnerable and in need of protection. Results suggest that the media is perceived to be perpetuating age-related biases and may be enhancing intergenerational discord at a time when generational unity is needed.

### PEOPLE INTERNALIZED MORE ATTITUDES TOWARD AGING DURING THE PANDEMIC

Chunyan Mai,[1] Hiu Ling Vivian Tsang,[2] and Helene Fung,[3], *1. the Chinese University of Hong Kong, Hong Kong,*

*Not Applicable, Hong Kong, 2. Hong Kong Metropolitan University, The Chinese University of Hong Kong, Hong Kong, 3. The Chinese University of Hong Kong, Shatin, N.T., Hong Kong, Not Applicable, Hong Kong*

Older adults are viewed as being vulnerable to COVID-19. Previous research revealed that individuals would internalize or dissociate with attitudes toward aging when they aged. In this study, data collected before the COVID-19 pandemic were compared with those collected during the pandemic to assess whether the pandemic might make older adults internalize or dissociate with attitudes toward aging to a greater extent.123 Hong Kong participants (50.4% females, M=60.59±13.28 years old) were recruited in a two-wave survey (wave 1 in 2018 and wave 2 in 2020) on attitudes toward aging and future self-views. After comparing the correlations between attitudes toward aging and future self-views in the two waves, we found stronger positive correlations between these 2 variables in wave 2 than in wave 1 in the personality and finance domains, but not in the family, independence, or health domains. These findings suggest that internalization of attitudes toward aging might be domain-specific. The pandemic might make older adults more likely to internalize positive personality attitudes toward aging and negative finance attitudes toward aging into their future self-views. Professionals may consider utilizing the internalization process to promote a positive attitude toward aging during the pandemic.

### Session 9020 (Poster)

### AGEISM AND PERCEPTIONS AND ATTITUDES ABOUT AGING

### "SPEND YOUR SPOONS WISELY": CONCEPTUALIZATIONS OF TIME, ENERGY AND AGING INVISIBLY WITH CROHN'S DISEASE.

Ginny Natale,[1] and Manacy Pai,[2], *1. Stony Brook University, Stony Brook, New York, United States, 2. Kent State University, Kent, Ohio, United States*

An increasing number of people with chronic medical disabilities are living longer and into old age due to the growing medical and technological advancements over the past half century. We used grounded theory to examine the lived experience of aging "with" a disability in a non-elderly population. On average, participants were 37 years of age at the time of interview. The average time since diagnosis was 17 years and ranged from 3 to 34 years. Many worked full-time outside of the home and some held advanced or graduate degrees. Of the 35 participants interviewed, three-quarters expressed worries about the future and aging, specifically related to physical limitations of having CD. The other 25% talked about learning to accept the diagnosis and 'moving forward' with their life as they age. All participants described the difficulties of fatigue and energy limitations. Planning of life was limited to 24 hours — a direct consequence of functional limitations of a relapsing-remitting disease. The most prominent theme that emerged from participants' narratives to explain aging invisibly with a chronic illness was quantifying energy into 'spoons', a way of measuring the stock of their energy on any given day. These findings translate into important insights into the process of aging for those who live and age "with" Crohn's as their everyday lives are immersed in managing the varying whims of this illness.

### AGE NORMS AND THE SPIRIT OF CAPITALISM

Jeremiah Morelock, *Boston College, Woburn, Massachusetts, United States*

Adulthood is often associated with hard work, in contrast to childhood and later life, which are associated with play, education, and leisure. Yet the work-fixated sense of adulthood is about more than just age norms. Like any such ethos, it is situated in socioeconomic history. Workers are forced to work hard, the work ethic framing their exploitation within an aura of moral righteousness. According to Weber the normative weight commonly associated with 'hard work' derives from the advent of Protestantism in the late middle ages. Weber says that this new worldview birthed the 'spirit of capitalism,' and set the stage for the modern world to take shape. In the seventeenth century—hence roughly coinciding with mercantilism and the Reformation—was the invention of the modern concept of childhood, i.e. the radical division of childhood from adulthood. This period also inaugurated the European Enlightenment, where reason was elevated as a supremely honorable aspect of humanity, in many ways as a new source of this-worldly pseudo-salvation. 'Adulthood' was infused with these values—the ideal [male] adult is rational, responsible, hard-working, self-sufficient, and financially secure. It was adulthood, more than and in contrast to other times of life (e.g., childhood and later life), that absorbed and normalized the new economic and cultural trends. The moral elevation of hard work, combined with the greater demarcation of adulthood in contrast to childhood and later life, set up children and older adults to take on a status of moral inferiority due to their exclusion from the working world.

### ALLOPHILIA AND EXPERIENCE: PREDICTORS OF FEAR IN DEVELOPING DEMENTIA

Amber Rusch, Michaela Clark, Moyosoreoluwa Jacobs, Carmen Thomassy, and Julie Hicks Patrick, *West Virginia University, Morgantown, West Virginia, United States*

Fear of developing dementia is common and has been linked to delays in seeking medical attention (Arlt et al., 2008). We used data from 320 adults (M age = 39.7, SD = 12.3, range 20 – 70) to examine the ways in which objective knowledge and subjective experience with dementia influence positive attitudes toward persons with dementia. We further examined how these constructs related to fear of developing dementia. A path analysis showed the model fit the data well, X2 (DF = 1) = 0.74, p = .39; RMSEA < .001. Objective knowledge and subjective experience were significantly associated with higher allophilia. Allophilia and subjective experiences were associated with personal fear. However, allophilia decreased fear, whereas subjective experiences were associated with increased fear of developing dementia. To clarify these findings, we conducted a moderated regression in which age was examined as a moderator of the relation between allophilia and fear as well as the relation between subjective experience and fear. Significant results were obtained [F (5, 294) = 10.41, p < .001; R2 = .15]. Age moderated the effect of personal experience on fear. Stronger effects emerged for adults in their 20s compared to those in their 40s; similarly, age exerted a stronger effect for those in their 40s than for those in their 50s. Regarding age effects on the relation between allophilia and fear of dementia, for adults in their 20s and 40s, allophilia reduced fear of dementia. For adults in their 50s, allophilia was associated with higher fear.

### ATTITUDES ABOUT DEMENTIA IN DIFFERENT STAGES OF ADULTHOOD

Melissa O'Connor,[1] Megan Pedersen,[2] and Susan McFadden,[3], *1. North Dakota State University, Moorhead, Minnesota, United States, 2. North Dakota State University, Fargo, North Dakota, United States, 3. University of Wisconsin Oshkosh, Oshkosh, Wisconsin, United States*

Research on attitudes toward dementia has often focused on younger and older adults; few studies have also included the age groups of established and middle adulthood. The current study utilized data from community-dwelling adults aged 18-95 (n=567) residing in two Midwestern states. Participants were divided into four age groups: emerging/young adulthood (ages 18-29), established adulthood (ages 30-45), middle adulthood (ages 46-64), and older adulthood (age 65+). ANOVA models were used to examine age group differences on the following outcomes: factual knowledge about dementia (total score on 14 true-false questions); attitudes toward dementia (total score on the 20-item Dementia Attitudes Scale); and a single item, “I am afraid of losing my memory” (rated on a 5-point scale). The effect of age group was significant in all models (p<0.01 for all). Emerging/young adults had significantly more knowledge about dementia, but less positive attitudes toward dementia, relative to established, middle-aged, and older adults. Attitudes and knowledge did not differ between established, middle-aged, and older adults. By contrast, older adults reported significantly more fear of memory loss than emerging/young, established, and middle-aged adults; fear did not differ between the latter three age groups. There were no significant interactions between age group and sex in any of the models. Implications of these findings are discussed.

### AWARENESS OF AGE-RELATED CHANGE AND ITS RELATIONSHIP WITH INDUCTIVE REASONING AND AGEISM

Abigail Voelkner,[1] and Grace Caskie,[2], *1. Lehigh University, Easton, Pennsylvania, United States, 2. Lehigh University, Bethlehem, Pennsylvania, United States*

Subjective aging is important due to its relationship with well-being. Diehl and Wahl (2010) proposed Awareness of Age-Related Change (AARC) as a measure of subjective aging; their theoretical model proposed that cognition’s relationship to AARC is mediated by ageist experiences. The current study tests this model and proposes an alternative model where cognition is hypothesized to mediate the relationship of ageist experiences to AARC. Inductive reasoning was used to measure cognition due to its susceptibility to ageism. Participants were 283 older adults aged 66-90 years (M=69.08, SD=3.36) without a dementia diagnosis or cognitive impairment. Inductive reasoning was measured by Word Series, Number Series, Letter Sets, and a composite score. AARC total losses, cognitive losses, total gains, and cognitive gains were used. Age, gender, and education covariates were included. Analysis of Diehl and Wahl’s (2010) model showed that the composite and individual reasoning measures had negative direct effects on all AARC measures. Ageism mediated the effect of the composite and individual reasoning measures on AARC total and cognitive losses. In the alternative model, ageist experiences had positive direct effects on AARC total and cognitive losses. The composite, Number Series, and Letter Sets mediated the effect of ageism on all AARC measures. Word Series mediated the effect of ageism on total and cognitive losses. Overall, inductive reasoning seems to play an important role in understanding the relationship of ageism with AARC. Thus, inductive reasoning abilities may be a potential intervention point to cultivate well-being. Future research should assess additional domains of cognition.

### C-REACTIVE PROTEIN MEDIATES THE ASSOCIATION BETWEEN SUBJECTIVE AGING AND INCIDENT HEART DISEASE

Hanamori Skoblow, and Christine Proulx, *University of Missouri, Columbia, Missouri, United States*

Recent studies have shown that negative perceptions of subjective aging are associated with a heightened risk of cardiovascular events (Stephan et al., 2020) and increased C-reactive protein (CRP), a biomarker associated with inflammation (Stephan et al., 2014). Because inflammation is deleterious to cardiovascular health, CRP might mediate the association between subjective aging and cardiovascular disease. The purpose of this study was to examine the association between subjective aging (i.e., negative self-perceptions of aging [SPA] and subjective age) and incident cardiovascular disease (e.g., heart attack, angina, congestive heart failure), and to determine whether CRP mediates this relation. We used up to five waves of repeated measures data from the Health and Retirement Study (HRS, 2008 - 2016) with adults aged 50 to 101 (n = 9,531). Two separate models were conducted in MPlus with bias-corrected bootstrap confidence intervals and controls for respondent age, sex, education, race, ethnicity, body mass index (BMI), diabetes, hypertension, depressive symptoms, and physical inactivity. There were significant indirect effects of both SPA and subjective age on incident cardiovascular disease through CRP (indirect effect SPA model = .02, CIs [.01, .03], $p < .05$; indirect effect subjective age model = .05, CIs [.02, .10], $p < .05$). In both models, CRP fully mediated the association between subjective aging and incident cardiovascular disease. Taken together, these findings underscore the importance of considering older adults' views of aging for understanding physical health and suggest that interventions aimed at improving views on aging may reduce inflammation and promote cardiovascular health.

### DAILY PERCEIVED STRESS AND PHYSICAL HEALTH SYMPTOMS: MODERATION BY SELF-PERCEPTIONS OF AGING

Dakota Witzel,[1] Shelbie Turner,[2] and Karen Hooker,[1], *1. Oregon State University, Corvallis, Oregon, United States, 2. Oregon State University, Oregon State University, Oregon, United States*

Research suggests increased daily perceived stress is related to worse physical health outcomes such as poor eating and exercise behavior (Li et al., 2019) and lower perceived health (Whitehead & Blaxton, 2020). While long-term implications for increased perceptions of stress on physical health are becoming clear (e.g., Rueggeberg et al., 2012), less is known about associations between daily perceptions of stress and subjective levels of physical health symptoms. Moreover, positive perceptions of one's own aging may buffer perceived stress's impact on physical health. Indeed, self-perceptions of aging (SPA) impact how people prepare for age-related stressors (Kornadt et al., 2015), and are associated with physical health trajectories (Luo & Li, 2020). Using a 100-day microlongitudinal study of 103 older adults, we examined the 1.) impact of both between- and within-persons associations of perceived stress on physical health symptoms and 2.) the potential moderating associations of SPA on perceived stress and physical health symptom associations. Preliminary models suggest that on days when people perceived more stress, they show a .03 increase in reporting physical health symptoms compared to days when they do not report more perceived stress ($p<.0001$). Further, people who experience more perceived stress on average, reported .06 more physical symptoms across the study period ($p <.0001$). While SPA did significantly predict physical health symptoms ($p=.004$), the association between perceived stress and physical health symptoms was not dependent on SPA ($p>.05$). Future directions may include exploring associations between daily stressful experiences, perceptions of stress, and valence of SPA.

### DIFFERENT STROKES FOR DIFFERENT FOLKS? EXAMINING A NEW MEASURE FOR AGE-RELATIVE SEXUAL ATTITUDES

Allyson Graf,[1] Kinsey Bryant-Lees,[1] Tracy Cohn,[2] and Maggie Syme,[3], *1. Northern Kentucky University, Highland Heights, Kentucky, United States, 2. Radford University, Radford, Virginia, United States, 3. Hebrew Senior Life, Boston, Massachusetts, United States*

Recent research suggests increasingly permissive attitudes towards sexual activity in later life. Harboring more conservative beliefs especially as one reaches older age, however, may translate into how one views and navigates sexual health changes. A sample of participants (N = 706; Mage = 52.72 years, SD = 9.57, range = 36-79; 60.8% male) was recruited through Amazon's Mechanical Turk to complete a survey on sexual beliefs about age and aging. Participants completed two versions of the Relative Sexual Attitudes Scale (RASA), wherein they were prompted to consider either "someone their own age" or "an older person" in response to items assessing sexual attitudes. Multi-group confirmatory factor analysis was used to confirm the original five-factor structure, reduce the total items from 31 to 25, and establish measurement equivalence for the 36-54 year-old and 55+ year-old samples. Through a series of profile analyses investigating each subscale, scores did not significantly differ between the two prompts, although significant age-group differences were found with the 36-54 year-old age group reporting more open attitudes than the 55+ year-old age group across all subscales, except the traditional mores subscale. Sexual attitude subscale scores also differed by gender, engagement in partnered sexual activity, and whether one had spoken to a health professional about their sexual health in the past year. The findings support use of the RASA for adults 36 and older and highlight applications to understanding differences in sexual health into later life.

### LACKING WARMTH AND COMPETENCE: HOW YOUNGER ADULTS UTILIZE "OK BOOMER" ACCORDING TO THE STEREOTYPE CONTENT MODEL

Katelyn Frey,[1] Michael Vale,[1] and Toni Bisconti,[2], *1. University of Akron, Akron, Ohio, United States, 2. The University of Akron, Akron, Ohio, United States*

Younger adults have coined the popular retort "OK Boomer," referring to the 76 million Baby Boomers born between 1946-1964. The Stereotype Content Model (SCM)

is a framework used to assess stereotypical perceptions of various groups, and older adults generally fall in the paternalistic "high warmth/low competence" quadrant. The stereotypes behind "OK Boomer" could correspond to any of the four quadrants of the SCM. The present study's goals were to determine the parameters for using the phrase, how hostile and benevolent ageism may underlie its use, and whether or not the eponymous "Boomer" fits into the same cluster in the SCM as older adults in general. In a sample of 316 participants (18-33; M = 23; SD = 5.25), we found that age was related to using "OK Boomer" such that being younger is associated with feeling more comfortable using the phrase in front of anyone (r = -.208, p < .01), using the phrase more frequently (r = -.218, p < .01), and sharing "OK Boomer" memes, pictures, and jokes online (r = -.203, p < .01). Hostile ageism, but not benevolent, was associated with an increased likelihood of saying "OK Boomer" in front of anyone (r = .256, p < .01), to use it more frequently (r = .242, p < .01), and to share "OK Boomer" jokes online (r = .301, p < .01). Content analysis results indicate that "OK Boomer" does not correspond to the paternalistic quadrant of the SCM due to Boomers' perceived low warmth.

### POSITIVE AND NEGATIVE AFFECT INFLUENCES ATTITUDES TOWARD AGING

R INTRIERI, and Paige Goodwin, *Western Illinois University, Macomb, Illinois, United States*

The Aging Semantic Differential (ASD; Rosencranz & McNevin, 1969) is one of the most widely used measures in the aging literature to measure attitudes rather than knowledge or beliefs about aging. Originally 32-items the ASD has been reduced through careful factor analysis to 20-items representing 4 factors representing: Instrumentality, Autonomy, Acceptability, and Integrity. Latent summary scores were created for each factor, with lower scores representing more positive attitude toward older adults. Despite its widespread use there are no published studies that examine the relationships between the four ASD factors and Positive and Negative affect. Positive and Negative affect are related to and represent the core aspects of Extraversion and Neuroticism. The prime objective of this study was to assess the relationships between Positive and Negative affect and the four ASD factors. The sample comprises 1189 undergraduate participants with a mean age of 22.02 (SD=6.27). The sample included 611 men and 578 women. Results showed the path model fitted the data well (CFI = 953, TLI = .944, RMSEA = 0.066, SRMR = 0.035). Positive affect was significantly related to Instrumentality, Acceptability, and Integrity (β= -0.073, (SE= 0.034); p=0.034; β= --0.141 (SE= 0.033), p=0.0001; β= -0.146 (SE= 0.032), p=0.0001). These results show that higher positive affect was related to more positive beliefs about Instrumentality, Acceptability, and Integrity. Negative affect was significantly related to Integrity (β= 0.079, (SE= 0.032); p=0.012) indicating that greater negative affect was related to more negative beliefs about bodily integrity.

### SELF-PERCEPTIONS OF AGING AMONG DEMENTIA CAREGIVERS: EVIDENCE FROM THE UK PROTECT STUDY

Shelbie Turner,[1] Serena Sabatini,[2] Helen Brooker,[3] Anne Corbett,[4] and Adam Hampshire,[5], *1. Oregon State University, Oregon State University, Oregon, United States, 2. University of Exeter, Exeter, England, United Kingdom, 3. Exeter, University of Exeter, England, United Kingdom, 4. University of Exeter, University of Exeter Medical School, England, United Kingdom, 5. Imperial College London, London, England, United Kingdom*

Contact with older adults impact the perceptions people have towards their own aging self (Jarrott & Savla, 2015) and how they prepare for their own age-related change (Kornadt et al., 2015). Caregivers have close, intimate contact with older adults, yet no research explores how that contact may impact caregivers' perceptions of their own aging. In this exploratory study, we compare perceptions of one's own aging between current and previous formal caregivers, non-formal caregivers, and never-caregivers. We utilized data from 1978 informal caregivers, 247 formal caregivers, and 5586 never-caregivers of the 2019 wave of the UK Protect Study. We conducted ANCOVA tests to compare global levels of Awareness of Age-Related Change (AARC) gains and losses, AARC gains and losses specific to cognition, attitudes towards one's own aging, and felt age across the three subgroups of participants with different caregiving roles. Omnibus results suggested that there were significant group differences (p<.05) in global levels of AARC gains and losses, AARC gains specific to cognition, and attitudes towards one's own aging (p<.05) for female, but not male, caregivers. However, effect sizes were either small or negligible. Therefore, despite frequent contact with older adults, dementia caregivers may not have better or worse self-perceptions of aging than non-caregivers. Such findings may be reflective of intergenerational ambivalence, and future work should consider how the nature of the caregiving situation (i.e. relationship quality, intensity of the care, caregiver burden) shapes caregivers' perceptions of their own aging, especially over time as caregivers navigate their own aging processes.

### SUBJECTIVE AGING IN CONTEXT: NEIGHBORHOOD SOCIAL ENVIRONMENT AND SELF-PERCEPTIONS OF AGING AMONG OLDER ADULTS

Eunyoung Choi,[1] Elizabeth Zelinski,[2] and Jennifer Ailshire,[2], *1. University of Southern California, University of Southern California, California, United States, 2. University of Southern California, Los Angeles, California, United States*

Self-perception of aging (SPA), one's attitude toward one's own aging, has been associated with health and well-being in later life. Whereas existing literature identifies individual-level predictors of SPA (e.g., education and health), little is known about the role of neighborhood context. The present study examines whether 1) neighborhood social environment is related to SPA and 2) age moderates this relationship. Our analytic sample includes 11,394 adults aged 50+ from the 2014 and 2016 waves of the Health and Retirement Study (Mean Age=68, SD=10.14, range 50-98). Indicators of neighborhood social environment include (a) perceived neighborhood social cohesion (the trust and social ties among community residents), (b) neighborhood friends, and (c) relatives living in the neighborhood. Regression analyses were performed to investigate the associations of each neighborhood-level indicator with the positive and negative dimensions of SPA. The models controlled for demographic, socio-economic, and health covariates. Greater neighborhood social cohesion (B=0.13, SE=0.01, p<.001) and having neighborhood friends (B=0.14, SE=0.02, p<0.001) were associated with higher levels of the positive SPA. As for the negative dimension of SPA, neighborhood social cohesion was the only significant predictor (B=-0.13, SE=0.01, p<0.001). Furthermore, we found significant interaction effects between neighborhood social cohesion and age: higher neighborhood cohesion was associated with more positive (B=-.003, SE=.00, p<.001) and less negative SPA ratings (B=-.003, SE=.00, p<.001) at younger ages than older ages. Our findings provide insights into how neighborhood social context shapes subjective aging, suggesting that a socially cohesive neighborhood may promote more favorable perceptions of aging, particularly for younger residents.

### THE IMPACT OF MENTAL HEALTH STIGMA AND AGEISM ON STUDENTS' INTENTION TO WORK WITH OLDER ADULTS: A MIXED METHODS DESIGN

Cindy Woolverton,[1] Katelind Biccum,[2] Aiping Yu,[2] Lanlan Xin,[2] and Jessica Strong,[3], *1. Michael E. DeBakey VA Medical Center, Houston, Texas, United States, 2. University of Prince Edward Island, University of Prince Edward Island, Prince Edward Island, Canada, 3. University of Prince Edward Island, Charlottetown, Prince Edward Island, Canada*

Approximately 20% of older adults have a mental or neurological disorder which can cause significant disability. With a growing older adult population, there is a need for providers receiving specialized training in aging to provide quality care. However, there continues to be shortages of students seeking careers in geriatrics and especially in working with older individuals with mental health (MH) concerns. The present study explored the relationship between MH stigma, ageism and intention to work with older adults among undergraduate students. Undergraduate students (N=188) completed a battery of questionnaires including intention to work with older adults, positive and negative attitude towards older adults, and open-ended questions exploring MH stigma views. Regression results indicated that MH stigma, positive, and negative attitudes significantly predicted intention to work with older adults, (F(3, 182) = 8.51, p = .000). Examination of the coefficients revealed that positive attitudes significantly predicted intention to work with older adults (t=4.38, p=.000), and MH stigma demonstrated a trend towards significance (t=1.90, p=.059). Open-ended responses were analyzed using qualitative description methods which revealed themes consistent with negative and positive stereotypes, MH problems going undetected, and need for additional support in recognizing and treating MH conditions among older adults. Positive attitudes are an important predictor in students' intention to work with older adults, and MH stigma may be an important factor to explore further. Qualitative themes also describe how MH concerns are an important area to focus on among older adults, although there continues to be evidence of aging stereotypes.

### THE ROLE OF AGING ANXIETY, AGEISM, AND HEALTH LOCUS OF CONTROL ON MIDDLE-AGED ADULTS HEALTH OUTCOMES AND BEHAVIORS

Hannah Bashian,[1] and Grace Caskie,[2], *1. Lehigh University, Quincy, Massachusetts, United States, 2. Lehigh University, Bethlehem, Pennsylvania, United States*

Older adults with more ageist attitudes and aging anxiety and who endorse an external health locus of control (HLOC) have poorer mental and physical health and less engagement in healthy behaviors than those who report less ageist attitudes, aging anxiety, and endorse an internal HLOC. However, middle-aged adults have not been examined in this literature. Using Terror Management Theory as a framework, this study examined the relationship of middle-aged adults' aging anxiety, ageist attitudes, and HLOC with health behaviors and mental and physical health outcomes. 391 middle-aged participants (40-55 years) completed measures of ageist attitudes, aging anxiety, HLOC (Internal, External, and Powerful Other), engagement in health behaviors, mental health, and physical health. The path analysis model demonstrated acceptable fit, $\chi 2(2)=7.794$, p=.02, CFI=.99, TLI=.92, RMSEA=.09). For health behaviors, eight of the 10 paths were significant; higher aging anxiety, higher ageist attitudes, and less endorsement of internal HLOC were related to less engagement in healthy behaviors. For mental health and physical health, five of the 10 paths were significant; in general, higher aging anxiety, higher ageist attitudes, and less endorsement of internal HLOC were related to poorer mental and physical health. This study demonstrated that middle-aged adults' aging anxiety, ageist attitudes, and health locus of control are related to their health behaviors and mental and physical health. Furthermore, higher endorsement of specific forms of ageist attitudes and aging anxiety were related to worse reported mental and physical health and to less engagement in health behaviors. Implications of these findings will be discussed.

### WHAT IS THE RELATIONSHIP BETWEEN FUNCTIONAL LIMITATIONS, PAIN, AND SELF-PERCEPTIONS OF AGING?

Jillian Minahan,[1] and Ashley Blasi,[2], *1. Fordham University, New Providence, New Jersey, United States, 2. Fordham University, Bronx, New York, United States*

Self-perceptions of aging (SPA) influences health and mortality during older adulthood (e.g., Kotter-Grühn et al., 2009; Sargent-Cox et al., 2012). Westerhof and Wurm (2015) found that increasing functional limitations (FL) worsened older adults' SPA. Additional research is needed to identify other factors that influence SPA. Although pain is common among older adults and is a frequent cause of disability (e.g., Brooks et al., 2019), it has not been examined as a factor influencing SPA. Pain is often misperceived as an inevitable part of aging because of widely held negative stereotypes about aging (Thielke et al., 2012). The experience of pain may activate internalized negative stereotypes about aging, which may worsen SPA. Thus, this study investigated: 1) the relationship between chronic and recent pain, FL, and SPA, and 2) the interactive effect of FL and pain on SPA within a sample of community-dwelling adults aged 65 years and older. This study included 5,126 participants from the 2014 wave of the Health and Retirement Study. Controlling for covariates, chronic pain (β = .09, p < .001) and recent pain (β = .12, p < .001) were associated with negative SPA and were stronger than FL (β = .04, p < .01). There was also a small interaction between FL and recent pain on SPA (β = -.03, p < .01) such that the negative impact of FL on SPA was stronger among individuals who reported low pain. These findings highlight the importance of pain in older adults' evaluation of their own aging.

### Session 9025 (Poster)

### AGING AND CHRONIC HEALTH CONDITIONS I

### BLOOD PRESSURE CONTROL AND CARDIOVASCULAR AND MORTALITY RISK IN VA NURSING HOME RESIDENTS

Xiaojuan Liu,[1] Sei Lee,[2] Michael Steinman,[2] Laura A. Graham,[3] Yongmei Li,[1] Bocheng Jing,[4] and Michelle Odden,[1], *1. Stanford University, Stanford, California, United States, 2. University of California San Francisco, San Francisco, California, United States, 3. VA Palo Alto Health Care System, Menlo Park, California, United States, 4. San Francisco VA Medical Center, San Francisco, California, United States*

Optimal blood pressure (BP) control in nursing home residents is controversial and this population has been excluded from trials. We evaluated the associations of BP level with cardiovascular (CV) events and all-cause mortality across antihypertensive medication categories in Veterans Affairs (VA) nursing home residents. Data for 18,589 residents aged 65 years and older was obtained from the VA Corporate Data Warehouse from October 2006 through September 2017. Baseline systolic BP (SBP) and diastolic BP (DBP) were divided into categories and analyses were stratified by antihypertensive therapy (0, 1, and ≥2 medications). Over a median follow-up of 1.8 years, CV events occurred in 3,519 (19%) residents and 15,897 (86%) residents died. In participants on no BP medications, high SBP (>150 mmHg) was associated with a greater risk of CV events (adjusted [cause-specific] hazard ratio, 1.39; 95% confidence interval, 0.94-2.06) compared with normal SBP (110-130mmHg). By contrast, in participants on ≥2 BP medications, the subgroup with low SBP (<110 mmHg) had a higher CV risk (1.38; 1.20-1.57). For DBP, in participants without BP medications, there were no differences in CV risk across DBP subgroups. Whereas among those on 1 or ≥2 medications, DBP <60 mmHg was associated with a higher CV risk (1.26; 1.03-1.55 and 1.35; 1.18-1.54, respectively) compared with normal DBP (70-80 mmHg). Participants with low SBP (<110 mmHg) and DBP (<70 mmHg) had an increased mortality risk regardless of the number of medications. These findings suggest a potential risk of low BP among nursing home residents on multiple antihypertensive medications.

### BONE-ACTIVE MEDICATION UTILIZATION FROM 2013-2017 AMID BENEFICIARIES AGED 65+ WITH MEDICARE PART D BY PROVIDER TYPE

Jennifer Kirk,[1] Sean Fleming,[2] and Denise Orwig,[3], *1. University of Maryland Baltimore, Laurel, Maryland, United States, 2. University of Maryland Baltimore, University of Maryland Baltimore, Maryland, United States, 3. University of Maryland, Baltimore, Baltimore, Maryland, United States*

As the United States' population increasingly consists of older adults aged 65+, an increase is expected in the prevalence of osteoporosis and the number of osteoporotic fractures. Bone-active medications (BAM) delay osteoporosis progression and prevent fragility fractures, but historically low treatment persistence rates and drug utilization for BAM exist among at-risk older adults. This research assessed for differences in the BAM utilization rates over five-years in Medicare Part D by provider type: geriatric specialists (GERO), generalists, specialists, nurse practitioners (NP), and physicians' assistants (PA). This longitudinal retrospective analysis included providers with at least one BAM prescription among beneficiaries aged 65+. An analysis of response profiles was used to model the mean BAM utilization rates overall and by provider group. Of the 50,249 providers included in this analysis, 88.15% were generalists, 5.76% specialists, 1.48% GERO, 2.73% NP, and 1.87% PA. From 2013-2017, the prevalence of BAM utilization was 6%. Over the five years, BAM utilization rates did not change significantly, but provider-specific rates were significantly different (F=12.53, p<.001). Provider-specific utilization rates were inconsistent with the highest utilization rates and most considerable variation observed among specialists (14.95%). PAs and NPs' BAM utilization rates were stable at around 9.02% and 9.20%, but GERO and generalists exhibited the lowest utilization rates, 4.86% and 5.79%, respectively. While specialists had the higher-than-expected utilization rates, the overall and provider-specific BAM utilization rates were low and did not increase over time. Further research is needed to identify how provider-related factors, like geographic region and clinical training, influence underutilization.

### CHANGES IN THE USE OF INTENSIVE AND SUPPORTIVE CARE WITH HEART FAILURE IN THE LAST MONTH OF LIFE BETWEEN 2001 AND 2013

Piling Chou,[1] and Peichao Lin,[2], *1. Kaohsiung Medical University, Kaohsiung, Taiwan, Kaohsiung, Taiwan (Republic of China), 2. Kaohsiung Medical University, Kaohsiung, Kaohsiung, Taiwan (Republic of China)*

**Background:** Heart failure (HF) is a global epidemic affecting the elder globally. It is uncertain what care patients with heart failure receive at their end of life and what care trends are in the last month of life. OBJECTIVES: This study's objective was to investigate the changes in the use of intensive and supportive procedures for Taiwanese patients with heart failure in their last month of life during 2001-2013.

**Methods:** Analysis of claims data of 25,375 patients with heart failure obtained from the National Health Insurance Research Database was performed to investigate the changes in the use of intensive and supportive procedures for Taiwanese patients with heart failure in their last month of life during 2001-2013.

**Results:** Over the whole study period, 53.3% of patients with heart failure were admitted to intensive care units in their last month of life. The percentages of patients receiving mechanical ventilation (54.3%-41.5%), cardiopulmonary resuscitation (41.5%-16.7%), decreased over time. The percentages of patients receiving artificial hydration and nutrition (52.5.9%-56.8%) and extracorporeal membrane oxygenation(ECMO) (0.52%-1.78%) increased over time. Patients under 75 years old were more likely to be admitted to intensive care units.

**Conclusion:** Over time, supportive procedures increased, and intensive procedures decreased in patients with heart failure in the last month of life. This study highlights a need for research, guidelines, and training in how to provide palliative care for end-stage patients with heart failure.

### COMMUNITY-ACADEMIC PARTNERSHIP TO IMPROVE NUTRITION AND BLOOD PRESSURE IN SENIORS: OUTCOMES & IMPACT OF COVID-19

Dozene Guishard,[1] Rhonda Kost,[2] Jonathan Tobin,[3] and Kimberly Vasquez,[4], *1. Carter Burden Network, New York, New York, United States, 2. Rockefeller University, New York, New York, United States, 3. Clinical Directors Network, New York, New York, United States, 4. The Rockefeller University, New York, New York, United States*

The Dietary Approaches to Stop Hypertension ("DASH diet") has been proven in research settings to lower blood pressure, but its implementation is untested among seniors in congregate meals settings. We report the planning, implementation, impact of COVID-19, and results of an Administration of Community Living-funded study to test whether two evidence-based interventions - DASH-alignment of congregate meals, and home blood pressure self-monitoring, can lower systolic blood pressure and increase blood pressure control among community-dwelling seniors.. Congregate meal menus were aligned with the DASH eating plan, through collaboration of Bionutrition professionals on the research team, CBN food services leadership, and the NYC Department for the Aging. Seniors provided feedback on the DASH-modified meal options. The intervention began on October 15, 2019 (Site 1) and February 3, 2020 (Site 2). The study was interrupted by the COVID-19 pandemic in March 2020, when congregate meals ceased, and when approximately 75% of primary outcome data were collected. Modified implementation permitted completion of modified study outcomes. Preliminary analyses suggest that some participants were able to lower their blood pressure in this program. The DASH diet implemented in the congregate meal setting, along with programs to support BP self-efficacy through modification of existing programs, may be a valuable and scalable model to reduce cardiovascular risk among community-living seniors.

### DEVELOPMENT AND VALIDATION OF MULTIMORBIDITY INDEX PREDICTING MORTALITY AMONG CHINESE OLDER ADULTS

Yan Luo,[1] Zi-Ting Huang,[2] Hui-wen xu,[1] zi-shuo Chen,[1] He-Xuan Su,[1] Hui Liu,[3] and Beibei Xu,[3], *1. Peking University School of Public Health, Beijing, Beijing, China (People's Republic), 2. Peking university School of Public Health, Beijing, Beijing, China (People's Republic), 3. Peking University Medical Informatics Center, Beijing, Beijing, China (People's Republic)*

This study aimed to construct a multimorbidity index among Chinese older adults. Participants aged 65-84 years (n=11,757) from the Chinese Longitudinal Healthy Longevity Survey (CLHLS). Fourteen self-reported chronic conditions were assessed at baseline. Outcome was all-cause mortality within five-year follow-up. We used restrictive association rules mining to identify the patterns of multiple chronic conditions associated with mortality. The weights of conditions and disease combinations were assigned using logistic regression adjusted by age and sex in training set. Multimorbidity index (MI) with individual diseases and multimorbidity index incorporating disease combinations (MIDC) were developed. We compared the performance of MI and MIDC with condition count and XGBoost algorithm in the validation set. There were no significant differences of c-statistics between condition count (0.687) and MI (0.692) or MIDC (0.689). The c-statistic of XGBoost algorithm (0.675) was the lowest among all models. The Integrated Discrimination Improvement (IDI) and categorical Net Reclassification Index (NRI) for MI (IDI: 0.01, $P < 0.001$; NRI: 0.01, $P = 0.127$), MIDC (IDI: 0.004, $p = 0.002$; NRI: 0.02, $P = 0.033$), and XGBoost model (IDI: 0.02, $P < 0.001$; NRI: 0.03, $P = 0.004$) were significantly positive compared with condition count. However, no significant differences for IDI and NRI were observed between MI and MIDC. Among Chinese older adults, weighted multimorbidity index with individual disease can better predict five-year mortality risk over condition count. There was little improvement in the predictive performance of the index after considering the joint effects of disease combinations.

### EVIDENCE-BASED INTEGRATIVE HEALTH CARE TO PROMOTE QUALITY OF LIFE IN OLDER ADULTS WITH CANCER

Robin Majeski,[1] and Delia Chiaramonte,[2] *1. University of Maryland, Baltimore County, Catonsville, Maryland, United States, 2. Greater Baltimore Medical Center, Towson, Maryland, United States*

Cancer disproportionately affects older adults and presents significant challenges to patients' quality of life. Use of complementary medicine is increasing among older adults with cancer and these modalities have the potential for both benefit and harm. Thus, it is important that health care professionals are knowledgeable about the evidence-supported benefits and risks of complementary and integrative health approaches in the care of older adults with cancer. Integrative cancer care provides a comprehensive approach to reducing symptom burden in patients suffering with cancer symptoms and side effects of cancer treatment. Symptoms such as pain, fatigue, nausea, sleep disturbance, mood disorder, perceived stress, and reduced quality of life are common in this population.This session will discuss an evidence-based integrative approach to cancer care which incorporates both pharmacologic and non-pharmacologic modalities to decrease symptom burden, enhance patient well-being, and improve quality of life. Non-pharmacologic modalities used in the integrative approach to care will be described and relevant evidence for risks, benefits and indications will be presented. Case studies will be discussed to demonstrate the integration of these techniques into conventional western medical treatment plans for older adults with cancer. Diversity and inclusion issues relevant to integrative medicine for underserved cancer patients will be addressed, as well as recommendations for future research to expand access of underserved populations to evidence-supported integrative cancer care. A resource list will be provided to participants.

### FACTORS RELATED TO THE LONELINESS OF OLDER WOMEN WITH HYPERTENSION IN TEHRAN

Ali Darvishpoor Kakhki, Sona Mohammadi, and Reza Miri, *Shahid Beheshti University of Medical Sciences, Tehran, Tehran, Iran*

Older women have longevity and face with common chronic diseases such as hypertension longer than men. In addition the refusal to accept older women into the mainstream of society can affect the loneliness of older women particularly in developing countries such as Iran. This study was conducted to describe factors related to loneliness of older women with hypertension in Tehran. This descriptive, correlational study was conducted on a sample of 300 older women above age 60 in five regions of Tehran in 2020. A socio-demographic questionnaire and the Russell Loneliness Scale were used for data collection. Content validity and Cronbach's alpha were used for evaluating the validity and reliability of questionnaires. 61% of older women were widowed and 37.3% lived alone with a mean age of 72.16(± 8.5) year. The mean score for loneliness was 66.26 (±13.44) on a 20 to 80-point scale. The scores of loneliness were influenced significantly by not having an income source, no living companion, chronic diseases, hospitalization in last year, family history of hypertension, and duration of hypertension. The best predictors of loneliness were hospitalization in last year, duration of hypertension, family history of hypertension, and chronic diseases. The findings of this study showed that loneliness is very common in older women with hypertension and is related to a number of factors. Monitoring modifiable factors such as hospitalization in the last year and non-modifiable factors such as duration of hypertension will help us to prevent or reduce loneliness in older women with hypertension.

### HEALTH TRAJECTORIES AFTER AGE 60: THE ROLE OF INDIVIDUAL BEHAVIOURS AND SOCIAL CONTEXTS

Amaia Calderón-Larrañaga,[1] Xiaonan Hu,[1] Miriam Haaksma,[2] Debora Rizzuto,[1] Laura Fratiglioni,[3] and Davide Vetrano,[1], *1. Karolinska Institutet, Solna, Stockholms Lan, Sweden, 2. Leiden University Medical Center, Leiden, Zuid-Holland, Netherlands, 3. Karolinska Institutet, 17175, Stockholms Lan, Sweden*

This study aimed to detect different health trajectories after age 60, and to explore to what extent individual and social factors may contribute to healthier ageing. Twelve-year health trajectories were identified in subjects from the Swedish National Study on Aging and Care-Kungsholmen (N=3108), integrating five indicators related to diseases, physical and cognitive function, and disability by means of nominal response models. Growth mixture models were applied to explore health trajectories in terms of rate and pattern of change. Baseline information about health-related behaviours and social context was collected through standardized questionnaires. The strength of the associations was estimated using logistic regression, and their impact through population attributable fractions (PAF). Three trajectories were identified grouping 78%, 18%, and 4% of people with respectively increasing rates of health decline Compared to the best trajectory, subjects in the middle and worst trajectories became functionally dependent 12.0 (95%CI:11.4-12.6) and 12.1 (95%CI:11.5-12.7) years earlier, respectively. Insufficient physical activity (OR:3.38, 95%CI:2.58-4.42), financial strain (OR:2.76, 95%CI:1.77-4.30), <12 years education (OR:1.53, 95%CI:1.14-2.04), low social connections (OR:1.45, 95%CI:1.09-1.94), low social participation (OR:1.39, 95%CI:1.06-1.83) and a body mass index ≥25 (OR:1.34, 95%CI:1.03-1.75) were associated with belonging to the middle/worst trajectories. The highest PAFs were observed for insufficient physical activity (27.1%), low education (19.3%) and low social participation (15.9%); a total PAF of 66.1% was obtained when considering all significant exposures together. Complementarily considering life-long factors belonging to the socioeconomic, psychosocial, and behavioural dimensions should be central to any strategy aimed at fostering health in older age.

### HYPERTENSION DIAGNOSIS, TREATMENT, AND CONTROL AMONG OLDER CHINESE: TRENDS IN THE HYPERTENSION CARE CASCADE

Erfei Zhao,[1] Qiao Wu,[2] Yuan Zhang,[3] and Eileen Crimmins,[2], *1. University of Southern California, Leonard Davis School of Gerontology, Los Angeles, California, United States, 2. University of Southern California, Los Angeles, California, United States, 3. University of North Carolina, University of North Carolina, North Carolina, United States*

Hypertension is a major risk factor for cardiovascular disease, which is the leading cause of death in China. Older persons are at higher risk of elevated blood pressure and are more likely to have insufficient hypertension care, including delayed diagnosis and poor management. However, we know little about hypertension care among older Chinese at a population level. We use a nationally representative sample of older adults from the China Health and Retirement Longitudinal Study (CHARLS) in 2011 and 2015 (n = 9,083), to clarify the hypertension care cascade for the older population in China by specifying the level of diagnosis, treatment, and control of hypertension. We then examine the characteristics of those (1) who received appropriate hypertension care and (2) whose care improved over time. Diagnosis and care improved between 2011 and 2015. Among those with hypertension, 55% and 67% were diagnosed in 2011 and 2015 respectively; 46% and 60% were treated with modern medication; and 20% and 29% were effectively controlled. Those who had higher income (OR=1.52; P<0.01) or obese (OR=2.43; P<0.001) were relatively more likely to be diagnosed, while those living in the western region (OR=0.65; P<0.01) or living in urban areas with a rural hukou (OR=0.54; P<0.01) were less likely. Persons age 75+ (OR=0.55; P<0.05) were less likely to have their blood pressure controlled, while those who had higher income (OR=1.50; P<0.05) were more likely. The improvement from 2011 to 2015 in hypertension care was concentrated among those that are obese or living in the West.

### IS CANCER HISTORY RELATED TO NEUROLOGIC SPECIALTY CARE IN PATIENTS WITH DEMENTIA?

Mackenzie Fowler, Kristen Triebel, Gabrielle Rocque, Ryan Irvin, Richard Kennedy, and Nicole Wright, *University of Alabama at Birmingham, Birmingham, Alabama, United States*

**Background:** The incidence and prevalence of aging-related diseases such as dementia and cancer are increasing, as are cancer survival rates. Cancer and its treatments have been associated with cognitive effects for those who later develop dementia. Guidelines have suggested that cancer patients return to follow-up in primary care following remission and be referred to specialists for cognitive complications, but it is unclear how well these guidelines are followed.

**Methods:** Electronic health record data at the University of Alabama at Birmingham were extracted from July 2003 May 2020. Rates of specialty care utilization on or after dementia diagnosis were compared by cancer history status in adults 50 years old or older at dementia diagnosis. Predictors of specialty care utilization were examined using logistic regression.

**Results:** Rate of specialty care utilization was lower for those with cancer history compared to those without on the date of dementia diagnosis (11.3% vs. 17.1%) and after diagnosis (13.5% vs. 19.2%). Older age at dementia diagnosis, non-Hispanic Black race, anticholinergic burden, socioeconomic status, and vascular risk factors were associated with lower odds of specialty care utilization. Dementia medication use was associated with higher odds of specialty care utilization on and after dementia diagnosis.

**Conclusions:** Cancer survivors with a dementia diagnosis are less likely to utilize specialty care than those with no history of cancer. Several factors predicted specialty care utilization. Additional studies should assess potential barriers in referring cancer survivors to specialty care for cognitive impairment.

### MARKERS OF GLUCOSE METABOLISM AND MUSCLE STRENGTH DECLINE AMONG THE OFFSPRING OF LONG-LIVED INDIVIDUALS

Iva Miljkovic,[1] Ryan Cvejkus,[1] Adam Santanasto,[2] Mary Feitosa,[3] Karen Schwander,[4] Mary Wojczynski,[5] Bharat Thyagarajan,[6] and Joseph Zmuda,[1], *1. University of Pittsburgh, Pittsburgh, Pennsylvania, United States, 2. University of Pittsburgh, Pittsburgh, Pittsburgh, Pennsylvania, United States, 3. Washington University School of Medicine in St. Louis, St. Louis, Missouri, United States, 4. Washington University, St. Louis, Missouri, United States, 5. Washington University School of Medicine, Washington University School of Medicine, Missouri, United States, 6. University of Minnesota, Minneapolis, Minnesota, United States*

Diabetes has been linked to accelerated muscle strength decline with aging. However, the association between glucose metabolism and muscle strength decline among individuals without diabetes is less clear. We tested whether fasting plasma markers of glucose and insulin metabolism (glucose, insulin, hemoglobin A1c, and soluble receptor for advanced glycation end products (sRAGE)) are associated with grip strength decline among 1415 non-diabetic offspring of exceptionally long-lived individuals who have a low diabetes risk (age range 36-88; mean age ± SD = 60 ± 8 years; mean BMI ± SD = 27 ± 4.7 kg/m2; 57% women). Grip strength was assessed using a hand-held dynamometer at two clinic visits over an average of 7.9 years. Multiple linear mixed models were adjusted for age, sex, field center, lifestyle, comorbidities, body weight, height, weight change, and family relatedness. Each standard deviation higher fasting insulin (7.3 mIU/L) was related to greater grip strength decline (-0.38 ± 0.16 kg; p=0.016), while each standard deviation higher fasting sRAGE (430 pg/mL) was related to slower grip strength decline (0.36 ± 0.18 kg; p=0.04). Our findings suggest that even among non-diabetic individuals from families with a clustering of "healthier" metabolic profiles - insulin metabolism and advanced glycation end products may be important biomarkers of muscle strength decline with aging. Potential mechanisms, including genetic and metabolic mediators underlying the observed associations, warrant further investigation.

### OLDER ADULT MAINTAINING AND IMPROVING HEALTH SELF-MANAGEMENT THROUGH PEER SUPPORTED SMART GOAL SETTING

Mary Hynes,[1] Nicole Anderson,[1] Monika Kastner,[2] and Arlene Astell,[1], *1. University of Toronto, Toronto, Ontario, Canada, 2. North York General Hospital, North York, Ontario, Canada*

Non-medical interventions to address risk factors (such as reducing smoking, increasing physical activity, and tackling limited social interaction) are needed to help tackle escalating social and financial health costs. Peer supported interventions have been used successfully to support persons' health self-management; however, there is limited evidence for group interventions facilitated by older adults. A proof-of-concept study by the first author demonstrated the potential of older adults meeting in groups to each create and follow through with a single SMART goal for any area of health over one-month. This study extends SMART goal setting to enhancing health management over six months. Older adult participants from across Ontario will attend virtual SMART goal setting group sessions followed by six monthly support group meetings where they are free to choose any goal, whether a mitigation or a new behavior. Each month the facilitator will assist participants to continue, modify, or set a new goal. At the end participants will complete surveys about their satisfaction with the method, their results and their desire to continue with SMART goals. They will also be asked if they would like to facilitate new groups to continue the spread of peer-supported SMART goal groups. This study is designed to empower older adults to maintain or improve management of their physical, psychological, and/or social health. It will reveal the impact of an older adult created and guided group health intervention on feelings of self-efficacy and well-being.

### OPPORTUNITIES AND BARRIERS TO MEDICATION SAFETY IN COMMUNITY-DWELLING OLDER ADULTS

Fatoumata Jallow,[1] Elisa Stehling,[2] Zara Sajwani,[3] Kathryn Daniel,[1] and Yan Xiao,[1], *1. University of Texas at Arlington, Arlington, Texas, United States, 2. University of Tx. at Arlington, Arlington, Texas, United States, 3. The University of Texas at Arlington, The University of Texas at Arlington, Texas, United States*

Community-dwelling multi-morbid older adults are a vulnerable population for medication safety-related threats. We interviewed a sample of these older adults recruited from local retirement communities and from primary care practices to learn their perceptions of barriers and enablers for their medication safety. The present study is part of the Partnership in Resilience for Medication Safety (PROMIS) study. One of the aims of this project is to identify barriers and opportunities to improve older adults' medication safety. These interviews were conducted during COVID-19 pandemic conditions. Results from this qualitative study suggest that trust between these older adults and their healthcare providers is an essential component of medication safety. Overarching themes include disruptions in medication management, caregivers caring for each other, patient safety practices or habits, and medication management literacy. Participants also shared strain due to lack of skills to navigate telemedicine visits, trust in Primary Care Providers (PCPs) and pharmacists to prescribe and dispense safely for them, reliance on PCPs and pharmacist to give essential information about medications without having to be asked. Our interviews illustrated large variations in older adults' perceived role in medication safety, with some developing expertise in understanding how medications work for them and how long-term medications should be periodically reviewed. The types of information needs and supports from PCPs were likely different. Understanding these barriers and enablers for safe medication management can help us develop medication safety improvements for this vulnerable population.

### PULMONARY AND PHYSICAL FUNCTION LIMITATIONS IN AGING MEN WITH AND WITHOUT HIV FROM THE MULTICENTER AIDS COHORT STUDY

Mona Abdo,[1] Ken Kunisaki,[2] Valentina Stosor,[3] Gypsyamber D'Souza,[4] Madiha Abdel-Maksoud,[1] Todd Brown,[5] Samantha MaWhinney,[1] and Kristine Erlandson,[6], *1. Colorado School of Public Health, Aurora, Colorado, United States, 2. University of Minnesota Medical School, Minneapolis, Minnesota, United States, 3. Northwestern University Feinberg School of Medicine, Chicago, Illinois, United States, 4. Johns Hopkins Bloomberg School of Public Health, Baltimore, Maryland, United States, 5. Johns Hopkins School of Medicine, Baltimore, Maryland, United States, 6. Colorado School of Medicine, Aurora, Colorado, United States*

We sought to determine effects of age, HIV serostatus, and smoking on the associations between pulmonary function and physical function impairments using Multicenter AIDS Cohort Study data. Associations between physical function outcomes gait speed (m/sec) and grip strength (kg) with normalized pulmonary function tests (diffusion capacity for carbon monoxide (DLCO, n=1,048) and forced expiratory volume in one second (FEV1, n=1,029)) were examined. Adjusted mixed-effects models included interaction terms to assess effect modification. 574(55%) were HIV+, with median age 57(IQR=48,64) and mean cumulative smoking pack-years 12.2(SD=19.0). 349(33%) had impaired DLCO (<80% of predicted) and 130(13%) had impaired FEV1 (<80% of predicted). Participants with impaired DLCO had weaker grip strength than those with normal DLCO (estimate= -3.5[95% CI=-4.6,-2.4]kg; p<0.001). Participants with impaired DLCO had slower gait speed than those with normal DLCO (estimate= -0.04[95% CI= -0.06,-0.02] m/sec; p=0.002). Age modified the DLCO effect on gait (p-interaction=0.01) but not grip (p-interaction=0.09). The association between decreased DLCO and slower gait was more pronounced in older participants. Smoking or HIV serostatus did not significantly modify the DLCO effect on gait (all p-interaction≥0.14) or grip (p-interaction=0.74, p-interaction=0.058, respectively). As with DLCO, participants with impaired FEV1 had weaker grip strength (estimate=-3.0[95% CI= -4.7,-1.3]kg; p<0.001) than those with normal FEV1. FEV1 was not associated with gait speed(p=0.98). Age, HIV serostatus or smoking did not modify the associations between FEV1 and gait speed or grip strength (all p-interaction>0.05). Associations between lower DLCO/FEV1 and decreased physical function suggest that interventions to improve pulmonary function may also preserve physical function with aging.

### THE IMPACT OF TOOTH RETENTION ON HEALTH AND QUALITY OF LIFE IN OLDER ADULTS

Adejare (Jay) Atanda,[1] Alicia Livinski,[2] Darien Weatherspoon,[2] Paul Fontelo,[2] and Shahdokht Boroumand,[2], *1. National Institutes of Health, Baltimore, Maryland, United States, 2. NIH, Bethesda, Maryland, United States*

America is aging rapidly, and older adults (age ≥65 y) are retaining more of their natural teeth, a trend expected to continue. Although much is known about the impact of complete tooth loss on overall health and well-being, less is known about the effect of partial tooth loss. We conducted a systematic review to advance our understanding of the impact of retaining ≥20 teeth on health and quality of life (QoL) in older adults using two tooth retention concepts – shortened dental arch (SDA) and functional dentition (FD). We searched seven scientific databases from 1981–2019 for publications on tooth retention and outcomes and impact on health and QoL. Ninety-six studies were included in this review. Most were assessed with low risk of bias (n=74) and of good quality (n=73) using the revised Cochrane Risk of Bias tool and Newcastle-Ottawa Scale. Tooth retention was defined as FD in 82 studies, SDA in 10 studies, and four studies used both. Most were cross-sectional and only seven were from the US. We found an increasing trend among published studies in using FD and SDA to describe natural dentition retention (50 articles in 2015-19 vs one in 1995-99). In general, having <20 teeth was associated with increased likelihood for functional dependence, onset of disability, declines in higher-level functioning, and lower QoL. New information is needed to facilitate clinical decision-making, care-giving, and to help health providers better meet the future oral health needs of an aging US population.

### USING HEALTH-RELATED QUALITY OF LIFE TO IDENTIFY THE INCIDENT CARDIOVASCULAR DISEASE RISK

Aung Zaw Zaw Phyo,[1] Joanne Ryan,[1] David A. Gonzalez-Chica,[2] Nigel P. Stocks,[2] Christopher M. Reid,[1] Andrew M. Tonkin,[1] Robyn Woods,[1] and Rosanne Freak-Poli,[1], *1. Monash University, Melbourne, Victoria, Australia, 2. University of Adelaide, Adelaide, South Australia, Australia*

Previous studies have revealed that poor health-related quality of life (HRQoL) is associated with a higher risk of hospital readmission and mortality in patients with cardiovascular disease (CVD). The association between HRQoL and incident CVD is still limited for general older people. This study explored the associations between baseline HRQoL and incident and fatal CVD in community-dwelling Australian and the United States older people enrolled in ASPREE clinical trial. A cohort of 19,106 individuals aged 65 to 98 years, who were initially free of CVD, dementia, or disability, were followed between March 2010 and June 2017. The SF-12 questionnaire was used to assess HRQoL, and the physical (PCS) and mental component scores (MCS) of SF-12 were derived using norm-based methods. Incident major adverse CVD events included fatal CVD (death due to atherothrombotic CVD), hospitalizations for heart failure, myocardial infarction or stroke. Analyses were performed using Cox proportional-hazard regression. Over a median 4.7 follow-up years, there were 922 incident CVD events, 203 fatal CVD events, 171 hospitalizations for heart failure, 355 fatal or nonfatal myocardial infarction and 403 fatal or nonfatal strokes. A 10-unit higher PCS, but not MCS, was associated with a lower risk of incident CVD (HR=0.86, 95%CI 0.79-0.92), hospitalization for heart failure (HR=0.72, 95%CI 0.60-0.85), and myocardial infarction (HR=0.85, 95%CI 0.75-0.96). Neither PCS nor MCS was associated with fatal CVD events or stroke. Physical HRQoL can be used in combination with clinical data to identify the incident CVD risk among community-dwelling older people.

### Session 9030 (Poster)

### AGING AND CHRONIC HEALTH CONDITIONS II

### ASSOCIATION OF BODY COMPARTMENT SHRINKAGE WITH SUBSEQUENT HEALTH CARE UTILIZATION IN OLDER MEN

John Schousboe,[1] Lisa Langsetmo,[2] Allyson Kats,[3] Brent Taylor,[3] and Kristine Ensrud,[3] *1. Park Nicollet Clnic / HealthPartners, Minneapolis, Minnesota, United States, 2. University of Minnesota, University of Minnesota, Minnesota, United States, 3. University of Minnesota, Minneapolis, Minnesota, United States*

Both height loss and weight loss among the very old are associated with adverse health outcomes including fractures and mortality. However, it is not clear whether the associations between weight loss and health outcomes are attributable to specific compartmental (fat vs. fat-free) loss or whether they are attributable to overall shrinkage. Our objective was to estimate the associations of compartmental loss and height loss with subsequent total health care costs, acute hospitalizations, and skilled nursing facility (SNF) stays over a three-year follow-up period, adjusted for each other and important covariates (age, race, multimorbidity, IADL impairment, depressive symptoms, walk speed). Our analytic cohort was 1505 older men (mean [SD] age 79.3 [5.2] years) who attended the 3rd Osteoporotic Fractures in Men (MrOS) study visit (V3) and who were enrolled in Medicare Fee for Service (FFS). Annualized changes in fat-free and fat mass (measured with dual-energy x-ray absorptiometry) and height were assessed over a mean (SD) 6.8 (0.3) years prior to V3. Total health care costs, acute hospital stays, and SNF stays were ascertained during 3 years after V3 using linked Medicare FFS claims files. Fat-free mass loss (per SD) was associated with total health care costs (cost ratio 1.10, 95% CI 1.01, 1.19), but not with acute hospital or SNF stays. Fat mass loss and height loss were not associated with health care utilization outcomes after multivariable adjustment. Loss of fat-free mass is modestly associated with higher total health care costs after accounting for age, race, multimorbidity, and IADL impairment.

### ASSOCIATION OF OBESITY, MULTIPLE CHRONIC CONDITIONS, AND FRAILTY: THE NATIONAL HEALTH AND AGING TRENDS SURVEY

John Batsis,[1] Christian Haudenschild,[2] Anna Kahkoska,[3] rebecca Crow,[4] David Lynch,[5] Curtis Petersen,[6] and Matthew Lohman,[7], *1. University of North Carolina at Chapel Hill, Chapel Hill, North Carolina, United States, 2. University of Minnesota, University of Minnesota, Minnesota, United States, 3. University of North Carolina at Chapel Hill, University of North Carolina at Chapel Hill/ Chapel Hill, North Carolina, United States, 4. VA White River Junction, White River Junction, Vermont, United States, 5. UNC Chapel Hill, University of North Carolina at Chapel Hill, North Carolina, United States, 6. Dartmouth College, Lebanon, New Hampshire, United States, 7. University of South Carolina, Columbia, South Carolina, United States*

As life expectancy increases, so does the risk of developing multiple chronic conditions (MCC). This is concerning as there is a growing obesity epidemic in older adults which is also associated with developing chronic diseases. Both obesity and MCC also increase the risk of frailty, yet the intersection of the three is not well understood. We evaluated the relationship between obesity, multimorbidity, and frailty using data from adults ≥65 years from the National Health and Aging Trends Survey. Obesity was classified using standard body mass index categories (e.g., ≥30kg/m2) and waist circumference (WC; females≥88cm; males≥102cm). MCC was classified as having ≥2 chronic conditions. Adjusted logistic regression models evaluated the association of BMI or WC categories on MCC (yes/no). An analysis limited to persons with obesity evaluated the relationship between frailty phenotypes (e.g, robust, pre-frail, frail) and MCC. Of the 4,967 participants (59.7% female), 79% resided in a private residence. The 70-79 age category was most prevalent. In those with MCC, there were 1,511 (30.4%) classified as having obesity using BMI, and 3,358 (67.6%) using WC. In those without MCC, there were 287 (17.6%) and 744 (51.7%). Compared to normal BMI, the odds of MCC was 0.71 [0.46,1.09], 1.25 [1.08,1.45] and 2.59 [2.15,3.11] in underweight, overweight and obesity. In pre-frailty and frailty, the odds of MCC were 2.52 [1.77,3.59] and 8.35 [3.7,18.85] in BMI-defined obesity. Using WC, the odds were 2.38 [1.94,2.91], and 5.89 [3.83,9.06]. Obesity using both BMI and WC are both strongly associated with multimorbidity and frailty.

### ASSOCIATIONS BETWEEN MEAL PROGRAM PARTICIPATION AND PROTEIN INTAKE IN PEOPLE OVER 65 (NHANES 2013-2018)

Sarah Collins,[1] Robert Blanco,[2] and Anika Hines,[3], *1. Virginia Commonwealth University, Richmond, Virginia, United States, 2. VCU, VCU, Virginia, United States, 3. VCU, VCU/Richmond, Virginia, United States*

Protein plays a critical role in healthy aging. Little research exists regarding the association between meal program participation and protein consumption among individuals 65 and older. The objective of this research is to provide health professionals with a better understanding of how meal program participation through delivery services or congregate sites may relate to nutritional status. We analyzed cross-sectional data on 2845 individuals ≥65 years old who participated in the National Health and Nutrition Examination Survey (NHANES) during 2013-2018. Using linear regression models, we explored relationships between meal participation and covariates ( sex, race, marital status, income, and age) on protein intake. Protein intake did not differ significantly between individuals who participated in meal programs and those who did not. However, among individuals who answered whether or not they participated in meal programs, race was significantly associated with decreased protein intake. Non-Hispanic Blacks experienced a two-day average 8.82 grams lower [SE:1.48; p<.0001] that their white counterparts. Similarly, Hispanic/Latinos' two-day protein average was 4.29 grams lower [SE:2.05; p=0.0426]. The association between earning an income of <$20,000 per year and protein intake was also statistically significant [β: -8.44. SE:2.4, p=0.0014]. Understanding protein intake among older adults who utilize meal programs is a gap in current literature. Results from this research may inform questions that health professionals should include in their assessments of older adults and provide guidance for nutrition policies and meal programs for people over 65.

### ASSOCIATIONS BETWEEN METFORMIN AND ASPIRIN USE ON CANCER INCIDENCE AND MORTALITY IN OLDER ADULTS.

Suzanne Orchard,[1] Jonathan Broder,[1] Jessica Lockery,[1] Peter Gibbs,[2] Robyn Woods,[1] Sara Espinoza,[3] Michael Ernst,[4] and John McNeil,[1], *1. Monash University, Melbourne, Victoria, Australia, 2. The Walter & Eliza Hall Institute of Medical Research, Parkville, Victoria, Australia, 3. University of Texas Health Science Center San Antonio, San Antonio, Texas, United States, 4. University of Iowa, Iowa City, Iowa, United States*

Diabetes increases risk of malignancies, and this association increases with age. Metformin may protect against cancer development and progression, but results are mixed and limited to younger cohorts. We examined whether metformin, in the presence or absence of aspirin, reduces incident cancer and cancer-related mortality in older adults. ASPirin in Reducing Events in the Elderly (ASPREE) was a primary prevention trial of daily aspirin vs placebo which enrolled community-dwelling adults from Australia (70+ years) and the US (65+ years for minorities) followed for a median of 4.7 years. Invasive cancer was adjudicated by an expert panel. Cox proportional-hazards models, controlling for age at randomization and known cancer risk factors, were used to analyse the relationship between baseline metformin use, randomized treatment arm, cancer incidence (first in-trial cancer) and mortality. For participants with controlled diabetes, there was a significant reduction in cancer mortality in metformin users compared to nonusers (Adjusted [Adj] HR=0.24, 95%CI=0.07, 0.80), but not for cancer incidence (Adj HR=0.61, 95%CI=0.29, 1.27). For participants with uncontrolled diabetes, there was no significant difference in cancer incidence (Adj HR=0.95, 95%CI=0.66, 1.38) or mortality (Adj HR=1.18, 95%CI=0.62, 2.26) between metformin and non-metformin users. Uncontrolled diabetes, irrespective of metformin use, increased risk of cancer incidence and mortality compared to non-diabetics. Aspirin did not modify the effect of metformin on cancer incidence or mortality. Our findings show that metformin may have protective effects against cancer-related mortality for those older persons whose diabetes is well-controlled, and underscores the importance of diabetes control to minimise cancer risk.

### CASE STUDY - CANDIDA AURIS IN SKILLED NURSING FACILITY

Nancy Vo,[1] John Chen,[2] Sayaka Tokumitsu,[1] and Armen Melik-Abramians,[1], *1. Kaiser Permanente Los Angeles Medical Center, Kaiser Permanente Los Angeles Medical Center, California, United States, 2. Kaiser Permanente Med Group/Southern California, Los Angeles, California, United States*

Candida Auris (C. auris), is a multidrug-resistant organism, first described in Japan 2009, and now a serious, emerging global health threat1. C. auris pathogen can potentiate morbidity and mortality, i.e. lifelong contact precaution isolation, intravenous antifungal treatment, hospitalization and mortality rate of 30-60%1. Los Angeles County (LAC) developed 15 new cases in May 2020, and 73 cases in July 2020, amidst COVID-19 pandemic2. A 88 year old Black female had a positive skin test for C. auris by LAC Department of Public Health (DPH) during skilled nursing facility (SNF) admission for hip fracture in September 2020. Patient's risk factors for C. auris included: age, kidney transplantation (1998) immunosuppression on tacrolimus, fungal infection on fluconazole, drug-drug interaction between tacrolimus-fluconazole including nephrotoxicity and neurotoxicity, malnutrition, bedbound, Stage 4 sacrococcyx pressure ulcer, osteomyelitis on broad-spectrum antibiotics, chronic indwelling catheter, and healthcare setting. Our multimorbid and frail patient remained asymptomatic with C. auris under an interdisciplinary team approach, including geriatricians, infectious disease, pharmacists, SNF team and local DPH. Our patient's psychosocial isolation and family distress with local DPH guidelines for COVID-19 SNF visitation restrictions were compounded by multifaceted coordination of patient-centered care between SNF team and specialists via telehealth. Further research in the prevention, detection, and management of C. auris is warranted to protect our vulnerable SNF residents. 1. Centers for Disease Control and Prevention. (2020). Tracking Candida auris. https://www.cdc.gov/fungal/candida-auris/tracking-c-auris.html 2. Los Angeles County Health Alert Network. (2020). CDPH Health Advisory: Resurgence of Candida auris in Healthcare Facilities in the Setting of COVID-19. http://publichealth.lacounty.gov/eprp/lahan/alerts/CAHANCauris082020.pdf

### CHARACTERISTICS AND PROGNOSES OF LONG-TERM HOME CARE PATIENTS

Takashi Yamanaka,[1] Maiko Mizuki,[1] Kiwami Kidana,[1] and Ryonosuke Yamaga,[2], *1. The University of Tokyo, Bunkyo-ku, Tokyo, Japan, 2. Tsubasa Home Care Clinic, Funabashi-city, Chiba, Japan*

With demographic aging, many older adults require home medical care. Although home-based primary care is promoted in the United States and Japan, there is insufficient evidence about it. We aimed to study the characteristics and prognoses of long-term home care patients. We prospectively registered 151 patients, estimated to receive physician home visits for more than six months, in a clinic in Chiba, Japan, in 2020. The mean (±SD) age was 83.9±10.0 years and ranged from 31 to 102 years. Most patients were men (60.3%) and aged 65 years or above (95.3%). We investigated clinical information, the Edmonton Symptom Assessment System Revised Japanese version (ESAS-r-J), Dementia Assessment Sheet in Community-based Integrated Care System 21 items (DASC-21), EuroQOL 5 dimensions 5-level (EQ-5D-5L) every six months, and the incidence of hospital admission, death, and patient transportation by ambulance. The most frequent diagnoses were dementia (31.1%), bone and articular diseases (17.2%), cerebrovascular diseases (11.9%), organ failure (9.3%), and neurological diseases (9.3%). Most patients (78.2%) showed more than 30 points on the DASC-21, suggesting cognitive impairment. Worse wellbeing, drowsiness, tiredness, anxiety, depression, and pain were the most prevalent symptoms. EQ-5D-5L index values were distributed around–0-0.2 and 0.4-0.7. During the first three months of physician home visits, 21.9% of patients had hospital admissions, 12.5% of them died, and 11.7% required hospital transportation by an ambulance. In this study, most long-term home care patients suffered from cognitive impairment. In addition to receiving care for daily life, these patients require intensive medical management.

### COST REDUCTION BEHAVIORS AND COST-RELATED MEDICATION NONADHERENCE IN OLDER ADULTS WITH ATRIAL FIBRILLATION

Benita Bamgbade,[1] David McManus,[2] Becky Briesacher,[1] Darleen Lessard,[2] Jordy Mehawej,[2] Mayra Tisminestzky,[2] Jerry Gurwitz,[2] and Jane Saczynski,[1] *1. Northeastern University, Boston, Massachusetts, United States, 2. University of Massachusetts Medical School, Worcester, Massachusetts, United States*

While factors such as forgetfulness may result in medication nonadherence, 2.7 million older adults in the US experience cost-related nonadherence (CRN). Limited research has explored CRN and associated cost-reduction behaviors (CRB) in older adults with atrial fibrillation. The objectives of this study were to 1) describe the prevalence of CRN, CRB and spending less on basic needs to afford medication and 2) examine factors associated with CRB among older adults with atrial fibrillation. Data were drawn from the Systematic Assessment of Geriatric Elements in Atrial Fibrillation (SAGE-AF), a prospective cohort of older adults with atrial fibrillation (>65 years). Using a self-administered survey, all participants completed a validated CRN measure. Chi-square and t-tests were used to evaluate differences in participant characteristics across CRB and significant characteristics ($p<0.05$) were entered into a logistic regression model. Participants (N=1244) were on average 76 years and 49% were female. Among all participants, 4.2% reported CRN; 69.1% reported CRB; and 5.9% reported spending less on basic needs. Compared to participants who did not engage in CRB, participants who engaged in CRB were less likely to be cognitively impaired and more likely to be a race/ethnicity other than non-Hispanic white; have Medicare insurance; and have comorbidities. CRB were common among older adults with atrial fibrillation and was associated with in-tact cognitive function, the presence of medical comorbidities and non-White race. Clinicians might consider providing patients with cognitive impairment additional support such as patient assistance programs or referrals to pharmacists for medication therapy management to assist with CRB.

### HEARING LOSS AND HEALTH CARE SEEKING

Nicholas Reed, *Johns Hopkins Bloomberg School of Public Health, Baltimore, Maryland, United States*

Hearing loss is common among older adults. Hearing loss is associated with increased health care expenditures, risk of 30-day readmission, and longer length of hospital stay. However, little is known about behaviors and attitudes in seeking care. In this cross-sectional analysis, we examined data from the 2016 Medicare Current Beneficiary Survey (MCBS) datasets. Participants are asked to describe their self-perceived trouble hearing. Health care seeking attitudes were assessed on all study participants in 2016 via self-report avoidance or delay of care, personal health concerns, and sharing health status. Multivariate regression models adjusted for demographic/socioeconomic characteristics and general health determinants were used to explore the association between trouble hearing and outcomes. In the 2016 MCBS, 12,140 Medicare beneficiaries, representing 51 million with survey weights, answered questions on help-seeking attitudes. In the sample, 55.6% reported no trouble hearing, while 38.8% and 5.5% reported a little trouble and a lot of trouble hearing, respectively. Those with a lot of trouble hearing were more likely to report avoiding doctors (Odds Ratio [OR] = 1.35; 95% Confidence Interval [CI] = 1.09 – 1.67) and delaying care (OR = 1.47; 95% CI = 1.19 – 1.82). However, no differences were found in personal health concerns or willingness to share health status with others. Poorer health care seeking behaviors may help explain higher costs associated with hearing loss as avoidance of care can exacerbate health problems. Further work is needed to understand underlying reasons and whether addressing hearing loss modifies the observed association.

### ILLNESS PERCEPTIONS AND HEALTH OUTCOMES AMONG COMMUNITY-DWELLING OLDER ADULTS WITH MULTIPLE CHRONIC CONDITIONS

Ayo Bankole, *George Washington University, Ashburn, Virginia, United States*

Illness perceptions (IP) has been associated with self-management and health outcomes in individuals with chronic diseases such as heart disease and diabetes; however, there is less research on the relationship between IP and health outcomes in individuals with multiple chronic conditions (MCC). Older adults with MCC are more likely to experience poor outcomes such as hospitalizations and poor self-rated health yet, there is less understanding of the processes associated with these outcomes. The purpose of this study was to (1) explore the relationship between IP and self-rated health among older adults with MCC (2) explore the relationship between IP and the number of hospitalization within the past year among older adults with MCC. Understanding these relationships may be instrumental to designing targeted interventions to improve health outcomes for this population. 116 participants (ages 65-90) completed the illness perception of multimorbidity scale, modified general health subscale of the SF-36 questionnaire, and self-reported number of hospitalizations within the past year. Ordinal logistic regression was used for analysis. Older adults who reported negative IP were likely to report worse self-rated health and this relationship remained significant after controlling for age and number of chronic conditions {-0.032 (95% CI (-0.050 to 0.014) p< 0.05}. There was no significant relationship between IP and the number of hospitalization within the past year. The study results study suggest that IP is associated with self-rated health in older adults with MCC. IP may be useful to design targeted interventions to improve self-rated health in this population.

### JOINT TRAJECTORIES OF MULTIMORBIDITY, FUNCTION, COGNITION, AND DEPRESSION IN THE HRS (1998-2016)

Corey Nagel,[1] Heather Allore,[2] Jason Newsom,[3] Anda Botoseneanu,[4] David Dorr,[5] Stephen Thielke,[6] Jeffrey Kaye,[5] and Ana Quiñones,[5], *1. University of Arkansas for Medical Sciences, Little Rock, Arkansas, United States, 2. Yale School of Medicine, New Haven, Connecticut, United States, 3. Portland State University, Portland, Oregon, United States, 4. University of Michigan - Dearborn, Dearborn, Michigan, United States, 5. Oregon Health & Science University, Portland, Oregon, United States, 6. University of Washington, University of Washington, Washington, United States*

There is substantial heterogeneity in the impact of multimorbidity on functional, cognitive, and emotional health. Few studies, however, have examined this simultaneously across these multiple domains. We used finite mixture modeling to identify latent clusters of individuals following similar joint trajectories of multimorbidity, functional ability, cognitive performance, and depressive symptoms among 11,841 HRS respondents aged 65+ from 1998 to 2014. We identified four distinct clusters of joint trajectories: (1) 32.5% of the sample were characterized by low multimorbidity (mean = 0.60 conditions at age 65; 2.2 conditions at age 90), minimal deterioration in functional or cognitive ability, and low depressive symptoms; (2) 33.5%, had increased multimorbidity compared with the first group (mean = 2.3 at age 65; 4.0 at age 90) but minimal functional or cognitive impairment and low depressive symptoms; (3) 19.9%, had relatively low multimorbidity (mean = 1.3 at age 65 increasing to 2.8 at age 90), but exhibited worsening cognitive performance, increasing functional limitations, and moderate depressive symptoms ; and (4) 14.1%, had higher multimorbidity (mean = 3.3 at age 65 increasing to 4.6 at age 90), worsening cognitive performance, substantial functional limitation, and high depressive symptoms. Black and Hispanic race/ethnicity, lower levels of income and education, male gender, and smoking history were significantly associated with membership in classes characterized by higher multimorbidity, cognitive and functional impairment, and greater depressive symptoms. This study provides insight into the heterogenous trajectories of aging and helps identify older individuals at higher risk for poor aging outcomes across multiple health domains.

### METABOLIC COMORBIDITIES AMONG PATIENTS WITH PERIPHERAL ARTERY DISEASE: A NATIONAL POPULATION-BASED STUDY

Gi Wook Ryu, Mona Choi, and Young Shin Park, *Yonsei University, Seoul, Seoul-t'ukpyolsi, Republic of Korea*

Peripheral artery disease (PAD) is a chronic disease which is associated with old age. PAD was known as an age-related chronic condition. Metabolic comorbid conditions which include hypertension, diabetes, and hyperlipidemia can have negative impacts on blood vessels aggravating PAD in elderly patients. Therefore, metabolic comorbidities need be considered in order to develop intervention for patients with PAD. The aim of this study is to find the characteristics of PAD patients with metabolic comorbidities. This is a retrospective study that used the national claim data of South Korea from 2009 to 2018. The inclusion criteria were adults (20+) and patients diagnosed with PAD as a primary or secondary diagnosis from 2011 to 2017. The frequency of hypertension, hyperlipidemia, diabetes, and metabolic comorbidities in PAD patients was examined. In addition, the difference in the number of metabolic comorbidities according to sex was identified using the chi-squared test. Among the total PAD adult patients(n=8,478,876), the number of elderly patients over 60 years old was 4,124,592(48.7%). Among the total patients, PAD patients with hypertension were the most common at 958,329 (11.30%). Sex was significantly related to having metabolic comorbidities and women showed higher proportion of metabolic comorbidities compared to men ($\chi 2$ =5.02, p<.001). As the frequency of PAD patients with hypertension were the highest, it is necessary to develop a health management program that considers metabolic comorbidities, especially hypertension, in order to manage PAD disease. In addition, there is a need for special interest in intervening metabolic conditions of female patients with PAD.

### OBESITY AND MULTIMORBIDITY IN THE USA: NATIONAL HEALTH AND NUTRITION EXAMINATION SURVEYS 2005-2014

David Lynch,[1] Curtis Petersen,[2] Hillary Spangler,[3] Anna Kahkoska,[4] and John Batsis,[5], *1. University of North Carolina, Chapel Hill, North Carolina, United States, 2. Dartmouth College, Lebanon, New Hampshire, United States, 3. UNC Hospitals, Chapel Hill, North Carolina, United States, 4. University of North Carolina at Chapel Hill, University of North Carolina at Chapel Hill/Chapel Hill, North Carolina, United States, 5. University of North Carolina at Chapel Hill, Chapel Hill, North Carolina, United States*

Declining mortality rates and an aging population have contributed to increasing rates of multimorbidity (≥2 chronic conditions) in the United States. Obesity is an important risk factor for the development of chronic diseases. We evaluated the association between obesity and multimorbidity, and how the prevalence of concomitant obesity has changed over time. We used data from 8,883 individuals aged ≥60 years with data on body mass index (BMI) and self-reported comorbidities from the National Health and Nutrition Examination Surveys 2005-2014. Logistic regression was used to quantify the association between BMI categories (<18.5, 18.5-24.9, 25-29.9, ≥30 kg/m2) and multimorbidity (yes/no). Change in proportions of obesity coexisting with multimorbidity by year was tested through linear regression. All analysis used NHANES survey design and weighting to be representative of the US population. The overall proportion of individuals with concomitant multimorbidity and obesity was 75%. As compared to a normal BMI (18.5-24.9 kg/m2), older adults with obesity (BMI ≥30 kg/m2) had higher odds of multimorbidity (OR 1.78, 95% CI 1.49,2.12). Persons with obesity had higher odds of decline in physical (1.41 [1.06,1.88]), basic (1.56 [1.13,2.15]), and instrumental activities of daily living (OR 1.58 [1.03,2.40]). The proportion of individuals with obesity and multimorbidity increased over time, but did not reach significance ($\beta = 0.008$, $p=0.051$). These results emphasize the role of obesity as a contributing factor to the burden of multimorbidity among older adults and underscore the importance of identifying and addressing obesity and multimorbidity via interventions to decrease obesity prevalence.

### PANDEMIC-INSPIRED STRATEGIES FOR CROSS-SECTOR COLLABORATION ON FOOD EQUITY ACROSS THE LIFESPAN

Joan Ilardo, and Angela Zell, *Michigan State University College of Human Medicine, East Lansing, Michigan, United States*

Early during the pandemic, access to food by residents across the lifespan was problematic in many communities. We observed well-intentioned responses by community organizations but a lack of centralized coordination across sectors, even as donations and resources significantly increased. Most of the organizations were in various sectors and not aware of the efforts and capabilities of others causing duplication or gaps in services. To prepare for future emergencies, our team created a project to develop and pilot a user-friendly, evidence-based roadmap to guide communities through the process of developing and sustaining effective collaborative partnerships for food and nutrition-related problems they could address together. We will describe the process through which we developed the roadmap structure and recruited stakeholders and content experts for our advisory board. To determine the effectiveness of our interventions, we designed methods with which we can analyze the organizations willing to use the roadmap and participate in the collaborative partnership; how they implement the roadmap; and ways they cope with challenges they face during implementation using strategies in the roadmap. We will describe elements of an effective, efficient roadmap development process using as many currently available evidence-based resources as possible and creating evidence-informed resources when we identify gaps. Expected outcomes are: 1) format of the final roadmap; 2) types of groups willing to use it; 3) how well the roadmap was implemented; 4) feasibility of continued use of the roadmap by groups over the long term; and 5) potential to expand roadmap use to other communities.

### PERCEIVED STRESS AND LIFE STRESSORS IN ADULTS WITH AND WITHOUT FIBROMYALGIA

Ha Nguyen, Courtney Buck, Barbara Cherry, and Laura Zettel-Watson, *California State University, Fullerton, Fullerton, California, United States*

Fibromyalgia (FM) is a widespread chronic pain condition often accompanied by comorbid conditions, such as depression, which may impact perception of stress severity. The current study examined perceived stress and life stressors in adults ages 50 and older with and without FM. It was hypothesized that individuals with FM and/or depression would subjectively rate stressors as more severe than those without. Ninety-four participants (52% with FM, 78% female) aged 50 to 93 (M = 67.72, SD = 9.26) were administered the Perceived Stress Scale (PSS) to measure perception of stress and an updated version of the Social Readjustment Rating Scale (SRRS) to assess stressors (i.e., major life events). The difference between the SRRS predetermined values and participants' subjective ratings was calculated. Difference scores indicated that self-reported severity exceeded standardized values. Hierarchical regression analyses revealed that older adults and men were less likely to report exaggerated stress severity. Controlling for age and gender, individuals with FM were significantly more likely to report stress severity far above standardized severity scores. Both depression and chronic pain impact stress ratings, but when controlling for the former, FM impact was no longer significant, suggesting that the impact is significantly greater for depression. Results also found a significant interaction between FM status and depression for perceived stress, but not for life event stressors, which may further emphasize the distinctions between the two measures. The findings underline the importance of assessing different types of stress and stressors in individuals with chronic pain and other related comorbidities.

### SARCOPENIC OBESITY IN OLDER ADULTS: FINDINGS FROM THE NATIONAL HEALTH AND AGING TRENDS STUDY

Kathleen Dondero,[1] Jason Falvey,[1] Brock Beamer,[2] and Odessa Addison,[3], *1. University of Maryland - Baltimore, Baltimore, Maryland, United States, 2. Baltimore Veterans Affairs Medical Center, BALTIMORE, Maryland, United States, 3. University of Maryland School of Medicine, Baltimore, Maryland, United States*

Sarcopenic obesity increases risk for dysmobility and loss of independence, (Gandham et al., 2021). However, the national burden of sarcopenic obesity and the resultant impacts for older adults has yet to be described. Within a nationally representative sample from the National Health and Aging Trends Study (NHATS), 2066 community-dwelling older adults were obese, representing 12,136,374 individuals in the United States, or 31.8% of all community dwelling older adults. Based on the European Working Group definition, 18% of the obese older adults were sarcopenic. Sarcopenic obese older adults were more likely to have fallen in the last month and been hospitalized over the prior year. After adjusting for age and sex, sarcopenic obese older adults were 3.7 times more likely (95% CI

2.2-5.0) to have 2 or more comorbid conditions and frailty was 6.4 times more likely (95% CI 4.4-9.5) compared to nonsarcopenic obese older adults. Sarcopenic obese older adults were also more likely to have 1+ ADL disabilities (OR 3.7; 95% CI 2.5-5.4). Further, they were more likely to be socially isolated (OR 2.1; 95% CI 1.3-3.2) and report food insecurity (OR 1.5; 95% CI 0.8-2.9). These findings suggest older adults with obesity and sarcopenia have higher rates of geriatric vulnerabilities, which might indicate a need for caution when recommending weight loss alone as an intervention. A more comprehensive intervention may be necessary to address social and physiological risks. Future studies should examine whether early intervention in sarcopenic obese older adults can reduce chronic health risk and preserve independence.

### SOCIAL SUPPORT AND LIVING SITUATION OF OLDER ADULTS WITH HIP-FRACTURE: A RETROSPECTIVE COHORT STUDY

Alexandra Krassikova,[1] Steven Stewart,[2] Jennifer Bethell,[3] Aileen Davis,[4] and Katherine McGilton,[5], *1. University of Toronto, Univerity of Toronto, Ontario, Canada, 2. Toronto Rehabilitation Institute, Toronto Rehabilitation Institute, Ontario, Canada, 3. Toronto Rehabiliitation Institute, Toronto Rehabilitation Institute, Ontario, Canada, 4. University of Toronto, University of Toronto, Ontario, Canada, 5. KITE-Toronto Rehabilitation, University Health Network, Toronto, Ontario, Canada*

Sustaining a hip-fracture is a life-changing event negatively affecting older adults. Although, social support is a known determinant of health outcomes, the relationship between social support and living situation of older adults with hip fracture remains under researched. For this study social support is conceptualized using the Finfgeld-Connett framework, where social support is seen as being composed of emotional and instrumental support. The objectives were to examine the relationship between two domains of social support and living situation: 1) after discharge; 2) 3-months after discharge; and 3) 6-months after discharge from an inpatient rehabilitation facility in a sample of older adults with hip fracture. Emotional support was measured as frequency of interaction with someone one week prior to hip fracture, whereas instrumental support was measured as help received in instrumental activities of daily living. Logistic regression was performed to examine the association between social support and living situation. Majority of study participants (N=139) were older (mean age 81.31), female (77.70%), had no cognitive impairment (68.35%), were not married (58.99%), and lived with someone (51.80%) in their own house (71.95%). Older adults with more emotional support were more likely to be discharged home, however little can be said about the effect of the association (OR 6.80, 95% CI 1.08, 22.31, P<.001). Persons receiving more instrumental support had less odds of living at home 3-months (OR 0.41, 95% CI 0.21, 0.78; P=.007) and 6-months after discharge (OR 0.59, 95% CI 0.38, 0.91, P=0.017). Social support is important for older adults during recovery.

### THE ASSOCIATION BETWEEN MEDICARE ANNUAL WELLNESS VISITS AND DETECTION AND MANAGEMENT OF DIABETES AMONG OLDER ADULTS

Abdulrahman Alsulami,[1] Kara Dassel,[2] Yao He,[3] and Nancy Allen,[3], *1. University of Utah, Jeddah, Makkah, Saudi Arabia, 2. University of Utah, Salt Lake City, Utah, United States, 3. University of Utah, Salt lake, Utah, United States*

The rising prevalence of diabetes mellitus (DM) among older adults is an increasing concern in the U.S. and is expected to nearly triple within the next 40 years. The purpose of this study is to investigate the effectiveness of Medicare Annual Wellness Visits (AWV) utilization on the management of DM among Medicare beneficiaries using data from 26,703 Medicare beneficiaries seen at 13 primary care community clinics (clinic visits between 2017 and 2019). A total of 34% of Medicare beneficiaries participated in an AWV. The total sample was, on average, 72.6 years old (SD=7.0), 57% female, 84% White, and 91% non-Hispanic and had between zero and three co-morbid conditions. The AWV group was significantly younger (mean difference 2.0 years; p<.001) and had fewer comorbid conditions (mean difference 0.1; p<.001) than the non-AWV group at their initial visits. Comparing AWV and non-AWV groups at the first patient visit and last patient visit, there were significantly fewer patients with DM in the AWV group compared to the non-AWV groups (19.2% vs. 24.7%; p<.001 and 53.5% vs. 59.2%; p<.001). DM management was better in the AWV group compared to the non-AWV group at both the first and last patient visits, as exhibited by lower A1C levels (M= 5.9(SD=0.8) vs. M=6.2(SD=1.1); p<.001 and M= 6.6(SD=0.8) vs. M=6.9(SD=1.4); p=.013), lower glucose levels (M=114.0(SD=34.0) vs. M=123.0(SD=51.0); p<.001), and fewer DM medications (M=0.1(SD=.4) vs. M=0.2(SD=0.5); p<.001 and M=0.2(SD=0.6) vs. M=0.3(SD=0.6); p<.001). These results suggest that AWV are effective managing diabetes in older adults Medicare beneficiaries.

### Session 9035 (Poster)

### AGING IN PLACE (BSS POSTER)

### DISPARITIES IN BASIC HOUSING NEEDS AS A PREDICTOR OF PSYCHOLOGICAL WELL-BEING AMONG OLDER ADULTS

Meghan Custis,[1] Jeongeun Lee,[2] and Natasha Peterson,[3], *1. Iowa State University, MUSCATINE, Iowa, United States, 2. Iowa State University, Ames, Iowa, United States, 3. Iowa State University, Iowa State University, Iowa, United States*

Adequate housing and safe environments are among older adults' foundational needs. Prior research suggests minority older adults face significant barriers to accessing affordable and appropriate housing. However, the effects of this environmental press on their psychological well-being are rarely addressed. This project examined racial disparities between minority and white older adults' housing and environment conditions and the differential impact on their psychological well-being. Using nationally representative data from the National Health & Aging Trends Study (NHATS), older adults' reported rating of the quality of housing conditions, financial security, neighborhood security, and the interviewer's rating of the home environment were analyzed. A total of 4,048 community-dwelling older adults aged 65 and over were selected for analysis. The sample demographics are predominantly white (77.5%), female (61.4%), and residing in the community (82%). Results found minority older adults reported poorer housing conditions, fewer home modifications, and lower financial and neighborhood security, compared to white counterparts. The impact of housing quality was more detrimental to minority older adults' psychological well-being, compared to white counterparts. These findings suggest a significant negative impact of home conditions on the psychological well-being of minority older adults. Home modifications are a viable option to increase or preserve functional status in the home, which could lessen the deleterious effects of environmental press on older adults' psychological outcomes, especially minorities. This study's findings provide information that bolsters our knowledge of housing and environment conditions, which are critical in efforts to reduce health disparities in late life.

### ENVIRONMENTAL FEATURES SUPPORTING NON-TRANSPORTATION WALKING IN OLDER DWELLERS IN A HILLY NEIGHBOURHOOD

Ryoichi Nitanai,[1] Ryogo Ogino,[2] Daisuke Umemoto,[3] Jun Goto,[4] and Junichiro Okata,[5], *1. the University of Tokyo, Tokyo, Tokyo, Japan, 2. Saga University, Saga, Saga, Japan, 3. The University of Tokyo, Bunkyo-ku, Tokyo, Japan, 4. Tokai University, Hiratsuka, Kanagawa, Japan, 5. Meiji University, Chiyoda-ku, Tokyo, Japan*

Walking is the basic mode of transportation; however, it is also considered as a recreational and physical activity. For elderly people, non-transportation walking (NTW) is necessary to maintain a good health; thus, irrespective of topography, living in an environment conducive to NTW is essential for the ageing community. This case study explores the features of the physical environment supporting NTW in older people, living in a hilly Japanese neighbourhood. We conducted semi-structured interviews with 23 older participants, with 6 being in their seventies, 13 in their eighties, and 4 in their nineties. We investigated the destinations and routes of their outings for a week, as well as their perception of walkability. Thereafter, we analysed the location of the NTW and the rationale behind the location choice. Consequently, four groups of people were identified based on their walking location: those who walked within a 1 km radius zone (N=6), those who walked outside the zone (N=8), those who walked both within and outside the zone (N=3), and those who did not walk (N=6). Moreover, each group had varied expectations regarding the physical environment, which is determined by their motivations and physical conditions, relating to the land use of the location of NTW. This implies the necessity of target identification and a suitable environmental approach for the target to promote NTW among older people in a hilly residential neighbourhood, such as improving comfort and connectedness by installing rest spots for the within-and-outside the zone walking group.

### IMPACT OF GREEN SPACE, AIR POLLUTION, AND WEATHER ON COGNITIVE FUNCTION IN MIDDLE AND OLD AGE IN CHINA

Lingling Zhang,[1] Ye Luo,[2] Xi Pan,[3] and Qing Wang,[4], *1. University of Massachusetts Boston, Boston, Massachusetts, United States, 2. Clemson University, Central, South Carolina, United States, 3. Texas State University, San Marcos, Texas, United States, 4. Shandong University, Shandong University, Jinan, Shandong, China (People's Republic)*

Population aging will inevitably bring an increasing burden of poor cognitive function. The risk factors for cognitive decline have been widely studied. Even though environmental hazards have the greatest adverse impacts on older adults and the existing evidence has shown that green space, air pollution, and weather have an impact on cognitive function, most of the studies were conducted in developed countries and limited to cross-sectional analyses. China has the largest aging population in the world so the research evidence from it can offer an insight to the study in other developing countries facing similar issues and inform future public health policy and disease control. Using the data from a nationally representative sample of adults aged 45 years and older from the three waves of China Health and Retirement Longitudinal Study (CHARLS 2011-2015) and China City Statistical Yearbook, this study estimated multilevel growth curve models for the effects of green space coverage, air pollution, and weather conditions on cognitive function and cognitive decline. It showed that after controlling for sociodemographic characteristics, built area green coverage rate was positively associated with cognition score at baseline, and higher annual minimum temperature was associated with faster decline in cognitive function. These effects did not substantially change after weekly total hours of physical activities and levels of social engagement were added and the interaction effects were examined between environmental conditions with them, respectively. More research on the mechanisms of the effects of environmental factors on cognition is needed such as the subgroup analyses.

### INCREASED SELF-EFFICACY AMONG OLDER ADULTS AGING-IN-PLACE DURING COVID-19.

Lee Lindquist,[1] Amber Miller,[1] Ruqayyah Muhammed,[1] Lauren Opsasnick,[2] Alaine Murawski,[1] and Vanessa Ramirez-Zohfeld,[3], *1. Northwestern University Feinberg School of Medicine, Chicago, Illinois, United States, 2. Northwestern University, Chicago, Illinois, United States, 3. Northwestern University Feinberg School of Medicine, Northwestern University Feinberg School of Medicine, Illinois, United States*

Self-efficacy is defined as a person's belief in their capacity to execute behaviors necessary to produce specific performance attainments. It also reflects confidence in the ability to exert control over motivation, behavior, and environment. During the COVID-19 pandemic, older adults were stressed with forced isolation, concerns over mortality, and finding alternate means of fulfilling their home-based needs. We sought to assess how COVID-19 pandemic affected the self-efficacy of a cohort of older adults aging-in-place. The LITCOG cohort is a group of community-dwelling older adults (65 years and older) who have had longitudinally assessment of cognition, health literacy, and functional skills over the past 15 years. As part of a larger study of the LITCOG cohort assessing decision making for aging-in-place, we assessed self-efficacy using validated PROMIS (Patient-Reported Outcomes Measurement Information System) measures with older adults prior to COVID-19 and during the COVID-19 pandemic. Survey results were obtained from 214 subjects (n=66 pre-COVID and n=148 during COVID). Nearly half of the sample (48.2%) had either marginal (25.5%) or low health literacy (22.7%). PROMIS General Self Efficacy was higher among those assessed during the COVID-19 pandemic (45.8 (7.7) pre-COVID vs 43.7 (8.0), p=0.07). PROMIS Self Efficacy for managing social interactions was higher during the COVID pandemic (45.0 (6.1) pre-COVID-19 vs. 48.7 (8.3) during COVID-19, p=0.02). During the stress and social isolation of the COVID-19 pandemic, older adults exhibited increased levels of self-efficacy. Ongoing longitudinal follow-up will determine how this self-efficacy evolves after the COVID-19 pandemic and impacts the ability to age-in-place.

### INTRINSIC, EXTRINSIC AND INTERACTION THEORIES ON FALLS AND AGING-IN-PLACE: A C-T-E SCOPING REVIEW

Bonnie Albright, *University of Massachusetts - Boston, Concord, Massachusetts, United States*

As aging in place increases in popularity, it is important to understand potential negative outcomes related to the trend. For this presentation, the conceptual-theoretical-empirical (C-T-E) scoping review technique was used to organize research on in-home falls of community-dwelling older adults. Research and theory were included from the fields of social gerontology, disability, policy, social justice, medicine, rehabilitation, and housing. While research from these multiple fields overlaps, an overarching conceptual framework for organizing this literature was found to categorize the theories into three main conceptual areas. The three conceptual areas are: intrinsic (related to the person only), extrinsic (related to external factors only), and interaction between intrinsic and extrinsic (related to the interaction between the person and external factors). This conceptual framework shares similarities with work by others in use of the terms intrinsic and extrinsic, and it draws on the larger influence of Bronfenbrenner's socio-ecological model. However, this review extends previous work by providing a framework for organizing the contributions to falls research across multiple disciplines.

### LIFE-SPACE CONSTRICTION IN AGING ADULTS

Tamatha Arms, *UNC Wilmington, Wilmington, North Carolina, United States*

Community-dwelling aging adults desire to maintain independence and prevent or delay a sequela of declining function and ultimate frailty. Early indicators of potential declines in function and frailty, such as life-space constriction (LSC), are important to identifying early. The purpose of this study was to examine factors associated with LSC and the influence of these factors and LSC on function and frailty. A cross-sectional study using a convenience sample of community dwelling persons 55 and older living in the South was conducted. Results indicated most participants (N = 72) were female (69%; n = 50) and half were White (53.5%; n = 38). LSC explained 34% variance in function (F = 3.805 (8, 59); p = .001) when environmental supports (social network), challenges (driving time it took the participant to get to the nearest full-service grocery store) and individual factors were controlled for. There was a significant difference between Black and White participants with environmental challenges (p = .001) and function (p = .001). Individual factors included challenges (age-related physiological changes, disease burden, and mental health limitations) and buoy (assistive devices), these explained 22% variance in self-reported frailty (F= 3.099 (6, 65); p = .01). Number of assistive devices was the only significant predictor of self-reported frailty.

### MOVES TO AGE-RESTRICTED HOUSING AND FUNCTIONAL HEALTH TRAJECTORIES AMONG INDEPENDENT LIVING OLDER ADULTS

Noah Webster, and Simon Brauer, *University of Michigan, Ann Arbor, Michigan, United States*

Where independent living older adults live has been found to have strong links with disability. For example, older adults living in age-restricted housing contexts (e.g., retirement communities) have been found to have worse functional health compared to those living in non-age-restricted settings. Theories and empirical research demonstrate positive and negative aspects of living in age-restricted housing. Recent availability of population-level longitudinal data with sufficiently large samples has made examination of this heterogeneity possible. In this study we examine whether a move to age-restricted housing is associated with functional health trajectories and whether age at time of move moderates this link. We examine these questions using nine waves of longitudinal data from a representative sample of 8,687 U.S. adults age 65 and older from the National Health and Aging Trends Study. Spline-like growth curve models were estimated to determine the intercept, slope prior to move to age-restricted housing, and slope after the move. We also tested whether these processes are conditional on age at time of move. Results indicate that regardless of age all respondents experienced a decline in functional health following a move to age-restricted housing. However, there is variation in the steepness of this decline by age at time of the move. People who move to age-restricted housing earlier experience a less steep decline in functional health post-move compared to those who move later. Findings suggest moving to age-restricted housing earlier may enable older adults to utilize resources often available in these settings to prevent steep health declines.

### NEIGHBORHOOD WALKABILITY AND PHYSICAL AND MENTAL HEALTH AMONG OLDER ADULTS LIVING IN A DEPRIVED COMMUNITY

Rie Suzuki,[1] Jennifer Blackwood,[2] Shailee Shah,[3] and Noah Webster,[4], *1. University of Michigan-Flint, Flint, Michigan, United States, 2. University of Michigan--Flint, Linden, Michigan, United States, 3. University of Michigan-Flint, University of Michigan-Flint, Michigan, United States, 4. University of Michigan, Ann Arbor, Michigan, United States*

**Background:** Identifying the factors to improve the quality of life (QOL) is vital to decrease morbidity and mortality rates among older adults. Although unfavorable neighborhood features have a significant negative impact on QOL, few studies have investigated these relationships in a deprived community. The purpose of the study was to understand how neighborhood walkability is associated with QOL using the SF-36 among urban-dwelling older adults.

**Methods:** This is a cross-sectional survey. Participants were recruited in 2018 and 2019 at regional health clinics in Flint, MI. To be eligible, participants had to be over 65 years old and Flint residents.

**Results:** Of the 132 participants, the majority were female (66%), African American (77%), single, divorced, or widowed (75%), and educated below GED level (84%). After adjusting for gender, assistive device use, medication, and the Supplemental Security Income receipt, multiple regression analysis revealed that those with better perceptions of land-mixed use and accessibility within their neighborhood were more likely to have better physical health ($\beta = .36, p<.05$). However, the perceptions of greater pedestrian safety were associated with the poor physical and mental health (PCS; $\beta = -0.19, p <.05$; MCS; $\beta = -0.25, p < .05$). Perceptions of the presence of walking hazards and crime were not significantly associated with QOL.

**Discussion:** Findings suggest that neighborhood walkability characteristics are associated with physical health. The development of walking programs with accessible neighborhoods will be urgent to improve the health-related QOL for older adults living in a targeted community.

### RACE/ETHNICITY MODERATION ON THE RELATIONSHIP BETWEEN NEIGHBORHOOD MINORITY COMPOSITION AND DEPRESSIVE SYMPTOMS

Seungjong Cho, *Texas Tech University, Lubbock, Texas, United States*

Research showed neighborhood of residence is an important determinant of depressive symptoms. However, the complex effects of neighborhood racial/ethnic composition and individuals' race/ethnicity on depressive symptoms were not fully explored in previous studies. This study tested whether individuals' own race/ethnicity moderates the relationship between neighborhood racial/ethnic composition and depressive symptoms. Applying social disorganization theory, this study investigates the relationships between neighborhood racial/ethnic composition (proportion of racial and ethnic minorities), individual race/ethnicity, and depressive symptoms. This study used a merged data from Health and Retirement Study 2016 and the American Community Survey 2014-2018 (N=5,241; all age 50 or older). This study applied a mixed-effects negative binomial regression model. It has four statistical models by race/ethnicity: (a) non-Hispanic Blacks only, (b) Hispanics only, (c) non-Hispanic Whites only, and (d) combined model. Covariates were included two individual-level variables (age and gender) and three census tract-level variables (the proportions of (a) population of income below the poverty level; (b) population of unemployed; (c) population of aged 65 and over). The results showed none of the neighborhood racial/ethnic minority composition was associated with depressive symptoms for the final combined model. The moderation effects of individuals' race/ethnicity were not significant. Not as a moderator but as the main effect, both non-Hispanic Blacks and Hispanics had higher depressive symptoms, compared to non-Hispanic Whites. Living in neighborhoods with higher poverty rates and females were also related to higher depressive symptoms. This study contributes to explore the subtle nature of depressive symptomatology and race both at individual-level and neighborhood-level.

### RURAL RESIDENTS' HOPES AND FEARS ABOUT AGING IN PLACE: THE NEED TO IMPROVE ACCESS TO AGING RESOURCES

Allyson Brothers, Yuquin Jiao, Sue Schneider, and Kaitlyn Wright, *Colorado State University, Fort Collins, Colorado, United States*

To support older adults' preferences to age in place, home and community-based aging-related resources are available, but are often under-utilized. Many barriers prevent individuals from accessing aging-related resources, especially in rural and geographically isolated locations. Therefore, we set out to better understand the perspectives of community members who plan to age in place in rural areas. We administered a survey as part of a broader university-community partnership called Senior Access Points (SAP), which addresses aging-related resource access. Participants were N = 210 individuals living in rural regions across Northern Colorado, ranging from 37 to 94 years old (mean age = 68.91, SD = 8.85). We assessed hopes and worries about growing older at home, and awareness of available resources. Two independent coders applied a pre-determined coding scheme, then achieved consensus ratings. An overwhelming majority of participants affirmed the importance of being able to remain in their current home (94.8%) or community (95.3%) as they age. Top hopes for aging in place centered around health/medical; housing/home services; and independent rural lifestyle. The top worries were related to health/medical; housing/home services, and transportation. Resource awareness was low: 43.3% of all participants were not aware of any available resources. Overwhelmingly, rural residents hope to grow older at home, but may not know how to connect to resources that support this goal. The resource needs we identified are being used to inform community-driven approaches to improve both awareness and availability of community resources in these rural communities.

### STAGES OF CHANGE IN PHYSICAL ACTIVITY AND NEIGHBORHOOD WALKABILITY AMONG OLDER ADULTS LIVING IN THE URBAN SETTING

Rie Suzuki,[1] Jennifer Blackwood,[2] Noah Webster,[3] and Shailee Shah,[4], *1. University of Michigan-Flint, Flint, Michigan, United States, 2. University of Michigan--Flint, Linden, Michigan, United States, 3. University of Michigan, Ann Arbor, Michigan, United States, 4. University of Michigan-Flint, University of Michigan-Flint, Michigan, United States*

Insufficient physical activity (PA) is considered an independent risk factor for chronic diseases. Although older adults living in lower-income areas often experience obstacles to walking locally, few studies have compared their walking experiences and the degree of readiness to change on engaging in PA. The purpose of this study was to compare perceptions of neighborhood walkability by the stages of change among older adults living in a lower-income community. Participants were recruited in 2018 at a regional health clinic in Flint, MI. To be eligible, participants had to be over 65 years old and Flint residents. Of the 132 participants, the mean age was 69.74 (SD=5.00) years old. The majority of respondents were female (66%); African American (77%); single, divorced, or widowed (75%); and educated below a GED level (84%). The results showed that older adults at the pre-contemplation/contemplation stage (PC/C) were less likely to perceive the availability of sidewalks on most streets and more likely to complain about much traffic along the street than those at the action/maintenance stage (A/M) ($p<0.05$). After controlling for covariates, multiple regression analysis showed that those at PC/C were less likely to state that their neighborhoods were accessible ($\beta = .17^*$) and to perceive the presence of walking hazard (e.g., lack of sidewalks) ($\beta = -.17^*$). Those who engaged in PA less than 30 minutes per day perceived the neighborhoods were accessible ($\beta = .23^*$). Findings suggest that it is essential to develop friendly support systems and accommodations to encourage walking in lower-income communities.

### TESTING FRAILTY, ACCESSIBLE HOUSING, AND CHANGES IN LIVING ARRANGEMENTS USING THE HEALTH AND RETIREMENT STUDY

Bonnie Albright, *University of Massachusetts - Boston, Concord, Massachusetts, United States*

This study examined housing accessibility elements of community-dwelling older adults using the Health and Retirement Study (HRS). Housing accessibility elements were tested as moderators in the relationship between prior frailty and later living arrangements. HRS physical measures were used to construct the Physical Frailty Phenotype and the Continuous Frailty Scale. The analytic method for the study was multinomial logistic regression. Latent class analysis was also used to identify housing accessibility element use-types. Study findings will be presented. Strengths and weaknesses of using the HRS to measure home accessibility and construct frailty scales will also be discussed.

### THE RELATIONSHIP BETWEEN FAMILY AND RESIDENTIAL SETTINGS: AGING IN PLACE VS LONG TERM CARE FACILITY IN SOUTH KOREA

Joohong Min,[1] Seung-eun Oh,[2] and Bon Kim,[3], *1. Jeju National University, Jeju, Cheju-do, Republic of Korea, 2. Jeju National University, Jeju National University, Cheju-do, Republic of Korea, 3. Jeju National University, Jeju-si, Cheju-do, Republic of Korea*

**Objective:** This study aims to explore the factors influencing long-term-care beneficiaries' decisions between home health care and institutional care in South Korea. This study further investigated the association between these two types of long-term care services and emotional, financial, and physical burden alleviation among the beneficiaries and their family members.

**Methods:** We analyzed data from 2019 Long-Term Care Survey. Out of 5,606 respondents, 4,079 long-term care beneficiaries and family members were included in this study. Logistic regression models were conducted to understand factors associated with types of long-term care services, and the association between types of long-term care services and burden alleviation.

**Results:** Having a spouse, having children, or a shorter geographic distance between the beneficiaries and their family members were factors associated with higher likelihood of receiving home health care rather than institutional care. Also, more sever mobility limitation, outpatient care, and fall experiences were associated with higher likelihood of receiving institutional care. Utilization of institutional care was significantly associated with higher alleviation of physical burden. No significant differences between home health care and institutional care were found on emotional and financial burden alleviation.

**Conclusions:** The findings suggest that family resources may be beneficial for older adults to pursue aging in place in later life. However, the physical toll of their family members may exist. Our findings provide evidence to inform public policy decisions on long-term care services for older adults and their family members.

### Session 9040 (Poster)

### AGING IN PLACE II

### GEOGRAPHIC DISTRIBUTION OF AVAILABILITY OF ADULT DAY SERVICES IN MISSOURI

Takashi Amano,[1] Yung Chun,[2] Sojung Park,[3] and Yi Wang,[4], *1. Rutgers University-Newark, Newark, New Jersey, United States, 2. Washington University in St. Louis, St. Louis, Missouri, United States, 3. Washington university, St.Louis, Missouri, United States, 4. University of Iowa, Iowa City, Iowa, United States*

Adult day service (ADS) is an important component of long-term supportive services. Geographic availability of ADS is an essential factor for aging in place especially for people with assistance needs. This study aims to examine the geographic distribution of availability of ADS and its relationship with the disadvantaged characteristics of neighborhoods. Data from the Missouri Department of Health and Senior Services and the American Community Survey were utilized. Geographic availability of ADS was measured as capacity (number of clients served) of ADS centers per week divided by the number of people who were 65 or older and under poverty at the census tract level. To examine neighborhood disadvantaged characteristics, principal component analysis was applied to construct a socioeconomic deprivation index (SDI). Using geographic information systems, we mapped ADS centers, geographic availability of ADS, and SDI scores. Pearson correlation coefficient was calculated between geographic availability of ADS and SDI scores. In 92.3% of the census tracts in Missouri, ADS centers are not available. Further, ADS centers are less likely to locate in rural areas or census tracts with higher numbers of residents 65 or older and poor. Also, lower availability of ADS was associated with higher levels of neighborhood disadvantage at a marginal level ($r = -0.163$). Our findings suggested that strategies should be identified to provide ADS in rural areas, especially in the areas with higher levels of neighborhood disadvantage. Further investigation on the geographic distribution of ADS accessibility and its association with neighborhood characteristics is warranted.

### HOUSING, NEIGHBORHOOD FACTORS, AND COGNITIVE STATUS OF OLDER ADULTS LIVING IN HOUSES VERSUS APARTMENTS

Ethan Siu Leung Cheung,[1] and Ada Mui,[2], *1. Columbia University, New York, New York, United States, 2. Columbia University, COLUMBIA UNIVERSITY, New York, United States*

This study examines associations between housing, neighborhood factors and cognitive status among community-dwelling older adults, and how the associations differ between older adults who live in houses and in apartment buildings. Specifically, using the neighborhood stressor theory, three research questions are examined: 1) What individual-level factors predict late-life cognitive status? 2) After controlling for individual-level factors, what housing and neighborhood factors are significant in predicting older adults' cognitive status? 3) How do individual, housing, neighborhood predictors of cognitive status differ between house and apartment residents? Using data from the Wave 3 NSHAP, multilevel linear regression analyses are conducted with the total sample. Results suggest that individual-level factors including young-age, female, white, and having a bachelor's degree are associated with better cognitive status. After controlling for individual-level factors, housing and neighborhood factors including quality maintenance and high level of community safety are associated with higher cognitive scores. In addition to the additive model, we also test the interactive effect between housing type and three level of factors –individual level, housing, and neighborhood factors. Findings suggest that the joint effect of depression and housing type on cognitive status is significant. To explore the last research question, we conduct parallel regression analyses by housing type. Findings suggest that quality maintenance and high level of community safety are associated with higher cognitive scores among house residents only. Findings highlight the predictors of cognitive health vary between older adults living in different residential environments.

### MOTIVATIONS TO JOIN AND STAY A MEMBER OF A VILLAGE FOR OLDER ADULTS

Joonyoung Cho, Ruth Dunkle, and Garrett Pace, *University of Michigan, Ann Arbor, Michigan, United States*

People join a customer-driven organization with motivations that may not be static over time, an important issue for long-term organizational viability. In this study, we examined motivations among members of ShareCare, the first Village for older adults in the U.S. Using qualitative data from a random sample of 91 members, we compared motivations for becoming a member and for continuing membership. Motivations to join and continue membership are not necessarily the same. Motivations were categorized as: instrumental, social, and altruistic motivation. We categorized length of membership as short-term: 8-years or less (51.63%) and long-term: 9-years and more (49.37%). While 36% of members joined only for instrumental motivation, 59% continued membership only for instrumental motivation. While about 52% joined with multiple motivations, only 35% of members mentioned multiple motivations when continuing their membership. Finally, 18% of short-term members mentioned altruistic motivation when continuing their membership, while 28% of long-term members mentioned altruistic motivation when continuing their membership. While people's motivation might change over time, altruistic motivation may be the greatest motivating factor for long-term memberships. Long-term members may identify themselves as supporters rather than users of the organization and cultivate stronger connections with other members over time. Our findings inform how to recruit and retain members in Villages, and customer-driven organizations for older adults more broadly.

### RESIDENTIAL ENVIRONMENT, DEPRESSIVE SYMPTOMS, AND ANXIETY SYMPTOMS AMONG COMMUNITY-LIVING OLDER ADULTS

Ethan Siu Leung Cheung,[1] and Jinyu Liu,[2], *1. Columbia University, New York, New York, United States, 2. Columbia University, Columbia University, New York, United States*

Past literature has suggested significant relationships between neighborhood environment and mental health of older adults. However, the effect of residential environments is underexplored. The present study aims to study: (Q1) how residential built environments are associated with depressive and anxiety symptoms among community-living older adults, and (Q2) whether the associations of their physical and cognitive health status with mental health vary by residential environments. We analyzed data from Round 9 of National Health and Aging Trends Study. Residential environments were indicated by home despair, cluttered home, and existence of entrance ramp. Covariates included age, gender, race, living arrangement, ADL limitations, physical capacity, and cognitive status. The logistic regression results show that higher levels of clutter at home and the lack of entrance ramp were significantly associated with more depressive symptoms and that levels of clutter were positively associated with anxiety symptoms. Residential environments significantly moderated the association between physical health and mental health. With similar physical capacity, older adults with higher levels of home despair and clutter had more depressive and anxiety symptoms. Older adults who had more cluttered home reported significantly higher levels of anxiety than those who had similar ADL limitations, but lived in a less cluttered housing environment. However, we didn't find any moderating effect of residential environments on cognitive impairment and mental health. Our findings promote the necessity for practitioners and policymakers to consider the effect of residential environments on mental health among both physically healthy and impaired older adults in the United States.

### SOCIAL ROLES OF FAMILY AND FRIENDS DIFFER IN SOCIAL NETWORKS OF OLDER ADULTS WHO LIVE ALONE

Sato Ashida,[1] Ellen Schafer,[2] and Lena Thompson,[3], *1. University of Iowa, Iowa City, Iowa, United States, 2. Boise State University, Boise State University, Idaho, United States, 3. University of Iowa College of Public Health, Iowa City, Iowa, United States*

Social networks consisting of family and friends tend to better facilitate older adults' emotional well-being than networks consisting of only family or only friends. This study assessed the heterogeneity of older adults' network compositions based on the network members' relationship (family vs. friends) and proximity (local vs. non-local) and evaluated the types of interactions between older adults and types of members. Adults 60 years and older living in a U.S. Midwestern city participated in a one-time structured survey (n=133), and reported about 1,730 social network members. Compared to participants who lived with others, those who lived alone reported more depressive symptoms and higher frequency of feeling lonely (p=0.002). Those who lived alone also had higher proportions of local friends in their networks than those who lived with others (p=0.02). Whereas the social roles of family and friends were similar in networks of older adults who lived with others, those who lived alone were less likely to identify family as who they co-engaged in social activities with (local family OR=0.55, non-local family OR=0.27) and who provided companionship (local family OR=0.33, non-local family OR=0.11) compared to their local friends. Having more members who co-engaged in activities was associated with lower depressive symptoms (p=0.05) and less frequency of feeling lonely (p<0.01). Providing supportive infrastructure for community-based older adults to develop and maintain co-engaging relationships with local friends may be beneficial. Network approaches can be used to identify existing network members who may be inspired to play this role.

### UNDERSTANDING MULTIDISCIPLINARY-TEAM PRACTICE IN DEVELOPING HOME ASSESSMENT TOOLS: A SYSTEMATIC REVIEW

Mengzhao Yan,[1] Kathleen Wilber,[2] and Jon Pynoos,[2], *1. University of Southern California, Los Angeles, California, United States, 2. University of Southern California, University of Southern California, California, United States*

As widely used instruments to identify risk factors and lay out preliminary plans of how to improve the built environment, home assessment tools play an important role in the process of modifying homes for older adults. Developed by a variety of disciplines and tailored to meet various needs, home assessment tools focus on features in homes and how they meet or hinder an older person's ability to accomplish tasks — in other words, person-environment fit. Based on a comprehensive review of ten evidence-based home assessment tools identified by researchers at the USC Leonard Davis School of Gerontology and the National Council on Aging, we found that a common assessment strategy is the use of multidisciplinary teams (MDTs) in developing and testing the assessment tools to ensure the reliability vs validity of different home modification programs. To understand the nature of MDT practice and derive a set of generalizable protocols for developing person-centered home assessment tools, we conducted a systematic analysis of the ten evidence-based home assessment tools and 41 peer-reviewed journal papers about how the tools were developed, used, and modified. In addition, we applied the RE-AIM framework (Reach, Effectiveness, Adoption, Implementation, and Maintenance) to examine the use of MDTs in developing the tools and carrying out the programs. Based on our analysis, we propose a set of preliminary protocols for developing home assessment tools and a logic model for conducting person-centered home modification programs.

### VALIDATION OF A MEASURE OF LONELINESS FOR HOMEBOUND OLDER ADULTS

Keith Chan,[1] Christina Marsack-Topolewski,[2] Sarah LaFave,[3] Maggie Ratnayake,[4] Jillian Graves,[2] and Diane Fenske[5], *1. Hunter College, New York, New York, United States, 2. Eastern Michigan University, Ypsilanti, Michigan, United States, 3. Johns Hopkins University, Baltimore, Maryland, United States, 4. Lori's Hands, Newark, Delaware, United States, 5. Eastern Michigan University, Eastern Michigan University, Michigan, United States*

The pandemic has disproportionately impacted older adults, highlighting the need to address social isolation for this population. Homebound older adults are at risk for loneliness, which is a correlate of poor mental and physical health. COVID-19 has exacerbated effects of social isolation by limiting contact with family and other visitors. In-depth empirical validation of loneliness scales is needed to examine the measurement of this construct for homebound older adults who are aging in place. This study examined the reliability and validity of the UCLA Loneliness Scale (v3) for a community-dwelling population of older adults who received home-based support services due to their homebound status or have chronic illness resulting in ADL limitations. Using in-home interviews, data were collected for 175 older adults using the UCLA Loneliness Scale. Reliability and confirmatory factor analyses were conducted to examine its psychometric properties. Findings demonstrated the scale had good internal consistency reliability ($\alpha = 0.91$). Confirmatory factor analyses indicated a two-factor solution, 1) disconnectedness and 2) connectedness, accounting for 92% of the variability in the 20 items. The lack of meaningful relationships ($\lambda = 0.73$, $p < 0.05$) or having someone to turn to ($\lambda = 0.68$, $p < 0.05$) substantively contributed to disconnectedness. Feeling that there were people to talk to ($\lambda = 0.67$, $p < 0.05$) and turn to ($\lambda = 0.76$, $p < 0.05$) contributed to connectedness. Future research can further examine how quality of relationships and benefits of being connected to others can address loneliness and isolation for this population.

### WHO AND WHERE? THE SPATIAL CONTEXT OF RACIAL AND ETHNIC DISPARITIES IN ECONOMIC SECURITY AMONG OLDER ADULTS

Yang Li,[1] Jan Mutchler,[2] and Nidya Velasco Roldan,[1], *1. University of Massachusetts, Boston, Massachusetts, United States, 2. University of Massachusetts Boston, Boston, Massachusetts, United States*

Many older adults experience challenging financial circumstances and do not have sufficient income to afford a basic budget in their home communities. Far higher proportions of racial and ethnic minority older adults live on incomes that fall short of what is needed to make ends meet relative to their White counterparts. We describe racial/ethnic disparities in late-life economic insecurity, which occurs when an older person lacks sufficient financial resources to cover necessary expenses in their home community. Although nationwide half of older singles are economically insecure, Massachusetts (62%), New York (65%), Vermont (57%), and Mississippi (57%) have the highest shares of older adults who experience economic insecurity. Compared to Whites, minority older adults have higher rates of economic insecurity in nearly every state, but racial/ethnic disparities are higher in some locations (Rhode Island, Massachusetts, Mississippi, Louisiana) and lower in others (Oregon, Arizona, Nevada, West Virginia). Disparities in economic insecurity reflect the precarious financial situations experienced by many older adults, rooted not only in risks and disadvantages accumulated over time, but also in the variable and uncertain social and economic contexts that accompany the aging experience. By situating older adults in their places of residence, we observe that the cost of remaining in community intersect with life-course experiences associated with social identities to produce disparities in economic security at older ages. The geographic variation in cost of living calls for context-specific assessment of economic security to evaluate the adequacy of economic resources and the associated risk of hardship.

### Session 9045 (Poster)

### AGING, DIVERSITY, AND HEALTH EQUITY

### A FOOD BOX INTERVENTION TO REDUCE BLOOD PRESSURE IN NATIVE AMERICAN ADULTS WITH HYPERTENSION: THE CHEERS STUDY

Valarie Blue Bird Jernigan,[1] Joy Standridge,[2] Tyra Shackleford,[2] Hilary Brookins,[2] Tvli Jacob,[1] and Tori Taniguchi,[1], *1. Oklahoma State University Center for Health Sciences, Tulsa, Oklahoma, United States, 2. Chickasaw Nation, Ada, Oklahoma, United States*

Diet-related chronic diseases, such as hypertension and obesity, are prevalent in Native American (NA) communities where poor food environments are prominent and healthy food access is limited. The Chickasaw Healthy Eating Environments Research Study (CHEERS) is an NIH-funded study aimed to improve Body Mass Index and blood pressure control among NA adults with uncontrolled hypertension. This multi-level randomized trial, guided by a community-based participatory research orientation, was co-created by tribal and university partners and is implemented within the Chickasaw Nation of Oklahoma. We created hypertension-specific food boxes that contained DASH diet foods, coupons for purchasing vegetables and fruits, educational materials, and heart-healthy recipes for supporting healthy eating. Food boxes were packed and shipped monthly to intervention participants with a 30-day supply of: one fruit serving/day, one vegetable serving/day, one serving of unsalted nuts or seeds/day, one serving of beans or lentils/day, and two servings of fatty fish/week. We will present our participatory approach in co-developing the CHEERS study methods, findings with a focus on older adults, and lessons learned. CHEERS is the first innovative food box intervention to be conducted in NA communities. Food box interventions show promise in improving dietary intake and reducing hypertension and obesity in rural and poor food environments.

### A RELATIONSHIP-ORIENTED MODEL OF RESEARCH PARTICIPATION: THE BRAIN HEALTH COMMUNITY REGISTRY

Andrea Gilmore Bykovskyi,[1] Haley Fuhr,[2] Shannon Mullen,[1] Laura Block,[1] Clark Benson,[1] Danielle Harris,[1] Quinton Cotton,[3] and Jessica Kendall,[2], *1. University of Wisconsin-Madison, Madison, Wisconsin, United States, 2. University of Wisconsin-Madison, University of Wisconsin-Madison, Wisconsin, United States, 3. University of Wisconsin - Medicine & Public Health, Fitchburg, Wisconsin, United States*

Historically excluded and minoritized populations are significantly under-included in health studies of Alzheimer's disease and related dementias (ADRD) despite bearing a disproportionate burden of disease—evidenced by higher incidence, prevalence, and poorer health outcomes. Meaningful progress toward identifying and alleviating causes of health disparities in ADRD necessitate effective and scalable approaches for broadening inclusion in research. Rigorous studies evaluating research participation among minoritized populations are limited and have predominantly focused on individual-level factors and behavioral change (i.e. religiosity, willingness). These approaches frequently overlook the influence of unequally distributed structural and social determinants on participation despite the compounded financial, social, emotional, and logistical consequences that result from ADRD. Using an intersectional and social justice lens, we developed the Participant and Relationship-Oriented Research Engagement Model, which characterizes research as a form of relationship and extends social determinants frameworks to the context of research participation. We report core components of the model and its application in the design and preliminary evaluation of the Brain Health Community (BHC) Registry, which features proactive and systematic evaluation of potential unmet needs among prospective participants, and connections to relevant services (i.e. respite care, adaptive devices). Preliminary testing of the model and participant feedback on the BHC suggest it is a feasible approach to research engagement, and that associated assessment tools and resource protocols are acceptable and sufficiently adaptable to heterogeneous sets of unmet needs. Primary challenges include ongoing assessment of engagement and routine changes in service ability, which can be addressed through community-based resource networks.

### ADDRESSING THE SOCIAL AND MEDICAL DETERMINANTS OF HEALTH FOR SAFE MEDICINES USE

June Simmons,[1] Raymond Woosley,[2] and Ester Sefilyan,[1], *1. Partners in Care Foundation, San Fernando, California, United States, 2. CredibleMeds, Inc., Oro Valley, Arizona, United States*

Both social and medical factors can negatively affect health outcomes, especially in vulnerable populations. To address these two types of factors in a hospital post-discharge population, two non-profit organizations collaborated to combine their novel decision support programs to address the question: could combined programs have greater potential for improved health outcomes? HomeMeds (HM), a social health program in which trained social services staff make home visits to vulnerable clients, was combined with MedSafety Scan (MSS), a medical health, clinical decision support tool. Data captured in the home visits were entered into the HM and MSS programs to detect those patients at greatest risk of adverse health outcomes due to medications. Patients received a post-discharge home visit by trained social services staff. The number of drugs reported as being taken was on average 10.4, which was less than prescribed at discharge in 62% of patients. Both programs detected serious risk of medication-induced harm, mostly from different causes such as drug-drug interactions or for use not recommended in older adult. Combined analysis of data from two novel decision support programs yielded complementary findings that together address both medical and social determinants of health. These have the potential to reduce medication-induced harm, costly re-hospitalization and/or emergency room visits and support the further evaluation of this combined approach in other vulnerable populations such as the seriously mentally ill, frail, those confined to home, opioid-dependent or otherwise impaired.

### AFRICAN-AMERICAN RACE PREDICTS 1-YEAR COGNITIVE DECLINE AMONG ADULTS WITHOUT MODERATE DEMENTIA

Niser Babiker,[1] Alan Gonzalez,[1] Jovany Soto,[2] Chengjian Shi,[3] Andrey Rzhetsky,[4] and Megan Huisingh-Scheetz,[1], *1. University of Chicago, Chicago, Illinois, United States, 2. Illinois Institute of Technology, Orland Park, Illinois, United States, 3. University of Chicago, chicago, Illinois, United States, 4. University of Chicacgo, Chicago, Illinois, United States*

Previous literature shows conflicting conclusions about the association between race and cognitive decline, particularly in early impairment. In this study, we aimed to test whether race predicted 1-year change in Montreal Cognitive Assessment (MoCA) score among older adults without moderate-severe dementia. We secondarily explored whether multimorbidity, polypharmacy, depressed mood, antidepressant use, body composition, or frailty changed the association. We analyzed data (n=122) from predominantly African American (AfA, 78.7%) community-dwelling older adults from the south side of Chicago. Participants underwent baseline and 1-year MoCA testing. Age, gender, race, education, monthly income, co-morbidities (Charlson Comorbidity Index), medication use (<5 vs ≥5), depression (PHQ-2), proportion lean mass (DEXA), and the frailty phenotype (range 0-5) were collected at baseline. In a multivariate linear model, we regressed 1-year MoCA score on baseline MoCA score, race, and demographics and then evaluated the impact of each covariate added separately to the model on the race-cognition relationship. The mean MoCA score at baseline was 25.2+/-0.2 (range 18-30) and 41.0% of participants experienced ≥1 point MoCA decline at 1 year. After adjusting for demographics, AfAs experienced a greater 1-year MoCA decline (β= -1.3, p=0.04) compared to other races. The effect size was unchanged after adjusting for multimorbidity and polypharmacy (β= -1.3, p=0.04), attenuated slightly after adjusting for frailty (β= -1.2, p=0.06), depressed mood (β= -1.2, p=0.05), lean mass (β= -1.2, p=0.04), and attenuated notably after adjusting for antidepressant use (β= -1.0, p=0.11). Findings support the need to further explore racial differences in cognitive decline and potentially related antidepressant underuse.

### ARE ANATOMICAL GIFT DONORS DEMOGRAPHICALLY REPRESENTATIVE OF THE AMERICAN AGING PATIENT POPULATION?

Amanda Collins,[1] and Yasmin Carter,[2], *1. University of Massachusetts Boston, Boston, Massachusetts, United States, 2. University of Massachusetts Medical School, Worcester, Massachusetts, United States*

Body donation for medical education is voluntary and open to all; however, it is undetermined if the donors studied at UMass Medical School (UMMS) are demographically representative of the national patient population. If not, medical students are missing the opportunity of experiencing normal variation within the population, which may promote bias in their clinical years. This cross-sectional study compared data from the UMMS Anatomical Gift Program (AGP) with the Health and Retirement Study (HRS) population data. This study examined sex, race, ethnicity, veteran status, and sexual orientation. 5 years (n=540) of AGP data and 3 waves (n=5,037) of HRS data were examined. The results demonstrate that sex differences between the AGP and HRS populations (55% for females vs. 45% for males; p=.10) are NOT significant. A significant racial difference between populations is noted (p=.000), with 98.3% of the AGP vs. 72.7% of the HRS identifying as white. Veterans are overrepresented in the AGP (22.6% AGP vs. 9.6% HRS; p=.000). 12.3% of HRS participants report Hispanic ethnicity compared to 0% in the AGP. In 2016, HRS included sexual orientation, with 92.7% of respondents identifying as heterosexual, 2.6% gay or lesbian, 1.0% bisexual and 1.3% other. No data were collected by the AGP pertaining to sexual orientation and neither database ask about gender identity. Aging populations are not represented in the anatomy labs at UMMS and likely nationally. Efforts are needed to improve this and enhance the education of the medical professionals, while expanding the end-of-life options for all community members.

### ASSOCIATION BETWEEN LONELINESS AND DISEASES SELF-MANAGEMENT IN OLDER ADULTS: SYSTEMATIC REVIEW

Emma Cho,[1] Ya-Ching Huang,[2] Alexandra Garcia,[3] and Hsuan-Ju Kuo,[3], *1. University of Texas at Austin School of Nursing, Austin, Texas, United States, 2. Texas State University, Round Rock, Texas, United States, 3. The University of Texas at Austin, Austin, Texas, United States*

**Purpose:** Older adults with chronic diseases are more at risk for loneliness, and loneliness has a negative impact on health behaviors, which are key to managing chronic diseases. However, little is known about the association between loneliness and self-management behaviors in older adults with chronic diseases. As societies worldwide experience the growth of aging populations who are at higher risk of having chronic diseases as they age, clinicians and researchers should assess and address loneliness of older adults with chronic diseases.

**Methods:** This systematic review synthesizes research found in PubMed, MEDLINE, PsychINFO, CINAHL, and SocINDEX.

**Findings:** fourteen studies were conducted in four countries and represented n= 128,610. Loneliness was measured by three different instruments. Reports of loneliness were frequent and ranged from 7.7% (in a report of severe loneliness) to 43.2% (moderate loneliness) of older adults. Older adults who experienced loneliness were less likely to be physically active, eat a healthy diet, or cope in positive ways and more likely to be female and seek healthcare.

**Conclusions:** This systematic review found that loneliness was moderately prevalent, and that loneliness was associated with negative disease self-management behaviors in older adults with chronic diseases. Gaps in the research include a need for studies guided by theoretical pathways, using a consistent, theoretically-based measure of loneliness, and conducted on among people with specific chronic diseases.

### ASSOCIATION OF SOCIAL DETERMINANTS, MULTIMORBIDITY, AND FUNCTIONAL STATUS WITH MORTALITY AFTER PNEUMONIA

Chan Mi Park,[1] Hye Chang Rhim,[2] Eun Sik Lee,[3] Wonsock Kim,[4] Jong Hun Kim,[5] and Dae Kim,[6] *1. Asan Medical Center, Songpa-gu, Seoul-t'ukpyolsi, Republic of Korea, 2. Harvard University, Orlando, Florida, United States, 3. Korea University Anam Hospital, Korea University Anam Hospital, Seoul-t'ukpyolsi, Republic of Korea, 4. Eulji Medical Center, Eulji Medical Center, Kyonggi-do, Republic of Korea, 5. CHA Bundang Medical Center, CHA Bundang Medical Center, Kyonggi-do, Republic of Korea, 6. Hebrew SeniorLife, Boston, Massachusetts, United States*

Social support, multimorbidity, and functional status are important determinants of health in older adults, but their prognostic implications remain unclear after an acute illness. We conducted a prospective cohort study of 201 patients 65 years or older who were hospitalized for pneumonia at a university hospital in Korea in 2019-2020. K-means cluster analysis was performed using social deprivation score (range: 0-5), activities of daily living (range: 0-7), instrumental activities of daily living (range: 0-7), physical limitation score (range: 0-7), and Gagne comorbidity index (range: 0-24) (higher scores indicate higher risk). Four groups were identified: 1) Group A: physically limited and non-disabled group with limited social support; 2) Group B: multimorbid but functional group with social support; 3) Group C: multimorbid and disabled group with social support; 4) Group D: multimorbid and disabled group with limited social support. For Groups A through D, the Kaplan-Meir estimates for 6-month mortality were 10.0%, 18.0%, 34.2%, and 43.6%, respectively, and the 6-month mean survival times were 166.4 days (95% CI: 156.1-176.6), 156.9 days (95% CI: 140.8-173.1), 145.2 days (95% CI: 126.6-163.8), and 125.9 days (95% CI: 107.7-144.1), respectively. After adjusting for sex, age, and pneumonia severity score, the hazard ratios for Groups B through D versus Group A were 2.07 (95% CI: 0.70-6.13), 3.14 (95% CI: 1.17-8.42), and 4.38 (95% CI: 1.73-11.04), respectively. Our results suggest that multimorbidity and disabilities were implicated in higher risk of 6-month mortality after pneumonia, and social support may mitigate this risk among those with multimorbidity and disability.

### CHRONIC DISEASES AND SELF-REPORTED HEALTH STATUS AMONG AMERICAN INDIAN/ALASKA NATIVE OLDER ADULTS

Ramona Danielson,[1] Collette Adamsen,[2] and Agnieszka Mason,[1], *1. North Dakota State University, Fargo, North Dakota, United States, 2. University of North Dakota, Grand Forks, North Dakota, United States*

**Background:** In the 1800s and 1900s, U.S. federal "Indian" policy (e.g., boarding schools, relocation) created historical trauma with impacts that reverberate today, such as the significant health challenges experienced among American Indian/Alaska Native (AI/AN) populations. Our study seeks to better understand the burden of chronic disease, and also resilience, among AI/AN older adults.

**Methods:** Data came from Cycle VII (2018-2020) of the National Resource Center on Native American Aging's "Identifying Our Needs: A Survey of Elders" survey of AI/AN adults ages 55+ from primarily rural tribal survey sites (N=20,642). Analysis explored self-assessed health status (very good/excellent, good, fair/poor) and looked for significant differences in prevalence of chronic conditions a doctor ever told them they had (e.g., high blood pressure, diabetes, depression, arthritis, asthma).

**Results:** Self-reported health among AI/AN adults age 55+ was: 26% very good/excellent, 39% good, and 35% fair/poor. 87% of respondents had 1+ chronic illness; 37% had 3+. Among those reporting very good/excellent health, 75% had 1+ chronic illness and 19% had 3+. High blood pressure was the most common chronic disease, at 56% (44% for very good/excellent compared to 67% for fair/poor), followed by diabetes, at 36% (24% for very good/excellent compared to 46% for fair/poor).

**Conclusions:** All of the chronic conditions examined showed significantly higher prevalence among AI/AN adults 55+ with fair/poor health. Notably, 1 in 5 respondents with 3 or more chronic conditions indicated very good/excellent health, reinforcing that successful aging can still be experienced by those with chronic health conditions.

### CONNECTING PATIENT AND PROVIDER BURNOUT TO EYE EXAM FREQUENCY AMONG LATINX OLDER ADULTS WITH DIABETES MELLITUS

Adrienne Martinez-Hollingsworth,[1] Theodore Friedman,[2] and Mohsen Bazargan,[2], *1. Samuel Merritt University, Los Angeles, California, United States, 2. Charles R. Drew University of Medicine and Science, Los Angeles, California, United States*

Among Latinx older adults, our current understanding of barriers to eye exam often fails to consider the impact of patient and provider burnout which can decrease treatment adherence and recommendation receptivity in this group. The purpose of this study was to examine correlates of eye exam frequency among Latinx older adults in South Los

Angeles and explore associations reflecting patient and/or provider burnout. Data analysis was informed by the Secret Self-Management Loop and the Burnout Dyad conceptual models. This secondary analysis used data collected from a convenience sample of non-institutionalized Latinx adults 55+ in South LA (n=165) and used multinomial regression analysis. Outcome variable is recency of eye exam, independent variables are self-reported health, including diabetes mellitus diagnosis, and either patient or provider burnout (that are functions of grouped demographic or quality of care variables). Variables associated with Provider Burnout, appear to represent a larger influence on eye examination frequency then variables associated with Patient Burnout, with the most influential factor being provider recommendation. A surprising finding was the number of participants who had never received this recommendation from a provider (21%). One-third (32%) of participants with diabetes mellitus had not had an eye examination within 12 months and almost one-fifth (13%) of participants with diabetes who had received this recommendation had not received the exam. Further exploration is needed to support a better understanding of how both patient and provider burnout impacts adherence to eye examination and other preventive care recommendations for diabetes mellitus among Latinx older adults.

### DIABETES PREVALENCE AMONG UNDERSERVED OLDER ADULTS IN THE US: THE CASE OF NATIVE HAWAIIANS AND PACIFIC ISLANDERS

Sela Panapasa,[1] William Herman,[2] and James McNally,[3], *1. Research Center for Group Dynamics, Ann Arbor, Michigan, United States, 2. University of Michigan Medical System, Ann Arbor, Michigan, United States, 3. University of Michigan, Ann Arbor, Michigan, United States*

The effects of chronic diabetes among older adults in the United States represent ongoing challenges in diagnosis, treatment, comorbidities, amputations, and the increased risk of death. These challenges are made more complicated among underserved populations due to limited access to healthcare, medication costs, and later diagnosis of the condition. These issues are particularly true for the NHPI population, which has high rates of lifetime diabetes, greater levels of poverty, and inadequate health insurance. Useful statistics about diabetes among the NHPI have been difficult to obtain due to their small population size and lack of inclusion in federal health surveys. While early work by Panapasa examined prevalence among NHPI males in California, no reliable measures of diabetes among older NHPI's existed at the national level. Released in 2017, the 2014 Native Hawaiian Pacific Islander National Health Interview Survey represents the first representative survey of the health and socio-economic correlates for this population, allowing the examination of health conditions such as diabetes at the national level. This presentation will examine the prevalence of diabetes among NHPI's aged 60 and older and the impacts of this disease on overall health and quality of life. The paper will use the NHPI-NHIS to examine the use and access to diabetic medications and overall access to affordable health care. The paper will examine differences by age group, gender, immigration status, and ethnicity. While we know the overall prevalence of diabetes is high, this paper will offer new information on differentials within the older NHPI population.

### DOES RACE/ETHNICITY INFLUENCE THE HEALTH OF LGBT OLDER ADULTS? AN ANALYSIS OF ADULTS AGED 50 AND OLDER

Adrielle Magdael,[1] Aditi Parashar,[2] Aisha Ozair,[2] and Christi Nelson,[3], *1. USF HPCC/USF School of Aging, TAMPA, Florida, United States, 2. University of South Florida, University of South Florida, Florida, United States, 3. University of South Florida, Tampa, Florida, United States*

Lesbian, gay, bisexual, and transgender (LGBT) health disparities have been well documented in previous research; however, limited research has been conducted on racial/ethnic differences in health among LGBT older adults. Past research suggests that LGBT adults from racial/ethnic minority groups may encounter more discrimination and stigma than white LGBT adults, resulting in poorer health. This study investigated differences in general health between racial/ethnic groups in LGBT adults aged 50 and older from the 2018 and 2019 Behavioral Risk Factor Surveillance System annual surveys. The average ages were 64.2 years for the lesbian, gay, and bisexual (LGB) participants (n=3636) and 65.4 years for the transgender participants (n=972). For self-rated general health, the chi-square analysis indicated that there were significant differences between the racial/ethnic groups for both LGB and transgender participants, $\chi2(4, n=3630)=46.47$, $p<.001$ and $\chi2(4, n=969)=19.03$, $p=.001$, respectively. Logistic regression analyses found that, compared to White LGB adults, Hispanic LGB adults had higher odds (aOR=1.8, 95% CI=1.2-2.5) and Asian LGB adults had lower odds of reporting fair or poor health, (aOR=0.43, 95% CI=0.2-0.9). For transgender participants, Hispanic and Other Race adults had approximately twice the odds of reporting fair or poor health compared to White adults (aOR=2.1, 95% CI=1.2-3.7, and aOR=1.9, 95% CI=1.2-3.0, respectively). In conclusion, the results of this study suggest that cultural differences in racial/ethnic groups may influence the health of the LGBT community, making it an important factor to consider in research on LGBT older adults.

### HEALTHCARE DISPARITY AND COMORBIDITY BURDEN IN HEART FAILURE PATIENTS OVER THE AGE OF 80

Anna Blach,[1] Amanda Pangle,[1] Jeanne Wei,[2] and Gohar Azhar,[2], *1. University of Arkansas for Medical Sciences, Little Rock, Arkansas, United States, 2. UAMS, UAMS, Arkansas, United States*

The healthcare industry is currently struggling with providing access and coverage for a rapidly ageing and increasingly diverse population with multiple co-morbid conditions. This retrospective study analyzed the electronic health records of elderly heart failure patients (age range 80-103; mean 87 ±4.9) for common co-morbid conditions of hypertension, hyperlipidemia, dementia and diabetes mellitus. Chart review analysis of 316 patients showed a racial distribution of 251 White vs. 65 Black patients (79% vs. 21%). Male patients were under-represented (B= 13.8% and W= 26.3%). Females patients predominated (B= 86.2% and W= 73.7%). Overall, the prevalence of all four comorbidities was approximately three times higher in Blacks (18.5%) vs. White (7.2%). The proportion of Blacks and Whites with HTN and was comparable at 98.5 and 92.4% respectively. Hyperlipidemia was present in 84.6% Black and 63.3% White. The diagnosis of diabetes was higher in Blacks, 41.5% compared to Whites, 21.9%. The greatest disparity was in the diagnosis of dementia which was higher in Blacks, 61.5% vs Whites, 44.6%. Our study is unique for studying healthcare disparity in octogenarian and nonagenarian residing in a rural setting. Our results also highlight the importance of making a special effort to engage older Black patients in seeking healthcare in addition to designing strategies to reduce barriers that impede access and availability of resources and clinical care, especially in economically underserved regions of the country.

### IMPACTS OF SOCIAL CAPITAL FACTORS ON BLOOD GLUCOSE CONTROL AND DEPRESSIVE SYMPTOMS

Tingzhong (Michelle) Xue,[1] Zahra Rahmaty,[2] Eleanor McConnell,[1] Yingzhi (Lindsay) Xu,[3] and Kirsten Corazzini,[2], *1. Duke University School of Nursing, Durham, North Carolina, United States, 2. University of Maryland School of Nursing, Baltimore, Maryland, United States, 3. Duke University, Durham, North Carolina, United States*

Social capital, conceptualized as resources arising from social networks, is receiving increased attention for its role in prevention and management of chronic conditions such as diabetes and depression that commonly co-occur. Although social capital has been linked to control of blood glucose and depression, previous research has not considered these two outcomes simultaneously while distinguishing between cognitive (i.e., perceived social support, shared values and trust in community) and structural (i.e., social connectedness and participation) domains. This study examined how these two domains of social capital relate to glucose control and depressive symptoms, and whether physical exercise and care access mediate those relationships, using structural equation modeling. The sample included 3,043 older adults aged 57 and above from wave 2 of the National Social Life, Health and Aging Project. Although a higher level of cognitive social capital was associated with higher levels of physical exercise (b=.38, p<.001), access to care (b=.40, p=.007), lower levels of blood glucose (b=-.43, p<.001) and depressive symptoms (b=-.84, p<.001), a higher level of structural social capital was associated only with a higher level of physical exercise (b=.16, p=.002). The mediating effects of physical exercise and access to care were not significant. Findings suggest that cognitive social capital may have greater influence on blood glucose and depressive symptoms than structural social capital, and therefore have different implications for practice, especially in the context of pandemic-related disruptions to social capital. Future research should examine other mediators and investigate how promotion of cognitive social capital might improve health outcomes.

### IS RESILIENCE PROTECTIVE OF MOVEMENT-EVOKED PAIN IN OLDER BLACK WOMEN WHO EXPERIENCE DISCRIMINATION?

Mayra Astencio,[1] Emily Bartley,[2] Staja Booker,[3] Kimberly Sibille,[4] Josue Cardoso,[5] Roger Fillingim,[6] Ellen Terry,[7] and Adriana Addison,[8], *1. University of Florida College of Nursing, Miami, Florida, United States, 2. University of Florida, Gainesville, Florida, United States, 3. University of Florida, GAINESVILLE, Florida, United States, 4. University of Florida, University of Florida, Florida, United States, 5. University of Florida, Pain Research and Intervention Center of Excellence (PRICE), Gainesville, Florida, United States, 6. University of Florida, gainesville, Florida, United States, 7. University of Florida College of Nursing, Gainesville, Florida, United States, 8. University of Alabama at Birmingham, BIRMINGHAM, Alabama, United States*

Older non-Hispanic black (NHB) individuals experience greater pain and more frequent experiences of perceived discrimination compared to non-Hispanic white individuals with knee osteoarthritis. The current study explored whether being resilient buffers against movement-evoked pain (MEP) in NHB women who report everyday experiences of discrimination. In a secondary analysis of the Understanding Pain and Limitations in Osteoarthritic Disease (UPLOAD-2) study, data were collected at the University of Florida and the University of Alabama at Birmingham. Participants were 58 community-dwelling older women who self-identified as NHB and reported knee osteoarthritis. Participants completed the Brief Resilience Scale, a self-report measure of trait resilience. MEP was assessed following the Short Physical Performance Battery. Moderation analyses were conducted to investigate whether resilience moderates the association between experiences of discrimination and MEP. Study site, age, body mass index, and income were included as covariates. Overall, neither everyday experiences of discrimination (b=.292, 95% confidence interval [CI]=-.415 to 1.000) nor trait resilience was associated with MEP (b=-11.540, 95% CI=-23.583 to .503). However, there was a significant interaction (b=1.037, 95% CI=.150 to 1.925) between experiences of discrimination and trait resilience in predicting MEP. Simple slopes analysis revealed that lower discrimination was associated with lower MEP, but only in women who reported high levels of resilience (b=1.100, p=.014), but this protective effect of resilience was absent in women reporting high discrimination. Our findings suggest that as discrimination increases, the protective effects of resilience on movement evoked-pain decreases. Therefore, high trait resilience may be protective when experiences of discrimination are low.

### LINKING SLEEP AND RACIAL HEALTH DISPARITIES: CHARACTERIZING SLEEP IN THE NATIONAL SLEEP RESEARCH RESOURCE

Alycia Sullivan Bisson,[1] Susan Redline,[2] and Shaun Purcell,[1], *1. Brigham and Women's Hospital, Boston, Massachusetts, United States, 2. Harvard Medical School, Boston, Massachusetts, United States*

To address the problem of racial health disparities, prior work has studied differences in environmentally-influenced and modifiable health behaviors, like nutrition and physical activity. Mounting evidence suggests that sleep plays a key role in health, including cardiometabolic and neurodegenerative disease. Thus, studies have begun to characterize sleep differences across racial groups. We aimed to better quantify differences in objective sleep that may contribute to racial health disparities. In preliminary analyses, we examined whole-night polysomnography from 728 individuals between the ages of 7 and 86 (M: 41.39, SD: 19.39) in the diverse Cleveland Family Study (45% males, 57% African Americans; AAs). Linear models examined racial differences in a battery of sleep metrics and tested interactions with age. Microarchitecture metrics included NREM spindle and slow oscillations, important to cognitive-aging and cardiometabolic health. AAs spent relatively more time in lighter N2 (b= 0.295, p<.001) and less time in deeper N3 (b= -0.364, p<.001) sleep. AAs also had lower NREM spectral power across multiple frequency bands (p<.001), and reductions in spindle characteristics including amplitude (b = -0.537, p<.001) and density (b = -0.341, p<.001). Metrics showed qualitatively different patterns of interaction with age: e.g., racial differences in N3 duration increased with age, and differences in spindle amplitude decreased with age (interactions p<.001), despite marked age-related reductions across all individuals. This work may help to identify specific modifiable aspects of sleep as targets for ameliorating health disparities. Patterns of racial differences over the lifecourse may illuminate different mechanisms being active at different points in development.

### MULTIMORBIDITY AMONG DIVERSE COMMUNITIES IN NEW ENGLAND: FINDINGS FROM THE HEALTHY AGING DATA REPORTS

Maki Karakida,[1] Chae Man Lee,[1] Taylor Jansen,[1] Shu Xu,[2] Frank Porell,[3] Nina Silverstein,[4] and Elizabeth Dugan,[1], *1. UMass Boston, Boston, Massachusetts, United States, 2. University of Masschusetts Boston, Dorchester, Massachusetts, United States, 3. University of Massachusetts Boston, University of Massachusetts Boston, Massachusetts, United States, 4. University of Massachusetts Boston, Boston, Massachusetts, United States*

The risk for multimorbidity increases with age. Community burden of comorbidities in New England (NE) was assessed by comparing state and community rates of two measures (having no comorbidities and having 4 or more) among Medicare beneficiaries age 65+ in CT, MA, NH, and RI. Data sources were the Medicare Current Beneficiary Summary File (2014-2017) and the American Community Survey (2014-2018). Small area estimation techniques were used to calculate age-sex adjusted community rates. Multimorbidity was measured as people with zero or with 4 or more of the following chronic conditions: Alzheimer's disease, asthma, atrial fibrillation, cancer (breast, colorectal, lung, and prostate), kidney disease, COPD, depression, diabetes, congestive heart failure, hypertension, hyperlipidemia, ischemic heart disease, osteoporosis, arthritis, and stroke. Rates for 4+ conditions: RI 63.8% (45.76-70.69%), CT 61.8% (47.82-70.05%), MA 60.7% (40-74.96%), NH 54.4% (36.67-62.99%). Results were mapped, showing the statewide and regional distribution of rates. Rates were much higher for having 4+ chronic conditions than not having any comorbidities. RI had the highest rates of 4+ and in MA the highest chronic disease rates were found in lower socioeconomic communities. CT has the highest number of diverse older residents and dual-eligible beneficiaries for Medicare and Medicaid in NE. The rates show late-life health disparities that have implications for independent living, quality of life, and mortality suggesting the need for policies to provide equitable access to care and resources to disadvantaged NE communities.

### NEEDS ASSESSMENT OF PERIMENOPAUSE RESOURCES AND SERVICES

Genna Losinski,[1] Hilary Hicks,[1] Pilar Thangwaritorn,[1] Ilana Engel,[2] Alexandra Laffer,[1] Amy Berkley,[3] and Amber Watts,[1], *1. University of Kansas, Lawrence, Kansas, United States, 2. University of Kansas, Kansas. City, Kansas, United States, 3. University of Kansas Medical Center, Kansas City, Kansas, United States*

Studies show that women lack knowledge about perimenopause and feel unprepared to make healthcare decisions during this life transition. Most women want to be involved in their healthcare decisions and need timely, free, and accurate information. We conducted a needs assessment in Douglas County, Kansas by systematically reviewing regional organizations that might offer services and resources related to women's health in midlife. We compared these resources to a benchmark for menopausal care available online nationwide (Gennev.com). We documented the primary purpose of each organization (e.g., cosmetic, wellness, medical care), services and resources offered (e.g., hormone therapy, counseling, non-pharmacological treatments), methods of outreach (e.g., blogs, classes), target audience, costs, and types of service providers (e.g., physician, counselor). We surveyed 9 regional websites: 5 offered medical care, 3 cosmetic and wellness services, 2 were municipal organizations, 2 offered mental health/social support. Four organizations offered services targeted specifically towards perimenopausal women. The most commonly offered services were hormone replacement therapy (44%), nutritional supplements (33%), and weight loss programs (33%). Very few offered educational resources (1) or menopause assessments (1) and none offered tailored psychosocial support for the perimenopause transition. The services offered were expensive with no free services and very few free resources. Organizations were primarily staffed by medical providers, only 1 organization had Menopause Practitioners certified by the North American Menopause Society. Our results demonstrate a need for comprehensive educational and support services for perimenopausal women to fulfill the need for timely, accessible, and accurate information during this understudied health transition.

### NOT OUT-OF-REACH: ENGAGING THE OLDER OLD ISOLATED AFRICAN AMERICANS WITH AND WITHOUT MCI

Laura Struble,[1] Kathleen Potempa,[2] Benjamin Hampstead,[3] Alexis Ellis,[4] Jesica Pedroza,[5] Peter Lichtenberg,[6] and Hiroko Dodge,[7], *1. University of Michigan School of Nursing, Ann Arbor, Michigan, United States, 2. University of Michigan, University of Michigan, Ann Arbor, Michigan,*

*United States, 3. University of Michigan, Ann Arbor, Michigan, United States, 4. University of Michigan, Ypsilanti, Michigan, United States, 5. University of Michigan, University of Michigan, Michigan, United States, 6. Wayne State University, Detroit, Michigan, United States, 7. Oregon Health & Science University, Portland, Oregon, United States*

The Internet-Based Conversational Engagement Clinical Trial (I-CONECT, ClinicalTrials.gov: NCT02871921) is a multi-center randomized, 12-month efficacy study. There is converging evidence that social isolation is a risk factor of cognitive decline and dementia. We hypothesized that increasing social interaction in older adults with normal cognition or mild cognitive impairment (MCI) could improve or sustain cognitive function through internet-based conversational engagement. African Americans (AA) are at higher risk for developing dementia but their participation in clinical trials is low. Objectives: (1) discuss the effective outreach process to recruit urban AA older old adults (mean targeted age of 80+); (2) describe how we retained participants in a yearlong study using technology-based interventions. The most successful outreach and recruitment sources were the voter registration mass mailings and the Healthier Black Elders Research Center. Successful recruitment methods included: hiring diverse staff, compensating participants' time, and adjusting research protocols for opting out of MRIs and genetic saliva samples. Technology intervention strategies included: providing user-friendly Chromebooks and free internet connections, simple instructions with pictures, vision and hearing correction, and in-home training with technology support backup. During the pandemic, we could assists participants in learning to use the laptop remotely. Over 12,000 subjects were contacted, which led to 39 randomized participants. Our retention rate thus far is over 75%. This demonstrates that AA older adults are reachable, willing to participate in research and able to use communication technology with appropriate supports for long-term sustainable interaction that may improve cognition and health equity.

### SELF-REPORTED PHYSICAL LIMITATIONS AMONG U.S. VETERANS COMPARED TO NON-VETERANS: FINDINGS FROM NHANES

Ronna Robbins,[1] Monica Serra,[2] and Odessa Addison,[3], *1. Audie Murphy VA Medical Center, San Antonio, Texas, United States, 2. UT Health San Antonio - Barshop Institute, San Antonio, Texas, United States, 3. University of Maryland School of Medicine, Baltimore, Maryland, United States*

Approximately 43% of males over the age of 65 years are Veterans. Veterans may be at elevated risk for functional declines due to barriers to health care access leading to accelerated loss of independence. This compared the prevalence of functional limitations in Veterans and non-Veterans. Data from two National Health and Examination Survey collection periods, administered 2013-2014 and 2015-2016, were used to compare physical functioning data between male Veterans (N=369) and non-Veterans (N=738) matched 1:2 for sex, race, and BMI. Individuals were considered a Veteran if they self-reported having "served in active duty in the U.S. Armed forces." Pearson's chi-square tests were used to assess differences in the prevalence of various self-reported functional limitations between groups. Veterans (mean±SEM: age: 64.5±0.54 years; BMI: 30.0±0.3 kg/m2) were disproportionately affected by self-reported functional limitations caused by long-term physical, mental, or emotional illnesses (8% vs. 3%, $p<0.004$). Twenty-five % of Veterans reported that these limitations kept them from working compared to 18% of non-Veterans ($p<0.003$). Veterans (38%) were also more likely to report being limited in the amount of work they could perform compared to non-Veterans (27%) ($p<0.01$). Additionally, Veterans (20%) were more likely to report the use of special healthcare equipment (i.e. cane, wheelchair) than non-Veterans (12%) ($p<0.001$). These data suggest that Veterans are at greater risk for functional limitations caused by self-reported long-term physical, mental or emotional illness. Therefore, further research is needed to determine if home- and community-based services could prevent further functional decline, ultimately allowing Veterans to maintain independence.

### STRENGTHENING STRATEGIES TO RECRUIT RACIAL/ETHNIC MINORITY POPULATIONS FOR HEALTH RESEARCH STUDIES

Roy Thompson,[1] Thomas Konrad,[2] and HANZHANG XU,[3], *1. Duke University, Durham, North Carolina, United States, 2. University of North Carolina at Chapel Hill, Chapel Hill, North Carolina, United States, 3. Duke University School of Medicine, Duke University, North Carolina, United States*

With the current spotlight on systemic racism and the need to address health inequities, it is critical to develop culturally appropriate strategies for recruiting research study participants from racial/ethnic minority groups. Empirical studies have highlighted that people from racial/ethnic minority groups have poorer health outcomes compared to non-Hispanic Caucasians. However, racial/ethnic minority groups remain underrepresented in healthcare research. Several factors may contribute to the lower participation of racial/ethnic minority groups. Sequelae of atrocities in healthcare research on African American/Black people in the US during slavery and Jim Crow eras were widespread and persistent. Discrimination against people of Hispanic descent and increased anti-Asian discrimination have also been documented. Fear and mistrust of the health system and researchers have been identified as critical barriers to participation in clinical research for these populations. Further, health research teams rarely reflect the racial/ethnic diversity of the US population, hindering diversity in recruiting study participants. Inadequate ethnic/racial minority groups participation in study populations not only weakens external validity of empirical studies, but research interventions and policies being implemented may not be culturally appropriate to all populations. Therefore, systemic strategies to improve recruitment of racial/ethnic minority groups should: 1) increase preferential funding to incentivize research teams becoming more racially/ethnically diverse; 2) increase recruitment of racial/ethnically diverse healthcare researchers; 3) use community-based participatory research designs to build trust among racial/ethnic minority populations; 4) provide training on culturally appropriate research study recruitment strategies to the academic communities; 5) apply a prism of intersectionality for representation throughout the research cycle.

### SUPPORTIVE HOMES AS MEDIATOR BETWEEN RURAL STATUS AND DISABILITY OF OLDER ADULTS

Bernard Steinman,[1] Bremen Whitlock,[2] Casandra Mittlieder,[2] Julie Overton,[3] and Jon Pynoos,[4], *1. University of Wyoming, University of Wyoming, Laramie, Wyoming, United States, 2. University of Wyoming, Laramie, Wyoming, United States, 3. University of Southern California, Los Angeles, California, United States, 4. University of Southern California, University of Southern California, California, United States*

By definition, older adults living in rural communities have fewer formal resources available to address aging-related functional needs. Supportive environments are frequently relied on in rural settings to help address this discrepancy. The purpose of this study was to assess the role of supportive housing features and home modifications in mediating the association between rurality and disability. We hypothesized that environmental supports would be more crucial in rural settings than non-rural settings. We analyzed data from the National Health and Aging Trends Study (NHATS). Variable selection was guided by the International Classification of Functioning, Disability and Health (ICF), including covariates for sociodemographics, chronic conditions, mobility functioning, and participation. A series of regression models tested mediation by environmental variables of the association between rurality (as determined by the metro/nonmetro file indicator) and ADL/IADL disability. Supportive home environments were operationalized using indicators of whether participants had access to homes from the outside without having to use stairs; presence of a bedroom, kitchen, and full bathroom with a shower or tub on the same floor; and whether bathroom fixtures had been modified with features such as grab bars. Results suggest a statistical relationship between rurality and disability that is explained in part by the presence or lack of supportive home features, and these effects were greater in rural settings. Implications are that older adults who live in rural settings can benefit greatly by supportive environments and modifications in areas of the home that are known to cause difficulty.

### Session 9050 (Poster)

### ALL THINGS TECHNOLOGY AND AGING

### ASSESSMENT OF SLEEP DURATION BASED ON ANKLE AND WRIST ACTIGRAPHY IN HOSPITALIZED OLDER PATIENTS.

Juliana Smichenko,[1] Tamar Shochat,[2] Nurit Gur-Yaish,[3] and Anna Zisberg,[4], *1. University of Haifa, Haifa, Hefa, Israel, 2. University of Haifa, University of Haifa, HaZafon, Israel, 3. Oranim Academic College of Education, Haifa, HaZafon, Israel, 4. University of Haifa, Haifa, HaZafon, Israel*

Poor sleep at time of hospitalization is associated with undesirable outcomes. Most studies performed in the hospital assess sleep by self-report, while only few rely on actigraphy. Although wrist actigraphy is commonly used for sleep assessment in field studies, in-hospital assessment may be challenging and cumbersome due to other more necessary monitoring devices that are often attached to patients' upper limbs, that may in turn affect interpretation of wrist activity-data. Placement on the ankle may be a viable solution. In this pilot study, we aimed to compare total sleep time (TST) using concomitant wrist and ankle actigraphy as well as self-report. Twenty-one older adults (65+) hospitalized in medical units wore ankle and wrist actigraphy devices and subjectively estimated their TST for an average of (2.15±1.01) nights. A total of 45 nights were available for analysis. Average TST in minutes was 332.06±81.58, 427.05±97.74 and 374.28±124.96 based on wrist, ankle, and self-report, respectively. Repeated measure mixed models analysis was performed adjusting for age, gender, and sleep medications. TST was significantly lower using wrist compared to ankle actigraphy ($F(2,102)=7.63$, $p=0.0008$), and both were not different from self-report. No significant within subjects variation and no interaction between device and repeated measures were found. Despite differences between ankle and wrist assessments, all three provide consistent TST estimation within subjects. Self-report provides a stable and accessible assessment of TST, representing a good approximation of ankle and wrist actigraphy. Findings provide preliminary support for the use of ankle actigraphy for sleep assessment in hospital settings.

### CIRCADIAN RHYTHM FOR FEMALE ENTERING OLD AGE: EXPLORATION ON THE EFFECT OF EATING BEHAVIOR

Hsiao-Han Tang,[1] and Ching-Ju Chiu,[2], *1. National Cheng Kung University, Institute of Gerontology, College of Medicine, Tainan, Taiwan (Republic of China), 2. National Cheng Kung University, Institute of Gerontology, College of Medicine, National Cheng Kung University, Tainan, Taiwan (Republic of China)*

Unhealthy lifestyle and eating behavior are associated with circadian rhythm disruption which contributes to numerous harmful outcomes. The relationship between circadian rhythm and eating behavior remains unclear. The study aims to investigate different types of eating behavior in middle-aged women and their variation in circadian rhythm. A descriptive, cross-sectional design was used. We recruited a convenience sample of 150 female aged 45 years or over from the community in southern Taiwan. Sociodemographic status, sleep diary and eating behavior were collected by questionnaires; behavioral circadian rhythm were monitored with the wrist-worn application. Four middle-aged participants were interviewed. Preliminary data show three main findings: (1) Sleep efficiency was decrease with age, (2) First meal within 2 hours after waking up was associated with higher amplitude (2.24 vs 1.43 log count), relative amplitude (0.92 vs 0.71), middle to vigorous physical activity time (101.22 vs 58.41 minute), lower lowest active 5 hr midpoint (2.63 vs 4.34 hour) and acrophase (13.67 vs 15.75), (3) Participants with morning chronotype have less sedentary behavior and higher most active 10 hr during wake time. Age and timing of first meal after waking up seem dominating circadian rhythm. Chronotype might be a significant factor for physical activity level. More data is needed to further confirm the association.

### DEVELOPMENT & TESTING OF A COMPREHENSIVE DIGITAL SELF-CARE SUPPORT SYSTEM FOR OLDER ADULTS WITH CHRONIC CONDITIONS

Priya Nambisan,[1] Kurt Stange,[2] Kalle Lyytinen,[2] Eva Kahana,[3] Edmund Duthie,[4] and Michael Potnek,[5], *1. University of Wisconsin- Milwaukee, Milwaukee, Wisconsin, United States, 2. Case Western Reserve University, Cleveland, Ohio, United States, 3. Case Western University, Case Western reserve University, Ohio, United States, 4. Medical College of Wisconsin, Milwaukee, Wisconsin, United States, 5. Outreach Community Health Center, Milwaukee, Wisconsin, United States*

This 3-phase study involves the conceptualization and design, development and usability testing of a Comprehensive Digital Self-care Support System (CDSSS) named myHESTIA for older adults with multiple chronic conditions (MCC). The objective of this study was to test whether a CDSSS can be developed for those who are dealing with MCC and whether such a system that is specifically developed for older adult patients will enable daily capture of self-care data. Participants for this 3-phase study included: 10 older adults (age>60) and 10 caregivers in Phase 1; 15 Geriatrics clinicians and 25 community-dwelling low-income older adults in Phase 2; and, 10 older adults (age>60) with MCC in Phase 3. Agile method of system development was used for the design and development of the system. The first two phases involved collecting data for designing and developing myHESTIA. The third phase involved small group usability and feasibility testing, in which the participants used myHESTIA trackers for 4 weeks. Results from phase 3 shows daily inputs were possible and the self-reported data shows that it was not at all difficult for older adults to track their symptoms daily. User experience data (n=10) shows overall positive experience along pragmatic (5.8 out of 7), hedonic (4.6 out of 7), sociability (5.5 out of 7) and usability (6.3 out of 7) experience dimensions. Finally, all the participants (n=10) who completed the phase 3 study reported intention to continue using myHESTIA. Results indicate that it is feasible to design a CDSSS for older adults with MCC.

### MEET CAREGIVERS WHERE THEY ARE: A REMOTE INTERVENTION CONNECTING CAREGIVERS TO COMMUNITY RESOURCES

Katherine Thompson,[1] Victoria Winslow,[1] El Pinkerton,[1] Elbert Huang,[2] Maria De Ornelas,[2] Soo Borson,[3] Jason Molony,[4] and Stacy Lindau,[2], *1. University of Chicago, Chicago, Illinois, United States, 2. The University of Chicago, Chicago, Illinois, United States, 3. University of Washington School of Medicine, Seattle, Washington, United States, 4. The University of Chicago Medicine, Chicago, Illinois, United States*

Informal caregivers of people with Alzheimer's disease and related dementias (ADRD) are a vulnerable, often isolated population with high rates of financial strain and need for community resource supports. Little is known about how best to connect these caregivers to resources, especially during the COVID-19 pandemic. CommunityRx-Caregiver is an evidence-based intervention that connects caregivers to community resources for basic needs, wellness, and caregiving. Using preliminary data from a randomized trial of CommunityRx-Caregiver (N=344), we examined caregivers' baseline confidence in finding community resources and their engagement in the CommunityRx-Caregiver intervention. Caregivers enrolled December 2020-February 2021 (n=26) received (1) personalized lists of community resources via text message (HealtheRx), (2) access to an online resource portal (FindRx) and (3) automated texts offering support for finding resources. Most caregivers were female (65%), Black (92%), >60 years old (64%) and 44% reported very good or excellent health. Nearly half of caregivers (46%) were completely confident in finding community resources. Overall, 81% of caregivers engaged with a text message or the FindRx. Nearly two-thirds (65%) of caregivers responded to at least one text message. More than a quarter (27%) used the FindRx tool; 5/7 of those shared FindRx resources with others. Caregivers sought resources including in-home personal care, exercise classes and support groups. Caregivers of people with ADRD, many of whom had low confidence in finding resources, engaged with a multi-modal information technology-based intervention to obtain community resource support. These preliminary findings suggest caregivers were receptive to a remotely-delivered community referral intervention during the COVID-19 pandemic.

### NOVEL TECHNOLOGY SUPPORT PROGRAM FOR OLDER ADULT PROGRAM WITH INTERPROFESSIONAL GERIATRICS STUDENTS

Bonnie Olsen,[1] Freddi Segal-Gidan,[1] Erin Thayer,[1] Yeini Guardia,[1] M. Christina Penate,[1] and Alexis Coulourides Kogan,[2], *1. University of Southern California - Keck School of Medicine, Alhambra, California, United States, 2. USC Keck School Of Medicine, Los Angeles, California, United States*

During the COVID-19 pandemic, many older adults were not receiving primary care services because they could not negotiate the technology for telehealth visits. Coupled with persisting pandemic physical distancing, increased social isolation in older adults was- and continues to be a significant problem. To combat these issues, we aimed to 1) prepare older adults for longitudinal isolation by encouraging social connectedness, and 2) enable older adults to safely access remote primary care services during the pandemic. We paired older adults from 9 housing sites in Los Angeles, CA with health professions graduate students from 9 programs at USC (N = 88 dyads) and provided iPhones to participants without a smartphone. Students educated and supported older adults about the use of technology to access primary care services and to socially connect with family/friends. When requested, 3 additional students provided enhanced 1:1 technology support. Among the 45 participating older adults who received iPhones (51.1%), 22 requests were made for enhanced technology assistance during the 6-month program. Most requests related to initial setup/navigation of iPhone (81.8%) or video calls (27.3%), where others requested help with Wi-Fi (13.6%), composing emails (4.5%), and adding language/translation features (4.5%). Nineteen (83%) technology support requests were successfully resolved; the remaining were unresolved due to loss to follow-up. Our findings demonstrate that older adults can successfully cross the digital divide when technology support is provided. Additionally, pairing older adults with health professions students is an effective strategy to enable access to remote primary care and social connectedness.

### PILOT STUDY OF A NEW TELEHEALTH PROGRAM FOR DEPRESSED OLDER ADULTS: THE POSITIVE EXPERIENCE PROJECT (P.E.P.)

Dolores Gallagher-Thompson,[1] Nancy Morioka-Douglas,[2] BJ Fogg,[3] and Mei-Le Keck,[4], *1. Stanford University School of Medicine, LOS ALTOS, California, United States, 2. Stanford University School of Medicine, Palo Alto, California, United States, 3. Stanford Medicine, Stanford, California, United States, 4. Stanford Health Care, Palo Alto, California, United States*

Social isolation, logistical challenges, and limited access to mental health providers who accept Medicare contribute to older adults having a higher risk for untreated depression. Primary care providers (PCPs) are strained due to time demands and lack of training in behavioral activation and similar therapies. This study was designed to reduce depression in older adult primary care patients without burdening their PCPs. We evaluated whether outpatients age 65+ with mild to moderate self-reported depressive symptoms (measured by PHQ-8) benefited from a brief evidence-informed program, the Positive Experience Project(PEP). This is derived from the tenets and practices of both behavioral activation and the "tiny habits" program. The former emphasizes the value of increasing engagement in everyday positive activities to lift mood; the latter provides a method to enhance success by encouraging patients to link these activities with existing habitual behaviors, and to celebrate completion. A script was written for each of four 30-minute sessions conducted in small groups using a telehealth platform. An analysis of patients of the first author yielded 50 eligible patients. 8 were invited to participate and 7 did. The group's mean PHQ-8 score pre-intervention was 10.1; post-intervention (4 weeks later), the average was 6.1 (P=.039). The use of "scripts" that guide the PCP through the visit enhances adoption. And PCPs can bill for these 30-minute sessions, making it feasible to help patients receive treatment for their depression. These promising results are currently being replicated by additional PCPs and their data will be included in the poster presentation.

### SPATIAL USAGE AND CIRCADIAN RHYTHM FOR OLDER ADULTS IN THE COMMUNITY IN TAIWAN

Chih-Liang Wang,[1] and Ching-Ju Chiu,[2], *1. National Cheng Kung University, Tainan City, Tainan, Taiwan, Province Of China, 2. National Cheng Kung University, Institute of Gerontology, College of Medicine, National Cheng Kung University, Tainan, Taiwan (Republic of China)*

Objective measure of lifestyle of the older adults living in the community is void in the literature. To obtain both objective and subjective measurements to ascertain mobile and day and night lifestyle of older adults living in the community, and to build lifestyle model of older adults in the community by sociodemographic character. This study is a cross-sectional research. 200 over-50-year older adults who own smartphone and live in southern Taiwan were interviewed. Wrist accelerometers to detect behavioral circadian rhythm, GPS app in smartphone to survey mobility, and questionnaire to assess psychological and social status. Preliminary finding of six participants (2 men and 4 women) was analyzed. Data show that participants about 60 years old have large discrepancies in comparison with participants in their 50s: lower sleep efficiency (73 vs 83), earlier Most active 10 hour midpoint (11.48vs 14.13 hour), higher interdaily variability (0.84 vs 0.75), wake after sleep onset (100.39vs 47.78 minutes), and higher exercise frequency (4.33 vs 1.66 times per week). In addition, men have more chronic disease, bigger waistline (103.5 vs 77.5 cm), higher BMI (30 vs 22.5), lower middle to vigorous physical activity time (39 vs 79 minutes), and more total sleep time (356 vs 317 minutes). Age and sex seem to be significant factors determining lifestyle of older adults. Other sociodemographic parameters will be further analyzed.

### STANDARDIZED SELF-REPORT TOOLS IN GERIATRIC MEDICINE PRACTICE: A QUALITY IMPROVEMENT STUDY

Melissa Northwood,[1] George Heckman,[2] Nicole Didyk,[1] Sophie Hogeveen,[1] and Amanda Nova,[2], *1. McMaster University, Hamilton, Ontario, Canada, 2. University of Waterloo, Waterloo, Ontario, Canada*

Comprehensive geriatric assessment (CGA)—a multidimensional diagnostic process to determine medical, cognitive, and functional capacity—has historically included a narrative history supplemented by use of tools to assess domains such as mood or cognition based on assessor preference. This approach to CGA likely works to assess individuals but with increasing clinical complexity and frailty among older adults, a non-standardized approach may mean that key issues are not assessed, and program quality cannot be determined. The COVID-19 pandemic added to these challenges as social distancing practices meant limited face-to-face appointments and use of phone and video assessments. This quality improvement study implemented the interRAI Check-Up Self-Report instrument through a software platform in a specialized geriatric services practice. The instrument can be used over the phone and summarizes specific health problems and needs as well as information about caregiver status and financial trade-offs. Focus groups were also conducted with specialized geriatric services interprofessional team to explore their experiences with implementation. The descriptive analysis of the self-report data revealed expected geriatric issues, such as cognitive and functional impairment, falls and pain. Clients were also commonly experiencing medical instability, cardiorespiratory symptoms, communication impairments, and elevated risk for emergency department visit. Staff found the self-report tool feasible, easy to use, efficient, and the program-level metrics helpful for program planning. In conclusion, introduction of a standardized self-report enhanced CGA by creating a systematic method to flag, track, and prioritize all areas of need for immediate and future care planning at both the client and program level.

### TECHNOLOGY FOR HEALTHY AGING: USE OF ELECTRONIC COMMUNICATION AMONG OLDER ADULTS

Rumei Yang,[1] Yan Du,[2] Haocen Wang,[3] Zuoting Nie,[4] Chumin Ji,[5] and Yun Jiang,[6], *1. Nanjing Medical University, Nanjing, Jiangsu, China (People's Republic), 2. School of Nursing, University of Texas Health Science Center at San*

*Antonio, San Antonio, Texas, United States, 3. Texas A&M University, College Station, Texas, United States, 4. School of Nursing, Nanjing, Jiangsu, China (People's Republic), 5. School of Nursing, Nanjing Medical University, Nanjing, Jiangsu, China (People's Republic), 6. University of Michigan, Ann Arbor, Michigan, United States*

In the digital era, many electronic platforms have been established to facilitate patient-provider communication, such as e-mail, text messaging, and patient portal. The use of these electronic platforms is termed as electronic-communication (e-communication). E-communication has a variety of personalized healthcare functions, such as exchanging information, reviewing lab results, and facilitating patient engagement. However, little is known about the actual use of e-communication among older adults who are potentially major users of e-communication considering their high-level health care needs. Understanding their use of e-communication is critical in improving the application of e-communication in older adults. Using data from American Health Information National Trends Survey (HINTS2019-Cycle3; n=1,961; meanage =74.10, range=65-98), we explored: 1) the prevalence of e-communication use among older adults, and 2) factors affecting their use of e-communication. Variables were measured by self-reports. Weighted logistic regression with replicate weights provided by the HINTS was performed for data analysis. We found that 50% older adults reported the use of e-communication in the last year. Factors associated with higher likelihood of older adults' e-communication use included younger age (OR=09.96, 95%CI=0.93-0.98, p<0.001), higher education (OR=4.82, 95%CI=2.32-10.02, p<0.001 for college graduate or higher), higher income (OR=1.58, 95%CI=1.05-2.38, p=0.030), comorbid conditions (OR=1.64, 95%CI=1.02-2.64, p<0.001), and having a regular provider (OR=2.06, 95%CI=1.31-3.22, p=0.002). This study provided nationally representative results demonstrating a great potential use of e-communication in older adults. Special attention is needed to focus on socially vulnerable older adults (e.g., those with older age, lower education and income, and having comorbidity).

### TELEMEDICINE UPTAKE IN A GERIATRIC ASSESSMENT CENTER DURING THE COVID CRISIS

Noelle Frye,[1] Margaret Doyle,[2] and Richard Marottoli,[3], *1. YNHH, Yale New Haven Hospital, Connecticut, United States, 2. Yale University, Yale University, Connecticut, United States, 3. Yale University, New Haven, Connecticut, United States*

The Yale New Haven Hospital Adler Geriatric Assessment Center is an outpatient consultative service that provides comprehensive assessment of older adults. As elsewhere, at Adler the COVID crisis necessitated a rapid shift in mode of care following a total cessation of in-person visits from late March 2020 to the end of May 2020. While our patients initially preferred telephone visits, video visits as a proportion of total scheduled increased from an average of 6% in the last full week of March to 24% in the last week in May possibly indicating increasing familiarity and comfort with the technology during that time. In addition, while video appointments as a proportion of total scheduled dropped rapidly in June 2020 as face-to-face appointments were reintroduced, we found a steady increase in the proportion of video visits from 3% in the first week of July 2020 to 7% in the second week of February 2021. To test for significance, we ran logistic regression models modelling the dichotomous video-appointment variable as the outcome and week and day of week as continuous variables. We found there was a significant increase in the proportion of appointments delivered over video both during the time when no face-to-face video appointments were allowed (OR=1.21, CI=1.13,1.30) and later in the pandemic (OR=1.04, CI=1.02,1.06). Durbin-Watson statistics were run to ensure that autocorrelation could be ignored. Sensitivity analyses limiting the sample to those with non-cancelled appointments gave similar results. Future analyses will examine patient clinical and demographic characteristics that might influence these trends.

### THE 4MS OF AGE-FRIENDLY CARE: SUCCESSFUL INFUSION INTO ELECTRONIC MEDICAL RECORDS LEADS TO IMPROVED CARE

Leah Tobey, and Robin McAtee, *University of Arkansas for Medical Sciences, Little Rock, Arkansas, United States*

Electronic Medical Records (EMR) and Health Information Technology (HIT) have changed the daily operations of the healthcare industry. For primary care systems/clinics, it has meant the purchase and tailoring of systems to fit specific needs of users and patients. As one of the HRSA funded Geriatric Workforce Enhancement recipients, the AR Geriatric Education Collaborative (AGEC) worked with a rural federally qualified healthcare clinic system for over a year to help them become IHI certified in Age-Friendly Care and the EMR has been a critical link. The system was crucial in identifying compliancy to the clinical Merit-based Incentive Payment System (MIPS) measures which helped identify the areas where most improvement was needed. Included in the process was a new geriatric screening tab in the EMR system. This tab contains 8 screenings including: depression, anxiety, alcohol use, prescription opioid use and recreational drug use, mentation, medication and mobility. This is in addition to asking "what matters" so that all four of the 4M's age friendly framework components were included. Clinicians are successfully using the systems and improvements in outcomes are beginning to be noted. The outcome MIPS measures are obtained quarterly from an EMR report and data is shared with the staff and new quality improvement projects are developed using PDSA (plan, do, study, act) cycles based on the outcomes of the data. Next steps with the EMR will be the development of flags that will notify the clinicians when a screening is needed.

### THE DIGITAL DIVIDE AMONGST HIGH-NEED HIGH-RISK VETERANS

Shirley Li,[1] Kiranmayee Muralidhar,[1] Fei Tang,[2] Willy Marcos Valencia,[3] and Stuti Dang,[4], *1. University of Miami Miller School of Medicine, Miami, Florida, United States, 2. Miami Veteran Affairs Healthcare System, Miami, Florida, United States, 3. Medical University of South Carolina, Miami, Florida, United States, 4. Miami Veterans Affairs Healthcare System, Miami, Florida, United States*

High-need high-risk (HNHR) veterans are medically complex and at the highest risk of hospitalization and long-term institutionalization. Technology can mitigate challenges these veterans have in accessing healthcare. Willingness to use technology as well as access and ability to use technology were assessed in this study. At the time of the survey, 2543 Miami VAHS veterans were listed as HNHR. 634 veterans ultimately completed the questionnaire, and 602 answered the "willingness to use video-visits" question. Of the 602 respondents, 327 (54.3%) reported they were willing for video-visits with the VA, while 275 (45.6%) were not. Those who were willing were significantly younger (P<0.001), with higher educational qualifications (P=0.002), and more health literate than those not willing (P<0.001). They were more also capable of using the Internet, more likely to use email and be enrolled in the VA's patient portal, My HealtheVet (P<0.001). However, of the veterans who were willing, 248 (75.8%) had a device with video-capable technology. Those with video-capable technology were younger (P=0.004), more health literate (P=0.01), and less likely to be Black or African American (P=0.007). They were more capable of using the Internet, more likely to use email, and be enrolled in My HealtheVet than those without (P<0.001). Half of the respondents were willing for video-visits but a quarter of those willing lacked requisite technology, thereby making only about 41.2% of the respondents willing and video-capable. To minimize the digital divide, especially during the ongoing COVID-19 pandemic, targeted measures need to address these disparities in this vulnerable population.

### USER PERCEPTIONS & SUBJECTIVE MEMORY CONCERNS ARE ASSOCIATED WITH BRIEF, REMOTE COGNITIVE ASSESSMENT PERFORMANCE

Samuel Lee,[1] Katherine Dorociak,[2] Nora Mattek,[3] Sarah Gothard,[3] Jonathan Lee,[4] Jeffrey Kaye,[3] and Adriana Hughes,[5], *1. University of Minnesota, Twin Cities, Minneapolis, Minnesota, United States, 2. Palo Alto VA Health Care System, Palo Alto, California, United States, 3. Oregon Health & Science University, Portland, Oregon, United States, 4. Oregon Center for Aging and Technology, Portland, Oregon, United States, 5. Minneapolis VA Health Care System, Minneapolis, Minnesota, United States*

Online cognitive tests offer a cost-effective, accessible means of cognitive screening and may prove especially important for individuals with memory complaints, a risk factor for cognitive impairment (Kaup et al., 2015). Although older adults' perceptions of everyday technologies impact their uptake and adoption, there is limited understanding about how perceptions of online cognitive screening tests impact test performance. The purpose of the current study was to examine relationships between performance on a brief, self-administered, web-based cognitive assessment tool (SMART) and user perceptions (e.g., ratings of challenge and length), technology confidence, brain health activities, and memory complaints. Participants were 1336 adults without a diagnosis of cognitive impairment (Mage=60.48 years, SD=15.18; 65.8% female; 81.8% White; 21.2% with subjective memory complaints). Most participants (97%) were willing to complete the SMART again, with over half (53.5%) willing to complete the SMART on at least a weekly basis. After adjusting for age and education, better SMART performance (i.e., faster completion time) was associated with user ratings of greater ease of completion, higher technology confidence, and greater participation in brain health activities (p<.05). In a subsample aged 60+, individuals with memory complaints took longer to complete certain SMART tasks (Trail Making Test B, Total SMART) than those without memory complaints (p<.05). Results suggest that the SMART is a well-accepted tool for frequent remote cognitive screening and highlight the importance of user perceptions, technology confidence, and memory complaints on online cognitive test performance.

### USING ARTIFICIAL INTELLIGENCE TO IMPROVE PAIN ASSESSMENT AND PAIN MANAGEMENT: A SCOPING REVIEW

Meina Zhang,[1] Linzee Zhu,[1] Shih-Yin Lin,[2] Keela Herr,[1] and Nai-Ching Chi,[3], *1. University of Iowa, Iowa City, Iowa, United States, 2. New York University, New York, New York, United States, 3. University of Iowa, university of Iowa, Iowa, United States*

Approximate 50 million U.S. adults experience chronic pain. It is a widely held view that pain has been linked to sleep disturbance, mental problems, and reduced quality of life. Uncontrolled pain has led to increased healthcare utilization, hospitalization, emergency visits, and financial burden. Recognizing, assessing, understanding, and treating pain can improve outcomes of patients and healthcare use. A comprehensive synthesis of the current use of AI-based interventions in pain management and pain assessment and their outcomes will guide the development of future clinical trials. This review aims to investigate the state of the science of AI-based interventions designed to improve pain management and pain assessment for adult patients. The electronic databases Web of Science, CINAHL, PsycINFO, Cochrane CENTRAL, Scopus, IEEE Xplore, and ACM Digital Library were searched. The search identified 2131 studies, and 18 studies met the inclusion criteria. The Critical Appraisals Skills Programme was used to assess the quality. This review provides evidence that machine learning, deep learning, data mining, and natural language processing were used to improve efficient pain recognition and pain assessment (44%), analyze self-reporting pain data (6%), predict pain (6%), and help physicians and patients to more effectively manage with chronic pain (44%). Findings from this review suggest that using AI-based interventions to improve pain recognition, pain prediction, and pain self-management is effective; however, most studies are pilot study which raises concerns about the generalizability of findings. Future research should focus on examining AI-based approaches on a larger cohort and over a longer period of time.

### Session 9055 (Poster)

### ALZHEIMER'S DISEASE AND THE BRAIN

### AGE-ASSOCIATED EPIGENETIC ALTERATIONS, SOMATIC MUTATIONS, AND THEIR CROSSTALK IN ALZHEIMER'S DISEASE

Yaroslav Markov, Kyra Thrush, and Morgan Levine, *Yale University, New Haven, Connecticut, United States*

Aging is the major risk factor for Alzheimer's Disease (AD), and as life expectancy increases, neurodegeneration will continue to afflict an ever-increasing proportion of the population. While numerous theories are attempting to explain the drivers behind AD pathology, what unites them is the observation that AD is reliably associated with a progressive buildup of age-related molecular changes. Because of the varying clinical presentations of AD in patients with similar genetic backgrounds, it has been postulated that epigenetics may be implicated in its etiology. Building on our prior work showing that AD pathology is linked to alterations in age-related DNA CpG methylation (DNAme) across various brain regions, we use state-of-the-art machine learning approaches to identify patterns of molecular damage in postmortem brain samples. We show that alterations in DNAme are associated with accelerated biological aging, AD, and the APOE e4 genotype, which is a major risk factor for AD. We also demonstrate that these associations are present in the PFC but not cerebellum -- in line with the current understanding of AD progression in the brain. Finally, we perform whole-exome sequencing and protein mass spectrometry on the same brain samples to test our hypothesis as to whether AD-associated alterations of DNAme are linked with the accumulation of somatic mutations that affect the structural and binding properties of protein epigenetic regulators.

### ANGIOTENSIN RECEPTOR BLOCKERS UPREGULATE ANGIOTENSIN TYPE 4 RECEPTOR IN BRAINS OF COGNITIVELY INTACT INDIVIDUALS

Caglar Cosarderelioglu,[1] Claudene J. George,[2] Qian-Li Xue,[3] Esther Oh,[3] Luigi Ferrucci,[4] David Bennett,[5] Jeremy D. Walston,[3] and Peter M. Abadir,[3], *1. Ankara University School of Medicine, Cankaya, Ankara, Turkey, 2. Albert Einstein College of Medicine/Montefiore Medical Center, New York City, New York, United States, 3. Johns Hopkins University School of Medicine, Baltimore, Maryland, United States, 4. National Institute on Aging, Baltimore, Maryland, United States, 5. Rush University, Chicago, Illinois, United States*

The primary dementia-protective benefits of Angiotensin receptor type 1 (AT1R) blockers (ARBs) are believed to arise from systemic effects on blood pressure. However, there is a brain-specific renin-angiotensin system (b-RAS) that acts mainly through three receptor subtypes: AT1R, AT2R, and AT4R. AT1R promotes inflammation and oxidative stress (OS). AT2R increases nitric oxide. AT4R is essential for dopamine release and mediates memory consolidation. Here, we aimed to investigate the effects of ARBs on b-RAS, OS, inflammation, PHF-tau, and beta-amyloid load. Postmortem frontal-cortex brains of age- and sex-matched cognitively intact (CI) individuals using (n=30) and not using ARBs (n=30) and Alzheimer's disease (AD) patients using (n=30) and not using ARBs (n=30) were studied. Protein levels of receptors were measured by Western blot. Protein carbonyl (PC) and cytokine levels were measured by ELISA. Tangle and amyloid-β scores were used as outcomes. In CI individuals, our data shows that ARB treatment was associated with higher protein levels of AT4R (median(range) 0.69(1.92) vs 0.17(1.18) CI+ARBs vs CI, p=0.02), lower level of OS marker PC (10.60(8.32) vs 11.26(7.44), CI+ARBs vs CI, p=0.03) and lower hippocampal and overall amyloid scores (0(5.45) vs 1.15(4.21) p=0.03, 0.79(12.75) vs 3.41(13.36) p=0.04, CI+ARBs vs CI, respectively). In AD group, ARB treatment was associated with lower AT1R protein levels (0.47(1.15) vs 0.59(1.99), AD+ARBs vs AD, p=0.02). No significant changes were observed in OS, inflammation, or PHF-tau and amyloid load in AD brains treated with ARBs. Our results highlight the impact of ARBs on the brains of cognitively intact and AD older individuals.

### COMPARING CORTICAL DEMYELINATION IN GERIATRIC MILD TRAUMATIC BRAIN INJURY AND ALZHEIMER'S DISEASE

Shania Wang,[1] Nahian Chowdhury,[1] Sean Mahoney,[1] and Andrei Irimia,[2], *1. University of Southern California, Los Angeles, California, United States, 2. University of Southern California, University of Southern California, California, United States*

Mild traumatic brain injury (mTBI) accelerates the rate of age-associated brain atrophy, whose pattern resembles the cortical neurodegeneration pattern observed in Alzheimer's disease (AD). Because the ratio R of T1-to-T2-weighted magnetic resonance imaging (MRI) intensities is a surrogate measure of cortical myelin concentration, mapping and quantifying changes in this ratio can improve our understanding of demyelination after geriatric mTBI and AD. T1- and T2-weighted MRIs were acquired acutely and ~6 months post-injury from 68 healthy controls (HCs, age (years, y): $\mu = 76$ y, $\sigma = 4$ y), 19 mTBIs (age $\mu = 70$ y, $\sigma = 5$ y), and 33 ADs (age $\mu = 77$, $\sigma = 6$). Volumes were co-registered using 3D Slicer's BRAINSFit module, and T2-constrained segmentations of T1 volumes were obtained using FreeSurfer. R and its time changes were computed at each cortical location. When comparing mTBI and AD patients to HCs, significant differences in R were found across ~10% and ~23% of the cortex, respectively ($p < 0.05$). When comparing mTBI to AD, the former exhibited significantly less myelin content in the lateral, medial, and ventral temporal lobes ($p < 0.05$), on the medial aspects of superior parietal lobules and superior frontal gyri ($p < 0.05$), and in orbital gyri ($p < 0.05$), whereas AD subjects had less myelin content on lateral aspect of the parietal lobe ($p < 0.05$). These results highlight demyelination differences in mTBI and AD. Future studies should examine the long-term trajectories to quantify the risk of neurodegenerative disease after mTBI.

### DECOUPLING OF GLOBAL BRAIN ACTIVITY AND CEREBROSPINAL FLUID FLOW IS LINKED TO ALZHEIMER'S PATHOLOGY

Feng Han,[1] Jing Chen,[2] Aaron Belkin-Rosen,[2] Yameng Gu,[2] Liying Luo,[3] Orfeu Buxton,[4] and Xiao Liu,[2], *1. the Pennsylvania State University, State College, Pennsylvania, United States, 2. The Pennsylvania State University, State College, Pennsylvania, United States, 3. The Pennsylvania State University, University Park, Pennsylvania, United States, 4. The Pennsylvania State University, The Pennsylvania State University, Pennsylvania, United States*

Alzheimer's disease (AD) is the most common cause of dementia in the old adult population. AD pathogenesis has been linked to the aggregation of toxic proteins, e.g., amyloid-β and tau. The glymphatic system may play an important role in clearing out these proteins via cerebrospinal fluid (CSF) flows through perivascular and interstitial spaces. Recent studies have suggested low-frequency (<0.1 Hz), sleep-dependent global blood-oxygenation-dependent-level (gBOLD; global resting-state functional MRI signal) during resting state is coupled with CSF movements, suggesting their potential link to glymphatic function. Here, we directly investigated whether the coupling between the gBOLD and CSF signals is related to AD-related pathology. By analyzing neuroimaging, neurobiological, and neuropsychological data from 118 human subjects (58-90 years of age; AD, early-stage AD, and control subjects included) collected in the Alzheimer's Disease Neuroimaging Initiative project, we found a strong coupling between the gBOLD and CSF signals. More importantly, the strength of this gBOLD-CSF coupling was significantly correlated with cortical amyloid-β level ($p = 0.019$), cognitive decline in the subsequent two years ($p = 0.013$), disease severity ($p = 0.035$), and several AD-related risk factors, including aging ($p = 0.011$), and gender ($p = 0.026$). These findings provide initial evidence for the critical role of resting-state low-frequency (<0.1 Hz) neural/physiological dynamics in AD pathology. They also suggest that the gBOLD-CSF coupling may serve as a non-invasive imaging marker for gauging the glymphatic function.

### LOCAL ATROPHY OBSERVED IN SUBJECTIVE COGNITIVE DECLINE VARIES BASED ON QUESTIONNAIRE EMPLOYED IN ADNI

Cassandra Morrison,[1] Mahsa Dadar,[2] Neda shafiee,[2] and Louis Collins,[3], *1. McConnell Brain Imaging Centre, Montreal Neurological Institute, McGill University, Montréal, Quebec, Canada, 2. McGill University, Montreal, Quebec, Canada, 3. McConnell Brain Imaging Centre, Montreal Neurological Institute, McGill University, Montreal, Quebec, Canada*

**Background:** Subjective cognitive decline (SCD) may be associated with increased risk for Alzheimer's disease. However, neither research nor clinical practices have implemented a universal approach to operationalize SCD. This study was designed to determine whether four different methods of defining SCD influence atrophy differences observed between SCD and normal controls (NC).

**Methods:** We included MRI scans from 273 participants (NC and SCD) from the Alzheimer's Disease Neuroimaging Initiative. We used four methods to operationalize SCD: Cognitive Change Index (CCI), Everyday Cognition Scale (ECog), Worry, and ECog+Worry. Deformation-based morphometry was performed to examine volumetric change at the lateral ventricles, amygdala, and superior temporal regions (CerebrA atlas; Manera et al., 2020)). A previously validated MRI analysis method (SNIPE) was used for volume and grading of the hippocampus and entorhinal cortex (Coupe et al., 2012). A logistic regression was completed to examine the association between diagnosis and atrophy in SCD and NC.

**Results:** Left hippocampal grading was lower in SCD than NC with the CCI (p=.041) and Worry (p=.021). When using ECog+Worry, smaller left entorhinal volume was observed in SCD than NC (p=.025). Both the right (p=.008) and left (p=.003) superior temporal regions were smaller in SCD than NC, with only the ECog.

**Conclusion:** Although SCD questionnaires are designed to measure the same construct, the results here suggest otherwise. These results suggest that the SCD questionnaire employed will influence whether atrophy is observed in SCD relative to NC. Future research is warranted to better understand how different methodologies result in inconsistent findings.

### OBESE BODY MASS INDEX IN LATE-LIFE AS A PROTECTIVE FACTOR FOR MILD COGNITIVE IMPAIRMENT, DEMENTIA AND DEATH

Ginny Natale,[1] Yun Zhang,[2] Douglas Hanes,[1] and Sean Clouston,[3], *1. Stony Brook University, Stony Brook, New York, United States, 2. Renaissance School of Medicine, Stony Brook University, Stony Brook, New York, United States, 3. Renaissance School of Medicine, Stony Brook University, Renaissance School of Medicine, Stony Brook University, New York, United States*

**Objective:** The goal of the current study was to estimate the hazards of conversion from unimpaired to mild cognitive impairment (MCI) to probable dementia and death for underweight, normal, overweight and obese older adults in an aging cohort where the timing of examinations may be associated with the severity of dementia.

**Methods:** We analyzed six waves of the National Health and Aging Trends Study (NHATS); a longitudinal aging cohort. Participants were classified into mutually exclusive cognitive statuses: cognitively unimpaired, MCI, probable dementia and death. Time-to-event ratios and cognitive transitions were examined with multistate survival models accounting for misclassification. BMI was computed from height and weight measurements and expressed in kg/m2 and categorized into underweight, normal, overweight and obese.

**Results:** Participants (n=6,078) were 77 years old, on average, and the majority were white, females and high school graduates. About one third (32.68%) of the sample has normal BMI, one third is overweight (35.59%), the rests are obese (26.41%) or underweight (5.33%). After adjusting for the effects of diabetes, CVD, vigorous physical activity, age and race/ethnicity, the protective effect of obesity in late-life against developing dementia (HR=0.44; 95%CI[0.29-0.67]) and dying from dementia (HR=0.63; 95%CI[0.42-0.95).

**Discussion:** Prior research shows the risk of dementia associated with obesity at older ages is either attenuated or reversed. Our findings support a protective factor of obesity in late-life against conversion to dementia and death.

### PDE4D AND HCN1 ULTRASTRUCTURE IN RHESUS MACAQUE ENTORHINAL CORTEX: RELEVANCE FOR AGING AND ALZHEIMER'S DISEASE

Dibyadeep Datta,[1] SueAnn Mentone,[2] and Amy Arnsten,[3], *1. Yale School of Medicine, New Haven, Connecticut, United States, 2. Yale University, New Haven, Connecticut, United States, 3. Yale University, Yale University, Connecticut, United States*

Tau pathology emerges in a distinct spatial and temporal pattern in Alzheimer's Disease (AD). Anatomical studies in AD subjects and rhesus macaques show earliest signs of tau pathology in the stellate cell islands in entorhinal cortex (ERC) layer II. However, the molecular mechanisms that confer vulnerability to ERC layer II cells early in AD is unknown. cAMP-PKA magnification of calcium release has been seen in prefrontal cortex, associated with HCN channel opening to dynamically regulate synaptic strength. This process is regulated by phosphodiesterases (PDE), regulation that is lost with age. The current study examined whether this "signature of flexibility" could also be seen in layer II ERC, underlying vulnerability to tau pathology with aging. We used high-spatial resolution immunoEM to localize PDE4D and HCN1 in young rhesus macaque (7-10y) ERC layer II. Our results suggest that PDE4D was concentrated on the SER-spine apparatus and in postsynaptic density, and HCN1 expressed in the membrane near excitatory synapses in dendritic spines. Within dendritic shafts, PDE4D labeling was observed along microtubules and near mitochondria, whereas HCN1 was organized in discrete clusters along the plasma membrane. These data suggest that PDE4D is optimally positioned to modulate cAMP microdomains and control calcium extrusion from the SER. HCN1 channels are localized in subcompartments to facilitate dynamic physiological representation of sensory experience and visual space governed by cAMP-PKA signaling. The anatomical patterns in ERC layer II corroborate our findings in vulnerable glutamatergic circuits in prefrontal cortex, suggesting conserved molecular features in association cortices most susceptible in AD.

### PHENOTHIAZINES TO TREAT ALZHEIMER'S DISEASE

Rachel Litke,[1] Bik Tzu Huang,[2] Damian Gonzalez,[2] Martine Rampanana,[2] Nicholas Grimaldi,[2] and Charles Mobbs,[3], *1. icahn school of medicine at mount sinai, New York, New York, United States, 2. Icahn School of Medicine at Mount Sinai, New York, New York, United States, 3. Icahn School of Medicine at Mount Sinai, new york, New York, United States*

Current treatments of Alzheimer's Disease (AD) are largely ineffective and do not address underlying pathophysiological processes. The model organism C. elegans has been successfully used to discover compounds to treat human diseases, some now in clinical trials. To develop novel drugs and explore pathways to treat AD, we took on a forward pharmacological approach with a C. elegans model for AD, completed with studies to expand results to lifespan as well as healthspan. We screened 2560 drugs from the Microsource Spectrum library for their ability to delay proteotoxicity (indicated by paralysis) in an Abeta transgenic C. elegans muscle model of AD (CL2006) in liquid medium. Among the most protective drugs were phenothiazines, which are orally active and cross the blood-brain barrier, desirable properties of drugs to treat AD. 80 phenothiazines congeners were further assessed; 60% were protective in CL2006 worms. 9/20 tested phenothiazines increased lifespan in N2 worms and 2/3 phenothiazines tested promoted significantly higher pharyngeal pumping rates compared with control till day 10 of adulthood in N2 worms. 2 of the drugs were protective in the C. elegans neuronal model of AD. This phenotypic screening approach led to the discovery of potential drugs to treat AD. These phenothiazines protect against Abeta toxicity, and assessment of efficacy to protect against other forms of proteotoxicity are ongoing. These studies suggest the utility of C. elegans to discover drugs to treat human diseases. Future studies will assess molecular mechanisms mediating the protective effects of these compounds.

### QUANTIFICATION OF SIMILAR NEURODEGENERATION ACROSS GERIATRIC CONCUSSIONS AND ALZHEIMER'S DISEASE

Andrei Irimia, and Kenneth Rostowsky, *University of Southern California, University of Southern California, California, United States*

Geriatric mild traumatic brain injury (mTBI) is a risk factor for Alzheimer's disease (AD), but few studies have studied how the neuroanatomic effects of these conditions can converge onto similar brain structure trajectories. Here we use magnetic resonance imaging to investigate similarities between mTBI and AD across both white and gray matter (WM and GM, respectively) using measures like fractional anisotropy (FA, a surrogate measure of WM integrity) and cortical thickness. We identify statistically significant similarities in neurodegeneration across mTBI ($N = 33$; age $\mu = 63$ years (y), $\sigma = 11$ y) and AD ($N = 66$; age $\mu = 76$ y, $\sigma = 9$ y) by testing for statistical equivalences of mean FA and cortical thickness. Both WM and GM are found to exhibit significant similarities in how mean FA and cortical thickness decrease, respectively, across mTBI and AD. For WM, the broadest spatial extent of statistical similarity between conditions, quantified as percentages of structures' volumes, is found within the superior fronto-occipital fasciculus (left (L): 91%, $p < 0.05$; right (R): 95%, $p < 0.05$), and in the crura of the fornix (L: 65%, $p < 0.05$; R: 80%, $p < 0.05$). Across mTBI and AD, cortical thinning trajectories are most similar in the superior precentral sulcus (L: 91%, $p < 0.05$; R: 100%, $p < 0.05$), and anterior lateral sulcus (L: 75%, $p < 0.05$; R: 86%, $p < 0.05$). Future studies should leverage such findings to identify AD risk factors in mTBI patients.

### REMYELINATION IMPROVES VOIDING DYSFUNCTION IN A MOUSE MODEL OF MULTIPLE SCLEROSIS

Ramalakshmi Ramasamy, Cara Hardy, Stephen Crocker, and Phillip Smith, *UConn Health, Farmington, Connecticut, United States*

Multiple sclerosis (MS) is a chronic inflammatory demyelinating disease of the central nervous system (CNS). Of note, over 80% of MS patients have urinary symptoms as one of their earliest symptoms. Since MS patients often live into older age, urinary incontinence and retention are significant problems affecting their quality of life. Although several studies show that inflammatory-demyelinating animal models of MS develop bladder dysfunction, the confounding influence of systemic inflammation in these models limits potential interpretation on the contribution of CNS-myelination to bladder dysfunction. We sought to address this knowledge gap using the cuprizone model of demyelination and remyelination. C57Bl/6 mice were treated with dietary cuprizone (0.2%w/w) for four weeks to induce demyelination. One group was allowed four additional weeks for recovery and remyelination. We performed voiding spot assay (VSA), urethane-anesthetized cystometry, and CNS-histology to assess demyelination-induced differences in urinary performance. We observed that cortical demyelination causes significant aberrance in voiding behavior (conscious cortical control) characterized by increased micturition frequency and reduced volume per micturition. Interestingly, remyelination restored healthy bladder function. However, there were no significant changes in the cystometric parameters (brainstem reflex) between the treatment groups. While MS is not classically considered a disease of aging, extending the longevity of these patients has not been reciprocated with improved treatments for their most-bothersome conditions, notably urinary symptoms that persist throughout life. Our data represent a novel compelling connection and strong correlation between CNS-myelination and cortical control of bladder function, which has potential implications in MS, aging, and aging-associated neurological disorders.

### SENOLYTICS IN A MODEL OF ALZHEIMER'S DISEASE

Ellen Wang, and Suckwon Lee, *Buck Institute for Research on Aging, Novato, California, United States*

The therapeutic effects of senescent cell killing with senolytics in neurodegeneration mouse models poise this strategy as an intervention candidate for Alzheimer's Disease (AD). However, it is unclear whether senolytic therapies for AD are translatable to human cells. To determine whether senolytics could be a viable therapeutic for AD, we have treated long-term mixed human neuron/astrocyte primary cultures with amyloid beta oligomers (ABO), which we have shown to induce a phenotype consistent with senescence in neurons. Fifteen days after ABO treatment, we administered Navitoclax (Nav) and the natural killer cell-line NK92, which are known to selectively kill senescent cells in the periphery. Following treatment, we assessed senescence markers in our cultures as well as senescent cell killing selectivity through cleaved Caspase 3 quantification. Our preliminary data show that Nav (8, 4, and 0.5uM) kills both control and ABO treated cells. NK92 cells (10 to 1 effector to target ratio) also kill some control cells, suggesting there is not a clear cut mechanism by which NK92 cells can distinguish senescent from non-senescent neurons or astrocytes. Although analysis of selective killing is ongoing, off-target killing indicates that we need more refined senolytic strategies to implement their safe human use.

### SHORT-TERM DIESEL EXHAUST EXPOSURE RESULTS IN NEUROINFLAMMATION AND WHITE MATTER INJURY

Krista Lamorie-Foote,[1] Kristina Shkirkova,[2] Qinghai Liu,[2] Constantinos Sioutas,[2] Todd Morgan,[2] Caleb Finch,[2] and William Mack,[3], *1. University of Southern California - Keck School of, Los Angeles, California, United States, 2. University of Southern California, Los Angeles, California, United States, 3. Keck School of Medicine of the University of Southern California, Los Angeles, California, United States*

Ambient air pollution (AAP) exposure is associated with white matter injury and cognitive decline in older adults(Chen et al. 2020,Erickson et al. 2020). Neuroinflammation and oxidative stress may contribute to this white matter injury. Diesel exhaust particulate matter (DEP) is a neurotoxic component of AAP.This study characterizes the time course by which neuroinflammation/oxidative stress occurs and results in white matter injury following DE exposure in a murine model. DEP (Sigma) was re-aerosolized for exposure. Mice were exposed to 100 µg/m3 DEP or filtered air (FA) for 5 hours (n=8/group), 100 hours (n=6/group), or 200 hours (n=6/group). Immunohistochemical analysis of degraded myelin basic protein (dMBP), a marker of myelin damage, was performed. Neuroinflammation and oxidative stress were assessed by histological analysis of complement C5a, an anaphylatoxin, and 4-Hydroxynonenal (4-HNE), a marker of lipid peroxidation.dMBP integrated density was increased in the corpus callosum of DEP mice at 5 ($p<0.01$), 100 ($p<0.01$), and 200 hours ($p<0.001$) compared to FA mice.C5a integrated density was increased in the corpus callosum of DEP mice at 5 ($p<0.01$), 100 ($p<0.01$), and 200 hours ($p<0.01$) compared to FA mice. 4-HNE integrated density was increased in the corpus callosum of DEP mice at 5 ($p<0.001$), 100 ($p=0.001$), and 200 hours ($p<0.001$) compared to FA mice. Neuroinflammation and oxidative stress are upregulated with associated white matter injury in the corpus callosum after 5 hours of DEP exposure.Short-term DEP exposure activates inflammatory/oxidative stress pathways, which may contribute to the pathogenesis of white matter injury.Erickson et al. 2020,PMID:32182984; Chen et al. 2020,PMID:32669395.

### SIGNIFICANT ASSOCIATIONS OF THE INTERPLAY BETWEEN STRESS RELATED GENES WITH ALZHEIMER'S DISEASE

Anatoliy Yashin,[1] Deqing Wu,[1] Konstantin Arbeev,[1] Olivia Bagley,[1] Igor Akushevich,[1] Arseniy Yashkin,[2] Matt Duan,[1] and Svetlana Ukraintseva,[1], *1. Duke University, Durham, North Carolina, United States, 2. Duke University, Morrisville, North Carolina, United States*

The lack of efficient medication against Alzheimer's disease (AD) is the most important problem for this health disorder today. One possible reason for this -- the implementing medical interventions "too late in the disease stage" – has been recently addressed in the initiative that defined the preclinical AD stage by measuring changes in preclinical AD biomarkers. According to this definition, beta amyloid (Aβ) is one of the key preclinical AD biomarkers. Experimental studies showed that Aβ results from proteolytic cleavage of APP by β- and γ-secretases. Production of β-secretase involves BACE1 gene, activated by cellular stress response. This suggest that AD might be initiated by cellular stressors and that multifactorial regulation of AD is likely to be driven by genes involved in cellular stress response. In this paper we investigate whether interplay between SNPs from the EIF2AK4 gene involved in sensing cellular stress signals and the APP gene dealing with Aβ production may be associated with AD in human data. For this, we evaluated association of the interactions of the pairs of SNPs from these genes with AD in the analysis of HRS data. We found that interactions between several SNPs have statistically significant associations with AD. The results of this analysis confirm that the interplay between gene served as a sensor of cellular stress and gene involved in production of preclinical AD biomarker in response to stress may influence human AD. This analysis illustrates an important step towards translation of the results of experimental AD studies to human applications.

### SYSTEMIC BIOENERGETIC CAPACITY CHANGES WITH COGNITIVE STATUS AND INSULIN SENSITIVITY IN OLDER ADULTS

Gargi Mahapatra,[1] Zhengrong Gao,[1] James Bateman,[2] Jenny L. Gonzalez-Armenta,[2] Ramon Casanova,[3] Suzanne Craft,[4] Anthony J. A. Molina,[5] and Allison Amick,[6], *1. Sticht Center for Healthy Aging and Alzheimer's Prevention, Winston-Salem, North Carolina, United States, 2. Wake Forest School of Medicine, Winston-Salem, North Carolina, United States, 3. Wake Forest University, Winston-Salem, North Carolina, United States, 4. Wake Forest School of Medicine, Winston Salem, North Carolina, United States, 5. University of California, San Diego, La Jolla, California, United States, 6. Wake Forest School of Medicine, Wake Forest School of Medicine, North Carolina, United States*

Systemic mitochondrial dysfunction is reported with AD progression, suggesting that peripheral blood cells may be used to investigate systemic mitochondrial alterations related to cognitive decline. We aimed to identify bioenergetic signatures associated with AD-related dementia and differences in insulin sensitivity associated with AD risk. We analyzed mitochondrial bioenergetics in peripheral blood cells collected from 365 older adults with varying cognitive status (normal, mild cognitive impairment, and AD) and insulin sensitivity. Normoglycemic individuals exhibited lower maximal bioenergetic capacity with AD (PBMCs: 239.6 pmol·min−1, p = 0.02; Platelets: 151.7 pmol·min−1, p = 0.06) compared to normal cognition (PBMCs: 271.5 pmol·min−1; Platelets: 171.7 pmol·min−1). Individuals with impaired insulin sensitivity exhibited lower maximal bioenergetic capacity in platelets with AD (171.6 pmol·min−1, p = 0.008) compared to normal cognition (210.6 pmol. min−1). Participants with impaired insulin sensitivity also exhibited unique bioenergetic profiles exemplified by overall higher levels of mitochondrial respiration, indicating that comorbidities such as diabetes can significantly influence bioenergetic capacity. We observed strong positive associations between maximal respiration in normoglycemic individuals with cognitive function, as measured by Modified Preclinical Alzheimer's Cognitive Composite (mPACC5) (p = 0.06), and fatty acid oxidation in individuals with impaired insulin sensitivity with cortical thickness (p = 0.05). This study demonstrates that circulating cells may provide a cost-effective and minimally invasive way to monitor systemic bioenergetic changes associated with AD risk, progression, and insulin sensitivity. These findings also suggest that blood-based bioenergetics are related to key features of AD development and progression and should be further developed as a potential biomarker.

### THE HUMAN GENES THAT LINK MIDDLE-AGE COMORBIDITIES AND ALZHEIMER'S DISEASE

Shin Murakami, *Touro University California, Vallejo, California, United States*

Advancements in biomedical research have identified the genes influencing life spans, stress resistance and age-related diseases, including Alzheimer's disease. Stress resistance includes resistance to multiple forms of stress, pathogens and toxic beta-amyloid which is tightly associated with Alzheimer's disease. We have investigated 431 human genes that are associated with co-morbidities (Vahdati Nia et al, 2017; Le et al., 2020). Those genes are involved in lipid metabolism, hemostasis, hemostasis, neuroendocrine and immune functions. The genes are relevant to middle-life health. We explore a wide variety of co-morbidities that could happen in middle to late life. I will give a brief review of increased stress resistance, and genetic markers associated with co-morbidities. I will discuss how the studies may benefit to fight against COVID-19. References: 1. Vahdati Nia B, Kang C, Tran MG, Lee D, Murakami, S. (2017) Front. Genet. 8:55. doi: 10.3389/fgene.2017.00055 2. Le D, Crouch N, Villanueva A, Phong Ta, Dmitriyev R, Tunzi M, and Murakami S. (2020) Journal of Neurology and Experimental Neuroscience. 6;S1.

### USING DROSOPHILA TO IDENTIFY NATURALLY-OCCURRING MODIFIERS OF ALZHEIMER'S DISEASE

Adrienne Wang,[1] Ming Yang,[2] Cecilia Fitzgerald-Cook,[2] Ben Harrison,[2] Akimi Green,[1] Kensington Hartman,[1] Matthew Zinkgraf,[1] and Daniel Promislow,[2], *1. Western Washington University, Bellingham, Washington, United States, 2. University of Washington, Seattle, Washington, United States*

Despite significant progress in identifying risk factors for late-onset Alzheimer's Disease (LOAD), much of the variance in disease pathogenesis remains unexplained, likely due to the contribution of many genes of small effect size. Model organisms such as Drosophila Melanogaster exhibit conservation in both disease-causing genes and cellular processes implicated in Alzheimer's Disease (AD), offering a genetically tractable model that can be statistically leveraged to identify causal variants. Here, we combine a Drosophila model of AD with the Drosophila Genetic Reference Panel (DGRP), a model of natural variation consisting of over 200 fully sequenced, isogenic lines derived from a wild-caught population. Expression of two proteins closely associated with AD pathogenesis, A□42 and Tau, in the Drosophila eye results in a "rough eye" phenotype, an easily quantifiable phenotype caused by degeneration of the ommatidial array. By quantifying the degree of A□42- and Tau-mediated degeneration across 164 lines of the DGRP and using a gene-based approach to map associations, we have identified and validated a subset of naturally occurring modifiers of degeneration in Drosophila. Enrichment analysis reveals that the set of genes identified in our screen show significant enrichment for genes identified as significant or suggestive (4x10-6>p>2x10-11) in human GWAS studies. The results presented here provide proof-of-principal for an approach that combines the strengths of forward genetic screens in model organisms with the power of human GWAS studies to identify and validate potential risk factors that have been difficult to detect in human studies alone.

### Session 9060 (Poster)

### ALZHEIMER'S DISEASE AND OTHER DEMENTIAS II

### COLLECTION OF DATA ON PERSONS LIVING WITH DEMENTIA WHO GO MISSING: FIRST RESPONDER PERSPECTIVES

Noelannah Neubauer,[1] Serrina Philip,[1] Samantha Dawn Marshall,[2] Christine Daum,[3] Hector Perez,[1] Antonio Miguel-Cruz,[4] and Lili Liu,[1], *1. University of Waterloo, Waterloo, Ontario, Canada, 2. University of Waterloo, University of Waterloo, Ontario, Canada, 3. University of Waterloo, Edmonton, Alberta, Canada, 4. University of Alberta, Edmonton, Alberta, Canada*

While it is commonly cited that 60% of persons living with dementia (PLWD) wander, it is unclear whether this number reflects global contexts. Population aging has created a pressing need for the development of programs to mitigate the risks of PLWD from getting lost and going missing. Such programs would require a national strategy for the collection and integration of data on missing incidents involving this population. This study is a first step to inform such a strategy. The purposes were to: 1) identify approaches to data collection on missing persons incidents involving PLWD among Canadian police and search and rescue (SAR) organizations; 2) describe the foreseeable challenges associated with developing a national data collection strategy. We used generic qualitative description to generate data with fifteen key informants. Virtual semi-structured interviews were completed and transcribed verbatim. Content analysis and trustworthiness strategies guided analysis and rigor. Our findings indicate that police and SAR organizations collect a multitude of data pertaining to missing incidents involving PLWD. However, there is a lack of standardization in data collection, entry and analysis. Privacy legislation, limited resources, and incompatible data management systems pose challenges to data sharing and interoperability. Underreporting of missing incidents to police results in an underestimation of missing incidents. An intersectoral, uniform approach to data collection would enable the storage, analysis and comparison of national data. Accurate data on critical wandering can inform prevention, search strategies, resource allocation and effectiveness of programs.

### DEMENTIA CARE MANAGEMENT: SOCIAL CAPITAL FOR PERSONS WITH DEMENTIA THAT LIVE ALONE

Adria Navarro,[1] Vickie Avila,[1] and Lynne Conger,[2], *1. Azusa Pacific University, Azusa, California, United States, 2. Alzheimer's Orange County, Irvine, California, United States*

Social connections are critical for healthy aging (Ahn et al., 2020). Consistent findings in research for persons with dementia that live alone show that a natural support network is the gold-standard for success, as social capital enhances well-being. Therefore a community-based intervention was piloted as part of a national 'special populations grant' in partnership with the local Meals-on-Wheels program. Dementia care management provided by graduate social work interns sought to provide weekly supportive contacts. Quantitative data was collected at baseline and following the program through administration of standardized measurement tools for “thriving” and for “dementia quality of life.” The sample to date (N=33) consists of a majority being white females (82.4%), with additional participants expected as the remaining enrolled complete the program. These early “completers” do not show significant changes in their specific “surviving to thriving” domains, yet they do report greater perceptions of quality in their life as a whole following the dementia care management intervention when comparing post data to their baseline (Chi Square 18.95, p=0.004). To date this intervention continues to be studied and suggests a positive impact is likely for older adults with dementia that live alone, as well as for the communities where they reside.

### DEVELOPING INFORMATION TECHNOLOGIES TO PROMOTE DEMENTIA E-FRIENDLY COMMUNITIES FOR COVID-19 AND BEYOND

Nicole Ruggiano,[1] Yan Luo,[2] Amy Hurd,[3] Kristen Lawlor,[3] Monica Anderson,[3] Zhe Jiang,[3] and Jeff Gray,[3], *1. University of Alabama, Hoover, Alabama, United States, 2. The University of Alabama, Tuscaloosa, Alabama, United States, 3. University of Alabama, Tuscaloosa, Alabama, United States*

People living with dementia (PLWD) and their caregivers often face barriers to education, support, and services that can improve their health and quality of life. Information technology (IT) has been suggested as a solution to overcoming such barriers, though the development of evidence-based IT for dementia care is still developing. This project gathered stakeholder (e.g., providers, caregivers) perspectives on the development of a proposed IT solution to support community asset mapping that would allow families to self-assess their dementia-related service needs, educate them about available services, and link them with services they need in their community. This proposed IT would create a dementia resource database that relies on crowdsourced data from community stakeholders as well as relevant data mined from existing sources (e.g., CMS certified nursing home data). As part of the planning process, this project conducted qualitative interviews with providers and caregivers in four metro areas in Alabama and their surrounding rural communities to learn more about the content and features that stakeholders perceive as being most effective for the proposed technology. Stakeholders also discussed their experience of utilizing IT solutions during the COVID-19 pandemic to promote access and continuum of care when barriers to service intensified. Thematic findings provide detail on: 1) motivating factors among stakeholders to contribute crowdsourced data that support community members affected by dementia; 2) potential barriers to implementing IT for dementia support, based on experiences with IT use during COVID-19; and 3) how stakeholders envision IT to better connect community members with needed services.

### EFFECT OF DANCE MOVEMENT THERAPY ON DEPRESSION AND ANXIETY FOR PERSONS WITH DEMENTIA: A SYSTEMATIC REVIEW

Ying Wang,[1] Mandong Liu,[2] Jing wu,[3] Zhixiao Dong,[4] Huan Cui,[4] Dianjun Shen,[3] and Youyou Tan,[4], *1. Lanzhou University, lanzhou, Gansu, China (People's Republic), 2. University of Southern California, Hangzhou, China (People's Republic), 3. Lanzhou University, Lanzhou Nniversity, Gansu, China (People's Republic), 4. Lanzhou University, Lanzhou, Gansu, China (People's Republic)*

Dance movement therapy ( DMT ) is a physical and psychological intervention, but there was no meta-analysis of RCTs in the systematic review of DMT for dementia, and the results of RCTs were inconsistent. The study aimed to assess the effectiveness of dance movement therapy on depression and anxiety of persons with dementia in comparison to no treatment, standard care. Six electronic databases (Cochrane, PsycINFO, Web of Science, PubMed, EBSCO, CNKI) were searched through January 2021. Randomized controlled trials (RCTs) in Chinese and English language were considered, including: (1) population is women and men of all age with dementia and MCI; (2) intervention is dance movement intervention; (3) comparison is no treatment or standard care; (4) outcome is depression or anxiety. The four review authors independently reviewed studies on an abstract/title level and again after reading the full paper, and we independently evaluated methodological quality. Six randomized controlled trials were identified on depression and anxiety symptoms in persons with dementia. The target sample size was 29-842 older adults. Meta-analysis showed there were significant differences in favor of dance in depression (SMD = 1.17, 95% CI: 0.39 to 1.95, P = 0.003), but not in outcomes of anxiety. Trials of high methodological quality, large sample sizes, and clarity in the way the intervention is put together and delivered are needed to assess whether dance movement therapy is an effective intervention on depression and anxiety for persons with dementia.

### INVOLVING PERSONS WITH DEMENTIA IN SOCIAL RESEARCH: A LITERATURE REVIEW

Pamela Manley,[1] and Candace Kemp,[2], *1. Georgia State University, BUFORD, Georgia, United States, 2. Gerontology Institute, Georgia State University, Atlanta, Georgia, United States*

Studies involving persons with dementia as research participants have increased over the years, due in part to an emphasis on patient and public involvement in health care and participatory action research. Recent studies indicate a growing trend toward engaging persons with dementia, not only as participants, but also as co-researchers. Further, studies involving persons with dementia as co-researchers and advisers have garnered increased attention due to the inclusion of this population's unique perspectives and lived experiences. Theoretically, frameworks such as person-centered- and relationship-centered care, also influence and shape the research process. This literature review examines empirical research conducted over the past decade that reports the involvement of persons with dementia as research participants ("research on") and co-researchers and advisers ("research with"). Among the 27 articles identified, 12 reported "research on," and 15 reported "research with" persons with dementia. "Research on" targeted participants' emotional expressions/responses, engagement in exercise/activities; social environment influences, and cognitive training; whereas "research with," which was mostly qualitative, focused on co-researchers' perspectives of and experiences with the research process; needs, priorities, and recommendations in research planning; partnering with persons with dementia on the design and development of research instruments, and the importance of critically evaluating the research process. Findings documenting research challenges, complexities, and ethical concerns are also discussed. Overall, findings demonstrate the feasibility of involving persons with dementia in a meaningful way and further affirms that including them as co-researchers is not only beneficial, but has the potential to enhance the entire research process.

### ONLINE HEALTH INFORMATION WANTS OF CAREGIVERS FOR PERSONS WITH DEMENTIA IN SOCIAL MEDIA

Alison Tang,[1] Jung Kwak,[1] Bo Xie,[1] Lu Xiao,[2] Sucheta Lahiri,[3] Olivia Flynn,[3] and Avinav Murugadass,[4], *1. The University of Texas at Austin, Austin, Texas, United States, 2. Syracuse University, Syracuse University, New York, United States, 3. Syracuse University, Syracuse, New York, United States, 4. Syracuse University, Syrcause, New York, United States*

Family caregivers of persons with dementia (PWDs) use social media to obtain and share health information. Yet relatively little is known about the types of information that these caregivers share. We address this gap by analyzing caregivers' posts (N = 401) in two subreddits, r/Alzheimers and r/dementia, which represent two subgroups on the social media platform Reddit. Our research questions were as follows: (1) What are these caregivers' main purposes in posting? (2) What types of health information do the caregivers exchange online? (3) What are the characteristics of online posts that receive the caregivers' greatest attention? Content and correlational analyses revealed that over half of users (57%) posted regarding specific types of health information that they desired to seek and share, whereas the remaining users posted information for sharing only. The health information most commonly posted fell into the following three categories: psychosocial health information, information about patients' daily care, and characteristics of health conditions. The more health information types that a post contained, the greater the number of people who participated in the subreddit discussions. Further research should examine how social media meet PWD caregivers' needs for information support.

### RISK FACTORS OF GOING MISSING IN PEOPLE LIVING WITH DEMENTIA: A SCOPING REVIEW

Noelannah Neubauer,[1] Hector Perez,[2] Antonio Miguel-Cruz,[1] Christine Daum,[3] Samantha Dawn Marshall,[4] Elyse Letts,[2] and Lili Liu,[2] *1. University of Alberta, Edmonton, Alberta, Canada, 2. University of Waterloo, Waterloo, Ontario, Canada, 3. University of Waterloo, Edmonton, Alberta, Canada, 4. University of Waterloo, University of Waterloo, Ontario, Canada*

Critical wandering is common in persons living with dementia, it is defined as wandering that results in an individual going missing. This exposes the missing vulnerable older adult to risks and dangers. Persons with dementia who become lost and go missing and get lost can face adverse outcomes, such as injury and death, yet the amount of information available on the risk factors associated with these incidents is scarce. The aim of this study was to identify the risk factors associated with critical wandering in persons living with dementia. We used Tricco et al.'s (2018) approach for scoping reviews and searched the following databases: Medline, EMBASE, CINAHL, and Scopus. We included studies that referred to critical wandering in persons with dementia, cognitive impairment, or Alzheimer, and published since 1980. We identified 3,376 publications, which was reduced to 1641 publications after we removed duplications. A total of 78 studies met the inclusion and exclusion criteria for analysis and extraction. A rigorous process to synthesize and categorize the research evidence was followed. We identified four different types of risk factors associated with going missing: (1) personal, (2) physical environment and geographical location, (3) cultural environment, and (4) social environment and support resources. Recognition of these risk factors can help persons living with dementia and their care partners identify interventions and proactive strategies to mitigate or prevent critical wandering. This will support persons with dementia, their care partners, and community organizations to balance safety, autonomy, and independence to maximize quality of life.

### STRATEGIES FOR IMPLEMENTING MUSIC-BASED INTERVENTIONS FOR PEOPLE WITH DEMENTIA: A SYSTEMATIC REVIEW

Takashi Amano,[1] Joe Strong,[2] Cole Hooley,[3] and Megumi Inoue,[4], *1. Rutgers University-Newark, Newark, New Jersey, United States, 2. University of Tennessee - Knoxville, Knoxville, Tennessee, United States, 3. Brigham Young University, Provo, Utah, United States, 4. George Mason University, Fairfax, Virginia, United States*

Previous research has found that music-based interventions are effective at improving several outcomes related to dementia for nursing home residents. Some studies have investigated the implementation of music-based interventions. However, to date, there is no synthesis available regarding strategies and outcomes of these implementation studies. This study aims to synthesize available evidence regarding implementation strategies and outcomes of music-based interventions for nursing home residents with dementia. We conducted a systematic literature search in PsychInfo, PubMed, MEDLINE, CINAHL, and The Cochrane Library. A total of 8 studies were included for the qualitative synthesis. Of the eight studies, half were studies of music therapy and the other half were on individualized music. We identified 49 implementation strategies. The most frequently reported category of strategies was planning (34.7%), followed by education (24.5%), quality management (24.5%), restructuring (12.2%), and finance (4.1%). No strategies under the category of attending to the policy context were reported. We identified 21 implementation outcomes. The most frequently reported category was adoption (33.3%), followed by fidelity (28.6%), appropriateness (19.0%), sustainability (9.5%), acceptability (4.8%), and cost (4.8%). No studies measured feasibility or penetration. Although various implementation strategies have been utilized, most of them are under the categories related to intervention methods. Less attention has been paid to strategies that aim at structural changes of intervention delivery systems. Future studies should investigate facilitators and barriers of implementing music-based interventions especially focusing on organizational and structural aspects.

### UNDERDIAGNOSIS OF DEMENTIA AMONG INDIGENOUS OLDER ADULTS WITH COGNITIVE IMPAIRMENT

Takashi Amano,[1] Carlos Andres Gallegos,[2] William Waters,[3] and Wilma Freire,[4], *1. Rutgers University-Newark, Newark, New Jersey, United States, 2. University of Vermont, Burlington, Vermont, United States, 3. Universidad San Francisco de Quito, Quito, Pichincha, Ecuador, 4. Universidad San Francisco de Quito, Universidad San Francisco de Quito, Pichincha, Ecuador*

Early and timely diagnosis of dementia has been recognized as key to improving health outcomes. However, underdiagnosis among the indigenous population has not been adequately investigated. This study examines the association between indigenous ethnic identity and receiving a diagnosis of dementia and identifies factors associated with receiving a diagnosis of dementia. Data were drawn from Ecuador's Survey of Health, Welfare and Aging (SABE) – 2009, based on a probability sample of households in Ecuador with at least one person 60 years or older. This is a robust data set that allows for understanding ethnic dimensions and disparities, especially because of the successful recruitment of indigenous households. The final sample consisted of 1,437 people who had cognitive impairment. Whether participants had ever been told that he/she had dementia was compared between the groups of people who identified themselves either as indigenous or others. Binary logistic regression analysis was utilized. Indigenous participants had a lower probability than others of receiving a diagnosis of dementia after controlling for health and function related covariates. This association became statistically insignificant after including socioeconomic factors in the model. The final model revealed that people who had higher educational attainment were more likely to receive a diagnosis of dementia. Findings suggest that underdiagnosis among indigenous older adults can be explained by their lower socioeconomic status rather than by health and functional status. This finding implies the possibility of developing strategies to promote timely diagnosis by targeting populations who are especially susceptible to underdiagnosis of dementia.

### Session 9065 (Poster)

### ALZHEIMER'S DISEASE I (HS POSTER)

### ALZHEIMER'S DISEASE RISK REDUCTION: AN EVIDENCE-BASED, MULTI-COMPONENT LIFESTYLE PROGRAM, PRE- & INTRA-COVID-19

Gina Touch, and Paul Bendheim, *University of Arizona College of Medicine Phoenix, Phoenix, Arizona, United States*

Alzheimer's disease (AD) is the 5th leading cause of death in the USA. With nearly 300 failed therapeutic trials to date, lifestyle modifications have been shown to reduce AD risk by as much as 50%. Preceding the FINGER Study by nearly a decade, the BrainSavers Brain+Body Total Fitness program was developed by an interdisciplinary team to reduce the risk of AD / all-cause dementia and promote healthy aging via education, exercise, and engagement. This evidence-based program utilizes the principles of neuroplasticity and cognitive reserve. Pre-Covid, BrainSavers was delivered live, led by certified instructors. Two years of curriculum were developed, comprised of six lifestyle components: cognitive exercise, physical exercise, healthful nutrition, socialization, stress reduction, and sleep hygiene. Results of a six month beta trial documented self-rated improvements in memory and general cognitive performance, quality of life, socialization, nutritional status, and physical fitness. Quantitative results showed statistically significant differences in physical fitness measures including cardio-respiratory endurance, lower body strength, balance, speed, and agility. Trends were seen in six of nine cognitive skills. During Covid the program was transformed into an online format as BrainSavers Synapse: Staying Connected, which has been enthusiastically received. Future research will compare longer-term outcomes of both formats. Based on results to date and extensive peer-reviewed literature on lifestyle as a modifier of dementia risk, we predict this program will contribute to better individual and societal outcomes, including substantial improvements in cognitive and overall health, and a significant reduction in healthcare costs.

### ANALYSIS OF SURVIVAL DATA WITH COMPETING RISKS IN ADRD (ALZHEIMER'S DISEASE AND RELATED DEMENTIAS) RESEARCH

Dianxu Ren,[1] Oscar Lopez,[2] and Jennifer Lingler,[3], *1. University of Pittsburgh, Pittsburgh, Pennsylvania, United States, 2. University of Pittsburgh, University of Pittsburgh, Pennsylvania, United States, 3. University of Pittsburgh, Pittsburgh, Virginia, United States*

Competing risk is an event that precludes the occurrence of the primary event of interest. For example, when studying risk factors associated with dementia, death before the onset of dementia serve as a competing event. A subject who dies is no longer at risk of dementia. This issue play more important role in ADRD research given the elderly population. Conventional methods for survival analysis assume independent censoring and ignore the competing events. However, there are some challenge issues using those conventional methods in the presence of competing risks. First, no one-to-one link between hazard function and cumulative incidence function (CIF), and Kaplan-Meier approach overestimates the cumulative incidence of the event of interest. Second, the effect of covariates on hazard rate cannot be directly linked to the effect of cumulative incidence (the risk). We will discuss two types of analyses in the presence of competing risk: Cause-specific hazard model and Fine-Gray subdistribution hazard model. Cause-specific hazard model directly quantify the cause-specific hazard among subjects who are at risk of developing the event of interest, while Fine-Gray subdistribution hazard model directly model the effects of covariates on the cumulative incidence function. The type of research questions (Association vs. Prediction) may guide the choice of different statistical approaches. We will illustrate those two competing risk analyses using the large national dataset from National Alzheimer's Coordinating Center (NACC). We will analyze the association between baseline diabetes status and the incidence of dementia, in which death before the onset of dementia is a competing event.

### ARE DRUGS THAT CAUSE DYSBIOSIS LONGITUDINALLY ASSOCIATED WITH COGNITIVE SCORES, COGNITIVE IMPAIRMENT, & DEMENTIA?

Nicholas Resciniti, Alexander McLain, Anwar Merchant, Daniela Friedman, and Matthew Lohman, *University of South Carolina, Columbia, South Carolina, United States*

Recent research has examined how the microbiome may influence cognitive outcomes; however, there is a paucity of research understanding how medication associated with dysbiosis may be associated with cognitive changes. This study used data from the Health and Retirement Study and the Prescription Drug Study subset for adults 51 and older (n=3,898). Continuous (0-27) and categorical (cognitively normal=12-27; cognitive impairment=7-11; and dementia=0-6) cognitive outcomes were used. Prescriptions utilized were proton pump inhibitors, antibiotics, selective serotonin reuptake inhibitors, tricyclic antidepressants, antipsychotics, antihistamines, and a summed dose-response measure. Linear mixed models (LMM) and generalized linear mixed models (GLMM) were used for continuous and binary outcomes. For the LMM model, the main effect for those taking one medication was insignificant; however, the interaction with time showed a significant decrease over time ($\beta$: -0.07; 95% confidence interval (CI): -0.14, 0.01). The mean cognitive score was lower for those taking two or more medications ($\beta$: -1.48; 95% CI: -2.70, -0.25), although the interaction with time was insignificant. GLMM results showed those taking two or medications had odds that are 612% larger (odds ratio (OR): 7.12; 95% CI: 3.03, 16.71) of going from cognitively healthy to dementia but the interaction with time showed decreased odds over time (OR: 0.92; 95% CI 0.86, 0.97). For cognitive impairment, those who took two or more medications had odds that were 45% larger (OR: 1.45; 95% CI: 1.05, 2.00) of going from cognitively healthy to cognitively impaired. This study indicated a dose-response aspect to taking medications on cognitive outcomes.

### ASSOCIATION BETWEEN BODY WEIGHT CHANGE IN LATE LIFE AND RISK OF DEMENTIA: A POPULATION-BASED COHORT STUDY

Jie Guo,[1] Anna Marseglia,[1] Ying Shang,[1] Abigail Dove,[1] Giulia Grande,[1] Laura Fratiglioni,[2] and Weili Xu,[1], *1. Karolinska Institutet, Solna, Stockholms Lan, Sweden, 2. Karolinska Institutet, 17175, Stockholms Lan, Sweden*

**Background:** Adiposity in midlife is a modifiable risk factor for dementia. However, the effect of adiposity in late-life on dementia remains unclear. We investigated the association of body mass index (BMI) and weight changes after age 60 with the incident dementia.

**Methods:** Within the Swedish National Study on Aging and Care-Kungsholmen, 1,673 dementia-free participants with data on BMI/weight both at baseline and the 6-year follow-up were followed to detect subsequent incident dementia cases. BMI change was assessed as the percentage of the difference between BMI at baseline and the initial 6-year follow-up and categorized into large (>10%) or moderate (5–10%) loss, stable (≤5%), and moderate (5–10%) or large (>10%) gain. Weight change (difference between weight at baseline and the 6-year follow-up) was categorized into large (>7.5 kg) or moderate (2.5–7.5 kg) loss, stable (≤2.5 kg), and moderate (2.5–7.5 kg) or large (> 7.5 kg) gain. Dementia was diagnosed following the DSM-IV criteria. Data were analyzed using Cox regression models.

**Results:** During the follow-up (median 5.78 years), 102 incident dementia cases developed. BMI/weight change showed U-shaped associations with dementia. Compared with stable BMI, the hazard ratios (95% confidence intervals) of dementia were 2.93 (1.72–4.91) for large BMI loss and 2.61 (1.09–5.54) for large BMI gain. Similar results were observed for a large weight loss (2.92 [1.67–5.07]) or gain (2.95 [1.16–6.53]). These associations became stronger among participants carrying an ApoE ε4 allele.

**Conclusion:** Both large bodyweight loss and gain are associated with a higher risk of dementia, especially among ApoE ε4 carriers.

### ASSOCIATION OF BLOOD CELL PARAMETERS OF PERIPHERAL INFLAMMATION WITH BRAIN IMAGING MEASURES

Yuan Fang,[1] Kathryn Lunetta,[1] Jesse Mez,[2] Michael Alosco,[2] Claudia Satizabal,[3] Wendy Qiu,[2] Margaret Doyle,[4] and Joanne Murabito,[5], *1. Boston University School of Public Health, Boston, Massachusetts, United States, 2. Boston University, Boston, Massachusetts, United States, 3. Glenn Biggs Institute for Alzheimer's & Neurodegenerative Diseases, University of Texas Health Science Center, San Antonio, Texas, United States, 4. University of Vermont, Colchester, Vermont, United States, 5. Boston University, Framingham, Massachusetts, United States*

Neutrophil to lymphocyte ratio (NLR), red cell distribution width (RDW), and mean platelet volume (MPV), are easily measured circulating blood cell parameters that reflect chronic peripheral inflammation which increases risk for dementia and Alzheimer's disease (AD). We investigated the cross-sectional association between these blood cell parameters and brain MRI measures, including total cerebral brain volume (TCBV) as percentage of total intracranial volume (TCV) to correct for differences in head size, hippocampal volume (HPV) and log transformed white matter hyperintensity (WMH) volume, in the Framingham Heart Study (FHS) cohorts. We identified 2882 FHS participants 25 to 92 years of age (mean 59 years), 53% women, who attended an exam that included a complete blood cell count sample and received a brain MRI within five years of blood draw. We used linear mixed effect models to examine associations, adjusting for age, age^2, sex, education, cohort, time between blood draw and MRI, prevalent cardiovascular disease, C-reactive protein, APOE-ϵ4 genotype and TCV for HPV and WMH, and accounting for familial correlation using a random effect. We observed significant (p≤0.01) associations between higher RDW and smaller TCBV, and between elevated NLR and larger WMH volume. Analysis on an older subgroup (age ≥60 years, mean 71 years, n=1357) demonstrated larger effect sizes and additional significance between increased RDW with smaller HPV. We conclude that chronic peripheral inflammation as measured by NLR and RDW associates with MRI measures of brain aging (TCBV, HPV) and vascular brain injury (WMH) in FHS, with stronger impact in participants ≥60 years.

### CHOOSING UNWISELY: DISSEMINATION NEEDS OF PRIMARY CARE PROVIDERS OF PATIENTS WITH ALZHEIMER'S DISEASE

Lee Lindquist,[1] Aylin Madore,[2] Stephanie Miller,[2] Theresa Rowe,[1] and Sara Bradley,[1], *1. Northwestern University Feinberg School of Medicine, Chicago, Illinois, United States, 2. DBC Pri-Med, LLC, Boston, Massachusetts, United States*

Choosing Wisely is a well-known campaign to disseminate evidence-based clinical practices to providers and patients to drive care decisions, with geriatrics recommendations released in 2013. In December 2019, we aimed to determine what the dissemination needs of primary care providers were towards these recommendations. We developed common clinical scenarios with follow-up survey questions, relative to the care of people with Alzheimer's disease (AD) and utilizing Choosing Wisely geriatrics recommendations. The survey was distributed online to a national cohort of providers. Providers were also asked to rate their confidence level and rationale for clinical choices. Results were analyzed used mixed methodology, with constant comparative analysis utilized for qualitative responses. Nationally from 41/50 states, 211 providers responded, 72% female, with occupations of physician (36%, 77), advanced practice nurse (50%, 106) and physician assistant (13%, 28), with family practice (63%, 142) and internal medicine (20%, 43) the most prominent fields. Results revealed erroneous geriatric practices, including 1.)checking urinalysis for mental-status changes (55%, 116), 2.)treating asymptomatic bacteria with unnecessary antibiotics (59%, 124), 3.)placement of gastric tubes in end-stage dementia (11%, 23). Qualitative analysis of rationale for incorrect responses revealed knowledge misconceptions (e.g.feeding tube would help avoid aspiration). Confidence levels were high among providers as 75.9% rated themselves as above average, yet did not correlate to clinical errors. Choosing Wisely geriatrics recommendations are not being followed by some providers. Highly confident providers made errors similar to lower confident providers. New ways to disseminate geriatric recommendations are needed to improve the care of patients with AD.

### DETECTING EARLY SIGNS OF ALZHEIMER'S DISEASE AND RELATED DEMENTIA ONSET FROM THE EHR

Karen Schliep,[1] Zachary Shepelak,[1] Nicolas Bitter,[1] Ramkiran Gouripeddi,[1] Truls Ostbye,[2] Ken Smith,[1] Samir Abdelrahman,[1] and Joanne Tschanz,[3], *1. University of Utah, Salt Lake City, Utah, United States, 2. Duke University, Durham, North Carolina, United States, 3. Utah State University, Logan, Utah, United States*

As dementia is widely under-detected, a predictive model using electronic health records (EHR) could provide a method for early screening to implement preventive strategies. There is limited research on using EHR to identify persons with Alzheimer's disease (AD) and related dementias (RD). In a data-driven approach, we used all ICD-9 diagnosis and CPT procedure codes from statewide inpatient, ambulatory surgery, and Medicare records, in addition to age at baseline and gender, to detect AD/RD from the Cache County Study on Memory in Aging (1995–2009). After removing participants diagnosed with dementia at baseline (n=335), 3882 (82%) Cache County Study participants could be linked to inpatient, ambulatory surgery, and/or Medicare EHR records; 484 (12.5%) of these 3882 had incident all-cause dementia, with 308 (7.9%) having AD/AD comorbid with RD; and 176 (4.5%) having RD without AD. We removed participant's ICD-9 codes occurring after first AD/RD diagnoses. EHR features (~2000) along with gold-standard diagnoses as class labels were then used to train and detect AD and/or RD using a Gradient Boosting Trees machine learning algorithm. Models evaluated with nested cross-validation yielded AUCs of 0.70 for all-cause dementia, 0.69 for AD/AD comorbid with RD, and 0.67 for RD without AD. Key factors detecting AD/RD included age at enrollment, cardiovascular, metabolic, and kidney disease, and sleep disturbances, with feature importance varying by record type and time frame prior to dementia onset. Our findings suggest that a patient's health status up to 12 years prior may be useful in identifying individuals at-risk for dementia development.

### EFFECTS OF CAREGIVER INTERVENTIONS FOR INFORMAL CAREGIVERS OF OLDER ADULTS WITH COGNITIVE DECLINE

Machiko Tomita, *University at Buffalo, Buffalo, New York, United States*

**Objectives:** To identify baseline factors and process factors, which indicate changes that are associated with caregiving confidence improvement attributed to caregiver support.

**Methods:** An intervention study using 35 informal caregivers (ICG) of older adults (≥65 years old) with cognitive decline. Recipients of ICGs belonged to the Programs of All Inclusive Care for the Elderly (PACE). Interventions were occupational therapy (OT) support or education about illness and effective caregiving methods, which took place in ICGs' homes. OT interventions included training to reduce physical strain, and improve time and task organizations, and providing assistive devices). Caregiver confidence was measured using a Visual Analog Scale. Data were divided into two groups: improved confidence and decreased/no-change confidence. Eleven baseline data of care recipients (CRs) and ICGs as well as five process data were analyzed using logistic regression.

**Results:** Baseline factors that differentiated the two groups were ICG's age, caregiving confidence level, and CR's cognitive status, of which classification accuracy was 94.3%. Only Zarit Buren Interview (ZBI) score was associated with caregiving confidence change, of which classification accuracy was 74.3%. Younger ICGs, lower cognition, and lower caregiving confidence among baseline factors, and improved ZBI among the process factors were associated with improved confidence.

**Discussion:** Although our interventions prevented 65.7% of caregivers form declining their caregiving confidence, improving caregiving confidence was difficult while CRs' cognition continued to decline. However, this positive change was possible even CRs had moderate dementia, on average. Personal interventions may be necessary to improve caregiving confidence and reduce ICG's burden.

### HORTICULTURE-BASED INTERVENTIONS TO ENHANCE HEALTH AND WELLBEING OF PEOPLE LIVING WITH DEMENTIA IN THE COMMUNITY

Theresa Scott,[1] Ying-Ling Jao,[2] Kristen Tulloch,[1] Yates Eloise,[1] and Nancy Pachana,[1] *1. The University of Queensland, St Lucia, Queensland, Australia, 2. Pennsylvania State University, University Park, Pennsylvania, United States*

The majority of people living with dementia in the early and middle stages are cared for at home by family caregivers. Participation in meaningful activities is important for good quality of life. Recreation based on horticulture is beneficial for people living with dementia in residential settings, yet evidence within community settings is less clear. The aim of this research was to examine the existing evidence for the impact of using contact with nature, gardens and plants to enhance well-being of people living with dementia in the community. Our secondary aim was to explore the outcome domains and instruments that were employed in the existing research studies, to inform future research efforts and guide clinical practice. A systematic search was conducted covering several databases and gray literature. Original studies that examined group or individual horticulture-based activities or interventions were included. Of 2127 articles identified through searching, 10 were selected for full review. The findings reveal that horticulture-based intervention showed positive impacts on food intake, social interaction, and well-being in older adults with dementia. Some evidence shows that horticulture-based activities may alleviate stressful symptoms associated with living with dementia. Future research may further evaluate the effect of the interventions on cognitive function, physical function, and behavioral symptoms in a more rigorous intervention design.

### IMPLICATIONS OF RACIAL DIFFERENCES IN THE SHIFTS IN THE SETTING OF CARE FOR ALZHEIMER'S DISEASE AND RELATED DEMENTIAS

Arseniy Yashkin,[1] Galina Gorbunova,[2] Anatoliy Yashin,[2] and Igor Akushevich,[2], *1. Duke University, Morrisville, North Carolina, United States, 2. Duke University, Durham, North Carolina, United States*

The prevailing setting of care has strong associations with the progression of a disease at time of first diagnosis, subsequent treatment, resulting health outcomes as well as both long-term and short-term costs. The care of Alzheimer's Disease (AD) and Related Dementias (ADRD) has been experiencing a shift from skilled nursing facility to home health care. However, changes in practice do not disseminate equally across the race/ethnicity spectrum of the U.S. and disadvantaged race/ethnicity-related groups often encounter differing conditions from those experienced by the majority. In this study, we calculated the race/ethnicity-related direct healthcare costs of individuals with AD and ADRD, stratified by care-provider structure (physician, inpatient, outpatient, skilled nursing facility, home health, hospice), and modeled the trends and the relative contributions of each setting over the 1991-2017 period using administrative claims from a 5% sample of Medicare beneficiaries. Inflation and the gradual switch of Medicare compensation to the HCC model between 2004 and 2007 were accounted for. We then applied an inverse probability weighting algorithm to propensity-score-match the AD/ADRD race/ethnicity-specific groups to Medicare beneficiaries to make them comparable in demographics and co-morbidity status but without AD/ADRD. Finally, we performed a comparison of the Medicare costs and associated survival within (AD/ADRD vs. No AD/ADRD) and between (Black vs. White vs. Hispanic) race/ethnicity-related groups. Comparisons were done for: i)1-year before; ii) 1-year after iii) years 2-11; iv) years 12-21 and v) years 22+ after an AD/ADRD diagnosis. We found significant race/ethnicity-related differences in costs and survival both before and after propensity score matching.

### INCIDENCE OF MILD COGNITIVE IMPAIRMENT, CONVERSION TO PROBABLE DEMENTIA, AND MORTALITY

Yun Zhang,[1] Ginny Natale,[2] and Sean Clouston,[3], *1. Renaissance School of Medicine, Stony Brook University, Stony Brook, New York, United States, 2. Stony Brook University, Stony Brook, New York, United States, 3. Renaissance School of Medicine, Stony Brook University, Renaissance School of Medicine, Stony Brook University, New York, United States*

**Background:** Few studies have jointly estimated incidence of MCI, conversion to probable dementia, and mortality.

**Methods:** We used data from six waves of the National Health and Aging Trends Study (2011-2016). Multivariable-adjusted multi-state survival models (MSMs) were used to model incidence upon accounting for misclassification.

**Results:** A total of 6,078 eligible NHATS participants 65 years of age and older were included (average age: 77.49 ±7.79 years; 58.42% females; 68.99% non-Hispanic White). Incidence of MCI was estimated to be 41.0 [35.5, 47.3]/1,000 person-years (PY). Participants converted to probable dementia at a high rate of 241.3 [189.6, 307.0]/1,000 PY, though a small number also reverted from MCI to cognitively normal. Education was associated with lower incidence of MCI and probable dementia, but increased mortality in those with MCI. There were also substantial racial and ethnic disparities in the incidence of MCI and dementia.

**Conclusions:** Our results underscore the relatively common incidence of and conversions between MCI and dementia in community-dwelling older Americans and uncover the beneficial impact of education to withstand cognitive impairment before death.

### MACHINE LEARNING-BASED PREDICTIVE MODELS OF BEHAVIORAL AND PSYCHOLOGICAL SYMPTOMS OF DEMENTIA

Eunhee Cho,[1] Sujin Kim,[2] Seok-Jae Heo,[3] Jinhee Shin,[1] Byoung Seok Ye,[2] Jun Hong Lee,[4] and Bada Kang,[1], *1. Yonsei University College of Nursing, Seoul, Seoul-t'ukpyolsi, Republic of Korea, 2. Yonsei University, Seoul, Seoul-t'ukpyolsi, Republic of Korea, 3. Yonsei University Graduate School, Seoul, Seoul-t'ukpyolsi, Republic of Korea, 4. National health insurance service Ilsan hospital, Goyang, Kyonggi-do, Republic of Korea*

Models predicting the occurrence of specific types of behavioral and psychological symptoms of dementia (BPSD) can be highly beneficial for its early intervention and individualized care planning. Using a machine learning approach, this study developed and validated predictive models of the occurrence of BPSD, categorized into seven subsyndromes, among community-dwelling older adults with dementia in South Korea. BPSD dairy was used to measure BPSD and the state of unmet needs daily. We measured sleep and activity levels using actigraphy, and stress and fatigue using a portable heart rate variability analyzer. We developed predictive models and conducted cross-validation using training data that consisted of the first two wave dataset, and then validated the models using wave 3 test data. To deal with imbalanced datasets, we used Synthetic Minority Oversampling Technique (SMOTE), an over-sampling method. Categorical variables were pre-processed using target encoding. We then compared the machine-learning models with logistic regression. The area under the receiver operating characteristic curve (AUC) scores of the support vector machine (SVM) models for the wave 3 test data showed a similar or greater value than logistic regression models across all BPSD subsyndromes. The SVM model (AUC = 0.899) had an AUC value greater than that of the logistic regression model (AUC = 0.717), particularly for hyperactivity symptoms. Machine learning algorithms, especially SVM models, can be used to develop BPSD prediction models to help identify at-risk individuals and implement symptom-targeted individualized interventions.

### MODIFIABLE RISK FACTORS OF BEHAVIORAL AND PSYCHOLOGICAL SYMPTOMS IN PEOPLE WITH DEMENTIA

Shinae Seo,[1] and Meghan Mattos,[2], *1. University of Virginia School of Nursing, Charlottesville, Virginia, United States, 2. University of Virginia, School of Nursing, Charlottesville, Virginia, United States*

Over ninety percent of people with dementia (PWD) experience behavioral and psychological symptoms, known to increase burden on care providers and healthcare systems. The purpose of this integrative review was to examine the modifiable risk factors of behavioral and psychological symptoms of dementia (BPSD) at the individual, caregiver, and environmental levels. An electronic database search was performed using PubMed, CINAHL[EBSCO], Web of Science, and PsycINFO from 2010-2020. Search terms included "dementia" AND ("Behavioral" OR "Behavioural" OR "Psychological" OR "Neuropsychiatric") AND "symptoms" AND ("Independent Living" OR "Community Dwelling" OR "Community Living" OR "Living at Home" OR "Ageing in Place." The search yielded 1,121 articles, and 14 articles were included in this review. Among the 14 articles, there were 11 modifiable risk factors presented across the individual, caregiver, and environmental levels. Individual-level factors included the presence of affective disorder, low quality of life, and leisure dysfunction. The modifiable caregiver-level factors included relationship with PWD, frequency of contact, caregiver burden, distress, frustration level, caregiver as a resource for PWD, and quality of dyadic relationship. One environmental factor, the presence of pandemic disease (e.g.,

COVID 19), was identified. This review presents the modifiable factors that contribute to the varied symptoms and multi domains of BPSD. Further research is necessary to determine whether, and to what degree, interventions targeting individual, caregiver, or environmental risk factors may reduce BPSD for PWD, caregivers, and providers within the community setting.

### MUSIC INTERVENTIONS FOR EARLY-STAGE COGNITIVE DECLINE: A SYSTEMATIC REVIEW AND META-ANALYSIS

Jennie Dorris,[1] Stephen Neely,[2] and Juleen Rodakowski,[1], *1. University of Pittsburgh, Pittsburgh, Pennsylvania, United States, 2. Carnegie Mellon University, Pittsburgh, Pennsylvania, United States*

Older adults in the early stages of Alzheimer's and dementia deserve effective modalities that support their cognition, emotional well-being, and social engagement. Music has demonstrated potential to support these critical outcomes through its ability to simultaneously stimulate multiple areas of the brain and induce neuroplasticity. We reviewed randomized controlled trials for studies that specifically utilized active music-making interventions for older adults with early-stage cognitive decline to assess their effects on cognition, emotional well-being, and social engagement. Additionally, this review categorized the specific music activities employed by each intervention. We conducted searches on Medline (Ovid), APA PsycInfo (Ovid) CINAHL (Ebsco), and Embase (Elsevier). Our search yielded 285 potential studies. We analyzed 19 studies with 1,387 participants for potential effect sizes and intervention ingredients. Of the 19 studies, eight studies, recruiting a total of 460 participants, were used to conduct a random-effects meta-analysis to assess the effect of music on cognition. Meta-analytic aggregration of effect sizes showed that music had a modest positive effect on cognition compare with the control conditions (SMD= 0.26; P= 0.008; 95% confidence interval, 0.07, 0.45; I2= 5%). The musical activities of 1) singing and/or playing pre-composed songs and/or 2) creating music in the moment were utilized in the protocols. This research demonstrates that active music-making supports cognition for older adults with early-stage cognitive decline. Future music programs should consider inclusion of pre-composed songs, as well as music creation, to better understand the power for music to provide critical support for a rapidly growing segment of the population.

### PREDICTORS OF BEHAVIORAL AND PSYCHOLOGICAL SYMPTOMS IN COMMUNITY-DWELLING OLDER ADULTS WITH DEMENTIA

Eunhee Cho,[1] Sujin Kim,[2] Sinwoo Hwang,[3] Eunji Kwon,[3] Seok-Jae Heo,[4] Jun Hong Lee,[5] Byoung Seok Ye,[2] and Bada Kang,[1], *1. Yonsei University College of Nursing, Seoul, Seoul-t'ukpyolsi, Republic of Korea, 2. Yonsei University, Seoul, Seoul-t'ukpyolsi, Republic of Korea, 3. Korea Armed Forces Nursing Academy, Daejeon, Taejon-jikhalsi, Republic of Korea, 4. Yonsei University Graduate School, Seoul, Seoul-t'ukpyolsi, Republic of Korea, 5. National health insurance service Ilsan hospital, Goyang, Kyonggi-do, Republic of Korea*

Although disclosing the predictors of different behavioral and psychological symptoms of dementia (BPSD) is the first step in developing person-centered interventions, the current understanding is limited as it considers BPSD as a homogenous construct, not accounting for its heterogeneity. Therefore, this study explored the predictors of BPSD subsyndromes, and built prediction models for these subsyndromes in community-dwelling older adults with dementia in Korea. This prospective study consisted of a two-wave dataset. We fit the generalized linear mixed models using Wave 1 data (N = 145) and then validated them using Wave 2 data (N = 59). BPSD and their proximal factors were assessed on a daily basis using diaries written by family caregivers. Sleep and activity levels were objectively measured using actigraphy. The amount of nighttime sleep hours was significantly associated with next-day sleep and nighttime behaviors (OR = 0.87; p = 0.005), with the amounts of energy expenditure showing significant association with euphoria/elation (OR = 0.02; p = 0.019). All subsyndromes except euphoria/elation were found to be significantly associated with either hunger, thirst, urination, or bowl movement; with all BPSD showing a significant association with environmental changes. We also found several background factors, including premorbid personality and taking sedatives as predictors for specific subsyndromes. The area under the receiver operating characteristic curve scores for the data were greater than 0.9 and 0.8 in Waves 1 and 2, respectively, across all subsyndromes. Prediction models for BPSD will help in the development of symptom-targeted, individualized interventions.

### PRESCRIPTION PATTERNS OF ANTI-DEMENTIA AND PSYCHOTROPIC DRUGS IN PEOPLE LIVING WITH DEMENTIA IN CHINA

Zhang Yingyang,[1] Hao Luo,[2] Gloria HY Wong,[2] Terry YS Lum,[2] Celine Chui,[2] Ian C.K. Wong,[2] and Huali Wang,[3], *1. The University of Hong Kong, Hong Kong, Hong Kong, 2. The University of Hong Kong, Hong Kong, Not Applicable, Hong Kong, 3. Peking University Institute of Mental Health (Sixth Hospital), Beijing, Beijing, China (People's Republic)*

Pharmacotherapy of dementia is a critical intervention for managing symptoms of and slowing progression of dementia. However, evidence on prescribing patterns of dementia medications and their associated factors in China is lacking. This study aimed to examine prescribing rates of anti-dementia and psychotropic drugs, and investigate factors associated with prescription of anti-dementia drugs and its co-prescription with psychotropic drugs in China. We used data from the Clinical Pathway for Alzheimer's Disease in China study, an eight-week multi-center registry study that was conducted in tertiary hospitals between Nov 12, 2012, and Jan 31, 2013. Anti-dementia and psychotropic drugs were coded according to the Anatomical Therapeutic Chemical codes. Logistic regressions were performed to examine factors associated with prescription patterns after controlling for demographic and clinical characteristics of people living with dementia and caregivers' characteristics. A total of 746 participants were included in this study, of which almost 80% of participants were prescribed anti-dementia drugs, and one-third were prescribed at least one psychotropic drug. The concomitant prescription rate of anti-dementia and psychotropic drugs was 24·3%. Logistic regression results showed that first consultation, dementia subtypes, dementia severity, functioning level, and having symptoms of psychosis and apathy were significantly associated with anti-dementia drug prescription. Frontotemporal dementia, worse functioning level, psychosis, agitation, and depression were significantly associated with co-prescription of anti-dementia and psychotropic agents. Practices of dementia prescriptions generally concurred with clinical guidelines in tertiary hospitals in China, while prescription of anti-dementia and psychotropic medications mainly depended on clinical symptoms of patients with dementia.

### THE EFFECTS OF MULTI-COMPONENT INTERVENTIONS ON COGNITION: A SYSTEMATIC REVIEW

Sangwoo Ahn, and Joel Anderson, *University of Tennessee at Knoxville, Knoxville, Tennessee, United States*

Given the lack of a cure for Alzheimer's disease (AD), the number of people with AD is expected to surge unless the onset is delayed. Although there have been efforts to examine the effects of single-domain neuroprotective interventions on cognition, no conclusive results have been found so far. Due to the multifactorial causes of AD, interventions combining multiple neuroprotective components may induce more beneficial effects. However, there are few comprehensive reviews evaluating the effects of multi-domain programs on cognition. Thus, the purpose of this systematic review was to evaluate the effects of currently available multi-component interventions on cognition such as global cognition, episodic memory, and/or executive function affected early in AD. The literature search was conducted using PubMed, CINAHL, Web of Science, Scopus, and PsycINFO up to September 2020. Of the 1,445 articles located, 17 met eligibility criteria (n = 10,056, mean age = 72.8 years). According to the Effective Public Health Practice Project Quality Assessment Tool for Quantitative Studies, 8 and 9 studies had strong and moderate overall quality, respectively. The effect sizes of each included study were calculated using Cohen's d. Multi-component interventions comprising physical activity, cognitive exercise, cardioprotective nutrition, and/or cardiovascular health consultation/education exerted beneficial effects on cognition (very small to moderate effect sizes; Cohen's d = 0.16 to 0.77). Clinically, health care providers are recommended to consider those elements to potentially stave off AD. There is a pressing need for researchers to identify optimally effective doses of neuroprotective multi-component interventions.

### TRAJECTORIES OF COGNITIVE FUNCTION AND ASSOCIATED FACTORS IN COMMUNITY-DWELLING OLDER ADULTS: A PROSPECTIVE STUDY

Zimu Wu,[1] Robyn Woods,[1] Elsdon Storey,[2] Trevor Chong,[2] Raj Shah,[3] Suzanne Orchard,[1] Anne Murray,[4] and Joanne Ryan,[1], *1. Monash University, Melbourne, Victoria, Australia, 2. Monash University, Monash University, Victoria, Australia, 3. Rush, Rush University Medical Center, Illinois, United States, 4. Hennepin HealthCare Research Institute, Hennepin HealthCare, Minneapolis, Minnesota, United States*

There is variability in cognitive aging between individuals. This study aimed to investigate cognitive aging trajectories, the associated modifiable factors, and the association of these trajectories with dementia. Community-dwelling older adults (n=19,114) without dementia or major cognitive impairment at inclusion were followed for up to 7 years, with regular standardized cognitive assessments. Group-based (multi-) trajectory modeling identified distinct cognitive trajectories. Structural equation modeling (n=16,018) was used to analyze the associated predictors. Four to seven trajectories were identified per cognitive domain, with generally stable trajectories. Improvement in verbal fluency and minor psychomotor slowing were common. Substantial decline in global cognition and episodic memory were observed in a small proportion of individuals. The highest proportions of dementia cases were in trajectories with major decline in global cognition (56.9%) and memory (33.2%). A number of sociodemographic characteristics, health behaviors and chronic conditions were either directly or indirectly associated with cognitive change in older adults. This study found that some individuals appear resilient to cognitive decline even with advancing age, and that factors that promote healthy cognitive aging are not simply the absence of factors which confer risk for decline.

### Session 9070 (Poster)

### ALZHEIMER'S DISEASE II (HS POSTER)

### A PHASE I TRIAL ASSESSING LOMECEL-B INFUSION IN INDIVIDUALS WITH ALZHEIMER'S DISEASE: STUDY DESIGN AND RATIONALE

Kevin Ramdas,[1] Ben Hitchinson,[2] Lisa McClain-Moss,[2] Keyvan Yousefi,[3] Liliana Diaz,[2] Barry Baumel,[4] Joshua Hare,[2] and Anthony Oliva,[2], *1. Longeveron LLC, Miami, Florida, United States, 2. Longeveron, Inc, Miami, Florida, United States, 3. Longeveron Inc., Miami, Florida, United States, 4. University of Miami, Miami, Florida, United States*

Alzheimer's disease (AD) is an irreversible neurodegenerative disorder characterized by memory loss and persistent cognitive dysfunction which significantly compromises quality of life. Brain inflammation is a prominent feature of AD pathology. Lomecel-B which is derived from culture-expanded medicinal signaling cells (MSCs) have immuno-modulatory capacity and control inflammation and the cytokine production of lymphocytes. The primary objective of this study was to evaluate the safety of Lomecel-B infused intravenously in individuals with AD. Safety was monitored by examining vital signs, physical and neurological exams, laboratory tests (hematology, coagulation, blood chemistry, and urinalysis). This was a multicenter phase 1 double-blinded, placebo controlled trial initiated with a safety run in phase of 3 individuals followed by a randomized phase of 28 individuals. During the safety run-in phase all subjects were treated with low dose Lomecel-B no less than 5 days apart, and evaluated for safety. In the randomized phase, subjects were treated with either low or high dose Lomecel- B or Placebo in a 2:2:1 randomization ratio. The study enrolled adults aged 50-80 years diagnosed with AD via confirmatory brain MRI and PET scan and a MMSE

score of 18-24. Safety and efficacy assessments were completed at 30, 90, 180, 270 and 365 days. We describe the design and rationale for this phase 1 trial with the primary objective of assessing the safety of Lomecel-B on adults with AD. The secondary efficacy measurements included ADAS-Cog 11, MMSE, TMT, UPSIT, GDS, blood biomarkers and numerous quality of life questionnaires.

### CARDIOMETABOLIC RISK FACTORS PREDICT EXECUTIVE FUNCTION SCORES IN HIGH-RISK INDIVIDUALS

Joshua Gills,[1] Megan Jones,[1] Anthony Campitelli,[1] Sally Paulson,[2] Erica Madero,[3] Jennifer Myers,[3] Jordan Glenn,[3] and Michelle Gray,[1], *1. University of Arkansas at Fayetteville, Fayetteville, Arkansas, United States, 2. University of Arkansas at Fayetteville, Cincinnati, Ohio, United States, 3. Neurotrack Technologies, Redwood City, California, United States*

Alzheimer's disease (AD) is expected to triple by 2050, affecting 16 million Americans. As a result, it is essential to combat this alarming increase in cognitive impairment through early detection. Cardiometabolic risk factors have shown to be associated with higher risk of AD. The purpose of this study was to determine if cardiometabolic risk factors could predict executive function scores in a high-risk population. Fifty (60.9±8.8 years) high-risk adults (classified by the Australian National University Alzheimer's Disease Risk Index) were enrolled in this study. Participants completed a 6-minute walking test, venous blood draw, blood pressure measurement, and the digit coding symbol test (DCS). Results were examined through a multiple linear regression with DCS as the dependent variable and age, sex, total cholesterol, high-density lipoprotein (HDL), low-density lipoprotein (LDL), glucose, 6-minute walking test, systolic blood pressure (SBP), and diastolic blood pressure (DBP) as predictor variables. The model explained 42% of the variance of DCS ($p = .04$) with SBP (45%; $p = .003$) as a significant predictor. LDL ($p = .087$) and DBP ($p = .123$) accounted for 24% and 22% of the variance for this model, respectively. These results suggest cardiometabolic risk factors predict executive function values in high-risk individuals. Higher SBP was significantly associated with lower DCS scores indicating SBP as a valuable tool for practitioners when evaluating cognitive decline. Further research should expand sample size and track values longitudinally to substantiate these claims.

### CAREGIVER SINGING INTERVENTION: DO EMOTIONS AND RESISTANCE DIFFER BETWEEN VASCULAR DEMENTIA AND ALZHEIMER'S?

Lena Hammar,[1] Gabriella Engström,[2] and Anna Swall,[2], *1. Mälardalen University / Dalarna University, Västerås, Vastmanlands Lan, Sweden, 2. Dalarna University, Falun, Dalarnas Lan, Sweden*

Persons with dementia in residential care commonly express resistance of aggressiveness. Caregivers Singing (CS) - when caregivers sing for or together with persons with dementia during caring, has shown to reduce these expressions and increase communication and cooperation. Previous studies of CS have included both persons with Alzheimer's disease (AD) and persons with Vascular dementia (VD), but no studies have been done focusing on possible differences regarding these diagnoses. As disabilities and symptoms differ between these diagnoses, the emotions and expressions, such as resistance may differ regarding response to CS. This polit study aims to describe emotions and resistiveness to care among persons with vascular or Alzheimer's disease. Participants were five persons with AD and five persons with VD living at two different nursing homes. Video observations (VIO) occurred with them and their caregivers during morning care situations four times without CS and four times with CS. In all, 80 VIOs were rated using the Observed Emotion Rating Scale and the Resistiveness to Care Scale. These were then analyzed with descriptive statistics. Results revealed that for both persons with AD and VD, the positive emotion pleasure were never observed without CS while with CS it increased for both groups. In contrast to the positive emotion effect of CS, the negative emotions and resistiveness decreased more for persons with VD than for persons with AD. For persons with VD, the number of observations without anger increased, while observation without anger or anxiety/fear for persons with AD remind the same.

### COMPARISON OF COGNITIVE INTERVENTION DELIVERY FORMATS IN PATIENTS WITH DEMENTIA-A NETWORK META-ANALYSIS

Yue Sun,[1] and Zhi-wen Wang,[2], *1. School of Nursing, School of Nursing, Peking University, Beijing, China (People's Republic), 2. Peking University Health Science Center, Beijing, Beijing, China (People's Republic)*

Cognitive intervention has been shown to be effective to delay cognitive decline in older adults with dementia. However, whether cognitive intervention could be effectively delivered in individual, group, telephone, guided self-help and unguided self-help formats remains unclear. Pubmed, Embase, Cumulative Index to Nursing and Allied Health Literature (CINAHL), CINAHL, the Cochrane Central Register of Controlled Trials, Web of science, China National Knowledge Infrastructure database, Chinese Biomedical Literature database and Wan Fang database were systematically searched. 3419 records were extracted, quality assessed, and double-blind screened by 2 authors. Totally 51 studies were included which enrolled 3388 participants. Network meta-analysis (NMA) was conducted to evaluate the relative effects and rank probability of different cognitive intervention delivery formats. For older adults with dementia, guided self-help, group and individual cognitive intervention delivery formats appeared effective in improving the cognitive function, while telephone and unguided self-help were not significantly inferior to control condition. Guided self-help had the highest probability of being the best treatment among the five cognitive intervention delivery formats. Health-care professionals should apply personalized cognitive intervention format based on individual condition and preferences.

### COMPARISON OF METHODS TO CLASSIFY ADRD IN CLAIMS AND CANCER REGISTRY DATA

Vinit Nalawade,[1] James Murphy,[1] Tim Schempp,[2] Melissa Yakuta,[2] Alison Moore,[1] Paul Gilbert,[2] and Melody Schiaffino,[2], *1. University of California, San Diego, La Jolla, California, United States, 2. San Diego State University, San Diego, California, United States*

Alzheimer's disease and related dementias(ADRD) affects 10.3% of older Americans (65+), among these 15-30% go on to be diagnosed with cancer. The highest burden of ADRD is experienced by Latino/a (12.2%) and African-American (13.8%) older adults. Older patients with pre-existing ADRD are less likely to receive guideline-concordant cancer care due to lack of consideration of cognitive status, and underestimation of ADRD diagnosis is an issue in secondary data. Our study compares two validated algorithms for classifying ADRD in a sample of cancer patients, the NCI-Charlson and CMS-Chronic Conditions Warehouse (CCW) index. We used existing claims from NCI's SEER-Medicare linked database (2004-2013, N=37,932). Patients were selected based on cancer diagnosis at any stage with at least 36 months of data prior to diagnosis to identify ADRD. We analyzed breast, lung, prostate, cervix, head & neck(HNC), and colorectal(CRC) cancers(CA). We found a prevalence of 2.8% (9549 cases of ADRD+CA) using the NCI-index compared with a prevalence of 5.6% (18989 cases) with the CCW-index. ADRD+CA numbers differed significantly in all cancers for all races, however, we observed the greatest magnitude of difference among Latino/a and African-American patients. The NCI index significantly underestimated prevalence compared with the CCW: 1.21% vs 3.28% Breast; 2.29% vs 4.60% CRC; 2.88% vs 6.44% Lung; 1.36% vs 8.62% Prostate, and 4.21% vs 11.61% HNC. Our findings suggest a need to develop validated algorithms for classification, using an evidence-base generated by incorporating information and decision-making theories from the expertise of clinicians currently diagnosing ADRD using clinical assessments in diverse populations.

### EATING DIFFICULTIES AMONG OLDER ADULTS WITH DEMENTIA IN SOUTH KOREAN LONG-TERM CARE FACILITIES: A SCOPING REVIEW

Eunju Choi,[1] Dukyoo Jung,[2] and Kyuri Lee,[1], *1. College of Nursing, Ewha Womans University, Seoul, Seoul-t'ukpyolsi, Republic of Korea, 2. Ewha Womans University, Seoul, Seoul-t'ukpyolsi, Republic of Korea*

This study aims to synthesize existing literature concerning eating difficulties among older adults with dementia in long-term care facilities. A scoping review, using the framework proposed by Arksey & O'Malley (2005) and improved and supplemented by Levac et al. (2010), was conducted. Literature was searched from five bibliographic databases—Research Information Sharing Service (RISS), Korean studies Information Service System (KISS), National Digital Science Library (NDSJ), Korean Medical Database (KMBASE), DataBase Periodical Information Academic(DBPia), Google Scholar, and gray literature. Literature selection and characteristics were approved by two independent reviewers, using pre-tested forms to determine final inclusion. Eventually, 111 articles from 2012–2020 were identified, and the 11 articles were used for the final analysis. We found that primarily utilized Eating behavior scale (EBS) and Edinburgh feeding evaluation in dementia scale (EdFED) had utilized as measurement tools for evaluating eating behavior. The most common factors related to eating behavior of older adults with dementia included cognitive and physical functions in the individual domain, the caregiver's attitude toward eating in the inter-individual domain, and types of meal in the environmental domain. Therefore, it is essential to develop measurement tools that reflect the eating behavior of older adults with dementia, a comprehensive understanding of the eating behavior of old adults with dementia, and create effective interventions that can be implemented in the specificity of long-term care facilities in Korea. The results of this analysis are intended to be used as basis to develop a meal support programs for older adults with dementia.

### FACTORS DRIVING THE TRANSITION OF ALZHEIMER'S DISEASE PATIENTS TO INSTITUTIONAL LONG-TERM CARE

Heather Davila,[1] Guneet Jasuja,[2] Lauren Moo,[3] Madhuri Palnati,[2] Qing Shao,[2] Quanwu Zhang,[4] Amir Abbas Tahami Monfared,[5] and Donald Miller,[2], *1. VA Boston Healthcare System, VA Boston Healthcare System, Massachusetts, United States, 2. VA Bedford Healthcare System, Bedford, Massachusetts, United States, 3. VA Bedford Health Care System, Bedford, Massachusetts, United States, 4. Eisai Inc, Woodcliff Lake, New Jersey, United States, 5. Eisai Inc., Woodcliff Lake, New Jersey, United States*

Progression of Alzheimer's disease (AD) may ultimately lead to costly institutional long term care (ILTC) so its avoidance is often a goal of care management. We studied predictors of AD patients transitioning to ILTC in the Veterans Affairs healthcare system (VA). We identified 30,017 Veterans at least 50 years old, with ≥2 ICD-9/10-CM diagnosis codes for AD on separate days, with first AD code in 2013-2018, at least 2 years of prior continuous VA service use, and no prior ILTC. Patients who subsequently transitioned to ILTC (cases) were matched to other AD patients with the same time since first AD code but no ILTC (controls) (median of 13 months; mean age of 80.2 years). The 8,261 matched sets were split randomly to a training sample, where logistic and random forest regressions were used to develop models, and a validation sample, where final models were evaluated. Predictors of ILTC initiation included measures of (1) poor health, such as high morbidity counts (Elixhauser score of 15+, odds ratio=1.31) and weight loss (1.29), (2) heavy service use, such as hospitalization (2.25) and home health care (1.54), and (3) dementia symptoms, such as a diagnosis code for dementia not-otherwise-specified recorded well before the AD code (1.93), functional/mobility difficulties (1.35), and lifestyle or psychosocial problems (1.53). The full model C statistic was 0.78. Transition to ILTC in AD patients is driven by many factors, including comorbidities, need for acute care, nonspecific symptoms of dementias, and functional challenges. Targeted interventions may delay transitions to ILTC.

### GAIT SPEED AND GRIP STRENGTH ARE PREDICTORS OF COGNITIVE DECLINE AND DEMENTIA IN OLDER INDIVIDUALS.

Suzanne Orchard,[1] Joanne Ryan,[1] Robyn Woods,[1] Galina Polekhina,[1] Elsdon Storey,[2] Raj Shah,[3] Trevor Chong,[2] and Anne Murray,[4] *1. Monash University, Melbourne, Victoria, Australia, 2. Monash University, Monash University, Victoria, Australia, 3. Rush, Rush University Medical Center, Illinois, United States, 4.*

*Hennepin HealthCare Research Institute, Hennepin HealthCare, Minneapolis, Minnesota, United States*

Lower gait speed and grip strength are common in older adults. However, the results of lower motor function on cognitive outcomes have been mixed. We examined the longitudinal association between baseline slow gait speed and weak grip strength, alone and in combination, with risk of incident dementia or cognitive decline in a cohort of older adults. Participants (n=19,114) aged 70 and over (65 if U.S. minority) without documented evidence of dementia, significant cognitive impairment, physical disability or previous cardiovascular disease at baseline, were recruited from community settings. Incident dementia was adjudicated by an expert panel using DSM-IV criteria. Incident cognitive decline was defined as a persistent intra-individual decline in score of >1.5 SD from baseline on any of the cognitive tests. Using cox proportional hazard models, slow gait speed at baseline was associated with an increased risk of dementia (63%) and cognitive decline (43%), over a median 4.7 years. Weak grip strength was not as strong a predictor, but was also associated with risk (43% and 11%, respectively). Both outcomes showed higher risk for dementia than cognitive decline. There was no gender-specific interaction. When considered together (adjusted for one another), gait speed and grip strength were both independently associated with cognitive decline and dementia. The synergistic association of these physical measures, each of which is readily administered in the clinic or home, serve as effective early markers of increasing risk of cognitive decline and incident dementia and thus, should be considered for routine health assessments for older adults.

### HIPPOCAMPAL VOLUME IS SMALLER IN FEMALE DOUBLE CARRIERS OF TWO STRONGEST AD GENETIC RISK FACTORS

Svetlana Ukraintseva,[1] Vladimir Popov,[2] Konstantin Arbeev,[1] Hongzhe Duan,[2] Olivia Bagley,[1] Alexander Kulminski,[2] and Anatoliy Yashin,[1], *1. Duke University, Durham, North Carolina, United States, 2. DUKE UNIVERSITY, Durham, North Carolina, United States*

Genetic risk factors for Alzheimer's disease (AD) may facilitate AD-related changes in the brain long before AD clinical manifestation. While APOE4 was linked to a reduced hippocampal volume (HV) in a number of studies, the impact of rs2075650, another polymorphism strongly associated with AD, on HV is less clear. The rs2075650 (in TOMM40) is only in moderate to low LD with APOE4, and may have independent effects on HV or interact with APOE4. We studied associations of rs2075650 (G allele, risk factor for AD), rs429358 (C allele, proxy for APOE4), and their combinations, with right HV measured by MRI, among 10,738 women and 9,775 men aged 60-75, from UK Biobank. We found that right HV was significantly ($p<0.02$) smaller in women who carry both AD risk variants (rs2075650(G) and rs429358(C)), than in non-carriers of both of these variants, while having only one risk variant (G or C) didn't clearly affect HV. The studied associations didn't reach statistical significance in men. Our results suggest that rs2075650(G) and rs429358(C) may contribute synergistically to a reduction in hippocampus volume, in females only, and support the role of interactions between genetic risk factors for AD in sex differences in preclinical biomarkers of AD pathology.

### HYPERTENSIVE DISORDERS OF PREGNANCY AND RISK OF ALZHEIMER'S DISEASE, VASCULAR DEMENTIA, AND OTHER RELATED DEMENTIA

Karen Schliep,[1] Yue Zhang,[1] Joanne Tschanz,[2] Jennifer Majersik,[1] Julio Facelli,[1] and Michael Varner,[1], *1. University of Utah, Salt Lake City, Utah, United States, 2. Utah State University, Logan, Utah, United States*

Several recent studies have examined whether hypertensive disorders of pregnancy (HDP) are associated with an increased risk for Alzheimer's disease (AD) and other related dementias (RD) with conflicting findings. Limitations to prior studies include lack of assessing risk by dementia subtype, inadequate sample sizes, and not fully exploring the role of mid-life factors. We performed a retrospective matched cohort study among women with >1 singleton pregnancy (1939–2013) using the Utah Population Database. HDP-exposed women (n=19,989) were one-to-two matched with unexposed women (n=39,679) by 5-year age groups, year of childbirth (within 1 year), and parity (1, 2, 3, 4, ≥5) at the time of the pregnancy. HDP pregnancies were complicated by preeclampsia (62%), gestational hypertension (34%), and eclampsia (4%). Women with a history of HDP had a higher hazard of all-cause dementia (HR=1.37; 95% CI: 1.26, 1.50) compared to women without a history of HDP after adjustment for maternal age, year of childbirth, and parity. The hazard doubled after additionally accounting for pre-pregnancy BMI (HR=2.31; 95% CI: 1.24, 4.32). Stratifying by dementia subtype, we found HDP to be associated with a higher hazard of vascular dementia (HR=1.64; 95% CI: 1.19, 2.26) and other related dementia (HR=1.49; 95% CI: 1.34, 1.65) but not Alzheimer's disease (HR=1.04; 95% CI: 0.87, 1.24) after accounting for competing risks. Mid-life hypertension and stroke were found to have the greatest mid-life impact, mediating 43% and 41% of dementia risk, respectively, highlighting women who may most benefit from close surveillance and early preventive and clinical interventions.

### INFORMATION AND COMMUNICATION TECHNOLOGY USE IN COMMUNITY-DWELLING PERSONS LIVING WITH DEMENTIA

Kyung Hee Lee, Ji Yeon Lee, and Bora Kim, *Yonsei University, Seoul, Seoul-t'ukpyolsi, Republic of Korea*

Although many persons living with dementia still remain living in the community, they encounter many difficulties due to cognitive and physical impairment. Information and Communication Technology (ICT) could be helpful to protect persons living with dementia from risky events and monitor changes in physical function. This study aimed to review studies regarding ICT usage to monitor physical activity and safety in community-dwelling persons living with dementia. We searched quantitative studies that utilized ICT to monitor physical activity and safety published from 2011 to 2020 through five databases; 24 studies were included in the systematic review. Most studies (79%) were observational studies and conducted in North America or Europe (75%). In terms of ICT usage, the most frequently used type was a wearable device (96%); data such as physical activity, gait, and circadian rhythms were gathered. The ICT data were utilized for: 1) comparing ICT data within dementia group or with normal cognition group; 2) exploring a relationship with other variables in observational studies; 3) measuring an outcome of the experimental studies; and 4) determining feasibility of a sensor itself. Less than half of the studies met all five criteria in quality assessment. We found the ICT is being used in various ways in research for community-dwelling persons living with dementia. However, we are uncertain about the effectiveness of ICT use and the quality of studies. Future studies with rigorous study design are needed to provide better evidence for ICT use in persons living with dementia.

### LONGITUDINAL ASSOCIATION OF FALL RISK FOR COMMUNITY DWELLING ELDERLY WITH AND WITHOUT ADRD

Pallavi Tyagi, *School of Public Health, University of Maryland, University of Maryland, College Park, Maryland, United States*

Falls amongst elderly with ADRD and are a major cause of functional impairment and increased mortality. The primary purpose of this study was to examine if fall status of community dwelling elderly residents with and without ADRD differs over a four year period and additionally does receipt of rehabilitation in any year modifies this association. National Health and Aging Trends Study (NHATS) data from 2015-2018 was used and sample consisted of participants age 65 and older who were community dwellers. Baseline characteristics of individuals in ADRD (n= 264) and non-ADRD group (n=2,971) was compared using chi square statistic. A generalized estimating equation model (multivariate logistic regression) was used to estimate odds ratio of falls in the two groups, adjusted for sociodemographic and clinical conditions that are predictors of fall risk. At baseline, ADRD group comprised of older, frail individuals who reported increased use of assistive device (AD) and presence of depression. Those in the non-ADRD group at baseline had significantly decreased odds for falls however no statistically significant change was observed for fall status over four years in the two groups (non-ADRD vs ADRD) and this association was not modified by receipt of rehabilitation. Those who used AD, had depression and received rehabilitation had increased odds for falls however no differences were found for those who lived alone and were frail. Future studies should examine the bi-directional interplay between falls and rehabilitation in elderly with ADRD with inclusion of other predictors of fall risk to study their unique characteristics.

### LUNG FUNCTION AND DEMENTIA RISK IN THE ATHEROSCLEROSIS RISK IN COMMUNITIES (ARIC) STUDY

Srishti Shrestha,[1] Xiaoqian Zhu,[1] Stephanie London,[2] Kevin Sullivan,[3] Pamela Lutsey,[4] B. Gwen Windham,[1] Michael Griswold,[3] and Thomas Mosley,[3], *1. University of Mississippi Medical Center, Jackson, Mississippi, United States, 2. National Institute of Environmental Health Sciences, Research Triangle Park, North Carolina, United States, 3. The University of Mississippi Medical Center, Jackson, Mississippi, United States, 4. School of Public Health, University of Minnesota, Minneapolis, Minnesota, United States*

Poor lung function has been linked with adverse neurocognitive outcomes including dementia, but evidence from well-designed prospective studies is limited. We therefore examined the association between lung function, as measured by forced expiratory volume in 1 second (FEV1) and forced vital capacity (FVC), and dementia risk in 12,688 participants of the ARIC study, a prospective study of adults aged 46-70 years (at index visit, mean age =57y, 45% male, 76% White) from four US communities. Lung function was assessed in 1991-1992 (index visit, 76% normal, 16% obstructive, and 8% restrictive lung function), and dementia was ascertained through 2019 via in-person assessments, telephone interviews, and medical record surveillance, with adjudication of dementia with all in-person exams. A total of 2452 developed dementia over 30 years of follow-up. We used Cox proportional hazards model to estimate hazard ratio (HR) and 95% confidence intervals (CI), adjusting for potential confounders (socio-demographics, behavioral factors, cardiovascular risk factors, APOE ε4). Higher FEV1 and FVC were associated with reduced dementia risk [(HR: 0.86, 95%CI: 0.78-0.98, per 1L increase in FEV1) and (HR: 0.86, 95%CI: 0.80-0.93 per 1L increase in FVC)]. Compared to normal lung function, restrictive disease was associated with elevated dementia risk [(HR: 1.19, 95%CI: 1.01-1.41), n=168 dementia cases]; HR for obstructive disease, though modestly elevated (1.09, 95%CI: 0.96-1.24, n=713 dementia cases), was not statistically significant. Our findings of decreased dementia risk with better lung function may have important implications in reducing burden of dementia that is attributable to environmental exposures and associated lung function impairment.

### PREVALENCE OF MCI AND DEMENTIA PRIOR TO INCARCERATION

Randall Kuffel,[1] Amy Byers,[2] Brie Williams,[2] Richard Fortinsky,[3] John Boscardin,[2] Yixia Li,[4] and Lisa Barry,[5], *1. San Francisco Veterans Affairs Health Care System, San Francisco, California, United States, 2. University of California, San Francisco, San Francisco, California, United States, 3. University of Connecticut School of Medicine, Farmington, Connecticut, United States, 4. San Francisco VA Health Care System, San Francisco, California, United States, 5. University of Connecticut Center on Aging, Farmington, Connecticut, United States*

As the number of older, incarcerated persons increases, prevalence of dementia and mild cognitive impairment (MCI) in this population will likely grow, with implications for healthcare costs and advance care planning within the prison setting. This study is the first to determine the prevalence of dementia or MCI in later-life adults prior to incarceration. We leveraged a national cohort of Veterans aged 50+ whose most recent incarceration was ≤10 years in length and who were released between 10/1/2012 and 9/30/2018 (N=17,962). We linked VA and CMS healthcare records to determine diagnosis of dementia or MCI within 3 years prior to incarceration. The overall sample had a mean age of 62.4 (±7.6) years at the start of their incarceration, were largely male (97%), and were 65% White and 30% Black. Before incarceration, 445 (2.5%) and 152 (0.8%) Veterans were diagnosed with dementia and MCI, respectively. Compared to those without a diagnosis of dementia or MCI prior to incarceration, those with dementia or MCI were older at incarceration start (66.1 [±8.3] vs. 62.3 [±7.9]), had more chronic medical conditions (4.0 [±2.2] vs. 2.2 [±1.9]), were more likely to experience homelessness (21.6% vs. 9.1%), have a TBI (24.5% vs. 9.1%), and have serious mental illness (81.7% vs. 49.1%). All comparisons were significant (p<.001). Our findings indicate that later-life adults with dementia or MCI are being incarcerated. Improved understanding of pathways linking cognitive impairment to later-life incarceration is needed to both prevent unnecessary incarceration and to help provide appropriate healthcare for this vulnerable group.

### RISK FACTORS OF SLEEP DISTURBANCE IN OLDER ADULTS WITH DEMENTIA: AN ACTIGRAPHY-BASED VALIDATION STUDY

Eunhee Cho,[1] Jinhee Shin,[1] Bada Kang,[1] Sujin Kim,[2] Sinwoo Hwang,[3] Eunji Kwon,[3] and Seok-Jae Heo,[4], *1. Yonsei University College of Nursing, Seoul, Seoul-t'ukpyolsi, Republic of Korea, 2. Yonsei University, Seoul, Seoul-t'ukpyolsi, Republic of Korea, 3. Korea Armed Forces Nursing Academy, Daejeon, Taejon-jikhalsi, Republic of Korea, 4. Yonsei University Graduate School, Seoul, Seoul-t'ukpyolsi, Republic of Korea*

Sleep disturbance is a common and significant symptom experienced by older adults with dementia. Early detection and timely treatment of sleep disturbance are critical to prevent adverse consequences including decreased quality of life for persons with dementia and increased caregiver burden. While direct observations and sleep diaries are often unreliable, actigraphy is a cost-effective method in measuring sleep problems in older adults with dementia and provides reliable and rich sleep data. Therefore, this study aimed to examine sleep disturbance objectively measured by actigraphy and its risk factors in community-dwelling older adults with dementia in Korea. This is a prospective study consisting of a two-wave dataset. The model was fitted using Wave 1 data (n=151) and then validated using Wave 2 data (n=59). Independent variables were demographics, cognitive and physical function, depressive symptoms, physical activity level, and neuropsychiatric symptoms measured by Neuropsychiatric Inventory(NPI), and clinical factors including dementia type, sedative use, and comorbidities. Sleep disturbance was defined as less than six nighttime sleep hours and sleep efficacy less than 75%. Using the Youden's Index, the sample was dichotomized into sleep disturbance group (n=83) and sound sleep group (n=68). The results of the generalized linear mixed model showed that the risk factors for sleep disturbance included vascular dementia, age, step count, and having three neuropsychiatric symptoms (i.e., delusions, depression, and disinhibition). Individuals with dementia at risk for sleep disturbance should be identified to prioritize early prevention strategies and individualized interventions. Particularly, management of delusion, depression, disinhibition is critical in preventing disturbed sleep.

### SOURCES OF POSITIVITY IN THE DAILY LIFE OF FAMILY CAREGIVERS OF PERSONS WITH DEMENTIA

Kyungmi Lee,[1] Breannlyn Archer,[2] Kaitlyn Cox,[2] and Carolyn Pickering,[2], *1. University of Alabama at Birmingham, Hoover, Alabama, United States, 2. The University of Alabama at Birmingham, Birmingham, Alabama, United States*

Family caregivers often experience fatigue, burnout, and health complications yet also enjoy many aspects of caregiving that may benefit their well-being. This study identifies positive aspects of caregiving in the daily life experiences of dementia family caregivers in order to inform interventions to support caregivers' well-being. This case study entails a secondary analysis of open-ended question data obtained from 165 family caregivers who answered daily diaries over 21 days (n = 2841 responses). We used content analysis to organize and elicit thematic categories from the data collected in response to the question "what was the best part of your day." A final 762 responses were selected as meeting the "care" criteria for the study, with an inter-rater reliability of 91.6%. Data analysis revealed three major sources of daily positive aspects including: caregiver-focused, patient-oriented, and support-system based. The analysis also revealed seven different kinds of daily positive aspects, such as getting to enjoy time with the care recipient or getting to accomplish other non-caregiving tasks. Many of the positive aspects of caregiving reported were enabled by social support, but they were ultimately from how they utilized that support (e.g., getting alone time) that provided the positivity. The findings of this study demonstrate the important role social support plays in caregiving, as well as highlights other possible intervention targets to create easier, more positive days for family caregivers.

### THE RELATIONSHIP BETWEEN NEUROPSYCHIATRIC SYMPTOMS, NIGHTTIME BEHAVIORS, AND ALZHEIMER'S DISEASE CSF BIOMARKERS

Meina Zhang,[1] Young-Eun Cho,[2] and Chooza Moon,[2], *1. University of Iowa, Iowa City, Iowa, United States, 2. University of Iowa, university of Iowa, Iowa, United States*

Alzheimer's disease (AD) commonly involves neuropsychiatric symptoms (NPS), such as nighttime behaviors (or sleep disturbance), hallucination, delusion, or mood changes. However, it is unclear how NPS and sleep disturbances are correlated with AD biomarkers. The purpose of this analysis was to examine how NPS and nighttime behaviors are associated with AD CSF biomarkers by cognitive status. A total of 1,667 subjects' (mean age = 69.4 SD=9.3, 48 % (808) were male) data from the National Alzheimer's Disease Coordinating Center (NACC) were used, including subjects with dementia (n = 577), mild cognitive impairment (MCI, n = 363), cognitive impairment but not MCI (n = 47), cognitive impairment due to Alzheimer's etiology (n= 608), and normal cognition (n = 680). The nighttime symptoms, number, and severity of NPS were assessed using the Neuropsychiatric Inventory Questionnaire Quick Version (NPI-Q). Cerebrospinal fluid (CSF) samples were analyzed for Aβ42,dft5 t-tau, p-tau. We used generalized linear models to explore the associations accounting for age, sex, APOE4

alleles, and BMI. We found the number of NPS were associated with Aβ42 (p = 0.042) in individuals with MCI, impaired, or dementia due to Alzheimer's etiology. Yet, the number of NPS were not associated with t-tau or p-tau in individuals with and without dementia. The severity of NPS including nighttime symptoms were not associated with biomarkers. Our results could suggest that the number of NPS can be reflected by higher CSF Aβ42 levels in the individuals with Alzheimer's etiology. Future longitudinal analyses are warranted to understand the causal relationships.

### TIME-VARYING INSOMNIA SYMPTOMS AND INCIDENCE OF COGNITIVE IMPAIRMENT AND DEMENTIA AMONG OLDER US ADULTS

Nicholas Resciniti, Matthew Lohman, Bezawit Kase, and Valerie Yelverton, *University of South Carolina, Columbia, South Carolina, United States*

There is conflicting evidence regarding the association between insomnia and the onset of mild cognitive impairment (MCI) or dementia. This study aimed to evaluate if time-varying insomnia is associated with the development of MCI and dementia. Data from the Health and Retirement Study (n = 13,833) from 2002 to 2014 were used (59.4% female). The Brief Insomnia Questionnaire was used to identify insomnia symptoms compiled in an insomnia severity index, ranging from 0 to 4. In the analysis, participants' symptoms could vary from wave-to-wave. Dementia was defined using results from the Health and Retirement Study (HRS) global cognitive assessment tool. Respondents were classified as either having dementia, MCI or being cognitively healthy. Cox proportional hazards models with time-dependent exposure using the counting process (start-stop time) were used for analysis. For each one-unit increase in the insomnia symptom index, there was a 5-percent greater hazard of MCI (HR = 1.05; 95% CI: 1.04–1.06) and dementia (HR = 1.05; 95% CI: 1.03–1.05), after fully adjusting. Using a nationally representative sample of adults aged 51 and older, this study found that time-varying insomnia symptoms are associated with the risk of MCI and dementia. This highlights the importance of identifying sleep disturbances and their change over time as potentially important risk factors for MCI and dementia.

### WHICH COGNITIVE BEHAVIORAL THERAPY FORMATS WORK FOR DEPRESSION IN DEMENTIA FAMILY CAREGIVERS ?

Yue Sun,[1] and Zhi-wen Wang,[2], *1. School of Nursing, School of Nursing, Peking University, Beijing, China (People's Republic), 2. Peking University Health Science Center, Beijing, Beijing, China (People's Republic)*

Cognitive behavioral therapy (CBT) has been shown to be effective to delay cognitive decline for family dementia caregivers (DCs). However, whether cognitive intervention could effectively reduce depression through internet, group, telephone, individual, unguided self-help and combined formats remains unclear. Pubmed, Embase, Cumulative Index to Nursing and Allied Health Literature (CINAHL), the Cochrane Central Register of Controlled Trials, Web of science, China National Knowledge Infrastructure database, Chinese Biomedical Literature database and Wan Fang database were systematically searched. A total of 34 studies were included in our analysis based on a series of rigorous screenings, which comprised 3577 DCs. We conducted a network meta-analysis (NMA) to evaluate the relative effects and rank probability of different CBT delivery formats. A series of analyses and assessments, such as the pairwise meta-analysis and the risk of bias, were performed concurrently. Compared with controls, internet, telephone, and individual showed the largest improvement on depressive symptoms, whereas the unguided self-help delivery format was less effective. Internet delivery formats had the highest probability among the five CBT delivery formats. Our study indicated that the internet might be the best delivery formats for reducing the depression of family DCs. The findings from our study may be useful for policy makers and service commissioners when they make choices among different CBT delivery formats.

### Session 9075 (Poster)

### ASSESSMENT

### AGING ACROSS THE LIFE COURSE: RESEARCH COLLECTIONS AVAILABLE FROM THE NATIONAL ARCHIVE OF COMPUTERIZED DATA ON AGING

James McNally, *University of Michigan, Ann Arbor, Michigan, United States*

The creation and maintenance of sustainable data archives can be challenging, but it offers clear advantages. Properly curated data can be used by multiple researchers, testing a variety of hypotheses, and increasing the return on investment to the expensive process of data collection. Having an internally managed archival system also provides greater control and autonomy in the equitable distribution of data resources. This process ensures all researchers will have full use of the data for original research, teaching, and new directions once the data leaves the control of the local investigator's control. This poster reviews the advantages of having a local strategy geared toward the preservation and sharing of gerontological research data. Using the National Archive of Computerized Data on Aging (NACDA) as a working example, the poster offers an overview of collections at NACDA. Using our metadata tools and variable search database, NACDA can identify studies in its collections that examine aspects of aging and health among adults during their lifecourse. Many of the studies are longitudinal or repeat measure cross-sectional studies. We are also able to identify studies that focus on aging that are not maintained by NACDA but which are available to interested researchers. Using a strategy of archival preservation combined with a strong focus on productive research Innovation in Aging, NACDA has amassed data and metadata covering a wide array of studies worldwide that address the aging lifecourse. Because our collections are multinational, we share these data at no cost to interested users worldwide

### COGNITIVE STATUS & DEMENTIA SEVERITY IN CVLT-II-SF FORCED CHOICE RECOGNITION: IMPLICATIONS FOR EFFORT ASSESSMENT

Karl Grewal,[1] Michaella Trites,[2] Megan O'Connell,[1] Andrew Kirk,[1] Stuart MacDonald,[2] and Debra Morgan,[1],

*1. University of Saskatchewan, Saskatoon, Saskatchewan, Canada, 2. University of Victoria, Victoria, British Columbia, Canada*

Effort testing is critical to neuropsychological practice, including dementia assessment. Questions exist around whether cognitive status or impairment severity impacts effort test performance in this population. Presently, we examined whether scores on an embedded effort test - the California Verbal Learning Test II Short Form (CVLT-II-SF) Forced Choice Recognition (FCR) - differed across diagnostic cognitive status groups and how severity of impairment modulated test performance. In a sample of memory clinic patients, three cognitive status groups were identified: subjective cognitive impairment (SCI; n = 92), amnestic mild cognitive impairment (a-MCI; n = 18), and dementia due to Alzheimer's Disease (AD; n = 70). Significant group differences in FCR performance were observed using one-way ANOVA ($p < .001$), with post-hoc analysis indicating the AD group performed significantly worse scores than the other groups. Using multiple regression, FCR performance was modelled as a function of cognitive status, impairment severity indexed MMSE, and their interaction, with a parallel analysis for the Clinical Dementia Rating Sum of Boxes (CDR-SOB) scores as an alternate severity measure. Results yielded significant main effects for MMSE ($p = 0.019$) and cognitive status ($p = 0.026$), as well as a significant interaction ($p = 0.021$). Thus, increases in impairment severity disproportionately impaired FCR performance for persons with AD, calling into question research-based cut scores for effort determination in dementia contexts. Corresponding CDR-SOB analyses were non-significant. Future research should examine whether CVLT-II-SF-FCR is an appropriately specific inclusion in a best-practice testing battery for evaluating effort in dementia populations.

### EXAMINING STEREOTYPE THREAT IN NEUROPSYCHOLOGICAL TESTING: A USABILITY AND USER EXPERIENCE PILOT STUDY

Jazlyn Armendariz,[1] Sarah Hwang,[1] Giovanna Garrido Blanco,[2] Maria Pena,[3] and Thomas Chan,[1], *1. California State University Northridge, Northridge, California, United States, 2. California State University Northridge, Chanhassen, Minnesota, United States, 3. California State University Northridge, Pacoima, California, United States*

Stereotype threat is defined as the situational predicament when people feel at risk of conforming to social stereotypes. Correspondingly, stereotype threat may negatively impair a persons' working memory and cognitive abilities during neuropsychological tests due to hyper awareness of negative stereotypes. Moreover, it is critical to test the usability and the user experience of application-based neuropsychological assessments within diverse aging adult populations. In this pilot study, verbal expressions of feeling pressure to succeed, within a diverse population of young adults, were examined while taking an application-based neuropsychological assessment. Data was collected from 15 self-identified respondents (i.e., 7 Latinx, 5 Asian, 3 Bi-racial). Before beginning the assessment, 8 out of 15 participants exhibited self-handicapping behaviors such as offering explanations of mental exhaustion due to work and lack of sleep. Literature suggests these expressions are related to the onset of anxiety prior to taking cognitive tests, and contribute to potentially offering an excuse in anticipation of poor performance. Additionally, 3 out of 15 participants noted that even though the tasks were simple, they felt unintelligent because they did not complete the tasks to their best abilities (e.g., "I felt stupid. It was simple"). Findings from this pilot support the negative impact stereotype threats have on feelings of inadequacy and increase of anxiety levels among ethnic minorities in testing settings. Further emphases on examining the usability and user experience of application-based tests are needed, particularly within a diverse population of aging adults to facilitate more culturally competent neuropsychological testing experiences.

### FINDING FACTORS IN FOOTFALLS: EXPLORING THE FACTOR STRUCTURE OF GAIT IN OLDER ADULTS.

Michael Willden,[1] Josh Palmer,[2] Kristina Kowalski,[3] Sandra Hundza,[1] and Stuart MacDonald,[1], *1. University of Victoria, Victoria, British Columbia, Canada, 2. University of Victoria, University of Victoria, British Columbia, Canada, 3. University of Calgary, University of Calgary, Alberta, Canada*

Gait is a reputed marker of global health spanning various bodily systems (MacDonald et al., 2017) and is a robust predictor of deleterious age-related outcomes (Van Kan et al., 2009). However, the sheer number of individual gait variables employed as predictors in the existing literature can obscure interpretations. To address this issue, researchers have explored the factor structure of gait indicators to explain variance in age-related gait performance, identifying disparate models characterized by three to five underlying latent gait constructs comprised of 8 to 23 indicators (Hollman et al., 2011; Lord et al., 2013). Beyond this heterogeneity, additional limitations characterizing this literature include solutions that assume statistical independence among gait constructs, as well as inclusion of severely multicollinear indicators. Using data from the Healthy Minds Healthy Bodies (HMHB) study, the present research focused upon replicating and contrasting previous factor analytic efforts. HMHB participants (n=128) were healthy community-dwelling adults ($M_{age}=72.81\pm5.24$ years; female=100). Gait indicators from a GAITRite computerized walkway were selected according to a priori theoretical rationale, compatibility with previous studies, and consideration of multicollinearity. Gait factor structure was initially analyzed using principal component analysis. Results indicate the presence of three latent gait domains reflecting pace, rhythm, and variability, accounting for over 82.4% of the variance in gait performance. Current proceedings involve implementing confirmatory factor analysis to compare competing gait models. Findings will address disparities across factor models in the gait literature, as well as discuss the optimal number of factors for describing the underlying dimensionality of gait.

### MEASUREMENT INVARIANCE IN THE ASSESSMENT OF MOOD BETWEEN AMERICAN AND MEXICAN COMMUNITY STUDIES

Manuel Herrera Legon,[1] and Daniel Paulson,[2], *1. University of Central Florida, Miami, Florida, United States,*

2. *University of Central Florida, Orlando, Florida, United States*

The Health and Retirement Study (HRS), a principal source for American public health research, has numerous global sister studies. Harmonization efforts seeking to establish measurement equivalence amongst these various datasets, is a critical prerequisite to cross-cultural research. Given well-known cultural variability in depressive symptom endorsement, the purpose of this study was to assess measurement invariance in a brief mood measure used in the HRS and the Mexican Health and Aging Study (MHAS). Total sample size using both groups was 15,319 participants (10,931 HRS; 4,388 MHAS) who were 65 and older from Waves 6 to 13 in the HRS and Waves 1 to 4 in the MHAS. MPlus Version 8.4 was used to conduct CFA analyses of measurement invariance. A contemporary approach with categorical data calls for examining threshold invariance first while establishing configural invariance, before examining invariance tests of thresholds, loadings, and intercepts in a second step. Results were that measurement invariance was not supported in this series of two steps with four out of six indices showing model fit in the first model and none of the indices showing model fit in the second model. These findings implied that there were differences in ways of responding to the brief mood measure between HRS and MHAS participants at the conceptual level. Thus, comparisons based on these measures may result in misleading findings and should be interpreted very conservatively. This study adds to the growing body of literature guiding harmonization efforts from the Program on Global Aging, Health and Policy.

### MEASUREMENT INVARIANCE OF NEGATIVE AFFECT IN AMBULATORY ASSESSMENTS OF YOUNG-OLD AND OLD-OLD ADULTS

Oliver Schilling,[1] Anna Lücke,[2] Martin Katzorreck,[3] Ute Kunzmann,[4] and Denis Gerstorf,[5], *1. University of Heidelberg, Heidelberg, Baden-Wurttemberg, Germany, 2. Heidelberg University, Heidelberg, Baden-Wurttemberg, Germany, 3. Humboldt University of Berlin, Berlin, Berlin, Germany, 4. Leipzig University, Leipzig, Sachsen, Germany, 5. Humboldt University of Berlin, Humboldt University of Berlin, Brandenburg, Germany*

Gero-psychological research increasingly considered intense longitudinal assessments of momentary affect to address affective aging. In particular, many studies employed negative emotion item lists for ambulatory assessments of negative affect. However, frequent self-reports on emotion items within short time intervals might change alertness towards and perception of one's emotional experiences. From an item-response-theoretic point of view, this might impair the stability of item functioning in terms of item discrimination between levels of affectivity and item severity (difficulty). Thus, we examined measurement invariance of negative emotion items commonly used for ambulatory assessments of negative affect. Ambulatory assessments from the EMIL study, obtained over seven consecutive days at six occasions per day from 123 young-old (aged 66-69) and 47 old-old (86-89) adults, were analyzed. Respondents self-reported on 13 negative emotion items, using a 0-100 slider to express the degree to which they felt the respective emotion. We ran multilevel structural equation models with Bayes estimation to analyze variability of negative affect factor loadings, item intercepts, and measurement error variances across repeated measures, thus checking for metric, scalar, and strict factorial invariance. For all sets of parameters, the findings do not strongly support measurement invariance, but point at partial invariance for item subsets. Taking on literature suggesting that criteria for invariance testing should not be too restrictive to meet pragmatic measurement equivalence requirements, further analyses and our conclusions focus on strategies that might allow for acceptable degrees of differential item functioning, enabling reliable analyses of intra-individual short-term variability in negative affect.

### MEASUREMENT OF CENTRE OF PRESSURE USING THE WII BALANCE BOARD IN OLDER ADULTS WITH SIMULATED VISUAL IMPAIRMENT

Berkley Petersen, Karen Li, Caitlin Murphy, and Aaron Johnson, *Concordia University, Concordia University, Quebec, Canada*

Postural stability is a complex skill dependent upon the coordination of motor, sensory and cognitive systems. The purpose of this project was therefore to explore how older adults' balance performance is impacted by increased cognitive load, hearing loss, and simulated vision loss. Twenty-eight older adults between the ages of 50 and 93 years ($M = 73.86$, $SD = 10.43$) were tested. Participants underwent standard sensory acuity and cognitive functioning tests. The balance trials varied as a function of cognitive load and visual challenge resulting in five conditions: (1) eyes closed, (2) normal vision clear goggles (NV) (3) simulated low vision (20/80) goggles (LV) (4) LV and math task, (5) NV and math task. Postural stability was assessed with three key center of pressure parameters: total path length (TPL), anterior-posterior amplitude (APA) and medial-lateral amplitude (MLA). A mixed-model ANOVA using hearing acuity as a covariate revealed significant effects of complexity in sway amplitude: (APA: $p < .017$; MLA: $p < .020$), while TPL approached significance ($p < .074$). T-tests revealed significant ($p < .05$) decreases in balance performance across all 3 centre of pressure parameters when comparing single task NV to dual-task NV, NV vs. eyes closed and single task NV vs. LV dual-task. There were significant positive correlations between hearing acuity and balance (MLA) under single task NV ($r = .491$) and LV conditions ($r = .497$). Results suggest the attentional demands from increased cognitive load and sensory loss lead to decreases in older adults' single- and dual-task balance performance.

### Session 9080 (Poster)

### ATTITUDES AND PERCEPTIONS TOWARD TECHNOLOGY

### ADAPTING TRAINING AND USE OF AN APPLICATION FOR COGNITIVELY IMPAIRED OLDER ADULTS AMIDST COVID-19

Francesca Falzarano,[1] Sarah Weingast,[2] Gabriel Gladstone-Groth,[3] Marco Ceruso,[4] and Sara Czaja,[5], *1. Weill Cornell Medicine, Douglaston, New York, United States, 2. Weill Cornell Medicine, Weill*

*Cornell Medicine, New York, United States, 3. Weill Cornell Medicine, New York, New York, United States, 4. Weill Cornell Med, New York, New York, United States, 5. Weill Cornell Medicine/Center on Aging and Behavioral Research, New York, New York, United States*

The emergence of COVID-19 and social distancing requirements have resulted in disruptions to daily life, reduced opportunities for social engagement, and diminished resource access for millions of older adults. Individuals with cognitive impairments (CI) are particularly vulnerable to risk for social isolation. This presentation will discuss the PRISM-CI pilot trial, which aims to examine the feasibility and potential efficacy of the PRISM-CI software system on enhancing connectivity and quality of life among a diverse sample of 50 older adults aged 65 and over with a CI. PRISM-CI, adapted from the PRISM system (developed by the Center for Research and Education on Aging and Technology Enhancement) for this population, is intended to support social engagement, memory, and access to resources and information. We will present data regarding the feasibility and perceived value of PRISM-CI and discuss the challenges, and strategies used, to adapt the PRISM-CI trial during the pandemic. We used a multi-modal approach to provide remote training and specialized tablet instruction that includes individualized training sessions tailored to individuals' learning needs, hobbies, and prior technology use. The adapted protocol also involves the use of remote access software for troubleshooting. We will also discuss how participant feedback guided the inclusion of additional features, such as Zoom videoconferencing and virtual library access, for the PRISM-CI application. Finally, we will demonstrate how the adaptation of the PRISM-CI protocol holds promise for the use of flexible, remote technology approaches to reach socially isolated older adults to foster psychosocial well-being.

### CO-DESIGN OF TECHNOLOGY DRIVEN SELF-MANAGEMENT WITH OLDER ADULTS: AN INTERNATIONAL APPROACH

Danielle Saney,[1] Alessandro Pollini,[2] Sandra Suijkerbuijk,[3] Regina Krijger,[4] and Arlene Astell,[5], *1. University of Toronto/Toronto Rehabilitation Institute, Toronto, Ontario, Canada, 2. BSD Design, Rome, Lazio, Italy, 3. Vilans, Utrecht, Utrecht, Netherlands, 4. Careyn, Utrecht, Utrecht, Netherlands, 5. University of Toronto, Toronto, Ontario, Canada*

There has been an emergence in technology applications (apps) aimed at addressing needs amongst older adults and persons with cognitive impairment (PwCI). Despite the ubiquity of these apps, utilization is low, primarily due to a lack of involvement of PwCI and the perception that these apps have little motivational value. Engaging PwCI in creative processes such as co-design could lead to the creation of apps that better meets the needs of this population. The current study applies a user-centered, participatory approach to involve PwCI in the design of a new self-management and coaching app, RESILIEN-T. Co-design workshops were held with 12 PwCI across Italy, Netherlands and Canada; structured as four modules: (1) introduction and expectation setting, (2) user analysis, (3) storytelling, and (4) collaborative design. Based on interviews with PwCI, stories of individuals which reflect the target population were created (personas) and the solutions to the needs of these personas were discussed. Information about participant's interests, computer proficiency and self-rated cognitive decline were collected. Participants were asked to try 10 existing apps and provided feedback on the design, usability and functionality. Lastly, participants were shown a prototype for RESILIEN-T and provided feedback based on the personas that they helped create. Co-design activities revealed that personalization is crucial for adherence. Aspects of physical and social activity, nutrition and cognition were most important to participants. Participants found many apps that are recommended for older adults do not appear age appropriate and seem condescending. These findings were common across PwCI from various nations.

### EXERCISE AND ATTITUDE FOR EXOSKELETONS: THE MEDIATION OF TECHNOLOGY ANXIETY AND PERCEIVED USEFULNESS

Seol Ah Lee,[1] Do Kyung Yoon,[1] Miseon Kang,[1] Kwang Joon Kim,[2] YoonMyung Kim,[1] Yun Mook Lim,[1] and Hey Jung Jun,[1], *1. Yonsei University, Seoul, Seoul-t'ukpyolsi, Republic of Korea, 2. Severance Hospital, Yonsei University College of Medicine, Seoul, Seoul-t'ukpyolsi, Republic of Korea*

The present study aimed to examine the effects of regular exercise on attitude towards using exoskeletons and the mediating effects of technology anxiety and perceived usefulness among Korean older adults. Data was collected through online recruitment in February 2021. The sample comprised 310 people (Age: 65-89, M=70.18, SD=4.58) who did not report dementia. The dependent variable was the attitude towards using technology, especially lower limb exoskeleton robots for exercise. The independent variable, regular exercise, was coded as a binary variable. The mediating variables were technology anxiety and perceived usefulness measured by the sum of three questions about exoskeleton robots, respectively. Gender, age, education level, and household income were included as control variables. The mediating effect was estimated by serial path analysis and bootstrapping (model fit indices: $\chi2=18.400$, df=8, $p<0.05$, RMSEA=0.065, CFI=0.973, TLI=0.940, SRMR=0.044). Results showed the total effect of regular exercise was significant (B=1.253, $p<0.01$) and the total indirect effect of it was significant(B=1.540, $p<0.001$). There was no significant direct effect of regular exercise on the attitude towards using technology. The association between regular exercise and the attitude of using technology was completely mediated by perceived usefulness (B=1.439, CI=0.569-2.358, $p<.01$). Although technology anxiety had no mediating effect alone, the serial mediating effect via the path from technology anxiety to perceived usefulness was significant (B=0.119, CI=0.004-0.332, $p<.05$). This study will be the first empirical study to examine the effects of health habits by expanding the senior technology acceptance model for older adults in Korea.

### FACTORS AFFECTING THE INTENTION TO USE EXOSKELETON FOR EXERCISE AND THE MODERATING EFFECT OF FRAILTY

Choi Bomi,[1] Susanna Joo,[2] Changmin Lee,[1] Chang Oh Kim,[3] Yun Mook Lim,[1] and Hey Jung Jun,[1],

*1. Yonsei University, Seoul, Seoul-t'ukpyolsi, Republic of Korea, 2. Yonsei University, Seodaemun-gu, Seoul-t'ukpyolsi, Republic of Korea, 3. Severance Hospital, Yonsei University College of Medicine, Seoul, Seoul-t'ukpyolsi, Republic of Korea*

This study aims to find relevant factors influencing the intention to use exoskeletons for exercise (IEE) among older adults and to analyze the moderating effect of frailty. The sample of this study is 310 people (65 or older) without cognitive impairment who completed an online survey. The intention to use exoskeletons was measured with three questions from the Senior Technology Acceptance Model (STAM). Potential relevant factors comprise sociodemographic characteristics, physical and psychological health, exercise, attitude towards aging, and social relationship. Linear regression analyses showed that depressive symptoms, regular exercise, attitude towards aging, and social participation were significantly related to IEE. People with more depressive symptoms and a negative attitude towards aging are more likely to have a higher level of IEE. People who exercise regularly and actively participate in social activities showed a higher level of IEE. Subgroup analyses were performed based on the frailty status measured with Korean Groningen Frailty Indicator (K-GFI). Among people without frailty (N=177), regular exercise, and social participation were positively related to IEE. The number of chronic diseases and social participation was positively related to IEE among people with frailty (N=133). The results of this study implied that poor health conditions lead to an increased need for exoskeletons. The results of this study also suggested that exercise and social participation work as facilitating factors in the context of gerontechnology acceptance. Results of subgroup analyses suggested that influencing factors on IEE can vary depending on the physical functional status.

### FRAILTY STATUS MODERATES THE ASSOCIATION BETWEEN SELF-EFFICACY AND THE INTENTION TO TECHNOLOGY USE

Miseon Kang,[1] Si Young Song,[1] Inhye Jung,[1] YoonMyung Kim,[1] Chang Oh Kim,[2] and Hey Jung Jun,[1], *1. Yonsei University, Seoul, Seoul-t'ukpyolsi, Republic of Korea, 2. Severance Hospital, Yonsei University College of Medicine, Seoul, Seoul-t'ukpyolsi, Republic of Korea*

This study examined how the status of frailty moderated the association between the self-efficacy about gerontechnology use and the intention to use gerontechnology (IUG) among Korean older adults. In this study, gerontechnology devices referred to exoskeleton robots for exercise. The data was collected through an online survey in February 2021, and 324 Korean older adults aged 65 and above were included in the analysis (Women: 50.9%, Men: 49.1%). The dependent variable was the intention to use gerontechnology from the Almere model (Heerink, 2010) and the independent variable was self-efficacy about gerontechnology use from the Senior Technology Acceptance Model(Chen & Chan, 2014). Both were measured as continuous variables. The moderating variable was the status of frailty (Non-frail=0, Frail=1). Age, gender, education level, and log-transformed household income were controlled for. Multiple linear regression to examine moderation effect was conducted using PROCESS Macro model 1. The findings showed that frailty status moderates the association between self-efficacy and IUG among Korean older adults. Concretely, the higher self-efficacy about gerontechnology use, the lower IUG for non-frail Korean older adults. However, the main effect of self-efficacy was non-significant for the frail sample. Even though self-efficacy has been known to affect the variables related to technology use or acceptance positively, the results suggest that there may exist differences in research results depending on participants' health status. The type of gerontechnology devices may also have affected the results. Further exploration is needed to the interaction effects of potential influencing factors on the gerontechnology acceptance model.

### GENDER DIFFERENCES IN ONLINE HEALTH-RELATED SEARCH BEHAVIORS AMONG OLDER ADULTS

Matthew Picchiello,[1] Payton Rule,[2] Tina Lu,[2] and Brian Carpenter,[3], *1. Washington University in St. Louis, Olivette, Missouri, United States, 2. Washington University in St. Louis, St. Louis, Missouri, United States, 3. Washington University in St. Louis, Saint Louis, Missouri, United States*

Nearly 60% of older adults use the internet for health-related reasons. Some studies have demonstrated differences in the frequency at which men and women perform various online activities. However, few studies have investigated gender differences in health-related search behaviors (HRSB). The purpose of this study was to examine differences in self-reported HRSB between older men and women. A total of 47 older adults (M age = 66.6, 55% female, 87% White) completed a survey assessing perceived usefulness and trust in the internet for health-care information, types of websites used, and reasons for looking up health information. Independent samples t-tests revealed that, compared to women, men regard the internet as more useful in helping them make health care decisions ($t(45) = 2.715$) and as a more trustworthy source ($t(45) = 2.24$, p's $< 0.05$). Men were more likely to get health information through sources affiliated with educational institutions ($\chi2(1) = 3.9$) and government agencies ($\chi2(1) = 8.8$), whereas women were more likely to use social media, ($\chi2(1) = 4.3$, p's $< 0.05$). Lastly, men were more likely to use the internet to learn about information on medical procedures ($\chi2(1) = 5.1$), while women were more likely to learn about alternative treatments ($\chi2(1) = 4.9$, p 's $< 0.05$) online. As 72.3% of participants indicated the internet as their first source for health information, interventions geared towards enhancing HRSB for older adults are needed, especially for older women whose HRSB may make them particularly vulnerable to misinformation.

### HUMAN FACTORS LINKED WITH INITIAL AND CONTINUOUS TRUST IN AUTONOMOUS SYSTEMS: A LITERATURE REVIEW

Maria Pena,[1] Jared Carrillo,[2] Nonna Milyavskaya,[3] and Thomas Chan,[3], *1. California State University Northridge, Pacoima, California, United States, 2. California State University Northridge, Van Nuys, California, United States, 3. California State University Northridge, Northridge, California, United States*

Many autonomous systems are being developed to assist older adults to age in place. However, there is little research related to the human factors associated with why older adults may initially and continuously trust these autonomous systems. More research in this area on older adults and trust in autonomy is needed to facilitate the technologies better everyday use. The current study conducted a literature review on the prevalent human factors that enable people to trust their interactions with smart technologies (e.g., artificial intelligence, navigational structures). Articles were collected from various disciplines on concepts such as trust in autonomy, human-computer interactions and teamwork. Thematic analysis revealed two convergent areas that were associated with initial and continuous trust: human and technological characteristics. Human characteristics are defined by a person's ability to understand and use autonomous systems. Generally, people with higher competency and abilities with autonomous systems demonstrated the ease of use to carry out desired actions with smart technology. Technological characteristics are defined by the system's performance, explainability, and its intended purpose between trust. Essentially, people were less critical of autonomous systems that were perceived to be useful, transparent, and predictable. Overall, the autonomous system's ability to perform its intended purpose and the users knowledge and technical qualifications dominate the relationship between initial and continuous trust with autonomous systems. These are the prevalent factors that need to be considered for the creation of trusted autonomous technologies for older adults to help them age in the approaching more advanced technological world.

### INTERACTION EFFECT OF SUBJECTIVE HEALTH AND ATTITUDE ABOUT AGING ON GERONTECHNOLOGY ACCEPTANCE

Hayoung Park,[1] Susanna Joo,[2] Kwang Joon Kim,[3] Chang Oh Kim,[3] Yun Mook Lim,[1] and Hey Jung Jun,[1], *1. Yonsei University, Seoul, Seoul-t'ukpyolsi, Republic of Korea, 2. Yonsei University, Seodaemun-gu, Seoul-t'ukpyolsi, Republic of Korea, 3. Severance Hospital, Yonsei University College of Medicine, Seoul, Seoul-t'ukpyolsi, Republic of Korea*

This study examined the interaction effect of subjective health and attitude about aging on gerontechnology acceptance among Korean older adults. The sample was 310 Korean older adults aged 65 and above without cognitive impairment who completed an online survey. The dependent variable was the attitude about gerontechnology, especially an exoskeleton robot for exercise. The independent variable was subjective health measured by the questions about self-reported health conditions. The moderating variable was the attitude about aging, which was measured by asking how much they feel less useful as they age. Covariates were age, gender, education level, employment status, income, and marital status. The results from the regression analyses using PROCESS macro and bootstrapping showed that the interaction effect of subjective health and attitude about aging is significant for gerontechnology acceptance; when older adults consider themselves less useful as they age, they tend to have positive attitudes about gerontechnology despite their subjective health. However, of those who consider that they are not less useful as they age, they tend to have positive attitudes about gerontechnology only when they consider themselves unhealthy. Thus, they tend to have negative attitudes about gerontechnology when they consider themselves healthy and useful. The findings imply that gerontechnology-based exercise programs or interventions could be welcomed by those who consider themselves unhealthy and think they are useless as they age. Also, the findings suggest that when applying an intervention program using gerontechnology, health status and self-assessment of aging should be considered in advance.

### OLDER KOREANS' INFORMATION SUPPORT AND INTERNET USE: INTERNET SKILLS AND TECHNOLOGY ATTITUDES

Hye Soo Lee, *Oregon State University, Corvallis, Oregon, United States*

While older Koreans have growing access to Internet, they still lag in actual utilization. This study examined effects of different information support sources on Internet utilization and whether these were mediated by Internet skills and technology attitudes among older men and women. This study used secondary data from 2019 Digital Divide Survey conducted by National Information Society Agency of Korea. The sample consisted of 1,031 Korean Internet users aged 60+, including 495 men and 536 women. Support sources included personal and professional. Skills were measured by ability to use specific features of mobile devices such as smartphones (seven items), while utilization was measured by the use of mobile devices for specific reasons (25 items). Serial mediation analyses using both skills and attitudes were conducted separately according to gender and support sources, covarying for demographics and health. In general, information support was positively associated with utilization. For men, personal informational support was mediated by technology attitudes only. For women, professional informational support was mediated by both Internet skills and technology attitudes, but the serial indirect effect was not significant for this model. The other two models showed significant serial mediation effects through Internet skills and technology attitudes, in this order. Only women had significant direct associations between information support and Internet utilization. Regardless of the source, informational support is positively associated with older Koreans' Internet utilization. Professional support for men and personal support for women may be most beneficial for greater Internet utilization.

### PRIVACY AND USABILITY IN COVID ENROLLMENT APPS

Laura Calloway,[1] Lesa Huber,[2] and L. Jean Camp,[1], *1. Indiana University, Bloomington, Indiana, United States, 2. School of Public Health, Indiana Univ, Bloomington, Indiana, United States*

Enrollment apps for COVID-19 vaccinations are meant to be privacy-enhancing, but poor design puts privacy at risk. We report on a qualitative exploration of the experiences of older adults attempting to register for vaccination. We engaged in a think-aloud protocol with six participants over age 65 over Zoom as they used the New York state vaccination portal. Authentication requirements were: Medicare

ID, DOB, address, and phone (optional). For this cohort, Social Security numbers were the default Medicare ID. We found that a privacy-enhanced authentication option exists, but efforts to use privacy-preserving enrollment were confounded by security-enhancing timeouts. Choosing to use the time-consuming privacy-preserving authentication increased the risk that available vaccines were taken. As a result, older adults reliant on volunteers to enroll revealed sensitive information and risked identity theft. A design that was meant to be privacy-enhancing by offering multiple avenues for authentication and ensuring logout via timeouts created a system where the more secure option was not effectively available due to a competing security mechanism. This was exacerbated by a counter counting down the number of vaccine sites available, similar to a well-known stress condition used to create cognitive load in laboratory experiments. All six participants used privacy-sensitive information to enroll; provided adequate information for identity theft; and all six encountered stop points. The countdown of available vaccination sites, the time required for insurance validation as an alternative to Medicare ID, and logging off after inactivity to prevent session theft each are good practices; but fail together.

### SOCIAL PARTICIPATION AND ATTITUDE TOWARDS GERONTECHNOLOGY: THE MODERATING ROLE OF FRAILTY STATUS

Ka Yeon Lee,[1] Sun Ah Lee,[2] Susanna Joo,[3] YoonMyung Kim,[1] Yun Mook Lim,[1] and Hey Jung Jun,[1], *1. Yonsei University, Seoul, Seoul-t'ukpyolsi, Republic of Korea, 2. The Pennsylvania State University, University Park, Pennsylvania, United States, 3. Yonsei University, Seodaemun-gu, Seoul-t'ukpyolsi, Republic of Korea*

The purpose of this study was to examine whether frailty status moderates the association between social participation and attitude towards using gerontechnology. The sample was Korean older adults without cognitive impairment (N = 310, aged 66-90, 51% women) who completed an online survey. The attitude towards using gerontechnology was measured with two questions from the Senior Technology Acceptance Model (Chen & Chan, 2014), asking whether using technology is a good idea and whether they like the idea of using technology. Social participation was assessed by asking whether the participants engage in social or community activities on a scale of 1–10. Frailty status was determined based on the Korean Groningen Frailty Indicator (K-GFI). Covariates were age, gender, marital status, employment status, education level, and household income. Results from regression analyses showed significant interaction between frailty status and social participation on attitude towards using gerontechnology. Specifically, social participation was associated with positive attitude towards using gerontechnology among non-frail older adults. This association was not significant among frail older adults. Our findings suggest that the relationship between social participation and attitude towards using gerontechnology might differ by physical health status. Among older adults who are physically healthy and actively participate in social activities, the attitude towards using gerontechnology might be more positive due to greater exposure to new technology-related information. Future studies need to address alternative ways to enhance technology-friendliness among older adults with poor physical health.

### TECHNOLOGY ANXIETY ON ATTITUDE TOWARDS TECHNOLOGY: THE MODERATING EFFECT OF AGE-FRIENDLY ENVIRONMENT

Do Kyung Yoon,[1] Seol Ah Lee,[1] DaeEun Kim,[1] Chang Oh Kim,[2] and Hey Jung Jun,[1], *1. Yonsei University, Seoul, Seoul-t'ukpyolsi, Republic of Korea, 2. Severance Hospital, Yonsei University College of Medicine, Seoul, Seoul-t'ukpyolsi, Republic of Korea*

The purpose of this study was to examine the moderating effect of an age-friendly environment on the relationship between technology anxiety and attitude towards technology among Korean older adults. We collected data by online surveys in February 2021, and the sample was 324 Korean older adults aged 65 and above. The dependent variable was the attitude towards technology, which meant the appraisal about using a wearable robot for exercise. The independent variable was technology anxiety, meaning an individual's apprehension of using a wearable robot. The moderating variable was age-friendly environment, which comprises domains of the physical environment, social environment, and municipal services. The higher the score is, the more age-friendly the environment was perceived. Control variables were age, sex, education, household income. The moderation effect was estimated by bootstrapping and PROCESS macro. Results showed that when older adults showed a higher level of technology anxiety, their attitude towards technology was less positive. Moreover, the moderation effect of an age-friendly environment was significant. Concretely, in the case of living in a less age-friendly environment, older adults with a higher level of technology anxiety were more likely to report a less positive attitude towards technology. However, the effect of technology anxiety on attitude towards technology was not significant among older adults living in a more age-friendly environment. It suggested that a practical intervention to reduce the level of technology anxiety is in need in order to promote a positive attitude towards technology, especially for older adults living in a less age-friendly environment.

### TECHNOLOGY USE, COMFORT, AND INTEREST: A COMPARISON BETWEEN CAREGIVER AND OLDER ADULT POPULATIONS

Sarah Hubner,[1] Akankshya Chataut,[1] Marcia Shade,[2] Ann Fruhling,[1] Natalie Manley,[2] Meaghan Fitzgerald Walls,[3] and Julie Blaskewicz Boron,[1], *1. University of Nebraska Omaha, Omaha, Nebraska, United States, 2. University of Nebraska Medical Center, Omaha, Nebraska, United States, 3. University of Nebraska Omaha, University of Nebraska at Omaha, Nebraska, United States*

Remaining community-dwelling is a goal for most aging adults; however, this may necessitate assistance from caregivers. To reduce burden and improve adult autonomy, recent technological advancements have provided various supports. These advancements may improve quality of life (QOL) while also enhancing psychological/physical well-being for adults and caregivers. To investigate relationships between technology, QOL, and caregiver burden, needs assessments with focus groups were utilized. Four older adult focus groups (N=20) and three caregiver focus groups (N=12) were convened. Older adult participants, aged 64-83 years (M=73.1,SD=5.3), were 50% female and generally white (90%). Caregiver participants, aged 31-78 years (M=61.9,SD=12.6), were majority female (83%) and generally white (92%). Because of the ongoing COVID-19 pandemic, focus groups were conducted via Zoom video-conferencing. Thematic analyses revealed major themes of privacy, transportation, and interest in streamlined technologies. Throughout groups, privacy was consistently described; participants were either 1) apathetic, noting absence of privacy or 2) hyper-vigilant about security, citing privacy as a major barrier to utilization. Transportation, specifically self-driving/enhanced vehicles, emerged as a focus for future technologies as a means to reduce care burden and improve personal autonomy/QOL. In general, participants noted that major barriers to technology use included complexity and cost; persons expressed interest in simpler/cheaper devices. This study indicates varied interest in technology while exposing barriers to use. Additionally, the methodology demonstrates the utility of technology (e.g., Zoom) in accessing vulnerable and/or isolated populations. Overall, understanding barriers to technology use and adoption informs upcoming developments and may improve accessibility and usefulness in future systems/devices.

### THE ROLE OF AGE-FRIENDLY ENVIRONMENT BETWEEN COGNITIVE ABILITY AND ATTITUDE TOWARDS GERONTECHNOLOGY

Inhye Jung, Miseon Kang, Si Young Song, Yun Mook Lim, and Hey Jung Jun, *Yonsei University, Seoul, Seoul-t'ukpyolsi, Republic of Korea*

This study examined the impact of the age-friendly environment on the association between cognitive ability and the attitude towards gerontechnology. The participants were 277 Korean young-old age 65 through 74. The data were collected by an online survey conducted in February 2021. The dependent variable was the attitude towards gerontechnology sum of perceived usefulness, perceived ease of use, and perceived adaptability (Heerink et al., 2010; Xu et al., 2015). The independent variable was the cognitive ability of participants to report on their own. The moderating variable was the age-friendly environment sum of physical, service-side, and cultural conditions. Multiple regression analysis was conducted to determine the relation between cognitive ability and the attitude towards gerontechnology. The moderating effect was then analyzed using PROCESS macro and bootstrapping. Results show that cognitive ability has a positive effect on the attitude towards gerontechnology, and the age-friendly environment has softened its effectiveness. When participants were living in a more age-friendly environment, their attitude toward gerontechnology was less affected by their cognitive ability. However, when age-friendly environmental condition scores above 62 (out of 75), the environmental aspect did not affect the association between cognitive ability and attitudes to gerontechnology. This study suggests that the age-friendly environment can narrow the disparity of the attitude towards gerontechnology depending on the cognitive capability levels under certain conditions. Regarding the attitude towards technology may affect the actual use, the possibility of environmental help is meaningful.

### TRUST WITH AUTONOMOUS INTELLIGENCE SYSTEMS TO PROMOTE ADOPTION IN ASSISTIVE TECHNOLOGIES: A LITERATURE REVIEW

Jared Carrillo,[1] Maria Pena,[2] Nonna Milyavskaya,[3] and Thomas Chan,[3], *1. California State University Northridge, Van Nuys, California, United States, 2. California State University Northridge, Pacoima, California, United States, 3. California State University Northridge, Northridge, California, United States*

While advancements in machine learning are increasing rapidly, very little progress has been made in its mass adoption despite its benefits in assistive technologies for older adults. By examining how users interact with smart technologies, characteristics of trust can be identified and enhanced to increase adoption of the next generation of assistive systems. The current study conducted a literature review to understand better how trust with autonomous systems is formed and maintained. Twenty-two pertinent articles were identified in which three themes emerged. First, people tend to forgive human errors more than errors made by machines -- meaning mistrust is exaggerated when systems make mistakes. Second, the development of trust depends on how the system solves the tasks it is assigned, for instance if a user does not believe the system acted in an "ethical way," distrust may form and the continuation of adoption is decreased. Lastly, trust depends on the situation and the risk/reward associated with using the system, for example the trust needed to board an autonomous plane differs from that for a simple grammar correction. Taken together, the black box ideology of autonomous systems may be an issue that prevents trust in them to be formed and maintained. Promising future directions are to create machine language translators that improve transparency of autonomous system behaviors (i.e., explainability). Even if assistive technologies are created to aid older adults -- the lack of focus on understanding the factors that foster trust may dampen their actual use.

### USING SMART SPEAKERS IN LOW-INCOME SENIOR HOUSING TO ENHANCE THE AGING IN PLACE EXPERIENCE: STAKEHOLDER VIEWS

Jane Chung,[1] Jodi Winship,[2] Katherine Falls,[2] Pamela Parsons,[2] and Michael Bleich,[2], *1. Virginia Commonwealth University, Glen Allen, Virginia, United States, 2. Virginia Commonwealth University, Richmond, Virginia, United States*

Smart speakers provide a platform that can integrate smart home technology and/or safety devices within the home to enhance quality of life and independent living for older adults. However, few attempts to utilize this technology specifically within low-income senior housing (LISH) residents have been documented. Our purpose was to explore different stakeholder perceptions about the use of smart speakers to support aging in place in older adults living alone in LISH. Smart speakers were deployed in individual LISH apartments, equipped with a voice technology-based aging in place solution for the facility. A qualitative analysis of semi-structured interviews using a constant comparative approach for emerging themes was conducted (n=10: older adult users, n=2: housing staff, n=2: voice technology developers). The three participant groups showed diverging perceptions in terms of benefits, uses, and stakeholder interests. Older adults found smart speakers useful in four main areas: assistance with daily tasks, feeling connected, safety measures, and emotional wellbeing. The two other groups showed a broader interest in the use of the smart speaker device, such as residential management tools and communication channels in addition to its potential use as safety and wellness tools. Older adults experienced significant difficulty setting up desired functions or finding instructions, which restricted utilization of the technology to a limited set of tasks. All stakeholder groups addressed a need for formal training or personalized tech support for older adult users. Findings indicate the importance of developing deployment strategies tailored to the needs and characteristics of the target user group.

### VOICES OF CAREGIVERS: KEY DEMANDS TOWARDS AI-DRIVEN HOME MONITORING IN COMMUNITY-BASED DEMENTIA CARE

Christian Wrede, Annemarie Braakman-Jansen, and Lisette van Gemert-Pijnen, *University of Twente, Enschede, Overijssel, Netherlands*

While most people with dementia prefer to live at home for as long as possible, this also puts more pressure on both their informal and formal care network. To provide support in home-based dementia care, there is growing interest in technology that allows caregivers to remotely monitor health and safety of people with dementia. Novel generations of these technologies are using non-wearable, pervasive sensors coupled with algorithms to continuously collect and model meaningful in-home information. However, while these self-learning monitoring systems develop rapidly, their target users' views and demands are still insufficiently mapped out. To identify possible barriers to acceptance and ways to overcome these, we conducted a scenario-based study, including semi-structured interviews with informal caregivers (n=19) and focus groups with home care professionals (n=16) of community-dwelling people with dementia. Inductive qualitative content analysis revealed that both groups of caregivers were concerned about the informational privacy of their care recipient with dementia, information overload, and ethical issues related to dehumanizing care. Identified demands mainly centered around how to overcome these barriers. We identified several demands related to specific functionalities, user experience factors, services surrounding the technology, and integration into the existing work context. Most notably, caregivers highlighted the importance of introducing AI-driven in-home monitoring technologies in a way it prevents them from feeling undervalued. In conclusion, our findings can help to inform the development of more acceptable and unobtrusive in-home monitoring technologies to support home-based dementia care.

### WHAT FACTORS ARE ASSOCIATED WITH FACILITATING CONDITIONS TO USE GERONTECHNOLOGY?

Hayoung Park,[1] Susanna Joo,[2] DaeEun Kim,[1] YoonMyung Kim,[1] and Hey Jung Jun,[1], *1. Yonsei University, Seoul, Seoul-t'ukpyolsi, Republic of Korea, 2. Yonsei University, Seodaemun-gu, Seoul-t'ukpyolsi, Republic of Korea*

The purpose of this study is to explore relevant factors associated with facilitating conditions to use gerontechnology among Korean older adults. The sample was 310 Korean older adults aged 65 and above without cognitive impairment who participated in an online survey. The facilitating conditions to use gerontechnology were measured by the sum of five questions about basic knowledge, available help, financial resources, accessibility, and social influences of using gerontechnology from the Senior Technology Acceptance Model (STAM). Possible relevant factors comprised socio-demographic characteristics, physical and mental health, environmental factors, and social relationships. The results from the linear regression analyses showed that employment status, household income, cognitive function, social activity participation, and support from friends or neighbors were significantly associated with facilitating conditions to use gerontechnology. Older adults who are employed, have higher household income, have better cognitive functions, participate more in social activities, and receive higher levels of support from friends or neighbors tend to be in more facilitating conditions to use gerontechnology. The findings from this study imply the necessity of facilitating conditions to use gerontechnology as social policies for older adults who are unemployed, have lower household income, have worse cognitive functions, and have fewer social resources. This study is meaningful in that it has empirically explored various factors related to facilitating conditions to use gerontechnology for older adults based on the STAM. Future studies are needed to explore significant factors associated with facilitating conditions to use gerontechnology via various contexts.

### WHO MATTERS FOR THE SUBJECTIVE PERCEPTIONS TOWARD GERONTECHNOLOGY?

Seong Hee Kim,[1] Susanna Joo,[2] Changmin Lee,[3] Chang Oh Kim,[4] Yun Mook Lim,[3] and Hey Jung Jun,[3], *1. Sungkyunkwan University, Suwon-si, Kyonggi-do, Republic of Korea, 2. Yonsei University, Seodaemun-gu, Seoul-t'ukpyolsi, Republic of Korea, 3. Yonsei University, Seoul, Seoul-t'ukpyolsi, Republic of Korea, 4. Severance Hospital, Yonsei University College of Medicine, Seoul, Seoul-t'ukpyolsi, Republic of Korea*

The present study aims to investigate how personal relationship satisfaction moderate the associations between types of social support providers and the subjective perceptions toward gerontechnology among Korean older adults. Data were collected by an online survey in February 2021. The sample was 256 older Koreans who have a partner and children (N=109 older adults with low personal relationship satisfaction, N=147 older adults with high personal relationship satisfaction, Age: 66-88, M=69.91, SD=4.19). The dependent variables for the subjective perceptions toward gerontechnology were attitude toward using gerontechnology and anxiety for gerontechnology. Independent variables were four types of social support providers (spouse, children, siblings/relatives, and friends/neighbor). Personal relationship satisfaction was a binary moderator, dividing the sample into low and high personal relationship satisfaction groups. We applied multigroup structural equation modeling. The results showed associations between social support providers and subjective perceptions toward gerontechnology differed by the quality of personal relationships. In detail, receiving support from spouses was associated with the lower level of anxiety of using gerontechnology among older adults in the low personal relationship satisfaction group. Moreover, receiving support from spouses was associated with a higher level of attitude toward using gerontechnology in the high personal relationship group. Receiving social support from other providers were not significant in both groups. The findings imply that the partner living with was salience for positive perception toward gerontechnology. Furthermore, support from spouses may differently work on the subjective perception toward gerontechnology by the quality of personal relationships.

### Session 9085 (Poster)

BIOLOGY OF AGING

### AGE-RELATED PHENOTYPES LINKED TO ABERRANT EXPRESSION AND LOCALIZATION OF A TELOMERIC PROTEIN

Amanda Stock,[1] Kun Wang,[1] Chengyu Liu,[2] Ross McDevitt,[1] Chongkui Sun,[1] Yi Gong,[1] and Yie Liu,[1], *1. National Institute on Aging, Baltimore, Maryland, United States, 2. National Heart, Lung, and Blood Institute, Bethesda, Maryland, United States*

Telomere attrition is associated with telomere biology disorders and age-related diseases. In telomere biology disorders, telomere uncapping induces a DNA damage response that evokes cell death or senescence. However, a causal mechanism for telomere attrition in age-related diseases remains elusive. Telomere capping and integrity are maintained by shelterin, a six-protein complex. Rap1 is the only shelterin member that is not required for telomere capping and is expressed at non-telomeric genomic and cytosolic regions. The objective of this study was to determine aberrant phenotypes attributed to non-telomeric Rap1. To test this, we generated a Rap1 mutant knock-in (KI) mouse model using CRISPR/Cas9 editing, in which Rap1 at telomeres is prevented, leaving only non-telomeric Rap1. Cell fractionation/western blotting of primary fibroblasts from Rap1 KI mice demonstrated decreased Rap1 expression and Rap1 re-localization off telomeres, with an altered cellular distribution. This same difference in Rap1 is also observed in human cells with telomere erosion, indicating that aberrant Rap1 in our model may recapitulate Rap1 in aging and human telomere biology disorders. Compared to wild-type control mice, Rap1 KI mice exhibited increased body weight, altered cytokine levels, behavioral deficits, and decreased lifespan. In conclusion, our results reveal a novel mechanism by which telomere shortening may contribute to age-related pathologies by disrupting Rap1 expression and cell localization.

### APOLIPOPROTEIN E IMPAIRS AGED BONE FRACTURE HEALING

Abhinav Balu,[1] Gurpreet Baht,[2] Rong Huang,[1] and Kristin Molitoris,[1], *1. Duke Molecular Physiology Institute, Durham, North Carolina, United States, 2. Duke University, Durham, North Carolina, United States*

Bone fracture healing and osteoblast differentiation are impaired with advanced age. Using a combination of parabiosis and proteomic models, we identified apolipoprotein E (ApoE) to be an aging factor in bone regeneration. Circulating levels of ApoE increased with age in patients and in mice. ApoE impaired bone fracture healing by decreasing bone deposition in the fracture callus which subsequently decreased the mechanical strength of healed tissue. Osteoblasts serve as the sole bone forming cells within the body. In tissue culture models, ApoE treatment decreased osteoblast differentiation and activity which led to decreased matrix formation and mineralization. This inhibition of osteoblast differentiation relied on down-regulation of the Wnt/β-catenin pathway. In mouse models, aged bone repair was rejuvenated when we lowered circulating ApoE levels using a hepatotropic AAV-siRNA model – serving as a proof of concept that ApoE can be targeted to improve bone repair in an older population. While promising, knockdown of circulating ApoE in such a fashion is likely not translatable to patient care. Thus, current work in our laboratory is focused on developing treatment strategies that temporally decrease circulating ApoE levels and consequently improve bone healing after acute injury and/or surgical orthopedic procedure in the geriatric population.

### BDNF SNP C270T MODIFIES THE ASSOCIATION BETWEEN HISTORY OF HEAD INJURY AND COGNITIVE STATUS IN OLDER ADULTS

Mikaela Drewel,[1] Hector Gonzalez,[1] Gail Rattinger,[2] Joshua Matyi,[1] Alexandra Hammond,[1] Kaitlyn Kauzor,[1] Mona Buhusi,[1] and JoAnn Tschanz,[1], *1. Utah State University, Logan, Utah, United States, 2. Binghamton University, Binghamton University, New York, United States*

Brain derived neurotrophic factor (BDNF), is a neurotrophin involved in neurogenesis and neuroplasticity. Several BDNF genes have been associated with cognitive function. Studies suggest head injury (HI) alters BDNF levels, and activities enhancing BDNF signaling promote better cognitive outcomes. We investigated the relationship between HI and BDNF single-nucleotide polymorphisms (SNPs) in predicting cognitive performance in a population-based sample of older adults. 4165 participants (56.7% female), dementia-free at baseline, were assessed triennially [follow-up years: mean (SD) = 5.85 (4.20), median = 7.33, maximum = 11.39]. Mean (SD) age was 75.36 (6.84). Cognition was assessed using the Modified Mini-Mental State Exam (3MS) and HI history from self-report. We examined interactions between BDNF SNPs [rs56164415 (BDNF C270T), rs6265 (Val66Met), rs2289656 (BDNF receptor trkB), and rs2072446 (NGF/BDNF receptor p75)] and history of HI (none, one, or multiple) in predicting cognitive decline. Covariates included age, education, sex, and apolipoprotein (APOE) E4 allele presence. Linear mixed-effect models indicated BDNF C270T significantly modified the association between HI and cognitive status ($p < .006$). Specifically, minor T allele carriers with single or multiple HI scored on average 2.08 and 3.21 points lower on the 3MS, respectively, than non-T carriers with no HI. Unexpectedly, there was a trend for APOE4*HI ($p = .078$) in that APOE E4 carriers with multiple HI scored higher than those lacking APOE E4 and HI. In this population-based sample, rs56164415 predicted cognitive outcomes that varied by history of HI. Factors influencing BDNF signaling may provide a potential avenue for intervention in recovery from HI.

### BIOLOGICAL AGING, MORTALITY, AND ALZHEIMER'S DISEASE RELATED BIOMARKERS FROM MIDLIFE TO OLD AGE

Carol Franz, Erik Buchholz, Amy Yongmei Qin, Xin Tu, and William Kremen, *University of California San Diego, La Jolla, California, United States*

People age at different rates and in different biological systems that may differentially contribute to accelerated decline. Better understanding of biological aging may contribute to identification of better targets for intervention. In 1005 VETSA participants we created 3 indicators of biological age: physiological age (PA), frailty, and brain age. PA included hemoglobin, glucose, lipids, height, weight, waist, systolic and diastolic blood pressure, and age. PA was calculated using the Klemera and Doubal (2006) method. The frailty index summed 37 health deficits (Jiang et al. 2017). A machine learning algorithm was used to estimate brain age across cortical and subcortical regions (Liem et al, 2017); predicted brain age subtracted from chronological age comprised the predicted brain age difference score (PBAD). Frailty and PBAD were calculated at waves 1, 2 and 3 when participants were average age 56, 62, and 68, respectively. PA markers were only available at waves 2 and 3. Outcome measures included mortality by wave 3 and scores on AD-related plasma biomarkers—Neurofilament light (NFL), Tau, and AB40 and AB42 at wave 3. Frailty at wave 1 and 2 predicted mortality. Frailty at wave 1 was significantly associated with wave 3 NFL, AB42 and AB40. Wave 2 & 3 frailty was associated with all biomarkers. Neither PA nor PBAD predicted biomarkers or mortality. The results are striking given the relatively young age of the sample. Even as early as one's 50s, frailty in a community-dwelling sample predicted accelerated decline and mortality when the outcome age was only 66-73.

### CAN WE SIMPLIFY AGING?

Svetlana Ukraintseva, and Anatoliy Yashin, *Duke University, Durham, North Carolina, United States*

Aging is indeed a complex process, but can it be simplified, so we could efficiently prioritize candidate anti-aging interventions and select those with largest impacts on key negative consequence of the aging, i.e., on increases in mortality risk and comorbidities with age? Here we argue that human aging and its negative consequences for health and lifespan are essentially driven by the interplay among three processes: (i) depletion of limited body reserves (e.g., of stem, immune, neural, muscle cells); (ii) inherent deficiency of cell/tissue repair mechanisms, which leads to accumulation of damage, allostatic load, and systems dysregulation; and (iii) general slowdown of physiological processes in the body (such as metabolism, proliferation and information processing) with age that results in slower responses to stressors and delayed recovery after damage (i.e., decline in resilience), which in turn contributes to increase in vulnerability to death with age. We show that the interplay among these processes can have ambivalent effects on health and longevity that should be taken into account to develop optimal anti-aging and pro-longevity strategies. In order to be efficient on the long-term, the anti-aging interventions may need to target the different causes of aging (reserve depletion, damage accumulation, and slowdown) simultaneously, to avoid undesirable trade-offs.

### DEMONSTRATION OF AGE-RELATED INCREASE IN BLOOD-BRAIN BARRIER PERMEABILITY BY LONGITUDINAL INTRAVITAL MICROSCOPY

Adam Nyul Toth,[1] Stefano Tarantini,[1] Jordan DelFavero,[1] Feng Yan,[2] Zoltan Ungvari,[1] Anna Csiszar,[1] Qinggong Tang,[2] and Priya Balasubramanian,[1], *1. University of Oklahoma Health Sciences Center, Oklahoma City, Oklahoma, United States, 2. University of Oklahoma, Norman, Oklahoma, United States*

Age-related blood-brain barrier disruption and cerebromicrovascular rarefaction contribute importantly to the pathogenesis of both vascular cognitive impairment and dementia (VCID) and Alzheimer's disease (AD). Recent advances in geroscience research enable development of novel interventions to reverse age-related alterations of the cerebral microcirculation for prevention of VCID and AD. To facilitate this research there is an urgent need for sensitive and easy-to-adapt imaging methods, which enable longitudinal assessment of changes in BBB permeability and brain capillarization in aged mice, that could be used in vivo to evaluate treatment efficiency. To enable longitudinal assessment of changes in BBB permeability in aged mice equipped with a chronic cranial window, we adapted and optimized two different intravital two-photon imaging approaches. By assessing relative fluorescence changes over the baseline within a volume of brain tissue, after qualitative image subtraction of the brain microvasculature, we confirmed that in 24 month old C57BL/6J mice cumulative permeability of the microvessels to fluorescent tracers of different molecular weights (0.3 kDa to 40 kDa) is significantly increased as compared to that of 5 month old mice. Real-time recording of vessel cross-sections showed that apparent solute permeability of single microvessels is significantly increased in aged mice vs. young mice. Cortical capillary density, assessed both by intravital two-photon microscopy and optical coherence tomography (OCT) was also decreased in aged mice vs. young mice. The presented methods have been optimized for longitudinal (over the period of 36 weeks) in vivo assessment of cerebromicrovascular health in preclinical geroscience research.

### DIFFERENTIALLY METHYLATED GENES IN MUSCLE ASSOCIATED WITH GAIT SPEED IN A NON-HUMAN PRIMATE MODEL OF AGING

Ellen Quillen, Brett Frye, Bethany Wildeman, Maggie Stainback, Thomas Register, and Carol Shively, *Wake Forest School of Medicine, Winston Salem, North Carolina, United States*

Age-related changes in DNA methylation are potent regulators of gene expression and may in part explain the onset of disease and disability. Vervet monkeys are a well-described model of neurocognitive and physical aging. Like humans, gait speed declines with age in vervets, and variability in gait speed in older animals is associated with age-related musculoskeletal and cognitive decline. To identify methylation patterns linked to aging-related physical decline, we investigated differentially methylated loci in vastus lateralis biopsies of 29 female vervets aged 8-28 years (~25-90 years in humans). We evaluated 107,490 loci on the Illumina Infinium Methylation EPIC Human Array that aligned with high fidelity to the vervet genome using the R package minfi and fit generalized linear mixed models to account for underlying genetic relatedness. We found 13 CpG methylation sites associated with 12 genes (CALCR, EBF4, GDNF, GMCL1, HAND1, HOXC10, IRX2, LBX2, MPPED2, SHISA6, SOX2, and WNT2) significantly differentially methylated with gait speed. Increased methylation was negatively associated with gait speed for all loci except GMCL1, reflecting the pattern of global hypermethylation of skeletal muscle tissue with age. Several of the associated genes are involved in development and differentiation including HOXC10 and LBX2, which regulates myoblast migration. CACNG8 regulates voltage-dependent calcium gated channels, and GDNF promotes motor neuron innervation of skeletal muscle. Most associations with muscle phenotypes are novel, but several have been linked to age-related bone diseases. We are currently evaluating the relationships of these differentially methylated loci with muscle mRNA expression and protein abundance.

### EFFECT OF COMBINED STRESSORS ON C. ELEGANS LIFESPAN

Bradford Hull,[1] and George Sutphin,[2], *1. University of Arizona, Tucson, Arizona, United States, 2. The University of Arizona, Tucson, Arizona, United States*

Cellular stress is a fundamental component of age-associated disease. Cells experience many forms of stress (oxidative, heavy metal, etc.), and as we age the burden of stress and resulting damage increases while our cells' ability to deal with the consequences becomes diminished due to dysregulation of cellular stress response pathways. By understanding how cells respond to stress we aim to slow age-associated deterioration and develop treatment targets for age-associated disease. The majority of past work has focused on understanding responses to individual stressors. In contrast, how pathology and stress responses differ in the presence of multiple stressors is relatively unknown; we investigate that here. We cultured worms on agar plates with different combinations of arsenic, copper, and DTT (which create oxidative/proteotoxic, heavy metal, and endoplasmic reticulum (ER) stress, respectively) at doses that result in 20% lifespan reduction individually and measured the effect on lifespan. We found that arsenic/copper and arsenic/DTT combinations created additive lifespan reductions while the copper/DTT combination created an antagonistic lifespan reduction when compared to controls ($p<0.05$). This antagonistic toxicity suggests an interaction either between the mechanisms of toxicity or the cellular response to copper and DTT. We are now evaluating the impact of copper and DTT individually and in combination on unfolded protein and heavy metal response pathways to understand the underlying mechanism of the interaction. Additionally, we are continuing to screen stressors to identify combinations that cause non-additive (synergistic or antagonistic) toxicity to build a comprehensive model of the genetic stress response network in C. elegans.

### EFFECTS OF FOXO3 ON MARKERS OF AGING IN BLOOD: AN OKINAWAN LONGEVITY COHORT STUDY

Richard Allsopp,[1] D. Craig Willcox,[2] Trevor Torigoe,[3] Mariana Gerschenson,[3] Randi Chen,[1] Tim Donlon,[3] Bradley Willcox,[4] and Michi Shimabukuro,[5], *1. Kuakini Medical Center, Honolulu, Hawaii, United States, 2. okinawa International university, International university of Okinawa, Okinawa, Japan, 3. JABSOM, Honolulu, Hawaii, United States, 4. Kuakini Medical Center, Kuakini Medical Center/Honolulu, Hawaii, United States, 5. Fukushima University, Fukushima Medical University, Okinawa, Japan*

FOXO3 is among only a few genes that demonstrate a consistently reproducible genetic association with human longevity. We previously demonstrated, in a cross-sectional study of Okinawan Japanese, that the principal longevity variant of FOXO3 (rs 2802292 "G allele") protects against age-associated attrition of telomere length. We now expand upon this initial observation in a more detailed cross-sectional analysis of the effect of FOXO3 on telomerase activity, FOXO3 expression and inflammatory cytokine levels in both men and women. In agreement with our initial study, we found the FOXO3 longevity variant conferred significant protection against telomere shortening to roughly the same degree in elderly (ages 55 and older) men and women. Carriers of the G - allele also had slightly higher levels of blood telomerase activity in young (ages 20 – 54) and elderly participants ($P\leq0.1$). The expression (mRNA) of FOXO3 increased steadily with age in young and old G – allele carriers (borderline $P\leq0.08$), in contrast to a lack of association with age in non-carriers. The FOXO3 G - allele was also observed to significantly impact levels of both interleukin 6 (IL 6) and IL10, but in a sex dependent manner ($P<0.05$). These sex-specific effects may point to different mechanisms by which FOXO3 exerts its effect on longevity in men and women.

### EPIGENETIC SIGNATURE OF CHILDHOOD ADVERSITY IN OLDER ADULTS

Eric Klopack, and Eileen Crimmins, *University of Southern California, Los Angeles, California, United States*

Past research has shown that childhood adversity (CA) affects the health of older adults; however, the biological mechanisms underlying this association are unclear. Though past research has implicated DNA methylation (DNAm), studies utilizing representative data from older adults and reliable DNAm measures are needed to answer key questions about how stable DNAm changes associated with CA are in later life. Methylation risk scores (MRSs) are an emerging tool that can be used as biomarkers of exposure and as a dimension reduction approach for mediation analyses. This study clarifies the association between CA and later life health by generating MRSs for childhood adversity based on an epigenome wide association study conducted in an independent sample and validating that measure in a nationally representative sample of older adults living in the US from the Health and Retirement Study (HRS), including 2016 methylation data from the HRS Innovative Subsample of the Venous Blood Study. For these 4,018 respondents, DNAm was assessed in whole blood using the Illumina Infinium Human Methylation EPIC BeadChip microarray. Results indicate that retrospective report of childhood SES is significantly associated with an MRS for CA after controlling for demographic factors (viz., race and ethnicity, age, gender, smoking status, and BMI), suggesting that DNAm signal from CA persists across the life course into older adulthood. This study helps clarify the biological processes underlying the association between CA and adult health.

### EPIGENETIC TRAJECTORIES OF AGING AND REPROGRAMMING

Peter Niimi,[1] Margarita Meer,[2] and Morgan Levine,[1], *1. Yale University, New Haven, Connecticut, United States, 2. Yale University, Yale University, Connecticut, United States*

The epigenetic landscape is remodeled with age, bringing about widespread consequences for cell function. With the revolutionary discoveries by Yamanaka and Takahashi, as well as those that built on this work, the transcription factors Oct4, Sox2, KLF4, and C-Myc (OSKM) can be expressed in a variety of cells, including fibroblasts, to make iPSCs. Once cells are reprogrammed, they show an erasure of epigenetic remodeling, suggesting an avenue to reverse aging. It has been recently shown that ectopic expression of three factors, OSK, can restore vision in mouse glaucoma model and reduces epigenetic age. It is not known the path epigenetic remodeling takes or whether all three factors, OSK, are required to remodel the epigenetic landscape. We hypothesize that during reprogramming, cells will reverse along a similar path they took during aging and eventually reverse along that path they took during differentiation. Alternatively, it may also be possible that cells take entirely new paths to reach a state of partial reprogramming or pluripotency. We used DNA methylation and RNA-seq as a multi-omics approach to map the trajectories cells make during aging, differentiation, and reprogramming. In human fibroblasts and hepatocytes, we tested the three-factor OSK mix, as well as pairwise factors OS, OK, and SK and individual Oct4, Sox2, and KLF4 for their effect on cell trajectories. This study provides a dynamic model for epigenetic changes in aging, differentiation, and reprogramming and highlights barriers and bottlenecks throughout the process.

### EPIGENOME WIDE ASSOCIATIONS OF SMOKING BEHAVIOR IN THE HEALTH AND RETIREMENT STUDY

Jonah Fisher,[1] Helen Meier,[1] Jessica Faul,[2] Colter Mitchell,[2] Eileen Crimmins,[3] and Bharat Thyagarajan,[4], *1. University of Michigan, Ann Arbor, Michigan, United States, 2. University of Michigan, University of Michigan, Michigan, United States, 3. University of Southern California, Los Angeles, California, United States, 4. University of Minnesota, Minneapolis, Minnesota, United States*

DNA methylation (DNAm) is an increasingly popular biomarker of health and aging outcomes. Smoking behaviors have a significant and well documented correlation with methylation signatures within the epigenome and are important confounding variables to account for in epigenome-wide association studies (EWAS). However, the common classification of individuals as 'current', 'former', and 'never' smokers may miss crucial DNAm patterns associated with other smoking behaviors such as duration, intensity, and frequency of cigarette smoking, resulting in an underestimation of the contribution of smoking behaviors to DNAm and potentially biasing EWAS results. We investigated associations between multiple smoking behavioral phenotypes (smoking pack years, smoking duration, smoking start age, and smoking end age) and single site DNAm using linear regressions adjusting for age, sex, race/ethnicity, education, and cell-type proportions in a subsample of individuals who participated in the HRS 2016 Venous Blood Study (N=1,775). DNAm was measured using the Infinium Methylation EPIC BeadChip. All 4 phenotypes had significant associations (FDR < 0.05) with many methylation sites (packyears=6859, smoking duration=6572, start age=11374, quit age=773). There was not much overlap in DNAm sites between the full set of models with only 6 overlapping between all 4. However, the phenotypes packyears and smoking duration showed large overlap (N=3782). Results suggest additional smoking phenotypes beyond current/former/never smoker classification should be included in EWAS analyses to appropriately account for the influence of smoking behaviors on DNAm.

### GENOMIC ANALYSIS OF NAD+ SYNTHESIS PATHWAYS INVOLVED IN AGING AND CANCER

Jeremy Meyers,[1] Raul Castro-Portuguez,[2] Luis Espejo,[3] and George Sutphin,[2], *1. University of Arizona, Tucson, Arizona, United States, 2. The University of Arizona, Tucson, Arizona, United States, 3. UNIVERSITY OF ARIZONA, TUCSON, Arizona, United States*

Cancer cells have elevated energy demands to sustain continuous growth and other malignant processes and undergo extensive metabolic reprogramming to meet these demands. One element of this reprogramming in many cancer subtypes is elevated synthesis of nicotinamide adenine dinucleotide (NAD+), a critical co-enzyme that supports energy production through both glycolysis and the TCA cycle. The kynurenine metabolic pathway is the evolutionarily conserved means by which cells produce NAD+ de novo from tryptophan. NAD+ levels drop with age, a contributing factor to many forms of age-related disease. While interventions that increase NAD+ have been shown to extend lifespan, previous work from our lab demonstrates that knockdown of several kynurenine pathway enzymes, thus decreasing de novo NAD+ production, results in increased longevity of Caenorhabditis elegans by 20-30%. To address this apparent contradiction, we propose that kynurenine pathway inhibition may produce metabolic feedback that results in upregulation of NAD+ recycling. Eukaryotic cells recycle NAD+ from nicotinamide (NAM) through one of two pathways: the Salvage pathway in mammalian cells and the Preiss-Handler pathway in C. elegans and related invertebrates species. We are using tools in C. elegans and human cell culture to examine the interaction between kynurenine/de novo NAD+ synthesis and NAD+ recycling through Salvage and Preiss-Handler. In particular, we are interested in how combining interventions between these pathways will influence activity throughout the NAD+ metabolic networks (measured via mass spectrometry), physiological phenotypes, and transcriptomic changes (via RNA sequence data) involved in aging and age-associated disease.

### INSERTION OF THE PROTECTIVE APP A673T MUTATION BY CRISPR/CAS9 BASE EDITING OR PRIME EDITING.

Jacques P. Tremblay,[1] Antoine Guyon,[2] Joël Rousseau,[1] Guillaume Tremblay,[1] Francis-Gabriel Begin,[1] and Gabriel Lamothe,[1], *1. Laval university, Québec, Quebec, Canada, 2. Laval university, Quebec, Quebec, Canada*

There is currently no treatment for Alzheimer disease (AD). However, the Icelandic mutation in the APP gene (A673T) has been shown to confer a protection against the onset and development of AD (Jonsson et al. Nature 2012). This single nucleotide mutation in APP exon 16 reduces the cleavage of the APP protein by the beta-secretase by 40% thus preventing the development of AD even in persons more than 95 years old. Our research group has initially shown that the presence of the A673T mutation in an APP gene reduced the secretion of beta-amyloid peptides even if there is also a FAD mutation in the gene. This is the case for 14 different FAD mutations. We have used CRISPR/Cas9 base editing and PRIME editing technologies to insert the A673T mutation in the APP gene. We have compared several different cytidine base editor complexes to achieve the most effective and accurate genome modification possible in HEK293T cells and in SH-SY5Y neuroblastomas. The insertion of the A673T mutation in cells containing the London mutation reduced the secretion of beta-amyloid peptides. We are currently using lentiviral vectors to infect neurons from a mouse model and human neurons induced from fibroblasts of a patient with the London mutation. The insertion of the protective Icelandic mutation in the APP gene using these editing technologies opens a new potential therapeutic avenue not only for Familial Alzheimer's diseases but also for sporadic Alzheimer's disease.

### LINKS OF SLEEP DURATION WITH BIOMARKERS OF ACCELERATED AGING: THE BALTIMORE LONGITUDINAL STUDY OF AGING

Emily Smail,[1] Adam Spira,[2] Brion Maher,[2] Ann Moore,[3] Pei-Lun Kuo,[4] Mark Wu,[5] Katie Stone,[6] and Dominique Low,[7], *1. Johns Hopkins University, Baltimore, Maryland, United States, 2. Johns Hopkins Bloomberg School of Public Health, Baltimore, Maryland, United States, 3. National Institute on Aging, Baltimore, Maryland, United States, 4. National Institute on Aging, National Institute on Aging, Maryland, United States, 5. Johns Hopkins University School of Medicine, Baltimore, Maryland, United States, 6. University of California, Berkley, San Francisco, California, United States, 7. Stanford University School of Medicine, Stanford, California, United States*

Sleep disorders and sleep deprivation have been linked to markers of biological aging, including methylation change and increases in white blood cell and neutrophil counts. However, little is known regarding the association of sleep duration with biological markers of aging. We investigated links of self-reported sleep duration with biological aging markers in 615 participants in the Baltimore Longitudinal Study of Aging (BLSA) aged ≥50 years (mean = 71.0 ± 11.2, 49.6% women, 68.8% white) with data on self-reported sleep duration in hours (i.e., ≤6 (n=131), >6 to 7 (n=234), >7 (n=250)), demographics, and genetic and methylation data (mDNA). Our aging biomarker outcomes were four epigenetic clocks (Horvath, Hannum, PhenoAge, and GrimAge), mDNA-estimated PAI1, and estimated granulocyte count. After adjustment for age, sex, and race, compared to those sleeping ≤6 hours, those reporting >7 hours of sleep had faster biological aging according to Hannum age-acceleration, PhenoAge, GrimAge, mDNA-estimated PAI1, and granulocyte count. In addition, sleep duration interacted with age, such that compared to individuals reporting ≤6 hours of sleep, individuals reporting >6 to 7 hours showed lower GrimAge with increasing age, and with sex, such that males with longer sleep duration (>6 to 7 and >7 hours) showed a lower granulocyte count compared to females. Findings suggest that both short and long sleep duration are associated with and may contribute to accelerated aging. Prospective studies in larger samples are needed to examine whether changes in sleep duration precede changes in aging biomarkers.

### LONGITUDINAL COURSE OF GDF15 LEVELS BEFORE ACUTE HOSPITALIZATION AND DEATH IN THE GENERAL POPULATION

Juliette Tavenier,[1] Ove Andersen,[1] Jan O. Nehlin,[1] and Janne Petersen,[2], *1. Copenhagen University Hospital Hvidovre, Hvidovre, Hovedstaden, Denmark, 2. Copenhagen University Hospital Frederiksberg, Frederiksberg, Hovedstaden, Denmark*

Growth differentiation 15 (GDF15) is a potential novel biomarker of biological aging. To separate the effects of chronological age and birth cohort from biological age, longitudinal studies investigating associations of GDF15 levels with adverse health outcomes are needed. We investigated changes in GDF15 levels over 10 years in an age-stratified sample of the general population and their relation to the risk of acute hospitalization and death. Serum levels of GDF15 were measured three times in 5-year intervals in 2176 participants aged 30, 40, 50, or 60 years from the Danish population-based DAN-MONICA cohort. We assessed the association of single and repeated GDF15 measurements with the risk of non-traumatic acute hospitalizations. We tested whether changes in GDF15 levels over 10 years differed according to the frequency of hospitalizations within 2 years, or survival within 20 years, after the last GDF15 measurement. The change in GDF15 levels over time was dependent on age and sex. Higher GDF15 levels and a greater increase in GDF15 levels were associated with an increased risk of acute hospitalization in adjusted Cox regression analyses. Participants with more frequent admissions within 2 years, and those who died within 20 years, after the last GDF15 measurement already had elevated GDF15 levels at baseline and experienced greater increases in GDF15 levels during the study. The change in GDF15 levels was associated with changes in C-reactive protein and biomarkers of kidney, liver, and cardiac function. Monitoring of GDF15 starting in middle-age could be valuable for the prediction of adverse health outcomes.

### METABOLIC SYNDROME AND NEUROCOGNITIVE FUNCTION AMONG OLDER HISPANICS/LATINOS WITH HIV

Maria Marquine,[1] Lily Kamalyan,[2] David Yassai-Gonzalez,[1] Mariana Cherner,[3] Ronald Ellis,[4]

Anya Umlauf,[4] Dilip Jeste,[1] and Robert Heaton,[4]
*1. University of California San Diego, La Jolla, California, United States, 2. San Diego State University/University of California San Diego Joint Doctoral Program in Clinical Psychology, San Diego, California, United States, 3. University of California San Diego, SAN DIEGO, California, United States, 4. University of California San Diego, San Diego, California, United States*

Neurocognitive impairment is prevalent among persons with HIV (PWH), particularly among Hispanics/Latinos/as/x (henceforth Hispanics). We examined disparities in HIV-associated neurocognitive function between older Hispanic and non-Hispanic White PWH, and the potential role of metabolic syndrome (MetS) in explaining these disparities. Participants included 116 community-dwelling PWH ages 50-75, who were enrolled in a cohort study in southern California (58 Hispanic [53% Spanish-speaking] and 58 age-comparable non-Hispanic White; Overall group: Age: M=57.9, SD=5.7; Education: M=13, SD=3.4; 83% male, 58% AIDS, 94% on antiretroviral therapy [ART], 4% detectable plasma RNA). A global neurocognition score was derived from T-Scores on a comprehensive neurocognitive battery, with separate demographic adjustments for English and Spanish-speakers. MetS was ascertained via standard criteria that considered central obesity, elevated triglycerides, low high-density lipoprotein cholesterol, and elevated fasting glucose, as well as current medical treatment for these conditions. Covariates examined included sociodemographic, psychiatric, substance use and HIV-disease characteristics. Hispanics had higher rates of MetS (56%) than non-Hispanic Whites (37%; p<.05). A stepwise regression model on global neurocognition including ethnicity and covariates that differed between ethnic groups, selected only Hispanic ethnicity as a significant predictor (B=-3.82, SE=1.27, p<.01). A comparable model also including MetS showed that both Hispanic ethnicity (B=-3.39, SE=1.31, p=.01) and MetS (B=-2.73, SE=1.31, p=.04), were significantly associated with worse global neurocognition. Findings indicate that MetS does not fully explain disparities in neurocognitive function between Hispanic and non-Hispanic White older PWH, but rather is an independent predictor of neurocognitive function along with Hispanic ethnicity.

### METHODS USED IN 'ALDH2*2 ASSOCIATION WITH LONGEVITY AND REDUCED RISK OF COGNITIVE DECLINE IN JAPANESE-AMERICAN MEN'

Anubhav Nikunj Singh Sachan,[1] Steven Edland,[1] Julia Chosy,[2] Lenore Launer,[3] Sudha Seshadri,[4] and Lon White,[2], *1. University of California San Diego, La Jolla, California, United States, 2. Pacific Health Research and Education Institute, Honolulu, Hawaii, United States, 3. National Institute on Aging, Bethesda, Maryland, United States, 4. UT Health San Antonio, San Antonio, Texas, United States*

ALDH2*2 is a loss of function mutation common in East Asian populations associated with facial flushing on exposure to alcohol and increased risk of certain cancers. Conversely, absence of the ALDH2*2 mutation is associated with increased risk of hypertension and cerebral microbleeds, and two recent studies report a higher frequency of ALDH2*2 alleles in nonagenarians compared to population control samples. We used survival analysis to investigate the association between ALDH2*2 and risk of cognitive impairment and death after controlling for midlife alcohol consumption and other covariates. Participants are 621 Japanese-American men (72 to 92) enrolled in the Honolulu Asia Aging Study (HAAS) and assessed for cognitive impairment for up to 20 years. Impairment was defined as crossing below a threshold score of 74 on the Cognitive Assessment Screening Instrument (CASI). Age at death was determined by Hawaii state death certificate. Ounces of ethyl alcohol consumed per month was assessed by structured interview (number, frequency, and type of beverage) conducted 25 years prior to baseline cognitive assessment. Persons heterozygous for the ALDH2*2 variant have reduced risk of cognitive impairment and reduced risk of death, compared to homozygote non-carriers. Covarying by alcohol exposure had no effect on observed associations. This study replicates previous findings associating ALDH2*2 with longevity, and provides evidence the protective effect extends to cognition. This poster details the statistical analysis carried out to obtain these results.

### MTOR INHIBITION ALTERS MIRNA PROFILE IN CULTURED BONE MARROW MESENCHYMAL STEM CELLS

Lisa Stukenborg,[1] Christian Sell,[1] and Manali Potnis,[2],
*1. Drexel University College of Medicine, Philadelphia, Pennsylvania, United States, 2. Drexel University, PHILADELPHIA, Pennsylvania, United States*

Progressive decline in adult stem cell function is a feature of aged tissues and contributes to multiple age-related diseases. Of particular interest are bone marrow mesenchymal stem cells, also known as bone marrow stromal cells (BMSCs), which are multipotent, and have significant therapeutic potential for age-related disease. BMSCs isolated from older individuals show decreased differentiation and characteristics of cell senescence. We and others find that inhibition of the mTOR pathway prevents senescence and extends BMSC proliferation. We have examined miRNA profiles in rapamycin-treated BMSCs to identify miRNAs that may be needed to maintain “stemness” and facilitate differentiation in this population in undifferentiated cell populations. The analysis reveals a distinct miRNA profile induced by rapamycin associated with enhanced differentiation capacity.

### PLASMA BIOMARKERS OF ANGIOGENESIS RELATED TO SMALL VESSEL BRAIN DISEASE IN SPRINT

Nicholas Pajewski,[1] Fanny Elahi,[2] Joachim Ix,[3] Ilya Nasrallah,[4] Jason Hinman,[5] Lenore Launer,[6] Jeff Williamson,[1] and Donna Wilcock,[7], *1. Wake Forest School of Medicine, Winston-Salem, North Carolina, United States, 2. University of California San Francisco, San Francisco, California, United States, 3. University of California San Diego, La Jolla, California, United States, 4. University of Pennslyvania, Philadelphia, Pennsylvania, United States, 5. David Geffen School of Medicine, University of California Los Angeles, Los Angeles, California, United States, 6. National Institute on Aging, Bethesda, Maryland, United States, 7. University of Kentucky, Lexington, Kentucky, United States*

Meta-analyses incorporating the Systolic Blood Pressure Intervention Trial (SPRINT) have shown a reduced incidence of dementia with blood pressure lowering. However, mechanistic explanations for this effect are lacking, apart from slowed progression of cerebral white matter lesions (WML). Here we examine possible biomarkers of angiogenesis related to small vessel brain disease including bFGF, FLT1, PLGF, TIE-2, VEGF, VEGF-C, and VEGF-D. The biomarkers were assayed in plasma at baseline and during follow-up (median follow-up = 3.8 years) in a subgroup of participants 60 to 89 years old from SPRINT (N=517). We modeled changes in each biomarker using robust linear mixed models accounting for treatment group, time since randomization, and kidney function. Participants were 69.8 ± 7.1 (standard deviation) years of age, 42.1% female, with a mean systolic blood pressure (SBP) of 138.2 ± 17.0 mm Hg. At baseline, none of the biomarkers were associated with WML lesion volume or total brain volumes adjusting for age (all p>0.05), while FLT1, PLGF, and TIE-2 were negatively associated with frontal gray matter cerebral blood flow (partial correlations of -0.11, -0.10, and -0.12 respectively, all p<0.05). For both intensive (target SBP<120 mm Hg) and standard (target SBP<140 m Hg) blood pressure control, mean levels for the majority of biomarkers increased during follow-up, with the exceptions of TIE-2 (decreased over follow-up) and VEGF-D (no change). We did not observe significant between-group differences for the change in these plasma biomarkers of angiogenesis comparing intensive to standard blood pressure treatment.

### SIRTUIN-1 ISOFORMS DIFFERENTIALLY REGULATE MITOCHONDRIAL FUNCTION

Xiaomin Zhang,[1] Fathima Ameer,[2] Jasmine Crane,[2] Gohar Azhar,[2] and Jeanne Wei,[2], *1. University of Arkansas for Medical Sciences, Little Rock, Arkansas, United States, 2. UAMS, UAMS, Arkansas, United States*

Alternative splicing generates multiple distinct isoforms that increase transcriptome and proteome diversity. Alternatively spliced isoforms may lose part of the protein domain and have different intracellular localization as well as distinct functions. The main form of the SIRT1 (SIRT1v1) protein contains 11 exons. We have identified two new isoforms, SIRT1v2 (lost 2 exons), and SIRT1v3 (lost 3 exons), but their effect on mitochondrial gene expression has not been reported. To study the effect of the three SIRT1 isoforms on mitochondrial gene expression and function, neuronal cells were transfected with SIRT1 isoforms v1, v2 or v3 plasmids, respectively. Gene expression was measured by quantitative reverse transcription PCR (RT-qPCR). Our data showed SIRT1 isoforms v1, v2 and v3 differentially regulated PCG-1alpha and PCG-1beta, which are the upstream regulators of mitochondrial structure and function. SIRT1v1 upregulated mitofusin-1 (MFN1), the mitochondrial dynamin-like GTPase (OPA1) gene, and the transcription factor A mitochondrial (TFAM) gene. In contrast, the SIRT1-v2 isoform repressed the MFN1, MFN2, and TFAM genes, while the SIRT1-v3 isoform repressed the MFN1 gene. In addition, the three SIRT1 isoforms differentially affected the mitochondrial respiratory complex I genes, including NDUFAB1, NDUFS1, NDUFV1, NDUFV2. The data indicates that SIRT1 regulates mitochondrial biogenesis and function through a signaling pathway involving PGC-1alpha, PCG-1beta, mitofusin 1 and 2, OPA1, and TFAM genes. Taken together, alternative splicing generated three SIRT1 isoform proteins with diverse functions. Age-related changes in the alternative splicing events are likely to impact sirtuin-regulated cellular functions and signaling pathways in aging and senescence.

### THE INTERPLAY BETWEEN STRESS RELATED GENES AND ITS ROLE IN HUMAN LONGEVITY: INSIGHTS FOR TRANSLATIONAL STUDIES

Anatoliy Yashin,[1] Deqing Wu,[1] Konstantin Arbeev,[1] Olivia Bagley,[1] Igor Akushevich,[1] Arseniy Yashkin,[2] Matt Duan,[1] and Svetlana Ukraintseva,[1], *1. Duke University, Durham, North Carolina, United States, 2. Duke University, Morrisville, North Carolina, United States*

Human lifespan is a multifactorial trait resulted from complicated interplay among many genetic and environmental factors. Despite substantial progress in clarifying many aspects of lifespan' variability the mechanism of its multifactorial regulation remains unclear. In this paper we investigate the role of genes from integrated stress response (ISR) pathway in such regulation. Experimental studies showed that persistent cellular stress may result in cellular senescence (for proliferating cells), or in apoptosis (for post-mitotic cells) which may affect health and lifespan in laboratory animals. These studies also showed which ISR genes are likely to interplay to produce joint effects on these traits. Note that in humans, the interplay between these genes does not necessarily influence these traits. This is because biological mechanisms regulating these traits in laboratory animals and humans may differ. This means that, when possible, the experimentally detected connections promising for human applications, should be verified using available human data before their testing in expensive clinical trials. In this paper we used HRS data to test connection between SNPs from the EIF2AK4 gene that senses cellular stress signals and the DDIT3 gene from the apoptosis regulation part of the ISR. We found genome wide significant associations between interacting SNPs from these genes and longevity. This result shows that available human data may be successfully used for making important steps in translation of experimental research findings towards their application in humans. Following this strategy may increase efficiency of clinical trials aiming to find appropriate medications to promote human health and longevity.

### USING THRESHOLD REGRESSION AS AN APPROACH TO INCORPORATE INFORMATIVE MISSINGNESS IN LONG LIFE FAMILY STUDY DATA

Ilya Zhbannikov,[1] Konstantin Arbeev,[1] Olivia Bagley,[1] Yuriy Loika,[1] Alexander Kulminski,[2] Svetlana Ukraintseva,[1] Eric Stallard,[1] and Anatoliy Yashin,[1], *1. Duke University, Durham, North Carolina, United States, 2. DUKE UNIVERSITY, Durham, North Carolina, United States*

Genetics of aging is important since aging is a major risk factor in most diseases. Variables describing physiological state and cognitive functioning that influence morbidity and mortality risks can serve as biomarkers of aging. They change with increasing age and the ways in which these variables change can also influence these risks. Missing data due to dropout or death create problems in longitudinal studies producing biased results especially if the gap between exams is relatively long, as is the case in the Long Life Family Study (LLFS). We applied the threshold regression model to LLFS data to investigate the vitality and its rate, which are conceptualized as latent variables characterizing health and longevity, and to cope with such a problem. We performed genome-wide association study by sex and age groups to discover genetic signals on these phenotypes. We found 11 variants from the DACT2 gene, p-values < 1E-6 and variants rs12151399 (p-value = 8.43E-8, intron variant, gene AGAP1, in females), rs27958 (p-value = 8.39E-8, intron variant, gene ARHGAP26, in males) showing associations with the vitality. Olfactory receptors showed significant enrichment among the group of males over 80 years for the rate of aging phenotype. Results showed that vitality and its rate differ among sex and age groups. This work is an important step toward understanding the processes of aging linking the vitality with individual genetics using data from deceased and living individuals.

### Session 9090 (Poster)

### BIOLOGY OF AGING AND BIOBEHAVIORAL HEALTH

### A NOVEL PROBIOTICS THERAPY FOR AGING-RELATED LEAKY GUT AND INFLAMMATION

Shaohua Wang,[1] Bo Wang,[2] Sidharth Mishra,[1] Shalini Jain,[1] Jingzhong Ding,[1] Stephen Krtichevsky,[3] Dalane Kitzman,[1] and Hariom Yadav,[4], *1. Wake Forest School of Medicine, Winston-Salem, North Carolina, United States, 2. NC A&T State University, Greensboro, North Carolina, United States, 3. Wake Forest School of Medicine, Wake Forest School of Medicine, North Carolina, United States, 4. Wake Forest Medical Center, Winston-Salem, North Carolina, United States*

Inflammaging characterized with increased low grade inflammation in older adults is common determinant of unhealthy aging; and is a major risk factor of morbidity and mortality in older adults. The precise origin of inflammation in older adults is not known, however, emerging evidence indicate that increased intestinal epithelial permeability (leaky gut) and abnormal (dysbiotic) gut microbiota could be one of the key source. However, no preventive and treatment therapies are available to reverse the leaky gut and microbiome dysbiosis in older adults. Here, we presented the evidence that a human-origin probiotics cocktail containing 5 Lactobacillus and 5 Enterococcus strains isolated from healthy human infant gut can ameliorate aging-related metabolic, physical and cognitive dysfunctions in older mice. We show that the Feeding this probiotic cocktail prevented high-fat diet–induced (HFD-induced) abnormalities in glycose metabolism and physical functions in older mice and reduced microbiota dysbiosis, leaky gut, inflammation. Probiotic-modulated gut microbiota reduced leaky gut by increasing tight junctions on intestinal epithelia, which in turn reduced inflammation. Mechanistically, probiotics increased bile salt hydrolase activity in older microbiota, which in turn increased taurine deconjugation from bile acids to increase free taurine abundance in the gut. We further show that taurine stimulated tight junctions and suppressed gut leakiness. Further, taurine increased life span, reduced adiposity and leaky gut, and enhanced physical function in Caenorhabditis elegans. Whether this novel human origin probiotic therapy could prevent or treat aging-related leaky gut and inflammation in the elderly by reversing microbiome dysbiosis requires evaluation.

### A PHASE 2B CLINICAL TRIAL ASSESSING LOMECEL-B INFUSION IN INDIVIDUALS WITH AGING FRAILTY: STUDY DESIGN AND RATIONALE

Kevin Ramdas,[1] Ben Hitchinson,[2] Lisa McClain-Moss,[2] Keyvan Yousefi,[3] Liliana Diaz,[2] Jorge Ruiz,[4] Joshua Hare,[2] and Anthony Oliva,[2], *1. Longeveron LLC, Miami, Florida, United States, 2. Longeveron, Inc, Miami, Florida, United States, 3. Longeveron Inc., Miami, Florida, United States, 4. VAMC, Miami, Florida, United States*

Frailty is a common and important geriatric syndrome characterized by age-associated declines in physiology and function across multiple organ systems, which lead to increased vulnerability to adverse health outcomes. A biological mechanism that underlies the decline in physical function associated with aging frailty is chronic inflammation. The MSCs in Lomecel-B have immuno-modulatory capacity and control inflammation and the cytokine production of lymphocytes. An individual's endogenous stem cell production decreases with age, this decrease likely contributes to reduced ability to regenerate and repair organs and tissues. Aging Frailty represents an exciting potential indication for cellular based therapies like Lomecel-B. This study is intended to evaluate the effects of Lomecel-B infusion compared to placebo on mobility and exercise tolerance, patient-reported physical function assessments and biomarkers for inflammation in individuals with Aging Frailty. This is a randomized, double-blind placebo-controlled, parallel multi-arm multicenter study enrolling adults aged 70-85 years identified as mildly or moderately frail per the CSHA Clinical Frailty Scale (CFS), with reduced six minute walk test (6MWT) and elevated Tumor Necrosis Factor-$\alpha$ (TNF-$\alpha$), at screening. 150 subjects (30 per group) were randomized to receive a single peripheral intravenous infusion of 25, 50, 100, or 200 million doses, or placebo. Safety and efficacy assessments were conducted at 30, 90, 180, and 270 days after infusion. A follow up telephone call to subjects was placed at 365 days. We describe the design and rationale in detail of this 2b study assessing the effects of Lomecel-B on older adults with Aging Frailty.

### CLAIMS-BASED NETWORK ANALYSIS OF DISEASE PROGRESSIONS IN COMPLEX AND NON-COMPLEX OLDER ADULTS

Peter Nicholas Onglao,[1] Ciara Janer,[1] Maria Eloisa Ventura,[1] and Lauren Bangerter,[2], *1. OptumLabs, Minnetonka, Minnesota, United States, 2. OptumLabs, Eden Prairie, Minnesota, United States*

Older adults are the fastest growing subset of complex patients with high medical, behavioral, and social needs. Understanding differences in disease progression patterns between complex and non-complex older adults is critical for understanding disease risk and tailoring patient-centered interventions. We identified complex patients as those having frequent medical encounters and multiple chronic conditions within the first year of the study period and non-complex patients as the converse. This study compares the disease progression patterns of (a) complex and (b) non-complex older adults by creating disease progression networks (DPN) from claims data of 762,362 patients (mean age = 73) from 2016 to 2020. We characterized the network size and density between the complex patient DPN (C-DPN) and non-complex patient DPN (NC-DPN), and compared disease progression incidence, time-to-progression, and age- and gender-related risk. Results show that the C-DPN was denser and had a wider range of values for risk of progression compared to the NC-DPN. This implies more varied disease progression patterns occurring in the complex adults. We were also able to compare (median) time-to-progressions of diseases relative to each subpopulation and found variation in disease progression time. Furthermore, k-means clustering on the network allowed us to identify highly connected diseases involved in many disease pathways that are prevalent among older adults. (e.g., lipoprotein disorders, hypertension, major depressive disorder). Our results suggest that DPNs can be used to identify important conditions and time-points for tailoring care to the complex and non-complex older adults.

### EFFECTS OF SMOKING CESSATION ON EPIGENETIC AGING

Brian Chen,[1] Weiye Wang,[1] Nichole Rigby,[1] Randal Olson,[2] and Steve Sabes,[3], *1. FOXO Technologies, Minneapolis, Minnesota, United States, 2. FOXO Technologies, FOXO Technologies, California, United States, 3. FOXO Technologies, FOXO Technologies, Minnesota, United States*

“Epigenetic clocks” have become widely used to assess individual rates of biological aging. However, experimental data are limited in humans to identify potential confounding factors that may influence one’s rate of epigenetic aging and multiple health outcomes. We examined multiple epigenetic aging measures among regular smokers who quit smoking for two weeks. DNA methylation markers were assessed in both whole blood and saliva at multiple time points using a customized DNA methylation microarray. Generally, no changes in epigenetic aging rates were detected in the two week observation period with the exception of pronounced decreases over time in rate of Hannum’s clock and Extrinsic Epigenetic Age Acceleration in blood DNA. In saliva DNA, decreases over time were detected in the rates of the GrimAge and DNAmPhenoAge clocks, but we saw an increase in the rate of the Skin and Blood Clock. Additional experimental studies of other common exposures may be useful to better characterize factors that may affect the observed “rate” of epigenetic aging.

### EXPLORING HEALTH IN THE UK BIOBANK: SOCIODEMOGRAPHIC, PSYCHOSOCIAL, LIFESTYLE AND ENVIRONMENTAL FACTORS

Julian Mutz,[1] Charlotte Roscoe,[2] and Cathryn Lewis,[1], *1. King's College London, London, England, United Kingdom, 2. Harvard T.H. Chan School of Public Health, Boston, Massachusetts, United States*

A greater understanding of factors associated with favourable health may help increase longevity and healthy life expectancy. We examined sociodemographic, psychosocial, lifestyle, and environmental exposures associated with multiple health indicators. The UK Biobank study recruited >500,000 participants, aged 37-73, between 2006–2010. Health indicators examined were 81 cancer and 443 non-cancer illnesses used to classify participants by health status; long-standing illness; and self-rated health. Exposures were sociodemographic (age, sex, ethnicity, education, income and deprivation), psychosocial (loneliness and social isolation), lifestyle (smoking, alcohol intake, sleep duration, BMI, physical activity and stair climbing) and environmental (air pollution, noise and residential greenspace) factors. 307,378 participants (mean age = 56.1 years [SD = 8.07], 51.9% female) were selected for cross-sectional analyses. Low income, being male, neighbourhood deprivation, loneliness, social isolation, short or long sleep duration, low or high BMI and smoking was associated with poor health. Walking, vigorous-intensity physical activity and more frequent alcohol intake was associated with good health. There was some evidence that airborne pollutants (PM2.5, PM10, and NO2) and noise (Lden) were associated with poor health, though findings were not consistent across all models. Our findings highlight the multifactorial nature of health, the importance of non-medical factors, such as loneliness, healthy lifestyle behaviours and weight management, and the need to examine efforts to improve health outcomes of individuals with low income.

### IMPLICATIONS OF BIOLOGICAL RATES OF AGING ON HEALTHCARE EXPENDITURES AND MORBIDITY

Alix Jean Santos,[1] Xavier Eugenio Asuncion,[1] Camille Rivero-Co,[1] Maria Eloisa Ventura,[1] Reynaldo II Geronia,[1] Lauren Bangerter,[2] and Natalie Sheils,[1], *1. OptumLabs, Minnetonka, Minnesota, United States, 2. OptumLabs, Eden Prairie, Minnesota, United States*

Understanding biological aging, which entails impeding the progressive decline of biological systems, is important in enabling older adults to live independently. However, the differences in how individuals evolve as they age suggest that aging is a process that does not progress on a single-dimensional trajectory. Moreover, longitudinal studies of aging that follow a cohort of individuals over the course of several years are commonly limited by cost, attrition, and subsequently small sample size. In this study, we used a variational autoencoder to estimate multidimensional rates of aging from cross-sectional routine laboratory data of 1.4 million Americans of at least 40 years of age, collected from 2016 to 2019. We uncovered four aging dimensions that represent the following bodily functions: 1) kidney, 2) thyroid, 3) white blood cells, and 4) liver and heart. We found that fast agers along these dimensions are more likely to develop chronic diseases that are related to these bodily functions. They also had higher health care expenditures compared to the slow agers. K-means clustering of individuals based on the different aging rates revealed that clusters with higher odds of developing morbidity had the highest cost across all types of health care services. Results suggest that cross-sectional laboratory data can be leveraged as an alternative methodology to understand rates of aging along different dimensions, and analysis of their relationships with future costs can aid in the development of interventions to delay disease progression.

### LONELINESS AND MARIJUANA USE AMONG OLDER ADULTS: AN EXAMINATION OF THE 2018 HEALTH AND RETIREMENT STUDY

Jie Yang,[1] and Andrew Yockey,[2], *1. East Carolina University, Greenville, North Carolina, United States, 2. University of North Texas Health Science Center, Denton, Texas, United States*

**Introduction:** There is substantial literature to suggest that loneliness is a risk factor for marijuana initiation, use, and continued use into adulthood. However, these relationships have yet to be investigated among older adults. Given that recent research suggests marijuana use is increasing among older adults, the purpose of the present study was to examine loneliness and other risk factors among a national sample of older adults ages 50 years or older.

**Methods:** A secondary data analysis was conducted on the 2018 Health and Retirement Study (HRS) was conducted (n = 1,431). The HRS is a national, biannual survey conducted in the United States to assess health, psychosocial, and demographic questions among adults ages 50 years or older. We created a loneliness scale from the available questions and assessed differences based on demographics, lifetime use, and past-year use of marijuana. Weighted analyses with cyclical tree-based hot-deck imputation were conducted.

**Results:** A sizeable percentage (23.5%) of older adults have ever used marijuana and a considerable amount (14.8%) of adults have used marijuana in the past year. Differences were found based on sex (p <.0001), age (p <.0001), race (p <.0001), and income (p <.0001). Loneliness significantly predicted marijuana usage, with adults who reported loneliness nearly 5 times more likely to use marijuana (aOR: 4.87, 95% CI 3.89, 6.10).

**Discussion:** The present study investigated loneliness and marijuana usage among a national sample of adults. Findings from the present study may inform behavioral health interventions, harm reduction, and gerontological health.

### OLDER ADULTS SHOW BIOMARKER EVIDENCE OF PICS AFTER SEPSIS

Robert Mankowski,[1] Stephen Anton,[2] Gabriela Ghita,[2] Christiaan Leeuwenburgh,[2] Lyle Moldawer,[2] Philip Efron,[2] Scott Brakenridge,[2] and Frederick Moore,[2], *1. University of Florida, Gainesville, Florida, United States, 2. University of Florida, University of Florida, Florida, United States*

**Background:** Hospital deaths after sepsis have decreased substantially and most young adult survivors rapidly recover (RAP). However, many older survivors develop chronic critical illness (CCI) with poor long-term outcomes. The etiology of CCI is multifactorial and the relative importance remains unclear. Sepsis is caused by a dysregulated immune response and biomarkers reflecting a persistent inflammation, immunosuppression and catabolism syndrome (PICS) have been observed in CCI after sepsis. Therefore, the purpose of this study was to compare serial PICS biomarkers in a) older (versus young) adults and b) older CCI (versus older RAP) patients to gain insight into underlying pathobiology of CCI in older adults.

**Methods:** Prospective longitudinal study with young (≤ 45 years) and older (≥ 65 years) septic adults who were characterized by a) baseline predisposition, b) hospital outcomes, c) serial SOFA organ dysfunction scores over 14 days, d) Zubrod Performance status at three, six and 12-month follow-up and e) mortality over 12 months. Serial blood samples over 14 days were analyzed for selected biomarkers reflecting PICS.

**Results:** Compared to the young, more older adults developed CCI (20% vs 42%) and had markedly worse serial SOFA scores, performance status and mortality over 12 months. Additionally, older (versus young) and older CCI (versus older RAP) patients had more persistent aberrations in biomarkers reflecting inflammation, immunosuppression, stress metabolism, lack of anabolism and anti-angiogenesis over 14 days after sepsis.

**Conclusion:** Older (versus young) and older CCI (versus older RAP) patient subgroups demonstrate early biomarker evidence of the underlying pathobiology of PICS.

### UROLITHIN A ENHANCES MUSCLE PERFORMANCE IN ELDERLY AND POSITIVELY IMPACTS BIOMARKERS LINKED TO CELLULAR HEALTH

Sophia Liu, and David Marcinek, *University of Washington, seattle, Washington, United States*

**Background:** Aging is associated with decline in mitochondrial function and reduced exercise capacity. Urolithin A (UA) is a natural gut metabolite shown to stimulate mitophagy and improve muscle function in aged animals, and induce mitochondrial gene expression in elderly.

**Purpose:** Investigate if oral administration of UA improved walking distance (6MWT), muscle fatigue resistance in hand (FDI) and leg (TA) muscles, and had an impact on plasma biomarkers. Method: We conducted a randomized, double-blind, placebo-controlled study (NCT03283462) in elderly subjects (65-90 yrs.) supplemented daily with 1000mg UA or placebo for 4 months. 128 subjects were screened and 66 randomized. 6MWT and ATPmax via MRS were assessed at baseline and at 4 months. Muscle fatigue tests and plasma analysis of biomarkers were assessed at baseline, 2 and 4 months.

**Results:** UA significantly improved muscle endurance (i.e., change in number of muscle contractions from baseline) in two different muscles (hand: PL 11.6 ±147.5, UA 95.3 ± 115.5; and leg: PL 5.7± 127.1, UA 41.4 ±65.5) compared with placebo at 2-months. Plasma levels of several acylcarnitines, ceramides and C-reactive-protein were decreased by UA at the end-of study. 6MWT distance (PL 42.5 ± 73.3 m, UA 60.8± 67.2 m) and ATPmax increased in both groups from baseline (PL 13.7±31.4%, UA19.4± 56.8%) with UA supplemented group exhibiting greater improvements, although these were not statistically different between groups.

**Conclusion:** UA supplementation improved muscle endurance, metabolic and inflammatory plasma biomarkers after 2-months, suggesting that UA can have a positive impact on muscle and cellular health in the elderly.

### Session 9095 (Poster)

### BIOLOGY OF AGING: COMPUTATIONAL AND SYSTEMS APPROACHES TO GEROSCIENCE

### ANALYSIS OF DGAT2 MUTATIONS REVEALS POTENTIAL LINKS BETWEEN CANCER AND LIPID DROPLET DEREGULATION

Meghan Graber, and Hayley Barta, *Xavier University, Cincinnati, Ohio, United States*

Diacylglycerol O-acyltransferase 2 is a transmembrane protein encoded by the DGAT2 gene that functions in lipid metabolism, triacylglycerol synthesis, and lipid droplet regulation. Since cancer cells exhibit altered lipid metabolism, it has been proposed that mutations in DGAT2 may contribute to this state. Using data from the Catalogue of Somatic Mutations in Cancer (COSMIC), we analyzed all reported DGAT2 mutations in human cancers. Bioinformatics analyses were performed to highlight the connections between age, pathogenicity, and cancer tissue type. Mutations are generally associated with samples from older individuals, except for those in glioblastomas which occur earlier. We also found that several DGAT2 mutations fall within the catalytic site of the enzyme and may affect enzyme function. Thus, these mutations may contribute to altered cancer metabolism. We identified D222V as a mutation hotspot neighboring a previously discovered Y223H mutation that causes Axonal Charcot-Marie-Tooth disease. Remarkably, Y223H has not been detected in cancers indicating it is inhibitory to cancer progression. Further analysis showed that most mutations do not affect DGAT2 gene expression suggesting this change is not a major contributor to cancer development. Intriguingly, although most cancers are characterized by low DGAT2 gene expression, some show high expression levels, indicating that, at least in certain cases, over-expression is not inhibitory to cellular proliferation. This work uncovers unknown roles of DGAT2 in cancers and suggests that its function may be more complex than previously appreciated.

### ASSOCIATION OF GRIMAGE DNA METHYLATION COMPONENTS AND 2-YEAR MORTALITY IN THE HEALTH AND RETIREMENT STUDY

Helen Meier,[1] Colter Mitchell,[2] Eileen Crimmins,[3] Bharat Thyagarajan,[4] and Jessica Faul,[2], *1. University of Michigan, Ann Arbor, Michigan, United States, 2. University of Michigan, University of Michigan, Michigan, United States, 3. University of Southern California, Los Angeles, California, United States, 4. University of Minnesota, Minneapolis, Minnesota, United States*

DNA methylation (DNAm) patterns related to age and aging phenotypes (i.e., epigenetic clocks) are of growing interest as indicators of biological age and risk of negative health outcomes. We investigated associations between the components of GrimAge, an epigenetic clock estimated from DNAm patterns for seven blood protein levels and smoking pack years, and 2-year mortality in the Health and Retirement Study (HRS) to determine if any of the DNAm subcomponents were driving observed associations. A representative subsample of individuals who participated in the HRS 2016 Venus Blood Study were included in this analysis (N=3430). DNAm was measured with the Infinium Methylation EPIC BeadChip. Deaths that occurred between 2016 and 2018 contributed to 2-year mortality estimates (N=159, 4.5% of the sample). Weighted logistic regression estimated the association first between GrimAge and 2-year mortality and second between the DNAm subcomponents and 2-year mortality. All models were adjusted for age, sex, race/ethnicity, education, current smoking status, smoking pack years and cell composition of the biological sample. The average GrimAge for participants with and without 2-year mortality was 77 years 68 years respectively. A one-year increase in GrimAge was associated with 17% higher odds of 2-year mortality (95% CI: 1.16, 1.17). Two of the seven DNAm blood protein subcomponents of GrimAge (TIMP metallopeptidase inhibitor 1, adrenomedullin) and DNAm smoking pack years were associated with 2-year mortality and DNAm smoking pack years appeared to drive the overall GrimAge association with 2-year mortality. GrimAge was a better predictor of 2-year mortality than the DNAm subcomponents individually.

### GLOBAL PHOSPHOPROTEOMIC PROFILING OF SKELETAL MUSCLE IN OVARIAN HORMONE-DEFICIENT FEMALE MICE

Mina Peyton,[1] Tzu-Yi Yang,[1] LeeAnn Higgins,[2] Todd Markowski,[1] Laurie Parker,[1] and Dawn Lowe,[1], *1. University of Minnesota, Minneapolis, Minnesota, United States, 2. University of Minnesota, University of Minnesota, Minnesota, United States*

Dynapenia, the age-related loss of skeletal muscle strength without the loss of muscle mass, significantly impacts the activities and quality of life of the aging population. Studies have shown that dynapenia occurs earlier in females than males in both human and rodent studies. Moreover, in females, estrogen deficiency has been shown to contribute to the loss of skeletal muscle strength as well as blunted recovery of strength after injury. The maintenance of skeletal muscle contractile function is vital to the overall health of women, especially as women live 1/3 of their life in an estrogen deficient state. Reversible protein phosphorylation is an indispensable post-translational modification, playing a key role in signal transduction pathways. Phosphorylation of skeletal muscle proteins have been shown to regulate sarcomeric function, excitation-contraction coupling, energy metabolism, and fiber-type composition. To define the physiological changes in the skeletal muscle phosphoproteome associated with estrogen deficiency, we used an ovariectomy model coupled with mass spectrometry. We identified, in total, 5,424 unique phosphorylation sites and 1,177 phosphoproteins in the tibialis anterior muscle. Ingenuity Pathway Analysis show decreased phosphorylation of contractile proteins and significant predicted inhibition of the upstream kinase, CDK6 (z-score -2.0) in ovariectomized compared to control muscles. Our results suggest that estrogen deficiency remodels the skeletal muscle phosphoproteome which may alter phosphorylation signaling that might contribute to the loss of strength in females.

### IN SILICO IDENTIFICATION OF ANTI-AGING PHARMACEUTICS FROM COMMUNITY KNOWLEDGE

Samuel Beck, Jun-Yeong Lee, and Jarod Rollins, *MDI Biological Laboratory, Bar Harbor, Maine, United States*

In this era of Big Data, the volume of biological data is growing exponentially. Systematic profiling and analysis of these data will provide a new insight into biology and human health. Among diverse types of biological data, gene expression data closely mirror both the static phenotypes and the dynamic changes in biological systems. Drug-to-drug or drug-to-disease comparison of gene expression signature allows repurposing/repositioning of existing pharmaceutics to treat additional diseases that, in turn, provides a rapid and cost-effective approach for drug discovery. Thanks to technological advances, gene expression profiling by mRNA-seq became a routine tool to address all aspects of the problem in modern biological research. Here, we present how drug repositioning using published mRNA-seq data can provide unbiased and applicable pharmaco-chemical intervention strategies to human diseases and aging. In specifics, we profiled over a half-million gene expression profiling data generated from various contexts, and using this, we screened conditions that can suppress age-associated gene expression changes. As a result, our analysis identified various previously validated aging intervention strategies as positive hits. Furthermore, our analysis also predicted a novel group of chemicals that has not been studied from an aging context, and this indeed significantly extended the life span in model animals. Taken together, our data demonstrate that our community knowledge-guided in silico drug-discovery pipeline provides a useful and effective tool to identify the novel aging intervention strategy.

### INTERPRETABLE MACHINE LEARNING OF HIGH-DIMENSIONAL AGING HEALTH TRAJECTORIES

Spencer Farrell,[1] Arnold Mitnitski,[2] Kenneth Rockwood,[3] and Andrew Rutenberg,[1], *1. Dalhousie University, Halifax, Nova Scotia, Canada, 2. Dalhousie Unversity, Dalhousie University, Nova Scotia, Canada, 3. Dalhousie University, Dalhousie Unversity, Nova Scotia, Canada*

We have built a computational model of individual aging trajectories of health and survival, that contains physical, functional, and biological variables, and is conditioned on demographic, lifestyle, and medical background information. We combine techniques of modern machine learning with an interpretable network approach, where health variables are coupled by an explicit interaction network within a stochastic dynamical system. Our model is scalable to large longitudinal data sets, is predictive of individual high-dimensional health trajectories and survival from baseline health states, and infers an interpretable network of directed interactions between the health variables. The network identifies plausible physiological connections between health variables and clusters of strongly connected heath variables. We use English Longitudinal Study of Aging (ELSA) data to train our model and show that it performs better than traditional linear models for health outcomes and survival. Our model can also be used to generate synthetic individuals that age realistically, to impute missing data, and to simulate future aging outcomes given an arbitrary initial health state.

### MAMMALIAN CO-METHYLATION NETWORK ANALYSIS OF SPECIES CHARACTERISTICS

Amin Haghani,[1] and Steve Horvath,[2], *1. Dept. of Human Genetics, David Geffen School of Medicine, University of California Los Angeles, Los Angeles, CA, USA, Los Angeles, California, United States, 2. UCLA, Los Angeles, California, United States*

The comparative cross-species analysis is a powerful tool to resolve the mysteries of evolution and phenotypic disparities among animals. This is the first network analysis of 10,000 DNA methylome data from 176 mammalian species to identify co-methylation modules that relate to individual (age, sex, tissue type) and species characteristics (e.g. phylogenetic order, maximum lifespan, adult weight). The unexpected correlation between DNA methylation and species were sufficiently strong to allow the construction of phyloepigenetic trees that parallel the phylogenetic tree. Weighted correlation network analysis identified 55 distinct co-methylation modules, i.e. sets of highly correlated CpGs. 31 of these modules are readily interpretable in terms of their relationship to age, maximum lifespan, tissue type etc. An age-related module was perturbed by gold standard anti-aging interventions in mice such as caloric restriction or growth hormone receptor knock outs. Our module-based analysis greatly enhances our biological understanding of age-related changes in DNA methylation across many species.

### NUCLEAR ARCHITECTURE DISRUPTION DURING AGING CAUSES MISEXPRESSION OF GENES LACKING CPG ISLANDS

Jun-Yeong Lee, Ian Davis, and Samuel Beck, *MDI Biological Laboratory, Bar Harbor, Maine, United States*

Global disorganization of chromatin architecture, characterized by disrupted nuclear lamina and associated heterochromatin, is commonly observed in various aging contexts, including premature aging diseases, cellular senescence, and normative aging. Although these conserved structural changes have been reported for over two decades, their impact on transcription and contribution to age-related degenerative changes remain unclear. Here we show that genes not associated with CpG islands (CGI- genes), which form heterochromatin when transcriptionally silent, are globally misexpressed in aged nuclei with disrupted chromatin architectures. Our data also show that CGI- gene misexpression is a direct outcome of nuclear architecture disruption. Notably, CGI- gene misexpression explains the molecular basis of various defects observed during aging, including loss of cellular identity and increased noises in transcription. We also show that uncontrolled secretory phenotypes commonly observed during aging are largely attributable to CGI- gene misexpression, which drives disruption of intercellular communication and fuel chronic inflammation in aged tissues. Our large-scale meta-analysis further demonstrates that CGI- gene misexpression is a common feature of mammalian aging and age-associated diseases. Interestingly, CGI- gene misexpression can be suppressed by anti-aging interventions. Our study suggests that age-associated CGI- gene misexpression is a novel biomarker of physiological aging which offers an effective therapeutic target for delaying or ameliorating degenerative changes associated with aging.

### PROFILING EPIGENETIC AGE IN SINGLE CELLS

Alexandre Trapp, Csaba Kerepesi, and Vadim Gladyshev, *Brigham and Women's Hospital, Boston, Massachusetts, United States*

DNA methylation of a defined set of CpG dinucleotides emerged as a critical and precise biomarker of the aging process. Multi-variate machine learning models, known as epigenetic clocks, can exploit quantitative changes in the methylome to predict the age of bulk tissue with remarkable accuracy. However, intrinsic sparsity and digitized methylation in individual cells have so far precluded the assessment of aging in single cell data. We developed scAge, a probabilistic approach to determine the epigenetic age of single cells, and validated our results in mice. scAge tissue-specific and multi-cell type single cell clocks correctly recapitulated the chronological age of the original tissue, while uncovering the inherent heterogeneity that exists at the single-cell level. The data suggested that while cells in a tissue age in a coordinated fashion, some cells age more or less rapidly than others. We showed that individual embryonic stem cells exhibit an age close to zero, that certain stem cells in a tissue showed a reduced age compared to their chronological age, and that early embryogenesis is associated with the reduction of epigenetic age in individual cells, the latter supporting a natural rejuvenation event during gastrulation. scAge is both robust against the low coverage that is characteristic of single cell sequencing techniques and is flexible for studying any cell type and mammalian organism of interest. We demonstrate the potential for accurate epigenetic age profiling at single-cell resolution.

### WIDESPREAD CORTICAL DEMYELINATION IN GERIATRIC CASES OF MILD TRAUMATIC BRAIN INJURY AND IN ALZHEIMER'S DISEASE

Andrei Irimia,[1] Nahian Chowdhury,[2] Shania Wang,[2] Sean Mahoney,[2] Van Ngo,[2] Kenneth Rostowsky,[1] Nikhil Chaudhari,[2] and Benjamin Hacker,[1], *1. University of Southern California, University of Southern California, California, United States, 2. University of Southern California, Los Angeles, California, United States*

Cortical demyelination is related to neurodegeneration after mild traumatic brain injury (mTBI) and Alzheimer's disease (AD). The ratio R of T1-to-T2-weighted magnetic resonance image (MRI) intensities is proportional to myelin content, and allows myelin changes to be mapped in vivo. T1 and T2 MRIs were acquired from mTBI patients (N = 97, age $\mu$ = 41 y; $\sigma$ = 19 y, range: 21-79) both acutely and chronically (~1 week and ~6 months post-injury, respectively), from AD patients (N = 80, age $\mu$ = 76 y; $\sigma$ = 8 y, range: 55-88), and from cognitively normal (CN) adults (N = 78, age $\mu$ = 75 y; $\sigma$ = 5 y, range: 12-90). AD and CN subjects' data were acquired less than a year apart. MRIs were analyzed using 3DSlicer's BRAINSfit (registration), FreeSurfer (segmentation), SPM12 (bias field correction) and custom MATLAB scripts to calculate myelin content and demyelination. The null hypothesis of no myelin change was tested at each cortical location for each pair of groups ($\alpha$ = 0.05), after accounting for age, sex and interscan interval. Compared to HCs, AD subjects featured significantly greater myelin loss in dorsolateral prefrontal cortex, lateral and medial temporal lobes (~52% of the cortex, $p < 0.05$). mTBI participants experienced significantly greater myelin loss across ~96% of the cortex ($p < 0.05$), suggesting that mTBI has dramatic impact upon cortical myelin content. Myelin loss magnitude was comparable across mTBI and AD, particularly within temporal lobes. Future research should study whether post-traumatic demyelination increases the AD risk.

### Session 9100 (Poster)

### BIOLOGY OF AGING: IMMUNITY AND AGING

### ANALYSIS OF SINGLE CELL DATA AS IT RELATES TO AGING AND LONGEVITY

Tanya Karagiannis,[1] Todd Dowrey,[1] Carlos Villacorta-Martin,[2] George Murphy,[2] Stefano Monti,[1] and Paola Sebastiani,[3], *1. Boston University, Boston, Massachusetts, United States, 2. Boston University School of Medicine, Boston, Massachusetts, United States, 3. Tufts Medical Center, Physician Organization, BOSTON, Massachusetts, United States*

Age-related disability and diseases are known to be delayed in people living to 100 years or more. Changes in the immune system with age are known, including in cell type composition and gene expression differences. To further explore changes in extreme longevity subjects, we investigated peripheral blood immune system cell subpopulations across age and extreme longevity at a single cell resolution. We performed an integrative analysis of public scRNA-seq datasets to define consensus cell types of longevity and age, and classified cell types in our novel New England Centenarian Study dataset. We integrated these datasets together to investigate cell type specific differences at a composition and gene expression level. Our findings identified higher cell type diversity in extreme longevity subjects compared to younger age groups, but no significant difference among younger age groups demonstrating that overall composition differences are unique to longevity. We identified novel differences in myeloid and lymphocyte populations; Extreme longevity subjects have higher composition of CD14+ Monocytes, Natural Killer cells, and T gamma delta populations and lower composition of CD16+ Monocytes and dendritic populations. We characterized gene expression differences between extreme longevity and younger age groups and differences in aging across younger age groups. We found that extreme longevity cell type specific signatures overlapped with the aging signatures by at least 50%. We identified unique genes to extreme longevity that are enriched for pathways specific to immune activation and inflammation, suggesting a protective mechanism for centenarians through efficient activation and regulation of immune subpopulations in peripheral blood.

### ASSOCIATION BETWEEN SERUM 25-HYDROXYVITAMIN D LEVELS AND INCIDENCES OF INFECTION IN LONG-TERM CARE

Ronna Robbins,[1] Monica Serra,[2] and Margaret Briley,[3], *1. Audie Murphy VA Medical Center, San Antonio, Texas, United States, 2. UT Health San Antonio - Barshop Institute, San Antonio, Texas, United States, 3. University of Texas at Austin, Austin, Texas, United States*

Insufficient serum 25-hydroxyvitamin D (25(OH)D) concentrations are associated with increased respiratory tract infections, influenza, and other infectious diseases. As the world deals with the COVID-19 pandemic, the interest of adequate serum levels to reduce the risk of infection has surfaced. This study determined if the number of infections per year are associated with serum 25(OH)D concentrations in long-term care (LTC). Participants (≥ 65 years) in a cross-sectional study were recruited across five LTC communities in Texas. Medical records were used to collect a one-year medical history using double-blind protocols. Blood draws were collected to measure serum 25(OH)D concentrations. Medical records were used to classify infections based upon documentation of signs and symptoms of infection concurrent with either a physician's note or antibiotic/antiviral medication prescription. Race, BMI, sex, age, and liver and renal disease diagnoses were used as confounders. Of the 177 participants (89% Caucasian, 63% female, mean age 83 years) 69% had ≥1 infection over year and 55% had insufficient serum 25(OH)D concentrations <30 mg/mL (mean 32.6 ng/mL). Linear regression did not show a significant association between serum 25(OH)D concentrations and number of infections (β 0.003; 95% CI -0.014, 0.018; p=0.760). Additionally, insufficient serum concentration did not increase the odds of having an infection (OR 1.02; 95% CI 0.05, -19.34; p=0.987). This study did not show a significant association between infection rates and serum 25(OH) D concentrations. However, further research is needed to determine if vitamin D supplementation could be an effective therapeutic intervention to reduce infection rates, including COVID-19.

### BIOLOGICAL AGING PREDICTS VULNERABILITY TO COVID-19 SEVERITY IN UK BIOBANK PARTICIPANTS

Chia-Ling Kuo,[1] Luke Pilling,[2] Janice Atkins,[3] Jane Masoli,[2] João Delgado,[4] George Kuchel,[1] David Melzer,[2] and Morgan Levine,[5], *1. University of Connecticut Health, Farmington, Connecticut, United States, 2. University of Exeter, Exeter, England, United Kingdom, 3. University of Exeter Medical School, University of Exeter, England, United Kingdom, 4. University of Exeter, Exerer, England, United Kingdom, 5. Yale University, New Haven, Connecticut, United States*

Age and disease prevalence are the two biggest risk factors for COVID-19 symptom severity and death. We therefore hypothesized that increased biological age, beyond chronological age, may be driving disease-related trends in COVID-19 severity. Using the UK Biobank England data, we tested whether a biological age estimate (PhenoAge) measured more than a decade prior to the COVID-19 pandemic was predictive of two COVID-19 severity outcomes (inpatient test positivity and COVID-19 related mortality with inpatient test-confirmed COVID-19). Logistic regression models were used with adjustment for age at the pandemic, sex, ethnicity, baseline assessment centers, and pre-existing diseases/conditions. 613 participants tested positive at inpatient settings between March 16 and April 27, 2020, 154 of whom succumbed to COVID-19. PhenoAge was associated with increased risks of inpatient test positivity and COVID-19 related mortality (ORMortality=1.63 per 5 years, 95% CI: 1.43-1.86, p=4.7x10E-13) adjusting for demographics including age at the pandemic. Further adjustment for preexisting disease s/conditions at baseline (OR_M=1.50, 95% CI: 1.30-1.73 per 5 years, p=3.1x10E-8) and at the early pandemic (OR_M=1.21, 95% CI: 1.04-1.40 per 5 years, p=0.011) decreased the association. PhenoAge measured in 2006-2010 was associated with COVID-19 severity outcomes more than 10 years later. These associations were partly accounted for by prevalent chronic diseases proximate to COVID-19 infection. Overall, our results suggest that aging biomarkers, like PhenoAge may capture long-term vulnerability to diseases like COVID-19, even before the accumulation of age-related comorbid conditions.

### COMBATING AGE-ASSOCIATED IMMUNE DECLINE USING KYNURENINE PATHWAY INTERVENTIONS

Luis Espejo,[1] Destiny DeNicola,[2] Sam Freitas,[2] Hope Dang,[3] Emily Turner,[3] Raul Castro-Portuguez,[2] Anne Haskins,[2] and George Sutphin,[2], *1. UNIVERSITY OF ARIZONA, TUCSON, Arizona, United States, 2. The University of Arizona, Tucson, Arizona, United States, 3. University of Arizona, Tucson, Arizona, United States*

Select kynurenine pathway interventions extend lifespan in invertebrate models and are of interest in treating age-associated diseases. Kynurenine pathway activity is responsive to inflammatory signaling, and we are evaluating the potential for these interventions to increase pathogen resistance and curtail age-associated immune decline in Caenorhabditis elegans and mammals. The kynurenine pathway facilitates the catabolism of tryptophan to nicotinamide adenine dinucleotide (NAD). Our lab has found that supplementing the kynurenine metabolite 3-hydroxyanthranilic acid (3HAA) or inhibiting the enzyme 3HAA dioxygenase (HAAO) extends lifespan in C. elegans. 3HAA has demonstrated pro/anti-inflammatory properties in mammals, suggesting a potential role in immune function. C. elegans have a primitive immune system that lacks an adaptive element, but it recapitulates aspects of innate immune signaling and pathogen response. I hypothesize kynurenine pathway interventions that impact C. elegans' lifespan similarly improve pathogen resistance and immunity. Interventions within the kynurenine pathway are capable of differentially impacting pathogenesis and lifespan of C. elegans challenged with Psuedomonas aeruginosa. C. elegans subjected to select lifespan-extending kynurenine pathway interventions fared better when challenged with P. aeruginosa at older ages. Additionally, fluorescent infection tracking has displayed decreased infection rates in worms with elevated 3HAA. Our data suggests pro-immune activity is facilitated by 3HAA acting downstream of the dbl-1 pathway in addition to directly inhibiting bacterial growth. Our goal is to discover the mechanism(s) through which the kynurenine pathway interacts with immune function in animals and identify potential targets for clinical therapy in aging populations.

### EARLY LIFE THYMECTOMY INDUCES GLUCOSE INTOLERANCE IN MIDDLE-AGED MICE

David Buckley, Daniel Trott, and Sunita Sharma, *University of Texas at Arlington, Arlington, Texas, United States*

Previously we have found that T cells contribute to age-related glucose intolerance. The purpose of this study was to test the hypothesis that early life thymectomy at 3wks of age induces T cell aging and subsequent impairments in glucose homeostasis in otherwise young animals. Male C57BL6 mice underwent thymectomy (thymex; n=7) or sham surgery (control; n=7) at 3wks of age. A glucose (2g/kg) tolerance test (GTT) was performed at 6 and 9mo via intraperitoneal injection. Following euthanasia at 9mo of age, splenic T cell phenotype was assessed by flow cytometry. Group differences were assessed by independent samples t-test or repeated measures ANOVA and Bonferroni post-hoc test. Data are presented as mean±SEM. At 6mo, the thymex animals had a significantly lower fasting glucose compared to controls (156.8±7.9mg/dl,174.1±5.8mg/dl, p=0.06). During the GTT, 6mo old thymex mice had a greater area under the curve (AUC) compared to controls (31893.8±612.3mg/dl, 28020.9±1112.9mg/dl, p=0.03). At 9mo, the thymex mice had greater fasting glucose compared to controls (215.6±11.6mg/dl, 176.3±7.9mg/dl, p=0.016), as well as a greater GTT AUC (61445.4±1949.2mg/dl, 41527.5±2530.3mg/dl, p=0.0001). The thymex group also had increased fasting and glucose stimulated insulin levels compared to controls (0=1.3±0.2ng/ml 0.3±0.1ng/ml, p=0.01; 15=1.7±0.2ng/ml,0.44±0.1ng/ml, p=0.0014). Thymex mice exhibited a blunted splenic CD4:CD8 ratio (0.5±0.2, 1.1±0.2, p=0.04) compared to controls and a trend toward a memory CD8+ T cell phenotype (23.1±11.6%, 7.1±2.6, p=0.08), both consistent with aging. This data indicates that early life thymectomy may accelerate T cell aging, resulting in impairments in glucose tolerance in otherwise young and middle aged mice.

### EXPERIENCING A NATURAL DISASTER ACCELERATES AGING OF THE IMMUNE SYSTEM

Marina Watowich,[1] Kenneth Chiou,[2] Michael Montague,[3] Melween Martínez,[4] James Higham,[5] Lauren Brent,[6] Michael Platt,[7] and Noah Snyder-Mackler,[2] *1. University of Washington, Tempe, Arizona, United States, 2. Arizona State University, Tempe, Arizona, United States, 3. University of Pennsylvania Perelman School of Medicine, Philadelphia, Pennsylvania, United States, 4. Medical Sciences Campus- University of Puerto Rico, Sabana Seca, Puerto Rico, United States, 5. NYU, New York University, New York, United States, 6. University of Exeter, Exeter, England, United Kingdom, 7. University of Pennsylvania, Philadelphia, Pennsylvania, United States*

Extreme adverse events such as natural disasters can accelerate disease progression and promote chronic inflammation. These phenotypes also increase in prevalence with age, suggesting that experiencing adversity might accelerate aging of the immune system. Adversity can induce persistent gene regulatory changes which may mechanistically explain the immune similarities between aging and adversity. To test how immune system aging is accelerated following a natural disaster, we measured the impact of Hurricane Maria on peripheral blood immune cell gene expression in a population of free-ranging rhesus macaques (Macaca mulatta) from before (n=435) versus after (n=108) Hurricane Maria. Experiencing Hurricane Maria altered the expression of 260 genes (FDR<10%), which were primarily involved in the inflammatory response. There was significant overlap in these hurricane-affected and age-associated genes with 40% (n=104) being associated with both the hurricane and aging, more than double the expected amount (Fisher's Exact Test OR=3.7, p=4.06 x 10–21). The effects of the hurricane and aging on gene expression were also significantly correlated (rho=0.23, p=1.33 x 10-84), suggesting that they alter similar molecular pathways in the immune system. Further, we found that animals that experienced the hurricane had a gene expression profile that was, on average, 1.6 years older than animals that did not experience the hurricane (the equivalent of 6–7 years in a human lifespan, p=0.003). Together, our results provide some of the first evidence that extreme natural disasters mechanistically accelerates aging in the immune system.

### HEMATOPOIETIC MOSAIC CHROMOSOMAL ALTERATIONS IN THE NEW ENGLAND CENTENARIAN STUDY.

Anastasia Leshchyk,[1] Giulio Genovese,[2] Stefano Monti,[3] Thomas Perls,[3] and Paola Sebastiani,[4] *1. Boston University, Brookline, Massachusetts, United States, 2. Broad Institute, Boston, Massachusetts, United States, 3. Boston University, Boston, Massachusetts, United States, 4. Tufts Medical Center, Physician Organization, BOSTON, Massachusetts, United States*

Mosaic chromosomal alterations (mCAs) are structural alterations that include deletions, duplications, or copy-neutral loss of heterozygosity. mCAs are reported to be associated with survival, age, cancer, and cardiovascular disease. Previous studies of mCAs in large population-based cohorts (UK Biobank, MGBB, BioBank Japan, and FinnGen) have demonstrated a steady increase of mCAs as people age. The distribution of mCAs in centenarians and their offspring is not well characterized. We applied MOsaic CHromosomal Alteration (MoChA) caller on 2298 genome-wide genotype samples of 1582 centenarians, 443 centenarians' offspring, and 273 unrelated controls from the New England Centenarian Study (NECS). Integrating Log R ratio and B-allele frequency (BAF) intensities with genotype phase information, MoChA employs a Hidden Markov Model to detect mCA-induced deviations in allelic balance at heterozygous sites consistent with genotype phase in the DNA microarray data. We analyzed mCAs spanning over 100 k base pairs, with an estimated cell fraction less than 50%, within samples with genome-wide BAF phase concordance across phased heterozygous sites less than 0.51, and with LOD score of more than 10 for the model based on BAF and genotype phase. Our analysis showed that somatic mCAs increase with older age up to approximately 102 years, but the prevalence of the subjects with mCAs tend to decrease after that age, thus suggesting that accumulation of mCAs is less prevalent in long-lived individuals. We also used Poisson regression to show that centenarians and their offspring tend to accumulate less mCA (RR = 0.63, p=0.045) compared to the controls.

### IMMUNE CELL AND ADIPOSE GENE EXPRESSION REPROGRAMMING IN JUVENILE MONKEYS BORN TO HEALTH AND WEIGHT-DIVERSE MOTHERS

Alistaire Ruggiero, Masha Block, Ravichandra Vemuri, Darla DeStephanis, Swapan Das, and Kylie Kavanagh, *Wake Forest School of Medicine, Winston-Salem, North Carolina, United States*

Over 93 million Americans are obese and 66 million suffer from metabolic disease. Roughly 40% of obese people do not have metabolic abnormalities (metabolically health obese [MHO]), while approximately 15% of lean do (metabolically unhealthy lean [MUL]). African green monkeys (AGMs) demonstrate naturally occurring obesity and metabolic syndrome (MetS) without diet manipulation, and MetS criteria are heritable. Age-matched maternal AGMs were classified by adjusted MetS criteria ([n=44]; waist >40cm, fasting glucose (FG) >100 mg/dL, SBP/DBP >135/85mmHg, and HDL-c <50mg/dL) and classified as metabolically healthy lean (MHL), MHO, MUL or metabolically unhealthy obese (MUO). Age, weight and sex-matched pre-pubertal juvenile offspring from these mothers were additionally selected (n=9-11/group; ages=1.1-3.4years) for evaluation. We assessed monocyte subtypes by flow cytometry, and subcutaneous adipose gene expression patterns by RNAseq. Non-classical monocytes were increased in obese and unhealthy mothers (MHO p=0.02, MUL p=0.003, MUO p=0.00002) compared to MHL. MUL and MUO juvenile offspring also had more non-classical monocytes compared to MHL (p=0.05 and p=0.07). Monocyte chemoattractant protein-1 (MCP)-1 was measured in plasma and found to be elevated in MUO juveniles (p=0.02). Patterns of increased cytokine and extracellular matrix gene expression were seen in MUL and MUO juveniles' adipose (6-7/group), mirroring obese and unhealthy mothers' adipose gene expression patterns. Maternal health and obesity influence offspring immune cells and adipose gene expression prior to weight gain and metabolic disease onset. Our data underscore maternal monocyte and adipose profiles as inherited phenotypes that present prior to adipose expansion and may be targets to improve intergenerational health trajectories.

### THE ROLE OF THE HUMAN IMMUNE SYSTEM IN THE AGING PROCESS: A MATHEMATICAL MODEL OF CELL AND CYTOKINE ACTIVATION

Kian Talaei,[1] Nuno Martins,[1] Elliott Suen,[2] Joshua Cho,[3] Julia Jahansooz,[3] Puneet Bhullar,[4] Walter Piszker,[3] and Steven Garan,[1], *1. University of California, Berkeley, Center for Research and Education in Aging, University of California, Berkeley, California, United States, 2. UC Berkeley, Berkeley, California, United States, 3. University of California, Berkeley, Berkeley, California, United States, 4. Mayo Clinic, Scottsdale, Arizona, United States*

The role of the human immune system as a factor in the aging process has led to extensive research in the field of infection biology and bioinformatics. Cell-based mathematical models have previously been used to simulate the immune system in response to pathogens. A variety of cells, such as activated and resting macrophages, plasma cells, antibodies, helper T cells, T-lymphocytes, and B-lymphocytes, have already been simulated by mathematical models. This work aims to incorporate cytokines in these mathematical models to create a more comprehensive simulation that can predict cytokine levels in response to a Gram-positive bacterium, S. aureus. To accomplish this, the cytokines Tumor Necrosis Factor Alpha (TNF-$\alpha$), Interleukin 6 (IL-6), Interleukin 8 (IL-8), and Interleukin 10 (IL-10) were studied to quantify the relationship between cytokine release from macrophages and the concentration of the pathogen, S. aureus ex vivo. The results of the simulation were compared to ex vivo human whole blood data to test its accuracy. The future expansion of this model may provide a clearer image of the various interactions within the immune system and this improved model of the immune system may also facilitate a better understanding of the mechanisms that lead to the degradation of the immune system during the aging process.

### Session 9105 (Poster)

### BIOLOGY OF AGING: INTERVENTIONS

### 3-HYDROXYANTHRANILIC ACID ADMINISTRATION DID NOT PREVENT AGE RELATED BONE LOSS

Carlos Isales,[1] Ke-Hong Ding,[2] Wendy Bollag,[3] Meghan McGee-Lawrence,[4] William Hill,[5] Xing-ming Shi,[3] Sadanand Fulzele,[1] and Mark Hamrick,[1], *1. Medical College of Georgia, Augusta, Georgia, United States, 2. Augusta University, augusta, Georgia, United States, 3. Augusta University, Augusta, Georgia, United States, 4. Medical College of Georgia, Augusta University, Augusta, Georgia, United States, 5. Medical University of South Carolina, Charleston, South Carolina, United States*

Aging is associated with accumulation of various tryptophan degradation products that may having either bone anabolic or catabolic effects. In epidemiologic studies, elevated levels of 3-hydroxyanthranilic acid (3-HAA) are associated with a higher bone mineral density (BMD). We have previously shown that the C57BL/6 mouse loses bone mass with age. Thus, we hypothesized that administering 3-HAA via a daily intraperitoneal (IP) injection would result in preserved or increased BMD. In an IACUC-approved protocol, we injected 26-month-old C57BL/6 mice with either a low dose (0.5 mg) or high dose (5 mg) of 3-HAA IP five days a week for eight weeks. At the end of this time mice were sacrificed and body composition and bone mineral density measured by DigiMus. BMD was significantly lower in the high dose 3-HAA group: 0.0570 + 0.004 vs 0.0473 + 0.006

vs 0.0432 + 0.0075 gm/cm2, (means+SD, Control vs 0.5 mg 3HAA vs 5 mg 3HAA, p=0.004, 0 vs 5.0 mg, n=6-9/group). 3-HAA had no significant impact on body composition (lean body mass: 86.7 + 1.7% vs 86.2 + 2.7% vs 86.1 + 2.0%, Control vs 0.5 mg vs 5.0 mg 3-HAA, p=ns; and fat mass 12.6 + 2.0% vs 13.8 + 2.7% vs 13.9 + 2.0% vs 0.2%, Control vs 0.5 vs 5 mg 3-HAA, p=ns). Thus, 3-HAA did not prevent bone loss in older mice but instead significantly worsened bone loss. 3-HAA is known to have both pro- and anti- oxidant effects depending on the environment. These data would suggest that at the higher concentrations 3-HAA is acting predominantly as a pro-oxidant molecule accelerating age-related bone loss.

### ASSOCIATION BETWEEN MEETING PHYSICAL ACTIVITY GUIDELINES WITH AMBULATION AND QUALITY OF LIFE IN CLAUDICATION

Andrew Gardner,[1] Polly Montgomery,[1] Ming Wang,[2] Biyi Shen,[2] Shangming Zhang,[2] and William Pomilla,[2], *1. Penn State College of Medicine, Hummelstown, Pennsylvania, United States, 2. Penn State College of Medicine, Penn State College of Medicine, Pennsylvania, United States*

We determined if meeting the 2018 physical activity guidelines was associated with better ambulatory function, health-related quality of life, and inflammation than failing to meet the guidelines in patients with peripheral artery disease and claudication. Secondly, we determined the optimal number of total daily steps that are needed to meet the physical activity guidelines. Five hundred seventy-two patients were assessed on their daily ambulatory activity for one week with a step activity monitor, and were grouped according to whether they achieved less than 150 minutes of moderate intensity physical activity per week (Group 1=Do Not Meet Guidelines; n=397), or whether they were above this threshold (Group 2=Meet Guidelines; n=175). Treadmill peak walking time (mean±SD) was significantly higher (p<0.001) in Group 2 (709±359 sec) than in Group 1 (427±281 sec). The health-related quality of life score for physical function was significantly higher (p<0.001) in Group 2 (61±22%) than in Group 1 (44±21%). High sensitivity C-reactive protein was significantly lower (p<0.001) in Group 2 (3.6±4.5 mg/L) than in Group 1 (5.9±6.1 mg/L). Finally, 7,675 daily steps was the optimal threshold associated with meeting the physical activity guidelines, with a sensitivity of 82.9% and a specificity of 88.4%. In conclusion, patients with claudication who meet the 2018 physical activity guidelines for US adults had better ambulation, HRQoL, and inflammation outcomes than those who failed to meet the guidelines. From a practical standpoint, patients with claudication best achieved the physical activity guidelines by taking a total of 7,675 daily steps.

### BENEFICIAL EFFECTS OF A PLASMA FRACTION ON INFLAMMATION AND SYNAPTIC DEFICITS FOR AGE-RELATED COGNITIVE DISORDERS

Ian Gallager, Jasmine Torres, Raniel Alcantara-Lee, Chirag Thadani, Rachel Britton, Eva Czirr, Steven P Braithwaite, and Viktoria Kheifets, *Alkahest Inc., San Carlos, California, United States*

The process of aging is multifactorial, and therefore single agent interventions would be unlikely to attenuate the myriad pathologies associated with advancing age. Plasma contains many beneficial factors which have been shown in animal models to ameliorate multiple age-related deficits across varied organ systems, including the brain. We confirmed that human plasma from young (18-22-year-old) donors reverses age-related cognitive decline and enhances hippocampal neurogenesis and cell survival in aged immunocompromised mice, while plasma from aged individuals (62-68 years old) has detrimental effects in young mice. We examined PF in a high-fat diet (HFD) mouse model, a surrogate for a western diet, which expresses many characteristics of aging within the CNS in an accelerated manner: decreased cell proliferation, synaptic connectivity and increased inflammation compared to normal diet (NC) controls. We demonstrate that PF administration in HFD mice resulted in decreased brain inflammation, increased synaptic connectivity, improved neural progenitor cell survival, as well as amelioration of behavioral endpoints without impacting the underlying metabolic changes induced by HFD. In summary, we demonstrate that PF is a multifactorial and multimodal intervention for the treatment of global changes induced by the process of aging.

### BOTH VITAMIN D SUPPLEMENTATION AND HIIT BOOST MUSCLE VDR EXPRESSION, WHICH MAY UNDERLIE BENEFITS FOR FRAILTY

Ken Seldeen, Ramkumar Thiyagarajan, Merced Leiker, Reem Berman, Yonas Redae, Carleara Weiss, Lee Chaves, and Bruce Troen, *University at Buffalo, Buffalo, New York, United States*

Frailty is a condition of poor response to stressors that increases susceptibility to adverse outcomes - including disability and death. Declining physical function is an important hallmark of frailty, and we previously published that long term vitamin D insufficiency from young to middle-age leads to declines in endurance and gait disturbances. Furthermore, we report that aged mice (24-months) made vitamin D insufficient for 4 months exhibit increased frailty, whereas those made hyper-sufficient do not. Exercise, including short session high intensity interval training (HIIT - 10 minutes/3x-week), also reverses frailty in aged mice. Here we investigate the impacts of aging, vitamin D, and exercise on underlying muscle quality and muscle stem cell activity. Our preliminary data reveal muscle vitamin D receptor (VDR) expression is lower in aged mice (24-28 months) relative to young mice (6 months). Yet HIIT, either one hour after a single session or following 6 weeks, increases VDR expression. HIIT also increases myonuclear accretion in muscle fibers – an indicator of in vivo stem cell activity – and stimulates progenitor cells proliferation ex vivo. Likewise, we observe that vitamin D hyper supplementation alone also increases muscle VDR expression and the number of satellite cells. These data indicate that both vitamin D supplementation and HIIT independently enhance VDR expression in skeletal muscle with associated greater satellite and muscle progenitor cell activity. These data critically link vitamin D physiology and HIIT in muscle, and thus provide a mechanistic basis for their benefits for muscle quality, function, and health during aging.

### CHRONIC EXPOSURE TO YOUTHFUL CIRCULATION LEADS TO EPIGENETIC REPROGRAMMING AND LIFESPAN EXTENSION

Bohan Zhang,[1] David Lee,[2] Alexander Tyshkovskiy,[3] Akshay Bareja,[2] Csaba Kerepesi,[1] Steve Horvath,[4] Vadim Gladyshev,[1] and James White,[2], *1. Brigham and Women's Hospital, Boston, Massachusetts, United States, 2. Duke University School of Medicine, Durham, North Carolina, United States, 3. Moscow State University, Moscow, Moskva, Russia, 4. UCLA, Los Angeles, California, United States*

Heterochronic parabiosis is a powerful rejuvenation model in aging research. Due to limitations in the duration of blood sharing and/or physical attachment, it is currently unclear if parabiosis retards the molecular signatures of aging or affects healthspan/lifespan in the mouse. Here, we describe a long-term heterochronic parabiosis model, which appears to slow down the aging process. We observed a “deceleration” of biological age based on molecular aging biomarkers estimated with DNA methylation clock and RNA-seq signature analysis. The slowing of biological aging was accompanied by systemic amelioration of aging phenotypes. Consistent with these findings, we found that aged mice, which underwent heterochronic parabiosis, had an increased healthspan and lifespan. Overall, our study re-introduces a prolonged parabiosis and detachment model as a novel rejuvenation therapy, suggesting that a systemic reset of biological age in old organisms can be achieved through the exposure to young environment.

### DIET AND STRESS IMPAIR OVARIAN FUNCTION IN MID-LIFE, INCREASING RISK OF CHRONIC DISEASES OF AGING IN PRIMATES

Brett Frye, Suzanne Craft, Thomas Register, Susan Appt, Mara Vitolins, Beth Uberseder, Marnie Silverstein-Metzler, and Carol Shively, *Wake Forest School of Medicine, Winston Salem, North Carolina, United States*

Ovarian dysfunction increases risk for chronic diseases of aging including cardiovascular disease, depression, cognitive impairment, and bone and muscle loss which promote frailty. Psychosocial stress disrupts ovarian function and recent observations suggest that Western diet may also. Determination of causal relationships among diet, psychosocial stress, and ovarian physiology is difficult in humans. Nonhuman primates provide relevant opportunities to investigate diet and psychosocial effects on ovarian physiology and aging because, like humans, they have monthly menstrual cycles and recapitulate many aging-related processes similar to humans. We examined ovarian function in 38 socially housed, middle-aged females fed either a Western or Mediterranean diet for 26 months (~ an 8-year period for humans). During the last 12 months, we examined cycle length, peak progesterone per cycle, and frequency of anovulatory cycles using blood sampling (3/week) and vaginal swabbing (6/week). Repeated measures analysis revealed that like middle-aged women, cycle length increased, and progesterone levels fell over time, suggesting that ovarian dysfunction generally increased in our sample with time. In addition, both Western diet and the stress of low social status reduced progesterone levels, disrupting ovarian function, and increasing risk of chronic diseases of aging. This study demonstrates the additive negative effects of poor diet and psychosocial stress on ovarian physiology in mid-life and lays the groundwork for future investigations to uncover associated metabolic signatures of accelerated aging. The results also suggest that a Mediterranean diet may exert a protective influence against ovarian dysfunction and its pathologic sequelae.

### EXERCISE DURING CHILDHOOD PROTECTS AGAINST CARDIAC DYSFUNCTION LATER IN LIFE

Danielle Bruns,[1] MacKenzie DeHoff,[1] Aykhan Yusifov,[1] Sydney Polson,[1] Ross Cook,[1] Emily Schmitt,[1] and Kathleen Woulfe,[2], *1. University of Wyoming, Laramie, Wyoming, United States, 2. University of Colorado-Denver, Aurora, Colorado, United States*

Cardiovascular disease continues to be a major cause of morbidity and mortality, particularly in aging populations. Exercise is amongst the most cardioprotective interventions identified to date, with early in life exercise such as during the juvenile period potentially imparting even more cardioprotective outcomes due to the plasticity of the developing heart. To test the hypothesis that juvenile exercise would impart later in life cardioprotection, we exercised juvenile male and female mice via voluntary wheel running from 3-5 weeks of age and then exposed them to cardiac stress by isoproterenol (ISO) at 4-6 and 18 months of age in adulthood and older age, respectively. We compared cardiac function and remodeling to sedentary control animals, sedentary animals who received ISO, and adult and aged mice that exercised for two weeks immediately before ISO exposure. Juvenile mice engaged in voluntarily wheel running, with male mice running 1.3 ± 0.8 km and female mice 2.8 ± 1.0 km a day. Echocardiography suggested that these juvenile animals underwent running-induced cardiac remodeling as evidenced by higher ejection fraction and stroke volume compared to sedentary controls. Exercise in the juvenile period attenuated ISO-induced cardiac hypertrophy and remodeling later in life compared to sedentary animals and those that exercised immediately before ISO administration. The mechanisms by which early versus late exercise is protective in adult and aged mice are under investigation. Further ongoing work will identify the adaptations induced by exercise in the juvenile heart that may help improve cardiac aging.

### EXERCISE-INDUCED TRANSCRIPTIONAL CHANGES IN AGED SKELETAL MUSCLE

Yori Endo,[1] Shayan Olumi,[2] Mehran Karvar,[3] Indranil Sinha,[4] and Yuteng Zhang,[3], *1. Harvard Medical School, Boston, Massachusetts, United States, 2. Northwestern University, Evanston, Illinois, United States, 3. Brigham and Women's Hospital, Harvard Medical School, Boston, Massachusetts, United States, 4. Birgham and Women's Hospital, Harvard Medical School, Boston, Massachusetts, United States*

Exercise is beneficial for physical functions across all ages. However, the response to exercise shifts from anabolism, resulting in limited gain of muscle strength and endurance. These changes likely reflect age-related alterations in transcriptional response underlying the muscular adaptation to exercise. The exact changes in gene expression accompanying exercise, however, are largely unknown, and elucidating them is of a great clinical interest for optimizing the exercise-based therapies for sarcopenia. In order to characterize the exercise-induced transcriptomic changes in aged muscle, a paired-end RNA sequencing was performed on the rRNA-depleted total RNA extracted from the gastrocnemius muscles of 24 months-old mice after 8 weeks of regimented exercise (exercise group) or sedentary activities (sedentary group). Differential gene expression analysis revealed upregulations in the group of genes involved in neurotransmission. In particular, genes encoding the transporters and receptor components of glutaminergic transmission were significantly upregulated in exercised muscles, as exemplified by Gria 1, Gria 2 and Grin2c encoding glutamate receptor 1, 2 and 2C respectively, Grin1 and Grin2b encoding N-methyl-D-aspartate receptors (NMDARs), Nptx1 responsible for glutaminergic receptor clustering, and Slc1a2 and Slc17a7 regulating synaptic uptake of glutamate. These changes were accompanied by an increase in post-synaptic NMDARs and acetylcholine receptors (AChRs), as well as their innervation at neuromuscular junctions (NMJs). These results suggest that neural responses predominate aged skeletal muscle following exercise, and indicate a possibility that glutaminergic transmission at NMJs may be responsible for synaptic protection and neural remodeling accompanying the exercise-induced functional enhancement in aged skeletal muscle.

### GENES CONTRIBUTING TO RESILIENCE AND SENSITIVITY TO LISINOPRIL AT OLD AGE: CLINICAL TRANSLATION OF GWA IN DROSOPHILA

Mariann M. Gabrawy,[1] Nick Khosravian,[2] George S. Morcos,[3] Meagan Jezek,[2] Jeremy D. Walston,[1] Peter M. Abadir,[1] and Jeff Leips,[2], *1. Johns Hopkins University School of Medicine, Baltimore, Maryland, United States, 2. University of Maryland Baltimore County, Baltimore, Maryland, United States, 3. University of Maryland School of Medicine, Baltimore, Maryland, United States*

Despite impressive results in restoring physical performance in rodent models, treatment with Renin-Angiotensin System (RAS) inhibitors such as Lisinopril have highly mixed results in humans, likely, in part, due to genetic variation in human populations. To date, the genetic determinants of responses to drugs such as RAS inhibitors remain unknown. Given the complexity of the relationship between physical traits and genetic background, genomic studies which predict genotype- and age-specific responses to drug treatments in humans or vertebrate animals are difficult. Here, using 126 genetically distinct lines of Drosophila, we tested the effects of Lisinopril on climbing speed and endurance at young and old age (N=14,310). Our data show that functional response and sensitivity to Lisinopril ranges from significant protection against physical decline (8–100% faster, $P < 0.0001$) to increased weakness ($P < 0.0001$) depending on both genotype and age ($P < 0.0001$). Genome-wide analyses revealed little to no overlap in candidate polymorphisms influencing sensitivity between ages nor between treatments within each age. Furthermore, network analyses led to identification of evolutionarily conserved genes in the WNT signaling pathway as being significantly associated with variations in sensitivity to Lisinopril. Genetic knockdown of Axin, frizzled, nemo, and wingless, genes with human orthologs AXIN1, FZD1, NLK, and WNT1, respectively, abolished the effects of Lisinopril treatment. Our results implicate these genes as contributors to the genotype- and age-specific effects of Lisinopril treatment and as potential therapeutic targets for improvement of resiliency. Our approach should be widely applicable for identifying genomic variants that predict age-dependent responses to pharmaceutical treatments.

### IMMATURE PEAR EXTRACT CONSTITUENTS EXERT MULTIFACETED ANTI-AGING EFFECTS

Akira Ogita,[1] Wakae Murata,[2] Ken Yamauchi,[3] Akiko Sakai,[1] Yoshihiro Yamaguchi,[1] Toshio Tanaka,[1] and Ken-ichi Fujita,[1], *1. Osaka City University, Osaka, Osaka, Japan, 2. Yonago National College of Technology, yonago, Tottori, Japan, 3. Keio University, Yokohama, Kanagawa, Japan*

Cellular senescence causes a gradual loss of physiological functions and induces chronic diseases, which negatively affect the quality of human life. Intervention in the cellular senescence process may reduce the incidence of these diseases while delaying the progression of age-related diseases, thereby prolonging human lifespan. In our previous study, we found that extending the chronological lifespan of budding yeast cells, a suitable cellular model for research on mammalian cells, could be achieved by adding immature pear extract (iPE). Moreover, at the 2020 GSA meeting, using a colony-counting method, we reported that both hydrophilic (WiPE) and hydrophobic (OiPE) iPE components exhibited a chronological lifespan prolongation on yeast cells. In this study, the expression of sirtuin-related genes, which regulate cellular senescence, was verified by quantitative real-time reverse-transcription polymerase chain reaction. Interestingly, sirtuin-related gene expression was significantly increased in the WiPE-treated cells only, and OiPE could extend the chronological lifespan of yeast cells through the mechanisms not involved in sirtuin-related gene expression. In general, hydrophobic and hydrophilic components exhibit different degradation and metabolism in cells. Since each component has a different strategy of absorption and excretion in the body, we hypothesize that iPE with multiple active components will have multifaceted effects on anti-aging. Our research on elucidating the mechanism of lifespan extension by OiPE and its application to mammalian cells is ongoing.

### KYNURENINE METABOLISM LIFESPAN EXTENSION MEDIATED BY OXIDATIVE STRESS RESPONSE AND HYPOXIC RESPONSE IN C. ELEGANS

Raul Castro-Portuguez,[1] Jeremy Meyers,[2] Sam Freitas,[1] Hope Dang,[2] Emily Turner,[2] Destiny DeNicola,[1] Luis Espejo,[3] and George Sutphin,[1], *1. The University of Arizona, Tucson, Arizona, United States, 2. University of Arizona, Tucson, Arizona, United States, 3. UNIVERSITY OF ARIZONA, TUCSON, Arizona, United States*

Aging is characterized by a progressive decline in the normal physiological functions of an organism, ultimately leading to mortality. Metabolic changes throughout the aging process disrupt the balance and homeostasis of the cell. The kynurenine metabolic pathway is the sole de novo biosynthetic pathway for producing NAD+ from ingested tryptophan. Altered kynurenine pathway activity is associated with both aging and a variety of age-associated diseases, and kynurenine-based interventions can extend lifespan in Caenorhabditis elegans. Our laboratory recently demonstrated knockdown of the kynurenine pathway enzymes kynureninase (KYNU) or 3-hydroxyanthranilic acid dioxygenase (HAAO) increases lifespan by 20-30% in C elegans. However, the mechanism of how these interventions may modulate response against different stressors during the aging process has yet to be explored. Fluorescent reporter strains show the stress-responsive transcription factors skn-1 (ortholog of NRF2/NFE2L2; oxidative stress response) and hif-1 (ortholog of HIF1A; hypoxic stress response) to be highly upregulated when the kynurenine pathway is inhibited. We also demonstrated the increase expression of gst-4 and gcs-1 (transcriptional targets skn-1), which are involved in production of the antioxidant glutathione (GSH), as well as upregulation of cysl-2 (transcriptional target of hif-1), a regulator of cysteine biosynthesis from serine. We hypothesize that lifespan extension resulting from kynurenine pathway inhibition is mediated, at least in part, by upregulation of these transcription factors, providing elevated defense against oxidative stress and hypoxic stress.

### LOSARTAN MITIGATES OXIDATIVE STRESS IN THE BRAINS OF AGED IL10-/- MICE

Nazaneen Saleh,[1] Caglar Cosarderelioglu,[2] Peter M. Abadir,[3] Ramya Vajapey,[4] and Jeremy D. Walston,[3], *1. University of Virginia, Springfield, Virginia, United States, 2. Ankara University School of Medicine, Cankaya, Ankara, Turkey, 3. Johns Hopkins University School of Medicine, Baltimore, Maryland, United States, 4. The Cleveland Clinic Foundation, Cleveland, Ohio, United States*

Chronic inflammation has been linked to frailty and declined cognition in older adults. Activation of the renin-angiotensin system (RAS) through the angiotensin Type1 receptor (AT1R) has been suggested as a contributory factor that links both inflammation and aging. Here we examined the impact of 4 weeks of oral Losartan treatment on IL10-/- mice brains, a mouse model of chronic inflammation and frailty. Frontal cortex, cerebellar, and hippocampal tissue of aged (100 weeks old) male IL10-/- mice were studied. Western blot techniques were employed to quantify changes in brain AT1R, nitrotyrosine (NT) as an oxidative stress marker, and Tau proteins. Our data show that aged IL-10 mice have significantly higher levels of AT1R in the cortex tissue but not in cerebellar or hippocampal tissue compared to age and sex-matched WT mice (0.63 + 0.35 vs 1.5 + 0.54, WT vs IL10, respectively, $P<0.004$). When treated with LOS, brain cortical tissue of IL10 -/- mice showed significant decreases in levels of AT1R (1.5 + 0.54 vs 0.98 + 0.50, IL10 vs LOS treated IL10, respectively, $P<0.04$), NT (0.72 + 0.12 vs 0.42 + 0.10, IL10 vs LOS treated IL10, respectively, $P<0.009$), and Tau protein (1.3 + 0.31 vs 0.15 + 0.08, IL10 vs LOS treated IL10, respectively, $P<0.004$) as compared to control IL10-/- mice. Losartan treatment had no significant effect on hippocampal AT1R or NT levels. Our results highlight the impact of Losartan, a drug commonly prescribed for the treatment of high blood pressure, on the brain-specific angiotensin system and its downstream effects on brain oxidative stress and Tau pathology.

### LOSS OF HYPOXIA SIGNALING LIMITS PHYSIOLOGIC AND MUSCLE ADAPTATIONS TO AEROBIC EXERCISE IN AGING

Yori Endo,[1] Mehran Karvar,[2] Yuteng Zhang,[2] Shayan Olumi,[3] and Indranil Sinha,[4], *1. Harvard Medical School, Boston, Massachusetts, United States, 2. Brigham and Women's Hospital, Harvard Medical School, Boston, Massachusetts, United States, 3. Northwestern University,*

*Evanston, Illinois, United States, 4. Birgham and Women's Hospital, Harvard Medical School, Boston, Massachusetts, United States*

To assess the differential effects of exercise with age, Young (Y, 10-12 weeks) and Old (O, 23-25 months) mice were subjected to regimented treadmill running or no regimented exercise. Y, trained mice experienced a significant increase in maximal distance running, maximal speed of running, and lean muscle mass in comparison to age-matched, untrained controls. O mice did not improve significantly in any of these measures following training. Transcriptome analysis of gastrocnemius from Y mice demonstrated differential regulation of 120 genes with exercise. None of these genes were similarly regulated in the O group. Genes most upregulated following exercise in Y mice were direct targets of the hypoxia signaling pathway. Immunoblotting demonstrated that aryl hydrocarbon receptor nuclear translocator (ARNT), a critical regulator of hypoxia signaling, increased 3-fold with exercise in Y mice, but this increase was absent in O mice following exercise. To assess whether this loss of ARNT in O muscle impaired the exercise response, we generated a mouse with inducible, skeletal muscle-specific knockout of ARNT (ARNT muscle (m) KO). Following regimented exercise, ARNT mKO mice did not improve maximal distance running, maximal running speed, or lean muscle mass in comparison to untrained ARNT mKO mice. Littermate, age-matched ARNT wild type mice increased significantly in all of these measures following training. Administration of ML228, an ARNT agonist, increased maximal running distance and speed in response to exercise training in O mice. These results suggest that restoration of ARNT and hypoxia signaling may restore the physiologic response to exercise in aging.

### MECHANISMS OF CELL NON-AUTONOMOUS LONGEVITY REGULATION

Scott Leiser,[1] Hyo Choi,[1] Ajay Bhat,[2] Marshall Howington,[2] Hillary Miller,[1] Shijiao Huang,[2] and Elizabeth Dean,[2], *1. University of Michigan, Ann Arbor, Michigan, United States, 2. University of Michigan, University of Michigan, Michigan, United States*

An organism's ability to respond to stress is crucial for long-term survival. These stress responses are coordinated by distinct but overlapping pathways, many of which also regulate longevity across taxa. Our previous work identified a cell non-autonomous signaling pathway led by the hypoxia-inducible factor and resulting in induction of flavin-containing monooxygenase-2 (fmo-2) to promote health and longevity. Our current work identifies a distinct cell non-autonomous pathway downstream of dietary restriction (DR) that also relies on fmo-2 induction to promote health and longevity. We now find that these cell non-autonomous pathways can be mimicked by small molecule interventions that increase longevity by inducing fmo-2. Based on the commonalities of these pathways, we hypothesized that fmo-2, a classically annotated xenobiotic enzyme, might play a key endogenous role in responding to metabolic stress. Our resulting data, using metabolic profiling and further epistatic analysis, both support this hypothesis and link fmo-2's mechanism to modifications in one-carbon metabolism (OCM), a key intermediate pathway consisting of the folate and methionine cycles. Using mathematical modeling and a labeled metabolomics approach, we were able to further identify the likely mechanism of fmo-2-mediated metabolic effects and connect them to both OCM and downstream components. We propose that fmo-2 is induced cell non-autonomously to modify systemic metabolism and longevity, and that fmo-2 is a key member of a conserved metabolic stress response.

### MODERATE CALORIE RESTRICTION ENHANCES HEPATIC GLUCAGON SENSITIVITY IN AGED MICE

Stephen Hutchison, Anastasiia Vasileva, Tyler Marx, Samantha Slavin, and Jennifer Stern, *University of Arizona, Tucson, Arizona, United States*

Chronic calorie restriction (CR) without malnutrition delays the onset of aging, extends lifespan, and improves metabolic function in many species. These CR-induced benefits have largely concentrated on the role of insulin signaling, while ignoring its counter-regulatory hormone, glucagon. Like insulin, hyperglucagonemia and decreased glucagon sensitivity are associated with impaired glucose homeostasis and decreased longevity. Conversely, activation of target molecules downstream of glucagon signaling such as AMPK and FGF21 are known to ameliorate these age-related impairments in metabolic function. To investigate the potential role of glucagon receptor signaling in CR-induced improvements in aging, we have implemented a moderate 15% CR in the mouse. Our studies show that a 15% calorie restriction initiated at 4 months of age enhances hypoglycemia-stimulated glucagon secretion ($P<.01$) and decreases basal serum glucagon ($P<.01$), while having no effect on glucagon receptor expression at the liver in 26-month-old mice. Consistent with enhanced hepatic glucagon sensitivity, CR increases glucagon-stimulated hepatic cyclic AMP production ($P<.05$). Glucagon is a primary regulator of AMPK activation and FGF21 release, both of which have been proposed as key molecules to account for CR-induced benefits to aging. CR increases both hepatic AMPK activation ($P<.05$) and FGF21 mRNA expression ($P<.05$). Additionally, CR reduces hepatic lipid accumulation ($P<.05$), and decreases fasting respiratory quotient ($P<.001$), indicating an increase in lipid oxidation. Our studies demonstrate that a moderate (15%) CR regimen enhances glucagon sensitivity and decreases hepatic lipid accumulation in aged mice. Thus, we propose glucagon signaling as a mediator of CR-induced improvements in aging.

### REDUCED MUSCLE OXIDATIVE CAPACITY DURING AND AFTER EXERCISE IN OLDER ADULTS WITH OBESITY

Fei Zhao,[1] Machiko Tomita,[2] and Anirban Dutta,[1], *1. State University of New York at Buffalo, Buffalo, New York, United States, 2. University at Buffalo, Buffalo, New York, United States*

**Objective:** Obesity and physical activity are two major factors affecting aerobic performance in older adults. The underlying mechanisms of the causes are still unknown. Oxidative capacity, muscles' maximal capacity to utilize oxygen, is a part of aerobic performance. Muscle oxygen level (SmO2), a measure of oxidative capacity, reflects the balance between oxygen delivery and oxygen demand. When oxygen delivery surpasses oxygen demand, SmO2 increases, which indicates higher oxidative capacity. This study aimed to identify the influence of obesity and physical activity level on SmO2 changes during and after short exercise.

**Methods:** This is a cross-sectional study. SmO2 changes during and after a bilateral heel-raise (BHR) test were measured in 60 community-dwelling older adults (≥ 60 years old) in four groups: inactive obese, active obese, inactive non-obese, and active non-obese. Inactive is defined as performing less than 150 minutes/week moderate-intensity activity.

**Results:** The inactive obese group took a longer time for SmO2 drop during the BHR test, had a lower SmO2 level at the end, and recovered slower after the test than all other groups. Especially, compared with the active non-obese group, this group exhibited significantly poorer SmO2 recovery one minute after the test, 5.3% less (p=.036). The active obese group's oxidative capacity was similar to the inactive non-obese group, although lower than the active non-obese group.

**Discussion:** To prevent physical decline in older adults with obesity, regular exercise has been recommended. This study substantiated this from a hemodynamics viewpoint. Older adults with obesity need 150+ minutes/week moderate-intensity exercise.

### REDUCING METHYLGLYOXAL RESCUES OBESITY RELATED PHENOTYPES AND LIFESPAN IN A LEPTIN RECEPTOR DEFICIENT MOUSE MODEL

Lauren Wimer,[1] Martin Valdearcos Contreras,[2] Muniesh Muthaiyan Shanmugam,[1] Jessica Ramirez,[1] Jennifer Beck,[1] Suneil Koliwad,[3] and Pankaj Kapahi,[1], *1. Buck Institute for Research on Aging, Novato, California, United States, 2. UCSF Diabetes Center, UCSF Diabetes Center, California, United States, 3. UCSF Diabetes Center, 94143, California, United States*

Obesity remains one of the leading risk factors for aging and age-related diseases such as Alzheimer's and type II diabetes, and effects 40% of the US population. The occurrence of obesity has been closely tied to an increase in sugar consumption with chronic hyperglycemia enhancing glycolysis. One of the byproducts of glycolysis is methylglyoxal (MGO), a reactive precursor for advanced glycation end-products (AGEs), which drives type II diabetes and its complications. We hypothesize that an MGO-derived AGE, MG-H1, affects hypothalamic regulation of food consumption and metabolism parallel to the leptin pathway in mice fed a high carbohydrate diet. Exogenous supplementation of MG-H1 increased food consumption rates and weight gain. Conversely, glycation byproduct lowering compounds (GLY-LOW), a customized chemical cocktail that blocks the production of MGO, rescued over-feeding phenotypes in wild-type mice. Furthermore, GLY-LOW treatment in a leptin receptor deficient mouse model rescued weight gain, diabetic phenotypes and lifespan. RNA sequencing of the hypothalamus of leptin receptor deficient mice treated with GLY-LOW showed significant downregulation of Rax, a gene responsible for tanycyte differentiation, and several genes involved in feeding and aging. We propose that specific cells in the hypothalamus both make and respond to MG-H1. We will also discuss evidence for the potential of GLY-LOW as a new class of therapeutics that reduce the effects of glycation to reduce food intake and slow aging.

### TARGETING CELLULAR SENESCENCE WITH NOVEL SENOTHERAPEUTICS BY DESIGN TO EXTEND HEALTHSPAN

Lei Zhang,[1] Brian Hughes,[2] Luise Angelini,[2] Ryan O'Kelly,[2] Matthew Yousefzadeh,[2] Ted Kamenecka,[3] Laura Niedernhofer,[2] and Paul Robbins,[2], *1. University of Minnesota, Saint Paul, Minnesota, United States, 2. University of Minnesota, Minneapolis, Minnesota, United States, 3. Scripps Florida, Scripps Florida, Florida, United States*

Senescent cells accumulate with age in various tissues and organs, leading to the decline in tissue function and deterioration of many age-related diseases and aging. Senolytics have emerged as an effective therapeutic approach to eliminate senescent cells to improve aging phenotypes and associated co-morbidities. Despite their promising potential, only a handful of senolytics have been reported, including a natural flavonoid fisetin discovered by our group. Fisetin has been shown to reduce senescence, suppress age-related pathology, and extend healthspan in aged mice. However, its moderate potency, potential mutagenic risk and poor bioavailability have limited its further clinical applications. By leveraging drug design, medicinal chemistry and high-content imaging analysis, we have successfully optimized the senolytic activity of fisetin, leading to the identification of two improved fisetin senolytic analogs (FAs) with reduced toxicity in non-senescent cells. The improved senolytic activity of these FAs was demonstrated in murine and human senescent cell models as well as in accelerated aging and naturally aged mouse models. The analysis of the senolytic activity of the FAs as well as several other recently identified senolytics, including a senolytic lipid, will be presented.

### TARGETING KYNURENINE METABOLISM TO REDUCE INFLAMMATION AND ENHANCE STRESS RESPONSE DURING AGING

George Sutphin,[1] Hope Dang,[2] Luis Espejo,[3] Raul Castro-Portuguez,[1] Bradford Hull,[2] Jeremy Meyers,[2] Destiny DeNicola,[1] and Emily Turner,[2], *1. The University of Arizona, Tucson, Arizona, United States, 2. University of Arizona, Tucson, Arizona, United States, 3. UNIVERSITY OF ARIZONA, TUCSON, Arizona, United States*

Aberrant kynurenine pathway metabolism is increasingly linked to aging and age-associated disease. Kynurenine metabolic activity increases with age and becomes dysregulated during various forms of age-associated pathology in humans. By manipulating one or more kynurenine pathway enzymes and metabolites, we have extended lifespan up to 40% in Caenorhabditis elegans. In particular, elevating physiological levels of the kynurenine pathway metabolite 3-hydroxyanthranilic acid (3HAA) by directly supplementing 3HAA or inhibiting the enzyme 3HAA dioxygenase (HAAO) extends C. elegans lifespan by ~30%. 3HAA delivered chronically in chow similarly extends lifespan in aged C57BL/6 mice. In ongoing work, we are investigating the mechanisms underlying the benefits of multiple kynurenine pathway interventions using tools in C. elegans, mice, and human cell culture. We have preliminary evidence for activation of broad-spectrum cellular stress response, enhanced immune function, and reduced inflammation. Among other roles, the kynurenine pathway is the sole metabolic route for de novo synthesis of nicotinamide adenine dinucleotide (NAD+) from tryptophan in Eukaryotic cells. We are examining the regulatory interaction between kynurenine metabolism and the two NAD+ recycling pathways, Salvage and Preiss-Handler, both as potential mechanistic mediators and as possible parallel targets for combined interventions with synergistic benefits in aging. We are further evaluating the impact of these interventions in several models of specific age-associated diseases, including sepsis, chronic inflammation, stroke, Alzheimer's disease, and cancer. Finally, we are developing pharmaceutical strategies to replicate key genetic and metabolic interventions within the kynurenine pathway that can be readily translated into clinical applications.

### THE INTERACTION OF OSMOTIC AND HEAVY METAL STRESS IN C. ELEGANS

Emily Turner,[1] Amanda Furtmann,[1] Hope Dang,[1] Destiny DeNicola,[2] and George Sutphin,[2], *1. University of Arizona, Tucson, Arizona, United States, 2. The University of Arizona, Tucson, Arizona, United States*

Cellular stress is an ever-present aspect of aging and a primary driver of many common age-associated diseases such as cancer, diabetes, or neurodegenerative diseases. As we age, stress-induced damage accumulates over time, along with reduced efficacy of stress response pathways at combatting such damage. Molecular stress response pathways are well studied in the context of individual stressors, but there is a lack of understanding of how these responses change when multiple stressors are encountered at the same time. The goal of our work is to explore the impact of multiple simultaneous stressors on health and survival, and to investigate the underlying molecular pathways involved. To accomplish this, we utilize the nematode Caenorhabditis elegans to monitor lifespan changes in response to various stressors. We simultaneously exposed C. elegans to high concentrations of sodium chloride and cadmium chloride, known to induce osmotic and heavy metal stress, respectively. We found that lifespan is drastically decreased by the combined stress, significantly more so than the reduction in lifespan caused by either individual stress. Our results show that glycerol levels, which are normally increased in response to osmotic stress, are significantly lowered when the two stresses are combined compared to levels detected for osmotic stress alone. This suggests that the presence of cadmium may sensitize worms to sodium and other osmotic stressors by blunting cells' ability to mount an appropriate molecular response. In ongoing work, we will continue to dissect the mechanisms through which cadmium influences glycerol production and other aspects of osmotic stress response.

### THE PROTECTIVE EFFECTS OF APIGENIN ON COGNITIVE FUNCTION AND THE BRAIN TRANSCRIPTOME IN OLD MICE

Alyssa Cavalier,[1] Zachary Clayton,[2] David Hutton,[2] Cali McEntee,[1] Douglas Seals,[2] and Thomas LaRocca,[1], *1. Colorado State University, Fort Collins, Colorado, United States, 2. University of Colorado Boulder, Boulder, Colorado, United States*

Age-related declines in cognitive function increase the risk of developing mild cognitive impairment and dementia, but select nutraceuticals (bioactive plant compounds) may hold promise for protecting the brain and improving cognitive function with age. Apigenin is a flavonoid nutraceutical found in chamomile and reported to inhibit multiple hallmarks of aging; however, it has not been studied in the context of brain aging specifically. We treated young (6 mo) and old (27 mo) C57BL/6N mice with apigenin (0.5 mg/mL in 0.2% carboxymethylcellulose) or control (0.2% carboxymethylcellulose) drinking water for 6 weeks. Then, we assessed cognitive function and performed RNA-seq to characterize global transcriptomic changes and potential mechanisms of action in the brain. We observed impaired novel object recognition (NOR) test performance (an index of learning/memory) in old vs. young control mice ($P<0.0001$), but old apigenin mice had ~3-fold higher NOR performance relative to old control mice ($P=0.02$). Transcriptomic analyses also showed age-associated gene expression changes related to immune function and inflammation, consistent with the established role of inflammation in brain aging. However, some of these key changes were reversed by apigenin. In fact, >300 genes were differentially expressed in old apigenin-treated mice vs. old controls, and the biological processes linked with these differences were related to innate and adaptive immune function, and cytokine and chemokine regulation. We are performing protein/signaling pathway analyses to elucidate downstream cellular changes associated with apigenin treatment, but our current results suggest apigenin may be a promising nutraceutical candidate for preventing brain aging.

### THE RELATIONSHIP BETWEEN PHYSICAL ACTIVITY INTENSITY AND VOLUME ON PROSPECTIVE FALLS IN OLDER ADULTS

Justin Whitten,[1] Dawn Tarabochia,[2] David MacDonald,[3] Rod Barrett,[3] Chris Carty,[3] and David Graham,[2], *1. Montana State University, BOZEMAN, Montana, United States, 2. Montana State University, Bozeman, Montana, United States, 3. Griffith University, Gold Coast, Queensland, Australia*

The benefits of Physical Activity (PA) for older adults have been well documented relative to several physiological and neuromuscular factors, but the direct relationship of PA to fall incidence is unclear. In particular, the influence of the intensity and volume of habitual activities of daily living is poorly understood. The purpose of this study was to evaluate the influence of general PA intensity and overall volume on prospective falls in older adults. The PA of 134 participants was recorded using accelerometers (ActiGraph-GT3X+) over 7 consecutive days. Intensity was classified as light, moderate and vigorous by step frequency. The activity of all participants was graded as sedentary to low intensity, no participant exhibited activity in the vigorous category. During the following 12-months, participants maintained a daily falls diary and completed monthly phone calls to monitor fall incidence. Responses were used to categorize participants as fallers or non-fallers. Eighteen participants experienced one or more falls during the 12-month period. There was no statistical difference between fallers and non-fallers in either total step count or the percentage of time spent in sedentary or light PA. While previous reports suggest that many falls occur during light PA, our results do not suggest that greater volumes of low intensity activities alone results in greater fall incidence. However, we suggest this result may be influenced by physical stimuli participants received within the larger overall study design including a session of repeated exposure to forward loss of balance.

### TOTAL TRANSCRIPTOME RESPONSES TO LOW AND HIGHER INTENSITY AEROBIC EXERCISE INTERVENTIONS IN OLDER ADULTS

Meghan Smith,[1] Gabriella McWilliams,[1] Angela Bryan,[2] Douglas Seals,[2] and Thomas LaRocca,[1], *1. Colorado State University, Fort Collins, Colorado, United States, 2. University of Colorado Boulder, Boulder, Colorado, United States*

Aerobic exercise is a universally recommended strategy for increasing healthspan, and recent advances in next-generation sequencing and bioinformatics (e.g., RNA-seq/transcriptomics) have made it possible to broadly profile the molecular transducers of exercise. However, most transcriptome studies of exercise have focused on coding genes only, and the transcriptomic response to different exercise interventions has not been characterized by RNA-seq in older adults. Therefore, we performed total RNA-seq (to capture both coding and non-coding gene expression) on peripheral blood mononuclear cells collected from healthy, previously sedentary older adults (males and females, aged 70 ± 1 years). Samples were collected before and after 16 weeks of either low-intensity continuous training (LICT, 50% maximum heart rate, 3 x 30 min/week) or moderate-intensity continuous training plus interval training (MICT+IT, 60-80% maximum heart rate, progressively increased to include IT, 3 x 30 min/week). We found that both interventions modified biological processes (transcriptome modules) related to oxygen transport and reduced inflammatory signaling/immune activation processes (more pronounced with LICT). Interestingly, transcriptome changes unique to LICT subjects included increased expression of genes linked with vascularization and endothelial cell migration, whereas MICT+IT was uniquely associated with a robust increase in antioxidant response gene expression. We also observed numerous changes in long non-coding RNAs and microRNAs that could be linked with these exercise-associated gene expression changes with both interventions. These data provide a first comprehensive look into transcriptomic changes associated with moderate vs. low intensity aerobic exercise in older adults, and they suggest distinct benefits of each exercise strategy.

### TREADMILL TRAINING IMPROVES AEROBIC CAPACITY IN AGED MALE MICE COMPARED TO VOLUNTARY WHEEL RUNNING

Emily Schmitt, Hunter Graves, and Danielle Bruns, *University of Wyoming, Laramie, Wyoming, United States*

Preclinical exercise studies typically use two forms of exercise training protocols: 1) voluntary wheel running and 2) forced treadmill running. Previous work from our group clearly demonstrates that older (18-month-old) male mice do not voluntarily engage in wheel running, especially compared to younger males or female mice. Therefore, we implemented a forced exercise treadmill training protocol to determine if treadmill training was superior to wheel running in improving aerobic capacity in older male mice.

**Purpose:** To determine if a 3-week treadmill training protocol improved time to exhaustion (TTE) in older male mice.

**Methods:** 18-month-old male mice (n=5) were provided a running wheel in their individual cage for 2 weeks or underwent daily treadmill training (n=6) for 3 weeks with increasing speed/incline. At the end of the training period we assessed TTE.

**Results:** Older male mice that trained on the treadmill demonstrated higher TTE compared to wheel (1382 □ 32 seconds versus 500 □ 99 seconds, respectively). In addition, older male mice that trained on the treadmill improved on average ~8% in their TTE test.

**Conclusion:** A 3-week treadmill training protocol improves aerobic capacity in older male mice to a greater extent than voluntary wheel running. Ongoing experiments will utilize this training protocol to understand age-related declines in cardiorespiratory fitness, circadian rhythm, and to test exercise as an intervention in the aging population.

### VALSARTAN AND SACUBITRIL COMBINATION TREATMENT ENHANCES COLLAGEN PRODUCTION IN OLDER ADULT HUMAN SKIN CELLS

Sela Marin,[1] Lolita S. Nidadavolu,[2] Jeremy D. Walston,[2] Daniele Gilkes,[3] Peter M. Abadir,[2] and Ines godet,[4], *1. Johns Hopkins University, Baltimore, Maryland, United States, 2. Johns Hopkins University School of Medicine, Baltimore, Maryland, United States, 3. Johns Hopkins School of Medicine, Baltimore, Maryland, United States, 4. Johns Hopkins University, 21287, Maryland, United States*

Collagen is a major component of the skin's support system, allowing for firmness, elasticity, and mechanical strength. In older adults, skin collagen production decreases significantly, and is associated with increased sagging, wrinkling, and thinning. The Renin Angiotensin System (RAS) is a key hormonal system that changes with age and affects multiple organ systems. While primary health benefits of Angiotensin (Ang) receptor type1 (AT1 R) blockers (ARBs) are believed to arise from systemic effects on blood pressure. There exists a skin-specific Renin Angiotensin System (RAS), but the impact of ARBs on older skin is unknown. Human skin fibroblasts from individuals aged 2 (young individual) and 57 (older individual) were treated with drugs that alter RAS: Valsartan (an ARB) and neprilysin inhibitor Sacubitril. Fibroblast proliferation and collagen production was quantified in response to the drug treatment using fluorescence microscopy. Fibroblasts from 57-year-old individuals were slower to proliferate and had less collagen content as compared to fibroblasts from young individual. Valsartan alone treatment had no effect on collagen production from young or old fibroblasts. In contrast, Sacubitril treatment increased collagen production by approximately three-fold in young (2.87 ± 0.27 RFU, P<.0001), and older (2.93 ± 0.53 RFU, P<.0001) fibroblasts. Concomitant treatment with Valsartan and Sacubitril increased collagen production by five-fold increase (5.36 ± 1.08 RFU, P<.0001) in young fibroblasts, and four-fold (4.18 ± 0.96 RFU, P=.003) in older cells. This study demonstrates a novel use for the widely prescribed drug combination, Sacubitril and Valsartan, which significantly improves collagen production in older adult fibroblasts.

### Session 9110 (Poster)

### BIOLOGY OF AGING: MITOCHONDRIA

### A STUDY OF C2C12 MYOBLAST BIOENERGETICS IN RESPONSE TO THE CCG-1423 RHO A INHIBITOR

Bachkhoa Nguyen,[1] Fathima Ameer,[2] Jasmine Crane,[2] Gohar Azhar,[2] Xiaomin Zhang,[1] and Jeanne Wei,[2], *1. University of Arkansas for Medical Sciences, Little Rock, Arkansas, United States, 2. UAMS, UAMS, Arkansas, United States*

CCG-1423 is a Rho A pathway inhibitor which has been reported to inhibit Rho/SRF-mediated transcriptional regulation. SRF and SRF cofactors, which include ternary complex factors (TCFs) and myocardin-related transcription factor (MRTF), regulate various cellular functions. The Rho/SRF signaling pathway also regulates the sirtuin 2 (SIRT2) gene that contains a classic serum response element (SRE) sequence. Current research on CCG-1423 focuses on gene expression levels of SRF in response to CCG-1423 and how SRF levels affect the cells; the studies are focused on cell morphology, migration, viability/reproduction, and overall function. The pathways of this inhibitor have yet to be fully elucidated, but several have been suggested with good evidence. Our goal is to study the effect of CCG-1423 on mitochondrial function and gene expression of cells. In this work C2C12 myoblast cells have been used as an in-vitro model to study cellular bioenergetics and variations in gene expressions induced by CCG-1423. The effect of CCG-1423 on mitochondrial function was determined by measuring the mitochondrial oxygen consumption rate and glycolysis rate after treating C2C12 cells with varying concentrations of CCG-1423 overnight. In C2C12 myoblast cells, CCG-1423 treatment significantly reduced mitochondrial oxygen consumption rate (OCR) in a dose-dependent manner. However, treatment of C2C12 cells with CCG-1423 overnight increased the extracellular acidification rate (ECAR) in a dose-dependent manner. By indicating that CCG-1423 represses mitochondrial respiration via the Rho-SRF signaling pathway, the results of this study may enable a better understanding of the bioenergetics of the cell in the aging body.

### DEPLETION OF THE MIR-34A “SPONGE” MALAT1 IN AGING SKELETAL MUSCLE: IMPLICATIONS FOR AGE-RELATED MUSCLE LOSS

Ling Ruan,[1] Mark Hamrick,[1] Bharati Mendhe,[1] Carlos Isales,[1] William Hill,[2] Meghan McGee-Lawrence,[3] and Sadanand Fulzele,[1], *1. Medical College of Georgia, Augusta, Georgia, United States, 2. Medical University of South Carolina, Charleston, South Carolina, United States, 3. Medical College of Georgia, Augusta University, Augusta, Georgia, United States*

We have recently shown that increased levels of reactive oxygen species (ROS) in aging skeletal muscle are associated with increased expression of the senescence-associated microRNA miR-34a-5p (miR-34a). The histone deacetylase Sirt1 is a validated target of miR-34a, and miR-34a expression is induced by the tumor suppressor p53 which is itself stimulated by ROS. Long noncoding RNAs (lncRNAs) are known to function as “sponges” for microRNAs, but the role of such competing endogenous RNAs (ceRNA) in muscle aging is not well understood. We therefore examined in skeletal muscles of young (4-6 mos) and aged (22-24) male and female mice the expression of several lncRNAs that are predicted to bind miR-34a-5p in silico and whose predicted binding has been validated experimentally. Results indicate a significant decrease in lncRNA MALAT1 expression with aging. MALAT1 is known to be highly expressed during the later stages of myoblast differentiation and myotube maturation. We therefore treated C2C12 cells at 48 hrs with hydrogen peroxide (10 uM) and examined changes in MALAT1 expression. MALAT1 was significantly decreased with H2O2 treatment, whereas miR-34a is increased in C2C12 cells after hydrogen peroxide exposure. Age-related muscle atrophy mediated by ROS may therefore result in part from related mechanisms involving miR-34a activity: an increase in miR-34a targeting Sirt1 resulting from p53 activation and an increase in miR-34a bioavailability resulting from a decline in miR-34a “sponging” due to ceRNA MALAT1 depletion. These findings suggest that therapeutic interventions increasing MALAT1 expression in muscle may potentially enhance the preservation of muscle mass with aging.

### EFFECTS OF GLYCINE AND N-ACETYLCYSTEINE ON GLUTATHIONE LEVELS AND MITOCHONDRIAL ENERGY METABOLISM IN HEALTHY AGING

Philipp Gut,[1] Giulia Lizzo,[1] Eugenia Migliavacca,[1] Leonidas Karagounis,[2] Tim Heise,[3] and Maximilian Eynatten,[2], *1. Nestlé Research, Lausanne, Vaud, Switzerland, 2. Nestlé Health Science, Vers-chez-les Blanc, Vaud, Switzerland, 3. Profile Institut for Metabolic Research, Neuss, Baden-Wurttemberg, Germany*

Glutathione is an intracellular antioxidant that neutralizes reactive oxygen species and prevents tissue damage. Dietary supplementation with the glutathione precursors glycine and n-acetylcysteine supports the maintenance of normal glutathione levels in several age-related diseases, but the optimal doses and their efficacy in healthy elderly are not established. We report results from a randomized controlled clinical trial in 114 healthy volunteers (mean age = 65 years) receiving glycine and n-acetylcysteine (GlyNAC) at three different doses for two weeks (1.2g/1.2, 2.4g/2.4g, 3.6g/.3.6g of each amino acid). Older subjects showed increased oxidative damage and a lower reduced-to-oxidized glutathione ratio (GSH:GSSG) compared to young subjects, but unchanged total glutathione levels. GlyNAC did not increase levels of circulating glutathione compared to placebo treatment, the primary study endpoint. However, stratification analyses suggest that subjects with high oxidative stress and low glutathione status responded with glutathione generation. We find that unrelated to glutathione status, healthy aging was associated with lower levels of fasting glycine that can be increased towards those observed in young subjects with supplementation. Using preclinical models, we find that tissue glycine depletion is a common feature of healthy aging. Supplementation of old mice with glycine efficiently improved age-related decline of mitochondrial respiratory function in skeletal muscle and prevented a gene program associated with protein catabolism observed in control-treated animals. In conclusion, GlyNAC is safe and well-tolerated and may selectively increase glutathione levels in older subjects with oxidative stress and glutathione demand. Our data further suggest that glycine may support mitochondrial function independently of NAC.

### LOSS OF ISCHEMIC TOLERANCE WITH AGE: CAN WE PROTECT AN OLD KIDNEY

Egor Plotnikov, *1. A.N. Belozersky Institute of Physico-Chemical Biology, Lomonosov Moscow State University, Moscow, Moskva, Russia*

The most abundant and vulnerable cohort of patients with acute kidney injury (AKI) is represented by the older people. It is well-known, the kidney tissue undergoes some changes with age, both at the morphological and molecular level. Therefore, when treating AKI in older patients, it is necessary to take into account the morphofunctional features of aging kidney tissue and metabolic alterations. We have shown that the kidney of old rats does not perceive signals from the most well-known protective approaches such as ischemic preconditioning (IPC) and caloric restriction (CR). Although the old kidney did not develop more severe AKI after ischemia, we found no pronounced effect on attempts to increase its resistance by IPC and CR. Analysis of the mechanisms underlying this loss of tolerance has shown that the most affected pathways are the mechanism of mitochondrial quality control, the effectiveness of autophagy, and the proliferative potential of kidney cells. However, several protective pathways activated in the young kidney were also active in the old one in response to the CR. In particular, an increase in SIRT1 deacetylase, antiapoptotic Bcl-xL, and a decrease in oxidative stress were observed. Our results show that some defense systems demonstrating their effectiveness in young organisms lose their beneficial effect in old organisms, while others still can be activated by protective approaches. Thus, it is necessary to carefully analyze the possibilities of increasing ischemic tolerance for old organisms. This work was supported by the Russian science foundation (grant #21-75-30009).

### METFORMIN PRESERVES MITOCHONDRIAL INTEGRITY AT OLD AGE IN MALE RATS.

Jonathan Wanagat,[1] Allen Herbst,[2] Austin Hoang,[1] Chiye Kim,[3] Judd Aiken,[4] Debbie McKenzie,[3] and Deena Goldwater,[1], *1. UCLA, Los Angeles, California, United States, 2. University of Alberta, Edmonton, Alberta, Canada, 3. University of Alberta, University of Alberta, Alberta, Canada, 4. UofA, Edmonton, Alberta, Canada*

Metformin is being deployed in clinical trials to ameliorate aging in older humans who do not have diabetes. In C. elegans, metformin treatment at old ages exacerbated mitochondrial dysfunction, led to respiratory failure, and shortened lifespan. Metformin is a commonly used, well-tolerated treatment for diabetes in older adults. Mitochondrial effects of metformin treatment in aged mammals has not been sufficiently investigated. We hypothesized that metformin treatment would not be toxic to older mammals. To define a therapeutic dose in aged hybrid rats, we evaluated two doses of metformin (0.1%, 0.75% of the diet) at 30-months of age. Body mass decreased at the 0.75% dose. Neither dose affected mortality between 30- and 34-months of age. We assessed mitochondrial quality, quantity, and function in aged rats treated with metformin at the 0.75% dose by measuring mitochondrial DNA copy number, deletion mutation frequency, and respirometry in skeletal muscle and heart. In skeletal muscle, we observed no effect of metformin on quadriceps mass, mtDNA copy number or deletion frequency. In the heart, metformin treated rats had higher mtDNA copy number, lower cardiac mass and no effect on deletion frequency. Metformin treatment resulted in lower mitochondrial complex I activity in both heart and quadriceps. Metformin did not compromise mitochondrial integrity, was well tolerated, and may have cardiac benefits to rats at old ages.

### SUCCESSFUL EXOGENOUS EXPRESSION OF ATP8, A MITOCHONDRIAL ENCODED PROTEIN, FROM THE NUCLEUS IN VIVO.

David Begelman,[1] Martin Brand,[2] Amutha Boominathan,[1] Caitlin Lewis,[1] Bhavna Dixit,[1] and Mark Watson,[2], *1. SENS Research Foundation Research Center, Mountain View, California, United States, 2. Buck Institute for Research on Aging, Novato, California, United States*

Replicative errors, inefficient repair, and proximity to reactive oxygen species production sites make the mitochondrial DNA (mtDNA) susceptible to damage with time. mtDNA mutations accumulate with age and accompany a progressive decline in organelle function. We lack molecular biology tools to manipulate mtDNA, thus we explore the possibility in vivo of utilizing allotopic expression, or the re-engineering mitochondrial genes and expressing them from the nucleus, as an approach to rescue defects arising from mtDNA mutations. This study uses a mouse model with a mutation in the mitochondrial ATP8 gene that encodes a protein subunit of the ATP synthase. We generated a transgenic mouse with an epitope-tagged recoded and mitochondrial-targeted ATP8 gene expressed from the nucleus. Our results show that the allotopically expressed ATP8 protein in the transgenic mice is robustly expressed across all tested tissues, successfully transported into the mitochondria, and incorporated into ATP synthase. We are currently evaluating if allotopic expression of ATP8 will functionally rescue the behavioral and bioenergetic defects in ATP8 mutant mice. Translating allotopic expression technology into a mammal and demonstrating systemic functional rescue will lend credence to utilizing allotopic expression as a gene therapy in humans to repair physiological consequences of mtDNA defects that may accumulate with age.

### Session 9115 (Poster)

### CANCER AND AGING

### EXPLORING SPIRITUALITY, LONELINESS AND HRQOL IN HISPANIC CANCER CAREGIVERS

Jennifer McElfresh,[1] Terry Badger,[1] Chris Segrin,[1] and Cynthia Thomson,[2], *1. University of Arizona, Tucson,*

*Arizona, United States, 2. University of Arizona, Mel and Enid Zuckerman College of Public Health, Tucson, Arizona, United States*

Providing care to an aging society in the new normal requires increased attention to the informal caregivers who support the health and well-being of older adults with chronic conditions. Hispanic caregivers carry a high caregiver-associated burden. Health disparities experienced by Hispanics, coupled with the emotional, social and physical demands of caregiving, may set an unprecedented risk for lower health-related quality of life (HRQoL). In a quantitative analysis, we investigated the relationship between spirituality, loneliness and HRQoL in Hispanic cancer caregivers using baseline data from the Support for Latinas with Breast Cancer study (N= 234 Hispanic caregivers). Findings suggested an indirect effect of spirituality on HRQoL through reduced loneliness among more spiritual caregivers, effects that were independent of age. The second study was conducted using qualitative semi-structured interviews (N= 10) with Hispanic caregivers. Interviews evaluated spirituality and HRQoL in Hispanic cancer caregivers who reported variable levels of loneliness. Five themes emerged: caregiver experience, coping strategies, loneliness, religion to gain strength or support, and spirituality to gain strength or support. Results supported the role of spirituality in promoting higher HRQoL in Hispanic cancer caregivers and elucidated pathways to intervene on HRQoL through spirituality. With Hispanics often underutilizing formal services, having an improved understanding of caregiving experiences, particularly related to spirituality, will support the development of culturally-relevant strategies and programming to promote HRQoL for Hispanic caregivers.

### GERIATRIC ONCOLOGY: COGNITION AND COMMUNICATION

Patricia Parker,[1] Yesne Alici,[2] Christian Nelson,[2] Smita Banerjee,[3] Charlotte Malling,[2] Nessa Coyle,[2] and Beatriz Korc-Grodzicki,[4], *1. Memorial Sloan Kettering Cancer Center, Memorial Sloan Kettering Cancer Center, NYC, New York, United States, 2. Memorial Sloan Kettering Cancer Center, New York, New York, United States, 3. Memorial Sloan Kettering Cancer Center, Memorial Sloan Kettering Cancer Center/New York, New York, United States, 4. Memorial Sloan Kettering Cancer Center, Memorial Sloan Kettering Cancer Center, New York, United States*

With the dramatic increase in the older adult population, assessment and care of chronic diseases of aging, notably cancer, cognitive impairment and functional decline, have increasing clinical importance. Most healthcare practitioners (HCPs) receive minimal education in geriatrics and/or communication skills and are not optimally prepared to treat older cancer patients. Geriatric Oncology: Cognition and Communication (Geri-Onc CC) trains HCPs to identify cognitive impairment and/or functional decline and improve communication with patients and caregivers. Geri-Onc CC is a 2-day virtual training. Day 1 covers depression, dementia, delirium, pharmacology, cognitive rehabilitation, language barriers, and decision-making capacity. Day 2 focuses on communication skills experiential practice in geriatric syndromes, cognitive syndromes and shared decision making. In addition, HCPs engage in 6 bimonthly web-based collaborative learning activities post-training. Thus far, three cohorts have participated (n=56). Participants were primarily female (88%), 68% non-Hispanic White, and represented multiple disciplines [psychologists (29%) social workers (25%), physicians (21%), others(25%)] and they work in various settings: comprehensive cancer centers (43%),community hospitals (18%), and others (33%). Most (48%) have been caring for older cancer patients for 1-5 years. All reported the training had value to them as a clinician, increased their knowledge of geriatrics, and helped them meet their training goals and 84% were extremely satisfied with the program. Recruitment has been successful. Participants have been diverse in terms of race/ethnicity, profession, practice characteristics and the populations they serve. Overall, participants have found the training valuable, future work will describe outcomes of the training.

### MEMORY AGING PHENOTYPES AMONG OLDER CANCER SURVIVORS: A LATENT GROWTH ANALYSIS OF THE HEALTH AND RETIREMENT STUDY

Ashly Westrick, Kenneth Langa, and Lindsay Kobayashi, *University of Michigan, Ann Arbor, Michigan, United States*

While cancer survivors experience many long-term health effects, there is limited evidence on the potentially heterogeneous memory aging of older cancer survivors. We identified memory aging phenotypes of older US cancer survivors, and determined sociodemographic and health-related predictors of membership. Data were from 2,755 survivors aged ≥50 in the U.S. Health and Retirement Study (1998 – 2016). Self-reported first incident cancer diagnosis (except non-melanoma skin cancer) and memory (composite immediate and delayed word-list recall score, combined with proxy-reported cognition) were assessed at biennial interviews. Memory aging phenotypes were identified using latent growth curve (LGC) models, with baseline being time of cancer diagnosis. Logistic regression evaluated predictors of group membership. 5 distinct memory aging groups were identified: low memory (n=165, 6.16%); medium-low memory (n=459, 17.1%); medium-high memory (n=733, 27.4%); high memory (n=750, 28.0%); and very high memory (n=571, 21.3%). The low memory group received less chemotherapy compared to the other groups (20.0% vs. 25.5%, 31.7%, 36.8%, 41.5%%, respectively), and had the shortest mean survival time after diagnosis (1.08 vs 2.10, 2.76, 3.37, 4.31 years, respectively). Older age at diagnosis (OR: 1.71, 95%CI: 1.61-1.82), being male (OR: 4.10, 95%CI: 2.82-6.51), having a history of stroke (OR: 4.62, 95%CI: 2.57-8.30) and depression prior to diagnosis (OR: 1.19, 95%CI: 1.05-1.34) were independently associated with being in the low memory group vs. the medium-high memory group. We identified distinct memory aging phenotypes among older cancer survivors. Further research should evaluate the influence of pre-cancer memory and how these phenotypes differ from the general population.

### OLDER BREAST CANCER SURVIVORS' COGNITIVE RESPONSE TO QIGONG/TAI CHI EASY: AN EXPLORATORY ANALYSIS

Dara James, Linda Larkey, and SeungYong Han, *Arizona State University, Phoenix, Arizona, United States*

Increasing rates of breast cancer coupled with improvements in treatment means the number of breast cancer survivors (BCSs) is growing. BCSs frequently report persistent cognitive deficits (i.e., “cancer-related cognitive impairment”) that impacts QOL and treatment compliance. Older (≥65 years old) BCSs are more likely to experience cognitive decline and impairment, partly due to the biological process of senescence. In the context of a larger RCT of BCSs (ages 45-75; stages 0-III), we evaluated cognitive function/performance effects on among the older participants (ages 65-75) of 8-weeks Qigong/Tai Chi Easy (QG/TCE) compared to education control (EdC). Cognitive function was measured using the Functional Assessment of Cancer Therapy-Cognitive Function (FACT-COG), including: perceived cognitive impairment (PCI), and perceptions of effects of cognitive function on quality of life (PCQOL). Cognitive performance was measured using the Wechsler Adult Intelligence Scale-Third Edition (WAIS-III): Digit Span (DS) and Letter-Number Sequencing (LNS). A multilevel model with random intercept was used to examine GroupXTime interactions: The majority of participants (N= 32) (M age= 69.7) were white (84%). Changes in WAIS-III DS, LNS and FACT-COG PCI were not statistically significant, but effect sizes were small to medium. The interaction between group and time was significant for FACT-COG PCQOL (p= 0.033) with a medium effect size, 0.14. Findings from this exploratory analysis of the larger study suggests that older BCSs' participation in QG/TCE may improve perceptions of effects of cognitive function on quality of life. Such improvements may increase cognitive-related self-efficacy, overall QOL and treatment compliance among older BCSs.

### PREDICTORS OF RACIAL DIFFERENCES IN DEPRESSION AND AFFECT AMONG OLDER ADULT, LONG-TERM CANCER SURVIVORS

Gary Deimling,[1] Dyanna Burnham,[2] and Gabrielle Beck,[2], *1. Case Western Reserve University, Cleveland, Ohio, United States, 2. Case Western Reserve University, CLeveland, Ohio, United States*

Research has long documented the psycho-social sequelae experienced by those who have been treated for and survived cancer. Depression, affect and other indicators of mood state have been an important focus of that research However, there is little research on racial differences in depression and affect outcomes or the specific cancer and age-related factors that predict them. The research to be presented is based on a 10 year, six wave NCI funded study of 471 older adult (age 60+), long-term cancer survivors randomly selected from the tumor registry of a comprehensive cancer treatment center. Key outcome measures were depression (CES-D) scale) and both positive and negative affect (PANAS). Covariance analyses and nested OLS Regression were used to identify Black-white differences these outcomes and the relative importance of both cancer and non-cancer predictors. Blacks reported lower levels of depression and negative affect when compared to whites. In a separate regression analysis of the black sub-sample, continuing cancer-related symptoms were by far the strongest predictors (beta =.16) of negative affect. In the white sub-sample, while cancer-related symptoms continued to be a significant predictor (beta=.16), non-cancer symptoms were substantially more important (beta=. 22). These results will hopefully help practitioners to have a better understanding of the nuanced racialized experiences and mental health among cancer survivors, and how these may impact after-care for older adult cancer survivors.

### SHAKEN IDENTITIES, RESERVE MASCULINE CAPITAL, AND THE LIVED EXPERIENCES OF AGING MEN WITH BREAST CANCER

Edward Thompson,[1] and Renee Beard,[2], *1. College of the Holy Cross, Broadview Heights, Ohio, United States, 2. College of the Holy Cross, College of the Holy Cross, Massachusetts, United States*

A question that begs to be examined is: How does aging men's discovery they have breasts as a result of their breast cancer diagnosis and having a breast removed through a mastectomy, affect their masculine subjectivities and practices, as they also go about also living with a life-threatening illness? The present study aimed to better understand how men come to live with the knowledge that they have both breasts and cancer. Interviews with seventeen men in the U.S. (mean age 62.8) with a breast cancer diagnosis, mastectomy, and, most often, post-surgical hormonal treatment uncovered stories of body-self disruption and identity dilemmas. All the men's identities had been shaken. After their mastectomy, they were reminded every morning that the body reflected in the mirror differed significantly from who they once were. Their stories revealed strategic themes: how they lived with cancer by slightly modifying conventional masculinities; and how others interacted with them, with the exception of mammography technologists, in terms of their gender, not their atypical illness. Only a few men initially felt their breast cancer was a gendered stigma. Noticeable was how the historical era when diagnosed and the age of the man at diagnosis contextualized their illness stories. In this presentation, three cases are used to exemplify the men's varied experiences with their non-normative bodies and their commonality in finding reserves of masculine capital to rebuke the existential loneliness of a man with breast cancer.

### TECHNOLOGY USE AMONG CANCER PATIENTS PRE- AND POST- COVID-19 PANDEMIC: THE ROLE OF DEMENTIA

Weixin Li,[1] Katherine Ornstein,[2] Yan Li,[3] and Bian Liu,[2], *1. Icahn school of Medicine at Mount Sinai, Long island city, New York, United States, 2. Icahn School of Medicine at Mount Sinai, New York, New York, United States, 3. Icahn School of Medicine at Mount Sinai, Icahn School of Medicine at Mount Sinai, New York, United States*

COVID-19 has highlighted increasing reliance on information and communication technology (ICT) and challenges in access and use. ICT access also provides resources that benefit users' mental health. Our study describes changes in the use of ICT before and during the COVID-19 pandemic among cancer patients with and without dementia. We identified 196 (1.6 million weighted population) older adults with a self-reported cancer history who participated in both 2019 and 2020 National Health and Aging Trends Study (NHATS). In 2019, cancer patients with dementia (9.9%) were less likely (adjusted OR 0.29; 95%CI, 0.11-0.78) to use information technology (IT) for health matters (contacting medical providers, handling health insurance matters, obtaining information about health conditions, and ordering prescription refills) compared to those without dementia. In contrast, dementia status was not associated with communication technology (CT) use (email or texts) or IT use for personal tasks (grocery shopping or online banking). IT use for personal tasks was inversely associated with anxiety symptoms (adjusted OR 0.22; 95%CI:0.06-0.83) and CT use was inversely associated with depressive symptoms (adjusted OR 0.25 (95%CI:0.07-0.97). In 2020, regardless of dementia status, all cancer patients increased their virtual (email/phone/video) contact with family, friends (3.4%-7.0%), and medical providers (17.2%-36.2%) while decreasing in-person contact (10.0%-15.7% and 21.8%-24.2%, respectively) during the pandemic. This study suggests that there are potential unmet daily needs for patients with comorbid cancer and dementia that may be met with improved ICT access. Such challenges are of increasing concern as COVID-19 has resulted in increased ICT reliance for older adults.

### Session 9120 (Poster)

### CHRONIC DISEASE MANAGEMENT (BSS POSTER)

### A QUALITATIVE INQUIRY ON SELF-MANAGEMENT DECISION MAKING AMONG RURAL AFRICAN AMERICANS WITH TYPE 2 DIABETES

Idethia Shevon Harvey, *Texas A & M University, Bryan, Texas, United States*

Living in a rural area has been recognized as a unique health disparity associated with higher rates of chronic disease. It is further compounded for those who are the most structurally vulnerable complicating access to care and negatively affecting health outcomes. Barriers to type 2 diabetes (T2DM) self-management remain a growing concern, particularly among minority communities living in underserved geographical areas. Much of the self-management research focused on compliance with medication regimens and modification of lifestyle choices. A less well-understood but arguably more critical aspect is the social factors in disease management decision-making. Purposive sampling was used to identify rural African Americans (n = 34). The mean age of participants was 65.9 years (SD = 12.3), and T2DM diagnosis was 15 years (SD = 12.4). The study utilized the consensual qualitative research methodology and the "Sort and Sift, Think and Shift" approach to identify themes. The participants reported an alternative way of integrating glucose monitoring through a "feedback loop" of body sensing. The longer they live with the condition (i.e., knowing my body), the more they can interpret whether they are hypoglycemic or hyperglycemic (i.e., deciphering the cues) to create and navigate their disease management strategy (i.e., body sensing). Self-management decision-making is a complex developmental process that includes disease trajectory and cultural and environmental factors. Findings from this study may provide a conceptual framework for ongoing inquiry and may provide insights to help T2DM educators and clinicians fully understand the complexity of long-term disease management among rural African Americans.

### FALL DETECTION: USING TECHNOLOGY TO PREVENT FALLS AND SAVE LIVES

Lydia Manning, *Dele Health Tech, River Forest, Illinois, United States*

Companies, providers, and consumers alike are increasing the use of technology in almost every industry. This increase in usage occurs simultaneously in a technology marketplace characterized by rapid evolution in design and products. Recent years have witnessed the surge of various technological products and solutions in the age-tech marketplace, particularly in senior living and many related to fall detection and prevention interventions. Falls can have a widespread and significant impact on health, can be deadly, and often result in high costs for individuals and older adult living facilities. One out of four older adults fall each year. Findings from a pilot project illustrate the total incident count for a 12-month period. Findings demonstrate the importance of having a complete solution for falls and a fall detection solution in place in an assisted living environment. Ideal environments for residents, families, staff, and those working in the facility with regard to smart tech are considered. It is important to consider how can these solutions empower residents and afford people autonomy and safety through dignified technology.

### RISK PERCEPTION AND COMMUNAL COPING IN FAMILIES AFFECTED BY TYPE 2 DIABETES

Jielu Lin,[1] Melissa Zajdel,[1] Melanie Myers,[2] and Laura Koehly,[1], *1. National Institutes of Health, Bethesda, Maryland, United States, 2. Cincinnati Children's Hospital Medical Center, Cincinnati, Ohio, United States*

Despite a recent decline, rates of type 2 diabetes remain high among older adults. Preventing and delaying the onset of the condition with lifestyle changes is key to reducing disease burden in the population. Type 2 diabetes is a complex disease, likely a result from the joint effect of genetic, socio-environmental and lifestyle risk factors that are clustered in families. As such, the prevention of type 2 diabetes is a communal coping process, where individuals communicate about risk and establish routines to facilitate one another's health habits and compliance with therapeutics. This poster investigates how such a process is affected by one's perception of risk based on his/her knowledge about family health history (FHH). We collected family network data from families of different racial backgrounds in the greater Cincinnati area (28 white and 17 black/ African American households; 127 participants). The analysis focuses on how the density of diabetes diagnosis in one's FHH affects communication about shared risk for type 2 diabetes and encouragement to maintain or adopt a healthy lifestyle. Results suggest a higher concentration of diabetes diagnosis in one's FHH is associated with a higher number of risk communication ties in all families. With regards to encouragement ties, high rates of diabetes diagnosis in FHH are associated with an increased number of encouragement ties only in families of black/African heritage. The findings highlight the need and promise of using FHH to motivate co-encouragement to maintain/adopt a healthier lifestyle in families of black/African heritage.

### THE EVALUATION OF SMART SPEAKER SKILLS FOR CHRONIC DISEASE MANAGEMENT OF OLDER ADULTS

Melissa Pighin,[1] and Yong Kyung Choi,[2], *1. University of California, Davis, Sacramento, California, United States,*

2. *University of California Davis School of Medicine, Sacramento, California, United States*

A voice-activated smart speaker is an emerging technology that presents unique opportunities to support the chronic disease management of older adults. We identified the available health-related smart speaker skills in Amazon Alexa platform that support chronic disease management and assessed their functionalities to inform the development of a home-based lifestyle intervention program for older adults with cardiovascular disease and type 2 diabetes. From January to March 2021, we searched Alexa Skills using keywords related to diabetes, medication, blood pressure and nutrition management. Our search produced total 156 potentially relevant skills (63 diabetes, 57 medication, 11 blood pressure and 25 nutrition related), of which 22 skills met inclusion criteria. Apps were excluded if it was only informational, not relevant to the topic, had zero user rating, available in language other than English, and required an external device or a subscription to a specific health plan or service. 22 skills (4 diabetes, 8 medication, 3 blood pressure and 7 nutrition) were evaluated with Echo Show 8 device. The skills were evaluated using the modified version of IMS Institute for Healthcare Informatics app functionality scores and the score (0 to 11) was calculated accordingly. The median number of functionalities was 3.5 and 68% of skills (15/22) had 4 or fewer functions. The highest rated skill was a medication management app named myNurseBot having 6 out of 11 functionalities. The poor functionality score highlights a need for a more robust and comprehensive smart speaker skill to support the health management of older adults.

### Session 9125 (Poster)

### CIVIC ENGAGEMENT AND VOLUNTARISM

### CIVIC ENGAGEMENT IN RETIREMENT AND THE SOCIOEMOTIONAL EXPERIENCE OF PANDEMIC TIME

Boroka Bo, *University of California, Berkeley, Berkeley, California, United States*

This research integrates literature from the sociology of the life course, sociology of emotions and the sociology of time to examine how Socioeconomic Status (SES) influenced retiree civic engagement during the COVID-19 pandemic. I find that SES framed both the social experience of time and the prevalent emotions experienced by retirees while physically distancing during the early days of the pandemic. These individual-level experiences translated to markedly different blueprints for civic engagement. High-SES retirees were more likely to 'go global', organizing to advocate for their interests. Conversely, low-SES retirees were more likely to 'turn in', minimizing their civic engagement. My findings reveal how existing sociopolitical inequalities may become further entrenched in public health crises. Policies aimed at combating inequalities in later life also need to consider socioemotional and sociotemporal factors.

### INTERNALIZED AGE STEREOTYPES AS A MEDIATOR BETWEEN VOLUNTEERING AND PSYCHOSOCIAL HEALTH FOR ADULTS 50+

Andrew Steward,[1] Leslie Hasche,[2] and Julie Anne Laser-Maira,[2], 1. *University of Denver, Lone Tree, Colorado, United States*, 2. *University of Denver, Denver, Colorado, United States*

The productive aging literature describes a wide range of psychosocial benefits of volunteerism for older adults. A growing, compelling body of literature drawing from stereotype embodiment theory identifies significant, negative public health impacts of internalized age stereotypes. Yet, little research has explored which activities may reduce internalized ageism and enhance psychosocial health as people age. This cross-sectional study examined whether internalized age stereotypes mediate the relationship between volunteering and social connectedness for adults 50+. A convenience sample of volunteers ($n = 112$) 50+ years of age residing in the U.S. Mountain West were recruited. A 15-minute, online survey was utilized. The independent variable was number of volunteer hours per week (mean = 6.25, SD = 4.85). The dependent variable was social connectedness measured by five items positively worded from the five-point, Likert-type UCLA loneliness scale ($\alpha = .85$; mean = 4.26, SD = 0.59). Drawing from the self-stereotypes of aging scale, the indirect effects of five internalized positive (e.g., "wise" and "capable") and five negative (e.g., "grumpy" and "helpless") age stereotypes were tested. Results indicate that increased internalized positive, not negative, age stereotypes partially mediated the relationship between volunteer hours and increased social connectedness, while holding constant age, gender, race, functional limitation, education, employment, length of volunteering, and previous volunteer experience. Although positive age stereotypes have long been considered a form of ageism, the results of this study suggest that internalizing positive age stereotypes may function as a form of esteem to promote enhanced psychosocial health as people age.

### THE MEDIATING EFFECT OF SOCIAL SUPPORT OF OLDER KOREAN ADULTS' VOLUNTEERING ON THE RELATIONAL AND LIFE SATISFACTION

Meeryoung Kim, *Daegu University, Daegu, Kyongsang-bukto, Republic of Korea*

For older adults wanting to maintain good health and stay active after retirement, volunteering is an important activity. Social capital is important factor for volunteering. Social support as a social capital, is a contributing factor that is important and needed by older adults who volunteer. Also as a result from volunteering, older adults can increase their social support through volunteering. This study examined whether emotional and instrumental social support mediate volunteering on both relational and life satisfaction. This study used the 6th additional wave of the Korean Retirement and Income Study (2016). Subjects for this study are over 60 years old and the sample size is 280. For data analysis Baron and Kenny's triangular regression analysis and the Sobel test were used for data analysis. Demographic variables were controlled. Volunteer variables such as volunteering asked by others or self-motivated, whether only one type of volunteering or more, professional volunteering, and volunteer hours were used as independent variables. Emotional and instrumental social support were used as mediators. Relationship satisfaction and life satisfaction variables were used as dependent variables. Emotional and instrumental support partially mediate volunteering asked by others to influence relational and life satisfaction. In addition, emotional support and instrumental support mediate "more than one kind of volunteering" to influence relationship satisfaction. As such, emotional and instrumental support through volunteering has a mediating effect on relationship satisfaction and life satisfaction.

### THE RELATIONSHIP BETWEEN OCCUPATION TYPES, EDUCATION, AND VOLUNTEER BEHAVIORS AMONG OLDER AMERICANS

Patrick Ho Lam Lai, Cal Halvorsen, and Christina Matz, *Boston College, Chestnut Hill, Massachusetts, United States*

Research shows that productive engagement in later life, such as paid work and volunteering, is associated with health and wellbeing. From a life-course approach, personal history and experiences developed from occupations earlier in life may affect individual's willingness and ability to volunteer in later life; reciprocally, volunteering tends to extend networks and roles from their previous work after retirement. Further, education, which influences career development, indirectly affects late-life volunteering. Using data from 329,938 individuals aged 50 to 85 in more than 700 occupations from the Volunteer Supplement of Current Population Survey since 2010, this study found that older adults had higher volunteer rates (40%) when their current or latest jobs require more human interactions, compared to those jobs were mainly characterized as office work (31%) and jobs not requiring much human interaction (21%). More specifically, occupations with higher volunteering rates were more likely to be related to social, educational, or spiritual sectors. Some careers seem to provide skills and networks needed in volunteer roles, such as accounting clerks (ranked 12th in volunteering) and museum technicians (ranked 13th). Diverse educational levels make the relationship between occupation and volunteering more complex. For instance, those older adults without a high school diploma and who have office work as their current or latest job (16%) have higher volunteering rates than those occupations requiring (14%) or not requiring much human interaction (10%). Policymakers can take the human interactions and skills needed in careers and educational levels into account when thinking of strategies to promote volunteering.

### THE RELATIONSHIP OF CAREERS WITH PEOPLE INTERACTIONS AND VOLUNTEERING BETWEEN GENDER-RACIAL GROUPS IN LATE-LIFE

Patrick Ho Lam Lai, *Boston College, Chestnut Hill, Massachusetts, United States*

Prior research showed that ethnic minorities in late-life tended to participate less in volunteering, compared to Whites. Older women, in general, spent more time volunteering than older men in the United States. Previous studies showed that occupational statuses, but have not yet discussed occupation categories, affected older adults' volunteering. The Current Population Survey dataset was utilized in this study to explore the relationship between careers with or without people interactions and volunteering of Americans aged 50 to 85 in an intersectionality lens. Regarding races, older African Americans who worked in occupations requiring human interactions, had almost double volunteering rate than those occupations not requiring these interactions. In respect of genders, compared to older men who worked in jobs requiring human interactions, the volunteer rate of those not requiring human interactions was 81% less. Either older African Americans or older men had more associations between their human interactions in career and their volunteering rate, than other racial groups or gender groups individually. Considering races and genders together, comparing to older Asian men who worked in fields needed interactions with others, the volunteering rate of those who did not work in these fields was 52% less. The association of older Asian men between fields requiring human interactions and volunteering rate was the least, among various gender-racial subgroups. Older adults with different racial-gender identities may face varying experiences in different types of occupations. Social and cultural factors among these identities are discussed to better understand the relationships between careers and volunteering in late-life.

### VOLUNTEERING AND CHRONIC INFLAMMATION IN LATER LIFE: IS SUSTAINED VOLUNTEERING BENEFICIAL FOR HEALTH?

Mallory Bell,[1] Madison Sauerteig,[2] and Kenneth Ferraro,[2], *1. Purdue, West Lafayette, Indiana, United States, 2. Purdue University, West Lafayette, Indiana, United States*

Although research on the health benefits of volunteering has proliferated in recent decades, most studies have focused on whether or not a person volunteers or the monthly frequency of volunteering. This study examines whether sustained volunteering has health benefits above and beyond occasional or short-lived volunteering. To investigate the salubrious effects of volunteering, the present study considers sustained volunteering engagement in terms of both formal and informal volunteering. Using four waves of data from the Health and Retirement Study, we assess the influence of sustained volunteering on chronic inflammation, measured by C-reactive protein (CRP). Results reveal that sustained engagement in formal and informal volunteering is related to lower CRP concentration, but this association is partly mediated by adult health and socioeconomic factors. Although sustained volunteering is associated with lower levels of chronic inflammation, older adults who maintain their volunteering over time are a select category of adults, characterized by higher education and wealth and better health.

### WHO VOLUNTEERS? RESULTS FROM A REGULAR COGNITIVE MONITORING STUDY

Britney Veal, Nasreen Sadeq, Taylor Atkinson, and Ross Andel, *University of South Florida, Tampa, Florida, United States*

Previous research indicates volunteering promotes well-being of individuals and communities. Volunteering in later-life may buffer some of the negative health effects experienced during retirement, facilitating opportunities for older adults to engage in meaningful activities and stay active. The current study examined characteristics of older adults who volunteered outside of participation in a regular cognitive monitoring study. All 124 members (M= 76.87, SD= 7.47; 80 volunteers, 44 non-volunteers) of a regular cognitive monitoring study, requiring completion of a 15-minute cognitive online test once a month, with complete data on personal characteristics, volunteer activities, as well as study adherence and dropout rates were included. ANCOVA and logistic regression analyses adjusted for sociodemographic characteristics were used to assess differences between volunteers and non-volunteers. Results indicated that volunteers were less educated (p<.05), and slightly more likely to be younger and women compared to non-volunteers. There were no differences in cognitive performance (ps>.05). Volunteers had lower scores for neuroticism (p=.02) and were marginally higher agreeable and extraverted (ps<.09). Volunteers needed more reminders to complete the monthly test (ps<.01) but had lower dropout rates (p=.001). The most frequent type of volunteer activity reported was religious. Volunteers were motivated mainly by altruism, although most reported multiple reasons such as building social relationships and feeling important. Findings provide information about characteristics that can help identify older adults who are likely to volunteer. Results regarding study adherence may have implications for promoting recruitment and retention among older adult volunteers.

### Session 9130 (Poster)

### COGNITION AND COGNITIVE IMPAIRMENT

### AN INNOVATIVE TRANSITIONS MODEL OF CARE FOR DELIRIUM: "DDEFY DELIRIUM" A PILOT FEASIBILITY RANDOMIZED TRIAL

Ariba Khan,[1] Marianne Klumph,[2] Alexander Schwank,[2] Sandy Hubatch,[2] Jonny Macias Tejada,[2] Colleen Galambos,[3] Michelle Simpson,[2] and Michael Malone,[2], *1. Advocate Aurora Health Care, Milwaukee, Wisconsin, United States, 2. Advocate Aurora Health, Milwaukee, Wisconsin, United States, 3. University of Wisconsin- Milwaukee, Milwaukee, Wisconsin, United States*

In current standard practice, without a structured process for delirium follow up, older individuals and their family caregivers seemed to be lost, as they transitioned from hospital to home. The aim of this study was to pilot test a theoretical post-hospital model of care (DDEFY delirium) to mitigate the complications in patients who had hospital delirium. This is a pilot feasibility randomized controlled trial for patients with hospital delirium. The intervention was carried out by a delirium transitions nurse with personalized interdisciplinary team recommendations. DDEFY delirium intervention encompasses: Diagnose cognitive disorder; review Drugs; Educate patient/family; assess Function; Your health goals. During COVID-19 pandemic a virtual intervention group was created. Thus, three groups were analyzed: control, intervention, and virtual intervention. Among the 35 participants (mean age 80 years (SD10), 40% Black, 46% female), 40% had a diagnosis of dementia, mean Charles Deyo score was 6.4, mean number of medications 11.4 (3.2), and a mean anticholinergic medication burden was 2.4. The intervention group and virtual intervention group rates were: recruitment: 44.6 %vs8.8%, feasibility: 97%vs97%, fidelity:100%vs100%, 30-day readmission 28.6%vs0%, and 30-day ED visits: 0 vs.1. There were no differences in 30-day readmission rates between control vs intervention (p=1.0), control vs virtual intervention (p=.53), nor comparing all 3 groups (p=.49). The results of this pilot study determined that delivering DDEFY intervention to patients with delirium is feasible. Lessons learned from conducting this study will help us design a larger trial with modifications for older patients with delirium who transition from hospital to home.

### BEST PRACTICES TO OVERCOME BARRIERS TO CAPACITY EVALUATIONS

Ronan Factora, and Saket Saxena, *Cleveland Clinic, Cleveland, Ohio, United States*

Because of the increasing incidence of elder abuse and financial exploitation, Adult Protective Services (APS) cases open for these individuals often relay on capacity evaluations conducted by a clinician to facilitate legal assignment of a surrogate decision maker. Despite this growing need, the number of physicians willing and capable of performing them is limited. Barriers reported by physicians reportedly impair their ability to conduct these evaluations include absence of relevant case information and lack of knowledge about the process itself. Geriatricians and related clinicians often perform these assessments. Sharing best practices with internists and family physicians may help overcome these barriers. A survey of geriatric medicine providers was conducted to identify essential components and questions necessary in the assessment of general decision making capacity. Twenty-nine providers at 6 academic institutions in Ohio responded to the survey and its follow-up inquiries. Though variability existed in evaluation styles and content between providers, a uniform set of recommendations was able to be generated. A total of 13 different summary recommendations were generated from this survey. Necessary components to these evaluations include (1) performance of cognitive testing (2) obtaining collateral information regarding functional status from another trusted individual (3) assessing the individual's insight into any reported functional impairments or safety concerns by explaining discrepancies between that individual's own observations and reported concerns from the trusted individual, and (4) using hypothetical situations to assess a person's judgment and reasoning in addressing any gaps in care or safety concerns raised during the interview.

### DAILY ALCOHOL USE COVARIES WITH DAILY CONCENTRATION PROBLEMS ACROSS THE LIFESPAN: FINDINGS FROM THE MIDUS REFRESHER

Jacqueline Mogle,[1] Ashley Linden-Carmichael,[2] Sara Miller,[3] and David Almeida,[3], *1. Penn State University, University Park, Pennsylvania, United States, 2. Penn State Univerity, State College, Pennsylvania, United States, 3. Pennsylvania State University, University Park, Pennsylvania, United States*

Alcohol use is typically associated with impaired cognitive functioning on tasks related to attention and concentration. However, it remains unclear whether these impairments persist across days in ways that are noticeable to the individual. We examined this using the daily diary project of the Midlife in the United States Refresher cohort. Participants (n=710; Mage=50.5; range 25-75) completed 8 nights of telephone-based diaries (Mdiaries=6.87) that included questions about daily alcohol use ("how many drinks did you have today?") and five items assessing concentration (e.g., "today, did you have difficulty concentrating?") rated on a scale (1=none of the time to 5=all of the time). Using autoregressive multilevel models, we examined how same and previous day alcohol use related to perceived difficulties with concentration. Greater total alcohol use over the diary period was related to reports of concentration problems (b=.31, SE=.10, p=.002) though current day (b=-.03, SE=.04, p=.49) and previous day alcohol use (b=.05, SE=.04, p=.23) were not. The association between previous day use and concentration problems was qualified by an interaction with total alcohol use (b=-.07, SE=.03, p=.002). Individuals who drank less alcohol in general, experienced greater perceived concentration problems following the days on which they did drink (b=.14, SE=.07, p=.03) relative to those who drank more alcohol across the diary period (b=-.04, SE=.04, p=.36). This relationship did not vary based on age, sex, or education. These results suggest that daily alcohol use could impair concentration across days, particularly for those adults who tend to consume less alcohol.

### DAILY COGNITIVE DIFFICULTIES AND SOCIAL EXPERIENCES AMONG OLDER ADULTS

Ruixue Zhaoyang,[1] Jacqueline Mogle,[2] Karra Harrington,[1] and Martin Sliwinski,[1], *1. The Pennsylvania State University, University Park, Pennsylvania, United States, 2. Penn State University, University Park, Pennsylvania, United States*

Self-reported cognitive difficulties are common in older adults and may be an early indicator of future cognitive decline or dementia. In past retrospective reports, cognitive difficulties have been linked with differences in social engagement or social relationships among older adults. However, little is known about how self-reported cognitive difficulties in daily life, such as memory lapses, relate to older adults' daily social experiences. This study examined how self-reported cognitive difficulties were related to older adults' daily social interactions and loneliness. Data were drawn from 312 community-dwelling older adults (aged 70 to 90 years) who reported their social interactions and loneliness throughout the day (five times) as well as cognitive difficulties (e.g., memory lapses, problems with attention) at the end of each day for 14 days. Multilevel models revealed that participants reported fewer memory lapses on days when they reported more frequent interactions with family members (p=.041). Higher levels of disruptions to daily activities caused by cognitive difficulties, in turn, predicted higher levels of loneliness the next day (p=.006), but not changes in social interactions the next day. At the between-person level, more memory lapses in daily life were associated with less frequent social interactions with friends, but more frequent unpleasant social interactions and higher levels of loneliness on average. These results suggest that older adults' self-reported cognitive difficulties were dynamically associated with their social interactions and loneliness at the daily level and played an important role in older adults' social life and well-being.

### DEVELOPMENT AND EVALUATION OF TREATMENT ADHERENCE INTERVENTIONS FOR OLDER ADULTS WITH MCI USING IOT DEVICES

Jinhee Shin,[1] Eunhee Cho,[1] Gwang Suk Kim,[1] Heejung Kim,[2] Byoung Seok Ye,[2] and Chang-Gi Pack,[3], *1. Yonsei University College of Nursing, Seoul, Seoul-t'ukpyolsi, Republic of Korea, 2. Yonsei University, Seoul, Seoul-t'ukpyolsi, Republic of Korea, 3. University of Illinois at Chicago, Seoul, Illinois, United States*

For older adults with mild cognitive impairment (MCI), treatment adherence is essential to prevent and delay dementia. Older adults with MCI should maintain treatment for chronic diseases, exercise regularly, and adhere to treatment to maintain health status. There is a lack of comprehensive interventions to promote treatment adherence (medication adherence and physical activity) for older adults with MCI. The purpose of this study was to develop an internet of things (IoT)-based real-time treatment adherence for old adults with MCI and examine the effectiveness of the program. This study was a randomized controlled trial. The patients were enrolled from the neurology outpatient department clinic at a hospital in Korea. The subjects were 18 in the experimental group and 20 in the control group. This study intervention was IoT-based medication adherence device and real-time monitoring sever plus wrist wearable device. The study consists of a 10-week intervention period. The intervention program was provided for only the experimental group and the control group with a wearable device and usual care. Assessments were conducted at baseline, 6 weeks, and 10-weeks. A mixed-effects model was used in the analysis to evaluate the program. The IoT-based treatment adherence intervention was effective in improving medication adherence over time ($\beta$ =11.465, p<.001), physical activity (K-PASE) ($\beta$ =27.376, p<.001) and average the number of steps per week ($\beta$=3202.53, p<.001). Health care providers can use this program to improve treatment adherence for chronic disease management and dementia prevention of older adults with cognitive impairment.

### DEVELOPMENT OF A LOCATOR DEVICE USABILITY SCALE FOR PERSONS WITH DEMENTIA AT RISK OF GETTING LOST

Noelannah Neubauer,[1] Christa Spenrath,[2] Serrina Philip,[1] Christine Daum,[3] Lili Liu,[1] and Antonio Miguel-Cruz,[2], *1. University of Waterloo, Waterloo, Ontario, Canada, 2. University of Alberta, Edmonton, Alberta, Canada, 3. University of Waterloo, Edmonton, Alberta, Canada*

There is an increasing number of persons living with dementia who live alone. Recent COVID-19 pandemic restrictions have resulted in more persons receiving care remote through information and communication technologies. Locating technologies can be a tool to help care partners monitor their loved ones living with dementia. These devices can also mitigate risks associated with going missing, by reducing time for search and returning the lost person home safely. However, there is no clear, standardized approach to assess the usability of these devices. The purpose of this study was to develop a locator device usability scale for persons living with dementia at risk of getting lost. A two-phase study that utilized a multi-method design and included participatory and iterative strategies was conducted. In the first phase, an item pool was generated through online focus groups with service providers, technology developers, care partners and persons living with dementia. The second phase refined the item pool using an online survey and online focus groups with the same stakeholder groups. Five overarching categories were identified as important for the usability of locating device: features, inclusivity, simplicity, aesthetic appeal, and ethics. Participants identified the need for multiple versions of the usability scale including one specifically for persons living with dementia. The newly developed locator device usability scale can enhance the acceptance of these devices, thereby supporting remote caregiving and promote the safety and autonomy of persons living with dementia.

### FACTORS THAT INFLUENCE THE EMOTIONAL IMPACT OF MEMORY PROBLEMS IN OLDER ADULTS: A MIXED-METHODS STUDY

Emily Bratlee-Whitaker,[1] Nikki Hill,[1] Jacqueline Mogle,[1] Rachel Wion,[2] Caroline Madrigal,[3] and Sakshi Bhargava,[1], *1. Penn State University, University Park, Pennsylvania, United States, 2. Indiana University, Indiana University, Indiana, United States, 3. Providence VA Medical Center, Providence VA Medical Center, Rhode Island, United States*

Older adults' experiences with memory problems may be an important indicator of current and future well-being; however, these experiences and their impacts are poorly characterized, particularly in those with co-occurring affective symptoms. The purpose of this mixed-methods study was to examine how the experience of memory problems influences emotional well-being in older adults without dementia, and whether this differs based on cognitive status and current depressive symptoms or anxiety symptoms. A convergent parallel mixed methods design was used in which quantitative and qualitative data were collected simultaneously, analyzed separately, and then integrated to determine how participants' experiences differed. Community-dwelling older adults (n=49, Mage = 74.5, 63% female) without severe cognitive impairment completed study questionnaires and two individual, semi-structured interviews. Five themes were identified that described the influence of memory problems on emotional well-being: Evoking Emotions, Fearing Future, Undermining Self, Normalizing Problems, and Adjusting Thinking. The extent to which memory problems impacted emotional well-being depended on multiple factors including current affective symptoms (primarily anxiety), characteristics of the experience (such as judgments of its importance), as well as personal experience with dementia. Notably, there were no thematic differences in the emotional impact of memory problems between older adults with normal cognition and those with evidence of mild cognitive impairment. Our findings suggest that thorough assessment of reports of memory problems, regardless of cognitive testing outcomes, should consider co-occurring subsyndromal affective disorders as well as older adults' evaluations of how memory problems influence their daily lives and well-being.

### FALL PREVENTION IN ADULTS WITH COGNITIVE IMPAIRMENT: SYSTEMATIC REVIEW AND META-ANALYSIS

Megan Racey,[1] Maureen Markle-Reid,[1] Muhammad Usman Ali,[1] Hélène Gagné,[2] Susan Hunter,[3] Jenny Ploeg,[1] Richard Sztramko,[1] and Diana Sherifali,[1], *1. McMaster University, Hamilton, Ontario, Canada, 2. Ontario Neurotrauma Foundation, Toronto, Ontario, Canada, 3. Western University, London, Ontario, Canada*

Cognitive impairment increases an individual's risk of falls due to the role cognition plays in gait control. Older adults with dementia fall 2-3 times more than cognitively healthy older adults and there is a lack of evidence for effective fall prevention interventions for community-dwelling cognitively impaired adults. We conducted a systematic review and meta-analysis to investigate the effectiveness of fall prevention interventions in improving falls, perceived risk of falls, gait, balance, and functional mobility. We searched 7 databases for interventions involving community-dwelling adults ≥50 years with mild to moderate cognitive impairment. Reviewers screened citations, extracted data, assessed risk of bias and certainty of evidence (GRADE). We performed a meta-analysis of 509 community-dwelling adults (mean age 67.5 to 84.0 years) with mild to moderate cognitive impairment from 12 randomized controlled trails (8 exercise interventions, 3 multifactorial, and 1 providing medication). Interventions had medium significant effects on perceived risk of falls (SMD -0.73 [-1.10, -0.36]), balance (SMD 0.66 [0.19, 1.12]), and timed up and go test (SMD -0.56 [-0.94, -0.17]) and small significant effects on gait speed and control (SMD 0.26 [0.08, 0.43]) with moderate certainty of evidence. There were no significant effects for falls. Sub-analysis showed that exercise and studies at low risk of bias remained significant for balance and perceived risk of falls. The effect of fall prevention interventions on falls remains unclear; exercise interventions are effective at addressing fall risk factors. However, high quality and longer studies with adequate sample sizes are needed to determine their effectiveness on falls.

### HIGHER SERUM BDNF LEVELS ARE ASSOCIATED WITH LOWER RISK OF COGNITIVE DECLINE IN OLDER ADULTS :THE OTASSHA STUDY

Yoshinori Fujiwara,[1] Kazushige Ihara,[2] Mitsugu Hachisu,[3] Hiroyuki Suzuki,[1] Hisashi Kawai,[1] Hirohiko Hirano,[1] Masahiro Hashizume,[4] and shuichi Obuchi,[1], *1. Tokyo Metropolitan Institute of Gerontology, Tokyo, Tokyo, Japan, 2. Hirosaki University, Aomori, Alabama, Japan, 3. Showa University, Tokyo, Alabama, United States, 4. Toho University, Tokyo, Tokyo, Japan*

**Introduction:** There has been growing interest in the use of circulating levels of brain-derived neurotrophic factor (BDNF) in the blood as a biomarker in the context of patients with Alzheimer's and other neurodegenerative diseases. Prospective data on cognitive decline in the broad older population, however, remain limited. We assessed the relationship of serum BDNF levels with short-term decline in cognitive functioning of community-dwelling older adults.

**Methods:** Prospective study of 405 adults 65-84 years old without dementia in Tokyo, Japan. The Montreal Cognitive Assessment-Japanese version (MoCA-J) and its subscales were used. Linear regression assessed standardized differences in test score differences between baseline (2011) and follow-up (2013) visits, according to baseline serum BDNF quartiles, with adjustment for baseline demographics, disease indicators, and cognitive scores.

**Results:** Among participants who performed on the MoCA-J at baseline (scores in bottom quartile), cognitive decline was .65 (95% CI: .08 - 1.2; p=.025) standard deviations (SD) more pronounced in those with lowest than highest BDNF levels. Decline in executive function, but not in other subdomains, was also most pronounced in those with lowest baseline serum BDNF levels (difference: .32 SD; 95%CI: .08-.55; p=.007)

**Conclusion:** Lower serum BDNF levels were associated with greater 2-year cognitive decline in community-dwelling older Japanese adults. Decline varied among cognitive subdomains, and baseline cognition. Research seeking to evaluate the added-value of serum BDNF for screening and/or health promotion initiatives involving physical activity, which has been linked to increment in BDNF levels, is warranted.

### HOW OLDER ADULTS REDEFINE ITEMS ON THE PROMIS-57 PROFILE PATIENT-REPORTED OUTCOME MEASURE

Ann Gruber-Baldini,[1] G. Jay Unick,[2] Veronica Fallon,[3] Philip Heiser,[3] Kimberly Bambarger,[3] and Lisa Shulman,[3], *1. University of Maryland School of Meidcine, Baltimore, Maryland, United States, 2. University of Maryland, Baltimore, Baltimore, Maryland, United States, 3. University of Maryland School of Medicine, Baltimore, Maryland, United States*

PROMIS® measures provide valid assessment of patient-reported outcomes, but have not been validated in older adults (especially aged 80+), including those with cognitive impairment. The objective of this project was to study how age-related role change effected the understanding of items on the PROMIS-57 Profile using cognitive interviews. Cognitive interviews were conducted with 38 adults, age 65+ with MoCA scores 10-30. Preliminary codes were created and then codes were added or modified as needed. Each interview was coded independently by two coders with differences resolved by consensus. The sample was 47% age 80+, 45% female, 18% African American, and 32% had a MoCA score between 10-17 (cognitively impaired). Thematic analysis of codes indicated that participants endorsed little or no impairment when they adapt to physical or cognitive disabilities by using economic means, instrumental support, physical aids, or by reducing activities. One respondent using grocery delivery services described no difficulty running errands or shopping. Another respondent reported no difficulty walking 15 minutes because they use a cane. Some reported no difficulty engaging in social roles when they restricted their activities due to disability or lack of appropriate social activities. Age-related changes effected responses on PROMIS-57 items. Findings suggest that age-related changes bias individuals to indicate less physical and cognitive impairment than their actual level of function. Physical functioning items show more bias for individuals with financial or instrument support, and social role items show more bias for those with restricted social networks.

### INFLAMMATION AND COGNITIVE FUNCTIONING IN OLDER ADULTS: THE ROLE OF GENDER AND OBESITY

Melody Moloci Noss,[1] Summer Millwood,[2] and Kate Kuhlman,[1], *1. University of California, Irvine, Irvine, California, United States, 2. University of Denver, Denver, Colorado, United States*

Systemic inflammation is associated with steeper cognitive decline over time. Identifying potential moderators of inflammation is crucial for understanding inflammation's contribution to abnormal cognitive decline. This study examined whether inflammation predicted changes in cognitive functioning over time and explored the moderating effects of sex and BMI on this association. Data was collected from a longitudinal nationally representative data set. (Health & Retirement Study). C-reactive protein (CRP) and global cognitive functioning assessments were collected from the 2006/2008 and 2010/2012 waves. Participants, n= 7,483, Age =71.39 years (SD = 9.24) , 60.2% female, were categorized into groups based on BMI (i.e. normal, overweight, and obese). Sex and BMI significantly moderated the association between increased hs-CRP and lower cognitive functioning, b = -.22 (SE = .09), p = .017. Women with high BMI exhibit twice the risk of low cognitive functioning, b = -.49 (SE = .07), p < .0001, compared to men with high BMI, b = -.21 (SE = .08), p = .01. Men with normal BMI exhibited twice the risk of low cognitive functioning, b = -.49 (SE = .08), p < .0001, compared to women with normal BMI, b = -.24 (SE = .06), p = .0001. Inflammation and BMI are modifiable factors that may prevent or slow -down abnormal cognitive decline. Understanding the potentially sex-dependent role of adipose tissue in the impact of inflammation on cognitive function may be critical to understanding the pathogenesis of cognitive impairment late in life as well as identifying efficacious intervention targets.

### INTERACTIONS BETWEEN PLASMA AMYLOID AND AGING MARKERS TO DETERMINE CLINICALLY MEANINGFUL COGNITIVE DECLINE

Wan-Hsuan Lu, Kelly Giudici, Bruno Vellas, and Philipe de Souto Barreto, *CHU Toulouse: Centre Hospitalier Universitaire de Toulouse, Gérontopôle de Toulouse, Institut du Vieillissement, Midi-Pyrenees, France*

**Background:** Brain amyloidosis is a well-known pathological hallmark of Alzheimer's disease (AD) and can be early identified by measuring plasma amyloid-β (Aβ) status. Growing evidence implicates the biological mechanisms of aging, including chronic inflammation, mitochondrial dysfunction and neurodegeneration, in AD pathogenesis. This study aims to investigate the interactions between plasma Aβ status and aging markers on clinically meaningful cognitive decline.

**Methods:** This secondary analysis from Multidomain Alzheimer Preventive Trial (MAPT) enrolled 401 community-dwelling older adults (mean age ± SD: 76.7 ± 4.6 years) who had clinical dementia rating (CDR) scale as 0 or 0.5, and who had their plasma biomarkers measured: amyloidosis: Aβ42/40 ratio; inflammatory: tumor necrosis factor receptor type 1 (TNFR-1), interleukin-6 (IL-6), monocyte chemoattractant protein-1 (MCP-1), C-reactive protein (CRP); mitochondrial dysfunction: growth differentiation factor 15 (GDF-15); neurodegeneration: neurofilament light chain (NfL). Cognitive decline was determined by diagnosed dementia and worsening CDR status. Cox regression and moderation modeling were applied to examine the interrelationships between biomarkers and risk of cognitive decline.

**Results:** Among 401 participants, 43.9% were cognitive normal (CDR=0) and 56.1% were mild cognitive impairment (CDR=0.5) initially. After 3.3 ± 1.1 years of follow-up, 7.0% of population evolved dementia and 34.2% had worsening CDR status. GDF-15 and NfL presented prospective associations with incident dementia. However, risk of dementia associated with plasma Aβ did not change after considering the serum level of GDF-15 and NfL.

**Conclusion:** The markers of mitochondrial dysfunction and neurodegeneration did not partially explain the associations between plasma Aβ status and cognitive decline in older adults.

### LONGITUDINAL ASSOCIATION OF EXECUTIVE FUNCTION AND BALANCE IN COMMUNITY-DWELLING OLDER ADULTS

Jennifer Blackwood,[1] Reza Amini,[2] Gerry Conti,[2] Quinn Hanses,[3] Rebekah Taylor,[4] Deena Fayyad,[5] and Rachelle Naimi,[2], *1. University of Michigan--Flint, Linden, Michigan, United States, 2. University of Michigan-Flint, Flint, Michigan, United States, 3. University of Michigan - Flint, University of Michigan-Flint, Michigan, United States, 4. University of Michigan - Flint, Flint, Michigan, United States, 5. University of Michigan-Ann Arbor, Ann Arbor, Michigan, United States*

Declines in Executive Function (EF) are associated with balance in community-dwelling older adults with Mild Cognitive Impairment (MCI). While this has been examined in cross-sectional studies, no longitudinal studies describe change over time. The purpose of this study was to examine how performance on the components of the Short Physical Performance Battery (SPPB) are associated with EF in community-dwelling older adults who transition into MCI. This secondary data analysis employed eight years of data from the National Health and Aging Trends Study dataset (2011 – 2018) with 1,225 participants in all eight waves (balanced). EF was measured with the Clock Drawing Test and SPPB balance tests included side-by-side, semi-tandem, full tandem, and single leg stance with eyes open or closed. Longitudinal ordered logistic regression was used to examine associations between each balance measure and EF while controlling for comorbidity, function, depression, gender, age, and ethnicity. EF was significantly associated with tandem, semi-tandem, and single leg stance after controlling for covariates. One point increase in SPPB can reduce the risk of EF impairment by 8.2% (Odds Ratio (OR)=0.918, $p<0.001$). Among SPPB components, semi-tandem (OR=0.468) and side-by-side (OR=0.472) were the strongest predictors of EF impairment. Declines in both EF and balance performance occurred over an eight-year period in adults. This may reflect common neural processes shared between the cognitive and motor areas of the central nervous system. Best practice suggests screening both balance (tandem, semi-tandem, or single leg stance) and EF in the clinical assessment of community-dwelling older adults.

### LONG-TERM VISIT-TO-VISIT BLOOD PRESSURE VARIABILITY AND COGNITION: A SYSTEMATIC REVIEW OF OBSERVATIONAL STUDIES

Tomiko Yoneda,[1] Jamie Knight,[1] Amelia Zhang,[2] Graciela Muniz-Terrera,[3] and Andrea Piccinin,[1], *1. University of Victoria, Victoria, British Columbia, Canada, 2. University of Toronto, Toronto, Ontario, Canada, 3. University of Edinburgh, Edinburgh, Scotland, United Kingdom*

Existing literature suggests that in comparison to a single blood pressure (BP) measurement, or the mean of multiple recordings, BP variability (BPV) may reflect dysfunction in cardiovascular regulatory mechanisms, leading to compromised cognitive health. No systematic review has yet synthesized observational reports examining the association between cognition and long-term visit-to-visit BPV. In response, a comprehensive literature search was executed in December, 2019, and updated in December, 2020. Methodological approach was pre-registered (https://osf.io/vmnuq/). Of 1385 reports, 27 met eligibility criteria. Most executed secondary analyses using existing longitudinal datasets of older adults (N=21). Intervals between measurement occasions ranged from 30 days to four years, and follow-up ranged from 0.5-25 years. Most studies computed more than one index of BPV (range=1-6), and all included at least three BP recordings (range=3-12). Given extensive between-study variability in analytic approach (e.g., BPV and cognition treated as continuous and/or categorical variables; number of covariates ranged 0-18), our team determined that meta-analyzing the results would be inappropriate. Despite heterogeneity in study characteristics, the majority (85.2%) reported that systolic BPV (sBPV) was negatively associated with cognition; specifically, higher sBPV was associated with cognitive impairment (N=9), cognitive decline (N=6), and/or risk of dementia (N=5). Four studies also revealed higher sBPV in individuals with dementia compared to controls. Three studies reported no association, while one reported a positive significant association between BPV and cognition. Results were similar for diastolic BPV. Despite considerable heterogeneity in study characteristics, greater variability in visit-to-visit BP appears to be consistently associated with adverse cognitive outcomes.

### MANIFESTATIONS OF AGING IN VIRTUAL REALITY IMPLEMENTATION OF ROD AND FRAME TEST

Michal Adamski,[1] Miroslaw Latka,[2] Anna Latka,[3] and Bruce West,[4], *1. Wrocław University of Science and Technology, Wrocław, Dolnoslaskie, Poland, 2. Wroclaw University of Science and Technology, Wroclaw, Dolnoslaskie, Poland, 3. Opole Neuropsychiatric Hospital, Opole, Opolskie, Poland, 4. Army Research Office, Research Triangle Park, North Carolina, United States*

Senior adults' reliance on the visual frame of reference for spatial orientation is a manifestation of an age-related shift in cognitive style from field independence to field dependence. We implemented a virtual reality rod and frame test (VR-RFT) to assess visual field dependence (VFD) in n=39 young adults (20-30 years old) and n=43 seniors (60 years old and above). The subjects were asked to determine subjective visual vertical (SVV) for 19 angles of frame tilt (running from -45 degrees to 45 degrees in steps of 5 degrees). The strong VFD of seniors was manifested not only by the increased error in the determination of SVV (SVVE) but also in its distribution. For small and large frame tilt angles, seniors' SVVE skewness and kurtosis were greater than those of young adults. The SVVE median dependence on frame tilt may be accounted for with a phenomenological model whose two parameters describe the strengths of primary (P) and secondary (S) visual attractors which subjects use to infer SVV:

the edges of the frame and its imaginary diagonals. For young adults, these parameters were: PY=14.91 and SY=12.51. For seniors, we observed an over 50% increase in the strength of the primary attractor PS=26.31 while the strength of the secondary one was only weakly affected by aging: SS=13.74. We demonstrate that the asymmetry between the strength of attractors significantly contributes to SVVE made by seniors at large frame tilts. We hypothesize that a variant VR-RFT may be used in rehabilitation to reduce excessive VFD.

### PREDICTING QUALITY OF LIFE WITH PHYSICAL AND COGNITIVE FUNCTIONING AMONG OLDER ADULTS WITH COGNITIVE IMPAIRMENT

Rhayun Song, Moonkyoung Park, Jisu Seo, Yuelin Li, Fan Xing, and Ahyun Ryu, *Chungnam National University, Dae Jeon, Taejon-jikhalsi, Republic of Korea*

**Purpose:** Older adults experience abnormal declines in physical and cognitive functioning that increase their risk of dependence, subsequently quality of life. This study aims to explore the relationship between physical and cognitive functioning, and to predict quality of life among older adults with mild cognitive impairment.

**Methods:** Survey was conducted with older adults registered at dementia support centers. Seventy-four older adults signed the consent form and participated in the study. physical functioning consisted of grip strength, balance (OLS), Timed up and go, and activities of daily living. Cognitive functioning was measured by K-MOCA. SF-12 was used to assess quality of life.

**Results:** The participants was 76 years old on average, more women (75.4%), and mostly elementary or less education level (60.9%). Physical functioning explained 22.1% of variance in cognitive functioning after controlling for age and gender (F change=4.789, p=.002). Balance (OLS: t=2.304, p=.024) and grip strength (t=2.207, p=.031) was significant predictors. Physical and cognitive functioning explained 36.7% of variance in quality of life after controlling for age and gender (F =5.466, p<.001). Indicators of physical functioning, TUG (t=-3.252) and grip strength (t=-2.633), were the most significant predictors of quality of life, while cognitive function explained additional 3.1% of variance in quality of life (F=3.216, p=.078).

**Conclusion:** Physical functioning were significant predictors of cognitive functioning, subsequently to quality of life among older adults with cognitive impairment. Health promoting strategies should focus on improving physical functioning of this population to maintain or prevent cognitive declining, and to promote quality of life.

### RELATIONSHIP BETWEEN TYPE 2 DIABETES CONTROL AND COGNITION IN OLDER ADULTS: FINDINGS FROM NHANES

Rozmin Jiwani,[1] Sara Espinoza,[1] Jing Wang,[2] and Monica Serra,[3], *1. University of Texas Health Science Center San Antonio, San Antonio, Texas, United States, 2. University of Texas Health Science Center at San Antonio, San Antonio, Texas, United States, 3. UT Health San Antonio - Barshop Institute, San Antonio, Texas, United States*

Cognitive health has emerged as an important public health concern for America's aging population. Type 2 Diabetes (T2D) may be associated with an exacerbated decline in cognitive performance. This study aimed to examine the relationship between T2D control and cognitive performance in older adults (≥60 years) using the 2013-2014 National Health and Nutrition Examination Surveys. Participants who completed the following cognitive assessments were included: 1) Consortium to Establish a Registry for Alzheimer's Disease Word List (CERAD-WL), 2) Animal Fluency (AF), 3) Digit Symbol Substitution Test (DSST) (higher scores associated with better cognition). Participants were stratified by four groups: no T2D (N=557), treated/controlled T2D (controlled; N=41), treated/uncontrolled T2D (uncontrolled; N=120), untreated T2D (N=86), based on self-reported T2D treatment, fasting plasma glucose, and hemoglobin A1c. Logistic regression was used to examine the relationship between T2D control and cognition. We observed that those with uncontrolled and untreated T2D each had ~15% lower DSST than those with no T2D (P<0.01). CERAD-WL and AF were similar across all groups. Unadjusted analyses showed that individuals with 1) lower CERAD-WL were more likely to have controlled and untreated T2D, 2) lower AF were more likely to have controlled and uncontrolled T2D, and 3) lower DSST were more likely to have uncontrolled and untreated T2D (P's<0.05). After adjusting for significant demographics and cardiovascular risk factors, only having uncontrolled T2D was associated with lower DSST (β=-3.164, P=0.04). These data indicate the need for longitudinal studies to further explore dynamic relationship and causal pathway between T2D control and cognitive impairment.

### RISK FACTORS FOR DEMENTIA ONSET IN OLDER ADULTS WITH METASTATIC RENAL CELL CARCINOMA

Samuel Miller,[1] Lauren Wilson,[2] Melissa Greiner,[3] Jessica Pritchard,[4] Tian Zhang,[5] Deborah Kaye,[2] Harvey Cohen,[2] and Michaela Dinan,[6], *1. Yale University, Hamden, Connecticut, United States, 2. Duke University School of Medicine, Durham, North Carolina, United States, 3. Duke University, Durham, North Carolina, United States, 4. Duke University, Duke University, North Carolina, United States, 5. Duke Cancer Institute, Duke Cancer Institute/Durham, North Carolina, United States, 6. Yale University, New Haven, Connecticut, United States*

Renal dysfunction is a driver of dementia. It is also associated with renal cell carcinoma, possibly the result of the tumor itself or from cancer treatment. This study evaluates metastatic renal cell carcinoma (mRCC) as a risk factor for developing mild cognitive impairment or dementia (MCI/D) as well as the impact of RCC-directed therapies on the development of MCI/D. We identified all patients diagnosed with mRCC in SEER-Medicare from 2007-2015. The main outcome was incident MCI/D within one year of mRCC diagnosis or cohort entry. Exclusion criteria included age <65 at mRCC diagnosis and diagnosis of MCI/D within preceding year of mRCC diagnosis. Patients with mRCC (n=2,533) were matched to non-cancer controls (n=7,027) on age, sex, race, comorbidities and year. Cox proportional hazards regression showed that having mRCC (HR 8.52, 95% MCI/D 6.49-11.18, p<0.001) and being older (HR 1.05 for 1-year age increase, 95% MCI/D 1.03-1.07, p<0.001) were predictive of developing MCI/D. A second Cox proportional hazards regression of only patients with mRCC revealed that neither those initiating treatment with oral anticancer agents (OAAs) nor those who underwent nephrectomy were more likely to develop MCI/D. Black patients had a higher risk of dementia compared to white patients (HR 1.92, 95% MCI/D 1.02-3.59, p=0.047). In conclusion, patients with mRCC were more likely to develop MCI/D than those without mRCC. The medical and surgical therapies evaluated were not associated with increased incidence of MCI/D. The increased incidence of MCI/D in older adults with mRCC may be the result of the pathology itself.

### SOCIAL DISENGAGEMENT AND COGNITIVE FUNCTION: DOES THE ASSOCIATION VARY BY GENDER?

Bada Kang, Eunhee Cho, and Sarah Oh, *Yonsei University College of Nursing, Seoul, Seoul-t'ukpyolsi, Republic of Korea*

Although social disengagement is considered to be a predictor of cognitive decline, and increase risk of Alzheimer's and related dementias, little is known regarding the gender-specific association between social disengagement and cognition among Korean middle-aged and older adults. Korea's Confucianism-based gender roles provide unique contexts to examine gender differences in the influence of social disengagement on cognition. This study investigated the association between social disengagement and cognitive function in a nationally representative sample of Koreans aged 45 years or older (N = 5,196 women and 2,707 men), using data from the Korean Longitudinal Study of Aging (2008-2018). Results from the generalized estimating equation model showed that compared to consistent social engagement, consistent non-engagement was significantly associated with lower cognitive function among both genders. Transitioning from social engagement to non-engagement was significant for males only. Of various types of social activities (religious, senior center, sport, reunion, voluntary, political), consistent non-engagement in a senior center was most associated with lower cognitive function among both genders, while consistent non-engagement in religious activities was significant for females only. While household arrangements were not associated with cognition in men, widowed women had increased risk of cognitive decline than married women, as did women living in households of three or more people. Depression was a predictor of cognitive decline among males only. In this gender-specific study, we found that consistent participation in social activities, especially via membership in a senior community center, is beneficial in preventing cognitive decline among both genders.

### SUBCLINICAL CARDIAC DYSFUNCTION AND COGNITIVE FUNCTION: A SYSTEMATIC REVIEW

Mario Maalouf,[1] Raymond Farah,[2] Sola Bahous,[3] Kamal Matli,[3] Adina Zeki Al Hazzouri,[4] and Christy Costanian,[3], *1. Lebanese American University, Lebanese American University, Beyrouth, Lebanon, 2. Lebanese University, Beirut, Beyrouth, Lebanon, 3. Lebanese American University, Beirut, Beyrouth, Lebanon, 4. Columbia University, New York, New York, United States*

**Background:** Cardiovascular disease, and more recently, subclinical cardiac dysfunction have both been implicated as important risk factors for cognitive decline. Several measures have been used to detect subclinical cardiac dysfunction, with global longitudinal strain (GLS) emerging as an important and more sensitive indicator than traditional measures. Yet, the association of GLS with cognitive function remains relatively unexplored.

**Objective:** The aim of this review is to systematically summarize the literature exploring the association between GLS and cognitive function.

**Methods:** We conducted a systematic review of the literature following PRISMA guidelines using the following databases: PubMed, OVID Medline, Embase, Web of Science, and CINAHL. Inclusion criteria were observational studies published in English, measuring GLS and assessing cognitive function through neuropsychiatric tests or brain imaging. Quality assessment was done using the Newcastle Ottawa Scale.

**Results:** The initial search revealed 394 studies, of which three met inclusion criteria and were included for final review. The three studies included were cross-sectional and of high quality. They all reported that lower GLS scores were associated? with worse cognitive function and more brain abnormalities in both bivariate and multivariable analysis.

**Conclusion:** Subclinical cardiac dysfunction, identified by GLS, was associated with worse cognitive function and presence of cerebral abnormality on brain imaging. The underlying mechanism could be attributed to dysfunctional autoregulatory and microvascular processes occurring in the brain vasculature. Further longitudinal studies are needed to better delineate the relationship between GLS and cognitive function.

### THE ASSOCIATION BETWEEN LEISURE GAMES AND COGNITIVE FUNCTIONING AMONG OLDER ADULTS

Tyran Terada,[1] and Yeonjung Lee,[2], *1. University of Hawai`i, Honolulu, Hawaii, United States, 2. University of Hawai'i at Mānoa, Honolulu, Hawaii, United States*

Stimulating leisure games have been shown to offer cognitive stimulation among older adults. This cross-sectional study examined the association between word games (crossword puzzles and scrabble) / cards and games (games such as chess) and cognitive functioning among adults aged 65 years and older (n=3271). All data were collected from the Health and Retirement Study (2016). Results from the hierarchical regression models suggest that higher levels of participation in word games ($p<.01$) and cards and games ($p<.01$) predicted higher levels of cognitive functioning. In the final model, after controlling for age, gender, ethnicity, marital status, education, and income, a total variance of 31 percent was explained. All covariates were statistically significant except marital status. Word games ($\beta$=.117, p<01) and cards and games ($\beta$=.054, $p<.01$) had a significantly positive association with cognitive functioning. These findings suggest that participation in word games and cards and games are associated with cognitive functioning among older adults.

**THE EFFECTS OF MILD COGNITIVE IMPAIRMENT ON FALL SEVERITY IN OLDER ADULTS**

Megan Jones,[1] Sally Paulson,[2] Joshua Gills,[1] Anthony Campitelli,[1] Jordan Glenn,[3] Erica Madero,[3] Jennifer Myers,[3] and Michelle Gray,[1], *1. University of Arkansas at Fayetteville, Fayetteville, Arkansas, United States, 2. University of Arkansas at Fayetteville, Cincinnati, Ohio, United States, 3. Neurotrack Technologies, Redwood City, California, United States*

Falls affect more than 30% of older adults and are one of the leading causes of injury, hospitalization, and mortality in this populations. Mild cognitive impairment (MCI) is one of the risk factors for falls in older adults. The purpose of this study is to determine if older adults with MCI have increased fall severity than older adults without MCI. Participants (n: 81: age: 79 ± 6) completed a Montreal Cognitive Assessment (MoCA) and a Hopkins Falls Grading Scale, a tool used to grade the severity of falls on a scale of 1-4 (1 = loss of balance without fall; 4 = fall requiring hospital admission). Participants were categorized as having MCI (score <26: N: 44: age: 81 ± 6.4) or non-MCI (score ≥26: n: 37: age: 77 ± 6). Groups were analyzed using a one-way ANOVA in SPSS to compare the severity of falls within the previous 12 months. There were no differences between groups for fall grade 1 (p =.22) or fall grade 2 (p =.45). There was a significant difference between groups for fall grade 3 (p =.04) and fall grade 4 (p =.05) with the MCI group having more of these falls compared to the non-MCI group. Older adults with MCI had a higher number of falls requiring medical attention than older adults without MCI. Although falls are a risk in all older adults, those with MCI may be at higher risk of more injurious falls than older adults without MCI.

**VITAMIN D, INSULIN-LIKE GROWTH FACTOR, AND COGNITIVE PERFORMANCE: AGE AND SEX VARIATIONS**

Magdalena Tolea,[1] Stephanie Chrisphonte,[2] and James Galvin,[2], *1. University of Miami Miller School of Medicine, Boca Raton, Florida, United States, 2. University of Miami Miller School of Medicine, Miami, Florida, United States*

Vitamin D has been consistently linked to better cognitive function in observational studies. This impact may be due in part through its influence on neurotrophins. Whether the relationships between vitamin D, neurotrophins, and cognition vary based on biological factors such as age and sex is unclear. Using data from a sample of 400 community-dwelling older (mean age=75.3±9.4; 47% female) participants in a cross-sectional study of cognitive aging, we assessed relationships between plasma 25-hydroxy-Vitamin D and performance on a neuropsychological battery modeled after the UDSv3.0. Moderation by age and sex and the impact of vitamin D on the relationship between Insulin-like Growth Factor-1 and cognitive performance were assessed by linear regression stratified by sex and age (median split at 76y). We found vitamin D to be positively linked to global cognition (MoCA: β=0.095±0.025SE, p<0.001), working memory (Number Span Forward: β=0.017±0.007SE, p=0.011; Number Span Backward: β=0.016±0.007SE, p=0.028), episodic memory (Immediate Recall : β=0.089±0.027SE, p=0.001; Delayed Recall: β=0.047±0.015SE, p=0.002), attention and processing speed (Trail Making A: β=-0.365±0.163SE, p=0.026), executive function (Trail Making B: β=-0.537±0.215SE, p=0.014; Number-Symbol Coding: β=0.139±0.057SE, p=0.016), and an overall measure of cognitive function (z score: β=0.049±0.018SE, p=0.007). Most of these relationships were observed in women and younger older individuals (<76y). In addition, vitamin D increased the effect of IGF-1 on global cognition and memory by 13% and 8%, respectively. Our findings suggest that vitamin D-focused dementia prevention efforts would benefit if targeted to women and younger segments of the senior population and/or as an adjuvant to cognitive enhancement interventions.

### Session 9135 (Poster)

### COGNITION I

**AGE-RELATED DECLINE IN PRAGMATIC REASONING OF OLDER ADULTS**

W.Quin Yow,[1] Jia Wen Lee,[2] and Xiaoqian Li,[3], *1. Singapore University of Technology & Design, Singapore, Not Applicable, Singapore, 2. Singapore University of Technology & Design, Singapore University of Technology & Design, Not Applicable, Singapore, 3. Singapore University of Technology and Design, Singapore, Not Applicable, Singapore*

As speech is often ambiguous, pragmatic reasoning—the process of integrating multiple sources of information including semantics, ostensive cues and contextual information (Bohn & Frank, 2019)—is essential to understanding a speaker's intentions. Despite current literature suggesting that certain social cognitive processes such as gaze-processing (Slessor et al., 2014) appear to be impaired in late adulthood, it is not well understood if pragmatic reasoning decline with age. Here, we examined young adults' (aged 19-25; n=41) and older adults' (aged 60-79; n=41) ability to engage in pragmatic reasoning in a cue integration task. In Experiment 1, participants had to integrate contextual (participants and speaker knew there were two novel objects but the latter could only see one), semantic ("There's the [novel-label]" or "Where's the [novel-label]"), and gaze (speaker looked at the mutually-visible object) cues to identify the referent (Nurmsoo & Bloom, 2008). In Experiment 2, participants received contextual and semantic cues less gaze cue. In both experiments, the target referent object for "There" and "Where" trials was the mutually-visible object and the object the speaker could not see respectively. Overall, young adults outperformed older adults, even in the simpler two-cue Experiment 2 (ps<.006). While older adults were significantly above chance in "There" trials for both experiments as well as "Where" trials in Experiment 2 (ps<.05), they had specific difficulty in integrating three cues in "Where" trials, where a more sophisticated interpretation of the multiple cues was required (p=.42). Our findings provide important insights into an age-related decline of pragmatic reasoning in older adults.

### COMPARING WORKING MEMORY AND VERBAL LEARNING IN OLDER ADULT MUSICIANS AND NON-MUSICIANS

Caroline Galloway,[1] and Jessica Strong,[2], *1. University of Prince Edward Island, University of Prince Edward Island, Prince Edward Island, Canada, 2. University of Prince Edward Island, Charlottetown, Prince Edward Island, Canada*

Previous research suggests that learning or playing an instrument may benefit working memory and executive functioning. The literature also suggests vocal training or singing ability may increase proficiency in verbal learning and working memory. Despite the benefits of musical training, the underlying mechanisms remain unclear. Older adult participants (N=38, Mean age =70.2) provided their music training history and completed a cognitive test battery. Musicians were either instrumentalists and/or vocalists (N=24) or non-musicians (N=14). Independent t test analyses were run with the current modest sample size to compare scores in basic and complex attention and working memory (Digit Span Forward (DSF) and Digit Span Backwards (DSB, and Digit Span Sequencing (DSS)), and verbal learning and memory (California Verbal Learning Test-3 (CVLT)). Results found that musicians/singers had higher scores compared to non-musicians on DSS (t(32)= -1.96, Cohen's d =.72, p =.058) and on CVLT delayed raw scores(t(32)= -1.98, Cohen's d=.71, p=.056), both with a medium-large effect size. There were no significant differences found between musicians and non-musicians in DSF and DSB or on CVLT immediate recall/learning. The results suggest that musical training, either instrumental or vocal, may contribute to working memory and verbal memory in older adults. Both the Digit Span task and CVLT rely heavily on executive functioning ability, which may act as a mechanism or mediator between instrumental and vocal training and scores on these cognitive tasks.

### DOES HBA1C INFLUENCE THE RELATIONSHIP BETWEEN STRESS AND COGNITION? FINDINGS FROM THE VA NORMATIVE AGING STUDY

Austin Brockmann,[1] Carolyn Aldwin,[1] and Avron Spiro,[2], *1. Oregon State University, Corvallis, Oregon, United States, 2. Boston University, Boston, Massachusetts, United States*

Type 2 diabetes has increased in prevalence globally, with potential adverse effects on cognition. Both high levels of hemoglobin A1c (HbA1c) and stressful life events (SLEs) are associated with impaired cognitive performance, but few studies have examined their synergistic effects. The present study examined direct effects of stress and HbA1c on several cognitive outcomes, and whether HbA1c moderated the relationship between SLEs and cognition. Utilizing a sample of 527 older men from the VA Normative Aging Study (Mage = 74.3, SD = 6.5), stress was inversely related to MMSE, verbal fluency, and pattern recognition; HbA1c was only inversely associated with MMSE. The moderation model was supported only for pattern recognition ($\beta = 1.64$, $p < .05$), with stress having worse effects in those high in HbA1c. Stratifying analyses by age group (<75, 75+) showed that stress predicted cognition only in the young-old, while HbA1c was inversely related to cognition only in old-old participants. Further, these age-group analyses yielded different effects of demographics on cognition. In the young-old, age was consistently inversely related to all cognitive outcomes, but in the old-old only with MMSE and word list recall. Among the young-old, education was associated with only word list recall but improved performance for most scales among the old-old. Finally, HbA1c intensified the effect of stress moderation on verbal fluency only in old-old ($\beta = 2.78$, $p < .05$). In summary, stress was more important for cognition in the young-old, while education and health status were more important in the old-old.

### EFFECTS OF AGE AND SELF-PERFORMANCE ON MEMORY FOR THE ACTIONS OF OTHER PEOPLE

Alan Kersten,[1] Julie Earles,[2] and Jennifer Brymer,[2], *1. Florida Atlantic University, Boca Raton, Florida, United States, 2. Florida Atlantic University, Jupiter, Florida, United States*

This research tested whether performing an action themselves leads young and older adults to have difficulty remembering which of a number of other people had performed that same action. It also tested whether observing another person perform an action leads to false memory for self-performance of that action. Young adults and healthy older adults 62 to 88 years of age viewed videos of actors performing actions. After viewing some of the actions, participants were instructed to perform those same actions themselves. Participants were tested one week later on their memory for their own actions and for the actions of the actors in the videos. Older adults were more strongly influenced by self-performance than were young adults when asked whether the actor in a test item had performed the same action previously. Young adults performed better than older adults at discriminating the correct and incorrect actors in the videos, although both groups showed reduced discrimination for actions that they had also performed themselves. The two groups were equally likely to falsely remember having performed an action that had only appeared in the videos, but young adults were better able than older adults to correctly identify the actions that they had in fact performed. Older adults thus have greater difficulty than young adults at distinguishing self-performed actions from actions performed by other people. This suggests the existence of common representations for the actions of oneself and others that must be bound to identity information to specify the correct source of the actions.

### EVALUATION OF EPISODIC AND LEXICAL METAMEMORY AND EXECUTIVE FUNCTION IN HEALTHY OLDER ADULTS

Atsuko Hayashi, *Kobe University, Kobe, Hyogo, Japan*

In older adults, it is important to maintain awareness of memory as well as memory performance. However, it is not clear whether the awareness of episodic and lexical memory changes with age and is related to self-evaluation of memory and executive function. Here age-related changes and the relationship between metamemory, executive function, and metamemory scale were investigated. Healthy old (n=40) and young (n=34) groups participated in this study. In the episodic memory task, participants were asked to memorize ten Kanji words and to estimate the number of words they could recall after ten minutes. In the lexical memory task, they rated the likelihood that they could write a target Kanji word written in hiragana and then wrote them down. They were also asked to complete the metamemory in adulthood(MIA) and the position stroop task. In the episodic and lexical memory and the position stroop task and MIA subscales, the performances of the younger group were significantly better than those of the older group. In the episodic memory task, there were correlations between the metamemory and MIA subscales in both groups, but in the lexical memory task, only in the old group. No correlation was found between the results of both memory tasks and the stroop test. These results suggest that older people overestimate memory performances in the episodic and lexical memory tasks and metamemory performances may be associated with self-evaluation of memory. In addition, metamemory might not be related to frontal lobe function as shown in executive function tasks.

### FRAILTY AND PROCESSING SPEED PERFORMANCE AT THE CUSP OF MIDLIFE IN CATSLIFE

Maria Luna,[1] Shandell Pahlen,[2] Robin Corley,[3] Sally Wadsworth,[3] and Chandra Reynolds,[4], *1. University of California, Riverside, Riverside, California, United States, 2. University of California, University of California, California, United States, 3. University of Colorado Boulder, Boulder, Colorado, United States, 4. University of California Riverside, Riverside, California, United States*

Frailty is an important multi-domain measure of health status and aging. Processing speed (PS) performance may be predictive of later frailty among older adults, but the interrelation between frailty and PS at the cusp of mid-adulthood is unclear. Using data from the ongoing Colorado Adoption/Twin Study of Lifespan Behavioral Development and Cognitive Aging (CATSLife; N = 1213; Mean age = 33.22 years; SD = 5.0), we constructed a 24-item frailty sum score across anthropomorphic, objective health, and perceived health and engagement measures based on the Accumulation of Deficits model. PS was measured using the Colorado Perceptual Speed (CPS) and WAIS-III Digit Symbol (DS) tests. All mixed-effects regression models accounted for clustering among siblings, and covariates included sex, age, race, ethnicity, and educational attainment. Intraclass correlations (ICCs) [95% CI] for frailty among siblings, adjusted for sex and age, ranged from near zero for siblings in adoptive families, .13 [.08-.30] for nonadoptive siblings/fraternal (DZ) twins, and .44 [.40-.48] for identical (MZ) twins, suggesting possible heritable influences. Poorer PS performance was associated with higher frailty, but was significant for DS only (B: DS = -0.43, p =.005). Furthermore, the DS-frailty association was magnified by age (B: DSxAge = -0.06, p =.025), suggesting that the associations between processing speed and frailty may increase with age. These findings help elucidate the interrelationship between indicators of frailty and cognitive performance for adults approaching midlife, a salient and understudied period within lifespan development.

### HIGH COGNITIVE LOAD SITUATIONS WITH DIFFERENT CONVERSATION TOPICS AFFECT WALKING SPEED AND COGNITIVE COMPLEXITY

Hyeon Jung Kim,[1] Julie Blaskewicz Boron,[1] Jennifer Yentes,[2] and Dawn Venema,[3], *1. University of Nebraska Omaha, Omaha, Nebraska, United States, 2. University of Nebraska at Omaha, University of Nebraska at Omaha, Nebraska, United States, 3. University of Nebraska Medical Center, Omaha, Nebraska, United States*

Walking and talking on the phone are common high-cognitive-load-situations (HCLS; e.g. dual-tasks), requiring extra attentional allocation and increasing perceived stress. We explored whether two load types, 1) single-task (ST) walking or talking on a phone and 2) HCLS walking while talking on a phone, influenced walking and/or cognitive performance among young (n=7; age=23.00±2.08yrs), middle-aged (n=14; age=44.79±7.42yrs), and older (n=15; age=74.47±3.91yrs) adults while controlling for perceived stress. Participants completed 3-minute trials of single-task walking (ST-W), single-task phone conversations with common (e.g., weather; ST-C) and uncommon topics (e.g., life experience; ST-U), and walking while talking on a phone (HCLS-C and HCLS-U). Walking speed was analyzed with 3(ST-W;HCLS-C;HCLS-U) x 3(Age) ANCOVA. HCLS resulted in slower walking speed (p<.001). Older adults exhibited slower speed across conditions compared to young (p=.015). Cognitive complexity (i.e., conversational tone and words greater than six letters (SIXLTR)) on the Linguistic Inquiry and Word Count (LIWC) were analyzed with 2(Cvs.U) x 2(STvs.HCLS) x 3(Age) ANCOVAs. Older age was associated with less cognitive complexity; positive tone (p=.014) and SIXLTR (p=.016), respectively in conversations. Uncommon topics reduced positive tone (p=.022) and SIXLTR (p=.003). Effects of HCLS on tone (p=.040) and SIXLTR (p=.005) varied with age. HCLS with different conversation topics resulted in reduced walking and cognitive complexity while controlling for perceived stress. The analysis of cognitive complexity using common/uncommon conversation topics is a novel method to assess the impact of HCLS. This research will disrupt the transformation of aging leading to a better understanding of attentional allocation and its effects on function.

### INTERACTIVE EFFECTS OF AGE AND INFLAMMATION ON CHANGE IN ECOLOGICALLY-ASSESSED COGNITIVE FUNCTIONING

Christopher Engeland,[1] Erik Knight,[2] Martin Sliwinski,[3] and Jennifer Graham-Engeland,[1], *1. Penn State University, University Park, Pennsylvania, United States, 2. University of Colorado Boulder, Boulder, Colorado, United States, 3. The Pennsylvania State University, University Park, Pennsylvania, United States*

Inflammation has been implicated as a precursor to steeper declines in age-associated cognitive decline. Here we investigated biomarkers of peripheral inflammation [basal cytokines, stimulated cytokines (ex vivo), C-reactive protein (CRP)] as moderators of age-related changes in cognitive functioning. As part of the Effects of Stress on Cognitive Aging, Physiology, and Emotion (ESCAPE) study, participants (N = 233; 65% female; 63% Black, 25% Hispanic; 25-65 years of age) completed up to four instances of ambulatory cognitive testing per day across two weeks, over three waves of annual assessments. After each 2-week ecological momentary assessment (EMA) burst, blood was collected and assayed for inflammatory biomarkers. Performance on spatial working memory (mean Euclidean distance errors), processing speed (mean symbol search reaction time), and working memory (n-back test accuracy) tasks were averaged across all instances within an EMA burst. CRP and age interactively predicted change in spatial working memory (B = 0.003, [0.000, 0.005], t(133.60) = 2.350, p = 0.020) such that higher CRP at older ages (~60 years) was associated with a loss of the expected practice effects across waves; at younger ages, CRP did not relate to change in spatial working memory. In a similar fashion, basal (B = -0.002, [-0.004, -0.000], t(103.26) = -2.399, p = 0.018) and stimulated cytokine levels (B = -0.002, [-0.004, -0.000], t(126.65) = -2.183, p = 0.031) interacted with age to predict change in processing speed across waves. These results indicate that inflammation may be critically associated with changes in cognitive functioning in older mid-life adults.

### INTERGENERATIONAL SOCIOECONOMIC MOBILITY AND COGNITIVE IMPAIRMENT IN CHINESE OLDER ADULTS: DOES GENDER MATTER?

Rong Fu,[1] and Yujun Liu,[2], *1. Siena College, Loudonville, New York, United States, 2. Northern Illinois University, Naperville, Illinois, United States*

The prevalence of dementia among older adults in mainland China is projected to increase rapidly in the next few decades. This study aimed to examine the impact of intergenerational socioeconomic mobility on the risk of cognitive impairment in a cohort of Chinese older adults, with a focus on potential gender differences. Data were derived from the 2011 wave of the Chinese Longitudinal Healthy Longevity Survey. Socioeconomic mobility in this study includes three dimensions: occupational mobility, educational mobility, and residential mobility. Cognitive impairment was assessed using the Chinese version of Mini-Mental State Examination. The final sample included 6,233 older adults aged 80 years and above. Logistic regression models were performed to assess the impact of the three dimensions of socioeconomic mobility on the risk of cognitive impairment in older men and women. For men, those with stable high occupational status across generations had the lowest risk of cognitive impairment. For women, those who received no education and lived in rural areas across generations had the highest risk of cognitive impairment. These findings lend support to the cumulative risk theory, which highlights the accumulation of risk factors that places individuals in jeopardy for negative health consequences in later life. The findings have implications for advancing supportive policies and practices related to maximizing the benefits of education and occupation for cognition in later life, especially for women in rural China.

### INVOLVEMENT IN DECISION-MAKING FOR DAILY CARE AND COGNITIVE DECLINE AMONG OLDER ADULTS WHO NEED CARE IN JAPAN

Ayane Komatsu,[1] Takeshi Nakagawa,[2] Taiji Noguchi,[2] and Tami Saito,[2], *1. Department of Social Science, Research Institute, National Center for Geriatrics and Gerontology, Obu, Aichi, Japan, 2. National Center for Geriatrics and Gerontology, Obu, Aichi, Japan*

Effective decision-making regarding daily care for older adults with needs could reduce the risk of dementia by preventing loss of motivation and improving care quality. However, empirical studies are scarce, particularly in non-Western countries with different socio-cultural backgrounds. By using 2-year longitudinal data of older Japanese adults aged 65 years and above who were receiving care at home, as well as of their family caregivers, we examined the association of involvement in decision-making with the onset of cognitive decline among older Japanese adults requiring care. The analysis included 219 cases of individuals with normal cognition and no missing variables at baseline and responded to the follow-up survey. An MMSE score of 23 or lower at follow-up was defined as the onset of cognitive decline. The level of involvement in decision-making was assessed by one item and dichotomized (not involved/involved). The covariates were age, gender, education, MMSE score, eligibility level for long-term care, and others at baseline. At baseline, 67.1% were 75 years or older, 58.9% were female, and 91.8% responded being "involved" in the decision-making. The incidence of cognitive decline at follow-up was 30.6%. The multivariable logistic regression analysis showed that involvement in decision-making (OR=0.298 [95% CI: 0.10-0.88], p=0.029) was negatively and significantly associated with the onset of cognitive decline. Our findings show the importance of involvement in the decision-making for daily care to reduce the risk of subsequent cognitive decline in older adults requiring care, even in a culture of familism.

### IS SUSCEPTIBILITY TO DEFAULT EFFECTS ASSOCIATED WITH AGE?

Julia Nolte, and Corinna Loeckenhoff, *Cornell University, Ithaca, New York, United States*

Older adults are more likely to avoid making decisions than younger adults are. Because the underlying reasons are poorly understood, the present study investigated the potential role of age differences in susceptibility to default effects. Defaults facilitate decision avoidance because decision makers are more likely to passively accept than to actively reject pre-selected default options. A representative lifespan sample (N = 500, Mage = 49.90, SDage = 19.34, 51% female, 67% non-Hispanic White) responded to a pre-registered online study. Participants completed one default effect task comprising two scenarios, one requiring opt-out and one requiring opt-in decisions (i.e., 15 vs. 0 pre-selected features each). Susceptibility to defaults was assessed through the discrepancy between scenarios. In addition, we collected data on known determinants of default effect compliance (i.e., perceived endowment, endorsement, ease, importance of the choice, and experience making similar choices) as well as post-decisional affect. Finally, participants responded to assessments of demographic background, personality, socioemotional and health status, and cognitive ability. Susceptibility to default effects was evident both at the individual and the group level (i.e., across and within scenarios). Unlike hypothesized, older age did not predict greater susceptibility, and older adults were less rather than more likely to endorse determinants of default effect compliance. Of the covariates assessed, only identifying as non-Hispanic White, greater perceived endorsement, and greater perceived ease predicted decision makers' susceptibility to default effects. Thus, results did not support our assumption that age differences in decision avoidance might reflect age-related increments in the acceptance of decision defaults.

### MUSIC ENGAGEMENT AND EPISODIC MEMORY AMONG MIDDLE-AGED AND OLDER ADULTS: A NATIONAL LONGITUDINAL ANALYSIS

Hillary Rouse,[1] Gizem Hueluer,[2] Mia Torres,[3] Yan Du,[4] Kyaien Conner,[2] and Hongdao Meng,[2], *1. University of South Florida, TAMPA, Florida, United States, 2. University of South Florida, Tampa, Florida, United States, 3. University of South Florida, University of South Florida, Florida, United States, 4. School of Nursing, University of Texas Health Science Center at San Antonio, San Antonio, Texas, United States*

Recent research suggests that engagement with particular activities, such as music, can influence age-related changes in episodic memory. However, it is unclear whether, and to what, extent music engagement is associated with the trajectory of episodic memory. The objective of this study is to examine how passive (i.e., listening to music) and/or active (i.e., singing or playing an instrument) music engagement influences episodic memory over a period of 12 years. Secondary data analysis of a sample (N=5095) of cognitively healthy adults from the Health and Retirement Study were used for this study. Linear mixed effects models were used to examine the independent effect of different levels of music engagement (i.e., low, medium, and high) on changes in performance on episodic memory tasks, while controlling for confounding factors. Compared to those with low engagement (i.e., neither listening nor singing/ playing an instrument), respondents who reported being engaged at the medium (i.e., either listening or singing/ playing an instrument) or high (i.e., both listening and singing/ playing an instrument) level performed 0.24 (p=0.003) and 0.52 (p<0.001) points better, respectively. We found evidence that music engagement attenuated the decline in episodic memory. The findings suggest that music engagement may be a protective factor against aged-related decline in episodic memory. Therefore, music engagement may offer a promising non-pharmacological intervention for dementia risk mitigation among community-living middle-aged and older adults. Future research should examine whether interventions to increase music engagement can affect the trajectories of aged-related decline in cognition in this large and growing population.

### NEUROANATOMICAL MODERATORS OF THE IMPACT OF MILD BEHAVIORAL IMPAIRMENT ON COGNITION

Hillary Rouse,[1] and Brent Small,[2], *1. University of South Florida, TAMPA, Florida, United States, 2. University of South Florida, Tampa, Florida, United States*

Older adults with mild behavioral impairment (MBI), or the presence of late-life neuropsychiatric symptoms, have a unique cognitive phenotype. However, the neural correlates associated with MBI-related cognitive changes is not well understood. The goal of this study is to examine if specific regions of the brain moderate the relationship between the presence of MBI and performance on tasks of cognition. Data from the National Alzheimer's Coordinating Center was utilized for this study. Participants (N=1,451) were included in our analyses if they were cognitively healthy or had mild cognitive impairment (MCI). Multiple domains of cognitive performance were evaluated. The neuroanatomical regions included hippocampus, caudal anterior cingulate (ACC), rostral ACC, entorhinal, and parahippocampal gray matter volume; and caudal ACC, rostral ACC, entorhinal, and parahippocampal mean cortical thickness. Hippocampal, entorhinal, and parahippocampal cortical gray matter volume moderated the relationship between MBI and performance on tasks of episodic memory. Left rostral ACC cortical gray matter volume and entorhinal and parahippocampal mean cortical thickness moderated the relationship between MBI and performance on language tasks. Hippocampi cortical gray matter volume also moderated the relationship between MBI and performance on processing speed tasks. Persons with smaller brain sizes in these areas were more negatively affected in these cognitive domains if they had MBI. These results suggest that the association between smaller brain volumes and cognition was stronger among persons with MBI. These findings suggest that older adults with MBI may perform worse on these tasks due to neurodegeneration that is present.

### PERSON, PLACE, AND TIME EFFECTS ON COGNITIVE FUNCTION AMONG OLDER PEOPLE IN TAIWAN

Hui-chuan Hsu, and Chyi-Huey Bai, *Taipei Medical University, Taipei, Taipei, Taiwan (Republic of China)*

**Purpose:** Individual's factors across time or combined with area characteristics related to cognitive function for older people have been widely explored, but little research examined person, place, and time effects altogether. The purpose of this study was to examine the effects of individuals, cities, and time on older people's cognitive function in Taiwan.

**Methods:** A nation-representative longitudinal individual data were from Taiwan Longitudinal Survey on Aging (TLSA) 1999-2015 panel data (analysis sample n=6349 persons, observations=12042). Cognitive function was scored 0-19. Individual's factors included demographics, health conditions and health behaviors, mental health and stress, social support and social participation, etc. Eleven city-level indicators were based on 22 cities and data were from the government open data sources. Mixed linear modeling analysis was applied.

**Results:** Better cognitive function was significantly related to individuals' working, ethnicity, younger age, better education level, better self-rated health, less psychological stress, receiving more emotional support, having higher economic satisfaction at the intercept. Sex, ethnicity, age, education, self-rated health, physical function, and social connectedness were significant at the time slope. When controlling for individuals' factors, population density and green land were significant at the intercept and at the time slope. Interactions of individual- and city-level factors were not significant.

**Discussion:** Individual's social participation and social support are protective factors of cognitive function for older adults. And an age-friendly environment providing appropriate cognitive stimulation and chances of social participation may be beneficial for cognitive function.

### RACE-DISCORDANT SCHOOL ATTENDANCE AND COGNITIVE FUNCTION IN LATER LIFE

Dawn Carr, and John Reynolds, *Florida State University, Tallahassee, Florida, United States*

Early schooling plays an important role in shaping cognitive development, both due to the level of academic rigor and the social environment of primary and secondary schools. This is reflected in current racial disparities in cognitive function in later life. Older minorities who attended predominantly White schools with more resources experienced significant cognitive benefits. This study explores whether there are benefits to cognitive functioning in later life from having attended socially diverse schools in early life. We examine the effects of having attended schools composed primarily of different race peers—race discordant schools (RDS)—among Black, Hispanic, and White older adults. Using retrospective and prospective data from the Health and Retirement Study, we examine the association between RDS exposure and four measures of cognitive function (working memory, episodic memory, mental status, overall cognitive function). We assess function at age 55 and 70, and examine change in functioning between age 55 and 70. We find that RDS exposed Blacks and Hispanics experience significant benefits in cognitive function at age 55, but only Blacks experience benefits at age 70. RDS exposed Whites reported higher overall working memory at age 70 relative to Whites in non-RDS schools, suggesting a cognitive benefit from diversity. Results suggest that exposure to more racially diverse school environments have potentially beneficial effects on cognitive function over the life course. Our findings suggest that the cultivation of diversity in schools could be an important long-term public health investment.

### RELATIONSHIP OF CANNABIS USE WITH IMMEDIATE, DELAYED, AND WORKING MEMORY: THE HEALTH AND RETIREMENT STUDY

Madison Maynard, Daniel Paulson, Michael Dunn, and Robert Dvorak, *University of Central Florida, Orlando, Florida, United States*

Past research has examined relationship between cannabis use and cognition among adolescents and young adults, but less is known about older adults despite rapidly increasing recreational and therapeutic cannabis use by this demographic. These relationships were explored cross-sectionally using data from the 2018 wave of the Health and Retirement Study (HRS). Dependent variables included immediate and delayed memory (10-item word list) and working memory (serial sevens; range 0-5). Cannabis use was categorized as non-user (n=886), past-user (n=334), current moderate (<52 uses/year; n=36), and current heavy (52+ uses/year; n=92). Mean age was 67.59 years (range: 50-98, SD=10.76). The sample was predominantly female (59%), and Caucasian (67%). Uncontrolled analyses found that cannabis use group was associated with immediate memory (F=6.14, p<.001), delayed memory (F=3.75, p=.01), and working memory (F=6.91, p<.001). Analyses controlled for gender, education, age, and race found that cannabis use group was no longer associated with delayed memory (F=1.74, p=.16) or working memory (F=1.66, p=.17); however, cannabis use was associated with immediate memory (F=3.75, p=.01) in controlled analyses. Current heavy users' (M=4.94, SE=.16) immediate memory worse than that of both non-users (M=5.48, SE=.06) and past users (M=5.49, SE=.09; p<.05 for both). Gender, education, age, and race significantly associated with immediate, delayed, and working memory, respectively (p<.05 for all). In conclusion, relative deficits in immediate memory, but not delayed memory or working memory, were associated with current heavy cannabis use among older adults. In combination with other findings, these results may inform development of safe-use guidelines for older adults.

### TECHNOLOGY SUPPORTING COGNITIVELY IMPAIRED OLDER ADULTS: A SCOPING REVIEW FOR THE ENHANCE CENTER

Edie Sanders, Robin Stuart, Alexander Exum, and Walter Boot, *Florida State University, Tallahassee, Florida, United States*

Cognitive impairments (CIs) result in difficulties with a wide range of daily activities. Older adults are especially at risk for CIs, and as the older adult population increases, so does the importance of understanding and supporting the needs of those with CIs. The Enhancing Neurocognitive Health, Abilities, Networks, and Community Engagement (ENHANCE) Center was established with a focus on developing technology-based support for socialization, transportation, and prospective memory needs of older adults with CIs due to mild cognitive impairment (MCI), traumatic brain injury (TBI), and stroke. The extent to which relevant literature in these domains existed was unknown. We conducted a scoping review to identify existing research meeting the following criteria: participants aged 50+ years classified as having a CI due to MCI, TBI, or stroke; and a focus on technology-based support for socialization, transportation, and/or prospective memory needs. Using PRISMA guidelines, we searched three electronic databases, and reviewers screened citations for inclusion and completed data charting. Following screening, only 11 studies met our inclusion criteria. Qualitative and quantitative data are reported for each study. In addition to few studies available, it was common for studies to include 20 or fewer participants. Most assessed technology interactions at one time and few studies examined longitudinal use and benefits. While each paper examined one aspect of user-centered design, no technologies were reported that underwent all stages of the user-centered design process, from needs assessment to iterative design and usability testing, to efficacy trial. Such gaps highlight the important role ENHANCE can play.

### THE RELATIONSHIP BETWEEN RELIGIOSITY AND COGNITIVE FUNCTION AMONG CHINESE OLDER ADULTS IN CHICAGO

Jacob Koppel Egierd,[1] Stephanie Bergren,[2] Lisa Lanza,[3] XinQi Dong,[3] and Dana Dytchwald,[3], *1. Rutgers University New, Monroe Township, New Jersey, United States, 2. Rutgers University, New Brunswick, New Jersey, United States, 3. Rutgers University, Rutgers Institute for Health, New Jersey, United States*

Evidence suggests religiosity may be related to cognitive decline in older adults living in the US and China. However, the relationship between religiosity and cognitive function has not been tested in a Chinese community in the US. Immigration and isolation often cause diasporas to differ from communities where they currently reside and their origin. This study aims to determine the relationship between religiosity, cognitive function, and demographic attributes in a sample of older Chinese adults age 60 to 105

living in the Chicago area (N = 3157). Regression analysis showed participation in organized religion significantly predicted higher global cognitive function ($\beta = 0.031$, $p < 0.001$, N = 3051). Of all cognitive function measures including episodic memory (East Boston Memory Immediate and Delayed Recall Test), perceptual speed (Symbol Digit Modalities Test), working memory (Digit Backwards Test), cognitive impairment (Mini Mental State Examination), and a composite measure of (global cognition), the importance of religion only significantly predicted greater working memory capacity ($\beta = 0.045$, $p = 0.003$, N = 3058). Practicing religion at home had a nonsignificant relationship with all measures of cognitive function. All analyses controlled for the following covariates: gender, education, income, number of children, marital status, and health insurance coverage status. Findings suggest that among aspects of religiosity, organized religious involvement may have a positive association with higher cognitive function. Future research should explore between-population differences in the relationships of social factors, religiosity, and cognition function to determine what practices can best benefit older adults in various communities.

### TRANSCRANIAL BRAIN STIMULATION IMPROVES COGNITION IN OLDER ADULTS WITH DEPRESSION AND ANXIETY

Mathieu Figeys,[1] Esther Kim,[2] Ada Leung,[1] Jim Raso,[3] Hubert Kammerer,[4] David Rawani,[3] and Glenrose Geriatggric Research Team (Alberta Health Services),[3], *1. University of Alberta, Edmonton, Alberta, Canada, 2. University of Alberta, University of Alberta, Alberta, Canada, 3. Alberta Health Services, Edmonton, Alberta, Canada, 4. Alberta Health Services, Alberta Health Services, Alberta, Canada*

Older adults admitted to hospital for rehabilitation often have some degree of concomitant cognitive impairment, which may be a barrier to optimizing rehabilitation approaches. Transcranial direct current stimulation (tDCS), a type of non-invasive brain stimulation, delivers a low electrical current across the brain. The neuromodulatory effects of tDCS can be of therapeutic benefit and has been shown to augment cognitive functions in both healthy and clinical populations. This study investigated the effects of tDCS on cognition in older adult inpatients with depression or anxiety. It was hypothesized that anodal tDCS over the left dorsolateral prefrontal cortex would increase cognitive performance compared to a placebo group. Twenty adults between 65 to 86 years of age admitted to the Glenrose Rehabilitation Hospital with underlying depression or anxiety were recruited. Anodal (n=10) or sham (n=10) tDCS stimulation was administered at 1.5mA over 20 minutes, for 10-15 sessions based on participant availability. Cognitive assessments were administered before and after the tDCS protocol. Anodal tDCS stimulation resulted in significant gains on the Symbol Digit Modality Test, Trail Making Test Part A, and Forward Digit Span. This study demonstrated a tDCS-invoked cognitive enhancement in the domains of attention, information processing speed, and short-term memory processes. With the rapidly ageing population, tDCS may be a potential therapeutic option for cognitive enhancement and may be beneficial in ageing-related cognitive-disorders including mild cognitive impairment and dementia.

### VASCULAR ILLNESS, COGNITION, AND SUBJECTIVE AGING: EXAMINING THE VASCULAR HYPOTHESIS OF AGING

Victoria Dunsmore, and Shevaun Neupert, *North Carolina State University, Raleigh, North Carolina, United States*

Cognition relates longitudinally and cross-sectionally to physical and psychological health among older adults. The Vascular Hypothesis of Aging (Drewelies & Gerstorf, 2020) suggests that illnesses of a vascular nature (e.g., stroke, hypertension, severe varicose veins) negatively affect cognitive abilities. Awareness of age-related change (AARC) is also related to cognition. What is not known is whether the presence of a vascular illness and daily cognitive abilities interact to predict daily awareness of age-related changes. The purpose of this study is to examine the daily fluctuations of cognition, (i.e., memory failures) and their interaction with vascular illness to predict daily awareness of age-related changes. Data were analyzed from 104 participants (M age = 64.67, 60-90 years) who completed online self-report questionnaires. On Day 1, participants answered baseline questionnaires regarding presence of vascular illness, and on Days 2-9 completed measures regarding AARC losses and memory failures. Multilevel models revealed main effects of daily memory failures on awareness of age-related losses, such that on days with more memory failures, older adults reported more age-related losses. We also found a main effect for vascular illness, such that those with a vascular illness reported higher levels of daily age-related losses. We did not find a significant interaction between vascular illness and daily memory failures on daily reported age-related losses. Our results provide preliminary evidence that the vascular hypothesis of aging may also extend to perceptions of age-related changes. Future research could consider examining daily symptoms of vascular illness as they unfold over time.

### VOICE BIOMARKERS AS POSSIBLE INDICATORS OF COGNITIVE AGING

Elizabeth Mahon,[1] and Margie Lachman,[2], *1. Brandeis University, Waltham, Massachusetts, United States, 2. Brandeis University, Brandeis University, Massachusetts, United States*

There is emerging evidence that measures of voice prosody are related to diagnoses of Alzheimer's Disease and Related Disorders. The goal of this study was to examine whether voice prosody measures (pitch, pulse, voice breaks, jitter, shimmer, and amplitude) are also related to individual differences in normal cognitive aging. Data are from the Midlife in the United States Wave 2 (M2) and Wave 3 (M3) for 2693 participants (ages 42-92 at M3) who completed the M2 and M3 Brief Test of Adult Cognition by Telephone (BTACT) and had M3 voice recordings. Voice variables were measured from cognitive interviews using three cognitive tests and averaged to create a composite for each voice variable. Voice prosody was related to age, sex, education, and health, which were included as covariates. Older adults, men, and those with more health conditions had higher jitter and shimmer. Older adults, women, and those with higher education and better health had more voice breaks. Hierarchical regression models, controlling for the covariates, examined the voice composites as predictors of each cognitive measure at M3

and change over 9 years from M2 to M3. As hypothesized, higher jitter predicted lower performance and greater decline on memory, category fluency, and attention. Contrary to predictions, a lower number of voice breaks predicted worse performance and greater declines on all cognitive tests. The results suggest that voice biomarkers are related to cognitive performance and decline, and they may offer a promising approach for identifying early signs of cognitive impairment or dementia.

### WHY DOES COGNITIVE RESERVE ALIGN WITH MODERN WESTERN IDEALS OF SUCCESS? THEORETICAL & METHODOLOGICAL PROPOSALS

Douglas Hanes, *Stony Brook University, Stony Brook, New York, United States*

Cognitive reserve (CR) is a framework that investigates discrepancies between brain pathology and cognitive decline. In explaining why individuals with similar levels of brain pathology display different levels of functional impairment, CR research focuses on factors that resemble modern, Western ideals of success: greater education, professional achievement, a self-directed life, and physically and intellectually stimulating leisure time. This theoretical paper documents this alignment between CR and modern, Western ideals of success to hypothesize different mechanisms by which CR may operate. The focus in the CR literature has been on investigating and operationalizing the direct cognitive changes that come from intellectual cultivation, and the native abilities that are hypothesized to produce differences in both education and cognitive outcomes. This paper argues that an attention to CR's relationship to current definitions of success presents alternative hypotheses about the mechanisms by which CR operates. Specifically, the paper outlines two potential mechanisms and frames alternative means of studying them: First, does the accrual of CR simply follow from being successful in conventional ways because of the material benefits of wealth and stability that success brings? Second, does a lack of success carry cognitive risks solely because of material deprivation, or are there additional psychosocial penalties that come from living a non-normative life—especially when that is not of one's choosing? This paper proposes both cross-cultural and intersectional methods to begin to better understand the relationship between normative success and cognitive health.

### Session 9140 (Poster)

### COGNITION II

### ASSOCIATION BETWEEN DIET AND COGNITIVE PERFORMANCE IN ADULTS AGED 60 AND OVER: NHANES, 2011–2014

Md Towfiqul Alam,[1] Elizabeth Vasquez,[2] and Sandra Echeverria,[3], *1. University of North Carolina at Greensboro, Greensboro, North Carolina, United States, 2. University of Albany, University of Albany, New York, United States, 3. University of North Carolina at Greensboro, University of North Carolina at Greensboro, North Carolina, United States*

There is limited evidence examining associations between diet and cognitive performance (CP) in older adults. We used the 2011-2014 National Health and Nutrition Examination Survey to determine if meeting dietary recommendations was associated with CP in adults 60+ years of age. Diet was based on the healthy eating index (HEI) 2015 and categorized into quintiles (higher quintiles indicating healthier diet). CP was based on word list learning, animal naming, and digit symbol substitution test, with scores above 25th percentile indicating adequate performance. Multivariate logistic regression modeling was conducted and adjusted for potential cofounders. A total of 3,068 participants completed the CP tests. A slightly higher percentage of participants were female (54.0%), predominantly White (80.5%) and the largest percentage (54.7%) was 60 to 69 years of age. The mean HEI-2015 score (0-100) was 54.89 (SE = 0.56). High CP scores increased with healthier dietary quintiles. However, results were only significant (p for trend <0.05) for digit symbol substitution test when comparing those in the highest quintile (82.53%) to those in the lowest (70.23%). Compared with participants in the lowest quintile of HEI-2015, participants in the highest quintile had a two-fold increased odds of better digit symbol substitution test scores, after adjusting for confounders (Odds Ratio [OR]: 1.96, 95% Confidence Interval [CI]: 1.28-3.01). Results showed that meeting healthy diet recommendations is associated with improved digit symbol substitution test, a marker of attention, processing speed and executive function. Future research should consider the role of diet in older adults to improve cognitive performance.

### CHRONIC STRESS PATTERNS AMONG OLDER ADULTS AND ASSOCIATIONS WITH COGNITIVE FUNCTIONING

Kun Wang,[1] Zainab Suntai,[1] and Xiayu Chen,[2], *1. University of Alabama, Tuscaloosa, Alabama, United States, 2. University of Illinois at Urbana-Champaign, New York, New York, United States*

Chronic stress has been associated with several adverse cognitive outcomes, including impaired judgement, executive functioning, and memory. Chronic stress has also been linked to several neurological conditions, including Dementia and Alzheimer's. While several biomedical measures of stress exist, stress is often subjective, and research has shown that the ability to cope with stress-known as stress reactivity-is more indicative of stress burden that the actual stressor itself. As such, this study aimed to identify the association between different patterns of stress and cognition among older adults. Data were derived from the 2016 Health and Retirement Study, a nationally representative study of older adults aged 50 and older living in the United States. Latent class analysis was used to identify different classes of stress and hierarchical linear regressions were conduced to identify the associations between identified stress classes and cognition. The latent class analysis resulted in four stress classes: high stress, financial stress, secondary stress, and low stress. The sequential logistic regression models revealed that while high stress and financial stress classes resulted in cognitive decline, the significance was mitigated after controlling for health and body functioning factors. This suggests that older adults are experiencing stressors mostly from health impairments and interventions should target improved health management and financial support for health conditions as an indirect way of reducing disparities in cognitive functioning resulting from chronic stress.

### DEPRESSIVE SYMPTOMS MODERATE THE ASSOCIATION BETWEEN LIFETIME DISCRIMINATION AND INHIBITION AMONG OLDER ADULTS

Regina Wright,[1] and Desiree Bygrave,[2], *1. University of Delaware, University of Deleware, Delaware, United States, 2. Benedict College, Benedict College, South Carolina, United States*

Discrimination has been identified as a potentially modifiable environmental stressor that reduces cognitive function. As the burden of discrimination can extend from early to late life, understanding its role in cognition in late life is critical. Further, understanding the potential moderating influence of depressive symptoms, which are common among older adults, on the linkage between discrimination and cognition, may provide further insight into the potential patterns of psychosocial stress and negative affect that may promote cognitive decline and dementia. Thus, we sought to examine whether depressive symptoms moderate linear relations of lifetime discrimination to cognitive function in the domains of visuospatial, verbal, and working memory, executive function, and psychomotor ability, adjusting for age, sex, race, and education. Participants were 165 older adults (34% male) with a mean age of 68.43y. Participants completed a health screening, a battery of cognitive tests, a psychosocial assessment, and cardiovascular testing relevant to the larger study. Linear regression results showed a significant interaction between lifetime discrimination and depressive symptoms ($p<.05$) related to the Stroop interference score, a measure of inhibition. A probe of the interaction showed that greater lifetime discrimination was associated with better inhibition among participants with fewer depressive symptoms. This paradoxical finding is consistent with scant research that shows exposure to discrimination may heighten performance, and is more common among individuals that have achieved more, both educationally and vocationally. Greater depressive symptomatology may reduce this paradoxical association. Future research should explore this question both longitudinally and in a larger sample.

### EDUCATION DIFFERENCES IN OLDER ADULTS' PERFORMANCE ON ONLINE ASSESSMENTS OF INDUCTIVE REASONING AND VERBAL MEMORY

Grace Caskie,[1] and Abigail Voelkner,[2], *1. Lehigh University, Bethlehem, Pennsylvania, United States, 2. Lehigh University, Easton, Pennsylvania, United States*

Paper-and-pencil measures of inductive reasoning and verbal memory administered in-person are well-established methods for measuring cognitive ability in adults. However, given recent increases in the use of online surveys, particularly during the COVID-19 pandemic when in-person research with older adults became difficult, we investigated whether these cognitive measures could be administered effectively online and whether older adults' performance on these measures of inductive reasoning and verbal memory might differ by education level. Data were collected online between mid-May and mid-June of 2020 from 292 individuals aged 66-90 years ($M=69.1$, $SD=3.3$). The sample was primarily White (91%) and had more women (62%) than men; 83 participants had a graduate-level education (master's/doctoral degree), 101 had an associate's or bachelor's degree, and 108 had less than an associate's degree. Three measures of inductive reasoning (Number Series, Letter Sets, and Word Series) and two measures of verbal memory (Immediate Recall and Delayed Recall of a list of 20 words) were completed by participants on an online platform. One-way MANOVA found a significant main effect for education group on the inductive reasoning measures (Wilks' lambda=.93, $p=.001$). However, follow-up univariate ANOVAs indicated significant differences by education group only for Number Series, with Tukey post hoc tests showing that the graduate-level and college-degree groups performed significantly better than the group with less than an associate's degree. Factorial repeated-measures ANOVA found a significant decline between immediate and delayed recall ($p<.001$) and that this difference varied by education group ($p=.003$). Implications of these findings will be discussed.

### EDUCATIONAL MOBILITY THROUGH MARRIAGE AND RISK OF COGNITIVE IMPAIRMENT IN LATE LIFE

Rong Fu, *Siena College, Loudonville, New York, United States*

**Objectives:** Marriage represents a long-term intimate relationship involving high levels of interaction and shared resources. Education, as an inter-individual resource, may influence the health status of an individual and his/her spouse. The aim of this study was to assess the impact of educational mobility through marriage on the risk of cognitive impairment in older adults.

**Methods:** Data were derived from the 2014 wave of the Chinese Longitudinal Healthy Longevity Survey. The final sample included 1,396 married men and 671 married women aged 65 years and older. Cognitive impairment was assessed using the Mini-Mental State Exam (MMSE). The gender-specific effect of educational mobility on the risk of cognitive impairment was tested by logistic regression analyses.

**Results:** Older men who experienced downward educational mobility through marriage had a higher risk of cognitive impairment, when compared to their upwardly mobile counterparts. This association was not observed in women. Having more years of schooling protected both men and women from being cognitive impaired in late life.

**Discussion:** These findings provide further evidence that downward socioeconomic mobility through marriage is associated with adverse health outcomes. Yet, the impact of spousal education on health must be understood through the lens of gender. Potential mechanisms that may link spousal education to cognition over the life course were discussed, including health literacy, health behaviors, and household resources.

### EVENT CENTRALITY OF POSITIVE AND NEGATIVE AUTOBIOGRAPHICAL MEMORIES IN OLDER ADULTHOOD

Justina Pociunaite, and Tabea Wolf, *Ulm University, Ulm, Baden-Wurttemberg, Germany*

Centrality of an event (CE) is a characteristic denoting how important a life experience is to one's identity. Usually, positive memories are more central than negative ones in the community samples. Nevertheless, there is emerging evidence showing substantial individual differences in how one perceives CE. Especially regarding age, one could expect pronounced differences due to age-related changes in personal goals. In this study, we investigated how older adults differ from young and middle-aged adults. Apart from age, we tested whether personality traits such as neuroticism and openness to experience influence the CE ratings among age groups. The sample comprised of 363 German participants, age ranging from 18 to 89 (M=49.57, SD=17.087), 67.2 % of the sample were women. Using multilevel analysis, we found the CE of positive memories to be higher in all age groups. The CE of positive events significantly differed for older adults compared to younger adults but not to the middle-aged group. With respect to personality, neuroticism had an impact only on the CE of negative memories in younger and middle-aged adults. For older adults, neither neuroticism, nor openness to experience had an impact on CE ratings. This shows that while older adults significantly differ from younger adults in the CE of positive memories, other individual differences characteristics do not have an impact on the way older adults perceive memories as central to their identity.

### EVIDENCE FOR A SPECIFIC ASSOCIATION BETWEEN SUSTAINED ATTENTION AND GAIT SPEED IN MIDDLE-TO-OLDER-AGED ADULTS

Courtney Aul,[1] Hannah Park,[2] Joseph DeGutis,[3] On-Yee Lo,[4] Victoria Poole,[5] Jonathan Bean,[6] Elizabeth Leritz,[1] and Michael Esterman,[7], *1. VA Boston Healthcare System, Boston, Massachusetts, United States, 2. Brandeis University, Waltham, Massachusetts, United States, 3. VA Boston Healthcare System/Harvard Medical School, Boston, Massachusetts, United States, 4. Hebrew SeniorLife/Harvard Medical School, Boston, Massachusetts, United States, 5. Rush University, Chicago, Illinois, United States, 6. VA Boston Healthcare System, VA Boston Healthcare System, Massachusetts, United States, 7. VA Boston Healthcare System/BU School of Medicine, Boston, Massachusetts, United States*

Although cognitive decline has previously been associated with mobility limitations and frailty, the relationship between sustained attention and gait speed is incompletely characterized. To better quantify the specificity of the sustained attention and gait speed association, we examined the extent to which this relationship is unique rather than accounted for by executive functioning and physical health characteristics. 58 middle-to-older-aged community-dwelling adults without overt illness or diseases (45-90 years old, 21 females) participated in the study. Each participant completed a 4-meter gait speed assessment and validated neuropsychological tests to examine various domains of executive functions including working memory (i.e., Digit Span), inhibitory control (i.e., Stroop Color Word Test), and task switching (i.e., Trail Making Test). Multiple physical and vascular risk factors were also evaluated. Sustained attention was assessed using the gradual onset continuous performance task (gradCPT), a well validated go/no-go sustained attention task. A series of linear regression models were created to examine how different aspects of cognition, including sustained attention and traditional measures of executive functioning, related to gait speed while controlling for a variety of physical and vascular risk factors. Among all predictors, gradCPT accuracy explained the most variance in gait speed ($R2 = 0.21$, $p < 0.001$) and was the only significant predictor ($\beta = 0.36$, $p = 0.01$) when accounting for executive functioning and other physical and vascular risk factors. The present results indicate that sustained attention may be uniquely sensitive and mechanistically linked to mobility limitations in middle-to-older adults.

### EXECUTIVE FUNCTIONING, EMOTION REGULATION, AND NEGATIVE EMOTION IN SUICIDAL OLDER ADULTS

Rachel Waldman,[1] Brian Liles,[2] Dimitris Kiosses,[3] and Richard Zweig,[4], *1. Yeshiva University, New York, New York, United States, 2. Weill Cornell Medicine, White Plains, New York, United States, 3. Weill-Cornell Medicine, White Plains, New York, United States, 4. Yeshiva University, Bronx, New York, United States*

Deficits in executive functioning, emotion regulation, and negative emotion have all been linked to suicidality. Yet, the complex interactions between these three factors and their relationships to suicidal behavior in older adults remain unclear. Participants (N = 39) were depressed middle and older adult (M = 62.0, SD = 9.41) inpatients with recent suicidal attempt or ideation, without psychotic depression or moderate or greater cognitive impairment (DRS>90). Participants were administered measures of executive functioning (Stroop and COWAT), emotion regulation (ERQ Suppression and Reappraisal; RRS-Brooding; UPPS- Premediation Scale), and negative emotion (PANAS-X), in addition to measures of depression (MADRS) and suicidality (C-SSRS). Results indicated that executive functioning was not significantly related to emotion regulation or negative affect, but measures of emotion regulation were related to negative emotion and frequency of suicidal ideation in bivariate analyses. Lower ERQ reappraisal tended to be associated with negative emotion (ß = -.392, p = .067) in multivariate analyses. Although comparisons were non-significant, effect sizes revealed that those who experienced daily suicidal ideation (C-SSRS) had lower reappraisal and higher brooding scores (Cohen's d = 1.014 - 1.456), as well as higher executive functioning (Stroop Color-Word trial) and overall cognition (DRS) scores (Cohen's d = 0.625 – 0.792) than less frequent ideators. Findings suggest that older inpatients with frequent suicidal ideation have poorer emotion regulation but may have more intact cognition and executive functioning than those with less frequent suicidal ideation.

### INTERGENERATION BONDING: AN ACCEPTABILITY AND FEASIBILITY STUDY

Lauren Rezac,[1] Miechelle McKelvey,[2] and Ladan Ghazi Saidi,[2], *1. University of Nebraska at Kearney,*

*Kearney, Nebraska, United States, 2. University of Nebraska at Kearney, University of Nebraska at Kearney, Nebraska, United States*

Social isolation and loneliness are detrimental risk factors to older adult's physical and psychological well-being and quality of life. Given the current situation of the COVID-19 pandemic, social isolation has risen. Social isolation affects younger adults as well and may increase the chances of depression and anxiety. In this study, we tested the acceptability and feasibility of an Intergenerational Bonding (IGB) Program with older and younger adults. Participants include students aged 19-29 at the University of Nebraska at Kearney and older adults above the age of 60 residing in the community. First, we surveyed younger and older adults to determine their interest level in participating in an IGB Program. Both groups of participants rated their interest in different activities. The most popular activities among both groups included engaging in conversations, board games, and an exchange of skills. Neither of the groups was in favor of participating in free housing opportunities or sports-related activities. Response rates were high in young adults but low in older adults, due to lack of trust. Then, in a pilot study, we measured the feasibility and acceptability of the IGB Program. Older adults residing in independent dwellings, assisted living environments, nursing homes and members of community groups were invited to participate in the intergenerational program. Response rates of older adults were low. Further, establishing collaboration with institutions such as nursing homes was not easy despite initial interest. Building trust and working with community activity group facilitators would be beneficial in recruiting older adults.

### INTERNET USE AND COGNITIVE FUNCTIONING AT THE INTERACTION OF AGE AND GENDER AMONG OLDER ADULTS

Kun Wang,[1] Zainab Suntai,[1] and Xiayu Chen,[2], *1. University of Alabama, Tuscaloosa, Alabama, United States, 2. University of Illinois at Urbana-Champaign, New York, New York, United States*

The positive relationship between internet use and cognition has long been documented in the gerontology literature, and researchers are consistently finding that internet use engages the brain in a way that improves cognitive functions such as multitasking, information processing and executive thinking. While there are numerous studies examining this association, few studies have explored the three-way interaction between age, gender, and internet use on cognition. This study aimed to examine the gendered moderation effect of age on the relationship between internet use and cognition among older adults. The study sample was derived from the 2016 Health and Retirement study, which is a biennial longitudinal panel study of adults aged 50 and older in the United States. Multilinear regression models were used to examine the three-way interaction of age, sex and internet use on cognition while controlling for other covariates. Results showed that women gained a greater increase in cognition as a result of internet use as they became older, while men had the same amount of increase in cognition as a result of internet use regardless of age. This indicates that internet use can be a positive agent in improving cognition among older adults regardless of age and sex, and interventions should focus on increasing internet use among older adults, to ensure equitable access to the benefits of internet use on cognition.

### MIND-BODY PRACTICE, PERSONALITY TRAITS, AND COGNITIVE PERFORMANCE: A 10-YEAR STUDY IN US ADULTS

Kallol Kumar Bhattacharyya,[1] Gizem Hueluer,[1] and Debra Dobbs,[2], *1. University of South Florida, Tampa, Florida, United States, 2. University of South Florida, School of Aging Studies, University of South Florida, Florida, United States*

It is widely established that physical activity is associated with better cognitive outcomes, and accumulating evidence suggests that mind-body practice (MBP) may yield similar benefits. Personality is related to both daily activities and cognition, but its role in the association between MBP and cognition is not well understood. The current study examines bidirectional temporal associations between personality traits, MBP, and cognition in healthy adults. We used data from waves 2 and 3 (2004-2014) of the Midlife in the United States (MIDUS) study from a total of 2,050 individuals (age: M=64 years, SD=11, range=42 to 92; 56% women). We applied a cross-lagged regression analysis to examine bidirectional effects between MBP, Big Five personality traits, and two cognitive domains (episodic memory and executive function) and controlled for sociodemographic factors, health, and functional status covariates in wave 2. After controlling for covariates, MBP was independently associated with a more favorable change in episodic memory, but not in executive function. Regarding cross-lagged effects of cognitive function, episodic memory was related to subsequent MBP and agreeableness, and executive function was related to subsequent MBP, openness, and conscientiousness. Agreeableness had a negative effect on subsequent executive function. The findings point toward bidirectional associations between cognitive function MBP, while there was no evidence for cross-lagged associations between personality and MBP. Future research should guide us whether MBP can counteract cognitive decline as an alternative and complementary practice and the role that personality can play in such interventions.

### MODIFIABLE LIFESTYLE FACTORS ASSOCIATED WITH COGNITIVE RECOVERY FROM MCI: A 12-YEAR LONGITUDINAL STUDY

Bingyu Li, *Shenzhen University, Shenzhen, Guangdong, China (People's Republic)*

**Introduction:** Many studies have investigated the risk factors associated with progression from mild cognitive impairment (MCI) to cognitive impairment, while it is unclear which lifestyle factors are associated with cognitive recovery among those who have mild cognitive impairment.

**Methods:** The study includes 7,422 participants above 65 years old with MCI from The Chinese Longitudinal Healthy Longevity Survey (CLHLS). Cox regression analysis was adopted to investigate the association between cognitive recovery and selected lifestyle factors. LASSO was applied to select the variables.

**Results:** Daily consumption of fresh fruits is associated with higher possibility of cognitive recovery (HR: 1.28, 95% CI: 1.15-1.42) while daily consumption of meat show opposite influence (HR: 0.90, 95% CI: 0.80-0.99). Smoking (HR: 0.99, 95% CI: 0.98-1.00) and alcohol consumption (HR: 1.00, 95% CI: 0.99-1.00) are both negatively associated with cognitive recovery. Daily engagement in reading (HR: 1.24, 95% CI: 1.00-1.54), housework (HR: 1.21, 95% CI: 1.08-1.35) as well as mahjong and other card games (HR: 1.23, 95% CI: 1.08-1.39) are associated with higher possibility of cognitive recovery.

**Conclusion:** This study has identified important modifiable lifestyle factors associated with natural cognitive recovery from MCI. The findings have considerable implications for dementia prevention.

### MULTITASKING IN OLDER ADULTS' DAILY ACTIVITIES: A PRELIMINARY INVESTIGATION

Abigail Stephan,[1] Junyan Tian,[2] and Lesley Ross,[1], *1. Clemson University, Clemson, South Carolina, United States, 2. Penn State University, State College, Pennsylvania, United States*

The ability to multitask, defined as conducting two or more activities simultaneously, is important in daily life. The majority of prior work has examined multitasking in laboratory settings. However, less is known about how multitasking in daily activities is related to older adults' executive functioning and perceptions of aging. The current study investigated these relationships in a sample of 33 older adults aged 65-81 (M=70.0, SD=3.6). Participants were asked to describe activities they did each day and estimate time spent in each activity across fourteen days; multiple activities reported in the same time frame were considered multitasking. Executive function was measured at baseline using the Trail Making Test Part B (TMTB), with higher scores indicating worse performance. Expectations regarding aging were measured at baseline using the Expectations Regarding Aging (ERA-12) survey, with higher scores indicating more positive perceptions. Twenty-seven participants (81.82%) reported at least one instance of multitasking in the fourteen-day period. Participants were divided into three groups based on the median number of reported multitasks: no multitasking (n=6), low multitasking (≤4; n=15), and high multitasking (>4; n=12). Although there were no significant differences within the ANOVA, participants who reported low multitasking trended towards poorer executive function and more positive expectations of aging (M_TMTB=100.28, M_ERA= 64.88) than both no multitasking (M_TMTB=82.12, M_ERA=50.46) and high multitasking groups (M_TMTB=94.90, M_ERA= 54.29). Additional research should investigate these possible relationships in larger samples and explore how covariates, such as gender and age, may moderate possible relationships.

### MUSICAL EXPERIENCE RELATES TO FUNCTIONAL CONNECTIVITY IN OLDER ADULTS

Meishan Ai,[1] Timothy Morris,[1] Laura Chaddock-Heyman,[2] Psyche Loui,[1] Susan Whitfield-Gabrieli,[1] Charles Hillman,[1] Edward McAuley,[3] and Arthur Kramer,[1], *1. Northeastern University, Boston, Massachusetts, United States, 2. University of Illinois at Urbana-Champaign, Urbana, Illinois, United States, 3. University of Illinois at Urbana-Champaign, Boston, Massachusetts, United States*

Previous studies have shown that engaging in musical activities throughout the lifespan may buffer age-related decline in auditory and motor function, as well as in general cognitive function. MRI studies have demonstrated that individuals with musical training and experience exhibited greater grey matter volume and functional connectivity in extensive brain regions, especially in auditory and motor systems, compared to matched controls with no particular musical training or experience. Therefore, musical activity is a potential protective factor for brain health across lifespan. However, how lifespan musical experience shapes functional connectivity in older adults is still unknown. The current analysis investigated whether general musical experience (Goldsmith Music Sophistication Index) is associated with functional connectivity in older adults (age=65.7±4.4, n=69), focusing on seed regions in primary motor areas (bilateral precentral gyrus) and primary auditory regions (bilateral anterior/posterior superior temporal gyrus) and their functional connectivity towards other areas throughout the whole brain. We found that older adults with more musical experience showed greater functional connectivity between anterior superior temporal gyrus and insula (R2=0.10, p=0.01), and between posterior superior temporal gyrus and cerebellum (R2=0.08, p=0.02). However, musical experience and music-related functional connectivity was not significantly correlated with general cognitive functions in our sample. Overall, our findings suggest that older adults with more musical experience might be more efficient in some aspects of auditory processing and auditory-motor skills, but this may not transfer towards domain-general cognitive tests. Our results support the notion that even non-professional engagement in musical experiences may afford benefits to the aging brain.

### PERCEPTUAL VERSUS CONCEPTUAL VALUE-DIRECTED STRATEGIC PROCESSING IN YOUNGER AND OLDER ADULTS

Lydia Nguyen,[1] Elizabeth Lydon,[2] and Raksha Mudar,[3], *1. iN 2. L, Denver, Colorado, United States, 2. University of Illinois Urbana-Champaign, Champaign, Illinois, United States, 3. University of Illinois-Urbana Champaign, Champaign, Illinois, United States*

Value-directed strategic processing involves selectively attending to and processing information deemed more important while ignoring or inhibiting less important information. What we selectively attend to can be driven by the value we ascribe to the information, often based on stimulus factors such as perceptual features that make the information stand out, or conceptual features that make it easy to group information. The current study investigated whether behavioral measures of value-directed strategic processing are differentially affected when value is defined by perceptual versus conceptual features, and how normal cognitive aging impacts processing. Cognitively normal younger (N = 16; mean age: 22.1 ± 2.9 years) and older adults (N = 16; mean age: 66.9 ± 7.3 years) completed two value-directed strategic processing tasks, where value was defined by either perceptual (i.e., uppercase and lowercase letters; Letter Case task) or conceptual (i.e., animals and household items; Categories task) features. Both groups had higher recall on the Categories task compared to the Letter Case task, and higher recall for high- than low-value words. However, older adults recalled fewer total words than younger adults, but the groups did not differ across task types. These findings indicate that manipulating perceptual and/or conceptual features to define value can be used to study value-directed strategic processing in younger and older adults. Furthermore, grouping information based on conceptual features may be more effective for promoting subsequent recall in both younger and older adults.

### PHYSICAL ACTIVITY AND EPISODIC MEMORY: AN ANALYSIS OF LONGITUDINAL ASSOCIATIONS OVER 12 YEARS

Rio Tate, and Gizem Hueluer, *University of South Florida, Tampa, Florida, United States*

With the increasing prevalence of Alzheimer's and related dementias, it is becoming a growing public health concern to identify modifiable risk factors to prevent cognitive decline. Previous research suggest that physical exercise may promote cognitive function in aging. However, most of this research is based on experimental or cross-sectional studies and fewer studies have studied longitudinal associations over longer time frames. In the present study, we examined how physical activity is related to cognition in older adults. To do so, we applied multilevel models to data from 29,740 participants (age at baseline: M = 63 years, SD = 11 years, 50 to 102 years) from years 2004-2016, measured biennially (waves 7-13) of the Health and Retirement Study. Our findings showed that at the between-person level, those who practiced light, moderate, and vigorous- physical activity more frequently than others showed higher levels of episodic memory than others at the age of 70. At the within-person level, participants performed better than usual on a test of episodic memory on occasions when they reported more frequent physical activity of light, moderate and vigorous intensity. More frequent vigorous physical activity was related to less age-related decline in episodic memory, while the frequency of moderate physical exercise was unrelated to age-related change in memory. Contrary to our expectations, more frequent light physical exercise was related to more age-related decline in episodic memory. We discuss the implications of these findings for future research and practice.

### RACIAL AND ETHNIC DIVERSITIES IN COGNITIVE HEALTH APPRAISALS: FINDINGS FROM THE HCAP

Yuri Jang,[1] Eunyoung Choi,[2] Yujin Franco,[2] Nan Sook Park,[3] David Chiriboga,[3] and Miyong T. Kim,[4], *1. University of Southern California, Los Angeles, California, United States, 2. University of Southern California, University of Southern California, California, United States, 3. University of South Florida, Tampa, Florida, United States, 4. University of Texas at Austin, Austin, Texas, United States*

The aims of the study were: (1) to investigate the relationship between cognitive performance and cognitive health appraisals across non-Hispanic White, non-Hispanic Black, and Hispanic older adults in the United States and (2) to explore within-group variations by examining interactions between cognitive performance and background and health variables. The sample (N = 3,099) included 2,260 non-Hispanic White, 498 non-Hispanic Black, and 341 Hispanic adults aged 65 or older, from the 2016–2017 Harmonized Cognitive Assessment Protocol. Regression models of cognitive health appraisals, indicated by self-rated cognitive health, were examined in the entire sample and in racial and ethnic subgroups to test direct and interactive effects of cognitive performance, indicated by the Mini-Mental State Examination (MMSE). The regression model for the entire sample showed direct effects of cognitive performance and race/ethnicity on cognitive health appraisals, as well as a significant interaction between cognitive performance and being non-Hispanic Black. Cognitive performance and cognitive health appraisals were positively associated in non-Hispanic Whites but not significantly associated in non-Hispanic Blacks. Our subsequent analysis within each racial/ethnic group showed that the effect of cognitive performance in non-Hispanic Blacks and Hispanics became either reversed or nonsignificant when background and health variables were considered. Modification by age or chronic medical conditions in each racial and ethnic group was also observed. Overall, these findings suggest that perceptions and appraisals of cognitive health vary by race and ethnicity and hold implications for how these differences should be considered in research and practice with diverse groups of older adults.

### RETIREMENT AGE MODIFIES THE ASSOCIATION BETWEEN EDUCATION/OCCUPATION AND COGNITION

Monica Nelson,[1] and Ross Andel,[2], *1. University of South Florida, Brandon, Florida, United States, 2. University of South Florida, Tampa, Florida, United States*

**Introduction:** According to the cognitive reserve and use-it-or-lose-it hypotheses, engagement in stimulating activities seems to benefit cognition, with engagement often associated with more education or higher occupational position. However, whether retirement may modify the association between education/occupation and cognition is unclear. We aimed to assess how age at retirement may modify the relationship between education/occupation and cognition.

**Methods:** Older adults (n=360) from the Alzheimer's Disease Neuroimaging Initiative who were cognitively normal and retired at baseline participated. Linear regression was used to assess how educational attainment (high/low) or occupational position (managerial, intermediate/clerical, routine/manual) related to executive functioning (EF) or memory, controlling for age, sex, depressive symptoms, and health status. Effect modification by retirement (early, on-time, late).

**Results:** High education (EF: b=0.37, SE=0.08, p<.001; memory: b=0.22, SE=0.05, p<.001), intermediate (EF: b=0.26, SE=0.11, p=.019; memory: b=0.18, SE=0.08, p=.018) and managerial (EF: b=0.23, SE=0.12, p=.045; memory: b=0.16, SE=0.08, p=.045) occupations (compared to routine/manual occupations) were associated with better EF and memory performance. High education was significantly associated with better EF and memory for participants who retired early (EF: b=0.43, SE=0.12, p<.001; memory: b=0.29, SE=0.10, p=.004) or on-time (EF: b=0.51, SE=0.15, p=.001; memory: b=0.24, SE=0.10, p=.014), but not for participants who retired late (EF: b=0.19, SE=0.15, p=.200; memory: b=0.09, SE=0.09, p=.334). Intermediate occupations were associated with EF only for participants who retired on-time (b=0.58, SE=0.21, p=.007).

**Conclusion:** Education and occupational position may influence cognition after retirement differently based on retirement timing, with effects most apparent for on-time retirement and substantially reduced for late retirement.

### SEX DIFFERENCES IN SPEECH AND HIGH-FREQUENCY HEARING LOSS ASSOCIATION WITH COGNITIVE IMPAIRMENT AMONG OLDER ADULTS

Wang Jingru, Yu Ying, and Guo Qi, *Shanghai University of Medicine and Health Sciences, Shanghai, Shanghai, China (People's Republic)*

**Objectives:** The purpose of this study was to investigate the relationship between speech-frequency hearing loss (SFHL), high-frequency hearing loss (HFHL), and cognitive impairment (CI). Then to determine whether there are any differences in gender among older community dwellers in China.

**Methods:** This study involved 1,012 adults aged ≥60 years (428 male; average age, 72.61±5.51 years). The participants had their hearing and cognition measured using pure tone audiometry and Mini Mental State Examination (MMSE), respectively. We used the audiometric definition of hearing loss (HL) adopted by the World Health Organization (WHO). Speech-frequencies were measured as 0.5 kHz, 1 kHz, 2 kHz, and 4 kHz; high-frequencies were measured as 4 kHz and 8 kHz. Pure tone average (PTA) was measured as hearing sensitivity.

**Results:** Our studies demonstrated a 37.6% prevalence of HL in males and a 36.0% prevalence of HL in females. Adjusted for confounding variables, the results from a multivariate analysis showed that SFHL was associated with CI in females (OR=2.400, 95% Confidence Interval=1.313–4.385) and males (OR=2.189, 95% Confidence Interval=0.599–2.944). However, HFHL was associated with CI only in females (OR=2.943, 95% Confidence Interval=1.505–5.754). HL was associated with poorer cognitive scores (P<0.05). "Registration" (P<0.05) in MMSE was associated with speech and high-frequency hearing sensitivity.

**Conclusion:** The associations between HL and CI varied according to gender in older community-dwellers, suggesting that different mechanisms are involved in the etiology of HL. Moreover, hearing sensitivity was negatively associated with cognition scores; therefore, early screening for HL and CI among older community-dwelling adults is advised.

### TELEPHONE-BASED WORD LIST RECALL AND HEARING ABILITY

Taylor Atkinson, and Ross Andel, *University of South Florida, Tampa, Florida, United States*

Certain consonant sounds called fricatives (e.g, "s" and "f") are difficult to hear over the telephone; phones exclude high-frequency sounds that affect their intelligibility. This may be problematic for older adults responding to phone-based memory tests. Many older adults have some degree of hearing loss, and older men have it more in the high-frequency range. Hearing loss, in combination with phone bandwidth restrictions, may reduce older adults' recall of fricative words. Participants (n=3,612, mean age=64.2, 60% women) in the 1998 wave of the Health and Retirement study (HRS) completed a word list immediate recall task over the phone. List 4 recall was examined because it was evenly split (5 each) between words with and without fricative consonant sounds. Subjective ratings of hearing and health, age, depression, and education were also measured. A Wilcoxon signed-rank test showed participants recalled fewer fricative (M=2.8) than nonfricative (M=3.0) words, Z=-8.47, p<.001. An ordinal regression for fricative word recall indicated a sex by hearing interaction; males with worse hearing were less likely to recall more fricative words, OR=.94, 95% CI [.88, 1.01], p=.076, after controlling for age, education, health, and depression. An ordinal regression for nonfricative word recall did not show a main effect for hearing or a hearing by sex interaction. For both models, age, education, and health were related to recall. Consonant sounds may influence phone-based word recall, particularly for older men. Attention should be paid to word selection when designing phone-based cognitive tests in order to avoid memory impairment overestimation.

### THE EFFECT OF CHILDHOOD EMOTIONAL ADVERSITY AND LATER LIFE FRIEND SOLIDARITY ON COGNITION

Kaleena Odd,[1] Julie Blaskewicz Boron,[1] Nicholas Turiano,[2] and Jonathan Santo,[1], *1. University of Nebraska Omaha, Omaha, Nebraska, United States, 2. West Virginia University, Morgantown, Virginia, United States*

Early life experiences can influence later life outcomes such as physical, mental, and cognitive health. Previous research investigated the effect of childhood socioeconomic status in relation to mid-to-later life cognition (Liu & Lachman, 2019); however, the effects of childhood emotional adversity on cognition have not been examined. Controlling for age, education, sex, and race, the current study investigated the influence of childhood emotional adversity and later life friend solidarity on change in later life episodic memory, executive functioning, and subjective memory (i.e., perceived memory compared to others same age). Utilizing the Midlife in the United States (MIDUS) database, we studied 2,752 participants (50-75 years, M=60.09, SD=6.97, 53% female, 84% White) with completed measures on MIDUS 1 retrospective childhood adversity, MIDUS 2 friend solidarity, and MIDUS 2/3 cognition. Multilevel modeling (Mplus) was used. Higher friend solidarity was associated with higher executive functioning (b=0.122, p<.01) and higher subjective memory (b=0.267, p<.001), suggesting the positive impact of supportive friendships. Higher childhood emotional adversity was associated with lower perceived subjective memory (b=-0.037, p<.05). There was no significant friend solidarity by emotional adversity interaction. Together, these findings suggest that later life friend solidarity may be important for objective and subjective cognition; whereas, childhood emotional adversity may play a role in subjective cognition. Given the associations in prior research between lower perceptions of memory and lower mental well-being and quality of daily life, experiencing childhood emotional adversity may increase risk of lower perceptions of well-being, including cognitive functioning.

### Session 9145 (Poster)

### COGNITION, COGNITIVE IMPAIRMENT, AND BRAIN INJURY

### DOES COGNITIVE STATUS MODERATE THE RELATIONSHIP BETWEEN ENVIRONMENTAL FACTORS AND SELF-REPORTED HEALTH?

Ethan Siu Leung Cheung,[1] and Ada Mui,[2], *1. Columbia University, New York, New York, United States, 2. Columbia University, COLUMBIA UNIVERSITY, New York, United States*

Using data from NHATS Round 9, the present study examines the relationships between environmental factors and self-reported health among older adults with dementia, mild cognitive impairment (MCI), and normal cognition. Based on neighborhood stress process theory, we investigate the following questions: 1) Are there associations between dwelling safety hazards and neighborhood environments and self-reported health? 2) Is cognitive status a moderator between the relationship? 3) How do these associations differ between older adults with varying cognitive status (i.e., dementia, MCI, and normal cognition)? A hierarchical linear regression analyses are conducted. Results indicate that better quality of sidewalk surface and neighborhood social cohesion are associated with better self-reported health, after taking into account sociodemographic, health, and social factors. Interaction terms are then used to examine the moderating effects of cognitive status on the associations; four interactions terms are found to be statistically significant. Lastly, separate linear regression analyses are implemented for the dementia, MCI, and normal cognition groups. Findings show that the predicting power of environmental factors vary by cognitive status of older adults. For individuals with dementia, tripping hazards, cluttered home, and community disconnectedness are associated with poor self-reported health. However, no significant relationship was found for older adults with MCI. For older adults with normal cognition, better quality of sidewalk surface and neighborhood social cohesion predict better self-rated health scores. Findings of this study illuminate the important role of a hazard-free home, community walkability, and socially cohesive neighborhood environments in predicting better health status of older adults.

### EMPLOYERS ’ RESPONSE TO WORKERS WITH PROGRESSIVE COGNITIVE IMPAIRMENT: A REVIEW OF POLICY IN CANADA

Josephine McMurray,[1] Kristina Kokorelias,[2] AnneMarie Levy,[1] Krista James,[3] Logan Reis,[4] Jennifer Boger,[4] and Arlene Astell,[5], *1. Wilfrid Laurier University, Waterloo, Ontario, Canada, 2. Sunnybrook Research Institute, Toronto, Ontario, Canada, 3. British Columbia Law Institute, Vancouver, Ontario, Canada, 4. Waterloo University, Waterloo, Ontario, Canada, 5. University of Toronto, Toronto, Ontario, Canada*

Longer lifespans, the gig economy, eligibility for government pensions, and more testing for age-related cognitive changes, increase the potential for workers developing mild cognitive impairment and/or early onset dementia (MCI|EOD) “on the job”. This critical analysis assesses Canada’s policy environment for employers when employees are diagnosed with MCI|EOD. Our search for policy literature included: a scoping review of academic literature involving Canadian-focused articles, and countries where novel or innovative policy had been evaluated and published; a search for Canadian court judgements and tribunal decisions; and a grey literature search in both Canadian and international sources, as innovation will often happen “at the margin” and updated policy may take years to be enacted and formalized. We used participatory research to obtain feedback from a broad group of stakeholders including employers, industry, professional organizations, and government, as well as people living with MCI/dementia, to ensure outputs were reflective of current policy. We found that: 1) Canadian federally-regulated employers are governed by similar Acts & Codes as the provinces and territories, with some notable exceptions, 2) Disability discrimination and accommodation case law in Canada is settled, however there are few cognitive impairment cases to provide specific guidance, 3) Scant empirical research in the scientific literature addresses policy that incents employers to build workspaces for employees with MCI|EOD that help them stay on the job longer. We conclude that engaging with employers to better understand their needs will help policy-makers to support them build workspaces that encourage productive engagement of all workers.

### EMPLOYERS’ RESPONSE TO WORKERS WITH PROGRESSIVE COGNITIVE IMPAIRMENT: A SYSTEMATIC LITERATURE REVIEW

Josephine McMurray,[1] AnneMarie Levy,[1] Logan Reis,[2] Kristina Kokorelias,[3] Jennifer Boger,[2] and Arlene Astell,[4], *1. Wilfrid Laurier University, Waterloo, Ontario, Canada, 2. Waterloo University, Waterloo, Ontario, Canada, 3. Sunnybrook Research Institute, Toronto, Ontario, Canada, 4. University of Toronto, Toronto, Ontario, Canada*

An aging workforce increases the risk of workers experiencing cognitive decline that may lead to a diagnosis of mild cognitive impairment or early onset dementia (MCI|EOD) while still employed. This systematic review explores the use of technologies (defined as any methods, processes, software, hardware or equipment) deployed by employers to accommodate, or build sustainable workspaces for, workers diagnosed with MCI|EOD. After screening 3,860 titles/abstracts and 67 full text reviews, we identified and analyzed eight articles that met our inclusion criteria. We found that: 1) The existing literature almost exclusively focuses on employees’ perspectives on the quality of work life when diagnosed with MCI|EOD, 2) Negative workspace culture toward employees’ cognitive decline, and the variability of disease onset and progression, may account for low employer awareness, 3) Employer responses focus on mitigation of risk associated with workers’ impairment. While this review demonstrates there is scant research exploring employers’ perspectives on employees diagnosed with MCI|EOD, there is even less that explores technologies designed to specifically address employers’ needs and challenges. Technology will increasingly facilitate early identification of progressive neuro-cognitive disorders, and tools to help employers respond to an employee’s MCI|EOD disclosure as a disability accommodation rather than a terminal performance management challenge. Empathic research, that engages organizations in the process of understanding the value of affordable, employer-side technologies that help build diverse, sustainable, productive workspaces is critical to a foundational understanding of our aging workforce and accommodating workers who develop MCI|EOD while still employed.

### GENDER DIFFERENCE IN UNMET NEEDS AMONG PEOPLE AGING WITH A TRAUMATIC BRAIN INJURY

Kelli Barton, and Emma Swinford, *University of Missouri-Kansas City Institute for Human Development, Kansas City, Missouri, United States*

Traumatic Brain Injury (TBI) can result in a myriad of short and long-term mental and physical changes and conditions. While fall-related brain injury prevention strategies and outcomes among older adults have been well-documented in previous literature, less is understood about the experiences and needs of those aging with a brain injury. The aim of this project is to explore gender differences in experiences and needs among people aging with a TBI. A Needs Assessment survey was conducted in early 2020 with adult TBI survivors and their family members in Missouri (n = 150). The mean age of respondents was 45.8 and 58% identified as male. Bivariate analyses reveal gender difference in unmet needs related to information and referral, recreation, and continuing education among TBI survivors. For example, more female respondents (43.1%) identified unmet needs associated with physical activity than their male counterparts (25.9%, $p < .05$). More females (61.3%) than males (43.4%) also identified unmet continuing education needs related to aging with brain injury ($p < .05$), whereas more males (10.8%) identified unmet continuing education needs on the topic of parenting (females: 1.6%, $p < .05$). Significantly more females (31.1%) than males (16.9%) identified lack of transportation as a barrier to accessing needed supports and resources ($p < .05$). Results will guide development of an Annual State Action Plan to maximize the independence, well-being, and health of Missourians aging with TBI and their families. A better understanding of needs and preferences can inform targeted policies, programs, and resources.

### GENDER DIFFERENCES IN MENTAL AND PHYSICAL HEALTH: IMPLICATIONS FOR AGING WITH A TRAUMATIC BRAIN INJURY

Emma Swinford, *University of Missouri-Kansas City Institute for Human Development, Kansas City, Missouri, United States*

Traumatic Brain Injury (TBI) is a major cause of disability and death in the U.S., and survivors often experience temporary or life-long health effects as a result of their injury. While risks and outcomes of fall-related TBI in older adults have been well-documented, the intersection of TBI-related health challenges and the experience of aging with a TBI is less well understood. This project explores gender differences in health outcomes among TBI survivors. A Needs Assessment survey was conducted in early 2020 with adult TBI survivors in Missouri (n=150). The mean age of respondents was 46 and 58% identified as male. Bivariate analyses reveal gender differences in health conditions among TBI survivors before and after injury. Significantly more males than females reported substance use disorder for alcohol (20.7% and 7.9%, $p < .05$) prior to injury, whereas twice as many females reported developing chronic pain after injury than males (68.3% and 31.0%, $p < .001$). Further, while about 21% of both male and female respondents reported experiencing other mental health conditions, such as anxiety, prior to injury, over 35% of males and almost 58% of females experienced mental health concerns after injury. Additionally, balance/mobility issues, sleep disorders, sensory issues, and cognitive challenges were frequently identified post-injury conditions. Co-morbidities impact our experiences, capabilities, and quality of life as we age. Policies and programs to support TBI survivors and their families may better address the co-occurring health conditions among TBI survivors by considering gender differences in the experience of aging with a TBI.

### GUARDIANSHIP AND SELF-SOVEREIGN IDENTITY: IMPLICATIONS FOR PERSONS LIVING WITH DEMENTIA

Noelannah Neubauer, and Lili Liu, *University of Waterloo, Waterloo, Ontario, Canada*

Self-sovereign identity (SSI), an identity management system where individuals own and manage their digital identity, can improve access and management of one's personal data. SSI is becoming feasible for the general public to use for their health and other personal data. Like any data system, when persons living with dementia no longer have capacity to provide informed consent, guardianship over their data is required. The purpose of this study was to examine the concept of guardianship within the context of SSI, specifically its application to persons living with dementia. This study followed a qualitative description approach. Seventeen semi-structured virtual interviews were conducted with persons living with dementia and care partners to elicit their perspectives on existing guardianship practices and guardianship within the context of SSI. Interviews were digitally recorded and transcribed verbatim. Conventional content analysis guided the analytic process. Participants had mixed impressions of existing guardianship practices. While some were positive, others thought existing practices failed to consider the complexity of caring for someone with dementia (e.g., presence of multiple guardians). Participants suggested that SSI has the potential to improve the security and safety of persons living with dementia who have had guardianship enacted (e.g., reduced risk of financial abuse.) Recommendations included ensuring that SSI guardianship processes are simple and flexible, building a user-friendly system that also considers the heterogeneity of persons living with dementia and their care partners. Overall, guardianship within the context of SSI was well received. Findings will be used to further inform the SSI guardianship processes.

### MARGINAL EFFECTS OF MULTIPLE YEARS OF VOLUNTEERING ON OBJECTIVE AND SUBJECTIVE MEASURES OF COGNITION

Yi Wang,[1] Takashi Amano,[2] Huei-wern Shen,[3] and Roger Wong,[4], *1. University of Iowa, Iowa City, Iowa, United States, 2. Rutgers University-Newark, Newark, New Jersey, United States, 3. University of North Texas, Denton,*

Texas, United States, 4. SUNY Upstate Medical University, Syracuse, New York, United States

Volunteering is conducive to older Americans' physical and mental health; however, the effect of volunteering on cognitive health is less studied. Using four waves (2010-2016) of the Health and Retirement Study, this study examined the incremental effect of volunteering engagement on older adults' cognitive health. We included10,718 cognitively unimpaired, community-dwelling individuals aged 51+ in 2010 and were alive through 2016. Volunteering engagement was measured by the number of times respondents participated in volunteering throughout the four waves. Objective cognition was assessed using the Telephone Interview for Cognitive Status (TICS), a standardized test of cognitive functioning. The TICS score was further categorized into three statuses: "No impairment," "Cognitive impairment no dementia (CIND)," and "Dementia." Subjective cognition referred to self-rated memory on a 5-point Likert scale. With sampling weights, ordered logit regression was performed controlling for health-related variables (e.g., health conditions, depression), SES (e.g., income, assets), contextual features (e.g., neighborhood safety, urbanicity), and sociodemographics. The average marginal effects (AMEs) were produced. Results show that more volunteering engagement significantly reduced the likelihood of CIND or dementia (OR=0.88, p<0.001). Specifically, every one-time increase in volunteering increased the probability of remaining cognitively normal by 0.01 (p<0.001), whereas it decreased the probability of CIND by 0.008 (p<0.001) and dementia by 0.001 (p<0.001). For subjective cognition, there was no significant relationship with volunteering. Our findings address gaps in literature by adding evidence of the incremental health benefits of volunteering on cognitive functioning. Differences in the findings for subjective and objective cognition warrant further investigation.

### SOCIALLY RELEVANT APPROACHES TO THE DETECTION OF DEMENTIA IN MINORITY OLDER ADULTS

Lana Sargent,[1] Sarah Lageman,[2] Leroy Thacker,[3] Sally Russell,[4] Marissa Mackiewicz,[5] and Elvin Price,[6], *1. VCU School of Nursing, Ashland, Virginia, United States, 2. VCU Parkinson's & Movement Disorders Center, Richmond, Virginia, United States, 3. Virginia Commonwealth University Wright Center for Clinical and Translational Reserach, Richmond, Virginia, United States, 4. VCU School of Nursing, Richmond, Virginia, United States, 5. VCU Institute for Inclusion, Inquiry, and Innovation (iCubed): Health and Wellness in Aging Populations, Richmond, Virginia, United States, 6. Virginia Commonwealth University/ School of Pharmacy, Richmond, Virginia, United States*

Substantial gaps remain in the scientific literature regarding low-income minority older adult populations with Alzheimer's disease and related dementias (ADRDs). Access to care and early cognitive screening are often barriers to advancing ADRD detection in low socioeconomic status (SES) minority older adults. Additionally, there is the need for demographically (age, education, sex, race, ethnicity, and income) corrected normative scores in cognitive measures. Our cross-sectional study evaluated the psychometrics of the Mini-Mental State Exam-2 (MMSE-2) and the NIH Toolbox Cognition Battery (NIHTB-CB). The sample consisted of n=80 community-based older adults without a diagnosis of dementia living in low-income high-rise housing units. Acceptability is assessed with a brief 6-item acceptability survey, multiple linear regression is used to get predicted cognitive scores adjusted for age, education, income, ethnicity, race, and sex, and t-test comparison of the adjusted scores found in this study to established norms. Results found a mean age of 73, 70% black, 48% with < 12th-grade education, 51% have a monthly income of < $1,000, and 49% with undiagnosed cognitive impairment (CI) by both measures. When applying demographic adjustments in the NIHTB-CB 1) standard scores; 2) age-corrected scores, and 3) demographically correct scores all remained significant (p > 0.0001). Participants reported high (80-95%) acceptability for the community-based cognitive screening, 18% reported concerns with cultural appropriateness of the questions in the NIHTB-CB as compared to 5% with the MMSE-2. This research lays the foundation for a community-based cognitive screening and care coordination program for the low SES minority older adult population.

### THE ROLE OF SOCIOECONOMIC INEQUALITIES IN TRANSITIONING TO NEUROCOGNITIVE DISORDERS IN ENGLISH POPULATION

Aswathikutty Gireesh,[1] Amanda Sacker,[1] Anne McMunn,[2] and Dorina Cadar,[1], *1. University College London, London, England, United Kingdom, 2. University College London (UCL), London, England, United Kingdom*

The association between socioeconomic position (SEP) and dementia is well studied. However, scant attention has been given to the relationship with mild cognitive impairment (MCI), often considered a transient state between normal cognition and dementia. The purpose of this study was to determine the role of various SEP markers such as education and wealth on transitioning to MCI and dementia over a four-year period using data from the English Longitudinal Study of Ageing, a national representative sample of the English population aged 50+. We ascertained MCI and dementia over four years, using a validated algorithm based on physician diagnosis and lower cognitive performance (1 standard deviation below the mean) on multiple standardised tests adjusted for age and education. A Multistate Markov survival model was utilised to investigate whether different SEP markers increased the risk of specific transitions between normal cognitive performance and MCI or dementia, with the latter being considered an absorbing state. During the study period, a quarter of participants progressed to MCI from the normal state. Being in the lowest quintile of wealth was associated with a lower probability of transitioning back to a normal cognitive state from MCI, compared with those in the highest quintile. Greater wealth was weakly associated with a lower risk of transitioning from normal cognitive state to MCI and from MCI to dementia. The overall results imply that socioeconomic advantage might be protective against rapid progression from mild to more severe neurocognitive disorders such as dementia in later life.

### Session 9150 (Poster)

### COVID-19 AND LONG-TERM SUPPORTS AND SERVICES

### BURNOUT, COMPASSION SATISFACTION, AND INTENT TO QUIT AMONG LONG-TERM CARE NURSING ASSISTANTS IN THE TIME OF COVID-19

Mallory Richert, *Xavier University, Cincinnati, Ohio, United States*

The COVID-19 pandemic has greatly exacerbated the stress and burden of those employed in long-term care (LTC) facilities due to staff shortages, increased risks on the job, and ever-changing COVID-19 protocol requirements. This study examines potential differences in pre-COVID-19 and current COVID-19 LTC facility employed nursing assistants on burnout, compassion satisfaction, job satisfaction, and intent to quit. The sample included 81 nursing assistants employed in LTC facilities across the United States, with data collected prior to (n= 42) and during COVID-19 related shutdowns (n= 39). Participants completed the Professional Quality of Life Scale 5 (ProQOL 5), a single-item self-report measure of job satisfaction, and a two-item self-report measure of intent to quit their current employment. Nursing assistants during COVID-19 reported a higher level of burnout and lower level of compassion satisfaction than nursing assistants Pre-COVID-19. However, there were no differences in job satisfaction or intent to quit. The results suggest there may be additional factors that influence an individual's decision to remain employed above and beyond the impacts of burnout and compassion satisfaction that may be unique to the caring professions. Future research might investigate factors that influence an individual's decision to remain employed as a nursing assistant during periods of increased stress and burnout. Additionally, the impact of COVID-19 related stress added to the already high levels of stress and burnout on nursing assistants calls for further attention and research devoted to psychological support of LTC staff during crisis and normal times.

### CHALLENGES IN CARING FOR PEOPLE WITH DEMENTIA DURING COVID-19: FINDINGS FROM LONG-TERM CARE FACILITIES ACROSS JAPAN

Tomoko Ikeuchi,[1] Mizue Suzuki,[2] Kazunori Kikuchi,[1] Akiko Kan,[1] and Chiho Shimada,[1], *1. Tokyo Metropolitan Institute of Gerontology, Tokyo, Tokyo, Japan, 2. Hamamatsu Universuty school of medicne, Hamamatsu, Shizuoka, Japan*

Long-term care facilities for older adults have been profoundly affected by the coronavirus disease 2019 (COVID-19) pandemic in Japan. This study investigated the challenges that care staff members faced by the height of the first wave (i.e., April 2020) and the height of the second wave (i.e., October 2020) of the pandemic at long-term care facilities in Japan. We mailed questionnaires in October 2020 to 5,895 care facilities throughout Japan. A total of 22.7% of the questionnaires were returned. Of those, 87.4% had at least one resident with dementia. Based on the results, 65.2% reported having restricted all visitors during the first wave. Although 42.8% reported continuing to restrict all visitors during the second wave, more than 54% allowed visitors while limiting the number of visitors or the time of each visit. Nearly 76% reported that restrictions on visitations may have exacerbated the behavioral and psychological symptoms of dementia (BPSD) among residents. In place of visitations, over 50% used video calls or phone calls to communicate with the family members, and 45.7% reported that the virtual visits were efficacious in alleviating the BPSD. However, more than 70% reported not having adequate Internet services and computer equipment at their facilities, and nearly 90% reported insufficient staffing. Our findings suggest that the pandemic may have pressed for a change of direction for dementia care with the introduction of virtual visitations and other initiatives. Nevertheless, facilities may face difficulties with implementing such changes due to inadequacies in the availability of resources.

### DNR LINKED TO REDUCED DEPRESSIVE SYMPTOMS IN NURSING HOME RESIDENTS DURING COVID-19 ILLNESS

Wingyun Mak,[1] Orah Burack,[1] Joann Reinhardt,[1] Himali Weerahandi,[2] Benjamin Canter,[3] and Kenneth Boockvar,[1], *1. The New Jewish Home, New York, New York, United States, 2. NYU Grossman School of Medicine, New York, New York, United States, 3. The New Jewish Home, The New Jewish Home, New York, United States*

Prior work shows that older adults who establish future care plans have a lower risk of depression. Residents in long-term care may benefit from establishing a do-not-resuscitate (DNR) order when cardiopulmonary resuscitation is unlikely to provide medical benefit. The current study examines whether having a DNR order in place prior to COVID-19 diagnosis was associated with fewer depressive symptoms during the illness course. Residents at a NYC skilled nursing facility with a positive COVID-19 PCR test between 3/1/2020 – 6/1/2020 were included (N=338). The Minimum Data Set (3.0) was used to examine residents' Patient Health Questionnaire-9 (PHQ-9) scores 1-30 days after diagnosis, functional status, cognition, age, and sex. A retrospective chart review was conducted to determine whether participants had an established DNR, DNI, and/or DNH order before developing COVID-19. Forty-eight percent, 46%, and 12% of participants had a DNR, DNI, or DNH order prior to COVID-19 illness, respectively. Average PHQ-9 score was 1.65 (SD=2.37). A hierarchical regression showed that after controlling for age ($\beta$=-.13, p=.06), sex ($\beta$=-.08, p=.28), cognition ($\beta$=.14, p=.04), and functional status ($\beta$=.23, p=.001; R2=.10, p=.001), having a DNR ($\beta$=-.22, p=.006) order in place prior to COVID illness was associated with lower endorsement of depressive symptoms during illness ($\Delta$R2=.04, p=.01). Results suggest that establishing a DNR in long-term care residents when appropriate may potentially buffer depressive symptoms during illness in nursing home residents regardless of their age, sex, cognitive abilities, and functional status. Future examination of the underlying mechanism is warranted.

### HOME CARE RESILIENCY DURING THE COVID-19 PANDEMIC: OLDER ADULT-HOME CARE AIDE DYADS' PERSPECTIVES

Naoko Muramatsu,[1] Lijuan Yin,[1] Maria Caceres,[1] and Jordan Skowronski,[2], *1. University of Illinois Chicago, Chicago, Illinois, United States, 2. University of Illinois Chicago, University of Illinois Chicago, Illinois, United States*

Homecare has increased its value as an alternative to nursing homes and adapted to evolving COVID-19 challenges. However, little is known about how COVID-19 has impacted community-dwelling older adults who need assistance with daily activities, including dressing, cooking, and shopping. Guided by the stress process framework, this mixed-method study examined how older homecare recipients experienced the acute and chronic stress during the first eight months of the pandemic, focusing on the role of home care aides (HCAs) in the context of Medicaid-funded in-home services. Thirty-five dyads of care recipients and HCAs participated in a COVID telephone survey as part of a larger study. Care recipients were typically older minority (40% African American, 31% Latinx) women (77%). Their COVID-related anxiety level, assessed by a 6-item Spielberger State Anxiety Inventory (1 "not at all" to 4 "very much"), was 2.2 (SD=0.9). While COVID-19 drastically reduced contacts with family members and healthcare providers, HCAs continued to provide care in person. One care recipient said, "Fortunately, I still have my HCA come and that keeps me sane." HCAs showed resilience while facing their own family- and work-related stress: "I have followed the rules and just adapted. (COVID) did not affect the activities for my client." Some dyads, however, experienced care disruptions because of COVID infection or fear in one or both parties. COVID-19 has demonstrated homecare resilience at the person-, dyad-, and organization-levels, calling for equitable, sustainable home-based care for a growing number of older adults who desire to stay in the home.

### PROVIDER COMMUNICATION AND LONELINESS IN SENIOR LIVING COMMUNITIES DURING THE COVID-19 PANDEMIC

Lindsay Wilkinson,[1] Julie Masters,[2] and Julie Blaskewicz Boron,[1], *1. University of Nebraska Omaha, Omaha, Nebraska, United States, 2. University of Nebraska, Lincoln, Nebraska, United States*

Prior research has demonstrated the impact of the COVID-19 pandemic on feelings of loneliness, but relatively little is known about loneliness in the context of senior living communities (SLCs). Indeed, the pandemic has led SLCs to enact new safety precautions, including visitor restrictions, intended to reduce the spread of COVID-19, which may have serious consequences for the psychosocial well-being of residents. Drawing on a sample of 733 adults ages 54 to 100 living in one of nine SLCs in December 2020 (response rate = 60%), linear regression models were used to examine whether perceived communication between SLCs and residents during the COVID-19 pandemic influenced feelings of loneliness. Analyses also considered whether this association varied as a function of education. Our findings reveal that 53% of respondents were very lonely during the pandemic. However, older adults who perceived that their SLC had been helpful to their understanding of the pandemic were significantly less lonely ($p < 0.01$), adjusting for sociodemographic and health characteristics. Moreover, we found that less educated older adults derived the greatest benefit from effective communication about the pandemic ($p < 0.05$). Those with less education reported feeling lonelier if they did not perceive that their SLC communicated in a way that helped them better understand the pandemic; there was no such association for those with higher education. The findings from this study provide support for the resource substitution hypothesis and demonstrate the importance of communication in alleviating feelings of loneliness during the COVID-19 pandemic.

### SHIFTING INFORMATION-SEEKING BEHAVIORS OF RURAL OLDER AMERICANS DURING THE ERA OF THE COVID-19 PANDEMIC

Brady Lund, *Emporia State University, Wichita, Kansas, United States*

This session reports findings on how older rural adults in the Midwest United States adapted their information seeking behaviors in the face of the COVID-19 pandemic. A series of nearly three-dozen interviews conducted during late-summer 2020 capture the experiences of members of this population in their own words. Findings indicate that the experiences of the rural older American during this period were often unique to each individual and cannot be easily explained by a single social or demographic factor. Those participants with greater educational attainment were more likely to use a variety of digital technologies (smartphones, tablets, at-home personal computers) prior to the pandemic and thus experienced fewer challenges finding reliable information when the pandemic began. Those who were married felt less socially-isolated, but were often more reliant on others to find information for them. Women were more likely than people with other gender identities to use social media to connect and find information, which helped abate feelings of isolation but also made them feel more susceptible to misinformation and information overload. All participants expressed some level of fear or concern that motivated them to seek health information, while many expressed the same motivation in seeking political and economic information. These findings suggest that the information seeking behaviors of rural older adults were dramatically altered by the COVID-19 pandemic, with most becoming more reliant on digital technology to find information, and all being motivated by fear, concern, and/or curiosity to find information about the pandemic.

### THE INTERPLAY OF COVID 19, REMOTE CONTACT, AND RACE AS RISK FACTORS FOR DEPRESSION

Youngjoon Bae,[1] Katherine Abbott,[2] and Mark Pachucki,[1], *1. University of Massachusetts, Amherst, Amherst, Massachusetts, United States, 2. Miami University, Oxford, Ohio, United States*

There have been concerns about how social distance policies and lockdowns due to COVID-19 have affected loneliness and depression among older adults in ways that may magnify racial disparities in health. We conducted panel logistic regression analyses with random effects using national data spanning 2004 to 2016 and the COVID-19 module (Wave 2020, administered in June and September) from the

Health & Retirement Study (n=15,504). Individuals living in a nursing home, diagnosed with dementia or Alzheimer's disease, and 64 years of age or younger were excluded from analyses. Age, gender, Hispanic status, education, marital status, working status, wealth, BMI, physical activity, smoking, drinking, and difficulty in meal preparation, eating without help, and grocery shopping were included as control variables. Findings suggest that older adults did not appear to experience increased loneliness during the pandemic relative to prior waves. However, Wave 2020 was an independent risk factor for depression. Greater in-person contact (OR: 0.97, CI: 0.95-0.99, p-val: 0.001) and remote contact (OR: 0.99, CI: 0.97-0.996, p-val: 0.008) were each independently associated with slightly decreased depression. Older Black Americans tended to be more depressed than their White counterparts (OR: 1.50, CI: 1.20-1.86, p-val: <0.001). However, a null interaction between race and wave suggested that Black Americans did not experience more increased depression in 2020 relative to prior waves. Analyses suggest that the COVID-19 pandemic might change at-risk groups for depression and communication by remote technology – often considered an inferior but necessary stopgap measure. Implications for practice and policy will be discussed.

### Session 9155 (Poster)

### COVID-19 AND MENTAL HEALTH AMONG OLDER ADULTS

### A COMPARISON OF MENTAL HEALTH IN A SAMPLE OF OLDER WOMEN BEFORE AND DURING THE COVID-19 PANDEMIC

Victoria Marshall,[1] Robina Sandhu,[2] Kathryn Kanzler,[3] Sara Espinoza,[1] Pamela Keel,[4] Andrea LaCroix,[5] Nicolas Musi,[3] and Lisa Kilpela,[3], *1. University of Texas Health Science Center San Antonio, San Antonio, Texas, United States, 2. University of Texas Health Science Center, San Antonio, Texas, United States, 3. UT Health San Antonio, San Antonio, Texas, United States, 4. Florida State University, Tallahassee, Florida, United States, 5. University of California at San Diego, San Diego, California, United States*

The COVID-19 pandemic has substantially impacted lives globally. Due to age-related risks, the older adult population has uniquely experienced negative changes caused by the pandemic. Research has also shown that the pandemic has disproportionately affected women. Therefore, it is important to understand how the mental health of older women has been impacted during this global crisis. This study aims to examine the differences in mental health indices in a sample of older women before versus during the COVID-19 pandemic. To date, participants include 201 women (aged 60-94) who completed an online survey of self-report measures assessing depression, anxiety, alcohol use, binge eating, positive affect, and emotional quality of life (QOL). We conducted one-way ANOVAs to compare each mental health construct in two samples of older women collected pre- and peri-pandemic. Results indicated that the peri-pandemic group reported significantly higher anxiety ($F = 5.25$, $p = .02$), with a trend for more role limitations due to emotional problems ($F = 2.79$, $p = .09$), than the pre-pandemic group. No significant differences emerged for levels of depression, alcohol consumption, binge eating, positive affect, or emotional wellbeing between groups. These findings point to the psychological resilience of older adults in the face of extreme adverse events, including this global crisis. Older women, while impacted differently during the COVID-19 pandemic, reported minimal exacerbations of mental health problems compared to older women pre-pandemic. Efforts to identify moderators that may either attenuate or promote further psychological resilience among older adults is warranted.

### BENEFIT-FINDING BUFFERS THE EFFECTS OF QUARANTINE ON OLDER ADULTS' MENTAL HEALTH DURING THE COVID-19 PANDEMIC

Fan Zhang,[1] and Sheung-Tak Cheng,[2], *1. Jinan University, Jinan University, Guangdong, China (People's Republic), 2. Education University of Hong Kong, The Education University of Hong Kong, Not Applicable, Hong Kong*

**Objective:** Older adults' health and well-being may suffer due to prolonged social isolation leading to loneliness and increased stress during the COVID-19 pandemic. The current study aimed to address the role of benefit-finding, defined as the capacity to derive meaning and positive aspects from stressful situations, in older adults' adaptation to the effects of quarantine.

**Methods:** 421 participants aged 50 or above in China participated in an online survey to study the effects of quarantine on loneliness, stress, anxiety, depression and life satisfaction, and the moderating role of benefit-finding.

**Results:** The results showed that quarantine was basically unrelated to any outcome. Further analysis showed, however, that the effect of quarantine varied by levels of benefit-finding. Only people with lower benefit-finding reported a higher level of loneliness, perceived stress, anxiety and depression, but no relationships were found at higher benefit-finding.

**Conclusions:** The findings extended our understanding of the role of benefit-finding in buffering the negative impact of adversity on older people. By mitigating the effects of prolonged social isolation, benefit-finding served as a protective factor in older people's adaptation to the sequelae of this pandemic.

### CHANGE IN STRESS AND POSITIVE EXPERIENCES AMONG OLDER OREGONIANS DURING THE COVID-19 PANDEMIC

Maria Kurth, Hye Soo Lee, Soyoung Choun, Carolyn Aldwin, and Dylan Lee, *Oregon State University, Corvallis, Oregon, United States*

Several cross-sectional studies have examined stressors and positive events among older adults during COVID-19. We extend these studies by examining changes across time in perceptions of stress and positive experiences. Older adults in Oregon (Mage = 71.1, SD = 7.3, range = 51-95) completed weekly surveys from April 28 to June 23, responding to an adaptation of the Daily Stress Inventory (DISE; Almeida et al., 2002). DISE examines stressors and positive experiences across six domains (health, spouse/partner, other relationships, work/volunteer, finances, and retirement) on a 7-point scale (1 = not at all to 7 = extremely). At baseline, those who felt more stressed were younger, female, and reported more chronic health conditions, while younger adults, especially males, reported more positive events. Positive and stress intensity scores were not correlated. Multilevel models found that for both positive, Blinear = -2.54, SE = 0.52, $p < .001$; Bquadratic = 0.21, $p < .05$, and stress, Blinear = -0.79, $p < .001$; Bquadratic = 0.07, $p <.01$, intensity showed decelerated decreases across time; residuals for both models were significant. Older adults had lower stress levels, while women and those with chronic health conditions had higher stress levels. Women also reported lower levels of positive events. In both models, neither age, gender, nor chronic health conditions predicted change. These results highlight the evolving experiences during COVID-19, as perceptions of stress and positive events decreased. Future studies should examine how the changing circumstances during COVID-19 affect adaptation, including perceived stress and positive events.

### CORRELATES OF RESILIENCE OF OLDER PEOPLE IN TIMES OF CRISIS

Isabelle Albert,[1] Martine Hoffmann,[2] Elke Murdock,[1] Josepha Nell,[3] and Anna Kornadt,[1], *1. University of Luxembourg, Esch-sur-Alzette, Diekirch, Luxembourg, 2. RBS, Itzig, Diekirch, Luxembourg, 3. Luxembourg Ministry of Education, Children and Youth, Luxembourg, Diekirch, Luxembourg*

Since the beginning of the Covid-19 pandemic, efforts have been made to shield older adults from exposure to the virus due to an age-related higher risk for severe health outcomes. While a reduction of in-person contacts was necessary in particular during the first months of the pandemic, concerns about the immediate and longer-term secondary effects of these measures on subjective well-being were raised. In the present study, we focused on self-reported resilience of older people in a longitudinal design to examine risk and protective factors in dealing with the restrictions. Data from independently living people aged 60+ in Luxembourg were collected via a telephone/online survey after the first lockdown in June (N = 611) and September/October 2020 (N = 523), just before the second pandemic wave made restrictions necessary again. Overall, results showed an increase in life-satisfaction from T1 to T2, although life-satisfaction was still rated slightly lower than before the crisis. Also, about a fifth of participants indicated at T2 difficulties to recover from the crisis. Participants who reported higher resilience to deal with the Covid-19 crisis at T2 showed higher self-efficacy, agreed more strongly with measures taken by the country and felt better informed about the virus. In contrast, participants who reported more difficulties in dealing with the pandemic, indicated reduced social contacts to family and friends at T2, and also felt lonelier. Results will be discussed applying a life-span developmental and systemic perspective on risk and protective factors in dealing with the secondary impacts of the pandemic.

### COVID-19 AND ITS IMPACT ON OLDER ADULTS' ROUTINE AND URGENT HEALTH CARE VISITS

Lindsay Wilkinson,[1] Julie Masters,[2] and Julie Blaskewicz Boron,[1], *1. University of Nebraska Omaha, Omaha, Nebraska, United States, 2. University of Nebraska, Lincoln, Nebraska, United States*

Older adults have not only been disproportionately affected by the COVID-19 pandemic, but relatedly, are also more vulnerable to other health problems. Moreover, previous research on epidemics shows that health care services are often disrupted during health crises—leading to less access to and lower utilization of health services unrelated to the health crisis. The primary aim of this research is to examine the impact of the COVID-19 pandemic on both routine and urgent health care visits. Using data collected in December 2020 on adults ages 54 to 100 living in one of nine senior living communities (SLCs) in the state of Nebraska (n = 723), logistic regression models were used to investigate correlates of missed health care visits, adjusting for sociodemographic and health characteristics. The results show that 58% of older adults in SLCs skipped or postponed a routine health care visit due to the pandemic, whereas 8% of older adults missed an urgent health care visit. The results further reveal that older adults with better perceived health ($p < 0.05$), higher life satisfaction ($p < 0.01$), and higher resilience ($p < 0.05$) all had lower odds of missing a routine health care visit due to the pandemic. In contrast, those who missed an urgent health care visit were more likely to have higher perceived financial strain ($p < 0.05$). The findings from this study demonstrate the consequences of the COVID-19 pandemic on older adults' health care utilization, which may have serious implications for their long-term health.

### EMERGENCY PREPAREDNESS IN 2010 AND CONCERNS ABOUT COVID-19: EVIDENCE FROM THE HEALTH AND RETIREMENT STUDY

Timothy Killian,[1] Betsy Garrison,[2] and Charleen McNeill,[3], *1. University of Arkansas, Fayetteville, Arkansas, United States, 2. University of Arkansas, University of Arkansas, Arkansas, United States, 3. East Carolina University, East Carolina University, North Carolina, United States*

Emergency preparations are particularly important for older persons as age-related vulnerabilities increase risk of morbidity and mortality associated with disasters. The novel COVID-19 virus combined with the ongoing efforts of the Health and Retirement Study (HRS) has provided researchers with the opportunity to examine how emergency preparedness was related to responses to the COVID-19 disaster approximately 10years later. The data for this study were generated from participants in the HRS.. This study only examined data from participants in both the disaster preparedness sub-sample of 2010 and COVID-19 sub-sample in 2020 resulting in data from 249 participants. In 2020, participants were asked how concerned they were about COVID-19 and whether or not they had been tested for the virus. This study found that disaster preparations in 2010 predicted lower levels of concern about COVID-19 in 2020. The differences in the means for all items, however, was not large enough to reach statistical significance with one exception, participating in an educational program about disaster preparedness (Wilcoxon-Z = -1.88, $p < .05$). Disaster preparations were associated with an increased likelihood of getting tested for COVID-19 for four of the six items. Statistical significance, however, was only achieved for a single item. Participants who had supplies for a three-day emergency in 2010 had higher rates of getting tested in 2020 (24.84%) as compared to those who did not have supplies for a three-day emergency (14.13%; $\chi2 = 4.03$, $p < .05$). If accepted for presentation, implications for an array of audiences will be developed.

### EXPLORING THE "NEW NORMAL": OLDER ADULTS' COPING STRATEGIES DURING COVID-19 PANDEMIC

Novia Nurain,[1] Lesa Huber,[2] Clara Caldeira,[3] and Kay Connelly,[3], *1. Indiana University, Indiana University, Indiana, United States, 2. School of Public Health, Indiana Univ, Bloomington, Indiana, United States, 3. Indiana University, Bloomington, Indiana, United States*

COVID-19 stay-at-home orders resulted in social isolation and psychosocial challenges for older adults around the world. To understand their lived experiences during the pandemic, we conducted a qualitative study using semi-structured interviews with 15 older adults living in community settings. Qualitative thematic analysis of the collected data identified themes and patterns of the "new normal" for these participants: ways of living, communication with family and friends, sense of autonomy, psychological responses, coping strategies, and perceived social support. This presentation focuses on participants' coping strategies. Participants used common coping strategies, customized to the unique challenges of stay-at-home orders. We categorized coping strategies as problem-focused, meaning-focused, and emotion-focused. Participants' problem-focused strategies aimed to reduce the risk of infection. Meaning-focused strategies included purposeful errands such as going to grocery stores. Emotion-focused strategies emphasized connecting with support networks (e.g., via Zoom) and efforts to maintain psychosocial and emotional well-being (e.g., seeking professional counseling). They also employed self-enhancing comparisons to increase self-concept and self-esteem. At the beginning of the pandemic, some temporarily used distraction/avoidance strategies such as eating comfort food and avoiding news about COVID-19 to maintain a positive emotional state. Our findings imply the applicability of frameworks such as life course perspective and selective optimization with compensation to highlight the successful adaptive strategies developed by older adults through experience. We argue against the ageist view of older adults as vulnerable. Rather, this study suggests that older adults can flexibly employ resilient coping strategies crafted over a lifetime of experiences in response to crises.

### LINKING COPING BEHAVIOR AND MENTAL WELL-BEING IN OLDER ADULTS DURING THE COVID-19 PANDEMIC

Dugan O'Connor, and Jennifer Smith, *Mather, Evanston, Illinois, United States*

In addition to being a significant source of stress, the COVID-19 pandemic required older adults to drastically alter their behaviors and routines. People cope with stress in various ways; however, the pandemic was a unique situation that warranted investigation of this topic. This study aimed to identify the ways older adults coped with the COVID-19 pandemic, and the relationship between specific coping behaviors and perceived stress and mental health. Two hundred thirty-one older adults, ages 53 to 90, completed an online survey about coping behaviors used to manage stress during the pandemic, as well as measures of loneliness, depression, perceived stress, and the negative impact of the COVID-19 pandemic on their lives. "Talking with friends and family" (83%) and "increased television watching or other screen-time" (68%) were the most common coping behaviors. A series of one-way analyses of covariance (ANCOVA), with race, gender, age, education, and income included as covariates, revealed "eating more often" and "drinking alcohol" were associated with greater loneliness, depression, and stress. "Increased screen time" was also associated with greater depression and stress. "Engaging in more family activities," was associated with a less negative impact of the pandemic, and "talking with friends and family" was associated with less loneliness. These findings suggest older adults who coped with stress of the COVID-19 pandemic through more eating, drinking, and the second most common behavior—watching TV—were more likely to report poorer well-being, and may benefit from programs to boost virtual social engagement.

### MENTAL HEALTH CONSEQUENCES OF THE COVID-19 PANDEMIC AMONG OLDER ADULTS WITH CHRONIC CONDITIONS

Courtney Polenick,[1] Diarratou Kaba,[2] Summer Meyers,[2] Annie Zhou,[2] Nikita Daniel,[2] Shreya Salwi,[2] and Vanessa Lee,[2], *1. University of Michigan, Ann Arbor, Michigan, United States, 2. University of Michigan, University of Michigan, Michigan, United States*

The COVID-19 pandemic may have a negative impact on mental health, especially among older adults with chronic conditions who are more vulnerable to severe illness. This cross-sectional qualitative study evaluated how the pandemic has impacted the ways that adults aged 50 and older with chronic conditions managed their mental health. Participants included a total of 492 adults ($M = 64.95$ years, $SD = 8.91$, range = 50 – 94) from Michigan (82.1%) and 33 other U.S. states who reported a diagnosis of at least one chronic condition and completed an anonymous online survey between May 14 and July 9, 2020. Participants provided open-ended responses to a question about the pandemic's impact on how they were taking care of their mental health. The data were coded to ascertain relevant concepts and were reduced to develop major themes. We determined four main themes. The pandemic impacted how participants took care of their mental health through: (1) pandemic-related barriers to social interaction; (2) pandemic-related routine changes; (3) pandemic-related stress; and (4) pandemic-related changes to mental health care. Taken as a whole, this study indicates that older adults with chronic conditions encountered a variety of challenges to managing their mental health in the early months of the COVID-19 pandemic, but also demonstrated considerable resilience. These findings identify potential risk and protective factors to target as part of personalized interventions to preserve their well-being during this pandemic and in future public health crises.

### OLDER ADULTS AND THE IMPACT OF COVID-19: LESSONS LEARNED FROM A TWO-STATE STUDY

Amanda Sokan,[1] and Tracy Davis,[2], *1. The University of Arizona, University of Arizona, Arizona, United States, 2. Rutgers University, New Brunswick, New Jersey, United States*

The COVID-19 pandemic has led to increased strains on the rapidly increasing aging population's mental, emotional, and physiological health. COVID-19, which belongs to a family of respiratory viruses, was first detected in China before spreading to other parts of the globe. Due to underlying health conditions and weakened immune systems, the aging population is at greater risk for contracting COVID-19. To better prepare for a future pandemic, it is necessary to explore the psychosocial impacts of limited human interactions to make the aging population feel safer while mitigating harm to their mental and emotional health. The purpose of this study is to highlight the experiences of the aging population with COVID-19, including psychosocial, behavioral responses to the pandemic, and older adults' overall well-being. We surveyed a total of 203 adults 55 and older regarding their experiences with the pandemic. Survey components included the COVID-19 Household Environment Scale (Behar-Zusman, Chavez, & Gattamorta, ND), selected items from the COVID-19 Impact Study and open-ended questions, the Generalized Anxiety Disorder Assessment (Williams et al., 2006), and the UCLA Loneliness Scale (Russell, Peplau, & Ferguson, 1978). Preliminary analyses indicate that most participants had not experienced any COVID-19 symptoms, nor did they know anyone who had passed away from the virus. However, participants did report loneliness and less family cohesion because of the pandemic. Findings from this study will be used to help older adults cope with the impact of the current pandemic and future pandemics.

### OLDER AGE AND OTHER PROTECTIVE FACTORS OF MENTAL HEALTH DURING THE COVID-19 PANDEMIC IN THE US

Kelly Smith,[1] Amanda Chappell,[2] Rachael Spalding,[1] Jenna Wilson,[1] Ilana Haliwa,[1] and JoNell Strough,[1], *1. WVU, morgantown, West Virginia, United States, 2. West Virginia University, Morgantown, West Virginia, United States*

Research conducted early in the COVID-19 pandemic (i.e., March 2020) suggested that older adults were less likely to experience negative pandemic-related mental health than younger people. We investigated whether this age-related advantage persisted during the July 2020 spike in COVID-19 cases and investigated links between coping strategies and mental health. We used data from the Understanding America Study (UAS) to conduct a secondary data analysis of participants (N = 5,753) aged 18-110 years (M=46.20, SD=12.88) who completed online self-report surveys twice—once immediately prior to the July spike in cases, and again during the spike. Surveys assessed engagement in protective behaviors (e.g., wearing a mask, washing hands), coping strategies (e.g., calling family/friends, getting extra exercise, meditating), and anxiety and depressive symptoms (using the Patient Health Questionnaire PHQ-4). Multiple regression analyses were used to identify predictors of anxiety and depression during the spike, controlling for previous anxiety and depression, race, ethnicity, income, education, and marital status. Older age and engaging in protective behaviors were associated with less anxiety, whereas coping by calling family/friends was associated with more symptoms, $R2 = .71$, $F(16, 5736) = 885.90$, $p < .001$. Coping by calling and getting extra exercise were associated with fewer depressive symptoms, whereas coping by using social media was associated with more symptoms, $R2 = .72$, $F(16, 5736) = 906.65$, $p < .001$. These findings highlight age as a protective factor for anxiety but not for depression and underscore the importance of social support as a protective factor for mental health.

### ONLINE LEISURE ACTIVITIES FOR SUSTAINED MENTAL HEALTH WELL-BEING IN OLDER ADULTS WITH COVID-19 MITIGATION

Solymar Rivera-Torres,[1] Elias Mpofu,[2] M. Jean Keller,[2] and Stan Ingman,[2], *1. University of North Texas, Denton, University of North Texas, Denton, Texas, United States, 2. University of North Texas, University of North Texas, Denton, Texas, United States*

Older adults (OA) experience psychosocial distress from the COVID-19 pandemic mitigations. While their participation in leisure and recreation activities (LRA) would be ameliorating, we do not know how LRA OA engages for their mental health (MH) well-being with COVID-19 mitigation. This scoping review aimed to trend the evidence on the types of LRA OA engage for their MH well-being across the young-old continuum (60-69 years) through to older-old (80 years and above) in the COVID-19 pandemic. We searched the following electronic databases (PubMed, EMBASE, CINAHL, Cochrane, JBI-ES, and Epistemonicos for LRA studies by OA with COVID-19 mitigation. To be included, we considered empirical articles published in English on LRA of OA 55+ years-old. Another criterion required articles describing those activities' qualities and the impact of LRA on MH and well-being during the COVID-19 pandemic. We resulted in seven empirical studies, two of which implemented in the USA and one from the USA and Canada, Spain, Israel, and Japan. Findings following narrative synthesis revealed trending evidence on OA to engage in online LRA for social, cognitive /intellectual, and emotional health. Leisure-time physical activity reduced negative MH symptoms as anxiety and depression in OA under COVID-19 threat. In conclusion, the present review's trending evidence suggests that OA engagement in social, physical, mental, and cognitive LRA enhanced their MH and overall well-being. Activities delivered by way of the Internet and television provided a cluster of beneficial opportunities for the OA mental health needs under the COVID-19 pandemic.

### PERCEIVED IMPACT OF COVID-19 ON OLDER ADULTS' MENTAL HEALTH AND BARRIERS TO MENTAL HEALTH CARE

Grace Caskie, Madison Tschauner, and Eve Root, *Lehigh University, Bethlehem, Pennsylvania, United States*

COVID-19 disproportionately impacted older adults in terms of fatalities, but also increased stress, isolation, and loneliness (Chen, 2020). We examined older adults' anxiety, depression, and barriers to mental healthcare during the COVID-19 pandemic and their perceptions of these variables prior to the pandemic. Further, we explored whether any perceived changes differed based on geographical location (rural, suburban, urban). Data were collected online between mid-June and mid-July of 2020 from 244 individuals aged 65-82 years (M=68.3, SD=3.5). The sample was primarily White (91%) and female (60%); most (n=119) lived in suburban settings, with 63 in urban and 60 in rural settings.

Repeated-measures ANOVAs at alpha=.01 showed that depressive symptoms, measured by the CESD-10 ($p<.001$), and anxiety symptoms, measured by the GAD-7 ($p<.001$), increased during the pandemic as did mental healthcare barriers related to transportation ($p=.004$) and beliefs that depression is a normal part of aging ($p<.001$). Only transportation concerns differed based on where older adults lived; those in rural ($p<.001$) and urban ($p=.004$) settings reported greater transportation barriers than those in suburban settings. No differences over time were found for barriers related to help-seeking ($p=.403$), stigma ($p=.156$), knowledge/fear ($p=.180$), finding a therapist ($p=.030$), ageism ($p=.302$), psychotherapist qualifications ($p=.265$), physician referrals ($p=.207$), or finances ($p=.818$). These findings highlight the impact of COVID-19 on older adults' perceptions of changes in their psychological well-being as well as their experience navigating mental health services.

### PERSONALITY AND CHANGE IN PERCEIVED CONTROL ACROSS THE FIRST FOUR MONTHS OF THE CORONAVIRUS PANDEMIC

Amanda Sesker,[1] Ji Hyun Lee,[1] Martina Luchetti,[1] Damaris Aschwanden,[2] Yannick Stephan,[3] Antonio Terracciano,[4] and Angelina Sutin,[1], *1. Florida State University College of Medicine, Tallahassee, Florida, United States, 2. Florida State University, Tallahassee, Florida, United States, 3. University of Montpellier, Montpellier, Languedoc-Roussillon, France, 4. FLORIDA STATE UNIVERSITY, Florida State University, Florida, United States*

**Objective:** This study examined change in perceived control (PC) across the first four months of the global coronavirus pandemic and whether change varied significantly by age and personality traits during the first four months of the pandemic.

**Methods:** Personality was assessed prior to the pandemic in a large national sample of 2,455 American adults (18-100 years) from a preregistered online survey (https://osf.io/q8cpd), first conducted between January 31, 2020 and February 10, 2020. Three additional follow-up waves were conducted: mid-March 2020 (following the World Health Organization declaration of the coronavirus a pandemic), late April 2020 (toward the end of the White House's 30 Days to Slow the Spread guidance), and late July 2020 (when patient deaths in the U.S. reached 132,918). PC was assessed in Waves 2-4.

**Results:** There were age differences in the trajectory of PC such that PC increase for middle-aged and older adults, whereas younger adults had lower PC and did not increase over the follow-ups. All personality traits but Openness were associated with PC at the first assessment. Conscientiousness, Extraversion, and Agreeableness positively predicted PC change over time. The association between Conscientiousness and Extraversion and increased PC over time was stronger at older ages.

**Conclusion:** Pre-pandemic personality predicted PC and PC change during the first four months of the pandemic, with middle-aged and older adults showing better adaption than younger adults. This study provides new evidence for PC change and associations between personality and PC during the COVID-19 pandemic.

### PHYSICAL ACTIVITY, SOCIAL SUPPORT, AND MOOD IN OLDER ADULTS DURING THE COVID-19 PANDEMIC

Morgan Inman,[1] Clare Murphy,[1] and Jessica Strong,[2], *1. University of Prince Edward Island, University of Prince Edward Island, Prince Edward Island, Canada, 2. University of Prince Edward Island, Charlottetown, Prince Edward Island, Canada*

Research shows that increased physical activity is associated with improved mood and reduced symptoms of depression in older adults. Prior research has also found that loneliness and social isolation have a significant impact on the mental and physical well-being of older adults, with higher amounts of social connectedness and social activity associated with more frequent positive mood states. Overall social isolation is increased due to the COVID-19 pandemic and this could have a large impact on the physical and mental health of older adults. A group of 36 community dwelling older adults (Mean age = 70.5) completed questionnaires measuring physical activity, social activity, and social support, during the COVID-19 pandemic. Analyses found that perceived social support and average social network size significantly predicted positive mood states ($F(2,33)=3.32$, $p<0.05$) accounting for 16.7% of the variance, with a large effect. After adding average number of hours of sedentary activity the model was not significant. Perceived social support was more predictive of positive mood ($\beta=0.32$) compared to network size ($\beta=0.17$). There was a trend for the same three variables to predict negative mood ($F(3,32)=2.76$, $p=0.06$) accounting for 22% of the variance. Sedentary behaviour was the most predictive ($t=2.68$, $p<0.05$, $\beta= 0.49$). This suggests that perceived social support is most predictive of positive mood, and sedentary behaviour is predictive of negative mood during the COVID-19 pandemic.

### PROTECTIVE AND RISK FACTORS OF DEPRESSION AMONG OLDER ADULTS DURING THE COVID-19 PANDEMIC

Yeonjung Lee,[1] and Tyran Terada,[2], *1. University of Hawai'i at Mānoa, Honolulu, Hawaii, United States, 2. University of Hawai`i, Honolulu, Hawaii, United States*

The coronavirus disease 2019 (COVID-19) pandemic adversely impacted the mental health of older adults. This study aims to explore the associations between protective/risk factors of depression during the pandemic and to examine the differences in these associations by marital status. Data from the Health and Retirement Study 2020 COVID-19 module, released in February, 2021, were used. The level of resilience during the pandemic was selected as a protective factor. The level of COVID-19 pandemic concern was selected as a risk factor. Among older adults aged 51 years and older, the weighted regression model found that higher levels of COVID-19 concern were associated with higher levels of depressive symptoms ($p<0.05$), whereas higher levels of resilience were associated with lower levels of depressive symptoms ($p<0.05$). Marital status moderated the association between COVID-19 concern and depressive symptoms. Never-married people were at higher risk of depressive symptoms than married people when COVID-19 concerns increased. It is important to enhance support for never-married people during the pandemic to protect their psychological well-being.

### SOCIAL ISOLATION, DEPRESSIVE SYMPTOMS AND PERCEIVED NEIGHBORHOOD ENVIRONMENT OF OLDER ADULTS DURING COVID-19

Ke Li, and Fengyan Tang, *University of Pittsburgh, Pittsburgh, Pennsylvania, United States*

Social isolation has been recognized as a social problem with negative effects on psychological well-being. Older adults are disproportionately affected by social isolation during the COVID-19 pandemic. Using data from the 2020 Health and Retirement Study COVID-19 Project, this study examined the relationship between social isolation and depressive symptoms among two groups of respondents differentiated by whether themselves or their social relationships were diagnosed with COVID-19. This study also explored the moderating role of perceived neighborhood environment. Depressive symptoms were measured using the eight-item CES-D. The index of social isolation was generated using five indicators, including living alone, no social participation, and less than monthly contact with children, family members, and friends. The moderator assessed two aspects of the neighborhood environment, including physical disorder and social cohesion. The results of bivariate analyses showed that respondents who were affected by COVID-19 were younger, more likely to be female, Hispanic, and Non-Hispanic Black, and with lower levels of social isolation. The results of multiple regression analyses indicated that social isolation was associated with more depressive symptoms, but this relationship was found to be only significant among respondents who were affected by COVID-19. Perceived neighborhood environment significantly moderated the relationship, as the effect of social isolation on depressive symptoms was stronger for respondents with more neighborhood physical disorders and less social cohesion. This study has implications for practice and policy, in that it underscored the importance of enacting strategies to improve the neighborhood environment, particularly for socially isolated older adults during the COVID-19.

### THE EFFECT OF VACCINES ON OLDER AFRICAN AMERICANS EMOTIONAL EXPERIENCES DURING THE COVID-19 PANDEMIC

Sophie Hanna,[1] Dwana Bass,[2] Sarah Shair,[3] Loraine DiCerbo,[1] Bruno Giordani,[4] and Voyko Kavcic,[1], *1. Wayne State University, Detroit, Michigan, United States, 2. Wayne State University, Sterling Heights, Michigan, United States, 3. University of Michigan, University of Michigan, Michigan, United States, 4. Neuropsychology/ Michigan Alzheimer's Disease Research Center, University of Michigan, Ann Arbor, Michigan, United States*

The COVID-19 pandemic is an unprecedented health emergency that has forced a change in the daily life of all individuals across the nation for over a year. As vaccinations have begun in Detroit, we examined their effect on older African Americans' emotional experiences and intent to get vaccinated during the pandemic to help understand how persons make decisions to accept vaccinations. For this study, 194 community-dwelling older African Americans (mean age = 75, age range = 64-94) were recruited from the Wayne State Institute of Gerontology Healthier Black Elders Center and general Detroit area. A telephone survey was administered to assess pandemic experience including demographics, emotional responses (e.g., gratitude, happiness, anger, fear), everyday stressors (e.g., economic problems, reduced privacy), and vaccination attitude (e.g., concern over safety, intent to vaccinate). Of the 194 participants, 149 completed the survey before the first vaccination occurred in the United States on December 15, 2020, and 45 completed the survey after. Participants had not yet been vaccinated, but 67% said they would as soon as available. Participants in the post-vaccination group, as compared to pre-vaccination group, showed increases in stress-related locus of control ($p=.03$) and reported being more likely to get vaccinated ($p=.02$). They showed decreased worry about availability of health and safety supplies ($p=.01$), reduced perceived stress ($p=.02$), and a decrease in fears of COVID-19 ($p=.05$) and vaccination safety ($p<.001$). The current study highlights the effect of vaccinations on the attitudes and emotions experienced by an older minority population living in an urban area.

### THE IMPACT OF COVID-19 CONCERNS ON THE MENTAL HEALTH OF OLDER ADULTS: A RAPID REVIEW

Sophia Perez, Alexandria Nuccio, and Ashley Stripling, *Nova Southeastern University, Fort Lauderdale, Florida, United States*

The Coronavirus (COVID-19) Pandemic continues to drastically impact older adults. Despite COVID-19 being linked to increased social isolation and loneliness, more research is needed on the psychological effects associated with older adults' concerns of the pandemic. The current review explores associations between the COVID-19 Pandemic and older adults' mental health to increase awareness and understanding. For this rapid review, empirical peer-reviewed source documents were identified through a computerized search using APA PsycInfo and Google Scholar bibliographical databases covering the years 2019 to 2021. The following keywords and combinations were used: "older adults," "COVID-19," and "mental health effects." Relevant exclusion criteria were applied, and all related English-language journal articles were read. 47 articles met inclusion criteria. Eight associated COVID-19 stress with loneliness, anxiety, depression, sleep disturbances, and poor psychological well-being, with three additional articles reporting elevations in women. Three articles revealed mixed findings regarding the impact of age on psychological variables. 13 articles evaluated changes among those with psychological/psychiatric diagnoses, and six explored physical activity and depression. Of the remaining articles, two concentrated on nutrition; seven examined routines, behaviors, and societal or risk perceptions; two evaluated coping mechanisms; and three examined emotional distress changes. Understanding COVID-19's psychological impact on older adults will take time. This rapid review revealed mixed findings regarding COVID-19 related concerns on older adults' psychological well-being, with multiple demographic variables uniquely impacting these outcomes. It is imperative that future research explore older adults' risks and develop interventions related to the psychological impact of COVID-19.

### THE MEDIATING ROLE OF RUMINATION AND PERSONAL GROWTH ON PANDEMIC DEPRESSION: A LONGITUDINAL STUDY

Teresa Paniagua,[1] Virginia Fernández-Fernández,[2] and MªÁngeles Molina Martínez,[3], *1. Universidad Europea de MAdrid, Majadahonda, Madrid, Spain, 2. Universidad Nacional de Educación a Distancia, Madrid, Madrid, Spain, 3. Universidad Francisco de Vitoria, Madrid, Madrid, Spain*

**Introduction:** The experience of recent months, caused by COVID-19 pandemic including strict home confinement, has required older people to implement coping strategies to combat the harmful effects of depression and associated loneliness.

**Method:** longitudinal study, including functionally and cognitively independent people over 65, three temporal evaluation measures: WAVE1 (6 months pre-COVID-19, N=305; M=73.63; 58.9% women), WAVE2 (during home confinement; N=151; M=73.14; 59.6% women) and WAVE3 (8 months post-WAVE2; N = 85; M=72.62; 64.70% women). Bivariate correlations and a multiple hierarchical regression model are performed to explain the variance of depression in WAVE3 from rumination and growth on general life events (in WAVE1) and rumination and personal growth associated with COVID-19, both in WAVE2 and WAVE3.

**Results:** statistically significant correlations are obtained between all the variables. The regression model explains 65.7% of the variance of depression (all steps significant). Both rumination (B=0.45;p=0.00) and growth (B=-0.40;p=0.00) on general life events (WAVE1) explain 54.5% of the depression in WAVE3. Rumination on COVID (B=.310;p≤0.01) in WAVE2 and the growth over COVID (B=-0.24;p≤0.01) in WAVE3, allow a significant explanation of 6.8 and 4.4% of the proposed model, respectively.

**Conclusions:** it seems clear the impact that emotional regulation strategies have on life events over time. In addition, rumination is an emotional process of maladaptive coping also in the face of the pandemic. However, the growth capacity of the person is a useful tool to combat the damaging effects of negative life events. In the elderly, it seems necessary to influence and bet on positive coping strategies.

### THE RELATIONSHIP BETWEEN COVID-19 ANXIETY AND WELL-BEING OUTCOMES IN OLDER VETERANS

Sara Kintzle, and Carl Castro, *University of Southern California, Los Angeles, California, United States*

Understanding the impacts of the COVID-19 pandemic on the well-being of vulnerable populations is essential in planning for care post pandemic and preparing for future pandemics. One such population often overlooked during the COVID-19 pandemic is older veterans, whose military service often results in physical and psychological effects that creates vulnerability to the impacts of COVID-19 beyond their age risks. The purpose of this research was to examine the relationship between COVID-19 anxiety and well-being in older veterans. Survey data was collected from the end of June 2020 through August 2020 and examined the well-being of veterans during the COVID-19 pandemic. This research looked at the 122 veterans in the sample who were aged 65 and older. Age ranged from 65 to 94 years (M=73, SD = 5.0). The sample was predominately Male (90%), White (83%), educated (65%) and married (69%). Moderate to high COVID-19 anxiety was reported in 38 percent of the sample. Older veterans who reported moderate to high COVID-19 anxiety were 5 times more likely to report loneliness, 11 times more likely to meet the diagnostic cut off for anxiety, 3 times for depression, 2 times for PTSD, and 3 times for moderate to severe insomnia. Results revealed a clear association between COVID-19 anxiety and the well-being of older veterans. Findings have implications for ensuring well-being outcomes are restored in older veterans as cause for pandemic anxiety is reduced. Results also demonstrate older veterans should be recognized as a vulnerable population in preparation for care future pandemics.

### Session 9160 (Poster)

### COVID-19 AND OLDER ADULTS

### CHALLENGES WITH CONDUCTING AN INVESTIGATIONAL DRUG STUDY IN OLDER ADULTS IN NURSING HOMES DURING A PANDEMIC

H Edward Davidson,[1] Lisa Han,[1] Melissa LaMantia,[1] Michelle Trout,[2] Joan Mannick,[3] and Stefan Gravenstein,[4], *1. Insight Therapeutics, LLC, Norfolk, Virginia, United States, 2. Insight Therapeutics, LLC, Insight Therapeutics, LLC, Virginia, United States, 3. Life Biosciences, Boston, Massachusetts, United States, 4. Brown University, Providence, Rhode Island, United States*

In the early months of the pandemic, SARS-CoV-2 infected nursing home residents in explosive and deadly outbreaks. Nursing home residents disproportionately accounted for over 40% of COVID-19 mortality nationally. This national emergency drove scientific and public health experts to develop and implement administrative, clinical, and research programs to limit the pandemic's impact, especially for high-risk individuals, such as those hospitalized or living in nursing homes. Nursing home policies, prompted by the Centers for Medicare & Medicaid Services (CMS) and the Centers for Disease Control and Prevention (CDC) guidance, severely restricted access beginning in March 2020 in an effort to limit disease exposure. In July 2020 we began the process to conduct an investigational SARS-CoV-2 post exposure prophylaxis study of nursing home residents, incorporating FDA guidance developed for conducting investigational drug trials in the context of COVID-19. Our research teams adapted our nursing home engagement, resident consenting and research data collection strategies accordingly. We remotely screened residents living in any of 28 nursing homes for eligibility to participate, ultimately consenting and randomizing individuals in 11 facilities. Of the 2,683 nursing home residents 65 years or older we screened, 48 (1.8%) agreed to consent individually or through proxy, most often a legally authorized representative. We will describe our research methods, with emphasis on how we addressed challenges presented due to performing all research tasks remotely and identify strategies that can qualitatively improve the remote nursing home research experience.

### CHARACTERIZING PATIENTS AND THEMES OF SARS-COV-2 VACCINE HESITANCY IN A NEW YORK CITY HIGH-RISK OLDER POPULATION

Stephanie Chow,[1] Yuji Yamada,[2] Lizette Munoz,[3] Susana Lavayen,[4] Jodi Payne,[2] Kavya Sreevalsan,[2] and Shamsi Fani,[2], *1. Icahn School of Medicine at Mount Sinai, New York, New York, United States, 2. Icahn School of Medicine at Mount Sinai, Icahn School of Medicine at Mount Sinai, New York, United States, 3. Mount Sinai Health System, New York, New York, United States, 4. Mount Sinai Hospital, Mount Sinai Hospital, New York, United States*

**Background:** Vaccines to prevent SARS-CoV-2 infection are deemed one of the most promising measures in controlling the devastating pandemic, yet there is significant vaccine hesitancy in some communities. Historic systemic health, discrimination, and structural inequities in specific racial and ethnic communities contribute to vaccine hesitancy with disproportionately negative impact. It is therefore critical to better understand vaccine hesitancy in this high-risk older population. The ALIGN (Acute Life Interventions, Goals, and Needs) program co-manages a panel of older patients with complex medical and psychosocial needs in an urban academic medical center.

**Methods:** ALIGN enrolled or graduated Patients or designated healthcare proxies were contacted by telephone to discuss SARS-CoV-2 vaccine willingness and hesitancy using a standardized web-based survey. Qualitative data was categorized into themes and subgroups. Demographic data was collected by chart review.

**Results:** Complete results are forthcoming and will include patient reported race and ethnicity baseline, vaccine hesitancy perceptions, with common overarching themes, and clinical team member debriefing. Iterative quality improvement actions taken based on elicited patient themes will also be included and assessed in telephone follow-up for changes in vaccine hesitancy.

**Conclusions:** We are conducting a qualitative and quality improvement study characterizing vaccine perceptions and hesitancy in a high-risk older group with focus on racial and ethnic disparities in this population. This preliminary data informs healthcare providers of potential health literacy, cultural and language, and other potential barriers in order to help further understand how to optimize SARS-CoV-2 vaccine acceptance and delivery in a patient population with elevated risk.

### COVID-19 IMPACTS ON MENTAL HEALTH CARE IN VETERANS HEALTH ADMINISTRATION COMMUNITY LIVING CENTERS

Jenefer Jedele,[1] Cameron Griffin,[2] Michele Karel,[3] and Kim Curyto,[4], *1. Veterans Health Administration, Ann Arbor, Michigan, United States, 2. VA, Ann Arbor, Michigan, United States, 3. Veterans Health Administration, Washington, District of Columbia, United States, 4. VA Western New York Healthcare System, Batavia, New York, United States*

COVID-19 forced VHA Community Living Centers (CLC) to adjust how mental health (MH) care is provided. Beginning March 2020, admissions and staff entering CLC space were restricted in response to the pandemic. Some care shifted from in-person to virtual. Veterans were more isolated due to visitor restrictions and cancellation of communal activities. Pre-COVID, CLC teams cared for an already complex population – 80% of residents had a MH diagnosis (24% with serious mental illness). Changing resident composition and increased isolation may intensify challenges in providing MH care. Using VHA administrative data, we assess the impact of the changing CLC environment during the pandemic by comparing monthly average rates of MH diagnoses and provision of MH care and as-needed psychotropics to CLC residents pre-COVID (Oct 2019 – Feb 2020) to the COVID period (Mar 2020 – Feb 2021). CLCs experienced a 26% decline in the monthly resident census. However, the monthly percentage of residents with a serious mental illness increased 13%. Pre-COVID, virtual MH encounters were received by 2% of residents; 35% received an in-person MH encounter. During COVID, 8% received a virtual MH encounter and 33% received in-person. As-needed antipsychotics remained unchanged, while as-needed benzodiazepine prescriptions decreased 15%. Despite increased MH concerns, CLC teams did not appear to respond with increased pharmacological interventions. Rather, teams seem to have maintained clinical service connection for those with MH concerns. Documenting successful approaches for addressing MH needs during this challenging time will be instructive for future care during times of crisis.

### DISCRIMINANT COVID-19 OUTCOMES BASED ON SAFETY ADHERENCE IN AN ACTIVE LIFESTYLE RETIREMENT COMMUNITY

Mitchell Roberts,[1] Erica Sappington,[2] Leonardo Guerra,[3] Lindsey Collins,[3] Ali Yalcin,[1] Carla VandeWerd,[3] and Jeff Lowenkron,[4], *1. University of South Florida, Tampa, Florida, United States, 2. University of South Florida, University of South Florida, Florida, United States, 3. University of Florida, University of Florida, Florida, United States, 4. The Villages Health, The Villages, Florida, United States*

Compliance with preventive behaviors recommended by public health officials plays a critical role in the control and prevention of COVID-19. Data were collected from those living in The Villages, FL, and surrounding communities via The Villages Health COVID-19 Rapid Testing Program in partnership with The UFHealth Precision Health Research Center. A descriptive ecological study was conducted to model COVID-19 positivity result variations by age, sex and adherence to CDC safety recommendations using chi-square tests. 9,993 tests were performed using Abbott's BinaxNOW™ COVID-19 Ag Card, and 931 (9.30%) positive cases were confirmed between 10/19/2020-2/26/2021. Median age was 69 years (range:12-103), and 5,578 (55.8%) individuals were female. No significant differences were found in positive test status (≥65=9.8%,<65=8.8%) amongst those over 65 (n=6567) and under 65 (n=3180) years old [X2 (1, N=9847)=2.49,p=.114]; however, positive test result differed by sex with males (10.6%) testing positive at higher rates than females [8.3%, X2(1, N =9993)=14.888, p< .001)]. A significant relationship between preventative behaviors and positive test status was also found. Not engaging in regular handwashing (p< .001) and failing to stay 6 feet or more away from others outdoors (p< .001) was significantly associated with positive test status. Further, not wearing a face mask in businesses/shops (p<.001) or indoors around 6+ people, (p<.001) was significantly associated with positive test status. In light of debate around the efficacy of mask wearing, these findings signal the importance of following CDC recommended public health behaviors for all ages across the lifespan to reduce the spread of COVID-19 infection.

### EARLY DOCUMENTATION OF LIFE-SUSTAINING TREATMENT ORDERS PRIOR TO DIAGNOSIS OF COVID-19 IN NURSING HOME RESIDENTS

David Henkin,[1] Brande Harris,[2] Sara Espinoza,[3] Hazel Caliz,[4] Kimberly Oakman,[4] Sandra Sanchez-Reilly,[5] and Marcos Restrepo,[5], *1. University of Texas HSC at San Antonio, Detroit , Michigan, United States, 2. South Texas Veterans Health Care System, South Texas Veterans Health Care System, Texas, United States, 3. University of Texas Health Science Center San Antonio, San Antonio, Texas, United States, 4. South Texas Veterans Health Care System, San Antonio, Texas, United States, 5. University of Texas HSC San Antonio, San Antonio, Texas, United States*

Coronavirus disease 2019 (COVID-19) has had a devastating impact on older adult nursing home residents (NHR). NHRs comprise greater than one-third of COVID-19 U.S. deaths, emphasizing the importance of engaging in end-of-life discussions. At South Texas Veterans Health Care System (STVHCS), we implemented early documentation of patient's Life-Sustaining Treatment (LST) or end-of-life goals-of-care preferences prior COVID-19 infection. We now aim to examine the association between early LST documentation (prior to COVID-19 diagnosis) and hospital admissions for COVID-19 by conducting a retrospective cohort study of Veteran NHRs at STVHCS from March 2020-January 2021. Inclusion criteria were NHRs with COVID-19 diagnosis, LST documentation, and clear timing of whether the LST documentation occurred before or after COVID-19 diagnosis. Logistic regression was used to determine the likelihood of hospitalization by whether LST was documented before or after COVID-19 diagnosis. 208 NHRs were diagnosed with COVID-19 and 160 (76.9%) had LST documentation. Of these, 148 were included in the analysis: 84 (56.8%) had a completed LST note prior to diagnosis and 64 (43.2%) after diagnosis. The hospitalization rate was 46% for those with LST prior to diagnosis compared to 78% in those with LST after diagnosis (OR = 0.24, 95% CI: 0.12-0.50, P<0.001), showing that early LST documentation was associated with 76% lower likelihood of hospitalization. Early interventions for LST documentation can reduce hospitalization in high-risk populations. These findings may have implications for reducing unnecessary hospitalizations, diminishing healthcare costs, and resolving ethical dilemmas related to potential resource allocation during a pandemic.

### EFFECT OF RACE AND DEMENTIA PREVALENCE ON A COVID-19 INFECTION CONTROL INTERVENTION IN MASSACHUSETTS NURSING HOMES

Alyssa Dufour,[1] Cyrus Kosar,[2] Vincent Mor,[2] and Lewis Lipsitz,[1], *1. Hebrew SeniorLife, Boston, Massachusetts, United States, 2. Brown University, Providence, Rhode Island, United States*

Nursing home (NH) residents, especially those who were Black or had dementia, had the highest infection rates during the COVID-19 pandemic. A 9-week COVID-19 infection control intervention in 360 Massachusetts NHs showed that adherence to an infection control checklist with proper PPE use and cohorting, was associated with declines in weekly infection rates. NHs were offered weekly webinars, answers to infection control questions, resources to acquire personal protective equipment, backup staff, and SARS-CoV-2 testing. We asked whether the effect of this intervention differed by racial and dementia composition of the NHs. Data were obtained from 4 state audits using infection control checklists, weekly infection rates, and Minimum Data Set variables on race and dementia to determine whether adherence to the checklist competencies was associated with decline in average weekly rates of new COVID-19 infections. Using a mixed effects hurdle model, adjusted for county COVID-19 prevalence, we found that the overall effect of the intervention did not differ by race, but proper cohorting of residents was associated with a greater reduction in infection rates among facilities with ≥20% non-whites (n=83). Facilities in the middle (50-61%; n=116) and upper (>61%; n=118) tertiles of dementia prevalence had the largest reduction in infection rates as checklist scores improved. Cohorting was associated with greater reductions in infection rates among facilities in the middle and upper tertiles of dementia prevalence. Thus, adherence to proper infection control procedures, particularly cohorting, can reduce COVID-19 infections, even in facilities with high percentages of high-risk residents (non-white and dementia).

### HOSPITAL IN HOME COVID-19 MONITORING: A NOVEL APPROACH TO KEEPING A WATCHFUL EYE ON OLDER ADULTS

Alissa Cooney,[1] Monica Serra,[2] Monica Lee,[3] Kimberly Oakman,[3] Laura Medlin,[3] Sunil Ahuja,[1] Marcos Restrepo,[1] and Sandra Sanchez-Reilly,[1], *1. University of Texas HSC San Antonio, San Antonio, Texas, United States, 2. UT Health San Antonio - Barshop Institute, San Antonio, Texas, United States, 3. South Texas Veterans Health Care System, San Antonio, Texas, United States*

Older adults suffering from severe acute respiratory syndrome coronavirus 2 (SARS-CoV-2) are at increased risk of death and hospitalization-related complications. The coronavirus disease 2019 (COVID-19) pandemic has forced adaptations in Telehealth, allowing COVID-19 patients to be managed at home. Traditionally, Hospital in Home (HIH) patients have better clinical outcomes and lower mortality compared to similar hospitalized patients. However, effectiveness of HIH for COVID-19 older adults remains unknown. This study examines the effect of age on rates of hospital readmission and overall mortality for patients enrolled in HIH after initial COVID-19 hospital discharge. A HIH COVID-19 monitoring program was developed to facilitate earlier hospital discharge and monitoring. Retrospective data between March 2020 and January 2021 were analyzed. Of the 402 subjects (age:26-99; mean:61.8), 13 (6.1%) subjects <65 years old vs 19 (10%) subjects □65 years old were readmitted to the hospital at least once. Two (0.94%) subjects <65 years old and 12 (6.3%) subjects □65 years old died. Older adults were 1.719 times more likely to be re-hospitalized (p=0.005) and 7.153 times more likely to die (p=0.017) compared to younger adults. Age remains a significant predictor of hospital readmission and mortality in subjects previously hospitalized for COVID-19 even when followed by monitoring programs like HIH. Further studies are needed to determine the best way to reduce hospital readmission and mortality rates for older adults after initial COVID-19 hospital discharge.

### HOW COVID-19 PANDEMIC CHANGED CONSUMPTION OF FRUITS AND VEGETABLES BY OLDER ADULTS

Lillie Monroe-Lord,[1] and Azam Ardakani,[2], *1. University of the District of Columbia, Washington, District of Columbia, United States, 2. Howard University, Howard University, District of Columbia, United States*

This study aims to determine the changes in consumption of fruits and vegetables of older adults before and since the COVID-19 pandemic. The data collection was administered by Qualtrics through an online survey conducted in August and September 2020. The total participants were 10,050. Differences between consumption of fruits (fresh, canned, frozen) and vegetables (carrots, sweet potatoes, broccoli, spinach) before and since COVID-19. Date were analyzed using the Wilcoxon's signed-rank test. Among participants, 5,767 females (57.4%) and 4,283 males (42.6%) and the average age of 62.09 (SD=11.22). 7.1% were Asian (N=701), 4.3% were Hispanic (N=429), 14.1% were African Americans (N=1393), and 74.5% were White (N=7,390). For total participants, consumption of fruits decreased significantly (p<0.001) since COVID-19. The decrease in consumption of fruits was larger in females (p<0.001) than males (p=0.026). It is likely because consumption of fruits by males was already low before the pandemic at 27% of the amount consumed by females. The decrease in consumption of fruits was not statistically significant in Asian (p=0.096) and African American (p=0.07), but significant in Hispanic (p=0.008) and White (p<0.001) participants. African American and Hispanic participants consumed a lower number of fruits before the pandemic compared to Asian and White participants. Consumption of vegetables had no significant change since COVID-19 for total participants regardless of gender and race. This study reported a significant decrease in the consumption of fruits, but not vegetables by older adults since COVID-19 pandemic.

### IMPACT OF COVID-19 ON OLDER ADULTS: CHANGES IN HEALTH ACCESS, HEALTH, SOCIALIZATION AND ADAPTIVE COPING ACTIVITIES

Judith Scott,[1] Sara Qualls,[2] and Stacy Yun,[1], *1. University of Colorado Colorado Springs, Colorado Springs, Colorado, United States, 2. University of Colorado at Colorado Springs, University of Colorado at Colorado Springs, Colorado, United States*

Indirect effects of stay-at-home guidelines may negatively affect mental health by reducing health self-care behaviors and engagement in social participation. This study reports on the impact of the COVID-19 pandemic on community-dwelling older adults' perceived physical and mental health and everyday health behaviors. 126 older adults participated in a county-wide telephone survey during June-July of 2020, asking about changes in mental and physical health, and adaptations in health behaviors. We investigated the effects of three negative everyday health behavior changes during the pandemic (changes in health services access, perceived changes in health, and increased social isolation) as well as two positive everyday health behaviors (adherence to stay-at-home guidelines to reduce risk, and adaptive coping activities) on mental health and COVID-related distress. Examples of active coping strategies were stockpiling resources, spiritual practices, or outreach to others. Descriptive statistics, bivariate correlations, and multiple regressions characterized the impact of COVID-19 on perceived mental health. Descriptive data included changes in health service access, changes in mental and physical health, reduced social engagement, increased adherence to guidelines, and increased adaptive coping activities. Significant predictors of mental health impact of the pandemic were changes in health service access ($\beta = .18$, $p < .05$), health changes ($\beta = .25$, $p < .01$), and adaptive coping activities ($\beta = .21$, $p < .05$). Findings suggest COVID-19 distress may be alleviated with improved health care access and increased social contact. Mental health challenges may also benefit from increased engagement in adaptive coping activities.

### IMPACT OF THE COVID-19 PANDEMIC ON PARTICIPATION IN COMMUNITY-DWELLING OLDER ADULTS: A CROSS-SECTIONAL ANALYSIS

Khang Nguyen,[1] Luciana Macedo,[2] Brenda Vrkljan,[2] Renata Kirkwood,[2] Jinhui Ma,[2] and Marla Beauchamp,[2], *1. McMaster University, Hamilton, Ontario, Canada, 2. McMaster University, McMaster University, Ontario, Canada*

Public health guidelines to prevent spreading COVID-19 place older adults at risk of loneliness and social isolation. Evidence suggests that participation protects older adults from such detrimental outcomes, therefore we aimed to identify the factors associated with participation in life roles among older adults living in the community during the COVID-19 pandemic. We conducted a telesurvey on a random sample of community-dwelling older adults living in Hamilton, Ontario, Canada, between May and July 2020. Outcome measures included participation in life roles, physical function, physical activity, mobility, mental health, nutrition, and demographics. We conducted two multivariate regression analyses with the Late Life Disability Instrument's (LLDI) frequency and limitations scales as the dependent variables. Candidate factors were organized by International Classification of Functioning, Disability, and Health (ICF) framework domains; personal factors, body functions and structures, activities, and environmental factors. A total of 272 older adults completed the telesurvey (mean age 78 ±7.3 yrs, 70% female). Age, using walking aids, driving status, household income, education, mental health, nutrition, physical function, and dwelling type explained 47.1% (p<0.001) of the variance observed in LLDI frequency scores. Using walking aids, driving status, receiving health assistance, mental health, and physical function explained 33.9% (p<0.001) of the variance observed in LLDI limitation scores. These findings highlight factors from multiple

ICF domains that are associated with participation limitation and frequency among older adults during the pandemic. Our findings have implications for developing public health initiatives to mitigate the effects of the pandemic on the participation of older adults.

### LONELINESS AMONG US ADULTS DURING THE EARLY PHASE OF THE COVID-19 PANDEMIC: FINDINGS FROM THE COVID-19 COPING STUDY

Brendan O'Shea,[1] Jessica Finlay,[1] Jasdeep Kler,[1] Carly Joseph,[2] and Lindsay Kobayashi,[1], *1. University of Michigan, Ann Arbor, Michigan, United States, 2. University of Michigan, Central Michigan University, Ann Arbor, Michigan, United States*

We aimed to estimate the prevalence of loneliness and identify the key sociodemographic, employment, living, and health-related risk factors for loneliness among middle-aged and older adults during the early COVID-19 pandemic in the US, when shelter-in-place and social distancing restrictions were in place for much of the country. Data were collected from online questionnaires in the COVID-19 Coping Study, a national study of 6,938 US adults aged 55-110 years, from April 2nd through May 31st, 2020. We estimated the population-weighted prevalence of loneliness (scores of ≥6/9 on the 3-item UCLA Loneliness Scale), overall and according to sociodemographic, employment, living, and health-related factors. We used population-weighted modified Poisson regression models to estimate prevalence ratios (PRs) and 95% confidence intervals (CIs) for the associations between these factors and loneliness, adjusted for age, sex, race, ethnicity, and education. Overall, 29.5% (95% CI: 27.9%, 31.3%) of US adults aged 55-110 were considered high in loneliness in April and May, 2020. In population-weighted, adjusted models, loneliness was most frequent among those with depression, those who were divorced or separated, those who lived alone, those diagnosed with multiple comorbid conditions, and individuals who were unemployed prior to the pandemic. In conclusion, we identified subpopulations of middle-aged and older US adults that were highly affected by loneliness during a period when COVID-19 shelter-in-place orders were in place across most of the country. These insights may inform the allocation of recourses to mitigate loneliness during times of restricted activity.

### THE EFFECT OF TESTING TURNAROUND TIME ON COVID-19 OUTBREAK SEVERITY WITHIN U.S. NURSING HOMES

Annie Rhodes, Leland Waters, Faika Zanjani, Tracey Gendron, and Rick Moore, *Virginia Commonwealth University, Richmond, Virginia, United States*

COVID-19 has brought renewed attention to infectious diseases in U.S. nursing homes (NHs). The Testing turnaround time (TAT) of SARS-CoV-2 is vital information, supporting staff ability to make decisions regarding resource allocation.

**Methods:** Using data obtained from the National Healthcare Safety Network's COVID-19 nursing home data set, we analyzed the TAT of laboratory polymerase chain reaction (PCR) testing on outbreak severity (number of people infected) for residents and staff. A MANOVA was performed on NHs submitting data over 26 weeks (May-November 2020). The independent variable was the average TAT for the two weeks prior (<24 hours, 1-2 days, 3-7 days, or 7+ days).

**Results:** N = 15,363 NHs. The TAT for the combined dependent variables of staff and resident COVID-19 cases. $F(10,781,354) = 3161.265$, Pillai's trace = .078, $p<.0005$, partial $\eta 2=.4$. The average outbreak severity for staff was 13.93 cases when TAT was < 24 hours, compared to 15.29 cases at 1-2 days. For residents, the difference was less pronounced but still significant. The average outbreak severity for residents was 17.07 cases when TAT was<24 hours, compared to 18.61 cases when the TAT was 1-2 days. Tukey post-hoc tests found significance for all levels of testing for residents and staff at $p<.0005$.

**Discussion:** Time differences to receive PCR test results from a laboratory are significant in outbreak severity for staff and residents. The most meaningful result positively impacting the ultimate spread and severity of gross cases is when the TAT for PCR results is < 1 day.

### THE IMPACT OF COVID-19: PERSPECTIVES OF RECREATIONAL THERAPISTS WORKING WITH OLDER ADULTS

Betsy Kemeny,[1] and Dawn DeVries,[2], *1. Slippery Rock University, Grove City, Pennsylvania, United States, 2. Grand Valley State University, Grand Rapids, Michigan, United States*

This study explored the perspectives of recreational therapists (RT) from Pennsylvania and Michigan and how COVID-19 has impacted older adults and their roles in various settings. COVID-19 safety restrictions limiting social interaction with both peers and families had the potential for negatively impacting the social and emotional well-being of older adults (Van Orden et al., 2020) and the roles of therapists who work with them. Because peer socialization and physical activity programs prevent falls (Cameron et al., 2018) and improve depressive symptoms (Harvey et al., 2015), a better understanding of COVID-19 impact is significant. From a qualitative viewpoint, 14 RTs from various levels of care were interviewed to better understand their perspective on the impacts of COVID on older adults and their own practice. Hour long interviews conducted via zoom focused on organizational changes, role changes, and impact on older adults. After recordings were transcribed, multiple researchers classified, summarized, and tabulated the data. A consensus method determined common themes. From the RT's perspective, older adults have not only been negatively impacted in the social domain. While many older adults showed resilience, others were impacted physically, emotionally, and cognitively. Moreover, an increased importance on meaningful engagement, recreation, and leisure emerged. Technology became an essential tool in interpersonal connection. Teamwork, personal self-care, and coping were integral to providing effective care. Post pandemic, RTs are concerned about the challenge of reengaging older adults in groups but are certain that technology will continue to be used in a more expansive way in programs.

### THE LIVED EXPERIENCE OF ASSISTED LIVING ADMINISTRATORS DURING THE COVID-19 PANDEMIC

Elizabeth Hill, Rebecca Davis, Paige Greer, and Susan Strouse, *Grand Valley State University, Grand Rapids, Michigan, United States*

Since March, 2020, administrators in assisted living (AL) residences have been challenged to provide the best care for their populations while undergoing a pandemic. Because nothing like this has happened in the recent past, AL administrators had to make many new decisions. The purpose of this phenomenological study is to reflect on the lived experiences of AL administrators during the COVID pandemic. Using a semi structured interview, individual interviews of four AL administrators from different AL communities were conducted via Zoom. The interviews contained questions related to the participants' experiences with the COVID-19 pandemic. The recorded interviews were transcribed verbatim into MAXQDA. Data analysis followed a modified Giorgi approach, by reviewing the recorded interviews, categorizing the data into meaning units, then situated units, generalized units, and then themes. Results indicated that AL administrators have been adapting to constantly changing and conflicting regulations. The pandemic incited fear, depression, moral distress, but also hope for the future. The data shows a multitude of feelings and actions related to the well-being of the staff, residents, and residents' families. The limitations of this study include a small sample size and the evolving nature of the pandemic in Michigan. Opportunities for future research would be to compare our findings to the experience of other AL's in the United States. The results show the complexity of AL administrators' lived experiences during the pandemic and highlight important considerations if an event like the pandemic were to occur again.

### TIMELINE OF COVID-19 INCIDENCE AND MORTALITY AMONG RESIDENTS AND STAFF OF SOUTH CAROLINA LONG-TERM CARE FACILITIES

Matthew Lohman,[1] Nicholas Resciniti,[1] Morgan Fuller,[1] and Joshua Sellner,[2], *1. University of South Carolina, Columbia, South Carolina, United States, 2. South Carolina Department of Health & Environmental Control, Columbia, South Carolina, United States*

The COVID-19 pandemic has disproportionately impacted older adults living in long-term care facilities (LTCFs), but little research has described parallel infection rates and mortality among LTCF residents and staff in relation to state-level mitigation measures. This study used comprehensive COVID-19 tracking data from the South Carolina Department of Health and Environmental Control (SCDHEC), including case report information on demographics, symptoms, comorbidities, and employment. We included all confirmed or probable COVID-19 cases and deaths among adult SC residents reported between 3/15/2020 and 1/2/2021. Residence or employment in LTCF, including nursing homes, assisted living, or skilled nursing facilities, were confirmed by SCDHEC. Cox proportional hazards models were used to compare mortality between residents/staff and counterparts in the community. Overall, 54,514 cases of COVID-19 were identified among older adults in SC. Of these, 13.5% (n = 7,366) resided in a LTCF. LTCF residents with COVID-19 were more likely to be hospitalized compared to older adults in the community and 74% more likely to die (HR= 1.74, 95% CI: 1.59-1.90), after controlling for age, gender, race, and chronic health conditions. LTCF staff had greater infection rates but lower risk of mortality (HR=0.58; 95% CI: 0.39-0.88) compared to the general population. Differences in COVID-19 incidence and mortality between residents/staff and the community decreased after statewide mitigation policies. This study indicates that LTCF residents are at increased risk of COVID infection and mortality, even accounting for pre-existing health conditions. LTCF settings are key sites for prioritizing prevention, vaccination, and training plans to prepare for future pandemics.

### TRANSFERS AMONG LONG-TERM NURSING HOME RESIDENTS DURING THE COVID-19 PANDEMIC

Ana Montoya,[1] Chiang-Hua Chang,[2] Pil Park,[2] and Julie Bynum,[1], *1. University of Michigan, Ann Arbor, Michigan, United States, 2. University of Michigan, Ann ARbor, Michigan, United States*

Transferring long-term nursing home residents between facilities can compromise the quality of life and be associated with functional decline, hospitalizations, and even death. This study aimed to examine transfer rates and identify risk factors associated with transfers among long-term nursing home residents before (2018-2019) and during the COVID-19 pandemic (2020). Using the Michigan state Minimum Data Set data 2018-2020, we identified long-term residents as those who stayed in nursing homes for at least 100 days each year (N=39,693, 39,454, and 35,575, respectively). We defined a facility-to-facility transfer as a direct transfer between two nursing homes. We first examined the likelihood of transfer by year using logistic regression models, adjusting for residents' age, sex, race, and marital status. We then examined two health statuses that could be associated with a transfer: activities of daily living (ADL) and cognitive impairment. Finally, we compared transfers that occurred before COVID-19 (2018-2019) and during COVID-19 (2020), adjusting for residents' demographic characteristics and health statuses. After adjustment, age was the only factor associated with transfers for all three years (Age>=80: AOR=0.61, 95% CI: 0.54-0.69; AOR=0.63, 95% CI: 0.55-0.72; AOR=0.71, 95% CI: 0.63-0.80, respectively). New risk factors in 2020 were Black race (AOR=1.22, 95% CI: 1.07-1.40) and requiring ADL assistance (AOR=1.24, 95% CI: 1.03-1.49). The COVID-19 period had higher transfer rate (unadjusted rates 2.9%, 2.7%, 3.5%, respectively) with 10% higher odds of transfer compared to before COVID-19 (AOR=1.10, 95% CI: 1.01-1.20). This finding suggests that COVID-19 has an impact on how nursing home transferred their long-term residents.

### TRENDS IN DELIRIUM RATE ACROSS 14 HOSPITALS DURING THE COVID-19 PANDEMIC: A COMPARATIVE STUDY.

Ariba Khan,[1] Asma Sabih,[2] Alexander Schwank,[2] Marianne Klumph,[2] and Michael Malone,[2], *1. Advocate Aurora Health Care, Milwaukee, Wisconsin, United States, 2. Advocate Aurora Health, Milwaukee, Wisconsin, United States*

During the COVID-19 pandemic, strategies to prevent delirium in the hospital were limited due to restrictions in staff and visitor policies. Thus, we suspected the delirium rate may increase during the pandemic. This study aimed to investigate the trends in delirium rate over past 2-years and compare this trend prior-to-and-during the COVID-19 pandemic in hospitalized older adults. Data was retrospectively obtained from the Acute-Care-for-Elders Tracker snapshot, an electronic health record tool to identify the presence of delirium within 48hrs of hospitalization for patients ≥65 years. Periods of interests were 3/2019-6/2019 (pre-COVID) and 3/2020-6/2020 (during-COVID). A weighted rate was calculated for each month by combining data from all hospitals for the total number of inpatients ≥65 years. The overall trend in the delirium rate was assessed with simple linear regression models and an ANCOVA. A $\chi 2$ and a Wilcoxon-Signed-Rank-Test were utilized to test for differences in the overall delirium rate between two time periods. Overall median delirium rate was 6.8% in 70,562 encounters of 42,878 patients (mean age= 78 years; mean length-of-stay= 6.5 days). The median delirium rate increased by 2.1% (6.6%to8.6%), for pre-COVID vs. during-COVID, respectively (Z=-3.044,p<0.001). There were no significant differences between actual and projected weighted delirium rates (p=0.18). However, the weighted delirium rate—for both the actual and projected trend lines—demonstrated significant changes over time (p<0.001).The trend in delirium rate increased over the study time period regardless of the pandemic. Further analyses with longer time-frame are crucial to understand the consequences of the pandemic on delirium rate.

### VARIABLES IMPACTING NURSING HOME GOALS OF CARE DISCUSSIONS AND ORDER IMPLEMENTATION DURING COVID-19

Orah Burack,[1] Joann Reinhardt,[1] Wingyun Mak,[1] Himali Weerahandi,[2] Benjamin Canter,[3] and Kenneth Boockvar,[1], *1. The New Jewish Home, New York, New York, United States, 2. NYU Grossman School of Medicine, New York, New York, United States, 3. The New Jewish Home, The New Jewish Home, New York, United States*

Nursing home (NH) residents are especially vulnerable to COVID-19, disproportionately suffering from severe illness and death. As such, resident Goals of Care (GOC) often had to be quickly established to ensure treatment preferences were known and respected. This study examined variables related to the occurrence of GOC discussions and added orders (Do Not Resuscitate, Do Not Intubate, and Do Not Hospitalize), including demographic, physical functioning, cognitive impairment, depression, number of diagnoses, and Optum participation (Optum provided added specialized care by nurse practitioners who routinely address GOC preferences). Subjects were 286 COVID positive residents from a large NYC NH. All data were obtained from the NH's electronic medical records. Patient median age was 81 n (interquartile range 71-88), 59% were female, 61% were long stay (stay >100 days) and 39% were short stay. Using bivariate correlations we found that older short stay residents were more likely to have GOC conversations. Additionally, older, cognitively impaired, Optum participants were more likely to have orders added. When all independent variables were entered into binary logistic regressions, only older age and being a primary English speaker were significantly related to the occurrence of GOC conversations (□2= 21.76**; N=278; Nagelkerke R2 = .10), while older age and being an Optum participant were related to added orders (□2=32.18**; N=164; Nagelkerke R2 = .24). Results have implications for (1) ensuring the GOC wishes of diverse populations are known and abided by and (2) improving the quality of clinician – resident GOC discussions.

### VIDEO CHAT USE AND MEALTIME BEHAVIORS IN OLDER ADULTS BEFORE AND DURING THE COVID-19 PANDEMIC

Laura Barre,[1] Tara Young,[2] and Sarah Coupal,[2] *1. Cornell University, Ithaca, New York, United States, 2. Cornell University, Cornell University, New York, United States*

Video chat allows people to connect when not physically together. Using video chat while sharing a meal (VideoDining) may decrease loneliness and improve older adults' nutritional intake. We conducted a cross-sectional online survey study using Amazon Mechanical Turk in June 2020. The objectives were to learn about eating with others, the use of video chat, and interest in VideoDining in older adults during the pandemic. There were 1331 survey attempts with 167 responses meeting the criteria for age (65 years of age or older), U.S. residency, and quality. Participants were 64% male, 77% white, 65% college-educated, and a median age of 67 years (IQR=2 years). Few participants lived alone (17%), yet 76% reported feeling isolated. Eating with others regularly, defined as several times a week or more, declined in the pandemic (44% vs. 59% pre-pandemic, p=0.0002). The use of video chat and eating when video chatting increased during the pandemic versus pre-pandemic (82% vs. 74%, p=0.003; 47% vs. 37%, p=0.0005). The majority of participants said they would VideoDine (50%) or consider trying it (37%). Interest in VideoDining did not vary by age, race, or gender. Participants who used video chat were more likely to say they would VideoDine than participants who had never used video chat (OR=3.1; 95% CI=1.25, 8.35; p=0.02). This data suggests most adults 65 years of age and older, already using the Internet, are experiencing isolation and decreased mealtime commensality during the pandemic. The vast majority are using video chat and are interested in trying VideoDining.

### VIRAL CULTURE IN HOSPITALIZED CONGREGATE CARE PATIENTS WITH PROLONGED SARS-COV-2 VIRAL RNA DETECTION

Thilaka Arunachalam,[1] Amit Singh,[2] Kathleen Stellrecht,[3] Sarah Elmendorf,[4] Tarani K. Barman,[5] Michael Robek,[1] Dennis W. Metzger,[1] and Michael Waxman,[6], *1. Albany Medical College, Albany, New York, United States, 2. Albany Medical Center, ALBANY, New York, United States, 3. Albany Medical Center, Albany, New York, United States, 4. Albany Medical Center, Albany Medical Center, New York, United States, 5. Albany Medical Disease, Albany, New York, United States, 6. Albany Medical College, Albany Medical College, New York, United States*

Prolonged detection of SARS-CoV-2 viral RNA has been observed in hospitalized congregate care patients following resolution of clinical symptoms. It is unknown whether patients with persistent PCR positivity pose a risk for COVID-19 transmission. The purpose of this study was to examine the results of serial PCR testing, viral load, and viral culture in patients awaiting discharge prior to a negative PCR test. We sampled 14 patients who were admitted from skilled nursing and/or rehabilitation facilities to a large academic medical center, had clinical signs and symptoms of COVID-19, and had multiple PCR-positive tests separated by at least 14 days.

PCR-positive nasopharyngeal swabs were obtained from each patient for viral load quantification and viral culture. The mean age of patients was 72.5 years (55 – 92), with a mean peak SOFA score of 5.6 (1 – 11). Patients were hospitalized for a mean of 37.0 days (25 – 60). RNA was detected by PCR for a mean of 32.9 days (19 – 47). Mean viral load for the first PCR-positive nasopharyngeal swab collected at our hospital was 5.81 genomic copies/mL (2.12 – 9.72). Viral load decreased significantly with days from clinical symptom onset (R = -0.69, 95% CI, -0.80 – -0.55). Four out of 28 samples grew active virus via culture, with no active virus isolates after 2 days of symptom onset. Our viral culture data suggests that persistent PCR positivity may not correlate with infectivity, which has important implications for COVID-19 infection control precautions among older congregate care patients.

### Session 9165 (Poster)

### COVID-19 PANDEMIC

### "HUNGER WOULD KILL US INSTEAD OF THE PANDEMIC:" ELDERS' RESPONSES TO COVID-19 IN ETHIOPIA

Messay Kotecho,[1] Margaret Adamek,[2] and Anduamlak Takele,[3], *1. Addis Ababa University, Addis Ababa, Adis Abeba, Ethiopia, 2. Indiana University, INDIANAPOLIS, Indiana, United States, 3. Debre Markos University, Debre Markos, Dire Dawa, Ethiopia*

The COVID-19 pandemic has posed unpredictable challenges globally. Urban elders in Global South nations are among the major population groups vulnerable to COVID-19. A qualitative case study design was used to uncover the challenges and sources of support for poor urban elders during COVID-19 lockdown in Ethiopia. Data were collected from 27 elders age 60 and above in Debre Markos Town via in-depth interviews and document review. Narrative data were analyzed using thematic data analysis. Four prominent themes were identified: 1. Food insecurity ("Hunger would kill us instead of COVID-19"), 2. Hopelessness ("Feeling hopeless and begging to die"), 3. Social isolation ("We prefer social support rather than food donations") and 4. Gratitude ("Feeling thankful"). The physical distancing program introduced to contain the pandemic isolated many elders and diminished their capacity to access support from others needed to perform their daily activities. An institutional welfare system is needed to ensure older adults in the Global South can live a joyful and dignified life, even through a global pandemic. Moreover, a special emergency fund to meet older adults' basic needs during a pandemic like COVID-19 should be introduced to minimize the effect of crises on vulnerable groups like destitute older adults in Ethiopia.

### ADAPTABILITY OF OLDER ADULTS AT THE ONSET OF THE COVID-19 PANDEMIC

Laurie Blackman,[1] Donna Wang,[2] Kathryn Krase,[1] Joyce Roberson-Steele,[1] Annette Clarke-Jones,[1] and Latoya Attis,[1], *1. Yeshiva University, Yeshiva University/ New York, New York, United States, 2. Springfield College, Springfield College/Springfield, Massachusetts, United States*

It is important to understand the unique experiences and perspectives of older adults who were required to incorporate critical adjustments to behavior during the onset of the COVID-19 pandemic. An anonymous, cross sectional survey was administered online through Qualtrics Survey Software in June 2020. The results of this study found that older adults utilized different sources of information than younger adults; they were more likely to read the newspaper or listen to the radio, and less likely to rely on social media for information. Older respondents in this study reported coping with the COVID-19 outbreak better than younger respondents, were less likely to report that they were personally affected by the virus, and less likely to feel overwhelmed by the amount of information about COVID-19. The findings of this study highlight resilience in older adults not found in younger adults, and provide an important step in identifying policy and practice suggestions to reduce negative repercussions for older adults experiencing the current crisis, as well as future generations of older adults who might experience similar events.

### EXPLORING CAREGIVER CHALLENGES AND COPING MECHANISMS IN THE MIDST OF COVID 19

Devyani Chandran,[1] and Marie Eaton,[2], *1. Western Washington University, Bellingham, Washington, United States, 2. Western Washington University, Western Washington University, Washington, United States*

This paper reports on the the challenges faced and coping strategies employed community-based care providers in a mid sized city in the Pacific Northwest in the midst of the Covid 19 pandemic. Researchers from a Palliative Care Institute conducted six online focus groups using the zoom platform. The project aimed at gathering important information on the experiences of caregivers as well as providing a virtual space where caregivers could support each other. A purposive sampling technique was used to gather data where participants were not chosen randomly but because they could best answer the research question. Membership of the focus groups included representatives from skilled nursing facilities, home-care agencies, elder law services, memory-care facilities, adult family homes, medical supply facilities, chaplains and nutritionists. Data gathered from the focus group were transcribed. A constant comparison method of analysis were employed and categories and themes were created from open coded data. Six key themes were identified which included: dealing with the impact of social isolation on caregiver-client relationships, assessing personal risk when dealing with clients living with dementia, facing challenges seeking continuing employment, struggling with social support and self care, using technology for professional and personal support and grappling with the challenges reentering a face to face environment once the pandemic is controlled.. Findings point to the importance developing and sustaining technological innovations that support workforce retention, fostering communication between the larger community, care providers and clients in various care settings and planning for safe reentry into a post Covid world.

### HIGHER STAR, BETTER PERFORMANCE IN US NURSING HOMES DURING THE COVID-19 PANDEMIC ?

Lei Yu,[1] Xiao Qiu,[1] and Tara Rose,[2], *1. Miami University, Oxford, Ohio, United States, 2. University of Southern California, Los Angeles, California, United States*

The Covid-19 pandemic has brought terrible difficulties to nursing homes, as they were locations with the highest number of confirmed Covid-19 cases and deaths in the US. The Centers for Medicare & Medicaid Services (CMS) applies the Five-Star Quality Ratings to indicate the quality of care in nursing homes based on health inspection survey, staffing, and resident outcome. Studies to date have contradictory findings regarding the relationship between nursing home reported Quality Ratings and Covid-19 cases and deaths based on US regional data. The purpose of this study is to examine whether nursing homes' Quality Ratings were related to the total number of resident Covid-19 cases and deaths at the US National level. The study examined US nursing homes (N=13,494) linked with CMS Nursing Home Compare data and Covid-19 Nursing Home data. Using multiple linear regression analyses, results showed nursing home Quality Ratings were significantly associated with Covid-19 residents' cases and deaths controlling for ownership type, size, occupation rate, and years of operation (p<.001; p<.001). Five-star nursing homes were less likely to have Covid-19 cases and deaths. Further, comparing lower Star Ratings nursing homes, 1-Star nursing homes showed no significant difference to 2-Star and 3-Star nursing homes when examining Covid-19 cases and deaths. Overall, the Five-Star Quality Ratings is a useful measure when investigating nursing homes' performance during the Covid-19 pandemic. Future policymakers and administrators should also focus on nursing homes with lower star ratings when improving the quality of nursing homes, particularly with regard to resident health.

### NO VISITORS ALLOWED! THE IMPACT OF COVID-19 RESTRICTIONS ON THE PSYCHOSOCIAL WELL-BEING OF NURSING HOME RESIDENTS

Shanae Shaw,[1] and Ellen Csikai,[2], *1. Alabama State University, Calera, Alabama, United States, 2. The University of Alabama, The University of Alabama, Alabama, United States*

The COVID-19 coronavirus pandemic changed life for everyone, but especially for nursing home residents. In March 2020, the Centers for Medicare and Medicaid Services enacted nursing home restrictions in response to the pandemic regarding visitation from outside family/friends and changes to facility activity programs. Despite the public health concern prompting these restrictions to prevent virus spread, these sudden changes affected nursing home residents' relationships with their spouses/partners. As part of a larger study to identify the nursing home policies and practices that preserve relationships among nursing home residents with spouses/partners, participants shared facilities' restrictions, social connection practices, and effect of coronavirus restrictions on residents' relationships with spouses/partners. The study utilized both an online survey (81 respondents) and ten telephone interviews with nursing home social workers in four Southern states. Twenty-eight percent of participants reported that no visitors were allowed; while 25% allowed couples to visit with one another as usual was reported by 25%. The most noted practices to maintain social connections were phone calls, video calls, and 'window' visits between residents and families/friends; however, "it's just not the same. It's affected them greatly". The interviews revealed further details about the detrimental effect of the COVID-19 restrictions on nursing home residents' overall mental health and attachment relationships with spouses/partners. These results highlight the importance of maintaining social connections between residents and spouses/partners. Nursing home social workers can develop policies and practices that enhance relationships and connections under all circumstances and work with other health care team members to ensure implementation.

### RELATIONSHIP BETWEEN SOCIAL DETERMINANTS OF HEALTH AND LONELINESS DURING COVID-19 PANDEMIC

Kalpana Padala,[1] Prasad Padala,[2] Christina Crawford,[2] Clinton Gauss,[2] Benjamin Wright,[2] Olive Phillips,[2] Richard Dennis,[2] and Hillary Lum,[3] *1. Central Arkansas Veterans Healthcare System, Central Arkansas Veterans Healthcare System, North Little Rock, Arkansas, United States, 2. Central Arkansas Veterans Healthcare System, North Little Rock, Arkansas, United States, 3. University of Colorado School of Medicine, Aurora, Colorado, United States*

**Background:** Older adults are most vulnerable to social isolation and loneliness during the COVID-19 pandemic compared to other populations. Risk factors for loneliness include old age, rural living, number of medical comorbidities, and poor social networks. The objectives of this study were to examine the prevalence of loneliness in older adults during COVID-19 and determine the correlation between social determinants of health and loneliness.

**Methods:** A cross-sectional study was conducted in community dwelling older Veterans (N=132). Demographic data were collected along with variables related to social determinants of health. Loneliness data were collected with the 3-item loneliness questionnaire, and social network was assessed using the 6-item Lubben social network scale.

**Results:** Demographic data included: mean age 73.3 (±7.5) years, 93.2% male, 53.5% rural, 84.1% Caucasian, and 13.6% African American. The majority of the participants reported loneliness (65.6%). Mean Lubben social network score was 14.6 (±6.6). There was a strong negative correlation between loneliness and social network (p<0.0001, r=-0.57; 95% CI: -0.67, -0.44). The prevalence of loneliness was significantly greater in those living alone compared to those not living alone (p=0.017; 83.9% vs. 60.6%) and those lacking internet access compared to those with internet access (p=0.025; 86.4% vs. 61.5%).

**Conclusion:** Loneliness was found to be highly prevalent in an older cohort during the COVID pandemic. Routine inquiry about loneliness is important. Social determinants of health are likely correlated with the presence of loneliness in older adults and could be greatly impacted by policy decisions made to control community disease transmission.

### SPATIAL DISTRIBUTION OF COVID-19 AND COMORBIDITIES IN NEW ENGLAND

Chae Man Lee,[1] Taylor Jansen,[1] Shu Xu,[2] Maki Karakida,[1] Frank Porell,[3] Nina Silverstein,[4] and Elizabeth Dugan,[1], *1. UMass Boston, Boston, Massachusetts, United States, 2. University of Masschusetts Boston, Dorchester, Massachusetts, United States, 3. University of Massachusetts Boston, University of*

*Massachusetts Boston, Massachusetts, United States, 4. University of Massachusetts Boston, Boston, Massachusetts, United States*

At the onset of the COVID-19 pandemic, the Centers for Disease Control and Prevention (CDC) identified chronic conditions which elevated the risk of COVID-19 complications: chronic obstructive pulmonary disease (COPD), diabetes, heart disease, kidney disease, and obesity. The aim of this study is to visualize the spatial distribution of confirmed cases of COVID-19 and local rates of comorbidities in CT, MA, NH, and RI and to identify the spatial clustering of hot spot between COVID-19 cases and rates of a summary measure of comorbidities across communities. This study collected data from state's departments of public health in 4 New England states of confirmed COVID-19 cases as of February 25th, 2021 and extracted community-level rates of comorbidities among adults age 65+ from recent Healthy Aging Data Report (www.healthyagingdatareports.org). Results showed that the cities Bridgeport, CT (n=14,637), Boston, MA (n=57,912), Manchester, NH (n=9,658), and Providence, RI (n=26,792) had the highest rates of COVID-19 and the highest population density. The GIS based map illustrated that the largest cities with the highest population densities had both relatively high incidences of COVID-19 and heavy burdens of comorbidities. This study found that the hot spot areas of COVID-19 were observed in communities with the highest chronic disease burdens. These hot spots of COVID-19 and comorbidities are areas where resources (testing, masks, vaccines) should be surged to protect the community. The identification of hot spots may motivate residents to take every mitigation step to prevent and control COVID-19.

### THE SEASONS OF COVID-19: UNDERSTANDING THE MOOD OF OLDER ADULTS IN THE UK THROUGH A BAROMETER SURVEY SPANNING 12 MONTHS

Nicola Palmarini,[1] Lesley Hall,[2] Alex Mitchell,[3] James McNaughton,[2] and Charlotte Nixon,[4], *1. Newscastle University, Newcastle Upon Tyne, England, United Kingdom, 2. Newcastle University, Newcastle upon Tyne, England, United Kingdom, 3. UK National Innovation Centre for Ageing, Newcastle upon Tyne, England, United Kingdom, 4. UK's National Innovation Centre for Ageing, Newcastle, England, United Kingdom*

The impact of COVID-19 on older adults has been analysed through different research approaches. However, with its sudden global spread, combined with uncertainty about which countermeasures would be employed, there was a lack of opportunity to systematically and continuously engage in a system of observing the moods of older adults forced to live in unexpected conditions. Ageist narratives, social distancing, the unending barrage of real and fake news, and the lockdowns, have given rise to what we define as a series of "seasons" of life, characterised not by the weather barometer, but by moods of people. How much did these external events, like the impact of weather, affect the mood of older adults? We immediately recognised the pandemic's long-term nature, and thanks to our position as an "observatory" of social dynamics, and because of our existing community of older adults (VOICE), we could involve our members to provide valuable insights about mood and wellbeing during the pandemic. We initiated a weekly pulse survey, based on the two same questions, starting in week 13 of 2020. Across the 50 weeks which followed, we received 2577 responses. They rated their mood on a scale of 1 (extra-stormy) to 5 (all sunshine), before we collated the data and mapped on key events related to media announcements and political decisions. Our research showed the impact of these events on the mood of participants, and the potential of this approach to identify trends in mood to help policy makers with informed decision-making during unprecedented times.

### TYPE 2 DIABETES MANAGEMENT AMONG OLDER BLACK AMERICAN MEN DURING THE COVID-19 PANDEMIC: A QUALITATIVE INVESTIGATION

Ledric Sherman,[1] DeLawnia Comer-HaGans,[2] and Sara Vera,[1], *1. Texas A&M University, College Station, Texas, United States, 2. Texas Department of State Health Services, Austin, Texas, United States*

As COVID-19 swept across the globe in 2020, it appeared to have infected and killed Black Americans at a disproportionately higher rate. However, few studies have focused specifically on the complications of managing diabetes, expressly type 2 diabetes (T2D), among Black men during the global pandemic. Therefore, the purpose of this qualitative study was to seek understanding of the experiences in managing T2D among Black men during the COVID-19 pandemic. One on one interviews were conducted via Zoom video conferencing with twenty-two (n=22) Black men regarding their experience of managing type 2 diabetes (T2D) in a pandemic environment. Four main themes emerged from the study, which are: (1) stress levels during the pandemic, (2) barriers to managing my diabetes, (3) who/what is helping the most, and (4) communication with health-care provider. As more information becomes available, it is apparent that having diabetes increases the risk for detrimental COVID-19 outcomes (i.e. increased lengths of hospital stays, the need for mechanical ventilation, and mortality. Future research efforts are crucially needed to provide an improved understanding of how individuals across all racial groups are managing diabetes during the COVID-19 pandemic.

### WE NEED TO PROTECT OURSELVES: THE MENTAL BURDEN OF MANAGING TYPE 2 DIABETES AMONG BLACK MEN DURING THE COVID19 PANDEMIC

Ledric Sherman,[1] Anthony Pattin,[2] Carla Pattin,[2] Michelle Strong,[1] and Sara Vera,[1], *1. Texas A&M University, College Station, Texas, United States, 2. The University of Toledo, Toledo, Ohio, United States*

As COVID-19 swept across the United States in 2020, it appeared to infect and kill Black Americans at a disproportionately high rate. When examining the literature pertaining to the pandemic, present COVID-19 research focuses on physical health, but research regarding mental health and stress during the COVID-19 pandemic are lacking, especially among Black men and minority men in general. Therefore, the purpose of this study was to learn more about the type 2 diabetes (T2D) management related stress among Black men (n=22) during the COVID-19 pandemic. One on one interviews were conducted via Zoom video conferencing to gain an understanding of the experiences of managing T2D in a pandemic environment. Three main themes emerged from the study, which are: (1) my stress levels during this time, (2) diabetes specific stressors, and (3) coping mechanisms. Black men with diabetes may need psychosocial support that holistically addresses general developmental and diabetes-specific stressors and their influences on one another. Recognizing how Black men react to stress is essential for understanding and addressing their disproportionally high prevalence of stress related to management of chronic conditions and avoiding additional complications, morbidity or mortality. It is imperative to examine how stress and coping frameworks relate to men's health, in general, but specifically in Black men.

### Session 9170 (Poster)

### COVID-19 PANDEMIC AND EDUCATION

### A REMOTE MUSIC-BASED SERVICE-LEARNING PROJECT TO SUPPORT ISOLATED OLDER ADULTS IN LONG-TERM CARE

Emma King, and Alaine Reschke-Hernandez, *University of Kentucky, Lexington, Kentucky, United States*

Growth of the aging population warrants a commitment to teaching students to work effectively with older adults. In an online university course focused on inclusive music practices, students engaged in an extensive service-learning project to enhance course learning objectives and increase sensitivity to disability, social justice, and inclusion issues across the lifespan. Based on community needs, one student aimed to reduce isolation among older adults living in a long-term care facility. The student selected this target and population because of the detrimental effects of isolation on psychosocial, cognitive, and physical well-being; and reduced access to interactive programs and services among older adults living in residential facilities during the COVID-19 pandemic. Components of the project included a service-learning proposal, an annotated bibliography, an interview with the community partner, three activity designs, and a reflective essay. Based on existing research regarding interventions used to reduce isolation among older adults, the three activities the student designed were music bingo, interactive singing, and stretches to music. Throughout the service-learning process, the student consulted the community partner to ensure relevance of the activities. Principles of Universal Design for Learning (UDL), such as providing multiple visual and auditory means to engage with materials, enhanced accessibility of the activities. In this presentation, the student and course instructor will reflect on this case experience and the value of service-learning to foster professional skill development, engaged citizenship in working effectively with older adults, and an appreciation of the need for justice, equity, and fairness for all community members.

### ASSESSING NEEDS, WELL-BEING, AND TELEHEALTH ACCESS OF OLDER ADULTS WITH LIMITED RESOURCES DURING COVID-19 PANDEMIC

Sashah Damier,[1] Assma Twahir,[2] Sweta Tewary,[3] Naushira Pandya,[2] and Nicole Cook,[2], *1. NSU, Davie,FL, Florida, United States, 2. NSU, Davie, Florida, United States, 3. Nova Southeastern University, Fort Lauderdale, Florida, United States*

The COVID-19 pandemic created new barriers and challenges to accessing primary care services, particularly among older adults who already faced barriers related to access to care, including transportation, health literacy, and self-management support. Nova Southeastern University South Florida Geriatric Workforce Enhancement Program (NSU SFGWEP) partnered with primary care clinics to conduct wellness calls to older adult patients identified through clinic EHR. The wellness calls' objectives were to 1) discuss COVID-19 protective measures; 2) assess wellness needs and access to care barriers; and 3) screen for telehealth support. From September 2020 to February 2021, the team (including medical students, public health students, and SFGWEP staff) contacted 200 patients via telephone and conducted a comprehensive wellness survey developed by the study team, informed by validated surveys. Among the 200 patients called, 60% (n=34) were very concerned about the Covid-19 pandemic, 33% (n=34) reported often feeling isolated from others, and 20% (n=34) expressed difficulty getting medical care. A smaller subset of patients reported concern about limited COVID-19 testing (n=1), lack of knowledge about seeing their provider via telehealth (n=7), lack of face masks (n=1), and challenges with obtaining medication refills (n=1). Following wellness calls, the NSU SFGWEP team provided education, referred to clinical resources, and, for low-income patients with access to care challenges, provided Samsung Tablets (n=50) with peer training to enable telehealth.

### COVID-19 AND AGE-FRIENDLINESS IN HIGHER EDUCATION

Celeste Beaulieu,[1] Nina Silverstein,[1] Lauren Bowen,[2] Susan Whitbourne,[3] and Joann Montepare,[4], *1. University of Massachusetts Boston, Boston, Massachusetts, United States, 2. University of Massachusetts, Boston, University of Massachusetts Boston, Massachusetts, United States, 3. University of Massachusetts Boston, University of Massachusetts Boston, Massachusetts, United States, 4. Lasell University, Newton, Massachusetts, United States*

During the COVID-19 pandemic, universities have changed to an online or hybrid format. These changes provide the opportunity for universities to be more accessible for all individuals. However, the logistics of university life during a pandemic has exposed significant and potentially enduring challenges and opportunities for designing and maintaining an Age-Friendly University. This study investigates perceptions of students, faculty, and staff in the lens of an age friendly university during the COVID-19 pandemic. This study draws on qualitative and quantitative data from over 10,000 faculty, staff, students, and life-long learners from 26 universities. Five items were asked to constituent groups about their perceptions on their university's response to COVID-19. Overall, students had the poorest average perception of satisfaction with their university's overall response to the pandemic, with 62% satisfied compared to 74% and 73% of faculty and staff, respectively. Further, 77% of faculty think the university's response to continuing education accommodated their needs, compared to 69% of students. Respondents from private universities reported more positive satisfaction than respondents from public universities ($M=4.23$, $SD=0.94$; $t(df=7405)=6.805$, $p<.001$). Qualitative data suggest that older students and faculty needed more technological assistance during this transition to primarily online learning to keep older members involved in the community. Older staff felt that they were more likely to be furloughed and were the group most likely to not have a choice in working on or off campus.

### FROM SURVIVING TO EXCELLING: ADAPTATION OF GERONTOLOGY SERVICE LEARNING DURING THE COVID-19 PANDEMIC

Donna Jensen,[1] Theresa Abah,[2] Carol Sewell,[3] and Terrence Ranjo,[3], *1. California State University, Sacramento, Sacramento, California, United States, 2. California State University, Sacramento, Sacramento State University, California, United States, 3. Sacramento State University, Sacramento, California, United States*

The COVID-19 pandemic has disproportionately impacted older adults, and the educational service-learning opportunities available to gerontology students. As an applied major, Sacramento State University's Gerontology Department heavily depends on service-learning. The pandemic affected existing gerontology placements and their ability to host student learning. In addition, at the outset of the pandemic, Sacramento State University immediately terminated in-person service learning. The Gerontology Department prioritized student and community safety while still valuing the need for students to have meaningful and relevant community-based learning experiences. Gerontology faculty worked with community partners to shift these vital learning experiences. This poster presentation focuses on the creative ways the department engaged community partners to continue quality learning opportunities for students while assisting community partners with the unrelenting shifts in operations. Three innovative service-learning and community engagement practices will be addressed, including a) Partnering with the California Office of Emergency Services (CalOES) to create and provide the statewide Social Bridging Project for older adults throughout California; b) Expanding the relationship with Sacramento State's Renaissance Society, a lifelong learning and community engagement program for older adults; and c) Partnering with a community-based Cardio-vascular Wellness Program to keep older adults active and engaged while remaining at home. The poster will review the pivot to virtual service learning and share how this shift enhanced student learning and community service. This will include expanding the use of technology and capitalizing on innovative methods to reach out and provide service to older adults, the local community, and the state of California.

### LOOKING FOR A BRIGHT SIDE: TALES FROM A RECREATIONAL THERAPY VIRTUAL SERVICE-LEARNING PROJECT DURING A PANDEMIC

Jennifer Taylor,[1] and Alyssa Doughty,[2], *1. University of Wisconsin-La Crosse, La Crosse, Wisconsin, United States, 2. University Wisconsin La Crosse, La Crosse, Wisconsin, United States*

The everchanging policies and inability to utilize university students due to COVID-19 impacted both residents living in long-term care as well as the next generation of students pursuing careers in the field. University Wisconsin-La Crosse (UWL) faculty strategized solutions as restrictions threatened to impact hands-on opportunities for students. Was there a safe and effective solution to offer residents evidence-based programming while also providing students with vital field experience? Simply stated, the answer was yes. Thus, the UWL Happiness Project was born. This session will outline the UWL Happiness Project, a ten-week, telehealth program implemented between a skilled nursing facility in rural Wisconsin and the UWL Therapeutic Recreation Program, an AGHE Program of Merit for Health Professions designated program. The evidence-based curriculum was developed by an emerging UWL graduate student scholar with faculty mentorship. The innovative curriculum focuses on increasing feelings of happiness using PERMA, a theoretical model grounded in positive psychology. During virtual sessions, older adult residents (ages 65-85) and students built connection while working through weekly focus areas (e.g. vitality, mindfulness, friendship). An overview of AGHE competencies addressed within the project, online course demonstration, and assignment development will be discussed along with information about how these connections fostered an opportunity for students to see aging from a different perspective. This is the first time we are presenting results from the newly developed program. In this, we look forward to sharing student measurements and outcomes, as well as lessons learned during this meaningful, stimulating, and insightful educational session.

### TRANSFORMING VIRTUAL TEAM-BASED LEARNING FOR RURAL HEALTHCARE STAFF: WHAT THE PANDEMIC TAUGHT US

Eve Gottesman,[1] Helen Fernandez,[2] and Judith Howe,[3], *1. US Department of Veterans Affairs, Bronx, New York, United States, 2. Icahn School of Medicine at Mount Sinai, New York, New York, United States, 3. James J. Peters VA Medical Center, Bronx, New York, United States*

During COVID-19, many training programs pivoted to virtual formats. For the Rural Interdisciplinary Team Training (RITT) Program, funded by the Veterans Health Administration as part of the Geriatric Scholars Program, there were unique challenges. Given a history of successful accredited in-person, team-based workshops for staff at rural and remote clinics, program developers needed to quickly devise a plan for an effective virtual training for team members working separately from each other. Without the ability to provide in-person education and training, rapid pivoting to virtual modalities was essential for ongoing education of those providing care for older adults. Using a web-based platform, team members and expert trainer facilitation, participants engaged in lively discussions and reflection using the chat feature. RITT adapted the curriculum to better meet the needs of busy healthcare providers working during the pandemic, including increased discussion of how COVID affects older Veterans. Three virtual RITT workshops were held between March 2020 and February 2021 with 64 participants from 12 rural clinics and medical centers. Over 90% of participants agreed or strongly agreed that they were satisfied with the virtual workshop, comparable to those participating in the in-person workshop in earlier years. Similar to others, we have found that the ability to flex a curriculum has benefits to both learners and educators and increases the reach of educational opportunities in gerontology and geriatrics. Particularly in rural areas where travel may be challenging, a virtual format may be a desirable long-term solution for the RITT program.

### Session 9175 (Poster)

### COVID-19, HEALTH SERVICES, BEHAVIORS, AND OUTCOMES

### CHANGES IN WELL-BEING DURING THE COVID PANDEMIC: A LONGITUDINAL STUDY OF OLDER ADULTS

Andrea Huseth-Zosel,[1] and Heather Fuller,[2], *1. North Dakota State University, Fargo, North Dakota, United States, 2. North Dakota State university, Fargo, North Dakota, United States*

The COVID-19 pandemic is a public health crisis the world has not seen in a century, with older adults faced with unique impacts due to their increased vulnerability and need to social distance. This research examines changes in physical and mental health and quality of life among older adults in the upper Midwest during the COVID-19 pandemic. Seventy older adults aged 70-97 participated in three phone interviews (April [Time 1], June [Time 2], and October [Time 3] 2020) focusing on experiences coping with the pandemic and understanding overall changes in well-being. Participants rated their quality of life, physical health, and mental health on a scale from 1 to 5 with 1 being "Poor" and 5 being "Excellent." Self-reported quality of life, mental health, and physical health initially declined between retrospective pre-COVID and Time 1 scores, with gradual increases seen across all three variables for Time 2 and Time 3 scores. Thematic analysis of qualitative responses for each interview wave identified salient themes of: 1) reduced quality of life, 2) distraction and routine, 3) loss and uncertainty, and 4) resilience and adaptation. The significance and meaning of these themes shifted across each time point. For example, the reduced quality of life theme initially encompassed loss of activities, later shifted to concerns about struggles to maintain relationships, and finally focused on hope for the future. Findings will be discussed in light of the significance of change over time as well as policy and practice implications for older adults.

### COVID-19 PANDEMIC ON SMOKING BEHAVIOR CHANGES AMONG AFRICAN AMERICAN SMOKERS ELIGIBLE FOR LDCT SCREENING

Tung-Sung Tseng,[1] Yu Hsiang Kao,[2] and Mirandy Li,[1], *1. LSUHSC, New Orleans, Louisiana, United States, 2. LSUHSc, New Orlans, Louisiana, United States*

Smoking has been observed to associate with an elevated severity of disease and risk of mortality among people with COVID-19. Additionally, African American smokers have higher rates of mortality from lung cancer than other racial/ethnic groups. Low dose computed tomography (LDCT) screening can detect lung cancer early to decrease lung cancer-specific mortality for current smokers but remains under-utilized among these population. However, we know little about the effect of COVID-19 pandemic on smoking behavior changes among African American smokers who qualify for LDCT screening. This study recruited 60 African American daily smokers seen in primary care clinics, who qualified to receive LDCT screening in a New Orleans, LA hospital. A total of 22 participants (36.7%) completed anonymous cross-sectional survey that collected demographic, disease history, tobacco use, and smoking cessation behaviors during the period of COVID-19 pandemic via phone interview. The majority were older (61.2 [SD=4.7]), female (77.3%), earned annual income less than $20,000 (100.0%), had Medicaid (63.6%), overweight/obesity (72.7%), planned to quit smoking within 6 months (52.4%), and would consider taking LDCT screening after COIVD-19 pandemic (95.2%). Half of smokers reported they have been diagnosed hypertension (47.6%), diabetes (52.4%), and arthritis (57.1%). Regarding health behavior changes, 42.9% smokers reported they smoked more, felt more stress (42.9%) and anxiety (33.4%) after COVID-19 outbreak. Smoking cessation programs may focus on this high-risk minority population in the post COVID-19 pandemic to help them decrease cigarette smoking and enhance their motivation to quit smoking.

### HEALTH BEHAVIORS IN TIMES OF COVID: DIFFERENT SOURCES OF SUPPORT FOR OLDER ADULTS

Elizabeth Zambrano Garza,[1] Christiane Hoppmann,[2] Denis Gerstorf,[3] Theresa Pauly,[4] Rachel Murphy,[1] Wolfgang Linden,[1] Maureen Ashe,[2] and Kenneth Madden,[1], *1. University of British Columbia, Vancouver, British Columbia, Canada, 2. University of British Columbia, University of British Columbia, British Columbia, Canada, 3. Humboldt University of Berlin, Humboldt University of Berlin, Brandenburg, Germany, 4. University of Zurich, University of Zurich, Zurich, Switzerland*

Eating a nutritious diet reduces vulnerability to common chronic diseases. Yet, older adults struggle to meet nutritional guidelines; many have found it particularly challenging to access fresh foods such as fruits and vegetables during the pandemic. Thus, it is vital to better understand how older adults may recruit the help of close others to support healthy dietary intake. This COVID-19 study examines the role of support for promoting fruit and vegetable consumption in daily life. Ninety-seven older adults participated with a close other of their choice (62 % spouse; 38% non-spouse Mage partner1 = 72, SD = 5.26, Mage partner2 = 62, SD = 16.38). Both partners completed two daily questionnaires for 10 days. In the morning they reported their intentions for eating fruit and vegetables. In the evening, they noted their consumed fruit and vegetable servings, the extent to which this matched their intentions, and their partners support in doing so. Consistent with previous research, the older participants were, the more they consumed fruits and vegetables. On days when participants received more support from their partner, they were more successful at reaching their dietary goals. Interestingly, initial findings suggest that associations were stronger when support was provided from a non-spouse than if the support came from spouse. Follow-up analyses, with a larger sample, will further examine some of the underlying mechanisms so as to better understand the role of different kinds of support providers during the pandemic and shed light on who may be best suited to provide support.

### HEALTH IMPACTS OF THE COVID-19 PANDEMIC AMONG OLDER ADULTS WITH CHRONIC CONDITIONS

Courtney Polenick,[1] Vanessa Lee,[2] Shreya Salwi,[2] Nikita Daniel,[2] Annie Zhou,[2] Summer Meyers,[2] and Diarratou Kaba,[2], *1. University of Michigan, Ann Arbor, Michigan, United States, 2. University of Michigan, University of Michigan, Michigan, United States*

The COVID-19 pandemic may have adverse health implications, particularly among older adults with chronic conditions who are at increased risk of severe illness. This cross-sectional study examined the early health impacts of the pandemic among adults aged 50 and older with chronic conditions. Participants included 700 adults (M = 64.60 years, SD = 8.85, range = 50 – 94) from Michigan (82.4%) and 33 other U.S. states who reported at least one chronic condition and completed an anonymous online survey between May 14 and July 9, 2020. Of these, 488 also provided open-ended responses. Individuals reported lower illness self-efficacy, less consumption of fruits, vegetables, and fried foods, and lower physical activity, along with greater alcohol use since the pandemic. About half (42.7%) reported worsened sleep. One in five (20.1%) reported at least some difficulty obtaining medications and over half (60.4%) reported at least some difficulty receiving routine care. Almost two-thirds (63.9%) had delayed preventative care and one in five (19.3%) had delayed essential medical treatment. Nearly half (42.6%) avoided contacting care providers about a physical health concern and one in eight (12.9%) avoided reporting mental health concerns. Qualitative data revealed that the pandemic has influenced how participants cared for their physical health through following guidelines related to COVID-19; coping with daily routine changes; greater awareness of self-care; mental health impacts; and health care disruptions. Older adults with chronic conditions report distinct pandemic-related challenges for self-care and health care that should be addressed in interventions to maintain their health and functioning.

### MEMORY PROBLEMS DURING COVID IN LOW-INCOME OLDER ADULTS

Faika Zanjani, and Annie Rhodes, *Virginia Commonwealth University, Richmond, Virginia, United States*

Prevention, with widespread lifestyle risk reduction at the community-level, is currently considered an effective method to decrease Alzheimer's disease (AD). As part of the Virginia Commonwealth University iCubed Health and Wellness in Aging Core, diverse older adults (60+) living in Richmond, VA, with incomes below $12,000/year and managing either diabetes/cardiovascular symptoms, were offered weekly lifestyle telephone-health coaching for 12-weeks, providing education, motivations, self-efficacy, and referral services for AD lifestyle risk. The study sample (n=40, mean age 68 years (range: 60-77 years) was 88% African American/Black (n=35), 100% Non-Hispanic, and 45% males (n=18)). Thirty-nine (95%) of subjects successfully participated in coaching sessions; on average 91.9% (11) sessions/subject were completed. Participants provided positive anecdotal feedback and the need for continued health coaching during COVID. N=30 (75%) of the original sample consented for continued health coaching during the Covid pandemic, 63% female, 88% African American/Black, 60-77 age range (mean age 69 years), and 47% reporting memory problems. Baseline Covid interviews indicated poorer health status associated with reporting memory problems for poor physical health days (F=7.03;p=.01); poor mental health days (F=6.88;p=.01); total mental/physical health poor days (F=2.76;p=.11); sad days (F=15.52;p=.001); worried days (F=6.27;p=.02); tired days (F=9.77;p=.004); feelings of emptiness (F=10.09;p=.004); feelings of rejection (F=3.382;p=.08); feelings of failure (F=7.58;p=.01); little interest/pleasure (F=7.84;p=.009); and feeling down (F=6.75;p=.02). In conclusion, this preliminary work creates the impetus for future large-scale AD prevention investigations to improve the lives of AD-risk, low-income, diverse older adults reporting memory problems. This research indicates the subjective reporting of memory problems requires health intervention.

### TALKING THROUGH TECHNOLOGY: MAINTAINING ESSENTIAL CONTACTS WITH OLDER ADULTS DURING THE COVID-19 PANDEMIC

Marcia Shade,[1] Sarah Hubner,[2] Julie Blaskewicz Boron,[2] Stephen Kotopka,[1] and Natalie Manley,[1], *1. University of Nebraska Medical Center, Omaha, Nebraska, United States, 2. University of Nebraska Omaha, Omaha, Nebraska, United States*

Older adults are at increased risk for contracting COVID-19 and are more vulnerable to poor outcomes. Public health efforts to prevent spread of COVID-19 resulted in widespread social/physical distancing; this changed adults' regular communication with their essential contacts, warranting development of solutions for socialization to reduce loneliness, bolstering quality of life. Essential contacts provide social/emotional/physical care for community-dwelling or institutionalized adults. This study aimed to explore how essential contacts of older adults utilize technology to maintain social connection in response to COVID-19. Participants (N=156) aged 55+ completed a Qualtrics questionnaire via Amazon Mechanical Turk; demographic, social contact, and technology use data were collected. Respondents (M Age=62.2±4.9) were generally female (72.4%), white (89.7%), and a contact for an institutionalized adult (59%). Data were analyzed descriptively with binary regressions. Results revealed that volumes of general [X2(4,N=156)=37.69,p<.001], in-person [X2(4,N=156=37.84,p<.001], and distanced [X2(4, N=156)=27.69,p<.001] social interaction were significantly associated with the older adult's environment (community-dwelling vs. institutionalized). In-person conversation was significantly associated with environment [X2(1,N=156)=29.38,p=0.001], while other technology-based communications (e.g., video-chat) were not. In-person conversation was positively predicted by the contact being a physical caregiver (B=2.324,p<.001), while smartphone use was positively predicted by being a social contact (B=1.287,p<.05). Findings suggest that although technology was used by participants to communicate with their older adult contacts across groups, environment and caregiver/contact type significantly influenced communication. It may be that, throughout COVID-19, dyads have relied on familiar methods of socialization, or that there is lack of access to more sophisticated technologies for communication. This warrants future investigation.

### TECHNOLOGY USE AMONG OLDER ADULTS TO MANAGE THEIR HEALTH DURING A GLOBAL PANDEMIC

Gashaye M. Tefera, Erin Robinson, and Geunhye Park, *University of Missouri, Columbia, Missouri, United States*

Risk of severe COVID-19 illness increases with age, and older adults are more likely to be hospitalized and die from COVID-19 and related complications as compared to their younger counterparts. This reality, combined with pandemic-related lockdown and social distancing policies, has increased in-home isolation for older adults. This includes cancelling in-person healthcare appointments and conducting many appointments via tele-health. As older adults have had to quickly pivot to learning new technologies, little is known about their experiences with navigating virtual healthcare during the pandemic. Therefore, this qualitative study aims to address that gap. One-on-one interviews (N=29) were conducted with older adults (Mean age=71.5; 86% female) via phone/Zoom. Participants were asked about their healthcare experiences during the pandemic and the role technology played. Interviews were transcribed and thematically analyzed using Nvivo12 software. Findings demonstrate that participants used technology to schedule medical appointments, engage in virtual visits with their providers, set reminders to take medications, and undertake their daily exercise routine. Post-lockdown, some participants preferred in-person visits due to the nature of their diagnosis, personal preference, or unfamiliarity with the needed technology. Older adults encountered challenges including cancelled appointments, miscommunication with providers, and lack of skill to use technologies. Cancellation of appointments and postponement of treatments affected the health of some of the participants. Implications of this research can inform tele-health approaches with older patients, as well as provider communication and coordination of care. Leveraging technology for preventative health approaches can also assist older adults in ongoing health maintenance and promote well-being.

### TELEHEALTH AS AN ELEMENT OF HOME AND COMMUNITY-BASED SERVICES IN A PANDEMIC: AN INTRINSIC CASE STUDY IN TWO RURAL AREAS

Nancy Karlin,[1] and Joyce Weil,[2], *1. University of Northern Colorado, Greeley, Colorado, United States, 2. University of Northern Colorado, University of Northern Colorado, Colorado, United States*

COVID-19 has changed the face of health care delivery. Using technology as a way to ensure Home and Community-Based Services (HCBS) as an option for older adults in rural areas is of increasing interest as a result of the pandemic. Literature suggests older adults do not adopt telehealth and/or medicine practices due to barriers (e.g., Internet and computer availability) and do not use telemedicine as a form of communication with medical staff. However, the combination of needing health care during the pandemic and having federal coverage via Medicare for telehealth virtual visit. Still studies suggest older adults may lack the necessary information about how to adopt telehealth and telemedicine and that they do not see their benefits. Additionally, the cost of technology, limited Internet access and rural connectivity issues persist. This study evaluates the potential for telehealth/medicine use in rural communities through two case studies of rural older persons in the Eastern Plains of Colorado and rural Western Nebraska. Results indicate, for older persons responding to the telehealth/medicine questions, there is support for its potential use with some using teleconferencing, health portals, along with the expectation that telehealth/medicine would be part of new health care systems. Resistance was met by some older adults in the Colorado sample who preferred face-to-face contact alongside other concerns about potential usage barriers such as the lack of Internet services or consistent connectivity. These participants indicated a lack of awareness in finding out how to access this form of medical support.

### THE IMPACT OF COVID-19 ON OLDER ADULTS

Rachel Ungar,[1] Rifky Tkatch,[1] Jane Huang,[1] Sandra Kraemer,[2] James Schaeffer,[1] and Charlotte Yeh,[3], *1. UnitedHealth Group, Minnetonka, Minnesota, United States, 2. UnitedHealthcare, Minnetonka, Minnesota, United States, 3. AARP Services, Inc., Washington, District of Columbia, United States*

**Background:** The onset of the COVID-19 pandemic has dramatically influenced the health and well-being of older adults. Changes in lifestyle patterns has required reframing communication habits and learning new skills to maintain social connections and access healthcare. Objectives: To assess 1) well-being measured prior to and during the COVID-19 era; and 2) use and comfort level of technology for social interactions and telehealth visits during this time.

**Methods:** A mailed survey to a randomly selected national sample (>65) during the summers of 2018, 2019, and 2020. Measures included mental and physical well-being and various psychosocial measures. For 2020, questions related to COVID-19 and the use of technology were included.

**Results:** A total of 4,696 (2018), 3,976 (2019) and 2,726 (2020) responded to these surveys (response rate ~27%). Overall, most constructs remained stable despite the ongoing pandemic. Most respondents reported average or high resilience (90%), high purpose (48%), stable social networks (76%), and low stress (55%). However, loneliness increased during 2020 (57%). Respondents who used technology were more likely to connect with family and friends. Only 43% reported high comfort with using technology, with older age (>75) less comfortable. At the time of the survey 37% had not seen a healthcare provider through telehealth services, and 15% felt their healthcare needs were not met by a telehealth experience.

**Conclusion:** Results demonstrate that respondents were doing well during COVID-19. Yet increases in loneliness and greater technology needs to stay socially connected and to access healthcare may result in negative long-term health outcomes.

### Session 9180 (Poster)

### COVID-19, SOCIAL SUPPORT, SOCIAL CONNECTEDNESS, AND LONELINESS

### AGING, EMPATHY, AND PROSOCIAL BEHAVIORS DURING THE COVID-19 PANDEMIC

Isu Cho,[1] Ryan Daley,[2] Tony Cunningham,[2] Elizabeth Kensinger,[2] and Angela Gutchess,[1], *1. Brandeis University, Waltham, Massachusetts, United States, 2. Boston College, Chestnut Hill, Massachusetts, United States*

Previous literature has shown age-related increases in prosociality (i.e., the tendency to engage in behaviors that benefit others). Can such age-related differences be observed during the COVID-19 pandemic, or would young adults' higher levels of COVID-19-related stress alter the relation between age and prosociality given the prior findings that stress may promote prosocial behaviors? Can empathy, one of the factors highly related to prosociality, explain any observed age-related differences? The current study examined the above questions, as well as whether age differences exist in target of prosocial behaviors (i.e., distant- versus close-others). To this end, participants (aged 18-89) enrolled in an ongoing study examining their experiences during the COVID-19 pandemic. They were asked to complete a series of surveys on dispositional empathy and prosocial behaviors during the pandemic. In the present analyses, the data were used from 330 participants from the USA who completed all of the surveys. Compared to younger adults, results indicate that older adults showed greater prosocial behaviors during the pandemic despite their higher risk of physical-health complications from COVID-19. Unexpectedly, empathy did not explain such age-related increases in prosocial behaviors even though it was positively related to individuals' prosociality. Interestingly, older adults reported increased prosocial behaviors towards close-others (i.e., family, friends) compared to young adults, suggesting that older adults seem to devote more resources into emotionally meaningful relationships. The current study contributes to our understanding of how prosociality differs with age during the stressful period of need that marks the COVID-19 pandemic.

### COVID-19 CONCERNS, DEPRESSION AND LONELINESS IN MIDDLE-AGE AND OLDER ADULTS

Angela Curl, and Katie Wolf, *Miami University, Oxford, Ohio, United States*

The coronavirus pandemic forced many people to restrict their activities and social interactions out of fear and due to local health regulations. This study examined whether one's self-reported level of concern related to COVID-19 was associated with loneliness and depressive symptoms. Using early release 2020 data from the Health and Retirement Study (N=2,759 adults over age 50), we conducted ordinary least-squares and logistic regressions, controlling for age, gender, education, marital status, self-rated health, and exercise. Higher levels of self-reported concern about the coronavirus pandemic were associated with more depressive symptoms (B=.05, p<.01) and increased odds of being lonely (OR=1.05, p<.01). Female sex, lower education, not being married, worse self-rated health, and lack of exercise were associated with more depressive symptoms and higher odds of being lonely, while older age was associated with lower depression but higher odds of loneliness. These results suggest that mental health assessments should include measures specifically asking about COVID-19 concerns and experiences (e.g., COVID-19 diagnosis, death of close friends or family due to COVID-19, unable to attend important events). The pandemic has raised public awareness of the negative consequences of social isolation and acted to destigmatize mental illness, and this could encourage middle-aged and older adults to seek professional help for depression.

### LONELINESS AND NEUROPSYCHIATRIC SYMPTOMS IN COGNITIVELY IMPAIRED OLDER ADULTS DURING COVID-19 PANDEMIC

Prasad Padala,[1] Kalpana Padala,[2] Ashlyn Jendro,[1] Clinton Gauss,[1] Kerrie Wilson,[1] Aparna Das,[3] Samuel House,[1] and Scott Mooney,[1], *1. Central Arkansas Veterans Healthcare System, North Little Rock, Arkansas, United States, 2. Central Arkansas Veterans Healthcare System, Central Arkansas Veterans Healthcare System, North Little Rock, Arkansas, United States, 3. University of Arkansas for Medical Sciences, Little Rock, Arkansas, United States*

**Background:** Cognitively impaired older adults living in the community have been vulnerable to the effects of COVID-19 confinement. The current study's objectives were to examine the prevalence of loneliness in such adults along with impact of COVID-19 on neuropsychiatric symptoms and functional status.

**Methods:** A cross-sectional study was conducted in community dwelling cognitively impaired older Veterans (N=41). Demographic data such as age, gender, race, and rurality were collected. Loneliness data were collected with the 3-item Loneliness Questionnaire. Cognition was assessed with the Tele-Montreal Cognitive Assessment (T-MoCA) and functional status of instrumental activities of daily living was assessed with the Functional Activities Questionnaire (FAQ). Neuropsychiatry symptoms including severity and distress were collected using the Neuropsychiatric Inventory (NPI), and change during COVID was also recorded for each symptom.

**Results:** Demographic characteristics included: mean age of 71.9 (±8.6) years, 95.1% male, 46.3% rural, 75.6% Caucasian, and 19.5% African American. Loneliness was prevalent in most participants (62.5%). T-MoCA and FAQ mean scores were 15.1 (±4.5) and 10.0 (±8.6), respectively. Mean NPI total severity and total distress were 8.4 (±5.9) and 11.4 (±8.5), respectively. Irritability was most frequently reported symptom (65%), followed by agitation (57.5%), anxiety (55%), depression (50%), and night-time behavior (50%). A majority of the participants reported worsening of neuropsychiatric symptoms during COVID (71.1%). Among those that reported worsening neuropsychiatric symptoms, 70.4% noted an increase in ≥ two symptoms.

**Conclusion:** Older adults with pre-existent cognitive impairment may be at high risk for loneliness and worsening of neuropsychiatric symptoms during the COVID pandemic.

### LONELINESS BEFORE AND DURING THE COVID-19 PANDEMIC: ASSOCIATIONS WITH CHRONIC ILLNESSES AND RELATIONSHIP QUALITY

Jay Kayser,[1] and Jacqui Smith,[2], *1. University of Michigan, Ann Arbor, Michigan, United States, 2. University of Michigan, University of Michigan, Michigan, United States*

While self-reported loneliness generally declines after age 65, the likelihood of experiencing chronic illnesses increases. During the Covid-19 pandemic, social isolation measures have changed the social context of many people. We address three research questions: 1) What is the predictive strength of chronic illnesses, relationship quality, and their interaction on loneliness? 2) Has Covid-19 altered experienced loneliness relative to pre-pandemic? 3) Was loneliness during Covid-19 associated with the number of prior chronic illnesses in 2016? To answer these questions, we have analyzed data from participants in the Health and Retirement Study (HRS) included in the early 2020 release who also completed the 2016 wave (N = 1106). On average, in 2016, these participants were age 74.64 (SD = 6.66) and reported 2.57 (SD = 1.39) chronic illnesses. In 2016, unadjusted multiple regression models revealed that chronic illnesses (β = .38) and relationship quality (β = -.41) were associated with loneliness (R2 = .28). When covariates were added, these values were attenuated but remained statistically significant. In 2020 during the pandemic, 8% of these participants reported they often felt lonely and 26% reported feeling lonelier since the start of the Covid-19 pandemic. People who had more chronic illnesses in 2016 reported feeling lonelier in 2020 as did people whose relationships were poorer quality (p < .05). Further analyses with final data from HRS are needed to confirm these trends. These findings highlight the importance of having longitudinal information to identify individuals at high risk and most likely to benefit from interventions.

### LONELINESS DURING COVID-19 AND MODES OF SOCIAL CONTACT USE AMONG OLDER ADULTS

Usha Dhakal, and Athena Koumoutzis, *Miami University, Oxford, Ohio, United States*

Due to social distancing and isolation recommendations, COVID-19 resulted in older adults' greater reliance on technology to contact friends and families. While the mental health of older adults during COVID-19 has been well explored, less is known about how changes in modes and frequency of social contact is associated with loneliness. Using the National Health and Aging Trends Study COVID-19 data, this study assessed how the frequency of varying modes of contact (e.g., phone/email/text, in-person visits, videocalls) during the pandemic was associated with feelings of loneliness during COVID-19 among community-dwelling Medicare beneficiaries (n = 2149). Participants were asked if they felt lonely "more often," "less often," or "about the same" compared to before the outbreak started. Multinomial regression analyses indicated that, compared to those who reported daily in-person visits, the odds of having more feelings of loneliness as compared to about the same as pre-COVID-19 was significantly higher among those who reported having in-person visits a few times (OR=2.17,CI=1.08-4.36), at least once (OR=2.37,CI=1.11-5.04), and never/less than once a week (OR=3.37, CI=1.59-7.16) while controlling for demographics, household, and social network size. Compared to daily use, use of phone/email/text at least once (OR=0.44, CI=0.201-0.965) or a few times (OR=0.76,CI=0.58-0.99) a week was associated with lower odds of reporting more feelings of loneliness versus about the same. Results suggest that greater use of technology that promote social engagement improves social connectedness and decreases COVID-19 related loneliness among older adults, and highlights the importance of older adults' access to technology, including reliable internet.

### OLDER ADULTS' WORRY ABOUT COVID-19: ASSOCIATIONS WITH EXPERIENCES OF COVID-19 AMONG SOCIAL CONNECTIONS

Amanda Leggett,[1] Hyun Jung Koo,[2] Lindsay Kobayashi,[2] Jessica Finlay,[2] Hannah Lee,[2] and Elaina Baker,[2], *1. The University of Michigan, Ypsilanti, Michigan, United States, 2. University of Michigan, Ann Arbor, Michigan, United States*

The COVID-19 pandemic has challenged the physical and mental health of older adults, yet it is unknown how much older adults worry about their own exposure. As older adults are at increased risk for severe complications from COVID-19, understanding patterns of worry may inform public health guidelines and interventions for this age group. We investigated older adults' worry about COVID-19 in the early months of the pandemic and associations with familial/friend's diagnosis or disease symptoms. Data comes from the baseline (April/May 2020), one-month, and two-month follow-up surveys from the COVID-19 Coping Study, a national longitudinal cohort study of US adults aged ≥55. We used linear regression models to investigate the association between self-reported familial/friend diagnosis or symptoms with pandemic worry, accounting for demographic factors and individual diagnosis or experience of COVID-19 symptoms. Participants (Baseline=4379, 1 month= 2553, 2 month=2682) were 67 years old on average, 72% were female, 5.7% were non-White, and 80.5% had a college degree. At baseline, 26.6% of participants had friends or family who had been diagnosed or experienced symptoms of COVID-19. Having friends or family diagnosed or with symptoms of COVID-19 (B=0.08, SE=0.04, p<.05), being female (B=0.42, SE=0.03, p<.001), and having higher educational attainment (B=0.06, SE=0.02, p<.001) were significantly associated with greater worry about COVID-19. These associations were consistent over 3 months. Understanding if worry about the pandemic correlates with following public health guidelines is a key next step so intervention strategies can prioritize older adults and their social networks.

### PREVALENCE OF SOCIAL ISOLATION BEFORE AND DURING THE COVID-19 PANDEMIC: A NATIONWIDE WEB-BASED SURVEY IN JAPAN

Hiroshi Murayama,[1] Ryo Okubo,[2] and Takahiro Tabuchi,[3], *1. Tokyo Metropolitan Institute of Gerontology, Tokyo, Tokyo, Japan, 2. National Center of Neurology and Psychiatry, Kodaira, Tokyo, Japan, 3. Osaka International Cancer Institute, Osaka, Osaka, Japan*

The coronavirus disease 2019 (COVID-19) pandemic is assumed to have increased the number of socially isolated older adults. Public health researchers and policymakers are concerned about the deleterious effects of social isolation on individuals' health. However, there is only limited evidence on the prevalence of social isolation. This study investigated the change in prevalence of social isolation caused by the spread of COVID-19 and examined various associated factors. Accordingly, data from the JACSIS study, a nationwide cross-sectional web-based questionnaire survey (N=28,000, age: 15–79 years) conducted in August–September 2020 (during the pandemic) were analyzed. The respondents who contacted family members, friends, or neighbors less than once a week were considered socially isolated. We examined individuals' frequencies of contact, including meeting in person, e-mail/text message, voice call, and video call, in January (before the pandemic; recall question) and August 2020. The weighted prevalence values of social isolation were 26.8% (26.0%–27.5%) in men and 15.8% (15.1%–16.4%) in women before the pandemic and increased to

34.4% (33.6%–35.2%) and 21.4% (20.7%–22.1%), respectively, during the pandemic. Further, compared to the younger age group, the increase in prevalence during the pandemic was greater for the older age group for both genders. Multinomial logistic regression analysis revealed that those who came to be socially isolated during the pandemic possessed a greater fear of COVID-19 than those who were not continuously socially isolated. These findings suggest the necessity of developing immediate measures for social isolation and risk communication regarding COVID-19.

### SOCIAL ISOLATION IN OLDER WOMEN DURING THE COVID-19 PANDEMIC: THE IMPACT ON QUALITY OF LIFE AND MENTAL HEALTH

Victoria Marshall,[1] Robina Sandhu,[2] Kathryn Kanzler,[3] Sara Espinoza,[1] Pamela Keel,[4] Andrea LaCroix,[5] Nicolas Musi,[3] and Lisa Kilpela,[3], *1. University of Texas Health Science Center San Antonio, San Antonio, Texas, United States, 2. University of Texas Health Science Center, San Antonio, Texas, United States, 3. UT Health San Antonio, San Antonio, Texas, United States, 4. Florida State University, Tallahassee, Florida, United States, 5. University of California at San Diego, San Diego, California, United States*

To mitigate the spread of COVID-19, countries worldwide enacted quarantines, particularly for older adults, as mortality from COVID-19 is inequitably distributed among this group. Notably, social isolation in older adults is associated with a heightened risk of cardiovascular, autoimmune, and mental health problems (e.g., depression, anxiety). Furthermore, the mental health of women in particular has been greatly impacted by the pandemic. Although previous research indicates that social isolation among older adults is a "serious public health concern", less is known about the extent to which the COVID-19 pandemic has exacerbated this issue. The primary objective is to investigate the effects of social isolation on mental health indices and health-related quality of life (HRQOL) in older women in the context of the COVID-19 pandemic. Participants include 77 postmenopausal women (aged 60+) who completed self-report measures online during the COVID-19 pandemic. Controlling for education and annual household income in all analyses, we used linear regression models to investigate the effects of social isolation on depression, anxiety, alcohol use, binge eating, and the 8 domains of the SF-36. Results indicate that, when controlling for education and income, social isolation significantly predicted depression, binge eating, and poorer HRQOL in all 8 domains of the SF-36 (all p's < .01) Social isolation did not predict anxiety and alcohol consumption when controlling for these sociodemographic variables. Enrollment is ongoing; this poster will report updated results. Results indicate the continued need for creative avenues to improve social connectedness during the COVID-19 pandemic.

### SOCIAL SUPPORT IS ASSOCIATED WITH BETTER HEALTH IN THE FACE OF COVID-19

Laura Bernstein, and Julie Hicks Patrick, *West Virginia University, Morgantown, West Virginia, United States*

The COVID-19 pandemic has the potential to influence the health of the nation, both directly and indirectly, though increased stress. As with other stressful crises, social support may buffer against the deleterious effects of the stress surrounding COVID-19 (Cohen & Wills, 1985). We were interested in how self-reported health changed during the first year of the COVID-19 pandemic in the United States and whether age or positive social exchanges influenced this potential change. We tested a latent growth curve model of change in SF12 scores over 4 points of measurement during the first year of the pandemic. Data from 237 adults (Mean age 40.7 yrs) were used to test whether SF12 scores changed over the 11 month period and whether age and initial positive social exchanges influenced both the intercept and trajectory of change over time. Results showed that the model fit the data well, X2 (DF = 13, N = 237) = 11.44, p = .57, RMSEA < .06. Of note, older age was associated with both better initial health (b = .036**) and a slower decrease over the year (b = -.005*). Initial positive social exchanges were associated with better initial health (b = .067*) but did not alter the trajectory of change over time. These findings suggest an age-related advantage for health in the face of COVID-19 and that positive social support is associated with better health, at least at the very beginning of the pandemic.

### VET TO VET MAINE: COVID 19 EFFECT ON A VETERAN COMPANION PILOT STUDY IN REDUCING SOCIAL ISOLATION AND LONELINESS

Marilyn Gugliucci,[1] Amy Lin,[2] and Shirley Weaver,[3], *1. University of New England College of Osteopathic Medicine, Kennebunk, Maine, United States, 2. University of New England, University of New England, Maine, United States, 3. University of New England College, University of New England, Maine, United States*

**Background:** Maine veterans represent 11.8% of Maine population, twice that of the United States. Veterans are at risk for social isolation, loneliness and suicide. The mission of Vet To Vet (V2V) Maine, a non-profit organization, connects trained veteran volunteers with fellow veterans (veteran friends) for companionship, assistance with benefits, and support independent living. This study: (1) assessed if V2V program fulfilled its mission; and (2) determined any care partner effects from the program.

**Methods:** Mixed methods research spanned 6-months, 2019-2020. Twenty-four participants; trained Veteran Volunteers (VV) were paired with Veteran Friends. Four Care Partners (CP) of Veteran Friends participated. Assessments included pre/post Veteran Rand Health Survey (VR-12), Patient Health Questionnaire (PHQ-9), and Late Onset Stress Symptomatology (LOSS) Short Form. CPs completed pre/post Zarit Burden Interview (ZBI-22) assessments. Qualitative interviews focused on visits/activities, relationship building, and program feedback. Data analyses included Wilcoxon Sign Test and NVivo 12+ Qualitative Data Analysis Software.

**Results:** Pre/post data failed to show significance (P=.05), however trends supported an improvement in mental and physical health scores. COVID-19 was a confounding variable as state stay-at-home orders occurred at the companion program study mid-point. Three key themes included; (1) Veteran Companionship; (2) Effects of COVID; and (3) Care Partners. The V2V Companion program was determined effective and reported highly successful relationship matches. CPs confirmed the importance and benefits of V2V.

**Conclusion:** The V2V Maine companion program pilot research supported success in connecting Veterans Volunteer and Veteran Friends; fostering companionship, friendships, mentoring, assisting with benefits, and supporting independent living.

### VILLANOVA REACHOUT FORMING CONNECTIONS WITH OLDER ADULTS

Christina Whitehouse,[1] Catherine Curley,[2] Caitlin Gomes,[2] Michelle McKay,[2] Christine Brewer,[2] and Melissa O'Connor,[3], *1. Villanova University, Villanova Univesity, Pennsylvania, United States, 2. Villanova University, Villanova University, Pennsylvania, United States, 3. Villanova University, M. Louise Fitzpatrick College of Nursing, Villanova, Pennsylvania, United States*

Older adults are at increased risk for loneliness and social isolation. Research on loneliness identifies increased rates of depression, increased cognitive decline, and poor cardiovascular health outcomes. The COVID-19 pandemic forced many older adults into social isolation for protection against this insidious virus. Mandated lockdowns and personal decisions to shelter-in-place produced a tremendous increase in rates of loneliness, especially among older adults. Identifying a need for communication and relationships, we created Villanova ReachOut, a program that partners interprofessional students (N= 66) with older adults (N=53) through weekly telephone or video calls. To assess the impact of our program we developed a five question survey administered via phone to older adults and a 13-item survey for volunteers to assess training, satisfaction, needs and impact of the program. Of the older adults (n=16) who completed the survey, 78.6% believe the program helped them feel less isolated throughout the pandemic and 93.8% indicated they looked forward to weekly calls with their partner . Volunteers who completed the survey (N=25), overwhelmingly stated they enjoy and look forward to their calls (100%) and their communication skills have improved (92%). Volunteers reported being paired up with an older adult for weekly conversation had a positive impact on their personal and professional development. Findings from our program evaluation provide rich data in descriptions of positive impact for both the older adult and volunteer. These findings also support the need for programs that engage in intergenerational dialogue, specifically targeting older adults and the potential older adult workforce.

### Session 9185 (Poster)

### DEMENTIA AND QUALITY OF LIFE

### ALZHEIMER'S PREVENTION LIFESTYLE RISK COACHING IN LOW-INCOME OLDER ADULTS

Faika Zanjani, and Annie Rhodes, *Virginia Commonwealth University, Richmond, Virginia, United States*

Prevention, with widespread lifestyle risk reduction at the community-level, is currently considered an effective method to decrease Alzheimer's disease (AD). As part of the Virginia Commonwealth University iCubed Health and Wellness in Aging Core, diverse older adults (60+) living in Richmond, VA, with incomes below $12,000/year and managing either diabetes/cardiovascular symptoms, were offered weekly lifestyle telephone-health coaching for 12-weeks, providing education, motivations, self-efficacy, and referral services for AD lifestyle risk. The study sample (n=40, mean age 68 years (range: 60-77 years) was 88% African American/Black (n=35), 100% Non-Hispanic, and 45% males (n=18)). Thirty-nine (95%) of subjects successfully participated in coaching sessions; on average 91.9% (11) sessions/subject were completed. On a scale of 0-100 (higher scores more positivity), rated their experience 93.3. On a scale of 0-10 (higher scores more improvement), rated their health improved 8.36. Pre/post-test analyses indicated lifestyle improvement trends over 4-months for total lifestyle risk (F=4.69;p=.037;effect=.12), social activity (F=4.63;p=.063;effect =.09), and improvement in certain psychological domains: AD knowledge (F=4.49;p=.041;effect=.11); cognitive functioning (short-term memory (F=4.99;p=.038;effect=.21); delayed memory (F=2.26;p=.154;effect=.11); Trails A (F=5.60;p=.0294;effect=.24); and Trails B (F=2.22;p=.154;effect=.11). Participants provided positive anecdotal feedback and the need for continued health coaching. In conclusion, this preliminary work creates the impetus for future large-scale lifestyle AD prevention investigations to improve the lives of AD-risk, low-income, diverse older adults. These findings demonstrate that telephone-based health coaching is feasible, based on participant engagement, and effective, based on positive trends in reducing AD risk factors.

### DEMENTIA CAREGIVER SURROGATE DECISION MAKING SELF-EFFICACY:DISTRESS, AND QUALITY OF LIFE

Giovanna Pilonieta,[1] and David Geldmacher,[2], *1. University of Alabama at Birmingham, Birmingham, Alabama, United States, 2. University of Alabama at Birmingham, University of Alabama at Birmingham, Alabama, United States*

We assessed the relationship between caregiver self-efficacy and caregivers' ratings of care recipient's health-related quality of life, the severity of neuropsychiatric symptoms, and associated caregiver distress for persons with Alzheimer's dementia (AD).

**Methods:** The 31-item DEMQOL-Proxy, Neuropsychiatric Inventory (NPI-Q), and the Self-Efficacy for Surrogate Decision-Making scale (SDM-SES) were collected from 26 family caregivers of people with AD expressing care resistant behaviors. We used Spearman correlations to assess relationships between SDM-SES, NPI-Q severity, and NPI-distress and DEMQOL-proxy. Among enrolled caregivers, 14 (54%) were women; mean age was 64.5 years, and 24 (92%) were college-educated. Their care recipients were 61% women, 77 % white, with a mean age of 76 years, and mostly college-educated (88%). Mean scores were DEMQOL-Proxy 91.27 (+/- 14.16), SDM-SES 16.38 (+/- 2.74), NPI-Q Severity score 14.23 (+/- 6.04), and NPI-distress 17.42 (+/-6.90). There were moderate correlations between DEMQOL-Proxy and SDM-SES (r=0.54), NPI severity (r= -0.42) and NPI-distress (r= -0.49). Secondary analysis showed a moderate correlation between SDM-SES and NPI-distress (r= -0.40). We identified associations between caregiver self-efficacy, quality of life, and caregiver distress. A higher baseline SDM-SES was associated with greater health-related quality of life for the care recipient. Lower self-efficacy scores were related to more caregiver distress related to neuropsychiatric symptoms. Higher NPI severity and caregiver distress were associated with lower quality of life for the care recipient. Interventions targeting self-efficacy may promote improved QOL and decrease caregiver distress in AD dyads.

### DEVELOPMENT AND PSYCHOMETRIC TESTING OF CODING SCHEME FOR DEMENTIA FAMILY COMMUNICATION USING VIDEO OBSERVATION

Sohyun Kim, *University of Iowa, Iowa City, Iowa, United States*

Understanding communication behaviors between persons living with dementia and family caregivers is essential for meaningful social interaction and decrease problematic behaviors and caregiving burden. The purpose of this study was to develop and test the psychometric properties of a coding scheme for dementia care interactions. The coding scheme items were developed from literature and expert review, and the pilot testing on 16 video-recorded interactions. A secondary analysis was conducted using 77 videos from 21 dyads of dementia family interactions naturally occurred in the participant's home. The final coding scheme consists of 11 codes for persons living with dementia (6 nonverbal and 5 verbal) and 12 codes for family caregivers (7 nonverbal and 5 verbal). Content validity was excellent (I-CVI = .93, S-CVI/UA = .71, S-CVI/Ave = .93 with 6 experts). Inter-item correlation was acceptable for both caregiver codes (positive nonverbal = .21, positive verbal = .15, negative nonverbal = .36, negative verbal = .29), and patient codes (positive nonverbal = .13, positive verbal = .27, negative nonverbal = .15, negative verbal = .18). Intra-rater reliability (Cohen's Kappa = .83, percentage of agreement = 83.88%) and inter-rater reliability (Cohen's Kappa = .81, percentage of agreement = 81.75%) were excellent. Findings suggest the preliminary psychometric properties of the newly developed coding scheme to assess dyadic interactions of persons living with dementia and their informal caregiver in-home care situations. Future testing of the coding scheme for application in communication interventions to improve quality social interaction in dementia care is discussed.

### EXPLORING PARADOXICAL LUCIDITY STORIES: WORKING TOWARDS PROMOTING MEANINGFUL ENGAGEMENT WHEN LEAST EXPECTED

Regula Robnett, and Thomas Meuser, *University of New England, Portland, Maine, United States*

Lucidity is the typical cognitive state of adults. However, conditions such as Alzheimer Disease and major neurocognitive disorders can rob people of their usual clarity. Episodes of "paradoxical lucidity" (PL) happen when there is a spontaneous return of lucidity in people who are assumed to have lost the capacity to engage and communicate. These often take place near the end of life. Anecdotal stories depicting PL have been shared for centuries, but the etiology and mechanisms of PL rarely have been examined scientifically. The purpose of this mixed-methods project was to "gather the stories," of witnessed episodes of PL, to describe them, including potential triggers and contextual supports. Sixty witnesses of episodes have shared their experiences. Two-thirds those displaying PL were female. Most had died within six months of the episode. Episodes generally lasted under an hour (range: "moments" up to 5+ hours). Illustrative descriptions about PL emerged: 1) A trusted person or professional, perhaps unknowingly, saying or doing the right thing could trigger an episode; 2) PL frequently took place in a sacred environment involving ritual spiritual practices (e.g., saying the rosary and singing hymns); 3) meaningful music was often a trigger; and 4) PL sometimes involved the return of one's professional or familial/friend role. Familiar sounds (e.g., music, voices) were the most common sensory antecedents. A deeper understanding of PL events could potentially drive interventions to promote these valued episodes. The possibility that end-stage neurocognitive impairments could be reversible, even for a short period, is thought-provoking and inspiring.

### EXPLORING WELL-BEING OUTCOMES AMONG PERSONS LIVING WITH DEMENTIA: SELF-REPORTS VERSUS CAREGIVER PROXY REPORTS

Morgan Minyo,[1] Sara Powers,[2] Alyssa Ciancibello,[2] Rachel Schaffer,[2] and David Bass,[2], *1. Cleveland State University, Cleveland, Ohio, United States, 2. Benjamin Rose Institute on Aging, Cleveland, Ohio, United States*

A vast amount of research has focused on the development of dementia caregiving evidence-based programs (EBPs) in order to mitigate the well-known negative impacts of providing care for persons living with dementia (PLWD). In order to support efficacious results, many programs utilize a variety of both objective and subjective outcomes as typically reported by the caregiver or collected via medical history and clinical assessments. However, more recent research has attempted to understand the subjective illness experience of PLWD through self-report measures. Using data obtained from an online database (i.e., Best Practice Caregiving), that currently provides in-depth information on 44 dementia caregiving EBPs, the current study aimed to examine the number of EBPs that incorporated PLWD self-report measures on a variety of well-being outcomes. Results indicate that out of the 44 EBPs included for analysis: 1) Four out of 19 programs evaluating PLWD symptom severity/difficulty/distress used a PLWD self-report measure; 2) Eight out of 10 programs evaluating PLWD symptoms of depression used a PLWD self-report measure; 3) Five out of 10 programs evaluating PLWD quality of life/care utilized a PLWD self-report measure; and 4) One out of 9 programs evaluating ADL/IADL dependencies utilized a PLWD self-report measure. Discussion will focus on potential gaps and limitations of primarily using caregiver proxy reports to collect personal well-being outcomes for PLWD and ways in which researchers can integrate more self-report measures into intervention and programmatic evaluations.

### FINDINGS FROM COMMUNITY WORKSHOPS DESIGNED TO HELP EXPAND ARIZONA'S DEMENTIA CAPABLE SYSTEM

DAVID Coon, Aylin Angulo, Marielysse Cortes, Berta Carbajal, Kassey Stotler, zenya Weatherall, and jami Goldman, *Arizona State University, Phoenix, Arizona, United States*

Among the 5.8 million people living with Alzheimer's disease (AD), there are three vulnerable groups where community partners can join efforts to serve the community more comprehensively. These include (a) people living alone with Alzheimer's disease and other related dementias (ADRD) who may or may not have a family caregiver, (b) people with Down Syndrome or another intellectual or developmental disability aging with ADRD and their family caregivers, and (c) people with ADRD and their family caregivers in the Latino community. Dementia capable systems are designed to address the needs and concerns of all individuals, families, and communities impacted by ADRD. The project develops and expands ADRD programs and services across Arizona through educational workshops, case management services, and evidence-based programs. Workshops (N=67) were provided to a variety of professionals and community members ranging from promotores/CHW's (community health workers) and case managers to family caregivers and people living with dementia (N=2,272). Workshops successfully attracted a substantial proportion of Hispanic or Latino/a participants (63%) and women (84.5%). Perception of benefit ratings were overwhelmingly positive with over 90% of participants agreeing or strongly agreeing that: the workshops met expectations; they were willing to attend other programs by us; and they learned something they could use. Moreover, based on their workshop experience, they felt more confident that they could help these three underserved populations. Overall, workshops were clearly acceptable to participants and feasible to deliver. In addition, they contributed to an increased awareness in ADRD related to the project's three target groups.

### FIRST: ENHANCING AGENCY DEMENTIA CAPABILITY THROUGH DEMENTIA-SPECIFIC INTERVENTIONS AND SUPPORTIVE SERVICES

Elizabeth Wellbrock, and Joanna Hutchinson, *County of San Diego Health and Human Services Agency, San Diego, California, United States*

Through the First Identify and Refer then Serve and Track (FIRST) Project, individuals and caregivers have critical connections to community organizations and resources to learn ways to handle living with memory problems. The FIRST project integrated new practices into existing programs to address gaps in service and piloted a new dementia-specific case management program. The initial intervention is a system-level change within the County of San Diego's Aging & Independence Services (AIS) department to identify, pilot, and implement a brief Alzheimer's Disease and Related Dementias (ADRD) screening tool. The tool was used by non-clinical personnel to identify potential ADRD cases. Individuals who screened positive for possible ADRD were referred to their physicians for an accurate diagnosis. The second intervention consisted of two components: a behavioral symptom management intervention for social workers to use in the home with caregivers and a dementia-specific case management program (including respite care) to improve quality of life and future planning for those with ADRD living alone or with a family caregiver. As of January 2021, 536 clients across several AIS programs have been screened for ADRD, of which, 60% screened positive. FIRST case management has served 196 clients, 70 who lived alone and 126 who lived with their caregiver. Respite was provided to 98 clients totaling to 3,666 hours. This poster evaluates the effectiveness of the program components in increasing dementia capability of an agency, and where applicable, its effect on caregiver burden and self-efficacy.

### INTEGRATING ALZHEIMER'S MESSAGES INTO CHRONIC DISEASE PROGRAMS

Leslie Best,[1] Rebecca Drewette Card,[2] Lisa Garbarino,[3] Christopher Taylor,[4] Kimberly Boim,[5] and Benjamin Olivari,[4], *1. National Association of Chronic Disease Directors, Decatur, Georgia, United States, 2. Public Health Partners, Topsham, Maine, United States, 3. Centers for Disease Control and Prevention (CDC), Atanta, Georgia, United States, 4. Centers for Disease Control and Prevention (CDC), Atlanta, Georgia, United States, 5. CDC, Atlanta, Georgia, United States*

The number of people in the United States with dementia is increasing, with nearly six million people living with Alzheimer's disease and related dementias. It is the fifth leading cause of death for those aged ≥65 years. Over 95% of people with dementia have another comorbid chronic condition. The Healthy Brain Initiative's State and Local Public Health Partnerships to Address Dementia: The 2018-2023 Road Map notes that public health agencies should raise awareness of the link between brain health and physical health, and specifically calls out tobacco prevention and control, cardiovascular health management diabetes prevention and management, obesity prevention and control, and injury prevention as intervention points. The National Association of Chronic Disease Directors developed brain health messages targeted to reduce risk for cognitive decline through the prevention and control of comorbid chronic conditions. These messages can be leveraged for public health action by integrating them into existing chronic disease programs.

### PSYCHOLOGICAL CHALLENGES AMONG OLDER ADULTS WITH EARLY STAGE DEMENTIA: A NARRATIVE LITERATURE REVIEW

Mijin Jeong, *University of Kansas, LAWRENCE, Kansas, United States*

Due to rapid expected growth in the population of older adults with dementia, exploring the issues and experiences related to early stage dementia (ESD) is a fundamental step toward helping individuals adjust to their diagnosis and transition into treatment. The purpose of this paper is to review the extant literature regarding how older adults adjust to and cope with the onset of dementia through major situations and difficulties. A narrative approach was applied to review 120 articles focused on ESD that were published in the U.S. and other western countries between 1995 to 2020. There were four apparent themes in the literature, which align with key chronological experiences related to ESD: diagnosis of dementia; stigma related to dementia; the development of identity with ESD; and social and service-related experiences of older adults with ESD. Stigma related to dementia was a powerful risk factor that hindered psychological adjustment to ESD. Varied cultural perspectives on dementia and a lack of knowledge of dementia symptoms among diverse older adults and their families were also major risk factors. In the U.S., there was a lack of literature, especially around the development of identity with dementia and older adults' perspectives on available services, Also, there were insufficient U.S.-based studies that explored the challenges of psychological adjustment among racial and ethnic minority groups. Future research could benefit from taking a life course perspective to assess ESD within the context of one's life and examine challenges associated with ESD across all four themes to promote empowerment.

### RELIGION AND SPIRITUALITY MEASURES IN DEMENTIA: AN INTEGRATIVE REVIEW OF THE LITERATURE

Katherine Britt,[1] Gayle Acton,[2] and Jung Kwak,[3], *1. University of Texas at Austin, Austin, Texas, United States, 2. University of Texas at Austin, The, Austin, Texas, United States, 3. The University of Texas at Austin, Austin, Texas, United States*

Literature documents positive effects of religion and spirituality on health including improved cognitive function, quality of life, and well-being. Extant research suggests that persons living with dementia (PWD) are more likely to have spiritual needs and rely on others to support their spiritual well-being than those without dementia. However, spiritual care is absent or minimally present in dementia care. To effectively address spiritual needs of PWDs, accurate screening and assessment is critical. We conducted an integrative review of the current literature on measures of religion and spiritualty for PWDs by searching five databases (ATLA Religion, CINAHL, PsychInfo, PubMed, and SocIndex) and identified 14 studies that were peer-reviewed original research articles focusing on assessment of religion/spirituality among PWDs and published between 2000-2020 in English. Most were conducted in Europe (n=7), included PWD in mild stage (n=68) from various settings, and were cross-sectional in design (n=8). Of a total of 17 measures identified, 6 were originally developed for the general population and then adapted for PWD, and only 3 were validated for PWD. A majority of the studies were limited in sample size, generalizability, methodological rigor, and measure validation. More research is needed using diverse samples and rigorous study designs to develop valid screening and assessment tools for this population. Improving religious and spiritual measures could greatly impact public health by improving quality of life for millions of individuals suffering from dementia and their caregivers who carry a heavy burden.

### SOCIAL DETERMINANTS OF HEALTH AND THE ONSET OF DEMENTIA IN LATER LIFE

Darren Liu,[1] Takashi Yamashita,[2] Betty Burston,[3] and Chetana Guthikonda,[1], *1. Des Moines University, Des Moines, Iowa, United States, 2. University of Maryland, Baltimore County, Baltimore, Maryland, United States, 3. University of Nevada, Las Vegas, Las Vegas, Nevada, United States*

Dementia is a debilitating neurodegenerative syndrome characterized by deterioration in memory, cognitive, behavioral, and physical capacity. Recent research has indicated that some early-life social determinants of health (SDH), which vary by race/ethnicity may hold clues to the onset of dementia. Although early life clinical risk factors of dementia have been identified, early-life SDH such as education, sociodemographic and socioeconomic characteristics are yet to be collated. This review study focused on early-life (less than18 years of age) SDH in relation to cognitive decline in later life and differences across racial/ethnic groups in the U.S. A systematic review of articles in and after January of 1999 was conducted using Scoping Reviews - an approach for evidence synthesis to determine the coverage of a body of literature. Studies that report the impact of early-life social determinants on late-life cognitive decline were identified through the searches of CINAHL, Global Health, PsycINFO, PubMed and Scopus databases. Our initial database search resulted in 823 studies, and of those, 102 studies satisfied the inclusion criteria. The systematic review identified the following risk factors: lower education (34%), lower socioeconomic status (25%), Adverse Childhood Experiences (ACEs) (14%), exposure to environmental toxins (11%), food insecurity (6.8%), and rural residence (4%). Although education and socioeconomic status are well-known risk factors of cognitive decline in later life, other understudied factors such as food insecurity and residing in rural areas are yet to be explored. Implications in terms of understanding the link between early life SDH and dementia in later life are discussed.

### VIRTUES AND CHARACTER STRENGTHS IN PERSONS WITH MILD ALZHEIMER'S DISEASE AND RELATED DEMENTIAS

Jocelyn McGee, and Michaela McElroy, *Baylor University, Waco, Texas, United States*

The Values in Action (VIA) framework, from positive psychology, consists of 6 virtues (wisdom, courage, humanity, justice, temperance, and transcendence) which can be broken down into 24 strengths of character (Peterson & Seligman, 2004). Although this framework has been used to understand virtues and strengths in various populations, persons with Alzheimer's disease and other progressive dementias have been largely excluded from such studies. The aims of our study were to utilize the VIA framework to: 1) examine the expression of virtues and character strengths in a sample of persons diagnosed with probable AD (who were clinically designated as having mild disease based on neuropsychological assessment); and 2) provide implications for further strengths-based research and clinical practice with this population. Twenty-eight older adults, between the ages of 56 and 93 (M = 77.88, SD = 9.88), participated in the study. All had a Clinical Dementia Reacting Scale (CDR) of 1 which suggested mild dementia. The Dimensions of Living with Dementia Interview (DLD; McGee & Carlson, 2013) was utilized for data collection. Interviews lasted approximately 45 minutes per participant. The interviews were professionally transcribed, audit checked, and uploaded to NVivo (2018) for analysis. Directed content analysis (Hsieh & Shannon, 2005) was utilized to extend the VIA framework to this sample. The virtues of wisdom, courage, humanity, temperance, and transcendence were demonstrated. We share how these virtues and corresponding character strengths can assist people with early-stage dementia in adaptation to the disease. Implications for clinical practice and further research are provided.

### Session 9190 (Poster)

### DEMENTIA PREVALENCE AND RISK FACTORS

### A PATH ANALYSIS OF AGITATION IN PERSONS WITH ALZHEIMER DISEASE

Katherine Kero,[1] Hossein Yarandi,[2] and Debra Schutte,[2], *1. Wayne State University, Northville, Michigan, United States, 2. Wayne State University, Detroit, Michigan, United States*

This study tested a theory-driven path model of predictors and outcomes of agitated behavioral symptoms in persons with Alzheimer Disease (AD) guided by the Nursing Theory of Unpleasant Symptoms. This secondary analysis included data from 48 persons with AD from institutional residences in Midwestern United States. Data included measures of physical, psychological, and situational antecedents of agitation, the symptom of agitation, and functional outcomes of agitation with 15 instruments and subscales. The data were a good fit for the empirical path model ($\chi2 = 1.049$, df = 2, p = .592). Statistically significant paths were found from measures of physical, psychological, and situational antecedents to agitation (B = 0.58, 0.446, and 0.51; $p \leq .001$) which explained 63% of the variance in agitation. Functional performance was influenced only by physical antecedents, which explained 66% of the variance in functional performance. While physical and psychological states may be comorbid to the AD disease process and therefore less modifiable, the situational measure was primarily defined by measures related to caregiver burden. Therefore, future agitation interventions should target strategies to improve caregiver burden.

### BEHAVIORAL CORRELATES OF SUBJECTIVE COGNITIVE DECLINE IN THE CANADIAN LONGITUDINAL STUDY ON AGING (CLSA)

Shawna Hopper,[1] Nicole Hammond,[2] and Arne Stinchcombe,[3], *1. Brock University, St Catharines, Ontario, Canada, 2. University of Ottawa, Ottawa, Ontario, Canada, 3. Brock University, St. Catharines, Ontario, Canada*

Subjective cognitive decline (SCD) is a self-reported decline in cognition among otherwise cognitively healthy older adults. It is believed that SCD may be a precursor to Alzheimer's Disease (AD). Analyzing data from the Canadian Longitudinal Study on Aging (CLSA), a large national sample of participants aged 45-85 at baseline, we sought to identify prospective relationships between health-related behaviors and SCD. Exposures were measured at baseline and SCD was measured three years later, with the question: "Do you feel like your memory is becoming worse?". A multivariable logistic regression model was used to estimate odds of SCD (analytic sample: n=35,680). Alcohol consumption was associated with increased odds of SCD, with regular drinkers (OR=1.13, 95% CI: 1.04, 1.22) and frequent drinkers (OR=1.17, 95% CI: 1.08, 1.27) more likely to report SCD than never drinkers. Compared to participants who never smoked, former smokers had increased odds of SCD (OR=1.13, 95% CI: 1.08, 1.18), whereas current smokers had reduced odds of SCD (OR=0.90, 95% CI: 0.83, 0.98). Participants who consumed five or more servings of fruits/vegetables had reduced odds of SCD (OR=0.95, 95% CI: 0.91, 0.99), when compared to those who consumed <5 servings. Lastly, we did not observe any associations between walking and SCD. This study identifies relationships between various health-related behaviors and SCD in a large population-based sample of older Canadians. Identification of modifiable risk factors may help with early prevention and intervention of SCD.

### ESTIMATING DEMENTIA FROM SELF-REPORTS OF DIAGNOSES AMONG ADULTS AGED 65 AND OVER: UNITED STATES, 2019

Ellen Kramarow, *National Center for Health Statistics, Hyattsville, Maryland, United States*

Prior research shows that, overall, about 10% of the population aged 65 and over in the U.S. has dementia. Estimating the prevalence of dementia from nationally representative surveys can be accomplished by asking respondents about a diagnosis, by administering a cognitive assessment, or, if available, by examining linked medical claims data. In 2019 for the first time, the National Health Interview Survey (NHIS) added "dementia, including Alzheimer's disease" to the questions asking about doctor-diagnosed health conditions. Although estimates derived from doctor-diagnosed questions usually underestimate the true prevalence of a condition, and estimating dementia from self-reports presents additional challenges, they are still useful for many surveillance and research objectives. Early diagnosis of dementia is encouraged to allow patients and their families to plan for future needs. The objective of this research is to describe the noninstitutionalized population aged 65 and over who have a dementia diagnosis, by selected socio-demographic, health, and healthcare utilization characteristics. Point estimates, standard errors, and 95% confidence intervals for percentages are calculated using NHIS adult sample weights and adjusted for the complex sample design of NHIS. Preliminary analyses show that, overall, 4% of the 65 and over noninstitutionalized population has a diagnosis of dementia. About 8% of respondents with less than a high school education reported a dementia diagnosis compared with 2% of those with more than a high school education. Those with a dementia diagnosis were more likely to report depression than those without a dementia diagnosis (44% vs. 14%).

### MOTORIC COGNITIVE RISK SYNDROME: ITS PROGNOSTIC VALUE FOR DEMENTIA AND OTHER HEALTH OUTCOMES. A SYSTEMATIC REVIEW

Donncha Mullin,[1] Alastair Cockburn,[2] Miles Welstead,[1] Michelle Luciano,[3] Graciela Muniz-Terrera,[1] and Tom Russ,[1], *1. University of Edinburgh, Edinburgh,*

*Scotland, United Kingdom, 2. NHS Lothian, Edinburgh, Scotland, United Kingdom, 3. University of edinburgh, Edinburgh, Scotland, United Kingdom*

Motoric cognitive risk syndrome (MCR) is a recently defined concept combining objective slow gait and subjective cognitive complaints, in the absence of dementia or significant functional impairment. MCR is associated with an increased risk for dementia but its prognostic value for other mental and physical health conditions is less studied. MCR is quick, inexpensive and easy to measure, making it a potentially useful clinical tool. This review aims to be the first to synthesise all mental and physical health outcomes associated with MCR since the term was coined in 2013. Results from multiple databases (MEDLINE, AMED, EMBASE, CINAHL, PsycINFO, and the Cochrane library; conception to November 2020) were screened independently and risk of bias was assessed. [Results are currently preliminary but definitive findings will be available in time for the conference] In total, 1057 references were screened, resulting in 34 studies being included, of which 14 will be meta-analysed. Eleven longitudinal studies examined MCR in relation to incident cognitive impairment or dementia conversion. Other prospective studies found that MCR predicted higher risk of mortality (n=2), falls (n=3), post-fall fractures (n=1), gait dysfunction (n=1), and cardiovascular risk factors and diseases (n=1). The results from the 23 cross-sectional studies reporting associations with MCR highlight areas for further study to better understand the biological mechanisms of MCR. By synthesising the latest evidence, this review reinforces the value of MCR for predicting incident dementia, but also adds weight to its value in relation to other important age-related health outcomes.

### MUSIC AND DEMENTIA: EXPLORING PROTECTIVE FACTORS FOR COGNITIVE FUNCTION

Sebastian Santana,[1] Michael Willden,[1] Debra Sheets,[1] Andre Smith,[1] Robert Stawski,[2] and Stuart MacDonald,[1], *1. University of Victoria, Victoria, British Columbia, Canada, 2. Oregon State University, Corvallis, Oregon, United States*

Ample research suggests that musical interventions have the potential to boost social connection, engender positive emotions, and potentially buffer against depression in people with dementia (PwD). Here, our focus concerns expanding the present body of knowledge by quantifying the benefits of a music-based nonpharmacological intervention. The Voices in Motion (ViM) choir is an intergenerational sociocognitive lifestyle intervention designed to support caregivers and PwD. Over the course of 18 months, the well-being of PwD and caregiver dyads (N = 32; mean age = 79.6 years; 53% female) were rigorously assessed using an intensive repeated measures design. This project set out to determine whether positive change in the social dimensions of health (i.e., social connection [SC] and psychological well-being [WB]) ameliorates depression in PwD. Multilevel modeling was employed to examine longitudinal change within and between individuals. SC significantly predicted intraindividual change (□20 = -0.48, p =.03), with a predictive trend for between person differences (□00 = -0.58, p =.08). On occasions when PwD reported more SC, relative to their own baseline, they also reported fewer depressive symptoms. The effect associated with WB was significant at the between-person level (□00 = -0.18, p =.01). Our analysis suggests that a lifestyle intervention targeting psychological health and wellbeing may also contribute to the depressive signs and symptoms in PwD. As the current health care system is forced to adapt to social distancing and constant precautionary measures, it is crucial to understand and potentially harness the protective effects of modifiable lifestyle factors.

### PREVALENCE OF DEMENTIA IN AMERICAN INDIAN/ ALASKA NATIVE MEDICARE BENEFICIARIES

Heehyul Moon,[1] Joseph Kaholokula,[2] Richard MacLehose,[3] Sunshine Rote,[4] and Soonhee Roh,[5], *1. University of Louisville, university of Louisville, Kentucky, United States, 2. John A. Burns School of Medicine, University of Hawaii at Manoa, Honolulu, Hawaii, United States, 3. University of Minnesota, Minneapolis, Minnesota, United States, 4. University of Louisville, Louisville, Kentucky, United States, 5. University of South Dakota, Sioux Falls,, South Dakota, United States*

Given the increase in life expectancy for American Indians and Alaska Natives (AIANs) due to many positive changes in social and environmental factors, the number of AIAN older adults with dementia is expected to grow from 23,850 in 2010 to over 100,000 by 2050. However, there have been few studies on the prevalence of dementia that have included AIANs . The purpose of the current study was to estimate the prevalence of dementia among AIANs over 65 years compared to non-Hispanic Whites (NHWs), Non-Hispanic Blacks (NHBs), Hispanics. The current study used survey data from Round 5 of the National Health and Aging Trends Study (NHATS, 2015) (N=7,449), a nationally representative study of Medicare beneficiaries 65 years and older. We estimated the age and gender-adjusted prevalence of dementia and 95% confidence intervals for each race/ethnicity. The majority of participants were between 65 and 74 years old. Slightly more than half of them were female. AIAN Medicare beneficiaries showed a significantly higher prevalence of dementia than NHWs after adjusting for age and gender (4% greater or higher prevalence). We also observed a significantly lower prevalence of dementia in AIAN Medicare beneficiaries than NHBs, Hispanics. While previous research has reported that AIANs shave a similar or lower prevalence of dementia than NHWs, our findings indicate significant dementia disparities in AIAN Medicare beneficiaries. Future research should focus on dementia prevalence and risk factors within/between AIAN tribes.

### RACIAL/ETHNIC DIFFERENCES IN TRAJECTORIES OF DEMENTIA ONSET

Sunshine Rote,[1] and Heehyul Moon,[2], *1. University of Louisville, Louisville, Kentucky, United States, 2. University of Louisville, university of Louisville, Kentucky, United States*

Racial and ethnic minority older adults—especially non-Latino Black and Latino older adults—continue to have a higher prevalence of dementia with longer delays in formal diagnosis compared to non-Latino Whites. Few studies have estimated racial/ethnic differences in trajectories of dementia onset using nationally representative data with representation from the three largest racial/ethnic groups in the U.S.: non-Latino White, non-Latino Black, and Latino older adults. Additionally, given the delays in formal diagnosis we rely on a measure of probable dementia that takes into account both formal diagnosis and cognitive function. Data from the National Health and Aging Trend Study (NHATS, 2011–2019) reveals three trajectories of dementia onset (early, late, and dementia-free) and we find that Latino and Black older adults are at greater risk for early dementia onset compared to non-Latino Whites. Our next step is to explore the role of social function for dementia disparities.

### RISING GEOGRAPHIC VARIATION IN ALZHEIMER'S DISEASE MORTALITY

Jessica Ho,[1] and Yujin Franco,[2], *1. University of Southern California, Los Angeles, California, United States, 2. University of Southern California, University of Southern California, California, United States*

The burden of Alzheimer's disease (AD) mortality has increased rapidly, growing by nearly 4- (men) and 6-fold (women) between 1990-2017. Limited attention has been paid to geographic inequalities in AD mortality. This study examines age-standardized AD mortality across 10 regions and the urban/rural continuum among adults aged 65+ using National Center for Health Statistics mortality and population data. We also examine mortality for a broader category, Alzheimer's disease and related dementias (ADRD), to address potential underreporting. The East South Central has the highest AD death rates and experienced larger increases–5-fold (men) and 7-fold (women)–than the nation as a whole. The Middle Atlantic consistently experienced the lowest AD mortality over the past quarter-century. Differences between the best- and worst-performing regions widened over time. AD mortality was 2.5 times higher in the East North Central than the Middle Atlantic region in 2017 (268 vs. 110 [men] and 374 vs. 147 [women] deaths per 100,000). Rural areas facing health care shortages and socioeconomic deprivation may encounter substantial challenges in addressing rising AD mortality. In several regions, rural disadvantages in AD mortality emerged and widened over time. The largest gaps between nonmetros and large central metros are in the East North Central, South Atlantic, and New England, as well as Appalachia (men) and West South Central (women), with nonmetros having 14-56% higher mortality than big cities. These findings identify the heavy burden of AD mortality in the Southern and rural U.S. and have important implications for health care, service, and caregiving provision.

### SELF-EXPERIENCED COGNITIVE FUNCTION IN THE DIGITAL ERA: ARE OLDER ADULTS AT RISK OF SUBJECTIVE COGNITIVE DECLINE?

Moriah Splonskowski,[1] Samantha Smith,[2] and Claudia Jacova,[2], *1. Pacific University, Forest Grove, Oregon, United States, 2. Pacific University, Hillsboro, Oregon, United States*

Older adults often find it difficult to use everyday technology proficiently. We hypothesized that these difficulties would be exacerbated in those with subjective cognitive decline (SCD), that is, self-perceived worsening of cognitive functions that has been associated with increased risk of future dementia. Here we investigated the relationship between SCD symptom burden and technology proficiency. A nation-wide sample of adults (N=483) ages 50-79 (66.5% female; 14.5% age >70) completed an online survey via a crowdsourcing website, Amazon Mechanical Turk. The survey included the Subjective Cognitive Decline Questionnaire (SCD-Q MyCog) (0-25, M=4.71, SD=5.77), questions about respondents' proficiency with computer, smartphone, and tablet (4-12, M=9.72, SD=1.97), and the PROMIS depression (M=13.18, SD=6.32) and anxiety (M=13.04, SD=5.68) scales. Linear regression was used to examine the ability of technology proficiency to predict SCD score. We also probed the interaction of technology proficiency with age (<70 vs. >70 years), and adjusted for covariates. We found that the age/technology interaction (B=-0.80), older age (B=7.49), lower education (B=-1.08), higher depression (B=0.20) and anxiety (B=0.16) symptoms predicted higher SCD burden (R-squared=.16). For respondents >70 years low technology proficiency predicted high SCD burden (B=-.79) whereas for those <70 years no relationship was found. Our study draws attention to older adults' self-experienced cognitive function in the digital era. The association between low technology proficiency and SCD may signal the adverse impact of the digital era on those who experienced technology only later in life. It is equally possible that declining technology proficiency is an indicator of emerging neurodegenerative disease.

### Session 9195 (Poster)

### DRIVING AND OLDER ADULTS

### DOES USEFUL FIELD OF VISION PREDICT ATTENTION WHILE DRIVING BETWEEN YOUNG AND OLDER ADULTS?

Rebecca Dunterman,[1] and R INTRIERI,[2], *1. Auburn University, Auburn, Alabama, United States, 2. Western Illinois University, Macomb, Illinois, United States*

The Insurance Information Institute (2017) reports drivers aged 65 and up are involved in the second highest rate of fatal car crashes. It is important that there is a fair and standardized assessment to test driving fitness. The prime objective was to assess the utility of the Useful Field of View (UFOV) across young and old groups to predict performance on a simulated driving exercise. Community-dwelling adults aged 65 and older (n=48) and students (n=48) recruited from an undergraduate research pool served as participants. They completed a series of demographic, health and cognitive measures, besides a Useful Field of Vison (UFOV) task and a driving simulation exercise. Results showed that collision avoidance and braking varied between age groups, with older adults appearing to be less likely to avoid collision (Older M = 12.46, SD = 10.25, Younger (M = 7.96, SD =4.92; n = 47), but quicker to brake (Older M = 3.64, SD = 3.41, Younger M = 9.79, SD =7.91). There were group differences for driving simulator performance, predicted by cognitive measures (Young; $R2$ = .099, p = 0.005; Old; $R2$ = 0.094, p = 0.038). UFOV scores did not predict group differences in driving simulator performance (Young; $R2$ = 0.009, p = 0.664; $\beta$ = 0.089, p = 0.437)

, (Older; R2 = 0.061, p = 0.522; UFOV-DA β = -0.074, p = 0.555; UFOV_SA β = 0.289, p = 0.194). These findings have implication for the development of pragmatic capacity to drive assessments.

### DRIVING FREQUENCY AND WELL-BEING IN OLDER ADULTHOOD

Zainab Suntai, Kefentse Kubanga, Abhay Lidbe, and Emmanuel Adanu, *University of Alabama, Tuscaloosa, Alabama, United States*

The activity theory of aging suggests that older adults age successfully when they remain active and engaged. While many older adults are still able to drive, not all are as engaged in social activities, despite having the transportation to be able to do so. As such, this study aimed to examine the association between the frequency of driving and overall well-being among older adults. The hypothesis is that older adults who drive more frequently would have higher well-being, as they are likely driving to engaging activities. A sample of 1,663 older adults who reported that they are able to drive were derived from the 2018 National Health and Aging Trends Study (NHATS). The NHATS is an annual longitudinal panel of survey of adults aged 65 and older living in the United States. Chi-square tests were used for bivariate analyses and a weighted multivariable logistic regression model was used to predict well-being based on driving frequency. Results showed that compared to those who drive every day, those who drive most days (OR=0.771, CI= [0.768-0.775]), some days (OR=0.495, CI= [0.492-0.497]), rarely (OR=0.558, CI= [0.555-0.562]) or never (OR=0.371, CI= [0.367-0.374]) were less likely to have high well-being. Interventions geared at improving well-being among older adults should therefore consider increasing awareness of social events, to ensure that older adults who are able to drive can have a good quality of life by driving to social activities.

### DRUGGED DRIVING AMONG SEXUAL MINORITY OLDER ADULTS IN THE UNITED STATES, 2015-2019

Jie Yang,[1] and Andrew Yockey,[2], *1. East Carolina University, Greenville, North Carolina, United States, 2. University of North Texas Health Science Center, Denton, Texas, United States*

**Introduction:** Drugged driving, the act of operating vehicles under the influence of one or more illicit substances is responsible for numerous emergency department visits, deaths, and increased medical costs. Despite higher instances of drug use, older sexual minority (LGB) adults are often neglected in prevention efforts. This study assessed inequities between sexual minority older adults and their heterosexual counterparts in drugged driving across three difference substances (alcohol, marijuana, other drugs).

**Methods:** Pooled data from individuals 50 years or older (n = 43,238; 1,115 sexual minority adults) in the 2015–2019 National Survey on Drug Use and Health were analyzed. Past-year driving under the influence of alcohol, marijuana, and illicit drugs were outcome variables, and survey-weighted frequencies with 95% confidence limits and crude ORs with 95% confidence intervals (CI) were estimated.

**Results:** In the past year, 4.82% of older adults drove under the influence of alcohol, 1.33% drove under the influence of marijuana, and 1.48% drove under the influence of illicit drugs. Sexual minority adults consistently showed higher odds of drugged driving than their heterosexual counterparts, with individuals who identify as bisexual being most at risk for driving under the influence of illicit drugs (aOR:4.49, 95%CI: 2.84, 7.08) and marijuana (aOR:3.95, 95%CI: 2.39, 6.51).

**Discussion:** There are differences drugged driving by sexual orientation across the three substances we assessed. These rates of substance use among older sexual minority adults warrant ongoing concern, and it is critical to consider differences across the life course in designing and evaluating interventions to address inequities.

### UNDERSTANDING DRIVING AVOIDANCE AMONG OLDER AFRICAN AMERICANS AND WHITES WITH DIABETES

Henrietta Armah,[1] Maya Martin,[2] Wesley Browning,[1] Ghislaine Atkins,[1] and Olivio Clay,[1], *1. University of Alabama at Birmingham, Birmingham, Alabama, United States, 2. Tuskegee University, Tuskegee, Alabama, United States*

Diabetes mellitus is one of the most common chronic diseases with half of the new diagnoses affecting adults aged 60 years and older. Although African Americans are more likely to develop the disease, they are also less likely to receive healthcare. Importantly, living with diabetes is likely to negatively impact mobility for aging adults as the disease is associated with lower physical functioning (e.g., ability to maintain one's balance). Further, diabetes could pose a significant threat to a person with diabetes' ability to drive and remain in the community. This study examines the relationships and influences of social determinants of health (e.g., race, gender, socioeconomic status) and cognition on avoiding driving maneuvers such as driving at night and in rush hour traffic among older adults with diabetes. Data from the University of Alabama at Birmingham (UAB) Diabetes and Aging Study of Health (DASH) were analyzed and of the 224 participants, 193 (86.16%) were current drivers. There was a gender difference with 94.12% of males and 79.51% of females being current drivers, p < .01. Within the sample of current drivers, 45% were African American and being female, not married, lower levels of education and cognition, low income, and being African American were associated with higher scores on driving avoidance. Cognition explained 30.44% of the racial difference in driving avoidance. Findings from this study will help identify individuals who are at-risk for reduced mobility and identify those who may need to be intervened upon to support a better quality of life.

### Session 9200 (Poster)

### DYADIC RESEARCH (BSS POSTER)

### A DYADIC STUDY OF DEPRESSION, CAPITALIZATION PATTERNS, AND LEISURE ACTIVITIES IN RETIREMENT

Ksenya Shulyaev,[1] Dikla Segel-Karpas,[2] and Nurit Gur-Yaish,[3], *1. Faculty of Social Welfare and Health Science, University of Haifa, Haifa, Hefa, Israel, 2. University of Haifa, Haifa, Hefa, Israel, 3. Oranim Academic College of Education, Haifa, HaZafon, Israel*

Late-life relationships, and specifically spousal relations, are increasingly recognized as an important factor shaping the wellbeing, health, social and emotional health of older people. Therefore, a better understanding of the health and well-being trajectories of older adults requires considering the characteristics of their spouses and couple dynamics. This study focused on the actual problem of engagement of recently retired older adults in the community and various leisure activities and examined how both older adults' and spouses' depression level influence their activities. We also consider the quality of relationships in a couple: how a partner generally responds when the participant discloses good news (capitalization). Fifty-three Israeli couples participated in the current study with one member of the couple 60 or older and retired within the last five years. Recently retired spouses rated their engagement in leisure activities, both spouses reported their level of depression, and partners of retired persons completed the Perceived Responses to Capitalization Attempts Scale. Results show that depression level of recently retired spouses had a direct negative effect (b(SE)=-7.8(3.38), CI(-14.65,-1.04), p=0.02) on their engagement in leisure activities, while the level of their partners' depression had no significant direct effect on retired persons' leisure activities. However, partners' depression associated (p=0.001) with negative capitalization patterns and mediation analysis showed an indirect effect of partners' depression via the capitalization (b(SE)=-2.77(1.7), CI(-6.41,-0.04), p=0.03). These results indicate that in encouraging newly retired people to participate in leisure activities it is important to consider both spouses' depression level and capitalization patterns in the couple.

### "I'M GETTING OLDER TOO": CHALLENGES AND BENEFITS EXPERIENCED BY VERY OLD PARENTS AND THEIR CHILDREN

Kathrin Boerner,[1] Elizabeth Gallagher,[2] Kyungmin Kim,[3] Daniela Jopp,[4] and Yijung Kim,[5], *1. University of Massachusetts Boston, Boston, Massachusetts, United States, 2. University of Massachusetts Boston, Boston, MA, Massachusetts, United States, 3. Seoul National University, Seoul, Seoul-t'ukpyolsi, Republic of Korea, 4. University of Lausanne, Lausanne, Vaud, Switzerland, 5. The University of Texas at Austin, Austin, Texas, United States*

Very old parents and their "old" children are a growing group in industrialized countries worldwide. Since most very old persons have outlived spouses and friends, their children, many of whom have reached old age themselves, are likely to become their primary social contact and to shoulder the care provision role. However, virtually nothing is known about the nature and implications of this relationship constellation. To fill this gap, the present study explored the challenges and rewards of the very old parent-child relationship. In-depth interviews were conducted with 114 parent-child dyads (parent age ≥ 90; child age ≥ 65). Narrative interview data on challenges and rewards were audiotaped, transcribed, and then systematically reviewed and coded, identifying recurrent themes and defining categories that reflected these themes. While both challenges and rewards were present, more rewards than challenges were reported overall. However, comparing parent and child perspectives revealed that the balance of challenges and rewards was less favorable for children. Narrative data further showed that the sense of burdening their children heavily weighed on at least a fourth of parents, reflecting this as a serious concern not only for children but also for parents. Challenges reported by children were often characterized by references to children's own advanced age and health problems, and the prolonged caregiving involvement due to their parents' longevity. Healthcare professionals, policy makers, and families should be made aware of this increasingly common phenomenon, and specific services and policies will be needed to adequately support very old adults and their families.

### CHARACTERISTICS OF OLDER ADULTS WHO RECEIVE ASSISTANCE WITH MANAGEMENT OF MULTIDRUG REGIMENS

Rachel O'Conor,[1] Julia Yoshino Benavente,[1] Mogan Eifler,[1] Lauren Opsasnick,[1] Laura Curtis,[1] Lee Lindquist,[2] and Michael Wolf,[1], *1. Northwestern University, Chicago, Illinois, United States, 2. Northwestern University Feinberg School of Medicine, Chicago, Illinois, United States*

Many older adults manage multiple chronic conditions requiring adherence to multidrug regimens, yet half are non-adherent, increasing their risk of hospitalization for poorly controlled chronic conditions. Few studies have investigated whether caregivers support medication-related behaviors of community-dwelling older adults. We interviewed 97 patient-caregiver dyads participating in a cognitive aging cohort study to identify factors associated with caregiver assistance in managing multidrug regimens. Patients completed a neuropsychological battery covering five cognitive domains. Health literacy and patient activation were measured using the Newest Vital Sign and Consumer Health Activation Index, respectively. Caregivers reported their medication-related involvement. Predictors of involvement in medication-related tasks were examined using logistic regression models. Patients were on average 71 years old, managing 4 comorbidities and prescribed 5 medications. The majority were female (73%) and identified as Black (46%) or White (47%). Caregivers' mean age was 65 years; half were female (53%), were predominantly spouses (57%) or children (26%), and lived with the patient (61%). 31% of caregivers ordered patients' prescribed medications, 40% helped manage their medications, and 50% spoke with the patient's clinician about their clinical care. Cognitive impairment (OR 2.60, 95% CI 1.08-6.25), limited health literacy (OR 2.97, 95% CI 1.26-6.97), and ≥3 comorbidities (OR 2.14, 95% CI 1.06-9.30) were associated with medication management assistance. Patient activation, gender, cohabitation, or relationship were not associated. These findings suggest that caregivers are assisting with older adults' medication management and should be included in clinical discussions about medication management, especially among patients with cognitive impairment, low health literacy or multimorbidities.

### DYADIC LONELINESS AND HBA1C LEVELS AMONG OLDER MARRIED COUPLES: EVIDENCE FROM THE IRISH LONGITUDINAL STUDY ON AGEING

Jeffrey Stokes,[1] and Adrita Barooah,[2], *1. University of Massachusetts Boston, Boston, Massachusetts, United States, 2. UMass Boston, Boston, Massachusetts, United States*

Loneliness is an important determinant of health and mortality among the aging population, including for cardiometabolic health. Yet research has largely focused on individual experiences of loneliness, rather than taking intimate relationships into account. However, recent studies have highlighted that the psychosocial well-being of one's partner may impact one's own health as well. Indeed, the stress generation hypothesis anticipates that loneliness in one partner may lead to more stressful interactions within relationships, and thus to worse health outcomes for both spouses. This is particularly true among older couples, as life events and shifting time horizons (e.g., retirement, socioemotional selectivity, reduced social networks) lead older persons to focus more time and energy on their closest relationships. Life events such as retirement may make adults' intimate relationships – and the experiences of their partner – more salient than ever before. In this study, we use dyadic structural equation modeling to examine associations between loneliness and HbA1c levels among 1,331 older married couples from The Irish Longitudinal Study on Ageing. Further, we test whether any such associations vary by age or employment status. Results indicate that one's own loneliness was not significantly linked with elevated HbA1c, irrespective of age or employment status. However, loneliness of a dyadic partner was significantly associated with elevated HbA1c among retired persons only. Further, this effect was not due to age, but rather to employment status itself. These findings suggest that relationship context is crucial when considering the dyadic health implications of loneliness among the older population.

### DYADIC SUCCESSFUL AGING AND THE LIMITS OF AGENCY

Markus Klingel, *Technical University Dortmund (Germany), Dortmund, Nordrhein-Westfalen, Germany*

With increasing life expectancy, late life has become a longer, crucial part of the individual and dyadic life course. New opportunities, tasks and decisions emerged. Successful aging norms emphasize agency and autonomy. This can be activating, but also alienating. With increasing constraints, agency is limited and ideals of autonomy become dysfunctional. This challenges also relationships. Aging, functional losses and approaching death threaten dyadic satisfaction and functionality. Potentially, successful aging norms could erode dyadic solidarity when needed the most: in late life. This mixed-methods longitudinal study combines interviews and questionnaires at three observations across five years. Its focus lies on change over time and findings at observation three. The sample consists of eight German couples (78-86 years old, 50-65 years married, high relationship satisfaction, white, urban). What does aging mean for individualized actors? How do aging couples negotiate, decide and act on aging, autonomy and death? How do successful aging norms modulate dyadic aging? Overall, actors have internalized successful aging and benefit by influencing their health positively. However, this has become ambivalent. Actors increasingly perceive their future as limited and beyond individual control. Acceptance of losses that challenge the self is difficult, autonomy ideals burdensome and death salient. As individual agency is constrained, the dyad is still a functional stronghold against aging. Yet, it has to adapt as well to – potentially differential - individual aging. Losses can and do threaten couples' functional and emotional unity. Four patterns of self-dyad dynamics emerged and exemplify tensions between individualized and dyadic successful aging.

### EXPLORING COLLABORATION ENJOYMENT AND DECISIONAL UNCERTAINTY: ACTOR-PARTNER EFFECTS IN ADVANCED CARE PLANNING

Shirin Hiatt,[1] Jia-Wen Guo,[2] Lee Ellington,[2] and Djin Tay,[2], *1. Oregon Health & Science University, Portland, Oregon, United States, 2. University of Utah, Salt Lake City, Utah, United States*

Caregivers are often engaged in decision making with and for patients. However, the role of patient-caregiver interpersonal processes on decisions about advance care planning (ACP) are not well known. This secondary data analysis examined the effects of patient-caregiver enjoyment about collaboration regarding choices for life-sustaining treatment on patients' and caregivers' decisional uncertainty following a dyadic ACP intervention. A purposive sample of 18 adult home health patients and their informal caregivers (N=36) participated in a one-group pretest posttest study. The Interpersonal Enjoyment subscale of the Perceptions of Collaboration Questionnaire and the Decisional Uncertainty subscale of the Decisional Conflict Scale were administered using parallel questionnaires. The Actor-Partner Interdependence Model (APIM) was used to examine actor and partner effects of patients and caregivers' interpersonal enjoyment on their uncertainty in decisions about ACP before and after the intervention. The mean age was 68.2±9.6 years for patients and 61.3±13.6 years for caregivers. The majority of patients (61.1%) and caregivers (72.2%) were female and married (55.6% and 66.7%, respectively). Almost all were non-Hispanic White (97.0%). Patients' and caregivers' interpersonal enjoyment and uncertainty scores were similar before the intervention. A significant partner effect between greater interpersonal enjoyment among caregivers at pretest and greater patient uncertainty at posttest ($\beta$=0.44, p=.037) was found. Previous analyses found that overall patients improved in decisional uncertainty at posttest. However, these findings suggest that for some dyads, interpersonal factors can negatively affect patients' decisional certainty. Future research is needed to verify this finding with a larger sample.

### FAMILY PROXIMITY AS A MODERATOR OF SPOUSAL ASSOCIATIONS OF DEPRESSION AMONG MEXICAN OLDER ADULTS

Katie Newkirk,[1] Maria Aranda,[2] Catalina Mourgues-Codern,[1] Ana Quiñones,[3] Rafael Samper-Ternent,[4] and Joan Monin,[5], *1. Yale University School of Medicine, New Haven, Connecticut, United States, 2. University of Southern California, Los*

Angeles, California, United States, 3. Oregon Health & Science University, Portland, Oregon, United States, 4. The University of Texas Medical Branch, Galveston, Texas, United States, 5. Yale School of Public Health, New Haven, Connecticut, United States

Depression among older adults is a public health issue, and a large literature highlights the importance of close relationships as both a risk and protective factor for depression. Research in U.S. samples suggests that one spouse's depressive symptoms can increase their partner's depressive symptoms, especially for women (Kouros & Cummings, 2010; Tower & Kasl, 1996). Little is known about interpersonal associations in depression, mitigating factors, and the role of gender among older couples in Mexico. This study examined (1) the effects of an individual's depressive symptoms on their spouse's symptoms and 2) whether living close to family buffered depression associations using data from the Mexican Health and Aging Study (n=4,071 dyads, age 50+ at initial interview). Depressive symptoms were measured in 2001, 2003, 2012, 2015, and 2018 using a modified 8-item version of the Center for Epidemiologic Studies-Depression Scale. Multilevel modeling was used to fit a dual-intercept growth model (centered at 2012) of husbands' and wives' depressive symptoms over time, controlling for age and education. Results showed a partner effect for husbands and wives, such that having a spouse with greater depressive symptoms in 2001 was associated with greater subsequent depressive symptoms, but not with rate of change in symptoms, in 2012. There was also a moderation effect such that the deleterious effect of husbands' depressive symptoms on wives' symptoms, as well as rate of increase in symptoms, was higher when family lived nearby, suggesting family may potentially exacerbate depression associations among spouses rather than a buffering them as hypothesized.

### NOT SEEING DOUBLE: DISCORDANCE IN DISEASE, FUNCTION, AND THEIR LONGITUDINAL ASSOCIATIONS IN MONOZYGOTIC TWINS

Elizabeth Teas,[1] Olivia Robertson,[2] Kristine Marceau,[2] and Elliot Friedman,[2], 1. Purdue University, West Lafayette, Indiana, United States, 2. Purdue University, Purdue University, Indiana, United States

Prior research on the causality and directionality between disease and functional limitations is ambiguous. The current study used longitudinal monozygotic twin data to test both directions linking disease burden and functional limitations in middle-aged and older adults, controlling for genetic and familial factors. We also examined potential moderation by psychological well-being. The Twins sub-sample from the first two waves of the longitudinal Midlife in the United States (MIDUS) study was used (Wave 1: 1995-1996, Wave 2: 2004-2006). Only monozygotic twins (N = 713) were included in analyses. In separate multi-level models, we examined disease burden at MIDUS 2 predicted by functional limitations at MIDUS 1 and MIDUS 2 functional limitations predicted by disease burden at MIDUS 1. Disease burden and functional limitations at MIDUS 2 varied substantially within families. There was no within-family association of earlier functional limitations with change in later disease burden ($b = .40$, $p = .39$), but there was a within-family association such that the twin with higher baseline disease burden had a greater increase in functional limitations than his/her co-twin ($b = .06$, $p = .02$). Well-being was not a moderator in either model. We found support for a potentially causal association between earlier disease burden and later increases in functional limitations, consistent with the Disablement Process Model. Sensitivity analyses confirm the detected within-family effect. Possible mechanisms linking disease burden and functional limitations are discussed as potential targets for future research.

### RELATIONSHIP FUNCTIONING AND GUT MICROBIOTA COMPOSITION AMONG OLDER ADULT COUPLES: FEASIBILITY OF DATA COLLECTION

Shelby Langer,[1] M. Aaron Guest,[2] Melissa Tolson,[2] Juan Maldonado Ortiz,[3] John DiBaise,[4] Helene Labonte,[4] and Rosa Krajmalnik-Brown,[3], 1. Arizona State University The College of Nursing & Health Innovation, Phoenix, Arizona, United States, 2. Arizona State University, Phoenix, Arizona, United States, 3. Arizona State University, Tempe, Arizona, United States, 4. Mayo Clinic, Scottsdale, Arizona, United States

An emerging area of research extends work on couple functioning and physical health to gut health, a critical marker of general health and known to diminish with age. As a foray into this area, we conducted a pilot study to determine feasibility of data collection (questionnaires and a stool sample) among older adult couples. Participants were recruited from the community using a variety of methods including social media. Among 41 persons responding with interest across recruitment sources, 32 were contacted for screening. Inclusion criteria were: age 60+, marriage or cohabiting partnership, and English speaking/understanding. Exclusion criteria were a gastrointestinal disorder, receiving enteric nutrition, use of antibiotics (past month), cancer treatment (past 6 months), and a +COVID-19 diagnosis (past 2 months). Among 31 eligible couples, 30 consented. All 60 participants completed questionnaires and provided a stool sample using DNAgenotek's OMR-200 collection kit, chosen for its ease and because samples can be stored at room temperature for 60 days. Sample characteristics were: M (SD) age = 66.57 (4.78); 53.3% female; 91.7% White; 1.7% Latinx; and 78.3% college-educated. 2 couples were same-sex. 43% reported at least one health condition and 25% reported use of a proton pump inhibitor (which can affect the gut microbiome), though none daily. Relational well-being was moderate-high on average per measures of dyadic adjustment and intimacy. Despite original plans to recruit couples in-person from a retirement community, remote operations were feasible via online assessment and study-coordinated shipping, a necessary yet fruitful shift due to the SARS-CoV-2 pandemic.

### THE IMPACT OF SELF- AND RELATION-INFERRED EFFICACY ON PHYSICAL ACTIVITY IN OLDER ADULT COUPLES

Tiana Broen,[1] Yoonseok Choi,[1] Christiane Hoppmann,[2] Kenneth Madden,[1] Maureen Ashe,[2] and Denis Gerstorf,[3], 1. University of British Columbia, Vancouver, British

*Columbia, Canada, 2. University of British Columbia, University of British Columbia, British Columbia, Canada, 3. Humboldt University of Berlin, Humboldt University of Berlin, Brandenburg, Germany*

Previous research has indicated that physical activity (PA) is a health-promoting behavior that is closely linked in couples. However, few studies have examined how PA is intertwined among couples in their everyday lives. For example, relation-inferred efficacy (RIE) is an individual perception that captures whether a close other believes in one's own abilities to perform specific behaviours; it originates from the sports literature on coach-athlete dyads and has been shown to shape athlete performance. Applying a repeated daily life assessment design, the current study targets self-efficacy (SE) and relation-inferred efficacy (RIE) as predictors of PA in older adult couples, as well as potential moderators when obstacles occur. We hypothesized that: (a.) There is a main effect of SE and RIE on PA. (b.) PA is lower on days when people anticipate barriers (c.) SE and RIE moderate the time-varying relationship between PA and barriers. Heterosexual couples (N=108 couples, Mage=70.5 years, SD=6.70) rated their SE and REI, completed daily electronic questionnaires asking about barriers and wore an accelerometer to capture indices of PA across seven days. In line with past work, SE (r(2438)=.13, p=<.001) and RIE (r(2438)=.14, p=<.001) were significantly related to total moderate-to-vigorous PA (MVPA) and step counts. A series of multilevel models were fit to examine the hypotheses. Preliminary analyses indicated that RIE (estimate=3.93, SE=1.49, p=.009) is a stronger predictor of MVPA than SE (estimate=2.83, SE=2.02, p=.16). Further analysis will be conducted to unpack daily life circumstances that create barriers to PA, including daily pain, anxiety, and tiredness.

### Session 9205 (Poster)

### EDUCATION AND ATTITUDES ABOUT AGING

### AN OUNCE OF PREVENTION: REDUCING AGEISM THROUGH THE LENS OF PUBLIC HEALTH

Erin Grinshteyn, *University of San Francisco, San Francisco, California, United States*

Ageism is pervasive. The negative consequences of ageism are vast, and the literature on the effects of ageism on health and health care is extensive. The perpetrators of ageism are equally vast. While it may be tempting to believe that those who go into the fields of gerontology and geriatrics are free from these attitudes and behaviors, this is untrue. It is reasonable to suspect that future public health professionals, even those interested in gerontology, may also carry ageist ideas and practices into their professional careers. This research was developed to determine whether teaching about aging and ageism in a public health course could reduce ageism among students. Participants were students in a class on aging and public health. All students were graduate students in a Master of Public Health (MPH) program. Multiple assessments were used to assess ageism including the Framboni Scale of Ageism (FSA), a validated 29-question measure used to assess ageism, and the Succession, Identity, and Consumption (SIC) scale, another scale assessing ageism. Students were enrolled in an elective course on aging and public health, which was taught through the public health lens of disease prevention and health promotion. Health topics related to aging are discussed with an emphasis on prevention. The contributions older adults make, and the resulting improved health and well-being of self, others, and community are promoted. And the class participates in activities with a variety of community-dwelling older adults. Results show that ageism among students is reduced after the semester long course.

### COLLEGE STUDENTS' IN JAMAICA OPINION ABOUT OLDER ADULTS AND THEIR ROLE AS FUTURE CAREGIVERS: CASE EXAMPLE

Sharon McKenzie,[1] and Sandra Latibeaudiere,[2], *1. Kean University, Union, New Jersey, United States, 2. University of the West Indies, Kingston, Kingston, Jamaica*

Ageing is a natural human experience, yet for many people getting old provoke fears of vulnerability, fragility, undesirability, and incapacity. Such perceptions are often attributed to various taboos, prejudices, discrimination and stereotype associated with old age. The purpose of this study was to assess college students' in Jamaica opinion about older adults and their role as future caregivers. Specifically, the study sought to: (1) identify whether college students hold ageist stereotype/negative image about older adults and whether they influence future role as caregiver, 2) assess their knowledge of the chronic conditions that affect older adults, and 3) assess whether they see themselves working or taking an active role in future caregiving. As we think about the future directions of healthcare provision for older adults, this small sample of college students provided a discourse about ageing and key elements that are important for educators in a developing country such as Jamaica to consider when building a gerontology curriculum.

### DEVELOPING AN EDUCATIONAL PROGRAM ON AGING FOR HIGH SCHOOL STUDENTS

Tracy Davis,[1] and Amanda Sokan,[2], *1. Rutgers University, New Brunswick, New Jersey, United States, 2. The University of Arizona, University of Arizona, Arizona, United States*

College students in disciplines that might provide services or work with older adults, such as medicine or social work, are usually the target of most educational programs on aging. High schools provide an untapped opportunity to engage students earlier. This project is the next step following a pilot study conducted in New Jersey and Kentucky to better understand high school students' attitudes and knowledge regarding aging. That study also reviewed current high school curriculum for aging-specific content and perceived barriers among teachers to incorporating aging education into the curriculum (Davis & Sokan, 2019). Study findings indicate inter alia, a need to educate high school students about aging, increase interactions among older and younger adults, incorporate education about careers on aging, and educate teachers on how to infuse more aging content into their courses. To that end, this project's goal was to develop both a training module and educational program on aging for high school students. Also, we propose a plan to develop, implement, and evaluate both the training module and the educational programs. We hypothesize that the training module will increase high school teachers' confidence in their ability to teach their students about aging. The educational program's delivery will increase students' knowledge of aging-related issues and awareness about careers in aging. Upon completing the project, we will use feedback from students and teachers to revise the educational program, for implementation among a larger sample of high schools.

### EVALUATING A GERIATRIC EDUCATIONAL PROGRAM: EXPLORING OPPORTUNITIES FOR INCREASING IMPACT AND SCALE

Shera Hosseini, Michelle Howard, and Allison Ward, *McMaster University, Hamilton, Ontario, Canada*

The geriatric population is rapidly growing, and this growth is beyond the pace of increase in the number of healthcare professionals who are qualified to care for and tend to the various needs of this significant subgroup of the population. The current university curricula have not been sufficient in terms of quantity as well as their ability to address the ageism inherent in the perspectives of students from across the educational spectrum. In recognition of the absence of standardized geriatric guidelines, medical associations across Canada and the United States have established geriatric learning competencies for medical programs. Nevertheless, there are exiting gaps regarding the development and evaluation of geriatric-focused didactic programs that adequately train and build competency among the students interested in pursuing careers with geriatric-specific elements. A university-wide program was developed to enhance aging education and build competency through sparking interest, providing better education related to aging, and building better relationships between future healthcare professionals and older adults. To evaluate the impact of this program, a logical framework was developed a-priori and revised through constant iterations and following discussion with the program's multidisciplinary stakeholder group. Quantitative measures are being augmented with in-depth qualitative interviews to explore elements influencing students' experiences with the program and the effect on their interests in and attitudes towards geriatrics. The results will inform our conclusions regarding program effectiveness in enhancing interest in geriatric-focused education among the students and trainees and assist with recommending future directions regarding impact and large-scale dissemination and implementation.

### PREDICTING STUDENTS' INTEREST IN AGING-RELATED CAREERS: SUGGESTIONS FOR PEDAGOGICAL INTERVENTION

Chih-ling Liou, *Kent State University, North Canton, Ohio, United States*

College students often hold negative attitudes toward elders and rank this area of practice at the bottom of their future professional life; colleges and universities have an important role to play in changing attitudes and attracting more professionals to work with older adults. This study examined factors contributing to students' attitudes toward older adults to provide suggestions for pedagogical intervention. Data was collected from 195 undergraduates participating in an online survey with questions on the quality of relationships with a grandparent and other nonfamilial older adults, previous experience and future interest in pursuing a career in an aging-related field, and The Fabroni Scale of Ageism (Fabroni et al., 2010). Path analyses using hierarchical multiple regression revealed that high quality relationships with older adults (i.e., both grandparents and nonfamilial elders) was associated with less negative attitudes and more interest in pursuing a future career in age-related jobs/internships. Although both types of relationship quality were significant in the model ($p<.05$), path coefficients demonstrated that relationships with nonfamilial elders have a greater impact on participants' attitudes ($\beta$= -.250, $p$=.001 versus $\beta$= -.146, $p$=.045). Previous working/internship experiences with older adults also predicted a greater willingness to pursue a future career in an aging-related field ($\beta$= .333, $p<.001$). Findings suggest that colleges could increase students' interest in pursuing aging-related careers with multiple interventions, such as developing opportunities to interact and build relationships with older adults in the community, updating information on job opportunities, pay scales, and advancement opportunities, and providing more gerontological course or modules.

### 'WE HAD MORE IN COMMON THAN I THOUGHT': SCAMMING AS AN UNDERGRADUATE SERVICE LEARNING TOPIC INVOLVING OLDER ADULTS

Marjorie Getz, *Methodist College, Peoria, Illinois, United States*

An ongoing activity that cuts across several courses in the Gerontology Certificate Program at our College is the completion of implicit association exercises focused on age. Most college students show a distinct preference for those who are younger adults. It is difficult to get across to these students that the construct of being an adult is appropriate for all people beyond adolescence without relevance to age. College students enrolled in healthcare programs often have distorted views of aging and may not fully appreciate that all adults may share common aspects of their current lives. We describe qualitative analyses of reflections taken from an undergraduate psychology course that included a service learning component involving older adult learners. The service learning lessons focused on victimization associated with fraud and scamming. The classroom structure involved round table discussions with direct contact between college students, older adults and local law enforcement personnel. Reflective practices were used to integrate course content (development in adulthood) into this service learning activity. We report on qualitative data taken from student reflections. Content analyses of reflective essays identified five themes which operated to produce stronger identification between age groups: frequency of being scammed across all 21 participants; insight that learning continues across the lifespan; understanding that broad learning challenges impact people (for different reasons) at both ends of the adult age spectrum; respect for adoption of strategies that facilitate learning/compensate for cognitive changes that occur with aging; acknowledgement that familiarity breaks down barriers between people.

### WHAT DO INTRODUCTION TO PSYCHOLOGY TEXTBOOKS HAVE TO SAY ABOUT OLDER ADULTS?

Zoe Hancock,[1] Matthew Wynn,[2] and Brian Carpenter,[1], *1. Washington University in St. Louis, Saint Louis, Missouri, United States, 2. Washington University in St. Louis, Washington University in St. Louis, Missouri, United States*

Introduction to Psychology is one of the most popular undergraduate courses, an entry course for psychology majors and also popular with students from other disciplines. Consequently, the content in introductory psychology textbooks has the potential to influence undergraduates' knowledge, attitudes, and interests, including those related to aging. The purpose of this study was to analyze aging-related content in introductory psychology textbooks to understand the topics to which students are exposed in this important course. We analyzed the indices of 21 best-selling Introduction to Psychology textbooks for both advanced and intermediate audiences, published between 2018 and 2020. We extracted and aggregated 275 unique, aging-specific index terms from the textbooks and analyzed their relative frequency. We identified 61 superordinate index terms corresponding to general terms (e.g., "aging," "death"). The indices also included 214 unique subordinate terms that were more specific (e.g., "aging, and cognition"). Across textbooks, the most frequent topics reflected negative consequences of aging (e.g., "Alzheimer disease" = appeared in 100% of textbooks, "death" in 52%). In contrast, positive aspects of aging appeared less often (e.g., "generativity" in 47%, "longevity" in 10%). Terms describing career opportunities were rare (e.g., "gerontology" in 5%, "geropsychology" in 5%), as were modern theories (e.g., "socioemotional selectivity theory" in 28%). Advocacy for comprehensive and balanced representation of aging in introductory psychology textbooks is critical for educating students and promoting interest in the field.

### Session 9210 (Poster)

### EDUCATION AND TRAINING

### A MASSIVE OPEN ONLINE COURSE TO EDUCATE HEALTHCARE PROFESSIONALS & CAREGIVERS ABOUT ALZHEIMER'S DISEASE

Mary DiBartolo, *Salisbury University, Salisbury, Maryland, United States*

Over 6 million Americans and 50 million persons worldwide are estimated to have Alzheimer's disease (AD) as it remains the major cause of dementia in the older adult population. Both healthcare professionals and family caregivers struggle with the complexities of caring for individuals with this progressive neurological disease. To address the ongoing knowledge and care gap regarding Alzheimer's disease among both healthcare professionals and family caregivers, a comprehensive massive open online course (MOOC) was developed and made available via the edX platform. MOOCs are open access and interactive courses offered via the web; they have emerged as a popular, self-paced mode of distance learning. Launched in 2020, the MOOC titled, Alzheimer's Disease & Dementia Care, consists of five modules reviewing symptoms, diagnosis, medications, communication and care tips, as well as a module outlining special considerations when the person with AD is hospitalized. While this educational strategy targets healthcare professionals (such as nurses, physical therapists and related practitioners), it is also designed for lay caregivers or anyone who wants to learn more the disease. The course utilizes a variety of teaching modalities and is free. To date over 7000 persons have enrolled from over 140 countries. MOOCs remain an innovative and engaging educational strategy to reach a global audience. More importantly, they can serve as an another outlet to enhance both the competence and confidence of both healthcare professionals and family caregivers by sharing best practices in caring for those with Alzheimer's disease and related dementias.

### IN THE EYE OF THE BEHOLDER: USING PHOTOGRAPHY TO TEACH GERONTOLOGY

Kristina Hash,[1] Matthias Naleppa,[2] and Anissa Rogers,[3], *1. West Virginia University, morgantown, West Virginia, United States, 2. Radford University, Radford, Virginia, United States, 3. CSUSB, Palm Desert, California, United States*

Due to the widespread access to smart phones and similar technology, photography and photographic images have become an ever-present part of contemporary social life. Photographic methods are also growing in their use in higher education pedagogy. As a specific application, photography can be a powerful tool to educate students about aging processes and issues that impact older adults. This poster will explore the use of photographic methods and tools to teach and integrate aging-related concepts into gerontology and social work courses, at both undergraduate and graduate levels. Specifically, the use of digital storytelling, photo mapping, photo voice, and photo therapy will be highlighted. Descriptions of and consideration for assignments along with example student projects will be displayed and discussed as will other potential projects and uses of photographic methods. Attention will be paid to how photographic methods can help students explore the diversity and intersection of individual characteristics and experiences with the aging process and how intersectional identities can influence, and be influenced by, aging and external factors and processes.

### MOVING TOWARD AN AGE-FRIENDLY UNIVERSITY: SURVEY PROCESS OF GSU 50+ STUDENTS

Grace da Rosa,[1] Jacqueline Laures-Gore,[2] Sarah Barber,[3] Mariam Qureshi,[3] and Elisabeth Burgess,[3], *1. Georgia State University, Suwanee, Georgia, United States, 2. Georgia State University, Georgia State University, Georgia, United States, 3. Georgia State University, Georgia State University - Atlanta, Georgia, United States*

The proportion of Georgia's population that is 60 years and older is growing rapidly. The 2010 U.S. Census Bureau predicted a growth of more than 20% of older adults by the year 2030. Georgia residents who are 62 and older are eligible to take courses at no or little cost at public state colleges. Due to the expected increase in Georgia's aging population, access to a free university education, and the large number of currently enrolled 62+ students at Georgia State University (GSU), it is crucial that GSU become an Age-Friendly University. During Fall 2019, a survey was distributed to 1046 students aged 50 years plus; 411 completed the survey (39% response rate). This presentation describes the process involved in designing and distributing the survey. Unique aspects of the survey's development include the cross-generational and interdisciplinary contributions of the student, faculty, affiliates, and staff from GSU's Gerontology Institute. The goal was to learn more about GSU students 50 years and older by assessing their motivation for attending school, challenges on campus, perceptions of how the university is currently addressing their needs, factors/resources that have helped/are helping them to adjust to school, and the extent to which they experience age discrimination on campus. The long-term goal of the survey is to use this information to direct GSU in becoming an Age-Friendly University.

### NOW WHAT? MAINTAINING MOMENTUM AFTER ACHIEVING DESIGNATION AS AN AGE-FRIENDLY UNIVERSITY

Diane Martin,[1] Nicole Brandt,[2] Denise Orwig,[1] Barbara Resnick,[3] Daniel Mansour,[4] Elizabeth Galik,[3] John Schumacher,[5] and Meredith Upton,[1], *1. University of Maryland, Baltimore, Baltimore, Maryland, United States, 2. University of Maryland School of Pharmacy, University of Maryland School of Pharmacy, Maryland, United States, 3. University of Maryland, Baltimore, Maryland, United States, 4. University of Maryand School of Pharmacy, Baltimore, Maryland, United States, 5. University of Maryland, Batlimore County, Baltimore, Maryland, United States*

The Age-Friendly University (AFU) designation in higher education recognizes the institution's commitment to a culture of age-inclusivity across programs and policies. While AFU institutions are embracing the demographic shifts in higher education and society at-large, effectively responding to the needs and desires of an increasingly older population requires ongoing acceptance and support from campus leaders to maintain momentum and stay relevant within a dynamic field. This session will describe the intentional and systematic approach utilized by an AFU steering committee to build interest in and develop meaningful collaborations in multiple domains across campus, including at the level of the president. Our initiatives focus on five themes that align with the 10 age-friendly university principles: support for workforce development, broadening community engagement, expanding engagement in aging research and dissemination, addressing barriers related to aging and our physical environment, and facilitating age-friendly efforts across our state. We will present our experiences in expanding awareness of and support for the AFU movement on our campus and share a model for institutions seeking ideas for sustainability of their own initiatives.

### SIMULATING TREATING IN PLACE IN LONG-TERM CARE: INTERPROFESSIONAL TEAM CARE VIDEO CASE FOR NURSE PRACTITIONERS

Laurie Kennedy-Malone, *UNCG School of Nursing, Greensboro, North Carolina, United States*

As a means of enhancing clinical simulation opportunities for adult-gerontology nurse practitioner students, a series of video simulations were created for use for nurse practitioner education. With funding through the Health Resources and Service Administration (HRSA) Advanced Nursing Education Workforce grant and partnering with nurse practitioner clinical educators from Optum Health Care, a video simulation focused on the concept of treating an older veteran within a long-term care facility rather than transferring to the acute care setting was developed. The case Treating in Place: Nurse Practitioner-Led Team Management of a Long-Term Care Patient Video involved a nurse practitioner collaborating with a physician, a registered nurse, a social worker, and a family member. The interactive simulation video was developed using the eLearning authoring tool H5P to create learning experiences for students that can be used either in face-to-face classroom experiences or embedded in learning management systems. H5P is a web-based authoring tool that helps faculty build interactive course content. H5P activities provide instant feedback to students, allowing them to self-assess their understanding of the dynamic video simulation case. A faculty handbook that describes the case scenario with the interactive questions and suggested discussion questions is available. The adult-gerontology primary care nurse practitioner competencies addressed for this case are identified in the faculty handbook. These videos have been widely disseminated and are being included in nurse practitioner curriculum across the country. A QR code with access to direct viewing of the video will be included in the presentation.

### STARTS AND STOPS: STRATEGIZING AN AGE-FRIENDLY UNIVERSITY COMMITMENT DURING A PANDEMIC

Paige Ebner,[1] Kenneth Ferraro,[1] Brian Pastor,[2] and Wendy Rogers,[2], *1. Purdue University, West Lafayette, Indiana, United States, 2. University of Illinois Urbana-Champaign, Champaign, Illinois, United States*

The Global Network of Age-Friendly Universities seeks to enhance age-inclusivity and engagement in higher education, but delivering age-friendly programming became very challenging during the COVID-19 pandemic. We examine how two land-grant universities adapted to the pandemic and draw some lessons from those experiences that may be useful for other universities seeking to implement or resume the AFU programming. The two main responses were to either pause many of the age-friendly initiatives at the university or adapt to virtual or online delivery platforms. To ensure the health and safety of older adults, colleges and universities paused many age-friendly initiatives such as intergenerational service-learning, technological assistance to older adults, and influenza vaccinations. Other programs continued but in a modified delivery format. Examples include: converting a face-to-face balance-training program to telehealth delivery; transitioning visitation programs to pen pal communication; and replacing face-to-face workshops offered by Extension Services with webinar delivery. Despite these challenges, we conclude that moving to virtual platforms and other methods of delivery, including conventional mail, has in some cases increased access for many older adults and became a lifeline during a time of social isolation for many older adults. Taken together, these experiences highlight the need for age-friendly universities to have contingency plans to ensure continuation of age-friendly programming in the event of pandemics or disasters. Finally, the pause in programming creates opportunities to re-launch or re-organize those initiatives in accord with federal and state safety guidelines.

### TEACHING IN GERIATRICS: IS STRUCTURED WRITTEN FEEDBACK EFFECTIVE FOR LECTURES

Theresa Pohlmann,[1] Klaus Hager,[2] Volker Paulmann,[1] and Sandra Steffens,[1], *1. Medical School Hannover, Hannover, Niedersachsen, Germany, 2. Medical School of Hanover, Hannover, Niedersachsen, Germany*

**Background:** Although there have been discussions about traditional lecturing, lectures are still largely a widespread concept of knowledge transfer. Therefore, it is important to constantly review and evaluate this format. The aims of this study were to analyze which effect a criteria-based written feedback has on the lecture course in geriatrics as an alternative to the conventional student evaluation. Furthermore, we wanted to investigate what kind of impact structured feedback has on lecturers in terms of content, organization and quality.

**Methods:** The study was a prospective longitudinal analysis. The 34 lectures on the subject of geriatrics were analyzed over two cohorts using a standardized evaluation sheet. The assessment was carried out on a 5-point-scale using a 22-item feedback instrument. After the first evaluation, each lecturer received an individual evaluation with strengths and suggestions for improvement. In the second cohort the lecture series was evaluated again, and individual feedback was sent.

**Results:** In six of 22 sub-categories the improvement was significant. The most significant improvement was made in terms of content/structure with an increase from 3.4 to 4.3 points.

**Conclusion:** This study shows that significant improvement in teaching is possible by means of individualized written feedback for the lecturers and that students perceive the resulting improvements positively. Our results suggest that the implementation of these feedback instruments in other modules might improve their teaching as well.

### THE 4MS PROVIDE A STRUCTURAL FRAMEWORK FOR ORGANIZING EDUCATIONAL MATERIALS

Edmund Duthie,[1] Deborah Simpson,[2] Amanda Szymkowski,[3] Kathryn Denson,[3] and Steven Denson,[3], *1. Medical College of Wisconsin, Milwaukee, Wisconsin, United States, 2. Advocate Aurora Health, Advocate Aurora Health/Milwaukee, Wisconsin, United States, 3. Medical College of Wisconsin, Medical College of Wisconsin/Milwaukee, Wisconsin, United States*

The John A Hartford Foundation and the Institute for Healthcare Improvement (IHI)'s 4Ms of mentation, mobility, medications and (what) matters most provide a much-needed framework for helping system leaders and frontline teams consistently deliver high-quality, age-friendly care. Geriatric Fast Facts (GFFs) is a virtual resource providing teachers/learners with peer-reviewed, evidence-based summaries on topics essential to older adult care via a searchable website [www.geriatricfastfacts.com]. To determine if GFFs can be classified by the 4Ms we initially did a free text search of all GFFs. That revealed GFFs whose foci were unrelated to the 4Ms (e.g., mobility emerged in a fluoroscopy GFF as a minor element related to patient positioning). Therefore, all GFFs were independently reviewed by a geriatrician and the website manager and classified according to the 4M rubric (a single GFF can be classified in multiple M's such as #93 on Age Friendly Health Systems). Any differences were adjudicated by the GFF editor. 64% (60/ 93) of GFFs strongly linked to one of the 4Ms. The number of GFFs dedicated to the 4Ms are as follows: 20 what matters most, 18 medications, 13 mentation, and 9 mobility. Those that were not coded within 4Ms were often very disease/specialty oriented. A total of 36 were not classified. For example, GGF #39 focuses on the etiologies of anemia among older adults. The 4M framework can be easily applied to educational materials to support consistent and clear conceptual model across learning conditions and materials.

### WHAT DO UNDERGRADUATES LEARN ABOUT ALZHEIMER DISEASE? AN ANALYSIS OF INTRODUCTION TO PSYCHOLOGY TEXTBOOKS

Zoe Hancock,[1] Matthew Wynn,[2] and Brian Carpenter,[1], *1. Washington University in St. Louis, Saint Louis, Missouri, United States, 2. Washington University in St. Louis, Washington University in St. Louis, Missouri, United States*

One of the most popular courses for undergraduate students, Introduction to Psychology, is often students' first exposure to scientific and clinical facts about Alzheimer disease

(AD). In order to learn how our current understanding of AD is presented to undergraduate psychology students, we analyzed passages related to Alzheimer disease that appear in contemporary Introduction to Psychology textbooks. We extracted and analyzed passages describing AD from twenty-four best-selling Introduction to Psychology textbooks for both advanced and intermediate undergraduate audiences, published between 2018 and 2020. We applied a standardized coding scheme to the passages to quantify what aspects of AD were most commonly described. Each textbook contained between 1 and 3 major passages regarding AD, most often appearing in the chapters on Memory or Human Development. Average word count for these passages was 409.1 words (SD = 194.8 words). Passages most often covered biological aspects of AD (87.5% of textbooks), symptoms (87.5%), prevalence (75%), and risk factors (75%). Disease prevention (62.5%) and illness course (62.5%) also appeared in the majority of textbooks, while aspects of treatment and management (25%), assessment and diagnosis (12.5%), and caregiving (25%) were mentioned less often. While the majority of books used contemporary and appropriate terminology to describe AD (e.g., "Alzheimer disease," "dementia," "neurocognitive disorder"), some textbooks maintained the use of out-of-date and inappropriate terminology (e.g., "senility" or "senile dementia" in 15%). Introductory psychology textbooks provide an opportunity to teach comprehensive, accurate information about AD and publishers and textbook authors could be guided in this effort.

### Session 9215 (Poster)

### EDUCATION AND TRAINING: WORKFORCE DEVELOPMENT

### ADAPTING THE ENVIRONMENTAL SCAN FOR GERONTOLOGY GRADUATE STUDENTS' CAREER EXPLORATION

Nasreen Sadeq, and Brianne Stanback, *University of South Florida, Tampa, Florida, United States*

Environmental scanning is a process that provides organizations with information about their internal and external strengths, challenges, and opportunities. Although traditionally used in business and strategic planning, environmental scanning is now being utilized in health care to evaluate currently available programs and services, identify gaps in patient care or research, and make educational, organizational, and policy recommendations. The current study explores adapting the environmental scan for students enrolled in a gerontology graduate program as a tool to facilitate career exploration. Students learned about environmental scanning and were instructed to perform an environmental scan on an important issue, program, or service relevant to their overall career goals. Because students completed their environmental scan while enrolled in an Alzheimer's Disease Management course, they were encouraged to factor in the increasing prevalence of Alzheimer's disease into their project. Students' environmental scan projects spanned several timely topics, including an evaluation of the services currently offered in assisted living facilities from the perspective of a geriatric social worker, a review of memory training interventions and a proposal for a new research study, and preliminary plans for opening an assisted living facility catering to older adults in the LGBTQ community that outlined financial considerations, staff training goals, and patient care plans. Completing the environmental scan project gave students an opportunity to investigate the current state of the career field they are planning to enter, and provided them with a product that they can build upon as they complete the graduate program and begin their careers.

### ALTERNATIVE PATHWAYS: THE USE OF FIELD-SPECIFIC MICRO-CREDENTIALS IN AGING SERVICES

M. Aaron Guest,[1] Leanne Clark-Shirley,[2] Cynthia Hancock,[3] Tina K. Newsham,[4] Katherine Alvarado,[3] Kristina Amirchian Renee DuMont,[5] Lauren Hackett,[5] Kaylah Jenkins J.R. Patton,[5] and Caylee Weaver, *1. Arizona State University, Tempe, Arizona, United States, 2. American Society on Aging, American Society on Aging, California, United States, 3. University of North Carolina Charlotte, Charlotte, North Carolina, United States, 4. University of North Carolina Wilmington, University of North Carolina Wilmington, North Carolina, United States, 5. University of North Carolina Wilmington, Wilmington, North Carolina, United States*

The rise of formal academic programs in gerontology at colleges and universities has been well documented over the last fifty years. Organizations such as AGHE and AGEC have been established to provide guidance, foster consistency, and advance formal gerontology education programs. Broadly, the purpose of these programs has been to develop a pipeline of trained gerontologists for the aging services workforce. What has been less documented is the rise of alternative pathways to gerontology and gerontological competence, including micro-credentialing. Micro-credentials are intended to provide quick-to-complete competency-based education around specific topics to demonstrate relevant skills to employers. To date, little is known about the prevalence of micro-credentialing in gerontology. Still, it may be that micro-credentials are sought in place of formal academic preparation due to their reduced cost, ease of completion, recognizability, and opportunity to quickly train employees in specific skills. To address this gap, we conducted a review of existing gerontological micro-credentialing opportunities. We identified a total of 51 micro-credentials with an explicit aging-focus and searched for associated competencies for these micro-credentials. In this poster, we describe findings on the emphases of micro-credentials, including dementia and care coordination, and review the programs' scope and nature of competencies. We argue that micro-credentialing can offer value for employees unable or unwilling to pursue formal academic training but should be differentiated from such training. Moving forward, it is critical to ensure alignment between gerontological micro-credentials and established gerontological competencies and standards and to differentiate micro-credentials from formal academic programs.

### AN 18-YEAR EXPERIENCE WITH AN INNOVATIVE GERIATRICS TRAINING MODEL: IMPLICATIONS FOR THE WORKFORCE

Cara O'Brien,[1] Evan Henricks,[2] Angela Beckert,[2] Kathryn Denson,[3] and Edmund Duthie,[2], *1. Medical College of Wisconsin, Wauwatosa, Wisconsin, United States,*

2. *Medical College of Wisconsin, Milwaukee, Wisconsin, United States,* 3. *Medical College of Wisconsin, Medical College of Wisconsin/Milwaukee, Wisconsin, United States*

Despite the growing population of older adults, the geriatrics workforce has not similarly expanded. The number of geriatrics fellows has declined by 14.3% from 2012-2017. Implementation of innovative training programs may improve this reality. In 2002, the Medical College of Wisconsin (MCW) created the first four-year combined medicine residency and geriatrics fellowship (Med-Ger). Similar programs are currently being developed. The aim of this study is to describe the outcomes of the MCW Med-Ger program.

**Primary endpoints:** American Board of Internal Medicine (ABIM) pass rates, ABIM Geriatric Medicine Certification pass rates, fellowship completion rates, and geriatric-focused practice.

**Results:** There was a Med-Ger program fill rate of 73.7% (n=38). There was equivalent ABIM pass rate of 100% between Med-Ger graduates (n=18) and traditional graduates (n=25). Med-Ger trainees were more likely to complete their geriatrics fellowship (94.4% vs. 88%) and practice in geriatric-focused careers (82.4% vs. 68.2%). These outcomes suggest the benefit of a combined program for training future geriatricians. The MCW Med-Ger fill rate exceeds the national geriatrics fellowship fill rate of under 50%. Additionally, graduates may be more likely to practice geriatric medicine. This may help address population needs for an increased geriatrics workforce. In 2020, the ACGME approved an Advancing Innovation in Residency Education (AIRE) Medicine-Geriatrics Integrated Residency and Fellowship national pilot program. Further investigation of why trainees choose Med-Ger training and are more likely to continue with careers in geriatrics is needed in order to replicate the success of the MCW Med-Ger program.

### AN INTER-UNIVERSITY VIRTUAL GERIATRIC CASE COMPETITION TO BUILD INTERPROFESSIONAL COLLABORATION SKILLS

Kristine Talley,[1] Marla Berg-Weger,[2] Devita Stallings,[3] Teresa Schicker,[1] Laura Pesja,[1] and Jean Wyman,[1],
1. *University of Minnesota, Minneapolis, Minnesota, United States,* 2. *Saint Louis University, Saint Louis, Missouri, United States,* 3. *Saint Louis University, St. Louis, Missouri, United States*

Developing positive learning experiences in team-based geriatric care is challenging. This presentation will highlight an inter-University geriatric case competition for developing interprofessional competencies in health professional students sponsored by the Geriatric Workforce Enhancement Programs at Saint Louis University and the University of Minnesota. The virtual competition involved teams of 4-5 undergraduate and graduate students from multiple health professions who designed a comprehensive care plan using a simulated complex geriatric patient case. Students were assigned to an interprofessional team with a faculty or community expert coach, attended an orientation, and developed a 20-minute recorded presentation. A panel of judges rated team presentations using a scoring rubric based on the Core Competencies for Interprofessional Collaborative Practice. Local competitions included a first and semi-final round, with the winning teams presenting at the inter-university competition held via live videoconferencing that involved a question-and-answer session. Prizes were given to the top teams. Students, coaches, and judges completed evaluation surveys focused on satisfaction with the competition format/procedures and achievement of interprofessional competencies. Twenty-one teams and 117 students from 12 disciplines participated. Team scores ranged from 2.2 to 4.3 (overall mean 3.1) on a 1-5 scale. Judge, coach, and student evaluations were positive, indicating students learned valuable lessons in group dynamics, team-based care, and geriatric care. Most students (82%) preferred the virtual competition format or had no preference on format. The virtual case competition provided a positive, engaging experience to introduce health professional students to geriatric team-based care and develop their readiness for collaborative practice.

### ART AND AGING: USE OF SOCIAL MEDIA FOR GERIATRIC EDUCATION

Gunjan Manocha, Casey Morton, Nicole Derenne, Heidi Bau, and Donald Jurivich, *University of North Dakota, Grand Forks, North Dakota, United States*

Social media as an educational tool for health care learning has untapped potential. Benefits of social media include peer-to-peer engagement, active learning and interprofessional training. Here we explored social media platforms as a vehicle to deliver short, pithy clinical pearls from evidence-based, peer-reviewed manuscripts. Key points from recent medical publications are paired with pre-existing artwork to provide visual reinforcement of the clinical pearl. Dubbed “Art and Aging”, the clinical pearl and artwork combination is posted on different social media platforms such as Instagram, Twitter and Facebook, thus allowing for an expansive audience. Different hashtags and tags are used to increase followers and engagement on each platform. Over a 9 months period learner engagement increased by 150% and includes a diverse learner profile. These curated social media platforms show considerable promise for disseminating Geriatrics best practices. As yet, we do not know subject matter retention or whether it changes clinical practices - both questions which are future research objectives.

### EXPERIENTIAL SERVICE LEARNING: PROMOTING COMPETENCY-BASED EDUCATION FOR GERONTOLOGY STUDENTS

Theresa Abah, *California State University, Sacramento, Sacramento State University, California, United States*

Service-learning is an effective pedagogical approach meant to deepen learners understanding of course content by linking scholarship and social action when structurally organized based on attainment of professional core competencies. The recent COVID-19 pandemic caused a rethinking of the way service learning practicum is delivered, especially as it relates to training health professionals who engage collaboratively with older persons (individuals aged 65 years and older). This poster examines the challenges faced by gerontology students as they transitioned to fully virtual learning and practicum format, the lessons learned, and how to close the gap between theory and practice for better programmatic processes. The data used to gather students experiences include; student reflective journals, class discussions and survey questions to students (n=44). The analytic framework utilized is the Social Cognitive Theory, (SCT)- which explains how individuals can master concepts through verbal and physical persuasion, including peer modelling. The goal is to promote leaners self- regulatory skills to achieve the course learning objectives, as obtained from the SCT six strategies for setting achievable goals, through: Feedback, self-instruction, self-monitoring, use of support and goal setting. Some of the lessons learned suggest students benefit more from service learning when they receive continuous feedback about how to develop intergenerational relationships with older adult partners assigned to in the community (68%), than from goal setting strategies (24%). The implication for practice is: there is a need to develop structured service-learning guidelines for undergraduate students in gerontology program to be prepared to better serve older adults.

### GERIPOP: OLDER ADULTS POPULATION HEALTH SERIOUS GAME

Gunjan Manocha,[1] Casey Morton,[1] Jeremy Holloway,[1] Scott Brewster,[2] Joseph Wood,[1] Richard Van Eck,[1] and Donald Jurivich,[1], *1. University of North Dakota, Grand Forks, North Dakota, United States, 2. Triad Interactive Media, Brooklyn, New York, United States*

Health professionals have limited opportunities to learn about population health in their curriculum. With a shortage of geriatricians nationwide, health care systems need different ways to provide evidence-based geriatric care. To address both these shortcomings, a serious game, called GeriPOP has been developed to allow trainees to explore the impact of assessment and management of principles of geriatric care (the 4Ms+) on quality of life, health, longevity, and health care costs by applying them to a virtual older adult population. Trainees assume the role of a system manager who is asked to explore ways to optimize health outcomes and lower costs. They develop their population health plan around a framework of Geriatric 4Ms+ and apply it in a virtual panel of older adult patients that move longitudinally into different age bands (65-74; 75-84; 85+). As the game progresses, a dashboard helps trainees track the impact of their treatment decisions across the population. Several levels of play allow trainees to explore various issues intersecting with aging such as gender, diversity, social determinants, and multiple chronic conditions. Periodic debriefings and explanatory pop ups during the game allow trainees to further explore evidence–based Geriatrics. The game engages health care trainees to strengthen their knowledge of Geriatrics through exploration of systems change. Future study is needed on whether Geri POP changes learner attitudes, future clinical practice or healthcare outcomes.

### IMPACT OF VIRTUAL REALITY ON HEALTHCARE PROVIDER EMPATHY FOR OLDER ADULTS WITH SENSORY IMPAIRMENT

Suzanne Dutton,[1] and Andrea Cimino,[2], *1. Sibley Memorial Hospital/Johns Hopkins, washington, District of Columbia, United States, 2. Danger Assessment Training and Technical Assistance Center, Johns Hopkins School of Nursing, Baltimore, Maryland, United States*

Virtual reality (VR) is an innovative technology that can simulate dual sensory impairment so that healthcare providers can experience this affliction common in older adults. The current study investigated whether VR simulation could increase empathy among healthcare workers. Empathetic care is linked with improved patient satisfaction, compliance, and outcomes. The study used a one-group pre/posttest study design implemented with healthcare providers at a hospital in the Mid-Atlantic region. All participants experienced a 7-minute VR scenario from the viewpoint of "Alfred", a 74-year-old with macular degeneration and high frequency hearing loss on a commercial VR headset (Oculus Rift). A survey assessed participants' self-reported knowledge, empathy, and behavior change. Empathy was measured using the validated tool Kiersma-Chen Empathy Scale (KCES). Analyses included descriptive statistics and paired t-tests. Survey results showed that participants increased their knowledge of macular degeneration and hearing loss, and that 9 of 14 empathy items had statistically significant increases (average absolute change = .41 points). Additionally, 97% of participants agreed or strongly agreed that they would utilize the information learned in their work with patients. Evidence suggests VR is an effective intervention to increase empathy and positively change behavior to support persons with sensory impairment.

### OLDER ADULTS' PERCEPTIONS OF COLLEGE CAMPUS ACCESSIBILITY

Ashley Ermer, and Nadine Verna, *Montclair State University, Montclair, New Jersey, United States*

The emerging Age-Friendly University Global Network encourages universities to engage older adults in university activities (Gerontological Society of America, 2019). As such, attention should be devoted to the accessibility of campus facilities to older adults as a potential mechanism to increase age diversity. Intergenerational interactions, which may take place on college campuses, promote better perceptions of other generations (Bertram et al., 2017), making campus accessibility for all age groups a priority. The present study sought to uncover older adults' perceptions of campus accessibility via an online survey. Participants were recruited through local newsletters, word of mouth, and included 81 community members (Age mean=71.58 years; 79% female; 89% White; 43% traveled to campus every few months). Descriptive analyses were conducted for closed-ended responses and two members of the research team used a constant comparative method (Corbin & Strauss, 2015) to code open-ended responses. Participants felt that campus was somewhat accessible (M = 2.72;1(very inaccessible) to 5(very accessible)), moderately easy to walk around (M=3.79;1(extremely difficult) to 7(extremely easy)), and felt somewhat welcome on campus (M=3.27; 1(strongly disagree) to 7(strongly agree). The following general themes emerged in the open-ended responses: 1)inaccessibility on campus was due to parking, drop-off locations, and topography (e.g., due to stairs, distance, hills) constraints; 2)feeling welcome on campus was due to people being helpful; and 3)difficulty in attending events was due to parking and lack of knowledge about events. Implications for campus initiatives that aim to attract older adults, especially for campuses that have topography constraints, will be discussed.

### SHINING A LIGHT INSIDE THE "BLACK BOX" OF NIH APPLICATION SUBMISSION AND REVIEW

Elia Ortenberg,[1] and Shalanda Bynum,[2], *1. National Institutes of Health, Bethesda, Maryland, United States, 2. Center for Scientific Review (CSR) National Institutes of Health (NIH), Bethesda, Maryland, United States*

What happens to applications after they are submitted to the National Institutes of Health, and how can you better prepare yourself and your application for the process of peer review? The Center for Scientific Review (CSR) works closely with the 24 funding institutes and centers at the National Institutes of Health that provide funding support for projects of high scientific merit and high potential impact. CSR conducts the first level of review for the majority of grant applications submitted to the NIH, which includes 90% of R01s, 85% of Fellowships, and 95% of Small Business Innovation Research (SBIR) applications as well as many other research and training opportunity activities. In this capacity, CSR helps to identify the most meritorious projects, cutting-edge research, and future scientists who will advance the mission of the NIH: to enhance health, lengthen life, and reduce illness and disability. The purpose of this project is to provide an overview of 1) what happens to NIH applications before, during, and after peer review at CSR; 2) a summary of new and current peer review policies and practices that impact investigators and their submitted applications; and 3) strategies for developing a strong NIH grant application. Peer review is the cornerstone of the NIH grant supporting process, and an insider's view can shine a light inside the "Black Box" of how the most meritorious projects are identified.

### STRENGTHENING COLLABORATIONS FOR GERIATRICS KNOWLEDGE: TWITTER JOURNAL CLUB FOR GERIATRIC FELLOWS

Gunjan Manocha,[1] Casey Morton,[1] Elizabeth Chapman,[2] Daniel Pellecer,[3] Kahli Zeitlow,[4] Tanjeev Kaur,[5] and Donald Jurivich,[1], *1. University of North Dakota, Grand Forks, North Dakota, United States, 2. University of Wisconsin, Madison, Wisconsin, United States, 3. Mayo Clinic, Rochester, Minnesota, United States, 4. Michigan Medicine, Ann Arbor, Michigan, United States, 5. University of Illinois at Chicago, Chicago, Illinois, United States*

The pandemic has challenged training programs in numerous ways, specifically in the ability to conduct group based teaching sessions. To overcome this challenge Twitter was examined as a vehicle for engaging Geriatric Fellows in education about critical appraisal of clinical research. A secondary objective was to develop educational synergy among university-based programs. To achieve these aims, 5 Midwestern Geriatric and Palliative Medicine Fellowship programs agreed to enroll their fellows into a monthly Geriatrics Twitter Journal Club, that commences on Twitter Tuesday and lasts a week. Each month, an assigned fellow selects an article to discuss and creates a short video to introduce it. A Twitter meister deliveres structured questions to guide fellows' collective input on the article being critiqued. Over a 3 month roll out of @GeriatricJC, the twitter account of the journal club has gained 144 followers that includes 20 fellows, 63 geriatricians/geriatric faculty, 28 organizational accounts, 5 students and around 28 other providers and experts. From December 2020- February 2021, account generated tweets resulted in an average of 397 impressions/day with 2548 visits to the account profile per month. Videos posted have averaged 73 views/video. Discussion in journal club using #GeriJC has garnered 178 tweets from participants. This project shows that Twitter is a feasible platform for a fellowship journal club among several training programs, thus expanding expertise in evidence-based medicine while lowering the administrative burden of preparing journal club within a single program and increasing both faculty and trainee convenience of learning.

### TO CLARIFY THE DURATION AND CHARACTERISTICS OF THE CONTINUATION OF HOME CARE FOR OLDER PEOPLE WITH DEMENTIA

Reiko Kanaya,[1] Asuka Oyama,[2] Hiroshi Toki,[3] Ryohei Yamamoto,[4] and Miyae Yamakawa,[1], *1. Osaka University Graduate School of Medicine, Suita City, Osaka, Japan, 2. Health and Counseling Center,Osaka University, Suita City, Osaka, Japan, 3. Osaka University Health and Counseling Center, Suita City, Osaka, Japan, 4. Osaka University, Suita City, Osaka, Japan*

As populations age worldwide, older people with dementia are increasing. Caregivers are also aging, necessitating arrangements like social services. How to prolong the home care desired by older people remains unclear. Using data from the Osaka National Health Insurance Database from 2012 to 2017 on insured persons' registers, medical notes, and care benefits, this study included 9591 people aged ≤74 years with first dementia drug prescription between April 2013 and December 2017. Using the prescription as baseline and hospitalization or nursing home admission as outcomes, home care duration and characteristics of medical and nursing care services during the year before baseline were evaluated. Survival was compared by Kaplan–Meier curves and the log-rank test. Multivariate analysis was performed using the Cox proportional hazards model. During follow-up, the outcomes were observed in 1473 patients : 317 admission , 1145 hospitalized and 11 both. Mean duration of home care in patients with the outcomes was 11.5 months, which differed significantly from patients without these outcomes. When patients were grouped by hospitalization in year before first prescription, the survival curves differed significantly. In multivariate analysis, sex, renin-angiotensin system agonists, hyperlipidemia drugs, hospitalization history in past year, care level, and diabetes drugs were significantly associated with the outcomes. Taken together, hospitalization history, female sex, and diabetes were associated with home care disruption. Those undergoing cardiovascular disease treatment continued to live at home. For people with dementia, it is important to intervene by focusing on past medical and nursing care to continue life at home.

### Session 9220 (Poster)

### EDUCATION AND TRAINING-PROGRAM EVALUATION-WORKFORCE DEVELOPMENT

### A STUDY ON TRENDS IN THE NUMBER AND CONTENT OF JOURNAL ARTICLE TITLES CONTAINING THE TERM "ELDER ABUSE"

Asako Katsumata,[1] and Noriko Tsukada,[2], *1. Rehabilitation Institution, Chiba, Chiba, Japan, 2. Nihon University, Tokyo, Tokyo, Japan*

Japan enacted the Elder Abuse Prevention Law on April 1, 2006; no amendments have been made since then. The purpose of this study is to examine trends in the number and content of journal article titles containing the term "elder abuse" and determine what further research is needed to identify where amendments to the law would be useful. We identified 986 articles using the CiNii database between the dates of 2005 to 2020. We categorized those titles by content, using a KJ Method. Preliminary analyses revealed that the average number of the articles published each year was 61.6, though a moving average of the numbers of articles on elder abuse has been steadily declining, as opposed to the average number for child abuse articles, 158, where the moving average staying the same. As for the analyses for the titles, they were categorized into 10 categories, including "law," "responses of professional personnel to elder abuse," "systems of government agencies," "responses of nurses," "institutional elder abuse," "support for caregivers," "reports on elder abuse in other countries," "dementia and elder abuse," "responses of medical institutions," and "others." It is suggested that more research needs to be done, especially in such areas as "verification of elder abuse cases," "psychological impacts on elder abuse victims," "empirical research conducted by medical doctors dealing with elder abuse cases," and "cooperation between police and professional institutions," many of which were found in research on child abuse where 4 law amendments have been made since its enactment of 2000.

### AN ASSESSMENT OF DEMENTIA CAREGIVERS' INTERACTION WITH COMMUNITY-BASED SERVICES

Deepika Pugalenthi Saravanan,[1] Nihal Satyadev,[2] Natashia Townsend,[3] Katherine Rose,[4] Harrison Ma,[5] Donna Benton,[6] and Lené Levy-Storms,[7], *1. UCLA, San Ramon, California, United States, 2. The Youth Movement Against Alzheimer's, Los Angeles, California, United States, 3. Youth Movement Against Alzheimer's, Culver City, California, United States, 4. Youth Movement Against Alzheimer's, Los Angeles, California, United States, 5. University of Southern California, Los Angeles, California, United States, 6. University of Southern California Family Caregiver Support Center, Los Angeles, California, United States, 7. UCLA Luskin School of Public Affairs/Social Welfare, West Hills, California, United States*

Respite care is an important service to address caregivers' stress and fatigue when caring for a person with dementia (PWD). YouthCare is a non-medical, at-home, intergenerational respite care program that partners trained student volunteers with PWDs. The Family Caregiver Survey was created and distributed to caregivers of PWDs in Los Angeles to better understand interactions with the community and its caregiver services. The survey assesses caregivers' demographics, daily activities, mental health, and the type of respite support needed. The survey findings (n=47) show that 53.2% of caregivers are 54 and older and 83% females. 40.4% of the caregivers listened to the radio primarily in the morning while 61.7% watched television in the afternoon to evening time. For transportation of PWDs to and from destinations, 78.3% of caregivers reported using their own vehicles. In regards to their mental health, 61.7% of the caregivers stated that they felt tired and unmotivated to complete daily activities. When asked why they sought respite services, 40% stated that they were overwhelmed by the responsibilities in addition to their own work. The groups that primarily support caregivers are family and professional respite services. Findings indicate that caregivers are most likely to trust resource recommendations from family and friends. Similar surveys should be administered in other cities and in rural locations to improve the generalizability of our findings.

### EARLY COLLABORATION MEASURES AMONG A CROSS-SECTOR STAKEHOLDER GROUP IN A STATEWIDE GWEP

Jennifer Crittenden,[1] Abigail Elwell,[2] David Wihry,[2] and Lenard Kaye,[2], *1. University of Maine, University of Maine, Maine, United States, 2. University of Maine, Bangor, Maine, United States*

The AgingME Geriatrics Workforce Enhancement Program (GWEP) uses collaboration across institutions of higher education, community-based organizations, and healthcare entities to imbed transformational healthcare practice change across Maine, a primarily rural state. To explore the factors that influence cross-sector collaboration among a diverse array of partners, a baseline anonymous electronic survey was distributed to the newly formed project steering committee. The survey consisted of the Wilder Collaborative Factors Inventory, an established measure of 22 research-based collaboration factors along with four open response questions on process-level challenges and opportunities for improvement. A total of eleven responses (N = 11) were received out of 20 Steering Committee members (55% response). Collaboration strengths noted in the assessment include unique purpose of statewide GWEP efforts (M = 4.41 out of 5 points), mutual trust among members (M = 4.32), favorable social and political environment (M = 4.27), and a history of collaboration among partners (M = 4.27). Lower scores were received on the multiple layers of participation (M = 3.45 out of 5 points), and ability to compromise factors (M = 3.45). Qualitative responses reinforced the need for a common understanding of the project's goals and outcomes early on in the collaboration. Barriers to collaboration included scheduling considerations and limited time and energy among partners due to heightened COVID-19 response efforts. Results elucidate: 1) Early collaboration strengths and needs of a newly formed statewide education collaborative; and 2) Strategic action steps and focal points informing early partnershipping among organizations engaged in interprofessional health education efforts.

### IMPLEMENTING A COMMUNITY-BASED COLLABORATIVE PROJECT DURING THE COVID-19 PANDEMIC: A PROCESS EVALUATION

Jarmin Yeh,[1] Leslie Ross,[1] Brittney Pond,[2] Brooke Hollister,[3] Tiffany Cheang,[4] Susie Yu,[5] and Corinne Eldridge,[5], *1. University of California, San Francisco, San Francisco, California, United States, 2. University of California, San Francisco, University of California, San Francisco, California, United States, 3. University of California, San Francisco, California, United States, 4. Alameda Alliance for Health, Alameda, California, United States, 5. Center for Caregiver Advancement, Los Angeles, California, United States*

In-Home Supportive Services (IHSS) caregivers are critical linchpins in our long-term care system, but little research exists to examine the strategies for enhancing their role working in the homes of persons living with dementia (PLWD). The aim of the IHSS+ Alzheimer's Disease and Related Dementias Training Project (IHSS+ ADRD Training Project) is to implement a competency-based dementia training program for 600 IHSS caregivers and their consumers; and evaluate the training program's impact on caregiving, long-term services and supports, and health systems. This project is a partnership between the University of California, San Francisco, Institute for Health & Aging; Center for Caregiver Advancement, a nonprofit organization founded by home care workers; and Alameda Alliance for Health, a non-profit managed care plan created by and for residents of Alameda County, California. The research design and funding of the project was established just prior to the COVID-19 pandemic. As the pandemic evolved, the IHSS+ ADRD Training Project underwent many revisions and pivoted to remote strategies to ensure progress could be made toward the aims. This poster describes a process evaluation of how various challenges were addressed and subsequent changes were made to the methodology. Using a community-based participatory research and program evaluation hybrid model, this project remained nimble, configuring an online dementia training program and evaluation methods that accommodated safety needs of community partners. Benefits and limitations of implementing the IHSS+ ADRD Training Project, using remote strategies, to ensure IHSS caregivers and their consumers could continue receiving education and support, are highlighted.

### OLDER MEN'S PARTICIPATION IN COGNITIVE TRAINING: A REVIEW.

Estela González,[1] Carmen Requena,[2] and PAULA ALVAREZ,[1], *1. Universidad de León, León, Castilla y Leon, Spain, 2. Universidad de León, Leon, Castilla y Leon, Spain*

**Background:** Cognitive training for healthy older adults living in a community is an essential resource that allows them to live at home for as long as possible.

**Objective:** The purpose of the review is to examine the degree of participation of males and females in longitudinal studies of cognitive training. Moreover, we want to identify if these studies include the gender variable in their analyzes or reflect on its importance. Method: This review considered longitudinal cognitive training studies were published in English and Spanish and conducted with healthy older adults living in a community.

**Results:** The Advanced Cognitive Training for Independent and Vital Elderly (ACTIVE) study (in English) and the Memoria Mejor (MM) Longitudinal Study (in Spanish), both illustrate the trend of sex/gender treatment of the studies reviewed: a) high participation of older people seventy and more years b) recruiting stratified by age and sex; c) males are disproportionately underrepresented in cognitive training studies [24% - 14%]; d) the evaluation measures (baseline, follow-up, and final) and dropout data are provided but not stratified by age and sex/gender.

**Conclusions:** Researchers demonstrate awareness about the impact of sex/gender differences but do not focus on it. Understanding sex/gender differences are necessary for understanding not only that these differences occur, but also why they occur; this will allow policies or intervention programs with approaches that are more equitable for both sexes/genders to be formulated.

### SOCIAL TRUST, LITERACY, AND LIFELONG LEARNING: A COMPARISON OF THE U.S. AND NORDIC COUNTRIES

Samuel Van Vleet,[1] Abigail Helsinger,[2] and Phyllis Cummins,[1], *1. Miami University, Oxford, Ohio, United States, 2. Scripps Gerontology Center, Miami University, Oxford, Ohio, United States*

Societal social trust has been shown to be related to economic growth and equality. Low levels of social trust are especially consequential in aging societies and can result in low levels of social capital and greater inequality at older ages. Nordic countries are known for their greater social trust, access to education, economic productivity, and social equality. To better understand social trust promoters, we explored data from the 2012/2014 Program for the International Assessment of Adult Competencies (PIAAC) to examine relationships among social trust, basic skills (i.e., literacy), and non-formal education (NFE) participation for adults ages 45 to 65, in the U.S., Denmark, Finland, Norway, and Sweden. Additionally, through 19 key informant interviews and a review of the literature, we investigated the structure and availability of NFE across the five nations. As compared to the U.S., adults ages 45 - 65 in Nordic countries have higher levels of social trust (all Nordic countries; $p < 0.001$), lower rates of poor literacy skills (Finland, Norway, and Sweden; $p < .001$), greater rates of participation in NFE (Denmark and Sweden; $p < 0.05$). Through the availability of NFE, such as folk high schools and learning circles in Nordic countries, adults can participate in NFE at little or no cost. Similar programs are not available in the U.S. This research informs policy and practice for the provision of NFE, which is critical to increase levels of social trust, and in turn, to promote economic development, social equality and positive aging in the U.S.

### STRATEGIES FOR DEVELOPING AN INCLUSIVE GERIATRIC PHYSICIAN WORKFORCE FOR OLDER ADULTS IN THE U.S.

Brandy Wallace,[1] Leanne Clark-Shirley,[2] and Pallavi Podapati,[3], *1. University of Maryland, Baltimore County, Baltimore, Maryland, United States, 2. American Society on Aging, American Society on Aging, California, United States, 3. Princeton University, Princeton University, New Jersey, United States*

The "geriatric imperative" has been part of the aging discourse for more than 30 years but neither geriatric practice nor older adults are homogenous. As the U.S. population ages, elders will become more racially and ethnically diverse; and, their health outcomes will be shaped by lifetime experiences with systemic discrimination and racism. Already, COVID-19 has made clear that older adults and non-Whites, particularly African Americans and Hispanics, disproportionately bear the burden of disease and illness. Research suggests health disparities will continue unless there is change within the health care system. The Institute of

Medicine (2001) reported on the problematic nature of the stark contrast between the diversity of patients and the physicians caring for them, including issues with patient trust and communication, yet no significant movement has been made to diversify the physician workforce. Despite being 13% and 16% of the U.S. population, respectively, African Americans and Hispanics make up just 5% and 6% of the practicing physician workforce. Further, practicing geriatricians represent less than 1% of physicians with very few physicians of color. There is a need for more African American and Hispanic geriatricians. In this systematic review, we examine recruitment and retention efforts targeting students of color, and curricula of geriatric medical programs in the U.S. We offer recommendations toward incentivizing physicians of color to enter geriatrics, strategies to support decolonization of geriatric medical curricula in undergraduate medical education programs, and the development of mentorship and pipeline programs to increase diversity in the geriatric physician workforce.

### TRAINING CLINIC PROVIDERS ON ADVANCE CARE PLANNING IMPROVES PROVIDER SELF-EFFICACY

Jeffrey Graupner,[1] Sandy Tun,[2] Carolyn Read,[3] Amena Qureshi,[3] Cassie Lee,[4] and Katherine Thompson,[1], *1. University of Chicago, Chicago, Illinois, United States, 2. University of Chicago Medicine, Chicago, Illinois, United States, 3. Oak Street Health, Oak Street Health, Illinois, United States, 4. Oak Street Health, Chicago, Illinois, United States*

Advance care planning (ACP) is a vital step to ensure patients receive and prioritize the care that best aligns with their end-of-life wishes, including discussion and documentation of an advance directive. Significant gaps in ACP among underserved populations have been well documented. Research suggests a successful strategy for increasing the communication between provider and patient about ACP is to educate clinicians on this important issue. Three, 2.5 hour training sessions were provided to healthcare staff of a large chain of older adult primary care clinics across three states. Lecture materials were created and presented by a palliative care (PC) physician and PC nurse practitioner. Presentations were held both in person and virtually. Participants were asked to complete a pre/post-training survey online which included a validated 17-item ACP Self-Efficacy Scale (Baughman, 2017), perceived barriers checklist, and additional quality improvement measures. A total of 131 providers attended one of three training sessions. 76 providers (58.0%) and 47 providers (35.9%) completed pre- and post-training surveys respectively. Scores on a 17-item validated ACP Self-Efficacy Scale were significantly higher after training (Wilcoxon signed rank test, $Z= 4.42$, $p <.001$). Participants ranked "lack of time" as the number one barrier to having ACP conversations both before and after the training, whereas "lack of training" ranked 2nd and fell to 7th after the training. These initial results suggest ACP self-efficacy among providers can be increased through a one-time training session. Previous literature has highlighted the importance of provider self-efficacy as factor in increasing ACP conversations with patients.

### Session 9225 (Poster)

### EDUCATION ON AGING AND OLDER ADULTS

### A RETROSPECTIVE STUDY OF RETIRED ACADEMICS: LONG-TERM CAREER BENEFITS OF PAST MENTORING BEHAVIORS

Giovanna Garrido Blanco,[1] Thomas Chan,[2] Jordan Boeder,[2] Veronica Fruiht,[3] Sarah Hwang,[2] and Kevin Erikson,[2], *1. California State University Northridge, Chanhassen, Minnesota, United States, 2. California State University Northridge, Northridge, California, United States, 3. Dominican University of California, Dominican University of California, California, United States*

While there is extensive literature on the benefits of mentoring for mentees, less is known about the impact of those relationships on mentors, particularly, after mentors complete their careers (i.e., retirement). For academics, the time and energy spent mentoring students can either be beneficial or costly to productivity. This study explores the associations between past mentoring and present evaluations of retired academics' careers, seeking to investigate the long-term career benefits of mentoring. Understanding the evaluation of older adults' careers at retirement in relation to their prior engagement in mentoring is critical, as mentoring is an integral component of careers in academia. Survey data were collected from a national sample of 277 retired academics averaging 73.6 (SD=6.2) years old and 7.7 (SD=5.8) years post-retirement. Results from structural equation models demonstrated that retired academics who reported having more protegees ($\beta=.19$, $p=.024$) and engaged in more mentoring behaviors ($\beta=.18$, $p=.027$) exhibited increased objective career benefits. Providing more mentoring functions was also associated with higher subjective career achievement ($\beta=.33$, $p<.001$). Interestingly, the number of mentees and mentoring behaviors were not correlated to career satisfaction. Findings from the current study demonstrate the association between past mentoring experiences with career success. Examining the link between mentoring behaviors and overall assessments of career in retirement offers important insight into the long-term benefits of mentoring in higher education training prompting further research into the realization of these benefits in later life.

### APPLYING THE NEW WORLD KIRKPATRICK MODEL TO EVALUATE AN ONLINE TRAINING COURSE INTRODUCING A MULTI-SENSORY DEVICE

Jennifer Perion,[1] Victoria Steiner,[2] Jennifer Kinney,[3] Kimberly McBride,[2] and Barbara Saltzman,[4], *1. University of Toledo, Temperance, Michigan, United States, 2. University of Toledo, Toledo, Ohio, United States, 3. Miami University, Oxford, Ohio, United States, 4. University of Toledo, University of Toledo, Ohio, United States*

Online education offers care providers flexibility and convenience. Applying the New World Kirkpatrick Model of training evaluation, this descriptive study evaluated the design and content of a 30-40 minute online training course that introduces direct-care workers to a multi-sensory device to help manage dementia symptoms in older adults. Following course completion, an online survey obtained ratings of engagement (i.e., aesthetics, ease of use, novelty, and involvement), relevance, and knowledge/skills gained from the training using a 5-point Likert scale. A convenience sample of 72 undergraduate students enrolled in health science and human service programs at a Midwestern university participated. The majority were white (83.3%), non-Hispanic (81.9%) females (88.9%). Most participants agreed or strongly agreed (median=4) with positive statements related to engagement with the course. Statements about relevance to their intended career were rated even higher (median=5). Wilcoxon signed-rank tests for matched pairs revealed statistically significant improvements on self-reported pre-post knowledge/skills scores ($p<0.005$). The results indicated that participants found the training aesthetically pleasing, easy to use, novel, and that it encouraged user involvement. Participants thought the topics covered were relevant to the professional career they are pursing, and they learned new knowledge/skills. Responses to open-ended questions suggested improvements to the design (e.g., color choice) and content (e.g., expanded topics and resources). Future research will evaluate a revised course with direct-care workers who will use the multi-sensory device in long term care facilities. Subsequently, an intervention study will determine the effectiveness of the device in increasing the well-being of people with dementia.

### EFFECT OF EXCHANGE THROUGH KENDO ON THE IMAGE OF THE ELDERLY OF JUNIOR HIGH SCHOOL KENDO CLUB MEMBERS

Daisuke Matsumoto, and Hayato Uchida, *University of Hyogo, Himeji-city, Hyogo, Japan*

This study targeted junior high school kendo club members who have practice experience with elderly kendo practitioners and examined the image of the elderly and the related factors that intergenerational exchange through kendo brings to the junior high school members. Kendo is one of the traditional Japanese martial arts. The subjects in this research were 193 players who practiced with the elderly kendo practitioners in Osaka prefecture. As a result of factor analysis to clarify the structure of the image, the "evaluation" factor and the "activity / competence" factor were extracted as in the previous research, and it was suggested that the junior high school players generally had positive image regarding the elderly practitioners. As a result of logistic regression analysis to clarify the factors related to the high/ low scores in the image of elderly kendo practitioners, “evaluation” factor showed a significant relationship between “intimacy with elderly practitioners” and “empathic interest”, and “activity / competence” factor indicated a significant relationship between “gender” and “intimacy with elderly kendo practitioners.” Although it is pointed out that the traditional style of kendo and the image of elderly kendo practitioners have a negative impact on children, it may be possible to control these factors through an appropriate guidance and approach depending on the children's stage of growth.

### EXAMINING THE EXPERIENCES OF ADULT LEARNERS IN HIGHER EDUCATION

Jyotsna Kalavar,[1] Kirsten Magda,[2] and Raquel Ariyo,[2], *1. Georgia Gwinnett College, Lawrenceville, Georgia, United States, 2. Georgia Gwinnett College, Georgia Gwinnett College, Georgia, United States*

In the last decade, there has been a shift of more non-traditional adult learners returning to pursue undergraduate education. Though traditional age students are in the majority, a rising population of adult learners has been steadily increasing. They are typically students who are 25 years and older, attend part-time, work full-time, and tend to juggle family or dependent demands with schoolwork. Studies show that these adult learners are at high-risk for academic underachievement and dropping out. However, educational institutions still operate with the same traditional learning paradigm that they previously used (without acknowledging the wealth of life experiences that adult learners bring), leading us to the question of how adult learners perceive their academic learning experiences. In this study, 171 adult learners (students aged 25 and older at the time of matriculation) at a regional college in Atlanta, participated in an online survey that examined their academic experiences, specifically meaningfulness of coursework, course delivery approaches, and the advantages as well as disadvantages of being an adult learner. Majority were females (83%), and the ethnic breakdown was as follows: White (41%), African American (26%), Hispanic (12%), and 22% reported other. Results of this study indicate that academic institutions need to pay attention to the learning experiences of this burgeoning student population. Understanding their perspectives on their academic experiences hold major implications for long-term meaningful change in academia.

### GRANDPARENTS AND PARENTS: AN ESSENTIAL PARTNERSHIP FOR LONGEVITY SOCIETIES

Paris Strom, *Auburn University, Auburn University/Auburn, Alabama, United States*

This presentation hypothesizes that an innovative collaboration by the adult generations will be necessary to enable conditions needed for family success in a longevity society. Unprecedented challenges of parents and grandparents are examined. Reasons why adults have to regard youth as a source of learning about their unique experiences in an age-separated society are explained. International curriculum development studies to support families of children from birth through adolescence are described. A curriculum that provides a common knowledge base about child and adolescent guidance is proposed to harmonize efforts of adults to support younger relatives. Curriculum for retirees should focus on continuing responsibilities other generations expect of them, learning about the lives of younger family members, and gaining awareness of parenting practices to reinforce lessons. Training volunteers in assisted living and long-term care facilities to be indigenous leaders of grandparent classes is discussed as a practical way to offer relevant learning and improve social support. Instruments are examined that assess ethnic relationships between adult generations, adults and adolescents, and track results of education intervention.

### THE FUTURE OF AGING IN A LONGEVITY SOCIETY: A COURSE FOR HIGH SCHOOL AND COLLEGE STUDENTS

Paris Strom, *Auburn University, Auburn University/Auburn, Alabama, United States*

Americans, on average, can anticipate living 85 years or perhaps 100 if born in this millennium. This extension of the lifespan has introduced a new stage of human development presenting unfamiliar challenges to policy makers, health care providers, employers, religious institutions, families, individuals, and schools. Education about longevity should begin in adolescence (ages 10-20) with the merger of science, experiences of older generations, and imagination of youth. Content of this online course focuses on the years after adolescence: early adulthood, middle age, retirement, and old age. After reading each of the 16 lessons, cooperative learning teams conduct structured interviews with older relatives, friends or neighbors who are further along in life's journey. All the lessons are augmented by 'what do you think? tasks used to motivate discussions, structure interviews, decide on reasoning and problem-solving scenarios, identify key concepts to apply, group lesson reviews, and self-evaluation for comparison with peers. If society wants to encourage adolescents to appreciate their national and ethnic heritage, benefit from learning how older generations see situations and interpret current events, and acknowledge the common need for maturity and spiritual development, then older people should become resources for education about longevity.

### Session 9230 (Poster)

### ELDER ABUSE AND NEGLECT

### CHARACTERISTICS OF FINANCIAL EXPLOITATION IN A SAMPLE OF ISRAELI OLDER ADULTS

Gali Weissberger,[1] S. Duke Han,[2] and Amit Shrira,[1], *1. Bar Ilan University, Ramat Gan, HaMerkaz, Israel, 2. University of Southern California, Los Angeles, California, United States*

Financial exploitation (FE) negatively affects wellbeing in older adulthood. However, characteristics of FE and its health correlates remain poorly understood. In this study, 138 Israeli older adults answered questions regarding FE history, and completed physical and mental health questionnaires. Of 138 participants, 23 reported a history of FE. FE participants were older (M birth year = 1950.35; sd = 9.65) than non-FE participants (M birth year = 1953.79; sd = 6.06; p = 0.028) and reported lower household income (p=0.001). Groups did not differ in education level or sex breakdown. The FE group reported older subjective age (p = 0.022), worse subjective cognition (p = 0.007), more depressive symptoms (p=0.002), and marginally higher anxiety symptoms (p = 0.099) than the non-FE group. Groups did not differ in reported levels of social support or number of medical conditions. When covarying for age, differences between groups in subjective cognition and depressive symptoms remained significant (ps ≤0.022), while subjective age differences became marginal (p = 0.07). The FE group responded to follow-up questions regarding FE experiences. Reported perpetrators included companies/businesses (most commonly reported, 30%), strangers, friends/neighbors, service providers, and family. Eleven reported losing 100 NIS to 10,000 NIS, and 10 reported losing 10,001 to over 100,000 NIS. Additionally, six FE participants reported that the FE is ongoing, and two reported additional FE experiences. Findings suggest that FE is related to mental and physical health of older adults. Findings also provide preliminary information regarding characteristics of FE in a sample of Israeli older adults.

### COGNITIVE OVERLOAD: HOW TO PROTECT OLDER ADULTS FROM DIMINISHED FINANCIAL DECISION-MAKING CAPACITY

Christopher Heye,[1] Elizabeth Loewy,[2] and Katie Wade,[3], *1. Whealthcare Planning, Belmont, Massachusetts, United States, 2. EverSafe, New yORK, New York, United States, 3. Whealthcare Planning, Belmont, Connecticut, United States*

The aging population in the US poses a major threat to the financial security of older adults and their families. Millions of older adults will need to successfully navigate a multitude of financial and legal issues if they are to safely manage their assets while they are alive, and then securely transfer trillions of dollars to their heirs in accordance with their wishes. But most older adults are less healthy than their younger counterparts, and 25% or more over 65 are likely to suffer from diminished decision-making capacity. In short, older adults in the US will have to make some of the most important financial decisions of their lives just as their decision-making capacity is in decline. We offer recommendations to make it easier for financial services firms, medical professionals, non-profit organizations, and technology companies to work together to find better solutions for managing the complex issues around diminished decision-making capacity that is only likely to worsen in the years ahead.

### ELDER FAMILY FINANCIAL EXPLOITATION: FAMILY RELATIONSHIPS ARE NEVER THE SAME

Athena Chung Yin Chan,[1] and Marlene Stum,[2], *1. University of Minnesota, St Paul, Minnesota, United States, 2. University of Minnesota -- Twin Cities, St. Paul, Minnesota, United States*

Millions of families are affected by elder family financial exploitation (EFFE), but the consequences for lifelong intergenerational family relationships and family functioning remain largely unexamined (i.e., parent/child, siblings). This study examines the consequences of experiencing EFFE on the quality of family relationships from the perspective of non-victim, non-perpetrator concerned family members (CFMs). Data from a larger EFFE mixed-methods study were utilized. A voluntary sample of 28 CFMs who had experienced EFFE participated in semi-structured, in-depth interviews and brief surveys. Analysis included identifying quality of family relationship themes from data related to differences EFFE has made in their family. Participating CFMs were primarily adult children of older victims, and siblings of the primary perpetrators. The findings reflect three patterns of changes in quality of family relationships between the CFMs and other family members, including: (a) Restoring trust and recovery within the family, (b) Alliances and taking sides, and (c) Estrangement and cutoff. While families were never the same after experiencing the exploitation, for some was a healing process to accept the family situation and restore and repair trust. Redefining who would be family moving forward was a focus for some families when perpetrators and CFMs took sides and formed allies with others. In the most dramatic changes, siblings, parents and others became cutoff and isolated from other family members. While a loss of relationship quality was common, strengthening of relationships also occurred. Understanding EFFE from an ecological family systems perspective can help to inform needed EFFE interventions, both processes and outcomes.

### EXPLAINING ONLINE FRAUD VICTIMIZATION OF OLDER ADULTS VIA CYBER ROUTINES AND LIFESTYLES

Katalin Parti, *Virginia Tech, Blacksburg, Virginia, United States*

The paper compares victim group characteristics: the differences between individuals under 55 and 55 and older, victimized by online fraud committed by a stranger. We test Cyber-Routine Activities Theory (cyber-RAT; Choi, 2008). In addition to active online lifestyle and computer familiarity, we include in the analysis independent variables such as living arrangements, occupation, and the willingness to report and ask for help. A representative sample of US citizens 18 or older was collected using a Dynata research panel in October 2020. We utilized one-way ANOVA on ranks for testing whether older and younger victims' characteristics can be derived from the same independent variables. We find that older victims differ in characteristics from younger victims. Those who live alone or in a marriage with no children, and retired are significantly less victimized by online fraud than those who live with a partner and children and having full-time jobs. Besides, they are less likely to report their victimization, and their online activities and computer familiarity make them more vulnerable to online fraud than younger adults. Our findings suggest that older victims need prevention and intervention solutions designed especially for them. In an aging society, individuals will be increasingly active online and in the job market. Employers must provide internet security training for employees on manipulative online scams. Crime prevention programs must include awareness-raising on the role of reporting and how to ask for help. Relatives, friends, and caretakers need to be trained in preventing and early recognizing online fraud impacting older adults.

### MULTI-METHOD EXAMINATION OF ELDER MISTREATMENT IN THE AGE OF COVID-19

Sonia Salari,[1] Sharon Talboys,[1] Annie Isabel Fukushima,[2] Heather Melton,[1] Seage Michelle,[1] and Megan Petersen,[1], *1. University of Utah, Salt Lake City, Utah, United States, 2. University of Utah, University of Utah, Utah, United States*

A multi-method study exposed COVID-19 influence on the pre-existing epidemic of elder mistreatment in Utah. We found changes in 1) abuse types, 2) service responses, 3) firearm access and 4) policy implications. Gun sales were tracked by news surveillance and FBI National Instant Criminal Background Check System (NICS) for pre-pandemic (2018/2019) and pandemic years (2020/2021). New requests for permits skyrocketed during the pandemic. The 2021 Utah State Legislature loosened restrictions on concealed permits. Domestic violence (DV) Fatality Tracker Data in pre-covid years were compared to 2020-2021. A figure illustrates the prevalence of DV fatalities, ages of victims by year and methods used. We conducted 15 in-depth interviews of stake holders who serve DV victims (shelters, police, etc.). DV shelters had a relative lack of children during the pandemic, but increased use by older persons 60+. Susceptibility to chronic respiratory distress syndrome, required social distance for older persons. DV shelters obtained CARES Act funds to adapt solutions, like placing victims in hotel rooms. Most victims stayed at home, confined with abuser(s), some without technology, so isolation decreased their safety. Evidence suggests some fatalities among elder adults. A case study during the pandemic described a 73-year-old mother's suspicious bank account activity. Bank employees sent police to her home. She was missing, but her co-resident adult son was in possession of her bank cards. She was later found in a shallow grave. Utah households have increased risks of DV fatalities in the wake of the pandemic and for years to come.

### SUSCEPTIBILITY TO COVID-19 SCAMS: DOES AGE MATTER?

Julia Nolte,[1] Yaniv Hanoch,[2] Stacey Wood,[3] and David Hengerer,[4], *1. Cornell University, Ithaca, New York, United States, 2. University of Southampton, Southampton, England, United Kingdom, 3. Scripps College, Claremont, California, United States, 4. Claremont Graduate University, Claremont, California, United States*

The COVID-19 pandemic has lead to a worldwide surge in COVID-19 related mass marketing scams. While COVID-19 poses higher health risks for older adults, it is unknown whether older adults are also facing higher financial risks as a result of such scams. Thus, the present study examined age differences in vulnerability to COVID-19 scams and factors that might help explain them. In June 2020, 68 younger (18 – 40 years, $M = 25.67$, $SD = 5.93$), 79 middle-aged (41 – 64 years, $M = 49.86$, $SD = 7.20$), and 63 older adults (65 – 84 years, $M = 69.87$, $SD = 4.50$) were recruited through Prolific. Participants responded to five COVID-19 solicitations, psychological measures, and demographic questions. Across solicitations, older adults perceived COVID-19 solicitations to offer significantly fewer benefits than both younger and middle-aged adults did. However, age groups neither differed in their perception of the solicitations' risks and genuineness nor in their willingness to act in response to COVID-19 solicitations. Overall, intentions to respond to COVID-19 solicitations were positively predicted by higher levels of educational attainment, a previous history of fraud victimization, and higher levels of positive urgency. As expected, stronger genuineness and benefit perceptions positively predicted action intentions, whereas stronger risk perceptions negatively predicted action intentions. Older adults did not exhibit greater vulnerability to COVID-19 solicitations: If anything, they were more skeptical of the benefits associated with these solicitations. Irrespective of age, risk, benefit, and genuineness perceptions were the key factors associated with intention to respond to solicitations.

### THE PSYCHOLOGICAL HEALTH CONSEQUENCES OF ELDER ABUSE AND NEGLECT IN SHELTERED HOMES

Khulood Alraddadi, *King Abdulaziz University, Jeddah, Makkah, Saudi Arabia*

**Background:** Psychological health effects of elder abuse and neglect in sheltered homes are unknown. Objectives:The objective of the present study was to determine if several types of elder abuse were related to an increased likelihood of negative psychological health.

**Methods:** Data were derived from the elder mistreatment in sheltered homes project, a cross-sectional study conducted in 2018 that included 446 older adults living in 43 sheltered homes.A face-to-face interview was carried out to complete structured questionnaires for participants to assess the psychological health effects of different subtypes of elder abuse.

**Results:** All subtypes of elder abuse were significantly associated with increased prevalence rates of depressive symptoms and sleep problems. Similarly, all subtypes except physical abuse were associated with higher rates of anxiety symptoms. In multivariate regression analysis, Older adults who reported physical mistreatment were three times more likely to experience suicidal ideation(OR=3.28, 95% CI=1.14 to 9.44), while no other subtypes were significantly associated with suicidal ideation.Older adults who report at least one type of abuse were eight times more likely to have depressive symptoms (OR=7.99, 95% CI=4.43 to 14.41), twice as likely to screen positive for anxiety symptoms(OR=2.05, 95% CI=1.20 to 3.49), three times more likely to have sleep problems (OR = 3.20, 95% (CI = 1.78 to 5.75). All subtypes of abuse were significantly associated with the presence of depressive symptoms, sleep problems, and anxiety symptoms except physical mistreatment.

**Conclusion:** Abuse is common among older adults living in sheltered homes and is associated with negative impacts on psychological health.

### Session 9235 (Poster)

### EMOTIONS IN OLDER POPULATIONS

### AGE DIFFERENCES IN AWE AMONG YOUNG, MIDDLE-AGED, AND OLDER ADULTS: EXTENSIONS OF SOCIOEMOTIONAL SELECTIVITY THEORY

Alexandria Ebert, Julie Hicks Patrick, Maya Huggins, Aradhita Yadava, and Sarah Collett, *West Virginia University, Morgantown, West Virginia, United States*

Increases in motivation for the pursuit of emotionally meaningful goals and activities as people age (socioemotional selectivity theory; SST) necessitates the investigation of means in which these goals and activities can be realized. In the present study adults (N = 130) aged 25 to 78 (M = 53.32; SD = 15.181) watched awe-inducing (view of space) and happiness-inducing (comedian Robin Williams interacting with Koko the "talking gorilla") videos and then completed measures associated with awe, affect, and well-being (measured via PGC Positive and Negative Affect Scales). Analyses of Variance (ANOVAs) were conducted to examine the effect of age (split by young, middle-aged, and older) on experiences of awe and positive affect in response to watching each video. There was a main effect of age on experiences of awe for both videos (ps < 05). Specifically, older adults experienced significantly higher levels of awe than young adults (p < .05) in response to video 1. They also experienced significantly higher levels of awe than young adults (p < .01) in response to video 2. Bivariate correlations among awe, well-being, and happiness were similar in strength and magnitude in each age group (ps < .05). Overall, consistent with Socioemotional Selectivity Theory, our findings suggest that affective reactions of awe and happiness, induced by videos, relate to well-being across age-groups. Future work should test whether the use of these awe-inducing videos can improve well-being.

### AGE GROUP DIFFERENCES IN RESPONSE TO REPEATED EXPOSURE TO LABORATORY STRESS TASKS

James Miller, and Gloria Luong, *Colorado State University, Fort Collins, Colorado, United States*

Research examining age differences in affect reactivity (i.e. how much affective experiences change in response to stressors) has produced mixed results, suggesting that there are areas of relative strength and weakness in regulatory processes across age-groups. The present study's goals were to examine potential age-group differences in affect reactivity and subjective task-appraisals across repeated exposures to a psychosocial laboratory stressor. In the Health and Daily Experiences (HEADE) study, younger (18-35 years old; n=107) and older adults (60-90 years old; n=90) were exposed to the Trier Social Stress Test on three occasions in a laboratory setting over a five-day period. Current affective experiences and task-appraisals were assessed at each session using validated self-report scales, with current affective experiences measured at baseline and task periods to determine affect reactivity. Repeated measures ANOVA analyses were conducted to examine age-group differences in affect reactivity and task-appraisals across sessions. In support of our hypotheses, younger adults showed greater reductions in their negative affect reactivity over time compared to older adults [F(2, 390)= 8.18, p<.001]. Additionally, younger adults' appraisals of task-difficulty decreased [F(2, 384)= 14.79, p<.001] and appraisals of task-performance increased [F(2,384)= 13.39, p<.001] across sessions, while older adults' task-appraisals remained stable. Age-group differences in negative affect reactivity and task-difficulty appraisals were not evident for the first session and only emerged after repeated exposure to the stressors. These results highlight the importance of identifying age-related vulnerabilities in adapting to repeated stressors, with implications for designing effective interventions aimed at improving health and well-being for older adults.

### IMMORAL INTENTIONS VS. ACCIDENTAL ACTIONS: AGE DIFFERENCES IN ENDORSEMENTS OF ANGER AND DISGUST

Alyssa Minton, Jason Snyder, Nathaniel Young, Verena Graupmann, and Joseph Mikels, *DePaul University, Chicago, Illinois, United States*

Given that older adults value social harmony and selectively avoid negativity (Carstensen & Mikels, 2005), we investigated whether older and younger adults differentially react to scenarios in which someone intends to harm others compared to someone who accidentally harms others. Younger (n = 112, M = 26.23) and older (n = 113, M = 66.42) adults read 8 scenarios in which a perpetrator intentionally acts to harm someone else but is unsuccessful (Desire condition) or a perpetrator accidentally harms someone else (Consequence condition; Giner-Sorolla & Chapman, 2017). Endorsements of anger and disgust toward the perpetrators were measured on 7-point scales (1 = Not at all, 7 = Extremely). Emotion endorsements were submitted to 2 (age) x 2 (condition) ANOVAs. Anger (M = 4.81, SD = 1.58) and disgust (M = 4.82, SD = 1.54) endorsements were higher in the Desire relative to Consequence condition (M = 2.64, SD = 1.33; M = 2.49, SD = 1.29, respectively), $F(2, 221) = 124.03$, $p < .001$; $F(2, 221) = 156.31$, $p < .001$, respectively. Moreover, older (M = 5.17, SD = 1.61) relative to younger (M = 4.45, SD = 1.37) adults were disproportionately disgusted in the Desire condition, $t(102) = 2.45$, $p = .016$, but no age differences emerged in the Consequence condition. Results indicate that older (relative to younger) adults are disproportionately disgusted when judging a person who intends to harm others. Older adults may respond more strongly than younger adults to malicious perpetrators, as they intentionally upset social harmony.

### INTERPERSONAL INTERACTIONS AND AFFECT IN DAILY LIFE AMONG MIDLIFE AND OLDER ADULTS

Robert Stawski,[1] Dakota Witzel,[1] Madeline Nichols,[1] and Susan Charles,[2], *1. Oregon State University, Corvallis, Oregon, United States, 2. University of California, Irvine, Irvine, California, United States*

Theories of age and emotional wellbeing posit that older age is associated with better affective well-being through avoidance or minimization of distressing experiences and prioritizing positive experiences and emotions. To test these theories, researchers have examined change in affect (i.e., reactivity) associated with negative interpersonal experiences in daily diary studies, given the compromising effects these interpersonal stressors exert on daily affect. In contrast, age differences in the potential affect-enhancing effects of positive interpersonal experiences have been comparatively neglected. Using the second wave of the National Study of Daily Experiences, we evaluated age differences in the frequency of daily negative and positive interpersonal interactions, as well as the affective responses to these interpersonal interactions. Positive and negative affect, as well as negative and positive interpersonal interactions were assessed on eight consecutive evenings. Analyses included 818 participants (Mage=53.3, SD=11.8, Range=34-83; 60% female) who experienced both negative and positive interpersonal interactions during the 8-day protocol. Preliminary results revealed increased frequency of negative interpersonal interactions and decreased frequency of positive interpersonal interactions with age (ps<.01). Further, negative interpersonal interactions were associated with increases in negative affect and decreases in positive affect (ps<.01), while positive interpersonal interactions were associated only with increased positive affect (p<.01). Finally, modest evidence of age-related reductions in the affective impact of negative, but not positive, interpersonal interactions emerged (p=.03). Discussion will focus on how studies of interpersonal interactions in daily life can inform theories of aging and promote emotional wellbeing throughout adulthood and later life.

### MARITAL CONFLICT, ANGER EXPRESSION, AND MARITAL INSTABILITY: ASSOCIATIONS BY AGE AND CULTURE

Dakota Witzel, Madeline Nichols, and Robert Stawski, *Oregon State University, Corvallis, Oregon, United States*

Positive social relationships, such as high-quality marriages, are associated with better health, especially among older adults. Moreover, negative components in marriages (i.e., disagreements and associated emotional responses) are linked to negative outcomes such as marital disruption (McGonagle et al., 1993) and divorce (Markman et al., 2010). Factors such as marital conflicts and emotion expression threaten marital stability and health and have been shown to decrease with age and in collectivist cultures (i.e., Japan compared to U.S.; Kitayama et al., 2015; Matsunaga & Imahori, 2009). While anger has featured as a specific emotion associated with compromised health and marital quality (Carrère et al., 2005), less is known about how marital conflict and the expression of anger may contribute to marital instability in later life, or differences in these links across age and culture. Using data from married respondents participating in the second wave of the Midlife in the United States (MIDUS, n=750, Mage=55.18, SD=11.5, %Female=50.13%) study or first wave of the Midlife in Japan (MIDJA; n=706, Mage=55.26, SD=13.68, %Female=47.73%) studies, we examined associations between disagreements, anger expression, and marital risk. Preliminary analyses revealed that marital disagreements and anger expression were each associated with increased marital instability in both the MIDUS and MIDJA samples (ps<.05). Additionally, the effects of marital disagreements and anger expression did not differ between the two samples or as a function of age. Discussion will focus on the relevance of expression and regulation of emotions for understanding marital (in)stability in midlife and aging and across cultures.

### NOSTALGIA FOR A PURPOSE? A COMPARISON OF THREE THEORETICAL FRAMEWORKS IN A LIFESPAN SAMPLE

Jennifer Turner,[1] Jessica Kerin,[2] Kenzie Delposen,[2] and Jennifer Stanley,[2], *1. Pennsylvania State University, State College, Pennsylvania, United States, 2. University of Akron, Akron, Ohio, United States*

Nostalgia is a common experience for most people, but the functions or motivations for nostalgia are unclear. Several theoretical arguments for the purpose of nostalgia have been offered: nostalgia may reduce anxiety or fear of mortality (i.e., Terror Management Theory), resolve developmental conflicts (i.e., ego-integrity vs. despair), or provide a touchstone to the past (i.e., self-continuity). The goal of this study was to compare these theoretical frameworks among young (YA), middle-aged (MA), and older adults'(OA) descriptions of nostalgia and explore whether content of nostalgia differs by age. We hypothesized that YA would report greater amounts of nostalgia related to self-continuity, MA would report more integrity-related nostalgia, and OA would report more Terror Management. Nostalgia recordings (N=593) were collected during a two-week daily diary study in 108 participants (ages 18-78 years; 60.2% women). Recordings were transcribed and then coded by two trained coders (Magreement=87.4%; $\kappa=.66$, $p<.001$) using a rubric containing three typologies of nostalgia: Terror Management, Integrity v. Despair, Self-Continuity. Only n=255 transcripts could be coded within these typologies. Supporting our hypotheses, YA reported more self-continuity (51.5%) than Integrity (39.4%) or Terror Management (9.1%), and MA reported more Integrity (42.0%), than self-continuity

(39.5%) or Terror Management (18.5%). Our third hypothesis was not supported: OA reported self-continuity most frequently (47.5%), followed by Integrity (31.9%) and Terror Management (20.6%). Nostalgia may provide a vehicle for self-reflection as people compare the past to the present, and future research should examine whether emphasis on different types of nostalgia has implications for psychological outcomes like wellbeing.

### RECOGNITION OF AFFECTIVE FACES OF DIFFERENT AGE GROUPS

Kim Jinhwi, Seunghee Park, and Jongwan Kim, *Jeonbuk National University, Jeonju, Cholla-bukto, Republic of Korea*

It has been found that valence and arousal are the core affect dimensions in emotional structure. In this study, we hypothesized that there might be differences between different age groups in emotional structure using six facial expression stimuli (angry, disgusted, fearful, neutral, happy, and sad) of three age groups (young, middle-aged, and old). Unlike previous studies asking participants to rate subjective ratings or similarities between stimuli, participants in this study were required to determine whether stimulus pairs were the same or different emotions and reaction time and accuracy were measured for further analyses. We assumed that it would be harder when the stimulus pair is similar whereas it would be easier when the pair is different. The results showed that for the same emotion pair condition, the sad-sad pair had the lowest accuracy and the longest reaction time, while the happiness-happiness pair had the highest accuracy and the shortest reaction time. For the different emotion pairs, angry-disgusted and disgusted-sad was the lowest accuracy and the longest reaction time. For age of the stimuli effect, responses to the old faces had the lowest accuracy and the longest reaction time. The results suggest that identification of emotional stimuli might be affected by emotion category and age. Further study may need to recruit various age groups, because participants in the current study were mostly young adults.

### SOCIOEMOTIONAL GOALS AND DEFINITIONS OF EMOTION TERMS: A QUALITATIVE ANALYSIS ACROSS AGE

Irina Orlovsky,[1] Rebecca Ready,[2] and Bruna Martins-Klein,[2], *1. University of Massachusetts Amherst, Northampton, Massachusetts, United States, 2. University of Massachusetts Amherst, Amherst, Massachusetts, United States*

Major theories of adult development posit that knowledge about emotion might evolve across the lifespan. Socioemotional Selectivity Theory (SST) and the Strength and Vulnerability Integration (SAVI) models imply that the manner in which older (OA) and younger adults (YA) conceptualize emotions may differ in valence, arousal, reference to social partners, time perspective, and the self. Quantitative accounts of age differences in conceptualizations of emotion-terms offer mixed support for theoretical expectations, but many predictions have yet to be tested qualitatively. In this study, 90 OA and 210 YA provided narrative descriptions of 11 (5 positive, 6 negative) emotion-terms. Responses were coded on valence, reference to self/others, and arousal. O/YA used similar synonyms to define emotion-terms. As predicted, YA used high arousal language in their definitions of negative (OR = 10.29, p = 0.018) and positive terms more than OA (i.e. Happy: OR = 1.27, p<0.001); OA referenced other persons such as family and friends (pos: OR = 0.13, p<0.001; neg: OA = 0.32, p=0.002) more than YA. Contrary to predictions, OA self-referenced more often than YA in positive (OR = 0.12, p=0.001) and negative definitions (OR= 0.11, p=0.004); this may be attributed to OA providing more situational examples in their responses than YA. Somewhat consistent with SAVI and SST, OA may reference high-arousal states less when conceptualizing emotions and associate their definitions more with social partners than YA. Future research should address OA greater use of situational examples when defining emotion terms, motivational factors and emotional impact of these age differences.

### THE EFFECT OF EVERYDAY STRESSORS AND THE PERCEIVED STRESS REACTIVITY SCALE ON VARIABILITY IN SYMPATHETIC AROUSAL

MacKenzie Hughes,[1] Christopher Hertzog,[1] Shevaun Neupert,[2] and Scott Moffat,[1], *1. Georgia Institute of Technology, Atlanta, Georgia, United States, 2. North Carolina State University, Raleigh, North Carolina, United States*

This ecological momentary assessment study examined the effect of naturally occurring stressors and perceived stress reactivity on alpha-amylase, a proxy of sympathetic nervous system arousal. There are age-related changes in physiological systems sensitive to stress, so the sample included 174 adults ages 20-78 (M=48.65, SD=19.28). At the beginning of the study, participants completed the Perceived Stress Reactivity Scale (PSRS; Schulz et al., 2005). For 10 consecutive days, participants were prompted five times per day to report exposure to stressors. During the same 10-day period, participants provided seven saliva samples per day, assayed for alpha-amylase. Multilevel modeling was used to examine daily and momentary associations between stressors, the PSRS, and alpha-amylase activity. On a daily basis, stressors did not predict changes in the diurnal alpha-amylase pattern, but higher perceived stress reactivity predicted steeper diurnal slopes and lower total daily output. A significant cross-level interaction emerged showing people higher in perceived stress reactivity had steeper awakening responses on days they experienced more stressors than usual. On a momentary basis, alpha-amylase levels were higher on occasions when participants reported stressors. In addition, higher levels of perceived stress reactivity predicted lower overall alpha-amylase levels. Findings suggest that 1) stressors are associated with elevations in momentary but not daily aggregate levels of alpha-amylase, and 2) the PSRS has prospective validity as a predictor of stress-related fluctuations in diurnal alpha-amylase patterns. Age was not a significant moderator of the relationship between stressors and alpha-amylase, potentially suggesting the effect of stressors on alpha-amylase activity is age invariant.

### Session 9240 (Poster)

### END OF LIFE, DEATH, AND DYING

### A CASE STUDY ON ANTICIPATED END-OF-LIFE CAREGIVING AMONG THE MILLENNIAL AMERICAN BORN CHINESE

Tongtong Li,[1] Aileen Zhang,[2] Ruotong Liu,[2] and Iris Chi,[3], *1. University of Southern California, Arcadia, California, United States, 2. University of Southern California, Los Angeles, California, United States, 3. University of Southern California, University of Southern California, California, United States*

Millennial American Born Chinese (ABCs) are in a double jeopardy position with end-of-life (EOL) care for their immigrant parents, because of both cultural and generational clashes. There is no existing empirical study about the millennial ABCs' attitudes or behaviors towards EOL caregiving. Our study is the first one to explore the millennial ABCs' anticipated EOL caregiving behaviors, support and resources needed, attitudes towards terminal illness disclosure and advance care planning (ACP) discussion with their parents, and how acculturation influences. A qualitative in-depth phone interview using a case study approach, with a scenario of caring for parents with Parkinson's disease and stage IV lung cancer, was adopted. Participants were recruited via convenience sampling, and a total of 27 (18 females and 9 males with an average age of 25) passed the screening and completed the interviews. Using the directed content analysis, researchers identified two themes: EOL caregiving and EOL decision making, which included five sub-themes: caregiving behaviors, needed supports and resources, care arrangement decision, terminal illness disclosure, and ACP. Both traditional Chinese culture of familism and filial piety, and western culture of autonomy and patients' rights to know were exhibited in every theme. Most participants did not fully understand ACP concept, but they were willing to initiate ACP conversation after comprehending ACP concept. This study constitutes an essential step towards understanding the millennial ABC EOL caregivers' financial, physical, and emotional needs from family, community and government, better establishing corresponding policies, and promoting public education in ACP to benefit this minority group.

### AWARENESS OF HOSPICE CARE AMONG RURAL RESIDENTS: FINDINGS FROM SOCIAL DETERMINANTS OF HEALTH FRAMEWORK

Yan Luo,[1] Hyunjin Noh,[2] Lewis Lee,[3] and Hee Lee,[4], *1. The University of Alabama, Tuscaloosa, Alabama, United States, 2. University of Alabama, Tuscaloosa, Alabama, United States, 3. Univeristy of Alabama, Tuscaloosa, Alabama, United States, 4. Univeristy of Alabama, Tuacaloosa, Alabama, United States*

Although the demand for hospice care increases as our society ages, the awareness of hospice care among adults in the southern rural region of the US has not been documented. This study aims to assess the rate of hospice care awareness among rural residents living in the Black Belt Region and examine social determinants of health (SDH) associated with the awareness. A cross-sectional survey was conducted among a convenient sample living in rural Alabama (N=182, age=18-91). Participants' awareness of hospice care, demographic characteristics (i.e., age, gender), and SDH (i.e., financial resources strain, food insecurity, education and health literacy, social isolation, and interpersonal safety) were assessed. Lastly, a binary logistic regression was used to examine the association between SDH and awareness of hospice care among participants while controlling for demographic characteristics. The majority of participants had heard of hospice care (82.4%), and older participants (over 50 years old) were more likely to report having heard of hospice care (OR=7.35, $p<0.05$). Participants reporting worries about stable housing (OR=0.05, $p<0.05$) and higher social isolation were less likely to have heard of hospice care (OR=0.53, $p<0.05$), while participants with higher health literacy had a higher likelihood to have heard of it (OR=2.60, $p<0.01$). Our study is the first study assessing the status of hospice care awareness among residents living in the Black Belt Region. This study highlighted that factors including age and certain SDH (i.e., housing status, health literacy, and social isolation) might be considered in the intervention to improve hospice care awareness.

### CAREGIVER TASK DIFFICULTIES AT THE END OF LIFE: A LATENT CLUSTER ANALYSIS

Kristin Litzelman,[1] Irene Kizza,[1] and Ashley Berghoff,[2], *1. University of Wisconsin-Madison, Madison, Wisconsin, United States, 2. University of Arkansas, Fayetteville, Arkansas, United States*

Caregivers engage in myriad tasks from household help to complex medical care. However, little information is available on how caregivers experience individual tasks – particularly key end-of-life tasks such as managing breathing problems or patients' sadness and anxiety. The purpose of this study was therefore to assess task difficulty. Using data from the National Health and Aging Trends Survey and the National Survey on Caregivers (2015-2017), we assessed eleven caregiving tasks in 241 primary caregivers of care recipients in their last month of life. A latent cluster analysis revealed three key clusters: 1) pervasive difficulties, in which caregivers reported difficulty across most or all of the tasks; 2) minimal difficulties; and 3) emotional management difficulties, in which caregivers reported difficulty with managing sadness and anxiety and lower levels of difficulty on the other tasks. Weighted frequency analyses revealed that caregivers in the pervasive difficulties cluster were most likely to be filial caregivers (85% versus 63% of the full sample, $p<0.05$) or co-residing with the care recipient (49% versus 37% of the full sample, $p<0.05$). Caregivers identified as having pervasive difficulties were also more likely to report providing intensive care, more than 100 hours per week (54% versus 36% of the full sample, $p<0.05$). Care recipient condition was not associated with cluster membership. The findings highlight the need to consider caregiver coping at the task-level and have implications for understanding unmet needs. Future research will assess predictors of cluster membership and how task difficulties are associated with symptoms and well-being outcomes.

### CHANGE IN MEDICAL AND HEALTH CARE DECISION-MAKING PATTERNS AT THE END-OF-LIFE: A COHORT OF THE VERY OLD PEOPLE

Xinran Liu, and Steven Albert, *University of Pittsburgh, Pittsburgh, Pennsylvania, United States*

How does medical and healthcare decision-making among the very old people change in their last year before death? We explored patterns of decision-making in the Health ABC cohort study in 2011-14 (years 15-17), which involved 12 waves of quarterly phone interviews. When the participant was unable to do the interview, a proxy completed it instead. We identified a sample of 291 decedents (aged 90.0±2.9 at death, 35.7% Black, 52.6% female) with at least 1-year follow-up before death. Percentages of decedents who have made medical or healthcare decisions in the last four quarters before death were 32.0%, 31.2%, 32.6%, 41.9%, respectively. Decedents made more healthcare decisions in the last quarter before death (P<0.01), compared to the baseline. Across all quarters, decision-making is most in need for medications (17.6%), hospital admission (13.2%), and ER/urgent care visit (13.2%). We matched a 1:1 sample of survivors at the time of death by race, sex, and age (within ±3 years). In random effects models with multiple imputations of missing data, we found that decedents were more likely to make healthcare decisions than survivors across all four quarters before death or censor (Odds ratio=1.99, 95%CI: 1.49-2.65, P<0.01). The significance still held after adjusting for age, female, race, education, and interview methods. Overall, compared to matched survivors, the frequency of making medical and healthcare decisions among the very old decedents has already been high in the last year before death. This frequency rose sharply in the last quarter before death.

### DEATH AND DYING: BOUNDARIES AND ROLES OF FAMILIES AND HEALTHCARE WORKERS DURING PATIENT END OF LIFE

Amber Thompson, and Rebecca Utz, *University of Utah, Salt Lake City, Utah, United States*

While death and dying often occur within or adjacent to the healthcare setting, grief & support of patients at end-of-life (EOL) remain largely within the realm of the family. Given this division of roles, healthcare workers intentionally set professional boundaries that balance their need to be empathetic and compassionate for patient and their families during EOL, while also maintaining a sense of objectivity and detachment which allows them to cope with patient loss and manage the competing demands of their workday. Tensions occur when healthcare workers are required to cross boundaries at EOL, either voluntarily or involuntarily. Using unobtrusive digital ethnography of a publicly accessible online forum for healthcare providers, this research investigates the boundaries set by families and healthcare workers at EOL, and how EOL circumstances sometimes require healthcare workers to cross or violate these professional boundaries. We suggest that the needs of the family at EOL (not necessarily the patient) serve as the catalyst for both boundary crossing & boundary violations for healthcare workers. Our data reveal that (1) boundary setting and training ought to address the patient-physician-family relationship (not just patient-physician), since the family members are such an integral part of EOL; (2) these EOL dynamics apply beyond the physician and should include all healthcare workers (nurses, etc.). As a result, patient & family centered care may not be fully achieved at EOL due to the ambiguity in the expected roles played by both families and healthcare workers during patient death and dying.

### EMBALMING, VIEWING AND THE SOCIAL CONSTRUCTION OF THE CORPSE: TIME FOR ANOTHER LOOK

Redmond Finney,[1] Lisa Shulman,[1] and Raya Kheirbek,[2], *1. University of Maryland School of Medicine, Baltimore, Maryland, United States, 2. University of Maryland School of Medicine, University of Maryland School of Medicine, Maryland, United States*

Embalming of the dead is more common in the United States than anywhere else in the world. Battles far from home during the Civil War with concern for contagion from dead bodies being shipped home, compelled President Lincoln to direct the troops to use embalming to allow the return of the Union dead to their homes. Viewings were common with war heroes and culminated with the viewing of Lincoln himself. In the 20th century embalming became a tradition despite substantial evidence indicating environmental and occupational hazards related to embalming fluids and carbon dioxide generated from manufacturing steel coffins before placing in concrete burial vaults. Embalming is promoted and considered helpful to the grieving process when families are comforted by a the appearance of a peaceful death. Embalmers are expected to produce an illusion of rest, an image that in some ways disguises death for the benefit of mourners. The dead are carefully displayed in a condition of liminal repose where the 'true' condition is hidden, and death is removed from the actual event. In this paper we highlight the spiritual and cultural complexities of embalming related- issues. We also provide data on the lack of grieving families' preparedness for the financial burden associated with the death of a loved one and the lack of knowledge of alternative options. We propose an innovative process to empower people facing serious illness, and their families to make shared and informed decisions, especially when death is the expected outcome.

### EMOTIONAL INTELLIGENCE SKILLS ENHANCE RESPONSIVE CARE OF HOSPITALIZED OLDER PATIENTS

Eva Kahana,[1] and Polina Ermoshkina,[2], *1. Case Western University, Case Western reserve University, Ohio, United States, 2. Case Western Reserve University, Cleveland, Ohio, United States*

Our previous research focused on the value of creative problem-solving in late life (Kahana, Kahana, & Ermoshkina, 2021). This paper considers the role of emotional intelligence in creative problem-solving for improving late-life medical care experiences. Emotional intelligence, which is situated at the intersection between the domains of intelligence and personality, can serve as a useful resource promoting personal and social adaptation to problems of old age. Our paper argues for the usefulness of emotional intelligence as older adults deal with challenges of the health care system. Emotional intelligence is rarely studied in gerontological research. Yet, it is required to overcome lack of attention to the patient experience and to provider–patient communication. Problems in communication with staff and in obtaining responsive care are prevalent for hospitalized older adults due to lack of continuity of care as unfamiliar "hospitalist" physicians typically offer care to frail elders. A key adaptive task of older patients involves securing investment by overworked health care providers. A friendly and extraverted self-presentation by older patients signals to the health care provider the social worth of the patient. Such demeanor can go a long way toward eliciting interest and caring responses from providers. Our paper offers a taxonomy of patient behaviors that can facilitate patient-centered care. Emotional intelligence shown by older patients reflected in expressions of appreciation for staff is likely to facilitate the interest of medical and nursing staff. Potential interventions to foster emotional intelligence among older patients will also be discussed.

### FACILATORS AND BARRIERS TO ADVANCE CARE PLANNING ENGAGEMENT AMONG CHRONIC KIDNEY PATIENTS IN TAIWAN

YuHsuan (Olivia) Wang,[1] and Susan Enguidanos,[2], *1. USC, Los Angeles, California, United States, 2. University of Southern California, Los Angeles, California, United States*

End-Stage Renal Disease (ESRD) patients experience high rates of cognitive impairment and lower quality of life at end of life. Having an advance care planning (ACP) conversation is associated with better care at end of life and improved care consistency with patient preferences. Taiwan ranks highest in ESRD and dialysis incidence worldwide, highlighting the importance of ACP among this population. Since 2019, the Patient Autonomy Act entitled Taiwanese to engage in ACP, but strategies to promote its uptake and the role of healthcare providers and caregivers in ACP conversations remains unclear. This study aimed to investigate facilitators and barriers to ACP engagment among Taiwanese with kidney disease and their caregivers. In 2020, we recruited 18 adults with stage 3—5 kidney disease and their caregivers. We conducted semi-structured interviews to elicit knowledges and perspectives on ACP. A grounded theory approach was used to guide the analysis of primary themes. Two researchers independently coded the transcripts and met to reconcile codes. The primary barrier to ACP was the misconception that it equated to funeral arrangements. Themes related to ACP facilitators included: trust in the nephrology care team (especially in case managers); straighforward communication; and in-person consultations. Additionally, transplant patients were more likely to have ACP discussions with their loved ones. While kidney disease patients preferred straightforward communication, caregivers preferred indirect communications. These findings emphasize the critical role of the nephrological care team members. Improving knowledge of ACP and facilitating communication between caregivers and kidney disease patients in Taiwan is needed.

### HOSPICE CARE FOR VETERANS: A COMMUNITY EDUCATION PROGRAM

Denise Kresevic, Barbara Heath, and muralidhar Pallaki, *VAMC, Cleveland VAMC, Ohio, United States*

The impact of trauma on care at end of life and the social isolation of the COVID-19 pandemic highlighted a critical gap in care of terminally ill veterans. It is estimated that 30% of Vietnam Veterans suffer from PTSD, suicide rates are 49% higher in older veterans than nonveterans, and 41% of veterans surveyed report post traumatic guilt. A survey of non-VA hospice agencies revealed many do not screen for PTSD, but desired training in this area. The VA responded to these challenges implementing an initiative to educate community caregivers on PTSD, suicide, and moral injury with expanded tele mental health services. Several VA sites were supported to participate in training focusing on PTSD, suicide, and moral injury and Telemental health. In Northeast Ohio, from 2019-20, 11 community hospice agencies participated in training, including 283 providers,120 (42%) nurses, 100 (35%) social workers, 29 (10%) volunteers, and 34 (12%) other. A majority of participants post-training (n=160) (84%-94%) reported enhanced knowledge, skills, or attitudes related to resources, education, and communication. Participants rated changes for assessment skills lowest for moral injury (34%), PTSD (41%), and suicide (56%). An analysis of telemental health visits (N=50) revealed that, 56% addressed spiritual support, (22%) family support, (10%) resources/referrals, and (8%) confusion. The majority of telehealth visits were VA initiated (84%), and 10% were hospice initiated Continued education regarding PTSD, suicide, and moral injury assessment skills is still needed for hospice care providers of veterans. These findings support the use of telemental health for care and consultation.

### INTENTION TO COMMUNICATE END-OF-LIFE WISHES AMONG RURAL AFRICAN AMERICANS: IS AWARENESS OF HOSPICE CARE IMPORTANT?

Yan Luo,[1] Lewis Lee,[2] Hyunjin Noh,[3] and Hee Lee,[4], *1. The University of Alabama, Tuscaloosa, Alabama, United States, 2. Univeristy of Alabama, Tuscaloosa, Alabama, United States, 3. University of Alabama, Tuscaloosa, Alabama, United States, 4. Univeristy of Alabama, Tuacaloosa, Alabama, United States*

The intention to communicate end-of-life wishes and its related factors among adults in the southern rural region of the US has not been studied. This study aims to: (1) assess the intention to communicate end-of-life wishes among rural residents living in the Black Belt Region; (2) controlling for demographics and social determinants of health (SDH), examine the relationship between awareness of hospice care and the intention to communicate end-of-life wishes. A convenient sample living in rural Alabama was collected to complete a cross-sectional survey (N=182, age=18-91). Univariate analyses were conducted to assess participants' intention to communicate end-of-life wishes, demographic characteristics, and SDH. Binary logistic regressions were used to examine the relationship between awareness of hospice care and the intention to communicate end-of-life wishes while controlling for demographics and SDH. The majority of participants were willing to communicate end-of-life wishes to their family (77.5%) or doctors (72.5%). Participants who were aware of hospice care were more likely to be willing to communicate end-of-life wishes to both their families (OR=10.08, $p<0.01$) and doctors (OR=7.20, $p<0.05$). Moreover, participants who were older were less likely to communicate end-of-life wishes to their doctors while participants with higher social isolation scores had lower intention to communicate end-of-life wishes to their families (OR=0.53, p<0.05). This is the first study assessing the intention of communicating end-of-life wishes among residents living in the Black Belt Region. This study demonstrated that awareness of hospice care is positively associated with the intention to communicate end-of-life wishes to both families and doctors.

### KNOWLEDGE OF HOSPICE CARE AMONG KOREAN AMERICAN IMMIGRANTS IN DEEP SOUTH

Eunyoung Choi,[1] Hee Lee,[2] Hyunjin Noh,[3] and Lewis Lee,[4], *1. University of Southern California, University of Southern California, California, United States, 2. Univeristy of Alabama, Tuacaloosa, Alabama, United States, 3. University of Alabama, Tuscaloosa, Alabama, United States, 4. Univeristy of Alabama, Tuscaloosa, Alabama, United States*

Despite the benefits of hospice care in end-of-life care, there is a dearth of research on the knowledge or perceptions of hospice care, particularly among immigrants. A handful number of existing studies with this population have mainly used qualitative research methods. The purpose of the current study was to investigate the knowledge about hospice care and identify its predictors. We used cross-sectional data from 256 Korean American immigrants living in Alabama (Mean age = 44.78, range 23–70, 50.4% female). The outcome variable was measured by whether the respondents had heard of hospice care. Independent variables included sociodemographic (age, gender, education, and income), health (functional limitation and chronic conditions), health care access (health literacy, health insurance, unmet medical needs due to the cost, and social isolation). Logistic regression analyses were performed. About 78% of the respondents reported that they had heard of hospice care. Older age (OR=1.05, 95% CI=1.01-1.09, p <.05), being female (OR=7.13, 95% CI=3.18-15.98, p <.001), and higher levels of education (OR=1.68, 95% CI=1.15-2.45) were significantly related to increased odds of knowledge about hospice care. There were no significant roles of health and health care access factors. Our findings suggest sociodemographic gradients present in immigrants' knowledge about hospice care, emphasizing the need for a targeted intervention to increase the hospice care knowledge.

### LENGTH OF STAY IN HOSPICE CARE ACROSS RACIAL/ETHNIC MINORITIES OVER 65 YEARS OF AGE: A DESCRIPTIVE ANALYSIS

Heshuo Yu,[1] and J. Scott Brown,[2], *1. Miami University in Oxford, Oxford, Ohio, United States, 2. Miami University, Oxford, Ohio, United States*

**Purpose:** This study aims to explore the relationship between race/ethnicity and length of stay in hospice care among adults over 65 years of age in the United States. This topic is understudied within a population-representative sample, particularly among non-White decedents.

**Methods:** Secondary analysis of data from the 2007 NHHCS (n=3,918). Race/ethnicity included Hispanics/Latinos, Non-Hispanic Whites, African Americans, and other races. Length of hospice stay was measured by the number of days that patients received hospice care from hospice agencies.

**Results:** The study found that African Americans have a longer length of stay in hospice agencies than Whites, even after controlling for all other factors in the model. Female gender, older age, and several diseases are covariates that significantly impact length of hospice stay.

**Discussion:** Compared to other races/ethnicities, the long length of stay in hospice among African Americans may negatively impact the quality of end-of-life care and quantity of skilled staff visits. Future research is recommended to further explore potential consequences of longer hospice stays, especially within African American communities. Studies with larger samples of minorities that integrate socioeconomic factors need to be done to better study the relationship between length of hospice stay and race/ethnicity.

### OLDER COUPLES' ADVANCE CARE PLANNING ENGAGEMENT PATTERNS AND ASSOCIATIONS WITH INDIVIDUAL AND SPOUSAL FACTORS

Hyo Jung Lee,[1] and Bon Kim,[2], *1. Nanyang Technological University, Singapore, Not Applicable, Singapore, 2. Jeju National University, Jeju-si, Cheju-do, Republic of Korea*

This study examines older couples' dyadic patterns of informal and formal advance care planning (ACP) and determines the associations of these patterns with their own and spousal characteristics. Using data from the 2014 and 2016 Health and Retirement Study, we performed a) latent class analysis to identify distinctive ACP engagement patterns and b) multinomial regression models to describe related characteristics of older couples (N = 1,545 couples). We identified four dyadic patterns of ACP engagement: a) high ACP engaging couple (45%); b) high engaging husband – low engaging wife (13%); c) high engaging wife – low engaging husband (11%); and d) low engaging couple (31%). Engagement in informal and formal ACP was associated with both individual and spousal factors: Older couples with advanced age or higher levels of education and wealth were more likely to engage in both informal and formal ACP, whereas only wife's high level of constrain or husband's greater number of depressive symptoms was associated with discordant ACP engagements. Couple-based approach to promote ACP merits older couples with limited resources or poorer psychological health in both or either spouse.

### PALLIATIVE CARE KNOWLEDGE AND PLANNING IN U.S. ADULTS

Amy Albright,[1] and Rebecca Allen,[2] *1. VA Maine HCS, Gorham, Maine, United States, 2. University of Alabama, Tuscaloosa, Alabama, United States*

Palliative care knowledge and health literacy are frequently underestimated in American adults; for example, as measured by the Newest Vital Sign (Weiss et al., 2005), 79.2% (n = 247) of participants within a Geriatrics Clinic sample displayed "adequate" functional health literacy, while 11.8% (n = 37) scored within the "possibly limited" range, and 9.0% (n = 28) scored within the "highly limited" range. There was additionally a significant association between health literacy and age (r = .15, p < .01) within this sample. The Palliative Care Knowledge Scale (PaCKS; Kozlov et al., 2018) was administered to participants, and higher scores indicated a greater knowledge of palliative care. This construct is particularly important to measure, as racial/ethnic disparities exist within this domain; for example, African Americans may have lower overall knowledge of palliative care services and advance care planning than non-Hispanic Whites (Noh et al., 2018). In the current study, knowledge of palliative care was measured using the PaCKS (Kozlov et al., 2018), and scores represented the widest possible range of 0 to 13 (M = 7.68, SD = 4.08). There was a significant correlation between age and PaCKS score (r = .12, p < .05), as palliative care knowledge increased with age. Females scored significantly higher (M = 8.29, SD = 3.91) than males (M = 6.81, SD = 4.18), t(309) = 3.18, p < .001. There was no main effect of race on palliative care knowledge, and post-hoc analysis using Tukey HSD did not demonstrate significant differences between groups.

### PATIENT PORTAL USE NEAR THE END-OF-LIFE

Jennifer Portz,[1] John David Powers,[2] David Bekelman,[1] Megan Baldwin,[3] Alejandra Casillas,[4] Jean Kutner,[1] and Elizabeth Bayliss,[3], *1. University of Colorado, Aurora, Colorado, United States, 2. Kaiser Permanente Colorado, Kaiser Permanente Colorado, Colorado, United States, 3. Kaiser Permanente Colorado, Denver, Colorado, United States, 4. UCLA David Geffen School of Medicine, Los Angeles, California, United States*

Use of patient portals, personal health information websites linked to electronic health records, in seriously ill populations is unknown, as is use by caregivers. We described portal use patterns among adults with serious illness nearing end-of-life and their caregivers within Kaiser Permanente Colorado. Inclusion criteria were: 1) seriously ill patients (defined by KP's "Care Group"), □18 years of age, who were registered for the portal, and died between 1/1/2016-6/30/2019; and 2) caregivers of these patients, □18 years of age, registered for a proxy account. Data included user characteristics and portal use metrics summarized monthly over the 12-month period prior to death. Models included an unadjusted linear trend of the days used by month using a generalized estimating equation Poisson model with a log link and an autoregressive correlation structure of order 1. We identified 6,517 seriously ill patients with portal registrations; 163 of these patients had proxy caregivers. Patient users were 77 years old, mostly frail and White, and caregivers were predominantly female. Average days of use among patients was 42.4 days and <1 day among their caregivers. Number of days used significantly increased by 0.7% per month from twelve months to one month prior to death (95% CI: 0.4%-1.0%; p-value <.0001) and peaked 3 months prior to the patient's death. Average use was high in comparison to previous portal research and suggests that as the patient approaches death portal use increases. Future research should explore how portals may serve as indicators for identifying and addressing end-of-life care needs.

### PREDICTORS OF ACP COMPETENCY AMONG CHAPLAINS BY SERVICE LINE

Soyeon Cho,[1] Jung Kwak,[2] Brian Hughes,[3] George Hands,[4] and Moon Lee,[5], *1. City University of New York/CityTech, Brooklyn, New York, United States, 2. The University of Texas at Austin, Austin, Texas, United States, 3. health Care Chaplaincy Network, health Care Chaplaincy Network, New York, United States, 4. HealthCare Chaplaincy Network, New York, New York, United States, 5. University of Texas, Austin, Texas, United States*

Healthcare chaplains have key roles in palliative care including facilitating advance care planning (ACP). However, little is known about chaplains' competency in ACP. We conducted an online survey with board-certified healthcare chaplains recruited from three major professional chaplains' organizations. We explored correlates of chaplains' competency in ACP facilitation among two groups of chaplains, general and special care (SC) chaplains (chaplains in oncology, intensive care, or palliative units) because SC chaplains are generally more involved in palliative care. The final sample included 481 chaplains with 89.8% reporting ACP as an important part of their work and 71.3% reporting to help patients complete advance directives. There was no significant difference in ACP competency between general chaplain group (n=240; M=39.61, SD=7.0) and SC chaplain group (n=241; M=40.65, SD=5.87). Hierarchical regression analyses revealed differences between the groups. General chaplains who practiced longer as a chaplain (b=1.02, p<.000), were more engaged in ACP facilitation (b=1.06, p<.05), had more positive attitude toward ACP (b=4.04, p<.000), and reported a higher level of participation in shared decision-making with other team members (b=.75, p<.000) were more competent in ACP facilitation. In the SC chaplain group, higher competency was associated with more positive attitude towards ACP (b=2.58, p <.05), and a higher level of participation in shared decision-making (b=1.05, p <.000). Overall, these findings suggest that healthcare chaplains, both general and special care, are competent and actively involved in ACP facilitation. Further systematic studies are warranted to examine the effects of chaplains facilitating ACP on patient and healthcare system outcomes.

### TILL DEATH DO US PART?: EXPLORING THE INFLUENCE OF DECEASED ROMANTIC PARTNERS ON THE LIVES OF OLDER WOMEN

Sara Hackett, *University of South Florida , Wesley Chapel, Florida, United States*

Recent research integrating the hierarchical mapping technique (HMT) and the continuing bonds framework has suggested that deceased individuals may be influential social convoy members. Building off this pilot work, the current qualitative descriptive study focused on how older women viewed the role of a longstanding deceased romantic partner in their current social network. Twenty women (Mean age = 78 years), recruited via social media and snowball sampling, participated in one 90-minute semi-structured interview. Each discussed their bereavement journey and completed a HMT diagram to comment on their social network and the presence or absence of the deceased within it. Nineteen participants described the deceased as being an active member of their convoy. Interestingly, 15 women placed them within the innermost circle of the diagram, separate from their other network members. Thematic analysis of interview transcripts expanded upon the HMT diagram exercise to reveal five major themes: "We're part of each other," "I think he supports me," "He would want me to be happy," "I just feel so grateful," and "I think about him every day but I don't talk about him every day". Perceptions that deceased romantic partners continue to play a key role in participants'

lives offers researchers and practitioners with a unique opportunity to examine how losses are experienced and carried into old age. Further, this study may assist with the development of interventions that help support bereaved individuals, specifically, interventions that focus on destigmatizing continuing bond expressions and provide assistance with communicating memorialization preferences.

### Session 9245 (Poster)

### ENVIRONMENT AND AGING, HOUSING

### AESTHETICS AND ENVIRONMENT: WHAT IS THE ROLE OF BEAUTY IN SUPPORTING AGEING WELL?

Holly Nelson-Becker,[1] and Eleanor van den Heuvel,[2], *1. Brunel University London, Uxbridge, England, United Kingdom, 2. Brunel University London, Brunel University London, England, United Kingdom*

Access to beauty is intrinsic to psychological, social, and spiritual health. Aesthetic sensibility includes awareness initiated in both mind and emotion accessed through nurturing environments (Caspari, Eriksson, & Naden, 2011). While individual tastes vary and aesthetic preferences are culturally conditioned, an appreciation of natural and constructed beauty is fundamental to human meaning-making, creativity, and innovation (Hillman 1998). Beauty is thus an instrumental tool that may support ageing well. We investigated the question of what aesthetics/beauty meant to older adults in England, how they experienced it, and whether experiencing beauty sustained them. Three focus groups were conducted with community dwelling participants aged between 60 and 93 (median age 75) for a total N of 14. Five themes emerged related to experience: an unexpected recognition; an evolving openness to experience; a universal perception available in micro and macro environments; a force that can alleviate depression; and a relational quality of some interactions. The value of beauty was identified through all groups: participants found it difficult to imagine a world with no beauty in it. They wondered if age made discernment capacity greater. Appreciation of beauty in unexpected places like a cracked pot led participants to identify happiness and wellbeing as outcomes of perception. This study suggests that beauty is essential for wellbeing and human flourishing and can emerge in unlikely ways. Implications are that professionals should assist older people to consider the role of beauty in life and develop interventions to consciously keep beauty awakened in normal and aesthetically-deprived environments.

### DECREASING BARRIERS TO CARE: VOICES OF RIDERS, DRIVERS, AND STAFF OF A RURAL TRANSPORTATION PROGRAM

Abby Schwartz,[1] Alice Richman,[2] Mallary Scott,[3] Haiyong Liu,[3] Weyling White,[4] and Caroline Doherty,[4], *1. East Carolina University, School of Social Work, Greenville, North Carolina, United States, 2. East Carolina University, East Carolina University, North Carolina, United States, 3. East Carolina University, Greenville, North Carolina, United States, 4. Roanoke Chowan Community Health Center, Ahoskie, North Carolina, United States*

Eastern North Carolina (eNC) is a rural, poor, and underserved region of the state with 1 in 5 adults living below the poverty level. Residents experience health disparities driven by limited access to healthcare and inequitable distribution of social determinants of health. Project TRIP (Transporting Residents with Innovative Practices) is a potential solution to barriers in accessing care in eNC. Results presented include the first phase of a multi-phase study evaluating and replicating TRIP's effectiveness. Data from qualitative interviews with TRIP riders, drivers, and staff (e.g., case managers) will be presented (n= 20). As a result of the COVID-19 pandemic, interviews were conducted by telephone with the goal of understanding both strengths and weaknesses of the transportation program from riders, drivers, and staff to gain a holistic understanding of TRIP. Of the riders interviewed, the majority (91%) were age 50 and over and African American. Themes that emerged from the data that highlighted strengths of the program included: improved health outcomes, no wait times for pick up or drop offs, cost free, and accommodating service. Themes related to areas of weaknesses or improvement included: needing more transportation vendors and a dedicated TRIP case manager and scheduling concerns. The presentation will conclude with considerations in translating the findings into a pilot and expansion of TRIP in another eNC county (study phases 2 & 3), and how the data can inform the development of transportation interventions in other states, with the goal of increasing access to healthcare for vulnerable rural populations.

### DISASTER PREPAREDNESS AMONG MIDDLE-AGED AND OLDER ADULTS: WHO IS THE LEAST PREPARED?

Caitlin Connelly,[1] Kathrin Boerner,[2] Natasha Bryant,[3] and Robyn Stone,[4], *1. University of Massachusetts Boston, Asheville, North Carolina, United States, 2. University of Massachusetts Boston, Boston, Massachusetts, United States, 3. LeadingAge LTSS Center @UMass Boston, Washington, District of Columbia, United States, 4. LeadingAge, Washington, District of Columbia, United States*

Adverse impacts of natural disasters are viewed as particularly concerning for older adults. Disaster preparedness is an important step towards offsetting potential harm. Research comparing different age groups with respect to their disaster preparedness has produced inconclusive evidence. Some studies found older adults more prepared than younger age groups, whereas others found them to be equally or less prepared. To shed light on this issue, we examined disaster preparedness among N = 16,409 adults age 40 and older from the American Housing Survey. Using logistic regression analyses, we compared preparedness levels of four groups – households of middle-aged adults (age 40-64), older adults (age 65-84), oldest old adults (age 85+), and mixed households comprised of both middle-aged and older adults. Findings showed that households of older adults and the oldest old had significantly higher preparedness levels compared to middle-aged and mixed households, accounting for demographics, living alone, and disability. However, the oldest old group appeared less prepared compared to the older adult group. Thus, while our findings suggest that older adults aged 65-84 may be better prepared for disasters than middle-aged adults, the oldest old group, who are likely at a higher risk of adverse impacts from natural disasters, may be less prepared than their relatively younger counterparts. Therefore, older adults should not be treated as a homogenous group when considering disaster preparedness. Rather, policies and interventions to improve disaster preparedness may benefit from focusing on specific high vulnerability groups.

### HIGHER CHILDHOOD ADVERSITY REDUCES ENVIRONMENTAL SATISFACTION AMONG OLDER ADULTS

Ramona Danielson, and Susan Ray-Degges, *North Dakota State University, Fargo, North Dakota, United States*

**Background:** Childhood adversity (CA) is associated with increased physiological and psychological challenges in adulthood. Normal aging comes with changes that may put adults with a history of CA at increased risk of a poor quality aging experience. Because suitable person-environment fit is an important component of well-being, the present study explored two hypotheses: 1) Higher CA is associated with lower perceived neighborhood quality, and 2) Higher CA is associated with higher perceived inequality in home.

**Methods:** Using MIDUS 2011-2014 Refresher Survey (for this study, N=1,017 adults ages 55-76), Spearman's rho correlation coefficients were run to explore the associations between a previously validated CA score and two environmental satisfaction indices. Perceived inequality in home comprised ratings on six items and perceived neighborhood quality comprised ratings on four items.

**Results:** Hypothesis 1: There was a significant positive association between the perceived inequality in home and cumulative CA, $r_s(998) = .200$, $p < .001$. Hypothesis 2: There was a significant negative association between the perceived quality of neighborhood index and cumulative CA, $r_s(998) = -.182$, $p < .001$. The strength of the associations were weak.

**Conclusions:** Higher cumulative experiences of CA were associated with increased feelings of home environment inequality, and decreased feelings of neighborhood quality. Significant differences were evident after decades had elapsed, demonstrating the long-lasting reach of CA. Further research exploring policy and practice approaches to mitigating the effects of CA is needed to allow aging adults to achieve better person-environment fit and equitable access to successful aging.

### MULTI-SECTOR COLLABORATION FOR IMPROVING HOME-BASED NUTRITION SUPPORTS: PROCESS FINDINGS FROM A CLINICAL TRIAL

David Wihry,[1] Jennifer Jain,[1] and Jennifer Crittenden,[2],
*1. University of Maine, Bangor, Maine, United States,*
*2. University of Maine, University of Maine, Maine, United States*

In-home supports and proper nutrition are critical to post-acute recovery and long-term health management for adults 60 and older. At the same time, such supports are often difficult to deploy in rural settings. To address these challenges, a unique multi-sector consortia was formed between a local Area Agency on Aging, a healthcare system, a health technology company, and a university to conduct a clinical trial of a novel in-home health technology program coupled with customized chronic care nutrition support. Early stage clinical trial development required coordination across health and community-based organizations to develop a pathway for older adults to access the in-home project supports. At the conclusion of the year one, six project partners were interviewed using a semi-structured interview protocol examining the strengths of early project design and challenges inherent in the early phases of a community-based clinical trial. Thematic analysis uncovered six themes instructive in formulating efficacious clinical trial methodologies: 1) Logistical challenges related to the pandemic, including reduced patient numbers and the curtailing of in-hospital recruitment; 2) Partner collaboration as essential to designing preferred project modifications; 3) The challenge of converting project referrals into project enrollees; 4) A new appreciation among community partners regarding institutional review board requirements; 5) Recommendations for addressing emerging staffing challenges; and 6) The overriding importance of engaging older adults in their own care and health promotion post-discharge. Results will inform construction of a replicable model for establishing novel research partnerships that span healthcare, social services, the business sector, and higher education.

### NATIONAL ESTIMATES OF CLIMATE-RELATED DISASTERS AMONG OLDER ADULTS WITH DEMENTIA

Katelyn Ferreira,[1] Katherine Ornstein,[1] Sue Anne Bell,[2] Mohammed Husain,[3] Cynthia Yee,[3] and Amy Kelley,[1],
*1. Icahn School of Medicine at Mount Sinai, New York, New York, United States, 2. University of Michigan, University of Michigan, Michigan, United States, 3. Icahn School of Medicine at Mount Sinai, Icahn School of Medicine at Mount Sinai, New York, United States*

Climate-related disasters can have devastating consequences, particularly for the growing population of older adults with dementia. Increasingly, older adults with dementia are aging in place at home, where they often receive assistance for mobility or self-care activities from family and/or paid caregivers. Understanding this population's experience with climate-related disasters is integral to mitigation, preparedness, and emergency response outside of institutional (e.g., nursing home) settings. We aimed to estimate the population of community-dwelling older adults—including those with dementia—who live in counties that experienced climate-related disasters. We used 2000-2016 data from the Health and Retirement Study (HRS), linked 2000-2018 with Federal Emergency Management Agency (FEMA) disaster data. We flagged each occurrence of climate-related disaster (e.g., hurricane, flood) and then identified HRS participants living in counties with a disaster declaration. Using survey weights, we obtained population-level estimates by disaster frequency and sample characteristics. In each two-year HRS period, we found that 14.26-20.50 million community-dwelling adults over age 65 in the contiguous US (32.2%-53.7%) resided in a county with at least one disaster. This includes 0.62 -1.12 million persons with dementia, 67.2%-76.8% of whom are reliant on caregivers. On average (per two-year period), more than one-third (36.3%) of community-dwelling older adults living with dementia lived in a county with a least one disaster. Policy makers and emergency planners should strongly consider the needs of older adults at the intersection of dementia and risk of climate-related disasters, with an eye towards ensuring access to mobility and self-care assistance.

### NEW SOLUTIONS FOR "OLD" PROBLEMS: IMPLICATIONS AND OPPORTUNITIES OF INTERGENERATIONAL HOMESHARING

Raza Mirza,[1] Jacalyn Tanner,[2] James Hull,[2] Taylor Hocking,[2] Anna Liu,[2] Jessica Hsieh,[1] and Christopher Klinger,[1], *1. University of Toronto, Toronto, Ontario, Canada, 2. National Initiative for the Care of the Elderly (NICE), Toronto, Ontario, Canada*

Across North America, many older adults have expressed their preference to live in their own homes and communities for as long as possible — and to 'age in place'. To address challenges faced by older adults living in the community, home-sharing - an exchange-based intergenerational housing approach, has empowered older adults to 'thrive in place' by providing additional income, companionship, and support with household tasks. In 2018, Toronto HomeShare was launched as an intergenerational home-sharing pilot program (n=22), matching older adults (55+) with postsecondary students intending to simultaneously address social isolation and the affordable housing crisis. In 2019, the pilot was adopted as a funded program in the City of Toronto with over 200 participants. Program results highlight unique benefits and challenges for older adults participating in home-sharing: (1) the capacity for intergenerational engagement to fulfill social needs, and (2) the importance of agency facilitation as a determinant of the experience for older adults. Survey findings indicate 88% of participants reported that participation in HomeShare positively impacted their general well-being, 88% reported improved financial security, 94% reported a delay in the need to move out of their community, and 72% felt that participation in HomeShare prevented the need for institutional care. These findings were used to transition Toronto HomeShare into a fully funded program as well as in the development of a national program. Beginning in January 2021 Toronto HomeShare transitioned to Canada HomeShare and will be scaling the program to Vancouver, Winnipeg, Halifax, Calgary, Montreal and other Canadian cities.

### OLDER ADULT BEHAVIORAL HEALTH ISSUES IN NEW ENGLAND: FINDINGS FROM THE HEALTHY AGING DATA REPORTS

Taylor Jansen,[1] Chae Man Lee,[1] Shu Xu,[2] Maki Karakida,[1] Frank Porell,[2] Nina Silverstein,[3] and Beth Dugan,[2], *1. UMass Boston, Boston, Massachusetts, United States, 2. University of Massachusetts Boston, University of Massachusetts Boston, Massachusetts, United States, 3. University of Massachusetts Boston, Boston, Massachusetts, United States*

Behavioral health issues in older adults are prevalent and have negative consequences on quality of life and overall health, yet are often untreated. The present study compares state and local community rates of behavioral health indicators of older adults age 60+ in Massachusetts (MA), New Hampshire (NH), Rhode Island (RI), and Connecticut (CT). For this study rates were calculated from the following data sources: Medicare Current Beneficiary Summary File 2014-2018 (2014-2015 MA, NH, and 2016-2017 RI, CT) and the Behavioral Risk Factor Surveillance System (2013-2015 MA, 2014-2016 NH, 2015-2017 RI, CT). Small area estimation techniques were used to calculate age-sex adjusted community rates for more than 170 health indicators (https://healthyagingdatareports.org/). This research examines disparities in rates across the 4 states for 4 behavioral health indicators: substance use disorder (SUD), tobacco use disorder (TUD), opioid use disorder (OUD), and excessive drinking. Results varied across states with RI reporting the highest rates of substance (7.0%) and tobacco use (10.8%) disorders, CT had the highest rate of opioid use disorder (2.2%), and MA and RI reporting the highest rates of excessive drinking (9.3%). Overall, MA had the greatest disparities in rates for all indicators (SUD: 6.6% (5.35-15.99%); TUD: 10.2% (2.67-24.20%); excessive drinking: 9.3% (5.63-19.98%)), indicating behavioral health disparities by community are most pronounced in MA. This study found behavioral health issues are prevalent among New England older adults and should no longer be overlooked. Furthermore, visualizing the community rates makes disparities evident and may guide resources and services to areas of highest need.

### POST-RECESSION HOUSING INSECURITY AND PHYSICAL AND MENTAL HEALTH OF MIDLIFE AND AGING ADULTS

Aarti Bhat,[1] David Almeida,[2] and Alexis Santos,[2], *1. Pennsylvania State University, State College, Pennsylvania, United States, 2. Pennsylvania State University, University Park, Pennsylvania, United States*

Housing insecurity, or limited/unreliable access to quality housing, is a powerful chronic stressor that can negatively affect individual health and well-being. This study extends prior research by examining the effect of multiple forms of housing insecurity on both the mental and physical health of aging adults using the Midlife in the United States study (MIDUS; N = 2532; M age = 63.42; 57% women; 16% black). Participants reported on experiences of anxiety/depression in the past year, number of chronic health conditions experienced in the last year, and experiences of housing insecurity since the 2008 recession (e.g., homelessness, threatened with foreclosure or eviction, missed rent or mortgage payment). 14% of participants reported experiencing one or more housing insecurity events in the aftermath of the recession. Higher levels of housing insecurity were experienced by midlife participants (ages 46-65) and black participants. Regression results showed that, even when controlling for prior health, housing insecurity was significantly associated with higher odds of experiencing anxiety/depression and additional chronic health conditions. These results suggest that housing insecurity experiences are fairly prevalent among midlife and aging adults, and that housing insecurity experiences leave these adults susceptible to compromised mental and physical health. This work has various implications for policy around addressing housing access and affordability issues for aging adults as a public health concern. Subsequent analyses will examine age, gender, and race/ethnic differences in these associations between housing insecurity and health outcomes.

### ROLE OF COMMUNITY CENTERS IN PROMOTING SUSTAINABLE REGIONAL LIFE OF COMMUNITY-DWELLING OLDER ADULTS WITH FRAILTY

Akiko Nishino,[1] Ryogo Ogino,[2] Takahiro Miura,[3] Ken-ichiro YABU,[4] Kanako TSUTSUMI,[5] Junichiro OKATA,[1] Kazuhiko Nishide,[1] and Tohru IFUKUBE,[1], *1. The University of Tokyo, bunkyo-ku, Tokyo, Japan, 2. Saga University, Saga, Saga, Japan, 3. National Institute of Advanced Industrial Science and Technology (AIST), Kashiwa, Chiba, Japan, 4. the University of Tokyo, the University of Tokyo, Tokyo, Japan, 5. Former The University of Tokyo, Bunkyo - Ｋｕ, Tokyo, Japan*

Japan's long-term care insurance system, which is a formal service, focuses only on older adults requiring care and support. Therefore, to create supportive communities for frail older adults, appropriate measures have been taken to establish community centers within their walking distance. However, the specific functions of these centers largely remain unknown. Accordingly, this study is aimed at clarifying the role of community centers by analyzing their services and management systems. In February 2020, we conducted a questionnaire survey (36% response rate) and four semi-structured interviews in O city, which has 36 community centers (81.45㎢, 36.4% elderly population). Results from the questionnaires revealed that the most frequent users of the community center were in their 70s (61.5%); such centers tended to provide informal services, such as exercises and cafes. Meanwhile, 57.2% of community centers collaborate with formal service providers. Community centers tend to be operated together with parent facilities, such as hospitals and nursing homes(61.2%). The results of the onsite survey showed that, in three cases, the community centers were situated within 200 meters of the parent facility. The findings show that these community centers facilitated creation of a supportive community that provides informal services to the frail elderly. Furthermore, they are operated in cooperation with formal service providers, hospitals, and nursing care facilities and are located in close proximity to one another. To summarize, the community centers continue to play a role in providing seamless services to the frail elderly even as their physical functions evolve.

### ROLE OF HOME-MODIFICATION TRAINING FOR CARE MANAGERS

Akiko Nishino, Yoritaka Harazono, Moeko Tanaka, Kazunori Yoshida, Toko Funaki, Ryosuke TAKADA, Takenori NASU, and Taketo TOBIMATSU, *The University of Tokyo, bunkyo-ku, Tokyo, Japan*

With the aging of society, the long-term care insurance system -which includes home modifications to continue living at home- was established in 2000. However, the quality of home modifications has been persistent issue, and effective training is expected to conclusively solve this problem. To this end, the purpose of this study is to clarify the rational for training care managers who plan home modifications. A survey comprising two sets of questionnaires was conducted; one set encompassed is all 62municipalities in Tokyo, whereas the other involved care manager who participated in training program. The results of the first questionnaire showed that, out of 62 municipalities, 9 (14.5%) provided training on home modification, of which 8 (88.9%) provided training on administrative procedures. In one municipality that provided training on practical aspects of home modification, we provide questionnaires to 59 care managers participating in the training. -Lectures on administrative procedures, physical conditions of invalids, and reading drawings were conducted by administrative staff, occupational therapists, and architects, respectively. Afterwards, the participants attended a planning workshop. According to the questionnaire conducted after the workshop, 80.4% of the participants could understand home modifications in the system, 85.5% understood how to modify homes based on the occupants' symptoms and physical conditions, 81.6% could interpret drawings, 90.2% could plan modifications, and 81.6% found the training useful. These findings indicate that the training of care managers has indeed been effective in actual practice. Improving the quality of home modifications through multidisciplinary cooperation is significant in maintaining home life.

### WEATHER-RELATED DISASTER IN A DIVERSE COHORT OF AGING ADULTS

Yvonne Michael,[1] Lauren Clay,[2] Kevin Smilely,[3] Rennie Joshi,[1] and Jana Hirsch,[4], *1. Drexel University, PHILADELPHIA, Pennsylvania, United States, 2. D'Youville College, Buffalo, New York, United States, 3. Louisiana State University, Baton Rouge, Louisiana, United States, 4. Drexel University, Philadelphia, Pennsylvania, United States*

As climate change contributes to increasing frequency and intensity of weather-related disasters, it is critical to define characteristics that increase risk of poor health outcomes during and after events. Given the aging of the United States (US) population and over-representation of older adults in disaster-prone areas, disaster-related impacts on older adults present a growing public health challenge. We linked data from the REGARDS study, a cohort of 30,107 Black and White adults (mean age 65 years at baseline, 2003-2007), with community data from the National Establishment Time Series database and longitudinal weather-related disaster data from the Spatial Hazard Events and Losses Database for the US. We calculated disaster exposure for each year for the county in which the respondents lived from 2003 – 2015: 84% of county-years showed at least some impacts, including 16% of counties experiencing medium impacts ($10- $50 property damage per capita or 2 fatalities) and 12% severe (greater than $50 per capital or 3 fatalities); this mirrors that of the continental US (77% some impact, 15% medium, 13% severe). REGARDS participants exposed to moderate or severe disasters were more likely to be Black and low socioeconomic status compared to those who were not exposed. For community characteristics, higher disaster exposure was associated with a greater density of resources including ambulatory care, food stores, social services, and destinations for daily living. Our approach showcases how disaster preparedness systems need better data about specific individual-and community-level factors that increase risk among older adults to better serve communities.

### Session 9250 (Poster)

### EXERCISE AND PHYSICAL ACTIVITY

### "ALL THEY DO IS WALK": SUCCESSFUL AGING AND SYMBOLIC BOUNDARIES AMONG A SELF-ORGANIZED MALL WALKERS CLUB

Jason Pagaduan, *University of Toronto, Scarborough, Ontario, Canada*

**Objectives:** This study examines how successful aging discourse manifests through physical and social participation among members of a self-organized mall walkers club. There is a paucity of research investigating successful aging in situ and theorizing the relationship between successful aging discourse and community participation. I draw on symbolic boundaries—a concept from cultural sociology—as a way to make sense of what mall walkers say and do.

**Methods:** I draw on data from 15 months of participant observations and interviews of mall walkers, all of whom are over 65 and predominantly Caribbean-Canadian women

**Results:** I identify three common boundaries: personal, interpersonal, and community, that mall walkers draw on to challenge narratives of decline and internalize dimensions of successful aging.

**Discussion:** These findings uncover the ways members in a self-organized community reinforce boundaries that highlight how certain dimensions of successful aging as something to be proud of and desirable. This article contributes to research on intersubjective experiences of aging by revealing how successful aging is rooted in community participation, rather than individual achievement.

### ACTIGRAPHY-BASED ASSOCIATIONS BETWEEN CHRONOTYPE AND PHYSICAL ACTIVITY IN OLDER ADULTS

Hilary Hicks, Genna Losinski, Alexandra Laffer, and Amber Watts, *University of Kansas, Lawrence, Kansas, United States*

Chronotype is a measure of the time of day people prefer to be most active or to sleep. There is a known relationship between chronotype and engagement in physical activity in young and middle-aged adults, such that individuals with a morning chronotype engage in more physical activity compared to those with an evening chronotype. Our study aimed to replicate this finding in an older adult sample. Actigraphy can be used to measure both physical activity and sleep. Because of its ability to capture information about bedtime and arise time, actigraphy can serve as an objective measurement of chronotype. Participants were 159 older adults (ages 60-89, M = 74.73) who wore an ActiGraph GT9X on their non-dominant wrist for 7 days in a free-living environment. Chronotype was measured continuously using the midpoint of the ActiGraph-calculated sleep interval. We used multiple regression to determine the relationship between physical activity and chronotype adjusting for sex, age, and body mass index. Results suggest that while these variables explain a significant amount of variance in physical activity, $R2 = 19.0\%$, $F (4, 152) = 8.921$, $p < .001$, there is no significant relationship between chronotype and total physical activity in our sample, ß= -.117, $p = .114$. These findings are inconsistent with what has been shown in younger samples and suggest that the relationship between chronotype and physical activity may change as one ages. Future research should consider whether particular physical activity intensities (vs. total activity) may have a relationship with chronotype in older adults.

### ADAPTING AN EVIDENCE-BASED PHYSICAL ACTIVITY PROGRAM FOR THE REJOIN TRIAL FOR OLDER BREAST CANCER SURVIVORS

Shirley Bluethmann,[1] Eileen Flores,[1] Meghan Grotte,[1] Jared Heitezenrater,[2] Cristina Truica,[3] and Kathryn Schmitz,[1], *1. Penn State College of Medicine, Hershey, Pennsylvania, United States, 2. Penn State College of Medecine, Penn State College of Medicine/ Hershey, Pennsylvania, United States, 3. Penn State College of Medicine, Penn State Cancer Institute, Pennsylvania, United States*

**Purpose:** Physical activity (PA) is a recommended part of breast cancer survivorship. PA promotes survival and mitigates symptoms in older breast cancer survivors (BCS), especially in reducing joint pain associated with adjuvant hormonal treatment. The purpose of this report is to describe adaptations to Fit & Strong!, an evidence-based curriculum, to meet the needs of older BCS.

**Methods:** First, we reviewed all educational materials with scientific experts, including specialists in breast and exercise oncology. Next, we conducted semi-structured phone interviews with 3 BCS for an in-depth review of educational materials for the trial. All interviews were recorded and transcribed. Constant comparative analysis was used to identify themes and specify required technical changes. Subsequently, we recruited 3 new BCS to pre-test adapted materials and exercise sessions, complete a follow-up interview to refine our final product and rate acceptability with older BCS.

**Results:** Overall, BCS found the materials and experience very acceptable (mean score of 9.5/10). Content changes included simplifying exercise instructions, prioritizing trial-specific content and updating photographs to be more age-appropriate. Due to COVID, the pre-test activity was conducted by Zoom and participants were given additional time and coaching to participate using this technology. BCS said they would prefer to exercise in person but reported the remote experience as very satisfactory.

**Conclusion:** Our multi-step adaptation process provided an acceptable intervention to meet the needs of older BCS. Lessons learned will be applied to the forthcoming clinical trial, which will also be conducted remotely to maximize safety and access.

### ARE MACHINE LEARNING MODELS USED TO REPRESENT ACCELEROMETRY DATA ROBUST TO AGE DIFFERENCES?

Mamoun Mardini,[1] Chen Bai,[1] Amal Wanigatunga,[2] Santiago Saldana,[3] Ramon Casanova,[3] and Manini Manini,[1], *1. University of Florida, Gainesville, Florida, United States, 2. Johns Hopkins Bloomberg School of Public Health, Baltimore, Maryland, United States, 3. Wake Forest University, Winston-Salem, North Carolina, United States*

Regular and sufficient amounts of physical activity (PA) are significant in increasing health benefits and mitigating health risks. Given the growing popularity of wrist-worn devices across all age groups, a rigorous evaluation for recognizing hallmark measures of physical activities and estimating energy expenditure is needed to compare their accuracy across the lifespan. The goal of the study was to build machine learning models to recognizing the hallmark measures of PA and estimating energy expenditure (EE), and to test the hypothesis that model performance varies across age-group: young [20-50 years], middle (50-70 years], and old (70-89 years]. Participants (n = 253, 62% women, aged 20-89 years old) performed a battery of 33 daily activities in a standardized laboratory setting while wearing a portable metabolic unit to measure EE that was used to gauge metabolic intensity. Participants also wore a Tri-axial accelerometer on the right wrist. Results from random forests algorithm were quite accurate at recognizing PA type; the F1-Score range across age groups was: sedentary [0.955 – 0.973], locomotion [0.942 – 0.964], and lifestyle [0.913 – 0.949]. Recognizing PA intensity resulted in lower performance; the F1-Score range across age groups was: sedentary [0.919 – 0.947], light [0.813 – 0.828], and moderate [0.846–0.875]. The root mean square error range was [0.835–1.009] for the estimation of EE. The F1-Score range for recognizing individual PAs was [0.263–0.784]. In conclusion, machine learning models used to represent accelerometry data are robust to age differences and a generalizable approach might be sufficient to utilize in accelerometer-based wearables.

### BARRIERS AND FACILITATORS IMPACTING PHYSICAL ACTIVITY AMONG RURAL AMERICAN INDIAN OLDER ADULTS

Maja Pedersen,[1] Kari Harris,[2] Jordan Lewis,[3] Mattea Grant,[2] Chelsea Kleinmeyer,[4] Ashley Glass,[4] Blakely Brown,[2] and Diane King,[5], *1. Stanford University, Stanford, California, United States, 2. University of Montana, Missoula, Montana, United States, 3. Memory Keepers Medical Discovery Team/University of Minnesota Medical School, Duluth campus, Duluth, Minnesota, United States, 4. Confederated Salish and Kootenai Tribal Health Department, St Ignatius, Montana, United States, 5. University of Alaska Anchorage, Anchorage, Alaska, United States*

**Background:** American Indian (AI) older adults experience pronounced health disparities and demonstrate among the lowest levels of physical activity (PA) of racial and ethnic groups. Nearly half of AI older adults live in rural areas, indicating distinct challenges to participation in PA. Research to identify factors influencing PA among this population is missing from the literature, yet is critical to inform culturally relevant PA intervention development and implementation.

**Purpose:** To identify barriers and facilitators to PA among rural AI older adults using the ecological model and qualitative methods.

**Methods:** A community-based approach was used to conduct semi-structured interviews with rural AI older adults. Interview questions were based on a multi-level ecological model. Content analysis was performed, using an iterative coding process to identify findings.

**Results:** Participants' (n=21) mean age was 66 years. Barriers and facilitators to PA were identified across ecological model levels. Barriers included factors such as caregiving and community responsibilities, lack of acceptable areas for walking, and overall lack of community-level support for older adult health. Facilitators included a personal connection to the land and ancestors through PA, multigenerational participation, and supportive tribal policies.

**Conclusion:** This study addressed a critical gap in the literature by identifying barriers and facilitators among rural AI older adults, which can inform PA intervention development. In this way, their voices are uplifted to shape efforts addressing longstanding health disparities through relevant public health interventions.

### CARDIO-DANCE EXERCISE TO IMPROVE COGNITION AND MOOD IN OLDER AFRICAN AMERICANS: A PROPENSITY-MATCHED COHORT STUDY

Bernadette Fausto, *Rutgers University-Newark, Newark, New Jersey, United States*

The current study sought to determine the influence of initial sleep quality and body mass index on the cognitive and mood outcomes of a community-based cardio-dance exercise program. Thirty-two older African Americans who participated in a five-month cardio-dance exercise program were propensity-matched to 32 no-contact controls (ages 60 to 88). Participants completed neuropsychological tests of attention, executive function, and memory and a self-reported depression measure at baseline and post-test. Among exercise participants, we observed significant improvements in depression ($\eta p2$=.12, p=.009) and attention ($\eta p2$=.12, p=.009) relative to controls. Improvements in executive function and attention were most pronounced among exercise participants with poor sleep quality ($\eta p2$=.41, p=.04) and with obesity ($\eta p2$ = .30, p=.001), respectively. This study provides novel evidence that cardio-dance exercise has the potential to improve depression in older African Americans. For those with poor sleep quality or obesity, exercise can also improve some cognitive outcomes.

### COGNITION AND SUBJECTIVE AGE PREDICT PHYSICAL ACTIVITY ENGAGEMENT: A LONGITUDINAL STUDY OF IMPAIRED OLDER ADULTS

Claire Growney,[1] Xianghe Zhu,[2] and Shevaun Neupert,[3], *1. Washington University in St. Louis, Saint Louis, Missouri, United States, 2. North Carolina State University, North Carolina State University, North Carolina, United States, 3. North Carolina State University, Raleigh, North Carolina, United States*

Physical activity is an important factor in preventing or slowing cognitive decline. However, the predictors of fluctuations in physical activity in a population that is already experiencing cognitive impairment is not well understood. Subjective age, such as how old one feels, has been tied to many health indicators in cognitively intact populations. Thus, we focused on the within-person associations between subjective age and physical activity as they unfold over time within a sample of cognitively impaired participants. The current study examined 400 reports from measurement burst data consisting of 5 weekly surveys conducted twice across 6 months from 68 cognitively impaired participants (M age = 70.14 (6.63), range = 60-92). Participants completed a battery of cognitive tests at baseline. At each weekly assessment, participants reported on their physical activity (e.g., exercise, outdoor, flexibility, and strength activities; Yes/No) and subjective age with respect to how old they feel overall and how old they feel mentally. There were longitudinal decreases in physical activity across the bursts, but on occasions when participants felt younger overall or younger mentally there were increases in physical activity. In addition, the effects of mental subjective age depended on cognitive ability, with those scoring lower in cognitive ability appearing to benefit the most from decreases (feeling younger) in mental subjective age. These findings suggest that perceptions of aging, especially within the domain of mental age, are tied to physical activity engagement for older adults with cognitive impairment.

### FACTORS ASSOCIATED WITH LATENT CLASSES OF EXERCISE MOTIVATION AMONG PHYSICALLY ACTIVE OLDER ADULTS

Choi Bomi,[1] Susanna Joo,[2] Changmin Lee,[1] Kwang Joon Kim,[3] DaeEun Kim,[1] YoonMyung Kim,[1] and Hey Jung Jun,[1], *1. Yonsei University, Seoul, Seoul-t'ukpyolsi, Republic of Korea, 2. Yonsei University, Seodaemun-gu, Seoul-t'ukpyolsi, Republic of Korea, 3. Severance Hospital, Yonsei University College of Medicine, Seoul, Seoul-t'ukpyolsi, Republic of Korea*

The objective of this study was to estimate the latent classes of exercise motivation and to find relevant factors in older adults. The sample comprises 179 people who reported practicing physical exercise regularly. We performed Latent Class Analysis (LCA) and multinomial logistic regression. Exercise motivation was observed with six indicators: medical advice, fun, weight loss, leisure, fitness, and socializing. Independent variables of regression analysis included sociodemographic characteristics (age, gender, marital status, education, and household income), health and well-being (subjective health, and life satisfaction), and satisfaction on neighborhood environment (physical, service, and sociocultural aspects respectively). Results of LCA indicated that the three-class model yielded optimal fit indices. Class 1 (7.5%) was labeled as 'mainly for medical advice and socializing'. Class 2 (46.5%) was labeled as 'mainly for fun', while class 3 (46.0%) was labeled as 'only fitness'. Results of multinomial logistic regression showed that males, people with lower education, and higher satisfaction with their sociocultural neighborhood were more likely to be categorized as 'mainly for fun' group compared to the reference group ('only for fitness'). Subjective health was marginally significant ($p<.10$): People with positive subjective health tend to be categorized as 'mainly for fun' than 'only for fitness' group. Satisfaction with their sociocultural neighborhood was marginally significant ($p<.10$) in distinguishing 'only for fitness' and 'mainly for medical advice and socializing' group. The results of this study emphasized the heterogeneity in exercise motivation. Significant factors of exercise motivation in this study implied the importance of individualized interventions to promote exercise participation.

### GET MOVING! YOUR BRAIN WILL THANK YOU.

Christina Nunez, Alexandria Nuccio, and Charles Golden, *Nova Southeastern University, Fort Lauderdale, Florida, United States*

Exercise and cardiovascular fitness are important for physical health and well-being. Recent studies show that exercise is associated with cognitive performance across multiple domains including memory, a common complaint for older adults. Data included a ten-word list of delayed recall, a clock drawing activity, and a three-meter walking course derived from the National Health & Aging Trends Study Database (NHATS Round 9). A total of 4977 participants were included in the analysis which was predominantly white (69.7%), non-Hispanic (94.5%), female (59.2%), and between the ages of 70-84 (62.7%). A hierarchical linear regression revealed that performance on the three-meter walking course positively predicted performance on delayed recall, $F(4,3999)=300.257$, $p<.001$, and on the clock drawing activity, which is a common screening task for cognitive decline, $F(4,3978)=156.433$, $p<.001$; accounting for 23.1% and 13.6% of the variability, respectively, over and above known demographic variables. Findings suggest that fitness may be one of many factors that is associated with memory and overall cognitive decline. These findings are timely as many individuals slowed down as a result of the COVID-19 pandemic, resulting in decreases in exercise and physical activity. Not being physically active or exercising may be related to poorer physical and cognitive health, with specific concerns regarding memory. Taking into consideration the fear and anxiety associated with declining memory in late life, it is crucial to explore this area further along with other factors that may contribute to the association and develop new ways for older adults to exercise safely during the COVID-19 pandemic.

### GET UP AND GO! AN EVALUATION OF FITNESS AND MEMORY.

Christina Nunez, Alexandria Nuccio, Sophia Perez, and Charles Golden, *Nova Southeastern University, Fort Lauderdale, Florida, United States*

As we age, exercise is increasingly important for physical health and well-being. Recent studies have shown that exercise is associated with cognitive performance across multiple domains, specifically memory, a common complaint for older adults. Data included a ten-word list of delayed recall, a clock drawing activity, and a sit-to-stand task (i.e., a low impact sub-maximal test of functional fitness) derived from the National Health & Aging Trends Study Database (NHATS Round 9). A total of 4977 participants were included in the analysis which was predominantly white (69.7%), non-Hispanic (94.5%), female (59.2%), and between the ages of 70-84 (62.7%). A hierarchical linear regression revealed that performance on the sit-to-stand task positively predicted performance on delayed recall, $F(4,3914)=245.141$, $p<.001$, and on the clock drawing activity, a common screening task for cognitive decline, $F(4,2893)=115.470$, $p<.001$; accounting for 20.1% and 10.6% of the variability, respectively, over and above known demographic variables. These findings indicate that exercise may be one of many factors that is associated with memory and cognitive decline. Given the continuation of quarantine procedures, these findings come at a time of significant clinical relevance. Research shows that many individuals slowed down because of the COVID-19 pandemic, and current findings suggest that not being physically active may be related to poorer physical and cognitive health, with specific concerns surrounding memory. Future research is essential in this area to tease out of other factor that may be contributing to this relationship and to develop new and innovated modalities for older adults to safely exercise.

### IS A LOW INTENSITY WALKING INTERVENTION SUCCESSFUL IN REDUCING DEPRESSION SYMPTOMS IN MIDDLE TO OLDER AGE WOMEN?

jingchuan wu, *Pennsylvania State University, State College, Pennsylvania, United States*

Depression is a common syndrome that has a strong negative impact on people's quality of life. Women, especially in middle age, are facing a greater risk for depressive disorders. Increasing one's physical activity level has shown great effects on reducing depression symptoms. The study evaluated the effect of a 12-week goal-directed walking intervention on walking engagement and depression symptoms among middle-aged women. A total of 55 middle to older age female participants (age range: 50–74) were included in the analysis. The 12-week intervention instructed participants to increase 15% of baseline steps every other week. Depression levels were measured pre-and post-intervention by the Center for Epidemiologic Studies Depression Revised Scale (CESD-R). A baseline measure-time structure- was considered as a possible mediator of association between walking and depression. Although no intervention effects were found in steps promotion and depression reduction in this walking program, we found that more depressed or younger participants had lower walking consistency It suggested that people with higher depression level or younger age require more instruction over time when engaging in physical activity interventions. Additionally, adherence of participants showed a over-time decreasing trend in this self-paced walking program. This inspired us to give timelier adjusts goals and exercise performance feedback to bolster participants' adherence in the future.

### PHYSICAL ACTIVITY AND AGING: EXPLORING MOTIVATIONS OF MASTERS SWIMMERS

Susan Brilliant,[1] Maria Claver,[2] Patti LaPlace,[3] and Cynthia Schlesinger,[3], *1. California State University, Long Beach, Redondo Beach, California, United States, 2. California State University, Long Beach, Whittier, California, United States, 3. California State University, Long Beach, Long Beach, California, United States*

Physical activity has many benefits for the optimal health of older adults (e.g., lowering risk of cardiovascular disease, depression, dementia and falls). However, less than one in four Americans meets guidelines for physical activity and evidence suggests inactivity increases with age. A growing number of unhealthy older adults will strain resources. Masters athletes are one segment of the population that some promote as exemplary because of their remarkable physical functioning. Swimming is a global, fast-growing sport in Masters athletics. This qualitative study explored the motivations and barriers of older adult (60+ years of age) members of a Masters swim club in Southern California. The researcher used purposive sampling to diversify the sample by selecting male and female participants (N=18) from multiple geographical areas. Guided by a primary research question: Why do Masters swimmers choose swim late in life, when most of their age cohort are becoming less physically active, the researcher conducted semi-structured interviews to delve into the lived experiences of the participants, solicited peer feedback and employed Interpretive Phenomenological Analysis. Four main themes emerged: Masters swimming provided an enjoyable way to slow physical decline; participants felt camaraderie; working out with a group provided challenge and achievement that reinforced their identity as swimmers; and their practice greatly reduced stress. A formidable barrier was revealed when most of the participants expressed longing and regret that they could not swim due to COVID risk and safety guidelines. These findings can be used to design community programs to promote lifelong physical activity participation.

### PILOTING THE EFFECTIVENESS OF A TEXT MESSAGING AND FITNESS TRACKING INTERVENTION WITHIN OLDER BLACK WOMEN

Olivio Clay,[1] Veronica Mixon,[2] William Opoku-Agyeman,[3] Meneka Johnson Nicholson,[4] and Pamela Bowen,[5], *1. University of Alabama at Birmingham, Birmingham, Alabama, United States, 2. UAB, Birmingham, Alabama, United States, 3. Healthcare Management, University of North Carolina at Wilmington, North Carolina, United States, 4. Rural Health Medical Program, Inc., Selma, Alabama, United States, 5. The University of Alabama at Birmingham, Birmingham, Alabama, United States*

Physical activity (PA) can help lower risk of obesity and type 2 diabetes, reduce anxiety, and reduce risk of Alzheimer's and other related dementias. Despite these benefits, older, obese Black women are not meeting CDC recommended PA guidelines at disproportionate rates. This study aims to identify whether a targeted intervention, Texting Older Sisters to Step (T.O.S.S.), can improve health-related outcomes within older Black women. A sample of 24 Black women (12 per group) age 60 and older who had a BMI > 30 were recruited. The treatment group received text messages previously validated to promote physical activity every day for 12-weeks and were placed in Fitbit communities. The control group received a general health or nutrition-related text message every Sunday. Participants ranged from 60 to 70 years of age with a mean of 64 and 90% had at least some college education. Overall, there was a significant reduction of 1.53 inches in waist circumference, $p < .01$. When the groups were compared, the treatment group showed a 2.16 inch reduction compared to a 0.91 inch reduction in the control group (Cohen's d=0.54, a medium effect size). Similarly, the treatment group lost 2.50 pounds on average compared to 1.33 in the control group (d=0.23). When the groups were compared on HgA1c, the treatment group was stable with a reduction of 0.01 unit whereas the control group reduction was 0.15 unit (d=0.23). Findings provide initial support for the T.O.S.S. intervention and suggest a modification of including nutrition information among the intervention messages.

### SILVER SNEAKERS IN CENTRAL PA: ASSESSMENT OF A COMMUNITY BASED EXERCISE PROGRAM IN A MIXED RURAL/URBAN CATCHMENT AREA

Eileen Flores,[1] Sage Nakagawa,[2] Robinn Moyer,[1] and Shirley Bluethmann,[1], *1. Penn State College of Medicine, Hershey, Pennsylvania, United States, 2. Queen's University, Ontario, Ontario, Canada*

Older cancer survivors present with unique challenges that may impact quality of life and increase physical dysfunction if not properly managed. Regular physical activity (PA) can help mitigate these effects. Silver Sneakers (SS), a free exercise program available to Medicare beneficiaries, has more than 16,000 US locations. To understand capacity of SS to serve older adults in our mixed rural/urban catchment area of Central Pennsylvania, we 1) identified all registered SS program locations in our 28-county catchment area and; 2) conducted phone questionnaires with SS program staff. Approximately 18 gyms closed during the pandemic, leaving a sample of 121 participating gyms. We talked to 80 gyms (66% response rate) to understand member and programming characteristics, training of staff and program marketing. Geographic locations of SS were mixed – 39% in rural and 61% in urban counties; the majority (43%) were located in private gyms or YMCAs. The majority of gyms reported membership was equally mixed by gender and described ages of members as 65-80 years (94%). Program staff said that many members exercised several times per week with friends/family. Program staff also reported that social opportunities (35%) were a primary reason participants remained active in SS. Most (89%) of the facilities were still able to offer SS during the pandemic, with the majority (60%) adapting format to Zoom and other video platforms to conduct classes. Overall, SS programs offer a sustainable option to facilitate access to exercise programs and reduce barriers to PA among older adults in our catchment area.

### THE ASSOCIATION BETWEEN LEISURE AND PHYSICAL ACTIVITY LEVEL WITH DEPRESSIVE SYMPTOMS AFTER 5-YEARS OF FOLLOW-UP

Milan Chang,[1] Hrafnhildur Eymundsdottir,[2] Alfons Ramel,[3] Sigurveig Sigurdardottir,[3] Vilmundur Gudnasson,[3] Lenore Launer,[4] and Palmi Jonsson,[5], *1. The Icelandic Gerontological Research Institute, Reykjavik, Hofuoborgarsvaoio, Iceland, 2. Landspitali University Hospitali, Landspitali University Hospitali, Hofuoborgarsvaoio, Iceland, 3. University of Iceland, Reykjavik, Hofuoborgarsvaoio, Iceland, 4. National Institute on Aging, Bethesda, Maryland, United States, 5. University hospital Landspitali, Reykjavik, Hofuoborgarsvaoio, Iceland*

**Background:** Depressive symptoms in older adults are associated with socioeconomic status (SES), medical care, and physical activity. However, there is little evidence on the longitudinal association between level of leisure activity (LA) and physical activity (PA) with depressive symptoms among community-dwelling older adults in Iceland. The study examined an association of LA and PA at baseline with high depressive symptoms (HGDS) assessed after 5 years of follow-up among community-dwelling older adults.

**Methods:** A large community-based population residing in Reykjavik, Iceland participated in a longitudinal study with 5 years of follow-up (n=2957, 58% women, 74.9±4.8 yrs). Those with HGDS or dementia at baseline were excluded from the analysis. The reported activity was categorized into 2 groups as no-activity versus any-activity. Depressive symptoms were assessed by the 15-item Geriatric Depression Scale (GDS) on average 5 years later.

**Results:** After adjusting for demographic and health-related risk factors, those who reported having any LA had significantly fewer HGDS after the follow-up of 5 years (6 or higher GDS scores, Odds Ratio (OR) = 0.46, 95% Confidence Interval (CI): 0.27 ~ 0.76, P = 0.003). However, reporting any PA at baseline was not significantly associated with HGDS (OR = 0.71, 95% CI: 0.51 ~ 1.00, P = 0.053).

**Conclusion:** Our study shows that any LA among older adults is associated with having less depressive symptoms 5 years later among community-dwelling older adults while having any PA was not associated with depressive symptoms after 5 years of follow-up.

### THE EFFECT OF LOW-INTENSITY DAILY WALKING ACTIVITY ON COGNITIVE AND BRAIN FUNCTION IN OLDER ADULTS

Dana Eldreth,[1] Vijay Varma,[2] Yi-Fang Chuang,[3] and Michelle Carlson,[4], *1. Johns Hopkins Bloomberg School of Public Health, Baltimore, Maryland, United States, 2. National Institute on Aging, Baltimore, Maryland, United States, 3. National Yang Ming Chiao Tung University, Taipei, Taipei, Taiwan (Republic of China), 4. Johns Hopkins University, Baltimore, Maryland, United States*

Physical activity is an effective intervention to prevent or delay cognitive decline and dementia in older adults; however, many have difficulty achieving recommended moderate- to vigorous-intensity guidelines. This study examined the impact of low-intensity daily walking activity on executive cognitive and brain function in 66 older adults (mean age=67.26 ; SD=6.04). Daily walking activity was measured using a step activity monitor and brain function was assessed using functional magnetic resonance imaging during the Flanker task. Analyses included whole and region of interest (ROI) in the right middle frontal gyrus (RMFG), occipital cortex (OCC) and anterior cingulate (ACC). Partial correlations were performed between step activity, behavioral performance, and ROI activation, adjusting for age and education. Most of the step activity was in the low-intensity range. No associations were observed between step activity and task performance ($p>.05$). Task-related activation occurred in the RMFG, lateral OCC and paracingulate ($p<.01$). Increased activation in the RMFG was associated with greater amount $r(62)=.390$, $p=.001$, duration $r(62)=.309$, $p=.013$ and frequency $r(62)=.327$, $p=.007$ of step activity. Stratification by sex revealed a positive association between amount of step activity and RMFG activation in women $r(44)= .360$, $p=.014$, but not men. Whole brain correlation revealed that amount of step activity was positively associated with precuneus activation ($p<.01$), an area impacted early in Alzheimer's disease. These results support the benefits of low intensity daily walking activity on prefrontal function in older adults and suggest the importance of designing attainable and sustainable physical activity interventions to promote brain health in older adults.

### THE EFFECTS OF EXERCISE ON COGNITIVE FUNCTION IN OLDER ADULTS WITH DIFFERENT TYPES OF DEMENTIA: A META-ANALYSIS

Guilherme Balbim,[1] Teresa Liu-Ambrose,[1] Ryan Falck,[1] Cindy Barha,[1] Jennifer Davis,[2] Samantha Starkey,[1] and Alexis Bullock,[3], *1. University of British Columbia,*

*Vancouver, British Columbia, Canada, 2. University of British Columbia-Okanagan, Kelowna, British Columbia, Canada, 3. the University of British Columbia, Vancouver, British Columbia, Canada*

Combating dementia is a public health priority, and exercise training is one promising strategy for dementia prevention. However, its efficacy in promoting cognitive outcomes in different types of dementia remains unknown. We conducted a systematic review (N = 27) and meta-analysis (N = 24) of randomized controlled trials with cognitive function as a primary or secondary outcome. We aimed to assess the effect of exercise interventions on the cognitive function of older adults (>60 years) diagnosed with different types of dementia. We synthesized data from 2,441 older adults with dementia. Eleven trials included older adults with multiple types of dementia, eight with Alzheimer's disease, six with unspecified types of dementia, and two with vascular cognitive impairment. We performed random-effects models using robust variance estimation (RVE) and tested potential moderators using the approximate Hotelling-Zhang test (HTZ). Results suggest a small effect of exercise on cognitive function for all-cause dementia (g = 0.18; 95% CI: 0.04, 0.33; p = 0.016); however, the effects did not differ by type of dementia. Moderation analyses showed that trials that did not specify participants' severity of dementia, applied individual-level randomization, and had higher intervention adherence demonstrated larger exercise effects on cognitive function for all-cause dementia. We conclude that exercise promotes small improvements in the cognitive function of older adults with all-cause dementia. More research including different types of dementia is needed if we hope to determine the precise effects of exercise for each type of dementia.

### THE INVENTORY OF PHYSICAL ACTIVITY BARRIERS FOR ADULTS 50 YEARS AND OLDER: REFINEMENT AND VALIDATION

Mariana Wingood,[1] Salene Jones,[2] Nancy Gell,[3] Denise Peters,[3] and Jennifer Brach,[4], *1. University of Vermont, University of Vermont, Vermont, United States, 2. Fred Hutchinson Cancer Research Center, Seattle, Washington, United States, 3. University of Vermont, Burlington, Vermont, United States, 4. University of pittsburgh, Pittsburgh, Pennsylvania, United States*

Addressing physical activity (PA) barriers is an essential component of increasing PA among the 56-73% of community-dwelling adults 50 years and older who are not performing the recommended 150 minutes of moderate-to-vigorous PA. As there is no feasible, multi-factorial tool to assess PA barriers among this population, we developed and validated a PA barrier assessment tool called the Inventory of Physical Activity Barriers (IPAB). We collected cross-sectional data on 503 adults (mean age 70.1), with 79 participants completing the scale twice for test-retest reliability and 64 completing a cross-over design examining the ability to use two administration formats interchangeably. Our analyses consisted of exploratory and confirmatory factor analysis, Cronbach alpha, intraclass correlation coefficient, Bland-Altman Plot, and t-tests. Using factor analysis, we identified and confirmed an eight-factor solution consisting of 27 items. The 27-item IPAB is internally consistent (alpha= 0.91), has a high test-retest reliability (intraclass correlation coefficient=0.99), and can differentiate between individuals who meet the recommended levels of PA and those who do not (p < 0.001). The IPAB scores ranged between 1.00-3.11 for the paper format (mean=1.78) and 1.07-3.48 for the electronic format (mean=1.78), with no statistical difference between the paper and electronic administration formats (p=0.94), resulting in the conclusion that the two administration formats can be used interchangeably. Participant feedback illustrates that the IPAB is easy to use, has clear instruction, and is an appropriate length. The newly validated IPAB scale can be used to develop individualized PA interventions that address PA barriers among patients 50 years and older.

### Session 9255 (Poster)

### FALLS: SCREENING, RISKS, AND PREVALENCE

### CAPTURING CHANGE IN BALANCE CONFIDENCE OVER 30 DAYS: INSIGHTS GAINED FROM A MICRO-LONGITUDINAL STUDY

Tai-Te Su,[1] Aileen Griffin,[2] Faith-Christina Washington,[1] Jacob Sosnoff,[3] and Shannon Meija,[4], *1. University of Illinois at Urbana-Champaign, Champaign, Illinois, United States, 2. University of Illinois at Urbana-Champaign, Aurora, Illinois, United States, 3. School of Health Professions, University of Kansas Medical Center, Kansas City, Kansas, United States, 4. University of Illinois, Champaign, Illinois, United States*

Balance confidence reflects one's estimate of their ability to maintain balance and avoid falls. Extensive literature has shown the relationships between balance confidence, functional limitations, and falls in later life. However, change in balance confidence, especially within short timescale, remains largely unknown and deserves further research. In this study, we aimed to investigate how older adults' balance confidence would change over 30 days and explore whether baseline characteristics would explain the individual differences in change. We used data from the Daily Balance Project that employed intensive-repeated measurements to examine the dynamics of subjective and objective fall risk across a month. Twenty-one participants (age=78.6±5.8, 48%female) were enrolled, and individual characteristics were measured upon recruitment. Throughout the study, participants self-reported their daily balance confidence using the Activity-Specific Balance Confidence (ABC) Scale. We performed growth modeling techniques to examine change within a multilevel framework. Our results showed that overall, ABC scores were high (79.9±17.4) at first, but the linear change was non-significant (b=0.03, SE=0.21, p=.89) on average. However, we found that balance confidence increased in individuals with higher educational attainment (b=0.37, SE=0.13, p<.01) and decreased among those with greater physical fall risk (b=-0.18, SE=0.07, p<.01) and accurate understanding of fall risk at baseline (b=-0.24, SE= 0.12, p=.04). Although ABC scores were stable within the period of one month, our study highlights the distinction of individual characteristics in the process of balance confidence appraisal. We suggest that these nuances should be taken into account when developing more fine-grained fall risk assessments and interventions.

### COMPARING THE SHORT AND ORIGINAL VERSIONS OF THE ACTIVITIES-SPECIFIC BALANCE CONFIDENCE (ABC) SCALE IN OLDER ADULTS

Chad Tiernan,[1] and Allon Goldberg,[2], *1. University of Michigan-Flint, grand blanc, Michigan, United States, 2. University of Michigan-Flint, Flint, Michigan, United States*

Balance confidence assessment in older adults has implications for falls and quality of life. It remains unclear whether the original Activities-specific Balance Confidence (ABC-16) scale or the shortened 6-item (ABC-6) scale is recommended. To further inform the decision-making process of balance confidence tool selection, a secondary analysis of an existing dataset consisting of 77 community-dwelling older adults was performed. ABC-16 and ABC-6 association and agreement, internal consistencies, and relationships with self-rated health (SRH) were assessed. Participants were primarily female (80.5%) between the ages of 60 and 87 years. Results indicated a strong association between the scales [$r = .97$, $p<.001$); ICC(2,1) = .80] but limited agreement (95% Limits of Agreement range = 22.1; mean difference of 7.2 points in the direction of the ABC-16). Cronbach's alphas were .95 (ABC-16) and .89 (ABC-6), suggesting high internal consistency for both scales but possible item redundancy with the ABC-16. Regression model 1 (ABC-6 = primary predictor) explained more of the variance ($R2=.36$) in SRH compared to model 2 (ABC-16 = primary predictor; $R2=.29$). Hotelling's t-test [$t(74)=2.4$, $p=.008$] indicated that the correlation coefficient (Multiple R) from the ABC-6 model was significantly higher than the correlation coefficient from the ABC-16 model. In conclusion, despite a high correlation, the two scales did not agree strongly and should not be considered interchangeable. Given that the ABC-16 takes longer to administer, does not relate to SRH as strongly, and could have redundant items, the ABC-6 should be considered for balance confidence assessment in older adults.

### DESCRIPTIVE EPIDEMIOLOGY OF FALL-RELATED INJURIES AMONG OLDER ADULTS IN ONTARIO, CANADA

Nicolette Lappan,[1] Aleksandra Zecevic,[2] Yu Ming,[1] Susan Hunter,[2] and Andrew Johnson,[2], *1. University of Western Ontario, University of Western Ontario, Ontario, Canada, 2. Western University, London, Ontario, Canada*

The number of older adults is growing rapidly in the province of Ontario meaning there will be more fall-related injuries (FRIs) in coming decades. Falls are the leading cause of injury-related hospitalizations in Canada. The purpose of this study was to describe the prevalence, circumstances, types, and locations of FRIs among older adults in Ontario. Using a population-based retrospective design, we analyzed secondary data from three health administrative databases (NACRS, DAD, RPDB) for 2010-2014. Older adults (≥ 65 years) admitted to an emergency department (ED) with a combined diagnosis of ICD-10-CA codes for a fall (W00-W19) and injury (S00-S99 or T00-T14) were selected. Descriptive statistics were performed in R and rates were reported per 100,000 population. There were 304,610 FRI ED admissions (3,089/100,000) and 143,210 patients (47.0%) were subsequently hospitalized (1,452/100,000). Females accounted for 63.0% ED and 61.2% hospital admissions. Age-specific rates increased with age at both ED (2,208/100,000 in 65-69 group, 6,552/100,000 in 90+ years old) and hospital (698/100,000 in 65-69 group, 4,364/100,000 in 90+ years old). Females had higher rates of ED (3,503 vs. 2,572/100,000) and hospital (1,598 vs. 1,270/100,000) admissions than males. The most common injury types at the ED were fractures (1,234/100,000), superficial injuries (719/100,000), other or unspecified injuries (572/100,000), open wounds (498/100,000), and sprains, strains, and tears (162/100,000). FRIs are a considerable problem for older adults and better injury prevention strategies are needed for all female age groups, the 90+ year age group of both genders, and fractures.

### DEVELOPMENT OF A SCREENING TOOL FOR FEET/FOOTWEAR- RELATED INFLUENCES ON FALL RISK

Mariana Wingood,[1] Elizabeth Peterson,[2] Christopher Neville,[3] and Jennifer Vincenzo,[4], *1. University of Vermont, University of Vermont, Vermont, United States, 2. University of Illinois Chicago, Champaign, Illinois, United States, 3. Upstate Medical University, Syracyse, New York, United States, 4. University of Arkansas for Medical Science, Fayetteville, Arkansas, United States*

The effectiveness of multifactorial fall risk assessment and intervention strategies is well documented. Although identifying feet/footwear-related influences on fall risk is a vital fall risk assessment component, few evidence-based resources or screening tools are available. To address this need, we developed the Screening Tool for Feet/Footwear-Related Influences on Fall Risk. Our tool is designed for older adults who are identified as at risk for falling, based on the CDC's Stopping Elderly Accidents, Deaths, and Injuries (STEADI) Algorithm for Fall Risk Screening, Assessment, and Intervention. Tool development was informed by results of our systematic review of lower-limb factors associated with balance and falls. Our initial tool was evaluated by an external group of 9 interprofessional content experts. Those experts recommended modification of 8 items and rated the tool's clarity as 81.2/100, appeal as 79.1/100, and clinical feasibility as 76.1/100. After incorporating recommended changes, we completed a modified Delphi study using 8 new interprofessional experts (average years of experience: 19.3). During Phase 1, Delphi participants recommended we combine items with similar treatment recommendations, add a question about orthoses, and increase the specificity of 9 items. This refinement resulted in a 20-item screening tool, which met approval after two rounds of consensus voting. Approval was defined based on the Item Content Validation Index, percentage of agreement > 80% on each item. The high level of agreement illustrates the tool's content validity. Using our tool, an older adult's feet/footwear-related risk factors can be identified and incorporated into an effective multifactorial fall prevention intervention.

### FALL-RELATED INJURIES IN OLDER ADULTS AND MEDICATIONS PRESCRIBED WITHIN 30 DAYS PRIOR TO THE INJURY

Yu Ming, Aleksandra Zecevic, Susan Hunter, Richard Booth, Andrew Johnson, and Rommel Tirona, *Western University, London, Ontario, Canada*

Fall-related injuries in older adults have serious consequences both for individuals and the public health care system. The purpose of this study was to identify medication classes prescribed within 30 days prior to the injury that were associated with fall-related injuries in older adults. This population-based, case-control study used secondary administrative health care data in Ontario, Canada. The cases were older adults, aged 66 years and older, who visited an emergency department for a fall-related injury. Controls were extracted from the Registered Person Database, and matched by same age, sex and residence area. Medication classes prescribed to both groups were recorded and logistic regression was conducted to examine the association between medications and fall-related injury. The case group included 255,270 older adults who experienced a fall-related injury over the five-year period (2010-2014). After adjustment for sex, age group, residence area, income level and number of medications prescribed, psychotropic medications (i.e., opioids, anti-epileptics, anti-Parkinson's drugs, and antidepressants), drugs for treatment of constipation, infection and benign prostatic hyperplasia, antithrombotic agents, statins and bronchodilators were identified to be related to increased risk of fall-related injuries. In addition to medications already on the list of fall-risk increasing drugs or FRIDs, this study uncovered that drugs for benign prostatic hyperplasia, cephalosporins, biphosphates and bronchodilators increased the risk of fall-related injury in older adults. Well-designed prospective cohort studies considering prescription indication and drug-drug interactions are needed to provide more convincing evidence on medications that may be associated with increased risks of fall-related injury in older adults.

### FALL-RELATED INJURY IN OLDER ADULT HOME CARE RECIPIENTS: A DESCRIPTIVE POPULATION STUDY

Danilla Xing,[1] Aleksandra Zecevic,[1] Nicolette Lappan,[2] and Yu Ming,[2], *1. Western University, London, Ontario, Canada, 2. University of Western Ontario, University of Western Ontario, Ontario, Canada*

Canada is experiencing a growing aging population leading to an increase in the number of individuals receiving home care. More needs to be known about home care clients who experience fall-related injuries. The purpose of this study was to describe the characteristics of Ontario home care recipients (65 and older) who experienced fall-related injury, and the characteristics of those injuries. We conducted a population-based descriptive study using secondary data from the IC/ES data repository for the period of 2010-2014. Person-level characteristics were extracted from the Resident Assessment Instrument - Home Care and injury characteristics from ICD-10 CA codes for falls (W00-W19) in combination with injuries (S00-S99 or T00-T14), available from the NACRS database. Descriptive statistics and rates were calculated using R. Results show the population (N= 88,731) was primarily female (67.0%), the largest age group was 85-89 years old (25.5%) and hypertension was the most prevalent (83.0%) chronic condition. Clinical Assessment Protocols (CAPs) indicated need for support in management of IADLs (75.4%), falls (72.3%) and pain (70.3%). Most patients (55.8%) used nine or more medications. In 90 days prior to home care assessment, 39.6% experienced no falls, 32.4% fell once, and 26.1% fell two or more times. Injuries primarily took place within the home (38.2%). Factures were the predominant injury type (40.8%), followed by superficial injuries (19.7%). These findings create a foundation for fall-related injury prevention in home care and further research on risk identification, the efficacy of CAPs, and home environment adjustments.

### SENSORY IMPAIRMENT IS ASSOCIATED WITH RECURRENT FALLS: STUDY OF WOMEN'S HEALTH ACROSS THE NATION

Carrie Karvonen-Gutierrez,[1] Michelle Hood,[1] Joshua Ehrlich,[2] Richard Neitzel,[3] and Kelly Ylitalo,[4], *1. University of Michigan, Ann Arbor, Michigan, United States, 2. University of Michigan, University of Michigan, Michigan, United States, 3. University of Michigan School of Public Health, Ann Arbor, Michigan, United States, 4. Baylor University, Waco, Texas, United States*

This study evaluated the relationship between individual and combined sensory impairments (vision, hearing, peripheral nerve (PN)) with recurrent falls in the past year among 1951 women (mean age 65.6 years) from the Study of Women's Health Across the Nation. Sensory impairments were defined as self-reported vision difficulty, hearing loss, or ≥4 on the Michigan Neuropathy Screening Instrument. Recurrent falls were defined as ≥2 self-reported falls. Hearing was the most commonly reported impairment (39.2%), followed by vision (22.1%) and PN (16.0%). Among those with any impairments, 7.0% of women reported impairments in all domains. Recurrent falls were more common among women with vision (19.4%), hearing (17.3%), or PN impairments (24.7%) as compared to women without sensory impairments (7.0%). The greatest burden of recurrent falls was among women with all three sensory impairments; one-third (34.6%) of women with vision, hearing and PN impairment were recurrent fallers. In an adjusted logistic regression model, vision, hearing, and PN impairments were associated with statistically significantly higher odds of recurrent falls in the past year (odds ratio (OR) = 1.58, 1.76, 2.11, respectively; all p<0.01), after adjustment for age, race/ethnicity, economic strain, and depressive symptoms. The presence of all three sensory impairments was associated with nearly 6-fold increased odds of recurrent falls (OR=5.65, 95% CI 3.25, 9.82) compared to women with no impairments. Sensory impairments often onset during midlife and early late adulthood. This work demonstrates that these impairments are associated with falls and that women with impairments across multiple sensory domains are at greatest risk.

### Session 9260 (Poster)

### FAMILY AND INTERGENERATIONAL RELATIONSHIPS

### ADULT CHILDREN'S MONITORING, KNOWLEDGE, AND INTERGENERATIONAL AMBIVALENCE

Noriko Toyokawa,[1] Nancy Darling,[2] and Teru Toyokawa,[3], *1. Southern Oregon University, Ashland, Oregon, United States, 2. Oberlin College and Conservatory, Oberlin, Ohio, United States, 3. California State University San Marcos, San Marcos, California, United States*

Monitoring aging parents' daily life is an essential task for adult children to ensure their parents' health and safety. The current study examined domains of parents' lives that adult children monitored as caregivers. Based on social domain theory (Smetana, 1999), we hypothesized that adult children would monitor parents' health and safety issues as respecting parents' autonomy in other issues. The study also examined how adult children's belief in need for monitoring and their perception of having actual knowledge of their parents' behaviors and thoughts would relate to the intensity of their intergenerational ambivalence. Adults who had at least one living parent (N=398, Mage=60, SD=7.7, range 45-77) participated in online surveys. Issues of parents' lives that adult children monitored were categorized into four domains by factor analysis: parents' financial safety, health and physical safety, substance use, and plans with other adult children. A series of regression analyses revealed that adult children's sense of need to know about parents' financial safety was associated with lower ambivalence, B=-.60, SE=.18, β=-.23, p=.001, whereas parents' physical safety was associated with greater ambivalence, B=.42, SE=.19, β =.17, p=.03. Adult children's perception of their knowledge about parents' financial safety was positively associated with their ambivalence, B=.58, SE=.20, β=.22, p=.004, whereas adult children's perception of parents' physical safety was negatively associated with their ambivalence, B=-.42, SE=.21, β=-.14, p=.05. Different meanings of different types of parents' safety issues for adult children as their caregivers and suggestions for future research will be discussed.

### AGREE TO DISAGREE: GENERATIONAL DIFFERENCES IN IDEOLOGICAL VALUES AND THE IMPACT ON DEPRESSIVE SYMPTOMS

Rachel Scott,[1] Acacia Lopez,[2] and Danielle Nadorff,[3], *1. Mississippi State University, Starkville, Mississippi, United States, 2. Mississippi State University, Starkvile, Mississippi, United States, 3. Mississippi State University, Mississippi State, Mississippi, United States*

Family Systems Theory states that values are transmitted between generations within families and, while many of these values are similar between immediate generations, there may be more differentiation in values between generation gaps. Ideological differences between generations may potentially cause subsequent tension and fluctuations in well-being. The current study sought to examine the moderating effect of generation on the relation between ideological values (political and religious) and depressive symptoms. Participants included 419 grandparents (age: M = 76, SD = 5.18) and 638 adult grandchildren (age: M = 29, SD = 5.57) from the 8th wave of the Longitudinal Study of Generations (LSOG) data set, collected in 2005. While violations in assumptions for the testing of religious ideology prevented additional analyses with that variable, significant mean differences were found between grandparents and grandchildren in political ideology scores, with grandparents expressing more conservative views. Generation also moderated the relation between political ideation levels and depressive symptoms. These relations trended in opposite directions for each generation (with non-significant conditional effects), such that when grandparents endorsed more conservative values, depressive symptoms decreased. Conversely, when grandchildren endorsed more conservative values, depressive symptoms increased. These findings indicate that while the older generation found a conservative viewpoint to be a protective factor for mental health, this was not true of younger generations, and indicates a need for age-specific cultural factors to be included in clinical intervention plans. Further implications for these findings will be discussed.

### AMBIVALENCE AMONG INTERGENERATIONAL RELATIONSHIPS IN OLD AGE

Sofia von Humboldt, Andrea Costa, Sara Silva, and Isabel Leal, *Instituto Superior de Psicologia Aplicada (ISPA), Lisboa, Lisboa, Portugal*

**Objective:** This study aims to analyze the perspectives of intergenerational relationships between older adults and adult children. For this purpose, a qualitative research was carried out, which analyzes these relations at a cross-national level.

**Methods:** Four hundred and twenty four older participants aged 65-97 years, were interviewed. Participants were of three different nationalities and lived in the community. All the interviews went through the process of verbatim transcription and subsequent content analysis.

**Results:** Two dimensions of generational ambivalence were revealed from the study; support and the conflict dimensions. Findings of content analysis produced six themes, which represent intergenerational relations between older adults and adult children: older adults-adult children interaction quality; family integration; care and support; definition of limits; distance and alienation; and communication difficulties.

**Conclusions:** This study highlighted the diversity of experiences in old age, in relation to intergenerational relationships and underlined the conflicting expectations from older adults in relation to their adult children.

**Keywords:** Adult children, ambivalence; conflict; intergenerational relationships; older adults; support.

### DEPRESSIVE COGNITIONS IMPACT ON A LATENT GROWTH CURVE MODEL OF DEPRESSIVE SYMPTOMS IN CAREGIVING GRANDMOTHERS

Christopher Burant, Carol Musil, Jaclene Zauszniewski, and Alexandra Jeanblanc, *Case Western Reserve University, Cleveland, Ohio, United States*

Grandmothers caring for grandchildren have elevated levels of depressive symptoms compared to grandmothers who do not provide care. While the CES-D measures the somatic, positive and negative affect, and interpersonal strain symptoms experienced with depression, the Depressive CognitionScale © captures the change in cognitive thinking that often precedes depression. Depressive symptoms, on the other hand, are state like in nature and describe depressive symptoms that have happened recently. While depressive cognitions, according to Beck's theory of depression, are the first negative thought processes to appear, these typically lead to other, more serious symptoms of depression. Specifically, depressive cognitions reflect negative thinking patterns and not depression. Data were collected on 343 participants in a longitudinal nationwide online research study of caregiving grandmothers. A latent growth curve model was used to track the trajectory of depressive symptoms at four time points (baseline, 2 weeks, 12 weeks, and 24 weeks). As depressive cognitions are the precursor to the development of depressive symptoms, a latent growth curve model was tested to gain an understanding of how depressive cognitions impacts the trajectory of depressive symptoms over time. The model fit the data well (Chi Square=21.025; df=9; p=.013; TLI=.976; CFI=.985; RMSEA=.063). Baseline depressive cognitions had a strong impact on the intercept (Standardized Beta=.76, p<.001) and the slope of depressive symptoms (Standardized Beta=-.67, p<.001). The continued impact of depressive cognitions over 24 weeks indicates the need for potential interventions to further address depressive cognitions as a way to decrease depressive symptoms in grandmother caregivers.

### DEVELOPMENT OF A NEW MEASURE OF INTERGENERATIONAL CONTACT

Karen Hooker,[1] Shelbie Turner,[2] Shannon Jarrott,[3] and John Geldhof,[2], *1. Oregon State University, Corvallis, Oregon, United States, 2. Oregon State University, Oregon State University, Oregon, United States, 3. The Ohio State University, Columbus, Ohio, United States*

Intergenerational relationships are a predictor of greater physical, mental, and emotional well-being; they can reduce ageism and facilitate feelings of purpose and meaning, shown to be important for health and mortality. Surprisingly there are no measures of intergenerational contact (IGC) shown to be reliable and valid across age groups. Therefore, we aimed to develop a psychometrically sound survey measure of IGC. We utilized a three-phase development process, which included a Delphi-style expert panel review of items, focus groups, and validation of the survey via Amazon MTurk. The resulting 18-item survey captures details on and comparisons between both familial and non-familial contact and is appropriate for adults of all ages. We conducted confirmatory factor analyses with the 9-item family and 9-item non-family subscales for 380 young and 256 middle-aged adults reporting on contact with older adults, and 348 older adults reporting on contact with younger adults. The family scale had good model fit across all three groups ($\chi2$ (78) = 245.74, p<.0001; RMSEA = 0.08, 90% CI = [0.07, 0.10]; CFI = .94; TLI = .92). After covarying for 2 pairs of items among the middle-age group, the non-family scale had good model fit across all groups ($\chi2$(75) = 217.21, p<.0001; RMSEA = 0.08, 90% CI = [0.07, 0.09]; CFI = .95; TLI = .93), indicating construct equivalence across age groups. The new IGC measure can be used in all adult age groups, making it useful for scientific projects as well as program evaluations. Funded by the RRF Foundation on Aging

### INTERGENERATIONAL CONFLICT AMONG ASIAN IMMIGRANT FAMILIES AND PSYCHOLOGICAL WELL-BEING: RESULTS FROM PIETY STUDY

Fatima Abdi,[1] Stephanie Bergren,[1] Lisa Lanza,[2] and XinQi Dong,[2], *1. Rutgers University, New Brunswick, New Jersey, United States, 2. Rutgers University, Rutgers Institute for Health, New Jersey, United States*

Research suggests that stress from migration and cultural adjustment may lead to intergenerational conflict (IC) within Asian immigrant families. Current research reports management of IC but fails to acknowledge the consequences it may have on offspring. The PIETY study, a longitudinal study of Chinese adult children (n = 547) in the greater Chicago area, aims to examine the relationship between IC and psychological wellbeing in children of Asian immigrant families. IC is assessed by the sum of items on conflicting opinions with parents based on finances, health, parenting, and lifestyle. Psychological wellbeing was measured by the Perceived Stress Scale with a cutoff value greater than or equal to 14, R-UCLA Loneliness Instrument scored on a binary scale, and Hospital Anxiety and Depression Scale (HADS) Anxiety Subscale with a cutoff value greater than or equal to 8. Logistic regression was conducted and controlled for age, gender, education, income, marital status, and household composition. Every one-point higher conflict with parents was associated with being 2.31 times more likely to experience stress for the adult child (OR: 2.31, 95% CI: 1.49-3.57, p<.001) and being 4.56 times more likely to experience loneliness (OR: 4.56, 95% CI: 2.79-7.43, p<.001). IC, however, had a nonsignificant positive association with anxiety in adult children. The association between IC and psychological wellbeing suggests that conflict is a result of complex factors, for which interventions could be developed to improve psychological wellbeing and resiliency in families who continue to navigate cultural changes in a foreign land.

### INTERGENERATIONAL FAMILY LEISURE IN THE COVID-19 PANDEMIC: SOME POTENTIALS, PITFALLS, AND PARADOXES

Tia Rogers-Jarrell, Deanna Vervaecke, and Brad Meisner, *York University, Toronto, Ontario, Canada*

COVID-19 has significantly changed the way we engage in leisure. The influence of public health measures and messaging on leisure put older and younger people alike at increased risk of stress, anxiety, loneliness, and isolation. Despite these similar experiences, ageism and tensions between generations intensified during the pandemic. Thus, it is imperative to encourage strategies that foster connections and solidarity between generations, such as participating in intergenerational family leisure. Intergenerational family leisure can both attenuate negative outcomes heightened or created by the pandemic (i.e., risk reduction) and increase positive experiences (i.e., wellness promotion). However, it is important to recognize that intergenerational family leisure may not be available, or ideal, for everyone, especially during the pandemic. There are longstanding and pandemic-specific pitfalls to engaging in intergenerational family leisure that need to be considered. Further, the conditions and handling of the COVID-19 pandemic have complicated family leisure in paradoxical ways. Many contradictions emerge as we navigate social systems and personal experiences when engaging in intergenerational family leisure during the pandemic. This paper critically presents some of the potentials, pitfalls, and paradoxes associated with connecting multiple generations in and through family leisure during the pandemic.

### INTERGENERATIONAL STRAIN AND SUBJECTIVE WELL-BEING: THE ROLE OF LEISURE ACTIVITY ENGAGEMENT

Amanda Collins,[1] Jeffrey Stokes,[1] and Elizabeth Dugan,[2], *1. University of Massachusetts Boston, Boston, Massachusetts, United States, 2. UMass Boston, Boston, Massachusetts, United States*

Family strain is associated with higher numbers of depressive symptoms and lower levels of life satisfaction. Leisure activities are observed to buffer the negative effects of family strain among younger adults, however, this phenomenon is understudied among older adults. This study examines the relationship between intergenerational strain and depressive symptoms and life satisfaction among persons aged 50 and older. The study also examines the moderating effects of gender and leisure activities. The analysis uses the Health and Retirement Study to addresses these questions. The results suggest that intergenerational strain (p=.000) and being female (p.=000), are associated with more depressive symptoms, while engagement in social leisure activities (p.=04) is associated with fewer. Intergenerational strain (p=.000) and being female (p=.03) are associated with lower levels of life satisfaction, while engagement in solitary (p=.000) and social leisure activities (p=.000) are associated with higher levels. Results from moderation models suggest that as intergenerational strain increases, women have lower life satisfaction and more depressive symptoms as compared to men (p=.000). Also, the association between intergenerational strain and life satisfaction is reduced among respondents who engaged in leisure activities (p=.002-social and p=.000-solitary). Further, the positive relationship between intergeneration strain and depressive symptoms is lower for persons who engage in leisure activities (p=.027-solitary and p=.013-social). Finally, women who engage in social and solitary leisure activities have fewer depressive symptoms than men (p=.037). The study findings imply that the subjective well-being of older persons may be improved in terms of intergeneration strain if they engage in leisure activities.

### PROGRAM PRACTICES PREDICT INTERGENERATIONAL INTERACTION AMONG YOUTH AND OLDER ADULTS

Shannon Jarrott,[1] Shelbie Turner,[2] Jill Juris Naar,[3] Rachel Scrivano,[1] and Raven Weaver,[4], *1. The Ohio State University, Columbus, Ohio, United States, 2. Oregon State University, Oregon State University, Oregon, United States, 3. Appalachian State University, Boone, North Carolina, United States, 4. Washington State University, Pullman, Washington, United States*

Non-familial intergenerational programs engage younger and older people in shared programming for mutual benefit, frequently involving senior centers or adult day programs and preschools. With growing interest in the potential benefits of intergenerational strategies, it is imperative to know their effects on participant interaction during intergenerational programming. To address this knowledge gap, activity leaders at five sites serving older adults and/or preschoolers received training to implement 14 evidence-based practices during intergenerational activities involving 109 older adult and 105 preschool participants over four years. We utilized multilevel modeling to test whether variations in implementation of practices were associated with variations in participants' responses to programming on a session-by-session basis. For both preschool and older adult participants, analyses revealed that the implementation of certain practices was associated with significantly more intergenerational interaction. Specifically, when person-centered best practices (e.g., leading activities that are age- and role-appropriate for older adults) were implemented, preschoolers (estimate=5.83, SD=2.11, p=0.01 and older adults (estimate=5.11, SD=.10, p=0.02) had more intergenerational interaction. Likewise, when environmental-centered best practices were implemented, such as pairing materials between intergenerational partners, preschoolers (estimate=6.05, SD=1.57, p=0.002) and older adults (estimate=6.50, SD=1.85, p=0.001) had more intergenerational interaction. Our findings reveal session-by-session variation in intergenerational interaction that can be impacted by implementation practices, which highlights the importance of training activity leaders to implement evidence-based practices. Researchers and practitioners should consider how session-by-session variation in program implementation affects participant response.

### SUBJECTIVE WELL-BEING, STATUS IDENTITY, AND INTERGENERATIONAL RELATIONS AMONG THE ELDERLY

Jieming Chen, *Texas A&M University - Kingsville, KIngsville, Texas, United States*

This study investigates the influences of intergenerational relations on the subjective wellbeing and status identity of the elderly population in China. The project draws insights from the studies of social mobility and stratification, and that of family relations and old age support. Because of widespread exchange of economic resources across generations and strong sense of connectedness among parent and adult children families that continue to exist in Chinese society today, we hypothesize that older parents' subjective sense of well-being and evaluation of their socioeconomic statuses are positively related with the socioeconomic conditions of their grown children, and the strength of the such relations with them. The study used the data from the 2013 China General Social Survey (CGSS), and the results provide fairly strong support to the hypotheses. The implications of the results on age-based stratification are discussed.

### SUPPORT EXCHANGES AMONG VERY OLD PARENTS AND THEIR CHILDREN: FINDINGS FROM THE BOSTON AGING TOGETHER STUDY

Kyungmin Kim,[1] Kathrin Boerner,[2] Yijung Kim,[3] and Daniela Jopp,[4], *1. Seoul National University, Seoul, Seoul-t'ukpyolsi, Republic of Korea, 2. University of Massachusetts Boston, Boston, Massachusetts, United States, 3. The University of Texas at Austin, Austin, Texas, United States, 4. University of Lausanne, Lausanne, Vaud, Switzerland*

Very old parents and their "old" children are a growing group in industrialized countries worldwide. Care needs of very old parents can be substantial, while children may also face their own age-related issues. However, little is known about support exchanges within very-old parent-child dyads. This study aimed to identify patterns of support exchanges occurring in these dyads, as well as to ascertain individual and relationship factors associated with these patterns. Participants were 114 very old parents (age ≥ 90) and their children (age ≥ 65) from the Boston Aging Together Study. Data were collected using comprehensive, semistructured in-person interviews with both dyad members, including standardized assessments of support exchanges, relationship quality, health, and perceptions of family norms. Actor-Partner Interdependence Models (APIM) were used to predict upward and downward support reported by children and parents. Both dyad members not only reported substantial upward support (given to parents by children) in all domains but also notable amounts of downward support (given to children by parents) in the domains of emotional support, listening, and socializing. Findings showed significant associations of parent functional impairment, parent and child relationship quality, and child perceptions of family obligation with upward support, and of relationship quality with downward support. Continued support exchanges among very old parents and their children indicated that intergenerational theories still hold up in very late life relationships. Healthcare professionals should be aware that attention to relationship quality and family norms might be vital to ensure that support needs are met.

### THE CONNECTION BETWEEN SOCIAL INEQUALITY AND INTERGENERATIONAL TRANSFERS BETWEEN THREE GENERATIONS IN EUROPE

Christian Deindl, *TU Dortmund University, Dortmund, Nordrhein-Westfalen, Germany*

Family members support each other across the entire family cycle. Parents help their adult children with financial transfers and hands-on-support and childcare, while children in mid-life often support their older parents with help and care. However, there is profound social inequalities linked to intergenerational transfers. While there is some research on inequality for some types of intergenerational transfers and some transfer directions, there is still no conclusive study bringing together all different support types between multiple generations from different social backgrounds over time. In our view, taking a longitudinal multi-generational perspective is essential to capture dependencies and negotiations within families from different socio-economic backgrounds within different regional contexts. If middle-aged parents have to take care of their own older parents, they have fewer resources for their(grand-)children, who might then receive less attention and support from them. This may differ according to access to support from public or private institutions. Here, country and regional specifics have a huge impact on support patterns within the family, which can only be captured when looking into developments and change. Using six waves of the Survey of Health, Ageing and Retirement in Europe (SHARE), we look at intergenerational transfers between multiple generations over time across European regions, considering mid-aged Europeans in the "sandwich" position between older parents and children and include multiple transfer directions and types over time to assess the links between social inequality and intergenerational solidarity in Europe's ageing societies. The impact of Covid 19 on this issue will also be considered.

### THE COSTS OF CONCERN: HEALTH IMPLICATIONS OF WORRIES ABOUT AGING PARENTS AND ADULT CHILDREN

Kelly Cichy,[1] and Athena Koumoutzis,[2], *1. Kent State University, Kent, Ohio, United States, 2. Miami University, Oxford, Ohio, United States*

As their parents age and their children enter adulthood, midlife adults need to manage their worries and concerns about both generations. In midlife, worries about aging parents' health and emerging needs for support co-occur alongside worries about adult children's relationships and prolonged need for support. Research reveals links between midlife adults' worry and sleep quality, underscoring how worries compromise health and well-being. In addition to compromising sleep, worries may also contribute to poor health behaviors, such as emotional eating. Emotional eating, where individuals eat in response to stressors and negative emotions, is a significant risk factor for overeating and obesity. Less is known; however, about how midlife adults' worries contribute to poor health behaviors. To address this gap, the current study considers how midlife adults' concurrent and previous day's daily worries about aging parents and adult children are associated with daily well-being and health behaviors. Respondents are midlife adults (40-60 years) from Wave II of the Family Exchanges Study (Fingerman et al., 2009). During 7 days of daily telephone interviews, respondents indicated if they worried about their adult children and their aging parent(s), if they ate food for comfort, and their daily negative mood. Controlling for demographics, on days when midlife adults worried about their adult child(ren), they reported more negative emotions than on days without these worries ($p <.05$). Respondents engaged in more eating for comfort the day after they reported worrying about their mother ($p < .05$). Implications for aging families will be discussed.

### THE EFFECTS OF SOCIAL SUPPORT ON THE PSYCHOLOGICAL WELL-BEING OF OLDER PARENTS: A LONGITUDINAL STUDY

Erik Blanco, *University of Southern California, Leonard Davis School of Gerontology, Los Angeles, California, United States*

This study examines whether parental support (the provision of social support by older parents to adult children) and filial support (older parents' receipt of social support from adult children) influence two orthogonal dimensions of older adults' psychological wellbeing: positive feelings and negative feelings. This study also highlights the importance of accounting for parental need as a mediator of social support. A longitudinal design is used to examine the effects of social support on the psychological wellbeing of older adults at Wave 6 (1998) and Wave 8 (2004) of the Longitudinal Study of Generations. Parental support significantly increases parents' positive feelings, which suggests that, when it comes to positive feelings, it is better to give support than to receive it. Filial support findings indicate that older adults with greater level of disability demonstrate a decrease in negative feelings when they received filial support. However, this effect does not hold for older adults with lesser levels of disability, suggesting that, when it comes to older adults' negative feelings, it is better to receive support (rather than to give it) when parents are in need. Although parental and filial support have the potential to buffer stressful life transitions in old age, most parents wish to remain independent, even in later life, making them reluctant to accept filial support. The parent-adult child relationship is crucial for psychological wellbeing, especially because of increased life expectancy.

### THE IMPACT OF LIVING ARRANGEMENTS AND INTERGENERATIONAL SUPPORT ON THE HEALTH STATUS OF OLDER PEOPLE IN CHINA

Yazhen Yang, Maria Evandrou, and Athina Vlachantoni, *University of Southampton, Southampton, England, United Kingdom*

Research to-date has examined the impact of intergenerational support in terms of isolated types of support, or at one point in time, failing to provide strong evidence of the complex effect of support on older persons' wellbeing. Using the Harmonised China Health and Retirement Longitudinal Study (2011, 2013 and 2015), this paper investigates the impact of older people's living arrangements and intergenerational support provision/ receipt on their physical and psychological wellbeing, focusing on rural/ urban differences. The results show that receiving economic support from one's adult children was a stronger predictor for higher life satisfaction among older rural residents compared to those in urban areas, while grandchild care provision was an important determinant for poor life satisfaction only for older urban residents. Receiving informal care from one's adult children was associated with a poor (I) ADL functional status and with depressive symptoms among older rural people. Meanwhile, having weekly in-person and distant contact reduced the risk of depression among older people in both rural and urban areas. The paper shows that it is important to improve the level of public economic transfers and public social care towards vulnerable older people in rural areas, and more emphasis should be placed on improving the psychological well-being of urban older residents, such as with the early diagnosis of depression.

### Session 9265 (Poster)

### FAMILY CAREGIVERS' PERCEPTIONS AND EXPERIENCES

### A QUALITATIVE ANALYSIS OF FAMILY CAREGIVER PERSPECTIVES FROM THE CAREGIVING TRANSITIONS STUDY

Marcela Blinka,[1] Chelsea Liu,[2] Orla Sheehan,[3] J. David Rhodes,[4] and David Roth,[1], *1. Johns Hopkins University, Baltimore, Maryland, United States, 2. Harvard School of Public Health, Brookline, Massachusetts, United States, 3. Johns Hopkins University School of Medicine, Johns Hopkins University, Maryland, United States, 4. University of Alabama at Birmingham, University of Alabama at Birmingham, Alabama, United States*

As people live longer, informal caregiving for family and friends is becoming increasingly common. Caregiver satisfaction with their role is now of greater importance to an increasing proportion of the U.S. population. Most research on caregivers has studied convenience samples, often restricted to caregivers of people with dementia. Various studies have examined the impact of caregiving on caregivers' health but to our knowledge there are no qualitative studies of caregiving experiences from caregivers in population-based samples. This study investigated the impact of caregiving on participants who transitioned into a caregiving role while participating in a national population-based study. Participants were from the Caregiving Transitions Study, which is ancillary to the Reasons for Geographic and Racial Differences in Stroke (REGARDS) Study. We thematically analyzed responses from 150 caregivers providing care for multiple different conditions to an open-ended question asked at the time of enrollment and designed to encourage caregivers to share additional details about their caregiving experience. Four major themes were identified: cultural/ family expectations; growth opportunities and reciprocity; stressors and challenges; and recommendations. Participants shared both positive and challenging experiences in their role as a family caregiver as well as the impact that these experiences had on their lives. Caregivers shared that one of the most important motivations for taking on this role was their sense of duty toward family. Caregivers also highlighted the positive impact of caregiving on their lives such as opportunities for personal growth, acquisition of new skills, and finding a sense of fulfillment and gratitude.

### AN INTEGRATIVE THEORETICAL MODEL TO PREDICT POSITIVE ASPECTS OF CAREGIVING IN DEMENTIA

Doris Yu,[1] Sheung-Tak Cheng,[2] and Timothy Kwok,[3], *1. University of Hong Kong, Hong Kong, Not Applicable, Hong Kong, 2. Education University of Hong Kong, The Education University of Hong Kong, Not Applicable, Hong Kong, 3. The Chinese University of Hong Kong, Shatin, N.T., Hong Kong, Not Applicable, Hong Kong*

Family caregiving for dementia is the crucial informal care resource to buffer the associated disease burden. Whereas substantial research focused on ameliorating the caregiving burden through increasing their coping resources, least attention is placed on how to promotive their positive aspects of caregiving (PAC). This longitudinal exploratory study aimed at testingWhereas the perceived self-efficacy was further enriched in the context of good social sup an integrative theoretical model which attempts to explain the evolvement of PAC from the paradigm of stress and coping and existentialism. From to June 2017 to April 2020, we have recruited a total of 403 dementia caregivers from the a geriatric clinic in Hong Kong (mean age = 56.2, SD = 12.2; child-caregiver: 73.9%). About 61% of them were taking care of PwD of moderate to severe dementia. Validated instruments were used to measure the hypothesized model constructs. By using path analysis, it was found that PAC was evolved from two conditions, including i) perceived self-efficacy developed through active coping strategies for carers with good to moderate social support and ii) meaning-focused coping in the context of high religiosity, better social support and active coping. Data-model fit was evident by RMSEA = 0.023, CFI = 0.994, NFI = 0.968 and AIC = 97.762. The findings suggested that PAC was evolved from the interaction of the stress-coping and meaning-making process. Empowering carers for successful caregiving experience, facilitating them to make meaning in the process, enhancing good dyadic relationship and social support are crucial to cultivate PAC.

### CAREGIVING ACROSS THE LIFE COURSE: LIFE HISTORY FINDINGS FROM THE HEALTH AND RETIREMENT STUDY (HRS)

Marina Larkina,[1] and Jacqui Smith,[2] *1. University of Michigan, Ann Arbor, Michigan, United States, 2. University of Michigan, University of Michigan, Michigan, United States*

Informal caregiving, defined as unpaid care provided to a relative or friend with some sort of special need, is a topic of research across different disciplines. Previous research highlights the prevalence and heterogeneity of caregivers in terms of their age, gender, relationship with the care recipient, and the duration of care provision. However, most research focuses on a specific episode of caregiving. Little is known about the people who provide care to multiple recipients throughout their own life. To fill this gap, we examined data from the HRS Spring 2017 Life History Mail Survey (N = 3520; age range 50-101 yrs). Participants reported their relationship with people to whom they had provided unpaid care for ≥ 6 months (max 5) and listed the start and end years of care. Compared with people who had not provided care, caregivers (N = 1000, 28%) were more likely to be women, white, and currently widowed. They cared for their parents (67%), spouses (22%), children (11%), or other relatives (16%) and 30% reported providing care two or more times (M = 1.44, SD = 0.81). Respondents, who reported multiple episodes of caregiving were more likely to be women, widowed, aged between 25 and 50 at the time of first providing care. People who first cared for their spouse were less likely to report multiple caregiving episodes comparing with those who cared for parents or children. Future research will examine the health and well-being consequences associated with caregivers' histories of providing unpaid care to others.

### CAREGIVING BETWEEN SPOUSE AND ADULT CHILD CAREGIVERS OF OLDER ADULTS WITH COGNITIVE IMPAIRMENT

Nicole Garcia,[1] Anna Papazyan,[2] Sarah Choi,[3] and Yeonsu Song,[1], *1. UCLA, Los Angeles, California, United States, 2. VA Greater Los Angeles Healthcare System, California, Los Angeles, California, United States, 3. University of California, Los Angeles, Los Angeles, California, United States*

Prior studies of caregiving characteristics by type of caregivers are inconsistent, particularly those who are spouses and adult children. This study examined caregiving characteristics between spouses and adult children of cognitively impaired older adults. We analyzed phone-screening data from an ongoing trial of a dyadic sleep intervention program for persons with dementia and their caregivers. Data included spouse caregivers (n=52) and adult child caregivers (n=24). Nearly all participants (95%) lived with their care recipients (91% with dementia). Types of caregiving activities were measured by activities of daily living [ADLs] and instrumental ADLs [IADLs] with their levels of intensity (0 [total independent] to 3 [total dependent]). Care recipients' sleep was measured by the Neuropsychiatric Inventory-Nighttime Behavioral Subscale (8 items). Analyses included Pearson correlations and t-tests. Adult child caregivers helped their care recipients at significantly higher levels as indicated by their measure of dependence in dressing (1.46±1.22 vs. 0.87±1.16, p=0.044), continence (1.22±1.38 vs. 0.54±1.04, p=0.021), laundry (2.87±0.46 vs. 2.13±1.24, p=0.007), and transportation (3.00±0.00 vs. 2.63±0.79, respectively; p=0.031) than spouse caregivers. Adult child caregivers also reported their care recipients having more difficulty falling asleep (56% vs. 19%, respectively; p=0.004) and having more numbers of sleep problems than spouse caregivers (3.54±2.08 vs. 2.48±1.51, respectively; p=0.014). The findings suggest that adult child caregivers may involve higher levels of caregiving responsibilities during daytime and nighttime, compared to spouse caregivers. Further research needs to explore complimentary ways to involve spouse and adult child caregivers in the care of this vulnerable population.

### DIFFERENT EXPERIENCES OF ADULT CHILD AND SPOUSAL CAREGIVERS WITH FAMILY CONFLICT

Stacy Yun,[1] Kendall Weber,[2] Geffen Ferszt,[1] JoAnna Dieker,[3] and Sara Qualls,[2], *1. University of Colorado Colorado Springs, Colorado Springs, Colorado, United States, 2. University of Colorado at Colorado Springs, University of Colorado at Colorado Springs, Colorado, United States, 3. University of Colorado Coloardo Springs, University of Colorado Colorado Springs, Colorado, United States*

Previous research indicates that different types of caregivers report distinct levels of family conflict (Dieker et al., 2019). However, as half (52%) of the participants in the previous study did not report family conflict, the purpose of this study was to examine the relationships among types of caregivers, family conflict, and caregiver burden in those who experience family conflict. Participants (N = 277; aged 19 to 87; M = 52.96) comprised of 197 adult child and 80 spousal caregivers. They completed the Caregiver Reaction Scale (O'Malley & Qualls, 2017) to assess different aspects of the caregiving experience. Hierarchical regressions (block 1: demographics, block 2: family conflict) were computed to predict caregiving burden. For spousal caregivers, the final model explained 2.2% of the variance in caregiving burden, $F(7, 69) = 0.22$, $p = .98$. None of the variables were significant. Additionally, family conflict did not uniquely influence caregiving burden beyond demographics, Fchange(2, 69) = 0.03, $p = .97$. For adult child caregivers, the set of predictors accounted for 20.5% of the variance in caregiving burden, $F(7, 183) = 6.75$, $p < .001$. Having more family beliefs and support conflict predicted greater caregiving burden ($ps < .01$). Family conflict scores also significantly explicated caregiver burden beyond demographics, Fchange(2, 183) = 17.60, $p < .001$. Results suggest that family conflict is a stronger driver of caregiver burden for adult child than spousal caregivers. Findings imply the need for clinicians to target appropriate interventions for adult child caregivers to reduce caregiver burden.

### EXAMINING SUBJECTIVE AGING THROUGH THE LENS OF THE CAREGIVER EXPERIENCE

Kristen Hardin-Sigler, Kaitlin Grelle, and Rebecca Deason, *Texas State University, San Marcos, Texas, United States*

Caregivers of individuals with Alzheimer's disease and related dementia (ADRD) often experience burden that has been associated with poor physical and psychological health outcomes (Andren & Elmstahl, 2007; Zimmerman et al., 2018). However, very little research investigates how the caregiving experience may impact an individual's subjective aging experience. Various aspects of subjective aging have been implicated in health outcomes and memory function (Brothers et al., 2017; Stephan, Sutin, Caudroit, & Terracciano, 2016). The purpose of this study was to investigate the differences in perceptions of subjective aging between caregivers and non-caregivers. Participants (N = 185) completed a survey assessing several aspects of subjective aging, including subjective age, or how old an individual feels, memory function, well-being, attitudes towards aging, and aging stereotypes. A series of independent t-tests indicated that there were significant differences between groups on subjective age (p = .013), and subjective memory function (p = .013). Caregivers (n = 93) reported feeling significantly older than their chronological age, reported significantly more subjective memory complaints, and also reported poorer subjective memory function when compared to the non-caregiver (n = 92) control group. Previous literature does suggest that older subjective age ratings are associated with poor subjective memory function, so these results are not necessarily surprising. However, these results suggest that caregiving for individuals with ADRD may negatively impact caregivers' perceptions of their own aging experience, but not necessarily their perceptions about aging in general.

### FAMILY CAREGIVING SUBTYPES IN THE CAREGIVING TRANSITIONS STUDY: A LATENT CLASS ANALYSIS

John Bentley,[1] Carly Lupton-Smith,[2] and David Roth,[3], *1. University of Mississippi, University of Mississippi, Mississippi, United States, 2. Johns Hopkins University, Johns Hopkins University, Maryland, United States, 3. Johns Hopkins University, Baltimore, Maryland, United States*

Family caregiving requires activities and experiences that have negative and positive features, producing stress but also providing benefits. The Caregiving Transitions Study (CTS) enrolled 283 caregivers from a national epidemiologic study, of which 32 were caregivers prior to enrollment in the parent study, and 251 became caregivers while participating in the parent study. Telephone interviewers were conducted after caregivers provided care for a minimum of 1.6 years (mean=7.7 years). Latent class analysis (LCA) was used to detect unobserved groups of caregivers. Number of problems (i.e., ADL, IADL, communication, emotional, disruptive behavior), average burden per problem, depressive symptoms, perceived stress, purpose in life, positive aspects of caregiving, hours of care, and duration providing care were used as indicators. Classes were subsequently compared on several external variables, including demographics, quality of life, leisure activities, and caregiving strain. The best-fitting model consisted of three classes (4.6% long-term, 27.6% high-distress, and 67.8% moderate-distress). Classes were similar with respect to sex, age, race, and primary caregiving status of the caregiver. Long-term caregivers had much longer caregiving durations and commonly provided care to a child. The high-distress class was noteworthy in terms of greater number of experienced patient problems; greater likelihood of caring for a person with dementia; higher levels of caregiving strain, depressive symptoms, perceived stress, and perceived burden; and lower levels of quality of life, purpose in life, positive aspects of caregiving, and leisure activities. These findings suggest that caregivers can be classified into distinct subtypes, with one subtype characterized as experiencing high distress.

### OLDER LATINOS' PERCEPTIONS OF THE CAREGIVING EXPERIENCE

Michelle Jaldin,[1] Guilherme Balbim,[2] Isabela Marques,[3] and David Marquez,[4], *1. University of Illinois at Chicago, Chicago, Illinois, United States, 2. University of British Columbia, Vancouver, British Columbia, Canada, 3. Universidade de Sao Paulo, Sao Paulo, Sao Paulo, Brazil, 4. Rush University, University of Illinois at Chicago, Illinois, United States*

There has been a rapid growth of Latinos age 65 and older in the United States and the population is projected to grow to 21.5 million by 2060. Latinos with Alzheimer's disease is expected to increase 832% by 2060. Caregiving for adults with Alzheimer's Disease and Related Dementias (ADRD) is physically, emotionally, and financially demanding, and has significant implications for caregivers' health, personal and social life, and overall well-being. This study aimed to describe the perceived experiences of middle-aged and older Latino who were primary caregivers of relatives with ADRD. We conducted semi-structured interviews with Latino caregivers to examine their perceived experiences of providing care for a relative with ADRD. Interviews were conducted in English and Spanish and were transcribed, translated into English when needed, and coded. We conducted direct content analysis. Participants were aged 50 to 75 years (n = 16), the majority were female (n = 12), and majority were caring for either their parent or spouse. We identified six reoccurring themes in the Latino caregiving experiences: (1) caregiver burden; (2) dealing with care recipient; (3) coping strategies; (4) social support; (5) cultural values; and (6) knowledge about services. The identified themes showed that Latino caregivers need support from their family and friends for caregiving. Latino family's structure plays an important role in caregiving experience. These themes are important to consider in future interventions that aim to reduce caregiver burden in Latinos as they influence the overall well-being of the caregiver.

### PERCEIVED APPRECIATION FOR CARE ASSOCIATES WITH HIGHER QUALITY CAREGIVING DAY-TO-DAY

Kylie Meyer,[1] Arielle Chinea,[2] Sara Masoud,[2] Kevin Hamilton,[2] Ashlie Glassner,[2] Jing Wang,[3] and Carole White,[2], *1. UT Health Sciences at San Antonio, San Antonio, Texas, United States, 2. UT Health San Antonio, San Antonio, Texas, United States, 3. University of Texas Health Science Center at San Antonio, San Antonio, Texas, United States*

Family members are critical to dementia care and the U.S. long-term services system. Yet, little is known about how to support the quality of care provided by family members, who often receive little training. We hypothesize that on days when caregivers feel more appreciated, they report providing a higher quality of care. To test this hypothesis, we asked spousal dementia caregivers (N=21) to complete 14 daily surveys that asked about their daily caregiving experiences. Our measure for "quality of care" was based on the

Exemplary Caregiving Scale, and included 3-items pertaining to provision of care (e.g., "You considered your spouse's wishes and opinions when providing assistance"). Response options included "Most of the time," "Some of the time," and "Never"; scores were summed (range 0 to 6). Caregivers were also asked to what extent their spouse appreciated the care provided ("Not at all," "Some," or "A lot"). We applied multi-level mixed models to the data, and controlled for age, gender, Hispanic ethnicity, number of behavioral symptoms of dementia each day and months since diagnosis. In adjusted models, we found that on days when caregivers believed care recipients appreciated care provided "Some" or "A lot," they reported providing higher quality care (B=0.52, p=0.010 and B=0.79, p<0.001, respectively) compared with days when caregivers believed care recipients appreciated care provided "Not at all". Preliminary results may inform programs to support caregivers' ability to provide high quality care (e.g., by helping caregivers to perceive rewards) and to identify caregivers at risk of providing low-quality care.

### RESEARCH PARTICIPATION AMONG COMMUNITY DWELLING DEMENTIA CAREGIVERS: REFLECTIONS AND SUGGESTIONS

Kiana Cruz,[1] Sama Joshi,[2] Taeyoung Park,[2] M. Carrington Reid,[1] Keela Herr,[3] Karl Pillemer,[2] and Catherine Riffin,[4], *1. Weill Cornell Medical College, New York, New York, United States, 2. Cornell University, Ithaca, New York, United States, 3. University of Iowa, Iowa City, Iowa, United States, 4. Weill Cornell Medical College, London, England, United Kingdom*

Clinical trials for dementia caregivers have suffered from small sample sizes that lack adequate power to detect treatment benefits. Addressing these methodological shortcomings is contingent upon successful recruitment and enrollment of caregiver participants, but major barriers impede their participation in research. This presentation describes the lessons learned from recruiting and enrolling dementia caregivers into a pilot randomized controlled trial designed to help caregivers recognize and communicate about pain in dementia care recipients. Using Bronfenbrenner's ecological model, we organize our discussion of challenges and opportunities into three levels: community (ecosystem), institution (microsystem), and individual. A key challenge at the community level was gatekeeping by organization leaders, including those from support groups, senior centers, and congregate living facilities. At the institutional-level, challenges included an absence of administrative mechanisms for identifying caregivers and a lack of caregiver research expertise on the Institutional Review Board. At the individual-level, challenges included time constraints and varying motivations for participating in research. Strategies for overcoming these challenges spanned the three levels and included establishing trust and rapport with various constituencies; adapting our recruitment approaches to meet the specific motivations of prospective participants; and refining recruitment scripts to allow for greater personalization. Employing these strategies, which can be generalized to recruit other hard-to-reach populations, helped to overcome recruitment challenges and expedite enrollment of caregivers from a diverse range of sociodemographic backgrounds. Further improvement will require coordinated changes at the institutional and community levels, including the development of central research registries and administrative mechanisms for identifying caregivers.

### ROLE-REVERSALS IN CAREGIVING: CASE STUDIES OF TWO WOMEN LIVING WITH LATE STAGE CANCER

Candice Reel,[1] J. Hunter Williams,[1] Emma Brennan,[1] Jonna Williams,[1] Kristen Payne,[2] and Rebecca Allen,[2], *1. The University of Alabama, Tuscaloosa, Alabama, United States, 2. University of Alabama, Tuscaloosa, Alabama, United States*

Many studies have examined the effects of caregiving burden and many others have focused on the effects of having a caregiver (Haynes-Lewis et al., 2018; Trevino, Prigerson, & Maciejewski, 2018; Semere et al., 2020). However, there is little data on the experience of role reversal, once responsible for caring for others and now being cared for while living with cancer. This project aims to identify ways in which women living with cancer cope with the internal struggles of receiving care. The current project is a case study of two females, one age 67, NHW, with a breast cancer diagnosis and one age 60, Black, with an ovarian cancer diagnosis, who once were caregivers and are now being cared for by family. Two semi-structured interviews were conducted that were approximately 60 minutes each. The study data are from a larger project focused on the self-perception of older women with late-stage cancer. Four independent researchers used thematic analysis to uncover common themes of coping between the two women receiving care. The themes uncovered were acceptance of the loss of autonomy, positive death attitudes, good relationships with their caregivers, and religiosity were identified and coded as coping strategies. The qualitative data showed that the use of these coping strategies helped the women be more accepting to care with less internal conflict. Future research should focus on generalizing these findings on a larger sample and use the data to help cancer patients better accept care from others.

### THE LIFE SATISFACTION OF INFORMAL CAREGIVERS IN EUROPE: REGIME TYPE, INTERSECTIONALITY, AND STRESS PROCESS FACTORS

Sean Browning, *University of Victoria, Victoria, British Columbia, Canada*

This research assessed the role of welfare state/family care regimes, intersecting social locations and stress process factors in influencing the life satisfaction of informal caregivers of care recipients with age-related needs or disabilities within a European international context. Empirical analyses were conducted with a sample of informal caregivers residing in Denmark, Sweden, France, Germany, Italy, Greece and the United Kingdom (n=6,007). Ordinary least squares and ordered logit regression models revealed that welfare state/family care regime, intersecting social locations, and stress process factors were independently associated with the life satisfaction of informal caregivers. Furthermore, there was some evidence to suggest that social location and stress process factors intervened in some of the relationships between regime type and life satisfaction. There was also some evidence that stress process factors intervened in the relationships between social location factors and life satisfaction. Overall, the results provide support for integrating welfare state/family care regime type and intersectionality factors into the stress process model as applied to the context of informal caregiving. The results also have policy and practice implications with regards to which social location and stress process factors explain specific disparities in life satisfaction between informal caregivers residing in different welfare state/family care regimes.

### THE OTHER CAREGIVERS: INFORMAL NON-SPOUSAL MALE CAREGIVERS FOR PERSONS WITH DEMENTIA

Gretchen Tucker, *University of Maryland, Baltimore, Columbia, Maryland, United States*

Informal caregivers for persons with Alzheimer's disease and related dementias (ADRD) have become an integral part of the long-term health care system. They are relied on to provided day-to-day care that is challenging, complex, and often spans several years. Most of the research on informal caregivers for persons with ADRD have focused on spousal caregiving, mother-daughter dyads, and daughters. There is sparse literature on informal non-spousal male caregivers for persons with ADRD. The objective of this research was to obtain an understanding of the experiences of informal non-spousal male caregivers for persons with ADRD. This descriptive qualitative pilot study consisted of in-depth one-on-one interviews with three informal non-spousal male caregivers for persons with ADRD. Four themes emerged through data analysis: 1) the male perspective and experience of caregiving, 2) relationship dynamics, 3) caregiving challenges, and 4) finding meaning within caregiving.

**Conclusion:** Similar to other caregivers, informal non-spousal male caregivers assisted with transportation, managing medical appointments, as well as bathing and personal care. Differences with other caregivers, specifically female caregivers, emerged in terms of descriptions of traditional versus non-traditional gender roles. The implications of this study are that public policies, support services and medical professionals need to understand and be able to address the different experiences and needs of informal non-spousal male caregivers.

### THE SHAPE OF CARE: PATTERNS OF FAMILY CAREGIVING AMONG CHINESE ADULTS IN THE MIDDLE TO LATER STAGE OF LIFE

Haoshu Duan, *University of Maryland, Arlington, Virginia, United States*

Due to the lack of institutional support, families have long been the primary caregivers in China. Most studies to date only focused on one single care activity during a particular life course stage. Nonetheless, older adults today are more likely to care for multiple family members concurrently or sequentially (serial caregivers). The studies on discrete snapshots of care activities failed to capture the patterns of family caregiving overtime. Utilizing four waves of longitudinal data from CHARLS (2011-2018, N=17,039), this study particularly focuses on care activities to grandchildren, parents, and spouse, and maps out the family caregiving patterns overtime. Using latent profile analysis, this study identifies five family caregiving patterns: 1). Light grandchild caregivers (27%), who on average provided 4.3 years' grandchild care mostly; 2). Heavy grandchild caregivers (11%), who on average on provided 7 years' grandchild care mostly; 3). Light caregivers for grandchildren and parents (7%), who sequentially provided 1-year care to grandchildren and parents; 4). Heavy serial caregiver (6%), who mostly provided care to spouse and grandchildren with higher overlapping years; 5). Overall light caregivers (49%), who on average provided less than one year of care to any recipient. The preliminary results suggest that heavy serial caregivers (6%) far worst in terms of depressive symptoms and more likely to report worsened self-rated health; and overall light caregivers (49%) have the lowest depressive symptoms and more likely to report good self-rated health.

### Session 9270 (Poster)

### FAMILY CAREGIVING (HS POSTER)

### AGING IMMIGRANT FAMILY CAREGIVERS HEALTH, SOCIAL ENGAGEMENT, AND HEALTH LITERACY

Mary Dioise Ramos, *Kennesaw State University, Kennesaw, Georgia, United States*

Family caregiving is evolving in multiple ways. There is an increasing recognition of the role of informal or unpaid family caregivers. Extensive body of research shows that family members who provide care to individuals with chronic or disabling conditions are themselves at risk. However, most evidence on family caregiving gear towards Caucasian middle-class populations. There is limited research that exists about aging immigrant family caregivers who are ill-prepared for their role and provide care with little or no support. The specific aim of this study was to assess and determine the association of health status, social engagement, and health literacy among aging immigrant family caregivers. This study utilized a non-experimental, cross-sectional, correlational design. Most of the participants are female married Asian women, who are retired, living with their spouses, and taking care of their family members more than 4 hours a day with Alzheimer's, Parkinson's, and cardiovascular disorder. Most participants have existing medical condition such as hypertension, diabetes, high cholesterol, and heart disease. Most participants experienced problem with sleep and difficulty falling asleep. While some participants experienced fatigue and having trouble doing regular leisure activities with others. There is an association between sleep disturbance and ability to participate in social roles and activities among aging immigrant family caregivers. Inclusion of people from different ethnicities, backgrounds, and socioeconomic position in caregiver research is vitally important. There is a need for a greater understanding of the contextual factors of family caregiving and recognizing the prevalence and characteristics of aging immigrant family caregivers.

### EXHAUSTED AND TRAPPED IN ISOLATION. CARING FOR A SPOUSE WITH DEMENTIA DURING THE COVID-19 PANDEMIC

Lena Hammar,[1] Marcus Johansson,[2] Lena Dahlberg,[2] Kevin J McKee,[2] and Martina Summer-Meranius,[3], *1. Mälardalen University / Dalarna University, Västerås,*

*Vastmanlands Lan, Sweden, 2. Dalarna University, Falun, Dalarnas Lan, Sweden, 3. Mälardalen University, Västerås, Vastmanlands Lan, Sweden*

Even before the Covid-19 pandemic, spouse carers of persons with dementia (PwDs) found their care responsibilities overwhelming and had little time to focus on their own lives. To minimize the risk of being infected with Covid-19, older persons are recommended to self-isolate in their homes, while formal support such as respite care and day care centers are withdrawn. This study involved semi-structured interviews with 24 spouse carers of community-living PwDs, with the aim of describing their situation during the pandemic. The interviews were analyzed with qualitative content analysis. Results revealed that they commonly declined help because of the perceived risk of their spouse being infected with Covid-19 and thus also possibly causing their death. They described feelings of being trapped in their situation, as they experienced having no choice than take all responsibility for the care of their partner themselves, with cost of being unable to take necessary breaks. This was described as making an already strained situation almost unbearable, which led to conflicts with their partner. However, the spouses also described positive aspects due to strategic changes in health and social care provision to prevent the spread of the virus, such as greater staff continuity in home care services, and patient transportation service. These made the PwD less stressed and influenced their everyday life positively. It could be concluded that the extent burden during the Covid-19 pandemic calls for extensive development of tailored support to better tackle the rapid changes that can occur in a society.

### FAMILY ADVOCACY FOR RESIDENTS IN NURSING HOMES DURING THE COVID-19 PANDEMIC

Cristina de Rosa, Yanjun ZHou, Amy Lyons, and Yu-Ping Chang, *University at Buffalo, Buffalo, New York, United States*

To protect one of the most vulnerable populations from COVID-19, nursing homes enacted and enforced visiting restrictions and other measures to limit the spread of this communicable disease. Family members, many of whom are former caregivers, were suddenly cut off from nursing home residents, and struggled to maintain connection with their loved ones residing in nursing homes. The purpose of this study was to describe the experiences of family members of residents in nursing homes in advocating for residents and themselves during a time of uncertainty and many challenges. This study used a qualitative descriptive approach to conduct individual interviews. Ten family members of residents of two nursing homes in a Northeastern state were interviewed by phone or videoconference using a semi-structured guide. Interviews were transcribed verbatim and analyzed using Braun and Clarke's Reflexive Thematic Analysis. Family members expressed concerns for the lockdown's impact on residents' psychosocial wellbeing in addition to the potential physical dangers of COVID-19. They explored creative means of meeting needs for information and interaction, but often felt that these efforts fell short of replicating the connectedness of in-person visits. Family members identified multiple missed opportunities for involvement in care, and voiced willingness to comply with infection prevention guidelines, such as maintaining distance, to be present with residents. Our findings indicate that family members advocated for residents' interests to ensure quality care. Future research and policy should consider family members as a potential resource for providing care and companionship during times of crisis.

### FAMILY CAREGIVER BURDEN OF MEDICATION ADMINISTRATION FOR OLDER ADULTS ADMITTED TO HOME HOSPICE

Jennifer Tjia,[1] Margaret Clayton,[2] Jennifer Smith,[2] Olivia Wood,[3] Susan Hurley,[3] Geraldine Puerto,[4] Vennesa Duodu,[4] and Susan DeSanto-Madeya,[5], *1. University of Massachusetts Medical School, University of Massachusetts Medical School, Massachusetts, United States, 2. University of Utah, The University of Utah, Utah, United States, 3. Care Dimensions, Care Dimensions, Massachusetts, United States, 4. UMass Medical School, UMass Medical School, Massachusetts, United States, 5. University of Rhode Island, University of Rhode Island, Rhode Island, United States*

**Objective:** To characterize FCG burden of medication administration for older adults in home hospice.

**Methods:** Pilot clinical trial of a hospice-staff level communication and medication review program to facilitate goal-concordant prescribing, including deprescribing, for older adults in home hospice. Patients newly admitted to hospice were eligible if >=65 years, prescribed >= 5 medications and had a FCG. Exclusion criteria included being non-English speaking or having a Palliative Performance Score<40. Measurements include 24-item FCG Medication Administration Hassle Scale (range 0-96) at hospice admission and at 2-, 4-, 6-, 8-weeks and monthly until death. Descriptive statistics characterize baseline FCG Hassle score.

**Results:** In this actively recruiting study, n=9 patient-caregiver dyads are enrolled to date. Mean patient age is 80.6 years (range 69-101). Of 9 caregivers, 7 were female, 5 children, and 3 spouses. The majority (67%) of caregivers were extremely involved in medication management. Mean FCG Hassle Score =17.1 (SE 5.9; range 2-58), and differed between spouses (mean =5 [SE 1.7; range 2-8]) and children (mean =31.4 [SE 9.53; range 3-58]). The highest burden concern was recognizing medication side effects, followed by feeling comfortable making medication decisions, arguing with the care-recipient about when to take medications, knowing why a medication is being given and whether it is effective, and knowing when to hold, increase, decrease a dose or discontinue the medication.

**Conclusion:** FCGs of older adults in home hospice report different levels of medication administration hassle depending on their relationship to the patient. The most bothersome concern is recognizing medication side effects.

### FRAUDULENT DATA DETECTION AND PREVENTION WITHIN THE NATIONAL CAREGIVER SURVEY

Jada Jackson,[1] Jessica Lipchin,[1] Rachel Zhang,[2] and Sheria Robinson-Lane,[1], *1. University of Michigan School of Nursing, Ann Arbor, Michigan, United States, 2. College of Engineering, University of Michigan Ann Arbor, University of Michigan Ann Arbor, Michigan, United States*

The National Caregiver Survey is an online, incentivized survey that aims to gather information about the health and coping strategies used by Black family caregivers of persons with dementia. The survey data will help elucidate the relationships between coping, health, and adaptation to family caregiving and facilitate the development of culturally responsive caregiver support programming. Virtually distributing this survey made it cost-effective, easily accessible, and quick to produce usable data. The online format also helped the team reach caregivers from across the nation, as well as keep participants safe during the COVID-19 pandemic. Unfortunately, because online surveys are advertised and administered digitally, they become targets for hacking, especially when the surveys are incentivized. The hacking attempts are executed through digital survey bots and threaten the integrity of the collected data. These corrupt responses also increase study costs by falsely rewarding the hackers for their survey responses and research team time in the investigation, detection, and removal of fraudulent responses. To detect potential bots, a reCAPTCHA bot system was incorporated into the survey, and survey questions were formatted specifically to thwart hacking attempts. Finally, data were cleaned to remove illogical, inconsistent, and duplicative surveys. Findings from this work may help researchers improve online survey design and data collection methods to provide greater confidence in conclusions drawn from virtually surveyed data.

### GENDER DIFFERENCES IN THE LINK BETWEEN INFORMAL CAREGIVING AND SUBJECTIVE WELL-BEING

Aeri Kim, and Kyungmi Woo, *Seoul National University, Seoul, Seoul-t'ukpyolsi, Republic of Korea*

Despite existing evidence on the negative association between informal caregiving and caregiver's well-being, three important knowledge gaps remain. First, the link warrants further scrutiny due to the possibility of individual heterogeneity. Second, less is known about how informal caregiving is related to caregiver's well-being. Third, there is little consensus in the literature regarding whether caregiver's gender matters. This study fills these gaps in the literature. Using seven waves of a large-scale, nationally representative longitudinal study of older adults in Korea between 2006 and 2018, this study employed generalized estimating equations models with a lagged dependent variable as well as fixed effects models. Findings from both models revealed that informal caregiving is negatively associated with subjective well-being, and this association is largely driven by female caregivers. To explore potential mechanisms undergirding this association, we examined the mediating roles of a number of health behaviors. We found that engaging in informal caregiving is associated with a reduction in regular exercise. Results from mediation analyses, however, suggested that regular exercise explains only a moderate amount of the observed association (12% for health-related life satisfaction and 8% for self-rated health). While informal caregiving is obviously a rewarding role, it poses a serious threat to caregiver's well-being. Findings of this study on gender differences in the well-being consequences of informal caregiving lend support to taking a gender-conscious approach in programs aiming to improve the well-being of informal caregivers.

### HOW FREQUENTLY ARE CAREGIVERS INCLUDED IN PATIENT EDUCATION FOR ONCOLOGY PATIENTS?

Daniel Hekman, Anne Mueller, and Beth Fields, *University of Wisconsin-Madison, Madison, Wisconsin, United States*

A growing body of literature shows that family and unpaid caregivers of older adults with cancer are assuming more care responsibilities, especially after discharge from an inpatient admission, and frequently report feeling unprepared to do so. Interprofessional collaborative practice can rectify this gap to help ensure caregivers are included in the care team and patient education in the hospital. This retrospective data analysis of electronic health record data examines the prevalence of caregiver involvement in education activities conducted by health care practitioners for older adult cancer inpatients at an academic medical hospital in the midwestern United States. Our dataset includes a total of 676 admissions of older adult cancer inpatients (565 unique patients) between 9/1/2018 and 10/1/2019. Descriptive statistical analyses were conducted to determine the prevalence of caregiver involvement in patient educational activities. The average patient was 75 years old (range: 66-89), white(95%) and male (57%). Approximately 5,720 educational topics were discussed with patients, and 88% of admissions included some patient education. Caregivers were included in 29.6% of educational topics discussed and at least one education session for 42.9% of all admissions. Caregivers are important collaborators in supporting the health and well-being of older adults with cancer, but they are often not included in patient educational activities prior to discharge. Practioners may need to evaluate barriers to including caregivers in patient education activities. A better understanding of this gap in education can help inform future interprofessional collaborative practice initiatives.

### INFORMAL CAREGIVERS' PERCEPTIONS OF BURDENS AND BENEFITS PREDICT GREATER CONFIDENCE IN THEIR ABILITIES

Cristina de Rosa,[1] Rebecca Lorenz,[1] and Suzanne Sullivan,[2], *1. University at Buffalo, Buffalo, New York, United States, 2. University at Buffalo, University at Buffalo, New York, United States*

Informal caregivers experience both burdens and benefits from caregiving. This analysis aimed to determine whether caregiver perceptions of burdens and benefits predicted feelings of confidence in their abilities. In the National Study of Caregiving (NSOC) Round II (2015), we identified 1,390 caregivers as "primary" for providing the greatest number of care hours in the past month to individuals age 65 and over. Logistic regression was performed to assess the influence of primary caregivers' gender, age, relationship to their care recipients, and self-reported indications of burdens and benefits on the odds that they would report confidence in their abilities. Caregivers were more likely to report confidence in their abilities when caregiving taught them to deal with difficult situations (OR=5.93, 95% CI [4.67, 7.54]), gave them satisfaction that their care recipient was well cared for (OR=1.97, 95% CI [1.26, 3.04]), and when caregiving brought them closer to their care recipient (OR=2.61, 95% CI [2.02, 3.36]). Caregivers were less likely to feel confident if they reported frequent changes in routine (OR=.78, 95% CI [.64, .96]). The final model predicted confidence (chi-square = 525.383 [4] p < .001) and correctly classified 78.7% of cases. All other variables were non-significant. These findings suggest that confidence in abilities is influenced by caregivers' perception of learning to handle difficult situations, satisfaction, closeness to the recipient, and burdens associated with changes in routine. Future research should further explore burdens and benefits of caregiving. Health care providers should routinely assess caregivers and provide referrals for additional resources.

### PATIENT AND CAREGIVER SYMPTOM TRAJECTORY: THE LAST 2 MONTHS OF CANCER HOME HOSPICE

Maija Reblin,[1] Miranda Jones,[2] Eli Iacob,[2] Djin Tay,[2] Kristin Cloyes,[3] Anna Beck,[4] Kathi Mooney,[2] and Lee Ellington,[2], *1. Moffitt Cancer Center, Tampa, Florida, United States, 2. University of Utah, Salt Lake City, Utah, United States, 3. University of Utah, Portland, Oregon, United States, 4. Huntsman Cancer Institute, Salt Lake City, Utah, United States*

Patient symptom management is a fundamental goal of cancer home hospice care. However, informal family caregivers, who are primarily responsible for daily patient care, also experience negative symptoms, especially at the end of the patient's life. While research has attended to patient symptom progression in home hospice, little research focuses on caregiver symptoms. To address this, we examined the frequency of both patient and caregiver symptoms to determine how these symptoms change in the last two months of the patient's life. Sixty-three cancer hospice caregivers from 4 US states prospectively reported daily patient and caregiver symptoms via an Interactive Voice Response phone system. We analyzed data from up to the last 60 days of the patient's life. Most caregivers were female (71.4%), Caucasian (88.9%), spouses of the patient (46%); average age was 59 years old (SD=13). Patients were mostly female (54%), with diverse solid tumor cancer diagnoses, and 72 years old (SD=11) on average. Most commonly reported moderate-to-severe patient symptoms were fatigue (67%), pain (47.5%), and loss in appetite (42.3%). Most common moderate-to-severe caregiver symptoms were fatigue (57.8%), trouble sleeping (45.1%), anxiety (52%), and depression (40.4%). Patient and caregiver symptoms were significantly correlated (Pearson r = .51, p<.001). Mixed-effects models found that both patient and caregiver symptoms (collapsed by week) worsened as patient death approached (ps <.01). Researchers and clinicians who are aware of the strong relationship between patient and caregiver symptoms are best able to address caregiver symptoms as part of hospice care, particularly as patient death approaches.

### PERCEIVED LIFE MEANING AND PURPOSE AND ITS ASSOCIATION WITH MENTAL AND PHYSICAL HEALTH AMONG FAMILY CAREGIVERS

Yu-Ping Chang,[1] Loralee Sessanna,[1] and Young Sik Seo,[2], *1. University at Buffalo, Buffalo, New York, United States, 2. Roswell Park Comprehensive Cancer Center, Buffalo, New York, United States*

Evidence suggests that having a sense of life meaning and purpose is related to physical health. However, the association between life meaning and purpose and physical and mental health among family caregivers remains unclear. This study aimed to examine whether family caregivers' perceived life meaning and purpose was associated with their physical and mental health (depression and anxiety). The National Study of Caregiving (NSOC) III cross-sectional survey (2017, N = 2,652) was utilized. One item was used to measure family caregivers' perceived life meaning and purpose and two composite variables were generated to measure depression and anxiety. Physical health was assessed by questions including pain, breathing problems, limited arm/leg strength, low energy, and sleep problems. Weighted logistic regression analyses with covariate adjustments (i.e., caregiver's age, sex, and race/ethnicity) were conducted to examine the association among family caregivers' perceived life meaning and purpose, mental and physical health. Results indicated that family caregivers' perceived life meaning and purpose was associated with a lower probability of having depressive symptoms (OR, .29, 95% Confidence Interval [CI], .15, .57) and anxiety (OR, .43, 95% CI, .23, .79). Furthermore, perceived life meaning and purpose was associated with a lower probability of having breathing problems (OR, .50, 95% CI [.25, .99]). Findings suggest that having a strong sense of life meaning and purpose is linked to better mental health and physical symptoms. Further research is needed to determine the mechanism regarding how life meaning and purpose may improve mental and physical health among family caregivers.

### PROLONGED MECHANICAL VENTILATION AT HOME VERSUS LONG TERM CARE: CAREGIVER CHARACTERISTICS AND STRAIN

Esther-Lee Marcus,[1] Jeremy Jacobs,[2] and Jochanan Stessman,[3], *1. Herzog Medical Center, Herzog Medical Center, Yerushalayim, Israel, 2. Hebrew University of Jerusalem, Jerusalem, Yerushalayim, Israel, 3. Hadassah Hebrew University Medical Center, Clalit Health Services, Yerushalayim, Israel*

Although the number of Prolonged Mechanical Ventilation (PMV) patients and their informal caregivers (CGs) is rising both at Home or Long Term Care (LTC), little is known concerning CG characteristics or strain. We enrolled 120 patients and 106 informal CGs: 34/46 and 72CGs/74 PMV patients from Home Hospital and LTC respectively. CGs were married (82%), female (60.4%), mean age 59 ±14 years; spouses (29%) or children (40%) of the PMV patient. The 13-item Modified Caregiver Strain Index (MCSI) (Maximum severity=26) was 13.6± 6.5, similar at Home vs. LTC (14.3±7.5 vs. 13.3±6.0, p=0.9). Most frequent complaints were distress concerning patient's changes (93%) or upsetting behaviours (82%), feeling overwhelmed (82%), sleep disturbance (69%) and emotional adjustments (67%). Home CGs reported significantly more physical and financial burden, confinement, and need for work adjustment, while LTC CGs reported greater emotional disturbance and upsetting patient behaviours. Hierarchical clustering identified three clusters of CG strain: burden (physical/time/financial), emotional (upsetting adjustment/ behaviours/overwhelmed) and disturbance (work/plans/confinement). Emotional strain was most frequent, irrespective of site of care; however CGs at Home vs. LTC experienced significantly higher burden and disturbance vs. higher emotional strain respectively. In multivariate models, after adjusting for numerous patient and CG variables, increasing CG strain was consistently associated with rising patient symptomatology. This relationship was pronounced among CGs of Home PMV patients, with a significant interaction variable of Home*Patient symptomatology. Our findings identify specific patterns of strain among caregivers of PMV patients whether at home or LTC, and highlight the importance of addressing their unique needs.

### SUPPORT NEEDS AS PERCEIVED BY GRANDPARENT-CAREGIVERS: A QUALITATIVE SYSTEMATIC REVIEW

Schola Matovu,[1] Deborah Whitley,[2] and Heather Young,[3], *1. University of California, Davis, Sacramento, California, United States, 2. Georgia State University, Atlanta, Georgia, United States, 3. University of California–Davis, Sacramento, California, United States*

Caregiving can have adverse mental and physical health outcomes. Older grandparents who are primary caregivers for their grandchildren report multiple health conditions such as depression, anxiety, hypertension, cardiac disease and chronic fatigue, which are caused by or otherwise exacerbated by the caregiving demands. We conducted this qualitative systematic review to identify support needs that contribute to such poor health outcomes and as perceived by grandparent-caregivers for minor grandchildren. We searched relevant databases (PubMed, PyschINFO, CINAHL, and Social Work Abstracts) using terms such as: child rearing, parenting, child custody, grandparents, support needs, and caregiving. Studies were included for review if they were written in the English language; used only qualitative methods; and were published from January 1990 to January 2020. Included studies were critically appraised using the Critical Appraisal Skills Programme checklist. Data were extracted from these studies and synthesized using meta-ethnography. Of the 2828 studies identified, 58 studies from 12 countries met all inclusion criteria for review. Three main themes emerged from the review: 1) grandparent-caregivers' personal needs, and 2) grandchildren needs. Both themes were further divided into subthemes of health (mental & physical), financial, social (interpersonal, cultural and environmental factors and services). Findings from this review have potential to: 1) inform design of comprehensive interventions and screening needed to address perceived support needs; and 2) identify gaps in and barriers to available support resources for older grandparent-caregivers. Further research is needed on comprehensive assessment of support needs and risk for poor health outcomes among grandparent-caregivers.

### TESTING PREDICTIVE FACTORS OF DEPRESSIVE SYMPTOMS AMONG GRANDPARENTS UNDER THE COVID-19 PANDEMIC

Yuqin Jiao, and Christine Fruhauf, *Colorado State University, Fort Collins, Colorado, United States*

An estimated 69.5 million Americans are reported to be grandparents. Among them, about 10% are raising grandchildren and the number of grandparents who are raising grandchildren (GRG) is increasing. Previous research on GRG suggests that the unexpected caregiving duties may lead to negative physical and mental health including more depressive symptoms when compared to non-caregiving grandparents (NGRG). Additionally, grandparent-grandchild relationships determined by emotional availability (EA) of the grandparent may be impacted. These factors might further be complicated, especially as it relates to the health and well-being of GRG, as a result of the COVID-19 pandemic. Thus, the overarching goal of this presentation is to use the biopsychosocial model to present a conceptual framework to test the mental well-being of GRG during the COVID-19 pandemic. In this presentation, we will 1) summarize appropriate literature on GRG; 2) share a COVID-19 health and well-being assessment survey designated for GRG in order to assess their health before and since the COVID pandemic; and 3) propose a conceptual model to investigate and test the protective role of physical activity and GRG's EA in the grandparent-grandchild relationship for the mental health of GRG. In our model, we argue that GRG experience more COVID-19 pandemic-related stress and more depressive symptoms when compared to NGRG. This proposed conceptual model offers one way to test the predictors of depressive symptoms on GRG. Future testing has the potential to shed new light on the development of appropriate intervention programs tailored to maintain the mental health of GRG.

### THE ASSOCIATION BETWEEN CAREGIVER EDUCATION ON ADULT T2DM AND PATIENT'S OUTCOMES IN COMMUNITY: A SYSTEMATIC REVIEW

Aluem Tark,[1] Jiyoun Song,[2] Jeong-Yeon Kim,[3] So Yeon Park,[3] and Kyungmi Woo,[3], *1. University of Iowa, University of Iowa, Iowa, United States, 2. Columbia University, Columbia University, New York, United States, 3. Seoul National University, Seoul, Seoul-t'ukpyolsi, Republic of Korea*

**Introduction:** Adult type 2 diabetes (T2DM) threatens public health and most patients manage their diabetic condition while in the community. As it is challenging for patients to properly manage diabetes alone, caregiver involvement in T2DM patient care is encouraged. This study aimed to examine the association between caregiver involvement in T2DM education within a community and the patients' diabetes care outcomes (e.g., glycated hemoglobin (HbA1c) level, behavior, or hospitalization).

**Methods:** The available scientific literature in PubMed, Cochrane, EMBASE, and CINAHL was searched. The methodological quality of bias was assessed using the Cochrane risk of bias tool.

**Results:** A total of 13 out of 741 published studies were synthesized in this review. There is evidence that caregiver involvement in T2DM education is effective in the reduction of HbA1C and BMI, but not necessarily effective in reducing lipids. Study results indicate that caregiver related interventions can significantly improve patient diabetes knowledge, physical activity, and self-efficacy, but results were more mixed regarding medication adherence. Risk of bias analysis classified the majority of studies (77%) to be moderate or high quality.

**Conclusion:** This review aimed to explore the association between caregiver involvement in adult T2DM education in the community and patients' diabetes care outcomes. The findings show an improvement in biological and behavioral self-management outcomes with caregivers involved in T2DM education, though no studies examined the direct association between complications or hospital readmission.

Future research focused on tailored interventions and longer follow-up of patient outcomes are recommended.

### THE EXPERIENCES OF FAMILY CAREGIVERS OF COMMUNITY-DWELLING OLDER ADULTS WITH DEMENTIA IN PROVIDING DAILY ORAL CARE

Abby Hellem, Kexin Zhou, Xi Chen, Jirakate Madiloggovit, Jennifer Nguyen, Rebecca Morris, and Sato Ashida, *University of Iowa, Iowa City, Iowa, United States*

Individuals with dementia increasingly rely on caregivers for daily oral care over time. This study explored the experience of family caregivers of community-dwelling individuals with dementia in providing oral care and their interest in caregiver oral education using the concepts of Social Cognitive Theory. Twenty-three caregivers ages 19-80 participated in a semi-structured qualitative interview that also included a structured questionnaire. Majority of caregivers were female (83%) with an average age of 56 years; 29% were spouses. Fifty-four percent of care recipients had natural teeth only, 42% had teeth and dentures, and 4% had dentures only. Caregivers were generally knowledgeable about the importance of oral health, but some expressed a lack of knowledge in how to perform oral care for others. Caregivers reported high levels of outcome expectation, agreeing that providing oral care would improve care recipient's oral health. Caregivers expressed mixed levels of self-efficacy; many cited reduced self-efficacy due to resistance or refusal of care. Quantitative data showed that higher confidence in knowledge and oral care skills was associated with greater confidence in providing oral care ($r=0.726$, $p<0.001$). Intent to participate in a caregiver oral health education program was associated with positive outcome expectations ($r=0.73$, $p=0.007$) and desire to learn the signs and symptoms of mouth pain and infection ($r=0.72$, $p=0.009$). Increasing family caregiver's oral health knowledge and skills, outcome expectations, and self-efficacy to provide care may help improve the oral health of persons with dementia. Additional qualitative and quantitative data and implications for practice will be presented.

### USE OF ACTIGRAPHY ON SLEEP OUTCOMES FOR DEMENTIA FAMILY CAREGIVERS: AN INTEGRATIVE REVIEW

Eunae Ju,[1] Melissa Pinto,[1] and Jung-Ah Lee,[2], *1. University of California Irvine, Irvine, California, United States, 2. University of California, Irvine, Irvine, California, United States*

Sleep difficulties are one of the foremost health problems that affect family caregivers of dementia patients increasing their risk for a host of mental health problems and hastening dementia patients' transitions to long-term care facilities. This integrative review aims to describe the objective measurement of sleep quality parameters of family caregivers using actigraphy and how well they are associated with self-reported subjective measures of sleep outcomes and psychological states. A search was performed using PubMed, CINAHL, and PsycInfo including articles from 2011 to 2020. Twenty studies met the inclusion criteria. Five sleep interventions (2 RCTs and 3 pre-post design) were found, including multi-component interventions (e.g., sleep hygiene, walking, daytime light therapy) that used actigraphy and other self-report measures. Duration of wearing actigraphy (wrist band/watch) varied in studies (3-days to 8-weeks). Most studies reported high accuracy and sensitivity of actigraphy. Sleep parameters measured by actigraphy included 'total sleep time', 'sleep efficiency', 'deep/light sleep', or 'wake time after sleep onset'. In eight studies, sleep parameters measured by actigraphy were significantly associated with sleep outcomes measured by sleep related self-reported scales (Epworth Sleepiness Scale, Pittsburgh Sleep Quality Index, all $Ps<.05$). Eleven studies used actigraphy to examine sleep measures associated with various mental health states (depression, burden, stress, positive/negative affect) and found significant relationships (All $Ps <.05$). Findings support that use of actigraphy for dementia family caregivers is a valid measure of sleep parameters when compared with their sleep self-reports. Furthermore, it was found that actigraphy sleep measures were significantly associated with psychological outcomes.

### UTILIZATION OF SOCIAL MEDIA PEER SUPPORT GROUPS AMONG FAMILY CAREGIVERS OF PATIENTS WITH DEMENTIA

Mary Dioise Ramos, *Kennesaw State University, Kennesaw, Georgia, United States*

Caring for a family member with dementia is particularly challenging. Unpaid family caregivers provide a significant amount of the care for aging relatives, and they provide the vast majority of long-term care. Family caregiving often results in negative effects, which compromises the caregiver's physical and psychosocial health. Social media support groups are an increasingly common venue for family caregivers supporting patients with dementia to exchange emotional, informational, and instrumental support. This study examined the utilization of social media support groups among family caregivers of patients with dementia during the pandemic. Using deductive thematic analysis, the use of social media support groups of family caregivers provide link to social interaction as a means when social distancing is enforced due to the pandemic. Family caregivers use social media support groups to share their personal experience, express their mood and feelings, offer prayers and positive quotes, keep up with the current events, gather information, and share feedback about dementia care services. Awareness of the potential advantages that social media support groups offer, healthcare providers can encourage family caregivers to use social media support groups as an empowering and practical platform. Further research is required about the long term benefit from social media support groups and the reliability and validity of the information that the family caregivers can get from the group.

### Session 9275 (Poster)

### FAMILY CAREGIVING DURING THE COVID-19 PANDEMIC

### "LOCKED OUT OF CAREGIVING": A CASE STUDY OF DEMENTIA CAREGIVING DURING THE COVID-19 PANDEMIC

Kristie Wood,[1] and Marie-Anne Suizzo,[2], *1. University of Texas, Austin, Austin, Texas, United States, 2. UT Austin, UT Austin, Texas, United States*

It is unclear how ambiguous loss in dementia caregiving is impacted by conditions of the Covid-19 pandemic.

Ambiguous loss describes situations in which closure is impossible and ambiguities within a family system ensue. Two situations of ambiguous loss exist. In the first type, one is psychologically absent, yet physically present, e.g. when one has dementia. In the second type, one is physically absent but psychologically present, e.g. moving to a nursing home. Ambiguous loss theory was applied to longitudinal interviews with an adult-child caregiver (age=52) of a mother with dementia, who resided in memory care during the Covid-19 pandemic. Theoretical analysis revealed both types of ambiguous loss were experienced in the dementia caregiving relationship. This was embedded within ambiguous loss type 2 due to the Covid-19 pandemic, e.g. social distancing and quarantine practices led to physical estrangement from others and ambiguity ensued about when, or if, estrangement would end before resulting in death. Further, the coping mechanisms defined in the ambiguous loss framework: restructuring identity, finding meaning, gaining mastery, increasing ambivalence capacity, reframing attachments, and gaining hope, were compromised due to overarching ambiguous loss attributed to the pandemic. Continued panic and frustration regarding lack of communication with and access to the memory care center instilled a sense of being "locked out of caregiving." Findings suggest dementia caregivers may experience both types of ambiguous loss compounded during the Covid-19 pandemic, suspending grief and coping processes, and inciting poorly understood needs and challenges that must be better understood to support dementia caregivers.

### A NATURAL EXPERIMENT CREATED BY PANDEMIC RESTRICTION: COMPARING IN-PERSON, HYBRID, & TELETHERAPY FORMATS

Tracy Wharton,[1] and Daniel Paulson,[2], *1. University of Central Florida, Winter Springs, Florida, United States, 2. University of Central Florida, Orlando, Florida, United States*

The FL-REACH intervention for families in early stage post-dementia diagnosis was originally adapted from the REACH II program for use in an outpatient clinic. Pandemic restrictions forced an adaptation to a teletherapy format. The timing of changes allowed comparison of caregivers who participated in clinic (n=10), switched modalities mid-treatment (n=7), and participated as teletherapy (n=14). Groups were similar in age range, gender, and relationship, with both spouses and adult children participating. Participants in the fully online group were more likely than others to have high school or trade school education than to have graduated from college. All participants in the in-person and hybrid groups had incomes over $40,000/year, while 36% of the online sample had less household income, were more likely to be Hispanic-identifying (43% versus 6%), and had higher burden scores (M=41.43 versus M=32.56 in person, M=29.86 hybrid) and lower preparedness scores (M=19.86 versus M=22.90 in person, M=28.14 hybrid) at baseline ($p<.05$). The intervention proceeded with the same intervention dosage (8 hours total), and outcomes were essentially comparable, with all groups showing statistically significant improvement on measures of preparedness, burden, and risk. While in-person intervention helped strengthen relationships with the medical team, inclusion of family via telehealth provided opportunity for a more culturally responsive and inclusive engagement, although there remain questions regarding reasons for differences at baseline. Identification of differences in key outcomes for direct comparisons between in-person, hybrid, and teletherapy interventions are limited in the evidence base, making this a unique study at an important moment in time.

### CAREGIVING DURING THE COVID-19 PANDEMIC: FACTORS ASSOCIATED WITH CAREGIVER STRESS AND COGNITION

Rachael Turner,[1] Celinda Reese-Melancon,[2] and Erin Harrington,[2], *1. Oklahoma State University, Stillwater, Oklahoma, United States, 2. Oklahoma State University, Oklahoma State University, Oklahoma, United States*

Caregivers are critical in helping persons with dementia (PWD) live at home longer, but the caregiving experience is associated with increased risk of physical (Vitaliano et al., 2003; Son et al., 2007; Fonareva & Oken, 2014) and cognitive decline among caregivers (Pertle et al., 2015; Lathan et al., 2016; Vitaliano et al., 2017). The present study examined the caregiver experience during the time of the Covid-19 pandemic including factors associated with caregiver stress, burden, and self-reported cognition (i.e., prospective and retrospective memory errors). In a sample of 56 caregivers of PWD, caregiver stress was positively associated with reports of greater life change resulting from Covid-19 and a greater frequency of care recipient depressive and disruptive behaviors; however, caregiver stress was not associated with care recipient memory problems. Additionally, caregiver burden was negatively associated with ratings of preparedness for the pandemic, but not with availability of support services or the amount of time spent caregiving. Further, frequencies of prospective and retrospective memory mistakes were positively associated with perceived stress, but not with caregiver burden. These findings reveal that caregivers of PWD report greater experiences of stress associated with the Covid-19 pandemic and other facets of their caregiving responsibilities (e.g., care recipient depressive and disruptive behaviors, frequency of memory mistakes). This work is a first step in identifying areas in which caregivers need assistance and expanding the literature on caregiver cognition by measuring self-reported everyday memory performance.

### CAREGIVING INTENSITY AND PSYCHOSOCIAL IMPACT OF COVID-19 IN DEMENTIA AND NON-DEMENTIA CAREGIVERS

Marika Humber,[1] Angeline Truong,[2] Madhuvanthi Suresh,[3] Josephine Jacobs,[4] Sam Thomas,[5] Trevor Lee,[6] Rashmi Risbud,[7] and Ranak Trivedi,[8], *1. VA Palo Alto Healthcare System, Menlo Park, California, United States, 2. Stanford University School of Medicine, Stanford, California, United States, 3. Palo Alto University, Atlanta, Georgia, United States, 4. VA Palo Alto Health Care System, Menlo Park, California, United States, 5. Stanford School of Medicine, Stanford, California, United States, 6. VA Palo Alto Health Care System, VA Palo Alto Health Care System, California, United States, 7. VA Palo Alto Health Care System, Palo Alto, California, United States, 8. Stanford University, Menlo Park, California, United States*

COVID-19 has adversely impacted the well-being of informal caregivers (CG) due to infection risk, changes to the home environment, and changes to resource availability. CG of persons living with dementia (PLWD) may be especially vulnerable due to the intensity of care provided. We compared CG activities and well-being among CG who did and did not care for PLWD during COVID-19. We conducted an anonymous online survey from April 2020-present. Respondents self-identified as 18+ years and CG to a child or adult with mental health or medical conditions. CG answered questions regarding hours of care provision and caregiving activities, and completed measures of CG burden (Zarit Burden Inventory-4), loneliness (UCLA Loneliness Scale), depressive symptoms (Patient Health Questionnaire), and anxiety (Generalized Anxiety Disorder-2). Of the 258 respondents within the United States, 86 cared for PLWD (33%; 88% female; 56±12 years) while 172 did not (66%; 87% female; 49±14 years). Compared to non-dementia CGs, more CGs of PLWD provided 40+ hours of caregiving/week (36% vs. 49%, p<.05), performed more caregiving activities (8.5 vs. 10.5, p<.01), and assisted with more activities of daily living (55% vs. 73%, p<.01). Compared to non-dementia CG, more dementia CG reported CG burden (53% vs. 67%; p<.05) and loneliness (7.3 vs. 9.1, p<.05). No differences in depressive symptoms or anxiety were found. Results suggest that existing needs of CG of PLWD may be exacerbated by the stressors and concerns of the pandemic, necessitating higher levels of support.

### DEVELOPMENT AND INITIAL EVALUATION OF THE CARING THROUGH COVID-19 PSYCHOTHERAPY GROUP FOR FAMILY CARE PARTNERS

Evan Plys,[1] Kadija N. Williams,[2] Caitlin J. Tyrrell,[3] and Rachel Weiskittle,[4], *1. University of Colorado Denver, Denver, Colorado, United States, 2. VA Boston Healthcare System, Brockton, Massachusetts, United States, 3. VA Pacific Islands Healthcare System, Honolulu, Hawaii, United States, 4. VA Boston Healthcare System, Jamaica Plain, Massachusetts, United States*

This project details the development and initial evaluation of a manualized psychotherapy support group for family care partners of persons living with dementia, specially designed to address pandemic-related stressors. The authorship team, consisting of clinical geropsychologists, developed a treatment manual based on existing protocols, such as: cognitive behavioral therapy for pandemic-related stress, grief management and ambiguous loss, and caregiver family therapy. The resulting 8-week Caring Through COVID-19 psychotherapy group was piloted in an outpatient mental health clinic via tele-mental health with six family care partners of persons living with dementia. All participants were women and spouses or partners of the care recipient; mean age was 70.5 (SD = 9.07). Preliminary data showed a non-significant and small reduction in depression (d = .22) and non-significant moderate reductions in caregiver burden (d = .52) and pandemic-related stress (d = .64). Moderate non-significant improvements were observed in general caregiver self-efficacy (d = .62) and self-efficacy for emotional regulation (d = .67). The majority of participants reported that the content of the group was novel (83%) and relevant (83%); the most utilized topics outside of the group were accepting emotions (100%) and challenging negative cognitions (83%). Overall, most participants were very or extremely satisfied with the group (67%). Additional data is currently being collected with another cycle of the group (n = 4). Preliminary findings suggest that the Caring Through COVID-19 group may be beneficial for supporting family care partners during the pandemic. Considerations for implementation and future plans for dissemination will be discussed.

### LEARNING NEEDS OF DEMENTIA FAMILY CAREGIVERS DURING THE PANDEMIC

Carolyn Clevenger,[1] Fayron Epps,[2] Molly Perkins,[3] Glenna Brewster Glasgow,[2] and Kenneth Hepburn,[2], *1. Emory University, Emory University, Georgia, United States, 2. Emory University, Atlanta, Georgia, United States, 3. Emory University of Medicine, Atlanta, Georgia, United States*

The COVID-19 pandemic has dominated and transformed all caregiving contexts and situations. In a time of COVID-19, caregivers now have to learn how to take all of the complicated precautions to keep themselves and their persons from being exposed to the virus given their population's mortality rate from COVID-19 infections exceed 40%. As part of a larger initiative to develop an asynchronous online education program for family caregivers of persons living with dementia illnesses (PLWD) to prepare them to master the new demands of their caregiving role in this extraordinary circumstance of the COVID-19 pandemic, we conducted three focus groups with 13 dementia family caregivers to inform the structure, content, and "feel" of the course. Focus groups were conducted with a lead interviewer, via Zoom, audio and video recorded and transcribed for analysis. Participants were asked two groups of questions: their lived experience over the past year and course content for caregiving during crisis. Caregivers identified 4 themes regarding their lived experience of caregiving during the pandemic: (a) mixed feeling about the stay-at-home orders; (b) positive adaptation to telemedicine, (c) vaccine risks and benefits; and (d) impact of social isolation on the PLWD. The groups also recommended specific course content based on their experiences. Participants recommended specific course content based on their experiences, such as health system navigation and the logistics of what to do following the death of a PLWD. Results from these groups have been incorporated into an asynchronous online course to be pilot tested in coming months.

### Session 9280 (Poster)

### FAMILY CAREGIVING II (SRPP POSTER)

### A SCOPING REVIEW OF YOUTH CAREGIVERS: ADDRESSING THE NEEDS OF THIS GROWING POPULATION

Heena Doshi,[1] Gracie Chen,[2] Norma Bostarr,[3] Debra Cherry,[4] Mary Mittelman,[5] and Nihal Satyadev,[6], *1. Care Generation, Los Angeles, California, United States, 2. University of California, San Diego, Temple City, California, United States, 3. Care Generation, SF, California,*

*United States, 4. Alzheimer's Los Angeles, Los Angeles, California, United States, 5. NYU Langone Health, NYU Grossman School of Medicine, New York, United States, 6. The Youth Movement Against Alzheimer's, Los Angeles, California, United States*

Generation Z, those who are born in 1997 or thereafter, account for six percent of the estimated 53 million family caregivers in the US – and this percentage is growing, especially given the health impacts of the pandemic. This review focuses on caregiving youth (ages 0-18) and young adult caregivers (ages 18-25) who assist household members who need regular assistance with ADLs and IADLs. This presentation will analyze key trends in the literature of this demographic and identify gaps in research. Our review broadens the knowledge base of how caregiving done by this population impacts brain development, and hence their long term physical, emotional, and mental health. In spite of the significant number of caregiving youth and young adults, current publications on these demographics remain sparse, with most studies being conducted on spousal and adult caregivers. Our group found that the existing literature reviewed the adverse implications on the health and education outcomes for young adult caregivers. This included increased anxiety/depression compared to non-caregivers and for those in post-secondary education, the contribution of caregiving to college incompletion. There are no known studies on this population who have entered the workforce and who also continue their caregiving role. Adequate understanding and characterization of caregiving youth and young adult caregivers will inform better interventions and future policy for them. Support for this demographic, in turn, may improve health outcomes for older adults, who are the majority of those in their care.

### "WE'VE ALL LOST SO MUCH": THE EXPERIENCES OF ESSENTIAL FAMILY CAREGIVERS' LONG TERM CARE VISITATIONS DURING COVID-19

Charlene Chu,[1] Amanda Yee,[2] and Vivian Stamatopoulos,[3], *1. University of Toronto, Toronto, Ontario, Canada, 2. University of Toronto, Calgary, Alberta, Canada, 3. University of Ontario Institute of Technology (UOIT), Oshawa, Ontario, Canada*

Family caregivers are integral to the care of long-term care (LTC) residents. COVID-19 public health policies initially restricted all essential caregivers from visiting LTC homes. In lieu of in-person visitations, caregivers were allowed technology-based visits then restrictive outdoor visits, followed by indoor visitations. This study aims to illuminate the experiences of essential caregivers' as they visited their loved ones in LTC during COVID-19's restrictive policies. We conducted seven caregiver focus groups (N=30) from Ontario and British Columbia, Canada. Thematic analysis and line-by-line coding were completed using NVivo. We found six themes that were common to all the visitation types: 1) "LTC Home disorganization" to facilitate visits and poor communication; 2) "Lack of staffing and resources"; 3) "Mistreatment from staff and management" as caregivers were seen as inconveniences; 4) "Shock and disbelief" when family members first saw their loved ones; 5) "Significant lack of person-centered or family-centered ethos" for example the residents' needs were ignored such that their cognitive and physical impairments sometimes made visitations impossible, as well as the burden of multiple weekly COVID-19 tests; and, 6) "Collateral damage" in the form of trauma and irreparable harm to the relationships between residents and families. These results emphasized caregivers who ultimately felt betrayed and ignored by the broader healthcare system. Our findings provide an in-depth understanding of how COVID-19 public health policies have impacted the essential caregivers and the long-lasting impacts on residents and caregivers alike. Understanding caregiver's experiences can inform future pandemic response policies and encourage more person-centered protocols.

### DIFFERENCES IN SUBJECTIVE AGE BY FILIAL CAREGIVING STATUS AMONG US ADULTS IN MID AND LATER LIFE

Hyojin Choi,[1] and Kristin Litzelman,[2] *1. University of Wisconsin--Madison, Madison, Wisconsin, United States, 2. University of Wisconsin-Madison, Madison, Wisconsin, United States*

Subjective age is an important indicator of age identity and is associated with both psychological and physical well-being. Previous studies have revealed that older adults who feel younger than their chronological age show better health status, better life satisfaction, and less risk of mortality. Considerable evidence shows that stress contributes to feeling older than one's chronological age. Given the fact that taking a caregiving role involves stress, it is expected that caregiving might accelerate subjective aging. This study examined the association between the stressor of caregiving and subjective age in mid and later life. Data were drawn from the Health and Retirement Study in 2014 and 2016. Participants aged 50 years and over (n=1,087) were identified according to adult-child caregiver status at across the two waves: those who provided care consecutively (long-term caregivers), those who became caregivers in 2016 (new caregivers), those who were no longer providing care in 2016 (recent caregivers), or those who did not report providing care in both 2014 and 2016 (non-caregivers). Linear regression analysis showed that new caregivers reported feeling older than their chronological age compared to non-caregivers. However, long-term or recent caregivers did not show significant differences in subjective age compared to non-caregivers. The finding is consistent with the stress process theory and adaptation hypothesis. Although the onset of caregiving stress may accelerate subjective aging, this deleterious effect may decrease over time due to family caregivers' adaptability. Future research will examine the role of support, resilience and mastery in this pathway.

### DIRECT-TO-CAREGIVERS RESEARCH DISSEMINATION: A NOVEL APPROACH TO TARGETING END-USERS

Lee Lindquist,[1] Shahla Baharlou,[2] Vanessa Ramirez-Zohfeld,[3] and Anna Liggett,[1], *1. Northwestern University Feinberg School of Medicine, Chicago, Illinois, United States, 2. Icahn School of Medicine at Mount Sinai, Icahn School of Medicine at Mount Sinai, New York, United States, 3. Northwestern University Feinberg School of Medicine, Northwestern University Feinberg School of Medicine, Illinois, United States*

Dissemination of geriatrics research usually occurs through conference presentations or publications viewed by colleagues in the same field. Older adults and their family caregivers have limited direct access to research findings. We sought to pilot a direct-to-caregiver workshop with the intent to disseminate geriatrics research directly to family caregivers of older adults. As part of an academic national conference, an 'Updates in Geriatrics Research' workshop is presented as a compilation of innovative research published in the prior year. We distilled workshop content into a lay format which was presented to family caregivers at two community-based caregiver symposiums. Mixed method surveys were completed by family caregiver attendees with open-ended responses analyzed using content and constant-comparative techniques. Of the 29 survey respondents, all were female, mean age 58.9 yrs. (range 52-72), providing care to older adults, mean age 87.2 years (range 66-97). Respondents unanimously identified learning information pertinent to their care recipient. When asked: Do you feel that direct-to-caregiver research dissemination is useful, all respondents selected yes. Open-ended responses for reasons why revealed two main themes: 1.) Creating informed caregivers: "Caregivers need this information in their toolbox." and 2.) Empowering caregiver-advocates: "The more we know, the better we can advocate for our loved ones and challenge their health care." Respondents all planned on sharing information with others, specifically family, friends, and physicians. In conclusion, disseminating geriatrics research direct-to-caregivers is feasible. Researchers, who present their work for scientific conferences, should consider translating their findings into presentations for community-based family caregivers.

### DYNAMICS OF UNMET NEED FOR SOCIAL CARE IN ENGLAND

Athina Vlachantoni, Maria Evandrou, Jane Falkingham, and Min Qin, *University of Southampton, Southampton, England, United Kingdom*

Meeting individuals' social care needs is a core element of UK social policy. However, the conceptualisation and operationalisation of 'unmet need' remain a challenge. This paper advances our understanding by incorporating a temporal dimension in the conceptual framework on unmet need to investigate the dynamics of met and unmet need for social care over time. Using data from Waves 6,7 and 8 of the English Longitudinal Study of Ageing, this paper examines five possible trajectories among individuals with a social care need at baseline: 1) no longer having such a need; 2) having continued needs met; 3) delayed needs met; 4) newly arisen unmet needs; and 5) persistent unmet needs. The results indicate that amongst those with need at baseline unmet need has decreased over time, indicating that some needs may be fulfilled with a delay. However, a significant proportion of older people experienced persistent unmet needs, particularly those who were younger, living alone, with educational qualifications, and with fewer difficulties with Activities of Daily Living at baseline. Understanding the dynamics of unmet need can support policymakers in ensuring that those facing an elevated risk of persistent unmet need over time do not fall through the social care safety net.

### EXAMINING PHYSICAL AND SOCIAL ENVIRONMENTS OF KOREAN AMERICAN FAMILY CAREGIVERS OF PERSONS LIVING WITH DEMENTIA

Kathy Lee,[1] Chang Hyun Seo,[2] Jon Shuffler,[2] Christina Miyawaki,[3] Joshua Grill,[4] and Hye-Won Shin,[4], *1. University of Texas at Arlington, Coppell, Texas, United States, 2. University of Texas at Arlington, University of Texas at Arlington, Texas, United States, 3. University of Houston, University of Houston, Texas, United States, 4. UC Irvine Institute for Memory Impairments and Neurological Disorders (UCI MIND), UC Irvine Institute for Memory Impairments and Neurological Disorders (UCI MIND), California, United States*

**Introduction:** East Asian Americans are considered a hard-to-reach population in the field. Existing resources are not inclusive of Korean family caregivers, and therefore, a community support system may be required for this ethnic group.

**Methods:** We conducted a telephone-based survey and in-depth interview with Korean family caregivers of persons with dementia (N=36; Mean age: 63.3) to examine their physical and social environments.

**Results:** Most caregivers (58%) co-resided with their care recipients. Those providing care with limited assistance from others showed greater financial hardship (p=0.03) and interference with employment (p=0.03). Job interference was further related to higher levels of caregiving burdens (p=0.01). The services used most were senior center services (25%) and in-home care services (17%), while desired services included health promotion and disease prevention services (50%) and culturally appropriate or medically tailored home-delivered meals services (31%). Caregivers socialized with others using a multi-messaging app (i.e., KakaoTalk) with others. About 74% of them addressed they used KakaoTalk always (52%) or often (22%), and nearly half of them (47%) said they searched for caregiving information online. Findings from our qualitative interviews confirmed positive attitude toward the use of technology. Korean family caregivers showed a lack of knowledge of not only existing community-based resources but also the disease-related information, particularly regarding early-stage support and home safety.

**Conclusion:** It is critical to develop a community education program that reflects their unique physical and social environment conditions, potentially through technologically delivered interventions, for outreach and engagement for Korean family caregivers of persons with dementia.

### FAMILY CAREGIVING AND DEPRESSION AMONG OLDER ADULTS IN JAPAN: A CROSS-SECTIONAL STUDY DURING THE COVID-19 PANDEMIC

Taiji Noguchi,[1] Takahiro Hayashi,[2] Yuta Kubo,[2] Naoki Tomiyama,[2] Akira Ochi,[2] and Hiroyuki Hayashi,[2], *1. National Center for Geriatrics and Gerontology, Obu, Aichi, Japan, 2. Seijoh University, Tokai, Aichi, Japan*

COVID-19 infections are particularly lethal in older adults; thus, social activities of older adults and their families in the community have been restricted. The threat of infection, restrictions on social activities, and limitations on the provision of care services for older adults could increase family caregivers' burden and impact their mental health. This cross-sectional study examined the association between family caregiving and change in depression during the COVID-19 pandemic. In October 2020, we conducted a mailed questionnaire survey on a random sample of functionally independent community-dwelling older adults in a semi-urban area of Japan. Based on the depression status between March and October 2020, participants were classified into four groups: “consistently non-depressed,” “depression onset,” “recovering from depression,” and “remained depressed.” Participants were assessed for providing care for their family members or not. Caregiver participants were also assessed on their caregiving role (primary or secondary), the severity of their care-recipient’s needs, and an increased caregiver burden. Data from 957 older adults were analyzed. The participants’ mean age (SD) was 80.8 (4.8) years, and 53.5% were female. Multivariable multinomial logistic regression analysis revealed that family caregiving was associated with depression onset (OR=3.17 [95%CI=1.57-6.40], p=0.001) and remaining depressed (2.53 [1.36-4.71], p=0.004). Particularly, primary caregivers, those providing care for family members with severer care need-levels, and those with an increased caregiver burden had a higher risk of depression onset and remaining depressed. Family caregivers could have severe mental health conditions during the pandemic. Developing a support system is essential to protect their mental health.

### HOPE, ACTIVE COPING AND WELL-BEING IN STROKE-SURVIVORS AND CAREGIVERS: AN ACTOR–PARTNER MODEL ANALYSIS

Esther Chow, and Sai-fu FUNG, *City University of Hong Kong, Kowloon, Not Applicable, Hong Kong*

**Purpose:** Stroke is a medical condition cause of suffering for both patients and their caregivers within the family. This paper aimed to assess the influence of the psychological construct of hope and active coping on hedonic wellbeing in patient with stroke and caregiver dyads by determining the dyadic patterns.

**Methods:** A baseline study including 200 stroke-survivor (Age 73.63; SD=7.22) and caregiver (62.49; SD=14.44) dyads with actor–partner interdependence model (APIM) estimated by structural equation modelling with maximum likelihood estimation. Herth Hope Index (HHI), Proactive Coping Inventory (PCI) and Personal Wellbeing Index (PWI) were used to measure hope, proactive coping and wellbeing, respectively.

**Results:** The APIM analysis revealed that both stroke patients and caregivers demonstrated double actor-only pattern. As such, stroke-survivors’ hope and active coping excreted an actor effect on their own wellbeing with beta = 0.48 ($p < 0.01$) and 0.16 ($p < 0.01$), respectively. Similarly, the caregiver also reported an actor effect between hope and wellbeing (beta = 0.84, $p < 0.01$) as well as active coping with wellbeing (beta = 0.37, $p < 0.01$). The overall SEM model also fulfilled the criteria for good model fit ($\chi 2$ (5.87), p = > 0.05, CFI = 0.98, TLI = 0.96 and RMSEA = 0.07).

**Conclusions:** The results suggest that both stroke patients and caregivers’ hedonic wellbeing are holding actor-oriented pattern with the hope and active coping. The implications for clinical practitioners, research and theoretical development are discussed.

### INNOVATIONS ON DEMENTIA ADVOCACY AMONG STUDENTS OF A MEDICAL UNIVERSITY IN TAIWAN

Tsuann Kuo, *Chung Shan Medical University, Chung Shan Medical University / Taichung City, Taichung, Taiwan (Republic of China)*

In 2017, Taiwan established Dementia Action Plans 2.0 to respond to the World Health Organization's call to increase dementia awareness and support for dementia carers. However, efforts have not yet been made to educate and increase dementia literacy on the younger generations. This paper addressed the outcomes to increase knowledge and information about dementia and caregiver resources to university students. 93 students participated in a two-day workshop on dementia literacy, followed by two months of advocacy in Taichung City, Taiwan. Students were divided into 14 advocacy groups and the outcome reports were categorized qualitatively using content analysis. The results showed that students were from nine departments and over one-quarter having a dementia loved one in the family. Four innovative categories were developed, including (1) dementia literacy for students and the public; (2) dementia friendly action plans; (3) dementia caregiver’s advocacy and (4) reducing dementia stereotypes. Highlights included students using social media to promote dementia literacy, face-to-face experiences to inform public education, dementia education on early onset dementia and using diverse bio-psycho-social angels to evaluate dementia. Students expanded advocacy to many social media, innovations and target areas, including Facebook, Instagram, Google spreadsheet, stickers, postcards, illustration of children’s books and public announcement. This paper revealed that younger generations used many advocacy methods that were thinking outside of the box. In conclusion, dementia is no longer an elder’s business but young adults can bring technological, inter-generational and cultural innovations into fulfilling the goals of Dementia Global Action Plans.

### KEY FACTORS IN INTERVENTION IMPLEMENTATION, FIDELITY, AND SUSTAINABILITY

Carey Sherman,[1] Kate Gordon,[2] and Kenneth Hepburn,[3], *1. Family Care Strategies, LLC, Ann Arbor, Michigan, United States, 2. Spilane Consulting, Columbia, Maryland, United States, 3. Emory University, Atlanta, Georgia, United States*

As part of an NIA-supported effort to develop an online course to train individuals to lead the evidence-based Savvy Caregiver program and to orient sponsoring organizations to the program, we conducted semi-structured interviews to assess success and sustainability “best practices”. Interviews were conducted with 17 leaders and trainers from eleven Savvy-providing organizations. Analysis of these interviews identified two main themes associated with successful program implementation: leadership commitment and trainer ownership. Paramount to success appears to be leaders’ clear understanding of and enthusiastic commitment to the value of the Savvy program to the organization’s constituents. This translated to careful selection, training, management and on-going development of Savvy program trainers. It contributed to leaders’ appreciation of Savvy as a gateway for clients to seek out other programs and services from the organization, while the gathering of meaningful evaluation data (using established outcome-assessment instruments)

contributed, in several cases, to garnering more lasting support to deliver the program. Organizations' commitment to the program was demonstrated by securing the kinds of adequate and appropriate training, typically involving both instruction and modeling, for Savvy program leaders. These efforts fostered a sense of ownership among the leaders – the sense that the program had positive value for the caregivers served. These findings should be of interest both to scholars engaged in the development of interventions and for organizations implementing them. Taken together, the themes highlight several factors for program implementation that maximize the chances of maintaining fidelity to core program principles and ensuring its sustainability.

### PIVOTING DURING THE PANDEMIC: A CASE STUDY OF THE SENIOR COMPANION PROGRAM PLUS

Noelle Fields,[1] Ling Xu,[1] Erin Roark,[2] Sruthi Sundar,[2] Ishan Williams,[3] and Joseph Gaugler,[4], *1. University of Texas at Arlington, Arlington, Texas, United States, 2. The University of Texas-Arlington, Arlington, Texas, United States, 3. University of Virginia, Charlottesville, Virginia, United States, 4. University of Minnesota, Minneapolis, Minnesota, United States*

**Introduction:** Growing research supports the use of older volunteers to provide respite and community-based assistance to persons with ADRD and their caregivers. This study explores the impact of COVID-19 on a face-to-face, peer-led psychoeducational intervention for African American ADRD family caregivers, the Senior Companion Program Plus (SCP-Plus), and its subsequent need to 'pivot' during the pandemic.

**Method:** The SCP-Plus was a randomized control trial across three states that assessed program impact on ADRD family caregiver stress/burden, coping, and social support. In spring 2020, the SCP-Plus intervention was halted because of the potential risk to participants due to COVID-19 (n = 20 enrolled dyads). In an effort to maintain rapport and trust, critical to retention in research studies, team members began weekly (March-April) and then bi-weekly calls (May-December) for the purpose of providing a social check-in and to provide updates on the status of the intervention.

**Results:** A total of 396 calls lasting approximately 10 minutes each were completed. Participants shared concerns around safety, access to food/supplies/masks/testing, feelings of stress and loss, concern for others, and the importance of technology as a means of social connection. Although the intervention aspect of the SCP-Plus ultimately ended due to COVID-19, information gleaned from these check-ins were used to pivot the study. The study moved forward by using a descriptive phenomenological approach to capture dyads' lived experiences during COVID-19.

**Discussion:** Overall, purposeful participant engagement through weekly/bi-weekly phone calls suggests that this is a promising strategy for participant retention as well as for pivoting research.

### PPA TELE-SAVVY: DEVELOPING AN ONLINE INTERVENTION WITH CAREGIVERS OF PERSONS WITH PRIMARY PROGRESSIVE APHASIA

Darby Morhardt,[1] Angela Roberts,[2] Alyssa Penn,[2] Allison Lindauer,[3] Emily Rogalski,[1] Sandra Weintraub,[1] and Kenneth Hepburn,[4], *1. Northwestern University, Chicago, Illinois, United States, 2. Northwestern University, Evanston, Illinois, United States, 3. Oregon Health Sciences University, Portland, Oregon, United States, 4. Emory University, Atlanta, Georgia, United States*

Primary progressive aphasia (PPA) typically results from a neurodegenerative disease such as frontotemporal lobar degeneration or Alzheimer's disease and is characterized by a progressive loss of specific language functions with relative sparing of other cognitive domains until later stages when widespread cognitive decline consistent with generalized dementia is more prevalent. PPA tends to appear earlier than most dementias, in late middle-age, and can result in a high degree of psychological and economic burden for the family. Thematic analysis of PPA caregiver studies reveal families are learning to adapt to not only declining language across communicative contexts and domains, but concomitant behavioral, social communication and personality changes over time. While there are several evidence-based dementia caregiver interventions, none are specifically designed for the PPA family caregiver. This pilot project, funded by the Emory University Roybal Center is the adaptation of an evidence-based on-line psychoeducation program (Tele-Savvy) to address the unique challenges facing informal caregivers of those living with PPA and to help these caregivers achieve mastery within this context. PPA caregivers have been engaged through focus groups to identify their most pressing caregiving challenges and how the existing Tele-Savvy curriculum should be adapted to meet their needs. Synchronous and asynchronous video modules have been designed to address: PPA education, the impact on dyadic connection and caregiving challenges and communication strategies specific to PPA. The Tele-Savvy central processes of coaching and de-briefing will also be pilot tested and refined.

### Session 9285 (Poster)

### FAMILY CAREGIVING: INTERVENTIONS AND SUPPORT

### FAMILY CAREGIVERS AND SUPPORT: IMPLICATIONS FOR WORK STRAIN AND FORMAL SERVICE USE

Joseph Svec, and Jeongeun Lee, *Iowa State University, Ames, Iowa, United States*

In the US, many employed caregivers make professional adjustments, exacerbating already tenuous balances between work and life. Using the framework of the Stress Process Model (SPM), current research examines the sources of support (both formal and informal) and the contextual factors that facilitate or impede caregiver support. In this research, we examine whether and to what extent caregiver work strain is ameliorated by the presence of additional family caregivers and formal service use. This study utilizes data provided by the National Study of Caregiving (NSOC) data. Using panel methods for the pooled waves, we analyze the associations between work-strain and the number of additional caregivers with utilization of formal support (such as paid service support). Preliminary analyses align with the Stress Process Model as additional caregivers for each respective care-recipient is associated with lower levels of work strain. On the other hand, utilization of formal services (paid help and Medicaid funding) is positively associated with work strain. These findings suggest that the number of additional caregivers can reduce the negative impact of caregiving on work related strain among employed caregivers. That is, multiple caregivers may be more reflective of cooperative arrangements which offset work disruptions that occur with the onset of caregiving. In addition, formal sources may more frequently be used as a last resort to address caregiver burnout. Ongoing analyses are examining changes in the number of caregivers and its impact on disruptive work event, which could lead to financial outcomes for caregivers.

### INTERDISCIPLINARY COMMUNITY-BASED SUPPORT FOR CAREGIVERS OF INDIVIDUALS LIVING WITH DEMENTIA

Gina Tucker-Roghi,[1] Sarah Tucker,[2] Jamie Escoubas,[2] and Renee Tolliver,[2], *1. Dominican University of California, SAN RAFAEL, California, United States, 2. Sonoma County Council on Aging, Santa Rosa, California, United States*

Evidence indicates family caregivers of individuals living with dementia (ILwD) are at risk for diminished physical and mental health; which may decrease their quality of life and directly impact their ability to provide care. An interdisciplinary approach to self-care and skill-building for caregivers is provided in a virtual support group offered by Council on Aging in Sonoma County, CA. As part of the nonprofit's Adult Day Program, the group is offered to client caregivers and has two goals: First, creating a community-based, long-term support system for ILwD who are aging-in-place; second, fostering a safe and supportive community for family caregivers, by providing opportunities to collaborate with peers and an interdisciplinary team that includes a Marriage and Family Therapist (MFT), an Occupational Therapist (OT), and a Recreation Therapist (the day program manager). The closed group model established through eight weekly sessions builds trusting relationships in a frame that combines: the OT client-centered and collaborative approach to problem-solving everyday challenges of caregiving, the MFT skills of creating a safe space for discussion and deeper exploration, and program staff insights regarding the ILwD's current interests and abilities exhibited during Day Program activities. Sessions include an emotional check-in by group members; a brief overview of best-practices and common caregiving concerns related to a weekly topic; and an opportunity for caregivers to explore the integration of best-practices into daily routines, while also attending to their well-being as caregivers. Program evaluation and results related to the program's effectiveness and implications for scalability will be discussed.

### PSYCHOSOCIAL INTERVENTIONS FOR PEOPLE WITH DEMENTIA AND THEIR CAREGIVERS IN PRIMARY CARE

Lou Frankenstein, and Georg Jahn, *Chemnitz Technical University, Chemnitz, Sachsen, Germany*

Psychosocial interventions, such as occupational and behavioral therapy are effective opportunities to support people with dementia and their caregivers in adapting to the cognitive and behavioral changes and the resulting challenges in everyday life they are facing. However, psychosocial interventions do not seem to have found their way into routine care yet. We wanted to get an insight into the knowledge and attitudes general practitioners have about occupational and behavioral therapy. In an online survey we asked medical students about the relevance of dementia, occupational therapy, and behavioral therapy during their studies. In another online survey we asked practitioners what they had learned about these topics and to what extent they are making use of psychosocial interventions. Then semi-structured interviews were carried out with general practitioners all over Germany, exploring their experiences with dementia and psychosocial interventions in primary care as well as their expectations regarding interdisciplinary cooperation. It became obvious that psychosocial interventions are not conveyed sufficiently within medical school. A lack of occupational therapy prescriptions for people with dementia seemed to result from uncertainties regarding the content of the approach and the budgeting of the prescriptions. Barriers for prescriptions of behavioral treatment were a lack of therapy places and the perceived inadequacy of the approach for this target group. General retentions to invest in people with dementia were expressed. These obstacles need to be overcome in order to provide optimal care for people with dementia and their family caregivers.

### QUALITATIVE STUDY OF AN MHEALTH MINDFULNESS INTERVENTION WITH CAREGIVERS OF OLDER ADULTS WITH MCI OR DEMENTIA

Danielle Llaneza,[1] XinQi Dong,[2] Paul Duberstein,[3] and Elissa Kozlov,[4], *1. Rutgers University, New Brunswick, New Jersey, United States, 2. Rutgers University, Rutgers Institute for Health, New Jersey, United States, 3. Rutgers University School of Public Health, Piscataway, New Jersey, United States, 4. Rutgers University, Rutgers University, New Jersey, United States*

Caregivers of patients with dementia experience high levels of emotional distress. mHealth interventions have the potential to feasibly address some needs of caregivers and reduce stress. This qualitative research study of (n = 15) caregivers of patients with dementia explored caregivers' experience using a mindfulness meditation mobile application. The qualitative interviews were guided and structured to allow participants to share their perceived benefits, drawbacks, likes, and dislikes of using mHealth strategies to manage stress and anxiety. We asked about the caregivers' experience with mindfulness before the study, use of the app, their positive/helpful and negative/unhelpful app experiences, the perceived value of the app, and potential enhancements of the app. Caregivers reported that the app was easy to use, engaging and that there were many perceived benefits. They also noted multiple barriers to using the app including time constraints and implementation of mindfulness techniques outside of direct app use. Most of the caregivers recommended using the app to increase knowledge of mindfulness and to reduce stress. Our findings support the growing body of literature on the practical use of mHealth strategies for caregivers. Future work should address the perceived barriers caregivers encounter when using mHealth strategies.

### SELF-EFFICACY IN THOUGHT CONTROL , NOT POSITIVE GAINS, MEDIATES EFFECT OF BENEFIT-FINDING INTERVENTION FOR CARERS

Sheung-Tak Cheng, *Education University of Hong Kong, The Education University of Hong Kong, Not Applicable, Hong Kong*

This study examines the therapeutic mechanism of the benefit-finding therapeutic (BFT) intervention that used cognitive reappraisal and alternative thinking to construct positive aspects of caregiving (PAC), in a cluster-randomized controlled trial for Alzheimer caregivers. 42 caregivers received BFT whereas 87 received psychoeducation as control. Both interventions were held in groups. Depressive symptoms and global burden were outcomes measured at baseline, postintervention, and 4- and 10-month follow-up. Mediators considered included PAC and three self-efficacies—controlling upsetting thoughts (SE-CUT), responding to disruptive behaviors, and obtaining respite. Using mixed-effects regression, we demonstrated that benefit-finding increased caregivers' PAC and SE-CUT, but that only SE-CUT uniquely predicted depressive symptoms and global burden longitudinally. Path analyses with bootstrapped confidence intervals showed that SE-CUT change from baseline to postintervention mediated intervention effects on depressive symptoms, but not global burden, at both follow-ups. No mediation effects were found for PAC and the other self-efficacies. As a conclusion, The BFT effect on depressive symptoms was partly accounted for by improvement in SE-CUT. The therapeutic mechanism for the effect on burden remained unknown. The study sheds light on the importance of actively promoting positive caregiver functioning.

### SUPPORTING OLDER FAMILY CAREGIVERS OF YOUNG ADULTS WITH IDD: A PILOT PROGRAM WITH SOCIALLY ASSISTIVE ROBOTICS

Ling Xu,[1] Julienne Greer,[2] Noelle Fields,[1] Priscila Tamplain,[2] John Bricout,[3] Bonita Sharma,[4] and Kristen Doelling,[5], *1. University of Texas at Arlington, Arlington, Texas, United States, 2. The University of Texas at Arlington, Arlington, Texas, United States, 3. University of Minnesota, twin cities, Minnesota, United States, 4. UT-San Antonio, San Antonio, Texas, United States, 5. UTA, Arlington, Texas, United States*

**Introduction:** The need for caregiver respite is well-documented for the care of persons with IDD. Social Assistive Robotics (SAR) offer promise in addressing the need for caregiver respite through 'complementary caregiving' activities that promote engagement and learning opportunities for a care recipient (CR) with IDD. This study explored the acceptability and usefulness of a SAR caregiver respite program responsive to feedback from both the CRs and their older family caregivers (age 55+).

**Methods:** Caregiver-CR dyads (N =11) were recruited. A mixed methods research design was deployed in three phases: Phase I with four focus groups to inform the program design; Phase II for program demonstration and evaluation with pre- and post-surveys; and Phase III with post-program interviews for feedback and suggestions.

**Results:** Quantitative data analysis in Phase II showed both caregivers and their CRs scored favorably the social presence of the robot (Pepper) and social engagement with Pepper. Caregivers also reported positive perceptions of Pepper in terms of anthropomorphism, animation, likeability, intelligence, and safety. Content analysis from Phase III interviews suggested that the SAR may offer physical/emotional respite to caregivers by providing companionship/friendship as well as promoting independence, safety/monitoring, and interactive engagement with children.

**Discussion:** SAR has potential in providing respite for older family caregiver demonstrated by the levels of CR engagement and learning with Pepper. Future studies need a longer program design and larger sample size to test the feasibility and efficacy of the intervention.

### THE IMPACT OF SOCIAL SUPPORT ON DEMENTIA CAREGIVERS' PSYCHOLOGICAL WELL-BEING: IS ONLINE COMPARABLE TO OFFLINE?

Eun-Hye Yi, *Indiana University, Los Angeles, California, United States*

Along with the groundbreaking development of communication technology, caregivers have migrated to online platforms to seek help. Social support theory, including main effect and moderation effect models, has provided a framework to understand the association among caregivers' stress, support from others, and their well-being. Despite the prevalence of online use for seeking help among caregivers, studies on the use of online social support within the context of caregivers' stressors and well-being are still underdeveloped. Guided by social support theory, this study aimed to examine the association of online social support (OnSS) and caregivers' mental health (MH) as compared with offline social support (OffSS). A subsample of caregivers of persons with Alzheimer's from the Health Information National Trends Survey from 2017-2018 was selected (n=264). For an analysis, ordered logistic regression models with Jackknife estimation methods were applied using Stata 15.1SE. First, OffSS had a positive direct association with caregivers' MH (Odds Ratio=12.48, p<.05) while OnSS did not. Next, the moderation effect model analysis found that OffSS interacted with caregiving burden while OnSS interacted with life stressors. The MH of caregivers who are in less favorable situations, such as working part-time while caring for a person with Alzheimer's, living with economic hardship, and having health problems, tended to be significantly affected by OnSS. Identifying the different roles of OnSS and OffSS for caregivers' MH, the findings of this study call for more attention to developing novel strategies and sensitive approaches to support family caregivers, especially those who fall in underserved groups.

### THE RELATIONSHIP BETWEEN BURDEN AND FINANCIAL FACTORS FOR HELP-SEEKING OLDER ADULT CAREGIVERS

Geffen Ferszt,[1] Stacy Yun,[1] Kendall Weber,[2] and Sara Qualls,[2], *1. University of Colorado Colorado Springs, Colorado Springs, Colorado, United States, 2. University of Colorado at Colorado Springs, University of Colorado at Colorado Springs, Colorado, United States*

Older adult caregivers often experience unique financial challenges (Schultz & Eden, 2016). The purpose of the present study was to explore the relationship between caregiver finances and caregiver burden for this sample. A sample of 131 caregivers seeking counseling services completed questionnaires assessing demographics, care recipient functioning (ADLs/IADLs), and finances (yearly income, impact of caregiving on finances). A majority, 74.8% of caregivers, indicated financial burden since caregiving. A hierarchical multiple regression was computed to predict caregiver burden. The caregiving characteristics block explained 8.1% of the total variance in caregiver burden, $F(7, 123) = 1.54$, n.s. Specifically, being younger was significantly associated with more caregiver burden. Adding the care recipient functioning block explained an additional 6.9% of the variance in caregiver burden, $F_{change}(2, 121) = 4.93$, $p < .01$. The caregiving characteristics and care recipient functioning model accounted for 15% of the total variance in caregiver burden, $F(9, 121) = 1.55$, $p < .05$. Again, younger age uniquely predicted greater caregiver burden. Lastly, caregiving finance factors contributed an additional 13.5% of the variance, $F_{change}(4, 117) = 5.54$, $p < .001$. Thus, the final caregiver characteristics, care recipient functioning, and caregiving finances model accounted for 28.5% of the total variance in caregiving burden, $F(13, 117) = 3.59$, $p < .001$. Specifically, having less income and greater monthly expenses related to caregiving predicted higher levels of caregiver burden. These findings imply that those with fewer resources may benefit from intervention for the heavier burden they perceive compared to peers with more financial resources.

### Session 9290 (Poster)

### FAMILY CAREGIVING: MENTAL AND PHYSICAL HEALTH

### A SCOPING REVIEW OF THE EXAMINATION OF SANDWICHED CAREGIVERS' PSYCHOLOGICAL WELL-BEING AND PHYSICAL HEALTH

Barbara Hodgdon, Jen D. Wong, and Patricia S. Pittman, *The Ohio State University, Columbus, Ohio, United States*

As numbers of sandwiched caregivers in the United States grow, it is essential to document the literature on the impact of dual care responsibilities on aspects of psychological well-being and physical health. This scoping review examined the literature on sandwiched caregivers' psychological well-being and physical health, identified gaps in the literature, and provided suggestions for future studies to advance the literature on sandwiched caregivers in the United States. Guided by the Arksey and O'Malley (2005) framework, this scoping review comprised of 15 peer-reviewed articles between 1980 and 2019, that examined aspects of the psychological well-being (e.g., depression, affect) and physical health (e.g., health behaviors, chronic conditions) of sandwiched caregivers in the United States. Findings showed that there was ambiguity surrounding the conceptualization of sandwiched caregivers, specifically how older and younger care recipients were defined. Also, most studies examined psychological well-being while physical health was understudied. The findings of this review also showed that, compared to non-sandwiched caregivers (e.g., spousal, filial caregivers) and non-caregivers, sandwiched caregivers exhibited greater depressive symptoms and psychological distress as well as poorer health behaviors. Furthermore, sandwiched caregivers who were female and employed were more susceptible to greater depressive symptoms than their employed male counterparts or employed non-caregivers. In considering future directions, more work is needed that examines physical health. Additionally, sandwiched caregivers of minority status merit attention as multigenerational care occurs at greater rates in these populations. Finally, caregiving during the pandemic may have a detrimental impact on sandwiched caregivers' lives which should be investigated.

### ACT-RELATED PROCESSES: VALUE-BASED LIVING ATTENUATES MEDITATING RELATIONSHIPS IN CAREGIVER STRESS OUTCOMES

Elizabeth Braungart Fauth,[1] Joshua Novak,[2] Ty Aller,[1] Heather Kelley,[1] and Michael Levin,[1], *1. Utah State University, Logan, Utah, United States, 2. Auburn University, Auburn, Alabama, United States*

Associations between behavioral and psychological symptoms of dementia (BPSD), caregiver burden, and depressive symptoms are well-established, and these constructs are often targeted in interventions. Increasingly, dementia caregiver interventions are informed by mindfulness- and acceptance-based approaches, such as Acceptance and Commitment Therapy (ACT). In addition to standard outcomes, like burden and depressive symptoms, these interventions/therapies seek improvements in individuals' psychological flexibility (e.g., cognitive defusion, present moment awareness, values-based living). Less is known how these constructs interact within well-established caregiver stress processes. We examined a moderated mediation model (N=161 dementia caregivers; PROCESS Procedure; SPSS Release 2.16.1), with BPSD frequency (Revised Memory and Behavior Problems Checklist) predicting depressive symptoms (10-item CES-D), mediated via caregiver burden (short Burden inventory). The moderator was the Values Questionnaire (Progress scale), and we controlled for gender, caregiver duration, age, income, and education. Results revealed that the indirect effect of BPSD on depressive symptoms through caregiver burden was weakened through higher progress toward values (moderated mediation significant at $p<.05$). In essence, greater levels of living according to values dampened the effect of BPSD on depressive symptoms, through care-related burden. These findings are important because caregivers often cannot leave this role, requiring them to learn to live with the caregiving role in healthy ways. Value-based living and committed action toward values signify caregivers' success at balancing care-related stress with other priorities, and psychologically adjusting to difficulties. Interventions that emphasize values-based living, like ACT, have promise for caregivers, offering healthy ways to psychologically adjust to, and live with, the experience.

### ANTICIPATORY CAREGIVING SCALE: DEVELOPMENT AND PRELIMINARY VALIDATION

Kendall Weber, and Sara Qualls, *University of Colorado at Colorado Springs, University of Colorado at Colorado Springs, Colorado, United States*

**Background:** As public awareness of family caregiving has grown, adults likely anticipate the role they may play as a caregiver for an aging loved one. Although anticipatory planning for caregiving has been studied, no measure of multiple dimensions of the anticipated caregiving experience exists. The purpose of the present study is to develop and validate the Anticipatory Caregiving Scale (ACS), an assessment of adult children's attitudes toward and level of expectation surrounding their potential role as an informal caregiver to a parent or parent-in-law.

**Method:** The ACS consists of six subscales that assess multiple factors that may influence willingness to or expectations about taking on a caregiving role: affect surrounding future caregiving, anticipated lifestyle interference, self-efficacy surrounding future caregiving, anticipated caregiving resources, endorsement of norms of family care which influence anticipated caregiving, and the relationship quality, current and anticipated, with the potential care recipient. A sample of 540 adults aged 18 and over recruited online completed the ACS, along with scales to assess convergent, discriminant, and concurrent validity.

**Results:** Overall, the ACS and its subscales demonstrated good reliability and validity, established through internal consistency, and convergent, discriminant, and concurrent validity.

**Discussion:** The ACS fills a gap in the current literature by providing a comprehensive, multidimensional assessment examining expectations about one's potential caregiving experience, specific to the adult child-parent dyad. Future studies should examine the psychometric properties of the ACS in a more diverse population of adults across different settings and assess the temporal stability and criterion validity of the scale.

### CAREGIVERS' MEDICAL CARE PROVISION AT PATIENT END-OF-LIFE: ASSOCIATIONS WITH EMOTIONAL DIFFICULTY AND GAINS

Amanda Leggett,[1] Hyun Jung Koo,[2] Elaina Baker,[2] and Hannah Lee,[2], *1. The University of Michigan, Ypsilanti, Michigan, United States, 2. University of Michigan, Ann Arbor, Michigan, United States*

Family caregivers play crucial roles in patient care and medical decision making, especially at end-of-life (EOL). Yet, most research focuses on caregivers' burden, with little attention to rewards that make for a fulfilling EOL care experience. We consider caregiver involvement at EOL and associations with caregiver stress and gains. Data are drawn from the 2017 National Study of Caregiving's last month of life and core interviews which includes caregivers (n=283) for a nationally representative sample of Medicare eligible older adults, and questions caregivers about their care provision and EOL experience. We consider indicators of caregivers' involvement in medical decision making and support received from providers as predictors of caregivers' emotional difficulty and gains at EOL utilizing linear regressions controlling for demographic characteristics. Caregivers were 60.7 years of age on average, 72.5% female, 21.3% non-white, and 11% were spousal partners. Making medical decisions was associated with increased emotional difficulty at EOL (B=0.93, SE=0.24, p<.001). In contrast, more caregiving gains were associated with having care decisions align with the CG's wishes (B=-0.64, SE=0.30, p<.05), being more informed by providers (B=0.41, SE=0.16, p<.05), helping the care recipient with anxiety or sadness (B=0.69, SE=0.28, p<.05), and surprisingly, feeling that care decisions were made without their input (B=0.82, SE=0.29, p<.01). Being more involved and informed in care was associated with both positive and negative caregiver outcomes at EOL. Understanding caregiver emotional difficulty and gains at EOL are critical for identifying how clinicians can better support caregivers at EOL and improving the caregiving experience.

### CAREGIVING IN OLDER ADULTHOOD AND MEANING IN LIFE

Zainab Suntai, *University of Alabama, Tuscaloosa, Alabama, United States*

While most of the literature on caregiving in adulthood focuses on grandparenting, there is an increasing trend of older adults providing care to an adult care recipient. Older caregivers are often females who are providing care to a spouse with medical conditions and are often doing so while coping with their own functional limitations, with limited support. Within the older adult literature, studies have captured the strain experienced by caregivers of older adults, who are often burdened by the loss of time and opportunity as a result of caregiving. For caregivers in older adulthood however, caregiving may be an avenue to remain engaged and active, and a way to have purpose in life. Therefore, the purpose of this study was to examine the association between caregiving in older adulthood and meaning in life. Data from the 2018 National Health and Aging Trends study were used, which is an annual longitudinal panel survey of Medicare beneficiaries in the United States. Chi square tests were used for bivariate analyses and a logistic regression model was used to predict meaning in life based on caregiving status. After accounting for all other explanatory variables, caregivers were 50% more likely to have meaning in life compared to non-caregivers (OR=1.501, CI= 1.493-1.510). This indicates that caregiving can be a form of meaningful/productive engagement for older adults and interventions should focus on providing adequate emotional and financial support to older caregivers.

### CAREGIVING SELF-EFFICACY AS A MEDIATOR OF NEGATIVE EMOTIONAL STATES AND POTENTIALLY HARMFUL BEHAVIORS

David Hancock,[1] Sara Czaja,[2] and Richard Schulz,[3], *1. Weill Cornell Medicine, New York City, New York, United States, 2. Weill Cornell Medicine/Center on Aging and Behavioral Research, New York, New York, United States, 3. University of Pittsburgh, Pittsburgh, Pennsylvania, United States*

Potentially harmful behaviors (PHB), such as wanting to scream at or hit a care recipient, are more likely when caregivers experience higher levels of stress. The current study expands on this research and identifies caregiving self-efficacy (SE) for dealing with disruptive behaviors as a mediator of the relationship between caregiver distress and PHB. Multilevel mediation models were tested using a sample of 244 caregivers of persons with dementia assessed three times over a one year period. In two separate models, SE mediated the relationship between caregiving burden/depression and the frequency of wanting to yell or scream at the care recipient in the past six months. Individuals with higher levels of depression and burden had lower levels of SE for dealing with disruptive behaviors. As SE decreased, the risk of potentially harmful behaviors increased. This mediation effect occurred at the within and between subject levels of the model. A significant indirect effect at the within-person level suggests that at timepoints where caregivers experienced more distress, they had lower self-efficacy and increased PHBs. Similar effects were observed at the between person level. These data suggest that both caregiver distress and self-efficacy are important intervention targets for minimizing PHBs.

### COGNITIVE-BEHAVIORAL DEMENTIA CAREGIVING STYLES: ASSOCIATIONS WITH CARE STRESS AND WELL-BEING

Amanda Leggett,[1] Cathleen Connell,[2] Laura Gitlin,[3] and Helen Kales,[4], *1. The University of Michigan, Ypsilanti, Michigan, United States, 2. University of Michigan School of Public Health, Ann Arbor, Michigan, United States, 3. Drexel University, College of Nursing and Health Professions, Drexel University, Pennsylvania, United States, 4. University of California, Davis, University of California, Davis, Sacramento, California, United States*

While Stress Process Models of family caregiving have been examined extensively, little focus has been placed on caregiver's actual management of care. We consider whether caregiving style classified previously through k-modes machine learning models and based on cognitive-behavioral approaches to care impact caregivers' experiences of care-related stress and well-being. The three previously identified styles include: Adapters- strong dementia understanding and adaptability, encouraging behavioral approach; Managers- poor dementia understanding and adaptability, critical behavioral approach; and Avoiders- moderate dementia understanding and adaptability, passive behavioral approach. Participants included 100 primary family caregivers for PWDs who were on average 64 years old, 74% female, and 18% non-White. Utilizing linear regressions, each caregiving style was considered a key predictor (reference: Adapters) of the Zarit Burden Interview (ZBI), Caregiver Assessment of Function and Upset (CAFU) upset score, Neuropsychiatric Inventory (NPI-C) distress scale, and Positive and Negative Affect scale (PANAS) controlling for dementia severity, care duration, co-residency, and demographics. Relative to Adapters, Managers had more CAFU upset ($\beta=0.4$, $p<.001$), more NPI-C distress ($\beta=0.4$, $p<.001$), and greater burden (ZBI) ($\beta=0.3$, $p<.001$). Avoiders showed significantly greater CAFU upset than Adapters ($\beta=0.2$, $p<.05$). Positive affect was not associated with caregiving style. Caregiving styles associated with less understanding and adaptability and a more critical behavioral approach showed worse caregiving outcomes accounting for dementia severity. Results can inform a nuanced approach to tailoring and targeting interventions based on caregiver styles with the goal of reaching caregivers at risk for poor outcomes and ultimately leading to significant public health impact.

### DAILY SLEEP, WELL-BEING, AND ADULT DAY SERVICES USE AMONG DEMENTIA CARE DYADS

Yin Liu,[1] Amanda Leggett,[2] Kyungmin Kim,[3] Courtney Polenick,[4] Susan McCurry,[5] and Steven Zarit,[6], *1. Utah State University, Logan, Utah, United States, 2. The University of Michigan, Ypsilanti, Michigan, United States, 3. Seoul National University, Seoul, Seoul-t'ukpyolsi, Republic of Korea, 4. University of Michigan, Ann Arbor, Michigan, United States, 5. University of Washington School of Nursing, Seattle, Washington, United States, 6. Pennsylvania State University, University Park, Pennsylvania, United States*

Sleep is critical for health and well-being among both persons with dementia (PwD) and their family caregivers (CG). This study described sleep characteristics for dementia family care dyads, then examined the associations with sleep quality, daytime functioning, and mood in the context of Adult Day Services (ADS) use. Caregivers (n = 173) reported daily bedtime, wake time, and sleep quality for themselves and the persons with dementia across 8 consecutive days (N = 1,359), where PwD attended ADS at least 2 days of the week. They also reported their own fatigue and affect and PwD's daytime and nighttime sleep and behavior problems on each day. Findings from multilevel models suggested that bedtime was earlier and total time in bed was shorter before an ADS day for the dyad, and also on an ADS day for PwD; wake time was earlier for the dyad on and following an ADS day. Using ADS related to better prior night sleep quality for PwD; it also weakened the association between nighttime sleep problems and higher daytime negative affect for CG. Yesterday's ADS use buffered the negative impact of shorter total time in bed on CG daytime fatigue; it also buffered the association between nighttime sleep problems and lowered CG daytime positive affect. Regular ADS use may protect against the adverse impact of sleep disturbances on daytime functioning and well-being for dementia care dyads.

### FAMILY CAREGIVERS' FEELINGS OF PREPAREDNESS FOR THE TRANSITION TO END-OF-LIFE CAREGIVING

Aimee Fox, Julia Sharp, and Christine Fruhauf, *Colorado State University, Fort Collins, Colorado, United States*

Family caregivers are vulnerable to emotional, psychological, physical, and financial stress during their care receiver's end-of-life (EOL), and often do not have the knowledge or skills to provide the care needed at EOL. Yet, few studies have examined how prepared caregivers feel for making the transition to EOL care. The purpose of this exploratory study was to investigate how family caregivers perceive their preparedness for the transition to providing EOL care, and factors that may be associated with feelings of preparedness. Family caregivers (N = 252) providing care to an adult family member for more than six months were recruited to complete an online, self-report survey. An exploratory factor analysis was conducted to break down the construct of caregiver preparedness. One-way ANOVAs and a multiple regression were conducted to examine factors associated with preparedness. Most caregivers feel less prepared for the transition to EOL care (M = 2.67, SD = 1.17) than for general caregiving responsibilities (M = 3.42, SD = 0.90). Factors associated with preparedness for EOL caregiving included the familial relationship between the family caregiver and care receiver, hours per week providing care, perceived burden of caregiving, and family conversations completed about advance care planning, legal and financial matters, and EOL wishes. Future studies should explore how to help family caregivers recognize the importance of seeking support from family members and community services and discussing EOL

care wishes and plans with aging family members so they can provide effective care and protect their own well-being.

### GENDER-SPECIFIC PREDICTORS OF POOR SLEEP QUALITY AND INSOMNIA AMONG CAREGIVERS OF PERSONS LIVING WITH DEMENTIA

Glenna Brewster,[1] Miranda McPhillips,[2] Kate Yeager,[3] Fayron Epps,[3] Kalisha Bonds Johnson,[3] Kenneth Hepburn,[3] and Donald Bliwise,[3], *1. Emory University, Stonecrest, Georgia, United States, 2. University of Pennsylvania, University of Pennsylvania, Pennsylvania, United States, 3. Emory University, Atlanta, Georgia, United States*

Caregiving for a person living with dementia (PLwD) may influence sleep patterns. Gaps exists about whether caregiver and PLwD factors impact sleep differentially based on caregivers' gender. The aim of this secondary data analysis was to identify predictors of sleep quality and insomnia in a sample of caregivers, stratified by gender, participating in a randomized controlled trial of a psychoeducational intervention. Outcome measures were sleep quality (Pittsburgh Sleep Quality Index, PSQI) and insomnia (Insomnia Severity Index, ISI). Participants (n=261) also completed measures about caregivers' perceived stress, burden, depression, and self-care, and PLwD's behaviors (i.e., apathy, sleep disorders). Linear regression modeling was used to identify the overall predictors of poor sleep quality (PSQI > 5; 52% of the sample) and insomnia (ISI > 7; 41% of the sample). Caregivers were primarily female (70.5%), White (73.6%), mean age of 64.6 (±11.2) years, and typically caring for a spouse (65.9%). For male caregivers, predictors of poor sleep quality were assisting the PLwD with instrumental activities of daily living and PLwD neuropsychiatric symptoms (F=4.45, p<.001); while caregiver self-care and PLwD neuropsychiatric symptoms predicted insomnia (F=4.49, p<.001). For female caregivers, the predictors of poor sleep quality were caregiver depressive symptoms and burden, and frequency of PLwD behavioral problems (F=4.46, p<.001); however, only perceived stress predicted insomnia (F=4.32, p<.001). Various factors related to caregiving appear to be more important than others in predicting sleep outcomes of male/female caregivers. Health care professionals should acknowledge gender differences when designing and implementing programs and interventions to improve sleep.

### PARENTING STYLE IN CHILDHOOD AND MENTAL HEALTH OUTCOMES OF CAREGIVING IN MIDDLE AND LATER ADULTHOOD

Yujun Liu,[1] M. Courtney Hughes,[2] and Abby Baumbach,[2], *1. Northern Illinois University, Naperville, Illinois, United States, 2. Northern Illinois University, DeKalb, Illinois, United States*

Objectives. This study examined the association between remembered parenting style of both mothers and fathers in childhood and mental health outcomes of caregiving in middle and later adulthood. Methods. Data were from the Midlife in the United States (MIDUS) study, a national survey that included 7,108 participants aged 24 to 75 years at baseline. The sample analyzed in the current study included 244 MIDUS participants who had given personal care to their mothers or fathers for one month or more during the last 12 months in the second and third waves. Parenting style variables, which included maternal/paternal affection and maternal/paternal discipline, were from the first wave; mental health outcome variables, which included emotional distress, depressive symptoms, and life satisfaction, were from the second and third waves. Multiple regression and multilevel modeling were applied using R. Results. Maternal affection was negatively associated with emotional distress. Paternal affection was negatively associated with depressive symptoms. The associations between maternal/paternal discipline and mental health outcomes were not significant. Among the caregivers who provided care for parents, those who had mothers with high affection in childhood experienced a lower level of emotional distress, those who had fathers with high affection experienced a lower level of depressive symptoms in middle and later adulthood. Discussion. Our findings have advanced the understanding of the long-term consequences of parenting style in childhood on mental health outcomes among family caregivers in later life. The results have implications in the development of interventions focusing on mental health outcomes among family caregivers.

### PREVALENCE OF BINGE DRINKING BY CAREGIVERS OF PERSONS WITH ALZHEIMER'S DISEASE OR RELATED DEMENTIA

Zachary Kunicki, and Richard Jones, *Brown University, Providence, Rhode Island, United States*

Some caregivers of persons with Alzheimer's Disease and related dementias (ADRD) are known to be under high levels of burden, which is associated with higher levels of anxiety, depression, and stress. Previous research has established anxiety, depression, and stress are associated with binge drinking, but little research has examined binge drinking rates among ADRD caregivers. Binge drinking could influence the ability of ADRD caregivers to provide care. The purpose of this study was to explore the prevalence and prevalence correlates of binge drinking among ADRD caregivers using the 2019 Behavior Risk Factor Surveillance Survey (BRFSS). We identified N = 1,642 persons who were the primary informal caregivers of a person with ADRD. Among them, the prevalence of binge drinking was 14 per 100 persons. Bivariable analyses suggested male caregivers and caregivers with 14 or more days of poor mental health in the past 30 days had the highest prevalence of binge drinking at 18 per 100 persons. Caregivers who were 65 or older or had the lowest prevalence at 3 per 100 persons. Caregiving characteristics revealed providing 20 to 39 hours of care per week had the highest prevalence of binge drinking (17 per 100) whereas spousal caregivers (9 per 100) had the lowest prevalence. Smoking status and hours per week providing care were associated with higher odds of binge drinking in multivariable analyses. Future research should examine if binge drinking by ADRD caregivers is related caregiver burden and the quality of care provided to the persons with ADRD.

### RESILIENCE IN FAMILY CAREGIVERS OF ADULTS WITH AUTISM SPECTRUM DISORDER: AN INTEGRATIVE REVIEW OF THE LITERATURE

Daphne Chakurian, *University of Missouri - Columbia, Roseville, California, United States*

Care of adults with Autism Spectrum Disorder (ASD) is a public health priority and costs are projected to be 549 billion US dollars by 2025. Middle and older adult FCGs of adults with ASD often provide lifelong care, experience chronic stress, consequently, are at risk of poor mental health and QOL. An integrative review examined factors associated with resilience in studies of middle and older adult FCGs of adults with ASD. A comprehensive literature search found 10 reports of 8 studies published in peer-reviewed scholarly journals before October 13, 2020. Studies and/or reports of factors associated with resilience in middle and older adult FCGs of adults with ASD were examined using PRISMA, and quality checklists. Some 340 articles met search criteria, 14 were fully reviewed, and 10 were included. Findings suggest FCGs of adults with ASD show capacity for resilience consistent with research on FCGs of children with ASD significant chronic stress. A broad range of resilience factors were studied, and resilience was associated with positive social support, higher QOL, self-efficacy, and problem and meaning-focused coping styles. There is a dearth of research on middle and older adult FCGs of adults with ASD. Increased reporting of social determinants of health and participation of underrepresented groups is needed. Future research must address FGC heterogeneity and specify theoretically grounded conceptual and operational definitions of resilience. Identifying resilience factors is necessary for intervention studies to enhance resilience.

### SLEEP QUALITY IN FAMILY CAREGIVERS AND MATCHED NON-CAREGIVING CONTROLS: THE REGARDS STUDY

Marcela Blinka,[1] Adam Spira,[2] Orla Sheehan,[3] Tansu Cidav,[4] J. David Rhodes,[5] Virginia Howard,[6] and David Roth,[1], *1. Johns Hopkins University, Baltimore, Maryland, United States, 2. Johns Hopkins Bloomberg School of Public Health, Baltimore, Maryland, United States, 3. Johns Hopkins University School of Medicine, Johns Hopkins University, Maryland, United States, 4. Johns Hopkins University School of Medicine, Baltimore, Maryland, United States, 5. University of Alabama at Birmingham, University of Alabama at Birmingham, Alabama, United States, 6. University of Alabama at Birmingham, Birmingham, Alabama, United States*

The high levels of stress experienced by family caregivers may affect their physical and psychological health, including their sleep quality. However, there are few population-based studies comparing sleep between family caregivers and carefully-matched controls. We evaluated differences in sleep and identified predictors of poorer sleep among the caregivers, in a comparison of 251 incident caregivers and carefully matched non-caregiving controls, recruited from the national REasons for Geographic and Racial Differences in Stroke (REGARDS) Study. Incident caregivers and controls were matched on up to seven demographic and health factors (age, sex, race, education level, marital status, self-rated health, and self-reported serious cardiovascular disease history). Sleep characteristics were self-reported and included total sleep time, sleep onset latency, wake after sleep onset, time in bed, and sleep efficiency. Family caregivers reported significantly longer sleep onset latency, before and after adjusting for potential confounders, compared to non-caregiving controls (ps < 0.05). Depressive symptoms in caregivers predicted longer sleep onset latency, greater wake after sleep onset, and lower sleep efficiency. Longer total sleep time in caregivers was predicted by employment status, living with the care recipient, and number of caregiver hours. Employed caregivers and caregivers who did not live with the care recipient had shorter total sleep time and spent less time in bed than non-employed caregivers. Additional research is needed to evaluate whether sleep disturbances contributes to health problems among caregivers.

### TELOMERE LENGTH AND THE TRANSITION TO FAMILY CAREGIVING IN THE REGARDS STUDY

William Haley,[1] Nicole Armstrong,[2] Ryan Irvin,[3] Marcela Blinka,[4] Rasika Mathias,[5] Jeremy D. Walston,[6] and David Roth,[4], *1. University of South Florida, Tampa, Florida, United States, 2. University of Alabama at Birmingham, University of Alabama at Birmingham, Alabama, United States, 3. University of Alabama at Birmingham, Birmingham, Alabama, United States, 4. Johns Hopkins University, Baltimore, Maryland, United States, 5. Johns Hopkins University, Johns Hopkins University, Maryland, United States, 6. Johns Hopkins University School of Medicine, Baltimore, Maryland, United States*

An increase in life expectancy and an aging population has resulted in increased risks and prevalence of age-related diseases. Previous studies have shown that factors, such as chronic stress, are associated with shorter telomere length. When telomeres become critically short, cells enter a state of senescence, which is a hallmark of aging. Several prior studies examining the relationship between caregiving and telomere length have reported mixed results. The present study utilized data from the Caregiving Transitions Study, an ancillary study to the Reasons for Geographic and Racial Differences in Stroke (REGARDS) study. The difference in telomere length across an average ~8.6 years was compared between 235 incident caregivers and 229 controls. Telomere length was determined using the qPCR telomere-to-single copy gene (IFNB1) ratio (T/S) for each participant at both baseline and follow-up timepoints. Regression models controlling for age, sex, race, and baseline telomere length examined the association between caregiving status (exposure) and the telomere length change (□T/S). Sensitivity models adjusted for potential lifestyle and socioeconomic factors, including income, education, BMI, cigarette smoking, and alcohol use. We did not observe a significant association between □T/S and caregiving (beta=0.041, p=0.615). Adding lifestyle and socioeconomic factors did not change the null relationship (beta=0.062, p=0.455). In conclusion, this study provides evidence against an association between caregiving and the change in telomere length. Ultimately, more research to address the complex relationship between caregiving and telomere attrition is needed in order to prevent or reduce adverse outcomes and improve the well-being of caregivers and care recipients.

### Session 9295 (Poster)

### FRAILTY

### A NOVEL ALGORITHM FOR ANALYSIS OF MULTIPLE ENDPOINTS USING RISK–BENEFIT PROFILES

Natalia Gouskova,[1] Dae Kim,[2] Sandra Shi,[3] and Thomas Travison,[2], *1. Hebrew SeniorLife, Marcus Institute for Aging Research, Hebrew SeniorLife, Boston, Massachusetts, United States, 2. Harvard Medical School, Boston, Massachusetts, United States, 3. Hebrew SeniorLife, Harvard Medical School, Roslindale, Massachusetts, United States*

Often it is necessary to evaluate effectiveness of an intervention on the basis of multiple event outcomes of variable benefit and harm, which may develop over time. An attractive approach is to order combinations of these events based on desirability of the overall outcome (e.g. from cure without any adverse events to death), and then determine whether the intervention shifts the distribution of these ordered outcomes towards more desirable (Evans, Follmann 2016). The win ratio introduced in Pocock et al 2012 was an earlier implementation of this approach. More recently Claggett et al 2015 proposed a more comprehensive method allowing nonparametric and regression-based inference in presence of competing risks. Key to the method is weighting observations by inverse probability of censoring (IPC) processes specific to participants and event types. The method has seemingly great practical utility, but computation of weights is a non-trivial challenge with real-life data when each event can have its own censoring time. We present a novel recursive algorithm solving this problem for an arbitrary number of events ordered by clinical importance or desirability. The algorithm can be implemented in SAS or R software, and computes IPC weights, as well as nonparametric or parametric estimates and resampling-based measures of uncertainty. We illustrate the approach using data from the SPRINT trial of antihypertensive intervention, comparing risk-benefit profiles for robust, pre-frail, and frail subpopulations, and in analysis of fall as a function of progressive risk factors. More general use of the software tools deploying the method is described.

### A NOVEL ELECTRONIC FRAILTY INDEX AS A PREDICTOR OF CLINICAL OUTCOMES AFTER TRANSCATHETER AORTIC VALVE IMPLANTATION

Maria Rangel,[1] Rahul Annabathula,[2] Kathryn Callahan,[1] Nicholas Pajewski,[1] Jeff Williamson,[1] and David Zhao,[2], *1. Wake Forest School of Medicine, Winston-Salem, North Carolina, United States, 2. Wake Forest Baptist Medical Center, Wake Forest School of Medicine, North Carolina, United States*

Transcatheter aortic valve implantation (TAVI) is becoming the preferred therapeutic approach for older adults with severe aortic valve disease. Frailty portends increase mortality and adverse outcomes after TAVI. We sought to evaluate an electronic Frailty Index (eFI) as a predictor for increased healthcare utilization, adverse clinical and functional outcomes. We retrospective studied 302 adults older than 65 years that underwent TAVI at our institution between October 2017 and September 2020. The mean age of the cohort was 79 ±6.94 years old; 43% were female. Frail individuals (eFI >0.20), as compared to Fit (eFI <0.10) and Prefrail (0.10>eFI<0.20), were more likely to have a higher society of thoracic surgeons score and a greater burden of comorbidities. Subjects classified as Prefrail/Frail had longer intensive care unit stay post-TAVI than fit individuals (>24 hours: 17% vs 4%, respectively, p 0.02); and trended toward longer hospitalization time and discharge to a setting different than home. The Prefrail/Frail group also had a higher proportion of subjects with persistent New York Heart Association Class III heart failure symptoms 30 days post-TAVI as compared to Fit (14% vs 2%, p 0.04), however both groups demonstrated significant symptomatic improvement post-procedure. No significant differences in 30 day mortality, major adverse cardiovascular events or readmissions were found. TAVI is an effective treatment with a low incidence of early adverse clinical outcomes in older adults regardless of frailty status; eFI could help in identifying and targeting susceptible adults that may require additional resources to recover post-TAVI.

### A RANDOMIZED PLACEBO-CONTROLLED TRIAL OF METFORMIN FOR FRAILTY PREVENTION IN OLDER ADULTS

Amir Tavabi,[1] Chen-pin Wang,[2] Joel Michalek,[3] Tiffany Cortes,[4] Ethan Leonard,[5] Becky Powers,[6] Nicolas Musi,[3] and Sara Espinoza,[1], *1. University of Texas Health Science Center San Antonio, San Antonio, Texas, United States, 2. UT Health San Antonio, UT Health San Antonio, Texas, United States, 3. UT Health San Antonio, San Antonio, Texas, United States, 4. University of Texas HSC at San Antonio, San Antonio, Texas, United States, 5. UT Health San Antonio - Barshop Institute, San Antonio, Texas, United States, 6. South Texas Veterans Health Care System, San Antonio, Texas, United States*

Frailty is a progressive physical decline leading to higher morbidity and mortality in older adults. Previous studies have demonstrated shared mechanisms between insulin resistance, inflammation, and frailty. The purpose of this trial is to determine whether metformin prevents frailty in non-frail, community-dwelling older adults (≥65 years) with pre-diabetes, determined by 2-hour oral glucose tolerance test (OGTT). Frail individuals (Fried criteria) and those with renal impairment (glomerular filtration rate <45 mL/min) are excluded. Eligible participants are randomized to metformin or placebo and followed for two years. The primary outcome is frailty; secondary outcomes include physical function (short physical performance battery), systemic and skeletal muscle inflammation (plasma and muscle inflammatory markers), muscle insulin signaling (muscle biopsy), insulin sensitivity (insulin clamp), glucose tolerance (OGTT), and body composition (dual-energy x-ray absorptiometry) measurements. Participants are followed every 3 months for safety assessments, every 6 months for frailty assessment and OGTT, and every 12 months for muscle biopsy. Currently, 99 participants, including 53 (53.5%) male and 91 (91.9%) white, are active (54) or have completed the study (35). At baseline, mean age was 72.3 ± 5.5 years, body mass index was 30.7 ± 5.9 kg/m2, and Hemoglobin A1c was 5.73 ± 0.37%. Mean frailty score was 0.5 ± 0.6 and the proportion of non-frail and pre-frail participants were 58.6% (n = 58) and 41.5% (n = 41), respectively. Findings of this clinical trial may have future implications for the use of metformin in older adults with pre-diabetes in order to prevent the onset of frailty.

### ASSOCIATION OF FRAILTY INDEX WITH CLINICAL BPH PROGRESSION AND SERIOUS ADVERSE EVENTS: THE MTOPS TRIAL

Scott Bauer,[1] Kristine Ensrud,[2] Louise C. Walter,[1] Anne M. Suskind,[1] William A. Ricke,[3] Teresa T. Liu,[3] Kevin T. McVary,[4] and Kenneth Covinsky,[1], *1. University of California, San Francisco, San Francisco, California, United States, 2. University of Minnesota, Minneapolis, Minnesota, United States, 3. University of Wisconsin School of Medicine and Public Health, Madison, Wisconsin, United States, 4. Stritch School of Medicine and Loyola University Medical Center, Maywood, Illinois, United States*

Lower urinary tract symptoms due to suspected benign prostatic hyperplasia (BPH) are increasingly treated with medications targeting obstruction among older men, but frailty may represent a novel risk factor for this condition. Our objective was to assess the associations between frailty and clinical BPH progression or serious adverse events (SAE) among 3047 men, age 50-89 years, enrolled in the Medical Therapy of Prostatic Symptoms Study, a placebo-controlled RCT of doxazosin, finasteride, or combination therapy on clinical BPH progression. We created a frailty index using 69 items collected at baseline and categorized men as fit (0-0.1), less fit (0.1-<0.25), or frail (0.25-1.0). The primary outcomes were time to 1) first composite event of clinical BPH progression, and 2) SAE requiring hospitalization. Cox proportional hazards models were adjusted for demographics, intervention, BPH surrogates, and comorbidities. At baseline, 28% men were fit, 58% were less fit, and 14% were frail. During follow-up (mean 4.5 years), the incidence rate of clinical BPH progression was 2.2/100p-y among fit, 3.0/100p-y among less fit (HR =1.28, 95% CI 0.98, 1.67), and 4.1/100p-y among frail men (HR=1.60, 95% CI 1.13, 2.26). Among men randomized to combination therapy, the SAE incidence rate was 3.4/100p-y for fit men versus 12.7/100p-y for frail men (HR=5.98, 95% CI 3.76, 9.52). In conclusion, frailty is independently associated with greater risk of both clinical BPH progression and SAE. The decision to initiate medical therapy for BPH among frail men should therefore include a discussion of both benefits and risks via shared decision making.

### BODY MASS INDEX AND FRAILTY AMONG OLDER MEXICAN AMERICANS: FINDINGS FROM AN 18-YEAR OF FOLLOW-UP

Megan Rutherford,[1] Brian Downer,[1] Chih-Ying Li,[1] and Soham Al Snih,[2], *1. The University of Texas Medical Branch, Galveston, Texas, United States, 2. University of Texas Medical Branch, Galveston, Texas, United States*

The objective of this study was to examine body mass index (BMI) as predictor of frailty among non-frail Mexican American older adults at baseline. Data are from an 18-year prospective cohort of 1,647 non-institutionalized Mexican American aged ≥ 67 years from the Hispanic Established Population for the Epidemiologic Study of the Elderly (1995/1996-2012/13). BMI (Kg/m2) was grouped according to the National Institutes of Health obesity standards (<18.5=underweight, 18.5–24.9=normal weight, 25.0–29.9=overweight, 30.0–34.9=obesity category I and ≥ 35=obesity category II and extreme obesity). Frailty was defined as meeting three or more of the following: unintentional weight loss of >10 pounds, weakness, self-reported exhaustion, low physical activity, and slow walking speed. Covariates included socio-demographics, comorbidities, cognitive function, depressive symptoms, and limitations in activities of daily living (ADL). General Estimating Equations were performed to estimate the odds ratio (OR) and 95% confidence interval (CI) of frailty as a function of BMI categories. All variables were analyzed as time varying except for gender and education. Participants in the underweight or obesity type II / morbidity obesity category had increased OR of frailty over time than those in the normal weight category (2.68, 95% CI=1.46-4.9 vs.1.55, 95% CI=1.02-2.35, respectively) after controlling for all covariates. Those who reported arthritis, hip fracture, depressive symptoms, or ADL disability had increased odds of frailty over time. This study showed a U-shaped relationship between BMI and frailty over an 18-year period of follow-up which has implications for maintaining a healthy weight to prevent frailty in this population.

### COGNITIVE FRAILTY AND RISK OF FUNCTIONAL DISABILITY IN OLDER JAPANESE ADULTS: A 4-YEAR PROSPECTIVE STUDY

Sanmei Chen,[1] Takanori Honda,[2] Tao Chen,[3] Hiro Kishimoto,[4] Shuzo Kumagai,[5] and Kenji Narazaki,[6], *1. Graduate School of Biomedical and Health Sciences, Hiroshima University, Hiroshima, Hiroshima, Japan, 2. Kyushu University, Fukuoka, Fukuoka, Japan, 3. Tongji University, Department of Physical Education, Tongji University, Shanghai, China (People's Republic), 4. Kyushu University, Kyushu University, Fukuoka, Japan, 5. Kumagai Institute of Health Policy, Kasuga City, Fukuoka, Japan, 6. Fukuoka Institute of Technology, Fukuoka, Fukuoka, Japan*

**Background:** Cognitive frailty is a newly proposed clinical entity, referring to concurrent cognitive impairment and physical frailty in the absence of dementia. The clinical significance of cognitive frailty remains poorly understood. We aimed to investigate the association between cognitive frailty and functional disability in older adults.

**Methods:** A total of 1,644 non-demented older adults aged ≥65 years (mean age: 73 ± 6 years; men: 41.8%) and without functional disability at baseline were followed-up for 4 years. Cognitive frailty was defined as the presence of both physical frailty (based on the modified Cardiovascular Health Study criteria) and cognitive impairment (Mini-Mental State Examination score of <24 points). Functional disability was identified using the database of Japan's Long-term Care Insurance System. Association between cognitive frailty and functional disability was assessed by using the Cox proportional hazard models.

**Results:** During the follow-up, 152 participants were identified as being functionally disabled. There was a significant interaction between physical frailty and cognitive impairment on the development of functional disability (P <0.1). Compared with being robust both physically and cognitively, the hazard ratio (95% confidence interval) of functional disability was 8.40 (4.05-17.42) for cognitively frailty, after adjustment for age, sex, education, living alone, smoking, drinking, number of comorbidities (hypertension, stroke, chronic heart disease, diabetes, chronic kidney disease, poor hearing, poor vision, osteoarthritis or rheumatism, minor trauma fracture, or cancer).

**Conclusion:** Cognitive frailty was associated with an increased risk of functional disability in community-dwelling older adults. Cognitive frailty could be an underrecognized risk factor for functional disability.

### EFFECTIVENESS OF TRANSITIONAL CARE FROM HOSPITAL TO HOME IN FRAIL OLDER ADULTS: A SYSTEMATIC REVIEW

Ji Yeon Lee,[1] Yong Sook Yang,[2] and Eunhee Cho,[3], *1. Yonsei University, Seoul, Seoul-t'ukpyolsi, Republic of Korea, 2. Yonsei University, Kwangju, Kwangju-jikhalsi, Republic of Korea, 3. Yonsei University College of Nursing, Seoul, Seoul-t'ukpyolsi, Republic of Korea*

Frail older adults are at high risk of negative consequences from hospitalization and are discharged without completely returning to their pre-existing health status. Transitional care is needed to maintain care continuity from hospital to home. This systematic review aimed to examine transitional care for frail older adults and its effectiveness. The Cochrane guidelines were followed, and search terms were determined by PICO: (P) frail older adults, not disease-specified; (I) transitional care initiated before discharge; (C) usual care; and (O) all health outcomes. Four databases were searched for English-written randomized controlled trials (inception to 2020), and eight trials were ultimately included. Frail older adults in eight trials (1996–2019) totaled 2,785, with a mean sample size of 310. The intervention components varied from hospital care (e.g., geriatric assessment, discharge planning, rehabilitation) to follow-up care after discharge (e.g., home visit, phone follow-up, community service). Most measured outcomes were readmission (n = 7), function (n = 4), quality of life (n = 4), self-rated health (n = 3), and mortality (n = 3). Statistical significance was reported in the following number of trials: readmission (n = 2), function (n = 2), quality of life (n = 1), self-rated health (n = 3), and mortality (n = 0). The effectiveness of the intervention on each outcome was inconsistent across the trials. Varied transitional care between hospital and home was implemented to improve health status; however, its effectiveness was controversial. A novel, yet evidence-based approach is needed to develop transitional care interventions for these vulnerable populations.

### FRAILTY AND DEMENTIA: DIFFERENCES IN HEALTH CARE UTILIZATION AND COSTS

Stephanie Denise Sison,[1] Gahee Oh,[2] Sandra Shi,[3] Brianne Olivieri-Mui,[4] Ellen McCarthy,[4] and Dae Kim,[5], *1. Beth Israel Deaconess Medical Center, Boston, Massachusetts, United States, 2. Hinda and Arthur Marcus Institute for Aging Research, Roslindale, Massachusetts, United States, 3. Hebrew SeniorLife, Harvard Medical School, Roslindale, Massachusetts, United States, 4. Hebrew SeniorLife, roslindale, Massachusetts, United States, 5. Hebrew SeniorLife, Boston, Massachusetts, United States*

Frailty and dementia are associated with poor health outcomes and increased health care utilization. A more nuanced understanding of this dynamic may be useful in improving care and developing policies. This retrospective cohort study was conducted using 5% random sample of Medicare fee-for-service beneficiaries (n=1,132,367; mean age 76.2 years; 57.9% female) in 2014-2016. We compared average 1-year home time (number of days alive outside of the hospital and SNF), mean total cost per beneficiary, and number of incident ICU stays per 100 person-years (PY) across four groups: frailty and dementia, dementia alone, frailty alone or neither. Frailty and dementia were identified using validated claims-based algorithms. We also determined differences in costs per group across different regions within the United States. Beneficiaries with both frailty and dementia had a high 1-year mortality rate of 21.9% (vs. dementia alone [9.7%], frailty alone [9.4%] or neither [2.1%]), while having less home time (306 days; difference of 36 days, 31 days, and 53 days, respectively), and more incident ICU stays per 100 PY (29.9 vs 9.5, 25.8, and 5.6, respectively). Mean total costs for beneficiaries with both was $26,030 compared to other groups ($12,096, $24,693, and $9,029, respectively). Across the United States, range of costs varied the most for beneficiaries with both frailty and dementia ($13,244-31,987 vs $4,621-15,364, $20,090-30,965, and $7,672-10,450, respectively). Increase in health care utilization and wide geographic variation in costs associated with patients with frailty and dementia suggests room for improvement in health care delivery to improve outcomes of this group.

### FRAILTY AND FUNCTIONAL STATUS IMPROVEMENT AFTER SKILLED NURSING FACILITY BASED POST-ACUTE CARE

Sandra Shi,[1] Brianne Olivieri-Mui,[2] Ellen McCarthy,[2] and Dae Hyun Kim,[2], *1. Hebrew SeniorLife, Harvard Medical School, Roslindale, Massachusetts, United States, 2. Hebrew SeniorLife, roslindale, Massachusetts, United States*

People admitted to a skilled nursing facility (SNF) for post-acute care undergo comprehensive evaluation and rehabilitation, potentially enabling prediction of future functional recovery. We identified the first SNF admission per beneficiary (n=250,159) between 07/01/2014 – 06/30/2016 in a 5% Medicare sample, using the Minimum Data Set (MDS) and the Outcome and Assessment Information Set (OASIS). Episodes were excluded for non-community discharge (n=43,397) or no OASIS admission assessment within 14 days of SNF discharge (n=77,989). A deficit accumulation Frailty Index (FI) was measured on admission MDS assessment and categorized into robust (MDS-FI<0.15), pre-frailty (MDS-FI0.15-0.24), mild frailty (MDS-FI0.25-0.34), and moderate or worse frailty (MDS-FI≥0.35). Outcomes were functional decline obtained from OASIS, readmission, or death after initiation of home care. Functional status was measured by activities of daily living from OASIS assessments. A total of 135,310 SNF episodes were matched to OASIS episodes. Of these, there were 6,472 (4.8%) robust patients, 38,923 (28.8%) pre-frail, 63,727 (47.1%) mildly frail and 26,053 (19.3%) moderately frail or worse. In a logistic regression after adjustment for OASIS admission function, compared to robust status, frailty was associated with hospital readmission or death within 30 days of OASIS admission, (mild frailty OR1.33 [95%CI 1.23-1.45] and moderate or worse OR1.81 [95%CI 1.66-1.97]). Frailty was also associated with functional decline at OASIS discharge, after adjustment for OASIS admission function (mild frailty OR1.50 [95%CI 1.38-1.63] and moderate or worse OR2.30 [95%CI 2.11-2.50]). Among those discharged from SNF with home services, a SNF-based MDS-FI is associated with increased likelihood of poor community outcomes.

### FRAILTY AND MACRONUTRIENTS INTAKE AMONG OLDER BRAZILIAN ADULTS

Carolina Freiria,[1] Graziele Silva,[2] Larissa Hara,[2] Tábatta Brito,[3] Flávia Arbex Silva Borim,[1] and Ligiana Corona,[2], *1. University of Campinas, Campinas, Sao Paulo, Brazil, 2. University of Campinas, University of Campinas, Sao Paulo, Brazil, 3. Federal University of Alfenas, Federal University of Alfenas, Sao Paulo, Brazil*

The adequate nutrition has an important role in the prevent and treatment of frailty, however, there are only few studies showing the relationship between macronutrients intake and this geriatric syndrome, especially in Latin countries. The aim of this study was to analyze the association between macronutrients intake and frailty among older adults in Brazil. This study included 521 community-dwelling individuals aged 60 years old or older. Frailty was assessed using a self-reported instrument and individuals were categorized in two groups: frail and non-frail (robust + pre frail). Food consumption was evaluated using the 24-hour recall and the software NDSR®. Differences between groups was assessed using the Mann Whitney test. The prevalence of frailty was 42.0%. Older adults considered frails presented lower intake of calories (1510.9 kcal vs 1639.3 kcal; p = 0.016), carbohydrates (196.8 g vs 213.3 g; p = 0.011), proteins (60.7 g vs 68.5 g; p = 0.016) and fiber (15.1 g vs 17.5 g; p= 0.002). They also had lower intake of protein per kilograms of weight (0.88 g/kg vs 0.99 g/kg; p= 0.010). The findings demonstrate high prevalence of frail in our sample, and that intake of most macronutrients was significantly lower among older adults with frail, indicating the importance of the screening of frail as well the evaluation of macronutrients intake among community-based older adults, to prevent malnutrition, sarcopenia and frailty in this population.

### FRAILTY IN A FRAILTY PREVENTION PROGRAM PARTICIPANTS DURING COVID-19 PANDEMIC: A CROSS-SECTIONAL JAPANESE STUDY

Takuya Kanamori,[1] Mizue Suzuki,[2] Tomoyoshi Naito,[3] Keigo Inagaki,[1] and Hiroyuki Umegaki,[4], *1. Hamamatsu University School of Medicine, Hamamatsu city, Shizuoka, Japan, 2. Hamamatsu Universuty school of medicne, Hamamatsu, Shizuoka, Japan, 3. Hamamatsu University School of Medicine, Hamamatsu city,, Shizuoka, Japan, 4. Nagoya University Graduate School of Medicine, Nagoya, Aichi, Japan*

**Objective:** Health conditions of older adults have deteriorated during the COVID-19 pandemic. Few studies have reported on the frailty of this group of people. The study aimed to investigate physical and social frailty in participants in a frailty prevention program during the COVID-19 pandemic.

**Methods:** A cross-sectional survey was conducted in Japan from January 2021. Further, 863 participants of a frailty prevention program completed the survey. The frequency of program attendance in 2020, physical frailty (5-item frailty screening index), social frailty ( diagnostic criteria of social frailty in NCGG-SGS), and depression (GDS-5) were assessed. A related factor of physical frailty was analyzed statistically by Welch's t test and the Chi-squared test.

**Results:** The study participants' mean age, proportion of women, and mean enrollment period in program were 86.8±4.9, 96.3%, 64.3±48.6 months, respectively. The program attendance ratio was 83.4% from January to March, 54.5% from April to June, 79.8% from July to September, and 80.0% from October to December. The prevalence of physical frailty was 20.3% non-frailty, 63.7% pre-frailty, and 15.6% frailty. The prevalence of social frailty was 10.0% non-frailty, 28.6% social pre-frailty, 61.8% social frailty, and the prevalence of depression was 36.8%. Participants with physical frailty were significantly older and showed higher prevalence of social frailty and depression, displaying significantly lower attendance program than non-frailty and pre-frailty older adults ($p<0.05$).

**Conclusions:** The study results suggest that more than half of the participants of a frailty prevention program have social frailty and a high risk of physical frailty due to COVID-19.

### FRAILTY PREVALENCE AND ASSOCIATION WITH MORTALITY ACROSS BIRTH COHORTS IN SWEDISH REGISTRY DATA

Alexandra Wennberg, and Karin Modig, *Karolinska Institutet, Stockholm, Stockholms Lan, Sweden*

Frailty is associated with poor health outcomes, reduced quality of life, and mortality. To understand how prevalence of frailty may have changed across birth cohorts, we investigated frailty prevalence at ages 75, 85, and 95 in people born in 1910, 1920, and 1930 in Swedish national registry data. Frailty was assessed with the Hospital Frailty Risk Score, a weighted sum of 109 ICD codes, which we calculated for each year leading up to the specified ages. We additionally investigated the association between frailty and mortality in these birth cohorts. We observed, at 75, a decrease in prevalence of frailty across birth cohorts (16.9%, 10.8%, and 8.8%, respectively). Interestingly, at 85, we found a U-shaped pattern, where those born in 1920 (14.1%) had lower prevalence of frailty than those born in either 1910 (27.7%) or 1930 (25.1%). At age 95, we saw a low prevalence of frailty in the 1910 (7.3%) and 1920 (3.8%) birth cohorts –potentially because of selective survival. There were not substantial differences in prevalence of frailty by sex or birth country. In Cox proportional hazard models adjusted for sex, frailty was consistently associated with mortality. We observed the greatest hazard ratios in the 1930 birth cohort at 75 (HR=2.79, 95% CI 2.62, 2.97) and 85 (HR=2.26, 95% CI 2.01, 2.53) and the 1920 birth cohort at 75 (HR=2.19, 95% CI 2.09, 2.29), where risk was double that of those who were not frail. Understanding changes in prevalence of frailty will help inform public health and intervention measures.

### FUNCTIONAL TRAJECTORIES AND QUALITY OF LIFE IN POST-ACUTE SKILLED NURSING FACILITY CARE AFTER HOSPITALIZATION

Sandra Shi,[1] Brianne Olivieri-Mui,[2] Ellen McCarthy,[2] and Dae Hyun Kim,[2], *1. Hebrew SeniorLife, Harvard Medical School, Roslindale, Massachusetts, United States, 2. Hebrew SeniorLife, roslindale, Massachusetts, United States*

Frailty predicts readmissions and mortality after acute hospitalizations. Understanding whether frailty predicts functional recovery after acute hospitalizations may help guide post-acute care and rehabilitation. This feasibility study enrolled 24 adults aged ≥65 years from a skilled nursing facility (SNF) after acute hospitalization. We calculated a deficit-accumulation frailty index (FI range: 0-1; non-frail [≤0.25], mild frailty [0.26-0.35], moderate [0.36-0.45], and severe [>0.45]) via in-person assessment on SNF admission. We measured weekly functional improvement with modified Barthel Index, as well as quality of life. Modified Barthel Index and quality of life were measured weekly by Patient-Reported Outcome Measurement Information System (PROMIS) (standardized score with mean 50 and SD 10, higher is better). The mean age was 83.3 years [SD 8.0], and 17 (71.8%) were female. Length of stay for those with severe frailty (FI>0.45) was 26.8 days [10.7] compared to those who were not frail, mildly frail, or moderately frail (13.3 [7.3], 9.4 [4.4], and 15.2 [4.9] respectively). Those with severe frailty also had delayed functional improvement (mean Barthel Index 48.6, 53.4, and 56.6 on admission, week 1, and week 2 of SNF admission respectively), compared to those with moderate frailty (mean Barthel Index 47.5, 69, 73) or mild frailty (68.3, 86, 90.5). Self-reported mental and physical health-related quality of life was relatively unchanged across SNF episode for all frailty categories. These findings suggest that older adults with moderate or severe frailty may experience a typical course of delayed functional recovery and that further monitoring may be necessary for prognostication.

### MALNUTRITION-SARCOPENIA SYNDROME AND ITS ASSOCIATED FACTORS AMONG OLDER ADULTS

Murad Taani, *University of Wisconsin-Milwaukee, Milwaukee, Wisconsin, United States*

Malnutrition and sarcopenia are present in parallel in older adults and characterized by a combination of inadequate nutrient intake and decreased muscle mass, strength, and/or function. The presence of both conditions has been termed Malnutrition-Sarcopenia Syndrome (MSS) and is associated with negative health outcomes. The objective of this correlational study was to identify the prevalence and factors associated with the malnutrition-sarcopenia syndrome among older adults living in continuing care retirement communities. A convenience sample of 104 older adults living in CCRCs participated in this study. Muscle mass, strength, and function were measured using bioimpedance analysis, Jamar digital hand dynamometer, and the Short Physical Performance Battery test, respectively. Physical activity, sedentary time, and nutritional status were measured using ActiGraph GT3X and Mini Nutritional Assessment, respectively. Questionnaires were used to measure self-efficacy for exercise and goal congruence for physical activity and protein intake. Of the 104 participants, 37 (35.2%) had sarcopenia, 19 (18.1%) had malnutrition, and 14 (13.5 %) had MSS. Compared with those without MSS, older adults with MSS were more than two times more likely to have a sedentary lifestyle (Odd ratio, 2.028; 95% confidence interval, 2.012–2.044). Findings showed that sarcopenia, malnutrition, and MSS are prevalent in older adults living in continuing care retirement communities. Older adults should be screened and assessed for both malnutrition and sarcopenia. The results also suggest that decreasing the sedentary time could help in preventing MSS among older adults living in continuing care retirement communities.

### OUTCOMES OF A TELEPHONE-BASED FRAILTY AND FUNCTIONAL ASSESSMENT

Stephanie Denise Sison,[1] Karla Tejada Arias,[2] Natalie Newmeyer,[3] Racheli Schoenburg,[2] Carolina Fonseca Valencia,[4] and Dae Kim,[5], *1. Beth Israel Deaconess Medical Center, Boston, Massachusetts, United States, 2. BIDMC, Boston, Massachusetts, United States, 3. Marcus Institute for Aging Research, Boston, Massachusetts, United States, 4. BIDMC, Providence, Rhode Island, United States, 5. Hebrew SeniorLife, Boston, Massachusetts, United States*

With the goal of increasing the clinical use of frailty, we piloted a quality improvement project to determine the feasibility and utility of a telephone-based frailty and functional assessment. We identified 122 established patients with serious medical illness from an academic geriatrics clinic. A geriatric fellow assessed the functional status and conducted the Mini Nutritional Assessment, telephone-MoCA, and Geriatric Depression Scale to generate a deficit-accumulation frailty index (FI) score which was automatically calculated through the electronic medical record. A note was then generated to inform the providers of the details of the assessment and to provide recommendations based on the findings. From November 2020 to March 2021, 104 out of 122 (85.2%) established patients (mean [SD]: 83.4 [7.1], 66% female, 81% White, and mean [SD] FI: 0.32 [0.17]) agreed and proceeded with the assessment. One month after the call, we found that the assessment was included in the clinical decision-making of 55 out of 100 patients seen by their primary care provider. The top 3 incorporated recommendations were chronic disease management based on frailty status (n=56), lifestyle change and counseling to prevent frailty progression (n=44), and management of cognition and mood (n=18). Management of physical status including referral to PT and OT were incorporated in 15 encounters. Our results suggest that a telephone-based frailty and functional assessment is feasible and has value-added contributions in improving the care of older adults through providing a holistic view of their health status.

### PHYSICAL FRAILTY AND COGNITIVE IMPAIRMENT IN OLDER NURSING HOME RESIDENTS: A LATENT CLASS ANALYSIS

Yiyang Yuan,[1] Kate Lapane,[2] Jennifer Tjia,[2] Jonggyu Baek,[2] Shao-Hsien Liu,[2] and Christine Ulbricht,[2], *1. University of Massachusetts Medical School, Burlington, Massachusetts, United States, 2. University of Massachusetts Medical School, University of Massachusetts Medical School, Massachusetts, United States*

Physical frailty (PF) has various clinical presentations and often co-occurs with cognitive impairment in older adults. In older adults in nursing homes (NHs), no research has examined the heterogeneous profile of PF and its association with cognitive impairment. Minimum Data Set 3.0 was used to identify older, long-stay, newly-admitted NH residents (2014-16; n=871,801). Latent class analysis was used to identify PF subgroups with FRAIL-NH items as indicators. Logistic regression was used to estimate the association between PF subgroups and cognitive impairment. The final model indicated three PF subgroups (prevalence): "mild PF" (7.6%), "moderate PF" (44.5%), and "severe PF" (47.9%). In all subgroups, residents had high probability of needing help with dressing. Older adults likely to belong to the "moderate PF" or the "severe PF" subgroups had high probabilities of requiring physical assistance to transfer between locations and inability to walk in a room. Additionally, residents likely to be in the "severe PF" subgroup had greater probability of bowel incontinence. Greater cognitive impairment was associated with increasingly higher odds to be in the "moderate PF" and "severe PF" subgroups: older residents with severe cognitive impairment were 20% more likely [adjusted odds ratio (aOR): 1.20, 95% confidence interval (CI): 1.17-1.23] and almost 7 times as likely (aOR: 6.86, 95%CI: 6.66-7.06) to belong to the "moderate PF" and "severe PF" subgroups, respectively. Findings provide new evidence for the interrelationship between PF and cognitive impairment in older NH residents and have implications for the development of interventions tailored to older residents' specific PF experience.

### PHYSICAL FRAILTY IS CORRELATED WITH WORSE QUALITY OF LIFE IN OLDER ADULTS WITH HYPERTENSION

Pan Liu, Yaxin Zhang, Shijie Li, Ying Li, Yumeng Chen, Ou Zhao, Yun Li, and Lina Ma, *Xuanwu Hospital, Capital Medical University, National Research Center for Geriatric Medicine, Beijing, Beijing, China (People's Republic)*

**Background:** Hypertension is one of the commonest chronic cardiovascular diseases in older adults. Frailty and hypertension often coexist in older people, but few studies have explored frailty in older hypertensive adults. We aimed to explore the correlation of frailty with quality of life in older hypertensive adults.

**Method:** We enrolled 291 patients with hypertension aged ≥60 years. Ambulatory blood pressure monitor was performed. Physical frailty was assessed by Fried phenotype. Quality of life was assessed by SF-36.

**Results:** Forty-eight (16.5%) patients were frail. Compared with non-frail older hypertensive patients, frail patients were older, had lower education levels, a higher rate of living alone, and a longer duration of hypertension. Moreover, they had lower diastolic blood pressure (DBP) and mean arterial pressure (MAP), and higher pulse pressure, more chronic diseases, a higher proportion of calcium channel blockers (CCBs) usage, and worse quality of life. Frailty scores were positively correlated with pulse pressure, and negatively correlated with DBP and MAP. The SF-36 score was negatively correlated with frailty scores and positively correlated with grip strength and walking speed. After adjusting for age, the SF-36 score was negatively correlated with frailty and positively correlated with walking speed. Frailty, when adjusted for age, duration of hypertension, DBP and comorbidity, had a significant effect on the SF-36 score.

**Conclusion:** Frailty was associated with worse quality of life of older adults with hypertension. Frailty prevention and intervention may help improve the quality of life of older hypertensive adults. Keywords: frailty, older adults, hypertension, quality of life

### PROMOTING EARLY ASSESSMENT OF FRAILTY IN THE NEW NORMAL: AN UPDATED EFI-CGA SOFTWARE TOOL

Katayoun Sepehri,[1] Hilary Low,[2] Grace Park,[3] and Xiaowei Song,[2], *1. University of British Columbia, Vancouver, British Columbia, Canada, 2. Surrey Memorial Hospital, Surrey, British Columbia, Canada, 3. Fraser Health, Surrey, British Columbia, Canada*

Frailty is a state of diminished physiological reserves. Being able to detect and manage frailty early is crucial for effective controlling of frailty-related adverse outcomes. Frailty can be assessed using the frailty index that counts the number of health deficits accumulated over time. Our previous research has enabled an electronic Comprehensive Geriatric Assessment (eCGA) and the calculation of the frailty index based on the eCGA (eFI-CGA). While the standalone eFI-CGA has been used by primary care providers in assessing home-living patients, its initial release was prior to the covid-19 pandemic; the associated new challenges were not targeted by the early version. In facilitating effective virtual assessment and care planning during the current "lockdown" and in the upcoming "new normal", most recently the eFI-CGA version 3.0 was released. In this paper, we 1) introduce the updated electronic frailty assessment tool and its usage, 2) describe the major updates of the software in dealing with challenges due to social isolation and remote assessment, and 3) evaluate the end-user experience with the upgraded methods in frailty assessment. These new developments and implementations allowed a search function to resume disrupted assessment sessions and quickly retrieve previously saved assessment records. The improved user interface promoted the clinicians to conveniently record detailed care plans and management details. The study provided a successful example of moving from disruption to transformation, benefiting the highly demanded healthcare of older adults in this challenging time.

### RISK FACTORS ASSOCIATED WITH COGNITIVE FRAILTY AMONG COMMUNITY-DWELLING OLDER ADULTS: A SCOPING REVIEW

Sumi Lee, and JuHee Lee, *Yonsei University, Seoul, Seoul-t'ukpyolsi, Republic of Korea*

**Objectives:** This study aimed to explore the risk factors associated with cognitive frailty(CF) among community-dwelling older adults, and to provide the impact of CF on health-related outcomes.

**Methods:** PubMed, EMBASE, Cochrane, PsycINFO, CINAHL, RISS, DBpia, NDSL, and KoreaMed databases were searched to retrieve studies. Two reviewers independently screened titles, abstracts and articles. The inclusion criteria are peer-reviewed articles written in English or Korean for community-dwelling older adults with both physical frailty and cognitive impairment present at the same time.

**Results:** A total of 3,513 were searched, and the final 33 were extracted according to the inclusion criteria. Physical factors affecting CF were the number of chronic disease, cardiovascular disease, activity of daily living(ADL), making telephone calls and shopping during instrumental ADL, and a Mini Nutrition Assessment–Short Form score. Psychological factor was depressive symptoms. Significant behavioral factors included self-reported physical activity, low vitamin D, smoking, frequent insomnia, and sedentary lifestyle. In social factors, social participation such as volunteering was identified as a protective factor. Mortality, followed by dementia was health related outcomes on CF, including ADL dependence, poor quality of life, and hospitalization. However, the CF-related fall was inconsistent.

**Conclusion:** A wide variety of factors have been presented in studies related to CF. In order to understand CF and improve health-related outcomes, older adults in CF should be screened as high-risk group. When the risk factors and protective factors of CF managed, better health-related outcomes will lead to successful aging of community-dwelling older adults.

### SKELETAL MUSCLE QUALITY OF NONAGENARIANS AND CENTENARIANS

Iva Miljkovic,[1] Adam Sterczala,[1] Emma Barinas-Mitchell,[1] Ryan Cvejkus,[1] Mary Feitosa,[2] Bharat Thyagarajan,[3] Mary Wojczynski,[4] and Joseph Zmuda,[1], *1. University of Pittsburgh, Pittsburgh, Pennsylvania, United States, 2. Washington University School of Medicine in St. Louis, St. Louis, Missouri, United States, 3. University of Minnesota, Minneapolis, Minnesota, United States, 4. Washington University School of Medicine, Washington University School of Medicine, Missouri, United States*

Skeletal muscle adipose tissue infiltration is hypothesized to lead to poorer muscle quality and function with aging. Indeed, skeletal muscle adiposity has emerged as a consistent, independent predictor of skeletal muscle strength, mobility, metabolic disorders, and survival among older adults. However, phenotypic features of skeletal muscle among the oldest-old remain poorly characterized. Herein, we evaluated the skeletal muscle characteristics of 54 nonagenarians and centenarians (mean age 98 years, range 90-110 years; 63% women) and 25 middle-aged individuals (mean age 54 years, range 40-59 years; 36% women) belonging to the Long Life Family Study (LLFS), an international, multicenter cohort of families with a clustering of longevity. Ultrasonography was used to measure echo intensity of the sternocleidomastoid muscle, which has a similar fiber type distribution to the rectus femoris. Greater echo intensity is indicative of lower muscle quality (greater adipose and fibrotic tissue). Current smoking, alcohol intake, and BMI were similar between the age groups. Nonagenarians and centenarians had lower grip strength (16.3 vs. 39 kg) and were less physically active (22.2% vs 66.7% exercised 1+ times per week) compared to younger individuals (P<0.001 for all). Mean±SE echo intensity, adjusted for gender, field center, BMI and physical activity was 52.1±1.7 among nonagenarians and centenarians compared to 44.2±2.4 among younger individuals (P=0.0098). Our preliminary findings suggest that nonagenarians and centenarians may have substantially lower skeletal muscle quality and strength compared to their younger aged counterparts. Additional research is needed to better understand the mechanisms leading to poorer muscle characteristics of the oldest-old.

### THE EFFECT OF LOW-DOSE ASPIRIN ON FRAILTY IN OLDER ADULTS IN THE ASPIRIN IN REDUCING EVENTS IN THE ELDERLY STUDY

Sara Espinoza,[1] A R M Saifuddin Ekram,[2] Robyn Woods,[3] Michael Ernst,[4] Galina Polekhina,[3] John McNeil,[3] Anne Murray,[5] and Joanne Ryan,[3], *1. University of Texas Health Science Center San Antonio, San Antonio, Texas, United States, 2. Monash University, Monash University, Melbourne, Victoria, Australia, 3. Monash University, Melbourne, Victoria, Australia, 4. University of Iowa, Iowa City, Iowa, United States, 5. Hennepin HealthCare Research Institute, Hennepin HealthCare, Minneapolis, Minnesota, United States*

There are no widely accepted pharmacologic treatments for frailty prevention. Since frailty is associated with inflammation, aspirin has the potential to reduce frailty. We investigated whether low-dose aspirin reduces incident frailty in participants of the ASPirin in Reducing Events in the Elderly (ASPREE) trial. In the U.S and Australia, 19,114 healthy community-dwelling individuals aged ≥70 years (U.S. minorities ≥65 years) were enrolled in ASPREE, a double-blind, placebo-controlled trial of 100mg daily low-dose aspirin vs. placebo. Frailty was defined according to a modified Fried frailty definition, and a frailty index which used a deficit accumulation model. Competing risk Cox proportional hazards models were used to compare time to incident frailty for aspirin vs. placebo. At baseline, 2.2% and 8.1% met criteria for frailty by Fried and frailty index criteria, respectively. Over a median of 4.7 years of follow-up, 2252 participants developed incident frailty according to Fried classification, and 4376 according to the frailty deficit accumulation index. There was no difference in the risk of incident frailty between individuals randomized to aspirin versus placebo according to either criteria (Fried frailty HR: 1.03, 95% CI 0.97-1.09, p=0.41; frailty index HR: 1.03, 95% CI 0.97-1.10, p=0.29). Change in frailty over time was not different between the aspirin and placebo treatment arms. The results were consistent across a series of sub-groups, including baseline frailty status. Based on these results, aspirin use in healthy older adults does not reduce incident frailty.

### Session 9300 (Poster)

### GERIATRIC-FOCUSED EDUCATION AND TRAINING

### CLINICAL GERIATRICS AS A TEAM SPORT: FOSTERING INTERPROFESSIONAL EXPERIENCES IN THE COMMUNITY

David Picella, and Diana Woods, *Azusa Pacific University, Azusa, California, United States*

Team-based care is necessary to provide better healthcare outcomes for the complex needs of older adults. Shared clinical learning experiences prepare practitioners to work in collaborative partnership to achieve optimal outcomes.

To promote collaborative partnership, we established interprofessional community based clinical experiences with older adults at home, in assisted living and in skilled nursing facilities. One nurse practitioner faculty member was paired with 2 students for each clinical experience day. Initially these were face-to-face encounters, however, with the onset of COVID-19, all high-risk encounters were converted to a virtual modality. The clinical encounters focused on the Age Friendly Model (4M). Post clinical discussions and recommendations focused on interprofessional treatment plans. A REDCap(TM) survey was completed by all student participants for program evaluation. Of the 14 surveys sent, 11 were completed; 10 (77%) females; 3 (23%) males; 7 (50%) family practitioner students; 7 (50%) adult-gerontology nurse practitioner students. Four had previous home health experience (14%), and 10 had none (86%). 4M Likert scale (1-5) means were "what matters" = 4.27, medications = 4.18, mentation = 4.09, and mobility = 4.09. Students found the overall experience valuable (mean = 4.27). Of 11 students, 3 (27%) were involved telehealth experiences. Students found real community based clinical experiences to be very enlightening, offering a different perspective, and altering their appreciation for the everyday life of the older adult. Future plans include adding social work and physical therapy students to these clinical experiences to enhance interprofessional education.

### EFFECTIVENESS OF THE DEMENTIA NURSING COMPETENCE E-LEARNING PROGRAM FOR NURSES IN ACUTE CARE HOSPITALS IN JAPAN

Mizue Suzuki,[1] Hiromi Yoshimura,[2] Soichiro Mimuro,[3] Keisuke Sawaki,[4] Nanayo Sasaki,[5] Kimiyo Mastushita,[6] Tetsuro Ishihara,[7] and Ikuko Sakai,[8], *1. Hamamatsu Universuty school of medicne, Hamamatsu, Shizuoka, Japan, 2. Institute for Graduate Nurses of Japanese Nursing Association, Tokyo, Tokyo, Japan, 3. Hamamatsu University School of Medicine, Hamamastu, Shizuoka, Japan, 4. Hamamatsu Shugakusha Junior High School & High School, Hamamastu, Shizuoka, Japan, 5. Hamamatsu University Hospital, Hamamastu, Shizuoka, Japan, 6. Seirei Mikatahara General Hospital, Hamamastu, Shizuoka, Japan, 7. Ishihara NeuroCognitive Clinic, seidai, Miyagi, Japan, 8. Graduate School of Nursing Chiba University, chiba, Chiba, Japan*

**Objective:** The number of older patients with dementia hospitalised in acute care hospitals increased and these patients underwent physical restrictions leading to a degeneration of essential mental and physical function. The dementia nursing competence e-learning program with audio-visual materials has been developed in acute care hospitals.

**Methods:** An application form that explained the research was distributed to 1,944 registered nurses from seven hospitals, and 110 people applied. Nurses used an e-learning program for a month in May and practiced applying the knowledge learned from programs June through November 2020. The nurses completed a questionnaire survey at four periods: first (before program/baseline), second (after program), third (three months later), and fourth (six months later). In the second, third, and fourth periods, 'Technical knowledge of the dementia nursing' and 'dementia nursing intervention' were significantly improved as compared with the first. In the fourth period, 'confidence of reduce of physical restriction' showed significant improvement compared to the first. In 'Ethical sensitivity scale of nurses', the first of the four sub-scales significantly increased as compared with the first period. In 'Self-assessment Scale of Nursing Practice for Elderly Patients with Cognitive Impairment with the Aim of Person-centred Care in Acute Care Hospitals', the fourth sub-scale showed significant improvement compared to the first. In the 'Personhood' sub- scale of the Japanese version of Approaches to Dementia Questionnaire, the fourth period showed a significant increase compared to the first.

**Conclusion:** The results suggest that this program was effective and led to reduced physical restrictions in nursing practice.

### END OF LIFE VIRTUAL REALITY TRAINING: MEDICAL STUDENT INCREASED EMPATHIC ABILITY

Marilyn Gugliucci,[1] Daniel Manukhin,[2] Elizabeth Dyer,[2] and Barbara Swartzlander,[2], *1. University of New England College of Osteopathic Medicine, Kennebunk, Maine, United States, 2. University of New England, University of New England, Maine, United States*

**Introduction:** It is unclear if medical student empathy declines by third year of clinical rotation trainings. Desensitization throughout the first two years may lead to decreases in empathy as a coping mechanism to avoid burnout in the clinical years. This study determined if self-assessed empathy increased after conducting an Embodied Labs, Inc. end of life virtual reality (VR) experience.

**Methods:** Mixed methods, quantitative/qualitative, research were applied for University of New England (UNE) College of Osteopathic Medicine (COM) 2nd year medical students (N=174). They completed the 3-part 30 minute Clay Lab VR experience. UNE IRB approved pre/post-tests focused on empathy. Data were collected using RedCap. Closed questions were analyzed applying frequency analysis and paired-sample t-test through excel. Open-ended questions were analyzed through N-VIVO 12+.

**Results:** The data included pre/post-tests from 146 students volunteers. Results indicated statistical significance (P=.01) in all closed questions except for question 7 (What is your view of conducting a full code on a patient with a DNR? (P=.14). The greatest difference seen between pre (23.97% agree or strongly agree) and post-test (64.38% agree or strongly agree) data was for question 3 (I gained knowledge about what hospice is by embodying Clay in this virtual reality lab); P= .00. Three qualitative themes included: Impact, Empathy, EOL Knowledge.

**Conclusion:** This VR Lab experience increased self-assessed empathy at the time of Clay Lab completion; however, enduring empathy and learning about hospice/EOL has not been measured. Further research is suggested to determine the longitudinal impact of virtual reality education.

### EXPERIENCE OF PHYSICAL THERAPY STUDENTS MENTORING OLDER ADULTS WITH HEALTH LITERACY TOOLS

Mary Milidonis,[1] Jane Keehan,[2] Katherine Montgomery,[3] Rebecca Deuley,[2] Sara Formoso,[2] and Karen Kopera-Frye,[4], *1. Cleveland State University, Hudson, Ohio, United States, 2. Cleveland State University,*

Cleveland, Ohio, United States, 3. Cleveland State University, Cleveland State University, Ohio, United States, 4. New Mexico State University, Las Cruces, New Mexico, United States

Health literacy is a top priority for Healthy People 2030. Healthy People 2030 defines personal health literacy as "the degree to which individuals have the ability to find, understand, and use information and services to inform health-related decisions and actions for themselves and others." The purpose is to understand the experience of physical therapy students using health literacy tools with older adults to promote the adoption of health literacy tools in healthcare encounters. This project analyzes the reflection responses from students using qualitative methods. The qualitative methods included student reflection papers, word clouds, and focus groups. Twelve students participated in focus groups/ reflections. Thirty-seven students participated in word clouds. Health literacy tools included plain language, teaching teach back and "Ask me 3"®. Students were taught by student leaders and faculty about the meaning of health literacy and oral communication tools. Pairs of students provided health education with health literacy tools to older adults. Students then participated in a small group reflection to create word clouds. Students answered questions and provided five words that best answer each question. Students believed the benefits of health literacy tools for older adults includes better learning, participation and engagement. Reasons to use health literacy in the future were improved older adult independence, education and adherence. Students completed reflections and interviews at the end of the year to detail their experience with the health literacy tools. The pedagogical approach highlighted the value of experiential learning for the students while mentoring older adults.

### GERIATRIC KNOWLEDGE GAPS OF COMMUNITY-BASED PROVIDERS: A NATIONAL STUDY OF ASKED QUESTIONS

Lee Lindquist,[1] Aylin Madore,[2] Stephanie Miller,[2] Alice Kerr,[3] and Sara Bradley,[3], *1. Northwestern University Feinberg School of Medicine, Chicago, Illinois, United States, 2. DBC Pri-Med, LLC, Boston, Massachusetts, United States, 3. Northwestern University Feinberg School of Medicine, Northwestern University Feinberg School of Medicine, Illinois, United States*

Primary care providers (PCP) - internists, family practitioners, nurse practitioners, physician assistants - play an integral role in the care of older adults, although many receive limited geriatrics education. We sought to examine what questions community-based PCPs had about geriatrics and clinical care of older adults. As part of large clinical continuing medical education (CME) conferences across 12 states (FL,GA,CA,IL,NY,MA,DC, PA,AZ,TX,TN,WA), PCPs attended a live in-person 60-minute geriatrics-focused lecture and entered questions into a mobile application. Questions were then qualitatively analyzed using constant-comparison and tie-break methodology. At all sites, 103 questions were asked with 158 upticks (PCPs could check off that they had similar question) with a range of 3-18 questions per lecture. PCPs asked questions on the following common themes: 1.) Medication-related (e.g. discontinuing medicines in asymptomatic patients, optimizing pain relief), 2.) Dementia (e.g. prevention, nutraceuticals, agitation) 3.) Medicare Coding 4.) Falls 5.) Weight loss, and 6.) Insomnia. There were a number of questions referencing incorrect practices (e.g. prescribing inappropriate medications such as benzodiazepines for sleep, placement of gastric tubes in late-stage dementia, antibiotics to treat asymptomatic bacteria). In conclusion, community-based PCPs nationally experience gaps in geriatric knowledge and several utilize practices that could jeopardize older adult health. While attending CME-based lectures is one means of overcoming these gaps, some PCPs may not find time or realize geriatrics as an educational need. PCPs need to be better supported with opportunities to ask geriatric care-related questions in order to improve the care of older adults.

### HOW 4MS AGE-FRIENDLY CARE LED TO IMPROVED COMPLIANCY OF OLDER ADULT FALL SCREENINGS IN RURAL PRIMARY CARE CLINICS

Leah Tobey, and Robin McAtee, *University of Arkansas for Medical Sciences, Little Rock, Arkansas, United States*

Past medical history of falls and fear of falling are reliable indicators of future fall risk of an older adult (OA). As one of the HRSA funded Geriatric Workforce Enhancement recipients, the AR Geriatric Education Collaborative (AGEC) worked with a rural federally qualified healthcare clinic system to help incorporate fall screens to satisfy the Mobility factor in the 4Ms age-friendly care framework. After consultation with the practitioners, it was decided to use the Timed-Up-And-Go (TUAG) screen because it is evidence-based and appropriate for OAs. Training on the use of the TUAG was completed next as was the addition of the screen into the EMR. Fall screens in one clinic were only completed 7% before training and 7 months after the training, this rose to almost 100%. In a second clinic, the screens were completed 22% of the time and this was increased to 66% after training. Training on mobility continues to occur on a regular basis as staff turns over and as new priorities arise, but the use of the TUAG as a mobility screen has been a critical component in the process of these rural clinics providing age-friendly care. Next steps with improving fall risks will be the development of flags within the EMR that will force practitioners to complete a full falls plan of care if the OA scored within the moderate or high fall risk categories. The plan will include home safety education and/or evaluation, PT or OT referrals to further support healthy aging for the OA.

### IMPLEMENTATION OF 4M AGE-FRIENDLY CARE IN ARKANSAS RURAL PRIMARY CARE CLINICS LED TO IMPROVED ADVANCE CARE PLANNING

Leah Tobey, and Robin McAtee, *University of Arkansas for Medical Sciences, Little Rock, Arkansas, United States*

The 4Ms Age-Friendly Framework has been introduced and implemented into nearly 2,000 primary care practices across the United States by Geriatric Workforce Enhancement Program's (GWEP) educational efforts. The AR Geriatric Education Collaborative, the GWEP in Arkansas, has provided monthly trainings to a rural federally qualified healthcare clinic system and educated clinicians about how to complete a Medicare Annual Wellness Visit (AWV) that was inclusive of an advance care plan. Specific educational training including the two main components of an ACP: living will preferences and medical power of attorney were reviewed as their role into “What Matters” was explained. Before 4Ms Age Friendly training, baseline data showed only 7% of older adults (OAs) had an established ACP in site 1 and 33% in site 2. After training, the rate of ACP rose to 47% in site 1 and 59% in site 2. During the training, not only were the two main components reviewed but case studies were provided about what questions to ask surrounding the “What Matters” question as a guide to further discuss an OAs wishes, priorities and end-of-life care. This project demonstrated that implementation of 4Ms Age-Friendly Care not only improves the completion of advance care plans but also further enhances the overall care of the older adult when “what matters” most to the older adults is known and communicated.

### MEDICAL/PHARMACY STUDENTS INTERPROFESSIONAL OLDER ADULT MEDICATION INTERACTIONS/CONTRAINDICATIONS PROJECT

Marilyn Gugliucci,[1] and Victoria Thieme,[2], *1. University of New England College of Osteopathic Medicine, Kennebunk, Maine, United States, 2. Univesity of New England College of Osteopathic Medicine, University of New England, Maine, United States*

The University of New England College of Osteopathic Medicine (UNECOM) Geriatrics Education Mentors [GEM] program, established in 2014, pairs UNECOM students with older community living adults. GEM assignments focus on health review, medical humanities, and geriatrics training. Each year approximately 90 older adults participate in GEMs. In 2019, the GEM program was expanded with Geriatrics Workforce Enhancement Program (GWEP) grant funding to: include first year medical students, include 2 additional assignments (4 assignments over 10 months to 6 assignments over 18 months), and to create interprofessional student collaboration. In the new GEM Assignment 4: Medication Interactions/Contraindications, UNECOM students with their GEM compiled details on the GEM’s medication list (prescriptions, herbal, OTC); one of 4 Ms of Age Friendly Health Care. UNECOM students (84 pairs) were then assigned to UNE School of Pharmacy (SOP) students (42 SOP students had 2 UNECOM pairs) to conduct a “Lexicomp” (App) medication interactions and Beers Criteria review. UNECOM students documented findings with the SOP student partner; discussed the processes of review with their GEM and the resultant findings; documented the GEM’s questions and how the UNECOM student answered those questions; and discussed next steps for the GEM regarding options for different medications - especially follow up with their prescribing physician(s) for any noted interactions/contraindications. For GEMs with few medications, a mock medication list was assigned to ensure student experiences with medication reviews and GEM discussion. Although time intensive preparation is required, UNECOM & SOP students attained significant learning as did the GEM mentors.

### RECOGNIZING AND RESPONDING TO ELDER MISTREATMENT: DEVELOPING AND TESTING AN ONLINE TRAINING FOR EMS PRACTITIONERS

Kristin Lees Haggerty,[1] Dana Wardlaw,[2] Melanie Miller,[3] Randi Campetti,[2] Athi Myint-U,[4] and Brad Cannell,[5], *1. Education Development Center, West Roxbury, Massachusetts, United States, 2. Education Development Center, Waltham, Massachusetts, United States, 3. Education Development Center, Jamaica Plain, Massachusetts, United States, 4. Education Development Center, Education Development Center, Massachusetts, United States, 5. University of Texas Health Science Center at Houston, Dallas, Texas, United States*

Elder mistreatment is an urgent and under recognized public health concern with devastating consequences for older adults, families, and health systems. Risk for elder mistreatment has increased during the COVID-19 pandemic, further highlighting the urgency to address it. Prehospital emergency medical service (EMS) practitioners have unique opportunities to recognize signs of elder mistreatment but often lack the training and tools required to facilitate consistent identification and intervention. To address this gap, Education Development Center collaborated with a team of expert advisors and EMS practitioners to develop and pilot test Recognizing and Responding to Elder Mistreatment: An Online Training for EMS Practitioners with funding from the RRF Foundation for Aging. This training aims to prepare EMS practitioners to recognize potential mistreatment and report suspected elder mistreatment in line with state laws and their professional code of ethics. In this presentation, we will describe the iterative development process, present results from a pilot test conducted with EMS practitioners in Massachusetts and share strategies and progress for disseminating the training nationally. The pilot study utilized a pre-post design to assess changes in knowledge, attitudes, and practices at baseline, immediately after and two months following participation in the training. Results indicate statistically significant improvements in knowledge related to elder mistreatment identification and response from pre- to post-training and maintenance of these improvements two months later. Participants reported feeling more prepared to address elder mistreatment in their work as EMS practitioners and applying their new knowledge and skills during the two months following the training.

### THE 4MS IN A PANDEMIC: A SURVEY OF TRAINING NEEDS AMONG HEALTHCARE PROFESSIONALS, OLDER ADULTS, AND COMMUNITY MEMBERS

Jennifer Crittenden,[1] Abigail Elwell,[2] David Wihry,[2] and Lenard Kaye,[2], *1. University of Maine, University of Maine, Maine, United States, 2. University of Maine, Bangor, Maine, United States*

The University of New England, in collaboration with the University of Maine, received a five-year grant from the Health Resources and Services Administration (HRSA) to improve the health and well-being of Maine’s older adults through enhanced training under HRSA’s Geriatrics Workforce Enhancement Program (GWEP). As part of these efforts, stakeholder training needs assessment data were collected via a statewide electronic survey that was distributed to community members and providers throughout Maine. The survey, which focused on the 4M's of Age-Friendly Healthcare, received 68 responses from older adults/community members (N = 26), program administrators (N = 12), along with community leaders, and those working in the public and non-profit sector (N = 13). A significant emphasis on social isolation, mental health, and grief and loss issues was noted and dominating themes centering on two dimensions of the 4M framework: "What Matters" and "Mentation." Findings reflect an overriding priority by providers and consumers to keep older adults socially connected (28%, N = 34) and maintaining mental health and well-being during the pandemic (21%, N = 14). Qualitative response analysis identified additional COVID-19-related training topics such as: what to do if you or a loved one contracts coronavirus, how to handle grief and loss related to COVID-19, strategies for supporting loved ones during COVID-19, and socially distanced bereavement support. Results indicate a need to focus on meeting the emotional and mental health needs of older adults, as well as the importance of encouraging connections and mitigating the effects of social isolation during COVID-19.

### USING THE 4 M OF THE AGE FRIENDLY HEALTH SYSTEM TO IMPROVE MIPS DOCUMENTATION IN PRIMARY CARE: A FEASIBILITY STUDY

Sweta Tewary,[1] Denise Kruszynski,[2] Naushira Pandya,[3] Nicole Cook,[3] Sashah Damier,[2] and Assma Twahir,[3], *1. Nova Southeastern University, Fort Lauderdale, Florida, United States, 2. NSU, Davie,FL, Florida, United States, 3. NSU, Davie, Florida, United States*

Age Friendly Health Systems (AFHS) commit to evidence-based, low-risk, coordinated care that is centered on what matters most to older adults, their families and caregivers. Nova Southeastern University South Florida Geriatric Workforce Enhancement Program (NSU SFGWEP) has partnered with multiple primary care clinics to provide dedicated AFHS training and support to increase AFHS transformation in Broward and Miami-Dade Counties. As part of the initiative, SFGWEP provide didactic training, clinic on-site brief demonstration, and infographic guidance for EHR documentation. NSU SFGWEP activities are conducted through training surveys, provider feedback, and e-clinical measures that align with CMS MIPS measures. Three participating health systems report annually on seven e-clinical measures that, collectively, provide indicators of the 4Ms of AFHS (what matters, medication management, mentation and mobility.) From baseline to Year 1, NSU SFGWEP saw improvement in controlled hypertension (54% to 94%), opioid screening (<1% to 11%), advance care planning (21% to 35%) and falls risk assessment (45% to 59%). Results demonstrate the need to continue and expand AFHS interventions for sustainability. In Year 2, SFGWEP will continue to expand awareness of best practices and benefits of the AFHS through education and training at NSU and at the various primary care sites. As mutual collaboration and implementation methods are shared among participating members, the expectation is that quality healthcare of our elder community adults will measurably improve.

### Session 9305 (Poster)

### GEROSCIENCE

### AGE, SEX, AND CEREBRAL MICROBLEEDS AFFECT WHITE MATTER INTEGRITY ACROSS ADULTHOOD AFTER MILD TRAUMATIC BRAIN INJURY

David Robles,[1] Ammar Dharani,[1] Nikhil Chaudhari,[1] Kenneth Rostowsky,[2] Layal Wehbe,[1] Michelle Ha,[1] Van Ngo,[1] and Andrei Irimia,[2], *1. University of Southern California, Los Angeles, California, United States, 2. University of Southern California, University of Southern California, California, United States*

The contributions of age, sex, and cerebral microbleeds (CMBs) to WM changes after mild traumatic brain injury (mTBI) have not been studied. We used diffusion tensor imaging (DTI) to map WM fractional anisotropy (FA) changes across the first ~6 months post-mTBI in 109 subjects aged 18-77 (46 females; age μ: 40 y, σ: 17 y) imaged within ~1 week post-injury and ~6 months later. After partialing out age, sex, and CMB counts, significant mean FA decreases were found in the anterior body, posterior body, and splenium of the corpus callosum (CC; p = 0.003, 0.009 and 0.015, respectively), left superficial frontal fasciculus (p = 0.008), and left branch of the corticospinal tract (CST; p = 0.007). Age contributed to mean FAs measured acutely in the CC body (p = 0.04), and chronically in the CC genu (p < 0.001), CC body (p = 0.01), and middle longitudinal fasciculi (p = 0.04), older adults exhibiting larger decreases. CMB counts were positively associated with mean FA decreases in the CC body (p = 0.04) and middle longitudinal fasciculi (p = 0.04). Significant age-by-sex and CMB count-by-age interactions mediated FA decreases in the CC genu (p = 0.02 and p = 0.03, respectively), older males exhibiting larger decreases. Thus, the CC, longitudinal fasciculi, superficial frontal WM and CST are particularly vulnerable to post-traumatic neurodegeneration moderated by age, sex and CMB count, men and older adults being at highest risk for adverse effects. Future research should investigate our findings relative to cognitive function.

### AGE-RELATED AORTIC STIFFNESS CAN BE TRANSFERRED AND AMELIORATED VIA FECAL MICROBIOTA TRANSPLANT IN MICE.

Nathan Greenberg,[1] Nicholas VanDongen,[2] Rachel Gioscia-Ryan,[2] Abigail Casso,[2] David Hutton,[2] Zachary Clayton,[2] Douglas Seals,[2] and Vienna Brunt,[2], *1. University of Colorado at Boulder, Boulder, Colorado, United States, 2. University of Colorado Boulder, Boulder, Colorado, United States*

Age-related increases in aortic stiffness contribute to the development of cardiovascular diseases (CVD). To determine whether the gut microbiome (GM) modulates age-related aortic stiffening, we performed fecal microbiota transplants (FMT) between young (Y; 3 month) and older (O; 25 month) male C57BL/6N mice. Following antibiotic treatment (to suppress endogenous microbiota), mice received weekly FMT (fecal samples collected at baseline) via oral gavage for 8-16 weeks from their own

(i.e., sham condition: Y-y, O-o [RECIPIENT-donor]) or opposite age group (Y-o, O-y) (N=8-12/group). In vivo aortic stiffness (pulse wave velocity [PWV]) was higher in older vs. young mice at baseline (382±8 vs. 328±7cm/sec, mean±SE, P<0.001). Arterial phenotypes were transferred such that old microbiota transplanted into young mice increased, while young into old decreased, PWV (Y-y: 325±10 vs. Y-o: 362±10cm/sec, P=0.022; O-o: 409±10 vs. O-y: 335±6cm/sec, P<0.001). Intrinsic mechanical stiffness of excised aortic rings (elastic modulus) increased after transplant of old into young (Y-y: 2141±223 vs. Y-o: 3218±394kPA, P=0.022), and decreased with young into old (O-O: 3263±217 vs. O-y: 2602±136kPA, P=0.016), indicating the GM mediates aortic stiffening by modulating structural changes in the arterial wall. Age-related increases in aortic abundance of advanced glycation end products (AGEs), which cross-link arterial structural proteins, tended to be transferred by the GM (Y-y: 0.022±0.001 vs. Y-o: 0.038±0.006 A.U., P=0.11; O-o: 0.120±0.029 vs. O-y: 0.038±0.009 A.U., P=0.06). The aging GM can induce aortic stiffening via promoting AGEs accumulation and crosslinking of arterial structural proteins, and thus might be a promising target for preventing/treating age-related aortic stiffening and CVD.

### GEROSCIENCE APPROACHES TO OBESITY

Alessandro Bitto, and Matt Kaeberlein, *University of Washington, Seattle, Washington, United States*

Besides aging, obesity is the greatest risk factor for numerous chronic pathologies, including metabolic syndrome, type 2 diabetes, cardiovascular disease, hypertension, and cancer. Preventing and treating obesity would greatly reduce healthcare costs and the impact of the aging process, with estimated savings up to $145,000,000,000. Pharmacological interventions identified by geroscience may prove effective against diet-induced obesity. To test this hypothesis, we fed a 66% kcal/fat diet to nine-month-old C57Bl6/N mice for 6 weeks and treated them with either rapamycin, acarbose, or a combination thereof. Rapamycin, and to a lesser extent acarbose, prevented weight gain and fat accumulation in these mice. We detected increased expression of the Liver Activating Protein (LAP) isoform of the transcription factor CCAT/Enhancer Binding Protein β (C/EBPβ) in the liver of mice treated with rapamycin. C/EBPβ-LAP mediates some of the effects of caloric restriction on nutrient metabolism and increases lifespan in a mouse transgenic model. We tested whether independent activation of C/EBPβ-LAP would recapitulate the effects of rapamycin by treating mice on a high-fat diet with adefovir dipivoxil, a reverse transcriptase inhibitor that can activate LAP in vitro independently of mTOR inhibition. Adefovir dipivoxil reduced weight and fat mass accumulation in mice over the course of 6 weeks. Mice treated with adefovir dipivoxil showed increased expression of genes involved in β-oxidation and lipids mobilization, and reduced activation of fatty acid biosynthesis and lipid storage pathways. Our results identify C/EBPβ-LAP as a potential new target to improve lipid homeostasis in both aging and obesity.

### PRE-OPERATIVE PHYSICAL FUNCTION PREDICTS POST-OPERATIVE SKELETAL MUSCLE GENE EXPRESSION IN OLD MICE

Samantha Asche-Godin,[1] Lauren Harlow,[1] Zachary Graham,[2] Weihua Huang,[3] Charles Mobbs,[4] Christopher Cardozo,[1] and Fred Ko,[1], *1. James J. Peters VA Medical Center, Bronx, New York, United States, 2. Birmingham VA Medical Center, Birmingham, Alabama, United States, 3. New York Medical College, Valhalla, New York, United States, 4. Icahn School of Medicine at Mount Sinai, new york, New York, United States*

In older adults, pre-operative physical function predicts post-operative outcomes. The biological mechanisms underlying vulnerability to physical decline remain poorly understood. Using a mouse model of laparotomy, we sought to identify biological correlates of post-operative function. 24-month-old male C57BL/6N mice were categorized as high functioning (HF) or low functioning (LF) based on pre-operative performance on the accelerating rotarod. On post-operative days (POD) 2 and 4, LF mice had lower rotarod latency to fall times than HF mice did. Forelimb grip strength was reduced after laparotomy in both HF and LF groups on POD 1 and 3 and did not differ significantly between these groups. Whole transcriptome sequencing analysis (RNAseq) of soleus muscles collected on POD 5 showed 224 and 228 differentially expressed genes (DEGs) for HF and LF, respectively, compared to their respective controls. Only 21 DEGs were observed in both groups, including Pparα, Fst and Pla2g15. Such changes may be hallmarks of the post-surgical response in aging. Pathway analysis of DEGs using Ingenuity Pathway Analysis software (Qiagen) revealed one pathway common to HF and LF (osteoarthritis) whereas activation of GP6 signaling and apoptosis signaling was observed in HF and inhibition of PPARα/RXR activation and PPARα signaling was noted in LF. We conclude that pre-operative performance on the accelerating rotarod correlates with differences in skeletal muscle gene expression, which may contribute to the differences in functional outcomes post-operatively in HF and LF mice. Further studies are needed to delineate the roles of these signaling pathways in physical resilience to surgery.

### SEX AND AGE DIFFERENCES IN DEFAULT MODE NETWORK FUNCTIONAL CORRELATION AFTER TRAUMATIC BRAIN INJURY

Anar Amgalan,[1] Alexander Mayer,[1] Michelle Ha,[1] and Andrei Irimia,[2], *1. University of Southern California, Los Angeles, California, United States, 2. University of Southern California, University of Southern California, California, United States*

The extent to which brain functional correlations (FCs) are modulated by age and sex is unknown. We studied default mode network (DMN) FC changes in 136 participants with mild traumatic brain injury (mTBI; 52 females, age range: 19 – 79 years, age $\mu$ = 42, age $\sigma$ = 17; 72 participants younger than 40). Structural and functional magnetic resonance images (MRIs) were acquired ~1 week and ~6 months post-injury; the FreeSurfer Functional Analysis STream (FS-FAST) was used for group-level FC comparisons across sexes and age groups (younger vs. older than 40). FC seeds were two sub-networks of the DMN, M1 and M2, defined by the standard Yeo parcellation scheme. For M1, clusters with significant FC differences across sexes were in the right paracentral lobule, central sulcus, postcentral gyrus, superior frontal gyrus, and precentral sulcus (p = 0.0001), and in the left paracentral lobule and central sulcus (p = 0.022). For M2, clusters spanned the right postcentral gyrus, middle occipital gyrus, transverse occipital sulcus, and central sulcus (p = 0.0001), the left precuneus and inferior parietal lobe (p = 0.0096). Females either exhibited no significant FC change or underwent FC increases. Males underwent significant FC decreases within all clusters, suggesting their increased vulnerability to mTBI-related effects. Clusters whose FCs differed significantly across age groups were localized to the left superior temporal gyrus (p = 0.0078), highlighting the vulnerability of temporal regions to age effects. Future studies should explore the age × sex interaction and uncover the mechanisms for these observed findings.

### THE ROLE OF OBESITY IN FRAILTY INCIDENCE: THE SAN ANTONIO LONGITUDINAL STUDY OF AGING

Tiffany Cortes,[1] Chen-pin Wang,[2] Helen Hazuda,[2] and Sara Espinoza,[3], *1. University of Texas HSC at San Antonio, San Antonio, Texas, United States, 2. UT Health San Antonio, UT Health San Antonio, Texas, United States, 3. University of Texas Health Science Center San Antonio, San Antonio, Texas, United States*

**Background:** Although initially conceptualized as a wasting syndrome, obesity has been associated with frailty in prior studies. The goal of this study was to examine the associations of obesity and waist circumference with frailty and determine whether they predict incident frailty in an ethnically diverse population of older Mexican Americans (MAs) and European Americans (EAs).

**Methods:** 749 MA and EA community-dwelling older adults (65+) participated in the baseline examination of the San Antonio Longitudinal Study of Aging (SALSA), and 474 participants completed the first follow up approximately 6 years later. Frailty was classified using Fried criteria. Baseline characteristics, including body mass index (BMI) and waist circumference (WC) were summarized by frailty category (non-frail, pre-frail, frail) using ANOVA. The odds of becoming frail at follow-up by baseline BMI and WC were estimated using separate logistic regression models, adjusting for age, sex, ethnicity, diabetes, comorbidity (presence of ≥2 chronic diseases not including diabetes), baseline frailty score, and follow-up time.

**Results:** At baseline, participants were 69 ±3 years old, 61% female, and 50% MA. BMI and WC increased with increasing frailty category (p <0.01 for both). BMI was a significant predictor of incident frailty (OR=1.08, 95% confidence interval [CI]: 1.02-1.14, p=0.011). WC also predicted frailty (OR=1.03, 95% CI: 1.01-1.05, p =0.017).

**Conclusion:** These results demonstrate that BMI and WC are significant predictors of frailty. Interventions which target obesity may reduce the incidence of frailty; however, more research in this area is needed.

### Session 9310 (Poster)

### GLOBAL AGING

### CENTENARIANS IN THE GLOBAL SOUTH: A VIEW FROM ETHIOPIA

Samson Chane,[1] and Margaret Adamek,[2], *1. Bahir Dar University, Bahir Dar, Oromiya, Ethiopia, 2. Indiana University, INDIANAPOLIS, Indiana, United States*

As global aging advances, the number of centenarians worldwide is greatly increasing. Most of what is known about centenarians comes the Global North. It is not clear what factors contribute to longevity of centenarians in impoverished, mostly rural areas of Global South nations that still lack basic amenities. Cultural differences in the profile, lifestyles, and needs of centenarians in Africa have yet to be documented. Using a case study design, this descriptive inquiry investigated the profiles of centenarians in Ethiopia including religion, marriage, education, occupation, income, and living arrangement. Data were generated through in-depth interviews with nine centenarians (1 woman, 8 men) and were analyzed using descriptive narrative analysis. Respondents were between 100 and 108 years old. All nine were adherents of Orthodox Christianity, had been married, and were great-grandparents. Their adult lives were marked by both residential and marital stability. The Ethiopian centenarians persevered through many losses and hardships with the help of strong community-based social networks. Unlike studies of centenarians in the Global North, most respondents were male and had strict religious upbringings. Understanding the unique profiles of centenarians in the Global South will help to inform research and practice with this growing population of the oldest-old.

### COMPARISON OF CATASTROPHIC OUT-OF-POCKET MEDICAL EXPENDITURES IN THE US AND SOUTH KOREA

Narae Kim, and Mireille Jacobson, *University of Southern California, Los Angeles, California, United States*

To date, relatively few studies have examined catastrophic out-of-pocket medical spending in the United States, especially in comparison to other high-income countries. We compared catastrophic out-of-pocket medical spending among adults age 65 and older in the United States versus South Korea, a high-income country with national health insurance that is often overlooked in cross-country comparisons. We defined catastrophic medical spending as health care expenditure for the past two years that exceeds 50% of one's annual household income. Using data from the 2016 Health and Retirement Study (HRS) and Korean Longitudinal Study of Aging (KLoSA), we performed a logistic regression to examine the factors affecting catastrophic out-of-pocket medical spending for older adults in both countries. We also performed a Blinder-Oaxaca decomposition to compare the contribution of demographics factors versus health system-level factors to catastrophic out-of-pocket medical spending. The proportion of respondents with catastrophic out-of-pocket medical expenditure was higher in the US; the proportion was 5.8% and 3.0% in the US and South Korea, respectively. Both in the US and South Korea, respondents who were in the lower-income quartiles, who had experienced a stroke or had diabetes, and who rated their health as poor had higher odds of catastrophic out-of-pocket medical expenditure. The Blinder-Oaxaca non-linear decomposition showed that the significant difference in the rate of catastrophic out-of-pocket medical spending between the two countries was attributable to unobservable system-level factors, not observed differences in the sociodemographic characteristics between the two countries.

### NON-RESPONSE TO POPULATION AGING IN SUB-SAHARAN AFRICA: A SURVEY OF GERONTOLOGY SCHOLARS

Margaret Adamek,[1] Messay Kotecho,[2] Samson Chane,[3] and Getachew Gebeyaw,[4], *1. Indiana University, INDIANAPOLIS, Indiana, United States, 2. Addis Ababa University, Addis Ababa, Adis Abeba, Ethiopia, 3. Bahir Dar University, Bahir Dar, Oromiya, Ethiopia, 4. University of Gondar, Gondar, Oromiya, Ethiopia*

Life expectancy is increasing globally, with the biggest gains expected in sub-Saharan Africa. In fact, most of the population growth globally in the next few decades will occur in sub-Saharan Africa. Using an online survey we investigated the perspectives of gerontology scholars on the challenges of aging in sub-Saharan Africa as well as the assets of elders. Respondents (n=72) from 17 countries, primarily in Africa, and representing 16 disciplines, identified the top issues facing African elders as: poverty, lack of trained professionals, food insecurity, disability/health issues, and long-term care. Older adults' unique strengths were noted as indigenous knowledge systems, being holders of cultural heritage, and their contributions to development. Respondents' biggest concerns about older adults in sub-Saharan Africa were the lack of government attention to aging issues (63%) and a lack of social services targeted to elders' needs (57%). Government funding (77.8%) and international partnerships (38.9%) were noted as resources needed to support aging research in sub-Saharan Africa. The response or non-response of governments in sub-Saharan Africa will determine whether the growing number of older adults will increasingly experience unmet needs and whether their assets will be considered in development efforts. Establishing professional networks of gerontology scholars in the region will help to document the challenges faced by elders, to plan for the coming demographic shift, and to empower elders to thrive as valued community members.

### OLDER CANCER SURVIVORS LIVING WITH FINANCIAL HARDSHIP IN CHINA: THE INFLUENCE OF CONFUCIAN FAMILY VALUES

Mingzhu Su, Li Liu, Hanlin Yue, Jiajun Zhang, and Nengliang Yao, *Shandong University, Jinan, Shandong, China (People's Republic)*

**Background:** Financial hardship has not been well studied among older cancer survivors, despite its debilitating effects on their health and well-being. Aim: To describe the lived experience of older Chinese cancer survivors and explore the financial impacts following a cancer diagnosis.

**Design:** A qualitative study conducted using semi-structured interviews with patients and family caregivers.

**Methods:** We individually interviewed twenty-one cancer survivors (aged □ 60) with financial hardship and twenty family caregivers in Shandong province between August 2020 and January 2021. A content analysis was performed by multiple coders.

**Findings:** Confucianism culture and the Chinese health system considerably impact the construct of financial hardship and its components. Four main categories were revealed:(1) healthcare providers were reluctant to discuss the diagnosis and costs of care with cancer patients; (2) financial transfer from adult children to older parents became prevalent after a cancer diagnosis;(3) cancer-related financial worries and stress spilled out into children's family; (4) coping and adjustment strategies were taken by the extended family.

**Conclusion:** Both older cancer survivors and their adult children experienced financial distress mediating through filial piety in China. Instruments are needed to screen for cancer-related financial hardship adapted to the healthcare system and Confucian family values.

**Key words:** Cancer survivors; older; financial hardship; qualitative; China

### ORGANIZATIONAL PRACTICES FOR THE AGING WORKFORCE: VALIDATION OF AN ENGLISH VERSION OF THE LATER LIFE WORKPLACE INDEX

Julia Finsel,[1] Anne Wöhrmann,[2] Mo Wang,[3] Max Wilckens,[1] and Jürgen Deller,[4], *1. Leuphana University Lüneburg, Lüneburg, Niedersachsen, Germany, 2. Federal Institute for Occupational Safety and Health (BAuA), Dortmund, Nordrhein-Westfalen, Germany, 3. University of Florida, Gainesville, Florida, United States, 4. Leuphana University of Lüneburg, Leuphana University of Lüneburg, Niedersachsen, Germany*

Due to aging workforces, research on organizational practices for older employees becomes more important for individuals and organizations. However, existing measures for such organizational practices tend to capture the construct with unidimensional scales, use single-item operationalizations, or focus on a specific area. Hence, Wöhrmann, Deller, and Pundt (2018) developed the Later Life Workplace Index (LLWI) to provide a multidimensional framework to measure organizational practices for older employees on nine dimensions, namely organizational climate, leadership, work design, health management, individual development, knowledge management, transition to retirement, continued employment after retirement, and health and retirement coverage. The LLWI has recently been operationalized and validated in Germany (Wilckens, Wöhrmann, Deller, & Wang, 2020). However, to utilize the index beyond German-speaking countries, a validated English version is required. Thus, we aimed to validate an English version of the LLWI using a sample of older U.S. employees (N = 279). Results support the domain level factor structure of the LLWI but show some redundancy among the 80 items for the overall nine domain factor structure. A comparison between the U.S. sample and a German sample (N = 349) confirmed configural and (partial) metric measurement invariance of the English version. Results further supported convergent, discriminant, criterion, as well as incremental validity. Researchers can utilize the new measure to gain a deeper understanding of organizational practices relevant for older employees, while practitioners are able to assess their organizational readiness for an aging workforce. We envision further translation and validation in other languages and cultural contexts.

### PERCEPTIONS OF GENERATIONAL CONFLICT AMONG THREE AGE GROUPS IN SOUTH KOREA

Ahyoung Lee,[1] Soondool Chung,[2] and Juhyun Kim,[3], *1. EWHA Womans University, Seoul, Seoul-t'ukpyolsi, Republic of Korea, 2. Ewha Womans University, Ewha Womans University, Seoul-t'ukpyolsi, Republic of Korea, 3. Chungnam National University, Daejeon, Taejon-jikhalsi, Republic of Korea*

After rapid industrialization during the past few decades, the gap between generations in South Korea has widened and the issue of generational conflict is being discussed as a social problem (Chung & Lim, 2018). The purpose of this study is to find out how each generation perceives generational conflict in the areas of family, politics, economy and social welfare, and culture. An online survey of 1,000 adults aged 20 and over was conducted nationwide in South Korea in January, 2021 with three age groups: the youngest group aged 20-39, mid-age group of 40- 64 and the oldest group of 65 and over. The questionnaire was created using the items developed by a previous research that used a Delphi technique (Chung, 2020). Participants answered how serious they perceive generational conflict in the dyadic relationship on 5-point Likert scales. Descriptive statistics were calculated, and t-tests have been performed to see the generational differences. Results show that the youngest group and the oldest group perceive the highest level of generational conflict each other in the areas of culture and politics. In cultural aspects, 'use of slang among the same group', 'Ability to utilize digital devices' were the items that had the highest level of conflict. In the political realm, progressive vs. conservative ideology was the area of the highest conflict. In addition, t-test results showed that the oldest group perceived generational conflict even deeper than the youngest group in the 'economy and social welfare' and cultural areas. Implications of these findings are discussed.

### POLICY DESIGN AND CONSUMER DIRECTION: CROSS-COUNTRY COMPARISONS ON CONSUMER-DIRECTED CARE PROGRAMS

Jinbao ZHANG,[1] Julia Shu-Huah WANG,[2] and Yu-Chih Chen,[3], *1. University of Hong Kong, Hong Kong, Hong Kong, 2. The University of Hong Kong, Hong Kong, Not Applicable, Hong Kong, 3. University of Hong Kong, Pok Fu Lam, Not Applicable, Hong Kong*

**Objectives:** The consumer-directed care (CDC) program aims to maximize health outcomes by offering older adults more control, choice, and flexibility over the care services they received. However, countries may operate CDC programs in different ways based on heterogenous sociostructural systems. We proposed a comparative framework to evaluate three dimensions of CDC—control and direct services, variety of service options, and information and support—and analyzed how countries varied in their policy design to achieve consumer direction.

**Methods:** Using cross-national document analysis, we analyzed eleven CDC programs from seven selected countries (Netherlands, United States (US), United Kingdom (UK), Germany, China, Australia, and Spain) with five CDC care regimes. A total of fourteen indicators capturing three dimensions of CDC programs was developed. We further used these indicators to evaluate and compare similarities and differences of policy features across countries using descriptive statistics and graphical approaches.

**Results:** CDC programs in the Netherlands, Arkansas, and the UK ranked at the top in consumer direction. All countries except Germany employed a "service-based" principle in determination of service type. Training care workers was in the most widespread use to assure quality of care. Merely the UK and Germany integrated CDC and conventional agency care without restrictions. Representative with relevant support was only available in the UK and Netherlands.

**Discussion and Implication:** CDC models involve multi-faced aspects, rather than dichotomies and discrete entities. Implications include the need for a systematic reflection with our developed framework and enriching variety of service options to promote consumer direction.

### UNDERSTANDING GLOBAL DEPRESCRIBING POLICY: OPPORTUNITIES AND CHALLENGES

Catherine Kim,[1] Allison Thompson,[2] Grace Kim,[2] Zachary Marcum,[3] Michelle Keller,[4] Andrea Sorensen,[5] and Nicole Brandt,[2], *1. University of Maryland School of Pharmacy, BALTIMORE, Maryland, United States, 2. University of Maryland School of Pharmacy, University of Maryland School of Pharmacy, Maryland, United States, 3. University of Washington, Seattle, Washington, United States, 4. Cedars Sinai, Cedars Sinai, California, United States, 5. UCLA, UCLA, California, United States*

The landscape of deprescribing, the planned process of dose reduction or stoppage for medications which are no longer of benefit, has been rapidly expanding with global efforts and the formation of regional and national deprescribing networks. The purpose of this qualitative study is to describe successes and challenges about deprescribing from thought-leaders across the world to inform future policy initiatives. We aim to conduct at least 15 key informant (KI) interviews; we have completed 13 to date. Codes were constructed to identify themes that depict the perspectives regarding deprescribing policy across the globe. The KIs primarily represent the fields of pharmacy and medicine from four global regions with years of deprescribing experience ranging from 5 to > 20. We identified two emerging overarching themes through our qualitative analysis: Regional Organization Support and Evidence & Knowledge gaps. Within these overarching themes, we further identified sub-themes and their representative quotes: Network Structure: "idea of the network was threefold: 1) To try and figure out what we need to activate healthcare providers to deprescribe; 2) To do work with community-dwelling seniors to motivate them and give them opportunities to deprescribe; 3) ...getting pharmacists to provide the education to the patients." Cost-effectiveness: "If we can show that it is cost-effective to deprescribe, that there is actually a return here, not just in health terms but in monetary terms, I think that would really push it along." This research will help to advance global efforts to optimize medication management.

### Session 9315 (Poster)

### GRANDPARENTS RAISING GRANDCHILDREN

### ACCEPTANCE COMMITMENT THERAPY INTERVENTION FOR CUSTODIAL GRANDFAMILIES: A VIRTUAL IMPLEMENTATION

Ella Faulhaber,[1] Meghan Custis,[2] Emily Heupel,[1] and Jeongeun Lee,[1], *1. Iowa State University, Ames, Iowa, United States, 2. Iowa State University, MUSCATINE, Iowa, United States*

Custodial grandfamilies often face challenges, such as psychological distress, parenting burden, and grandchildren showing internalizing and externalizing behaviors. Given their circumstances, effective training and education is critical to provide a supportive family environment for both custodial grandparents and custodial grandchildren. The aim of this study was to develop, pilot and evaluate a psychosocial intervention for custodial grandfamilies. The program, interACT, was implemented virtually due to restrictions related to COVID-19. It is an intervention for custodial grandparents (CGPs) and custodial grandchildren (CGC) to improve psychological wellbeing and life skills. Participant eligibility was determined by grandfamilies having Iowa residency and legal guardianship or custody of an 8-12 year old grandchild. The program utilizes the Acceptance and Commitment Therapy (ACT) framework, focusing on psychological flexibility, acceptance, and psychosocial resilience for CGPs and CGC. The program used techniques such as mindfulness, self-compassion, and decision-making skills. During the pilot program stage, participants were divided into CGPs and CGC groups, and completed self-paced weekly 45 minute long modules through a program website. To enhance online session experience, Extension staff facilitated hour-long Zoom calls to discuss module contents and foster peer connection for both groups. Findings will be available at the conclusion of the pilot program. We expect to find increased psychological well-being and improved life skills for both groups right after the implementation of the ACT. Findings and limitations will be discussed with practical implication for program implementation via virtual delivery for the current custodial families. Future studies could extend current curriculum to other populations.

### GRANDPARENTS RAISING GRANDCHILDREN: APPLYING BIOECOLOGICAL THEORY TO SCHOOL RELATIONSHIPS AND GRANDPARENT NEEDS

Katharine Black,[1] and Nancy Mendoza,[2], *1. The Ohio State University, Ottawa, Ohio, United States, 2. The Ohio State University, The Ohio State University, Ohio, United States*

This paper examines the development, sustainability and importance of positive working relationships between grandparents raising grandchildren ("grandparents") and school district staff through the application of Bronfenbrenner's bioecological theory of human development. Grandparents have unique needs and often lack adequate resources, knowledge, and support when engaging in their new and often sudden role as primary caregiver of a grandchild. Access to educational resources, adequate information, and school district support is critical as a grandchild transitions into their grandparent's home. This inquiry aims to develop a conceptual framework for understanding how forming and maintaining positive working relationships between grandparents and school district staff will systematically and adequately address the educational needs of grandparents and their grandchild's academic success. Tenets of the bioecological model include the application of proximal processes that outline the need for frequent and regular interactions between a person and their environment over extended periods of time. The purpose of this study is to develop positive pathways of support through the application of the interconnected elements of proximal processes of the bioecological theory including process, person, context, and time, and the five bioecological interactive (micro-, meso-, exo-, macro-, chrono-) systems of human development. More specifically, educational needs of grandparents as caregivers are addressed through tenets of the bioecological theory to inform school districts and encourage the development of positive working relationships and effective education navigation protocols to better serve this unique and growing population.

### IS AGE JUST A NUMBER? INFLUENCE OF CAREGIVER AGE ON INJURIES OF CHILDREN RAISED BY GRANDPARENTS

Acacia Lopez,[1] Rachel Scott,[2] and Danielle Nadorff,[3], *1. Mississippi State University, Starkvile, Mississippi, United States, 2. Mississippi State University, Starkville, Mississippi, United States, 3. Mississippi State University, Mississippi State, Mississippi, United States*

Unintentional injuries are the most common cause of death for children in the United States. One factor linked to their occurrence is parenting style (i.e., a collection of factors such as physical health, mental health, and possible cohort differences in parenting role expectations). Differences in parenting behaviors may be evident in grandparents caring for their grandchildren, due to cohort differences and age-related declines in cognitive and physiological processes. This may impact their abilities to monitor, supervise, and respond to children. Further, Hayslip & Kaminski report custodial grandparents are less likely than parents to understand and respond to the psychological and emotional needs of children but are more likely to enforce discipline. This study sought to explore the ways in which parenting styles are associated with unintentional injury behaviors in children (via caregiver age) for grandchildren raised by grandparents. Participants were grandparents raising their grandchildren, recruited via Qualtrics Panel Service (N = 323). Conditional process analyses were conducted using Model 1 of SPSS PROCESSv3.5. Age moderated the relation between consistency of discipline and child unintentional injury (F (1, 231) = 12.67, p <.001) as well as level of supervision and child unintentional injury (F (1,146) = 6.23, p = .01). Age did not moderate the relation between positive parenting and unintentional injuries. These results imply that children being raised by older grandparents were especially at risk for increased injuries when their grandparents used less consistent discipline or lower rates of supervision. Pathways are suggested for age-specific psychoeducation interventions for custodial grandparents.

### PREDICTORS OF CUSTODIAL GRANDPARENTS' PERCEIVED BARRIERS TO THE USE OF SERVICES

Julian Montoro-Rodriguez,[1] Bert Hayslip Jr,[2] and Jennifer Ramsey,[3], *1. Charlotte, North Carolina, United States, 2. University of North Texas, Denton, Texas, United States, 3. Case Western Reserve University, Cleveland, Ohio, United States*

Getting timely access to help, information, and a variety of services is paramount among the challenges of raising a grandchild, and grandparents face a variety of internal and external barriers in getting such help. The present pilot exploratory study focused on caregiving-related and personal resource variables best predicting grandparent caregivers' perceptions of barriers to receiving services. Fifty-two grandparents (M age = 59.1) raising their grandchildren completed measures assessing caregiver strain, social support, resilience, self-care, psychosocial adequacy, health, depression, and grandchild relationship quality. They also completed measures of the extent to which they faced personal and caregiving-related difficulties giving rise to the need for services (e.g. health, grandchild well-being, support from others) as well as the extent to which they had experienced barriers to service (health/financial limitations, isolation, transportation, respite care, lack of knowledge of services) in the past 3 months. Correlations (p < .05) suggested that psychosocial adequacy (r = -.32), depression (r = .27), caregiver strain (r = .42) and difficulties (r = .48) were all related to greater perceived barriers. Regression analyses (F7, 40 = 2.81, p < .02) indicated that caregiver strain (Beta = .33, p < .05) and difficulties giving rise to the need for services (Beta = .32, p < .04) emerged as most salient in predicting barriers. These findings underscore the fact that personal, caregiving-related, and interpersonal factors exacerbate the barriers associated with grandparents' accessing needed services and reinforce such factors' impact on grandparents as targets for overcoming impediments to accessing services among them.

### SERVING KINSHIP FAMILIES DURING COVID: PIVOTAL MOMENTS

Andrea Smith,[1] MIchelle Mongeluzzo,[2] and Tawyna Drente,[2], *1. Western Michigan University, Ada, Michigan, United States, 2. Kids Central Inc, Wildwood, Florida, United States*

Kinship caregivers, who are relatives or non-family members providing care to children when biological parents are unable to do so, comprise over 2.5 million adults in the United States. The vast majority are grandparent caregivers. The 7.8 million children in their care make up approximately 10.5 percent of all children in the United States under the age of 18 (Generations United, 2017: State of Grandfamilies). Navigating daily life is often challenging. Kinship caregivers routinely face difficulties in multiple aspects of their lives, including finances, physical health, mental health, education, employment, parenting, and family relationships. The COVID pandemic heightened existing challenges and stimulated new issues for many kinship providers and the children in their care. This poster will highlight actions taken by one Family Service agency, annually serving approximately 225 kinship families, to meet the unprecedented needs of family members and kinship program staff during COVID. A timeline of decision-related rationales, specific actions taken and results related to these actions will be presented. Data summarizing results for kinship families (n =32) related to COVID-impacted programmatic responses and changes, including level of involvement with group services, recidivism, perceived isolation, and efficacy related to their caregiving roles will be presented. Results summarizing the impact of the agency's COVID-related responses on kinship staff (n = 6) will also be presented, including data on staff members' level of stress, perceived support, perceptions of programmatic effectiveness, and prioritized importance of changes will also be shared.

### VIRTUAL EMPOWERMENT TRAINING: REACHING GRANDPARENT CAREGIVERS DURING COVID19

Carole Cox, *Fordham University, Fordham University, New York, United States*

COVID-19 has had a devastating impact on minority populations in the United States who have disproportionately been at risk of getting the virus, having severe illness, and dying from it, with these risks most pronounced for older adults. The impact has been particularly severe on the more than on the more than 2, 7 million grandparents raising their grandchildren in the United States. Covid-19 has added profound strains to these families as they struggle with resources and isolation, frequently without assistance as well as the challenge of helping children to navigate online learning. The Virtual Empowerment Training Project, developed as a 7 session pilot program for low income grandparent caregivers in New York includes classes that strengthen parenting, communication, coping with loss and grief, and community empowerment. Participants were given ipads and computer training prior to classes, all conducted through Zoom. Initial data from the 56 participants (M age = 62, Race/ethnicity=Black, 79%, Hispanic, 16%, Income annual 41%<$15,K ) indicate improvement, i.e. a lessening of Negative Affect regarding the grandchild (p < .01) in the quality of their relationships with their grandchildren while also experiencing a decline (p < .01) in their sense of parental efficacy, possibly reflecting their becoming more critical of their own parental skills. Participant evaluations of the course were overwhelmingly positive, with more than 90% finding it extremely helpful, valuable, and eager to share it with others and to become more involved in improving the lives of grandfamilies.

### Session 9320 (Poster)

### HEALTH CARE AND SERVICES

### A CROSS SECTIONAL SURVEY OF FACTORS INFLUENCING HEALTHCARE ACCESS IN OLDER WOMEN OF SOUTH INDIA

Sreejith Sudhakar,[1] Justin Jose,[2] and Shanuga Cherayi,[3], *1. Manipal Academy of Higher Education, Manipal, Karnataka, India, 2. Dream A Dream, Cherupuzha, Kerala, India, 3. Dharmagiri jeevas Social Center, Cherupuzha, Kerala, India*

We examined the determinants of healthcare access barriers, treatment-seeking, and self-medication in older women aged 60 years and more, using a cross-sectional survey design. Using a structured interview format, we interviewed 1005 older women from 7 out of 14 districts in the state through a stratified random sampling procedure. Multiple linear regression analysis results reveal that older women's healthcare access barriers significantly increased when they experienced a long duration of multimorbidity alongside poor recognition of autonomy and basic amenities available at health facilities. However, confidentiality, the ability to pay for healthcare expenditure, and the type of health care significantly improved healthcare access. In factors influencing older women's delay in treatment-seeking, optimal instrumental functionality in daily living, optimal quality of life and access to healthcare services significantly reduced delay in treatment initiation. Whereas poor health-seeking behaviors, long duration of multimorbidity, and the quality of basic amenities at hospitals significantly increased treatment initiation delay and explained 13.6% of the variance. In factors influencing older women's use of self-medication, advancing age, living in rural areas, optimal functionality, perception of providers' respect for confidentiality were associated with increased self-medication frequency. Whereas, better wealth status, prompt attention to older women's health needs, and basic amenities at hospitals significantly reduced their self-medication practice. Therefore, the optimal functional abilities, fewer morbidities, and optimal health system responsiveness significantly reduce healthcare access barriers and self-medication while improving older women's treatment-seeking behaviors.

### A WINDOW TO TELEHEALTH GERIATRIC SOCIAL WORK JOB MARKET LANDSCAPE: A CROSS-SECTIONAL STUDY

Lihua Huang, *Grand Valley State University, Grand Rapids, Michigan, United States*

The purpose of this research study is to explore current Telehealth geriatric social work by describing its most recent job market characteristics. As part of a larger longitudinal research, data for this cross- sectional study was collected in January 2021. Three top job search engines, Google, ZipRecruiter, and Indeed, were used to collect data on Telehealth social work job openings. On each search engine, five searches were completed with the five key words: “social work” Telehealth jobs, LCSW Telehealth jobs, remote LCSW jobs, Telehealth “social work” jobs, and Telehealth "social worker” jobs. It analyzed 112 Telehealth geriatric social work job ads, 12.8% of total 873 ads from these fifteen searches. Results from descriptive and thematic data analysis show large, for-profit organizations are dominating the Telehealth geriatric social work field while small private practices are emerging during the pandemic. The study found Telehealth geriatric social work is providing vital continuum services to older people in communities, hospitals, and long-term care facilities at individual, family, and group levels. The results document innovative technological tools present new methods to engage, assess, and intervene, particularly with mental health needs. The Telehealth organizations are making the pitch to attract competitive professionals. While nearly 1/3 of the sampled organizations stated they intended to make Telehealth/remote positions temporary, the study concludes Telehealth has built its infrastructure and workforce to become an indispensable and ongoing part of gerontological and geriatric social work. Social work education, research, and practice must pay close attention to its implications in skill building.

### CONTINUE OR NOT TO CONTINUE? ATTITUDES TOWARDS DEPRESCRIPTION AMONG COMMUNITY-DWELLING OLDER ADULTS IN CHINA

Jie Tan,[1] Chenkai Wu,[1] Li Zhang,[2] Ying Wang,[1] and Lihui Chen,[1], *1. Duke Kunshan University, Kunshan, Jiangsu, China (People's Republic), 2. Suzhou Municipal Hospital, Kunshan, Jiangsu, China (People's Republic)*

Inappropriate prescribing of medications and polypharmacy among older adults could lead to avoidable harms. It is hence vital to stop potentially inappropriate medications in this vulnerable group. An approach coined ‘deprescribing’ has been used to describe a patient-centerd process of optimizing medication regimens. But patient resistance to discontinuing medication use is a significant barrier to deprescribing. The present study aims to describe attitudes towards deprescribing and to examine individual-based characteristics that might be associated with these attitudes among community-dwelling older adults in China. We conducted a cross-sectional study through in-person interviews using the validated Patients’ Attitudes Towards Deprescribing questionnaire in two communities through the community-based physical examination platform. Participants were 65 years and older and had at least one chronic disease and one regular prescription medication. Of the 1,897 participants in the study, the average age was 74 years and 1,023 (53.9%) were women. The majority had one chronic disease (n=1,364 [71.9%]) and took 1-2 medications (n=1,483 [78.2%]). A total of 947 (50.0%) older adults reported being willing to stop taking one or more of their medicines if their physician said it was possible, and 1204 (63.5%) older adults wanted to stop a medicine been taking for a long time. Chronological age, marital status, number of chronic diseases, and self-rated health status were associated with the attitudes towards deprescribing. This study showed that half of the participants were willing to cease a medication that their physician though was no longer required. Individual-level factors were associated with attitudes towards deprescribing.

### OLDER ADULTS' PERCEPTIONS AND USE OF PATIENT PORTALS: A COMPARATIVE ANALYSIS OF TWO SAMPLES

Hyojin Son,[1] Eun-Shim Nahm,[2] Shijun Zhu,[2] Elizabeth Galik,[2] Barbara Van de Castle,[2] Kristin Seidl,[2] and Vince Russomanno,[3], *1. National Institutes of Health Clinical Center, Bethesda, Maryland, United States, 2. University of Maryland, Baltimore, Maryland, United States, 3. University of Maryland Medical System, Linthicum Heights, Maryland, United States*

Older adults can benefit from using patient portals. Little is known whether the perceptions and use of patient portals differ among diverse older adult populations. The aim of this study was to assess the difference in perceived usability of patient portals, self-efficacy for using patient portals, and patient portal use between two adult samples aged 65 years or older. One sample was recruited from a health care system, including hospitals and clinics (n = 174), and the other sample was recruited from nationwide communities (n = 126). Conducting a secondary data analysis using two survey datasets, this study performed a series of linear and ordinal logistic regression analyses. The health care system sample had a higher mean number of chronic diseases and proportion of recent hospitalization than the community sample. The health care system sample showed higher perceived usability, self-efficacy, and usage frequency of patient portals compared to the community sample. eHealth literacy was a significant predictor of perceived usability and self-efficacy. Perceived usability was another significant predictor of self-efficacy. Self-efficacy and health condition variables significantly predicted the more frequent use of patient portals. Compared to the health care system sample, the relationship between perceived usability and use of patient portals was stronger and significant in the community sample. These findings suggest that approaches for promoting patient portal use should consider personal characteristics and health conditions of diverse older adult populations. Future research needs to focus on assessing the impact of using patient portals on older adults' health care outcomes.

### PATTERNS OF HEALTHCARE COSTS AMONG OLDER ADULTS: DEMOGRAPHICS, HEALTH PERSONALITY, AND RESILIENCE

Rotem Arieli, and Peter Martin, *Iowa State University, Ames, Iowa, United States*

The purpose of this study was to examine healthcare costs of older adults in relation to demographic characteristics, individual health personality traits, and resilience. Data included 3,907 participants, 65 and older, collected by a large provider of Medicare Supplemental Health Insurance. The Health Personality Assessment*, Brief Resilience Scale, total healthcare cost, and demographic information were used. In our sample, the average healthcare cost was $13,283.69 (SD=30,784.87), ranging from $0–$989,084, and higher healthcare costs were found among older, male, and less health-neurotic (i.e., lower health-related anxiety) adults. Configural frequency analyses were conducted to identify "types" and patterns of healthcare costs by age and gender. The following significant patterns emerged: Women in the oldest group with high healthcare costs and women in the young-old age group who had low healthcare costs occurred significantly more than expected by chance, $p<.01$. Next, we hypothesized configuration patterns for resilience, health personality, and healthcare costs. Results confirmed the following "types" or patterns occurring more often than expected by chance: less-resilient individuals with high health neuroticism and high healthcare costs, $p<.001$, and less-resilient, less-health-conscientious adults with high healthcare costs, $p<.001$. The results suggest higher healthcare costs for individuals who are less resilient, more neurotic about their health, and less disciplined in their health practices. Future intervention programs may benefit from promoting resilience, reducing health neuroticism, and increasing health conscientiousness. *The Health Personality Assessment (HPA) is © 2021 United HealthCare Services, Inc. All rights reserved.

### PSYCHOLOGICAL INFLEXIBILITY AND GERIATRIC PRIMARY CARE: TRANSFORMING PERI-URBAN AND RURAL AGING

Lindsey Jacobs,[1] Rebecca Allen,[2] Timothy Ly,[3] John Bell,[4] Dana Carroll,[5] and Anne Halli-Tierney,[4], *1. The University of Alabama, The University of Alabama, Alabama, United States, 2. University of Alabama, Tuscaloosa, Alabama, United States, 3. University of Alabama, University of Alabama, Alabama, United States, 4. The University of Alabama, Tuscaloosa, Alabama, United States, 5. Auburn University Harrison School of Pharmacy, Tuscaloosa, Alabama, United States*

Based on the acceptance and commitment therapy (ACT) framework, human suffering is thought to be caused by psychological inflexibility. Psychological inflexibility is characterized by rigid avoidance of unpleasant experiences, fusion with unhelpful thoughts, lack of contact with the present moment, fusion with a narrow self-narrative, and lack of clarity and contact with one's core values in life. Psychological inflexibility captures the unhelpful or unworkable ways in which individuals respond to emotional discomfort. Research using samples of adults under age 65 indicate that psychological inflexibility is associated with poorer quality of life and mental well-being; however, the literature on psychological inflexibility in older adults is limited. Patients (N=129) ages 65 and older presenting to a Geriatric Primary Care clinic in the Deep South completed measures of depression, anxiety, subjective health literacy, and psychological inflexibility. Our team used the Acceptance and Action Questionnaire-II (AAQ-II), which is the most commonly used measure of psychological inflexibility. Anxiety (r = 0.66, p < .001) and depression (r = 0.70, p < .001) were moderately correlated with psychological inflexibility, which is consistent with the existing literature on psychological inflexibility in adults under the age of 65. Subjective health literacy significantly predicted psychological inflexibility, b = –.058, t(127) = -4.07, p < .001. This finding provides additional support for the importance of increasing health literacy among older adults in the Deep South, as it has implications in level of psychological flexibility and, thus, quality of life and mental well-being.

### SELF-REPORTED UTILIZATION OF NUTRITION-RELATED RESOURCES IN VETERANS COMPARED TO NON-VETERANS

Monica Serra,[1] Ronna Robbins,[2] and Odessa Addison,[3], *1. UT Health San Antonio - Barshop Institute, San Antonio, Texas, United States, 2. Audie Murphy VA Medical Center, San Antonio, Texas, United States, 3. University of Maryland School of Medicine, Baltimore, Maryland, United States*

As they age, Veterans are at elevated risk for developing nutrition-associated chronic diseases compared to their Non-Veteran counterparts. This is despite Veterans often being eligible for a variety of nutrition-related resources. This project compared self-reported utilization of community and government nutrition-related resources in male Veterans compared to Non-Veterans participating in the 2013-2014 and 2015-2016 National Health and Nutrition Examination Surveys. Veterans (mean: age: 59 years; BMI: 29 kg/m2; N=135) self-reported “yes” and Non-Veterans (age: 61 years; BMI: 30 kg/m2; N=230) self-reported “no to “ever having served on active duty in the U.S. Armed Forces.” A similar percentage of Veterans (3%) and Non-Veterans (4%) reported utilizing meal delivery programs (i.e., “Meals on Wheels) and eating at community or senior centers (both 7%) in the past year. Veterans were less likely than Non-Veterans to report receiving emergency food (i.e. from church or food bank) in the past year (7% vs. 12%; P<0.01) and tended to be less likely to report ever having received benefits from a nutrition assistance program (NAP; i.e., Food Stamps) (27% vs. 32%; P=0.08). Veteran also reported their household receiving more financial assistance the last time they received support from a NAP ($281 vs. $188; P=0.02). These data suggest that Veterans have less reliance on NAP than Non-Veterans; however, when they do receive assistance, Veterans appear to receive greater financial support. Future studies are needed to identifying ways to improve access to nutrition-related resources in those at risk for food insecurity.

### THE IMPACT OF A WHOLE HEALTH MODEL OF CARE ON PATIENT OUTCOMES

Anna Faul,[1] Pamela Yankeelov,[2] Joseph D'Ambrosio,[2] Sam Cotton,[3] Barbara Gordon,[1] and Maria Hall-Faul,[4], *1. University of Louisville, Louisville, Kentucky, United States, 2. University of Louisville, University of Louisville, Kentucky, United States, 3. University of Louisville Trager Institute, University of Louisville Trager Institute, Louisville KY, Kentucky, United States, 4. University of Connecticut, University of Connecticut, Connecticut, United States*

The FlourishCare Model (FCM) transforms primary care sites by addressing all determinants of health and focusing on helping patients to flourish. The FlourishCare Index (FCI) is a clinical measure to assess the effectiveness of the FCM to address all determinants of health. We will present data on the effectiveness of the FCM serving 159 older adults with MCCs. The sample was mostly female (77%), White (64%), retired (54%), married (30%) or widowed (20%) and living in urban areas (64%). The mean age was 69 (SD=15), with 13 years education (SD=3). Patients changed significantly over time on total FCI scores (57%-72%;□=3.80,SE=0.63). Results show significant growth over time for individual health behaviors (58%-67%;□=2.14,SE=0.84), health care access (71%-89%;□=4.43,SE=1.00) and social determinants (62%-85%;□=5.54,SE=1.02) with psychological determinants (54%-61%;□=1.74,SE=0.95) and environmental determinants (70%-81%;□=2.81,SE=1.62) showing a trend to significance. Interaction effects with time show that the FCM supported patients with lower education attainment to improve at a higher rate than those with higher education attainment, for the total FCI score (□=-0.59,SE=0.24) and health care access (□=-0.94,SE=0.38). Receiving mental health counseling resulted in more improvement in psychological determinants than those who did not receive counseling (□=3.43,SE=2.04). The FCM was able to support rural patients at a higher rate than urban patients to gain access to health care (□=4.13,SE=2.02). The FCM supported Hispanic patients the most in improving social determinants of health (□=8.40,SE=3.93). This study showed the importance of a systems approach to care using measures that focus on what matters most to older adults who value quality-of-life outcomes.

### Session 9325 (Poster)

### HEALTH CARE UTILIZATION

### EXPLORING DEMENTIA CARE IN AN ACUTE CARE SETTING: PERSPECTIVES OF SOCIAL WORKERS AND NURSES.

Ruth Dunkle,[1] Katherine Cavagnini,[1] Joonyoung Cho,[1] Laura Sutherland,[2] Helen Kales,[3] Cathleen Connell,[4] and Amanda Leggett,[5], *1. University of Michigan, Ann Arbor, Michigan, United States, 2. Wayne State University, Ann Arbor, Michigan, United States, 3. University of California, Davis, University of California, Davis, Sacramento, California, United States, 4. University of Michigan School of Public Health, Ann Arbor, Michigan, United States, 5. The University of Michigan, Ypsilanti, Michigan, United States*

Nurses and social workers in acute care settings have unique perspectives about providing care to persons living with dementia (PLwD) who experience behavioral and psychological symptoms of dementia (BPSD). Their distinctive roles and training have important implications for the recovery and well-being of PLwDs during hospital stays. This study utilized the "rigorous and accelerated data reduction" (RADaR) technique to compare perspectives of social workers (n=12) and nurses (n=5) in a Midwestern tertiary care facility about their caring for PLwds with BPSD. Three major themes were identified: 1) patient engagement and coordination with family and professionals, 2) treatment and medical management, and 3) barriers to care. Similarities between social workers and nurses emerged within the themes, including the importance of family involvement and providing person centered care. Differences emerged particularly within the treatment and medical management theme, as nurses utilize medications to treat BPSD and social workers were more likely to use redirection. While there is distinctive training for nurses and social workers, both identified similar barriers to providing care to PLwDs with BPSD, including time constraints, competing demands, and lack of training on BPSD management. Results demonstrate how an understanding of the critical and complementary roles that nurses and social workers play in dementia care and work together to build a care team can inform best practices to support symptom management and quality of life in PLwDs. Continuing education and training could be beneficial for both professionals to improve the quality of care for PlwDs.

### INSUFFICIENT SLEEP AND HEALTH CARE UTILIZATION: A SCOPING REVIEW

Kolade Olatunde,[1] and Susan Patton,[2], *1. University of Arkansas, University of Arkansas, Arkansas, United States, 2. University of Arkansas, Fayetteville, Arkansas, United States*

Insufficient sleep is a common problem among older adults with 26% over the age of 65 reporting less than seven hours sleep in a 24 hour time period. Evidence indicates that untreated sleep disorders are associated with osteoarthritis, heart disease, hypertension, diabetes, obesity, falls, decreased cognitive performance, and decreased health related quality of life in older adults. A scoping review was undertaken to determine what is known about the association between insufficient sleep or insomnia and health care utilization. The Joanna Briggs Institute, Methodology for JBI Scoping Reviews was used to guide the review. Searches were conducted in PubMed, HINARI, Google Scholar and Cochrane databases. Twenty nine studies were included. Overall, the review indicates that reduced sleep is associated with a greater odds of difficulties in daily activities, higher rates of health care utilization and costs, and poly pharmacy. Findings also reveal sociodemographic and geographic variations in prevalence of healthy sleep duration. Although the majority of studies focused on the causes and consequences of insomnia and recommended clinical and behavioral health promotion interventions, there is a gap in studies related to the public health or economic impact of insufficient sleep. Research in this area will provide perspectives on the need to raise awareness of the importance of sleep and to incorporate the awareness into policies that improve sleep health.

### OLDER NONMEDICAL AND MEDICAL CANNABIS USERS: HEALTH CHARACTERISTICS, CANNABIS USE PATTERNS, AND CANNABIS SOURCES

Namkee Choi,[1] and Diana DiNitto,[2], *1. University of Texas, Austin, Texas, United States, 2. University of Texas at Austin, Austin, Texas, United States*

Despite rapidly growing number of older medical cannabis users, research on them is scant. In this study, we examined medical and nonmedical cannabis users aged 50+ on health-related characteristics and cannabis use patterns and sources. Hypotheses were that compared to nonmedical users, medical users are more likely to have physical and mental health problems, use healthcare services, discuss their drug use with a healthcare professional, use cannabis more frequently, and purchase cannabis from a medical dispensary and other sources rather than obtain it as a gift, share someone else's, or use other means. We used the 2018 and 2019 National Survey on Drug Use and Health data (N=17,685 aged 50+; male=8,030; female=9,655) and multivariable logistic regression analysis to test hypotheses. Of the sample, 8.9% reported past-year cannabis use. Of past-year users, 18.5% reported any medical use. Of medical users, 70.9% reported exclusive medical use and 29.1% reported using medically and nonmedically. A large proportion obtained cannabis from private/informal sources. Any medical use, compared to nonmedical use, was associated with lower odds of alcohol use disorder but higher odds of discussing drug use with a healthcare professional (AOR=4.18, 95% CI=2.53-6.89), more days of use (AOR=2.56, 95% CI=1.35-4.86 for 200-365 days), and purchase at a medical cannabis dispensary (AOR=4.38, 95% CI=2.47-7.76). Medical and nonmedical users did not differ on physical health, and both had high behavioral health problem rates. However, only a small portion discussed their drug use with a healthcare professional. More healthcare professional attention to older cannabis users is needed.

### SOCIAL WORKERS IN PRIMARY CARE INCREASE ACCESS TO PALLIATIVE CARE

Portia Cornell,[1] Christopher Halladay,[1] Pedro Gozalo,[2] Caitlin Celardo,[3] James Rudolph,[1] and Anna-Rae Montano,[1], *1. Providence VA Medical Center, Providence, Rhode Island, United States, 2. Brown University, Providence, Rhode Island, United States, 3. Northport VA Medical Center, Northport VA Medical Center, New York, United States*

Clinical trials show that palliative care improves patient experiences and reduces costs, and use of palliative care and hospice care have been increasing over the past three decades. In the Veterans Administration health care system (VA), Veterans may receive palliative care concurrently with other treatments. However, many barriers exist to the use of palliative care, such as patients' misperceptions. Social workers in primary care teams may increase use of this valuable service by establishing trust between patient and care team, educating patients and caregivers, and coordinating services. Leveraging a national social-work-staffing program as a natural experiment, we evaluated the effect of hiring one or more social workers to the primary-care team on use of palliative or hospice care among Veterans with a recent hospital stay. Our data included 91,675 episodes of care between 2016 and 2018. 1.45 percent of episodes were followed by use of palliative care or hospice within 30 days. The addition of one or more social workers through the staffing program was associated with an increase of 0.53 percentage points ($p<0.001$) in the probability of any palliative or hospice care, i.e., a more than 30% increase relative to the mean. Policy makers and health system leaders who seek to improve patient experience and reduce costs through increased access to palliative and hospice care could consider social work staffing as a policy tool to achieve those aims.

### TALKING ABOUT COMPLIMENTARY AND ALTERNATIVE MEDICINE: A CONVERSATION ANALYSIS STUDY

Sean Halpin,[1] Kathleen Len,[2] and Michael Konomos,[3], *1. University of Georgia, Decatur, Georgia, United States, 2. University of California Los Angeles, University of California, Los Angeles, California, United States, 3. Emory University, Atlanta, Georgia, United States*

Multiple Myeloma (MM) is a clonal plasma cell malignancy characterized by low blood counts and increased risk of infection, and primarily afflicts older adults. Although MM is incurable, advances in treatment, including autologous stem cell transplant (ASCT) has improved the lifespan of patients. MM patients commonly use over-the-counter complementary and alternative medicines (CAM) alongside conventional cancer therapies which, often without recognition by health care practitioners, may impact their treatment. Using data from an 18-month ethnographic study, we applied conversation analysis to examine 1180 minutes of audio-recordings to describe how patients and nurses interacted about CAM during ASCT education visits. Patients (n=12) had a median age of 62 years (IQR= 54-73), were mostly white (n=12, 75%), male (n=9, 56%), and had a moderate score on the FACT-G7 of 15 (IQR= 10-20). All patients had a caregiver present during their visit. Nurses (n=3) were aged 39 (IQR= 29-49) all with at least five years providing care to patients with blood cancers. Results suggested that nurses rarely provided direct feedback about CAM modalities, instead providing brief responses, and moving on to other topics. Excerpts were categorized into three groups, (1) demonstration of implicit epistemic authority, (2) demonstration of deferred epistemic authority in patient-initiated conversations, and (3) demonstration of deferred epistemic authority in nurse-initiated conversations. Understanding how conversations surrounding CAM are navigated can provide insights into patient-communication in general, and methods for improving ASCT education.

### THE EFFECT OF HOME- AND COMMUNITY-BASED SERVICES ON ACTIVITY ENGAGEMENT

Peter Sun, *Washington University in St. Louis, St. Louis, Missouri, United States*

This study examined the association between home- and community-based services (HCBS) and social, cognitive, and physical engagement among community-dwelling older adults in the U.S. Data were drawn from the 2012 Health and Retirement Study (HRS). The sample consisted of respondents ages 50 and over who answered questions on HCBS utilization and activity engagement (n = 567). Genetic matching and propensity score weighting were used to mimic randomized control and treatment groups, in order to estimate the population average treatment effect on the treated (PATT). HCBS utilization was found to be significantly associated with social engagement (PATT = 0.17, SE = 0.05, $p < 0.05$) and physical (PATT = -0.20, SE = 0.07, $p < 0.05$) engagement but not significantly associated with cognitive engagement (PATT = -0.04, SE = 0.12, $p > 0.05$). Sensitivity analyses found that the results were robust to the estimation model. These findings suggest that HCBS utilization is a promising model for increased activity engagement, and future policies aimed at targeting these outcomes are warranted.

### Session 9330 (Poster)

### HEALTH PROMOTION (AGHE POSTER)

### DEVELOPMENT OF DIET SCORE FOR REFLECTING DIET PATTERNS AND DIETARY GUIDELINES: A CASE OF SINGAPORE

Yuezhong Liu,[1] Rakhi Verma,[2] Ringo Ho,[3] and Yin-Leng Theng,[4], *1. Nanyang Technological University, Singapore, Not Applicable, Singapore, 2. Nanyang Technological University, Singapore, 3. Nanyang Technological University, Singapore, Singapore, Singapore, 4. Nanyang Technological University, Singapore, Singapore*

Foods and dietary patterns substantially affect health outcomes. The overall dietary assessment score associated with dietary guidelines in Singapore has not been assessed previously. This study aimed to develop and evaluate diet score for identifying the relationship between dietary patterns and dietary guidelines in Singapore. Using a localised diet score survey collaborated with the Commonwealth Scientific and Industrial Research Organisation (CSIRO), we conducted a cross-sectional study of 600 Singapore persons in two-generational cohorts (40-64: 300 and > 65 years: 300). The proposed local diet score was calculated to reflect their overall compliance with the Dietary Guidelines in Singapore. ANOVA analysis was used to identify the significant difference among socio-demographic variables associated with diet score and comparison analysis was performed to compare the diet patterns and diet score. There are significant differences among age, education, housing, residency associated with diet score. Diet score of older cohort (M= 67.71, SD= 13.38) is significantly higher than young cohort (M= 60.73, SD= 14.71). The highest education level (University or tertiary) obtain the lowest diet score (M= 58.58, SD= 14.41). The participants who live in the landed property (M= 69.45, SD= 14.43) are higher than those who live in Condominium and Public House. And the participants who live alone (M= 67.26, SD= 14.66) have a higher average diet score. Two-generational cohorts are not compliant with recommendations about dietary guideline well in Singapore. The present findings suggest that dietary patterns need improvement in aspects such as vegetables and extra food components.

### FOOD INSECURITY IN OLDER ADULTS: GERONTOLOGY STUDENTS AND RSVP A PARTNERSHIP THAT WORKS

Phyllis Greenberg,[1] and Jessica VanderWerf,[2], *1. Saint Cloud State University, St. Cloud, Minnesota, United States, 2. St. Cloud State University, Saint Cloud State University, Minnesota, United States*

A gerontology course related to policies /programs each year researches, develops and designs a service-learning project related to an issue/concern for older adults and their quality of life. Students wanted to work with vulnerable older adults and after research and discussion decided on tackling the issue of food insecurity in older adults. Food insecurity is a growing issue for older adults which has been exasperated by COVID-19. According to Meals on Wheels America (2020) there has been a 22% increase in the number of older adults needing food assistance. In addition, while the need for food banks has increased donations have declined (Next Avenue, 2020). Students partnered with RSVP, which had previously conducted a food donation project.. Students took on the responsibility for advertising, soliciting grocery stores to allow us to set up and engage shoppers in purchasing items for the project. In addition, they reached out to the university community and set up food donation stations. RSVP sent out emails to their constituents to encourage them to volunteer and do their shopping on the date of the project. Students were paired with RSVP volunteers at two stores and provided shopping lists and information about food insecurity in older adults to shoppers. Students collected 566 pounds of food. The food was distributed equally between Catholic Charities, which has a senior shopping program and the Somali Elder Community. Students sorted the food by categories and removed any foods with pork/gelatin products for the Somali Community.

### GERIATRIC ORAL HEALTH FOR DENTAL AND NON-DENTAL PRIMARY CARE PROVIDERS

Maryam Tabrizi, *The University of Texas School of Dentistry at Houston, Houston, Texas, United States*

Age-Friendly Health System is the innovation that provides care to older adults within 4Ms structure comprehensively. The 4Ms structure is the clinicians' guideline for providing treatments for each individual based on what matters most which is the first M. The 4Ms structure creates a person focus approach for the health of the elderly on every visit, as what matters most may change over time based on other health conditions of life situation. We must consider our patients come from all kinds of life walks, based on his/ her lifestyle, health beliefs, and cultural background. Consequently, older adults may define health and wellness differently and they might have different needs, oral health is no exception. However, evaluating both primary care office workflow and hospital workflow is missing the health element of oral care. The current health flow designed by the Institute of Health Improvement (IHI) in a collaborative effort with John A. Hartford may create a larger gap between oral health and overall health. In the light of integrating oral health to the overall health, the best place and most feasible in both primary care and hospital workflow is at the time of Check history for a baseline on 4Ms. This poster will clearly illustrate how oral health can integrate to overall health leading by the non-dental profession who usually take history for a baseline. As oral health is an integrated factor in the health of the geriatric population in the Age-Friendly Health System.

### GLOBAL UNBIASED DETERMINATION OF COMORBIDITY (GUDC) IN U.S. OLDER ADULTS

Beth Hogans,[1] Leslie Katzel,[1] Bernadette Siaton,[2] Raya Kheirbek,[3] and John Sorkin,[4], *1. VAMHCS, Baltimore, Maryland, United States, 2. University of Maryland School of Medicine, Baltimore, Maryland, United States, 3. University of Maryland School of Medicine, University of Maryland School of Medicine, Maryland, United States, 4. University of Maryland, Baltimore VA Medical Center, Maryland, United States*

With age, many adults develop multiple comorbid conditions; and resulting clinical complexity increases markedly so that identifying how specific conditions effect others remains important. Here, our primary objective was rapid unbiased appraisal of pair-wise condition-specific comorbidity; our second objective was identification of common conditions with highest and lowest rates of such comorbidity. In 2016, utilization of ICD-10 codes became mandatory for providers rendering care to Medicare beneficiaries. Universal adoption of ICD-10 coding ensued and concomitantly, all patients had ICD-9 codes replaced with new codes, so that 2017 data represent an opportunity to examine massive amounts of 'freshly' coded patient claims data. Evaluating ICD-10 coding data at individual and population levels, we appraised how often two codes were utilized together, i.e. estimated pair-specific comorbidity. Expanding this computationally, we determined the extent to which any given condition was co-coded with all other utilized diagnostic codes, i.e., estimated global, unbiased pair-wise comorbidity. We term this metric the global unbiased dyadic comorbidity (GUDC) value. Based on 40 million claims for a representative sample of 1.5 million older adults across the U.S., GUDC values varied with age and gender but were highly stable across varying comorbid condition prevalence, e.g., common (>1%) vs. less common (1/1000-1/100) prevalence. GUDC values for HIV in older adults were modest, compared to high values for ARDS, we infer substantive progress in HIV

management among older adults. We discuss the interpretation and potential applications of GUDC and conclude that access to comorbidity appraisals may advance geriatric care, more study is needed.

### PAEAN: PAIN IN AGING, EDUCATIONAL ASSESSMENT OF NEED - AN INTERPROFESSIONAL COLLABORATION

Beth Hogans,[1] Bernadette Siaton,[2] Lana Brown,[3] Laura Frey-Law,[4] Chris Herndon,[5] Luis Buenaver,[6] Les Katzel,[2] and Patricia Thomas,[7], *1. VAMHCS, Baltimore, Maryland, United States, 2. University of Maryland School of Medicine, Baltimore, Maryland, United States, 3. Central Arkansas Veterans Healthcare System, Little Rock, Arkansas, United States, 4. University of Iowa Health Care, Carver College of Medicine, Iowa City, Iowa, United States, 5. School of Pharmacy Southern Illinois University Edwardsville, Edwardsville, Illinois, United States, 6. Johns Hopkins University, Baltimore, Maryland, United States, 7. Case Western Reserve School of Medicine, Cleveland, Ohio, United States*

Pain is prevalent in older adults limiting independence directly and through comorbidity-related effects on functional domains such as mobility, well-being, sleep, productivity, and oligo-pharmacy. Improved outcomes for older adults with pain depends on provider knowledge and competence; concomitantly, Veterans, women, and others at socioeconomic disadvantage may face increased pain, comorbidities, and complications of treatments. Previous guidance for educational programs, from pre-licensure to post-graduate training, in geriatrics and pain have focused on expert opinion, whereas an evidence-based approach is preferred. Our working group is conducting a structured needs assessment regarding comorbidities of common pain-associated conditions in older adults.

**Methods:** To capture expertise in medicine, nursing, pharmacy, clinical psychology, and physical therapy, we extended an open invitation to members of the VA Geriatric Research, Education, Clinical Centers Associate Director-Education network and selected, nationally-recognized clinical education experts outside VA.

**Results:** An eight-member working group, interprofessional in composition, through multiple remote meetings has defined goals of the program, evaluated preliminary evidence addressing the clinical needs of older adults with pain, and posed 'curious questions' about the available large-scale data. The overarching goal is evidence-based needs assessment of gaps in education about pain in older adults, with purposeful attention to risks of healthcare inequities for older adult women, Veterans, persons of color, those at socioeconomic disadvantage, and caregivers.

**Conclusions:** Interprofessional collaboration is effective in framing a broad needs assessment regarding pain and common comorbidities in older adults with the intent of meeting the educational needs of clinical trainees. More study is needed.

### SAVING SENIOR SMILES: A COMMUNITY OUTREACH EDUCATIONAL PROGRAM AND PILOT RESEARCH PROJECT

Prajakta Joshi,[1] and Kadambari Rawal,[2], *1. Boston University Henry M. Goldman School of Dental Medicine, Boston, Massachusetts, United States, 2. BU Henry M Goldman School of Den, Boston university, Massachusetts, United States*

Globally, poor oral health has been evidenced more frequently among older adults. Thus, it is imperative to develop strategies for improving the oral health knowledge and access to dental care amongst the older adult population. The Saving Senior Smiles (S3) pilot outreach program was launched as an oral health education and awareness program for community-dwelling older adults across senior centers in the greater Boston area (Massachusetts, USA). The outreach consisted of oral health educational seminars presented by pre- doctoral dental students from three dental schools in the Boston area. The presentations highlighted the significance of oral health, and the importance of seeking routine dental care. Pre and post-test surveys were administered to assess the participants' utilization of oral health services and oral health knowledge. The surveys were completed by 85 older adults (Female= 58.8%) across five senior centers. Questions pertaining to utilization of dental services revealed that 78.8% of the participants had a dentist. Expectedly, the center that reported the greatest number of missing teeth (Fenway center= 70.6%) had the least number of individuals who had a dentist (58.3%). With regard to oral health knowledge, before the seminars, less than half of the participants (42.2% ) across all the senior centers were aware of the common oral conditions that affected older adults ( dry mouth, gum recession and changes in oral bacteria) and after the presentation over 60% of the participants responded correctly to these knowledge questions. Overall, these findings emphasize the value of simple community-based interventions for older adults.

### Session 9335 (Poster)

### INTERVENTION RESEARCH

### COMPUTER-MEDIATED FOCUS GROUPS TO ADVANCE AGING RESEARCH: CHALLENGES AND OPPORTUNITIES.

DAVID Coon, Abigail Gomez-Morales, phil Carll, Lourdes Cordova, allison Glinka, and Socorro Gonzalez-Piles, *Arizona State University, Phoenix, Arizona, United States*

When compared to in-person offerings, fewer focus groups to date have been conducted with user-friendly technologies to help reach diverse communities of older adults with chronic health conditions (e.g., Alzheimer's disease, Type II diabetes, Parkinson's) and the family caregivers and professional providers who assist them. The current project describes the adaptations needed to deliver successful computer-mediated focus groups via videoconference, thereby providing solutions to barriers faced by participants who often cannot attend in-person because they are house-bound due to transportation or financial barriers, live in rural areas or reside too far from focus group offerings, or work full or part-time and face scheduling conflicts. During the pandemic, we successfully recruited diverse groups of family caregivers, care recipients, and professional providers into computer-mediated focus groups. Caregivers (83%) and care recipients (17%) between 34 to 90 years old (N=47) took part in the series of focus groups facilitated in English and Spanish (25.5%). Over 40% of participants self-identified as Hispanic or Latinx, Native American, or African American with roughly 15% attending from rural areas. Similarly, professional providers age 18 to 80 (N=25) attended separate groups in either English or Spanish (48%). Our results suggest that computer-mediated focus groups offer a unique opportunity to reach diverse samples of older adults, family caregivers, and their providers. These computer-mediated focus groups also offer the chance to learn novel ways to break barriers to health access by providing virtual reach capabilities for those facing health, transportation, work, or geographic barriers.

### DEVELOPMENT OF ONLINE CHILD SUPPORT ACTIVITIES BY OLDER ADULTS; AN ACTION RESEARCH DURING THE COVID-19 PANDEMIC

Mai Takase,[1] Ryogo Ogino,[2] Ryoichi Nitanai,[3] and Jun Goto,[4], *1. The University of Tokyo, Bunkyo-ku, Tokyo, Tokyo, Japan, 2. Saga University, Saga, Saga, Japan, 3. the University of Tokyo, Tokyo, Tokyo, Japan, 4. Tokai University, Hiratsuka, Kanagawa, Japan*

Japanese communities have been attempting a novel type of childcare support, wherein community-dwelling older adults form a specialized group (support group) that aims to provide child support activities. Before the COVID-19 pandemic, the group gathered children and mothers in community spaces and conducted events. However, on-site support had to be halted due to the pandemic. In this study, we report a case of action research aimed at shifting the activities online. First, a suitable online support plan was explored by hosting several discussions with child-rearing mothers. A questionnaire survey was then conducted to determine the most-sought intervention contents (N=19). Finally, based on the results, an intervention was conducted. As a result of the discussions, the hosting of online programs was set as the main goal. Out of the ten activities studied, the three most popular activities were programming (n=17), English conversation (n=16), and science workshop (n=15). Based on the results, an online science workshop that built a Bottleium, a small aquarium using a bottle, was hosted. Eight children participated in the event. A post-activity survey revealed that all participants attended the online activity for the first time, and the parents were happy to have joined the activity that entertained their child during the quarantine period. Furthermore, focus group interviews were conducted with the support group; they reported being satisfied with the outcome and recognized the importance of their role as member of support group. The results suggest the positive effect of this project on both older adults and the children.

### EFFECTS OF A 6-WEEK INTEGRATED DEMENTIA PREVENTION INTERVENTION IN COMMUNITY-DWELLING OLDER ADULTS

Sunghee Tak,[1] Ji-yeon Kim,[2] Hana Ko,[3] SuJung Jung,[4] and Jaegyeong Lee,[1], *1. Seoul National University, Seoul, Seoul-t'ukpyolsi, Republic of Korea, 2. Eulji University, Uijeongbu, Seoul-t'ukpyolsi, Republic of Korea, 3. Gacheon University, Incheon, Seoul-t'ukpyolsi, Republic of Korea, 4. Semyung University, Jecheon, Seoul-t'ukpyolsi, Republic of Korea*

This study examined the week of a 6-session integrated dementia p program on dementia knowledge, attitude toward dementia, fear of dementia, and dementia prevention behaviors among community dwelling elders. Using a nonequivalent control group, pre-posttest design, study participants were recruited from a senior center in Seoul, Korea. A total of 40 participants completed the study while half of them were in the experimental group and another half were in the control group. They completed survey questionnaires before and after the program. The findings showed that the program was effective only to decrease the fear of dementia of the older adults. Knowledge, attitude toward dementia, and healthy behaviors may be difficult to change in a short period of time. However, the integrated dementia prevention program may be effective to decrease negative emotions, particularly, fear toward dementia among older adults. The fear of dementia needs to be further assessed individually in order to identify particular causes and triggers and provide tailored interventions.

### EXPLORING FACTORS THAT SHAPE ADHERENCE TO TECHNOLOGY-BASED COGNITIVE INTERVENTIONS

Erin Harrell,[1] Nelson Roque,[2] Walter Boot,[3] and Neil Charness,[3], *1. University of Alabama, Tuscaloosa, Tuscaloosa, Alabama, United States, 2. University of Central Florida, University of Central Florida, Florida, United States, 3. Florida State University, Tallahassee, Florida, United States*

A cognitive intervention study was conducted to explore methods to improve adherence to a technology-based cognitive intervention and uncover individual differences that predict adherence (N = 120). The study was divided into two phases: (1) in which participants were asked to follow a prescribed schedule of training that involved gamified neuropsychological tasks administered via a tablet, and (2), in which participants were asked to play as frequently as they wished. Positively and negatively framed messages about cognitive health were delivered via the software program, and measures of cognition, technology proficiency, self-efficacy, technology attitudes, and belief in the benefits of cognitive training were collected. We computed an aggregate measure of adherence during each of the two phases, as well as a measure of daily engagement. Across data modeling approaches, the finding was consistent: only during Phase 2, was there evidence that positively-framed messages encouraged greater adherence over negatively-framed messages. Measures of memory and self-efficacy demonstrated some, but limited, ability to predict individual differences in adherence.

### FEASIBILITY AND BARRIERS OF AN OLFACTORY TRAINING INTERVENTION

Natalia López-Bago Cid,[1] Tomiko Yoneda,[1] Nathan Lewis,[1] Michael Willden,[1] Anna Nelson,[1] Nadia Semenoff,[2] Andrea Piccinin,[1] and Jamie Knight,[1], *1. University of Victoria, Victoria, British Columbia, Canada, 2. University of Calgary, Calgary, Alberta, Canada*

Olfactory dysfunction is a common issue in late-life and can be an early indicator for neurodegenerative diseases. Further, olfactory interventions not only improve olfaction but have shown promise for the delay and treatment of dementia. This study aimed to better understand the feasibility and barriers of implementing an olfactory intervention. Participants (N=23) between the ages of 52-86 (mean=71) years were recruited from the community. A demographic questionnaire showed participants were all non-smokers and identified as women (70%), men (26%), and transgender (4%). The majority were married (61%), while some were separated or divorced (17%), widowed (13%), or single (9%). Four focus groups, guided by both structured and open-ended questions, were conducted and audio-recorded with 3-7 unique participants per group. Data were transcribed, thematically analyzed, and independently coded, which resulted in three overarching themes: (1) cognitive, genetic, and environmental factors of smell, (2) methods to reduce barriers and increase the feasibility of an intervention, and (3) flexibility with technology use. Findings suggest that implementing an olfactory intervention is feasible and of interest to older populations especially when provided with detailed training protocols that have flexibility in the amount of technology used within the study. Barriers included sensitivity to smells, allergies, and dexterity issues. Reducing these barriers will facilitate implementation and decrease the likelihood of attrition. Consulting the target population provides insights into barriers, participant interest, and can assist with the development of training and intervention programs.

### RESEARCH WITH OLDER ASIAN AMERICAN FAMILY CAREGIVERS PRE- AND DURING THE PANDEMIC: CHALLENGES AND LESSONS LEARNED

Jung-Ah Lee,[1] and Hannah Nguyen,[2], *1. University of California, Irvine, Irvine, California, United States, 2. California State University, Dominguez Hills, Carson, California, United States*

Research with hard-to-reach, monolingual adults from ethnic minority communities can present a multitude of challenges throughout the research process. This presentation will highlight challenges and lessons learned from two pilot studies with Vietnamese-, Cambodian-, and Korean-American family caregivers aged 50 and older. The first study (n=9) implemented a one-on-one, telephone-based psychosocial intervention before the COVID-19 pandemic; the second is an ongoing study (n=12) consisting of a group-based intervention via Zoom. Throughout recruitment, the following challenges arose: addressing the lack of familiarity with research among caregivers, earning the trust of caregivers, and identifying creative ways to recruit caregivers to participate. During study implementation, common challenges included: caregivers' unpredictable daily schedule that made it difficult to participate in the scheduled classes, caregivers feeling apprehensive about technology and Zoom, access to reliable internet, and facilitating participation and engaging the voices of caregivers over the phone or via Zoom. Strategies were identified to address these barriers: engaging the support and collaboration of trusted, bilingual and bicultural community-based providers, building culturally-responsive rapport with caregivers, and seeking continuous feedback from caregivers to improve the appeal of the project implementation. The COVID-19 pandemic added an additional layer of difficulty to the research, requiring creativity and flexibility in implementation that took into consideration caregivers' heightened anxiety, distress, lack of participation due to around-the-clock care, and loss and grief. The challenges and lessons learned from these studies could guide the development of future research efforts and strategies to effectively engage older hard-to-reach, monolingual Asian American caregivers.

### THE FOCUS GROUP MUST GO ON: LESSONS LEARNED FROM CONDUCTING VIRTUAL FOCUS GROUPS

Ashlee Cordell,[1] Christa Wilk,[2] Silvia Orsulic-Jeras,[1] Sara Powers,[1] Farida Ejaz,[1] and Lisbeth Sanders,[3], *1. Benjamin Rose Institute on Aging, Cleveland, Ohio, United States, 2. Benjamin Rose Institute, Cleveland, Ohio, United States, 3. LifeBio, Marysville, Ohio, United States*

The Covid-19 pandemic has presented a multitude of challenges in conducting research with human subjects. In response, researchers have found creative ways to complete these studies using alternative methods that incorporate social distancing. Fortunately, numerous technologies exist today that allow individuals to connect with one another over short and long distances. The current study describes the development of LifeBio Memory: an app-based product that utilizes artificial intelligence and machine learning to improve an existing life story intervention designed for persons living with dementia (PWD). Seven focus groups (n=35), originally planned in-person, were successfully converted to a virtual setting. Groups were hosted using a Zoom platform, lasted 75-90 minutes (Mean = 85; SD = 5.3), and consisted of participants from 14 different states: One group of community-dwelling PWDs with early-stage dementia (n=5); two groups of current and former users of the original LifeBio program (n=12); and four groups of residential care staff and directors (n=18). Virtual focus group delivery was determined to be an acceptable and feasible alternative to traditional in-person formats. Topics discussed in this poster will include: 1) recruitment procedures, 2) screening protocols, 3) methods for sharing materials, 4) guidance for providing technology support, and 5) communication strategies to increase retention. Further discussion will focus on challenges faced when collecting data in a virtual setting, tips for successful facilitation, advantages to using virtual alternatives, and other lessons learned from the virtual field.

### USING LIFE STORY TECHNIQUES WITH DIVERSE COMMUNITIES: LESSONS LEARNED FROM A FOCUS GROUP STUDY

Christa Wilk,[1] Ashlee Cordell,[2] Silvia Orsulic-Jeras,[2] Sara Powers,[2] Farida Ejaz,[2] and Lisbeth Sanders,[3],*5*
*1. Benjamin Rose Institute, Cleveland, Ohio, United States, 2. Benjamin Rose Institute on Aging, Cleveland, Ohio, United States, 3. LifeBio, Marysville, Ohio, United States*

Providing high quality, cost-effective dementia care remains a major health challenge. Life story work, used in residential care settings, helps engage persons living with dementia (PWD) at a low cost with minimal staff burden. LifeBio, one such intervention, is designed to elicit life history data and care preference information through comprehensive life story interviews. LifeBio Memory, an adaptation of LifeBio, utilizes novel speech-to-text technology to process life story data more efficiently. Seven focus groups were conducted to evaluate the acceptability and feasibility of LifeBio Memory. Three types of focus groups were held (n=35) and audio recorded: 1) One group of early-stage PWDs (n=5); 2) Two groups of current and former users of original LifeBio (n = 12); and 3) Four groups of residential care staff and directors (n=18). Sessions were transcribed and thematic analyses were conducted. Findings indicated high levels of acceptability and feasibility of LifeBio Memory. Further, a secondary theme emerged signaling the need to prioritize the emotional safety of PWDs participating in life story work. This poster will discuss: 1) the life story interview process, 2) identifying PWDs who would most benefit from a life story program, 3) involving family and staff care partners to identify sensitive topics, 4) interview question design, selection and order, and 5) expanding life story work across diverse communities. Discussion will highlight the importance of protecting the emotional well-being of marginalized communities by identifying potential underlying traumas that could impact the safe delivery of otherwise effective life story interventions.

### WHERE ARE ALL THE MEN? A REVIEW OF THE BARRIERS AND FACILITATORS TO PARTICIPATION IN HEALTH PROMOTION INTERVENTIONS

Britteny Howell,[1] Sage Corbett,[1] and Jennifer Peterson,[2], *1. University of Alaska Anchorage, Anchorage, Alaska, United States, 2. University of Alaska Fairbanks, Fairbanks, Alaska, United States*

Research shows that men in the U.S. experience significant morbidity and earlier mortality than women and are less likely to access, interpret, and apply health information to improve their outcomes. Although evidence-based health promotion programs have proven successful at increasing healthy lifestyle behaviors and reducing morbidity among older adults, older males are still significantly less likely to enroll and sustain participation in such health interventions. While studies have shown the barriers and facilitators to older adult participation in health programs in general, it is largely unknown why older male recruitment and participation in health promotion interventions remains so low. In this poster presentation, we conducted a thorough review of the last 20 years of existing research across a variety of academic search databases to outline the barriers, facilitators, and recommendations for increasing older male participation in health promotion programs. Of 1,194 initial search results, 383 article abstracts were thoroughly screened for inclusion, and 26 articles met all inclusion criteria. Included studies were coded and analyzed using Grounded Theory and reveal that masculine gender roles, as well as program scope, environment, and gender of the instructors and other participants, were important factors for male participation. Interventions should include men in all aspects of program planning and implementation, take into account men's existing relationships and interests to create gender-sensitive programming, and clearly delineate the benefits to participation. Lastly, the field of public health would benefit by helping to normalize men's participation in health promotion interventions.

### Session 9340 (Poster)

### LIFE COURSE AND DEVELOPMENTAL CHANGE

### AGE, PERIOD, AND COHORT EFFECTS ON LITERACY SKILLS ACROSS LIFE STAGES

Takashi Yamashita,[1] Thomas Smith,[2] and Phyllis Cummins,[3], *1. University of Maryland, Baltimore County, Baltimore, Maryland, United States, 2. Northern Illinois University, DeKalb, Illinois, United States, 3. Miami University, Oxford, Ohio, United States*

Literacy skills are essential adult competencies for economic, social, political and cultural participation, which are linked to higher quality of life. Literacy skills are known to be lower for older age groups. However, relatively little is known about cohort and period effects, which provide clues to the sociohistorical impacts on literacy, in addition to the well-known age effects, over the life course. This study analyzed three nationally representative cross-sectional survey data of the U.S. adults at five time points, from the 1994 International Adult Literacy Survey (IALS), 2003 Adult Literacy and Life Skills Survey (ALL), and the 2012/2014/2017 Program for International Assessment of Adult Competencies (PIAAC). The total analytic sample was 17,450 adults age between 18 and 65 years old. The literacy measures were re-scaled (0-500 points) to be comparable across the surveys. An age-period-cohort hierarchical linear model (i.e., cross-classified random effects model) was constructed using the Bayes estimator. Individuals were cross-classified based on 14 five-year birth cohort and 5 periods (survey years) information. Results showed that literacy skills improved [95% credibility-interval (CI) for linear effect of age = (0.31, 1.07), but the rate of improvement declined over time, faster rates of decline in later life stages [95% CI for quadratic effect of age = (-0.17, -0.09)]. Additionally, the notable variability across the cohorts and periods [95% CI variances = (5.34, 52.52) and (2.30, 172.01), respectively] were identified. Possible explanations for the identified age, period and cohort effects on literacy and implications for adult competencies in later life are evaluated.

### DAILY STEPS BUFFER THE IMPACT OF DAILY STRESS ON MOOD IN YOUTH AND MIDDLE-AGED/OLDER ADULTS

Daniel Fleming,[1] Yin Liu,[1] Myles Maxey,[2] Elizabeth Braungart Fauth,[1] and Troy Beckert,[2], *1. Utah State University, Logan, Utah, United States, 2. Utah State University, Utah State University, Utah, United States*

Physical activity has known associations with lower stress and improved well-being. These studies often include samples from one developmental phase at a time, which is helpful for researchers in those developmental areas, but less informative for identifying predictors of health and well-being across the lifespan. The current study examined whether protective aspects of physical activity (steps) on stress and mood worked similarly in widely different age cohorts. We also examined these relationships at the daily level, as opposed to global/macro levels. Participants (n = 119, 67% female) were 44 adolescents between 13-18 years (Mage (SD) = 15.73 (1.48) years, 57% female) and 77 middle-aged/older adults between 55-76 years (Mage (4.97) = 59.67, 74% female). They self-reported global life satisfaction and demographic characteristics at baseline and completed ecological momentary assessments (three per day for three consecutive days, across six measurement bursts, each spaced two weeks apart) via smart phones, reporting on their mood, stressor exposures/types, and end-of-day pedometer step count. Multilevel models showed that daily steps had protective effects against social network stressors on both daily mood and life satisfaction, such that more steps weakened the negative relationship between network-related stressors, mood, and life satisfaction. This protective effect was uniform for both older and younger adults, and across boys/men and girls/women. Overall, the present study suggested the importance of physical activity, even that of general step count, on buffering daily stress on daily mood and general life satisfaction for participants at multiple phases of the lifespan.

### DEPENDENCY, MORTALITY, INVISIBILITY: LINKING CHILDHOOD DISABILITY WITH LIFE COURSE HEALTH OUTCOMES

Sarah Laditka, James Laditka, and Jessica Hoyle,
*University of North Carolina at Charlotte, Charlotte, North Carolina, United States*

People who experienced disability in childhood are living longer. It is not clear if longer lives indicate better health and less dependency, or if longer life is accompanied by increased dependency. We addressed that question by studying the joint dynamics of mortality and dependency. This population is "invisible" in most national surveys, which do not ask about childhood disability. We evaluated special education history as an indicator of childhood disability, and used that indicator to estimate dependency and life expectancy throughout adult life. Data: Panel Study of Income Dynamics and the Health and Retirement Study (n=20,563). Activities of daily living (ADLs), instrumental ADLs, and cognition defined five functioning levels including dependency and death. Multinomial logistic Markov models estimated probabilities for transitioning among the levels, with or without a history of childhood disability, adjusted for demographics. We used the probabilities in microsimulations, creating large populations of completed lives, identifying dependency at each age for each individual. Analysis showed special education history was a valid indicator of childhood disability; 13% had such history. With parent education less than high school, remaining life at age 20 was 46.0 years for people with that history, 58.3 for others; corresponding results with parent's bachelor's degree: 48.3 and 60.7 ($p<0.05$). Corresponding population percentages dependent 5+ years were: 15.2% and 3.8%, 13.1% and 3.8% (all $p<0.05$). Special education history can indicate childhood disability. People with that history had significantly more dependency than others, and shorter lives. Accommodations and interventions can improve their health and functioning.

### DISCREPANCIES IN OBJECTIVE AND SUBJECTIVE FINE MOTOR ABILITIES IN OCTOGENARIANS

Elizabeth Braungart Fauth,[1] Andrew Hooyman,[2]
Sydney Schaefer,[2] Anna Hall,[1] and Marie Ernsth-Bravell,[3]
*1. Utah State University, Logan, Utah, United States,*
*2. Arizona State University, Tempe, Arizona, United States,*
*3. Jönköping University, Jönköping, Jonkopings Lan, Sweden*

Older individuals may have discrepancies between self-reported and performance-based abilities on activities of daily living (ADL). We examined objective and self-reported fine motor abilities (FMA). FMA are required for many ADLs, but are examined less frequently than gross-motor tasks in this population. We used two waves of the population-based OCTO-Twin study including mono-/dizygotic Swedish twins, aged 80+. One twin was randomly selected for analyses (baseline N=262; wave 2 N=198; Meanage =83.27; SDage=2.90; 66.4% female). Participants self-reported their ability to manipulate things with hands (cannot do, some problem, no problem) and completed a timed FMA assessment including five everyday tasks (e.g. inserting a key in a lock). Slow performance was coded as 1+ SD from the mean (=80+ seconds). At baseline, 65.8% of slow performers reported 'no problems' with hand manipulation. Over two waves (two years), a two-factor ANOVA (including slowness-by-perception interaction) supported a significant difference in total motor task performance between slow performers reporting 'no problems' and fast performers reporting 'no problems', for both rate of change (diff = -26 seconds, $p<.0001$) and wave 2 level (diff = 50 seconds, $p < .0001$). 82% of slow performers at wave 2 reported 'no problems', which is surprising given that they had become even slower over the past two years. Findings suggest that objective FMA measures are needed, as self-report is inaccurate and not prognostic. Future work will examine if discrepancies in performance/perceived FMA predict poorer outcomes, and/or if reporting 'no problems' despite slower performance is protective against cognitive adaptation to slowing.

### HEALTH BEHAVIORS IN THE LGB+ POPULATION: VARIATION ACROSS ADULTHOOD

Jessica Noblitt, and Anne Barrett, *Florida State University, Tallahassee, Florida, United States*

Health behaviors, which predict physical and mental health, are patterned by social factors, with some groups engaging in more health-enhancing behaviors than others. LGB+ people face more economic and social barriers to participation in healthy behaviors, along with the stress of discrimination that could lead to unhealthy behaviors to cope. Although some studies have examined variation in health behaviors by sexual identity, they focus almost exclusively on adolescents and young adults. However, such differences may decline across adulthood, as stress related to sexual identity declines with age among LGB+ individuals. Addressing this issue, we use data from the National Health Interview Survey (2016-2018) to examine differences by sexual identity in substance use, weight-related behaviors, healthcare utilization, and sleep. We compare the patterns across three age groups – young, middle-aged, and older adults. Results for each health behavior reveal that differences by sexual identity are indeed greatest among young adults. The magnitude is smaller in middle age, and no significant differences by sexual minority status are observed at older ages.

### THE POSITIVE AND NEGATIVE AFFECT RELATION IN THE CONTEXT OF STRESS AND AGE

Jessica Blaxton,[1] Niccole Nelson,[2] and Cindy Bergeman,[2],
*1. Metropolitan State University, Saint Paul, Minnesota, United States, 2. University of Notre Dame, University of Notre Dame, Indiana, United States*

Research suggests that the within-person inverse relationship between negative affect (NA) and positive affect (PA) indicates poorer emotional well-being, and this interaffect correlation fluctuates in relation to the context of the individual. Specifically, age, stress, and global PA all relate to changes in the interaffect correlation. The current study uses comprehensive data from the Notre Dame Study of Health & Well Being (NDHWB), which allows us to uniquely examine between-person differences in within-person change and variability in the interaffect correlation, thereby examining these constructs from a process-oriented perspective. Midlife and later life participants (N = 965) completed daily questionnaires assessing stress, NA, and PA. Three-level multi-level models illustrated that the interaffect correlation becomes more negative during times of stress, adults with greater global PA experience a stronger inverse interaffect correlation during times of stress, and days of higher stress relate to a stronger inverse interaffect correlation for older adults compared to midlife adults. The findings illustrate the idiographic nature of these relationships and suggest that later life adults and adults with high levels of global PA undergoing higher than typical stressful situations experience stronger inverse interaffect correlations.

### TRAJECTORIES OF FUNCTIONAL HEALTH FOLLOWING STROKE: THE ROLE OF SOCIAL RESOURCES

Takeshi Nakagawa,[1] Taiji Noguchi,[1] Ayane Komatsu,[2] Masumi Ishihara,[1] and Tami Saito,[1], *1. National Center for Geriatrics and Gerontology, Obu, Aichi, Japan, 2. Department of Social Science, Research Institute, National Center for Geriatrics and Gerontology, Obu, Aichi, Japan*

Stroke is one of the major causes of disability in old age. Predictors for the functional prognosis have been studied, but the role of social resources in recovery has not studied as much. We examined whether social resources available before and after stroke onset improved functional prognoses. Data was derived from longitudinal data collected between 1987 and 2006 from Japanese adults aged 60 years and older. We identified 396 people who had experienced their self- or proxy-reported first stroke during follow-up (age at stroke onset: M = 76.0, SD = 6.9; 74.2% women). Functional health was measured by self- or proxy-reported activities of daily living. Social resources were indexed as residential status, contact with non-coresident children, social participation, and perceived support. Analyses were adjusted for age at stroke onset, gender, and education. A multiphase growth model showed that functional health typically deteriorated surrounding stroke and gradually declined thereafter. There were also individual differences in the trajectories of functional health. Individuals who more frequently participated in social groups prior to stroke and those who came to participate more frequently thereafter exhibited less functional decline immediately following stroke. Our findings indicate that social participation plays a protective role against adverse prognoses following stroke regardless of when individuals start participating. Inclusive communities would enable older adults to remain independent. Our study was limited in that crucial information about stroke, such as objective measures of initial severity, was not available and that individuals with more severe stroke may have dropped out after the onset.

### Session 9345 (Poster)

### LONG-TERM CARE POLICY II

### ASSET TRANSFERS AMONG THOSE ACCESSING THE MEDICAID PROGRAM: ARE WEALTHY OLDER ADULTS GAMING THE SYSTEM?

Jane Tavares,[1] and Marc Cohen,[2], *1. LeadingAge LTSS Center @UMass Boston, Boston, Massachusetts, United*

*States, 2. University of Massachusetts Boston, University of Massachusetts Boston, Massachusetts, United States*

Medicaid is the largest payer of long-term services and supports and millions of older Americans rely on the means-tested program for health care coverage. There has been longstanding concern that wealthy older adults are taking advantage of the program by divesting assets in order to qualify for coverage. The existing research on the issue is somewhat dated, does not focus on the question of asset transfer, and often lacks a significant longitudinal view. Thus, questions remain about whether states need to tighten asset eligibility rules to prevent the wealthier older adults from accessing the program. This analysis explores longitudinal data from the Health and Retirement Study (1998 to 2016) to determine the extent to which wealthier Americans age 50 and older engage in asset transfer to access Medicaid. Our findings demonstrate that this may occur among a relatively small proportion of wealthy older adults, and that tightening Medicaid eligibility criteria would likely have a small to modest impact on the financial status of the program.

### DIFFERENTIAL EFFECTIVENESS OF THE MINNESOTA SAFE PATIENT HANDLING ACT BY HEALTH CARE SETTING

Christina Rosebush,[1] and Katherine Schofield,[2], *1. University of Minnesota, Twin Cities, Minneapolis, Minnesota, United States, 2. University of Minnesota Dultuh, Duluth, MN, Minnesota, United States*

The Minnesota Safe Patient Handling (SPH) Act requires nursing homes, hospitals, and outpatient facilities to develop comprehensive SPH programs and acquire mechanical lifts. The law was designed to prevent the adverse outcomes of manual patient handling among workers (e.g., musculoskeletal injuries) and care recipients (e.g., falls, skin tears). Reducing manual handling is of particular concern in nursing homes where residents' care needs necessitate frequent lifts and transfers. To date, research has focused on the effects of SPH laws separately in nursing homes and hospitals. Our study aimed to assess whether change in worker injury rate differed between nursing homes and other health care settings following enactment of the 2007 Minnesota law. We used 2005-2017 claims data from a large workers' compensation insurer and assessed the effects of time, health care setting, and their interaction on claim rate using negative binomial regression models. The claim rate for patient handling injuries was highest in nursing homes (2.8/million payroll), followed by hospitals (1.4/million payroll), and outpatient facilities (0.04/million payroll). Across settings, patient handling claims declined by 38% (95% CI 19-53%) between pre-law (2005-2007) and post-implementation (2014-2017). The decline in claims over time did not differ by health care setting (Wald $\chi 2$ for interaction=3.40, p=0.758). Our results suggest that nursing homes are successfully addressing the unique mobility needs of their residents in their mandated SPH programs. Future work should seek to describe the magnitude and nature of care recipient injuries caused by lifting and transferring and evaluate trends in care recipient injuries over time.

### FAILING TO COMPLAIN: DO NURSING HOMES WITH MORE RESIDENTS WITH DEMENTIA HAVE FEWER COMPLAINTS?

Kallol Kumar Bhattacharyya,[1] Lindsay Peterson,[2] John Bowblis,[3] and Kathryn Hyer,[2], *1. University of South*

*Florida, Tampa, Florida, United States, 2. University of South Florida, University of South Florida, Florida, United States, 3. Miami University, Oxford, Ohio, United States*

The majority of nursing home (NH) residents have Alzheimer's Disease or Related Dementias (ADRD). However, the association of ADRD prevalence and NH quality is unclear. The objective of the current study is to understand the association of NH characteristics, including the proportion of ADRD residents, with the prevalence of NH complaints as an indicator of quality of care and quality of life. We merged data from the ASPEN Complaints/Incident Tracking System with national NH data from the Certification and Survey Provider Enhanced Reports, the Minimum Data Set, the Area Health Resource File, and zip-code level rural-urban codes in 2017. Three groups of NHs were created, including those whose proportion of residents with ADRD was in the top decile (i.e., high-dementia NHs (N=1,473)) and those whose proportion of ADRD residents was in the lowest decile (i.e., low-dementia NHs (N=1,524)). Bivariate results revealed high-ADRD NHs had higher percentages of Medicaid-paying residents, were less likely to be for-profit and chain-affiliated, had lower staffing hours and lower percentages of Black, Hispanic, and Asian residents. Using NHs in the middle deciles as reference, negative binomial regression models showed that having a low proportion of ADRD residents was significantly associated with higher numbers of total complaints ($p<.001$) and substantiated complaints ($p<.001$), whereas having a high proportion of ADRD residents was significantly associated with lower numbers of substantiated complaints ($p=.001$). The findings suggest the proportion of residents with ADRD in NHs is associated with quality, as measured by complaints. Policy implications of these findings will be discussed.

### MEANINGFUL ASSESSMENT OR MINIMUM COMPLIANCE: PASRR FOR NURSING HOME RESIDENTS WITH MENTAL ILLNESS

Taylor Bucy,[1] Kelly Moeller,[1] John Bowblis,[2] and Tetyana Shippee,[1], *1. University of Minnesota, University of Minnesota, Minnesota, United States, 2. Miami University, Oxford, Ohio, United States*

The Omnibus Budget Reconciliation Act (OBRA) of 1987 included provisions for the Preadmission Screening and Resident Review (PASRR) program, which requires states to create and maintain systems to assess persons with serious mental illness (SMI) seeking NH care. The prevalence of SMI in NHs is increasing, and little is known about the effectiveness of the PASRR program intervention. We conducted 20 interviews with state and national PASRR stakeholders, including assessors, hospital discharge planners, mental health advocates, geriatricians and geriatric psychiatrists. Interview data were triangulated with state provided materials on PASRR collection and implementation. Based on these interviews, we identified four themes: 1) variation in the implementation of federal PASRR legislation across states and jurisdictions, 2) the need for investment in professional development and workforce capacity, 3) lack of usefulness of PASRR in ongoing care planning, and 4) the need to consider the role of age, race/ethnicity, and stigma on quality of care for NH residents with SMI. Stakeholders agree that PASRR legislation was well intentioned, but also expressed concern regarding the completion of PASRR as an issue of compliance versus meaningful assessment. More work is needed to determine how best to develop and support the care needs of people with SMI, while being mindful of the original goals of deinstitutionalization that prompted OBRA passage. In order to assess the impact of the PASRR program on quality of care and mental health outcomes, further research should take an evaluative approach through meaningful use of PASRR data.

### PREVENTABLE? LONG-TERM CARE POLICY SUCCESSES AND FAILURES DURING COVID-19 PANDEMIC: A SCOPING LITERATURE REVIEW

CHAORAN WU,[1] Aleksandra Zecevic,[2] Maxwell Smith,[3] and Shannon Sibbald,[1], *1. University of Western Ontario, London, Ontario, Canada, 2. Western University, London, Ontario, Canada, 3. University of Western Ontario, University of Western Ontario, Ontario, Canada*

The number of older adults who live in long-term care (LTC) is expected to increase worldwide. The COVID-19 pandemic has caused serious consequences in Canadian LTC homes, while homes in China and Japan reported minimal infection and death rates in residents. The differences in LTC policies may be one of the contributors. The purpose of this literature review was to identify elements of the LTC policies that might have impacted COVID-19 outcomes in LTC homes in Canada, China, and Japan. A scoping review was conducted following the framework proposed by Arksey and O'Malley. Scholarly articles and grey literature published between January 2015 and June 2020 were identified in six databases, four in English (CINAHL, Scopus, ProQuest, and PubMed), one in Chinese (CNKI), and one in Japanese (CiNii), using MeSH terms for LTC and health policy. Grey literature was identified using Google. Data were extracted, summarized and common themes identified through content analysis. A total of 52 articles and 26 grey sources were included in the review based on determined inclusion criteria. They were research articles, reviews, government or association reports, policy briefs, policy documents, and guides. Four common themes of challenges emerged: caregiver workforce, service provision, funding, and physical environments. Three sub-themes were identified for caregiver workforce and service provision. Differences in COVID-19 consequences in LTC homes in the three countries seem to be related mainly to the challenges with the caregiver workforce and the lack of funding. The result suggests Improvements of LTC policies are required, especially in Canada.

### SCOPING REVIEW: HOME AND COMMUNITY-BASED SERVICE WAIVER PROGRAMS AND PERSON-REPORTED OUTCOMES

Taylor Bucy,[1] John Mulcahy,[1] Eric Jutkowitz,[2] and Tetyana Shippee,[1], *1. University of Minnesota, University of Minnesota, Minnesota, United States, 2. Brown University, Brown University, Rhode Island, United States*

State Medicaid programs are rebalancing their long-term care spending from nursing home to home and community-based services (HCBS). Emphasis on person-centered and person-directed care warrants investigation into models of HCBS delivery that promote quality of life. We performed a scoping review of the literature to catalogue the breadth of the studies describing HCBS waiver programs targeting adults (18+). We identified 757 articles, and after duplicate removal and reconciliation, we excluded articles on children or adolescents, non-peer reviewed reports, international studies, and articles that did not describe HCBS waiver programs. After abstract and title review, 292 articles met our inclusion criteria. Most included articles (22.3%) were single state descriptive evaluations or evaluations of service use patterns among participants. 17.8% of included articles examined multi-state or national variation in program trends, while 17.1% made national program conclusions without a major focus on interstate comparison. Less common were studies examining integrated care or dual-eligibles (7.5%), PACE (3.4%), medication management (3.1%), quality and satisfaction of both consumer and caretaker perspectives (3.8%) and consumer-only perspectives (5.1%). The remaining articles focused on HIV (4.1%), TBI (1.4%) or ID/DD (14.4%) waiver programs. The 8.9% of articles addressing quality and satisfaction consisted mostly of interviews, either with state Medicaid administrators or with care recipients and/or caregivers. Consumer reported satisfaction and unmet care needs were the primary outcomes examined. Given the heightened focus on long-term care as a result of the ongoing coronavirus pandemic, this review justifies further exploration into the delivery and outcomes of state-directed HCBS waiver programs.

### THE DEVELOPMENT OF A SELF-APPRAISAL TOOL FOR THE LEVEL OF LONG-TERM CARE SERVICE INTEGRATION.

Yu-Chien Chang,[1] and Ya-Mei Chen,[2], *1. National Taiwan University, Taiwan, Taipei, Taiwan (Republic of China), 2. National Taiwan University, Taipei, Taipei, Taiwan (Republic of China)*

Introduction Taiwan is the fastest aging countries in the world. In 2016, Taiwan implemented Long-Term Care Plan 2.0 (LTC Plan 2.0), aims to provide coordinated and integrated LTC services. However, how to assess the level of integration and which integration mechanisms are better applied are still unclear in the literature. This study intended to address a research question regarding "How can agencies measure their level of service integration?" and, therefore, aimed to develop an integration assessment tool—the Taiwanese Self-Assessment for LTC Systems Integration (TwSASI)- for LTC agencies to use to self-evaluate their current "level" of providing integrating LTC services. Methods TwSASI was first developed base on Connie J. Evashwick's (2005) framework and literature review, including four domains: inter-entity planning and management, care coordination, integrated information system, and integrated financing, and 11 dimensions with 51 items. Through the Delphi method, with two rounds of investigation and feedback from 26 experts, RAND/UCLA Appropriateness Method (RAM) was used to assess the consensus regarding the dimensions and items developed and refined the tool content accordingly. Results After two rounds of investigation, four domains remained with 10, 11, 4, and 5 items in each domain respectively. All items reached good experts' consensus with medians of the 30 items' importance, feasibility, and appropriateness all over 8. The Scale Content Validity Index (SCVI) of the 4 dimensions all over than 0.9. Conclusion The TwSASI can be feasible for evaluating the level of LTC service integration in Taiwan. LTC agencies can improve their level of service integration accordingly.

### WHO IS COMING TO JAPAN UNDER THE NEW STATUS OF RESIDENCE "SPECIFIED SKILLED CARE WORKERS"?

Noriko Tsukada, *Nihon University, Tokyo, Tokyo, Japan*

The Japanese government created a new status of residence called "Specified Skilled Workers" encompassing 14 job categories that have laborer shortages, including long-term care (LTC) workers on April 1, 2019. A survey of students (N=79) at a language institute in Manila, Philippines was conducted in February, 2020 to explore characteristics of future foreign LTC workers in Japan under this new status of residence and identify factors that may help facilitate worker retention. At the time of data collected, these students were studying both Japanese and LTC practices in order to pass skill evaluation tests to enter Japan. Students were asked their perceptions of long-term care, planned lengths of stay, concerns about staying in Japan, and future career plans. Preliminary analyses revealed that 67 (88%) were single, 74(93.7%) had graduated from universities, and a half of them had a nursing background. Most students (93.7%) showed interest in an extended stay in Japan, with some interested in taking the national certified care worker examination which permits extended work in Japan. The most cited concern about being in Japan was "weather, climate and typhoons" (28.1%), followed by "language proficiency" and "living expenses" (21.9%) respectively. As Japan has limited immigration policies but a large need for LTC workers, it is imperative to understand how to attract and retain foreigners who obtain the Specified Skilled Worker status. This includes addressing both work-related and living-related concerns and needs.

### Session 9350 (Poster)

### LONG-TERM CARE PRACTICE

### DIRECT CARE WORKERS EMPLOYED AS MEDICAL AIDES: HOME HEALTH AIDES, NURSING ASSISTANTS, AND ORDERLIES

Christopher Kelly, and Jerome Deichert, *University of Nebraska at Omaha, Omaha, Nebraska, United States*

**Purpose:** This study describes the differences among direct workers (DCWs) employed as medical aides in three occupations: home health aides, nursing assistants, and orderlies. Design and

**Methods:** Data were from the 1% Public Use Microdata Sample (PUMS) of the 2019 American Community Survey (ACS). Logistic regression was used to compare demographic and employment characteristics of DCWs employed as medical aides in three occupations: home health aides, nursing assistants and orderlies.

**Results:** Compared to orderlies and psychiatric aides, home health aides are more likely to be foreign born, more likely to be female, less likely to work in institutional settings, less likely to be under age 25, less likely to work year-round full-time, less likely to have more education, and less likely to receive insurance from their employers.

**Implications:** Since 2018, the PUMS of the ACS separates nursing, psychiatric, and home health aides (previously one occupational category) into three: home health aides, nursing assistants, and orderlies. This affords researchers a more precise understanding of this part of the direct care workforce. Home health aides represented more than 2/3 of DCWs employed as medical aides in 2019. Further, these workers are distinct among medical aides. More than nursing assistants and orderlies, home health aides are older, female, underinsured, foreign-born, and with limited education. This reflects both the barriers home health aides face to other occupations and also the preferences of their employers (which include private households). These findings have implications for the recruitment and retention of medical aides across all three occupations.

### DO PERSONALITY AND BELIEF INFLUENCE THE OWNERSHIP OF PRIVATE LONG-TERM CARE INSURANCE?

Shu-Chuan Jennifer Yeh,[1] Hsueh-Chih Chou,[2] and Shih-Hua Sarah Chen,[3], *1. National Sun Yat-sen University, Kaohsiung, Kaohsiung, Taiwan (Republic of China), 2. Kaohsiung Veterans General Hospital, Kaohsiung Veterans General Hospital, Kaohsiung, Taiwan (Republic of China), 3. The University of Chicago, Chicago, Illinois, United States*

Long-term care is the largest out-of-pocket expenditure risk for the elderly. Private long-term care insurance is one crucial source to cover the gap. The study aims to investigate (1) whether wealthy people are more likely to purchase private long-term care insurance (LTCI); (2) whether personality traits are associated with ownership of LTCI; and (3) whether individual experience influences the ownership of private LTCI. We surveyed 375 participants for three groups, including nurses, executive MBA, and MBA students. EMBA group had highest percentage of personal discretionary income (defined as greater than 1,780 US dollar per month) (77.6%), followed by MBA group (17.6%) and nurse group (8.8%). The nurse group had highest in long-term caring experience for sick family members (46.4%), self-experience catastrophic diseases (11.2%), and number of family/relatives/friends share responsibilities of caregivers (2.33 persons). The nurse group had lowest risk propensity score as well as openness to experience traits. The percentage of ownership in private LTCI was 48%, 45.6%, and 28.8% for nurse, EMBA, and MBA groups, respectively. After controlling for age and tenure, results from logistic regression indicated that MBA group was less likely to own LTCI compared with nursing group. Compared with less than 30,000, only personal discretionary income 30,001~ 50,000 had higher odds ratio to own LTCI. Wealthy is inconclusive in determining ownership of private LTCI. Nurses who were lowest risk propensity and openness traits and have more sickness experience were more likely to purchase private LTCI. Believe needing LTCI is the strongest predictor in ownership of LTCI.

### DO YOUNG ADULTS SEE VALUE IN ADVANCE DIRECTIVES?

Yuchi Young,[1] Arianna Stone,[2] Taylor Perre,[3] Kuo-Piao Chung,[4] and Ya-Mei Chen,[5], *1. SUNY at Albany, Rensselaer, New York, United States, 2. University at Albany, University at Albany, New York, United States, 3. University at Albany, Rensselaer, New York, United States, 4. National Taiwan University, School of Public Health, Taipei, Taipei, Taiwan (Republic of China), 5. National Taiwan University, Taipei, Taipei, Taiwan (Republic of China)*

Many Americans avoid end-of-life care planning; only 26% have completed an advance directive (AD). An AD promotes end-of-life care with dignity allowing individuals to make end-of-life treatment and care decisions before they are unable to do so. Previous studies related to ADs are focused on older adults with serious illness or people with functional/mental disability. The objective of this survey is to better understand young adults' knowledge of and attitude toward ADs and their preferences for ADs related to treatment and care options. Methods. Participants include graduate students (n=25) attending a state university in New York State (NYS). Data were collected using two ADs (Five Wishes; Medical Orders for Life-Sustaining Treatment (MOLST)) and one survey questionnaire. Summary statistics and multivariate models will be used to address the study aims. Results. Preliminary results show the average age was 23 years, 72% were female, 48% White, and 44% Black. The majority of young adults hadn't completed an AD; however, their attitude toward ADs was positive; the majority believe it is important to have an AD prepared at their current age; and they believe young adults would willing to fill out ADs. Young adults can make difficult treatment and care decisions when the situation requires it. Conclusion. The study findings can be useful to policy makers, healthcare providers and other stakeholders in promoting population-based healthcare decision-making. Limitation. Participants were recruited from one university in NYS; thus, the study results may be generalized to a population sharing similar characteristics.

### EFFECTIVENESS OF SUPERVISION ON WORK ENGAGEMENT AND TURNOVER INTENTION OF CARE MANAGERS IN JAPAN

Ryosuke HATA, *Hokusei Gakuen University, Sapporo, Hokkaido, Japan*

In Japan, care managers engage frail older adults to support their assisted living in long term care insurance system. However, due to the lack of some or all supervision, many care managers face problems such as low work engagement and high turnover rate. This study aims to examine what types of supervision have positive effects on work engagement and turnover intensions of care managers in Japan. The sample of 241 care managers were asked whether they have received individual supervision in the workplace (ISVW), individual supervision in the community (ISVC), group supervision in the workplace (GSVW), or group supervision in the community (GSVC). Independent samples t-tests and one-way ANOVAs were conducted to examine the effectiveness of each types of supervision on work engagement and turnover intension. T-tests showed that only GSVW was significantly related to work engagement ($t=-2.06$, $p<0.05$). Whereas, only ISVW had a significant effect on turnover intensions ($t=2.37$, $p<0.05$). One-way ANOVAs revealed that 28 care managers receiving GSV had significantly higher work engagement than 92 care managers who did not receive any SV (F=5.33, p<0.01). 40 care managers receiving both ISV and GSV showed significantly lower turnover intentions than 92 care managers who received neither ISV nor GSV (F=2.84, p<0.05). Since the results have implications for the importance of supervisions to enhance work engagement or to reduce turnover intension of care managers, a larger sample will need to confirm these effects.

### EVIDENCE-BASED RECOMMENDATIONS TO INFORM BEST PRACTICES FOR LGBTQ OLDER ADULTS IN LONG-TERM CARE SETTINGS

Katherine Fasullo,[1] Erik McIntosh,[1] Todd Ruppar,[2] Sarah Ailey,[1] and Susan Buchholz,[3], *1. Rush University, Chicago, Illinois, United States, 2. Rush University College of Nursing, Chicago, Illinois, United States, 3. Michigan State University, East Lansing, Michigan, United States*

Lesbian, gay, bisexual, transgender, and queer (LGBTQ) older adults are more likely to live alone and have less familial support, which disproportionately contributes to a reliance on long-term care facilities as they age. Best-practice guidelines supported by scholarly literature to care for LGBTQ older adults in long-term care settings do not exist. This review synthesizes literature about LGBTQ older adults in long-term care facilities and provides recommendations for best practice guideline development. Four electronic databases were searched in June 2019 for studies conducted between 2000 – 2019 related to caring for LGBTQ older adults in long-term care settings. An integrative literature review was completed on the twenty eligible studies. Findings showed that LGBTQ participants fear discrimination in long-term care leading to the invisibility of their identities. They recognize a need for increased staff training and the importance of community networks and facility preferences. Long-term care staff have mixed experiences with inclusive practices and complex views of LGBTQ older adults. They experience training deficits and have a need for more expansive training modalities. The recommendations offered by both LGBTQ participants and long-term care staff are to revise policies and forms as well as provide widespread training and education. LGBTQ participants recommend that their unique identities be recognized within long-term care while long-term care staff recommend leadership involvement to change culture and practice. This review provides evidence-based recommendations to promote equitable healthcare to the LGBTQ older adult population and calls to attention the need for long-term care settings to uniformly follow best-practices.

### INTERGENERATIONAL ENGAGEMENT IN RESIDENTIAL SETTINGS: A SCOPING REVIEW OF THE LITERATURE

Andrea Gardiola,[1] Raza Mirza,[1] Amanda Bull,[1] Christopher Klinger,[1] Jessica Hsieh,[1] and Samantha Peck,[2], *1. University of Toronto, Toronto, Ontario, Canada, 2. Family Councils of Ontario, Toronto, Ontario, Canada*

Intergenerational engagement provides a rich environment for people of different ages to come together and exchange life stories, skills, and knowledge. Today, intergenerational interactions are decreasing, however, these exchanges can have positive implications for seniors in residential care homes (RCHs) and younger persons. A scoping review following Arksey and O'Malley's five-step framework was conducted to investigate the impact of intergenerational engagement and programs (IGPs) on older adults in RCHs. A systematic search of ten electronic databases and hand search of references was carried out; thematic content analysis to established key themes. A total of 1,183 academic and grey literature sources were reviewed, with 66 full-text studies assessed for eligibility. Of these sources, 35 studies met inclusion criteria. Studies highlighted four main themes: 1. Types of IGPs, 2. Psycho-social benefits for older adults and improved status among elders with cognitive impairments, 3. Younger person benefits, suggesting reduced ageism and improved social and communication skills, and 4. Program recommendations, including the need for enthusiastic program facilitators, coordination between facilities, sensitivity training for younger persons, detailed advertisements, and appropriate activities for different age groups. Findings inform future practice and research, highlighting that IGPs are an effective strategy to alleviate negative health outcomes for seniors in RCHs. Future research is needed to evaluate long-term effects and further health outcomes. IGPs provide an opportunity to facilitate purposeful and reciprocal relationships between generations, fostering intergenerational understanding. By studying IGPs and intergenerational interactions, we can better determine practices that meaningfully engage elders in RCHs in Canada.

### IS HIGHER NURSE AIDE RETENTION ASSOCIATED WITH FEWER NURSING HOME ALLEGATIONS AND COMPLAINTS?

Katherine Kennedy, *Miami University, Oxford, Ohio, United States*

Consumer voices are often left out from assessments of nursing home (NH) quality. For this reason, consumer allegations and complaints against nursing homes were studied in relation to facility rates of nurse aide retention. Analyses involved means and frequencies, correlations, ANOVAs with Tukey correction to examine the independent and dependent variables (N=690). Four quartiles of retention were created. In the final models, medium, high, and extremely high retention facilities are compared to the low retention facilities. Negative binomial regressions were estimated on total, substantiated, and unsubstantiated counts of allegations and complaints. All regressions controlled for the same characteristics, including nurse aide empowerment, consistent assignment, administrator turnover, director of nursing turnover, average age of residents, and percent female. The correlation between retention and the dependent variables was negative and statistically significant (r=-0.11, p<.01). The ANOVAs showed that high retention NHs (61-72%) received significantly fewer allegations than low (0-48%) and medium (49-60%) retention NHs; they also received fewer unsubstantiated allegations, and fewer complaints, both substantiated and unsubstantiated. After controlling for other variables, each retention group was significantly related to having fewer allegations and complaints compared to the low retention NHs. Notably, high retention NHs received between 29 and 35% fewer allegations and complaints of all types. Unexpectedly, extremely high retention NHs had more allegations, complaints, and unsubstantiated allegations than high retention NHs. Policy and practice have a role to promote nurse aide retention, improve job quality, and ensure adequate support for this critical, in-demand workforce.

### SUPPORT FOR ADJUSTING THE INTENTIONS OF FAMILY MEMBERS AND USERS REGARDING CARE SERVICE USE: AIMED AT CARE MANAGEMENT

Kazutaka Masuda, *Mukogawa Women's University, Nishinomiya, Hyogo, Japan*

In Japan, there are key healthcare professionals for home nursing care for elderly people called Care Managers. The care manager coordinates the service while adjusting the family situation and the user's intentions. The purpose of this study was to examine the practical structure of support for adjusting the intentions of family members and users regarding care service use. Data from seven cases, where family members and users have different intentions regarding care service use, were analyzed using the grounded theory approach. The phenomenon of "confirmation of discrepancies" was discovered with six sub-categories: adjusting the intentions of users and their families, effort to restore relationships, expression of intention to refuse involvement, expression of desire for adjustment, arrangement of opportunities for adjustment of intentions, and appropriate service adjustment. Four patterns occurred in the process of "confirmation of discrepancies": smooth adjustment, restoration and promotion of mutual relationships, failure to reach an agreement, and negative feedback loops. These patterns were based on a combination of the care managers' degree of understanding strength, the managers' degree of insistence, the managers' degree of representation of mutual feelings, the degree of managers' prediction of life prospects, the degree of trust in care managers, and the degree of expression of family anxiety.

### THE PRESERVATION OF SPOUSAL AND PARTNER RELATIONSHIPS AMONG NURSING HOME RESIDENTS

Shanae Shaw,[1] and Ellen Csikai,[2], *1. Alabama State University, Calera, Alabama, United States, 2. The University of Alabama, The University of Alabama, Alabama, United States*

The decision to seek placement in a nursing home may be especially difficult for spouses or partners of the potential nursing home residents. Disruption of the attachment relationship following placement may influence the psychosocial well-being of nursing home residents. Although the responsibility of nursing home staff is to ensure psychosocial well-being, including awareness of the influence that separation can have on a spousal or partner relationship, little is known about services offered for the maintenance of spousal and partner relationships. This study was conducted to identify nursing home practices that had preservation of spouse/partner relationships as the goal. A mixed methods approach utilized both an online survey (81 respondents) with nursing home social workers in four Southern states and ten telephone interviews (from among the respondents). Survey results revealed that 49% of respondents' facilities had a written policy to preserve these relationships, however, only 22% reported having a specific formal program to carry this out. In the interviews, participants expanded on survey topics and shared that facilitating outings and encouraging participation in routine facility activities were commonly used to preserve relationships. Privacy was discussed as being important in maintaining relationships and also found to be a barrier to doing so. Nursing home social workers can utilize the results in the design and implementation of specific services to preserve relationships and maintain strong attachment between residents and their spouses/partners. These will be best carried out in a coordinated, collaborative manner among multiple disciplines within the nursing home setting.

### WHEN ADVOCATES ARE FORCED TO GO VIRTUAL: THE LONG-TERM CARE OMBUDSMAN RESPONSE TO COVID 19

H. Wayne Nelson,[1] F. Ellen Netting,[2] Mary W. Carter,[1] and Bo Kyum Yang,[1], *1. Towson University, Towson, Maryland, United States, 2. Virginia Commonwealth University, Richmond, Virginia, United States*

This study explores strategies used by the nation's Long-Term Care (LTC) Ombudsman Programs (LTCOP) to perform their grass roots, investigatory, sentinel defense advocacy during the near total COVID LTC lock out from March 13th 2020 through September 17th, when the "ban" was conditionally lifted. Our layered systematic searches by title, subject, and total text for unrestricted allusions to the LTCOP used the multi-disciplinary database Academic Search Ultimate that includes mass media. Selection criteria included print and broadcast news. Search keywords were "ombudsman" singly and with 10 other terms. This was augmented by reviewing the National LTC Ombudsman Resource Center (NORC) clearinghouse information website and by interviewing NORC staff. Resulting (172) media entries (92% print) were manually coded independently by a team of five, and iteratively reconciled according to a simple flat frame format to identify key words and associated themes. Four main LTCOP lock out strategies emerged: (1) virtual resident interventions (via phone, Skype, Zoom, in-facility allies); (2) public outreach (services provided, sharing COVID data and best practices, social isolation threats and mitigation efforts [window visits], need for volunteers); (3) systems advocacy (state/federal; CMS, legislative and other testimony about social isolation, CARES Act check problems, visitation issues; and (4) partnering with others (multi-agency planning groups, task forces, Zoom town halls, interstate information sharing). NORC interviews revealed that older LTCOP volunteers are seizing the COVID lock-out to retire undermining an already short-staffed network—so calls for volunteers were evident in about 25% of all stories regardless of any other focus.

### WHEN IS A NURSING HOME COMPLAINT REALLY A COMPLAINT? MAKING SENSE OUT OF INCREASED COMPLAINTS IN U.S. NURSING HOMES

Xiao Qiu, Jane Straker, and Katherine Abbott, *Miami University, Oxford, Ohio, United States*

Official complaints are one tool for addressing nursing home quality concerns in a timely manner. Similar to trends nationwide, the Ohio Department of Health (ODH) has noticed a trend in increasing nursing home complaints and has partnered with the Scripps Gerontology Center to learn more about facilities that receive complaints. Greater understanding may lead to proactive approaches to addressing and preventing issues. This study relies on two years of statewide Ohio nursing home complaint data. Between 2018 and 2019, the average complaint rate per 100 residents went from 6.59 to 7.06, with more than 70% of complaints unsubstantiated. Complaint information from 629 Ohio nursing homes in 2018 was linked with Centers for Medicare and Medicaid Services Nursing Home Compare data, the Ohio Biennial Survey of Long-Term Care Facilities, and Ohio Nursing Home Resident and Family Satisfaction Surveys. Using ordered logistic regression analyses, we investigated nursing home providers' characteristics using different levels of complaints and substantiated complaints. Findings suggest that providers with higher complaint rates are located in urban areas, had administrator and/or director of nursing (DON) turnover in the previous 3 years, experienced decreased occupancy rates, had reduced nurse aide retention, and received lower family satisfaction scores. Additionally, providers with administrator and/or DON turnover, and low family satisfaction scores are more likely to have substantiated complaints. Because increasing numbers of complaints are accompanied by relatively low substantiation rates, policy interventions targeted to specific types of providers may improve the cost-effectiveness of complaint resolution, as well as the quality of care.

### Session 9355 (Poster)

### LONG-TERM SUPPORTS AND SERVICES

### BURNOUT, COMPASSION SATISFACTION, AND PERSONALITY AMONG NURSING ASSISTANTS: WHO IS AT RISK?

Mallory Richert, *Xavier University, Cincinnati, Ohio, United States*

Nursing assistants often experience high rates of turnover and burnout, which may lead to poor resident care outcomes and quality of life, as well as continued staff shortages and increased workload for nursing assistants. This study examined personality correlates of burnout and compassion satisfaction among 100 nursing assistants employed as nursing assistants in long-term care (LTC) and hospitals throughout the United States. Participants completed the Professional Quality of Life Scale 5 (ProQOL 5) and the Big Five Inventory – 2 Short (BFI-2-S). There was a significant positive correlation between compassion satisfaction and agreeableness, and extraversion, and between burnout and neuroticism. Additionally, there were significant negative correlations between compassion satisfaction and neuroticism, and between burnout and agreeableness, and extraversion. These results indicate which nursing assistants may be more likely to experience burnout and may be at an increased risk of turnover. Specific interventions may be developed for such individuals to increase compassion satisfaction, reduce burnout, and reduce staff turnover. Furthermore, information regarding personality types of individuals at greater or lesser risk for burnout may be helpful for LTC administrators in the recruitment and hiring of nursing assistants, and thus may reduce rates of turnover. Resident care outcomes may also improve as nursing assistant hiring efforts are focused more on individuals who are less likely to experience burnout. Future researchers might investigate potential risk and protective factors for burnout and compassion satisfaction in nursing assistants.

### CARE AIDES' PERCEPTIONS OF CARING FOR NURSING HOME RESIDENTS WITH PAST PSYCHOLOGICAL TRAUMA

Trina Thorne,[1] Heather Titley,[1] Peter Norton,[2] Ruth Lanius,[3] and Carole Estabrooks,[1], *1. University of Alberta, Edmonton, Alberta, Canada, 2. University of Calgary, University of Calgary, Alberta, Canada, 3. University of Western Ontario, London, Ontario, Canada*

The dynamic interplay between dementia and psychological trauma can exert powerful effects on nursing home residents' behavioral symptoms and quality of life. Our objectives in this exploratory study were to assess care aides' perceptions of how often they worked with residents with past psychological trauma, the types of trauma encountered, and reasons for these beliefs. We conducted semi-structured cognitive interviews (n = 10) with care aides in June 2019 to inform the development of a trauma needs assessment (4 questions) that we included in a large survey of nursing staff (2019 - 2020). Care aides (n = 3761) were sampled from 91 randomly selected urban nursing homes stratified by health region, owner operator model, and size. We completed basic statistics and content analyses. Care aides identified residents they believed to have psychological trauma histories and provided reasons for their beliefs. Approximately 12% of the reported traumatic events were disclosed to staff. The most common, broad categories of trauma to emerge during analysis were abuse (40%) and war exposure (30%). Each had sub-categories. The most common categories of signs of trauma were re-experiencing symptoms such as flashbacks and nightmares (28%), and avoidance of specific triggers, such as water or intimate care (24%). The majority of the reported signs of trauma were persistent and distressing for staff and residents. Some behaviours assumed to be responsive behaviours of dementia may relate to traumatic stress symptomatology. Implementing trauma-informed supports for residents and care aides is essential to person-centred care and optimal quality of life.

### DEMENTIA CARE COACHING: A PILOT TO EVALUATE ACCEPTABILITY AND FEASIBILITY IN CARE COMMUNITIES

Lorna Prophater,[1] Boeun Kim,[2] Basia Belza,[2] Sarah Cameron,[3] and Sam Fazio,[1], *1. Alzheimer's Association, Chicago, Illinois, United States, 2. University of Washington, Seattle, Washington, United States, 3. Alzheimer's Association, Datyon, Ohio, United States*

The Alzheimer's Association (AA) Dementia Care Practice Recommendations (DCPR) outline ten recommendations to achieve quality care with a person-centered focus. The AA has developed tools to assist care communities (CC) to evaluate their status within the recommendations by working with a trained coach to maximize adoption and implementation of these recommendations. The purpose of this pilot was to evaluate the acceptability and feasibility of pairing trained DCPR coaches with CC teams to implement the DCPR tools.

Seven CCs were recruited and four received the DCPR overview and self-assessment. Of the four CC, one withdrew and did not receive the intervention. The remaining three were located in a suburban area, nonprofit, and with memory care units. Data was collected from November 2019 through March 2020. Nine CC staff participated with a mean age 35.8 years and had worked for 11.8 years. Baseline mean scores on the Organizational Readiness to Implementing Change (ORIC) scale were 4.6 for the commitment domain and 4.4 for the efficacy domain. Mean scores on the Nursing Home Employee Satisfaction Survey were high. Sixty-nine percent of CC participants were satisfied with their jobs (greater than 4). Findings from mid-project interviews with the coaches revealed difficulty with scheduling appointments, significant efforts needed to get the "right" people at the table and need for the DCPR tools to be more user-friendly. No post-intervention results were collected due to closing of the CCs to visitors due to COVID. The DCPR tools shows promise and are being evaluated in additional CCs.

### DEVELOPMENT OF AN ANIMAL-ASSISTED ACTIVITY/THERAPY DOG CHECKLIST FOR LONG-TERM CARE FACILITIES

Karen Dunn,[1] Amy Johnson,[1] and Melissa Winkle,[2], *1. Oakland University, Rochester, Michigan, United States, 2. American Occupational Therapy Organization, North Bethesda, Maryland, United States*

Animal-assisted activities (AAA) and therapy standards of practice have been published to protect the well-being of animals, animal handlers, and the special populations of patients that benefit from this mode of treatment. Inconsistencies among practice standards with concerns surrounding the topics of dog welfare, human well-being, and zoonotic transmission have been reported. The purpose of this qualitative research study was to review published AAA and therapy standards with older adult populations for best practices, conduct focus group sessions with caregivers from long-term care facilities that allow therapy dog visitation, and synthesize findings into an AAA checklist to be used by long-term care facility decision-makers when interviewing or bringing in therapy dog teams. Comparative analyses utilizing a systematic and sequential approach was used to analyze the data from the focus group sessions. Due to the COVID-19 pandemic, only two focus group sessions at one long-term care facility were conducted resulting in a total of 15 caregivers. Four themes emerged from the data: promotes positive mood, essential resident screenings, caregiver roles, and memory aides. Relevant themes and AAA and therapy standards and guidelines were then combined in the development of the AAA/Therapy Dog Checklist. Administrators may find having a user-friendly AAA/therapy dog checklist a useful tool that can be used when interviewing therapy dog teams to ensure future dog therapy experiences will be positive and safe. The safety and well-being of residents in long-term care facilities and animals are essential to promote positive health outcomes for both populations.

### DISTRESS BEHAVIOR CONVERSATIONS: SUPPORTING WHOLE PERSON WHOLE TEAM RESPONSES IN VA COMMUNITY LIVING CENTERS

Julia Loup,[1] Kate Smith,[1] Susan Wehry,[2] Sharon Sloup,[3] Jennie Keleher,[3] Princess Nash,[3] Christine Hartmann,[4] and Andrea Snow,[3], *1. University of Alabama, Tuscaloosa, Alabama, United States, 2. University of New England College of Osteopathic Medicine, Biddeford, Maine, United States, 3. Tuscaloosa VA Medical Center, Tuscaloosa, Alabama, United States, 4. VA Bedford Healthcare System, VA Bedford Healthcare System, Massachusetts, United States*

Resident distress behavior, a prevalent challenge in long-term care, contributes to resident morbidity, staff burden, and turnover. We describe an education model developed in the Veterans Administration (VA) Community Living Centers (CLC) through a CONCERT (VA CLCs' Ongoing Center for Enhancing Resources & Training) quality improvement series. The Distress Behavior Conversation (DBC) uses a team meeting structure and process. Informed by unmet need and relational coordination theories, it guides the whole team, inclusive of interdisciplinary team members and front-line staff with resident contact, through a collaborative problem-solving action-planning discussion. DBC uses facilitated round-robins to identify potential resident behavior causes and individualized solutions. DBC supports the team in maintaining whole person and whole team mindsets, thus challenging the narrower medical model of discipline-specific clinical mindsets and staff level hierarchies. Over two years we have co-created and refined DBC through trainings and team debriefings with over 80 CLCs. Care teams reported "aha" moments during DBCs their thinking shifted ("we are now looking at the REAL why"; "we went from asking, how did he fall? to, why did he fall?; "tended to try to treat falls in a standardized way, [but] when you focus on a specific person you get to focus on HIS needs"; "personal information about the Veteran is the 5th vital sign!"). Teams additionally reported reduced strain and improved collaborative thinking ("I feel better about what I'm doing...more motivated to keep going!; "Now I see it is a team approach – don't have to do it by myself.").

### LEARNING BEST PRACTICES FOR EDUCATING A CAREGIVING WORKFORCE

Sweta Tewary,[1] Yumna Indorewala,[2] Nicole Cook,[3] Naushira Pandya,[3] Sashah Damier,[4] and Assma Twahir,[3], *1. Nova Southeastern University, Fort Lauderdale, Florida, United States, 2. NSU, NSU, Florida, United States, 3. NSU, Davie, Florida, United States, 4. NSU, Davie,FL, Florida, United States*

It is well established that the health professional workforce is not adequately prepared to meet the demands of an aging older population. Caregivers are often the backbone supplemental workforce for seniors, providing daily care with assistance with activities of daily living, with little training. Part of the mission of the South Florida Geriatric Workforce Enhancement Program (SFGWEP) is to support and empower caregivers through community based training programs. Between January 1 2020 to January 31, 2021 SFGWEP provided education to more than 340 caregivers on topics related to opioid use, effective communication with individuals with dementia and other topics. Attendees responded to a short evaluation survey, which included three multiple-choice questions on if attending was a good use of their time, if they gained knowledge and if they plan to apply material, and two open-ended questions to identify opportunities for improvement in future trainings. Responses were overwhelmingly positive (>98% for multiple-choice questions.) There were also three open-ended questions that were analyzed using a modified thematic approach. The three questions covered what attendees learned, what they wanted to learn more about and suggestions for improvement. Analysis suggests that attendees plan to be more mindful about communication (e.g. improve eye contact, listen more) and that they want more information on neurocognitive disorders and resent research, including psychological changes due to disease and medication side effects. In terms of improvement, attendees said the program should allow more time for questions and should use more engaging materials (polls, posters, flyers and case studies).

### MULTIPLE STREAMS ANALYSIS OF THE LONG-TERM CARE INSURANCE IN SOUTH KOREA: UNDERSTANDING POLICY CHANGES (2008 - 2014)

Mijin Jeong, *University of Kansas, LAWRENCE, Kansas, United States*

The Long-Term Care Insurance (LTCI) Act in South Korea was enacted in 2008 to improve the quality of life of older adults by promoting better health and to mitigate the burden of care on family members. In 2014, the Enforcement Decree for the LTCI Act was revised to broaden criteria for eligible recipients of LTCI-related services and care. This policy analysis seeks to explore the political circumstances under which the Act was formed and how social environmental factors had evolved to revise the LTCI Act using a multiple streams policy analysis framework. A combination of factors influenced the status of LTCI policy agenda, including shifts in aged demographic structure and increasing medical expenditures. From the Korean National Dementia Plan, a pilot project of dementia care was conducting to prove the efficiency of dementia care service. While the Korean Senior Citizens Association (KSCA) was less successful gaining press attention around dementia care, the presidential election and candidates' election pledges were key factors to suddenly open the opportunity to extend the recipients for dementia care. The process through which the LTCI Act was revised and expanded showed the importance of the political environment associated with the election. Based on the recognition of LTIC policy agenda and already testing the efficiency of dementia care services, the election leaded to revision of LTCI Act and it quickly diffused by the new administration. From the revision of LTCI, international policymakers and scholars should recognize how the political events might use the policy for older adutls.

### PERSONAL CARE AIDES IN RESIDENTIAL CARE AND ADULT DAY CENTERS: DIFFERENCES IN TRAINING, BENEFITS, AND ROLES

Manisha Sengupta,[1] and Farida Ejaz,[2], *1. CDC/NCHS, Hyattsville, Maryland, United States, 2. Benjamin Rose Institute on Aging, Cleveland, Ohio, United States*

Personal care aides (PCAs), along with other direct care workers, provide the hands-on care, including help with activities of daily living for individuals receiving care in residential care communities (RCC) and adult day services centers (ADSC). Recruitment and retention of such workers is a challenge as low pay, inadequate training, unsatisfactory roles and lack of benefits contribute to turnover. Using data from the 2018 National Study of Long-Term Care Providers, the only nationally representative data about PCAs in RCCs and ADSCs, this study will assess differences in training hours, benefits, and work roles among PCAs in these settings. About 76% of RCCs and 66% of ADSCs employed aides. On average, PCAs received 32 hours and 51 hours of initial training in ADSCs and RCCs, respectively. Results from bivariate analyses (accounting for complex survey design), showed that benefits received by PCAs varied by sector. A higher percentage of PCAs in ADSCs than in RCCs received health insurance for employees (60% vs. 46%), and pension (51% vs. 40%). About 51% of ADSCs and 46% of RCCs reported that PCAs rarely or sometimes attended care plan meetings. Further, 11% of RCCs and 15% of ADSCs reported that aides rarely or sometimes worked with the same care recipient. This overview of PCA activities, training and benefits may provide insights into approaches to improve the retention of PCAs and subsequently the quality of care provided across sectors.

### RESILIENCE AND MENTAL HEALTH AMONG MALE OLDER ADULTS IN EXTREME POVERTY IN LONG-TERM CARE FACILITIES IN RURAL CHINA

Ying Ma,[1] and Patrick Leung,[2], *1. University of Houston, Richmond, Texas, United States, 2. University of Houston, Houston, Texas, United States*

Older adults in extreme poverty refer to "three-noes people": no working ability, no income source, and no children or legal supporters. They are eligible for a government-funded welfare system called "Five Guarantee system". The majority of residents in rural welfare institutes are male older adults in extreme poverty. Research demonstrates that resilience is a critical factor in shaping health. This study aims to examine the association between resilience and mental health for male older adults in extreme poverty, and its differences in young-old (60-69 years), old-old (70-79 years), and oldest-old groups (≥80 years). A cross-sectional study was conducted with 1,427 eligible subjects in rural long-term care facilities from Anhui province in China during 2019, with a response rate of 77.4%. Resilience was measured by the Chinese version of Connor-Davidson Resilience Scale, including three subscales of optimism, strength and tenacity. Mental health was assessed using General Health Questionnaire-12. A MANOVA test revealed a significant difference among age groups on three subscales of resilience [Pillai's Trace=.023, F (6,1486) =2.709, p=.013, ηp2=.012]. Tukey Post hoc indicated the oldest reported significantly lower levels of strength, optimism, and tenacity compared to the other two groups. A multiple logistic regression identified a significant negative association between resilience and mental disorder for the old-old (OR=0.95, 95%CI:0.93-0.97) and oldest-old (OR=0.93, 95%CI: 0.88-0.99) groups. Our findings identified th differences in the link between resilience and mental health within the three different age groups. Resilience-training programs to improve mental health would require targeting specific subscales of resilience for each group.

### ROBOTIC-ASSISTED VIRTUAL CARE

Lillian Hung, *University of British Columbia, Vancouver, British Columbia, Canada*

Heightened isolation during the pandemic has exacerbated the stress, anxiety, and adverse consequences through the loss of family connections older people experience in LTC. Heavy workload and staffing shortage limit staff's capacity to assist residents in accessing regular virtual visits. Using a Collaborative Action Research (CAR) approach, this project aims to assess the implementation of a telepresence robot, Double 3 to help residents connect with their families. CAR allows careful planning of implementation with stakeholders (patient and family partners, staff, and decision-makers), tailoring adaption to the complex LTC environment. We will program path planning to allow efficient movement between target destinations (residents' rooms) and the charging dock. For example, the robot will go to a resident's room every morning or evening to help the resident to make a virtual call with family. The project involves three phases (a) Observe and Reflect, (b) Act and Adapt, (c) Evaluate. We work with two Canadian LTC homes in British Columbia to investigate feasibility and acceptability. CAR emphasizes research with, rather than research on people. Meaningful engagement with patient and family partners, frontline staff, and decision-makers at each site throughout the whole project will ensure the project will meet the local needs. Anticipated resident outcomes include improved quality of life, mood, perceived loneliness, perceived social support, and acceptance. Anticipated staff outcomes include perceived ease of use, and acceptability.

### ROOM TYPE AND SOCIAL COHESION IN SENIOR-LIVING FACILITIES

ZHE WANG, *Henan University, Henan University, Kaifeng, Henan, China (People's Republic)*

Private rooms are generally preferred by senior-living residents. However, having roommates may help residents to build and maintain social networks in the facility, leading to promoted social cohesion and mental health. The differences in social cohesion among senior-living residents who resided in private or shared rooms need investigation. This research collected empirical data from eight senior-living facilities in Beijing and Shanghai, China. Focusing on social cohesion, room type, and personal factors, on-site questionnaire surveys recruited 345 residents receiving independent living, assisted living, or nursing care services. Facility environments were measured and rated by researchers through on-site observation. Controlling for personal and facility factors, ANOVA tests were employed to investigate the differences in social cohesion among residents who resided in private, double, or triple rooms. Room type was found significant to the social cohesion in assisted-living and nursing care residents. At the assisted-living level, compared with private and double rooms, triple rooms were more likely to contribute to social cohesion ($p<=0.01$). At the nursing care level, residents with less numbers of roommates (private versus double and double versus triple) had stronger social cohesion ($p<=0.05$). At the independent-living level, no factors significant to social cohesion were found. These findings can be used to guide the new design, renovation, and modification of senior-living environments to promote social cohesion. Recommendations for future research and practice implications for senior-living professionals and facility designers are discussed. Senior-living facilities should be built to be social-friendly through design and planning and within the context of its cultural characteristics.

### THE INFLUENCE OF STAFF-RESIDENT INTERACTIONS ON RESISTIVENESS TO CARE BEHAVIOR IN ASSISTED LIVING

Rachel McPherson,[1] Barbara Resnick,[2] and Elizabeth Galik,[2], *1. University of Maryland Baltimore and Baltimore County, Catonsvile, Maryland, United States, 2. University of Maryland, Baltimore, Maryland, United States*

Resistiveness to care (RTC) is a behavioral and psychological symptom of dementia that is common among dementia residents in assisted living facilities. RTC encompasses verbal and nonverbal behaviors that oppose care, such as crying, grabbing, hitting, or yelling, among many other resistive behaviors. The quality of care interactions which can be positive, neutral or negative, have been associated with increased RTC. The purpose of this study was to test the association between quality of care and RTC. This was a secondary data analysis using baseline data from the Function-Focused Care for Assisted Living Using the Evidence Integration Triangle (FFC-AL-EIT) implementation study. Controlling for cognition, age, gender, medication use, and comorbidities, it was hypothesized that quality of care interactions would be associated with resistiveness to care. A linear regression analysis was conducted to test the hypothesis. The sample included 794 participants the majority of whom were white women with a mean age of 89.48 (SD=7.61). The mean RTC was .09 (SD=.41, range 0-13) and the mean quality of care interactions were 5.96 (SD=1.44, range 0-7). Based on the regression analysis there was no significant association between quality of care and RTC. These findings may be due to the high quality of care provided and limited RTC in this sample. Ongoing research is needed, however, to continue to explore these relationships and assure that all RTC is being reported among staff and that there is no evidence of negative quality of care interactions in these settings.

### Session 9360 (Poster)

### MENTAL HEALTH (BSS POSTER)

### AGE, EMOTION REGULATION, AND WELL-BEING AFTER THE 2016 FLOOD

Katie Cherry,[1] Piper Bordes,[2] Matthew Calamia,[3] and Emily Elliott,[3], *1. Louisiana State University, Louisiana State University/Baton Rouge, Louisiana, United States, 2. Louisiana State University, Louisiana State University, Louisiana, United States, 3. Louisiana State University, Baton Rouge, Louisiana, United States*

In 2016, catastrophic flooding in south Louisiana claimed 13 lives with billions of dollars in damage to homes and communities in the decade after Hurricanes Katrina and Rita devastated the US Gulf Coast. In this study, we tested the inoculation hypothesis which predicts that older adults will be less distressed than younger adults due to their prior experience with severe weather events. Participants were 218 predominately middle-aged and older adults who varied in current and prior flood experience: less than half (40%) did not flood in 2016, 31% had flood damage, and 29% had relocated permanently inland after catastrophic losses in the

2005 Hurricanes Katrina and Rita and they flooded again in 2016. Depression symptoms were assessed with the 9-item Patient Health Questionnaire (PHQ-9). Emotion regulation strategies were measured using the Cognitive Emotion Regulation Questionnaire-Short Form. Results indicated that the older adults had fewer symptoms of depression and were less likely to report self blame for flood-related adversities compared to younger adults. The two age groups did not differ significantly on the emotion regulation strategies of acceptance, reappraisal, positive refocusing, other blame, and perseveration. Age was inversely associated with symptoms of depression and the maladaptive strategies of self blame for flood-related misfortune and perseveration over losses. These data support the inoculation hypothesis and suggest that prior severe weather experiences, which are likely for older adults living in hurricane prone areas, are important for post-flood resilience. Implications of these findings for disaster planning and age-sensitive interventions to mitigate adversity are considered.

### BEST PRACTICES FOR WORKING WITH LATINX OLDER ADULTS IN MENTAL HEALTHCARE

Kendall Weber,[1] and Lisa Stone,[2], *1. University of Colorado at Colorado Springs, University of Colorado at Colorado Springs, Colorado, United States, 2. University of Colorado Colorado Springs, Colorado Springs, Colorado, United States*

**Background:** The number of older adults in the United States is growing rapidly. The percentage of individuals from ethnic minority groups that make up this population is also rapidly increasing, with Latinx older adults comprising the fastest growing subgroup. However, Latinx older adults historically underutilize mental health services, in large part due to the lack of culturally sensitive and informed care provided by mental health professionals (de Guzman et al., 2015). However, to date, comprehensive, evidence-based best practices for mental healthcare for Latinx older adults do not exist.

**Method:** A literature review was conducted of research on the developmental, social, cognitive, biological, and affective bases of behavior among Latinx older adults.

**Results:** Taking an integrated, evidence-based psychological approach with cultural considerations, we found that the literature could broadly be organized into six best practice guidelines. We propose assessing for and incorporating the following topics into mental health treatment of Latinx older adults: immigration status, acculturation, attitudes towards mental health, physical and cognitive health disparities, discrimination, and unique preferences for care structure in later life.

**Discussion:** These guidelines are intended to represent basic principles to incorporate into practice and do not represent an exhaustive list of factors to consider for a heterogenous group of older adults. Instead, the six, empirically-based guidelines proposed in this study can serve as a starting point for increasing mental health providers' awareness of the unique experiences of Latinx older adults, with the aim of improving the experience of this historically underserved population in mental healthcare treatment.

### COGNITIVE CHANGES IN OLDER ADULTS FOLLOWING A STEPPED CARE INTERVENTION FOR LATE-LIFE DEPRESSION

Anna Y Zhang,[1] Shiyu Lu,[2] Tianyin Liu,[2] Dara KY Leung,[2] Gloria HY Wong,[2] and Terry YS Lum,[2], *1. The University of Hong Kong, The University of Hong Kong, Not Applicable, Hong Kong, 2. The University of Hong Kong, Hong Kong, Not Applicable, Hong Kong*

Older adults with depression may manifest cognitive decline and treating depression may maintain or improve cognition. However, cognitive outcomes could be overlooked in non-pharmacological interventions for depression. This analysis investigated cognitive changes in a stepped-care intervention (Clinical Trial ID: NCT03593889) and the potential association with individual depressive symptom change. The community-dwelling older adults at risk of or with depressive symptoms without significant cognitive impairment (n=802) were assigned to intervention group (n=644) and control group (n=138). Depressive symptoms and cognitive functions were measured using Patient Health Questionnaire-9 and Cognitive Montreal Assessment-5 minutes protocol, respectively. Paired-t-Test showed significant improvements in overall cognition and attention in both intervention and control groups, but the improvements of language fluency (Intervention: MD=-0.51 $p<0.01$; control: MD=0.14, $p=0.500$) and orientation (Intervention: MD=-0.22 $p<0.05$; control: MD=-0.11, $p=0.229$) only displayed in intervention group. As control group had better cognition at baseline, linear mixed-effects model analysis was used to compare between-group difference. Intervention group had no significant cognitive improvement after adjusting the covariates but a potential improvement in language fluency (Coef. =0.442, SE=0.247, $p=0.074$). A linear regression analysis in intervention group indicated that reduction of concentration problem ($\beta=0.106$, $p<0.05$) and retardedness ($\beta=0.117$, $p<0.01$) under the symptomatology of depression were associated with the improvement of language fluency. In this group of older persons without significant cognitive impairment, there is no clear evidence of global cognitive benefits in a stepped care depression intervention, although there may be improvements in certain cognitive domains, which may be related to improvements in cognitive aspects of depression.

### COMPARING COGNITIVE AND PHYSICAL LIMITATIONS AS PREDICTORS OF DEPRESSION AMONG OLDER ADULTS

Clara Scher,[1] Takashi Amano,[2] and Lenna Nepomnyaschy,[3], *1. Rutgers University, New York, New York, United States, 2. Rutgers University-Newark, Newark, New Jersey, United States, 3. Rutgers University, New Brunswick, New Jersey, United States*

Depression in older adults is associated with loss of functioning and increased mortality. While many factors contribute to depression among this population, activities of daily living (ADL) limitations and cognitive impairment have been identified as key risk factors. However, no study, to our knowledge, has examined the extent to which physical and cognitive limitations independently and jointly contribute to the risk of depression. The current study describes the prevalence and compares the independent and joint associations of these limitations with depression in a nationally representative sample of adults aged 51 and older in the US. Analyses are based on a sample of 17,044 repeated observations on 6,636 unique primary respondents from three waves of pooled data from the Health and Retirement Study. We estimate linear and logistic multivariate regression models investigating the association between ADL limitations (any limitation on Katz ADL scale), cognitive impairment (<12 on the TICS-27 scale), and depressive symptoms (8-item CES-D), controlling for a standard set of socioeconomic and health factors. First, we find that 66% of respondents report no limitations, 16% report only cognitive impairment, 11% report only ADL limitations, and 7% report both types of limitations. Multivariate analyses suggest that ADL limitations have a much stronger association with depression compared to cognitive impairment, and this association is robust across alternative specifications. In next steps, we will take advantage of the longitudinal nature of these data to estimate changes in these characteristics over time and within individuals and explore heterogeneity in associations across relevant groups.

### DEPRESSIVE AND ANXIETY DISORDERS IN ALCOHOL USE AND RELATED PROBLEMS AMONG OLDER AFRICAN AMERICANS

Tomorrow Arnold, *University of Tennessee at Chattanooga, chattanooga, Tennessee, United States*

The older African American (AA) population is expected to triple by 2050; however, research on depression, anxiety, and alcohol use among older AAs is lacking. Current mental health and substance use studies involving older AAs often focus on between-race differences, frequently comparing AAs to Whites, without addressing within-group variation in the former. As such, little is known about the associations between depression, anxiety, alcohol use and related disorders in this population. The present study used data from 2016-2017 Midlife in the United States (MIDUS) Milwaukee 2 to examine whether depressive and anxiety symptoms and disorders are associated with alcohol use and alcohol problems among 274 African Americans aged 50 to 93 (62.8% women). Of the sample, 9.5%, 3.6%, and 6.6% met criteria for depression, generalized anxiety (GAD), and panic disorders, respectively. About 8.1% had drank heavily and 18.5% binge drank in the past month with 10.2% reporting alcohol problems in the past year. Those with depression and those with panic disorder were more likely to be heavy drinkers; while only those with panic disorder were more likely to be binge drinkers. Meeting criteria for depression or panic disorder but not GAD disorder were both more likely to have had alcohol-related problems than those not meeting criteria. Only panic disorder was associated with increased likelihood of drinking more than intended in the past year. Preliminary exploratory findings suggest that the associations between depressive symptoms, type of anxiety disorder, alcohol use, and problems varied by gender.

### DEPRESSIVE SYMPTOMS MEDIATE THE INFLUENCE OF FIBROMYALGIA STATUS ON PHYSICAL PERFORMANCE AND BMI IN AGING ADULTS

Dylan Serpas,[1] Laura Zettel-Watson,[2] and Barbara Cherry,[2], *1. California State University, Fullerton, Bonita, California, United States, 2. California State University, Fullerton, Fullerton, California, United States*

Fibromyalgia is a chronic pain condition that is frequently accompanied by comorbid conditions, including depression. Depression is associated with reduced physical functioning and health disproportionately affecting middle-aged and older adults with fibromyalgia. This study examined depressive symptoms as a mechanism through which FM status is associated with BMI and physical performance among adults in mid-to-late-life. Participants included 250 community-dwelling middle-aged and older adults (82% female) with (59%) or without (41%) fibromyalgia (M age = 64.44, SD = 9.16). Depressive symptoms were measured using the Beck Depression Inventory-II, BMI was objectively assessed, and physical performance was measured using the Fullerton Advanced Balance scale, 6-Minute Walk Test, 30-Second Chair Stand, and 8-Foot Up and Go Test. Physical performance measure analyses were adjusted for age. Asymptotic mediation analyses revealed that fibromyalgia status was indirectly associated with higher BMI (95% CI [.18, 16.74]), and poorer performance in the Fullerton Advanced Balance (CI [-2.93, -1.24]), 6-Minute Walk Test (CI [-73.75, -35.35]), 30-Second Chair Stand (CI [-2.45, -1.16]), and 8-Foot Up and Go test (CI [.35, .92]) via depressive symptoms. Participants with fibromyalgia reported greater depressive symptoms which was subsequently associated with greater BMI and reduced physical performance. Findings support depressive symptoms as one factor through which fibromyalgia status is associated with higher obesity risk and reduced physical function in middle-aged and older adults with fibromyalgia. This study supports fibromyalgia status as a critical consideration when evaluating the health and disability risk of aging adults.

### DIFFERENT STATISTICAL APPROACHES TO DEVELOP A GUIDELINE FOR IMPROVEMENT OF CAREGIVER'S MENTAL HEALTH

Amanda Leggett,[1] and Hyun Jung Koo,[2], *1. The University of Michigan, Ypsilanti, Michigan, United States, 2. University of Michigan, Ann Arbor, Michigan, United States*

Caregiver burden is common, and improvement of caregivers' mental health could lead to better quality of care and well-being for both caregivers and care recipients. We investigate ways to develop a guideline to enhance caregiver's mental well-being by applying and comparing regression tree and ensemble tree models. Data comes from the 2017 National Health and Aging Trends Study and National Study of Caregiving. Dementia caregivers' (n=945) aspects of caregiving, care activities, support environment, and participation along with basic demographics and health are considered. First, insignificant predictors are preselected using linear regression with backward selection, which will not be included in the tree models. Using the predetermined predictors that are not excluded in the backward selection method, regression tree and ensemble tree models are generated to predict emotional difficulty of caregivers. The regression tree with the preselected predictors predicts caregivers with low to moderate levels of overload and high levels of joy being with their care recipient associated with the lowest level of emotional difficulty. On the other hand, if caregivers have high levels of overload and low to moderately high levels of positive affect, this is linked with the highest level of emotional difficulty. Ensemble tree models showed similar results with lower error measures. Using tree-based methods can help determine the most important predictors of caregiver mental health. Easily interpretable results with applicable decision rules can provide a guideline for intervention developers.

### EFFECTIVE RECRUITMENT STRATEGIES FOR HOME-LIVING VULNERABLE OLDER ADULTS WITH DEPRESSION INTO A PSYCHOTHERAPY RCT

Eva-Marie Kessler,[1] Fee Hoppmann,[1] Julie L. O'Sullivan,[2] Paul Gellert,[3] and Christina Tegeler,[4], *1. MSB Medical School Berlin, Berlin, Berlin, Germany, 2. Charité – Universitätsmedizin Berlin, Charité – Universitätsmedizin Berlin, Berlin, Germany, 3. Charité – Universitätsmedizin Berlin, 10117, Berlin, Germany, 4. MSB Medical School Berlin, MSB Medical School Berlin, Berlin, Germany*

Objectives: Vulnerable older adults, such as physically impaired or care-dependent individuals, are vastly underrepresented in psychotherapy research. Improving their inclusion in randomized controlled trials is necessary to determine the effectiveness of psychotherapy in this population. This study is the first to systematically evaluate strategies to recruit home-living vulnerable older adults with clinically significant depression into a large randomized controlled psychotherapy trial. Potential participants were approached directly (self-referral) or via cooperation with gatekeepers (gatekeeper-referral).

**Methods:** The initiator of the first contact with the study team and successful recruitment strategies were recorded. Referral strategies were compared with respect to number of inquiries and inclusion rates; study personnel's time investment; and participant characteristics (sociodemographics, functional and cognitive status, depression and anxiety scores).

**Results:** Most of the N=197 participants were included via gatekeeper-referral (80.5%, 95%CI=[74.9%,86.1%], but time investment for gatekeeper-referrals was five times higher than for self-referral by media reports. Clinical psychologists and medical practitioners referred the largest proportion of participants (32.3% each) and referral by medical practitioners led to highest inclusion rates (55.6%; $\chi^2(3)=8.964$, $p<.05$). Most participants were referred from a hospital setting (50.3%), whereas referral numbers by medical practices were low (15.9%). Participants who initiated the first contact themselves had higher inclusion rates and were less functionally and cognitively impaired.

**Conclusions:** Including home-living vulnerable older adults into psychotherapy trials requires simultaneous implementation of diverse recruitment strategies. Medical practitioners and psychologists, especially in hospitals, are the most effective recruitment strategy, but self-referral via media is most cost-efficient in terms of time investment.

### EMOTION REGULATION PROFILES OF DEVELOPMENT OF DEPRESSIVE SYMPTOMATOLOGY: A LONGITUDINAL STUDY.

Teresa Paniagua,[1] Virginia Fernández-Fernández,[2] and MªÁngeles Molina Martínez,[3], *1. Universidad Europea de MAdrid, Majadahonda, Madrid, Spain, 2. Universidad Nacional de Educación a Distancia, Madrid, Madrid, Spain, 3. Universidad Francisco de Vitoria, Madrid, Madrid, Spain*

**Introduction:** COVID-19 pandemic has had a psychological impact on the eldest population. The aim is to analyse whether there are differences depending on the emotional regulation profile shown by a group of older people 6 months before the pandemic and the depressive symptomatology of these people at the same time, during home confinement and 8 months later.

**Method:** Longitudinal study, sample of people over 65, three evaluation measures: WAVE1 (6 months before COVID-19,N=305;M=73.63;58.9% women), WAVE2 (house confinement;N=151;M=73.14;59.6% women) and WAVE3 (8 months later;N=91;M=72.62;64.70% women). We measured depressive symptomatology (CES-D; Radloff, 1977) and nine emotional regulation strategies (CERQ-S; Garnefski et al., 2001; Carvajal et al., 2020), with which 3 clusters were preset (after dendogram inspection and K means analysis). Three mean difference analyses (one-factor ANOVA) were performed taking as factor profiles and as outcomes variables depression in each wave.

**Results:** profile 1, people use adaptive cognitive-emotional regulation strategies; profile 2, those with low levels of strategies (adaptive and maladaptive); profile 3, high scores in maladaptive strategies. Statistically significant differences between profiles 1 and 3, in the pre-confinement depression variable (F'2,91=6.18;p=.00) and during confinement (F'2,91=4.02;p=.02). Profile 3 higher depressive symptomatology ($\bar{x}1=17.16;\bar{x}2=16.80$) than 1 ($\bar{x}1=8.41;\bar{x}2=9.65$). Differences between profile 1 and 2 and 3 in depression 8 months after confinement (F'2,91=4.02;p=.02). Profile 1 lower levels of depression ($\bar{x}3=98.00$) than 2 ($\bar{x}3=15.78$) and 3 ($\bar{x}3=14.20$). Profiles explain 12.3%, 8.4% and 12.5% of the depression variance in each wave.

**Conclusions:** a "protected profile" (1), a "medium-term vulnerable profile" (2) and a "vulnerable profile" (3) to the development of depressive symptomatology.

### EMPIRICALLY EVALUATED SUICIDE PREVENTION PROGRAM APPROACHES FOR OLDER ADULTS: A SYSTEMATIC REVIEW

Vivian Miller,[1] Noelle Fields,[2] Ling Xu,[2] Marta Mercado-Sierra,[3] and Marissa Wallace,[2], *1. Bowling Green State University, Bowling Green, Ohio, United States, 2. University of Texas at Arlington, Arlington, Texas, United States, 3. Texas A&M Commerce, Commerce, Texas, United States*

Suicide is a serious public health concern, particularly for individuals in later life. Studies suggest that greater attention to suicide prevention programs for older adults is needed as well as continued research related to interventions with older adults at risk of attempting suicide. A systematic review of the literature on suicide prevention treatment and effectiveness is fundamental to assessing existing services and developing new programs and practice standards. This systematic review of the literature extends an earlier and well-cited systematic review (1966-2009) by examining articles published between 2009 and 2021 with a focus on what types of empirically evaluated suicide prevention programs effectively prevent and reduce suicidality in older adults. The Preferred Reporting Items for Systematic Reviews and Meta-Analyses guidelines were used to gather the appropriate extant research and improve reporting accuracy. A three-stage review guided the selection of the articles. At stage one, titles were screened, which excluded 284 articles based on the inclusion criteria. Second, after a full review of each abstract, a final 14 articles remained for full-text review. Lastly, three independent researchers reviewed each of the full-text articles, and six articles were excluded. The final sample includes eight articles (N=8). The articles were categorized into three types of programs: 1) primary and home health care, 2) community-based outreach, and 3) counseling. Following a description of the articles, the authors assessed each study using the GRADE rating system. Findings underscore the critical need for evidence-based suicide prevention programs for older adults. Implications for future research are offered.

### EXAMINING MEASUREMENT INVARIANCE OF DEPRESSION AMONG MALE AND FEMALE IN CHINESE OLDER ADULTS

Roberto Melipillán,[1] and Mengyao Hu,[2], *1. Universidad del Desarrollo, Concepcion, Bio-Bio, Chile, 2. University of Michigan, Ann Arbor, Michigan, United States*

Depression of older adults is an important public health concern. With the increasing popularity of cross-cultural research and comparison studies, researchers are facing a difficult problem: responses to the depression scales obtained from different population groups may not always be comparable. This study examines the measurement invariance of the 10-item version of the Center for Epidemiological Studies Depression (CES-D) Scale across male and female in Chinese older adults. Data are drawn from the baseline wave of the China Health and Retirement Survey (CHARLS), a national survey conducted biennially with a sample of the Chinese population who are 45 years of age or older. The final sample size includes 15,977 respondents; 53.2% of whom are female. The mean age for the sample is 58.3 (SD = 10.2). Measurement invariance (MI) tests based on Multiple Group Categorical Confirmatory Factor Analyses (MGCCFA) was performed. Results show that full scalar model was not supported, and question items invariant across groups were identified. These results indicate that any mean comparisons of CES-D across Chinese male and female older adults not accounting for the noninvariance in the items could be biased, highlighting the importance of performing MI tests before conducting mean comparisons across groups.

### HOW DO ALCOHOL USE AND DEPRESSION PREDICT GRIP STRENGTH AMONG MIDDLE-AGED AND OLDER ADULTS?

Song Ge,[1] Linda Dune,[2] Desiree' Frantz,[2] Laurel Laviolette,[2] Mary Njuguna,[3] xianping tang,[4] and Changwei Li,[5], *1. University of Houston-Downtown, University of Houston-Downtown, Texas, United States, 2. University of Houston Downtown, Houston, Texas, United States, 3. University of Houston downtown, Richmond, Texas, United States, 4. Xuzhou Medical University, Xuzhou, Jiangsu, China (People's Republic), 5. Tulane University School of Public Health and Tropical Medicine, New Orleans, Louisiana, United States*

**Background:** Physical performance is an important indicator that reflects current and predicts future health. In this study, we examined the association of alcohol use and depression with grip strength a national sample of middle aged and older Chinese adults.

**Methods:** We used the baseline data from the China Health and Retirement Longitudinal Study (CHARLS) and constructed a multivariate linear regression using SAS 9.4 to examine the independent association of alcohol use (never, former, moderate, and at-risk drinkers) and depression with grip strength controlling for socio-economic factors and domestic partner status.

**Results:** The study population consisted of 12,488 Chinese adults ( mean age 59 ) . The prevalence of ever drinking during lifetime and current at-risk drinking (>14 standard drinks [one standard drink contains 14 grams of pure alcohol] per week) in this population was 25.7% and 15.2% respectively. 28.4% of the study population had depression. Compared with never drinkers, moderate and at-risk alcohol use were independently associated with better grip strength ($P<0.0001$). Depression was independently negatively associated with grip strength ($P<0.0001$).

**Conclusions:** We found that current alcohol use might be protective of grip strength while depression might be detrimental to grip strength among middle-aged adults. However, the underlying mechanism is unclear. Given the negative impact of alcohol and depression on adults' overall health, clinicians should assess alcohol use and depression in middle-aged and older patients using validated tools and provide resources. Clinicians should counsel patients that if depression is not managed, patients may suffer from depression associated health consequences such as declined grip strength.

### INFLUENCE OF DEPRESSION AND PERSONALITY ON SOCIAL FUNCTIONING IN OLDER ADULTS

David Freedman,[1] George Lederer,[2] Lauren Atlas,[3] Richard Zweig,[4] Dimitry Francois,[5] Rachel Waldman,[6] Jennifer Ho,[4] and Helene Geramian,[4], *1. Yeshiva University, Brooklyn, New York, United States, 2. Yeshiva University, Scarsdale, New York, United States, 3. New York-Presbyterian/Columbia University Irving Medical Center, NEW YORK, New York, United States, 4. Yeshiva University, Bronx, New York, United States, 5. NY, White Plains, New York, United States, 6. Yeshiva University, New York, New York, United States*

Among older adults there is significant comorbodity between depression and personality pathology and both are associated with poorer social functioning. Personality pathology is associated with greater prevalence, poorer recovery, and a higher likelihood of recurrence of depression in older adults. This study is a secondary analysis examining the relationships between personality traits associated with personality pathology (i.e. high neuroticism and low agreeableness), depression, and social functioning across older adults surveyed in primary care and psychiatric inpatient settings (N = 227). Individual variable as well as interaction models were examined. Higher neuroticism (FChange $[1,217] = 40.119$, $p < .001$), lower agreeableness (FChange $[1,217] = 20.614$, $p <$

.001), and clinical status (i.e. primary care vs. psychiatric inpatient) (FChange [1,217] = 19.817, p < .001) were associated with poorer social functioning. Clinical status moderated the relationships between neuroticism and social functioning (B = -.0147, p = . 0341) and between agreeableness and social functioning (B = .0268, p = .0015). Interaction effects were not observed between neuroticism and depression or agreeableness and depression as they relate to social functioning. However, depression severity was observed to mediate the relationship between neuroticism and social functioning [Indirect effect = .0212, 95% CI = .0141, .0289]. These findings highlight the importance of accounting for depression and clinical status in the assessment and treatment of older adults with personality pathology. Findings warrant future research focused upon mechanisms through which personality pathology and depression influence functional status in older adults.

### MOBILE INTERVENTION OUTCOMES COMPARED PRE AND POST-SHELTER-IN-PLACE FOR MIDDLE AGED AND OLDER ADULTS

Chalise Carlson,[1] Ana Alfaro,[1] Christina Chick,[2] Martha Bruce,[3] Valerie Forman Hoffman,[4] and Christine Gould,[1], *1. VA Palo Alto Health Care System, Palo Alto, California, United States, 2. Stanford University School of Medicine, Stanford University, California, United States, 3. The Dartmouth Institute, Geisel School of Medicine at Dartmouth, New Hampshire, United States, 4. Meru Health, Meru Health, California, United States*

The Meru Health Program (MHP), a mobile app-based intervention targeting depressive symptoms, teaches mindfulness and cognitive behavioral skills through video lessons and practices. MHP includes integrated asynchronous therapist and peer support. Our aim was to examine whether using the MHP reduced loneliness and improved mental health quality of life (QoL) in middle-aged and older adults with depressive symptoms (Patient Health Questionairre-9 [PHQ-9] ≥ 7). The timing of this study partially overlapped with the emergence of the SARS-CoV-2 (COVID-19) pandemic resulting in California's shelter-in-place (SIP) orders. Fifty participants (42 enrolled prior to SIP) completed baseline assessments with a mean age of 57.06 (SD = 11.26; Range: 40-81 years) exhibiting mild to moderate depression symptoms (PHQ-9: M = 12.28, SD = 5.47). Participants enrolled pre-SIP exhibited significant improvements in QoL, F(1,38) = 12.61, p = .001, η2 = .25, and significant declines in loneliness, F(1,38) = 5.42, p = .03, η2 = .13. Improvements in QoL were found for post-SIP participants as well, F(1,44) = 6.02, p = .02, η2 = .12. In contrast, loneliness did not improve for the post-SIP cohort, perhaps alluding to the increased impact of social isolation during SIP. Our findings indicate MHP can improve QoL symptoms before and during SIP. It is possible that middle-aged and older individuals may require more individualized support during SIP to help alleviate loneliness when social connection is severely restricted. MHP remains a promising and scalable solution for those middle-aged and older adults struggling with mental health symptoms.

### OLDER ADULTS' EXPERIENCES IN AN ONLINE INTERVENTION FOR MANAGING SUBJECTIVE DEPRESSIVE SYMPTOMS

Janella Hudson,[1] Rachel Ungar,[2] Laurie Albright,[3] Rifky Tkatch,[2] James Schaeffer,[2] and Ellen Wicker,[4], *1. Optum/United Healthcare, Tampa, Florida, United States, 2. UnitedHealth Group, Minnetonka, Minnesota, United States, 3. UnitedHealth Group, Minneapolis, Minnesota, United States, 4. AARP Services, Inc., Washington, District of Columbia, United States*

**Background:** Many older adults struggle with late-life depression, stress, and anxiety, especially when facing age-related transitions including retirement, relocation, and the death of a spouse. Given the consequences of depression among older adults, which include higher rates of suicide, timely interventions that help to manage depressive symptoms are essential.

**Objective:** The primary purpose of this study was to explore the perceived efficacy of an online program in improving subjective depressive feelings.

**Methods:** Older adult participants were recruited for semi-structured interviews (n = 24) in a web-based intervention that included interactive games and activities undergirded by a cognitive behavioral therapy (CBT) approach. Participants were asked to provide feedback about program features, including weekly module content, games, interactive activities and community interactions, and any perceived effects on their health behaviors and/or emotional well-being. Participants' responses were analyzed using qualitative content analysis.

**Results:** Participants reported several gains, including developing the habit of forming ongoing, incremental goals, achieving wellness-related goals, and experiencing an overall positive shift in perspective. In addition, participants reported feeling greater gratitude, increased positivity, and improvement in mood. Featured games and activities helped to promote stress relief and entertainment, and mindfulness exercises were cited as the most helpful and/or enjoyable among participants. Participants expressed a preference for program content related to aging and aging-related transitions.

**Conclusions:** This study demonstrated feasibility of an interactive web-based intervention for older adults with subjective depressive feelings, while also providing important findings about users' preferences for personalized, aging-related feedback.

### PARENTING STRESS AND MENTAL HEALTH IN MIDLIFE ADULTS: EVALUATING THE ROLE OF GENDER

Jean Choi,[1] Elizabeth Munoz,[2] Robin Corley,[3] Sally Wadsworth,[3] and Chandra Reynolds,[4], *1. The University of Texas at Austin, Austin, Texas, United States, 2. The University of Texas at Austin, The University of Texas at Austin, Texas, United States, 3. University of Colorado Boulder, Boulder, Colorado, United States, 4. University of California Riverside, Riverside, California, United States*

Parenthood is a major source of stress in midlife that can have adverse consequences for long-term mental health trajectories. Yet, little research asks how parenting stress impacts mental health for both mothers and fathers in midlife. The current study examined (a) whether parenting stress was associated with parental depressive and anxiety symptoms and (b) whether these associations vary by gender. We utilized data from the ongoing Colorado Adoption/Twin Study of Lifespan behavioral development and cognitive aging (CATSLife); participants were aged 28 to 49 who reported having child(ren) (N = 520). Participants completed surveys that encompassed measures of demographics, relationships, health, and well-being. Overall, multilevel models accounting for non-independence among siblings and with relevant covariates (e.g., number of children, marital status) showed that higher levels of parenting stress were associated with greater depressive (b = .47 (.12), p<.001) and anxiety (b = .27 (.09), p<.05) symptoms. An evaluation of the individual parenting stress items indicated that feeling less happy and more overwhelmed in the parental role were significantly associated with higher levels of anxiety and depressive symptoms. Parents who reported feeling less close to their children were also significantly more likely to report greater levels of depressive symptoms. These effects were consistent across mothers and fathers. Our study provides further insight into the negative associations between parenting stress and mental health among both mothers and fathers, and warrants further investigation into resources that may buffer these negative effects prior to late life.

### PROTECTIVE EFFECTS OF PERCEIVED CONTROL ON PRESCRIPTION DRUG MISUSE 10-YEARS LATER IN THE MIDUS STUDY

Sara Miller, and David Almeida, *Pennsylvania State University, University Park, Pennsylvania, United States*

The current study examined if control beliefs predict prescription drug misuse (PDM) 10-years later and how problem drinking status moderates this relationship. PDM refers to taking medications without a prescription or in a manner not intended by the prescriber. Older adults are especially vulnerable to PDM due to drug sensitivity, comorbid health conditions, and high rates of polypharmacy. Participants were adults (n=2162, 56% female, mean age=54, range=30-84) who completed Waves 2 and 3 of the Midlife Development in the United States (MIDUS) study. At Wave 2, participants reported on two subscales of perceived control (personal mastery and constraints) and past 12-month problem drinking behaviors. At Wave 3, participants reported past 12-month PDM of five substances (painkillers, sedatives, stimulants, tranquilizers, and depression medications). Results indicated that at Wave 3, 10% of the sample reported misusing at least one prescription drug in the past year. Logistic Regression analysis revealed that problem drinking was associated with higher odds of PDM (p<0.001), and perceived control was associated with lower odds of PDM (p<0.05) after controlling for previous PDM and sociodemographic, health behavior, and health status covariates. However, there was an interaction effect such that perceived control was not protective for those individuals who engaged in problem drinking at Wave 2 (p<0.05). Future analyses will explore the meaning of this interaction. Identifying psychosocial protective factors, such as perceived control, predicting PDM will be critical for designing interventions that prevent the adverse consequences of PDM among this population.

### THE EFFECT OF COMPUTERIZED COGNITIVE TRAINING FOR ADULTS OVER 40 WITH DEMENTIA-RELATED ANXIETY

Jennifer Roberts,[1] and Molly Maxfield,[2], *1. University of Colorado Colorado Springs, Colorado Springs, Colorado, United States, 2. Arizona State University, Phoenix, Arizona, United States*

Dementia-related anxiety (DRA) may occur when cognitive lapses are appraised as threatening. Individuals with DRA may seek activities to improve cognitive function, including popular computerized cognitive training programs like Lumosity©. We evaluated if DRA changed after eight weeks of Lumosity© use and whether changes were maintained over time. Participants aged 40 and older with preexisting DRA participated via Amazon's Mechanical Turk (T1 N = 395; age M = 52.49, SD = 8.71) and were randomly assigned to the experimental (Lumosity© software), active control (Lumosity© crossword puzzles), or no treatment group. Participants completed measures of DRA at T1 and at four follow-up points (T2 = 8 weeks; T3 = 12 weeks; T4 = 16 weeks; T5 = 20 weeks). Repeated measures ANOVAs were used to evaluate the change in DRA. A significant T1-T2 reduction in DRA occurred for the Lumosity© group only (p = .01, partial-eta2 = .03). Longitudinal changes were observed for the Lumosity© group only: DRA scores at T1 were significantly greater than at T2, T4, and T5 (ps < .05). A step-up test procedure was conducted to determine minimum treatment dose effects. A greater reduction in DRA occurred between the Lumosity© and crossword puzzle groups between 25.00 and 29.99 hours of software use (p = .05, partial-eta2 = .19). Lumosity© software outperformed crossword puzzles in DRA reduction from T1 to T2, which was maintained for 12 weeks post-software use. Independent of Lumosity's intended purpose of supporting cognitive functioning, participants subjectively believe it helps and experience associated benefits.

### THE ILLNESS EXPERIENCE OF VETERANS WITH DEMENTIA: PERCEIVED MEASURES RELATED TO DEPRESSIVE SYMPTOMS

Amanda MacNeil,[1] Katherine Judge,[2] Kate McCarthy,[3] and David Bass,[4], *1. Cleveland State University, Fairview Park, Ohio, United States, 2. Cleveland State University, Cleveland, Ohio, United States, 3. Benjamin Rose Institute on Aging, Benjamin Rose Institute on Aging, Ohio, United States, 4. Benjamin Rose Institute on Aging, Cleveland, Ohio, United States*

Recent work has examined how individuals with dementia (IWDs) experience their illness, although few studies have looked at IWDs who report heightened depressive symptoms, a key well-being outcome. Stressing the ability of IWDs to self-report and guided by the Stress Process Model for Individuals with Dementia, this study examined the relationships between depressive symptoms and various aspects of the illness experience including objective cognition, perceived memory difficulty, perceived functional difficulty, and dyadic relationship strain. The sample includes IWDs with mild to severe dementia who are veterans (N=69). Significant positive correlations emerged between depressive symptoms and several measures of the illness experience: perceived cognition (r=.48, p<.001), perceived function (r =.43, p<.001), and dyadic relationship strain (r=.32, p=.01). In contrast, objective cognition, measured by a modified version of the Blessed Orientation Memory Concentration test, was not significant (r =-.06, p=.63). A multiple regression found the total variance explained by all independent variables was 32%

(R2=.32, F(4,68)=7.58, p<.001), with perceived memory difficulty (B=.26, p<.01) and dyadic relationship strain (B=.25, p=.04) accounting for unique and significant variance in depressive symptoms. A mediation analysis indicated perceived memory difficulty fully mediated the relationship between perceived functional difficulty and depressive symptoms. Findings highlight the importance of IWDs perceptions of their illness experience for psychosocial well-being outcomes, such as depressive symptoms. Findings add to the literature by showing the importance of IWDs perceptions of their illness and their impact on well-being outcomes. Results also demonstrate the utility and feasibility of including self-reported data from IWDs in research studies.

### THE LONG-TERM IMPACT OF CHILDHOOD DISABILITY ON MENTAL HEALTH IN MID- TO LATE-ADULTHOOD

Jessica West, *Duke University, Nantucket, Massachusetts, United States*

For decades, life course and stress process scholars have documented that negative, stressful experiences in childhood have consequences for health across the life course. One aspect of the childhood adversity that deserves more research attention is childhood disability. Children with disabilities experience higher levels of psychological distress compared to their peers and having a disability can negatively impact traditional markers of the transition to adulthood (e.g., education, employment, family status). At present, there is limited evidence regarding the impact of childhood disability on mental health over multiple years of adulthood. This study applies random effects models to nationally representative data from five waves (2008-2016) of the Health and Retirement Study (n=15,380; n=590 with a childhood disability), to examine how experiencing disability before the age of 16 shapes depressive symptoms over multiple years of adulthood. Given known gender differences in mental health, the models are stratified by gender to examine how the association between childhood disability and adult mental health varies by gender. Preliminary results suggest that experiencing a childhood disability is associated with different patterns of depressive symptoms in adulthood. Men who experienced childhood disability report more depressive symptoms in adulthood, net of sociodemographic, adult health, and childhood disadvantage covariates. Women who experienced childhood disability report more depressive symptoms in adulthood, net of all covariates except for childhood depression. Next steps are to conduct age-based growth curve models using Stata's mixed function to estimate whether childhood disability influences baseline and growth of depressive symptoms in adulthood.

### TIME USE, GENDER, AND MENTAL HEALTH OF OLDER ADULTS IN RURAL CHINA

Zhiyong Lin,[1] and Feinian Chen,[2] *1. The University of Texas at Austin, Austin, Texas, United States, 2. University of Maryland College Park, University of Maryland, Maryland, United States*

Time use is considered a valuable descriptor of people's lifestyles, and studying how people spend their time is critical for understanding the determinants and consequences of individual well-being. In this study, we first develop a time use typology to characterize how older adults in rural Chinese families allocate their time in later life, and then examined how older adults' time allocation influenced their mental health, with a special focus on differential implications for older women and men. Data derived from 2015 and 2018 waves of a longitudinal study of 1,007 older adults, aged 60 and older, living in rural areas of Anhui Province, China. We specifically focused on how social and solitary dimensions of time use, as well as time spent within and outside households, impacted depressive symptoms of older adults. Using the K-means cluster analysis, we identified four time use categories: "work-oriented," "socially-active," "homemaker/caretaker," and "socially-isolated." Results from fixed-effects regression analysis demonstrated that older women involved in "socially-active" time-use category tended to report better mental than those in other time-use types, while the time spent on housework and caregiving was harmful to their mental health. For older men, more time spent on paid activities outside households ("work-oriented") was associated with better psychological outcomes while solitary leisure time ("socially-isolated") was associated with higher levels of depressive symptoms. These findings will be helpful for health policymakers and practitioners who seek to better identify vulnerable subpopulations and to design effective intervention strategies to reduce mental health problems.

### Session 9365 (Poster)

### MENTAL HEALTH (HEALTH POSTER)

### DENTAL ISSUES AND DEPRESSIVE SYMPTOMS IN INSTITUTIONALIZED OLDER ADULTS

Alexandre Bulgarelli,[1] Luiza Guilhermina Lopes,[2] Karla Frichembruder,[2] and Camila Santos,[3], *1. Federal University of Rio Grande do Sul, Porto Alegre, Rio Grande do Sul, Brazil, 2. University of Rio Grande do Sul, Porto Alegre, Rio Grande do Sul, Brazil, 3. Federal University of Rio Grande do Sil, Porto Alegre, Rio Grande do Sul, Brazil*

**Objective:** To map and discuss scientific knowledge involving the research object "Oral health conditions and depression in institutionalized elderly people".

**Method:** Systematic scoping review. The mapping of the selected data was carried out using Summative Content Analysis from Manifest Themes' perspective in the texts. After the exclusions, 27 articles were selected.

**Results:** With the analysis of the articles, it was possible to divide them into two themes named: 'Oral health condition: Long-term institution, depressive disorders and pluralities' and 'Depression in institutionalized older adults: medicalization, oral health conditions and subjectivities'. Heterogeneity was found in the sub-themes of the articles accessed and in the characteristics of the published studies as well. All continents have publications on the topic. Many studies with deductive methods have been carried out when dealing with its methodology, and less research has been carried out with inductive methods.

**Conclusion:** The present study identified that there is an association between some oral health conditions (dry mouth and tooth loss) and prevalence of depressive disorders in institutionalized polder people. The knowledge produced about oral health and mental health in long-term care facilities for the elderly is relatively recent, reflecting the contemporary nature of the theme. Besides, the construction of this knowledge is associated with the diversity of epidemiological and qualitative studies seeking to answer questions that involve technical and subjective plurality involving mental health and oral health of institutionalized older adults.

### EFFECTS OF PARTICIPATING IN A VOLUNTEER DRIVING PROGRAM ON MENTAL HEALTH IN SERVICE RECIPIENTS AND VOLUNTEERS

Machiko Tomita,[1] and Sutanuka Bhattacharjya,[2],
*1. University at Buffalo, Buffalo, New York, United States,*
*2. Georgia State University, Atlanta, Georgia, United States*

**Objectives:** To assess the effects of a volunteer transportation program on mental health in riders and volunteers.

**Methods:** A cross-sectional study (N=133, age ≥60) compared pre- and existing (≥2 years) riders, pre- and existing (≥2 years) volunteer drivers, and riders (Rs) and pre-volunteers (PreVs), representing general older adults. The cohorts belonged to a large, community volunteer organization. Outcome measures, depression and quality of life, were analyzed using ANCOVA. Percentages of people who wanted to go to specific destinations with available transportation were identified for pre-riders (PreRs) and Rs.

**Results:** Rs had better depression scores (p<.001), no longer exhibited depressive symptoms (p=.005), and were better in quality of life (p=.002) than PreRs. Rs were similar to PreVs. PreRs' major needs were going to medically related places (doctors' offices – 74.4%, drug stores - 44.2%, hospitals – 37.2%) and basic living (grocery – 60.5%, clothing -37.2%). In Rs, these had significantly lowered, but still 40% wanted to go to doctors' offices and 30%, grocery stores. Volunteer driver's (Vs)' depression (p=.009), health (p=.006), and social relationships (p=.004) were significantly better than PreVs'.

**Discussion:** Although the use of free transportation up to four times a month may not be enough to improve perceived health for Rs, it was beneficial to prevent depression and increase quality of life. Since many PreVs were doing volunteer work other than driving, the type of volunteer work matters. Regularly helping people, in person, with their core needs for living resulted in positive outcomes for Vs.

### GROUP-BASED SMOKING CESSATION PROGRAM FOR OLDER ADULTS WITH SERIOUS MENTAL ILLNESS

Heather Leutwyler,[1] and Erin Hubbard,[2], *1. University of California, San Francisco, San Francisco, California, United States, 2. UCSF, UCSF, California, United States*

Smoking is one of the most important modifiable risk factors for excess morbidity and mortality in older adults with serious mental illness (SMI). Many older smokers with SMI are reportedly motivated to quit, however evidence-based treatment targeting this vulnerable group is limited. To address an urgent need to identify interventions that assist smoking cessation efforts, we are conducting a pilot two-arm randomized controlled trial (RCT) targeting adults with SMI. Our VIdeogame-based Physical activity ("VIP") smoking cessation intervention includes: a) group videogame-based physical activity intervention (50 minutes, 3X/week for 12 weeks), b) pharmacotherapy (bupropion or nicotine replacement therapy), and c) smoking cessation counseling. Upon completion of the 12 week program, participants in the VIP and control groups completed a semi-structured interview in order to determine how the program impacted their smoking cessation. To date, six participants completed an interview. Participants described how the program helped with smoking cessation because it allowed them to "face their addiction" and learn more about why they smoke and how to quit. The program provided the structure, resources, and encouragement needed to start the process of quitting. Finally, they enjoyed having the game time as a distraction from smoking. Older adults with SMI need support, resources, and group-based exercise as they begin quitting and practice the skills needed to quit.

### INCREASING MIXED-BERRY FLAVONOID INTAKE MAY REDUCE DEPRESSIVE SYMPTOMS IN OLDER ADULTS: FRAMINGHAM HEART STUDY

Courtney Millar,[1] Alyssa Dufour,[2] Marian Hannan,[1] and Shivani Sahni,[1], *1. Hebrew SeniorLife, Roslindale, Massachusetts, United States, 2. Hebrew SeniorLife, Boston, Massachusetts, United States*

Depression affects more than 250 million people worldwide. Although epidemiological studies have linked higher dietary flavonoids with depression prevention in older women, it is unknown if increasing dietary flavonoids could effectively reduce depression. Mixed berries (blueberry, blackberry, and raspberry) are a rich source of flavonoids, particularly anthocyanin, flavanol, and flavan-3-ol subclasses. Our aim was to determine the association of mixed-berry flavonoid intake with change in depressive symptoms over ~8 years in older adults from the Framingham Heart Study. This community-based prospective longitudinal study included 1,278 adults with assessments on diet (food frequency questionnaire) and depressive symptoms (Center for Epidemiologic Studies Depression, CES-D) at baseline (1998-2001) and follow-up (2005-2008). Absolute change in mixed-berry flavonoid intake (defined as sum of anthocyanin, flavanol, and flavon-3-ols, mg/day) and change in CES-D scores were calculated. Linear regression estimated beta and standard error (SE) for change in CES-D scores per 250 mg/day increase in mixed-berry flavonoids (obtained from ~3/4 cup of mixed berries), adjusting for baseline age, sex, energy-intake, current smoking, body mass index, physical activity, cardiovascular disease, and non-melanoma cancer. Mean age was 59±9 years (range: 33-81), 57% female and mean change in mixed-berry flavonoid intake was 15.0±72.8 mg/day over ~8 years. In adjusted models, each 250 mg/day increase in mixed-berry flavonoid intake was associated with a 1-point reduction in depressive symptoms (beta: -1.06, SE: 0.61, p=0.08) over ~8 years, although this was not statistically significant. These data highlight the need for randomized clinical trials of flavonoid-rich berries to target depressive symptoms in older adults.

### MINIMALLY ADEQUATE MENTAL HEALTH TREATMENT AND MORTALITY IN PRIMARY CARE OLDER ADULTS WITH DEPRESSION AND ANXIETY

Helen-Maria Vasiliadis,[1] Catherine Lamoureux-Lamarche,[1] Sébastien Grenier,[2] and Pasquale Roberge,[1], *1. Université de Sherbrooke, Université de Sherbrooke, Quebec, Canada,*

2. *Université de Montréal, Institut universitaire de gériatrie de Montréal, Quebec, Canada*

Receipt of quality mental health (MH) care can influence mortality. Given the scarce literature on the topic, the aim was to assess the 3-year risk of mortality in older adults (OA) associated with receiving adequate MH treatment for depression/anxiety in an epidemiologic context. The study sample included 358 OA with depression/anxiety recruited in primary care practices and followed prospectively for 3 years. Mortality was assessed from vital statistics data. Adequate care was based on receipt of pharmacotherapy, follow-up care and psychotherapy. Propensity score analysis was carried out where the inverse probability (IPW) of receiving adequate treatment was calculated. Time to event analyses with IPW was used to assess the effect of receipt of adequate MH treatment on the risk of mortality controlling for individual and health system factors. The results showed that receipt of adequate MH treatment reduced the risk of mortality (HR0.44; 95% CI: 0.22 – 0.99). Individual factors that increased mortality were male sex, being single, reduced functional status and cognitive functioning, # physical disorders, current smoking; while exercise reduced risk. Health system factors such as past # of hospitalizations increased the risk; while # of emergency department visits and continuity of care reduced mortality. Finally, treating depression/anxiety with minimal follow-up care and pharmacotherapy or psychotherapy has a significant impact on reducing mortality in OA. Primary care physicians should recognize the important potential impact of years of lives saved when providing quality MH care to OA.

### SLEEP HEALTH, DEPRESSION, PAIN AND QUALITY OF LIFE AMONG OLDER ADULTS DURING THE FIRST MONTHS OF THE COVID-19 PANDEMIC

Rebecca Lorenz,[1] Devita Stallings,[2] Janice Palmer,[3] and Helen Lach,[4], *1. University at Buffalo, Buffalo, New York, United States, 2. Saint Louis University, St. Louis, Missouri, United States, 3. Webster University, St. Louis, Missouri, United States, 4. Saint Louis University, Saint Louis, Missouri, United States*

To slow the spread of Covid-19, many states instituted restrictions on group size for religious services, exercise, and social engagement. We are beginning to understand the effect of these mandates on older adults. The purpose of this study was to examine the relationships between sleep health, depression, pain, and quality of life (QOL) among older adults during the initial months of the Covid-19 pandemic. Older adults completed an anonymous online survey to collect data including personal characteristics, behaviors, and health conditions during May-September 2020. Sleep Health was assessed with a survey of satisfaction, timing, efficiency, and duration of sleep along with daytime alertness. Pearson correlations were used to explore relationships between age, education, socioeconomic status, pain, depression, and QOL. Participants (N=509) were primarily female (n=392, 77%), white (n=466; 92%), college educated (n=471, 93%) and with a mean age of 75.6 years (SD=5.0; range 63-93 years). Mean Sleep Health score was 7.4 (SD=2.1; range 0-10). Higher (better) Sleep Health scores were associated with education (r=.15, p<01) and socioeconomic status (r=.17, p<.01) and lower scores with depression (r= -.35, p<.01), pain (r= -.23, p<.01), and QOL (r= -.26, p<.01). Poorer Sleep Health among older adults during the initial months of the pandemic were associated with depression, pain, and reduced QOL. Sleep, depression, and pain have reciprocal relationships that may have lasting consequences on physical and mental health among older adults. These findings suggest that poor sleep health should be identified and treated to improve QOL among older adults.

### Session 9370 (Poster)

### MENTAL HEALTH AND TECHNOLOGY

### CONNECTING OLDER ADULTS WITH MENTAL HEALTH APPS: A SURVEY OF PROVIDER APP AND EDUCATION MATERIAL USE

Priyanka Mehta,[1] Chalise Carlson,[2] Jason Anderson,[2] Ana Alfaro,[2] Erin Sakai,[2] and Christine Gould,[2], *1. VA Palo Alto Health Care System, Palo Alo, California, United States, 2. VA Palo Alto Health Care System, Palo Alto, California, United States*

Many older veterans have access to mobile devices and are interested in using apps for mental health self-management, but few have ever downloaded health apps. To address the need for awareness of and access to VA mental health apps, we developed patient educational materials aimed towards older (or novice) users of mobile devices. The present study explored health care providers' and staff's perceptions about use of mental health mobile applications (apps) with older veterans and examined potential utility of these patient educational materials. Requestors of mobile device education materials (N = 90) were surveyed when ordering materials and again 4 months later. Baseline and follow-up surveys assessed frequency of app recommendation, and comfort recommending apps. Baseline surveys examined perceived advantages of apps; follow-up surveys examined perceived utility of the educational materials. Descriptive statistics and qualitative analysis were conducted. Most requesters (68.5%) initially were not comfortable using apps, yet perceived many advantages to using apps and hoped materials could facilitate app use. At follow-up, requestors felt more comfortable recommending apps alongside our materials. Qualitative analysis revealed perceived advantages to using the education materials. The benefits of developing and disseminating educational materials for providers to share with older veterans helped support older veterans' app use, and potentially increased providers' comfort with and frequency of recommending apps to their older patients. Access to educational materials can mitigate discomfort among providers in recommending apps to older users and may bring about valuable discussions about apps which support mental health.

### EMENTAL HEALTH LITERACY AND PSYCHOLOGICAL DISTRESS AS PREDICTORS OF BARRIERS TO MENTAL HEALTH SERVICES

Eve Root, and Grace Caskie, *Lehigh University, Bethlehem, Pennsylvania, United States*

Since the COVID-19 pandemic, psychologists have begun to rely heavily on technology to provide mental health information and services (APA, 2020). As the older adult population increases, the number of older adults in need of mental health services also increases; however, little is known about the way older adults might utilize technology to inform mental health-related decisions. This study expands on the construct of eHealth Literacy by examining eMental Health Literacy, which is defined as the degree to which individuals seek, find, understand, and appraise basic mental health information and services online that are needed to inform mental health-related decisions. A sample of 244 older adults (M=68.34, range=65-82 years) were recruited online through Amazon Mechanical Turk. A structural equation model was estimated specifying eMental Health Literacy and psychological distress as predictors of extrinsic and intrinsic barriers to mental health services. After adding three correlated errors, the model achieved good fit ($\chi 2(110)=329.20$, $p<.001$, SRMR=.08, CFI=.93, TLI=.91, GFI=.86, RMSEA=.09). All indicators were significantly related to their latent construct ($p<.001$). The results indicated that, controlling for psychological distress, higher eMental health literacy was significantly related to fewer reported intrinsic ($b=-.386$, $p<.001$) and extrinsic barriers ($b=-.315$, $p<.001$) to mental health services. Higher distress was also significantly related to more intrinsic ($b=.537$, $p<.001$) and extrinsic barriers ($b=.645$, $p<.001$) to mental health services. These findings suggest that, as we move towards a more digital world, eMental health literacy could play a significant role in the way older adults navigate through the mental healthcare system.

### MOOD LIFTERS: A PEER-LED MENTAL HEALTH PROGRAM FOR OLDER ADULTS VIA VIDEO CONFERENCING

Courtney Funk,[1] Rebecca Ferber,[2] Cathleen Connell,[1] Susan Maixner,[2] Scott Roberts,[2] Jennifer Porte,[3] and Anne Harrington,[4], *1. University of Michigan School of Public Health, Ann Arbor, Michigan, United States, 2. University of Michigan, Ann Arbor, Michigan, United States, 3. Mood Lifters, LLC, Ann Arbor, Michigan, United States, 4. The University of Michigan, ANN ARBOR, Michigan, United States*

Effective and scalable mental health programs are greatly needed for older adults, given the vast majority in need do not receive formal mental health services. In this study, we adapted Mood Lifters—a peer-led, community-based program promoting mental well-being—to address the unique needs of older adults. The 14 weekly program sessions were delivered via Zoom. Twelve older adults (mean age = 69.7 years; 4 men, 8 women) enrolled; 9 completed the program (2 of 3 dropouts were due to health issues). A battery of validated measures administered within one week before and after the program assessed domains including depression and anxiety, stress management, and health behaviors. Compared to baseline, participants who completed the program showed significant improvements in perceived stress ($p=0.03$), sleep quality ($p=0.02$), and emotion regulation via cognitive reappraisal ($p=0.06$). Depression and anxiety symptoms (assessed by the Geriatric Depression and Anxiety Scales, respectively) were lower at program completion, although improvements were not statistically significant. No significant changes from pre- to post-test were reported in loneliness and health behaviors. Participant ratings of program satisfaction were very high (mean = 4.78/5, with 1=poor, 5=excellent). Results from this pilot test of Mood Lifters for Seniors suggest it is feasible and acceptable for outreach to older adults, with preliminary evidence of benefits in several domains related to mental health and wellness. Future randomized trials with larger, more diverse samples will be necessary to confirm program benefits.

### PILOTING AN AUGMENTED REALITY LIFE REVIEW EXPERIENCE TO PROMOTE MENTAL HEALTH OUTCOMES IN AGING ASIAN AMERICAN WOMEN

Sarah Hwang,[1] Jazlyn Armendariz,[1] Jeremy Argueta,[1] Veronica Fruiht,[2] and Thomas Chan,[1], *1. California State University Northridge, Northridge, California, United States, 2. Dominican University of California, Dominican University of California, California, United States*

Asian-American older women report the highest prevalence of suicidal ideations and rates of completed suicide compared to other racial groups. Ironically, Asian-American communities report disproportionately low rates of formal mental health utilization—this may be attributed to the lack of culturally-relevant services and negatively ingrained perceptions of mental health aid. One potential solution that has not been widely investigated is the use of technology to help older Asian-American women engage in mental health interventions. This study leverages innovations in augmented reality (AR) technology (i.e., overlaying of digital holograms onto the real world) to create a life review intervention aimed at promoting mental health well-being. The application, Tell-Being, is a personalized holographic life review experience that facilitates older adults to foster a sense of coherence and wholeness within their lives. Pilot data collection was amassed from four aging Asian-American female participants averaging 51.3 (SD=8.61) years of age. Initial pre/post analyses showcased mean differences that trend towards a higher presence of emotion regulation from pre-test (M=4.88, SD=1.08) to post-test (M=5.21, SD=1.17). Although data collection was prematurely halted due to COVID-19, results trended in promising directions. The technological innovations and findings from this study may lead to promising novel avenues to address barriers for older Asian-American women in seeking mental health assessment and treatment in a "new normal" world.

### TECHNOLOGY BASED COGNITIVE BEHAVIORAL THERAPY ON PSYCHOLOGICAL DISTRESS: EXPLORING HEALTH, PAIN, AND ACTIVITY

Avani Shah,[1] Martin Morthland,[2] and Forrest Scogin,[2], *1. University of Alabama, Tuscaloosa, Alabama, United States, 2. University of Alabama, University of Alabama, Alabama, United States*

This randomized controlled trial investigates two technologically based self-administered cognitive behavioral depression treatments (CBT) on psychological distress in older adults. Health may change the ability to participate in types of activities, thereby impacting mental well-being and treatment response. The aims of this research are 1) to understand the impact of technologically based cognitive behavioral treatment on psychological distress 2) explore how health, pain, and activity engagement may affect treatment response. Fifty one participants recruited were randomized to one of 3 groups: audio-based cognitive behavioral therapy, computer-based cognitive-behavioral therapy, and control group. The combined treatment groups are compared to the control group. Health was examined in multiple ways; the Vulnerable Elders Scale-13 score ; (Saliba et al., 2001); and a reported chronic pain condition. For overall psychological distress, improvement on the Brief Symptom Inventory General Severity Index (GSI; Derogatis & Spencer, 1983) scores from baseline to post-treatment indicated treatment response. The California Older Person's Pleasant Events Scale (COPPES; Rider, Gallagher-Thompson, & Thompson, 2004) measured activity engagement. While controlling for the Time 1 GSI score, an ANOVA revealed a significant difference in psychological distress between the CBT treatment group and control group $F(1, 43) = 4.22$, $p = .046$. A linear regression analysis with the VES-13 score and GSI baseline score as predictors and the GSI posttreatment score as the dependent variable, found that health did not significantly predict psychological distress outcomes. Observation of the descriptives and these analyses suggest that CBT can impact psychological distress, potentially even with variations in health and pain.

### Session 9375 (Poster)

### MENTAL HEALTH, SOCIAL CONNECTION, AND ISOLATION

### DOG PARK MEMBERSHIP AND LIFE SATISFACTION AMONG OLDER ADULTS

Betz King,[1] Adam Duberstein,[2] and McGlinn Maureen,[3], *1. King & Associates, King & Associates, Michigan, United States, 2. King & Associates, Hazel Park, Michigan, United States, 3. Southfield Mental Health, Southfield, Michigan, United States*

Dog park members initially join and attend dog parks for the wellbeing of their dogs, but often experience their own biopsychosocial benefits. This mixed methodology (Quantitative n=44, Qualitative n=11) ) pilot study utilized qualitative heuristic interviewing (Moustakas, C., 1990) and the Satisfaction with Life Survey (Pavot, W., & Diener, E. 2013). Data gathered from interviews and surveys administered to participants of a members-only dog-park indicate a high satisfaction with life. Members 60 years and older reported feelings of life satisfaction almost 7 points over the total respondent average, placing them in the "highly satisfied" range. All members experience the dog-park as a supportive social environment that benefits their physical health, mental health and the well-being of their canine companions. Five qualitative themes were identified: Canine Well-being, Community, Mental Health Benefits, Physical Health Benefits and Fights, Falls & Frustrations. These findings demonstrate the need for more research into the impact and importance of pet ownership, community dog parks and outdoor green spaces on older adults and life satisfaction.

### FOSTERING INTERGENERATIONAL CONNECTIONS IN THE TIME OF COVID-19: A FRIENDLY CALLER PROGRAM

Maria Claver,[1] Alexandra Wilkinson,[2] Tatia Clark,[3] and Chan Park,[4], *1. California State University, Long Beach, Whittier, California, United States, 2. California State University, Long Beach, California State Univ Long Beach, California, United States, 3. California State University Long Beach, California State University, Long Beach, California, United States, 4. Covia - Lutheran Towers, Long Beach, California, United States*

Social isolation affects one in five older adults, significantly increases the risk of premature death from all causes and is associated with higher rates of depression, anxiety and suicide. Covid-19 has exacerbated social isolation, including among older adults that reside in senior apartments. In response, a Friendly Caller Program was developed to foster intergenerational social connections among university students and residents in a large housing community that serves older adults aged 62 and older who have limited income and have mobility impairments. This study aimed to evaluate the Friendly Caller Program from the perspective of the older adult. An online survey includes questions about the participants' demographic characteristics, physical and mental health self-assessment, social support, and ways in which the Friendly Caller Program has affected these areas of their life. An open-ended question assesses older adult participant expectations of the Friendly Caller Program. Results describe the population currently being served by this program and indicate that the program has a positive influence on participants' feelings of safety, support and ability to function. Suggestions for future research include assessing university student perceptions about the benefits of participation as callers in the program and creating a toolkit that can guide other universities to create similar programs in partnership with housing communities that serve older adults.

### SAVING FALL-INJURED OLDER ADULTS FROM DEPRESSIVE SYMPTOMS: THE MEDIATING ROLE OF SOCIAL PARTICIPATION

Yalu Zhang, Lei Zhang, Jiling Sun, Xinhui Zhang, Jingjing Sun, Xinming Song, and Gong Chen, *Peking University, Beijing, Beijing, China (People's Republic)*

Falls are the second primary cause of unintentional injury deaths globally. Prior studies found that fall incidences are associated with depressive symptoms among older adults, which could reversely lead to repeated fall incidences. However, few have investigated the role of social interventions in saving fall-injured older adults from experiencing depressive symptoms among older adults. Using the Chinese Health and Retirement Longitudinal Study (CHARLS) 2011-2018 data and multiple levels of fixed-effect analysis, this study examined the potential mediating role of social participation in alternating the effect of fall injuries on depressive symptoms. For the first time, this study specified the fall-injured older adults among those who had fall incidences. It also implemented the current literature by removing the bias caused by unobservable confounding variables at provincial and city levels. The descriptive results show that 22.2% and 20.6% of rural (n=4,972) and urban (n=3,258) older adults (65+), respectively, experienced fall incidences, among whom 45.1% needed one or more times of medical treatment. The fixed-effect results show that for urban older adults, social participation accounted for partial effects (17.2%) of fall injuries on their depressive symptoms. For rural older adults, fall injuries are significantly associated with more depressive symptoms, but social participation no longer functions as the mediator. Findings from this study emphasize the necessity of collecting efforts from multiple levels to improve the social engagement of urban older adults who had fall injuries. Future studies could further specify what types of social participation would be more helpful in buffering the intervention effects.

### TECHNOLOGY ACCEPTANCE AND DEPRESSIVE SYMPTOMS AMONG COMMUNITY-DWELLING OLDER PEOPLE

Suk-Young Kang,[1] Jeungkun Kim,[2] Jeffrey Winthal,[3] and Rosemarie Lenz,[3], *1. Binghamton University, State University of New York, Binghamton, New York, United States, 2. Kangnam University, Yong-In, Kyonggi-do, Republic of Korea, 3. Binghamton University, Binghamton, New York, United States*

Depression is a major public health issue among older adults, with an estimated prevalence between 5% and 10%. The aim of this study is to explore the possible benefits technology acceptance has in reducing depression among older people. Mail-survey data were collected from community-dwelling adults over the age of 65. This method was chosen over face-to-face surveys due to Covid-19. There were 192 total participants. The GDS-5 was used to measure the level of depressive symptoms. Among the participants, 25 participants (13%) scored higher than 2, indicating the presence of depression. Using a hierarchical regression analysis revealed that the equation explained 42.4 % of the variance (adjusted R square =.382) in levels of depression (F (3,176) = 9.973, p <.000). Variance inflation factor (VIF) values were smaller than 10, indicating that multicollinearity among the correlates was not an issue. The correlates of the level of depression were: level of education, overall physical health, level of loneliness, perceived ease of technology use, attitude toward technology use, and intent to purchase new technology for older people. Results indicate that a positive attitude toward technology use might be inversely associated with depression levels. This shows how quality of life related to mental health may be improved by a change in attitude in favor of technology use. Participants were interested in learning to use new technology, and would like more opportunities to do so. Policy changes that increase lifelong learning options would help to make this happen.

### THE IMPACT OF ORAL HEALTH ON PSYCHOLOGICAL DISTRESS AMONG OLDER ADULTS IN CALIFORNIA

Esmeralda Melgoza,[1] Jennifer Archuleta,[1] Emma Tran,[2] Gabino Abarca,[2] and Anna Fiastro,[3], *1. UCLA Fielding School of Public Health, Los Angeles, California, United States, 2. UCLA, UCLA, Los Angeles, California, United States, 3. University of California, Los Angeles, Los Angeles, California, United States*

In California, the population of adults ages 65 and older is projected to increase from 11% in 2010 to 19% in 2030. The aging of the population requires modifications in public health to ensure that people are not only living longer, but also healthier lives. Oral health is an important, but often overlooked factor that affects the overall health of older adults. Poor oral health increases the risk of physical comorbidities, decreases chewing performance, limits food choices, and exacerbates weight loss. Furthermore, poor oral health disrupts social health via decreases in verbal communication and facial expressions, such as smiling. This study examines the effects of oral health, assessed by tooth condition, on psychological distress among adults ages 65 and older in California. The study uses the 2019 California Health Interview Survey (CHIS), an annual survey of a representative sample of the state's residential, noninstitutionalized population. Logistic regression models are used to determine the association between tooth condition on psychological distress controlling for gender, race, and elderly poverty index. Worsening tooth condition increases the odds of having psychological distress with lower odds among individuals 85+, and higher odds among women, and non-Hispanic whites compared to Hispanics. Public health programs and interventions are required in California to prevent and mitigate the impacts of poor oral health on psychological distress among the increasing and diverse older adult population.

### THE INTERPLAY BETWEEN RETIREMENT TRANSITION SEQUENCES AND MENTAL HEALTH: FOCUSING ON GENDER DIFFERENCES

Oejin Shin,[1] Sojung Park,[2] and BoRin Kim,[3], *1. University of Illinois at Urbana Champaign, Savoy, Illinois, United States, 2. Washington university, St.Louis, Missouri, United States, 3. University of New Hampshire, University of New Hampshire, New Hampshire, United States*

Although retirement has been given a substantial amount of attention, there are gaps in the literature on 1) the various forms of retirement (nature= voluntary/involuntary, timing= early/ late, type= full/ partial) using with previous employment history, and 2) gender differences in retirement transition. Drawing on the life course perspective, this study examined the gender differences in retirement transition sequences using the labor participation history and various forms of retirement. Data are from the 2004 to 2016 HRS with 1,653 older workers. Sequence analysis was used to answer how individuals experienced retirement in the extended time frame. OLS regression analysis was used to estimate the relationship between retirement transition sequences and depression. For both genders, eight clusters of retirement transition sequences were identified. However, the most prevalent group for males was those who experience voluntary retirement transition from full-time work in mid-time point (19%), while the most prevalent group was a gradual involuntary retirement (21%) for females. Regarding the association with depression, those who experienced voluntary retirement after full-time work in all different time points (early, mid, late) and those who retired from self-employment were less likely to have depressive symptoms for males. For females, only those who experienced voluntary retirement from full-time work in mid-time point were less likely to have depressive symptoms. This result contributes to identifying the heterogeneity of retirement transition sequences and their association with depression. The result suggests important implications of gender-specified intervention programs to prevent involuntary retirement and mental health support program for involuntary retirees.

### Session 9380 (Poster)

### MINORITY AND DIVERSE POPULATIONS: BETWEEN-GROUP DIFFERENCES AND WITHIN-GROUP EXPERIENCES

### BMI CHANGE AFTER PARENT'S DEATH IN ADULTHOOD: EXAMINING AGE AND RACE DIFFERENCES WITH TIME-VARYING EFFECTS MODELING

Hye Won Chai, Hyungmin Cha, and Debra Umberson, *The University of Texas at Austin, Austin, Texas, United States*

Parental bereavement in adulthood is a stressful event that can have adverse health consequences for middle and older adults, including weight gain. Considering that the impact of bereavement is found to vary depending on the timing of death as well as across race/ethnicity, changes in weight after a parent's passing may also be contingent on the timing of parent's death and the bereaved individual's race/ethnicity. Using Time-Varying Effects Modeling (TVEM), this study examined whether changes in BMI following a parent's death differed across respondent's age when their parent passed away. We also tested whether these age differences varied by race. Data came the Health and Retirement Study (HRS) Waves 1 – 13 and we selected respondents who experienced passing of either parent while participating in HRS. Analyses were run separately for mother's death (n = 6,191) and father's death (n = 3,301). Results showed significant racial/ethnic differences in BMI change following a mother's death, particularly during late midlife to early late life. Specifically, non-Hispanic White and Black adults showed a greater increase in BMI compared to Hispanic adults. These race differences were consistent for father's death as well, but to a lesser extent compared to mother's death. Results suggest that White and Black adults who lost their parents between late midlife and early late life gained more weight compared to their Hispanic counterparts. This may be attributed to the racial/ethnic differences in health behaviors in response to parent's death.

### CARING FOR THE STONEWALL GENERATION: ASSESSMENT OF STAFF TRAINING FOR TRANSGENDER PERSONS LIVING IN LONG TERM CARE

Sara English, *University of South Carolina, Blair, South Carolina, United States*

Transgender persons who came of age in the late 1960s are considered LGBTQ+ elders - The Stonewall Generation. These persons experience unique bio-psychosocial challenges, often complicated by a history of a lack of access to good medical care and social supports. Discrimination and bias can influence the provision of care and the protection of privacy for transgender or gender non-conforming persons. Staff training is essential to provide ethical care for aging trans persons who require residential care. This presentation examines current staff training modules of 100 Long Term Care (LTC) facilities, assessing training needs to provide affirming, culturally competent, and ethical care for sexual and gender minorities.

**Keywords:** cultural competence; Long Term Care; staff training; transgender

### CULTURAL SAFETY WITHIN THE INDIGENOUS HEALTH CONTEXT: FINDINGS FROM A REVIEW OF REVIEWS

Juanita-Dawne Bacsu,[1] Christina Chakanyuka,[2] Andrea DesRoches,[3] Jennifer Walker,[4] Jessy Dame,[5] Megan O'Connell,[1] Paisly Symenuk,[6] and Lisa Bourque Bearskin,[7], *1. University of Saskatchewan, Saskatoon, Saskatchewan, Canada, 2. University of Victoria, Victoria, British Columbia, Canada, 3. University of Saskatchewan, University of Saskatchewan, Saskatchewan, Canada, 4. Laurentian University, Sudbury, Ontario, Canada, 5. UBC, Vancouver, British Columbia, Canada, 6. Independent, Edmonton, Alberta, Canada, 7. Thompson Rivers University, Kamloops, British Columbia, Canada*

First Nations, Inuit, and Métis older adults often face systemic barriers to accessing culturally safe and equitable healthcare, including racism, structural injustice, and a historical legacy of colonialism. However, there is a paucity of knowledge on cultural safety interventions and implementation strategies in care for older adults. This presentation aims to: 1) explore persistent barriers to achieving health equity and advancing cultural safety in healthcare; and 2) identify cultural safety interventions to improve healthcare for Indigenous older adults. Guided by Arksey and O'Malley's scoping review framework, we conducted a review of reviews published between January 2010 to December 2020 on Indigenous cultural safety in healthcare. We searched five databases (CINAHL, PubMed, Scopus, Web of Science, and Google Scholar) and hand-searched reference lists of relevant articles. We conducted a thematic analysis to identify patterns and themes in the literature. Key barriers to achieving health equity and advancing cultural safety in healthcare included care providers lacking knowledge of Indigenous culture, power imbalances, racism, and discrimination. A range of cultural safety interventions were identified, from education and training initiatives for healthcare providers (emergency physicians and occupational therapists) to collaborative partnerships with First Nations, Inuit, and Métis communities. As First Nations, Inuit, and Métis populations age, there is a growing need for safe healthcare services for Indigenous older adults, and these findings suggest focusing on healthcare providers knowledge and attitudes is key. Research is necessary to develop, implement, and evaluate cultural safety interventions aimed at healthcare providers to improve healthcare for Indigenous older adults.

### CUMULATIVE COVID-19 DISPARITIES IN NURSING HOME: FOCUSING ON GEOGRAPHICAL FACTORS AND RACIAL COMPONENTS

Sungjae Hong,[1] and Shannon Meija,[2], *1. University of Illinois--Urbana-Champaign, Champaign, Illinois, United States, 2. University of Illinois, Champaign, Illinois, United States*

The impact of COVID-19 has been greatest in vulnerable US populations. This study examines the cumulative geographical and racial disparities of COVID-19 cases in nursing homes. Analysis of COVID-19 Nursing Home Data from Centers for Medicare & Medicaid Services was limited to weekly reports from the nursing homes that reported the ratio of black residents, from 2020-05-31 to 2021-01-17 (N=268,222 from 8,026 nursing homes). The outcomes were weekly COVID-19 cases and death per 1,000 occupied beds.

Nursing homes were categorized by a geographic (rural vs. urban) and racial composition (>50% of residents are black vs. else). Elapsed time and county-level weekly COVID-19 cases and deaths/1,000 people were the key covariates. Multilevel zero-inflated negative binomial regression revealed evidence of cumulative COVID-19 disparity between rural and urban nursing homes. At the earliest time, COVID-19 incidence was lower in rural nursing homes than in urban nursing homes (IRR=0.406 for cases, 0.034 for death). The significant interaction with time implied that, over and above evolving disease prevalence, rural nursing homes became more likely than urban nursing homes to experience COVID-19 over time (IRR=1.057 for cases, 1.193 for death). Nursing homes, with >50% black residents, were more likely to experience COVID-19 than their counterparts at the earliest time (IRR=1.339 for cases, 5.630 for death), but independent of local disease prevalence, this disparity decreased over time (IRR=0.973 for cases, 0.972 for death). Our findings suggest that racial and geographic factors contribute to the cumulation of disadvantage during the COVID-19 crisis at the second half of 2020.

### EXPERIENCES OF BLACK AND WHITE FAMILY HOSPICE CAREGIVERS: ANXIETY, DEPRESSION, QOL, BURDEN, HOSPICE COMMUNICATION

Lauren Starr,[1] Karla Washington,[2] Subhash Aryal,[3] Debra Parker Oliver,[2] and George Demiris,[4], *1. University of Pennsylvania, Philadelphia, Pennsylvania, United States, 2. Washington University in St. Louis, St. Louis, Missouri, United States, 3. University of Pennsylvania School of Nursing, Philadelphia, Pennsylvania, United States, 4. School of Nursing, University of Pennsylvania, University of Pennsylvania, Pennsylvania, United States*

Although hospice care benefits seriously ill patients and their families, growing evidence suggests anxiety, depression, and altered quality of life are prevalent among family hospice caregivers. It is unknown if Black and white family hospice caregivers experience differences in mental health, quality of life, caregiver burden, or quality of hospice communication. In this secondary analysis of baseline data collected from 717 family hospice caregivers in two randomized clinical trials, we compared anxiety (GAD-7), depression (PHQ-9), quality of life (CQLI-R), caregiver burden (Zarit), and caregiver-reported quality of hospice team communication (CCCQ) between Black and white caregivers. Black and white caregivers differed demographically across multiple variables. In bivariate analysis, we found no differences in depression (P=0.3536), anxiety (P=0.0733), caregiver burden (P=0.6680), and perceptions of caregiver-centered hospice communication (P=0.4549). White caregivers reported lower quality of life than Blacks (P=0.0386), specifically in emotional (P=0.0321) and social (P=0.0002) domains. Financial and physical quality of life did not differ. In multivariate regression analyses controlling for caregiver and patient factors, we found no racial differences in depression (P= 0.5071), anxiety (P = 0.7288), quality of life (P=0.0584), caregiver burden (P=0.9465), or hospice communication (P=0.8779). Variables explained 7.7% to 20% of variability in outcomes, suggesting research is needed to understand which other factors contribute to hospice caregiver coping and communication experiences. Results suggest Black and white informal hospice caregivers experience similar levels of anxiety, depression, burden, and perceptions of hospice team communication quality. Interventions to support hospice caregivers across racial groups are needed.

### EXPLORING THE IMPACT OF ALCOHOL AND OTHER DRUG USE ON HIV CARE AMONG AFRICAN AMERICAN OLDER ADULTS LIVING WITH HIV/AIDS

Lesley Harris,[1] Sydney Silverstein,[2] Timothy Crawford,[2] Jelani Kerr,[1] and Diana Ball,[1], *1. University of Louisville, Louisville, Kentucky, United States, 2. Wright State University, Dayton, Ohio, United States*

For people living with HIV, there are multiple barriers to engagement with care. This study qualitatively examines the role of use of alcohol and other drugs (AOD) on the health and management of Human Immunodeficiency Virus (HIV) disease among older African Americans (≥50 years). It draws on interviews conducted with twenty-seven older African Americans living with HIV in the Louisville, Kentucky area. Interviews were transcribed verbatim and then analyzed using constructivist grounded theory analytic techniques. Participants' understandings of their AOD use fell on a continuum of problematic use to use for self-care. Regardless of where participants fell on this continuum, they faced a) environmental impacts of AOD use and b) current or historic discrimination from the health care system. The analysis focused on gaining a deeper understanding of the intersection of AOD use and engagement in the HIV care continuum. This revealed six major phases, which occurred at various stages of the continuum: (1) Linking AOD use as the cause of HIV diagnosis (2) Having AOD use facilitate denial of HIV, (3) Experiencing problematic use, (4) "Testing the Waters," (5) Relying on AIDS Service Organizations (ASO) and medical providers and (6) Maintaining health and/or using AOD for self-care. We discuss the ways that stigma along the lines of race, gender, and age intersect with co-occurring conditions such as substance use disorders in complex and multifaceted ways. Recommendations include assessing a patients' AOD use in relationship to the HIV care continuum to assess patients' experiences and barriers within systems of care.

### GEOGRAPHIC VARIATION IN GENDER GAP AMONG OLDER ADULTS IN INDIA AND CHINA: APPLICATION OF A NEW AGEING MEASURE

Arun Balachandran,[1] and Feinian Chen,[2], *1. University of Maryland College Park, Hyattsville, Maryland, United States, 2. University of Maryland College Park, University of Maryland, Maryland, United States*

A continuous rise in the life expectancy of females above that of the males among older adults in India and China may give an impression that the gender gap in health is decreasing. However, given the systemic bias against females in these countries across multiple facets, and the diversity across provinces, a fuller understanding of gender gap calls for (a) understanding the gender gap in multiple dimensions of health, and (b) understanding the variations across provinces. We estimate a multi-dimensional old-age threshold (MOAT) across provinces in India and China, that specifies different old-age thresholds for female and male populations after simultaneously accommodating for multiple dimensions related to their health. These aspects of health include remaining life expectancy, intellectual and functional health. We estimate the gender gap across provinces in these countries by differencing the MOAT of males against that of females. In addition, we also illustrate the gender gap across individual dimensions of health. Our results show that females in almost all the provinces of India and China have a lower MOAT than their male counterparts, showing an earlier advent of 'old-age' among females compared to males. The estimates based on remaining life expectancy shows gender gap in favor of females, but the estimates of multi-dimensional gender gap are higher and biased against females. A huge variation is seen across provinces, with Karnataka and Hubei showing lower levels of gender gap and Rajasthan and Yunnan showing higher gender gaps in India in China respectively.

### HEALTH LITERACY AND HEALTH CONDITIONS AT THE INTERSECTIONS OF GENDER AND RACE IN LATER LIFE

Takashi Yamashita,[1] Darren Liu,[2] Betty Burston,[3] and Jennifer Keene,[3], *1. University of Maryland, Baltimore County, Baltimore, Maryland, United States, 2. Des Moines University, Des Moines, Iowa, United States, 3. University of Nevada, Las Vegas, Las Vegas, Nevada, United States*

The benefits of health literacy are well-documented. Health literacy is a set of skills to locate, understand, and use health-related information to make optimal health decisions. However, relatively less is known about the long-term relationship between health literacy and overall health conditions among older adults. Additionally, health literacy and health at the intersection of gender and race/ethnicity, rather than gender and race separately, are yet to be investigated. This study analyzed sub-samples (n = 1,260 adults age 50+) of the 2010 Health and Retirement Study (HRS) health literacy module data, and the 2012, 2014, and 2016 HRS data to examine the trajectories of health based on eight physical and mental conditions (0-8 points: better-worse) among older adults. Latent growth curve mixture models were used to investigate the changes in health and six groups defined by gender (women and men) and race/ethnicity (White, Black, and Hispanic). Results showed that overall health deteriorated over time (latent-slope = 0.19, $p < 0.001$) but the trajectories were diverse (latent-slope variance = 0.06, $p < 0.001$). Greater health literacy (0-5 points: worse-best scaling), which was measured with a validated scale, was associated with better overall health only among White women and men. Notably, White women received the baseline health benefits ($b = -0.20$, $p < 0.05$) from health literacy whereas Black women ($b = 0.09$, $p > 0.05$) did not [$\Delta b = 0.09 -(-0.20) = 0.29$, $p < 0.05$]. Other detailed comparisons, theoretical explanations, and public health policy implications for diverse older populations were evaluated.

### I KNOW I'M HEALTHY BECAUSE: QUALITATIVE DEFINITIONS OF HEALTH IN OLDER RURAL AFRICAN-AMERICANS

Carolyn Adams-Price,[1] and Muhammed Riaz,[2], *1. Mississippi State University, Mississippi State, Mississippi, United States, 2. Mississippi State University, Mississippi State University, Mississippi, United States*

In recent years, there has been attention to health disparities between racial groups in the US, and between urban and residents. Older rural African Americans are at high risk, but have historical reasons tor distrust the health care system. This study examined qualitative definitions of health in older rural African Americans. Our sample included 47 African Americans aged 52-79 (20 male, 27 female, median age = 66 ) from non metropolitan counties in northeast Mississippi, at least 10 miles from the nearest town of more than 1000. Participants rated their health on a 5-point scale; only 1 person rated their health as a 5 for excellent. On average, they rated their health as fair. Most reported significant health problems, (mean=2) including 17 (36%) who reported having been diagnosed with diabetes. Participants were asked by interviewers "how they knew they were healthy." Their responses were transcribed. Using phenomenological methods, participants' responses were sorted into naturally-occurring categories, which were retested against the data. The categories that emerged were Performing Basic Tasks is Enough/I'm OK (12), Good Health Due to Healthy Behaviors (8), Healthy Due to Social Support or God (11), Healthy Despite One Problem (6), and I'm Not OK (7). Given that our sample is somewhat younger than most gerontological samples, participants seemed to have relatively low expectations about their health, which might not be surprising considering the health problems in the sample. Interventions to improve the health of this group should concentrate on increasing health self-efficacy and expectations.

### INTERSECTIONS OF GENDER AND RACE AMONG OLDER WORKERS DURING THE COVID-19 PANDEMIC

Takashi Yamashita,[1] Wonmai Punksungka,[1] Samuel Van Vleet,[2] Abigail Helsinger,[3] and Phyllis Cummins,[2], *1. University of Maryland, Baltimore County, Baltimore, Maryland, United States, 2. Miami University, Oxford, Ohio, United States, 3. Scripps Gerontology Center, Miami University, Oxford, Ohio, United States*

The novel coronavirus/COVID-19 pandemic in 2020 has impacted the aging workforce. In addition to local data and case studies that are rapidly increasing, baseline national-level inquiries are needed for investigating relevant social inequalities. Also, the intersections of gender and race/ethnicity among older adults are critical yet understudied areas. For example, older minority women's experience in the pandemic, compared to older men, are yet to be investigated. We analyzed the nationally representative 2020 Health and Retirement Study (HRS) COVID-19 module data. Based on the sample of 2,086 adults aged 50 years and older, employment during the pandemic as well as psychosocial measures, including social support and attitudes toward work, are examined. We used survey-weighted binary logistic regression models. Results showed that older Black women (Odds-ratio = 0.52, $p = 0.02 < 0.05$) were less likely to work for pay compared to White women during the pandemic. Also, older Hispanic men (Odds-ratio = 2.82, $p = 0.03 < 0.05$) were more likely to work for pay than older White men. Older Hispanic women (Odds-ratio = 2.41, $p = 0.03 < 0.05$) were more likely to worry about getting social support during the pandemic than White women. However, there was no significant differences in the changes in attitudes toward work across gender and racial/ethnic groups during the pandemic. Based on the baseline national data analysis, we discussed possible policy changes and interventions that consider the intersections of gender and race/ethnicity to help older adults re-adjust to post-pandemic work environments and labor markets.

### KEEP ON KEEPIN' ON: INVESTIGATING ACES AND POSITIVITY AMONG BEREAVED BLACK MIDDLE AGED AND OLDER ADULTS

Danielle McDuffie, *The University of Alabama, Tuscaloosa, Alabama, United States*

Many of the most damaging life events are more prevalent among Black older adults. Black people have been found to have higher amounts of adverse childhood experiences (ACEs), which are linked to detrimental life impacts. Additionally, bereavement occurs at a higher rate among Black people and older adults. Despite these challenges, Black older adults have been repeatedly cited overcoming these challenges. Accordingly, the present study sought to investigate whether Black middle to older aged adults who encountered two of life's most difficult challenges (i.e. bereavement and ACEs) would still maintain positivity. 103 middle to older Black adults (M=44.72, SD=5.48, 67% male) from a larger online grief study were probed about factors including time since loss, positive outlook, and ACES. A linear regression and mediation analysis were used to analyze the data. ACES were found to significantly predict positive outlook among bereaved middle to older Black adults (F=11.46, p=.001), such that as the number of ACES increased, so did positivity in spite of bereavement. Notably, this association was not mediated by time since loss. Results from this study provide evidence that even when faced with some of life's most difficult events, Black middle to older adults were still able to reframe their situation with a positive focus. The ability for Black middle to older aged adults to reframe their tragedies into positivity could provide a basis for the use of Positive Psychological techniques specifically within this population. Additionally, this study provides further evidence that Black people exhibit exceeding resilience.

### MANIFESTATIONS OF RACIAL TRAUMA IN BEREAVED MIDDLE TO OLDER AGED BLACK ADULTS

Danielle McDuffie, *The University of Alabama, Tuscaloosa, Alabama, United States*

Black adults have a higher likelihood of experiencing bereavement and increased negative implications of systemic racism compared to other groups. The effects of racism have also been suggested to have an impact on how bereaved Black individuals conceptualize their loss and the deceased. However, there is limited literature on how direct and indirect childhood experiences with racial violence and viewing racially violent deaths impact bereaved Black adults later in the lifespan. The current study seeks to explore the impacts childhood engagement with racial violence might have on bereaved middle to older Black adults. 103 middle to older aged Black adults (M=44.72, SD=5.48, 67% male) from a larger online grief study were probed about factors including somatization, depression, affect, grief, and the prevalence and intensity of exposure to race-based violence during their childhoods. Linear regressions and bivariate correlations were used for data analysis. Childhood racial violence significantly predicted grief (F=6.348, p=.013). Additionally, experiencing childhood racial violence was significantly associated with somatization (r=.197, p=.047), depression (r=.198, p=.045), and negative affect (r=.256, p=.010). Endorsed intensity of racial violence was significantly associated with depression and negative affect (r=.464, p=.000; r=.440, p=.000, respectively). Bereaved Black middle to older adults seem greatly impacted by childhood experiences of racial violence. It is important to consider the role outside cultural influences such as racial trauma might have on other deleterious mental health experiences such as bereavement. Furthermore, in the assessment of ACEs among Black and other people of color, it could be important to include childhood racial violence.

### MINORITY STRESS IN THE CONTEXT OF THE DISABLEMENT PROCESS MODEL

Jeffrey Lentz, *University of North Georgia, Oakwood, Georgia, United States*

The changing demographics and growing diversity in the United States pose significant challenges for researchers, particularly scholarship involving sexual minority adults' health and aging processes. Not much is known about how all minority stressors could lead to a disability. Sexual minority adults are at a greater risk of developing a disability later in life than their heterosexual counterparts (Fredriksen-Goldsen, Kim, & Barkan, 2012). Drawing from critical components of the disablement process model (Verbrugge & Jette, 1994), this dissertation sought to understand the relationship between minority stress and disability status among sexual minority adults 50 years and older. Minority stress in the context of the disablement process model is a social condition. While exploring the relationship between minority stress and disability status, intra-individual factors and extra-individual factors were assessed to see if they mediated the relationship between minority stress and disability status among sexual minorities 50 years and older. Discrimination is significantly associated with having a disability. None of the intra-individual factors and extra-individual factors mediated the relationship between minority stress and disability; however, several intra- and extra-individual were associated with greater or lesser odds of experiencing a disability. This dissertation concluded that discrimination is associated with disability status among sexual minority adults 50 years and older. On the other hand, the disablement process model does not support minority stress as a social condition leading to a disability. On the other hand, this dissertation's results support the ideology that experiencing discrimination is associated with a disability.

### MOTIVATION TO LEARN AT THE INTERSECTIONS OF AGE, GENDER, AND RACE

Takashi Yamashita,[1] Thomas Smith,[2] Shalini Sahoo,[1] and Phyllis Cummins,[3], *1. University of Maryland, Baltimore County, Baltimore, Maryland, United States, 2. Northern Illinois University, DeKalb, Illinois, United States, 3. Miami University, Oxford, Ohio, United States*

Continuing adult education and training, or lifelong learning, has become increasingly important to fully engage in rapidly changing technology and information-rich societies. However, without motivation to learn (MtL), lifelong learning participation is unlikely to occur. Although previous research has identified lifelong learning gaps by various demographic characteristics, including age, gender, and race/ethnicity, little is known about the intersectionality or differences in MtL across specific sub-groups (e.g., older Black women vs. older Black men) at the national level. The current study analyzed U.S. data from the 2012/2014/2017 Program for International Assessment of Adult Competencies (PIAAC) to examine MtL at the intersections of age (five 10-year age groups), gender (women vs. men), and race (White vs. Black). The previously established 4-item latent MtL construct was evaluated for twenty sub-groups using the alignment optimization method, which is a machine learning algorithm for latent mean estimation and simultaneous multiple group comparisons. Results showed that the latent MtL construct was validly measured across the sub-groups, and the estimated sub-group means were then used to develop a national MtL profile. Overall, older adults tended to have lower MtL than younger age groups. Notably, compared to than older Black men age 66+ years, older White men aged 55-65 and 66+ years old had lower MtL (latent mean differences of -0.29 and -0.41, respectively, $p < .05$). Additionally, older Black women had significantly lower MtL than older Black men (latent mean difference = -0.50, $p < .05$). The national MtL profiles, the intersectionality and policy implications were discussed.

### MULTIMORBIDITY TRAJECTORY CLASSES AS PREDICTED BY RACE, ETHNICITY, AND SOCIAL RELATIONSHIP QUALITY

Jason Newsom,[1] AnneMarie O'Neill,[1] Emily Denning,[1] Anda Botoseneanu,[2] Heather Allore,[3] Corey Nagel,[4] David Dorr,[5] and Ana Quiñones,[5], *1. Portland State University, Portland, Oregon, United States, 2. University of Michigan - Dearborn, Dearborn, Michigan, United States, 3. Yale School of Medicine, New Haven, Connecticut, United States, 4. University of Arkansas for Medical Sciences, Little Rock, Arkansas, United States, 5. Oregon Health & Science University, Portland, Oregon, United States*

Growth mixture modeling was used to classify multimorbidity (≥2 chronic conditions) trajectories over a 10-year period (2006-2016) in the Health and Retirement Study (N = 7,151, mean age = 68.6 years). Race/ethnicity (non-Hispanic Black, Hispanic, non-Hispanic White) and social relationship quality (positive social support and negative social exchanges, such as criticisms) were then used to predict trajectory class membership, controlling for age, sex, education, and wealth. We identified three trajectory classes: initial low levels and rapid accumulation of multimorbidity (increasing: 12.6%), initial high levels and gradual accumulation of multimorbidity (high: 19.5%), and initial low levels and gradual accumulation of multimorbidity (low: 67.9%). Blacks were more than twice as likely to be in the increasing (OR = 2.04, CI[1.29,3.21]) and high (OR = 2.28 CI[1.58,3.206]) multimorbidity groups compared with Whites, but there were no significant differences between Hispanics and Whites for either trajectory class (OR = .84 CI[.47,1.51]and OR = .74 CI[.41,1.34], respectively). Increments in perceived support were associated with significantly lower risk of membership in the increasing (OR = .59, CI[.46,.78]) and high classes (OR = .54 CI[.42,.69]), and increments in negative exchanges were associated with significantly higher risk of membership in the increasing (OR = 1.64 CI[1.19,2.25]) and high classes (OR = 2.22 CI[1.64,3.00]). These results provide important new information for understanding health disparities and the role of social relationships associated with multimorbidity in middle and later life that may aid in identifying those most at risk and suggesting possible interventions for mitigating that risk.

### PERSPECTIVES ON AGING WELL AMONG OLDER BLACK WOMEN IN THE US

Jacquelyn Coats, *Brown School, Washington University in St. Louis, Saint Louis, Missouri, United States*

Despite changes in the demography of older Black women who are living longer, there is limited research on how older Black women conceptualize and understand successful and healthy aging. The objective of this presentation is to interrogate the meaning and cultural aspects of aging among older Black women, gaining insight into how gender and race operate and intersect to shape experiences and perceptions of aging. Using an intersectionality framework, this qualitative study was conducted with three older Black women. The women ranged in age from 58-65 years old, each residing in an US urban city (Detroit, St. Louis, Atlanta). Data were collected between October and November 2020, using a semi-structured, open-ended interview protocol to encourage participants to provide in-depth descriptions of how they conceptualized aging. Interviews were between 2 – 2.5 hours, conducted via videoconferencing, and audio-recorded for transcription. Participants discussed: the life experiences that have shaped their ability to age well; what it means to age well, and factors that might hinder someone from aging well. Transcripts were coded using a constructivist grounded theory approach. Results revealed six themes Black women in later life identify: aging well, aging as a mindset, independence and freedom, authenticity, personal control and preparation, and aging role models. This extends the knowledge base on how older Black women view aging and factors that enhance or diminish their ability to age well. Results from this study can be used to enhance the development of public health and social work interventions with older Black women.

### RACE/ETHNICITY, VASCULAR AGING, AND MORTALITY RISK: EVIDENCE FROM THE HEALTH AND RETIREMENT STUDY

Kevin Heffernan, Janet Wilmoth, and Andrew London, *Syracuse University, Syracuse, New York, United States*

Vascular aging, which is associated with cardiovascular disease risk and mortality, is characterized by increasing arterial stiffness. The gold standard method for the assessment of arterial stiffness is carotid-femoral Pulse Wave Velocity (cfPWV). An emerging body of research suggests that cfPWV can be reasonably estimated from two commonly measured clinical variables—age and blood pressure. Thus, estimated Pulse Wave Velocity (ePWV) holds promise as a novel and easily obtained measure of arterial stiffness that can be used to study vascular aging, particularly with nationally representative datasets that collect biomarker data on sufficiently large sample sizes to examine race/ethnic differences. This analysis uses data from the 2006-2016 Health and Retirement Study to examine race/ethnic variation in the relationship between ePWV and mortality risk. We estimate logistic regression models predicting mortality over an eight-year period for four racial/ethnic groups: White, Black, Other, and Hispanic. Controls are included for sociodemographic characteristics, health status and behaviors, and blood biomarkers such as C-reactive protein, cystatin-C, hemoglobin A1c, total cholesterol and high-density lipoprotein (HDL) cholesterol. The results indicate ePWV increases the risk of mortality in the total sample and among each race/ethnic group, net the effect of age, systolic blood pressure, and diastolic blood pressure. Mechanisms that mediate this relationship are explored. The findings provide insight into vascular aging processes that influence mortality risk among race/ethnic groups.

### RACIAL/ETHNIC DIFFERENCES IN THE LONGITUDINAL EFFECTS OF FEAR OF FALLING ON FALLS

Tuo Yu Chen,[1] and Giyeon Kim,[2], *1. Taipei Medical University, Taipei, Taipei, Taiwan (Republic of China), 2. Chung-Ang University, Seoul, Seoul-t'ukpyolsi, Republic of Korea*

Research suggests that the effects of fear of falling on falls may differ by race/ethnicity. We investigated whether race/ethnicity (White, Black, and Hispanic) moderated the longitudinal effects of fear of falling on the incidence of falling and having a repeated fall among community-dwelling older adults. We used data from 2011-2018 of the National Health and Aging Trends Study (NHATS). These included a total of 19,516 person-intervals from 5,113 respondents. Self-reported any fall in the past year was the outcome variable with baseline fear of falling as the predictor and race/ethnicity as the moderator. Known risk factors for falls were included as covariates. Results showed that among respondents without the experience of falling at baseline, baseline fear of falling significantly increased the odds of a new-onset of fall at 1-year follow-up among Blacks, compared to Whites. Among respondents who already fell at baseline, baseline fear of falling significantly increased the odds of having a repeated fall later on among Hispanics, compared to Whites. Clear evidence of racial/ethnic differences was found in the relationship between fear of falling and falls among community-dwelling older adults in the U.S. Special attention should be paid to Black older adults with a fear of falling but have not fallen down recently and Hispanics with fear of falling and have fallen in the past year. Readily available educational programs should be actively advertised to older adults to reduce the fear of falling and culturally tailored educational programs should be developed for older adults from racial/ethnic minority backgrounds.

### RELATIONSHIP BETWEEN MEANINGFUL ACTIVITY ENGAGEMENT AND WELL-BEING MODERATED BY RACE

Dugan O'Connor, and Jennifer Smith, *Mather, Evanston, Illinois, United States*

Engaging in meaningful activities has benefits for health and well-being in older adults; however, racial differences in meaning from activities have been observed. The current study examined how the relationship between engagement in a variety of meaningful activities and well-being differs for Black and White older adults. Participants (130 White and 101 Black older adults), ages 53 to 90 (M=69), completed an online survey that measured the extent to which engaging in various activities provided meaning or fulfillment as well as measures of purpose in life, positive affect, negative affect, and loneliness. Overall, the most meaningful activities included “spending time with family,” “reading,” and “being outdoors/experiencing nature.” The least meaningful activities included “job/career,” “caring for pets/animals,” and “supporting a social or political cause.” Black older adults reported greater meaning from “religious faith,” “spiritual practices/meditation,” “spending time with family,” and “listening to music,” and less meaning from “caring for pets/animals,” compared to White older adults. A series of multiple regression analyses (with age, gender, education, and income as covariates) revealed that greater meaning across activities was associated with lower loneliness, regardless of race. Significant Meaning x Race interactions revealed meaning was positively associated with sense of purpose and positive affect for Black but not White older adults. These findings suggest that finding meaning in leisure activities is a psychological resource that may contribute to Black older adults’ well-being. The racial differences in sources of meaning further support the importance of considering the target population when designing activity programs for older adults.

### THE EFFECTS OF LATENT CLASSES OF STRESS ON HEALTH OUTCOMES IN KOREAN MIDDLE-AGED ADULTS: A FOCUS ON GENDER DIFFERENCES

Kyuyoung Cho, *Dong-A University, Busan, Pusan-jikhalsi, Republic of Korea*

This study indicated the effect of the latent classes of stress on the physical and psychological health outcomes in Korea. Using the 2010 Korea Health Panel Study, 1,689 middle-aged adults (women: n=793, men: n=896) were analyzed to identify the latent classes of stress by gender using Latent Profile Analysis (LPA). After the determination of the number of latent classes, health outcomes (anxiety/depression and health status) were also regressed on the latent classes including covariates (age, marital status, and education level). The perceived stresses (financial diversity, disease of self or family, children’s education, and family conflicts) are classified as the 2-class model for women and the 3-class model for men. The classes of women are named ‘high stress and ‘low stress; however, the classes of men are named ‘family-related stress’, ‘disease stress’, and ‘low stress.’ The different combinations of stress are associated with anxiety/depression and health status respectively. This study will discuss the difference of latent stress classes by gender and extend the understanding of stress groups and health outcomes.

### THE INTERACTIVE EFFECTS OF EDUCATION AND SOCIAL SUPPORT ON BLOOD PRESSURE IN AFRICAN AMERICANS

DeAnnah Byrd,[1] Yanping Jiang,[2] Samuele Zilioli,[3] 5Roland Thorpe,[4] Peter Lichtenberg,[2] and

Keith Whitfield,[5], *1. Arizona State University, Phoenix, Arizona, United States, 2. Wayne State University, Detroit, Michigan, United States, 3. Wayne state university, Detroit, Michigan, United States, 4. Johns Hopkins Bloomberg School of Public Health, Baltimore, Maryland, United States, 5. University of Nevada Las Vegas, Las Vegas, Nevada, United States*

This study examined whether the effects of received and provided social support on blood pressure (BP) would differ by education. Data from 602 African American adults (48-95 years) enrolled in the Baltimore Study of Black Aging—Patterns of Cognitive Aging were analyzed using multiple linear regression. We found no main effects of received and provided social support on BP. However, a significant moderation effect was observed for systolic BP, such that greater received social support was positively associated with higher systolic BP among individuals with low levels of education, adjusting for age, sex, chronic health conditions, and depressive symptoms. The findings demonstrate that social support and education have joint effects on blood pressure, which highlights the importance of considering psychosocial determinants of adverse cardiovascular health outcomes that disproportionately affect African Americans.

### THE RELATIONSHIP BETWEEN METABOLIC HEALTH AND MORTALITY AMONG OLDER HISPANICS IN THE US AND MEXICO

Maria Carabello, *University of Michigan, Ann Arbor, Michigan, United States*

Studies consistently show that Hispanics, especially first-generation Mexican immigrants, face lower mortality risks in mid-to-late life than US-born non-Hispanic whites. This extended lifespan defies expectations given Hispanics' disadvantaged socioeconomic status relative to whites and thus is referred to as the Hispanic paradox. However, it remains an open question as to whether the Hispanic paradox in mortality mirrors a lower chronic disease burden. To address this gap, this study will combine and leverage two harmonized longitudinal population-based data sources of late-middle-aged and older adults in the United States and Mexico; the Health and Retirement Study and the Mexican Health and Aging Study. First, I evaluate differences in the association between metabolic syndrome (MetS) and mortality risk for older adults living in Mexico, first-generation Mexican immigrants to the US, US-born Mexican Americans, and US-born whites. Second, I explore the extent to which the proportion of deaths attributable to MetS in each of these groups can be explained by differences in socioeconomic and health/behavioral characteristics. This study uses Cox proportional hazards models to estimate the mortality risks of MetS across groups, as well as the associated population attributable fractions (PAFs) to investigate potential differences within a decomposition framework. Developing this detailed understanding of metabolic health and the associated mortality risks across multiple generations of older Mexican immigrants may help us identify modifiable lifestyle and behavioral factors to better manage these conditions and alleviate possible complications as current and future generations of Mexican immigrants age in the US.

### THE UTILITY OF SOCIAL MEDIA RECRUITMENT TO ACHIEVE A MORE DIVERSE PARTICIPANT SAMPLE

Faith-Christina Washington,[1] Tai-Te Su,[1] Aileen Griffin,[2] Jacob Sosnoff,[3] and Shannon Meija,[4], *1. University of Illinois at Urbana-Champaign, Champaign, Illinois, United States, 2. University of Illinois at Urbana-Champaign, Aurora, Illinois, United States, 3. School of Health Professions, University of Kansas Medical Center, Kansas City, Kansas, United States, 4. University of Illinois, Champaign, Illinois, United States*

The COVID-19 pandemic created an immediate, lasting impact on recruitment methods in academic research, most notably in the field of gerontology. To protect older adult participants' health during the COVID-19 crisis, the Daily Balance Project, a 30-day micro-longitudinal study of older adults' awareness of balance in daily life, shifted to complete remote administration. Our new remote protocol included developing new methodologies to recruit participants with varying degrees of fall risk and educational attainment. In this study, we present our approach to remote online recruitment and compare educational attainment, objective and subjective fall risk, and alignment of objective/subjective fall risk across three samples recruited via a) Fall Clinic registry (16 participants); b) University e-newsletter to faculty and staff (5 participants); c) social media recruitment (7 participants). Eligibility included being 65+ and wireless internet at home. For samples a and b, screening assessments were conducted via phone while baseline assessments were conducted in-person. For sample c, screener and baseline assessment were virtual. Analysis of recruitment methods aims to determine whether recruitment via social media platforms may provide a sample of participants with more variation in fall risk or alignment of subjective versus objective balance. Results demonstrate no significant differences in educational attainment ($p=0.7949$) or balance confidence ($p=0.213$), despite significant differences in the alignment of objective and subjective fall risk ($p=0.031$). Participants from samples a and b proved more able to accurately assess fall risk, while sample c had the most misalignment between subjective and objective fall risk assessments.

### Session 9385 (Poster)

### MINORITY AND DIVERSE POPULATIONS

### ARE AGE DIFFERENCES BETWEEN PARTNERS RELATED TO GENDER AND GENERATIONS AMONG MIDDLE-AGED AND OLDER ASIAN AMERICANS?

Minzhi Ye,[1] and Di Mei,[2], *1. Benjamin Rose Institue on Aging, Benjamin Rose Institue on Aging, Ohio, United States, 2. The University of Hong Kong, The University of Hong Kong, Not Applicable, Hong Kong*

Previous research has shown that women who immigrate to the United States tend to partner with much older spouses. However, most studies have focused on young people and first-generations. Spousal age differences among older Asian Americans with different generations have not been well studied. Using data from the Annual Social and Economic Supplement survey (2013-2019), we employed the segmented assimilation theory to test 7,064 married middle-aged and older (50+) Asian Americans. Logistic regression analyses were conducted to understand the association between spousal age differences and individual background, including gender, ethnicity, socioeconomic status, generation, and marriage types. Of the 3,342 men, 20% were married to wives at least 6 years younger and 2% were married to wives at least 6 years older. Men who were Japanese or had inter-ethnical marriages were more likely to marry women at least 6 years older. Men who were Indian, Vietnamese, or having an interracial marriage were more likely to marry women at least 6 years younger. Of the 3,722 women, 3% were married to husbands at least six years younger and 19% were married to husbands at least six years older. Women who were Indian or Vietnamese were more likely to marry men at least six years older. Women who had a high school diploma or were third-plus generation were more likely to marry men at least 6 years younger. The findings reflect the complexity of Asian senior marriage and provide insight for policymakers to design new or improved social integration programs for senior immigrants.

### BARRIERS THAT CONTRIBUTE TO THE DIGITAL DIVIDE AMONG OLDER COMMUNITY DWELLING ADULTS IN LATER LIFE

Mai See Yang,[1] Haowei Wang,[2] and Yong Kyung Choi,[3], *1. UC Davis, UC Davis, California, United States, 2. The Pennsylvania State University, University Park, Pennsylvania, United States, 3. University of California Davis School of Medicine, Sacramento, California, United States*

Research demonstrates that race and health literacy contribute to the digital divide, which is a major public health concern for older adults in the U.S. However, we still lack information about what types of barriers older adults have through a comprehensive examination using population -based data. This study focuses specifically on barriers to technology use among older adults. We use data from the Health and Retirement study 2012 Module "Technology Use: Barriers and Benefits" (N = 1,416). About 42% of participatnts did not use any technology (e.g., emails, social media, smart phone) (n = 501). The mean age for this non-user group was 72 years old (SD 10.3). 13% were foreign born, over half were female (56%), and the majory were somewhat educated (72% with a high school education or lower). About 23% of non-users were self-reported black, 16% Hispanic, 3% other race, and 58% non-Hispanic white. Barriers for adopting the use of technology included too difficult to keep up with the changes in technology (78%), too complicated (69%), not interested (65%), too much time required to learn (53%), too hard to learn (52%), expensive (43%), not easily available (24%), and opposed to using new technologies (27%). Results suggest that barriers were significantly correlated with more depressive symptoms among older adults who did not use technology. Compared to users, non-users were also more likely to have health conditions (e.g., hypertension, diabetes, lung disease, stroke, and arthritis). Findings of this study provide directions to address digital divide among older adults.

### DOES LEVEL OF EQUALITY IN STATE OF RESIDENCE RELATE TO LGBT HEALTH? AN ANALYSIS OF ADULTS AGED 50+ YEARS FROM 34 US STATES

Christi Nelson, and Ross Andel, *University of South Florida, Tampa, Florida, United States*

Lesbian, gay, bisexual, and transgender (LGBT) health disparities have been well documented in previous research. This study examined whether the level of equality in state of residence (high, medium, fair, poor, or negative), determined by tallied LGBT-related laws and policies, was associated with health outcomes for LGBT adults. This study consisted of 3486 LGB and 959 transgender adults ages 50+ as well as 1:1 propensity matched heterosexual and cisgender participants from the 2018 and 2019 Behavioral Risk Factor Surveillance System (BRFSS) surveys. Separate logistic regression analyses for the LGB, transgender, heterosexual, and cisgender groups were conducted to assess health differences by state equality ranking. Results indicated that LGB participants in fair ranked states were more likely to report fair/poor general health (aOR=1.4, 95% CI=1.1-1.8) and 14 or more days of poor mental health in the past 30 days (aOR=1.4, 95% CI=1.1-1.9) compared to LGB in high ranked states. LGB participants in a low or negative ranked state were more likely to report fair/poor health (aOR=1.6, 95% CI=1.3-2.0), 14 days or more of poor physical health (aOR=1.5, 95% CI=1.1-1.8), and 14 or more days of poor mental health (aOR=1.3, 95% CI=1.0-1.7) in the past 30 days. Transgender participants in medium and low/negative ranked states were more likely to report fair/poor health (lowest aOR=1.75, 95% CI=1.3-2.5) compared to transgender individuals in high equality states. Similar results were not found for the matched heterosexual and cisgender groups. These results suggest that LGBT-related laws and policies may play a role in LGBT health.

### EVALUATING THE ECONOMIC IMPACT OF MARRIAGE VERSUS COHABITATION IN SAME-SEX COUPLES AGE 50+

Setarreh Massihzadegan,[1] and Jan Mutchler,[2], *1. UMASS Boston, Boston, Massachusetts, United States, 2. University of Massachusetts Boston, Boston, Massachusetts, United States*

Utilizing the first set of 5-year American Community Survey data available since the United States' legalization of same-sex marriage in mid-2015, this poster investigates the economic security of older adults (age 50+) in same-sex marriages compared to those in same-sex partnerships who are cohabiting but not married. Viewed through the lens of cumulative disadvantage theory, we consider differences in the economic circumstances of same-sex couples by gender and by geographic location. Findings point to gender differences in economic well-being, but relatively few differences based on marital status. For example, rates of low income are somewhat higher among female couples than among their male counterparts, but marital status differences are not substantial. These findings suggest that the benefits of being married that have long been recognized among older adults may not extend equally to same-sex couples. Findings are discussed with respect to the emerging salience of marriage within the LGBTQ older community, future research opportunities, and important policy implications.

### THE RELATIONSHIP BETWEEN SOCIAL ISOLATION AND SENSE OF COMMUNITY AMONG OLDER ADULTS IN PUERTO RICO

Thomas Buckley,[1] and Denise Burnette,[2], *1. Virginia Commonwealth University, NORFOLK, Virginia, United*

States, 2. Virginia Commonwealth University, Virginia Commonwealth University, Virginia, United States

Psychological sense of community (SOC) is linked to key health and wellbeing outcomes for older adults and among Latin American populations. Prior research shows that social factors may affect SOC, but this has yet to be studied among Puerto Rican older adults. This study draws on Social Resource Theory to test the hypothesis that social isolation is associated with SOC among older adults in Puerto Rico. We collected data through face-to-face interviews in a non-probability sample of community dwelling adults aged 60+ throughout Puerto Rico in 2019-2020 (N = 154). We measured social isolation with the Spanish translation of the LSNS-6 (range 0-30, mean= 14.00, SD= 5.99), where higher scores indicate less isolation, and SOC with the Spanish translation of the Brief Sense of Community Scale (range 0-32, mean= 24.75, SD= 6.04). This cross-sectional study used multiple linear regression to test the association between social isolation and SOC, while controlling for gender, age, income and living arrangement. Higher scores on the LSNS-6 were associated with higher SOC (β=0.31, SE=0.08, p<0.001). Among the sociodemographic covariates, increased age was associated with higher SOC (β=0.12, SE=0.05, p<0.05). This study demonstrates that older adults in Puerto Rico who are more socially isolated have lower SOC, and that SOC increases with age. In order to promote SOC in this population, interventions should focus on reducing social isolation and may benefit from targeting young-old older adults. Future research should continue to examine these relationships and extend to other Latin American cultures.

### WHAT'S RACE GOT TO DO WITH IT? HOW PALLIATIVE CARE CONSULTATION MAY MITIGATE RACIAL DISPARITIES IN FUTURE CARE

Lauren Starr,[1] Connie Ulrich,[2] G. Adriana Perez,[3] Subhash Aryal,[2] Paul Junker,[4] Nina O'Connor,[5] and Salimah Meghani,[2], *1. University of Pennsylvania, Philadelphia, Pennsylvania, United States, 2. University of Pennsylvania School of Nursing, Philadelphia, Pennsylvania, United States, 3. University of Pennsylvania School of Nursing, Phildelphia, Pennsylvania, United States, 4. University of Pennsylvania Health System, Philadelphia, Pennsylvania, United States, 5. Penn Medicine, Perelman School of Medicine, Philadelphia, Pennsylvania, United States*

It is unknown if care and cost outcomes differ by race and ethnicity following discharge from a hospitalization involving palliative care consultation to discuss goals-of-care (PCC). In this secondary analysis of 1,390 seriously-ill patients age 18+ alive at discharge who self-identified as Black, Hispanic, Asian, white, or other race and received PCC at an urban, academic medical center, we used binomial logistic regression and multiple linear regression controlling for demographic and clinical variables to identify factors associated with care experiences and costs following discharge from a hospitalization with PCC. In adjusted analyses, discharge to hospice was associated with Medicaid (p=0.016). Thirty-day readmission was associated with age 75+ (P=0.015), Medicaid (P=0.004), admission 30 days prior (P<0.0001), and Black race compared to white (P=0.016). Number of future days hospitalized was associated with Medicaid (P=0.001), admission 30 days prior (P=0.017), and Black race compared to white (P=0.012). Having any future hospitalization cost was associated with patient ages 65-74 (P=0.022) and 75+ (P=0.023), Medicaid (P=0.014), admission 30-days prior (P<0.0001), and Black race compared to white (P=0.021). Total future hospitalization costs were associated with female gender (P=0.025), Medicaid (P=0.009), admission 30 days prior (P=0.040), and Black race compared to white (P=0.037). Race or ethnicity was not a predictor of hospice enrollment. Randomized controlled trials are needed to understand if PCC is an intervention that reduces racial disparities in end-of-life care. Qualitative insights are needed to explain how PCC and socioeconomic factors such as Medicaid may mitigate future acute care use among racial and ethnic groups.

### Session 9390 (Poster)

### MOBILITY AND DISABILITY

### ADULTS AGING WITH SPINAL CORD INJURY: PREVALENCE AND ASSOCIATED RISK FACTORS FOR DIAGNOSIS OF DIABETES MELLITUS

Junha Park, and Lisa Bratzke, *University of Wisconsin-Madison, Madison, Wisconsin, United States*

With the increased life expectancy, people aging with spinal cord injury (SCI) are more likely to experience chronic conditions, including diabetes mellitus (DM). The results of previous literature related to the prevalence of DM are mixed and risk factors associated with diagnosis of DM after SCI are not well defined. This study aims to investigate the prevalence of DM and explore associated risk factors for diagnosis of DM among adults aging with long-standing spinal cord injury in the United States. This is a secondary data analysis using the National Spinal Cord Injury Model Systems Database. Participants included 516 people age 45 and older who have been living with SCI for more than 10 years. The prevalence of DM in this sample was 13.2%. Multivariate logistic regression, controlling for confounding variables, was conducted to identify risk factors associated with DM diagnosis in this sample. The multivariate logistic regression model found that the participants who responded with less severe SCI measured by the ASIA impairment scale were less likely to be diagnosed with DM (OR=0.332, p=.017). Also, DM was found to be significantly associated with BMI (OR=1.043, p=.010) and age (OR=1.038, p= .010) respectively. Duration of disability was not significantly associated with DM. Future research is needed to validate these findings and identify other common risk factors for DM such as diet/nutrition. Further, exploration of the effect size of risk factors is also warranted. Such findings will inform interventions to aid prevention and early detection of DM.

### AN INTERNATIONAL REVIEW OF MOTOR VEHICLE COLLISION RISK WITH MEDICAL CONDITIONS IN OLDER ADULTS

Mark Rapoport,[1] Joanne Wood,[2] Jamie Dow,[3] Desmond O'Neill,[4] Judith Charlton,[5] and Sjannie Koppel,[5], *1. University of Toronto, Toronto, Ontario, Canada, 2. Queensland University of Technology, Brisbane City, Queensland, Australia, 3. SAAQ, Quebec City, Quebec,*

Canada, 4. Trinity College Dublin, Dublin, Dublin, Ireland, 5. Monash University, Clayton, Victoria, Australia

The objective was to examine the impact of seven categories of medical illness on risk of Motor Vehicle Collisions (MVC) in older adults. In late 2019, a systematic review of the MVC risk associated with alcohol use disorders, psychiatric disorders, epilepsy, diabetes, hearing loss, vision disorders and sleep disorders was conducted. A total of 64,720 titles were screened, and 138 articles were included. Of these, only thirteen pertained to older adults, only six showed increased MVC risk in at least one condition, and only seven were rated of "Good" quality. Hearing impairment was associated with MVC only if associated with visual acuity or contrast sensitivity impairments (RR 1.52, 95% CI 1.01-2.3 and RR 2.41, 95% CI 1.62-3.57, respectively). A high depression score was associated with increased MVC (RR 1.5, 95% CI 1.1-2.1) in one study, but a similar relationship was not found in two other studies. Glaucoma increased at-fault MVC risk (RR 1.65, 95% CI 1.20-2.28) in one study, but no relationship was found in another. Visual field loss increased MVC risk in three of four studies (RR or HR ranging from 1.31 to 2.32). One negative study each were identified for alcohol use disorders, age-related macular degeneration, any eye disease, or any psychiatric disorder, and four negative studies were identified for reduced visual acuity. No studies of older adults were found for epilepsy or sleep disorders. Interpretation of MVC risk in older drivers with medical illness is rendered challenging by the paucity and quality of studies.

### BRAIN MAGNETIC SUSCEPTIBILITY IS ASSOCIATED WITH SLOWER GAIT IN COMMUNITY-DWELLING OLDER ADULTS

Victoria Poole,[1] Robert Dawe,[1] Sue Luergans,[1] David Bennett,[1] Aron Buchman,[1] and Konstantinos Arfanakis,[2], *1. Rush University, Chicago, Illinois, United States, 2. Rush University, Rush Alzheimer's Disease Center, Illinois, United States*

Age-related slowing of gait is exceedingly common and a robust predictor of various adverse health outcomes in older age. Prior neuroimaging studies have documented diverse non-specific structural brain abnormalities which are related to slow gait; however, the extent to which quantitative susceptibility mapping (QSM), which measures regional magnetic susceptibility in the brain, associates with gait speed remains unexplored. In the current study, 415 non-demented community-dwelling older adults (91 males; 81+/- 7 years) underwent an MRI (Siemens 3T TIM Trio) and in-home motor assessment. Gait speed was measured and averaged across 2 timed 8-ft walks. MR-acquired QSM data were pre-processed, registered to ICBM template, and spatially smoothed with a 5mm FWHM Gaussian kernel. When these maps entered group-level GLMs, voxel-wise associations with gait speed were of interest, after adjusting for demographics. We observed very strong negative associations between gait speed and magnetic susceptibility, such that those with slower gait had higher susceptibility in bilateral inferior frontal, superior temporal, and angular gyri (corrected p<.0005). Robust associations were also observed in the middle frontal, precentral, and postcentral gyri of the right hemisphere. These novel findings suggest that reduced myelination or increased iron accumulation in these brain regions may contribute to impaired gait. Future work will need to determine to what extent these cross-sectional QSM metrics are independent predictors of incident adverse health outcomes when controlling for other common brain imaging abnormalities observed in older adults.

### DIFFERENTIAL INFLUENCE OF COVID-19 PANDEMIC ON LIFE-SPACE MOBILITY OF OLDER ADULTS

Anna Zisberg,[1] Ksenya Shulyaev,[2] Nurit Gur-Yaish,[3] and Efrat Shadmi,[4], *1. University of Haifa, Haifa, HaZafon, Israel, 2. Faculty of Social Welfare and Health Science, University of Haifa, Haifa, Hefa, Israel, 3. Oranim Academic College of Education, Haifa, HaZafon, Israel, 4. University of Haifa, Mount Carmel, Israel, Haifa, Hefa, Israel*

Life-space mobility (LSM) is critical to aging successfully since it is essential to maintain independence, affecting the health and quality of life of older adults. During the COVID-19 pandemic older adults, who are at high-risk of serious illness and complications, are restricted by stay-at-home orders, limiting their outdoor activities. This study evaluated differences in LSM before and during the pandemic and factors related to limited pre-hospitalization mobility. We used a natural experiment design comparing LSM one month prior to hospitalization and its related factors on two subsamples of hospitalized older adults: recruited before and after February 2020 (pandemic outbreak). No significant differences were observed in LSM between pre-pandemic (N=141, M(SD)=54.9(33,5)) and during-pandemic (N=186, M(SD)=55.3(32.9)) samples, even after adjustment for cognitive, functional, and demographic characteristics (F=2.281, p=0.13). Of those who participated during the pandemic, a total of 94 (50.5%) declared that their mobility was strongly affected by the pandemic and had significantly lower LSM (F=4.626, p<0.01) comparing both to those who declared not being affected (N=92) and to the pre-pandemic group, controlling for potential cofounders. In the "during-pandemic" group older adults with higher basic physical functioning, higher economic status, and those with lower levels of education were more likely to indicate that their pre-hospital mobility was affected by the pandemic. These results show that the pandemic period has a differential effect on life-space mobility of older adults. Functional, socio-economic, and educational factors need to be considered in planning how to maintain older adults' mobility during the ongoing pandemic.

### DIGITAL MOBILITY ASSESSMENT FOR REGULATORY AND CLINICAL ENDORSEMENT IN HIP FRACTURE PATIENTS

Carl-Philipp Jansen,[1] Kristin Taraldsen,[2] Hubert Blain,[3] and Clemens Becker,[4], *1. Robert-Bosch-Hospital Stuttgart, Karlsruhe, Baden-Wurttemberg, Germany, 2. Norwegian University of Science and Technology, Trondheim, Sor-Trondelag, Norway, 3. Centre Hospitalier Universitaire de Montpellier, Montpellier, Aquitaine, France, 4. Robert-Bosch-Hospital, Stuttgart, Baden-Wurttemberg, Germany*

Hip fracture is the most frequent non-intentional injury of older persons leading to hospital admission in Europe and North America. Until recently, in regulatory submissions no attention was given to patients' mobility after sustaining/recovering from a hip fracture. To better evaluate efficacy and effectiveness of new drugs and treatments, it is necessary to develop mobility biomarkers since failure to recover and regain pre-fracture mobility is considered the single most important disability symptom experienced by hip fracture patients, often leading to care home admission. However, regularly used measures of mobility capacity are not representative of individuals' performance in real life, intermittent in nature, and require visiting study centers. Digital technology has the potential to revolutionize mobility assessment in a real-life setting. With this presentation we build a case for a valid solution for real-world digital mobility assessment in hip fracture patients as carried out in the "Mobilise-D" clinical validation study.

### DUAL-TASK GAIT SPEED AND MOBILITY ARE POSITIVELY ASSOCIATED WITH DECLARATIVE MEMORY

Michelle Gray,[1] Sally Paulson,[2] Joshua Gills,[1] Erica Madero,[3] Jennifer Myers,[3] Anthony Campitelli,[1] Megan Jones,[1] and Jordan Glenn,[3], *1. University of Arkansas at Fayetteville, Fayetteville, Arkansas, United States, 2. University of Arkansas at Fayetteville, Cincinnati, Ohio, United States, 3. Neurotrack Technologies, Redwood City, California, United States*

In the US, it is not recommended to perform routine screening assessments for cognitive function or impairment among older adults, due to the lack of effective pharmacological treatments. These common practices result in delayed identification and treatments for slowing cognitive decline progression. Thus, the purpose of the present investigation was to determine the ability to predict cognition from common measures of physical function. Seventy-five community-dwelling older adults (80.7±5.4 years) completed physical function and cognitive assessments. Physical function was assessed using the Short Physical Performance Battery (SPPB), peak velocity during a power sit-to-stand task, and dual-task walking test. Cognition (declarative memory) was assessed using a validated Visual Paired Comparison test. 38% of the variance in cognition was accounted for by the predictor variables (age, sex, education, SPPB, dual-task, peak velocity). Significant predictors included dual-task walking ($p = .03$), SPPB ($p = .02$), and education ($p = .02$). For each 1 second faster during the dual-task performance, cognition increased by 4 percentile units. Likewise, each 1 unit increase in SPPB resulted in an increase of 4 percentile points in cognition. The results indicate more than a third of the variance in declarative memory can be predicted by commonly assessed measures of physical function. This information is useful when identifying older adults that may have cognitive impairment before overt signs are realized. With the lack of recommended cognitive testing, using physical function declines to identify possible cognitive decline is promising. These results are preliminary in nature and longitudinal determination is warranted.

### EYE AND VISUAL HEALTH IN NEW ENGLAND: FINDINGS FROM THE HEALTHY AGING DATA REPORTS

Shu Xu,[1] Taylor Jansen,[2] Chae Man Lee,[2] Maki Karakida,[2] Frank Porell,[3] Elizabeth Dugan,[2] and Nina Silverstein,[4], *1. University of Masschusetts Boston, Dorchester, Massachusetts, United States, 2. UMass Boston, Boston, Massachusetts, United States, 3. University of Massachusetts Boston, University of Massachusetts Boston, Massachusetts, United States, 4. University of Massachusetts Boston, Boston, Massachusetts, United States*

Eye and visual health issues in older adults are prevalent, often undetected and untreated, but can contribute to poor physical and mental health issues, and higher mortality rates. The study describes state and local community rates of eye and visual health indicators (cataract, glaucoma, self-reported vision difficulty, and clinical diagnosis of blindness or visual impairment) of older adults 65+ in MA, NH, RI, and CT. Data sources used to calculate rates were: the American Community Survey (2014-2018 RI, 2012-2016 MA and NH, 2014-2018 CT) and the Medicare Current Beneficiary Summary File (2016-2017 RI, 2015 MA and NH, 2016-2017 CT). Small area estimation techniques were used to calculate age-sex adjusted community rates for more than 150 health indicators (https://healthyagingdatareports.org/). Disparities in rates were examined for 4 eye and visual health indicators: cataract, glaucoma, self-reported vision difficulty, and clinical diagnosis of blindness or visual impairment. Results showed variability in rates across states. MA had the highest rates of self-reported vision difficulty (5.8%) and blindness or visual impairment (1.5%), and the greatest differences in rates of self-reported vision difficulty (0.00-40.91%). CT had the highest rates of glaucoma (28.3%), and the greatest differences in rates of glaucoma (19.51-41.91%) and blindness or visual impairment (0.44-4.39%). RI had the highest rates of cataract (67.5%). Understanding the distribution of community rates makes disparities evident, and may help practitioners and policymakers to allocate resources to areas of highest need.

### METABOLIC SYNDROME AND OBJECTIVE PHYSICAL PERFORMANCE MEASURES IN MID-TO-EARLY LATE LIFE WOMEN: SWAN

Elsa Strotmeyer,[1] Robert Boudreau,[1] Brittney Lange-Maia,[2] Samar El Khoudary,[1] Kelly Ylitalo,[3] Andrea Kriska,[1] Carrie Karvonen-Gutierrez,[4] and Jenna Napoleone,[1], *1. University of Pittsburgh, Pittsburgh, Pennsylvania, United States, 2. Rush University Medical Center, Chicago, Illinois, United States, 3. Baylor University, Waco, Texas, United States, 4. University of Michigan, Ann Arbor, Michigan, United States*

Metabolic syndrome (MetS) is a cluster of factors (hypertension, abdominal obesity, impaired fasting glucose, low high-density lipoprotein, hypertriglyceridemia). How midlife MetS impacts future physical functioning is uncertain. We hypothesized that higher midlife MetS components are associated with poorer physical performance in early late life for multi-ethnic women. MetS status from 1996-2011 (8 visits) and objective physical performance (2015/16; short physical performance battery (SPPB; 0-12), 40-foot walk (m/s), 4-meter gait speed (m/s), chair stands (sec), stair climb (sec)) were assessed in the Study of Women's Health Across the Nation (SWAN; n=1722; age 65.4±2.7 years; 26.9% Black, 10.1% Chinese, 9.8% Japanese, 5.5% Hispanic). Poisson latent class growth modeling identified trajectory groups distinguishable by number of MetS components: none (23.9%),

1=low-MetS (28.7%), 2=mid-MetS (30.9%), >3=high-MetS (16.5%). High-MetS versus none had higher body mass index, pain, financial strain, and lower physical activity and self-reported health (p < 0.0001). Black and Hispanic women were more likely to be in high-MetS and had worse physical functioning, along with Chinese women, versus White (all p<0.05, except gait speed in Hispanic). Adjusted linear regression related MetS groups to physical performance. High-MetS versus none demonstrated adjusted worse 40-ft walk (β:-0.08; 95% CI:-0.13, -0.03), gait speed (β:-0.09; 95% CI:-0.15, -0.02), SPPB (β:-0.79; 95% CI: -1.15, -0.44), and chair stands (β:0.69; 95% CI: 0.09, 1.28), but not stair climb, with race significantly related to all except 40-ft walk. Midlife MetS trajectories related to poor physical performance in early late life multi-ethnic women. Managing midlife metabolic function may improve physical performance in late life.

### MOBILIZING ELDERS: AN INTERPROFESSIONAL EFFORT

Andrew de la Paz,[1] David Lynch,[2] Carissa Lau,[1] Kimberly Mournighan,[1] John Batsis,[3] Laura Hanson,[4] Maureen Dale,[4] and Kimberly Young,[4], *1. UNC, Chapel Hill, North Carolina, United States, 2. University of North Carolina, Chapel Hill, North Carolina, United States, 3. University of North Carolina at Chapel Hill, Chapel Hill, North Carolina, United States, 4. University of North Carolina, University of North Carolina, North Carolina, United States*

Older adults often experience functional decline during hospitalization as a result of immobility. Such decline has associated adverse outcomes, including gait instability, falls, pressure injuries, delirium, and new nursing home admissions. Our objective was to create an effective and sustainable in-hospital mobility program through enhanced interdisciplinary cooperation in an Acute Care of the Elderly (ACE) unit. An interdisciplinary team at UNC's 25-bed ACE unit planned and delivered enhanced patient mobility beginning in July 2020. We used an input-process-output model to design and analyze an intervention based on enhanced collaboration. Inputs included a mobility taskforce which was comprised of physicians, nurses, physical and occupational therapists, and quality improvement specialists. Through regular meetings, each taskforce member contributed to the study design and were empowered to identify barriers to implementation. Outputs included stakeholder engagement and mobility rates. Early results show a doubling in mobility rates over a 6-month period with consistent and enthusiastic stakeholder engagement. Observations of such benefits include: a) stakeholder inclusion from each discipline ensured implementation that was pragmatic and easily incorporated into the daily workflow; b) mobility champions regularly disseminated information to their respective disciplines, leading to changes using a quality improvement process; and c) barriers to implementation were rapidly identified, and mobility champions were motivated to find solutions, allowing cohesive incorporation of a broad spectrum of priorities. An interprofessional team model is effective to mobilize hospitalized older adults, potentially reducing adverse hospital outcomes. Successful implementation of such programs is dependent on interprofessional collaboration.

### MODERATORS OF DUAL TASK GAIT EFFECTS IN MILD COGNITIVE IMPAIRMENT AND DEMENTIA

Edward Ofori,[1] Dara James,[2] Olivia Kaczmarek,[3] and Mark Gudesblatt,[4], *1. ASU, Arizona State University, Arizona, United States, 2. Arizona State University, Phoenix, Arizona, United States, 3. SouthShore Neurologic, Southshore Neurologic, New York, United States, 4. South Shore Neurologic, Islip, New York, United States*

Spatiotemporal gait parameters may provide indication about the cognitive status of individuals. Dysfunction in specific gait features has been associated with increased risk of cognitive decline. Here we use spatiotemporal gait patterns to determine whether specific cognitive domain scores moderate the effects during dual-tasking on individuals with mild cognitive impairment (MCI) and dementia. Participants (n=46; mean age: 77.0±8.9 years) with a diagnosis of cognitive impairment (n=16), or dementia (n=30) were included. They performed validated computerized cognitive assessment battery (CAB, NeuroTrax BrainCare) to obtain cognitive domain measures of executive function (EF), attention, memory, visual-spatial processing (VSP), information processing speed (IPS), and a global cognitive score (GCS) measure. Using the Zeno Walkway Gait Analysis System (Protokinetics), measures of velocity, stride width (SW), stride time (ST), stride length, cadence, double support (DS), and gait variability were obtained for both single-task and DT gait. Data analysis was conducted using SPSS 26 and PROCESS 3.5. As expected, the dementia group had lower cognitive domain scores and slower walking speed than MCI group. Results also indicated that visual-spatial processing skills was the only cognitive domain that did have a moderation effect on gait velocity (F=4.2, p<0.05, R-square change 10%). Our results indicate that differences between walking speed in MCI and dementia groups are moderated by visual spatial skills. Improvement in visual spatial skills could improve the dual task effects of individual gait measures.

### PERSISTENT DISABILITY SIX MONTHS AFTER INITIAL DISABILITY LESS LIKELY IN OLDER WOMEN

Raj Shah,[1] Katherine Webb,[2] Joanne Ryan,[2] Rory Wolfe,[2] Michael Ernst,[3] Sara Espinoza,[4] and Robyn Woods,[2], *1. Rush, Rush University Medical Center, Illinois, United States, 2. Monash University, Melbourne, Victoria, Australia, 3. University of Iowa, Iowa City, Iowa, United States, 4. University of Texas Health Science Center San Antonio, San Antonio, Texas, United States*

Many community-dwelling older adults develop activity of daily living (ADL) disability and subsequently regain function. Using data from the ASPirin in Reducing Events in the Elderly (ASPREE) clinical trial, we examined the relationship of gender, incident disability, and persistent disability 6 months after the incident disability. Walking, bathing, dressing, transferring, toileting, and eating were assessed as ADLs, at bi-annual interviews. ADL disability was defined as requiring help with or inability to do or severe difficulty with ≥1 ADL; persistent disability was an ADL loss at 6 months after a first (incident) ADL disability. Discrete time, multivariable Cox proportional hazards regression was utilized to estimate associations with developing incident ADL disability described as cause-specific hazard ratios, with death as a competing outcome. For persons with incident ADL disability, odds of developing persistent disability at 6 months as compared to recovery was determined using multivariable logistic regression. These analyses included 18,414 (51.6% women) ASPREE participants in the United States and Australia aged 70+ years (65+ years if U.S. ethnic minority) without ADL disability at trial entry. During a median follow-up of 4.7 years, 1,485 participants (63.2% women) developed an incident ADL disability, and, of those, 272 (57.0% women) met criteria for persistent disability at 6 months. Women had an increased risk (HR=1.17, 95% CI=1.05 to 1.32) of developing incident ADL disability; however, women were less likely to have persistent disability versus recovery 6 months later (OR=0.66, 95% CI=0.49 to 0.89). Why persistent disability development is lower in older women needs further exploration.

### PHYSICAL FUNCTION TRANSITIONS AND HEALTHCARE UTILIZATION AMONG OLDER MEXICAN AMERICANS.

Amy Givan, Lin-Na Chou, Yong-Fang Kuo, and Soham Al Snih, *University of Texas Medical Branch, Galveston, Texas, United States*

The aim of this study was to examine the relationship between 2-year physical function transitions and one-year healthcare utilization among Mexican American Medicare beneficiaries. The sample consisted of 429 Mexican Americans ≥75 years old from the Hispanic Established Population for the Epidemiologic Study of the Elderly linked with Medicare claims data from the Centers for Medicare and Medicaid Services. Short Physical Performance Battery (SPPB) from Wave 5 (2004/05) to Wave 6 was used to create physical function transition groups. The outcomes were physician visits (<6, 6-12, >12 visits), number of emergency room visits, and number of acute hospitalizations one-year after physical function transitions. Multinomial logistic regression and Generalized Estimating Equation with negative binomial distribution were used to estimate the odds ratio of healthcare utilization as a function of physical function transition groups, controlling for socio-demographics and comorbidities. Participants who improved or remained moderate-high physical function had lower odds ratio (OR) of being hospitalized (0.40, 95% Confidence Interval [CI]=(0.18, 0.90)) or visiting the emergency room (OR=0.52, 95% CI=0.32-0.84) one-year later compared to participants who remained in the low physical function group. No difference in physician visits across physical function transition groups was found. This study showed healthcare utilization differed by physical function transition groups among Mexican American Medicare beneficiaries. Physical function improvement or maintenance of moderate-high physical function should be targeted in older Mexican Americans, a population at great risk of developing disability, to reduce or delay dependency and healthcare burden.

### RELATIONSHIP BETWEEN CLUSTERS OF CHRONIC CONDITIONS AND DISABILITY TRAJECTORIES

Tara Klinedinst, Lauren Terhorst, and Juleen Rodakowski, *University of Pittsburgh, Pittsburgh, Pennsylvania, United States*

Recent evidence shows that more complex clusters of chronic conditions are associated with poorer health outcomes. Less clear is the extent to which these clusters are associated with different types of disability (basic and instrumental activities of daily living (ADL, IADL) and functional mobility (FM)) over time. This was a longitudinal analysis using the National Health and Aging Trends Study (NHATS) (n = 6,179). Using latent class analysis, we determined the optimal clusters of chronic conditions, then assigned each person to a best-fit class. Next, we used mixed-effects models with repeated measures to examine the effects of group (best-fit class), time (years from baseline), and the group by time interaction on each of the outcomes in separate models over 4 years. We identified 5 chronic condition clusters: “multisystem morbidity” (13.9% of the sample), “diabetes” (39.5%), “osteoporosis” (24.9%), “cardio/stroke/cancer” (4.5%), and “minimal disease” (17.3%). Group by time interaction was not significant for any outcome. For ADL outcome, only time was significant ($F_{3,16249} = 224.72$, $p < .001$). For IADL, both group ($F_{4,5403} = 6.62$, $p < .001$) and time ($F_{3,22622} = 3.87$, $p = .009$) were significant. For FM, both group ($F_{4,5920} = 2.96$, $p = .02$) and time were significant ($F_{3,16381} = 213.41$, $p < .001$). We did not find evidence that any cluster experienced greater increases in disability over time, but all clusters containing multiple chronic conditions had risk of IADL and FM disability. Increased screening for IADL and FM disability could identify early disability and prevent decline.

### RISK FACTORS OF WALKING WHILE TALKING DECLINE IN OLDER ADULTS: CENTRAL CONTROL OF MOBILITY AND AGING STUDY

Oshadi Jayakody,[1] Joe Verghese,[2] Helena Blumen,[2] and Emmeline Ayers,[2], *1. Albert Einstein College of Medicine, The Bronx, New York, United States, 2. Albert Einstein College of Medicine, New York, New York, United States*

**Background:** Slow gait speed during walking while talking (WWT-speed) is associated with an increased risk of falls and dementia. Age-related changes in WWT-speed and associated risk factors, however, are poorly understood. This study examined 1) change in WWT-speed over time 2) factors associated with change in WWT-speed.

**Methods:** A total of 431 older participants (M Age=76.8±6.4 years; M follow-up 4.5±2.3 years) enrolled in the Central Control of Mobility in Aging study were examined. WWT-speed was measured with a computerized walkway while participants recited alternate letters of the alphabet while walking. The following baseline measures were examined as risk factors: demographic [age, sex, education], medical [hypertension, diabetes, cardiac arrhythmias, history of stroke, Parkinson’s disease, kidney disease, arthritis, depression], cognitive [global cognition, executive function, processing speed], sensorimotor [balance, grip strength, vision], falls and frailty. Linear mixed effect models were used to examine 1) change in WWT-speed over time 2) risk factors of WWT-speed change.

**Results:** WWT-speed declined over time (b -1.06, 95%CI -1.45, -.68) independent of baseline age, sex and education. Rate of WWT-decline was modified by age (b -.10, 95%CI -.17, -.03) and poorer balance (b -1.12, 95%CI -1.95, -.28).

Lower scores in tests of global cognition and processing speed and, kidney disease predicted slow WWT-speed on average.

**Conclusion:** Greater age and poorer balance accelerate WWT-speed decline while poorer global cognition, slow processing speed and kidney disease predicts slow WWT-speed. These factors may provide potential targets for future interventions to prevent decline in WWT-speed and associated adverse health outcomes.

### UTILITY OF THE LATE LIFE FUNCTION AND DISABILITY INSTRUMENT (LLFDI) IN REHABILITATION SERVICES FOR OLDER ADULTS

Pamela Toto,[1] and Anne Stankiewicz,[2], *1. University of Pittsburgh, Pittsburgh, Pennsylvania, United States, 2. Capable Care Solutions LLC, Lancaster, Pennsylvania, United States*

**Introduction:** The Late Life Function and Disability Instrument (LLFDI) is a valid self-report tool that quantifies disability based on activity limitations and participation restrictions in everyday life. Both the original longer tool (LLFDI) and the shorter computer adaptive version (LLFDI-CAT) offer practitioners a method for measuring function independent or in conjunction with performance-based assessment. Objectives: Examine scores of the LLFDI and LLFDI-CAT for measuring disability in older adults who are receiving rehabilitation services in community and institution settings.

**Method:** A secondary data analysis was conducted comparing scores from occupational therapy evaluations with older adults from 3 groups: 1) older adults in primary care using the LLFDI; 2) older adults in primary care using the LLFDI-CAT; older adults in a skilled nursing facility (SNF) using the LLFDI-CAT.

**Results:** Mean scores for Activity Limitation and Participation Restriction were lowest for older adults in a SNF indicating greater disability. A one-way Analysis of variance on ranks showed a main effect for Activity Limitation, $\chi2$ (2) = 22.267, $p < 0.001$, and Participation Restriction, $\chi2$ (2) = 60.372, $p < 0.001$. Post-hoc analyses revealed significant differences between groups based on tool (i.e. LLFDI vs. LLFDI-CAT) for Activity Limitations and setting (i.e. primary care vs. SNF) for Participation Restriction.

**Conclusion:** The LLFDI-CAT may be the preferred instrument to measure disability in older adults across treatment settings. Additional research is warranted to understand how personal and environmental factors influence LLFDI-CAT outcomes.

### Session 9395 (Poster)

### MULTIDIMENSIONAL ASPECTS OF LONG-TERM CARE

### CULTIVATING A PATIENT-CENTERED ENVIRONMENT (CAPE): RENOVATIONS IN LONG-TERM CHRONIC CARE

Elizabeth Howard,[1] and Tammy Retalic,[2], *1. Boston College, Dover, Massachusetts, United States, 2. Hebrew SeniorLife, Boston, Massachusetts, United States*

Achieving institutional goal of full, person-centered care was encumbered by an outdated structural "hospital model" at one long-term care facility that undertook building renovations, transforming long hallways into "neighborhood" of compact households. Quality of Life Survey and Long-term Care Minimum Data Set generated data at baseline and 1-year follow-up, comparing renovated(RU) and non-renovated unit(NRU) residents (n=36) to evaluate achievement of person-centered care. RU residents indicating they could "eat when I want" increased 75% to 81% at follow-up and decreased 17% for NRU residents. Sixty-seven percent of RU residents reported bathing "when they want" in contrast to 40% of NRU residents. Most RU residents agreed, "staff act on my suggestions."More RU residents (68% vs 53%) agreed: "I spend time with other like-minded residents" and more RU residents (86% vs 43%) reported opportunity to explore new skills, interests. RU residents more often reported (50% vs 37%) "people ask for my help or advice." Similar differences were observed with "it is easy to make friends here," 67% RU residents responding affirmatively. RU residents reporting "feeling down" improved, moving from 46% to 50% disagreeing with this item with while increased number of NRU residents (18% to 22%) reported "feeling down" at follow-up.Improvement with independent performance of bed mobility, transfer, walking, and dressing among RU residents was observed while NRU residents had decreased percentages of independence. Evaluation of resident outcomes demonstrated improvement with personal choice, activities, personal relationships, functional independence and mood. Physical unit renovations appear to enhance implementation of person-centered care model.

### DO DAYTIME ACTIVITY, MOOD AT BEDTIME AND UNIT TUMULT PREDICT NIGHTTIME SLEEP QUALITY OF LONG-TERM CARE RESIDENTS?

Murad Taani,[1] and Christine Kovach,[2], *1. University of Wisconsin-Milwaukee, Milwaukee, Wisconsin, United States, 2. University of Wisconsin Milwaukee, Milwaukee, Wisconsin, United States*

Sleep quality declines in old age and is particularly poor for long-term care (LTC) residents with dementia. Compromised sleep quality is associated with severe cognitive and neuropsychiatric symptoms, agitation, aggressiveness, and poor quality of life. Based on the premise that stressors can have a cumulative effect on people with dementia throughout the day that contributes to negative consequences later in the day, we examined if daytime activity, mood, and unit tumult were associated with sleep quality. A convenience sample of 53 LTC residents with dementia participated in this correlational study. Objective sleep quality and activity variables were measured using Actigraphy, and mood was measured by the Observed Emotion Rating Scale. Unit tumult was defined as events in the residents living area that are deviations from the typical day (i.e., census changes, being cared for by a certified nursing assistant from a temporary staffing agency, and lower than usual staffing level). Comorbid illness and level of dementia were control variables. Half of the sample had a sleep efficiency that was less than .85 and were awake for more than 90 minutes at night. Comorbid illness, negative mood at bedtime, and daytime activity level accounted for 26.1% of the variance in total sleep minutes. Census changes and the use of temporary agency staff were associated with poor total sleep time and sleep efficiency. Findings suggest that daytime activity, mood at bedtime, and unit tumult should be considered when designing and testing interventions to improve sleep quality among LTC residents with dementia.

### FACTORS INFLUENCING RETENTION INTENTION OF NURSES AT LONG-TERM CARE HOSPITALS IN KOREA

So Young Shin,[1] and Joo Hee Kim,[2], *1. Inje University, Busan, Pusan-jikhalsi, Republic of Korea, 2. Busan Hangun Hospital, Busan, Pusan-jikhalsi, Republic of Korea*

**Purpose:** This study aimed to investigate the levels and correlations of role conflict, nursing professionalism, nursing work environment and retention intention, and the factors influencing retention intention of nurses at long-term care hospitals.

**Methods:** The subjects included 183 nurses at nine long-term care hospitals in one metropolitan city in Korea. A set of self-reported questionnaires was administered to assess general characteristics, role conflict, nursing professionalism, nursing work environment, and retention intention of the subjects. Collected data was analyzed using descriptive statistics, t-tests, one-way ANOVA, Pearson correlation coefficients, and multiple linear regression.

**Results:** 183 subjects with a mean (±SD) age of 41.66 (±12.29) years were included in the final analyses. Retention intention had a significant positive correlation with nursing professionalism ($r=.39$, $p<.001$) and nursing work environment ($r=.51$, $p<.001$). Nursing work environment had a significant negative correlation with role conflict ($r=-.30$, $p<.001$) and a significant positive correlation with nursing professionalism ($r=.48$, $p<.001$). In the final multiple regression analysis, the factors influencing retention intention of subjects were number of beds ($\beta=-.15$ $p<.026$), nursing professionalism ($\beta=.19$, $p=.007$) and nursing work environment ($\beta=.36$, $p<.001$). The explanatory power of number of beds, nursing professionalism and nursing work environment on retention intention was 34.0% ($F=16.66$, $p<.001$).

**Conclusion:** Improving nursing professionalism and nursing work environment of nurses at long-term care hospitals will ultimately enhance their retention intention and positively impact on the quality of gerontological nursing service.

### FAILURE TO THRIVE: NURSING HOME STAFF EXPERIENCES IN CARING FOR RESIDENTS DURING THE COVID-19 PANDEMIC

Yu-Ping Chang,[1] Audrieanna Raciti,[2] Cristina de Rosa,[1] Margaret Doerzbacher,[1] Yanjun ZHou,[1] Chia-Hui Chen,[1] and Amy Lyons,[1], *1. University at Buffalo, Buffalo, New York, United States, 2. University at Buffalo, 7168292015, New York, United States*

Nursing home residents and staff have accounted for roughly 40% of Coronavirus-related deaths in the U.S. The burden of caring for vulnerable residents coupled with isolation policies has taken a significant emotional toll among direct health care staff in long term care facilities. This study explores nursing home staff's experiences in caring for residents during the COVID-19 pandemic. A qualitative descriptive approach with a semi-structured guide was used to conduct individual interviews. We recruited nursing home staff employed during the COVID-19 pandemic in long term care facilities located in New York State. Interviews were recorded, transcribed verbatim, and then analyzed using Braun and Clarke's Reflexive Thematic analysis. Twelve nursing home staff were interviewed. Participants consistently refer to failure-to-thrive as an extremely concerning problem because many residents demonstrate decreased appetite and poor nutrition, inactivity, and depressive symptoms due to social isolation. They also often feel frustrated and overwhelmed due to uncertainty and shortages of staff. Five main themes were identified, including doing their best to manage residents' failure-to-thrive, working as a team, keeping family members informed and connected, struggling to balance competing personal and professional demands, and needing support to reduce stress and build strength. Our study findings indicate that nursing home staff experienced a high level of stress and identified failure-to-thrive caused by isolation and loneliness as a common phenomenon among nursing home residents during the COVID-19 pandemic. Interventions are urgently needed to reduce isolation and loneness in nursing home residents and to provide support for staff.

### HUMAN CAPITAL AND EMPLOYMENT OUTCOMES AMONG FOREIGN EDUCATED AND US NURSES WORKING IN LONG TERM CARE

Roy Thompson,[1] Susan Silva,[1] Kirsten Corazzini,[2] Thomas Konrad,[3] Michael Cary, Jr.,[1] and Eleanor McConnell,[4], *1. Duke University, Durham, North Carolina, United States, 2. University of Maryland School of Nursing, Baltimore, Maryland, United States, 3. University of North Carolina at Chapel Hill, Chapel Hill, North Carolina, United States, 4. Duke University School of Nursing, Durham, North Carolina, United States*

Employing Foreign Educated Nurses (FENs) helps address Registered Nurse (RN) shortages in long-term care (LTC) in the United States (US). However, examination of factors explaining differences in their employment outcomes relative to US Educated Nurses (USENs) is limited. This study uses 2018 National Sample Survey of Registered Nurses data to compare income, work hours, job satisfaction, and human capital, defined as personal characteristics (knowledge, work experience) and behaviors (job mobility), of FENS and USENs working full-time in LTC. A human capital score, consisting of highest nursing education, skill certifications, state licensures, years of experience, multi-state employment history, and multi-lingual status was constructed. Covariates included nurse demographics, direct care role, and ability to practice to full scope. Covariate-adjusted group differences in employment outcomes and human capital were compared using ANCOVA and logistic regression. Mediation analyses explored whether human capital explained FEN vs USEN differences. FENs earned higher hourly wages ($p=0.0169$), worked fewer hours annually ($p=0.0163$), and reported greater human capital ($p<.0001$) compared to USENs. FENs and USENs, however, had similar annual salaries ($p=0.3101$) and job satisfaction ($p=0.1674$). Human capital mediated FEN vs USEN effects on hourly wages but not annual work hours. FENs' higher levels of human capital partially account for FEN vs USEN differences in hourly wages. Application of the human capital concept advanced our ability to examine differences in employment outcomes and highlight aspects of the value that FENs contribute to LTC settings.

### INDICATORS OF ELDER MISTREATMENT: CORRELATES AMONG VETERANS RECEIVING CARE IN THE VETERANS HEALTH ADMINISTRATION

Jenefer Jedele,[1] Cameron Griffin,[2] and Julie Weitlauf,[3], *1. Veterans Health Administration, Ann Arbor, Michigan, United States, 2. VA, Ann Arbor, Michigan, United States, 3. VAPAHCS/Stanford, Palo Alto, California, United States*

Among community-dwelling adults ages 65 and older, approximately 11% have experienced elder mistreatment (EM), including physical, emotional or sexual abuse, neglect, or financial exploitation. EM research typically focuses on this age group; however, Veterans receiving Veterans Health Administration (VHA) care have increased earlier morbidity, which may accelerate the impacts of EM. Using a cohort of all VHA Veterans 50 years and older with VHA use in 2018-2020, we examined correlates of EM. ICD-10 codes from clinical encounters identified Veterans with indications of EM (n=4,427). A 10% sample of Veterans without indications of EM was selected for comparison (n=530,535). Logistic regression compared EM+ Veterans to the comparison sample and assessed overall demographic and clinical differences as well as differences by age, i.e. 50-64 versus 65 and older. Overall, female gender (OR=5.3, 95% CI=4.3-6.5), non-white race/ethnicity (OR=1.7, CI=1.5-1.9), dementia (OR=3.0, CI=2.6-3.5), PTSD (OR=2.0, CI=1.6-2.5), anxiety (OR=1.3, CI=1.0-1.5), military service connected disability status (OR=1.3, CI=1.1-1.5), and higher Elixhauser medical morbidity scores (OR=1.1, CI=1.1-1.1) were associated with EM. Prior year ER visits (OR=28.0, CI=23.6-33.4), inpatient stays (OR=14.0, CI=11.5-17.0), and mental health visits (OR=26.1, CI=22.2-30.6) also predicted EM+ status. Forty-six percent of VHA Veterans with indicators of EM were aged 50-64. For these Veterans, female gender, PTSD, service connection, and mental health visits were associated with increased risk of EM compared to Veterans 65+. Findings highlight clinical correlates of EMs among Veterans in VHA care. Increased awareness of EM risk factors is warranted and may inform VHA efforts for EM prevention, detection and intervention.

### LIVING IN THE NEW NORMAL: EFFECT OF RESIDENTIAL SETTING ON PERCEPTION OF A MEANINGFUL LIFE AMONG OLDER WOMEN

Judith Scott,[1] Helen Graham,[1] Ann Mayo,[2] and Melissa Benton,[1], *1. University of Colorado Colorado Springs, Colorado Springs, Colorado, United States, 2. University of San Diego, San Diego, California, United States*

Perception of a meaningful life is related to depression, anxiety, and general well-being. The sense that one's life is meaningful influences overall quality of life, which influences aging well. It is not clear whether differences in residential setting influence perception of a meaningful life. This study evaluated the effect of residential setting (community versus assisted living) on perception of a meaningful life in 48 older (79.7 ± 1.0 years) women living in the community (n=24) or assisted living (n=24) who were pair matched by age. They completed a one-time questionnaire regarding self-rated health and whether life has meaning. Both questions were scored on a 5-point scale with 0 indicating poor health or no life meaning and 4 indicating excellent health or strong life meaning. There were no significant differences in age between women in community living (CL) and assisted living (AL) (78.0 ± 09 vs. 81.5 ± 1.6 years, respectively; p=0.7). Both groups also reported similar self-rated health scores (CL: 2.4 ± 0.2; AL: 2.2 ± 0.2; p=0.4), indicating good-very good health. However, there were significant differences between groups in their perception of a meaningful life. Women in CL reported significantly lower scores compared to women in AL (2.9 ± 0.2 vs. 3.6 ± 0.1; p=0.006), indicating that women in CL perceived a less meaningful life. Based on our findings, it appears that the supportive infrastructure provided by AL residential settings may promote quality of life and successful aging by enhancing the perception of a more meaningful life.

### NURSING HOME FACTORS AND THEIR IMPACT ON COVID-19 CASES: A STUDY OF WISCONSIN STATE

Mohammed Abahussain,[1] Priya Nambisan,[2] Colleen Galambos,[2] Bo Zhang,[2] and Elizabeth Bukowy,[3], *1. University of Wisconsin- Milwaukee, Wauwatosa, Wisconsin, United States, 2. University of Wisconsin-Milwaukee, Milwaukee, Wisconsin, United States, 3. Medical College of Wisconsin, Wauwatosa, Wisconsin, United States*

COVID-19 has been devastating for Nursing Homes (NHs). The concentration of older adults with underlying chronic conditions inevitably made the setting highly vulnerable leading to high rates of mortality for residents. However, some nursing homes fared better than others. This study examines several quality measures and organizational factors to understand whether these factors are associated with COVID-19 cases in Wisconsin. We combined three datasets from Centers for Medicare & Medicaid Services (CMS) – the Star Rating dataset, Provider Information dataset and COVID-19 Nursing Home dataset. Data used is from the period of Jan 1 – Oct 25, 2020 for the state of Wisconsin. The analysis includes 331 free-standing NHs with no missing values from the data sets. The variables used were self-reported information on nursing home ratings, staff shortage, staff reported hours, occupancy rate, number of beds and ownership. Of the 331 NHs examined, shortages were reported of 25.4%, 31.1%, 3.2% and 15.6% of licensed nurse staff (25.4%), nurse aides (31.1%), clinical staff, (3.2%) and other staff (15.6%) Additionally, there was a significant (p<.05) positive correlation between number of beds and COVID-19 cases, and there was no statistically significant association between occupancy rate and COVID-19 cases. NHs with better star ratings were also found to have less COVID-19 cases. Interestingly, private NHs had significantly higher COVID-19 cases than for-profit and government owned NHs, a finding that is congruent with other studies in this area. Recommendations for practice will be discussed.

### POTENTIAL SOURCES OF RACIAL AND ETHNIC DISPARITIES IN NURSING HOME INFLUENZA VACCINATION

Melissa Riester,[1] Elliott Bosco,[1] Barbara Bardenheier,[2] Patience Moyo,[3] Rosa Baier,[1] Melissa Eliot,[1] and Andrew Zullo,[1], *1. Brown University, Providence, Rhode Island, United States, 2. Brown University School of Public*

*Health, Smithfield, Rhode Island, United States, 3. Brown University School of Public Health, Providence, Rhode Island, United States*

Racial and ethnic disparities in influenza vaccination among nursing home (NH) residents are well-documented and have persisted over time, suggesting that new strategies are necessary to reduce disparities. We conducted a retrospective cohort study to examine the degree to which observable characteristics drove influenza vaccination disparities. We linked Minimum Data Set (MDS) assessments to facility-level data for short- and long-stay NH residents aged ≥65 years. We included residents with six-month continuous enrollment in Medicare and an MDS assessment during the influenza season (October 1, 2013 through March 31, 2014). Using nonlinear Oaxaca-Blinder decomposition, we decomposed the disparities in vaccination between White versus Black and White versus Hispanic residents. We analyzed short- and long-stay residents separately. Our study included 630,373 short-stay and 1,029,593 long-stay residents. Among short-stay residents, 67.2% of Whites, 55.1% of Blacks, and 54.5% of Hispanics were vaccinated against influenza; among long-stay residents, 84.2% of Whites, 76.7% of Blacks, and 80.8% Hispanics were vaccinated against influenza. Across the four comparisons, the crude disparity in influenza vaccination ranged from 3.4-12.7 percentage points. By equalizing 27 characteristics, these disparities could be reduced by 37.7%-59.2%. Living in a predominantly White facility and proxies for NH quality were important contributors to the disparity, although characteristics unmeasured in our data (e.g., NH staff attitudes and beliefs) contributed 40.8%-62.3% to the disparity across comparisons. Intervening on factors associated with NH quality may reduce racial/ethnic disparities in influenza vaccination. Qualitative research is essential to explore potential contributors not captured in our administrative data.

### RACIAL AND ETHNIC DISPARITIES IN PAIN MANAGEMENT FOR NURSING HOME RESIDENTS: A SCOPING REVIEW

Cassandra Dictus,[1] Youngmin Cho,[2] Tamara Baker,[3] and Anna Beeber,[4], *1. University of North Carolina - Chapel Hill, Carrboro, North Carolina, United States, 2. University of North Carolina at Chapel Hill, Chapel Hill, North Carolina, United States, 3. University of North Carolina, Chapel Hill School of Medicine, University of North Carolina at Chapel Hill (School of Medicine), North Carolina, United States, 4. University of North Carolina at Chapel Hill, UNC Chapel Hill, North Carolina, United States*

Within nursing homes, residents commonly experience pain that unfortunately goes underrecognized and undertreated, having a dramatic negative impact on residents' quality of life. Nursing homes are becoming more racially and ethnically diverse, and there is concerning evidence documenting disparities in the quality of nursing home care. In other healthcare settings, people of diverse race groups often receive less optimal pain management, but the evidence regarding racial disparities has not been synthesized for nursing homes. Thus, the purpose of this review was to investigate what is known about racial disparities related to pain management (e.g. assessment, treatment, preferences) in US nursing homes. We completed a scoping literature review using PRISMA-ScR guidelines and searching PubMed, CINHAL, and Scopus for peer-reviewed, empirical studies. Most studies were older large retrospective cohort studies of administrative data documenting that White residents were more likely than residents of diverse race groups to have pain documented and treated. Only a few studies looked at possible reasons to explain the disparities; differences were not found to be related to nursing staff racial bias nor differences in pain-related diagnoses. However, there was evidence of racial differences in resident behavior and attitudes related to pain management. None of the studies examined systemic factors related to differences among nursing homes, which has been implicated in studies looking at other outcomes including COVID-19. More research is needed which examines the causal mechanisms behind the documented racial disparities in pain management so that gaps in care can be reduced.

### SPIRITUALITY AS AN ESSENTIAL ELEMENT OF PERSON-CENTERED CARE

Rachel Nathan,[1] Deborah Zuercher,[2] Steven Eveland,[3] Anjana Chacko,[3] and Raya Kheirbek,[4], *1. University of Maryland School of Medicine, University of Maryland, Maryland, United States, 2. University of Maryland Medical Center, University of Maryland, Maryland, United States, 3. University of Maryland, Baltimore, Maryland, United States, 4. University of Maryland School of Medicine, University of Maryland School of Medicine, Maryland, United States*

Data demonstrate that the majority of patients with serious or chronic illness would like their clinicians to address their spirituality but that the majority of clinicians do not provide such care. Reasons cited include lack of training. Palliative Medicine, built on the biopsychosocial-spiritual model of care, has long recognized the critical role of spirituality in the care of patients with complex, serious, and chronic illness. There is mounting evidence that spiritual care is a fundamental component of all high-quality compassionate health care, and it is most effective when it is recognized and reflected in the attitudes and actions of both patients and health care providers. We conducted focus groups as a first step in the process to arrive at a consensus definition of “spiritual care.” A second step involved collecting and comparing frameworks and models that recognize that providers cannot be made compassionate simply through the imposition of rules; methods were needed to achieve behavior change. The study group developed and piloted curriculum to train health care providers. The created curricula covered the definitions of a spiritual care, self-awareness, cultural sensitivity, assessment, and skills. As part of ongoing curriculum development processes, training included evaluation tools to accompany skill development . Our work demonstrated the need for compassionate presence during encounters, for applying the spirituality in professional life; and for identifying ethical issues in inter-professional spiritual care. We concluded that it is feasible to train clinicians to address spirituality and provide holistic and patient-centered care in an effort to minimize suffering.

### TECHNOLOGIES AND THE EFFECTS ON SOCIAL ENGAGEMENT IN LONG-TERM CARE FACILITIES DURING COVID-19: A SCOPING REVIEW

Timothy Wood,[1] Shannon Freeman,[2] and Alanna Koopmans,[3], *1. University of Northern British Columbia, Canmore, Alberta, Canada, 2. University of Northern British Columbia, Prince George, British Columbia, Canada, 3. UNBC, University of Northern British Columbia, British Columbia, Canada*

During the COVID-19 pandemic, the sense of loneliness and social isolation felt by older adults in long-term care facilities has been exacerbated. Although there has been an increase in the number of digital solutions to mitigate social isolation during COVID-19, facilities in northern British Columbia do not have sufficient information regarding the technologies to support social connectedness. To support evidence-based policy decisions, a scoping review was conducted to identify existing virtual technology solutions, apps, and platforms that promote social connectedness among older adults residing in long-term care. A combination of keywords and subject headings were used to identify relevant literature within PubMed, CINAHL EBSCO, PsychINFO EBSCO, Embase OVIDSP, and Web of Science ISI databases. DistillerSR was used to screen and summarize the article selection process. Twenty-three articles were identified for full-text analysis. A variety of technologies are described which can be used to mitigate the impacts of social isolation felt by long-term care residents. However, many of these digital solutions require stable highspeed internet. This remains a challenge for facilities in northern areas as many have limited access to reliable internet. Metrics used to evaluate social engagement in the context of long-term care are also outlined. This research provides the preliminary groundwork necessary to better inform policy decisions about which technologies are available and, of these, which are effective at enhancing social connectedness for older adults in long-term care.

### TECHNOLOGY GUIDED ASSESSMENT FOR URINARY TRACT INFECTION: CREATING A COMMON INTERPROFESSIONAL LANGUAGE

Donna Owen,[1] Alyce Ashcraft,[2] Kyle Johnson,[2] Huaxin Song,[2] and John Culberson,[2], *1. Texas Tech University Health Sciences Center, Houston, Texas, United States, 2. Texas Tech University Health Sciences Center, Lubbock, Texas, United States*

The Shared Meaning Model (SMM) is a grounded theory, derived in a previous study. This model demonstrates pathways for communication between nurse and primary care providers (PCPs) in the nursing home (NH), In this study we used the SMM for feasibility testing of a clinical decision support app (CDS app) using a descriptive, structured observational design. This study also provided a forum for initial testing of the SMM. The CDS app algorithm provided a common language to assess a resident with the goal of sharing this information with a PCP. The CDS app guided licensed vocational nurses (LVNs) (N=10) in assessing a standardized nursing home resident in a simulation setting experiencing symptoms of a potential urinary tract infection (UTI). Interviews with LVNs provided details of CDS app usability and concerns about using the CDS app with NH residents. Videos recorded LVNs interacting with the resident while using the CDS app on an iPad®. Time-stamps logged duration of the assessment. Bookmarked segments were used for discussion in LVN interviews. Videos were coded for eye contact, conversation, and touch between LVN and resident and documented personalized interactions. Findings indicated areas (lab values, drug names) for changes to language in the algorithm. In less than 12 minutes the CDS app enabled LVNs to collect information based on language used by PCPs to make decisions about the presence of a UTI. Relationships between initial constructs in the SMM were supported. This CDS app holds promise for building a common language to enhance interprofessional communication.

### THE IMPACT OF UNMET ADL NEED ON THE SELF-RATED HEALTH AND LIFE SATISFACTION OF CHINESE OLDER ADULTS

Jing Huang, Edmond Pui Hang Choi, and Pui Hing Chau, *The University of Hong Kong, Hong Kong, Not Applicable, Hong Kong*

This study aims to examine the associations of change in unmet need for assistance with Activities of Daily Living (ADL) with the self-rated health and life satisfaction of community-dwelling Chinese older adults. Using national longitudinal data from the Chinese Longitudinal Healthy Longevity Study, we examined the associations of unmet ADL need with self-rated health and with life satisfaction from baseline (T1) to a 3-year follow-up (T2) among 1,914 older adults with ADL limitation. Change in unmet ADL need was categorized into “Persistently Unmet”, “Unmet at T1 Only”, “Unmet at T2 Only”, and “Never Unmet”. Self-rated health and life satisfaction were rated by 5-point Likert scales. Linear mixed models were performed to examine the associations, controlling for sociodemographic factors, health conditions, and social support. The results showed that older adults whose ADL needs were persistently unmet, those unmet at T2 only, and those never unmet, experienced a significant decline in self-rated health from baseline to follow-up, but those unmet at T1 only experienced a significant rise in self-rated health. While the life satisfaction was stable from baseline to follow-up among older adults whose ADL needs were persistently unmet or never unmet, it significantly decreased among those unmet at T2 only and significantly increased among those unmet at T1 only. The effects of unmet ADL need on self-rated health and life satisfaction appeared to be short-term rather than long-term. These findings facilitate a better understanding of unmet ADL need and emphasize the importance to fully meet the ADL needs of older adults.

### WHAT IS KNOWN ABOUT CYCLING WITHOUT AGE: A SCOPING LITERATURE REVIEW

Nicole Kaufman,[1] Aleksandra Zecevic,[2] Suna Christensen,[3] and Cotnam Victoria,[4], *1. University of Western Ontario, Toronto, Ontario, Canada, 2. Western University, London, Ontario, Canada, 3. Independent Researcher, Copenhagen, Hovedstaden, Denmark, 4. Western University, Western University, Ontario, Canada*

The Cycling Without Age (CWA) program provides residents of long-term care homes with a bike ride experience, as a volunteer pedals them around the community in a specially designed trishaw. There is limited evidence of the program's effectiveness on older adults, pilots, and communities. The purpose of this literature review is to scope and summarize contemporary CWA discourses to generate future research questions that will provide evidence for future implementation of CWA. Data collection and analysis followed Arksey and O'Malley's 2005 framework. A systematic search was conducted in PubMed, OMNI, and Ebscohost databases. A grey literature search strategy incorporated: grey literature databases, customized Google searches, targeted websites, consultation with expert librarians, and a social media analysis on Twitter, Facebook and LinkedIn. Content analysis was used to identify the key themes. A total of 165 sources (2 peer-reviewed, 103 grey literature, 60 social media) were included in the final analysis. The three main themes were (a) meaning from being on a bike, (2) impacts of CWA, and (3) formation of relationships. Findings suggest that the CWA program brought valuable meaning to the participants' lives, significantly improved their happiness, and was associated with the formation of new and diverse intergenerational relationships. A large amount of anecdotal evidence, social media chatter, and global adoption of CWA indicate its importance and potential to satisfy the need of older adults to engage with society. Future research on the physical and mental health benefits of CWA is required to support further implementation of the program.

### WOMEN WITH OBESITY ARE MORE LIKELY TO HAVE LONG-TERM INDWELLING BLADDER CATHETERIZATION IN U.S. NURSING HOMES

John Harris,[1] Mary Ackenbom,[1] Alison Trinkoff,[2] Steven Handler,[1] John Engberg,[3] David Wolf,[4] and Nicholas Castle,[5], *1. University of Pittsburgh, Pittsburgh, Pennsylvania, United States, 2. University of Maryland, Baltimore, Maryland, United States, 3. RAND, Pittsburgh, Pennsylvania, United States, 4. Bellarmine, Louisville, Kentucky, United States, 5. West Virginia University, Allison Park, Pennsylvania, United States*

Reducing indwelling catheters and increasing clean intermittent catheterization is a key element of effective infection control and maintaining functional independence in nursing homes. Nursing care is often more difficult as obesity increases, leading to more nursing care or equipment to provide care. We hypothesized that nursing homes are more likely to use indwelling catheters for people with obesity because indwelling catheterization likely eases the nursing burden of toileting and personal hygiene care for residents with obesity. The study design was a retrospective cohort study of U.S. nursing home female residents in Minimum Data Set in 2013. Obesity and normal weight (the reference group) were categorized using National Institutes of Health criteria. Indwelling and intermittent bladder catheterization was defined during periodic assessment of residents. We modeled the outcomes using logistic regression using a robust variance estimator. Model covariates included obesity category, resident age, dementia status, comatose status, Stage 3 or 4 pressure ulcers, and the number of activities of daily living deficits. The study cohort included 1,068,388 female residents in 15,230 nursing homes. Obesity (BMI ≥ 30 kg/m2) prevalence was 31.9%. The prevalence of indwelling catheterization was 5.2% and of intermittent catheterization was 0.4%. The odds ratio of indwelling catheter use for obese residents varied from 1.05 to 1.74 (all with p-values <0.001), whereas the odds ratio. of intermittent catheter use varied from 0.84 to 0.46 (all with p-values <0.01) compared to residents of normal weight. Increasing obesity is independently associated with increased long-term indwelling bladder catheterization and decreased intermittent catheterization.

### Session 9400 (Poster)

### OLDER ADULTS' EXPERIENCES AND PERCEPTIONS OF THE COVID-19 PANDEMIC

### "IT IS THE SCOURGE OF GOD" MISCONCEPTIONS OF COVID 19 PANDEMIC AMONG OLDER MARKET TRADERS IN IBADAN, NIGERIA

Olubukola Omobowale,[1] and Adeola Fowotade,[2], *1. University of Ibadan, Nigeria, Ibadan, Oyo, Nigeria, 2. College of Medicine,, Ibadan, Oyo, Nigeria*

**Background:** Community perception on COVID-19 can influence the development of the right attitude towards mitigating the spread of the Sars CoV 2 virus. Older adults are at risk of severe infections and mortality is high among them. Objectives: This study was conducted to document the knowledge, perceptions and misconceptions of COVID-19 among older market traders in Ibadan, Oyo State, Nigeria.

**Methods:** A cross-sectional study conducted in two densely populated markets in Ibadan. An interviewer-administered semi-structured questionnaire was used to collect data on the knowledge and perception of COVID-19. Data were analyzed using SPSS version 23. Level of significance was set at $p<0.05$.

**Results:** A total of 321 respondents were sampled. All participants were aware, source was mainly through radio (93.5%), and 65.8% believed COVID-19 was as a scourge from God for punishments of sins. Only 41.1% had good knowledge of spread with personal contact (95.3%) mostly reported. On knowledge of symptoms and preventive measures, dry cough (84.7%) and frequent hand washing (95.6%) were mostly reported. Knowledge of cause was significantly associated with age ($p=0.04$) and marital status ($p=0.001$), while level of education ($p=0.012$) was significantly associated with knowledge of spread.

**Conclusion:** Misconceptions about the knowledge of the cause and spread of COVID-19 were prevalent among the study population. The implication of this finding among older adults and the significant effect of some sociodemographic factors on the knowledge of the cause and spread of COVID-19 calls for urgent health-promoting interventions that would dispel the misconceptions.

### AGE AND GENDER DEMOGRAPHICS PREDICT COMPLIANCE WITH COVID-19 PUBLIC HEALTH MEASURES: DATA FROM A GLOBAL SAMPLE

Amber Heemskerk,[1] Tian Lin,[1] Elizabeth Harris,[2] Natalie Ebner,[1] and Jay Van Bavel,[3], *1. University of Florida, Gainesville, Florida, United States, 2. New York University, New York City, New York, United States, 3. New York University, New York, New York, United States*

The COVID-19 global pandemic has brought far-reaching consequences on individual and societal levels. Social distancing and physical hygiene constitute effective public health measures to limit the spread of the virus. The current study investigates individual age and gender demographics, in interaction with a country's human development index (HDI), as crucial factors influencing compliance with public health measures in a large multi-national adult lifespan sample. This report leverages data from a large-scale international collaboration (Van Bavel et al., 2020; https://psyarxiv.com/ydt95/) comprising 45,576 individuals from 66 countries/territories. Participants provided self-reports of their compliance/agreement with three public health measures (i.e., spatial distancing, physical hygiene, policy support). Older age, female gender, and lower HDI were independently associated with greater compliance with public health measures. In addition, a significant three-way interaction between participant age, participant gender, and a country's HDI revealed that compliance was lowest in younger adults from well-developed countries, while compliance was highest among females across all ages from less-developed countries. Compliance with public health measures is crucial in effectively reducing coronavirus spread. Our findings suggest that age and gender as individual-level demographics, in tandem with HDI as a country-level predictor, affect individuals' willingness to comply with public health measures. These results highlight the potential of data-driven, tailored (i.e., towards specific demographics) health campaigns and public policies in the fight against a global pandemic.

### AGE DIFFERENCES IN POSITIVE EVENT APPRAISALS DURING COVID-19: EVIDENCE FROM A DAILY DIARY STUDY

Patrick Klaiber, Lydia Ong, Anita DeLongis, and Nancy Sin, *University of British Columbia, Vancouver, British Columbia, Canada*

Multiple studies suggest that community-dwelling older adults are psychologically resilient in the face of the COVID-19 pandemic. Notably, during the initial weeks of the COVID-19 outbreak, older age was associated with engaging in more daily positive events (Klaiber et al., 2021, Journal of Gerontology: Psychological Sciences). We followed up on these findings by exploring age differences in positive event appraisals during the COVID-19 pandemic. During the 7-day diary study conducted between March and August 2020, 1036 participants (mean age = 45.95, SD = 16.04, range = 18-91) reported their positive events in nightly surveys. If at least one positive event occurred, participants rated their appraisals of the event on the following dimensions: importance, calmness, happiness, gratitude, personal responsibility, and control. Older adults (60 years+) rated their positive events to be more personally important and felt more calm and happy during these events, compared to younger (18-39 years) and middle-aged adults (40-59 years). Furthermore, older adults felt more grateful during positive events compared to younger but not middle-aged adults. There were no age differences in feelings of control or personal responsibility for positive events. These findings highlight the importance of daily positive events for older adults during a time of major stress. In line with theories on adult development, daily positive event processes in older adults are characterized by valuing positive and meaningful social connections, as well as a greater degree of positive event-specific emotions such as feeling calm, happy, and grateful.

### ATTITUDES AND BELIEFS OF OLDER BLACK AMERICANS TOWARD THE COVID-19 VIRUS AND VACCINE

Dwana Bass,[1] Sophie Hanna,[2] Sarah Shair,[3] Loraine DiCerbo,[2] Bruno Giordani,[4] and Voyko Kavcic,[2], *1. Wayne State University, Sterling Heights, Michigan, United States, 2. Wayne State University, Detroit, Michigan, United States, 3. University of Michigan, University of Michigan, Michigan, United States, 4. Neuropsychology/ Michigan Alzheimer's Disease Research Center, University of Michigan, Ann Arbor, Michigan, United States*

The COVID-19 pandemic, an unprecedented health emergency, has devastated the nation, and disproportionately affected persons of color, especially Black Americans. It has forced health officials to rapidly develop and distribute COVID-19 vaccines, resulting in the importance of understanding Black Americans' attitudes and beliefs about COVID-19. We analyzed experiences of 167 Black Americans, ages 65 and older, recruited from Wayne State Institute of Gerontology Healthier Black Elders Center and surrounding communities. Participants were telephoned starting September 2020 and given the GAD-7 anxiety scale and a COVID-19 questionnaire measuring demographics, stressors, and emotional responses associated with the COVID-19 pandemic. A scale was also designed, adapted from the Health Belief Model, to measure fear of getting COVID-19, beliefs about the origins of COVID-19, uncertainty about vaccine safety, and intent to be vaccinated (5-point Likert scale). Of the 167 participants, 112 (67%) said they would agree to vaccination, 24 (14%) were ambivalent, and 31 (19%) said they would decline. T-tests comparing pro- and anti-vaccine participants showed that those not planning to get vaccinated expressed lower generalized anxiety ($p=.002$), COVID-19 fear ($p<.001$), and concerns about vaccine safety ($p=.01$), but greater belief that COVID-19 is man-made ($p=.05$). The current study provides a snapshot of urban Black American older adults who are in general eager to get vaccinated for COVID-19. Counterintuitively, those unwilling to accept the COVID-19 vaccine also had lower concerns for vaccine safety. More research is needed to fully understand the attitudes and beliefs of this underserved population regarding the COVID-19 virus and vaccine.

### EXPLORING OLDER ADULTS' LIVED EXPERIENCES OF COVID-19: A NARRATIVE INQUIRY STUDY

Tia Rogers-Jarrell, and Brad Meisner, *York University, Toronto, Ontario, Canada*

COVID-19 dramatically changed daily life for older adults in numerous and complex ways. Research is calling for an understanding on how COVID-19 has and will impact aging, and older adults' lived experiences with aging, within the context of the pandemic. Social and physical distancing guidelines have put older adults at an increased risk for social isolation. Intergenerational tensions have also intensified during the pandemic, and early research states the labeling of older adults as a homogenous and "vulnerable" group can lead to an increased risk of ageism in their communities.

Therefore, the purpose of this study is to explore how community-dwelling older adults (ages 65 and greater) experience daily life amid the COVID-19 pandemic using a biopsychosocial approach. This study employs a critical qualitative narrative inquiry design. Data will be collected through solicited diaries and semi-structured individual interviews (via telephone and video conferencing software). Data will be analyzed thematically and involve a re-storying of the findings. Preliminary results will be presented and discussed. This study aims to inform new and critical perspectives that broaden our understanding of how the overall health, wellness, and quality of life of older adults can be supported. Findings contribute to the current and developing knowledge of older adults' first-person accounts of their experiences within the COVID-19 pandemic.

### FACTORS PREDICTING COMMUNITY-DWELLING OLDER ADULTS' COVID-19 EXPERIENCES IN CENTRAL TEXAS

Atami Sagna De Main,[1] Bo Xie,[1] Kristina Shiroma,[2] and Nathan Davis,[1], *1. The University of Texas at Austin, Austin, Texas, United States, 2. The University of Texas at Austin, The University of Texas at Austin, Texas, United States*

Older adults, disproportionally affected by the COVID-19 pandemic, face health, social and structural vulnerabilities. Their experiences require systematic examination. Our study aimed to examine factors predicting community-dwelling older adults' experiences during COVID-19. We collected data via the telephone between June-August 2020 from a convenience sample of older adults in Central Texas (N= 200; age range=65-92 years; Mean=73.6, SD=6.33). We conducted multinomial logistic regression analyses to model relationships between self-reported COVID-19 experiences (positive, mixed, negative) and age, gender, race, income, education, frequency of communication with family and friends, feelings of loneliness and amount of COVID-19 information obtained. Factorial analysis revealed no statistically significant interaction effect. Multinomial logistic regression analysis revealed statistically significant main effects of annual household income, feelings of loneliness and amount of COVID-19 information obtained on predicting COVID-19 experiences. Age, gender, race, education, and frequency of communication with family and friends were not significant predictors. The odds of having a positive COVID-19 experience rather than negative experiences increased by 6.94 for an annual household of $60,000- $99,999, and by 6.02 for not feeling lonely. The odds of having a positive experience during COVID-19 rather than mixed increased by 9.90 for an annual household income of $100,000 or more. Participants who reported having "too much information" about COVID-19 were more likely to have mixed experiences compared to those with positive experiences. Our findings underscore the crucial role of financial security and social connections in reducing economic and emotional challenges older adults are facing during this crisis.

### FRAMING THE COVID-19 PANDEMIC THROUGH A FAMILY LENS: RESULTS OF A QUALITATIVE THEMATIC ANALYSIS

Lauren Gilbert,[1] Omolola Adepoju,[2] LeChauncy woodard,[2] and Daniel Howard,[3], *1. University of Houston College of Medicine, Houston, Texas, United States, 2. Humana Integrated Health System Sciences Institute, Houston, Texas, United States, 3. Texas A&M University at College Station, Texas A&M University at College Station, Texas, United States*

**Background:** The growing proportion of older adults in the U.S. population tends to be most vulnerable to the effects of natural disasters such as pandemics. To date, little has been done to counteract the impacts of public health emergencies and disasters on the aging populations, particularly in African American and Latinx communities.

**Methods:** We administered a survey to community-dwelling minority older adults, 55+, in the Houston metroplex, between 11/2020 and 01/2021. The survey assessed how the COVID-19 pandemic has impacted minority older adults. This thematic analysis focused on open-ended questions regarding daily health management, biggest health concerns, and personal experiences with COVID-19.

**Results:** A total of 575 older adults completed the survey. The mean age was 69 years, 74% were female, 71% reported English as their primary language and 27% were college educated. Three main themes of COVID-19 related concerns emerged from the thematic data analysis: 1) Fear of contracting COVID-19 from family members and fear of passing COVID-19 on to family members. 2) Social needs, including prolonged isolation from family/friends to stay safe, obtaining basic necessitates such as food, medications, and transportation . (3) Personal experiences focused on COVID-19 cases, hospitalizations, and deaths of family/community members.

**Conclusions:** These older minority adults framed their experiences and concerns regarding the COVID-19 pandemic through the lens of family and their community. Their personal relationships permeated their responses and demonstrate the importance of integrating a family lens into future disaster planning, response and recovery efforts for minority older adults.

### LISTENING TO OLDER ADULTS: A QUALITATIVE ANALYSIS OF ADVICE GIVEN DURING COVID-19

Bryce Van Vleet,[1] Heather Fuller,[2] and Andrea Huseth-Zosel,[1], *1. North Dakota State University, Fargo, North Dakota, United States, 2. North Dakota State university, Fargo, North Dakota, United States*

The life experience of older adults offers a unique perspective of coping through historical crises. Specific advice offered by older adults is generally underrepresented in the literature. This qualitative study explores advice offered by older adults to others on how to cope through the COVID-19 pandemic as well as advice for the community about the needs of older adults. A Midwestern sample of 67 older adults aged 70-97 completed one phone interview in June of 2020 as part of a larger study about their experiences with social distancing and isolation. Participants were asked what advice they would give to others during the pandemic and what advice they would give to communities and families about the needs of older adults during the pandemic. Transcripts of these conversations were coded using in vivo and holistic coding as first-cycle methods. These codes were then analyzed using pattern coding as a second-cycle method. Results indicated older adults offered advice along three domains: fostering physical and mental wellbeing, promoting positive life perspectives, and maintaining connections. Advice to communities regarding the needs of older adults included having a selfless attitude and taking intentional actions like grocery shopping and writing letters. However, older adults also recommended avoiding extremes to allow them to maintain their independence and preserve physical distance for safety. Older adults utilized their life perspective and their own coping strategies when offering advice. Future research should evaluate the effectiveness of the advice given and how likely that the advice will be utilized by others.

### NATIVE AMERICAN ELDERS' EXPERIENCES DURING THE COVID-19 PANDEMIC: CASE STUDIES

Bo Xie,[1] Kristina Shiroma,[2] John Lowe,[1]
Atami Sagna De Main,[1] and Nathan Davis,[1], *1. The University of Texas at Austin, Austin, Texas, United States, 2. The University of Texas at Austin, The University of Texas at Austin, Texas, United States*

The COVID-19 pandemic has affected community-dwelling elder adults' experiences during the COVID-19 pandemic. We report 4 case studies of Native American elders' pandemic experience. Participants were recruited from community-dwelling older adults in Central Texas. Data collection took place via in-depth, semi-structured telephone interviews during June-August 2020. Four of the participants self-identified as Native American. Three of them were male; between the ages of 74 and 75; had at least some college education. The fourth Native American elder was a 68 year-old female with some college education. All four participants were coping well with everyday life during the pandemic. Connectedness emerged as the overarching theme among the 4 cases. Regular communication with their families was expressed as most important. A variety of communication technology was used to maintain contact with family members such as phone calls, texting, email, and video chat services particularly Zoom, FaceTime, and Facebook Video Chat. Challenges with using these technologies were also frequently reported. The participants expressed they did not feel a sense of increased loneliness or loss of being connected. Another theme emerged related to surviving the impact of the pandemic. Having the vaccine accessible along with financial resources necessary to sustain essential needs were most frequently expressed by the participants. These findings have implications for community interventions and policies that support the provision of mechanisms for Native American elders to maintain a sense of connectedness, including the adoption and use of communication technology, during times of crises such as pandemics and natural disasters.

### PERCEIVED FEAR OF COVID-19 AMONG NEPALI OLDER ADULTS

Saruna Ghimire,[1] Uday Narayan Yadav,[2]
Om Prakash Yadav,[3] and Janardan Subedi,[4], *1. Miami University, Oxford, Ohio, United States, 2. University of New South Wales, SYDNEY, New South Wales, Australia, 3. Ministry of Health and Population, Kathmandu, Bagmati, Nepal, 4. Miami University, Miami University, Ohio, United States*

Although coronavirus-disease-2019 (COVID-19) impacted everyone in some ways, it disproportionally impacted the older population. Given their increased vulnerability to severe illness and mortality, the ongoing pandemic has created greater distress, anxiety, and fear among the older population. In Nepal, a South Asian country nestled in the Himalayas between India and China, most stories of older adults are untold– both in the pre-COVID-19 and the COVID-19 era. This study aimed to explore the perceived fear of COVID-19 among Nepali older adults. A cross-sectional study was conducted between July-September 2020 among 847 older adults (≥60 years) residing in three districts of eastern Nepal. The seven-item Fear of COVID-19 Scale assessed the perceived fear of COVID-19; higher scores on the scale (ranges 7 to 35 indicated greater fear. A sizeable proportion of the participants' reported being afraid (35%), anxious (32%), uncomfortable (24%), clammy (14%), and sleepless (12%), while 28% were fearful of losing their life due to COVID-19. In adjusted regression analysis, older age group, Dalit (minority) ethnicity, and remoteness to the health facility were associated with greater fear of COVID-19. Surprisingly, pre-existing health conditions were inversely associated with fear of COVID-19. Greater fear of COVID-19 amidst the pandemic, although anticipated, urges us to reflect on the most vulnerable groups' psychological needs not just during COVID-19 but in the future events of pandemics and public health emergencies. Fear during emergencies could be battled with accurate and effective information as well as better preparedness and psychosocial interventions.

### Session 9405 (Poster)

### PAIN ASSESSMENT, MANAGEMENT, AND PALLIATIVE CARE

### INTER- AND INTRA-RACIAL DIFFERENCES IN PAIN AND PERFORMANCE-BASED FUNCTION IN OLDER ADULTS WITH OSTEOARTHRITIS

Staja Booker,[1] and Roger Fillingim,[2], *1. University of Florida, GAINESVILLE, Florida, United States, 2. University of Florida, gainesville, Florida, United States*

Osteoarthritis (OA) contributes to movement-evoked pain, impaired function and mobility, and reduced quality of life among older adults. Assessment of pain has not traditionally considered the dynamic changes that occur with gross motor movement, and thus self-reports of pain often reflect static or resting pain. This case-control pilot study examined inter- and intra-racial differences in movement-evoked pain and performance-based function in older adults (N= 28) with knee OA. Cases consisted of Blacks and Whites with OA; controls included Blacks without OA. The Biodex Pro System 4, an isokinetic and isometric dynamometer commonly used in rehabilitative medicine, measured knee muscle function. Pain intensity was assessed pre-, ante-, and post- completion of 2 repetition sets of five alternating knee flexion and extension maximum voluntary contractions at angular velocities of 90° (greater resistance) and 180° (lower resistance). Repeated Measures Analysis of Variance with Bonferroni correction identified statistically significant differences in pain for within- and between-subjects at 90° and 180°. Pain increased during the repetitions and decreased after completion of both repetition sets; this non-linear relationship was significant (p= .004). One-way ANOVA demonstrated peak torque (extension), a muscle's maximum strength capability, was significantly higher in White cases and Blacks controls compared to Blacks cases. Novel findings revealed that baseline pain is much higher and functional performance is significantly lower in Blacks with OA compared to White cases and Black controls. This research advances precision pain measurement and our understanding of the biological mechanisms uniquely involved in the experience of knee OA and mobility.

### MEDICAL COMPLEXITY, MORTALITY AMONG HIGH-COST MEDICARE ADVANTAGE ENROLLEES: PALLIATIVE, HOSPICE IMPLICATIONS

Samuel Amodeo,[1] Henrik Kowalkowski,[2] Halley Brantley,[3] Lauren Bangerter,[4] David Cook,[5] and Nicholas Jones,[6], *1. OptumLabs at UnitedHealth Group, Eagan, Minnesota, United States, 2. UnitedHealth Group, Minneapolis, Minnesota, United States, 3. UHG, Minnetonka, Minnesota, United States, 4. OptumLabs, Eden Prairie, Minnesota, United States, 5. United Health Group, Optum Labs, Minnesota, United States, 6. Brown University School of Public Health, Brown University, Rhode Island, United States*

Older adults with high medical spend require tailored interventions and care delivery to meet their complex needs. Palliative is a high-value solution for high-cost patients because it provides relief from the symptoms, pain, and stress associated with multiple conditions. Likewise, other high-cost patients may be closer to end-of-life and therefore benefit from hospice care. For Accountable Care Organizations (ACOs) and hospitals to implement palliative care, these programs must identify and target the high-need patient populations. This study explored patterns of spending and mortality across 4 years (2016-2019) using claims from 1,701,647 patients continuously enrolled in UnitedHealth Group Medicare Advantage (mean age=73.7; S.E.=0.01). Patients with healthcare spend in the top decile were segmented into three subgroups based on health conditions and spend patterns. Analyses identified a subgroup of patients (mean age=76.6; S.E.=0.04), with the highest rate of mortality, and significantly more chronic conditions and frailty, indicating their cost and mortality was driven by medical complexity. Odds ratios from a multinomial logistic model tie blood formulation drugs (OR XX), medicative procedures (OR XX), and nonhospital-based care (OR XX) to members of this subgroup may be connected to short-term mortality. There is a critical need to identify patients who stand to benefit from palliative and end of life care, this is particularly true for high-cost high-need patients. Our study suggests that patterns of medical complexity and morality within high-cost patient subpopulations can be used to identify high-cost patients who would benefit from palliative or hospice care.

### MISSED OPPORTUNITIES FOR COMPASSION: A CALL TO ACTION FOR INCARCERATED INDIVIDUALS DENIED COMPASSIONATE RELEASE

Claire Morton,[1] Rachel Nathan,[2] Anjana Chacko,[3] and Raya Kheirbek,[4], *1. Une, Baltimore, Maryland, United States, 2. University of Maryland School of Medicine, University of Maryland, Maryland, United States, 3. University of Maryland, Baltimore, Maryland, United States, 4. University of Maryland School of Medicine, University of Maryland School of Medicine, Maryland, United States*

In 2016, a total of 4,117 state and federal prisoners died in publicly or privately operated prisons. Each year from 2001 to 2016, an average of 88% of deaths in state prisons were due to natural causes, with more than half of those due to cancer, heart disease or liver disease, conditions for which non-incarcerated citizens often benefit from palliative care and hospice. Prisoners age 55 and older are the fastest-growing segment of the population residing in prisons, as well as those with the highest mortality rate. Compassionate release of seriously ill prisoners became a matter of federal statute in 1984 and has currently been adopted by the majority of U.S. prison jurisdictions. The spirit of the mandate is based on the idea that catastrophic health conditions ie terminal illness affect the four principles of incarceration: retribution, rehabilitation, deterrence, and incapacitation. Concerned about an aging prison population, overcrowded facilities, and soaring costs, many policy makers are calling for a wider use of compassionate release for persons with terminal illness as well as broader prison reform. The prognosticating criteria of compassionate release guidelines are clinically flawed, and the application and procedural barriers are prohibitive. In this paper we review cases of patients who qualified for compassionate release but had their applications denied. We will discuss the urgent need for access to quality palliative medicine for incarcerated persons with advanced illness and call healthcare providers to action with the aim of reducing suffering and promoting social justice for those in need.

### PREVALENCE OF MUSCULOSKELETAL PAIN AND ANALGESIC TREATMENT AMONG HOME-DWELLING OLDER ADULTS: CHANGES 1999–2019

Tuuli Lehti, and Kaisu Pitkälä, *University of Helsinki, Helsinki, Uusimaa, Finland*

Pain has been shown to be undertreated in the older population. At the same time, the increased opioid use is of concern in the Western world. This study analyzes temporal trends in pain management among home-dwelling people aged 75 to 95 using cross-sectional cohort data spanning 20 years. The Helsinki Aging Study recruited random samples aged 75, 80, 85, 90, and 95 in 1999, 2009, and 2019. In total, 5,707 community-dwelling people participated in the questionnaire survey. Participants reported their medical diagnoses, regular prescription medications, and the presence of back pain or joint pain within the last 2 weeks (never, sometimes, or daily). We compared analgesics use in people reporting musculoskeletal pain and in people not reporting pain in 1999, 2009, and 2019. Of participants, 57–61% reported intermittent or daily musculoskeletal pain. The percentage of people taking a daily analgesic increased from 9% in 1999 to 16% in 2019. The use of NSAIDs decreased from 1999 to 2019, while the use of paracetamol increased from 2% to 11%. Of participants, 3% took daily opioids in 2019. Of those reporting daily musculoskeletal pain, 20% in 1999, 35% in 2009 and 32% in 2019 took regular pain medication. Pain remains undertreated in the older population, although the use of regular prescribed analgesics increased from 1999. The use of NSAIDs diminished, while the use of paracetamol increased. Daily opioid use remained modest from 1999 to 2019.

### QUALITATIVE DOCUMENT ANALYSIS OF PATIENT'S HEALTHCARE TRAJECTORY WITH AND WITHOUT PALLIATIVE CARE

Melissa Steffen,[1] Kenda Stewart Steffensmeier,[2] Bruce Alexander,[3] and Maresi Berry-Stoelzle,[3], *1. VA Office of Rural Health, Veterans Rural Health Resource Center-Iowa City (VRHRC-IC), Iowa City, Iowa, United States, 2. Iowa City VA Health Care System, Iowa City, Iowa, United States, 3. Iowa City VA Medical Center, Iowa City, Iowa, United States*

Palliative care is important to the care of seriously ill patients to support the patient and family. Palliative care is often timely in the inpatient setting, but delayed in outpatient care, leading to missed opportunities. Identifying when to engage patients with palliative care in outpatient settings has been challenging. As part of a larger quality improvement project to increase access to palliative care, a qualitative sub-study was completed to identify missed palliative care engagement opportunities in patient's healthcare trajectories. A document analysis of patients notes from a convenience sample of 20 recently deceased patients who received care within the Veteran Affairs healthcare system (VAHCS) was completed. Patients were sorted into four categories that emerged from initial analysis: cancer/palliative, non-cancer/palliative, cancer/non-palliative, and non-cancer/non-palliative. Two qualitative analysts reviewed the notes, paying particular attention to notes preceding or following seminal healthcare events. Patients in the cancer/non-palliative category were more likely to decline preventive care, engage less with the VAHCS health care or only interacted with the VAHCS for specific needs (e.g., determine VA health benefits). Similarly, non-cancer/non-palliative care patients were more likely to use a mix of VAHCS and outside healthcare, with inpatient care occurring outside of the VAHCS. For non-palliative care patients, seminal healthcare events were less likely to occur in the VAHCS. Thus, identifying opportunities to engage patients with palliative outside of seminal healthcare events may be important to increasing patient access within the VAHCS.

### RELIABILITIES OF MEAN AND VARIABILITY OF AMBULATORY PAIN AMONG COMMUNITY DWELLING OLDER ADULTS

Jinshil Hyun, Jiyue Qin, Cuiling Wang, Mindy Katz, Jelena Pavlovic, Carol Derby, and Richard Lipton, *Albert Einstein College of Medicine, Bronx, New York, United States*

Individual's pain experiences vary substantially over time periods, and the variability in pain may be an important metric to predict health consequences. However, research on its reliability is lacking among older adults. We aimed to examine the reliabilities of both intra-individual mean (IIM) and intra-individual variability (IIV) of subjective pain reports assessed using ecological momentary assessments (EMA) among racially diverse, community dwelling older adults. Participants were from the Einstein Aging Study (N=311, age=70-91) and completed a 14-day EMA protocol which included self-reports of pain intensity 6 times a day. Pain IIV was quantified using intraindividual standard deviation (iSD). We followed Wang and Grimm(2012)'s approach to calculate the reliability of IIM and IIV. Over a 2-week period, we found excellent reliabilities for both pain IIM (.99) and pain IIV (.91), showing that these measures are reliable and can be used to link with various health outcomes among community dwelling older adults. We also estimated the average number of assessments that produce acceptable levels of reliability. The average of 2 assessments for pain IIM and 23 assessments for pain IIV produced values that exceeded reliability score of .80, suggesting that a briefer study design may be used to reduce participants' burden with reliable pain metrics. Future studies need to examine whether pain IIV is associated with cognitive, emotional, and physical health among older adults and whether intervention studies that target to reduce pain IIV improve health consequences.

### SUPPORTING INNOVATIVE STRATEGIES TO REDUCE OPIOID-RELATED HARMS AMONG OLDER ADULTS IN PRIMARY CARE

Rosanna Bertrand,[1] Nicole Keane,[2] Sarah Shoemaker-Hunt,[2] Clarissa Hsu,[3] Michael Parchman,[3] Sebastian Tong,[4] and Arlene Bierman,[4], *1. Abt Associates, Abt Associates, Massachusetts, United States, 2. Abt Associates, Cambridge, Massachusetts, United States, 3. Kaiser Permanente of Washington Health Research Institute, Seattle, Washington, United States, 4. Center for Evidence and Practice Improvement (CEPI), Agency for Healthcare Research and Quality (AHRQ), Rockville, Maryland, United States*

Older adults are more likely to be prescribed opioids and to suffer from opioid-related harms. Despite growing concerns about opioid misuse in older adults, providers and health care systems often struggle with approaches that would effectively manage opioid use and reduce opioid misuse in older adults. To address this issue, the Agency for Healthcare Research and Quality funded a four-year project to work with primary care practices in developing and testing innovative strategies for opioid management in older adults. To develop a change package that will inform learning collaboratives where primary care practices will be encouraged to test new or modified strategies in managing opioids in older adults, Abt, the contractor, first completed an environmental scan to identify existing resources/tools. Identified resources/tools were vetted by an expert panel and appropriate items were used to develop a change package consisting of nine high-leverage change (HLC) strategies (e.g., Develop processes/workflows that clearly define roles/responsibilities and promote coordinated team-based care). In the change package, multiple key activities that accompany each HLC strategy are presented as examples of strategies that could be implemented to bring about the selected HLC. Primary care practices participating in learning collaboratives will use the change package to guide the development and testing of strategies to manage opioids in their older adults, which will inform the development and refining of a compendium of strategies to best reduce harms of opioid use in older adults.

### Session 9410 (Poster)

### PAIN, PAIN MANAGEMENT, AND PALLIATIVE CARE

### AGE DIFFERENCES IN THE USE/EFFICACY OF EMOTION COPING STRATEGIES AMONG ADULTS WITH CHRONIC PAIN: A SCOPING REVIEW

Gillian Fennell,[1] Elaine Wethington,[2] M. Carrington Reid,[3] Erica Sluys,[1] Kelsey Donovan,[4] Valeria Cardenas,[1] Elizabeth Zelinski,[1] and Susan Enguidanos,[1], *1. University of Southern California, Los Angeles, California, United States, 2. Cornell University, Ithaca, New York, United States, 3. Weill Cornell Medical College, New York, New York, United States, 4. University of Massachusetts Amherst, Amherst, Massachusetts, United States*

Active coping strategies (e.g., exercise and pharmacological treatments) typically do not leave chronic pain patients completely pain-free. Therefore, individuals turn to emotion-focused strategies to cope with associated impairment and psychosocial consequences. General coping strategy use has been shown to differ by age. This scoping review explored age differences in the use and effectiveness of emotion-focused strategies in adults experiencing chronic pain. Studies were located via advanced searches in PubMed, PsycINFO, CINAHL, Embase, Web of Science, and Proquest Dissertations and Theses Global and referral. Two reviewers independently conducted abstract screenings and full-text extractions. Conflicts were discussed and resolved by the PI. We identified 15 studies that met our inclusion criteria, of which 14 met criteria for high methodologic quality. The majority of studies utilized the Coping Strategies Questionnaire to assess differential use of pain-coping strategies. The remaining studies used one of five other questionnaires. Only one study examined the differential effect of age on the efficacy of emotion-focused strategies. Five of the eight studies that examined hoping/prayer coping reported the strategy's positive association with age. Age was not associated with ignoring pain or reinterpreting pain sensations in any of the eight studies in which these strategies were measured. We concluded that older age was associated with the use of praying/hoping as a means of coping with pain. No other consistent associations between age and other measured coping strategies were identified. Future research should account for auxiliary stressors and pain characteristics while investigating the differential effect of age on pain coping efficacy.

### ASSOCIATIONS OF PAIN SEVERITY AND MOBILITY WITH AGE IN CHRONIC LOW BACK PAIN: DOES THE TYPE OF ASSESSMENT MATTER?

Taylor Buchanan, Deanna Rumble, Kristen Allen-Watts, Katie O'Neal, Tammie Quinn, Thomas Buford, and Burel Goodin, *University of Alabama at Birmingham, Birmingham, Alabama, United States*

Chronic low back pain (cLBP) can lead to severe pain symptoms as well as disability in adults. As individuals age, pain symptoms and mobility outcomes can become increasingly debilitating. However, current findings regarding the influence of age on symptoms and outcomes are mixed and may be attributed to the assessment methodologies for pain and mobility. Therefore, we sought to examine the association of age with broad and specific assessments of pain severity and mobility commonly implemented in adults with cLBP. cLBP participants (n = 158) completed questionnaires regarding pain intensity and disability including demographics, Clinical Pain Assessment (CPA) and the Oswestry Low Back Pain questionnaire (OLBP). Participants also completed assessments of movement-evoked pain and difficulty by performing the Short Physical Performance Battery (SPPB). Pearson's chi-square tests and regression-based analyses were conducted using SPSS version 26.0. Among cLBP participants, age was associated with pain-related disability indexed by section one of the OLBPS regarding pain intensity ($F= 5.0$, $p<.05$), and mobility via total SPPB score ($F= 11.7$, $p<.05$). Interestingly, age predicted greater self-reported difficulty climbing stairs ($F= 21.7$, $p<.05$), performing chores ($F= 17.0$, $p<.05$), walking ($F= 14.0$, $p<.05$), and running errands ($F= 13.4$, $p<.05$) from the CPA. Further, age predicted total balance ($F= 3.2$, $p<.05$), gait speed ($F= 7.8$, $p<.05$), and chair stand ($F= 6.5$, $p<.05$) scores of SPPB. Age is associated with questionnaires assessing cLBP pain severity and is also associated with mobility outcomes. Future research should seek to understand the influence of age on movement-evoked pain in cLBP.

### CHALLENGES WITH PAIN TREATMENT FOR RURAL OLDER ADULTS: FAMILY CAREGIVERS' VIEWS

Hyunjin Noh,[1] Cho Rong Won,[2] and Zainab Suntai,[1], *1. University of Alabama, Tuscaloosa, Alabama, United States, 2. University of Alabma, Tuscaloosa, Alabama, United States*

Family caregivers face various challenges in assisting older adults experiencing pain and difficult symptoms. Living in rural areas poses additional obstacles to their caregiving. The purpose of this study was to explore family caregivers' lived experiences in caring for older adults with pain and discomfort in rural communities. A qualitative research design was adopted to capture the common essence of participants' experiences through a phenomenological method. Purposeful sampling was used, and the participant criteria was: age 18+, have good thinking skills, resident of Alabama, provide unpaid assistance to a family/relative who has chronic/serious health conditions and experienced pain/discomfort in the last 3 months. Ten participants were recruited from rural counties of Alabama. Individual semi-structured interviews were conducted via phone and were recorded and transcribed verbatim. Inductive, thematic analysis of the data revealed themes in five categories: 1) impact of pain (physical and psychological/emotional toll), 2) coping strategies (faith/contentment with life/logistical adaptation), 3) impact of Covid-19 (physical health/social interaction/mental health/added caregiving), 4) challenges in pain treatment (transportation (time/distance/driver/cost) and non-transportation related problems (healthcare provider issues/health insurance/financial burden)), and 5) suggestions (transportation-related (more transportation options/tailored services) and non-transportation-related support (home-based services/better health insurance coverage)). Findings of this study highlight rural family caregivers' unique experiences in assisting older adults' access to pain treatment, particularly during the Covid-19 pandemic. Policy- and program-level intervention is called for to increase individualized transportation options, improve health insurance coverage, and expand financial support for rural older adults experiencing pain and their caregivers.

### CONNECTING THE DOTS: PAIN MENTAL MODELS OF SPOUSAL CAREGIVERS OF VETERANS WITH DEMENTIA AND PAIN

Phoebe Block,[1] Srijana Shrestha,[2] Tracy Evans,[3] Michelle Hilgeman,[4] Julia Loup,[5] Mark Kunik,[3] and A. Lynn Snow,[5], *1. Battle Creek VA Medical Center, Battle Creek, Michigan, United States, 2. Wheaton College Massachusetts, Norton, Massachusetts, United States, 3. Baylor College of Medicine, Houston, Texas, United States, 4. Tuscaloosa VA Medical Center, Tuscaloosa, Alabama, United States, 5. University of Alabama, Tuscaloosa, Alabama, United States*

Pain is prevalent among persons with dementia (PWDs), yet often goes underrecognized and undertreated. Exploring caregivers' pain mental models may provide valuable insight into how they conceptualize pain, and how such conceptualizations affect their identification of and response to PWDs' pain. We identified and described the pain mental model(s) of spousal caregivers of community-dwelling veterans with dementia and pain through a secondary qualitative thematic analysis of recordings of a psychosocial intervention aimed at preventing aggression in PWDs with pain. Thirty female spousal caregivers (11 Black, 10 non-Hispanic White, and 9 Hispanic) comprised the present sample. Two themes were identified: Pain Assessment Beliefs and Knowledge (PA) and Pain Management Beliefs and Knowledge (PM). In our proposed mental model framework, PA and PM affect the ways caregivers answer two PA-related questions (Is there a problem?, Is this problem pain?) and three PM-related questions (Is the pain treatable?, Is it worth treating?, How do I prefer to treat it?). Caregivers are moved to action when they "connect the dots" by identifying a problem in PWDs' behavior, labeling the problem as pain, and identifying a response (i.e., a treatment approach) they consider worth trying. Disconnects in caregiver understanding of PWDs' behavior are common in this sample, and predictably lead to inaction. The proposed mental model provides further explanation about how caregivers do or do not synthesize and apply pain knowledge and experience, allowing for the identification of potential areas of intervention (e.g., pain psychoeducation) to improve pain treatment for the PWDs under their care.

### DEVELOPMENT OF A MULTI-MODAL, DYADIC INTERVENTION FOR PERSISTENT PAIN: A QUALITATIVE STUDY

Aimee Fox,[1] Christine Fruhauf,[1] Jennifer Portz,[2] Marieke Van Puymbroeck,[3] Julia Sharp,[1] Heather Leach,[1] and Arlene Schmid,[1], *1. Colorado State University, Fort Collins, Colorado, United States, 2. University of Colorado, Aurora, Colorado, United States, 3. Clemson University, Clemson, South Carolina, United States*

People who experience persistent pain often require help from a family member, partner, or friend. These caregivers frequently have pain, but are often not included in interventions. Caregivers and care-receivers who both experience pain are more likely to be socially isolated, experience communication conflict, and have decreased quality of life. Interventions should target caregiving dyads to help them manage their pain together. Feasibility studies that include manual development, intervention evaluation, and refinement of intervention manuals support randomized controlled trials and help move interventions from research to practice. Thus, the purpose of this qualitative study was to explore (a) the needs of caregiving dyads, (b) input from medical and allied health experts, and (c) feedback from intervention facilitators and evaluators, informing the development and refinement of an intervention manual for people with persistent pain. A total of 16 caregiving dyads and one individual (caregiver couldn't participate) experiencing pain participated in focus groups. Eight experts then participated in a focus group or one-on-one interview. Lastly, after the intervention ended, 15 intervention facilitators and fidelity evaluators participated in one focus group. Data were uploaded into NVivo software and analyzed using constant comparison. Findings identified the importance of interventions to focus on pain interference, novel and modifiable approaches to managing pain as a dyad, and addressing the emotional and psychological effects of experiencing pain. Using qualitative approaches to develop, test, and refine an intervention manual enhances the relevancy, acceptability, and translation of our intervention to meet the needs of caregiving dyads experiencing pain.

### GENDER DIFFERENCES IN PAIN: EXAMINING EXPLANATIONS FOR THE ASSOCIATION

Cherish Michael, and Anne Barrett, *Florida State University, Tallahassee, Florida, United States*

Physical pain is a gendered experience: Women report higher levels of it than do men. This pattern may stem from differences in experiences of the body. Women are socialized to be attentive to its functioning, appearance, and sensations, while men are discouraged from paying much attention to their bodies. Little is known, however, about the precise social and economic pathways leading to gender differences in pain, especially in middle and later life when pain is most prevalent. We examine this issue using data from Wave 3 of Midlife in the United States (2013-2014). We consider four possible explanations for women's more frequent reports of pain: economic security, physical and mental health, social relationships, and discrimination. Results indicate that women are more likely than men to report experiencing chronic pain, as well as greater effects of it on their everyday lives. However, only two of the explanations contributed to explaining this association. Economic security and physical and mental health accounted for substantial portions of the association between gender and pain – 57 and 73 percent, respectively. In contrast, no mediating role was observed for either women's social relationships, in particular the greater strain they experience in them, or their more frequent reports of everyday and lifetime discrimination. The final model including all the possible explanations revealed that gender was no longer significant, suggesting that middle-aged and older women's greater pain is explained by their worse health and economic circumstances.

### GENDER DIFFERENCES IN THE RELATIONSHIP BETWEEN COPING STYLE AND ATTITUDES TOWARDS PALLIATIVE CARE

Ilana Engel,[1] Amber Watts,[2] Tamara Baker,[3] and Christian Sinclair,[4], *1. University of Kansas, Kansas. City, Kansas, United States, 2. University of Kansas, Lawrence, Kansas, United States, 3. University of North Carolina, Chapel Hill School of Medicine, University of North Carolina at Chapel Hill (School of Medicine), North Carolina, United States, 4. University of Kansas Medical Center, University of Kansas Medical Center, Kansas, United States*

Palliative care (PC) is becoming more widely available and its benefits, including improved quality of life for patients, have been demonstrated. Studies on patient-level barriers to PC access focus on knowledge and misperceptions. This study aimed to explore, among a community sample, whether more approach-focused coping styles may be associated with more positive attitudes towards PC and whether more avoidant coping styles are associated with more negative attitudes towards PC. Two linear regression analyses (an approach model and an avoidance model) were conducted to determine predictors of attitudes towards PC, controlling for potential confounds. The sample consisted of 87 community-dwelling adults ages 65+ (mean age=72.72 (5.88); 56.32% = women; 86.21% = White). In both models, more knowledge of PC was associated with more positive attitudes towards PC ($\beta = .71$, $p<.01$). Coping by engaging more social support was significantly associated with more positive attitudes towards PC ($\beta = .54$, $p<.05$). Results demonstrated a significant interaction ($\beta = -1.24$, $p<.01$) such that women who endorsed high levels of disengaged coping reported more favorable attitudes towards PC than men who endorsed high levels of disengaged coping. Results indicate the need for a tailored approach to PC education for patients and families. Men who often cope with a stressor via distraction, self-blame, denial, or giving up may be less receptive to acceptance of PC. Future research on educational interventions tailored for individuals with distinct coping styles may be beneficial, particularly for men who frequently rely on disengaged coping styles.

### IMPROVING QUALITY OF CARE VIA EFFECTIVE PAIN MANAGEMENT

Jennifer Rose, *The College of St. Scholastica, Foxboro, Wisconsin, United States*

Pain is neither a vital sign nor a normal part of aging. Yet, older adults frequently experience pain chronically or from an acute event. Pain was identified as a gap per the Centers for Medicare & Medicaid Services Quality Measures report (2019). The purpose of this quality improvement project was to improve the assessment of pain at a skilled nursing facility (SNF) by using a standardized tool. The Comprehensive Pain Assessment Tool for the Cognitively Intact evaluates the complex sensation and emotional reaction of the pain experience. Nurse managers (N=7) received 1:1 education on pain, pain assessment, use of the pain assessment tool, and took a post-test. Chart audits were conducted to identify tool use and evaluate the patient response. Additional data were collected from nurse managers via a questionnaire. All nurse managers received education and completed the post-test. Pain assessments and care plans were completed for 100% of the SNF residents in the cohort (N=22). Follow-up assessments were completed on only 75% of the cohort. Of the cohort, 95% demonstrated improved physical ability and functioning in activities of daily living as their pain experience improved. Only 4.5% of the cohort participated in the anticipated level of minutes of therapy as a result of facility infection control limitations due to the COVID-19 pandemic. This project demonstrated improved pain management through use of a tool to comprehensively assess pain. An organizational policy to comprehensively assess pain at this SNF could promote a higher level of independence and functioning for older adults.

### INSTITUTIONAL EFFECTS ON EARLY PALLIATIVE CARE AMONG MEXICAN-HERITAGE ELDERS

Susan Miller,[1] and Ester Carolina Apesoa-Varano,[2], *1. UC Davis, 2. UC Davis, Sacramento, California, United States*

This paper addresses Mexican-heritage older people's experiences with early palliative care (EPC). EPC is the early provision of medical, social and spiritual reports to relieve suffering. Empirically, Mexican-heritage older people are known to have less access to EPC and, when they access it, to receive care of lower quality. However, little work has explored how Mexican-heritage older people think about and access such care. The paper addresses this gap. Methods are longitudinal: 36 Mexican-heritage people ranging in age from 55 to 90 years completed longitudinal semi-structured qualitative interviews, for a total of 69 interviews. Results explore how respondents' participation in social institutions may mediate the effects of larger social structural constraints on their health and access to care.

### KEEP YOUR HOPES UP: AN EXAMINATION OF RACIAL DIFFERENCES IN THE ASSOCIATION BETWEEN HOPE AND PAIN

Kyrsten Hill,[1] Emily Behrens,[2] Dylan Smith,[3] Brian Cox,[4] Patricia Parmelee,[2] and Jason DeCaro,[4], *1. The University of Alabama, Tuscaloosa, Alabama, United States, 2. University of Alabama, University of Alabama, Alabama, United States, 3. Stony Brook University, Stony Brook, New York, United States, 4. University of Alabama, Tuscaloosa, Alabama, United States*

Hope has been associated with increased pain tolerance (Snyder et al., 2005) and has been incorporated in interventions targeting chronic pain (Howell et al., 2015; Katsimigos et al., 2020). Research suggests that African Americans with osteoarthritis (OA) pain experience greater pain severity and disability compared to non-Hispanic White individuals (Vaughn et al., 2019). Although the literature is limited, there is some evidence to suggest racial/ethnic differences in hope (Chang & Banks, 2007). The current study examined race as a moderator of the association between hope and pain in a sample of older adults. Experience sampling (ESM) data was used from a multi-site study examining non-Hispanic White and African American individuals with knee osteoarthritis (OA). Participants completed the Adult Hope Scale (Snyder et al., 1991) during baseline interviews and self-reported momentary pain during 28 ESM calls. Multilevel models revealed a significant interaction between hope and race ($p = .04$). Specifically, greater hope was associated with decreased momentary pain, and this association was stronger for African American compared to non-Hispanic White individuals. Results suggest that high levels of hope may be particularly protective for African American chronic pain patients. These findings can help inform existing and future interventions focused on enhancing hope in chronic pain populations. (Supported by AG041655, P. Parmelee and D. Smith, Co-PIs)

### LONELINESS PREDICTS DEVELOPMENT OF PAIN, FATIGUE, AND DEPRESSION IN OLDER ADULTS

Victoria Powell, Navasuja Kumar, Mohammed Kabeto, Andrzej Galecki, Daniel Clauw, and Maria Silveira,
*University of Michigan, Ann Arbor, Michigan, United States*

Pain, fatigue, and depression form a well-recognized symptom cluster that is posited to have a shared mechanism. It is possible that chronic psychosocial stressors such as loneliness may impact the central nervous system and immune system, potentially leading to symptom cluster development. Loneliness is an increasingly recognized type of psychosocial stress, especially among older American adults. Thus, we investigated whether loneliness increased risk of developing the symptom cluster of pain, fatigue, and depression over time. Using Health and Retirement Study data from 2006 – 2016, we examined self-respondents ≥50 years-old for the presence of co-occurring pain, fatigue, and depressive symptoms surpassing threshold levels (ie., the symptom cluster). Loneliness (measured by the 3-item UCLA Loneliness Scale) at baseline was used as the predictor of interest. Of the total sample (n=11,766), n=5,956 (50.6%) had up to two complete sets of follow-up symptom cluster measurements. Logistic regression models for longitudinal data were fitted using a generalized estimating equation (GEE). After adjusting for demographic and clinical variables, loneliness strongly predicted the development of the symptom cluster (adjusted OR=3.16 (95% CI 2.77, 3.61)) as well as each component symptom (pain adjusted OR=1.54 (95% CI 1.42, 1.67); fatigue adjusted OR=1.88 (95% CI 1.74, 2.03); depression adjusted OR = 3.87 (95% CI 3.55, 4.21). Further research should investigate whether interventions targeting loneliness or other psychosocial stressors may have a role in prevention of this symptom cluster.

### PAIN INTERFERENCE AND CONSCIENTIOUSNESS IN OLDER ADULTS

Stephanie Judge,[1] Suzanne Segerstrom,[1] and Kaitlyn Meyr,[2], *1. University of Kentucky, Lexington, Kentucky, United States, 2. University of Kentucky, University of Kentucky, Kentucky, United States*

Pain interference increases with age and occurs when pain interrupts daily activities. Individuals vary in their amount of interference at a given level of pain. Conscientiousness is a personality trait characterized by diligence, perseverance, and goal-directedness, and is associated with fewer unhealthy behaviors and better health, including less pain and fewer functional limitations. This study tested three hypotheses on the relationships between pain, Conscientiousness, and pain interference among community-dwelling older adults (N=210) in a longitudinal study. At semi-annual interviews, participants reported their pain and interference. Conscientiousness was measured at baseline and follow-up. Multilevel models tested the between- and within-person relationships among study variables. Greater pain predicted more interference (person: $\gamma01=.541$, SE=.042, p<.0001; visit: $\gamma10 =.495$, SE=.014, p<.0001) but higher Conscientiousness decreased interference ($\gamma02=-.156$, SE=.064, p<.025). There was an interaction between Conscientiousness and pain: At higher levels of pain, older adults higher in Conscientiousness experienced much less interference than their less conscientious peers ($\gamma11=-.199$, SE=.089, p<.025). Older age at baseline predicted a greater decrease in Conscientiousness over the study period (b=-0.013, t(91)=-2.07, p<.05). Conscientiousness reduces the negative impact of pain on daily function. This protective effect may reflect perseverance and commitment to valued activities, consistency with proactive health behaviors, or other attitudes and behaviors that reduce the likelihood of psychosocial sequelae of pain. Overall, the sample decreased in Conscientiousness over time; however, the direction and amount of change varied considerably. These results refine existing knowledge of personality in old age and implicate personality factors as a potential target for pain management.

### SELF-REPORTED PAIN AND SYMPTOMS IN COMMUNITY DWELLING OLDER ADULTS: RESULTS FROM THE STUDY OF AGING II

Kristen Allen-Watts,[1] Deanna Rumble,[1] Cynthia Brown,[2] and Richard Kennedy,[1], *1. University of Alabama at Birmingham, Birmingham, Alabama, United States, 2. Louisiana State University Health Sciences Center - New Orleans, New Orleans, Louisiana, United States*

Pain is a common concern for community-dwelling older adults. There are a range of symptoms that may occur with pain that can be recurring and severe, which are associated with decreased quality of life. This study aims to characterize overall symptom load by utilizing the Brief Symptom Scale in community dwelling older adults who experience mild to severe pain. Data were extracted from the UAB Study of Aging II, a prospective, population-based study of mobility among community-dwelling older adults 75 years and older. Self-reported pain in the past 4 weeks and symptoms (e.g., pain, tired, nausea, depression, anxiety, shortness of breath) were included. The SPSS version 27.0 statistical package was used for analysis. Sixty-six percent were Non-Hispanic White, 58% were female, 40% lived in housing designed especially for the disabled, 49% were widowed, and 30% had a High School degree or GED. The mean age was 81 years (standard deviation 4.8). Of interest, over one third of the sample (38.1%) experienced moderate to severe pain, upper back pain was the most common area where pain occurred and feeling tired was the most common symptom. As the aging population continues to increase, so will the prevalence rates for pain. Findings suggests older adults with pain have multiple concomitant symptoms. Because the elderly represents a fragile and large group of the population, it is important to pay close attention to these symptoms.

### THE MODERATING ROLE OF DEPRESSION ON MOMENTARY PAIN-AFFECT ASSOCIATIONS IN OSTEOARTHRITIS

Emily Behrens,[1] Kyrsten Hill,[2] Dylan Smith,[3] Jason DeCaro,[4] Brian Cox,[4] and Patricia Parmelee,[1]

*1. University of Alabama, University of Alabama, Alabama, United States, 2. The University of Alabama, Tuscaloosa, Alabama, United States, 3. Stony Brook University, Stony Brook, New York, United States, 4. University of Alabama, Tuscaloosa, Alabama, United States*

Previous research has found a reciprocal relationship between pain and depression, in which each influences the severity of the other (Chou, 2007; Hawker et al., 2011, Kroenke et al., 2011; Schieir et al., 2009). Studies have found that depressed individuals exhibit stronger pain-mood associations than never-depressed individuals (Conner et al., 2006; Tennen et al., 2006). The current study investigated main and interactive effects of depressive symptoms on the momentary associations between pain and mood. Experience sampling (ESM) data was used from a multi-site study examining individuals with knee osteoarthritis (OA). Participants completed self-report measures of global depression and momentary pain, negative affect (NA), and positive affect (PA). Cross-sectional associations among momentary pain and affect were examined in a series of hierarchical multilevel models that nested the 28 ESM calls (Level 1) within participants (Level 2). A parallel set of multilevel models tested lagged associations among momentary variables. Depression significantly moderated the contemporaneous ($p < .001$) and lagged ($p < .003$) associations between pain and NA, suggesting that depression intensifies the momentary pain-NA linkage. There were no significant interaction effects for PA. These findings extend existing knowledge by illustrating how depressive symptoms influence the everyday experience of OA pain and its impact on affective well-being. (Supported by AG041655, P. Parmelee and D. Smith, Co-PIs)

### Session 9415 (Poster)

### PERSONALITY

### A COORDINATED ANALYSIS EXAMINING THE ASSOCIATION BETWEEN PERSONALITY TRAITS AND COGNITIVE DISPERSION

Tomiko Yoneda,[1] Alejandra Marroig,[2] Emily Willroth,[3] Eileen Graham,[3] Scott Hofer,[1] Daniel Mroczek,[3] and Graciela Muniz-Terrera,[4], *1. University of Victoria, Victoria, British Columbia, Canada, 2. Universidad de la República, Uruguay, Instituto de Estadística, Universidad de la República, Montevideo, Uruguay, 3. Northwestern University, Chicago, Illinois, United States, 4. University of Edinburgh, Edinburgh, Scotland, United Kingdom*

Cognitive dispersion is the degree of within-person variation in performance across cognitive tasks at the same testing occasion. Existing literature indicates that cognitive dispersion may be an early marker of poor brain health, dementia and mortality. Limited research, however, has examined individual differences in cognitive dispersion. Although personality traits are associated with individual differences in cognitive functioning, no research has examined personality and cognitive dispersion. In this project, we execute a pre-registered, coordinated analysis of seven diverse, international longitudinal studies of aging (Ntotal=33,581; mean age range=56.4-71.2) to investigate the extent to which the Big Five personality traits are associated with cognitive dispersion. For methodological approach, see /osf.io/wrnjq/. Cognitive dispersion scores were derived from cognitive test results, and independent linear regression models were fit independently in each study to examine personality traits as predictors of dispersion scores, adjusting for mean cognitive performance and socio-demographics (age, sex, education). Results from individual studies were synthesized using random-effects meta-analyses. Results revealed minimal evidence for associations between cognitive dispersion and personality traits in independent analyses or in meta-analyses. Based on the meta-analytic estimates, only higher levels of openness were associated with greater cognitive dispersion. Mean cognitive scores were negatively associated with cognitive dispersion across the majority of studies, indicating that individuals with higher mean performance had less dispersed cognitive scores. Our study contributes to the replicability and transparency efforts characteristic of open science by pre-registering our study and drawing on the collaborative network of the Integrative Analysis of Longitudinal Studies of Aging and Dementia (IALSA).

### IMPACT OF PERSONALITY FEATURES AND INTERPERSONAL PROBLEMS ON ANXIETY AMONG OLDER ADULTS

Olivia Noel,[1] Katie Granier,[2] Daniel Segal,[3] Marissa Pifer,[2] and Lisa Stone,[2], *1. Peak View Behavioral Health, Colorado Springs, Colorado, United States, 2. University of Colorado Colorado Springs, Colorado Springs, Colorado, United States, 3. University of Colorado at Colorado Springs, University of Colorado at Colorado Springs, Colorado, United States*

**Introduction:** Anxiety is a significant mental health problem among older adults and is associated with multiple other mental disorders, poor psychosocial functioning, and reduced quality of life. Personality traits and disorders, along with interpersonal problems, may play a significant role in anxiety, but these relationships are not well understood among older adults. This study examined relationships between anxiety with normative personality traits, personality disorder (PD) features, and interpersonal problems.

**Method:** Community-dwelling older adults (N = 130) completed the Geriatric Anxiety Scale (GAS), Coolidge Axis Two Inventory (CATI), Big Five Inventory-2 (BFI-2), and Circumplex Scales of Interpersonal Problems (CSIP).

**Results:** Anxiety was positively correlated with 13 of 14 CATI PD scales, ranging from .23 (Narcissistic) to .61 (Depressive). Regarding normative personality, anxiety was associated with Agreeableness (-.23), Conscientiousness (-.30), Extraversion (-.31), and Negative Emotionality (.56). Regarding interpersonal problems, anxiety was positively related to all eight CSIP scales: Self-Sacrificing (.30), Domineering (.31), Exploitable (.40), Intrusive (.41), Self-centered (.47), Nonassertive (.50), Socially Inhibited (.60), and Distant/Cold (.62). Regression analyses indicated that PD features accounted for the most variance in anxiety (53%), followed by interpersonal problems, (46%) and normative personality traits (33%).

**Discussion:** Anxiety appears to be meaningfully associated with PD features, several aspects of normative personality, and interpersonal problems, suggesting that these variables may play a role in the development of anxiety, or vice versa. Our findings especially speak to the growing awareness of the deleterious impact of PD features on clinical syndromes in later life, as evidenced by strong comorbidities with anxiety.

### PATHOLOGICAL TRAITS AND INTERPERSONAL DIFFICULTIES IN DEPRESSED OLDER ADULTS: CLINICAL VERSUS COMMUNITY SAMPLING

George Lederer,[1] David Freedman,[2] Lauren Atlas,[3] Shira Kafker,[4] Ira Yenko,[4] Angel Mak,[4] Dimitry Francois,[5] and Richard Zweig,[4], *1. Yeshiva University, Scarsdale, New York, United States, 2. Yeshiva University, Brooklyn, New York, United States, 3. New York-Presbyterian/Columbia University Irving Medical Center, NEW YORK, New York, United States, 4. Yeshiva University, Bronx, New York, United States, 5. NY, White Plains, New York, United States*

Personality pathology, represented by high neuroticism and low agreeableness in the Five Factor Model of Personality, has been identified as a predictor of depression in mixed-age samples and preliminary studies of older adults. Research on older people, however, has not examined the differential impact of pathological personality traits and processes on depression or examined them across treatment settings. This secondary analysis examined personality traits and processes as predictors of depression, evaluated the moderating effect of interpersonal problems, and assessed stratification of these personality variables across community and clinical settings. Older adults (N=395) ranging in age from 55 to 99 (M = 72.06; SD = 10.10) from inpatient psychiatric, outpatient medical, and community settings completed self-report measures of personality traits (NEO-FFI Agreeableness and Neuroticism), processes (Inventory of Interpersonal Problems), and depression (GDS-30). Higher neuroticism predicted worsened depressive symptoms ($\beta = .765$, $p < .001$), as did lower agreeableness ($\beta = -.163$, $p = .002$) and more interpersonal problems ($\beta = .459$, $p < .001$). Findings partially supported the stratification of personality traits and processes by setting. Interpersonal problems moderated neither the neuroticism-depression or agreeableness-depression relationships. Personality traits and processes predict depression in older adults across care settings but do not significantly interact. Levels of pathological traits and processes vary across community and clinical settings.

### PERSONALITY CHANGE PROFILES AND CHANGES IN COGNITION AMONG MIDDLE-AGED AND OLDER ADULTS

Mirjam Stieger,[1] Yujun Liu,[2] Eileen Graham,[3] Jenna DeFrancisco,[1] and Margie Lachman,[4], *1. Brandeis University, Waltham, Massachusetts, United States, 2. Northern Illinois University, Naperville, Illinois, United States, 3. Northwestern University, Chicago, Illinois, United States, 4. Brandeis University, Brandeis University, Massachusetts, United States*

Previous research on the relationship between personality traits and cognitive abilities has primarily focused on cross-sectional studies or on specific personality traits in relation to selected cognitive dimensions. The present study extends existing research by exploring associations among 20-year personality change profiles and 10-year cognitive change in middle-aged and older adults. The present study included 2,652 participants of the Midlife in the United States study (MIDUS) ranging in age between 20 - 74 years (M = 46.61, SD = 11.26) at the first of the three measurement occasions. Latent Profile Analysis (LPA) was used to capture profiles of change across the Big Five personality traits of extraversion, conscientiousness, agreeableness, openness, and emotional stability combined. Results of the LPA identified three personality change subgroups: Decreasers, Maintainers, and Increasers. Across the 20 years, the Decreasers showed greater decreases on the Big Five personality traits, the Maintainers remained mostly stable, and the Increasers showed greater personality trait increases. Also, the Maintainers and Decreasers were significantly older than the Increasers. Longitudinal multilevel models were used to examine the relationship between these three personality change profiles and cognitive change. Age, sex, education, physical activity, functional health, and self-rated health were added as covariates. Results show that cognitive decline was greater for the Decreasers and less for the Increasers compared to the other personality change profiles. The results have implications for developing interventions to target personality trait change in middle and later adulthood as a potential means for reducing declines in cognitive functioning.

### THE INTERPERSONAL CIRCUMPLEX AND THE ALTERNATIVE MODEL OF PERSONALITY DISORDERS: RELATIONSHIPS AMONG OLDER ADULTS

Lisa Stone,[1] and Daniel Segal,[2], *1. University of Colorado Colorado Springs, Colorado Springs, Colorado, United States, 2. University of Colorado at Colorado Springs, University of Colorado at Colorado Springs, Colorado, United States*

**Introduction:** The interpersonal circumplex model measures interpersonal dysfunction along two axes (communion and agency), resulting in eight unhealthy patterns: Domineering, Vindictive, Cold, Socially Avoidant, Nonassertive, Exploitable, Overly Nurturant, and Intrusive. It is unclear how the circumplex model applies to older adults and their unique biopsychosocial contexts. This study examined relationships between the circumplex and personality disorder features, using the Alternative Model of Personality Disorder's (AMPD) personality functioning and pathological personality trait constructs.

**Method:** Older adults (N = 202) completed the Inventory of Interpersonal Problems-Short Circumplex (IIP-SC), the Levels of Personality Functioning Scale-Self-Report (LPFS-SR), and the Personality Inventory for DSM-5 (PID-5) to measure pathological personality traits.

**Results:** Correlations were computed between the IIP-SC's eight circumplex scales with the LPFS-SR's four personality functioning domains and with the PID-5's five domains. All circumplex scales significantly ($p < .001$) and positively correlated with all LPFS-SR and PID-5 domains, with large effect sizes (> .45). Next, regressions were conducted, with the LPFS-SR and PID-5 domains predicting each IIP-SC scale. Across the eight regressions, the AMPD constructs accounted for significant variance in the IIP-SC scales, ranging from 38% (Nonassertive) to 64% (Domineering and Cold).

**Discussion:** Significant overlap between the interpersonal circumplex and the AMPD was demonstrated, but patterns are distinct from previous research among younger adults.

The circumplex was limited in its relation to the AMPD's personality functioning, but the pathological personality trait model was well represented through the circumplex. Results indicate that the circumplex may have some validity among older adults and warrants further investigation.

### Session 9420 (Poster)

### PHYSICAL ACTIVITY AND EXERCISE

### ACTIGRAPHY MEASURED PHYSICAL ACTIVITY ON COGNITIVE FUNCTIONING IN OLDER ADULTS

Pilar Thangwaritorn, and Amber Watts, *University of Kansas, Lawrence, Kansas, United States*

Physical activity may preserve cognitive functioning in older adults. This study examined associations between objectively measured physical activity and cognitive functioning. We recruited participants (Mage = 75.38 years, SD = 5.99) with (N=26) and without (N=181) cognitive impairment from the University of Kansas Alzheimer's Disease Center (KU-ADC). We collected cognitive data representing verbal memory, attention, and executive function. Accelerometers (Actigraph GT9X) were used to measure physical activity 24 hours a day for 7 days in a free-living environment. Physical activity was categorized as moderate to vigorous physical activity (MVPA) based on the Freedson (2011) Adult Vector Magnitude cut points. The association between cognitive functioning and total MVPA was evaluated by using multiple regression. We used factor analysis to create three composite scores (verbal memory, attention, executive function) from 11 individual cognitive tests. Compared to verbal memory and attention, results indicate that total MVPA was more strongly associated with executive function ($\beta = 0.001$, $p = .024$). These findings are consistent with the literature suggesting that executive function in older adults may benefit from physical activity. Future research should investigate the physiological mechanisms by which MVPA benefits executive function in contrast to types of activity that might benefit verbal memory and attention.

### AGE AND GENDER DIFFERENCES IN LONG-TERM EXERCISE BEHAVIOR FOR OLDER ADULTS WITH HEART DISEASE

Helen Graham,[1] Yuki Asakura,[2] and Kathy Prue-Owens,[1], *1. University of Colorado Colorado Springs, Colorado Springs, Colorado, United States, 2. Centura Health Penrose - St. Francis, Colorado Springs, Colorado, United States*

Exercise decreases mortality and hospital admissions. Exercise adherence is challenging, and little is known about exercise adherence especially in older adults with heart disease. To gain an understanding of long-term exercise behaviors in older adults we conducted a cross-sectional study of individuals diagnosed between 2016-2020 with myocardial infarction (MI)/angina. Emails were sent in 2020 to recruit participants. Exercise adherence was measured using the Exercise Adherence Rating Scale (EARS), Godin's Leisure-Time Activity Scale (GLTEQ) for exercise intensity, and self-report for impact of COVID-19. Descriptive statistics and t-tests were used to analyze data. Eight-hundred and seven individuals ($\bar{x}$ age 67.3) responded to the on-line survey. The majority were males (68.8%), married, (68.9%), and retired (59.3%). Co-morbidities included hypertension (32%), hyperlipidemia (21%), diabetes (12%), and depression (6.2%). Long-term exercise behaviors were independently observed in participants ≥65yr (n=526) and <65yr (n=281). Females ≥65yo demonstrated higher exercise adherence scores compared with males ≥65yo (1.66 ± 1.1 vs. 1.30 ± 21.7; t = -2.59, p=.010). Conversely, males scored higher in exercise intensity (34.4 ± 24.7 vs. 22.6 ± 21.7; t = 3.84, p=.000). Gender related exercise adherence and exercise intensity did not differ significantly in <65yo (p=.278 & p=.282, respectively). Exercise frequency decreased in both age groups after COVID-19 Pandemic started, however the decrease was significant only in older adults (p=.014) indicating they were at greater risk for exercise problems when faced with environmental barriers. Additional research is recommended as to the impact of environmental factors on exercise adherence in older adults and potential interventions.

### BIOMECHANISM AND EXERCISE EFFECT OF FITNESS WALKING USING TWIN WALKING STICKS

Ken Yamauchi,[1] Tsutomu Ichikawa,[2] Akira Ogita,[3] Hironori Yoshida,[4] Hiromichi Hasagawa,[5] Hiroshi Matsui,[6] and Shota kagawa,[7], *1. Keio University, Yokohama, Kanagawa, Japan, 2. Department of Early Childhood Education and Care, Matsuyama, Ehime, Japan, 3. Osaka City University, Osaka, Osaka, Japan, 4. Liberal Arts Education Center, Ashikaga, Tochigi, Japan, 5. Fitness center, Nagakute, Aichi, Japan, 6. Kyoto Medical Center, Kyoto, Kyoto, Japan, 7. Makuhari Human Care, Chiba, Chiba, Japan*

In Japan, walking poles with pairs of sticks developed exclusively for fitness walking have been designed. A new concept of walking style (WS) has been conceived using these walking sticks to "effectively" walk around the city, comprehensive sports parks, or at rehabilitation hospitals. Stick manufacturers are promoting its health benefits; however, evidence supporting these claims is lacking. Hence, this study aimed to measure the influence of walking sticks and evaluate the exercise effect based on functional physical fitness related to WS characteristics. The participants were 12 WS instructors. They engaged in WS at a comfortable speed after walking normally at the same speed (WN) for ~5 m (seven times), followed by WS again. The walking speed, step length, stride width, walk ratio, one-leg support time, and trajectory of the center of gravity (CG) (in the horizontal and vertical directions of one walking cycle) calculated from the whole-body skeleton model were analyzed. The gait of WS increased the step length, step width, and walking ratio as compared with that of WN (p<0.05). WS likely reduce cadence and one-leg support time (p<0.05). The CG locus in the left-right direction showed no significant differences between WS and WN. The maximum value of the CG locus in the vertical direction was high in WS (p<0.05). WS can be used as a navigation training tool that improves a walker's exercise efficiency and left-right leg coordination, thereby improving walking posture. This may help reduce the anxiety due to injuries and pain that may occur while walking.

### CAREGIVERS' PERCEPTION ABOUT THEIR PARTICIPATION IN A COMMUNITY-BASED EXERCISE INTERVENTION - BODY & BRAIN PROJECT

Oscar Ribeiro,[1] Pedro Marques,[2] Duarte Barros,[3] Paula Silva,[3] Joana Carvalho,[3] and Flávia Borges-Machado,[3], *1. University of Aveiro, University of Aveiro, Aveiro, Portugal, 2. Department of Education and Psychology, University of Aveiro, University of Aveiro, Aveiro, Portugal, 3. Research Centre in Physical Activity, Health and Leisure (CIAFEL), Faculty of Sports, University of Porto, University of Porto, Porto, Portugal*

Evidence is scarce on caregivers' perception regarding their participation in exercise interventions targeting individuals with neurocognitive disorder (NCD). This study aims to investigate the views of family caregivers of people with NCD about taking part in a community-based physical exercise intervention with their care-recipients. Twenty caregivers (N Male: 13; 66.5 ± 14.39 years old; age range: 36-88) answered to a semi-structured interview conducted by telephone about their perception on participating or not as class members of a 6-month multicomponent training intervention. Transcribed data from the interview were analyzed through thematic analysis. Main themes regard their perceived key-role in the care recipients' participation, which included knowing their limitations, making them feel accompanied and motivated, and the possibility of providing comfort and tranquility throughout the intervention. Caregivers also mentioned the possibility of fulfilling own needs for physical activity and being engaged in new experiences. Disturbing the care recipients' involvement and performance, the opportunity for respite during the sessions' time, and being enrolled in the program only in specific moments or by telephone were also mentioned. Findings highlight the inclusive perspective of caregivers to take part of exercise programs designed for people with NCD, not only due to their decisive role on care-recipients engagement but also due to the associated (in-/)direct personal benefits. This data may be useful for planning and prescribing future community-based exercise interventions for NCD caregiving dyads. Trial registration: ClinicalTrials.gov - NCT04095962. Supported by FCT: "Body and Brain" (POCI-01-0145-FEDER-031808), CIAFEL (FCT/UIDB/00617/2020), and Ph.D. Grants (SFRH/BD/136635/2018) to FM (2020.05911.BD) to DB.

### CREATION AND PILOT TESTING OF THE CONFIDENCE TO ENGAGE IN PHYSICAL THERAPY IN OLDER ADULTS (CEPT) MEASURE

Patricia Bamonti,[1] Rebekah Harris,[2] Jennifer Moye,[1] and Jonathan Bean,[2], *1. VA Boston Healthcare System, Boston, Massachusetts, United States, 2. VA Boston Healthcare System, VA Boston Healthcare System, Massachusetts, United States*

The measurement of self-efficacy is important in physical therapy (PT) settings where patients face barriers and adoption of new behavior is critical for recovery. However, existing measures of exercise self-efficacy do not account for the internal (i.e., fatigue) and external (i.e., scheduling) barriers to self-efficacy observed in older adults. We developed a self-report measure assessing an individual's confidence to engage in PT despite barriers. Qualitative ratings from patients (N = 75; age M =78.26 ± 11.2; 80% white; 20% African American) enrolled in PT at a Veterans Affairs Medical Center (VAMC) and their physical therapist were used to create a 21-item pool for the Confidence to Engage in Physical Therapy in Older Adults (CEPT). We next evaluated item characteristics and scale reliability and validity of the CEPT in a new sample of patients (N = 19; age M = 81.11± 8.93; 88.8% white, 11.1% African American) enrolled in an outpatient PT program who also received the Geriatric Depression Scale-15 (GDS-15), and the Activities-specific Balance (ABC) Scale. Response choice ranged from 0% (not confident at all) to 100% (highly confident) with higher scores indicating greater self-efficacy. Item analyses indicated adequate response variability across items (M = 55.9 ± 24.80, range 10-85). The scale demonstrated evidence of internal consistency reliability (Cronbach's alpha) = 0.98. Construct validity was demonstrated by positive association between CEPT and the ABC ($r = .74$, $p < .001$) and negative association with GDS-15 ($r = -.64$, $p < .01$). The CEPT requires further evaluation with larger sample sizes.

### DOES A 6-MONTH MULTICOMPONENT TRAINING IMPROVE FUNCTIONAL CAPACITY OF INDIVIDUALS WITH A NEUROCOGNITIVE DISORDER?

Oscar Ribeiro,[1] Flávia Borges-Machado,[2] Duarte Barros,[2] Laetitia Teixeira,[3] and Joana Carvalho,[2], *1. University of Aveiro, University of Aveiro, Aveiro, Portugal, 2. Research Centre in Physical Activity, Health and Leisure (CIAFEL), Faculty of Sports, University of Porto, University of Porto, Porto, Portugal, 3. ICBAS.UP, ICBAS.UP, Porto, Portugal*

Regular physical activity and exercise have been proposed as non-pharmacological therapeutic approaches to prevent and manage neurocognitive disorders (NCD). Multicomponent training (MT) combining aerobics, strength, postural and balance exercises seem to be effective at improving individuals with NCD in their ability to independently perform activities of daily living (ADL). This quasi-experimental controlled trial aims to analyze the effects of a 6-month MT intervention on functional capacity of individuals diagnosed with NCD. Forty-three subjects (N Major NCD: 36) participated in the Body&Brain Project and were subdivided in exercise group (EG; N: 23; 75.09 ± 5.65 years; age range: 61-83) or a control group (CG; N:20; 81.90 ± 5.95 years; age range: 70-89). The EG was submitted to bi-weekly exercise sessions, and the CG received monthly recreation sessions. At baseline and at post-intervention Timed-Up-and-Go (TUG), 6-meters Walk Speed and Handgrip tests were applied to evaluate lower body mobility, walking speed and handgrip strength, respectively. Results from linear mixed models revealed a statistically significant interaction between group (intervention vs. control) and time for TUG and walk speed test, but not for handgrip strength. The 6-month MT intervention improved lower body mobility and walking speed of older adults diagnosed with NCD, which might potentially impact ADL independence and quality of life. Trial registration: ClinicalTrials.gov - NCT04095962. Supported by FCT: "Body and Brain" (POCI-01-0145-FEDER-031808),

CIAFEL (FCT/UIDB/00617/2020), and Ph.D. Grants (SFRH/BD/136635/2018) to FM and [2020.05911.BD] to DB.

### DUAL-TASK COSTS IN GAIT SPEED DIFFERS ACROSS AGE GROUPS

Sally Paulson,[1] Michelle Gray,[2] Joshua Gills,[2] Anthony Campitelli,[2] Megan Jones,[2] Joohee Sanders,[3] Erica Madero,[4] and Jordan Glenn,[4], *1. University of Arkansas at Fayetteville, Cincinnati, Ohio, United States, 2. University of Arkansas at Fayetteville, Fayetteville, Arkansas, United States, 3. Shippensburg University of PA, Shippensburg, Pennsylvania, United States, 4. Neurotrack Technologies, Redwood City, California, United States*

With age, there are simultaneous reductions in gait speed (GS). This decrease in GS has been associated with an increased fall risk and negatively impacts independence. Further, GS naturally declines with the addition of a secondary stimulus (i.e., cognitive requirements). Combined, these decrements can be additive in nature potentially leading to robust declines with advancing age. Therefore, the aim of this study was to examine age-related effects of dual-task cost (DTC) while walking. Adults (N = 145), over the age of 45 years, completed two walking trials for each GS condition: habitual (HAB) and fast (FST), with and without a DT (i.e., counting backwards by serials of three). Subjects were classified into four age groups: youngest-old (YG ≤ 64 years, n = 24), young-old (YO, 65-74 years, n = 46), middle-old (MO = 75-84 years, n = 54), and oldest-old (OO ≥ 85 years, n = 21). DTC was calculated and ANOVAs were used to assess differences between the groups. There was no difference in HAB DTC between the age groups (p=.61). However, there was a significant difference in FST DTC (p=.04) between the YO (M±SD: -14 ± -11%) and OO (M±SD: -24 ± -12%). These data indicate there was an age-related affect for fast dual-task cost, but not for habitual dual-task cost while walking. An increase in dual-task cost among the oldest-old may be associated with an inability to properly maintain a faster cadence while performing an arithmetic task which may be related to task prioritization.

### EFFECT OF HIGH-INTENSITY INTERVAL TRAINING ON RHEUMATOID ARTHRITIS CD4+ T CELL OXIDATIVE METABOLISM

Brian Andonian,[1] David Bartlett,[2] Nancie MacIver,[2] William Kraus,[2] and Kim Huffman,[2], *1. Duke University School of Medicine, Durham, North Carolina, United States, 2. Duke University, Durham, North Carolina, United States*

Persons with rheumatoid arthritis (RA) have poor cardiorespiratory fitness and accelerated biological aging driven by systemic impairments in metabolism and inflammation. In this study of older RA participants, our goal was to identify the effects of a high-intensity interval training (HIIT) program on cardiorespiratory fitness and peripheral CD4+ T cell metabolism. We isolated CD4+ T cells from peripheral blood mononuclear cells in sedentary female RA participants (n=6; age=64.0+/-6.3 years) who underwent cardiopulmonary exercise testing and phlebotomy before and after 10 weeks of HIIT. HIIT improved RA cardiorespiratory fitness by 6.5+/-6.0% (pre-HIIT VO2 peak=25.1+/-5.1 ml/kg/min, post-HIIT VO2 peak=26.7+/-5.0; p=0.05). As measured by Seahorse XF Mito Stress Test, there were no significant mean changes in CD4+ T cell oxidative (oxygen consumption rate (OCR); pmol/min) or glycolytic (extracellular acidification rate (ECAR); mpH/min) metabolism, however there was large interindividual variability. RA peripheral CD4+ T cells preferred glycolytic metabolism (pre-HIIT mean basal OCR/ECAR ratio=0.78+/-0.13 pmol/mpH), while HIIT non-significantly shifted cellular preference toward oxidative metabolism (post-HIIT mean basal OCR/ECAR ratio=0.86+/-0.16; p=0.30). Increases in RA cardiorespiratory fitness following HIIT were significantly associated with increases in RA peripheral CD4+ T cell OCR/ECAR ratio (Spearman's rho=1.0, p<0.001) and basal and maximal respiration (rho=0.89, p=0.02 for both). Additionally, increases in CD4+ T cell mitochondrial ATP-linked respiration were significantly associated with increased quantities of circulating naïve CD4+CCR7+CD45RA+ T cells (rho=0.89, p=0.02). Our findings suggest that targeting cardiorespiratory fitness may be key in modulating T cell specific oxidative metabolism and function to prevent immunosenescence in older patients with chronic inflammatory diseases.

### EFFECT OF VIDEO ASSISTED HOME-BASED EXERCISE INTERVENTION ON FALL RISK AND GAIT PARAMETERS IN OLDER ADULTS IN INDIA

Snehal Kulkarni, and Aarti Nagarkar, *Savitribai Phule Pune University, Pune, Maharashtra, India*

Countries across the globe recommended isolation to protect older adults from COVID-19 infection. However, this led to decreased mobility and physical inactivity potentially increasing their risk of fall. The study was conducted in a group of 88 older adults between 60-74 years with known gait impairments and high fall risk. The participants were part of our cohort study on fall prevention program. Fall risk and gait impairments were measured using wearable sensors during the Timed-up and go test (TUG) at baseline. Using technology, a 16-week video assisted home based exercises intervention was delivered to reduce fall risk and improve gait parameters. The intervention consisted of flexibility, strengthening, balance and gait training exercises given progressively through one video session per week. The participants performed these exercises at home for the rest of the week. A home visit immediately after 16th week was arranged to collect post intervention parameters. Results showed an average 20% decrease in fall risk post intervention. An overall large effect size with Cohen's d of 0.90 was reported for fall risk. Significant difference in TUG time (Z = -4.610, p< 0.000), stride velocity (Z= -5.035, p<0.000), stride length (Z = -5.867, p<0.000), time taken to stand (Z = -7.363, p<0.000) and time taken to turn (Z = -6.079, p<0.000) was observed in the post-test measurements as compared to pre-test measurements. Therefore, we conclude that video assisted exercise programs can be highly beneficial as alternatives to in person exercise intervention to prevent falls during COVID-19 isolation.

### EFFECTS OF DIFFERENT 16-WEEK EXERCISE INTERVENTIONS ON BONE MINERAL DENSITY OF SEDENTARY OLDER WOMEN

Amy Ellis,[1] Kristi Crowe-White,[2] and Gary Hunter,[3], *1. University of Alabama, Tuscaloosa, Alabama, United States, 2. The University of Alabama, Tuscaloosa, Alabama,*

United States, 3. University of Alabama at Birmingham, Birmingham, Alabama, United States

Multicomponent exercise that includes both resistance and aerobic training is recommended to prevent loss of bone mineral density (BMD) in postmenopausal women. However, optimal training frequency has not been determined. Sixty-three non-osteoporotic sedentary women ages 60y and older were randomized to one of three exercise groups for sixteen weeks: 1) one resistance and one aerobic session per week, 2) two resistance and two aerobic sessions per week, or 3) three resistance and three aerobic sessions per week. Resistance exercise included supervised sessions on weight machines, and aerobic exercise was treadmill walking. BMD of the hip and lumbar spine (L1-L4) was assessed by dual energy X-ray absorptiometry (Prodigy, GE Medical Systems Lunar, Madison, WI, software version 6.10.029), and z scores were calculated from a reference population adjusted for age and sex. Among the total cohort with BMD measurements at week 16 (n=58; 83% white), z scores improved for the trochanter, Ward's triangle, total hip, L1 and L4. Within-group comparisons showed improvement at the trochanter, total hip, and L1 for group 2 only, while only group 1 demonstrated an increase at L4 ($p<0.05$ for all). However, no time-by-group interactions were observed. Sixteen weeks of combined resistance and aerobic training is effective for improving BMD of older adult women. Results suggest training frequency of two sessions per week may be optimal. Postmenopausal women should be encouraged to do aerobic exercise such as walking plus resistance training at least once weekly to prevent osteoporosis.

### EFFECTS OF TAI CHI ON BIOMARKERS AND THEIR IMPLICATION TO NEUROREHABILITATION – A SYSTEMIC REVIEW

Hao (Howe) Liu,[1] and Yasser Salem,[2], *1. University of North Texas Health Science Center, Fort Worth, Texas, United States, 2. University of North Texas Health Science Center/, Fort Worth, Texas, United States*

**Introduction:** As an effective holistic therapeutic exercise program, Tai Chi (TC) has been widely used for patients with a variety of neurological disorders. In last 1-2 decades, there has been an increase in the number of research studies that examined the TC effects on biomarkers including inflammatory cytokines, oxidative stressors, and neurotrophic factors. Thus, the purpose of this article is to review such effects and their possible implications to neurorehabilitation.

**Method:** In this systematic review, we searched TC-related articles from the last 15 years until July 2020 that had investigated changes of biomarkers after TC practice. The search identified 24 studies that were included in our analysis.

**Results:** It is found that TC practice is able to 1) reduce pro-inflammatory and increase anti-inflammatory cytokines (including Interleukins -1, 6, 10, 12, tumor necrosis factor, the nuclear factor kappa-light-chain-enhancer of activated B cells, and the C-reactive protein); 2) decrease oxidative stress factors (like plasma 8-isoprostane, malondialdehyde, and protein carbonylation); and 3) increase neurotrophic factors (brain-derived neurotrophic factor (BDNF), and N-Acetylaspartate).

**Conclusions:** TC may take effect on patients with neurological dysfunctions through anti-inflammation, anti-oxidative stress, and neural health promotion.

### FIT FOR THE NEXT FIFTY EXERCISE PROGRAM: 25 YEARS OF REFLECTIONS, RESULTS, AND PARTNERSHIPS

Mary Pagan, *SUNY Oswego, Baldwinsville, New York, United States*

Consistent exercise provides a multitude of physical, social, and emotional benefits. Common barriers to regular exercise for older adults include time, transportation, risk of injury, existing limitations, and negative experiences or attitudes about exercise. Fit for the Next Fifty is a comprehensive exercise and wellness program designed to address barriers and excuses. The program , based in CNY, has an impressive 25 year history of providing a unique mix of aerobic, strength training, yoga, and balance-based ballet. Participants (100-120) attend up to 5 classes per week at no charge during summer months and continue through winter months for a small fee. Developing and sustaining funding partnerships has been critical to the long-term success of Fit for the Next Fifty. Participants, ages 60-96, are active providers of feedback and suggestions, a key component to keeping the music, movements, and fellowship enjoyable and meaningful for over two decades. Program details, participant pictures and testimonials, research results, surveys across the years, and partnering/funding strategies provided. Of special interest is the social support dimension of the program. Participants have developed a sophisticated network to support each other outside of the exercise and wellness programs provided by Fit for the Next Fifty.

### PERCEIVED EXERTION IN PHYSICAL ACTIVITY MEASUREMENT ACROSS THE LIFECOURSE: RESULTS FROM SWAN

Kelly Ylitalo,[1] Carrie Karvonen-Gutierrez,[2] Barbara Sternfeld,[3] and Kelley Pettee Gabriel,[4], *1. Baylor University, Waco, Texas, United States, 2. University of Michigan, Ann Arbor, Michigan, United States, 3. Kaiser Permanente, Oakland, California, United States, 4. The University of Alabama at Birmingham, Birmingham, Alabama, United States*

Physical activity (PA) guidelines recommend 150 minutes of moderate-intensity or 75 minutes of vigorous-intensity PA, in addition to muscle strengthening activities, each week. Many questionnaires ascertain PA frequency, duration, and intensity to benchmark achievement of PA recommendations. However, most scoring algorithms utilize absolute intensity estimates when exertion may be influenced by age or other sociodemographic or health characteristics. This study compared PA estimates with and without adjustments for perceived exertion and determined if that difference was associated with individual characteristics. Women (n=2,711) from the longitudinal Study of Women's Health Across the Nation who completed ≥3 Kaiser Physical Activity Surveys (KPAS) across 8 biennial visits were included (baseline age: 46.4±2.7 years). KPAS responses were converted to metabolic equivalent of a task (METs) using the Compendium of Physical Activities to estimate absolute and perceived intensity-adjusted MET values. Latent class growth modeling identified subgroups of participants following similar patterns of change in the difference between absolute intensity-based and perceived intensity-adjusted estimates across time. Four major trajectory classes emerged with patterns reflecting: (1) lessening high-intensity exercise (2.2%); (2) increasingly high-intensity exercise (3.1%); (3) consistently moderate-intensity exercise (92.0%); and (4) consistently low-intensity exercise (2.8%). Consistently low-intensity exercisers, for whom absolute intensity-based estimates exceeded perceived intensity-adjusted estimates, were more likely to be Japanese or Chinese (p<0.001) and have lower BMI (p=0.05). However, for most participants, absolute intensity-based estimates approximated perceived intensity-adjusted estimates over time, suggesting that traditional PA scoring techniques may provide sufficient estimates for PA in longitudinal cohort studies of mid-life and older adult women.

### PHYSICAL ACTIVITY IMPACTS WALKING DISTANCES AND ENERGY CONSUMPTION OF PATIENTS WITH PERIPHERAL ARTERY DISEASE

Nathaniel Evans,[1] Sara Myers,[2] Mahdi Hassan,[1] Danae Dinkel,[2] Jason Johanning,[3] and Iraklis Pipinos,[3], *1. University of Nebraska Omaha, Omaha, Nebraska, United States, 2. University of Nebraska at Omaha, Omaha, Nebraska, United States, 3. University of Nebraska Medical Center, Omaha, Nebraska, United States*

Lower extremity peripheral artery disease (PAD) is attributed to buildup of atherosclerotic plaques preventing adequate blood flow, leading to pain during walking, and ultimately physical inactivity. Normal day-to-day levels of physical activity may impact the distance a subject can walk before claudication pain onset, as well as their energy consumption capabilities. This study compared walking performance (initial claudication distance (ICD) and absolute claudication distance (ACD)), and energy consumption (EC) between active and inactive subjects with PAD. The distinction between groups was made using previous research that declared the average PAD patient walks 3586 steps/day. Ten subjects were classified as active (□3586 average steps/day) and sixteen participants as inactive (<3586 steps/day) based on a 7-day accelerometer measurement. The Gardner progressive treadmill test was used to asses ICD, ACD, and EC. EC was measured using a metabolic cart and calculated from the second minute of walking and the last minute prior to stopping due to claudication pain. The average ICD and ACD for the active group were 130.6±106.7 meters and 306.0±184.7 meters, respectively and 143.8±119.0 meters and 248.0±156.0 meters, respectively for the inactive group. The average EC for the second minute and last minute were 9.6±1.9 mlkg-1min-1 and 11.5±2.4 mlkg-1min-1 respectively for active group and 7.0±3.1 mlkg-1min-1 and 8.1±3.8 mlkg-1min-1 respectively for inactive group. The data suggests that the active group had better walking performance and greater energy consumption indicating increased efficiency of oxygen transport and extraction capability in the leg muscles.

### PRESCRIBING PERSONALIZED EXERCISE PROGRAMS USING SMARTPHONE SENSORS FOR REMOTE FITNESS ASSESSMENT

Jeremy Jacobs,[1] Ziv Yekutiel,[2] Mical Arnon,[3] Esther Argov,[4] Keren Tchelet Karlinsky,[5] Eti Benmoha,[3] and yael Netz,[6], *1. Hebrew University of Jerusalem, Jerusalem, Yerushalayim, Israel, 2. Montfort, Montfort Brain Monitor, HaMerkaz, Israel, 3. Academic College at Wingate, Academic College at Wingate, HaMerkaz, Israel, 4. Academic College at Wingate, Wingate Academic College, HaMerkaz, Israel, 5. Montfort Brain Monitor, Montfort Brain Monitor, HaMerkaz, Israel, 6. Wingate Academic College, Wingate Academic College, HaMerkaz, Israel*

Guidelines for physical activity emphasize multiple fitness components among people aged >65. The age-related increase in variability of fitness components necessitates accurate individualized assessment prior to optimal prescription for personalized exercise program. Accordingly, we tested feasibility and effectiveness of a novel tool designed to remotely assess balance, flexibility, and strength using smartphone sensors (accelerometer/gyroscope), and subsequently remotely deliver personalized exercise programs via smartphone. This pilot study enrolled 52 healthy volunteers (34 females) aged 65+, with normal cognition and low fall-risk. Baseline preliminary data from smartphone fitness assessment were analyzed to generate 42 fitness digital-markers, used to generate personalized exercise programs (5 times/week for 6 weeks). Programs included graded exercises for upper/lower body, flexibility, strength, and balance (dynamic, static, vestibular). Fitness was remotely assessed at baseline and after six weeks. Average age was 74.7±6.4 years; adherence was 3.6±1.7 exercise sessions/week. Significant improvement for pre/post testing was observed for 10/12 digital-markers of strength/flexibility for upper/lower body (sit-to-stand repetitions/duration; arm-lift duration; torso-rotation; arm-extension/flexion). Balance improved significantly for 6/10 measures of tandem-stance, with consistent (non-significant) trends observed across 20 balance digital-markers of tandem-walk and one leg-stance. Balance showed greatest improvement among the 37 participants exercising ≥3/week. These preliminary results serve as proof of concept among people aged >65: high adherence and improved fitness confirm the potential benefits and niche for remote fitness assessment used to generate personalized exercise programs. Future research is required to confirm the benefits among specific patient groups, such as those with frailty, deconditioning, cognitive and functional impairment.

### RELIABILITY OF GRIP STRENGTH AS A PREDICTOR OF HAND LIMITATION AMONG U.S. OLDER ADULTS: HOW GOOD IS GRIP STRENGTH?

Rachel Logue, Susan Brown, Rebecca Hasson, and Matthew Davis, *University of Michigan, Ann Arbor, Michigan, United States*

Grip strength is commonly used to assess hand function in older adults and is associated with health outcomes including muscle strength, cognition, and mortality. However, the degree to which grip strength predicts an actual hand limitation is unknown. This study evaluated grip strength as a predictor of hand limitations associated with activities of daily living. Using the 2011-14 National Health and Nutrition Examination Survey (NHANES), we selected five self-reported hand-related functional limitations to classify older adults reporting one or more limitations versus those with no limitations. We identified 2,064 older adults (age≥65), 31% of whom reported a hand-related limitation. Odds ratios were used to assess the association between grip strength quartile and the likelihood of a hand limitation while controlling for sex, race/ethnicity, education level, income, and pain. Receiver operator curves were used to evaluate the degree to which grip strength discriminates between those with limitations versus those without. Older adults with very low grip strength (lowest quartile) were more likely to have at least one limitation (OR:6.1, 95% CI:3.2,11.8) than those with high grip strength (highest quartile). However, receiver operator curves suggested grip strength only modestly discriminated hand limitations (area under curve:0.71). While self-reported hand limitations were associated with lower grip strength, it was a relatively poor predictor of hand impairments among older adults. This study suggests grip strength may not predict hand function as well as previously thought. Better assessments are needed to adequately evaluate upper extremity impairments to help older adults maintain functional independence.

### RESISTANCE TRAINING IMPROVES MOBILITY DISABILITY IN COMMUNITY-DWELLING OLDER ADULTS: A META-ANALYSIS

Christina Prevett,[1] Kevin Moncion,[2] Stuart Phillips,[2] Julie Richardson,[2] and Ada Tang,[2], *1. McMaster University, Perth Road, Ontario, Canada, 2. McMaster University, McMaster University, Ontario, Canada*

Mobility disability is the impairment in function that affects the performance of daily tasks due to declines in physical function. Exercise interventions, particular resistance training, may have a positive impact on mobility disability, but the evidence for the effects of resistance training in older adults with mobility disability has not been previously systematically reviewed. This study was a systematic review of evidence related to resistance training on physical function for adults over 65 years of age with mobility disability. Four databases (PEDro, MedLine, Ovid, Web of Science) were searched from inception to February 2, 2021 for randomized controlled trials. Twenty-four articles from 22 studies (3,656 participants) were included in the review. Mean participant age ranged from 63-87 years and exercise interventions ranged from 10 weeks to 12 months in duration. Greater changes in 6-minute Walk Test (6MWT) distance (n=638, p<0.0001; mean difference (MD) 16.1 metres; 95%CI 12.3-19.9), lower extremity strength (n=785, p<0.0001; standard MD 2.01; 95%CI 1.27-2.75) and usual gait speed (n=2,106, p<0.001; MD 0.05 metres/second, 95%CI 0.03-0.07) were seen with resistance training as compared to control. These results were maintained if resistance training was a sole intervention or a component of a multi-component program. Sensitivity analysis based on risk of bias concerns did not change results. This review demonstrates that resistance training improves walking capacity, strength and walking speed in community-dwelling older adults and may facilitate aging in place. Since improvements in strength and gait speed contribute to independence, our results indicate highly beneficial outcomes for older persons.

### SEX DIFFERENCES IN POWER DECREMENT IDENTIFIED ACROSS THE LIFESPAN

Anthony Campitelli,[1] Sally Paulson,[2] Jennifer Vincenzo,[3] Jordan Glenn,[4] Megan Jones,[1] Melissa Powers,[5] Joshua Gills,[1] and Michelle Gray,[1], *1. University of Arkansas at Fayetteville, Fayetteville, Arkansas, United States, 2. University of Arkansas at Fayetteville, Cincinnati, Ohio, United States, 3. University of Arkansas for Medical Science, Fayetteville, Arkansas, United States, 4. Neurotrack Technologies, Redwood City, California, United States, 5. University of Central Oklahoma, Edmond, Oklahoma, United States*

Muscular power has been shown to be a significant predictor of physical function in older adults, but assessments of power have traditionally been performed in movements not specific to activities of daily living (ADLs). Recent research examined power in the context of ADL-specific movements, but it is unclear how ADL-specific lower-body power differs over lifespan in males and females. This investigation sought to describe ADL-specific power decline across the lifespan and analyze differences between the sexes. Adults (n = 557) aged 18-89 volunteered and were divided into age cohorts (18-30, 50-59, 60-69, 70-79, and 80-89 years). Participants performed a sit-to-stand (STS) task with as much velocity as possible while connected to a linear position transducer (LPT). The LPT calculated average and peak power. The average result of 5 individual STS trials was analyzed for each of the power variables. The first significant decrement in average STS power (p < .01) was observed at an earlier age cohort in males than females (60-69 in males vs. 70-79 in females). The per decade magnitude of power decrement after age 60 was larger in males than females in both absolute magnitude and percent decrease (11.74 vs. 10.09% decrease per decade). As power and physical function are correlated, this may have implications for the rate and age of functional decline in males. Additionally, understanding the differences in ADL-specific power decline between males and females gives clinicians and health professionals valuable information for developing preventative fitness paradigms specific to members of a given sex.

### STIMULATING A MATURE BODY'S DEFENSE SYSTEM BY MAINTAINING PHYSICAL ACTIVITY: A LITERATURE REVIEW

Mary Murphy, *Fairfield University, Fairfield, Connecticut, United States*

This review provides summary of research findings on the effects of exercise for changes in the immune system most associated with aging. Immunosenescence is identified as an immune dysregulation with aging that leaves an older adult susceptible to infections and a host of immune-related disorders. Extrinsic modulators of immunosenescence include pathogens, mental stress, nutrition, and exercise. Moderate short acute exercise over time enhances the immune system. Heavy exertion or prolonged exercise bouts may contribute to immunosenescence. In one study, a J-curve result was identified for upper respiratory tract infection. A moderate exercise workload was associated with a 40-50% decrease in upper respiratory tract infections while a 2-6-fold increase was identified among individuals consistently completing heavy exertion. Transient increases of the inflammatory markers of C-reactive protein and Interleukin-6 are noted after excessive exercise. The older adult should consider small increments of change in an exercise load to limit exercise-induced inflammation. These same inflammatory markers are chronically expressed in obese individuals in a resting state. Strategies to manage weight within recommended range to avoid obesity will limit activation of proinflammatory immune cells. In conjunction with physical activity, the lifestyle behaviors that most support immune system health include adequate sleep, nutrition, hydration, and avoidance of excessive alcohol intake. When planning a safe moderate exercise workload, additionally consider hygienic practices to lower transmission of pathogens. Transmission decreases with hand washing, limited hand-to-face contact, distance from large crowds or those with cough, avoiding spaces with poor ventilation and update vaccinations.

### TEXTING OLDER SISTERS TO STEP (TOSS) USING FITBITS TO PROMOTE PHYSICAL ACTIVITY: A FEASIBILITY STUDY

Pamela Bowen,[1] William Opoku-Agyeman,[2] Veronica Mixon,[3] Olivia Affuso,[1] and Olivio Clay,[4], *1. The University of Alabama at Birmingham, Birmingham, Alabama, United States, 2. Healthcare Management, University of North Carolina at Wilmington, North Carolina, United States, 3. UAB, Birmingham, Alabama, United States, 4. University of Alabama at Birmingham, Birmingham, Alabama, United States*

Black women are disproportionately diagnosed with obesity (BMI ≥ 30 kg/m2). Obesity is a preventable but complex, public health problem that is multifaceted, chronic, and approximately 58% of Black women 60 years and older are classified as obese, compared to 38% of their White counterparts. This 12 week, pre/post, 2-group study aimed to determine if a peer-informed physical activity (PA) intervention with peer support would be feasible among community- dwelling, obese, older Black women to promote regular PA. Forty-eight potential participants were screened, 24 categorized as obese were enrolled and completed the study. The mean age was 64 (SD 3.0) years. Steps were measured by a Fitbit-Inspire with data successfully collected on 98% of days with the treatment group averaging a daily increase of 700-steps more than the control. Evaluation of intervention's acceptability revealed that 100% enjoyed the study and using the Fitbit device. Text message readability was 100% and 95% said the study was motivational. Additionally, 8.3% said daily prompts were too frequent, 12% indicated that future studies should include additional social support, and 88% did not comment on the Fitbit community option for support, suggesting that this feature was not practical. Findings demonstrated that this intervention meets the criteria of being scalable, low cost, feasible, and acceptable for the older Black women. Using self-monitoring techniques in combination with at least one other behavioral strategy, such as our TOSS messages (cues for motivation) as the delivery channel for health promotion messages are a promising approach to increase PA behaviors.

### THE ASSOCIATION BETWEEN VISION ACUITY, SLEEP DURATION, AND PHYSICAL ACTIVITY AMONG US ADULTS AGED 50 YEARS AND OLDER

Shu Xu, *University of Masschusetts Boston, Dorchester, Massachusetts, United States*

Studies suggested that people with low vision are more likely to have worse sleep quality and less frequent participation in physical activities compare with people with better vision. Studies also showed that physical activities is a very important factor for one's sleep. However, there is relatively little research on the association between vision acuity, sleep, and physical activity. This study examines the relationships between vision acuity and sleep duration among middle-aged and older adults in the US, and the role of leisure-time physical activity in this relationship. Using nationally representative data from the National Health and Nutrition Examination Survey 2007-2008, a cross-sectional analysis on adults age 50 years and older was conducted (n=2.247). Visual acuity was assessed by participant's vision of better-seeing eye (i.e., none, mild, moderate, and server visual impairment), and we measured sleep duration (i.e., short, average, and long duration) and leisure-time Physical Activity (i.e., inactive/insufficiently active and sufficiently active). Descriptive analysis showed that 31.06% of older adults experienced moderate or severe visual impairment, and 46.81% respondents experienced abnormal sleep duration. Multinomial logistic regression analyses showed that compared to people without visual impairment, people with moderate or severe visual impairment were more likely to have longer sleep duration than normal sleep duration (OR, 1.62, $p<0.05$). Leisure-time physical activity was not found to significantly mediate the relationship between visual acuity and sleep duration. Other variables were controlled in the models. Findings suggest that US adults age 50+ with low vision are at greater risk of experiencing abnormal sleep duration.

### Session 9425 (Poster)

### POLICY, FINANCING, AND SOCIAL SERVICE DELIVERY

### A POLICY MAPPING ANALYSIS OF THE U.S. CONGRESSIONAL APPROACH TO MEDICAL AID-IN-DYING

Nancy Kusmaul,[1] Ji Hyang Cheon,[2] and Allison Gibson,[3], *1. University of Maryland, Baltimore County, Baltimore, Maryland, United States, 2. University of Maryland Baltimore, University of Maryland Baltimore, Maryland, United States, 3. University of Kentucky, University of Kentucky, Kentucky, United States*

Oregon was the first state to legalize medical aid-in-dying (MAID), in 1994. Since then eight states and Washington, DC have legalized MAID through legislation. Despite literature exploring the legal and ethical aspects of MAID, very little research examines MAID policy at the federal level. This study aimed to 1) examine the objectives of MAID legislation introduced to the US Congress, and 2) investigate whether these bills increase or decrease access to MAID. This study used the congress.gov website to search for bills related to MAID introduced by the US Congress between 1994 and 2020. From the 98 bills identified, we excluded bills that were not directly related to MAID or were introduced in subsequent congresses. In total, 23 bills were retained and analyzed. The greatest number of bills aimed to restrict funds for MAID, followed by bills that sought to regulate the drugs used for MAID. Other bills prohibited the development of policies supporting MAID, regulated penalties for practitioners related to the drugs used for MAID, and restricted legal assistance for accessing MAID. These bills intended to block or limit patient access to MAID by restricting drugs, funds, health care services, legal assistance, policy, and research. These findings suggest that the federal approach is incongruous with the growing numbers of states that have legalized MAID. Federal policymakers must develop policies to 1) prevent discrimination against vulnerable groups, 2) support funds to study MAID, and 3) build a system to allows eligible individuals to access MAID equally.

### ASSISTED LIVING ADMINISTRATORS' VIEWS OF PALLIATIVE CARE

Elise Abken,[1] Molly Perkins,[2] and Alexis Bender,[3], *1. Emory University School of Medicine, Atlanta, Georgia, United States, 2. Emory University of Medicine, Atlanta, Georgia, United States, 3. Emory University, Atlanta, Georgia, United States*

As many older adults with progressive chronic conditions choose to age-in-place in assisted living (AL) communities, external healthcare workers (e.g., those who provide palliative care) increasingly support AL staff in caring for residents with complex health needs. Palliative care is a branch of healthcare dedicated to preserving quality of life by attending to the physical, mental, and spiritual needs of individuals with chronic, life-threatening diseases and is well suited to manage AL residents' progressive medical conditions. However, AL residents and their care partners often face barriers to accessing palliative care. Using data from a larger 5-year NIA-funded study, we examined AL administrator knowledge and use of palliative care in seven AL communities around the Atlanta metropolitan area that were racially, ethnically, and socioeconomically diverse. Findings from thematic analysis of semi-structured interviews with 16 administrators indicated that 15 of 16 administrators were familiar with palliative care. A minority of administrators clearly distinguished palliative care from hospice services and conceptualized it as a "bridge" to hospice services. Administrators emphasized how palliative care assists communities in caring for health concerns in-house rather than having to send residents to the hospital. Despite their positive view of palliative care, administrators described infrequent use of palliative services in their communities. Findings show that although none of the AL communities integrate palliative care with their service offerings, AL administrators see value in palliative care for their residents. We provide recommendations for improving palliative care access and quality of life for AL residents at end of life.

### DO STATE AGENCY ON AGING STRATEGIC PLANS INCLUDE TERMS RELATED TO MALNUTRITION?

Mary Beth Arensberg,[1] Jaime Gahche,[2] and Johanna Dwyer,[3], *1. Abbott, Columbus, Ohio, United States, 2. National Institutes of Health, Bethesda, Maryland, United States, 3. National Institutes of Health, National Institutes of Health, Maryland, United States*

Demand for federal nutrition assistance programs is increasing as the older population grows and further accelerated with the COVID-19 pandemic. Older adult nutrition programs are based on federal nutrition guidelines that have traditionally focused on healthy populations, yet many older adults have multiple chronic conditions/advanced age. Some guidelines are changing; the 2020 Dietary Guidelines for Americans recognize older adults' risk for malnutrition and also need for adequate protein to prevent lean muscle loss with age. The 2020 Older Americans Act (OAA) reauthorization included reduction of malnutrition in OAA's official purpose and added program participant screening for malnutrition. The OAA requires State Agencies on Aging submit multiyear strategic plans to receive program funding, but it is unknown how the plans address risks for malnutrition, including overweight, underweight, and muscle loss (sarcopenia/frailty). We searched 51 State Agency on Aging strategic plans posted at advancingstates.org to determine their frequency of mentioning nutrition, malnutrition/underweight/undernutrition, obesity/overweight, frail/frailty, sarcopenia, and dietary supplements/oral nutrition supplements (DS/ONS)/meal replacements. Every state plan included nutrition but less than a third included malnutrition. There was wide variability in how nutrition and malnutrition were incorporated into state goals and strategies. Very few plans included obesity, frailty, and DS/ONS terms; none included sarcopenia. Although there has been some movement, there is need for many State Agencies on Aging plans to address all aspects of malnutrition including overweight, underweight/other factors related to muscle loss (sarcopenia/frailty) that adversely impact healthy aging. Wide disparities in plan structure/use of terms create opportunities for more common approaches/definitions.

### DOES CLOSING THE DONUT HOLE REDUCE FINANCIAL BURDENS AMONG MEDICARE PART D BENEFICIARIES?

Yalu Zhang, Lan Liu, Jingjing Sun, Xinhui Zhang, Jiling Sun, Xinming Song, and Gong Chen, *Peking University, Beijing, Beijing, China (People's Republic)*

The Medicare Part D donut hole has been gradually closed since 2010. But it is still unclear how it has impacted the beneficiaries' relative financial burdens, especially in the later stage of the closing plan. The measurement of catastrophic health expenditure induced by prescription drugs (CHE-Rx) reflects the relative financial burdens to beneficiaries' household income, which bears more information than the measure of dollar-value expenses or the absolute poverty line used in prior studies. Using the Medical Expenditure Panel Survey 2008-2017 longitudinal national representative data and the method of difference-in-differences, this study found that the donut hole closing policy was associated with more usage of prescription drugs (b=2.84, p=0.023) and a higher likelihood of experiencing CHE-Rx (b=2.4%, p=0.011) among those who fell in the donut holes. Besides, the results show that the donut hole closing policy did not generate any immediate effects on prescription drug usage, CHE, and CHE-Rx. For the first time, this paper examined both the aggregated and marginal impact of the policy implementation, which had closed by an additional 35% between 2013 and 2017, on the relative financial burden among the beneficiaries.

### HOME- AND COMMUNITY-BASED SERVICES USE PATTERNS AND FUNCTIONAL IMPROVEMENT AMONG OLDER CARE RECIPIENTS IN TAIWAN

Hsiao-Wei Yu,[1] Shih-Cyuan Wu,[2] and Ya-Mei Chen,[2], *1. Chang Gung University of Science and Technology, Taoyuan City, Taoyuan, Taiwan (Republic of China),*

2. *National Taiwan University, Taipei, Taipei, Taiwan (Republic of China)*

The new version of Taiwan's 10-Year Long-Term Care Plan launched in 2016 aims to reinforce the integration of home- and community-based services (HCBS). The underlying HCBS use patterns and effectiveness of functional improvement among care recipients merit investigation. The purpose of the study was to examine the association of HCBS and changes in ADLs among care recipients with different levels of disabilities in Taiwan. We accessed the sub data of Taiwan's Long-Term Care Services Management Online System. Samples were aged 65 and over and had completed records of baseline and reassessment information during 2018 (N = 4787). Latent class analysis and multivariate linear regression were applied to examine the relationship of HCBS and functional changes. Four HCBS subpatterns were found: home-based personal care services (home-based PS) (59.16%), home-based reablement services (home-based RS) (23.90%), home-based multiple services (home-based MS) (11.93 %), and community-based services (5.01%). In the cases with mild disabilities at baseline, recipients receiving home-based RS had higher probabilities of improving in ADLs among four HCBS subgroups (for example: β = 2.65, SE = 1.19 in comparison to home-based PS). Care recipients with moderate-to-severe disability at baseline, ADLs improvement was only found in home-based PS (β = 1.63, SE = 0.82 in comparison of home-based MS). In the cases with profound disabilities, home-based PS showed positive effects on ADLs improvement (β = 2.45, SE = 0.80 in ADLs, compared to home-based RS). The study suggested that HCBS subpatterns had different impacts on older adults with different disability levels.

### LEADERSHIP AMONG DIRECTOR OF SOCIAL WORK AT SKILLED NURSING FACILITIES (SNFS)

John Paul Abenojar, *Walden University, Arlington, Virginia, United States*

Skilled Nursing Facilities (SNFs) provide ongoing care to the seniors and chronically ill. To maximize the quality of this care, SNF staff must be trained to respond to patient care crises and communicate across departments. Although researchers have studied the leadership styles, strategies, and interactions of facility administrators and nursing directors, little was known about the leadership styles and strategies imployed by the director of social worker (DSW). The aim of this phenomenological study was to explore how DSW influenced leadership policies, prepared subordinated for crisis intervention and management, perceived that social workers influenced patient care decisions, and believed that communication amongst SNF staff about patient care could be improved. The conceptual framework was based on the multilevel leadership model construct, the situational leadership model construct, and the complex adaptive leadership model construct. Participants included a purposive sample of 10 DSW working in a large, corporate SNFs in Virginia. Data were collected via in person, semi structured interviews consisting of open-ended questions. Data were analyzed via Hycner's phenomenological approach. Findings from this investigation helped clarify roles and responsibilities of DSW, thereby improving the leadership they provide to subordinate social workers. Findings may be used to improved communication across professionals within SNFs and in their role in patient decisions.

### LEADERSHIP AMONG DIRECTORS OF SOCIAL WORK IN LONG TERM CARE FACILITIES

John Paul Abenojar, *Walden University, Arlington, Virginia, United States*

Long term care facilities (LTC) provide ongoing care for seniors and chronically ill. To maximize the quality of the care, LTC staff must be properly trained to respond to patient care crises and communicate across departments. Although researchers have studied the leadership styles, strategies and interactions of facility administrators and nursing directors there is a substantial gap in the literature on the leadership styles and strategies employed by Directors of Social Work (DSW). The aim of this phenomenological study was to address this gap in research by exploring how DSW influenced leadership policies, prepared subordinates for crisis intervention and management, perceived that social workers influence decision making in patient care, and believed that communication amongst LTC staff about patient care could be improved.

### PREPARING EFFECTIVE INTERPROFESSIONAL TEAMS TO MEET THE NEEDS OF OLDER ADULTS IN INTEGRATED HEALTHCARE SETTINGS

Karen Bullock,[1] Kim Stansbury,[1] David Fitzpatrick,[2] and Gloria Anderson,[1], *1. North Carolina State University, Raleigh, North Carolina, United States, 2. NCSU, RALEIGH, North Carolina, United States*

**Background:** Gerontology education has evolved from focusing primarily on the individual practitioner outcomes to promoting integrated, inter-professional team approaches to integrated care. Practicum training and service learning are effective pedagogy for paraprofessionals in integrated care settings to support clinicians and advance their effectiveness in meeting the needs of older adults. Interprofessional education (IPE) aims to enhance the capacity of practitioners to work collaboratively as integrated team members. Yet, little is known about the implementation of IPE in colleges and universities that are not affiliated with a medical center or medical school. This presentation will describe the implementation of IPE in a School of Social Work without a medical school system. We will explore students' experiences with the implementation, facilitation, and evaluation of this workforce development model, and will discuss both challenges and successes.

**Methods:** Narrative data collected over the past two-year period with social work student participants in IPE reflect their perspectives on the selection process for the specialized training, placement in an interprofessional integrated care setting, and the learning experience. Participants represented cohorts of more than 200 trainees.

**Results:** Challenges of effective interprofessional health care teams include the approach taken to integrating the teams, level of knowledge and skills required to be an effective team member, and the need for supervision within integrated care experiences. Successes include increased professional self-efficacy. Participants reported post-completion of IPE, (1) desirable outcomes achieved for their patients, such as care satisfaction and role satisfaction for themselves as practitioners.

### THE MEDICARE CURRENT BENEFICIARY SURVEY (MCBS): PROVIDING UNIQUE ACCESS TO DATA ON THE MEDICARE POPULATION

Hildie Cohen,[1] Sarah Hoyt,[2] Sara Navin,[3] Jennifer Titus,[3] and Mia Ibrahim,[4], *1. NORC at the University of Chicago, Phoenix, Arizona, United States, 2. NORC at the University of Chicago, Bethesda, Maryland, United States, 3. NORC at the University of Chicago, Chicago, Illinois, United States, 4. NORC at the University of Chicago., Bethesda, Maryland, United States*

The Medicare Current Beneficiary Survey (MCBS) is a continuous, multipurpose survey of a nationally representative sample of the Medicare population, conducted by the Centers for Medicare & Medicaid Services (CMS). It collects data on demographics, health insurance, health status, health care expenditures, satisfaction with care, and access to care for Medicare beneficiaries. The MCBS provides a unique source of information regarding beneficiaries aged 65 and over and beneficiaries aged 64 and below with disabilities residing in the United States that cannot be obtained solely through CMS administrative sources. For researchers interested in issues of health care utilization and cost, CMS releases two Limited Data Set (LDS) files for each data year and a Public Use File (PUF) freely available for download and use. Also, special topic based PUFs have been released on the impact of COVID-19 on Medicare beneficiaries. This presentation will demonstrate the importance of the MCBS for research on the Medicare population, discuss how researchers can access the data, where researchers can find published MCBS estimates, what content areas have recently been added, such as food insecurity, limited English proficiency, and COVID-19 vaccination uptake and what new content is on the horizon. The presentation will also discuss the operational challenges posed by the COVID-19 pandemic, and the content enhancement opportunities created by the public health emergency. It will conclude with a review of the suite of materials and documentation available for data users to enhance their research and utilize more timely data.

### Session 9430 (Poster)

### PSYCHOLOGICAL WELL-BEING AND SUCCESSFUL AGING

### AGE DIFFERENCES IN EXPERIENCING MEANING IN LIFE: A MULTIDIMENSIONAL APPROACH

Nicole Long Ki Fung,[1] and Helene Fung,[2], *1. The Chinese University of Hong Kong, Hong Kong, Hong Kong, 2. The Chinese University of Hong Kong, Shatin, N.T., Hong Kong, Not Applicable, Hong Kong*

Coherence, purpose and significance were defined as the three facets of the presence of meaning in life (Martela & Steger, 2016). This study investigated the age differences in the three facets of meaning in life between younger and older adults. We recruited 241 younger adults (Mage=18.72, SD=1.50, 36.5% male) and 114 older adults (Mage=64.93, SD= 5.94, 52.6% male) from Hong Kong to fill out a one-hour online survey. We adapted the Multidimensional Existential Meaning Scale, which measured sense of coherence, purpose, significance. In specific, the adapted version measured significance in two sub facets: the naturalistic significance (important to other people) and cosmic significance (impact lasts beyond lifetime and space). The results showed that older adults had higher sense of coherence (t=3.47, p<.001), higher sense of cosmic significance (t=6.29, p<.001) but lower sense of purpose than younger adults (t=-2.02, p<.05). There were no significant differences in naturalistic significance between the two age groups (t=1.24, p=.22). Within both age groups, participants had the highest score in purpose, followed by coherence and naturalistic significance. They had the lowest score in cosmic significance. This study illustrated that younger and older adults have different absolute levels of meaning facets but are similar in the relative levels of meaning facets. Further studies can investigate how changes in absolute levels of meaning facet and preservation of the relative levels may affect well-being across age.

### CORRELATES OF PSYCHOLOGICAL WELL-BEING AMONG KOREAN VIETNAM WAR VETERANS

Hyunyup Lee,[1] Sungrok Kang,[1] Soyoung Choun,[2] and Carolyn Aldwin,[2], *1. Korea Military Academy, Seoul, Seoul-t'ukpyolsi, Republic of Korea, 2. Oregon State University, Corvallis, Oregon, United States*

Prior research on Veterans' mental health has largely focused on identifying risk and protective factors for negative psychological symptoms such as PTSD. However, mental health indicates not merely absence of psychopathology, but also the existence of positive psychological well-being (Keyes, 2005). Thus, the current study aimed to examine the correlates of psychological well-being, which is less studied, in an Asian sample, Korean veterans. Data for this 2017 study were from Korean Vietnam War Veterans Study. Participants were 348 male veterans, and their mean age was about 72 years old (SD = 2.7, range = 65-84). Using Keyes' (2002) classification criteria, psychological well-being was divided into three types: flourishing (9.5%), moderately health (59.95%), and languishing (25.3%). Own-way analyses of variance showed that the groups did not differ in demographic variables (age, marital status, education, and income). Further, there were no differences in combat exposure, negative appraisals of military service, smoking, and alcohol consumption. However, significant group differences were found for resources; Scheffé's post-hoc analyses indicated that optimism, positive appraisals of military service, four types of social support (family, significant others, friend, and military peer), and self-rated health were significantly different among the groups, and highest in the flourishing group. The moderately health group showed higher levels of positive appraisals of military service and four types of social support than the languishing group. Thus, the majority (about 60%) of Korean Vietnam veterans were moderately psychologically healthy in this sample, but those with positive psychosocial resources were more likely to be healthiest.

### DAILY ACTIVITY ENGAGEMENT PREDICTS MORE POSITIVE AFFECT ESPECIALLY ON DAYS WHEN A PERSON FEELS YOUNG

Erica O'Brien,[1] and Shevaun Neupert,[2], *1. Pennsylvania State University, University Park, Pennsylvania, United States, 2. North Carolina State University, Raleigh, North Carolina, United States*

Engagement in a wide array of mental, social, and physical leisure activities confers several health benefits. Indeed, theories of successful aging argue that an active lifestyle serves as an important criterion for maintaining high levels of psychological, functional, and physical well-being in old age. Findings from parallel studies also show that people who hold positive (self-)views of aging exhibit higher and maintained levels of well-being over time. Yet, whether views of aging enhances the link between activity engagement and well-being - and whether they do so on a daily basis – remains unknown. This study therefore sought to extend prior literature by examining the relationship between activity engagement, subjective age, and affective ratings within-person over several days. Old adults (N = 115; Age: Range = 60 – 90, M = 64.65, SD = 4.86) in the Mindfulness and Anticipatory Coping Every Day (MACED) study completed an 8-day daily diary. Participants reported on their positive and negative affect, the age they subjectively felt compared to their actual age, and the number and types of leisure activities in which they engaged. Results from multilevel analyses indicate that people felt more positive on days when they also engaged in more activities (total across mental, social, physical types) than usual. Moreover, the effect of activity engagement was most pronounced on days when people felt younger than usual. No effects were found for negative affect. Preliminary findings suggest that people benefit psychologically from daily leisure activities and a positive self-view of aging.

### OLDER PORTUGUESE AND MEXICAN ADULTS AND SEXUAL WELL-BEING? A CROSS-CULTURAL QUALITATIVE STUDY

Sofia von Humboldt,[1] Neyda Ma. Mendoza-Ruvalcaba,[2] José Alberto Ribeiro-Gonçalves,[1] Gail Low,[3] and Isabel Leal,[1], *1. Instituto Superior de Psicologia Aplicada (ISPA), Lisboa, Lisboa, Portugal, 2. University of Guadalajara CUTONALA, Guadalajara, Jalisco, Mexico, 3. University of Alberta, Edmonton, Alberta, Canada*

**Objectives:** Sexual well-being (SWB) refers to the subjective emotional and cognitive evaluation of the quality of the individual's sexuality, it plays a relevant role in quality of life and health promotion on old age and has cross-cultural implications. The aim of this study is to analyse comparatively the perspectives of older adults on their SWB in Portugal and Mexico.

**Methods:** Data were collected from 86 Portuguese and 80 Mexican community-dwelling participants aged 65 years and older, using a semi-structured interview protocol. Older adults were inquired about their perceptions on what contributes to their sexual well-being. Socio-demographic data were also enquired. Content analysis was used to identify key themes.

**Results:** Outcomes indicated eight themes: eroticism, supportive relationship, positive self-concept, health and self-care, romance, active life, tenderness and care, and no pain and no pregnancy restrictions, for both samples. Eroticism was the most frequent theme reported by Portuguese participants (31.4%) and health and self-care were the most frequent theme reported by Mexican participants (26.5%).

**Conclusions:** The empirical results of this study indicated that SWB is strongly influenced by socio-cultural and psychosocial values. This cross-cultural comparison between Portugal and Mexico contributes to understand this concept in old age with different perspectives and place a scenario for future culture-adapted interventions and comprehensive policies. Keywords: Mexican, older adults; Portuguese; qualitative study; sexual well-being

### PROACTIVE COPING AND MINDFULNESS ARE ASSOCIATED WITH LESS STRESS IN OLDER ADULTS

Lyndsey Graham, and Shevaun Neupert, *North Carolina State University, Raleigh, North Carolina, United States*

We examined the consequences of both chronic and life-event stressors for older adults, as well as antecedent strategies, such as proactive coping and mindfulness, that may mitigate stress. Given the potential negative outcomes associated with stress in older adulthood, exploring strategies to reduce or mitigate the negative impact of stress may be useful in promoting well-being in adulthood. Proactive coping involves an accumulation of resources that leads to reduced or avoided stressors in the future (Aspinwall & Taylor, 1997). Mindfulness calls an individual's attention to the present moment, or may be characterized as an open, accepting attitude (Brown & Ryan, 2003). Using data from the Mindfulness and Anticipatory Coping Everyday study (English et al., 2019; Neupert & Bellingtier, 2017), 296 older adults in the United States, aged 60-90 years (M = 64.67, SD = 4.36), participated in relevant online survey measures. Results from multiple regression analyses suggested that people high in both chronic stress and life event stress had worse health, and that people high in proactive coping and mindfulness reported less stress. Study results underscore the impact of stress on health outcomes, and provide support for the use of antecedent strategies to address negative impacts of stress.

### RECALLING MEMORIES OF LIFE'S CHALLENGES WITH PURPOSE AND REDEMPTION: RESOURCES TO FOSTER RESILIENCE?

Shubam Sharma,[1] Joshua Perlin,[1] and Susan Bluck,[2], *1. University of Florida, Gainesville, Florida, United States, 2. University of Florida, University of Florida, Florida, United States*

Unique life challenges occur across life phases, including later life. Life story research suggests that the way challenges are narrated has consequences for multiple domains of well-being. Two factors for positively reframing challenges are one's sense of purpose in life (Windsor et al., 2015) and redemption (McAdams et al., 2001). This study used moderated-mediation analyses to investigate whether: 1) challenge relates to psychosocial and cognitive well-being, differentially by age, and 2) narrating with greater purpose and redemption ameliorates effects of challenges on well-being, by age. Participants (N = 99 young, 88 older adults) rated self-disruption of challenging events from their lives (IV1) and reported number of recent life challenges experienced (IV2). Eudaimonic well-being (DV1) and cognitive well-being (DV2) were assessed. Purpose (M1) and redemption (M2) were reliably content-analyzed from participants' narratives of autobiographical challenges (e.g., illness, loss of other). For Aim 1, young and older adults who experienced more challenges showed lower eudaimonic well-being but higher cognitive well-being. Perceived self-disruption was unrelated to well-being. For Aim 2, results showed that how individuals narrate (i.e., with purpose, with redemption) the challenges that have occurred in their lives mediates effects of challenge. Specifically, exhibiting a sense of purpose mediated the relation between perceived self-disruption and cognitive well-being. Redemption buffered negative effects of both self-disruption and number of challenges on eudaimonic well-being. Mediation results held regardless of age. Findings suggest purpose and redemption are two narrative mechanisms that act as psychological resources to support well-being in the face of life's inevitable challenges.

### RESILIENCE AMONG OLDER ADULTS WITH TYPE 2 DIABETES FROM THE LOOK AHEAD TRIAL

KayLoni Olson,[1] Marjorie Howard,[2] Jeanne Mccaffery,[3] Gareth Dutton,[4] Mark Espeland,[5] Felicia Simpson,[6] Karen Johnson,[7] and Rena Wing,[8], *1. Alpert Warren Medical School of Brown University, Providence, Rhode Island, United States, 2. Wake Forest University Health Sciences, Winston Salem, North Carolina, United States, 3. University of Connecticut, Storrs, Connecticut, United States, 4. University of Alabama at Birmingham (UAB), Birmingham, Alabama, United States, 5. Wake Forest School of Medicine, Winston-Salem, North Carolina, United States, 6. Department of Mathematics, Winston-Salem State University, Winston-Salem, North Carolina, United States, 7. University of Tennessee Health Science Center, Memphis, Tennessee, United States, 8. The Miriam Hospital, Providence, Rhode Island, United States*

There is growing interest in identifying factors protecting against aging-related decline. This cross-sectional study evaluated associations of self-reported resilience (ability to bounce back) with factors linked to aging-related decline among older adults with Type 2 diabetes (T2DM). Participants were 3,199 adults (72.2±6.2 years, 61% female, 61% white, BMI 34.2±8.2 kg/m2) enrolled in Look AHEAD (a multi-site RCT comparing weight loss to diabetes education among individuals with T2DM), who were followed observationally after the 10-year intervention was discontinued. The following items were assessed approximately 14.4yrs post-randomization: Brief Resilience Scale; overnight hospitalizations in past year; physical functioning measured objectively (gait speed, grip strength) and via self-report (Pepper Assessment Tool for Disability; Physical quality of life (QOL; SF-36)); a composite measure of phenotypic frailty based on having ≥3 of unintentional weight loss, low energy, slow gait, reduced grip strength, physical activity. Depressive symptoms (PHQ-9) and mental QOL (SF-36) were also measured. Logistic/linear regression was used to evaluate the association of these variables with resilience adjusted for age, race, and gender. Greater resilience was associated with lower BMI (p=.01), fewer hospitalizations (p=.02), better physical functioning (i.e., lower self-reported disability, better self-reported physical QOL, faster gait speed, greater grip strength and lower likelihood of meeting criteria for frailty; all p<.001), fewer depressive symptoms and greater mental QOL. Resilience is associated with better performance on indicators of overall functioning and risk for decline among older adults. Findings correspond with efforts to shift narrative on aging beyond 'loss and decline' to highlight opportunities to facilitate healthy aging.

### THE EFFECT OF BEHAVIORAL ACTIVATION ON OLDER ADULTS' ENGAGEMENT AND WELLBEING: A RANDOMIZED CONTROL TRIAL PROTOCOL

Julia Scott,[1] Alexis Howard,[2] Mary Luszcz,[2] Trevor Mazzucchelli,[3] Ruth Walker,[2] and Tim Windsor,[2], *1. Flinders University, Brompton, South Australia, Australia, 2. Flinders University, Adelaide, South Australia, Australia, 3. Curtin University, Perth, Western Australia, Australia*

Meaningful activity engagement in later life is widely recognized as crucial for ageing well, but age-related changes and transitions can impede such participation. A behavioral activation framework can provide a person-centred, value-consistent therapeutic approach to increasing activity engagement that is both easy to administer, cost effective and accessible to a broad audience. Although there is evidence supporting the utility of behavioral activation as a treatment for depression in older adults, this study will be the first to examine whether a behavioral activation is more effective in increasing activity engagement and psychological wellbeing among a non-clinical sample of older adults, compared to a multi component positive psychology intervention. This randomized controlled trial will examine the impact of two therapeutic approaches on activity engagement and wellbeing among older adults. One hundred and fifty adults aged 65+ who have relatively lower scores on a measure of engagement with life will be randomized to either a behavioral activation-based intervention, or a multi-component positive psychology intervention. The interventions will involve six individual weekly sessions conducted via telephone or video conference. Participants will be assessed pre-, post-intervention, and at three months follow-up. Outcome measures will include activity engagement, positive affect, and psychological wellbeing. Intra-individual variability will also be assessed via micro-longitudinal data in the behavioral activation condition. This study will be the first to provide evidence to the effectiveness of behavioral activation as an intervention to increase activity engagement and wellbeing among older adults, compared to other therapeutic approaches to increase psychological wellbeing.

### THE MODERATION ROLE OF ACCULTURATION ON DIETARY PATTERNS AND STRESS AMONG U.S. CHINESE IMMIGRANTS

Qun Le,[1] and XinQi Dong,[2], *1. Rutgers University, New Brunswick, New Jersey, United States, 2. Rutgers University, Rutgers Institute for Health, New Jersey, United States*

Studies have demonstrated that healthy dietary patterns are related to diminished stress. However, the potential moderation role of acculturation on dietary patterns and stress is unclear among the those whose eating habits are impacted by immigration. The aim of this study is to explore the moderation role of acculturation on dietary patterns and stress among Chinese elder immigrants in the United States. Data were conducted from the PINE Study with 3053 Chinese adults aged over 60 years in the Great Chicago area. Dietary patterns were measured via a 48-items Food-frequency questionnaire with frequency and size weighted. Items were identified into different food groups based on Dietary Guidelines. Acculturation was assessed by a 12-item short-scale among the population. Stress was measured via a 10-item Perceived Stress Scale with cutoff 14 indicating either low or high stress.

Multiple logistic regression was used to examine the moderation effects on the associations with demographic characteristics, medical comorbidities, and BMI adjusted. After controlling covariates, one unit increasing in fruit consumption (OR: 0.61 (95%CI: 0.52 -0.72)) or coffee (OR: 0.49 (95%CI: 0.36 - 0.67)) was associated with lower odds of stress. However, after adding acculturation as an interaction term, the negative relationship between fruit or coffee consumption with stress was moderated by a higher level of acculturation (fruit: OR: 1.05 (95%CI: 1.02 - 1.08), coffee: OR: 1.05 (95%CI: 1.01 - 1.09) respectively). The associations between dietary patterns and stress may differentiate based on acculturation level among the elder immigrants. Further longitudinal studies should investigate potential causality.

### THE ROLE OF AGE, RELIGION/SPIRITUALITY, AND HUMOR IN THE OPTIMISM OF COMMUNITY-DWELLING OLDER ADULTS

Bonnie Kenaley,[1] Eun hae Kim,[2] Kimberly McClive-Reed,[3] and Zvi Gellis,[4], *1. Boise State University, Boise, Idaho, United States, 2. Texas State University, San Marcos, Texas, United States, 3. University of Pennsylvania, NISKAYUNA, New York, United States, 4. University of Pennsylvania, Philadelphia, Pennsylvania, United States*

Current literature reveals that perceived optimism decreases with age (D'Argembeau et al., 2011; Newby-Clark & Ross, 2003). However, replication of these studies is limited. In particular, a lack of investigations exists in examining optimism as individuals transition across older adulthood. Considering the dearth of literature that examines the influence of religion/spirituality and humor on the optimism of older adults, 203 members of Osher Lifelong Learning Institutes, age 65 years and older, from Idaho and California completed pen and paper or electronic surveys. The study used hierarchical multiple regression analysis to examine the impact of age, positive religion/spirituality coping skills, and humor on the optimism of community-dwelling older adults. The participant's gender significantly explained a 19.3% variance in the optimism scores, whereas age did not significantly contribute to the model (R Change =.000). Positive religious or spiritual coping skills and humor significantly contributed to the variance in optimism scores, explaining a 2.3% and 21.6% variance, respectively. In the final model (F (1, 202, = 13.78, p = .000), all variables except age significantly contributed to the model with humor revealing the highest beta value (beta = .467, p = .000). The findings suggest that optimism is perceived differently by gender but does not change with age. While positive religious coping skills influence optimism, internal or external humor strategies may be more helpful to instill, promote or maintain optimism in older adults. The addition of humor assessment items in wellness evaluations and humor-infused interventions may foster optimism in community-dwelling older adults.

### THE ROLE OF LAUGHTER FOR SUCCESSFUL AGING OF ALASKA NATIVE ELDERS

Jordan Lewis, *Memory Keepers Medical Discovery Team/ University of Minnesota Medical School, Duluth campus, Duluth, Minnesota, United States*

During a visit over tea and pilot bread, discussing successful aging and well-being, an Inupiat Elder shared that "It's better to live a happier life, enjoy life all you can." These wise words inspired the theme of this chapter which explores the role of laughter in the resilience and wellbeing of Alaska Native Elders. Based on a decade of research, this chapter introduces the concept of Alaska Native Eldership and provides an overview of the five strands of Alaska Native successful aging that contribute to their ability to remain resilient. Laughter is a universal emotional experience, and Alaska Native Elders have taught us that is has different functions that enable us to be resilient and age successfully. Laughter is woven throughout the strands of successful aging as a key to health and well-being and this chapter discusses the four types of laughter used to protect themselves, including redirecting conversations, practicing humility, taking away the power of stressful topics, as well as sharing happiness and laughter with others. This chapter concludes with reflections on how laughter contributes to our ability to find meaning and purpose in life and strengthening our relationships with family and community so we can remain resilient as we age.

### Session 9435 (Poster)

### RETIREMENT, FINANCIAL WELL-BEING, AND ECONOMICS OF AGING

### BENEFITS OF WISE ORGANIZATIONS ON EMPLOYEES' WELL-BEING: A COMPARISON OF YOUNGER AND OLDER WORKERS

Monika Ardelt, and Bhavna Sharma, *University of Florida, Gainesville, Florida, United States*

Research has shown a positive relation between personal wisdom and well-being, particularly in old age. Yet, it is unknown whether wisdom in the workplace also has a positive impact on workers' well-being. We created a wise organization index for nine organizations based on 74 to 390 average employees' ratings of perceived flexibility at work, work opportunities for training and development, satisfaction with work benefits, absence of time pressure at work, work-life balance, job fulfillment, and job security. We predicted a stronger relation of wise organization on well-being for older workers (N=269; age range 50-74, M=56.08, SD=5.04) than for younger workers (N=552; age range 19-49, M=35.10, SD=8.17) who can more easily change jobs. Results of multigroup analyses in LISREL 9.30 showed that the wise organization index had significant indirect effects on employees' physical and subjective well-being at the second wave of data collection, mediated by employees' perception of wise (fair and supportive) leadership assessed six months earlier and overall work satisfaction (career as calling, satisfaction with career progress, enthusiasm at work, and great workplace) at Wave 2. Contrary to expectations, the effects were not statistically different between the two age groups. However, physical well-being had a statistically stronger association with subjective well-being among younger rather than older workers, possibly indicating a positive health selection effect in the older age group. It appears that wise organizations encourage wise leadership and enable workers to work longer by contributing to their work-related, physical, and subjective well-being.

### CHANGES IN CONSUMPTION EXPENDITURES AMONG BABY-BOOMERS AND YOUNG-OLDS: LATENT TRANSITION ANALYSIS

Si Young Song,[1] Hey Jung Jun,[1] Susanna Joo,[2] and Do Kyung Yoon,[1], *1. Yonsei University, Seoul, Seoul-t'ukpyolsi, Republic of Korea, 2. Yonsei University, Seodaemun-gu, Seoul-t'ukpyolsi, Republic of Korea*

The aim of this study was to examine the longitudinal transition of consumption expenditures among both baby-boomers and young-olds in South Korea. We used data from the 6th (2016) and the 7th (2018) waves of the Korean Longitudinal Study of Ageing (KLoSA). The final sample comprised 1,806 baby-boomers (age range=53-61 in 2016) and 1,483 young-olds (age range=65-74 in 2016). Consumption expenditures were observed with nine types of expenses: food, eating out, public education, private education, housing, health-care, clothing, cultural entertainment, and savings. According to the results from latent transition analysis (LTA), three consumption subgroups were identified among baby-boomers: “non-expenditure for education (NE, 69.7%)” group, “high-public education expenditures (PE, 10.7%)” group, and “high-public and private education expenditures (PPE, 19.6%)” group. For baby-boomers, NE and PE were more likely to remain the same type throughout the two waves, and PPE was most likely to move to NE two years later. Meanwhile, the consumption expenditures of young-olds were divided into “low-saving (LS, 63.7%)” group, “high-saving (HS, 40%)” group, and “education cost-centered (EC, 5.3%)” group. In the case of young-olds, the transition between the groups was unlikely to occur across the two waves which can be interpreted as having fewer life cycle changes than baby-boomers. This study suggests that it is necessary to take into account the difference between the generations when understanding longitudinal transition of consumption expenditures.

### FINANCIAL PLANNING FOR RETIREMENT: DEVELOPING AN INTEGRATED MODEL

Dannii Yeung,[1] Alvin Ho,[2] and Alfred Lam,[2], *1. City University of Hong Kong, Hong Kong, Not Applicable, Hong Kong, 2. City University of Hong Kong, City University of Hong Kong, Not Applicable, Hong Kong*

With higher life expectancy of the aging population, retirees nowadays will spend a prolonged period of time after retirement. Yet, past studies have consistently revealed a lack of retirement savings among working adults, implying an inadequacy to maintain the quality of life in late adulthood. This study therefore aims to identify the factors influencing the working adults' intention to purchase financial products for retirement (such as deferred annuity and voluntary contribution to retirement fund) and develop an integrated model of financial planning for retirement. A total of 598 Hong Kong Chinese working adults from diverse age and income groups completed an online survey on intentions to save and purchase specific financial products for retirement. The results of MANCOVA reveal that compared to older workers, younger workers had lower intentions to save and purchase financial products for their retirement even after controlling for their monthly income [F(16,1797)=2.24, p=.003, partial n2=.015]. An integrated model of financial planning for retirement is proposed by incorporating the concepts of the interdisciplinary psycho-motivation model and Theory of Reasoned Actions ($\chi 2$ = 40.42, p<.001, CFI = .99, RMSEA = .07, SRMR = .02). In particular, the positive effects of financial literacy, subjective norms, social support, and future time perspective on intention to save and purchase financial products for retirement have been found to be mediated by retirement goal clarity but not attitudes towards retirement. Future direction on promoting younger and older workers' retirement planning especially in the financial domain will also be discussed in the presentation.

### MISMATCH BETWEEN PHYSICAL HEALTH AND JOB DEMANDS AMONG OLDER WORKERS BY FULL RETIREMENT AGE

Theresa Andrasfay, *University of Southern California, Los Angeles, California, United States*

Gradual increases to the Social Security full retirement age (FRA) from 65 to 67 were justified by improvements in the health of the older population and a general shift toward less physically demanding jobs. These two trends have been studied independently, but it is important to consider the agreement of these two factors—job demands and health—to understand whether those expected to work longer to receive full benefits have compatible health and job characteristics to do so. Using data from the 1992-2018 waves of the Health and Retirement Study, I observe 19,383 working individuals with FRA ranging from 65-67 while they are approaching retirement (ages 51-60). I compare the prevalence of person-work mismatch—defined by the co-occurrence of physical health conditions and self-reported physical job demands—by FRA. I find that individuals with an older FRA are less likely to be employed in physically demanding jobs while having arthritis. However, they are more likely to be employed in physically demanding jobs while having pain or fair/poor self-assessed health and are more likely to be employed in jobs requiring frequent stooping, kneeling, or crouching while simultaneously having difficulty with these activities. The co-occurrence of physically demanding work while having multiple mobility limitations has remained stable across the FRA cohorts. These findings indicate that older workers expected to work longer to receive full benefits have not experienced substantial improvements in the compatibility between their physical health and job demands that would facilitate working longer, and by some measures compatibility has declined.

### RACIAL AND ETHNIC DIFFERENCES IN RETIREMENT SATISFACTION AMONG OLDER US ADULTS

Samuel Van Vleet, and Sara McLaughlin, *Miami University, Oxford, Ohio, United States*

Along with population aging, the diversity of the older US population is increasing. Research suggests that racial and ethnic minorities experience disadvantages over the life course that can negatively impact later life. Despite this, little research has examined racial and ethnic differences in satisfaction with retirement. Using data from the 2016 wave of the Health and Retirement Study, we compared retirement satisfaction among Black (n = 1,068), Hispanic (n = 674),

Other (n=161), and White (n = 4,833) older adults (age 65+). Retirement satisfaction was measured with the following item: "All in all, would you say that your retirement has turned out to be very satisfying, moderately satisfying, or not at all satisfying?" Responses were categorized as very satisfied vs. all others. Approximately 43% of Black, 35% of Hispanic, 39% of Other, and 56% of White Americans reported being very satisfied with retirement ($\chi2(2.4)=58.9$; $p < .0001$). In multivariate logistic models controlling for age, educational level, gender, household income, marital status, and functional limitations, the odds of being very satisfied with retirement were 32% lower for Hispanic (OR=0.68; 95% CI= 0.55, 0.85) and 37% lower for Other Americans (OR=0.63; 95% CI= 0.43,0.92) relative to their White counterparts. No significant difference was evident for Black and White Americans in adjusted analysis (OR=0.96; 95% CI=0.76,1.20). Our findings indicate that inequalities in the retirement experience exist by race and ethnicity in the United States. More research is needed to understand the factors responsible for lower retirement satisfaction among Hispanic and Other Americans.

### RETIREMENT, HAPPINESS, AND HEALTH IN JAPAN

Shohei Okamoto, and Erika Kobayashi, *Tokyo Metropolitan Institute of Gerontology, Itabashi-ku, Tokyo, Japan*

While health effects of retirement have been well studied so far, previous findings remain inconclusive, and mechanisms underlying the linkage between retirement and health are unclear. This can be driven by regional or cohort heterogeneity as well as methodological differences, such as outcome measures and identification strategies; thus, much evidence needs to be accumulated. Utilising a national household survey conducted every year in 2004-2019 in Japan (the Japan Household Panel Survey), we evaluate the effects of retirement among Japanese adults aged 50-75 on their happiness and health in addition to other outcomes that could attribute to happiness or health changes (e.g. health behaviours, time use for some activities, and the expenses by item). As outcomes are not measured every year, we analyse 4,340-7,902 person-year observations by 756-1,389 individuals with the necessary information from 2009. To deal with the potential endogeneity of retirement, we adopt an instrumental variable approach utilising changes in retirement policy and public pension eligible age. Consequently, instruments seem valid only for men, and we find that retirement increases male retirees' happiness and decreases psychological stress while effects on other health measures are not observed. Although their satisfaction with their income decline, perhaps because of the loss of their wage income, they tend to increase the proportion of expenses for cultural and recreational activities. Enhancement in personal life quality by more leisure activities and stress reduction from work, rather than improvements in health behaviours and physical health, may be key to understanding health benefits in retirement.

### SOCIOECONOMIC STATUS, TIME SCARCITY AND WELL-BEING IN RETIREMENT

Boroka Bo, *University of California, Berkeley, Berkeley, California, United States*

We tend to think of retirement as a great equalizer when it comes to relief from the pernicious time scarcity characterizing the lives of many individuals in the labor force. Puzzlingly, this is not entirely the case. Using data from the MTUS (N=15,390) in combination with long-term participant observation (980 hours) and in-depth interviews (N=53), I show that socioeconomic characteristics are important determinants of retiree time scarcity. Neighborhood disadvantage gets under the skin via time exchanges that are forged by both neighborhood and peer network characteristics. The SES-based 'time projects of surviving and thriving' undergirding the experience of time scarcity lead to divergent strategies of action and differing consequences for well-being. For the advantaged, the experience of time scarcity is protective for well-being in later life, as it emerges from the 'work of thriving' and managing a relative abundance of choices. For the disadvantaged, the later life experience of time scarcity is shaped by cumulative inequality, further exacerbating inequalities in well-being. The final section of the article offers an analysis and interpretation of these results, putting retiree time scarcity in conversation with the broader literature on socioeconomic status and well-being.

### THE ASSOCIATION BETWEEN SUBJECTIVE AND OBJECTIVE FINANCIAL KNOWLEDGE: PATH ANALYSIS TO INVESTOR BEHAVIOR BY AGE

Rain Lee,[1] Jin Sil,[2] and Euijin Jung,[3], *1. Yeshiva University, New York, New York, United States, 2. UC San Diego, San Diago, California, United States, 3. University of Kansas, Kansas, Kansas, United States*

Financial literacy affects stock market participation, as well as individuals' age, gender, income, and education level. However, measuring financial literacy is more appropriate to identify individuals with strong knowledge of finance rather than average individuals with general knowledge. This could be problematic to identify general participation of the stock market and investment as more individuals are now participating without having to have such knowledge. This study explored how individuals' subjective financial skills and well-being affect investment participation by age. Overall, males are likely to participate more in both retirement and non-retirement investment. In between the boomer generation and younger group, the younger generation who reported participating in a non-retirement investment, such as stock market were having a higher score on subjective financial well-being (STDYX = .052, 95% CI [.07, .08]; $p < .05$). Importantly, among the older group, subjective financial skill score becomes a predictor of participating stock market (STDYX = .09, 95% CI [.01, .17]; $p < .05$). As the result suggest, while younger participants focus more on financial well-being, such as having security on finances, when they are participating in a non-retirement investment, whereas older adults are likely to invest based on their beliefs on financial skills regardless of secured finances. A retirement plan has shifted toward less on savings and more on investing. Older adults are now interested more in participating in investments, such as the stock market than the young population, and the proper preparedness for those older adults in participating in the investment is needed.

### THE SOCIAL RELATIONS AND ECONOMIC PREPARATION FOR RETIREMENT OF ONE-PERSON HOUSEHOLDS

Heayoung Sung, *National Pension Research Institute, Jeon-Ju si, Jeollabuk-do, Cholla-bukto, Republic of Korea*

**Objectives:** The purpose of this study is to find out the effect of middle-aged one-person households on social relations and economic preparation for retirement. In addition, even within one-person households, I attempted to determine the difference according to whether the cause of the households' composition was separation after marriage or a one-person household without a spouse due to being unmarried, bereavement, or divorce.

**Methods:** As a result of the analysis using the data of the 2019 Korean Social Survey of the National Statistical Office, single-person households had weaker economic conditions, social relations, and economic preparation for retirement when compared to multi-person households. One-person households lacked a social network to receive help when they face difficulties compared to multi-person households, but there was no effect on group activities or the satisfaction with human relationships. However, households with no spouse had a negative impact on the social network, group activities, and the satisfaction with human relationships.

**Conclusions:** Middle-aged one-person households and one-person households with no spouse had a negative effect on economic preparation for retirement, when labor activity and asset variables were not considered. However, statistical significance was not found when these variables were considered.

### WHAT ARE WE WORRIED ABOUT? MID-LIFE COUPLES' FINANCIAL CONCERNS ABOUT THEIR RETIREMENT

Jeremy Yorgason,[1] Dikla Segel-Karpas,[2] Ashley Ermer,[3] Hailey Weller,[4] Shenan Owens,[5] and Brandan Wheeler,[6], *1. Brigham Young University, Provo, Utah, United States, 2. University of Haifa, Haifa, Hefa, Israel, 3. Montclair State University, Montclair, New Jersey, United States, 4. Brigham Young University--Provo, Provo, Utah, United States, 5. Brigham Young University, Brigham Young University - Provo, Utah, United States, 6. Mississippi State University, Mississippi State, Mississippi, United States*

Retirement is an expected stage of life that couples plan for far in advance. Despite knowing that years of life without regular income are anticipated, some underprepare, leading to financial uncertainty in later years. In this study we explore financial concerns for retirement expressed by a sample of 335 midlife (Mage=44) couples that participated in the Flourishing Families study. We also examined predictors of those concerns across a 1-year period. Results suggested that both husbands and wives worried about insufficient income, excess spending, and heavy debt in retirement. Minor concerns included being worried about paying for their children's education, net worth, and general expenses. Lower income was predictive of both husbands and wives being worried about having insufficient income in retirement. Higher income was predictive of husbands having concerns about excess spending. Although having retirement benefits was not predictive of any worries, having retirement savings was associated with wives having a greater likelihood of reporting worries about heavy debt and net worth in retirement. Better financial communication was associated with fewer husbands reporting concerns about excess spending and fewer wives reporting concerns about heavy debt. Having concerns about a spouse not being financially responsible were associated with more husbands reporting worries about excess spending and heavy debt in retirement. When wives reported higher social connection with a child, they also were more likely to report worries about expenses. Findings suggest that saving for retirement, communicating well about finances, and being financially responsible are associated with fewer financial concerns in retirement.

### Session 9440 (Poster)

### SLEEP

### DAILY EVENING ELECTRONIC MEDIA USE AND SLEEP IN LATER LIFE

Yijung Kim, Shiyang Zhang, and Karen Fingerman, *The University of Texas at Austin, Austin, Texas, United States*

Sleep complaints and disorders are one of the most common disturbances to health and well-being in later life. Evening electronic media use has been shown to influence the subsequent quantity and quality of sleep, but most research focused on younger age groups who are more likely to use new media (e.g., social media) to replace or complement traditional mass media such as television. To investigate how different types of evening media use is related to sleep in later life, we used ecological momentary assessment data from the Daily Experiences and Well-being Study (N = 231; Mage = 73.61) to examine how evening computer use and television viewing affect subsequent sleep hours and perceived sleep quality. Across all evening assessments, 43% of the evenings were spent using computers, and 80% of the evenings were spent watching television. Findings from a series of within-between random effects models indicated that evening computer use and television viewing had independent associations with sleep quantity and quality. That is, older adults reported fewer hours of sleep, more difficulty falling asleep, and worse overall sleep quality on nights following the evening computer use. In contrast, evening television viewing was associated with feeling less tired the next day morning. The results highlight the continued presence of television viewing in older adults' daily lives and their distinction from general computer use. The social context in which older adults watch television in the evening may potentially explain how different electronic media use influences sleep in later life.

### MAKING THE MOST OF ADVERSITY: A FULLY REMOTE ECOLOGICAL MOMENTARY ASSESSMENT AND ACTIGRAPHY STUDY OF HOSPITAL NURSES

Taylor Vigoureux, Christina Mu, and Soomi Lee, *University of South Florida, Tampa, Florida, United States*

The COVID-19 pandemic has created challenges and opportunities for research. This is especially true for research on essential workers, such as hospital nurses. In adaptation to the pandemic, the current study aimed to assess the feasibility and acceptability of a fully remote study to collect data on psychological and behavioral measures such as daily stress and sleep, utilizing ecological momentary assessments (EMA) and sleep actigraphy. Our remote study protocol was conducted through a web platform that provided detailed video and written instructions regarding the study and facilitated virtual onboarding meetings with participants. Outpatient day shift nurses (n=86) responded to a background survey, 84 of whom completed 14 days of EMA and sleep actigraphy. Feasibility was assessed by compliance rates to the 14-day study protocol. Acceptability was assessed by analyzing qualitative feedback provided during onboarding meetings (n=82). The compliance rates of EMA (91.8%) and actigraphy (97.9%) were high. The EMA compliance was higher than that from a pre-COVID, non-remote study of inpatient day shift nurses from the same hospital (86.6%, p=.030). Themes from content analysis were mostly positive with 51.2% reporting "easy, clear, simple onboarding process" and 16.3% reporting "helpful website". Only six participants provided solely negative feedback (e.g., "communication problems" or "technical difficulties/preferences"). Our remote study protocol was feasible and well-accepted by nurses. A similar methodology could be used in studies on broader healthcare workers and those caring for aging populations to better understand their unique challenges and develop effective strategies to help them, both during and after the pandemic.

### POOR SLEEP MODERATES THE ASSOCIATION BETWEEN GENERALIZED ANXIETY AND HIPPOCAMPAL VOLUME AMONG OLDER ADULTS

Desiree Bygrave,[1] and Regina Wright,[2], *1. Benedict College, Benedict College, South Carolina, United States, 2. University of Delaware, University of Deleware, Delaware, United States*

Poor sleep is common among older adults, and associated with hippocampal atrophy -- a strong predictor of memory decrements. Underlying this association are psychosocial risk factors, such as generalized anxiety, that may further exacerbate poor sleep and brain pathology. Given that poor sleep and generalized anxiety are often comorbid, there is a critical need to establish whether generalized anxiety is related to hippocampal volume among poor sleepers. To address this gap, this cross-sectional study examined the relationship between generalized anxiety (GAD-7), and total hippocampal volume, and whether it varied as a function of sleep quality (PSQI Total < 5 good sleepers; PSQI Total ≥ 5 poor sleepers). Data were analyzed from 165 older adults (mean age = 68.48y, 33% male, 41% African American), free of major disease. Linear regression analysis, adjusting for sex, race, education and depression, showed a statistically significant Generalized Anxiety x Sleep interaction for hippocampal volume (p=.02). Further probing of this interaction revealed that among poor sleepers, greater generalized anxiety was associated with lesser hippocampal volume (p=.01). Findings suggest generalized anxiety may influence hippocampal volume in the context of poor sleep among older adults. As poor sleep is associated with age-related neurodegeneration, our findings suggest that improvements in sleep quality may reduce the impact of generalized anxiety on hippocampal volume in older adulthood. Future research should examine whether generalized anxiety mediates relations of sleep quality to specific memory outcomes.

### RURAL/URBAN DIFFERENCES IN SLEEP DISORDERS AMONG OLDER ADULTS IN GHANA

Abolade Oladimeji, *case western reserve university, cleveland, Ohio, United States*

Sleep plays a significant role in the maintenance of the body, and experiences of sleep disorders may have adverse health outcomes on older adults. While a substantial number of studies have documented rural and urban differences in sleep quality and duration, none has examined rural/urban differences in sleep disorders among older adults in Ghana. The analytical sample included 2,142 older adults (65 years and above) who are part of the cross-national study of the Research on Early Life and Aging Trends and Effects (RELATE) conducted in Ghana in 2007 (µage = 74, female 54%). The analysis was based on multivariate logistic regression in predicting the odds of experiencing sleep difficulty among older adults and how this may differ based on rural/urban residence. Results reveal that net of other factors, older adults in rural Ghana are more likely to experience sleep disorders than their urban counterparts (OR=1.29, P<.05). Possession of Jewelry in Africa is a symbol of higher social status, the result shows that net of other factors, those who own jewelry are more likely to experience sleep disorder than those who do not possess jewelry (OR=1.69, P<.001), those who are obese show greater odds of sleep disorder than those with normal weight (OR=1.59, P<.01). Other significant differences were education, self-rated health, and feeling of happiness. These findings show that causal factors of sleep disorder among older adults are multifactorial and their underlying mechanisms may vary by geography(rural/urban)

### SLEEP DISTURBANCE AMONG OLDER KOREAN IMMIGRANTS

Yeonsu Song,[1] Haesook Kim,[2] Diane Lee,[3] Gery Ryan,[4] B Josea Kramer,[3] Jennifer Martin,[5] Ron Hays,[1] and Sarah Choi,[6], *1. UCLA, Los Angeles, California, United States, 2. Holy Names University, Holy Names University, California, United States, 3. VA Greater Los Angeles Healthcare System, North Hills, California, United States, 4. Kaiser Permanente, Pasadena, California, United States, 5. VA Greater Los Angeles, Los Angeles, California, United States, 6. University of California, Los Angeles, Los Angeles, California, United States*

Little is known about sleep disturbance among older Korean immigrants living in the United States. In this exploratory study, we conducted focus groups with 6 and phone interviews with 22 older Korean immigrants to explore and understand their sleep experiences. Three members of the research team fluent in Korean independently coded each focus group and phone interview transcript to identify underlying themes. The following themes emerged: Daytime naps were acceptable behaviors, particularly among those who were retired or not employed. Many reported worry and anxiety rumination behaviors that interfered with their sleep. In addition, praying, reading the bible, and singing hymns often occurred while in bed, and smartphone or tablet use was a frequent activity as well. Drinking alcohol and taking sleeping pills were used as a last resort to help with sleep, particularly among those who lived alone. Internal (e.g., biological urge, snoring, medical conditions) and external distractions (e.g., noise) contributed to nighttime awakenings. Living alone and worrying about the future made it difficult for some to return to sleep after awakening. Seeking advice from peers was a common behavior but did not seem to help with sleep. Calming activities such as reading a book, taking a shower, watch television, or writing a diary were routinely performed before bedtime. These findings are consistent with unhealthy sleep behaviors shown in other racial/ethnic minority older adults with poor sleep. Sleep education programs in Korean-speaking churches may be used to target those who are socially isolated and may benefit older Korean immigrants.

### TRENDS IN SHORT SLEEP DURATION IN THE NATIONAL HEALTH AND INTERVIEW SURVEY 2004-2018: A DECOMPOSITION ANALYSIS

Jiahui Xu,[1] Orfeu Buxton,[2] Liying Luo,[3] Alyssa Gamaldo,[3] Ashton Verdery,[3] Melissa Hardy,[2] and Qian Xiao,[4], *1. The Pennsylvania State University, Pennsylvania, United States, 2. The Pennsylvania State University, The Pennsylvania State University, Pennsylvania, United States, 3. The Pennsylvania State University, University Park, Pennsylvania, United States, 4. The University of Texas Health Science Center at Houston, Houston, Texas, United States*

Since the early 2000s, it is increasingly common that people have short sleep durations (<=6 per 24-hour), making this a high-prevalence public health issue, especially among Black individuals. We investigate how trends in short sleep duration are influenced by changes in population aging, socioeconomic factors (e.g., education, labor force participation, marital status), and health conditions and behaviors (e.g., pain, smoking, drinking, obesity, psychological distress). We use the pooled cross-sectional data from the 2004-2018 National Health and Interview Survey, a large and nationally representative study. We partition overall trends in short sleep duration into (a) compositional effects due to distributional changes in age structure, socioeconomic and health factors and (b) unexplained rate effects. Subsequently, we compare the relative contribution of each factor to the total compositional effects among Black and White participants. Results demonstrate that greater education is associated with greater prevalence of short sleep duration among Black Americans, but reduced prevalence among White Americans. For both racial groups, population aging contributes relatively little to temporal patterns in short sleep duration; by contrast, higher distress and lower labor force participation in recent years are associated with the increasing prevalence of short sleep duration.

### WILL I FEEL THIS IN MY DREAMS? EXAMINING TEMPORAL ASSOCIATIONS BETWEEN DAYTIME AND DREAM AFFECT

Cassandra Richardson, Taylor Vigoureux, and Soomi Lee, *University of South Florida, Tampa, Florida, United States*

Despite the theory that dreams function to process emotions, few studies have examined how emotional experiences during daytime ("daytime affect") are associated with the emotional tone of dreams ("dream affect") that night, and vice versa. This study examined bidirectional associations between dream affect and daytime positive and negative affect. Participants were 84 nurses who completed two weeks of ecological momentary assessments. If participants remembered the previous night's dreams (nparticipants=68; ndays=391), they reported the dream's emotional tone upon waking ('0'=very negative to '100'=very positive). Participants also responded to the Positive and Negative Affect Scale three times/day. Multilevel modeling simultaneously tested two temporal directions (daytime affect→dream affect, dream affect→daytime affect) at the within- and between-person levels. After adjusting for socio-demographic and work characteristics, at the within-person level, dream affect was more positive than usual on nights following more positive daytime affect (B=0.25, p=.003). In the other temporal direction, dream affect was not associated with the following day's positive affect. At the between-person level, nurses who reported more positive dream affect also reported more positive daytime affect (B=0.24, p=.025). No associations emerged with negative affect. Findings suggest that daytime affect is associated with the emotional tone of that night's dreams, but only in the context of positive affect. Importantly, negative affect was relatively low in this sample, so different patterns may emerge for people more prone to negative affect. Overall, these novel findings support the theory that dreams serve to process emotions, providing insight into the mystery of the function of dreams.

### Session 9445 (Poster)

### SOCIAL DETERMINANTS OF HEALTH AND AGING (BSS POSTER)

### AGING MEN AND MASCULINITY IDEOLOGIES: VARIANCE COMPOSITION OF THE MRNS-BF AND MEASUREMENT INVARIANCE BY MEN'S AGE

Edward Thompson,[1] and Ronald Levant,[2], *1. College of the Holy Cross, Broadview Heights, Ohio, United States, 2. University of Akron, University of Akron, Ohio, United States*

The construct traditional masculinity ideology (TMI, Levant & Richmond, 2016), like the construct dominant masculinities (Coles, 2009; Messerschmidt, 2019), refers to culturally-based principles about proper gender practices for men. The present study aimed to create a briefer and psychometrically stronger form of the Male Role Norms Scale (MRNS), a long-standing and important measure of TMI. Using an archival data set (N = 626) with men age 25 and older, the MRNS was shortened using a set of recommended practices. Confirmatory factor analysis and assessment of measurement invariance showed the resulting MRNS-BF satisfies good fit principles with configural, metric, yet not reliable scalar invariance for age. It is a 6-item measure whose common factor measures TMI through two dimensions that mimic tenets Brannon (1976) and Connell (1995) theorized as underlying principles of masculinity ideologies in Western cultures: Earning and maintaining Respect/status, and No sissy stuff/avoidance of femininity in gender practices. Mean scores importantly show perhaps generational, certainly age cohort differences – aging men differed from established and middle-aged men by more moderately endorsing norms that specify men must strive to earn others' respect and the cultural emphasis on no sissy stuff. Younger age cohorts strongly adopted principles of gender equality. These observed differences must be interpreted with great caution, since the men in the three age groups did not seem to have a common zero point. Still, the MRNS-BF has good psychometric properties, and its brevity can assist future research on how gender guidelines influence aging men's health decisions and behavior.

### ASSOCIATIONS BETWEEN NEIGHBORHOOD SES DISADVANTAGE AND FEELINGS OF DEPRESSION AND LONELINESS IN OLDER ADULTS

Dextiny McCain,[1] Adrienne Aiken Morgan,[2] and Regina Wright,[3], *1. North Carolina Central University, Durham, North Carolina, United States, 2. North Carolina A&T State University, Greensboro, North Carolina, United States, 3. University of Delaware, University of Deleware, Delaware, United States*

Previous research suggests depressive symptoms and loneliness are increasingly prevalent among older adults living in lower-income neighborhoods. The purpose of this study was to examine the extent to which neighborhood socioeconomic status (SES) was associated with depressive symptoms and loneliness among a sample of older adults from the Healthy Heart and Mind Study (N = 165; mean age = 68.48 (SD = 6.26); 66.7% women; 40.6% African American). It was hypothesized that older adults living in neighborhoods with greater socioeconomic disadvantage would report more depressive symptoms and loneliness than those residing in neighborhoods with less socioeconomic disadvantage. Depression was assessed with the Beck Depression Inventory-II (BDI-II), and loneliness was assessed using the Revised University of California, Los Angeles (UCLA) Loneliness scale. Neighborhood SES was measured with the Area Deprivation Index (ADI), which allows rankings of neighborhoods by SES disadvantage both statewide and nationally. After controlling for demographic variables (age, sex, and race), linear regression analyses showed that greater neighborhood SES disadvantage was associated with higher depression scores ($\beta$ = -.094; p = .041) and higher loneliness scores ($\beta$ = -.258; p = .003). These findings highlight the importance of neighborhood context on mental health in older adults, as underserved populations are more likely to experience declines in mental health under strenuous circumstances. Future research should investigate the impact of neighborhood SES on mental health in aging adults.

### FACTORS ASSOCIATED WITH THE USE OF TELEHEALTH IN MIDDLE-AGED AND OLDER ADULTS DURING THE COVID-19 PANDEMIC

Yan Du,[1] Rumei Yang,[2] Qingwen Xu,[3] and Bo Xie,[4], *1. School of Nursing, University of Texas Health Science Center at San Antonio, San Antonio, Texas, United States, 2. Nanjing Medical University, Nanjing, Jiangsu, China (People's Republic), 3. New York University, New York University, New York, United States, 4. The University of Texas at Austin, Austin, Texas, United States*

The COVID-19 pandemic has promoted the adoption and use of telehealth, particularly in the early months of the pandemic. However, people with diverse characteristics may, or may not, be able to use telehealth, presenting digital divide in health care and potential health equity-related issues. This study aimed to assess the use of telehealth among middle-aged and older adults during COVID-19, and to explore factors associated with their telehealth utilization. We used publicly available data from the California Health Interview Survey collected during January 2019 and December 2020 (N=15, 279; mean age= 64.23±11.59; female: 52.7%). Approximately 11.0% of the sample used telehealth at least once. Bivariate and multivariate logistical regression analyses found that, compared with non-users, telehealth users were more likely to be having higher numbers of chronic conditions, with self-reported mental distress, living in urban areas, born in the US, with higher English proficiency, higher education, and having higher incomes. Age, race/ethnicity, and gender were not significantly correlated with telehealth usage. Logistic regression revealed that having mental distress (OR=1.48, 95% CI=1.29-1.71, p<0.01), more chronic conditions (OR=1.48, 95% CI=1.29-1.71, p<0.001) and living in an urban area (OR=1.93, 95% CI=1.36-2.74, p<0.001) were independently related to telehealth use. These findings suggest that telehealth, while being beneficial during the pandemic, might also introduce new challenges that exacerbate existing health inequity and disparities. Policy and community-based interventions are needed to promote the use of telehealth among middle-aged and older adults with diverse characteristics.

### RACIAL/ ETHNIC AND GENDER DIFFERENCES IN OLDER ADULTS' CHRONIC STRESS PATTERNS

Kun Wang, and Zainab Suntai, *University of Alabama, Tuscaloosa, Alabama, United States*

Chronic stress has been associated with several adverse psychological, physical, and cognitive outcomes. While there exists a baseline level of stress among all individuals, certain groups of people are at risk of developing chronic stress due to existing hardships and stressors. Black Indigenous and/ or people of color (BIPOC) and women have historically experienced several inequities including higher rates of certain chronic illnesses, interpersonal discrimination, socioeconomic disparities, and several other adverse outcomes. In addition to stressors from racial/ethnic and gender identities, older adulthood is a major transitional period marked by changes in physical, emotional, and cognitive well-being, which have been shown to affect overall well-being and mental health. As such, this study aimed to examine the association between chronic stress and cognition among older adults, using the intersectionality of race and sex. Data from the 2016 Health and Retirement Study were used, resulting in a final sample of 6,015 adults aged 50 and older. Latent class analysis was used to determine chronic stress patterns by sex and race, and a three-step method was used to examine the effects of covariates on stress class memberships by race and sex subgroups. Results indicated that compared to White men, the high stress classes among White women, BIPOC men and BIPOC women contained more stressors. Interventions targeted towards the mitigation of chronic stress among older adults should consider how intersectional identities combine to create increased hardships and stressors.

### TRAJECTORIES OF K-12 SCHOOLS AND DEMOGRAPHIC DIFFERENCES: EVIDENCE FROM 2017 LIFE HISTORY MAIL SURVEY

Wenshan Yu,[1] Xuefei Li,[2] and Jacqui Smith,[3], *1. Institute for Social Research, University of Michigan (Ann Arbor), Ann Arbor, Michigan, United States, 2. Institute for Social Research, University of Michigan, Ann Arbor, Michigan, United States, 3. University of Michigan, University of Michigan, Michigan, United States*

Besides information about the highest degree, little information about early-life education is available in most population surveys. This study identified the trajectories of K-12 education history among older adults in the Health and Retirement Study born between 1930 and 1960, and examined the associations with demographic variables. Drawing on 2017 Spring and Fall Life History Mail Survey (LHMS; n = 3,206), we used sequence analysis to determine and classify trajectories of school types across the education history. We identified five trajectories: 1) always private school with White students, 2) always public school with White students, 3) always public school with Non-White students, 4) mostly private school with Non-White students, and 5) no report of school types. The trajectories showed that changes in school type (i.e. private to public) often happened in grade 9. Changes rarely happened across race/ethnicity groups (i.e. mostly White to mostly non-White). We used multinomial logistic regression to examine the relationship between demographic variables and education trajectories. We found that compared to Black participants, White participants were significantly less likely to be in mostly Non-White schools (public and private schools, $p<0.001$). The 1940s and 1950s cohort were more likely to join mostly White private schools than the 1930s cohort (odds ratio: 1.70 for 1940s and 1.62 for 1950s separately, $p<0.005$). Our findings illustrate a novel application of sequence analysis with life history data, as well as new evidence on recial segregation in early-life education within the last century.

### Session 9450 (Poster)

### SOCIAL DETERMINANTS OF HEALTH AND AGING (SRPP POSTER)

### AREA DEPRIVATION SHARE: A NEW MEASURE OF SOCIAL NEED FACED BY HOSPITALS SERVING OLDER ADULTS

Kellia Hansmann,[1] Amy Kind,[2] and Ryan Powell,[2], *1. University of Wisconsin--Madison, Madison, Wisconsin, United States, 2. Geriatrics Division, Madison, Wisconsin, United States*

Medicare's Hospital Readmissions Reduction Program (HRRP) places disproportionate penalties on hospitals serving populations with complex medical and social needs. Without measures to identify the social need intensity of populations cared for by these hospitals, the HRRP cannot account for these risk factors, leading to burdensome penalties that may inadvertently hinder the ability of such hospitals to care for vulnerable populations. The objective of this study is to characterize the social need intensity of US hospital acute care populations. Using the Area Deprivation Index (ADI), a validated measure that ranks neighborhood socioeconomic disadvantage based on income, employment, housing, and education factors, we determined an "Area Deprivation Share" (ADS) for hospitals with 25 or more discharges using 100% of national Medicare claims data from 2013-2014. Hospital ADS is the proportion of qualifying discharges residing in the most disadvantaged neighborhoods (ADI ≥ 80th percentile) out of all qualifying discharges during the study period. Of 4,603 hospitals, median ADS was 17% (Interquartile Range: 6% - 34%). Hospitals in the highest quintile of ADS (39% to 100%), were more frequently located in small towns or isolated rural areas (52.6%, comparted to 24.2% in lower quintiles) and served a higher percentage of Black patients (19.0%, comparted to 9.7% in lower quintiles). ADS is a potential tool to inform future Medicare policy decisions. Additional research will inform how hospitals target care processes to meet the needs of older adults with complex social needs. Further study can also explore overlapping disadvantage domains of socioeconomic status, race, and rurality.

### ASSOCIATION OF HOME BASED PRIMARY CARE ENROLLMENT WITH SOCIAL DETERMINANTS OF HEALTH FOR OLDER VETERANS

Anna-Rae Montano,[1] Augustus Ge,[2]
Christopher Halladay,[1] Samuel Edwards,[3] James Rudolph,[1] and Portia Cornell,[1], *1. Providence VA Medical Center, Providence, Rhode Island, United States, 2. Department of Veterans Affairs, Providence, Rhode Island, United States,*

*3. VA Portland Health Care System, Portland, Oregon, United States*

The Veterans Administration (VA) Home-based Primary Care (HBPC) program provides comprehensive primary care to older Veterans with multiple chronic conditions who may be at risk of adverse health outcomes due to their social determinants of health. Area Deprivation Index (ADI) can be used as a surrogate measure of a Veteran's social needs. The objective of this study was to estimate the effect of neighborhood disadvantage, as measured by ADI, on HBPC enrollment for a sample of older Veterans. We estimated a linear multivariate model in which the exposure was ADI and the outcome was enrollment in HBPC. Controls included clinical and demographic characteristics. In a final sample of 12,005,453 observations (total Veteran months) on 353,485 individual Veterans, 18.4% lived in high-deprivation neighborhoods (ADI greater than or equal to 80). Mean monthly probability of new HBPC enrollment was 0.0061. Controlling for clinical characteristics, housing instability, and distance from the medical center, Veterans residing in high-deprivation neighborhoods were 1.4% to 14.8% less likely to enroll in HBPC, though the association was not statistically significant. The VA HBPC program provides beneficial comprehensive, primary care services to Veterans at risk of poor health outcomes. However, a Veteran's social determinants of health could prevent enrollment. More research is needed to determine the relationship between Veterans' social needs and HBPC enrollment.

### ENGAGING STAKEHOLDERS IN A PRAGMATIC TRIAL OF HOME-DELIVERED MEALS FOR PERSONS WITH DEMENTIA

Jill Harrison,[1] Kathleen McAuliff,[2] and Kali Thomas,[2], *1. Brown University, Brown University, Rhode Island, United States, 2. Brown University, Brown University/Providence, Rhode Island, United States*

Gathering stakeholder feedback is essential to designing and implementing relevant and actionable research. Additionally, stakeholders, particularly those directly impacted by an intervention, bring unique insights and experiences. This paper presents the process and findings of a research endeavor to co-design a pragmatic clinical trial with a Stakeholder Advisory Panel (SAP) in an effort to understand facilitators and barriers to conducting the research and implementing study findings. The proposed trial compares the impact of frozen, drop-shipped meals versus daily home-delivered meals provided by Meals on Wheels (MOW) programs on the ability of individuals living with dementia to age in place. We recruited nine SAP members, who were compensated for their time. The SAP is composed of a) MOW clients with dementia, b) family members of MOW clients with dementia, c) paid or volunteer MOW drivers, and d) MOW staff. A research team member facilitated two 90-minute meetings with the SAP members via Zoom. The topics of the meetings included potential benefits and challenges with each mode of meal delivery, the importance of the primary outcome (time to nursing home placement), topics of interest to include in interviews with clients and caregivers, and how participants would explain the study to a friend. Audio of the Zoom meetings was transcribed, and meeting summaries were shared with the SAP. Benefits of forming and engaging a SAP, as well as key lessons learned from SAP members and how recommendations were reflected in changes to the study protocol will be discussed.

### INTERGENERATIONAL EDUCATION AND LATE-LIFE COGNITIVE DECLINE AMONG LATINOS AND NON-HISPANIC WHITES

Erika Meza,[1] Yea-Hung Chen,[1] Isabel Allen,[1] Hector Gonzalez,[2] M Maria Glymour,[3] and Jacqueline Torres,[3], *1. University of California, San Francisco, San Francisco, California, United States, 2. University of California, San Diego, San Diego, California, United States, 3. University California San Francisco, San Francisco, California, United States*

Latinos face a growing burden of Alzheimer's Disease and related dementia (ADRD). Although education has been established as a strong predictor of ADRD, evidence to date is primarily for non-Latino cohorts. Few studies have assessed the relationship between intergenerational education and one's cognitive decline. Using the US Health and Retirement Study (N=20,860) we evaluated the joint effect of parental and own educational attainment on immediate and delayed verbal memory scores (range 0-10) from 1998 to 2016. The exposure was a 4-category variable based on parents' (highest of mother's or father's) and participant's own high school attainment: first-generation (parents' education <12; own ≥12); multi-generation (both ≥12: REF); neither graduated high school (both <12) and parent(s) graduated high school but not respondent (parents ≥12; own <12). Linear mixed effects models with subject-specific random intercepts and random slopes were stratified by race/ethnicity and tested for a 3-way interaction term (exposure x Latino x time). Models controlled for age, sex, place of birth and retest effects. Baseline verbal memory scores did not differ for first-generation compared to multi-generation high school graduates. Verbal memory decline was faster for first- compared to multi-generation high school graduates among non-Hispanic whites (e.g., $\beta$=-0.04; 95% CI: -0.05, -0.03, delayed verbal recall); among Latinos, first and multi-generation high school graduates had similar rates of decline (e.g. $\beta$=0.00; 95% CI: -0.03, 0.04, delayed verbal recall; p<0.001 for three-way interaction). Our findings suggest social and economic policies that facilitate educational achievement, particularly for important population subgroups, may reduce ADRD risk.

### TYPOLOGY OF TRAUMATIC EVENTS AND PHYSICAL FUNCTION

Gabriella Dong, and Mengting Li, *Rutgers, The State University of New Jersey, New Brunswick, New Jersey, United States*

Individuals experience various traumatic events over the life course, but little is known about the patterns of lifetime exposure to traumatic events. This study aims to identify traumatic event typology and examine its relationship with physical function. Data were from the 2017-2019 PINE study (N= 3,125). Traumatic events were evaluated by earthquake, typhoon, tornado, residential fire, physical assault, robbery, sexual assault, divorce, bereavement, cancer, homeless, imprisonment, and falsely accused. Physical function was measured by activities of daily living (ADL), with lower scores indicating better physical function. Analysis was conducted using latent class analysis and the four-class model fits the data best. We identified four typologies: limited trauma, severe trauma, natural disaster, and mild-to-moderate trauma. The "limited trauma" (33.8%) has the lowest exposure to all traumatic events except typhoon and homeless. In contrast, an equivalent "severe trauma" (33.3%) has the highest exposure to all traumatic events except natural disasters. A small "natural disaster" (4.8%) has the highest exposure to natural disaster and moderate exposure to other traumatic events. The "mild-to-moderate trauma" (28.2%) has mild-to-moderate trauma exposures. The mild-to-moderate trauma group (M=0.38, SD=2.12) has better physical function than limited trauma (M=0.69, SD=3.08), severe trauma (M=0.61, SD=2.81), and natural disaster (M=0.71, SD=3.22) groups. After controlling confounding variables, the mild-to-moderate trauma group has lower risks of ADL impairment than the limited trauma group (OR=0.66, 95%CI=0.47-0.93). The findings suggest mild-to-moderate exposure to traumatic events might benefit older adults' health, while limited trauma might not be able to develop resilience and severe trauma overwhelms coping strategies.

### Session 9455 (Poster)

### SOCIAL SUPPORT, SOCIAL CONNECTEDNESS, AND LONELINESS I

### CUMULATIVE EFFECT OF LONELINESS AND SOCIAL ISOLATION ON HEALTH OUTCOMES AMONG OLDER ADULTS

Timothy Barnes,[1] Stephanie MacLeod,[2] Rifky Tkatch,[1] Manik Ahuja,[1] Laurie Albright,[3] James Schaeffer,[1] and Charlotte Yeh,[4], *1. UnitedHealth Group, Minnetonka, Minnesota, United States, 2. UnitedHealth Group, United States, 3. UnitedHealth Group, Minneapolis, Minnesota, United States, 4. AARP Services, Inc., Washington, District of Columbia, United States*

Loneliness and social isolation are described similarly yet are distinct constructs. Numerous studies examine each construct separately; however, less research has been dedicated to exploring their impacts together. Using survey and claims data among adults age 65+ (N=6,994), the cumulative effects of loneliness and social isolation on late-life health outcomes were examined using Chi-square and multivariate regression models. Loneliness and social isolation were measured using the UCLA-3 Loneliness Scale and the Social Network Index. Participants were grouped into four categories of loneliness and social isolation based on overlap, including: lonely only (L), socially isolated only (SI), both lonely and socially isolated (LSI), or neither (N). Outcomes included quality of life and healthcare utilization and costs. Among participants, 9.8% were considered L, 20.6% SI, 9.1% LSI, and 60.5% N. Respondents were primarily female (55.0%) and 70-74 years of age (27.1%). Those considered LSI were more likely to be older, female, less healthy, depressed, with lower quality of life and greater healthcare utilization patterns. Participants who were L or LSI had higher rates of emergency room visits compared to the N group; LSI had the highest medical costs. Results demonstrate the cumulative effects of loneliness and social isolation among older adults. Findings not only fill a gap in research exploring the impacts of these constructs later in life, but also confirm the need for approaches targeting older adults who are both lonely and socially isolated. As the COVID-19 pandemic continues, this priority will continue to be urgent for older adults.

### EVALUATION OF A WEB-BASED WELLNESS PROGRAM TO REDUCE LONELINESS AMONG OLDER ADULTS

Catherine Zaidel,[1] Lizi Wu,[2] Rifky Tkatch,[2] Laurie Albright,[3] and Charlotte Yeh,[4], *1. UnitedHealth Group, Lafayette, Colorado, United States, 2. UnitedHealth Group, Minnetonka, Minnesota, United States, 3. UnitedHealth Group, Minneapolis, Minnesota, United States, 4. AARP Services, Inc., Washington, District of Columbia, United States*

**Background:** Loneliness has been associated with adverse health outcomes, including increased mortality risk. Interventions aimed at addressing maladaptive social cognition have documented efficacy in reducing loneliness among older adults.

**Purpose:** The purpose of this study was to determine the feasibility and efficacy of a web-based wellness program in reducing loneliness and improving psychosocial health among older adults with an AARP® Medicare Supplement plan insured by UnitedHealthcare Insurance Company.

**Methods:** Eligible individuals were 65 years and older, who self-identified as lonely on a prior survey, and indicated that they had access to the internet through a computer or smartphone. Participants completed up to eight online modules comprised of a short lesson on an aspect of maladaptive social cognition, followed by a comprehension quiz, selection of a short-term goal, and a phone or text chat with a program coach. Four surveys were administered to assess the effects of program participation: (1) prior to the start of the program, (2) after completion of four online modules, (3) after completion of all eight modules, and (4) 30-60 days later.

**Results:** Attrition was high. Overall, 220 (42%) program participants completed both T1/T2 surveys, 193 also completed a T3 survey, and 177 also completed a T4 survey. Post-survey data indicated that loneliness and perceived stress decreased while mental wellbeing, resilience, and perception of aging improved.

**Conclusion:** Digital interventions aimed at addressing maladaptive social cognition offer potential to reach lonely older adults and support psychosocial wellbeing through reduced loneliness.

### EXPLORING SOCIAL SUPPORT SYSTEMS AND EXPERIENCES OF OLDER WOMEN IN TEDE COMMUNITY, OYO STATE, NIGERIA.

Kafayat Mahmoud, *University of Kansas, Lawrence, Kansas, United States*

Rural communities in Nigeria are rapidly aging due to the massive movement of young adults to the cities, especially after marriage and/or in search of employment. This has adversely affected the social supports for older persons. The current study explored the experiences of older adults living in rural communities in Southwestern Nigeria. The study adopted an exploratory qualitative research design, in-depth interview techniques were adopted, and results were analyzed using thematic analysis. For this pilot study, conducted in Tede community in Oyo state in Nigeria, 10 older women, aged 70+ years were purposively selected for the study. The fact that all participants were women was due to the fact that older persons found in the community were mostly women. Consonant with previous research, this pilot study found that there was limited formal and informal support systems for older women in the community. The study additionally revealed that older women expressed feelings of abandonment by adult children, having insufficient funds, as well as inability to access health care. Consequently, these women resorted to alternative means to fend for themselves. For instance, despite having functional limitations, older women in the community would engage in physically demanding tasks such as going into the bushes to cut and gather firewood to sell, as well as engaging in other forms of petty trading, while others resorted to begging for alms for sustenance. This pilot study highlights the experience of poverty among older women and the need for more structural interventions for older persons in Nigeria.

### LIVING/EATING ARRANGEMENT, LONELINESS, AND MENTAL DISTRESS AMONG OLDER KOREAN IMMIGRANTS: GENDER DIFFERENCE

Nan Sook Park,[1] Yuri Jang,[2] Soondool Chung,[3] David Chiriboga,[1] and William Haley,[1], *1. University of South Florida, Tampa, Florida, United States, 2. University of Southern California, Los Angeles, California, United States, 3. Ewha Womans University, Ewha Womans University, Seoul-t'ukpyolsi, Republic of Korea*

Structural isolation such as living alone poses a mental health risk in diverse groups of older adults, including older immigrants. Given that those living with others might also be disengaged, the present investigation included eating alone as another source of isolation and examined the impact of the combination of living and eating alone. The proposes of the study were to examine (1) how living and/or eating alone would impact mental distress, (2) whether the impact would be mediated by feelings of loneliness, and (3) if there would be gender differences in the mediation effect. The data were drawn from the Study of Older Korean Americans (SOKA), which surveyed older Korean immigrants in five states during 2017–2018. The living/eating arrangement was classified into four-groups: living/eating with others (57%), living with others/eating alone (12.4%), living alone/eating with others (7.3%), and living/eating alone (23.1%). Using the PROCESS macro, we tested the mediation effect of loneliness and the moderation effect of gender in the relationship between the typology and mental distress controlling for background/health characteristics and social capital related variables. Two groups (living with others/eating alone and living/eating alone) had sociodemographic, health, and social capital disadvantages. Analyses demonstrated that mental distress was linked with living with others/eating alone and living/eating alone, of which relationships were mediated by loneliness only among women. Findings suggest that not only structural isolation (e.g., living alone) but also disengagement with others (e.g., eating alone) need to be considered to understand emotional well-being in older immigrant population and gender difference.

### LONGITUDINAL RELATIONSHIP BETWEEN LIVING ALONE AND ACTIVITY ENGAGEMENT OF CHINESE OLDER ADULTS

Fengyan Tang, and Ke Li, *University of Pittsburgh, Pittsburgh, Pennsylvania, United States*

The living arrangement of older adults plays a key role in their health status and psychological well-being. Yet the relationship between living arrangement and activity engagement remains unclear. Using data from three waves of the nationally representative China Health and Retirement Longitudinal Study (CHARLS) with a study sample of 7,479 respondents aged 60 or older, this study examined the effect of living alone on the frequency of activity engagement among Chinese older adults. Two types of activity engagement were examined in this study, including social interaction with friends and leisure activity (i.e., play chess, go to a sport or club). The multinomial logistic regression analyses were performed using generalized structural equation modeling (GSEM). Compared with those living with others, older adults living alone were older, more likely to be female and living in urban areas, and with fewer years of education and more functional limitations. The results also indicated that after controlling for a set of covariates, living alone status was significantly associated with the increased likelihood of high and medium frequency of both social interaction and leisure activity in reference to no engagement. This study not only improves the understanding of activity engagement preference of Chinese older adults who are living alone but also indicates the importance of improving community facilities and outdoor spaces to promote activity engagement among older adults.

### MARITAL SUPPORT RECIPROCITY AND LIFE SATISFACTION AMONG OLDER KOREANS

Hye Soo Lee, *Oregon State University, Corvallis, Oregon, United States*

The importance of reciprocity in social support for well-being has been shown, but few studies have investigated marital support reciprocity in older Korean samples. This study examined the associations between three types of marital support reciprocity and life satisfaction, stratified by age and gender. The sample consisted of 1,578 men and 1,464 women from the 2017 National Survey of Older Koreans, divided into young-old (65-74) and old-old (75+) groups (M age = 75.06, SD = 6.35). Participants self-reported emotional, instrumental, and physical support provided to and received from spouses, and life satisfaction (LS). Regression models controlling for covariates showed that results varied by age and gender. For young-old males, received emotional and provided instrumental support were positively associated with LS. For young-old females, both received and provided emotional support, and received instrumental support, were positively associated with LS, but provided physical support showed negative associations. For old-old males, providing emotional support was positively associated with LS; for old-old females, only received emotional support was significant. Using interaction terms to assess reciprocity, young-old females and old-old males showed reciprocity effects for instrumental support. When participants provided and received high levels of support, life satisfaction levels were high. However, when participants provided low levels of support, received support was not significant. Thus, the effects of receipt and provision of support on LS varied by age and gender among older Koreans, but reciprocity of instrumental support was only important for young-old women and old-old men.

### POSITIVE PERIPHERAL SOCIAL NETWORK MEMBERS IN OLDER AGE: GLOBAL AND DAILY APPRAISALS OF SOCIAL PARTNERS

Claire Growney,[1] and Tammy English,[2], *1. Washington University in St. Louis, Saint Louis, Missouri, United States, 2. Washington University in St. Louis, St. Louis, Missouri, United States*

Socioemotional selectivity theory suggests older adults maintain relationships with close social partners with whom they experience positive emotions. It is unclear how age and closeness predict social partner appraisals in different contexts. We examine semantic and experiential appraisals of positivity, as well as emotional outcomes. Participants (N = 258) aged 25-85 (M = 52.05, SD = 16.31) reported their general experience of enjoyment and conflict with social partners of varying closeness. In an experience sampling procedure (6x/day for 10 days), participants reported their current experience of emotions and information about their most recent social interaction: pleasure, discomfort, and relationship closeness with their social partner. Semantic (global) appraisals of relationships positively predicted experiential (daily) appraisals, and this association was stronger among relatively older adults. Results revealed older adults gave less negative appraisals compared to younger adults, regardless of closeness. Older adults reported more positive appraisals than younger adults for non-close relationships, whereas close relationships were evaluated positively regardless of age. For younger adults, interaction pleasure with non-close partners was less strongly linked to subsequent positive emotions than pleasure with close partners. For older adults, however, interaction pleasure predicted greater subsequent positive emotions regardless of relationship closeness. Overall, these findings suggest older adults' positive appraisals of partners are not simply the result of emotionally gratifying memory distortions. Older adults may be able to derive emotional benefits from a wider variety of social interactions than younger adults, suggesting peripheral social network members can be leveraged to enhance emotional well-being in later adulthood.

### PRELIMINARY IMPACT OF A SOCIAL CONNECTION PROGRAM ON OLDER ADULTS

Rachel Ungar,[1] Rifky Tkatch,[1] Yan Cheng,[1] Sandra Kraemer,[2] Michael McGinn,[1] and Ellen Wicker,[3], *1. UnitedHealth Group, Minnetonka, Minnesota, United States, 2. UnitedHealthcare, Minnetonka, Minnesota, United States, 3. AARP Services, Inc., Washington, District of Columbia, United States*

**Background:** Research demonstrates social connections decrease loneliness and improves life satisfaction among older adults. Unfortunately, the COVID-19 pandemic has limited social connectedness, specifically for older adults. Thus, programs aiming to increase social connectedness among older adults are imperative.

**Purpose:** The primary objective of this study was to determine if the telephonic Peer-to-Peer (P2P) program can improve social connectedness and loneliness among older adults. A secondary objective was to determine whether additional improvements in life satisfaction and perception of aging were achieved.

**Methods:** Eligible older adults (age 65+) were recruited via outbound calls and/or a mailer. Participants were mailed a T1 survey, completed intervention training, and matched into a dyad. The matched dyad engaged in weekly telephone calls for 12 weeks. Post 12 weeks, participants completed a T2 survey, and a T3 four weeks later.

**Results:** Overall, 7,544 individuals were contacted to participate, and 759 expressed interest in participation. A total of 475 participants (62%) completed a T1, 372 (78%) completed training, and 348 (94%) were matched. Gender distribution was skewed towards females (74%), and most were 65-74 years old (53%). Preliminary results show significant differences between lonely and not lonely participants, with lonely participants reporting more negative health associations across all measures.

**Conclusion:** Once agreeing to participate, results showed a high likelihood of continuing in P2P, thus demonstrating a social connectedness opportunity for older adults. Delay in mailing and scheduling training may contribute to challenges in attrition. However, developing automated processes utilizing technology may decrease lag time for future phases.

### SOCIAL CONNECTEDNESS AND COMMUNICATION TECHNOLOGY AMONG PAID AND UNPAID CAREGIVERS OF MIDDLE-AGED AND OLDER ADULTS

Shinduk Lee,[1] Marcia Ory,[1] Deborah Vollmer Dahlke,[2] and Matthew Smith,[3], *1. Texas A&M University, College Station, Texas, United States, 2. DVD Associates, LLC, DVD Associates, LLC, Texas, United States, 3. Texas A&M School of Public Health, College Station, Texas, United States*

**Objective:** To examine differential relationships between communication technology use and social connectedness among paid and unpaid caregivers providing care for middle-aged and older adults.

**Methods:** This nationwide study utilized a cross-sectional Qualtrics panel survey from caregivers, ages 50 years and older (n=504) of middle-aged and older adults. About 17% were paid (n=86) and 83% (n=418) were unpaid caregivers. Primary outcomes were caregivers' sense of belonging to their local community and social bonds. The primary predictor of interest was caregivers' use of communication technology (texting/communication applications). A multivariable regression analysis was performed to predict each outcome based on communication technology use, separately among paid and unpaid caregivers. Multivariable regression analyses were repeated after including the caregivers' payment status (paid/unpaid) and the interaction term between the caregivers' payment status and the use of communication technology. The models were adjusted for caregivers' age, education, financial status, place of residence, and total weekly hours of caregiving.

**Results:** Use of communication technology had a statistically-significant positive association with sense of belonging only among paid caregivers (b=25.8, p=.005). The relationship between use of communication technology and sense of belonging was significantly different between paid and unpaid caregivers (p=.005). Use of communication technology was significantly associated with social bonds only among unpaid caregivers (b=0.4, p<.001). There was no statistically-significant differential association between communication technology use and social bonds.

**Conclusion:** Communication technology may play differential roles in linking paid and unpaid caregivers with their community and interpersonal groups. Additional efforts should examine mechanisms that provide meaningful caregiver support.

### SOCIAL SUPPORT AND WEIGHT OUTCOMES OVER A SIX-MONTH WEIGHT LOSS INTERVENTION FOR RURAL OLDER ADULTS

Anna Kahkoska,[1] Curtis Petersen,[2] David Lynch,[3] Hillary Spangler,[4] Karen Fortuna,[2] and John Batsis,[5], *1. University of North Carolina at Chapel Hill, University of North Carolina at Chapel Hill/Chapel Hill, North Carolina, United States, 2. Dartmouth College, Lebanon, New Hampshire, United States, 3. University of North Carolina, Chapel Hill, North Carolina, United States, 4. UNC Hospitals, Chapel Hill, North Carolina, United States, 5. University of North Carolina at Chapel Hill, Chapel Hill, North Carolina, United States*

Rural older adults aged ≥65 years with obesity (body mass index (BMI) ≥30 kg/m2) showed an overall favorable response to a six-month, technology-based weight-management intervention. Our objective was to characterize how friends or family support for eating and exercise behaviors at baseline was associated with baseline weight and intervention response. We analyzed data from six subscales of the Social Support and Exercise Survey from 44 participants. Six-month weight change (≥5% of baseline) was considered clinically-significant. For each subscale, continuous and categorial outcomes were modeled with linear and logistic regression models, respectively, adjusted for sex and age. Crude associations of social support clusters, generated in an exploratory hierarchical cluster analysis, and weight outcomes were evaluated. The sample was 73.2 ± 3.9 years, 73% female, with mean baseline weight 97.8±16.3 kg and BMI 36.5±5.2 m/kg2. Family encouragement for healthy eating was negatively associated with baseline weight (β=-0.53, p=0.046). Social support scores were not associated with either six-month weight loss outcome (p>0.10). Two exploratory clusters were found: Cluster 1 (C1) (n=34) and Cluster 2 (C2) (n=9). C2 had higher mean social encouragement and discouragement, with lower mean baseline weight (90.0±11.7 vs 99.8±16.8kg C1; p=0.10). Weight loss was comparable (C1 4.6±3.7 versus C2 4.8±2.6kg; p=0.89), with no differences in clinically-significant weight loss (C1 45% versus C2 67%; p=0.46). These pilot data suggest that family member social support may act as collaterals to support clinical outcomes in the community. Evaluating different types within family support may elucidate associations with physiological outcomes in larger samples.

### THE ROLE OF SOCIAL CONNECTION/ENGAGEMENT PREDICTS CHANGES IN DEPRESSIVE SYMPTOMS AND IADL IN STROKE

Joanne Elayoubi,[1] and Monica Nelson,[2], *1. USF, land o lakes, Florida, United States, 2. University of South Florida, Brandon, Florida, United States*

Social connections/engagement have been found to be potentially protective against depression and declines in physical functioning. We examined whether social connection/engagement was protective against depression and functional decline after stroke. Participants were 898 individuals with incident stroke from the Health and Retirement Study between 1998-2012. Multilevel modeling was used to examine how social connection/engagement were associated with trajectories of depressive symptoms and limitations with instrumental activities of daily living (IADLs). Models controlled for age, gender, education, and race. In addition, analyses with depressive symptoms as outcome controlled for functional limitations with ADLs. Participants who were lonely and did not have friends in their neighborhood pre-stroke had more depressive symptoms at the time of stroke. Participants with close children pre-stroke showed less increase in depressive symptoms over time. Within-person increase in loneliness and within-person decline in providing help were related to more depressive symptoms post-stroke. Participants who felt lonely and did not provide help pre-stroke had more IADL limitations at the time of stroke. Smaller pre-stroke household size and pre-stroke volunteering were associated with less increase in IADL limitations with stroke. Within-person increase in having friends and providing help after stroke were associated with fewer IADL limitations post-stroke. Taken together, these findings suggest that social connection/engagement may buffer the negative psychological and physical outcomes of a stressful event such as stroke.

### WHO NEEDS A FRIEND? HOW AGE AND HAVING SOMEONE ONE CAN COUNT ON EXPLAIN SUBJECTIVE WELL-BEING IN INDIA

Erika Sanborne, *University of Minnesota--Twin Cities, West Saint Paul, Minnesota, United States*

Subjective well-being is now considered a reliable predictor of many desirable outcomes not only at the individual level, in terms of one's personal health, but also at the national level, in terms of a nation's per capita gross domestic product, progress towards reaching sustainable development goals, and other metrics. Subjective well-being has several known causes that reliably predict well-being across time and place, such as income, education, prosociality, and perceived corruption (i.e. REF). Given the benefits of well-being to both individuals and nations, and that subjective well-being is often predicted by variables that are not easily altered, this study aims to better understand the relationships between subjective well-being and some of its known predictors, in the context of India. Three hypotheses were tested and found significant with nationally representative samples of a total of 57,077 survey respondents in India, using data from Gallup World Poll for 2006-2019. Hypothesis #1 tests for having someone one can count on as a mediator. Hypothesis #2 tests for age as a moderator. Hypothesis #3 is a moderated mediation that best explains how the known predictors of subjective well-being make their influence, and with whom. This study's findings give insights into the ways in which subjective well-being in India can be better understood and thus improved. Such understanding may also help local Indian nonprofit organizations, as well as other researchers and mental health providers, with shared interests in the growing prevalence of suicide in India.

### Session 9460 (Poster)

### SOCIAL SUPPORT, SOCIAL CONNECTEDNESS, AND LONELINESS II

### BIRTH OF THE SOCIETY OF DIVOREES – CHANGING PATTERNS OF CIVIL STATUS IN LATER LIFE

Torbjorn Bildtgard,[1] and Peter Öberg,[2], *1. Stockholms University, Stockholm, Stockholms Lan, Sweden, 2. University of Gävle, Gävle, Gavleborgs Lan, Sweden*

Half a century ago Lopata used the concept "society of widows" to describe the gendered reality of late life singlehood, where widowed women were excluded from coupled social life, depended on a community of other widows for social integration, and refrained from initiating new relationships due to "sanctification" of their former husbands. We use Swedish, American and EU census data and a national survey to Swedes 60-90 years old (n=1225; response rate 42%) to illustrate a substantial change in the demographic landscape of late life singlehood. More people enter later life as divorcees or become divorced at a high age. Among Swedes 60+ divorcees outnumber widowed people, and the incidence of late life divorce has more than doubled since the millennium in what has been called the "grey divorce revolution". Many other Western countries follow the same demographical trend, posing important questions about the transformation of late life singlehood. Based on two Swedish studies we will show that the structure of the late life single community is becoming less gender skewed as a consequence of the emerging society of divorcees, and that in this society relationship careers are increasingly complex, attitudes to repartnering increasingly liberal and partner sanctification seldom an issue. We conclude by proposing the concept "society of divorcees" for this new demographic landscape of late life singlehood, argue that research is needed to capture this new reality, and discuss the implications of this change for access to social support later life.

### EFFECTS OF LONELINESS AND STRESS MANAGEMENT ON PERCEIVED STRESS IN OLDER ADULTS

Angela Gifford,[1] Hayley Fouche,[2] and Janelle Beadle,[2], *1. University of Wisconsin-Madison, Madison, Wisconsin, United States, 2. University of Nebraska at Omaha, Omaha, Nebraska, United States*

In older adulthood, individuals may experience acute and chronic stressors, such as the loss of independence, mobility, or the experience of chronic diseases. Loneliness is also a concern in older adulthood as many experience the loss of close others and smaller social networks. Loneliness is well-established as being associated with higher stress levels in younger adults, but there are mixed findings on the impact in older adulthood. Furthermore, while older adults may engage in behaviors designed to reduce and manage stress, it is not known whether these behaviors modulate the relationship between loneliness and chronic stress. The current study examined the relationship between loneliness and stress in older adulthood and the degree to which stress-management moderated this relationship (note: data collected prior to COVID-19 pandemic). We hypothesized that loneliness and perceived stress would be positively associated, but that stress-management would moderate this relationship. Participants included 40 healthy older adults (Mage= 69.18, Range:55-86yrs; 29 females) who completed the UCLA-Loneliness Scale, the Perceived Stress Scale, the NEO-FFI (to assess neuroticism), and demographic information about participation in stress management activities. We found that loneliness was significantly associated with chronic stress, $r(38) = .539$, $p < .001$. Furthermore, loneliness and neuroticism were significant predictors of perceived stress, $F(2, 37) = 10.657$, $p < .001$, $R2 = .366$. These findings demonstrate that loneliness is a significant predictor of perceived stress in older adulthood and point to a need for further exploration of effective stress-management tools in later life.

### ENHANCING GROUP BONDING IN AN ACTING CLASS OF OLDER ADULTS IN URBAN SUBSIDIZED HOUSING

Laura Sutherland,[1] Ruth Dunkle,[2] and Garrett Pace,[2], *1. Wayne State University, Ann Arbor, Michigan, United States, 2. University of Michigan, Ann Arbor, Michigan, United States*

Creative arts such as acting can promote social contact and bonding among socially isolated populations. Yet the benefits of art programming among older adults in low-income urban settings remain unexplored. A professionally administered theater group comprised of older adults living in urban low-income housing met for 12 weeks to learn acting skills and perform a play. The purpose of this study was to identify: 1) why participants enroll in a residence-based acting and improvisation course, and 2) what aspects of the course contribute to group bonding. Participants (n=14) were African American. The average age was 63 years, 14% were men, 57% had a high school degree or less, 79% reported good to excellent health, and the mean ADL score was 1.45 (range: 1-2.5). A researcher was present at each class session to observe and take field notes. Pre-post interviews included closed and open-ended questions. The researchers reviewed field notes and interview transcripts for a priori themes and emergent themes through independently coding data, discussing similarities and discrepancies, and coming to consensus on themes. Results showed that participants were motivated to enroll to meet new people, come out of themselves, learn something new, and gain artistic skills. Participants indicated the course contributed to group bonding via teaching techniques, course structure, the teacher's use of self, the expression of self, and mutually enhanced coping skills. Results from this study provide guidance for the design of theater groups in similar settings and inform recruitment efforts of older adults in creative arts programs.

### EVALUATING THE VALIDITY OF THE COLUMBIA-SUICIDE SEVERITY RATING SCALE FOR LONELY OLDER ADULTS

Jacqueline Mai,[1] Emily Bower,[1] and Kimberly Van Orden,[2], *1. Pacific University, Hillsboro, Oregon, United States, 2. University of Rochester Medical Center, University of Rochester Medical Center, New York, United States*

The risk of suicide death represents a significant problem facing older adults. They are less likely to disclose suicidal ideation (SI) and more likely to die from a suicide attempt compared to younger populations. Accurate screening tools for suicide risk are necessary to identify high-risk individuals who could benefit from intervention. The Columbia-Suicide Severity Rating Scale (C-SSRS), considered the gold standard for clinician-administered suicide risk assessments, was not developed for use with older adults. The purpose of this investigation is to evaluate the C-SSRS's sensitivity in capturing previous suicidal behavior (behavior subscale) and current intent (severity subscale), both of which are highly predictive of suicide in older adults. 105 adults 60 years and older (M=72.10, SD=9.16; 68.6% female) who endorsed loneliness or feeling like a burden in the past two weeks were enrolled in a larger controlled trial and completed baseline C-SSRS, Quick Inventory of Depressive Symptoms (QIDS), and Geriatric Suicide Ideation Scale (GSIS). Exclusion criteria included significant cognitive impairment. Concurrent validity will be evaluated using random-effects mixed linear regression to test associations between C-SSRS scores and GSIS and QIDS scores, respectively. Baseline responses indicate that 14.9% of participants reported at least one lifetime suicide attempt. Within the last month, 66.7% wished to be dead, 20% had active SI without a plan, 6.7% had active SI with some intent to act, and 6.7% had active SI with a specific plan and intent. Findings from this project will help guide safety assessment recommendations and inform interventions targeting older adult suicide risk.

### LONELINESS, SOCIAL ISOLATION AND ALL-CAUSE MORTALITY IN OLDER ADULTS

Timothy Barnes,[1] Rifky Tkatch,[1] Manik Ahuja,[1] Laurie Albright,[2] James Schaeffer,[1] and Charlotte Yeh,[3], *1. UnitedHealth Group, Minnetonka, Minnesota, United States, 2. UnitedHealth Group, Minneapolis, Minnesota, United States, 3. AARP Services, Inc., Washington, District of Columbia, United States*

As distinct constructs, loneliness and social isolation have both been associated with mortality in older adults. Many studies have examined each construct separately; however, few have examined their impact together, especially within the U.S. Using data from a large sample of U.S. adults age 65+ (N=7,982), the effect of loneliness and social isolation on all-cause mortality was examined considering their separate and joint effects. Measures were based on the UCLA-3 Loneliness Scale and the Social Network Index (SNI). Loneliness was categorized as: Severe, moderate, or no loneliness. Social isolation (defined by the SNI) was categorized as: Limited, medium, or diverse social networks (SN). Cox proportional hazards regression models were performed. Among participants, there were 328 deaths after data collection (4.1%). In separate, adjusted models, loneliness (severe, HR=1.86, 95% CI: 1.43-2.41 and moderate, HR=1.51, 95% CI: 1.16-1.98) and social isolation (limited SN, HR=2.37, 95% CI: 1.72-3.27 and moderate SN, HR=1.55, 95% CI: 1.12-2.14) were both associated with mortality. Modeled together, loneliness (severe, HR=1.55, 95% CI: 1.18-2.04 and moderate, HR=1.40, 95% CI: 1.07-1.83) and social isolation (limited SN, HR=2.08, 95% CI: 1.49-2.89 and moderate SN, HR=1.46, 95% CI: 1.05-2.02) both remained significantly associated with all-cause mortality with limited SN as the stronger indicator. Results demonstrate that both loneliness and social isolation contribute to greater risk of mortality among older adults. Furthermore, individuals with limited SN are at greatest risk. As the COVID-19 pandemic continues, loneliness and social isolation should be targeted safely in efforts to reduce mortality risk among older adults.

### LOW FRIEND CONNECTION LEADS TO LOW FUNCTIONAL CAPACITY OF OLDER ADULTS: FINDINGS FROM A 2-YEAR LONGITUDINAL STUDY

Mai Takase,[1] Kyo Takahashi,[1] Ryogo Ogino,[2] Tomoki Tanaka,[3] Jun Goto,[4] and Katsuya Iijima,[3], *1. The University of Tokyo, Bunkyo-ku, Tokyo, Tokyo, Japan, 2. Saga University, Saga, Saga, Japan, 3. The University of Tokyo, Bunkyo-ku, Tokyo, Japan, 4. Tokai University, Hiratsuka, Kanagawa, Japan*

Maintaining personal connections in geriatric years is important to sustain the functional capacity of older adults. In this study, we define the term functional capacity as the ability to perform tasks and activities necessary for one's independent life. Meanwhile, the type of personal connection and its effect on functional capacity remain unclear. We classified personal connections into connections with family or friends and examined their association with the functional capacity of independently living Japanese older adults. A two-year longitudinal study (2016 and 2018) was conducted using data from the Kashiwa Cohort Study. The amount of personal connections was determined using the Lubben Social Network Scale. The Japan Science and Technology Agency Index of Competence was used to assess participants' functional capacity. The change in functional capacity was analyzed by binomial logistic regression analysis (N=638, reference: group with high family and friend connection). The results showed a decline in the functional capacity of the group with low friend and family connections (odds ratio (OR): 0.48, 95%CI: 0.29–0.82). This trend was also seen for the group with a high family connection but low friend connection (OR: 0.43, 95%CI: 0.24–0.78). Meanwhile, there was no significant difference between the reference group and the group with low family but high friend connection (OR: 0.85, 95%CI: 0.47–1.56). The results imply the importance of having a friend for sustainable functional capacity in old age. Interventions that facilitate friend-to-friend interactions or promote new friendships among older adults can be considered as support strategies.

### NEED TO BELONG AND SOCIAL TIES IN LATE LIFE

Zexi Zhou, Yijung Kim, Shiyang Zhang, and
Karen Fingerman, *The University of Texas at Austin, Austin, Texas, United States*

According to socioemotional selectivity theory, older adults are more selective and tend to shrink their social network to their closest ties. However, a heightened need to belong, which is characterized by a stronger desire for acceptance and motivation to affiliation, may alter this common pattern. We know little about how the need to belong shapes social network structure, especially in late life. This study investigated the associations between the need to belong, size of social network, and engagement with social ties among older adults. Participants (N = 314) aged over 65 years from the Daily Experiences and Well-being Study completed a baseline interview regarding their close ties (i.e., social convoy members), and weak ties, as well as a self-report measure of need to belong. They completed ecological momentary assessment (EMA) surveys reporting their social encounters every 3 hours over 5 to 6 days. Need to belong was unrelated to the number of close ties. In contrast, participants with a higher need to belong reported more less close (but still important) ties and weak ties than those with a lower need to belong, but spent a similar amount of time (i.e., proportion of EMA involving social encounters) with either their close ties or weak ties. These results suggest that the need to belong may motivate older adults to go beyond their closest ties to weaker ties, and highlight the discrepancies between the sense of being connected to social partners and the actual engagement with them in this process.

### NURSING STUDENTS VISITING PEOPLE WITH DEMENTIA ONLINE DURING COVID-19: A QUALITATIVE STUDY

Michelle Kimzey,[1] Jodi Patterson,[2] and Beth Mastel-Smith,[3], *1. Harris College of Nursing & Health Sciences (Texas Christian University), Fort Worth, Texas, United States, 2. TCU, Fort Worth, Texas, United States, 3. University of Texas at Tyler, Tyler, Texas, United States*

The coronavirus disease 2019 (COVID-19) crisis has impacted the daily routines of students, people living with dementia, and their care partners. Social distancing results in fewer interpersonal interactions and enjoyable activities which makes life more challenging for those living with dementia. The purposes of this multiple case study were to understand how nursing students, people with dementia, and care partners (a) describe online visits between nursing students and people with dementia during stay-at-home directives in response to COVID-19 and (b) the perceived visit benefits. Nursing students participated in online visits to socially engage with their mentor (person living with dementia). During the visits it was anticipated that care partners would enjoy a brief respite. After 12 visits, investigators completed one-to-one online interviews with students (n = 10), care partners (n = 8) and mentors (n = 8). All cases reported a positive experience, perceptions of the conversations, improved social connection and meaning and purpose, mentor's enhanced cognition and planned future connections. Relationships were formed between students, people with dementia, and care partners during online visits, an activity that might be implemented outside of a crisis to prevent social isolation across generations. Future efforts to engage people with dementia in residential facilities should be formally integrated into the care plan and staff dedicated to help with technology assigned.

### OLDER ADULT'S MARITAL STATUS, SUPPORT EXCHANGES AND PSYCHOLOGICAL WELL-BEING IN EVERYDAY LIFE

Yee To Ng,[1] and Karen Fingerman,[2], *1. University of Texas at Austin, Austin, Texas, United States, 2. The University of Texas at Austin, Austin, Texas, United States*

Social support exchanges are an integral part of older adults' well-being. Yet, we know little about how older adults' marital status may influence their support exchanges with different social partners in everyday life, and whether the effect of support exchanges on daily well-being vary by marital status. Adults aged 65+ (N = 278) completed an initial interview about their background and close social networks; then, participants reported whether they provided or received support from their close social partners and rated their psychological well-being for 5 to 6 days. Multilevel logistic models revealed that married older adults were more likely to provide or receive daily support from their close partners than widowed or divorced older adults. However, with respect to specific non-spousal ties, married older adults were less likely to provide support to siblings, friends or others (acquaintances, neighbors) compared to divorced older adults. Although married older adults were more likely to receive support from children than divorced older adults, they were less likely to receive support from siblings and friends compared to widowed or divorced older adults. Furthermore, receiving support from other familial ties (grandchild, other relatives) was associated with reduced daily well-being for widowed older adults whereas married older adults were able to maintain their daily well-being in such situation. Findings highlight the central role siblings and friends play in unmarried older adults' daily support networks and suggest that receiving support could have differential impact on daily well-being depending on older adults' marital status.

### SENSORIMOTOR, COGNITIVE AND AFFECTIVE BARRIERS TO SOCIAL RELATIONS IN AGING: A DYNAMICAL FRAMEWORK

Emmanuelle Tognoli,[1] Alice Wead,[2] Joseph McKinley,[1] Christopher Beetle,[1] and Christine Williams,[1], *1. Florida Atlantic University, Boca Raton, Florida, United States, 2. Florida Atlantic University, BOCA RATON, Florida, United States*

Social interactions of all sorts (e.g. conversing, playing tennis, singing, strolling, etc.) rely on information flows between participants. The process of aging, however, can alter individuals' sensorial, motor, cognitive and affective functioning in ways that may compromise their affinity for social interactions. For instance, hearing deficits or cognitive difficulties associated with word retrieval may contribute to disengagement from conversation and other forms of social interaction, which can lead to social retreat of the affected individuals. Strategies for mitigating such effects must take into account not only individuals' own functional capacities, but also those of their partners in varying social contexts. Indeed, varied social contexts and diversity in partners can offer a beneficial balance of relational effort and comfort. For example, instead of comfortably strolling exclusively with partners of comparable cognitive and motor capabilities, strolling with faster partners can improve social engagement and long-term prospects for a wider range of social interactions. This work reviews an array of possible changes in individual abilities arising from both normal healthy aging and complications due to medical conditions, with an emphasis on their impact on interactions in varying social contexts and diverse groups of social partners. We incorporate theoretical models to explore a wide range of potential mitigation strategies, both for affected individuals and for other members of the social groups surrounding them. Our work focuses on healthy social aging over the long term, which is known to protect physical wellbeing, cognition and brain function.

### SOCIAL SUPPORT MODERATES THE IMPACT OF MARITAL TRANSITIONS ON DEPRESSION FOR OLDER ADULT WOMEN

Julia Tucker,[1] Nicholas Bishop,[2] Kaipeng Wang,[3] and Farya Phillips,[4], *1. Texas State University, New Braunfels, Texas, United States, 2. Texas State University, San Marcos, Texas, United States, 3. University of Denver, Denver, Colorado, United States, 4. The University of Texas at Austin, Austin, Texas, United States*

Given the rapid growth of older Americans and the increased incidence of divorce among this population, it is paramount to identify negative health outcomes following marital transition and investigate the potential protective role of social support. Our study aims to identify relationships between change in depression and marital transitions, test whether social support moderates this association, and to examine variation by gender. The sample included 3,705 participants from the Health and Retirement Study, who reported being married or partnered in 2012. Changes in marital status were measured between 2012 and 2014 (remained married/partnered (reference), divorced/separated, and widowed). Depression was measured using the Center for Epidemiological Studies Depression short form (CESD-8). Three types of social support from family, friends, and children were assessed: social support, social strain, and social contact. Autoregressive multiple regression was used to examine the relationship between change in depression, marital transitions, social support, and gender. Widowhood and social strain were independently associated with an increase in CESD-8 scores between 2012 and 2014. Significant interactions between social support and social strain, and separation/divorce were identified, and the relationship between social support, depression, and divorce varied by gender. Change in depression was positively associated with social support for separated/divorced females, but not separated/divorced males. These results indicate that social support may modify the influence of divorce on changes in depression among recently divorced older females. These findings can help mental health service providers more effectively target older adults at the greatest risk of depression after experiencing a marital transition.

### STRESS-BUFFERING FACTORS OF SOCIAL INTEGRATION ON DEPRESSIVE SYMPTOMS OVER TIME IN LATE-LIFE

Emily Kinkade,[1] and Heather Fuller,[2], *1. North Dakota State University, Fargo, North Dakota, United States, 2. North Dakota State university, Fargo, North Dakota, United States*

The negative impacts of stress on older adults' well-being are well documented, and social integration is posited as protective against such detrimental effects. Previous research illustrates the stress-buffering effect of social relationships on both physical and mental health, such as depressive symptoms, in older adults. The purpose of this study was to expand on prior findings by investigating the longitudinal stress-buffering effect of various dimensions of social integration on depressive symptoms among an older sample. Four waves of data were drawn from the Social Integration and Aging Study, including 416 older adults (ages 60-100). Subscales of the Social Integration in Later Life Scale measuring frequency and satisfaction with social ties and community interaction were used to assess distinct dimensions of social integration. Multilevel modeling demonstrated that two facets of social integration—satisfaction with social ties and frequency of community interaction—moderated the relationship between perceived stress and trajectories of depressive symptoms over time. Participants who reported high levels of stress reported fewer depressive symptoms if they had high satisfaction with social ties and high frequency of community involvement. Interestingly, frequency of contact with social ties and satisfaction with community interaction did not similarly buffer negative effects for depressive symptoms. These findings indicate the value of remaining actively engaged in the community and maintaining meaningful relationships as older adults age. Future research should investigate programs to foster relationships and engagement between older adults and their communities, with particular consideration of populations at a greater risk for isolation.

### THE EFFECT OF INTERPERSONAL RELATIONSHIP AND SOCIAL ACTIVITY ON THE PHYSICAL AND MENTAL HEALTH OF OLDER KOREAN ADULTS

Meeryoung Kim,[1] and Linda Park,[2], *1. Daegu University, Daegu, Kyongsang-bukto, Republic of Korea, 2. University of Wisconsin-Madison, Madison, Wisconsin, United States*

Maintaining interpersonal relationships and social activities are important as you get older. Activity theory indicates that social activities and human relations are important factors for older adults' physical and mental health. However, the effects between the quantity and quality of interpersonal relationships and social activities will be different. This study compared which of the effects has a greater impact between interpersonal and social activities on physical and mental health. This study used the 6th additional wave (2016) and 7th wave (2017) of the Korean Retirement and Income Study. The subjects of this study were older adults who are aged 65 and older and the sample size was 2,152. Multiple regression was used for data analysis. Demographic variables were controlled. Independent variables were interpersonal relationships, social activities, satisfaction with interpersonal relationships, and satisfaction with social activities. Dependent variables were physical health and mental health, with depressive symptoms used as a proxy for mental health. βs was used to determine the relative influence on dependent variables. Interpersonal relationships, satisfaction with interpersonal relationships, and satisfaction with social activities significantly influenced physical health. Among them, interpersonal satisfaction was found to be the most influential factor on physical health. In addition, interpersonal satisfaction was found to be the most influential factor on mental health than interpersonal relationships. Satisfaction with social activities only affected physical health. The implications of this study were that the quality of interpersonal relationships and social activities of older adults affected physical and mental health more than quantity.

### UNDERSTANDING TECHNOLOGY ANXIETY BY THE INTERACTION BETWEEN SOCIAL SUPPORT AND EDUCATIONAL CONTEXT

Susanna Joo,[1] Changmin Lee,[2] YoonMyung Kim,[2] Chang Oh Kim,[3] Yun Mook Lim,[2] and Hey Jung Jun,[2], *1. Yonsei University, Seodaemun-gu, Seoul-t'ukpyolsi, Republic of Korea, 2. Yonsei University, Seoul, Seoul-t'ukpyolsi, Republic of Korea, 3. Severance Hospital, Yonsei University College of Medicine, Seoul, Seoul-t'ukpyolsi, Republic of Korea*

The purpose of this study was to examine the interaction effects of social support from family and educational contexts on technology anxiety among Korean older adults. We collected data by online recruiting in February 2021, and the sample was Korean older adults without dementia (N=310; 65-89 years old). The dependent variable was technology anxiety, which meant the expected degree of worry under the assumption that a wearable robot for exercise was used. Independent variables were four types of social support (emotional, instrumental, physical, and financial support) provided by family members, such as spouse, children, or siblings. The moderating variable was the binary educational context (high school and under=0; college level and over=1). Interaction effects were estimated by bootstrapping and PROCESS macro with four regression models about each type of social support. Results showed the interaction effect between physical support and educational context was significant on technology anxiety. Concretely, getting more physical support was significantly associated with a lower level of technology anxiety for highly educated older adults, while it was not significant for less-educated older adults. There was no additional type of social support which had not only significant interaction effects with educational context but also main effects on technology anxiety. It suggested that providing direct physical help, including daily care or assistance, could decrease feeling technology anxiety, especially not for less-educated seniors but for highly educated Korean older adults.

### Session 9465 (Poster)

### SPIRITUALITY AND RELIGION

### CAN WORKING TOGETHER BUFFER DEPRESSION AMONG OLDER, RELIGIOUS AFRICAN AMERICAN COUPLES?

Antonius Skipper,[1] Andrew Rose,[2] Ethan Jones,[2] Alex Reeves,[1] and Jhazzmyn Joiner,[1], *1. Georgia State University, Atlanta, Georgia, United States, 2. Texas Tech University, Lubbock, Texas, United States*

Depression is a growing concern among older African Americans, as many within this group hesitate to seek professional help from psychiatrists or counselors. Instead, existing literature notes that older African Americans frequently utilize informal social support networks (e.g., church leaders) to respond to stress and buffer the negative effects of depression and depressive symptoms. Yet, little is known about the shared coping practices of older African American couples in relation to depression. Given the commonly noted high levels of religiosity among African Americans, this study examined communal coping as a mediator between sanctification and depression for older African American couples. This study utilized the dyadic data of 194 (146 married and 48 cohabiting) African American couples between the ages of 50 and 86 years. Capturing data with the Revised Sanctification of Marriage scale, the Communal Coping scale, and the Major Depression Inventory, bias-corrected bootstrap analysis revealed that men's relationship sanctification and women's depression was partially mediated by men's, as well as the sum of men's and women's, communal coping in married couples. Further, men's relationship sanctification and men's depression was partially mediated by men's, as well as the sum of men's and women's, communal coping. In addition, women's sanctification was positively associated with men's depression, directly. These findings are valuable in understanding the complex buffers, and contributors, to depression among older African American couples who may identify closely with religion but prefer the support of a partner over professional care.

### CROSS-LEVEL MEDIATING EFFECTS OF SOCIAL RELATIONSHIPS BETWEEN RELIGIOSITY AND SUCCESSFUL AGING

Hayoung Park,[1] Hey Jung Jun,[1] and Susanna Joo,[2], *1. Yonsei University, Seoul, Seoul-t'ukpyolsi, Republic of Korea, 2. Yonsei University, Seodaemun-gu, Seoul-t'ukpyolsi, Republic of Korea*

This study aimed to analyze the cross-level mediating effects of social relationships on the association between religiosity and successful aging. The data was the 7th Korean Longitudinal Study of Ageing and the sample was 1,191 couples aged 65 and above. Independent variables were the level of participation in religious activities at the individual level and religious similarity between couples at the couple level. The dependent variable was successful aging at the individual level, which consisted of physical, cognitive, social, and psychological dimensions. Mediating variables were two social relationships: frequency of social interaction at the individual level and marital satisfaction at the couple level. We applied Full Information Maximum Likelihood estimation to include 8% of the sample with missing values in data. According to the multi-level mediation path analysis, both frequency of social interaction and marital satisfaction had mediating effects on the association between the level of participation in religious activities and successful aging; the more they participate in religious activities, the higher the frequency of social interaction and marital satisfaction, and this had positive effects on successful aging. Also, marital satisfaction had a mediating effect on the association between religious similarity and successful aging; when married couples have the same religion, marital satisfaction was higher than when they do not, and this had a positive effect on successful aging. This study is meaningful in presenting multi-dimensional discussions on religiosity and social relationships in later life and a new empirical model to promote successful aging at both individual and couple levels.

### RELATIONAL SANCTITY AS A CONTRIBUTOR TO FORGIVENESS: DYADIC DATA FROM OLDER AFRICAN AMERICAN COUPLES

Antonius Skipper,[1] Andrew Rose,[2] Alex Reeves,[1] Jhazzmyn Joiner,[1] and Ethan Jones,[2], *1. Georgia State University, Atlanta, Georgia, United States, 2. Texas Tech University, Lubbock, Texas, United States*

Although research finds that healthy romantic relationships can provide several benefits in older adulthood, few studies examine the relational characteristics of older African American couples. Further, despite positive associations between religiosity and age, particularly among African Americans, a dearth of dyadic data consider the importance of religious constructs within the relationships of older African Americans. To address this gap, this study utilized dyadic data from the Strong African American Couples Project to examine the interconnection between relational sanctity and forgiveness among married and cohabiting older African American couples. A total of 194 African American couples (146 married and 48 cohabiting) aged 50 to 86 years were included in the analysis, and Actor Partner Independence Models were used to test the relational effects of sanctity and forgiveness. Findings revealed that no significant effects existed when women's relational sanctity was the predictor variable. However, men's relational sanctity had a significant positive association with both his own forgiveness of his partner and his perception of his partner's forgiveness. These findings have valuable implications for professionals engaging older African American couples. First, this study helps to counter the often deficit-focused literature on African American couples by highlighting the potentially stabilizing influence of viewing one's relationship as sacred. Second, this study offers a rare glimpse into the aspects of men's religiosity that may be more consequential than women's. Both practitioners and clergy could use this information to inform counseling efforts that seek to build on the strengths of married and cohabiting older African American couples.

### RELIGIOSITY AND PERCEIVED SOCIAL SUPPORT ENHANCE OLDER ADULTS' RESILIENCE AFTER THE 2016 FLOOD

Katie Cherry,[1] Matthew Calamia,[2] Emily Elliott,[2] and Angelina Cantelli,[2], *1. Louisiana State University, Louisiana State University/Baton Rouge, Louisiana, United States, 2. Louisiana State University, Baton Rouge, Louisiana, United States*

In 2016, catastrophic flooding destroyed homes and property across south Louisiana. This study is part of a larger program of research that addresses the role of prior hurricane and flood experiences on current health and well-being in later life. Participants were predominately middle-aged and older adults who varied in current and prior severe weather experiences (M age=49.6 years, age range 18-88 years). All were tested during the immediate aftermath of the 2016 flood (Wave 1; N=223) and most participated in a follow-up assessment 9 (+/- 3) months later (Wave 2; N=202). Cherry et al. (2021) reported that greater flood stressors at Wave 1, such as displacement, flood-related losses, and damage to homes and property, were associated with more symptoms of post-traumatic stress disorder (PTSD). In this study, we tested the hypothesis that age, religiosity, and perceived social support would be positively associated with post-flood resilience at the Wave 2 follow-up. Results indicated that age was positively associated with religiosity and resilience, and negatively correlated with symptoms of PTSD. Additionally, faith community involvement, non-organizational religiosity, and religious beliefs and practices were all significantly correlated with post-flood resilience. Perceived social support was positively associated with resilience, and inversely correlated with PTSD symptoms. These data suggest that religiosity and perceived social support are valuable resources that foster post-disaster resilience among middle aged and older adults. Implications of these data for current views on age-related strengths and vulnerabilities after severe weather events are discussed.

### THE IMPACT OF HEALTH LITERACY AND RELIGIOSITY ON ATTITUDES TOWARDS PHYSICIAN-ASSISTED DEATH

Amy Albright,[1] Joshua Tutek,[2] and Rebecca Allen,[3], *1. VA Maine HCS, Gorham, Maine, United States, 2. Stanford University, Stanford University School of Medicine, California, United States, 3. University of Alabama, Tuscaloosa, Alabama, United States*

The aim of the current study was to examine the relationship between functional health literacy and religiosity regarding attitudes towards physician-assisted death (PAD). Of participants, the majority were female (62.6%) and non-Hispanic White (79.6%), and ages ranged from 19 to 83 ($M = 37.81$, $SD = 12.55$). As measured by the Newest Vital Sign, 82.6% ($n = 219$) of individuals within the current sample had adequate functional health literacy, while 10.6% ($n = 28$) scored within the "possibly limited" range, and 6.8% ($n = 18$) scored within the "highly limited" range. There was a positive association between religiosity and age ($r = .21$, $p < .001$), and older participants were more likely to endorse religious beliefs and/or activities. There was a significant association between greater acceptance of attitudes towards PAD and functional health literacy ($r = .17$, $p < .01$), indicating that those with higher health literacy have more positive attitudes towards PAD. There was no significant association between attitudes towards PAD and age ($r = -.02$, $p > .05$) or education ($r = -.05$, $p > .05$). Similarly, attitudes did not differ by gender ($t(256) = -.66$, $p > .05$) or by race/

ethnicity, ($F(5, 253) = .73$, $p > .05$). Of note, functional health literacy may be particularly important to monitor in this context, as several studies (i.e., Kobayashi et al., 2015) have shown that health literacy may decrease with mild cognitive impairment and may therefore provide important information regarding older adults with this condition.

### TRANSFORMATIONS AND TRANSMISSION – A CONTEXTUAL LOOK AT RELIGIOUS BIOGRAPHIES OF OLDER ADULTS

Jenni Spannari,[1] Kati Tervo-Niemela,[2] and Laura Kallatsa,[2], *1. University of Eastern Finland, Helsinki, Pohjois-Karjala, Finland, 2. University of Eastern Finland, Joensuu, Pohjois-Karjala, Finland*

Examination of religious biographies tend to show increase of religiosity towards old age, most often in the context of a previously familiar religious community. These changes in individuals do not happen in a vacuum. Religious landscapes are also in transformation, characterized by a steady decline of institutional religiosity and religious practice in most European countries and more recently in the US and Canada, too. However, there is a dire lack of detailed knowledge on how these changes in individuals and societies are intertwined. This paper presents findings of the Finnish subproject of the five-country research project “Transmission of religion across generations.” The paper utilizes both three-generation interviews, and the contextual information gathered in the families about the past and present generations. The narratives about religious biographies of the oldest (gen.1) interviewees are discussed, and set in the context of the changes in the surrounding social sphere and the interviewees’ role in the family. Key findings include a general trend of increased flexibility, openness and communication over time – both in the religious views of the individuals, the roles different generations take in the family, and the cultural atmosphere in the society. Also, the results suggest that this flexibility is an essential factor in successful transmission of religion or other convictions across generations. The findings illustrate the complexity and contextuality of building and researching narratives of religious biographies. Thus, the results contribute to future examinations on how changes in societies and families affect the religious styles and convictions of older adults.

### Session 9470 (Poster)

### TECHNOLOGY, SOCIAL SUPPORT, AND CONNECTEDNESS

### COMMUNITY COLLABORATIONS & TECHNOLOGY SUCCESSFULLY CONNECT ISOLATED OLDER ADULTS TO EDUCATION & ACTIVITIES

Laura Spradley, *UAMS Arkansas Geriatric Education Collaborative (AGEC), Little Rock, Arkansas, United States*

Providing programs, activities and education to older adults (OA) is a challenge under normal circumstances. The Arkansas Geriatric Education Collaborative (AGEC) is a HRSA Geriatric Workforce Enhancement Program with a mission to “provide high quality programs that support healthy aging in Arkansas.” Prior to the pandemic, AGEC educators provided face-to-face programs to OA’s through senior centers, places of worship and other public venues. The pandemic changed all that. In-person programs were replaced with zoom presentations, social media events and pre-recorded programs placed on websites and patient-learn platforms for 24/7 viewing. Gaining viewership proved difficult and after collaborative research, it was determined the major barrier was a digital divide between access, usage and knowledge of digital platforms. To overcome this barrier, AGEC utilized TV, radio, libraries, digital infographics, newsletters and video tips addressing Wi-Fi and technology training. Videos, distributed via multiple routes, addressed basic topics such as “Creating and Utilizing Zoom and Facebook accounts” and “how to improve telehealth visits”. After establishment of a regular audience, AGEC engaged new and established partners and hosted a plethora of educational programs and activities further expanding the viewing audiences. In addition, with personalized emails and targeted marketing, AGEC engaged OA audiences in caregiving workshops, on-line caregiver support groups, telephone check-ins and exercise programs. Many OAs have found ways to bridge the digital divide and are engaged and active with educational and program activities and have used their new skills to connect with other OAs, grandkids, friends and even their spiritual communities.

### EFFECT OF THE SARS-COV-2 PANDEMIC ON TECHNOLOGY USE: IN-PERSON VERSUS VIDEO-CHAT COMMUNICATION

Sarah Hubner,[1] Alex Swanson,[2] Akankshya Chataut,[1] Stephen Kotopka,[3] Natalie Manley,[3] Marcia Shade,[3] and Julie Blaskewicz Boron,[1], *1. University of Nebraska Omaha, Omaha, Nebraska, United States, 2. University of Nebraska at Omaha, Omaha, Nebraska, United States, 3. University of Nebraska Medical Center, Omaha, Nebraska, United States*

COVID-19 risk-reduction efforts have protected high-risk individuals (including older adults) but have significantly altered life; persons now face reduced socialization. Advancing technologies (e.g., video-chat) may be useful in alleviating consequences of risk-reduction efforts, including loneliness, by improving access to alternative connection/communication across the lifespan. The purpose of this study was to investigate the relationships between technology use and individuals as this may contribute to well-being among older adults during COVID-19 and future isolating events. Participants (N=652) aged 19+ completed a questionnaire via Amazon Mechanical Turk; demographic, socialization, and technology-use data were collected. Respondents (MAge=45.15±15.81) were generally male (50.1%) and white (77.3%). In-person communication and video-chat were analyzed descriptively and with binary regressions. Results of a Wilcoxon Signed-Ranks Test indicated that video-chat (mean rank=228.45) was reported at higher frequency of use versus in-person conversations (mean-rank=202.48), $Z=-4.8, p<.001$). Additionally, being female positively predicted use of video chat ($B=0.42, p<.05$) while increasing age negatively predicted use (B=-0.01,p<.05). Regression results suggest that populations reporting higher video-chat communication (e.g., females, younger adults) may be motivated by maintaining social connectedness despite distancing and/or are committed to healthy behaviors, increasing aversion to in-person experiences. In contrast, it may be that persons reporting low video-chat use (e.g., males, older adults) may be less interested in distanced communication or may have lower technology comfort/access. Notably, sampling bias may influence results as data was collected online; future investigation is warranted. Ultimately, understanding interest in and barriers to using technology is vital to developing systems/services which support connection/communication when in-person contact is limited.

### INVESTIGATION OF COMMUNICATION TYPE AND INDIVIDUAL CHARACTERISTICS : A LONGITUDINAL HRS ANALYSIS

Kaleena Odd, Sarah Hubner, Julie Blaskewicz Boron, and Hyeon Jung Kim, *University of Nebraska Omaha, Omaha, Nebraska, United States*

Older adults are at increased risk for loneliness/isolation, particularly with new COVID-19 recommendations; however, communication may help mitigate these negative perceptions. Reductions in loneliness/isolation may also significantly improve quality of life and well-being for vulnerable populations. Thus, the purpose of this study was to investigate the relationships between communication, individual characteristics, and time, to provide a clearer understanding of communication patterns in a longitudinal cohort. Participants (N=2351) with no missing data on any variables of interest (across time-points) were pulled from the Health and Retirement Study's Consumption and Activity's Mail Survey (waves collected: 2013, 2015, 2017). When last reported (2016/17), respondents were an average age of 70.14(SD=9.9), were generally female (63.0%) and white (75.7%). Analyses included longitudinal investigation, normality tests, and regression. Assumptions were violated in ANOVA; results of a Kruskal-Wallis test revealed that there were no significant changes in the distribution of in-person or distanced communication across the three waves. Individual responses were then averaged and standardized across waves (per participant for each outcome variable). In-person communication regression results reveal that being female positively predicted in-person conversation volume (B=0.23,p<.001) as did increasing number of years in school (B=0.03,p<.001), while being non-white negatively predicted in-person conversation (B=-0.301,p<.001). Distanced communication regression results reveal being female positively predicted volume of distanced communication (B=0.381,p<.001); however, being non-white and younger positively predicted increased volume of distanced communication (B=0.241,p<.001; B=0.005,p<.001, respectively). Given the varied communication patterns, future research should consider explanatory mechanisms in addition to investigating changes as a result of the ongoing pandemic.

### SOCIAL MEDIA USE AND EMOTIONAL WELL-BEING: THE ROLE OF PHYSICAL ACTIVITY

Xin Yao Lin,[1] and Margie Lachman,[2], *1. Brandeis University, Waltham, Massachusetts, United States, 2. Brandeis University, Brandeis University, Massachusetts, United States*

There are mixed findings as to whether social media use (SMU) is positively or negatively related to well-being (positive/negative affect), and this relationship varies by age. The current study seeks to further explore this relationship by examining physical activity (PA) as a potential mediator at both a within (intraindividual) and between-person (interindividual) level across adulthood. The data are from the Midlife in the United States Refresher eight-day daily diary study (N=782, ages 25-75) with self-reported frequency of SMU, PA, and well-being (positive/negative affect). Multilevel structural equation modeling simultaneously tested how the relationships between the variables differed at both the between- and within-person levels. Between-person results showed that across the week, those who reported less SMU reported engaging in more PA, and more PA was associated with more positive affect. PA significantly mediated the relationship between SMU and positive affect for midlife and older adults, but not for younger adults. Effects for negative affect were not significant. Within-person results indicated that days with more PA were associated with more positive affect; however, PA did not mediate the relationship between SMU and positive or negative affect. These findings suggest the benefits of engaging in PA on one's positive emotional well-being at both the between- and within-person levels. However, for midlife and older adults, more SMU across the week may take away time from engaging in PA, which in turn lowers their positive affect. Implications of the effects of SMU on PA and well-being across adulthood are discussed.

### TECHNOLOGY AND SOCIAL ISOLATION, LONELINESS, AND HEALTH INEQUITIES AMONG OLDER ADULTS

Jeffrey Jutai, and Joshua Tuazon, *University of Ottawa, Ottawa, Ontario, Canada*

Because of the COVID-19 pandemic, older adults have been advised to stay-at-home to reduce the risk of infection. Social distancing and quarantine measures increase their vulnerability to adverse health outcomes like depression and cardiovascular disease. Technology is an effective tool to promote social connectedness among older adults affected by the pandemic; however, its role in reducing loneliness and health inequities is not well understood. The goal of this project was to construct a model for how technologies may be deployed to mitigate the impact of a pandemic on social isolation, loneliness, and health inequities for older adults. PubMed, SCOPUS, and PsychINFO were searched for the following keywords: "social isolation," "loneliness," "social support," "resilience," "technology," "pandemic," and "health inequities." Articles selected for full analysis attempted to understand or observe how technology alleviates social isolation and/or loneliness among older adults. Research evidence indicates that using technology reduces loneliness directly and indirectly (by reducing social isolation) and can strengthen social support, which in turn promotes resilience among older adults. Video-based technologies encourage care-seeking behaviors in this population. There is insufficient evidence to determine technology's relationship to health inequities experienced by older adults. The model we have proposed should help advance research on the relationship between technology and health inequities among older adults that may be aggravated by pandemic-like situations. We hypothesize that technology interventions for social support and functional competence should be sequenced in order to have the best effects on reducing health disparities.

### THE ASSOCIATION BETWEEN SOCIAL SUPPORT AND ATTITUDE TOWARD TECHNOLOGY DIFFERS BY EDUCATIONAL LEVEL

Susanna Joo,[1] Changmin Lee,[2] Kwang Joon Kim,[3] DaeEun Kim,[2] and Hey Jung Jun,[2], *1. Yonsei University, Seodaemun-gu, Seoul-t'ukpyolsi, Republic of Korea, 2. Yonsei University, Seoul, Seoul-t'ukpyolsi, Republic of Korea, 3. Severance Hospital, Yonsei University College of Medicine, Seoul, Seoul-t'ukpyolsi, Republic of Korea*

The present study aimed to examine the moderating effects of the educational context on the association between social support from family members and attitude toward using gerontechnology among Korean older adults. The sample was Korean older adults without dementia (N=310, Age: 65-89, M=70.18, SD=4.58). Data were collected by online recruiting in February 2021. The dependent variable was the attitude toward using gerontechnology, especially, an exoskeleton robot for exercise. Independent variables were four types of social support (emotional, instrumental, physical, and financial support) from family members. Moderating variable was the binary educational group (high school and below, or college and over). We analyzed four regression models including each interaction term between education and a type of social support using PROCESS macro and bootstrapping. Results showed educational context had a single significant moderating effect on the association between emotional support and attitude toward using gerontechnology. Specifically, emotional support had a significant effect on having a positive attitude toward using gerontechnology among older adults who graduated high school or were less educated. However, it was not significant among older adults who were highly educated. Moreover, other types of social support did not have significant main effects as well as interaction effects with education on the attitude toward using gerontechnology. Findings of the present study implied emotional support from family members, such as spouse, children, or siblings, was useful to enhance having a positive attitude toward using new technology, especially for older adults who did not experience college-level educational context.

### THE ASSOCIATION BETWEEN SPOUSAL SUPPORT AND ATTITUDE FOR GERONTECHNOLOGY VIA FACILITATING CONDITIONS

Seong Hee Kim,[1] Susanna Joo,[2] Changmin Lee,[3] Kwang Joon Kim,[4] DaeEun Kim,[3] YoonMyung Kim,[3] and Hey Jung Jun,[3], *1. Sungkyunkwan University, Suwon-si, Kyonggi-do, Republic of Korea, 2. Yonsei University, Seodaemun-gu, Seoul-t'ukpyolsi, Republic of Korea, 3. Yonsei University, Seoul, Seoul-t'ukpyolsi, Republic of Korea, 4. Severance Hospital, Yonsei University College of Medicine, Seoul, Seoul-t'ukpyolsi, Republic of Korea*

This study examined the mediating effects of facilitating conditions (FC) on the association between types of social support providers and attitude toward using gerontechnology (AUG) or between types of social support and AUG. The sample was 256 older Koreans having a partner and children (N=256; 66-88 years old; M=69.91; SD=4.19). The dependent variable was AUG in terms of an exoskeleton robot for exercise. There were two kinds of independent variables: 1) four types of social support provider (spouse, children, siblings/relatives, and friends/neighbors), and 2) four types of social support (emotional, instrumental, physical, and financial support). Mediating variable was calculated as the mean of FC from five questions. There were two analytic steps: 1) structural equation modeling with four latent variables about types of social support provider, and 2) path analysis with four types of social support if only for significant providers at the first analysis. The results from the first step of analysis showed that only social support from spouses had a significant effect on AUG via FC to use gerontechnology. In the second step of analysis, emotional support from spouses was associated with the higher level of AUG via FC to use gerontechnology. The findings could shed light on the salience of emotional support from spouses in terms of the positive attitude on technology usage in later life through enhancing FC for technology acceptance.

### THE ASSOCIATION BETWEEN THE TYPES OF EXERCISE MOTIVATION AND SOCIAL PRESENCE ABOUT GERONTECHNOLOGY

Si Young Song,[1] Inhye Jung,[1] Miseon Kang,[1] Kwang Joon Kim,[2] DaeEun Kim,[1] and Hey Jung Jun,[1], *1. Yonsei University, Seoul, Seoul-t'ukpyolsi, Republic of Korea, 2. Severance Hospital, Yonsei University College of Medicine, Seoul, Seoul-t'ukpyolsi, Republic of Korea*

The purpose of this study was to identify the types of exercise motivation and examine the association between the types of exercise motivation and social presence about exercise-related gerontechnology among Korean young-olds. In this study, social presence about gerontechnology implies the degree of perception of a robot that helps exercise functions as human-like socially interacting entities (Heerink, 2010). Online survey data collected from the Korean older adults over the age of 65 in February 2021 was used, and the subjects of this study were 154 young-olds aged 65 to 74 who exercise regularly. Latent class analysis (LCA) was conducted to classify the types of exercise motivation, followed by multiple regression analysis. As a result of LCA, the types of exercise motivation was classified with two groups. These groups were named 'for pleasure and leisure (PL, 77.2%)' and 'for maintenance of health (MH, 22.8%)', respectively. The result of multiple regression showed that compared to the second group (MH), the social presence about gerontechnology was high for the first group (PL) after controlling age, gender, education level, marital status, household income and chronic disease. These results indicate the Korean young-olds' exercise motivation may vary and expectations for social presence toward exercise-related gerontechnology differ depending on the exercise motivation. To date, the importance of social presence in gerontechnology has tended to be emphasized mainly in the care field. This study suggests that exercise-related gerontechnology devices also need to consider the aspect of social presence especially for young-olds who exercise for pleasure and leisure.

THE EFFECTIVENESS OF AN INTERGENERATIONAL TECHNOLOGY PROGRAM FOR OLDER ADULTS: A PILOT STUDY

Dolapo Adeniji,[1] Margaret Adamek,[2] and Sally Catlin,[3], *1. IUPUI, Avon, Indiana, United States, 2. Indiana University, INDIANAPOLIS, Indiana, United States, 3. Indiana University IUPUI, Indianpolis, Indiana, United States*

While an increasing number of services and opportunities are available through technology devices such as smartphones and iPad, older adults often lack the technology skills and know-how to access such services. The use of social media, email, and texting can also lessen social isolation of older adults. In this project, nine undergraduate Computer Information Technology students enrolled in a service-learning course served as mentors for older adults. A total of 33 older adults (MAge= 77.9 SDAge= 8.62) participated in a 14 weeks intergenerational technology education at two community senior centers in an urban Midwest city. Fourteen participants completed both pre- and post-surveys. Significant improvement was found between pre- and post- surveys outcomes in technology anxiety and social engagement of the older adults. Findings from our qualitative data revealed that intergenerational program enables older adult to benefit from individual and group learning, make new friends among peers, experience intergenerational interactions, and have confidence in technology use. However, the intergenerational technology program helped to decrease technology anxiety and improve the participants' social engagement. Engaging technology students in mentoring older adults in small group at a community center proved to be mutually beneficial to both the students and the older adults. The program boosted older adults' comfort with technology use as well as encouraging social engagement with peers, mentors, and the virtual world.

UNDERSTANDING RACIAL AND RURAL DISPARITIES IN THE RELATIONSHIP BETWEEN SOCIAL ISOLATION AND SOCIAL TECHNOLOGY USE

Kaileigh Byrne, Reza Ghaiumy Anaraky, Hannah Barfield, and Summerlin Nickel, *Clemson University, Clemson, South Carolina, United States*

Social isolation is characterized by lack of social contacts and high degrees of loneliness. Feelings of loneliness and social isolation are linked to declines in cognitive functioning and increased risk of dementia. Previous research suggests that loneliness is more prevalent among Black and rural older adults compared to White and urban-dwelling older adults. Given these disparities, it is important to identify methods that reduce social isolation and loneliness among this population. Social technology, such as Facebook and Skype, is one possible way to connect with others. This study uses the Health and Retirement Study (HRS) dataset to examine racial and rural disparities in the relationship between social technology use and social isolation, loneliness, and social support among individuals age 50 and older. The overarching hypotheses are that (1) rural-dwelling older adults and older Blacks will report less social technology use compared to urban-dwelling and older White adults, and (2) there will be a negative relationship between loneliness and social technology use, and (3) a positive relationship between perceived positive social support and social technology use. Racial or rural disparities in these latter potential relationships are exploratory. Multiple linear regression analysis will be performed to assess these relationships. Preliminary correlational results indicate that, consistent with prior work, greater use of social technology was associated with higher social support (N=6,029; r=.29, p<.001). However, contrary to our hypothesis, greater self-reported loneliness was associated with greater social technology (r=.09, p<.001). Examination of potential racial and rural disparities in these relationships are currently underway.

### Session 9500 (Late Breaking Poster)

LATE BREAKING POSTER SESSION I

A DIGITAL INTERVENTION TO ALLEVIATE LONELINESS AND DEPRESSION AMONG OLDER PERSONS DURING THE COVID-19 OUTBREAK

Stav Shapira,[1] Ella Cohn-Schwartz,[2] Daphna Yeshua-Katz,[3] Limor Aharonson-Daniel,[4] A. Mark Clarfield,[3] and Orly Sarid,[3], *1. Ben-Gurion University of the Negev, Ben-Gurion University of the Negev, HaDarom, Israel, 2. Ben-Gurion University, Beer-Sheva, HaDarom, Israel, 3. Ben-Gurion University of the Negev, Beer Sheva, HaDarom, Israel, 4. Ben-Gurion University of the Negev, Beer Shave, HaDarom, Israel*

Social distancing has been proven to be effective in reducing infections but may cause ill effects on the mental health of older adults. We evaluated the effects of a short-term virtual group intervention that provided tools to promote better coping, and mitigate adverse mental health effects during the outbreak of the covid-19 pandemic. A Randomized controlled trial tested the effects of a guided intervention comprised of seven online group sessions in which cognitive-behavioral techniques targeting maladaptive beliefs and appraisals were learned and practiced via ZOOM. A total of 82 community-dwelling adults from Israel, aged between 65 - 90 were randomized to either an intervention group (n=64) or a wait-list control group (n=18). Loneliness (UCLA loneliness scale) and depressive symptoms (PHQ-9) were measured pre-intervention, post-intervention, and at 1-month follow-up. The findings showed a significant decrease in loneliness and depression scores in the intervention group with results maintained at 1-month follow-up. There were no significant changes in the wait-list control group. In addition, ten participants (16%) from the intervention group demonstrated a clinically meaningful decrease in depression between baseline and post-intervention, and this was maintained among 7 participants (10%) at 1-month follow-up, compared to only 1 participant (5%) in the control group. Our intervention presents a simple and easy-to-implement tool. Its relevance extends beyond the current pandemic as the skills acquired can be applied in other forms of social crises and during routine life, in order to promote the mental health of older adults who live alone and/or reside in remote areas.

### A FAT-PROMOTING PLANT EXTRACT FROM ARTEMISIA SCOPARIA EXERTS GEROPROTECTIVE EFFECTS ON C. ELEGANS HEALTH & LIFESPAN

Bhaswati Ghosh, Hayden Guidry, Maxwell Johnston, and Adam Bohnert, *Louisiana State University, BATON ROUGE, Louisiana, United States*

Like other biological processes, aging is not random, but subject to molecular control. Natural products that act on conserved metabolic pathways may provide entry points to extend animal lifespan and promote healthy aging. Here, we show that a botanical extract from Artemisia scoparia (SCO), which promotes fat storage and metabolic resiliency in mice, exerts pro-longevity effects on the nematode Caenorhabditis elegans, even when administered in mid-adulthood. SCO-treated worms exhibit significantly higher levels of fat compared to controls but live up to 40% longer, with signs of improved stress resistance in late age. Molecularly, SCO links elevated fat to enhanced longevity and stress resistance via activation of the transcription factor DAF-16/FOXO and upregulation of DAF-16-targeted Δ9 desaturases, lifespan-extending metabolic enzymes that oversee the biosynthesis of monounsaturated fatty acids. These findings identify SCO as a natural product that can modify fat regulation for longevity benefit and add to growing evidence indicating that elevated fat can be pro-longevity in some circumstances.

### A META-ANALYSIS OF THE EFFECTS OF TAILOR ACTIVITY PROGRAM (TAP) FOR PEOPLE WITH DEMENTIA

Jiin Jeong,[1] Eun-Young Yoo,[2] Byoung-Ho Kang,[1] and Yae-Na Ha,[1], *1. Graduate school, Yonsei University, Republic of Korea, Kangwon-do, Republic of Korea, 2. College of Software and Digital Healthcare Convergence, Yonsei University, Republic of Korea, Kangwon-do, Republic of Korea*

Ninety eight percent of people with dementia are accompanied by neuropsychiatric symptoms (NPS). NPS is an important predictor of the negative prognosis of dementia. It also increases the burden on caregivers and lowers the quality of life. The tailored activity program (TAP), which is occupation-based intervention, have a positive effect on reducing NPS through meaningful activities. The aim of this study was to provide an integrated effectiveness of the TAP on NPS in people with dementia and caregiver burden through meta-analysis. We searched for studies that indicated the effectiveness of TAP through Embase, ProQuest, Pubmed, and RISS. We included a total of seven TAP studies written in Korean and English. Of these seven study designs, five were randomized control trials (RCTs) and two were one group non-RCTs. The result of meta-analysis shows that the effect size of the NPS was 0.62 (95% confidence interval [CI]=0.40-0.83, $p<0.001$), the caregiver burden was 0.68 (95% CI=0.29-1.07, $p=0.001$). Both variables indicated moderate effect. These results indicate that the TAP is an effective intervention for reducing NPS of people with dementia and the burden of caregivers. Therefore, TAP is clinically useful approach, we expect TAP to be actively applied to people with dementia in the community.

### ACTION PLANNING CHECKLIST FOR SOCIAL DETERMINANTS OF HEALTH: OLDER ADULTS WITH CHRONIC CONDITIONS

Joan Ilardo, and Angela Zell, *Michigan State University College of Human Medicine, East Lansing, Michigan, United States*

Medical residents need training to assess social determinants of health (SDOH) related to chronic conditions. We created a checklist to identify SDOH affecting residency clinic patients' ability to manage chronic conditions. The tool: 1) involves resident training; 2) provides decision support checklist; 3) influences patient activation; and 4) increases provider and patient communication through shared decision making. Action Planning Guide checklist (APG) includes questions pertaining to SDOH preventing patients from managing their chronic conditions and actions patients will take. Areas identified are discussed between patient and resident, increasing patient activation. The clinic's nurse care facilitator guides referrals to community-based resources. Fifty-two patients were enrolled, with 75% of patients responding they would like to be better managers of their chronic conditions. This information is used to develop patient's goals of care. Over 90% of patients said their conditions affect their lives and discussed ways better to care for themselves. Over 80% discussed medication management, health goals to improve their quality of life, and made a plan that maps out ways to reach their goals. All of these are essential for achieving positive health outcomes for older patients with chronic conditions. These attributes promote effective patient/provider partnerships. Seventy referrals were made; food through 2-1-1 (47%); monthly commodity food program (30%); utility payments (11%), and transportation (9%). Twenty-seven referrals were made to agencies serving older adults; 25 to the local AAA information and assistance services, and 2 to Senior Project Fresh Voucher Program.

### ADDRESSING PHYSICAL, FUNCTIONAL, AND PHYSIOLOGICAL OUTCOMES IN OLDER ADULTS VIA INTEGRATED MHEALTH INTERVENTION

Melba Hernandez-Tejada,[1] Sundaravadivel Balasubramanian,[2] John Bian,[2] Mohan Madisetti,[2] Alexis Nagel,[2] Samantha Bernstein,[2] and Teresa Kelechi,[2], *1. University of Texas HSC at Houston, Houston, Texas, United States, 2. Medical University of South Carolina, Charleston, South Carolina, United States*

**Objective:** We evaluated components of an integrated mobile (m)Health-based intervention "Activate for Life" (AFL) on health outcomes in lower-income older adults (65 years and older). Method: AFL incorporates balance (Otago; OG), physical strength (Gentle Yoga and Yogic Breathing; GYYB), and mental engagement (Behavioral Activation; BA) components. Thirty participants were randomly allocated to one of three Arms (n=10 per each arm): OG (Arm 1), (OG+GYYB (Arm2), or OG+GYYB+BA (Arm 3, or full AFL). Groups were evaluated for physical, functional and physiological endpoints at baseline, and posttreatment (12-weeks and/or 3-month follow up).

**Results:** Improvements over time in pain interference and 1,5 Ag biomarker were noted for all groups. No significant changes were observed in other physical, functional and physiological measures. DiscussionThis study illustrated potential benefits of the AFL intervention on the health of lower-income older adults and lessons learned from this pilot will be used to make improvements for a large-scale randomized controlled trial.

### ADVANCE CARE PLANNING AND HOSPICE USE AMONG PEOPLE WITH DEMENTIA: A REPORT FROM THE HEALTH AND RETIREMENT SURVEY

Kathryn Coccia, *Saint Louis University, St. Louis, Missouri, United States*

People with Alzheimer's disease and related dementias (ADRD) frequently receive sub-optimal end-of-life care (EOLC), often enduring invasive procedures such as tube feeding, resuscitation, and surgery within days of their death. While advance care planning (ACP) has shown effectiveness in improving EOLC for those with ADRD, there are many barriers to ACP specific to the ADRD population. Research suggests that hospice care is optimal in reducing end of life suffering for ADRD patients. This study aimed to empirically assess hospice utilization and ACP for individuals with ADRD compared to individuals without ADRD, and to assess the impact of ACP on hospice utilization for individuals with ADRD. Data came from the 2016-2018 wave of the Health and Retirement Study (HRS), a national longitudinal study collecting health and demographic data on older Americans. This analysis evaluated survey responses from 1,224 proxy respondents for individuals who died during this period. In this sample, people with ADRD were both significantly more likely to have utilized hospice care (OR=1.37) and to have written EOLC instructions in place (OR=1.19). Those with ADRD were 22% less likely to have discussed their EOLC wishes with their proxy than those without ADRD. Having a written EOLC plan in place significantly increased the odds of hospice utilization (OR=1.37) but discussion around EOLC preferences increased odds of hospice utilization at a higher rate (OR=1.59). These results support policy to advance earlier ACP conversations around EOLC preferences and the implementation of written EOLC instructions to reduce suffering for individuals with ADRD diagnoses .

### AN ENVIRONMENTAL CONTRIBUTOR TO PARKINSON'S DISEASE CAUSES A HORMETIC LIFESPAN EFFECT IN C. ELEGANS

Jennifer Thies, Guy A Caldwell, Kim A. Caldwell, and Hanna Kim, *The University of Alabama, Tuscaloosa, Alabama, United States*

Only 5-10% of Parkinson's Disease (PD) cases have a direct genetic origin; however, exposure to herbicides, pesticides, and interactions with soil are potential risk factors. PD is characterized by the loss of dopaminergic (DA) neurons and the formation of protein inclusions that contain α-synuclein (α-syn). Conversely, a soil bacterium, Streptomyces venezuelae (S. ven), produces a secondary metabolite that causes age- and dose- dependent DA neurodegeneration in C. elegans; it also exacerbates α-syn-induced DA neurodegeneration. Previous studies from our lab determined that exposure to the S. ven metabolite caused oxidative stress, mitochondrial fragmentation and enhanced reactive oxygen species (ROS). Here we report that exposure to S. ven metabolite causes a hormetic effect on C. elegans lifespan, where low concentrations (5X) extend lifespan in N2 animals, but at higher concentrations (20X) lifespan is decreased. To further examine this hormetic response, we examined daf-16 mutants in this assay. daf-16 mutants displayed no significant differences between solvent and metabolite at both high and low concentrations, suggesting the hormetic response is daf-16 dependent. We also studied S. ven metabolite on C. elegans aging mutants. We investigated mutants in the AMPK signaling pathway and found when exposed to the 20X concentration of S. ven metabolite, aak-2 mutants displayed no significant difference between solvent and metabolite over lifespan. However, when aak-2 mutants were exposed to solvent control and the 5X concentration, mutants displayed a decreased lifespan. This suggests that functional aak-2 might be important for increased lifespan when combating toxicants following chronic exposure.

### ARE LONELINESS AND SOCIAL ISOLATION EQUAL THREATS TO HEALTH AND WELL-BEING? AN OUTCOME WIDE LONGITUDINAL APPROACH

Tatiana Henriksson,[1] Julia Nakamura,[2] and Eric Kim,[1], *1. University of British Columbia, Vancouver, British Columbia, Canada, 2. University of British Columbia, University of British Columbia/Vancouver, British Columbia, Canada*

The detrimental effects of loneliness and social isolation on health and well-being outcomes are well documented. In response, governments, corporations, and community-based organizations have begun leveraging emerging tools to create interventions and policies aimed at reducing loneliness and social isolation at-scale. However, these efforts are frequently hampered by a key knowledge gap: when attempting to alleviate specific health and well-being outcomes, decision-makers are unsure whether to target loneliness, social isolation, or both. Participants (N=13,752) were from the Health and Retirement Study- a diverse nationally representative, and longitudinal sample of U.S. adults aged > 50 years. We examined how changes in loneliness and social isolation over a 4-year follow-up period (from t0:2008/2010 to t1:2012/2014) were associated with 32 indicators of physical-, behavioral-, and psychosocial-health outcomes 4-years later (t2:2016/2018). We used, multiple logistic-, linear-, and generalized-linear regression models, and adjusted for sociodemographics, personality traits, pre-baseline levels of both exposures (loneliness and social isolation), and all outcomes (t0:2008/2010). After adjusting for a wide range of covariates, we observed that both loneliness and social isolation have similar effects on physical health outcomes and health behaviors, whereas loneliness is a stronger predictor of psychological outcomes. In particular, behavioral dimensions of the social isolation measure (i.e., participation in social/religious activities, social interaction frequency) were most strongly associated with the largest number of health and well-being outcomes, including all-cause mortality. Loneliness and social isolation have independent effects on various health and well-being outcomes, thus, should be distinct targets for interventions aimed at improving the health and well-being.

### ARE SUBACUTE CARE PATIENTS LIVING LONGER?

Nidhi Kejriwal,[1] Samantha Tello,[2] Brooke Davis,[3] Mira Kubba,[4] David Evans,[3] Norma Gonzales,[5] and J. Robert Evans,[6], *1. University of California Los Angeles, Redlands, California, United States, 2. Western University of Health Sciences, Western University of Health Sciences, California, United States, 3. Brigham Young University, Brigham Young University, Utah, United States, 4. University of San Diego, University of San Deigo, California, United States, 5. Community Hospital of San Bernardino, Community Hospital of San Bernardino, California, United States, 6. Community Hospital of San Bernardino, evansgi, California, United States*

In order to provide prognostic information for gerontologists who regularly counsel families, we determined to measure the longevity of subacute patients who have feeding tubes and tracheostomies. This study compares two cohorts of patients: 2002-2006 and 2015-2019. T-tests were performed to compare the total days in acute care, the total survival days, and the number of hospital admissions between the two groups. Results revealed (2002-2006, 2015-2019), some variance in the acute care days between the two groups (M= 15.4186, 21.49438) and p= .66. There is a wide difference in the total survival days between the two groups with individuals from 2015-2019 living longer than 2002-2006 (M= 229.8198, 644.0449), p< .001. However, there is no statistically significant difference in the number of hospital admissions between the two groups (M= 0.994186, 0.7752809), p= .09754. We hypothesize that advances in technology, medicine, and care over the span of 17 years contribute to increased longevity. On average, patients in the 2015-2019 group survived 414 days longer than the first group. Yet, even with such advances, more days were spent in acute care in the second group (2015-2019). Our data show subacute longevity has nearly tripled in the last decade. Although patients are living longer, they are often in a vegetative state; in most instances, there is no apparent improvement in quality of life. This study provides current data which will help gerontologists improve prognostication and allow them to form a more realistic long view of care.

### ASSOCIATION BETWEEN ANTICHOLINERGIC BURDEN AND DEMENTIA IN UK BIOBANK

Jure Mur, Simon Cox, Riccardo Marioni, Tom Russ, and Graciela Muniz-Terrera, *University of Edinburgh, Edinburgh, Scotland, United Kingdom*

Previous studies on the association between the long-term use of anticholinergic drugs and dementia report heterogenous results. This variability could be due to, among other factors, different anticholinergic scales used, and differential effects of distinct classes of anticholinergic drugs. Here, we use 171,775 participants of UK Biobank with linked GP prescription records to calculate the cumulative annual anticholinergic burden (ACB) and ascertain dementia diagnoses through GP- and inpatient records. We then use Cox proportional hazards models to compare 13 anticholinergic scales and anticholinergic burden (ACB) due to different classes of drugs in their association with dementia. We find dementia to be more strongly predicted by ACB than by polypharmacy across most anticholinergic scales (standardised ORs range: 1.027-1.125). Furthermore, not only the baseline ACB, but the slope of the longitudinal trajectory of ACB (HR=1.094; 95% CI: 1.068-1.119) is predictive of dementia. However, the association between ACB and dementia holds only for some classes of drugs – especially antidepressants, antiepileptics, and high-ceiling antidiuretics. Moreover, we do not find a clear relationship between reported anticholinergic potency and dementia risk. The heterogeneity in findings on the association between ACB and dementia may in part be due to different effects for different classes of drugs. Future studies should establish such differences in more detail and further examine the practicality of using a general measure of anticholinergic potency as it relates to the risk of dementia.

### ASSOCIATION OF SHOULDER DYSFUNCTION WITH MOBILITY LIMITATION IN OLDER ADULTS OF THE BLSA

Derik Davis,[1] Kai Sun,[2] Laurence Magder,[2] and Eleanor Simonsick,[3], *1. University of Maryland School of Medicine, Ellicott City, Maryland, United States, 2. University of Maryland School of Medicine, Baltimore, Maryland, United States, 3. National Instute on Aging/NIH, Baltimore, Maryland, United States*

Mobility limitation affects one-third of older adults; yet, the impact of shoulder dysfunction which effects roughly 20%, is inadequately documented. As arm swing is a fundamental component of ambulation, we investigated the cross-sectional association between shoulder range of motion (ROM) and walking endurance using time to walk 400m as quickly as possible and lower extremity performance using the expanded Short Physical Performance Battery (e-SPPB). Data are from 614 men (50.5%) and women aged ≥ 60 years (mean 71.8 ±8 years) in the Baltimore Longitudinal Study of Aging (BLSA) who performed bilateral shoulder elevation and/or bilateral shoulder external rotation (ER) during nurse-administered physical examination. We examined odds of poor 400m-walk and e-SPPB performance defined as the worst quartile associated with abnormal shoulder elevation (≤9%) relative to bilateral normal shoulder elevation and abnormal shoulder external rotation (≤5%) relative to bilateral normal shoulder external rotation (ER) in separate analyses. Analyses were adjusted for age, sex, weight and height. Adjusted odds (95% confidence interval) of poor 400m-walk performance associated with abnormal shoulder elevation (N=254) were 4.7 (1.1-19.5;p=0.035) and with abnormal shoulder ER (n=401) were 4.8 (1.4-16.7;p=0.010). Adjusted odds of poor e-SPPB performance associated with abnormal shoulder elevation (N=462) were 3.5 (1.6-7.7;p=0.002) and with abnormal shoulder ER (n=457) were 2.9 (1.1-7.4;p=0.030). Results suggest abnormal shoulder ROM is associated with poorer walking endurance capacity and lower-extremity functional performance in the relatively healthy older adults. Future research is warranted to develop novel screening paradigms that mitigate mobility limitation and functional decline in older adults with shoulder dysfunction.

### BRAIN CELLULAR SENESCENCE IN MOUSE MODELS OF ALZHEIMER'S DISEASE

Ruben Riordan, *Linus Pauling Institute, Corvallis, Oregon, United States*

The accumulation of senescent cells contributes to aging pathologies, including neurodegenerative diseases, and its selective removal improves physiological and cognitive function in wild type mice as well as in Alzheimer's disease (AD) models. AD models recapitulate some, but not all components of disease and do so at different rates. Whether brain cellular senescence is recapitulated in some or all AD models, and whether the emergence of cellular senescence in AD mouse models occurs before or after the expected onset of AD-like cognitive deficits in these models is not yet known. The goal of this study was to identify mouse models of AD and AD-related dementias that develop measurable markers of cellular senescence in brain and thus may be useful to study the role of cellular senescence in these conditions. We measured levels of cellular senescence markers in brains of P301S(PS19), P301L, hTau, and 3xTg-AD mice that model amyloidopathy and/or tauopathy in AD and related dementias, and in wild type, age-matched control mice for each strain. Expression of cellular senescence markers in brains of transgenic P301L and 3xTg-AD mice was largely indistinguishable from that in WT control age-matched mice. In contrast, markers of cellular senescence were significantly increased in brains of transgenic P301S and hTau mice as compared to WT control mice at the expected time of onset of AD-like cognitive deficits. Taken together, our data suggest that P301S(PS19) and hTau mice may be useful for the study of brain cellular senescence in tauopathies including, but not limited to, AD.

### CARE EXPERIENCES AND EXPECTATIONS OF OLDER SEXUAL MINORITY ADULTS

Mekiayla Singleton, and Susan Enguidanos, *University of Southern California, Los Angeles, California, United States*

Sexual minority (SM) adults have unique care needs and experiences, partially because they receive and give care by and to "chosen family". This study examines the care experiences and expectations of diverse SM adults. Using data from the 2018 AARP Survey "Maintaining Dignity: Understanding and Responding to the Challenges Facing Older LGBT Americans," logistic and ordinal regressions were conducted to examine associations with care experiences (i.e., provided caregiving and received caregiving) and care expectations (i.e., likelihood of having to provide care and need care) among SM respondents. Gender was highly associated with care experiences, with female respondents being 70% and 74% more likely to have provided caregiving [OR:1.71, SE=.26; $p<0.001$] and received caregiving [OR:1.74, SE=.22; $p< 0.001$]. Relationship status was significantly associated with care expectations, with those who were married/civil union/domestic being 4 times [OR:4.0, SE=.52; $p<0.001$] and those in a relationship being 3 times [OR:3.3, SE=.51; $p<0.001$] more likely to expect that they will provide care in the future. Those same respondents had a 64% [OR:1.64, SE=.21, $p<0.001$] and 55% [OR:1.55, SE=.23, $p<0.01$] greater odds of reporting being "very likely" that they will need care in the future. Additionally, older age, being a racial minority, having higher education, and being employed were significantly and positively associated with care experiences and expectations. These findings provide a deeper insight into how SM individuals of different backgrounds experience and anticipate different aspects of caregiving. Moreover, we will discuss how our findings compare to non-SM individuals and implications of these findings.

### CAREGIVING AND COVID-19: PERSPECTIVES FROM A CARE COACH

Kelly Marnfeldt,[1] Lilly Estenson,[1] Julia Rowan,[1] and Kathleen Wilber,[2], *1. University of Southern California, Los Angeles, California, United States, 2. University of Southern California, University of Southern California, California, United States*

Family caregivers of community-dwelling older adults have faced unprecedented caregiving challenges during the COVID-19 pandemic. Examining the accumulated impact on family caregivers can help health and aging service providers design resources and supports that are resilient to emergency situations, and reduce negative psychological and physical consequences and risk of abuse within caregiving dyads. Data was collected as part of a pilot intervention in which "Care Coaches" provided telephonic coaching sessions to family caregivers of older adults. We examined Care Coach observations documented after coaching sessions with 24 family caregivers between March 2020 and February 2021. Two coders employed thematic analysis to generate codes and themes. The sample was 70% female, 80% were the spouse or significant other of their care receiver, the mean age was 61, and 53% were Non-Hispanic White. Themes and sub-themes included: (1) increased caregiver burden and diminished care networks due to fear of exposure to or contraction of COVID-19, (2) barriers to accessing in-home personal assistance services and home-delivered meals despite intervention efforts, and (3) the exacerbation of caregiver social isolation due to COVID-19 lockdown policies. Findings highlight the ways in which COVID-19 has amplified caregiver burden through the breakdown of formal and informal support systems. Potential adaptations of community-based services for older adults and their caregivers include remote service liaisons and need assessment of caregiver dyads to assure access to home-based personal assistance services and nutrition support for those at greatest risk of negative consequences during emergency service lapses.

### CBOS' CAPACITY AND RESILIENCE ON SERVING OLDER AA AND NHPI ADULTS DURING THE PANDEMIC

Eun Jeong Lee,[1] KeliAnne Hara-Hubbard,[2] Sonia Bishop,[3] Jae Lim,[4] and Linda Ko,[2], *1. Asian American Resource and Information Network, Wood Ridge, New Jersey, United States, 2. University of Washington, Seattle, Washington, United States, 3. Fred Hutchinson Cancer Research Center, Seattle, Washington, United States, 4. Swedish Health Services, Seattle, Washington, United States*

Community-based organizations (CBOs) are essential settings for older Asian American (AA) and Native Hawaiian

Pacific Islander (NHPI) adults for accessing culturally and linguistically appropriate services and connecting with and support each other. This study examined the impact of the COVID-19 pandemic on CBOs' ability to serve older AA & NHPI adults. This mixed methods study (survey and semi-structured interviews) used a sequential exploratory design. We recruited 65 leaders and staff members from 40 CBOs serving older AA & NHPI adults nationally. Descriptive analysis was conducted with the survey data followed by thematic analysis of the interview data. Many CBOs were impacted by the increased demands for services (80%) and created new services (75%) while experiencing programming disruption (69%), decreased staffing (55%), and loss of revenue (38%). Some CBOs temporarily closed their organizations (38%), while others closed permanently (3%). To remain in operation, many CBOs (65%) increased their online presence, hired staff (52%), and recurred to financial reserves (20%). The semi-structured interviews identified four themes: 1) CBOs resourcefulness to acquire and share resources, 2) technology as a connector for CBOs and an isolator for older adults, 3) heightened racial discrimination against Asians, and 4) emergence of multi-level resilience (personal/ community/organizational). CBOs experienced disruption in their operation, and heightened racial discrimination during the pandemic. Yet, CBO's ability to remain resilient was critical to continue to provide key programs for older adults. Future studies may want to examine evolving needs of CBOs as they adjust to new public health challenges during the pandemic.

### CHALLENGE OR OPPORTUNITY? IMPACTS OF FALLS AMONG OLDER ADULTS LIVING WITH DEMENTIA ON THEIR CARE PARTNERS

Yuanjin Zhou,[1] Emily Ishado,[2] and Tatiana Sadak,[2], *1. The University of Texas at Austin, Austin, Texas, United States, 2. University of Washington, Seattle, Seattle, Washington, United States*

Previous studies suggest that falls among community-dwelling older adults living with dementia (OLWD) harm the health and wellbeing of their family/friend care partners. However, little is known about the process through which falls impact care partners. We conducted a grounded theory analysis using 59 semi-structured interviews with care partners of OLWD who were recently hospitalized and had a history of falls. We identified several areas of care partners' functioning that were affected by falls in positive and negative ways: everyday life, health management for OLWD, and interactions with healthcare providers. Both the fall events and fall risks had negative consequences of reducing care partners' self-care activities and work productivity. Other adverse consequences of fall risks were (1) care partners' fatigue and conflicts with OLWD due to the intense requirement of daily monitoring, and (2) hesitance to ask healthcare providers for assistance because clinicians frequently did not teach care partners how to address fall risks and might recommend institutionalization. However, OLWD's fall events became a transition point for some care partners to seek support and gain more information and skills about managing OLWD's health conditions, which might reduce care partners' burden in the long term. Because OLWD's falls may have negative and positive consequences for care partners, both problem-solving and strength-based fall management approaches are needed. These strategies focus on developing and sustaining care partners' self-care, developing collaborative relationships with OLWD, enhancing successful capacity for OLWD's health management, and cultivating partnerships with healthcare providers.

### CHALLENGES AND BENEFITS: VOLUNTEERISM AMONG OLDER ADULTS DURING COVID-19

Alicia Sellon,[1] Tina K. Newsham,[2] Renee DuMont,[1] Claire Hollifield,[3] and Alicia Thomas,[3], *1. University of North Carolina Wilmington, Wilmington, North Carolina, United States, 2. University of North Carolina Wilmington, University of North Carolina Wilmington, North Carolina, United States, 3. University of North Carolina Wilmington, wilmington, North Carolina, United States*

Social distancing restrictions and regulations, put in place to reduce the spread of COVID-19, disrupted the daily lives of active older adult volunteers. One year into the pandemic, we used a mixed-methods approach to explore how these regulations had impacted the quality of life, loneliness, and volunteer behavior of 26 older adults who were active volunteers (i.e., at least an hour a week) prior to the start of the pandemic. All the participants were white and non-Hispanic, and the majority were female (65.4%). The average age was 71, with a range from 53 to 87 years old. On average, participant scores on the UCLA loneliness scale (4.23 ±1.39) indicated a low amount of loneliness and high scores on the Brunnsviken Brief Quality of Life (BBQ) scale (83.54 ±10.97) indicated a high quality of life. Thematic findings from the interviews conveyed that, despite the challenges and risks associated with volunteering during a pandemic, participants valued volunteer work enough to make adjustments or seek out new volunteer activities. The research team identified two overarching themes related to participants' discussions of volunteering during the pandemic: Challenges and changes and Benefits of volunteering during a pandemic. Participants' discussions of how volunteer work changed and why they continued to or sought out new volunteer activities during a pandemic can guide organizations seeking to support or recruit older volunteers, particularly as the pandemic continues. These findings also provide further evidence of the important role that volunteerism can play in the well-being of older adults.

### CLINICAL AND UTILIZATION OUTCOMES OF MATCHED PEOPLE WITH AND WITHOUT HIV AGED 65+

Brianne Olivieri-Mui,[1] Sandra Shi,[2] Gahee Oh,[3] Ellen McCarthy,[1] Ira Wilson,[4] Monty Montano,[5] and Dae Hyun Kim,[1], *1. Hebrew SeniorLife, roslindale, Massachusetts, United States, 2. Hebrew SeniorLife, Harvard Medical School, Roslindale, Massachusetts, United States, 3. Hinda and Arthur Marcus Institute for Aging Research, Roslindale, Massachusetts, United States, 4. Brown University, Providence, Rhode Island, United States, 5. Brigham and Women's Hospital, Boston, Massachusetts, United States*

The prevalence of age-standardized comorbidities is significantly elevated for PLWH across an array of cohorts. However, healthcare needs of older people living with

(PLWH) and without (PWOH) HIV may be similar if they have similar geriatric conditions. PLWH and PWOH aged 65+ and eligible for Medicare from 7/1/2014-1/1/2015 were matched 1:1 on age, sex, race, and census region (n=7654). Cox regression assessed count of prevalent geriatric conditions (dementia, depression, falls, hip fracture, sensory deficits, osteoporosis, orthostatic hypotension, urinary incontinence, frailty, and polypharmacy), and risk for clinical or utilization outcomes (cancer, kidney disease, muscle wasting, hepatitis C, liver disease, myocardial infarction, stroke; hospitalization, nursing home and home health admission) during follow-up between 1/1/2015-12/31/2016. PLWH and PWOH are similar in count of geriatric conditions. Compared to those with none, those having 2+ geriatric conditions were similar across PLWH and PWOH in their risk of ≥1 clinical outcome (PLWH: HR 1.57 95% CI [1.29-1.90]; PWOH: HR 1.31 [1.02-1.67]), hospitalization (PLWH: HR 2.35 [1.96-2.83]; PWOH: HR 2.07 [1.65-2.60]), and home health admission (PLWH: HR 2.09 [1.58-2.76]; PWOH: HR 2.20 [1.55-3.12]). Having 2+ geriatric conditions, PWOH had 4.45 times the risk (95% CI 3.16-6.26) and PLWH had 2.88 times the risk (95% CI 2.18-3.81) of NH admission compared to no geriatric conditions. In this study, PLWH use nursing homes less than PWOH despite having a similar number of geriatric conditions and clinical outcomes. Further research to understand this apparent discrepancy will be critical to achieve equity in nursing home access.

### CLOSING THE LOOP: AN ENVIRONMENTAL SCAN OF APS-REPORTER FEEDBACK POLICIES AND PRACTICES

Olanike Ojelabi,[1] Randi Campetti,[1] Kathy Greenlee,[2] and Kristin Lees Haggerty,[3], *1. Education Development Center, Waltham, Massachusetts, United States, 2. Greenlee Global LLC, Greenlee Global LLC, Kansas, United States, 3. Education Development Center, West Roxbury, Massachusetts, United States*

Abuse, neglect, and exploitation of older adults are prevalent and underreported in the United States. Pathways to identifying and resolving cases of abuse against older adults depend on mandated and non-mandated reporters bringing attention to these cases through reports to Adult Protective Services (APS). However, existing research points to several barriers to reporting. One significant barrier is a lack of communication from APS to reporters about reports they have made (e.g., whether the report is appropriate for APS, the investigation outcome, and services provided by APS). This lack of reciprocal communication likely serves as a disincentive for future reporting. This study aims to promote improved communication between APS and reporters by examining the legal, ethical, and practical barriers and facilitators to communication at key points in the reporting and response pathways. In this first phase of the project, we conducted an environmental scan of policies and practices related to reporting, investigation, and feedback. Early results from the environmental scan suggest most APS agencies (81%) do not currently provide feedback to reporters. Among those providing feedback, 20% provide feedback only to mandated reporters, and 50% provide only procedural feedback, which focuses on the process of receiving and screening reports for investigation and not on the outcome of the investigation. In the next phase of this study, we will supplement these findings through interviews with APS leaders across the U.S. These early results will begin to fill an important gap in the understanding of feedback loops between APS and reporters.

### COGNITIVE STIMULATION THERAPY IN OUTPATIENT SETTINGS FOR PERSONS WITH DEMENTIA

Max Zubatsky,[1] Marla Berg-Weger,[2] Matthew Amick,[3] Debbie Blessing,[4] Katelyn Sansone,[3] Anna Brende,[2] and Janice lundy,[5], *1. Medical Family Therapy Program, Saint Louis University, Missouri, United States, 2. Saint Louis University, Saint Louis, Missouri, United States, 3. Saint Louis University, Saint Louis University, Missouri, United States, 4. A.T. Still University of Health Sciences, A.T. Still University, Missouri, United States, 5. Perry County Memorial Hospital, Perry County Memorial Hospital, Missouri, United States*

Cognitive Stimulation Therapy (CST) is a non-pharmacologic evidence-based intervention for persons living with mild to moderate dementia. This clinical intervention therapy follows a structured protocol designed to connect people with memory loss to others by providing opportunities for social engagement and group discussion of current events and a different themed activity each session. This presentation will include findings from a multi-site study of the group intervention conducted in an urban and two rural out-patient settings with community-dwelling older adults. Pre- and post-assessments captured data on cognitive function, depression, quality-of-life, and mobility. While CST is offered in over 30 countries, this is the first large-scale CST study conducted in the U.S. Implications for future practice and research will be presented.

### COMPARISON OF OLDER ADULT AND HEALTHCARE PROVIDER BELIEFS ABOUT FALL PREVENTION STRATEGIES

Gwen Bergen, Ankita Henry, and Yara Haddad, *Centers for Disease Control and Prevention (CDC), Atlanta, Georgia, United States*

Older adults reported about 36 million falls in 2018. Although effective strategies are available to minimize fall risk, little is known about older adults' and healthcare providers' awareness of these strategies. This study describes and compares older adults' and healthcare providers' beliefs about fall prevention strategies. Demographic and fall-related data for older adults were obtained from the 2019 fall cohort of Porter Novelli ConsumerStyles. Similar data from primary care practitioners, nurse practitioners, and physician assistants, were gathered from the 2019 cohort of DocStyles. Percentages and 95% confidence intervals were calculated to compare older adults and providers. Most providers (91.3%) and older adults (85.1%) believed falls can be prevented. High percentages of providers and older adults identified strength and balance exercises (90.7% and 82.8% respectively) and making homes safer (90.5% and 79.9% respectively) as strategies that help prevent falls. More providers reported that Tai Chi (45.7%) and managing medications (84.2%) can prevent falls compared to older adults

(21.7% and 24.0% respectively; p<0.0001). Sizable percentages of providers and older adults endorsed less evidence-based strategies including aerobic exercise (70.7% and 58.4% respectively) and being more careful (69.3% and 81.6% respectively). Among older adults, lower endorsement of evidence-based strategies (e.g., Tai Chi, medication management) coupled with higher endorsement of limited evidence-based strategies (e.g., being careful, aerobic exercise) suggest some older adults lack awareness of effective fall prevention interventions. Increased patient and provider communication can increase awareness about the benefits of evidence-based strategies for fall prevention.

### COMPARISON OF PHYSICAL ACTIVITY LEVEL FROM OSTEOPOROSIS, PARKINSON AND HEALTHY SUBJECTS

Seong Hyun Moon,[1] Thurmon Lockhart,[1] and Krupa Doshi,[2], *1. Arizona State University, Tempe, Arizona, United States, 2. MAYO Clinic, Scottsdale, Arizona, United States*

Lifestyle at the habitation immensely affects the progression of various illnesses, such as Osteoporosis and Parkinson's disease (PD). These disorders lead patients to a sedentary lifestyle and result in significantly less movement compared to the average healthy individual. The combination of these backgrounds escalates the percentage of fall incidents. Quantifying physical activity levels from longitudinal Activities of Daily Living (ADL) data of these disease patients could stipulate intuition of their fall mechanisms. The objective of this study is to compare the osteoporosis, Parkinson's disease, and healthy group's physical activity level from their ADL. For this study total of eighteen subjects participated (healthy=6, osteoporosis=6, PD=6). The result indicated that the dynamic physical activity level for the healthy subject was 13.2%, the osteoporosis subject was 7.9%, and the PD subject was 7.0%. This indicates that there was a significant decline in physical activity level for the PD compared to healthy subjects (P=0.0024*). Also, a comparison between healthy and osteoporosis subjects showed a significant difference (P=0.0066*). Lastly, the physical activity level of PD and osteoporosis subjects did not have a significant difference among them (P=0.6276). The aim of this study was to evaluate the physical activity level of the osteoporosis, PD, and healthy subjects. The systematic approach of collecting physical activity levels with the Inertial Measurement Unit (IMU) device allowed researchers to collect the quantitative data of ADL. In this experiment, healthy subjects were significantly more physically active compared to osteoporosis and PD patients.

### COMPARISONS OF FACTORS ASSOCIATED WITH SUICIDAL IDEATION OF OLDER INDIVIDUALS BY HEALTH STATUS

Hyeyeon Sung,[1] Jihun Na,[2] Sungkyu Lee,[1] and Sehyun Baek,[1], *1. Soongsil University, Seoul, Seoul-t'ukpyolsi, Republic of Korea, 2. Korea Center on Gambling Problems, Seoul, Seoul-t'ukpyolsi, Republic of Korea*

This study examined the factors associated with suicidal ideation among older individuals and compared those factors by their objective and subjective health status. Data were obtained from the 13th wave of the Korean Health Panel Survey in 2018. The sample of 6,283 older individuals, who are 55 years and older, was classified into four groups by their objective and subjective health status. Objective health status was measured by the number of chronic health conditions, and subjective health status was defined by an individual's self-reported health status. To examine the factors associated with suicidal ideation among four groups of older individuals, logistic regression analyses were conducted after controlling for socio-demographic characteristics, physical health and mental health characteristics. The results show that depression and anxiety were found as common factors associated with suicidal ideation for all four groups. As for the group of older individuals who reported bad objective health and bad subjective health, younger age, being male, and low educational attainment were found to be associated suicidal ideation. For those with bad objective health regardless of their subjective health status, the level of stress was found to be related to suicidal ideation among older individuals. Based upon those results, the present study discussed practical and policy implications for suicide prevention among older individuals by reflecting their objective health and subjective health status.

### COMPLEMENTARY AND INTEGRATIVE HEALTH APPROACHES AND OPIOID PRESCRIPTIONS AMONG OLDER VETERANS WITH CHRONIC PAIN

Ling Han,[1] Joseph Goulet,[2] Melissa Skanderson,[2] Doug Redd,[3] Cynthia Brandt,[4] and Qing Zeng,[5], *1. Yale School of Medicine, New Haven, Connecticut, United States, 2. VA Connecticut Healthcare System, West Haven, Connecticut, United States, 3. Washington DC VA Medical Center, Washington DC, District of Columbia, United States, 4. Yale School of Public Health, West Haven, Connecticut, United States, 5. George Washington University, Washington DC, District of Columbia, United States*

Complementary and integrative health (CIH) approaches are recommended in national guidelines as viable options for managing chronic pain and de-prescribing opioids. We followed 1,993,455 Veterans with musculoskeletal disorders for two years who were not using opioids at study entry. CIH exposure was ascertained from primary care visits for acupuncture, massage and chiropractic care via natural language processing and structured data. Opioid prescriptions during the 2-year follow-up were abstracted from Veterans Health Administration (VHA) electronic pharmacy records. Propensity score (PS) was used to match CIH recipients with non-recipients with most comparable baseline characteristics. Overall, 140,902 (7.1%) Veterans received CIH, with a prevalence of 2.7% for Veterans aged ≥ 65y, comparing to 6.3% and 10.5% for those aged 50-64y and ≤ 49y, respectively. Among the 1:1 PS-matched sub-cohort (136,148 pairs), Cox proportional hazard model revealed that time to fill first opioid prescriptions was significantly longer for CIH recipients (mean: 587 days) than non-recipients (mean: 491 days), with adjusted Hazard Ratio of 0.48 (95% Confidence Interval (CI): 0.45-0.51) for Veterans ≥ 65y, 0.44 (95% CI: (95% CI: 0.43-0.45) for 50-64y and 0.47 (95% CI: 0.46-0.48) for age ≤ 49y group (p value for interaction, 0.003).

Sensitivity analyses among full cohort or modeling total supply of first opioid prescriptions derived consistent results. These findings suggest potential benefit of CIH use in delaying and reducing opioids prescriptions for patients with chronic pain and may have implication for older Veterans ≥ 65y who have been found less likely to seek CIH therapies than their younger counterparts.

### COUPLING EFFECTS OF DEPRESSION AND FUNCTIONAL DISABILITY: USING BIVARIATE LATENT CHANGE SCORE MODEL

Gina Lee, and Peter Martin, *Iowa State University, Ames, Iowa, United States*

The purpose of the study was to examine the coupling effect of depression and functional disability over four time points using the data from the Health and Retirement Study (HRS). The sample included participants who survived to 98 years or older (N = 458). Four alternative latent change score models were computed to examine the univariate and bivariate effects among depressive symptoms (CES-D) and functional disabilities (ADL): No-coupling, univariate model of ADL to change in CES-D, univariate model of CES-D to change in ADL, and bivariate model. As hypothesized, the no-coupling model did not fit the data well, $\chi 2$ (26) = 164.86, CFI = 0.85, RMSEA = 0.11. Model 2, ADL predicting change in CES-D, did not fit the data well, $\chi 2$ (25) = 164.18, CFI = 0.85, RMSEA = 0.11. Model 3, CES-D predicting change in ADL, also did not fit the data, $\chi 2$ (25) = 148.06, CFI = 0.87, RMSEA = 0.10. The bivariate model fit the data well, $\chi 2$ (21) = 66.94, CFI = 0.95, RMSEA = 0.07, and was the best fitting model. All level to change effects were significant in model 4. One's CES-D at prior waves was positively associated with change in ADL at subsequent waves, and ADL at prior waves was positively associated with change in CES-D at subsequent waves. In conclusion, there is a significant coupling effect between depressive symptoms and ADL over time. Future health policies should monitor older adults' mental and functional health simultaneously for their possible spillover effects.

### COVID-19 IN HOSPITALIZED PATIENTS ADMITTED FROM SKILLED NURSING FACILITIES: FACTORS ASSOCIATED WITH MORTALITY

Rongfang Zhan,[1] Cheng Yin,[1] and Liam O'Neill,[2],
*1. University of North Texas, Corinth, Texas, United States, 2. University of North Texas, University of North Texas, Texas, United States*

**Background:** Nursing home residents were impacted disproportionately by the coronavirus because of their vulnerabilities. Although many studies concentrated on risk factors associated with mortality of hospitalized patients, there were limited studies epitomizing them from skilled nursing facilities to hospitals. The study aims to identify inpatients' characteristics on demographics, hospital admission types, insurance types, and chronic diseases associated with mortality among our cohort patients in Texas.

**Methods:** Individuals above 50 years, diagnosed with Covid-19, and admitted from skilled nursing facilities were included in the retrospective cohort study. Pearson's Chi-Square and Mann-Whitney tests were applied to measure four major perspectives between survivors and non-survivors. Then, a binary logistic regression was employed to determine the association between independent variables and mortality.

**Results:** A total of 218 patients were included in the study, of which 54 (24.8%) died during hospitalization. According to the univariate analysis, expired patients were more likely to be emergency admission (p = 0.001), elective admission (p = 0.02), Medicaid as primary payment (p = 0.034), heart disease (p = 0.027), CKD (p = 0.03), and hypertension (p = 0.002). The binary logistic regression revealed that hypertension (OR = 3.176, 95% CI: 1.200-8.409, p = 0.02) and Medicaid (OR = 2.637, 95% CI: 1.287-5.405, p = 0.008) as primary payment had significantly high odds of mortality.

**Conclusion:** Hypertension and Medicaid as primary payment are the strongest predictive factors associated with mortality and suggest that hospitals in Texas distribute critical care and resources while prevent and treat them to increase survival rates.

### CULTURAL CONSIDERATIONS TO THE LIFE PARTICIPATION APPROACH IN APHASIA: A FILIPINO CASE STUDY

Tricia Olea Santos,[1] Hanna Ulatowska,[2] and Carla Krishan Cuadro,[3], *1. University of Texas at Dallas, Dallas, Texas, United States, 2. University of Texas, Dallas, Texas, United States, 3. St. Luke's Medical Center, Quezon City, National Capital Region, Philippines*

Stroke is among the common causes of chronic disability (Feigin, 2014). Around one-third of stroke survivors are affected by aphasia, a communication disorder affecting the ability to comprehend and express oneself (Dickey, et. al., 2010). Culture is essential to understanding aphasia and providing person-centered care. Philippine cultural identity is reflected via respect for older persons, collectivism in family and community, and devotion to religion (Pe Pua & Protacio-Marcelino, 2000). The Filipino family is a primary support system, and cultural values directly influence caregiving approaches in chronic disability. This single case study examines the life of a Filipino man who has successfully lived with aphasia for over 25 years. Having finished a doctorate from Harvard University, served as the youngest University president, and member of the Philippine government, he suddenly had a stroke and was able to communicate only via single words, gestures, and facial expressions. His life is discussed in the context of the unique, multi-modal communication system which developed through the years with his family. Music and symbolism via watercolor paintings also define his aphasia journey. The value of religion in Filipino culture (Cruz, et. al., 2019) and its role in fostering positivity in his aphasia journey is examined. This study also highlights Filipino collectivism through the support of family and community in addressing communication needs and facilitating meaningful relationships at various stages in life. Cultural values deeply rooted in Filipino caregiving, such as debt of gratitude and filial devotion to parents (Enriquez, 1992), are discussed.

### DEVELOPMENT AND VALIDATION OF A MEASURE OF SUPERVISORY BEST PRACTICES IN GERONTOLOGICAL SOCIAL WORK

Daniel Kaplan,[1] and Keith Chan,[2], *1. Adelphi University, Cortlandt Manor, New York, United States, 2. Hunter College, New York, New York, United States*

Agency-based supervision is essential to skilled practice and staff retention, directly impacting the quality of services. The Supervisory Leaders in Aging (SLA) program was designed to strengthen supervision of the aging services workforce. SLA was implemented in four states and trained 134 social work supervisors. The purpose of this study was to develop a novel, comprehensive, and practice-informed measure of supervisory best-practices in gerontological social services. The primary outcome for the SLA program was the Practice Inventory for Supervision in Aging Services (PISAS), which evaluated the frequency of recent use of best practices as identified by the project team and vetted by instructors and an interdisciplinary advisory board. Reliability and confirmatory factor analyses were conducted to examine its psychometric properties. Findings demonstrated the scale had good internal consistency reliability ($\alpha = 0.88$). Confirmatory factor analyses indicated a three-factor solution, 1) Gerontological Social Work Skills, 2) Program Development Skills, and 3) Supervision and Leadership Skills, accounting for 72% of the variability in the 27 items. Gerontological Social Work Skills captured best practices regarding mental health in late life, heightening awareness of elder abuse, and working with families. Program Development Skills captured assessment, measuring outcomes, and translating evidence into practice. Supervision and Leadership Skills captured individual supervision, group supervision and leadership in interdisciplinary practice. Results indicated that PISAS is a reliable and valid measure of use of best practices in gerontological social work supervision. Further implications and limitations of this measure in assessing outcomes of gerontological education and training programs are discussed.

### DEVELOPMENT OF AN ADL-PRACTICE GUIDELINE: THE NEXT STEP TOWARDS GUIDANCE IN ESSENTIAL NURSING CARE ACTIVITIES

Svenja Cremer,[1] Michel Bleijlevens,[1] Silke Metzelthin,[1] Janneke de Man-van Ginkel,[2] and Sandra Zwakhalen,[3], *1. Maastricht University, Maastricht, Limburg, Netherlands, 2. UMC Utrecht, Utrecht, Utrecht, Netherlands, 3. Maastricht University, Maastrichjt, Limburg, Netherlands*

Supporting and respecting care receivers in Activities of Daily Living (ADL) lies at the essence of nursing care, irrespective of diagnosis or healthcare setting. ADL-care is an intimate form of caring, and therefore close and personal to the care receiver, aiming to enhance their independence and comfort. Even though ADL-care is indispensable and highly valued by care receivers, the scientific foundation of ADL-care is weak. This leaves nursing professionals with insufficient guidance as to what constitutes quality ADL-care and what activities are necessary and effective. Therefore, we developed an ADL practice guideline according to the framework of the Dutch Institute for Health (AQUA-guideline) and AGREE II. The guideline was developed over three stages: (1) Determination of the target population and scope, (2) Analysis of problems leading to guiding questions and answering these based on literature search and consensus, (3) Testing and validation. A multidisciplinary working group determined the purpose, target group, and five clinical questions. We used literature search and consensus procedures to answer these questions in close collaboration with care receivers and professional care providers. This guideline provides guidance for nursing professionals to choose appropriate ADL-care options in five modules covering recommendations: (1) Involving care receivers in ADL-related care choices, (2) Identifying ADL-care needs, (3) Choosing effective interventions to enhance ADL-independence and comfort, (4) Supporting informal caregivers in ADL-care, and (5) Using ADL-care for early detection of health problems. These modules are considered leading for future developments in essential nursing care and will be evaluated in a pilot implementation.

### DISCOURSE ANALYSIS UNCOVERS COMPULSORY ALTRUISM AND POWER PARADOXES FOR FAMILY CAREGIVERS OF THOSE WITH DEMENTIA

Candace Harrington, *University of Louisville, Louisville, Kentucky, United States*

Rural female family caregivers are under-represented, under-reported, and under-studied in rural caregiving and Alzheimer's disease-related dementias (ADRD) research. Caregivers' power struggles are often invisible and unknown. These constructs have social, policy, and practice implications for both family caregivers and their care recipients with ADRD. The purpose of this study was to explore how Foucauldian discourse analysis (FDA) elucidates rural female family caregivers' acquisition of caregiving roles for those with ADRD. FDA focuses on power structures and relationships in society as expressed through language and practices that affect marginalized groups. Textual data for this secondary analysis consisted of 157 pages of interview transcripts with 10 rural female caregivers. The systematic discourse analysis elucidated two socially constructed power and relationship structures. Compulsory altruism described complex socially constructed caregiving and gender role expectations, grounded in reciprocity, duty, and filial piety. A power paradox occurred when filial piety, duty, and reciprocity were in direct opposition to the caregivers' beliefs and value systems. In this sample, the subsequent sense of ambiguity about violating personhood and autonomy delayed black and white caregivers' responses to intervene with family members with ADRD. These delays resulted in near misses from wandering, driving-related accidents, cooking-related fires, financial exploitation by other family members, mistreatment that involved both caregiving dyad partners, and one tragic incident of a parent's death resulting from wandering-related exposure to elements. FDA was a valuable qualitative approach to elucidate family caregivers' power struggles that were previously invisible and unknown. These findings have broad implications for clinicians and researchers.

### DIVERSITY, EQUITY AND INCLUSION IN AN AGE-FRIENDLY HEALTH SYSTEM

Elizabeth Eckstrom,[1] Emily Morgan,[2] Bryanna De Lima,[3] and Anna Pleet,[4], *1. Oregon Health & Science University, Hood River, Oregon, United States, 2. Oregon Health and*

*Science University, Portland, Oregon, United States, 3. Oregon Health & Science University, Portland, Oregon, United States, 4. Vita-Salute San Raffaele University, Milan, Lombardia, Italy*

The Age-Friendly Health Systems (AFHS) initiative uses a 4Ms framework to encourage patient-centered care for older adults. Many health systems have implemented the core elements of AFHS – What Matters, Mentation (Cognition and Depression), Medications, and Mobility – with the goal to uniformly apply these elements to all patients 65 years and older. However, equity in AFHS delivery has not yet been examined. Five diversity, equity, and inclusion (DEI) factors, including gender, race, ethnicity, preferred language and MyChart activation, were cross-sectionally analyzed against the 4Ms framework for patients seen (in person or virtual visit) in an academic internal medicine clinic between April 2020 and April 2021 (N= 3370) using two-way contingency tables. Preferred language, gender, and MyChart activation yielded significant pairings with the 4M metrics. For the AFHS What Matters metric, females were 1.10 times more likely than males and English-speaking patients were 1.67 times more likely than non-English speaking patients to receive advance care planning ($p < 0.01$). Females and patients with MyChart activation were about 2.0 times more likely to have a high-risk medication on their medication list compared to males and patients without MyChart activation ($p < 0.01$). MyChart activation was also significantly associated with cognitive screening. Patients with MyChart activation were 1.09 times more likely than patients without MyChart activation to get cognitive screening ($p < 0.001$). This study, the first to incorporate demographic data, into AFHS quality measures, suggest a need to develop best practices for equitable Age-Friendly care at the clinical team and the institutional policy level.

### DO FAMILY CAREGIVERS OF DEMENTIA PATIENTS FEEL COMPETENT IN PAIN MANAGEMENT?

Hui Zhao,[1] Pamela Kulbok,[2] Ishan Williams,[2] Carol Manning,[2] and Rafael Romo,[3], *1. James Madison University, Harrisonburg, Virginia, United States, 2. University of Virginia, Charlottesville, Virginia, United States, 3. Dominican University of California, San Rafael, California, United States*

Older adults with dementia rely on others to recognize and treat their pain and will ultimately become dependent. Family caregivers (FCGs) play a crucial role in pain management, yet limited data is available regarding the factors that impact their abilities. This qualitative descriptive study sought a deep understanding of FCGs perception of their abilities to manage pain for a loved-one with dementia. A sample of 25 adult family caregivers of community-based older adults with dementia was recruited in central Virginia. Participants were 29 to 95 years old, predominantly white, married, female, and high school graduates. We conducted semi-structured interviews that were audio recorded and analyzed using constant comparative analysis. Participants' who perceived greater competence with pain management reported less pain for their loved-one, and their level of confidence was influenced by 3 factors: progress and stage of dementia: this increases the complexity of care, affecting FCGs ability to manage pain and engendering a self-perception of incompetence; developing adaptive mechanisms: built self-efficacy and improved FCGs perceived competence;, and support from professionals: a greater degree of support alleviated FCGs concerns and instill new skills, Effective pain management depended on family caregivers' belief in their own abilities, and perceived competence could be improved by learning new skills or making adaptations. Professional care givers need to routinely assess FCGs abilities and provide adequate interventions.

### EFFECTS OF A WEB-BASED INTERVENTION ON CARDIOVASCULAR AND PHYSICAL HEALTH OF KOREAN OLDER ADULTS DURING THE PANDEMIC

SOONHYUNG KWON,[1] and Oejin Shin,[2], *1. University of Illinois at Urbana-Champaign, Savoy, Illinois, United States, 2. University of Illinois at Urbana Champaign, Savoy, Illinois, United States*

During the lockdown, 97.5% of Korean senior centers in South Korea were closed to prevent the spread of the coronavirus disease 2019 (COVID-19). The threat of the COVID-19 presented the need for alternative interventions for Korean older adults to maintain cardiovascular and physical health. Korean senior centers implemented web-based interventions to provide physical health services, but their effectiveness was not yet assessed. Thus, our study aimed to identify the effects of a web-based intervention using a smartwatch and mobile app in older adults when compared to center-based intervention during the pandemic. This study collected 117 Korean older adults (≥ 60) who participated in the 12-week web-based and center-based physical interventions using a smartwatch and mobile app. This quasi-experimental study was conducted between August and December in 2020. We analyzed the pre-posttest of cardiovascular and physical health across two intervention types. Our regression results indicated that participants in the 12-week web-based intervention reported better cardiovascular (systolic blood pressure: $b = -13.77$, $p < .001$; cholesterol: $b = -11.71$, $p < .05$) and physical health (muscular function: $b = 2.99$, $p < .001$; body balance: $b = -1.31$, $p < .001$; cardiopulmonary endurance: $b = 33.33$, $p < .001$) than those in center-based intervention at posttest. The findings imply a web-based intervention is likely to become an innovative therapeutic strategy for older adults' health to respond to the rapidly changing social service systems amid the pandemic.

### EFFECTS OF NON-PHARMACOLOGICAL INTERVENTIONS ON ELDERLY IN NURSING HOMES WITH SLEEP DISORDER: A META-ANALYSIS

Sunok Jung, Eunju Choi, and Hyeyoung Kim, *College of Nursing, Ewha Womans University, Seoul, Seoul-t'ukpyolsi, Republic of Korea*

**Purpose:** This study aimed to examine the effects of nonpharmacological sleep intervention programs to improve sleep quality among the elderly in long-term care facilities.

**Methods:** A literature search and selection was performed on nine different databases using the Preferred Reporting Items for Systematic Review and Meta-Analysis Statement. In total, 14 studies met the inclusion criteria and were systematically reviewed. For the meta-analysis, the effect size was estimated using the random-effects model on Review Manager (RevMan) desktop version 5.4 of the Cochrane Library.

**Result:** The meta-analysis of nonpharmacological interventions obtained a total effect size of 1.0 (standardized mean difference [SMD] = 1.0, 95% confidence interval [CI]: 0.64–1.35), which was statistically significant (Z = 5.55, p < .001). The most frequent nonpharmacological interventions identified were the interventions using aroma; the effect size was 0.61 (SMD = 0.61, 95% CI: 0.14–1.08), which was statistically significant (Z = 2.55, p = .01). In subgroup analysis, group-specific interventions, interventions for >4 weeks, and untreated control studies were more effective.

**Conclusion:** This study confirms that nonpharmacological interventions are effective in improving sleep quality among the elderly in long-term care facilities. However, the small sample size and risk of bias in assessing the interventions of individual studies are unclear or high, thereby limiting the generalizability of the results. Further studies based on randomized control trials and the development of evidence-based interventions that consider the elderly participants' physical activity levels, intervention methods and duration, and control group selection are needed to obtain more conclusive evidence.

### END-OF-LIFE CARE FACTORS PREDICT AFFECTIVE SEQUENCES IN OLDER ADULTS' FINAL MEMORIES OF SPOUSAL LOSS

Emily Mroz,[1] Nerea Anaya-Dominguez,[2] and Susan Bluck,[3], *1. Yale University, New Haven, Connecticut, United States, 2. University of Florida- Life Story Lab, Gainesville, Florida, United States, 3. University of Florida, University of Florida, Florida, United States*

Memories from the dying days of a deceased spouse are vividly recalled and can guide grief adjustment in older adulthood (Mroz & Bluck, 2018). End-of-life factors (e.g., place of death, quality of death) likely impact the nature of recall of such memories over time. Intersecting psychology and palliative care perspectives, the current study employs mixed-methods to examine relations between end-of-life care factors and affective sequences in older adults' final memories of spousal loss. Fifty-three participants (Mage = 81.59; M = 6.81 years since loss) completed a Final Memory Interview, provided place of spousal death (in hospital, outside of hospital), and completed the Good Death Inventory (GDI; Miyashita et al., 2008). GDI responses were organized into four quality of death categories. Final memories were reliably content analyzed for affective sequences (i.e., positive and negative affect themes; interrater agreements > .70): redemption (bad mitigated by good, McAdams 1999), contamination (good spoiled by bad; McAdams, 1998), positive stability, and negative stability. Loss of a spouse in hospital, compared to outside of hospital, related to narrating final memories with contamination, $F = 4.05$, $p < .05$. Quality of death predicted narration of final memories with positive affective sequences: lower reported comforting environment related to redemption ($t = -3.05$; $p < .01$) and higher reported appropriate medical care related to positive stability ($t = 2.60$; $p < .05$) in memories. As healthcare provision continues to adjust to improve end-of-life circumstances across care environments, the impact of circumstances on close others should factor into initiative development.

### ENGAGING UNDERREPRESENTED OLDER ADULTS IN ADRD AND AGING RESEARCH: A SCOPING REVIEW

Nisha Godbole,[1] Jeannette Beasley,[2] Simona Kwon,[3] Timothy Roberts,[4] Julie Kranick,[5] Scott Sherman,[2] Chau Trinh-Shevrin,[2] and Joshua Chodosh,[2], *1. NYU Grossman School of Medicine, South Setauket, New York, United States, 2. NYU Grossman School of Medicine, New York, New York, United States, 3. NYU School of Medicine, New York, New York, United States, 4. NYU Langone Health, New York, New York, United States, 5. NYU Langone Medical Center, NYU Langone Medical Center, New York, United States*

The rapidly aging and diversifying U.S. population coincides with increases in prevalence of Alzheimer's disease and related dementias (ADRD) and other aging-related disorders. Unfortunately, older adults and racial and ethnic minorities are often underrepresented in research studies. The differing barriers that underrepresented older adults face in research engagement indicate that results from studies conducted on younger and majority populations may not maintain external validity outside of those groups. Therefore, efforts to engage diverse older adults in research is imperative. The goal of this scoping review was to summarize findings of the current state of National Institute on Aging (NIA) sponsored research, identifying extant literature on engaging diverse older adult populations in aging and ADRD research. Among 566 articles screened for inclusion, 436 were included in the final analysis. Results showed that African Americans were represented in over half the studies (63.5%), but Native Hawaiian/Pacific Islander and American Indian or Alaska Native populations were not well represented. Community- and convenience-based recruitment and retention strategies that have demonstrated prior success in research engagement were widely utilized. Racial, ethnic, and income status breakdowns were not included in 30.0%, 57.1%, and 53.4% of studies respectively, making it difficult to assess the applicability of findings for particular groups. Inclusion of Alzheimer's disease patients or those with mild cognitive impairments was also poorly defined in most studies. Findings highlight gaps in existing literature that can be used to inform future research, and recruitment and retention strategies for engaging racial and ethnic minority older adults in research.

### EVALUATING THE CONVERSATION STARTER KIT IN LONG TERM CARE: A CANADIAN PERSPECTIVE

Sharon Kaasalainen,[1] and Tamara Sussman,[2], *1. McMaster University, Hamilton, Ontario, Canada, 2. McGill university, McGill University, Quebec, Canada*

This study evaluated an advance care planning intervention, the Conversation Starter Kit (CSK) booklet, for use in long term care (LTC) homes. This study used a quasi-experimental, one group pre/post design. Quantitative surveys were administered before and after a 3-month advance care planning intervention (CSK booklet). Data were collected at three LTC homes in southern Ontario. We collected data from 55 resident who were able to make decisions on their own paired with 11 family members of these residents. We also collected data from 24 family members of residents who were not able to make decisions on their own.

Quantitative surveys were administered before and after the intervention. An additional structured interview was completed at the end of the intervention period, which included both closed and open-ended questions to assess perceptions about the CSK booklet's use or non-use. Residents reported higher engagement in advance care planning after having completed the CSK booklet than before, particularly related to asking questions to health care providers about health care decisions. Family members reported feeling very certain that they would be able to make decisions on behalf of the resident but they felt less certain after completing the CSK booklet, implying that the CSK booklet raised their awareness of the types of decisions that they might need to make, hopefully triggering them to become more prepared for these decisions in the future. The CSK appears acceptable, easy to use for residents and family members/friends in LTC, and can improve resident engagement in ACP.

### EXPERIENCES AND PERCEIVED ORIGINS OF COMPASSIONATE AGEISM AMONG OLDER ADULTS DURING THE COVID-19 PANDEMIC

Catherine Ju,[1] Meghan McDarby,[2] Matthew Picchiello,[3] and Brian Carpenter,[4], *1. Washington University St. Louis, St. Louis, Missouri, United States, 2. Washington University, St. Louis, Missouri, United States, 3. Washington University in St. Louis, Olivette, Missouri, United States, 4. Washington University in St. Louis, Saint Louis, Missouri, United States*

During the COVID-19 pandemic, there was a rise in media messages (MMs) and interpersonal behaviors (IBs) that could have been considered as reflecting compassionate ageism (i.e., ageism that stems from perceptions of older adults [OAs] as warm but incompetent). However, it is unclear how OAs experienced these MMs and IBs during the pandemic. The current study examined how OAs perceived pandemic-related MMs and IBs. We recruited 74 community-dwelling OAs (Mage = 73.18, 58% female). Participants completed a survey in which they reported the extent to which they had encountered five MMs and nine IBs throughout the COVID-19 pandemic. Then, participants rated whether they believed each MM and IB was motivated by care and how offended they were by it. Nearly all participants had encountered MMs about OAs' vulnerability to COVID-19 (e.g., more likely to contract COVID-19, 97%; more likely to die from COVID-19, 97%). Furthermore, most participants experienced IBs emphasizing their vulnerability to COVID-19 (e.g., told by another person they had a higher likelihood of contracting COVID-19, 64%; someone had checked in on them unprompted, 63%). However, across MMs and IBs, most participants (59–100%) perceived them as motivated by care and concern, and a relatively small proportion (0–20%) reported being offended by them. Our findings underscore the importance of understanding nuances of ageism from the perspective of OAs themselves. Different forms of ageism (i.e., compassionate ageism, hostile ageism) rooted in certain stereotypes about older adults (i.e., high warmth-low competence) may uniquely shape the lived experiences of OAs.

### EXPERIENCES AND PERCEPTIONS OF USING A LOW-COST PET ROBOT FOR OLDER ADULTS AND PEOPLE WITH DEMENTIA

Wei Qi Koh,[1] Pascale Heins,[2] Sally Whelan,[1] Elaine Toomey,[3] Dympna Casey,[1] and Rose-Marie Dröes,[4], *1. National University of Ireland Galway, Galway, Galway, Ireland, 2. Maastricht University, Maastricht, Limburg, Netherlands, 3. University of Limerick, Limerick, Limerick, Ireland, 4. Vrije Universiteit Amsterdam (Amsterdam UMC), Amsterdam, Noord-Holland, Netherlands*

Pet robots are a practicable substitute for animal-assisted therapy. They have been shown to have positive impacts on older adults, including people with dementia, such as providing companionship and facilitating social interaction. However, the issue of affordability can hinder equal access to such technology. The purpose of our study was to understand the perceptions and experiences of using a low-cost, commercially available pet robot with older adults and people with dementia. We used a novel methodology of analysing a large volume of user reviews that were collected from 15 consumer websites. A total of 1,327 user reviews that met our pre-specified inclusion criteria were included. Descriptive statistics was applied to characterise demographic data, and inductive qualitative content analysis was used to identify themes in the textual data. Most reviews were obtained from consumer sites in the United States, and most reviewers were family members of the users (i.e., older adults and people with dementia). We found that circumstantial reasons, such the inability to own live animals, prompted reviewers to purchase the pet robot. Most reviewers had positive perceptions of the pet robot, and described various activities that users engaged in with it. Impacts of using the pet robot, such as positive emotions, were also described. Finally, experiences about practical aspects of its use, such as durability and hygiene, were discussed. Overall, this study provides useful knowledge that can help researchers, robot developers and clinicians understand the viability of using low-cost pet robots to benefit older adults, including people with dementia.

### EXPLORING THE PATIENT-PROVIDER RELATIONSHIP IN OLDER ADULT PAIN MANAGEMENT

Sophia Sheikh,[1] Natalie Spindle,[2] Jennifer Brailsford,[3] Jason Beneciuk,[4] Monika Patel,[1] Brittany Johnson,[5] Robin Moorman Li,[5] and Phyllis Hendry,[2], *1. University of Florida College of Medicine – Jacksonville, Jacksonville, Florida, United States, 2. UF College of Medicine Jacksonville, Jacksonville, Florida, United States, 3. UF Health Jacksonville, Jacksonville, Florida, United States, 4. Brooks Rehabilitation and the College of Public Health & Health Professions at the University of Florida, Jacksonville, Florida, United States, 5. University of Florida College of Pharmacy, Jacksonville, Florida, United States*

Successful health outcomes in older patients are linked to the quality of the patient-provider relationship. Our study objective was to further understand the role of this relationship specific to pain management through perspectives from older adults and healthcare providers. Semi-structured interviews and focus groups were conducted with 9 older adults and 11 multidisciplinary healthcare providers. Transcripts were analyzed using a thematic analysis. Three main concepts emerged: (1) defining pain management goals — differences in providers and patients' goals for pain and function, with sub-themes of realistic goal setting and a shift in pain treatment to minimize opioids as a first-line medication; (2) communication — perceived gap in providers communicating and coordinating across disciplines and with patients, with sub-themes of improving positive communication and inconsistent messaging among providers; and (3) therapeutic alliance — all parties feel that developing a relationship is built on consistent trust and open dialogue. Although providers and older adults often expressed similar perspectives, there were several areas of misalignment identified within each concept, representing areas of disconnect within the patient-provider pain management relationship. Our findings indicate providers could benefit from education on improving communication around realistic goals and patient-centered outcomes and incorporation of more holistic pain management approaches when working with older adult patients. Further study should focus on developing educational interventions to address the identified shortcomings.

### FACTORS INFLUENCING BRAIN HEALTHY DIETS AMONG OLDER MIDWESTERN AFRICAN AMERICANS

Ashley Shaw,[1] Samantha Fikru,[1] Jannette Berkley-Patton,[2] Chris Goode,[3] Jaime Perales,[4] and Eric Vidoni,[4], *1. University of Kansas Medical Center, Kansas City, Kansas, United States, 2. University of Missouri-Kansas City, Kansas City, Missouri, United States, 3. Ruby Jean's Juicery, Kansas City, Missouri, United States, 4. University of Kansas Medical Center, Fairway, Kansas, United States*

Emerging evidence suggests that up to half of Alzheimer's disease (AD) cases are a result of modifiable risk factors related to poor diet such as blood pressure and diabetes, disproportionately affecting African Americans. Adherence to brain healthy diets remains low among African Americans. Therefore, it is important to understand needs, preferences, and barriers to inform intervention designs aimed to reduce AD risk among African Americans. Using a qualitative community-based research approach, we conducted six semi-structured focus group discussions (n= 30) to explore older African American experiences with brain healthy diets, preferences, and barriers to eating healthy. Secondly, we assessed the feasibility and acceptability of collecting biomarkers of health status and dietary behaviors among African Americans. A thematic analysis was conducted to identify emerging themes and biomarkers of health status were analyzed using descriptive statistics. We identified four themes: "Knowledge and Beliefs about Brain Healthy Diets," "Eating Practices," "Ways to Improve Dietary Practices," and "Preferences for Enhancing Awareness about Brain Healthy Diets." Themes indicated that older African Americans are receptive to healthy dietary consumption but perceived lack of education, cost, access, and perception of losing one's culture as barriers to adherence. A total of 87.5% of participants completed biomarker assessments and 82% tested positive for 1 or more cardiovascular risk factor. Our study identified ways to enhance brain healthy dietary practices to reduce dementia risk. Also, our study demonstrated feasibility and acceptability in collecting AD related biomarkers in the African American community.

### FACTORS INFLUENCING IMPLEMENTATION OF EHEALTH TECHNOLOGIES TO SUPPORT INFORMAL DEMENTIA CARE: AN UMBRELLA REVIEW

Sofia Bastoni,[1] Christian Wrede,[1] Marcia C. da SIlva,[2] Robbert Sanderman,[1] Andrea Gaggioli,[3] Annemarie Braakman-Jansen,[1] and Lisette van Gemert-Pijnen,[1], *1. University of Twente, Enschede, Overijssel, Netherlands, 2. University of Twente, Esnchede, Overijssel, Netherlands, 3. Universitá Cattolica del Sacro Cuore, Universitá Cattolica del Sacro Cuore, Lombardia, Italy*

The increase of People with Dementia (PwD) living at home underscores the need for innovative eHealth technologies that support both patients and informal caregivers (IC). Sustainable implementation of eHealth technologies within this target group can, however, be difficult. Our study aims at providing an overview of (1) technologies employed in the context of informal dementia care (IDC) and (2) factors influencing the implementation of these technologies. Five databases were searched for (systematic) reviews. 21 reviews were included. A combination of deductive and inductive thematic analysis was performed, using the NASSS Framework to organize the findings. We identified technologies used "by IC", "by PwD" and "with PwD". Most represented technologies included: (i) devices for in-home monitoring (ii) technologies for supporting memory, orientation, and day structure, and (iii) communication technologies. Most factors influencing implementation related to the condition of dementia, characteristics of the technology, the expected/perceived value by users, and the characteristics of the IC. Considerably less has been reported on factors related to the implementing organization, the technology supplier, the wider institutional and sociocultural context of policy and regulations, and the adaptation of technology over time. Our study 1) created a comprehensive overview of eHealth technologies employed in the context of IDC and contributes to a better understanding of factors influencing their implementation, and 2) uncovered a knowledge gap regarding success factors for implementation related to the wider context. Although future research is needed, these findings can help researchers improving the development and implementation of eHealth technologies to support IDC.

### FACTORS RELATED TO THE CARE MANAGEMENT PRACTICE FOR OLD PUBLIC ASSISTANCE RECIPIENTS IN OSAKA CITY OF JAPAN

Takako Ayabe,[1] Yoshihito TAKEMOTO,[2] Shinichi OKADA,[3] Johannes Kiener,[4] and Masakazu SHIRASAWA,[5], *1. BAIKA Women's university, ibaraki, Osaka, Japan, 2. Okayama Prefectural University, Soja, Okayama, Japan, 3. Osaka City University, Osaka, Osaka, Japan, 4. Saitama University, Saitama, Saitama, Japan, 5. International University of Health and Welfare, Minato City, Tokyo, Japan*

The research was conducted between January 22 and February 25, 2021. The data was collected by self-administered questionnaires mailed to the participants at

800 care management centers and comprehensive community support centers in Osaka City. The centers were randomly selected. The response rate was 19.1%. The independent variables were: obtaining the qualification of a Senior Care Manager (SCM), who was a qualified person that acquired advanced knowledge and skills in care management by advanced training; experience years in Social Work (SW); experience years in care management; experiences in training programs for team approach; and experiences in training programs for supporting Old Public Assistance Recipients (OPAR). The dependent variables were the categorized contents in the Care Management Practice for old public assistance recipients. They were: Care planning and Implementation (CI); Assessment; Financial Support and Evaluation (FSE); Contract and Explanations in care management; Coordinating Informal support and Formal services in Care planning; and Arrangements in Financial supports for Formal service costs. The Structural Equation Modeling was performed for the examinations of the relationships. As a result, the goodness of the fit indices was acceptable, and we retained the models. In correlational analyses, CI and Assessment were significantly correlated with SCM ($p<.05$). FSE was significantly correlated with SW ($p<.001$) and OPAR ($p<.05$). In conclusion, the results implied that advanced qualification of a Senior Care Manager and a specified training program for supporting old public assistance recipients were effective in providing appropriate care management services.

### FAMILY CAREGIVING IN THE SOMALI-AMERICAN COMMUNITY DURING COVID-19

Rajean Moone,[1] Kamal Suleiman,[2] and Elizabeth Lightfoot,[3], *1. University of Minnesota, Woodbury, Minnesota, United States, 2. University of Pennsylvania, Woodbury, Minnesota, United States, 3. Arizona State University, Woodbury, Minnesota, United States*

This poster describes a study of Somali American family caregiving during the COVID-19 pandemic, specifically investing the unique caregiving challenges faced by Somali caregivers. The findings from this study, which was part of a larger study related to family caregiving, were drawn from in-depth interviews of ten Somali family caregivers in Minnesota. All interviews were conducted in Somali during the summer of 2020 and translated and transcribed by a certified translator and research assistant. The major themes that emerged from this study related to Visitation, Hospital Accompaniment, and Self Sacrifice. As Somali culture is centered around extended family connections, isolation places particular strain. Second, family members typically serve as advocates, translators and guides during hospital visits. The restrictions on hospital accompaniment due to COVID-19 caused increased stress and poorer care of family caregivers and their loved ones. Third, in the Somali community, family placement is a last resort. Caregivers reported great sacrifices in time, comfort and opportunities to care for family members during COVID-19. Most of the caregivers described their coping with these sacrifices in terms of radical acceptance and God consciousness. These findings have important implications for providing supports for Somali family caregivers.

### FEASIBILITY OF AN INNOVATIVE REHABILITATION PROGRAM ADAPTED FOR THE POST-ACUTE NURSING HOME SETTING

Addie Middleton,[1] Jane Driver,[2] Marcus Ruopp,[2] Lindsay Lefers,[2] Jessica Rawlins,[2] Rebekah Harris,[3] and Jonathan Bean,[3], *1. VA Boston, Boston, Massachusetts, United States, 2. VA Boston Healthcare System, Brockton, Massachusetts, United States, 3. VA Boston Healthcare System, VA Boston Healthcare System, Massachusetts, United States*

Live Long Walk Strong is a rehabilitation program that produces large clinically meaningful improvements in mobility when implemented as an outpatient program for older adults. We adapted Live Long Walk Strong for the post-acute nursing home setting within the Veterans Health Administration as a clinical demonstration project. The adapted version includes novel elements and bridges the inpatient stay and three months post-discharge. The inpatient phase focuses on maximizing functional recovery and includes activities focused on timing and coordination of gait, leg strength and power, and trunk muscle endurance. The care transition and virtual (telehealth) post-discharge phase focuses on case management and engagement in physical activity programs. Coaching and behavior change are a consistent focus throughout the program. To date, 13 Veterans (mean age 67.9, SD 11.7 years) have completed the inpatient phase, and of those Veterans, six have completed the entire program, five are still active, one was lost to follow-up, and one was rehospitalized. The program demonstrates feasibility, 91% of all inpatient sessions and 81% of all post-discharge sessions were completed. Regarding preliminary efficacy, 83% of Veterans who completed the program exceeded the minimal detectable change score (4 points) on the Activity Measure for Post-Acute Care (AM-PAC) Mobility scale from program enrollment to completion (mean change 6.5, SD 6.9 points). Based on findings from this clinical demonstration project, the program is feasible. However, future research is needed to further examine the program's impact on mobility and other outcomes important to older Veterans receiving post-acute nursing home care.

### FEASIBILITY TRIAL OF AN INTEGRATED TREATMENT "ACTIVATE FOR LIFE" FOR PHYSICAL AND MENTAL WELL-BEING IN OLDER ADULTS

Melba Hernandez-Tejada,[1] Sundaravadivel Balasubramanian,[2] Alexis Nagel,[2] Mohan Madisetti,[2] and Teresa Kelechi,[2], *1. University of Texas HSC at Houston, Houston, Texas, United States, 2. Medical University of South Carolina, Charleston, South Carolina, United States*

This study describes the feasibility and patient satisfaction for an integrated treatment to address multiple health outcomes in a sample of older adults living in a low-income independent residence facility and their own homes in the community. Specifically, 30 older adults were offered the opportunity to participate in a feasibility study of different components of Activate for Life treatment targeting balance and physical strength (Otago Exercise Program), breathing retraining (Gentle Yoga and Yogic Breathing), and mental health (Behavioral Activation for Depression). Three treatment combinations were compared in a randomized repeated measures design to determine if adding components to the existing Otago program were feasible and if this affected patient satisfaction. Arm1: the Otago strength and balance program alone (n = 10); Arm 2: Otago + Gentle Yoga and Yogic Breathing (n = 10); and Arm 3: Otago + Gentle Yoga and Yogic Breathing + Behavioral Activation (we named this combination 'Activate for Life' n = 10). Dependent measures included recruitment rate, session completion characteristics, and satisfaction with the program. Overall, study and treatment components proved feasible, and participants reported high satisfaction with all 3 Arms.

### FOOD ACCESS, DIET QUALITY, AND NUTRITIONAL STATUS OF OLDER ADULTS DURING COVID-19: A SCOPING REVIEW

Emily Nicklett,[1] Kimson Johnson,[2] Lisa M. Troy,[3] Maitreyi Vartak,[1] and Ann Reiter,[1], *1. The University of Texas at San Antonio, San Antonio, Texas, United States, 2. University of Michigan, Ann Arbor, Michigan, United States, 3. University of Massachusetts Amherst, University of Massachusetts Amherst, Massachusetts, United States*

COVID-19 has imposed challenges for older adults to access food, particularly in minority, lower income, and rural communities. However, the impact of COVID-19 on food access, diet quality, and nutrition of diverse older adult populations has not been systematically assessed. The objective of this study is to examine changes in food access, diet quality, and nutritional status among older adults during the COVID-19 pandemic and the potential differential impacts of the COVID-19 pandemic on these nutrition-related outcomes using the framework of the socio-ecological model. An electronic search was conducted using three databases (PubMed, CINAHL, and Web of Science) on March 7, 2021. Original, peer-reviewed English-language studies published 10/1/2019-3/1/2021 were considered for which the age of participants was 50 years and older (average age range 50-98). In order to be considered, studies must have examined food access, food security, or nutrition constructs as an outcome. The initial search yielded 13,628 results, of which 9,145 were duplicates. Of the remaining 4,483 articles, 13 articles were in scope and therefore selected in the final analysis, which can be characterized as descriptive (n=5), analyt-ical (n=6), and correlational (n=2). Studies were conducted among community-dwelling older adult populations (n=7) as well as those temporarily residing in hospital settings (n=6) in 10 countries. More research is needed to examine the impact of COVID-19 on food access/security and the differential barriers experienced by older adult populations.

### GENERATIONAL MULTIMORBIDITY DISEASE CLUSTERS FOR BRITISH COHORTS BORN 1921 – 1960

Stacey Voll,[1] Graciela Muniz-Terrera,[2] and Scott Hofer,[1], *1. University of Victoria, Victoria, British Columbia, Canada, 2. University of Edinburgh, Edinburgh, Scotland, United Kingdom*

The aim of this study is the first step in our understanding of the uniqueness and stability of multmorbdity disease patterns for different generations. The unique historical context that each generation has been exposed to is thought to have systemic health impacts and differences in epidemiological make-up (Clouston et al. 2021). Literature suggests that multimorbidity disease patterns, are similar across countries (Hernandez et al, 2021 – in press) and observational points, and that migration into complex disease clusters is more common as people age (Cassell et al, 2018, Kingston et al. 2018). Most commonly reported are Cardiovascular and Metabolic disease clusters which lead to lower quality of life, mortality and morbidity (Kudesia, 2021). We asked: Do multimorbidity disease patterns differ for unique generations? Using the ELSA, the disease clusters of three cohorts were examined; an older cohort, born 1921-1930, a middle cohort born 1931-1940 a younger cohort born 1941-1950 and the "newest" cohort, born 1951-1960. Self-reported dementia and memory problems lead a specific cluster for the middle cohort, those born in 1931-1940, but not for the other cohorts. While disease patterns were different between sex for other clusters, the disease cluster of dementia and memory problems held similar disease patterns for males and females, with a prevalence of 3%. The dementia/memory problem cluster loaded with cardio/metabolic diseases. This suggests that complex multimorbidity for the British 1931-1940 cohort has had an impact related to dementia and memory problem diagnoses for this specific generation, for males and females alike.

### HEALTH AND FINANCIAL RISK-TAKING PROPENSITY DURING THE COVID-19 PANDEMIC: DIFFERENCES BY AGE AND TIME

Barış Sevi, and Natalie Shook, *University of Connecticut, Storrs, Connecticut, United States*

The COVID-19 pandemic has presented a global health threat of unprecedented magnitude and had a devastating impact on the world's economy. Accordingly, the riskiness of decisions related to health and finance may have increased. However, health and financial threats have differentially affected different age groups. For example, COVID-19 posed a greater health threat to older adults (65+ years) than younger or middle-aged adults, whereas financial threat due to the pandemic affected younger and middle-aged adults more than older adults. This study examined differences in the levels of health and financial risk-taking propensity by time of the pandemic and age group: young (18-39 years), middle-aged (40-64 years), and older adults (65+ years). A sample of 488 individuals residing in the US (245 Woman; Mage = 51.07, SD = 15.99) completed three waves of surveys in March, April, and May 2020. We found that risk-taking propensity for both health and financial decisions decreased over time. The risk-taking propensity was significantly lower in April and May than March, but risk-taking propensity in April and May did not significantly differ. The three age groups were all significantly different than each other in both health and financial risk-taking propensity at all three waves. Younger adults reported higher risk-taking propensity than older and middle-aged adults, and middle-aged adults reported higher risk-taking propensity than older adults. The findings indicate that the pandemic may have influenced all individuals to take less risks in the fields of health and finance regardless of their age.

### HOUSING COST BURDEN AND WELL-BEING IN OLDER ADULTS MODERATED BY NEIGHBORHOOD COHESION AND DISORDER

Ji Hyang Cheon,[1] Min Kyoung Park,[2] and Todd Becker,[3], *1. University of Maryland Baltimore, University of Maryland Baltimore, Maryland, United States, 2. University of Maryland Baltimore County, Baltimore, Maryland, United States, 3. University of Maryland, Baltimore, Baltimore, Maryland, United States*

Although aging in the community promotes well-being in older adults, contextual factors (e.g., housing cost burden, neighborhood cohesion, neighborhood disorder) may impact this relationship. Identifying such risk factors represents a first step toward improving older adult well-being. NHATS data (Rounds 5–8) were used to answer two research questions (RQs). RQ1: “Is housing cost burden significantly associated with well-being?” RQ2: “Is this association further moderated by neighborhood cohesion and neighborhood disorder?” Participants were 18,311 adults ≥ 65 years old. Well-being was assessed by summing 11 commonly identified indicators. Two items were merged to assess housing cost burden (categories: “no burden,” “no money for utilities,” “no money for rent,” and “no money for utilities or rent”). Neighborhood cohesion and disorder were combined (categories: “no cohesion, no disorder,” “yes cohesion, no disorder,” “no cohesion, yes disorder,” and “yes cohesion, yes disorder”). Both RQs were assessed through a random coefficient model controlling for established covariates. RQ1 results revealed that, compared to “no burden,” “no money for utilities or rent” ($B = -1.22$, $p = .003$) and “no money for rent” ($B = -1.50$, $p = .007$) were significantly associated with well-being. RQ2 results revealed that “no cohesion, no disorder” significantly moderated the association between “no money for utilities or rent” and well-being ($B = -2.44$, $p = .011$). These results indicate that increased housing cost burden is associated with decreased well-being, especially for those reporting no neighborhood cohesion. Future research should examine neighborhood-level protective factors promoting cohesion for older adults to support well-being.

### HOW DO FAMILY CAREGIVERS’ VALUES INFLUENCE PAIN MANAGEMENT FOR OLDER ADULTS WITH DEMENTIA?

Hui Zhao,[1] Pamela Kulbok,[2] Ishan Williams,[2] Carol Manning,[2] and Rafael Romo,[3], *1. James Madison University, Harrisonburg, Virginia, United States, 2. University of Virginia, Charlottesville, Virginia, United States, 3. Dominican University of California, San Rafael, California, United States*

Professional caregivers rely on formal training when managing pain among patients with dementia, but family caregivers (FCGs) lack this foundation. Instead, FCGs use informal sources that may reflect a values-driven decision-making process. Few studies have examined how FGCs’ personal values impact pain management for dementia patients. We sought to examine the influence of personal values on pain management among FCGs for community-dwelling older adults with dementia using qualitative descriptive methods. Twenty-five adult FCGs, aged from 29 to 95, were recruited in central Virginia. Participants were predominantly white, married, female, and high school graduates. We conducted semi-structured interviews that were audio recorded and analyzed using constant comparative analysis. Four themes emerged: 1) Priority for pain management: when quality of life is valued over other factors (i.e., length of life), priorities focused on no pain, leading to better pain management; 2) Moral perspectives: negative views toward drugs, especially opioids, led to less use and greater report of pain; 3) Beliefs about alternative therapy: negative views led to less likely use of non-traditional approaches and reports of more pain, and 4) Personal experience of pain: past personal experiences of pain (negative or positive) influenced the priority placed on pain management and the FCG’s ability to provide effective pain management. The diverse views held by FCGs demonstrate a value-based process and suggest a modifiable factor in pain management. Helping FCGs reflect biases while reinforcing values that improve pain management would lead to improve pain and quality of life for older adults with dementia.

### HOW LONELY ARE OLDER AMERICANS ACT NATIONAL FAMILY CAREGIVER SUPPORT PROGRAM PARTICIPANTS?

Claire Pendergrast,[1] and Heather Menne,[2], *1. Syracuse University, Syracuse, New York, United States, 2. RTI, RTI, District of Columbia, United States*

Older Americans Act (OAA) family caregiver services connect family members caring for older adults with a diversity of community-based resources and supports. Social isolation and loneliness are known public health threats, and family caregivers may face greater vulnerability to loneliness given the often-intensive time demands of care provision. Policy stakeholders and aging services providers are increasingly focused on combating loneliness among older adults and family caregivers. To inform efforts to reduce loneliness, we conducted descriptive analyses to identify characteristics of the participants in the OAA National Family Caregiver Support Program associated with higher rates of loneliness, measured with the 3-item UCLA Loneliness Scale. Using data from the 2019 National Survey of Older Americans Act Participants, we examined how caregiver loneliness varied based on caregiver age, gender, income, race and ethnicity, living alone, rurality, and self-reported health, as well as care recipients’ health status and difficulties with ADLs. Among our sample of 1,824 family caregivers, rates of loneliness were high overall (70%). Loneliness was significantly higher for caregivers with poor health (71.4%), incomes less than $20,000 (75.3%), living alone (75.4%), aged 65 or older (73.2%), Hispanic caregivers (82.2%) and caregivers for care recipients with 3 or more ADLs (76.0%). Findings underscore the importance of increasing social engagement opportunities for family caregivers. Policies and programs focused on reducing caregiver loneliness should be accessible to all family caregivers but should prioritize outreach and engagement for groups at higher risk of loneliness.

### IMPACT OF IN2L TABLETS ON LONELINESS AND WELL-BEING: FINDINGS OF AN INNOVATIVE INDUSTRY-AAA PROGRAM

Lydia Nguyen,[1] Karen O'Hern,[2] Adam Siak,[2] Kristi Stoglin,[2] and Charlotte Mather-Tayor,[3], *1. iN2L, Denver, Colorado, United States, 2. iN2L, Greenwood*

*Village, Colorado, United States, 3. Area Agency on Aging of Broward County, Sunrise, Florida, United States*

Area Agency on Aging (AAA) senior and adult day centers closed due to COVID-19, causing many older adults to lose an important source of connection and engagement, leading to social isolation. To combat negative consequences, iN2L and a Florida AAA partnered on an innovative program providing iN2L tablets to AAA-supported older adults to use at home. The tablets have a simple interface, content specifically designed for older adults (e.g., games; music; movies), and video call capability. Participants included 51 independent older adults (mean age 77) and 39 family caregivers (mean age 59) of people with dementia. Participants completed phone surveys with AAA case managers at baseline and 3 months, including UCLA Loneliness Scale (3 item) and questions about their tablet experiences. Findings show positive trends for loneliness and well-being in both groups. At 3 months, lonely participants decreased from baseline by 25% for independent older adults and 18% for family caregivers. Over 80% of independent older adults agreed the tablet engages them in meaningful activities, provides daily enjoyment, and helps with relaxation. For family caregivers, 79% agreed the tablet is another tool in their caregiver toolkit and about 70% agreed the tablet adds daily enjoyment, helps with relaxation, and provides engagement in meaningful activities for their family member. Approximately 50% of caregivers felt happier, less stressed, and less irritable since using the tablets. This work has implications for the utility of technology in promoting engagement and connection, alleviating negative effects of social isolation, and the effectiveness of industry-AAA partnerships.

### IMPACTS OF THE PANDEMIC ON OLDER ADULTS' SOCIAL ISOLATION

Stephanie MacLeod, *UnitedHealth Group, United States*

The risk of COVID-19 exposure and likelihood of severe illness have been critical concerns among older adults during the pandemic. Meanwhile, social distancing has worsened social isolation, with severe impacts on connectedness among seniors. Effects of the pandemic may lead to an extended crisis, with impacts on health outcomes. Our primary purpose was to summarize emerging research describing impacts of the pandemic on social isolation and related health outcomes among older adults. A streamlined search was conducted to fit the scope of this review, with key terms determined to identify relevant publications. Common research databases and mainstream resources were utilized. We focused on research published or released since the start of 2020, primarily rapidly reviewed studies, to align with the timing of the pandemic. Early research suggests that the pandemic has worsened social isolation among older adults. Social isolation is now more urgent, as many seniors lost their usual connections due to social distancing. While these measures help to prevent virus exposure, this approach must be balanced with maintaining social connectedness. Thus, a "COVID-19 paradox" has emerged: safety protocols protect older adults but concurrently place them at risk of social isolation. Adapted approaches are urgently needed to safely address the consequences of a potential long-term social recession.

### IN VIVO QUASI-ELASTIC LIGHT SCATTERING EYE SCANNER DETECTS MOLECULAR AGING IN HUMANS AND MICE

Douglas Parsons,[1] Olga Minaeva,[1] Srikant Sarangi,[1] Danielle Ledoux,[2] Juliet Moncaster,[1] John Clark,[3] David Hunter,[2] and Lee Goldstein,[1], *1. Boston University School of Medicine, Boston, Massachusetts, United States, 2. Boston Children's Hospital, Boston, Massachusetts, United States, 3. University of Washington, Seattle, Washington, United States*

The absence of clinical tools to evaluate individual variation in the pace of aging represents a major impediment to understanding aging and maximizing health throughout life. The lens is an ideal tissue for quantitative assessment of molecular aging in vivo. Long-lived proteins in lens fiber cells are expressed during fetal life, do not undergo turnover, accumulate molecular alterations throughout life, and are optically accessible in vivo. We used quasi-elastic light scattering (QLS) to measure age-dependent signals in lenses of both healthy human subjects and wild-type C57BL/6 mice. Age-dependent QLS signal changes detected in vivo in humans and mice recapitulated time-dependent changes in hydrodynamic radius, protein polydispersity, and supramolecular order of human lens proteins during long-term incubation (~1 year) and in response to sustained oxidation (~2.5 months) in vitro. Our findings demonstrate that QLS analysis of lens proteins provides a practical technique for noninvasive assessment of molecular aging in vivo.

### INTERGENERATIONAL TUTORING: OLDER ADULTS SUPPORTING CHILDREN'S ACADEMIC NEEDS VIA VIDEO CONFERENCING

Jessica Hoffman,[1] Edward Miller,[2] Jeffrey Burr,[3] Jan Mutchler,[2] James Hermelbracht,[4] Robert Volpe,[1] Robin Codding,[5] and Amy Briesch,[6], *1. Northeastern University, Boston, Massachusetts, United States, 2. University of Massachusetts Boston, Boston, Massachusetts, United States, 3. McCormack Graduate School, Boston, Massachusetts, United States, 4. University of Massachusetts Boston, University of Massachusetts Boston, Massachusetts, United States, 5. Northeastern University, Northeastern University, Minnesota, United States, 6. Northeastern University, Northeastern University, Massachusetts, United States*

COVID-19 resulted in societal disruptions across the lifespan. School (K-12) closures were among the most challenging impacts of the virus, leaving many parents with the burden of schooling their children at home. Another major impact of the virus was the social isolation and loneliness felt by many retired, older adults, who were sheltering at home. The disruptions of COVID-19 led our inter-professional team to develop the Intergenerational Tutoring program. Intergenerational Tutoring addresses a service delivery gap in schools because tutors expand schools' capacity to implement evidence-based instruction with students in need of individual support. At the same time, research shows that meaningful volunteering supports the well-being of older adults across physical, psychosocial and cognitive dimensions of health. The aim of the Intergenerational Tutoring program is to pair older adults with kindergarten children in high needs schools to implement early literacy interventions remotely via Zoom.

Our poster will describe the Intergenerational Tutoring program including tutor training and tutoring implementation. We will summarize the initial findings from our pilot study conducted in spring and summer 2021 with tutors and children. Data will include (1) themes from tutor interviews regarding the personal meaningfulness of the program and the program's associated benefits and challenges; (2) implementation fidelity data; (3) impact of tutoring on children's early literacy skills; and (4) parent feedback. We will summarize lessons learned and next steps for the program.

### INTERSECTIONS OF AGEISM AND GENDER STIGMA: EXPLORING LONG-TERM CARE EMPLOYEES' ATTITUDES TOWARDS AGING

Tarah Loy-Ashe,[1] and Brent Hawkins,[2], *1. Southern CT State University, New Haven, Connecticut, United States, 2. University of North Carolina Wilmington, Wilmington, North Carolina, United States*

The purpose of this mixed methods, single case study was to explore long-term care (LTC) employees' attitudes towards age and gender. The intersection of Rosemary Garland-Thomson's theory of feminist disability (2001) and Hailee Gibbons' compulsory youthfulness theory (2016) provided the conceptual framework for this project. The sample consisted of 60 LTC direct care employees, all employed at the same organization, who completed an on-line survey during the COVID-19 pandemic. The survey consisted of demographic questions and the Fraboni Scale of Ageism. Twenty-one of these employees participated in a 30-45 minute phone interview. Cultural artifacts were also collected. All data were collected during the COVID-19 pandemic. Although quantitative results showed no statistical significance, qualitative results suggest that employees do exhibit some ageist attitudes towards the residents for whom they care. Despite evidence that most employees felt a deep connection with residents, they detached themselves from the aging process. The theoretical framework lends hand in explaining how both ableism and ageism appeared to contribute to this detachment. Findings indicated employees' interactions and attitudes towards residents were influenced by themes compassionate ageism, ableism, and identity, which resulted in meta theme caregiver validation and reward. Employees received validation and altruistic reward from positive interactions with what they perceived to be "ideal" residents; those who fit the stereotype of a nursing home resident, such as older, pleasant, and dependent. The perceived "ideal" residents varied by gender. Generally, female residents were expected to be more independent and at times viewed negatively when requesting assistance.

### INTERVENTIONS ADOPTING BODY-MIND-SPIRIT MODEL TO IMPROVE THE HOLISTIC HEALTH OF OLDER ADULTS: A SYSTEMATIC REVIEW

Xinyue Hu,[1] Tongtong Li,[2] and Iris Chi,[3], *1. University of California, Los Angeles, Los Angeles, California, United States, 2. University of Southern California, Arcadia, California, United States, 3. University of Southern California, University of Southern California, California, United States*

This systematic review aims to summarize 5 key information from non-pharmaceutical intervention studies which adopt Body-Mind-Spirit (BMS) model for older adults: (1) definition of BMS, (2) types and formats of the interventions, (3) background and BMS training of the interventionists, (4) activities included in the interventions, and (5) effect of these interventions on the holistic health of older adults. We conducted a systematic search of 9 databases (ProQuest, Web of Science, PsycINFO, PubMed, Cochrane, Wanfang, AIRITI, CADAL, CNKI) for studies published in English or Chinese through May 31, 2021. Inclusion criteria were: (1) Must be empirical studies; (2) Participants must be aged 55 and above; and (3) Must adopt the BMS model or contain BMS in full-text. We found 15 studies (7 RCTs, 1 cluster randomized trial, 3 mixed-method studies, and 4 qualitative studies). Ten studies (66.67%) adopted Chan's BMS model. Thirteen studies (86.67%) adopted in-person group interventions. Only five studies (33.33%) provided BMS training to the interventionists. Six articles (40%) categorized the activities as body-, mind- or spirituality-related. Ten studies (66.67%) reported effectiveness in all 3 dimensions of BMS. Of the 7 RCTs, 5 were rated as medium-quality, and 2 were rated as low-quality according to the Cochrane's Risk of Bias tool. Most interventions based on the BMS model claimed to be effective in improving the holistic health of older adults. In order to improve the internal validity, future RCT studies should be more prudent about the randomization process and adhere to the BMS model when designing the interventions.

### INTRODUCING THE ATTITUDES TOWARD TECHNOLOGY LONGITUDINAL AGING STUDY (ATLAS): INITIAL WAVE 1 FINDINGS

Robin Stuart, *Florida State University, Tallahassee, Florida, United States*

Differences between younger and older adults' use and adoption of technology have declined over the past two decades, though the mechanisms behind observed trends are uncertain. Few longitudinal studies have tried to capture detailed changes in technology attitudes, adoption, and usage over time among older adults. This presentation presents newly collected data from the first wave of the Attitudes toward Technology Longitudinal Aging Study (ATLAS), a 5-wave questionnaire-based longitudinal study of older adults' attitudes toward technology and levels of technology use ($N = 88$; Men = 30; Women = 58; Mage = 69.7 years). We present baseline characteristics of Wave 1 and explore predictors of technology use, adoption, and proficiency. Waves 2 through 5 will assess changes in these domains. Wave 1 results replicated previous findings in that older age was associated with lower computer and mobile device proficiency (computer: $r = -.219^*$, $p < .05$ , mobile device: $r = -.291^{**}$, $p < .01$). However, there was variability among both types of proficiency (McomputerProf = 27.39, SD = 3.57 ; MmobileProf = 31.52, SD = 9.21), indicating room for change over time. Both types of proficiency were correlated with level of technology use (computer: $r = -.219^*$, $p < .05$ , mobile device: $r = -.572^{***}$, $p < .001$). Taken together, these initial relationships suggest the possibility that future waves will see changes in technology use predicted by changes in age-related differences in technology proficiency and attitudes.

### LEVERAGING MEDICAL CLAIMS TO PREDICT LONG-TERM CARE TRANSITIONS AMONG OLDER ADULTS IN THE UNITED STATES

Megan Backhaus,[1] An-Ting Jhuang,[1] Ben Griffith,[1] and Lauren Bangerter,[2], *1. UnitedHealth Group, Minnetonka, Minnesota, United States, 2. OptumLabs, Eden Prairie, Minnesota, United States*

Most older adults prefer to age in place rather than moving to a long-term care (LTC) facility, but little is known about the factors that predict entry into LTC. This study sought to utilize administrative claims data to understand the predictors of LTC transitions using de-identified claims data from Medicare Advantage members in the UnitedHealth Group Clinical Discovery Database. We investigated LTC transitions of 250,587 adults (Mean age = 77, standard deviation = 7.75) between January 1, 2016 and December 31, 2019. Types of predictors for these transitions include aggregated medical data surrounding chronic conditions and frailty indices, as well as healthcare utilization and demographics in 2016 and 2017. We then fit data of these types to an extreme gradient boosting (XGBoost) model to predict long-term care transitions in 2018 and 2019 (ROCAUC = 0.84, accuracy = 0.84, precision = 0.68, and recall = 0.42). Frailty indicators, such as falls and fractures, mobility problems, dementia, and delirium, as well as osteoporosis are strong predictors of LTC transitions. These findings can be used to design interventions aimed at preventing LTC transitions and enabling older adults to age in place.

### LONG-LASTING EFFECTS OF THE PANDEMIC ON OLDER ADULTS' FOOD INSECURITY: THE URGENT NEED FOR ENHANCED SNAP BENEFITS

Lauren Popham,[1] Jane Tavares,[2] and Marc Cohen,[3], *1. National Council on Aging, Arlington, Virginia, United States, 2. LeadingAge LTSS Center @UMass Boston, Boston, Massachusetts, United States, 3. University of Massachusetts Boston, University of Massachusetts Boston, Massachusetts, United States*

Despite the start of COVID-19 pandemic recovery in the U.S., food insecurity remains at elevated levels with 10% of American adults reporting food insecurity nearly three times higher than pre-pandemic (Census Bureau's Household Pulse Survey, June 2021). To gain insight into the long-term impacts of the pandemic on older adults, we examined food insecurity patterns during the last economic recession and the role that the Supplemental Nutrition Assistance Program (SNAP) played in mitigating food insecurity and skipped meals. We analyzed data on adults age 60+ from the Health and Retirement Study, looking at the Great Recession (2008) as a predictor of what to expect in the next decade of pandemic recovery. A key finding was that food insecurity more than doubled among older adults during the Great Recession and remained elevated even 10 years later. Regression analyses showed that SNAP use among older adults weakened the relationship between poverty and food insecurity, but didn't eliminate it—17% of older adults still reported food insecurity two years after enrolling in SNAP. The data indicates that a growing share of older SNAP users' benefits have not kept up with rising food costs. In fact, 85% of beneficiaries had monthly benefit amounts below the USDA 'Thrifty Plan" budget. Congress recently passed the American Rescue Plan which increases SNAP benefits temporarily, yet these enhancements are about to run out. This study underscores the need for permanent SNAP enhancements to help prevent long-lasting hunger facing millions of older Americans.

### MANAGEMENT CHALLENGES FACED BY ADMINISTRATORS AT DEMENTIA CARE NURSING HOMES IN CHINA

Lin Jiang,[1] Fei Sun,[2] Robin Bonifas,[3] and David Hodge,[4], *1. The University of Texas Rio Grande Valley, Edinburg, Texas, United States, 2. Michigan State University, East Lansing, Michigan, United States, 3. Indiana State University, Terre Haute, Indiana, United States, 4. Arizona State University, Phoenix, Arizona, United States*

Currently, more than 10 million Chinese older adults have been diagnosed with dementia, a number that is expected to increase as the population in China rapidly ages. Yet, little research exists on dementia care in Chinese long-term care facilities. Our research addresses this gap in the knowledge-base by examining the challenges nursing home directors encounter as well as the coping strategies they employ to deal with these challenges. Twenty-one facility directors employed by faith-based nursing homes across 14 provinces in China participated in this study. Semi-structured interviews were conducted in respondents' native language via phone or in person. Most respondents reported a religious affiliation, and about half were female and had been facility directors for more than four years. Two researchers fluent in Mandarin and English coded interview transcripts; thematic analysis was conducted to identify patterns in the data. The results indicated four primary challenges, which pertained to recruiting and retaining nursing staff, funding, lacking support from the government, and conflicts with family members. The coping strategies included obtaining and using external resources such as volunteer visitors, operationalizing personal spiritual beliefs, and providing training to improve skills and empathy among employees. This study contributes to nursing home practice by expanding our knowledge of culturally relevant dementia management strategies in China. Suggestions to address management challenges from a policy and practical perspective include clear and sustainable financial support from the government, staff training, and staff-resident ratio regulations, seeking external resources, and integrating spiritual strategies into problem management and service quality improvement.

### MOTIVATION TO LEARN AND MULTILINGUALISM ACROSS THE ADULT LIFE STAGES IN THE U.S.

Shalini Sahoo,[1] Takashi Yamashita,[2] Roberto Millar,[3] and Phyllis Cummins,[4], *1. University of Maryland, Baltimore, Hanover, Maryland, United States, 2. University of Maryland, Baltimore County, Baltimore, Maryland, United States, 3. The Hilltop Institute, Baltimore County, Maryland, United States, 4. Miami University, Oxford, Ohio, United States*

Lifelong learning or continuing education over the life course has become necessary to navigate a rapidly changing technological landscape. Motivation to learn (MtL) is essential for facilitating lifelong learning. In the U.S., most of the educational opportunities are available in English. Moreover, little is known about associations between being multilingual and MtL across the life stages. This study analyzed nationally representative data from the 2012/2014/2017 Program for International Assessment of Adult Competencies (PIAAC) restricted use file (RUF). Using a previously established latent MtL construct, structural equation models were estimated by four age groups --- 25-34 (n = 2,310); 35-44 (n = 1,610); 45-54 (n = 1,670); and 55 and older (n = 2,620). Results showed that being multilingual was associated with greater MtL among younger age groups, including age 25-34 ($b = 0.20$, $p = 0.01$) and 35-44 ($b = 0.28$, $p < 0.001$), after adjusting for the demographic, socioeconomic and health characteristics of individuals. Multilingualism was not associated with MtL among older age groups, including 45-54 ($b = 0.06$, $p = 0.50$) and 55 and older ($b = 0.13$, $p = 0.19$). Findings suggest that education policies that target younger multilingual adults are likely to be effective while enhancing MtL of monolingual (i.e., English-speaking only) adults seems to be a necessary first step. Yet, a similar approach may not be effective for older adults, arguably due to more diverse life circumstances, educational needs, and learning style preferences. More detailed interpretations of empirical results and theoretical explanations are needed.

### MOTIVATIONS AND EXPERIENCES OF OLDER ADULT VOLUNTEERS IN A TELEHEALTH NURSING SIMULATION ACTIVITY

Jennifer Crittenden,[1] and Kayla Thompson,[2], *1. University of Maine, University of Maine, Maine, United States, 2. University of Maine, Bangor, Maine, United States*

The COVID-19 pandemic has posed challenges to safely engaging older adults in volunteer activities. This research explored a unique partnership between a Retired Senior and Volunteer Program (RSVP) and a school of nursing to administer a telehealth virtual simulation training for nurse practitioner students. Semi-structured interviews were carried out with nursing simulation coordinators and volunteers after the telehealth simulation exercise. The purpose of this research was to identify principles of successful virtual volunteer engagement for telehealth simulations. This initial pilot study encompassed debriefing interviews with volunteers (N = 3) and interviews with simulation coordinators (N = 2). Three major themes emerged within the response coding: 1) the benefits of virtual simulation volunteering, 2) technology as a facilitating factor and challenge, and 3) unique volunteer management considerations. Both volunteers and coordinators noted that volunteers derived positive emotional benefits and new insights from their participation. Coordinators discussed the “authenticity” factor that older adults brought to the simulation experience as a benefit to engaging older adult volunteers. Technology sub-themes included accessibility considerations, experience with the online format, and other logistical considerations in conducting telehealth simulation. Volunteer management sub-themes encompassed volunteer skills and motivations, the perceived successful aspects of training, and improvements for future simulations. Volunteers discussed an interest and connection to healthcare and education as a motivating factor for their participation in the telehealth simulation. This small scale pilot research will be expanded through future simulation activities to continue to identify principles of practice for engaging older adults in virtual volunteerism.

### NATIONAL FAMILY CAREGIVER SUPPORT PROGRAM PARTICIPANTS' RECOMMENDATIONS TO BOOST CAREGIVER SUPPORTS

Heather Menne,[1] Natalie Mulmule,[2] Angela Gasdaska,[2] Emily Costilow,[2] and Kristen Robinson,[3], *1. RTI International, Washington, District of Columbia, United States, 2. RTI International, RTI International, North Carolina, United States, 3. DLH/SSS, Silver Spring, Maryland, United States*

For more than 20 years, family caregivers have been supported through the National Family Caregiver Support Program (NFCSP) of the Older Americans Act (Title IIIE). The NFCSP provides information to caregivers about available services; assistance in gaining access to services; counseling, support groups and caregiver training; respite care; and supplemental services. In the 2019 National Survey of Older Americans Act Participants, 1,909 NFCSP caregivers were asked “What recommendations do you have for improving the service?” The resulting 748 open-ended responses were thematically coded. The thematic analysis yielded six major themes: Additional Resources, Staffing, Communication, Care Coordination, Quality of Services, and Eligibility. Sub-themes were identified for Additional Resources and Staffing. The most common sub-themes for Additional Resources were requests for more help or services (e.g., grocery shopping), increased funding or financial assistance, and more service hours (e.g., overnight or holiday care). The most common sub-theme for Staffing was the need for consistent staffing due to high turnover of staff. Chi-Squared tests and Fisher’s Exact tests indicated that there were no significant associations between any of the recommendation themes and the gender of the caregiver, employment status of the caregiver, or whether the care recipient has Alzheimer’s or dementia. Many of the themes align with results from a recent RAISE Family Caregiving Advisory Council Report. Recommendations from both sets of findings indicate ways that programs, services, and policies can be enhanced to support the needs of care recipients and caregivers.

### NEPALI OLDER ADULTS WITH PRE-EXISTING CONDITIONS AND THEIR HEALTHCARE ACCESS AMID COVID-19 PANDEMIC

Aman Shrestha,[1] Saruna Ghimire,[1] Uday Narayan Yadav,[2] Bunsi Chapadia,[1] Om Prakash Yadav,[3] Sabuj Kanti Mistry,[4] and Mark Fort Harris,[4], *1. Miami University, Oxford, Ohio, United States, 2. University of New South Wales, SYDNEY, New South Wales, Australia, 3. Ministry of Health and Population, Kathmandu, Bagmati, Nepal, 4. University of New South Wales, Kensington, New South Wales, Australia*

COVID-19 has greatly impacted older adults with pre-existing non-communicable conditions (hereafter called pre-existing conditions) in terms of their access to essential healthcare services. Based on the theory of vertical health equity, this study investigated access to healthcare by Nepali older adults with pre-existing conditions during the COVID-19 pandemic. A cross-sectional study surveyed 847 randomly selected older adults (≥60 years) in three districts of eastern Nepal. Survey questionnaire, administered by trained community health workers, collected information on participants reported difficulty obtaining routine care and medications during the pandemic, in addition to questions on demographics, socioeconomic factors, and pre-existing conditions. Cumulative scores for pre-existing conditions were recoded as no pre-existing condition, single condition, and multimorbidity for the analyses. Chi-square tests and binary logistic regressions determined inferences. Nearly two-thirds of the participants had a pre-existing condition (43.8% single condition and 22.8% multimorbid) and reported experiencing difficulty obtaining routine care (52.8%) and medications (13.5%). Participants with single (OR: 3.06, 95%CI: 2.17-4.32) and multimorbid (OR: 5.62, 95%CI: 3.63-8.71) conditions had three and five-fold increased odds of experiencing difficulty accessing routine care. Findings were similar for difficulty obtaining medication (OR single: 3.12, 95%CI: 1.71-5.69; OR multimorbid: 3.98, 95%CI: 2.01-7.87) where odds were greater than three-folds. Older adults with pre-existing conditions in Nepal, who require routine medical care and medication, faced significant difficulties obtaining them during the pandemic, which may lead to deterioration in their pre-existing conditions. Public health emergency preparedness should incorporate plans for both managing the emergency and providing continuing care.

### NUTRITIONAL RISK AND HEALTH-RELATED QUALITY OF LIFE IN OLDER ADULTS AGING WITH HIV

Nicole Viviano,[1] Ann Gruber-Baldini,[2] Sarah Schmalzle,[3] Kristen Stafford,[3] Sarah Chard,[4] Kareshma Mohanty,[3] Elizabeth Parker,[3] and Uzoamaka Eke,[3], *1. University of Maryland, Baltimore, Gaithersburg, Maryland, United States, 2. University of Maryland School of Meidcine, Baltimore, Maryland, United States, 3. University of Maryland School of Medicine, Baltimore, Maryland, United States, 4. UMBC, Baltimore, Maryland, United States*

Due to antiretroviral treatment success, individuals with HIV are living longer. People aging with HIV (PAWH, 50+) may be more likely to experience nutritional risk compared to their HIV-negative counterparts due to biopsychosocial factors. The DETERMINE checklist measure accounts for social and economic factors as well as aspects of the aging process that are not typically considered when examining nutritional risk and are important for PAWH. The current study examined nutritional risk and health-related quality of life (HRQoL) in PAWH using the DETERMINE checklist and PROMIS t-scores (mental and physical HRQoL) through secondary analyses of 158 participants in the Strengthening Therapeutic Resources in Older patients agiNG with HIV (STRONG) study. DETERMINE nutritional risk scores (0-21) were separated into 4 groups (low-risk [0-2, n=13], moderate-risk [3-5, n=28], high-risk [6-12, n=78], very high-risk [13-21, n=39]). The sample was 55% male, 94% Black/African American and had a mean age=59 (SD=5.5). Most of the sample (74%) were at high or very high nutritional risk and low HRQoL t-score: physical M=43.7 (SD=9.5), and mental M=45.7 (SD=10.1). Mental and physical HRQoL were significantly (p<.001) associated with nutritional risk group as tested through linear regressions. Means were as follows: physical HRQoL low-risk M=53.4 (SD=10.6), moderate-risk M=47.4 (SD=8.9), high-risk M=43.5 (SD=8.1), very high-risk M=38.4 (SD=8.9); mental HRQoL low-risk M=54.0 (SD=8.9), moderate-risk M=49.1(SD=7.9), high-risk M=46.1(SD=9.5), and very high-risk M=39.5 (SD=9.7). These associations remained significant after controlling for age and sex. Higher nutritional risk as measured by the DETERMINE checklist in PAWH was associated with poorer physical and mental HRQoL.

### OLDER ADULTS PLACE GREATER IMPORTANCE ON A PURPOSEFUL RETIREMENT

Rachel Best,[1] Gabrielle Pfund,[2] M. Teresa Cardador,[3] Victor Strecher,[4] and Patrick Hill,[2], *1. Yeshiva University, Bronx, New York, United States, 2. Washington University in St. Louis, St. Louis, Missouri, United States, 3. University of Illinois at Urbana-Champaign, Champaign, Illinois, United States, 4. University of Michigan, Ann Arbor, Michigan, United States*

Sense of purpose is associated with desirable health and well-being measures in older adults. Unfortunately, existing research points to complexity in the connection between purpose and retirement: some but not all people decline in sense of purpose following retirement, and some view it as nonessential to maintain a purpose specifically during retirement. These findings suggest there may be individual differences both in the importance placed on being purposeful specifically during retirement, and that there may be a discrepancy in purpose importance before retirement and during retirement. In this study, we examined whether perceived purpose importance correlates with age and personality, as well as working status. Data were collected from a U.S sample (N = 2,009), aged18-93 (M =48.51). Participants completed a survey assessing the Big Five personality traits and were asked to rate the importance of purpose before and after retirement. Findings suggest that, overall, people believe it wasrbe important to have a purpose and direction during retirement (M = 3.86). Perceived purpose importance during retirement was greater among older, conscientious, and less neurotic adults, but working status did not appear to play a role. Moreover, when comparing perceptions of purpose importance before and during retirement, age was the distinguishing factor differentiating who perceives during-retirement purpose as more important than before-retirement purpose, such that older adults placed greater importance on sense of purpose during retirement. Results indicate that older adults do value having a purpose during retirement, suggesting that purpose-focused interventions may be well-received by this population.

### ORAL HEALTH FOR OLDER ADULTS: POLICY BARRIERS AND OPPORTUNITIES

Stephanie de Sam Lazaro,[1] Anchee Nitschke Durben,[1] and Juliette Kline,[2], *1. St. Catherine University, Saint Paul, Minnesota, United States, 2. St. Catherine University, St. Paul, Minnesota, United States*

Tooth decay and gum disease are two of the most common chronic health conditions in the United States, are reversible and preventable, and impact approximately 68% of older adults nationwide (CDC, 2021; World Health Organization, 2020). While the Affordable Care Act added provisions to health prevention services, oral health prevention coverage was only included for children, leaving many adults and older adults without coverage (Nasseh & Vujicic, 2017). The research team used a rapid review process using 17 key search term combinations to identify literature in three medical databases (PubMed, CINAHL, and Consumer Health Complete) to identify system and policy level barriers and opportunities to address oral health equity issues for older adults in the United States. 40 articles met inclusion criteria for thematic analysis. Findings revealed three barrier categories: 1) poor oral health literacy of patients and health care providers, 2) reimbursement variability contributing to access and utilization barriers, 3) workforce and scope of practice variability. In addition, four opportunity categories were identified: 1) community-based oral health programming for older adults, 2) new reimbursement models, 3) medical-dental collaborations, and 4) policy and practice act updates. The COVID-19 public health crisis has impacted the implementation of some system and policy level opportunities. However, new health care initiatives specific to Medicare in discussion at the national level provide an opportunity to make some headway on the policy updates needed to address the oral health of older Americans. Findings and implications will be shared with the audience.

### PAID CARE SERVICES AND TRANSITIONING OUT OF THE COMMUNITY AMONG DIVERSE OLDER ADULTS WITH DEMENTIA

Maria Roche-Dean,[1] Sol Baik,[2] Heehyul Moon,[3] Norma Coe,[4] Anna Oh,[5] and Laura Zahodne,[6], *1. Western Michigan University, Battle Creek, Michigan, United States, 2. University of Maryland School of Social Work, Baltimore, Maryland, United States, 3. University of Louisville, university of Louisville, Kentucky, United States, 4. University of Pennsylvania, Philadelphia, Pennsylvania, United States, 5. San Francisco VA Health Care System, San Francisco, California, United States, 6. Clinical Science, Ann Arbor, Michigan, United States*

**Objectives:** Paid care provided in the home or through community organizations includes important support services for older adults with dementia such as cleaning and personal care assistance. These services could delay the transition to long-term care, but access may differ across sociodemographic groups. This study examined the relationship between paid care and transitioning out of the community among diverse older adults with dementia.

**Methods:** Using data from 303 participants (29.4% Black) with probable dementia in the National Health and Aging Trends Study (2011-2019), subdistribution hazard models estimated the association between receiving paid care at baseline and the probability of transitioning out of the community over the next eight years. Covariate selection was guided by the Andersen model of healthcare utilization.

**Results:** Paid care was associated with lower risk of transitioning out of the community (SHR = 0.70, 95% CI [0.50, 0.98]). This effect was similar after controlling for predisposing factors and most prominent after controlling for enabling and need for services factors (SHR = 0.63, 95% CI [0.42, 0.94]) and was only evident among Whites. There were no racial differences in the use of paid care, but Black participants were less likely to transition out of the community than Whites despite evidencing greater care needs.

**Discussion:** Paid care services may help delay transitions out of the community. Future research should seek to explain racial differences in access to and/or preferences for home-based, community-based, and residential care.

### PASSIVE INFRARED MOTION SENSORS IMPROVED THE DETECTION ACCURACY OF NOCTURNAL AGITATION

Wan-Tai Au-Yeung, Lyndsey Miller, Zachary Beattie, and Jeffrey Kaye, *Oregon Health & Science University, Portland, Oregon, United States*

Actigraphy has been used to detect agitation in persons with dementia, although this technology must be worn by participants. Another promising sensing methodology is passive infrared (PIR) motion, which provides continuous, low-cost, and unobtrusive data, and may also improve the detection of agitated periods. Using data from the MODERATE (Monitoring Dementia-Related Agitation Using Technology Evaluation) study, we compared the predictive value of detecting agitation in a male participant, who was 64 years old with Alzheimer's disease (AD), living in a memory care unit, and monitored with actigraphy on his wrist and four PIR motion sensors within his living quarters. The participant's medical record indicated that he experienced agitation during 17 nights over 96 consecutive days. 929,037 data points were captured for analysis. From each night, the features extracted from the actigraphy wearable included total and standard deviation of activity counts, activity counts in the most and the least active hours, and median activity counts in one hour. Features extracted from the PIR motion sensors included dwell durations in the areas around bed, sofa, front door and bathroom, and the number of transitions between these areas. Using logistic regression to predict agitated periods, comparable classification performances were achieved using these two sets of features (AUC = 0.74 for wearable and AUC = 0.71 for PIR motion sensors). When these two sets of features were combined, the classification performance showed notable improvement (AUC = 0.83). This study points to the value of utilizing PIR motion sensors for detecting dementia-related agitation.

### PATHWAYS TO REDUCED OVERNIGHT HOSPITALIZATIONS: EVALUATING 62 PHYSICAL, BEHAVIORAL, AND PSYCHOSOCIAL FACTORS

Jean Oh,[1] Julia Nakamura,[2] and Eric Kim,[1], *1. University of British Columbia, Vancouver, British Columbia, Canada, 2. University of British Columbia, University of British Columbia/Vancouver, British Columbia, Canada*

As healthcare costs rise steadily and rapidly, researchers and policymakers are increasingly interested in reducing healthcare utilization costs. Growing evidence documents many factors that may influence healthcare utilization; however, less is known about how changes in candidate predictors influence subsequent healthcare utilization. Using data from 11,374 participants in the Health and Retirement Study

(HRS)—a diverse, longitudinal, and nationally representative sample of older adults in the United States, we evaluated a large range of candidate predictors of overnight hospitalizations. Using generalized linear regression models with a lagged exposure-wide approach, we evaluated if changes in 62 predictors over four-years (between t0;2006/2008 and t1;2010/2012) were associated with subsequent hospitalizations during the two years prior to t2 (2012-2014 (Cohort A) or 2014-2016 (Cohort B)). After adjustment for a rich set of baseline covariates, changes in some health behaviors (e.g., frequent physical activity), physical health conditions (e.g., no physical functioning limitations), and psychosocial factors (e.g., higher purpose in life, lower anxiety, more volunteering) were associated with decreased hospitalizations four years later. However, there was little evidence that other factors (e.g. smoking, obesity) were associated with subsequent hospitalizations. Notably, some psychosocial factors had effect sizes as large as some physical health conditions. Several indicators of physical health, health behaviors, and psychosocial well-being may predict subsequent hospitalizations, and these factors may be novel targets for interventions and policies aiming to reduce healthcare costs in older adults.

### PERSONAL AND ENVIRONMENTAL CHALLENGES OF AGING IN PLACE WITH LONG-TERM MOBILITY DISABILITIES

Widya Ramadhani,[1] and Wendy Rogers,[2], *1. University of Illinois Urbana-Champaign, Urbana, Illinois, United States, 2. University of Illinois Urbana-Champaign, Champaign, Illinois, United States*

Maintaining independence while aging in place at home requires support, especially for older adults aging with long-term mobility disabilities. As age-related changes progress, individuals with long-term mobility disabilities experience more challenges engaging with daily living activities (ADLs) and instrumental activities of daily living (IADLs). To understand the activity challenges of these older adults, we analyzed the interview data from the Aging Concerns, Challenges, and Everyday Solution Strategies (ACCESS) study, a comprehensive user needs assessment of 60 older adults who have had mobility disabilities for at least ten years (Koon et al. 2019). We selected interview data that focused on the conduct of ten activities at home: bathing, dressing, moving around, toileting, transferring, doing hobbies, housekeeping, home maintenance, managing diet and nutrition, and caring for others. This archival study used the coding schemes from the ACCESS study that were developed using both conceptual- and data-driven approaches (Koon et al., 2019). The ecological theory of adaptation and aging (Nahemow and Lawton, 1973) was the underlying framework to identify the challenges related to older adults' functional capacity (personal) and physical environmental barriers (environmental). We identified five main challenges: physical strength, general health limitations, mobility limitations, physical access, and transferring. Older adults' responses to overcoming the challenges involved personal, environmental, and person-environment interaction strategies. This study provides insights into the relationship between the source of environmental barriers and personal coping strategies to guide the design of appropriate aging in place supports for older adults with mobility disabilities.

### PREDICTORS OF FOOD INSECURITY BEFORE AND DURING COVID-19 AMONG MIDDLE-AGED AND OLDER ADULTS

Emily Nicklett,[1] and Jianjia Cheng,[2], *1. The University of Texas at San Antonio, San Antonio, Texas, United States, 2. School of Public Health, Ann Arbor, Michigan, United States*

The indirect impact of COVID-19 on food security of middle aged and older adults is not well understood. This study examines changes in risk factors for food security from 2018-2020 in a population-based sample. Using data from the Health and Retirement Study (2018 and 2020 waves), we utilized generalized estimating equations (GEE) with repeated measures to examine factors associated with food insecurity among US adults aged 50 and older (n=3170) before COVID-19 and since COVID-19. The prevalence of food insecurity doubled from 2018 (4.83%) to 2020 (9.54%). In multivariate analyses, the population-averaged odds of experiencing food insecurity was 81% higher in 2020 compared to 2018. Other factors significantly associated with higher odds of food insecurity included being female (OR: 1.29), Black (OR: 1.46), lowest quintile for wealth (OR: 1.82), not working due to a disability (OR: 3.29), renting (OR: 2.04), greater IADL limitations (OR: 1.32), and greater number of chronic illness comorbidities (OR: 1.14). Factors significantly associated with lower odds of food insecurity included older age (65-74: OR: 0.73; 75+: OR: 0.56) and being above the median income level (OR: 0.47). Partnership status, education level, and ADL limitations were not significantly associated with the population-averaged odds of experiencing food insecurity. This study identified factors related to food insecurity among a community-dwelling sample of middle aged and older adults in the U.S. Future research should examine the impact of policies and intervention strategies to address the disproportionate impact of COVID-19 on populations at increased risk of experiencing food insecurity.

### PREDICTORS OF POSITIVE OR NEGATIVE REACTIONS TO LEARNING ALZHEIMER'S BIOMARKER RESULTS

Claire Erickson,[1] Nate Chin,[2] Erin Jonaitis,[2] Fred Ketchum,[2] Carey Gleason,[2] and Lindsay Clark,[1], *1. University of Wisconsin-Madison, Madison, Wisconsin, United States, 2. UW Madison, Madison, Wisconsin, United States*

With improved detection of Alzheimer's disease and biomarker accessibility, more adults with no or mild symptoms may learn their AD biomarker results. Yet, potential psychosocial impact of learning AD biomarkers is not well understood. In a phone survey, we assessed potential reactions after learning about a hypothetical positive AD biomarker result. Data were collected from cognitively healthy participants (n=334, mean age=64.8±7.7) enrolled in longitudinal AD studies. Exploratory factor analysis identified five latent factors following a hypothetical positive biomarker result: advanced care planning, lifestyle changes to reduce dementia risk factors, psychological distress, subjective cognitive complaints, and stigma. Using linear regression, we found that predictors of potential pessimistic reactions (distress, cognitive complaints, stigma) included higher trust in research (Distress:b:0.04, p:0.04), no dementia family history (Stigma:b:-0.30,p:0.04), poorer memory self-rating (Cognitive complaints:b:-0.19,p:0.02), and Black racial identity (Cognitive complaints:b:0.30,p:0.02, Stigma:b:0.40,p:0.003). Predictors of potential optimistic reactions (advanced care planning, lifestyle changes) included more trust in research (Planning:b:0.07,p<0.0001) and Black racial identity (Planning:b:0.38,p:0.003), as well as younger age (Lifestyle:b:-0.02,p:0.02) and belief in AD controllability (Planning:b:0.22,p:0.003, Lifestyle:b:0.23,p:0.002). Concern about developing AD was associated with increased likelihood of all potential reactions. While AD concern associates with optimistic and pessimistic potential reactions, specific factors of family history, racial identity, trust, belief in AD controllability, and memory rating differentially predict each of the potential outcomes of learning AD biomarker results. These findings may help target education efforts to prepare and reduce risk of negative reactions for cognitively healthy adults who learn their AD biomarker results.

### PROTECTION AGAINST APOE4-ASSOCIATED AGING PHENOTYPES WITH A LONGEVITY-PROMOTING INTERVENTION

Cassandra McGill,[1] Amy Christensen,[2] Wenjie Qian,[2] Caleb Finch,[3] Berenice Benayoun,[2] and Christian Pike,[2], *1. University of Southern California, Gardena, California, United States, 2. University of Southern California, University of Southern California, California, United States, 3. University of Southern California, Los Angeles, California, United States*

Two of the primary risk factors for late onset Alzheimer's Disease (AD) are aging and APOE genotype. While the causal relationship between aging and AD is not well defined, there are strong leads from shared phenotypes such as decreased metabolic function and increased inflammation. APOE genotype may be linked to AD phenotypes through the regulation of aging processes. The NIA Interventions Testing Program (ITP) recently found that 17α-estradiol (17αE2) treatment increases rodent lifespan. Since 17αE2 acts upon systemic and neural pathways associated with AD pathology, we propose that 17αE2 may be a pleiotropic intervention strategy. Further, because APOE4 is associated with a senescent phenotype, 17αE2 may have APOE genotype-specific effects. Using 10-month-old APOE3 or APOE4 targeted replacement male mice maintained on normal chow with and without 14.4 ppm 17aE2 for 20 weeks, our initial results indicate genotype differences in the efficacy of 17αE2 across multiple outcomes. APOE4 mice exhibited an aged phenotype compared to APOE3, with APOE4 mice having a higher frailty index; however, 17αE2 treatment reduced the frailty index most strongly in APOE4 mice. APOE4 mice were impaired across multiple metabolic measures including body weight, plasma leptin, and hepatic steatosis. 17αE2 significantly attenuated the APOE4 metabolic phenotype. These data confirm and extend prior findings that APOE4 is linked to progeroid effects both peripheral and neural outcomes associated with AD risk. Importantly, 17αE2 significantly improved a range of measures, but showed the strongest effects in the APOE4 genotype. This research was funded by the Cure Alzheimer's Fund.

### RACIAL DISPARITIES IN HOSPITALIZATION EXPENDITURES OF OLDER ADULTS IN SINGAPORE

Cynthia Chen,[1] Xueying Guo,[2] Hwee Lin Wee,[2] and Kelvin Bryan Tan,[3], *1. National University of Singapore, Singapore, Not Applicable, Singapore, 2. National University of Singapore, Singapore, Not Applicable, Singapore, 3. Ministry of Health, Singapore, Not Applicable, Singapore*

The World Population Prospects 2019 reports that the proportion of people who aged 65 and above takes up 9 per cent globally in 2019, reaching up to 16 per cent by 2050. Asia has the fastest rise: from 1 in 9 people aged 65 and above to 1 in 4 in 2050. Rapid growth in older adults has strong implications for diseases and healthcare expenditure. For Singapore, the transition from 'ageing society' (7% seniors) in 1999 to 'super-aged society' (20% seniors) in 2026 is projected to take 27 years, much faster than Japan's 36 years. We used the Singapore Multi-Ethnic Cohort (MEC) of 14,465 subjects aged 21 to 94, and the Future Elderly Model (FEM) microsimulation model to project disease burden and hospitalization expenditures to 2050. We found that Chinese females had the highest life expectancy of 86.0 years, followed by Indian and Malay females with 80.4 and 75.6 years respectively. In all racial groups, women lived longer than men by 5-7 years. Cumulative hospitalization expenditures of older adults aged 51+ was US$69,500 for Chinese, US$67,600 for Malays and US$86,100 for Indians; US$71,200 for males and US$70,700 for females. The increased hospitalization spending for all three ethnic groups was due to the underlying manifestation of chronic diseases, including diabetes, hypertension, heart disease and stroke. Variations in environmental risk factors such as diet, cigarette smoking and physical activity across ethnic groups may contribute to racial differences in chronic diseases and disability. Therefore, targeted interventions are needed to reduce racial disparities.

### RACIAL SEGREGATION AND MENTAL HEALTH SERVICE USE BY OLDER ADULTS

Kyeongmo Kim,[1] and Denise Burnette,[2], *1. Virginia Commonwealth University, Henrico, Virginia, United States, 2. Virginia Commonwealth University, Virginia Commonwealth University, Virginia, United States*

Older adults living in racially segregated neighborhoods often lack access to mental health care. This study assessed the role of racial segregation in mental health service use and examined whether the relationship between segregation and mental health service use differs by race/ethnicity. We linked residential segregation data from the National Neighborhood Change Database to the 2015 Medical Expenditure Panel Survey. The sample included 4,023 adults aged 65 and older. We measured mental health service use as visit(s) to a mental health professional and/or use of prescribed medication for mental health (1=yes, 0=no) during the past year. Residential segregation was assessed using a combined measure of isolation (level of interaction with the same racial and ethnic group members) and dissimilarity (evenness of distribution of racial groups). Indices ranged from 0 (integrated) to 1 (segregated). We adjusted for age, sex, race/ethnicity, marital status, education, income, attitude toward health care, health insurance, and mental health status. Multiple logistic regression analyses showed that older adults living in more segregated counties were less likely to use a mental health service than those living in more integrated counties (OR=0.77, p=.04). The relationship did not differ by race/ethnicity. As expected, Blacks and

Hispanics underused mental health services compared to Whites. The findings highlight that racial segregation limits access to mental health care. Practitioners and policy-makers should identify mental health needs and service use patterns to target services effectively and efficiently. Future research should explore the intersection of income and mental health care resources in segregated neighborhoods.

### RELATIONSHIP QUALITY PREDICTS ADVANCE CARE PLANNING ENGAGEMENT IN FAMILIES EXPERIENCING COGNITIVE DECLINE

Rachel Bloom,[1] and Celia Fisher,[2], *1. Fordham University, New York, New York, United States, 2. Fordham University, Bronx, New York, United States*

Although clinical guidelines recommend that people showing signs of cognitive decline engage in Advance Care Planning (ACP) while they still have decision-making capacity, too often this opportunity to clarify values and treatment preferences is missed among patients and their families. In reflection of the paucity of empirical data on factors influencing how this planning process begins for families experiencing cognitive decline, this study explored facilitators and barriers to ACP among adult children of parents showing signs of early- to mid-stage dementia, with a particular focus on relationship quality. Among this sample ($N = 315$), relationship quality positively and significantly predicted advance care planning engagement ($r = .349$, $p < .001$). Financial burden weakly and positively predicted ACP engagement ($r = .123$, $p <.05$), while both psychological burden (-.614, $p < .001$) and financial burden (-.290, $p < .001$) negatively and significantly predicted relationship quality. This study validates the use of the ACP Engagement Survey (ACPES) adapted for surrogates among adult children of people experiencing cognitive decline and contributes to a scarce literature on the impact of relationship quality on ACP engagement.

### RELYING ON MYSELF: THE LIVED EXPERIENCE OF BEING AT RISK FOR FALLING IN THE HOSPITAL AMONG OLDER ADULTS

Hanne Dolan, Cindy Rishel, Jessica Rainbow, and Ruth Taylor-Piliae, *University of Arizona, University of Arizona, Arizona, United States*

Inpatient falls are a persistent problem and despite research efforts during the last decade, inpatient fall rates have not significantly decreased. Older adults have an estimated 50% greater inpatient fall rate than younger adults. How older adults perceive their own fall risk affects their adherence to fall prevention recommendations. The aim of this phenomenological study was to gain a deeper understanding of the lived experiences of being at risk for falling in the hospital among older adults aged 65 years and older (N=9). Participants (female=55%, age range=67 – 86) were interviewed twice using video conferencing within two weeks of hospital discharge. The audio-recorded interviews were transcribed, and then analyzed using van Manen's interpretive phenomenological method. The Health Belief Model expanded with the concepts of independence, fear of falling, embarrassment, dignity, and positivity effect served as the theoretical framework. Five major interpretive themes emerged: Relying on Myself, Managing Balance Problems in an Unfamiliar Environment, Struggling to Maintain Identity, Following the Hospital Rules, and Maintaining Dignity in the Relationships with Nursing Staff. These themes describe how the participants thoughtfully planned their mobilization to avoid falls. This process was influenced by their struggling to remain independent, following the hospital fall prevention rules out of politeness, and experiencing both positive and negative relationships with nursing staff. Hospitalized older adults employed their self-efficacy to manage balance problems in the hospital. These findings have not been previously documented in the literature. Fall prevention interventions supporting hospitalized older adults' self-management of fall risk are needed.

### RESEARCH CONCERNING THE CARE PLANNING BY CARE MANAGERS IN HOME-BASED COMMUNITY CARE SUPPORT CENTERS OF JAPAN

Takako Ayabe,[1] and Shinichi OKADA,[2], *1. BAIKA Women's university, ibaraki, Osaka, Japan, 2. Osaka City University, Osaka, Osaka, Japan*

The research was conducted between February 9 and 28, 2017. The care management centers were randomly selected from the national list of the centers. The data were collected by self-administered questionnaires mailed to the care managers at 500 care management centers in six prefectures in the Kinki area of Japan. The independent variables were gender, age, experience years of care managers and/or social workers, Clients' Physical and Mental conditions (CPM), Client's Lifestyle (CL), Physical and Mental conditions of the Caregivers (PMC), and Human and Financial resources for Clients and their Caregivers (HFCC). The dependent of variables were the categorized contents in the care planning. They included the Approach for exploring Client's needs (AC), Coordination among Care services within the program in accordance with the needs of client (CC), Coordination among Formal services and informal supports without the program in accordance with the needs of clients (CF). We examined the relationships between the dependent and independent variables by using the Structural Equation Modeling. The results indicated that the goodness of the fit indices was acceptable, and we retained the models. In correlational analyses, AC was significantly correlated with PMC ($p<.01$), CPM ($p<.05$), and CL ($p<.001$). CC was significantly correlated with PMC ($p<.001$), CPM ($p<.001$), and CL ($p<.01$). CF was significantly correlated with PMC ($p<.05$), HFCC ($p<.05$), and CL ($p<.05$). In conclusion, our findings suggest that care managers should recognize that information concerning the clients' and their caregivers' conditions is significant in making appropriate care planning for the clients and their caregivers.

### RESPITE SERVICE USE AMONG DEMENTIA AND NONDEMENTIA CAREGIVERS: FINDINGS FROM THE CAREGIVING IN THE U.S. 2015 SURVEY

Yura Lee,[1] Wonchan Choi,[2] and Min Sook Park,[2], *1. Helen Bader School of Social Welfare, Milwaukee, Wisconsin, United States, 2. University of Wisconsin-Milwaukee,*

*University of Wisconsin-Milwaukee/Milwaukee, Wisconsin, United States*

Caring for persons with dementia places a significant physical, emotional, and financial burden on caregivers. Although dementia caregivers may be exposed to more challenging caregiving environment than caregivers of those with other chronic conditions, little is known about how specific factors related to respite service use differ between dementia and nondementia caregivers. Thus, this study first examined factors related to respite service use among caregivers and further tested the moderating effect of dementia caregiver status in these relationships using nationally representative U.S. data. Logistic regression analyses were conducted among 1,203 caregivers (276 dementia and 927 nondementia caregivers) from the national Caregiving in the U.S. 2015 data. Caregivers' race and ethnicity as a predisposing factor, caregivers' self-rated health as an enabling factor, and care recipients' living arrangement and functional limitations as need factors were significantly related to respite service use among caregivers. Moreover, dementia caregiver status moderated the association between enabling factors (i.e., household income, work status, and self-rated health) and respite service use. Our findings imply that dementia caregivers may be more in need of respite service use than nondementia caregivers when they have limited enabling factors (e.g., lower household income, nonworking status, poorer health). Policy and practice efforts that specifically support enabling factors are suggested to promote more respite service use among dementia caregivers.

### ROLE OF NATIVITY IN END OF LIFE CARE PLANNING

Kay Thwe Kyaw,[1] Elizabeth Helzner,[2] Carl Rosenberg,[3] and Michael Reinhardt,[3], *1. SUNY Downstate Health Sciences University, Peekskill, New York, United States, 2. SUNY Downstate Health Sciences University, Brrooklyn, New York, United States, 3. SUNY Downstate Health Sciences University, Brooklyn, New York, United States*

Given the rapidly aging population, optimal end-of-life (EOL) consistent with individual wishes is a public health priority. Advanced Care Planning (ACP) involves Advanced Directives (AD) and establishing a Power of Attorney (POA). AD describe EOL Care preferences including options to limit treatment, withhold treatment, provide comfort care, and prolong treatments. Nativity can provide meaningful guidance in decision-making at the end of life. Data from this study came from the Health and Retirement Study, nationally representative longitudinal study of U.S. residents. The sample included 4,015 older adults, 65 and above years of age who died during study follow-up. Nativity was categorized as U.S born and Foreign born. ACP variables included presence of AD and POA, and EOLC preferences included provide comfort care, limit, withhold, or prolong treatment. Covariates included age, gender, race, marital status, education, and subjective health at baseline. Cox Proportional Hazards (Cox PH) and Weibull Models were used to identify associations between nativity and end of life care.

**Results:** Compared to U.S born, Foreign born participants were less likely to have POA (HR: 0.75; 95% CI:0.64-0.89) in Cox PH and POA (HR: 0.63; 95 % CI:0.53-0.75) Weibull models in unadjusted models, limited treatment (HR: 1.58; 95 % CI: 1.2, 2.1), and prolong treatment (HR: 0.23; 95 % CI:0.06-0.99) and Cox PH and (HR: 0.20; 95 % CI: 0.05-0.83) in Weibull modes.

**Conclusion:** There are differences in Advanced Care Planning by nativity. Country of origin should be considered when helping individuals plan for end-of-life care.

### ROLES OF SOCIAL ISOLATION AND RURALITY ON FUNCTIONAL LIMITATIONS IN OLDER ADULTS LIVING IN THE US

Kaleigh Ligus,[1] Keith Bellizzi,[1] and Greg Rhee,[2], *1. UCONN, Storrs, Connecticut, United States, 2. UCONN, Farmington, Connecticut, United States*

Social isolation is a growing problem among adults aged ≥65. Using 2019 data from the National Health and Aging Trends Study (NHATS) (n=4,603), we examined the associations of social isolation and rurality with functional limitations in US older adults. We hypothesized that rural older adults would report social isolation more than non-rural adults, and social isolation and rurality would have an interaction effect on difficulty in performing activities of daily living (ADLs). Stress buffering theory guided this research suggesting individuals who have greater social connections also have greater coping skills to buffer against health-related stress. We assessed rural and non-rural older adults' social isolation (measured by a composite score of engagement in community activities and social connections) and difficulty in completing ADLs (e.g., difficulty in dressing, bathing, and eating in the past month). Our results supported one of the hypotheses that there were differing levels of social isolation among both rural and non-rural older adults. In both rural and non-rural groups, oldest-old (85+), non-White adults, those with poor health or had multiple comorbidities were significantly isolated or experienced severe isolation. These results support (1) the premise that specific demographic characteristics are associated with social isolation as well as (2) a growing body of research showing rural adults have unique characteristics that are protective against social isolation. Our findings are related to demographic predictors which could help target interventions toward specific at-risk groups. Policymakers and healthcare practitioners should be aware of risks for social isolation and prepare to discuss these issues.

### SEDENTARY BEHAVIOR, SLEEP QUALITY, AND SUBJECTIVE MEMORY FUNCTION IN MIDDLE-AGED ADULTS

Yen Chen,[1] Carole Holahan,[2] and Darla Castelli,[2], *1. University of Michigan, Ann Arbor, Michigan, United States, 2. The University of Texas at Austin, Austin, Texas, United States*

While there is evidence that prolonged sedentary behaviors (SB) are associated with poor memory performance, less is known about the effect of SB on subjective memory. Poor subjective memory could be an early symptom of mild cognitive impairment or dementia. Besides SB, sleep quality has been identified as an essential component of cognitive health. Yet little is known about the effects of different types of SB on sleep quality and how such effects could, in turn, affect middle-aged adults' subjective memory. The sample included 306 adults ranging in age from 40 to 60 (M = 44.42) from

Amazon MTurk. Mediation analysis with PROCESS (Hayes, 2013) was used to examine the mediational path, controlling for demographic variables. Exploratory factor analysis categorized 10 different sedentary activities into Common Engaging SB (e.g., sitting in cars), High Engaging/Leisure SB (e.g., doing hobbies when sitting), and Less Engaging/Passive SB (e.g., watching TV). Common Engaging SB included a variety of behaviors, including transportation, reading, talking on the phone, and socializing. Common Engaging SB had a significant direct effect on sleep quality (B = 0.19, p < .001) and frequency of forgetting (B = -1.61, p < .001), and sleep quality had a significant direct effect on frequency of forgetting (B = -1.90, p < .001). Sleep quality mediated the association between Common Engaging SB and frequency of forgetting (indirect effect = -0.05, 95% bootstrap CI = -0.09, -0.02). Reducing sitting time, particularly Common Engaging SB, could be a useful strategy to improve subjective memory functioning.

### SELF-REPORTED FRAILTY CONCERNS IN OLDER BREAST CANCER SURVIVORS DURING THE COVID-19 PANDEMIC

Adele Crouch,[1] and Diane Von Ah,[2], *1. University of Pennsylvania, Philadelphia, Pennsylvania, United States, 2. Indiana University, Indianapolis, Indiana, United States*

Frailty among older adults is common, especially those who have undergone breast cancer treatment; however, we do not know how frailty among this group presented during the COVID-19 pandemic. The purpose of this descriptive, cross-sectional study was to examine self-reported frailty among older breast cancer survivors (BCS) during the pandemic. This IRB-approved study recruited BCS who were at least 1-year post-treatment and 60 years of age or older, via online advertisements (e.g., Dr. Susan Love Foundation). BCS completed demographic and Tilburg Frailty Indicator (TFI) RedCap questionnaires from 11/2020 to 05/2021. The TFI, is a 15-item measure with 3 sub-scales with published cut points indicating frailty: total (5), physical (3), psychological (2), and social (2). Descriptive statistics were used. Older BCS (n=203) who were on average 65.5 (SD=4.7) years of age, white (93.6%; n=190) and had stage II breast cancer at diagnosis (39.9%; n=81) participated. The average total (M=5.4, SD=2.5) and physical (M=3.2, SD=1.5) frailty scores were above the threshold for frailty. Overall, 58.6% (n=119) and 63.1% (n=128) scored at or above the threshold on the total and physical sub-scales, respectively. In addition,78.8% (n=160) responded that they 'missed having people around' on the social frailty sub-scale. Research has shown that higher TFI scores (more frailty) are associated with increased healthcare utilization, poorer quality of life, and even mortality. Thus, frailty among older BCS is an important health concern within the context of the pandemic. Further research is needed to understand the lasting effects of self-reported frailty for BCS including COVID-19 survivors.

### SHOULDER FUNCTION AND FATTY INFILTRATION ON MRI IN OLDER ADULTS DURING REHABILITATION FOR ROTATOR CUFF TEAR

Derik Davis,[1] Ranyah Almardawi,[2] Omer Awan,[2] Lawrence Lo,[3] Sagheer Ahmed,[4] Shams Jubouri,[2] and Rao Gullapalli,[2], *1. University of Maryland School of Medicine, Ellicott City, Maryland, United States, 2. University of Maryland School of Medicine, Baltimore, Maryland, United States, 3. Perelman School of Medicine at the University of Pennsylvania, Philadelphia, Pennsylvania, United States, 4. Geisel School of Medicine at Dartmouth, Lebanon, New Hampshire, United States*

Rotator cuff tear is highly prevalent in older adults, with supraspinatus tendon tear (STT) the most common. Shoulder rehabilitation is a major treatment strategy, but supraspinatus-muscle-fatty infiltration (FI) and shoulder function in older adults with rotator cuff tear primarily managed by physical therapy (PT) is inadequately documented. We tested the hypothesis that older adults receiving usual-care PT when stratified by supraspinatus tear-status differ in supraspinatus FI [by quantitative Dixon fat fraction (FF) and semi-quantitative Goutallier grade (GG) on MRI] and shoulder function [by the American Shoulder and Elbow Surgeons score (ASES-score)] over time. Longitudinal cohort study (pilot): adults 60-85 years, PT-cohort (n=15) and control-cohort (n=25). Participants completed both shoulder MRI and ASES survey at baseline and follow-up visits. Kruskal-Wallis test compared within cohort among 3 groups: no tear (no-STT), partial-thickness tear (pt-STT), full-thickness tear (ft-STT). Mann-Whitney U test compared equivalent groups between cohorts. Baseline PT-cohort groups differed for GG (p=0.033) [no tear, 0.50±0.50;pt-STT, 1.11±0.22;ft-STT, 1.50±0.50] without difference in age, BMI, comorbidity, or ASES-score. Baseline control-cohort groups differed for FF (p=0.034) [no-tear, 5.77%±1.16%;pt-STT, 7.14%±6.26%;ft-STT, 21.44%±10.44%], without difference in age, BMI, comorbidity, or ASES-score. Baseline no-tear groups for ASES-score (p=0.049) differed between cohorts: PT-cohort (58.87±8.21) versus control-cohort (83.98±21.89). Both cohorts showed no difference in Δ-FF or Δ-GG over time. PT-cohort groups differed for Δ-ASES-score over time (p=0.042)[no-tear, 16.65±4.69;pt-STT, -7.24±0.94;ft-STT, 4.48±3.45], but control-cohort groups did not (p>0.050). Our results suggest differences exist for supraspinatus FI and self-reported shoulder function among older adults receiving PT for rotator cuff tear when stratified by supraspinatus tear-status.

### SIX-MONTH LOWER-LEG SENSORY STIMULATION AUGMENTS NEURAL NETWORK CONNECTIVITY ASSOCIATED WITH IMPROVED GAIT

Chun Liang Hsu,[1] Ikechukwu Iloputaife,[2] Lars Oddsson,[3] Brad Manor,[4] and Lewis Lipsitz,[5], *1. Hinda and Arthur Marcus Institute for Aging Research, Harvard Medical School, Burnaby, British Columbia, Canada, 2. Hebrew Senior Life, Boston, Massachusetts, United States, 3. RxFunction, Eden Prairie, Minnesota, United States, 4. Hinda and Arthur Marcus Institute for Aging Research, Harvard Medical School, Boston, Massachusetts, United States, 5. Hebrew SeniorLife, Boston, Massachusetts, United States*

Foot sole somatosensory impairment associated with peripheral neuropathy (PN) is prevalent and a strong independent risk factor for gait disturbance and falls in older adults. A lower-limb sensory prosthesis providing afferent input related to foot sole pressure distributions via lower-leg vibrotactile stimulation has been demonstrated to improve gait in people with PN. The effects of this device on brain function related to motor control, however, remains equivocal. This study aimed to explore changes in brain network connectivity after six months of daily use of the prosthesis among individuals with diagnosed PN and balance problems. Functional Gait Assessment (FGA) and resting-state functional magnetic resonance imaging were completed before and after the intervention. Preliminary analysis on participants who have completed the study to date (N=5; mean age 76 years) indicated altered connectivity of the sensorimotor network (SMN), frontoparietal network (FPN), and the default mode network (DMN) post-intervention ($Z>3.11$, unadjusted $p<0.05$). Participants displayed an average improvement of 5.5 point in the FGA (Minimal Clinically Important Differences>4 for community-dwelling older adults) that was correlated with connectivity changes (unadjusted $p<0.05$). Specifically, improved FGA was associated with: 1) increased connectivity between the SMN, cerebellum, and occipital cortex; 2) increased connectivity between the FPN, cerebellum, calcarine and intracalcarine; and 3) decreased connectivity between DMN and intracalcarine. These early findings suggest that long-term use of a lower-limb sensory prosthesis may induce neuroplastic changes in brain network connectivity reflecting enhanced bottom-up sensory-attentional processing and suppression of the DMN that are relevant to gait improvements among older adults with PN.

### SOCIAL CONNECTEDNESS, MEALS ON WHEELS SERVICES AND HEALTHCARE UTILIZATION AMONG HIGH-NEED OLDER ADULTS

Jennifer Chubinski,[1] Sarah Walsh,[2] and France Weaver,[1], *1. Xavier University, Cincinnati, Ohio, United States, 2. Eastern Michigan University, Ypsilanti, Michigan, United States*

Homebound vulnerable adults 65+ are at an increased risk for social isolation and loneliness. The adverse consequences of loneliness are profound – including increased health care utilization, burden of dementia, chronic diseases, and mortality. Meals on Wheels (MOW) is a familiar source of nutritional support for homebound individuals who wish to stay in their homes and has additional important benefits. A growing body of evidence demonstrates that MOW provides mental and social health benefits beyond nutrition, but less is known about the interplay between MOW, social cohesion, and health services use. This project will address this gap in the literature using data from the 2013-2020 National Health and Aging Trends Study (NHATS), a nationally-representative panel study of 65+ Medicare enrollees. Using matching and longitudinal multivariate techniques, the risks of hospitalization and permanent nursing home entry are compared between MOW users and non-users. Our longitudinal dataset includes 11,266 observations. Of those, 12.8% rely on MOW or other food assistance (N= 1,488) and 16.6% experience low social cohesion (N= 1,936). Some 6.6% of participants are nursing home residents (N= 748) and the 39.1% report an overnight hospital stay in the prior year (N= 4,560). MOW is a comparatively low-cost intervention to help homebound older adults retain their independence and limit costlier healthcare utilization. This work extends our understanding of MOW services beyond simple nutrition benefits to its potential impact on social health.

### SOCIAL DETERMINANTS OF HEALTH AMONG OLDER KOREAN IMMIGRANTS IN THE UNITED STATES: A SYSTEMATIC REVIEW

Sojeong Lee,[1] and VICTORIA RIZZO,[2], *1. University at Albany, State University of New York, Albany, New York, United States, 2. University at Albany, SUNY, Albany, New York, United States*

The visible impact of the SDoHs on health and behavioral health as well as health disparities among minority populations is heightened due to COVID-19. One group about which little is known in relation to SDoHs is the older Korean immigrant population in the U.S. To examine the impact of SDoHs on the health, mental health, and health care utilization, a systematic review of studies focused on SDoHs for this population was conducted. Using multiple indexing terms, databases were searched for articles published in English between January 1, 2011 and December 2020. Articles were included in the search if they examined social determinants of health of older Korean immigrants defined as foreign-born Koreans aged 60 or older who live in the United States regardless of citizenship or legal immigration status. A total of 1090 articles were identified in the search. A review of abstracts for inclusion criteria resulted in 118 articles for review. Seventy-one articles were excluded during the review process. A total of 47 articles met inclusion criteria and were evaluated. The review revealed that SDoHs, including education level, financial resources, access to health insurance, level of acculturation and level of social support, influenced cognitive status, depressive symptoms, health status and quality of life. These findings validate the need for interventions to address the social care needs of older Korean immigrants and can be used to identify the role of social workers in addressing the SDoHs that result in health disparities for older Korean immigrants.

### STIGMA OF DEMENTIA DURING COVID-19: FIRST INSIGHTS FROM A TWITTER STUDY

Juanita-Dawne Bacsu,[1] Megan O'Connell,[1] Alison Chasteen,[2] and Sarah Fraser on behalf of CCNA's SSI Working Group,[3], *1. University of Saskatchewan, Saskatoon, Saskatchewan, Canada, 2. University of Toronto, Toronto, Ontario, Canada, 3. University of Ottawa, Ottawa, Ontario, Canada*

Stigma is a critical issue that reduces the quality of life for people living with dementia and their care partners. Despite this knowledge, little research examines stigma of dementia, especially within the context of the COVID-19 pandemic. This presentation aims to: 1) identify the contributing factors of stigma against dementia during the COVID-19 pandemic; and 2) describe actions to challenge stigma of dementia. Using Twitter data, tweets were compiled with Python's GetOldTweets application from February to September 2020. Search terms included keywords for dementia (e.g., Alzheimer's) and COVID-19 (e.g., coronavirus). From the 20,800 tweets, filters were used to exclude irrelevant tweets. The remaining 5,063 tweets were analyzed by a group of coders with 1,743 tweets identified for further stigma-related coding. The 1,743 tweets were exported to Excel for thematic analysis and divided among 13 coders. Each tweet was coded independently by two reviewers to ensure intercoder reliability (e.g., 86%). Contributing factors of stigma of dementia included: ageism and devaluing the lives of people with dementia (e.g., 'old and dying anyways'); misinformation and false beliefs (e.g., 'COVID-19 vaccine causes dementia'); political dementia-related slander and ridicule (e.g., 'dementia Joe'); and stigma within healthcare and long-term care organizations (e.g., pushing DNR orders). Globally, there is an urgent need for more dementia education and awareness targeted towards the general public, healthcare workers, and policymakers to reduce stigma against people living with dementia. Further research is necessary to explore the contributing factors and interventions to reduce stigma of dementia during the COVID-19 pandemic and beyond.

### THE (UN)WILLINGNESS TO KEEP LIVING AT VERY ADVANCED AGES

Oscar Ribeiro,[1] Lia Araujo,[2] Laetitia Teixeira,[3] and Rosa Marina Afonso,[4], *1. University of Aveiro, University of Aveiro, Aveiro, Portugal, 2. Polytechnic Institute of Viseu, Viseu, Viseu, Portugal, 3. ICBAS - University of Porto, ICBAS.UP, Porto, Portugal, 4. University of Beira Interior, University of Beira Interior, Castelo Branco, Portugal*

A long life is a general desire that will be reached by more and more people, particularly in developed countries. But the delay of mortality raises important questions about quality of life in the later years. Centenarians have received attention from different disciplines, particularly from demography and genetics, but a psychological approach on whether life at age 100 is perceived as worth living is still very limited. This study explores centenarians' will to live and associated factors in a sample of 121 centenarians (mean age 101 years; SD 1.63 years; 84.3% female), who answered to a questionnaire comprising sociodemographic characteristics, health status, social functioning, and well-being as well as open questions on their will to live and end-of-life issues. Of the total sample, 31.4% expressed willingness to live longer, 30.6% did not, and 38% presented no clear positioning. From the qualitative thematic analysis, annoyance, uselessness, loss of meaning, disconnection, and loneliness were the most common justifications for being reluctant to live longer. Positive valuation of life and good self-rated health, followed by having a confidant and reduced pain frequency, were the reasons for being willing to live longer. From the quantitative analysis, associated factors of will to live include pain frequency, self-rated health, having a friend confidant and valuation of life. This study provides researchers suggestions for further investigation and highlight the importance of inquiring and understanding very old people's values and views on their will to live, future wishes, and meaning in life.

### THE DAILY CHALLENGES OF CARING FOR GRANDCHILDREN: A DAILY ANALYSIS OF EMOTIONAL AND PHYSICAL REACTIVITY TO STRESSORS

Elizabeth Hahn Rickenbach, and Janelle Fassi, *Saint Anselm College, Manchester, New Hampshire, United States*

Grandparents are increasingly providing extensive and custodial care for their grandchildren. Many factors have contributed to a societal rise in caregiving among grandparents, including addiction, incarceration, dual-income families, and the cost of childcare. Past work has highlighted positive effects of grandparenting (e.g. reduced dementia risk); however, research is limited that examines the day to day challenges grandparent caregivers experience. The goal of this research was to examine daily experiences of stressors, positive events, physical symptoms, and daily mood of grandparent caregivers. Participants (n=18 grandparent caregivers) filled out a diary survey for five consecutive days that measured daily stressors and positive events. A total of 90 diaries were completed. Stressors were reported on 97.6% of days. Multilevel analysis examining emotional and physical reactivity to daily events showed that, controlling for age and gender, on days when participants reported more stressors than average, they reported higher negative affect (p=.019), lower positive affect (p=.003) and more physical symptoms (p=.002). Positive events were not significantly associated with daily mood or daily physical symptoms. Overall, the findings supported the hypothesis that grandparent caregivers experience emotional and physical reactivity to the daily challenges they experience. Future research should examine resources and supports to reduce the impact of daily stressors, as well as the particular challenges among underrepresented groups, particularly Black and Latino grandparents, who provide disproportionate levels of care for their grandchildren. The current study highlights the potential vulnerability and daily needs for support among grandparents who provide regular and custodial care for their grandchildren.

### THE FINANCIAL AND OPERATIONAL EFFECTS OF COVID-19 ON OHIO'S LONG-TERM CARE RESIDENTIAL SETTINGS

Katherine Kennedy,[1] Robert Applebaum,[1] and Kathryn Brod,[2], *1. Miami University, Oxford, Ohio, United States, 2. LeadingAge Ohio, Columbus, Ohio, United States*

The COVID-19 pandemic has had a drastic impact on Ohio's long-term care facilities. Yet, months into the crisis, the financial ramifications and workforce shortage were unknown. In partnership with the Scripps Gerontology Center at Miami University, LeadingAge Ohio and the Ohio Health Care Association developed an online survey that was launched in July 2021. Response rates were 46.4% for skilled nursing facilities (SNFs; N=446) and 35.8% for residential care facilities (RCFs; N=287). Core questions compared the first quarters of 2020 and 2021. Declines in operating revenues (-11.7% SNFs; -10% RCFs) and rising labor costs per patient day (17.9% SNFs; 16.1% RCFs) contributed to most providers experiencing a financial loss in the most recent month (78% SNFs; 66% RCFs). The increased documented use of agency staff is an important finding of this work; 62% of SNFs and 34% of RCFs spent money on agency staff. Despite increases in starting wages, the labor crisis remains severe. As of July 2021, SNFs had an average of 19.51 open positions, of which 9.82 were for state-tested nurse aides and 5.65 were for nurses. RCFs had an average of 8.83 open positions, of which 4.24 were for resident care assistants and 1.89 were for nurses. The challenges faced by the long-term care industry have rightly focused on the deleterious impacts of COVID on residents and staff. But these data also suggest that the financial impacts on the industry are serious and will likely shape access and provision of care in the future.

### THE GROWING BURDEN OF INFORMAL CAREGIVERS DURING COVID-19

Stephanie MacLeod, *UnitedHealth Group, United States*

Caregiver burden has negative effects on health outcomes and quality of life. Meanwhile, safety protocols during the

COVID-19 pandemic created immediate impacts on informal caregiving with increasing burden on family caregivers. Our primary purpose was to describe the impacts of the pandemic on caregiver burden among informal caregivers, and their sudden shift in roles as a result. This review describes emerging effects on various aspects of health and explores future directions to support informal caregivers. A streamlined search was conducted to fit the scope of this review, with key terms determined to identify relevant publications. Common research databases and mainstream resources were utilized. We focused on research published since March 2020 to align with the timing of the pandemic in the US. Early research suggests that the pandemic has worsened caregiver burden among informal family caregivers. Reported health impacts include greater stress, pain, depression, sleep problems, and irritability, decreased social connectedness and quality of life. Informal family caregivers face negative health outcomes and distress as a result of greater caregiver burden and intensity during the COVID-19 pandemic. Immediate solutions are needed to alleviate this growing burden and provide ongoing support. Future work should explore the potential of boosting positive resources such as resilience and purpose to ease caregiver burden.

### THE LIVE LONG WALK STRONG REHABILITATION PROGRAM: WHAT FEATURES IMPROVE MOBILITY SKILLS? VIRTUAL PILOT

Rebekah Harris,[1] Jonathan Bean,[1] Elisa Ogawa,[2] Addie Middleton,[2] and Catherine Kelly,[2], *1. VA Boston Healthcare System, VA Boston Healthcare System, Massachusetts, United States, 2. VA Boston, Boston, Massachusetts, United States*

To evaluate the feasibility of delivering the Live Long Walk Strong (LLWS) rehabilitation program among community dwelling, mobility limited older Veterans in the VA Boston Healthcare System. Community dwelling Veterans 50 years and older identified as being at high risk for mobility decline based on self-report task modification and AM-PAC mobility questions. All Veterans received 10 sessions over 8 weeks of LLWS Physical Therapy care focusing on novel impairments related to mobility decline and behavioral change strategies. Sessions were delivered 1:1 with a Physical Therapist over 45 minutes. To assess feasibility, we tracked recruitment and retention metrics. We assessed length of each session, number of sessions attended, and any reason for withdrawal. To examine technological feasibility, we recorded number and type of issue along with resolution of the issue. A total of 178 Veterans were contacted to participate. Twenty Veterans were enrolled into the LLWS virtual pilot between October 2020 – May 2021. Among our 20 enrolled, 5 did not complete the program. Reasons for not completing included: being enrolled in another exercise study simultaneously and non-related medical complications. Among those completing, an average of 9.7 out of 10 intervention sessions were completed. An average of 1.8 technology difficulties per Veteran was experienced within the intervention. The most frequent technology issues experienced were related to camera positioning and Wi-Fi bandwidth resulting in delayed video and audio. LLWS is feasible to deliver as a virtual mode of care in middle and older aged Veterans at high risk for mobility decline.

### THE MEDIATION EFFECTS OF PERCEIVED HEALTHCARE DISCRIMINATION ON RACIAL AND ETHNIC DISPARITIES IN END OF LIFE CARE

Junghee Han, *University of Southern Indiana, Evansville, Indiana, United States*

Numerous studies demonstrate racial and ethnic differences in end-of-life (EOL) care, including place of death and hospice use. Experiencing discrimination in healthcare is often cited in the literature as a potent source explaining the pathways of the racial and ethnic disparities in EOL care. However, none of the studies have tested its mediating effects on racial and ethnic disparities in EOL care. The study examines if the effects of race and ethnicity on place of death and hospice use are mediated by perceived healthcare discrimination. This is a secondary analysis of 2008-2014 data from the nationally representative Health and Retirement Study, including oversampling of Blacks and Hispanics. Samples included 1,446 decedents aged 65 or older who completed an exit interview by proxy. Perceived healthcare discrimination was measured by a self-report of receiving poorer service or treatment than other people from doctors or hospitals. In multivariate analyses, Blacks were more likely to die at hospitals ($OR=1.57$, $p < .05$) than Whites. Those who experienced discriminatory healthcare were more likely to die at hospitals than those who never experienced discriminatory healthcare ($OR=1.44$, $p < .05$). However, the Karlson-Home-Breen (KHB) method showed no significant mediating effects of perceived healthcare discrimination on racial and ethnic disparities in place of death. Race and ethnicity did not affect hospice use. Although there is no mediating effect of healthcare discrimination on racial and ethnic disparities in EOL care, its direct impact is observed. The research contributes to evidence on the significant role of discrimination in healthcare choices.

### THE RELATIONSHIP BETWEEN FAMILY CAREGIVERS' ANTICIPATORY GRIEF AND SEVERITY OF DEMENTIA.

Nicole Gavin,[1] Mu Shan,[2] Shelly Johns,[3] Katherine Judge,[4] and Nicole Fowler,[1], *1. Indiana University School of Medicine, IU Center for Aging Research, Indianapolis, Indiana, United States, 2. Indiana University, Indianapolis, Indiana, United States, 3. Indiana University School of Medicine, Center for Health Services Research, Indianapolis, Indiana, United States, 4. Cleveland State University, Cleveland, Ohio, United States*

Anticipatory grief is the process of experiencing normal bereavement before the physical death of a significant person. To date, anticipatory grief has been related to higher levels of caregiver depression, anxiety, subjective burden, and poorer problem solving. Additionally, higher levels anticipatory grief are observed in caregivers of those with Alzheimer's Disease and Related Dementias (ADRD) compared to caregivers of those with mild cognitive impairment, implying a relationship between disease severity and caregiver anticipatory grief. Analyses were performed on data for ADRD caregivers (n=56) enrolled in the IU Telephone Acceptance and Commitment Therapy for Caregiver (TACTICs) trial; an RCT evaluating an ACT intervention for ADRD caregivers with anxiety. Inclusion criteria included identifying as the primary caregiver of an ADRD patient, and clinically significant anxiety (GAD7 score >10). The average age of caregivers was 61.9 years, 41.1% were spouses, 83.9% were white and 14.3% were black. Mean anticipatory grief scores were notably higher (84.6) compared to the previously reported means across the literature (74.9). Using multiple regression models, we determined a caregivers' anticipatory grief, as measured by the anticipatory grief scale, is not significantly associated with the patients' dementia severity, as measured by the Dementia Severity Rating Scale (DSRS) (p=0.66), Results revealed that higher levels of caregiver burden, as measured by the Zarit Burden Index, are significantly associated with more anticipatory grief (p< 0.01). Understanding these relationships contributes to a better understanding of ADRD caregivers, identifying "high-risk" caregivers, and informing future interventions and care.

### UNDERSTANDING FUNCTIONAL MOBILITY AND QUALITY OF LIFE AMONG ISOLATED AGING POPULATION IN THE US

Jessika Dayrit,[1] Ana maria Pasatiempo,[2] and Kevin Hitosis,[3], *1. Washington University in St. Louis, Tamuning, Guam, United States, 2. Department of Veterans Affairs, Department of Veterans Affairs, Alaska, United States, 3. Department of Veterans Affairs, Agana Heights, Guam, United States*

**Background:** Social isolation among older adults linked to serious health conditions. However, little is known if functional mobility among isolated aging adults impacts quality of life. Objectives: This study will examine the association between levels of functional mobilities and risks for developing depression, poor health status, and physical inactivity by controlling socioeconomic factors.

**Methods:** This is a cross-sectional study, using Behavioral Risk Factor Surveillance System (BRFSS) data 2017. Target population are over age 65 who are living alone (N=50,784). Outcome variables are depression, self-reported health status, and physical inactivity whereas main predictors are activities of daily living (ADL) and instrumental activities of daily living (IADL) controlling for gender, race, marital status, employment status, annual income, and educational level. Weight adjustment analysis and logistic regression were conducted.

**Results:** Depression, self-reported poor health status, and physical inactivity are higher among isolated aging adults who have limitations with ADL/IADL. Specifically, those with difficulties concentrating/decision making (OR=3.62; CI=3.35-3.91) have higher chance of developing depression than those who do not have this limitation. Female are at risk for developing depression (OR=1.41-1.61; CI=1.51), yet they are likely to report for better health status (OR=1.54; CI=1.45-1.64) than males. Asian (OR=0.56; CI=0.39-0381), Black or African American (OR=0.54; CI=0.48-0.60) are less likely to be diagnosed with depressive disorder than White.

**Discussion/Recommendation:** Physical and cognitive changes that come with aging can pose challenge, as functional capacity diminishes in home environment. Further research should be explored in longitudinal studies on mobility and improving quality of living among isolated aging population.

### VIRTUAL TESTING OF SENIORS: THE FEASIBILITY OF RESEARCH WITH ONLINE NEUROPSYCHOLOGICAL TESTS (FRONT)

Chris Pauley,[1] Linda Yetman,[2] Jody-Lynn Lupo,[3] Penelope Slack,[4] Diane Jacobs,[5] Chris McGibbon,[6] Manuel Montero Odasso,[7] and Pamela Jarrett,[2], *1. Horizon Health New Brunswick, Saint John, New Brunswick, Canada, 2. Horizon Health Network, Horizon Health Network, New Brunswick, Canada, 3. University of California San Diego, Alzheimer's Disease Cooperative Study (ADCS), UC San Diego, Alzheimer's Disease Cooperative Study (ADCS), California, United States, 4. University of British Columbia, Vancouver, British Columbia, Canada, 5. University of California San Diego, La Jolla, California, United States, 6. University of New Brunswick, Fredericton, New Brunswick, Canada, 7. Lawson Research Institute, London, Ontario, Canada*

Synergic@Home is a feasibility study evaluating the effects of exercise and cognitive interventions for the prevention of dementia in at-risk individuals over age 60. The COVID-19 pandemic changed the study's methods, with standardized neuropsychological tests needing to be administered virtually. Experience and research into the viability of neuropsychological assessments administered virtually is limited. After receiving permission to adapt the tests for virtual administration, a neuropsychologist, project managers, and research coordinators developed their approach. A PowerPoint presentation using text and visual stimuli from the tests was developed with on-screen instructions for the raters. An iterative development process involved feedback from the team in order to maximize the fidelity of these methods compared to in-person administration. Mock assessments supervised by a neuropsychologist further refined the methods and confirmed rater adherence to standardized procedures. A secure videoconferencing platform meeting privacy requirements was used. Dual monitors for the raters provided instructions on one monitor while stimuli for the participant was on the second monitor. The participant could only see the stimuli. This method of administering neuropsychological assessment, the Feasibility of Research with Online Neuropsychological Testing (FRONT), is being used to evaluate older adult participants in Synergic@Home. Results from this feasibility study may set the stage for new research methodologies and/or clinical evaluations in the future. This project is funded by the New Brunswick Healthy Seniors Pilot Project and the Canadian Consortium on Neurodegeneration in Aging (with grants from Public Health Agency of Canada and the Canadian Institutes of Health Research, with additional funding partners).

### WHAT WORKS AND WHAT DOESN'T: GERONTOLOGY FOCUSED PHD/ DSW GRADUATES SPEAK OUT

Matthew Myrick,[1] and Lauren Snedeker,[2], *1. Widener University, PHILADELPHIA, Pennsylvania, United States, 2. Rutgers University, New Brunswick, New Jersey, United States*

Lin et al. (2015) projected there would be a shortage of approximately 195,000 social workers in the United States by 2030. In the next twenty years, it is estimated that Americans over the age of 65 will actually outnumber children under the age of 18 (US Census, 2018). With a longstanding reputation for being less "glamorous", social work with older adults will continue to experience deficits in the amount of those who commit to this field of practice unless more lasting change occurs (Cummings et al., p. 645, 2005). We must take a closer look at what takes place in the classroom at schools of social work to understand why social workers are not interested in working with older adults (Scharlach et al., 2000). Berkman et al. (2016) described in their work that a critical shortage of gerontology-focused social work faculty exists in schools of social work. Thus, we cannot expect more social workers to work with older adults unless they are exposed to this work in their educational programs. The purpose of this study is to report on the academic experience, research agenda, professional experiences (practice and teaching), and future goals of social work PhD/ DSW graduates. Ten social work doctoral graduates were interviewed in order to understand the impact their academic programs had on their commitment to older adults in their field and to learn their recommendations for schools of social work in an effort to sustain and grow the gerontological workforce.

### Session 9505 (Late Breaking Poster)

### LATE BREAKING POSTER SESSION II

### A PLATFORM TO STUDY THE EFFECTS OF HOME ENVIRONMENT ON HEALTH AND WELLBEING OF OLDER ADULTS

Amir Baniassadi,[1] Lewis Lipsitz,[2] Alvaro Pascual-Leone,[3] Brad Manor,[1] and Wanting Yu,[2], *1. Hinda and Arthur Marcus Institute for Aging Research, Harvard Medical School, Boston, Massachusetts, United States, 2. Hebrew SeniorLife, Boston, Massachusetts, United States, 3. Harvard Medical School, Boston, Massachusetts, United States*

While older adults' living environment is rarely well-tuned to their specific needs, technological advances provide new opportunities to understand, and ultimately optimize, the relationship between the home environment and health outcomes. We aimed to establish proof-of-concept and feasibility of a platform enabling real-time, high-frequency, and simultaneous monitoring of environment, biological variables, and outcomes related to health and wellbeing in older adults. We recruited 7 participants (6 females, 1 male, aged 78-90, MoCA scores 14–28), installed environmental sensors measuring temperature, humidity, and CO2 inside their homes, provided them with wearables that measure sleep, activity, body temperature, and heart rhythms, and asked them to use a tablet to complete four sets of questionnaires and cognitive tests per day for three consecutive weeks. Environmental sensors collected data with no disruption or complaint from participants. Average compliance with the wearables was 81% (ring) and 60% (watch). All participants preferred the ring due to ease-of-use. Compliance was better in those with higher MoCA scores. Three participants were able to use the tablet successfully and completed 90% of prescribed questionnaires and cognitive tests. Cognitive and/or motor issues prevented the other participants from using the tablet. Exit interviews revealed that participants would prefer to complete a maximum of two sets of daily questionnaires and cognitive tests (five minutes each) in longer-term studies. These results suggest that it is feasible to study the impact of the environment on biological rhythms, cognition, and other outcomes in older adults and provide recommendations for ensuring long-term compliance with the protocol.

### A SCOPING REVIEW OF PSYCHOLOGICAL SENSE OF COMMUNITY AMONG COMMUNITY DWELLING OLDER ADULTS

Thomas Buckley, *University of Pittsburgh, NORFOLK, Virginia, United States*

Psychological sense of community (PSOC) is an important construct for health and well-being outcomes for older adults. Drawing on the Ecological Theory of Aging and the Age-Friendly Cities (AFC) framework, this scoping review explored how PSOC has been used in research with community dwelling older adults. I followed Arksey and O'Malley's (2005) scoping review guidelines. Initial database searches yielded 860 articles. I included 33 in the final sample. I grouped articles based on study populations and conceptualization and operationalization of PSOC. I used thematic analysis to explore topic areas and main findings. The AFC framework guided development of themes and others emerged during analysis. Results show most studies used Asian or White samples and focused on geographic community or neighborhoods. Among the several measures of PSOC, the Brief Sense of Community Scale performed best with older adults. Topical research areas in the thematic analysis were built (1) built environment and neighborhoods, (2) social participation and connection, (3) civic participation, (4) PSOC as a protective factor, (5) health and well-being, (6) relocation, and (7) scale development. PSOC was a consistent predictor of health and well-being and served as a mediator to link neighborhood or environmental characteristics with health and well-being. Future research needs to examine PSOC in geographically and culturally diverse samples and conduct further psychometric testing of PSOC scales with older adults. PSOC is conceptually related to the AFC framework and serves as a mechanism that links AFC features and well-being outcomes. These results can inform practice and refine theory.

### A STUDENT-LED COMMUNITY OUTREACH TELEHEALTH PROGRAM FOR COVID EDUCATION AND HEALTH PROMOTION (COACH)

Michelle Yang,[1] Cam Clayton,[2] Devin Harris,[3] Chelsea Pelletier,[4] Julia Schmidt,[2] Jill Zwicker,[2] and Brodie Sakakibara,[5], *1. University of British Columbia, Richmond, British Columbia, Canada, 2. University of British Columbia, Vancouver, British Columbia, Canada, 3. Interior Health Authority, Kelowna, British Columbia, Canada, 4. University of Northern British Columbia, Prince George, British Columbia, Canada, 5. University of British Columbia, Kelowna, British Columbia, Canada*

While public health measures of quarantining, socially isolating, and physical distancing are important to minimize the spread of coronavirus (COVID-19), these actions may also compromise the ability to manage one's own health, thereby increasing the risk of adverse health events. The purpose of this study was to evaluate a student-delivered Community Outreach teleheAlth program for Covid education and Health promotion (COACH) to community-living adults (age ≥65 years). We hypothesized that COACH would improve health promoting behaviour as measured by the Health Directed Behaviour subscale of the Health Education Impact Questionnaire. We also anticipated COACH would improve secondary outcomes of perceived stress, depressive and anxiety (Depression, Anxiety, and Stress Scale-21), social support (Medical Outcomes Study Social Support Survey), health-related quality of life (Short Form-36), and health promotion self-efficacy (Self-Rated Abilities for Health Practices Scale). In this single-group pre-post study, we recruited 75 community-living adults with access to telephone/video-conferencing technology to participate in six 30-45 minute sessions with trained medical students over a two-month period. The mean age of participants was 72.4 years (58.7% female), with 80% reporting two or more chronic conditions. No participants were diagnosed with COVID-19 during participation. Paired sample t-tests showed significant improvement in health directed behaviour ($p < .001$, $d = 0.45$) and self-efficacy ($p <.001$, $d = 0.44$), but significant decrease in mental health-related quality of life ($p < .001$, $d = -1.69$). Overall, COACH may help improve health directed behaviour and health promotion self-efficacy, despite decreases in mental health possibly associated with COVID-19 restrictions.

### A VIRTUAL COMPANIONSHIP INTERVENTION REDUCES LONELINESS DURING THE COVID-19 PANDEMIC

Ellen Rudy, Kelsey McNamara, Rajiv Patel, and Corey Sturm, *Papa Inc., Miami, Florida, United States*

Loneliness and social isolation are established risk factors for many clinical conditions yet few scalable interventions exist. Papa Inc. is a national service that pairs older adults with "Papa Pals" (empathetic, laypeople) who provide companionship and assistance with everyday tasks. Participants have free access if their Medicare Advantage plan offers it. During the COVID-19 pandemic, Papa provided virtual companionship visits via telephone or video. This study evaluated the impact of virtual companionship visits on loneliness status (UCLA 3-item Loneliness Scale) during the COVID-19 pandemic. The sample (N=894) included adults ages 65+ who identified as lonely at baseline and who completed at least one virtual visit between March 18, 2020 and December 31, 2020. Virtual visits were classified into four categories based on participants' total number of visit minutes: Low (124 ave min), Medium Low (ML) (305 ave min), Medium High (MH) (567 ave min), and High (1360 ave min). Lonely and severely lonely participants engaged a mean of 573 and 673 minutes in the program, respectively. Improvement in loneliness status was associated with greater use of minutes for the ML and MH participants compared to Low participants (ML OR: 1.46 95CI: 1.00 - 2.11, MH OR 1.65 95CI: 1.13 - 2.40). These findings indicate that a virtual companionship intervention can be an impactful and scalable tool for older adults who want to age at home and have limited social support, especially during the uncertain COVID landscape. Further research is warranted to understand persistent loneliness.

### ACETYLATION OF TAU INDUCES ALZHEIMER'S DISEASE-ASSOCIATED TAU IN TRANSGENIC MICE

Addison Ali,[1] Kristeen Pareja,[2] and Tara Tracy,[1], *1. Buck Institute for Research on Aging, Novato, California, United States, 2. Buck Institute, Buck Institute, California, United States*

Alzheimer's disease (AD) is a neurodegenerative disorder that is characterized by neurofibrillary tangles (NFTs) and amyloid beta plaques. These NFTs are made up of aggregated tau proteins. Tau is involved in stabilizing microtubules and does not usually display aggregation. Acetylation of tau protein causes an increase in tau aggregation but its role in AD progression is still not well understood. I hypothesized that enhanced acetylated tau results in an increase in AD-like tau pathology. To test this, a murine prion promoter-tauKQ transgene was injected into the mouse fertilized oocyte. The tauKQ mutation alters lysine to glutamine to mimic acetylation of tau. Nontransgenic mice were used as controls. AT8 and GT-38 antibodies were used in immunohistochemistry (IHC) to target phosphorylated tau and AD-associated tau, respectively. GT-38 is conformation-dependent and requires 3R and 4R tau isoforms which makes it specific to AD. Through immunofluorescence, increased phosphorylated tau was observed in the hippocampus of the tauKQ mice compared to the nontransgenic mice. I focused on the dentate gyrus, CA1 region, and the mossy fibers of the CA3 region since they are involved in many memory processes. Through chromogenic IHC, the tauKQ mice exhibited more 3R+4R tau isoform pathology in the mossy fibers than the nontransgenic mice. This data suggests that an acetylation mimic is sufficient to stimulate an abundance of AD-related tau pathology in transgenic mice which is consistent with my hypothesis. The tauKQ mouse model can assist in understanding the role of tau acetylation and tau progression for AD.

### ADULT DAY SERVICE USE AMONG ETHNIC MINORITY OLDER ADULTS: AN UPDATED INTEGRATIVE REVIEW

Yawen Li,[1] Jinyu Liu,[2] Fei Sun,[3] and Ling Xu,[4], *1. California State University San Bernardino, San Bernardino, California, United States, 2. Columbia University, Columbia University, New York, United States, 3. Michigan State University, East Lansing, Michigan, United States, 4. University of Texas at Arlington, Arlington, Texas, United States*

Adult day services (ADS) are a preferred care option for racial and ethnic minorities compared to other types of long-term care services in the United States. However, there is limited knowledge on minority ADS users. Focusing on minority older adults, this study aims to (a) identify facilitator and barriers of ADS use, and (b) examine ADS's effect on health and wellbeing. Using Whittemore and Knafl's methodology of integrative reviews, we searched relevant studies published between 2010 to 2020 in Ageline, PubMed, PsycINFO, CINAHL, Web of Science and Google Scholar and included 8 articles in this review after extensive screening and critical appraisal. Crowe Critical Appraisal Tool (CCAT) was used to assess methodological rigor of the studies included in this review. This review showed that individual factors of ADS use among minority older adult included functional impairment, diabetes, race, gender, and degree of loneliness. Organizational characteristics, such as availability of transportation services, bilingual nurses, peer support, and cultural activities, and structural factors including for-profit status and source of payment were also related to ADS use among minority older adults. Positive outcomes associated with ADS use were improved quality of life and sense of fulfillment. Better understanding of minority older adults' experience with ADS will help tailor the services to better fit their cultural preferences and needs. Future research should move beyond individual-level factors to identify and address organizational and structural factors such as institutional structure, organization culture and practice impact on disparities and discrimination in services access and quality.

### ADVERSE CHILDHOOD EXPERIENCES ASSOCIATED WITH EPIGENETIC AGE AND DEPRESSIVE SYMPTOMS IN OLDER ADULTS

Eric Klopack,[1] Eileen Crimmins,[1] Steve Cole,[2] Teresa Seeman,[2] and Judith Carroll,[2], *1. University of Southern California, Los Angeles, California, United States, 2. University of California, Los Angeles, Los Angeles, California, United States*

Adverse childhood experiences (ACEs) increase risk for depression in adolescents and older adults and have been linked to accelerated biological aging. We hypothesized that accelerated epigenetic aging may partially explain the link between ACEs and depression. This study examines second-generation epigenetic clocks (viz., GrimAge, PhenoAge, and DunedinPoAm38) as mediators of the link between ACEs and depressive symptoms in older adulthood. We utilize structural equation modeling to assess mediation in the Innovative Subsample of the Venous Blood Study from the Health and Retirement Study (N = 2672). Results indicate that experiencing more than 1 ACE is significantly associated with greater GrimAge and DunedinPoAm38, with limited evidence of increasing aging with increasing ACEs. GrimAge and DunedinPoAm38 were also significantly associated with more depressive symptoms. These associations were partially reduced by lifestyle factors. GrimAge explained between 9 and 13% of the association between ACEs and adult depressive symptoms, and DunedinPoAm38 explained between 2 and 7% of the association between ACEs and adult depressive symptoms. Findings indicate that accelerated aging, as measured by GrimAge and DunedinPoAm38, is strongly associated with ACEs in older Americans, that these clocks are highly associated with depressive symptoms in older Americans, and that these clocks mediate a proportion of the association between ACEs and adult depressive symptoms. Epigenetic clocks may represent a physiological mechanism underlying the link between early life adversity and adult depression. Lifestyle factors are a potential area for intervention.

### AGE-RELATED CHANGES IN ONGOING THOUGHT RELATE TO EXTERNAL CONTEXT AND INDIVIDUAL COGNITION

Adam Turnbull,[1] Giulia Poerio,[2] Feng Lin,[3] and Jonathan Smallwood,[4], *1. University of Rochester Medical School, Rochester, New York, United States, 2. University of Essex, Colchester, England, United Kingdom, 3. Stanford, Stanford, California, United States, 4. Queen's Universtiy, Kingston, Ontario, Canada*

Understanding how age-related changes in cognition manifest in the real world is an important goal for aging research. One means of capturing these changes involves “experience sampling” participant’s self-reported thoughts as they go about their daily lives. Previous research using this method has shown age-related changes in ongoing thought: e.g., older adults have fewer thoughts unrelated to the here-and-now. However, it is currently unclear how these changes reflect cognitive aging or lifestyle changes. 78 younger adults and 35 older adults rated their thought contents along 20 dimensions and the difficulty of their current activity in their daily lives. They also performed cognitive tasks in the laboratory. In a set of exploratory analyses using Principal Component Analysis (PCA), we found that older adults spent more time thinking positive, wanted thoughts, particularly in demanding contexts, suggesting they may use different strategies to regulate their emotions. In line with previous research, older adults spent less time mind wandering about their future selves. Past-related thought related to episodic memory differently in older and younger adults. Additionally, PCA analyses performed separately in older and younger adults showed high similarity to an analysis performed on the combined sample, suggesting a similar structure to ongoing daily life thought in older and younger adults. These findings inform the use of experience sampling to understand cognitive aging, highlighting the need to consider content along multiple dimensions as well as the context in which thoughts are reported when analyzing aging ongoing thought.

### AGE-RELATED CHANGES IN POSTURE STEADINESS IN THE COMPANION DOG

Alejandra Mondino Vero,[1] Grant Wagner,[1] Edgar Lobaton,[1] Katharine Russell,[2] and Natasha Olby,[3], *1. North Carolina State University, Raleigh, North Carolina, United States, 2. NCSU, NCSU, North Carolina, United States, 3. North Carolina State University, NCSU, North Carolina, United States*

Aging is associated with changes in the sensory-motor system that could lead to dynamic instability. In fact, postural control deficits have been proposed as an early indicator of frailty. Measurements of the displacement of the center of pressure (COP) using pressure mat data are useful tools to determine postural steadiness. Companion dogs represent a powerful model to study aging in people because they share our environment and experience similar age-related diseases. To date, the effect of aging on postural control in dogs has not yet been evaluated. The aim of this study was to determine the correlation between age and the displacement of the COP in dogs during quiet standing. Due to the diversity of life expectancy in dogs according to their body size, age was normalized as a fraction of the predicted life expectancy. Dogs older than 75% of their life expectancy (n=18) were asked to stand on a pressure mat for 8 seconds per trial during at least five trials. Only the frames where the dogs were standing still and facing forward were analyzed. Age as a fraction of life expectancy was significantly correlated ($p<0.05$) with the Medio-lateral Range, Root-Mean-Square Distance, 95% Confidence Ellipse, and Total Sway Area of the COP. These results show that, as in humans, aging in dogs is associated with postural control deficits and therefore reinforce the dog as a suitable model for translational studies of aging and postural steadiness.

### AGING SERVICES WORKERS IN THE PANDEMIC: VOICED EXPERIENCE OF SENIOR CENTER STAFF & CASE WORKERS

Esther Okang,[1] Siobhan Aaron,[2] Katherine Supiano,[2] and Abdul Osman,[2], *1. University of Utah, Salt Lake City, Utah, United States, 2. University of Utah, Salt Lke City, Utah, United States*

The pandemic necessitated immediate shutdown of senior centers, requiring a rapid pivot in the delivery of services to older adults by direct care workers. We provided psychosocial support to older adult service personnel-including Aging and Adult Services case workers and Senior Center Staff, and conducted focus groups with staff at intervals to capture the mid-point of the pandemic (peak of older adult deaths), onset of vaccine availability and the re-entry phase as programs re-opened. We evaluated coping and self-efficacy of workers and discerned sustained high levels of coping and perceived job performance. Using a phenomenological lens, we analyzed transcribed recordings, generated codes, and created categories of experiences. Several themes emerged: personal and professional resilience, passion for serving older adults, motivation to perform their job well, stress of not having face-to-face contact with clients, insufficient resources-especially in rural areas, lack of essential training, feeling disjointed as a team, and work-life balance. Over the course of the pandemic, workers expressed increasing resiliency and skills to navigate the pandemic, oscillations in their fears for their clients' well-being, and gratitude that they kept their jobs and gained additional State resources. As the vaccine was available and utilized, and as senior centers were reopening, senior center staff were enthusiastic, yet case workers remained apprehensive about long-term consequences of the pandemic. This study affirms the role of direct care workers as essential and valuable. Yet, their expressed need for more education, psychosocial support, and community awareness of their service remains to be addressed.

### AN ONLINE COMMUNITY INTERVENTION FOR OLDER PERSONS WITH PRE-FRAILTY AND FRAILTY: PILOT STUDIES

Oleg Zaslavsky,[1] Frances Chu,[2] Shaoqing Ge,[3] Andrew Teng,[2] Shih-Yin Lin,[4] George Demiris,[5] and Annie Chen,[2], *1. University of Washington, Seattle, Washington, United States, 2. University of Washington, University of Washington, Washington, United States, 3. University of Washington School of Nursing, Seattle, Washington, United States, 4. New York University, New York, New York, United States, 5. School of Nursing, University of Pennsylvania, University of Pennsylvania, Pennsylvania, United States*

Online community interventions can support self-management in older populations but have rarely targeted symptomology of pre-frailty and frailty. To support older adults' pre-frailty/frailty symptom management, we iteratively refined an approach entitled Virtual Online Community for Aging Life Experience (VOCALE) in three consecutive pilot studies (2018-2020). These studies employed asynchronous online discussions in which participants were asked to respond to weekly prompts. A study facilitator moderated the discussion, encouraging participants to respond to both the prompts and comments of other participants. In the first pilot (n=8), participants engaged in a collective exploration of different symptoms of pre-frailty and frailty. The second (n=10) and third (n=10) pilots employed a hybrid approach including collaborative exploration and learning of different problem-solving therapy skills over eight weeks. The mean age of participants of the three pilots combined was 80.6 (SD = 7.0). Most participants were female (71%). Participant attrition ranged from 20-25%. Many participants who completed the study noted that they enjoyed the discussions. The participants also found the moderators' follow-up questions and support timely and engaging. Additionally, we observed small but positive changes in self-efficacy measures. These pilot studies have confirmed that older adults with pre-frailty and frailty are interested, and can successfully engage in online community interventions, with the technical support and moderation provided, even during the initial stages of the COVID-19 pandemic, when lockdown policies were rolled out. Participation in the intervention was also associated with increased awareness of the need to be proactive in self-management concerning frailty-related symptoms.

### ASSESSING CARDIOMETABOLIC HEALTH RISK IN CHILDREN LIVING WITH GRANDPARENT PRIMARY CAREGIVERS: INTERIM ANALYSIS

MinKyoung Song,[1] Laura Hayman,[2] Karen Lyons,[3] Hannah Bessette,[1] Mary Roberts Davis,[1] Kerri Winters-Stone,[1] and Carol Musil,[4], *1. Oregon Health & Science University, Portland, Oregon, United States, 2. University of Massachusetts Boston, Boston, Massachusetts, United States, 3. Boston College, Chestnut Hill, Massachusetts, United States, 4. Case Western Reserve University, Cleveland, Ohio, United States*

Minimal research has been conducted on the effect that grandparents as primary caregivers have on the cardiometabolic health of children who live with them, even though a number of studies have examined the influence of parent caregivers. As a first step towards filling that gap, we examined physiological and behavioral indicators of cardiometabolic health risk among children (aged 7 to 12 years) living with grandparent primary caregivers in Oregon and Washington. We measured body mass index and total cholesterol/glycohemoglobin (HbA1c), as well as physical activity/sleep and diet. In this preliminary analysis of our findings with 10 dyads (mean age $64.2 \pm 4.0$ years for grandparents; $9.3 \pm 1.9$ years for grandchildren), we report that on most of the indicators - obesity, physical activity, sleep, and diet - these children's levels were comparable to national averages across all household types (not differentiated by type of family structure). However, 25% of the grandchildren (n=2) participating in our study had a total cholesterol level $\geq 200$, compared to 7.4% of children from a nationally representative dataset. Similarly, 14% of the grandchildren (n=1) participating in our study had HbA1c $\geq 6.5\%$, compared to $< 0.5\%$ of children from a nationally representative dataset. Our findings suggest that these children may be at higher cardiometabolic health risk (e.g., hyperlipidemia). Further investigations with a larger sample and more examination of cardiometabolic risk profiles including lipids/blood glucose assessment are required to validate our preliminary findings.

### ASSOCIATION OF DUAL SENSORY IMPAIRMENT WITH INCIDENT MOBILITY AND ADL DIFFICULTY

Colby Gendron,[1] Nicole Armstrong,[2] Camila Vieira Ligo Teixeira,[3] Willa Brenowitz,[4] Frank Lin,[5] Bonnielin Swenor,[6] Richard Jones,[7] and Eleanor Simonsick,[8], *1. Brown University, Brown University, Rhode Island, United States, 2. Warren Alpert Medical School of Brown University, Providence, Rhode Island, United States, 3. NIA/NIH Biomedical Research Center, Baltimore, Maryland, United States, 4. UCSF, UCSF, California, United States, 5. Johns Hopkins University, Johns Hopkins University, Maryland, United States, 6. Johns Hopkins University, Baltimore, Maryland, United States, 7. Brown University, Providence, Rhode Island, United States, 8. National Institute on Aging, National Institute on Aging, Maryland, United States*

Hearing and vision impairment are each independently associated with incident mobility disability and disability in activities of daily living (ADL). Whether dual sensory impairment (DSI) in both hearing (pure-tone average >25 dB) and vision (impaired visual acuity and/or impaired contrast sensitivity) is associated with greater risk of incident mobility and ADL difficulty, as compared to single or no sensory impairments, has not been well-studied. To examine these associations, we used data from 2,020 Health Aging and Body Composition Study participants aged 70-79 years without mobility limitations. Incident mobility difficulty was defined as the first instance of a lot of problems or inability to walk ¼ mile and/or climb 10 steps, and incident ADL difficulty was defined as the first instance of problems with any ADL. Cox proportional hazards models adjusted by demographic covariates, diabetes, hypertension, and depressive symptoms were used to model these associations. Approximately 22.7% of the study had DSI. DSI was associated with increased risk of both incident mobility (Hazard Ratio [HR]=2.43, 95% Confidence Interval [CI]: 1.60, 3.69) and ADL difficulty (HR=2.39, 95% CI: 1.60, 3.56). Vision impairment only was associated with risk of incident mobility difficulty (HR=1.74, 95% CI: 1.09, 2.78), but not incident ADL difficulty (HR=1.45, 95% CI: 0.91, 2.32). Hearing impairment only was not associated with risk of either outcome. Synergistic effects of DSI on the additive scale were present. Sex and race did not modify associations. Monitoring of DSI may be beneficial in delaying incident difficulty.

### ASSOCIATION OF DUAL SENSORY IMPAIRMENT WITH LONG-TERM DEPRESSIVE AND ANXIETY SYMPTOMS

Colby Gendron,[1] Camila Vieira Ligo Teixeira,[2] Willa Brenowitz,[3] Bonnielin Swenor,[4] Danielle Powell,[4] Eleanor Simonsick,[5] Richard Jones,[6] and Nicole Armstrong,[7], *1. Brown University, Brown University, Rhode Island, United States, 2. NIA/NIH Biomedical Research Center, Baltimore, Maryland, United States, 3. UCSF, UCSF, California, United States, 4. Johns Hopkins University, Baltimore, Maryland, United States, 5. National Institute on Aging, National Institute on Aging, Maryland, United States, 6. Brown University, Providence, Rhode Island, United States, 7. Warren Alpert Medical School of Brown University, Providence, Rhode Island, United States*

Hearing (HI) and vision impairment (VI) are each independently associated with long-term depressive and anxiety symptoms, but the joint effects of both (DSI) may be associated with a greater risk of belonging to long-term chronically high depressive and anxiety trajectory classes. Multinomial logistic regression models adjusted by demographics and depressive symptoms were used to examine the associations of dual hearing (pure-tone average >25 dB) and vision impairment (impaired visual acuity and/or contrast sensitivity) with long-term depressive and anxiety symptom trajectory classes among 2,102 participants of the Health, Aging and Body Composition Study, a cohort of older adults without mobility difficulty aged 70-79 years. An additional model evaluated the two-way interaction between DSI and social contact. Elevated depressive symptoms were defined as ≥8 on the 10-item Center for Epidemiologic Studies-Depression Scale, and anxiety symptoms were defined as present on the Hopkins Symptom Checklist. DSI was associated with increased risk of being chronically depressed (Risk Ratio, RR=1.86, 95% Confidence Interval, CI: 1.19, 2.92), not periodically depressed (RR=1.24, 95% CI: 0.91, 1.69). Those with DSI were at an increased risk of belonging to the periodically anxious (RR=1.56, 95% CI: 1.14, 2.13) and chronically anxious (RR=1.79, 95% CI: 1.02, 3.12) groups, as compared to the other groups. Single sensory impairments were not associated with increased risk of being periodically or chronically anxious. Social contact did not modify any associations. Synergistic effects between HI and VI were present. Those with DSI may be at greater risk for mood disorders, so sensory evaluations may mitigate these.

### ASSOCIATION OF SEDENTARY AND ACTIVE BOUT FREQUENCY WITH MORTALITY IN OLDER MEN USING ACCELEROMETRY

Lauren Roe,[1] Stephanie Harrison,[2] Kyle Moored,[1] Kristine Ensrud,[3] Katie Stone,[4] Kelley Pettee Gabriel,[5] Jane Cauley,[1] and Peggy Cawthon,[2], *1. University of Pittsburgh, Pittsburgh, Pennsylvania, United States, 2. California Pacific Medical Center, Research Institute, San Francisco, California, United States, 3. University of Minnesota, Minneapolis, Minnesota, United States, 4. University of California, Berkley, San Francisco, California, United States, 5. The University of Alabama at Birmingham, Birmingham, Alabama, United States*

**Background:** Time spent sedentary increases with age and has several negative health consequences. We sought to examine associations between daily sedentary and active bout frequency with all-cause mortality.

**Methods:** Data are from 2,918 men in the Osteoporotic Fractures in Men (MrOS) study (mean age at Visit 3±SD: 79.0±5.1 years) with valid activity monitor data (5.1±0.3 days worn>90%) at Year 7 visit (Visit 3, 2007-2009). Sedentary and active bout frequencies are defined as the daily transition frequency from a sedentary bout lasting 5+ minutes to activity of any intensity, and the transition frequency from an active bout lasting 5+ minutes to sedentary. Deaths were centrally adjudicated using death certificates. Cox proportional hazard models were used to examine associations between quartiles of sedentary (Q1 referent, <13.6 bouts/day) or active (Q1 referent, <5 bouts/day) bout frequency and mortality. Models were repeated, stratifying by median daily total time spent sedentary and active.

**Results:** After 9.4±3.7 years of follow-up, 1,487 (51.0%) men died. Men averaged 16.9±5.1 and 8.2±4.2 sedentary and active bouts/day, respectively. After full covariate adjustment, each quartile reflecting a higher sedentary (Q4 vs Q1 HR: 0.68, 95%CI: 0.58-0.81, p-trend<0.001) and active bout (Q4 vs Q1 HR: 0.57, 95%CI: 0.48-0.68, p-trend<0.001) frequency was associated with lower mortality risk. There was no evidence that effects differed by total sedentary time (p-interaction for sedentary bout frequency and total sedentary time>0.05).

**Conclusions:** More frequent, prolonged sedentary and active bouts are associated with a lower mortality risk in older men and is not moderated by total sedentary time.

### ASSOCIATIONS BETWEEN HIV STIGMA AND MENTAL HEALTH AMONG OLDER HISPANICS AND WHITES LIVING WITH HIV

Kayle Karcher,[1] Lily Kamalyan,[2] Veronica Gonzalez,[3] Lilla Brody,[4] Robert Heaton,[5] Raeanne Moore,[6] Dilip Jeste,[7] and Maria Marquine,[7], *1. University of California-San Diego, San Bernardino, California, United States, 2. San Diego State University/University of California San Diego Joint Doctoral Program in Clinical Psychology, San Diego, California, United States, 3. UCSD, San Diego, California, United States, 4. Weill Cornell Medicine, New York, New York, United States, 5. University of California San Diego, San Diego, California, United States, 6. UC San Diego, San Diego, California, United States, 7. University of California San Diego, La Jolla, California, United States*

Hispanics/Latinos/as/x (henceforth Hispanics) have higher rates of HIV infection than non-Hispanic (NH) Whites, particularly in older age. People living with HIV (PWH) are at increased risk of stigma and poor mental health, but these associations have not been thoroughly examined in older PWH. We investigated ethnic differences in HIV stigma and its association with mental health in older Hispanic and NH White PWH. Participants included 116 PWH ages 50-75 (58 Hispanic and 58 NH White) from southern California (for the overall cohort: 82.7% male; 57.7% AIDS, 93.9% on antiretroviral therapy). Participants completed self-report measures of HIV-stigma, depression (Beck Depression Inventory-II; BDI-II), and cumulative alcohol use (i.e., lifetime total quantity/total days). Covariates examined included sociodemographic and HIV-disease characteristics. An independent sample t-test showed no significant ethnic differences in HIV stigma (p=.82). Separate multivariable linear regression models on mental health outcomes (adjusting for significant covariates) showed no significant interaction between HIV stigma and ethnicity on BDI-II scores (p=.83) or cumulative alcohol use (p=.51). Follow up models removing the interaction term, showed that increased HIV stigma was associated with higher BDI-II scores (B=0.34, 95% Cl=0.21-0.48; p<.001), but not with cumulative alcohol use (p=.49) in the overall sample. Findings indicate a significant link between HIV stigma and depression symptoms in older PWH, with comparable associations among Hispanics and NH Whites. Future studies examining factors that may moderate the link between HIV stigma and depression in diverse older PWH would help guide the development of interventions aimed at improving mental health in this population.

### BIOPSYCHOSOCIAL CORRELATES OF COGNITIVE FUNCTION AMONG KOREAN OLDER ADULTS: HISTORY OF HYPERTENSION AND DIABETES

Kyuyoung Cho,[1] and Hye Won Chai,[2], *1. Dong-A University, Busan, Pusan-jikhalsi, Republic of Korea, 2. The University of Texas at Austin, Austin, Texas, United States*

Based on biopsychosocial perspectives on health, this study examined risk and protective factors of cognitive function among Korean older adults. Specifically, we focused on comparing the role of these factors based on the respondents' history of having hypertension or diabetes. This study used 2009 Korean National Health Insurance Service data that included a sample of older adults who maintained qualification for health insurance and medical aid in 2002 (n=26,242). Cognitive function was measured using KDSQ-C and biopsychosocial factors included metabolic syndrome, drinking, smoking, and walking. The sample was divided into two groups based on their medical history, and thus four sets of linear regression models were analyzed to explore the associations between biopsychosocial factors and cognitive functioning. Among individuals with a history of hypertension, metabolic syndrome, drinking, and walking were associated with cognitive functioning. For those without a history of hypertension, only drinking and walking were associated with cognitive functioning. For diabetes, smoking and walking were associated with cognitive functioning among older adults with a history of diabetes. For those without a history of diabetes, drinking and walking were associated with cognitive functioning. In sum, metabolic syndrome was a particularly significant correlate of cognitive function among Korean older adults with a history of hypertension. Walking was a consistently significant factor regardless of medical history. These results highlight the importance of considering medical history of chronic conditions such as hypertension and diabetes in identifying factors associated with older adults' cognitive function and further developing tailored prevention programs for cognitive decline.

### CAN A DATA-DRIVEN MEASURE OF NEUROANATOMIC DEMENTIA RISK BE CONSIDERED A MEASURE OF BRAIN AGING?

Ramon Casanova,[1] Andrea Anderson,[2] Jamie Justice,[2] Gwen Windham,[3] Rebecca Gottesman,[4] Thomas Mosley,[5] Lynne Wagenknecht,[6] and Stephen Kritchevsky,[2], *1. Wake Forest University, Winston-Salem, North Carolina, United States, 2. Wake Forest School of Medicine, Wake Forest School of Medicine, North Carolina, United States, 3. University of Mississippi Medical Center, Jackson, Mississippi, United States, 4. NIH, Bethesda, Maryland, United States, 5. The University of Mississippi Medical Center, Jackson, Mississippi, United States, 6. Wake Forest School of Medicine, Winston Salem, North Carolina, United States*

There is an increasing interest in identifying aging-related factors which may be permissive of Alzheimer's Disease (AD) emergence. We previously used machine learning to derive an index of neuroanatomic risk of dementia called

AD pattern similarity (AD-PS) score using MRIs obtained in the Atherosclerosis Risk in Communities (ARIC) study. Here, we investigate the potential of the AD-PS scores as a brain-focused measure of biologic age. Among 1970 ARIC participants with MRI collected at ARIC Visit 5, we related AD-PS scores to three measures of aging: mortality (n=356) over 8 years of follow-up; an a priori panel of 32 proteins related to aging (N=1647); and a deficit accumulation index (DAI) based on 38 health-related measures. We found lower AD-PS scores associated with significantly lower mortality (HR=0.58, CI-95%, [0.45 - 0.75], p < 0.001) after adjusting for age, race, smoking and hypertension. Among the 32 proteins, nine were significantly associated to AD-PS scores (p < 0.05) with 4 remaining significant adjusting for multiple comparisons (Growth/differentiation factor 15, Tumor necrosis factor receptor superfamily member 1A and 1B and Collagen alpha-1(XVIII) chain). Finally, in a linear regression model after adjusting for age, race, sex, hypertension and smoking, AD-PS scores were associated with the DAI (p < 0.001). The consistent patterns of associations suggest that a data-driven measure of AD neuroanatomic risk may be capturing aspects of biologic age in older adults.

### CARE RECIPIENT DIAGNOSIS MODERATES THE RELATIONSHIP BETWEEN CAREGIVER WORRY AND VIGILANCE

Katherine Craig,[1] Shirit Kamil-Rosenberg,[1] and J. Kaci Fairchild,[2], *1. Veterans Affairs Palo Alto Health Care System, Palo Alto, California, United States, 2. VA Palo Alto Health Care System, Palo Alto, California, United States*

Family members of persons diagnosed with dementia or a traumatic brain injury (TBI) are often relied upon to provide daily support to their care recipients. However, research on the differing experiences of caregivers based on care recipient diagnosis is limited. The aim of this study was to examine the impact of worry and feelings of vigilance among caregivers of people with cognitive impairment due to either TBI or dementia. This sample included 61 caregivers (88.5% female, mean age 57.3±15.5) of persons with either a TBI (n = 32) or dementia (n = 29). Worry was assessed with the Penn State Worry Questionnaire and Vigilance was assessed with the Caregiver Vigilance Scale. Linear regressions revealed that after controlling for age, care recipient diagnosis moderated the relationship between worry and caregiver vigilance. Specifically, worry was significantly associated with caregiver vigilance in those caring for someone with dementia; however, a similar relationship was not seen in those caring for someone with a TBI. This suggests caregivers of people with TBIs have a different experience of worry and vigilance than caregivers of people with dementia. These findings demonstrate the need for more research on the unique needs of caregivers of people with TBIs. Additionally, this research suggests interventions targeting worry may be particularly effective in supporting caregivers of people with TBIs.

### COGNITIVE DECLINE AND RETIREMENT: FINDINGS FROM THE CANADIAN LONGITUDINAL STUDY ON AGING.

Catherine Gosselin,[1] Benjamin Boller,[2] and Meghan Désilets-Jutras,[3], *1. Université du Québec à Trois-Rivières, Trois-Rivières, Quebec, Canada, 2. Université du Québec à Trois-Rivières, Université du Québec à Trois-Rivières, Quebec, Canada, 3. Université du Quebec à Trois-Rivières, Université du Québec à Trois-Rivières, Quebec, Canada*

Since increasing life expectancy leads to a longer period of retirement, several studies have been investigating the possible impact of retirement on cognitive health. Several epidemiological studies with cross-sectional designs have reported a negative association between retirement and cognitive capacities. However, very few studies with longitudinal designs have confirmed the negative effect of retirement on cognitive functioning. The present study was conducted to investigate the impact of retirement on cognitive capacities among older Canadians. We used data from the Comprehensive cohort of the Canadian Longitudinal Study on Aging (CLSA) to compare performance retirees and workers (N = 1442), 45 to 85 years of age at baseline. Memory and executive functioning were assessed using standardized assessment tools at baseline and at three-year follow up. Retirees and workers were matched for age, gender and education using the nearest neighbor propensity score method with a caliper of 0.02. Mixed ANOVA and post hoc analyses were conducted separately for the English- and French-speaking samples. Results for the English-speaking sample showed a significant decline on both the Stroop and the Mental Alternation Task for retirees compared to workers from baseline to follow-up. These results support previous cross-sectional studies that have demonstrated a negative effect of retirement on executive functioning. The absence of significant results in the French-speaking sample will be discussed in terms of sample size and professional occupation.

### COMBATING SOCIAL ISOLATION AMONG OLDER IMMIGRANT ADULTS: A QUALITATIVE INTERPRETIVE META-SYNTHESIS

Vivian Miller,[1] Betty Tonui,[2] and Dolapo Adeniji,[3], *1. Bowling Green State University, Bowling Green, Ohio, United States, 2. Oakland University, Oakland University Rochester, Michigan, United States, 3. IUPUI, Avon, Indiana, United States*

Older immigrants totaled 7.3 million in 2018, representing 13.9 percent of the population of seniors in the U.S. While this population is found to contribute significantly to society, along with new opportunities comes circumstantial challenges. Of these, one of the most salient issues for foreign-born older adults is social isolation. Additionally, this population may be at an increased risk for social isolation with poor mental health because migrating to a new country might results in resettlement challenges. Despite these concerns, less is known about the consequences of social isolation among older immigrant adults. Guided by the Population Interest Context (PICO) framework and the Qualitative Interpretive Meta-Synthesis (QIMS) guidelines, this study seeks to explore consequences of social isolation among older immigrant, as well as interventions to combat isolation. The final sample of six full text articles were published between 2011 and 2021, totaling 180 participants with ages ranging from 61 to 93 years old. Findings from the study indicated that older immigrants are at risk of social isolation and loneliness because they have fewer social connections due to leaving behind their familiar social group in the home country, encounter linguistic challenges that negatively contribute to greater social isolation and poor mental health. Despite these difficulties older immigrants reported various social interventions, i.e., access to senior centers, community programs and services to be of greater importance in building social networks. Authors discuss opportunities for future research, such as exploring evidence-based studies on interventions for social isolation and loneliness of older immigrant populations.

### CONCEPTUALIZING THE VALUE OF AGING: WHAT IS IT LIKE TO BE AN OLDER WOMAN IN THE 21ST CENTURY?

Karen Shilvock-Cinefro, *George Williams College/Aurora University, Aurora, Illinois, United States*

The basis for this hermeneutic phenomenological research was to identify common themes in women 65 years of age and older and determine whether these women felt valued in ageing. The best description of this research becomes the study of the phenomena as real living or "entering the lifeworld "(Finlay, 2012). The research sought to increase the knowledge of how older women feel about their own ageing and the effect of society's response to them. The participants covered a large age range of 66 to 93 years of age all of which experienced physical, emotional, and social changes involving age. The participants' response to these changes of ageing and society's response to them ranged from very positive to very distressing. Twelve women were interviewed from a vast range of locations throughout the United States through zoom due to COVID 19. Their responses reflect four main categories: ageism, successful ageing, active engagement, and social support. The interviews focused on seven main questions: Tell me about being your age? Is there a time you can recall when you first felt older? Have you ever felt mistreated as an older adult? Have you ever felt you were discounted or ignored as being an older woman? Have you done anything to maintain your youth? Have you done anything to feel youthful? Have you ever felt dismissed related to your age? Utilizing seven questions and the conversation with these questions brought forth this study. This became a platform for these women to tell their stories.

### CORESIDENCE INCREASES RISK OF TESTING POSITIVE FOR COVID-19 AMONG OLDER BRAZILIANS

Flavia Andrade,[1] Nekehia Quashie,[2] and Luisa Schwartzman,[3], *1. University of Illinois--Urbana-Champaign, Urbana, Illinois, United States, 2. Technical University of Dortmund, Dortmund, Nordrhein-Westfalen, Germany,*
*3. University of Toronto, Mississauga, Ontario, Canada*

Brazil is among the countries hit hardest by COVID-19, and older adults are among the vulnerable groups. Intergenerational coresidence and interdependence among family members, both prevalent in Brazil, likely increase social and physical contact. Using nationally representative data from the COVID-19 module of the Brazilian National Household Sample Survey, collected from July to November of 2020, we examined the association between living arrangements and exposure to and testing for COVID-19 among 63,816 Brazilians 60+. Our multivariate analyses utilize multilevel mixed-effects Poisson regression to examine the association between living arrangements and the COVID-19 outcome measures. Results show that those living alone were more likely to report having symptoms and having had a test for COVID-19. However, older adults in multigenerational (PR=1.532, 95% CI 1.15, 2.04, $p<0.001$) and skipped generation households (PR=1.607, 95% CI 1.04, 2.48, $p<0.001$) were more likely than solo-dwellers to test positive for COVID-19. Those with symptoms were more likely to test, regardless of their living arrangement. Among older adults without symptoms, those living alone had a higher probability of testing than those living in multigenerational or skipped-generation households. Overall, our findings suggest that coresidence with younger family members is a risk factor for older adults' health due to the higher COVID-19 positivity. As younger Brazilians are increasingly vulnerable to COVID-19 and experiencing severe outcomes, policy makers need to be more attentive to the health needs of households that comprise older and younger cohorts, which are also more prevalent in poor and marginalized segments of the population.

### DEMENTIA READY: COMPARING APPROACHES FOR BUILDING CAREGIVER READINESS AND KNOWLEDGE OF DEMENTIA

Michael Lepore,[1] Kate Keefe,[2] Erica DeFrancesco,[2] Julie Robison,[3] Alis Ohlheiser,[4] Christine Bailey,[5] and Kathy Kellett,[4], *1. LiveWell Alliance, Southington, Connecticut, United States, 2. LiveWell, Plantsville, Connecticut, United States, 3. University of Connecticut, University of Connecticut, Connecticut, United States, 4. UConn Health, Farmington, Connecticut, United States, 5. University of Connecticut Health Center, Farmington, Connecticut, United States*

Despite the rising prevalence of dementia and the high cost and complexity of care for people with dementia, most dementia care is provided at home by informal caregivers who are not clinically trained. Building caregiver readiness and knowledge of dementia is key to supporting quality care and desirable health outcomes, such as preventing falls and reducing nursing home admissions. We sought to determine and compare the impact of two interventions—Resilient Living with Dementia (RLWD) and Care of Persons with Dementia in their Environments (COPE)—and of their combined delivery (both RLWD and COPE) on increasing caregiver readiness and knowledge of dementia. Between January 2019 and March 2021, 77 caregivers of people with dementia in Connecticut participated in RLWD and/or COPE and completed the Alzheimer's Disease Knowledge Scale (ADKS) and the Preparedness for Caregiving Scale (PCGS) at baseline and at four-month and ten-month follow-ups. Analyses were conducted to compare outcomes by intervention(s). From baseline to four months and to ten months, we observed statistically significant ($p < .05$) improvement on the ADKS among participants in RLWD, and on the PCGS among participants in COPE and among participants in RLWD. The most substantial impact on PCGS was observed among participants in both COPE and RLWD. No improvement in the ADKS was observed among participants in only COPE, but ADKS improvement was observed at four months among participants in COPE and RLWD. Findings suggest that the benefits of COPE and RLWD for building dementia caregiver readiness are complementary and mutually reinforcing.

### DEMENTIA RISK IN OLDER VETERANS WITH FRAILTY: A CROSS-SECTIONAL STUDY

Christian Gomez Hernandez,[1] Alma Diaz,[2] Ahmadou Sow,[2] Gauty Athouriste,[2] Ezekiel Ijaopo,[3] and Jorge Ruiz,[4], *1. Miami VA Healthcare System, Miami, Florida, United States, 2. Miami VAMC, Miami, Florida, United States, 3. University of Miami/Jackson Health System, Miami, Florida, United States, 4. VAMC, Miami, Florida, United States*

Frailty, a clinical syndrome characterized by vulnerability to stressors resulting from multisystemic loss of physiological reserve, predicts future cognitive decline. However, frailty has also been proposed as a dementia risk factor, predicting future cognitive impairment. The study aim was to determine frailty in older veterans and its association with risk of dementia. Community-dwelling Veterans ≥50 years completed a mailed socio-demographic questionnaire and Self-Administered Gerocognitive Examination (SAGE), July 2019-May 2020. The information was complemented with EHR data. We calculated the CAIDE score, a validated tool predicting dementia (≥6 points= high risk 20 years later) and the 31-item VA frailty index data (frail ≥.20, non-frail ≤.20). After adjusting for socio-demographic characteristics, smoking, alcohol/substance abuse, OSA and anticholinergic use, odds ratio (OR) and 95% CI were calculated using BLR to assess the cross-sectional association between frailty and dementia risk (CAIDE ≥6 points and MCI). The survey response rate was 19.75% (1,073 of 5,432). Participants mean age was 68.38 (SD=8.49) years, 57.50% (n=617) Caucasian, 69.34% (n=744) non-Hispanic, 95.81% (n=1,028) male, and 36.72% (n=394) frail. 11.84%(n=127) screened positive for MCI and 15.38% (n=165) for dementia. 689 (75.88%) veterans were at high risk for dementia of whom 426 (61.83%) were non-frail and 263 (38.17%) were frail. Frailty was cross-sectionally associated with higher risk for dementia in older Veterans, adjusted OR:1.45 (95%CI:1.016-2.070), p=.041. The mailed screening was a feasible and practical approach to screen for dementia risk. Early identification of patients with frailty can help in the implementation of interventions aimed at preventing or delaying dementia.

### DEPRESSION MEDIATES THE CAUSAL EFFECT OF FOOD INSECURITY TRANSITION ON COGNITIVE FUNCTION

Peiyi Lu,[1] Katrina Kezios,[2] and Adina Zeki Al Hazzouri,[2], *1. Columbia University, Fort Lee, New Jersey, United States, 2. Columbia University, New York, New York, United States*

Despite substantial evidence on the relationship between food insecurity and worse cognition, few studies have examined the underlying mechanisms. This study examined whether and to what extent depression mediated the casual effect of food insecurity on cognition in midlife. Longitudinal data from the Coronary Artery Risk Development in Young Adults Study were used. The 2000 survey was used as our study's baseline, when respondents aged 33-45. We lagged the explanatory and mediator variables to set up temporality. Food insecurity transition from 2000-2005 surveys was recoded (persistent food secure, persistent food insecure, became food insecure, and became food secure). Depression was measured in 2010, while cognitive functions were measured in 2015. Causal mediation analysis examined and decomposed the direct and indirect effect. Compared to persistent food security, both persistent and became food insecure were linked to worse cognition, after adjusting demographic and cardiovascular risk factors. Further, for both exposure groups, depression had significant and strong mediation effects (natural indirect effect ranged from -0.037 to -0.018). Specifically, as respondents experienced persistent food insecurity or became food insecure, their depression increased, which, in turn, reduced their cognition. The proportion of the total effect of food insecurity transition on cognition mediated by depression ranged from 7-24% for processing speed, verbal memory, cognitive, and language functions. Persistent and became food insecure reduced cognitive function directly or indirectly through increasing depression among middle-aged adults. Interventions targeting either food insecurity or treatment of depression among food insecure individuals may have the potential to slow premature cognitive aging.

### DESIGNING A GENETIC SCREEN TO IDENTIFY FACTORS REQUIRED FOR MEIOTIC NUCLEAR REJUVENATION

Chelsey Jones, *Howard University, Hyattsville, Maryland, United States*

During the natural cycle of life, most eukaryotic organisms grow old, age, and die. A common natural mechanism by which organisms "reset" their lifespan is through sexual reproduction; however, how this rejuvenation takes place remains unknown. My lab has found that meiosis in budding yeast, the developmental program that forms sex cells, eliminates age-induced damage. This involves the formation of a novel nuclear compartment, the Gametogenesis Uninherited Nuclear Compartment (GUNC), which acts as a trash can for accumulated age-induced damage. To understand the molecular details of this process, I worked on designing a screen for genes involved in GUNC formation. My mentor and I fused three different proteins targeted to the GUNC and a protein that is able to bind to a drug-resistance plasmid, in order to couple the inheritance of a selectable DNA marker with the elimination of age-induced damage. Initial testing of these three fusion proteins suggested that they were unable to successfully target the plasmid to the GUNC; as such, testing of additional candidate proteins is necessary. We plan to eventually use this system to identify mutations that disrupt GUNC formation and cause inheritance of the drug-resistance plasmid. By identifying and perturbing proteins involved in GUNC formation, we are hoping to be able to drive the inheritance of specific types of age-induced damage, allowing for the determination of what a symptom versus a cause of aging is.

### DIABETES, OBESITY, AND OSTEOPOROSIS IN AN ETHNICALLY DIVERSE POPULATION OF WOMEN RECEIVING OSTEOPOROSIS SCREENING

Feven Kahsay,[1] Wendy Yang,[2] Malini Chandra,[3] Catherine Lee,[4] Nailah Thompson,[4] and Joan Lo,[4], *1. McGovern Medical School, Houston, Texas, United States, 2. The Permanente Medical Group, San Jose, California,*

*United States, 3. Kaiser Permanente Northern California, Oakland, California, United States, 4. The Permanente Medical Group, Oakland, California, United States*

Osteoporosis screening by bone density (BMD) testing is recommended for women aged 65-75 years. However, patients with diabetes, a risk factor for fracture, often have higher body mass index (BMI) which contributes to higher BMD. These factors may vary by race/ethnicity. The relationship of diabetes (≥2 diagnoses and treatment), obesity (BMI ≥30), and BMD-defined osteoporosis (femoral neck BMD T-score ≤ -2.5) was examined in a diverse primary care population of 44,313 non-Hispanic White, 6,103 Black, 7,777 Hispanic, and 12,634 Asian women aged 65-75 years who underwent BMD screening. Those with recent fracture, osteoporosis treatment, bone disorders, and metastatic cancer were excluded. Modified log-Poisson regression was used to examine the association of diabetes and BMD-osteoporosis. Among 70,827 women, 18% had diabetes. The prevalence of diabetes was 2-fold higher in Black, Hispanic and Asian women compared to White women. Overall, women with diabetes (versus no diabetes) were more likely to be obese and, except for Hispanic women, less likely to have BMD-osteoporosis. In unadjusted analyses, diabetes was associated with lower risk of BMD-defined osteoporosis in White, Black, and Asian women, but not Hispanic women. However, the association was attenuated or no longer evident after adjusting for BMI, suggesting that the lower burden of BMD-osteoporosis in women with diabetes is mediated in part by higher BMI. These findings support consideration of diabetes when assessing fracture risk in women undergoing osteoporosis screening. However, more studies in non-White populations with a high burden of diabetes are important since these relationships appear to differ by race/ethnicity.

### DO INFLAMMATION MARKERS MODERATE ASSOCIATIONS BETWEEN CAREGIVING AND QUALITY OF LIFE, HEALTH, AND DEPRESSION?

Elizabeth Orsega-Smith,[1] Marie Kuczmarski,[2] Adam Davey,[1] Brianna Wolfle,[1] Alan Zonderman,[3] and Michele Evans,[2], *1. University of Delaware, Newark, Delaware, United States, 2. National Institutes of Health, Baltimore, Maryland, United States, 3. NIA, NIA, Maryland, United States*

Caregiving responsibilities can put stress and strain on older adults including emotional distress, depression, decline in physical functioning, and decreased self- reported quality of life. Chronic stress such as from caregiving may be related to chronic inflammation, but this has been less widely examined. Therefore, the purpose of this study is to examine whether the association between caregiving and outcomes including quality of life indicators, self-rated health, and depressive symptoms are moderated by physical activity, stress coping, diet quality inflammatory index, and selected biomarkers of inflammation. We used data from waves 3 and 4 of the Healthy Aging in Neighborhoods of Diversity across the Life Span (HANDLS) study, from which 1945 participants identified as caregivers. Caregiver participants were mostly African American (59%) and female (55%) with a mean age was 47.7 years at the start of the study. In linear regression models, caregiving for grandchildren (CGC) predicted higher depressive symptoms and lower quality of life and self-rated health. Results demonstrated that the association between erythrocytes sedimentation rate (ESR) was a significant moderator between CGC and quality of life, self-rated health, and depressive symptoms (p<.05). Serum magnesium levels were significant moderators between caregiving for parents and both quality of life and self-rated health (p<0.05). These results suggest that inflammatory factors may influence the health of diverse older adult caregivers. Further research may evaluate the effect of these moderators over time.

### DOES FRAILTY INFLUENCE INHOSPITAL MANAGEMENT AND OUTCOMES OF COVID-19 IN OLDER ADULTS IN THE US?

Soko Setoguchi,[1] Richard Kennedy,[2] Nathaniel Kuhrt,[3] Timothy Bergquist,[4] Jessica Islam,[5] Heidi Spratt,[6] Vithal Madhira,[7] and Joseph Siu,[8], *1. Rutgers Robert Wood Johnson Medical School, New Brunswick, New Jersey, United States, 2. University of Alabama at Birmingham, Birmingham, Alabama, United States, 3. New Jersey Medical School, Newark, New Jersey, United States, 4. Sage Bionetworks, Kent, Washington, United States, 5. H. Lee Moffitt Cancer Center and Research Institute, Tampa, Florida, United States, 6. University of Texas Medical Branch, Galveston, Texas, United States, 7. Palila Software LLC, Palila Software LLC, Nevada, United States, 8. University of Nebraska Medical Center, Omaha, Nebraska, United States*

Older age has been consistently associated with adverse COVID-19 outcomes. Frailty, a syndrome characterized by declining function across multiple body systems is common in older adults and may increase vulnerability to adverse outcomes among COVID-19 patients. However, the impacts of frailty on COVID-19 management, severity, or outcomes have not been well characterized in a large, representative US population. Using the National COVID Cohort Collaborative, a multi-institutional US repository for COVID-19 research, we calculated the Hospital Frailty Risk Score (HFRS), a validated EHR-based frailty score, among COVID-19 inpatients age ≥ 65. We examined patient demographics and comorbidities, length of stay (LOS), systemic corticosteroid and remdesivir use, ICU admission, and inpatient mortality across subgroups by HFRS score. Among 58,964 inpatients from 53 institutions (51% male, 65% White, 18% Black, 9% Hispanic, mean age 75, mean Charlson comorbidity count 3.0, and median LOS 7 days), 38,692 (66%), 4,180 (7%), 3,531 (6%), 3,525 (6%) and 7,862 (13%) had HFRS scores of 0-1, 2, 3, 4, and >=5 , respectively. Frailty was only moderately correlated with age and comorbidity (□=0.178 and 0.348, respectively, p<0.001). Overall, 34% received systemic corticosteroid and 19% received remdesivir. We observed 4% ICU admissions and 16% inpatient death. Among non-ICU admissions, after adjusting for demographics and comorbidities, frailty (HFRS ≥ 2) was associated with 79% greater systemic corticosteroid use and 22% greater remdesivir use, whereas a higher HRFS score was marginally associated with higher rates of severe COVID disease, inpatient death, or ICU admission.

### DURATION OF REPRODUCTIVE PERIOD AND RISK OF TRANSITIONING TO MILD COGNITIVE IMPAIRMENT AND DEMENTIA

Jamie Knight,[1] Tomiko Yoneda,[1] Nathan Lewis,[1] Graciela Muniz-Terrera,[2] David Bennett,[3] and Andrea Piccinin,[1], *1. University of Victoria, Victoria, British Columbia, Canada, 2. University of Edinburgh, Edinburgh, Scotland, United Kingdom, 3. Rush University, Chicago, Illinois, United States*

Decreasing estrogen levels have been hypothesized to be associated with increased risk of dementia, yet the current literature reveals conflicting results. This study aimed to determine whether a longer reproductive period, as an indicator of longer exposure to endogenous estrogens, is associated with risk of transitioning to MCI and dementia. Women 65 and over (N=1507) from the Rush Memory and Aging Project met eligibility for the current analysis. The average length of reproductive period (menopause age minus menarche age) was 35 years (range=16-68 years), and 64% had natural menopause. Multistate survival modeling (MSM) was used to estimate the influence of reproductive period on risk of transitioning through cognitive states including mild cognitive impairment (MCI) and clinically diagnosed dementia, as well as death. Multinomial regression models estimated total and cognitively unimpaired life expectancies based on the transition probabilities estimated by the MSM. Results suggest that women with more reproductive years were less likely to transition from no cognitive impairment (NCI) to MCI, and were more likely to return to NCI from MCI. Analyses also suggest two additional years free of cognitive impairment for women with 45 vs 25 years of reproduction, though reproduction period did not significantly impact overall life expectancy. This study suggests that the number of years of reproductive duration is not associated with the transition to dementia, but is possibly associated with delayed cognitive decline, reduced risk of MCI, increased likelihood of returning to NCI from MCI, and increased lifespan free of cognitive impairment.

### EFFECTIVENESS OF A STAFF TRAINING PROGRAM TO STIMULATE PHYSICAL ACTIVITY IN HOMECARE: A CLUSTER RCT

Silke Metzelthin,[1] Teuni H Rooijackers,[1] GA Rixt Zijlstra,[1] Erik van Rossum,[2] Annemarie Koster,[1] Silvia Evers,[3] Valeria Lima Passos,[1] and Gertrudis IJM Kempen,[1], *1. Maastricht University, Maastricht, Limburg, Netherlands, 2. Zuyd University of Applied Sciences, Maastricht, Limburg, Netherlands, 3. m, Maastricht, Limburg, Netherlands*

Reablement encourages older adults to do things themselves rather than having things done for them. To implement reablement in practice homecare staff needs the right knowledge, attitude, skills and support. This study evaluated the effectiveness of the “Stay Active at Home” reablement training program. A 12-month cluster-RCT was conducted, involving staff (n=313) and clients (n=264) from 10 homecare teams, five of which were trained. Effects were evaluated using data from accelerometers, physical performance tests, questionnaires and electronic patient records. No beneficial effects were observed in older adults for sedentary behavior; daily, physical, and psychological functioning; and falls. In homecare staff there were no statistically significant differences between study groups for self-efficacy and outcome expectations scores except for higher self-efficacy scores in more compliant staff (adjusted mean difference: 1.9 [95% CI 0.1, 3.7]). No differences were observed for any cost category except for domestic help costs in the intervention group (adjusted mean difference: €-173 [95% CI -299, -50]). The probability that “Stay Active at Home” is cost-effective compared to usual care at a willingness-to-pay of €20,000 was 19.7%/ daily minute of sedentary time averted, 19.2%/ percent of sedentary time averted as proportion of wake/wear time, and 5.9%/QALY gained, respectively. The reablement training program needs further development based on the lessons learned before wider implementation.

### EFFECTS OF TINNITUS ON COGNITION AND DEPRESSIVE SYMPTOMS OVER TIME

Jennifer De Anda,[1] Teresa Warren,[2] Tyler Bell,[3] William Kremen,[3] and Carol Franz,[3], *1. San Diego State University, La Jolla, California, United States, 2. San Diego State University, San Diego, California, United States, 3. University of California San Diego, La Jolla, California, United States*

Evidence suggests links among tinnitus, depression, and cognition. We examined these associations over time. We hypothesized baseline tinnitus would predict poorer cognitive performance and more depressive symptoms an average of 11.4 years later. We examined 839 men at two timepoints (baseline age M=55.94; follow-up age M=67.56). At each time point participants responded yes/no if they had tinnitus. We created three tinnitus status groups – no tinnitus at either time, tinnitus at both, and no tinnitus at baseline but tinnitus at follow-up. At both time points we measured cognitive performance with tests of episodic memory, processing speed, executive function, and verbal fluency. Depressive symptoms were based on the Center for Epidemiological Studies Depression scale. There was no association between tinnitus and any measure of cognitive performance. Depressive symptoms declined from baseline to follow-up. In separate mixed models predicting depressive symptoms, there was a significant main effect for tinnitus status at baseline ($p = .003$) and follow-up ($p < .001$). Those with tinnitus at both times had significantly higher depressive symptoms than the “No tinnitus” group ($p < .001$). This association remained significant after accounting for baseline depressive symptoms ($p = .011$) at follow-up. Results did not support the hypothesis that tinnitus would be associated with poorer cognitive function. However, depressive symptoms declined among those with no tinnitus than the other groups. The relationship between tinnitus and depressive symptoms may have implications for future cognitive performance among older adults, given previous evidence that depressive symptoms are risk factors for cognitive decline.

### ENGAGING WITH AGING: A QUALITATIVE STUDY OF AGE-RELATED CHANGES AND ADAPTATIONS

Shaoqing Ge,[1] Kuan-Ching Wu,[2] Hillary Frey,[3] Maryam Saudagaran,[3] Derick Welsh,[3] Janet Primomo,[4] and Basia Belza,[5], *1. University of Washington School of*

*Nursing, Seattle, Washington, United States, 2. University of Washington School of Nursing, University of Washington School of Nursing, Washington, United States, 3. University of Washington School of Nursing, University of Washington, Washington, United States, 4. University of Washington Tacoma School of Nursing & Healthcare Leadership, University of Washington Tacoma, Washington, United States, 5. University of Washington, Seattle, Washington, United States*

Engaging with Aging is an emerging framework proposed by Carnevali which provides a new lens to understand an active, conscious daily living process of coping with age-related changes (ARCs) taken on by older adults. Study aims were to 1) describe the ARCs experienced by community-dwelling older adults; 2) identify the strategies and resources used by older adults to accommodate the daily living challenges caused by the associated ARCs; and 3) evaluate the framework of EWA based on findings from aims 1 and 2. We conducted semi-structured interviews with 29 participants aged 64 to 98 online due to COVID-19 restrictions. We used a virtual card sort to assist data gathering. Fifteen ARCs (e.g., changes in hearing, changes in stability, changes in sleep, etc.) were mentioned by participants and their corresponding adaptations were discussed. We found that older adults linked their adaptations to their ARCs based on their changing capacities and needs. Commonly used adaptations included conserving energy, utilizing tools or technology, and being more conscious before and while taking actions. The challenges caused by COVID-19 in implementing the adaptations were also discussed (e.g., increased difficulty in understanding others due to mask-wearing). Our study substantiates the EWA framework by showing the commonality among older adults in linking ARCs with adaptations. Implications for clinicians and researchers include using EWA to help older adults identify personalized solutions that fit their capacities. Our study is late-breaking as we recently finished data analysis and the information included was not yet available by the previous submission deadline.

### EXPERIENCES OF AFRICAN AMERICAN DEMENTIA CAREGIVERS DURING THE COVID-19 PANDEMIC

Sloan Oliver, Karah Alexander, and Fayron Epps, *Emory University, Atlanta, Georgia, United States*

African American caregivers are often confronted with the complexities of caregiving through the lens of race and associated health disparities. The COVID-19 pandemic has both exacerbated the systemic disparities and deeply rooted inequities experienced by African Americans and laid bare their effects on the community of caregivers. The purpose of this project was to explore the experiences of African American dementia caregivers during the COVID-19 pandemic. Nineteen African American caregivers of persons living with dementia were recruited by primary investigators and community partners with purposeful sampling techniques to participate in semi-structured focus groups that were held April 2021. Four overarching themes were constructed during thematic analysis: social isolation, decreased well-being, the good and bad of telehealth, and challenges in fulfilling the caregiver role. Caregivers expressed that they became socially isolated from family and friends, which led to them becoming depressed and mentally strained. Several caregivers felt they could not carry out their caregiver duties due to the constraints surrounding the pandemic. The varying levels of interaction with and the comfort level of physicians utilizing telehealth led to caregivers having mixed reviews on the popularized service. The results of this study will be used to culturally adapt caregiving education courses and programs promoting mastery and competency during a pandemic. In preparations for future public health crises, healthcare professionals will be able to use the results of this study to address the specific needs and improve the experiences of African American dementia caregivers.

### FACTORS ASSOCIATED WITH OLDER PEOPLES´ PERCEPTIONS OF DIGNITY AND WELL-BEING AT RESIDENTIAL CARE FACILITIES.

Charlotte Roos,[1] Moudud Alam,[2] Anna Swall,[1] Anne-Marie Boström,[3] and Lena Hammar,[4], *1. Dalarna University, Falun, Dalarnas Lan, Sweden, 2. Dalarna University, Borlänge, Dalarnas Lan, Sweden, 3. Karolinska Institutet, Karolinska Institute, Stockholms Lan, Sweden, 4. Mälardalen University / Dalarna University, Västerås, Vastmanlands Lan, Sweden*

Dignity and well-being should be promoted in care of older people living at residential care facilities (RCFs). In addition, care should be person-centred. Dignity and well-being can be interpreted as person-centred outcomes. Older people living at RCFs experience a lack of dignity and well-being. To promote this, it is important to understand the associated factors to target. The aim of this study was to examine the associations between perceived dignity and well-being and factors related to attitudes of staff, the care environment and individual issues (age, gender, self-rated health and dementia) among older people living at RCFs. A national cross-sectional study was conducted retrospectively. All older people 65 years and older (n=71,696) living at RCFs in 2018 were invited to respond to the survey. The survey included the areas: self-rated health, indoor-outdoor-mealtime environment, performance of care, treatment from staff, safety, social activities, availability of staff and care in its entirety. Age, gender and diagnosed dementia were collected from two national databases. Data was analysed using ordinal logistic regression models. The result indicated that respondents who had experienced disrespectful treatment, who did not thrive in the indoor-outdoor-mealtime environment, who rated their health as poor and respondents with dementia had higher odds of being dissatisfied with dignity and well-being. There is a need to improve the prerequisites of staff regarding respectful attitudes and to improve the care environment. The Person-centred Practice framework, targeting the prerequisites of staff and the care environment, can be used as a theoretical framework for designing future improvements.

### FAKE NEWS DETECTION IN AGING DURING THE ERA OF INFODEMIC

Didem Pehlivanoglu,[1] Tian Lin,[1] Kevin Chi,[1] Eliany Perez,[1] Rebecca Polk,[1] Barian Cahill,[1] Nichole Lighthall,[2] and Natalie Ebner,[1], *1. University of Florida, Gainesville, Florida, United States, 2. University of Central Florida, Orlando, Florida, United States*

Increasing misinformation spread, including news about COVID-19, poses a threat to older adults but there is little empirical research on this population within the fake news literature. Embedded in the Changes in Integration for Social Decisions in Aging (CISDA) model, this study examined the role of (i) analytical reasoning; (ii) affect; and (iii) news consumption frequency, and their interplay with (iv) news content, in determining fake news detection in aging during the COVID-19 pandemic. Young (age range 18-35 years, M = 20.24, SD = 1.88) and older (age range 61-87 years, M = 70.51, SD = 5.88) adults were randomly assigned to view COVID or non-COVID news articles, followed by measures of analytical reasoning, affect, and news consumption frequency. Comparable across young and older adults, fake news detection accuracy was higher for news unrelated to COVID, and non-COVID fake news detection was predicted by individual differences in analytic reasoning. Examination of chronological age effects further revealed that detection of fake news among older adults aged over 70 years depended on interactions between individual CISDA components and news content. Collectively, these findings suggest that age-related susceptibility to fake news may only be apparent in later stages of older adulthood, but vulnerabilities are context dependent. Our findings advance understanding of psychological mechanisms in fake news evaluation and empirically support CISDA in its application to fake news detection in aging.

### FALLING BETWEEN THE CRACKS: EXPERIENCES OF BLACK DEMENTIA CAREGIVERS NAVIGATING U.S. HEALTH SYSTEMS

Karah Alexander, Sloan Oliver, and Fayron Epps, *Emory University, Atlanta, Georgia, United States*

In addition to numerous care responsibilities, family caregivers are expected to navigate health systems and engage in healthcare management tasks on behalf of their persons living with dementia (PLWD). These challenging tasks pose additional difficulties for Black dementia caregivers. Due to the centuries-old, disadvantaged social history of Black Americans, several unique stressors, vulnerabilities, and resources have emerged which inform and affect Black dementia caregivers' experiences and well-being. Focus groups were held with Black caregivers (N = 19) from the United States (U.S.) to explore the unique experiences and perspectives of this population navigating the U.S. health system on behalf of their PLWD. Five overarching themes were developed during thematic analysis: Forced Advocacy, Poor Provider Interaction, Payor Source Dictates Care, Discrimination, and Broken Health System. Black dementia caregivers unanimously concurred that the health system that they experience in America is "broken." Gaps in the health system can lead to people [as one caregiver passionately expressed] "falling between the cracks," in terms of care, services, and resources needed. Caregivers agreed that class, sex, utilizing public health insurance, and being a "person of color" contribute to their difficulties navigating the health system. Caregivers perceived being dismissed by providers, forcing them to advocate for both themselves and their PLWD. Healthcare providers and researchers can utilize these findings to improve the experiences and healthcare outcomes of Black patients with dementia and their caregivers. Additionally, these findings can lead to the development of culturally tailored caregiver education programs.

### FEAR, RESISTANCE, OR ANTICIPATION? OLDER TRUCK DRIVERS' REACTIONS TO THE ADOPTION OF AUTOMATED VEHICLES

Amy Schuster,[1] Jenna Van Fossen,[2] Danielle Sperry,[1] and Shelia Cotten,[1], *1. Clemson University, Clemson, South Carolina, United States, 2. Michigan State University, East Lansing, Michigan, United States*

The forecasted adoption of autonomous vehicles (AVs) will lead to major changes to the job of truck driving. These changes may be particularly challenging for drivers, as the population of truck drivers skews much older than that of other occupations. In this study we sought to understand truck drivers' attitudes towards AVs and the longevity of their job. We conducted focus groups with truck drivers, their supervisors, and upper-level managers of trucking companies. We relate supervisors' and managers' experiences working with drivers through the rollout of new technologies to further understand drivers' initial reactions to automation and how their attitudes may develop. Based on qualitative open coding our analysis uncovered two overarching themes. The first theme is the unknown. With AVs, companies expect that experience will be less important, so they can hire younger workers. In response, drivers have expressed fear of being displaced and anxiety over the uncertainty of not knowing how their jobs will be affected. The second theme is adaptability, and desire to adapt. Older drivers have expressed resistance to adapting to AVs and to their job changing. Concerningly however, managers envision the need for a driving workforce that has experience working with technology and is adaptable. Our study identifies key challenges concerning older workers' reactions and career decisions in response to automation. Accounting for driver reactions to AVs is necessary not only to build theory and understanding on worker reactions to automation, but also for workforce planning and to support employees, particularly older workers.

### FEASIBILITY, ACCEPTABILITY, AND IMPACT OF A SELF-GUIDED E-LEARNING MEMORY PROGRAM FOR OLDER ADULTS

Danielle D'Amico,[1] Iris Yusupov,[2] Lynn Zhu,[3] Jordan Lass,[3] Cindy Plunkett,[4] Brian Levine,[3] Angela Troyer,[3] and Susan Vandermorris,[3], *1. Ryerson University, Toronto, Ontario, Canada, 2. York University, Toronto, Ontario, Canada, 3. Baycrest, Toronto, Ontario, Canada, 4. Baycrest, North York, Ontario, Canada*

Clinician-led memory interventions have been shown to increase knowledge, reduce anxiety, promote memory-strategy use, and increase brain-healthy lifestyle behaviours in older adults with normal age-related memory changes. A self-guided, e-learning version of the Baycrest Memory and Aging Program® was recently developed to increase accessibility to memory interventions. The objectives of the current study were to assess program feasibility (retention rate), acceptability (satisfaction), and participant-reported impact (memory concerns, behaviour change, goal attainment). As part of a larger study, participants were 139 healthy older adults (mean age: 73±7, 73% female). Ninety-two individuals completed the program (retention rate=66%). Anonymous feedback data indicated a high level of satisfaction with the program overall (98%), the pace and clarity of the learning modules (100%), and the organization and navigation of the interface (92%). Suggested improvements included offering more interaction with others and addressing minor platform glitches. There was a decrease in the level of concern about memory change, with 64% expressing concern at a level consistent with the Jessen et al. (2014) criteria for Subjective Cognitive Decline at baseline, and 23% expressing the same at post-test. The majority of participants reported increases in using memory-strategies (63-97%) and lifestyle-promoting behaviours (40-72%). All participants reported moderate to high satisfaction with personal goal attainment. Results support feasibility, acceptability, and impact of a self-guided e-learning adaptation of memory intervention. E-learning tools may be a promising avenue to deliver accessible brain health promotion in later life, especially in the context of the shift to virtual care during and beyond COVID-19.

### FORMATIVE EXAMINATION OF A MULTI-FOCUS EDUCATIONAL EXERGAME DESIGNED FOR OLDER ADULTS

Laurie Ruggiero,[1] Elizabeth Orsega-Smith,[2] and Roghayeh Barmaki,[2], *1. University of Delaware, University of Delaware, Delaware, United States, 2. University of Delaware, Newark, Delaware, United States*

Exergames and digital health games have shown promising outcomes in older adults. Most games have had one focus (e.g., physical activity, cognitive functioning). We developed a demonstration version of a multi-focus educational exergame (i.e., healthy eating, physical activity, cognition) that builds on healthy aging theory. Community-engaged and mixed methods (e.g., surveys, focus groups) research approaches were used to examine preliminary game acceptability and usability. The game was demonstrated with 20 senior center members (95% female; 48% African American; 52% White; average age 64 years) and participants were able to play the game. The post-gameplay survey results support acceptability/usability of the game. For example, 87% of participants “agreed” or “strongly agreed” that they felt comfortable playing; the game instructions were clear; the text was readable; and gameplay was enjoyable. The majority also “agreed”/“strongly agreed” that the audio was appealing/helpful in playing the game (86%); sound quality was appropriate (78%); hand tracking was precise (57%), feedback on correct/incorrect responses was motivating (73%); they felt excited to get the correct answers (80%); they would play the game again (87%); and they would recommend it to a friend/family member (80%). When asked how often they would play it, the responses were: 33% five or more times/week; 27% three-four times/week; 20% one-two times/week; and 20% never. Observations and focus groups further clarified acceptability and identified areas for improvement (e.g., game instructions). Preliminary results support acceptability of this multi-component educational exergame with older adults and suggest the potential for future tailoring of this game.

### FUNCTIONAL STATUS IN OLDER ADULTS WITH PERIPHERAL ARTERY DISEASE IN KOREA: A CROSS-SECTIONAL STUDY

Yesol Kim, Mihui Kim, Gi Wook Ryu, and Mona Choi, *Yonsei University, Seoul, Seoul-t'ukpyolsi, Republic of Korea*

Peripheral artery disease (PAD) is a chronic, progressive atherosclerotic disease resulting in worse functional status. It is an important factor that affects mobility and quality of life in older adults with PAD. This study aimed to identify the functional status and its associated factors of older adults diagnosed with PAD. We conducted a cross-sectional study among older adults aged 65 above diagnosed with PAD at a tertiary hospital in Seoul, Korea. Participants' functional status was measured using a Walking Impairment Questionnaire (WIQ) which consisted of distance, speed, and stair-climbing. We measured cardiac health behavior, social support, health perception, and clinical manifestation through self-administered questionnaires. Among 94 participants, the mean age was 74.98±6.21 years, and 91.5% were male. The mean score of WIQ was 0.59±0.30 out of 1; the mean scores for distance, speed, and stair-climbing of WIQ were 0.67±0.40, 0.45±0.27, and 0.64±0.37, respectively. Participants' functional status was significantly associated with age ($\beta$=-0.012, P=.002), sex ($\beta$=-0.284, P=.001), ulcers of the lower extremity (LE) ($\beta$=-0.242, P=.031), using a walking-assist device ($\beta$=-0.240, P=.002), walking difficulty due to pain of LE ($\beta$=-0.142, P=.006), and health behavior about physical activity ($\beta$=0.099, P=.021). This regression model predicted 53.5% of participants' functional status (F=8.63, P<.001). This study indicated that younger age, female, independent walking, no ulcers of LE, no walking difficulty, and higher physical activity behavior were significantly associated with better functional status in older adults with PAD. Therefore, healthcare professionals should develop and provide interventions to promote physical activity and alleviate symptoms to enhance functional status.

### GENERATIVITY IN LIFE REVIEW: DISCOVERING LIFE LESSONS AND WISDOM IN NATURALISTIC INTERGENERATIONAL CONVERSATIONS

Patricia Chilton,[1] Cindy Woolverton,[2] Elizabeth Glisky,[1] Matthias Mehl,[1] and Matthew Grilli,[1], *1. University of Arizona, Tucson, Arizona, United States, 2. Michael E. DeBakey VA Medical Center, Houston, Texas, United States*

According to the theory of generativity, one would expect older adults to inherently feature life lessons in naturalistic conversations with younger adults. Little though, is known about the process of these conversations, and to what extent they convey wisdom characteristics. In this project, intergenerational conversations between university students and older adults living in assisted and independent living communities were analyzed to identify life lessons within older adults’ informal life reviews. In the original study, 37 young and 52 older adults engaged in an intergenerational interaction as part of an undergraduate course. These conversations were recorded with participants’ consent, and transcribed with identifying information removed. For the current project, we analyzed

15 of these recorded conversations, averaging 46 minutes each between 10 students and 5 older adults to (1) develop a coding scheme and procedure to examine life lessons in intergenerational conversations, and (2) investigate whether wisdom characteristics are embedded into life lessons shared in this context. On average, each older adult referenced 4 life lessons (SD = 2) per conversation, which were coded for the following constructs: meaning making, personal growth, emotional valence, wisdom characteristics, life lesson type, and autobiographical memory type. Exploratory analyses suggest life lessons are inherently integrated into naturalistic intergenerational conversations, and that reflectivity is the most frequently expressed wisdom characteristic. This supports previous research identifying reflectivity as key to wisdom, and to the process of generativity. Further analysis is needed to illuminate the value of intergenerational conversations, particularly in a time of age segregation and ageism.

### IMPACT OF APOE GENOTYPE ON DIET-INDUCED MITOCHONDRIAL ADAPTATIONS IN MOUSE SKELETAL MUSCLE

Niaya James,[1] Oyinkansola Shonde,[2] Nahdia Jones,[3] G. William Rebeck,[4] Joanne Allard,[1] and verona Mulgrave,[5], *1. Howard University, Washington, District of Columbia, United States, 2. District of Columbia, United States, 3. Columbia University, Washington, District of Columbia, United States, 4. Georgetown University Medical Center, washington, District of Columbia, United States, 5. Delaware State University, Howard University, District of Columbia, United States*

Apolipoprotein E (APOE), a component of lipoproteins that facilitates cholesterol transportation, has three variants in the human genome: APOE2, E3, and E4. Prior research found that carriers of APOE4 are more susceptible to developing Alzheimer's disease (AD) and other brain disorders than those who possess other APOE alleles, and that these carriers are also predisposed to mitochondrial impairment– an early characteristic of neuronal dysfunction. Peroxisome proliferator-activated receptor gamma coactivator-1 alpha (PGC-1α) is a major biomarker for mitochondrial biogenesis and function and cytochrome c oxidase subunit IV (COX4) is the terminal enzyme of the mitochondrial respiratory chain. Decreased measures of these proteins indicate reduced mitochondrial function. Aside from genetic inheritance, lifestyle factors such as diet and exercise significantly impact one's risk for mitochondrial dysfunction and AD. In these studies, we examined the impact of APOE variance on physiological adaptations induced by either exercise or a high-fat diet, with a focus on biomarkers of mitochondrial function. Western blots were used to measure COX4 and PGC-1α levels in skeletal muscle tissue from female APOE3 and APOE4 knock-in transgenic mice. Based on performance on a motorized rotating rod and voluntary wheel-running, we deduced that female APOE4 mice exhibit reduced motor coordination and activity relative to APOE3 mice. APOE4 mice also had reduced COX4 levels that were increased by the high-fat diet. In contrast, COX4 levels in APOE3 mice were reduced in the high-fat diet group. Our data show that diet and APOE genotype interact to produce adaptations in mitochondrial proteins in skeletal muscle.

### IMPACT OF COVID-19 PANDEMIC ON MENTAL HEALTH, SOCIAL CONNECTEDNESS AND COGNITIVE PERFORMANCE OF OLDER ADULTS

Juliana Souza-Talarico,[1] Fernanda Silva,[2] Maria Clara Jesus,[3] Breno JAP Barbosa,[4] Ricardo Nitrini,[5] and Sonia MD Brucki,[4], *1. The University of Iowa, Iowa City, Iowa, United States, 2. School of Nursing, University of Sao Paulo, Sao Paulo, Sao Paulo, Brazil, 3. School of Nursing, Universit of Sao Paulo, Sao Paulo, Sao Paulo, Brazil, 4. Faculty of Medicine, University of Sao Paulo, Sao Paulo, Sao Paulo, Brazil, 5. Faculty of Medicine, University of Sao Paulo, Sao PAulo, Sao Paulo, Brazil*

The COVID-19 pandemic has profoundly impacted older adult's health and well-being worldwide. We explored the impact of the COVID-19 pandemic on daily activities and mental health and its relationship with cognitive performance in older adults.

**Methods:** One-hundred individuals 60 years and older, without cognitive impairment and enrolled in the Brazilian Memory Study (BRAMS), a longitudinal study, were applied the UCLA Loneliness Scale, Perceived Stress Scale (PSS), Geriatric Depression Scale (GDS), and Mini-Mental State Examination (MMSE). Participants were asked whether they had changes in daily routine and social connectedness during the pandemic.

**Results:** Almost half of the participants (48.4%) reported that the COVID-19 pandemic significantly affected their lives, 38.9% lost a relative or friend because of COVID-19, and 60% had daily routine changes. Relationships (40.5%) and emotion (22%) were reported as the most impacted area. Stopping physical activities and stay at home represented the main routine changed for 78% of participants. The use of voice messages through mobile phones to maintain social connectedness increased from 24.2% to 42.1%. For 38% of participants, their autonomy to daily decisions decreased, and 40% complained that memory got worse during the pandemic. More than 30% felt more stress, loneliness, or depression than in the pre-pandemic period. Controlling for age, sex, and education, higher loneliness scores were significantly associated with low MMES scores ($p = 0.018$).

**Conclusion:** Significant changes in life, daily routine, social connectedness, and mental health-related to the COVID-19 pandemic were reported by older adult participants. Loneliness was associated with lower cognitive performance.

### IMPACT OF NEIGHBORHOOD SOCIOECONOMIC DISADVANTAGE ON STAFFING HOURS IN US NURSING HOMES

Jason Falvey,[1] Erinn Hade,[2] Steven Friedman,[2] Rebecca Deng,[3] and Jasmine Travers,[4], *1. University of Maryland - Baltimore, Baltimore, Maryland, United States, 2. NYU Grossman School of Medicine, New York City, New York, United States, 3. NYU Wagner School of Public Health, New York City, New York, United States, 4. New York University, New York, New York, United States*

Severe socioeconomic disadvantage in neighborhoods where nursing homes (NH) are located may be an important contributor to disparities in resident quality of care. Disadvantaged neighborhoods may have undesirable attributes (e.g., poor public transit) that make it challenging to recruit and retain qualified staff. Lower NH staffing could subsequently leave residents vulnerable to adverse events. Thus, the purpose of this study was to evaluate whether NHs located in socioeconomically disadvantaged neighborhoods had lower healthcare provider staffing levels. We linked publicly available NH data geocoded at the Census block-group level with the Area Deprivation Index, a measure of neighborhood socioeconomic factors including poverty, employment, and housing quality (percentiles: 1-100). Consistent with prior literature on threshold effects of neighborhood poverty on outcomes, we characterized NHs as being located in a disadvantaged neighborhood if the census-block group ADI score was ≥85/100. We used generalized estimating equations clustered at the county level with fixed effects for state and rural location to evaluate relationships between ADI score and staffing. NHs located in socioeconomically disadvantaged neighborhoods had 12.1% lower levels of staffing for registered nurses (mean: 5.8 fewer hours/100 resident-days, 95% CI: 4.4-7.1 hours), 1.2% lower for certified nursing assistants (2.9 fewer hours/100 resident days; 95% CI 0.6-5.1 hours), 20% lower for physical therapists (1.4 fewer hours/100 resident-days; 95% CI 1.1-1.8 hours), and 19% lower for occupational therapists (1.3 fewer hours/100 resident-days; 95% CI 1.0-1.6 hours). These findings highlight disparities that could be targeted with policy interventions focused on recruiting and retaining staff in socioeconomically disadvantaged neighborhoods.

### INFECTION CONTROL IN SMALL RESIDENTIAL CARE SETTINGS: INSIGHTS FROM A NATIONAL SURVEY AND WASHINGTON STATE DATA

Carolyn Ham, and Anna Unutzer, *Washington State Department of Health, Shoreline, Washington, United States*

Infection control is a vital issue in long-term care, and the increasing popularity of small residential care facilities (SRCF) raises questions about the effectiveness of this model for preventing facility-acquired infections. In SRCF, care is provided in a residential home to a small number of residents. The setting lacks common terminology, and states license SRCF under various titles including Adult Family Homes, Adult Foster Homes and Family Care Homes. To better inform infection control efforts in this unique setting type, DOH staff conducted a comprehensive search to locate states that license SRCF. A total of 24 states were identified and approached to participate in a qualitative research study; 21 responded, three declined and nine were unable to participate due to staff time constraints. Between March 12th and April 15th, 2021, ten public health and regulatory staff from nine states completed semi-structured telephonic interviews on infection control in SRCF. Infection control licensing requirements and public health oversight for SRCF varied significantly across participating states. Data from these interviews was analyzed and compared with two Washington State Adult Family Home (AFH) sources: 1) online survey of AFH providers 2) Infection Control Assessment and Response evaluations conducted by public health staff. Four themes were identified in all three data sets: access to personal protective equipment, environmental safety, staffing issues and knowledge deficits. SRCF are valued by states that license them. Despite the challenges of implementing infection control in the home-like environment, extraordinary opportunities exist for improving care and preventing infections in this setting.

### INFLUENCES OF PREJUDICE AND STEREOTYPING IN THE DIRECT CARE WORKFORCE

Jennifer May, *Duke University, Gaston, North Carolina, United States*

Direct Care Workers (DCW; nursing assistants, personal care aides, home health aides) have the most one on one care with sexual and gender minority (SGM) older adults who reside in residential care facilities or use home health services. DCWs make up a vast majority of the healthcare workforce, holding almost five million jobs in 2019, with approximately 70% of the positions held being in residential care facilities. In a qualitative design study, 11 DCWs were interviewed using an open-ended, semi-structured format to describe their perceptions of care provided to SGM older adults in residential care facilities and the home health setting. These results were part of a larger qualitative study which found there were cues of stereotyping and prejudice in DCW narratives toward SGM older adults. The category DCWs' care and social system referred to characteristics of the DCWs' work environment and the perspectives, attitudes, and reported care toward SGM older adults and diverse populations. It was determined that there are synergies among SGM older adults' care and DCW along with DCW workforce issues (short staffed, low wages, lack of health benefits) that may prevent the DCW from being accepting of implicit bias training or culture change within these facilities/agencies. Implications for practice, policy, and future research are discussed.

### INSIGHT INTO THE FEASIBILITY AND ACCEPTABILITY OF A MULTI-LIFESTYLE DEMENTIA RISK INTERVENTION

Laura Dodds, and Joyce Siette, *Macquarie University, Macquarie Park, New South Wales, Australia*

Lifestyle interventions based on behaviour change principles may provide a useful mechanism in reducing dementia risk amongst older adults, however intervention acceptability remains relatively unexplored. We assessed the feasibility and acceptability of BRAIN BOOTCAMP, an Australian initiative aiming to improve dementia literary and reduce dementia risk by delivering a brain health box addressing multiple lifestyle factors through education, physical prompts and an individualised brain health profile. Semi-structured phone interviews were conducted with participants (N=94) at completion of the program (3-months) using a theoretical sampling approach to select a range of participants with varying brain health scores, age, gender, education and locality. Interview topics included participants' overall experience and suggestions for program improvement. Interviews were transcribed and analysed using thematic analysis. Participants were mostly female (79%), with a mean age of 72.6 years (SD=5.4), from an English-speaking background (89.4%) and resided in metropolitan areas (76.6%). Participants positively perceived the program, resulting in high usability and acceptability. Valuable aspects included building dementia awareness in an innovative way, and having re-assessments which identified areas for personal improvement. Participants further discussed how the program prompted lifestyle change, including setting goals (e.g., physical activity) and facilitated a general awareness of their brain health. Suggested improvements included shorter surveys, regular check-ins, and specific tailoring of the program to be more inclusive for older adults with varying levels of health. Our study demonstrated that a simple, innovative program could be a promising medium for delivering comprehensive educational resources and induce lifestyle change for older adults.

### INTERDISCIPLINARY INNOVATIONS UTILIZING PET ROBOTS TO MEET RESEARCH, EDUCATION, AND CARE NEEDS

Meredith Troutman-Jordan,[1] and Boyd Davis,[2], *1. University of North Carolina Charlotte, Charlotte, North Carolina, United States, 2. UNC Charlotte, Charleston SC, South Carolina, United States*

Studies of the impact of robotic companion pets are proliferating, authored by several disciplines, each with different concerns. Roboticists focus on technology design and artificial emotional intelligence as opposed to general preferences for soft, furry, interactive animals. Others worry that as people interact with potentially deceptive technology, they may think the pet is alive. While aware of these serious concerns, gerontologists have focused on how lonely older persons without cognitive impairment respond to social 'helper' robots. More recent studies emphasize the possible impact of animatronic pets on persons with dementia (PWD). Therapeutic benefits of these pets are just being established. Our current pilot study is timely in that it now involves semi-structured interviews with formal/ informal caregivers of PWD who have been given a robot pet. We are eliciting perceptions, opinions, and observations of the PWD's response to robotic pets. We recruited 8 gerontology students as much-needed assistants for a research-driven topics course to afford them field exposure to PWD, caregivers, and direct research experience. Because students seldom have experience either with robotic pets or PWD, they read selected articles and received training/practice in semi-structured interviewing techniques. Students next conducted interviews with caregivers of PWD who have interacted with the pets. All interviews are audio-recorded, transcribed and deposited in the Carolinas Conversations Collection. Content and thematic analysis of transcriptions, student activity logs and bi-weekly reflective discussions will inform next steps in intervention research, testing therapeutic outcomes such as agitation reduction by pet robots for PWD.

### LOW COGNITIVE PERFORMANCE INCREASES THE RISK OF HOSPITAL-ASSOCIATED COMPLICATIONS IN OLDER ADULTS

Juliana Souza-Talarico,[1] Siomara Yamaguti,[2] Adriana Dutra,[3] and Daniel Apolinario,[3], *1. The University of Iowa, Iowa City, Iowa, United States, 2. Hospital do Coracao HCor, Hospital do Coracao (HCor), Sao Paulo, Brazil, 3. Hospital do Coracao HCor, Hospital do Coracao HCor, Sao Paulo, Brazil*

Considering the limited evidence regarding the factors that contribute to long-term consequences after hospitalization of older people, we analysed the relationship between cognitive performance and hospital-associated complications (HAC). One thousand, three hundred Individuals aged 60 and older (mean age 82.3, 53.3% female), not assigned to palliative care and admitted in medical and surgical wards from a private hospital, were followed up from admission to 30 days after discharge. HAS was evaluated using a multicomponent measure that combines 12 hospital-associated complications (delirium, functional decline, falls, pressure injuries, bronchoaspiration, non-planned ICU transfer, physical restraints, hospital stay > 30 days, death, long-term care transfer, and readmission). Cognitive performance was assessed using the "10-point cognitive screener (10-CS)", which combines temporal orientation, category fluency, and word recall evaluation.

**Results:** Overall, 464 (35.7%) participants had one or more HAC during their admission. Patients with HAC showed lower 10-CS scores than those with in HAC ($p < 0.001$). Adjusting for sociodemographic data, medication, chronic diseases, delirium screening, functional performance, each 10-CS point decreased the HAC changes by 19.2% (odds ratio = 0.808; 95% CI = 0.660 – 0.990).

**Conclusion:** These findings show that low cognitive performance was significantly associated with the risk of developing HAC during hospitalization and within 30 days after discharge. That evidence forms the critical foundation for the next steps towards validating the accuracy of these models in predicting vulnerability to HAC and developing screening tools to be used at the point of care.

### LUNG FUNCTION RESERVE AND PHYSICAL FUNCTION IN HEALTHY OLDER ADULTS: FINDINGS FROM BLSA

Joey Saavedra,[1] Ajoy Karikkineth,[2] Luigi Ferrucci,[2] and Eleanor Simonsick,[3], *1. Iowa State University, Ames, Iowa, United States, 2. National Institute on Aging, Baltimore, Maryland, United States, 3. National Instute on Aging/NIH, Baltimore, Maryland, United States*

Forced Expiratory Volume in 1-second (FEV1) that falls below the lower limit of normal (LLN) is a well-established correlate of functional limitation and disability. However, less is known about the functional implications of gradations of lung function above the LLN. We examined the cross-sectional association between gradations of healthy lung function and usual gait speed, reported walking ability, and fast 400m walk performance in 750 persons (50.7% men) aged 55-95 free from respiratory disease and mobility limitations, participating in the Baltimore Longitudinal Study of Aging (BLSA). The 2012 Global Lung Initiative (GLI) reference equations were used to calculate FEV1 Z-scores, with healthy lung function categorized as follows: $-1.6 < Z \le -1.0$ (pre-clinical), $-1.0 < Z \le -0.3$ (low normal), $-0.3 < Z \le 0.3$ (normal), $0.3 < Z \le 1.0$ (high-normal), and $Z > 1.0$ (high). Associations between gradations of healthy lung function and physical function were evaluated using multivariate linear regression, adjusting for age, sex, height, weight, and waist circumference. Compared to the ‘pre-clinical’ category, the difference in 400m walk time was 0.71 (p>.05), -6.60 (p>.05), -12.21 (p<0.05), and -15.52 (p<0.01) seconds for the ‘low normal’, ‘normal’, ‘high-normal’, and ‘high’ categories, respectively. No associations between gradations of healthy lung function and normal gait speed or walking ability were found (p>0.05). Higher levels of lung function reserve are associated with better 400m walking performance, thus efforts to promote and/or reduce loss of lung function reserve may help individuals maintain high functional capacity in later life.

### MAINTENANCE OF PHYSICAL FUNCTION IN ADULT AND OLDER ADULT MICE USING AEROBIC EXERCISE

Ted Graber, Megan Pajski, Christopher Byrd, Nainika Nadigama, Alyssa Fennel, Emily Seguin, and Anna Seguin, *East Carolina University, Greenville, North Carolina, United States*

As we age, physical and neuromuscular function declines gradually. Exercise is a therapy to improve neuromuscular ability. Pre-clinical models are needed to understand molecular mechanisms contributing to age-associated functional decline and how exercise affects that downward trajectory. Our goal was to compare the differences in effects of two validated mouse models of endurance exercise designed to mimic human training studies: high intensity interval training (HIIT) and voluntary wheel running (VWR). We hypothesized that both adult and older adults (10 and 26 months old at end, respectively: 10m and 26m) would respond to both exercise regimens by improving or maintaining exercise/physical capacity, but that adult mice would benefit more. We randomly assigned male C57BL/6 mice into experimental groups: 10m: (VWR, HIIT, sedentary control, CON, n=8 per group), and 26m (VWR, n=8, HIIT, n=10). We measured functional ability (pre- and post-intervention) using CFAB (comprehensive functional assessment battery), our composite scoring system (grip strength, inverted cling, treadmill endurance, activity rate, rotarod), tracked body composition (EchoMRI), and measured muscle wet mass. We found that significant retention of ability (CFAB difference, repeated measures ANOVA, p<0.05) and fat percentage (ANOVA., %change: 10m: CON +125%, HIIT +101%, VWR +52%; 26m: VWR -42%, HIIT +26%, p<0.05) was promoted by both exercise modalities compared to control, and furthermore HIIT may have better efficacy in the adult versus the older mice. In conclusion, both exercises are valid models with derived benefits as expected in similar human studies. We anticipate future work using these models to undertake mechanistic investigations.

### MAPPING THE ENDOGENOUS KETOGENIC SYSTEM ACROSS AGES, SEX AND DIETS

Brenda Eap,[1] Mitsunori Nomura,[1] Oishika Panda,[2] Thelma Garcia,[1] and John Newman,[3], *1. Buck Institute for Research on Aging, Buck Institute for Research on Aging, California, United States, 2. Buck Institute for Research on Aging, Buck Institute of Research on Aging, California, United States, 3. Buck Institute for Research on Aging, Novato, California, United States*

Understanding how our cells maintain energy homeostasis has long been a focus of aging biology. A decline in energy metabolism is central to many age-related diseases such as Alzheimer’s disease, heart failure, frailty, and delirium. Intervening on pathways involved in energy homeostasis can extend healthy lifespan. When our primary energy substrate glucose, is scarce, our bodies use ketone bodies (i.e. beta-hydroxybutyrate, acetoacetate, acetone). Aging is associated with glucose intolerance and insulin insensitivity, yet what role ketone body metabolism might play in compensating for impaired glucose utilization in age-related diseases is understudied. Here, we investigated how the body’s endogenous ketone body production and utilization pathways are modulated by age across the lifespan of female and male C57BL/6 mice (4 mo old, 12 mo old, 22 mo old). We show how different ages have different metabolic and gene expression responses to 1-week ketogenic diet (KD) or ketone ester diet. We observed an apparently compensatory ketogenic response in older animals fed normal diet, with a stronger compensatory response driven by KD. We observed tissue-specific changes, including induction of ketone body production enzymes in the aging heart. When comparing the ketogenic capacity between sexes, females had a higher basal level and less variation with age, underscoring the importance of sexual dimorphism in metabolism. Overall, these findings suggest that older animals use ketone bodies to meet energetic demands in a normal diet context. This study supports the potential roles of ketogenic therapies such as exogenous ketones to improve energy homeostasis in conditions of aging.

### MENTORSHIP PROGRAM CONTENT RATINGS: ADVANCING DIVERSITY IN AGING AND ALZHEIMER’S DISEASE RESEARCH AND CAREERS

Sheri Thompson,[1] and Lauren Pineda,[2], *1. University of California San Diego, encinitas, California, United States, 2. UCSD, UCSD, California, United States*

MADURA is an R25 Advancing Diversity in Aging Research (ADAR) undergraduate training program funded by the National Institute on Aging and based at the University of California San Diego. Its objectives are to improve retention and academic success of underrepresented minority (URM) trainees, and to increase graduate/medical school applications and/or entry into Aging/ADRD careers. This multi-component program includes paid research lab experience, research and academic skills training, peer support, faculty-led small groups, guest seminars, and additional professional development opportunities. The Year 1 Cohort of 32 students consisted of Hispanic/Latinx and other students under-represented in collegiate MSTEM studies. Almost ¾ of the mentees also came from disadvantaged backgrounds, per current NIH criteria. Evaluation data have been collected through brief, weekly, anonymous online student surveys, plus more comprehensive quarterly mentee and mentor surveys. This poster will describe the Program structure and 20+ group activities, trainings, seminars, and modalities, and report student ratings of satisfaction and utility. 92% of trainees rated the overall program value as Excellent, and 92% also rated effectiveness in promoting Aging/ADRD

careers among underrepresented students as Excellent. Quality ratings of components such as pay, research placements, group training and group mentorship were rated as Excellent or Good by 96%. Relative student component rankings, specific suggestions for improvements, plus Mentee and Mentor perspectives on research-specific training needs, will also be presented. Based upon these experiences and data, the authors will make specific suggestions for future URM research training, mentorship program content, and expanded delivery modalities.

### NEURON-SPECIFIC MECHANISMS CONTROL THE MITOCHONDRIAL REGULATOR PGC-1A

Eric McGregor,[1] Dylan Souder,[1] Josef Clark,[2] Timothy Rhoads,[2] Kevin Elicieri,[2] Mark Beasley,[3] Darcie Moore,[2] and Rozalyn Anderson,[4], *1. University of Wisconsin--Madison, Madison, Wisconsin, United States, 2. University of Wisconsin-Madison, Madison, Wisconsin, United States, 3. University of Alabama at Birmingham, University of Alabama at Birmingham, Alabama, United States, 4. University of Wisconsin College of Medicine, University of Wisconsin, Madison, Wisconsin, United States*

Mitochondrial dysfunction has been proposed as a hallmark of the aging process. Specifically, as a function of aging, mitochondria appear to have decreased enzyme activity and respiratory capacity and increase reactive oxygen species production. Brain aging is associated with morphological and homeostatic changes, including alterations in brain size, cognitive impairment, and white and grey matter integrity. However, the causes of these changes remain an open and actively pursued field of study. The ubiquitously expressed transcriptional coactivator peroxisome proliferator-activated receptor gamma-coactivator 1 (PGC-1a) has been described as the master regulator of mitochondrial function. Despite the emerging connections between PGC-1a and disease vulnerability, the regulation of PGC-1a outside of the skeletal muscle, liver, and adipose tissue is not well defined. This is particularly true in the brain, where PGC-1a is enriched in neurons, and alterations in expression levels have been linked to neurodegenerative disorders. Here we report that astrocytes and neurons differ substantially in mitochondrial status and the transcript variants of PGC-1a expressed, including using a neuron-specific promoter. Taking advantage of the ability of the tau-kinase GSK-3b to influence PGC-1a expression, we investigate how transcript variants are differentially regulated in primary neurons and astrocytes. Neuronal PGC-1a responds robustly to GSK3b inhibition by lithium, switching the dominant promoter, leading to changes in isoform distribution and abundance, while astrocytes are refractory to lithium treatment. The data presented here highlight key mechanisms for neuron-specific metabolic regulation that are likely to be relevant to neurodegenerative diseases that have a link to mitochondrial dysfunction.

### NIA COORDINATED ANALYSIS OF DYNAMIC SOCIOEMOTIONAL EXPERIENCES AND WELL-BEING DURING THE PANDEMIC

Shevaun Neupert,[1] Sumbleen Ali,[2] Isabella Bouklas,[3] Rita Hu,[4] Reilly Kincaid,[5] Niccole Nelson,[6] Shiyang Zhang,[7] and Stacey Scott,[8], *1. North Carolina State University, Raleigh, North Carolina, United States, 2. Stony Brook University, State University of New York, New York, United States, 3. Duke University, Durham, North Carolina, United States, 4. Social Work, Ann Arbor, Michigan, United States, 5. Purdue University, West Lafayette, Indiana, United States, 6. University of Notre Dame, University of Notre Dame, Indiana, United States, 7. The University of Texas at Austin, Austin, Texas, United States, 8. Stony Brook University, Stony Brook, New York, United States*

We conducted a coordinated analysis of eight longitudinal studies with data collected during the COVID-19 pandemic. Our overarching aim was to examine within-person fluctuations in health and well-being during the pandemic that may differ across the adult lifespan. The studies are from different regions of the U.S. with data collected during different periods of the pandemic. These studies sampled heterogenous age groups, used diverse methods, and were harmonized on constructs. Four longitudinal studies (Notre Dame Study of Health & Well-being, Within-Family Differences Study, Social Relations Study, and the Einstein Aging Study [EAS] Covid Telephone Interviews) and four intensive, microlongitudinal studies (Daily Experience and Well-being Study, Daily COVID-19 Spring, Daily COVID-19 Fall, EAS), with data collected between March 2020 and August 2021 were analyzed. In three studies, older adults were consistently less variable (i.e., lower within-person standard deviation) in negative emotional well-being such as negative affect and depressive symptoms compared to younger adults. In four studies older adults were also less variable in stress. Evidence of better outcomes associated with social interactions was found in three studies, where within-person variability in social interactions was positively correlated with variability in positive affect across age. These findings point toward the complexities of dynamic socioemotional experiences that unfold across historical periods and across the lifespan. These within-person fluctuations could be used as a benchmark to examine long-term trajectories of well-being.

### NON-PHARMACOLOGICAL INTERVENTIONS TO MANAGE TYPE 2 DIABETES IN OLDER HISPANICS: A SYSTEMATIC REVIEW

Edgar Vieira,[1] Kayleigh Sherbutt,[1] Madison Scanlan,[2] and Heather Frederick,[1], *1. Florida International University, Miami, Florida, United States, 2. Florida international University, Miami, Florida, United States*

Type 2 diabetes is a serious public health problem that affects millions of Americans. Hispanics are disproportionately affected and have high incidence of type 2 diabetes. Lifestyle modifications in diet and increased physical activity are recommended in addition to medication. The purpose of this systematic review was to analyze the scientific literature concerning the effects of exercise, nutrition, and combined diet and exercise interventions on type 2 diabetes management in older Hispanics. We searched three databases for studies that included dietary interventions, exercise interventions, or a combination to manage type 2 diabetes in older Hispanics. A total of 653 studies were screened and reviewed, with seven being included in the review. Our findings indicate that physical activity interventions significantly reduce glycosylated hemoglobin, and diet interventions also led to decreased levels of HbA1c. There is a significant effect in HbA1c levels on individuals receiving a combination of diet and exercise interventions compared to control groups. Implementing diet or exercise interventions in older Hispanics with Type 2 diabetes leads to significantly reduced glycosylated hemoglobin levels; the effects of combined diet and exercise interventions were not superior to the effects of single interventions in HbA1c levels. Exercise and diet seem to be effective non-pharmacological interventions to manage type 2 diabetes in older Hispanics, but additional research is needed.

### NORMALIZED GRIP STRENGTH IS INVERSELY ASSOCIATED WITH DNAM AGE IN MIDDLE AGE AND OLDER ADULTS.

Mark peterson,[1] Stacey Collins,[2] Helen Meier,[2] and Jessica Faul,[1], *1. University of Michigan, University of Michigan, Michigan, United States, 2. University of Michigan, Ann Arbor, Michigan, United States*

There is a large body of evidence linking muscular weakness, as determined by low grip strength, to a host of negative aging-related health outcomes. Given these links, grip strength has been labeled a "biomarker of aging"; and yet, this metric provides no biological plausibility. The objective of this study was to determine the association between grip strength and DNA methylation (DNAm) age acceleration. Middle age and older adults from the 2006-2008 waves of the Health and Retirement Study with 8-years of follow-up were included. Cross sectional and longitudinal modeling were performed to examine the association between grip strength (normalized to body mass: NGS) and DNAm age acceleration, adjusting for cell composition, sociodemographic variables, and smoking. Three DNAm clocks were incorporated for estimating age acceleration including the established DunedinPoAm, Levine, and GrimAge clocks. There was a robust and independent cross sectional association between NGS and DNAm age for men (β:-0.36; p<0.001) and women (β:-0.36; p<0.001) using the DunedinPoAm clock, and for men only using the Levine (β:-8.04; p=0.01) and GrimAge (β:-4.76; p=0.01) clocks. There was also an independent longitudinal association between baseline NGS and DNAm age for men (β:-0.27; p<0.001) and women (β:-0.36; p<0.001) using the DunedinPoAm clock, and for women only using the Levine (β:-8.20; p<0.001) and GrimAge (β:-6.04; p<0.001) clocks. Our findings provide some evidence of age acceleration among men and women with lower NGS. Future research is needed to understand the extent to which DNAm age mediates the association between grip strength and chronic disease, disability, and mortality.

### OLDER PEOPLE'S PERCEPTIONS OF WHAT IS NEEDED TO EXPERIENCE DIGNITY AND WELL-BEING AT RESIDENTIAL CARE FACILITIES.

Charlotte Roos,[1] Anna Swall,[1] Lena Hammar,[2] Anne-Marie Boström,[3] and Bernice Skytt,[4], *1. Dalarna University, Falun, Dalarnas Lan, Sweden, 2. Mälardalen University / Dalarna University, Västerås, Vastmanlands Lan, Sweden, 3. Karolinska Institutet, Karolinska Institute, Stockholms Lan, Sweden, 4. University of Gävle, Gävle, Gavleborgs Lan, Sweden*

Dignity and well-being are central values in care of older people living in residential care facilities. In addition, care of older people living in residential care facilities should be person-centred. Dignity and well-being can according to the person-centred practice framework be interpreted as person-centred outcomes. Despite this older people living in residential care facilities have described that they not fully experience dignity and well-being and improvements are needed. To improve care it is important to know what to target. The aim of this qualitative study was therefore to describe residents' perceptions and experiences of what is needed to live with dignity and a sense of well-being. Interviews were carried out with older people living at residential care facilities (n=20). Inductive content analysis was used to analyse data and one overarching theme and three categories emerged. The result revealed the importance of, and that staff and the care environment supported, to manage daily life by oneself, to be shown respect and to belong to a social context. For older people to experience the person-centred outcomes dignity and well-being managers at residential care facilities need to develop and support the staff prerequisites related to knowledge, skills and attitudes and to improve the care environment. According to the person-centred practice framework, the staff prerequisites and the care environment must be taken into account to achieve the person-centred outcomes dignity and well-being.

### PEAK EXPIRATORY FLOW PREDICTS INCIDENT DEMENTIA IN A U.S. SAMPLE OF OLDER ADULTS

Patrick Donahue,[1] Qian-Li Xue,[2] and Michelle Carlson,[3], *1. Johns Hopkins Bloomberg School of Public Health, Baltimore, Maryland, United States, 2. Johns Hopkins University School of Medicine, Baltimore, Maryland, United States, 3. Johns Hopkins University, Baltimore, Maryland, United States*

Dementia is an increasingly important public health problem with known vascular contributors. Respiratory function, measured by peak expiratory flow (PEF), may be a novel modifiable risk factor in reducing the risk of dementia along the vascular pathway. We investigated the association between PEF and incident dementia in older adults from the National Health and Aging Trends Study (NHATS). Using NHATS criteria, participants were categorized as having or not having probable incident dementia during NHATS Rounds 2-4, spanning three years. Of 3,622 participants with available PEF and covariate data, 543 (15.0%) had incident cases of dementia. Quartile of baseline PEF was analyzed as a predictor of incident dementia using logistic regression models, while controlling for several health and sociodemographic covariates. The fourth quartile of PEF had statistically significantly decreased odds of incident dementia when compared to the first PEF quartile (OR = 0.27; 95% CI [0.19, 0.40]). Significantly reduced odds of incident dementia were found when comparing the third and second PEF quartiles to the first PEF quartile, as well (OR = 0.35; 95% CI [0.26, 0.47] and OR = 0.62; 95% CI [0.48, 0.80], respectively). These relationships were dose-dependent so that increasing PEF quartile levels were more protective against incident dementia. PEF may be considered as an easily administered, low-cost measure of respiratory function and a possible screening tool for dementia risk. Improving PEF may reduce dementia risk through vascular mechanisms

(e.g., increased blood circulation and brain oxygenation). Future research should explore these potential causal pathways between PEF and dementia.

### PERCEIVED PARTNER RESPONSIVENESS AND DEPRESSION: THE MODERATING ROLE OF AGE AND GENDER

Betul Urganci, *Cornell University, Ithaca, New York, United States*

A growing body of research suggests that greater perceived partner responsiveness- the extent to which individuals feel cared for, understood, and validated by their romantic partner- leads to longer, healthier, and happier life in adulthood, yet little is known about possible moderating factors between responsiveness and well-being. Using a longitudinal design, the current study tested the moderating roles of age and gender in association between perceived partner responsiveness and depression symptoms. The data for the present study came from the National Survey of Midlife in the United States (MIDUS I and II) which is a longitudinal study on health and aging. A life span sample of 2856 married or cohabiting individuals (1402 Female, Mage= 47.16) completed measures of perceived partner responsiveness, depression, age, and gender in two waves (T1 and T2). The results showed that greater perceived partner responsiveness at T1 predicted lower depressive symptoms at T2 controlling for depressive symptoms at T1. This finding remained when controlling for potential confounders including demographics and health covariates. The moderation analysis demonstrated that participants' age was not a significant moderator in the association between perceived partner responsiveness and depression. Yet, gender significantly was a significant moderator such that the association of perceived partner responsiveness and depression was significant for female but not for male participants. These findings can have implications for mental health and relational well-being.

### PILOT TESTING OF THE UB-CAM DELIRIUM SCREENING APP

Ashley Kuzmik,[1] John Joseph Hannan,[2] Long Ngo,[3] Marie Boltz,[4] Priyanka Shrestha,[5] Sharon Inouye,[6] Donna Fick,[4] and Edward Marcantonio,[7], *1. Penn State University, University Park, Pennsylvania, United States, 2. Pennsylvania State University, State College, Pennsylvania, United States, 3. Harvard T.H. Chan School of Public Health, Brookline, Massachusetts, United States, 4. Pennsylvania State University, University Park, Pennsylvania, United States, 5. Pennsylvania State University, 6. Harvard Medical School, Boston, Massachusetts, United States, 7. Beth Israel Deaconess Medical Center, Boston, Massachusetts, United States*

Systematic screening improves delirium detection among hospitalized older adults. This poster describes the development and pilot testing of an iOS-based app that incorporates the Ultra-Brief Confusion Assessment Method (UB-CAM), a two-step, delirium detection protocol that combines the UB-2 (2-item screener) and 3D-CAM. Previous work tested a RedCAP-based UB-CAM app in 527 patients with 399 physicians, nurses, and certified nursing assistants (CNAs) showing it can be successfully completed by all three disciplines in 97% of eligible patients in 80 seconds on average with over 85% accuracy relative to a gold standard. To improve accessibility to the clinical setting, our research team now collaborated with a computer scientist to develop and refine an iOS-based UB-CAM app for the iPhone and iPad through iterative "laboratory" testing. The app was piloted by non-clinician, research testers in hospitalized older adults (age $\bar{x}$ =83, SD= 8.0) with dementia (Clinical Dementia Rating Scale $\bar{x}$ =1.1, SD= .30); 64% were assessed to be delirium positive. The app demonstrated preliminary efficiency (90 seconds on average), high acceptability (100% satisfaction of users), and reliability (100% inter-rater). This project underscores the need for close collaboration between researchers, clinicians, and computer scientists with iterative testing of bedside-facing apps prior to testing with patients. Next steps include testing effectiveness in a pragmatic trial with clinician users (physicians, nurses, CNAs), integrating the UB-CAM app into the routine hospital care of all older patients. Having rapid, accurate bedside delirium detection has the potential to transform care.

### PILOTING THE "ANTI-OPPRESSIVE PRACTICE & RESEARCH WITH DIVERSE OLDER ADULTS" TRAINING PROGRAM DURING COVID-19

David Camacho,[1] Kelly Pacheco,[2] Sabrina Feldman,[2] Usha Kaul,[2] Patricia Kim,[3] and M. Carrington Reid,[4], *1. University of Maryland Baltimore, Baltimore, Maryland, United States, 2. New York University, New York University, New York, United States, 3. Weill Cornell Medicine, Weill Cornell Medicine, New York, United States, 4. Weill Cornell Medical College, New York, New York, United States*

There is a critical need to develop our gerontological-sensitive workforce. Social workers (SW) frequently provide services to older adults yet there are few opportunities for them to train as gerontological clinicians and/or researchers. To provide an opportunity for SW students to gain gerontological knowledge, clinical practice, and research skills, we developed, and pilot tested the "Anti-Oppressive Practice and Research with Diverse Older Adults" virtual training program at a major medical facility in Manhattan, NY. We explored the feasibility, implementation, and impact of this novel program. The 21-hour weekly MSW field placement program followed an anti-oppressive framework and included: 1) supervision and training sessions; and 2) direct clinical and research practice (e.g., theory, funding, assessment, data collection etc.) and aging topical seminars (e.g., depression, loneliness, pain etc.). Racially diverse supervisors and graduate SW students engaged in reflective writing exercises, iterative discussions (recorded & transcribed) and a thematic analysis of data. All interns successfully completed the program and reported enhanced skills related to SW core competencies and research (e.g., standardized assessments), research and practice gap awareness (e.g., minority aging) and plans to pursue advanced research training and/ or gerontological clinical work. Intern challenges included: 1) disconnect between MSW curricula and research placements; and 2) managing minority and contextual stressors (e.g., imposter syndrome, covid-19, civil unrest). Supervisory challenges included: 1) humanizing sensitive discussions via virtual communication and 2) resource constraints. Future research should systematically assess program effects (e.g., SW core competencies) and how to facilitate interprofessional collaborations to develop diverse gerontological SWs and researchers.

### PLEASE DO SOMETHING: WHAT CAREGIVERS FOR FAMILY MEMBERS WITH FTD NEED

Samantha Smith,[1] Allison Lindauer,[2] and Leslie Tran,[1], *1. Oregon Health & Science University, Portland, Oregon, United States, 2. Oregon Health Sciences University, Portland, Oregon, United States*

Frontotemporal dementia (FTD) often presents with pronounced behavioral symptoms that contribute to family Care Partner (CP) burden and psychological strain. FTD-specific interventions that support the unique challenges of FTD-CPs are lacking. The present focus group study (Phase 1), elicited feedback from twelve CPs of persons with FTD on the multi-component video-based STELLA intervention (Support via TEchnology: Living and Learning with Advancing ADRDs), to inform the revision and adaptation of STELLA for FTD-CPs (Phase 2). Using Thomas's (2006) analytic approach to evaluation data, the investigators reviewed the raw text from two focus groups and used an inductive approach to create categories that informed future STELLA adaptation and revision. To address trustworthiness, each investigator independently analyzed the transcripts and CP-annotated STELLA booklets. Six commonalities emerged. Three address the caregiver experience: Burden and living with complex behaviors; Difficulties in getting a diagnosis; and Barriers to participation. The other three reflect specific intervention adaptations: Make a Roadmap, STELLA-FTD Structure, and Booklet edits. The focus group findings offer practical suggestions to create a revised STELLA intervention to address the needs of families living with FTD. The suggestions are nested in the intense caregiving experience of living with complex behavioral symptoms, feeling burdened, isolated, and "living in darkness." Both the investigators and caregivers appreciate the difficulty in crafting an intervention that meets the needs of all families living with FTD, but the caregivers encouraged the team to develop "something": "…You won't be able to solve every problem or meet every need… [but] please, please do something."

### PLEIOTROPY OF ALZHEIMER'S DISEASE AND EDUCATIONAL ATTAINMENT: INSIGHTS FROM THE SUMMARY STATISTICS

Yury Loika,[1] Elena Loiko,[1] Irina Culminskaya,[1] and Alexander Kulminski,[2], *1. Duke University, Durham, North Carolina, United States, 2. DUKE UNIVERSITY, Durham, North Carolina, United States*

Epidemiological studies report beneficial associations of higher educational attainment (EDU) with Alzheimer's disease (AD). Prior genome-wide association studies (GWAS) also reported variants associated with AD and EDU separately. The analysis of pleiotropic predisposition to these phenotypes may shed light on EDU-related protection against AD. We examined pleiotropic predisposition to AD and EDU using Fisher's method and omnibus test applied to summary statistics for single nucleotide polymorphisms (SNPs) associated with AD and EDU in large-scale univariate GWAS at suggestive-effect (5×10-8

### PREDICTIVE ABILITY OF SELF COMPASSION IN PSYCHOSOCIAL OUTCOMES OF CAREGIVERS OF PERSONS WITH DEMENTIA

Claire Grant,[1] and Katherine Judge,[2], *1. Cleveland State University, South Euclid, Ohio, United States, 2. Cleveland State University, Cleveland, Ohio, United States*

Caregivers of Individuals with Dementia (IWDs) often face increased rates of depression, anxiety, and burden because of their role as caregiver. Self-compassion, a construct centered around self-kindness and understanding has not been well studied within the caregiving population. The present study was aimed at understanding the relationship between self-compassion and the psychosocial outcomes of burden, depression, and anxiety. Strong relationships between self-compassion and these outcomes have been established in other populations, but these relationships have not been studied with the dementia caregiving population. A diverse sample of dementia caregivers providing over 5 hours of care per week were recruited through CloudResearch and MTurk ($N = 99$). Participants were aged 18 to 69 years ($M = 38.61$) and 66.7% were female. 67.7% were White, 13.1% were Black, and 8.1% were Asian. 73% were children/in law or grandchildren/in law of the individual with dementia and 12% were a close friend of the individuals with dementia. The individuals with dementia had an average age of 73.88 years. Results of multiple regression models showed that self-compassion was a significant predictor of depression ($\beta = -.25$, $p = .025$), anxiety ($\beta = -.36$, $p = .001$), and burden ($\beta = -.25$, $p = .023$) even while controlling for other constructs including self-esteem, types of coping, and IWD impairment level. Self-compassion will be discussed as a novel contribution to the caregiving literature in furthering our understanding of well-being predictors and how to target self-compassion as a modifiable factor for offsetting the negative impacts of caregiving.

### PREDICTORS OF SLEEP AMONG SPOUSAL CARE DYADS LIVING WITH CHRONIC CONDITIONS

Florence Johnson,[1] Sheria Robinson-Lane,[2] Lianlian Lei,[3] Yin Liu,[4] Yeonsu Song,[5] Seung-won Emily Choi,[6] and Toni Antonucci,[3], *1. University Of Michigan School of Nursing, Ann Arbor, Michigan, United States, 2. University of Michigan School of Nursing, Ann Arbor, Michigan, United States, 3. University of Michigan, Ann Arbor, Michigan, United States, 4. Utah State University, Logan, Utah, United States, 5. UCLA, Los Angeles, California, United States, 6. Texas Tech University, Lubbock, Texas, United States*

Sleep health relates closely to physical health and well-being among older adults with chronic health conditions. However, little is known about the dyadic sleep patterns of these individuals and their spousal caregivers. Secondary analyses of the 2015 National Health and Aging Trends Study and National Study of Caregiving (N= 62

care dyads, mean age 78.59 years for care recipients and 75.77 years for caregivers) were completed to examine the sleep patterns, and related factors, of spousal dyads at both the individual and dyadic levels. Sleep measures included frequency in trouble falling back asleep and insomnia symptoms. Predictors included demographics, depressive symptoms, and positive affect for dyads and contextual factors such as dementia caregiving, care burden and support, neighborhood cohesion, and relationship quality. Multilevel dyadic and actor-partner interdependence models were used to complete analyses. Though intraclass correlation was poor (dementia care dyads ICC=0.123, non-dementia care dyads ICC=0.043), persons with dementia/spousal caregiver dyads (n=102) had more similar sleep and insomnia patterns than dyads with other chronic conditions. Poor sleep among dyads was correlated with higher care burden ($\beta$ = -0.31, p <.0001), however, better relationship quality marginally enhanced the association ($\beta$ = -0.23, p = .08). Individual depressive symptoms negatively affected dyadic sleep patterns. However, positive affect only had an actor effect and was related to better individual sleep. Other contextual factors did not affect sleep patterns. These findings suggest the importance of both caregiver and care recipient characteristics on sleep at dyadic levels, particularly those with dementia.

### PREFRONTAL ACTIVATION IS ASSOCIATED WITH GAIT QUALITY DURING AN ATTENTIONAL TASK IN OLDER ADULTS

Emma Baillargeon,[1] Anisha Suri,[1] Nemin Chen,[1] Xiaonan Zhu,[1] Caterina Rosano,[1] Ervin Sejdic,[2] Theodore Huppert,[1] and Andrea Rosso,[1], *1. University of Pittsburgh, Pittsburgh, Pennsylvania, United States, 2. Swanson School of Engineering, University of Pittsburgh, Pittsburgh, Pennsylvania, United States*

Prefrontal cortical activation varies by walking task and is a marker of attentional demand. We compared prefrontal activation by functional near-infrared spectroscopy (fNIRS) to accelerometry-derived gait quality. We hypothesized greater activation with lower gait quality (greater step-time coefficient-of-variation, decreased cadence, smoothness, regularity, and signal variability). Participants (n=114; age 74.4±6.0 years, 59.6% female) were independently ambulating individuals >64 years. Attentional (reciting every-other alphabet letter) and physical (uneven surface) challenges mimicked community mobility and provided four 15m walking conditions: even, uneven, ABC-even, and ABC-uneven. fNIRS data were referenced to quiet standing and averaged within left and right hemispheres. Gait metrics from a tri-axial accelerometer at the lower-back included cadence (steps/min), step-time coefficient-of-variation, signal variability (standard deviation), smoothness (harmonic ratio), and regularity (entropy). Associations between fNIRS and gait were quantified using Pearson correlations ($\alpha$=0.05). Results were consistent across hemispheres, gait axes, and robust to adjustment for age and gait speed; we report unadjusted coefficients for left hemisphere and anterior-posterior gait direction. Greater prefrontal activation was associated with slower cadence (r=-0.220, p=0.019), lower signal variability (r=-0.228, p=0.015), and reduced smoothness (r=-0.194, p=0.039) during ABC-even. No relation was observed for step-time coefficient-of-variation or regularity. Results were similar for the ABC-uneven condition, except there was no association with gait smoothness but was with step-time coefficient-of-variation (r=0.25, p=0.007). Prefrontal activation was not correlated to gait quality in non-ABC conditions. Our findings support our hypothesis only during the ABC challenge, suggesting that older adults may rely on prefrontal activation to complete attentional but not physical challenges during gait.

### PREVENTING ELDER MALTREATMENT: IDENTIFICATION OF HIGH RISK FACTORS FROM LTC ADMINISTRATIVE DATA

Shiau-Fang Chao,[1] Chen-Wei Hsiang,[2] Kuan-Ming Chen,[3] Ya-Mei Chen,[4] Ji-Lung Hsieh,[4] Yu-Hsuan Chou,[4] Ming-Jen Lin,[4] and Shih-Cyuan Wu,[4], *1. National Taiwan University, Taipei City, Taiwan (Republic of China), 2. National Taiwan University, National Taiwan University, Taipei, Taiwan (Republic of China), 3. National Bureau of Economic Research, CAMBRIDGE, Massachusetts, United States, 4. National Taiwan University, Taipei, Taipei, Taiwan (Republic of China)*

Elder maltreatment is a serious problem endangering physical, emotional, and material well-being of older persons, especially those with physical and cognitive impairment. However, detecting the incident of elder maltreatment is difficult and its prevalence has been seriously underestimated. This study explores how LTC use relates to elder maltreatment report, using government LTC service records in Taiwan. A total of 88,633 reported cases in adult protection system in 2019 were merged with 443,952 valid cases in LTC service system. Descriptive statistics were firstly performed to examine the proportion and characteristics of repeated cases in both systems. Linear probability modeling was then used for analyses. 1. In 2019, 3,413 elder maltreatment clients can be identified in LTC service system, accounting for 27.3% of the elder maltreatment cases. 2. Older persons who used LTC service first and being reported as elder maltreatment cases later had a higher prevalence of being discovered by social workers and care attendants. 3. These group of clients also had higher proportion of being reported as neglected by others, abandonment, and self-neglected. 4. Characteristics in LTC service system, such as being older, low severity of disability, high cognitive impairments, low income status, and with a LTC service use record, were related to high probability of being detected with elder maltreatment problems. Characteristics in LTC service system could be effective indicators in discovering potentially abusive situations of disabled older persons. Training and education are essential for LTC service providers to enhance their literacy and ability of assessing elder maltreatment.

### PSYCHOACTIVE SUBSTANCE USE AMONG MIDDLE-AGED AND OLDER ADULTS WITH VISUAL IMPAIRMENT

Francisco Lopez,[1] Jason Leddy,[2] Benjamin Han,[2] and Joseph Palamar,[3], *1. UCSD MADURA R25 Advancing*

*Diversity in Aging Research Program, La Jolla, California, United States, 2. UC San Diego, La Jolla, California, United States, 3. New York University, New York, New York, United States*

Older adults with visual impairment may be at risk for developing substance use disorder (SUD) as psychoactive substance use is often used to cope with the stressors of vision loss. This study estimates the national prevalence and risk of psychoactive substance use among older adults with visual impairment. We analyzed data of respondents age ≥50 from the 2015-2019 National Survey on Drug Use and Health, an annual cross-sectional survey of a nationally representative sample of non-institutionalized individuals in the U.S. (N=43,886). We estimated and compared prevalence of past-year use of cannabis, cocaine, misuse of prescription opioids, sedatives, stimulants/tranquilizers, alcohol use disorder (AUD), any SUD, and nicotine dependence between adults with visual impairment to those without. Comparisons were conducted using chi-square and we used multivariable generalized linear models using Poisson and log link to estimate adjusted prevalence ratios (aPRs) for adults with visual impairment relative to those without, controlling for demographics and diagnosis of ≥2 chronic diseases. An estimated 6.1% experienced visual impairment. Those with visual impairment had higher prevalence of AUD, nicotine dependence, misuse of prescription opioids, tranquilizers, and stimulants, and SUDs. In adjusted analyses, vision-impaired adults had higher risk of AUD (aPR=1.71, 95% CI: 1.40-2.09), nicotine dependence (aPR =1.53, 95% CI:1.35-1.73), opioid misuse (aPR =1.54, 95% CI:1.26-1.90), and SUD (aPR=1.67, 95% CI:1.40-2.01). Psychoactive substance use adds unique health risks for older adults with vision loss, who may suffer significant psychological stress and loss of independence. Screening for substance use among all older adults with visual impairment should be considered.

### PSYCHOLOGICAL EFFECT OF ANTI-ASIAN ATTITUDES BY COVID-19 ON ASIAN AMERICANS IN COASTAL ALABAMA

Hosik Min,[1] Roma Hanks,[1] and Denise Lewis,[2], *1. University of South Alabama, Mobile, Alabama, United States, 2. University of Georgia, Athens, Georgia, United States*

This study aimed to understand how the anti-Asian attitude due to the COVID-19 affected Asian American communities in Alabama. We asked whether Asian Americans were worried about going out due to the anti-Asian attitude due to COVID-19. This study conducted online surveys to Cambodians or Laotians, who were 18 years and older, were living in Coastal Alabama, in May 2020. To avoid in-person contact, respondents answered questions online. A total of 353 respondents participated in the survey. In the Cambodian community, more younger adults participated in the survey, while more middle-aged adults participated from the Laotian community. Laotians had longer educational attainment and watched multiple media to obtain COVID-19 related information. Cambodians (72%) were afraid of COVID-19 infection more than Laotians (53%). More Cambodians (73%) were afraid to go out because of the anti-Asian attitude than Laotians (52%). The logistic regression analysis presented that people worried more about the COVID-19 infection were less likely to go out due to anti-Asian attitudes. Educational attainment did not have a protective effect. Watching multiple media sources decreased the worry about the anti-Asian attitude for Laotians. The age cohort showed both a protective and exacerbate the effect. Cambodians, who were in their thirties, were worried about going out. However, Laotian fifties and over did not worry about going out. This difference might be related to the length of the stay in the U.S. Hanks et al. found that Cambodians, compared to Laotians, had more new immigrants who recently came to the community to marry.

### PSYCHOSOCIAL MENTORSHIP NEEDS: PROMOTING DIVERSITY IN AGING AND ALZHEIMER'S DISEASE RESEARCH AND CLINICAL CAREERS

Sheri Thompson,[1] Lauren Pineda,[2] Savannah Gamboa,[3] and Gisselle Santiago,[3], *1. University of California San Diego, encinitas, California, United States, 2. UCSD, UCSD, California, United States, 3. University of California San Diego, La Jolla, California, United States*

The UC San Diego MADURA Mentorship Program, funded by an R25 National Institute on Aging grant, has goals to improve diversity in Aging and Alzheimer's Disease (ADRD) research and clinical practice. MADURA strives to improve retention and academic success of its URM trainees, and to increase graduate/medical school applications and/or entry into Aging/ADRD careers. Mentees receive paid research experience, skills training, peer support, seminars, and additional professional development opportunities. Given local demographics and Co-Investigator expertise, MADURA recruits primarily Hispanic/Latinx (H/L) students, and other URM students. Non-exclusive racial identifications of 32 Year 1 mentees were: 69% H/L, 9% Black/African American, 22% Asian and 31% other race. 72% met NIH criteria for Disadvantaged Backgrounds. As of June 2021, 100% of students completed surveys assessing Racial Microaggressions and Imposter Syndrome experiences, and Professional Self-efficacy. This poster will define the concepts, discuss relevance for URM student success and retention, and present student data on each topic. As examples, 56% of trainees experienced overt discrimination and 72% reported having experienced microaggressions at least occasionally, as undergraduates. As related to Imposter Syndrome, 62% secretly worried that others would find out that they are not as bright and capable as others think. Half attributed accomplishments to being a "fluke," or due to reasons other than true merit. Still, in terms of Professional Self-efficacy, 62% of mentees thought it moderately or very likely that they would succeed as an Aging/ADRD researcher. The authors will make specific suggestions for optimized URM training and mentorship program content, and provide resources.

### RECRUITING RESIDENT PARTICIPANTS FROM NURSING HOMES DURING THE COVID PANDEMIC: CHALLENGES AND SOLUTIONS

Leena Almasri,[1] Barbara Carlson,[1] Julie Myers,[1] Rebecca Koszalinski,[1] Melissa Franklin,[1] Alysa Kelsh,[1]

and Diana Sturdevant,[2], *1. University of Oklahoma Health Sciences Center, Oklahoma City, Oklahoma, United States, 2. University of Oklahoma HSC, Oklahoma City, Oklahoma, United States*

Recruiting nursing home residents as participants in research is challenging. In early 2021, Covid-19 cases rose rapidly in nursing homes, prompting the rapid deployment of infectious disease protocols and ultimately, facility lockdowns to control the spread of the virus. By September, 2020, many research projects were delayed or cancelled, and future research was jeopardized. During this period, as well as prior to and after the administration of the COVID vaccine, we enrolled residents in a complex protocol involving administration of two Shingles vaccines (0- and 90 days) and three separate blood samples. Here, we present the strategies we used to recruit 216 residents, from 23 homes, over a 9-month period. We faced many challenges. Our research staff faced weekly COVID-19 antigen tests prior to entering the facility, adhering to strict protocols on travel, as well as packaging of materials that entered and left the facility. N95 masks and face shields further made it difficult to communicate with residents. For homes, COVID protocols required residents to be transported to specified areas to meet with research staff. Daily monitoring of COVID and Shingrix vaccine symptoms became part of daily care. To minimize resident harm and interruption of workflow in nursing homes, we utilized principles of stakeholder engagement, healthcare leadership, infectious disease/immunology, and staff (research and nursing homes) empowerment. In the face of crisis, like the COVID-19 pandemic, we have gained the trust and commitment of these facilities; thus, establishing a sustainable partnership that is prepared for what comes next.

### REMOTE ASSESSMENT PREFERENCES OF NIA ALZHEIMER'S DISEASE RESEARCH CENTER PARTICIPANTS DURING COVID-19 PANDEMIC

Michael Nunnerley,[1] Nora Mattek,[2] Zachary Beattie,[3] and Jeffrey Kaye,[3], *1. Oregon Health & Science University, Vancouver, Washington, United States, 2. Oregon Health and Science University, Portland, Oregon, United States, 3. Oregon Health & Science University, Portland, Oregon, United States*

**Background:** In a progressively technology-infused world, older adults are becoming accustomed to the digitization of research participation. During the COVID-19 pandemic, it became necessary to conduct remote study visits typically carried out over the telephone or using video calling programs (e.g. Zoom).

**Methods:** The National Alzheimer's Coordinating Center developed a COVID-19 Technology Accessibility Survey to determine preferred types of study visits for study participants and to understand how this older population accesses the internet.

**Results:** Seven Alzheimer Disease Research Centers collected 1282 survey responses (mean age: ±71.8 years, mean education: 16.5±2.5 years, race/ethnicity: 86% White/non-Hispanic). In respondents aged 65-80, internet access was endorsed by 73% regarding smartphones, 61% for laptops, and 46% for tablets. Interest in wearable devices was expressed by 21% of respondents and interest in smart homes by 6%. Study visit preference among this age group was 42% in favor of in-person, 25% via phone, and 33% via video. In respondents aged >80 internet access was endorsed by 47% regarding smartphones, 47% for laptops, and 34% for tablets. Interest in wearable devices was expressed by 9% of respondents and interest in smart homes by 3%. Study visit preference among this age group was 38% in favor of in-person, 34% via phone, and 29% via video.

**Discussion:** In light of the COVID-19 pandemic, the survey suggests many study participants are interested in remote visits. Significant age group differences are present regarding internet access. Further studies are required to increase the understanding of the efficacy of this research visit format.

### RESILIENCE IN RELATION TO MULTIMORBIDITY IN OLDER ADULTS: A SCOPING REVIEW

Hohyun Seong,[1] Heather Lashley,[1] Katherine Bowers,[1] and Kirsten Corazzini,[2], *1. University of Maryland Baltimore, Baltimore, Maryland, United States, 2. University of Maryland School of Nursing, Baltimore, Maryland, United States*

Multimorbidity is widespread, costly, and associated with a range of deleterious symptoms, affecting 70-80 % of older adults. Resilience in late life has been the focus of considerable research to understand differences in vulnerabilities and recovery from stressors relevant to multimorbidity. Despite this, previous reviews have not focused on resilience in relation to multimorbidity in older adults; therefore, this study synthesized relevant literature. The study design was a scoping review following JBI methodology. Searched electronic databases included PubMed, Embase, CINAHL, and PsycINFO. Data were extracted by two independent reviewers and charted using Garrard's review matrix method. Gough's weight of evidence criteria were used to appraise quality. Of 468 retrieved studies, 14 met inclusion criteria, primarily from the US, UK, and Canada. Most resilience in multimorbidity frameworks operationalize resilience as dependent on the socio-environmental context of older adults. Resilience was commonly considered a dynamic process, but only one study was longitudinal. Measures were primarily psychological or psycho-social in nature and did not include biological or physical measures of resilience. Quality of life and quality of care were common outcomes; resilience significantly related to these outcomes. Findings indicate both the important relationships of resilience with outcomes of multimorbidity, as well as multiple gaps in our current understanding of resilience in relation to multimorbidity. Results highlight the need for studies with diverse populations across diverse cultures, studies that incorporate multidimensional measures, with attention to physiological or physical properties of resilience, and longitudinal studies that capture the dynamic process of resilience in multimorbidity.

### SAFETY AND EFFICACY OF REPEAT DOSES OF ALLOGENEIC MESENCHYMAL STEM CELLS IN PATIENTS WITH AGE RELATED FRAILTY

Russell Saltzman,[1] Ivonne Schulman,[2] Aisha Khan,[2] and Joshua Hare,[2], *1. University of Miami (UM), Miller School*

*of Medicine - Interdisciplinary Stem Cell Institute (ISCI), Miami, Florida, United States, 2. University of Miami (UM), Miami, Florida, United States*

Age-related frailty is a common geriatric condition characterized by a decline in physical and immunological capacity that is associated with depletion of endogenous stem cells and leads to increased vulnerability for adverse health outcomes. Allogeneic mesenchymal stem cells (allo-MSCs) exert immunomodulatory effects and promote tissue repair, which may be able to impede the negative effects of the aging process. The objective of this study was to explore the safety and efficacy of repeated infusions of allo-MSCs in subjects with aging-frailty. Mean age at time of first and second infusions was 75.5 and 77 years of age, respectively. In this open-label clinical trial, 24 participants received two intravenous infusions of allo-MSCs with an average interval of 17.6 months between doses. Safety endpoints included incidence of treatment-emergent serious adverse events (TE-SAEs) within 1-month post-infusion and increase in Panel Reactive Antibodies (PRAs) at 6-months post-infusion. Primary efficacy endpoint was change in 6-minute walk test (6MWT) distance at 6-months post-infusion. No TE-SAEs occurred within 1-month post-infusion. PRAs remained stable throughout the study, indicating no evidence of immune rejection. 6MWT increased by 42 meters after the first infusion (P=0.018). Eighteen months later elevation persisted (P=0.026), but did not increase further after the second infusion. In summary, repeated intravenous infusions of allo-MSCs were safe in participants with age-related frailty and showed remarkable improvement in physical performance. Given the excellent safety and efficacy profiles demonstrated in this study, larger clinical trials are warranted to further quantify the efficacy of repeated dosing of allo-MSCs in this multisystem disorder.

### SOCIAL ISOLATION, CAREGIVING ALONE, AND CAREGIVING STRESS IN FAMILY CAREGIVERS OF OLDER ADULTS IN KOREA

Sol Baik,[1] and Jiweon Jun,[2], *1. University of Maryland, Baltimore, Baltimore, Maryland, United States, 2. Seoul National University, Center for Transnational Migration and Social Inclusion, Seoul National University, Seoul-t'ukpyolsi, Republic of Korea*

The tendency of caregiving alone is increasing, and these solo caregivers often perceive caregiving responsibilities as a burden. Still, literature on positive aspects of caregiving shows that not all caregivers experience severe distress. Little is known on which factors make a difference in experiencing caregiving distress among solo caregivers. We focused on the empirical findings on the negative impact of social isolation on caregiver's mental health, examining if and how the intersection of solo caregiving and social isolation is related to severe caregiving stress among caregivers of older adults in Korea. We analyzed 501 family caregivers of older adults in Korea using survey data from the Care Work and the Economy research project (2018). We conducted ordinal logistic regression analysis. The findings show that solo caregivers with a lack of social time fall under the most at-risk group of caregivers in terms of experiencing severe stress (OR=3.72, SE=0.93) whereas solo caregivers with enough social time did not show significantly higher stress compared to the reference group (OR=1.50, SE=0.43). Being socially isolated caregivers still had high levels of stress despite the division of care (OR=2.16, SE=0.55), implying the need to provide caregivers more time for social interaction with others. The current public long-term care insurance in Korea provides limited hours of in-home care aide services to enable aging in place of older adults. To reduce the social isolation of caregivers, it is necessary to extend the service hours and provide support, such as creating online caregiver networks.

### SOCIOECONOMIC STATUS ACROSS THE LIFE COURSE AND SMOKING TRAJECTORIES OF OLDER ADULT SMOKERS IN THE U.S.

Jaqueline Avila,[1] Sangah Lee,[2] Ezinwa Osuoha,[3] Dale Dagar Maglalang,[2] Alexander Sokolovsky,[2] and Jasjit Ahluwalia,[2], *1. Brown University, Providence, Rhode Island, United States, 2. Brown University, Brown University, Rhode Island, United States, 3. Cornell University, Cornell University, New York, United States*

The objective of this study is to assess how SES over the life course impacts smoking cessation among older adult smokers in the U.S. 6,058 current smokers 50 years and older were identified from the 1998-2018 Waves of the Health and Retirement Study (HRS). The outcome of interest was smoking cessation. The main independent predictor was lifetime SES, categorized as low child and low adult SES (persistent low); low child, high adult SES; high child, low adult SES; and high child, high adult SES (persistent high). Multilevel mixed-effect logistic model was used to examine how lifetime SES predicts smoking cessation at age 65 and over time, adjusted by health and smoking covariates. The majority of older smokers had persistent high lifetime SES (60.3%), followed by high child/low adult SES (18.7%), persistent low SES (15.5%) and low child/high adult SES (5.6%). Compared to those with persistent high SES, those with persistent low SES were more likely to be Hispanic (25.9% vs. 3.0%, p<0.001) or non-Hispanic Black (22.7% vs. 8.7%, p<0.001), respectively. The adjusted results showed that at age 65, compared to those with persistent high SES, those with persistent low SES, low child/high adult SES, and low adult/high child SES were less likely to quit (OR: 0.42, 95%CI:0[.31-0.56]; OR:0.37, [0.24-0.55]; OR:0.53, [0.40-0.70], respectively). Similar results were observed over time for those with persistent low SES and low adult/high child SES. However, there was no significant difference for those with low child/high adult SES.

### STAYING CONNECTED DURING THE COVID-19 PANDEMIC: EXPERIENCES OF OLDER PEOPLE IN MEXICO AND SCOTLAND

Louise McCabe,[1] Verónica Montes de Oca,[2] Nereide Curreri,[3] Marissa Vivaldo,[4] Alison Dawson,[1] Miguel Rivera,[2] Maria Arroyo,[5] and Elaine Douglas,[1], *1. University of Stirling, Stirling, Scotland, United Kingdom, 2. National Autonomous University of Mexico (UNAM), National Autonomous University of Mexico (UNAM), Distrito Federal, Mexico, 3. North-West University, South Africa, Vanderbijlpark, North-West, South Africa, 4. National Autonomous University of Mexico, Mexico City, Distrito Federal, Mexico, 5. Universidad Juarez del Estado*

*de Durango, Universidad Juarez del Estado de Durango, Durango, Mexico*

During the pandemic older people saw transformations in their social connections due to lockdowns and other restrictions. Technology provided one mechanism for them to stay connected with others, but technology may not be accessible or desirable for everyone. Gender, socioeconomic status, ethnicity, age and other factors enhance or limit engagement with technology. This project explored experiences of older people in Mexico and Scotland during the pandemic and examined the potential of everyday technology to help maintain social connectedness. A mixed methods approach included secondary analysis of large-scale datasets alongside primary data. Online semi-structured interviews and focus groups were carried out with 36 older people in Mexico and 23 older people in Scotland. Sampling was purposeful creating a diverse sample across age, gender, ethnicity and socioeconomic status. The findings demonstrate that advantages and disadvantages accumulated in the life course determine how older people select, optimize and compensate for new ways of staying socially connected during the pandemic in both countries. The use of technologies among older people is further mediated by structural inequalities with differences found between Mexico and Scotland in specific patterns identified. Further, stereotypes about older age and technology use are obstacles to the use of technology, as they affect the perception of self-efficacy by older people. Despite the obstacles, this study has shown that older people have a broad range of resources that have enabled them to cope with the pandemic and utilise technology to maintain social connections. The project offers recommendations to support older people's human rights.

### THE EXPERIENCES OF OLDER ADULTS WITH CANNABIS AND MENTAL HEALTH: A SCOPING REVIEW OF THE LITERATURE

Raza Mirza,[1] Amanda Bull,[1] Andrea Gardiola,[1] Sabina Mirza,[2] Mary Hynes,[1] and Jessica Hsieh,[1], *1. University of Toronto, Toronto, Ontario, Canada, 2. York University, Woodbridge, Ontario, Canada*

Following the 2018 federal legalization of cannabis in Canada, there was a drastic increase in older adults reporting marijuana use. Most cannabis research today focuses on children and young adults, however, it is important to acknowledge the potential harms in seniors as well. Aging and substance use presents unique considerations, such as the interactions between cannabis and chronic conditions, multiple comorbidities, polypharmacy, and mental health. The goal of this scoping review was to analyze the literature that addresses mental health outcomes of seniors who use cannabis, in order to answer the main research question: What is the relationship between older adults' use of cannabis and mental health? Following Arksey and O'Malley's five-stage framework, 10 electronic databases were searched along with a hand search of references. The search revealed 7000+ peer-reviewed and grey literature sources. 233 full-text sources were assessed for eligibility, with a total of 25 articles included. Thematic content analysis produced four major themes which addressed: (1) Usage characteristics; (2) User characteristics; (3) Outcomes; and (4) Physical and mental health considerations. Findings from this scoping review are positioned in terms of their implications for research, practice, and policy. While more in-depth, qualitative methods are required to develop further research, several harm-reduction strategies may be immediately utilized by both users and healthcare practitioners. It is critical that older adults and their physicians are able to make cannabis-related decisions with evidence-informed guidance to prevent problematic cannabis use and ensure positive mental health outcomes.

### THE INFLUENCE OF COGNITIVE FUNCTION ON ADHERENCE TO BREAST CANCER SCREENING AMONG OLDER AMERICAN WOMEN

Chien-Ching Li,[1] Fritzi Flores,[1] Alicia K. Matthews,[2] Bryan James,[3] and Raj Shah,[4], *1. Rush University, Rush University, Illinois, United States, 2. University of Illinois at UIC, Chicago, Illinois, United States, 3. Rush University, Chicago, Illinois, United States, 4. Rush, Rush University Medical Center, Illinois, United States*

Cognitive decline and impairment among older adults have become an important public health issue. Previous research shows older women have a greater prevalence of Alzheimer's disease than Men. Among women, breast cancer is one of the most common types of cancer. Over half of breast cancer deaths occur in women aged 65 and older. Therefore, early detection of breast cancer through mammogram screening is important among older women. This study aimed to examine the influence of cognitive function on adherence to mammogram breast cancer screening among older American women aged 65 and older. Data from the Health and Retirement Study (2012-2016) was obtained and analyzed. The independent variable of the study was cognitive function (normal, not normal). Adherence to mammogram (low, moderate, high) was the dependent variable. Multinomial regression was performed to examine the association between cognitive function and adherence to mammogram after controlling for demographic covariates. In the study, 33.3% of respondents had impaired cognitive function and 21.7% showed low adherence to mammogram screening. Regression results found that older women with impaired cognitive function were more likely to be in low adherence group (OR=1.30, $p=0.01$) or moderate adherence group (OR=1.47, $p<0.001$) relatively to be in high adherence group compared to older women with normal cognitive function. The development and implementation of interventions are needed for reducing barriers to accessing cancer screening services in high-risk vulnerable populations. This submission is considered late-breaking research because study findings were obtained from a recently completed student's master's project.

### THE LINK BETWEEN ENVIRONMENT, AGE, AND HEALTH IN A LARGE COHORT OF COMPANION DOGS FROM THE DOG AGING PROJECT

Brianah McCoy,[1] Layla Brassington,[1] Greer Dolby,[1] Kelly Jin,[2] Devin Collins,[3] Matthew Dunbar,[4] and Noah Snyder-Mackler,[1], *1. Arizona State University, Tempe, Arizona, United States, 2. Allen Institute for Brain Science, Allen Institute for Brain Science, Washington, United States, 3. University of Washington, University of Washington, Washington, United States, 4. University of Washington, UNiversity of Washington, Washington, United States*

Exposure to social environmental adversity strongly predicts health and survival in many species such as non-human primates, wild mammals, and humans. However, little is known about how the health and mortality effects of these social determinants vary across the lifespan. Using the companion dog, which serves as a powerful comparative model for human health and aging due to our shared biology and environment, we examined which components of the social environment impact health, and how the effects vary with age, in dogs. We first drew on detailed survey data from owners of 27,547 dogs from the Dog Aging Project and identified six factors that together explained 35% of the variation in dog's social environment. These factors all predicted measures of health, disease, and mobility, when controlling for dog age and weight. Factors capturing measures of financial and household adversity were linked to poorer companion dog health, while factors associated with the social companions, like dogs and adults, were linked to better health. Interestingly, some of these effects differed across a dog's lifespan: for instance, the effect of neighborhood disadvantage on disease instances was strongest in older dogs. Together, our findings point to similar links between adversity and health in companion dogs, and set up future work on the molecular and biological changes associated with environmental variation in order to identify ways to mitigate or even reverse the negative environmental effects.

### THE MODIFIED MINI MENTAL STATE (3MS) TEST - DEVELOPMENT OF AN ONLINE TRAINING PROGRAM

Tara Rose,[1] Evelyn Teng,[1] Chia Ying Chen,[1] Elyse Manzo,[2] Katherine Erickson,[3] and Helena Chui,[1], *1. University of Southern California, Los Angeles, California, United States, 2. University of Southern California, Huntington Beach, California, United States, 3. University of Southern California, Moraga, California, United States*

The Modified Mini Mental State (3MS), a screening test for cognitive abilities, can be administered in approximately 10 minutes and has a score range of 0-100. Early detection of cognitive impairment is important for clinical care. An individual's score change over time can also help assess disease progression and treatment effects. The Web of Science, an online database, reports 1,864 publications using the 3MS, including 412 in the last 5 years. Articles came from 52 countries in 7 continents, with languages in English (98.7%), French (0.5%), Spanish (0.5%), German (0.2%), Czech (0.1%), and Korean (0.1%). The fields that publish with 3MS data included Geriatrics/Gerontology, Neurosciences/Neurology, Psychiatry, Psychology, and General/Internal Medicine. One reason for the popularity of the 3MS is its detailed manual for test administering and scoring; however, to date the manual is available only in print form. Here we present an online training program. It includes detailed directions on administering and scoring, video of a 3MS administration, and post-training tests on a trainee's competency. This training material also covers clinical benefits of cognitive screening and a comparison of the 3MS with other measures. The 3MS helps clinicians with early detection of cognitive impairment, allowing time to address its underlying and potentially reversible causes, and aids in disease management. This online training program will help ensure accuracy and reduce between-tester variability of the obtained scores in both clinical care and research studies.

### THE RELATIONSHIP BETWEEN DEMENTIA SEVERITY & CAREGIVER PREFERENCES FOR DECISION MAKING ROLE REGARDING MAMMOGRAPHY

Molly Frank,[1] Seho Park,[2] Kathleen Lane,[3] Alexia Torke,[1] Mara Schonberg,[4] Greg Sachs,[1] Peter Schwartz,[5] and Nicole Fowler,[1], *1. Indiana University School of Medicine, IU Center for Aging Research, Indianapolis, Indiana, United States, 2. Department of Biostatistics, School of Medicine, Department of Biostatistics, School of Medicine, Indiana, United States, 3. Indiana University School of Medicine, Indianapolis, Indiana, United States, 4. Beth Israel Deaconess Medical Center, Boston, Massachusetts, United States, 5. Center for Bioethics, Indianapolis, Indiana, United States*

The incidence of Alzheimer's disease and related dementias (ADRD) and breast cancer increases with age. Despite being one of the most effective ways to diagnose breast cancer early, mammography in ADRD patients comes with an increased risk of treatment complications and false-positive results. Family caregivers are often involved in the decision-making process, and this study evaluates the relationship between dementia severity and caregiver preferences when making decisions about mammography with the patient alone, and with the patient and doctor. We included 181 caregivers from the Decisions about Cancer screening in Alzheimer's Disease trial, which uses the Dementia Severity Rating Scale (DSRS) to assess dementia severity and a modified Control Preferences Scale (CPS) to assess each caregiver's preferred decision-making approach. Multinomial logistic regression models evaluated the relationship between DSRS and CPS categories (active, passive, and collaborative), while controlling for the caregivers' age, sex, race, education, and relationship to patient. Model 1 examined the caregivers' preferred role with the patient, and it found a significant association between increased dementia severity and preference for a collaborative approach ($p<0.001$) or passive approach ($p<0.05$) compared to an active approach. Model 2 did not find a significant association between dementia severity and the caregivers' preferred role when making decisions with the patient and doctor; however, those with increased age and education were more likely to prefer an active role. The association between dementia severity, caregiver characteristics, and decision-making preferences supports the need for approaches to support ADRD caregivers with medical decision making.

### THE RELATIONSHIP BETWEEN PROTEIN INTAKE AND GUT MICROBIOTA IN COMMUNITY-DWELLING OLDER MEN FROM THE MROS STUDY.

Samaneh Farsijani,[1] Jane Cauley,[1] Shyamal Peddada,[2] Lisa Langsetmo,[3] James Shikany,[4] Kristine Ensrud,[5] Eric Orwoll,[6] and Anne Newman,[1], *1. University of Pittsburgh, Pittsburgh, Pennsylvania, United States, 2. NIH, BETHESDA, Maryland, United States, 3.*

*University of Minnesota, University of Minnesota, Minnesota, United States, 4. University of Alabama at Birmingham, Birmingham, Alabama, United States, 5. University of Minnesota, Minneapolis, Minnesota, United States, 6. Oregon Health & Science University, Portland, Oregon, United States*

Despite growing evidence supporting the role of protein consumption in promoting muscle health, the possible mediation by gut microbiota remains unclear. Here, we determined the association between the quantity of dietary protein and gut microbiome composition in community-dwelling older adults. We performed a cross-sectional analysis on 775 older men from the Osteoporotic Fractures in Men (MrOS) study with available dietary information and stool samples at visit 4 (2014-16). Protein intake extracted from a brief Food Frequency Questionnaire and adjusted to total energy intake using the residual method. Gut microbial taxa were determined by 16S (v4) sequencing (Greengenes references). 11,534 Operational Taxonomic Units were identified and assigned to 21 phyla with dominance of Firmicutes (45%) and Bacteroidetes (43%). We performed distribution-based analysis ($\alpha$-diversity), distance-based Permutation Multivariate Analysis of Variance ($\beta$-diversity), and taxa abundance (by ANCOM-BC R-package) to determine associations between protein intake and gut microbiome. Mean energy-adjusted protein intake was 62.0±10.8 g/d [0.8±0.3 g/kgBW/d]. Participants with higher protein intake had higher Shannon and Chao1 $\alpha$-diversity indices ($P<0.05$). For $\beta$-diversity analysis, participants with higher protein intake had a different center in weighted and unweighted UniFrac PCoA vs. those with lower intake ($P<0.05$) adjusted for age, race, clinical center, energy intake, weight, height, and medications. Tenericutes phylum and several genus-level OTUs, including Klebsiella, Tyzzerella, Christensenellaceae, Ruminococcaceae, Blautia, and Veillonella were differentially abundant between quartiles of protein intake (FDR corrected $P<0.05$). Our data support an association between dietary protein and gut microbiota diversity, a relationship that could potentially influence physical function and sarcopenia development.

### THE ROLE OF OCCUPATIONAL SWITCHING ON RETIREMENT OF AMERICAN MIDLIFE WORKERS

Yun taek Oh, *University of Minnesota, Minneapolis, Minnesota, United States*

An increasing number of midlife Americans are financially unprepared for retirement. This is a problem because of the increasing life expectancy that prolongs the need for financial resources. One way to resolve this problem is to postpone full retirement by having bridge jobs that provide more time to work and accumulate retirement savings. While having a bridge job means numerous labor market behavior such as working for different employers and reducing work hours and intensity, there is a limited number of studies focused specifically on how switching occupations can contribute to retirement decisions with a longer time frame. This study investigated the association between occupational switching and retirement patterns of American midlife workers aged between 50 to 59 years using the Health and Retirement Study longitudinal data from 2004 to 2016, Occupational Information Network data, and American Community Survey from 2003 to 2016. The changes in occupational demandingness before and after switching occupations were reflected by using mover design event study regression with fixed effects. In general, occupational switching is associated with later retirement until two to three years after switching occupations for both genders, yet this association varies by the directions of the change in occupational demandingness.

### TOUCHSCREEN TECHNOLOGY TO IMPROVE DEMENTIA CARE MANAGEMENT FOR OLDER ADULTS LIVING IN THE COMMUNITY

Ellen Brown,[1] Nicole Ruggiano,[2] mariateresa H. Munoz,[3] lisa Roberts,[3] C. Victoria Framil,[3] Bernardo Arroyo,[3] and Michelle Bourgeois,[4], *1. Florida International University, Miami, Florida, United States, 2. University of Alabama, Hoover, Alabama, United States, 3. Florida International University, Florida International University, Florida, United States, 4. Unviersity of South Florida, Tampa, Florida, United States*

Chronic disease management challenges for persons living with dementia (PLWD) often includes difficulties communicating how their feeling or describing symptoms to caregivers. To address this issue, an interdisciplinary team is developing a touchscreen mobile technology that will allow the assessment of clinical information about the PLWD, such as sleep quality, appetite, pain level, and mood. The mobile technology also allows caregivers to share this information with providers in real-time from their homes. In the team's effort to incorporate a graphic measure to assess mood into the technology, a literature review revealed no such measurement tool has been established and validated for assessment with PLWD. This study focused on developing a graphic clinical assessment tool that can be used to assess mood for PLWD and establishing the face and content validity for this assessment tool. A team that included multidisciplinary clinical experts and a graphic artist designed a series of pictures of facial expressions and body language depicting a continuum of mood states (i.e., happy through depressed). The research team consulted with existing artistic depictions of sadness expression, including medical illustrations depicting depression. Multiple iterations have resulted in a series of images that represent moods from happy to very sad. The team considered a number of factors in developing the images including gender, ethnicity, and race. Consensus on the construct validity was achieved by the expert clinical panel. Details of a follow-up study to evaluate construct validity will be presented.

### TRANSLATING AND ADAPTING THE MODIFIED MINI MENTAL STATE (3MS) TEST FOR MULTIPLE CULTURAL POPULATIONS

Tara Rose,[1] Evelyn Teng,[1] Helena Chui,[1] Katherine Erickson,[2] Chia Ying Chen,[1] and Elyse Manzo,[3] *1. University of Southern California, Los Angeles, California, United States, 2. University of Southern California, Moraga, California, United States, 3. University of Southern California, Huntington Beach, California, United States*

Within healthcare settings, screening of cognitive abilities in older adults is routinely conducted for the detection, early intervention, and management of cognitive impairments. The Modified Mini Mental State (3MS) test takes approximately 10 minutes to administer and has a score range of 0 - 100. It can provide an estimated MMSE score, and has been used in multiple countries since 1987 with approximately 1,900 publications. The United States has many diverse populations with different languages and cultural backgrounds. How to appropriately translate and adapt the original 3MS test in English for each minority group in order to better serve them is an important issue. Cross-cultural assessment involves much more than accurate translation of test items across languages. One needs to know not only the oral and written languages involved, but also the life experiences and circumstances of the target populations. This presentation first covers some general considerations in test translation and adaptation, including attention to cultural, ecological, and language specifics. We shall then present Chinese, Korean, and Hindi 3MS record forms to illustrate the reasons and ways for modification of some of the test items. To accommodate different writing systems, for example, 3MS test versions with an alphabet are different from ones with logographic character representations. Modifications of the 3MS items include those on temporal orientation, spatial orientation, naming, and repetition. In summary, cultural, ecological, and linguistic differences must be taken into account for cognitive screening in order to enhance cross-population comparability and be more inclusive for aging ethnic minorities.

### TRANSNATIONAL CAREGIVING FOR OLDER ADULTS AND CAREGIVERS' WELLNESS IN JAPANESE AMERICANS DURING THE PANDEMIC

Kazumi Hoshino,[1] Winston Tseng,[1] and Kei Kamide,[2], *1. The University of California at Berkeley, Berkeley, California, United States, 2. Osaka University, Suita, Osaka, Japan*

Global migration has greatly affected intergenerational family support beyond national borders, in particular adult children's transnational family caregiving for elderly parents. Specifically, the COVID-19 pandemic has largely influenced transnational caregiving due to the travel restrictions. Transnational caregiving for older adults includes adult children's periodical returning to their home country and/or adult children's caregiving for their parents in their settled country. The goal of this study was to identify trajectories between adult children's transnational caregiving for their parents and caregivers' wellness in Japanese Americans before and during the pandemic. We conducted semi-structured interviews with Japanese Americans 40 to 59 years of age (N=20) in California before the lockdown and during the increasing number of patients infected with the Delta variant. The qualitative data analysis showed some Japanese Americans periodically returned to Japan to provide caregiving for their parents before the pandemic, while others didn't. However, the former group currently relied on their families in their home country more than before. The limitations led to not only distress over uncertainty but also release from a strong sense of reciprocity and filial responsibility, by changing from physical support to emotional and financial support via online. They also enhanced cultural identity as Japanese Americans, by thriving from discrimination against Asian Americans. Thus, our findings demonstrate important factors that impacted on transnational caregiving and caregivers' wellness, including cultural identity, family norms, beliefs and practices of intergenerational support, social and historical contexts, financial remittance, ICT use, and healthcare policies among the underrepresented populations across the Pacific.

### TREHALOSE ENHANCES LONG-TERM RECOVERY IN 18-MONTH-OLD MICE BY INCREASING AUTOPHAGY AFTER TRAUMATIC BRAIN INJURY

Rodney Ritzel, Yun Li, Jordan Carter, Niaz Khan, Junyun He, Samantha Allen, Alan Faden, and Junfang Wu, *University of Maryland School of Medicine, Baltimore, Maryland, United States*

Older patients with traumatic brain injury (TBI) have higher mortality and poorer long-term outlook compared to younger individuals. This may contribute to the assumption that aggressive management of geriatric TBI is futile. The present study examined the long-term recovery potential and underlying mechanisms associated with advanced age in male C57BL/6 mice using a controlled cortical impact model of TBI. Older (18 mos) mice had higher mortality compared to younger (10 wks) mice at 12 weeks post-injury. While aging alone had a profound impact on behavioral ability, the recovery slope in some, but not all, neurobehavioral tests was relatively similar between young and old injured mice. NanoString analysis identified several age- and injury-specific genes that were differentially expressed, including those involved with the complement, phagocytosis, and autophagy pathways. Flow cytometry demonstrated dysregulation of autophagic function in microglia with normal aging which was exacerbated after TBI. Given the critical role for autophagy in promoting the cellular degradation of cytoplasmic materials, we reasoned that treatment with the autophagic inducer, trehalose, may be a viable therapeutic strategy. Trehalose was administered in the drinking water (3%) starting at d1 post-injury up to 8 weeks. Older TBI mice treated with trehalose exhibited either delayed deficits or enhanced recovery in cognitive and motor tasks. Trehalose modified expression of autophagy markers and reprogrammed the microglial response to TBI. Our data indicate that microglia undergo chronic changes in autophagic regulation that are associated with poor outcome. Boosting autophagy may be a promising therapeutic strategy for older TBI patients.

### UNDERSTANDING OLDER ADULTS MOTIVATIONS AND PREFERENCES FOR A MEDICATION ADHERENCE APP

Olivia Kupiec,[1] Maurita Harris,[2] and Wendy Rogers,[3], *1. University of Illinois Urbana Champaign, Champaign, Illinois, United States, 2. University of Illinois at Urbana-Champaign, Champaign, Illinois, United States, 3. University of Illinois Urbana-Champaign, Champaign, Illinois, United States*

As the percentage of older adults with hypertension continues to increase, medication adherence remains low. However, medication adherence can potentially be improved through the use of medication reminder apps. Medication reminder apps contain numerous features that enable older adults to remember to take their medication, such as providing alerts to take their medication, reminding them when to refill their prescription, and more. Despite being aware of these apps, many older adults lack the motivation needed to use them continuously. We recruited 9 participants (60 years or older) who currently take medication for a chronic condition. Using a mixed-methods approach, we gathered quantitative survey data using the TechSAge Demographic Background, Motivation, and Behavior Change Technique Questionnaires). Qualitative data were gathered through a semi-structured interview that asked questions about general motivations and preferences in addition to engaging participants in co-designing a medication adherence app. Results from the interview were analyzed through a thematic analysis that identified comprehension and preferences of older adults in medication reminder app usage. We tested five different intrinsic motivation factors, and results indicate older adults are most motivated intrinsically due to perceived choice, perceived competence, value/usefulness, effort/importance, and pressure/tension. We also tested five factors of extrinsic motivation, and results indicate older adults are most motivated extrinsically due to introjected regulation, reward-driven, external regulation, compliance, and identification. These data provide insights to guide the design of medication reminder apps to support older adults in the self-management of their chronic conditions.

### UNDERSTANDING THE PREDICTIVE FACTORS THAT INFLUENCE THE PERCEIVED BURDEN OF DEMENTIA CAREGIVERS

Kaitlyn Lucas,[1] and Katherine Judge,[2], *1. Cleveland State University, Westlake, Ohio, United States, 2. Cleveland State University, Cleveland, Ohio, United States*

Caregivers of individuals with dementia have increased levels of burden because of their caregiving duties which in turn results in negative effects on their physical, emotional, and mental wellbeing. Few studies have examined individual difference-based constructs such as positive and negative affect in relation to caregiver burden. Positive affect is characterized by pleasant emotions and expressions and higher levels of overall life satisfaction whereas negative affect is characterized by more pessimistic emotions and expressions and lower overall life satisfaction. Analyses were performed on dementia caregivers (n=102) using the online data collection tool MTurk Prime. The average caregiver provided 21 or more hours of care with 76.9% of caregivers living with their loved one with dementia. Significant correlations were found between caregiving burden and positive affect (r= -.31, p=.002) and negative affect (r= .56, p=<.001), with higher positive affect related to lower burden and higher negative affect related to greater burden. Results of multiple regression models showed that positive affect ($\beta$ = -.24, p = .03) and negative affect ($\beta$ = .35, p = .01) were significant predictors of burden even while controlling for other constructs including types of coping, compassion towards other, and assessment of family dynamics. Understanding the relationship between these constructs will allow for more individualized interventions to be created to help reduce the level of burden that dementia caregivers experience.

### UNDERSTANDING THE ROLE OF COMMUNICATION TECHNOLOGY IN FACILITATING SOCIAL CONNECTIVITY AND ADDRESSING LONELINESS

George Mois,[1] Kerstin Emerson,[2] Tiffany Washington,[2] and Jenay Beer,[2], *1. University of Illinois, Champaign, Illinois, United States, 2. University of Georgia, Athens, Georgia, United States*

In the United States, two out of five adults report feelings of loneliness. The evolvement of communication technologies presents a promising potential in helping improve social connectivity and address the experience of loneliness. However, the sense of presence (embodiment) users are able to achieve through the technologies can vary depending on their abilities and functions. The present study identified user characteristics associated with an interest to adopt telepresence technologies (e.g., videoconferencing, smart displays, robots) across various levels of embodiment. The data for this study were collected using a Qualtrics survey which was distributed via Amazon Mechanical Turk. The participants recruited for this study were between the ages of 18-78 years old, constituting a total sample size of 384 participants. The data were analyzed using four logistics regression models. The dependent variables aimed to identify participants' interest to adopt telepresence technologies across varying embodiment levels. Across the lifespan older adults were significantly more likely to report lower rates of overall loneliness than young and middle-aged adults. Our findings indicate that those interested in adopting communication technologies with higher levels of embodiment had significantly higher odds of reporting being divorced or widowed (OR=4.12, p<.05), reside in a rural community (OR=2.20, p<.05), and report higher rates of emotional loneliness (OR=1.20, p<.05). Across the four models, there was no significant difference in participants' interest to adopt telepresence technologies. These results suggest that the sense of presence achieved across the various types of communication technologies may help address feelings of loneliness and support healthy aging.

### USE OF LONG-TERM CARE DECREASED OVER TIME AMONG THE OLDEST-OLD WITH AND WITHOUT DEMENTIA - A REGISTER-BASED STUDY

Mari Aaltonen,[1] Leena Forma,[2] Jutta Pulkki,[1] Jani Raitanen,[1] and Marja Jylhä,[1], *1. Tampere University, Tampere University / Tampere, Pirkanmaa, Finland, 2. University of Helsinki, University of Helsinki / Helsinki, Uusimaa, Finland*

Care policies for older adults emphasize aging-in-place and home care over residential long-term care (LTC). We explore how the use of residential LTC in the last five years of life among people with and without dementia changed between those who died in 2001, 2007, 2013, and 2017 in Finland. Retrospective data drawn from the national health and social care registers include all those who died aged 70+ in 2007, 2013, and 2017, plus a 40% random sample from 2001 (N=128 050). Negative binomial regression analysis was used to estimate the association of dementia with LTC use during the last five years of life (1825 days). The independent variables included dementia, age, marital status, annual income, education, and chronic conditions. In the total study population, the proportion of LTC users and the mean number of days in LTC increased until 2013, after which it decreased. Changes in LTC use differed between different age groups and by dementia status. Over time, the decrease in round-the-clock LTC use was steep in those aged 90≤ with dementia and in people aged 80≤ without dementia. The individual factors related to morbidity and sociodemographic factors did not explain these results. The changes in LTC care policy may have contributed to the decrease in LTC use among the oldest. However, according to national statistics, the availability of formal home care has not increased. This development may suggest that the oldest-old and those with dementia – a highly vulnerable group – are left without proper care.

### USING DIAGNOSTIC ULTRASOUND TO SUPPORT THE DIAGNOSE SARCOPENIA IN OLDER ADULTS: A SYSTEMATIC REVIEW

Edgar Vieira, Lily Charles, Monica Cortes, and Tabitha Lees, *Florida International University, Miami, Florida, United States*

Dual-energy x-ray absorptiometry (DXA) is currently the gold standard for diagnosing loss of muscle mass in older adults (a component of sarcopenia diagnosis). Magnetic resonance imaging (MRI) and computed tomography (CT) have also been used successfully. Due to elevated costs, limited access, exposure to radiation, and increased difficulty of operation, other methods have been explored as alternatives. We reviewed the literature on the use of diagnostic ultrasound to assist in the diagnose sarcopenia in older adults by searching MEDLINE, Embase, and CINAHL using a variation of terms related to "ultrasound", "sarcopenia", and "older adults". We included studies that included older adults over the age of 60. Eighteen studies were included after screening for eligibility and conducting full-text reviews. The most common transducer head frequency utilized in the studies were 5-12 and 8 MHz (three studies each), followed by 5, 6, and 7.5 MHz (two studies each). The most common musculature examined was anterior thigh musculature, followed by muscles of the lower leg, upper extremity, abdominals, and head/neck. Measurements most taken were muscle thickness/cross sectional area (18 studies), followed by muscle echogenicity (9 studies), and pennation angle (3 studies). Ultrasound is a reliable and valid tool to examine muscle thickness to assist in diagnosing sarcopenia. However, echogenicity measures of a muscle were not reliable. Further research is needed with increased sample size and variance amongst subjects to generalize and create normative data. In addition, standardized protocols for the use of ultrasound to assist in the diagnosing sarcopenia need to be established.

### VISUALIZING PERSON-CENTERED LONG-TERM CARE: AN EXPLORATORY SCOPING REVIEW AND EVIDENCE MAPPING

Mary Lou Ciolfi,[1] Rocky Coastlines,[2] Catherine Taylor,[3] Kayla Thompson,[4] Jennifer Crittenden,[5] Lenard Kaye,[4] Angela Hunt,[6] and Paul Nebenzahl,[7], *1. University of Maine, Orono, Maine, United States, 2. University of Maine Center on Aging, Bangor, ME 04401-4324, Maine, United States, 3. University of Maine Center on Aging, Bangor, Maine, United States, 4. University of Maine, Bangor, Maine, United States, 5. University of Maine, University of Maine, Maine, United States, 6. The Cedars, Portland, Maine, United States, 7. The Mayer-Rothschild Foundation, Chicago, Illinois, United States*

Despite decades of professional, academic, and policy interest in person-centered long-term care (LTC), the field continues to be challenged by the absence of a comprehensive depiction of the concept and a lack of consistency reflected across studies and measures. In response, a participatory action, research-focused, partnership between an institution of higher education and an LTC community (the University of Maine Center on Aging and The Cedars), with funding from The Mayer-Rothschild Foundation, is identifying and mapping the landscape of person-centered LTC in nursing homes and assisted living communities. A collaborative, ongoing, exploratory scoping literature review and evidence mapping has compiled a database of 663 academic and 115 grey literature articles through 65 systematic searches reviewing over 4,296 articles. An iterative process from both the resident and organizational perspectives revealed nine core domains (e.g. dining, resident care, environment, quality improvement, identity and personhood, etc.) and two substantive research gaps (the intersection of person-centered long term care with diversity, equity, and inclusion issues, and pandemic considerations). For mapping purposes, domain content was analyzed categorically based on concept, information revealed about resident, family, and staff experience, and operations applicability. The identified person-centered LTC domains, categorical analysis, identified gaps, and visual representation via mapping will contribute to generating research ideas, supporting the development of effective operationalization for LTC settings, and contributing to an understanding of the theoretical scope and concrete elements of a person-centered care model that aims to improve the wellbeing and quality of life of older adults in long-term residential settings.

### WHAT HAPPENS WHEN THE PATIENT DOESN'T DIE? UNDERSTANDING LIVE DISCHARGE FROM HOSPICE CARE

Stephanie Wladkowski,[1] Kathryn Coccia,[2] Anna Wingo,[3] Ruaa Al-Juboori,[4] and Cara Wallace,[2], *1. Eastern Michigan University School of Social Work, Eastern Michigan University, Michigan, United States, 2. Saint Louis University, St. Louis, Missouri, United States, 3. Saint Louis University, Saint Louis University, Missouri, United States, 4. Saint Louis University, Saint Louis, Missouri, United States*

Hospice has been shown to improve end-of-life outcomes, yet with eligibility limited to a six-month prognosis, the hospice system is not structured to meet longer-term needs. Though hospice is strongly associated with death, some enrolled patients do not decline as predicted leading to what is referred to as a 'live discharge.' In 2018, 6.3% of all hospice discharges were patients discharged alive due to decertification, or no longer meeting eligibility requirements. The aim of this presentation is to review current literature surrounding live discharge, discuss policy and practice challenges within current discharge practices, and present new research directions from two current NIH-funded studies. Studies of live discharge often do not differentiate between revocation and decertification, yet these are very different phenomena, particularly regarding decision making. Patients discharged from hospice are often referred to as "not dying fast enough," or "failure to die on time," yet, they are still dying from chronic illness, just outside the prescribed six-month framework. Affected patients lose access to important supportive services and resources, still require substantial care, and can struggle to process feelings of abandonment and uncertainty. Further, an increased burden is placed on primary caregivers who may be unprepared for this transition. Clinicians across agencies report great variability in managing live discharges with no standardized protocols. These findings demonstrate the complexities of live discharge, the need for more research to support a standardized and reimbursable discharge process and to define unmet needs for both patients and caregivers affected by live discharge.

### WORKING AND CARING DURING THE CORONAVIRUS PANDEMIC: THE IMPACT OF WORKPLACE POLICY ON WORKING FEMALE CAREGIVERS

Jessica McLaughlin,[1] Ashley Taeckens-Seabaugh,[2] Amy Kennicutt,[3] and Taylor Capellaro,[4], *1. University of Denver, Fort Collins, Colorado, United States, 2. University of Denver, Denver, Colorado, United States, 3. Data 4 Equity, LLC, Miami Beach, Florida, United States, 4. Connally Counseling, Garden City, Michigan, United States*

The unprecedented nature of the coronavirus pandemic created significant socioenvironmental changes for working caregivers who found themselves juggling a new landscape of working and caring. Changes in workplace policy were often intended to accommodate those with caring responsibilities, however, there is little information available on how working female informal caregivers of older adults (defined as individuals age 50 or older) received, interpreted, and experienced those policy changes. Given this, it is necessary to gather a complete picture of workplace policy in the daily lives of working female caregivers during the pandemic. This qualitative study involved interviews held between February and April 2021 via video conferencing technology with 29 working female caregivers, ranging in age from 27 to 75 years old. Using a Role Conflict framework and descriptive, structural, and emotion coding strategies, analysis of written transcripts revealed that, while many caregivers were grateful that their workplaces had become more accommodative during the pandemic, apprehension and uncertainty about the future, both with caregiving and with work, also weighed heavily on many of them. The most positively endorsed workplace policy changes were flexibility in work schedules and the ability to work remotely during the pandemic. This research elucidates policy implications for working female caregivers outside of the pandemic context, as many of these policies enabled caregivers to provide care while working with greater ease.

### Session 9510 (Late Breaking Poster)

### LATE BREAKING POSTER SESSION III

### A QUALITATIVE ANALYSIS OF DIETARY PRACTICES AND MOTIVATORS AMONG OLDER ADULTS LIVING WITH PARKINSON DISEASE

Christine Ferguson,[1] Jeannine Lawrence,[2] Joy Douglas,[2] Seung Jung,[2] Anne Halli-Tierney,[3] Chuong Bui,[4] Kyndal Oden,[2] and Amy Ellis,[2], *1. University of Alabama at Birmingham, Tuscaloosa, Alabama, United States, 2. University of Alabama, Tuscaloosa, Alabama, United States, 3. The University of Alabama, Tuscaloosa, Alabama, United States, 4. University of Alabama, University of Alabama, Alabama, United States*

Research supports the role of diet in the onset and progression of Parkinson disease (PD); however, there is no specific dietary pattern recommended for PD. This is partially due to a paucity of in-depth data on the dietary practices of this population. Therefore, the purpose of this study was to qualitatively explore the dietary practices and motivators of older adults with PD. Eleven dyadic semi-structured interviews with older adults and their care-partners were conducted via Zoom about their dietary practices and motivators. Interviews were audio-recorded and transcribed verbatim, and data were thematically analyzed using NVivo 12 software. The following themes were identified: 1) Intentionally making healthier choices on a regular basis; 2) Following a specific dietary pattern; for example, the Mediterranean diet, ketogenic diet, vegetarian diet, and/or intermittent fasting; 3) Limiting or avoiding certain foods, food components, and/or food groups, such as dairy, gluten, sugar, animal meat, and/or alcohol; 4) Purchasing or growing organic produce; and 5) Adjusting the timing of their protein intake to their medications. Their PD diagnosis and symptoms were reported as the primary motivators for following their respective diets. Overall, older adults with PD may be motivated by their diagnosis to modify their dietary intake; however, there are a variety of patterns or restrictions they may be adhering to. These results support the need for a consensus on the dietary recommendations for this patient population.

### A REMOTE TAI CHI PROGRAM FOR DIVERSE OLDER ADULTS WITH MULTISITE PAIN DURING THE COVID-19 PANDEMIC

Yael Koren,[1] Suzanne Leveille,[1] Catarina Ambrizzi Moraes,[1] William Butts,[1] Peter Wayne,[2] Gloria Yeh,[3] and Tongjian You,[1], *1. University of Massachusetts Boston, University of Massachusetts Boston, Massachusetts, United States, 2. Harvard Medical School, Chestnut Hill, Massachusetts, United States, 3. Harvard Medical School, Boston, Massachusetts, United States*

The goal of this study was to evaluate feasibility and acceptability of a remote Tai Chi program in diverse older adults with multisite pain and risk for falls during the COVID-19 pandemic. Adults aged ≥65y living in diverse Boston neighborhoods were invited through mailed letters to participate in a recruitment and screening survey. Eligible adults were re-contacted to join a 4-week Tai Chi or light exercise program offered online twice weekly. We conducted pre- and post-interviews to assess pain characteristics, fall risk, computer use, and satisfaction with the program. Primary outcomes were class attendance, experience, and program safety. Among 335 survey respondents, 105 (31%) were eligible based on multisite pain and fall history or cane/walker use. Of the eligible respondents, average age was 74y, 75% were women, 62% were Black, and 31% had high school education or less. We assigned 32 participants to 4 Tai Chi (Yang-style Tai Chi tailored to older adults with pain) or 2 light exercise (stretching and strength exercise) groups conducted via zoom; of these, 24 (75%) completed the program. Overall, 79% attended ≥6 of 8 classes. There were no adverse events reported. Regarding experiences with remote exercise, 67% reported it was very easy to join, 88%, very easy to see the instructor and 83%, very easy to participate. For future planning, 29% prefer remote classes, 33% prefer in-person classes, and 38% could do either. In conclusion, remote exercise programming is safe and feasible for diverse older adults who have multisite pain and risk of falls.

### A WALK IN THE COUNTRY: STORYING RURAL JOURNEYS OF DEMENTIA CARE

Jami Horne, and Katie Aubrecht, *St. Francis Xavier University, St. Francis Xavier University, Nova Scotia, Canada*

Dementia and dementia caregiving are increasingly recognized as significant public health issues. Dementia may be more prevalent in rural communities; in part due to higher rates of population aging. In Canada the Nova Scotia provincial dementia strategy Towards Understanding (2015) emphasizes the need to address the unique realities of rural dementia care as a priority issue; however, research remains limited on this demographic in the province, and Atlantic Canada more broadly. This presentation shares findings from the Royal Bank of Canada Foundation funded study, Rural Dementia Caregiving: A Community Life Story, conducted in 2021 to address this critical knowledge gap. The qualitative research design involved a narrative review, archival research and narrative analysis of interviews that yielded rich stories of family/friend dementia caregiving in rural Nova Scotia. Stories illustrate how history, culture and identity inform dementia caregiver realities, experiences and self-perceptions. Study results also suggest that rural dementia caregiving is characterized by factors that include strong community networks and deep-rooted connections to land, culture, and heritage, which can be experienced as supportive as well as constraining. The conditions of life in rural communities, including restricted access to internet, transportation, essential services and paid care providers, pose challenges to dementia caregiving. They also provide opportunities in which networks and connections become more visible and may even be strengthened. Findings demonstrate the lived realities of rural dementia caregivers and the people they care for are unique. Addressing their needs require a distinct approach that acknowledges and can appropriately respond to these differences.

### "FOR ME, IT'S CONNECTION": OLDER ADULTS EXPERIENCE WITH TECHNOLOGY DURING COVID-19

Vivian Miller,[1] HeeSoon Lee,[1] and Erin Roark,[2], *1. Bowling Green State University, Bowling Green, Ohio, United States, 2. Augusta University, Augusta, Georgia, United States*

As a result of COVID-19, older adults have experienced isolation, lost social contacts, and a decrease in connections. A recent study found that "approximately one-quarter of community-dwelling older adults are considered to be socially isolated, and 43% of them report feeling lonely." Various innovative interventions have emerged, including technology-based interventions as a means to reduce social isolation in older adults, particularly as information communication technology (ICT) use is on the rise among this population. However, it remains to be known how these connections are faring for older adults in the pandemic and whether these ICT connections lead to greater or lesser feelings of social connectedness. Thirty-nine (N=39) in-depth semi-structured interviews were conducted to explore the lived experiences of technology use among older adults during COVID-19. Participants experiences with ICT ranged from illiterate to savvy. Most participants indicated Zoom was the primary means to stay socially connected to family and friends. Participants emphasized that ICT may be a possible solution to deal with loneliness for those older adults who are especially isolated due to COVID-19 restrictions. Barriers and challenges to ICT use included taking too much time to use and needing help to fix any problems that arose. Finally, participants shared essential aspects of ICT use, revealing that it was 'technology or nothing.' Findings from this study indicate a need for a simple ICT for the older adult population. Moreover, findings suggest opportunities for peer-support ICT training programs for older adults.

### ADAPTING PSYCHOTHERAPY FOR COMORBID SUBSTANCE USE AND BIPOLAR DISORDER IN OLDER SEXUAL MINORITIES: A CASE STUDY

Sarah Nanami Morehouse,[1] Kirenia Brunson,[1] Ashley Stripling,[2] Jodie Maccarrone,[1] Jessica Choe,[1] Julian Garcia,[1] and Nicholas Boston,[1], *1. Nova Southeastern University, Davie, Florida, United States, 2. Nova Southeastern University, Fort Lauderdale, Florida, United States*

Approximately 65 to 95% of individuals with bipolar disorder (BD) are diagnosed with an additional psychiatric condition (Kessler, 1999). Alcohol, the most commonly abused substance amongst individuals with BD (Xiao et al., 2016), has been linked to significant increases in suicide attempts, disability, hospitalizations, and mortality (Baldessarini et al., 2008; Goldberg et al., 1999; Mitchell et al., 2007; Nery & Soares, 2011). Despite these ill effects, little is known about how to effectively treat, or adapt existing treatment appropriately, for the growing numbers of individuals who are dually diagnosed with BD and alcohol use disorder (AUD) and hold the identity of lesbian, gay, bisexual, transgender, or queer (LGBTQ) in late life. Thus, the purpose of this study is to demonstrate how treatment was adapted to a self-identified gay man with comorbid BD and AUD from a relational, culturally sensitive perspective while simultaneously implementing two short-term interventions: cognitive behavioral therapy (CBT) and a behavioral substance use program. In line with Knight & Poon's (2008) Contextual Life Span Theory for Adapting Psychotherapy with Older Adults (CALTAP) and a multicultural lens that incorporates relevant research on older LGBTQ individuals, modifications were made to the content, structure, language, and duration of therapy while cultivating a safe and empathic space. Idiographic data and progress monitoring measures suggests treatment resulted in substance use and distress reduction, as well as mood stabilization. However, additional booster sessions may be advantageous given the risk for substance abuse relapse and the compounding effect it may exert on persons with BD.

### ADRD CAREGIVING EXPERIENCES AND HEALTH BY RACE, ETHNICITY AND CARE RECIPIENT GEOGRAPHIC CONTEXT

Tiffany Kindratt,[1] Dominigue Sylvers,[2] Aya Yoshikawa,[3] Mónika López Anuarbe,[4] Noah Webster,[5] and Erin Bouldin,[6], *1. University of Texas at Arlington, Arlington, Texas, United States, 2. University of Michigan School of Public Health, University of Michigan Institute for Social Resarch, Michigan, United States, 3. Texas Woman's University, Texas Woman's University, Texas, United States, 4. Connecticut College, New London, Connecticut, United States, 5. University of Michigan, Ann Arbor, Michigan, United States, 6. Appalachian State University, Boone, North Carolina, United States*

Few studies have examined how the intersectionality of geographic context and race/ethnicity influences Alzheimer's disease and related dementia (ADRD) caregiving. Our aims were to determine whether 1) caregiver experiences and health differed across urban and rural areas; and 2) these links were moderated by caregiver race/ethnicity. We used data from the 2017 National Health and Aging Trends Study and National Study of Caregiving. The sample included caregivers (n=808) of care recipients ages 65+ with 'probable' ADRD (n=482). Geographic context was defined as care recipient's residence in metro (urban) or non-metro (rural) counties. Outcomes included caregiving experiences (burden, gains, life impacts, service/resource use) and health (self-rated, anxiety, depression symptoms, chronic health conditions). Bivariate analyses indicated that non-metro ADRD caregivers were less racially/ethnically diverse (82.7% white) and more were spouses/partners (20.2%). Among racial/ethnic minority ADRD caregivers, non-metro context was associated with having more chronic conditions (p<.01), providing less care (p<.01), and not co-residing with care recipients (p<.001). Amid white ADRD caregivers, non-metro context was associated with not reporting caregiving was more than they could handle (p<.05) and finding financial assistance for caregiving (p<.05). Multivariate regression analyses demonstrated that non-metro minority ADRD caregivers had 3.09 times higher odds (95% CI=1,02-9.36) of reporting anxiety in comparison to metro minority ADRD caregivers. Geographic context shapes ADRD caregiving experiences and caregiver health differently across racial/ethnic groups. Despite higher rates of ADRD and ADRD-related mortality in non-metro areas, findings suggest both positive and negative aspects of caregiving among White, Black, and Hispanic ADRD caregivers.

### AGE DIFFERENCES IN ANTICIPATED USE OF VIRTUAL HEALTHCARE SERVICES AFTER THE PANDEMIC

Matthew Murphy,[1] William Hills,[1] and Karen Hills,[2], *1. Coastal Carolina University, Conway, South Carolina, United States, 2. Beaufort Jasper Hampton Comprehensive Health Services, Conway, South Carolina, United States*

Healthcare has undergone a significant transformation during the pandemic, with virtual services being rapidly developed and implemented to keep pace with societal needs. This study documented this change in healthcare by examining access and use of video-based, virtual service use before and during the pandemic. Participants for the study (n = 685) included three groups, including retirement-aged persons, middle-aged adults, and traditional college-aged students. Measures for the study included access to and utilization of physical and mental health services, satisfaction with services accessed, and anticipated access and use of virtual services following the pandemic. Results showed that most participants (94.2%) believed that virtual healthcare would persist after the pandemic; three-quarters of adults (75.2%) but only half of college-aged (52.8%) and retirement-aged (57.6%) participants anticipated using virtual healthcare in the future. Prior use and satisfaction with virtual healthcare services mediated anticipated future use for retirement-aged participants (p < .001), but only satisfaction with virtual healthcare was a marginal predictor for college-aged participants (p = .051), and neither were predictors for adult-aged participants. These results support that people believe virtual healthcare will persist after the end of the pandemic, but that there are age-related differences in who anticipates using these services in the future, and which factors will make the most difference in attracting clients. These differences can impact how healthcare providers market and develop further tele-health services to increase the likelihood of use by retirement-aged participants, and suggests that client satisfaction is a key mediator for different age groups.

### AGE DIFFERENCES IN THE ASSOCIATION BETWEEN CARDIOVASCULAR DISEASE, DEPRESSION, AND SUICIDE RISK

Ruifeng Cui,[1] Alaa Shalaby,[2] Armando Rotondi,[2] Amy Albright,[3] and Judith Callan,[2], *1. West Virginia University, Morgantown, West Virginia, United States, 2. VA Pittsburgh Healthcare System, Pittsburgh, Pennsylvania, United States, 3. VA Maine Healthcare System, Lewiston, Maine, United States*

Cardiovascular disease (CVD) is prevalent among older adults aged 60+ (75%). The literature shows a strong bidirectional association between risk for CVD and risk for depression, although there is limited research regarding whether the strength of this association differs by age. CVD may also be related to suicide risk; however, the literature is both limited and mixed, with studies inconsistently finding an association. Additionally, no known studies have investigated age differences in this relationship. The present study examined the association between CVD (assessed via diagnostic checklist), depression (PHQ-8), and suicide risk (SBQ-R), as well as whether these associations differed by age. The current sample consisted of 301 younger adults (aged 18-40) and 432 older adults (aged 60+) recruited online through Mechanical Turk (younger adults: 78.1% white, 46.5% female; older adults 91.4% white, 56.3% female). Older adults had more CVD diagnoses (M=0.9) than younger adults (M=0.3). The association between CVD (i.e., 1+ CVD diagnoses vs. 0 diagnoses) and mental health was moderated by age (depression interaction p<.001; suicide risk interaction p=.033). Among younger adults, presence of CVD diagnosis was associated with 85% higher depression symptoms (M=6.1 vs 11.3) and 48% higher suicide risk scores (M=5.8 vs 8.6) when compared to no diagnoses. CVD had less of a negative impact among older adults and was associated with 64% higher depression symptoms (M=3.1 vs 5.1) and only 14% higher suicide risk scores (M=4.3 vs 4.9). Providers treating CVD may consider assessing and addressing depression and suicide risk, especially among younger patients with CVD.

### AGE, BODY MASS INDEX (BMI) AND COGNITIVE DIFFICULTIES IN APPALACHIAN WEST VIRGINIA

Camila Romero,[1] Moyosoreoluwa Jacobs,[2] and Bernard Schreurs,[3], *1. WVU, West Virginia University, West Virginia, United States, 2. West Virginia University, Morgantown, West Virginia, United States, 3. WVU, Rockefeller Neuroscience Institute, WVU, West Virginia, United States*

Several known factors exacerbate the risk of cognitive difficulties among older adults. In addition to place-based disparities, body weight also predicts cognitive health. In fact, overweight and Obese BMI (Benito-Leon et al., 2013) and underweight BMI (Xiang & An, 2015) are risk factors for cognitive difficulties. Whether the effect of BMI operates similarly across age among adults facing place-based disparities is not clear. In order to better understand the role of BMI among adults already at-risk for health disparities, we used the 2018 BRFSS data to examine the relations among these variables among the 4817 West Virginian adults in the BRFSS database. Approximately 16.5% had difficulty making decisions or remembering. Approximately 73% were overweight or obese, 26% were healthy BMI, and 1% were underweight. A multinomial logistic regression was conducted to examine cognitive difficulties. Healthy BMI and ages 25 – 44 years were the reference groups. The omnibus test was significant, $\chi 2$ (5, N = 4817) = 38.71, p < 0.0001. Age and BMI uniquely contributed to the classification. Post hoc inspection of the Odds Ratios showed that adults ages 60 years and older with obesity were 1.27 times more likely to report cognitive difficulties, while those who were underweight were 3.74 times more likely to report cognitive difficulties. That individuals over age 60 with an obese or underweight BMI report more cognitive difficulties highlights the intersection among age, obesity and location on cognitive health disparities in West Virginia.

### AGEISM IN HIRING DECISIONS: SENSE OF PURPOSE AS A MITIGATING STRATEGY

Megan Wilson,[1] and Patrick Hill,[2], *1. Washington University in St. Louis, Saint Louis, Missouri, United States, 2. Washington University in St. Louis, St. Louis, Missouri, United States*

Discrimination against older adults in the workplace is a pervasive issue that has important consequences for older adults, leading to lower well-being (Stokes & Moorman, 2020) and worse job outcomes (Macdonald & Levy, 2016). One area where discrimination manifests is in hiring practices, and thus research is needed to understand factors that impact willingness to hire older adults. One potential intervention target to reduce age discrimination in hiring is sense of purpose. Sense of purpose manipulations have previously been successful in increasing one's comfort with diversity (Burrow & Hill, 2013), and thus may prove successful in combatting age discrimination in the workplace. Therefore, the current studies sought to understand whether sense of purpose was related to ageist attitudes and hiring decisions, and how a purpose manipulation might serve as a tool to combat discrimination in hiring. Across two studies (n = 594, MAge = 27.20), participants were shown the resumé of either an older adult applicant (62-years-old) or younger adult applicant (32-years-old), and were asked how hireable they would rate the applicant. The research found that the purpose manipulation did effectively increase individuals' sense of purpose. In addition, the research found that sense of purpose was negatively related to ageist attitudes. However, the purpose manipulation was unsuccessful in reducing ageist attitudes, and had no effect on ageist discrimination in hiring. These results suggest that while sense of purpose is negatively related to ageist attitudes, manipulating purpose may not be an effective tool to reduce ageist attitudes or discrimination.

### APPLYING INTERSECTIONALITY FRAMEWORK TO EXPLORE THE DEVELOPMENT OF FRAILTY IN OLDER ADULTS

Shamatree Shakya, Susan Silva, and Michael Cary, Jr., *Duke University, Durham, North Carolina, United States*

**Background:** Frailty is a state of compromised homeostasis reserve that makes older adults susceptible to adverse health outcomes. Frailty is highly prevalent among women, racial and ethnic minorities. We aimed to investigate the combined influence of gender, race, and ethnicity on the development of frailty in older adults without frailty at baseline using the intersectionality framework.

**Methods:** The data came from the Health and Retirement Study, a nationally representative US older adults of 65 years and older (2006-2012). Logistic regressions were used to examine the relationships.

**Results:** 18.7% of older adults without frailty at baseline developed frailty after a 4-year follow-up. Females were likely to develop frailty than males (aOR 1.66, 95% CI 1.32-2.09, p < 0.0001). In comparison to Non- Hispanic Whites, Non-Hispanic Blacks (aOR 2.66, 95% CI 1.89, 3.74, p < <.0001) and Hispanics or others (aOR 1.73, 95% 1.16, 2.58, p < .0068) had the greater likelihood of developing frailty, after adjusting for age and clinical morbidities, such as lung disease and cardiometabolic diseases. The intersectionality approach showed that both Non-Hispanic Black females (aOR 1.82, 95% CI 1.12-2.99, p = 0.0185) and males (aOR 3.30, 95% CI 1.85-5.91, p < .0001) had the highest likelihood of developing frailty than Non-Hispanic Whites at 4-year post-baseline, adjusting for age and chronic clinical conditions.

**Conclusion:** Our findings highlight the importance of taking the intersectionality approach to examining the frailty risk in later life, which will help in providing precision-based care.

### ARE GENETIC AND ENVIRONMENTAL CONTRIBUTIONS TO VERBAL FLUENCY AND EPISODIC MEMORY SOLELY MODERATED BY SLEEP?

Tina Vo,[1] Shandell Pahlen,[2] William Kremen,[3] Matthew Mc Gue,[4] Marianne Nygaard,[5] and Chandra Reynolds,[1], *1. University of California Riverside, Riverside, California, United States, 2. University of California, University of California, California, United States, 3. University of California San Diego, La Jolla, California, United States, 4. University of Minnesota, Minneapolis, Minnesota, United States, 5. Department of Public Health, University of Southern Denmark, Odense, Syddanmark, Denmark*

Decreases in sleep duration and cognitive functioning often occur and co-occur in aging although these patterns are not universal. Underlying etiologies, i.e., genetic and environmental factors, contribute to why people differ on cognitive functioning at shorter versus longer sleep durations. The current study tested whether sleep duration alters the genetic and environmental contributions to why middle-aged and older adults vary on cognitive functioning. Using 4 twin studies from the Interplay of Genes and Environment Across Multiple Studies (IGEMS) consortium (Mage=56.5, range=35.0-91.2, N=5,210, 1,083 complete MZ pairs, 1,522 complete DZ pairs) we tested quantitative genetic twin models considering sleep, depressive symptoms, and age as moderators of verbal fluency (i.e., Animal Naming) and episodic memory (i.e., Word List). For verbal fluency, sleep duration and depressive symptoms were significant when dropped together from the model ($\chi2(6)=15.22$, p=0.02) but not individually ($\chi2sleep(3)=7.17$, p=0.07; $\chi2dep(3)=5.81$, p=0.12), indicating that both moderators may affect differences in verbal fluency performance. For episodic memory, sleep duration moderation was only significant via the shared environmental factor ($\chi2(1)=5.26$, p=0.02), indicating that sleep may affect differences in episodic memory performance via environmental influences that make siblings more similar to one another. Overall, results illustrate patterns of higher genetic influences on cognitive function at short sleep (4 hours) and higher shared environmental influences on cognitive function at long sleep (10 hours). These findings may align with associations of upregulation of neuroinflammatory processes at short sleep and common reporting of mental fatigue at long sleep, both of which are associated with poorer cognitive functioning.

### ASSOCIATION OF FIRST EMPLOYMENT CHARACTERISTICS AND HOSPITALIZATION IN THE MAYO CLINIC BIOBANK

Paul Takahashi, Euijung Ryu, Nicole Larson, Gregory Jenkins, Kirt Christine, Kathleen Yost, and Janet Olson, *Mayo Clinic, Rochester, Minnesota, United States*

Work history is associated with long term health outcomes We hypothesize that characteristics of the first work experience, such as age at first job and length of work (hereafter job) are associated with future risk of hospitalization. We further hypothesize that the length of work will be associated with hospitalization. We conducted a survey of adults >60 years using a nested case-control approach within the Mayo Clinic Biobank. We collected job related variables including age at job start, reason for ending, and length of time. To test associations between each variable and hospitalization, we used age and gender adjusted logistic regression models. Our study included 4,024 subjects: 1,801 cases and 2223 controls. The mean age at time of match was 77.3 years (SD 7.2 years) with 49.2% males. Older age at the first full-time job was associated with lower chance of hospitalization later in life (OR=0.81 [0.67, 0.97] for those who started the job over 22 compared to those started at 18 or less). Cases were more likely to have stopped working because of illness (OR=2.04 [95% CI 1.29,3.27]). Cases were less likely to have stopped working because of retirement (OR=0.82 [95% CI: 0.72, 0.93]). We found cases were employed with a slightly shorter time (20.5 yrs. (SD 16.6)) compared to controls (21.8 yrs. (SD 16.3)) (p=0.005). Cases started work earlier and stopped work more frequently because of illness/disability compared to controls. This could reflect educational attainment in controls. This study highlights work history as potential predictor of future hospitalization.

### ASSOCIATIONS AMONG FALL RISK APPRAISAL, BODY COMPOSITION, AND PHYSICAL ACTIVITY IN OLDER ADULTS

Ladda Thiamwong,[1] Rui Xie,[2] Renoa Choudhury,[2] Joon-Hyuk Park,[2] Oscar Garcia,[2] Aleatha Rossler,[2] and Jeffrey Stout,[2], *1. College of Nursing, University of Central Florida, Orlando, Florida, United States, 2. University of Central Florida, Orlando, Florida, United States*

One-third of older adults have maladaptive fall risk appraisal (FRA), a condition in which there is a discrepancy between perceived fall risk or levels of fear of falling (FOF) and physiological fall risk (balance performance). We aimed to examine the associations among FRA, body composition, and physical activity (PA) using Assistive Health Technology, including the Bioelectrical Impedance Analysis, BTrackS Balance System, and activity monitoring devices. We evaluated 124 older adults with a mean age of 74.81 (SD=7.31, range 60 to 96), 77% were female, and 72% had no history of falls. The multinomial logistic regression was used to analyze the data. FRA was classified into 4 quadrants, and we found 47% of rational FRA (low FOF and normal balance), 19% of incongruent FRA (low FOF despite poor balance), 18% of irrational FRA (high FOF despite normal balance), and 16% congruent FRA (high FOF and poor balance). We found these following variables are associated with FRA: accelerometer-based moderate to vigorous physical activity (MVPA: mins), self-reported PA score (strength & flexibility), had difficulty walking up 10 steps without resting (resistance), had difficulty walking several city blocks (ambulation), left-hand average handgrip strength, CDC fall risk score, Senior Technology Acceptance (STA) score and body composition including Body Fat Mass (BFM), Percent Body Fat (PBF), Body Mass Index (BMI), Whole Body Phase Angle, Skeletal Muscle Mass (SMM) and Skeletal Muscle Index (SMI). Our results support the efficacy of using Assistive Health Technology on screening individuals with maladaptive FRA with targeted interventions to reduce fall risk.

### ASSOCIATIONS BETWEEN VISUAL, HEARING FUNCTIONING AND COGNITIVE FUNCTIONING AMONG HISPANICS/LATINOS

David Lee,[1] D. Diane Zheng,[1] Laura McClure,[2] Karen Cruickshanks,[3] Charlotte Joslin,[4] Hector Gonzalez,[5] Neil Schneiderman,[6] and Byron L Lam,[1], *1. University of Miami Miller School of Medicine, Miami, Florida, United States, 2. University of Miami Miller School of Medicine, University of Miami Miller School of Medicine, Florida, United States, 3. McPherson Eye Research Institute, University of Wisconsin-Madison, Madison, Wisconsin, United States, 4. University of Illinois at Chicago, Chicago, Illinois, United States, 5. University of California, San Diego, La Jolla, California, United States, 6. University of Miami, Miami, Florida, United States*

Findings that visual impairment (VI) and hearing impairment (HI) are associated with cognitive functioning are drawn from studies that involved few Hispanic/Latino participants. We utilized data from the Miami Ocular SOL ancillary study to the Hispanic Community Health Study/Study of Latinos (HCHS/SOL) with 1056 participants aged 45 and older. The outcomes were neurocognitive performances assessed by the Digit Symbol Substitution Test (DSST, executive function), Word Frequency Test (verbal fluency), Brief Spanish-English Verbal Learning Test-recall (B-SEVLT recall, episodic memory), and the Six-Item Screener (global cognitive functioning). Visual functioning was measured by National Eye Institute Visual Function Questionnaire (NEI-VFQ). Hearing function which was measured by Hearing Handicap Inventory for Adults and Elderly (HHIA/HHIE) was available for all HCHS/SOL participants (n=9343). Multiple regression was performed for each cognitive outcome while controlling for age, gender, education, Hispanic/Latino ethnicity background, cardiovascular risk factors, depression and complex design. NEI-VFQ was associated with 3 of the 4 cognitive outcomes: DSST ($\beta$ =0.14, se=0.027, $p<0.01$), Word Frequency Test ($\beta$=0.042, se=0.016, $p<0.01$), B-SEVLT-recall ($\beta$=0.021, se=0.007, $p<0.03$). HHIA/HHIE was not associated with any of the cognitive measures examined. The HHIA/HHIE analysis was repeated using data from all sites; similar results were observed. Visual functioning but not hearing functioning is associated with worse cognition in Hispanics/Latinos, although previous HCHS/SOL analysis indicated that hearing loss as assessed by pure tone audiometry was associated with worse cognitive functioning. Longitudinal assessment of both clinical and functional measures is needed to understand the impact of sensory impairment on cognition in Hispanics/Latinos.

### BETTER BRAIN HEALTH THROUGH EQUITY: ADDRESSING HEALTH AND ECONOMIC DISPARITIES IN DEMENTIA

Rajiv Ahuja, and Cara Levy, *Milken Institute, Washington, District of Columbia, United States*

Dementia disproportionately impacts the health and financial security of women and certain minority groups. Long-standing inequities create distrust of the medical system, fewer treatment options, and reduced access to care. Research predicts that from 2020 to 2060, the number of African Americans and Latinx living with dementia will grow by nearly 200 percent and 440 percent, respectively, while prevalence among non-Hispanic Whites will increase by 69 percent. As the prevalence of dementia rises, so will the costs associated with dementia care. African Americans bear 1/3 of the costs associated with dementia. And the costs for Latinx living with Alzheimer's disease are expected to exceed $100 billion by 2060. To mitigate these growing health and economic concerns, efforts to improve dementia care must put equity front and center. This presentation highlights five actionable recommendations to build health equity by reducing disparities in dementia prevention, detection, diagnosis, and care. These recommendations center around two overarching themes: (1) Strengthening the infrastructure among healthcare, long-term care, and community-based organizations to achieve greater health equity for people living with dementia and their caregivers and (2) Expanding dementia-friendly networks and workplaces in racially and ethnically diverse communities. The recommendations discussed in this presentation will offer guidance for policymakers, health services researchers, businesses, health systems, and communities to reduce the inequitable impact of dementia on African Americans and Latinx, which is even more vital amid demographic trends showing a population growing older and more racially and ethnically diverse.

### BIAS AGAINST OLDER ADULTS IN YOUNG CHILDREN AND THEIR PARENTS

Jenny Bauer, and Jennifer Bellingtier, *Friedrich Schiller University Jena, Jena, Thuringen, Germany*

Parents are an important source of social learning for their children. However, little is known about whether they play a role in shaping ageist attitudes in children. We investigated how parents' biases against older adults would relate to those of their children and how preferences would differ depending on the child's age. Participants were 56 parent-child dyads with the children's age ranging from four to eight years (parents mean age = 36.95, SD = 5.49). Children were divided into three age groups, preschool (n = 18), early school-aged (n = 18), and middle school-aged (n = 20). Children and parents completed a picture rating task, which included the evaluation of 28 images of younger and older adults faces. Children used a smiley-face rating scale on a touch-screen computer, and parents used a sliding preference scale for their ratings. It was found that both, children ($t(55) = 5.47$, $p < .001$, $d = 0.73$) and their parents ($t(55) = 2.05$, $p = 0.045$, $d = 0.27$), gave significantly more positive ratings to younger than to older adults, which is consistent with an underlying bias for younger adults. Contrary to our expectations, this preference in children held across age groups and was not associated with parental preferences. Nevertheless, it has been shown that ageist preferences can already be detected in childhood. Further longitudinal research is needed to track the development of ageism from childhood on, and efforts to combat ageism should be addressed not only to adults, but to children as well.

### BUTTERFLY GARDEN CONNECTS COMMUNITY LIVING CENTER RESIDENTS, FAMILY, AND STAFF DURING THE COVID-19 PANDEMIC

Maribel Rodriguez-Gonzalez,[1] Maura Miller,[2] Gelda Pratt,[3] Micheal A Silverman,[4] and Sandra DiScala,[5]

*1. Florida Atlantic University- West Palm Beach VA Medical Center, West Palm Beach VA Medical Center/West Palm Beach, Florida, United States, 2. West Palm Beach VA Medical, West Palm Beach VA Medical Center/ West Palm Beach, Florida, United States, 3. West Palm Beach VA Medical Center, West Palm Beach VA Medical Center, Florida, United States, 4. West Palm Beach VA Medical Center, West Palm Beach VA Medical Center/ Geriatric Extended Care, Florida, United States, 5. West Palm Beach VA Medical Center, West Palm Beach VA Medical Center/ West Palm Beach, Florida, United States*

The COVID-19 Pandemic has led to significant morbidity and mortality in older residents of long-term care facilities. In addition, the stringent restrictions on visitation of family and loved ones has further socially isolated residents leading to an increase in depression, loneliness, and spiritual distress. The Community Living Center (CLC) staff at West Palm Beach VA Medical Center wanted to address this dilemma and created a unique "Butterfly Garden" (BG) visitation space. This space is a therapeutic garden adjacent to the CLC that can be accessed by families without having to enter the facility. Participants in the BG reported feelings of peace, undisturbed reflection, and tranquility as they observed and experienced nature's life cycle. This show of nature's beauty promotes visual, tactile, and olfactory sensory stimulation while attracting hummingbirds, bees, butterflies, and peace to this calm garden space. The BG visitations offers residents, family, and staff opportunities to experience the health benefits of nature during their visits under strict CDC social contact guidelines. From September through December 2020 and from January through March 2021 there were 67 and 184 visits respectively as families became more involved. The feedback from residents, families, and staff indicated that the spirits of all participants were raised by the BG visits despite the difficult challenges of social distancing and limited CLC visitations. This BG concept could serve as a model for other long-term care facilities to allow socially distant family visits to loved ones in a safe nature-based environment of care with or without a pandemic.

### CAN REMOTE MONITORING MEASURE LIFE ACTIVITY AND CAREGIVER EXPERIENCE? EARLY RESULTS OF MULTI-MODAL ASSESSMENTS

Allison Lindauer,[1] Walter Dawson,[2] Sarah Gothard,[2] Leslie Tran,[2] Zachary Beattie,[2] and Jeffrey Kaye,[2], *1. Oregon Health Sciences University, Portland, Oregon, United States, 2. Oregon Health & Science University, Portland, Oregon, United States*

Subjective assessments of dementia caregiver burden are vulnerable to recall and recency biases. Objective continuous home assessment using passive technologies (e.g., bed mats, actigraphy watches) can provide ecologically valid detail on caregiver stress and family function. We tested the utility of objective assessment of activity before, during and after the behavioral intervention of STELLA (Support via Technology: Living and Learning with Advancing AD) which facilitates effective online management of behavioral symptoms of dementia. We present preliminary data on objective measures of sleep and step counts, and subjective measures of burden. We captured data from three caregivers caring for a family member with dementia. Each family lives in home with unobtrusive monitoring devices that recorded data on sleep (Emfit sleep mat) and daily steps (Withings watch). Self-report assessments of burden, depression and grief were collected prior to and after the 2-month intervention. Objective data was collected continuously. Pre/post subjective assessments suggest that the STELLA intervention has the potential to reduce behavioral symptom frequency and caregiver reactivity to symptoms (pre-STELLA behavior frequency=44.9, post=39.2; pre-STELLA reactivity=50.5; post=38.5). Step count ranged from 775 steps/day to 5065, with each participant trending fewer steps during the intervention. Mean sleep time ranged from 6.3 to 8.6 hours and didn't change during the intervention. The small sample size limits interpretation but provides evidence that it is feasible to collect continuous objective life-activity data during caregiver interventions. This digital data has the potential to inform the validity of subjective findings by limiting recall and recency biases.

### CARDIOVASCULAR MONITORING SYSTEMS FOR AGING IN PLACE: CURRENT PERSPECTIVES AND A NOVEL TOILET SEAT-BASED SYSTEM

Carla VandeWeerd,[1] Mitchell Roberts,[2] Lindsey Collins,[3] Erica Sappington,[4] Lydia Poon,[4] and Jeff Lowenkron,[5], *1. UF Health Precision Health Research Center-The Villages, The Villages, Florida, United States, 2. University of Florida, Tampa, Florida, United States, 3. University of Florida, University of Florida, Florida, United States, 4. The University of Florida, The Villages, Florida, United States, 5. The Villages Health, The Villages, Florida, United States*

Cardiovascular disease (CVD) is a leading cause of death. Questions remain as to how older adults, providers and researchers can harness remote patient monitoring (RPM) to maintain/improve cardiovascular health--especially in light of COVID-19 and increased reliance on telehealth. The objective of this study was to understand the perceptions of older adults with cardiovascular challenges and providers surrounding a novel RPM device. The Heart Seat (THS) developed by Casana, is a toilet-seat-based cardiac monitoring device. Focus groups, stratified by gender, were conducted in 2021 by the UF Health Precision Health Research Center (UFIRB202100290) with older (55+) adults (n=36) in The Villages, Florida. Adoption, benefits/concerns, usability, utility and gender differences were explored. One-on-one provider interviews (n=6) explored future utility of THS. The primary benefit of THS noted by providers and older adults was ease-of-use and passive data collection, promoting adherence. Providers considered THS 'easy-to-use' and a positive alternative to current RPM devices. While genders' sentiments towards cardiac monitoring devices were similar, males reported having more experience with RPM. Despite this, females reported using cardiac monitoring devices more consistently than males. Therefore, passive RPM may be beneficial for increasing adherence in males. Participants' largest concern surrounding RPM was information sharing, including data monitoring, and security. Providers were also concerned about information sharing, specifically who would receive/monitor and interpret data from RPM. RPM devices should focus on enhancing ease-of-use, catering to user and provider information sharing and data monitoring/ interpretation preferences and privacy.

### CAREGIVING PATTERNS AND THEIR ASSOCIATION WITH CAREGIVING BURDEN AND GAINS

Talha Ali,[1] Gail McAvay,[1] Joan Monin,[2] and Thomas Gill,[1], *1. Yale School of Medicine, New Haven, Connecticut, United States, 2. Yale School of Public Health, New Haven, Connecticut, United States*

Family and friend caregivers play a critical role in helping older adults live long and healthy lives. Using the construct of "care types" we examine whether caregivers providing more intense care experience higher burden and lower gain compared to caregivers providing less intense care. Data are from the 2015 and 2017 rounds of the National Study of Caregiving (N=2,146), a study of the caregivers of older adults enrolled in the National Health and Aging Trends Study. In a previous analysis, we derived five care types at baseline (2015), that varied in the number and type of care activities, care duration, and regularity of care provided. Caregivers reported whether caregiving was financially, emotionally, and physically difficult for them. Participants were also asked whether caregiving made them more confident, taught them to deal with difficult situations, brought them closer to the recipient, and gave them satisfaction that the recipient was well cared for. We estimated the association between care types derived at baseline and caregiver burden and gains at follow-up using logistic regression. Compared to caregivers in the least intense care type, those in the more intense care types were significantly more likely to report financial and physical burden, as well as increased ability to deal with difficult situations and increased satisfaction that their loved one was well cared for. These associations remained significant after adjusting for confounders. Care types can be used to predict burdens and gains experienced by caregivers and to effectively target caregiver support services and interventions.

### CHANGES IN DAILY LIFE CONDITIONS AMONG KOREAN OLDER ADULTS AFTER COVID-19 OUTBREAK

Jiyoung Lyu, Young Bum Kim, and Yeon Ok Lim, *Hallym University, Chuncheon, Kangwon-do, Republic of Korea*

The pandemic of COVID-19 has had a significant impact on peoples' daily life conditions. Therefore, it is worth understanding changes in daily life conditions among older adults after COVID-19 outbreak. This study was aimed to explore the impact of the pandemic on daily life conditions among Korean older adults. To achieve this aim, an online survey was conducted during April, 2021 to include 184 Korean adults aged 60 years or older nationwide. The results are as follows. First, about 54.9% older Koreans reported that their daily life has stopped since COVID-19. Second, the top 3 increased daily life conditions were spending time at home (77.2%), using internet (60.3%), and shopping online (51.6%). Third, the top 3 decreased daily life conditions were traveling (90.2%), meeting with friends (90.2%), and meeting with family members (80.4%). Fourth, the top 3 daily life conditions with no change were sleeping time (72.3%), eating instant food (58.2%), and household income (53.8%). These findings suggest that several daily life conditions changed after COVID-19 outbreak. Further study is needed to examine the impact of these changes on physical and mental health among older adults.

### CHILD MALTREATMENT AND THE PSYCHOLOGICAL CONSEQUENCES OF COVID-19 FOR OLDER ADULTS

Tyler Bruefach, Dawn Carr, and Natalie Sachs-Ericsson, *Florida State University, Tallahassee, Florida, United States*

Traumatic experiences in early life impact adults' well-being and their abilities to respond to adversities over the life course. Child maltreatment is a particularly salient stressor in childhood and scholars have noted the psychological implications of such experiences that extend into late life. People who experienced maltreatment in childhood have more difficulty maintaining and developing high quality relationships, regulating their emotions, and they engage in poorer coping behaviors amidst major stressors. Our study focuses on how child maltreatment (i.e., emotional abuse; physical abuse; sexual abuse; emotional neglect) shaped older adults' changes in depression during the early stages of the COVID-19 Pandemic. Using a dataset released in 2021, based on a community sample of older adults collected in September 2018 and June 2020, we found that exposures to emotional neglect (1.630; $p < 0.001$) and emotional abuse (0.670; $p < 0.05$) in childhood were both associated with increases in depression scores in association with the pandemic, relative to those without such exposures. In addition, the more forms of maltreatment that individuals were exposed to in childhood, the more they experienced negative psychological health consequences in association with the pandemic. Our results suggest that early life traumas play a role in how older adults respond to stressful situations. Clinical treatments for depression may be more effective if they take into consideration how these early life experiences influence exposures to new stressors in later life.

### CO-DESIGNING A VIRTUAL REALITY APPLICATION TO ENHANCE REMINISCENCE THERAPY FOR PERSONS WITH DEMENTIA

Winnie Sun, and Alvaro Quevedo, *Ontario Tech University, Oshawa, Ontario, Canada*

Reminiscence therapy for persons with dementia is often being conducted by employing analog media including pictures and videos organized and presented by a caregiver. However, such media lacks the immersive experience to support patient engagement and successful recollection of reminiscence events. Recently, Virtual Reality (VR) is gaining momentum as a potential technological tool to support dementia care due to its increased immersion, presence, and embodiment. Haptic artifacts can be used to enrich reminiscence therapy as part of the multi-sensory stimuli to increase immersion and patient engagement, as well as improving social connectedness and cognitive health. The purpose of this project is to explore the use of VR application to advance reminiscence therapy for persons with dementia. We have prototyped an immersive and non-immersive VR framework that allows caregivers to deliver reminiscence therapy for persons with dementia with varying stages in their disease progression. These reminiscence therapy sessions are built by employing a narrative storyboard and content management through a series of co-designing sessions with content experts at the Geriatric Dementia Unit in Ontario, Canada. A caregiver-led VR framework will be adopted to enable the caregiver to guide the persons with dementia to safely navigate through the interactive VR environment, while allowing the patients to engage with the interactive VR elements using a point-and-pinch gesture approach. We anticipate that the VR experiences hold the potential for improving the interactions between persons with dementia and caregivers, as well as enhancing the reminiscence experiences to promote the maximal therapeutic benefit of patient's recovery.

### COLLECTION OF FREE-LIVING ACCELEROMETRY DATA IN LARGE CLINICAL STUDIES BEFORE AND DURING THE COVID-19 PANDEMIC

Jacek Urbanek,[1] Kunihiro Matsushita,[2] Robert Kaplan,[3] Josef Coresh,[2] and Jennifer Schrack,[2], *1. Johns Hopkins School of Medicine, Baltimore, Maryland, United States, 2. Johns Hopkins University, Baltimore, Maryland, United States, 3. Albert Einstein College of Medicine, New York, New York, United States*

The recent COVID-19 pandemic had a substantial impact on clinical research, including recruitment and follow-up visits in new and ongoing studies, especially affecting ones focused on older, at-risk adults. As the objective assessment of physical activity with wearables is usually initiated during in-person visits, the collection of these data experienced substantial, unplanned gaps. We report the frequency of data collection (visits-per-month) in studies collaborating with the Accelerometry Resource Core (ARC) at Johns Hopkins Center on Aging and Health. We focus on two, NIH-funded, studies that implemented the ARC accelerometry protocol. The Atherosclerosis Risk in Communities that stopped visit 8 enrollment in early 2020 and reinstated in 2021 for visit 9, and the Peripheral Artery Disease Study of SOL that started the data collection in early 2021, first via the mail-in protocol, then shifting towards in-clinic visits. Through March 2020, ARC processed an average of 125 new accelerometry per month (SD = 54). There was no new data collected for the remainder of 2020. The collection restarted in January 2021 with an average of 55 (SD = 43) files a month in the first and 112 (SD = 53) in the second quarter of 2021. A total of 573 new accelerometry observations were collected across both studies since the first wave of COVID-19 in March 2020 including 282 observations collected exclusively using a mail-in protocol. This recovery of data collection demonstrates that wearable devices allow for safer, remote assessment of physical activity, function, and sleep eliminating the need for in-person visits.

### COMMUNICATION DIFFICULTIES AND THEIR ASSOCIATION WITH CAREGIVING BURDENS IN APHASIC DEMENTIA

Angela Roberts,[1] Haylie Santos,[1] Nathan Gill,[2] Hui Zhang,[2] Elizabeth Salley,[2] and Emily Rogalski,[2], *1. Northwestern University, Evanston, Illinois, United States, 2. Northwestern University, Chicago, Illinois, United States*

Primary progressive aphasia (PPA) is a clinical dementia syndrome for which there is no effective disease-modifying treatment. Impairments in language are the primary and persistent symptoms, and severely limit participation in everyday activities and family conversations. Despite this, there are no published studies examining the objective relationship between conversation difficulties and caregiving burden in PPA. We tested the hypothesis that the severity of care partner perceived conversation difficulties predicts caregiving burden using the Perception of Conversation Difficulty-Dementia Alzheimer's Type and the Montgomery Borgatta Caregiving Burden Scale. The analysis included baseline data from 78 care partners (62% female) enrolled in the Communication BridgeTM-2 randomized control clinical trial of a speech-language intervention for PPA. Care partners had a mean age of 64.5 years (SD=10.76) and a mean relationship duration with the PPA participant of 38.6 years (SD=15.29). Eighty-six percent were spouses, 5% were adult children, and the remaining 9% were friends or siblings. Higher ratings of conversation difficulties were associated with increased caregiving burden for both objective burden ($p < 0.001$) and subjective stress burden ($p < 0.001$). The relationship between conversation difficulties and objective burden was mediated by dependence in activities of daily living and care partner depression, whereas the relationship with subjective stress burden was mediated by depression only. This is the first large scale study of care partner reported conversation difficulties and caregiving burden in PPA. The finding that conversation difficulties have a direct relationship with caregiving burden is an important consideration for interventions and outcome measurement in PPA.

### CONCURRENT AND PREDICTIVE ASSOCIATIONS BETWEEN THE LIFE'S SIMPLE 7 & BRAIN STRUCTURE IN MIDDLE-AGE AND EARLY-OLD AGE

Teresa Warren,[1] McKenna Williams,[2] Christine Fennema-Notestine,[2] Jeremy Elman,[2] Jennifer De Anda,[3] William Kremen,[2] and Carol Franz,[2], *1. San Diego State University, San Diego, California, United States, 2. University of California San Diego, La Jolla, California, United States, 3. San Diego State University, La Jolla, California, United States*

American Heart Association's (AHA) Life's Simple 7 (LS7), an index of cardiovascular health risks, has been associated with worse brain outcomes but few examined this relationship in midlife. We examined whether LS7 scores at midlife were associated with brain morphometry in early old age. Participants were 471 men who participated in the Vietnam Era Twin Study of Aging. The LS7 index was assessed at mean age 62 (range 55-66) and 68 (range 61-71) and included smoking, physical activity, diet, body mass index, cholesterol, glucose, and blood pressure. Each factor was coded, per AHA criteria, on a 3-point scale (0/poor-2/ideal) and summed to create a composite score (0-14). At mean age 68, participants underwent structural magnetic resonance imaging, which was used to create the previously validated brain measures. Scores included: the ratio of abnormal white matter to white matter, and two Alzheimer's disease brain signatures (cortical thickness/volume signature and a mean diffusivity (MD) signature). Analyses controlled for age, education, income, ethnicity, and APOE genotype. Concurrently at mean age 68, the LS7 was associated with cortical thickness/volume ($F=4.85$, $p = .028$), MD ($F=10.89$, $p = .001$) signatures and abnormal white matter ratio ($F=14.04$, $p < .001$). Prospectively, the LS7 at mean 62 was significantly associated with age 68 cortical thickness/volume ($F=5.08$, $p = .025$) and MD ($F=5.54$, $p = .019$) signatures but not with abnormal white matter ratio. These results suggest that prevention strategies that promote heart healthy behaviors could have implications for healthy brain aging.

### CONSEQUENCES OF GENOMIC DNA MONO-RIBONUCLEOTIDES FOR CHROMOSOMAL STABILITY

Tavia Roache, *Howard University, Washington, District of Columbia, United States*

Mono-ribonucleotides are building blocks for poly-nucleotide RNA chains (e.g., messenger RNA), but if mis-incorporated into duplex DNA can cause mutagenesis and chromosomal instability. During DNA synthesis by Pol γ, remnants of unremoved RNA primers contribute to elevated mono-ribonucleotide triphosphates resulting in nucleotide pool imbalance, ultimately favoring mis-incorporated ribonucleotides during replication. Moreover, although polymerases generally replicate DNA with high fidelity, the steric gate occasionally allows a mis-incorporated ribonucleotide. Thus, a mono-ribonucleotide is one of the most abundant lesions in genomic DNA of eukaryotes. If unremoved from double-stranded DNA, the ribonucleotide exerts negative effects on replication, transcription, and genomic maintenance, with lasting effects on cellular homeostasis. Even a single ribonucleotide in telomeric DNA comprises shelterin binding and telomere capping causing vulnerability to spontaneous hydrolysis which potentiates telomere shortening. Consistent with this, a ribonucleotide positioned in double-helical DNA alters its structure by torsinally distorting the sugar-phosphate backbone. Fortunately, cellular response and repair pathways exist to help cells cope with mis-incorporated mono-ribonucleotides. The Ribonucleotide Excision Repair (RER) or a Topoisomerase 1 (Top1)-mediated pathway remove embedded ribonucleotides. For RER, RNase H2 incises 5' of a mono-ribonucleotide, creating an access point for its removal. If cells are deficient in RNase H2, Top1 initiates removal of the ribonucleotide. However, Top1 is less accurate than RNase H2, which can lead to mutagenesis. Studying the mechanisms in which ribonucleotides are incorporated into DNA or further metabolized should provide insight to their negative consequences for chromosomal integrity, cancer, and auto-immune disease attributed to a genetic deficiency of RNase H2.

### CONSEQUENTIAL IMPACTS OF TOBACCO USE ON COGNITIVE PERFORMANCE

Shandell Pahlen,[1] Michael Stallings,[2] Robin Corley,[3] Sally Wadsworth,[3] and Chandra Reynolds,[4], *1. University of California, University of California, California, United States, 2. University of Colorado, Boulder, Boulder, Colorado, United States, 3. University of Colorado Boulder, Boulder, Colorado, United States, 4. University of California Riverside, Riverside, California, United States*

Tobacco use represents a pernicious lifestyle factor that may influence processes of aging, including cognitive functioning. As individuals tend to start smoking before adulthood, it may serve as an important factor in cognitive development and maintenance. We explored smoking history-cognition associations in a sample approaching midlife. Study data was derived from the Colorado Adoption/Twin Study of Lifespan behavioral development and cognitive aging (CATSLife 1; N = 1195 [53% F]; $\bar{x}$age = 33.2 years, SD = 5.0). All cognitive measures were t-scored covering working memory, spatial reasoning, processing speed (WAIS-III Digit Span, Block Design, and Digit Symbol, and Colorado Perceptual Speed) and episodic memory domains (Picture Memory, immediate and delayed). Tobacco use measures included ever-smokers, current-smokers, and log-transformed packyears. Mixed-effects regression models were applied, accounting for sex, age, race, ethnicity, and clustering among siblings. Tobacco use was associated with worse episodic memory, spatial and speed performance, but not working memory. When educational attainment was included, patterns remained consistent though attenuated. Results suggested current-smokers scored 0.27 to 0.36 SD lower than non-smokers on speed and spatial reasoning tasks. Episodic memory performance was reduced by approximately 0.07 to 0.1 SD per log packyear. In a sample approaching midlife, the harmful impacts of tobacco use on cognitive performance may be already apparent with cumulative impacts of packyears on episodic memory and current smoking associated with spatial and speed performance. This work helps to elucidate the temporal associations of an important lifestyle factor that may influence cognitive functioning prior to midlife.

### COVID-19 RELATED MEDIA CONSUMPTION AND MENTAL HEALTH IN OLDER ADULTS

Erta Cenko,[1] Christopher Kaufmann,[2] and Todd Manini,[1], *1. University of Florida, Gainesville, Florida, United States, 2. University of Florida, San Diego, California, United States*

At the beginning of the COVID-19 pandemic, consuming media was critical to identify precautionary behaviors to reduce the spread of the virus, particularly for older adults. Media consumption leads to heightened awareness, but may also negatively affect mental health. We examined whether non-social and social media consumption impacted anxiety and depression relative to pre-COVID-19 symptoms. We conducted an anonymous, cross-sectional survey in May and June 2020. Participants (n=1,168, 73.2 years, 56.8% women, 94.9% White), were asked to estimate their amount of time spent consuming pandemic-related media each day, and to report on anxiety and depressive symptoms both before and after the pandemic onset. We characterized change in anxiety and depression by subtracting scores on current anxiety and depressive symptoms from their recalled symptoms prior to the pandemic. Respondents with high pandemic-related media consumption (>3hrs) were more likely to have increased anxiety, compared to those with low (<1hr) media consumption (OR:1.57, 95%CI:1.09-2.23). Similarly, respondents with increased social media consumption during the pandemic were 64% more likely to have depression, compared to those who did not use social media. This association was bi-directional— those who reduced their social media use were 45% less likely to have depression and 26% less likely to have anxiety, compared to those who never used social media. Older adults consuming more pandemic-related media had increased anxiety. Increased social media consumption was associated with elevated depression symptoms. The potential benefits of media consumption about the COVID-19 pandemic may have unintended negative consequences on mental health.

### COVID-19 TELECONFERENCING EXPERIENCES OF CAREGIVERS & SPOUSES WITH DEMENTIA FROM QA/QI SUPPORT GROUP STUDY

Roscoe Nicholson,[1] Maureen O'Connor,[2] Andrew Nguyen,[3] Rebecca Salant,[4] Tiffany Donley,[5]

Steven Shirk,[6] Cynthia Epstein-Smith,[7] and Mary Mittelman,[8], *1. University of Chicago, Evanston, Illinois, United States, 2. VA Bedford Healthcare System, Bedford, Massachusetts, United States, 3. Boston University School of Medicine, Boston, Massachusetts, United States, 4. NYU School of Medicine, NYU School of Medicine New York City, New York, United States, 5. NYU School of Medicine, New York, New York, United States, 6. Bedford Veterans Administration Medical Center, Bedford, Massachusetts, United States, 7. NYU Langone Health, New York, New York, United States, 8. NYU Langone HealthNYU Grossman School of Medicine, NYU Grossman School of Medicine, New York, United States*

In summer 2020, researchers conducted a Quality Assurance and Quality Improvement (QA/QI) assessment of the NYU Langone Alzheimer's Disease and Related Dementias Family Support Program's adaptations in response to COVID by interviewing 10 participating spouse caregivers of persons with dementia (PWD). The primary adaptations were shifting from in-person to online services, changing support groups from biweekly to weekly, and offering an arts-based group for PWD daily rather than weekly . In the course of these interviews, all respondents described their adaptation to remote teleconferencing programming, and five also contrasted their experiences with those of the PWD.

**Methods:** After transcription and de-identification, a codebook was created from the transcript content that included a priori topics of interest as well as emergent themes using framework analysis. These transcripts were then coded by two independent coders through an iterative process and consultation with the codebook creator, who also resolved any discrepancies between coders.

**Results:** Respondents reported largely successful transitions to teleconferencing for themselves, though missing the physical contact afforded by meeting in-person. However, they also described some interactional challenges related to participants talking over one another, and suggested more active moderating to facilitate greater turn-taking. The respondents' descriptions of the PWD's response suggested a much less successful transition to teleconferencing. Challenges and barriers included lack of interest, difficulty following or participating in conversation, and teleconferencing creating confusion, such making it "hard for her to separate out when everybody's in the same place or not."

### CREATION OF A LATIN-AMERICAN DEMENTIA ADVANCE CARE PLANNING GUIDE

Lauren Solkowski,[1] Kara Dassel,[2] Nancy Aruscavage,[2] Ana Sanchez-Birkhead,[1] Katherine Supiano,[1] Rebecca Utz,[2] and Eli Iacob,[2], *1. University of Utah, University of Utah, Utah, United States, 2. University of Utah, Salt Lake City, Utah, United States*

The LEAD Guide (Life-Planning in Early Alzheimer's and Dementia) is an advance care planning conversation guide for use within the context of dementia (Dassel et al., 2019). Considering that Latino adults have the highest risk of ADRD, a culturally sensitive and translated Spanish version of the LEAD Guide was needed. Therefore, the objective of this study was to: 1) translate the LEAD Guide into a Latin-American Spanish version (i.e., LA LEAD) and 2) assess the applicability and acceptability of the LA LEAD through focus groups with Latino older adults. First, the LEAD Guide was translated into a "neutral" Spanish version. Second, forward and backward translation was conducted to create the LA LEAD. Third, two 1.5-hour focus groups with Spanish-speaking Latino adults age 50+ who were: a) healthy adults (N=7) or b) current or previous dementia caregivers (N=7) were held. The focus groups were recorded, translated, and transcribed for descriptive analysis, which revealed three domains regarding the LA LEAD: 1) Family Dynamics: the guide could help prevent family conflict, designate a health care proxy, and reduce burden; 2) Cultural Expectations: acknowledgement of cultural nuances between LA countries, the familial responsibility of caring for family in the home, and the influence of religion on end-of-life care decisions, and 3) Health Literacy: lack of knowledge about advance care planning conversations, documentation, and dissemination. This process resulted in the creation of a validated LA LEAD Guide, which is a culturally and linguistically appropriate and acceptable advance care planning tool for Latino older adults.

### DEMENTIA-RELATED CARE IN ACUTE CARE SETTINGS: A QUALITATIVE META-SYNTHESIS OF PATIENT AND FAMILY PERSPECTIVES

Richard Chunga, *University of Massachusetts Boston, Kew Gardens, New York, United States*

Researchers have long emphasized the importance of a person-centered approach to health care, especially regarding the treatment of individuals living with dementia. However, the fast pace of acute care settings can be a difficult place to provide such care to patients, where there are tensions between the emphasis on efficient treatment of acute medical co-morbidities and person-centered dementia care. This paper is a meta-synthesis of qualitative studies examining perspectives of patients and their family members regarding their acute care experiences. It takes an interpretive approach, using primarily inductive reasoning to generate themes across available studies' findings. Emergent themes are organized under two major dimensions of the hospital environment: the physical environment, including sensory and tangible elements, and the social environment, including the hospital atmosphere and communication practices. Persons with dementia feel overly stimulated by the busy physical environment of the hospital, yet they are often left to languish alone, sometimes even physically restrained. Patients reported feeling lonely, fearful, and confused, identifying diverse physical and social environmental attributes like physical clutter, noise, and lack of empathy from care providers as contributors. Based on acute care experiences and reports from patients and family members, although the acute condition is treated, persons with dementia often leave the acute care environment in worse functional condition than when they entered. Given the increasing prevalence of persons with dementia in acute care settings, it is clear that we must prioritize innovations and programs aimed at improving hospital practices, educating staff, and creating more dementia-friendly environmental designs.

### DEVELOPING BEHAVIORAL MARKERS OF SOCIAL FUNCTION IN AGING: A METHODOLOGY USING PITCH MIMICRY

Christine Williams,[1] Emmanuelle Tognoli,[1] Alice Wead,[2] Christopher Beetle,[1] and Joseph McKinley,[1], *1. Florida Atlantic University, Boca Raton, Florida, United States, 2. Florida Atlantic University, BOCA RATON, Florida, United States*

The Covid pandemic brought to the forefront the crucial role of social interactions for society at large and in gerontological practice. Social interactions play a paramount role in preserving cognitive reserve in older adults. They rely on neurobehavioral processes that are complex (engage large parts of the brain and demand integrity of multiple perceptuomotor, attentional, cognitive and memory functions). Pitch mimicry is a well-known and spontaneously arising social phenomenon that requires the integrity of numerous processes of the brain, and we hypothesize that it constitutes a potentially sensitive behavioral marker of neurodegeneration in Alzheimer's Disease and Related Dementias (ADRD). We developed and validated a series of algorithms to parse verbal exchanges between people and quantify the level of mimicry that each exhibit with their partners. Those algorithms are based on silence thresholding, carefully parametrized CEPSTRAL algorithms for automatic pitch estimation and Synchrosqueezing Transform for validation. We introduce a theoretical model to compare our estimates of pitch mimicry with model's expectations based on the null hypothesis that its neurobehavioral pathways retain their integrity. Our method will allow researchers to study the evolution of pitch mimicry in aging individuals and its sensitivity to diverse social contexts, including those preserving lasting social engagement. Our method will also allow us to test the hypothesis that Pitch Mimicry is a sensitive behavioral marker of dementia, a condition characterized by a breakdown in social relatedness.

### DEVELOPMENTAL ANALYSIS OF PROSPECTIVE EFFECTS OF PROBLEM DRINKING AND HEALTH PROBLEMS IN THREE-AGE GROUPS

Thomas Kwan,[1] Douglas Bowlby,[2] and Matthew Lee,[3], *1. Rutgers University, Manalapan, New Jersey, United States, 2. Rutgers University, Rutgers University, New Jersey, United States, 3. Rutgers University, Piscataway, New Jersey, United States*

Past research has clearly demonstrated interrelations between drinking and health. However, little research has investigated this from a lifespan-development perspective, which is the objective of the current study. Our hypotheses predicted results consistent with the familiar "J-shaped curve" of drinking effects on health, including that health problems would be (1) lower in moderate drinkers than abstainers and (2) higher in excessive drinkers than moderate drinkers. We also hypothesized that these protective effects of moderate drinking would increase with age across the lifespan. The current study used two waves of data from a large U.S.-representative sample. Analyses used 3*3 between-persons ANCOVAs that tested a three-level Wave-1 drinking-group factor and a three-level Wave-1 age-group factor. Of particular importance were the drinking-group-by-age interactions. Various Wave-2 health outcomes were predicted in different ANCOVAs, and each ANCOVA controlled for Wave-1 levels of the Wave-2 health outcome. Across nearly all health outcomes, young adults did not show significant differences between abstainers and moderate drinkers, whereas midlife and older adults consistently showed better health for moderate drinking versus abstainers. This suggests that protective effects of moderate drinking apply more-so to midlife and older adults than young adults. Surprisingly, excessive drinkers generally did not show poorer health than moderate drinkers, except for mixed evidence for such effects only among older adults. Thus, only older adults showed patterns entirely consistent with our hypothesized "J-shaped curve." A next analytic step we will conduct in advance of this poster presentation will assess if alternative excessive-drinking operationalizations more consistently signal health problems.

### DIETARY PATTERNS OF US CHINESE OLDER ADULTS

Alexander Dong,[1] Dana Dychtwald,[1] Lisa Lanza,[2] Qun Le,[1] and Stephanie Bergren,[1], *1. Rutgers University, New Brunswick, New Jersey, United States, 2. Rutgers University, Rutgers Institute for Health, New Jersey, United States*

A westernized diet, characterized by intake of foods high in fat and sugar, has been associated with several chronic conditions. However, the dietary pattern of US Chinese older adults is not well understood. The primary objective of this study was to determine the relationship between years of US residence and other demographic factors and the intake of foods high in fats and sugar. As part of the Population Study of Chinese Elderly, participants were given a 48-item food frequency questionnaire, which were further placed into primary food groups. Each group was then categorized into whether they consumed the food group at least once a week. The total sample was 59% female with an average age of 75, with 49% consuming fatty foods and 85% consuming sweets in the past week. Using logistic regression (N=3053), each additional year of US residence (range of 0-93 years) was associated with a higher dietary intake of fats (OR: 1.01 (95%CI:1.01-1.02)) and sweets (OR: 1.01 (95%CI:1.00-1.02)). Additionally, higher education was associated with lower consumption of fats (OR: 0.98 (95%CI:0.96, 0.99)) and higher income was associated with higher consumption of fats (OR: 1.11 (95%CI: 1.04, 1.18)). For sweets, women compared to men were 54% less likely to consume sweets in the last week (OR: 0.46 (95%CI:0.36, 0.59)), and higher education was associated a greater likelihood of consuming sweets (OR: 1.07 (95%CI: 1.05, 1.10)). Study findings suggest that immigration related factors and demographic factors may influence consumption of a westernized diet high in fats and sugars.

### DISCRIMINATION AND SLEEP IN CHINESE AMERICAN OLDER ADULTS

Alexander Dong,[1] Dana Dychtwald,[1] Stephanie Bergren,[1] Qun Le,[1] and Lisa Lanza,[2], *1. Rutgers University, New Brunswick, New Jersey, United States, 2. Rutgers University, Rutgers Institute for Health, New Jersey, United States*

Since the start of COVID-19, reports of discrimination in the US against Asian Americans have increased approximately 150%. Prior research has demonstrated that victims of discrimination are more likely to experience physiological health concerns, possibly linked to sleep. The objective of this study was to determine if there was a relationship between disordered sleep and discrimination among Chinese older adults using data collected from the Population Study of ChINese Elderly (N=3124, 59% female). To assess, the experience of discrimination in nine settings (school, hiring, work, housing, medical, service, finance, public, and authority) and four indicators of sleep quality (duration, trouble falling asleep, insomnia, and self-reported sleep quality) were evaluated using logistic- and multinomial logistic regression. With an average age of 75 years, discrimination was experienced by 7.2% of participants. Experiencing any discrimination was associated with lower odds of longer sleep durations (>8 hours) compared to those sleeping 6-8 hours. Experiences of discrimination in housing (OR: 5.51 (95%CI:1.08-27.98)) and with authority figures (OR: 6.02 (95%CI:1.16-31.31)) were significantly associated with shorter sleep durations (<6 hours), compared to those sleeping 6-8 hours. Those who experienced discrimination in a school setting were less likely to have trouble falling asleep (OR: 0.28 (95%CI:0.09-0.88)), while discrimination in medical settings were more likely to experience insomnia (OR: 2.29 (95%CI:1.13-4.63)). All other relationships between discrimination and sleep measures were non-significant. Given mixed evidence and the increased relevancy of discrimination against Asian Americans, further research on how discrimination may impact health outcomes and sleep quality is warranted.

### DRAG EXPRESSION AMONG OLDER GAY MEN: EXPLORATORY FINDINGS OF A PILOT STUDY

Brian Chapman,[1] Laura Donorfio,[2] and Debra Tomasino,[3], *1. University of Connecticut, WATERBURY, Connecticut, United States, 2. University of Connecticut, Waterbury, Connecticut, United States, 3. UConn, Mansfield, Connecticut, United States*

This poster presentation highlights pilot findings of how older gay male drag queens define drag expression and its associated positive and negative attributes. While drag has become more mainstream, little is known about this sexual and gender minority (SGM) and what it means to be an older drag queen and how it interfaces with societal gender expectations. (Knutson, Koch, Sneed, Lee, & Chung, 2020; O'Brien, 2018). Research to date reports that while sexual minority youth experience bullying, anxiety, lower self-esteem, and suicidal ideation at higher rates than their heterosexual and gender conforming peers, research has not examined the role drag expression plays as a healthy coping mechanism and, in particular, what role it may play for older drag queens. (Levasseur, Kelvin, Rosskopf, 2013; Mueller, James, Abrutyn, & Levin, 2015). Several studies have found that familial support and connectedness offers valuable protective factors for sexual minority youth in their sexual identity development, but again, little is known about the benefits this may provide older drag queens (Brandon-Friedman & Kim, 2016; Eisenberg & Resnick, 2006). Utilizing Grounded Theory, in-depth interviews were conducted with gay males over the age of fifty (n=5) who identified as drag queens to understand how drag expression is integrated into one's persona, how it may serve as a healthy coping mechanism, and how it interfaces with dragism, generativity, and family relationships over their lifespan (Donorfio, 2020). In addition to sharing the qualitative findings, demographic and data measures of personality, coping, resilience, and mood are also be reported.

### EARLY CLINICAL UTILITY DATA OF A BLOOD BIOMARKER TEST IN THE EVALUATION OF MILD COGNITIVE IMPAIRMENT

Mark Monane,[1] Joy Snider,[2] Jonathan Drake,[3] Tim West,[4] Ilana Fogelman,[5] Joel Braunstein,[5] David Holtzman,[2] and Randall Bateman,[2], *1. Monane Group Clinical Consulting, Monane Group Clinical Consulting, New Jersey, United States, 2. Washington University, Washington University, Missouri, United States, 3. The Warren Alpert Medical School of Brown University, The Warren Alpert Medical School of Brown University, Rhode Island, United States, 4. C 2. N, St. Louis, Missouri, United States, 5. C2N Diagnostics, St. Louis, Missouri, United States*

There is an important unmet need for timely, noninvasive, low-burden evaluation of patients presenting with mild cognitive impairment (MCI). The PrecivityAD™ blood test quantifies plasma concentrations of amyloid-β 42 and 40 and apolipoprotein E-specific peptides that are combined with age in an algorithm to identify the likelihood of amyloid plaques in the brain as measured by amyloid PET scans. This test has previously demonstrated 92% sensitivity and 77% specificity. The study objective was to evaluate the clinical utility of the test and the Amyloid Probability Score (APS), the test output value. Eighteen patients (median age 72, 55% male, 88% white) with MCI received the PrecivityAD blood-based biomarker test. The mean APS was 31 (range 0-91): 13 patients had low scores (APS 0-35), 2 had high scores (58-100) and 3 had intermediate (inconclusive) scores (APS 36-57). The mean probability of Alzheimer's disease (AD) diagnosis was rated by clinicians as 63% (range 25-80) pre-test and 31% post-test (range 5-95) ($p < 0.0005$). AD probability rated by clinicians was decreased in 12/13 low APS patients and increased in 2/2 high APS patients. Donepezil was discontinued in 5/8 low APS patients on therapy and initiated in 1/1 high APS patients not on therapy, representing a change in treatment management in 33% (6/18) of study patients. In summary, this blood-based biomarker test showed clinical utility in its association with physician decision-making around diagnostic certainty and drug therapy management in MCI patients.

### EFFECTIVENESS OF HOME- AND COMMUNITY-BASED SERVICES IN DECREASING HEALTH CARE SERVICE AND EXPENDITURE IN TAIWAN

Ya-Mei Chen,[1] Shih-Cyuan Wu,[1] Shiau-Fang Chao,[2] Kuan-Ming Chen,[3] Chen-Wei Hsiang,[4] Ming-Jen Lin,[1] Ji-Lung Hsieh,[5] and Kuan-Ju Tseng,[4], *1. National Taiwan University, Taipei, Taipei, Taiwan (Republic of China), 2. National Taiwan University, Taipei City, Taiwan (Republic of China), 3. National Bureau of Economic Research, CAMBRIDGE, Massachusetts, United States, 4. National Taiwan University, National Taiwan University, Taipei, Taiwan (Republic of China), 5. Graduate Institute of Journalism, Taipei City, Taipei, Taiwan (Republic of China)*

Background Whether long-term care service use decreases older adults' health care service use and cost has been a strong interest among aging countries, including Taiwan. The current study examined the impact of continuous use of HCBS offered by Taiwan's LTC plan 2.0 on older adults' health service utilization and cost overtime. Methods This study used the LTC Plan 2.0 database and the National Health Insurance Plan claim dataset, and included 151,548 clients who had applied for and were evaluated for LTC services for the first time from 2017 through 2019 and continuously used any LTC Plan 2.0 services for six months. Outcome variables were users' health service utilization and health care cost 12 months before and after starting to continuously use HCBS. Latent class analysis and generalized estimating equations were used to investigate the influences of different service use patterns on the changes in physical functions. Results Three subgroups of LTC recipients with different use patterns, including home-based personal care (home-based PC) services (n = 107324, 70.8%), professional care services (n = 30466, 20.1%), and community care services (n = 13794, 9.1%) were identified. When compared to care recipients in the community care group, those in the home-based PC group had more emergency room expenditures (1 point/month, p< 0.05) but less hospitalization expenditures (38 points/month, p<0.001), while the professional care group had less emergency room and hospitalization expenditures (3 and 138 points/month, p< 0.001). Conclusion Those receiving professional care and home care services spent less on health care service utilization.

### EFFECTS OF GLYCINE SUPPLEMENTATION ON MITOCHONDRIAL FUNCTION AND PROTEIN DEGRADATION IN SKELETAL MUSCLE OF OLD MICE

Giulia Lizzo,[1] Kamila Muller,[1] Jonathan Thevenet,[1] Stefan Christen,[1] Kim Zarse,[2] Michael Ristow,[2] Nabil Bosco,[1] and Philipp Gut,[1], *1. Nestlé Research, Lausanne, Vaud, Switzerland, 2. ETHZ, Zurich, Zurich, Switzerland*

Glycine is the simplest amino acid and it has a pivotal role in different metabolic processes, such as being a building block of glutathione, collagen and purine bases, or taking part in methylation reactions, detoxication and ammonia metabolism. Although considered for many years a non-essential amino acid, glycine levels are decreased in certain conditions, as the endogenous synthesis cannot fulfill the needs required to sustain all the cellular processes in which glycine is involved. Here we describe that glycine levels are significantly lower in skeletal muscle of aged zebrafish and mice and in plasma of humans compared to young subjects. We therefore fed healthy old mice for 6 weeks with a glycine-supplemented diet and observed a significant restoration of glycine levels in skeletal muscle and liver towards young mouse levels. Moreover, old mice showed decreased mitochondrial function in glycolytic and oxidative fibers, and a significant increase in oxygen consumption was observed in glycolytic fibers after glycine supplementation. The improvement of mitochondrial function is not associated to an increased mitochondrial biogenesis or an increased antioxidant capacity, but glycine supplementation increases both total GSH and GSSG levels, suggestive of a pro-oxidant environment. Overall, glycine supplementation induced an increase in the cross-sectional area of fibers. Finally, we carried out RNA-Seq study to decipher the impact of higher glycine intake. Our results suggest that age-associated glycine deficiency plays an important role in atrophy of muscle, especially in glycolytic fibers, and is reversible with a dietary supplementation.

### EFFECTS OF RELATIONSHIP TYPE ON QUALITY OF LIFE IN OLDER ADULTS WITH COGNITIVE IMPAIRMENT AND THE INFORMAL CAREGIVERS

Aiping Lai,[1] Julie Richardson,[2] Lauren Griffith,[1] Ayse Kuspinar,[1] and Jenna Smith-Turchyn,[1], *1. McMaster University, Hamilton, Ontario, Canada, 2. McMaster University, McMaster University, Ontario, Canada*

**Purpose:** The purpose of this study was to assess whether there was an association between care-recipient relationship type and the QoL of older adults and their informal caregivers, and whether this association pertained to older adults' cognitive function.

**Methods:** This was a secondary data analysis. Older adults (n=1230) and their informal caregivers (n=1871) were identified from participants in the National Health and Aging Trends Study (NHATS) Round 5 and the National Study of Caregiving (NSOC) II. A series of bivariate and multivariable regression models examined the associations among the care-recipient relationship type and QoL in older adults and their informal caregivers, adjusted for socio-demographic variables as well as cognitive functioning.

**Results:** Both older adults and caregivers' QoL outcomes varied by the type of relationship. Recipients cared for by adult-child caregivers or multiple caregivers experienced higher functional limitation than those cared by spousal caregivers ($\beta$=.79, CI [.39, 1.19]; $\beta$=.50, CI [.17, .82], respectively). "Other" caregivers, such as siblings, friends, etc., had lower odds of experiencing negative emotional burden than spousal caregivers (OR=.26, CI [.13, .52]; OR=.53, CI [.35, .81], respectively). "Other" caregivers were also 51% less likely to experience social strain than spousal caregivers. Lower odds of experiencing negative emotional burdens were also found with multiple caregivers. The association between adult-child caregivers and social strain was explained by the recipients' cognitive function.

**Conclusions:** Care-recipient relationship type impacts the QoL in both recipients and their informal caregivers. This association appears to be affected by care recipients' cognitive function level.

### EGG CONSUMPTION AND 4-YEAR CHANGE IN COGNITIVE FUNCTION: THE RANCHO BERNARDO STUDY

Donna Kritz-Silverstein, and Ricki Bettencourt, *University of California San Diego, La Jolla, California, United States*

The role of dietary cholesterol in cognitive decline is unclear. Eggs are a rich source of nutrients and dietary cholesterol. This study examines the association of egg consumption with 4-year change in cognitive function in 890 older, community-dwelling adults. Participants were 357 men and 533 women aged □55y (means=70.1□8.4 and 71.5□8.8, respectively, p=0.016), from the Rancho Bernardo Cohort who attended a 1988-91 clinic visit. Egg intake was obtained with a food frequency questionnaire. Cognitive function was assessed with the Mini-Mental Status Exam (MMSE), Trails B and category fluency, and reassessed in 1992-96. In this sample, rates of egg consumption ranged from never (14.0% of men, 16.5% of women) to □5/week (7.0% of men, 3.8% of women; p=0.0013). Mean 1988-91 cognitive function scores for men vs. women were 27.5 vs. 27.7 on the MMSE (p=0.08), 105.9 vs. 121.6 on Trails B (p<0.0001), and 20.2 vs. 18.2 on category fluency (p<0.0001). Sex-specific regression analyses examined associations of egg consumption with change in cognitive function. In women, after adjustment for age and education, egg intake was associated with less decline over time in category fluency (beta=-.10, p=0.01), which remained significant after adjustment for smoking, alcohol, exercise, cholesterol, calorie intake, and protein intake (p=0.02). No other associations were found in women, and no associations were observed in men before and after adjustment for covariates. Results suggest that while high in dietary cholesterol, egg consumption is not associated with decline in cognitive function. For women, there may be a small beneficial effect for verbal memory.

### ERECTILE FUNCTION, SEXUAL SATISFACTION, AND COGNITIVE DECLINE IN MEN FROM MIDLIFE TO OLD AGE

Riki Slayday,[1] Tyler Bell,[2] Michael Lyons,[3] William Kremen,[2] and Carol Franz,[2], *1. The Pennsylvania State University, State College, Pennsylvania, United States, 2. University of California San Diego, La Jolla, California, United States, 3. Boston University, Boston, Massachusetts, United States*

We investigated how changes in erectile function and sexual satisfaction relate to cognitive decline in men from midlife into early old age. This is a major transitional period for increased incidence of erectile function and for cognitive decline. We examined 833 men from the Vietnam Era Twin Study of Aging whose mean ages were 56, 61, and 68 at the time of assessment. Erectile function and sexual satisfaction were measured using scores from the International Index of Erectile Function. Individuals with erectile dysfunction at baseline were excluded. Cognitive performance was measured using factor scores for separate domains of episodic memory, executive function, and processing speed. We tested linear mixed models hierarchically adjusted for demographics, sexual activity, as well as physical and mental health confounders to examine how changes in erectile function and sexual satisfaction related to changes in cognitive function. Declines in erectile function were associated with declines in episodic memory (p=.004, d=.25), while declines in sexual satisfaction were associated with declines in processing speed (p=.006, d=.19). Decreasing erectile function and sexual satisfaction may be indicative of individuals also likely to be facing cognitive decline. Possible mechanisms accounting for these changes may include white matter microvascular disease and/or various lifestyle influences. Discussing and tracking sexual health with middle aged men may be a crucial step in identifying those likely to face cognitive decline.

### EXAMINING THE IMPACT OF COVID-19 ON LONELINESS AND SOCIAL ISOLATION AMONG AFFORDABLE HOUSING RESIDENTS

Marilyn Mock,[1] Roisin Goebelbecker,[1] Sherry Pomerantz,[2] Jennifer DeGennaro,[2] and Elyse Perweiler,[2], *1. Fair Share Support Services, Inc./Fair Share Housing Development, Camden, New Jersey, United States, 2. Rowan School of Osteopathic Medicine, Stratford, New Jersey, United States*

Loneliness and social isolation are serious public health concerns associated with higher risks of clinical depression, suicidal ideation, coronary artery disease, stroke, functional decline, an increased risk of developing dementia and cancer mortality. Recent reports indicate the prevalence and dangers of loneliness and social isolation have increased as a result of the COVID-19 pandemic, especially among older populations. In order to address these concerns among residents living at Northgate II (NGII), a 302-unit affordable housing development in Camden, NJ, Fair Share Support Services, Inc. (FSSS), the non-profit arm of Fair Share Housing Development, collaborated with the New Jersey Institute for Successful Aging (NJISA) and the DHHS-funded Geriatric Workforce Enhancement Program (GWEP) to develop a loneliness/social isolation survey using two evidenced-based tools, the UCLA Loneliness Scale and the Steptoe Social Isolation Index. FSSS piloted the loneliness and social isolation survey with 192 low-income minority older adults residing at NGII. Results indicate that 49% of the NGII residents surveyed fall into 5 "at-risk" categories: 1) lonely and isolated (9%), 2) lonely/somewhat isolated (8%), 3 ) lonely/not isolated (9%), 4) isolated/somewhat lonely (9%), and 5) isolated/not lonely (14%). FSSS, will utilize survey results and follow-up interviews to tailor social service/other interventions to meet the needs and preferences of residents with the goal of preventing serious health problems associated with loneliness and social isolation, allowing residents to age in place.

### EXAMINING THE IMPACT OF INDIVIDUAL AND SHARED BIOLOGICAL RISKS ON HEALTH AMONG OLDER MARRIED COUPLES

Tai-Te Su,[1] Shannon Meija,[2] and Richard Gonzalez,[3], *1. University of Illinois at Urbana-Champaign, Champaign, Illinois, United States, 2. University of Illinois, Champaign, Illinois, United States, 3. University of Michigan, Ann Arbor, Michigan, United States*

Relationship research has suggested that health among spouses is interdependent and should be considered jointly. Using data from the 2008/2010 and 2016/2018 waves of the Health and Retirement Study (3858 qualified couples; age=67.0±9.6), we investigated the joint influence of married partners' individual and shared cumulative biological risk on future health outcomes. Two risk indicators were constructed to indicate biological health in different domains. Individual grip strength, walk speed, lung function, and cystatin-C were biomarkers selected to construct frailty risk whereas blood pressure, pulse, waist circumference, C-reactive protein, glycohemoglobin, high-density lipoprotein cholesterol, and total cholesterol were biomarkers used to construct cardiometabolic risk. Shared risk was calculated as the number of risks the partners shared. We employed multilevel Poisson regression models to nest partners within couples and examine the effects of individual and shared cumulative risks on future functional limitations. Heckman correction was performed to correct potential selection bias. Our unadjusted models showed individual (frailty: b=0.22, p<.001; cardiometabolic: b=0.10, p<.001) and shared (frailty: b=0.17, p<.001; cardiometabolic: b=0.08, p<.01) risks are associated with greater future functional limitations. Further, shared cardiometabolic risk moderated the effect of individual risk (b=-0.01, p<.05). In the adjusted models, the direct associations between shared risks and future functional limitations were explained by indicators of partner selection and shared experiences. In the fully adjusted model, the cross-level interaction for frailty risk became statistically significant. The unique set of dynamics shown in our study offered new insights into understanding how couples influence one another in the context of multisystem biological health.

### EXAMINING THE RELATIONSHIP BETWEEN HOSPICE AGENCY CHARACTERISTICS AND COMPLAINT DEFICIENCIES

Xiaochuan Wang, Susanny Beltran, Denise Gammonley, Norma Conner, and Milo Leon, *University of Central Florida, Orlando, Florida, United States*

The U.S. hospice industry has expanded over the last decade. Similar to nursing homes, research guided by the Donabedian framework has documented quality differences in hospice based on agency characteristics, including profit status and rural status. Yet, compared to nursing homes, quality oversight and transparency in hospice remain limited. When families report substandard care, a complaint survey is launched to investigate allegations. Using publicly available regulatory oversight data (e.g., CMS QCOR, Medicare PACPUF, CAHPS HIS, Hospice Compare), and guided by the Donabedian framework, this study describes hospice agency structure and process characteristics associated with care complaint deficiencies (outcome). Of the 4,415 hospice facilities examined, 453 (or 10.3%) have had complaint survey deficiency citations between January 2018 and December 2020. Chi-square and ANOVA tests were conducted to compare facility characteristics (e.g., ownership status, percentage of Medicare beneficiaries in rural zips), nursing and social work involvement, and CAHPS scores between hospices with and without complaint survey deficiencies. Results indicated that the average proportion of beneficiaries with a rural zip for Medicare correspondence was significantly lower in hospices with deficiencies (p<.001). Finding also suggested that weekly total nursing and social work minutes were significantly higher in hospices with deficiencies. Additionally, family ratings of hospice team communication, symptom management, and overall satisfaction were higher in facilities without complaint survey deficiencies. Future research and practice implications will be discussed.

### EXPERIENCES OF RURAL AND URBAN ASSISTED LIVING COMMUNITIES IN OREGON DURING COVID-19 PANDEMIC

Ozcan Tunalilar,[1] Jason Kyler-Yano,[1] Sheryl Elliott,[1] Sarah Dys,[2] and Paula Carder,[3], *1. Portland State University, Portland, Oregon, United States, 2. Vital Research, LLC, Portland, Oregon, United States, 3. OHSU-PSU School of Public Health, Portland, Oregon, United States*

This study presents findings on the impact of the COVID-19 pandemic as reported by a representative sample of Oregon assisted living communities (AL) between December 2020 and March 2021. Of the 559 AL eligible to participate, 346 completed eleven questions related to their experiences since March 2020. These questions covered topics such as access to personal protective equipment (PPE) and accurate information, communication with and support from government agencies, ability to find staff and new residents, ability to address pandemic-related concerns of residents' families and staff, use of virtual visits and telehealth for residents, and visitor restrictions. Response categories ranged from 0 (strongly disagree) to 4 (strongly agree) and we coded "agree" and "strongly agree" responses as having experienced that issue. Among responding AL, 42% were located in rural or frontier areas. We present three findings. First, most AL experienced adverse impact due to COVID-19, especially regarding issues likely to be outside of their control compared to those within their control. Second, while almost all urban-based AL reported that their residents used virtual communication technologies and tools for telemedicine/telehealth (96%) or virtual social visits (96%), rural AL were less likely to report so (90% and 92%, respectively). Finally, rural AL experienced significantly greater staffing difficulties (75%) compared to their urban counterparts (82%). In sum, while all AL would benefit from better regulatory guidance on policies and access to PPE, rural AL might especially benefit from additional, context-specific resources.

### FACTORS ASSOCIATED WITH ATTACHMENT AND CARE DECISIONS

Kimberly Cassie, *University of Oklahoma, Tulsa, Oklahoma, United States*

It is widely accepted that remaining in the community for as long as possible is preferable to placement in a care facility. For many, this can only be realized with the support of a family caregiver. Previous research on the relationship between attachment and caregiving decisions is sparse, but tends to suggest there is a relationship between attachment and the decision to assume caregiving responsibilities, but more information is needed to better understand this unique relationship. This exploratory research seeks to address gaps in our understanding by asking is attachment related to the decision to care for a parent and what factors are associated with attachment. A convenience sample of 128 individuals caring for older parents was surveyed to answer these questions. Results indicate lower attachment related avoidance was associated with greater odds of caring for a recipient in the community rather than placing the recipient in a care facility. No relationship between attachment related anxiety and placement decisions was observed. Additionally, greater levels of attachment related avoidance were observed among caregivers reporting lower levels of filial responsibility, more adverse childhood experiences, less perceived support, and greater financial stability. Findings from this study can be used to support the development of interventions to strengthen attachment between adult children and their parents before care decisions are necessary.

### FACTORS DRIVING HIGH-NEED HIGH-RISK VULNERABLE VETERANS USE OF OUTPATIENT HEALTHCARE

Ali Vaeli Zadeh,[1] Fei Tang,[2] Carlos Gomez,[1] Luci Leykum,[3] Orna Intrator,[4] Bruce Kinosian,[5] Willy Marcos Valencia,[6] and Stuti Dang,[7], *1. Miami VA Healthcare System, Miami, Florida, United States, 2. Miami Veteran Affairs Healthcare System, Miami,*

*Florida, United States, 3. South Texas Veterans Health Care System, San Antonio, Texas, United States, 4. Canandaigua VA Medical Center, Canandaigua, New York, United States, 5. Philadelphia VAMC, Philadelphia, Pennsylvania, United States, 6. Medical University of South Carolina, Miami, Florida, United States, 7. University of Miami, Miami, Florida, United States*

Using predictive analytic modeling, the Veterans Affairs (VA) Geriatrics and Extended Care Data Analysis Center (GECDAC) identified vulnerable “High-Need High-Risk” (HNHR) Veterans, as requiring more support and services. We sought to identify variables linked with utilization of our outpatient HNHR C4 clinic offering Comprehensive Geriatric Assessment, Care Planning, Care Coordination, and Co-management". Of 724 HNHR Veterans contacted, 531 were reached and invited to participate; 193 were not reached, 326 were reached but declined the C4 clinic, 205 attended the clinic. We compared these groups. Independent variables were organized using Anderson’s behavioral model into predisposing (age, gender, race, ethnicity), enabling (drive time, service eligibility, Area Deprivation Index, marital status), and need factors (mental health cognitive condition, ambulatory care sensitive conditions, NOSOS, JFI, CAN, etc.). C4 enrollment acceptance was the outcome. Results showed that compared to patients who declined, HNHR veterans who attended C4 clinic had more chronic health conditions($p<0.01$), more service eligibility($p=0.01$), more driving time to the closest VA clinic($p=0.01$), and more were married ($p=0.01$). Patients who declined C4 clinic might have greater barriers to care access. Accessing needed healthcare among HNHR older adults maybe impacted more by enabling factors that allow the individual to seek care if needed and are the resources that may facilitate access to services, rather than need factors, which include individuals' perceptions of their health and functional state, and healthcare needs assessed by professionals. More social and intermediary determinants of health should be incorporated as enabling factors into models striving to understand drivers of healthcare use.

### FAMILY CAREGIVER HEALTH IN A PANDEMIC

Kimberly Cassie, *University of Oklahoma, Tulsa, Oklahoma, United States*

Each year family caregivers provide care and services worth billions of dollars to support the needs of older Americans. Their support is invaluable to keep individuals in the community for as long as possible and to allow individuals to attain and maintain their highest practicable level of well-being. But what impact does caregiving have on one’s health? Does caregiver health decline with the assumption of caregiving duties? Did caregiver health change during the pandemic? If so, how and what factors are associated with declines in caregiver health? To answer these questions, an exploratory survey was conducted among a convenience sample of 195 family caregiver. Almost a third of those sampled reported excellent or very good health, while 44% reported good health, and almost a quarter reported poor or fair health. Forty-eight percent reported their health had declined since they assumed caregiving duties and 29% reported their health had declined during the pandemic. Employed caregivers and those experiencing less depression/anxiety reported better health. Those experiencing a decline in health with caregiving were more likely to be female, not employed, experienced more stress and more depression/anxiety. Those experiencing a decline in health during the pandemic reported less spirituality, greater attachment related avoidance, and greater depression/anxiety. Findings from this research can be used to inform future research on the effect of the pandemic on family caregiving and to plan interventions to protect caregiver health as they provide vital services to maintain individuals in the community for as long as possible.

### FEASIBILITY AND ACCEPTABILITY OF THE TECHNOLOGY-BASED FALL RISK ASSESSMENTS FOR OLDER ADULTS

Ladda Thiamwong,[1] Oscar Garcia,[2] Renoa Choudhury,[2] Joon-Hyuk Park,[2] Jeffrey Stout,[2] and Rui Xie,[2], *1. College of Nursing, University of Central Florida, Orlando, Florida, United States, 2. University of Central Florida, Orlando, Florida, United States*

Promising technologies, which are simple, portable, quick, non-invasive, and inexpensive, may open new horizons on fall risk assessments and provide important information for older adults. We used a mixed-methods approach to examine the feasibility and acceptability of technology-based fall risk assessments, including the BTrackS Balance System, Bioelectrical Impedance Analysis, and activity monitoring devices among older adults. Data were collected via a Qualtrics survey and interviews. The acceptability was measured by the Senior Technology Acceptance (STA) and semi-structured interviews with 15 participants. The STA consists of four domains with 14 items, and the semi-structured interview includes three main questions related to experiences about balance performance tests, body composition, and activity monitoring. One hundred twenty-four community-dwelling older adults completed the online survey, and 15 older adults participated in the interviews. The majority of participants were female, and 72% had no history of falls. Race and ethnicity were 17% Hispanic, 7% African Americans, and 3% Asian Americans. About 7% had COVID-19 positive, 31% reported fear of COVID, and 14.5% were afraid of losing their life to COVID. The word-of-mouth strategy and key person approach were used and had an incredible impact on the recruitment process. None of the participants had ever had their fall risk and fear of falling assessed before agreeing to participate in this study. The technology-based fall risk assessments were feasible and acceptable. About 78% of participants liked the idea of using technology to assess falls risk, and 79% agreed that using technology would enhance their effectiveness in daily activities.

### FEASIBILITY AND ACCEPTABILITY OF VIDEOCHAT DURING MEALS IN ADULTS AGING IN PLACE OF AVERAGE AGE 88

Alexa Lyman, and Laura Barre, *Cornell University, Ithaca, New York, United States*

Older adults in the United States prefer to age-in-place. However, living and eating alone are risk factors for malnutrition. Using videochat during mealtimes, i.e., VideoDining, can provide commensality and social facilitation to improve nutritional intake. The objective of this study was to determine if older adults aging-in-place can independently VideoDine with family or friends. We recruited eleven older adults from Full Circle America Steuben, a virtual assisted living program for adults aging-in-place in rural New York. All participants had Amazon EchoShow devices for videochat. Participants were instructed on VideoDining and asked to independently schedule four VideoDine sessions with a family/friend in four weeks. Surveys were collected at baseline, after VideoDine sessions, and end-of-study. Participants were 91% female and 100% white. The average age was 88 years. All participants were widowed and living alone except for one married couple. Overall, 45% of participants VideoDined four times, 36% of participants VideoDined two to three times, and 27% not at all, for an average of 2.7 sessions in a month. Participants VideoDined during all meals, although dinner was most common (66% of meals), and breakfast least common (12% of meals). Average comfort was rated 7.6/10 (1=not comfortable, 10=comfortable), median enjoyment was 9.3/10 (1=not enjoyable, 10=enjoyable), and median ease of VideoDining was 4.1/5 (1=very difficult, 5=very easy). On average, participants rated their VideoDining meal experience a 7.6/10 (1=poor, 10=excellent). With access to videochat technology, older adults can connect with a dining partner and have a favorable experience sharing a meal over videochat.

### FEASIBILITY OF IN-HOME SALIVA COLLECTION OF CORTISOL AND DHEA-S AS A BIOMARKER OF STRESS IN DEMENTIA CARE DYADS

Azita Emami,[1] Gabriella Engström,[2] and Hyejin Kim,[3], *1. University of Washington School of Nursing, University of Washington, Washington, United States, 2. Dalarna University, Falun, Dalarnas Lan, Sweden, 3. University of Washington School of Nursing, Seattle, Washington, United States*

Dementia afflicts affected individuals and their family caregivers worldwide. Although a non-pharmacological intervention has been recommended as a first-line approach to minimize adverse outcomes (e.g., stress) in dementia care dyads (persons with dementia [PWD] and their family caregivers), most evaluations of such interventions have relied on subjective (e.g., self- or proxy-report) rather than objective (e.g., biomarkers) measures. We aimed to explore the feasibility of saliva collection of cortisol and dehydroepiandrosterone sulfate (DHEA-S) as a non-intrusive method in dementia care dyads. Dementia care dyads living at home were recruited from the memory center in Sweden. Prior to the saliva collection, participants received a one-hour education session with a hands-on demonstration led by a trained study coordinator. Participants were instructed to collect saliva three times (two for morning, one for evening)/day, five days/week for eight consecutive weeks. Out of 32 care dyads (32 PWD and 32 family caregivers), 24 (75.0%) completed the saliva collection. On average, 105.5 (87.92%) and 105.9 (88.25%) samples were collected from PWD and family caregivers during eight weeks. There were no statistically significant differences (p>0.05) in the average number of saliva samples (i.e., total samples, morning or evening samples) between PWD and family caregivers. The findings of this pilot study showed that saliva collection of cortisol and DHEA-S as a stress measurement was feasible in dementia care dyads living at home. Robust and person-centered procedures, tailored educational materials, and effective communication with dementia care dyads should be considered in future biomarker research on stress in dementia care dyads.

### FINANCIAL IMPACTS OF THE COVID-19 PANDEMIC ON U.S. OLDER ADULTS: ASSESSING PANDEMIC-INDUCED JOB AND INCOME LOSS

Walter Dawson,[1] Nora Mattek,[2] Sarah Gothard,[1] and Jeffrey Kaye,[1], *1. Oregon Health & Science University, Portland, Oregon, United States, 2. Oregon Health and Science University, Portland, Oregon, United States*

The COVID-19 pandemic has greatly impacted the economic security of millions of older adults. Job loss and reductions in personal income were significant in 2020 stemming from pandemic-induced shutdowns that temporarily closed large swaths of the U.S. economy. Yet, the specific financial impacts of the pandemic on older adults, including family care partners, are not well understood. To understand the COVID-19 pandemic's effects on the health and financial well-being of older adults, we gathered data from the Research via Internet of Technology and Experience (RITE) Study, a longitudinal survey panel providing data from thousands of participants of various ages and backgrounds in the U.S. on their use of healthcare and technology (N=1,365). We measured by population strata including age, sex, and education and other characteristics including caregiver status. Adults between 20-40 years of age experienced the highest rate of job loss and reduction in wages (33%) as a result of the pandemic, while adults aged >70 years experienced the lowest rate (12.5%). However, adults aged 50-60 and 60-70 also experienced relatively high levels of job loss at (28.4% and 25.7%, respectively). Behavior changes and disruptions to typical routines to avoid COVID-19 infections may have contributed to job and personal income loss amongst Individuals aged 50-60 and 60-70. However, these findings suggest potentially high levels of economic insecurity amongst individuals who continue to work into late-life. These results may help policymakers understand how to better tailor interventions and policies to mitigate economic insecurity, particularly for populations disproportionately impacted by the pandemic.

### FOCUS GROUP FINDINGS OF TRIAL PARTICIPANTS IN A CAREGIVER PSYCHOSOCIAL INTERVENTION: SAVVY CAREGIVER PROGRAM

Maria Aranda, Iris Aguilar, and Rene Maldonado, *University of Southern California, Los Angeles, California, United States*

Previous work highlights the importance of sociobehavioral interventions to address dementia caregiving health and wellbeing outcomes. By empowering caregivers to become objective managers of their loved one's illness, and own self-care, they are less likely to exhibit negative outcomes. We are conducting a mixed-method, randomized trial to test manualized, multi-family psychoeducational group interventions: Savvy Caregiver Express, and Savvy Caregiver Program. This poster describes the qualitative findings of four focus group interviews recently conducted to elucidate the study participation experiences of family caregivers enrolled in the parent study. Twenty-five racially and ethnically diverse participants (21 women, 4 men) caring for a family member with cognitive decline participated in focus group interviews conducted via videoconferencing methods. We captured more nuanced experiences from the perspective of study participants with regards to the caregiver interventions and their research participation. Interviews were conducted by trained research personnel, lasted 60-75 minutes, and followed an open-ended questioning route. Based on thematic analyses, we identified the following themes: 1) Changing one's mindset: Seeing life through their shoes; 2) Getting information in one place; 3) Expanding the personal experience; 4) Fears and vulnerability; 5) Time constraints vs. wanting more; 6) Not everyone is at the same place; 7) Technology: It's going to be part of our lives; and 8) Research: Not always in sync. Our findings indicate high satisfaction with most components of the program while specific recommendations were offered to improve the intervention and study experience such as tailoring materials to stage-specific needs.

### GENOMIC SEQUENCING OF SOD1D YEAST THAT ESCAPE SPORE DEATH

Temiloluwa Adanlawo,[1] and Helen Vander Wende,[2], *1. Howard University, Washington, District of Columbia, United States, 2. University of California Berkeley, Berkeley, California, United States*

Amyotrophic lateral sclerosis (ALS) is a devastating neurodegenerative disease that impacts nerve cells and the spinal cord, which in some cases are linked to mutations in the Superoxide Dismutase 1 (SOD1) gene. Sod1 is an antioxidant within cells that converts reactive oxygen from superoxide into water using a copper and zinc ion to deactivate the oxygen. When the SOD1 gene is deleted, yeast cells are still able to undergo meiotic divisions and generate four spores, but the spores that are produced are inviable. However, we see that randomly, sod1Δ spores can grow on rich media. This leads us to hypothesize that somewhere in the genome, there is a suppressor mutation that allows these cells to grow. We tested this hypothesis by preparing samples for whole genome sequencing. By comparing the genomic sequences from our suppressor mutants to wild-type controls, we're able to identify a single point mutation within a gene called NCA2, which codes for a protein that regulates expression of Fo-F1 ATP synthase subunits 6 and 8. Given this result, we are now working to try and understand the relationship between the sod1Δ spore death phenotype and the modulation of ATP synthase activity. In summary, the results from our work have the potential to further help us understand what role Sod1 plays in yeast meiosis and may be able to give us a deeper understanding for ALS cases that are linked to Sod1.

### GOALS OF CARE CONVERSATIONS IN NURSING HOME AND ASSISTED LIVING CARE PLAN MEETINGS

Gail Towsley,[1] Djin Tay,[2] and Linda Edelman,[1], *1. University of Utah College of Nursing, Salt Lake City, Utah, United States, 2. University of Utah, Salt Lake City, Utah, United States*

Me & My Wishes is a novel systematic approach for long-term care residents living with dementia to record videos about their care preferences that can be shared with staff and families in care plan meetings. To understand how the videos were utilized in Goals of Care (GOC) conversations, we coded and analyzed transcripts of recorded care plan meetings at the time of sharing the video using a priori codes derived from GOC conversation elements. Coding discrepancies were resolved in team meetings; finalized codes were summarized to derive themes. Thirty-four care plan meeting conversations between residents (n=34), family members (n=29) and staff (n=35) were analyzed. Residents appreciated sharing personal histories and preferences via video, while staff members appreciated deeper understanding of residents' care preferences. Two themes described care plan meeting conversations: Everyday Care - a checklist-style assessment of the resident's daily care (e.g., help with activities of daily living), activities engaged in and satisfaction with care; and Clarifying Care Goals - checking the resident's treatment preference (e.g., pain management, CPR), explaining hospice, or confirming the resident's contact person. Several elements of GOC were not discussed (e.g., disease progression) and conversations lacked depth and comfort evidenced by apologetic language and abrupt transitions of topics rather than exploring alignment of goals with care preferences. Me & My Wishes videos are a mechanism for residents to voice preferences. Standardized guidance, which is lacking in long-term care, is needed to help care teams engage in meaningful conversations to ensure alignment of goals and treatment preferences.

### HEALTH EQUITY IMPLICATIONS OF COVID-19 ON DEMENTIA CARE IN COMMUNITY FOR PEOPLE OF AFRICAN DESCENT

Mary Lukindo,[1] Barbara Hamilton-Hinch,[1] OmiSoore Dryden,[1] and Katie Aubrecht,[2], *1. Dalhousie University, Dalhousie University, Nova Scotia, Canada, 2. St. Francis Xavier University, St. Francis Xavier University, Nova Scotia, Canada*

This presentation shares results from an analysis of interviews conducted to understand the health equity implications of COVID-19 responses on dementia care in the community for people of African descent in Nova Scotia, Canada. Interviews were embedded within a larger multi-method rapid research project that aimed to assess the impact of COVID-19 on dementia care for geographically and socially marginalized groups in the province. Data from the interviews was analyzed using a constructivist thematic analysis method, guided by an intersectional theoretical scaffold. Three themes were identified related to systemic barriers, mental health, system navigation and self-care, and collected under the overarching theme of 'facing the unknown with dementia'. Results emphasized the value and notable absence of community driven, culturally specific dementia programs, resources and navigators for people living with dementia, family caregivers and care workers of African descent. Participants identified lack of health system and care provider knowledge and understanding of the cultures and histories of people of African descent as a barrier to quality care and to addressing dementia-related stigma. Conversations focused on the need for practical and accessible tools, strategies and perspectives responsive to the actually lived realities and needs of people in community, and for research that actually contributes to individual and collective life in tangible, timely and culturally meaningful ways. Recommendations focus on the importance of centering community in dementia care programs, policy, practice and research to improve services and supports for people of African descent.

### HOPE AS A MOTIVATOR FOR HEALTHY BEHAVIORS IN OLDER ADULTS: FINDINGS FROM A CROSS-SECTIONAL SURVEY

Mushira Khan,[1] Patrick Hill,[2] Catherine O'Brien,[3] and Dugan O'Connor,[3], *1. University of Victoria, Plainfield, Illinois, United States, 2. Washington University in St. Louis, St. Louis, Missouri, United States, 3. Mather, Evanston, Illinois, United States*

Hope can be understood as a motivational state that enables people to move toward their goals. Yet, how hope may act as a motivator for healthy behaviors in older adults is not well-understood. Further, the extant literature utilizes varied conceptualizations of hope, and a better understanding of the constructs that underlie the relationship between hope and health behaviors is needed. This study examined the relationship between hope and health behaviors, explored how this relationship may differ across different socio-demographic groups, and considered how hope relates to perceived future selves among older adults. Community-dwelling adults 55 years and older (n = 711; mean age 67.38 years; 280 men, 431 women) completed an online, cross-sectional survey. Survey measures included, along with the Adult Hope Scale (AHS) and the Herth Hope Index (HHI), a health behaviors checklist, self-reported health, and a future self scale. We found a moderately strong positive correlation between hope and healthy behaviors in older adults (AHS $r = 0.46$, $p < .01$; HHI $r = 0.50$, $p < .01$). Participants with higher levels of hope also reported more positive future selves and better health. The associations were similar across different racial/ethnic groups and the magnitude of this effect held even after controlling for gender, education, marital status, and income. Of the two hope scales, we recommend the AHS measure given its relative parsimony, greater use in the field, and the fact that the associations were fairly similar to the HHI with respect to health and health behavior.

### HOSPITAL, EMERGENCY DEPARTMENT, AND HOME HEALTH USE IN OLDER ADULTS WITH SENSORY IMPAIRMENT

Laura Wallace,[1] Karen Hirschman,[1] Mary Naylor,[1] Liming Huang,[2] and Pamela Cacchione,[3], *1. University of Pennsylvania, Philadelphia, Pennsylvania, United States, 2. University of Pennsylvania, University of Pennsylvania, Pennsylvania, United States, 3. University of Pennsylvania, University of Pennsylvania School of Nursing, Pennsylvania, United States*

Hearing, vision, and dual (combined hearing and vision) sensory impairments (HI, VI, and DSI) are common in older adults and associated with adverse health outcomes. However, it is not clear how sensory impairments impact healthcare utilization in older adults. This study aims to examine hospital, emergency department (ED), and home health care use amongst adults 65 and older diagnosed with HI, VI, and DSI in an urban academic health system. This secondary analysis (N=45,000) used a limited data subset of older adult primary care patients' EHR data from a parent study examining medical complexity, healthcare use, and social vulnerability. Using logistic regression and controlling for participant demographics and comorbidities, results show HI, VI, and DSI increase the likelihood of having an ED visit (OR 1.29, $p<.0001$; OR 1.28, $p=0.0011$; OR 1.50, $p=.0328$, respectively) and a home health episode (hearing OR 1.41, $p<.0001$; vision OR 1.42, $p=.0002$) compared to those without sensory impairment (SI). No significant difference was found in hospital use and home health use for DSI. This is the first known study to examine ED use for older adults with VI and DSI, and home health use for older adults with SI in the US. Findings suggest older adults with SI have greater utilization and dependence on healthcare services. Older adults with SI may benefit from outpatient assessments and interventions to mitigate risks of ED use. Findings also support research into the drivers of healthcare use amongst this population, financial implications, and intervention development to prevent avoidable healthcare use.

### HOSPITAL-INDUCED DELIRIUM AMONG MEDICAL OLDER ADULTS: EVALUATING THE VERACITY OF PROGNOSTIC MODELS

Urszula Snigurska,[1] Ragnhildur Bjarnadottir,[1] and Robert Lucero,[2], *1. University of Florida College of Nursing, Gainesville, Florida, United States, 2. University of California Los Angeles, Los Angeles, California, United States*

Several prognostic models have been developed and validated for delirium prediction among older adults. However, model development and validation studies need to be evaluated for risk of bias to establish the veracity of the prognostic models. This is a critical step before they can be implemented in clinical practice. Multiple systematic reviews have evaluated prognostic models of hospital-induced delirium. However, none of the existing systematic reviews evaluated the validity of models for non-surgical, medical hospitalized older adults. We conducted a scoping review to evaluate the validity of existing prognostic models of hospital-induced delirium in medical older adults. CINAHL, PsycINFO, PubMed, and Web of Science were searched for original studies. The database search yielded 4,312 records. Five studies were included in the qualitative synthesis. All the studies claimed to have developed valid prognostic models. However, the risk of bias assessment revealed that existing prognostic models of hospital-induced delirium in medical older adults are at a high risk of bias. Collectively, the statistical analysis was the greatest source of bias. Notably, while we have seen a proliferation of prognostic models for use in the surgical older adult population, efforts at developing prognostic models in the medical older adult population seem to have declined since the early 1990s. Newer methods of data collection, such as data mining of electronic health records, and statistical analysis, such as machine learning, have shown promise in accurate prediction of hospital-induced delirium while overcoming many challenges associated with manual data collection and traditional statistical analyses.

### HOW CAN WE ENCOURAGE OLDER ADULTS TO ADOPT DIGITAL SERVICES?

Yutong Xie,[1] Jiayi Wu,[1] and W.Quin Yow,[2], *1. River Valley High School, Singapore, Not Applicable, Singapore, 2. Singapore University of Technology & Design, Singapore, Not Applicable, Singapore*

Majority of older adults (OA) do not use technology frequently. For example, 84% of Singapore OA own a smartphone, however, only 22% have tried apps like online shopping. Similarly, 42% of US OA own a smartphone, yet one-third of them have never used the internet. Past research indicated poorly designed user interfaces as a barrier to technology use. Based on the Technology Acceptance Model (TAM), we hypothesize that OA-friendly user interfaces can increase perceived usefulness (PU) and perceived ease of use (PEOU) of technology, which would translate into increased digital services adoption rate. Forty healthy participants aged 65 to 86 (6 males, 34 females, mean age = 72.3) were recruited through OA activity centers in Singapore and assigned to either control or experimental groups. Both groups used an online website to perform specific shopping tasks and answered a TAM questionnaire. The control group used a website modeled after current websites while the experimental group used an OA-friendly website designed based on literature review and pre-survey, with features like OA-friendly mode, tutorial and online helpdesk. Data analysis was conducted using an independent sample t-test and bootstrapping. The experimental group showed a significantly higher proficiency in performing tasks (p=0.037), PEOU (p=0.049), and PU (p=0.044) than the control group. This suggests that user interfaces friendlier to OA increases their acceptance of technology, which could translate into a higher digital service adoption rate. Therefore, this research sets the basis for further research on digital services guidelines for older adults, including for telehealth and others.

### IMPACT OF RESTRICTED CONTACT BETWEEN GRANDPARENTS AND GRANDCHILDREN DURING THE COVID-19 PANDEMIC

Verena Klusmann, Sara Cengiz, Carina Materna, and Christian Spreckels, *University of Hamburg, University of Hamburg, Hamburg, Germany*

To combat the COVID-19 pandemic strict contact restrictions have been imposed on institutions for both older and younger people, social structures have been locked down, families have been urged to reduce contact with older relatives, and people over 65 have been temporarily banned from their workplaces and from attending events, both in public and private spaces. These measures are assumed to have a number of psychosocial consequences. For this questionnaire study, 268 pupils (7-to-10 years-old) of nine different schools in Hamburg, Germany, with different social index were asked about how they experienced, perceived, and behaved during the COVID-19 pandemic. 75% of the children reported on restricted contact to their grandparents: While 41% did not meet their grandparents at least for a certain time at the beginning of the pandemic, 34% did not meet their grandparents during the whole first year of the pandemic. Of those who met their grandparents, 25% kept physical distance to them. These contact restrictions were significantly higher in schools with a lower social index, chi2(8)=15.49, p=.05. Those children who never met their grandparents also reported on higher perceived stress, t(220)=-2.37, p=.019, d=-.33, tended to have lower subjective well-being, t(223)=-1.73, p=.09, d=-.24, and had higher risk perceptions concerning COVID-19 infections, t(223)=-2.18, p=.03, d=-.31. Hence social isolation and loneliness is not only an issue for older people themselves, but contact restrictions also potentially increase the stress load and impair the well-being of children who have to do without support and care of their grandparents in sensitive developmental phases.

### INFLAMMATORY BIOMARKERS AND SEX HORMONES INTERACT TO PREDICT ECOLOGICALLY-ASSESSED COGNITIVE PERFORMANCE

Erik Knight,[1] Erin Harrington,[2] Martin Sliwinski,[3] Jennifer Graham-Engeland,[4] Jelena Pavlovic,[5] and Christopher Engeland,[4], *1. University of Colorado Boulder, Boulder, Colorado, United States, 2. Pennsylvania State University, University Park, Pennsylvania, United States, 3. The Pennsylvania State University, University Park, Pennsylvania, United States, 4. Penn State University, University Park, Pennsylvania, United States, 5. Albert Einstein College of Medicine, Bronx, New York, United States*

Inflammatory biomarkers and sex hormones have been investigated as independent risk and resilience factors for cognitive decline in older adults. Many sex hormones are anti-inflammatory and there is emerging evidence that sex hormones may buffer the risk for cognitive decline associated with higher inflammation. However, few studies have included concurrent examination of inflammation and sex hormones in studies of cognitive performance and cognitive aging. A diverse sample of older adults (N = 245; 65% female, 42% Black, 13% Hispanic; mean age = 76.8 years) had blood drawn before and after a two-week measurement burst that included three cognitive tests (6x per day) assessing working spatial memory, perceptual speed, and feature binding. Testosterone, estradiol, estrone, and six basal cytokine concentrations were quantified. Composite scores of basal inflammation were calculated. Multilevel modeling indicated that heightened inflammation related to poorer spatial working memory performance (B = 0.213, 95%CI[0.11, 0.414], p = .040). In addition, sex hormones moderated the association of cytokine concentration with perceptual speed (e.g., basal cytokines x testosterone: B = 0.13, [-0.24, -0.03], p = 0.013; similar effects evident for estrogens). Decomposition these interactions revealed that heightened inflammation predicted poorer performance, but only among individuals with lower sex-hormone concentrations. This study provides evidence of immune and hormonal-by-immune associations with performance in two cognitive domains in older adults. Examining the functional crosstalk between immune and sex hormone functioning will improve understanding of risk and resilience factors related to cognitive performance and help predict cognitive decline in older adults.

### IN-HOME ONLINE MUSIC THERAPY FOR PSYCHOLOGICAL HEALTH AMONG CAREGIVERS OF PERSONS WITH DEMENTIA: A PILOT STUDY

Hyejin Kim,[1] Gabriella Engström,[2] Töres Theorell,[3] and Azita Emami,[4], *1. University of Washington School of*

*Nursing, Seattle, Washington, United States, 2. Dalarna University, Falun, Dalarnas Lan, Sweden, 3. Karolinska Institutet, Stockholm, Stockholms Lan, Sweden, 4. University of Washington School of Nursing, University of Washington, Washington, United States*

Family caregivers who provide care to persons with dementia (PWD) in a home setting may feel greater stress, depressive symptoms, or struggle to deal with issues in their lives. Indeed, PWD often require 24-hour care that includes ensuring safety, providing emotional support, and assistance with activities of daily living and multiple instrumental activities of daily living. This study examined the short-term effects of an online music intervention on stress, coping, and depression among caregivers of PWD. We included 35 caregivers (n=24 [intervention group], n=11 [comparison group]) living at home with their family member with dementia. Mann-Whitney U test and Wilcoxon signed-ranked test were performed to examine between- and within-group differences from baseline to eight weeks after the intervention. The coping subscale yielded a significant difference between the groups at post-test (U=76.50, Z= -1.978, p=0.048), indicating the intervention group had better coping than the comparison group at post-test. Significant within-group differences in overall stress (Z= -2.200, p= 0.028) and coping subscale (Z= -1.997, p=0.046) in the comparison group at post-test suggest that overall stress and coping were maintained from baseline to post-test in the intervention group, whereas the comparison group had significantly higher overall stress and lower coping at post-test. This home-based online music program showed potential benefits for caregivers of PWD by taking into account participants' musical preferences and ease of access to the program. A randomized controlled trial with a larger sample size and objective measurements of stress and depressive symptoms (e.g., biomarkers) should be conducted in the future.

### INTAKE OF FLAVONOIDS AND ODDS OF FRAILTY ONSET IN ADULTS IN THE FRAMINGHAM OFFSPRING COHORT

Thuy Nga Nguyen,[1] Courtney Millar,[2] Douglas Kiel,[3] Marian Hannan,[2] and Shivani Sahni,[2], *1. University of Arizona, Tucson, Arizona, United States, 2. Hebrew SeniorLife, Roslindale, Massachusetts, United States, 3. Hebrew SeniorLife, Hebrew SeniorLife, Massachusetts, United States*

Polyphenols (antioxidants derived from plant-foods) could play a role in inhibition of oxidative stress and frailty reduction, yet data on the polyphenol subclass of dietary flavonoids is limited. This study sought to determine the association between dietary flavonoids and frailty onset in middle-aged and older adults. This prospective cohort study included non-frail individuals from the Framingham Offspring Cohort (FOC) with total flavonoid intake (mg/day; defined as sum flavonols, flavan-3-ols, flavonones, flavones, and anthocyanins via Harvard Food Frequency Questionnaire), frailty (via Fried phenotype), and covariate information measured at baseline (1998-2001). Follow-up frailty was evaluated in 2011-2014. Logistic regression estimated odds ratio (OR) and 95% confidence intervals (95% CI) adjusting for relevant confounders. Participants (n=1,701; 55.5% female) had a mean age of 58.4 years (SD ± 8.3). Mean flavonoid intake was 309 mg/d (SD ± 266). After 12.4 years (SD ± 0.8), 224 (13.2%) individuals exhibited frailty. In age and sex adjusted models, every 50 mg/day of higher total flavonoid intake was associated with 3% reduced odds of frailty [OR (95%CI): 0.97 (0.94-1.00), p-value: 0.05). Further adjustment for smoking, energy and protein intake, and disease indicators did not appreciably change the association, and associations became non-significant (p-value=0.12). Thus, there was no association between flavonoid intake and odds of frailty onset in adults in the FOC. This could be due to participants' higher intake of flavonoids compared to average intake of ~200 mg/d in Americans.

### INTERGENERATIONAL CONNECTIONS TO IMPROVE SOCIAL WELL-BEING OF OLDER ADULTS WITH ADRD: A RESEARCH PROTOCOL

Ling Xu,[1] Noelle Fields,[1] Kathryn Daniel,[1] Brooke Troutman,[2] and Daisha Cipher,[3], *1. University of Texas at Arlington, Arlington, Texas, United States, 2. USAF Academy, USAF Academy, Colorado, 5United States, 3. UTA, Arlington, Texas, United States*

There is growing concern about social isolation, loneliness, and diminished emotional well-being among persons with ADRD who live in the community. Research suggests that reminiscence strategies, especially with the production of a digital story book, combined with an intergenerational approach may yield significant benefits for older adults. Reminiscence approaches are typically implemented by trained professionals. However, the use of trained volunteers is of growing interest due to the costs associated with reminiscence programs. This research protocol describes the development and testing of an intergenerational, telephone-based reminiscence program offered by trained, young adult volunteers that includes the use of digital storytelling (DST). Specifically, the study addresses three aims: 1) Quantitatively test the effectiveness in improving social and emotional well-being of older adults with ADRD; 2) Quantitatively test attitudes towards aging and knowledge and awareness of ADRD disease among the young adults; and (3) Qualitatively evaluate the usefulness of this intervention from perspectives of the dyads. A randomized control trial is used to assess the effects of the intervention using an explanatory sequential mixed methods design. The older and younger participants (n = 92 dyads) are randomly assigned to intervention (reminiscence) or sham (social visit) group, and then randomly matched. Data are collected at baseline, mid-intervention, post- intervention, 3 months-post intervention and through qualitative interviews (selected dyads). The proposed evaluation activities will allow us to determine the applicability of intergenerational reminiscence with DST as an effective intervention for supporting the social and emotional needs of persons with ADRD.

### IS "CULTURE" NEEDED IN ABORIGINAL ELDER CARE STATION? AN STUDY FROM THE PERSPECTIVE OF SERVICE PROVIDERS

Li-Chuan Liu, *National Taitung University, Taitung, Taitung, Taiwan (Republic of China)*

Many studies show that cultural perspective is an important factor in caring for elderly tribal adults. To understand the level of attention that the Cultural Health Station of Indigenous People attaches to culture during its operation, this study selected Taitung County as the region of study. A qualitative focus group research method and quantitative questionnaire, we try to understand "What are the demands of elderly tribal adults?" "Do services provided by the Tribal Cultural Health Station satisfy the demands of elderly tribal adults?" and "What are the gaps between the service demands and provided to elderly tribal adults?" The results showed that service providers believe that culture is markedly important to elderly tribal adults, that culture-based care designs offered by the Tribal Cultural Health Station is currently insufficient, and that to enhance the capacity of the multiethnic Tribal Cultural Health Station, the cognition and understanding of policy makers and enforcers must be elevated.

### LARGE SCALE SURVEY RESEARCH WITH OLDER ADULTS/PERSONS WITH DISABILITIES IN A PUBLIC HEALTH CRISIS

Sarah Dys,[1] Hannah Huebner,[2]
Norma Carrillo-Van Tongeren,[1] Courtney Sirk,[1]
Harold Urman,[1] and Cathy Coddington,[1], *1. Vital Research, LLC, Los Angeles, California, United States, 2. Vital Research, LLC, Vital Research, California, United States*

Best practice for measuring quality improvement and consumer satisfaction of health and human services for older adults and people with disabilities relies on in-person survey administration. This poster highlights adaptation strategies undertaken across three large-scale evaluation studies of program/service delivery conducted during the COVID-19 pandemic, necessitating a departure from in-person techniques: 1) Integrated Satisfaction Measurement for the Program of All-Inclusive Care for the Elderly (I-SAT-PACE), 2) National Core Indicators-Aging and Disabilities/Intellectual and Developmental Disabilities (NCI-AD/IDD), and 3) Assisted Living Resident Quality of Life (AL-QOL). Data collection for these projects occurred from September 2020 to August 2021, providing an opportunity to showcase project adaptation over the course of the pandemic. Using project implementation examples across 15 states and approximately 10,100 participants, we discuss implications for successful survey coordination, interviewer training, data collection, and participant/stakeholder engagement during a public health emergency. Strategies included pivoting to phone, Zoom, and paper-based data collection and increasing technical assistance for field staff and participants. Project teams were able to increase access to participation by implementing multimodal survey delivery, mitigate coronavirus exposure, continue collecting older adults and people with disabilities' experiences, and compare results based on method of delivery. Technology barriers, field staff dropout, need for larger sample sizes, and inclusion of participants with dementia, hearing, and speech impairments present important tradeoffs to consider. These examples indicate it is possible to administer hybrid data collection methods across populations with varying cognitive and physical abilities without compromising data quality.

### LIKE A DEW DROP ON A LOTUS LEAF: PERCEPTIONS OF AGING WELL IN SOUTH ASIAN AMERICAN OLDER ADULTS

Mushira Khan,[1] Sheetal Shah,[2] and Ajla Basic,[3],
*1. University of Victoria, Plainfield, Illinois, United States, 2. University of California (Davis), Sacramento, California, United States, 3. Mather, Evanston, Illinois, United States*

Past research has underscored four key themes prevalent in popular and scientific discourse on successful aging in North America – the emphasis on individual agency and control; continuing productive activity into old age; the value of independence in late life; and an ideal construction of permanent personhood, wherein the realities of mortality and decline are inadequately addressed (Lamb, 2014). Yet, the meanings attached to successful aging differ across cultures and are not very well-understood. The Perceptions of Aging Well in Diverse Populations study aims to acquire a holistic understanding of the attitudes and beliefs around aging well across cultures and to identify the similarities and differences in these perceptions within diverse racial and ethnic groups. This presentation highlights preliminary findings from in-depth, semi-structured qualitative interviews with South Asian Americans 50 years and older (n=19; 9 men, 10 women). Participants shared that a sense of inevitability and aging with "grace", "dignity", and "wisdom" were key components of successful aging. Maintaining good health, keeping a positive attitude, and remaining independent in later life appeared motivated primarily by a desire to remain connected to, but not necessarily "burden" adult children with caregiving responsibilities. Religious faith and spiritual well-being, availability of support systems, and a sense of community were key facilitators. Limited English proficiency and loneliness posed challenges to aging well, particularly in late-life immigrants. These findings provide unique insights into subjective perceptions of successful aging and may help inform programs and policies that support the health and well-being of older South Asian Americans.

### LONG-TERM CARE AND WIDE EFFECTS ON CHILDREN CAREGIVERS -- NEW EVIDENCE FROM TAIWANESE ADMINISTRATIVE DATA

Kuan-Ming Chen,[1] Chen-Wei Hsiang,[2] Yu-Hsuan Chou,[3]
Shiau-Fang Chao,[4] Kuan-Ju Tseng,[2] Ming-Jen Lin,[3]
Ya-Mei Chen,[3] and Shih-Cyuan Wu,[3], *1. National Bureau of Economic Research, CAMBRIDGE, Massachusetts, United States, 2. National Taiwan University, National Taiwan University, Taipei, Taiwan (Republic of China), 3. National Taiwan University, Taipei, Taipei, Taiwan (Republic of China), 4. National Taiwan University, Taipei City, Taiwan (Republic of China)*

Long-term care (LTC) needs have profound impacts on the care-receivers and their children. Past research has focused on primary caregivers' short-term responses to LTC needs on limited aspects. This study brings new Taiwanese administrative data on health insurance, LTC program usages, and tax records. Rich information allows this study to explore long-term impacts on care-receivers' extended families. Event study combined with various regression analyses is the main framework of this study. Using the longitudinal record of more than 23 million individuals older than 65 over 18 years, the present study examines extended family members' various outcomes along the LTC needs trajectory. Among others, these outcomes include labor market participation and health expenditures. There are several findings in this study. Parents' LTC needs decrease all children's average full-time labor force participation by 2.5 percentage points even 10 years after the needs incurred. These needs do not directly increase children's health expenditure. A precise zero effect is found on children's health expenditure before, during, and after parents' LTC needs. Nevertheless, parents' health status or LTC risks, in general, may still pass on to children through other channels. The inter-generational health association is found to be approximately 0.25, indicating some degree of transmission. The results suggest that the impacts of LTC needs on family members are profound and widespread. Policy needs to address multiple aspects to cater to potential difficulties for care-receivers' family members.

### MEASUREMENT INVARIANCE OF A LATENT DEMENTIA INDEX BY GENDER IN THE AGING, DEMOGRAPHICS, AND MEMORY STUDY

Joseph Saenz,[1] Alice Kim,[2] and Christopher Beam,[2], *1. UNIVERSITY OF SOUTHERN CALIFORNIA, Los Angeles, California, United States, 2. University of Southern California, University of Southern California, California, United States*

Population-based aging studies allow researchers to study dementia and its correlates. Few include dementia diagnoses. Latent variable models have been used to create latent dementia indexes (LDI) using cognitive and functional ability to approximate dementia. The LDI is applied across diverse populations, but it is unclear whether gender affects its measurement properties. We assess whether the LDI can be used to measure dementia equivalently for men and women. We use the 2001-2003 Aging, Demographics, and Memory Study (n=856, 355 men, 501 women). Cognitive ability was assessed using memory, executive function, attention, spatial ability, orientation, and language tasks. Functional ability was informant-reported. We used confirmatory factor analysis to test factorial invariance across gender and compare latent means to determine which group had lower means, consistent with greater dementia likelihood. Model fitting results suggest metric invariance of the LDI but only partial scalar invariance across gender. Latent mean differences in the LDI were observed (Mdiff = .39, SE = 0.19, p = .042), with women lower, on average, than men. Correlations between LDI and dementia diagnosis were stronger for both men (r=-.82) and women (r=-.85) than correlations between dementia and Mini-Mental Status Exam scores (-.69 and -.73, respectively). The LDI may be reliably and validly used to measure and compare dementia likelihood in men and women. Results suggest lower LDI scores in women, indicating greater dementia likelihood. Gender differences may be partially attributed to differences in measurement properties of items, possibly due to gender differences in educational returns and employment factors.

### MEASURING COVID-RELATED AGEISM

Mindy Fain,[1] Amanda Sokan,[2] Jake Harwood,[3] Kathie Insel,[4] Zhao Chen,[5] and Linda Phillips,[6], *1. University of Arizona Center on Aging, Tucson, Arizona, United States, 2. Mel & Enid Zuckerman College of Public Health, University of Arizona, Arizona, United States, 3. University of Arizona, University of Arizona, Arizona, United States, 4. Behavioral and Social Science, Tucson, Arizona, United States, 5. The University of Arizona, Tucson, Arizona, United States, 6. College of Nursing, University of Arizona, Arizona, United States*

Prejudice, discrimination, and negative stereotypes based on age (ageism) are long-standing and strongly implicated in poor health outcomes and limited access to health care for older adults. Recent writings suggest the COVID-19 pandemic raised the specter of ageism to an entirely new level. Do these observations reflect an exaggeration of "usual" ageism or a unique manifestation of intergenerational tension rooted in resentments of younger people concerning COVID-related disruptions in their lives believed to be primarily a function of older people's vulnerability to the disease phenomenon? To address this question, the purpose of this study was to develop and test an instrument to measure ageist tendencies associated with the COVID-19 pandemic. Scale items, written to reflect attitudes about paternalism, inconvenience, and sacrifice, were assessed for content validity. Then the 12-item scale was administered to 227 undergraduate and graduate students in the health and social sciences. Analysis showed items have strong internal consistency and concurrent and discriminant validity. Importantly the scale explained unique variance over and above other standard measures of ageism. Ageism is deeply embedded in global and U.S. culture and strongly related to negative outcomes. This scale will assist researchers investigating the ageist consequences of the current pandemic and help us to monitor what could be long-term residual ageist effects of the COVID pandemic.

### MONTANI SEMPER LIBERI: AGE AND DEPRESSION AMONG VETERANS IN APPALACHIA

Alex Dang,[1] Maxwell Nimako,[2] and Amy Fiske,[3], *1. West Virginia University, West Virginia University, West Virginia, United States, 2. WVU, West Virginia University, West Virginia, United States, 3. West Virginia University, Morgantown, West Virginia, United States*

Depression is higher in rural areas and military veterans (Kimron et al., 2019; Bedard-Gilligan et al., 2018). West Virginia, the only state contained entirely within Appalachia, has a higher percentage of military service among its citizenry than other states. Thus, the purpose of the current study was to examine the association between veteran status and depression among adults in WV. Using 2018 WV data from the CDC's Behavioral Risk Factor Surveillance System, we examined depression as a function of veteran status and age, among 612 younger adults, 1813 middle-aged adults, and 2445 older adults (N = 4,870; 12.4% veterans). Our ANOVA revealed a significant overall effect, $F(5, 4864) = 14.64$, $p < .001$, a main effect for veteran status (18.8% of veterans and 26% of non-veterans reported depression), and an age effect emerged, with more younger (28.6%) and middle-aged adults (30.5%) reporting depression than did older adults (20.3%). No significant interaction between age and veteran status emerged, $F(2, 4864) = 1.75$, $p = .175$. Of note, 25% of the sample reported having depression. Given that place-based mental health disparities exist, this finding is not unexpected. But fewer older adults and fewer veterans reported depression. At least three possibilities warrant further investigation. Future studies should examine whether these age and veteran status differences in depression reflect differences in resilience, differences in reporting, and/or differences in selective survival.

### OLDER ADULTS PERFORMED WORSE ON COGNITIVE ASSESSMENT AT LONELIER MOMENTS: USING AMBULATORY ASSESSMENT APPROACH

Jee eun Kang, Martin Sliwinski, and Karra Harrington, *The Pennsylvania State University, University Park, Pennsylvania, United States*

This study focused on investigating the short-term effect of loneliness on older adults' cognitive performances in daily life. Loneliness is suggested as a risk factor for cognitive health, but results in previous studies are inconsistent due to the lack of valid measures and limited research design. The attention-depletion hypothesis highlights that acute stress could immediately compromise cognitive ability by consuming attentional resources. Accordingly, this study examined whether loneliness, as one of the stressors related to one's social relationship, was immediately associated with worse daily cognitive performances in older adults. Using an ecological momentary assessment approach, 311 community-dwelling older adults (Mage=77.5 (range=70-90), 67% female, 45% white) reported their level of loneliness as well as performed cognitive assessment five times a day for 16 days. Multilevel modeling showed that on occasion when participants reported a higher level of loneliness than normal, they performed worse in the processing speed test ($p<.01$) and the short-term memory binding test ($p<.01$) during those moments, controlling for age, gender, education, ethnicity, IADL, and retest-practice effect. Moreover, those momentary associations between loneliness and cognitive performances remained significant after controlling for the momentary level of feeling depressed. Unlike the concurrent effect, there was no lagged effect of loneliness on daily cognitive performances. These results suggest that transient but intense feelings of loneliness can function as acute stress and thus, compromise daily cognitive functioning short-term. Results will be discussed in terms of the potential benefit of momentary real-time interventions to lessen feelings of loneliness to maintain older adults' cognitive functioning.

### OLDER ADULTS' ACCEPTANCE OF TECHNOLOGY DURING THE PANDEMIC: THE COVID TECHNOLOGY ACCEPTANCE MODEL (TAM)

Megan O'Connell,[1] Kristen Haase,[2] Allison Cammer,[1] Shelley Peacock,[1] Theodore Cosco,[3] and Lorraine Holtslander,[1], *1. University of Saskatchewan, Saskatoon, Saskatchewan, Canada, 2. University of British Columbia, Vancouver, British Columbia, Canada, 3. Simon Fraser University, Vancouver, British Columbia, Canada*

During the pandemic, technology-mediated communication was one of the few ways to maintain social and community connections. We explored how the pandemic impacted older adults' use and appraisal of technology. In a random sample of 407 older adults (M age = 81.1 years; range 65-105 years) almost half (n = 161) reported they changed how they used technology to virtually connect with others during the pandemic, and 78 of these reported that this was new technology for them. We adapted the technology acceptance model (TAM) for the pandemic, the COVID-TAM, and describe how physical distancing led to new acceptance of technology due to an increased perception of usefulness of technology for maintaining community and social connections. The 71 older adults who denied using technology were asked about the reasons underlying their reluctance to use technology to access social networks and community events during the pandemic. Thematic analysis revealed factors consistent with a double-digital divide; lack of physical exposure to technology creates an additional psychological barrier to adoption of new technology. Of the technology-reluctant subgroup of older adults, few reported lack of perceived usefulness of technology during the pandemic. Instead, most reported lack of self-efficacy or fear of technology underlying their lack of technology use for social and community connections during the pandemic, which we incorporate into the COVID-TAM. Findings indicate that technology training can help mitigate this fear and increase social and community connections that are technology-mediated in circumstances where physical distancing is necessary.

### OUTCOMES AND TREATMENT COSTS OF SKILLED NURSING FACILITY PATIENTS WITH PRESSURE INJURIES AND MALNUTRITION

Kirk Kerr,[1] Cory Brunton,[1] and Mary Beth Arensberg,[2], *1. Abbott Nutrition, Columbus, Ohio, United States, 2. Abbott, Columbus, Ohio, United States*

Skilled nursing facilities (SNF) provide care for individuals requiring skilled care while transitioning to a more permanent residence post hospitalization. This analysis shows that diagnosed malnutrition and pressure injuries (PI) adversely impact SNF patients' health and recovery. Length of SNF stay, total charges, and discharge disposition were analyzed using SNF claims from 2016-2020 Centers for Medicare & Medicaid Services (CMS) Standard Analytical File databases. An average of 4.5% SNF patients had diagnosed PIs, and 4.9% had diagnosed malnutrition. Patients with diagnosed malnutrition were more likely to have PIs than patients without diagnosed malnutrition (11.9% vs 4.1%). Patients with PIs had higher charges ($12,304 vs. $10,937), were less likely to be discharged home (11.1% vs 18.9%), and more likely to be discharged to a hospital (15.8% vs 11.0%) or deceased (2.8% vs 1.6%). Patients with diagnosed malnutrition displayed a similar pattern for charges ($11,587 vs $10,969), and discharge to home (14.5% vs 18.8%), hospital (13.5 vs 11.1%) or deceased (2.8% vs 1.6%). Length of SNF stay did not differ between patients with and without PIs (18.5 vs 18.6) and was slightly shorter for patients with diagnosed malnutrition (17.3 vs 18.9). While higher probability of rehospitalization or death could impact these results, drivers behind these differences need further investigation. Because malnourished patients were more likely to have PIs and both PI and malnutrition are associated with poorer patient discharge outcomes and higher costs, efforts to identify malnutrition and implement proper nutrition interventions should be prioritized as part of SNF quality improvement initiatives.

### PAVING THE WAY FOR AGEIST ATTITUDES THROUGH CHILDREN'S BOOKS

Lena-Emilia Schenker, and Jennifer Bellingtier, *Friedrich Schiller University Jena, Jena, Thuringen, Germany*

Older adults are underrepresented and rarely appear in major roles in children's literature. According to developmental intergroup theory, numerically smaller groups are likely to become targets of stereotypes and prejudice. Because parental ageist attitudes are related to those of their children, and parents typically choose their children's literature, we investigated parental preferences for books featuring older and younger adults and what factors might predict this preference. In an online survey, 176 parents of children aged 12 or younger rated children's book covers featuring a child and a prominent younger or older adult. There were two identical versions of each book cover on which only the age of the adult varied. Each respondent viewed covers featuring older and younger adults, but only saw one version of each cover (i.e., counterbalanced design). Parents indicated their preference for the books by stating how much they and their children would like the book and how likely they would be to buy it. Stereotypical expectations regarding the books' storylines were rated on a semantic differential scale (e.g., modern vs. old-fashioned). Results revealed that there were no significant differences in preferences for books featuring younger, compared to older adults. However, a stronger difference in preference for books featuring younger, over older adults was predicted by the extent of stereotypical expectations regarding the storylines. In particular, this preference was stronger in parents who expected stories with older adults to conform to prevailing ageist stereotypes, suggesting that ageist expectations may deter some parents from books featuring older adults.

### PHYSICAL INDICATORS OF AGING ARE RELATED TO CELLULAR SENESCENCE SIGNAL P16INK4A IN MIDLIFE ADULTS

Kelly Rentscher, Teresa Seeman, Steve Cole, and Judith Carroll, *University of California, Los Angeles, Los Angeles, California, United States*

Cellular senescence signal p16INK4a has been identified as a biomarker of aging that accumulates with chronological age across several tissues in mice and humans and may be potentially modifiable by interventions. This study examined whether physical indicators of aging were associated with p16INK4a and other markers of the aging process in midlife adults. Participants were 543 adults aged 26–78 years (Mage=54.0; 50.5% female) in the Midlife in the United States Refresher cohort. Interviews, questionnaires, and performance tests measured physical indicators of aging, including the Fried frailty index, limitations in daily activities, and age-related comorbidities. RNA sequencing of whole blood assessed biomarkers of aging: p16INK4a (CDKN2A), the DNA damage response (DDR), and the senescence-associated secretory phenotype (SASP). Older age was associated with enhanced p16INK4a ($r=.11$, $p=.01$), DDR ($r=.34$, $p<.001$), and SASP ($r=.38$, $p<.001$) expression. Multiple regression models that adjusted for age, sex, race/ethnicity, BMI, comorbidities, and time between assessments revealed that frailty (pre-frail/frail vs. non-frail) was associated with greater p16INK4a ($B=0.13$, $p=.048$) and marginally greater DDR ($B=0.06$, $p=.06$) expression. Limitations in daily activities were also associated with p16INK4a ($B=0.12$, $p=.045$). History of heart disease, stroke, arthritis, and cancer were associated with DDR and SASP expression in unadjusted models only ($ps<.05$). In summary, senescence indicator p16INK4a was elevated in whole blood samples from middle-aged adults who showed signs of frailty and limitations in daily activities. Findings suggest that whole blood p16INK4a expression might potentially be used to detect early signs of aging and target interventions to reduce biological aging and frailty.

### POOR PERFORMANCE IN SNF-VBP PROGRAM IS ASSOCIATED WITH WORSE COVID-19 OUTCOMES IN NURSING HOMES

Jennifer Gaudet Hefele,[1] Matt Aldag,[2] Riad Elmor,[2] Charanya Kaushik,[2] and Jessica Simpson Ballard,[2], *1. Booz Allen Hamilton, Hollis, New Hampshire, United States, 2. Booz Allen Hamilton, Bethesda, Maryland, United States*

Skilled Nursing Facility Value-Based Purchasing (SNF-VBP) was a new Medicare payment program when COVID-19 began. SNF-VBP aims to improve care through payment bonuses and penalties. However, studies have shown that minority-serving nursing homes (NHs) tend to fare worse under SNF-VBP (more likely to receive penalties, less likely to receive bonuses). This study sought to examine whether SNF-VBP performance prior to the pandemic was associated with COVID-19 outcomes and whether associations varied in NHs where the majority of residents are Black/African American (majority-Black/AA). Using publicly available data on COVID-19 outcomes and vaccinations, SNF-VBP performance, and NH characteristics, we found that majority-Black/AA NHs were less likely to have zero infections; had higher case fatality rates; and had lower resident and staff vaccinations rates compared to NHs where the majority of residents are White. Across all NHs, worse SNF-VBP performance was associated with worse COVID-19 outcomes (the bottom quintile of SNF-VBP performers were more likely to experience COVID-19 infections and had lower vaccination rates; the highest performers had higher vaccination rates). However, in stratified analyses, SNF-VBP performance was not significantly associated with COVID-19 outcomes in majority-Black/AA NHs compared with majority-White NHs. The association between poor SNF-VBP performance and poor COVID-19 outcomes is concerning. Overall findings suggest that SNF-VBP performance prior to the pandemic is an important indicator of subsequent COVID-19 outcomes. However, it is unclear whether poor SNF-VBP performance is signaling overall poor quality or whether it is signaling a financial disadvantage caused by the program itself that in turn impacted COVID-19 outcomes.

### PRECARIOUS AGING: A WORKING DEFINITION

Amanda Grenier,[1] Christopher Phillipson,[2] Grace Martin,[3] Abiraa Karalasingam,[3] Karen Kobayashi,[4] Patrik Marier,[5] and Debbie Laliberte-Rudman,[6], *1. University of Toronto and Baycrest Hospital, Toronto, Ontario, Canada, 2. The University of Manchester, Manchester, England, United Kingdom, 3. University of Toronto, Toronto, Ontario, Canada, 4. University of Victoria, Victoria, British Columbia, Canada, 5. Concordia University, Montreal, Quebec, Canada, 6. Western University, London, Ontario, Canada*

Until recently, studies of precarity have overlooked aging and late life. This poster presents a snapshot of conceptual work in progress on a Canadian Social Sciences and Humanities Research Council (SSHRC) Insight Grant on precarity and aging. The poster outlines existing definitions and theoretical perspectives, key results, a current evolving conceptual model, and a working definition of Precarious Aging. It situates existing knowledge and definitions of precarity, highlights crucial intersectional locations of gender, im/migration and (dis)ability, and clarifies the concept of precarity in later life. Results at this point in the study are based on conceptual reviews, reviews of literature on precarity and aging, and the consideration of allied concepts. In conclusion, the concept of precarity offers a promising lens to guide research in the field of social and critical gerontology, providing a foundation for an enhanced understanding of the lives and realities of older people with regards to aging, disadvantage, and inequality.

### PREDICTING CAREGIVER REACTIONS TO CHALLENGING BEHAVIORS IN THE CONTEXT OF DEMENTIA

Darby Simon, and Benjamin Mast, *University of Louisville, Louisville, Kentucky, United States*

Challenging behaviors exhibited by people living with dementia have been associated with a variety of negative outcomes including greater caregiver burden, nursing home placement, and lower quality of life. Although there has been considerable research on psychological and behavioral changes in dementia, little research has explored family caregiver reactions to these changes and what caregiver characteristics are associated with stronger emotional reactions. This research examined the relationship between established indicators of caregiver mental health (depression, burden, grief, well-being) and caregiver reaction scores on the Revised Memory and Behavior Problems Checklist (RMBPC). The sample consisted of 76 family caregivers for people living with dementia, aged 25 to 93, who participated in a study on caregiver burden and grief. Multiple regression was used to predict RMBPC caregiver reaction scores from the Zarit Burden Inventory, Geriatric Depression Scale, Ryff Psychological Well-Being Scale, and Anticipatory Grief Scale while controlling for RMBPC total behavior frequency scores. RMBPC total behavior frequency scores and Zarit Burden Inventory were significant predictors of caregiver reaction scores ($F(2,74) = 87.559$, $p < .001$, $R2 = .703$). More frequent, challenging behaviors were associated with more distressing reactions and higher caregiver burden also predicted more distress on the RMBPC reaction scores. Psychological well-being was associated with lower reactions at the bivariate level but was not significant in the full regression model. Future research is needed to better understand these relationships and implement this knowledge to benefit family caregivers.

### PREDICTORS OF NURSING HOME COVID-19 CASES: A COMMUNITY VULNERABILITY APPROACH

Matthew Peterson,[1] and Larry Lawhorne,[2], *1. UNC Wilmington, Wilmington, North Carolina, United States, 2. Wright State University, Fairborn, Ohio, United States*

It is well known that the Covid-19 pandemic has placed considerable burden on nursing homes, including from resident, facility, and community perspectives, among others. This study examined facility and community factors that were related to incident Covid-19 cases in nursing home facilities. N=12,473 US nursing homes were included in this study. Data from June 2020 - January 2021 from several publicly available sources were combined to create a dataset that included facility name, size, ownership, mortality rate, Covid case rate, personal protective equipment (PPE) and staff shortages, % white residents, and % Medicaid residents. Community factors included core-based statistical area (CBSA) Covid case rates, urban/rural, CBSA death rates, and the CDC's Social Vulnerability Index (SVI). Zero-inflated Poisson regression models were used to determine predictors of 8-month Covid case counts, normalized by facility size. Results indicated that higher staff shortages, poorer facility rating, for-profit ownership, proportionally more Medicaid and non-white residents were all significantly associated with higher Covid case rates over 8 months (all $P < 0.0001$). Significant community level predictors of higher cases included urban setting and higher SVI. PPE shortages was not associated with higher case counts. Of all the factors included, SVI was the strongest predictor of Covid case counts. This large US study assists in determining critical facility and community factors that predict increasing Covid burden in nursing homes. Particularly, SVI is an important factor in determining facility and public health policy, and targeting resources in large scale health crises such as the Covid-19 pandemic.

### PROJECT ECHO-AGE FRIENDLY COMMUNITY CARE: APPLYING THE ECHO® MODEL WITH AREA AGENCY ON AGING STAFF

Katherine Bennett,[1] Mary Pat O'Leary,[2] and Elizabeth Phelan,[1], *1. University of Washington, Seattle, Washington, United States, 2. Aging and Disability Services - Seattle/King County, Aging and Disability Services - Seattle/King County, Washington, United States*

The Extension for Community Healthcare Outcomes (ECHO®) model originated at the University of New Mexico to improve access to specialty care in underserved communities via telehealth and has spread worldwide. It has been utilized to improve geriatrics care with interprofessional participants but not specifically to train community case managers in Age-Friendly care and community initiatives. This is a program evaluation of an ECHO developed exclusively for Area Agency on Aging (AAA) case managers via a community-campus partnership. Twenty-eight AAA staff (including 15 case managers) participated in a 6-session series (March through April 2021) focused on the 4Ms of Age-Friendly care (What Matters, Medication, Mentation, and Mobility). Each one-hour session included a mini-didactic and at least one case presented by participants utilizing a novel age-friendly case form. Participants completed pre and post surveys to assess self-efficacy in applying Age-Friendly principles on 5-point scales. Surveys were anonymous and analyzed comparing pre-post using Mann-Whitney U tests. Of the 28 participants, 86% completed pre and 65% post-surveys. Self-efficacy for providing Age-Friendly care improved from 2.58 pre to 4.5 post ($p<0.001$) and for providing recommendations to colleagues on Age-Friendly care from 3.4 pre- to 4.5 post ($p<0.001$). Participants showed significantly increased self-efficacy ($p<0.05$) pre vs. post on 14/15 Age-Friendly care items. One hundred percent would recommend this ECHO program to a colleague. This pilot utilizing Project ECHO with AAA staff increased their self-efficacy for providing Age-Friendly care and suggests this may be an effective tool to bridge the Age-Friendly Health Systems and Age-Friendly Community initiatives.

### SOCIAL SUPPORT AND SOCIAL STRAIN AS PREDICTORS OF DEPRESSIVE SYMPTOM TRAJECTORIES FOLLOWING A MARITAL TRANSITION

Emily Denning,[1] AnneMarie O'Neill,[1] Sheila Markwardt,[2] Anda Botoseneanu,[3] Heather Allore,[4] Corey Nagel,[5] David Dorr,[2] and Ana Quiñones,[2], *1. Portland State University, Portland, Oregon, United States, 2. Oregon Health & Science University, Portland, Oregon, United States, 3. University of Michigan - Dearborn, Dearborn, Michigan, United States, 4. Yale School of Medicine, New Haven, Connecticut, United States, 5. University of Arkansas for Medical Sciences, Little Rock, Arkansas, United States*

We investigated trajectories of depressive symptoms over 10 years following a marital transition (widowed or divorced) using data from the Health and Retirement Study (N = 377, mean age = 67.55 years; years 2006-2016). Piecewise growth curve models were estimated to investigate whether social support and strain from one's spouse, measured prior to transition, predicted depressive symptom trajectories following the transition. Covariates included sex, age, education, race/ethnicity, wealth, and chronic conditions. Overall, trajectories of depressive symptoms after a marital transition were significantly negative, indicating a decrease in depressive symptoms over time. Findings did not differ significantly between participants who were widowed and those who were divorced. Spousal social support was associated with an increase in depressive symptoms and spousal social strain was associated with a decrease in depressive symptoms following a marital transition in separate models: support: b = .250, SE = .062, p <.001; strain: b = -.209, SE = .095, p <.05. However, social strain was not significant when examined jointly with social support. Depressive symptoms declined significantly for low (b = -.370, SE = .100, p <.001) and average levels of social support (b = -.113, SE = .037, p <.01), but not for high levels of spouse social support (b = .143, SE = .109, p = .188). These results suggest those with the highest levels of spousal support prior to the transition, whether divorced or widowed, experienced a more difficult recovery and may be targets for additional mental health support following a marital transition.

### SPOUSES' EMOTION REGULATION SKILLS AND DEPRESSIVE SYMPTOMS IN THE CONTEXT OF EARLY STAGE DEMENTIA

Amanda Piechota,[1] and Joan Monin,[2], *1. Yale School of Public health, New Haven, Connecticut, United States, 2. Yafle School of Public Health, New Haven, Connecticut, United States*

In older adults, the suffering of a relative with chronic disease or illness can severely impact their caregiver. However, older adults also tend to have better emotional regulation than younger age groups. Very little is known about these dynamics in the context of early-stage dementia in which both spouses are struggling with caregiving or experiencing dementia and need one another for support. In the present study, we hypothesized that when both partners have better emotion regulation skills, each partner's depressive symptoms would be the lowest. We made the same hypothesis for both the person with dementia and their spouse without dementia. We used self-report baseline data from an intervention study of 70 older adult married couples (N=140) where one spouse has early-stage dementia. Spouses completed questionnaires that measured their emotional regulation habits (Difficulties in Emotion Regulation Scale) and their recent depressive symptoms (CESD). Results from the actor partner interdependence model showed that when both partners had high emotional regulation skills scores there were the lowest levels of depressive symptoms for each dyad member (B=4.31, SE=.94, t(49.9)=4.61, p<.001). Findings suggest couple-focused interventions to enhance emotion regulation skills are important for coping in the early stages of dementia.

### STATE VARIATION IN POTENTIALLY BURDENSOME TRANSITIONS AMONG ASSISTED LIVING RESIDENTS AT END OF LIFE

Xiao (Joyce) Wang,[1] Joan Teno,[2] Pedro Gozalo,[1] David Dosa,[1] Kali Thomas,[3] and Emmanuelle Belanger,[4], *1. Brown University, Providence, Rhode Island, United States, 2. Oregon Health & Science University, Portland, Oregon, United States, 3. Brown University, Brown University/ Providence, Rhode Island, United States, 4. Brown University School of Public Health, Providence, Rhode Island, United States*

Little is known about the quality of end-of-life care in assisted living (AL), particularly transitions at end of life. This study aims to provide a first national look at potentially burdensome transitions at end of life among AL residents and to examine how those vary by state. This is a retrospective cohort study of Medicare beneficiaries who died in 2018 and resided at a validated 9-digit ZIP code for an AL with 25 or more beds4 on the 120th day before death (N=37,668). Three types of potentially burdensome transitions were considered: 1) healthcare transitions during last 3 days of life, 2) three or more all-cause hospitalizations during the last 90 days of life, and 3) two or more hospitalizations for urinary tract infections, sepsis, pneumonia, and dehydration during last 120 days of life. Hospitalizations and decedents' locations were obtained from multiple administrative claims records. Out of the 37,668 AL decedents, 7,015 (18.6%, 95% CI: 18.2%-19.0%) experienced at least one potentially burdensome transition. States varied widely in the rate of burdensome transitions, ranging from 30.9% in North Dakota to 8.9% in Wyoming. Our results support quality concerns for end-of-life care among AL residents. Future studies are needed to explain state variation and how it relates to factors such as residents' co-morbidities, end-of-life care practices in AL, and state regulations. This study had two limitations. First, multiple hospitalizations for Medicare Advantage beneficiaries might be under-reported. Second, the results are not generalizable to persons in ALs with fewer than 25 beds.

### STEREOTYPES OF OLDER ADULTS, OLDER MEN, AND MALE LEADERS PREDICT EXPECTATIONS, STANCE, AND VOTING INTENTIONS

Caitlin Monahan,[1] Ashley Lytle,[2] Elizabeth Inman,[3] Marybeth Apriceno,[4] Jamie Macdonald,[5] and Sheri Levy,[4], *1. Stony Brook University, Brooklyn, New York, United States, 2. Stevens Institute of Technology, Hoboken, New Jersey, United States, 3. Stony Brook University, Stony Brook University, New York, United States, 4. Stony Brook University, Stony Brook, New York, United States, 5. St. Francis College, Brooklyn, New York, United States*

The 2020 U.S. Presidential Election offered a unique opportunity to examine how stereotypes of older adults, older men, and male leaders impact expectations of candidate job performance and intentions to vote for Biden or Trump. This online study involved 500 college students from two universities from September 30th until November 3 (Election Day). A Biden and Trump model were tested for the relationships among (a) stereotypes from public discourse with (b) expectations of candidates/ presidential performance with (c) voting stance (pro- and anti-Biden vs pro- and anti-Trump) and (d) intentions to vote for Biden/Trump. As expected, for the Biden model, endorsement of older adult (lesser endorsement of senile, unhealthy), male leadership (greater endorsement of assertive and collaborative, lesser endorsement of uncaring), and older male stereotypes (greater endorsement of elder statesman and family-focused) predicted greater expectations of Biden's performance, which predicted pro-Biden and anti-Trump stances and ultimately voting intentions for Biden. As expected, for the Trump model, endorsement of older adult (lesser endorsement of senile), male leadership (greater endorsement of assertive, collaborative, lesser endorsement of immoral and uncaring), and older male stereotypes (greater endorsement of elder statesman) predicted greater expectations of Trump's performance, which predicted pro-Trump and anti-Biden stances and ultimately voting intentions for Trump. Taken together, these results suggest examining relevant categories of stereotypes associated with candidates and voting stances provides a fuller picture of voting behavior toward multiple candidates vying for office in addition to political ideology and voting intentions.

### SYSTEMATIC REVIEW OF PSYCHOLOGICAL AGING RESEARCH IN NEPAL

Kezang Tshering,[1] Srijana Shrestha,[2] and Barbara Kamholz,[3], *1. Wheaton College, Wheaton College, Massachusetts, United States, 2. Wheaton College, Norton, Massachusetts, United States, 3. UCSF, UCSF, California, United States*

Nepal faces unprecedented levels of aging similar to trends in many less well-resourced countries. It has limited capacity to address the medical, social and psychological needs of older persons. Difficult choices regarding allocation of resources will be needed. In this review, we hope to clarify what is already known in aging research in Nepal. The databases APA PsychINFO and PubMed were searched. The inclusion criteria were peer-reviewed articles on i) psychological constructs and mental illnesses, ii) use of original data, iii) inclusion of senior participants and iv) studies conducted in Nepal. Studies that included mixed age group and cross-country comparisons were excluded from this review. The initial search resulted in 76 articles from APA PsychINFO and 590 articles from PubMed. Articles were reviewed independently for inclusion and exclusion criteria. A total of 49 articles were included in the final list. Preliminary results showed that the largest share of articles focused on depression (32.1%), followed by quality of life/life satisfaction or loneliness(18.9%). A large number of studies also examined prevalence rates of psychiatric and neurocognitive disorders (22.6%). Common conditions, like dementia and delirium were studied only in 1.9% and 3.8% of published studies respectively. All of the studies were cohort-based and none focused on evaluations of psychosocial/medical interventions. Robust intervention studies are needed to help improve the lives of seniors in Nepal. To our knowledge this is the first comprehensive review of published articles on psychological construct in aging populations in Nepal.

### TELE-HEALTHCARE IN RESEARCH AND EDUCATION: AGE DIFFERENCES IN ACCESS AND UTILIZATION OF HEALTHCARE RESOURCES

William Hills,[1] Matthew Murphy,[1] and Karen Hills,[2], *1. Coastal Carolina University, Conway, South Carolina, United States, 2. Beaufort Jasper Hampton Comprehensive Health Services, Conway, South Carolina, United States*

Societal needs highlighted during the pandemic have led to significant changes in healthcare, including the rapid development and implementation of tele-care consumer options. This study examined video-based, virtual healthcare access and utilization before and during the pandemic. Participants included traditional college-aged students, middle-aged adults, and retirement-aged persons (n = 685); measures included access to physical and mental health services, consumer satisfaction with type of services accessed, and anticipated use of virtual healthcare following the pandemic. Results showed that approximately half of participants (49.2%) had experience with virtual healthcare, with most of these cases during the pandemic. Virtual healthcare was more often used for physical compared to mental healthcare services, with college-aged participants more likely to use mental healthcare services than adult and retirement-aged participants. Laptop computers were most widely used to access services, with smartphone use proportionally lower in retirement-aged participants (31.4%). Overall satisfaction with virtual services was high (Mdn = 5 on a 6-point Likert scale), but college-aged participants trended toward a lower satisfaction (Mdn = 4.25) than other age categories. These results support that virtual healthcare service development and access experienced significant growth during the pandemic. Age differences in the types of services, types of devices, and satisfaction with virtual services all suggest a similar theme for age-related considerations of life stage, life responsibilities, and comfort and familiarity with technology that must be addressed for virtual healthcare to reach its full potential and reach equitably across the lifespan.

### THE EFFECT OF A VIRTUAL REALITY DELIVERED PHYSICAL ACTIVITY INTERVENTION ON THE PHYSICAL FUNCTION OF OLDER ADULTS

Kyle Kershner,[1] Joy Furlipa,[2] Peter Brubaker,[2] Amber Brooks,[3] Lindsey Page,[1] Diane Ehlers,[1] and Jason Fanning,[2], *1. University of Nebraska Medical*

Center, Omaha, Nebraska, United States, 2. Wake Forest University, Winston-Salem, North Carolina, United States, 3. Department of Anesthesiology, Wake Forest School of Medicine, North Carolina, United States

COVID-19 public health recommendations have prohibited many older adults from attending in-person physical activity (PA) programs that improve physical function and promote functional independence. Most PA programs have shifted towards a video conference (VC) format, but this modality has been noted to “flatten” the social experience which is fundamental for lasting behavior change. Virtual reality (VR) is now designed for immersion and place-presence and may be better suited for instilling a feeling of social connection, which will likely improve physical function. The purpose of this study was to evaluate differences in physical function after a 4-week in-home VR or VC based PA intervention. Low-active adults (66.8±4.8 years) were randomized to VR (n=5) or VC (n=4) based PA counselling and instructed to find activities that were intrinsically motivating. VR participants were asked to select pre-approved available active games in addition to enjoyable real-world activities. ANCOVA models were used to explore group differences in six-minute walk distances across time. Results are reported using $\eta^2$ effect sizes based on the small sample size. After controlling for baseline values, the ANCOVA models revealed a moderate-to-large magnitude effect for distance traveled during the six-minute walk test ($\eta^2=.10$). Additionally, the VR group participants walked 42.63 meters further, which approaches a clinically meaningful difference. These promising early findings suggest there is value to exploring the impact of VR-delivered, group-mediated activity promotion on physical functioning in older adults. Future research should investigate aspects of VR that promote increased social connection and physical function in the older adult population.

### THE HERITABILITY OF COGNITIVE AGING: A SYSTEMATIC REVIEW OF LONGITUDINAL TWIN STUDIES

Alice Kim,[1] Alyssa Kam,[1] Maxwell Kofman,[2] and Christopher Beam,[1], *1. University of Southern California, University of Southern California, California, United States, 2. University of Southern California, Los Angeles, California, United States*

Heritability of cognitive ability changes across late adulthood, although whether genetic variance increases or decreases in importance is not understood well. We performed a systematic review of the heritability of cognitive ability derived from longitudinal twin studies of middle-aged and older adult twins. Using the Preferred Reporting Items for Systematic Reviews and Meta-Analyses (PRISMA) guidelines, articles were identified in APA PsycINFO and Clarivate Web of Science electronic databases. Identified articles were screened by title and abstract; remaining full-text articles were then fully evaluated. Reference sections served as an additional method for identification of relevant articles. In total, 3,106 articles were identified and screened, 28 of which were included and were based on data from 10 longitudinal twin studies published from 1994-2021. There are large genetic influences on an initial level of cognitive performance across domains whereas there are small to moderate genetic influences on change in performance with age. Evidence was less definitive about whether the same or different genetic factors contribute to both level and change. Non-shared environmental influences appeared to drive individual changes in cognitive performance. Heritability tended to either be stable or decline after 65 years, possibly because of the increasing importance of non-shared environmental influences on cognitive ability. Recent studies report increases in heritability across specific subtests and domains. Shared environmental variance accounted for little variance in cognitive ability. Emerging research questions and future directions for understanding genetic and environment influences in the context of gene-environment interplay are highlighted in this review.

### THE IMPACT OF AGE ON MOBILE TECHNOLOGY USE DURING THE COVID-19 PANDEMIC

Yi Lin, and Graham Rowles, *University of Kentucky, Lexington, Kentucky, United States*

The COVID-19 pandemic led to quarantines and mandatory spatial distancing; people of all ages were encouraged to use technologies instead of actual human contact as part of COVID-19 prevention. The promotion of mobile applications (apps) during the pandemic influenced mobile technology use behavior. This study explored age differences in mobile technology use during the COVID-19 pandemic. A pilot-tested survey was distributed using online survey software. Persons surveyed were 35 years of age or older, currently living in the United States of America with experience using mobile technology. Survey questions pertained to mobile technology use frequency and factors influencing the decision to use mobile technology. The nationwide response included 1212 individuals. The average age of participants is 56.12±12.26 years old (female: male = 1.24:1). Responses were categorized participants into three age groups, 35 to 49, 50 to 64, and 65 or older. Daily mobile technology use frequency increased significantly ($p<0.01$) for all groups during the COVID-19 pandemic, with participants 35 to 49 having a significantly higher ($p<0.01$) use frequency than other groups. Regarding factors influencing the decision to use mobile technology, 64.1% of respondents aged 50 to 64 identified the necessity of using this technology during the pandemic as a significant factor, and 64.0% of participants in age 65 or older reported that the availability of functions on mobile devices is critical. Overall, the COVID-19 pandemic led to a significant increase in mobile technology use with people in different age groups differentially valuing the factors that affected their user behavior.

### THE IMPACT OF HCBS USE PATTERNS ON OLDER ADUTS' PHYSICAL FUNCTION AMONG CONTINUOUS SERVICE USERS

Ya-Mei Chen,[1] Shih-Cyuan Wu,[1] Kuan-Ming Chen,[2] Chen-Wei Hsiang,[3] Shiau-Fang Chao,[4] Ming-Jen Lin,[1] Ji-Lung Hsieh,[5] and Yu-Hsuan Chou,[1], *1. National Taiwan University, Taipei, Taipei, Taiwan (Republic of China), 2. National Bureau of Economic Research, CAMBRIDGE, Massachusetts, United States, 3. National Taiwan University, National Taiwan University, Taipei, Taiwan (Republic of China), 4. National Taiwan University, Taipei City, Taiwan (Republic of China), 5. Graduate Institute of Journalism, Taipei City, Taipei, Taiwan (Republic of China)*

Background Taiwan, one of the fastest aging countries in the world, provides home-and community-based services (HCBS) care recipients through the second 10-Year Long-Term Care Plans (LTC Plans 2.0). The current study aimed to examine the impact of continuous use of HCBS offered by LTC plan 2.0 on older adults' physical function change overtime. Methods This study used the LTC Plan 2.0 database and the National Health Insurance Plan claim dataset, and included 157,682 clients who had applied for and were evaluated for LTC services for the first time from 2018 through 2020 and continuously used any LTC Plan 2.0 services for six months. Outcome variables were measured by activities ofdaily living (ADLs) and instrumental activities of daily living (IADLs) between baseline assessment and the assessment after the six months of serivce utilization. Latent class analysis and generalized estimating equations were used to investigate the influences of different service use patterns on the changes in physical functions. Results Three subgroups of LTC recipients with different use patterns, including home-based personal care (home-based PC) services (n = 111,976; 71.01%), professional care services (n = 31,584; 20.03%), and community care services (n = 14,122; 8.96%) were identified. When compared to care recipients in the community care group, those in the home-based PC group improved more in IADL scores, and the professional care group improved more ADL scores. Conclusion Those receiving more professional services made more progress in ADL ability, but those in home-based PC made more progress in IADL ability.

### THE IMPACT OF SUPERVISOR SUPPORT ON THE JOB SATISFACTION OF IMMIGRANT AND MINORITY LONG-TERM CARE WORKERS

Frances Hawes,[1] and Shuangshuang wang,[2], *1. University of Wisconsin-Eau Claire, eau claire, Wisconsin, United States, 2. Shandong University, School of Public Health, Cheeloo College of MedicineJinan, Shandong,, Shandong, China (People's Republic)*

The need for long-term care workers (LTCW) will grow significantly as the American population ages. Understanding the factors that impact job satisfaction of this workforce has important implications for policy and practice. Previous research has demonstrated the effect of supervisor support on the job satisfaction of these workers; however, much less is known about how this effect differs among different race/ethnicity or immigration groups. This study examined how supervisor support mediates the associations between race/ethnicity, immigration status, and job satisfaction among nursing assistants (NAs). Data of 2,763 NAs were extracted from the National Nursing Assistant Survey (2004). Race/ethnicity groups included White (54%), African American (30%), Asian (2%), Hispanic (10%), and others (4%). Immigration status included U.S.-born citizens (87%), naturalized (7%) and resident/alien (6%). Bivariate analyses showed that Asian NAs perceived higher levels of supervisory support than other races, whereas U.S.-born NAs reported lower levels of supervisory support than naturalized and residents/aliens. Findings from multivariate analyses indicated that non-Hispanic Asians and Resident/Alien workers reported significantly higher levels of job satisfaction than their counterparts, and the associations were fully mediated by NAs' perceived supervisor support. These findings support prior research that supervisor support is important to improving job satisfaction and contribute to the literature that Asians/Residents/Aliens long-term care workers may be more sensitive to supervisory support and may be more grateful if they received support from supervisors. Managers should be aware of these racial differences and by being supportive they may improve NAs job satisfaction and reduce turnover rates.

### THE ROLE OF RELATIONSHIP QUALITY FOR SOLITUDE EXPERIENCES DURING THE PANDEMIC

Yoonseok Choi,[1] Theresa Pauly,[2] Elizabeth Zambrano Garza,[1] Tiana Broen,[1] Denis Gerstorf,[3] and Christiane Hoppmann,[4], *1. University of British Columbia, Vancouver, British Columbia, Canada, 2. University of Zurich, University of Zurich, Zurich, Switzerland, 3. Humboldt University of Berlin, Humboldt University of Berlin, Brandenburg, Germany, 4. University of British Columbia, University of British Columbia, British Columbia, Canada*

As time spent at home has significantly increased during the pandemic, reports of household conflict has also risen among people living with others (Usher et al., 2020). One solution to alleviate the potential stress of increased time with others could be carving out time to oneself. The present study investigated how living conditions (e.g., with others vs. alone) are associated with everyday desire for solitude and whether daily solitude experience comes with improved daily emotional well-being in people living with others. Furthermore, it also explored whether relationship quality is associated with solitude experience in a similar manner as living conditions. To do so, we used repeated daily life assessments from a lifespan sample (N = 215; M age = 38.3 years, SD age = 17.5; 78 % female) collected during the early pandemic (April to August 2020). Findings indicate that neither living conditions nor relationship quality were directly associated with daily desire for solitude, but higher relationship well-being was related to low preference for solitude when measured as an individual trait. In addition, relationship quality significantly moderated everyday solitude–affect links: higher relationship quality was related to reduced negative affect during solitude, and conflict was related to increased positive and decreased negative affect on solitude as compared to non-solitude days. The results imply that it is the subjective experience of relationships rather than objective living conditions that shape daily affective quality during solitude.

### UNDERSTANDING BARRIERS TO HEALTHCARE ACCESS FOR HEART FAILURE PATIENTS DURING THE COVID-19 PANDEMIC

Hanzhang Xu,[1] Julie Miller,[2] Roy Thompson,[1] Bradi Granger,[3] and Matthew Dupre,[2], *1. Duke University, Durham, North Carolina, United States, 2. Duke University School of Medicine, Duke University School of Medicine, North Carolina, United States, 3. Duke University, Duke University School of Nursing, North Carolina, United States*

Early outpatient follow-up within two weeks after hospital discharge is an effective strategy for improving transitions of care in older patients with heart failure (HF). However, implementing timely follow-up care for HF

patients has been challenging, especially during the COVID-19 pandemic. This convergent mixed-methods study identified patients' barriers to accessing care and ascertained their recommendations for addressing these barriers. We enrolled 264 HF patients admitted to the Duke Heart Center between May 2020 and August 2021. A standardized survey and electronic health records (EHR) were used to collect patients' sociodemographic, psychosocial, behavioral, and clinical data. For patients who reported some difficulty accessing their healthcare (n=30), semi-structured interviews were conducted to understand these barriers. Data were analyzed using rapid analysis techniques. Barriers to accessing care varied across participants, with scheduling an appointment being the most common barrier (12 of the 30 responses). Participants indicated that job-related conflicts, providers' availability, or COVID-19 contributed most to the difficulty in scheduling an appointment. Some participants experienced more difficulties during the pandemic due to fewer appointments available for non-acute and non-COVID-19 related needs. Transportation was another critical barrier, which was often associated with the participants' physical functional status. Participants identified the benefits of using telemedicine to address access to care barriers; however, they shared their concerns that telemedicine visits may not be sufficient to assess their HF conditions. Study findings highlight the need for more continual, tailored, and patient-centered interventions to improve access to care in older HF patients.

### USING TAIWANESE UNIVERSAL HEALTH INSURANCE DATA TO ESTIMATE LTC NEEDS WITH MACHINE LEARNING

Kuan-Ming Chen,[1] Chen-Wei Hsiang,[2] Shiau-Fang Chao,[3] Ming-Jen Lin,[4] Kuan-Ju Tseng,[2] Yu-Hsuan Chou,[4] Ji-Lung Hsieh,[5] and Ya-Mei Chen,[4], *1. National Bureau of Economic Research, CAMBRIDGE, Massachusetts, United States, 2. National Taiwan University, National Taiwan University, Taipei, Taiwan (Republic of China), 3. National Taiwan University, Taipei City, Taiwan (Republic of China), 4. National Taiwan University, Taipei, Taipei, Taiwan (Republic of China), 5. Graduate Institute of Journalism, Taipei City, Taipei, Taiwan (Republic of China)*

One of the core issues in long-term care (LTC) policy is the growing imbalance between demand and supply of LTC services due to aging population. To estimate the imbalance and allocate LTC resources, the government regularly conducts surveys. These surveys are expensive given the sample size requirements and imprecise given their subjective nature. This study links the administrative records of the universal health insurance database with LTC program usage records in Taiwan to explore this issue. Machine learning algorithms are used in projecting LTC needs from administrative records. LTC program usage records provide detailed LTC needs information and the amount of service each individual used. In addition, health insurance claim data provides rich health information. Specific LTC needs are predicted for each individual. By further extrapolating to future demographics, long-term LTC needs could be projected. There are several findings in this study. Prediction of difficulties in activities of daily livings (ADL), measured by Barthel index, works best using the Gradient Boosting algorithm. The mean absolute error is 17.67 out of a 0 to 100 scale. In addition to dementia and stroke, diagnosis of pressure ulcer (ICD 10 code: L89) and pneumonia (ICD 10 code: J18) have high predictive power for LTC needs. Prediction of Instrumental ADL (IADL) also performs well with a mean absolute error 1.31. The prediction model suggests high LTC needs and excess demand as the demographics changing. Our study provides a reliable way of using rich information to estimate future LTC needs without conducting additional costly surveys.

### VACCINE ACCESS SHRINKS DISPARITIES BETWEEN LONG-TERM CARE AND COMMUNITY RATES OF COVID-19 MORTALITY.

Nicholas Resciniti,[1] Daniel Kaplan,[2] Joshua Sellner,[3] and Matthew Lohman,[4], *1. University of Southern California, Los Angeles, California, United States, 2. Adelphi University, Cortlandt Manor, New York, United States, 3. Arnold School of Public Health, University of South Carolina, Columbia, South Carolina, United States, 4. University of South Carolina, Columbia, South Carolina, United States*

This longitudinal secondary data analysis examines differences in COVID-19 incidence and mortality among long-term care facility (LTCF) residents with those living in the community in South Carolina (SC) throughout the pandemic, including the time of vaccine availability. Data came from the SC Department of Health and Environmental Control (SCDHEC). Descriptive statistics and trends for cases of infections and deaths were calculated. Cox proportional hazards were used to compare COVID-19 mortality in LTC residents to community dwelling older adults, controlling for age, gender, race, and pre-existing chronic health conditions. Until early January of 2021, significantly greater incidence rates of infection (116.2 per 10,000 per month) and hazard of death after infection (HR=1.83, 95% CI: 1.70-1.98) were experienced among LTC residents as compared to older adults in the community even after statewide mask mandates and visitation guidance. Since vaccine availability, COVID incidence rates among LTC residents fell by half (59.5 per 10,000 per month after vaccines), and the relative hazard of death compared to older adults in the community was diminished (HR=1.44, 95% CI:1.29-1.61). Reducing the gap between LTCF and community-wide infection and mortality rates suggests that vaccination against COVID-19 is correlated with reduced disease spread in the greater community and in LTCF. Results indicate that policies and regulations addressing LTC resident and staff vaccination may effectively protect the most vulnerable older adults and the workforce providing their care while mask mandates and visitation guidance do not.

### VIRTUAL CARE FOR CAREGIVERS OF PWD: ADAPTING TO THE NEW NORMAL

Steven Shirk,[1] Maureen O'Connor,[2] Jaye McLaren,[3] Kendra Pugh,[2] Andrew Nguyen,[4] Sarah Boucher,[2] and Lauren Moo,[5], *1. VA Bedford Healthcare System, Pepperell, Massachusetts, United States, 2. VA Bedford Healthcare System, Bedford, Massachusetts, United States, 3. VA Bedford Healthcare System, VA Bedford Healthcare System, Massachusetts, United States, 4. Boston University, Boston, Massachusetts, United States, 5. VA Bedford Health Care System, Bedford, Massachusetts, United States*

Caregivers of persons with dementia (PWD) often experience increases in depression, anxiety, and burden as the disease progresses. In fact, as the PWD's neuropsychiatric symptoms increase and independence in ADLS decrease, caregivers psychological and physical health outcomes worsen. The literature suggests that caregiver interventions that teach specific skills are more beneficial than psychoeducational interventions, particularly regarding the amelioration of the psychological impacts of informal caregiving. However, because of caregiving demands, caregiver's own physical limitations, and competing obligations, it can be difficult to attend caregiver support or education programs outside the home. With the emergence of the COVID-19 pandemic, arranging such interventions became more complex, Therefore, we report on preliminary qualitative outcomes of a study investigating the feasibility and acceptability of converting an in-person, group dementia caregiver education intervention, CARE, to a telehealth platform. We report the findings of two objectives: 1) lessons learned when attempting to convert an in-person group intervention to telehealth and 2) experience and perceived benefit of attending a virtual group from the perspective of the participants of our first two groups. Briefly, our findings demonstrate the strong need for technological support. Participants report positive experience regarding the convenience of attending the group from their home, the benefits of the assigned exercises, and the support they found from other group members. The COVID-19 pandemic has forced many to embrace the virtual option as they adapt to a new normal. There are undoubtedly hurdles to overcome, but there are also advantages to be leveraged.

### Session 9515 (Late Breaking Poster)

### LATE BREAKING POSTER SESSION IV

### A CLOUD INFRASTRUCTURE FOR LARGE SCALE HEALTH MONITORING IN OLDER ADULT CARE FACILITIES

Yeonsik Noh,[1] and Uchechukwu David,[2], *1. University of Massachusetts, Amherst, Massachusetts, United States, 2. University of Massachusetts Amherst, Amherst, Massachusetts, United States*

Technology development in the sub-field of older adult care has always been on the back-burner compared to other healthcare areas. But with increasing life expectancy, this is poised to change. With the increasing older adult population, the current older adult care facilities and personnel are struggling to keep up with demand. To address this matter, we proposed a distributed system infrastructure that will enable large-scale monitoring of vital signals and early detection of emergency situations in nursing homes and assisted living communities in this study. The system is mainly comprised of node devices and cloud infrastructure. The node device collects data from multiple users and forwards this data to the cloud service. Communication between the vital-sensing devices on the users and the node device is accomplished using Bluetooth Low-Energy (BLE). The Node device is implemented on a Raspberry Pi Model 3b+ which has built-in BLE capability. A cloud server consists of back-end and front-end components. The back-end handles all the data processing and logical decisions that drive the front-end. The front-end is the interface provided to the end-users, which can monitor all patients in nursing homes at the same time. We examined our system in terms of scalability, real-time operation, cost, and usability and then found that our system provides not only a smart remote monitoring solution that can provide a better aging experience for older adults and their families, but also increase automation in nursing homes leading to a reduction in running cost and an increase in capacity.

### A LONGITUDINAL TRAJECTORY OF LONELINESS AND THE COVID-19 PANDEMIC AMONG THE OLDEST-OLD POPULATION

Jinmyoung Cho,[1] Shinae Choi,[2] Gelareh Rahimighazikalayeh,[3] Peter Martin,[4] Melinda Heinz,[5] and Yeon Ji Ryou,[4], *1. Baylor Scott & White Health Research Institute, Temple, Texas, United States, 2. The University of Alabama, Tuscaloosa, Alabama, United States, 3. Baylor Scott & White Health Research Institute, Dallas, Texas, United States, 4. Iowa State University, Ames, Iowa, United States, 5. Upper Iowa University, Fayette, Iowa, United States*

Loneliness is significantly associated with health and well-being among oldest-old adults. Due to the outbreak of the COVID-19, physical and social distancing policies might elevate loneliness among the oldest-old population. This study examined the trends and changes in the prevalence of feeling lonely using the 2020 HRS COVID-19 module merged to the 15 waves of the HRS RAND longitudinal datasets from 1992 to 2018. A total of 14,371 respondents, including 614 respondents aged 80 years and older were included. Generalized linear models compared age group differences within the 2020 module. Generalized estimating equations assessed the longitudinal change at the individual level and the trend of feeling loneliness among oldest-old adults from 1992 to 2020. Loneliness was assessed with one item of the CES-D scale (i.e., during the past week, felt lonely). After adjusting for demographic characteristics and health, the results showed that oldest-old adults were more likely to feel lonely compared to younger age groups (18% for 80's vs. 14% for 50's) during the early months of the pandemic. A longitudinal trajectory also showed that they feel lonelier than in prior years (19% in 2020 vs. 14% in 2018). However, compared to same-age groups from earlier years, a significantly lower prevalence of feeling lonely was observed (18% in 2020 vs. 27% in 1994). The results show that the outbreak of the COVID-19 may elevate feeling lonely, but the recent cohorts be less lonely than earlier cohorts. Future research should continue to explore protective factors for loneliness among oldest-old adults.

### A QUALITATIVE STUDY OF THE IMPACT OF COVID-19 ON HOSPICE AND HOME-BASED PALLIATIVE CARE IN TAIWAN

YuHsuan (Olivia) Wang,[1] and Susan Enguidanos,[2], *1. USC, Los Angeles, California, United States, 2. University of Southern California, Los Angeles, California, United States*

COVID-19 has had a tremendous impact on individuals and health care, particularly among those with serious illnesses. Although Taiwan initially reported low rates of COVID-19 (20.7 cases per million in 2020), by early 2021

the Alpha variant began spreading in Taiwan, which went into a soft lockdown from May to July. This study aimed to investigate the impact of COVID-19 on Hospice and Home-Based Palliative Care (HBPC) patients, family, and medical team member in Taiwan during different COVID-related time points. From November 2020 to May 2021, we conducted semi-structured interviews with 2 patient, 5 caregivers, and 8 medical team members in the hospice and HBPC setting to elicit their views about the impact of COVID-19 on care. Content analysis was used to guide the analysis of themes. Two researchers independently coded transcripts and met to reconcile codes. Results showed varying impact across the time points. In 2020, patients, caregivers, medical team members felt little impact of COVID-19. However, in 2021, HBPC caregivers reduced the frequency of HBPC visits to lower the household COVID-19 infection risk. Hospice and HBPC team members reported that more caregivers selected HBPC over hospice. They also observed an increase in complicated grief among family survivors following the soft lockdown. The impact of the pandemic on homecare provision increased as prevalence of COVID-19 increased, impacting care and quality of hospice and HBPC. Further research is needed to better understand the impact of the pandemic on the quality of hospice /HBPC and grief experiences.

### A TREND OF KOREAN OLDER ADULTS' AWARENESS AS A THREAT TO SOCIETY DURING THE COVID-19 PANDEMIC

SOONHYUNG KWON,[1] Jaesung An,[2] and Oejin Shin,[3], *1. University of Illinois at Urbana-Champaign, Savoy, Illinois, United States, 2. California State University East Bay, Hayward, California, United States, 3. University of Illinois at Urbana Champaign, Savoy, Illinois, United States*

**Background:** Unlike ageism that consists of one's prejudice, stereotype, and discrimination toward older adults, age-based threats are one's negative cognition (Levy, 2001). Previous studies indicated that the younger generation stigmatizes the older generation as unworthy during the COVID-19 pandemic (Meisner, 2021). However, there is no study looking at how older adults perceive themselves as threats to society during this time of the pandemic. Thus, our study aimed to understand the varying trend of older adults' awareness as a threat to society in association with socio-economic profiles before and during the pandemic.

**Method:** This study included 637 Korean older adults who answered the older generation's threats to society from 2018 to 2020. We used Latent Class Analysis (LCA) to categorize participants into different subgroups that shared distinct patterns of threats to society. Multinomial logistic regression examined how the subgroups in threats to society were associated with socio-demographic characteristics in each year.

**Results:** For three waves, three clusters of threats to society (low, mid, and high) were identified. Although the mid-level of threat remained the same (60% of the sample for three years), the high level of threats has been doubled in 2019 (25%) compared to 2018 (11%) and 2020 (13%). Regarding the associated socio-demographic characteristics with threats to society, those who being female in 2018 and younger age in 2020 were more likely to be associated with mid-level of threats to society.

**Discussion:** Further study needs to identify the relationship between awareness as a threat to society and health outcomes.

### ACCELEROMETRY-BASED ASSESSMENT OF PHYSICAL ACTIVITY IN OLDER ADULTS DURING COVID-19 PANDEMIC

Renoa Choudhury,[1] Ladda Thiamwong,[2] Oscar Garcia,[1] Rui Xie,[1] Jeffrey Stout,[1] and Joon-Hyuk Park,[1], *1. University of Central Florida, Orlando, Florida, United States, 2. College of Nursing, University of Central Florida, Orlando, Florida, United States*

COVID-19 pandemic has caused a severely detrimental effect on the physical, psychological, and functional well-being of older adults by forcing them to limit their social activities. This study investigates the amount and intensity of daily physical activity (PA) in older adults, living under the social distancing guidelines during COVID-19 pandemic. In this cross-sectional study, 124 community-dwelling older adults (Age:60–96 years, mean:75±7.14) were recruited via word-of–mouth and key person approach between March 2021-August 2021. Participants completed an online survey on COVID-19 questionnaires and wore an ActiGraph GT9X accelerometer on their non-dominant wrist for consecutive 7 days in free-living conditions. Euclidean Norm Minus One cut-points were used to estimate the total time spent in sedentary behavior (SB), light PA (LPA) and moderate-to-vigorous PA (MVPA). Results showed that, 7% had COVID-positive, 55% perceived moderate severity of COVID in their area, 31% reported fear of COVID, and 14.5% were afraid of losing their life to COVID. On average, participants obtained 12.43±2.1 hours/day of SB, 3.47±1.05 hours/day of LPA and 42.71±29.71 minutes/day of MVPA. MVPA minutes/day was significantly higher (P = 0.006) in participants aged 60-85 years than those aged 85+ years (45.38 minutes/day vs 14.25 minutes/day). When age-adjusted data was compared to pre-COVID-19 studies, we found COVID-19 pandemic had negatively impacted the physical activity level in older adults (29.33% decrease in MVPA and 39.2% increase in SB). These findings can be useful in developing guidelines and/or interventions to promote physical activity and healthy aging among older adults, particularly those in social isolation.

### ACCEPTABILITY OF A NEWLY DEVELOPED FINANCIAL PROGRAM FOR CAREGIVERS OF INDIVIDUALS LIVING WITH A CHRONIC ILLNESS

Claire Grant,[1] Sam Fazio,[2] Monica Moreno,[2] Beth Kallmyer,[2] Kerry Lanigan,[2] and Katherine Judge,[3], *1. Cleveland State University, South Euclid, Ohio, United States, 2. Alzheimer's Association, Chicago, Illinois, United States, 3. Cleveland State University, Cleveland, Ohio, United States*

Over 40 million informal caregivers provide care to adults aged 50 or older with a chronic illness. In addition to the negative health and well-being impact, caregivers experience financial difficulties including lost income, retirement benefits, future earnings, and unanticipated out-of-pocket costs. Few evidence-informed programs exist to assist caregivers in understanding and managing these financial tasks. This poster presents preliminary acceptability data from 71 caregivers who completed the newly developed Managing Money: A Caregiver's Guide to Finances program. The 60-minute program was delivered online by trained community educators and addressed caregiving costs/impacts; future planning; initiating conversations; avoiding financial abuse/fraud; and identifying needs. Participants (Mage = 59.45, SD = 11.31) were 87.32% female with 77.46% self-identified as White, 9.86%, as Black, and 8.45% as Hispanic/Latino. Using a Likert scale (1=strongly disagree to 5=strongly agree), participants indicated the program: 1) identified financial challenges (M= 4.11 ; SD = 0.73); 2) provided information for managing money (M = 3.99; SD = 0.64); 3) content was easy to read/understand (M = 4.49; SD = 0.61); 4) program length was appropriate (M = 4.18; SD= 0.68); and 5) activities were helpful (M = 4.13; SD = 0.69). Participants rated the program as very important (M = 4.54; SD = 0.82) and would highly recommend (M = 4.52; SD = 0.53). Results indicated the program was well-received by participants and highly acceptable. Discussion will highlight key program features designed to promote acceptability along with the importance of measuring acceptability for large-scale implementation along with next research steps.

### ACCULTURATION MODERATES COGNITIVE PERFORMANCE IN ADULTS AT RISK OF AUTOSOMAL DOMINANT ALZHEIMER'S DISEASE.

Kayla Tureson,[1] Christopher Beam,[2] and John Ringman,[3], *1. University of Southern California, Los Angeles, California, United States, 2. University of Southern California, University of Southern California, California, United States, 3. Keck School of Medicine at USC, Keck School of Medicine at USC, California, United States*

Acculturative processes via persistent exposure to a set of cultural practices and behaviors can influence cognitive functioning (Park & Huang, 2010). The impact of acculturation on populations at risk for Alzheimer's disease (AD), however, remains understudied. Persons with or at-risk for early-onset autosomal dominant AD (ADAD) offer a known AD pathogenesis and the opportunity to study whether acculturation moderates their cognitive performance. The present study used a latent variable model to test whether effects of latent cognitive ability on observable cognitive performance depend on acculturation. Participants included 119 adults with or at-risk for ADAD, the majority of whom were of Mexican origin with various levels of U.S. acculturation. Participants completed the Cognitive Abilities Screening Instrument (CASI) and the Acculturation Rating Scale for Mexican Americans-II (ARSMA-II). Confirmatory factor analysis was used to estimate a latent general cognitive ability factor from nine domains (e.g., attention, abstraction and judgment). The ARSMA-II was used to test whether factor loadings depended on level of acculturation, covarying for mutation status. Results revealed ARSMA-II scores nearly significantly moderated the effects of general cognitive ability on abstraction and judgment ($\lambda = 0.20$, SE = 0.11, p = .070). Individual differences in general cognitive ability at lower levels of acculturation likely predict lower abstraction and judgment performance. Cognitive assessments may not equally represent true cognitive ability in Mexican-Americans. Although the CASI was developed as a cross-cultural measure of cognitive functioning, caution should be exercised in inferring true cognitive functioning in Mexican-Americans who may not be acculturated to the U.S.

### ADVANCE CARE PLANNING AMONG HEALTHCARE PROFESSIONALS DURING COVID-19

Jessica Hahne,[1] and Brian Carpenter,[2], *1. Washington University in St. Louis, St. Louis, Missouri, United States, 2. Washington University in St. Louis, Saint Louis, Missouri, United States*

The COVID-19 pandemic is bringing healthcare professionals face to face with gravely ill patients in complex clinical situations. Caring for patients experiencing lengthy intubation, heavy sedation, rapid decline, and significant distress at the end of life has the potential to shift the perspectives of healthcare professionals regarding their own end-of-life care. This study explored advance care planning (ACP) among medical professionals and whether COVID-19 experiences altered their healthcare preferences and planning. Ninety-eight professionals (mean age = 45.6, 75% female) completed an online survey about ACP conversations, behavioral intentions to pursue ACP, openness to life-prolonging interventions, and ACP resource needs. ACP conversations were most extensive with spouse/partner (89% had talked about care preferences "some" or "a lot") and to a lesser extent with parents (64%) and other healthcare providers (69%). Two-thirds (67%) of respondents had an ACP conversation since the start of COVID. Among respondents who had not completed ACP documents, 64% had taken some step toward ACP. When asked whether their preferences for life-prolonging medical interventions had changed, 70% reported no change, 16% reported being less open, and 14% reported being more open. A majority (60%) requested resources to help them pursue ACP on their own, although many (42%) were interested in assistance at their workplace. Given that only 37% of our sample had themselves completed an advance directive, our results suggest now may be a critical moment to engage professionals in ACP, considering how their experience during the pandemic has motivated ACP conversations and a reconsideration of preferences.

### ADVERSE NEIGHBORHOOD ENVIRONMENTS AND HEALTH BEHAVIORS AMONG OLDER AMERICANS

Yeon Jin Choi, *University of Southern California, Los Angeles, California, United States*

Maintaining healthy lifestyle, including healthy diet and physical activity, in adverse neighborhood environments may be more difficult for older adults because of changes linked to aging, which make them more vulnerable to their environments. This study aims to investigate the association of neighborhood disorder with diet quality and physical activity in a national sample of older Americans. For this study, we used data from the Health and Retirement Study. Neighborhood disorders include vandalism, boarded houses, abandoned cars, demolished houses, trash, litter, or junk, poorly kept communal areas, homeless people, prostitution, winos or junkies, and drug use or drug dealing near residents' housing unit (range: 0-11). Diet quality and physical activity were assessed using the Healthy Eating Index 2015 (HEI-2015; range:0-100) and the metabolic (MET) equivalent activity points (range: 0-31 in this sample). Ordinary least squares regression models were estimated to examine an association between neighborhood disorder, diet quality, and physical activity. Neighborhood disorder was associated with poor diet and physical inactivity. For one additional negative neighborhood feature, HEI-2015 scores and MET-equivalent activity points decreased by 0.55 (95% CI: -1.09. -0.01) and 0.69 (95% CI: -1.05, -0.33). Findings of this study suggest that older adults living in adverse neighborhoods are at a greater risk of poor diet and physical inactivity, which are important risk factors for poor health and chronic diseases. Promoting neighborhood environments and perceived neighborhood safety would increase access to health food, encourage healthy diet and physical activity, and support healthy aging.

### AGE DISCRIMINATION AND PERCEIVED WORK ABILITY: THE MEDIATING EFFECT OF SELF-EFFICACY

Hyun Kang,[1] and Hansol Kim,[2], *1. George Mason University, Fairfax, Virginia, United States, 2. National Rehabilitation Research & Training Center on Family Support, Pittsburgh, Pennsylvania, United States*

As the number of older workers in the U.S. workforce increases, perceived work ability, which indicates a worker's capacity to perform job-related tasks or to remain employed, becomes increasingly important. However, age discrimination may undermine the ability of older adults to remain active in the workplace as it poses a significant barrier to their work ability. The purpose of this study was to examine how age discrimination affects perceived work ability among older workers. We also evaluated the role of self-efficacy as a potential mediator between age discrimination and perceived work ability. Self-efficacy can contribute to older adults' productive aging since it helps them view age-related situations more positively. Using 2,011 respondents (aged 50+) data from the 2018 Health and Retirement Study, structural equation modeling analysis was conducted. Our findings indicated that age discrimination had a direct negative effect on perceived work ability (B = -.230, p < .001). Older workers who experienced more age discrimination were more likely to have low levels of perceived work ability. The indirect effect of self-efficacy (B = -.177; 95% CI = -.240, -.135) was significant. Older workers who experienced more age discrimination were more likely to have low levels of self-efficacy, and this relationship led to lower levels of perceived work ability. These results suggest that greater efforts are required to reduce age discrimination and its negative consequence on perceived work ability and self-efficacy among older workers. Furthermore, age discrimination laws should be more explicitly enforced in the policy direction for older workers.

### AGE-RELATED ETHNIC DIFFERENCES IN SELF-REPORTED HAND FUNCTION

Ejlal Bin Mulayh,[1] Rachel Logue,[2] and Susan Brown,[2], *1. University of Michigan, University of Michigan, Michigan, United States, 2. University of Michigan, Ann Arbor, Michigan, United States*

Racial/ethnic differences in the prevalence of disability based on self-reported activities of daily living (ADLs) exist in older Americans, particularly in Hispanic adults. Such studies have relied on large data sets in which disability is measured across a broad range of functional tasks. While useful in generating a global measure of disability, it precludes an ability to differentiate, for example, deficits in upper versus lower extremity function which can provide useful information in targeting therapeutic interventions. Despite known age-related declines in hand function and concomitant ADLs, racial/ethnic differences in hand-related ADL limitations have not been addressed. Using 2011-2018 data from the National Health and Nutrition Examination Survey (NHANES), we identified 3,189 non-Hispanic White and Hispanic adults aged 65 and older, and classified responses based on perceived difficulty to five self-reported tasks requiring hand dexterity. Compared to non-Hispanic Whites, Hispanic males reported 2.2 times higher rates of difficulty for dressing tasks (p<0.01) and 3.3 times higher rates for difficulties preparing meals (p<0.01). Similar rates of difficulty were also observed in females. Additionally, Hispanic females reported 4.6 times higher rates of difficulty in tasks requiring grasping small objects (p<0.01). Deficits in hand function are often under-reported in older adults despite the role of dexterity in maintaining functional independence. The results presented here indicate that difficulties in hand-related ADLs are more prevalent in the Hispanic population and warrant greater attention in health care settings. Future work will include identifying factors contributing to these observed differences in self-reported difficulties in hand-related ADLs.

### ALLOSTATIC LOAD FOLLOWING SHORT-TERM INTERVENTION: COGNITION IN OLDER HYPERTENSIVE ADULTS

Anthony Cirilo,[1] Jordan Kohn,[2] Gavrila Ang,[2] Meredith Pung,[2] Emily Troyer,[2] Christopher Pruitt,[2] Laura Redwine,[3] and Suzi Hong,[2], *1. UCSD MADURA R25 Advancing Diversity in Aging Research Program, San Diego, California, United States, 2. University of California, San Diego, San Diego, California, United States, 3. University of South Florida, Tampa, Florida, United States*

Allostatic load (AL), a measure of cumulative effect of prolonged stressors across physiological systems, is consistently associated with adverse health outcomes. Greater AL is correlated with functional decline in aging, but effects of behavioral interventions, such as Tai Chi (TC), on AL in older adults in a short-term is unknown. To investigate the effects of TC practice on AL and cognitive function and an AL-cognition relationship, older adults (60-95 years) with hypertension were recruited and randomly assigned to 12-week TC or Healthy Aging Practice-centered Education (HAP-E) classes. The AL index (ALI) included: SBP and DBP; urinary epinephrine and norepinephrine; plasma inflammatory biomarkers (CRP, IL-6); metabolic biomarkers (HDL, total cholesterol, triglycerides, HbA1c); and BMI. The Montreal Cognitive Assessment (MoCA) was administered to assess cognitive function. Generalized linear mixed-effects models, adjusted for age, race, education, and intervention attendance, was used. Pre- and post-intervention ALI did not change significantly in TC (2.61 (1.48) to 2.76 (1.62)) or HAP-E (2.84 (1.61) to 2.66 (1.86)). High ALI was associated with lower MoCA scores, indicating poorer cognitive performance (IRR=0.96; 95% CI: 0.93-0.98; p=0.002) across the time points. Of note, the MoCA scores did not significantly change across time (25.4 (3.2) to 26.0 (3.0)). 12-week

TC or HAP-E interventions did not lead to a significant change in ALI or cognitive performance in our population. However, our findings show greater AL theoretically attributed to chronic stress is associated with cognitive functioning in older adults consistently over about 4 months.

### ANALYZING THE SPECIFIC ROLE OF COGNITIVE FUNCTIONING ON SUCCESSFUL AGING

Neyda Ma. Mendoza-Ruvalcaba,[1] Elva Dolores Arias-Merino,[2] Maria Elena Flores-Villavicencio,[3] and Maria Elena Rodriguez-Diaz,[3], *1. University of Guadalajara CUTONALA, Guadalajara, Jalisco, Mexico, 2. Universidad de Guadalajara, Zapopan, Jalisco, Jalisco, Mexico, 3. Universidad de Guadalajara, Universidad de Guadalajara, Jalisco, Mexico*

**Introduction:** The cognitive functioning, as a general measure, is a criterion commonly used to define and operationalize successful aging. (Project-Conacyt-256589) The aim of this study is to analyze specific domains of cognitive function and its relationship with the successful aging in older adults.

**Methods:** Population based, random sample included n=453 community-dwelling older adults 60-years and older (mean age=72.51,SD=8.11 years,59.4% women). Cognitive functioning was assessed by a comprehensive battery including working memory(Digit Span Backward WAIS-IV), episodic memory, meta-memory(self-report), processing speed(Symbol Digit WAIS-IV), attention(TMT-A), executive functioning(TMT-B), learning potential(RAVLT), language(FAS), visuospatial skills(Block Design WAIS-IV). Successful aging was operationalized as no important disease, no disability, physical functioning, cognitive functioning, and being actively engaged. Sociodemographic and health data were also asked. Data were analyzed in SPSSv24, MANOVAs and size effects were calculated.

**Results:** In total 11.2% were successful agers and 11.4% had Mild Cognitive impairment. Global cognitive functioning was significantly related to the achievement of successful aging criteria. Cognitive functioning had a significant effect on successful aging, specifically executive functions (F=1.07,p=.000) explained 32.7% of the variance, attention explained 29.8% (F=1.19,p=.006), processing-speed 21% (F=1.38,p=.000), and learning potential 21.5% ((F=1.12,p=.005). Language, visuospatial skills, working memory and meta-memory had a very small effect.

**Conclusion:** Knowledge generated by this study reveals the specific role of cognitive domains on successful aging, and sets a scenario to promote successful aging, through alternatives centered in the improvement of cognition in the older adults.

### AS WOMEN LIVE LONGER, WHAT DO THEY NEED MOST?

Jennifer Yahner,[1] and Jeanette Hussemann,[2], *1. Urban Institute, Washington, District of Columbia, United States, 2. NORC at the University of Chicago, Chicago, Illinois, United States*

Women in the United States can live into their 80s, 90s, and even 100s—outliving men nearly five years on average. Over the next four decades, the number of women aged 85 years and older will nearly triple in size. Many will live alone and in poverty, with increasingly fewer supports on which to rely as they age. Although women can spend their lives caring for children, partners, and parents, often while working multiple jobs, as they grow older, many find their physical, emotional, and financial needs cannot be met. Using data recently collected for the Urban Institute's EMPOWER: Building Late-Life Resilience study, with funding from the National Institute of Justice, we examine the needs of low-income women aged 85 years and older (N=35) living alone in Arizona communities. We explore issues of home safety perceptions and social isolation and study their relationship to women's physical, emotional, and financial wellbeing.

### ASSISTED LIVING ADMINISTRATORS' MENTAL AND EMOTIONAL HEALTH DURING THE COVID-19 PANDEMIC

Jaclyn Winfree,[1] Ozcan Tunalilar,[1] Jason Kyler-Yano,[1] Serena Hasworth,[2] and Paula Carder,[3], *1. Portland State University, Portland, Oregon, United States, 2. Portland State University, Portland State University, Oregon, United States, 3. OHSU-PSU School of Public Health, Portland, Oregon, United States*

Little is known about assisted living (AL) administrators' mental and emotional health, particularly during a global pandemic in which most of their residents are highly vulnerable to infection, hospitalization, and death. Considering that administrator turnover and burnout have been associated with negative outcomes such as decreased quality of resident care, low staff morale, and reduced financial solvency, this study examined how AL administrators described their mental and emotional state throughout the first year of the COVID-19 pandemic. Using thematic analysis, our team coded 18 qualitative interviews conducted from May-August 2021. The themes included declining physical health due to stress, feelings of inadequacy and self-doubt, and increased burnout. Many administrators described increased staffing challenges as directly impacting their daily stress levels. Some administrators described feeling guilty and doubting their interpretation or implementation of regulations, particularly in incidents that further distanced residents from peers and loved ones. A few administrators described their disposition or personality changing due to what they experienced during the pandemic. One administrator stated, "I'm not an anxiety person, but I feel anxiety about a lot of things. In fact, my doctor has talked to me about starting some medications to help with that." Multiple administrators made comments such as, "I don't know that there could be a more stressful position than executive director of assisted living…the COVID pandemic reinforced that. This is rough." Understanding AL administrators' mental and emotional health during a public health crisis allows for understanding, supporting, and retaining critical leaders in long-term care communities.

### ASSOCIATION BETWEEN LENGTH OF RESIDENCE IN THE U.S. AND INSURANCE COVERAGE WITHIN U.S. CHINESE OLDER ADULTS

Natalie Tuseth,[1] XinQi Dong,[2] Stephanie Bergren,[3] and Michael Eng,[4], *1. Rutgers, The State University of New Jersey, New Brunswick, New Jersey, United States, 2. Rutgers, New Brunswick, New Jersey, United States, 3. Rutgers University, New Brunswick, New Jersey, United States, 4. NYU Grossman School of Medicine, New York City, New York, United States*

Barriers to affordable insurance may worsen disparities among underserved populations. Immigrants with <5 years of residence are not eligible for Medicare and Medicaid and are potentially without affordable alternatives. This study aims to look at the relationship between length of residence in the U.S. and insurance coverage within U.S. Chinese older adults ages 65+.This study used data from a representative sample of 2,365 community-dwelling U.S. Chinese older adults age 65+. The association between length in the U.S. (<5, 6-10, 10+) and insurance status was analyzed using chi squared test and logistic regressions. Within this sample, 58 (2.78%) participants had coverage outside Medicare and Medicaid, with 279 participants reporting no coverage. The vast majority of participants living in the U.S. <5 years had no insurance (81.48%). In a fully adjusted model, participants who were older and female were positively associated with having insurance coverage (OR:1.11 [1.07,1.15] and OR:1.29 [0.88,1.90]). Conversely, both living in the U.S. <5 years (OR:0.009 [0.006, 0.014]),and between 5-10 years (OR:1.20 [0.13,0.30]) were negatively associated with insurance coverage. When including coverage outside of Medicaid and Medicare, residence <5 years and 5-10 years were still negatively associated with insurance coverage ((OR:0.13 [0.009,0.02]), and (OR:0.19 [0.13,0.30])). Vulnerable populations such as older immigrants may not have access to insurance outside of public options, making a 5-year waiting period an additional barrier to quality health care.

### ASSOCIATION OF DEMENTIA SEVERITY AND ASSISTANCES NEEDS ON MISSED HOME HEALTH VISITS.

Sara Knox,[1] Brian Downer,[2] Allen Haas,[2] and Kenneth Ottenbacher,[2], *1. Medical University of South Carolina, Charleston, South Carolina, United States, 2. University of Texas Medical Branch, Galveston, Texas, United States*

Individuals with Alzheimer's Disease and Related Dementias (ADRD) experience barriers to accessing health care services, including services provided during home health care. Additionally, it is not clear if people with ADRD who are admitted to home health care receive all the services needed to maximize their outcomes. Barriers to receiving the optimal care can include the presence or absence of a care giver, behavioral and psychological symptoms of ADRD, or therapists lacking the skills needed to effectively engage patients in therapy sessions. These barriers may vary dependent on the patient's ADRD severity. The purpose of this study was to examine the relationship between dementia severity and early discharge from home health care. This was a retrospective study of 142,376 Medicare beneficiaries with ADRD who received home health care between October 2016 and September 2017. Early discharge was defined as discharge from home health with more than two missed visits. Early discharge rates were calculated, and multilevel logistic regression was used to estimate the relative risk (RR) of early discharge, by dementia severity level, adjusted for patient and clinical characteristics. 10.4% of beneficiaries had an early discharge. Dementia severity was not associated with risk of early discharge. However, level of medication assistance needed was found to be associated with risk of early discharge (RR=0.849; 95% CI 0.759 - 0.948). Medication management may impact a patient's ability to adequately attend and engage in home health therapy services. Further studies are needed to better delineate the interaction between medication management and early discharge.

### ASSOCIATIONS BETWEEN PACS SYMPTOMS AND RISK FACTORS AMONG LONG HAULERS IN THE RUTGERS POST-COVID RECOVERY PROGRAM

Alice Dawson,[1] and William Hu,[2], *1. Rutgers University--New Brunswick, New Brunswick, New Jersey, United States, 2. Rutgers University, New Brunswick, New Jersey, United States*

At least 2/3 of people with mild to moderate COVID-19 infection will experience long-haul COVID symptoms that persist for weeks or months, however, risk factors that modify the likelihood that one develops these symptoms are unknown. Patients referred to the Post-COVID Recovery Program at Rutgers in New Brunswick (n= 108) through primary care referral or self-submitted online request and experiencing a wide variety of Post-Acute COVID-19 Syndrome (PACS) symptoms were stratified by those without self-reported cognitive complaints (n=54), those with self-reported cognitive complaints who scored well on cognitive testing (n=29), and those with self-reported cognitive complaints who scored poorly on cognitive testing (n=25). Comparisons between groups were made using ANOVAs and Chi Squared: for COVID-19 disease severity, COVID-19 disease treatment, comorbid COVID-19 symptoms during infection, comorbid PACS symptoms post-infection, pre-existing health conditions, levels of depression and anxiety, level of fatigue, and social determinants of health (access to healthcare, economic stability, housing stability). Preliminary analyses indicated that whereas people without complaints were normally distributed according to age (p>0.200 for Kolmogorov–Smirnov test), people with complaints and deficits were skewed towards the older age group (p<0.001 for K-S test) suggesting age to be a risk factor for cognitive impairment in PACS. Participants that reported cognitive complaints also reported increased symptoms of depression, anxiety, and fatigue, compared to participants without cognitive complaints. These data provide insight into associations between PACS symptoms and risk factors relevant in understanding this novel disease.

### ASSOCIATIONS OF ADVERSE CHILDHOOD EXPERIENCES WITH EXECUTIVE FUNCTION AND BRAIN-DERIVED NEUROTROPHIC FACTOR

Cindy Tsotsoros,[1] Madison Stout,[2] and Misty Hawkins,[2], *1. Oklahoma State University, Stillwater, Oklahoma, United States, 2. Oklahoma State University, Oklahoma State University, Oklahoma, United States*

Adverse childhood experiences (ACEs) may predict markers of neurocognitive performance (i.e., executive function; EF) and brain health/plasticity (i.e., brain-derived neurotropic factor; BDNF). This pilot examined the magnitude of effects between: 1) ACES and EF performance, 2) ACEs and BDNF levels, and 3) EF performance and BDNF levels. We hypothesize that higher ACEs will be associated with poorer EF scores and lower BNDF levels and that lower EF scores will be associated with lower BDNF levels. Given the pilot nature of the study, an emphasis is placed on effect size vs.

significance. Participants were 36 middle-aged women enrolled in the NICE SPACES trial (age=31.4 years, BMI=34.2, racially minoritized=37.9%). ACES were quantified using the 10-item Adverse Childhood Experiences Scale. EF was measured using the fluid cognition composite from the NIH Toolbox – Cognition Battery. BDNF was estimated using proBDNF levels estimated from serum collected via venipuncture. Higher ACEs levels were not directly associated with EF scores (b = 0.03, p = .854); but did show a meaningful negative beta coefficient with BDNF levels (b = -0.34, p = .053). EF scores and BDNF showed a positive coefficient that did not reach significance (b = .26, p = .122). In a modest pilot of middle-age women, higher ACEs were associated with lower BDNF, indicating greater adversity in childhood is linked to lower neurotrophins levels in adulthood. The lower BDNF levels may help explain poorer performance on cognitive tasks. Larger follow-up studies in more powered samples are warranted given the size of detected coefficients.

### ATTACHMENT STYLE DIFFERENCES IN THE AFFECTIVE EXPERIENCE OF CHRONIC PAIN IN OSTEOARTHRITIS

Katherine Cheesman,[1] Patricia Parmelee,[2] and Dylan Smith,[3], *1. The University of Alabama, Tuscaloosa, Alabama, United States, 2. University of Alabama, University of Alabama, Alabama, United States, 3. Stony Brook University, Stony Brook University, New York, United States*

**Objective:** To examine the role of adult attachment style in the daily affective experiences of older adults with physician-confirmed knee osteoarthritis (OA).

**Methods:** As part of a larger study of racial/ethnic differences in everyday quality of life with OA, 292 persons over the age of 50 completed a baseline interview including the Revised Adult Attachment Scale (RAAS; Collins, 1996). Dimensional RAAS attachment scores were coded into the secure, preoccupied, fearful, and dismissing groups (Bartholomew & Horowitz, 1991). Positive affect (PA), negative affect (NA), and pain were assessed using an experience sampling methodology consisting of 4 daily phone calls over 7 days. These analyses used ANCOVAs to examine 28-call means and SDs for PA, NA, and pain.

**Results:** After controlling for demographics, results indicated significant group differences in average PA, NA, and pain. Pairwise comparisons indicated that participants endorsing a stable attachment style reported significantly more PA and less NA than those with a fearful attachment style. Group differences for pain were marginal and less clear cut. Significant differences also emerged for variability of NA and pain. Individuals with a secure attachment style were significantly less variable in NA than those in the fearful and preoccupied groups. For pain variability, the preoccupied group showed more variability than those with secure or dismissive styles.

**Implications:** Results contribute to a growing understanding of how individual attachment style may underlie the day-to-day affective experience of chronic pain. (Supported by R01-AG041655, D. Smith and P. Parmelee, PIs.)

### BEST PRACTICES FOR LIVING LABS WHEN STUDYING OLDER ADULTS LIVING IN RURAL COMMUNITIES

Ashley Nakagawa,[1] Shannon Freeman,[2] Alanna Koopmans,[3] Chris Ross,[4] and Richard McAloney,[2] *1. University of Northern British Columbia, Honolulu, Hawaii, United States, 2. University of Northern British Columbia, Prince George, British Columbia, Canada, 3. UNBC, University of Northern British Columbia, British Columbia, Canada, 4. University of Northern British Columbia, University of Northern British Columbia, British Columbia, Canada*

There are two core concepts that make living labs distinguishable: involvement of users as co-creators and evaluation in a real-world setting. Living labs increase the potential for product acceptance and adoption due to testing and tailoring with target users. Currently, there is a lack of a universally accepted guideline for best practices. The objective of this review is to identify the best practices of living labs that can be recognized by the scientific community and followed in future labs. A 5-stage scoping review, following Arksey and O'Malley's (2005) framework, was used to map out the coverage of different aspects of living lab methodology. A systematic search for articles involving living lab framework and older adults published between 2016-2021, was conducted in seven databases. Nine articles were included after review, the majority of which were published in health journals and were from Italy and the United States. An overview of consistent user involvement in the innovation process, real-world testing vs. laboratory testing, and participant scope findings will be shared. Multiple rounds of user feedback, real-world testing, and a small but diverse participant group were the most successful in increasing positive sentiments about the products tested in a living lab environment. The lack of published articles on living lab frameworks studying older adults indicate a gap in the literature. Creating a universally accepted definition for living labs and guidelines for best practices will allow for scientific validity and comparisons of studies and may increase the use and popularity of living labs.

### BREST CANCER SCREENING AND MOBILE WEB APP INTERVENTION: PERCEPTIONS, KNOWLEDGE, AND NEEDS AMONG NATIVE WOMEN

Soonhee Roh,[1] and Yeon-Shim Lee,[2], *1. University of South Dakota, Sioux Falls,, South Dakota, United States, 2. San Francisco State University, San Francisco, California, United States*

American Indian (AI) women have the highest breast cancer mortality and lowest breast cancer screening rates in the U.S. The present study, in collaboration with the Yankton Sioux Tribe (YST) in South Dakota, sought to (1) identify the general public/professionals' knowledge, attitudes, and needs for a mobile web app for breast cancer screening (wMammogram) intervention, and (2) inform development of the wMammogram intervention to improve Breast Cancer screening among YST women. Following a community-based participatory research approach, two focus groups were conducted in October 2020 with a total of 22 YST women aged 40-70 years, including 17 elderly women. Each group consisted of 11 community leaders, members, healthcare professionals. Qualitative analysis was conducted using grounded theory. Participants in both groups were generally favorable toward the wMammogram intervention, and noted a potential health benefit, particularly for women in their 50s to 60s. Key areas identified by participants for intervention include: (1) needs for better knowledge of breast cancer, screening, and prevention/early detection, (2) culturally tailored educational materials rooted in AI cultural values and beliefs (e.g., holistic wellness approach, Native lifestyles), (3) barriers (e.g., fear), (4) motivators (e.g., reminders), and (5) suggestions for acceptability (e.g., content and structure of messages). These results suggest that the wMammogram intervention, which is culturally tailored and addresses the community's concerns, can be a feasible, acceptable, and effective tool to promote breast cancer screening among YST women. The results informed the development of an innovative health intervention to help reduce health disparities experienced in Indian Country.

### CAREGIVING FOR PERSONS LIVING WITH DEMENTIA IN THE NEW NORMAL: FAMILY CAREGIVER'S PERSPECTIVE.

Priyanka Shrestha,[1] Donna Fick,[2] Marie Boltz,[2] Susan Loeb,[3] and Andrew High,[3], *1. Pennsylvania State University, 2. Pennsylvania State University, University Park, Pennsylvania, United States, 3. Pennsylvania State University, State College, Pennsylvania, United States*

Persons living with dementia (PLWD) are at increased risk for COVID-19 and associated poor outcomes, including: incident delirium, hospitalization, severe symptoms, intensive care admission, and even death. PLWD are likely to rely on family caregivers to maintain their health and well-being in the community. Under normal circumstances, caregiving can be stressful and complex, and the COVID-19 pandemic has the potential to change and exacerbate the stresses of family caregiving. As a part of a larger study using descriptive qualitative methodology to explore the family caregiver understanding and experiences related to delirium in caring for a person with dementia, 14 participants (age $\bar{x}$ =67, SD= 13.8) were asked about the impact of COVID-19 on their caregiving for PLWD. Thematic analysis of the transcribed interviews using Dedoose generated four overarching themes associated with the family caregiver's perspective of changes in caregiving during the COVID-19 pandemic: 1) Cautious of COVID-19 exposure, 2) We can't go in, 3) Feeling of isolation for both the PLWD and caregiver, and 4) Six-feet distance. The current study highlights the importance of understanding the needs of PLWD during a pandemic based upon the perspective of their family caregivers and will inform the development of ways to safely incorporate family caregivers in the interdisciplinary care team. Caregivers are integral to the care of PLWD across settings of care and should be partners even during a pandemic. Solutions for care include integrating technology for individualized approaches. Finally, future areas for research will be discussed.

### CAREGIVING, LONELINESS, AND STRESS: THE ROLE OF COVID-19

Sumaiyah Syed,[1] Iris Yang,[2] and Stephanie Wilson,[2], *1. Southern Methodist University, Dallas, Texas, United States, 2. Southern Methodist University, DALLAS, Texas, United States*

According to many prior studies, informal caregivers are at risk for heightened loneliness and distress. Moreover, the COVID-19 pandemic has introduced new challenges that may be accentuated among caregivers. This study examined caregiving frequency and its relation to loneliness, perceived stress, and negative affect during the COVID-19 pandemic. We then investigated the moderating roles of COVID-19 concerns and impacts, anticipating magnified effects among caregivers. Participants included respondents from the Health and Retirement Study (HRS) COVID-19 project sample (n=2108, mean age=69) who reported their caregiving frequency, general concern about COVID-19, related concerns about family members' health, and social disruptions caused by the pandemic. Controlling for age, gender, and health status, daily caregivers reported significantly greater distress during COVID-19 compared to non-caregivers (p=.036). Higher levels of concern about family members' health during COVID-19 was significantly associated with greater loneliness among daily caregivers (p = .009), but not among non-caregivers, such that daily caregivers with greater concerns had the highest levels of loneliness. On the other hand, unexpectedly, daily caregivers who experienced fewer social disruptions due to the pandemic reported higher levels of loneliness (p = .002); however, the association was null for non-caregivers. Findings suggest that daily caregivers may be particularly vulnerable to greater loneliness and stress during the COVID-19 pandemic. These experiences may exacerbate existing mental health disparities for those providing daily care.

### COHORT DIFFERENCES IN DAILY LIFE: OLDER ADULTS HAVE HIGHER AFFECT VARIABILITY THAN 18 YEARS AGO

Johanna Drewelies,[1] and Rachel Koffer,[2], *1. Humboldt University of Berlin, Humboldt University Berlin, Berlin, Germany, 2. Arizona State University, Phoenix, Arizona, United States*

Lifespan psychological and life course sociological perspectives have long acknowledged that individual functioning is shaped by historical and socio-cultural contexts. Secular increases favoring later-born cohorts are widely documented for general well-being (among older adults). However, little is known about secular trends in short-term fluctuations in daily affective well-being and whether historical changes have occurred in young, middle-aged, and older adults alike. To examine these questions, we compared data from two independent national samples of the NSDE survey obtained 18 years apart (1995/96 vs. 2013/14) and identified case-matched cohorts (per cohort, n = 782, aged = 23–75 years) based on age and gender. We additionally examine the role of economic and health resources for cohort differences in affective variability. Results revealed that later-born cohorts report higher variability in daily negative affect than did those 18 years ago. In contrast, no cohort differences emerged in variability in daily positive affect. We conclude from our national US sample that secular trends in affect variability do not generalize unanimously to different timescales across adulthood. We discuss possible underlying mechanisms and practical implications.

### CONFIRMING A DROSOPHILA MELANOGASTER MODEL OF HUNTINGTIN AGGREGATION

Akosua Biritwum,[1] Simon Levy,[2] Bess Frost,[2] and Atanu Duttaroy,[1], *1. Howard University, Washington, District of Columbia, United States,*

*2. UT Health San Antonio, San Antonio, Texas, United States*

For decades, doctors, psychologists, and psychiatrists alike have struggled to treat the symptomatic effects of Huntington's disease. Huntington's disease is an autosomal dominant brain disease that results in the deterioration of a person's physical and mental state. Once a person inherits the disease, they end up dying from it more often than not. At present, there are 41,000 Americans with symptomatic Huntington's disease, and 200,000 more are currently at-risk of inheriting the disease. Given its 50/50 chance of inheritance, there seems to be no end in sight to this degenerative ailment. My research study, however, will show that with a more robust approach, finding a cure for this disease is possible. Ultimately, the aim of this project was to test an already established model in Drosophila melanogaster regarding the "huntingtin" protein responsible for Huntington's disease. This was achieved by first demonstrating that the flies which were modified to produce huntingtin could, in fact, produce the protein. Secondly, an experimental process was created to configure a system through which the amount of protein produced by each fly could be quantified. This quantification was vital in creating a baseline that would allow for the identification of potential therapeutic treatments in the future. In short, by establishing a quantifiable model for huntingtin, this study will pave the way to new insights on huntingtin aggregation and the identification of possible treatments for Huntington's disease in the future.

### CONVERGENT AND CONSTRUCT VALIDITY OF A CONVERSATION DIFFICULTIES OUTCOME MEASURE IN PRIMARY PROGRESSIVE APHASIA

Haylie Santos,[1] Angela Roberts,[1] Nathan Gill,[2] Elizabeth Salley,[2] Hui Zhang,[2] and Emily Rogalski,[2], *1. Northwestern University, Evanston, Illinois, United States, 2. Northwestern University, Chicago, Illinois, United States*

Cognition and language changes, and their impacts on functional communication, are central to many dementias. Thus, functional communication, including conversation difficulties, is an important endpoint for clinical trials. To develop robust outcomes in primary progressive aphasia (PPA), a dementia characterized by communication impairments, we examined the convergent and construct validity of the Perception of Conversation Difficulties-Dementia Alzheimer's Type (PCI-DAT; Orange et al., 2009). The PCI-DAT is a care partner reported measure of conversation difficulties. Eighty-two care partners with a mean age of 64.8 years (SD=10.61; 85% spouses, 5% adult children, 10% friends/siblings) whose mean relationship duration to the person with PPA was 39.1 (SD=15.1) years completed the study. Pearson's correlation indicated a significant, modest correlation ($r=-0.54$, $p<0.0001$) between the PCI-DAT Perception of Conversation Difficulties subscale and the Communication Effectiveness Index (Lomas et al., 1989) suggesting strong convergent validity. A Rasch analysis conducted on the same PCI-DAT subscale showed high person (0.92) and item (0.95) reliability indicating a robust overall scale structure that adequately evaluates various levels of conversation difficulty severity in PPA. Six items (27%) had minor 'fit' issues (defined by Wright and Linacre, 1994 as having infit statistics < 0.6 or > 1.4) relative to the underlying construct. Results suggest strong convergent and construct validity of the PCI-DAT in PPA and indicate items that will benefit from further development. Overall, our results suggest that the PCI-DAT holds promise for use as a functional communication endpoint in PPA clinical trials. Data for all five PCI-DAT subscales will be presented.

### COPING WITH THE SUBJECTIVE IMPACT OF COVID-19 AMONG OLDER ADULTS IN PUERTO RICO

David Camacho,[1] Yamile Marti,[2] Sunghwan Cho,[3] Thomas Buckley,[4] Julia Vazquez,[5] Denise Burnette,[6] and Humberto Fabelo,[6], *1. University of Maryland Baltimore, Baltimore, Maryland, United States, 2. Columbia University, Columbia University School of Social Work, New York, United States, 3. VCU, Richmond, Virginia, United States, 4. Virginia Commonwealth University, NORFOLK, Virginia, United States, 5. University of Maryland, University of Maryland, Baltimore, Maryland, United States, 6. Virginia Commonwealth University, Virginia Commonwealth University, Virginia, United States*

The COVID-19 pandemic poses serious physical and mental health risks for older adults worldwide. To develop culturally and contextually congruent services to mitigate these risks requires understanding their stress and coping processes, which remain understudied in Latin America. This study examines qualitative data from 51 adults aged 60 and over who participated in an ongoing study of older Puerto Ricans' knowledge, attitudes, and practices about COVID-19. Trained interviewers collected the data by telephone from January to August, 2021. Two-thirds of participants were female, 60% had less than high school education and 90% had poverty-level incomes. Drawing on Lazarus and Folkman's Stress and Coping Theory, we conducted a thematic analysis of responses to open-ended questions about the nature and extent of COVID-related stressors, stress management, and meanings and guidance they had gleaned from their experience. Participants perceived the pandemic as an added threat to ongoing chronic stressors (e.g., Hurricane Maria, poverty, political instability); disruptions in daily routines, family cohesion, and grief and loss processes; and increased isolation and loneliness. They reported using cognitive, behavioral, socioemotional and spiritual coping, including positive thinking, keeping occupied, relaxation, religious practices and, in a few cases, social media. Participants highlighted a revitalized appreciation for emotional qualities of relationships, freedom and life in general. Consistent with our guiding theory, cultural, contextual, religious, and socio-political factors shaped their appraisals of stress and their coping strategies. Future research should examine how these practices relate to health outcomes and quality of life and how they can inform effective, appropriate interventions.

### COVID-19 SILVER LININGS - EXPERIENCE OF SPOUSAL CAREGIVERS OF PERSONS WITH DEMENTIA ENGAGED IN SUPPORT PROGRAM

Andrew Nguyen,[1] Elizabeth Stevenson,[2] Mary Mittelman,[3] Roscoe Nicholson,[4] Tiffany Donley,[5] Rebecca Salant,[6] Steven Shirk,[7] and Maureen O'Connor,[1], *1. Boston University School of Medicine, Boston, Massachusetts, United States, 2. NYU Langone Health, New York, New York, United States, 3. NYU Langone HealthNYU*

*Grossman School of Medicine, NYU Grossman School of Medicine, New York, United States, 4. University of Chicago, Evanston, Illinois, United States, 5. NYU School of Medicine, New York, New York, United States, 6. NYU School of Medicine, NYU School of Medicine New York City, New York, United States, 7. Bedford Veterans Administration Medical Center, Bedford, Massachusetts, United States*

Caring for a person with dementia (PWD) has been consistently associated with negative effects on health, including increases in caregiver depression, anxiety, and burden. Emerging studies have shown that the COVID-19 pandemic has increased these factors due to reported increases in caregiver workload and cognitive and behavioral symptoms of the PWD. We interviewed 10 spousal caregivers of PWD from the NYU Langone Alzheimer's Disease and Related Dementias Family Support Program in Summer 2020 during the COVID-19 pandemic in order to gain feedback about their experiences during the pandemic and the transition from in-person to videoconferencing that could be used to improve services and support. Caregivers discussed the challenges faced during the pandemic but also the unique opportunities the situation presented. We report here on those positive aspects of COVID-19 from the perspective of the caregivers we interviewed.

**Methods:** Interviews of caregivers residing with their spouses in the New York City area were conducted via videoconferencing, transcribed, deidentified, and analyzed using framework analysis methods.

**Results:** We found that caregivers reported some positive reaction to videoconferencing that included increased support group cohesion, increased convenience, feeling less obligated to participate in events, and new opportunities for social contact. Participants also discussed positive intercouple relationship changes such as increased quality time spent together. Our findings resonate with a body of literature focused on understanding the positive aspects of caregiving. Understanding the full presentation of the caregiver experience, including both positive and negative aspects, is important for developing interventions and resources for this unique group.

### COVID-19 VACCINES IN SKILLED NURSING FACILITIES: RECURRING INTERVIEWS WITH ADMINISTRATORS

Amy Meehan,[1] Joan Brazier,[1] Caroline Madrigal,[2] Renee Shield,[1] and Emily Gadbois,[1], *1. Brown University, Providence, Rhode Island, United States, 2. Providence VA Medical Center, Providence VA Medical Center, Rhode Island, United States*

Skilled nursing facilities (SNFs) are on the front lines of changing policies regarding the COVID-19 pandemic. The most recent development is a potential vaccine mandate for staff working in SNFs. We use ongoing findings from 130 of 160 in-depth, semi-structured interviews in progress with administrators at 40 SNFs in eight diverse healthcare markets across the United States to understand the current landscape of COVID-19 in SNFs. Four repeated interviews at 3-month intervals provide a unique longitudinal perspective on the impact of COVID-19 and SNFs' response to vaccinations, including the vaccine mandate. Rigorous thematic analysis reveals insights into administrator responses and creative approaches to address vaccine hesitancy, and future expectations for SNF operations in light of the vaccine and the mandate. Administrators express cautious hope that the vaccine will allow SNFs to return to a new normal of daily life for residents in terms of family visitations, communal dining, and resident activities. Overriding questions include how to overcome persistent vaccine hesitancy from SNF staff who cite fear of side effects despite education initiatives and how to stem staff retirement or transition to other healthcare settings. SNFs represent a microcosm of the country's concerns as a whole. Insight into the evolving and complex dynamics shed important light on national trends and help provide solutions for moving forward. Findings from this study have implications for policymakers and SNF leadership as they consider ways to promote vaccination and retain staff amid vaccine mandates.

### CROSS-VALIDATION OF THE FINANCIAL EXPLOITATION VULNERABILITY SCALE

Juno Moray, and Peter Lichtenberg, *Wayne State University, Detroit, Michigan, United States*

Most of the available clinical tools that detect the experience of financial exploitation (FE) are not practical for use by the many professionals who work with older adults. The available measures are often time-consuming, require specialized training to administer, or focus exclusively on the cognitive aspects of financial decision-making. The need for a brief, standardized measure of contextual risk prompted the development of the Financial Exploitation Vulnerability Scale (FEVS; Lichtenberg et al., 2020). The purpose of this study was to cross-validate the FEVS as a psychometrically sound measure of contextual risk for FE. Participants were recruited from the community (n=95), and through a financial coaching service for older adults who had experienced a financial scam (n=21). A total of 114 older adults were recruited for the study, 33 who had experienced FE and 81 who had not. An independent samples t-test demonstrated that the FEVS total score successfully differentiated older adults based on the experience of financial exploitation. The FEVS total score was correlated with age, but no other demographic factors or collected neurocognitive measures. A ROC curve detecting FE analysis revealed an area under the curve of 0.68. Internal consistency of the FEVS was a=0.80. In a logistic regression model, only the FEVS and word-reading ability were related to FE. The results of this cross-validation study are very similar to the initial study, demonstrating that the FEVS is an accessible, theory-based tool that detects the experience of FE.

### DECLINING TREND IN USE OF SLEEP MEDICATIONS AMONG OLDER ADULTS IN THE UNITED STATES

Christopher Kaufmann,[1] Adam Spira,[2] Emerson Wickwire,[3] Ramin Mojtabai,[2] Sonia Ancoli-Israel,[4] Constance Fung,[5] and Atul Malhotra,[4], *1. University of Florida, San Diego, California, United States, 2. Johns Hopkins Bloomberg School of Public Health, Baltimore, Maryland, United States, 3. University of Maryland School of Medicine, Baltimore, Maryland, United States, 4. University of California San Diego School of Medicine, La Jolla,*

*California, United States, 5. VA Greater Los Angeles Healthcare System, North Hills, California, United States*

Recent initiatives to discourage over-prescription of sleep medications have increased awareness of their potential adverse effects in older adults; however, it is unknown whether these efforts translated into a decline in use of these medications in the United States. We assessed recent national trends in the use of medications for sleep disorders. Data came from the 2013-2018 National Health and Nutrition Examination Survey. At each of three waves of in-person assessments, participants presented prescription bottles for all medications used in the prior month. Interviewers recorded each medication and participants self-reported duration and indications for use. We identified all medications used for a sleep disorder and categorized medications into two groups: FDA-approved sleep medications, and medications used off-label (i.e., any other medication reported to be used for sleep disorders). We examined changes in the prevalence in use of these medications across the study period. The odds of using medications for sleep disorders decreased 31% between 2013 and 2018 (odds ratio [OR]=0.69, 95% confidence interval [CI]=0.51-0.92). This trend was driven by declines in use of FDA-approved sleep medications (OR=0.45, 95% CI=0.34, 0.59). Of note, among those age 80+ years, we observed an 86% decline (OR=0.14, 95% CI=0.05-0.36) in the odds of using FDA-approved sleep medications over the study period. Results suggest a possible effect of efforts to curb over-prescription and encourage judicious use of these agents. Future research needs to examine whether these changes have coincided with improved sleep health in the growing population of older adults.

### DELIRIUM DURATION PREDICTS 1-MONTH AND 6-MONTH MORTALITY IN SEPTIC PATIENTS FROM AN ACUTE GERIATRICS UNIT

Paolo Mazzola,[1] Antonella Zambon,[2] and Giuseppe Bellelli,[3], *1. Università degli Studi di Milano-Bicocca, Monza, Lombardia, Italy, 2. University of Milano-Bicocca, Milano, Lombardia, Italy, 3. University of Milano-Bicocca, Monza, Lombardia, Italy*

Sepsis is highly prevalent in the older population compared to younger age groups. We showed that the SOFA score predicts the risk of death at 30 days in patients discharged from an acute geriatrics unit (AGU). We aim at comparing the ability of both delirium duration and SOFA to predict 1-month and 6-month mortality among septic patients. We performed an observational cohort study recruiting all patients consecutively admitted to San Gerardo hospital AGU (Italy) between March 2017 and January 2020, aged ≥70, who were diagnosed with sepsis according to 2016 Sepsis-3 criteria. All patients underwent a comprehensive geriatric assessment, including delirium twice a day with the 4AT. Outcomes were 1- and 6-month mortality rates. From 3,326 hospitalized patients, 235 were included in the study (median age 84 years, 42% females). Delirium accounted for 71.9% (169 patients, median duration 3 days). One-month and 6-month mortality rates were 32.3% and 55.3%, respectively. Age, albumin, hemoglobin, and PCR levels were associated with mortality and included as covariates in our Base Model. We performed pairwise comparison between c-indexes of the Base Model vs. Base+delirium duration (days) vs. Base+SOFA. The increment of predictive performance of model including delirium duration was statistically significant (c-index: 0.67 vs. 0.75 when considering 1-month mortality; 0.70 vs. 0.75 for 6-month mortality). Base+delirium duration performed better than Base+SOFA, but the difference not significant. Delirium duration performs as well as SOFA score in predicting 1- and 6-month mortality, with practical implications for the management of these patients in the continuum of care.

### DESCRIPTIVE EVIDENCE OF COVID-19'S IMPACT ON BLACK LGBTQ ADULTS AMIDST ONGOING HEALTH INEQUITIES

charleigh Flohr,[1] and Cassandra Burton,[2], *1. Human Rights Campaign Foundatiom, Washington, District of Columbia, United States, 2. AARP, Atlanta, Georgia, United States*

Black LGBTQ people significant challenges and discrimination as they face the barriers of living at the intersection of multiply marginalized identities, which have worsened during the coronavirus pandemic. At a baseline, LGBTQ people exhibit elevated risk of being negatively impacted by the pandemic across health, economic and other social outcomes (Cahill et al., 2020; Heslin & Hall, 2021; Human Rights Campaign, 2020). Black LGBTQ people have also suffered significant economic losses (Human Rights Campaign Foundation, 2020) .

**Methods:**The Human Rights Campaign Foundation and AARP supported and partnered with Community Marketing & Insights to conduct a survey of 1,815 Black LGBTQ adults in the United States. The online survey was fielded between September 21 and October 30, 2020. Many Black LGBTQ adult participants report healthcare discrimination in the last three years, with 19% reporting racial discrimination and 11% reporting sexual orientation-based discrimination. Furthermore, 31% of Black transgender adults report healthcare discrimination in the last three years. Preventing or treating COVID-19 is an important health concern to 67% of Black LGBTQ adults aged fifty-five and older, 63% of Black LGBTQ adults aged thirty-five to fifty-four and 53% aged eighteen to thirty-four. Overall, Black LGBTQ adults report being significantly impacted by the pandemic, including negative impacts on their social health (60%), mental health (44%), the physical health of their close friends and family (33%), their finances (30%), and their employment status (22%). Overall, one-quarter (25%) of Black LGBTQ adults had at least one close friend or family member die from coronavirus illness.

### DISABILITY & SLEEP QUALITY OF CUSTODIAL GRANDPARENTS DURING FALL 2020 OF THE COVID-19 PANDEMIC

Kellie Mayfield,[1] Karen Clark,[2] and Raeda Anderson,[3], *1. Georgia State University Byrdine Lewis College of Nursing and Health, Atlanta, Georgia, United States, 2. Southern University and A&M College, Baton Rouge, Louisiana, United States, 3. Shepherd Center, Atlanta, Georgia, United States*

Disability of custodial grandparents, grandparents who are the primary caretakers of their grandchildren often in parent absent households, are not frequently examined. One in four adults in the U.S. lives with a disability with the highest percentage of disabilities reported in the South. Quality sleep is integral for overall wellbeing and is altered with age. Sleep complaints of older adults are associated with multiple adverse health outcomes such as dementia, stroke and obesity. The objective of this study was to examine the relationship between disability and sleep quality amongst custodial grandparents during the COVID-19, Fall 2019 in Georgia. Thirty-four custodial grandparents were recruited from the Georgia Division of Aging Kinship Care Support Groups, ages 42 to 78, with most identifying as African American. Disability status and the Pittsburgh Sleep Quality Index were measured. Results showed a significant negative relationship for custodial grandparents' disability status and sleep quality ($\chi 2$= 9.167, p=0.027; $\Gamma$=-0.683, p=0.002), sleep disturbance ($\chi 2$= 12.150, p=0.002; $\Gamma$=-0.897, p<.001), and use of sleeping medication ($\chi 2$= 9.645, p=0.022; $\Gamma$=-0.785, p<.001). Custodial grandparents with a disability had worse sleep quality, more sleep disturbances, and took more sleeping medication compared to custodial grandparents without a disability. Results have implications for kinship care providers and medical practitioners when engaging with custodial grandparents about their health, disability and impacts on their sleep quality.

### DO CAREGIVER INTERVENTIONS IMPROVE OUTCOMES IN RELATIVES WITH DEMENTIA? A META-ANALYSIS

Sheung-Tak Cheng, *Education University of Hong Kong, The Education University of Hong Kong, Not Applicable, Hong Kong*

Despite plenty of reviews on the benefits of nonpharmacological interventions for dementia informal caregivers, large-scale review on the effects of these interventions on the care-recipients (CRs) is lacking. We searched PsycINFO, CINAHL with Full Text, MEDLINE, and PubMed from inception to end of 2020 and found 144 articles that reported randomized controlled trials of caregiver interventions using CR outcomes. Interventions were found to reduce neuropsychiatric symptoms and mood disturbance, enhance cognition and quality of life, and delay institutionalization and mortality, with care coordination/case management, educational intervention with psychotherapeutic components, and direct training of the care-recipient (with caregiver involvement) being the more potent interventions. However, the effects were generally small to very small. Together with existing findings on caregiver outcomes, a tripartite scaffolding model of caregiver support is proposed. Future directions in terms of developing consensual guidelines, a registry of intervention manuals, and family-centered programs are discussed.

### DOES RETIREMENT AGE IMPACT FUNCTIONAL LIMITATIONS IN LATER LIFE?

Ziyao Xu, *Miami University, Oxford, Ohio, United States*

The US government is gradually shifting the full retirement age in Social Security to age 67. However, previous studies suggest that this shift could negatively impact the mental and physical health of retirees. To understand the potential impact of raising the full retirement age on the functional health of retirees, this longitudinal study examined changes in physical functioning over time in retirees by age at retirement. Twelve waves of the Health and Retirement Study (1994 – 2018) were used. A total of 8,261 retirees was included. The retirement age was a categorical variable: very early age (<62), early age (62-64), traditional age (65-67), and late age (>67). Physical functioning was measured using 15 Activities of Daily Living and Instrumental Activities of Daily Living. A GEE model was used to assess the relationship between the retirement age category and the number of functional limitations. In the adjusted model, after controlling for all the other variables including baseline health and functioning, late retirement was associated with an 8.9% increased risk of functional limitations compared to traditional age retirement (IRR: 0.91, 95% CI:0.84 –0.98). Compared to late retirees, the risk of functional limitations was increased by 28.6% in very early age retirees (IRR: 1.29, 95% CI:1.21–1.36). Compared to those retiring at traditional retirement age, those retiring late, after 67, have increased the risks of functional limitations. Although levels of disability could influence age of retirement, these results suggest that for some workers efforts to increase age of full retirement, could have negative effects.

### DYADIC PERCEPTIONS OF COVID-19 PANDEMIC IMPACT ON EVERYDAY LIFE

Jennifer Margrett,[1] Celinda Reese-Melancon,[2] 5Dan Russell,[1] Lauren Stratton,[1] Erin Harrington,[3] and Jyoti Savla,[4], *1. Iowa State University, Ames, Iowa, United States, 2. Oklahoma State University, Oklahoma State University, Oklahoma, United States, 3. Pennsylvania State University, University Park, Pennsylvania, United States, 4. Virginia Tech, Blacksburg, Virginia, United States*

It is important to understand the effects of the COVID-19 pandemic not only on individuals' daily lives, but also their close partners. Current literature suggests that the COVID-19 pandemic has impacted older adults' lives in several ways, including the frequency of social interactions and change in various life habits (Lesbrasseur et al., 2021). Data from 42 middle-aged and older, long-term married or cohabitating dyads were collected as part of an ongoing study of everyday cognition and functioning among couples. Participant age ranged from 40-85+, and couples were partnered for 9-60+ years. During this study, COVID-19 pandemic impact was assessed using six items (1 = No change to 4 = Severe change) examining daily routines, medical and mental health access, social contacts, and pandemic and family-related stress; reports ranged from six to 19. On average, women reported significantly higher COVID-19 pandemic impact compared to men. For both partners, the greatest disruptions reported related to routines and social contacts. Further analysis examined COVID-19 pandemic impact in dyads. For eight dyads, both partners reported relatively lower COVID-19 impact (6-11), whereas for six dyads, both partners reported higher impact scores (14-19). Discussion focuses on within-dyad and between-dyad differences related to perceptions of the pandemic's impact.

### EFFECTS OF A MULTIPLE CHRONIC CONDITION (CC) REMOTE MONITORING PROGRAM ON CLINICAL OUTCOMES AMONG OLDER ADULTS

Eldin Dzubur,[1] Roberta James,[2] Jessica Yu,[2] Julia Hoffman,[2] and Bimal Shah,[2], *1. Teladoc Health,*

*pasadena, California, United States, 2. Teladoc Health, Mountain View, California, United States*

Older adults are faced with an increased risk of comorbid chronic disease such as diabetes. While multiple health behavior change interventions (MHCIs) are known to improve clinical outcomes more than targeted interventions, less is known whether such effects persist in older populations. The objective of the study was to examine the effects of multiple chronic condition (CC) remote monitoring program enrollment and mental health program enrollment on glucose and blood pressure reduction, adjusting for self-monitoring behaviors. In a sample of 594 older adults (age 55+, 14% 65+ years, 46.8% female) evaluated over a 12-month period, statistical models showed that older adults with uncontrolled diabetes (A1c >= 7.0%) had a 7.9 pt. reduction in blood glucose for each additional program enrolled and a 22.7 pt. reduction in blood glucose when enrolled in mental health compared to those not enrolled. Similarly, older adults with uncontrolled hypertension (BP >= 130/80) had a 4.8 pt. reduction in systolic blood pressure for each additional program enrolled and a 7.2 pt. reduction in systolic blood pressure when enrolled in mental health compared to those not enrolled. The findings indicate the potential for multiprogram digital health interventions that incorporate mental health to further improve clinical outcomes in older adults suffering from multiple chronic diseases, namely diabetes and hypertension.

### EFFECTS OF OBESITY REDUCTION ON PHYSICAL FUNCTION, INFLAMMATION AND OSTEOARTHRITIS IN OLDER ADULTS

Michael Borack,[1] Kathryn Porter Starr,[2] Connie Bales,[1] Jamie Rincker,[3] Lou DeFrate,[3] Amy McNulty,[3] James Coppock,[3] and Abigail Holt,[3], *1. Duke University School of Medicine, Durham, North Carolina, United States, 2. Duke University School of Medicine, Hillsborough, North Carolina, United States, 3. Duke University, Durham, North Carolina, United States*

Age-related increases in chronic inflammation lead to reduced physical function via damage to muscle and joints and contribute to osteoarthritis (OA) risk. Obesity in older adults with OA further exacerbates inflammatory damage. Whether obesity reduction can lessen inflammation and improve OA is unknown; however, novel biomarkers may provide an answer. We completed a 6-mo. weight loss intervention (-500 kcal/day), studying blood biomarkers of inflammation and cartilage damage along with physical function in obese older adults with (OA+; n=39) and without an OA diagnosis (OA-; n=20). Participants were aged > 60 yrs (mean = 70.2±6.0) and obese (BMI =34.6±4.7 kg/m2). At endpoint, weight loss was -6.3±4.0% and -5.8±4.1% in OA+ and OA-, respectively, with no group difference. Change scores for function for OA+ and OA- were: Short Physical Performance Battery score (+1.7±1.3 and +2.1±1.5), 8 ft up and go (-0.7±1.0 and -0.9±1.12 sec) and 6 min walk (+31.4±105.1 and +39.5±57.4 meters). All improved from baseline ($p<0.05$), with no group difference. Concerning blood biomarkers, there was a decrease ($p<0.05$) in cartilage oligomeric matrix protein (COMP: OA biomarker), indicating a potential benefit for OA. Change in COMP also differed between groups; OA- had a greater ($p<0.05$) reduction than OA+. Pooled results showed improved adiponectin ($p<0.05$), with no group difference. There were no changes for CRP, CTX-1, IL-6 and TNF-α. Our novel findings link early intervention with better reduction of OA risk and inflammation in obese older adults and also show important benefits for improved physical function regardless of OA status.

### EXAMINING DIFFERENT TYPES OF SLEEP AMONG CUSTODIAL GRANDPARENTS DURING COVID-19

Karen Clark,[1] Kellie Mayfield,[2] and Raeda Anderson,[3], *1. Southern University and A&M College, Baton Rouge, Louisiana, United States, 2. Georgia State University Byrdine Lewis College of Nursing and Health, Atlanta, Georgia, United States, 3. Shepherd Center, Atlanta, Georgia, United States*

Sleep is associated with healthy living. With increased age, sleep is harder to initiate and maintain. Currently, over two million grandparents have become primary caregivers to their grandchildren and are at risk for poor sleep outcomes. Research shows that grandparent caregivers are at risk for depression due to poor sleep quality. Thus, this study aimed to identify the sleep quality of custodial grandparents to gain a better understanding of sleep patterns during COVID-19 in 2020. Thirty-four custodial grandparents were recruited from the Georgia Division of Aging Kinship Care Support Groups from September through October 2020. Participants were between 42 to 78 years old with a mean age of 57. Participants completed the Pittsburgh Sleep Quality Index. Stata statistical software was used to analyze the relationship between the sleep quality subscales. Results showed a significant positive relationship for custodial grandparents between sleep quality and daytime dysfunction ($\chi2=25.993$, $p=0.002$; $\Gamma=0.495$, $p=0.039$) as well as sleep quality and sleep disturbance ($\chi2=11.129$, $p=0.084$; $\Gamma=0.751$, $p<0.001$). There is a significant positive relationship between daytime dysfunction and sleep duration ($\chi2=14.984$, $p=0.091$; $\Gamma=0.681$, $p<.001$), where grandparents with daytime dysfunction have longer sleep duration. Findings suggest grandparents with poor sleep quality are more likely to experience daytime dysfunction and have more sleep disturbances in the COVID-19 environment. Our study will benefit researchers and practitioners caring for custodial grandparents and contribute to future research focused on custodial grandparents and sleep quality.

### EXPLORE THE ROLE OF ABETA IN AXONAL TRAFFICKING DEFICITS INDUCED BY ALPHA SYNUCLEIN IN PARKINSON DISEASE MOUSE MODEL

Alfredo Castro, *UCSD MADURA R25 Advancing Diversity in Aging Research Program, La Jolla, California, United States*

Alpha synuclein (ASYN) is a neuronal protein that is observed in significant amounts in the brain and is encoded for by the SNCA gene, it functions as a regulator for the trafficking of synaptic vesicles. It has been noted that the buildup of alpha synuclein has been found in the form of Lewy bodies in studies involving patients with Parkinson's diseases (PD). Gathering an understanding for the manner in which alpha synuclein affects the synaptic structure and the movement of axonal trafficking will help further our understanding towards the formation of Lewy bodies. Experimenting with the way in which ASYN affected the intervention of Abeta was important, to see the toxicity of Abeta in axonal trafficking. The PD and SynKO mouse models treated with Abeta both showed an effect on the anterograde moving speed of both the PD and SynKO neurons. Synaptic formation was examined, and it was found that ASYN had a large negative influence on the synapse formation in PD neurons. This was due to the significantly reduced colocalization that was found in the treated neurons. It was confirmed that ASYN caused neuronal atrophy through the over expression of GFP-ASYNWT wild type or the GFP-ASYNA53T. Comprehending ASYN effect on the axonal trafficking and the synaptic structure of PD neurons can help understand the mechanism that may be present which possibly stimulates Alzheimer's Disease in PD patients.

### EXPLORING THE ECOLOGY OF THIRD AGE INFORMAL LANGUAGE LEARNER GROUPS

Gareth Barnes, *Macquarie University, Sydney, New South Wales, Australia*

This research explores the factors involved in the emergence of an independently organized Third Age informal language learner group in a community centre in Japan. The methodology applies PPCT (Process-Person-Context-Time) from Bronfenbrenner's bioecological approach to provide a detailed perspective of the people, the environment and settings over time to show how these factors interact to construct an emergent learner group. The analysis looks at how and why this specific learner ecology emerges and ultimately, how it can benefit the Third Age and inform healthy ageing policy. The findings show that by engaging in second language learning, the participants find meaningful and active involvement in the group by creating a setting that welcomes self-expression, while balancing limiting and facilitating factors of resilience and reciprocal support, self-management, sage-ing, interest, agency, and responsibility. The result is the creation of a multilingual, multicultural, and multigenerational place of inclusion within the community. The study highlights the heterogeneity of the 3rd Age and illustrates the interplay of contexts outside of the learner group from micro to macro, individual and group resources, and the influence of the specific social time period. It also shows the social importance of creating opportunities for autonomous informal language learning settings in the community while highlighting the impact of Third Age agency.

### FALLS AMONG OLDER ADULTS IN THE PHILIPPINES AND VIETNAM: RESULTS FROM NATIONALLY REPRESENTATIVE SAMPLES

Tuo Yu Chen,[1] Grace Cruz,[2] Cong Nguyen Vu,[3] and Yasuhiko Saito,[4], *1. Taipei Medical University, Taipei, Taipei, Taiwan (Republic of China), 2. University of Philippines, Quezon City, Quezon, Philippines, 3. Institute of Population, Health and Development, Ha Noi, Ha Noi, Vietnam, 4. Nihon University, Tokyo, Tokyo, Japan*

Falls are a major public health issues globally. However, no study with nationally representative samples, previously, had been done to understand falls among older adults in the Philippines and Vietnam. Using a biopsychosocial perspective, this study investigated the prevalence of falls and their associated factors among community-dwelling older adults in these countries. Cross-sectional data were drawn from the Longitudinal Study of Ageing and Health in the Philippines (N = 4,606) and the Longitudinal Study of Ageing and Health in Vietnam (N = 4,378). The outcome variables were any falls in the past year. Independent variables included biophysical factors (vision/chronic conditions/functional impairments/pain locations/insomnia symptoms/sleep medications/grip strength/walking speed/postural control), psychological factors (depressive symptoms), and sociodemographic factors (age/sex/education/living in urban area/living alone/social network size). Descriptive analysis and logistic regression analysis were used to analyze data. The results showed that 17.7% of the Filipino older adults fell in the past year and it was 7.3% among Vietnamese older adults. Significant factors that increased the odds of any falls among Filipino older adults were having a higher level of education, living in urban area, living with others, experiencing more functional impairments, reporting one or more pain locations, and having poor grip strength. In Vietnam, having more chronic conditions, experiencing more functional impairments, and reporting two or more pain locations were found to increase the odds of any falls. Population in the Philippines and Vietnam are aging rapidly. Findings from this study are timely in identifying at-risk individuals and preparing for effective falls prevention strategies.

### FEAR OF FALLING, FALL RISK, DEPRESSION, AND ANXIETY IN COMMUNITY-DWELLING OLDER ADULTS

Aleatha Rossler,[1] Ladda Thiamwong,[2] Rui Xie,[1] Jeffrey Stout,[1] Joon-Hyuk Park,[1] Oscar Garcia,[1] and Renoa Choudhury,[1], *1. University of Central Florida, Orlando, Florida, United States, 2. College of Nursing, University of Central Florida, Orlando, Florida, United States*

Fear of falling is common in the older adult population with an estimated 43% being affected. We aimed to examine the associations among fear of falling, fall risk, depression, and anxiety in community-dwelling older adults. For this study 124 participants ranging from 60 to 96 years of age were recruited from the community settings in Central Florida. Fear of falling, fall risk, depression and anxiety were assessed using the Falls Efficacy Scale International (FES-I), the CDCSTEADI fall risk assessment, the Patient Health Questionnaire (PHQ) for depression, and the Geriatric Anxiety Inventory Short form (GAI-SF) for anxiety respectively. Data was collected via the Qualtrics survey. Comparisons were made for those below age 75 and those aged 75 and older, with 51.6% being under 75. Four ethnicity categories were also used: African American (8.1%), Asian (2.4%), Hispanic (14.5%), and non-Hispanic white (75%). All participants scored above 4 on the STEADI scale indicating fall risk. 42 scored positive for fear of falling on the FES-I scale and of that 42, 35.7% had a history of one or more falls in the last year ($p < .01$). 46.8% of the participants screened positive for depression and 100% of participants were positive for anxiety. Using one-way ANOVA analysis, we found significant relationships between (1) depression ($p<.01$); (2) STEADI ($p<.01$) and FES-I.

### FEASIBILITY OF COMBINING NONINVASIVE BRAIN STIMULATION AND PERSONALIZED COUNSELING TO INCREASE PHYSICAL ACTIVITY

On-Yee Lo,[1] Connor Mulvey,[2] Christine Lee,[2] Margaret Gagnon,[2] Lewis Lipsitz,[2] and Brad Manor,[3], *1. Hebrew SeniorLife/Harvard Medical School, Boston, Massachusetts, United States, 2. Hebrew SeniorLife, Boston, Massachusetts, United States, 3. Hinda and Arthur Marcus Institute for Aging Research, Harvard Medical School, Boston, Massachusetts, United States*

Few older adults meet recommended physical activity guidelines. Behavioral interventions may be more effective when combined with other modalities to promote activity. Transcranial direct current stimulation (tDCS) designed to increase the excitability of the left dorsolateral prefrontal cortex (dlPFC) — a brain region subserving motivation and executive function — has the potential to augment behavioral interventions. We designed a randomized, double-blinded trial to examine the feasibility of combining personalized behavioral counseling and tDCS targeting the left dlPFC to improve physical activity and related outcomes in sedentary older adults living within the supportive housing. Participants wore a Fit-Bit throughout the study period. Baseline step counts were determined for two weeks, then participants completed four bi-weekly personalized counseling sessions over eight weeks. They were also randomized to receive 10 sessions of tDCS or sham stimulation over the two weeks after the baseline. Physical, cognitive, and patient-reported outcomes were assessed at baseline, after ten brain stimulation sessions, and after four behavioral sessions. 33 individuals were screened and 16 enrolled (age=80±7, 13 females). 13 participants completed the study, including 100% of study assessments, 99±5% of brain stimulation sessions, and 98±7% of behavioral sessions. Fit-Bit adherence rate was 93±13%. Daily step counts were 3197±1480 at baseline and 4722±2553 over the last two weeks of the intervention. While the study is ongoing and blinded, these preliminary results indicate that it is feasible to conduct a controlled study of tDCS combined with personalized behavioral counseling to increase physical activity in sedentary older adults living within supportive housing.

### FEASIBILITY OF USING A COMMERCIAL BOARD GAME TO ASSESS UPPER EXTREMITY FUNCTION IN OLDER ADULTS

Allison Niemiec,[1] Yareli Lopez Hernandez,[2] Rachel Logue,[1] Ejlal Bin Mulayh,[2] and Susan Brown,[1], *1. University of Michigan, Ann Arbor, Michigan, United States, 2. University of Michigan, University of Michigan, Michigan, United States*

Upper extremity function, particularly the hand, declines with aging and is predictive of executive ability and independence. Standard assessments typically focus on strength partly due to a lack of easily administered functional tasks requiring multi-joint coordination and precision grasp. This study aimed to determine the feasibility of using an inexpensive board game to assess upper extremity function in older adults. Six healthy older adults (77 +/- 5.1 years) completed reaching tasks using the Connect4® game that requires grasping and placing small discs into a vertical board. Tasks included different hand configurations (unilateral, bilateral), and two dual-task conditions (serial subtraction by 7s and placing colored discs to match specific color patterns). The time to complete each task was recorded. For comparison purposes, participants completed a standardized pegboard test (Purdue Pegboard) using one or both hands. Connect4 results were similar to age-normative findings reported for the Purdue Pegboard. Dominant versus non-dominant hand performance did not differ while bilateral coordination tasks were slower than unilateral tasks for both the Purdue Pegboard (p<0.05) and Connect4 (p<0.01). Pegboard and Connect4 times were moderately to strongly correlated for all hand configurations. Dual-task conditions using Connect4 led to longer completion times (p<0.05). Preliminary results support the use of Connect4 as a functional upper extremity assessment tool for older adults. It is inexpensive, engaging, easy to use, and allows for cognitive-motor assessment using dual-task protocols, a critical factor in maintaining functional independence in older individuals. Further research will include a formal validation study across a wider age range.

### FOOT MECHANICS DEFINE DIRECTIONAL CHANGES IN CURVED-PATH WALKING: NEW METHODS TO ASSESS THE MOTOR SKILL OF WALKING

Haley Hicks,[1] Anthony McBroom,[2] Patrick Roscher,[3] Jessie VanSwearingen,[4] and Kristin Lowry,[5], *1. Broadlawns Medical Center, Des Moines, Iowa, United States, 2. Des Moines University, Des Moines, Iowa, United States, 3. Protokinetics, Inc, Havortown, Pennsylvania, United States, 4. School of Health and Rehabilitation Sciences, Pittsburgh, Pennsylvania, United States, 5. University of Pittsburgh, University of Pittsburgh, Pennsylvania, United States*

Although it is essential to navigating the world, curved path walking is a challenge to mediolateral balance control. The focus of previous curved-path walking research was in spatiotemporal characteristics. We quantified the foot-ground interaction, center of pressure (COP) characteristics during non-linear (eg curved-path) walking important to understand the functional mechanics of directional changes for curved paths. We hypothesized the foot mechanics differ between older adults with better versus poorer curved-path walking (Figure of 8 Walk Test, F8W). Twenty-five older adults (mean age 71.8 ± 8.9 years) completed the F8W on an instrumented walkway (Protokinetics, LLC.) The derived metrics of the foot mechanics included medial/lateral movement of the COP for inside and outside steps, maximum medial and lateral COP excursions, and total medial/lateral COP range. Pearson correlations were used to examine relations F8W (time and steps) and COP metrics; ANOVAs were used to examine differences in COP metrics between older adults grouped by median-split of F8W time. Longer F8W time and more steps were related to lesser total COP range and outside foot lateral maximum excursion (r range -0.415 to -0.706, p<0.04). Older adults with stronger F8W performance compared to poorer F8W performance had larger outside foot total COP ranges (3.61cm vs 4.39cm, p=0.016) and greater lateral excursion (1.60cm vs 2.12cm, p=0,003). Foot-ground interactions offer new insights into control of curved path walking and methods for evaluating efficacy of interventions focused on improving walking skill in older adults.

### HETEROGENEOUS PATTERNS OF OLD-AGE VULNERABILITY AND LONELINESS FROM THE CROSS-NATIONAL COMPARATIVE PERSPECTIVE

Oejin Shin,[1] sojung park,[2] Ji young Kang,[3] Minyoung Kwak,[4] and Jihye Baek,[5], *1. University of Illinois at Urbana Champaign, Savoy, Illinois, United States, 2. Washington university, St.Louis, Missouri, United States, 3. Chungnam National University, Daejeon, Ch'ungch'ong-namdo, Republic of Korea, 4. Daegu University, Gyeongsan, Gyeongbuk, Kyongsang-bukto, Republic of Korea, 5. Gwanak Senior Welfare Center, St. Louis, Missouri, United States*

Loneliness has been extensively examined as a major risk factor for mortality and morbidity among older adults. In this research, we identified multi-dimensional patterns of old-age vulnerability in five countries (U.S., Korea, Sweden, Germany, Italy) guided by the welfare regime framework and examined how the patterns of vulnerability are associated with loneliness in old age in each country. Data were drawn from comparative national aging data: HRS for the U.S. (n=4331), KLOSA for Korea (n=3721), SHARE for Sweden (n=2746), Germany (n=2271), and Italy (n=2988). We used three vulnerability constructs: 1) material vulnerability (poverty, high out-of-pocket expenses for health care, unaffordability for housing), 2) health vulnerability (multiple chronic condition, depression), and 3) social vulnerability (living alone, contact with children and friend, social participation). Latent Class Analysis and regression analyses were used for data analysis. The relative proportion of the least vulnerable group varies substantially (from 11.88% in Italy to 59.33% in the U.S). In Sweden and Germany, around 7% of the sample belongs to the most vulnerable group, while in other countries the most vulnerable group was not found. Notably, in Italy, older people in the social vulnerable group were more likely to feel lonely when compared to least vulnerable, suggesting the country-specific significance of social supportive environment. This study is the first research to examine the empirical structure of the concept and how it may vary across countries related to their loneliness. A full discussion of country-specific discussion and policy implication will be presented.

### HIPPOCAMPAL KNOCKOUT OF P300 AFFECTS LEARNING AND MEMORY IN MICE

Chase Rector,[1] and Lin Wang,[2], *1. Howard University, Brandywine, Maryland, United States, 2. NIH, NIH, Maryland, United States*

Aging has been associated with cognitive decline, as seen in various learning and memory processes. Specifically, p300, a lysine acetyltransferase, has been shown to decrease with age, which could have an effect on cognition. In a series of behavioral tests, the effect of the knockout of p300 was studied in mice. In the water T maze test and the object recognition test, the results conveyed that the mice's learning skills had not been impacted by the knockout of p300. But the water T maze test results further showed that the p300 knockout mice had a decline in their cognitive flexibility to new information. These findings suggest that the knockout of p300 has a negative impact on cognition. We expect that the overexpression of p300 in older mice will restore the cognition that might have been lost with aging.

### HOME CARE PHYSICAL THERAPISTS' VIEWS ON INTER-PROFESSIONAL SHARED DECISION MAKING

Cindy Moore, *Rutgers University, Hamilton, New Jersey, United States*

Shared decision making (SDM) has been associated with increased patient satisfaction and engagement and improved process and care outcomes. However, SDM use by physical therapists (PTs) working with older adults in the home care setting is not known. Understanding home care PTs' views about and experiences with SDM, including inter-professional SDM, can inform SDM research and strategies for increasing its use. This presentation highlights inter-professional aspects of SDM described by home care PTs in the qualitative arm of a mixed methods, implementation science frameworks-informed study exploring PTs' perspectives on SDM in home care. Semi-structured interview data from twenty PTs (80% female; mean age = 50.6, SD=12.7, range 28-73) representing all geographic regions of the continental United States and having varied levels of geriatric (mean 24.2 years, SD=11.6, range 3-40) and home care (mean 15.7, SD=11.5, range 2-40) experience were analyzed using thematic content analysis. A trustworthiness plan guided data collection and analysis, and reflexive journaling, memo, audit trail, peer debriefing, data triangulation, and member checking were used to support study rigor. Three inter-professional SDM-related themes were identified: team members facilitating SDM for all disciplines; within-team sharing of and competition for patient care resources; and limited patient/caregiver participation in team treatment planning decisions. Findings support the need for including inter-professional issues in home care SDM implementation and measurement research and suggest topics to include in future studies.

### HOSPICE CAREGIVER BURDEN AND WORK PRODUCTIVITY: AN EXPLORATORY ANALYSIS

Megan Thomas Hebdon,[1] Jiayun Xu,[2] Djin Tay,[3] Maija Reblin,[4] Kristin Cloyes,[5] and Lee Ellington,[3], *1. University of Texas at Austin, University of Texas at Austin, Texas, United States, 2. Purdue University, Purdue University, Indiana, United States, 3. University of Utah, Salt Lake City, Utah, United States, 4. University of Vermont, Burlington, Vermont, United States, 5. University of Utah, Portland, Oregon, United States*

Hospice caregivers experience burden that impacts their physical, emotional, and social well-being. Little is known about how caregiver burden impacts occupational well-being through employment and work productivity. Thus, our purpose was to explore the relationships between caregiver burden and dimensions of work productivity/impairment (absenteeism, presenteeism, activity impairment, and overall impairment) among working hospice cancer caregivers. This is a secondary data analysis of baseline data from a larger study of caregiver communication and bereavement. Study data including demographics, preparedness for caregiving, caregiver burden, and work productivity/impairment were analyzed using descriptive statistics, correlation analysis, and stepwise multiple linear regression. Dimensions of work productivity/impairment were dependent variables, and age and preparedness for caregiving were covariates. Working caregivers (N=54) had an average age of 52 (SD: 12.65), and were primarily White (83%), female (70%), married (69%), employed full-time (70%) and had a household income of $50,000 or more (65%). Greater caregiver burden was significantly associated with lower preparedness for caregiving (r=-.41, p<.01), and greater activity impairment (r=.50, p<.01), presenteeism (r=.44, p<.01), and overall impairment (r=.36, p<.05), but not absenteeism. Caregiver burden predicted activity impairment (b=1.72 [.72, 2.71], p<.01), presenteeism (b=1.44 [.33, 2.55], p<.01), and overall impairment (b=1.42 [.09, 2.74], p<.05), even after controlling for age and preparation for caregiving. More burdened caregivers may be having more challenges with work productivity. Additional research is needed to examine these relationships in a larger, more diverse sample. Future research should also investigate how supportive work environments and leave policies reduce caregiver burden and promote work productivity.

### IMPACT OF RECREATIONAL PHYSICAL ACTIVITY AND BODY COMPOSITION ON INSULIN RESISTANCE IN OLDER ADULT POPULATION

Pao-Feng Tsai, Chih-Hsuan Wang, Caralise Hunt, Sarah Watts, and Katilya Ware, *Auburn University, Auburn, Alabama, United States*

**Purpose:** The study was to identify the impact of physical activity (PA) and body composition on insulin resistance in older adults and investigate racial/ethnic difference in these relationships.

**Method:** This secondary data analysis project used 717 participants who were 55 years and older without diabetes from the 2017-2018 National Health and Nutrition Examination Survey. Major variables included homeostatic model assessment of insulin resistance (HOMA-IR), body mass index (BMI), waist circumference (WC), and recreational PA. The covariates included were demographics, nutrition intake, and lifestyle (smoking and alcohol usage). Hierarchical multiple regressions were conducted to analyze the data.

**Results:** Body composition and recreational PA had interaction and main effects on HOMA-IR in the overall sample. Level of HOMA-IR decreased with increasing levels of recreational PA in individuals with lower levels of BMI, but in individuals with higher levels of BMI, HOMA-IR increased. Level of HOMA-IR increased with increasing levels of recreational PA across the range of WCs, but the rate of increase was slightly more pronounced at higher level of WCs. These results varied across racial/ethnic groups.

**Conclusion:** Overall, both recreational PA and body composition are important factors that affect level of HOMA-IR, but body composition seems to be the dominant factor. PA is only beneficial for older adults with low BMI. Interventions for reducing insulin resistance should be tailored to specific racial/ethnic groups.

### IT TAKES A VILLAGE - ATYPICAL PRESENTATION WARRANTS TEAMWORK FOR SEPSIS CARE

Teresa Cranston, and Nicole Bailey, *Penn Medicine Lancaster General Health, Lancaster, Pennsylvania, United States*

Sepsis is the body's overwhelming response to infection that can lead to tissue damage, organ failure, and death. Sepsis, disproportionately, affects older adults due to an aging immune system and chronic illness. Older adults present atypically delaying diagnosis and intervention. This delay has been associated with increased morbidity and mortality. However, with early detection and rapid treatment there can be a reduction in poor outcomes. It is key, that clinicians practice collaboration and communication when diagnosing & treating patients that may have a different presentation. Early detection, monitoring, and intervention are crucial to survival of the older septic patient. The Continuous Monitoring Unit (CMU) consists of RNs, who monitor telemetry, video, and the Sepsis BPA/Checklist 24 hours/7 days a week. These nurses evaluate all active ED and acute care BPA data points. With their specific training, they dismiss the irrelevant and align the significant data while looking for possible notes of infection. They do not act upon the BPA unless the patient meets criteria. If there is confirmation of the BPA, the nurse collaborates with the primary provider to initiate recommended sepsis care. Since the beginning of August 2021, the CMU RN is utilizing the Sepsis Checklist, a customized tool built into the EMR that allows the CMU nurse to monitor the key elements of the Sepsis Bundle and intervene concurrently to complete the CMS SEP1 recommendations. Recommendations are based on the Surviving Sepsis Campaign.

### LATENT CLASSES OF FIRST INCIDENT DIFFICULTY IN INSTRUMENTAL ACTIVITIES OF DAILY LIVING

Danielle Feger,[1] Jennifer Deal,[1] and Alden Gross,[2], *1. Johns Hopkins University, Baltimore, Maryland, United States, 2. Johns Hopkins Bloomberg School of Public Health, Baltimore, Maryland, United States*

Ability to perform instrumental activities of daily living (IADLs) deteriorates during prodromal Alzheimer's disease (AD), eventually leading to impaired everyday functioning and dementia. Ordering and timing of IADL difficulty onset may identify individuals at greater risk of cognitive impairment, but most studies only consider total number of difficult tasks. Leveraging longitudinal data from the Advanced Cognitive Training in Independent and Vital Elderly (ACTIVE) Study who entered free of any IADL difficulty (N=1266), we hypothesized that a latent class analysis based on timing of first reported IADL task difficulty would reveal class differences in cognitive functioning . Participants were followed until they self-reported at least one IADL difficulty, study completion (10 years), or loss to follow-up. Discrete-time multiple event process survival mixture (MEPSUM) models were used to simultaneously estimate hazards of incident IADL task difficulty across 7 task groups. Two, 3, 4, and 5 latent class models were fit to the data. Both unadjusted and covariate-adjusted models (adjusted for age, sex, race, education, marital status, and general health rating) were fit. Using the 2-class solution as the most parsimonious model, model entropy was 0.855. The model was able to distinguish a class of participants with lower global cognitive factor scores at baseline (Cohen's D = 0.23, P = 0.04). We conclude that first incident IADL difficulty may be a useful measure in identifying individuals with worse cognitive functioning.

### LIFESPAN DEVELOPMENTAL ANALYSIS OF HEALTH PROBLEMS AND PROBLEM DRINKING ACROSS WHITE AND UNDERREPRESENTED ADULTS

Willard Boyd,[1] Yimei Li,[2] Mohammed Ahmed,[3] Dania Mohammed,[4] Thomas Kwan,[5] and Matthew Lee,[6]

*1. Rutgers The State University of NJ, Weehawken, New Jersey, United States, 2. Rutgers The State University of NJ, Piscataway, New Jersey, United States, 3. Rutgers The State University of New Jersey, Rutgers Center of Alcohol & Substance Use Studies, New Jersey, United States, 4. Rutgers Center of Alcohol & Substance Use Studies, Rutgers Center of Alcohol & Substance Use Studies, New Jersey, United States, 5. Rutgers University, Manalapan, New Jersey, United States, 6. Rutgers University, Piscataway, New Jersey, United States*

Research has illustrated potential health benefits from moderate drinking, but also health risks from excessive drinking. Few studies have contrasted drinking effects on health across different periods of the lifespan, and how such contrasts may vary across sociodemographic subpopulations. In this study, we investigated underrepresented racial and ethnic group status as a moderator of drinking effects on health across the lifespan. Analyses used data from two waves of a large U.S.-representative sample. We estimated a series of 3*3 between-persons ANOVAs testing effects of Wave-1 drinking group (abstainer, moderate drinkers, and excessive drinkers), age (young adulthood, midlife, and older adulthood), and drinking-group-by-age interactions in White versus underrepresented status. The outcome variable was Wave-2 hypertension (controlling for Wave-1 hypertension). In the older-adult White group, results reflected the familiar "j-shaped" curve of alcohol effects on health. Specifically, abstainers experienced higher hypertension than moderate drinkers (with marginal significance: p=.054), and excessive drinkers experienced higher hypertension than moderate drinkers (p= .002). In contrast, among underrepresented older adults, hypertension levels did not vary significantly by drinking group. Graphical results clarified that the lack of drinking effects among underrepresented older adults reflected that they had similarly elevated hypertension across all three drinking groups, whereas the White older adults only had comparably elevated hypertension in the excessive-drinker group. These findings suggest that the positive health effects of moderate drinking apply primarily to White older adults. Our poster will discuss potential explanations for the apparent lack of health benefits of safe-drinking practices among underrepresented older adults.

### LONELINESS AND ISOLATION AS BARRIERS TO MENTAL HEALTH SERVICES IN A RURAL COMMUNITY

Robert Maiden,[1] Danielle Gagne,[2] and Bert Hayslip,[3], *1. Alfred University, Alfred, New York, United States, 2. Alfred University, Alfred University, New York, United States, 3. University of North Texas at Denton, Denton, Texas, United States*

As America grapples with COVID-19, issues regarding mental health have been of rising concern, particularly among those who are isolated. According to the May 2021 American Perspectives Survey, "Americans report having fewer close friendships than they once did, talking to their friends less often, and relying less on their friends for personal support1." Additionally, 49% have three or fewer close friends in 2021, compared to 27% in 1990. 17% have no friends in their core social network. Loneliness has been associated with physical and mental health risks. We sought to explore whether loneliness was also a barrier to seeking mental health services. 90 surveys were collected from rural New York. Respondents were aged 51 to 90, Caucasian (96.6%), and female (73.3% vs, 26.7%). Overall, 34.8% said they lived alone. 29.2% would seek mental health services for feelings of loneliness, while 75.4% would do so if isolated from family. Those who felt detached or isolated from others were significantly less likely to seek help from a counselor (r = - 0.25) or MD (r = - 0.37). Isolation also negatively related to measures on the resiliency scale (

**Purpose:** - 0.22, Perseverance: - 0.33, being ok alone: - 0.32), and positively related to depression (r = .65). Those scoring higher on the "okay with being alone" scale had an increased likelihood of seeking counseling (r = 0.22). Thus, isolation and loneliness are complex topics. Intervention ought to be based on perceptions of being alone. Further research is needed.

### LONELINESS, SOCIAL SUPPORT, AND PSYCHOLOGICAL WELLBEING AMONG OLDER ADULTS

Poshan Dahal,[1] Eva Kahana,[2] Tirth Bhatta,[3] and Polina Ermoshkina,[4], *1. Case Western Reserve University, Case Western Reserve University, Ohio, United States, 2. Case Western University, Case Western reserve University, Ohio, United States, 3. University of Nevada, Las Vegas, Nevada, United States, 4. Case Western Reserve University, Cleveland, Ohio, United States*

Social support in old age has been linked to psychological wellbeing outcomes, such as depressive symptoms. However, insufficient attention has been paid to implications of social support for different domains of psychological wellbeing. In this study, we explored these associations among 797 older adults (mean age = 78.61 years) living in a retirement community in Florida from the ECRC study. Our findings show that measures of social support and connectedness have varying influences on psychological wellbeing. Loneliness was associated with lower life satisfaction (b=- -1.12, p<0.001) and higher depressive symptoms (b=3.52, p<0.001). Higher self-rated social support was associated with higher life satisfaction (b= 1.66, p<0.001) but did not predict depressive symptoms. Depressive symptoms, however, were significantly higher (b=-1.45) among individuals who reported that they don't have anyone who they can turn to if they feel lonely and want to talk. Feeling lonely also predicted lower positive affects among these older adults (b=-0.65, p<0.001). Similarly, loneliness also predicted higher negative affects (b=1.28, p<0.001). Negative affects were also significantly higher among women (b=-1.15, p<0.001) but lower among those who were living alone (b=-1.06, p<0.001). Overall, our findings underscore the importance of social support and connectedness for psychological wellbeing in later life. This finding is consistent with prior research demonstrating significance of social support in later life for the overall psychological wellbeing of the older adults.

### LONGITUDINAL ASSOCIATIONS OF PHYSICAL TOUCH WITH LONELINESS AMONG OLDER ADULTS

Taylor Brown,[1] Reese Giddens,[2] and Stephanie Wilson,[3], *1. Southern Methodist University, Dallas, Texas, United States, 2. Southern Methodist University, Southern*

*Methodist University, Texas, United States, 3. Southern Methodist University, DALLAS, Texas, United States*

Older adults in the US face heightened risks for social disconnection, and the COVID-19 pandemic has further exacerbated this crisis. Physical touch is a key dimension of social connection that uniquely predicts physical and mental health benefits. However, most studies have been limited by cross-sectional designs, and no prior work has examined the long-term effects of physical touch on loneliness. To investigate the prospective association between physical touch and loneliness among older adults, this study utilized data from 1626 older adults (Mean age = 68, range = 57-85) who participated in Waves 1 and 2 of the National Social Life, Health, and Aging Project (NSHAP). Participants reported on their loneliness and physical contact with family and friends, as well as with pets, at both waves. Results revealed that more frequent physical contact with family and friends predicted larger decreases in loneliness over the subsequent five years (p<.0001), controlling for age, race, gender, health conditions, marital status, frequency of social interaction, and baseline levels of loneliness. Physical contact with pets had no unique effect (p=.136). To further assess directionality, models tested whether lonelier people experienced decreased touch over time, and the effects were null (p>.250). Taken together, this longitudinal study is the first to identify the unique contribution of human physical touch to prospective changes in loneliness, beyond the well-established effects of covariates, including social interaction frequency. Touch represents a compelling mechanism by which social isolation may lead to loneliness, which in turn raises risks for poor health and premature mortality.

### MEASURING MINDFULNESS: ASSESSING THE UTILITY OF THE FFMQ IN THE OLDER VETERANS WITH DEMENTIA AND THEIR CAREGIVERS

Brian Clawson,[1] Phoebe Block,[2] Daniel Durkin,[3] Amber Collins,[1] Lindsey Jacobs,[1] and Michelle Hilgeman,[4], *1. Tuscaloosa VA, Tuscaloosa, Alabama, United States, 2. Battle Creek VA Medical Center, Battle Creek, Michigan, United States, 3. The University of Mississippi, University, Mississippi, United States, 4. Tuscaloosa VA Medical Center, Tuscaloosa, Alabama, United States*

Mindfulness is increasingly popular as a low cost, convenient, and accessible way to address mental health and chronic health conditions. Despite its popularity, best practices in measuring mindfulness in clinical settings and intervention research are still being defined. The Five Facet Mindfulness Questionnaire (FFMQ-15) measures mindfulness traits; however, its use has been limited in older adults, those with dementia, and caregivers. Method: Caregivers (N=82) and veterans with dementia (N=62) enrolled in a randomized pilot intervention study completed the 15-item FFMQ at baseline, 6-month, and 12-month assessments. Veterans were mostly male (98%), White/Caucasian (65%), and living with a partner/spouse (79%). Caregivers (M=65 years old) were mostly female (89%) and White/Caucasian (66%). FFMQ response options were simplified to a 3-point Likert-scale for individuals with dementia (0=rarely true to 2=often true).

**Results:** Internal consistency statistics (Cronbach's alphas) at the scale-level were acceptable among caregivers at baseline and 6-months (.71-.75) but questionably reliable at 12-months (.59, N=46). For individuals with dementia, the simplified version of the FFMQ (with 3 response options) achieved questionable reliability at baseline (.57, N=56) and 6-months (.67, N=32), but improved to acceptable at 12-month assessments (.75, N=15), after significant attrition.

**Conclusion:** Researchers should apply caution when using the FFMQ total score with caregivers and those with cognitive impairments. Though simplified response options eased administration, utility of the tool may be limited in those who are more impaired. Before mindfulness measures can be used meaningfully, reliability of available tools like the FFMQ-15 need to be examined in more diverse samples.

### MEASURING UTILITY OF VASS ELDER MISTREATMENT SCREENER WITHIN COMMUNITY DWELLING U.S. CHINESE OLDER ADULTS

Natalie Tuseth,[1] Stephanie Bergren,[2] and XinQi Dong,[3], *1. Rutgers, The State University of New Jersey, New Brunswick, New Jersey, United States, 2. Rutgers University, New Brunswick, New Jersey, United States, 3. Rutgers, New Brunswick, New Jersey, United States*

Elder mistreatment (EM) is often underreported, making potential screening a valuable tool. There is limited literature on the screening utility, especially for minority populations. This abstract aims to study sensitivity and specificity of a commonly used 10-point EM screener compared to a detailed EM questionnaire among Chinese older adults. This study used data from a representative sample of 3,157 community-dwelling U.S. Chinese older adults 60+. Chi-squared test was conducted between VASS 10-questionare screener and EM measured by 56 items on psychological, physical and sexual mistreatment, caregiver neglect and financial exploitation subtypes. Sensitivity and specificity was calculated using the Bayes Theorem. In this sample, average age was 72 and 59% female. 637 (20.30%) reported any EM while 475 (15.14%) older adults screened positive for EM. Of participants reporting any EM, 365 (57.30%) did not screen positive for EM. The screener had a sensitivity of 42.70% and specificity of 91.88% for all EM subtypes. Gaps between reported EM and negative EM screener is smaller in psychological (sensitivity 72.85%, specificity 91.07%) and physical (sensitivity 63.64%, specificity 86.66%) EM subtypes, but much larger in financial exploitation (sensitivity 34.60%, specificity 86.85%) and neglect (sensitivity 14.11%, specificity 84.75%). The VASS screener demonstrates poor sensitivity but acceptable specificity rate for any EM. The screener showed better sensitivity and specificity for physical and psychological mistreatment, but performed worse for more common forms of mistreatment like financial exploitation and neglect. Modifying this screener may improve sensitivity and specificity in identifying EM.

### MULTIFACETED SLEEP HEALTH SCORE IS ASSOCIATED WITH FRAILTY IN A NATIONAL SAMPLE OF OLDER ADULTS IN TAIWAN: SEX MATTERS

Tuo Yu Chen,[1] Soomi Lee,[2] and Orfeu Buxton,[3], *1. Taipei Medical University, Taipei, Taipei, Taiwan (Republic of China), 2. University of South Florida, Tampa, Florida, United States, 3. The Pennsylvania State University, The Pennsylvania State University, Pennsylvania, United States*

Although individual sleep characteristics are related to frailty, these characteristics do not occur separately. A multi-dimensional measure of sleep might provide a better estimation of frailty compared to isolated sleep characteristics. This study investigated the association of a multidimensional measure of sleep health with frailty and examined whether such relationship differed by sex. Data were from the Taiwan Longitudinal Study on Aging (2011), a survey with a nationally representative sample of Taiwanese older adults (N=2,015). Frailty was defined using the Fried-criteria. Self-reported sleep during the past month was used to conceptualize the five sleep health dimensions in the SATED model (satisfaction-alertness-timing-efficiency-duration; higher scores representing better sleep health). Their relationship was estimated using logistic regression analysis adjusting for sociodemographic (age, sex, education), health (chronic conditions, cognitive function, pain, depressive symptoms [excluding items overlapping with frailty and sleep]), and lifestyle (drinking, smoking, exercise) characteristics. The results showed that having a better sleep health composite score was significantly related to lower odds of being frail in both sexes adjusting for sociodemographic information. Such effect remained significant among females but not males after adding health and lifestyle characteristics to the models. Sleep satisfaction and daytime alertness in both sexes and sleep duration among females were significantly associated with frailty adjusting for sociodemographic information. Only alertness among males was significantly related to frailty in model with all covariates. Our findings show that having a better sleep health across multiple dimensions is related to a lower risk of being frail, and the association differs by sex.

### NUTRITION PROGRAM IMPROVES HEALTH-RELATED OUTCOMES OF NON-DIABETIC ELDERLY AT NUTRITIONAL RISK

Carlos Cano-Gutierrez,[1] Luis Carlos Venegas-Sanabria,[1] Gabriel Gomez,[2] Juan Diego Misas,[2] Cory Brunton,[3] and Suela Sulo,[4], *1. Pontificia Universidad Javeriana, Bogota, Distrito Capital de Bogota, Colombia, 2. Abbott Nutrition, Bogota, Distrito Capital de Bogota, Colombia, 3. Abbott Nutrition, Columbus, Ohio, United States, 4. Abbott Nutrition, Chicago, Illinois, United States*

Malnutrition or its risk affects up to 1 in 3 older adults receiving outpatient care post a hospitalization or for chronic disease management. Although malnutrition poses a negative burden on someone's recovery and health preservation, it can be effectively addressed through cost-effective nutrition interventions delivered as comprehensive quality improvement programs (QIPs) aiding to advance healthcare professional's nutrition education/training and improve quality of care for at-risk/malnourished individuals. Although evidence from US and Europe demonstrates nutrition-focused QIPs are effective in delivering high-quality nutrition care and improve health outcomes of outpatients at-risk/malnourished, to date, no evidence has been reported from Latin American countries. We assessed effectiveness of a comprehensive, nutrition-focused QIP in a Colombian outpatient clinic. Between 09/2019-03/2020, 504 (of total 618) QIP participants were classified at-risk/malnourished and non-diabetics. Participants were followed for 90-days either in-person or via telehealth mechanisms (during COVID-19-imposed lock-down period). QIP interventions included healthcare professional nutrition education; QIP participant continuous nutrition and exercise counselling and 60-day supply of oral nutrition supplement (Ensure®, Abbott). QIP participants were 69% female, with >2 comorbidities, and mean age of 73. Improvement or maintenance of good mental health/well-being, frailty status, cognition and quality of life was reported for 90.7% (456/503), 87.3% (407/466), 86.7% (405/467) and 47% (237/504) participants, respectively (p-values<0.05). Results support QIP effectiveness in driving improved health-related outcomes for non-diabetic, at-nutritional-risk participants. These findings highlight the importance of nutrition-focused QIPs with ONS for older adults during their recovery phase post a recent hospitalization and/or for chronic disease management.

### OLDER ADULTS AND FAMILY PERSPECTIVE ON INTERACTION WITH NURSES IN HOSPITAL: THE ROLE OF MUTUAL UNDERSTANDING

Orly Tonkikh,[1] Nurit Gur-Yaish,[2] Ksenya Shulyaev,[3] Amos Rogozinski,[4] and Elena Siegel,[5], *1. University of California Davis, Sacramento, California, United States, 2. Oranim Academic College of Education, Haifa, HaZafon, Israel, 3. Faculty of Social Welfare and Health Science, University of Haifa, Haifa, Hefa, Israel, 4. University of Haifa, Nesher, HaZafon, Israel, 5. University of California, Davis, Sacramento, California, United States*

Optimal nurse-patient-family interaction is required to provide effective family-centered care for hospitalized older adults and their families. This qualitative descriptive study explored nurses' interactions with older adult patients and their family members during acute hospitalization. We used semi-structured interviews to collect data from a convenience sample of nine dyads of older adults (aged 62-85) and family members (7 children and 2 spouses) who accompanied them during an acute hospitalization in medical or surgical units. Interviews were performed via Zoom beginning in December 2020 until August 2021, 1-12 months after the hospitalization. Thematic analysis was used to inductively capture key patterns in data. Both patients and family members revealed three factors contributing to the way nurses interact with patients and families: (1) nurses' recognition and understanding of patients' needs for family members' presence and participation in care; (2) nurses recognition that family members expect dedication of attention, beyond nurses' focus on patient's care (3) patient and family members' recognition of the extreme workload of nursing staff. Participants described a range of informal approaches used by both nurses and families to address each other's needs. Both patients and families emphasize the benefits and costs of nurses engaging in "exceptional" interactions with patients and families considering structural characteristics such as establishing a personal relationship or accepting family visits beyond the rules. The findings provide direction for further exploration of hospitalization structures and processes needed to support optimal nurses' interactions with families accompanying older adults and family-centered approach training in acute care context.

### OLDER BLACK AMERICANS AND DEPRESSIVE SYMPTOMS DURING THE COVID-19 PANDEMIC

David Chae,[1] Connor Martz,[2] Kara Chung,[1] Roland Thorpe,[3] and Karen Lincoln,[4], *1. Tulane Universtiy, Tulane University, Louisiana, United States, 2. Tulane University, New Orleans, Louisiana, United States, 3. Johns Hopkins Bloomberg School of Public Health, Johns Hopkins Bloomberg School of Public Health, Maryland, United States, 4. University of Southern California, Los Angeles, California, United States*

Black Americans have experienced multiple health threats during the COVID-19 pandemic, including greater risk of infection compared to Whites. In addition, older adults are more susceptible to worse disease consequences including hospitalization and mortality compared to those who are younger. Racism and economic costs are additional public health crises during this time that have disproportionately impacted Black Americans. Using data from the Uncovering COVID-19 Experiences and Realities (UnCOVER) Study, we examined depressive symptoms in relation to: (1) worry/fear of COVID-19; (2) work loss among household members (being laid off, reduced work hours); and (3) vicarious racism, a particularly salient source of psychosocial stress during the COVID-19 pandemic, including hearing about or seeing acts of racism committed against other race group members. Participants were Black Americans aged 50 years or older (n=300) from five cities (Atlanta, Chicago, Los Angeles, New Orleans, and New York) from May-July 2020. Depression was assessed using the Patient-Reported Outcomes Measurement Information System Short Form. In multivariable linear regression models, all three public health threats were significantly associated with depressive symptoms. When in the model simultaneously, worry/fear of COVID-19 ($b=0.30$, $SE=0.12$, $p<0.01$) and vicarious racism ($b=0.62$, $SE=0.15$, $p<0.001$) showed positive associations; work loss was no longer statistically significant ($b=0.62$, $SE=0.43$, $p=0.15$). When added, the corresponding three-way interaction term was significant ($b=0.12$, $SE=0.04$, $p<0.01$). Synergistic epidemics ("syndemics") among older Black Americans amplify mental health tolls. Multi-pronged public health strategies are required to address depression in this population.

### PERCEIVED EVERYDAY DISCRIMINATION AND C-REACTIVE PROTEIN INFLUENCE ON COGNITION OF OLDER BLACK ADULTS

Sydney Kirven,[1] Amy Thierry,[2] and Heather Farmer,[3], *1. Xavier University of Louisiana, New Orleans, Louisiana, United States, 2. Xavier University of Louisiana, Xavier University of Louisiana, Louisiana, United States, 3. University of Delaware, Newark, Delaware, United States*

Black adults and women are more likely to experience serious cognitive decline in older age than their white and male counterparts. Evidence suggests perceived discrimination is associated with poor cognition in older adults, though the mechanisms remain unclear. Perceived discrimination has been linked to elevated inflammatory markers, such as C-reactive protein (CRP), which increases risk for worse cognitive functioning. Yet, little research has investigated whether CRP is implicated in the association between discrimination and cognition among Black older adults or if this relationship differs by gender. Using 2006-2016 data from Black adults ≥65 years old(N=1343) in the nationally representative Health and Retirement Study, random effects linear regression models (1) tested the association between discrimination and cognitive functioning; (2) explored whether this relationship differed for women and men; and (3) assessed whether elevated CRP mediated the association between discrimination and cognitive functioning. More frequent discrimination was associated with worse cognitive functioning ($b= -0.24$, $SE=0.11$, $p<0.05$), though gender did not moderate this relationship. Elevated CRP was significantly associated with worse cognitive functioning ($b= 0.40$, $SE=0.18$, $p<0.05$). Discrimination remained statistically significant in this model, indicating no mediation by CRP. Of note, inclusion of depressive symptoms and cardiometabolic conditions accounted for the association between both discrimination and CRP with cognitive functioning. These findings demonstrate the need for more within-group research on older Black adults documenting the complex relationship between discrimination, inflammation, and cognitive health. This approach will provide greater understanding of the biopsychosocial mechanisms underlying disparities in cognitive functioning in Black adults.

### PERCEIVED NEIGHBORHOOD DISORDER, SOCIAL COHESION, AND DEPRESSIVE SYMPTOMS AMONG SPOUSAL CAREGIVERS

Yeon Jin Choi, and Jennifer Ailshire, *University of Southern California, Los Angeles, California, United States*

Most prior research on caregivers' mental health focused on individual or household factors, we know much less about the influence of neighborhood factors on mental health of spousal caregivers. The current study fills the gap in our knowledge by examining the association of neighborhood characteristics (i.e., perceived neighborhood disorder and neighborhood social cohesion) and depressive symptoms among spousal caregivers. We used data from 2006 to 2016 waves of the Health and Retirement Study, which includes 2,362 spousal caregivers. Negative binomial regression models were estimated to examine the association of perceived neighborhood disorder and neighborhood social cohesion with depressive symptoms. A greater perceived neighborhood disorder was associated with higher CES-D scores, which indicates more depressive symptoms. On the other hand, a higher level of neighborhood social cohesion was associated with lower CES-D scores. When they were included in the same model, the association between neighborhood disorder and depression disappeared, while respondents who reported higher levels of neighborhood social cohesion continue to exhibit lower CES-D scores than those lived in less cohesive neighborhoods. This study highlights the importance of neighborhood contexts in understanding caregivers' well-being. Findings of this study suggest that neighborhood social cohesion may attenuate the negative effects of neighborhood disorder. Therefore, enhancing positive characteristics of the neighborhood may promote well-being of spousal caregivers.

### PERCEPTIONS OF MEDICAL CANNABIS PACKAGING AND LABELING AMONG MIDDLE-AGED AND OLDER CANADIANS

Melissa O'Connor,[1] Vanessa Christiuk,[2] and Megan Pedersen,[3], *1. North Dakota State University,*

*Moorhead, Minnesota, United States, 2. Eloski Health Centre, Fargo, North Dakota, United States, 3. North Dakota State University, Fargo, North Dakota, United States*

The use of cannabis for therapeutic purposes is becoming more popular in many countries, including the United States and Canada. In Canada, middle-aged and older adults make up the largest proportion of medical cannabis users. Canadian legislation mandates that medical cannabis be packaged in plain-looking containers with small labels, childproof caps, and required health warnings. This is meant to standardize the way cannabis products are distributed, as well as protect children from accidental ingestion. However, there is limited research on how these regulations affect cannabis users over age 45. In the present study, residents of Winnipeg, Manitoba, Canada aged 45 and older (n=40) were surveyed regarding their experiences with medical cannabis packaging and labeling. Half of the participants (50%) felt they had a hard time opening their medical cannabis container. A majority (60%) thought having an easy-open lid would be helpful. Most participants (78%) reported experiencing difficulties reading the label on their container, and 75% thought it would be helpful to have a printout of the label in a larger font. In addition, 89% of participants who took more than one kind of medical cannabis favored a symbol on their medication bottle that would indicate the type of medical cannabis contained inside. Implications for policy makers and future research are discussed.

### PERCEPTIONS OF PARTNER COGNITIVE ABILITY DURING THE COVID-19 PANDEMIC

Celinda Reese-Melancon,[1] Jennifer Margrett,[2] Dan Russell,[2] Rachael Turner,[3] Erin Harrington,[4] Lauren Stratton,[2] and Jyoti Savla,[5], *1. Oklahoma State University, Oklahoma State University, Oklahoma, United States, 2. Iowa State University, Ames, Iowa, United States, 3. Oklahoma State University, Stillwater, Oklahoma, United States, 4. Pennsylvania State University, University Park, Pennsylvania, United States, 5. Virginia Tech, Blacksburg, Virginia, United States*

Media reports provide anecdotal evidence of increased forgetfulness during the COVID-19 pandemic (Cushing, 2021; Purtill, 2020). Scientific evidence suggests social isolation can impact on cognition (Evans et al., 2018), but the question remains whether those living with a partner experience similar deficits. The present study examined whether middle-aged and older adults' perceptions of their own and their partner's memory abilities were related to self-reported impact of the pandemic on daily life (e.g., limited social interactions, delayed health care, and disruption to routine). In a sample of 80 married individuals (49% female; age range 40-86 years), we found that participants' beliefs about the impact of the pandemic on daily life and their depression ratings significantly predicted ($p<0.05$) their perceptions of their partner's prospective memory abilities. Specifically, pandemic impact on daily life predicted 9.3% of the variance in participants' reported perceptions of their partners' prospective memory abilities, and participant depression ratings predicted an additional 5.1% of the variance. Surprisingly, these variables did not predict perceptions of participants' own cognition or perceptions of partners' retrospective memory abilities. In sum, people who reported greater impact of the pandemic on their lives were more likely to believe that their partner frequently forgot to carryout prospective memory intentions (e.g., failed to pass along a message or take medication), and depression further clouded their perception of their partner's cognition. These findings should be extended to consider relationship quality and whether individuals consider their partners a reliable source of external memory support during times of life disruption.

### PERCEPTIONS ON OWN AGING: COMPARISONS BETWEEN YOUNG ADULTS WITH AND WITHOUT CAREGIVING EXPERIENCE

Phoebe Clark,[1] Vanessa Cuppari,[2] Matthew Picchiello,[3] Michiko Iwasaki,[4] and Andrew Futterman,[5], *1. Loyola University MD, Gaithersburg, Maryland, United States, 2. Loyola University Maryland, Baltimore, Maryland, United States, 3. Washington University in St. Louis, St. Louis, Missouri, United States, 4. Loyola University MD, Department Of Psychology, Maryland, United States, 5. Loyola University MD, Loyola University MD, Maryland, United States*

Although informal caregiving for older adults (OAs) can increase knowledge and awareness about one's own aging (Pope, 2013), it can also negatively impact caregivers' physical health and emotional wellbeing (AARP & NAC, 2020) and have spillover effects on school, work, and marriage (Dellmann-Jenkins & Blankemeyer, 2009). Despite the recent trend of family caregiving for OAs by young adults (YAs), research about these young caregivers is scarce. The present study focused on YAs' perceptions on aging. We hypothesized that YAs who provided at least three months of caregiving tasks for OAs would hold more awareness and negative perceptions on their own aging, as measured by a modified version of the Brief Aging Perceptions Questionnaire (Sexton et al., 2014), compared to those who did not. We recruited 234 YAs between the ages of 18 - 40 (Mage = 29.78, SD, age = 4.83; 59% White; 65.4% male) and had them complete a survey via Amazon Mechanical Turks. About one third (32.1%) had caregiving experience. Results of independent t-tests revealed that caregivers scored higher on awareness of aging [$t(229) = 6.950.$, $p < .001$, $d = .865$] and negative consequences/control [$t(231) = 6.528.$, $p < .001$, $d = .927$]. Scores of positive consequences/control did not differ between the two groups. Our findings indicate the need for psychological interventions designed to help young caregivers integrate their caregiving experiences with less negative aging perceptions. Future research should examine the direct effects of caregiving experience on perceptions of aging between young and middle-aged adults.

### PHASE 2B STUDY OF AN AD26.RSV.PREF VACCINE FOR PREVENTION OF RSV-MEDIATED RESPIRATORY TRACT DISEASE IN OLDER ADULTS

Ann Falsey,[1] Kristi Williams,[2] Efi Gymnopoulou,[3] Stephan Bart,[4] John Ervin,[5] Els De Paepe,[3] Christy Comeaux,[6] and Esther Heijnen,[6], *1. University of Rochester School of Medicine, Rochester, NY, USA, Rochester, New York, United States, 2. Janssen Research and Development, Spring House, PA, USA, Spring House, Pennsylvania, United States, 3. Janssen Infectious Diseases, Beerse, Belgium, Beerse, Antwerpen, Belgium, 4. Optimal*

*Research, LLC/Synexus Clinical Research/AES, Woodstock, MD, USA, Rockville, Maryland, United States, 5. AMR Kansas City, Kansas City, MO, USA, Kansas City, Missouri, United States, 6. Janssen Vaccines & Prevention BV, Leiden, The Netherlands, Leiden, Zuid-Holland, Netherlands*

Respiratory syncytial virus (RSV) may cause serious lower respiratory tract disease (LRTD) in older adults, and there is currently no licensed vaccine. CYPRESS (NCT03982199) is a randomized, double-blind, placebo-controlled Phase 2b proof-of-concept trial of an Ad26.RSV.preF-based vaccine for the prevention of RSV-mediated LRTD in older adults. Adults aged ≥65 years were randomized 1:1 before the RSV season to receive Ad26.RSV.preF-based vaccine or placebo. Acute respiratory infection symptoms were collected through a patient eDiary and/or clinician assessment until the end of the RSV season. The primary endpoint was the first occurrence of RTPCR-confirmed RSV-mediated LRTD according to any of 3 case definitions: (1) ≥3 symptoms of lower respiratory tract infection (LRTI), (2) ≥2 symptoms of LRTI, or (3) ≥2 symptoms of LRTI or ≥1 symptom of LRTI with ≥1 systemic symptom. Immunogenicity was assessed in a subset of approximately 200 participants. A total of 2891 participants in each study arm received study treatment. Vaccine efficacy was 80% (94.2% CI, 52.2-92.9%), 75% (50.1-88.5%), and 69.8% (43.7-84.7%) for case definition 1, 2, and 3, respectively (all P <0.001). In the vaccine arm, geometric mean fold increase in antibody titers 14 days after vaccination was 13.5 for RSV neutralizing antibodies and 8.6 for RSV prefusion F-specific binding antibodies, and median frequency of RSV-F-specific INFγ T-cells increased from 34 to 444 SFC/10^6 PBMC; no relevant changes were observed in the placebo arm. The Ad26.RSV.preF-based vaccine was highly effective against RSV-mediated LRTD through the first RSV season and elicited robust immune responses in older adults.

### PHYSICAL ACTIVITY PROGRAMMING FOR OLDER ADULTS IN ASSISTED LIVING: CONTEXTUAL FACTORS TO CONSIDER

Katelyn Webster, and Janet Larson, *University of Michigan School of Nursing, Ann Arbor, Michigan, United States*

Sedentary behavior may adversely affect physical and cognitive health of older adults in assisted living (AL). Replacing sedentary behavior with light physical activity (PA) could help them maintain functional abilities and independence. We interviewed AL residents to obtain their guidance regarding the implementation of an intervention to reduce sedentary behavior. Here we report the results of a thematic analysis exploring contextual factors that may influence intervention implementation. We interviewed 20 residents (mean age 83.1; 60% women) and identified 7 themes. The first was attitudes and beliefs about PA. Most residents believed PA was important, but some lacked motivation or confidence to perform PA. Another theme was attitudes and beliefs about aging, as some residents felt discouraged about aging and uncertainty about how much PA they could safely perform. Abilities of AL residents was seen as an important consideration. It was noted that residents have a wide range of abilities and this could present challenges in planning a PA program appropriate for all residents. Social influences for PA should be considered, as residents may find encouragement from family or other residents. Space for being active is another factor because it is typically limited within AL. We found that some residents wanted more challenging exercise classes than currently provided by their facility. Finally, residents described limited opportunities for PA due to the nature of the AL environment. This thematic analysis brings attention to important factors that could influence the implementation of PA interventions with the AL population.

### PREDICTION OF COVID-19 STRESS AMONG COMMUNITY DWELLING OLDER ADULTS: THE ROLE OF ANXIETY AND RESILIENCY

Geffre Jean Francois, Dawn Carr, and Natalie Sachs-Ericsson, *Florida State University, Tallahassee, Florida, United States*

Worldwide, the COVID-19 pandemic has been an unparalleled source of stress. Older adults with anxiety are vulnerable to higher levels of stress during the pandemic. However not all older adults with anxiety will experience severe stress; resiliency may decrease such negative outcomes. There have been few, if any, longitudinal studies that followed older adults before and during the pandemic. Our data of community dwelling older adults (aged 60-92) is unique in that it allowed for an investigation of psychological variables that increase and decrease negative outcomes during the pandemic. Our longitudinal study examined the influence of pre-pandemic anxiety and resiliency on the severity of COVID related stress.

**Methods:** The pre-pandemic data was obtained in September 2018, and the pandemic data was collected in June 2020. In the baseline survey we obtained measures of anxiety and resiliency. During the pandemic we measured the severity of COVID related stressors. We hypothesized that anxiety would predict higher level of COVID-stress, whereas resiliency would be associated with decreased severity of COVID-stress. Further we predicted that resiliency would attenuate the association between anxiety and COVID-stress.

**Results:** Using OLS regression, we found that anxiety predicted higher COVID-stress, whereas resiliency predicted lower COVID-stress. However, resiliency did not moderate the association between anxiety and COVID-stress.

**Conclusion:** Older adults are subject to many unavoidable negative life events, such as death of family members and chronic health problems. Resiliency may help buffer against such adversities. Development of intervention programs to enhance resiliency may increase psychological resources and foster healthy aging.

### PROBLEM-DRINKING ACROSS THE LIFESPAN: CROSS-SECTIONAL VERSUS LONGITUDINAL EFFECTS AMONG MIDLIFE AND OLDER ADULTS

Thomas Britton,[1] Annabel Kady,[2] Yimei Li,[3] Angelo DiBello,[3] and Matthew Lee,[3], *1. Rutgers University, Piscataway, New York, United States, 2. Rutgers University, Baltimore, Maryland, United States, 3. Rutgers University, Piscataway, New Jersey, United States*

When considering problem drinking from a lifespan-developmental perspective, an often-stated premise is that problem drinking escalates during adolescence, peaks around early young adulthood, and then declines throughout the remainder of the lifespan. However, while there is a strong empirical basis for such changes throughout adolescence and young adulthood, the notion of continued declines throughout midlife and older adulthood is less firmly established and based primarily on cross-sectional data. Thus, this study contrasted cross-sectional versus longitudinal age effects on problem-drinking changes across the lifespan, with particular focus on midlife and older adulthood. Analyses used data from a large, two-wave, U.S.-representative sample. We generated descriptive "porcupine figures" graphically depicting both cross-sectional and longitudinal age effects simultaneously, and we estimated mixed-ANOVAs to partition, test, and contrast cross-sectional versus longitudinal age effects. As expected, analyses confirmed the well-known rise and fall of problem drinking across young adulthood in both cross-sectional and longitudinal age effects. In contrast, in midlife and older adulthood, only cross-sectional age effects were consistent with the notion of continued age-related declines throughout these ages, whereas the longitudinal data showed a mixture of stability and escalation at these ages. Age-confounded cohort effects are one plausible explanation for how cross-sectional data can lead to spurious conclusions about developmental change. By potentially yielding a more accurate understanding of lifespan-developmental change in midlife and older adulthood, findings like ours could help guide lifespan-developmentally-informed interventions for midlife and older-adult problem drinkers; an objective of increasing importance in light of the ongoing aging of the U.S. population.

### PSYCHOLOGICAL IMPACT OF COVID-19 PANDEMIC ON FRONTLINE RESPONDERS AND STUDENTS IN TRAINING

Andrew Revell,[1] and Mitchell Gauvin,[2], *1. University of Massachusetts Dartmouth, University of Massachusetts Dartmouth, Massachusetts, United States, 2. University of Massachusetts Dartmouth, North Dartmouth, Massachusetts, United States*

Medical personnel have been in the frontlines of the pandemic leading to increased levels of stress and an impact on mental health. Risks may include, but are not limited to, pronounced burnout (Shechter et al., 2020), vicarious trauma, and post-traumatic stress disorder. The goal of this investigation was to gain insight on the psychological effects that the pandemic had on both frontline responders (EMTs and emergency room staff) and students in clinical training. Emerging adults and adult participants (N=150; ages 18-46; 70.4% ages 18-24) were recruited through the introductory psychology subject pool, community healthcare, and social media. Linear regression and means testing were employed to assess differences between current frontline workers and future workers on the Depression, Anxiety, and Stress Scale (DASS-21; Lovibond, 1995) on irritability, sleep, covid-19 positive presence, concentration, and other mental health factors. Hierarchical linear regression, controlling for age, indicated higher anxiety subscale scores (b=2.49, p=.008) and higher stress subscale scores (b=2.25, p=.035) were present on the DASS-21 for women. Dichotomous means testing indicated higher anxiety, stress, and depression levels for those who also reported a significant change in sleep habits (p <.001) and for those who reported being more irritable on their days off (p <.001) during the pandemic. Students in training (37.7%) indicated interest in considering a different career path (r = .302, p = .02). Future studies should examine these dynamic relationships among mental health factors among healthcare professionals and the implications for training the next generation.

### PSYCHOSOCIAL PREDICTORS OF PSYCHOLOGICAL WELL-BEING DURING THE COVID-19 PANDEMIC

Yeon Ji Ryou,[1] Gina Lee,[1] Rotem Arieli,[1] Peter Martin,[1] Shinae Choi,[2] Jinmyoung Cho,[3] and Melinda Heinz,[4], *1. Iowa State University, Ames, Iowa, United States, 2. The University of Alabama, Tuscaloosa, Alabama, United States, 3. Baylor Scott & White Health Research Institute, Temple, Texas, United States, 4. Upper Iowa University, Fayette, Iowa, United States*

People worldwide have been largely affected by the COVID-19 outbreak. In addition to worries about physical health, it also causes concerns about psychological and mental health. This research aims to explore predictors affecting psychological well-being during the pandemic using the 2018 Health and Retirement Study (HRS) RAND longitudinal data (N = 42,233) and the 2020 HRS COVID-19 module (N = 3,266). Demographics (i.e., gender, age, and education), psychosocial (i.e., personality traits), and health (i.e., comorbidity) variables were included in multivariate logistic and ordinary least square regression analyses predicting feeling "overwhelmed," "stressed," and "lonely" during the pandemic. Our results indicated that neuroticism was positively associated with all outcomes. Women were more likely to feel overwhelmed, stressed, and lonely compared to men. Age negatively predicted the overwhelmed and stressed feelings. Furthermore, the effect of depressive symptoms in 2012, 2014, 2016, and 2018 on psychological well-being was assessed by conducting a latent growth curve model. Findings indicate that initial level and increasing change of depressive symptoms over four-time points (waves 11-14) were positively related to psychological feelings. A higher level of depressive symptoms at the initial level of 2012 and increasing reports of depression symptoms predicted higher rates of being stressed, feeling overwhelmed, and lonely during the COVID-19 pandemic. The results have implications for future research and interventions that should target the emotional antecedents and consequences of pandemics.

### QUALITY OF LIFE AND WELLBEING IN PERIPHERAL ARTERIAL DISEASE: A QUALITATIVE STUDY

Mary Byrnes,[1] Heather Dillaway,[2] Bryan Aaron,[3] Alisha Heximer,[4] Valeria Valbuena,[5] Matthew Corriere,[6] and Nicholas Osborne,[6], *1. University of Michigan, Ann Arbor, Michigan, United States, 2. Wayne State University, Wayne State University, Michigan, United States, 3. University of Michigan Medical School, University of Michigan Medical School, Michigan, United States, 4. University of Michigan, University of Michigan Medical School, Michigan, United States, 5. University of MIchigan, University of Michigan, Michigan, United States, 6. University of Michigan, University of Michigan, Michigan, United States*

Peripheral arterial disease (PAD) is a vascular condition disproportionately affecting adults > 60 and the leading cause of disability for adults > 50. Because PAD is marked by severe leg pain and sometimes lower extremity amputation, quality of life (QOL) and wellbeing may be compromised however, we understand little about these constructs in this population. Furthermore, surgical care providers lack a comprehensive understanding of how individuals think about wellbeing and what is important to individuals during surgical care. We conducted a qualitative photographic elicitation study (n = 60) in one academic multidisciplinary PAD clinic to understand specific aspects of QOL of older individuals with PAD. Guided by interpretive description, a methodology pioneered in nursing, we analyzed data within and across five clinical symptom severity categories to examine for QOL constructs, impact on everyday life, understanding of disease, and desired treatment. Results demonstrate that individuals do not fully understand PAD diagnosis or its implications (e.g., "[I] have never heard of it. Do I have that?"). Disease-specific knowledge was prevalent among patients experiencing lower extremity amputation but those suffering from wounds or gangrene had limited understanding. Furthermore, patients' descriptions of QOL conflicted with the conceptualization of QOL in clinical practice and research. That is, many participants describe QOL based on activities they are capable of performing despite limitations. Results demonstrate the need for integrating gerontological knowledge into clinical care to improve quality of care for older adults.

### REALITY OR A DREAM: BARRIERS AND FACILITATORS FOR NURSING ASSISTANTS PURSUING A NURSING CAREER

Stephanie Trotter, and Leah Richardson, *University of Arkansas for Medical Sciences, Little Rock, Arkansas, United States*

Certified nursing assistants (CNAs) serve a critical role in the care of older adults. However, CNAs often experience significant professional and personal burdens related to caregiving work. Professionally, CNAs experience exorbitant workplace stress (e.g., physical injury, burnout, emotional exhaustion, staffing shortages, turnover). Personally, CNAs may have only a high school education, are subjected to low-paying jobs, and little opportunity for career advancement. Further, CNAs are disproportionately of minority race. Clearly, CNAs are negatively impacted by many social determinants of health. Ultimately, these burdens negatively impact older adults' care provision and quality of life. Transitioning to a nursing career may alleviate some of these complex problems, but this has scantly been explored. A qualitative descriptive study was designed to 1) identify interest in a nursing career, and 2) explore perceived barriers and facilitators of transitioning into a nursing profession. CNAs from nursing facilities participated in private, semi-structured interviews. Recorded interviews (n = 6) were transcribed verbatim. Preliminary thematic analyses yielded two overarching themes: The Dream and The Reality. Rich subthemes began emerging from both overarching themes. Example subthemes from The Dream were: family legacy in healthcare, and finding purpose. Example subthemes from The Reality were: versus (CNAs vs. nurses; nurses vs. nursing care), and work-life balance. These preliminary findings suggest that CNAs express desire in becoming a nurse, although a range of personal and professional barriers and facilitators exist. Making the nursing dream become reality may improve CNAs' social determinants, workplace outcomes, and resident outcomes, but further exploration is warranted.

### RELATIONSHIP BETWEEN SEVERITY OF COMORBIDITIES AND SELF-PERCEPTIONS OF AGING

DaJung Chang, *Miami University, Oxford, Ohio, United States*

An older adult with negative self-perceptions of aging (SPA) can lead to lower self-rated health and a higher risk of mortality. To stay a positive SPA, keep a healthier status is very important. However, evidence also proved that health conditions, like a physical limitation, could predict the level of SPA. Older adults usually have a higher prevalence rate of chronic diseases than the younger population, which can adversely impact them. The purpose of this study is to determine the relationship between the severity of comorbidities and the change of SPA during a time. I examined data in 7,034 people from the 2012 wave Health and Retirement Study (HRS) and followed the respondents who have answer the SPA scale in the leave-behind questionnaire in 2016. The generalized estimating equation was used to analyze the relationship between the severity of comorbidities and SPA in different waves. To measure the severity of comorbidities, a reduced index of the comorbidities severity scale (CmSS) was created to collect the health condition from HRS. Results statistical model shows that an individual with more severity of comorbidities may have a more negative SPA. However, the relationship does not follow through with the times. These findings enhance the previous study that there are relationships between severity of comorbidities and SPA. The benefit of this study is to use a different measurement to identify the severity of comorbidities and extend to more in-depth research.

### REPRESENTATION OF OLDER ADULTS IN COVID-RELATED NEWSPAPER COVERAGE

Mijin Jeong,[1] Sarah Jen,[2] Hyun Kang,[1] Michael Riquino,[1] and Jaime Goldberg,[3], *1. University of Kansas, LAWRENCE, Kansas, United States, 2. University of Kansas, Lawrence, Kansas, United States, 3. University of Wisconsin-Madison, Madison, Wisconsin, United States*

Based on clinical and epidemiological evidence, COVID-19 infection can occur in people of all ages; however, the media typically focuses its attention on the vulnerability of older adults and individuals with chronic illnesses. This study aims to explore the representation of older adults during the first month of the pandemic in the U.S. by comparing the narratives of older adults and younger adults in national media sources. A systematic search identified 115 articles published in four major newspapers in the U.S. included USA Today, The New York Times, Los Angeles Times, and The Washington Post between March 11 and April 10, 2020 in which older adults and younger adults were quoted on topics related to the intersection of COVID-19 and aging. Quotes were inductively reviewed using thematic content analysis. In 115 articles, there were 265 quotes from older adults (n=104, 39%) and younger adults (n=161, 61%). When comparing patterns that were common or distinctive between older and younger individuals quoted, three key themes emerged: 1) impacts of COVID-19 on older adults and resulting vulnerability, 2) debated perspectives over the value of older adults' lives, and 3) a counternarrative of resiliency among older adults. This study provides the opportunity to understand how the pandemic may impact representations of older adults and findings emphasize the importance of voice among older adults to combat ageist messaging and promote counternarratives to assumptions of vulnerability. Also, it suggests for policymakers and practitioners to insight into how the representation of older adults is disseminated by media.

### RESIDENTIAL CARE IN CALIFORNIA: SPATIAL AND TEMPORAL TRENDS IN FACILITY DEVELOPMENT AND CARE CAPACITY

Stephen Frochen,[1] Jennifer Ailshire,[2] Seva Rodnyansky,[3] and Connor Sheehan,[4], *1. VA Greater Los Angeles Healthcare System, Costa Mesa, California, United States, 2. University of Southern California, Los Angeles, California, United States, 3. Occidental College, Los Angeles, California, United States, 4. ASU, Tempe, Arizona, United States*

The U.S. is aging, and the older adult population and number of long-term care services are growing but not at corresponding rates and concentrations depending on location. Insufficient research has analyzed residential care at the neighborhood or city level of analysis, where geographical trends in growth often reveal notable patterns of long-term care unobserved at county and state levels of analyses. We merged the California Department of Social Services Residential Care for the Elderly Dataset with census place and tract data to chart the growth of facilities and beds per older adults in all of California and its three largest cities, including 805 facilities licensed from 1996 to 2015. During the study timeframe, residential care steadily increased in California by the number of facilities and beds relative to older adults. However, due to a consistently increasing older adult population, the Cities of San Diego and San Jose experienced gradual and intermittent decline in capacity per older adults, respectively, even as they added many beds to their inventories from the sporadic development of large assisted living and continuing care retirement communities. Additionally, San Jose and Los Angeles exhibited the most overlap in mapping densities of facility development and oldest old adults, with San Diego showing less intersection in cartographic analysis. Understanding facility development and care capacity trends can help local agencies and jurisdictions in the U.S. and other countries discern whether planning policies and other geographical and development factors appropriately encourage the development of residential care and other long-term care facilities.

### SAFETY AND TOLERABILITY OF AN AD26.RSV.PREF-BASED VACCINE IN A PHASE 2B STUDY IN OLDER ADULTS

Ann Falsey,[1] Kristi Williams,[2] Efi Gymnopoulou,[3] Stephan Bart,[4] John Ervin,[5] Els De Paepe,[3] Esther Heijnen,[6] and Christy Comeaux,[6], *1. University of Rochester School of Medicine, Rochester, NY, USA, Rochester, New York, United States, 2. Janssen Research and Development, Spring House, PA, USA, Spring House, Pennsylvania, United States, 3. Janssen Infectious Diseases, Beerse, Belgium, Beerse, Antwerpen, Belgium, 4. Optimal Research, LLC/Synexus Clinical Research/AES, Woodstock, MD, USA, Rockville, Maryland, United States, 5. AMR Kansas City, Kansas City, MO, USA, Kansas City, Missouri, United States, 6. Janssen Vaccines & Prevention BV, Leiden, The Netherlands, Leiden, Zuid-Holland, Netherlands*

Respiratory syncytial virus (RSV) may cause severe lower respiratory tract disease in older adults and there is currently no approved vaccine. We assessed the safety and reactogenicity of an Ad26.RSV.preF-based vaccine in a randomized, double-blind, placebo-controlled Phase 2b proof-of-concept trial in adults aged ≥65 years (CYPRESS; NCT03982199). Prior to the RSV season, participants were randomized 1:1 to receive an Ad26.RSV.preF-based vaccine or placebo. Solicited adverse events (AEs; fatigue, headache, nausea, myalgia, fever, injection site reactions) and unsolicited AEs were assessed from time of vaccination to Day 8 and Day 29, respectively, in a safety subset of 695 participants (vaccine, n=348; placebo, n=347). All participants were followed for serious AEs (SAEs) until the end of the RSV season or 6 months after vaccination, whichever occurred later. A total of 5728 participants were randomized and received vaccine or placebo (n=2891 in each group). In the safety subset, the frequency of solicited AEs and Grade ≥3 solicited AEs was 51.4% and 3.2% in the vaccine group and 20.2% and 0.6% in the placebo group, respectively. The most frequent solicited AEs in the vaccine group were fatigue, myalgia, headache, and injection site pain/tenderness. The rates of unsolicited AEs and Grade ≥3 unsolicited AEs were similar between the vaccine (16.7% and 1.7%) and placebo (14.4% and 1.4%) groups. In the overall study population, the rate of SAEs was similar between groups (vaccine, 4.6%; placebo, 4.7%); none were considered related to the vaccine. The Ad26.RSV.preF-based vaccine was safe and well tolerated in adults aged ≥65 years.

### SELF- AND INFORMANT-REPORTED LONELINESS AND COGNITIVE FUNCTION: EVIDENCE FROM THREE HCAP STUDIES

Ji Hyun Lee,[1] Martina Luchetti,[2] Amanda Sesker,[2] Damaris Aschwanden,[3] Antonio Terracciano,[4] and Angelina Sutin,[2], *1. University of Michigan, 2. Florida State University College of Medicine, Tallahassee, Florida, United States, 3. Florida State University, Tallahassee, Florida, United States, 4. FLORIDA STATE UNIVERSITY, Florida State University, Florida, United States*

Loneliness is defined as a distressing feeling that arises from the discrepancy between one's desired and perceived social relationships. Theorists have posited that loneliness involves affective, cognitive, and behavioral components that can be noticed by close family or friends. Little is known about whether social withdrawal, a behavioral marker of loneliness observed by knowledgeable others, shares similar associations with cognition as self-reported loneliness. The present study examined whether self-reported and informant-rated loneliness are related to cognitive function in older adulthood. Data come from Harmonized Cognitive Assessment Protocol subsample included in three national aging studies from the US (HRS; N = 2,821, Mage = 75.8), England (ELSA; N = 896, Mage = 73.6), and India (LACI-DAD; N = 2,994,

Mage = 69.3). Respondents reported on 1-item loneliness and informants rated the respondent's withdrawal behavior. Regression models were used to analyze the association between both measures of loneliness and cognition, controlling for respondent (age, gender, education, race/ethnicity) and informant (age, gender, education, spouse, known years, frequency of contact) covariates. Meta-analysis showed that both respondent-reported and informant-rated loneliness were independently associated with lower cognitive functioning (memory, speed–attention–executive, visuospatial ability, and fluency) and global cognition (MMSE). The associations between observed withdrawal and cognition did not vary by informant-level characteristics nor the respondent's cognitive impairment status. The present study indicates that withdrawal behavior observed by informants is associated with cognitive function, supporting psychosocial observations provided by knowledgeable others can be utilized in detecting cognitive function.

### SEX DIFFERENCES IN CARDIOVASCULAR MARKERS AND BDNF BETWEEN CEREBRAL PALSY AND MILD COGNITIVE IMPAIRMENT

Kheng Siang (Ted) Ng,[1] James Carollo,[2] Alex Tagawa,[3] ZHAOXING PAN,[1] and Patricia Heyn,[4], *1. University of Colorado Anschutz Medical Campus, Aurora, CO, Colorado, United States, 2. University of Colorado Anschutz Medical Campus, University of Colorado Anschutz Medical Campus-Center for Gait and Movement Analysis (CGMA), Colorado, United States, 3. University of Colorado Anschutz Medical Campus, Denver, Colorado, United States, 4. University of Colorado Anschutz Medical Campus, Arvada, CO, Colorado, United States*

Adults with cerebral palsy (CP) have higher risk of developing geriatric syndromes. Mild cognitive impairment (MCI) is an intermediate stage between healthy aging and dementia, often co-morbid with cardiovascular disease (CVD). We recently showed an "accelerated aging model", where CP shares similar CVD risk factors with MCI, potentially accounting for CP's increased risk of dementia. In this study, we further examined sex differences between CP and MCI (aim 1) and within CP (aim 2). From an accredited clinical motion analysis laboratory at Children's Hospital Colorado (CP) and a university in Singapore (MCI), we recruited 72 adults with CP [mean (SD) of age=20 (5.3), Sex: men=47.2% and women=52.8%] and MCI [mean (SD) of age=71.28 (6.03), Sex: men=29.2% and women=70.8%]. We analyzed blood Pressure (BP), Framingham Heart Study Score (FHSS), and brain-derived neurotrophic factor (BDNF). Compared to MCI, women with CP had lower BDNF ($\beta$=-3.550, 95% CI=-5.659 to -1.441, p=0.001), while men with CP had lower diastolic BP ($\beta$=-28.204, 95% CI=-52.148 to -4.260, p=0.022). Both women and men with CP also had lower FHSS, compared to MCI ($\beta$=-2.515, 95% CI=-3.721 to -1.309, p<0.001; $\beta$=-3.724, 95% CI=-5.561 to -1.888, p<0.001, respectively). Women in the CP cohort showed lower FHSS ($\beta$=-0.172, 95% CI=-0.310 to -0.033, p=0.016). We found sex-related differences in BDNF and CVD markers. Comparing across and within cohorts, although having lower BDNF levels, women with CP had better FHSS. These findings support our accelerated aging hypothesis, and further suggest sex differences in aging-related risk factors in CP, supporting sex-related precision medicine approach.

### SINGLE CELL ANALYSIS REVEALS STOCHASTIC CELL-TO-CELL VARIATION IN STRESS RESPONSE AND SENESCENCE PROGRAM

Nikolay Burnaevskiy,[1] and Alexander Mendenhall,[2], *1. University of Washington, University of Washington, Washington, United States, 2. University of Washington School of Medicine, University of Washington School of Medicine, Washington, United States*

Cells have various means to respond to molecular stress. Upon stress, proliferating cells can adopt different fates, e.g. commit to apoptosis, go into senescence or recover from stress and resume proliferation, depending on severity of the stress. Proper balance between these modes of response is critical for maintaining tissue homeostasis with age, as both exacerbated and insufficient response can result in pathology. Remarkably, even genetically identical cells of the same type in the controlled environment can exhibit a spectrum of responses to the same stress challenge. We hypothesized that analyzing response of individual cells in controlled environment can help better understand the mechanisms that ensure a balanced response to molecular stress. We used large scale single cell RNA-sequencing to analyze response of individual human fibroblasts to oxidative stress. Consistent with various fates adopted by individual cells upon stress, we observed different transcriptional signatures, that correspond to those fates. Surprisingly, when we specifically analyzed ß-gal+ senescent cells, we still observed transcriptional heterogeneity, with only a subset of cells exhibiting pro-senescent transcriptional signature (e.g. activated p53 and TNF-a pathways) while another subset exhibits a gene expression signature of senescent-like arrest. Hence, we find that in addition to known stress-related fates (apoptosis, senescence, recovery) senescence-like response is heterogeneous with only subset of cells exhibiting expected pro-senescent gene expression signature. Further characterization of heterogeneity of stress response and senescent-like fates will help better understand the mechanisms of homeostatic control in the face of molecular stress and aging.

### STRATEGIES TO MAINTAIN SKILLED NURSING FACILITY STAFFING DURING COVID: ADMINISTRATOR PERSPECTIVES

Amy Meehan,[1] Renee Shield,[1] Caroline Madrigal,[2] Joan Brazier,[1] and Emily Gadbois,[1], *1. Brown University, Providence, Rhode Island, United States, 2. Providence VA Medical Center, Providence VA Medical Center, Rhode Island, United States*

As COVID-19 has resulted in a skilled nursing facility (SNF) staffing crisis, administrators attempt to maintain adequate staffing and stem decreasing patient census levels. We conducted four repeated interviews to date (n=130) at 3-month intervals with administrators from 40 SNFs in eight diverse healthcare markets across the United States. We used thematic analysis to examine their perspectives over time, including the perceived impact on staffing. Results include: 1) the impact of COVID-19 on staffing levels, and 2) strategies used in response to this crisis. Staffing levels have decreased throughout the pandemic, and struggles to maintain adequate staffing levels and patient census numbers have continued as the pipeline of potential new staff constricts. Facilities turned to agencies, many for the first time. Since agencies offer higher salaries, staff are drawn away from employment by SNFs, leading to a cycle of wage wars, and agencies are also challenged to provide staff. SNF administrators describe their responses to this crisis, such as flexible schedules, increased paid time off, sharing of non-direct-patient-care tasks, financial incentives (referral, sign-on, "no-call out", and other general bonuses); wage analyses, and enhanced employee benefit packages. Some hire recruitment specialists, collaborate with nearby administrators, use creative advertising, or work with local schools. The vaccine mandate worries administrators; as one stated: "I can't afford to lose one person, let alone 20 because of this mandate...". Given the dwindling pool of potential employees, we present NH administrators' strategies to attract and retain staff.

### STRUCTURAL RACISM IS ASSOCIATED WITH ASSISTED LIVING LOCATION

Lindsey Smith,[1] Paula Carder,[2] Kali Thomas,[3] Robin Baker,[1] and Neal Wallace,[1], *1. Oregon Health & Science University - Portland State University School of Public Health, Portland, Oregon, United States, 2. OHSU-PSU School of Public Health, Portland, Oregon, United States, 3. Brown University, Brown University/Providence, Rhode Island, United States*

Our objective was to measure the association between structural racism, a previously unmeasured but theoretically causal factor, and assisted living communities (ALCs) location as fewer ALCs are located in counties with a greater percentage of the population reported as Black (PPB). We used a recently developed measure of structural racism—the racial opportunity gap (ROG), which compares the economic mobility of Black and White people who grew up in the same area with parents who had similar incomes. We estimated a multilevel mixed-effects bivariate regression model to examine the factors contributing to the presence of ALC. We relied on state and county random effects. The likelihood of an assisted living being located in a census tract in 2019 was significantly positively associated with the percent of the population over the age of 65 (OR=150.1573, p=<0.001), the PPB (OR=2.9916, p=0.004), and higher median incomes (OR=1.0, p=<0.001). In contrast, rurality (OR=0.5656, p=<0.001), unemployment rates (OR=0.0288, p=<0.001), and census tracts that have a high PPB in addition to a high county ROG (OR=.0058, p=0.0137) are all associated with a lesser likelihood of an ALC. The interaction coefficient between the ROG and PPB reverses the previously documented negative association between the PPB and ALC presence. This result empirically supports the premise that structural racism, not population race alone, is a negative determinant of where an ALC is located within a county.

### SUCCESSFUL AGING: MEMORY AND SMART TECHNOLOGY IMPLICATIONS.

Neyda Ma. Mendoza-Ruvalcaba,[1] Elva Dolores Arias-Merino,[2] Karla Patricia Vázquez Núnez,[3] and Marlene Alvarado Rodriguez,[4] *1. University of Guadalajara CUTONALA, Guadalajara, Jalisco, Mexico, 2. Universidad de Guadalajara, Zapopan, Jalisco, Jalisco, Mexico, 3. Universidad de Guadalajara, Doctorado en Investigación Multidisciplinaria en Salud, Universidad de Guadalajara, Jalisco, Mexico, 4. Universidad de Guadalajara, Maestría en Gerontología, Universidad de Guadalajara, Jalisco, Mexico*

The cognitive functioning as a general measure, is a criterion commonly used to define and operationalize successful aging(SA). The aim of this study is to analyze the specific role of memory (objective and subjective) and its relationship with the use of smart technology (ST) and SA.(Project-Conacyt-256589) Population based, random sample included n=453 community-dwelling older adults 60-years and older (mean age=72.51,SD=8.11 years,59.4%women). Memory was assessed through working memory(Digit Span Backward WAIS-IV), episodic memory, metamemory(self-report), subjective memory, and learning potential(RAVLT). SA was operationalized as no important disease, no disability, physical functioning, cognitive functioning, and being actively engaged. Participants were asked if they use cellphone, computer, or tablet. Pearson´s correlation test and linear regression models were performed. In total 11.2% were successful agers.53.6% used cellphone,14% computer,8% tablet, 44.1% any devise.Results show significant correlation between SA and subjective memory, learning potential and the use of ST. Results of the multiple regression analysis emerged on a significant model using the entered method:F=26.05,p>.000, explaining 21.4% of the variance of SA. Although objective memory measurements were no significant for SA, all memory measurements were related to the use of ST. Knowledge generated by this study reveals the specific role of the metamemory on the SA, underlining the relevance of subjectivity on aging. We need to reflect about the limitations of older adults to access to a digital world in order to achieve a SA.

### SYSTEMS AGING CLOCK: A NOVEL EPIGENETIC AGING CLOCK MODELED FROM ORGAN & BODILY FUNCTION BASED MORTALITY INDICES

Raghav Sehgal, and Morgan Levine, *Yale University, New Haven, Connecticut, United States*

A diverse array of aging clocks, derived from a variety of omics data and clinical biomarkers, have been developed to describe aging and predict age-related disease. As such, these biomarkers are particularly applicable for use in observational studies, basic science and clinical trials focused on tackling biological aging. However, ongoing research suggests significant heterogeneity in aging, with deterioration and disease occurring in different organ systems or functional domains at various rates across individuals. Existing aging clocks only measure heterogeneity in the degree of aging, not in the manner of aging (e.g. different organ systems or functional domains). We hypothesize these unique trajectories exist and that they can be captured using a systems based approach. In our work, using clinical chemistry biomarkers from participants in the Health and Retirement Study (HRS), Framingham Heart study (FHS) and Women's Health Initiative (WHI) , we modeled unique epigenetic aging trajectories from distinct groups of biological processes (such as Immune function, metabolic function, hepatic function, cardiac function, renal function and more). Interestingly, these biological system specific scores when combined gave an aging clock with superior mortality prediction than any published aging clock. We further validate the system aging scores and aging clock in different clinical studies to show the added advantage of such a measure, such as the fact that people with similar epigenetic age may have very different system scores. Overall, this method introduces the potential for quantitative and multi-dimensional, personalized aging scores that are indicative of an individual's disease and disorder risk.

### THE BUFFERING EFFECT OF RESILIENCE ON THE SOCIAL CONSEQUENCES OF COVID IN OLDER WOMEN

Nacia Goldberg, Dawn Carr, Miles Taylor, and Natalie Sachs-Ericsson, *Florida State University, Tallahassee, Florida, United States*

Prior to the pandemic, public health experts argued that loneliness was among the most significant threats facing women's health and wellbeing. As the COVID-19 pandemic brought our social lives to an abrupt pause in March, 2020, older adults were encouraged to remain isolated from friends and family. Social distancing guidelines led many older people to decrease social interactions with others. Using a community-based longitudinal study of women age 60+, we examined how changes in feelings of social connections with others influenced loneliness in October 2020 relative to prior to the pandemic (in September 2018). Our previous research has shown that psychological resilience decreases the negative consequences of major life stressors in later life. We hypothesized that women with high social consequences of the pandemic would experience increased loneliness, but resilience would buffer these effects. In line with our hypotheses, results showed that those who reported significant declines in social connectedness with others during the pandemic (i.e., high social consequences) experienced significant increases in loneliness (beta=0.125; $p<0.001$). Resilience, alternatively, was significantly associated with decreased loneliness (beta=-0.05; $p<0.05$), and buffered the social consequences of the pandemic. That is, as resilience increased, the social consequences of COVID-19 significantly declined ($p<0.01$), and resilience attenuated the negative consequences of high levels of social consequences of COVID-19 on loneliness, while those with high social consequences and low resilience experienced significant increases in loneliness in association with the pandemic. Based on our findings, we discuss potential clinical implications for resilience-based interventions for older adults.

### THE IMPACT OF RETIREMENT RESOURCES ON U.S. OLDER FEMALE WORKERS' RETIREMENT TIMING: THEORY OF PLANNED BEHAVIOR MODEL

Hansol Kim,[1] David Ekerdt,[2] Tamara Baker,[3] Amber Watts,[2] Tracey LaPierre,[2] and Yan Bing Zhang,[2] *1. University of Pittsburgh, Pittsburgh, Pennsylvania, United States, 2. University of Kansas, Lawrence, Kansas, United States, 3. University of North Carolina, Chapel Hill School of Medicine, University of North Carolina at Chapel Hill (School of Medicine), North Carolina, United States*

For older workers, having a retirement plan is important for a successful transition. Social awareness of the problems encountered by older women during retirement remains low. Women have limited retirement resources due to their unequal work experience, and older women with access to fewer retirement resources often postpone their retirement. This research examined how the timing of older women's retirement was influenced by their retirement resources as well as their marital status. The study used 2014 HRS and RAND data, and collected sample of women aged 50-62 years old who worked either full or part time (n=3,593). Respondents were female (56%), white (63%), married (70%), and working full time (82%). Guided by the theory of planned behavior (TPB), multiple regression analysis examined gender differences in predicting older adults' retirement timing. TPB included three sub factors: attitudes toward retirement, subjective norm, and perceived behavioral control. Logistic regression analyzed the effects of respondents' expectations of retirement (i.e., with vs without expected timing). The findings indicated that the TPB model works similarly for men and women but there is a difference according to marital status. Unmarried women are less likely to have accumulated financial resources and more likely to anticipate a later retirement (1.4 years) than married women and are also less likely to set an expected timing for retirement ($p<05$). Such a robust research agenda would provide key information for government agencies and policymakers and contribute to the development of retirement planning models or retirement education programs for older women.

### THE IMPACTS OF SOCIO-ECONOMIC CHALLENGE ON LONG-TERM PHYSICAL AND MENTAL HEALTH IN RETIREMENT AGE

ILAN KWON,[1] sojung park,[2] BoRin Kim,[3] and ByeongJu Ryu,[4], *1. Our Lady of the Lake University, San Antonio, Texas, United States, 2. Washington university, St.Louis, Missouri, United States, 3. University of New Hampshire, University of New Hampshire, New Hampshire, United States, 4. Brown School, Washington University in St. Louis, St. Louis, Missouri, United States*

Despite consistent evidence on the negative effect of social and economic challenges on health, little is known about the pattern of economic difficulties people experience and the impact of those challenging patterns on long-term health in later life. This study used the national data, Mid Life in the United States (MIDUS 3 in 2013-2014), to identify the different patterns of socio-economic challenges that older Americans (50-64 ages old) experienced during the Recession in 2008 and to examine the impact of past challenging experiences on physical and mental health in their later life. Socio-economic challenges included twenty-six items such as losing or moving a job, missing rent, selling or losing a home, bankruptcy, having debts, and cutting spending. We conducted the latent class analysis and regression while controlling other social determinant factors (e.g., education, employment status, poverty, etc.). The latent class analysis result found five patterns during the Recession: people who experienced various difficulties during the Recession, who moved their jobs, who experienced financial difficulties, who bought a home with decreased debts, and who experienced no difficulty. Compared to people with no challenging experience, those who needed to move their jobs but could make debt off during the Recession reported physically healthier, but not mentally healthier in later life. Interestingly, among this group, women reported more long-term physical health problems than men. The findings suggest the close connection between physical and mental health and the importance of long-term care for mental health among older adults in recovering from socio-economic challenges.

### THE MORTALITY OF OLDER ADULT INPATIENTS WITH KIDNEY CANCER DURING THE PANDEMIC: A RETROSPECTIVE COHORT STUDY

Kendall Brune,[1] Cheng Yin,[2] Rongfang Zhan,[2] and Liam O'Neill,[3], *1. Meharry Medical College, Nashville, Tennessee, United States, 2. University of North Texas, Corinth, Texas, United States, 3. University of North Texas, University of North Texas, Texas, United States*

**Background:** Elderly patients are a vulnerable group during the Covid-19 pandemic, especially those with cancer. Our study aims to identify how Covid-19 impacts elderly inpatients with kidney cancer and determine risk factors associated with increased mortality.

**Methods:** Our retrospective cohort study utilized the PUDF dataset and included inpatients over 60-year-old, diagnosed with kidney cancer, and hospitalized within 30-day. Person's Chi-Square was used to measure the differences between survivors and non-survivors, and the Mann-Whitney test was for non-normality distribution for continuous variables. Then, a binary logistic regression was employed to identify the association between independent variables and mortality.

**Results:** Five hundred and twenty-two patients were included in the study, of which 7 (1.4%) died during hospitalization. According to the univariate analysis and Mann-Whitney test, expired patients were more likely to experience older age ($p = 0.005$), longer length of stay ($p = 0.009$), ICU ($p = 0.012$), HMO Medicare Risk ($p = 0.005$), Covid-19 ($p < 0.001$), paralysis ($p < 0.001$), and higher illness severity ($p < 0.001$). The binary logistic regression revealed that older age (OR = 1.120, 95% CI: 1.004-1.249, $p = 0.042$) and the SOI (OR = 4.635, 95% CI: 1.339-16.052, $p = 0.016$) had significantly high odds of mortality.

**Conclusion:** The retrospective cohort study reveals that although Covid-19 was not a predictive factor associated with increased mortality, there was a statistically significant difference between the survivor and non-survivor groups. Further studies need to assess its association with kidney cancer or other various types of cancer.

### THE MYTH OF CARE COORDINATION: WHETHER PROFESSIONAL CARE COORDINATION IMPROVED OLDER ADULTS' HEALTH OUTCOMES?

Kedong Ding, *Case Western Reserve University, Cleveland, Ohio, United States*

**Background:** Current evidence on the effects of Care Coordination (CC) on older adults' well-being and health service utilization is inconsistent. Previous studies are mostly limited to regional data and focus mostly on nurse-led CC instead of layperson Care Coordinators like family caregivers. This study explores the effects of having CC in a national sample of U.S. older adults and whether the coordinators' professionalism impacts the effect of having CC on multidimensional health outcomes (Health outcomes were conceptualized as physical health, healthcare utilization, and care encounters).

**Methods:** Data were from the 2016 and 2018 waves of the Health and Retirement Study (HRS) (n=1,372). Multivariate regression models were used to examine the effects of CC on multidimensional health outcomes in 2016 and the longitudinal effects of having CC. We also tested the effect of Care Coordinators' professionalism on the multidimensional health outcomes. All models controlled for sociodemographic characteristics and health status.

**Results:** Findings suggest that having CC doesn't have a positive effect on older adults' health outcomes. Having CC was associated with an increased number of acute diseases ($\beta = 0.16$, $p < .001$) and nonacute diseases ($\beta = 0.21$, $p < .01$) in longterm. The results regarding cross-sectional effects show that receiving care from a Coordinator was related to increased health service utilization. Participants with professional Care Coordinators were more likely to report receiving person-centered care (OR=1.60, $p<.05$).

**Conclusion:** This study demonstrates the limited effects of CCs on older adults' physical health outcomes, and emphasized the importance of care coordinators' qualifications.

### THE RELATIONSHIP BETWEEN COVID 19 ANXIETY AND DEMENTIA CAREGIVERS BURDEN AND SUFFERING

Kaitlyn Lucas,[1] Eleanor Batista-Malat,[2] Seho Park,[3] Shelly Johns,[4] Nicole Fowler,[5] and Katherine Judge,[6], *1. Cleveland State University, Westlake, Ohio, United States, 2. Indiana University School of Medicine, Regenstrief Institute, Indiana, United States, 3. Indiana University School of Medicine, Department of Biostatistics, School of Medicine, Indiana, United States, 4. Indiana University School of Medicine, Center for Health Services Research, Indianapolis, Indiana, United States, 5. Indiana University School of Medicine, IU Center for Aging Research, Indianapolis, Indiana, United States, 6. College of Sciences and Health Professions, Cleveland State University, College of Sciences and Health Professions, Ohio, United States*

The impact of COVID-19 on dementia caregivers is gaining new interest. It is unknown how the pandemic has impacted caregivers' burden and existential suffering. Analyses were performed on data for dementia caregivers (n=89) enrolled in the Indiana University Telephone Acceptance and Commitment Therapy for Caregivers (TACTICs) pilot trials. Individuals were primary caregivers of a family member with dementia and had clinically significant anxiety measured by a GAD-7 score >10 or between 5-9 with reported interference in life. COVID-19 anxiety was measured using the NIH CoRonavIruS Health Impact Survey (CRISIS) questions. Caregivers were on average 55.2 years of age with 56.2% being child or child-in-law, 71.9% were white and 24.7% were Black. Mean burden scores, measured by the Zarit Burden Index, were higher (44.29) compared to means reported across the literature (26.7) indicating the sample experienced higher than normal levels of burden. Mean existential suffering scores measured by the subscale of Experience of Suffering Scale were lower (9.37) compared to means across the literature (11.5) indicating that overall participants experienced lower levels of existential suffering compared to those in previous studies. A significant relationship was found between COVID-19 anxiety and burden levels (x2= 9.07, p<0.05), with higher levels of COVID-19 anxiety associated with greater burden. A non-significant relationship was found between COVID-19 anxiety and existential suffering (x2=5.99, p=0.11). Results highlight the impact of COVID-19 anxiety as an external stressor on dementia caregiving. and the importance of considering context of external stressors when implementing intervention protocols for caregivers of individuals with dementia.

### THRIVING-IN-PLACE: EXAMINING THE IMPACT OF INTERGENERATIONAL LIVING IN THE TORONTO HOMESHARE PROGRAM

Raza Mirza,[1] Melissa Macri,[1] Deirdre Kelly-Adams,[1] Carley Moore,[1] Andrea Austen,[2] Jacalyn Tanner,[3] Jessica Hsieh,[1] and Christopher Klinger,[1], *1. University of Toronto, Toronto, Ontario, Canada, 2. City of Toronto, City of Toronto, Ontario, Canada, 3. National Initiative for the Care of the Elderly (NICE), Toronto, Ontario, Canada*

Most global cities, like Toronto, have rapidly aging populations who want to remain in homes and communities of their choice. Concurrently, seniors face vulnerabilities associated with low income, ageism, social isolation and loneliness. These vulnerabilities inhibit many seniors' desires to age-in-place. The Toronto HomeShare Program, an intergenerational homesharing program facilitates aging-in-place by matching seniors with post-secondary students. The program, with an implementation focus and a research study, was developed to address and understand the needs of seniors seeking assistance, light supports and companionship at home, in exchange for reduced-rent housing for students. A mixed methods research design was employed. Seniors and students (n=22) completed a 167 question survey (n=22) and in-depth interviews (n=18). Quantitative data yielded descriptive statistics and qualitative data was subject to thematic content analysis. Participants agreed that homesharing programs could address risk for social isolation (95%), the need to move from their community (96%), and reduce risks of economic and social exclusion for young and old (97%). From the qualitative data, six benefits were apparent for all participants: (1) reduced social isolation and loneliness, (2) increased intergenerational exchange, (3) increase financial security, (4) household assistance, (5) increased general wellbeing; (6) enhanced companionship/safety. In 2020, Toronto HomeShare (now Canada HomeShare) was recognized by the World Health Organization as an age-friendly best practice, and has been scaled nationally in 16 cities. Intergenerational homesharing programs could be a catalyst for policy and cultural reform and to support older adults to not only remain in their communities, but to thrive-in-place.

### TOO CLOSE FOR COMFORT? COVID-19-RELATED STRESS AMONG OLDER COUPLES AND THE MODERATING ROLE OF CLOSENESS

Kira Birditt, Angela Turkelson, and Angela Oya, *University of Michigan, Ann Arbor, Michigan, United States*

Married and cohabiting couples have important influences on one another's stress and well-being. Pandemic-related stress may influence the extent to which couples' stress levels are coregulated. This study examined the experience of nonspecific stress and pandemic-related stress and the moderating role of closeness among couples aged 50 and over in which at least one member had hypertension. A total of 30 couples reported their feelings of closeness to one another in a baseline interview and their feelings of nonspecific stress and pandemic-related stress every three hours for 5 days. There was no difference in closeness and nonspecific stress between husbands and wives. Wives reported greater pandemic-related stress than husbands. Actor-partner interdependence models revealed that wives' nonspecific stress predicted husbands' nonspecific stress ($b = 0.17$, $SE = 0.04$, $p < .001$) and that husbands' nonspecific stress predicted wives' nonspecific stress in each three hour period ($b = 0.19$, $SE = 0.04$, $p < .001$) and these associations were not moderated by closeness. Coregulation in pandemic-related stress among husbands and wives was moderated by wives' feelings of closeness such that when wives' feelings of closeness were lower, greater husband pandemic-related stress predicted lower pandemic-related stress for wives ($b = -0.16$, $SE = 0.07$, $p < .05$) whereas when wives' feelings of closeness were higher, greater husband pandemic-related stress predicted greater pandemic-related stress for wives ($b = 0.22$, $SE = 0.09$, $p < .05$). These findings indicate that closeness may have detrimental effects especially when considering emotional coregulation in couples regarding the pandemic.

### TREATING CAREGIVER GRIEF WITH NARRATIVE THERAPY

Tara Matta, *University of South Florida, Tampa, Florida, United States*

Dementia, a devastating neurodegenerative disease with over 10 million new diagnoses each year, is characterized by many symptoms including memory loss .Individuals with memory less experience changes in mood, personality, behavior, cognition and activities of daily living which affect their daily lives. These monumental life shifts often occur rapidly, leaving caregivers unprepared to deal with the changes. Caregivers face a unique situations navigating anticipatory grief and changes in their relationships with their loved ones. Current psychological intervention for caregivers includes utilization of cognitive-behavioral therapy and psychoeducation. More recently, intriguing research has emerged regarding the efficacy of narrative therapy for couples where one partner experiences memory loss. However, treating the anticipatory grief component specifically for caregivers has been largely overlooked in these studies. Narrative therapy revolves around identifying the current story that caregivers utilize as their cognitive framework, helping to find alternative plotlines and to process their newly-built cognitive framework. It involves externalizing the problem (in this case, dementia) and locating strengths that the caregiver and their care receiver share to "fight" the problem. Insights from both the current literature and the field have demonstrated a promising outlook on the use of narrative therapy. Such insights imply a need for more research regarding this modality specifically for caregivers, as its core ideas can be easily disseminated to gerontologists, mental health professionals and caregivers.

### USER PERCEPTIONS OF A TECHNOLOGY-BASED SOCIAL MEMORY AID FOR PERSONS WITH MEMORY CONCERNS

Brenna Horn,[1] Elizabeth Albers,[1] Eric Jutkowitz,[2] Jessica Finlay,[3] Lauren Mitchell,[4] Ashley Millenbah,[5] and Jude Mikal,[1], *1. University of Minnesota, Minneapolis, Minnesota, United States, 2. Brown University, Brown University, Rhode Island, United States, 3. University of Michigan, Ann Arbor, Michigan, United States, 4. Emmanuel College, Boston, Massachusetts, United States, 5. University of Minnesota, University of Minnesota, Minnesota, United States*

People with memory concerns (PWMC) are likely to experience social withdrawal and isolation. Although assistive technologies and memory aids are available to support PWMC and their family caregivers, few have been shown to improve social engagement. This study aimed to gain perspectives of PWMC and their family caregivers on the feasibility and utility of a technology-based social memory aid. We recruited 20 dyads of people with mild to moderate memory concerns and family caregivers to evaluate Smartwatch Reminder (SR), a notification system that provides a name, relationship, and photograph of nearby social contacts to aid in recognition. Dyads viewed a demonstration of the SR prototype, and then participated in semi-structured interviews over Zoom video conferencing from June to August, 2020. Interview transcripts were analyzed using thematic analysis, with analyses completed in August 2021. Our findings indicate that participants anticipated important benefits from using the technology, and thought the technology would be easy to use. Participants perceived that the memory aid could benefit them now and in the future by alleviating socialization-related stress for both members of the care dyad; however, certain features of SR restricted the potential benefits, such as the requirement that social contacts have the SR application, and that prompts are only provided during social encounters. Our findings will inform future technology-enabled memory aid development to improve social engagement for PWMC and support family caregivers.

### WITHIN-PERSON VARIATION IN EMOTIONAL EXHAUSTION AMONG CAREGIVERS FOR OLDER ADULTS

Tomoko Wakui, *Tokyo Metropolitan Institute of Gerontology, Itabashi, Tokyo, Japan*

Caregiving is everyday life for family members of older adults. Care recipients' care requirements, service usage, and caregivers' physical and emotional conditions differ day by day. Little is known how the differences and variances relate to informal caregivers' mental health. This study aimed to examine informal caregivers' day-to-day fluctuation in emotional exhaustion and discuss the within-person effects on mental health among informal caregivers. We developed the Caregiving Visualization Project toolkit (Care VIP), a software program for tracking on daily basis components of care experiences such as care task of Activity of Daily Living (ADL) and Instrumental Activity of Daily Living, service usages, and caregiving burden, as well as eight items of caregivers' emotional exhaustion. We recruited study participants between May 2018 to March 2019 who provided instrumental help to community-dwelling older adults. A total of 75 participants, who accessed the Care VIP every day by using tablets or computers and completed a one-month study, were analyzed in this study. Females comprised 80.0%, and the average age of caregivers was 52.7 years (SD=9.1). The majority were those who provided care to parents (69.3%), and those who provided to parents-in-law and spouses were 16.0% and 5.3%, respectively. The average score of the eight items on the emotional exhaustion scale, with a 4-point Likert scale, was 23.4 (SD=4.9); however, each question showed different variations. Within-person effects on mental health among informal caregivers will be discussed.

### YOUR SUFFERING IS ALSO MINE: OLDER AND YOUNGER COUPLES' RESPONSES TO THE PARTNER'S UPSETTING MEMORY.

Jared Isaac Cortez,[1] Stephanie J Wilson,[2] M. Rosie Shrout,[3] and Janice Kiecolt-Glaser,[4], *1. Southern Methodist University, Dallas, Texas, United States, 2. South Methodist University, Dallas, Texas, United States, 3. Purdue University, Purdue University, Indiana, United States, 4. Ohio State University, Columbus, Ohio, United States*

Aging theories posit that older adults maximize their well-being by regulating their emotions and investing in their closest relationships. Most research has examined these mechanisms using study confederates rather than close dyads. The existing work on couples has focused on marital conflict; none has examined responses to the spouse's emotional suffering. To address this, 107 married couples ages 40-86 listened to their partner disclose an upsetting personal memory. Afterward, listeners rated their own and their partner's emotions and perspective-taking; observers reliably coded listeners' engagement and disclosers' emotional intensity. Aging theories offer competing predictions: older listeners may disengage from their partner's disclosure to avoid experiencing negative emotions. Alternatively, older adults may be more engaged and thus more reactive, given the increased investment in their close relationships. Findings showed that older listeners rated their disclosing partner as less sad compared to younger counterparts ($p < .05$). However, this effect was attenuated ($p = .077$) by observed emotional intensity, as older disclosers exhibited less intense emotions. There were no age differences in listeners' own reactivity, perspective-taking, or observed engagement. Taken together, older adults disclosed with less emotional intensity, consistent with theory. By contrast, older listeners' ratings were validated by external coders, not driven by positivity biases. Further, older listeners were no more or less engaged or reactive to their spouse's disclosure than younger listeners. This study highlights a context wherein social and emotional motivations are at odds. Teasing these motivations apart will help us to better understand how social-emotional processes develop across adulthood.

### Index

Arabic numbers refer to session numbers.

Duplicate numbers indicate the participant is listed as an author on multiple abstracts within a session.

A

(Alberta Health Services), Glenrose Geriatric Research Team: 9135
Aaron, Bryan: 9515
Aaron, Siobhan: 9505
Aarts, Sil: 4335
Abadir, Peter M.: 4390, 9055, 9105
Abah, Theresa: 9170, 9215
Abahussain, Mohammed: 9395
Abarca, Gabino: 9375
Abbey, Enoch J.: 4160
Abbott, Katherine M.: 2065, 2460, 2510, 3015, 4095, 9150, 9350
Abbott-Enz, Pamela S.: 2050
Abdel-Maksoud, Madiha: 9025
Abdelrahman, Samir: 9065, 9025
Abdur-Rahman, Ihsan: 1240
Abenojar, John Paul T.: 9425
Öberg, Peter: 1080, 3530, 9460, 9425
Abner, Erin: 2015
Abraham, Alison G.: 2160
Abraham, Jesse M.: 4500
Abramson, Tobi A.: 4280
Abshire Saylor, Martha: 4340
Abujarad, Fuad: 2070
Abushakrah, Jan: 1160
Abushousheh, Addie: 2505
Achterberg, Wilco P.: 2120
Ackenbom, Mary: 9395
Acosta-Rodriguez, Victoria: 1440
Acton, Gayle: 9185
Adamek, Margaret: 9165, 9310, 9470
Adams, Dorinda A.: 3240
Adamsen, Collette: 9045
Adamski, Michal: 9130
Adams-Price, Carolyn E.: 1050, 9380
Adanlawo, Temiloluwa: 9510
Adanu, Emmanuel: 1485, 9195
Addison, Adriana S.: 9045
Addison, Odessa: 9030, 9045, 9320
Adelman, Toby: 3255
Adeniji, Dolapo O.: 9470, 9505
Adepoju, Omolola E.: 9400, 3115, 3470
Adesanya, Ikmat: 2315
Adeyemi, Oluwaseun J.: 1485
Adhikari, Samrachana: 2280
Adjognon, Omonyele: 4415
Adriana, Adriana: 4035
Affuso, Olivia: 9420
Aflatoony, Leila: 3535
Afonso, Rosa Marina: 9500
Agarwal, Mansi: 2410
Agboji, Aderonke O.: 3055
Aggarwal, Neelum: 1260, 2075
Agharazidermani, Maedeh: 4470
Aghjayan, Sarah L.: 4115
Agmon, Maayan: 1095, 4435
Agrawal, Yuri: 4040
Aguila, Emma: 4185, 4265
Aguilar, Iris: 9510
Aharonson-Daniel, Limor: 9500
Ahearn, Eileen: 4430
Ahluwalia, Jasjit: 9505
Ahmed, Mohammed: 9515
Ahmed, Sagheer: 9500, 2120, 9065
Ahuja, Manik: 9455, 9460
Ahuja, Rajiv: 9510
Ahuja, Sunil: 9160, 9140
Aichele, Stephen: 3175
Aiken, Judd: 9110
Aiken Morgan, Adrienne T.: 1435, 9445
Ailey, Sarah: 9350
Ailshire, Jennifer: 2435, 3520, 3525, 4100, 4205, 4265, 4635, 9020, 9515
Aitken, Sarah: 3245, 4590
Ajrouch, Kristine J.: 1295, 4605
Akishita, Masahiro: 3490
Akosah, Kwame O.: 4130
Akosionu, Odichinma: 1275
Akushevich, Igor: 1230, 2060, 2115, 3040, 3160, 4585, 9055, 9065, 9085
Al Snih, Soham: 1230, 9295, 9390, 9140
Alam, Moudud: 9505
Alarid-Escudero, Fernando: 2360
Albadawi, Amani: 2365
Albers, Elizabeth A.: 1030, 1420, 2325, 3185, 9515
Albert, Isabelle A.: 4630, 9155
Albert, Steven M.: 2250, 9240
Alberth, Andrew G.: 1245
Albright, Amy E.: 4195, 9240, 9465
Albright, Amy: 9510
Albright, Bonnie: 9035
Albright, David L.: 3030
Albright, Laurie: 2175, 9360, 9455, 9460
Alcantara-Lee, Raniel: 9105
Alcorn, Kathleen: 4430
Aldag, Matt: 9510, 1500, 9135, 9155, 9430
Alexander, Bruce: 9405
Alexander, Chuck: 1340
Alexander, Karah: 9505
Alexander, Lacy: 3550
Alfaro, Ana J.: 1110, 9360, 9370
Alfaro, Ana Jessica: 1110
Ali, Addison: 9505
Ali, Muhammad Usman: 9130
Ali, Sumbleen: 9505
Ali, Talha: 9510
Ali, Yumna: 2120
Alici, Yesne: 9115
Alix, Andrew: 9115, 0, 3270, 4510
Al-Juboori, Ruaa: 9505
Alkema, Gretchen: 2090, 2460
Allan, Alexa C.: 3520
Allard, Joanne S.: 9505
Allemand, Mathias: 2305
Allen, Isabel E.: 9450
Allen, Laura: 3305, 4090
Allen, Nancy: 9030
Allen, Rebecca S.: 3370, 3405, 4195, 4275, 4370, 9240, 9265, 9320, 9465
Allen, Samantha: 9505, 9410
Aller, Ty: 9290
Allgood, Kristi: 3320
Allison, Theresa A.: 1495, 2325
Allore, Heather G.: 3040, 9030, 9380, 9510

Allsopp, Richard: 3415, 9085
Almardawi, Ranyah: 9500, 9505
Almeida, David M.: 1035, 1065, 1165, 1430, 2310, 2450, 3520, 3550, 9130, 9245, 9360
Almeida-Meza, Pamela: 1460
Almirola, Carlos: 3170
Alosco, Michael: 9065
Alpert, Naomi: 3075
Alraddadi, Khulood: 9230
Al-Saleh, Stacy: 2365, 9030
Alvarado, Katherine: 9215
Alvarado Rodriguez, Marlene: 9515
Alvarez, Emily: 4045
Alvarez, Paula: 9220
Aly, Iman M.: 4190, 3490, 9040, 9060, 9145, 9360
Ambrizzi Moraes, Catarina: 9510
Ambrosius, Walter T.: 1305
Ameer, Fathima: 9085, 9110
Amgalan, Anar: 9305
Amick, Allison: 9055
Amick, Matthew: 9500
Amin, Ravindra: 3095
Amini, Reza: 9130
Amirchian, Kristina: 9215
Amodeo, Samuel J.: 9405
An, Yang: 2105, 2285, 4040, 4065
An, Jaesung: 9515
An, Ping: 4585
An, Yang: 2285
Anaya-Dominguez, Nerea A.: 9500
Ancoli-Israel, Sonia: 9515, 3295
Andel, Ross: 2200, 3080, 9125, 9140, 9385
Andersen, Ove: 1095, 9085
Andersen, Stacy L.: 2015, 2110
Andersen, Troy: 1405
Anderson, Andrea: 9505
Anderson, Ellen: 3595
Anderson, Gloria T.: 9425
Anderson, Jason R.: 9370
Anderson, Joel G.: 3570, 9065
Anderson, Keith A.: 1325, 1420, 2470, 3475, 2235, 4030
Anderson, Monica: 9060
Anderson, Nicole: 9025
Anderson, Raeda K.: 9515
Anderson, Rozalyn: 1440, 2250, 4105
Anderson, Ruth A.: 2140, 3055, 3325, 3605, 3610, 3250, 4225, 4465
Anderson, Stacy L.: 4585
Anderson, Wayne L.: 2090
Andersson, Björn: 1045
Andes, Laura: 2010
Andonian, Brian J.: 9420
Andrade, Flavia C.D.: 4185, 9505
Andrasfay, Theresa: 9435
Andreoletti, Carrie: 2345, 3500, 4490
Andrieu, Sandrine: 1280
Andringa, Ronald: 2225
Anema, Jason: 2020, 4585
Ang, Gavrila: 9515
Angel, Jacqueline L.: 4185
Angel, Ronald J.: 4185
Angelini, Luise: 9105
Angelo, Sahil: 3090
Angulo, Aylin: 9185
Anker, Lauren: 4535
Ankuda, Claire K.: 1450
Annabathula, Rahul: 9295
Annis-Brayne, Kristen: 3245
Ansello, Edward F.: 2350, 4020, 9010
Anton, Stephen: 9090
Antonescu, Corina: 4390, 3245, 4340, 4450
Antonucci, Toni C.: 1295, 2135, 4605, 4620, 9505
Anunsen, Roger: 2050
Anuskiewicz, Blake: 3300
Apesoa-Varano, Ester Carolina: 9410
Apolinario, Daniel: 9505
Appel, Lawrence J.: 3290
Appel, Michael: 3455, 3545
Applebaum, Allison J.: 3165
Applebaum, Robert: 2460, 4095, 4270, 9500
Applewhite, Ashton: 4010
Apriceno, Marybeth: 9510
Aqajari, Seyed Amir Hossein: 2075
Aquirre, Annabella S.: 2030
Aranda, Maria P.: 4210, 4235, 9200, 9510
Arany, Szilvia: 3610
Araujo, Lia: 9500
Arbaje, Alicia: 2360, 4405
Arbeev, Konstantin: 3040, 4585, 9055, 9070, 9085
Arbex Silva Borim, Flávia: 9295
Archambault, Dennis: 3455, 3545
Archer, Breannlyn M.: 9070
Archibald, Paul: 3640
Archuleta, Jennifer: 9375
Ardakani, Azam: 9160
Ardelt, Monika: 1505, 9435
Arensberg, Mary Beth: 9425, 9510
Arevalo, Sandra P.: 1295
Arfanakis, Konstantinos: 9390
Argov, Esther: 9420
Argueta, Jeremy: 9370
Arias-Merino, Elva Dolores: 1300, 9515
Arieli, Maya: 4435, 9320, 9515
Ariyo, Raquel: 9225
Ariyoshi, Mie: 4090
Arling, Greg: 3585, 4425
Armah, Henrietta: 9195
Armbruster, William: 4495
Armendariz, Jazlyn A.: 9075, 9370
Armendariz, Marina: 1370
Armenti, Karla P.: 3255
Arms, Tamatha E.: 2355, 9035
Armstrong, Nicole: 4040, 9505
Armstrong, Nicole D.: 9290
Arnette, Shannon: 4240, 4260
Arnold, Tomorrow D.: 9360
Arnon, Mical: 9420
Arnsten, Amy: 9055
Arora, Kanika: 2055, 4310
Arpawong, Thalida: 4195, 4345
Arpino, Bruno: 3225
Arrieta, Martha: 2260
Arroyo, Bernardo: 9505
Arroyo, Maria C.: 9505
Arterburn, David: 3300
Arunachalam, Thilaka: 9160
Aruscavage, Nancy: 9510
Aryal, Subhash: 4035, 4445, 9380, 9385
Asadi, Neda: 3440
Asakura, Yuki: 9420
Asante, Samuel: 3345, 9305
Aschwanden, Damaris: 3185, 9155, 9515
Asghar-Ali, Ali: 1110
Ashcraft, Alyce: 9395
Ashe, Maureen C.: 9175, 9200
Ashida, Sato: 4200, 9040, 9270
Astell, Arlene: 9025, 9080, 9145
Astencio, Mayra: 9045
Asthana, Sanjay: 2335
Asuncion, Xavier Eugenio: 9090, 9025
Atherton, Olivia E.: 2305, 4515
Athouriste, Gauty: 9505
Atkins, Ghislaine: 9195
Atkins, Janice L.: 3320, 9100
Atkinson, Taylor: 9125, 9140
Atlas, Lauren S.: 9360, 9415
Attis, Latoya: 9165
Au, Rhoda: 2015
Au, Walker Siu Hong: 3470, 4280
Auais, Mohammad: 3485
Aubertin-Leheudre, Mylène: 3485

Aubrecht, Katie: 3395, 9510
August, Kristin J.: 3580, 9140
Austen, Andrea: 9515
Austin, Hannah L.: 4055
Au-Yeung, Wan-Tai M.: 9500
Avent, Elizabeth S.: 2040, 2475, 4350
Avidor, Sharon: 4080
Avila, Jaqueline C.: 4635, 9505
Avila, Vickie: 9060
Avila-Rieger, Justina F.: 4265
Awan, Omer: 9500
Ayabe, Takako: 9500
Ayalon, Liat: 4080, 4630
Aycock, Dawn M.: 3640
Ayedun, Adeola A.: 4140, 4270
Ayers, Emmeline: 9390
Azhar, Gohar: 9045, 9085, 9110

**B**

B. Ferdows, Nasim: 1060
Béland, François: 2145, 2405
Bültmann, Ute: 1200
Babenko-Mould, Yolanda: 3590
Babiker, Niser A.: 4065, 9045
Bacci, Jennifer: 3285
Bachner, Yaacov G.: 2260, 3345
Backer, Ryan: 4010
Backhaus, Ramona: 3585
Backhouse, Daniel: 3460
Backman, Annica: 2045, 3440
Bacsu, Juanita-Dawne R.: 4005, 9380, 9500
Badea, Alexandra: 2085
Badger, Terry: 9115
Badji, Atef: 1210
Badovinac, Lisa: 3555
Bae, Suyeong: 4330, 4430
Bae, Youngjoon: 9150
Baek, Jihye: 9515
Baek, Jonggyu: 9295
Baek, Sehyun: 9500
Baernholdt, Marianne: 2140, 3325
Bae-Shaaw, Yuna H.: 4455
Bagley, Olivia: 4585, 9055, 9070, 9085
Baharlou, Shahla: 3060, 9280
Bahnson, Judy: 4550
Bahous, Sola: 9130
Baht, Gurpreet: 9085
Bai, Chen: 9250
Bai, Chyi-Huey: 9135
Bai, Xue: 4380
Baier, Rosa: 9395
Baik, Dawon: 3535
Baik, Sol: 4180, 9505, 9500
Bailey, Abigail: 4140
Bailey, Christine: 9505
Bailey, Nicole E.: 9515
Bailey Jr., Donald E.: 3610
Baillargeon, Emma M.: 9505
Bair, Woei-Nan: 4395
Bajracharya, Rashmita: 2115
Bakare, Hameed: 2390
Baker, Elaina: 9180, 9290
Baker, Felicity: 4530
Baker, Robin: 9515
Baker, Tamara: 2300, 3320, 4290, 9395, 9410, 9515
Baker, Zachary G.: 1005, 3010, 4130, 4350, 9380
Balasubramanian, Priya: 9085
Balasubramanian, Sundaravadivel: 9500
Balaswamy, Shantha: 2430
Balbim, Guilherme M.: 9250, 9265
Balboni, Tracy: 4135
Baldwin, Julie A.: 2410
Baldwin, Megan: 9240
Bales, Connie W.: 9515, 4120
Ball, Diana: 9380
Balls-Berry, Joyce E.: 1310
Balmuth, Alexa: 1315, 2190
Balu, Abhinav: 9085
Bambarger, Kimberly: 9130
Bamgbade, Benita A.: 9030
Bamonti, Patricia: 4535, 9420
Banda, Andrew: 3630
Banda, Joseph A.: 1010
Bandeen-Roche, Karen: 3290, 3485, 4070
Bandiera, Frank: 3400
Banegas, Jasmine: 4245, 2300
Banerjee, Smita C.: 9115
Banerjee, Sube: 3605
Bangerter, Lauren R.: 1180
Bangerter, Lauren: 9090, 9405
Baniassadi, Amir: 9505
Bankole, Ayomide: 9030
Bao, Yuhua: 2070
Bar, Sudipta: 4510
Barba, Cheyanne: 1230
Barbabella, Francesco: 4005
Barbee, Harry: 4460
Barber, Justin: 2405
Barber, Sarah: 9210
Barbieri, Alessandro: 4510
Barbieri, Magali: 2390
Barbosa, Breno JAP: 9505
Barchi, Daniel: 4590
Bardach, Shoshana: 2015, 2405
Bardenheier, Barbara H.: 1055, 9395
Bareham, Bethany K.: 3090
Bareja, Akshay: 9105
Barfield, Hannah: 9470
Barg, Frances: 3450
Barha, Cindy K.: 9250
Barinas-Mitchell, Emma: 1210, 9295
Barker, Anna: 4070
Barlow, Meaghan: 4560
Barmaki, Roghayeh: 9505
Barman, Tarani K.: 9160
Barnard, Juli: 4570
Barnes, Laura: 1125
Barnes, Alexandra L.: 1065
Barnes, Deborah: 3095
Barnes, Gareth T.: 9515
Barnes, Lisa L.: 1310, 1435, 2495, 4065
Barnes, Stacy L.: 1400
Barnes, Timothy L.: 2175, 9455, 9460
Barnes-Farrell, Janet: 4055
Barnet, Jodi H.: 1130
Barnett, Melissa A.: 2500
Barnstaple, Rebecca: 3065
Barooah, Adrita: 9200
Barr, Erik: 2315
Barragan, Cassandra L.: 4360, 9160, 9510
Barrenetxea, Jon: 4590
Barrera Caballero, Samara: 1005, 2166, 3050, 4110, 1485, 9340, 9410
Barrett, Donna: 4325
Barrett, Kendall: 3555
Barrett, Rod: 9105
Barros, Duarte: 9420
Barry, Lisa C.: 4200, 9070
Barsness, Sonya: 4575
Bart, Stephan: 9515
Barta, Hayley: 9095
Bartholomae, Suzanne: 9005
Bartlett, David B.: 4115, 9420, 3410
Bartley, Emily J.: 3095, 9045
Bartley, Jenna: 3120
Bartoldus, Victoria: 2140, 3325
Barton, Kelli N.: 9145
Barton, sheila: 3060
Bartres-Faz, David: 2130
Barzilai, Nir: 3495
Basaria, Shehzad: 4160
Bashian, Hannah M.: 9020
Basic, Ajla: 9510
Bass, David M.: 1025, 3235, 4085, 9185, 9360
Bass, Dwana: 9155, 9400
Bastoni, Sofia: 9500
Basu, Avinandan: 3650
Basu, Rashmita: 1075, 1465
Batchelor, Melissa: 3565, 4215
Bateman, James R.: 9055
Bateman, Randall J.: 9510
Bates, Laurel G.: 2090
Bates, Timothy: 1270
Batista-Malat, Eleanor: 9515
Batsis, John A.: 9030, 9390, 9455, 1200, 1490, 3000

Bau, Heidi: 9215
Baucom, Brian: 4600
Bauer, Juergen: 4335
Bauer, Michael: 2150
Bauer, Scott R.: 9295
Baughman, Amy W.: 1075
Bauldry, Shawn: 2300
Baumann, Ana: 1190
Baumbach, Abby: 9290
Baumel, Barry: 9070
Baumgart, Matthew: 3160
Bauste, Grant: 1175
Bayles, Megan A.: 3145, 3650
Baylin, Ana: 1210
Bayliss, Elizabeth: 3565, 9240
Bayne, Alycia: 2375, 4045
Bazargan, Mohsen: 9045
Bazzano, Lydia: 2015
Beach, Scott: 1285, 1330, 3380, 3535, 4085
Beadle, Janelle N.: 2166, 9460
Beam, Christopher R.: 9510, 9515
Beamer, Brock: 2130, 9030
Bean, Jonathan F.: 9140, 9420, 9500
Beard, Renee L.: 9115
Beasley, Jeannette: 9500
Beattie, Zachary: 4065, 9500, 9505, 9510
Beauchamp, Mark: 1165
Beauchamp, Marla: 9160
Beaudreau, Sherry A.: 1110, 4535
Beaulieu, Celeste C.: 1040, 9170
Beauregard, Lisa K.: 2025, 2095, 2445
Beavers, Kristen M.: 1305
Beavers, Daniel P.: 1305, 3310
Becantur, stephanie: 2140
Beck, Anna: 9270
Beck, Emorie D.: 2305, 4515
Beck, Gabrielle: 9115
Beck, Jennifer N.: 4510, 9105
Beck, Samuel: 9095
Beckel, Julia: 2370
Becker, Clemens: 4115, 9390
Becker, Todd D.: 9500, 3375, 4545
Beckert, Angela: 9215
Beckert, Troy: 9340
Beckstein, Kassidy: 4330
Beckwith, Ellen: 1135
Beeber, Anna: 2140, 3325, 3605, 9395
Beech, Bettina M.: 3640
Beer, Jenay: 1140, 9505
Beerman, Isabel: 4315
Beetle, Christopher B.: 9460, 9510, 9110
Begin, Francis-Gabriel P.: 9085
Behrens, Emily: 9410
Behrens, Liza L.: 1390, 2080, 3015
Beilin, Lawrence: 4395
Bekelman, David: 9240
Belanger, Emmanuelle: 4375, 9510
Belgrave, Melita: 2385
Belkin-Rosen, Aaron: 9055, 2215, 4015
Bell, John G.: 4195, 9320
Bell, Mallory: 9125
Bell, Sue Anne: 9245
Bell, Tyler R.: 9505, 9510, 1435, 2200, 2400
Bella, Jesse: 2065
Bellelli, Giuseppe: 9515
Bellen, Hugo J.: 4510
Bellettiere, John: 2130, 3300, 4070
Bellingtier, Jennifer A.: 9510, 4630
Bellizzi, Keith: 9500
Belsky, Daniel: 1120
Beltran, Susanny J.: 9510, 2485, 3515
BeLue, Rhonda: 3445
Belza, Basia: 3330, 9355, 9505
Benayoun, Berenice: 9500
Bench, Shaylee: 3465
Bender, Alexis A.: 2470, 3090, 9425
Bendheim, Paul E.: 9065
Beneciuk, Jason: 9500
Benmoha, Eti: 9420
Bennett, David A.: 2495, 9055, 9390, 9505
Bennett, Katherine: 1340, 9510
Benson, Clark: 1180, 3535, 9045
Benson, Jade: 3460
Benson, Karen: 4415
Benson, Lizbeth: 1165
Bentley, John P.: 9265
Benton, Donna: 9220
Benton, Melissa J.: 9395
Bentur, Netta: 1410
Benz, Jennifer: 4045
Berdahl, Carl T.: 1390
Berg, Kristen A.: 1230
Bergeman, Cindy: 9340
Bergen, Gwen: 9500
Berghoff, Ashley: 9240
Bergman, Elizabeth J.: 2345
Bergman, Sky: 3410
Bergmann, Johannes M.: 2510
Bergmans, Rachel: 1105
Bergquist, Timothy: 9505
Bergren, Stephanie: 9510, 2255, 9135, 9515
Berg-Weger, Marla: 4175, 9215, 9500
Berical, Erin: 3035
Beringer, Robert: 2000, 2375, 3035, 4600
Berish, Diane: 2120, 2180, 3180, 3205
Berkan Hozempa, Chandra: 1495
Berkley, Amy S.: 1130, 9045
Berlin, Melissa: 2015
Berlowitz, Dan: 1075
Berman, Jacquelin: 4255, 4280
Berman, Rebecca: 2065
Berman, Reem: 9105
Bernays, Florence: 4430
Bernier-Carney, Catherine: 1405
Bern-Klug, Mercedes: 1070, 1445, 9180
Bernstein, Samantha: 9500
Berridge, Clara: 1020, 3000
Berry-Stoelzle, Maresi: 9405
Bert, Waters: 4260
Berta, Whitney: 3440, 3605, 4425
Bertisch, Suzanne: 3295
Bertrand, Rosanna: 9405
Besser, Lilah M.: 3160
Bessette, Hannah C.: 9505
Best, Irene: 3180, 3445
Best, John: 2435, 3210, 3465
Best, Leslie A.: 9185
Best, Rachel D.: 9500
Bethell, Jennifer: 9030
Bettencourt, Ricki: 9510
Bettiol, Silvana: 3405
Beynon, Cynthia: 2425
Bezerko, Mara: 9000
Bhagianadh, Divya: 2055
Bhargava, Sakshi: 9130, 4560
Bhasin, Shalendar: 3120
Bhat, Aarti C.: 9245
Bhat, Ajay: 9105
Bhatt, Jem: 4610, 1360, 1505, 3075, 4280, 9515
Bhattacharjya, Sutanuka: 9365
Bhattacharya, Aditya: 4470
Bhattacharyya, Kallol Kumar: 9140, 9345
Bhattarai, Gandhi: 2175
Bherer, Louis: 1210
Bhullar, Puneet: 9100
Bial, Martha C.: 4620
Bian, John: 9500
Bian, Xueli: 2140
Bibbo, Jessica: 2315, 4325
Biccum, Katelind: 9020
Bieber, Dawn: 4455
Bierman, Alex: 1395
Bierman, Arlene: 9405
Biggar, Virginia: 1180, 1080, 3530, 9460
Bilgel, Murat: 2285, 3430
Bin Mulayh, Ejlal: 9515
Binder, Ellen F.: 3120
Birchfield, Thomas R.: 1010
Birditt, Kira: 4320, 9515
Birkeland, Robyn: 1030, 3185, 4305
Bisconti, Toni L.: 9015, 9020
Bishop, Alex: 1505
Bishop, Kathleen: 4020, 2480
Bishop, Nicholas: 9460

Bishop, Sonia: 9500
Bitter, Nicolas: 9065
Bitto, Alessandro: 9305
Bjarnadottir, Ragnhildur: 9510
Bjerkan, Jorunn: 2485
Blöchl, Maria: 4560
Blach, Anna: 9045
Black, Kathy: 1375, 4495
Blackberry, Irene: 1090
Blacker, Deborah: 1005, 9165
Blackshear, Chad: 1280
Blackwell-Hoge, Terri: 3295
Blackwood, Jennifer: 9035, 9130
Blain, Hubert: 9390
Blair, Elizabeth: 4140, 4230
Blancato, Robert B.: 3505
Blanchard, Jeanine: 1125, 4075
Blanco, Erik: 9260
Blanco, Robert: 9030, 1150, 2170, 4085, 4420, 9020
Blaskewicz Boron, Julie: 9080, 9135, 9140, 9150, 9155, 9175, 9470
Blatter, Catherine: 2045
Blaxton, Jessica: 9340
Bleich, Michael: 9080
Bleijlevens, Michel H.: 9500
Blessing, Debbie: 9500
Blieszner, Rosemary: 1030
Blinka, Marcela D.: 9265, 9290
Bliwise, Donald: 9290
Block, Laura: 1180, 3535, 9045
Block, Masha: 9100, 9410, 9515
Blocker, Kenneth A.: 2365
Blomberg, Ben: 4240
Bloom, Rachel F.: 9500
Bluck, Susan: 9430, 9500
Blue Bird Jernigan, Valarie: 9045
Bluestein, Dan: 4240
Bluethmann, Shirley M.: 9250
Blumen, Helena: 9390
Blumenthal, Jacob: 2130
Bo, Boroka B.: 9125, 9435
Boafo, Jonelle: 1325, 3260
Boateng, Augustine C.O.: 4445
Bobitt, Julie: 1190, 4310
Bobroff, Stella: 9000
Boe, Meredith: 4605
Boeder, Jordan: 9225
Boerner, Kathrin: 1415, 2025, 2150, 2425, 9200, 9245, 9260
Boger, Jennifer: 4465, 9145
Bohnert, Adam: 9500
Boim, Kimberly F.: 9185
Bolin, Salli: 1420
Bolkan, Cory: 1300, 1330
Bollag, Wendy: 9105
Boller, Benjamin: 9505
Boltz, Marie: 1270, 2080, 3015, 3180, 3205, 3315, 3445, 3585, 9505, 9515
Bomi, Choi: 2435, 9080, 9250
Bonds Johnson, Kalisha: 3460, 3620, 4340, 9290
Bonifas, Robin P.: 9500
Bonner, Alice: 4230
Bonner, Gloria J.: 2075
Boockvar, Kenneth: 2445, 9150, 9160
Boogaerts, Garner: 9000
Booker, Staja Q.: 9045, 9405
Bookwala, Jamila: 1215
Boominathan, Amutha: 9110
Boot, Walter R.: 2225, 3000, 3170, 3655, 4145, 4470, 9135, 9335
Booth, Richard G.: 9255
Boots, Lizzy M.M.: 4610
Borack, Michael S.: 4120, 9515
Boratgis, Gabriel D.: 1180
Bordes, Piper: 9360
Bordone, Valeria: 3225
Borges-Machado, Flávia: 9420
Boroumand, Shahdokht: 9025
Borrero, Lisa: 3360
Borson, Soo: 9050
Bortone, Ilaria: 2085
Boscardin, John: 4550, 9070
Boscia, Francesco: 2085
Bosco, Elliott: 9395
Bosco, Nabil: 9510
Bostarr, Norma: 9280
Boston, Nicholas: 9510
Boström, Anne-Marie: 9505
Bosun-Arije, Stella: 4565
Botoseneanu, Anda: 9030, 9380, 9510
Bouchard, Lauren M.: 2230
Boucher, Nathan A.: 4080
Boucher, Sarah: 9510
Boudreau, Robert M.: 2085, 3290, 9390
Bouklas, Isabella: 1050, 9505
Bouldin, Erin D.: 1350, 3235, 9510
Bourgeois, Michelle: 2225, 9505
Bourgeois-Guerin, Valérie: 3105
Bournias, Themistokles: 4115
Bourque Bearskin, Lisa: 9380
Bourquin, Jennifer: 4450
Bowblis, John R.: 1275, 4095, 9345
Bowen, Lauren M.: 1040, 9170, 1155
Bowen, Pamela G.: 9250, 9420
Bower, Emily S.: 1135
Bowers, Katherine: 9505
Bowie, Janice: 4340
Bowlby, Douglas: 9510
Bowles, Kathy: 2200
Bowling, William: 2450
Boyd, Cynthia: 2010, 3565, 4525
Boyd, Willard L.: 9515
Boyle, Patricia: 2495
Braakman-Jansen, Annemarie: 9080, 9500
Brach, Jennifer: 2105, 4520, 9250
Bradley, Dana: 1265, 3305, 4090
Bradley, Lauren: 2040
Bradley, Sara: 9065, 9300
Bradshaw, Patrick: 3295
Bradt, Joke: 2385
Brailsford, Jennifer: 9500
Brais, Hannah: 3105
Braithwaite, Steven: 1015, 9105
Brakenridge, Scott: 9090
Brand, Martin D.: 9110
Brander, Danielle M.: 4115
Brandt, Martina: 3525
Brandt, Cynthia: 9500
Brandt, Nicole: 1345, 3500, 9210, 9310
Brantley, Halley: 9405
Brassington, Layla: 9505
Bratlee-Whitaker, Emily: 9130
Bratzke, Lisa: 9390
Brauer, Simon G.: 4605, 9035
Brauner, Daniel J.: 1395, 4045
Braunstein, Joel B.: 9510
Bravo, Diamond: 3175
Brazier, Joan: 9515
Brem, Rachel B.: 4510
Brende, Anna: 9500
Brennan, Emma: 9265
Brennan, Maura J.: 3420, 4250
Brennan-Ing, Mark: 2330
Brenowitz, Willa: 4190, 9505
Brent, Lauren: 4390, 9100
Brewer, Christine: 9180
Brewster, Glenna S.: 2295, 4340
Brewster, Amanda: 4140, 4270, 3460, 4340, 9290
Brewster, Scott: 9215
Brewster Glasgow, Glenna: 9275
Brick, Timothy: 1165
Brickman, Adam M.: 3320
Bricout, John: 9285
Bride, Jessica: 4605
Briesacher, Becky: 1345, 9030
Briesch, Amy: 9500
Briley, Margaret: 9100
Brill, Shai: 4335
Brilliant, Susan: 9250
Brito, Tábatta: 9295, 9185
Britton, Maggie: 4130

Britton, Rachel: 9105
Brocklebank, Laura: 1460
Brockmann, Austin D.: 9135
Brod, Kathryn: 9500
Broder, Jonathan C.: 9030
Brody, Abraham A.: 1325, 3055, 3260, 3475, 4405, 4410
Brody, Lilla: 9505
Broen, Tiana: 9200, 9510
Bronstein, Laura: 2500
Brooker, Helen: 9020
Brookins, Hilary: 9045
Brooks, Amber K.: 3065, 9510
Brothers, Allyson F.: 9035
Brottman, Barbra: 1350
Broussard, jordan: 2005
Brown, Amy: 3650
Brown, Blakely: 9250
Brown, Candace S.: 1265, 3620
Brown, Candace: 3025
Brown, Charlotte: 1425
Brown, Colette: 4580
Brown, Cynthia J.: 4290, 4590, 9000, 9410
Brown, Diane K.: 1000
Brown, Ellen: 9505
Brown, J. Scott: 9240
Brown, Jocelyn L.: 3240
Brown, Katherine A.: 4450
Brown, Lana: 9330
Brown, Lauren: 2210
Brown, Lisa M.: 2260, 3080
Brown, Maria T.: 3225, 3530, 4295
Brown, Monique J.: 3645
Brown, Rebecca: 2010
Brown, Sandra C.: 1030
Brown, Susan H.: 9420, 9515
Brown, Todd: 9025
Brown-Borg, Holly M.: 4105
Brown-Hughes, Travonia: 1370, 3075
Browning, Sean D.: 9265
Browning, Wesley: 9195
Brownridge, George W.: 4510
Broyles, Ila Hughes: 2090
Brubaker, Peter: 9510
Bruce, Marino A.: 3640
Bruce, Martha L.: 9360
Brucki, Sonia MD: 9505
Bruefach, Tyler: 9510
Brune, Kendall R.: 9515
Brunker, Cherie S.: 1405
Bruno, Michelle: 2200
Bruns, Danielle R.: 9105
Brunson, Kirenia: 9510
Brunt, Vienna: 9305
Brunton, Cory: 9510, 9515
Bryan, Angela: 9105
Bryant, Alison: 2165
Bryant, Natasha: 2320, 2445, 9245
Bryant-Lees, Kinsey B.: 9020
Brymer, Jennifer: 9135
Buchanan, Brooke: 4225
Buchanan, Taylor: 9410
Buchholz, Erik: 9085
Buchholz, Susan W.: 9350
Buchman, Aron S.: 2495, 9390
Buck, Courtney N.: 9030
Buck, Harleah G.: 2000, 3200, 3450
Buckley, David J.: 9100
Buckley, Thomas D.: 9385, 9515, 9505
Bucy, Taylor I.: 9345
Buenaver, Luis: 9330
Bueno, Michael V.: 1345
Buford, Thomas: 4590, 9410
Buhrmann, Anna S.: 3190
Buhusi, Mona: 9085
Bui, Chuong: 3405, 9510
Bukowy, Elizabeth: 9395
Bulanda, Jennifer R.: 3580
Bulgarelli, Alexandre F.: 9365
Bull, Amanda C.: 2005, 9350, 9505
Bullock, Alexis: 9250, 9425
Buma, Lise E.: 3625
Bunck, Leah: 2215, 4305
Bunger, Alicia C.: 1190
Burack, Orah: 2445, 9150, 9160
Burant, Chris: 2500, 9260, 1345, 2200, 4405
Burgess, Elisabeth: 3090, 9210
burke, James R.: 3055, 4620
Burman, Bonnie K.: 4325
Burnaevskiy, Nikolay: 9515, 1335, 3240
Burnette, Denise: 9385, 9500, 9515
Burnette, Lauren: 4565
Burnham, Dyanna: 9115
Burns, Annette: 4570
Burns, Edith: 4150
Burns, Jacquelyn: 1355
Burns, Jeffrey: 4015
Burns, Kathryn: 3250
Burns, Robert: 3380
Burr, Jeffrey: 2425, 3030, 3390, 4555, 9500
Burris, Jessica L.: 2450
Burston, Betty: 1470, 9185, 9380
Burton, Cassandra: 9515
Butt, Hamza: 1250
Butters, Meryl A.: 4345
Butts, Donna: 3560
Butts, William: 9510
Buxton, Orfeu: 1130, 9055, 9440, 9515
Buys, David R.: 3630
Bybee, Sara: 4600
Byers, Amy: 4550, 9070
Bygrave, Desiree C.: 9140, 9440
Bynum, Julie: 9160
Bynum, Keyanna: 4595
Bynum, Shalanda A.: 9215
Byrd, Christopher: 9505
Byrd, DeAnnah R.: 9380
Byrne, Kaileigh A.: 9470
Byrnes, Mary E.: 9515
Byrnes, Tru: 9000

**C**

Caban-Holt, Allison: 1370
Cabrera, Isabel: 3580, 4085, 4110
Cacchione, Pamela: 1155, 2420, 9510
Caceres, Maria: 9150, 1460, 9145
Cadet, Tamara: 3130
Cafeo, Christina: 3500
Cagle, John G.: 3375, 4545
Cagney, Kathleen A.: 1050, 4265
Cahill, Barian: 9505
Cahill, Kevin E.: 3615
Cai, Yurun: 3290, 4040
caiola, courtney: 3610
Calamia, Matthew: 9360, 9465
Calasanti, Toni: 4055, 4235
Caldeira, Clara: 9155
Caldeira de Mello, Ruth: 2100, 1200, 1280, 3460, 4435, 9025
Caldwell, Elizabeth Perry: 1210
Caldwell, Guy A: 9500
Caldwell, Kim A.: 9500
Calhoun, Melanie: 2475
Caliz, Hazel: 9160
Calkins, Margaret: 2505
Callahan, Kathryn E.: 1095, 9295
Callan, Judith: 9510
Calloway, Laura: 9080
Camacho, David: 4605, 9505, 9515
Camell, Christina: 2440
Cameron, Sarah: 9355
Camillo, Cheryl: 2205
Cammer, Allison: 9510
Camp, L. Jean: 9080
Campbell, Grace: 3535
Campbell, Maura: 2275
Campetti, Randi: 9300, 9500
Campitelli, Anthony: 9070, 9130, 9390, 9420
Canada, Kelli E.: 4475
Canham, Daryl: 3255
Canham, Sarah: 3105, 4385
Cankurtaran, Sadiye: 3590
Cannell, Brad: 9300
Cannon, Melissa L.: 3185, 3210
Cano, Carlos A.: 4635
Cantelli, Angelina: 9465
Canter, Benjamin E.: 9150, 9160
Cantu, Phillip: 4185
Cao, Guofeng: 3355
Cao, Jian: 1015
Cao, Jiawei: 1485
Cao, Qiuchang: 4495
Cao, Qiuchang (Katy): 1140
Cao, Xian: 3340

Capellaro, Taylor J.: 9505
Caprio, Thomas V.: 2120, 3610, 4240
Capuano, Ana W.: 1435, 2495
Carabello, Maria: 9380
Carbajal, Berta: 9185
Carbonell, Ellen: 3005, 3165
Cardador, M. Teresa: 9500
Cardellini, Jennifer: 4325
Cardenas, Valeria: 2125, 9410
Carder, Paula: 1220, 3015, 4375, 9510, 9515
Cardoso, Josue S.: 9045
Cardozo, Christopher P.: 9305
Carino, James A.: 4220
Carll, phil: 9335
Carlson, Barbara: 9505
Carlson, Chalise: 1110, 4535, 9360, 9370
Carlson, Michelle C.: 1095, 4520, 9250, 9505
Carmel, Sara: 2260
Carmichael, Alicia: 2165
Carmichael, Pierre-Hugues: 1045
Caroll-Haskins, Katrina: 4530
Carollo, James: 9515
Carpenter, Brian D.: 9500, 9515, 2000, 3100, 9080, 9205, 9210
Carpenter, Joan G.: 1390
Carpenter, Kyrie: 4010
Carpenter, Rebekah: 2370
Carr, Alan: 1290
Carr, Dawn C.: 9135, 9510, 9515, 1085, 1355, 2370, 4470
Carr, Deborah: 2135, 2265, 2415
Carrillo, Jared: 9080
Carrillo-Van Tongeren, Norma: 9510
Carrington-Lawrence, Stacy: 3400
Carrion, Iraida: 1090
Carroll, Dana G.: 4195, 9320
Carroll, Judith: 3130, 9505, 9510
Carstensen, Laura L.: 4440
Carter, Jordan: 9505
Carter, Mary W.: 9350
Carter, Yasmin: 9045
Carthron, Dana L.: 3550
Carty, Chris: 9105
Carvajal, Scott: 4167
Carvalho, Joana: 9420
Cary, Jr., Michael P.: 9395, 9510
Casanova, Ramon: 9055, 9250, 9505
Casey, Dympna: 9500
Casillas, Alejandra: 9240
Caskie, Grace: 9020, 9140, 9155, 9370
Cassidy, Jessica: 2470
Cassie, Kimberly M.: 9510
Casso, Abigail: 9305
Castaneda, Salvador: 3005
Castellano, Joseph: 2245
Castelli, Darla M.: 9500
Castillo, Aaron: 1060
Castle, Nicholas G.: 1275, 9395
Castro, Alfredo: 9515
Castro, Carl A.: 9155
Castro, Jose: 4510
Castro, Saul: 1320
Castro-Portuguez, Raul: 4390, 9085, 9100, 9105
Catic, Angela G.: 4250
Catlin, Sally: 9470
Cato, Kenrick: 2120
Cattaneo, Gabriele: 2130
Cauley, Jane A.: 4520, 9505
Cavagnini, Katherine: 9325
Cavalier, Alyssa N.: 9105
Cavalli, Stefano: 1415
Cawthon, Peggy M.: 4160, 4520
Cawthon, Peggy: 9505
Cekic, Sezen: 3175
Celardo, Caitlin: 9325
Celly Martins Ribeiro de Souza, Marina: 3345
Cengiz, Sara: 9510
Cenko, Erta: 9510
Cerino, Eric S.: 2310, 3520
Ceruso, Marco: 2225, 9080
Cha, Hyungmin: 9380
Cha, Jinhee: 1030, 4350
Chacko, Anjana: 9395, 9405
Chaddock-Heyman, Laura: 9140
Chae, Joosuk: 3150
Chahine, Lana: 2195
Chai, Hye Won: 1430, 9380, 9505, 4050
Chakanyuka, Christina: 9380
Chakraborty, Shayok: 4470
Chakurian, Daphne: 9290
Chamberlain, Stephanie: 3325, 3605
Chan, Angelique W.: 3050, 4075
Chan, Athena Chung Yin: 4120, 4245, 9230
Chan, Kai Nin Joseph: 2465
Chan, Kathryn E.: 3045, 9040
Chan, Keith: 9500
Chan, Patrick: 3460
Chan, Thomas: 9075, 9080, 9225, 9370
Chan, Ya-Ning: 2140, 3610
Chandel, Navdeep S.: 4105
Chandra, Malini: 9505
Chandran, Devyani: 9165
Chandy, Jasmine: 3005
Chane, Samson: 9310
Chaney, Cassandra: 3640
Chang, Chiang-Hua: 9160
Chang, DaJung: 9515
Chang, E-Shien: 1285, 1335, 2170, 3235, 4630
Chang, Milan: 1045, 3075, 9250
Chang, Yu-Chien: 9345
Chang, Yu-Ping: 9270, 9395
Chao, Tony C.: 2295
Chao, Ying-Yu: 2180, 3660
Chapadia, Bunsi: 9500
Chapleski, Elizabeth: 4230, 9510
Chapman, Elizabeth: 9215
Chapman, Karen R.: 2295
Chapman, Susan: 3370, 9155
Chard, Sarah: 9500
Charles, Lesley: 3250
Charles, Lily: 9505
Charles, Susan: 1065, 2310, 2450, 3520, 4440, 4580, 9235
Charlesworth, Georgina: 4610
Charlton, Judith: 9390
Charness, Neil H.: 1330, 2225, 3170, 4470, 9335
Chase, Jo-Ana: 1345
Chase-Cantarini, susan: 1405
Chasteen, Alison: 9500
Chataut, Akankshya: 9080, 9470
Chatterjee, Samir: 3550
Chatters, Linda M.: 1185, 2460
Chau, Pui Hing: 9395
Chaudhari, Nikhil N.: 3435, 9095, 9305
Chaudhry, Aisha: 3015
Chaudhury, Habib: 3540, 4385
Chaves, Lee: 9105
Chayaput, Prangtip: 9000
Cheang, Tiffany: 9220, 1005, 9515
Chelgren, Britta: 3535
CHEN, HSIU-HSI: 3340
Chen, Huashuai: 3110
Chen, Zibei: 2415
Chen, Annie: 9505
Chen, Chia Ying: 9505
Chen, Chia-Hui: 9395
Chen, Cynthia: 4590
Chen, Diefei: 1290
Chen, Dorothy: 3295
Chen, Feinian: 4245, 4555, 9360, 9380
Chen, Gong: 9375, 9425
Chen, Gracie: 9280
Chen, Hui: 1200
Chen, Hui-Ching: 2030, 4345
Chen, Jieming: 9260
Chen, Jing: 9055
Chen, Jiu-Chiuan: 3310
Chen, John S.: 9030, 3370, 4275
Chen, Lei-Shih: 1195

Chen, Lihui: 9320
Chen, Lin: 4225
Chen, Nemin: 2105, 9505
Chen, Randi: 3415, 9085
Chen, Sanmei: 9295
Chen, Shang-Ti: 2180
Chen, Shih-Hua Sarah: 9350
Chen, Tao: 9295
Chen, Tuo Yu: 9380, 9515
Chen, Xi: 1115, 3610, 9270
Chen, Xiaomeng: 4070, 4395
Chen, Xiayu: 9140
Chen, Ya-Mei: 9345, 9350, 9425
Chen, Yea-Hung: 9450
Chen, Yen T.: 9500
Chen, Yi: 1060
Chen, Yu-Chih: 9310
Chen, Yumeng: 9295
Chen, Zhao: 1085, 1250, 9510
Chen, Zhirui: 3355, 4555
Chen, Zi-shuo: 4435, 9025
Cheng, Jianjia: 1145, 9500
Cheng, Jie: 1100, 4295
Cheng, Mengling: 4205
Cheng, Michael: 4360
Cheng, Sheung-Tak: 9155, 9265, 9515
Cheng, Yan: 2175, 9455, 3160
Cheon, Ji Hyang: 1355, 4180, 9425, 9500
Cherayi, Shanuga: 9320
Cherlin, Emily J.: 4140, 4270
Cherner, Mariana: 9085
Cherniack, Martin: 4055
Cherry, Barbara J.: 9030, 9360
Cherry, Debra: 1060, 9280
Cherry, Katelin: 4335
Cherry, Katie E.: 2260, 9360, 9465
Cheung, Ethan Siu Leung: 2360, 9040, 9145
Chevalier, Stéphanie: 3485
Chew, Felicia: 4455
Chi, Iris: 1070, 3225, 3610, 4350, 9240, 9500
Chi, Kevin: 4440, 9505
Chi, Nai-Ching: 9050
Chiang, Hsueh-Sheng: 1435
Chiaramonte, Delia: 9025
Chick, Christina: 9360
Chilton, Patricia D.: 9505
Chin, Jessie: 1475, 2485
Chin, Nate: 9500
Chinea, Arielle: 9265
Chiong, Cloie: 3325
Chiou, Kenneth L.: 9100
Chiri, Giuseppina: 3275
Chiriboga, David A.: 3150, 9140, 9455
Chiu, Ching-Ju: 9050
Cho, Emma S.: 9045
Cho, Eunhee: 9065, 9070, 9130, 9295
Cho, Isu: 9180
Cho, Jinmyoung: 9515, 1010, 1205, 4205
Cho, Joonyoung: 9040, 9325
Cho, Joshua W.: 9100
Cho, Kyuyoung: 9380, 9505
Cho, Seungjong: 9035
Cho, Soyeon: 9240
Cho, Sunghwan: 9515, 3115
Cho, Young-Eun: 9070
Cho, Youngmin: 3325, 9395
Cho, Youngshin: 4430
Choate, Sarah E.: 1030
Chodosh, Joshua: 9500
Choe, Jessica: 9510
Choi, Edmond Pui Hang: 9395
Choi, Eunju: 9070, 9500
Choi, Eunyoung: 9020, 9140, 9240
Choi, HwaJung: 2265
Choi, Hyo S.: 9105
Choi, Hyojin: 4280, 9280
Choi, Jean: 3520, 9360
Choi, Jimmy: 2060
Choi, Julie: 1135
Choi, Mi Sun: 2095
Choi, Mona: 9030, 9505
Choi, Namkee G.: 9325
Choi, Sarah E.: 9265, 9440
Choi, Seung-won Emily: 9505
Choi, Shinae L.: 9515, 2265
Choi, Soo Chan: 2025
Choi, Tsai-Chin: 2265
Choi, Won: 3035
Choi, Wonchan: 9500
Choi, Yeon Jin: 4255, 4500, 9515
Choi, Yong Kyung: 4255, 9120, 9385
Choi, Yoonseok: 9200, 9510
Choi, Youngmin: 2140
Chong, Trevor T.: 9065, 9070
Chong, Wayne F.: 2030, 2430
Choo, Esther: 2070
Chopik, William J.: 2305, 3070, 3185, 3575, 4205, 4515
Chosy, Julia: 9085
Chou, Hsueh-Chih: 9350
Chou, Lin-Na: 1230, 9390, 9025
Choudhury, Renoa: 1100, 9510, 9515
Choula, Rita: 1395
Choun, Soyoung: 1500, 9155, 9430
Chow, Esther O.: 9280
Chow, Stephanie W.: 4450, 9160
Chowdhury, Nahian F.: 3435, 9055, 9095
Chrisphonte, Stephanie: 9130
Christen, Stefan: 9510
Christensen, Amy: 9500
Christensen, Kaare: 2020, 2110, 2285, 4585
Christensen, Suna: 9395
Christian, Thomas: 1240
Christine, Kirt: 9510
Christiuk, Vanessa: 9515
Chu, Charlene H.: 2045, 9280
Chu, Frances: 9505
Chu, Li: 1410
Chu, Nadia M.: 4070, 4395
Chua, Kevin: 4590
Chuang, Yi-Fang: 9250
Chubinski, Jennifer: 9500
Chui, Celine: 9065
Chui, Helena C.: 9505
Chun, Yung: 9040
Chung, Edwin K. H.: 2315
Chung, Jane: 9080
Chung, Joon: 3295
Chung, Kuo-Piao: 3340, 9350
Chung, Kyuhyung: 3115
Chung, Misook Lee: 4235
Chung, Shang-En (Michelle): 4025
Chung, Soondool: 9310, 9455, 9510
Chura, Lindsay R.: 1080, 4215
Churchill, Linda C.: 2030, 1025, 9185
Ciarleglio, Adam J.: 3320
Cicero, Ethan: 3570
Cichy, Kelly E.: 9260
Cidav, Tansu: 9290, 2445, 3090, 3365
Cimino, Andrea N.: 9215
Ciofi, Joy: 2470
Ciolfi, Mary Lou: 9505
Cipher, Daisha: 9510
Cirilo, Anthony: 9515
Ciułkowicz, Marta: 3585
Civiletto, Gabriele: 3285
Clarfield, A. Mark: 9500
Clark, Christi: 2385
Clark, Phillip: 3420
Clark, Amy G.: 4080
Clark, John I.: 9500
Clark, Karen C.: 9515
Clark, Lindsay: 9500
Clark, Michaela: 9020
Clark, Patricia C.: 3640
Clark, Phillip G.: 2350, 4020, 9005, 9515
Clark, Sunday: 2070
Clark, Tatia: 9375
Clark, Valerie: 1075, 1270
Clarke, Anthony: 1100
Clarke, Philippa: 4180, 4520
Clarke-Jones, Annette: 9165
Clark-Shirley, Leanne: 9215, 9220
Clason, Caitlin: 3450
Clauw, Daniel: 9410
Claver, Maria L.: 9250, 9375
Clawson, Brian R.: 9515
Clay, Lauren A.: 9245

Clay, Olivio J.: 9195, 9250, 9420
Clayton, Cam: 9505
Clayton, Margaret: 9270
Clayton, Zachary S.: 9105, 9305
Cleary, Jennifer: 4605
Clevenger, Carolyn K.: 4340, 9275
Clouston, Sean: 1295, 2450, 3070, 4120, 9055, 9065
Cloutier, Denise: 3455
Cloyes, Kristin: 9515, 3375, 4600, 9270
Clutton, Jon: 4015
Coastlines, Rocky: 9505
Coates, Martha C.: 2295, 4595
Coats, Heather: 3535
Coats, Jacquelyn: 9380
Cobb, Peter J.: 4360
Cobb, Ryon J.: 2210, 3320
Coblenz, Clara: 1080
Coburn, Andrew: 3605
Coccia, Kathryn: 9500, 9505
Cochran, Lelah: 3650, 4165
Cockburn, Alastair: 9190
Coco, Laura: 4167
Codding, Robin: 9500
Coddington, Cathy: 9510
Coe, Christopher: 3550
Coe, Norma B.: 9500
Cohen, Alan A.: 3485
Cohen, Harvey J.: 9130
Cohen, Hildie: 9425
Cohen, Kara: 1435, 3170, 3650
Cohen, Marc: 1245, 4275, 4450, 9345, 9500
Cohen, Rachel B.: 9005
Cohen, Rebecca W.: 3430
Cohen, Rinat: 4335
Cohen, Tara: 1390
Cohen-Mansfield, Jiska: 4335
Cohn, Tracy J.: 9020
Cohn-Schwartz, Ella: 2260, 4630, 9500
Cole, Stephanie: 3410
Cole, Steve: 9505
Cole, Steve W.: 9510, 3125
Cole, Thomas R.: 4260
Coleman, Bernice: 1390
Coleman, Carissa K.: 4190
Coleman, Max: 4555
Colina, Sonia: 4167
Collett, Sarah: 9235
Collinge, William: 2075, 9045, 9260
Collins, Amber: 9515
Collins, Devin: 9505
Collins, Lindsey: 9160, 9510
Collins, Louis: 9055
Collins, Sarah: 9030
Collins, Stacey: 9505
Collins-Fletcher, Kadesha: 2060
Colman, Ricki: 3600
Coman, Emil: 4200
Comas-Herrera, Adelina: 3585
Comeaux, Christy A.: 9515
Comer-HaGans, DeLawnia: 9165
Comito, Anthony: 4335
Compernolle, Ellen: 1050, 3530
Condon, David: 4515
Cone, Nicholas: 1135, 1205
Cong, Zhen: 3355
Conger, Lynne: 9060
Conn, David K.: 4365
Connell, Cathleen M.: 1205, 2265, 3320, 9290, 9325, 9370
Connell, LaShauna M.: 4445
Connelly, Caitlin: 1205, 9245
Connelly, Kay: 9155
Conner, Kyaien O.: 9135
Conner, Norma: 9510
Conroy, David: 1165
Conte, Kathleen: 2380
Conti, Gerry: 9130
Convery, Kimberly: 3055
Conway, Emma: 4465
Conwell, Sara: 3500
Conwell, Yeates: 2120, 2270, 4365
Conyers, Christian: 4215
Cook, Andrea: 3300
Cook, David J.: 9405
Cook, Heather G.: 3425
Cook, Kelley: 2500
Cook, Nicole: 9170, 9300, 9355
Cook, Ross: 9105
Cooke, Steph: 2195
Coon, DAVID W.: 3490, 9185, 9335
Cooney, Alissa M.: 9160
Cooney, Julene: 3470
Cooney, Teresa M.: 4245
Cooper, Julie: 3300
Coppock, James A.: 9515
Corazzini, Kirsten N.: 9505, 2045, 2100, 3015, 3100, 9045, 9395
Corbett, Anne: 9020
Corbett, Sage: 9335
Cordell, Ashlee: 9335
Cordova, Lourdes: 9335
Coresh, Josef: 2085, 9510
Corley, Robin P.: 9135, 9360, 9510
Cormier, Émilie: 3105
Corneau, Emily: 1075, 3275
Cornelison, Laci J.: 3015
Cornell, Portia Y.: 1075, 3275, 4375, 9325, 9450
Cornwell, Tom: 2205
Corona, Ligiana P.: 9295
Corpora, Miranda: 3015
Corriere, Matthew: 9515
Cortes, Constanza J.: 3435
Cortes, Marielysse: 9185
Cortes, Monica: 9505
Cortes, Tiffany M.: 9295, 9305
Cortez, Jared Isaac: 9515
Cory, Theodore J.: 3270, 9055, 9105
Cosco, Theodore: 9510
Cosentino, Stephanie: 2015
Costa, Andrea: 9260
Costanian, Christy: 9130, 4235
Costello, Alexander: 4075
Costello, Peter R.: 3540
Costilow, Emily: 9500
Cotten, Shelia: 2325, 3145, 9505
Cotter, Valerie T.: 1327, 4167, 4340
Cotton, Samantha G.: 4240
Cotton, Quinton: 3535, 9045
Cotton, Sam: 3245, 4175, 9320
Coughlin, Joseph: 2400
Coulourides Kogan, Alexis: 2125, 9050
Coupal, Sarah: 9160
Courser, Rebecca: 3320
Couve, Amanda: 3070
Covinsky, Kenneth: 4550, 9295
Cox, Brian: 9410
Cox, Carole B.: 4620, 9315
Cox, Elizabeth D.: 3015
Cox, Kaitlyn: 9070
Coyle, Caitlin: 3210, 4230
Coyle, Nessa: 9115
Craft, Suzanne: 9055
Craft Morgan, Jennifer: 2470, 3475, 9505
Crandall, Carolyn: 2130
Crane, Breanna M.: 1095, 4520
Crane, Jasmine: 9085, 9110
Cranston, Teresa A.: 9515
Crawford, Christina G.: 9165
Crawford, Timothy N.: 9380
Cremer, Svenja: 9500
Crimmins, Eileen: 1055, 2435, 3520, 3575, 9025, 9085, 9095, 9505
Crittenden, Jennifer: 3630, 9220, 9245, 9300, 9500, 9505
Crnek-Georgeson, Kylie T.: 4365
Crocker, Stephen J.: 9055
Crocombe, Leonard: 3405
Croker, J Alton: 4310
Croll, Zachariah: 3285, 9500
Crouch, Maria C.: 2380
Crow, Rebecca: 9030
Crowe, Michael: 2200, 2400
Crowe-White, Kristi: 9420
Crowley, Max: 1320
Cruz, Grace: 9515
Cruz, Kiana: 9265
Cruz, Yanira: 1395

Crystal, Stephen: 4300
Csikai, Ellen L.: 9165, 9350
Csiszar, Anna: 9085
Cuadro, Carla Krishan: 9500
Cudjoe, Thomas: 1465, 2010, 3620, 4420, 4525
Cuervo, Ana Maria: 4315
Cuevas, Adolfo G.: 3595
Cuevas, Heather E.: 2515
Cui, Huan: 9060
Cui, Ruifeng: 4365, 9510
Culberson, John: 9395
Cullen, John: 3245
Culminskaya, Irina: 9505
Cummins, Phyllis: 2050, 2055, 3515, 9220, 9340, 9380, 9500
Cunningham, Tony J.: 9180
Cuppari, Vanessa: 9515, 3580, 9180
Curley, Alexaandra: 2010
Curley, Catherine: 9180
Curreri, Nereide Alhena: 3630, 9505
Currey, Katherine: 3060
Curry, Leslie A.: 4140, 4270
Curtis, Laura: 9200, 4415, 9160
Custis, Meghan: 9035, 9315
Cutchin, Malcolm P.: 4060
Cvejkus, Ryan K.: 1210, 2020, 4585, 9025, 9295
Czaja, Sara J.: 1010, 2225, 3170, 3235, 4480, 9080, 9290
Czirr, Eva: 9105, 4180, 9210

**D**

da Silva, Marcia C.: 9500
Désilets-Jutras, Meghan: 9505
Díaz-González, Ma. Guadalupe: 1300
Dabelko-Schoeny, Holly: 1140, 1420, 4495
Dadar, Mahsa: 9055
Dagerman, Karen: 1125
D'Agostino, Carol S.: 4150
Dahal, Poshan: 3075, 9515
Dahl Aslan, Anna: 2435
Dahlberg, Lena: 2075, 9270
Dale, Maureen C.: 9390
Daley, Ryan T.: 9180
Dallo, Florence J.: 1295, 1230
D'Ambrosio, Joseph: 4175, 9320, 1315, 2190, 2400, 3045
Dame, Jessy: 9380, 1045, 4015, 9505
Damier, Sashah: 9170, 9300, 9355
Dams, Judith: 4115
Danesh, Valerie: 1050
Dang, Alex: 9510
Dang, Hope: 9100, 9105
Dang, Linh: 4485
Dang, Stuti: 9050, 9510
Daniel, Gaea: 4340
Daniel, Kathryn M.: 9025, 9510
Daniel, Nikita R.: 9155, 9175
Danielson, Ramona A.: 9045, 9245
Danilovich, Margaret: 2025, 2065, 4075, 4195, 4565, 4300
Dansereau, Lisette: 3395
D'Antonio, Patricia M.: 2395, 4010
Danzo, Amy: 3500
Dao, Elizabeth: 3460
D'Arcy, Marjory: 4120
Dardess, Pam: 3325
Darin-Mattsson, Alexander: 3460
Darling, Nancy: 9260
Darvishpoor Kakhki, Ali: 9025
Das, Aparna: 9180
Das, Swapan K.: 9100, 4230
Dassel, Kara B.: 1405, 2035, 3025, 9030, 9510
Datta, Dibyadeep: 9055
Daudt, Helena: 2000, 3410
Dault, Helena: 2375, 3035
Daum, Christine: 9060, 9130, 2040
Davey, Adam: 9505
David, Daniel: 4405
David, Uchechukwu: 9515
Davidson, Richard J.: 1065
Davies, Megan: 2340
Davila, Heather: 1075, 2275, 9070
Davis, Eric M.: 1125
Davis, Aileen: 9030
Davis, Anne H.: 4230
Davis, Boyd H.: 9505
Davis, Brooke: 9500
Davis, Elizabeth: 1355, 3175, 3005
Davis, Glenn: 4335
Davis, Ian: 9095
Davis, Jennifer C.: 9250
Davis, Laura: 3285
Davis, Leslie L.: 2140
Davis, Matthew A.: 9420
Davis, Nathan: 1050, 3145, 9400, 2095, 9160
Davis, Simon: 2085
Davis, Tracy: 9155, 9205
Davitt, Joan: 3240
Davlyatov, Ganisher: 4355
Davy, Philip M.: 3415
Daw, E. Warwick: 2020, 4585
Daw, Jonathan: 3550
Daw, Warwick E.: 4585
Dawe, Robert J.: 9390
Dawn Marshall, Samantha: 9060
Dawson, Alice L.: 9515
Dawson, Alison: 9505
Dawson, Walter: 9510, 9500
De Anda, Jennifer: 9505, 9510
de Boer, Bram: 2340, 3585
de Cabo, Rafael: 1440
De Fries, Carson: 3115, 3560, 3595, 4545, 9005
de Klerk-Rubin, Vicki: 4225
de la Paz, Andrew: 9390
De Lima, Bryanna: 9500
De Main, Atami S.: 1050, 9400, 3145
de Man-van Ginkel, Janneke M.: 9500, 3625
De Marchi, Umberto: 3285
de Medeiros, Kate: 3385, 4260
De Ocampo, Ricardo: 9000, 9050
De Paepe, Els: 9515
de Rosa, Cristina: 9270, 9395
de Sam Lazaro, Stephanie L.: 9500
de Souto Barreto, Philipe: 1280, 9130
De Stampa, Matthieu: 2205
de Vries, Brian: 2375, 3035, 4235
de Vugt, Marjolein E.: 4610
de Waal, Margot W.: 2120
Deal, Jennifer A.: 1130, 2085, 4030, 4040, 4190, 9515
Deal, Mike: 4115
Dean, Elizabeth S.: 9105
DeAnda-Duran, Ileana: 2015
Dearing, James: 3440
Deason, Rebecca G.: 9265
DeCaro, Jason: 9410
Decker, Autumn: 1300, 2300
DeFrancesco, Erica: 9505
DeFrancisco, Jenna: 9415
DeFrate, Lou E.: 9515
Degenholtz, Howard: 4095, 4180
DeGennaro, Jennifer: 9510
DeGroot, Lyndsay: 4340
DeGutis, Joseph: 9140
DeHoff, MacKenzie: 9105
Deichert, Jerome: 9350
Deimling, Gary T.: 9115
Deindl, Christian: 9260
Dekhtyar, Serhiy: 1280
Delaney, Scott W.: 2465
DelFavero, Jordan: 9085
Delgado, Ferdinand: 4110
Delgado, João: 9100, 3320
DeLiema, Marguerite: 3240
Deller, Jürgen: 9310

DeLongis, Anita: 2170, 9400
DelPo, Amy: 3560
Delposen, Kenzie: 9235
DeMarco, Kayla: 3055
Demaria, Marco: 3135
Demiris, George: 4145, 9380, 9505
DeMott, Andrew: 1080, 4355
Deng, Rebecca: 9505
DeNicola, Destiny: 9100, 9105
Denning, Emily C.: 9380, 9510
Dennis, Richard A.: 9165
Denson, Kathryn: 9210, 9215
Denson, Steven: 9210
DePalma, Alexandra: 4200
Depp, Colin A.: 3295
Der Ananian, Cheryl: 1080, 1150, 2325, 4110
Derby, Carol A.: 9405
Derenne, Nicole: 9215
Desai, Smit: 1475, 2485
DeSanto-Madeya, Susan: 9270
DesRoches, Andrea: 9380
DeStephanis, Darla D.: 9100
Detels, Roger: 2160
DeTurck, Alex: 4225
Deuley, Rebecca: 9300
Devanand, Davangere P.: 1180
Devier, Andrea: 2215, 4305
deVries, Brian: 2000
DeVries, Dawn: 9160
Dey, Judith: 2090
Dhakal, Usha: 9015, 9180
Dharani, Ammar: 3435, 9305
Di Gessa, Giorgio: 1360, 2390, 3225
Diallo, Ana: 4360
Diaz, Alma: 9505
Diaz, Ana: 2065
Diaz, Liliana: 9070, 9090
DiBaise, John: 9200
DiBartolo, Mary C.: 9210
DiBello, Angelo M.: 9515
diCardi Hladek, Melissa D.: 4525
DiCarlo, Nicholas: 2025
DiCerbo, Loraine M.: 9155, 9400
Dick, Andrew W.: 2410, 3260
Dickerson, Anne E.: 2515, 2060
Dickson, Daniel: 2425
Dictus, Cass: 2140, 9395
Didyk, Nicole: 9050
Dieciuc, Michael: 4470
Diederich, Freya: 4000
Diehl, Manfred: 4025
Dieker, JoAnna: 9265
Diekmann, Rebecca: 4335
Diener, Ed: 2465
Dietch, Jessica: 1430
DiGuiseppi, Carolyn: 2515
Dijkstra, Karin: 4610
Dikhtyar, Oksana: 2055, 3515
Dilanchian, Andrew: 4470
Dillaway, Heather: 9515
Dim, Eugene Emeka: 1150
DiMaria-Ghalili, Rose Ann: 2295, 4595
Diminich, Erica D.: 1295
Dinan, Michaela A.: 9130
DiNapoli, Pamela P.: 3255
Ding, Ding: 1255
Ding, Jingzhong: 9090
Ding, Kedong: 9515
Ding, Ke-Hong: 9105
Ding, Yew Yoong: 3405
DiNitto, Diana M.: 9325
Dinkel, Danae: 9420
Dinov, Ivaylo D.: 3055
DiScala, Sandra: 9510
Dixit, Bhavna: 9110
DiZazzo-Miller, Rosanne: 1395
Do, Quyen: 2390
Dobbs, Debra: 2035, 4170, 4480
Dobbs, Bonnie: 3250, 4225
Dobbs, Debra: 2000, 2035, 2260, 2460, 3080, 4575, 9140
Dobert, Rebecca M.: 3420, 4250
Dodds, Laura: 9505
Dodds, Michael: 2455
Dodge, Hiroko H.: 3055, 3200, 4065, 9045
Dodge, Jeffrey E.: 3610
Doebbeling, Brad N.: 1080, 2325, 1150, 4045
Doelling, Kristen: 9285
Doerzbacher, Margaret: 9395
Doherty, Caroline: 9245, 9500, 3405
Dolbin-MacNab, Megan: 1320
Dolby, Greer: 9505
Dombeck, Carrie: 4080
Domhoff, Dominik: 3585
Donahue, Patrick T.: 9505, 9030
Donelle, Lorie: 3590
Dong, Alexander: 9510
Dong, Fanghong: 4035
Dong, Gabriella: 3660, 9450
Dong, XinQi: 1155, 1195, 2060, 2255, 3375, 3610, 3660, 9135, 9285, 9430, 9515
Dong, Zhixiao: 9060
Donley, Tiffany: 9510, 9515
Donlon, Tim: 3415, 9085
D’Onofrio, Gail: 2070
Donofry, Shannon: 4115
Donohoe, Maria: 4200
Donorfio, Laura K.: 9510, 3360, 4490
Donovan, Heidi: 3535, 4085
Donovan, Kelsey: 9410
Doran, Kelly: 1270, 3180
Dorneo, Allison: 3275
Dorociak, Katherine E.: 9050
Dorr, David: 9030, 9380, 9510
Dorris, Jennie L.: 9065
Dosa, David: 1220, 2260, 2460, 3080, 4375, 9510
Doshi, Heena: 9280
Doshi, Krupa: 9500
Dougherty, Ryan J.: 2285, 3430
Dougherty Wood, Sarah: 1045
Doughty, Alyssa: 9170
Douglas, Elaine: 9505
Douglas, Joy: 9510
Douglas, Natalie F.: 1190, 2065
Doupe, Malcolm: 3440, 3605, 4425
Dove, Abigail: 9065
Dow, Jamie: 9390
Dow, William H.: 4265
Dowling, Maritza: 2200, 4605
Downer, Brian: 9295, 1230, 2145, 2200, 2400, 9515
Downer, Paige B.: 2145, 4280
Dowrey, Todd: 9100
Doyle, Margaret: 9065, 9050
Dröes, Rose-Marie: 9500, 4610
Drace, Melanie: 3565
Drake, Anna: 3185
Drake, Jonathan: 9510
Drazich, Brittany F.: 4525, 9085
Drewelies, Johanna: 3085, 9515
Drewette Card, Rebecca: 9185
Driesse, Tiffany: 3000
Driver, Jane: 9500
Driver, Tim: 4230
Drost, Jennifer C.: 1000, 2315, 4325, 1455
Dryden, OmiSoore: 9510
D’Souza, Gypsyamber: 9025
D’Souza, Stephanie: 2180
D’Souza, Violet: 3610
Du, Chenguang B.: 1330
Du, Pang: 2390
Du, Yan: 9050, 9135, 9445
Duan, Haoshu: 9265
Duan, Hongzhe: 4585, 9070
Duan, Matt: 9055, 9085
Duan, Yinfei: 1275
Dubal, Dena: 2245
Duberstein, Adam: 9375
Duberstein, Paul: 1155, 3465, 9285
Dubowitz, Tamara: 4345
Duchaine, Caroline S.: 1045
Dudley, Nancy E.: 3255
Duezel, Sandra: 3085

Duff, Laurie: 1325
Duffy, Karen: 4145
Dufour, Alyssa B.: 9160, 9365
Dugan, Beth: 9245
Dugan, Elizabeth: 9045, 9165, 9260, 9390
Duggan, Katherine A.: 1130
Dukhovnov, Denys: 2390
Dumaresq, Ella: 4530
DuMont, Renee: 9010, 9215, 9500
Dunbar, Matthew: 9505
Duncan, Dustin T.: 3645
Dune, Linda: 9360
Dunkle, Ruth E.: 9040, 9325, 9460
Dunlap, Pamela M.: 4520
Dunn, Karen S.: 9355
Dunn, Michael: 9135
Dunphy, Kim: 4530, 9135
Dunterman, Rebecca A.: 9195
Duodu, Vennesa: 9270
Dupre, Matthew E.: 3055, 4145, 9510
Dupuis, Kate: 4465
Dupuis, Sherry: 4465
Duque, Gustavo: 1170
Duran, Miriana: 1005
Durga, Aditi: 3055, 3260
Durkin, Daniel W.: 4535, 9515
Duthie, Edmund H.: 9050, 9210, 9215
Dutra, Adriana: 9505
Dutta, Anirban: 9105
Duttaroy, Atanu: 9515
Dutton, Gareth: 9430
Dutton, Suzanne: 1235, 2230, 9215
Duval, Sue: 4305
Dvorak, Robert: 9135
Dworakowski, Olenka: 4505
Dwyer, Johanna: 9425
Dychtwald, Dana K.: 9510
Dyer, Elizabeth: 9300
Dys, Sarah: 1220, 3220, 4375, 9510
Dytchwald, Dana: 9135
Dziura, James: 2070
Dzubur, Eldin: 9515

E
E, Jian-Yu: 3290
Eaker, April: 3365
Eap, Brenda: 9505
Earles, Julie L.: 9135
Eastman, Marissa: 2115
Eaton, Jacqueline: 1265, 3050, 4360
Eaton, Marie: 9165
Eaton, Nicholas R.: 2450
Ebert, Alexandria R.: 9235
Ebner, Natalie: 9400, 9505, 9210
Eby, David: 2515
Echeverria, Sandra: 9140
Eckenrode, John: 1335
Eckstrom, Elizabeth: 9500
Edelman, Linda S.: 9510, 1340, 1405, 2425
Edelstein, Offer E.: 3345
Edland, Steven: 9085
Edmonds, Natali N.: 4285
Edvardsson, David: 3440, 4275
Edwards, Chelsea: 2070
Edwards, Samuel T.: 9450
Efron, Philip: 9090
Egbert, Anna R.: 2330, 3590
Ehlers, Diane: 9510
Ehlinger, Peter: 3550, 2195, 3430
Ehrlich, Joshua R.: 1045, 2085, 2145, 3465, 4030, 9255
Eickholt, Jasmin: 4530
Eifler, Mogan: 9200
Eilers, June: 2065
Einstein, Gilles: 2365
Eising, Carla: 4430
Ejaz, Farida K.: 3035, 9335, 9355
Eke, Uzoamaka: 9500
Ekerdt, David J.: 9515, 3190, 4170
Ekram, A R M Saifuddin: 4395, 4550, 9295
El Khoudary, Samar R.: 1210, 9390
Elahi, Fanny: 9085
Elaut, els: 2150
Elayoubi, Joanne: 9455
Eldadah, Basil A.: 1180, 2285, 9250
Eldridge, Corinne: 9220
Elfassy, Tali: 1195
Elfenbein, Pamela: 1300, 2035, 3500
Eliot, Melissa: 9395
Elkarif, Talia N.: 4530
Elkes, Shelby: 3105, 4385
Ellerby, Lisa M.: 4510
Ellington, Lee: 3375, 4600, 9200, 9270, 9515
Elliott, Cerise L.: 3400
Elliott, Emily: 9360, 9465
Elliott, Sheryl: 9510
Ellis, Alexis J.: 9045
Ellis, Amy C.: 9420, 9510
Ellis, Jennifer L.: 2050
Ellis, Julie: 1180
Ellis, Katrina R.: 1185
Ellis, Ronald: 9085
Elman, Alyssa: 2070
Elman, Jeremy: 1435, 9510
Elmansi, Ahmed: 1170
Elmendorf, Sarah L.: 9160
Elmor, Riad: 9510
Eloise, Yates: 9065
Elwell, Abigail: 9220, 9300
Elzie, Carrie A.: 1235
Emami, Azita: 9510
Emerson, Kerstin G.: 9505, 1140
Emery-Tiburcio, Erin: 3005, 4250
Emlet, Charles A.: 1425, 4010
Emmons, Russell S.: 3270
Endo, Yori: 9105
Eng, Brittany: 4075
Eng, Michael: 9515
Engberg, John: 9395
Engel, Ilana: 9045, 9410, 9135, 9510
Engelman, Michal: 2335
Engle, Ryann: 1270, 9380
English, Tammy: 9455
Engström, Gabriella: 9070, 9510
Enguidanos, Susan: 1240, 2125, 4545, 9240, 9410, 9500, 9515
Ensrud, Kristine E.: 9030, 9295, 9505
Epps, Fayron: 3215, 3460, 3605, 4135, 4340, 9275, 9290, 9505
Epstein-Smith, Cynthia: 9510
Erb, Tara: 1495
Ercole, Maya: 4530, 9500
Erickson, Katherine: 9505
Erickson, Kirk: 4115
Ericsson, Malin: 2435
Erikson, Kevin: 9225
Eriksson, Erik: 4060
Erlandson, Kristine: 9025
Erler, KImberly S.: 2410
Ermer, Ashley E.: 3125, 9215, 9435
Ermoshkina, Polina: 3075, 9240, 9515
Ernst, Michael E.: 4070, 4395, 4550, 9030, 9295, 9390
Ernsth-Bravell, Marie: 9340
Ersek, Mary: 1390
Ertle, Emilee: 2505
Ervin, John: 9515
Erving, Christy L.: 1185
Escoubas, Jamie: 9285
Escrig-Pinol, Astrid: 2045
Espejo, Luis S.: 9085, 9100, 9105
Espeland, Mark A.: 1305, 3310, 4160, 4550, 9430
Espinoza, Aura: 4075
Espinoza, Sara E.: 4395, 4550, 9030, 9130, 9155, 9160, 9180, 9295, 9305, 9390
Estabrooks, Carole: 9355, 3440, 4425
Estape, Tania: 1090
Estenson, Lilly: 9500
Esterman, Michael: 9140
Estes, Carroll: 2025, 4300
Estrada, Leah V.: 3260
Estus, Erica: 3560
Etokebe, Ime: 2385
Ettema, Teake P.: 4610
Evandrou, Maria: 9260, 9280
Evans, David: 9500
Evans, J. Robert: 9500
Evans, Joni K.: 4550
Evans, Michele K.: 9505, 4180
Evans, Molly R.: 1245
Evans, Nathaniel: 9420
Evans, Tracy: 9410

Eveland, Steven: 9395
Exum, Alexander J.: 9135
Eyler, Lisa T.: 3295
Eymundsdóttir, Hrafnhildur: 1045, 3075, 9250
Eynatten, Maximilian: 9110

**F**

Fabelo, Humberto: 9515
Faber, Michael: 2050, 4490
Fabius, Chanee: 1345, 2320, 3220, 3535, 4070, 4340
Facelli, Julio: 9070, 9130
Faden, Alan: 9505
Fain, Mindy J.: 1250, 9510
Fairchild, J. Kaci: 9505
Fakha, Amal: 2340
Fakhoury, Nour: 1475
FakhrHosseini, Shabnam: 1315
Falcón, Luis: 3595
Falck, Ryan S.: 3460, 9250
Falgas Bague, Irene: 4167
Falkingham, Jane: 9280
Fallon, Veronica T.: 9130
Falls, Katherine: 9080
Falsey, Ann R.: 9515
Falvey, Jason: 9030, 9505
Falzarano, Francesca: 2445, 3235, 3365, 4085, 9080
Fan, Erica K.: 4345
Fan, Li: 4395
Fang, Yuan: 4215, 9065
Fani, Shamsi: 9160
Fanning, Jason: 9510, 3065, 3085
Farah, Kimberly S.: 2345, 3500
Farah, Raymond: 9130
Farina, Mateo: 4635
Farmer, Bridget: 3155
Farmer, Heather R.: 1370, 9515
Farrell, Spencer: 9095
Farrell, Timothy W.: 1405
Farrier, Lauren: 1200, 9505
Fass, Marjorie: 3500
Fassi, Janelle: 9500
Fasullo, Katherine: 9350
Faul, Anna C.: 9320
Faul, Anna: 3245, 4175, 4240
Faul, Jessica: 9085, 9095, 9505
Faulhaber, Ella: 9315
Fausto, Bernadette A.: 9250, 2145, 3465
Fauth, Elizabeth B.: 2166, 9290, 9340
Fayyad, Deena: 9130
Fazio, Elena: 1010
Fazio, Sam: 2015, 2505, 9355, 9515
Federman, Alex: 3475, 4410
Federwitz, Chad: 1505
Fedichev, Peter: 3270
Feger, Danielle M.: 9515
Fei, Zhe: 3600
Feifer, Richard: 1055
Feige, Jerome: 3285
Feinhals, Gretchen: 2475
Feitosa, Mary F.: 1210, 2020, 2285, 9025, 9295
Feldman, Sabrina: 9505
Felix, Mariana: 1250
Felsted, Katarina F.: 1300, 4360
Feng, Guanggang: 3355
Feng, Qiushi: 3110
Feng, Zhanlian: 2090
Fenley, Robin C.: 4620
Fennel, Alyssa: 9505, 9410
Fennema-Notestine, Christine: 9510
Fenske, Diane: 9040
Fenstermacher, Erika A.: 4365
Ferber, Rebecca A.: 9370
Ferguson, Christine C.: 9510
Ferguson, Giselle A.: 1035, 1050
Ferguson, Leah E.: 1355, 3175, 4345
Ferland, Guylaine: 1045
Fernández-Fernández, Virginia: 9155, 9360, 3050, 3580, 4110
Fernandez, Camilo: 2015
Fernandez, Helen: 9170
Fernandez Cajavilca, Moroni D.: 9510
Ferrari, Michel J.: 1505
Ferraro, Kenneth F.: 2300, 9125, 9210, 9245
Ferrell, Abigail R.: 4270
Ferrone, Christine: 3420
Ferrucci, Luigi: 1015, 1120, 1200, 2105, 2285, 4040, 4065, 4160, 9055, 9505
Ferszt, Geffen M.: 9265, 9285
Fiastro, Anna: 9375
Fick, Donna: 3205, 9505, 9515, 2180, 3100, 9270
Fields, Noelle L.: 2470, 2475, 3475, 4495, 4555, 9280, 9285, 9360, 9510
Figeys, Mathieu: 9135
Figueiredo, Sabrina: 3485
Filips, Julie: 1110
Fillingim, Roger: 3095, 9045, 9405
Finch, Caleb: 9055, 9500
Finch, Laura: 1050, 4420
Findley, Patricia: 4195
Fingerman, Karen: 1050, 2450, 3145, 4320, 4580, 9440, 9460
Finkel, Deborah: 2435, 4420
Finlay, Jessica: 1005, 1145, 2055, 2325, 3185, 3210, 4570, 9160, 9180, 9515
Finley, Katrina: 4015
Finney, Redmond: 9240
Finnie, Dawn M.: 1180
Finsel, Julia S.: 9310
Finucane, Erin T.: 2010
Fiocco, Alexandra: 1045
Fiore, Julio F Jr.: 3485
Firestone, Monica: 3155
Fiscella, Andrew: 9005
Fischl, Margaret A.: 2160
Fisher, Celia B.: 9500
Fisher, Gwen: 2370, 4345
Fisher, Jonah: 9085
Fisher, Kathleen: 4595
Fisher, Lauren: 3450
Fisher, Mary M.: 3610
Fiske, Amy: 4365, 9510
Fitch, Dan: 1065
Fitch, John: 1010
Fitzgerald, Kelly: 3305
Fitzgerald Walls, Meaghan: 9080
Fitzgerald-Cook, Cecilia: 9055, 3160, 3330
Fitzpatrick, David C.: 9425
Flaherty, Ellen: 3420
Flanagan, Jane: 4610
Flapper, Marinke: 1345, 3570
Fleming, Daniel J.: 2166, 9340
Fleming, Sean: 1055, 2160, 9025
Flemons, Kristin: 3230, 4465
Flett, Gordon L.: 4365
Flohr, Charleigh: 9515
Flood, Sarah: 4265
Florea, Liliana: 4390
Flores, Eileen S.: 9250
Flores, Erica: 4015
Flores, Fritzi: 9505
Floresca, Ysabel Beatrice A.: 2365
Flores-Villavicencio, Maria Elena: 9515
Floriancic, Natalie: 3590
Floridi, Ginevra: 3525
Flynn, Olivia A.: 9060
Fofanova, Tatiana: 4335
Fogelman, Ilana: 9510
Fogg, BJ: 9050, 3095
Fong, Melanie: 2025
Fonseca Valencia, Carolina: 9295
Fontelo, Paul: 9025
Force, Lawrence T.: 4425
Forchuk, Cheryl: 3590
Ford, Ariel: 3260
Ford, Cassandra: 4570
Ford, Gregory: 4360
Ford, John: 2060, 2040
Ford-Robertson, Joanne: 4135
Forman Hoffman, Valerie: 9360
Formoso, Sara: 9300
Forrester, Sarah N.: 1370
Forsyth, Garrett: 4025
Fortes Villas Boas, Paulo José C.: 2100
Fortinsky, Richard H.: 1060, 3245, 4055, 4075, 9070

Fortuna, Karen: 1200, 1490, 2275, 9455
Fortune, Emma: 2130
Fosnight, Susan M.: 1000
Foster, D. Brian: 4390
Foster, Liam: 4005
Foster, Zane: 1010
Fouche, Hayley A.: 9460
Fout, Betty: 1240
Fowler, Mackenzie E.: 1380, 9025
Fowler, Nicole R.: 9505, 9500, 9515
Fowotade, Adeola: 9400
Fox, Aimee L.: 9290, 9410
Fox, Jonathan: 9005
Fox-Grage, Wendy: 1245, 2165
Fréel, Stéfanie: 4180
Framil, C. Victoria: 9505
Francis, Jess: 1475
Franco, Yujin: 4100, 4460, 9140, 9190
Francois, Dimitry: 9360, 9415
Frangiosa, Theresa: 1180
Frank, Lori: 1225
Frank, Molly: 9505, 9285
Franklin, Melissa: 9505
Frantz, Desiree': 9360
Franz, Carol: 1435, 9505, 9510
Franz, Carol E.: 9085
Franzosa, Emily: 2320, 2445, 3475, 4410
Frase, Robert T.: 1350, 2300
Fraser on behalf of CCNA's SSI Working Group, Sarah: 9500
Fratiglioni, Laura: 1280, 9025, 9065
Frazier, Robbin: 4130
Freak-Poli, Rosanne: 9025
Frechman, Erica: 2000
Frederick, Heather: 9505
Fredericksen, Jessica: 2040
Fredriksen Goldsen, Karen I.: 1425, 4010
Freed, Christopher R.: 2260, 9360, 9415
Freeman, Hyun: 4590
Freeman, Shannon: 3055, 3630, 9395, 9515
Freire, Wilma B.: 9060, 9295
Freitag, Callie: 1020
Freitas, Sam: 9100, 9105
Frey, Hillary: 9505, 9020
Frey-Law, Laura: 9330
Frichembruder, Karla: 9365
Frick, Susan: 3005
Fried, Linda P.: 3485
Friedman, Daniela: 9065
Friedman, Elliot: 9200
Friedman, Mackey R.: 2160
Friedman, Steven: 9505
Friedman, Theodore: 9045
Friedmann, Erika: 2315
Fritz, Elizabeth: 4475
Friz, Amanda: 3535
Frochen, Stephen: 9515
Frost, Bess: 9515
Fruhauf, Christine: 9270, 9290, 9410
Fruhling, Ann: 9080
Fruiht, Veronica: 9225, 9370
Frye, Brett M.: 9085
Frye, Noelle: 9050
Fu, Rong: 9135, 9140
Fu, Wenhao: 3470
Fua, Imogen: 1330
Fuhr, Haley: 9045
Fujita, Ken-ichi: 9105
Fujiwara, Yoshinori: 9130
Fukaya, Taro: 3635
Fukui, Chie: 3375
Fukuoka, Brooke MO: 3610
Fukushima, Annie Isabel: 9230
Full, Kelsie M.: 1130
Fuller, Heather: 2400, 3630, 9175, 9400, 9460
Fuller, Kristi: 1140
Fuller, Morgan: 9160
Fuller, Shannon A.: 1020
Fuller-Thomson, Esme R.: 3190
Fulmer, Terry: 3205, 4230
Fulzele, Sadanand: 1170, 9105, 9110
Funaki, Toko: 9245
Fung, Constance H.: 9515
Fung, Helene H.: 1410, 2220, 9015, 9430
Fung, Kathy: 2020, 3285, 9430
FUNG, Sai-fu: 9280, 9370
Furlipa, Joy: 9510
Furtado, Maxine: 1100
Furtmann, Amanda: 9105
Furuno, Jon: 3285, 4125
Futterman, Andrew: 9005, 9515
Gómez, Jennifer M.: 3550

G
Gabrawy, Mariann M. M.: 9105
Gadbois, Emily: 9515
Gaggioli, Andrea: 9500
Gagné, Hélène: 9130
Gagne, Danielle: 9515
Gagnon, Margaret: 9515
Gahche, Jaime J.: 9425
Galambos, Colleen: 9130, 9395
Gale, John: 3285
Galecki, Andrzej: 9410
Galik, Elizabeth: 1270, 2080, 3180, 3315, 3625, 9210, 9320, 9355
Gallager, Ian: 9105
Gallagher, Elizabeth A.: 2025, 9200
Gallagher-Thompson, Dolores E.: 3165, 9050
Gallego-Alberto, Laura: 3580, 4085
Gallegos, Carlos Andres: 9060, 2055
Gallopyn, Naomi: 4410
Galloway, Caroline E. M: 9135
Galoso, Leonardo: 3650
Galvin, Angeline: 2085
Galvin, James E.: 1125, 9130
Galvin, Kathleen T.: 2485
Gamaldo, Alyssa: 1370, 9440
Gamboa, Savannah: 9505
Gammonley, Denise: 1445, 9510
Gan, Daniel R. Y.: 2435, 3210, 3385, 3465, 3540
Ganzel, Barbara: 4240
Gao, Manjing: 3190, 4255
Gao, Qin: 3370
Gao, Xiang: 2430
Gao, Ye: 4080
Gao, Zhengrong: 9055
Garan, Steven A.: 9100
Garbarino, Jason: 2230
Garbarino, Lisa T.: 9185
Garcia, Alexandra A.: 9045
Garcia, Catherine: 2210, 2240, 4265
Garcia, Emmanuel E.: 1130
Garcia, Julian: 9510
Garcia, Marc A.: 2240, 2410, 4100
Garcia, Nicole: 9265
Garcia, Oscar: 1100, 9510, 9515
Garcia, Thelma: 9505
Garcia Morales, Emmanuel E.: 1327, 2145, 4030, 4055
Garcia-Cifuentes, Elkin: 4635
Gardiola, Andrea M.: 2005, 9350, 9505
Gardner, Andrew W.: 9105
Gardner, Graham: 3395, 3590
Garrido Blanco, Giovanna: 9075, 9225
Garrison, Betsy: 9155
Garrow, George: 3205
Gartner, Allie: 4330
Gary-Webb, Tiffany L.: 4345
Garza, Jennifer: 4055
Gasdaska, Angela: 9500
Gaskin, Darrell: 4340
Gassoumis, Zachary: 2040, 2475, 4350
Gaster, Barak: 3330
Gates, Madison L.: 4255, 4280
Gatz, Margaret: 3310, 4195, 4345, 9510
Gaudig, Trisha K.: 1175
Gaudreau, Pierrette: 1045, 3485, 1005, 1030, 1180, 1420, 2325, 2340, 2360, 3010, 3185, 4130, 4210, 4305, 4350, 9280
Gauss, Clinton H.: 9165, 9180
Gautam, Ramraj: 2030, 3500

Gauvin, Mitchell J.: 9515
Gavin, Nicole: 9500
Ge, Augustus: 9450
Ge, Shaoqing: 9505
Ge, Song: 9360
Ge, Yiran: 4135
Geary, Carol R.: 2065
Gebeyaw, Getachew: 9310
Gee, Nancy R.: 2315
Geiger, Jennifer R.: 1030
Gelder, Michael: 4355
Geldhof, John: 1500, 9260
Geldmacher, David S.: 1380, 9185
Gell, Nancy M.: 3065, 3300, 9250
Gellert, Paul: 9360
Gellis, Zvi: 3410, 9430
Gendron, Colby: 9505
Gendron, Tracey: 4010, 4260, 4360, 9160
Geng, Jing: 3370
Genovese, Giulio: 9100
George, Claudene J.: 9055
Gerald, Melissa S.: 1295
Geramian, Helene: 9360
Geronia, Reynaldo II: 9090
Gerritsen, Debby: 3585
Gerschenson, Mariana: 9085
Gerstorf, Denis: 3085, 3265, 4560, 9075, 9175, 9200, 9510
Getz, Marjorie A.: 9205
Gewirtz, Abigail: 4245
Ghachem, Ahmed: 3485
Ghaiumy Anaraky, Reza: 9470
Ghazi Saidi, Ladan: 9140
Ghezzi, Elisa: 3610
Ghiasi, Akbar: 4355
Ghilarducci, Teresa: 2415
Ghimire, Saruna: 9400, 9500
Ghisletta, Paolo: 3175
Ghita, Gabriela: 9090
Ghosh, Bhaswati: 9500
Ghosh-Dastidar, Bonnie: 4345
Giandrea, Michael D.: 3615
Gibbs, Lisa: 1340, 2075, 4175
Gibbs, Peter: 9030
Gibson, Allison: 1125, 2015, 2235, 2405, 3055, 9425
Giddens, Reese: 9515, 9460
Gil, Efrat: 1095, 4435, 9400
Gilbert, Marissa: 4300
Gilbert, Paul E.: 9070
Gilbertson-White, Stephanie: 3610
Gilkes, Daniele: 9105
Gill, Jeanine: 2410
Gill, Kenneth: 3595
Gill, Nathan: 9510, 9515
Gill, Paneet: 2000, 2375, 3035
Gill, Thomas: 2070, 9510
Gillespie, Suzanne: 4145
Gillespie, Brenda: 1045
Gilligan, Megan: 1350, 2135, 2300, 3530, 4125, 9070, 9130, 9390, 9420
Gilmore Bykovskyi, Andrea: 1180, 3495, 3535, 9045
Gimm, Gilbert: 1465
Gingerich, Stephanie: 1300
Ginsburg, Liane: 3440, 3605, 4425
Giordani, Bruno: 9155, 9400
Gioscia-Ryan, Rachel A.: 9305
Gireesh, Aswathikutty: 1460, 9145
Girling, Laura: 3385
Girouard, Hélène: 1210
Gitlin, Laura N.: 1205, 1327, 1420, 1450, 2235, 2385, 2505, 3215, 4210, 9290
Giudici, Kelly: 9130
Giuliani, Gianluigi: 2085
Giulioni, Jenna: 2125, 9390
Gladstone-Groth, Gabriel: 9080
Gladyshev, Vadim N.: 3270, 4510, 9095, 9105
Glaser, Karen: 3525
Glass, Ashley: 9250
Glassner, Ashlie: 9265
Gleason, Carey E.: 3365, 9500
Gleason, Hayley: 2425
Gleason, Kathy S.: 3565, 4230
Glenn, Jordan M.: 9070, 9130, 9390, 9420
Gliatto, Peter: 4410
Glicksman, Allen: 4000
Glinka, allison: 9335
Glisky, Elizabeth: 9505
Glose, Susan: 2355
Glotzer, David: 4150
Glover, Crystal M.: 2495
Glymour, M Maria: 1270, 9450
Glynn, Nancy W.: 1200, 2085, 2195, 2285, 3290, 3430, 4395, 4520, 4585
Gmelin, Theresa: 2085, 3430, 4520
Godbole, Nisha: 9500
Goderis, Geert: 2340
Godet, Ines: 9105
Godina, Sara L.: 3160
Goebelbecker, Roisin: 9510
Goidel, Kirby: 4420
Goins, R. Turner: 2380
Goldberg, Allon: 9255
Goldberg, Jaime: 1290, 3225, 9515
Goldberg, Lynette R.: 3405
Goldberg, Nacia R.: 9515
Golden, Adam: 3475
Golden, Charles: 9250
Golden, Robyn: 3005, 3165, 4250
Goldman, jami: 9185
Goldstein, Lee E.: 9500
Goldstein, Mary K.: 4535
Goldwater, Deena: 9110
Goler, Tim: 1505
Goler, Timothy D.: 1360, 4425
Golub, Justin S.: 2085, 3320
Gomes, Caitlin: 9180
Gomes Da Rocha, Carla: 1415
Gomez, Carlos: 9510
Gomez, Gabriel: 9515, 9505
Gomez-Morales, Abigail: 9335
Gong, Xianmin: 1410
Gong, Yi: 9085
Gonyea, Judith G.: 2010
González, Estela: 9220
González Guardia, Jorge: 3580
Gonzales, Ernest: 1085, 3220
Gonzales, Norma: 9500
Gonzalez, RIchard: 1295, 2165
Gonzalez, Alan: 9045
Gonzalez, Damian: 9055
Gonzalez, Hector: 9085
Gonzalez, Hector M.: 9450
Gonzalez, Richard: 9510
Gonzalez, Rose I.: 2195
Gonzalez, Veronica: 9505
Gonzalez-Armenta, Jenny L.: 9055
Gonzalez-Bautista, Emmanuel: 1280
Gonzalez-Chica, David A.: 9025
Gonzalez-Kruger, Gloria: 4595
Gonzalez-Lopez, Paloma M.: 1180
Gonzalez-Piles, Socorro: 9335
Gonzalez-Ulloa, José Rosario: 1300
Gonzolez, Alan: 4065
Goodin, Burel: 9410
Goodman, Matthew O.: 3295
Goodwin, Paige E.: 9020
Gooneratne, Nalaka S.: 2295, 2385
Gopalakrishnan, Chandrasekar: 2290
Gorbenko, Ksenia: 3475, 4410
Gorbunova, Galina A.: 1230, 9065
Gordon, Barbara: 1340, 4175, 4240, 9320
Gordon, David: 3550
Gordon, Jean K.: 4590
Gordon, Kate: 9280
Gordon, Kristina C.: 4080
Gordon, Lily M.: 3080
Gordon, Lily: 3080

Gore, Janelle: 4135, 4340
Goroncy, Anna: 1340
Gosdin, Melissa: 4050
Gosselin, Catherine: 9505
Gothard, Sarah: 3000, 9050, 9510
Goto, Jun: 9035, 9335, 9460
Gottesman, Eve M.: 2320, 9170
Gottesman, Rebecca: 9505, 1280
Gottschalk, Sophie: 4115
Gotwals, Amy E.: 3140
Gould, Christine E.: 1110, 4535, 9360, 9370
Goulet, Joseph: 9500
Gouripeddi, Ramkiran: 9065
Gouskova, Natalia: 9295
Gove, Dianne: 2065
Gowrishankar, Saahithya: 3650
Gozalo, Pedro: 1240, 4375, 9325, 9510
Graber, Meghan: 9095, 9505
Grabowski, David: 1450, 2410
Grady, James: 1060, 4055
Graetz, Elena: 2480
Graf, Allyson S.: 3500, 9020
Graf, Emily: 2090
Graf, Sylvie: 2220
Graham, David: 9105
Graham, Eileen K.: 1035, 2305, 2450, 9415
Graham, Helen L.: 9395, 9420
Graham, James: 4330
Graham, Kirsten L.: 9015
Graham, Laura A. A.: 2020, 3285, 9025
Graham, Lyndsey N.: 9430
Graham, Zachary A.: 9305
Graham-Engeland, Jennifer: 9135, 9510
Gran, Brian: 4280
Granbom, Marianne: 4060
Grande, Giulia: 9065
Granger, Bradi B.: 4145, 9510, 9415
Grant, Claire E.: 9505, 9515
Grant, Mattea: 9250
Grant, Molly: 2380
Grasso, Stephanie M.: 4185
Graupmann, Verena: 9235
Graupner, Jeff: 1340, 9220
Gravenstein, Stefan: 1055
Graves, Hunter: 9105
Graves, Jillian: 9040
Gray, Jeff: 9060
Gray, Michelle: 9070, 9130, 9390, 9420
Gray, Nicholas: 2225, 4470
Green, Akimi: 9055
Green, Ariel: 3565
Greenberg, Nathan T.: 9305
Greenberg, Phyllis A.: 9330
Greenberg, Sherry A.: 3250, 3405
Greenberg, Steven: 3415
Greene, Katelyn A.: 1305
Greenfield, Emily: 1470, 2460, 4345, 4495
Greenlee, Kathy: 9500
Greenwald, Margaret: 1400
Greenwood-Hickman, Mikael Anne: 3300
Greer, Julienne: 9285
Greer, Paige: 9160
Gregg, Jeffrey J.: 1110
Gregorio, Mario: 3180
Greimel, Susan: 4115
Greiner, Melissa: 9130
Grelle, Kaitlin: 9265
Grenier, Amanda: 3395, 4385, 9510
Grenier, Sébastien: 9365
Gresham, Lauren K.: 1065, 9075
Greyson, Sylvan: 2360
Grieff, Abigail: 2410
Griffin, Aileen M.: 1475, 3085, 9255, 9380
Griffin, Cameron: 1175, 9160, 9395
Griffin, Joan M.: 1180
Griffith, Lauren: 9510
Griffiths, Patricia C.: 1435
Grill, Joshua: 9280
Grilli, Matthew: 9505
Grimaldi, Nicholas J.: 9055
Grimm, Kevin J.: 3520
Grinshteyn, Erin: 9205
Griswold, Michael: 1280, 9070
Groenvynck, Lindsay: 2340
Groom, Lisa: 4405
Groover, Kim: 1240
Groskaufmanis, lauren: 4450
Gross, Alden: 1290, 2285, 4040, 4070, 4190, 4205, 9515
Grosse, Scott D.: 2090
Grossman, Molli: 4195, 4345
Grotte, Meghan: 9250
Growney, Claire M.: 9250, 9455
Gruber-Baldini, Ann L.: 9130, 9500
Gruenewald, Tara L.: 4195, 4345
Gu, Yameng: 9055
Guadamuz, Jenny: 3030
Gualtieri, Lisa: 2275
Guardia, Yeini C.: 9050
Gudnason, Vilmundur: 1045
Gudnasson, Vilmundur: 3075, 9250
Guerbaai, Raphaëlle: 4475
Guerra, Leonardo: 9160
Guerrero, Lourdes: 2240
Guess, Aundaray: 4600
Guest, M. Aaron: 1080, 1160, 4045, 9010, 9200, 9215
Guglielmo, Dana: 3235
Gugliucci, Marilyn R.: 1235, 2230, 2345, 4490, 9180, 9300
Guida, Jennifer: 3130
Guidry, Hayden: 9500
Gulati, Mangla: 3500
Gullapalli, Rao: 9500
Gum, Amber: 4080
Gunzler, Douglas D.: 1230
Guo, Fangqi: 3310
Guo, Jia-Wen: 9200
Guo, Jie: 1200, 9065
Guo, Man: 1195, 2060
Guo, Xueying: 9500
Guralnik, Jack: 2115, 4160
Gurinovich, Anastasia: 2110
Gurses, Ayse: 2360
Gurung, Shreemouna: 3105, 4385
Gurwitz, Jerry: 9030
Gur-Yaish, Nurit: 3050, 9050, 9200, 9390, 9515
Gusmano, Michael: 2025, 2095, 2445, 3390
Gustafson, Deborah: 2160, 2315
Gut, Philipp: 3285, 9110, 9510
Gutchess, Angela: 9180
Guthikonda, Chetana: 9185
Gutierrez, Angela: 2210
Gutman, Gloria M.: 2000, 2375, 3035
Gutman, Roee: 1055
Guyon, Antoine: 9085
Gvili, Racheli Lital: 4530
Gwyther, Lisa P.: 3095
Gymnopoulou, Efi: 9515

H

Ha, Jinkyung: 2075
Ha, Jung-Hwa: 1070
Ha, Michelle Y.: 9305
Ha, Yae-Na: 9500
Haaksma, Miriam: 9025
Haas, Allen: 9515
Haas, Maximilian: 4505
Haas, Steven A.: 2480
Haase, Claudia M.: 4505
Haase, Kristen: 9510
Hachisu, Mitsugu: 9130
Hack, Samantha: 2275, 4070, 4120
Hacker, Benjamin: 9095
Hackett, Lauren: 9010, 9215
Hackett, Sara E.: 1385, 9240
Hacking, Coen: 4335
Haddad, Yara: 9500
Hade, Erinn M.: 9505
Hadley, Evan: 3020, 4585
Hagedorn, Aaron: 3355
Hagemann, Lauren P.: 1410
Hagen, Erika W.: 1130

Hager, Klaus H.: 9210
Hagg, Sara: 1015, 9095
Hahn, Sarah J.: 1385
Hahn Rickenbach, Elizabeth: 9500
Hahne, Jessica: 9515
Haibach-Beach, Pamela: 2145
Haigis, Marcia: 4105
Hale, Timothy M.: 2365
Halek, Margareta: 2505
Haley, William E.: 2000, 2465, 9290, 9455
Halfacre, Katie: 3630
Haliwa, Ilana: 9155
Hall, Anna: 9340
Hall, Charles B.: 2020
Hall, Lesley: 9165
Halladay, Christopher W.: 9325, 9450
Hall-Faul, Maria A.: 9320
Halliday, Drew: 1495
Halli-Tierney, Anne: 4195, 9320, 9510
Halmo, Rebekah: 3130, 1090, 3645, 9325
Halvorsen, Cal J.: 1085, 2475, 9125
Ham, Carolyn R.: 2095, 9505
Hamers, Jan: 2340, 3585, 4335
Hamilton, Kevin: 9265
Hamilton-Hinch, Barbara: 9510, 4560
Hammar, Lena M.: 2075, 9070, 9270, 9505
Hammash, Muna: 4235
Hammond, Alexandra: 9085
Hammond, Nicole G.: 9190
Hampshire, Adam: 9020
Hampstead, Benjamin M.: 9045
Hamrick, Mark: 1170, 9105, 9110
Han, Hae-Ra: 4167
Han, Shuyu: 1115
Han, Benjamin H.: 9505
Han, CHENGMING: 4280, 4300
Han, Daikwon: 3115
Han, Feng: 9055
Han, Gang: 1010
Han, Haera: 4167, 4340
Han, Junghee: 9500
Han, S. Duke: 9230
Han, Sae Hwang: 1155
Han, Sangyoon: 2475, 2485
Han, SeungYong: 9115
Hancock, Cynthia R.: 9215
Hancock, David W.: 1335, 9290
Hancock, Zoe: 9205, 9210
Hand, Carri: 3590
Hande, Mary Jean: 3425
Handler, Steven: 9395
Hands, George: 9240, 3070, 9055, 9135
Hanks, Roma S.: 9505, 2260
Hanlon, Alexandra: 3380
Hanna, Gay P.: 4575, 9155, 9400
Hannan, John Joseph: 9505
Hannan, Marian: 9365, 9510
Hannans, Jaime: 1235
Hannestad, Jonas: 1015
Hanoch, Yaniv: 1335, 9230
Hanratty, Barbara: 3090
Hansen, Malene: 3495
Hansen, Kelly: 1005, 2015
Hanses, Quinn: 9130, 9450
Hanson, Erik D.: 4115
Hanson, Laura C.: 9390
Happel, Christine: 1140
Haq, Fajr: 3440
Haque, Raza u.: 3250, 9000
Hara, Larissa M.: 9295
Hara-Hubbard, KeliAnne: 9500
Harazono, Yoritaka: 9245
Harden, J. Taylor: 3640
Harding, Alyson B.: 4520
Hardin-Sigler, Kristen: 9265
Hardy, Cara C.: 4390, 9055
Hardy, Melissa: 9440
Hare, Joshua M.: 9070, 9090, 9505
Harel, Dovrat: 4530
Harezlak, Jaroslaw: 3290
Harkins, Kristin: 2410
Harkness, Audrey: 3645
Harlow, Lauren M.: 9305
Harootyan, Bob: 3280
Harper, Alexandra E.: 4455, 9335
Harrington, Anne W.: 9370
Harrington, Candace C.: 9500
Harrington, Christina: 4305
Harrington, Erin E.: 9510, 9515, 9275
Harrington, Karra: 4125, 9130, 9510
Harrington Meyer, Madonna: 2415
Harris, Maurita T.: 1475, 2225, 2365
Harris, Brande: 9160
Harris, Danielle: 9045
Harris, Devin: 9505
Harris, Elizabeth: 9400
Harris, Emily: 2015
Harris, John A.: 1275, 9395
Harris, Kari: 9250
Harris, Lesley M.: 9380
Harris, Mark Fort: 9500
Harris, Maurita T.: 9505
Harris, Melissa L.: 2075
Harris, Orlando: 1020, 2375
Harris, Rebekah: 9420, 9500
Harrison, Ben: 9055
Harrison, Jill S.: 9450
Harrison, Jordan: 3260
Harrison, Stephanie: 9505
Harrison, Tracie C.: 1225
Harrison, Zinnia: 1240
Hart, Alysha: 2075
Hart, Sara E.: 2425
Hartman, Kensington: 9055
Hartmann, Christine: 1075, 1270, 3275, 4415, 9355
Harvath, Theresa: 3165, 3555
Harvey, Idethia Shevon: 9120
Harwood, Jake: 9510
Hasagawa, Hiromichi: 9420
Hasche, Leslie: 3115, 3560, 9005, 9125
Hash, Kristina M.: 9210
Hashim, Nawal: 3050
Hashizume, Masahiro: 9130
Haskins, Anne: 9100
Hasler, Brant: 1130
Hassan, Mahdi: 9420
Hassing, Linda B.: 4485
Hasson, Rebecca E.: 9420
Hastings, Mary K.: 2295
Hasworth, Serena: 9515
HATA, Ryosuke: 9350
Hatcher, Simon: 4365
Hathaway, Zachary: 2295, 4595
Haudenschild, Christian: 9030
Hausdorff, Jeffrey: 1280, 2260, 4145, 4570
Havlik, Richard: 3590
Havrilla, Sera: 2275
Hawes, Frances M.: 4545, 9510
Hawkins, Brent: 9500
Hawkins, Misty: 9515
Hawkley, Louise: 1050, 3000, 4420
Hay, Lexy: 4330
Hayashi, Atsuko: 9135
Hayashi, Hiroyuki: 9280
Hayashi, Takahiro: 9280
Hayden, Kathleen M.: 2085, 4550
Hayman, Laura L.: 9505
Hays, Ron D.: 9440
Hayslip, Bert: 9515, 9315
Hayward, Mark: 4350
Hazuda, Helen: 9305, 2145, 3410, 4150
He, Junyun: 9505
He, Ranran: 4380
He, Yao: 9030
He, Zhe: 4470
Heath, Barbara: 9240
Heaton, Robert: 9085, 9505
Heckman, George: 9050
Hedberg, Eric: 3530
Heemskerk, Amber: 9400
Hefele, Jennifer: 2425
Heffernan, Kevin: 9380
Heffernan, Kristin: 3410
Heffner, Kathi: 2120
Heijnen, Esther: 9515

Hein, Andreas: 4335
Hein, Maria: 3565
Heins, Pascale: 4610, 9500
Heinz, Melinda: 1400, 9515
Heinze, Franziska: 3585
Heise, Tim: 9110
Heisel, Marnin J.: 3620, 4365
Heiser, Philip: 9130
Heitezenrater, Jared: 9250
Helfand-Green, Emma: 3455
Helgeson, Michael J.: 3610
Hellem, Abby K.: 9270
Heller, Tamar: 2350
Helm, Faith D.: 2350, 4020
Helppie-McFall, Brooke: 1105, 2370
Helsinger, Abigail: 2055, 3515, 9220, 9380
Helzner, Elizabeth: 9500
Henage, Cristine B.: 4240
Henderson, Charles R.: 4605
Henderson, David AG: 3585
Henderson, Stuart: 4050
Hendricksen, Meghan: 2360, 3605
Hendry, Phyllis: 9500
Hengenius, James: 2105, 2195, 3430
Hengerer, David: 1335, 9230
Henkens, Kène: 1200, 9160
Hennessa, Alex: 2320
Henning-Smith, Carrie: 4570
Henreid, Andrew: 1390
Henricks, Evan: 9215
Henriksson, Tatiana I.: 9500
Henry, Ankita: 9500
Heo, Seok-Jae: 9065, 9070
Hepburn, Kenneth: 3515, 3535, 4340, 9275, 9280, 9290
Heppner, Alex: 3015
Herbey, Ivan I.: 1075
Herbst, Allen: 9110
Herd, Pamela: 2335, 2460
Herman, William H.: 9045
Hermant, Aurélie: 3285
Hermelbracht, James: 9500
Hernandez-Tejada, Melba: 9500
Herndon, Chris: 9330
Herr, Keela: 4590, 9050, 9265
Herrera, Luz: 3115
Herrera, Manuel J.: 9075
Herrmann, François: 1415
Herron, Carson: 4115
Hertzog, Christopher: 9235
Her-Xiong, Youhung: 4295
Hess, Thomas M.: 2220
Hess, Thomas M. M.: 4580
Hester, Kamil: 2025
Heston-Mullins, Jennifer: 4095, 4270
Heupel, Emily: 9315
Hew, Brian: 3415
Heximer, Alisha: 9515, 9230
Heyn, Patricia C.: 9515, 2015, 2350, 2395, 3465, 9200
Hickey, Johanna vTS: 1220, 3015
Hicks, Gregory E.: 2420
Hicks, Haley: 9515
Hicks, Hilary J.: 1155, 9045, 9250
Hicks, Nytasia M.: 2055, 3395, 3515
Hicks Patrick, Julie: 3390, 1505, 9015, 9020, 9180, 9235
Hideki, Hashimoto: 1460
Higgins, LeeAnn: 9095
Higgins-Chen, Albert T.: 1015, 3435
High, Andrew: 9515
Higham, James: 4390, 9100
Highfill, Christine: 4495
Hilgeman, Michelle M.: 4135, 4535, 9410, 9515
Hill, Melanie S.: 3465
Hill, Andrea M.: 2470
Hill, Carl V.: 1020, 2210, 2375, 3040
Hill, Daniel: 1020
Hill, Elizabeth: 9160
Hill, Kyrsten C.: 4370, 9410
Hill, Marcus: 1260
Hill, Nikki: 4560, 9130
Hill, Patrick L.: 9500, 9510, 2305, 2470
Hill, Sarah: 3640
Hill, William D.: 1170, 9105, 9110
Hill-Jarrett, Tanisha: 1185
Hillman, Charles H.: 9140
Hills, Karen T.: 9510
Hills, William E.: 9510
Hilmer, Sarah: 3245, 4590
Hiltz, Megan: 4475
Hindmarch, Whitney: 3230, 4465
Hines, Anika: 9030
Hinman, Jason: 9085
Hinrichsen, Gregory A.: 3060, 4010
Hinton, Ladson: 3595, 4050
Hirano, Hirohiko: 9130
Hirayama, Ryo: 3635
Hirsch, Jana A.: 3160, 4520, 9245
Hirschey, Rachel: 2140, 3610
Hirschman, Karen B.: 9510, 2000, 2390, 3380, 3450
Hisler, Garrett: 1130
Hitchinson, Ben: 9070, 9090
Hitosis, Kevin: 9500
Hitzig, Sander L.: 3455
Hives, Benjamin: 1165
Hiyoshi, Ayako: 3615
Hladek, Melissa deCardi: 4525
Ho, Alvin: 9435
Ho, Jennifer: 9360
Ho, Jessica Y.: 4460, 9190
Ho, Ringo M-H: 2430, 9330
Hoang, Austin N.: 9110
Hobday, John: 3515
Hoben, Matthias: 3325, 3440, 3605, 4425
Hocking, Taylor: 9245
Hodes, Richard: 3020, 3195
Hodgdon, Barbara T.: 9290
Hodge, David R.: 9500
Hodgson, Nancy: 1390, 2295, 2385, 3010, 4035, 4445, 4050
Hoel, Viktoria: 4610
Hofer, Matthias: 2305
Hofer, Scott M.: 9415, 9500
Hoffman, Elise: 3065
Hoffman, Geoffrey: 2075
Hoffman, Jaco R.: 3630, 9500
Hoffman, Julia: 9515
Hoffmann, Martine: 4630, 9155
Hogan, Cainnear K.: 3320
Hogans, Beth B.: 9330
Hogans, Beth: 2130
Hogeveen, Sophie: 9050
Hohman, Jessica: 4430
Holahan, Carole K.: 9500
Holley, Lyn M.: 2395, 3360
Hollifield, Claire: 9500
Hollis, NaTasha D.: 2090
Hollis-Sawyer, Lisa A.: 4055, 1060, 9220
Holloway, Jeremy: 9215
Holmes, Megan: 3630
Holmes, Sarah D.: 1345, 2100, 2235
Holstad, Marcia: 2315, 3645
Holt, Abigail G.: 9515
Holt-Lunstad, julianne: 2135
Holtsalnder, Lorraine: 9510
Holtzman, David: 9510
Honda, Takanori: 9295
Honeycutt, Amanda A.: 2090
Hong, Ickpyo: 4330, 4430
Hong, Michin: 3150
Hong, Soyun: 4015, 9380, 1475
Hong, Suzi: 9515
Honig, Lawrence S.: 2285
Hood, Michelle M.: 1045, 9255
Hooker, Karen A.: 1500, 3050, 9020, 9260
Hooley, Cole: 9060

Hooyman, Andrew: 9340
Hopp, Faith P.: 4230
Hopper, Shawna: 9190
Hoppmann, Christiane: 1165, 4560, 9175, 9200, 9510
Hoppmann, Fee: 9360
Horan, Jaime: 2400
Horn, Andrea B.: 4505
Horn, Brenna: 2325, 9515
Horne, Jami: 9510
Horowitz, Amy: 3365
Horton, Kelly: 1400
Horvath, Steve: 3600, 9095, 9105
Hoselton, Jill: 3105, 9505
Hossain, Lubna: 4200
Hosseini, Shera: 9205
Hou, Su-I: 1100, 2030, 3340, 4215
Hou, Wei: 4120
Hou, Yifei: 1350, 3530
Houchins, Michelle: 1080, 2325
House, Samuel J.: 9180
Houston, Denise K.: 1095, 1305
Hovland, Cynthia A.: 1000
Howard, Christopher: 2495
Howard, Daniel L.: 3115, 9400
Howard, Elizabeth P.: 9395
Howard, Jennifer: 4270
Howard, Khoa Phan: 3530
Howard, Marjorie: 9430
Howard, Michelle: 9205
Howard, Virginia J.: 2465, 9290
Howe, Judith: 9170
Howe, Judith L.: 3305, 4290
Howe, Melissa J.: 3035
Howell, Britteny M.: 9335
Howington, Marshall B.: 9105
Howlett, Susan E.: 1120
Hoyle, Jessica: 9340
Hoyt, Emily: 1030, 1330
Hoyt, Sarah: 9425
Hreha, Kimberly: 2145
Hsieh, Jessica: 2005, 9245, 9350, 9505, 9515
Hsieh, Katherine L.: 1305
Hsu, Amy T.: 3585
Hsu, Chun Liang: 9500, 3460, 4435
Hsu, Clarissa: 9405, 9135
Hu, Bo: 1465
Hu, Mengyao: 9360, 1255
Hu, Rita X.: 9505, 1145
Hu, William T.: 9515
Hu, Xiangyou: 4390
Hu, Xiao: 4360
Hu, Xiaonan: 1200, 9025
Hu, Xinyue: 9500
Hu, Yixin: 4395, 3595
Hua, Cassandra L.: 3080, 4375, 4570
Hua, Yingxiao: 2255
Huang, Alison: 4030
Huang, Liming: 4035, 4445
Huang, Alison: 2070
Huang, Bik Tzu: 9055
Huang, Elbert: 9050
Huang, Jane: 9175
Huang, Jing: 9395
Huang, Lihua: 9320
Huang, Liming: 9510
Huang, Rong: 9085
Huang, Shijiao: 9105
Huang, Tianyi: 3295
Huang, Weihua: 9305
Huang, Ya-Ching: 9045
Huang, Yufang: 4590
Huang, Zi-Ting: 4435, 9025
Hubatch, Sandy: 9130
Hubbard, Erin: 9365
Hubbard, Jennifer L.: 4240
Huber, Gail: 1080
Huber, Lesa: 9080, 9155
Huber, Zilla M.: 4505
Hubley, Emily: 3425
Hubner, Sarah: 9080, 9175, 9470
Hudson, Janella: 2175, 9360
Hudzinski, Taylor K.: 4110
Huebner, Hannah: 9510
Hueluer, Gizem: 3070, 4580, 9135, 9140
Huertas, Cristina: 4085
Huffman, Derek: 2110
Huffman, Kim M.: 9420
Huffman, Taylor: 4335
Hugenschmidt, Christina: 3065, 4160
Huggins, Maya: 9235
Hughes, Adriana M.: 9050
Hughes, Brian: 9240, 9105, 1190
Hughes, Laura: 3605
Hughes, M. Courtney: 9290
Hughes, MacKenzie L.: 1080, 9235
Hughes, Susan: 1080, 4355
Hughes, Timothy: 1280
Hughes Rinker, Cortney: 4045
Huh-Yoo, Jina: 4595
Huisingh-Scheetz, Megan J.: 3000, 4065, 9045
Huisman-de Waal, Getty: 3625
Hull, Bradford: 9085, 9105
Hull, James D.: 9245
Humber, Marika B.: 3200, 9275
Hundza, Sandra: 9075
Hung, William: 1455
Hung, Lillian: 3180, 3510, 9355
Hung, Man: 4540
Hunt, Angela P.: 9505
Hunt, Caralise: 9515
Hunt, Jennifer A.: 1365
Hunter, David G.: 9500
Hunter, Gary: 9420
Hunter, Susan W.: 9130, 9255
Huo, Hua (Poppy): 4320
Hupcey, Judith: 3205
Huppert, Theodore J.: 2105, 3430, 9505
Hurd, Amy: 9060
Hurley, Susan: 9270
Husain, Mohammed: 9245
Hu-Seliger, Tina: 1015
Huseth-Zosel, Andrea: 3630, 9175, 9400
Hussain, Sultana Monira: 4070
Hussemann, Jeanette: 9515
Husser, Erica K.: 3205
Hutchinson, Joanna: 9185
Hutchins-Wiese, Heather L.: 4120
Hutchison, Stephen M.: 4390
Hutton, David A.: 9105, 9305
Hwang, Ann: 4450
Hwang, Philip: 2015
Hwang, Sarah S.: 9075, 9225, 9370
Hwang, Sinwoo: 9065, 9070
Hwang, Woosang: 3530, 4295
Hwang, Yeji: 4035, 4445
Hyde, Eric: 3300
Hyer, Kathryn: 2260, 2460, 3080, 9345
Hynes, Mary T.: 9025, 9505
Hytman, Lauren: 4320
Hyun, Jinshil: 9405

I

Iaboni, Andrea: 3565
Iacob, Eli: 9270, 9510
Ibrahim, Mia: 9425
Ichikawa, Tsutomu: 9420
Ifukube, Tohru: 9245
Igarashi, Ayumi: 3375, 4275
Igarashi, Heidi: 1500
Ignatowicz, Agnieszka: 3615
Ihara, Emily S.: 2040, 2385, 3555, 4045, 4240
Ihara, Kazushige: 9130
Iida, Masumi: 3075
Iijima, Katsuya: 9460
Ijaopo, Ezekiel: 9505
Ikeuchi, Tomoko: 4425, 9150
Ilardo, Joan: 3250, 9030, 9500
Illes, Judit: 3330
Iloputaife, Ikechukwu: 9500
Inagaki, Asa: 3375
Inagaki, Keigo: 9295
Inderhees, Kacie: 4190
Indorewala, Yumna: 9355
Infurna, Frank: 1320, 2325, 3520
Ingle, M. Pilar: 3115
Ingman, Stan: 9155
Ingram, Maia: 4167, 4260
Inman, Elizabeth: 9510

Inman, Morgan: 9155
Innella, Nancy: 3250
Inoue, Megumi: 2385, 3490, 3555, 4045, 4240, 9060
Inouye, Sharon: 9505
Insel, Kathleen: 1250, 2365, 9510
Intrator, Orna: 9510
INTRIERI, R C.: 9020, 9195
Inzitari, Marco: 3460
Ione, Adrienne: 1350
Iosepovici, Skylar L.: 1290
Ip, Edward: 1095, 3065
Irimia, Andrei: 3435, 9055, 9095, 9305
Ironson, Gail: 3645
Irvin, Ryan: 1380, 9025, 9290
Irwin, Pamela: 3425
Isaacowitz, Derek: 2250
Isaacs, Lauren: 2375
Isales, Carlos M.: 1170, 9105, 9110
Isenberg, Naomi: 3235
Ishihara, Masumi: 9340
Ishihara, Tetsuro: 9300
Islam, Jessica Y.: 9505
Ismirle, Jennifer: 2070
Israel, Sarah M.: 1050
Isralowitz, Richard: 3345
Issah, Moshood: 2390
Itoh, Sakiko: 4425, 1075
Iwarsson, Susanne: 4060
Iwasaki, Michiko: 9005, 9515
Ix, Joachim: 9085

J

Jabbour, Jason: 4080
Jack, Clifford R.: 2085
Jackson, Jeannette: 2165
Jackson, Barbara L.: 4195
Jackson, Elizabeth A.: 1210
Jackson, Jada: 9270
Jackson, Joshua: 4515
Jackson, Kathleen S.: 2250
Jackson, Kathryn K.: 1035, 2305
Jackson, Phyllis D.: 4150
Jacob, Tvli: 9045
Jacobs, Diane M.: 9500
Jacobs, Elliot B.: 3435
Jacobs, Jeremy M.: 9270, 9420
Jacobs, Josephine C.: 9275
Jacobs, Lindsey: 9515, 4370, 4535, 9320
Jacobs, Moyosoreoluwa: 9020, 9510
Jacobson, Mireille: 9310
Jacot, Guillaume Eric: 3285
Jacova, Claudia: 9190
Jahan, Momana: 2010
Jahanian, Omid: 2130
Jahansooz, Julia R.: 9100
Jahn, Georg: 2170, 9285
Jain, Elizabeth: 2465
Jain, Felipe A.: 2166
Jain, Jennifer: 9245
Jain, Mohit: 4585
Jain, Shalini: 9090
Jaldin, Michelle A.: 9265
Jallow, Fatoumata: 9025
James, Bryan D.: 1035, 2495
James, Bryan: 9505
James, Dara: 9115
James, Katherine: 4050
James, Krista: 9145, 9505
James, Roberta: 9515
Janer, Ciara: 9090
Janevic, Mary: 3320
Jang, Heejung: 1465
Jang, Yuri: 3150, 4045, 4460, 9140, 9455
Janicki, Matthew P.: 2350
Jankowski, Catherine: 3535
Jansen, Carl-Philipp: 4115, 9390
Jansen, Taylor: 1245, 9045, 9165, 9245, 9390
Janssen, Leah M.: 1385, 2050, 3475, 4380
Jantzi, Katie: 3140
Jao, Ying-Ling: 2120, 2180, 3180, 9065
Jaquet (née Bauer), Jenny: 4630, 9510
Jarrett, Pamela: 9500
Jarrott, Shannon E.: 1190, 1500, 3560, 9260
Jason, Kendra: 1085, 1250, 1355
Jasuja, Guneet: 9070
Jauregui, Elena: 3460
Javier, Noelle Marie A.: 4450, 9390
Jayasuriya, Darren: 1090
Jean Francois, Geffre: 9515
Jeanblanc, Alexandra B.: 1320, 2500, 4245, 9260
Jedele, Jenefer M.: 1175, 4415, 4430, 9160, 9395
Jeffers, Eva M.: 1350, 2375, 3235
Jeffery, Bonnie: 4005
Jeffries-Silmon, Brenda: 3365
Jen, Sarah: 2150, 9515
Jendro, Ashlyn: 9180
Jeng, Philip: 2070
Jenkins, Gregory: 9510
Jenkins, Kaylah: 9215
Jensen, Christine J.: 4350, 4565
Jensen, Donna: 9170
Jensen, Per H.: 4220
Jeon, Sangha: 1065, 4580
Jeong, Jiin: 9500
Jeong, Mijin: 2150, 9185, 9355, 9515
Jernigan, Cheryl: 2065
Jespersen, Brooke V.: 4305
Jeste, Dilip V.: 3645, 4440, 9085, 9505
Jester, Dylan J.: 1430, 3080
Jesus, Maria Clara F.: 9505
Jezek, Meagan: 9105
Jha, Shaili: 3595
Jhamba, Tapiwa: 4265
Ji, Chumin: 9050
Ji, Ming: 1155, 9005
Ji, Yoonjung: 2115, 4050
Jiang, Kening: 1130, 2085
Jiang, Lin: 9500
Jiang, Shangqing: 3285
Jiang, Yanping: 9380, 3550
Jiang, Yufu: 3530
Jiang, Yun: 9050
Jiang, Zhe: 9060
Jiao, Yuqin: 9270
Jiao, Yuquin: 9035
Jicha, Gregory: 2015, 2405
Jicha, Greg: 1125
Jiménez-Gonzalo, Lucía: 3050, 3580, 1490
Jin, Huajie: 2205
Jin, Kelly: 9505
Jin, Zhezhen: 4585
Jing, Bocheng: 2020, 3285, 9025
Jingru, Wang: 9140
Jinhwi, Kim: 9235
Jiwani, Rozmin B.: 9130
Johanning, Jason: 9420
Johansson, Marcus F.: 2075, 9270
Johns, Shelly: 9500, 9515
Johnson, Aaron: 9075
Johnson, Amy: 9355
Johnson, Andrew M.: 9255
Johnson, Brittany: 9500
Johnson, Charla: 2195
Johnson, Elma B.: 4210
Johnson, Florence U.: 3055, 9505
Johnson, Ian M.: 1020, 3455, 3545
Johnson, Jennifer: 3155
Johnson, Julene K.: 1020, 1270, 2375
Johnson, Julia K.: 1125, 2405
Johnson, Justin: 2315
Johnson, Karen C.: 4550, 9430, 9500
Johnson, Kyle: 9395
Johnson, Lee Ann: 2180
Johnson, Matilda: 4110, 4200
Johnson, Shanthi: 4005
Johnson II, Theodore M.: 3645
Johnson Nicholson, Meneka C.: 9250
Johnston, Deirdre: 2320, 3245, 4340, 4450
Johnston, Maxwell: 9500
Joiner, Jhazzmyn: 4135, 9465
Jonaitis, Erin: 9500
Jones, Tessa: 3055
Jones, Chelsey E.: 9505
Jones, Ethan: 4135, 9465
Jones, Megan D.: 9070, 9130, 9390, 9420

Jones, Miranda: 3375, 9270
Jones, Nahdia: 9505
Jones, Nicholas W.: 9405
Jones, Patricia: 2410, 3020, 3400
Jones, Richard: 4205, 9290, 9505
Jones, Salene: 9250
Jones, Sarah E.: 3655
Jones, Vanya: 2515, 3285, 3605, 4150
Jonsson, Palmi: 1045, 3075, 9250
Joo, Susanna: 1430, 2435, 9080, 9250, 9435, 9460, 9465, 9470
Jopp, Daniela S.: 1415, 2025, 4205, 9200, 9260
Jose, Justin P.: 9320
Joseph, Carly A.: 1145, 9160
Joseph, Lyndon J.: 3400
Joshi, Prajakta: 9330
Joshi, Rennie: 9245
Joshi, Sama: 9265
Joyce, Geoffrey: 3030, 4455, 9500
Ju, Eunae: 2075, 9270
Jubanyik, Karen: 2070
Jubouri, Shams: 9500
Juckett, Lisa: 2215, 4305
Judge, Katherine: 9515
Judge, Katherine S.: 3235, 9360, 9500, 9505, 9515
Judge, Stephanie T.: 9410
Judon, Kimberly: 2320
Jun, Hey Jung: 2435, 9080, 9250, 9435, 9460, 9465, 9470
Jun, Jiweon: 9505
June, Andrea M.: 2345, 3500
June, Joseph: 2260, 2460, 3080, 4570
Jung, Karen: 2250, 9070
Jung, Euijin: 9435
Jung, Inhye: 9080, 9470
Jung, Seung: 9510
Jung, SuJung: 9335
Jung, Sunok: 9500
Jung, Wonkyung: 1345
Jung Kim, Hyeon: 9470
Junker, Paul: 9385
Juraschek, Stephen P.: 3290
Juris Naar, Jill: 1190, 3560, 9260
Jurivich, Donald: 9215
Justice, Amy C.: 3645
Justice, Jamie N.: 9505, 1305, 2410, 3335, 4550
Jutai, Jeffrey W.: 9470
Jutkowitz, Eric: 1290, 2325, 2360, 3010, 4270, 4305, 9345, 9515
Juul, Filippa: 1195

**K**

K. Newsham, Tina M.: 1470, 3360, 9010, 9215, 9500
Kåreholt, Ingemar: 4110
König, Stefanie: 3615
Kürüm, Esra: 1355, 3175, 4345
Kaasalainen, Sharon: 9500
Kaba, Diarratou: 9155, 9175
Kabeto, Mohammed: 9410, 2115
Kady, Annabel: 9515
Kadylak, Travis: 3145, 3650
Kaeberleien, Matt: 4170
Kaeberlein, Matt: 9305
Kafker, Shira: 9415
Kagawa, Shota: 9420
Kahan, Fay: 3060
Kahana, Eva: 1230, 1360, 1505, 2265, 3075, 4280, 9050, 9240, 9515
Kahana, Jeffrey s.: 4425
Kahkoska, Anna R.: 1200, 9030, 9455
Kaholokula, Joseph K.: 9190
Kahsay, Feven: 9505
Kahya, Melike: 1280
Kai, Ichiro: 3635
Kaimal, Girija: 4530
Kaiser, Anica Pless: 1500
Kalavar, Jyotsna M.: 9225
Kale, Helen: 1205, 9290, 9325
Kalicki, Alex: 4410
Kallatsa, Laura: 9465
Kallianpur, Kalpana J.: 3415
Kallmyer, Beth: 9515
Kam, Alyssa: 9510
Kamalyan, Lily: 9085, 9505
Kamdar, Neil: 2360, 4180, 4450, 4565
Kamenecka, Ted: 9105
Kamer, Angela: 2280
Kamholz, Barbara: 9510
Kamide, Kei: 9505
Kamil-Rosenberg, Shirit: 9505
Kammerer, Hubert: 9135
Kampen, Ruth: 1495
Kan, Chen: 3355
Kan, Akiko: 9150
Kanamori, Takuya: 9295, 9215
Kane, Heidi: 1430
Kane, Kevin: 1100
Kaner, Eileen: 3090
Kang, Bada: 9065, 9070, 9130
Kang, Byoung-Ho: 9500
Kang, Hyojung: 4310
Kang, Hyun: 9515
Kang, Jee eun: 9510
Kang, Ji young: 9515
Kang, Miseon: 9080, 9470
Kang, Suk-Young: 4460, 9375
Kang, Sungrok: 9430
Kanzler, Kathryn: 9155, 9180
Kao, Hsueh-Fen Sabrina: 4225
Kao, Yu Hsiang: 9175
Kapahi, Pankaj: 4510, 9105
Kaplan, Daniel B.: 9500, 9510
Kaplan, Robert: 2270, 9510
Kapur, Mira: 1090, 9100
Karagounis, Leonidas: 9110
Karakida, Maki: 9045, 9165, 9245, 9390
Karalasingam, Abiraa: 9510
Karas, Marta: 3290
Karasik, Rona J.: 1470, 4490
Karbakhsh, Mojgan: 3035
Karcher, Kayle D.: 9505
Karel, Michele J.: 1110, 4415, 9160, 3345
Karikkineth, Ajoy: 2285, 9505
Karlawish, Jason: 1180, 2410
Karlin, Nancy J.: 9175
Karlsson, Ida: 2435
Karmarkar, Amol: 2410
Karon, Sarita: 2090, 4270
Karpiak, Stephan: 2330
Karpiak, Stephen: 2330, 3590
Karvar, Mehran: 9105
Karvonen Gutierrez, Carrie: 1045, 1210, 9255, 9390, 9420
Kase, Bezawit: 9070, 4310
Kasper, Judith D.: 4030
Kastner, Monika: 9025
Kats, Allyson M.: 9030
Katsumata, Asako: 9220
Katz, Benjamin: 2195
Katz, Ira: 4430
Katz, Mindy J.: 2020, 9405
Katz, Rain: 2285, 3430
Katzel, Les: 2130, 9330
Katzel, Leslie: 9330
Katzorreck, Martin: 3265, 9075
Kaufman, Jennifer e.: 2330
Kaufman, Nicole: 9395, 9510, 9515, 1130, 3295
Kaul, Usha: 9505
Kaup, Migette: 2505, 3015
Kaur, Ravneet: 2195
Kaur, Tanjeev: 9215
Kaushal, Navin: 1210
Kaushik, Charanya: 9510
Kaushik, Vibha: 3105
Kauzor, Kaitlyn: 9085
Kavanagh, Kylie: 9100
Kavcic, Voyko: 9155, 9400
Kawai, Hisashi: 9130
Kaya, Alaattin: 1120
Kaye, Deborah R.: 9130
Kaye, Jeffrey: 3000, 3055, 3200, 4065, 9030, 9050, 9500, 9505, 9510

Kaye, Len: 3210, 9220, 9300, 9505, 9180, 9405
Keating, Norah C.: 3630
Keating, Norah: 4000
Keck, Mei-Le Z.: 9050
Keefe, Bronwyn: 4225
Keefe, Francis: 3095, 3425, 3440
Keefe, Kate: 9505
Keehan, Jane: 9300
Keel, Pamela: 9155, 9180
Keenan, Teresa: 2165
Keene, Jennifer: 9380
Keepper, Susie: 2280
Keezer, Mark R.: 3485
Kehoe, Priscilla: 2075
Keisari, Shoshi: 4530
Keiser, Sahru: 1020, 1270, 2375
Keita-Fakeye, Maningbe: 2360
Kejriwal, Nidhi: 9500
Kelechi, Teresa: 9500
Keleher, Jennie: 9355
Kelfve, Susanne: 1150
Keller, Abiola: 4235
Keller, Heather: 4465
Keller, James: 1360
Keller, M. Jean: 9155
Keller, Michelle S.: 1390, 9310
Keller, Sara: 2360
Kellett, Kathy G.: 9505, 3395
Kelley, Susan J.: 2500
Kelley, Amy: 4450, 9245
Kelley, Heather: 9290, 1185, 3390, 4300
Kelley, Megan: 3015
Kelley, Melinda: 3020, 3195
Kelly, Catherine: 9500
Kelly, Christine: 3395
Kelly, Christopher: 9350
Kelly, Kathleen: 4015
Kelly, Kimberly: 1430
Kelly-Adams, Deirdre: 9515
Kelsh, Alysa: 9505, 3410, 9160, 4100
Kemp, Candace: 2470, 3090, 9060
Kempen, Gertrudis I.J.M.: 2340, 3625, 9430
Kendall, Jessica: 9045
Kennedy, Brian: 4315
Kennedy, Katherine A.: 1350, 9350, 9500
Kennedy, Mallory: 4045
Kennedy, Mary: 1495
Kennedy, Richard E.: 9505, 1380, 4590, 9000, 9025, 9410
Kennedy, Teri: 4565
Kennedy-Malone, Laurie M.: 9210
Kennelty, Korey: 1390
Kennicutt, Amy: 9505
Kenning, Heather: 1250
Kensinger, Elizabeth A.: 9180
Kepczynska, Paulina: 1055
Kerdic, Richard: 3245
Kerepesi, Csaba: 9095, 9105
Kerin, Jessica: 9235
Kerkhof, Yvonne J. F.: 4610
Kermah, Dulcie: 3640
Kernan, Laura: 1455
Kero, Katherine: 9190
Kerr, Alice: 9300
Kerr, Jelani C.: 9380, 9510
Kershner, Kyle I.: 9510
Kersten, Alan W.: 9135
Kessler, Eva-Marie: 9360
Kessler, Penny: 1300
Ketchum, Fred: 9500
Key, Kent: 3155, 3550
Keyes, Laura M.: 4495
Keys, Fay: 4230
Kezios, Katrina: 9505
Khadka, Narayan: 3035
Khadka, Sangeeta: 3405
Khamzina, Madina: 3655
Khan, Aisha: 9505
Khan, Anam M.: 2360, 4180, 4450, 4565, 9130, 9160, 3225, 9510
Khan, Niaz: 9505
Khavjou, Olga A.: 2090
Kheifets, Viktoria: 9105
Kheirbek, Raya E.: 1505, 2130, 3500, 9240, 9330, 9395, 9405
Khoo, Yit Mui: 1070
Khor, Wei Lynn: 4120
Khosravian, Nick: 9105
Kidana, Kiwami: 9030
Kiecolt-Glaser, Janice K.: 3125, 9515
Kiefe, Catarina I.: 1370
Kiel, Douglas: 9510
Kiener, Johannes: 9500
Kietzman, Kathryn: 3370, 4275
Kikuchi, Kazunori: 9150
Kilaberia, Tina: 4015
Kilgore, Zach: 3455, 3545, 9155
Kilpela, Lisa: 9155, 9180, 9080, 9470
Kim, Dae: 2290
Kim, Suk-Hee: 3500
Kim, Aeri: 9270
Kim, Alexander S.: 1327
Kim, Alice J.: 9510
Kim, Boeun: 9355
Kim, Bon: 9035, 9240
Kim, Bora: 9070
Kim, BoRin: 9375, 9515, 9310
Kim, Chang Oh: 9080, 9460
Kim, Chiye: 9110
Kim, Dae: 9295
Kim, Dae H.: 2290, 9045, 9295
Kim, Dae Hyun: 9000, 9295, 9500
Kim, DaeEun: 9080, 9250, 9470
Kim, Deborah: 1140
Kim, Eric S.: 2465, 3185, 9500
Kim, Esther: 9135
Kim, Eun hae: 9430
Kim, Eunbea: 1350
Kim, Eunkyung: 2115, 4050, 3175
Kim, Giyeon: 1490, 9380
Kim, Grace: 9310
Kim, Gwang Suk: 9130
Kim, Gyeonghui: 3175
Kim, Ha Neul: 4180, 4215
Kim, Haesook: 9440
Kim, Hanna: 9500
Kim, Hansol: 9515, 4085
Kim, Heejung: 4015, 4430, 9130
Kim, Hyein: 4015
Kim, Hyejin: 9510
Kim, Hyeon Jung: 9135
Kim, Hyeyoung: 9500
Kim, Hyun-Jun: 1425
Kim, Jeong-Yeon: 9270, 3530, 4460, 9375
Kim, Jiwon: 4185
Kim, Ji-yeon: 9335
Kim, Jong Hun: 9000, 9045
Kim, Jongwan: 9235
Kim, Jongwoong: 4500
Kim, Joo Hee: 9395
Kim, Juensung J.: 1505
Kim, Juhyun: 9310
Kim, Jung Hyun: 2480
kim, junpyo: 4500
Kim, Kwang Joon: 9080, 9250, 9470
Kim, Kyeongmo: 4500, 9500
Kim, Kyungmin: 1180, 1205, 2025, 2150, 3390, 9200, 9260, 9290, 3150, 9125, 9460
Kim, Mihui: 9505
Kim, Mijung: 4015, 4430
Kim, Miyong T.: 3150, 4167, 9140, 9310
Kim, Patricia: 9505, 4410
Kim, Sage: 4355
Kim, Seon: 4500, 4045
Kim, Sohyun: 4380, 9185
Kim, Steffi M.: 2380
Kim, Sujin: 9065, 9070
Kim, Wonsock: 9000, 9045
Kim, Yesol: 9505
Kim, Yijung K.: 1155, 2025, 3145, 9200, 9260, 9440, 9460
Kim, Yoon Chung: 4575
Kim, YoonMyung: 9080, 9250, 9460, 9470
Kim, Young Bum: 9510
Kim, Yuntae: 4015, 4430
Kim-Knauss, Yaeji: 2220
Kimminau, Kim: 2065
Kimzey, Michelle: 9460
Kincaid, Reilly: 9505
Kind, Amy: 9450
Kindratt, Tiffany: 1295, 9510
King, Alyssa P.: 4120
King, Anna: 3405
King, Betz: 9375
King, Diane: 9250
King, Emma G.: 9170
King, Jennifer J.: 9115

King, Justine: 3655
King, Katherine: 9015
King, Mackenzie: 4330
King, Madeline: 2040, 4350
Kinkade, Emily E.: 9460
Kinney, Jennifer M.: 1385, 9225
Kinosian, Bruce: 9510, 4410
Kintzle, Sara: 9155
Kiosses, Dimitris: 3595, 9140
Kirch, Matthias: 2165
Kirk, Andrew: 9075
Kirk, Jennifer M.: 9025
Kirk, Laura N.: 1300
Kirkwood, Renata: 9160
Kirsch, Katherine E.: 1030
Kirsch, Julie: 2300
Kirven, Sydney: 1370, 9515
Kirvin-Quamme, Andrew: 1065
Kishimoto, Kyoko: 1470
Kishimoto, Hiro: 9295
Kistler, Christine: 3325
Kitko, Lisa: 2120, 3205, 2150, 3570
Kitzman, Dalane: 1305, 9090
Kivipelto, Miia: 1260
Kizony, Racheli: 4435
Kizza, Irene: 9240
Klaiber, Patrick: 2170, 9400
Klatt, Brooke N.: 4520
Klee, Matthias: 1290
Kleinmeyer, Chelsea: 9250
Klenk, Jochen: 4115
Kler, Jasdeep: 2115, 9160
Kliegel, Matthias: 4505
Kline, Juliette J.: 9500
Klinedinst, Tara C.: 3535, 4085, 9390
Klingel, Markus: 9200
Klinger, Christopher: 2005, 9245, 9350, 9515, 3530, 9085, 9505
Klumph, Marianne: 9130, 9160, 9510
Knapp, Kenneth A.: 3235
Knebl, Janice: 4240
Knecht, Jenny: 3205
Knight, Erik: 9135, 9510
Knight, Jamie E.: 9130, 9335, 9505
Knollman-Porter, Kelly: 2510
Knowles, Molly: 2090, 9515
Knurek, Sean: 3155
Ko, Eunjung OS: 3365, 4080
Ko, Fred C.: 9305
Ko, Hana: 9335
Ko, Linda: 9500
Ko, Michelle E.: 1390
Kobayashi, Erika: 3075, 3635, 9435
Kobayashi, Karen M.: 3225, 3455, 9510
Kobayashi, Lindsay: 1145, 2055, 2115, 3185, 4570, 9115, 9160, 9180
Koch, Melissa: 4465
Koehly, Laura: 9120
Koenen, Karestan C.: 3595, 4285
Koffer, Rachel E.: 9515
Kofman, Maxwell: 9510
Kogan, Yulia: 4150
Koh, Wei Qi: 4610, 9500
Koh, Woon-Puay: 4590
Kohanski, Ronald A.: 3020, 3400, 4315
Kohl, Gianna J.: 4610
Kohn, Jordan N.: 9515
Koizumi, Naoru: 4045
Kok, Rob: 1345
Kokorelias, Kristina: 9145
Kolachalama, Vijaya B.: 2015
Kolanowski, Ann: 2080, 2505, 3015, 3180, 4415
Koliwad, Suneil: 9105, 1230, 3160, 9135, 9340
Kondrikov, Dmitry: 1170
Konetzka, R. Tamara: 1395, 4045, 2060, 2255, 3375, 3525, 3590, 3660, 4200
Kong, Jooyoung: 1290, 2305, 3225, 4205
Kong, Xiangjing: 1255
Konomos, Michael: 1090, 9325
Konrad, Thomas R.: 9045, 9395
Koo, Hyun Jung: 9180, 9290, 9360, 1205
Koon, Lyndsie: 3650
Koopmans, Alanna: 9395, 9515
Koopmans, Raymond: 3585
Kopera-Frye, Karen: 2380, 4490, 9300
Koppel, Sjannie: 9390
Koppel Egierd, Jacob I.: 9135
Korc-Grodzicki, Beatriz: 9115
Kordomenos, Cleanthe: 2090
Koren, Yael: 9510, 3085, 4630, 9155
Kornblith, Erica: 4535
Kosar, Cyrus: 9160
Koschate, Jessica: 4070, 4120
Kosick, Lawrence: 1365
Koss, Catheryn S.: 3515
Kossowska-Kuhn, Dorota: 2225
Koszalinski, Rebecca: 9505
Kotarba, Joseph A.: 3070
Kotecho, Messay M.: 9165, 9310
Kotopka, Stephen: 9175, 9470
Kotwal, Ashwin A.: 1020, 2070, 2325
Kotz, David: 3000
Koufacos, Nicholas: 2320, 1485, 4095, 4270, 9180, 9260
Kovach, Christine R.: 9395
Kowalkowski, Henrik F.: 9405
Kowalski, Kristina: 9075
Kozlov, Elissa: 9285
Kozlov, Elissa K.: 1155, 3375, 3590, 4335
Kraal, A. Zarina: 4580
Kraemer, Sandra: 2175, 9175, 9455
Krajmalnik-Brown, Rosa: 9200
Kramarow, Ellen A.: 9190
Kramer, Arthur: 9140
Kramer, B Josea: 9440
Kranick, Julie: 9500
Krase, Kathryn: 9165
Krassikova, Alexandra: 9030
Kraus, William E.: 9420
Kravchenko, Julia: 1230, 2060, 2115, 3040, 3160
Kremen, William: 1435, 9085, 9505, 9510
Kresevic, Denise: 9240
Krieger, Abba M.: 2410
Krieger, Nikolas I.: 1230
Krijger, Regina: 9080
Kriska, Andrea M.: 9390
Kritchevsky, Stephen B.: 1305, 4160, 9505
Kritz-Silverstein, Donna: 9510
Kroemer, Guido: 4315
Kroenke, Candyce: 3130
Krok-Schoen, Jessica L.: 3130
Kropp, Denise J.: 1000
Krtichevsky, Stephen: 1095, 4550, 9090
Kruse, Florien: 3585
Kruszynski, Denise: 9300
Krutchen, Rachel: 1085
Kuball, Tim: 2170
Kubanga, Kefentse: 1485, 9195
Kubba, Mira: 9500
Kubo, Yuta: 9280
Kubzansky, Laura D.: 3595
Kuchel, George A.: 3245, 3320, 4390, 9100
Kuczmarski, Marie: 9505
Kuerbis, Alexis: 3090
Kuffel, Randall L.: 4550, 9070
Kuhlman, Kate R.: 9130
Kuhrt, Nathaniel: 9505
Kuipers, Allison L.: 1210
Kulbok, Pamela: 9500
Kulkarni, Snehal S.: 9420
Kullgren, Jeffrey: 2165
Kulminski, Alexander: 4585, 9070, 9085, 9505
Kumagai, Shuzo: 9295
Kumar, Amit: 2410
Kumar, Navasuja: 9410, 9290

Kuniholm, Mark: 2160
Kunik, Mark E.: 9410
Kunisaki, Ken: 9025
Kunkel, Suzanne R.: 4140, 4230, 9015
Kunzmann, Ute: 3085, 3265, 4560, 9075
Kuo, Chia-Ling: 3320, 9100
Kuo, Hsuan-Ju: 9045
Kuo, Pei-Lun: 1015, 2105, 2285, 4030, 4040, 9085
Kuo, Tsuann: 9280, 4215
Kuo, Yong-Fang: 1230, 9390
Kupiec, Olivia: 9505
Kurth, Maria: 1500, 9155
Kurzen, Crystal: 2215, 4305, 1225, 1355, 2100, 3475, 9425
Kuspinar, Ayse: 9510
Kutner, Jean: 9240
Kutz, Alexander: 2290
Kuzmik, Ashley: 3180, 3445, 9505
Kuzucan, Aida: 1345
Kwak, Jung: 1445, 9060, 9185, 9240
Kwak, Minyoung: 9515
Kwan, Rick: 2030
Kwan, Crystal: 1395
Kwan, Thomas D.: 9510, 9515
Kwan, Thomas: 3090
Kwok, Timothy: 1410, 9265
Kwok, Wai-Wai: 3095
Kwon, Eunji: 9065, 9070
KWON, ILAN: 9515
Kwon, Simona: 9500, 3395, 9515
Kwong, Connie: 3370
Kwong, Yanyee: 4380
Kyaw, Kay Thwe: 9500
Kyeong, Yena: 1355
Kylén, Maya: 4060
Kyler-Yano, Jason Z.: 9510, 9515
Kymre, Ingjerd G.: 2485
Kyprianou, Natasha: 3075

L
La Fazia, David: 1425
Lövheim, Hugo: 3440
Lücke, Anna J.: 3265, 9075
López Anuarbe, Mónika: 9510
López-Bago Cid, Natalia: 9335
López-Ortega, Mariana: 4185
Labonte, Helene: 9200
Labouvie-Vief, Gisela: 2465
Labudek, Sarah: 4115
Laceulle, H.E.: 4260
Lacey, Samantha R.: 4055
Lach, Helen W.: 3590, 9365
Lachman, Margie: 3085, 3655, 9135, 9415, 9470
Lachs, Mark S.: 1335
LaCroix, Andrea: 2130, 3300, 4070, 9155, 9180
Ladda, Shawn: 3245
Laditka, James: 9340
LaFave, Sarah: 9040
Laffer, Alex: 1155
Laffer, Alexandra: 9045, 9250
Lageman, Sarah: 9145
Laha-Walsh, Kirsten: 4420
Lahiri, Sucheta: 9060
Lai, Aiping: 9510
Lai, Daniel: 1335, 1085, 9125
Lake, Derek: 4270
Laliberte-Rudman, Debbie: 9510
Lam, Alfred: 9435
Lam, Emily: 3105
Lamar, Melissa: 2495
Lamarre-Cliche, Maxime: 1210
Lamb, Raza: 3160
Lambrou, Nickolas H.: 3365, 3570
Lamont, Helen: 2090
LaMonte, Michael: 2130, 4070
Lamorie-Foote, Krista: 9055
Lamothe, Gabriel P.: 9085
Lamoureux-Lamarche, Catherine: 9365
Lampkin, Cheryl: 2165, 1415
Lamura, Giovanni: 4005
Landers, Steven: 2055
Landman, Bennett A.: 4065
Landry, Amy: 1075
Lane, Kari: 1360
Lane, Kathleen: 9505
Lang, Frieder: 2220
Lang, Sabine: 1015
Langa, Kenneth M.: 2085, 2265, 9115
Lange-Maia, Brittney S.: 2495, 9390, 9200
Langsetmo, Lisa: 9030, 9505
Langton, Lynn: 3240
Lanigan, Kerry: 9515
Lanius, Ruth: 9355
Lanza, Lisa: 9135, 9510
Lapane, Kate: 9295
Lapham, Susan: 4195
Lapid, Maria I.: 1180
Lapierre, Sylvie: 4365
LaPierre, Tracey: 9515
LaPlace, Patti: 9250
Lappan, Nicolette: 9255
LaQuaglia, Maria: 3345
Largent, Emily A.: 2410
Larkey, Linda: 9115
Larkina, Marina: 1105, 9265
LaRocca, Thomas J.: 9105
Larson, Janet L.: 9515
Larson, Nicole: 9510
Laser-Maira, Julie Anne: 9125
Lashley, Heather: 9505
Lass, Jordan: 1045, 4015, 9505
Latib, Aisyah: 3405
Latimer, Abigail: 4235
Latka, Anna: 9130
Latka, Miroslaw: 9130
Latkin, Carl: 2010, 4525
Lau, Carissa: 9390
Lau, Sandra: 4070, 4120, 4335
lau, Wallis C.Y.: 4050
Lauderdale, Diane: 3460
Launer, Lenore: 1045, 9085, 9250
Laures-Gore, Jacqueline: 9210
Laurin, Danielle: 1045
Laurita-Spanglet, Jessie: 3065
Lavayen, Susana: 9160
Laver, Gary: 4225
Laviolette, Laurel S.: 9360
Lawhorne, Larry: 9510
Lawlor, Brian: 4570
Lawlor, Kristen: 9060
Lawrence, Jeannine: 9510
Lawson, Katherine M.: 1035
Lay, Jennifer C.: 1410
Layman, Shannon: 2385
Lazarus, Rachel: 4215
Le, Anne: 4390
Le, Ocean: 4085, 2255, 3660, 9430, 9510
Le, Tham: 1055
Leach, Heather: 9410
Leak Bryant, Ashley: 2140, 3610
Leal, Isabel: 9260, 9430
Leanage, Allison: 4540
Leavey, Gerry: 4570
LeBlanc, Raeann G.: 3230, 9010
LeBrasseur, Nathan: 2110, 2440
Leddy, Jason F.: 9505
Lederer, George: 9360, 9415
Ledoux, Danielle M.: 9500
Lee, Dylan: 1500
Lee, Hochang Ben: 4167
Lee, Jane: 1085
Lee, Joseph H.: 2285, 4585
Lee, Laura: 3630
Lee, Ahyoung: 9310
Lee, Cassie: 9220
Lee, Catherine: 9505
Lee, Chae Man: 9045, 9165, 9245, 9390
Lee, Chaiwoo: 1315, 2190
Lee, Changmin: 9080, 9250, 9460, 9470
Lee, Changsook: 1070
Lee, Chioun: 3190, 4255
Lee, Chiyoung: 4330
Lee, Christine: 9515
Lee, David: 9105
Lee, Diane: 9440
Lee, Dylan: 1500, 9155
Lee, Ellen E.: 3295
Lee, Eun Jeong: 9500
Lee, Eun Sik: 9000, 9045
Lee, Gina: 9500, 9515

Lee, Haena: 1105, 2435, 3190
Lee, Hannah: 9180, 9290
Lee, Heather: 1075
Lee, Hee: 9240
Lee, HeeSoon: 9510
Lee, Hye Soo: 1500, 9080, 9155, 9455
Lee, Hyo Jung: 9240, 9430
Lee, Jaegyeong: 9335, 2365
Lee, Jeenkyoung: 1350
Lee, Jeongeun: 1135, 1205, 2265, 9035, 9285, 9315
Lee, Ji Hyun: 3185, 9155, 9515
Lee, Ji Yeon: 9070, 9295
Lee, Jia Qi: 3405
Lee, Jia Wen: 4190, 9135
Lee, Jin: 4125
Lee, Jonathan: 9050, 3190
Lee, JuHee: 9295
Lee, Jun Hong: 9065
Lee, Jung-Ah: 1340, 2075, 4175, 9270, 9335
Lee, Jun-Yeong: 9095
Lee, Ka Yeon: 9080, 2475, 9280
Lee, Kyuho: 2425
Lee, Kyung Hee: 9070
Lee, Kyungmi: 9070
Lee, Kyuri: 9070
Lee, Lewis: 9240
Lee, Mark: 1290, 3190
Lee, Matthew: 3090, 9510, 9515
Lee, May-Ling June: 4075
Lee, Monica: 9160
Lee, Moon: 9240
Lee, Rain: 9435
Lee, Samuel Y.: 9050
Lee, Sangah: 9505
Lee, Sangeun: 4015
Lee, Sei J.: 2020, 3285, 9025
Lee, Seolah: 9080
Lee, Shinduk: 9455
Lee, Sojeong: 9500
Lee, Soomi: 1065, 1430, 2450, 3295, 4580, 9440, 9515
Lee, Suckwon: 9055
Lee, Sumi: 9295
Lee, Sun Ah: 1165, 9080
Lee, Sunghee: 4430
Lee, Sungkyu: 9500
Lee, Sun-Kyung: 4245
Lee, TaeWha: 2115, 4050
Lee, Trevor: 9275
Lee, Vanessa Y.: 9155, 9175
Lee, Vincent: 1335
Lee, Yeonjung: 1395
Lee, Yeonjung J.: 9130, 9155
Lee, Yeon-Shim: 9515
Lee, Yooumi: 3150
Lee, Youjung: 2500
Lee, Yu-Chien: 2290
Lee, Yunhwan: 3175, 9500
Leedahl, Skye N.: 3360, 3560
Leeman, Jennifer: 3325
Lees, Tabitha: 9505, 9300, 9500
Leeuwenburgh, Christiaan: 9090
Lefers, Lindsay: 9500
Leff, Bruce: 4410
Lefler, Lisa: 2380
Legault, Véronique: 3485
Leger, Kate A.: 2450
Leggett, Amanda N.: 9180, 9290, 9325, 9360
Leggett, Amanda: 1205, 2165, 2305, 4205
Lehallier, Benoit: 1015
Lehning, Amanda J.: 4180
Lehti, Tuuli E.: 9405
Lei, Lianlian: 9505
Leiker, Merced: 9105
Leips, Jeff: 9105
Leipzig, Rosanne: 3060
Leirbekk Mjøsund, Hanne: 3625
Leis, Aleda M.: 1210
Leiser, Scott F.: 9105
Leist, Anja: 1290, 2040, 2480, 4195
Lekhak, Nirmala: 1360, 1505
Lekovitch, Cara A.: 4455
Leland, Natalie E.: 4455, 9000
Lemieux, Catherine M.: 2355
Lemke, Kelsey: 3550
Lemme, Holly: 3105
Lemmon, Elizabeth: 3585
Len, Kathleen: 9325
Lendon, Jessica P.: 1325
Lentz, Jeffrey: 9380
Lenz, Rosemarie: 9375
Lenze, Eric: 3100
Leon, Milo: 9510
Leonard, Natalie: 2165
Leonard, Ethan R.: 9295
Leonard, John: 4330
Leonardo, Juliette: 2515
Leontowitsch, Miranda: 4385
Lepore, Michael J.: 2045, 2100, 4275, 9505
Leritz, Elizabeth: 9140
Leroi, Iracema: 1327, 9100
Leslie, Myles: 3250, 4225
Lessard, Darleen: 9030
Lessem, Rachel N.: 2065
Letts, Elyse G.: 2040, 9060
Leung, Reynold: 2430
Leung, Ada: 9135
Leung, Dara KY: 2215, 3095, 9360
Leung, Patrick: 9355
Leung, Tung Ming: 3075
Leurgans, Sue E.: 2495, 9365
Levant, Ronald F.: 9445
LeVasseur, Michael T.: 4595
Leveille, Suzanne: 4075, 9510
Leveille, Suzanne G.: 9000
Levesque, Laryssa: 4015
Levi, Bonnell: 3300
Levin, Michael: 9290
Levine, Brian: 1045, 4015, 9505
Levine, Morgan E.: 1015, 3435, 3495, 9055, 9085, 9100, 9515
Levinson, Abbie R.: 4270
Levkoff, Sue E.: 4245, 3380
Levy, AnneMarie: 9145
Levy, Becca R.: 1285, 1335, 2170, 3235, 4010, 4630
Levy, Cara: 9510
Levy, Cari: 1075, 3275
Levy, Sheri: 9510
Levy, Simon: 9515
Levy-Storms, Lené: 9220
Lewinson, Terri D.: 3455, 3475
Lewis, Caitlin J.: 9110
Lewis, Cathryn: 4050, 9090
Lewis, Denise: 9505, 1395
Lewis, Jordan P.: 2315, 2380, 9250, 9430
Lewis, Nathan A.: 9505, 2470, 9335
Lewis, Teresa: 4425
Leykum, Luci: 9510
Li, Juan: 1255
Li, Kaigang: 4025
Li, Xia: 4215
Li, Bingyu: 9140
Li, Changwei: 9360
Li, Chien-Ching: 3340, 9505
Li, Chih-Ying: 9295
Li, Fangyu: 3430
Li, Guohua: 2515
Li, Jiajia: 3470
Li, Junxin: 1130, 2295, 4015
Li, Karen: 9075
Li, Ke: 9155, 9455
Li, Lun: 9005
Li, Lydia W.: 1145
Li, Mengchi: 4015
Li, Meng-Hao: 2385
Li, Mengting: 1195, 2255, 3660, 9450
Li, Mirandy: 9175
Li, Qinghua: 2090
Li, Qiwei: 1450, 4525
Li, Ruike: 1195
Li, Shijie: 9295
Li, Shirley: 9050
Li, Shuzhuo: 4555, 9240, 9500
Li, Wei: 4540, 9115
Li, Wenjun: 1100, 2030
Li, Xiaoli: 1055
Li, Xiaoqian: 4190, 9135
Li, Xuefei: 9445
Li, Yan: 9115
Li, Yang: 3310, 9040
Li, Yawen: 9505
Li, Yimei: 9515
Li, Ying: 9295
Li, Yixia: 4550, 9070
Li, Yongmei: 9025
Li, Yong-Zhen: 1395
Li, Yue: 3410, 2120, 4405
Li, Yuelin: 9130

Li, Yun: 2200, 4395, 9295, 9505
Liang, Jersey: 3635
Liang, Daan: 3355, 4235
Liang, Xiaohui: 3000, 2120, 2180, 3180
Lichtenberg, Peter A.: 9515, 1020, 1330, 1335, 2375, 3155, 9045, 9380
Lidbe, Abhay: 1485, 9195
Lieberman, Lauren: 2145
Liebreich, Rob: 2040
Liggett, Anna: 9280
Light, Michael A.: 3545
Lightfoot, Elizabeth: 9500
Lighthall, Nichole: 9505
Ligus, Kaleigh: 9500
Liles, Brian K.: 9140
Lim, Emily: 4555
Lim, Jae: 9500
Lim, Kyuhee: 4430
Lim, Yeon Ok: 9510
Lim, Yun Mook: 9080, 9460
Lin, Amy: 9180
Lin, Feng V.: 9505, 2270
Lin, Frank: 1130, 2085, 4030, 4040, 4167, 4190
Lin, Honghuang: 2015, 9120
Lin, John: 1290
Lin, Paul: 2360, 4180, 4565
Lin, Peichao: 9025
Lin, Shih-Yin: 3055, 3260, 4405, 9050, 9505
Lin, Tian: 9400, 9505
Lin, Xin Yao: 3655, 9470, 9510
Lin, Zhiyong: 4555, 9360
Lincoln, Karen: 1470, 2240
Lind, Lisa: 2260
Lindau, Stacy: 9050
Lindauer, Allison: 9280, 9505, 9510
Lindberg, Brian W.: 1340, 2165, 3140, 3350, 3505, 4290
Lindeman, David: 4145
Linden, Wolfgang: 9175
Lindenberg, Jolanda: 1360
Lindenberger, Ulman: 3085
Linden-Carmichael, Ashley: 9130
Lindkvist, Marie: 3440
Lindquist, Lee A.: 3060, 9035, 9065, 9200, 9280, 9300
Lindsey, Ronald W.: 2295
Lingler, Jennifer H.: 1125, 9065
Links, Paul S.: 4365
Linscott Lowe, Bethany: 1220
Linville, Tabitha: 3325
Lio, Shoon: 2260
Lion, Katarzyna: 3585
Liou, Chih-ling: 9015, 9205
Lipchin, Jessica: 9270
Lipsitz, Lewis A.: 9500, 9505, 9515, 1280, 2130, 3320, 4435, 9160
Lipton, Richard B.: 9405
Lise, Kristi T.: 2170
Lisha, Nadra: 2070
Litke, Rachel: 9055
Littlejohn, Jenna L.: 1327
Litzelman, Kristin: 9240, 9280
Liu, Anna: 9245
Liu, Bian: 9115, 4380
Liu, Chelsea: 2465, 9265
Liu, Chengyu: 9085
Liu, Darren: 1470, 3340, 9185, 9380
Liu, Fangyu: 2285, 4065
Liu, Haiyong: 9245
Liu, Hui: 9025
Liu, Jing: 4100, 4220
Liu, Jingwen: 4380
Liu, Jinyu: 1070, 2360, 9040, 9505
Liu, Lan: 9425
Liu, Li-Chuan: 9510
Liu, Li-Fan: 3340
Liu, Lili: 2040, 9060, 9130, 9145, 1070
Liu, Minhui: 3380, 4110
Liu, Pan: 2200, 4395, 9295, 1285, 1335, 3380
Liu, Qinghai: 9055
Liu, Ruotong: 4350, 9240
Liu, Shao-Hsien: 9295
Liu, Shelley Smith: 9420
Liu, Sophia: 9090
Liu, Teresa T.: 9295
Liu, Tianyin: 3095, 3470, 4280, 9360
Liu, Wen: 3565, 4380
Liu, Wenlin: 1255
Liu, Xiao: 9055
Liu, Xiaojuan: 9025, 9240
Liu, Yanqin: 4545
Liu, Yi: 4395
Liu, Yie: 9085
Liu, Yin: 1130, 1205, 2166, 2305, 4205, 9290, 9340, 9505
Liu, Yingling: 3580
Liu, Yu: 4215
Liu, Yuezhong: 9330
Liu, Yujun: 9135, 9290, 9415
Liu, Yumeng: 3395
Liu, Zhijian: 1255
Liu-Ambrose, Teresa: 3460, 9250
Livinski, Alicia: 9025
Lizzo, Giulia: 9110, 9510
Llaneza, Danielle H.: 1155, 9285
Lo, Joan: 9505
Lo, Lawrence: 9500
Lo, On-Yee: 1280, 9140, 9515
Lobaton, Edgar: 9505, 3230
Lock, Sarah L.: 1080, 4215
Lockery, Jessica E.: 9030
Lockhart, Thurmon: 9500
Lodi-Smith, Jennifer: 4320
Loeb, Susan: 9515
Loeckenhoff, Corinna E.: 9135
Loewenstein, David: 1010
Loewy, Elizabeth: 9230
Logue, Rachel N.: 9515, 9420
Lohman, Matthew: 9510
Lohman, Matthew C.: 9030, 9065, 9070, 9160
Loika, Yuriy: 9085
Loika, Yury: 9505
Loiko, Elena: 9505
Lokanathan, Tharshini: 2030, 4345
Lokon, Elizabeth J.: 3410
Lomeli, Sindy: 2125
London, Andrew: 9380
London, Stephanie: 9070
Long, Monica: 1340
Lopes, Luiza Guilhermina O.: 9365
Lopez, Acacia: 9260, 9315
Lopez, Francisco A.: 9505
Lopez, Oscar: 9065
Lopez, Ruth Palan: 2360, 3605
Lopez Hernandez, Yareli: 9515
Lord, Justin: 1075, 4355
Lorenz, Rebecca A.: 3590, 9270, 9365
Losada-Baltar, Andrés: 1005, 2166, 3050, 3580, 4085, 4110
Losinski, Genna: 1155, 9045, 9250
Lothary, Allura: 2220, 3655
Lou, Vivian, W. Q.: 2030, 2430, 4360
Lou, Yifan: 1070, 2360
Loui, Psyche: 9140
Loup, Julia R.: 9355, 9410
Louwagie, Katie: 4350
Lovasi, Gina S.: 3160
Low, Carissa: 1165
Low, Dominique V.: 9085
Low, Gail: 9430
Low, Hilary: 9295
Lowe, Dawn: 9095
Lowe, John: 9400
Lowe, Sarah: 4630
Lowe, Scott W.: 2440
Lowenkron, Jeff: 9160, 9510
Lowry, Kristin: 9515
Loy-Ashe, Tarah: 9500
Loyd, Christine M.: 9000
Loyen, Anne: 4435
Lu, Ake T.: 3600, 3525, 3590, 4200, 9505
Lu, Shiyu: 9360
Lu, Tina: 9080, 9130
Lubben, James: 2405
Lubetsky, Sara: 4410
Luby, Pam: 3240
Lucantoni, Davide: 4005
Lucas, Kaitlyn: 9505, 9515
Lucero, Robert: 9510
Luchetti, Martina: 3185, 9155, 9515

Luchsinger, José A.: 1180, 3320
Luci, Katherine: 1410
Luciano, Michelle: 9190
Lucksted, Alicia: 2275
Luecke, Anna J.: 3085
Luergans, Sue E.: 9390
Luitjens, Emily: 1175
Luk, Joanne: 4380
Lukindo, Mary: 9510
Luk-Jones, Susanna: 4240
Lum, Hillary D.: 9165
Lum, Terry YS: 1045, 2215, 3095, 3470, 4280, 9065, 9360
Lumley, Mark: 2465
Luna, Maria G.: 9135
Lund, Brady: 9150
Lundy, Janice: 9500
Lunetta, Kathryn: 9065
Luo, Minxia: 4580, 1045, 4050, 9065
Luo, Huabin: 1075, 1115, 1465, 2280
Luo, Jing: 1035, 2305
Luo, Liying: 9055, 9440, 1290
Luo, Meng Sha: 1145
Luo, Minxia: 4580
Luo, Yan: 4435, 9025, 9060, 9240
Luo, Ye: 2435, 9035
Luong, Gloria: 3265, 9235
Lupo, Jody-Lynn: 9500
Lupton-Smith, Carly: 9265
Lustria, Mia: 4470
Luszcz, Mary: 9430
Luth, Elizabeth: 3260
Luthar, Suniya S.: 3520
Lutsey, Pamela: 1130, 9070
Lutz, Julie: 1135
Lv, Siman: 4215
Ly, Timothy K.: 4195, 9320, 1435, 2225, 9140
Lyketsos, Constantine G.: 1327
Lyman, Alexa P.: 9510
Lynch, David: 9030, 9390, 9455
Lynch, Jennifer: 4610
Lyons, Amy G.: 9270, 9395
Lyons, Karen S.: 3200, 9505
Lyons, Michael J.: 9510
Lytle, Ashley: 9510
Lyytinen, Kalle: 9050

**M**

Ma, Chenjuan: 3260, 4405
Ma, Harrison: 9220
Ma, Jinhui: 9160
Ma, Lina: 2200, 4395, 9295
Ma, Ping: 1195
Ma, Ying: 9355
Ma, Yuan: 1200
Márquez-González, María: 3580, 4085, 4110
Mérida-Herrera, Laura: 4085, 4110
Maćkowiak, Maria: 3585, 9130
Macaluso, Suzie: 4490
Maccarrone, Jodie: 9510
Macdonald, Birthe: 4580
MacDonald, David: 9105
MacDonald, Grace: 4115
Macdonald, Jamie: 9510, 1495, 9075, 9190
MacDougall, Luke S.: 3555
MacEachern, Lauren: 3605, 4425
Macedo, Luciana: 9160
Macias Tejada, Jonny: 9130
Macinko, James: 4255
MacInnes, Roddy: 3560
MacIver, Nancie J.: 9420
Mack, William J.: 9055
Mackenstadt, Darby: 1050
MacKenzie, Blair: 3060
Mackiewicz, Marissa: 4360, 9145
MacLehose, Richard: 9190
MacLeod, Stephanie: 9455, 9500
MacNeil, Amanda M.: 9360
MacPhail, Catherine: 2330
MacPherson, Andrew: 2165
Macri, Melissa: 9515
Madden, Connie: 2425
Madden, David: 2085
Madden, Kenneth M.: 9175, 9200
Madero, Erica: 9070, 9130, 9390, 9420
Madhira, Vithal: 9505
Madiloggovit, Jirakate: 3610, 9270
Madisetti, Mohan: 9500
Madore, Aylin: 9065, 9300
Madrid, Diana A.: 1305
Madrigal, Caroline: 3015, 9130, 9515
Maercker, Andreas: 1290, 4430
Magaziner, Jay: 2115, 3120
Magda, Kirsten: 9225
Magdael, Adrielle F.: 9045
Magder, Laurence: 9500
Magid, Hoda: 2115
Magid, Kate: 1075, 3275
Maglalang, Dale Dagar: 9505
magnerelli, Jacki: 2005
Magwood, Gayenell: 3640
Mahapatra, Gargi: 9055
Mahapatra, Neely: 3115
Maher, Alexander S.: 3435
Maher, Brion: 9085
Maheshwari, Sfurti: 1360
Mahmood, Atiya: 3105, 4385
Mahmoud, Kafayat: 2390, 9455
Mahmoudi, Elham: 2360, 4180, 4450, 4565
Mahon, Elizabeth: 9135
Mahoney, Sean O.: 9055, 9095
Mahroum, Naim: 2205
Mai, Chunyan: 2220, 9015
Maiden, Robert J.: 1160, 4490, 9515
Mair, Christine A.: 4125, 4180
Maixner, Susan: 9370
Majersik, Jennifer: 9070
Majeski, Robin: 3555, 9025
Mak, Angel: 9415
Mak, Hio Wa: 4440
Mak, Wingyun: 2445, 9150, 9160
Makaroun, Lena K.: 1285
Makris, Evie: 2400
Malani, Preeti: 2165
Maldonado, Rene H.: 9510
Maldonado Ortiz, Juan: 9200
Malecki, Kristen C.: 3590
Malecki, Maya: 3650
Malhotra, Atul: 9515
Malhotra, Rahul: 3050, 4075
Malle, Bertram: 1155
Malling, Charlotte: 9115
Malone, Michael: 9130, 9160
Mammen, Jennifer S.: 4160
Mamo, Sara K.: 1327
Man, Kenneth K.C.: 4050
Manalel, Jasmine: 4605
Mancino, Mairin: 4355
Manful, Adoma: 3260
Mangialasche, Francesca: 1260
Manheim, Chelsea: 4145, 4570
Manietta, Christina: 2510
Manini, Todd: 3290, 9250, 9510
Manis, John: 4510
Mankowski, Robert: 9090
Manley, Natalie A.: 9080, 9175, 9470
Manley, Pamela: 9060
Manly, Jennifer: 4195
Mann, Jim: 3540
Manning, Carol A.: 1125
Manning, Carol: 9500, 1505, 9120
Manocha, Gunjan D.: 9215
Manor, Brad: 1280, 2130, 3460, 4435, 9500, 9505, 9515
Mansour, Daniel Z.: 9210
Manukhin, Daniel: 9300
Manzo, Elyse C.: 9505
Mao, Weiyu: 1115, 3610
Marani, Husayn: 4350
Marcantonio, Edward R.: 9505
Marceau, Kristine: 9200
Marchildon, Gregory: 2040, 4350
Marcinek, David: 9090
Marconi, Vincent C.: 3645

Marcum, Zachary A.: 3285, 9310, 9270, 9250
Margolis, Rachel: 1360
Margrett, Jennifer: 1030, 9515, 2425, 9510, 9105
Marini, Christina M.: 1130, 3125, 3525
Marino, Victoria R.: 2000, 2465
Marion, Cecelia: 3250
Mariotti, Marco: 3270
Markides, Kyriakos S.: 1230, 3380, 4450
Markland, Alayne: 4590
Markle-Reid, Maureen: 9130
Markov, Yaroslav: 3435, 9055
Markowski, Todd: 9095
Marks, Loren: 3640
Markwardt, Sheila: 3285, 9510
Markwood, Sandy: 1025
Marmo, Suzanne: 3115, 9500
Marottoli, Richard A.: 9050
Marques, Isabela G.: 9265
Marques, Pedro: 9420
Marques Borges, Carolina: 3345
Marquez, David X.: 1310, 2485, 9265
Marquez, Isabela: 4635
Marquine, Maria J.: 9085, 9505
Marroig, Alejandra: 9415
Marrone, Nicole: 4167
Marrs, Sarah: 4260, 9010
Marsack-Topolewski, Christina: 1395, 9040
Marsalis, Scott: 4245
Marseglia, Anna: 9065
Marsh, Anthony P.: 1305
Marshall, Gillian L.: 3640
Marshall, Teresa: 2455, 9155, 9180
Marsland, Anna: 4115
Martínez, Melween I.: 9100, 4455
Marti, Yamile: 9515
Martin, Mike: 4505
Martin, Damali N.: 3400
Martin, Diane J.: 9210
Martin, Grace K.: 9510
Martin, Jennifer: 9440, 4120
Martin, Maya: 9195
Martin, Peter: 4330, 9320, 9500, 9515, 3415
Martin, Roy C.: 1380, 4590
Martindale-Adams, Jennifer: 3380
Martinez, Catherine: 2060
Martinez Amezcua, Pablo: 4040
Martinez-Hollingsworth, Adrienne: 9045
Martins, Nuno: 9100
Martins-Klein, Bruna: 9235
Martire, Lynn M.: 1130, 3125
Marx, Katherine A.: 1420, 4210, 4390, 9105
Marzan-Rivera, Nicole: 4390
Masaki, Kamal: 3415
Masliah, Eliezer: 3020
Maslow, Katie: 2505
Masoli, Jane: 3320, 9100
Mason, Agnieszka: 9045
Masotti, Barbara: 1415
Masoud, Sara: 9265
Masse, Sybil: 3475
Massihzadegan, Setarreh: 9385
Massimo, Lauren M.: 3450
Massuda, Cindy: 1240
Mast, Benjamin T.: 9510, 2505
Mastel-Smith, Beth: 9460
Masters, Julie: 4490, 9150, 9155
Mastushita, Kimiyo: 9300, 9350
Mate, Kedar KV: 3485
Mateos, José Tomás: 2045
Materna, Carina: 9510
Mather-Tayor, Charlotte: 9500
Mathews, Jenni: 4020
Mathias, Rasika A.: 9290
Matli, Kamal: 9130
Matovu, Schola: 9270
Matsui, Hiroshi: 9420
Matsumoto, Daisuke: 9225
Matsushita, Kunihiro: 9510
Matta, Tara D.: 9515
Mattek, Nora: 3000, 9050, 9505, 9510
Matthews, Alicia K.: 9505
Matthews, Kathleen C.: 1175
Matthews, Kevin: 2375
Mattos, Meghan K.: 1125, 4145, 9065
Matus, Austin M.: 3450
Matyi, Joshua: 9085
Matz, Christina J.: 1085, 9125
Mauney, Karen: 3605
Maureen, McGlinn: 9375
Mausbach, Brent T.: 1005, 2166
Mavandadi, Shahrzad: 3365
MaWhinney, Samantha: 9025
Maxey, Myles: 9340
Maxfield, Molly: 4045, 9360
May, Jennifer T.: 9505
Mayberry, Pamela: 4270
Maydan, Gal: 4335
Mayer, Alexander S.: 9305
Mayfield, Kellie E.: 9515
Maynard, Madison: 9135
Mayo, Ann M.: 9395
Mayo, Nancy E.: 3485
Mazor, Kathleen: 2360
Mazzara, Nicholas: 3055
Mazzola, Paolo: 9515
Mazzucchelli, Trevor: 9430
Mc Gue, Matthew: 9510
McAdams-DeMarco, Mara A.: 4070, 4395
McAiney, Carrie: 4465
McAloney, Richard: 9515
McAtee, Robin: 4175, 9050, 9300
McAuley, Edward: 9140
McAuliff, Kathleen E.: 9450
McAvay, Gail: 9510
McBride, J. Marvin: 4240
McBride, Kimberly: 9225
McBroom, Anthony: 9515
McCabe, Louise: 9505
Mccaffery, Jeanne: 9430
McCain, Dextiny: 9445
McCall, Matthew D.: 4080
McCann, Brandy: 1030
McCarthy, Ellen P.: 2360, 3605, 9295, 9500
McCarthy, John: 4430
McCarthy, Kate: 3235, 9360
McCarty, Matthew: 4590
McCaughey, Deirdre: 3230, 4465
McCauley, Kathleen: 2390
McClain-Moss, Lisa: 9070, 9090
McClive-Reed, Kimberly P.: 9430
McColloch, Elizabeth: 1085, 2475
McConnell, Eleanor S.: 2510, 4330, 9045, 9395
McCoy, Brianah: 9505
McCullough, Wayne: 3550
McCurry, Susan: 1005, 2015, 9290
McDaniel, Justin: 3030
McDarby, Meghan: 2230, 9500
McDevitt, Ross: 9085
McDonald, Kimberly K.: 2050
McDonold, Siobhan: 3130
McDonough, Carol C.: 3500
McDowell, Cynthia: 1495
McDuffie, Danielle L.: 9380
McEachron, Don: 4595
McElroy, Michaela: 9185
McEntee, Cali M.: 9105
McFadden, Susan: 9020
McFarland, Mary M.: 4545
McGaffigan, Erin: 2065
McGee, Jocelyn: 1210, 4135, 4295, 9185
McGee-Lawrence, Meghan: 1170, 9105, 9110
McGhan, Gwen: 3230, 3250, 4225, 4465
McGibbon, Chris: 9500
McGill, Sharon: 1300
McGilton, Katherine S.: 2045, 3565, 9030

McGinn, Michael: 9455, 2175
McGovern, Justine: 1265
McGready, John: 4160
McGuire, Catherine: 3605
McGuire, Lisa C.: 1350, 2180, 3160, 3235, 3330
McHugh, Molly: 2390, 3380
McInnes, D. Keith: 3645
McIntosh, Tom: 4005
McIntosh, Erik: 9350
McIntyre, Maddie: 4260, 4075, 9180
McKay, Tara: 4460
McKee, Kevin J: 2075, 9270
McKelvey, Miechelle: 9140
McKenna, Kevin: 4080
McKenzie, Glenise: 1425
McKenzie, Camille S.: 4195
McKenzie, Debbie: 9110
McKinley, Joseph C.: 9460, 9510
McLain, Alexander: 9065
McLaren, Jaye: 9510, 9505
McLaughlin, Sara J.: 9435
McLeish, Sammy: 4330, 2040
McManus, David: 9030
McMunn, Anne: 9145
McMurray, Josephine: 9145
McNally, James W.: 9045, 9075
McNamara, Kelsey C.: 9505
McNaughton, James A.: 9165
McNeil, John J.: 4070, 9030, 9295
McNeill, Charleen: 9155
McNulty, Amy L.: 9515
McPheron, Chelsea: 4225
McPherson, Rachel: 3625, 9355
McPhillips, Miranda V.: 2295, 4015, 4035, 4445, 9290
McSorley, V. Eloesa: 2495
McVary, Kevin T.: 9295
McWilliams, Gabriella T.: 9105
Means, Samuel: 4340
Medina-Walpole, Annette: 3610
Medlin, Laura M.: 9160
Medoff, Deborah: 2275
Meehan, Amy: 9515
Meeks, Suzanne: 2250
Meer, Margarita: 1015, 3435, 9085
Meghani, Salimah H.: 9385
Mehawej, Jordy: 9030
Mehl, Matthias: 9505
Mehmedi, Liberta: 4110
Mehmood, Yasir: 3455, 2145, 2405, 1110
Mehta, Priyanka S.: 9370
Mei, Di: 9385
Meier, Helen CS: 9085, 9095, 9505
Meier, Tabea: 4505
Meija, Shannon: 1475, 3085, 9255, 9380, 9510
Meisner, Brad A.: 9260, 9400, 1150, 2325
Mejia-Arango, Silvia: 4185
Melekin, Amanuel: 1325
Melgoza, Esmeralda: 9375
Melik-Abramians, Armen: 9030
Melillo, Karen Devereaux: 3500
Melipillán, Roberto: 9360
Melton, Heather: 9230
Meltzer, Gabriella: 3185, 4570
Melzer, David: 3320, 9100
Mendenhall, Alexander: 9515
Mendes de Leon, Carlos F.: 2495
Mendez, Jennifer C.: 1400
Mendez, Kyra W.: 4167
Mendhe, Bharati: 9110
Mendo, Christian W.: 3485
Mendoza, Nancy: 2500, 1300, 9430, 9515
Meng, Hongdao: 9005, 9135
Mengel-From, Jonas: 2110, 9500
Menne, Heather: 9500
Mensinger, Janell L.: 4075
Mentone, SueAnn: 9055
Meraz, Rebecca: 1210, 4135
Mercado-Sierra, Marta: 9360
Merchant, Aditi: 4555
Merchant, Anwar: 9065
Merickel, Jennifer: 1485
Merten, Natascha: 3590
Mesa, Robert: 1195
Messelis, Els: 2150
Metoxen, Elijah: 3365
Metzelthin, Silke F.: 2340, 3625, 9500
Metzger, Dennis W.: 9160
Meuser, Thomas: 9185
Meyer, Julienne E.: 4275
Meyer, Kylie: 3215, 9265, 9085, 9105
Meyers, Summer N.: 9155, 9175
Meynadasy, Melissa: 3595
Meyr, Kaitlyn: 9410
Mez, Jesse: 9065
Meza, Erika: 9450
Mezuk, Briana: 3155, 3395, 4485
Miao, Hongyu: 2120
Michael, Cherish: 1485, 9410
Michael, Yvonne L.: 3160, 9245
Michaelis, Elias: 3175
Michalek, Joel E.: 9295
Michel, Christoph: 1280
Michelle, Seage: 9230
Michener, Alyson R.: 3245
Middleton, Addie: 9500
Middleton, Laura: 4465
Midgett, Sharnita: 1310
Mielenz, Thelma: 2515
Mielke, Michelle: 2130
Migliaccio, John N.: 4615
Migliavacca, Eugenia: 9110
Migneault, Deborah: 1060
Miguel-Cruz, Antonio: 2040, 9060, 9130
Mikal, Jude: 2325, 9515, 4440, 9235
Mikrut, Mikala: 9015
Milani, Sadaf A.: 2200, 2400, 4635
Miles, Toni: 1225, 2135, 9300
Miljkovic, Iva: 9025, 9295
Millar, Courtney: 9365, 9510
Millar, Roberto: 9500
Millenbah, Ashley N.: 2325, 4350, 9515
Miller, Debra: 3365
Miller, Lyndsey M.: 3200
Miller, Amber: 9035
Miller, Donald: 9070
Miller, Edward A.: 9500, 2025, 2095, 2425, 2445, 3310, 3390, 4275, 4545
Miller, Hillary A.: 9105
Miller, Jacqueline: 3370
Miller, James W.: 9235
Miller, Jennifer: 4235
Miller, Julie B.: 1315, 2190, 2400, 3045
Miller, Julie: 9510
Miller, Lyndsey M.: 3200
Miller, Lyndsey: 9500
Miller, Marshall G.: 4120
Miller, Maura: 9510
Miller, Melanie: 9300
Miller, Michael E.: 1095
Miller, Ryan M.: 1305
Miller, Samuel M.: 9130
Miller, Sara E.: 9130, 9360
Miller, Stephanie: 9065, 9300
Miller, Susan: 9410
Miller, Virginia: 2130
Miller, Vivian J.: 9505, 9510, 1355, 3475, 3515, 9360
Mills, Whitney: 1075
Millstein, Joshua: 3310
Millwood, Summer N.: 9130
Milman, Sofiya: 3135
Milne, Barry: 2180
Milyavskaya, Nonna: 9080
Mimuro, Soichiro: 9300
Min, Hosik: 9505, 9035
Min, Se Hee: 3055
Minaeva, Olga: 9500
Minahan Zucchetto, Jillian: 3365
Ming, Yu: 9255
Mingo, Chivon: 2240, 2395, 3215
Minor, Lisa: 1270, 4415

Minteer, Christopher J.: 1015, 3435
Minton, Alyssa R.: 4440, 9235
Minyo, Morgan: 1025, 3235, 9185
Mirajkar, Shalmali: 2166
Miri, Reza: 9025
Mirza, Raza M.: 2005, 9245, 9350, 9505, 9515
Mirza, Sabina: 9505
Misas, Juan Diego: 9515
Mishra, Sidharth: 9090
Mistler, Lisa A.: 3255
Mistry, Sabuj Kanti: 9500
Mitchell, Jamie: 3155
Mitchell, Alex: 9165
Mitchell, Barbara A.: 9005
Mitchell, Colter: 9085, 9095
Mitchell, Kimberly M.: 3555
Mitchell, Lauren L.: 1005, 1030, 2325, 3185, 4305, 9515
Mitchell, Mary M.: 3090
Mitchell, Natalie: 9500
Mitchell, Susan: 2360
Mitchell, Uchechi A.: 1370, 2210, 2480
Mithani, Sameer: 3230
Mitnitski, Arnold: 9095
Mittelman, Mary S.: 9510, 9515, 9280
Mitzner, Tracy L.: 1435, 2365, 3170, 3650
Miura, Takahiro: 9245
Miwa, Hiroyasu: 4425
Mixon, Veronica: 9250, 9420
Miyawaki, Christina: 9280
Miyawaki, Christina E.: 1350, 3380, 4450
Mizuki, Maiko: 9030
Mlinac, Michelle: 2275
Mobbs, Charles: 9055, 9305
Mochel, Amy: 3275
Mochizuki, Satoru: 3490
Mock, Marilyn: 9510
Modara, Gelareh: 3105
Modig, Karin: 9295
Moeller, Kelly: 9345
Moen, Phyllis: 4265
Moffat, Scott D.: 9235, 2310, 3180, 3445, 3520, 4560, 9130
Mohamed, Nihal E.: 1370, 3075
Mohamed Nassur, Abubakar: 3440
Mohammadi, Sona: 9025
Mohammed, Dania: 9515, 4335
Mohanty, Kareshma: 9500
Mohr, David C.: 1075
Mois, George: 1140, 9505
Mojdeh, Heavner: 3500
Mojtabai, Ramin: 9515
Mola, Giada: 4530
Moldawer, Lyle: 9090
Molina, Anthony J. A.: 9055
Molina Martínez, MªÁngeles: 9155, 9360
Molinari Ulate, Mauricio: 4610
Molitoris, Kristin: 9085
Molnar, Lisa: 2515
Moloci Noss, Melody: 9130
Molony, Sheila: 2505
Molony, Jason: 1340, 9050
Monaghan-Geernaert, Pamela: 2380, 9510
Monaro, Sue R.: 3245
Moncaster, Juliet A.: 9500
Moncion, Kevin: 9420
Mondino Vero, Alejandra: 9505
Mongoven, Jennifer M.: 3555, 4015
Monin, Joan K.: 9510, 1335, 2170, 3125, 3235, 9200
Moniz-Cook, Esme: 2505
Monroe, Todd: 2410, 9160
Montague, Michael J.: 9100, 9325, 9450
Montano, Monty: 9500, 1040, 1375, 2345, 3500, 9170
Montero Odasso, Manuel: 9500
Montes de Oca, Verónica Z.: 9505, 4185
Montgomery, Katherine: 9300
Montgomery, Kymberlee: 4595
Montgomery, Polly: 9105
Monti, Stefano: 9100
Montoro-Rodriguez, Julian: 9315
Montoya, Ana: 9160
Montoya, Yadira: 3000
Monturo, Cheryl: 2095
Moo, Lauren: 1455, 3275, 4535, 9070, 9510
Moody, Kate A.: 4410
Moon, Chooza: 9070
Moon, Heehyul: 9190, 9500
Moon, Seong Hyun: 9500
Moone, Rajean P.: 1225, 9500
Mooney, Kathi: 4600, 9270
Mooney, Scott: 9180
Moonie, Sheniz: 3570
Moore, Alison: 9070
Moore, Ann: 2285, 9085
Moore, Carley: 9515
Moore, Cindy L.: 9515
Moore, David J.: 3645
Moore, Frederick: 9090
Moore, Katelyn: 4595
Moore, Raeanne C.: 9505
Moore, Rick: 9160
Moore-Bembry, Natalie: 1470
Moored, Kyle D.: 1095, 2085, 3290, 3430, 4520, 9505
Moorman, Sara: 3225, 4345
Moorman Li, Robin: 9500
Mor, Vincent: 1005, 1055, 1075, 3275, 9160
Mora, Maria Juanita: 3105
Morais, Calia A.: 3095
Morais, José A.: 3485
Morano, Carmen: 3035
Moray, Juno: 9515, 1335
Morcos, George S.: 9105, 9510
Morelli, Alexandra: 3420
Morelock, Jeremiah: 9020
Moreno, Monica: 9515
Moreton, Elizabeth: 3325
Morgan, Brianna: 1390, 2390
Morgan, Damian: 4220
Morgan, Davie: 4135, 4295
Morgan, Debra: 9075
Morgan, Emily: 9500
Morgan, Stephanie: 2515
Morgan, Todd: 9055
Morhardt, Darby J.: 9280
Morin, Ruth: 4550
Morioka-Douglas, Nancy: 9050
Morley, John E.: 4175, 1045
Morris, Brian: 3415
Morris, Rebecca: 9270
Morris, Timothy: 9140
Morrison, Cassandra: 9055
Morrison, Charles Tyler: 2295
Morrow, Daniel G.: 1475, 2365
Morrow, Melissa: 2130
Morrow-Howell, Nancy: 1190, 2345, 3100
Morselli, Marco: 1015
Morthland, Martin: 9370
Morton, Casey: 9215
Morton, Claire: 9405, 2265
Moscirella, Marybeth: 4455
Moser, Debra: 4235
Mosley, Thomas: 1280, 2085, 9070, 9505
Mosqueda, Laura: 2040, 2475, 4350
Moss, Karen O.: 2410, 3365
Motel-Klingebiel, Andreas: 1150
Mouchaers, Ines: 2340
Moulder, Robert G.: 4580
Mountain, Gail: 2505
Mourany, Lyla: 1230
Mourgues-Codern, Catalina V.: 9200
Mournighan, Kimberly: 9390
Moxley, Jerad: 3170, 3235
Moye, Jennifer: 9420
Moyer, Robinn: 9250, 4330

Moyo, Patience: 1075, 3275, 9395
Mpofu, Elias: 9155
Mroczek, Daniel: 1035, 2305, 2310, 2450, 3185, 9415, 9500
Mu, Christina X.: 1430, 9440
Muñoz, Elizabeth: 4505
Mudar, Raksha A.: 1435, 2225, 3655, 4190, 9140
Mudge, Alison: 4590
Mueller, Anne: 9270, 3100
Mueller, Kim: 1180
Mueller-von Aschwege, Frerk: 4335
Muhammed, Ruqayyah: 9035
Mui, Ada C.: 9040, 9145
Mulcahy, John F.: 9345
Mulgrave, verona E.: 9505
Mullen, Shannon: 9045
Muller, Kamila: 3285, 9510
Mullin, Donncha: 9190
Mullins, Megan: 2115
Mulmule, Natalie S.: 4270, 9500
Mulvey, Connor: 9515
Muniz-Terrera, Graciela: 9130, 9190, 9415, 9500, 9505
munjal, arshi: 3610
Munly, Kelly: 1385, 2350, 4020
Munoz, Elizabeth: 1295, 3520, 9360
Munoz, Lizette: 3060, 9160
Munoz, Mariateresa H.: 9505
Murabito, Joanne M.: 1210, 4585
Murabito, Joanne: 9065
Murakami, Shin: 9055
Muralidhar, Kiranmayee: 9050
Muralidharan, Anjana: 2275
Muramatsu, Naoko: 2485, 9150
Murata, Wakae: 9105
Murawski, Alaine: 9035
Murayama, Hiroshi: 9180
Murchie, Peter: 4120
Murdock, Elke: 4630, 9155
Murphy, Ian: 4495
Murphy, Caitlin: 9075
Murphy, Calliope C.: 2295
Murphy, Clare: 9155
Murphy, George: 9100
Murphy, James D.: 9070
Murphy, Mary M.: 9420
Murphy, Matthew S.: 9510
Murphy, Rachel: 9175
Murray, Anne: 4550, 9065, 9070, 9295
Murry, Logan: 1390
Murugadass, Avinav: 9060
Muruthi, Bertranna: 3550
Muruthi, James: 3550
Musch, David: 1045
Musi, Nicolas: 9155, 9180, 9295
Musil, Carol: 9260, 9505, 1320, 2500, 4245
Muskat, Mary: 1335
Mutchler, Jan E.: 3210, 3310, 4230, 9040, 9385, 9500
Muthaiyan Shanmugam, Muniesh: 9105
Mutter, Justin B.: 4145
Mutz, Julian: 4050, 9090
Myer, Deanna: 2100
Myer, Dillon: 3655
Myers, Dennis: 4135, 4295
Myers, Janet A.: 1020
Myers, Jennifer: 9070, 9130, 9390
Myers, Julie: 9505
Myers, Melanie: 9120, 9420
Myers Hurst, Laurel: 2410
Myint-U, Athi: 9300
Myrick, Matthew A.: 9500

**N**

Na, Annalisa: 2295
Na, Jihun: 9500
Nabors, Emily B.: 4230
Nadash, Pamela: 1245, 2025, 2095, 2445, 3390
Nadigama, Nainika: 9505
Nadorff, Danielle K.: 2500, 9260, 9315
Naganathan, Vasi: 3245, 4590
Nagarkar, Aarti: 9420
Nagel, Alexis: 9500
Nagel, Corey: 9030, 9380, 9510
Nah, Suyoung: 3125
Nahm, Eun-Shim: 9320
Naimi, Rachelle: 9130
Naito, Tomoyoshi: 9295
Najt, Charles: 4105, 9515
Nakagawa, Kazuma: 3415
Nakagawa, Sage: 9250
Nakagawa, Takeshi: 4205, 9135, 9340
Nakamura, Julia S.: 9500, 2465
Nakamura, Mikiko: 2095
Nakashima, Taeko: 2375
Nalawade, Vinit V.: 9070
Naleppa, Matthias: 9210
Nam, Sanghun: 4330, 4430
Nam, Seok In: 2475, 2485, 3150
Nambisan, Priya: 9050, 9395
Namkung, Eun Ha: 2265
Nanasi, Tibor: 1015
Nancy, Hoffart: 9000, 9390
Narazaki, Kenji: 9295
Nash, Paul: 2330
Nash, Princess: 1270, 9355
Nasrallah, Ilya: 9085
NASU, Takenori: 9245
Natale, Ginny L.: 9020, 9055, 9065
Nathan, Rachel: 9395, 9405
Naughton, Michelle: 3130
Navarro, Adria E.: 9060
Navin, Sara: 9425
Naylor, Mary: 9510, 2390, 3380
Neal, David P.: 4610
Neal, Vanessa L.: 2510
Nearing, Kathryn: 1455
Nebenzahl, Paul R.: 9505
Neelamegam, Malinee: 1090
Neely, Stephen: 9065
Nehlin, Jan O.: 9085
Nehrkorn-Bailey, Abigail: 4025
Neiberg, Rebecca H.: 1305
Neighbors, Harold: 3550
Neitzel, Richard: 9255
Nell, Josepha: 4630, 9155
Neller, Sarah A.: 4545
Nelson, Anna: 9335
Nelson, Christi: 9045, 9385
Nelson, Christian: 9115
Nelson, H. Wayne: 9350
Nelson, Matt: 4095
Nelson, Monica E.: 9140, 9455
Nelson, Niccole: 9340, 9505, 9245
Nembhard, Ingrid: 3245
Nepomnyaschy, Lenna: 9360
Nerz, Corinna: 4115
Netting, F. Ellen: 9350
Netz, yael: 9420
Netzer, Giora: 3500
Neubauer, Andreas: 1035, 2450
Neubauer, Noelannah: 9060, 9130, 9145, 2040
Neumann, Johannes: 4070
Neupert, Shevaun D.: 3085, 9135, 9235, 9250, 9430, 9505
Neven, An: 4610
Neville, Christopher: 9255
Newkirk, Katie: 9200
Newman, Anne B.: 1200, 1305, 3335, 9505
Newman, Aurora: 1135
Newman, John: 3335, 9505, 3005, 4250
Newmark, Rebecca L.: 2325
Newmeyer, Natalie M.: 9295
Newsom, Jason T.: 9030, 9380
Newton, Nicky: 1215, 4320
Ng, Cindy SY: 2430
Ng, Kheng Siang (Ted) K.: 2405, 3465, 9515
Ng, Weiwen: 1275
Ng, Yee Sien: 3405
Ng, Yee To: 4580, 9460
Ngandu, Tiia: 1260
Ngo, Long: 9505
Ngo, Van: 9095, 9305

Nguyen, An T.: 1390
Nguyen, Andrew: 9510, 9515
Nguyen, Annie L.: 2330
Nguyen, Bachkhoa: 9110, 9030
Nguyen, Hannah: 9335
Nguyen, Hoang: 4450
Nguyen, Jennifer: 9270
Nguyen, Khang: 9160
Nguyen, Lydia T.: 1435, 4190, 9140, 9500, 2130, 9510
Nguyen, Uyen-Sa: 3030
Ni, Cuiping: 4215
Ni, Pengsheng: 3275
Nicholas, J. Skye: 2365
Nichols, Emma: 4205, 3380
Nichols, Madeline J.: 9235
Nicholson, Roscoe F.: 3000, 9510, 9515
Nickel, Summerlin: 9470
Nicklas, Barbara J.: 1305, 3065
Nicklett, Emily J.: 9500
Nicolay, Sarah: 4325
Nidadavolu, Lolita: 4390, 9105
Nie, Jing: 4215
Nie, Qiong: 1475, 2365
Nie, Zuoting: 9050
Niedernhofer, Laura: 2440, 9105
Nielsen, Lisbeth: 3020
Nieman, Carrie L.: 1327, 2235, 4167
Niemiec, Allison C.: 9515
Niemiec, Matthew: 4075, 9085
Nikitin, Jana: 2220
Nilsen, Charlotta: 4110
Nimako, Maxwell: 9510
Nirenberg, Anita: 3395
Nishide, Kazuhiko: 9245
Nishikitani, Mariko: 4090
Nishino, Akiko: 9245
Nishizuka, Chiharu: 3075, 9035, 9335
Nitrini, Ricardo: 9505
Nitschke Durben, Anchee M.: 9500
Nixon, Charlotte J.: 9165
Njuguna, Mary N.: 9360
Nkimbeng, Manka: 1005, 3010, 4130, 4210
Noble, Brie: 3285
Noblitt, Jessica: 1485, 9340
Noel, Olivia R.: 9415
Noel, Polly: 3395
Noguchi, Taiji: 9135, 9280, 9340
Noguchi-Watanabe, Maiko: 3375
Noh, Hyunjin: 3095, 9240, 9410
Noh, Yeonsik: 9515
Nolte, Julia: 3510, 9135, 9230
Nomura, Mitsunori: 9505
Nord, Elizabeth: 1065
Noriega de la Colina, Adrian: 1210, 4075, 4195
Norris, Keith C.: 3640
Northwood, Melissa: 9050
Norton, Edward: 2265
Norton, Jonathan: 3565
Norton, Peter: 3440, 9355
Noujaim, Deborah: 4200
Nova, Amanda: 9050
Novak, Josh R.: 3125
Novak, Joshua: 9290
Novakovic-Agopain, Tatjana: 4535
Novik, Nuelle: 4005
Nuccio, Alexandria G.: 9155, 9250
Nuckols, Teryl K.: 1390
Nunez, Christina A.: 9250, 9505
Nurain, Novia: 9155
Nwakasi, Candidus C.: 4565
Nygaard, Marianne: 2020, 2110, 4585, 9510
Nyul Toth, Adam: 9085
O'Kelly, Ryan: 9105
O'Sullivan, Julie L.: 9360

O

Oakman, Kimberly: 9160
Oaks, Dawn: 1125
Oates, Gabriela: 1380
O'Brien, Tara: 3365
O'Brien, Cara C.: 9215
O'Brien, Catherine: 9510, 1165, 9430
O'Brien, Kelly: 2200
Obuchi, shuichi: 9130
Obuchon, Kathleen: 3245
Ochi, Akira: 9280
O'Connell, Carrie: 2485
O'Connell, Megan E.: 9075, 9380, 9500, 9510
O'Connor, Deborah: 3385, 9155, 9380, 9510
O'Connor, Heidi L.: 3285, 4150
O'Connor, Maureen: 9510
O'Connor, Maureen K.: 9515
O'Connor, Melissa: 4075, 9180
O'Connor, Melissa L.: 9020, 9515
O'Connor, Nina: 9385
O'Conor, Rachel: 9200
Odd, Kaleena: 9140, 9470
Odden, Michelle C.: 2020, 2115, 3285, 3335, 9025
Oddsson, Lars: 9500
Oden, Kyndal: 9510
O'Donnell, Arden E.: 2010
Oestreich, Alicia: 2180
Ofotokun, Igho: 2315
Ogawa, Elisa: 9500
Ogino, Ryogo: 9035, 9245, 9335, 9460, 9105, 9420
Ogle, Destiny: 3530, 2195
Oh, Anna: 9500
Oh, Esther: 1327, 9055
Oh, Gahee: 9295, 9500
Oh, Jean: 9500
Oh, Jeewon: 3185
Oh, Patricia A.: 3210, 3630, 4495
Oh, Sarah: 9130
Oh, Seung-eun: 9035
Oh, Yun taek: 9505
O'Hara, Courtney: 9010
O'Hara, Ruth: 4535
O'Hern, Karen: 9500
Ohlheiser, Alis: 9505
Ojelabi, Olanike: 9500
Okada, Shinichi: 9500
Okamoto, Shohei: 9435, 3075
Okang, Esther N.: 9505
Okata, Junichiro: 9035, 9245
O'Keefe, Hannah: 3090
Okoye, Safiyyah M.: 1130, 4070, 4180
Okubo, Ryo: 9180
Oladimeji, Abolade: 9440
Olatunde, Kolade: 9325
Olazarán, Javier: 1005, 2166, 3050
Olby, Natasha: 9505
O'Leary, Mary Pat: 9510
Oliva, Anthony A.: 9070, 9090
Olivari, Benjamin S.: 2180, 2375, 3160, 3330, 9185
Oliveira, Iara: 2090
Oliver, Debra Parker: 9380
Oliver, Haley: 2215, 4305
Oliver, Sloan: 9505
Olivieri-Mui, Brianne: 9295, 9500
Olmos, Ricardo: 2166
Olness, Gloria S.: 1410
Olopoenia, Abisola: 1345
Olsen, Bonnie J.: 9050
Olson, Janet: 9510
Olson, Jennifer M.: 4080
Olson, KayLoni: 9430
Olumi, Shayan: 9105
Omobowale, Olubukola C.: 3405, 9400
O'Neal, Katie: 9410
ONeill, Liam: 1055
O'Neill, Lisa: 1250
O'Neill, AnneMarie S.: 9380, 9510, 1265, 9390
O'Neill, Liam: 9500, 9515
Ong, Anthony D.: 1065, 2305, 4525, 2170, 9400
Onglao, Peter Nicholas S.: 9090
Opoku-Agyeman, William: 9250, 9420
Opsasnick, Lauren: 9035, 9200
Opur, Fredrika A.: 4215
Oravecz, Zita: 1165, 9030, 9065, 9070
O'Reilly, Maria: 2095
Orewa, Gregory: 4355, 9235
Ornstein, Katherine: 1450, 3075, 3475, 4410, 9115, 9245
O'Rourke, Hannah: 3605

Orsega-Smith, Elizabeth: 9505
Orsulic-Jeras, Silvia: 1005, 9335, 9215
Orwig, Denise: 2115, 9025, 9210
Orwoll, Eric S.: 9505
Ory, Marcia G.: 1010, 9455
Osborne, John: 4590
Osborne, Nicholas: 9515
O'Shea, Brendan Q.: 1145, 9160
Osinska, Magdalena: 2045
Osman, Abdul: 9505
Ostbye, Truls: 3055, 9065
Osteen, Kathryn: 1210
O'Sullivan, David: 2060
O'Sullivan, Roger: 4570
Osuna, Margarita M.: 3520, 4100, 4635
Osuoha, Ezinwa: 9505, 3545, 4600
Oswald, Frank: 4060, 4385
Otero, Marcela C.: 1110
Ott, Corilyn: 1270
Ottenbacher, Kenneth: 1230, 9515
Ottosen, Terri: 3325
Oudshoorn, Abe: 3590
Overton, Julie: 4230, 9395
Owen, Kylie: 1090
Owens, Eric: 4605
Owens, Jesse B.: 3415
Owens, Shenan: 9435
Owino, Jonix: 2400
Owsiany, Montgomery T.: 4365
Oya, Akari: 4320
Oya, Angela: 9515
Oyama, Asuka: 9215
Öylü, Gülin: 1150, 3175
Ozair, Aisha: 9045
Ozturk, Sirinnaz: 3590

**P**

P de Paula Couto, Maria Clara: 2220
Pérez, Laura M.: 3460
Paasche-Orlow, Sara: 4135
Pabiniak, Chester: 1005
Pabst, Keegan: 1240
Pace, Garrett T.: 9040, 9460
Pachana, Nancy A.: 9065
Pacheco, Kelly: 4605, 9505
Pachucki, Mark: 9150
Pack, Chang-Gi: 9130
Padala, Kalpana P.: 9165, 9180
Padala, Prasad R.: 9165, 9180
Pagan, Mary: 3555, 9420
Page, Kyle S.: 4415
Page, David: 3055
Page, Fannisha: 2215, 4305
Page, Lindsey: 9510
Pagedar, Nitin A.: 1090
Pahlen, Shandell: 9135, 9510
Pai, Manacy: 9020
Painter, Mairead: 3505
Pajewski, Nicholas: 1305, 9085, 9295
Pajski, Megan L.: 9505
Pak, Victoria: 4340
Palamar, Joseph J.: 9505
Palaniappan, Latha: 1195
Pallaki, muralidhar: 9240
Palmarini, Nicola: 9165
Palmer, Janice L.: 3590, 9365
Palmer, Jennifer: 4135
Palmer, Josh: 9075
Palmer, Lauren: 2090, 3275, 4270
Palmertree, Stephanie: 3325
Palms, Jordan D.: 3190
Palnati, Madhuri: 9070
Palta, Mari: 1130
Pamplin, John: 2210
Pan, Nicol: 4360
Pan, Wei: 4330
Pan, Xi: 2435, 9035, 4395
Pan, Zhaoxing: 9515
Panapasa, Sela V.: 9045
Panda, Oishika: 9505
Pandit, Sonia G.: 2010, 3245
Pandya, Naushira: 9170, 9300, 9355
Pangle, Amanda: 9045
Paniagua, Teresa: 9155, 9360
Pankow, James S.: 2085
Pantalon, Michael V.: 2070
Pantel, Johannes: 4385
Panza, Francesco: 2085, 4075
Papas, Athena: 2455
Papazyan, Anna: 9265
Parajuli, Jyotsana: 2180
Parashar, Aditi: 9045
Parchman, Michael: 9405
Pardasani, Manoj: 3115, 4255
Pareja, Kristeen A.: 9505
Parham, Lori: 3210
Parisi, Jeanine M.: 2235
Park, Chan: 9375
Park, Chan Mi: 2205, 9000, 9045
Park, Cystal: 1500
Park, Geunhye: 1360, 2325, 9175
Park, Grace: 9295
Park, Hannah E.: 9140
Park, Hayoung: 9080, 9465
Park, Hyeyoung: 3230
Park, Jeongsoo: 1105, 2220
Park, Joon-Hyuk: 1100, 9510, 9515
Park, Junha: 9390
Park, Juyoung: 4115, 3150
Park, Linda: 9460
Park, Min Kyoung: 9500
Park, Min Sook: 9500
Park, Min-Kyoung: 4125
Park, Moonkyoung: 9130
Park, Nan Sook: 3150, 9140, 9455
Park, Pil: 9160
Park, Seho: 9515, 9505
Park, Seunghee: 9235
Park, So Yeon: 9270
Park, Sojung: 9040, 9375, 9515
Park, Soojin: 4255
Park, Sung S.: 2135, 2240
Park, Sung-Ji: 4330
Park, Taeyoung: 9265
Park, Yaegin: 4595
Park, Young Shin: 9030
Parker, Elizabeth: 9500
Parker, Lauren: 1325, 1420, 3010, 3320, 4210
Parker, Laurie: 9095
Parker, Patricia A.: 9115
Parker, Susan W.: 4265
Parker Oliver, Debra: 4145
Parmar, Jasneet D.: 3250, 4225, 4465
Parmelee, Patricia A.: 9410, 9515
Parodi, Jose F.: 3630
Parsey, Carolyn: 1020
Parsons, Kelly: 1125, 2405
Parsons, Douglas S.: 9500
Parsons, Matthew: 3625
Parsons, Pamela: 4360, 9080
Parti, Katalin: 9230
Pasatiempo, Ana maria: 9500
Pascual-Leone, Alvaro: 1280, 2130, 9505
Paskett, Electra: 3130
Pasquini, Giancarlo: 1035, 1050, 2450, 3520
Pastor, Brian M.: 1375, 9210
Pastore, Valentina: 2085
Patel, Janvi: 4595
Patel, Krishna: 1130
Patel, Kushang: 3065
Patel, Monika: 9500
Patel, Rajiv: 9505
Patel, Roshni: 3160, 3235
Patorno, Elisabetta: 2290
Patskanick, Taylor: 1315, 2400, 3045
Patterson, Deijah: 9010
Patterson, Jodi: 9460
Pattin, Anthony: 9165
Pattin, Carla: 9165
Patton, J.R.: 9010, 9215
Patton, Susan K.: 9325
Paudel, Anju: 1270, 3180
Paul, Sudeshna: 4340
Pauley, Chris R.: 9500
Paulmann, Volker: 9210
Paulo, João: 4510
Paulson, Daniel: 9075, 9135
Paulson, Sally: 9070, 9130, 9390, 9420
Pauly, Mark V.: 2390
Pauly, Theresa: 3085, 4560, 9175, 9510
Paumer, Linda: 9215
Pavez-Fox, Melissa: 4390
Pavlovic, Jelena M.: 9405
Pavlovic, Jelena: 9510
Paxton, Raheem: 3405
Payne, Jodi: 9160
Payne, Kristen: 9265

Peacock, Shelley: 9510
Pearman, Ann: 1080
Pearsall, Vesselina: 2440
Pearson, Caroline: 4355
Pearson, Karen: 3285
Peck, Samantha: 9350
Peckham, Allie: 1080, 2040, 4045, 4350
Peddada, Shyamal: 9505
Pedersen, Maja: 9250
Pedersen, Megan: 9020, 9515, 1095
Pedersen, Nancy L.: 2435
Pedroso Chaparro, María del Sequeros: 1005, 3050, 4085, 4110
Pedroso-Chaparro, María del Sequeros: 3580
Pedroza, Jesica M.: 9045
Pedtke, Joseph H.: 4265
Peeples, Amanda: 2275
Peerman, Carey H.: 3440
Pehlivanoglu, Didem: 9505
Pei, Yaolin: 1115, 3110
Pellecer, Daniel: 9215
Pellegrini, Matteo: 1015
Pelletier, Chelsea: 9505
Pelton, Leslie: 3005, 3165, 4230
Peltonen, Markku: 1260
Pena, Maria C.: 9075, 9080
Penate, M. Christina: 9050
Pence, Brandt D.: 3270
Pendergast, Jacquelyn: 2275, 4415, 3220, 9500
Pendleton, Chandler: 3610
Penfold, Robert B.: 1005, 2015
Peng, Changmin: 3390, 4555
Peng, Man-Man: 3470
Peng, Siyun: 2480, 4555
Peng, Yisheng: 3380
Penn, Alyssa: 9280
Pennell, Michael: 3130
Penney, Lauren S.: 3395
Penning, Margaret J.: 1395
Peppard, Paul E.: 1130, 3590
Peralta, Carmen: 2020, 3285
Perek-Białas, Jolanta: 4005
Perera, Gayani: 2410
Perera, Subashan: 2105, 4520
Perez, Eliany: 9505
Perez, G. Adriana Adriana: 2200, 2295, 4035, 4445, 9385
Perez, Hector: 9060
Perez, Janelle: 2140, 3325
Perez, Sasha: 4410, 4450
Perez, Sophia G.: 9155, 9250
Perez, Viviana I.: 4315
Perion, Jennifer: 9225
Perissinotto, Carla: 1020, 2325
Perkhounkova, Yelena: 3565
Perkins, Molly M.: 2330, 3090, 3535, 3645, 9275, 9425
Perkinson, Margaret A.: 1265
Perlin, Joshua D.: 9430
Perls, Thomas: 2015, 2110, 4585, 9100, 4275
Perre, Taylor: 9350
Perrin, Nancy: 2235, 4525
Perry, Brea: 2480, 4555
Perry, Tam E.: 1020, 2375, 3155, 3455, 3545
Pervratil, Michael: 2225
Perweiler, Elyse: 9510
Perzynski, Adam T.: 1230
Pesja, Laura: 9215
Pestine-Stevens, Althea: 1470, 4495
Pestritto, Taylor N.: 9015
Peters, Denise: 9250
Peters-Beumer, Lisa: 1325, 2470
Petersen, Berkley J.: 9075
Petersen, Curtis L.: 1200, 9030, 9455
Petersen, Janne: 1095, 9085
Petersen, Megan: 9230
Peters-Nehrenheim, Viktoria: 2510
Peterson, Andrew: 1180
Peterson, Colleen M.: 1005, 3185, 4305, 4350
Peterson, Elizabeth: 9255
Peterson, Eric D.: 4145
Peterson, Jennifer R.: 9335
Peterson, Lindsay J.: 2260, 2460, 3080, 4570, 9345
Peterson, Mark: 2360, 4450, 9505, 9510
Peterson, Natasha L.: 1205, 2265, 9035
Peterson, Roberta: 2040
Petkus, Andrew J.: 3310
Petri, Florence: 3365
Petrie, Carol: 3420
Petros, Ryan: 1425, 2295, 2385, 4035, 4290, 4445
Pettee Gabriel, Kelley: 9420, 9505
Pettigrew, Corinne: 1370
Pevnick, Joshua M.: 1390, 9095
Pfoh, Elizabeth R.: 1230, 4430
Pfund, Gabrielle N.: 9500, 2305
Pham, K. Linh: 4595
Pham, Van: 3030
Pharr, Jennifer: 3570
Phelan, Elizabeth A.: 9510
Philip, Serrina: 9060, 9130
Phillips, Dianna: 4420
Phillips, Farya: 9460
Phillips, Judith Robertson R.: 2050, 4145, 4570
Phillips, Karon: 2460
Phillips, Linda R.: 9510
Phillips, Olive A.: 9165
Phillips, Stuart: 9420
Phillipson, Christopher: 9510
Phinney, Alison: 3180
Phyo, Aung Zaw Zaw: 9025
Piatt, Gretchen: 3550
Piazza, Jennifer R.: 2310, 3520
Picavet, Susan: 2130, 4435
Picchiello, Matthew: 9515
Picchiello, Matthew C.: 9500, 9080
Piccinin, Andrea M.: 9130, 9335, 9505
Picella, David V.: 9300
Pickering, Carolyn E.: 9070
Pidgeon, Dawna M.: 3420
Piechota, Amanda: 3125, 9510
Piedra, Lissette M.: 3035
Pieper, Carl: 4115
Pierce, Grey: 3035
Pierpaoli, Christina: 3460
Piersol, Catherine V.: 4455
Pietrobon, Ricardo: 3040
Piette, John D.: 3200
Pifer, Marissa: 9415
Pighin, Melissa: 9120
Pignolo, Robert: 1170
Pike, Christian: 9500
Pike, Kenneth C.: 4165
Pilkington, Bryan: 3250
Pillemer, Karl: 1335, 2070, 3235, 3280, 9265
Pilling, Luke C.: 3320, 9100
Pilonieta, Giovanna: 9185, 1380
Pimentel, Camilla B.: 1075, 1455
Pineda, Lauren: 9505
Pinkerton, El: 9050
Pinto, Melissa: 9270
Pinto Taylor, Emily: 3645
Piol, Silvia: 4530
Piper, Rosie: 4167
Pipinos, Iraklis: 9420
Pishko, Claire: 1080, 2325
Pisu, Maria: 1380
Piszker, Walter: 9100
Pitkälä, Kaisu H.: 9405
Pittman, Corrine: 1327
Pittman, Patricia S.: 9290
Pituch, Keenan A.: 4045
Pizzi, Laura: 2360
Plankey, Michael: 2160
Plasencia, Melanie Z.: 2400
Plassman, Brenda: 2280, 3610
Platt, Jonathan: 3160
Platt, Michael L.: 9100
Platts, Loretta G.: 3615
Pleet, Anna R.: 9500
Ploch, Emily: 3535
Ploeg, Jenny: 9130
Plotnikov, Egor: 9110, 1240
Plude, Dana: 3170
Plummer, Katherine: 3200
Plunkett, Cindy: 9505, 9275
Pociunaite, Justina: 9140

Podapati, Pallavi: 9220
Poerio, Giulia: 9505
Pohlmann, Theresa: 9210
Pohnert, Anne: 3405
Polekhina, Galina: 9070, 9295
Polenick, Courtney A.: 2305, 4205, 4320, 9155, 9175, 9290
Polivka, Larry: 1075, 4095
Polivka-West, LuMarie: 1075
Polk, Rebecca: 9505
Pollack, Harold: 1395, 4045
Pollard, Cheryl: 3250, 4225
Pollini, Alessandro: 9080
Pollmanns, Claas: 2170
Pollock, Hannah: 3590
Pollock, Kiera: 3570
Polson, Sydney: 9105
Pomerantz, Sherry: 9510
Pomeroy, Mary Lou: 1465
Pomilla, William: 9105
Pond, Brittney L.: 9220
Pongan, Lauren: 1395, 4085
Poobalan, Amudha: 4120
Poole, Meredith: 3015
Poole, Victoria N.: 9140, 9390
Poon, Lydia: 9510
Pope, Caitlin N.: 1125, 2200, 2400
Pope, Natalie E.: 4495
Popejoy, Lori L.: 4475
Popescu, Mihail: 1360
Popham, Lauren E.: 9500
Popov, Vladimir: 9070
Porell, Frank: 9045, 9165, 9245, 9390, 1020, 1270, 2375
Porte, Jennifer L.: 9370
Porter, Kristen e.: 2330
Porter, Laura S.: 3095
Porter, Martha: 3395
Porter, Michelle M.: 2345
Porter Starr, Kathryn N.: 9515, 4120
Portz, Jennifer D.: 9240, 9410
Potempa, Kathleen: 9045
Potnek, Michael F.: 9050
Potnis, Manali: 4510, 9085
Powell, Daniel: 4120
Powell, Danielle: 4190, 9505
Powell, Mebane: 4255
Powell, Ryan: 9450
Powell, Tia: 1005
Powell, Victoria D.: 9410
Power, Melinda: 4605
Powers, Becky: 9295
Powers, James: 1265, 4175
Powers, John David: 9240
Powers, Melissa: 9420
Powers, Sara: 1025, 4085, 9185, 9335
Prabhu, Sharmila: 4565
Prather, Christina: 4605
Pratt, Gelda: 9510
Prendergast, Alice: 1140
Prescott, Carol: 4195, 4345
Presse, Nancy: 1045, 3485
Preuss, Benedikt: 3585
Prevett, Christina: 9420
Price, Carrie: 1327
Price, Debora: 1360
Price, Elvin: 4360, 9145
Price, Pollie: 4540
Prichett, Laura: 2010
Prieto, Lucas: 4180, 4215
Prigerson, Holly: 3260
Primomo, Janet: 9505
Principi, Andrea: 4005
Prinelli, Federica: 1280
Pritchard, Jessica E.: 9130
Prizer, Lindsay: 2505
ProcterGray, Elizabeth: 1100, 2030
Prohaska, Thomas R.: 4570
Promislow, Daniel: 9055
Prophater, Lorna: 9355
Proulx, Christine: 9020
Province, Michael A.: 2020, 4585
Provolo, Natalia: 4630
Pruchno, Rachel: 1320, 2390
Prue-Owens, Kathy: 9420
Pruitt, Christopher: 9515
Prusaczyk, Beth: 1190, 3100
Pryor, Hanna: 1300
Puerto, Geraldine: 9270
Pugalenthi Saravanan, Deepika: 9220
Pugh, Jacqueline A.: 3395
Pugh, Kendra: 9510
Pullen, Carly: 9015
Pung, Meredith A.: 9515
Punksungka, Wonmai: 9380
Purcell, Shaun: 9045
Purol, Mariah F.: 3185
Purser, Gregory: 2355
Purwins, Daniel: 2510
Puterman, Eli: 1165
Puts, Martine: 3565
Pyatt, Tabitha: 4340
Pynoos, Jon: 4230, 9040
Pyrkov, Timothy: 3270

**Q**

Qato, Danya: 1055, 1345
Qato, Dima M.: 3030
Qi, Guo: 9140
Qi, Xiang: 1115, 2280, 3110
Qian, Wenjie: 9500
Qiao, Yujia (Susanna): 3290, 3430, 4395, 4520
Qin, Amy Yongmei: 9085
Qin, Jiyue: 9405
Qin, Min: 9280
Qin, Weidi: 1410, 3340, 3655
Qiu, Fengxian: 4220
Qiu, Jiayuan: 4215
Qiu, Wendy: 9065
Qiu, Xiao: 9165, 9350
Qiushi, Feng: 4590
Qu, Xiaomin: 1115
Quach, Emma: 3275
Quach, Lien T.: 1100, 3030
Qualls, Sara H.: 9160, 9265, 9285, 9290
Quaranta, Nicola: 2085
Quashie, Nekehia T.: 3525, 9505
Quevedo, Alvaro: 9510
Quiñones, Ana: 2480, 3285, 9030, 9200, 9380, 9510
Quigg, Mark S.: 1125
Quillen, Ellen E.: 9085
Quinn, Joseph F.: 3615, 2485
Quinn, Tammie: 9410
Quintanilla Salinas, Isaac: 3175
Qureshi, Muhammad: 4385
Qureshi, Amena: 9220
Qureshi, Mariam: 9210
Röcke, Christina: 4580

**R**

Rabbitt, Patrick: 3175
Rabin, Laura: 4560
Rabinowitz, Jill A.: 4065
Racey, Megan: 9130
Raciti, Audrieanna: 9395
Rackman, Alexander: 3005
Raetz, Jaqueline G.: 3330
Rahimighazikalayeh, Gelareh: 9515
Rahman, Anna: 2125
Rahman, Momotazur: 4270
Rahmani, Amir: 2075, 2100, 9045
Rainbow, Jessica: 9500
Raj, Ken: 3600
Raja, Moonika: 2485
Raji, Mukaila: 1230
Ralston, James: 1005, 2015
Ram, Nilam: 1165, 3085, 3265, 4560
Ramadhani, Widya A.: 2225, 9500
Raman, Rema: 1260
Ramasamy, Ramalakshmi: 4390, 9055, 9070, 9090
Ramel, Alfons: 1045, 3075, 9250
Ramirez, Jessica: 9105, 1005, 1020, 2015
Ramirez, Mildred: 1180
Ramirez Gomez, Liliana A.: 2166
Ramirez-Zohfeld, Vanessa: 9035, 9280
Ramos, Mary Dioise: 9270
Rampanana, Martine: 9055
Ramsay, Jessica E.: 3320
Ramsey, Jennifer: 9315
Ramsey-Klawsnik, Holly: 1330
Randall, Kevin: 1505
Randall, Phillip M.: 1160

Rangel, Maria O.: 9295
Ranjo, Terrence: 9170
Ranta, Tuula: 4145
Rantz, Marilyn: 4475
Rao, Rani: 3095
Rapoport, Mark: 9390
Rasekaba, Tshepo: 1090
Raso, Jim: 9135
Rasoal, Dara: 3615
Ratanatharathorn, Andrew: 3595
Rathbun, Alan: 2115
Ratliff, Kathryn B.: 1330
Ratnayake, Maggie: 9040
Rattinger, Gail B.: 9085
Rauch, Lisa: 3255
Raue, Martina: 1315
Rauer, Amy: 3125, 3200
Rauktis, Mary: 2375
Raveis, Victoria H.: 3395, 4150
Rawal, Kadambari: 9330
Rawani, David: 9135
Rawlins, Jessica: 9500
Ray, Midge N.: 1075, 3050
Ray-Degges, Susan: 9245
Raymo, James: 1140, 4125
Razzaghi, Hilda: 2090
Read, Carolyn A.: 9220
Ready, Rebecca E.: 9235
Rebeck, G. William: 9505
Reblin, Maija: 9515, 3375, 9270
Rebok, George W.: 3175, 4025, 4167, 4345, 1155
Reckrey, Jennifer: 2320, 4410
Rector, Chase: 9515
Redae, Yonas: 9105
Redd, Doug: 9500
Redfern, Mark: 2105
Redlich, Kim: 4575
Redline, Susan: 3295, 9045
Redwine, Laura S.: 9515
Reed, Ashley: 1400
Reed, Lai: 3515
Reed, Marilyn: 1327
Reed, Nicholas: 1130, 1327, 2145, 4030, 4040, 4055, 9030
Reel, Candice D.: 4370, 9265, 9275, 9515
Reeve, Emily: 3565
Reeves, Alex: 4135, 9465
Reeves, Alexis: 2210
Regan, Carol: 1245
Regier, Natalie G.: 2235, 4110
Register, Thomas: 9085
Reich, Amanda: 3260
Reid, Carolina: 2010
Reid, M. Cary: 4605
Reid, Christopher M.: 4070, 9025
Reid, M. Carrington: 9265, 9410, 9505
Rein, David B.: 4355
Reinhard, Susan C.: 3165
Reinhardt, Joann P.: 2445, 9150, 9160
Reinhardt, Michael: 9500
Reis, Logan: 9145
Reistetter, Timothy: 2145
Reiter, Ann: 9500
Reither, Eric N.: 1130
Rejeski, W. Jack Jack: 1305, 3065
Remillard, Elena: 3650
Ren, Dianxu: 9065
Rencher, Patricia A.: 4230
Rentscher, Kelly E.: 9510
Reohr, Paige: 1135, 4055
Requena, Carmen: 9220
Reschke-Hernandez, Alaine E.: 2385, 9170, 9065, 9070, 9160, 9510
Resnick, Susan M.: 2105, 2285, 4040, 4065
Resnick, Barbara: 1270, 2010, 2080, 2315, 3180, 3315, 3445, 3500, 3625, 9210, 9355
Restorick Roberts, Amy: 1445
Restrepo, Marcos: 9160
Retalic, Tammy: 9395
Reuben, David: 1005
Reuland, Melissa: 3245, 4340, 4450
Revell, Andrew J.: 1375, 9515
Reyes, Laurent: 3545, 4345
Reyes-Farias, David: 2010
Reynolds, Christina: 4065
Reynolds, Addam S.: 4345
Reynolds, Chandra A.: 4420, 9135, 9360, 9510
Reynolds, Courtney: 3035
Reynolds, John: 9135, 9140
Reznik, Alexander: 3345
Rhee, Greg: 9500
Rhim, Hye Chang: 9000, 9045
Rhodes, Anne: 4240
Rhodes, Annie: 9010, 9160, 9175, 9185
Rhodes, J. David: 9265, 9290
Rhodes, Shana A.: 4175
Rhodes, Shanae: 3215
Rhodus, Elizabeth: 1125, 2015, 2405
Riaz, Muhammed: 9380
Ribeiro, Fabiana: 4195
Ribeiro, Oscar: 9420, 9500
Ribeiro-Gonçalves, José Alberto: 9430
Richards, lorie: 4540
Richardson, Stephanie: 3465
Richardson, Arlan: 1440
Richardson, Cassandra: 9440
Richardson, Julie: 9420, 9510
Richardson, Leah: 9515
Richardson, Michaela: 4190
Richardson, Virgina: 2235
Richert, Mallory: 9150, 9355
Richman, Alice R.: 9245
Richmond-Rakerd, Leah: 2180
Ricke, William A.: 9295
Riegel, Barbara: 3450
Riester, Melissa R.: 9395, 9265, 1345, 3010
Riley, Kiernan: 2080
Rillamas-Sun, Eileen: 4165
Rimm, Eric B.: 3595
Rincker, Jamie: 9515
Ring, Lauren: 4000
Ringman, John: 9515
Riordan, Ruben: 9500
Ripp, Jonathan: 3475
Riquino, Michael: 9515
Risbud, Rashmi: 3200, 9275
Rishel, Cindy: 9500
Ristow, Michael: 9510
Ritchie, Christine: 3095, 4410
Ritter, Andre V.: 4150
Ritterband, Lee M.: 1125
Ritzel, Rodney: 9505
Rivera, Eleanor: 3380
Rivera, Miguel: 9505, 1060, 2410, 9155
Rivero-Co, Camille: 9090
Rizer, Sandra: 2015
Rizzo, Matthew: 1485
RIZZO, VICTORIA: 9500
Rizzuto, Debora: 9025
Roach, Ashley: 3605
Roach, Lauren: 2400
Roache, Tavia: 9510
Roan, Carol: 2335
Roark, Erin: 9280, 9510
Robbins, Paul: 9105
Robbins, Ronna: 9045, 9100, 9320
Robek, Michael D.: 9160
Roberge, Pasquale: 9365
Roberson-Steele, Joyce: 9165
Roberto, Karen A.: 1030, 1330
Roberts, Amy Restorick: 1445, 3580
Roberts, Andrea L.: 3595
Roberts, Angela C.: 9280, 9510, 9515
Roberts, Brent W.: 1035
Roberts, Glenda: 4235
Roberts, Jennifer: 9360
Roberts, Lisa: 9505
Roberts, Max T.: 1130, 9160, 9510
Roberts, Scott: 9370
Roberts, Timothy R.: 9500
Roberts, Tonya J.: 3015
Roberts Davis, Mary C.: 9505
Robertson, Olivia: 9200
Robillard, Julie: 3000
Robin, Rachel: 3230
Robinette, Jennifer: 3310
Robins, Richard W.: 1035, 4515, 1360, 2325, 3645, 4600, 9175
Robinson, Kristen: 9500
Robinson, Michael: 3095

Robinson-Lane, Sheria G.: 3055, 9270, 9505
Robison, Julie: 1060, 3245, 3390, 3395, 9505
Robles, Dagoberto: 1250
Robles, David J.: 3435, 9305, 9185, 9500
Rochford, Grace: 4190
Rockwood, Kenneth: 9095
Rocque, Gabrielle B.: 1380, 9025
Rodakowski, Juleen: 9065, 9390
Rodnyansky, Seva: 9515
Rodriguez, Diana: 4025
Rodriguez, Rachel L.: 1110
Rodriguez-Diaz, Maria Elena: 9515, 9510
Rodriguez-Mañas, Leocadio: 1280
Rodriguez-Morales, Grisel: 3005
Rodriguez-Stanley, Jacqueline: 3550
Roecke, Christina: 4505, 4580
Roehr, Susanne: 1260
Roerig, Monika: 2040, 4350
Roes, Martina: 2065, 2510
Rogalski, Emily: 9280, 9510, 9515
Rogers, Anissa: 9210
Rogers, Anita H.: 3605
Rogers, Anna: 4225
Rogers, Wendy A.: 9500, 9505, 1375, 1475, 2225, 2365, 3145, 3170, 3650, 3655, 9210
Rogers-Jarrell, Tia: 9260, 9400
Rogina, Blanka: 1120
Rogozinski, Amos: 9515
Roh, Soonhee: 9190, 9515
Rohner, Shauna L.: 1290, 4430
Rojo-Wissar, Darlynn M.: 1130
Rolland, Yves: 1280
Rollins, Jarod A.: 4510
Romatz, Courtney: 4330
Romero, Camila: 9510
Romero, Lindsey: 3550
Romero Moreno, Rosa: 3050
Rommerskirch-Manietta, Mike: 2510
Romo, Rafael: 9500
Ronneberg, Corina: 4545
Rooks, Ronica N.: 2240, 2395, 4540
Roopsawang, Inthira: 2030
Roos, Charlotte V.: 9505
Root, Eve Z.: 9155, 9370
Roque, Nelson A.: 9335, 2020, 4470
Rorai, Vanessa: 3155
Rosano, Caterina: 2105, 2195, 3430, 4520, 9505
Rosati, Robert J.: 2055
Roscher, Patrick: 9515
Roscoe, Charlotte: 9090
Rose, Andrew: 4135, 9465
Rose, Jennifer: 9410
Rose, Karen: 2410, 3365, 4080
Rose, Katherine: 9220, 3035
Rose, Savannah: 2260
Rose, Tara: 9165, 9505
Rosebush, Christina: 1005, 4130, 9345
Rosen, Tony: 4590, 1285, 2070
Rosenberg, Carl: 9500
Rosenberg, Dawn A.: 4390
Rosenberg, Dori E.: 3300
Rosenberg, Walter: 3005
Rosenkranz, Susan J.: 3375
Rosich, Rosellen M.: 1340, 2380
Rosland, Ann-Marie: 1285, 4085
Ross, Brielle N.: 3655
Ross, Chris: 9515
Ross, G. Webster: 3415
Ross, Lesley: 9140
Ross, Leslie: 1060, 9220
Ross, Sarah E.: 4240
Rossler, Aleatha M.: 9510, 9515
Rosso, Andrea L.: 9505, 1095, 2105, 2195, 3160, 4345, 4520
Rostowsky, Kenneth A.: 3435, 9055, 9095, 9305, 4185, 9190
Roth, Adam R.: 2480, 4555
Roth, David L.: 2465, 3235, 3290, 4025, 9265, 9290
Roth, Robert: 3000
Rothberg, Michael: 4430
Rothermund, Klaus: 2220
Rothgang, Heinz: 3585
Rotondi, Armando: 9510
Rouch, Stephanie: 4455, 9000
Roulston, Kathryn: 1090
Rouse, Hillary J.: 9135
Rouse, Paul: 3625
Rousseau, Joël: 9085
Rousseau, Julie: 1340, 4175
Rowan, Julia: 9500
Rowan, Noell L.: 2355
Rowe, Meredeth: 1155
Rowe, Theresa: 9065
Rowles, Graham: 3210, 3540, 9510
Roy, Indrakshi: 2410
Rozario, Philip A.: 2460, 4290
Ruan, Ling: 9110
Rubeo, Chris: 4140, 4270
Rubin, Leah: 2160
Rubinsky, Anna: 2020, 3285
Rubtsova, Anna: 2315, 3645
Ruby, Joseph: 1160
Rudnik, John: 1315, 3045
Rudolph, James L.: 1075, 2410, 9325, 9450
Rudy, Ellen T.: 9505
Ruggero, Camilo: 1430, 9060, 9505, 9100
Ruggiero, Laurie: 9505
Ruissen, Geralyn: 1165
Ruiz, Jorge: 9090, 9505
Rule, Payton: 9080
Rumble, Deanna: 9410
Runels, Tessa: 2320
Runge, Katharina: 1200
Runge, Katherine: 2010
Ruopp, Marcus D.: 9500
Rupasinghe, Viraji: 3440
Ruppar, Todd: 3285, 9350
Rurka, Marissa: 4425
Rusch, Amber V.: 9020
Rush, Jonathan: 2310, 3520
Russ, Tom C.: 2435, 9190
Russell, Wynfred: 4130
Russell, Dan: 9515
Russell, David: 1345
Russell, Katharine: 9505
Russell, Sally: 9145
Russell, Wynfred: 4210
Russomanno, Vince: 9320
Rutenberg, Andrew: 9095
Rutherford, Megan: 9295
Ryan, Cara: 4320
Ryan, Carrie: 1460
Ryan, Gery W.: 9440
Ryan, Joanne: 4395, 4550, 9025, 9065, 9070, 9295, 9390
Ryan, Lindsay: 1215, 4320
Rybarczyk, Bruce D.: 4370
Ryff, Carol D.: 3590
Rymaszewska, Joanna: 3585
Ryou, Yeon Ji: 9515
Ryu, Ahyun: 9130
Ryu, ByeongJu: 9515
Ryu, Euijung: 9510
Ryu, Gi Wook: 9030, 9505
Rzhestsky, Andrey: 4065, 9045

S

S. Roe, Lauren: 9505
Saadeh, Marguerita: 1280, 4435
Saathoff, Elizabeth: 4200
Saavedra, Joey M.: 9505
Sabat, Steven R.: 3540
Sabatini, Serena: 9020
Sabbagh, Marwan: 2200
Sabih, Asma: 9160
Sacco, Lawrence B.: 3615
Sachs, Greg: 9505
Sachs-Ericsson, Natalie: 3595, 9510, 9515
Sacker, Amanda: 9145
Saczynski, Jane: 9030
Sadarangani, Tina: 1325, 1420, 3260
Sadeq, Nasreen A.: 9125, 9215
Sadler, Tonie E.: 1395, 4045
Saenz, Joseph: 4460, 9510

Safayan, Luke: 9105
Safren, Steven A.: 3645
Sahay, Amar: 2245
Sahni, Shivani: 9365, 9510
Sahoo, Shalini: 9380, 9500
Sain, Debaleena: 4345
Sainz, Mayra: 4135, 4340
Saito, Tami: 9135, 9340
Saito, Yasuhiko: 9515
Sajnani, Nisha: 4530
Sajwani, Zara: 9025
Sakai, Akiko: 9105
Sakai, Erin Y.: 9370
Sakai, Ikuko: 9300
Sakakibara, Brodie M.: 9505
Sakib, Nazmus: 3080, 4570
Sakka, Mariko: 3375
Salant, Rebecca: 9510, 9515, 9230
Salazar, Laura: 3640
Saldana, Santiago: 9250
Saleh, Nazaneen D.: 9105
Salem, Yasser: 9420
Salinas-Rodriguez, Aaron: 1280
Salisu, Margaret: 3550
Salley, Elizabeth: 9510, 9515
Salmon, Adam: 2110
Saltzman, Barbara: 9225
Saltzman, Russell G.: 9505
Salwi, Shreya M.: 9155, 9175
Samper-Ternent, Rafael: 2145, 9200
Sampson, Laura: 3595
Samuel, Laura J.: 4180, 4525
Samuel, Preethy: 1395
Samus, Quincy M.: 2360, 3245, 4340, 4450
San Souci, Lance: 2015
Sanborne, Erika: 9455
Sanchez-Birkhead, Ana: 9510
Sanchez-Reilly, Sandra: 9160, 4390
Sanderman, Robbert: 9500
Sanders, Edie C.: 2225, 9135
Sanders, Joohee I.: 9420
Sanders, Lisbeth: 9335
Sanders, Margaret: 2315, 4325
Sandhu, Robina: 9155, 9180, 2390
Saney, Danielle: 9080
Sanford, Claudia: 3455, 3545
Sanford, Jon a.: 3650
Sanon, Martine: 4450
Sansone, Katelyn: 9500
Santana, Leticia: 3005
Santana, Sebastian: 1495, 9190, 2020, 4585, 9025
Santiago, Gisselle: 9505
Santo, Jonathan: 9140
Santora, Kenneth: 3020, 3195
Santos, Alexis R.: 3550, 9245
Santos, Alix Jean: 9090
Santos, Camila M.: 9365
Santos, Flavia H.: 2350
Santos, Haylie: 9510, 9515
Santos, Tricia Olea: 9500
Sappington, Erica: 9510, 9160
Sapra, Mamta: 1410
Saquib, Nazmus: 3130
Saragosa, Marianne: 2040
Sarangi, Srikant: 9500
Sardone, Rodolfo: 2085
Sargent, Lana: 4360, 9145
Sarid, Orly: 9500
Sariol, Carlos A.: 4390
Sarkar, Neil: 1055
Sarma, Sisira: 4365
Sasaki, Nanayo: 9300
Sasser, Jennifer (Jenny) R.: 2050
Satizabal, Claudia: 9065
Satyadev, Nihal: 9220, 9280
Saudagaran, Maryam: 9505, 2300, 9125
Saunders, Michele J.: 2280, 2455
Saunders, Pamela A.: 1235, 3385, 4575
Savage, Amber M.: 3440
Savell, Anita D.: 1110
Saville, Neika: 1340, 4175
Savla, Tina: 1030
Savla, Jyoti: 1030, 1330, 1410, 9515
Sawaki, Keisuke: 9300
Saxena, Saket: 9130
Sayer, Janis: 3005
Sazonov, Edward: 3405
Sborgia, Giancarlo: 2085
Scanlan, Madison: 9505
Scarpa, Philip: 4390
Scatliffe, Terika: 4110, 4200
Sceppa, Carmen C.: 4090
Schaap, Laura: 2130, 4435
Schaefer, Stacey M.: 1065
Schaefer, Sydney: 9340, 2175, 9175, 9360, 9455, 9460
Schafer, Donna: 1160
Schafer, Ellen: 9040
Schafer, Marissa J.: 2440
Schafer, Markus: 1150, 4125
Schaffer, Rachel: 1025, 4085, 9185
Schall, Arthur: 4385
Schalm, Corinne: 3440
Schempp, Tim: 9070
Schenker, Lena-Emilia: 4630, 9510
Schepens Niemiec, Stacey L.: 1125, 4075
Scher, Clara: 4495
Scher, Clara J.: 9360
Schiaffino, Melody K.: 9070
Schicker, Teresa: 9215
Schiller, Martha: 1400
Schilling, Oliver K.: 3085, 3265, 4560, 9075
Schilz, Matthew: 3115, 3560, 9005
Schlegel, Emma C.: 4080
Schlener, Susan D.: 2020
Schlesinger, Cynthia: 9250
Schlesinger, Jennifer: 1060, 9065, 9070
Schmader, Kenneth: 3095
Schmalzle, Sarah: 9500
Schmid, Arlene: 9410
Schmidt, Steven M.: 4060
Schmidt, Julia: 9505
Schmidt, Jurgen C.: 2060
Schmidt, Steven M.: 4060, 9105
Schmitz, Kathryn: 9250
Schneider, Emily: 3275
Schneider, Haley: 4200
Schneider, Lon: 1125
Schneider, Sue: 9035
Schnipper, Jeffrey L.: 1390
Schoenberg, Nancy: 1125
Schoenburg, Racheli: 9295
Schofield, Katherine: 9345
Schonberg, Mara: 3130, 9505
Schousboe, John T.: 9030
Schrack, Jennifer A.: 4030, 9510, 1015, 2105, 2285, 3290, 3430, 4025, 4040, 4065
Schreurs, Bernard G.: 9510
Schroepfer, Tracy: 1240
Schuler, Brittany: 4245
Schulman, Ivonne H.: 9505
Schultz, Amy A.: 3590
Schulz, Richard: 3010, 4085, 9290
Schumacher, Benjamin T.: 3300, 4070
Schumacher, John: 2345, 9210
Schupf, Nicole: 3320, 4585, 2325, 3145, 9010, 9505
Schutte, Debra: 9190
Schutter, Natasja: 1345
Schutz, Vivian: 2100
Schwab-Reese, Laura M.: 1285
Schwander, Karen: 9025
Schwank, Alexander: 9130, 9160, 9245
Schwartz, H. Andrew: 1050
Schwartz, Lindsay: 1220, 3325
Schwartz, Mark: 2280
Schwartz, Peter: 9505
Schwartz, Sara: 1020
Schwartz, Todd: 3605
Schwartzman, Luisa: 9505
Schweizer, Sara: 4535
Schwenk, Michael: 4115
Sciarratta, Lena: 1130
Sciegaj, Mark: 3015
Scior, Katrina: 4610
Scmidt, Andrea E.: 3585
Scogin, Forrest: 9370, 9160, 9395
Scott, Mallary: 9245
Scott, Rachel K.: 9260, 9315

Scott, Stacey: 1035, 1050, 1295, 2450, 3520, 4125, 4505, 9505
Scott, Susan C.: 3485
Scott, Theresa L.: 9065
Scrivano, Rachel: 9260
Scrivano, Rachel M.: 1190, 3560
Seabury, Seth: 4455
Seals, Douglas R.: 9105, 9305
Seaman, Aaron T.: 1090
Sebastiani, Paola: 2015, 2110, 4585, 9100
Sedivy, John: 4510
Seeher, katrin: 4180
Seeley, Janet: 2330
Seeman, Teresa E.: 9505, 9510
Sefcik, Justine S.: 2295, 2385, 4445, 4595
Seferian, Ed G.: 1390
Sefilyan, Ester: 9045
Segal, Daniel L.: 9415
Segal-Gidan, Freddi: 9050
Segel-Karpas, Dikla: 9200, 9435
Segelman, Micah: 2090
Segerstrom, Suzanne C.: 9410
Segev, Dorry L.: 4070, 4395
Segosebe, Kebafe: 9010
Segrin, Chris: 9115
Seguin, Anna: 9505
Seguin, Emily: 9505
Sehgal, Raghav: 1015, 3435, 9515
Sehgal, Sonia: 1340, 4175
Seibert, Kathrin: 3585
Seidl, Kristin: 9320, 4255
Sejdic, Ervin: 2105, 9505, 9105
Sell, Christian: 4510, 9085
Sellner, Joshua: 9510, 9160
Sellon, Alicia: 9500
Semco, Robert: 3260
Semenoff, Nadia: 9335
Senczyszyn, Adrianna: 3585
Sengupta, Manisha: 1325, 9355
Senko, Enejda: 2015
Seo, Chang Hyun: 9280
Seo, Jisu: 9130
Seo, Shinae: 9065
Seo, Young Sik: 9270
Seoh, Thomas C.: 3135
Seong, Hohyun: 9505, 9295
Serpas, Dylan G.: 9360
Serra, Monica C.: 9045, 9100, 9130, 9160, 9320
Serrano, Diandra: 3105
Seshadri, Sudha: 9085
Sesker, Amanda A.: 9515, 3185, 9155
Sessanna, Loralee: 9270, 9505, 1340, 4240, 9500
Sewell, Carol: 9170
Sexson, Kathryn: 3555
Shackleford, Tyra: 9045
Shade, Marcia: 9080, 9175, 9470
Shadmi, Efrat: 1095, 9390
Shadovitz, Sydney S.: 3515
Shadyab, Aladdin: 3130
shafiee, Neda: 9055
Shah, Avani A.: 9370
Shah, Bimal: 9515
Shah, Raj C.: 9505, 2495, 3005, 9065, 9070, 9390
Shah, Shailee: 9035
Shah, Sheetal: 9510
Shaid, Elizabeth C.: 2390
Shair, Sarah: 9155, 9400
Shakya, Shamatree: 9510
Shalaby, Alaa: 9510
Shan, Liang: 1380
Shan, Mu: 9500
Shang, Ying: 9065
Shao, Qing: 9070, 9500
Shapiro, Jasmine: 4225
Shapiro, Lindsey S.: 4355
Shardell, Michelle: 1015, 2115, 2285, 4160
Sharieff, Shanaz: 4410
Sharifian, Neika: 4580
Sharit, Joseph: 3170
Sharkey, Michelle: 3450
Sharma, Anita: 3345
Sharma, Anjali: 2160
Sharma, Bhavna: 9435
Sharma, Bonita: 9285, 4540
Sharma, Rahul: 4590
Sharma, Rhea: 2360, 9430
Sharma, Sunita: 9100
Sharp, Julia L.: 9290, 9410
Shaw, Benjamin: 3615, 4590, 9165, 9350
Shea, Adam M.: 9015
Sheean, Hope: 4555
Sheehan, Connor: 4635, 9515
Sheehan, Orla: 3565, 4410, 9265, 9290
Sheen, Angelica M.: 3175
Sheets, Debra: 1495, 9190
Sheffler, Julia L.: 3595
Sheffler, Pamela: 1355, 3175
Sheftel, Mara Getz: 2215, 4100
Sheikh, Sophia: 9500
Sheils, Natalie: 9090
Shelley, Mack: 3525, 3590, 4200
Shen, Biyi: 9105
Shen, Dianjun: 9060
Shen, Huei-wern: 9145
Shen, Xia: 3270
Shende, Shraddha: 1435, 3650, 3655, 4190
Shepelak, Zachary: 9065
Shepherd-Banigan, Megan: 4080
Sheppard, Christine: 3455
Sher, Kenneth: 3090
Sherbutt, Kayleigh: 9505
Sherifali, Diana: 9130, 1215
Sherman, Carey: 3515, 9280
Sherman, Ledric D.: 4420, 9165
Sherman, Scott: 9500
Sherman-Wilkins, Kyler J.: 1370
Shervani, Saira: 3000
Shetterly, Susan: 3565
Shewmaker, Peter: 2360, 4270
Shi, Chengjian: 4065, 9045, 9295, 9500
Shi, Xing-ming: 1170, 9105
Shield, Renee: 9515
Shier, Victoria: 4455
Shieu, Bianca: 2140, 3605
Shigemoto, Yuki: 1195
Shikany, James M.: 9505
Shim, Minjung: 4595
Shimabukuro, Michi: 9085
Shimada, Chiho: 9150
Shin, Hye-Won: 9280
Shin, Jinhee: 9065, 9070, 9130
Shin, Jonggak: 3175, 9375, 9500, 9515
Shin, So Young: 9395
Shindyapina, Anastasia: 3270, 4510
Shinohara, Masumi: 3375
Shippee, Tetyana P.: 1275, 4130, 4375, 9345
Shippee-Rice, Raelene: 3255
Shirai, Yumi: 4020
SHIRASAWA, Masakazu: 9500
Shirk, Steven D.: 9510
Shirk, Steven: 9510, 9515
Shiroma, Kristina: 1050, 3145, 9400
Shively, Carol: 9085
Shkirkova, Kristina: 9055
Shochat, Tamar: 9050
Shoemaker-Hunt, Sarah: 9405
Shonde, Oyinkansola: 9505
Shook, Natalie J.: 9500
Shovali, Tamar E.: 3560, 4490
Shrader, Cho-Hee: 3645
Shrestha, Aman: 9500
Shrestha, Priyanka: 9505, 9515
Shrestha, Srijana: 9410, 9510
Shrestha, Srishti: 9070
Shrira, Amit: 9230
Shrout, M. Rosie: 3125, 9515
Shue, Jody: 4230
Shuffler, Jon: 9280
Shuldiner, Jennifer: 1410
Shulman, Lisa M.: 9130, 9240
Shultz, Kenneth: 4345
Shultz, Tanja: 4610
Shulyaev, Ksenya: 1095, 9200, 9390, 9515
Shum, Angie KY: 3095
Shuman, Stephen K.: 3610
Siak, Adam: 9500, 2130, 4435

Siaton, Bernadette C.: 2130, 9330
Sibbald, Shannon: 9345
Sibille, Kimberly T.: 9045
Sicinski, Kamil: 2335
Siden, Hannah E.: 2030
Siedlecki, Karen: 2170
Siegel, Elena: 2425, 9515
Sierra, Felipe: 3135, 4290
Siette, Joyce: 9505
Sigurdardottir, Sigurveig: 1045, 3075, 9250
Sil, Jin: 9435
Silbert, Lisa C.: 3055, 4065
Silva, Caroline: 1490
Silva, Fernanda C.: 9505
Silva, Graziele M.: 9295
Silva, Paula: 9420
Silva, Sara: 9260
Silva, Susan: 9510
Silva, Susan G.: 9395
Silveira, Maria J.: 9410
Silver, Stephanie A.: 1180
Silverman, Micheal A: 9510
Silverstein, Merril: 3225, 3530, 4295, 4460
Silverstein, Nina M.: 1040, 2345, 4490, 9045, 9165, 9170, 9245, 9390
Silverstein, Sydney: 9380
Simelane, Sandile: 4265
Simmons, Janine M.: 2270
Simmons, June: 9045
Simning, Adam: 4240
Simon, Michael: 2045, 4475, 9510
Simon, Melissa A.: 1195, 2060
Simon, Neta: 3065
Simone, Mark J.: 3245, 4290
Simons, Kelsey V.: 1445
Simonsick, Eleanor: 9505
Simonsick, Eleanor M.: 9500, 9505, 2105, 2285, 2315, 3290, 3430, 4040, 4065, 4160
Simpson, Deborah: 9210
Simpson, Elizabeth: 2025, 2095, 2445, 3390, 4275
Simpson, Felicia R.: 4550, 9430
Simpson, Michelle: 9130
Simpson Ballard, Jessica: 9510, 3160
Sin, Nancy L.: 1430, 2170, 9400
Sinclair, Christian T.: 9410
Sindi, Shireen: 3460
Singer, Dianne: 2165
Singer, Rachel F.: 2375
Singh, Amit: 9160
Singh, Tanya: 2400, 9085
Singleton, Jenny: 3650
Singleton, Mekiayla: 9500, 9105
Sion, Katya: 4335
Sioutas, Constantinos: 9055
Sirikun, Jatuporn: 9000
Sirk, Courtney: 9510, 9295
Sitlinger, Andrea: 4115
Siu, Joseph: 9505
Sivanandam, Shalini: 4045
Sizzano, Federico: 3285
Sjöberg, Linnea: 3460
sjögren, karin: 3440
Skanderson, Melissa: 9500
Skarha, Julianne M.: 3080
Skidmore, Elizabeth: 9000
Skipper, Antonius D.: 3640
Skipper, Antonius: 1185, 3640, 4135, 9465
Skoblow, Hanamori F.: 9020
Skolasky, Richard L.: 4110, 4340
Skowronski, Jordan M.: 9150
Skubic, Marjorie: 1360
Skytt, Bernice: 9505
Slack, Penelope: 9500
Slade, Martin D.: 4630
Slaton, Cassie E.: 1030
Slattum, Patricia: 1340
Slaug, Björn: 4060
Slavin, Maggie: 3430
Slavish, Danica: 1430, 9510
Sliwinski, Martin: 1295, 2020, 3520, 4125, 4505, 9130, 9135, 9510
Sloan, Danetta H.: 3245, 4340
Sloan, Frank: 2280
Sloane, Philip D.: 1220, 3015, 4375
Sloup, Sharon: 9355
Sluys, Erica: 9410, 9085, 4030
Small, Brent: 1430, 9135
Smallwood, Jonathan: 9505
Smetanka, Lori: 1330, 3505
Smichenko, Juliana: 9050
Smilely, Kevin T.: 9245
Smith, Chelsea: 3000
Smith, Gregory C.: 1320
Smith, Masey: 3630
Smith, Alexander: 1020, 2325
Smith, Andre: 1495, 3225, 9190
Smith, Brian N.: 1500
Smith, Carson: 2365
Smith, Claire E.: 3295
Smith, Dylan: 9515
Smith, Dylan M.: 9410
Smith, Eric: 4430
Smith, Erica: 1180
Smith, Genee S.: 3640
Smith, Jacqui: 1105, 3085, 9180, 9265, 9445
Smith, Jennifer L.: 9155, 9380
Smith, Jennifer: 9270
Smith, Kate: 9355
Smith, Kelly: 9155
Smith, Ken: 9065
Smith, Kendra: 3115
Smith, Kevin M.: 1445
Smith, Lenora: 3055
Smith, Lindsey: 1220, 4375, 9515
Smith, Marie: 3245
Smith, Matthew L.: 1010, 4420, 9455
Smith, Maxwell: 9345
Smith, Meghan E.: 9105
Smith, Phillip P.: 4390, 9055
Smith, Samantha: 9190, 9505
Smith, Thomas: 9340, 9380
Smith, Veronica: 4075
Smith-Greenaway, Emily: 1360, 2400
Smithroat, Noemi: 4450
Smith-Turchyn, Jenna: 9510
Smock, Pamela: 2415
Snedeker, Lauren: 9500
Sneed, Rodlescia S.: 3155, 3395, 4120
Snider, Joy: 9510
Snigurska, Urszula A.: 9510
Snow, A. Lynn: 4415, 1270, 9410
Snow, Andrea L.: 9355
Snow, Rachel: 4265
Snyder, Jason: 9235
Snyder, Rani E.: 1245, 3005, 4230
Snyder-Mackler, Noah: 4390, 9100, 9505, 2465
Socci, Marco: 4005
Sokan, Amanda E.: 9155, 9205, 9510, 1250
Sokolovsky, Alexander: 9505
Sol, Ketlyne: 1185, 4605
Soleimani, Laili: 3060
Solkowski, Lauren: 9510
Sollinger, Ann: 1125
Solomon, Alina: 1260
Solomon, Phyllis: 3410
Solorzano, Nelly: 4145, 4570
Solway, Erica: 1365, 2165
Somerville, Ceara: 3210
Sommet, Nicolas: 4205
Son, Hyojin: 9320
Song, Huaxin: 9395
Song, Jiyoun: 3535, 9270
Song, Kijun: 4015
Song, MinKyoung: 9505, 9130
Song, Si Young: 9080, 9435, 9470
Song, Xiaowei: 9295
Song, Xinming: 9375, 9425
Song, Yeonsu: 3570, 9265, 9440, 9505, 3440, 3605, 4425
Song, Zeyuan: 2110
Sonnega, Amanda J.: 1105, 2370
Sood, Neeraj: 4455
Sorensen, Andrea: 9310
Soriano, Christina T.: 3065
Sorkin, John: 2130, 9330

Sorocco, Kristen: 4250
Sorocco, Kristen H.: 4250
Sosnoff, Jacob: 1475, 3085, 9255, 9380
Soto, Jovany: 4065, 9045
Sourdet, Sandrine: 1280
Souza-Talarico, Juliana: 9505
Sow, Ahmadou: 9505
Spännäri, Jenni: 4260
Spalding, Rachael: 9155
Spangler, Hillary B.: 3000, 9030, 9455, 9465
Speiser, Jaime L.: 1095
Spencer, Liam: 3090
Spenrath, Christa: 9130
Sperry, Danielle: 9505
Spetz, Joanne: 1270, 3370
Spiers, Jude A.: 3440, 3605
Spilsbury, Karen: 2045
Spini, Dario: 1415, 4205
Spira, Adam P.: 1130, 4030, 4065, 9085, 9290, 9515
Spiro, Avron: 1500, 9135
Spliedt, Morgan: 3245, 4340, 4450
Splonskowski, Moriah A.: 9190
Spradley, Candace: 2375
Spradley, Laura: 9470
Sprague, Briana N.: 3215
Spratt, Heidi: 9505
Spreckels, Christian: 9510
Spurlock, Wanda J.: 1030
Squires, Allison: 1255
Sreenivas, Ramavarapu S.: 3650
Sreevalsan, Kavya: 9160
Srinivasan, Malathi: 1195
Staben, Omar: 3520
Stabler, Henry: 1030
Stacke, Tobias I.: 2510
Stafford, Kristen: 9500
Stagg, Brian: 3465
Stainback, Maggie: 9085
Stajduhar, Kelli: 3605
Stallard, Eric: 9085
Stallings, Devita T.: 3590, 9215, 9365
Stallings, Michael: 9510
Stallworth, Nicole: 1100
Stamatopoulos, Vivian: 9280
Stanback, Brianne M.: 9215
Standridge, Joy: 9045
Stange, Kurt C.: 9050
Stankiewicz, Anne: 9390
Stanley, Padraic: 3005
Stanley, Jennifer: 9235, 4335
Stansbury, Kim L.: 9425
Stapleton, Gerald R.: 1080
Stark, Susy: 1390, 3490, 3650
Starkey, Samantha Y.: 9250, 9380, 9385
Staudacher-Preite, Sandra: 2340
Stawski, Robert: 1495, 2115, 2310, 3520, 9190, 9235, 2060
Steel, Peter: 4590
Steele, Joel S.: 3200
Steele, Jenessa C.: 9015
Steer, Christopher: 1090
Steffen, Melissa J.: 9405
Steffens, David: 1060
Steffens, Sandra: 9210
Stehling, Elisa S.: 9025
Steiner, Adrienne: 1465
Steiner, Victoria: 9225
Steinert, Lars: 4610
Steinman, Bernard A.: 4230
Steinman, Michael: 2020, 3285, 9025
Stelfox, Kevin: 4120
Stellman, Jeanne M.: 1500
Stellman, Steven D.: 1500
Stellrecht, Kathleen A.: 9160, 9140
Stephan, Yannick: 3185, 9155
Stepniak, Catherine: 1350, 3530
Steptoe, Andrew: 1460, 4205
Sterczala, Adam: 9295
Stern, Jennifer: 4390, 9105
Stern, Michael: 4590
Sternfeld, Barbara: 9420
Sterns, Harvey: 1160
Stessman, Jochanan: 9270
Stevens, Alan B.: 1010, 1465
Stevens-Lapsley, Jennifer: 3120
Stevenson, Elizabeth: 9515
Steward, Andrew: 3115, 3560, 9005, 9010, 9125
Stewart, Steven: 9030
Stewart Steffensmeier, Kenda: 9405
Stieger, Mirjam: 9415
Stillman, Chelsea: 4115
Stinchcombe, Arne: 3545, 9190
Stites, Shana D.: 1310, 2410, 3570, 9085
Stocks, Nigel P.: 4395, 9025
Stoeckel, Kim: 3305
Stoglin, Kristi: 9500
Stokes, Jeffrey E.: 9200, 9260
Stone, Patricia W.: 2410, 3260
Stone, Arianna: 3340, 9350
Stone, Arthur A.: 4440
Stone, Katie: 3295, 9085, 9505
Stone, Lisa E.: 9360, 9415
Stone, Robyn: 2010, 2320, 2445, 9245
Storey, Elsdon: 9065, 9070
Stosor, Valentina: 2160, 9025
Stotler, Kassey: 9185
Stout, Jacques: 2085
Stout, Jeffrey R.: 1100, 9510, 9515
Stout, Madison: 9515
Straker, Jane K.: 2095, 4140, 4270, 9350
Stratton, Sara K.: 1285
Stratton, Lauren: 9515
Strecher, Victor J.: 9500
Street, Lara: 3250
Strelkova, Olga: 4510
Strickland-Hughes, Carla M.: 1355, 3175, 4345
Stringer, Cody: 4450
Stripling, Ashley M.: 9155, 9510
Stroebel, Armin M.: 2510
Strogatz, David: 2515
Stroik, Brady: 3535
Strom, Paris S.: 9225
Strong, Jessica V.: 9015, 9020, 9135, 9155
Strong, Joe: 9060
Strong, Michelle: 9165
Strotmeyer, Elsa S.: 2420, 9390
Strough, JoNell: 9155
Strouse, Susan: 9160
Struble, Laura M.: 9045
Stuart, Robin K.: 2225, 9135, 9500
Stubblefield, Alexander: 3395
Studenski, Stephanie A.: 4520
Stukenborg, Lisa: 9085
Stum, Marlene: 9230
Sturdevant, Diana: 2095, 9505
Sturm, Corey: 9505
Su, He-Xuan: 4435, 9025
Su, Tai-Te: 1475, 3085, 9255, 9380, 9510
Su, Yan-Jhu: 2230
Suarez, Piedad: 4635
Subedi, Janardan: 9400
Sublett, Jennifer F.: 9015
Sudha, S.: 3035, 9320
Suen, Jonathan J.: 4167
Suen, Elliott K.: 9100
Suen, Johan: 3050
Sugawara, Ikuko: 3075, 3635
Sugihara, Yoko: 3635
Sugisawa, Hidehiro: 3635
Suijkerbuijk, Sandra: 9080, 1350, 2135, 2300, 3530
Suizzo, Marie-Anne P.: 9275
Suleiman, Kamal: 9500
Sullivan, Jennifer L.: 2275, 4135, 4415
Sullivan, Kevin: 1280, 9070
Sullivan, Suzanne S.: 9270
Sullivan Bisson, Alycia N.: 9045
Sulo, Suela: 9515
Summerhayes, Emily: 1390
Summer-Meranius, Martina: 2075, 9270
Sun, Chongkui: 9085
Sun, Fei: 2430, 4180, 4215, 4545, 9500, 9505
Sun, Haosen: 4125
Sun, Jiling: 9375, 9425

Sun, Jingjing: 9375, 9425
Sun, Kai: 9500
Sun, Peter C.: 9325, 2480
Sun, Winnie: 9510
Sun, Yue: 9070
Sundar, Sruthi: 9280
Sung, Heayoung: 9435, 9500, 3050, 4075
Suntai, Zainab: 1240, 1485, 2485, 3030, 3095, 4420, 9140, 9195, 9290, 9410, 9445
Supiano, Katherine: 9505, 2425, 4600
Supiano, Katherine: 9510
Surachman, Agus: 3550
Suresh, Madhuvanthi: 3200, 9275
Suri, Anisha: 2105, 9505
Suskind, Anne M.: 9295
Sussman, Tamara: 3105, 9500
Sutherland, Laura: 9325, 9460
Sutin, Angelina R.: 1035, 3185, 4515, 9155, 9515
Sutphin, George L.: 9085, 9100, 9105
Suyasith, Pornthip: 9000
Suzuki, Hiroyuki: 9130
Suzuki, Mizue: 9150, 9295, 9300
Suzuki, Rie: 9035
Svec, Joseph J.: 1135, 4125, 9285
Swall, Anna: 9505, 9070
Swanson, Alex: 9470
Swartzlander, Barbara: 9300
Swenor, Bonnielin: 9505, 2085, 3290, 4030, 4040
Swensen, Kira L.: 1010
Swenson, Tami: 2055
Swerdlow, Russell: 3175
Swierenga, Sarah J.: 2070
Swinford, Emma L.: 9145
Swinton, John: 3385
Syed, Sumaiyah: 3125
Syed, Sumaiyah U.: 9515
Sylvers, Dominigue L.: 9510
Sylvestre, Marie-Pierre: 3485
Syme, Maggie: 9020
Symenuk, Paisly M.: 9380
Szabo-Reed, Amanda: 4015
Szanton, Sarah: 1130
Szanton, Sarah L.: 1450, 3100, 4015, 4110, 4167, 4180, 4340, 4525
Szcześniak, Dorota: 3585
Szerszen, Anita: 4150
Sztramko, Richard: 9130
Szymanski, Benjamin R.: 4430
Szymkowski, Amanda: 9210

T

Taani, Murad H.: 9295, 9395
Tabrizi, Maryam: 9330
Tabuchi, Takahiro: 9180
Taeckens-Seabaugh, Ashley: 9505
Tagawa, Alex: 9515
Tahami Monfared, Amir Abbas: 9070
Tahmaseb McConatha, Jasmin: 2005
Taioli, Emanuela: 3075
Taitel, Michael: 2400
Taiwo, Abiola: 3550
Tak, Sunghee H.: 9335
Takada, Ryosuke: 9245
Takahashi, Kyo: 9460, 9510
Takase, Mai: 9335, 9460
Takele, Anduamlak: 9165
Takemoto, Yoshihito: 9500, 9100
Talboys, Sharon: 9230
Talley, Kristine M.: 9215
Talwar, Sonia: 1390, 4035
Tamburri, Nicholas B.: 1495
Tamplain, Priscila: 9285
Tan, Chenxin: 2280, 3110
Tan, Jie: 4050, 9320
Tan, Kelvin Bryan: 4590, 9500
Tan, Kok Yang: 4075
Tan, Youyou: 9060
Tanaka, Moeko: 9245
Tanaka, Tomoki: 9460
Tanaka, Toshio: 9105
Tang, Ada: 9420
Tang, Alison Y.: 9060
Tang, Fei: 9050, 9510
Tang, Fengyan: 2375, 9155, 9455
Tang, Hsiao-Han: 9050
Tang, Qinggong: 9085
tang, xianping: 9360
Taniguchi, Tori: 9045
Tanner, Corinna: 3465, 9245, 9515
TANNOU, Thomas: 4305
Tarabochia, Dawn: 9105
Taraldsen, Kristin: 9390
Tarantini, Stefano: 9085
Tark, Aluem: 3535, 9270
Tarraf, Wassim: 2410, 3155
TATE, JUDITH A.: 3445
Tate, Karen: 9000
Tate, Rio S.: 9140
Tavabi, Amir A.: 9295
Tavares, Jane: 4450, 4545, 9345, 9500, 9085
Tay, Djin L.: 9200, 9270, 9510, 9515
Tay, Laura: 3405
Taylor, Harry O.: 2460
Taylor, Philip: 2050
Taylor, Beth: 2060
Taylor, Brent C.: 9030
Taylor, Catherine: 9505, 3560
Taylor, Christopher A.: 2180, 3160, 3235, 9185
Taylor, Daniel: 1430
Taylor, Deanne: 3425
Taylor, Janiece L.: 4110, 4340, 4525
Taylor, Jennifer: 9170
Taylor, John: 3590
Taylor, Miles G.: 9515, 1355
Taylor, Philip: 4220
Taylor, Prince: 2275
Taylor, Rebekah: 9130
Taylor, Robert J.: 1185, 2460, 3155
Taylor, Ronald S.: 4230
Taylor, Rosemary: 3255
Taylor, Tonya N.: 2315, 3645
Taylor-Piliae, Ruth: 9500
Tchelet Karlinsky, Keren: 9420, 9200
Teaster, Pamela B.: 1285, 1330
Teeling, Emma: 3600
Tefera, Gashaye M.: 1360, 2325, 9175
Tegeler, Christina: 9360
Teixeira, Laetitia: 9420, 9500
Teixeira-Santos, Ana Carolina: 4195
Tejada Arias, Karla: 9295
Tell, Eileen J.: 1245
Tello, Samantha: 9500
Telonidis, Jacqueline: 1405
Templeman, Maureen E.: 2000
ten Brinke, Lisanne F.: 3460
Teng, Andrew: 9505
Teng, Chiao-Hsin: 2140
Teng, Evelyn L.: 9505
Teno, Joan: 9510, 1240, 4375
Teodoro, Liberty: 1125
Tepsa, Hannah L.: 3015
Terada, Tyran M.: 9130, 9155
Teresi, Jeanne A.: 1180
Terhorst, Lauren: 9390
Terhune, Katherina N.: 3500, 9010
Teri, Linda: 1005, 1425
Terracciano, Antonio: 3185, 4470, 4515, 4630, 9155, 9515
Terrill, Alexandra: 4540
Terry, Ellen L.: 9045
Terry, Linda: 2015
Tervo-Niemela, Kati: 9465
Terzaghi, Damon: 4230
Testoni, Ines: 4530
Tewary, Sweta: 9170, 9300, 9355
Tezcan-Guentekin, Huerrem: 2510
Tha, Soe Han A.: 1020
Thacker, Leroy: 9145
Thadani, Chirag: 9105
Thangwaritorn, Pilar: 9045, 9420
Thao, Le Thi Phuong: 4070
Tharpe, Rita: 2075
Thayer, Deborah: 3605, 9050
Theilheimer, Lucy: 4045

Theng, Yin-Leng: 9330
Theorell, Töres: 9510
Thevenet, Jonathan: 9510
Thiamwong, Ladda: 9510, 9515, 1100, 2030, 4000
Thielke, Stephen: 9030
Thieme, Victoria: 9300
Thierry, Amy D.: 2240, 9515, 1370
Thies, Jennifer: 9500, 3245, 4590
Thiyagarajan, Ramkumar: 9105
Thoma, Myriam V.: 1290, 4430
Thomas, Alicia: 9500
Thomas, Kali S.: 9510, 9515, 1220, 3015, 3080, 3495, 4375, 9450
Thomas, Kali: 3495
Thomas, Patricia: 9330
Thomas, Sam: 3355
Thomas, Sam C.: 9275
Thomas, Shirley A.: 4230, 1405, 3375, 9515
Thomas Tobin, Courtney S.: 2210
Thomassy, Carmen: 9020
Thompson, Katherine: 4025
Thompson, Alisha R.: 1030
Thompson, Allison: 9310
Thompson, Amber: 9240
Thompson, Casidee: 3375
Thompson, Edward H.: 9005, 9115, 9445
Thompson, Hilaire J.: 1345
Thompson, Kat: 4025
Thompson, Katherine: 1340, 4250, 9050, 9220
Thompson, Kayla: 9500, 9505
Thompson, Lena: 4200, 9040
Thompson, Nailah: 9505
Thompson, Roy A.: 9510, 9045, 9395, 9505
Thompson Cañas, Reid: 3550
Thomsen, Timothy A.: 3610
Thomson, Cynthia A.: 9115
Thorne, Trina: 3440, 9355
Thornton, Marleen: 3250
Thorp, Julia: 3015
Thorp, Jr, Roland J.: 1370
Thorpe, Roland J.: 9380
Thorpe, Jr., Roland J.: 1185, 1370, 2210, 2410, 3155, 3320, 3640
Thorvaldsson, Valgeir: 4485
Thosingha, Orapan: 9000
Thrush, Kyra: 1015, 3435, 9055
Thumuluri, Deepthi: 3065
Thunell, Johanna: 3030
Thyagarajan, Bharat: 2020, 4585, 9025, 9085, 9095, 9295
Tian, Junyan: 9140, 2105, 2195, 3430, 4040, 4065
Tian, Shubo: 4470
Tibbits, Lauren: 9000
Tiernan, Chad: 9255
Timko, Christine: 3200
Tingvold, Maiken: 4630
Tirona, Rommel G.: 9255
Tisminestzky, Mayra: 9030
Titler, Marita G.: 2075
Titley, Heather K.: 9355, 3440
Titus, Jennifer: 9425, 9270, 9295
Tkatch, Rifky: 2175, 9175, 9360, 9455, 9460
Toba, Kenji: 3490, 9050, 9300
TOBIMATSU, Taketo: 9245
Tocco, Maggie: 1335
Tognoli, Emmanuelle: 9510, 9460
Toki, Hiroshi: 9215
Tokumitsu, Sayaka: 9030
Tolea, Magdalena I.: 9130
Toles, Mark: 3605
Tolliver, Renee: 9285
Tolson, Melissa: 9200
Tomasino, Debra: 9510
Tomita, Machiko R.: 9065, 9105, 9365
Tomiyama, A. Janet: 1165
Tomiyama, Naoki: 9280
Tompkins, Catherine J.: 2040, 2385, 3555, 4240
Tong, Rachel: 4590
Tong, Sebastian: 9405
Tong, Weiqun: 2160
Tonkikh, Orly: 3050, 9515
Tonkin, Andrew M.: 4070, 9025
Tonui, Betty: 9505
Toomey, Elaine: 9500
Tooze, Janet A.: 1095
Topper, Rachel: 2510
Torezani, Silvia A.: 1505
Torigoe, Trevor: 9085
Torke, Alexia: 9505
Tormos, Jose: 2130
Torres, Jacqueline: 1270, 4185, 9450
Torres, Jasmine: 9105
Torres, Mia D.: 9135
Torres, Sandra: 4000
Torres, Stacy: 2415
Toth, Matt: 3275
Toto, Pamela E.: 4455, 9390
Touch, Gina: 9065
Touchett, Hilary: 4535
Townsend, Natashia: 9220
Towsley, Gail L.: 1405, 4545, 9510
Toyama, Itsuko K.: 2375
Toyama, Masahiro: 2465
Toyokawa, Noriko: 9260
Toyokawa, Teru: 9260
Tracy, Elizabeth: 4245
Tracy, Tara: 9505
Tran, Christopher: 3415
Tran, Emma: 9375
Tran, Leslie: 9505, 9510
Tran, Sydney J.: 1215
Tran, Thi: 1270, 2375
Tran, Thuy Dan N.: 3015
Tranah, Gregory J.: 3295
Trani, Jean-Francois: 1310
Trapp, Alexandre: 9095
Travers, Jasmine: 2410, 3535, 9505
Travis, Alexis: 1365
Travison, Thomas G.: 4340, 9295
Tremblay, Guillaume P.: 9085
Trevedi, Ranak: 1110
Trevino, Kelly M.: 4010, 4330
Triebel, Kristen L.: 1380, 9025
Trinh, Mimi: 2365
Trinh-Shevrin, Chau: 9500
Trinkaus, Eva-Maria: 9010
Trinkoff, Alison: 9395
Triolo, Federico: 3460
Trites, Michaella: 1495, 9075
Trivedi, Amal: 1060
Trivedi, Ranak: 3200, 9275
Troen, Bruce: 9105
Trott, Daniel: 9100
Trotter, Stephanie: 9515
Troutman, Brooke: 9510
Troutman-Jordan, Meredith L.: 9505
Troxel, Wendy M.: 4345
Troy, Lisa M.: 9500
Troyer, Angela: 1045, 4015, 9505
Troyer, Emily: 9515
Truica, Cristina: 9250
Trumbo, Jami: 4167
Truong, Angeline A.: 9275, 9515
Tsang, Hiu Ling Vivian: 2220, 9015
Tschanz, JoAnn: 9085
Tschanz, Joanne: 9065, 9070
Tschauner, Madison E.: 9155
Tse, Dwight: 2220
Tseng, Heidi: 4025
Tseng, Tung-Sung: 9175
Tseng, Winston: 9505
Tshering, Kezang: 9510
Tsotsoros, Cindy: 9515
Tsui, Emma K.: 2320
Tsui, Jenny: 4595
Tsukada, Noriko: 9220, 9345
Tsumura, Hideyo: 4330
TSUTSUMI, Kanako: 9245
Tu, Xin: 9085
Tuazon, Joshua: 9470
Tucker, Gretchen G.: 9265
Tucker, Gretchen: 4090
Tucker, Julia E.: 9460
Tucker, Sarah M.: 9285

Tucker-Roghi, Gina C.: 9285
Tucker-Seeley, Reginald: 3310
Tulloch, Kristen: 9065
Tumosa, Nina: 3420, 4240
Tun, Sandy: 9220
Tunalilar, Ozcan: 9510, 9515
Tung, Yi-Hsuan: 3225
Turcot, Valerie: 3485
Tureson, Kayla: 9515
Turiano, Nicholas: 9140
Turiano, Nicholas A.: 2450
Turkelson, Angela: 4320, 9515
Turker, Hamid B.: 3510
Turnbull, Adam: 9505
Turner, Emily A.: 9100, 9105
Turner, Jennifer R.: 4560, 9235, 9275, 9515
Turner, Robert W.: 3055, 4605
Turner, Rose L.: 4455
Turner, Shelbie: 1500, 3050, 9020, 9260, 9430
Tur-Sinai, Aviad: 1410
Tuseth, Natalie: 9515
Tutek, Joshua: 9465
Twahir, Assma: 9170, 9300, 9355
Twisk, Johannes: 2130
Tyagi, Pallavi: 9070
Tyrrell, Caitlin J.: 9275
Tyshkovskiy, Alexander: 9105

U
Uchida, Hayato: 9225
Uhrenfeldt, Lisbeth: 2485
Uittenhove, Kim: 1415
Ukhanova, Maria: 3285
Ukraintseva, Svetlana: 4585, 9055, 9070, 9085, 9500, 1410
Ulbricht, Christine: 9295
Ulrich, Connie: 9385
Umberson, Debra J.: 1430, 2135, 9380
Umegaki, Hiroyuki: 9295
Umemoto, Daisuke: 9035
Umlauf, Anya: 9085
Ungar, Rachel: 2175, 9175, 9360, 9455
Ungvari, Zoltan: 9085
Unick, G. Jay: 9130
Unnikrishnan, Archana: 1440
Unroe, Kathleen: 1225
Unutzer, Anna: 9505
Unutzer, Jurgen: 4050, 3580, 4135
Upton, Meredith L.: 9210
Urban, Karl: 1330
Urbanek, Jacek K.: 3290, 4065, 9510
Urbaniak, Anna: 3510
Urban-Wojcik, Emily: 1065
Urganci, Betul: 9505
Urlings, Judith: 3585
Urman, Harold: 9510
Utz, Rebecca: 9240, 9510
Uygur, Mehmet: 3230
Vázquez Núnez, Karla Patricia: 9515

V
Vacek, James: 4015
Vaeli Zadeh, Ali: 9510
Vajapey, Ramya: 9105
Vakharia, Nirav: 4430
Valadares, Kevin J.: 4250
Valbuena, Valeria: 9515, 9000
Valdearcos Contreras, Martin: 9105
Vale, Michael T.: 9015, 9020
Valencia, Willy Marcos: 9050, 9510
Valencia-Laver, Debra: 4225
Valenti, Korijna: 4380
Valenzano, Dario: 3600
Van Vleet, Samuel C.: 1150, 9220, 9435
Van Aartjik, Alexa: 4010
van Achterberg, Matheus: 2340
Van Bavel, Jay: 9400
van Campen, Jos: 1345
Van de Castle, Barbara: 9320
van den Heuvel, Eleanor: 9245
van der Steen, Jenny T.: 2120
Van Eck, Richard: 9215
Van Fossen, Jenna A.: 9505
van Gemert-Pijnen, Lisette: 9080, 9500
van Haastregt, Jolanda C.M.: 2340
Van Haitsma, Kimberly S.: 2080, 2510, 3015
van Heumen, Lieke: 2350
van Landingham, Suzanne W.: 3590
van Melick, Els: 1345
van Oostrom, Sandra: 2130, 4435
Van Orden, Kim A.: 1135, 2275, 4365
Van Puymbroeck, Marieke: 9410
van Rossum, Erik: 2340
Van Treese, Katherine: 3365, 9400
Van Vleet, Samuel: 9380
van Zon, Sander K.R.: 1200
Vander Stichele, Elisabeth: 2150
Vander Wende, Helen: 9510
Vandermorris, Susan: 1045, 4015, 9505
VanderWeele, Tyler J.: 2465
VanderWerf, Jessica: 9330
VandeWeerd, Carla: 9510
VandeWerd, Carla: 9160
VanDongen, Nicholas: 9305
Vandorden, Kimberly: 1135
VanSciver, Angela: 4015
VanSwearingen, Jessie: 2105, 9515
Varadaraj, Varshini: 4030
Vara-García, Carlos: 1005, 2166, 3050, 4110
Varlamova, Maria: 4005
Varma, Vijay: 9250
Varner, Michael: 9070
Varrasse McPhillips, Miranda: 2385
Vartak, Maitreyi: 9500
Vasileva, Anastasiia: 4390
Vasiliadis, Helen-Maria: 9365, 4350
Vasquez, Elizabeth: 2160, 9140
Vazquez, Julia: 9515
Vazquez-Arias, Martha Elena: 1300
Veal, Britney: 9005, 9125
Vega, Irving E.: 1295
Vega, Marilisa: 3375
Vega, William: 4185
Velasco Roldan, Nidya: 9040, 3310
Vellani, Shirin: 2045, 3565
Vellas, Bruno: 1280, 3135, 9130
Vemuri, Prashanthi: 1280
Vemuri, Ravichandra: 9100
Venegas-Sanabria, Luis Carlos: 9515
Venema, Dawn M.: 9135
Ventura, Maria Eloisa: 9090
Vera, Sara: 9165
Verbeek, Hilde: 2340, 3585, 4335
Verdery, Ashton M.: 1360, 9440
Verghese, Joe: 9390
Verhage, Miriam: 1360
Verhey, Frans R.J.: 4610
Verma, Rakhi: 9330
Verna, Nadine: 9215
Verschuren, Monique: 2130, 4435
Vervaecke, Deanna: 9260
Vetrano, Davide L.: 1200, 1280, 3460, 4435, 9025
Victor, Christina: 2405, 4570
Victoria, Cotnam: 9395
Videon, Tami: 2055
Vieira, Edgar R.: 9505
Vieira, Rute: 4120
Vieira Ligo Teixeira, Camila: 9505, 9000
Vigen, Cheryl L.: 4075
Vigoureux, Taylor F.: 1430, 9440
Villacorta-Martin, Carlos: 9100
Villalonga Olives, Ester: 2100
Villeda, Saul: 2245
Vincent, David: 3115

Vincenzo, Jennifer: 9255, 9420
Vinoo, Deepa: 3095
Vinson, Latrice: 1175
Vipperman, Andrew: 1220
Visser, Marjolein: 2130, 4435
Vivaldo, Marissa: 9505
Viveiros, Jennifer A.: 1375, 9500
Vivoda, Jonathon: 1485, 2515
Vlachantoni, Athina: 9260, 9280
Vlaeyen, Ellen: 2340
Vluggen, Stan: 3625
Vo, Nancy: 9030
Vo, Tina T.: 9510
Voelkner, Abigail R.: 9020, 9140
Vogel, Carlyn E.: 2000
Vogel, Mia T.: 1345
Vogelsmeier, Amy L.: 4475
Voll, Stacey: 9500
Vollmer Dahlke, Deborah: 9455
Volpe, Robert J.: 9500
Voltmer, Helena: 4270
Von Ah, Diane: 9500
von Gunten, Armin: 1415
von Humboldt, Sofia: 9260, 9430
von Känel, Roland: 1005, 2166
von Oertzen, Timo: 4560
Von Rosen, Philip: 4435
Voss, Maren Wright: 4540
Vredevoogd, Mindy: 4050
Vrkljan, Brenda: 9160
Vu, Cong Nguyen: 9515
Vu, Huy: 1050
Vu, Thi: 3325
Wöhrmann, Anne M.: 9310

**W**

Wachholz, Patrick A.: 2100
Wacht, Oren: 3345
Wada, Tadashi: 2205
Wade, Katie: 9230
Wadsworth, Lori: 4540
Wadsworth, Sally J.: 9135, 9360, 9510
Wagas, Rafael: 1125
Wagenknecht, Lynne: 9505
Wager, Mark: 1250
Wagg, Adrian: 3605, 4425
Wagner, Grant H.: 9505
Wagner, Jenny: 4050
Wagner, Laura: 1270, 3370
Wahl, Hans-Werner: 1475, 3085, 3265, 4560
Wakefield, Dorothy: 3395, 4075, 4200
Waktins, Michele: 3455, 3545
Wakui, Tomoko: 3635, 4425, 9515
Waldman, Rachel E.: 9140, 9360
Waldron, Danielle A.: 3620, 2460
Waldrop, Drenna: 3535
Waldstein, Shari R.: 4180
Walke, Lisa M.: 3245
Walker, Rod L.: 3300
Walker, Anne: 3560
Walker, Jennifer D.: 9380
Walker, Jessica: 1110
Walker, Ruth: 9430
Walkup, Michael P.: 1305, 9220
Wallace, Cara L.: 1070, 9505
Wallace, Julianne: 3030
Wallace, Laura: 9510
Wallace, Marissa: 9360
Wallace, Meredith L.: 3295
Wallace, Neal: 9515
Wallace, Susan: 1420
Wallem, Alexandra: 1055
Wallner, Lauren: 2115
Walsemann, Katrina M.: 1105
Walsh, Christine A.: 3105
Walsh, Edith G.: 3275
Walsh, Matthew C.: 3590
Walsh, Sarah: 4360, 9500
Walston, Jeremy D.: 3290, 3335, 4390, 4395, 9055, 9105, 9290
Walter, Louise C.: 9295
Walton, Jannell: 1495
Wanagat, Jonathan: 9110
Wang, Hang: 4040, 4065
Wang, Junqiao: 2140
Wang, Adrienne M.: 9055
Wang, Aining: 1285, 1335
Wang, Bo: 9090
Wang, Chen-pin: 9295, 9305
Wang, Chih-Hsuan: 9515, 9050
Wang, Cuiling: 9405
Wang, Donna: 9165
Wang, Ellen: 9055
Wang, Fei: 1395, 3340
Wang, Haocen: 9050
Wang, Haowei: 1360, 4255, 9385
Wang, Huali: 4180, 9065
Wang, Jing: 1255, 2100, 2140, 3325, 2480, 9130, 9265, 2120, 4405
Wang, Joyce: 3275
Wang, Julia Shu-Huah: 9310
Wang, Kaipeng: 2430, 3115, 3595, 4545, 9460
Wang, Katherine: 1115, 3110
Wang, Ke: 4080
Wang, Kuan H.: 2270
Wang, Kun: 9140, 9445, 9085
Wang, Lihua: 2020, 4585
Wang, Lin: 9515, 9005
Wang, Ming: 9105
Wang, Mo: 9310
Wang, Qin: 1255
Wang, Qing: 9035
Wang, Shania H.: 9055, 9095
Wang, Shaohua: 9090
Wang, Shuangshuang: 9510
Wang, Siyue: 4395
Wang, Xiao (Joyce): 2425, 9510
Wang, Xiaochuan: 1445, 9510
Wang, Xinhui: 3310
Wang, Xueqing: 1140
Wang, Yi: 1195, 9040, 9145
Wang, Ying: 9060, 9320
Wang, Yixiao: 3275
Wang, YuHsuan (Olivia): 9240, 9515
Wang, Yunzhang: 1015
Wang, Yushu: 4015
Wang, Zhe: 9355, 9070
Wanigatunga, Amal A.: 4030, 2105, 2285, 3290, 3430, 4040, 4065, 9250
Wanigatunga, Sarah K.: 4065
Wanka, Anna: 3510, 4060, 4385
Ward, Allison: 9205
Ward, Caitlin: 4590
Ward, Rachel: 2260
Wardlaw, Dana M.: 9300
Ware, Katilya: 9515
Warren, David E.: 2166
Warren, John Robert: 3190
Warren, Teresa S.: 9505, 9510
Washington, Erin: 1235
Washington, Faith: 3085
Washington, Faith-Christina I.: 9255, 9380
Washington, Karla T.: 4145, 9380
Washington, Tarisha: 3005
Washington, Tiffany R.: 9505, 3475
Wastila, Linda: 1055, 1345, 2160
Waszynski, Christine: 2060
Watanabe, Kentaro: 4425
Waters, Leland H.: 1340, 4240, 9160
Waters, William F.: 9060
Watkins, Yashika J.: 2075, 2195
Watman, Deborah: 2320
Watowich, Marina M.: 4390, 9100
Watson, Amanda: 2010
Watson, Mark: 9110
Watts, Amber: 1155, 3175, 4015, 9045, 9250, 9410, 9420, 9515
Watts, Sarah: 9515
Waxman, Michael J.: 9160
Wayne, Peter: 9510
Wazinski, Karla: 4060
Wead, Alice M.: 9460, 9510
Weatherall, zenya: 9185
Weatherspoon, Darien: 9025

Weaver, Ashley A.: 1305
Weaver, Caylee: 9215
Weaver, France: 9500
Weaver, Jen: 4605
Weaver, Raven H.: 1300, 2300, 3630, 9260
Weaver, Shirley: 9180
Webb, Katherine: 9390
Webb, Nicole: 1090, 3070
Weber, Kathleen: 2160
Weber, Kendall L.: 9265, 9285, 9290, 9360
Webster, Noah J.: 2010
Webster, Britney A.: 1320, 9515
Webster, Noah J.: 1295, 1475, 2010, 4605, 9035, 9510
Wee, Hwee Lin: 9500
Weech-Maldonado, Robert: 1075, 2425, 4355
Weeks, Lori: 3440
Weerahandi, Himali: 9150, 9160
Wehbe, Layal: 9305
Wehry, Susan: 9355
Wei, Jeanne Y.: 9045, 9085, 9110, 4330
Weidmann, Rebekka: 3070
Weil, Joyce: 3105, 9175
Weinberg, J. Brice: 4115
Weingast, Sarah: 1010, 9080
Weinreich, Donna M.: 1160
Weinstein, Andrea M.: 4345
Weinstein, Barbara E.: 1327
Weinstein, Elliott R.: 3645
Weintraub, Sandra: 9280
Weiskittle, Rachel E.: 2275, 4535, 9275
Weiss, David: 3085
Weiss, Carleara: 9105, 9230
Weissman, Joel: 3260
Weitlauf, Julie C.: 9395
Weldrick, Rachel: 1465, 3105, 4385
Wellbrock, Elizabeth: 9185
Weller, Hailey: 9435
Welmer, Anna-Karin: 1280, 4435
Welsh, Derick: 9505
Welstead, Miles: 9190
Wen, Katherine: 2070
Wennberg, Alexandra: 2235, 4030, 9295
Wenzel, Jennifer: 4015
Werner, Nicole: 1180
Werner, Kelsey: 1085, 2475
Werthman, Emily H.: 2315
West, Bruce J.: 9130
West, Jessica S.: 9360
West, Tim: 9510
Westerlund, Hugo: 3615, 9115
Wetherell, Julie L.: 1110, 4535
Wethington, Elaine: 4605, 9410
Wetle, Terrie: 3495
Wettstein, Markus: 3085
Wetzel, Sarah: 4595
Wharton, Whitney: 3570
Wheaton, Felicia V.: 4110, 4200
Wheeler, Brandan: 9435
Wheelock, Sunshine: 3365
Whelan, Sally: 9500
Whetung, Cliff: 1085
Whitbourne, Susan K.: 9170, 1040
White, Avalon: 3465
White, Carole: 9265
White, Elizabeth: 1055
White, James: 9105
White, Katie: 1140, 4495
White, Lon: 9085
White, Weyling: 9245
Whitehead, Samantha: 1300
Whitehouse, Christina R.: 2390, 9180
Whitehouse, Emma: 3545
Whitfield, Keith E.: 1370, 2395, 3640, 9380
Whitfield-Gabrieli, Susan: 9140
Whiting, C. Grace: 1245, 1395, 2165, 3165, 1005
Whitley, Deborah M.: 2500, 9270
Whitson, Heather: 1327, 2085, 2145, 9105
Whittington, Caroline: 4590
Whitton, Alaina: 2375
Wicker, Ellen: 2175, 9360, 9455
Wicker, La Cheryl: 4230
Wickrama, Kandauda: 3530
Wickwire, Emerson M.: 9515
Wieck, Cornelia: 3265
Wiese, Lisa: 1125
Wihry, David C.: 9220, 9245, 9300
Wijnhoven, Hanneke: 2130, 4435
Wilber, Kathleen: 9040, 9500
Wilckens, Max R.: 9310
Wilcock, Donna: 9085
Wild, Katherine: 3055
Wildeman, Bethany: 9085
Wiley, Joshua: 1430
Wilk, Christa: 9335
Wilkie, Rachel Z.: 3520
Wilkinson, Alexandra: 9375
Wilkinson, Lindsay R.: 9150, 9155
Wilks, Scott E.: 1030
Willcox, Bradley J.: 3415, 9085
Willcox, D. Craig: 9085, 3415, 9075, 9190, 9335
Williams, Adelle: 3410
Williams, Allison: 3395
Williams, Brie: 9070
Williams, Christine L.: 2075, 9460, 9510
Williams, Ishan C.: 9500, 1125, 1340, 3220, 9280
Williams, J. Hunter: 9265
Williams, Jamia: 2145
Williams, Jonna: 9265
Williams, Kadija N.: 9275
Williams, Kristi: 9515
Williams, Kristine: 3565, 4190, 4590
Williams, McKenna E.: 9510
Williams, Shellie: 1340
Williams, Victoria: 2335
Williams-Farrelly, Monica M.: 1105
Williamson, Jeff: 9085, 9295
Williman, Martha R.: 4325
Willis, Celia E.: 2410
Willroth, Emily C.: 1035, 2305, 9415
Wilmoth, Janet M.: 3150, 9380
Wilson, Kimberley: 3545
Wilson, Stephanie J.: 3125
Wilson, Ashley: 1010
Wilson, Christina: 1405
Wilson, Ira: 9500
Wilson, Jenna: 9155
Wilson, Kenneth A.: 4510
Wilson, Kerrie: 9180
Wilson, Kimberly: 4365
Wilson, Lauren E.: 9130
Wilson, Leigh Ann: 4365
Wilson, Megan E.: 9510
Wilson, Paige: 4190
Wilson, Robert S.: 1435, 2495
Wilson, Stephanie J.: 3125, 3525, 9515
Wilson, Traci L.: 4140, 4230, 4270, 4460, 9105
Windham, B. Gwen: 1280, 9070
Windham, Gwen: 9505
Windsor, Tim: 4460, 9430
Wine, Hallie: 1080, 2325
Winfree, Jaclyn: 1220, 9515
Wing, David: 3295
Wing, Rena: 9430
Wingo, Anna: 9505
Wingood, Gina: 2315
Wingood, Mariana: 3300, 9250, 9255
Winkle, Melissa Y.: 9355
Winship, Jodi: 9080
Winslow, Victoria: 9050
Winters-Stone, Kerri: 9505
Winthal, Jeffrey: 9375
Wion, Rachel: 9130
Wister, Andrew: 2435, 3465, 9005
Witt, Catherine: 1405
Witzel, Dakota D.: 9020, 9235, 1240, 2125, 9505

Wojczynski, Mary K.: 2020, 2085, 2285, 4585, 9025, 9295
Wold, Loren E.: 3365
Wolf, David G.: 1275, 9395
Wolf, Douglas: 3225
Wolf, Katie E.: 9180
Wolf, Michael: 9200
Wolf, Tabea: 9140
Wolfe, Rory: 9390
Wolff, Jennifer L.: 1345, 2320, 3010, 3100, 4070, 4180
Wolfle, Brianna D.: 9505
Wolf-Ostermann, Karin: 3585, 4610
Won, Cho Rong: 3095, 9410
Wong, Aileen: 4167
Wong, Frankie HC: 2215
Wong, Gloria HY: 1045, 2215, 3095, 3470, 4280, 9065, 9360
Wong, Ian C.K.: 4050, 9065
Wong, Jaclyn: 2070
Wong, Jen D.: 9290
Wong, Rebeca: 4280, 4635
Wong, Roger: 9145
Woo, Esther: 4360
Woo, Hee-Soon: 4330, 4280
Woo, Kyungmi: 9270
Wood, Joanne: 9390
Wood, Joseph: 9215
Wood, Kristie A.: 9275
Wood, Olivia: 9270
Wood, Stacey: 1335, 9230
Wood, Timothy: 9395
Woodard, LeChauncy: 3115, 9400
Woodhouse, Mark: 1275
Woods, Diana L.: 9300
Woods, Nancy F.: 4165
Woods, Robyn L.: 4395, 4550, 9025, 9030, 9065, 9070, 9295, 9390
Woodward, Amanda: 4305
Woodward, Jennifer: 9000
Woolverton, Cindy B.: 9505, 4535, 9020
Woosley, Raymond L.: 9045
Worrilow, Kathryn: 2020
Woulfe, Kathleen: 9105
Wray, Laura O.: 3365, 4080, 4415
Wrede, Christian: 9080, 9500
Wretman, Christopher J.: 1220
Wright, Aidan: 1165
Wright, Benjamin C.: 9165
Wright, Bernadette: 4045
Wright, Kaitlyn: 9035
Wright, Kathy D.: 2410, 3365
Wright, Nicole C.: 1380, 9025, 9140, 9440, 9445
Wrosch, Carsten: 4560
Wrzus, Cornelia: 3265
Wu, Chao-Yi: 3200
Wu, Deqing: 4585
Wu, Bei: 1075, 1115, 1195, 1255, 1325, 2100, 2140, 2280, 2360, 2410, 3110, 3260, 3610, 9345
Wu, Chao-Yi: 4065
Wu, Chenkai: 2115, 4050, 9320
Wu, Deqing: 9055, 9085
Wu, Di: 4030
Wu, Jiayi: 9510
Wu, Jing: 9060
Wu, Jingchuan: 9250
Wu, Junfang: 9505
Wu, Kuan-Ching: 9505
Wu, Lizi: 2175, 9455
Wu, Mark: 4065, 9085
Wu, Qiao: 1055, 9025
Wu, Rachel: 1355, 3175, 4345
Wu, Ruiqian: 1485
Wu, Shih-Cyuan: 9425
Wu, Shinyi: 3225, 4350
Wu, Wenlong: 1360
Wu, Yan Yan: 2280, 3110
Wu, Zimu: 9065
Wurm, Susanne: 3085
Wylie, Molly: 2025, 2445, 3365, 4275
Wyman, Jean: 4115, 9215
Wyman, Mary F.: 3365, 4080
Wynn, Matthew: 9205, 9210
Wyte-Lake, Tamar M.: 4145, 4570

**X**

Xiang, Qingyan: 4585
Xiao, Jing Jian: 3310
Xiao, Lu: 9060
Xiao, Qian: 9440
Xiao, Yan: 9025
Xie, Xianjin: 3610
Xie, Bo: 1050, 3145, 9060, 9400, 9445
Xie, Kristal: 3415
Xie, Rui: 9510, 9515
Xie, Yutong: 9510
Xin, Lanlan: 9020, 9255
Xing, Fan: 9130
Xu, Beibei: 4435, 9025, 4425
Xu, Emily: 4410
Xu, Hanzhang: 1195, 3055, 4145, 9045, 9510
Xu, Hui-wen: 4435, 9025
Xu, Jiahui: 9440
Xu, Jiayun: 9515, 3380
Xu, Lili: 4540
Xu, Ling: 9360, 4555, 9280, 9285, 9505, 9510
Xu, Qingwen: 3595, 9445
Xu, Shu: 9045, 9165, 9390, 9420, 9245
Xu, Tongling: 4380
Xu, Weihao: 4395
Xu, Weili: 1200, 1280, 9065
Xu, Wenqian: 2170
Xu, Yanfeng: 2500, 4245
Xu, Yangyi: 3650
Xu, Yingzhi (Lindsay): 9045
Xu, Yunnan: 2390
Xu, Ziyao: 9515
Xue, Qian-Li: 4070, 9055, 9505
Xue, Tingzhong (Michelle): 4330, 9045

**Y**

YABU, Ken-ichiro: 9245
Yadav, Hariom: 9090
Yadav, Om Prakash: 9400, 9500
Yadav, Uday Narayan: 9400, 9500
Yadava, Aradhita: 9235
Yaffe, Kristine: 3295, 4190, 4535
Yahirun, Jenjira: 4350
Yahner, Jennifer: 9515
Yakuta, Melissa: 9070
Yalcin, Ali: 9160
Yamada, Yuji: 9160
Yamaga, Ryonosuke: 9030
Yamaguchi, Yoshihiro: 9105
Yamaguti, Siomara T.: 9505
Yamakawa, Miyae: 9215
Yamamoto, Ryohei: 9215
Yamamoto-Mitani, Noriko: 3375
Yamanaka, Takashi: 9030
Yamashita, Takashi: 2050, 2115, 9185, 9340, 9380, 9500
Yamauchi, Ken: 9105, 9420
Yan, Elsie: 1335
Yan, Feng: 9085
Yan, Kevin: 1395, 4045
Yan, Li: 1460
Yan, Mengzhao: 9040
Yan, Qin: 1015
Yancura, Loriena: 2500
Yang, Wei: 3610
Yang, Bo Kyum: 9350
Yang, Emmy: 2070
Yang, Iris S.: 9515
Yang, Jeanne: 1055, 9090, 9195
Yang, Mai See: 4255, 9385
Yang, Michelle C.: 9505
Yang, Ming: 9055
Yang, Rumei: 9050, 9445
Yang, Sophie: 3180
Yang, Tzu-Yi: 9095
Yang, Wei: 1465
Yang, Wendy: 9505
Yang, Yazhen: 3525, 9260
Yang, Yong Sook: 9295
Yang, Yunjia: 1250
Yankeelov, Pamela: 3245, 4175, 4240, 9320
Yankner, Bruce: 3435, 2205
Yao, Yujing: 4585
Yap, Yang: 1430
Yarandi, Hossein: 9190
Yarbro, Johnathan R.: 3270

Yasaka, Taisuke: 3375
Yashin, Anatoliy: 9085, 1230, 4585, 9055, 9065, 9070
Yashkin, Arseniy P.: 1230, 2115, 3040, 3160, 9055, 9065, 9085
Yassai-Gonzalez, David: 9085
Yau, Jessie Ho-Yin: 4280
Ye, Byoung Seok: 9065, 9130
Ye, Jing: 4245
Ye, Minzhi: 4225, 9385
Yeager, Kate: 3460, 9290
Yeager, Reid: 3355, 9280
Yee, Cynthia: 9245
Yeh, Yen-Po: 3340
Yeh, An-Yun: 2180
Yeh, Charlotte: 2175, 9175, 9455, 9460
Yeh, Gloria: 9510
Yeh, Jarmin: 1060, 2025, 3545, 9220
Yeh, Shu-Chuan Jennifer: 9350
Yekutiel, Ziv: 9420
Yelverton, Valerie: 9070
Yelvington, Miranda: 9010
Yenko, Ira: 9415
Yenokyan, Gayane: 4390
Yentes, Jennifer: 9135
Yeo, Hyesu: 9005
Yeshua-Katz, Daphna: 9500
Yetman, Linda: 9500
Yeung, Dannii Y.: 2315, 4205, 9435
Yeung, Ellen W.: 3090
Yi, Stella S.: 1195
Yi, Eun-Hye G.: 9285
Yi, Julie: 1327
Yin, Cheng: 1055, 9500, 9515
Yin, Lijuan: 9150
Ying, Kejun: 3270
Ying, Meiling: 4405
Ying, Yu: 9140
Yingyang, Zhang: 9065
Ylitalo, Kelly R.: 9255, 9390, 9420
Yockey, Andrew: 9090, 9195
Yokota, Fumihiko: 4090
Yonashiro-Cho, Jeanine: 2040, 2475, 4350, 9130, 9335, 9415, 9505
Yoo, Eun-Young: 9500
Yoo, Jennifer: 1070
Yoo-Jeong, Moka: 2160
Yoon, Hyunwoo: 3150, 9080, 9435
Yoon, Heewon: 3520
Yoon, Ho-Kyoung: 3295
Yoon, Hyunwoo: 4045
Yoon, Jihye: 3175
Yoon, Joonsik: 4295
Yoon, Sukyung: 2025, 3115
Yorgason, Jeremy B.: 3465, 9435
Yoshida, Hironori: 9420
Yoshida, Kazunori: 9245
Yoshikawa, Aya: 9510
Yoshimura, Hiromi: 9300
Yoshino Benavente, Julia: 9200
Yost, Kathleen: 9510
You, Tongjian: 9510
Younan, Diana: 3310
Young, Heather M.: 4015, 9270
Young, Kenlyn: 4120
Young, Kim: 1090
Young, Kimberly: 9390, 3410
Young, Nathaniel: 4440, 9235
Young, Ruth Anne: 9010
Young, Sandra: 3440
Young, Tara: 9160
Young, Yuchi: 3340
Yousefi, Keyvan: 9070, 9090
Yousefzadeh, Matthew: 9105
Yow, W.Quin: 2030, 4190, 4345, 9135, 9510
Yu, Gary: 1325, 3260
Yu, Aiping: 9015, 9020
Yu, Bin: 2060, 2115, 3040, 9265
Yu, Fang: 2120, 4115
Yu, Heshuo: 9240, 9425
Yu, Jessica: 9515
Yu, Jiao: 3340
Yu, Jinxiao: 3555, 2145, 2405, 3055, 3465, 9165
Yu, Susie: 9220
Yu, Wanting: 2130, 9505, 9445
Yuan, Changzheng: 1200
Yuan, Nicole: 1250
Yuan, Yiyang: 9295
Yun, Stacy W.: 9160, 9265, 9285
Yusifov, Aykhan: 9105
Yusupov, Iris: 1045, 9505

Z

Zaccaria, Daniele: 1415
Zahodne, Laura B.: 9500, 1295, 3190, 4580
Zaidel, Catherine: 2175
Zajdel, Melissa: 9120
Zalakain, Joseba: 3585
Zamani, Mohammadzaman: 1050
Zambon, Antonella: 9515, 9175, 9510
Zaninotto, Paola: 2390
Zanjani, Faika A.: 2240, 4360, 4565, 9160, 9175, 9185
Zúñiga, Franziska: 2045, 2340, 4475
Zare, Hossein: 3640
Zarit, Steven: 1205, 9290
Zarowitz, Barbara: 1345
Zarse, Kim: 9510
Zaslavsky, Oleg: 9505
Zauszniewski, Jaclene A.: 9260
Zauszniewski, Jaclene: 4245
Zavala, Catalina: 4195, 4345
Zavala, Daisy V.: 4505
Zecevic, Aleksandra: 9255, 9345, 9395
Zechner, Michelle: 3595
Zeitlow, Kahli: 9215
Zeitzer, Jamie: 3295
Zeki Al Hazzouri, Adina: 9130, 9505
Zelinski, Elizabeth: 9020, 9410
Zell, Angela M.: 3250, 9030, 9500
Zelterman, Daniel: 1335, 2170, 3235
Zeng, Qing: 9500
Zettel-Watson, Laura: 9030, 9360
Zhai, Ranran: 3270
Zhan, Heying: 4220
Zhan, Rongfang: 1055, 9500, 9515
Zhang, Wenhan: 1220, 4375
Zhang, Xu: 2440
Zhang, Aileen: 9240
Zhang, Amelia: 9130
Zhang, Anao: 3595
Zhang, Anna Y: 4280, 9360
Zhang, Bo: 9395, 1035
Zhang, Bohan: 9105
Zhang, Dan: 4555
Zhang, Fan: 9155, 1410
Zhang, Hangming: 4395
Zhang, Hui: 9510, 9515
Zhang, Jinbao: 9310
Zhang, Jingchen: 4245
Zhang, Keqing: 3110
Zhang, Lei: 9105, 9375
Zhang, Li: 9320
Zhang, Lin: 4115
Zhang, Lingling: 2435, 9035
Zhang, Meina: 9050, 9070
Zhang, Meng: 4410
Zhang, Qian: 3370
Zhang, Quanwu: 9070
Zhang, Rachel W.: 9270
Zhang, Sainan: 4265
Zhang, Shangming: 9105
Zhang, Shenghao: 4470
Zhang, Shiyang: 2450, 9440, 9460, 9505
Zhang, Tian: 9130
Zhang, Wei: 2280, 3110
Zhang, Weiwei: 3175
Zhang, Wencheng: 3225
Zhang, Wenhui: 2195
Zhang, Xiaochen: 3130, 9085, 9110, 4460
Zhang, Xinhui: 9375, 9425
Zhang, Yalu: 3370, 9375, 9425
Zhang, Yan Bing: 9515
Zhang, Yaxin: 2200, 9295
Zhang, Ying: 1485
Zhang, Yiye: 2070, 4590
Zhang, Yuan: 4205, 9025
Zhang, Yue: 9070, 9000

Zhang, Yun: 4120, 9055, 9065
Zhang, Yuteng: 9105
Zhang, Zhenmei: 2480
Zhao, David: 9295
Zhao, Erfei: 9025
Zhao, Fei: 9105, 9500
Zhao, Ou: 9295
Zhao, Qianhua: 1255
Zhao, Qianwei: 4245
Zhao, Shibei: 1270, 3275
Zhao, Xiaopeng: 3555
Zhao, Yaohui: 1460
Zhaoyang, Ruixue: 1050, 3125, 4125, 9130, 9085, 3240
Zhong, Selena: 3035
Zhou, Jing: 3300
Zhou, Yuanjin: 3000
Zhou, Yun: 3110
Zhou, Yunshu: 2085
Zhou, Allen: 4555
Zhou, Annie N.: 9155, 9175
Zhou, Anthony: 4555
Zhou, Junhong: 1280, 2130
Zhou, Kexin: 9270
Zhou, Tianjing: 1200
Zhou, Yanjun: 9270, 9395
Zhou, Zexi: 9460
Zhu, Linzee: 9050, 1045, 4015, 9505
Zhu, Shijun: 1270, 3180, 9320
Zhu, Xianghe: 3085, 9250
Zhu, Xiaonan: 2195, 3430, 9505
Zhu, Xiaoqian: 9070
Zhu, Yujun J.: 2125, 4545
Zhuang, Jie: 2085
Ziade, Garnelle: 1415
Zieschang, Tania: 4070, 4120, 4335
Zigman Suchsland, Monica L.: 3330
Zilioli, Samuele: 3550, 9380
Ziller, Erika: 4150
Zimmerman, Sheryl: 1220, 2100, 2445, 2505, 3015, 4375
Zimmermann, Jaroslava: 4550
Zinkgraf, Matthew: 9055
Zipunnikov, Vadim: 4065
Zisberg, Anna: 1095, 9050, 9390
Zissimopoulos, Julie: 3030
Zitter, Jessica: 2215
Zmora, Rachel: 1005, 4305
Zmuda, Joseph M.: 1210, 2020, 2285, 4585, 9025, 9295
Zonderman, Alan: 9505
Zonderman, Alan B.: 4180
Zuñiga, Julie: 2515
Zubatsky, Max: 4175, 9500
Zuber, Jeffrey: 3380
Zuercher, Deborah: 9395
Zulka, Linn Elena: 4485
Zullo, Andrew R.: 9395
Zullo, Joseph: 3435
Zulman, Donna: 3200
Zurek, Johanna: 2350
Zurlo, Karen A.: 2415
Zwakhalen, Sandra M.G.: 3625, 9500
Zwanziger Elsinger, Summer: 1400
Zweig, Richard: 9140, 9360, 9415
Zwetchkenbaum, Samuel: 3610
Zwicker, Jill G.: 9505
