# Correction to: Program Abstracts from The GSA 2021 Annual Scientific Meeting, “Disruption to Transformation: Aging in the ‘New Normal’”

**DOI:** 10.1093/geroni/igac013

**Published:** 2022-04-04

Due to an administrative error with the export of abstracts to production, the authors on some of the published abstracts were listed in an incorrect order. The affected abstracts have been resupplied online with the correct author listings.

